# Metagenomics Analysis Reveals an Extraordinary Inner Bacterial Diversity in Anisakids (Nematoda: Anisakidae) L3 Larvae

**DOI:** 10.3390/microorganisms9051088

**Published:** 2021-05-19

**Authors:** Susana C. Arcos, Felipe Lira, Lee Robertson, María Rosa González, Noelia Carballeda-Sangiao, Isabel Sánchez-Alonso, Laura Zamorano, Mercedes Careche, Yolanda Jiménez-Ruíz, Ricardo Ramos, Carlos Llorens, Miguel González-Muñoz, Antonio Oliver, José L. Martínez, Alfonso Navas

**Affiliations:** 1 Museo Nacional de Ciencias Naturales, Dpto Biodiversidad y Biología Evolutiva, CSIC, 28006 Madrid, Spain; scobacho@mncn.csic.es (S.C.A.); robertson.lee@inia.es (L.R.); mrosaglopez@mncn.csic.es (M.R.G.); yjr@mncn.csic.es (Y.J.-R.); 2 Centro Nacional de Biotecnología, Departamento de Biotecnología Microbiana, CSIC, 28049 Madrid, Spain; felipelira3@gmail.com (F.L.); jlmtnez@cnb.csic.es (J.L.M.); 3 Departamento de Protección Vegetal, INIA, 28040 Madrid, Spain; 4 Servicio de Immunología, Hospital Universitario La Paz, 28046 Madrid, Spain; noeliacarba@hotmail.com (N.C.-S.); mgonzalez_munoz@hotmail.com (M.G.-M.); 5 Instituto de Ciencia y Tecnología de Alimentos y Nutrición, CSIC, 28040 Madrid, Spain; isabel.sanchez@csic.es (I.S.-A.); mcareche@ictan.csic.es (M.C.); 6 Servicio de Microbiología y Unidad de Investigación, Hospital Son Espases, (IdISPa), 07120 Palma de Mallorca, Spain; laura.zamorano@ssib.es (L.Z.); aoliverp@yahoo.es (A.O.); 7 Unidad de Genómica, “Scientific Park of Madrid”, Campus de Cantoblanco, 28049 Madrid, Spain; ricardo.ramos@fpcm.es; 8 Biotechvana, “Scientific Park”, University of Valencia, 46980 Valencia, Spain; carlos.llorens@biotechvana.com

**Keywords:** anisakids, microbiota, nematode-bacteria association

## Abstract

L3 larvae of anisakid nematodes are an important problem for the fisheries industry and pose a potential risk for human health by acting as infectious agents causing allergies and as potential vectors of pathogens and microrganisms. In spite of the close bacteria–nematode relationship very little is known of the anisakids microbiota. Fresh fish could be contaminated by bacteria vectored in the cuticle or in the intestine of anisakids when the L3 larvae migrate through the muscles. As a consequence, the bacterial inoculum will be spread, with potential effects on the quality of the fish, and possible clinical effects cannot be discarded. A total of 2,689,113 16S rRNA gene sequences from a total of 113 L3 individuals obtained from fish captured along the FAO 27 fishing area were studied. Bacteria were taxonomically characterized through 1803 representative operational taxonomic units (OTUs) sequences. Fourteen phyla, 31 classes, 52 orders, 129 families and 187 genera were unambiguously identified. We have found as part of microbiome an average of 123 OTUs per L3 individual. Diversity indices (Shannon and Simpson) indicate an extraordinary diversity of bacteria at an OTU level. There are clusters of anisakids individuals (samples) defined by the associated bacteria which, however, are not significantly related to fish hosts or anisakid taxa. This suggests that association or relationship among bacteria in anisakids, exists without the influence of fishes or nematodes. The lack of relationships with hosts of anisakids taxa has to be expressed by the association among bacterial OTUs or other taxonomical levels which range from OTUs to the phylum level. There are significant biological structural associations of microbiota in anisakid nematodes which manifest in clusters of bacteria ranging from phylum to genus level, which could also be an indicator of fish contamination or the geographic zone of fish capture. Actinobacteria, Aquificae, Firmicutes, and Proteobacteria are the phyla whose abundance value discriminate for defining such structures.

## 1. Introduction

Nematodes of the family Anisakidae are common parasites of marine mammals, where these nematodes complete their biological cycle reaching the adult stage. With worldwide geographic distribution, the L3 larvae of these nematodes (larvae in the third stage) are an important problem for the fisheries industry [1] due the commercial fishes being very important paratenic hots. The L3 larvae of anisakids pose a risk for human health by acting as agents causing allergies [2,3], as potential vectors of allergens in foods (fish and fish products) [4,5] and pathogenic microoganisms [6]. These nematodes can infect humans an other hosts as fishes and cephalopods which are considered accidental and paratenic host, respectively. After the first clinical case in 1960 associated with the consumption of fresh raw fish infected by *Anisakis* [7], the incidence of these parasites is today considered an international public health problem. Although the parasite does not develop and complete the biological cycle in the humans [8], the infestation by these nematodes or their excreted-secreted products in fishes may cause severe allergies [4,9] in an ever increasing, more sensitized worldwide population. According to the “Rapid Alert System for Food and Feed of the European Union” *A. simplex sensu lato* is considered responsible for more than 33% of the biological risk alerts in Europe [10]. The epidemiological studies on the incidence of Anisakiasis (or Anisakiosis) in the Spanish population conclude that it is one of the effects of nematodes with the highest prevalence in Spain with some regional variations ranging from 0.43% to 22% [11]. It is considered a problem associated with Spanish customs and habits given that Spain is one of the main consumer countries of fish and seafood products. The family Anisakidae (Nematoda: Rhabditida: Ascaridomorpha) is represented by at least 24 genera split into three subfamilies: Goeziinae (parasites of Teleostei), Raphidascaridinae (fish parasites in general and occasionally birds) and Anisakinae (parasites of mammals, birds, reptiles and elasmobranch fish). Seven genera of Anisakidae are paratenic or obligatory parasites of marine fishes for human consumption (generally teleostii) [*Anisakis*, *Paranisakis*, *Contracaecum* (*Thynascaris*), *Phocanema* (*Pseudoterranova*), *Raphidascaris* (*Hysterothylacium*), *Sulcascaris* and *Terranova*] [12]. The best known are *Phocanamea* (*Pseudoterranova*), *Contracaecum* (*Thynascaris*), *Raphidascaris* (*Hysterothylacium*) and *Anisakis*. Although the real incidence of other species of the genus *Anisakis* in humans is not known, the species complex *Anisakis simplex sensu lato* (formed by three species *A. simplex sensu stricto*, *A. pegreffii* and *A. berlandi* [13]) is considered responsible for the majority of parasitosis and allergies by means of the consumption of raw or insufficiently processed or cooked fish in Spain and worldwide [14,15]; the main reason could be due to it being the most frequent species of the family Anisakidae. The infestation of fishes by other Anisakidae is considered restricted to certain geographic areas and associated mainly with cod (infestation by *Pseudoterranova decipiens*) or herring (*Clupea* spp.); however, the distribution of anisakid species is universal and having “multi hosts”, with the common knowledge of the presence of L3 larvae of other anisakids in commercial fishes, which are not members of the genus *Anisakis.*

Nematodes are able to provide valuable information on bacterial virulence mechanisms which are difficult to obtain with traditional animal models [16,17]. In the review of Wekell, et al., [6] at least, 69 pathogenic microorganisms were considered to be mainly propagated through seafood products, suggesting that control of seafood is a relevant aspect in the sanitary alert in the public national health system. When *Caenorhabditis elegans* have been used as animal model [18] it has been observed that numerous bacteria infect the intestine and cuticular surface, a situation that may ground the hypothesis of dispersing infections among populations in contact with nematodes. It has also been demonstrated that bacteria that may be a risk for human health are also associated to nematodes, including: *Aeromonas hydrophila* [19], *Enterococcus faecalis* [20], *Escherichia coli* [21], *Salmonella* enterica [22], *Serratia marcescens* [23], *Staphylococcus aureus* [24], *Streptococcus* spp. [25] and *Yersinia* spp. [26]. Despite this close bacterial–nematode relationship very little is known about the role of nematodes in general in the spread of infectious diseases, a feature particularly relevant in the case of anisakids given the cosmopolitan nature of the family Anisakidae. It is worth mentioning that fresh fish containing anisakids could be contaminated by bacteria vectored in the cuticle or in the intestine of these nematodes when the L3 larvae migrate through the muscles. As a consequence, the bacterial microbiota associated to anisakids will be extended through all fish meat, affecting the shelf life and quality of the product, as well as acting as transmitters of potential pathogens. Indeed, to the known allergenic processes there are other symptomatic effects linked to anisakids intake when raw or poor cooked fish include, diarrheas, vomits, fever and abdominal pain, with all possible combinations [27], all symptoms of bacterial infections.

In this work we have studied the microbiome of Anisakidae based on the examination of fishes parasitized by larval specimens in the same development stage (third stage larvae, L3) captured in waters from the FAO 27 fishing area (a very important commercial area for Europe) in a general survey carried out in the Central Fish Market of Madrid (Mercamadrid).

## 2. Material and Methods

### 2.1. Anisakids Collection and Taxonomic Identification

Anisakids were collected during a general survey carried out in Mercamadrid Central Fish Market, related to the incidence of these nematodes in commercial fish species. All specimens studied were captured in the FAO 27 fishing area of distribution. Pools of L3 larvae specimens (12–20) were randomly obtained from each of 113 different individual fishes hosts in which the presence of anisakids were evident in a visual inspection (Additional file 1: Table S1); they were removed from each fish following published procedures [28,29,30]. The 113 larvae pools were rinsed in 0.9% saline solution and placed in an antibiotic-antimicotic solution (80 mg gentamicin sulphate, 0.625 mg amphotericine B, 10,000 IU penicillin G, 10 mg streptomycin sulphate, 4.5 mL of saline Hank´s solution making up to 10 mL of volume with bi-distilled water; Sigma Aldrich, St. Louis, MO, USA). After 40 min in this disinfectant solution the larvae pools were rinsed in bi-distilled water for 1 h. From each of 113 larvae pools (from each fish host), a specimen was isolated and for each of these larvae specimens, the caudal part was used for DNA extraction and polymerase chain reaction (PCR) amplification for species identification; this caudal part and the rest of the body (used for 16S rRNA gene amplification) were separately stored at −80 °C until required. anisakids species identification was performed following the taxonomic criteria of [31,32] by using the ITS1 region of the nuclear ribosomal DNA (rDNA). Briefly, individual anisakids L3 stage juveniles were placed in an Eppendorf tube after previously having a small part of the caudal region removed to allow molecular identification of each individual. DNA was extracted and purified using the Speedtools Tissue DNA Extraction Kit (Biotools) following manufactures instructions. Molecular identification was carried out for each individual using PCR-RFLP. The forward primer A 5′-GTCGAATTCGTAGGTGAACCTGCGGAAGGATCA-3′ and reverse primer B 5′-GCCGGATCCGAATCCTGGTTAGTTTCTTTTCCT -3′ [32] were used in reactions containing, 10 mM Tris-HCl (pH 8.3), 1.5 mM MgCl2, 50 mM KCl, 200 µM each of dATP, dCTP, dGTP and dTTP and 1 unit of DNA polymerase (Biotools B and M labs, S.A. Madrid, Spain). Initial denaturalization was carried out for a period of 2 min at 94 °C followed by 35 cycles of 94 °C for 1 min, 58 °C for 1 min, 72 °C for 1 min followed by a final 7 min extension at 72 °C. Amplified DNA fragments were digested with the restriction enzymes HhaI and HinfI (New England Biolabs, Massachusetts, MA, USA) following the manufacturer’s instructions. Restriction fragments were separated by electrophoresis in aTris- Borate-EDTA (TBE) buffered 2.5% agarose gel, stained with SYBER Safe DNA gel stain and visualized with ultraviolet (UV) illumination.

### 2.2. 16 S rRNA-rRNA Amplicons, Preparation and Illumina Sequencing

Total DNA of L3 individuals without caudal parts previously used for taxonomic anisakids identification were extracted as above [Speedtools Tissue DNA Extraction Kit (Biotools, B and M labs, S.A. Madrid, Spain)]. Purified DNAs were quantified by Picogreen and 3ng of input DNA were used for specific amplification using the primers.

5’-ACACTGACGACATGGTTCTACACCTACGGGNGGCWGCAG-3’ and

5’-TACGGTAGCAGAGACTTGGTCTGACTACHVGGGTATCTAATCC-3’,

These amplify the V3-V4 region of 16S rRNA gene [33] and include an extension tail that allows further processing. As standard, the first PCR was done for 20 cycles using Q5^®^ Hot Start High-Fidelity DNA Polymerase (New England Biolabs, Massachusetts, MA, USA) in the presence of 100nM primers. Negative non-template controls were included in every round of PCR amplification made to check the possible presence of contaminants at the reagent level, showing in all cases completely negative results. Positive amplifications were assessed by gel electrophoresis and positive samples were used for a second PCR of 15 cycles using Q5^®^ Hot Start High-Fidelity DNA Polymerase (New England Biolabs, Massachusetts, MA, USA) in the presence of 400nM of second-step primers:

5′-AATGATACGGCGACCACCGAGATCTACACTGACGACATGGTTCTACA-3′ and 5′-CAAGCAGAAGACGGCATACGAGAT-[10 nucleotides barcode]-TACGGTAGCAGAGACTTGGTCT-3′) which anneal over the extension of the first primer pair and serve also for sample barcoding, were taken from the Access Array Barcode Library for Illumina Sequencers (Fluidigm). The final amplicons were validated and quantified in a Bioanalyzer (Agilent) so that an equimolecular pool could be prepared. The pool of amplicons was further purified by agarose gel extraction and was titrated after purification and checking by quantitative PCR, using the “Kapa-SYBR FAST qPCR kit forLightCycler480” and a reference standard for quantification. This pool was finally denatured and seeded on an Illumina flow cell at a density of 10pM, where clusters were formed and sequenced using a “MiSeq Reagent Kit v3”, in a 2 × 300 pair-end sequencing run on a MiSeq sequencer (illumina).

### 2.3. Bioinformatics Analysis and Data Availability

To analyze the structure of the microbiota, the V4 variable region of the microbial 16S rRNA RNA was amplified using the standard Illumina primers (Illumina Inc., San Diego, CA, USA). The resultant libraries were sequenced at the facilities from the “Fundación Parque Científico de Madrid” (FPCM, Campus de Cantoblanco, Spain) using the Illumina platform in a pair-end reads with 280 cycles. Approximately 25 Mb were sequenced from the 113 microbiota with an average of ~250,000 reads for each sampled microbiota (151,171 bp to 412,396 bp) and were analyzed using MOTHUR v.1.35.1 software [34] following the standard procedure (SOP) for Illumina MiSeq sequences (http://www.mothur.org/wiki/MiSeq_SOP, accessed on 16 March 2017) to check the raw data using the FASTQC tool [35]. The sequences were filtered by quality using PRINSEQ-lite v.0.20.4 [36]. To this work, some modifications at the SOP were implemented [37]. Quality control of sequences eliminated low-quality reads (shorter than 200 bp and average quality lower than 25) as described in [38]. The paired-end reads were assembled and sequences were removed if they did not have a length ranging between 280 and 320 base pairs. After assembling, all sequences were trimmed to start and finish at the same coordinates [39]. Unique sequences from all samples were merged and aligned to the SILVA (https://www.arb-silva.de, accessed on 28 January 2017) bacteria reference database [40,41]. Operational taxonomic units (OTUs) were identified and grouped using the software package MOTHUR v.1.31.2 following the recommendations of the standard operating procedure for Illumina MiSeq platform (https://www.mothur.org/wiki/MiSeq_SOP, accessed on 16 March 2017).

According the pipeline followed, paired-end reads were merged and chimeric reads were removed using USEARCH v6.1 [42,43] implemented in MOTHUR. After, resultant sequences were clustered into taxonomic units (OTUs). The resulting sequences that were not classified at least at the kingdom level or identified as Archaea, Eukaryota, chloroplast or mitochondria were removed from the analysis. After, the sequences were classified into groups corresponding to their taxonomy at different levels (kingdom, order, family and genus) and then assigned to OTUs using a phylotype threshold of equal or greater than 97% with further taxonomic classification using the Silva database [44]. After OTU assignation, the data were corrected to fix the misallocated taxa adjusting it to the right level using a custom Perl script. All taxa were counted with respect to their presence in each sample and the taxa with less than 20 sequences counted in all 113 samples were considered less representative and then were culled to avoid misunderstood analysis. Taxa with ≥20 sequences were maintained for further analysis.

### 2.4. Statistical Analysis

MOTHUR was also used to generate diversity indices (Simpson and Shannon) and rarefaction curves of samples. The Renyi diversity index was used to explore differences in OTU diversity among L3 anisakids individuals. This index provided the profile of four diversity indices (total richness, Shannon–Weiner, Simpson–Yule, and Berger–Parker) according a value (α). The Renyi index has the advantage that allows in a glance to compare independence of samples diversity; that is, when different profiles intercept, their diversity are non-comparable [45]. Calculation of Renyi index was carried out using PAST software [46] (http://folk.uio.no/ohammer/past, accessed on 4 April 2020).

MOTHUR was also used for quantitative analysis by means of correlations (Pearson and Spearman) according to number of sequences in each individual (samples). The programme also provides cluster analysis based on correlations indices in order to detect nematode taxon or host (fish) influence. For detection of bacterial biota structure (clusters or bacterial association), we have used Statistica v.6 programme (STATISTICA data analysis software system, version 6. 2001, www.statsoft.com, accessed on 19 July 2019) [47].

Cluster analysis of samples and factorial analysis, including hierarchical analysis option for classification clusters of variables into de factorial space was performed. Microbiota comparison of clusters was made by the discriminant analysis option after logarithm transformation of abundance [log (x + 1)]. When needed, graphic representation of most significant microbiota was referred to according to those values and clusters of samples. Linkage of clusters of samples with inferred microbiota structure was performed by projection of the active and supplementary variables option through the principal component option into the factor analysis programme; this was used due the facilities for hierarchical analysis of oblique factors which allow the number of clusters in the set of variables to be detected.

## 3. Results

The 113 larva L3 individuals used for this study were taken from ten different fish species (Additional file 1: Table S1): *Micromesistius poutassou* (9), *Merluccius merluccius* (76), *Conger conger* (2), *Gadus morhua* (2), *Lepidorhombus boscii* (9), *Lophius budegassa* (5), *Lophius piscatorius* (5), *Phycis blennoides* (2), *Scomber scombrum* (1), *Thunnus thynnus* (2). The anisakids taxa resulted as: *Pseudoterranova* sp. (5), *Anisakis simplex* (100), *A. pegreffii* (7) and hybrid haplotypes from *A. simplex* and *A. pegreffii* (1). The asymmetric representation of these variables in the survey avoid the use of standard parametric statistic. However, the use of a multifactorial approach made it possible to detect the importance of each of the variables related to microbiota, even if they are spurious.

A total of 2,689,113 sequences were generated (average of 23797 sequences per analyzed sample) at the different taxonomical level (phyla, class, order, family, and genus) of associated microbiota based on the 16S rRNA gene sequences for OTUs (Supplementary Materials Table S2). The bacterial lineages which have been detected based on 16S rRNA gene sequencing follow a conservative taxonomy. Fourteen phyla have been taxonomically characterized through 1803 different sequences quoted to OTUs of which, 105 are “unclassified” (5.82% of the whole sequences). In total, 14 phyla, 31 classes and 52 orders were unambiguously identified (Table 1) except four orders from the phylum Cyanobacteria (from classes MLE1-12, Subsection IV and Subsection III in addition to other unclassified classes). Table 2, Table 3 and Table 4 shows the systematics of orders, families (129) and genera (187) (only unambiguous genera are considered; 6 with very scarce representation and 13 unclassified genera were excluded). For most of the Cyanobacteria it was not possible to classify them at phylum level. Proteobacteria (Table 2) is represented by 92 genera (40% are members of the Class Alphaproteobacteria), Firmicutes (75 genera, from which 68% are members from the order Clostridales) and Actinobacteria (Table 3) (46 genera, from which 74% are from the order Actinomycetales) are the most diverse phyla detected in this study. The genera of these three phyla represent 81% of the total associated genera of anisakids. Taking this into account, the class Alphabacteria represented by 7 orders and 55 genera should be the most important from Proteobacteria; Rhizobiales (20 genera), Rhodospirales (14 genera) and Sphingomonadales (at least 9 genera) are the most important orders from Proteobacteria. Class Clostridia would be the most important from Firmicutes, especially the order Clostridiales (51 genera) and class Actinobacteria represented by order Actynomycetales (34 genera) from the phylum Actinobacteria. The phyla Acidobacteria, Aquificae, Clamydiae, Deinococcus-Thermus, Fusobacteria, Planctomycetes, Synergetes and Verrcomicrobia have a low genera’s diversity in anisakids (Table 4).

The presence (frequency) and abundance of phyla and orders in the individuals of anisakids (samples) is summarized in Table 1: Actinobacteria, Bacteroidetes, Firmicutes, Fusobacteria, Proteobacteria and Cyanobacteria are present in all anisakids specimens. Other important presence is for Acidobacteria (present in 76% of samples), Chloroflexi (74.33%), Planctomycetes (63.72%) and Deinococcus-Thermus (61.94%). There is a minor presence of Verrucomicrobia (23.9%), Chlamydiae (15.93%), Synergistetes (10.62%) and Aquificae (2.65%). The most abundant phyla into the anisakids are Proteobacteria (with an average of 9291 bacteria per L3 larvae), Firmicutes (average 5659) and Actinobacteria (average 2838). Fusobacteria (average 755), Bacteroidetes (average 705) and Cyanobacteria (average 297) could be considered of medium abundance in the L3 larvae compared with the rest of the phyla which range from 9 to 76 bacteria per L3 larva: Aquificae (average 76), Synergistetes (average 73), Chloroflexi (average 66), Acidobacteria (average 59), Planctomycetes (average 35), Deinococcus-Thermus (23), Verrucomicrobia (average 17) and Chlamydiae (average 9).

Orders with higher presence (frequency) range from 75% to 100% considering the total individuals (samples). These are: Acidobacteriales, Actinomycetales, Bacteroidales, Flavobacteriales, Sphingobacteriales, Deinoccocales, Bacillales Lactobacillales, Clostridiales, Fusobacteriales, Caulobacterales, Rhizobiales, Rhodobacterales, Rhodospirillales Sphingomonadales, Burkholderiales, Neisseriales, Campylobacteriales Enterobacteriales, Pasteurellales, Pseudomonadales and Vibrionales. Orders present in the samples (anisakids individuals) with a frequency between 40% to 75% are: Acidimicrobiales, Bifidobacteriales, Coriobacteriales, Erysipelothrichales, Planctomycetales, Aeromonadales, Alteromonadales and Xanthomonadales. Orders with a frequency between 10% to 40% are: Rubrobacterales, Chlamydiales, Anaerolineales, Chloroflexales, Bdellovibrionales, Myxococcales, Cardiobacteriales and Oceanospirillales, Finally, orders with a frequency below 10% in the total of samples are: Aquificales, Caldilineales, Thermales, Mycoplasmatales, Thermoanaerobacteriales, Phycisphaerales, Parvularculales, Rickettsiales, Desulfobacterales, Desulfovibrionales, Desulfuromonadales and Cardiobacteriales. The most abundant orders (number of bacteria per L3 larva) are clearly differentiated from the rest: Actinomycetales (2625), Clostridiales (4450), Rhizobiales (2472) and Sphingomonadales (2692). There are other group of orders whose abundance into the anisakids individuals range between 300 to 800 bacteria per juvenile L3 such as: Lactobacillales, Fusobacteriales, Caulobacterales and Pseudomonadales. The rest of the orders have an average abundance under 300 bacteria per L3 larva.

### 3.1. Microbial Richness and Diversity of Samples (Anisakids Individuals)

In order to avoid redundancy, the statistical analysis was applied to phylum, order, family and genus levels where taxonomy was well established.

**Figure 1:**
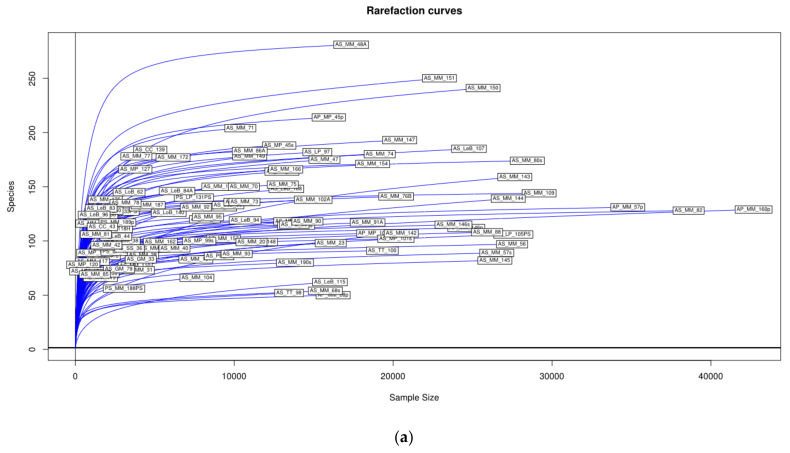

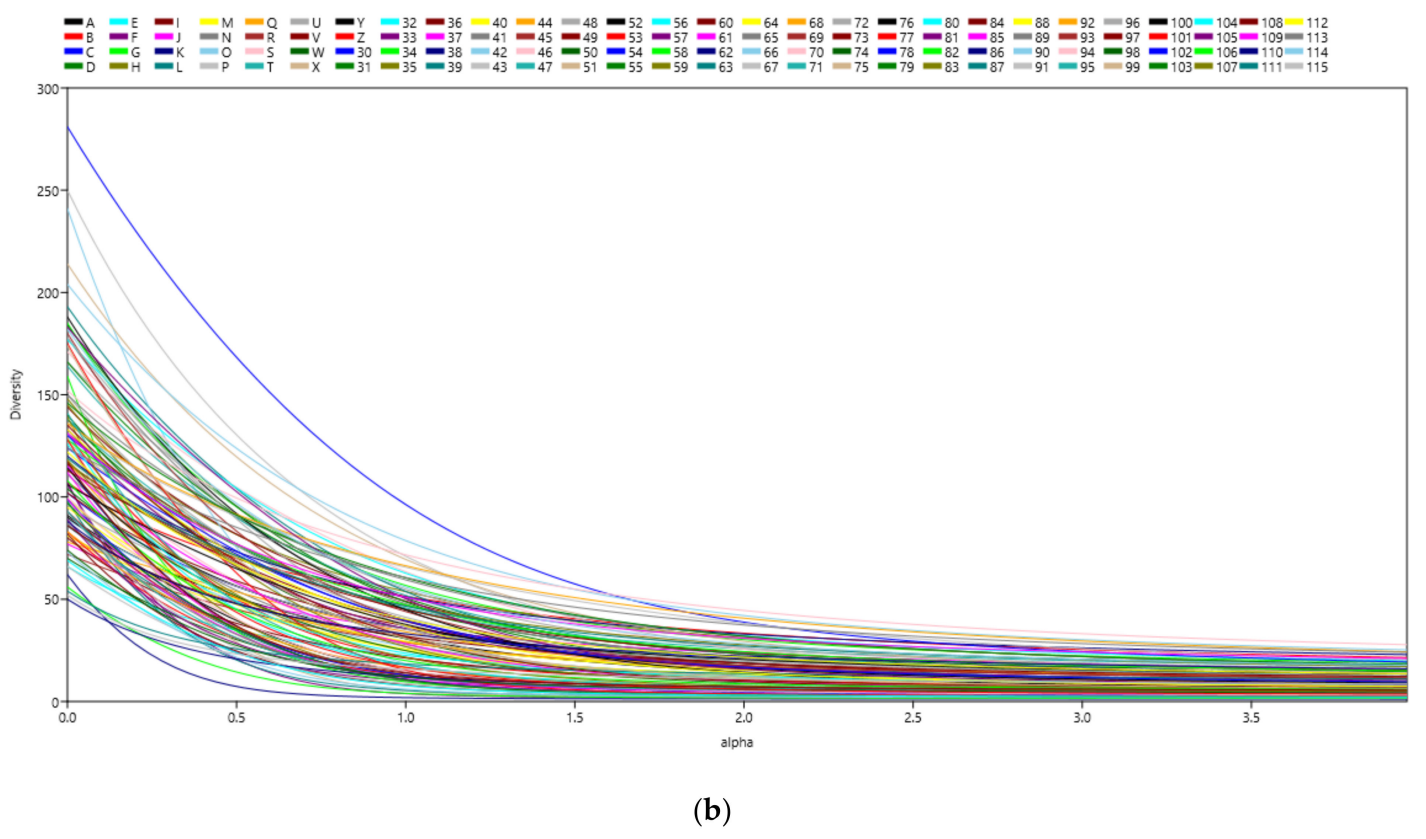
Diversity profiles. (**a**) Rarefaction curves of operational taxonomic units (OTUs) which are clustered at 97% sequence identity across the 113 samples of anisakids. All samples are represented. None fish host or anisakids species seems to be related with the bacterial OTUs diversity structure. (**b**) Diversity ordering by mean of Renyi´s index family curves showing differences in OTUs diversity among each anisakid individuals. The Renyi index estimates total richness for a = 0, Shannon-Weiner index for α = 1, the inverse Simpson-Yule index for α = 2 and 1/Berger-Parker index for α = Infinite. Most of the anisakids individuals are non-comparable because their diversity profiles are intersecting.

The rarefaction curves per each sample indicate that most of the OTUs in the “sampled community” have not been observed because new OTUs would be found with additional sampling. According to our data, the “asymptotic stage” would be reached in 80% if we would have taken 2500 samples (Figure 1a) (values in Supplementary materials Table S3). In the present study, based only in 113 samples (L3 individuals), we found an average of 123 OTUs per L3 individual (minimum 20 OTUs, maximum 281 OTUs). All of this indicates the extraordinary diversity of bacteria associated to anisakids nematodes and also that this study can be considered representative of that diversity. The diversity ordering by mean of Renyi´s index family curves (Figure 1b) shows independent differences in OTUs diversity comparing all anisakids individuals demonstrating than diversity among them are non-comparable (most of the diversity profiles intersect among them). The use of Shannon and Simpson diversity indices scatter graphically the samples (Supplementary materials Figure S1a,b) indicating again an extraordinary diversity of bacteria at OTUs level. These values range from 0.3 to 4.7 for the Shannon and from 2 to 45 for the Simpson diversity indexes (the inverse option better represents diversity). Both indices clearly detected clusters of samples which are not significantly related with fish hosts or anisakid taxa at the phyla or order levels. This implies that any relationship would exist at a genus or family level without the influence of fish species. The lack of relationships of the microbiome composition with hosts of anisakids taxa has to be expressed by the association among bacterial OTUs or other taxonomical levels which are ranging from OTUs to phylum level.

### 3.2. Quantitative Analysis (Correlation) Does Not Support Inference of Microbiota Structure

Little correlation was obtained at the OTUs level considering the total number of samples if Pearson or Spearman correlation indices are used (Additional file 3: Figure S2a,b). The average number of sequences per phylum, anisakids and fish hosts are calculated for the most frequent bacteria phyla according to the frequency and are represented in Supplementary materials Figure S3a–c, Table S4. At a bacterial phyla level, Pearson correlation index express better than Spearman some quantitative relationship among anisakids and fish hosts (Supplementary materials Figure S4a,b). Hosts (fish and Anisakids) seems to have some influence in the abundance of each phyla within the microbiome according to the Pearson index. Using this analysis, we found two positive correlations among *A. simplex*, *A. pegreffii*, their hybrids and *Pseudoterranova* sp. when their hosts are SS (*Scomber scombrum*), MM *(Merluccius merluccius)*, TT (*Thunnus thynnus*), LoB (*Lophius budegassa*), GM (*Gadus morhua*), MP (*Micromesistius poutassou*) and CC (*Conger conger*) and another positive correlation between *A. simplex* and *A. pegreffii* when the hosts are LP (*Lophius piscatorius*), MP (*Micromesistius poutassou*), PB (*Phycis blennoides*) and LeB (*Lepidorhombus boscii*). This (the Pearson correlation) could be considered the unique quantitative relationships among bacterial phyla, anisakids and fishes. No other taxonomical level of bacteria below phylum showed any significance. Supplementary materials Table S5 shows the abundance values of the most representatives orders according to anisakids and hosts, while their frequency profiles are represented in Supplementary materials Figure S5a–c. Order composition and their relationships according to anisakids species and fish hosts does not support also the influence of hosts in the microbiota relationships (Supplementary materials Figure S6a,b) above order level, but little further information could be obtained according the data reflect the lower taxonomical level, as is the family and genus level. For this reason we think than an analysis of the microbiome structure, in other words the bacterial clusters that are associated across the different samples, would clarify the factors, which lead the associated microbiome structure to anisakids nematodes.

### 3.3. Microbiota Structure Associated to Anisakids

**Figure 2:**
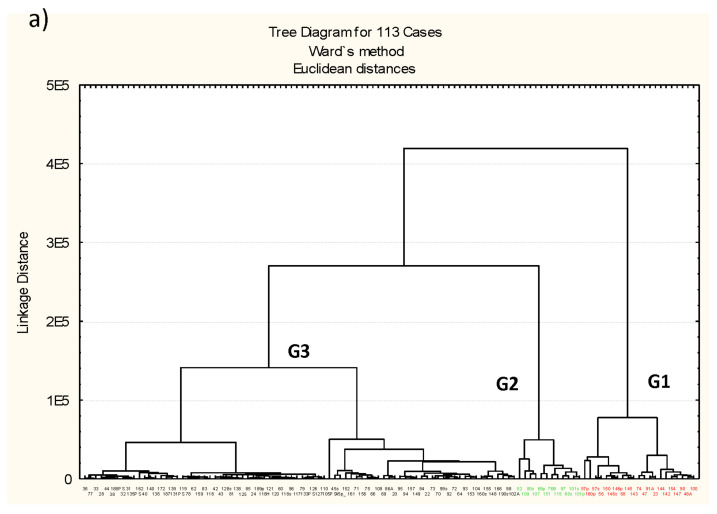

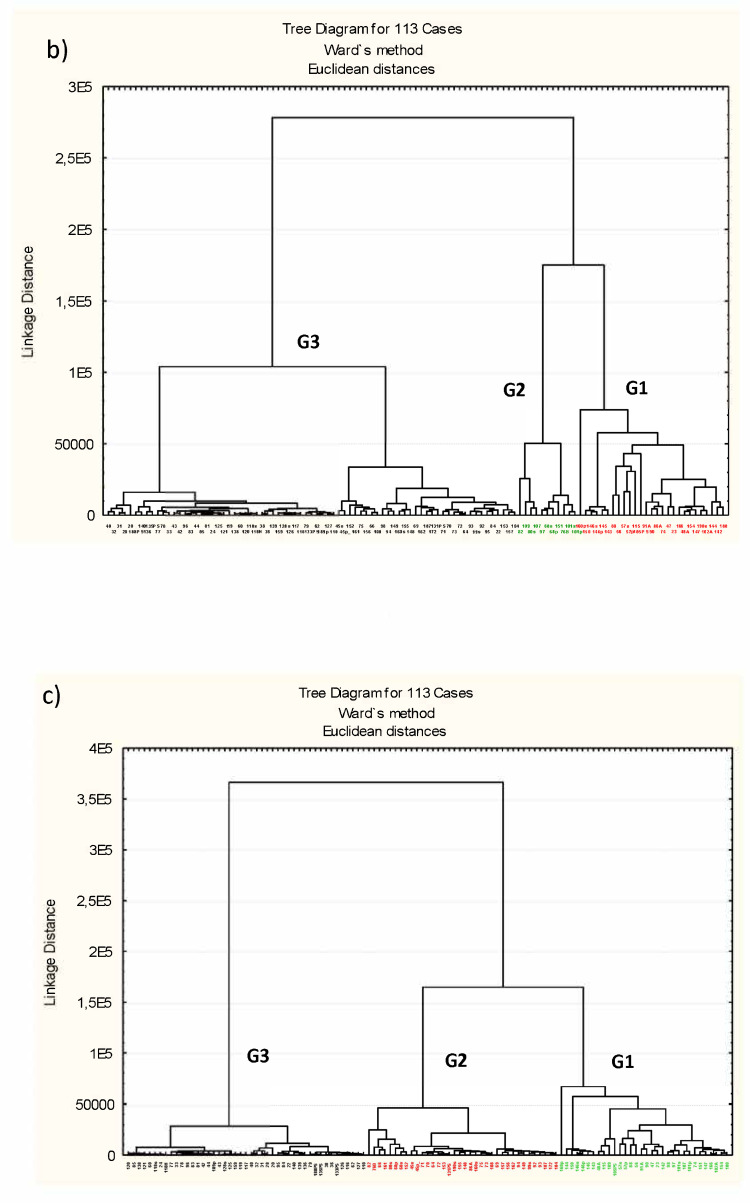
Cluster analysis of anisakids samples L3 considering three different taxonomic levels of bacteria; phyla (**a**), order (**b**) and genus (**c**). Substantially the same structure is maintained from phyla to order level. At genus level a new significant cluster (green) is formed joining samples from red and green clusters from higher taxonomic levels, while the extended black cluster from phyla and order levels is split in two (black and red). This structure is manifested by ordination methods through correspondence and detected by the factorial analysis (Figure 3, Figure 4 and Figure 5).

Discriminant cluster analysis (Figure 2a–c) of samples based in microbiome composition reflects almost an identical structure of samples through phylum and order levels (Figure 2a,b). At a class level the cluster distribution of the samples is identical to phylum (data at class level not shown); both are practically the same as at order level. The only difference of cluster based in order level with that based on phylum level is that some samples formerly present in a group (G2 or G3) in a phylum cluster are “moving” to other group in order cluster (sample 115 from G2 to G1 and samples 86A, 102A and 105PS from G3 to G1). At the family and genus level, it is almost identical (data at family level is not shown), however the structure of the cluster at family and genus levels (Figure 2c) is very different to the former clusters phylum to order (Figure 2a,b).

Groups of samples are statistically compared by means of discriminant analysis reflecting the differences among them and determining which variables (bacteria) are the most important included in the analysis (Table 5). Actinobacteria (total average = 2,838,283), Aquificae (2,017,699), Firmicutes (total average = 5,659,035) and Proteobacteria (total average = 9,291,938) are the phyla whose abundance determines the sample groups in the clusters. Aquificae, exclusively represented by the genus *Hydrogenobacter* (total average = 20,176), is absent in G1, with very scarce representation in G3. The statistical values are very significant and also the percentage of classification for each group of samples; total percent of correct classification is 96.46% (Table 5a). When orders are included (Table 5b) the statistical significance as well as the percent of correct classification is also very high (97.34%). Actinobacteria is exclusively represented by the order Actinomicetales (34 genera, including some unclassified), Firmicutes is represented by the orders Bacillales (5 genera including some unclassified) and Clostridiales (51 genera including some unclassified) and Proteobacteria is represented by the orders Campylobacterales (3 genera: *Arcobacter*, *Campylobacter* and *Sulfurospirillum*), Caulobacterales (4 genera: *Caulobacter*, *Brevundimonas*, *Phenylobacterium* and *Asticcacaulis*). Table 5b also includes the orders Anaerolineales (one unclassified genus) and Caldilineales (genus *Caldilinea*) from the phylum Cloroflexi and the order Bacteroidales (6 genera) from phylum Bacteroidetes which did not reach the established level of significance (*p* < 0.05) in Table 5a. At the family and genus levels (Figure 2c) the clusters of samples maintain three different groups (G1, G2 and G3). But the new G1 is mainly formed with samples which were formerly at Order level from G1, five samples of G2 (97, 101s, 101p, 107, 109, 151) and one sample from from G3 (98). The discriminant analysis was not applied at this level due the number of “empty cells” in the data matrix.

**Figure 3:**
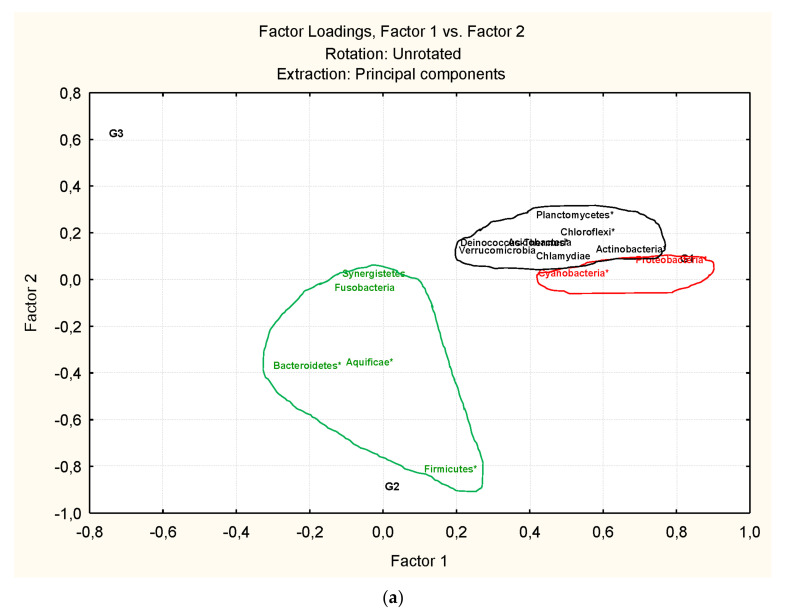

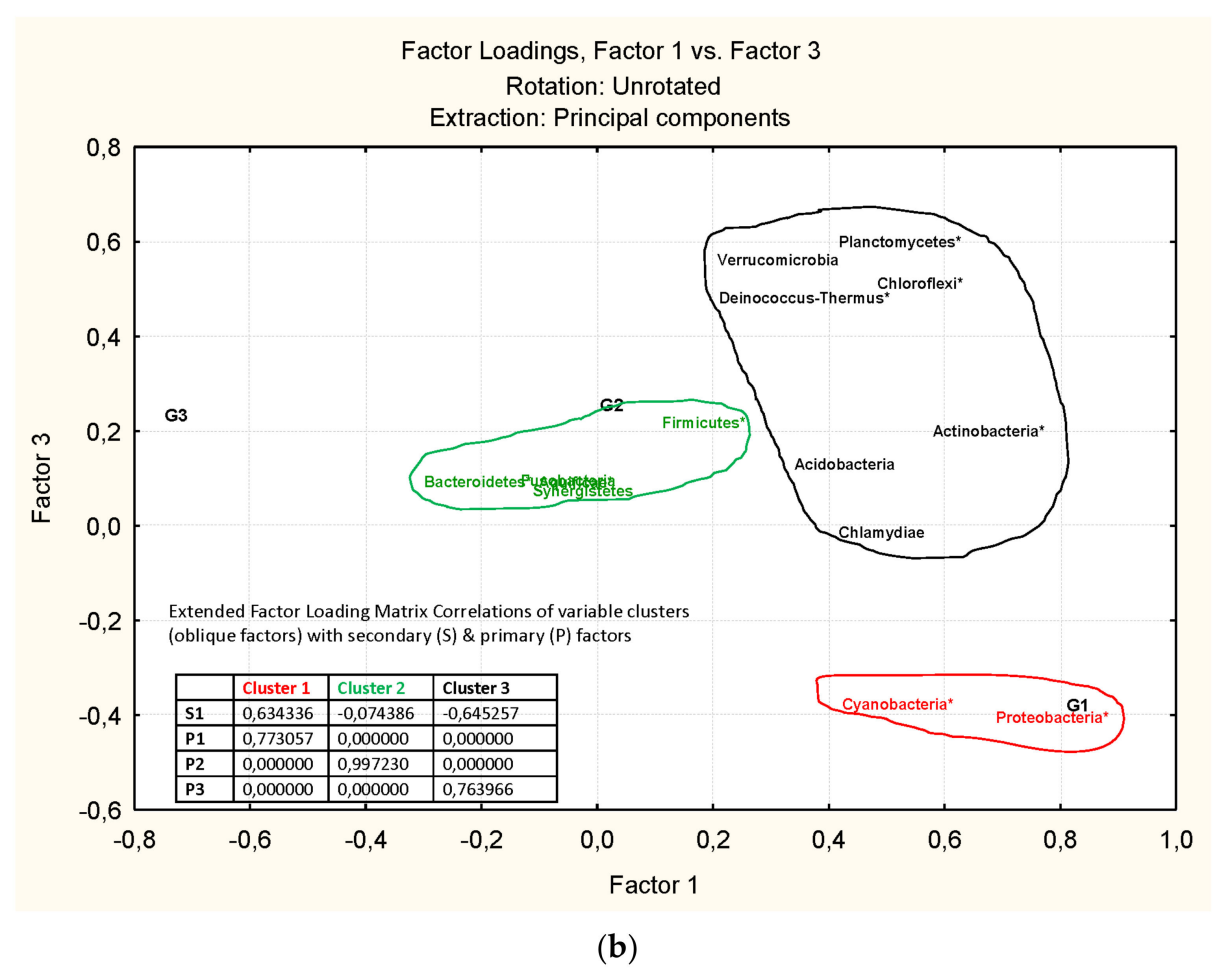
Factorial Analysis considering the anisakids samples and bacteria at phyla level. In the factorial space defined by Factor 1 vs. Factor 2 (**a**) and Factor 1 vs. Factor 3 (**b**) can be seen the correspondence with the classified clusters of Figure 2a.

Factor analysis allows us to define the number of clusters of bacteria in addition to their association (microbiota association) in spite of the fact that the percentage of representation is low when the number of variables is high as is the case at the genus level. This method combines the bacteria (active variables) explaining the total variability of the factorial space, with the bacteria which explain the variability into each cluster. At the phylum level (Figure 3) the percentage of explanation for the first three factors is 46.62% of the total. The correlations of variables clusters with what are the primary and secondary factors are very high indicating the existence of at least three clusters of phyla: cluster 1(Cyanobacteria and Proteobacteria), cluster 2 (Bacteriodetes, Aquificae, Firmicutes, Fusobacteria and Synergistetes) and Cluster 3 (Verrucomicrobia, Planctomicetes, Chlofoflexi, Deinococcus-Thermus; Acidobacteria, Actinobacteria, Chlamidiae;). The association defined by cluster 1 (Cyanobacteria, Proteobacteria) should be more specific of samples from group 1 (G1); the association defined by cluster 2 (Bacteriodetes, Aquificae, Firmicutes, Fusobacteria and Synergetes) is more related to samples from group 2 (G2) while the other association from cluster 3 is mainly related to Group 1 and 2. Group 3 of samples gather all the associations. The most important phyla to establish the association among them are: Proteobacteria and Cyanobacteria (Cluster 1); Aquificae, Bacterioidetes and Firmicutes (Cluster 2) and Actinobacteria, Chloroflexi, Deinococcus-Thermus and Planctomycetes (Cluster 3).

**Figure 4:**
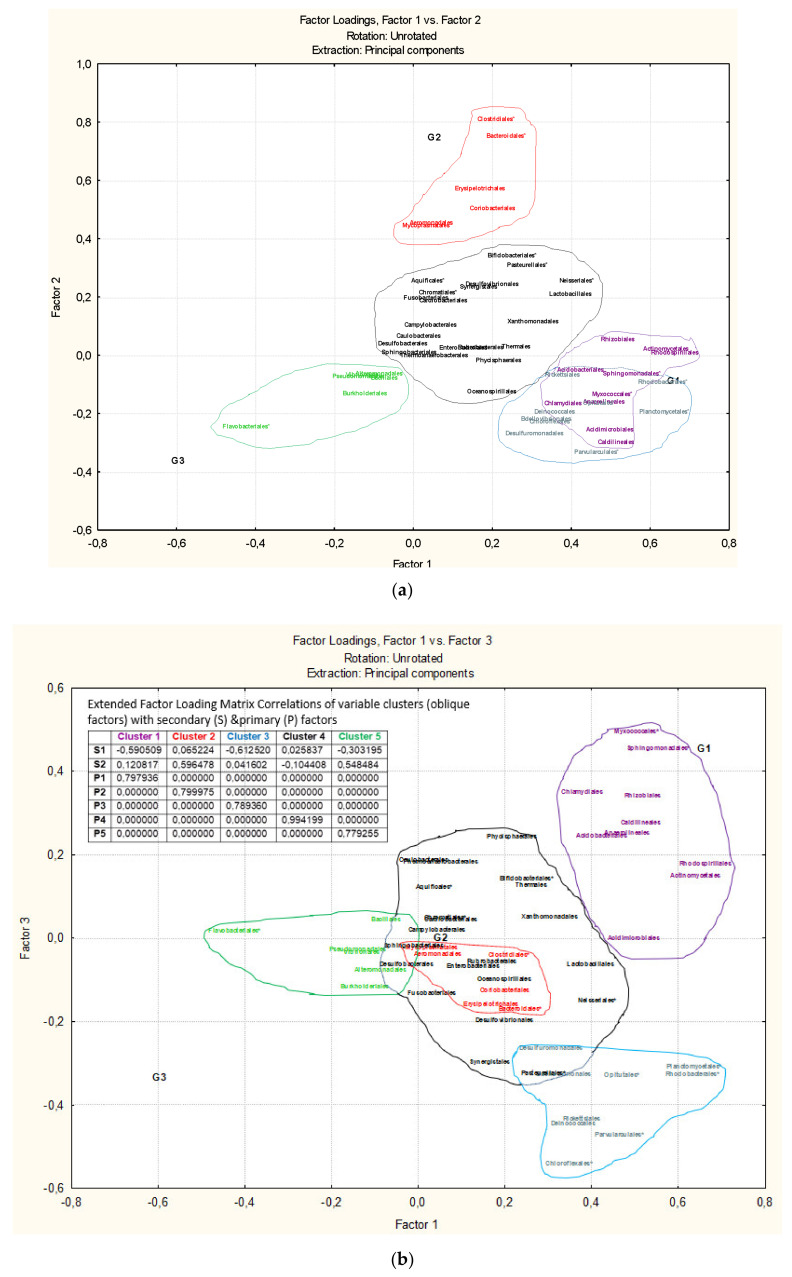
Factorial analysis considering the anisakids samples and bacteria at order levels. In the In the Factorial Space defined by Factor 1 vs. Factor 2 (**a**) and Factor 1 vs. Factor 3 (**b**) is disposed the correspondence with the classified clusters of Figure 2b.

The total variability at the order level (Figure 4) is explained by 25.75% for the three first factors. One of the reasons could be that it was not possible to classify any order from Cyanobacteria. However, five bacterial clusters can be detected in the hierarchical analysis that involves at least five main factors for ordering the microbiota in the factorial space (36.78% of total explanation). The clustering of associated microbiota detected at phylum level is also detected at order level confirming the relationships. The main influence for conforming the five associated clusters of orders are due to 14 of them: two orders of Bacteroidetes (Flavobacteriales and Bacteroidales), one of Firmicutes (Clostridiales), the only order of Aquificae (Aquificales, due to its central position in the factorial space), one of Planctomycetes (Planctomycetales), six of Proteobacteria (Chromatiales, Neisseriales, Parvularculales, Pasteurellales, Sphingomonadales, Rhodobacterales), one of Actinobacteria (Bifidobacteriales), one of Verrucomicribia (Opitutales) and one of Chloroflexi (Chloroflexales). Regarding the clusters of orders (Figure 4), they can be related with groups of samples (Figure 2b): cluster 1 is mainly associated to samples in group 1, cluster 2 is mainly associated to samples in group 2, clusters 3 and 4 could be associated to any group of samples, and cluster 5 is mainly related to samples of group 3.

**Figure 5:**
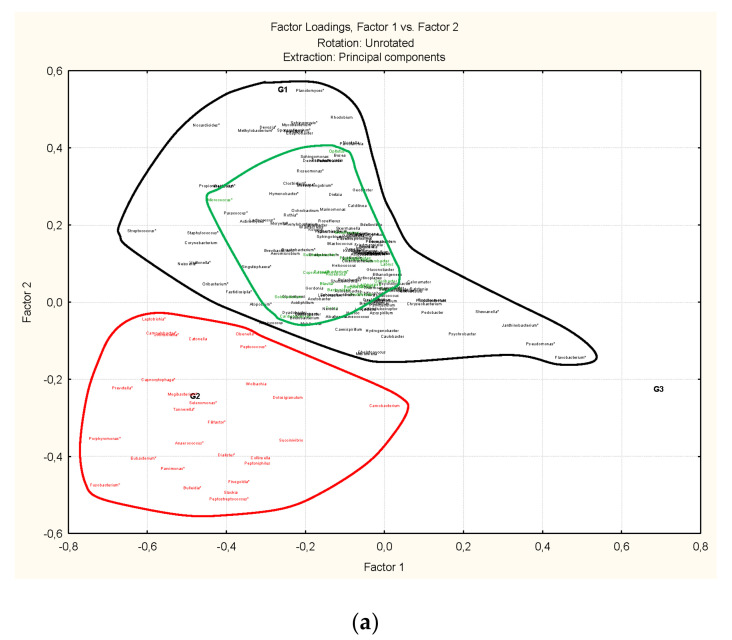

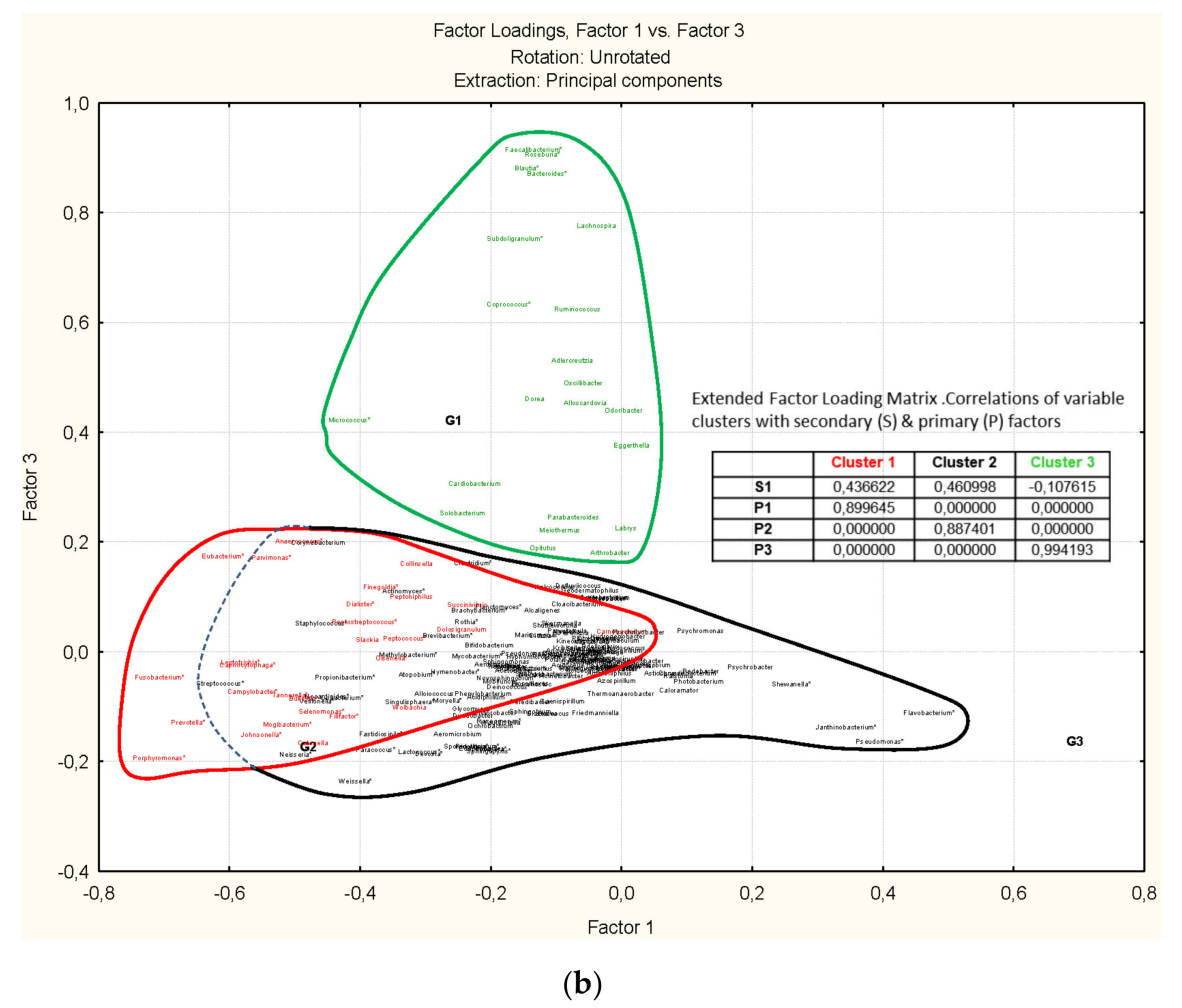
Factorial analysis considering the anisakids samples and bacteria at genus level. In the Factorial Space defined by Factor 1 vs. Factor 2 (**a**) and Factor 1 vs. Factor 3 (**b**) is disposed the correspondence with the classified clusters of Figure 2c.

Factor analysis and its hierarchical analysis also identified three clusters of bacterial association when it is applied to the genus level (Figure 5). In spite the large number of variables (187 genera), the clusters are quite well defined with very high correlations (values ranging from 0.88 to 0.948) among clusters and variables. Comparison with Figure 2c, Cluster 1 is clearly associated to samples in group 2, cluster 2 with samples of group 1 and 3 and cluster 3 with samples of group 1. In Table 2, Table 3 and Table 4 the genera which conform the three clusters are highlighted by three colors (black, green and red) and those which are of highest influence in the factorial space are marked with an asterisk.

### 3.4. Genus-Level Associations (Clusters Description): The Ultimate Test Is the Significant Structure which Represents the Microbiota Association at Genus Level

Cluster 1 merges independently 28 genera which are thermophiles or mesophiles, anaerobes (facultative or strict). This cluster is mainly related with the set of samples from G2 (Figure 2c). In this cluster is located *Wolbachia*, genus of Rickettsiales, which is an endosymbiont of arthropods and nematodes. It is estimated to infect more than 65% of insect species and considered an extensive symbiont in Nematoda [48]. At least 60.71% of the cluster members are considered typical periodontal and oral bacteria of mammals (some of them include species causing gingival or dental disease in humans) such as: *Campylobacter*, *Capnocytophaga*, *Eubacterium*, *Fusobacterium*, *Porphyromonas*, *Prevotella*, *Tannerella* [49]; *Selenomonas*, *Leptotrichia* and *Peptococcus* [50,51]; *Bulleida* [52]; *Mogibacterium* [53]; *Catonella*, *Johnsonella* and *Dialister* [54]; *Filifactor* [55] and *Parvimonas* [56,57]. Species of genus *Carnobacterium* are not known to be pathogenic in humans; *C. divergens* and *C. maltaromaticum* may cause disease in fish [58]. *Succinivibrio* is an obligate anaerobe which was firstly considered inhabitants of the rumen of cattle and sheep [59]. The rest of the bacteria are considered part of the Human gastrointestinal microbiome: *Olsonella* [60], *Slackia* [61], *Collinsella* [62] and *Peptococcus* [63]. From what can be considered to be members of a generalistic human microbiome, we found: *Anaerococcus* [64] and other opportunistic human pathogens causing nosocomial infections such as *Finegoldia* [65], *Peptoniphilus* [66] and *Dolosigranulum* with only one known species, *D. pigrum* [67].

Cluster 2, associated to samples which have defined the Figure 2c, is characterized by 158 genera (unclassified genera has not been included in factorial analysis). Except three genera (*Wolbachia*, *Campylobacter* and *Succinivibrio*) all the recorded genera from phylum Proteobacteria are represented in this Cluster by: classes Alphaproteobacteria (37 genera), Betaproteobacteria (4), Epsiloproteobacteria (2), Deltaproteobacteria (6) and Gammaproteobacteria (10).

Alphabacteria are represented by 7 orders: Rhizobiales (13 genera) some of which are endosymbionts of animals [68] such as *Devosia* [69], endosymbionts of plants such as *Bradyrhizobium*, *Bosea* and *Mesorhizobium* [70], but most of the Rhizobiales which have been found in this study are common components of soil and water microbiota: *Hyphomicrobium* used in the denitrification of sewage [71], *Methylobacterium* [72], *Angulomicrobium*, *Pedomicrobium* [73], *Rhodobium* [74], *Chelatococcus* [75], *Parvibaculum* [76], *Nordella* [77] and *Ochrobactrum* (also isolated from the intestinal tract in human) has been associated to nosocomial infections in immunocompromised patients by Holmes et al. [78] and Kern et al. [79].

In this cluster 2 there are the four genera of the order Caulobacterales (*Asticcacaulis*, *Brevundimonas*, *Phenylobacterium* and *Caulobacter*) usually described as freshwater bacteria [80]. The order Parvularculales is represented by the marine genus *Parvularcula* [81]. Order Rhodobacterales is represented by water eutrophic bacteria *Paracoccus* and *Roseobacter sensu lato* clade, with important functions in the marine biogeochemical cycle (25% of coastal marine bacteria are members of the *Roseobacter* clade [82]). All the genera found in this study from the order Rhodospirillales are members of Cluster 2. They are typical bacteria of aquatic and soil environment-producing acetic acid (*Gluconobacter*, *Roseomonas*, *Acetobacter*, *Acidocella*, *Acidiphilium*) or what are considered purple non sulfur bacteria (*Skermanella*, *Azospirillum*, *Caenispirillum*, *Magnetospirillum* and *Defluviicoccus* [83]). Although they are considered mainly as environmental bacteria (air, soil and water), some of these species such as *Roseomonas* are isolated frequently from wounds, abscesses and the genitourinary tract [84]. The Order Rickettsiales is represented by the genus *Rickettsia*, which is a symbiont of Eukaryota and can induce disease in humans [85]. Sphingomonadales (*Sphingomonas*, *Novosphingobium*, *Sphingobium*, *Sphingopyxis* and *Erythrobacter*) are common aerobic bacteria isolated from a wide range of environments, including temperate and polar soils, marine sediments and plant surfaces and tissues [86]. 

Genera of the class Betaproteobacteria are *Janthinobacterium*, *Alcaligenes*, *Ralstonia* and *Neisseria*. Some species have clinical importance for humans [such as *Neisseria* [87], can be agents of nosocomial and opportunistic infections (*Alcaligenes* [88]), plant pathogens (*Ralstonia* [89]) or have antifungal properties able to control *Batrachochytrium dendrobatidis* when the bacteria is a symbiont of amphibians (i.e., *Janthinobacterium* [90]).

*Arcobacter* and *Sulfurospirillum* are the genera of class Epsiloproteobacteria which are in this cluster; *Arcobacter* comprises some species which can be human and animal pathogens widely distributed in many environment including marine ones [91] while *Sulfurospirillum* is found in contaminated sediments, wastewater plants, marine environments or on biocathodes [92]. Class Deltaproteobacteria is represented by the following orders and genera: Bdellovibrionales (*Peridibacter* and *Bdellovibrio*), Desulfobacterales (*Desulfobulbus*), Desulfuromonadales (*Geobacter*) and Myxococcales (*Halianqium*). All of these genera are quite uniform from an ecological and physiological point of view (sulfur-reducing bacteria). These genera are very common in anaerobic aquatic and marine sediments [93]. 

Finally, class Gammaproteobacteria (excluding the unclassified genera) is represented by orders Aeromonadales (*Aeromonas*), Alteromonadales (*Shemanella*, *Psychromonas*), Enterobaceriales (Enteric Bacteria cluster), Oceanospirillales (*Marinomonas*), Pseudomonadalles (*Pseudomonas*, *Psychrobacter*, *Acinetobacter*), Vibrionales (*Photobacterium*) and Xanthomonadales (*Nevskia*). *Aeromonas* is an ubiquitous genus present in continental and marine water, in which most of their species have been associated with human diseases (as opportunistic in immunocompromised patients, gastroenteritis and infected injuries); it is also considered a pathogen in fishes, amphibians and reptiles [94]. *Shewanella* and *Psychromonas* are marine genera from cold waters [95,96]. Bacteria from “Enteric_Bacteria_cluster” [97] (order Enterobacteriales) includes medically important genera and species which are part of human and animal microbiota. Its distribution is widespread along any environment. *Marinomonas* is a marine bacteria [98] with important ecological implication. Order Pseudomonadales is represented by three genera (*Psychrobacter*, *Acinetobacter* and *Pseudomonas*). *Psychrobacter* is recorded frequently in marine environments [99] but also in terrestrials and some species have been isolated from foods [100]. *Acinetobacter* is a typical bacteria from soils [101]. *Pseudomonas* is a well-known genus with at least eight different phylogenetic groups of species [102]; its distribution is ubiquitous and present in all type of ecological niches from terrestrial to marine. Some species are pathogens of animals and plants. *Pseudomonas* and *Acinetobacter* are considered pathogens for immunocompromised patients [103]. Vibrionales (*Photobacterium*) and Xantomonadales (*Nevskia*) are present in this cluster. Most of the species of *Photobacterium* are symbionts of marine organisms [104] and it is demonstrated that also some of them are pathogens of fishes [105]. *Nevskia* is an aquatic bacteria living at the air–water interface (the epineuston) forming hydrophobic surface films [106] and constitutes a clade of sister species into Xantomonadales [107]. 

The phylum Actinobacteria is represented in this cluster 2 by two Classes and 33 genera (unclassified genera are not included): Rubrobacteria [order Rubrobacterales (1 genus)], Actinobacteria [orders Actinomycetales (29) and Bifidobacteriales (2)] and Coriobacteria [order Coriobacteriales (1)]. Rubrobacterales is represented by the extremophilic genus *Rubrobacter* considered to be radiotolerant [108]. From Coriobacteriales we have recorded the genus *Atopobium* [109] a common member of human pathogenic biota [110,111]. The Order Actinomycetales has a complex taxonomy; they can be found mostly in soil and organic matter and also in animals. In addition, they constitute symbiotic nitrogen-fixing associations with more than 200 species of plants; they can serve as biocontrol agents, or be plant pathogens. Genera of this order are also component of the human urogenital tract or the oral digestive system. They also have wide medicinal (antibiotic production; i.e., *Streptomyces* spp.) and botanical applications [112]. In cluster 2 is represented by the 29 genera recorded in Table 3. It can be considered as the most important genera: *Brachybacterium* which has been isolated from sea water [113]; *Dietzia* considered as opportunistic human pathogen [114]; *Rothia* which causes a wide range of serious infections, especially in immunocompromised hosts [115]; *Mycobacterium*, a genus free-living in soil and water widely distributed but the major habitat for some species is the diseased tissue of warm-blooded hosts (*M*. *tuberculosis* and *M. leprae* are the most known pathogens of this genus for humans) [116]; some species of *Nocardia* cause disease in immunocompromised patients [117].

The phylum Bacteroidetes is represented in cluster 2 by orders Flavobacteriales (*Flavobacterium, Cloacibacterium, Chryseobacterium, Polaribacter, Wautersiella, Amoebinatus*) and Sphingobacteriales (Pedobacter, Sphingobacterium, Hymenobacter, Dyadobacter, Chitinophaga). Flavobacteriales have been isolated in many environments, including soil, marine water, plants, and animal gut. They play an important role in in aquatic and terrestrial environments, accounting for more than 20% of microbial communities. Some flavobacterial species (*Flavobacterium* spp.) are also responsible for severe fish disease [118,119]. Sphingobacteriales are all considered as “environmental” bacteria with a wide ecological niche, from water to soil [120]. The phylum Cloroflexi is represented by the orders Caldilineales (*Caldilinea*) and Cloroflexales (*Roseiflexus*). Both genera are filamentous thermophilic and found in hot springs [121,122]. All members of phylum Cloroflexi are considered to be the origin of photosynthesis [123]. 

The phylum Firmicutes is represented by the classes Bacilli (14 genera), and Clostridia (22 genera). The genera of Bacilli are members of two orders [orders Bacillales (*Listeria*, *Ammoniphillus*, *Kurthia*, *Staphylococcus* and *Laceyella*) and Lactobacillales (*Desemzia*, *Alloiococcus*, *Streptococcus*, *Lactococcus*, *Leuconostoc*, *Weissella*, *Lactobacillus*, *Flacklamia*, *Aerococcus*)]. From Bacillales *Listeria, Kurthia* and *Ammoniphillus* can be found in soil, but the former also is found in vegetables and animals where it can be considered a pathogen [124,125,126]. Eleven species of *Staphylococcus* can be isolated from humans as commensals, some of them pathogenic in persons debilitated by chronic illness, traumatic injury, burns or immunosuppression [127]; *Laceyella* is considered to be an environment thermophilic bacteria [128]. From Lactobacillales, *Desemzia* is a lactic acid bacteria (as the rest of the order) common in fish gut considered to be sometimes abundant in the intestine, notably in freshwater fish [129]; *Alloiococcus* has only one known species with implications as a secondary pathogen in human otitis [130]. The rest of Lactobacillales of this cluster 2 are ubiquitous bacteria, usually found in decomposing plants, milk products and foods in fermentation (including fish) contributing to the healthy microbiota of animals and humans [131].

The genera of Clostridia is represented by two orders [Thermoanaerobacterales (*Caldicellulosiruptor* and *Thermoanaerobacter*) and Clostridiales (*Helcococcus, Eubacterium, Sedimentibacter, Shuttleworthia, Oribacterium, Moryella, Butyrivibrio-Pseudobutyrivibrio, Fastidiosipila, Ethanoligenens, Acetivibrio, Sporacetigenium, Desulfosporosinus, Veillonella, Mitsuokella Megamonas, Clostridium, Caloramator, Acetobacterium*)]. Both species of Thermoanaerobacterales are thermophilic and anaerobic showing adaptation to survive in elevated temperatures (up to 80–90 °C) without oxygen [132]. Regarding Clostridiales, all genera are strict anaerobic bacteria; the ability to form spores makes them highly stable in the environment being natural soil inhabitants as saprophytic bacteria [133]. However, genera of this order have also common presence as members of gut microbiota with clinical and health functions [134].

The rest of the genera from this cluster 2, are members of the phyla Acidobacteria (*Chloroacidobacterium*, *Acidobacterium*, *Edaphobacter*, *Koribacter* and *Solibacter*), Aquificae (*Hydrogenobacter*), Deinococcus-Thermus (*Truepera* and *Deinococcus*), Fusobacteria (*Psychrilyobacter* and *Sneathia*), Planctomycetes (*Singulisphaera*, *Planctomyces*, *CL500-3* and *Phycisphaera*) and Synergistetes (*Jonquetella*). The genera of Acidobacteria are considered soil inhabitants with a ubiquitous distribution along many ecosystems [135,136]. Aquificae (*Hydrogenobacter*) encompass a set of bacteria able to live in harsh environments (these bacteria have been found in springs, pools, and oceans) [137,138]. *Truepera* and *Deinococcus* from the phylum Deinococcus-Thermus are considered as typical extremophiles bacteria [139,140,141]. From Fusobacteria, *Psychrilyobacter* was isolated from marine sediment from the Atlantic Ocean [142] and *Sneathia* appears to be a significant, emerging opportunistic pathogen that may play a significant role in urogenital track for humans (male and female); as a common component of the vaginal microbiota that can affect vaginal and reproductive health [143]. Planctomycetes (*Singulisphaera*, *Planctomyces*, *CL500-3* and *Phycisphaera*) are aquatic bacteria (brackish water mass, fresh water and marine) [144,145] but also present in wastewater and terrestrial soils [146]. Genus *Jonquetella* is considered to be a bacterium of anaerobic environments including soil, oil wells, and wastewater treatment plants and animal gastrointestinal tracts; as other members of Synergistetes they are also associated to human pathology such as cysts, abscesses, and periodontal diseases [147].

Cluster 3, associated with samples from G1 (Figure 2c), is formed by 22 genera from phyla Proteobacteria (*Labrys* and *Cardiobacterium*), Deinococcus-Thermus (*Meiothermus*), Verrucomicrobia (*Opitutus*), Actinobacteria (*Micrococcus*, *Arthrobacter*, *Alloscardovia*, *Adlercreutzia* and *Eggerthella*), Bacteroidetes (*Bacteroides*, *Parabacteroides* and *Odoribacter*) and Firmicutes (*Solobacterium*, *Roseburia*, *Coprococcus*, *Blautia*, *Dorea*, *Lachnospira*, *Faecalibacterium*, *Subdoligranulum*, *Ruminococcus* and *Oscillibacter*). This cluster groups bacteria mesophiles as aerobic or anaerobic. The genus *Labrys* (mesophile, 30 °C) contains soil-inhabiting bacteria species (Rhizobiales) including aerobes or facultative anaerobes bacteria [148]. *Cardiobacterium* is a part of normal human microbiome (especially oropharyngeal zone) (aerobes and enriched CO_2_ atmosphere) [149]. *Meiothermus* (thermophilic, 35–68 °C) aerobic and facultative anaerobes genus [150]. *Opitutus* behaves as an obligate anaerobe in an oxygen-tolerance test growing at temperatures of 10 to 37 °C [151]. The genus *Micrococcus* (strict aerobe), includes species that are generally mesophile but some may be also cryophiles; although this genus have been isolated from human skin and animals, it is considered to be a normal environmental inhabitant, especially in water, dust, and soil [152]. *Arthrobacter* is a strict aerobe genus, usually found in soil. Although *Alloscardovia*, *Adlercreutzia* and *Eggerthella* have been isolated from human and animal digestive tracts [61,153,154], could potentially be present in soil and water as the other Actinobacteria. *Alloscardovia*, shows high aerotolerance while some species of *Adlercreutzia* and *Eggerthella* are anaerobic. As Bacteriodites (order Bacteroidiales) *Bacteroides*, *Parabacteroides* and *Odoribacter* are widely distributed in the environment, including sea, soil, water and digestive tract of humans and animals [155]; they are anaerobic genera. Firmicutes of this cluster are represented by orders Erysipelotrycales by means of *Solobacterium* (anaerobe, family Erysipelotrichaceae) considered to be a common member of the microbiome of human salivary glands [156] and two families of the order Clostridiales (Lachnospiraceae and Ruminococcaceae). In general bacteria of the family Lachnospiraceae are considered to be very abundant in rumens [157] and the human gut microbiota [158]: *Roseburia* (anaerobe) is associated to the digestive tract (cecum) of mammals [159,160]; *Coprococcus*, anaerobic, is part of the human faecal microbiota [161]; *Blautia* from humans, cattle and chickens [162], *Dorea* isolated in human faeces [163] and *Lachnospira* found in the rumen of bovine animals [164] and intestine of others such as pigs [165]. Regarding Ruminococcaceae, all members of this family are obligate anaerobes and mesophiles [166]. *Faecalibacterium prausnitzii* the unique species of the genus is very abundant in the human gut microbiota [167]. Members of *Ruminococcus* and *Subdoligranulum* are also found in the human gut and or faeces [168,169] while the genus *Oscillibacter*, first isolated from the clam *Corbicula japonica* [170], has been also recorded in the rumen of cattle [171]. 

## 4. Discussion

In this study, we report the first detailed composition of the bacterial diversity associated to anisakid nematodes based on the analysis of bacterial rRNA sequences belonging to the microbiome of anisakids present in paratenic hosts from a large geographical area (FAO 27). The association of microbial organisms with metazoa is now recognized to be inter-dependent, and has been a new frontier in zoology [172]. This association has been denominated metaorganism first and holobiont afterwards [173] and has been considered as a “unit of natural selection” [174]. Following these assumptions, [175] the first systematic analysis of the native microbiome of *Caenorhabditis elegans*, which is mainly characterized by Proteobacteria including the genera *Ochrobactrum*, *Pseudomonas*, *Stenotrophomonas* and *Sphingomonas* was performed. In that study the native microbiome of wild type naturate *C. elegans* strains in comparison with the congeneric species *C. briggsae* and *C. remanei* was described. However, it is necessary to assume than while any microbiome can present some biological associations with the host, these are not necessarily symbionts. Therefore, the bacterial communities in nematodes can be composed by associated and symbiotic bacteria. 

Our results are obtained after an individualized canonical disinfection of the external cuticle of all Anisakids individuals which were used for the experiment. Consequently, the metagenomics analysis reports the symbionts and associated microbial communities. Because the individuals were in the L3 stage, many of the bacterial communities need to be related to the bacteria which are present in the fish host since the time it was parasitized by the anisakid larvae and the time the fish reached the market. This is sufficient time to obtain a realistic panorama about the microbiota composition of anisakids nematodes in the FAO 27 fishing area. Also, it is worth considering the ability of Proteobacteria to colonize the nematode gut in accordance with experiments carried out by [175]; the plant and fungal endosymbiont bacteria family Burkholderiaceae (represented in our study by genus *Ralstonia*) has been also demonstrated as a symbiont in the plant parasitic nematode *Xiphinema americanum* [176]. This would confer possible beneficial fitness, increasing the resistance of nematodes against bacterial pathogens [177] and even antifungal activity as is known from [178]. Mutualism among bacteria and nematodes are well known [179]. Recently *Oncholaimus dyvae* (sp. nov.) was described in the Lucky Strike vent field on the Mid-Atlantic Ridge [180,181] which was previously considered to harbor bacterial symbionts typical of hydrothermal vent fauna, such as sulfur-oxidizing bacteria related to classes Epsilonproteobacteria and Gammaproteobacteria which are frequently found in our study.

No reports are available regarding the microbiota associated to anisakids on such a large geographic area as FAO 27. Despite the scarce number of samples (111) and the asymmetric representation in the survey of anisakids and fish taxa (not equally represented), the diversity present in the total studied populations is rather high; however it only represents part of the possible diversity according the rarefaction curves of the samples (Figure 1a). Logically it is impossible to discover all the OTUs or species of microbial communities, but the observed bacterial OTUs are compared among them by direct comparison of different samples sizes. Rarefaction (Figure 1a), as a significant general method of significant value [182], suggests than the characterization of most of the diversity would reach between 5000 and 10,000 anisakids individuals where each individual can considered as a “microbial population” [183] although our results are statistically representative. Microbiome composition is not substantially affected by the studied fish hosts and their diversity, and in general is non-comparable according to Renyi´s index (Figure 1b). Proteobacteria, Firmicutes and Actinobacteria are the most diverse compared with the other eleven phyla detected in this study. All of this indicates an extraordinary bacterial diversity associated with these nematodes. Neither evenness (Shannon index) nor dominance (inverse of Simpson´s index) [184] show any fish host trend at OTU-level analysis (Additional file 2: Figure S1a,b) but clusters of samples (individuals) are detected, indicating bacterial associations over OTU level and independent of fishes’ influence. This fact, added to the high level of diversity values suggests a “functional redundancy” in the sense of [185] (within an ecosystem, different species contribute in equivalent ways to an equal function) as detected in gut microbiota when carnivores and herbivores are compared [186] providing support for detecting the microbiome structure, and grounding the hypothesis that bacterial communities associated to anisakids are developed independently of what could be a priori considered the major influence variable such as fish host (or even anisakids taxa).

Within the metazoans, the nematodes occupy third place in number of species, after arthropods and mollusks. However, the abundance of their populations is greater that of any other multicellular organism. They are extraordinarily versatile (parasites of marine and terrestrial animals, plants, and human beings), and of great trophic diversity occupying all the niches in all ecosystems on the planet. Therefore, they are a very diverse group from taxonomic, ecological, and geographical points of view [187,188,189]. The bacterivory is a common and unspecific phenomenon in many species of nematodes. Bacterivores are members of at least seven of the 16orders of Nematoda from marine and terrestrial habits (Rhabditida, Chromodorida, Plectida, Monhysterida, Desmoscolecida, Araeolaimida and Enoplida) [190]. Also specific and mutualistic cases include *Steinernema carposae* and *Xenorhabdus nematophila* [191] or symbionts as is the case for *Wolbachia* and filarial nematodes [48]. Finally, pathogenic bacteria have been demonstrated as an evolutionary force for nematodes [16,179].

The water temperature where samples were taken for this study ranges from −2 °C to +18 °C, and therefore cryophiles are represented in the bacterial communities (typically: *Aeromonas*, *Alcaligenes*, *Alteromonas*, *Bacillus*, *Clostridium*, *Flavobacterium*, *Lactobacillus*, *Leuconostoc*, *Listeria*, *Pseudomonas*, *Serratia*, *Bacillus*, *Micrococcus*, *Lactobacillius* and *Corynebacterium*) [192,193]. Regarding fish hosts, except for the internal organs and the muscles, the scales, the skin, the gills and the digestive tract, harbor a high numbers of bacteria [194] which is increased by the intestinal emissions of fish during fishery manipulation [193] and by L3 anisakids larvae which contaminate the fish muscle by means of post migration after death of fish hosts [195,196].

Many of the bacteria genera we have found in this study encompass non-typical marine bacterial that might cause infections in humans through contaminated fish products due to handling during the processing and distribution chains before reaching consumers. According to some authors [197,198] human pathogen bacteria in the marine environment have been demonstrated to be vectored by fish and 69 human pathogenic microorganisms are considered to be the main source of contamination in sea food products [6]. However the real incidence of bacteria related to seafood illnesses might be dependent on gastronomic tradition (high incidence in countries where there is a high consumption of seafood [199]).

Our results suggest a geographical or ecological influence of bacterial communities association in addition to fish contamination after the capture and during the fish processing manipulations until its arrival to the consumer. At least in this study the fish paratenic host seems a secondary variable as determinant of bacterial community.

Methodologically, in addition to the classical cluster analysis, the factorial approach allows significant clusters to be defined in spite of the percentage of representation (related to the number of variables) being low. This implies, but does not explain, a general variability in the whole set of OTUs, genera etc.; that is, there is detected variability in each cluster but that variability is due to a not considered or studied variable. The number of bacterial genera found in this study is at least 187 (excluding spurious and unclassified OTUs at a genus level). The structure of bacterial communities associated with the samples was substantially maintained and clusters’ similarities were generally the same along the taxonomic scale from phyla to families. Statistically it was not possible to test this at the genus level owing to gaps in the samples which corrupt the statistics (due the unclassified OTUs). It seems that bacterial OTUs are a mixture of environmental and fish intestinal microbiota. Firmicutes, Actinobacteria, Bacterioidetes and Proteobacteria were the core of the seafood bacterial community in what is an important study [200]. Comparing our results with those of these authors, we have obtained the same phyla plus Acidobacteria (3%) as the core of associated bacteria to anisakids, but with different representations, that is: in our case Bacteriodites has a scarce representation (5%) while previous authors recorded 40%; Proteobacteria (70% vs. 50%); Actinobacteria (15% vs. 4.5%) Firmicutes (40% vs. 5%). Interestingly Chaillou et al [200] considered than 83% of the associated microbiota in sea food is from water, sediment and soil origin.

Following that, we think that regarding our study, in most of the cases the associations within groups of bacteria could be linked to the ecological or geographical niches in which the fishes were captured or dependent on the time since capture and fishing manipulation.

## 5. Conclusions

There are significant biological structural associations of microbiota in anisakid nematodes which can only be explained or determined by geographic and human influence. This is manifested in clusters and association of bacteria ranging from plylum to genus level which could be also an indicator of degree of fish contamination or from the geographic zone of fishing. Actinobacteria (Actinomycetales), Aquificae (Aquificales), Firmicutes (Bacillales and Clostridiales), Proteobacteria (Campylobacterales and Caulobacterales) are the phyla whose value of abundance enable us to define such a structure.

## Figures and Tables

**Table 1:** Order of bacteria distributed to classes and phyla, which have been recorder in 113 L3 individuals of anisakids. Colors are in accordance to the association they forms in Figure 3 and Figure 4. Asterisks denote the importance the taxa have in factorial analysis. Also is reported the frequency and the average mean of phyla and orders in 113 L3 individuals of anisakids.

	Acidobacteria	Actinobacteria*	Aquificae*	Bacteroidetes*	Chlamydiae	Chloroflexi*	Deinococcus-Thermus*	Firmicutes*	Fusobacteria*	Planctomycetes*	Proteobacteria*	Synergistetes*	Verrucomicrobia	Cyanobacteria*	Unclassified (105 seqs)	
Acidobacteria	Acidobacteriales															(75.2%), 59.80
Rubrobacteria		Rubrobacterales														(13.3%), 8.93
Actinobacteria		Acidimicrobiales Actinomycetales Bifidobacteriales *														(54%), 75.79 (100%), 2625.95 (57.52%), 79.78
Coriobacteriia		Coriobacteriales														(65.48%), 76.01
Aquificae			Aquificales*													(2.65%), 76.00
Bacteroidia				Bacteroidales*												(97.34%), 211.62
Flavobacteria				Flavobacteriales*												(99.11%), 254.03
Sphingobacteria				Sphingobacteriales												(91.15%), 129.55
Chlamydiae					Chlamydiales											(15.93%), 9.55
Anaerolineae						Anaerolineales										(13.27%), 21.13
Caldilineae						Caldilineales										(7.08%), 23.87
Chloroflexia						Chloroflexales*										(10.62%), 5.41
Deinococci							Thermales Deinoccocales									(6.19%), 30.14 (59.30%), 21.50
Bacilli								Bacillales Lactobacillales								(99.11%), 112,41 (99.11%), 323.06
Mollicutes								Mycoplasmatales								(8.10%), 16.78
Erysipelotrichia								Erysipelothrichales								(52.21%), 22.76
Clostridia								Clostridiales* Thermoanaerobacteriales								(100%), 4450.98 (8.85%), 21.90
Fusobacteria									Fusobacteriales							(100%), 755.18
Planctomycetya										Planctomycetales*						(45.13%), 16.43
Phycisphaerae										Phycisphaerales						(5.31%), 32.00
Alphaproteobacteria											Caulobacterales Parvularculales* Rhizobiales Rhodobacterales* Rhodospirillales Rickettsiales Sphingomonadales*					(94.70%), 667.46 (0.89%), 31.00 (100%), 2472.23 (91,15%), 192.87 (96,46%), 197.50 (7,68%), 15.11 (100%), 2692.78
Betaproteobacteria											Burkholderiales Neisseriales*					(100%), 189.26(83,18%), 28.25
Epsiloproteobacteria											Campylobacteriales					(95.60%), 540.42
Deltaproteobacteria											Bdellovibrionales Desulfobacterales Desulfovibrionales Desulfuromonadales Myxococcales					(14.16%), 25.31 (1.76%), 15,50 (6.2%), 6.28 (7.96%), 7.22 (25.66%), 10.93
Gammaproteobacteria											Aeromonadales Alteromonadales Cardiobacteriales Chromatiales* Enterobacteriales Oceanospirillales Pasteurellales* Pseudomonadales Vibrionales Xanthomonadales					(44.24%), 77.56 (53,09%), 22.10 (19,46%), 11.77 (8.85%), 7.30 (87.61%), 35,56 (30.97%), 7.65(80.53%), 37,61 (100%), 301.72 (82,30%), 199.86 (57,52%), 10.41
Synergistia												Synergistales				
Opitutae													Opitutales			
unclassified														unclassified		
MLE1-12														unclassified		
Subsection IV														unclassified		
Subsection III														unclassified		
(Phyla % in total samples) Mean of sequences	(76%) 59.60	(100%) 2838.28	(2.65%) 76.00	(100%) 705.41	(15.93%) 9.55	(74.33%) 66.13	(61.94%) 23.87	(100%) 5659.03	(100%) 755.18	(63.72%) 35.56	(100%) 9291.94	(10.62%) 73.75	(23.9%) 17.59	(100%) 297.74		

**Table 2:** Genera of Proteobacteria members of anisakids microbiota. Colors represent the clusters in which they are in Figure 5. Asterisks denote the most contributive in the ordination of Factorial Space.

				Alphaproteobacteria				Betaproteobacteria		Epsiloproteobacteria			Deltaproteobacteria							Gammaproteobacteria					
	Caulobacterales	Parvularculales	Rhizobiales	Rhodobacterales	Rhodospirillales	Rickettsiales	Sphingomonadales	Burkholderiales	Neisseriales	Campylobacterales	Bdellovibrionales	Desulfobacterales	Desulfovibrionales	Desulfuromonadales	Myxococcales	Aeromonadales	Alteromonadales	Cardiobacteriales	Chromatiales	Enterobacteriales	Oceanospirillales	Pasteurellales	Pseudomonadales	Vibrionales	Xanthomonadales
Caulobacteraceae	*Caulobacter* *Brevundimonas* *Phenylobacterium* *Asticcacaulis*																								
Parvularculaceae		*Parvularcula*																							
Bradyrhizobiaceae			*Bradyrhizobium* unclassified *Bosea*																						
Methylobacteriaceae			Methylobacterium*																						
unclassified			unclassified																						
Hyphomicrobiaceae			Devosia* unclassified *Hyphomicrobium* *Angulomicrobium* *Pedomicrobium*																						
Rhodobiaceae			RhodobiuM*																						
Phyllobacteriaceae			*Mesorhizobium* unclassified																						
Xanthobacteraceae			*Labrys*																						
Beijerinckiaceae			*Chelatococcus*																						
Methylocystaceae			*unclassified*																						
Rhodobiaceae			*Parvibaculum*																						
Brucellaceae			*Ochrobactrum*																						
Nordella cluster			*Nordella*																						
Rhizobiaceae			unclassified																						
Rhodobacteraceae				Paracoccus* unclassified *Roseobacter_clade*																					
Acetobacteraceae				*Gluconobacter* unclassified Roseomonas* Acetobacter *Acidocella* *Acidiphilium*																					
Rhodospirillaceae				*Skermanella* *Azospirillum* *Caenispirillum* *Magnetospirillum* unclassified *Defluviicoccus*																					
unclassified				unclassified																					
wr0007				unclassified																					
Candidatus_Alysiosphaera cluster				unclassified																					
Anaplasmataceae						*Wolbachia*																			
Rickettsiaceae						Rickettsia*																			
Candidatus_Odyssella cluster						unclassified																			
SM2D12						unclassified																			
Sphingomonadaceae							Sphingomonas* Novosphingobium* unclassified Sphingobium Sphingopyxis*																		
unclassified							unclassified																		
Erythrobacteraceae							unclassified *Erythrobacter*																		
GOBB3-C201							unclassified																		
Oxalobacteraceae								Janthinobacterium*																	
Alcaligenaceae								*Alcaligenes*																	
Comamonadaceae								*unclassified*																	
Burkholderiaceae								*Ralstonia*																	
Neisseriaceae									Neisseria* unclassified																
Campylobacteraceae									*Arcobacter* Campylobacter* * Sulfurospirillum*																
Bacteriovoraceae											*Peredibacter*														
Bdellovibrionacea											*Bdellovibrio*														
Desulfobulbaceae												*Desulfobulbus*													
Desulfovibrionaceae													unclassified												
Geobacteraceae														*Geobacter*											
Polyangiaceae												unclassified													
Haliangiaceae												*Haliangium*													
Unclassified															*unclassified*										
Aeromonadaceae																*Aeromonas*									
Succinivibrionaceae																*Succinivibrio*									
Shewanellaceae																	Shewanella*								
Psychromonadaceae																	*Psychromonas*								
Cardiobacteriaceae																		unclassified *Cardiobacterium*							
Chromatiaceae																			Unclassified						
Enterobacteriaceae																				unclassified *Enteric_Bacteria_cluster*					
Oceanospirillaceae																					*Marinomonas*				
Pasteurellaceae																						unclassified			
Pseudomonadaceae																							Pseudomonas* unclassified		
Moraxellaceae																							*Psychrobacter* *Acinetobacter* unclassified		
Vibrionaceae																								unclassified *Photobacterium*	
Sinobacteraceae																									*Nevskia*
Xanthomonadaceae																									unclassified

**Table 3:** Genera of Actinobacteria, Bacterioidetes Cloroflexi and Firmicutes members of anisakids microbiota. Colors represent the clusters in which they are in Figure 5. Asterisks denote the most contributive in the ordination of Factorial Space.

		Actinobacteria					Bacteroidetes		Chloroflexi						Firmicutes		
	Rubrobacteria		Actinobacteria		Coriobacteria	Bacteroidia	Flavobacteria	Sphingobacteria	Anaerolineae	Caldilineae	Chloroflexia	Bacilli		Mollicutes	Erysipelotrichia	Clostridia	
	Rubrobacterales	Acidimicrobiales	Actinomycetales	Bifidobacteriales	Coriobacteriales	Bacteroidales	Flavobacteriales	Sphingobacteriales	Anaerolineales	Caldilineales	Chloroflexales	Bacillales	Lactobacillales	Mycoplasmatales	Erysipelotrichales	Clostridiales	Thermoanaerobacterales
Rubrobacteriaceae	*Rubrobacter*																
Acidimicrobiaceae (5)	unclassified																
Actinomycetaceae (2)			Actinomyces* *Mobiluncus*														
Brevibacteriaceae (2)			Brevibacterium*														
Cellulomonadaceae			unclassified														
Corynebacteriaceae			Corynebacterium*														
Dermabacteraceae			Brachybacterium*														
Dermatophilaceae			unclassified														
Dietziaceae			*Dietzia*														
Geodermatophilaceae			*Blastococcus* *Geodermatophilus*														
Glycomycetaceae			*Glycomyces*														
Intrasporangiaceae			*Ornithinicoccus*														
Kineosporiaceae			*Kineococcus*														
Microbacteriaceae			unclassified														
Micrococcaceae			Micrococcus* Rothia * *Kocuria* *Arthrobacter*														
Micromonosporaceae			*Actinoplanes*														
Mycobacteriaceae			Mycobacterium*														
Nocardiaceae			*Rhodococcus Gordonia* *Nocardia*														
Nocardioidaceae			Nocardioides* Aeromicrobium *Kribbella*														
Propionibacteriaceae			Propionibacterium* *Friedmanniella*														
Pseudonocardiaceae			*Pseudonocardia* *Amycolatopsis*														
Sporichthyaceae			*hgcI_clade*														
Streptomycetaceae			*Streptomyces*														
Streptosporangiaceae			*Nonomuraea*														
Thermomonosporaceae			*Actinomadura*														
Bifidobacteriaceae				*Alloscardovia* *Bifidobacterium* *Gardnerella*													
Coriobacteriaceae					Atopobium* Olsenella* *Slackia* *Unclassified* * Collinsella* *Adlercreutzia* *Eggerthella*												
Prevotellaceae						Prevotella*											
Bacteroidaceae						Bacteroides*											
Porphyromonadaceae						Porphyromonas* *Parabacteroides* Tannerella* *Odoribacter*											
Flavobacteriaceae							Flavobacterium* unclassified *Cloacibacterium* Capnocytophaga* Chryseobacterium Polaribacter *Wautersiella* *Amoebinatus*										
Sphingobacteriaceae							*Pedobacter* unclassified *Sphingobacterium*										
Cytophagaceae							Hymenobacter* *Dyadobacter*										
unclassified							unclassified										
Chitinophagaceae							unclassified *Chitinophaga*										
Anaerolineaceae									Unclassified								
Caldilineaceae										*Caldilinea*							
Chloroflexaceae											*Roseiflexus*						
Listeriaceae											*Listeria*						
Paenibacillaceae											*Ammoniphilus*						
Planococcaceae											*Kurthia*						
Staphylococcaceae											Staphylococcus*						
Thermoactinomycetaceae											*Laceyella*						
unclassified												unclassified					
Carnobacteriaceae												unclassified *Desemzia* *Dolosigranulum* *Alloiococcus* *Carnobacterium*					
Streptococcaceae												Streptococcus* Lactococcus*					
Leuconostocaceae												*Leuconostoc* Weissella*					
Lactobacillaceae												unclassified *Lactobacillus*					
Aerococcaceae												*Flacklamia* *Aerococcus*					
Mycoplasmataceae														unclassified			
Erysipelotrichaceae															Bulleidia* Solobacterium unclassified		
Family_XI_Incertae_Sedis															Anaerococcus* Finegoldia * *Peptoniphilus* Parvimonas* Mogibacterium* *Helcococcus* *unclassified* *Eubacterium* *Sedimentibacter*		
Lachnospiraceae															Roseburia* Johnsonella* Catonella* *unclassified* *Incertae_Sedis* Coprococcus* *Shuttleworthia* Blautia* Oribacterium* Moryella* *Butyrivibrio-Pseudobutyrivibrio* *Dorea* *Lachnospira*		
Ruminococcaceae															Faecalibacterium* Subdoligranulum unclassified Fastidiosipila* Incertae_Sedis *Ruminococcus* *Ethanoligenens* *Acetivibrio* *Oscillibacter*		
Peptostreptococc aceae															*Incertae_Sedis* Peptostreptococcus* Filifactor* Peptococcus* Sporacetigenium* *Desulfosporosinus*		
Veillonellaceae															Veillonella* Selenomonas* *unclassified* *Mitsuokella* *Megamonas* Dialister*		
Clostridiaceae															unclassified Clostridium* Caloramator		
unclassified															unclassified		
Eubacteriaceae															Eubacterium* *Acetobacterium*		
Family_III_Incertae_Sedis																	*Caldicellulosiruptor* *Thermoanaerobacter*

**Table 4:** Genera of Acidobacteria, Aquificae Clamydiae, Deinococcus-Thermus, Fusobacteria, Planctomycetes, Synergetes and Verrcomicrobia members of anisakids microbiota. Colors represent the clusters in which they are in Figure 5. Asterisks denote the most contributive in the ordination of Factorial Space.

	Acidobacteria	Aquificae	Chamydiae	Deinococcus-Thermus		Fusobacteria	Planctomycetes		Synergistetes	Verrucomicrobia
	Acidobacteria	Aquificae	Chlamydiae	Deinococci		Fusobacteria	Planctomyceta	Phycisphaerae	Synergistia	Opitutae
	Acidobacteriales	Aquificales	Chlamydiales	Thermales	Deinococcales	Fusobacteriales	Planctomycetales	Phycisphaerales	Synergistales	Opitutales
Acidobacteriaceae	*Chloroacidobacterium* *Acidobacterium* Edaphobacter* *Koribacter Solibacter*									
Aquificaceae		*Hydrogenobacter*								
Unclassified			unclassified							
Thermaceae			*Meiothermus*							
Trueperaceae					Truepera*					
Deinococcaceae					Deinococcus*					
Fusobacteriaceae						Fusobacterium* unclassified *Psychrilyobacter*				
Leptotrichiaceae						Leptotrichia* Sneathia unclassified				
Planctomycetaceae							Singulisphaera* Planctomyces* Unclassified			
Phycisphaeraceae								*CL500-3* *Phycisphaera*		
Synergistaceae									unclassified *Jonquetella*	
Opitutaceae										Opitutus*

**Table 5:** Discriminant analysis values for significant bacteria at phylum (**a**) and order (**b**) level and according group of sample classification (* = *p* < 0.05; ** = *p* < 0.01; *** = *p* < 0.001).

**F (28.194) = 20.998 *p* < 0.0000**	**F-Remove** **(2.97)**	***p*-Level**	**Sample Group 1**	**Sample Group 2**	**Sample Group 3**	**All Groups**
**Actinobacteria**	6.0925 **	0.003218	5324.208	2505.167	2115.364	2838.283
**Aquificae**	4.4817 **	0.013752	0.000	18.000	0.1558442	2.017699
**Firmicutes**	126.8766 ***	0.000000	5412.583	24247.08	2839.013	5659.035
**Proteobacteria**	76.6841 ***	0.000000	23561.5	5097.167	5498.013	9291.938
**Percent correct classification**			87.50	91.66	100.00	96.46
(a)						
**F (104.118) = 5.7435 *p* < 0.0000**	**F-Remove** **(2.59)**	***p*-Level**	**Sample Group 1**	**Sample Group 2**	**Sample Group 3**	**All Groups**
**Actinomycetales**	6.736752 ***	0.002467	5007.208	2131	1960.87	2625.947
**Anaerolineales**	3.30950 *	0.043427	8.833333	0.00000	1.363636	2.80531
**Bacillales**	3.98242 *	0.023859	91.08334	158.8333	110.3636	111.4159
**Bacteroidales**	6.13735 **	0.003789	177.4167	858.75	113.1948	206.0089
**Caldilineales**	3.22566 *	0.046832	7.75	0.08333334	0.05194805	1.690266
**Campylobacterales**	9.84095 ***	0.000205	1903.167	95.58334	149.9091	516.5132
**Caulobacterales**	8.75310 ***	0.000469	1726.917	109.6667	372.1688	632.0266
**Clostridiales**	6.00333 *	0.004235	4161.167	20972.33	1966.558	4450.982
**Sphingomonadales**	13.72837 ***	0.000013	60.54167	184.8333	125.6234	118.0885
**Percent correct classification**			91.66	91.66	100	97.34
(b)						

## Data Availability

Gene sequences have been deposited at GenBank under the accession numbers file SUB8561078: MW335712-MW336932.

## Supplementary Materials

The following are available online at https://www.mdpi.com/article/10.3390/microorganisms9051088/s1, All data generated or analyzed during this study are included in this article and its Additional information files. The 16S rRNA gene sequences have been deposited at GenBank under the accession numbers file SUB8561078: MW335712-MW336932; Table S1. Samples used in the survey (project: Anavas 16S); Table S2. The different taxonomical level (phyla, class, order, family, genus) of associated microbiota to anisakids based in the 16S rRNA sequences for OTUs; Figure S1. Analysis of biota diversity in anisakids; Table S3. Rarefaction values per OTUs used for obtaining the Figure 1; Figure S2. Quantitative analysis: correlations between samples based and bacterial OTUs composition; Figure S3. Distribution of most representative microbiota phyla according anisakids and Fish; Figure S4. Pearson and Sperman Correlation based on the bacteria phylum composition of the four anisakids variant; Figure S5. Distribution of most representative microbiota orders according anisakids and Fish; Figure S6. Pearson and Sperman correlation based on the order composition of the anisakid variants and fishes; Table S4. Average number of sequences per anisakids and fish hosts calculated for the most frequent bacteria phyla; Table S5. Average number of sequences per anisakids and fish hosts calculated for the most frequent bacteria order.
