# Supplementary material for: Metagenomics Analysis Reveals an Extraordinary Inner Bacterial Diversity in Anisakids (Nematoda: Anisakidae) L3 Larvae
Source: Microorganisms. 2021 May 19;9(5):1088. doi: 10.3390/microorganisms9051088 (PMC8158776; doi:10.3390/microorganisms9051088)
Supplement: Supplementary file 1 [file Supporting file Seqs OTUs (Mothur output).pdf › Supporting file Seqs OTUs (Mothur output).pdf]

OTUNumber 100_S55_1.fastq 101P_S57 101S_S56 102A_S58 104_S59 105PS_S111 107_S60 108_S61 109_S62 110_S63 115_S64 116_S65 117_S66
118H_S67 118S_S68 119_S69 120_S70 121_S71 125_S72 126_S73 127_S74 128S_S75 131PS_S108 133PS_S109 135PS_S110 136_S76 138_S77
139_S78 140_S79 142_S81 143_S82 144_S83 145_S84 146P_S85 146S_S86 147_S87 148_S88 149_S89 150_S90 151_S91 152_S92
153_S93 154_S94 155_S95 156_S96 157_S97 159_S98 160P_S99 160S_S100 161_S101 162_S102 166_S103 172_S104 187_S105 188PS_S112
189P_S106 190S_S107 20_S1 22_S2 23_S3 24_S4 28_S5 31_S6 32_S7 33_S8 36_S9 38_S10 40_S11 42_S12 43_S13 44_S80 45p_S14 45s_S15 47_S16 48a_S17 56_S18 57p_S20
57s_S19 60_S21 62_S22 64_S23 66_S24 68p_S26 68s_S25 69_S27 70_S28 71_S29 72_S30 73_S31 74_S32 75_S33 76B_S34 77_S35 78_S36 79_S113 80S_S37 81_S38 82_S39 83_S40
84A_S41 85_S42 86A_S43 88_S44 90_S45 91A_S46 92_S47 93_S48 94_S49 95_S50 96_S51 97_S52 98_S53 99S_S54 repSeqName repSeq OTUConTaxonomy

Otu0001 3866 239 731 2181 1268 938 1323 1513 273 439 0 0 7 388 94 7 5 19 33 78 323 43 708 0 28 236 60 213
668 6012 13180 11215 14855 11753 11939 3799 355 1148 11680 3636 698 329 2816 503 197 1264 16 339 1487 20 318 932 293 159 45 137 3464
2661 331 9320 292 2211 2509 961 344 819 1295 1766 54 77 750 477 226 7984 1113 1467 4222 3960 179 40 384 294 1402 241 128 177
358 356 629 2463 288 91 29 41 2 679 16 43 21 109 0 1477 5585 2860 3370 148 585 570 866 31 232 298 200
M02255_77_000000000-ADUT3_1_1101_13422_10668 T----AC---GG-AG-GGA--------GCT-A-G------C--G--T---T--GT-T-CGG-AA------TT-A--C-T--GG-GC-------GT--A-----AA-GC-GC-AC-------G-TA-G-G-C-G------------G--C-TT-T-G-T-----
------AA----G-T-T-A--------G-A-G--G--TG--A-AA-GC--C-T-GG-A-G-----CT-C-AA------C-T-C-C-A-G-A--A-T----T-G--C-C---T--T-------T--AA-G-A---C-------T--G-C--AT--C-G-C----T-T-G-A-A-T--C---C-AG-----GA-G-A--------G-G-T-G---AG-T------GG--A--ATT----C-
C-G-A-GT--GT-A-G-AG-GT--G---A--A-A---TT-C-GT-AG--AT-A-TT--C-G--G-A-A-G-A-AC-A-CC---AG--T--G--GC-GAA-G--G-C--G-------G--C-T-C-A---CTG--G--AC-T-G--------------------G-T---A-T-T--GA--CG-C-T-G--A-GG--T-G-CG-A--AA-G-C---G-TG--GG-
G--AG-C-A-AA--------CA--GG Bacteria(100);Proteobacteria(100);Alphaproteobacteria(100);Sphingomonadales(100);Sphingomonadaceae(91);Sphingomonas(89);unclassified(89);unclassified(89);unclassified(89);

Otu0002 0 0 0 6 0 0 0 0 0 0 11 52 14 11 9 98 34 27 196 91 10 58 0 2 0 1 20 0
0 0 0 7 0 5 0 1 5 0 1 1 14 5 6 1 10 0 162 3 7 1 392 15 200 633 36 2 62
18 65 141 383 3 441 104 22 532 219 4 54 0 224 8 17 0 79 8 0 0 0 0 0 0 0 0 4 0
0 0 0 0 8 0 61 32 324 0 240 0 46 9 671 0 0 0 6 0 4 0 0 1 0 0 1
M02255_77_000000000-ADUT3_1_1115_27848_17294 T----AA---GC-AC-CGG--------CTA-A-C------T--A--C---G--TG-C-CAG-CA------GC-C--G-C--GG-TA-------AT--A-----CG-TA-GG-GT-------G-CA-A-G-C-G------------T--TAAT-C-G-G-----
------AA----T-TAC-T--------G-A-T--G--TG--A-AA-TC--C-C-CG-G-G-----CT-C-AA------C-C-T-G-G-G-A--A-T----T-G--C-A---T--T-------G--GA-G-A---C-------T--G-C--AA--G-G-C----T-A-G-A-A-T--C---T-GG-----CA-G-A--------G-G-G-G---GG-T------AG--A--ATT----
C-C-A-C-GT--GT-A-G-CA-GT--G---A--A-A---TG-C-GT-AG--AT-A-TG--T-G--G-A-G-G-A-AC-A-CC---GA--T--G--GC-GAA-G--G-C--A-------G--C-C-C-C---CTG--G--GT-C-A--------------------A-G---A-T-T--GA--CG-C-T-C--A-TG--C-A-CG-A--AA-G-C---G-TG--GG-
G--AG-C-A-AA--------CA--GG Bacteria(100);Proteobacteria(100);Betaproteobacteria(100);Burkholderiales(99);Oxalobacteraceae(97);Janthinobacterium(81);unclassified(81);unclassified(81);unclassified(81);

Otu0003 750 499 463 1187 500 474 146 400 181 80 4 1 43 31 8 24 5 18 40 98 339 50 35 75 13 69 41 120
43 45 1512 4406 680 918 545 1158 1619 1069 1041 460 445 1784 541 922 430 522 39 428 900 19 90 1169 272 183 1 279 1769
27 33 20 4 5 4 4 0 22 1 4 2 59 43 71 281 221 1780 93 241 144 16 10 157 92 0 1 80 28
105 259 345 233 245 56 14 52 0 5 0 112 4 206 0 127 472 505 98 122 1011 441 271 34 455 1603 142
M02255_77_000000000-ADUT3_1_1101_27555_6886 T----AC---GT-AG-GGC--------GCA-A-G------C--G--T---T--GT-C-CGG-AA------TT-A--T-T--GG-GC-------GT--A-----AA-GA-GC-TC-------G-TA-G-G-C-G------------G--T-TT-G-T-C-----------GC-
---G-T-C-T--------G-C-T--G--TG--A-AA-GC--C-C-GG-G-G-----CT-T-AA------C-C-C-C-G-G-G--T-G----T-G--C-A---G--T-------G--GG-T-A---C-------G--G-G--CA--G-A-C----T-T-G-A-G-T--G---C-AG-----TA-G-G--------G-G-A-G---AC-T------GG--A--ATT----C-C-T-G-
GT--GT-A-G-CG-GT--G---A--A-A---TG-C-GC-AG--AT-A-TC--A-G--G-A-G-G-A-AC-A-CC---GA--T--G--GC-GAA-G--G-C--A-------G--G-T-C-T---CTG--G--GC-T-G--------------------T-T---A-C-T--GA--CG-C-T-G--A-GG--A-G-CG-A--AA-G-C---A-TG--GG-G--AG-
C-G-AA--------CA--GG Bacteria(100);Actinobacteria(100);Actinobacteria(100);Actinobacteridae(100);Actinomycetales(100);Micrococcineae(100);Micrococcaceae(99);unclassified(59);unclassified(59);

Otu0004 0 35 75 97 0 120 48 143 0 72 3 14 0 11 11 0 5 0 7 25 72 12 77 4 6 11 0 23
0 2280 4839 2044 2658 3219 2733 487 149 317 2067 459 438 93 24 49 66 139 0 98 828 0 15 0 27 0 4 8 0
411 324 3265 104 211 95 216 124 137 67 452 69 56 89 268 223 704 895 15271 5379 2005 41 29 91 52 0 0 69 0
131 169 99 61 0 62 7 4 0 2038 9 93 17 75 0 2109 5908 4202 3735 673 613 1064 1044 0 47 0 1
M02255_77_000000000-ADUT3_1_1103_12778_10339 T----AC---GA-AG-GGG--------GCT-A-G------C--G--T---T--GC-T-CGG-AA------TC-A--C-T--GG-GC-------GT--A-----AA-GG-GT-GC-------G-TA-G-G-C-G------------G--G-TC-T-T-T-----
------AA----G-T-C-A--------G-G-G--G--TG--A-AA-TC--C-T-GG-A-G-----CT-C-AA------C-T-C-C-A-G-A--A-C----T-G--C-C---T--T-------T--GA-T-A---C-------T--G-A--AG--A-T-C----T-T-G-A-G-T--T---C-GG-----GA-G-A--------G-G-T-G---AG-T------GG--A--ACT----G-
C-G-A-GT--GT-A-G-AG-GT--G---A--A-A---TT-C-GT-AG--AT-A-TT--C-G--C-A-A-G-A-AC-A-CC---AG--T--G--GC-GAA-G--G-C--G-------G--C-T-C-A---CTG--G--CC-C-G--------------------A-T---A-C-T--GA--CG-C-T-G--A-GG--C-A-CG-A--AA-G-C---G-TG--GG-G-
-AG-C-A-AA--------CA--GG Bacteria(100);Proteobacteria(100);Alphaproteobacteria(100);Rhizobiales(100);Bradyrhizobiaceae(100);Bradyrhizobium(95);unclassified(95);unclassified(95);unclassified(95);

Otu0005 0 0 0 28 0 85 1 1 0 0 14307 950 104 47 213 261 84 57 402 87 163 159 70 107 0 154 28 32
245 1 26 0 0 12 8 46 0 16 540 23 0 0 20 0 50 97 682 50 213 58 1 0 0 0 1 43 0
379 10 69 12 9 4 15 0 760 0 285 17 3 4 66 0 46 121 135 21 17 32 829 46 29 1 0 0 0
89 0 64 34 0 98 217 249 243 397 10 0 128 117 0 38 83 0 439 19 42 19 19 35 8 0 0
M02255_76_000000000-ADUNE_1_1112_23139_22017 .....AC----T-AC-GT---------GCC-A-G------C--A--G---C--CG-C-GGT-AA------TA-C--G-T--AG-GT-------GG--C-----AA-GC-G--TT-------G----T-C-C-G------------G--A-TC-T-T-T----------
AA----G-T-C-T--------G-A-T--G--TG--A-AA-GC--C-C-CC-G-G-----CT-C-AA------C-C-G-G-G-G-A--G------G-GT-C-A---T--T-------G--GA-A-A---C-------T--G-G--AG--A-A-C----T-T-G-A-G-T--G---C-AG-----AA-G-A--------G-G-A-G---AG-T------GG--A--ATT----C-C-
A-C-GT--GT-A-G-CG-GT--G---A--A-A---TG-C-GT-AG--AT-A-TG--T-G--G-A-G-G-A-AC-A-CC---AG--T--G--GC-GAA-G--G-C--G-------A--C-T-C-T---CTG--G--TC-T-G--------------------T-A---A-C-T--GA--CG-C-T-G--A-GG--C-T-CG-A--AA-G-C---G-TG--GG-G--
AG-C-A-AA--------CA--GG Bacteria(100);Firmicutes(100);Bacilli(100);unclassified(100);unclassified(100);unclassified(100);unclassified(100);unclassified(100);unclassified(100);

Otu0006 60 760 923 458 0 21 1427 562 871 232 5 0 0 16 5 1 0 3 2 2 51 3 830 13 8 9 10 3
0 2 181 385 0 29 5 198 175 31 511 595 1168 630 786 54 112 90 0 103 193 0 326 1414 379 38 3 1 0
112 11 0 3 43 8 15 0 4 39 8 6 8 18 84 94 87 34 326 80 29 68 21 1030 219 0 0 127 1090
0 1030 875 39 153 66 19 18 0 125 9 113 13 97 0 116 96 586 8 224 1 37 134 37 944 1692 484
M02255_77_000000000-ADUT3_1_2114_8513_21629 G----AC---GG-GG-GGG--------GCA-A-G------T--G--T---T--CT-T-CGG-AA------TG-A--C-T--GG-GC-------GT--A-----AA-GG-GC-AC-------G-TA-G-G-C-G------------G--T-GA-A-T-C----------
GG----G-T-T-G--------A-A-A--G--TG--A-AA-----G-T-CG-C-C-----AA-A-AA------C-T-G-G-T-G-G--A-A----T-G--C-T---C--T-------C--GA-A-A---C-------C--A-A--TT--C-A-C----T-T-G-A-G-T--G---A-GA-----CA-G-A--------G-G-A-G---AG-T------GG--A--ATT----T-C-G-
T-GT--GT-A-G-GG-GT--G---A--A-A---TC-C-GG-AG--AT-C-TA--C-G--A-A-G-G-A-AC-G-CC---AA--A--A--GC-GAA-G--G-C--A-------G--C-T-C-T---CTG--G--GT-C-C--------------------C-T---A-C-C--GA--CG-C-T-G--G-AG--T-G-CG-A--AA-G-C---A-TG--GG-G--
AG-C-G-AA--------CG--GG Bacteria(100);unclassified(100);unclassified(100);unclassified(100);unclassified(100);unclassified(100);unclassified(100);unclassified(100);unclassified(100);

Otu0007 1 0 0 22 36 83 0 0 0 97 25 0 2 13 31 0 0 0 0 6 3 1 142 0 1243 837 1 56
1 8 4 10 1 0 0 1 30 9 20 2 0 110 0 0 14 130 0 0 18 10 0 13 0 0 0 0 11
0 0 0 0 0 0 1 0 0 0 0 0 0 0 0 0 0 0 0 0 0 35 53 76 0 20 2 0 1
67 0 0 0 3 0 0 0 0 10 0 0 0 0 0 7 0 0 2 32 86 0 4 0 24 0 1
M02255_76_000000000-ADUNE_1_1101_12689_9279 G----AA---GC-AC-CG---------GCT-A-A------C-----T---C--CG-T--GC-CA------GC-A--G-C--CG-CG-------GT--A-----AT-AC-GG-A--------G-GG-T-G-C-G------------A--G-CT-G-T-T-----------AA----
T-C-A-G--------G-A-A--G--TG--A-AA-GC--C-C-GG-G-G-----CT-C-AA------C-C-T-C-G-G-A--A-C----A-G--C-A---T--T-------T--TG-A-A---C-------T--G-G--CA--G-A-C----T-A-G-A-G-T--C---T-TG-----TA-G-A--------G-G-G-G---GG-T------AG--A--ATT----T-C-A-G-GT-
-GT-A-G-CG-GT--G---A--A-A---TG-C-GT-AG--AG-A-TC--T-G--A-A-G-G-A-AT-A-CC---GG--T--G--GC-GAA-G--G-C--G-------G--C-C-C-C---CTG--G--AC-A-A--------------------A-G---A-C-T--GA--CG-C-T-C--A-GA--T-G-CG-A--AA-G-C---G-TG--GG-G--AG-C-A-
AA--------CA--GG Bacteria(100);Proteobacteria(100);Gammaproteobacteria(100);Vibrionales(100);Vibrionaceae(100);unclassified(95);unclassified(95);unclassified(95);unclassified(95);

Otu0008 0 0 0 0 1 0 0 0 0 4 0 7 72 28 5 9 54 14 4 4 4 23 0 0 0 0 5 0
4 0 0 0 7 0 0 1 0 1 0 25 7 2 5 4 11 10 101 0 1 7 21 0 5 91 55 75 68
9 3 13 8 20 0 4 211 22 234 69 19 34 86 45 80 0 0 0 0 3 1 0 0 0 0 0 0 0
0 0 0 0 12 4 5 13 73 4 104 3 122 0 6 53 10 0 1 0 0 0 0 1 0 0 0
M02255_76_000000000-ADUNE_1_2114_23043_11222 G----AC---AG-AA-TAA--------GCA-C-C------G--G--C---T--AACT-CTG-TG------CC-A--G-C--AG-CC-------GC--G-----GT-AA-TA-CA-------G-AG-G-G-T-GC------------A--A-GC-G-T-T---
--------AA----G-T-C-G--------G-A-T--G--TG--A-AA-TC--C-C-CG-G-G-----CT-C-AA------C-C-T-G-G-G-A--A-C----T-G--C-A---T--T-------C--AA-A-A---C-------T--G-T--CG--A-G-C----T-A-G-A-G-T--A---T-GG-----TA-G-A--------G-G-G-T---GG-T------GG--A--ATT----
T-C-C-T-GT--GT-A-G-CG-GT--G---A--A-A---TG-C-GT-AG--AT-A-TA--G-G--A-A-G-G-A-AC-A-CC---AG--T--G--GC-GAA-G--G-C--G-------A--C-C-A-C---CTG--G--AC-T-G--------------------A-T---A-C-T--GA--CA-C-T-G--A-GG--T-G-CG-A--AA-G-C---G-TG--GG-
G--AG-C-A-AA--------CA--GG Bacteria(100);Proteobacteria(100);Gammaproteobacteria(100);unclassified(84);unclassified(84);unclassified(84);unclassified(84);unclassified(84);unclassified(84);

Otu0009 408 0 0 0 0 30 0 0 0 0 0 0 0 13 0 0 0 1 0 29 29 6 24 57 43 25 0 312
2 1 202 96 0 2 236 32 219 52 4 15 5 18 125 0 0 0 0 18 319 0 30 231 42 696 0 72 0
52 30 16 4 0 10 8 0 16 27 20 4 67 3 432 131 128 379 161 92 0 44 27 0 160 0 0 76 35
89 317 87 373 107 54 7 60 0 60 8 0 0 67 2 311 158 57 30 39 172 123 78 4 225 0 134
M02255_77_000000000-ADUT3_1_1101_11476_14959 T----AC---GG-AG-GGG--------GCT-A-G------C--G--T---T--GT-T-CGG-AA------TT-A--C-T--GG-GC-------GT--A-----AA-GC-GC-AC-------G-TA-G-G-C-G------------G--A-CC-G-G-A----
-------AA----G-T-T-G--------G-G-G--G--TG--A-AA-TC--C-C-GG-G-G-----CT-C-AA------C-C-T-C-G-G-A--A-C----T-G--C-C---T--T-------C--AA-A-A---C-------T--A-T--TG--G-T-C----T-G-G-A-G-T--T---C-GA-----GA-G-A--------G-G-T-G---AG-T------GG--A--ATT----
C-C-G-A-GT--GT-A-G-AG-GT--G---A--A-A---TT-C-GT-AG--AT-A-TT--C-G--G-A-G-G-A-AC-A-CC---AG--T--G--GC-GAA-G--G-C--G-------G--C-T-C-A---CTG--G--CT-C-G--------------------A-T---A-C-T--GA--CG-C-T-G--A-GG--T-G-CG-A--AA-G-C---G-TG--GG-
G--AG-C-A-AA--------CA--GG Bacteria(100);Proteobacteria(100);Alphaproteobacteria(100);Rhodobacterales(99);Rhodobacteraceae(99);Paracoccus(86);unclassified(86);unclassified(86);unclassified(86);

Otu0010 2425 268 803 1275 0 128 391 419 0 110 0 3 0 11 5 4 7 17 34 1 72 18 0 0 13 91 43 102
98 3 53 403 162 326 57 180 308 97 435 181 7 115 1475 13 181 227 11 72 109 50 347 116 207 12 0 70 0
41 28 75 8 17 15 23 7 0 11 0 0 0 50 229 27 30 219 93 476 0 140 46 0 376 0 0 852 319
36 0 0 1098 169 66 12 6 0 568 0 29 9 26 0 114 1159 152 1083 352 127 529 203 16 296 364 462
M02255_77_000000000-ADUT3_1_1101_25799_6575 T----AC---GA-AG-GGG--------GCT-A-G------C--G--T---T--GC-T-CGG-AA------TC-A--C-T--GG-GC-------GT--A-----AA-GG-GC-GC-------G-TA-G-G-C-G-------------G--C-GT-T-T-T-----------AA----G-T-C-G--------G-G-G--G--TG--A-AA-GC--C-T-GT-G-G-----CT-C-AA------C-C-A-C-A-G-A--A-T----G-G--C-C---T--T-------C--GA-T-A---C-------T--G-G--GA--C-G-C----T-T-G-A-G-T--A---T-GG-----TA-G-A--------G-G-T-T---GG-T------GG--A--ACT----G-C-G-A-GT--GT-A-G-AG-GT--G---A--A-A---TT-C-GT-AG--AT-A-TT--C-G--C-A-A-G-A-AC-A-CC---GG--T--G--GC-GAA-G--G-C--G-------G--C-C-A-A---CTG--G--AC-C-A---------------------T-T---A-C-T--GA--CG-C-T-G--A-GG--C-G-CG-A--AA-G-C---G-TG--GG-G--AG-C-A-AA--------CA--GG Bacteria(100);Proteobacteria(100);Alphaproteobacteria(100);Rhizobiales(100);Methylobacteriaceae(100);Methylobacterium(100);unclassified(100);unclassified(100);unclassified(100);

Otu0011 0 0 92 375 163 130 750 79 212 246 2 0 4 20 11 0 6 0 2 2 25 5 0 3 0 8 8 62
33 43 418 0 59 36 114 25 12 87 39 125 0 41 158 307 35 78 6 199 514 0 14 99 0 24 0 6 103
12 314 93 17 36 19 0 59 22 4 13 4 7 0 2192 3100 20 141 167 173 6 25 9 216 255 0 0 40 287
138 182 192 186 463 299 15 40 0 245 0 484 3 172 0 132 24 85 33 405 746 306 81 14 59 0 554
M02255_77_000000000-ADUT3_1_1106_6762_5073 T----AC---GT-AT-GTC--------ACG-A-G------C--G--T---T--AT-C-CGG-AT------TT-A--T-T--GG-GC-------GT--A-----AA-GC-GC-GT-------C-TA-G-G-T-G-------------G--T-TA-T-G-T-----------AA----G-T-C-T--------G-A-T--G--TG--A-AA-AT--G-C-AG-G-G-----CT-C-AA------C-T-C-T-G-T-A--T------T-G--C-G---T--T-------G--GA-A-A---C-------T--G-T--AT--A-A-C----T-A-G-A-G-T--A---C-TG-----GA-G-A--------G-G-T-A---AG-C------GG--A--ACT----A-C-A-A-GT--GT-A-G-AG-GT--G---A--A-A---TT-C-GT-AG--AT-A-TT--T-G--T-A-G-G-A-AT-G-CC---GA--T--G--GG-GAA-G--C-C--A-------G--C-T-T-A---CTG--G--AC-A-G---------------------A-T---A-C-T--GA--CG-C-T-G--A-AG--C-G-CG-A--AA-G-C---G-TG--GG-T--AG-C-A-AA--------CA--GG Bacteria(100);Fusobacteria(100);Fusobacteria(100);Fusobacteriales(100);Fusobacteriaceae(100);Fusobacterium(100);unclassified(100);unclassified(100);unclassified(100);

Otu0012 384 386 5 1 1 36 812 349 463 92 12 5 8 50 0 1 3 1 1 14 33 4 36 7 21 17 3 16
5 11 3 39 0 196 0 382 0 179 111 659 269 278 0 48 558 0 8 238 89 119 4 429 58 7 0 0 0
293 275 15 54 7 26 5 119 12 23 152 102 37 50 520 90 74 52 284 1484 448 46 13 268 994 0 1 83 288
196 57 505 195 606 1333 92 133 14 2198 61 2913 79 353 23 0 216 0 30 24 0 327 341 22 152 1 162
M02255_76_000000000-ADUNE_1_1102_16502_18697 T----AC---GT-AA-GGT--------CCG-A-G------C--G--T---T--GT-C-CGG-AA------TC-A--T-T--GG-GC-------GT--A-----AA-GG-GT-AC-------G-TA-G-G-C-G-------------G--A-TA-A-G-C-----------AA----G-T-T-A--------G-A-A--G--TG--A-AA-TC--C-T-AT-A-G-----CT-C-AA------C-T-A-T-A-G-T--A------A-G--C-T---T--T-------T--AA-A-A---C-------T--G-C--TC--A-T-C----T-T-G-A-G-G--T---A-TG-----GA-A-G--------G-G-A-A---AG-T------GG--A--ATT----C-C-T-A-GT--GT-A-G-CG-GT--G---A--A-A---TG-C-GC-AG--AT-A-TT--A-G--G-A-G-G-A-AT-A-CC---GG--T--G--GC-GAA-G--G-C--G-------A--C-T-T-T---CTG--G--CC-A-T---------------------A-A---A-C-T--GA--CG-C-T-G--A-GG--T-A-CG-A--AA-G-C---G-TG--GG-T--AG-C-A-AA--------CA--GG Bacteria(100);Firmicutes(100);Clostridia(100);Clostridiales(100);Family_XI_Incertae_Sedis(100);Anaerococcus(100);unclassified(100);unclassified(100);unclassified(100);

Otu0013 136 264 0 89 538 11 0 70 316 56 1 0 0 0 0 0 0 0 4 30 55 6 20 8 24 11 8 118
4 4 0 0 0 77 0 0 219 172 20 1 186 125 73 23 0 62 0 13 1 0 3 177 46 23 0 19 459
7 0 0 0 0 0 0 0 0 0 0 2 0 1 14 67 90 401 69 0 3 9 0 0 134 0 0 0 0
17 206 103 19 128 109 7 0 0 62 0 0 11 0 0 215 504 0 0 0 0 0 0 0 14 0 32
M02255_77_000000000-ADUT3_1_1114_3340_13414 T----AC---GG-AG-GGG--------GCT-A-G------C--G--T---T--GT-T-CGG-AA------TT-A--C-T--GG-GC-------GT--A-----AA-GC-GC-AC-------G-TA-G-G-C-G-------------G--C-TT-T-G-T-----------AA----G-T-T-A--------G-A-G--G--TG--A-AA-GC--C-C-GG-A-G-----CT-C-AA------C-T-C-C-G-G-A--A-T----T-G--C-C---T--T-------T--AA-G-A---C-------T--G-C--AT--C-G-C----T-T-G-A-A-T--C---A-TG-----GA-G-A--------G-G-T-G---AG-T------GG--A--ATT----C-C-G-A-GT--GT-A-G-AG-GT--G---A--A-A---TT-C-GT-AG--AT-A-TT--C-G--G-A-A-G-A-AC-A-CC---AG--T--G--GC-GAA-G--G-C--G-------A--C-T-C-A---CTG--G--AC-A-T---------------------G-T---A-T-T--GA--CG-C-T-G--A-GG--T-G-CG-A--AA-G-C---G-TG--GG-G--AG-C-A-AA--------CA--GG Bacteria(100);Proteobacteria(100);Alphaproteobacteria(100);Sphingomonadales(100);Sphingomonadaceae(93);Sphingomonas(69);Sphingomonadaceae(56);Sphingomonas(56);unclassified(56);

Otu0014 0 0 0 5 0 1 1 1 3 1 273 6 0 13 1 732 3 70 68 31 27 42 20 21 0 8 11 3
73 0 0 0 0 0 0 1 0 1 0 9 2 0 0 0 0 3 207 8 40 7 0 0 0 0 0 1 6
23 40 7 3 6 0 36 0 1 0 33 6 0 3 0 0 1 39 1 6 0 5 0 0 0 0 0 0 0
1 0 0 0 2 5 0 11 61 0 0 0 3 0 0 0 0 39 0 0 0 0 0 0 0 0 2
M02255_76_000000000-ADUNE_1_2105_12717_24892 A----AG---CC-AC-G-G--------CTA-A-C------T--A--C---G--TG-C-CAG-CA------GC-C--G-C--GG-TA-------AT--A-----CG-TA-GG-TG-------G-CA-A-G-C-G-------------T--T-GTCC-G-G-----------AA----G-T-C-T--------G-A-T--G--TG--A-AA-GC--C-C-CC-G-G-----CT-C-AA------C-C-G-G-G-G-A--G------G-GT-C-A---T--T-------G--GA-A-A---C-------T--G-G--GA--G-A-C----T-T-G-A-G-G--A---C-AG-----AA-G-A--------G-G-A-G---AG-T------GG--A--ATT----C-C-A-A-GT--GT-A-G-CG-GT--G---A--A-A---TG-C-GT-AG--AT-A-TT--T-G--G-A-G-G-A-AC-A-CC---AG--T--G--GC-GAA-G--G-C--G-------G--C-T-C-T---CTG--G--TC-T-G---------------------T-T---A-C-T--GA--CG-C-T-G--A-GG--C-G-CG-A--AA-G-C---G-TG--GG-G--AG-C-A-AA--------CA--GG Bacteria(100);Firmicutes(100);Bacilli(100);Bacillales(100);unclassified(100);unclassified(100);unclassified(100);unclassified(100);unclassified(100);

Otu0015 0 0 0 1 18 117 1 0 0 60 198 0 2 42 39 0 0 0 0 21 17 0 327 0 241 50 2 35
0 0 9 9 4 0 2 1 28 26 42 0 1 246 6 3 38 314 1 21 35 39 0 58 0 2 0 0 24
0 0 0 0 0 0 0 7 0 0 0 0 0 0 0 0 0 0 0 0 0 42 49 51 0 0 0 0 1
110 1 0 2 8 1 0 0 0 2 0 0 0 0 0 23 0 0 0 26 32 0 0 1 13 0 0
M02255_76_000000000-ADUNE_1_2112_17062_19534 G----AA---GA-AG-CAC--------CGG-C-T------A--A--C---T--CC-G-TGC-CA------GC-A--G-C--CG-CG-------GT--A-----AT-AC-GG-A--------G-GG-T-G-C-G------------A--G-CT-G-T-T-----
------AA----G-C-A-G--------G-A-T--G--TG--A-AA-GC--C-C-GG-G-G-----CT-C-AA------C-C-T-C-G-G-A--A-C----A-G--C-A---T--T-------T--TG-A-A---C-------T--G-G--CA--G-A-C----T-A-G-A-G-T--C---T-TG-----TA-G-A--------G-G-G-G---GG-T------AG--A--ATT----
T-C-A-G-GT--GT-A-G-CG-GT--G---A--A-A---TG-C-GT-AG--AG-A-TC--T-G--A-A-G-G-A-AT-A-CC---GG--T--G--GC-GAA-G--G-C--G-------G--C-C-C-C---CTG--G--AC-A-A--------------------A-G---A-C-T--GA--CG-C-T-C--A-GA--T-G-CG-A--AA-G-C---G-TG--
GG-G--AG-C-A-AA--------CA--GG Bacteria(100);Proteobacteria(100);Gammaproteobacteria(100);Vibrionales(100);Vibrionaceae(100);unclassified(94);unclassified(94);unclassified(94);unclassified(94);

Otu0016 241 288 0 43 0 33 0 75 187 136 0 51 238 70 47 13 10 128 219 159 46 4 3 767 0 45 147 83
384 2 7 2 164 190 0 19 0 38 90 35 0 16 134 3 125 205 0 54 121 0 5 38 13 14 0 2 17
0 0 0 7 0 0 4 0 0 0 0 1 13 6 0 1 96 16 0 0 0 60 88 1213 0 0 0 25 15
60 79 98 25 52 33 1 8 916 46 4 0 15 15 0 0 150 0 21 67 0 0 42 4 36 231 26
M02255_77_000000000-ADUT3_1_1102_12712_17990 T----AC---GT-AG-GGC--------GCA-A-G------C--G--T---T--AT-C-CGG-AA------TT-A--T-T--GG-GC-------GT--A-----AA-GA-GC-TC-------G-TA-G-G-C-G------------G--T-TT-G-T-C------
-----GC----G-T-C-T--------G-C-C--G--TG--A-AA-GT--C-C-GG-G-G-----CT-C-AA------C-C-C-C-G-G-A--T-C----T-G--C-G---G--T-------G--GG-T-A---C-------G--G-G--CA--G-A-C----T-A-G-A-G-T--G---A-TG-----TA-G-G--------G-G-A-G---AC-T------GG--A--ATT----
C-C-T-G-GT--GT-A-G-CG-GT--G---A--A-A---TG-C-GC-AG--AT-A-TC--A-G--G-A-G-G-A-AC-A-CC---GA--T--G--GC-GAA-G--G-C--A-------G--G-T-C-T---CTG--G--GC-A-T--------------------T-A---A-C-T--GA--CG-C-T-G--A-GG--A-G-CG-A--AA-G-C---A-TG--
GG-G--AG-C-G-AA--------CA--GG Bacteria(100);Actinobacteria(100);Actinobacteria(100);Actinobacteridae(100);Actinomycetales(100);Micrococcineae(100);Micrococcaceae(100);unclassified;unclassified;

Otu0017 0 0 0 0 0 21371 0 0 0 0 0 0 0 0 0 0 0 0 0 0 0 0 1878 15 0 3 0 0
0 0 0 0 0 0 0 0 0 0 0 0 0 0 0 0 0 0 0 0 4 0 0 0 0 0 4 0 0
0 0 0 0 0 0 0 0 0 0 0 0 0 0 0 0 0 0 0 0 0 0 6 0 0 0 0 0 0
0 0 0 0 0 0 0 1 1 0 0 0 0 0 0 0 0 0 0 0 0 0 5 0 0 0
M02255_77_000000000-ADUT3_1_1101_7636_3682 T----AC---AT-AT-GTC--------GCA-A-G------C--G--T---T--AT-C-CGG-AT------TT-A--T-T--GG-GC-------GT--A-----AA-GC-GC-GT-------C-TA-G-G-C-G------------G--T-TT-A-C-T-----------AA---
-G-T-C-T--------G-A-T--G--TG--A-AA-AT--G-C-AG-G-G-----CT-C-AA------C-T-C-T-G-T-A--T------T-G--C-G---T--T-------G--GA-A-A---C-------T--G-G--TA--G-A-C----T-A-G-A-G-T--A---T-TG-----GA-G-A--------G-G-T-G---GG-C------GG--A--ACT----A-C-A-A-GT-
-GT-A-G-AG-GT--G---G--A-A---TT-C-GT-AG--AT-A-TT--T-G--T-A-G-G-A-AT-G-CC---GA--T--G--GA-GAA-G--T-C--A-------G--C-T-C-A---CTG--G--AC-A-T--------------------A-T---A-C-T--GA--CG-C-T-A--A-AG--C-G-CG-A--AA-G-C---G-TG--GG-G--AG-C-A-
AA--------CA--GG Bacteria(100);Fusobacteria(100);Fusobacteria(100);Fusobacteriales(100);Fusobacteriaceae(100);unclassified(100);unclassified(100);unclassified(100);unclassified(100);

Otu0018 549 126 0 51 0 0 0 68 87 0 0 5 0 1 0 0 0 0 0 4 3 1 11 0 0 1 0 1
0 0 0 0 0 0 15 87 0 22 0 48 0 9 49 0 11 0 0 0 0 0 0 101 9 115 0 0 0
0 0 0 7 0 2 0 7 0 0 0 0 5 0 41 0 10 49 66 0 0 1 23 0 0 0 0 23 12
0 179 162 39 0 0 0 11 0 0 0 27 0 48 0 56 45 30 0 21 22 21 0 0 4 1 0
M02255_77_000000000-ADUT3_1_1105_10928_6952 T----AC---GT-AG-GGT--------GCG-A-G------C--G--T---T--GT-C-CGG-AA------TT-A--T-T--GG-GC-------GT--A-----AA-GG-GC-TC-------G-TA-G-G-C-G------------G--T-TT-G-T-C-----------GC-
---G-T-C-G--------G-G-A--G--TG--A-AA-AC--T-C-AG-G-G-----CT-T-AA------C-C-C-T-G-A-G--C-C----T-G--C-T---T--C-------C--GA-T-A---C-------G--G-G--CA--G-A-C----T-A-G-A-G-G--T---A-TG-----CA-G-G--------G-G-A-G---AA-C------GG--A--ATT----C-C-T-G-
GT--GT-A-G-CG-GT--G---A--A-A---TG-C-GC-AG--AT-A-TC--A-G--G-A-G-G-A-AC-A-CC---GG--T--G--GC-GAA-G--G-C--G-------G--T-T-C-T---CTG--G--GC-A-T--------------------T-A---C-C-T--GA--CG-C-T-G--A-GG--A-G-CG-A--AA-G-T---G-TG--GG-G--AG-
C-G-AA--------CA--GG Bacteria(100);Actinobacteria(100);Actinobacteria(100);Actinobacteridae(100);Actinomycetales(100);Propionibacterineae(100);Nocardioidaceae(94);unclassified(94);unclassified(94);

Otu0019 0 0 0 0 0 41 418 481 0 0 0 0 0 0 10 0 0 0 5 0 0 0 29 0 0 0 0 0
10 0 0 0 0 0 35 0 15 3 9 44 0 112 0 2 0 76 1 0 0 0 0 0 18 1 1 0 0
4082 489 669 173 424 194 2561 1260 257 174 1814 200 49 131 241 165 955 135 604 5894 4625 0 9 0 0 0 0 0 2
0 0 0 175 0 20 0 3 0 50 0 0 4 51 0 291 464 270 4263 187 127 327 401 8 17 0 0
M02255_77_000000000-ADUT3_1_1109_7316_17338 T----AC---GA-AG-GGG--------GCT-A-G------C--G--T---T--GC-T-CGG-AA------TT-A--C-T--GG-GC-------GT--A-----AA-GG-GA-GC-------G-TA-G-G-C-G------------G--A-CT-G-T-T-----------
AA----G-T-T-A--------G-A-G--G--TG--A-AA-GC--C-C-AG-G-G-----CT-C-AA------C-C-T-T-G-G-A--A-T----T-G--C-C---T--T-------T--GA-T-A---C-------T--G-G--CA--G-T-C----T-T-G-A-G-T--A---C-GG-----AA-G-A--------G-G-T-A---TG-T------GG--A--ACT----C-C-G-
A-GT--GT-A-G-AG-GT--G---A--A-A---TT-C-GT-AG--AT-A-TT--C-G--G-A-A-G-A-AC-A-CC---AG--T--G--GC-GAA-G--G-C--G-------A--C-A-T-A---CTG--G--TC-C-G--------------------T-T---A-C-T--GA--CG-C-T-G--A-GG--C-T-CG-A--AA-G-C---G-TG--GG-G--AG-
C-A-AA--------CA--GG Bacteria(100);Proteobacteria(100);Alphaproteobacteria(100);Caulobacterales(100);Caulobacteraceae(100);Caulobacter(100);unclassified(100);unclassified(100);unclassified(100);

Otu0020 0 1 0 0 0 30 0 0 0 0 0 1 1 0 4 0 0 0 13 7 51 0 61 5 55 47 8 51
52 2 668 250 786 1010 424 122 0 133 357 36 0 0 181 27 0 0 2 0 0 8 30 196 173 0 3 0 0
0 0 12 5 0 0 0 0 0 0 0 0 18 10 0 10 79 419 0 0 0 0 11 0 78 0 0 39 77
58 283 225 244 78 45 59 5 5 777 12 0 19 166 0 388 1614 322 454 0 0 0 165 0 1 0 0
M02255_77_000000000-ADUT3_1_2113_15045_19476 T----AC---GG-AG-GGA--------GCT-A-G------C--G--T---T--GT-T-CGG-AA------TT-A--C-T--GG-GC-------GT--A-----AA-GC-GC-GC-------G-TA-G-G-C-G------------G--C-TA-T-T-C-----
------AA----G-T-C-A--------G-A-G--G--TG--A-AA-GC--C-C-GG-G-G-----CT-C-AA------C-C-C-C-G-G-A--A-C----T-G--C-C---T--T-------T--GA-A-A---C-------T--A-G--GT--A-G-C----T-A-G-A-A-T--C---T-TG-----GA-G-A--------G-G-T-C---AG-T------GG--A--ATT----C-
C-G-A-GT--GT-A-G-AG-GT--G---A--A-A---TT-C-GT-AG--AT-A-TT--C-G--G-A-A-G-A-AC-A-CC---AG--T--G--GC-GAA-G--G-C--G-------A--C-T-G-A---CTG--G--AC-A-A--------------------G-T---A-T-T--GA--CG-C-T-G--A-GG--T-G-CG-A--AA-G-C---G-TG--GG-
G--AG-C-A-AA--------CA--GG Bacteria(100);Proteobacteria(100);Alphaproteobacteria(100);Sphingomonadales(100);Sphingomonadaceae(100);Novosphingobium(96);unclassified(96);unclassified(96);unclassified(96);

Otu0021 0 0 0 434 0 0 0 93 75 0 0 0 0 0 0 0 0 0 3 5 1 0 98 0 6 0 0 69
3 0 0 0 0 0 35 106 0 3 0 98 73 78 190 134 0 0 0 0 0 2 1 0 52 137 0 1 0
50 0 0 0 0 0 0 0 0 0 0 0 5 10 143 3 0 180 0 0 0 0 3 245 0 0 0 36 0
8 0 121 32 239 0 10 0 0 27 0 43 0 0 0 79 0 0 76 0 0 0 48 0 9 0 0
M02255_77_000000000-ADUT3_1_2113_17589_17344 T----AC---GG-AG-GGG--------GCT-A-G------C--G--T---T--GT-T-CGG-AA------TT-A--C-T--GG-GC-------GT--A-----AA-GC-GC-AC-------G-TA-G-G-C-G------------G--C-TT-T-G-T-----
------AA----G-T-T-A--------G-A-G--G--TG--A-AA-GC--C-C-GG-G-G-----CT-C-AA------C-T-C-C-G-G-A--A-T----T-G--C-C---T--T-------T--AA-G-A---C-------T--G-C--AT--C-G-C----T-T-G-A-A-C--G---T-CG-----GA-G-A--------G-G-T-A---AG-T------GG--A--ATT----C-
C-G-A-GT--GT-A-G-AG-GT--G---A--A-A---TT-C-GT-AG--AT-A-TT--C-G--G-A-A-G-A-AC-A-CC---AG--T--G--GC-GAA-G--G-C--G-------A--C-T-T-A---CTG--G--AC-G-A--------------------C-T---G-T-T--GA--CG-C-T-G--A-GG--T-G-CG-A--AA-G-C---G-TG--GG-G-
-AG-C-A-AA--------CA--GG Bacteria(100);Proteobacteria(100);Alphaproteobacteria(100);Sphingomonadales(100);Sphingomonadaceae(99);Sphingomonas(88);Sphingomonadaceae(88);Sphingomonas(88);unclassified(88);

Otu0022 1 377 376 238 1 2 674 143 0 0 3 8 11 1 38 3 0 11 0 31 96 25 2 27 6 29 21 25
27 1418 103 1257 565 476 40 1151 409 280 271 766 1767 281 473 360 999 470 0 431 835 2507 20 556 123 25 10 227 305
365 649 102 179 38 44 52 693 39 69 382 268 82 172 1022 226 22 111 951 2574 1000 17 30 743 3915 4796 1976 47 116
534 459 452 1049 857 7103 166 926 25 7004 133 14637 286 627 168 12 488 249 20 0 307 34 285 16 157 0 137
M02255_77_000000000-ADUT3_1_1114_24010_5547 T----AC---GT-AT-GGA--------GCG-A-G------C--G--T---T--GT-C-CGG-AA------TT-A--T-T--GG-GC-------GT--A-----AA-GG-GT-AC-------G-CA-G-G-C-G------------G--T-TT-A-A-T-----------AA-
---G-T-C-G--------A-A-T--G--TT--A-AA-GA--T-C-GG-G-G-----CT-C-AA------C-C-C-C-G-T-A--A------A-G--C-A---T--T-------G--GA-A-A---C-------T--G-A--TA--A-A-C----T-T-G-A-G-T--A---G-TG-----GA-G-A--------G-G-A-A---AG-T------GG--A--ATT----C-C-T-A-
GT--GT-A-G-TG-GT--G---A--A-A---TA-C-GT-AG--AT-A-TT--A-G--G-A-G-G-A-AT-A-CC---AG--T--A--GC-GAA-G--G-C--G-------A--C-T-T-T---CTG--G--AC-A-C--------------------A-A---A-C-T--GA--CG-C-T-G--A-GG--T-A-CG-A--AA-G-C---G-TG--GG-G--AG-C-
A-AA--------CA--GG Bacteria(100);Firmicutes(100);Clostridia(100);Clostridiales(100);Family_XI_Incertae_Sedis(100);Finegoldia(100);unclassified(100);unclassified(100);unclassified(100);

Otu0023 174 80 0 3 0 131 12 0 0 0 0 1 0 144 14 0 1 19 23 76 388 24 27 0 46 887 277 310
1688 99 44 0 211 0 16 891 1719 1298 505 855 179 44 253 56 22 0 1 40 0 40 163 1314 63 0 0 117 153
1 0 11 0 8 2 0 0 40 0 0 0 13 9 16 28 14 470 85 0 0 0 5 0 238 292 0 0 920
103 66 194 2202 57 3 6 0 0 26 0 0 0 0 0 0 2334 289 1392 374 0 865 218 0 63 1174 0
M02255_77_000000000-ADUT3_1_1104_20260_18697 T----AC---GA-AG-GGG--------GCT-A-G------C--G--T---T--GC-T-CGG-AA------TC-A--C-T--GG-GC-------GT--A-----AA-GG-GC-GC-------G-TA-G-G-C-G------------G--T-CT-T-T-T-----
------AA----G-T-C-G--------G-G-G--G--TG--A-AA-GC--C-T-GT-G-G-----CT-C-AA------C-C-A-C-A-G-A--A-T----T-G--C-C---T--T-------C--GA-T-A---C-------T--G-G--GA--G-A-C----T-T-G-A-G-T--T---C-GG-----AA-G-A--------G-G-T-T---GG-T------GG--A--ACT----
G-C-G-A-GT--GT-A-G-AG-GT--G---A--A-A---TT-C-GT-AG--AT-A-TT--C-G--C-A-A-G-A-AC-A-CC---GG--T--G--GC-GAA-G--G-C--G-------G--C-C-A-A---CTG--G--TC-C-G--------------------A-C---A-C-T--GA--CG-C-T-G--A-GG--C-G-CG-A--AA-G-C---G-TG--
GG-G--AG-C-A-AA--------CA--GG Bacteria(100);Proteobacteria(100);Alphaproteobacteria(100);Rhizobiales(100);Methylobacteriaceae(100);Methylobacterium(100);unclassified(100);unclassified(100);unclassified(100);

Otu0024 0 0 0 0 0 0 0 0 0 0 2 9 34 8 5 28 2 39 24 62 8 99 1 23 6 0 25 2
17 0 0 1 0 0 0 0 0 0 0 0 0 0 0 0 0 0 0 0 0 0 0 0 0 0 0 0 0
29 1 0 4 99 37 22 2 10 0 11 154 181 39 6 2 3 2 1 1 0 9 7 0 0 0 0 0 0
0 0 0 1 0 0 0 12 2 0 26 0 32 0 37 0 0 0 0 0 0 0 0 0 1 0 1
M02255_77_000000000-ADUT3_1_2102_8514_23781 T----GC---CA-GC-AGC--------CGC-G-G------T--A--A---T--AC-G-GAG-GA------TC-C--A-A--GC-GT-------TA--T-----CC-GG-AA-TC-------A-TA-G-G-C-G------------G--T-CA-G-A-T-----------AA-
---G-T-C-A--------G-T-G--G--TG--A-AA-GC--C-C-AT-C-G-----CT-C-AA------C-G-G-T-G-G-A--A-C----G-G--C-C---A--T-------T--GA-T-A---C-------T--G-T--CT--G-A-C----T-T-G-A-A-T--T---A-TT-----AG-G-A--------A-G-T-A---AC-T------AG--A--ATA----T-G-T-A-
GT--GT-A-G-CG-GT--G---A--A-A---TG-C-TT-AG--AG-A-TT--A-C--A-T-G-G-A-AT-A-CC---AA--T--T--GC-GAA-G--G-C--A-------G--G-T-T-A---CTA--C--TA-A-T--------------------A-T---A-T-T--GA--CG-C-T-G--A-TG--G-A-CG-A--AA-G-C---G-TG--GG-T--AG-C-
G-AA--------CA--GG Bacteria(100);Bacteroidetes(100);Flavobacteria(100);Flavobacteriales(100);Flavobacteriaceae(100);Flavobacterium(100);unclassified(100);unclassified(100);unclassified(100);

Otu0025 0 1487 2083 0 1 29 2176 401 3591 0 0 0 6 14 0 0 0 0 0 0 1 0 18 11 0 0 0 9
0 0 101 251 0 19 0 1 0 0 0 525 0 72 0 43 0 0 0 48 0 0 0 0 0 1 0 0 0
0 0 0 0 0 0 0 0 0 0 0 1 11 1 50 22 69 323 0 0 0 5 4 1 0 0 0 57 57
0 0 0 22 0 30 40 0 1 37 0 144 0 0 0 0 0 0 0 1 1 37 48 69 1277 1230 37
M02255_77_000000000-ADUT3_1_1105_11899_8025 T----AC---GT-AT-GGT--------GCA-A-G------C--G--T---T--AT-C-CGG-AT------TT-A--C-T--GG-GT-------GT--A-----AA-GG-GA-GC-------G-CA-G-G-C-G------------G--T-GC-G-G-C-----------AA-
---G-T-C-T--------G-A-T--G--TG--A-AA-GC--C-C-GG-G-G-----CT-C-AA------C-C-C-C-G-G-T--A-C----T-G--C-A---T--T-------G--GA-A-A---C-------T--G-T--CG--T-A-C----T-A-G-A-G-T--G---T-CG-----GA-G-G--------G-G-T-A---AG-T------GG--A--ATT----C-C-T-A-
GT--GT-A-G-CG-GT--G---A--A-A---TG-C-GT-AG--AT-A-TT--A-G--G-A-G-G-A-AC-A-CC---AG--T--G--GC-GAA-G--G-C--G-------G--C-T-T-A---CTG--G--AC-G-A--------------------T-A---A-C-T--GA--CG-C-T-G--A-GG--C-T-CG-A--AA-G-C---G-TG--GG-G--AG-
C-A-AA--------CA--GG Bacteria(100);Firmicutes(100);Clostridia(100);Clostridiales(100);Lachnospiraceae(100);Roseburia(97);unclassified(97);unclassified(97);unclassified(97);

Otu0026 0 0 0 66 0 46 41 1 0 0 0 0 0 0 0 10 0 0 0 16 112 2 0 15 13 9 0 7
0 15 174 60 41 0 236 79 0 283 171 316 103 0 0 0 5 116 0 125 0 33 0 124 0 0 0 46 0
165 90 0 45 0 9 4 45 8 34 23 34 1 20 298 6 0 0 873 894 291 0 10 47 68 0 1052 67 109
92 0 0 122 843 741 83 74 0 876 28 1042 9 96 2 0 199 58 0 1 0 0 189 0 0 0 0
M02255_77_000000000-ADUT3_1_1107_19234_16567 T----AC---GT-AT-GGA--------CCG-A-G------C--G--T---T--GT-C-CGG-AA------TC-A--T-T--GG-GC-------GT--A-----AA-GG-GT-AC-------G-TA-G-G-C-G------------G--C-CT-A-G-T-----
------AA----G-T-T-A--------G-A-A--G--TG--A-AA-TA--A-T-AT-A-G-----CT-C-AA------C-T-A-T-A-T-A--A------A-G--C-T---T--T-------T--AA-A-A---C-------T--G-T--TA--G-G-C----T-T-G-A-G-A--G---A-TG-----AA-A-G--------G-G-A-A---AG-T------GG--A--ATT----C-
C-T-A-GT--GT-A-G-CG-GT--G---A--A-A---TG-C-GC-AG--AT-A-TT--A-G--G-A-A-G-A-AT-A-CC---GG--T--G--GC-GAA-G--G-C--G-------A--C-T-T-T---CTG--G--TC-A-T--------------------C-A---T-C-T--GA--CG-C-T-G--A-GG--T-A-CG-A--AA-G-C---G-TG--GG-T--
AG-C-A-AA--------CA--GG Bacteria(100);Firmicutes(100);Clostridia(100);Clostridiales(100);Family_XI_Incertae_Sedis(100);Anaerococcus(98);unclassified(98);unclassified(98);unclassified(98);

Otu0027 42 0 75 9 153 0 7 55 0 0 1 0 0 0 3 0 6 1 1 6 21 0 0 0 4 0 0 0
6 6 5 36 42 65 0 67 126 98 68 5 54 82 45 61 18 19 0 19 58 0 1 0 14 0 0 8 0
0 9 0 0 0 0 0 5 0 2 1 4 5 1 37 0 36 31 0 25 22 18 18 90 54 0 1 19 0
67 24 84 40 101 0 0 12 0 10 2 0 0 38 0 47 49 103 0 7 0 146 21 7 9 0 0
M02255_77_000000000-ADUT3_1_1108_17404_7563 T----AC---GT-AG-GGT--------GCG-A-G------C--G--T---T--GT-C-CGG-AA------TT-A--T-T--GG-GC-------GT--A-----AA-GA-GC-TT-------G-TA-G-G-C-G------------G--T-TT-G-T-C-----------GC--
--G-T-C-T--------G-C-T--G--TG--A-AA-AT--C-C-GG-G-G-----CT-C-AA------C-C-C-C-G-G-A--C-T----T-G--C-A---G--T-------G--GG-T-A---C-------G--G-G--CA--G-A-C----T-A-G-A-G-T--G---T-GG-----TA-G-G--------G-G-A-G---AC-T------GG--A--ATT----C-C-T-G-
GT--GT-A-G-CG-GT--G---A--A-A---TG-C-GC-AG--AT-A-TC--A-G--G-A-G-G-A-AC-A-CC---GA--T--G--GC-GAA-G--G-C--A-------G--G-T-C-T---CTG--G--GC-C-A--------------------C-T---A-C-T--GA--CG-C-T-G--A-GA--A-G-CG-A--AA-G-C---A-TG--GG-G--AG-
C-G-AA--------CA--GG Bacteria(100);Actinobacteria(100);Actinobacteria(100);Actinobacteridae(100);Actinomycetales(100);Micrococcineae(100);Intrasporangiaceae(98);unclassified(62);unclassified(62);

Otu0028 0 0 0 0 0 0 0 0 0 0 1 0 45 12 1 2 5 6 0 1 4 13 0 0 0 0 1 0
2 1 0 2 2 1 1 6 0 1 1 3 2 0 0 0 1 0 10 0 0 0 7 1 6 70 69 27 0
21 2 19 1 11 0 8 22 8 40 18 10 18 76 1 2 0 1 0 1 0 0 0 1 0 0 0 0 0
0 0 0 0 0 0 3 13 19 0 12 0 13 0 0 17 1 0 0 0 0 0 0 0 0 0 0
M02255_77_000000000-ADUT3_1_1114_17442_14716 T----AC---AG-AG-GGT--------GCA-A-G------C--G--T---T--AA-T-CGG-AA------TT-A----------C--------T----------G--G--GC-------G-TA-G-G-T-G------------G--T-TT-G-T-T----------
AA----G-T-T-G--------G-A-T--G--TG--A-AA-TC--C-C-CG-G-G-----CT-C-AA------C-C-T-G-G-G-A--A-C----T-G--C-A---T--T-------C--AA-A-A---C-------T--G-A--CT--G-A-C----T-A-G-A-G-T--A---T-GG-----TA-G-A--------G-G-G-T---GG-T------GG--A--ATT----T-C-C-
T-GT--GT-A-G-CG-GT--G---A--A-A---TG-C-GT-AG--AT-A-TA--G-G--A-A-G-G-A-AC-A-CC---AG--T--G--GC-GAA-G--G-C--G-------A--C-C-A-C---CTG--G--AC-T-G--------------------A-T---A-C-T--GA--CA-C-T-G--A-GG--T-G-CG-A--AA-G-C---G-TG--GG-G--
AG-C-A-AA--------CA--GG Bacteria(100);Proteobacteria(100);Gammaproteobacteria(100);Pseudomonadales(100);Pseudomonadaceae(100);Pseudomonas(99);unclassified(99);unclassified(99);unclassified(99);

Otu0029 778 799 1120 622 585 302 247 605 497 249 10 11 15 122 14 4 6 5 6 12 76 5 91 60 10 23 7 32
32 184 306 48 287 216 205 248 136 255 240 364 201 227 390 737 268 89 0 157 108 4 15 202 76 10 8 59 21
16 29 9 0 0 6 1 0 7 10 0 0 12 7 19 7 19 240 112 195 129 231 141 171 1136 309 0 329 213
28 208 24 133 175 122 1 39 0 58 9 86 15 572 1 6 344 264 194 378 953 588 407 58 551 220 182
M02255_77_000000000-ADUT3_1_1108_10283_17146 T----AC---GT-AG-GGT--------GCG-A-G------C--G--T---T--AT-C-CGG-AA------TT-A--T-T--GG-GC-------GT--A------AA-GA-GC-TC-------G-TA-G-G-C-G------------G--T-TT-G-T-C------
-----GC----G-T-C-T--------G-C-C--G--TG--A-AA-GT--C-C-GG-G-G-----CT-T-AA------C-T-C-C-G-G-A--T-C----T-G--C-G---G--T-------G--GG-T-A---C-------G--G-G--CA--G-A-C----T-A-G-A-G-T--G---C-AG-----TA-G-G--------G-G-A-G---AC-T------GG--A--ATT----
C-C-T-G-GT--GT-A-G-CG-GT--G---G--A-A---TG-C-GC-AG--AT-A-TC--A-G--G-A-G-G-A-AC-A-CC---GA--T--G--GC-GAA-G--G-C--A-------G--G-T-C-T---CTG--G--GC-T-G--------------------T-A---A-C-T--GA--CG-C-T-G--A-GG--A-G-CG-A--AA-G-C---A-TG--
GG-G--AG-C-G-AA--------CA--GG Bacteria(100);Actinobacteria(100);Actinobacteria(100);Actinobacteridae(100);Actinomycetales(100);Micrococcineae(100);Micrococcaceae(100);Micrococcus(78);unclassified(78);

Otu0030 53 65 64 294 154 129 120 168 40 19 0 2 0 0 1 0 0 3 2 12 47 4 1 0 0 12 0 72
21 281 174 130 174 25 184 48 60 115 163 0 30 106 162 73 14 40 0 6 200 180 1 146 30 76 0 56 26
26 69 37 5 22 7 0 0 7 6 0 0 8 1 86 68 133 94 60 101 9 12 4 41 31 0 0 201 248
137 134 166 229 333 62 11 33 0 129 3 13 5 106 0 16 84 255 27 266 375 152 115 9 76 103 324
M02255_76_000000000-ADUNE_1_1109_26206_17835 .....AC----T-AC-GT---------GCC-A-G------C--A--G---C--CG-C-GGT-AA------TA-C--G-T--AG-GT-------GG--C-----AA-GC-G--TT-------G----T-C-C-G-------------G--A-TC-C-T-T----------
AA----G-T-C-T--------G-A-T--G--TG--A-AA-GC--C-C-CC-G-G-----CT-C-AA------C-C-G-G-G-G-A--G-G----G-T--C-A---T--T-------G--GA-A-A---C-------T--G-G--GG--A-A-C----T-T-G-A-G-T--G---C-AG-----AA-G-A--------G-G-A-G---AG-T------GG--A--ATT----C-C-
A-T-GT--GT-A-G-CG-GT--G---A--A-A---TG-C-GT-AG--AT-A-TA--T-G--G-A-G-G-A-AC-A-CC---AG--T--G--GC-GAA-G--G-C--G-------A--C-T-C-T---CTG--G--TC-T-G--------------------T-A---A-C-T--GA--CG-C-T-G--A-GG--C-T-CG-A--AA-G-C---G-TG--GG-T--
AG-C-A-AA--------CA--GG Bacteria(100);Firmicutes(100);Bacilli(100);Lactobacillales(100);unclassified(100);unclassified(100);unclassified(100);unclassified(100);unclassified(100);

Otu0031 0 0 0 0 0 169 51 0 0 0 2 0 7 0 0 0 0 2 0 2 8 1 0 0 0 8 0 0
2 2 0 184 0 77 0 0 248 32 1 128 483 0 83 50 92 0 0 120 0 763 13 204 23 5 0 0 834
94 76 0 19 0 6 3 115 0 2 91 46 3 18 322 38 0 36 323 1009 263 0 1 0 146 0 1376 0 110
38 82 0 80 97 716 50 61 4 1087 16 1476 0 48 5 0 0 0 0 45 0 0 115 4 85 0 0
M02255_76_000000000-ADUNE_1_1108_22434_20789 T----AC---GT-AG-GGG--------GCG-A-G------C--G--T---T--GT-C-CGG-AA------TT-A--C-T--GG-GC-------GT--A-----AA-GG-GT-TC-------G-CA-G-G-C------------------A-T--G-G-C-------
----AA----G-T-C-C--------G-A-T--G--TA--A-AA-GG--C-G-AA-A-G-----CT-C-AA------C-T-T-T-C-G-T--A------A-G--C-A---T--C-------G--GA-A-A---C-------T--G-T--CA--A-G-C----T-T-G-A-G-T--G---A-AG-----GA-G-A--------G-G-C-A---AG-T------GG--A--ATT----C-C-
T-A-GT--GT-A-G-CG-GT--G---G--A-A---TG-C-GT-AG--AT-A-TT--A-G--G-A-G-G-A-AT-A-CC---GG--T--G--GC-GAA-G--G-C--G-------A--C-T-T-G---CTG--G--AC-T-T--------------------C-A---A-C-T--GA--CG-C-T-G--A-GG--A-A-CG-A--AA-G-C---G-TG--GG-T--
AG-C-A-AA--------CA--GG Bacteria(100);Firmicutes(100);Clostridia(100);Clostridiales(100);Family_XI_Incertae_Sedis(100);Peptoniphilus(100);unclassified(100);unclassified(100);unclassified(100);

Otu0032 0 0 0 0 0 0 0 0 0 20 0 0 0 0 0 0 0 0 75 2 0 0 0 0 0 0 29 0
0 0 0 0 0 0 0 1 0 0 0 0 0 0 38 0 0 0 0 0 0 0 2353 0 563 109 4 0 0
0 0 0 0 0 0 0 0 0 0 0 1 0 0 0 0 4 8 0 0 0 0 0 0 18 0 0 0 0
17 0 0 0 0 9 0 0 435 0 15 0 0 0 0 0 0 0 0 0 0 0 0 0 0 0 0
M02255_76_000000000-ADUNE_1_1111_9116_17641 T----CG---GC-TA-ACT--------CCG-T-GC-----C--A--G---C--AG-C-CGC-GG------TA-A--T-A--CG-GA-------GG--A-----TC-CA-AG-CG-------T-TA-T-C-C-G-------------GA-T-TT-A-T-T-----------AA-
---G-T-C-A--------G-G-G--G--TG--A-AA-GA--C-G-GT-G-G-----CT-C-AA------C-C-A-T-C-G-C--A-G----T-G--C-C---C--T-------T--GA-T-A---C-------T--G-A--TG--A-G-C----T-T-G-A-A-T--A---C-AC-----TA-G-A--------G-G-T-A---GG-C------GG--A--ATG----T-G-A-C-
AA--GT-A-G-CG-GT--G---A--A-A---TG-C-AT-AG--AT-A-TG--T-C--A-C-A-G-A-AC-A-CC---GA--T--T--GC-GAA-G--G-C--A-------G--C-T-T-A---CTA--T--GG-T-G--------------------T-T---A-T-T--GA--CG-C-T-G--A-GG--C-A-CG-A--AA-G-C---G-TG--GG-G--AT-C-
A-AA--------CA--GG Bacteria(100);Bacteroidetes(100);Sphingobacteria(100);Sphingobacteriales(100);Sphingobacteriaceae(100);Sphingobacteriaceae(100);Pedobacter(96);unclassified(96);unclassified(96);

Otu0033 0 0 0 565 145 86 315 581 188 240 3 0 0 0 15 2 0 0 0 9 41 0 0 6 0 0 0 20
0 0 87 84 0 0 28 506 267 79 142 365 1479 44 0 90 555 0 0 17 365 45 8 65 40 2 1 0 0
209 171 18 9 24 36 26 131 19 11 91 105 6 29 148 9 1 3 612 1509 412 12 5 187 162 0 1946 43 320
268 1 224 197 75 1125 47 126 6 1451 24 2600 38 220 25 133 299 124 0 52 1 0 99 7 37 0 50
M02255_77_000000000-ADUT3_1_1101_17889_12243 T----AC---GT-AG-GGG--------GCT-A-G------C--G--T---T--GT-C-CGG-AA------TC-A--C-T--GG-GC-------GT--A-----AA-GG-GT-TC-------G-CA-G-G-C-G-------------G--A-AA-T-G-C----
--------AA----G-T-C-A--------G-A-T--G--TA--A-AA-GG--C-A-GT-A-G-----CT-T-AA------C-T-A-C-T-G-T--A------A-G--C-A---T--T-------T--GA-A-A---C-------T--G-C--AT--A-T-C----T-T-G-A-G-A--A---G-AG-----TA-G-A--------G-G-T-A---AG-T------GG--A--ATT----T-
T-T-A-GT--GT-A-G-CG-GT--G---A--A-A---TG-C-GT-AG--AT-A-TT--A-A--A-A-A-G-A-AT-A-CC---GG--T-G--GC-GAA-G--G-C--G-------A--C-T-T-A---CTG--G--GC-T-C--------------------A-T---T-C-T--GA--CG-C-T-G--A-GG--A-A-CG-A--AA-G-C---G-TG--GG-T--
AG-C-A-AA--------CA--GG Bacteria(100);Firmicutes(100);Clostridia(100);Clostridiales(100);Family_XI_Incertae_Sedis(100);Peptoniphilus(100);unclassified(100);unclassified(100);unclassified(100);

Otu0034 0 0 247 0 0 0 0 0 82 0 0 0 2 7 0 0 0 0 4 0 0 0 96 16 3 1 0 87
0 0 63 66 0 9 0 56 75 3 39 0 0 0 216 0 0 67 0 1 133 0 4 0 30 10 0 56 420
0 0 0 0 14 8 70 8 0 4 21 0 5 0 10 0 0 158 0 67 0 0 0 91 0 0 0 46 129
39 36 0 71 40 12 0 0 2 0 0 0 1 0 0 28 273 18 0 0 0 62 0 2 0 0 0
M02255_77_000000000-ADUT3_1_1107_5421_15173 T----AC---GA-AG-GGG--------GCT-A-G------C--G--T---T--GT-T-CGG-AA------TT-A--C-T--GG-GC-------GT--A-----AA-GC-GC-AC-------G-TA-G-G-C-G-------------G--A-TA-T-T-T-----------AA-
---G-T-C-A--------G-G-G--G--TG--A-AA-TC--C-C-AG-A-G-----CT-C-AA------C-T-C-T-G-G-A--A-C----T-G--C-C---T--T-------T--GA-T-A---C-------T--G-G--GT--A-T-C----T-T-G-A-G-T--A---T-GG-----AA-G-A--------G-G-T-G---AG-T------GG--A--ATT----C-C-G-A-
GT--GT-A-G-AG-GT--G---A--A-A---TT-C-GT-AG--AT-A-TT--C-G--G-A-G-G-A-AC-A-CC---AG--T--G--GC-GAA-G--G-C--G-------G--C-T-C-A---CTG--G--TC-C-A--------------------T-T---A-C-T--GA--CG-C-T-G--A-GG--T-G-CG-A--AA-G-C---G-TG--GG-G--AG-C-
A-AA--------CA--GG Bacteria(100);Proteobacteria(100);Alphaproteobacteria(100);Rhizobiales(100);unclassified(92);unclassified(92);unclassified(92);unclassified(92);unclassified(92);

Otu0035 24 0 0 54 0 0 0 33 0 0 0 14 0 13 1 0 0 0 5 2 0 1 18 0 0 0 2 2
0 0 5 65 0 0 0 22 0 10 23 50 0 45 90 0 0 29 0 27 0 0 0 51 7 12 0 0 0
0 0 0 0 12 0 0 0 0 10 0 0 0 2 13 26 51 15 0 0 0 7 21 0 0 0 0 0 0
0 0 36 0 0 0 14 0 146 0 0 0 9 0 0 0 0 39 11 0 0 0 20 5 0 0 0
M02255_77_000000000-ADUT3_1_2118_18625_11352 T----AC---GT-AG-GGT--------GCA-A-G------C--G--T---T--AT-C-CGG-AA------TT-A--T-T--GG-GC-------GT--A-----AA-GA-GC-TC-------G-TA-G-G-C-G------------G--T-TT-G-T-C------
-----GC----G-T-C-T--------G-C-T--G--TG--A-AA-AC--T-G-GA-G-G-----CT-C-AA------C-C-T-C-C-A-G--C-C----T-G--C-A---G--T-------G--GG-T-A---C-------G--G-G--CA--G-A-C----T-A-G-A-G-T--G---C-GG-----TA-G-G--------G-G-A-G---AT-T------GG--A--ATT----C-
C-T-G-GT--GT-A-G-CG-GT--G---G--A-A---TG-C-GC-AG--AT-A-TC--A-G--G-A-G-G-A-AC-A-CC---GA--T--G--GC-GAA-G--G-C--A-------G--A-T-C-T---CTG--G--GC-C-G--------------------T-A---A-C-T--GA--CG-C-T-G--A-GG--A-G-CG-A--AA-G-C---A-TG--GG-
G--AG-C-G-AA--------CA--GG Bacteria(100);Actinobacteria(100);Actinobacteria(100);Actinobacteridae(100);Actinomycetales(100);Micrococcineae(100);Microbacteriaceae(100);unclassified(100);unclassified(100);

Otu0036 555 713 112 474 0 462 262 521 1 60 5 0 10 54 8 4 2 14 10 46 153 11 20 35 18 33 5 48
23 267 303 121 500 484 29 441 193 543 598 241 203 307 470 443 940 398 16 167 87 23 69 197 143 13 0 47 849
22 311 115 39 11 39 27 0 29 5 16 4 10 7 65 30 18 63 65 78 3 38 11 368 383 0 461 150 0
43 174 138 111 291 405 22 48 5 498 25 408 156 225 0 44 0 223 0 320 0 137 19 17 264 2 47
M02255_77_000000000-ADUT3_1_1101_14023_13075 T----AC---GT-AA-GGA--------CCG-A-G------C--G--T---T--GT-C-CGG-AA------TC-A--T-T--GG-GC-------GT--A-----AA-GG-GT-AC-------G-TA-G-G-C-G------------G--C-TA-G-A-A-----
------AA----G-T-T-A--------G-A-A--G--TC--A-AA-GG--C-T-AT-A-G-----CT-C-AA------C-T-A-T-A-G-T--A------A-G--C-T---T--C-------T--AA-A-A---C-------T--A-T--TT--A-G-C----T-T-G-A-G-A--A---A-TG-----GA-A-G--------G-G-A-A---AG-T------GG--A--ATT----C-
C-T-A-GT--GT-A-G-CG-GT--G---G--A-A---TG-C-GC-AG--AT-A-TT--A-G--G-A-A-G-A-AT-A-CC---GG--T--G--GC-GAA-G--G-C--G-------A--C-T-T-T---CTG--G--CC-A-T--------------------T-A---T-C-T--GA--CG-C-T-G--A-GG--T-A-CG-A--AA-G-C---G-TG--GG-T--
AG-C-A-AA--------CA--GG Bacteria(100);Firmicutes(100);Clostridia(100);Clostridiales(100);Family_XI_Incertae_Sedis(100);Anaerococcus(100);unclassified(100);unclassified(100);unclassified(100);

Otu0037 27 0 0 9 0 0 2 0 3 0 0 3 0 15 0 0 0 0 0 3 6 1 13 24 0 0 0 0
0 70 37 0 48 34 36 85 430 34 21 19 30 28 49 494 126 116 0 7 37 0 0 26 2 0 4 8 0
0 6 6 0 8 0 0 0 1 1 0 2 0 0 0 0 0 7 9 10 0 11 6 30 63 0 0 41 42
0 15 0 17 7 15 0 2 0 29 0 29 5 1 0 13 2 0 0 0 0 13 0 1 0 0 0
M02255_77_000000000-ADUT3_1_1114_21315_17096 T----AC---GT-AG-GGT--------GCG-A-G------C--G--T---T--GT-C-CGG-AA------TT-A--C-T--GG-GC-------GT--A-----AA-GA-GC-TC-------G-TA-G-G-C-G------------G--T-TT-G-T-C------
-----AC----G-T-C-G--------G-C-T--G--TG--A-AA-TC--C-T-AG-G-G-----CT-T-AA------C-C-C-T-G-G-A--C-G----T-G--C-A---G--G-------C--GA-T-A---C-------G--G-G--CT--G-A-C----T-T-G-A-G-T--A---C-TA-----CA-G-G--------G-G-A-G---AC-T------GG--A--ATT----T-
C-T-G-GT--GT-A-G-CG-GT--G---G--A-A---TG-C-AC-AG--AT-A-TC--A-G--G-A-A-G-A-AC-A-CC---GA--T--G--GC-GAA-G--G-C--A-------G--G-T-C-T---CTG--G--GT-A-G--------------------T-A---A-C-T--GA--CG-C-T-G--A-GG--A-G-CG-A--AA-G-C---A-TG--GG-
T--AG-C-G-AA--------CA--GG Bacteria(100);Actinobacteria(100);Actinobacteria(100);Actinobacteridae(100);Actinomycetales(100);unclassified(100);unclassified(100);unclassified(100);unclassified(100);

Otu0038 0 0 22 0 0 0 14 0 0 0 0 0 0 0 0 0 0 0 0 0 0 0 0 0 0 0 0 0
0 0 0 0 0 1 29 0 0 0 1 0 0 44 0 0 16 0 0 16 264 0 1 0 0 0 0 0 69
0 14 0 0 0 0 0 9 0 0 0 0 0 0 5 4 0 0 0 11 0 0 0 0 18 0 0 15 61
0 0 0 0 0 13 2 0 0 0 0 0 0 23 0 0 0 16 5 13 235 26 0 0 13 0 0
M02255_76_000000000-ADUNE_1_2106_12740_17352 T----AC---GT-AG-GGG--------GCA-A-G------C--G--T---T--AT-C-CGG-AT------TC-A--T-T--GG-GC-------GT--A-----AA----G---C-------------G-C------------------T-CT-G-T-T-----------
AG----G-T-C-G--------G-G-A--G--TT--A-AA-TC--C-G-GG-G-G-----CT-C-AA------C-C-C-C-C-G-T--T------C-G--C-T---C--C-------C--GA-T-A---C-------C--G-G--CA--G-A-C----T-T-G-A-G-T--T---T-GG-----TA-G-G--------G-G-A-A---GG-T------GG--A--ATT----C-C-T-
A-GT--GT-A-G-CG-GT--G---G--A-A---TG-C-GC-AG--AT-A-TT--A-G--G-A-A-G-A-AC-A-CC---AG--T--G--GC-GAA-G--G-C--G-------G--C-C-T-T---CTG--G--GC-C-A--------------------T-A---A-C-T--GA--CG-C-T-G--A-GG--A-G-CG-A--AA-G-C---T-AG--GG-G--
AG-C-A-AA--------CA--GG Bacteria(100);Actinobacteria(100);Actinobacteria(100);Coriobacteridae(100);Coriobacteriales(100);Coriobacterineae(100);Coriobacteriaceae(100);Atopobium(100);unclassified(100);

Otu0039 139 1552 1895 22 0 18 2068 178 2804 0 0 0 4 4 0 0 0 0 3 1 0 0 22 0 5 0 9 4
0 0 0 535 0 0 0 0 0 0 0 1100 0 0 102 69 0 0 0 0 0 0 4 0 34 0 0 1 0
0 0 0 2 0 0 0 0 0 0 0 0 5 0 24 21 63 167 0 0 0 0 0 0 0 0 0 0 0
0 95 0 0 0 0 8 0 0 0 0 55 0 0 0 47 0 0 1 0 0 0 77 28 1442 1 37
M02255_77_000000000-ADUT3_1_1101_17405_17843 A----AC---GT-AG-GTC--------ACA-A-G------C--G--T---T--GT-C-CGG-AA------TT-A--C-T--GG-GT-------GT--A-----AA-GG-GA-GC-------G-CA-G-G-C-G------------G--G-AA-G-A-C----
-------AA----G-T-T-G--------G-A-A--G--TG--A-AA-TC--C-A-TG-G-G-----CT-C-AA------C-C-C-A-T-G-A--A-C----T-G--C-T---T--T-------C--AA-A-A---C-------T--G-T--TT--T-T-C----T-T-G-A-G-T--A---G-TG-----CA-G-A--------G-G-T-A---GG-C------GG--A--ATT----C-
C-C-G-GT--GT-A-G-CG-GT--G---G--A-A---TG-C-GT-AG--AT-A-TC--G-G--G-A-G-G-A-AC-A-CC---AG--T--G--GC-GAA-G--G-C--G-------G--C-C-T-A---CTG--G--GC-A-C--------------------C-A---A-C-T--GA--CG-C-T-G--A-GG--C-T-CG-A--AA-G-T---G-TG--GG-
T--AG-C-A-AA--------CA--GG Bacteria(100);Firmicutes(100);Clostridia(100);Clostridiales(100);Ruminococcaceae(100);Faecalibacterium(100);unclassified(100);unclassified(100);unclassified(100);

Otu0040 136 0 0 32 36 264 495 302 117 0 0 0 0 0 0 2 0 0 3 8 21 0 30 7 0 0 0 0
8 0 227 246 76 41 91 69 172 43 172 119 249 25 0 151 9 0 0 85 52 324 0 0 0 14 0 0 0
124 0 6 0 12 13 0 12 10 7 6 8 0 19 72 10 0 0 0 50 72 16 4 0 105 36 1 0 48
97 19 261 47 0 231 6 37 0 275 48 903 42 13 12 99 0 170 56 24 391 0 105 4 15 0 137
M02255_76_000000000-ADUNE_1_1104_15576_11618 T----AC---GT-AA-GGA--------CCG-A-G------C--G--T---T--GT-C-CGG-AA------TC-A--T-T--GG-GC-------GT--A-----AA-GG-GT-AC-------G-TA-G-G-C-G------------G--G-TT-T-T-T------
-----AA----G-T-T-A--------G-A-A--G--TC--A-AA-GG--C-T-AT-A-G-----CT-C-AA------C-T-A-T-A-G-T--A------A-G--C-T---T--C-------T--AA-A-A---C-------T--G-G--GG--A-C-C----T-T-G-A-G-T--A---A-TG-----GA-A-G--------G-G-A-A---AG-T------GG--A--ATT----C-
C-T-A-GT--GT-A-G-CG-GT--G---G--A-A---TG-C-GC-AG--AT-A-TT--A-G--G-A-G-G-A-AT-A-CC---GG--T--G--GC-GAA-G--G-C--G-------A--C-T-T-T---CTG--G--CC-A-T--------------------T-A---A-C-T--GA--CG-C-T-G--A-GG--T-A-CG-A--AA-G-C---G-TG--GG-T-
-AG-C-A-AA--------CA--GG Bacteria(100);Firmicutes(100);Clostridia(100);Clostridiales(100);Family_XI_Incertae_Sedis(100);Anaerococcus(100);unclassified(100);unclassified(100);unclassified(100);

Otu0041 0 0 0 0 0 0 0 0 0 0 0 3 9 0 0 2 2 3 1 8 0 31 0 5 0 0 8 0
0 0 0 0 0 0 0 0 0 0 0 0 0 0 0 0 0 0 0 0 0 0 0 0 0 0 0 0 0
1 0 0 0 16 0 0 0 0 0 0 34 34 0 0 0 0 0 0 0 0 1 0 0 0 0 0 0 0
0 0 0 0 0 0 0 1 4 0 3 0 3 0 2 0 0 0 0 0 0 0 0 0 0 0 0
M02255_76_000000000-ADUNE_1_1106_12350_5324 C----GG---CT-AA-CTC--------CGT-G-C------C--A--G---C--AG-C-CGC-GG------TA-A--T-A--CG-GA-------GG--A-----TC-CA-AG-CG-------T-TA-T-C-C-G------------G--T-CA-G-A-T-----------AA--
--G-T-C-A--------G-T-G--G--TG--A-AA-GC--C-C-AT-C-G-----CT-C-AA------C-G-G-T-G-G-A--A-C----G-G--C-C---A--T-------T--GA-T-A---C-------T--G-T--CT--G-A-C----T-T-G-A-A-T--T---A-TT-----AG-G-A--------A-G-T-A---AC-T------AG--A--ATA----T-G-T-A-GT-
-GT-A-G-CG-GT--G---A--A-A---TG-C-TT-AG--AG-A-TT--A-C--A-T-G-G-A-AT-A-CC---AA--T--T--GC-GAA-G--G-C--A-------G--G-T-T-A---CTA--C--TA-A-T--------------------A-T---A-T-T--GA--CG-C-T-G--A-TG--G-A-CG-A--AA-G-C---G-TG--GG-T--AG-C-G-
AA--------CA--GG Bacteria(100);Bacteroidetes(100);Flavobacteria(100);Flavobacteriales(100);Flavobacteriaceae(100);Flavobacterium(100);unclassified(100);unclassified(100);unclassified(100);

Otu0042 0 0 0 0 0 13 0 0 0 0 0 0 0 0 0 0 0 0 0 0 0 0 15 0 6 0 0 0
0 0 0 0 0 8 1 114 15 0 0 0 7 0 0 13 7 0 0 0 3 1 0 22 63 2 2254 81 0
0 0 0 0 0 4 18 0 4 0 0 0 0 0 9 0 16 50 0 0 0 0 0 249 0 0 0 0 0
0 0 0 0 0 0 0 0 0 0 0 0 0 0 0 0 0 0 0 0 0 0 0 0 0 0 0
M02255_77_000000000-ADUT3_1_1104_20268_19099 T----AC---GA-AG-GGG--------GCT-A-G------C--G--T---T--GT-T-CGG-AT------TT-A--C-T--GG-GC-------GT--A-----AA-GC-GC-AC-------G-TA-G-G-C-G------------G--A-CT-A-T-T-----
------AA----G-T-C-A--------G-G-G--G--TG--A-AA-TC--C-C-GG-G-G-----CT-C-AA------C-C-C-C-G-G-A--A-C----T-G--C-C---T--T-------T--GA-T-A---C-------T--G-G--TA--G-T-C----T-T-G-A-G-T--T---C-GA-----GA-G-A--------G-G-T-G---AG-T------GG--A--ATT----C-
C-G-A-GT--GT-A-G-AG-GT--G---A--A-A---TT-C-GT-AG--AT-A-TT--C-G--G-A-G-G-A-AC-A-CC---AG--T--G--GC-GAA-G--G-C--G-------G--C-T-C-A---CTG--G--CT-C-G--------------------A-T---A-C-T--GA--CG-C-T-G--A-GG--T-G-CG-A--AA-G-C---G-TG--GG-
G--AG-C-A-AA--------CA--GG Bacteria(100);Proteobacteria(100);Alphaproteobacteria(100);Rhizobiales(94);unclassified(94);unclassified(94);unclassified(94);unclassified(94);unclassified(94);

Otu0043 0 0 0 0 81 0 4 32 54 0 0 0 0 0 0 0 3 0 3 0 0 2 30 0 11 2 4 0
0 0 0 22 0 85 42 0 0 0 17 76 18 20 199 103 35 0 0 32 79 0 0 49 18 1 0 0 61
0 21 0 0 0 0 0 0 0 0 0 3 6 0 19 6 17 54 52 0 0 8 7 0 0 0 0 0 26
27 91 0 17 53 0 0 0 0 0 0 28 2 0 0 37 32 100 10 0 0 4 26 7 0 0 0
M02255_76_000000000-ADUNE_1_2106_6073_11214 T----AC---GT-AG-GGT--------GCG-A-G------C--G--T---T--GT-C-CGG-AA------TT-A--T-T--GG-GC-------GT--A-----AA-GG-GC-TC-------G-TA-G-G-C-G------------G--T-TT-G-T-C-----------GC-
---G-T-C-G--------G-G-A--G--TG--A-AA-AC--A-C-CG-G-G-----CT-T-AA------C-T-C-G-G-T-G--C-T----T-G--C-T---T--T-------C--GA-T-A---C-------G--G-G--CA--G-A-C----T-A-G-A-G-G--T---A-TG-----CA-G-G--------G-G-A-G---AA-C------GG--A--ATT----C-C-T-G-
GT--GT-A-G-CG-GT--G---A--A-A---TG-C-GC-AG--AT-A-TC--A-G--G-A-G-G-A-AC-A-CC---GG--T--G--GC-GAA-G--G-C--G-------G--T-T-C-T---CTG--G--GC-A-T--------------------T-A---C-C-T--GA--CG-C-T-G--A-GG--A-G-CG-A--AA-G-T---G-TG--GG-G--AG-
C-G-AA--------CA--GG Bacteria(100);Actinobacteria(100);Actinobacteria(100);Actinobacteridae(100);Actinomycetales(100);Propionibacterineae(100);Nocardioidaceae(100);unclassified(65);unclassified(65);

Otu0044 0 0 0 0 0 0 21 0 0 0 2 6 6 450 204 0 2 0 2 0 19 0 0 0 2 0 3 10
0 0 0 145 40 0 0 0 0 0 0 0 45 85 0 0 0 0 59 0 0 32 0 0 1 0 0 0
36 0 0 0 0 0 9 0 0 0 0 2 3 0 0 0 0 88 0 0 0 8 5 0 0 2724 1689 4 0
0 0 0 24 0 20 0 0 2 0 10 6 7 0 0 0 33 0 26 0 0 0 0 0 1 0 75
M02255_77_000000000-ADUT3_1_1105_25050_22992 T----AC---GA-AG-GGG--------GCT-A-G------C--G--T---T--GT-T-CGG-AT------TT-A--C-T--GG-GC-------GT--A-----AA-GC-GC-AC-------G-TA-G-G-C-G------------G--A-CT-T-T-T------
-----AA----G-T-C-A--------G-G-G--G--TG--A-AA-TC--C-C-AG-A-G-----CT-C-AA------C-T-C-T-G-G-A--A-C----T-G--C-C---T--T-------T--GA-T-A---C-------T--G-G--AA--G-T-C----T-T-G-A-G-T--A---T-GG-----TA-G-A--------G-G-T-G---AG-T------GG--A--ATT----C-
C-G-A-GT--GT-A-G-AG-GT--G---A--A-A---TT-C-GT-AG--AT-A-TT--C-G--G-A-G-G-A-AC-A-CC---AG--T--G--GC-GAA-G--G-C--G-------G--C-T-C-A---CTG--G--AC-C-A--------------------T-T---A-C-T--GA--CG-C-T-G--A-GG--T-G-CG-A--AA-G-C---G-TG--GG-G-
-AG-C-A-AA--------CA--GG Bacteria(100);Proteobacteria(100);Alphaproteobacteria(100);Rhizobiales(100);unclassified(92);unclassified(92);unclassified(92);unclassified(92);unclassified(92);

Otu0045 0 0 0 0 0 0 0 0 0 0 0 0 0 0 0 0 0 0 0 0 0 0 0 0 0 0 0 0
0 0 0 0 0 0 0 0 0 0 0 0 0 0 0 0 0 0 0 0 0 0 0 0 14 0 0 0 0
0 0 0 5 0 0 0 0 0 0 0 0 0 0 0 0 0 0 0 0 8502 5 0 0 88 0 0 0 0
0 108 145 0 0 0 0 2 0 71 0 0 0 161 0 0 0 0 0 0 0 0 0 0 0 0 0

M02255_77_000000000-ADUT3_1_1101_7055_12150 T----AC---GA-AG-GGG--------GCT-A-G------C--G--T---T--GC-T-CGG-AA------TT-A--C-T--GG-GC-------GT--A-----AA-GG-GC-GC-------G-TA-G-G-C-G------------G--A-TC-G-T-T-----------
AA----G-T-C-A--------G-A-G--G--TG--A-AA-TC--C-C-AG-G-G-----CT-C-AA------C-C-C-T-G-G-A--A-C----T-G--C-C---T--T-------T--GA-T-A---C-------T--G-G--CG--A-T-C----T-T-G-A-G-T--A---T-GA-----GA-G-A--------G-G-T-A---TG-T------GG--A--ACT----C-C-G-
A-GT--GT-A-G-AG-GT--G---A--A-A---TT-C-GT-AG--AT-A-TT--C-G--G-A-A-G-A-AC-A-CC---AG--T--G--GC-GAA-G--G-C--G-------A--C-A-T-A---CTG--G--CT-C-A--------------------T-T---A-C-T--GA--CG-C-T-G--A-GG--C-G-CG-A--AA-G-C---G-TG--GG-G--AG-
C-A-AA--------CA--GG Bacteria(100);Proteobacteria(100);Alphaproteobacteria(100);Caulobacterales(99);Caulobacteraceae(99);Brevundimonas(95);unclassified(95);unclassified(95);unclassified(95);

Otu0046 303 0 0 19 62 11 0 0 0 0 0 0 0 0 1 0 0 0 2 0 5 0 0 0 0 0 0 0
0 1 0 0 0 0 0 29 0 28 0 0 49 0 0 18 26 0 0 0 0 0 0 19 2 41 0 25 0
0 0 1 4 0 5 0 0 1 3 0 9 5 0 5 56 26 136 0 12 0 1 7 0 0 0 0 0 0
76 137 107 27 58 0 0 1 0 0 0 0 0 0 0 40 0 3 0 0 0 58 0 0 0 0 0

M02255_77_000000000-ADUT3_1_1104_25016_13795 T----AC---GT-AG-GGC--------GCA-A-G------C--G--T---T--GT-C-CGG-AA------TT-A--T-T--GG-GC-------GT--A-----AA-GA-GC-TC-------G-TA-G-G-C-G------------G--T-TT-G-T-C------
-----GC----G-T-C-T--------G-C-T--G--TG--A-AA-AC--T-C-AA-G-G-----CT-C-AA------C-C-T-T-G-G-G--C-T----T-G--C-A---G--T-------G--GG-T-A---C-------G--G-G--CA--G-A-C----T-A-G-A-G-T--G---C-GG-----TA-G-G--------G-G-T-G---AC-T------GG--A--ATT----C-
C-T-G-GT--GT-A-G-CG-GT--G---G--A-A---TG-C-GC-AG--AT-A-TC--A-G--G-A-G-G-A-AC-A-CC---GA--T--G--GC-GAA-G--G-C--A-------G--G-T-C-A---CTG--G--GC-C-G--------------------C-A---A-C-T--GA--CG-C-T-G--A-GG--A-G-CG-A--AA-G-C---A-TG--GG-
G--AG-C-G-AA--------CA--GG Bacteria(100);Actinobacteria(100);Actinobacteria(100);Actinobacteridae(100);Actinomycetales(100);Micrococcineae(100);Cellulomonadaceae(59);unclassified;unclassified;

Otu0047 0 0 0 0 0 0 0 0 0 18 0 0 0 10 0 3 0 0 4 0 0 0 31 0 41 0 0 45
0 0 0 0 0 0 0 93 0 0 0 71 0 0 0 0 0 0 0 0 234 0 4 58 107 6 0 0 0
0 0 0 0 0 0 0 0 0 0 0 0 0 4 0 0 43 26 160 0 0 0 0 0 0 0 0 13 0
0 77 0 73 0 0 0 15 0 16 0 0 5 0 0 111 0 0 0 0 0 0 0 0 0 0 0

M02255_77_000000000-ADUT3_1_1106_10931_10709 T----AC---GG-AG-GGG--------GCT-A-G------C--G--T---T--GT-T-CGG-AA------TT-A--C-T--GG-GC-------GT--A-----AA-GC-GC-AC-------G-TA-G-G-C-G------------G--C-TT-T-G-T-----
------AA----G-T-T-A--------G-A-G--G--TG--A-AA-GC--C-C-GG-G-G-----CT-C-AA------C-T-C-C-G-G-A--A-T----T-G--C-C---T--T-------T--AA-G-A---C-------T--G-C--AT--C-G-C----T-A-G-A-A-T--T---G-TG-----GA-G-A--------G-G-T-A---AG-T------GG--A--ATT----C-
C-G-A-GT--GT-A-G-AG-GT--G---A--A-A---TT-C-GT-AG--AT-A-TT--C-G--G-A-A-G-A-AC-A-CC---AG--T--G--GC-GAA-G--G-C--G-------A--C-T-T-A---CTG--G--AC-A-C--------------------A-T---A-T-T--GA--CG-C-T-G--A-GG--T-G-CG-A--AA-G-C---G-TG--GG-G-
-AG-C-A-AA--------CA--GG Bacteria(100);Proteobacteria(100);Alphaproteobacteria(100);Sphingomonadales(99);unclassified;unclassified;unclassified;unclassified;unclassified;

Otu0048 0 22 0 30 0 0 0 0 15 0 0 0 0 107 46 0 1 0 0 0 3 0 0 4 3 8 17 0
0 69 11 39 24 129 188 1 33 14 42 0 7 21 40 9 26 22 0 28 59 0 0 50 0 0 0 7 0
0 0 0 0 0 0 0 0 0 0 0 0 0 2 21 0 0 192 0 46 0 0 0 0 8 0 0 13 20
16 26 40 19 0 17 2 0 0 38 0 0 0 11 0 3 293 10 39 11 0 68 0 0 0 0 28

M02255_77_000000000-ADUT3_1_1113_11213_20972 T----AC---GT-AG-GGT--------CCG-A-G------C--G--T---T--GT-C-CGG-AA------TT-A--C-T--GG-GC-------GT--A-----AA-GA-GC-TC-------G-TA-G-G-T-G------------G--T-TT-G-T-C------
-----GC----G-T-T-G--------T-T-C--G--TG--A-AA-AC--T-C-AC-A-G-----CT-C-AA------C-T-G-T-G-G-G--C-G----T-G--C-G---G--G-------C--GA-T-A---C-------G--G-G--CA--G-A-C----T-G-G-A-G-T--A---C-TG-----CA-G-G--------G-G-A-G---AC-T------GG--A--ATT----C-
C-T-G-GT--GT-A-G-CG-GT--G---G--A-A---TG-C-GC-AG--AT-A-TC--A-G--G-A-G-G-A-AC-A-CC---GG--T--G--GC-GAA-G--G-C--G-------G--G-T-C-T---CTG--G--GC-A-G--------------------T-A---A-C-T--GA--CG-C-T-G--A-GG--A-G-CG-A--AA-G-C---G-TG--GG-
G--AG-C-G-AA--------CA--GG Bacteria(100);Actinobacteria(100);Actinobacteria(100);Actinobacteridae(100);Actinomycetales(100);Corynebacterineae(100);Mycobacteriaceae(100);Mycobacterium(100);unclassified(100);

Otu0049 0 0 0 0 0 0 0 0 0 0 0 0 110 5 0 4 0 0 5 0 0 0 0 31 0 0 0 0
0 0 73 0 0 0 37 35 0 0 0 0 1 0 92 0 49 2 0 30711 240 0 0 54 19 0 0 142 0
69 67 9 0 0 0 0 0 28 0 1 8 0 0 0 10 45 0 0 0 43 6 0 16 0 1 0 0 0
0 83 0 0 56 0 0 8 0 0 0 0 0 0 0 1 0 0 0 0 0 0 0 0 0 0 0

M02255_77_000000000-ADUT3_1_1106_9970_16182 T----AC---GG-GG-GGT--------GCA-A-G------C--G--T---T--AC-T-CGG-AA------TC-A--C-T--GG-GC-------GT--A-----AA-GA-GC-AT-------G-TA-G-G-C-G------------G--A-TT-A-A-T-----------
AA----G-T-T-T--------G-A-A--G--TG--A-AA-TC--C-T-AT-A-G-----CT-T-AA------C-T-A-T-A-G-A--A-C----T-G--C-T---T--T-------G--AA-A-A---C-------T--G-T--TA--A-T-C----T-A-G-A-A-T--G---T-GG-----GA-G-A--------G-G-T-A---GA-T------GG--A--ATT----T-C-T-G-
GT--GT-A-G-GG-GT--A---A--A-A---TC-C-GT-AG--AG-A-TC--A-G--A-A-G-G-A-AT-A-CC---GA--T--T--GC-GAA-G--G-C--G-------A--T-C-T-A---CTG--G--AA-C-A--------------------T-T---A-T-T--GA--CG-C-T-G--A-GA--T-G-CG-A--AA-G-C---G-TG--GG-G--AG-C-
A-AA--------CA--GG Bacteria(100);Proteobacteria(100);Epsilonproteobacteria(100);Campylobacterales(100);Campylobacteraceae(100);Arcobacter(100);unclassified(100);unclassified(100);unclassified(100);

Otu0050 0 0 0 0 0 0 0 0 0 0 0 0 0 0 0 0 0 0 0 62 8 4 0 0 8 0 4 2
0 0 0 2 0 23 42 0 0 0 14 0 0 0 55 0 0 51 0 0 1 0 0 40 12 4 0 17 0
0 0 0 0 0 0 0 0 0 0 0 0 0 0 0 0 17 224 156 0 0 1 0 26 43 0 0 0 0
23 42 43 0 0 19 0 0 1 0 0 0 0 16 0 19 0 22 0 0 0 128 1 0 0 0 0
M02255_77_000000000-ADUT3_1_2112_13839_10069 T----AC---GG-AG-GGG--------GTT-A-G------C--G--T---T--GT-T-CGG-AA------TG-A--C-T--GG-GC-------GT--A-----AA-GC-GC-GC-------G-TA-G-G-C-G------------G--A-TC-A-G-A----
-------AA----G-T-T-G--------G-G-G--G--TG--A-AA-TC--C-C-GG-G-G-----CT-C-AA------C-C-C-C-G-G-A--A-T----G-G--C-C---T--T-------C--AA-G-A---C-------T--C-C--TG--G-T-C----T-T-G-A-G-T--T---C-GA-----GA-G-A--------G-G-T-G---AG-T------GG--A--ATT----
C-C-G-A-GT--GT-A-G-AG-GT--G---A--A-A---TT-C-GT-AG--AT-A-TT--C-G--G-A-G-G-A-AC-A-CC---AG--T--G--GC-GAA-G--G-C--G-------G--C-T-C-A---CTG--G--CT-C-G--------------------A-C---A-C-T--GA--CG-C-T-G--A-GG--T-G-CG-A--AA-G-C---G-TG--GG-
G--AG-C-A-AA--------CA--GG Bacteria(100);Proteobacteria(100);Alphaproteobacteria(100);Rhodobacterales(89);Rhodobacteraceae(89);unclassified(89);unclassified(89);unclassified(89);unclassified(89);

Otu0051 0 0 0 0 0 0 0 0 0 0 0 2 0 0 0 1 0 0 6 0 0 0 8 3 0 4 12 3
1 0 0 0 0 0 0 0 0 0 0 0 0 0 0 0 0 0 8 0 0 0 0 0 0 0 0 4 0
0 0 0 1 0 0 3 0 5 0 0 0 0 0 0 0 0 0 0 0 0 0 1 0 0 0 0 0 0
0 0 0 0 0 0 34 0 34 0 0 0 6 0 0 0 0 0 0 0 0 0 0 0 0 0 0
M02255_76_000000000-ADUNE_1_1118_19756_15711 G----AA---GA-AG-GAC--------CGG-C-T------A--A--C---T--CC-G-TGC-CA------GC-A--G-C--CG-CG-------GT--A-----AT-AC-G--GA-------G--G-G-T-C-G------------G--A-GC-G-T-T------
-----AA----T-C-G-A--------G-A-T--G--TG--A-AA-GC--C-C-CG-G-G-----CT-C-AA------C-C-T-G-G-G-A--A-T----T-G--C-A---T--T-------T--CG-A-A---C-------T--G-G--CG--A-A-C----T-A-G-A-G-T--C---T-TG-----TA-G-A--------G-G-G-G---GG-T------AG--A--ATT----C-
C-A-G-GT--GT-A-G-CG-GT--G---A--A-A---TG-C-GT-AG--AG-A-TC--T-G--G-A-G-G-A-AT-A-CC---GG--T--G--GC-GAA-G--G-C--G-------G--C-C-C-C---CTG--G--AC-A-A--------------------A-G---A-C-T--GA--CG-C-T-C--A-GG--C-A-CG-A--AA-G-C---G-TG--GG-
G--AG-C-A-AA--------CA--GG Bacteria(100);Proteobacteria(100);Gammaproteobacteria(100);unclassified(100);unclassified(100);unclassified(100);unclassified(100);unclassified(100);unclassified(100);

Otu0052 0 0 0 0 0 0 0 0 0 0 0 3 3 0 0 3 2 23 23 4 0 0 0 8 0 0 7 0
0 0 0 0 0 0 0 0 0 0 0 0 0 0 0 0 0 0 0 0 0 0 0 0 0 0 0 0 0
0 0 9 11 0 0 51 0 1 0 0 0 0 0 0 0 5 0 0 0 0 0 0 0 0 0 0 0 0
0 0 0 0 0 0 0 0 0 0 16 0 0 0 5 0 0 0 0 0 0 0 0 0 0 0 0
M02255_76_000000000-ADUNE_1_1119_10793_12203 T----AA---GG-AT-CGG--------CTA-A-C------T--C--C---G--TG-C-CAG-CA------GC-C--G-C--GG-TA-------AT--A-----CG-GA-GG-AT-------C-CA-A-G-C-G------------G--TATT-A-G-T-----
------AA----G-T-C-A--------G-T-G--G--TG--A-AA-GC--C-C-AT-C-G-----CT-C-AA------C-G-G-T-G-G-A--A-C----G-G--C-C---A--T-------T--GA-T-A---C-------T--G-C--TA--A-A-C----T-T-G-A-A-T--T---A-TT-----AG-G-A--------A-G-T-A---AC-T------AG--A--ATA----T-
G-T-A-GT--GT-A-G-CG-GT--G---A--A-A---TG-C-TT-AG--AG-A-TT--A-C--A-T-G-G-A-AT-A-CC---AA--T--T--GC-GAA-G--G-C--A-------G--G-T-T-A---CTA--C--TA-A-T--------------------G-G---A-T-T--GA--CG-C-T-G--A-TG--G-A-CG-A--AA-G-C---G-TG--GG-T--
AG-C-G-AA--------CA--GG Bacteria(100);Bacteroidetes(100);Flavobacteria(100);Flavobacteriales(100);Flavobacteriaceae(100);Flavobacterium(99);unclassified(99);unclassified(99);unclassified(99);

Otu0053 0 0 0 0 0 0 88 6 0 0 0 0 0 0 0 0 0 0 0 1 9 0 0 0 0 0 0 0
0 0 0 0 0 0 0 0 0 0 0 0 171 0 0 0 151 0 0 0 0 402 1 0 17 0 0 0 0
33 0 0 18 0 0 0 0 0 0 99 34 0 13 0 21 0 0 0 554 0 0 7 0 0 0 0 0 0
0 0 0 0 0 420 10 64 0 238 0 832 4 0 0 0 0 0 0 0 0 0 0 0 0 0 0
M02255_77_000000000-ADUT3_1_1101_17676_12768 T----AC---GT-AG-GGG--------GCG-A-G------C--G--T---T--GT-C-CGG-AA------TC-A--C-T--GG-GC-------GT--A-----AA-GG-GT-TC-------G-CA-G-G-C-G------------G--T-CT-A-C-C-----
------AA----G-T-C-C--------G-A-T--G--TG--A-AA-GG--C-A-TG-G-G-----CT-C-AA------C-C-C-A-T-G-T--A------A-G--C-A---T--T-------G--GA-A-A---C-------T--G-G--TA--G-A-C----T-T-G-A-G-T--T---A-AG-----GA-G-A--------G-G-T-A---AG-T------GG--A--ATT----C-
C-T-A-GT--GT-A-G-CG-GT--G---A--A-A---TG-C-GT-AG--AT-A-TT--A-G--G-A-A-G-A-AT-A-CC---GG--T--G--GC-GAA-G--G-C--G-------A--C-T-T-A---CTG--G--AC-T-T--------------------A-A---A-C-T--GA--CG-C-T-G--A-GG--A-A-CG-A--AA-G-C---G-TG--GG-G-
-AG-C-A-AA--------CA--GG Bacteria(100);Firmicutes(100);Clostridia(100);Clostridiales(100);Family_XI_Incertae_Sedis(100);Peptoniphilus(100);unclassified(100);unclassified(100);unclassified(100);

Otu0054 0 82 0 41 0 0 86 0 0 0 0 0 0 0 1 3 0 0 0 0 0 0 0 0 0 0 0 16
0 0 184 0 130 0 25 47 0 0 0 0 0 0 0 25 0 0 2 0 115 0 0 0 0 0 0 0 52
0 11 0 0 4 0 0 0 1 0 0 0 0 1 91 86 0 12 0 0 1 0 6 0 24 1 0 23 60
36 78 0 41 89 0 0 6 0 20 0 13 0 0 0 0 0 0 0 28 0 29 15 0 52 0 0
M02255_77_000000000-ADUT3_1_1119_25074_13210 T----AC---GT-AT-GTC--------GCG-A-G------C--G--T---T--AT-C-CGG-AA------TT-A--T-T--GG-GC-------AT--A-----AA-GG-GC-AT-------C-TA-G-G-C-G------------G--C-CT-T-T-C-------
----AA----G-T-C-A--------G-G-G--G--TG--A-AA-AC--C-T-GC-G-G-----CT-C-AA------C-C-G-C-A-G-G--C-C----T-G--C-C---T--T-------T--GA-A-A---C-------T--G-A--TA--G-G-C----T-G-G-A-G-T--A---C-CG-----GA-G-A--------G-G-T-G---GA-C------GG--A--ACT----G-
C-A-C-GA--GT-A-G-AG-GT--G---A--A-A---TT-C-GT-AG--AT-A-TG--T-G--C-A-G-G-A-AT-G-CC---GA--T--G--AT-GAA-G--A-T--A-------G--T-T-C-A---CTG--G--AC-G-G--------------------T-A---A-C-T--GA--CG-C-T-G--A-AG--T-G-CG-A--AA-G-C---C-GG--GG-G-
-AG-C-G-AA--------CA--GG Bacteria(100);Fusobacteria(100);Fusobacteria(100);Fusobacteriales(100);Leptotrichiaceae(100);Leptotrichia(100);unclassified(100);unclassified(100);unclassified(100);

Otu0055 336 80 0 40 101 228 1 150 18 141 4 0 0 155 33 0 2 0 18 7 19 11 0 0 26 225 8 31
905 0 4 0 90 63 63 58 0 51 37 27 23 78 362 0 15 0 0 0 0 26 15 247 20 2 0 22 0
47 70 43 0 11 15 18 8 14 0 0 0 0 15 113 10 86 246 0 0 0 8 0 0 153 0 0 30 25
0 39 0 2154 0 7 0 1 0 68 0 0 6 41 0 87 336 48 95 259 123 48 229 0 33 0 0
M02255_77_000000000-ADUT3_1_1101_7238_12025 T----AC---GA-AG-GGG--------GCT-A-G------C--G--T---T--GC-T-CGG-AA------TC-A--C-T--GG-GC-------GT--A-----AA-GG-GC-GC-------G-TA-G-G-C-G------------G--C-TG-A-T-T----------
TA----G-T-C-G--------A-G-G--G--TG--A-AA-GC--C-T-GT-G-G-----CT-C-AA------C-C-A-C-A-G-A--A-T----T-G--C-C---T--T-------C--GA-T-A---C-------T--G-G--TT--G-G-C----T-T-G-A-G-A--C---C-GG-----AA-G-A--------G-G-A-C---AG-C------GG--A--ACT----G-C-G-
A-GT--GT-A-G-AG-GT--G---A--A-A---TT-C-GT-AG--AT-A-TT--C-G--C-A-A-G-A-AC-A-CC---AG--T--G--GC-GAA-G--G-C--G-------G--C-T-G-T---CTG--G--TC-C-G--------------------G-T---T-C-T--GA--CG-C-T-G--A-GG--C-G-CG-A--AA-G-C---G-TG--GG-G--AG-
C-A-AA--------CA--GG Bacteria(100);Proteobacteria(100);Alphaproteobacteria(100);Rhizobiales(100);Methylobacteriaceae(100);Methylobacterium(100);unclassified(100);unclassified(100);unclassified(100);

Otu0056 0 0 0 0 0 0 0 0 0 0 0 7 21 13 29 4 50 8 3 9 24 7 0 0 0 0 3 0
0 0 0 0 0 0 0 1 0 0 0 0 0 0 0 0 0 0 46 0 2 0 39 0 10 8 0 16 1
0 0 2 29 0 0 14 50 5 25 0 6 3 2 0 0 0 0 0 0 1 0 0 0 0 0 0 0 0
0 0 1 0 0 4 13 8 5 0 7 0 11 0 8 0 0 0 0 0 0 0 0 0 0 0 0
M02255_76_000000000-ADUNE_1_1112_20802_18049 G----AC---AG-AA-TAA--------GCA-C-C------G--G--C---T--AACT-CTG-TG------CC-A--G-C--AG-CC-------GC--G-----GT-AA-TA-CA-------G-AG-G-G-T-GC-----------A--A-GC-G-T-T---
--------AA----T-C-G-G--------A-A-T--G--TG--A-AA-TC--C-C-CG-G-G-----CT-C-AA------C-C-T-G-G-G-A--A-C----T-G--C-A---T--C-------C--AA-A-A---C-------T--G-G--CA--A-G-C----T-A-G-A-G-T--A---T-GG-----TA-G-A--------G-G-G-T---AG-T------GG--A--ATT----
T-C-C-T-GT--GT-A-G-CG-GT--G---A--A-A---TG-C-GT-AG--AT-A-TA--G-G--A-A-G-G-A-AC-A-CC---AG--T--G--GC-GAA-G--G-C--G-------A--C-T-A-C---CTG--G--AC-T-G--------------------A-T---A-C-T--GA--CA-C-T-G--A-GG--T-G-CG-A--AA-G-C---G-TG--GG-
G--AG-C-A-AA--------CA--GG Bacteria(100);Proteobacteria(100);Gammaproteobacteria(100);unclassified(89);unclassified(89);unclassified(89);unclassified(89);unclassified(89);unclassified(89);

Otu0057 297 0 0 39 0 0 0 0 0 0 0 0 0 0 0 0 0 1 0 0 12 0 0 0 0 0 0 3
0 0 0 87 0 0 0 122 0 28 38 0 0 0 16 0 0 0 0 0 0 0 95 0 0 0 0 0 102
0 0 0 1 0 0 5 9 0 8 0 0 0 0 14 0 0 24 0 33 0 0 0 0 0 0 0 24 0
0 0 0 0 0 0 0 0 0 0 0 0 0 0 0 0 0 73 10 0 0 0 0 0 0 0 0
M02255_77_000000000-ADUT3_1_1102_11614_3024 T----AC---GA-AG-GGG--------GCT-A-G------C--G--T---T--GC-T-CGG-AA------TT-A--C-T--GG-GC-------GT--A-----AA-GG-GC-GC-------G-TA-G-G-C-G------------G--C-AC-T-G-T----------
TA----G-T-C-A--------G-G-C--G--TG--A-AA-TT--C-C-TG-G-G-----CT-C-AA------C-C-T-G-G-G-G--A-C----T-G--C-G---C--T-------T--GA-T-A---C-------G--G-C--AG--A-G-C----T-A-G-A-G-G--A---T-GG-----AA-G-A--------G-G-C-T---CG-C------GG--A--ATT----C-C-
C-A-GT--GT-A-G-AG-GT--G---A--A-A---TT-C-GT-AG--AT-A-TT--G-G--G-A-A-G-A-AC-A-CC---GG--T--G--GC-GAA-G--G-C--G-------G--C-G-A-G---CTG--G--TC-C-A--------------------T-T---A-C-T--GA--CG-C-T-G--A-GG--C-G-CG-A--CA-G-C---G-TG--GG-G--
AG-C-A-AA--------CA--GG Bacteria(100);Proteobacteria(100);Alphaproteobacteria(100);Rhodospirillales(100);Acetobacteraceae(100);unclassified(100);unclassified(100);unclassified(100);unclassified(100);

Otu0058 0 0 0 0 0 0 0 0 0 0 0 1 7 0 1 19 3 2 0 5 1 22 0 5 0 0 3 0
1 0 0 0 0 0 0 0 0 0 0 0 0 0 0 0 0 1 0 0 2 0 0 0 0 0 0 0 0
5 0 0 2 21 5 7 1 2 0 1 39 61 21 0 0 0 0 0 0 1 1 0 0 0 0 0 0 0
0 0 0 0 0 0 4 0 0 0 13 0 8 0 4 0 0 0 0 0 0 0 0 0 0 0 0
M02255_77_000000000-ADUT3_1_2112_25668_13708 T----AC---GG-AG-GAT--------CCA-A-G------C--G--T---T--AT-C-CGG-AA------TC-A------------------TT----------GG-GT----------G-TA-G-G-C-G------------G--T-CA-G-A-T----------
AA----G-T-C-A--------G-T-G--G--TG--A-AA-GC--C-C-AT-C-G-----CT-C-AA------C-G-G-T-G-G-A--A-C----G-G--C-C---A--T-------T--GA-T-A---C-------T--G-T--CT--G-A-C----T-T-G-A-A-T--T---A-TT-----AG-G-A--------A-G-T-A---AC-T------AG--A--ATA----T-G-T-
A-GT--GT-A-G-CG-GT--G---A--A-A---TG-C-TT-AG--AG-A-TT--A-C--A-T-G-G-A-AT-A-CC---AA--T--T--GC-GAA-G--G-C--A-------G--G-T-T-A---CTA--C--TA-A-T--------------------A-T---A-T-T--GA--CG-C-T-G--A-TG--G-A-CG-A--AA-G-C---G-TG--GG-T--AG-
C-G-AA--------CA--GG Bacteria(100);Bacteroidetes(100);Flavobacteria(100);Flavobacteriales(100);Flavobacteriaceae(100);Flavobacterium(100);unclassified(100);unclassified(100);unclassified(100);

Otu0059 0 0 0 0 0 0 0 0 0 0 0 4 39 11 1 0 2 0 4 0 1 3 0 0 0 0 0 0
0 0 0 0 0 0 0 0 1 0 0 0 1 0 0 0 0 0 4 0 0 0 0 0 0 14 0 16 0
0 0 2 0 1 0 0 2 0 18 0 1 0 42 1 0 0 0 0 0 0 0 0 0 0 0 0 0 0
0 0 0 0 0 0 5 2 0 0 0 0 4 0 0 7 0 0 0 0 0 0 0 0 0 0 0
M02255_77_000000000-ADUT3_1_1115_18276_4051 C----TA---AC-TC-TGT--------GCC-A-G------C-----A---G--CC-G-CGG-TA------AT-A--C-A--GA-GG-------GTG-C-----AA-GC-GT-TA-------A-TC-G-G---A------------A--T-TA-C-T-T----------AA---
-G-T-T-G--------G-A-T--G--TG--A-AA-TC--C-C-CG-G-G-----CT-C-AA------C-C-T-G-G-G-A--A-C----T-G--C-A---T--T-------C--AA-A-A---C-------T--G-A--CT--G-A-C----T-A-G-A-G-T--A---T-GG-----TA-G-A--------G-G-G-T---GG-T------GG--A--ATT----T-C-C-T-GT-
-GT-A-G-CG-GT--G---A--A-A---TG-C-GT-AG--AT-A-TA--G-G--A-A-G-G-A-AC-A-CC---AG--T--G--GC-GAA-G--G-C--G-------A--C-C-A-C---CTG--G--AC-T-G--------------------A-T---A-C-T--GA--CA-C-T-G--A-GG--T-G-CG-A--AA-G-C---G-TG--GG-G--AG-C-A-
AA--------CA--GG Bacteria(100);Proteobacteria(100);Gammaproteobacteria(100);Pseudomonadales(98);Pseudomonadaceae(98);unclassified(98);unclassified(98);unclassified(98);unclassified(98);

Otu0060 0 0 0 0 0 0 0 0 0 0 0 0 0 0 0 0 0 0 0 11 18 0 0 0 0 0 0 27
0 4 32 0 0 0 18 0 0 62 66 68 0 13 0 0 0 0 0 0 0 0 6 49 0 4 0 34 0
0 0 0 0 0 0 0 0 0 0 0 0 0 0 0 0 0 167 0 0 0 0 6 0 0 0 0 0 0
0 0 0 0 0 0 0 0 0 18 0 0 0 0 0 0 0 0 0 0 0 0 70 3 0 0 0
M02255_77_000000000-ADUT3_1_1112_6665_11056 T----AC---GA-AG-GGG--------GCT-A-G------C--G--T---T--GT-T-CGG-AA------TT-A--C-T--GG-GC-------GT--A-----AA-GC-GC-AC-------G-TA-G-G-C-G------------G--A-TA-C-T-T----------AA-
---G-T-C-A--------G-G-G--G--TG--A-AA-TC--C-C-GG-G-G-----CT-C-AA------C-C-C-C-G-G-A--A-C----T-G--C-C---T--T-------T--GA-T-A---C-------T--G-G--GT--A-T-C----T-C-G-A-G-T--C---C-GG-----AA-G-A--------G-G-T-G---AG-T------GG--A--ATT----C-C-G-A-
GT--GT-A-G-AG-GT--G---A--A-A---TT-C-GT-AG--AT-A-TT--C-G--G-A-G-G-A-AC-A-CC---AG--T--G--GC-GAA-G--G-C--G-------G--C-T-C-A---CTG--G--TC-C-G--------------------G-T---A-C-T--GA--CG-C-T-G--A-GG--T-G-CG-A--AA-G-C---G-TG--GG-G--AG-
C-A-AA--------CA--GG Bacteria(100);Proteobacteria(100);Alphaproteobacteria(100);Rhizobiales(100);unclassified(100);unclassified(100);unclassified(100);unclassified(100);unclassified(100);

Otu0061 0 0 0 0 0 0 0 0 0 0 0 1 6 4 3 0 12 0 0 3 6 4 0 0 0 0 0 0
0 0 0 0 0 0 0 0 0 0 0 0 0 0 0 0 0 0 18 0 0 0 23 0 3 0 0 9 0
0 0 0 9 0 1 2 4 0 4 0 0 0 0 0 0 0 0 0 0 0 0 0 0 0 0 0 0 0
0 0 0 1 0 2 2 2 0 0 1 0 0 0 3 0 0 0 0 0 0 0 0 0 0 0 0
M02255_76_000000000-ADUNE_1_2101_7129_19100 T----AC---CG-AC-AGA--------ATA-A-G------C-----A---C--CG-G-CTA-AC------TC-T--G-T--GC-CA-------GC--AG----CC-GC-GG-TA-------A-TA-C-A-G-A------------G--G-GT-G-C-A----------AG-
---C-G-T-T--------A-A-T--C--GG--A-AA-TC--C-C-CG-G-G-----CT-C-AA------C-C-T-G-G-G-A--A-C----T-G--C-A---T--C-------C--AA-A-A---C-------T--G-G--CA--A-G-C----T-A-G-A-G-T--A---T-GG-----TA-G-A--------G-G-G-T---AG-T------GG--A--ATT----T-C-C-T-
GT--GT-A-G-CG-GT--G---A--A-A---TG-C-GT-AG--AT-A-TA--G-G--A-A-G-G-A-AC-A-CC---AG--T--G--GC-GAA-G--G-C--G-------A--C-T-A-C---CTG--G--AC-T-G--------------------A-T---A-C-T--GA--CA-C-T-G--A-GG--T-G-CG-A--AA-G-C---G-TG--GG-G--AG-
C-A-AA--------CA--GG Bacteria(100);Proteobacteria(100);Gammaproteobacteria(100);unclassified(96);unclassified(96);unclassified(96);unclassified(96);unclassified(96);unclassified(96);

Otu0062 0 0 0 0 0 0 0 0 0 0 0 0 0 0 0 0 0 0 0 1 0 0 0 0 3 1 0 66
0 2 0 0 0 0 12 0 0 55 60 14 23 20 1 18 0 0 12 55 0 0 0 0 0 5 0 20 0
0 0 0 0 0 0 0 0 0 0 0 0 4 0 10 0 10 117 51 0 0 0 0 0 0 0 0 0 0
22 0 0 10 0 0 0 0 0 34 0 0 5 25 0 15 0 68 0 17 0 0 32 0 0 0 42
M02255_77_000000000-ADUT3_1_2101_18422_14147 T----AC---GA-AG-GGG--------GCT-A-G------C--G--T---T--GT-T-CGG-AA------TC-A--C-T--GG-GC-------GT--A-----AA-GG-GC-GC-------G-TA-G-G-C-G------------G--C-TT-T-A-T-----
------AA----G-T-C-G--------G-G-G--G--TG--A-AA-GC--C-T-GT-G-G-----CT-C-AA------C-C-A-C-A-G-A--A-T----T-G--C-C---T--T-------C--GA-T-A---C-------T--G-T--AT--G-G-C----T-T-G-A-G-A--C---C-GG-----AA-G-A--------G-G-T-A---AG-T------GG--A--ACT----
G-C-G-A-GT--GT-A-G-AG-GT--G---A--A-A---TT-C-GT-AG--AT-A-TT--C-G--C-A-A-G-A-AC-A-CC---AG--T--G--GC-GAA-G--G-C--G-------G--C-T-T-A---CTG--G--TC-C-G--------------------G-T---T-C-T--GA--CG-C-T-G--A-GG--C-G-CG-A--AA-G-C---G-TG--GG-
G--AG-C-A-AA--------CA--GG Bacteria(100);Proteobacteria(100);Alphaproteobacteria(100);Rhizobiales(100);unclassified(100);unclassified(100);unclassified(100);unclassified(100);unclassified(100);

Otu0063 145 0 12 74 0 0 0 0 0 0 0 0 0 25 2 0 0 0 0 0 1 0 84 8 0 0 4 1
0 0 0 0 0 32 0 27 0 4 0 0 0 0 31 0 20 0 4 0 0 0 1 0 0 3 4 0 0
0 0 0 0 0 0 0 0 0 0 0 0 0 0 18 0 28 0 0 0 0 2 0 0 14 0 0 0 0
0 0 62 22 0 0 0 0 0 0 0 0 0 0 0 0 0 14 13 31 0 0 8 1 0 0 28
M02255_77_000000000-ADUT3_1_2119_19457_16458 T----AC---GT-AG-GGT--------GCA-A-G------C--G--T---T--GT-C-CGG-AA------TT-A--T-T--GG-GC-------GT--A-----AA-GA-GC-TC-------G-TA-G-G-C-G------------G--T-TT-G-T-C------
-----GC----G-T-C-T--------G-C-T--G--TG--A-AA-TC--C-C-GA-G-G-----CT-C-AA------C-C-T-C-G-G-G--C-C----T-G--C-A---G--T-------G--GG-T-A---C-------G--G-G--CA--G-A-C----T-T-G-A-G-T--G---C-GG-----TA-G-G--------G-G-A-G---AT-T------GG--A--ATT----C-
C-T-G-GT--GT-A-G-CG-GT--G---G--A-A---TG-C-GC-AG--AT-A-TC--A-G--G-A-G-G-A-AC-A-CC---GA--T--G--GC-GAA-G--G-C--A-------G--A-T-C-T---CTG--G--GC-C-G--------------------T-T---A-C-T--GA--CG-C-T-G--A-GG--A-G-CG-A--AA-G-C---A-TG--GG-
G--AG-C-G-AA--------CA--GG Bacteria(100);Actinobacteria(100);Actinobacteria(100);Actinobacteridae(100);Actinomycetales(100);Micrococcineae(100);Microbacteriaceae(89);unclassified(86);unclassified(86);

Otu0064 231 0 272 0 0 115 365 77 731 0 0 0 0 1 0 0 0 3 2 0 0 2 0 93 0 4 0 1
7 0 282 0 263 0 0 0 0 1 0 193 0 0 71 12 0 0 0 0 0 1 7 152 39 0 0 0 0
0 0 0 0 0 0 0 0 0 0 0 0 13 0 0 0 12 215 0 0 0 0 0 0 0 0 0 0 0
0 188 0 185 73 0 0 10 0 0 0 0 0 0 0 271 228 1 59 0 0 53 0 10 46 0 0
M02255_77_000000000-ADUT3_1_1103_5800_7439 T----AC---GT-AG-GGG--------GCT-A-G------C--G--T---T--AT-C-CGG-AA------TT-A--C-T--GG-GC-------GT--A-----AA-GG-GT-GC-------G-TA-G-G-T-G------------G--T-TT-C-T-T----------AA--
--G-T-C-A--------G-A-G--G--TG--A-AA-GG--C-T-AC-G-G-----CT-C-AA------C-C-G-T-A-G-T--A------A-G--C-C---T--T-------T--GA-A-A---C-------T--G-G--GA--A-A-C----T-T-G-A-G-T--G---C-AG-----GA-G-A--------G-G-A-G---AG-T------GG--A--ATT----C-C-T-A-
GT--GT-A-G-CG-GT--G---A--A-A---TG-C-GT-AG--AT-A-TT--A-G--G-A-G-G-A-AC-A-CC---AG--T--T--GC-GAA-G--G-C--G-------G--C-T-C-T---CTG--G--AC-T-G--------------------T-A---A-C-T--GA--CA-C-T-G--A-GG--C-A-CG-A--AA-G-C---G-TG--GG-G--AG-C-
A-AA--------CA--GG Bacteria(100);Firmicutes(100);Clostridia(100);Clostridiales(100);Peptostreptococcaceae(100);Incertae_Sedis(63);unclassified(63);unclassified(63);unclassified(63);

Otu0065 0 0 0 0 0 0 0 0 0 12 5 0 0 0 0 0 0 0 8 0 0 0 6 0 0 0 0 0
0 64 3 0 0 0 75 103 0 0 42 47 0 0 79 78 31 89 4 1 0 14 0 0 0 0 2 2 0
33 52 0 4 0 8 0 73 0 1 37 41 0 1 258 308 38 16 33 534 370 0 5 0 51 0 0 80 0
44 9 54 34 345 1184 13 70 3 830 1 2185 23 33 12 0 0 21 17 0 0 0 82 0 0 0 0
M02255_77_000000000-ADUT3_1_1116_24372_14063 T----AC---GT-AG-GGG--------GCT-A-G------C--G--T---T--AT-C-CGG-AT------TT-A--C-T--GG-GC-------GT--A-----AA-GG-GT-GC-------G-TA-G-G-T-G------------G--T-CC-T-T-C------
-----AA----G-T-C-G--------G-T-G--G--TT--A-AA-GG--C-T-AC-G-G-----CT-C-AA------C-C-G-T-A-G-T--A------A-G--C-C---G--C-------C--GA-A-A---C-------T--G-G--AG--G-A-C----T-T-G-A-G-T--G---C-AG-----GA-G-A--------G-G-A-A---AG-T------GG--A--ATT----C-
C-C-A-GT--GT-A-G-CG-GT--G---A--A-A---TG-C-GT-AG--AT-A-TT--G-G--G-A-G-G-A-AC-A-CC---AG--T--A--GC-GAA-G--G-C--G-------G--C-T-T-T---CTG--G--AC-T-G--------------------C-A---A-C-T--GA--CA-C-T-G--A-GG--C-A-CG-A--AA-G-C---G-TG--GG-T-
-AG-C-A-AA--------CA--GG Bacteria(100);Firmicutes(100);Clostridia(100);Clostridiales(100);Peptostreptococcaceae(100);Peptostreptococcus(100);unclassified(100);unclassified(100);unclassified(100);

Otu0066 0 0 0 0 0 3 0 0 0 0 2 26 3 0 2 8 2 0 42 1 0 0 0 83 4 0 34 7
16 0 0 0 0 0 0 0 0 0 0 0 0 0 0 0 2 0 0 0 0 0 0 0 0 0 0 0
0 0 0 0 0 0 0 0 0 0 0 0 0 0 0 0 0 0 0 0 0 6 0 0 0 0 0 0
0 0 0 0 0 0 0 0 1 0 1 0 1 0 0 0 0 0 0 0 0 0 0 2 0 0 0
M02255_77_000000000-ADUT3_1_2102_8361_17127 T----AC---GG-AG-GAT--------CCA-A-G------C--G--T---T--AT-C-CGG-AA------TC-A-------------------TT-----------GG-G-----------G-TA-G-G-C-G------------G--C-CT-A-G-C-----------AA----G-T-
C-A--------G-T-G--G--TG--A-AA-TC--T-C-CC-C-G-----CT-C-AA------C-G-G-G-G-A-A--A-C----G-G--C-C---A--T-------T--GA-T-A---C-------T--A-C--TA--G-G-C----T-T-G-A-A-T--T---A-TT-----AG-G-A--------A-G-T-A---AC-T------AG--A--ATA----T-G-T-A-GT--GT-
A-G-CG-GT--G---A--A-A---TG-C-TT-AG--AT-A-TT--A-C--A-T-G-G-A-AT-A-CC---AA--T--T--GC-GAA-G--G-C--A-------G--G-T-T-A---CTA--C--TA-A-T--------------------G-G---A-T-T--GA--CG-C-T-G--A-TG--G-A-CG-A--AA-G-C---G-TG--GG-G--AG-C-G-AA-----
---CA--GG Bacteria(100);Bacteroidetes(100);Flavobacteria(100);Flavobacteriales(100);Flavobacteriaceae(100);Flavobacterium(100);unclassified(100);unclassified(100);unclassified(100);

Otu0067 0 0 0 82 0 23 0 0 0 0 0 0 0 0 0 0 0 0 0 1 6 0 0 0 0 0 0 0
0 2 0 11 0 0 0 165 0 21 1 32 0 30 41 0 0 0 0 0 0 0 0 21 7 0 0 0 0
0 0 0 0 0 0 0 0 0 0 0 0 0 0 0 0 14 52 0 0 0 0 0 0 0 0 0 0 0
0 0 0 0 0 0 0 0 0 0 0 0 0 0 0 0 0 51 41 0 0 0 0 2 17 0 0
M02255_77_000000000-ADUT3_1_1101_19067_10246 T----AC---GT-AG-GTG--------GCA-A-G------C--G--T---T--GT-C-CGG-AT------TT-A--C-T--GG-GC-------GT--A-----AA-GA-GC-GC-------G-CA-G-G-C-G------------G--T-CG-T-T-C-----
------AA----G-T-C-G--------A-G-T--G--TG--A-AA-GC--C-C-CC-G-G-----CT-C-AA------C-T-G-G-G-G-A--G-G----G-T--C-A---C--T-------C--GA-T-A---C-------T--G-A--TC--G-A-C----T-C-G-A-A-G--G---C-AG-----GA-G-A--------G-G-G-A---AG-T------GG--A--ATT----
C-C-C-G-GT--GT-A-G-TG-GT--G---A--A-A---TG-C-GT-AG--AT-A-TC--G-G--G-A-G-G-A-AC-A-CC---AG--T--G--GC-GAA-G--G-C--G-------A--C-T-T-C---CTG--G--CC-T-G--------------------T-T---C-T-T--GA--CG-C-T-G--A-GG--C-G-CG-A--AA-G-C---T-AG--GG-
G--AG-C-A-AA--------CG--GG Bacteria(100);Chloroflexi(100);KD4-96(100);unclassified(100);unclassified(100);unclassified(100);unclassified(100);unclassified(100);unclassified(100);

Otu0068 0 0 0 1 15 18 1 0 0 21 11 18 6 1 0 0 0 2 7 2 3 5 2 21 0 2 4 2
16 0 0 0 1 0 0 0 0 0 1 0 0 0 1 0 15 0 0 0 0 0 0 0 0 0 0 0
0 0 0 0 0 0 0 0 0 0 0 0 0 0 0 0 0 0 0 0 37 30 9 0 0 0 0 1
0 0 0 1 0 1 13 0 0 5 2 1 9 18 0 13 0 3 34 1 4 15 17 92 0 0 0
M02255_77_000000000-ADUT3_1_1112_17482_4688 C----TA---AC-TC-TGT--------GCC-A-G------C-----A---G--CC-G-CGG-TA------AT-A--C-A--GA-GG-------GTG-C-----AA-GC-GT-TA-------A-TC-G-GAATT------------A--C-TC-T-A-T-----------AA-
---G-T-C-A--------G-A-T--G--TG--A-AA-TC--C-C-CG-G-G-----CT-T-AA------C-C-T-G-G-G-A--A-C----T-G--C-A---T--C-------T--GA-A-A---C-------T--G-T--AG--A-G-C----T-A-G-A-G-T--A---T-GT-----GA-G-A--------G-G-A-A---GG-T------AG--A--ATT----C-C-A-G-
GT--GT-A-G-CG-GT--G---A--A-A---TG-C-GT-AG--AG-A-TC--T-G--G-A-G-G-A-AT-A-CC---GA--T--G--GC-GAA-G--G-C--A-------G--C-C-T-T---CTG--G--CA-T-A--------------------A-T---A-C-T--GA--CA-C-T-G--A-GG--C-T-CG-A--AA-G-C---G-TG--GG-T--AG-C-
A-AA--------CA--GG Bacteria(100);Proteobacteria(100);Gammaproteobacteria(100);Pseudomonadales(100);Moraxellaceae(100);Psychrobacter(98);unclassified(98);unclassified(98);unclassified(98);

Otu0069 0 0 0 0 0 0 4 0 0 0 2 7 0 0 0 0 0 0 4 0 0 0 0 0 0 0 0 0
0 17 0 0 0 3 709 50 0 0 0 0 0 0 0 0 0 0 0 0 0 0 53 0 0 0 0 0
0 0 0 0 0 0 0 0 0 1 0 0 0 0 0 0 33 23 220 0 0 11 4 0 0 0 0 0 0
82 0 0 0 181 0 0 0 0 0 0 0 0 0 0 0 0 0 0 0 0 0 0 0 0 0 0
M02255_77_000000000-ADUT3_1_1106_5539_20798 T----AC---GG-AG-GGG--------GCT-A-G------C--G--T---T--GT-T-CGG-AA------TT-A--C-T--GG-GC-------GT--A-----AA-GC-GC-AC-------G-TA-G-G-C-G------------G--A-CC-G-G-A-----------
AA----G-T-C-A--------G-A-G--G--TG--A-AA-TC--C-C-AG-G-G-----CT-C-AA------C-C-T-T-G-G-A--A-C----T-G--C-C---T--T-------T--GA-A-A---C-------T--C-C--TG--G-T-C----T-T-G-A-G-G--T---C-GA-----GA-G-A--------G-G-T-G---AG-T------GG--A--ATT----C-C-G-
A-GT--GT-A-G-AG-GT--G---A--A-A---TT-C-GT-AG--AT-A-TT--C-G--G-A-G-G-A-AC-A-CC---AG--T--G--GC-GAA-G--G-C--G-------G--C-T-C-A---CTG--G--CT-C-G--------------------A-T---A-C-T--GA--CG-C-T-G--A-GG--T-G-CG-A--AA-G-C---G-TG--GG-G--
AG-C-A-AA--------CA--GG Bacteria(100);Proteobacteria(100);Alphaproteobacteria(100);Rhodobacterales(100);Rhodobacteraceae(100);unclassified(69);unclassified(69);unclassified(69);unclassified(69);

Otu0070 0 0 0 1 0 0 0 0 0 0 1 0 1 0 1 4 3 3 8 10 0 4 0 0 0 0 1 0
0 0 0 0 0 0 0 0 0 0 0 0 0 0 0 0 0 0 19 0 0 0 27 0 7 40 2 0 0
0 1 19 25 0 34 8 0 33 7 0 0 0 29 0 0 0 3 1 0 0 0 0 0 0 0 0 1 0
0 0 0 0 0 0 0 5 30 0 16 0 10 0 42 0 0 0 0 0 0 0 0 0 0 0 0
M02255_76_000000000-ADUNE_1_1118_14414_20727 C----GG---CT-A--ACT--------ACG-T-G------C--C--A---G--CA-G-CCG-CG------GT-A--A-T--AC-GT-------AG--G-----GT-GC-AA-GC-------GTTA-A-T-C-G------------G--T-TT-T-G-T------
-----AA----G-T-C-T--------G-A-T--G--TG--A-AA-TC--C-C-CG-G-G-----CT-C-AA------C-C-T-G-G-G-A--A-T----T-G--C-A---T--T-------G--GA-G-A---C-------T--G-C--AA--G-G-C----T-A-G-A-A-T--C---T-GG-----CA-G-A--------G-G-G-G---GG-T------AG--A--ATT----C-
C-A-C-GT--GT-A-G-CA-GT--G---A--A-A---TG-C-GT-AG--AT-A-TG--T-G--G-A-G-G-A-AC-A-CC---GA--T--G--GC-GAA-G--G-C--A-------G--C-C-C-C---CTG--G--GT-C-A--------------------A-G---A-T-T--GA--CG-C-T-C--A-TG--C-A-CG-A--AA-G-C---G-TG--GG-G-
-AG-C-A-AA--------CA--GG Bacteria(100);Proteobacteria(100);Betaproteobacteria(100);Burkholderiales(100);Oxalobacteraceae(100);Janthinobacterium(93);unclassified(93);unclassified(93);unclassified(93);

Otu0071 1 8 0 8 38 0 24 72 22 10 1 1 0 3 0 0 0 14 3 3 10 0 46 16 6 55 13 17
6 133 41 146 24 277 13 44 64 38 28 76 11 76 99 214 31 35 3 105 96 186 4 68 30 5 0 0 39
12 24 29 2 0 7 5 2 0 0 0 3 0 4 32 34 17 5 40 90 58 8 24 16 138 0 0 2128 12
226 164 72 20 47 14 26 6 0 0 7 7 4 32 2 46 16 46 0 0 0 0 19 0 0 0 77
M02255_76_000000000-ADUNE_1_1111_8553_7151 T----A----AG-CA-CCG--------GCT-A-A------C-----T---C--TG-T-GCC-AG------CA-G--C-C--GC-G--------GT--A-----AT-AC-AG-AG-------G-GT-G-C-G-A------------G--C-TT-G-T-T-----------AA----
G-T-C-G--------G-A-T--G--TG--A-AA-TC--C-C-TG-A-G-----CT-T-AA------C-T-T-A-G-G-A--A-T----T-G--C-A---T--T-------C--GA-T-A---C-------T--G-G--GA--A-G-C----T-A-G-A-G-T--A---T-GG-----GA-G-A--------G-G-A-T---GG-T------AG--A--ATT----C-C-A-G-GT--
GT-A-G-CG-GT--G---A--A-A---TG-C-GT-AG--AG-A-TC--T-G--G-A-G-G-A-AT-A-CC---GA--T--G--GC-GAA-G--G-C--A-------G--C-C-A-T---CTG--G--CC-T-A--------------------A-T---A-C-T--GA--CG-C-T-G--A-GG--T-A-CG-A--AA-G-C---A-TG--GG-G--AG-C-A-
AA--------CA--GG Bacteria(100);Proteobacteria(100);Gammaproteobacteria(100);Pseudomonadales(100);Moraxellaceae(100);Acinetobacter(100);unclassified(100);unclassified(100);unclassified(100);

Otu0072 0 0 0 0 0 0 0 0 0 0 0 0 7 1 0 0 6 0 0 1 2 0 0 0 0 0 0 0
0 0 0 0 0 0 0 0 0 0 0 0 0 0 0 0 3 0 14 0 0 1 0 0 0 7 0 5 11
2 0 0 0 8 0 1 36 1 37 15 1 7 5 0 12 0 0 0 0 0 0 0 0 0 0 0 0 0
0 0 0 0 1 0 0 1 8 0 15 0 15 1 0 3 0 0 0 0 0 0 0 0 0 0 0
M02255_76_000000000-ADUNE_1_2106_6491_11604 T----AA---GC-AC-CGG--------CTA-A-C------T--C--T---G--TG-C-CAG-CA------GC-C--G-C--GG-TA-------AT--A-----CA-GA-GG-GT-------GCAA-GCG-T-T------------A--A-TC-G-G-T-----------
AA----G-T-T-G--------G-A-T--G--TG--A-AA-TC--C-C-CG-G-G-----CT-C-AA------C-C-T-G-G-G-A--A-C----T-G--C-A---T--T-------C--AA-A-A---C-------T--G-T--CG--A-G-C----T-A-G-A-G-T--A---T-GG-----TA-G-A--------G-G-G-T---GG-T------GG--A--ATT----T-C-C-
T-GT--GT-A-G-CG-GT--G---A--A-A---TG-C-GT-AG--AT-A-TA--G-G--A-A-G-G-A-AC-A-CC---AG--T--G--GC-GAA-G--G-C--G-------A--C-C-A-C---CTG--G--AC-T-G--------------------A-T---A-C-T--GA--CA-C-T-G--A-GG--T-G-CG-A--AA-G-C---G-TG--GG-G--
AG-C-A-AA--------CA--GG Bacteria(100);Proteobacteria(100);Gammaproteobacteria(100);Pseudomonadales(100);Pseudomonadaceae(100);unclassified(100);unclassified(100);unclassified(100);unclassified(100);

Otu0073 0 0 0 0 0 0 0 0 0 0 0 0 0 0 0 0 0 0 0 0 14 0 7 0 0 4 0 0
0 0 1 69 0 0 0 113 92 55 1 0 0 0 0 16 8 0 0 0 0 0 0 0 30 0 0 0 0
0 0 0 0 0 0 0 0 0 0 0 0 0 0 27 0 0 29 0 0 0 0 0 0 0 0 0 0 0
0 0 0 16 0 0 10 12 0 9 0 41 1 0 0 38 167 0 189 71 116 81 33 3 0 0 0
M02255_77_000000000-ADUT3_1_1103_15036_9999 T----AC---GA-AG-GGG--------GCT-A-G------C--G--T---T--GC-T-CGG-AA------TT-A--C-T--GG-GC-------GT--A-----AA-GG-GC-GC-------G-TA-G-G-C-G------------G--A-CA-G-T-T-----------
TA----G-T-C-A--------G-A-G--G--TG--A-AA-GC--C-C-AG-G-G-----CT-C-AA------C-C-T-T-G-G-A--A-T----T-G--C-C---T--T-------T--GA-T-A---C-------T--G-G--CT--G-T-C----T-T-G-A-G-T--T---C-GG-----GA-G-A--------G-G-T-G---AG-T------GG--A--ATG----C-C-G-
A-GT--GT-A-G-AG-GT--G---A--A-A---TT-C-GT-AG--AT-A-TT--C-G--G-C-G-G-A-AC-A-CC---AG--T--G--GC-GAA-G--G-C--G-------A--C-T-C-A---CTG--G--CC-C-G--------------------A-T---A-C-T--GA--CG-C-T-G--A-GG--C-G-CG-A--AA-G-C---G-TG--GG-G--
AG-C-A-AA--------CA--GG Bacteria(100);Proteobacteria(100);Alphaproteobacteria(100);Caulobacterales(100);Caulobacteraceae(100);Phenylobacterium(87);unclassified(87);unclassified(87);unclassified(87);

Otu0074 0 0 0 0 0 53 0 0 0 21 96 97 6 10 28 0 0 0 0 5 0 0 15 107 0 354 76 61
200 0 0 0 0 0 0 0 0 0 0 0 0 0 0 0 21 0 0 0 0 0 0 0 0 0 0 0 0
0 19 0 8 0 0 0 0 2 0 0 0 0 0 0 0 0 0 0 0 0 0 30 429 0 0 0 0 0 0 0
295 0 0 0 0 0 18 0 22 124 3 26 1 0 0 0 29 0 23 11 44 0 14 1 19 0 0
M02255_76_000000000-ADUNE_1_1113_18355_14483 T----GG---CT-A--ACT--------ACG-T-G------C--C--A---G--CA-G-CCG-CG------GT-A--A-T--AC-GT-------AG--A-----TG-GC-AA-GC-------G-TT-GTC-C-G------------G--A-TC-T-T-T-------
----AA----G-T-C-T--------G-A-T--G--TG--A-AA-GA--C-C-CC-A-G-----CT-C-AA------C-T-G-G-G-G-A--A------G-GT-C-A---T--T-------G--GA-A-A---C-------T--G-G--AG--A-A-C----T-T-G-A-G-T--G---C-AG-----AA-G-A--------G-G-A-G---AG-T------GG--A--ATT----C-
C-A-T-GT--GT-A-G-CG-GT--G---A--A-A---TG-C-GT-AG--AT-A-TA--T-G--G-A-G-G-A-AC-A-CC---AG--T--G--GC-GAA-G--G-C--G-------A--C-T-C-T---CTG--G--TC-T-G--------------------T-A---A-C-T--GA--CG-C-T-G--A-GG--C-T-CG-A--AA-G-C---G-TG--GG-G-
-AG-C-A-AA--------CA--GG Bacteria(100);Firmicutes(100);Bacilli(100);Lactobacillales(100);Carnobacteriaceae(100);unclassified(98);unclassified(98);unclassified(98);unclassified(98);

Otu0075 0 0 0 0 0 0 0 0 0 0 0 0 0 0 0 0 0 0 0 6 7 0 0 0 5 0 3 0
0 0 0 0 0 0 3 26 0 48 4 0 0 0 63 0 0 0 0 0 0 0 0 53 0 2 0 15 0
0 0 0 0 0 3 0 0 0 0 0 0 0 0 0 0 25 0 0 0 0 0 0 0 0 0 0 0 0
0 38 0 0 0 0 0 5 0 0 0 0 0 0 0 0 0 0 27 29 93 0 0 0 0 0 0
M02255_77_000000000-ADUT3_1_1109_8776_6952 T----AC---GT-AG-GGT--------CCG-A-G------C--G--T---T--GT-C-CGG-AA------TT-A--T-T--GG-GC-------GT--A-----AA-GG-GC-TC-------G-TA-G-G-C-G------------G--T-TT-G-T-C----------GC--
--G-T-C-G--------G-G-A--G--TG--A-AA-AC--T-C-AG-G-G-----CT-C-AA------C-C-C-T-G-A-G--C-G----T-G--C-T---T--C-------C--GA-T-A---C-------G--G-G--CA--G-A-C----T-A-G-A-G-G--T---A-TG-----CA-G-G--------G-G-A-G---AA-C------GG--A--ATT----C-C-T-G-
GT--GT-A-G-CG-GT--G---G--A-A---TG-C-GC-AG--AT-A-TC--A-G--G-A-G-G-A-AC-A-CC---GG--T--G--GC-GAA-G--G-C--G-------G--T-T-C-T---CTG--G--GC-A-T--------------------T-A---C-C-T--GA--CG-C-T-G--A-GG--A-G-CG-A--AA-G-C---A-TG--GG-G--AG-
C-G-AA--------CA--GG Bacteria(100);Actinobacteria(100);Actinobacteria(100);Actinobacteridae(100);Actinomycetales(100);Propionibacterineae(100);Nocardioidaceae(100);Aeromicrobium(79);unclassified(79);

Otu0076 0 0 0 0 0 0 0 0 0 0 0 7 9 0 3 6 8 4 6 194 43 150 0 0 0 0 3 0
22 0 0 0 0 0 0 0 0 0 0 0 0 0 0 2 0 0 6 0 0 0 0 0 0 0 0 0 0
0 5 0 16 0 0 8 0 1 0 0 0 175 27 7 2 0 11 6 2 0 0 0 0 0 0 0 0 0
0 1 0 0 0 1 0 17 6 0 3 0 0 4 32 0 0 0 0 1 0 0 0 0 0 0 0
M02255_76_000000000-ADUNE_1_2114_23364_21203 C----TC---GC-AG-AAT--------AAG-CACC-----G--G--C---T--AACT-CTG-TG------CC-A--G-C--AG-CC-------GC--G-----GT-AA-TA-CA-------G-AG-G-G-T-GC-----------A--A-GC-G-T-T--
---------AA----G-T-C-G--------G-A-T--G--TG--A-AA-TC--C-C-TG-A-G-----CT-T-AA------C-T-T-A-G-G-A--A-T----T-G--C-A---T--A-------C--GA-T-A---C-------T--G-G--GA--G-A-C----T-A-G-A-G-T--A---T-GG-----GA-G-A--------G-G-A-T---GG-T------AG--A--ATT----
C-C-A-G-GT--GT-A-G-CG-GT--G---A--A-A---TG-C-GT-AG--AG-A-TC--T-G--G-A-G-G-A-AT-A-CC---GA--T--G--GC-GAA-G--G-C--A-------G--C-C-A-T---CTG--G--CC-T-A--------------------A-T---A-C-T--GA--CA-C-T-G--A-GG--T-A-CG-A--AA-G-C---A-TG--GG-
G--AG-C-A-AA--------CA--GG Bacteria(100);Proteobacteria(100);Gammaproteobacteria(100);Pseudomonadales(98);Moraxellaceae(98);unclassified(93);unclassified(93);unclassified(93);unclassified(93);

Otu0077 9 11 2 24 3 7 14 29 13 2 0 0 0 0 0 0 1 0 0 1 2 1 0 3 0 0 0 2
0 25 4 0 7 14 9 2 8 1 15 17 4 9 33 20 0 7 0 8 18 8 1 4 2 3 0 1 8
1 43 23 1 1 0 0 0 1 0 0 0 0 0 8 3 163 8 0 8 2 2 0 5 19 13 0 31 43
12 34 28 45 25 7 0 11 0 3 0 1 0 12 0 6 2 23 0 35 32 29 15 1 13 25 62
M02255_77_000000000-ADUT3_1_1106_26298_15966 T----AA---CT-AC-GTG--------CC--A-G------C--A--G---C--CG-C-GGT-AA------TA-C--G-T--AG-GT-------CC--C-----GA-GC-GT-TG-------T-CC-G-G-C-G------------G--T-TA-G-A-T-------
----AA----G-T-C-T--------G-A-A--G--TT--A-AA-GG--C-T-GT-G-G-----CT-T-AA------C-C-A-T-A-G-T--A------C-G--C-T---T--T-------G--GA-A-A---C-------T--G-T--TT--A-A-C----T-T-G-A-G-T--G---C-AA-----GA-G-G--------G-G-A-G---AG-T------GG--A--ATT----C-C-
A-T-GT--GT-A-G-CG-GT--G---A--A-A---TG-C-GT-AG--AT-A-TA--T-G--G-A-G-G-A-AC-A-CC---GG--T--G--GC-GAA-A--G-C--G-------G--C-T-C-T---CTG--G--CT-T-G--------------------T-A---A-C-T--GA--CG-C-T-G--A-GG--C-T-CG-A--AA-G-C---G-TG--GG-G--
AG-C-A-AA--------CA--GG Bacteria(100);Firmicutes(100);Bacilli(100);Lactobacillales(100);Streptococcaceae(100);Streptococcus(100);unclassified(100);unclassified(100);unclassified(100);

Otu0078 0 0 0 0 0 0 33 1 0 44 0 0 0 0 0 0 3 0 0 0 0 0 0 0 0 0 0 11
0 0 2 0 0 0 0 0 0 12 8 0 0 0 40 0 0 117 0 0 1 0 0 0 0 0 0 1 0
0 0 0 0 0 0 0 0 0 0 0 0 0 0 0 0 18 84 0 0 0 0 9 0 0 0 0 0 0
0 0 0 0 66 0 0 0 0 0 0 0 0 0 0 23 67 55 0 0 0 0 0 0 18 0 0
M02255_77_000000000-ADUT3_1_1109_14119_19360 T----AC---GG-AG-GGG--------GCT-A-G------C--G--T---T--GT-T-CGG-AA------TT-A--C-T--GG-GC-------GT--A-----AA-GC-GC-AC-------G-TA-G-G-C-G------------G--A-TC-A-G-C----
-------AA----G-T-T-A--------G-G-G--G--TG--A-AA-TC--C-C-GG-G-G-----CT-C-AA------C-C-C-C-G-G-A--A-C----T-G--C-C---T--T-------T--AA-A-A---C-------T--G-T--TG--G-T-C----T-G-G-A-G-T--T---C-GA-----GA-G-A--------G-G-T-G---AG-T------GG--A--ATT----
C-C-G-A-GT--GT-A-G-AG-GT--G---A--A-A---TT-C-GT-AG--AT-A-TT--C-G--G-A-G-G-A-AC-A-CC---AG--T--G--GC-GAA-G--G-C--G-------G--C-T-C-A---CTG--G--CT-C-G--------------------A-T---A-C-T--GA--CG-C-T-G--A-GG--T-G-CG-A--AA-G-C---G-TG--GG-
G--AG-C-A-AA--------CA--GG Bacteria(100);Proteobacteria(100);Alphaproteobacteria(100);Rhodobacterales(54);Rhodobacteraceae(54);unclassified(54);unclassified(54);unclassified(54);unclassified(54);

Otu0079 0 151 88 106 0 24 215 80 0 0 0 0 0 0 0 0 0 0 0 0 0 0 0 0 0 0 0 0
0 0 2 0 165 0 0 0 0 0 18 1 0 37 0 21 0 0 0 86 43 0 5 82 0 2 0 0 0
0 0 15 0 10 0 0 0 15 0 0 0 0 0 177 253 0 18 0 0 0 0 0 0 20 0 0 0 14
0 46 66 0 0 0 3 23 0 35 0 0 0 0 0 0 0 54 10 42 434 0 0 10 0 0 119
M02255_77_000000000-ADUT3_1_2111_6463_13104 T----AC---GT-AT-GTC--------GCA-A-G------C--G--T---T--AT-C-CGG-AA------TT-A--T-T--GG-GC-------AT--A-----AA-GG-GC-AT-------C-TA-G-G-C-G------------G--C-CA-G-G-C----------AA--
--G-T-C-T--------G-G-G--G--TG--A-AA-AC--T-T-GC-G-G-----CT-C-AA------C-C-G-C-A-A-G--C-C----T-G--C-C---C--T-------G--GA-A-A---C-------T--G-C--CT--G-G-C----T-A-G-A-G-T--G---C-TG-----GA-G-A--------G-G-T-G---GA-C------GG--A--ACT----G-C-A-C-
GA--GT-A-G-AG-GT--G---A--A-A---TT-C-GT-AG--AT-A-TG--T-G--C-A-G-G-A-AT-G-CC---GA--T--G--AT-GAA-G--A-T--A-------G--T-T-C-A---CTG--G--AC-G-G--------------------C-A---A-C-T--GA--CG-C-T-G--A-AG--T-G-CG-A--AA-G-C---T-GG--GG-G--AG-
C-G-AA--------CA--GG Bacteria(100);Fusobacteria(100);Fusobacteria(100);Fusobacteriales(100);Leptotrichiaceae(100);Leptotrichia(100);unclassified(100);unclassified(100);unclassified(100);

Otu0080 0 410 34 0 0 0 82 132 269 0 0 0 0 0 0 0 0 0 0 0 0 0 0 0 0 0 0 0
0 0 48 0 0 0 0 0 0 0 0 0 0 0 0 0 0 0 0 0 0 0 0 0 0 0 0 0
0 0 0 0 0 0 0 0 0 0 0 0 0 1 0 0 0 101 0 0 0 0 0 0 0 0 0 0
0 0 0 0 0 0 1 0 0 0 0 0 0 0 0 0 81 0 0 0 0 0 33 0 108 0 84
M02255_77_000000000-ADUT3_1_1113_23437_13088 T----AC---GT-AG-GGG--------GCA-A-G------C--G--T---T--AT-C-CGG-AA------TT-A--C-T--GG-GT-------GT--A-----AA-GG-GT-GC-------G-TA-G-G-T-G------------G--T-AT-G-G-C----
-------AA----G-T-C-A--------G-A-A--G--TG--A-AA-AC--C-C-AG-G-G-----CT-T-AA------C-T-C-T-G-G-G--A-C----T-G--C-T---T--T-------T--GA-A-A---C-------T--G-T--CA--G-A-C----T-G-G-A-G-T--G---C-AG-----GA-G-A--------G-G-T-A---AG-C------GG--A--ATT----
C-C-T-A-GT--GT-A-G-CG-GT--G---A--A-A---TG-C-GT-AG--AT-A-TT--A-G--G-A-G-G-A-AC-A-TC---AG--T--G--GC-GAA-G--G-C--G-------G--C-T-T-A---CTG--G--AC-T-G--------------------A-A---A-C-T--GA--CA-C-T-G--A-GG--C-A-CG-A--AA-G-C---G-TG--GG-
G--AG-C-A-AA--------CA--GG Bacteria(100);Firmicutes(100);Clostridia(100);Clostridiales(100);unclassified(100);unclassified(100);unclassified(100);unclassified(100);unclassified(100);

Otu0081 322 155 0 323 1201 87 95 292 61 88 1 0 3 8 5 1 7 10 2 30 158 10 129 6 5 10 0 58
12 5323 765 306 949 469 1117 694 501 387 269 398 650 159 484 232 12 0 0 490 1711 0 48 138 165 72 0 11 0
0 148 160 4 33 0 0 25 0 4 10 32 11 8 143 169 377 175 0 105 55 12 0 54 154 298 0 71 239
144 204 0 24 172 51 13 61 1 49 0 0 7 100 0 18 180 286 3 434 63 174 96 22 160 1 1139
M02255_77_000000000-ADUT3_1_1101_11002_21397 T----AC---GT-AG-GGC--------GCG-A-G------C--G--T---T--GT-C-CGG-AA------TT-A--T-T--GG-GC-------GT--A-----AA-GA-GC-TT-------G-TA-G-G-C-G------------G--T-TG-G-T-C-----
------GC----G-T-C-T--------G-C-T--G--TG--A-AA-GG--C-T-GG-G-G-----CT-T-AA------C-C-C-T-G-G-T--T-T----T-G--C-A---G--T-------G--GG-T-A---C-------G--G-G--CT--A-A-C----T-A-G-A-G-T--G---C-AG-----TA-G-G--------G-G-A-G---AC-T------GG--A--ATT----
C-C-T-G-GT--GT-A-G-CG-GT--G---G--A-A---TG-C-GC-AG--AT-A-TC--A-G--G-A-G-G-A-AC-A-CC---GA--T--G--GC-GAA-G--G-C--A-------G--G-T-C-T---CTG--G--GC-T-G--------------------T-A---A-C-T--GA--CG-C-T-G--A-GA--A-G-CG-A--AA-G-C---A-TG--
GG-G--AG-C-G-AA--------CA--GG Bacteria(100);Actinobacteria(100);Actinobacteria(100);Actinobacteridae(100);Actinomycetales(100);Micrococcineae(100);Micrococcaceae(100);Rothia(100);unclassified(100);

Otu0082 0 0 0 0 0 0 0 0 0 0 23 5 6 0 0 0 0 0 0 0 0 0 31 11 0 387 111 55
33 168 0 0 0 0 65 0 0 0 0 0 0 0 0 0 0 0 0 0 0 0 0 0 0 0 0 0
0 0 0 0 0 0 0 0 0 0 0 0 0 0 0 0 14 0 0 0 0 24 486 0 0 0 0 0 0
0 0 0 0 0 0 1163 2 62 106 45 0 11 0 0 0 0 0 0 0 0 0 0 0 0 0 0
M02255_77_000000000-ADUT3_1_1116_14118_9798 T----AC---GG-AG-GGT--------GCA-A-G------C--G--T---T--AC-T-CGG-AA------TC-A--C-T--GG-GC-------GT--A-----AA-GA-GC-GT-------G-TA-G-G-C-G------------G--A-TA-G-G-T----------
AA----G-T-C-A--------G-A-A--G--TG--A-AA-TC--C-A-AT-A-G-----CT-C-AA------C-T-A-T-T-G-A--A-C----T-G--C-T---T--T-------T--GA-A-A---C-------T--G-C--TT--A-T-C----T-A-G-A-A-T--A---T-GG-----GA-G-A--------G-G-T-A---GA-T------GG--A--ATT----T-C-T-G-
GT--GT-A-G-GG-GT--A---A--A-A---TC-C-GT-AG--AG-A-TC--A-G--A-A-G-G-A-AT-A-CC---GA--T--T--GC-GAA-G--G-C--G-------A--T-C-T-A---CTG--G--AA-C-A--------------------T-T---A-T-T--GA--CG-C-T-G--A-GA--C-G-CG-A--AA-G-C---G-TG--GG-G--AG-C-
A-AA--------CA--GG Bacteria(100);Proteobacteria(100);Epsilonproteobacteria(100);Campylobacterales(100);Campylobacteraceae(100);Arcobacter(100);unclassified(100);unclassified(100);unclassified(100);

Otu0083 0 0 0 0 0 0 6 113 0 0 0 0 0 0 1 0 0 0 1 0 0 0 0 0 0 13 0 6
0 0 0 0 0 0 0 0 21 0 0 0 0 0 0 0 0 0 0 0 0 0 1 0 0 0 0 0
0 0 0 0 0 0 0 0 0 0 0 0 0 0 0 0 0 203 0 0 0 0 0 0 0 0 0 0
0 0 0 0 0 0 0 0 0 0 0 0 0 50 0 48 0 0 36 0 0 0 0 0 0 0 0
M02255_77_000000000-ADUT3_1_2108_24908_18432 T----AC---GG-AG-GGA--------GCT-A-G------C--G--T---T--GT-T-CGG-AA------AT-A--C-T--GG-GC-------GT--A-----AA-GC-GC-AC-------G-TA-G-G-C-G------------G--C-GC-A-G-C----
-------AA----G-T-C-A--------G-G-G--G--TG--A-AA-TC--C-C-GG-G-G-----CT-C-AA------C-C-C-C-G-G-A--A-C----T-G--C-C---C--T-------T--GA-A-A---C-------T--G-C--AG--T-G-C----T-A-G-A-A-T--C---T-TG-----GA-G-A--------G-G-C-G---AG-T------GG--A--ATT----
C-C-G-A-GT--GT-A-G-AG-GT--G---A--A-A---TT-C-GT-AG--AT-A-TT--C-G--G-A-A-G-A-AC-A-CC---AG--T--G--GC-GAA-G--G-C--G-------A--C-T-C-G---CTG--G--AC-A-A--------------------G-T---A-T-T--GA--CG-C-T-G--A-GG--T-G-CG-A--AA-G-C---G-TG--GG-
G--AG-C-A-AA--------CA--GG Bacteria(100);Proteobacteria(100);Alphaproteobacteria(100);Sphingomonadales(100);unclassified(51);unclassified(51);unclassified(51);unclassified(51);unclassified(51);

Otu0084 79 0 61 288 0 203 0 0 0 0 0 0 0 11 0 0 0 0 0 2 0 0 0 23 0 5 0 0
15 0 0 30 111 59 24 150 0 14 17 44 0 38 468 98 28 122 0 77 0 0 28 0 62 133 0 3 0
64 0 52 41 18 5 0 117 15 219 25 6 123 32 705 205 116 0 174 110 0 3 18 332 68 1 0 205 0
0 201 0 396 76 0 55 3 0 0 0 0 0 0 0 90 0 0 201 52 59 0 0 4 0 0 0
M02255_77_000000000-ADUT3_1_1115_4791_20758 T----AC---GA-AG-GGG--------GCT-A-G------C--G--T---T--GC-T-CGG-AA------TC-A--C-T--GG-GC-------GT--A-----AA-GG-GC-GC-------G-TA-G-G-C-G------------G--C-CA-T-T-C----------
AA----G-T-C-G--------G-G-G--G--TG--A-AA-GC--C-T-GT-G-G-----CT-C-AA------C-C-A-C-A-G-A--A-T----T-G--C-C---T--T-------C--GA-T-A---C-------T--G-T--TT--G-G-C----T-T-G-A-G-T--A---T-GG-----TA-G-A--------G-G-T-T---GG-T------GG--A--ACT----G-C-G-
A-GT--GT-A-G-AG-GT--G---A--A-A---TT-C-GT-AG--AT-A-TT--C-G--C-A-A-G-A-AC-A-CC---GG--T--G--GC-GAA-G--G-C--G-------G--C-C-A-A---CTG--G--AC-C-A--------------------T-T---A-C-T--GA--CG-C-T-G--A-GG--C-G-CG-A--AA-G-C---G-TG--GG-G--
AG-C-A-AA--------CA--GG Bacteria(100);Proteobacteria(100);Alphaproteobacteria(100);Rhizobiales(100);Methylobacteriaceae(100);Methylobacterium(100);unclassified(100);unclassified(100);unclassified(100);

Otu0085 280 122 56 189 178 112 173 130 118 99 0 0 3 28 9 4 8 4 21 37 145 23 142 116 35 13 16 37
8 691 455 664 409 563 478 1111 1282 520 793 363 537 610 575 1935 859 1298 22 264 389 0 50 592 147 24 6 70 432
42 200 45 15 26 27 29 0 31 16 16 12 22 8 95 14 166 80 193 229 181 138 96 464 624 183 70 322 381
197 604 358 212 408 129 33 37 4 161 5 115 19 70 0 273 274 211 117 320 293 390 603 18 145 180 115
M02255_77_000000000-ADUT3_1_1104_10775_17601 T----AC---GT-AG-GGT--------GCG-A-G------C--G--T---T--GT-C-CGG-AT------TT-A--T-T--GG-GC-------GT--A-----AA-GG-GC-TC-------G-TA-G-G-T-G-------------G--T-TG-A-T-C------
-----GC----G-T-C-G--------G-A-A--G--TG--T-AA-TC--T-T-GG-G-G-----CT-T-AA------C-C-C-T-G-A-G--C-G----T-G--C-T---T--T-------C--GA-T-A---C-------G--G-G--TT--G-A-C----T-T-G-A-G-G--A---A-GG-----TA-G-G--------G-G-A-G---AA-T------GG--A--ATT----C-
C-T-G-GT--GG-A-G-CG-GT--G---G--A-A---TG-C-GC-AG--AT-A-TC--A-G--G-A-G-G-A-AC-A-CC---AG--T--G--GC-GAA-G--G-C--G-------G--T-T-C-T---CTG--G--GC-C-T--------------------T-T---C-C-T--GA--CG-C-T-G--A-GG--A-G-CG-A--AA-G-C---G-TG--GG-
G--AG-C-G-AA--------CA--GG Bacteria(100);Actinobacteria(100);Actinobacteria(100);Actinobacteridae(100);Actinomycetales(100);Propionibacterineae(100);Propionibacteriaceae(100);Propionibacterium(100);unclassified(100);

Otu0086 0 0 0 0 0 0 0 0 0 0 54 1 0 7 4 129 0 9 11 9 5 2 6 6 0 0 0 0
5 0 0 0 1 0 0 0 0 0 0 1 0 15 0 0 1 0 30 1 5 0 0 0 0 0 0 0 1
0 1 0 0 5 0 11 0 0 0 0 0 0 1 0 0 0 0 0 0 0 1 0 0 0 0 0 0 0
0 0 0 0 0 0 0 0 5 0 0 0 0 0 0 12 0 7 0 0 1 0 0 0 0 0 0
M02255_76_000000000-ADUNE_1_2104_26899_16941 .....AC----T-AC-GT---------GCC-A-G------C--A--G---C--CG-C-GGT-AA------TA-C--G-T--AG-GT-------GG--C-----AA-GC-G--TT-------G----T-C-C-G-------------G--T-CT-C-T-T-----------
AA----G-T-C-T--------G-A-T--G--TG--A-AA-GC--C-C-CC-G-G-----CT-C-AA------C-C-G-G-G-G-A--G------G-GT-C-A---T--T-------G--GA-A-A---C-------T--G-G--GA--G-A-C----T-T-G-A-G-G--A---C-AG-----AA-G-A--------G-G-A-G---AG-T------GG--A--ATT----C-C-
A-A-GT--GT-A-G-CG-GT--G---A--A-A---TG-C-GT-AG--AT-A-TT--T-G--G-A-G-G-A-AC-A-CC---AG--T--G--GC-GAA-G--G-C--G-------G--C-T-C-T---CTG--G--TC-T-G--------------------T-T---A-C-T--GA--CG-C-T-G--A-GG--C-G-CG-A--AA-G-C---G-TG--GG-G--
AG-C-A-AA--------CA--GG Bacteria(100);Firmicutes(100);Bacilli(100);Bacillales(93);unclassified(93);unclassified(93);unclassified(93);unclassified(93);unclassified(93);

Otu0087 8 8 0 8 0 2 0 4 0 1 0 0 0 0 0 0 1 0 0 0 0 0 1 0 0 0 0 0
0 0 3 0 0 0 16 0 0 4 0 2 2 0 3 8 7 9 0 0 2 0 0 2 1 0 0 0 0
0 0 0 0 0 0 0 0 0 0 0 0 0 0 1 0 2 0 0 0 0 0 0 0 0 14 1 26 2
3 3 0 5 6 2 0 0 0 3 1 0 0 3 0 0 3 1 0 10 38 0 0 1 0 0 3
M02255_77_000000000-ADUT3_1_1115_27318_14317 C----GG---CT-AA-CT---------ACG-T-G------C--C--A---G--CA-G-CCG-CG------GT-A--A-T--AC-GT-------AG--G-----TC-CC-GA-GC-------G-CT-GTG-C-G-------------G--T-TT-G-A-T------
-----AA----G-T-C-T--------G-A-A--G--TT--A-AA-GG--C-T-GT-G-G-----CT-C-AA------C-C-A-T-A-G-T--T------C-G--C-T---T--T-------G--GA-A-A---C-------T--G-T--CA--A-A-C----T-T-G-A-G-T--G---C-AG-----AA-G-G--------G-G-A-G---AG-T------GG--A--ATT----C-
C-A-T-GT--GT-A-G-CG-GT--G---A--A-A---TG-C-GT-AG--AT-A-TA--T-G--G-A-G-G-A-AC-A-CC---GG--T--G--GC-GAA-A--G-C--G-------G--C-T-C-T---CTG--G--TC-T-G--------------------T-A---A-C-T--GA--CG-C-T-G--A-GG--C-T-CG-A--AA-G-C---G-TG--GG-G-
-AG-C-G-AA--------CA--GG Bacteria(100);Firmicutes(100);Bacilli(100);Lactobacillales(100);Streptococcaceae(100);Streptococcus(100);unclassified(100);unclassified(100);unclassified(100);

Otu0088 0 0 0 121 0 3 13 0 0 0 0 0 0 1 0 0 0 0 0 3 5 0 8 0 6 10 0 0
3 0 0 7 0 0 0 0 18 11 6 0 6 78 0 1 0 0 0 0 47 0 17 3 21 44 0 0
0 0 0 0 0 3 1 0 8 0 0 0 1 1 40 5 0 0 0 0 0 0 0 0 0 0 0 1
792 29 0 7 0 28 0 1 0 9 1 0 0 0 0 0 16 0 0 12 0 0 4 0 0 0 0
M02255_76_000000000-ADUNE_1_1107_8076_11701 C----GG---CT-AA-AT---------ACG-T-G------C--C--A---G--CA-G-CCG-CG------GT-A--A-T--AC-GT-------AT--G-----TC-CC-GA-GC-------GTTA-T-C-C-G-------------G--T-TG-G-T-T-----------AA---
-G-T-C-T--------G-A-T--G--TG--A-AA-GC--C-C-GG-A-G-----CT-C-AA------C-T-C-C-G-G-A--A-A----G-G--C-A---T--T-------G--GA-A-A---C-------T--G-G--TT--A-A-C----T-T-G-A-G-T--G---C-AG-----TA-G-A--------G-G-T-A---AG-T------GG--A--ACT----C-C-A-T-
GT--GT-A-G-CG-GT--G---G--A-A---TG-C-GT-AG--AT-A-TA--T-G--G-A-A-G-A-AC-A-CC---AG--C--G--GC-GAA-G--G-C--G-------G--C-T-T-A---CTG--G--AC-T-G--------------------T-A---A-C-T--GA--CG-T-T-G--A-GG--C-T-CG-A--AA-G-T---G-TG--GG-T--AG-C-
A-AA--------CA--GG Bacteria(100);Firmicutes(100);Bacilli(100);Lactobacillales(100);Leuconostocaceae(100);Leuconostoc(100);unclassified(100);unclassified(100);unclassified(100);

Otu0089 34 0 0 0 0 0 0 0 0 0 1 89 9 11 10 14 2 43 15 95 7 36 31 23 0 0 10 0
18 0 0 0 0 0 0 0 0 0 5 0 15 33 0 25 0 0 0 1 3 0 0 18 0 0 4 0
7 0 0 3 0 0 29 0 1 0 49 2 1 2 61 68 0 5 55 0 1 0 0 0 0 0 0 1 0
7 0 39 0 0 24 7 10 12 0 7 0 6 5 3 10 0 0 4 1 0 0 0 0 0 10 0
M02255_76_000000000-ADUNE_1_1102_10222_22814 C----TA---AC-TC-TGT--------GCC-A-G------C-----A---G--CC-G-CGG-TA------AT-A--C-A--GA-GG-------GT--G-----CA--A-GC-G--------T-TA-A-T-C-G-------------G--A-CA-A-T-T---------
--AA----G-T-C-A--------A-A-T--G--TG--A-AA-TC--C-C-CG-A-G-----CT-T-AA------C-T-T-G-G-G-A--A-T----T-G--C-A---T--T-------C--GA-T-A---C-------T--G-G--TT--G-G-C----T-A-G-A-G-T--A---T-GG-----GA-G-A--------G-G-A-T---GG-T------AG--A--ATT----C-C-
A-G-GT--GT-A-G-CG-GT--G---A--A-A---TG-C-GT-AG--AG-A-TC--T-G--G-A-G-G-A-AT-A-CC---GA--T--G--GC-GAA-G--G-C--A-------G--C-C-A-T---CTG--G--CC-T-A--------------------A-T---A-C-T--GA--CA-C-T-G--A-GG--T-A-CG-A--AA-G-C---A-TG--GG-G--
AG-C-A-AA--------CA--GG Bacteria(100);Proteobacteria(100);Gammaproteobacteria(100);Pseudomonadales(100);Moraxellaceae(100);Acinetobacter(100);unclassified(100);unclassified(100);unclassified(100);

Otu0090	12	0	0	3	0	0	0	5	0	0	0	0	0	0	0	0	0	0	0	13	0	0	0	0	0	0	0	0
0	0	0	0	0	0	0	0	0	54	1	0	0	1	0	0	0	1	0	0	0	0	0	0	0	0	0	5	29
0	10	0	0	0	3	0	0	0	0	0	0	0	0	1	0	0	2	0	3	13	0	0	0	0	0	103	0	0
0	0	2	0	5	0	1	0	0	0	0	0	0	0	0	0	0	16	0	13	22	0	0	1	5	0	0		

M02255_77_000000000-ADUT3_1_2114_20734_8193 T----AC---GT-AG-GGC--------GCG-A-G------C--G--T---T--GT-C-CGG-AA------TT-A--T-T--GG-GC-------GT--A-----AA-GA-GC-TT-------G-TA-G-G-C-G------------G--T-TT-G-T-C-----------GC-
---G-T-C-T--------G-C-T--G--TG--A-AA-GG--C-C-GG-G-G-----CT-T-AA------C-C-C-C-GTG-T--A-T----T-G--C-A---G--T-------G--GG-T-A---C-------G--G-G--CA--G-A-C----T-A-G-A-G-T--G---C-AG-----TA-G-G--------G-G-A-G---AC-T------GG--A--ACT----C-C-T-G-
GT--GT-A-G-CG-GT--G---G--A-A---TG-C-GC-AG--AT-A-TC--A-G--G-A-A-G-A-AC-A-CC---GA--T--G--GC-GAA-G--G-C--A-------G--G-T-C-T---CTG--G--GC-T-G--------------------T-A---A-C-T--GA--CG-C-T-G--A-GA--A-G-CG-A--AA-G-C---A-TG--GG-G--AG-
C-G-AA--------CA--GG Bacteria(100);Actinobacteria(100);Actinobacteria(100);Actinobacteridae(100);Actinomycetales(100);Micrococcineae(100);Micrococcaceae(53);unclassified;unclassified;

Otu0091	0	1	0	100	0	0	41	0	0	0	0	0	0	0	0	3	0	0	0	8	17	0	36	0	0	0	0	0
0	2	220	0	0	0	37	0	0	67	5	91	1027	49	0	0	0	0	0	2	0	1524	22	38	0	0	7	0	463
46	134	16	5	0	0	6	4	0	0	18	23	0	10	0	0	0	0	0	118	0	5	0	0	95	1142	0	0	79
209	63	0	0	113	356	0	20	0	498	19	1296	18	53	3	54	0	0	0	0	0	0	12	0	36	0	0		

M02255_76_000000000-ADUNE_1_2101_8786_21344 T----AC---GT-AA-GGA--------CCG-A-G------C--G--T---T--GT-C-CGG-AA------TC-A--T-T--GG-GC-------GT--A-----AA-GG-GT-AC-------G-TA-G-G-C-G------------G--T-TA-A-A-A-----------AA-
---G-T-T-A--------G-A-A--G--TG--A-AA-GG--C-T-AT-A-G-----CT-C-AA------C-T-A-T-A-G-T--A------A-G--C-T---T--T-------T--AA-A-A---C-------T--G-T--TT--A-A-C----T-T-G-A-G-A--G---A-TG-----GA-A-G--------G-G-A-A---AG-T------GG--A--ATT----C-C-T-A-GT-
-GT-A-G-CG-GT--G---A--A-A---TG-C-GC-AG--AT-A-TT--A-G--G-A-G-G-A-AT-A-CC---GG--T--G--GC-GAA-G--G-C--G-------A--C-T-T-T---CTG--G--CC-A-T--------------------T-A---T-C-T--GA--CG-C-T-G--A-GG--T-A-CG-A--AA-G-C---G-TG--GG-T--AG-C-A-
AA--------CA--GG Bacteria(100);Firmicutes(100);Clostridia(100);Clostridiales(100);Family_XI_Incertae_Sedis(100);Anaerococcus(100);unclassified(100);unclassified(100);unclassified(100);

Otu0092	0	0	0	0	0	0	0	0	0	0	0	0	0	0	0	0	0	0	0	7	18	0	57	0	0	5	0	9
4	0	0	88	0	0	89	56	3	77	0	0	0	0	0	66	29	0	0	0	1	0	1	0	134	0	0	0	0
0	0	0	0	0	0	0	0	0	0	0	0	0	0	0	0	0	0	0	0	0	0	0	0	0	0	0	0	63
0	0	79	0	0	0	25	0	0	0	0	0	0	0	0	60	137	19	0	0	0	0	0	0	0	0	0		

M02255_77_000000000-ADUT3_1_1101_2238_13804 T----AC---GA-AG-GGG--------GCT-A-G------C--G--T---T--GT-T-CGG-AA------TT-A--C-T--GG-GC-------GT--A-----AA-GC-GC-AC-------G-TA-G-G-C-G------------G--A-TC-G-A-T-----------CA-
---G-T-C-A--------G-G-G--G--TG--A-AA-TC--C-C-AG-A-G-----CT-C-AA------C-T-C-T-G-G-A--A-C----T-G--C-C---T--T-------T--GA-T-A---C-------T--G-T--CG--A-T-C----T-A-G-A-G-T--A---T-GG-----AA-G-A--------G-G-T-G---AG-T------GG--A--ATT----C-C-G-A-
GT--GT-A-G-AG-GT--G---A--A-A---TT-C-GT-AG--AT-A-TT--C-G--G-A-G-G-A-AC-A-CC---AG--T--G--GC-GAA-G--G-C--G-------G--C-T-C-A---CTG--G--TC-C-A--------------------T-T---A-C-T--GA--CG-C-T-G--A-GG--T-G-CG-A--AA-G-C---G-TG--GG-G--AG-C-
A-AA--------CA--GG Bacteria(100);Proteobacteria(100);Alphaproteobacteria(100);Rhizobiales(100);unclassified(56);unclassified(56);unclassified(56);unclassified(56);unclassified(56);

Otu0093	0	0	0	0	0	0	0	0	0	0	0	0	0	3	2	0	0	0	0	0	0	0	0	0	0	0	0	0
0	0	0	0	0	0	0	0	0	0	0	0	0	0	0	0	0	0	0	0	0	0	1	0	0	0	26	0	0
2	0	0	0	0	0	0	0	0	0	0	0	0	0	0	0	0	0	0	0	184	0	0	0	0	288	207	0	9
0	0	0	0	0	0	0	0	0	0	0	1	0	0	0	0	0	0	0	0	0	0	0	0	0	0	0		

M02255_76_000000000-ADUNE_1_1113_14717_16707 T----AA---GC-AC-CGG--------CTA-A-C------T--A--C---G--TG-C-CAG-CA------GC-C--G-C--GG-TA-------AT--A-----CG-TA-GG-GT-------G-CA-A-G-C-G------------T-TAATC-G-G-A----
-------AA----G-A-A-A--------G-A-T--G--TG--A-AA-TC--C-C-AG-A-G-----CT-T-AA------C-T-T-T-G-G-A--A-C----T-G--C-A---T--T-------T--TT-A-A---C-------T--A-C--CG--A-G-C----T-A-G-A-G-T--G---T-GT-----CA-G-A--------G-G-G-A---GG-T------GG--A--ATT----C-
C-G-C-GT--GT-A-G-CA-GT--G---A--A-A---TG-C-GT-AG--AT-A-TG--C-G--G-A-G-G-A-AC-A-CC---GA--T--G--GC-GAA-G--G-C--A-------G--C-C-T-C---CTG--G--GA-T-A--------------------A-C---A-C-T--GA--CG-C-T-C--A-TG--C-A-CG-A--AA-G-C---G-TG--GG-G-
-AG-C-A-AA--------CA--GG Bacteria(100);Proteobacteria(100);Betaproteobacteria(100);Burkholderiales(100);Alcaligenaceae(100);unclassified(100);unclassified(100);unclassified(100);unclassified(100);

Otu0094	0	0	0	0	3	3	0	0	0	5	0	0	0	0	0	0	0	0	0	0	0	0	15	0	0	4	10	133
8	0	0	0	0	0	0	0	0	1	0	0	0	0	2	0	1	22	0	4	0	0	0	1	0	0	0	0	0
0	0	0	0	0	0	0	9	0	0	0	0	0	0	0	0	0	0	0	0	0	0	10	0	0	0	0	0	0
2	0	0	0	0	0	0	0	0	1	0	0	0	0	0	5	0	0	0	0	0	0	5	0	11	0	0		

M02255_76_000000000-ADUNE_1_2111_10614_24517 TG---AC---AG-AA-GAA--------GCA-C-C------G--G--C---T--AACT-CCG-TG------CC-AG-C-A-GCC-GC-------GG--T-----AA-TA-CG-GA-------G-GG-T-G-C-G------------G--T-TT-G-T-T--
---------AA----T-C-G-A--------G-A-A--G--TG--A-AA-GC--C-C-CG-G-G-----CT-C-AA------C-C-T-G-G-G-A--A-C----T-G--C-A---T--T-------T--CG-A-A---C-------T--G-G--CA--A-A-C----T-A-G-A-G-T--G---T-GA-----TA-G-A--------G-G-G-T---GG-T------AG--A--ATT---
-T-C-A-G-GT--GT-A-G-CG-GT--G---A--A-A---TG-C-GT-AG--AG-A-TC--T-G--A-A-G-G-A-AT-A-CC---GA--T--G--GC-GAA-G--G-C--A-------G--C-C-A-C---CTG--G--GT-C-A--------------------A-C---A-C-T--GA--CG-C-T-C--A-TG--T-A-CG-A--AA-G-C---G-TG--
GG-G--AG-C-A-AA--------CG--GG Bacteria(100);Proteobacteria(100);Gammaproteobacteria(100);Alteromonadales(100);unclassified(99);unclassified(99);unclassified(99);unclassified(99);unclassified(99);

Otu0095 0 0 0 0 0 0 20 0 0 0 0 0 0 0 0 0 0 0 0 6 0 1 0 0 0 0 0 0
3 0 0 1 20 0 0 0 0 0 7 0 24 0 29 0 1 22 0 0 0 0 0 0 14 0 0 0 0
0 0 0 1 0 0 0 0 0 0 0 0 0 0 2 0 27 93 0 0 0 0 0 0 0 0 0 0 0
36 0 127 22 0 42 0 0 0 36 0 0 0 77 0 26 0 34 12 12 0 3 0 0 0 0 0
M02255_77_000000000-ADUT3_1_1112_9263_9387 T----AC---GT-AG-GGT--------GCG-A-G------C--G--T---T--GT-C-CGG-AA------TT-A--T-T--GG-GC-------GT--A-----AA-GA-GC-TT-------G-TA-G-G-C-G------------G--C-TT-G-T-C-----------GC-
---G-T-C-T--------G-C-T--G--TG--A-AA-GC--C-C-GG-G-G-----CT-T-AA------C-C-C-C-G-G-G--T-C----T-G--C-A---G--T-------G--GG-T-A---C-------G--G-G--CA--G-G-C----T-A-G-A-G-T--G---T-GG-----TA-G-G--------G-G-A-G---AC-T------GG--A--ATT----C-C-T-G-
GT--GT-A-G-CG-GT--G---G--A-A---TG-C-GC-AG--AT-A-TC--A-G--G-A-G-G-A-AC-A-CC---GA--T--G--GC-GAA-G--G-C--A-------G--G-T-C-T---CTG--G--GC-C-A--------------------T-T---A-C-T--GA--CG-C-T-G--A-GA--A-G-CG-A--AA-G-C---G-TG--GG-G--AG-
C-G-AA--------CA--GG Bacteria(100);Actinobacteria(100);Actinobacteria(100);Actinobacteridae(100);Actinomycetales(100);Micrococcineae(100);Intrasporangiaceae(70);unclassified(66);unclassified(66);

Otu0096 0 0 0 0 0 0 0 0 0 0 0 0 0 0 0 0 0 0 0 0 0 0 16 5 0 0 0 0
0 0 0 74 0 0 0 0 0 0 0 0 0 0 137 43 0 0 0 0 0 0 0 71 7 0 0 0 0
0 0 0 0 0 0 0 0 0 0 0 0 0 0 19 0 12 16 0 0 0 0 6 0 0 0 0 0 0
0 0 0 0 0 0 6 0 0 0 0 0 0 0 0 34 0 0 32 0 0 0 0 0 0 0 0
M02255_77_000000000-ADUT3_1_1108_16420_15451 T----AC---GA-AG-GGG--------GCT-A-G------C--G--T---T--GT-T-CGG-AT------TT-A--C-T--GG-GC-------GT--A-----AA-GC-GC-AC-------G-TA-G-G-C-G------------G--A-TT-G-T-T-----
------AA----G-T-C-A--------G-G-G--G--TG--A-AA-TC--C-T-GG-A-G-----CT-C-AA------C-T-C-C-A-G-A--A-C----T-G--C-C---T--T-------T--GA-T-A---C-------T--G-G--CA--A-T-C----T-C-G-A-G-T--C---C-GG-----AA-G-A--------G-G-T-A---AG-T------GG--A--ACT----C-
C-T-A-GT--GT-A-G-AG-GT--G---G--A-A---TT-C-GT-AG--AT-A-TT--A-G--G-A-A-G-A-AC-A-CC---AG--T--G--GC-GAA-G--G-C--G-------G--C-T-T-A---CTG--G--TC-C-G--------------------G-T---A-C-T--GA--CG-C-T-G--A-GG--T-G-CG-A--AA-G-C---G-TG--GG-G-
-AG-C-A-AA--------CA--GG Bacteria(100);Proteobacteria(100);Alphaproteobacteria(100);Rhizobiales(100);Hyphomicrobiaceae(100);Devosia(100);unclassified(100);unclassified(100);unclassified(100);

Otu0097 0 0 0 0 0 63 16 106 195 30 0 0 0 0 0 0 0 6 5 0 21 0 0 0 13 0 0 0
5 0 3 1 0 0 0 33 0 0 58 0 0 0 66 0 0 0 0 1 0 0 0 37 17 23 0 0 115
20 0 0 0 6 0 0 0 3 0 4 0 25 0 0 17 21 128 0 0 0 0 17 0 0 0 1 12 88
0 0 0 0 124 0 11 3 0 0 0 18 0 0 0 64 0 0 0 0 0 0 0 1 0 0 0
M02255_77_000000000-ADUT3_1_1110_27089_11132 T----AC---GG-AG-GGG--------GTT-A-G------C--G--T---T--GT-T-CGG-AA------TG-A--C-T--GG-GC-------GT--A-----AA-GC-GC-GC-------G-TA-G-G-C-G------------G--A-TC-A-T-C----
-------AA----G-T-C-G--------G-G-G--G--TG--A-AA-TC--C-C-GG-G-G-----CT-C-AA------C-C-C-C-G-G-A--A-T----G-G--C-C---T--T-------C--GA-G-A---C-------T--G-G--TG--G-T-C----T-T-G-A-G-T--T---C-GA-----GA-G-A--------G-G-T-G---AG-T------GG--A--ATT----
C-C-G-A-GT--GT-A-G-AG-GT--G---A--A-A---TT-C-GT-AG--AT-A-TT--C-G--G-A-G-G-A-AC-A-CC---AG--T--G--GC-GAA-G--G-C--G-------G--C-T-C-A---CTG--G--CT-C-G--------------------A-C---A-C-T--GA--CG-C-T-G--A-GG--T-G-CG-A--AA-G-C---G-TG--GG-
G--AG-C-A-AA--------CA--GG Bacteria(100);Proteobacteria(100);Alphaproteobacteria(100);Rhodobacterales(95);Rhodobacteraceae(95);unclassified(95);unclassified(95);unclassified(95);unclassified(95);

Otu0098 0 0 0 1 0 0 0 0 0 1 232 10 0 0 2 0 0 0 4 1 0 0 0 0 0 0 0 0
0 0 0 0 0 0 0 0 0 0 7 0 0 0 0 0 1 1 12 0 2 0 0 0 0 0 0 0 0
0 0 0 0 0 0 0 0 6 0 0 0 0 0 0 1 0 2 0 0 0 1 17 0 0 0 0 0 0
0 0 0 0 0 0 2 4 1 6 0 0 0 0 0 0 0 0 0 0 0 0 0 0 0 0 0
M02255_77_000000000-ADUT3_1_1112_19794_4386 .....AC----T-AC-GT---------GCC-A-G------C--A--G---C--CG-C-GGT-AA------TA-C--G-T--AG-GT-------GG--C-----AA-GC-GT-TG-------T-CC-G-G---A------------T--T-TC-T-T-T-----------AA----G-
T-C-T--------G-A-T--G--TG--A-AA-GC--C-C-CC-G-G-----CT-C-AA------C-C-G-G-G-G-A--G------G-GT-C-A---T--T-------G--GA-A-A---C-------T--G-G--AG--A-A-C----T-T-G-A-G-T--G---C-AG-----AA-G-A--------G-G-A-G---AG-T------GG--A--ATT----C-C-A-C-GT--
GT-A-G-CG-GT--G---A--A-A---TG-C-GT-AG--AT-A-TG--T-G--G-A-G-G-A-AC-A-CC---AG--T--G--GC-GAA-G--G-C--G-------A--C-T-C-T---CTG--G--TC-T-G--------------------T-A---A-C-T--GA--CG-C-T-G--A-GG--C-T-CG-A--AA-G-C---G-TG--GG-G--AG-C-A-
AA--------CA--GG Bacteria(100);Firmicutes(100);Bacilli(100);unclassified(100);unclassified(100);unclassified(100);unclassified(100);unclassified(100);unclassified(100);

Otu0099 0 22 53 18 0 0 56 69 42 0 0 0 0 0 0 0 0 0 0 0 0 0 1 0 0 0 0 0
0 0 0 0 22 0 0 29 22 0 0 7 0 36 25 0 0 0 0 0 73 0 0 14 0 0 0 0 0
20 0 0 0 0 0 0 0 0 0 0 0 0 0 0 0 4 16 0 0 0 0 0 0 0 0 0 0 12
0 0 38 0 0 0 0 0 0 0 0 0 0 68 0 0 4 15 0 0 0 17 0 0 7 0 0
M02255_76_000000000-ADUNE_1_1116_10454_8040 .....AC----T-AC-GT---------GCC-A-G------C--A--G---C--CG-C-GGT-AA------TA-C--G-T--AG-GT-------GG--C-----AA-GC-G--TT-------G----T-C-C-G------------G--A-CT-T-T-T-----------AA----G-
T-C-T--------G-A-T--G--TG--A-AA-GC--C-C-CC-G-G-----CT-T-AA------C-C-G-G-G-G-A--G------G-GT-C-A---T--T-------G--GA-A-A---C-------T--G-G--GA--G-A-C----T-T-G-A-G-T--G---C-AG-----AA-G-A--------G-G-A-A---AG-C------GG--A--ATT----C-C-A-T-GT--
GT-A-G-CG-GT--G---A--A-A---TG-C-GT-AG--AT-A-TA--T-G--G-A-G-G-A-AC-A-CC---AG--T--G--GC-GAA-G--G-C--G-------G--C-T-T-T---CTG--G--TC-T-G--------------------T-A---A-C-T--GA--CG-C-T-G--A-GG--C-T-CG-A--AA-G-C---G-TG--GG-G--AG-C-A-
AA--------CA--GG Bacteria(100);Firmicutes(100);Bacilli(100);Lactobacillales(72);unclassified(72);unclassified(72);unclassified(72);unclassified(72);unclassified(72);

Otu0100 0 0 0 0 0 0 0 0 0 0 0 0 0 0 0 0 0 0 0 0 0 0 8 0 0 0 7 0
0 0 0 1 0 19 0 0 33 9 15 44 0 0 0 0 0 0 0 0 0 0 0 28 5 1 0 0 0
0 0 0 0 0 0 0 0 0 0 0 0 0 0 28 0 6 3 0 0 0 0 0 0 5 0 0 0 0
17 0 0 0 0 0 0 0 0 0 0 0 0 0 0 0 0 0 0 0 0 0 0 4 0 0 0
M02255_77_000000000-ADUT3_1_1113_14554_17806 T----AC---GT-AG-GGC--------GCA-A-G------C--G--T---T--GT-C-CGG-AA------TT-A--T-T--GG-GC-------GT--A-----AA-GA-GC-TC-------G-TA-G-G-C-G-------------G--C-TT-G-T-C------
-----AC----G-T-C-G--------G-A-T--G--TG--A-AA-GC--C-C-GG-G-G-----CT-T-AA------C-C-C-C-G-G-G--T-C----T-G--C-A---T--T-------C--GA-T-A---C-------G--G-G--CT--A-G-C----T-A-G-A-G-T--G---T-GG-----TA-G-G--------G-G-A-G---AT-C------GG--A--ATT----C-
C-T-G-GT--GT-A-G-CG-GT--G---A--A-A---TG-C-GC-AG--AT-A-TC--A-G--G-A-G-G-A-AC-A-CC---GG--T--G--GC-GAA-G--G-C--G-------G--A-T-C-T---CTG--G--GC-C-A--------------------T-T---A-C-T--GA--CG-C-T-G--A-GG--A-G-CG-A--AA-G-C---G-TG--GG-
G--AG-C-G-AA--------CA--GG Bacteria(100);Actinobacteria(100);Actinobacteria(100);Actinobacteridae(100);Actinomycetales(100);Streptomycineae(100);Streptomycetaceae(100);Streptomyces(52);unclassified(52);

Otu0101 0 0 0 0 0 53 27 0 0 0 0 0 0 7 0 0 0 0 0 0 0 0 0 5 0 4 2 23
9 0 5 0 0 0 26 191 0 0 39 25 0 0 0 0 0 0 0 0 0 13 0 0 20 0 0 154
0 0 0 0 0 0 0 0 0 0 0 0 0 0 0 9 0 39 0 52 0 0 14 0 0 1 0 43 0
0 90 0 0 0 0 0 0 0 0 0 0 0 2 0 104 162 0 14 0 0 559 52 3 34 488 0
M02255_77_000000000-ADUT3_1_1111_8657_12448 T----AC---GA-AG-GGG--------GCT-A-G------C--G--T---T--GT-T-CGG-AA------TT-A--C-T--GG-GC-------GT--A-----AA-GG-GC-GC-------G-TA-G-G-C-G-------------G--T-AC-T-T-C-----------AA-
---G-T-C-A--------G-G-C--G--TG--A-AA-GC--C-C-CG-G-G-----CT-C-AA------C-C-T-G-G-G-A--A-C----C-G--C-G---C--T-------T--GA-G-A---C-------T--G-G--AG--A-A-C----T-A-G-A-G-T--T---C-GG-----GA-G-A--------G-G-A-T---GG-T------GG--A--ATT----C-C-C-A-
GT--GT-A-G-AG-GT--G---A--A-A---TT-C-GT-AG--AT-A-TT--G-G--G-A-A-G-A-AC-A-CC---GA--T--G--GC-GAA-G--G-C--A-------G--C-C-A-T---CTG--G--AC-C-G--------------------A-C---A-C-T--GA--CG-C-T-G--A-GG--C-G-CG-A--AA-G-C---G-TG--GG-G--AG-
C-A-AA--------CA--GG Bacteria(100);Proteobacteria(100);Alphaproteobacteria(100);Rhodospirillales(100);Rhodospirillaceae(100);Skermanella(100);unclassified(100);unclassified(100);unclassified(100);

Otu0102 73 23 2 99 0 0 7 1 0 0 0 1 0 0 0 0 0 0 0 0 11 0 0 33 0 0 0 0
0 98 7 36 0 11 9 0 0 35 16 0 5 11 14 0 30 35 0 0 0 68 0 58 0 0 0 0 0
0 0 0 0 0 0 0 8 0 1 0 0 0 1 0 50 9 4 0 0 0 0 1 0 24 0 47 1 65
84 35 16 23 64 0 0 3 0 0 0 0 2 30 0 0 26 0 0 23 0 0 0 4 13 400 0
M02255_77_000000000-ADUT3_1_1113_20397_14953 T----AC---GT-AG-GGC--------GCA-A-G------C--G--T---T--GT-C-CGG-AA------TT-A--T-T--GG-GC-------GT--A-----AA-GA-GC-TT-------G-TA-G-G-T-G-------------G--C-TT-G-T-C------
-----GC----G-T-C-T--------G-C-C--G--TG--A-AA-AC--C-C-GA-G-G-----CT-C-AA------C-C-T-C-G-G-G--C-G----T-G--C-G---G--T-------G--GG-T-A---C-------G--G-G--CA--G-G-C----T-A-G-A-G-T--G---T-GG-----TA-G-G--------G-G-A-G---AC-T------GG--A--ACT----
C-C-T-G-GT--GT-A-G-CG-GT--G---A--A-A---TG-C-GC-AG--AT-A-TC--A-G--G-A-A-G-A-AC-A-CC---GA--T--G--GC-GAA-G--G-C--A-------G--G-T-C-T---CTG--G--GC-C-A--------------------T-T---A-C-T--GA--CA-C-T-G--A-GA--A-G-CG-A--AA-G-C---A-TG--GG-
T--AG-C-G-AA--------CA--GG Bacteria(100);Actinobacteria(100);Actinobacteria(100);Actinobacteridae(100);Actinomycetales(100);Micrococcineae(100);Dermabacteraceae(100);Brachybacterium(100);unclassified(100);

Otu0103 0 0 0 0 0 0 0 0 62 0 0 0 0 0 0 0 0 0 0 2 10 6 0 0 0 0 0 5
0 0 0 0 0 0 0 0 0 50 3 0 0 0 172 0 0 0 0 0 0 0 0 0 0 0 0 0 0
0 0 0 0 0 3 0 0 0 0 0 0 0 0 0 0 0 30 0 0 0 0 0 149 0 0 0 0 0
52 0 224 0 0 0 0 0 0 0 0 0 0 0 0 0 43 0 25 0 0 0 0 0 0 0 0
M02255_77_000000000-ADUT3_1_1103_13413_21182 T----AC---GA-AG-GGG--------GCT-A-G------C--G--T---T--GC-T-CGG-AA------TC-A--C-T--GG-GC-------GT--A-----AA-GG-GC-GC-------G-TA-G-G-C-G-------------G--C-CG-A-T-C----
-------AA----G-T-C-A--------G-A-G--G--TG--A-AA-GC--C-C-AA-G-G-----CT-C-AA------C-C-T-T-G-G-A--A-T----T-G--C-C---T--T-------T--GA-T-A---C-------T--G-T--TC--G-G-C----T-C-G-A-G-A--C---C-GG-----AA-G-A--------G-G-T-T---AG-T------GG--A--ACT----
G-C-G-A-GT--GT-A-G-AG-GT--G---A--A-A---TT-C-GT-AG--AT-A-TT--C-G--C-A-A-G-A-AC-A-CC---AG--T--G--GC-GAA-G--G-C--G-------G--C-T-A-A---CTG--G--TC-C-G--------------------G-T---T-C-T--GA--CG-C-T-G--A-GG--C-G-CG-A--AA-G-C---G-TG--GG-
G--AG-C-A-AA--------CA--GG Bacteria(100);Proteobacteria(100);Alphaproteobacteria(100);Rhizobiales(56);unclassified(56);unclassified(56);unclassified(56);unclassified(56);unclassified(56);

Otu0104 0 0 0 0 17 16 0 0 0 14 11 7 1 4 3 0 0 1 1 3 0 0 2 8 0 3 10 0
18 0 0 1 1 0 0 0 0 0 0 0 0 0 0 0 18 0 0 0 2 0 0 0 0 0 0 0 0
0 0 0 0 0 0 0 0 0 0 0 0 0 0 0 0 0 0 0 0 0 19 13 3 0 0 0 2 0
9 0 0 0 0 5 9 0 1 4 0 0 0 12 0 6 4 0 30 0 2 10 23 41 6 0 0
M02255_77_000000000-ADUT3_1_1113_3223_18395 C----TG---TG-CC-AGC--------AGC-C-G------C--G--G---T--AA-TACAGAGG------GT-G--C-A--AG-CG-------TT--A----ATC-GG-AATTA-------C-TA-G-G-T-G-------------G--C-TC-T-A-T-----------
AA----G-T-C-A--------G-A-T--G--TG--A-AA-TC--C-C-CG-G-G-----CT-T-AA------C-C-T-G-G-G-A--A-C----T-G--C-A---T--C-------T--GA-A-A---C-------T--G-T--AG--A-G-C----T-A-G-A-G-T--A---T-GT-----GA-G-A--------G-G-A-A---GG-T------AG--A--ATT----C-C-A-
G-GT--GT-A-G-CG-GT--G---A--A-A---TG-C-GT-AG--AG-A-TC--T-G--G-A-G-G-A-AT-A-CC---GA--T--G--GC-GAA-G--G-C--A-------G--C-C-T-T---CTG--G--CA-T-A---------------------A-T---A-C-T--GA--CA-C-T-G--A-GG--C-T-CG-A--AA-G-C---G-TG--GG-T--AG-
C-A-AA--------CA--GG Bacteria(100);Proteobacteria(100);Gammaproteobacteria(100);Pseudomonadales(100);Moraxellaceae(100);Psychrobacter(100);unclassified(100);unclassified(100);unclassified(100);

Otu0105 0 0 0 0 178 0 0 0 29 0 0 0 0 0 0 0 0 0 0 0 5 0 0 0 0 0 0 0
0 2 0 0 0 0 0 105 0 47 2 0 0 0 0 0 0 0 0 0 0 0 0 0 0 0 0 0
0 0 0 0 0 0 0 0 0 0 0 0 4 0 78 8 0 129 0 0 0 0 0 106 0 0 0 0 0
0 0 0 0 0 0 0 0 0 0 4 0 0 0 0 36 0 0 0 0 0 0 0 0 0 0 0
M02255_77_000000000-ADUT3_1_1101_15477_10244 T----AC---GG-AG-GGA--------GCT-A-G------C--G--T---T--GT-T-CGG-AA------TT-A--C-T--GG-GC-------GT--A-----AA-GC-GC-AC-------G-TA-G-G-C-G------------G--C-TT-T-G-T-----
------AA----G-T-T-A--------G-A-G--G--TG--A-AA-GC--C-C-GG-G-G-----CT-C-AA------C-C-C-C-G-G-A--A-T----T-G--C-C---T--T-------T--AA-G-A---C-------T--G-C--AT--C-G-C----T-T-G-A-A-T--C---C-GG-----GA-G-A--------G-G-T-G---AG-T------GG--A--ATT----C-
C-G-A-GT--GT-A-G-AG-GT--G---A--A-A---TT-C-GT-AG--AT-A-TT--C-G--G-A-A-G-A-AC-A-CC---AG--T--G--GC-GAA-G--G-C--G-------G--C-T-C-A---CTG--G--AC-C-G--------------------G-T---A-T-T--GA--CG-C-T-G--A-GG--T-G-CG-A--AA-G-C---G-TG--GG-
G--AG-C-A-AA--------CA--GG Bacteria(100);Proteobacteria(100);Alphaproteobacteria(100);Sphingomonadales(100);unclassified(97);unclassified(97);unclassified(97);unclassified(97);unclassified(97);

Otu0106 0 0 0 0 0 0 0 0 0 0 0 0 0 8 3 0 4 1 0 0 0 1 0 0 0 0 0 0
0 0 0 0 0 0 0 0 0 0 0 0 0 0 1 0 0 0 10 1 0 0 0 0 0 5 0 3 0
0 0 0 0 0 0 1 19 0 19 1 6 8 8 7 11 0 0 0 0 0 0 0 0 0 0 0 0 0
0 0 0 0 0 0 0 1 2 0 3 0 26 0 0 0 0 0 0 0 0 0 0 0 0 0 0
M02255_76_000000000-ADUNE_1_2104_5482_20190 G----AC---AG-AA-TAA--------GCA-C-C------G--G--C---T--AACT-CTG-TG------CC-A--G-C--AG-CC-------GC--G-----GT-AA-TA-CA-------G-AG-G-G-T-G------------C--A-AG-C-G-T----------
TA----A-T-C-G--------G-A-A--G--TG--A-AA-TC--C-C-CG-G-G-----CT-C-AA------C-C-T-G-G-G-A--A-C----T-G--C-A---T--T-------C--AA-A-A---C-------T--G-A--CG--A-G-C----T-A-G-A-G-T--A---T-GG-----TA-G-A--------G-G-G-T---GG-T------GG--A--ATT----T-C-C-
T-GT--GT-A-G-CG-GT--G---A--A-A---TG-C-GT-AG--AT-A-TA--G-G--A-A-G-G-A-AC-A-CC---AG--T--G--GC-GAA-G--G-C--G-------A--C-C-A-C---CTG--G--AC-T-G--------------------A-T---A-C-T--GA--CA-C-T-G--A-GG--T-G-CG-A--AA-G-C---G-TG--GG-G--
AG-C-A-AA--------CA--GG Bacteria(100);Proteobacteria(100);Gammaproteobacteria(100);unclassified(88);unclassified(88);unclassified(88);unclassified(88);unclassified(88);unclassified(88);

Otu0107 0 0 0 0 0 0 89 0 0 0 0 0 0 0 0 0 0 0 0 0 0 0 0 0 0 0 0 25
1 0 71 0 46 0 0 180 0 0 0 17 49 49 101 136 0 0 0 0 0 0 0 0 73 0 0 49 0
0 55 203 0 76 32 0 0 54 28 0 0 0 0 32 5 162 29 0 12 0 0 28 0 0 0 1 440 31
118 72 204 0 51 0 0 32 0 60 0 0 0 61 0 0 0 0 0 35 0 0 0 0 0 0 51
M02255_77_000000000-ADUT3_1_2104_9065_11564 T----AC---GA-AG-GGG--------GCT-A-G------C--G--T---T--GC-T-CGG-AA------TT-A--C-T--GG-GC-------GT--A-----AA-GG-GA-GC-------G-TA-G-G-C-G------------G--A-CA-T-T-T----------
AA----G-T-C-A--------G-G-G--G--TG--A-AA-TC--C-C-GG-G-G-----CT-C-AA------C-C-T-C-G-G-A--A-T----T-G--C-C---T--T-------T--GA-T-A---C-------T--G-G--GT--G-T-C----T-T-G-A-G-T--A---T-GA-----GA-G-A--------G-G-T-A---TG-T------GG--A--ACT----C-C-G-
A-GT--GT-A-G-AG-GT--G---A--A-A---TT-C-GT-AG--AT-A-TT--C-G--G-A-A-G-A-AC-A-CC---AG--T--G--GC-GAA-G--G-C--G-------A--C-A-T-A---CTG--G--CT-C-A--------------------T-T---A-C-T--GA--CG-C-T-G--A-GG--C-T-CG-A--AA-G-C---G-TG--GG-G--AG-
C-A-AA--------CA--GG Bacteria(100);Proteobacteria(100);Alphaproteobacteria(100);Caulobacterales(100);Caulobacteraceae(100);Brevundimonas(100);unclassified(100);unclassified(100);unclassified(100);

Otu0108 0 0 0 0 0 0 0 0 43 0 0 0 0 0 0 0 0 0 0 0 0 0 0 0 0 0 0 0
0 0 54 0 0 0 0 83 0 0 0 0 0 0 0 0 0 0 0 0 0 0 0 0 0 0 0 0 271
0 0 0 0 0 0 0 0 0 0 0 0 0 0 0 2 0 18 0 0 0 0 0 0 0 0 0 0 0
0 0 0 0 24 0 0 0 0 10 0 0 0 0 0 0 0 0 0 0 0 0 0 0 0 0 0
M02255_77_000000000-ADUT3_1_1102_20113_10044 T----AC---GT-AG-GGG--------TCG-A-G------C--G--T---T--GT-C-CGG-AG------TT-A--C-T--GG-GC-------GT--A-----AA-GC-GT-GC-------G-CA-G-G-C-G------------G--C-TC-A-T-T-----
------GC----G-C-C-C--------G-G-C--G--TG--A-AA-GC--C-C-CC-G-G-----CT-C-AA------C-C-G-G-G-G-A--G-G----G-T--C-G---T--C-------G--GG-G-A---C-------G--G-G--TG--A-G-C----T-T-G-A-G-G--G---T-AT-----CA-G-G--------G-G-C-T---GG-T------GG--A--ATT----
C-C-C-G-GT--GT-A-G-TG-GT--G---A--A-A---TG-C-GT-AG--AG-A-TC--G-G--G-A-G-G-A-AC-A-CC---CG--T--G--GC-GAA-G--G-C--G-------G--C-C-A-G---CTG--G--GA-T-A--------------------C-A---C-C-T--GA--CG-C-T-G--A-GG--C-A-CG-A--AG-G-C---G-TG--
GG-G--AG-C-G-AA--------CG--GG Bacteria(100);Chloroflexi(100);Thermomicrobia(100);JG30-KF-CM45(100);unclassified(100);unclassified(100);unclassified(100);unclassified(100);unclassified(100);

Otu0109 36 17 0 39 18 9 11 15 11 3 0 0 0 1 0 0 0 0 3 0 2 0 0 0 0 1 0 8
4 0 27 16 9 2 16 0 4 19 24 8 17 9 36 52 20 29 0 40 4 1 0 9 2 0 1 1 0
4 1 13 0 0 0 0 1 0 0 2 0 0 0 6 0 15 2 0 9 0 0 4 0 0 83 8 133 26
17 27 14 31 66 15 5 4 0 15 1 0 1 37 0 2 14 46 1 94 143 10 5 0 11 15 16
M02255_77_000000000-ADUT3_1_1105_10953_11523 T----AA---CT-AC-GTG--------CC--A-G------C--A--G---C--CG-C-GGT-AA------TA-C--G-T--AG-GT-------CC--C-----GA-GC-G--TT-------G----T-C-C-G------------G--T-TT-G-A-T----------
-AA----G-T-C-T--------G-A-A--G--TT--A-AA-GG--C-T-GT-G-G-----CT-C-AA------C-C-A-T-A-G-T--T------C-G--C-T---T--T-------G--GA-A-A---C-------T--G-T--CA--A-A-C----T-T-G-A-G-T--G---C-AG-----AA-G-G--------G-G-A-G---AG-T------GG--A--ATT----C-C-A-
T-GT--GT-A-G-CG-GT--G---A--A-A---TG-C-GT-AG--AT-A-TA--T-G--G-A-G-G-A-AC-A-CC---GG--T--G--GC-GAA-A--G-C--G-------G--C-T-C-T---CTG--G--TC-T-G--------------------T-A---A-C-T--GA--CG-C-T-G--A-GG--C-T-CG-A--AA-G-C---G-TG--GG-G--AG-
C-G-AA--------CA--GG Bacteria(100);Firmicutes(100);Bacilli(100);Lactobacillales(100);Streptococcaceae(100);Streptococcus(100);unclassified(100);unclassified(100);unclassified(100);

Otu0110 0 0 0 0 0 0 0 0 0 0 0 0 0 0 0 0 0 0 0 0 0 0 0 0 0 0 0 0
222 0 1 1 0 0 0 63 0 0 13 1 0 0 1 0 0 87 0 0 0 0 0 0 0 0 0 1 0
0 0 0 0 18 0 0 0 0 2 0 6 0 9 71 25 0 0 0 388 183 0 1 0 93 0 0 0 93
0 0 0 0 36 1771 6 50 0 405 0 1401 3 7 7 0 0 0 0 0 0 0 29 0 0 0 36
M02255_77_000000000-ADUT3_1_1105_24267_16865 T----AC---GT-AT-GTC--------GCA-A-G------C--G--T---T--AT-C-CGG-AT------TT-A--T-T--GG-GC-------GT--A-----AA-GC-GC-GT-------C-TA-G-G-C-G------------G--C-AA-G-G-A------
-----AA----G-T-C-T--------G-A-T--G--TG--A-AA-AT--G-C-GG-G-G-----CT-C-AA------C-T-C-C-G-T-A--T------T-G--C-G---T--T-------G--GA-A-A---C-------T--G-C--CT--T-A-C----T-A-G-A-G-T--A---C-TG-----GA-G-A--------G-G-T-A---GG-C------GG--A--ACT----A-
C-A-A-GT--GT-A-G-AG-GT--G---A--A-A---TT-C-GT-AG--AT-A-TT--T-G--T-A-G-G-A-AT-G-CC---GA--T--G--GG-GAA-G--C-C--A-------G--C-C-T-A---CTG--G--AC-A-G--------------------A-T---A-C-T--GA--CG-C-T-A--A-AG--C-G-CG-A--AA-G-C---G-TG--GG-T--
AG-C-A-AA--------CA--GG Bacteria(100);Fusobacteria(100);Fusobacteria(100);Fusobacteriales(100);Fusobacteriaceae(100);Fusobacterium(100);unclassified(100);unclassified(100);unclassified(100);

Otu0111 0 0 0 0 0 0 0 0 0 0 2 11 58 17 10 0 0 7 3 0 6 0 0 1 2 1 0 0
0 0 0 0 0 0 0 0 0 1 0 0 0 0 0 0 1 0 5 0 0 0 0 0 0 25 2 22 0
0 1 4 0 0 0 0 9 2 39 0 0 2 60 0 3 0 0 0 0 0 0 0 0 0 0 0 0 0
0 0 0 1 0 0 5 8 5 0 11 1 13 0 0 12 0 0 0 0 0 0 0 0 1 0 0
M02255_76_000000000-ADUNE_1_1110_23811_11194 C----TA---AC-TC-TGT--------GCC-A-G------C-----A---G--CC-G-CGG-TA------AT-A--C-A--GA-GG-------GT-----------GC-AA-GC-------G-TT-A-A-T-C------------G--G-AA-G-T-T---------
--AA----G-T-T-G--------G-A-T--G--TG--A-AA-TC--C-C-CG-G-G-----CT-C-AA------C-C-T-G-G-G-A--A-C----T-G--C-A---T--T-------C--AA-A-A---C-------T--G-A--CT--G-A-C----T-A-G-A-G-T--A---T-GG-----TA-G-A--------G-G-G-T---GG-T------GG--A--ATT----T-C-
C-T-GT--GT-A-G-CG-GT--G---A--A-A---TG-C-GT-AG--AT-A-TA--G-G--A-A-G-G-A-AC-A-CC---AG--T--G--GC-GAA-G--G-C--G-------A--C-C-A-C---CTG--G--AC-T-G--------------------A-T---A-C-T--GA--CA-C-T-G--A-GG--T-G-CG-A--AA-G-C---G-TG--GG-G--
AG-C-A-AA--------CA--GG Bacteria(100);Proteobacteria(100);Gammaproteobacteria(100);Pseudomonadales(100);Pseudomonadaceae(100);unclassified(76);unclassified(76);unclassified(76);unclassified(76);

Otu0112 0 0 0 0 0 0 0 0 0 0 0 1 8 2 0 1 0 3 0 1 3 0 0 0 0 0 0 0
0 0 0 0 0 0 0 0 0 0 0 0 0 0 1 0 0 0 0 0 0 0 0 2 13 24 8 0
4 0 0 0 1 0 0 0 6 1 0 3 2 11 0 0 0 0 0 0 0 1 0 0 0 0 0 0 0
0 0 0 0 0 0 0 1 2 0 8 0 3 0 0 2 0 0 1 0 1 0 0 0 0 0 0
M02255_77_000000000-ADUT3_1_1112_9961_8135 G----CC---AG-CA-GCC--------GCG-G-T------A--A--T---A--CA-G-AGG-GT------GC-A--A-G--CG-TT-------AA--T-----CG-GA-AT-TA-------C-TA-G-G-T-G------------G--T-TT-G-T-T----------AA--
--G-T-T-G--------G-A-T--G--TG--A-AA-TC--C-C-CG-G-G-----CT-C-AA------C-C-T-G-G-G-A--A-C----T-G--C-A---T--T-------C--AA-A-A---C-------T--G-A--CA--A-G-C----T-A-G-A-G-T--A---T-GG-----TA-G-A--------G-G-G-T---GG-T------GG--A--ATT----T-C-C-T-
GT--GT-A-G-CG-GT--G---A--A-A---TG-C-GT-AG--AT-A-TA--G-G--A-A-G-G-A-AC-A-CC---AG--T--G--GC-GAA-G--G-C--G-------A--C-C-A-C---CTG--G--AC-T-G--------------------A-T---A-C-T--GA--CA-C-T-G--A-GG--T-G-CG-A--AA-G-C---G-TG--GG-G--AG-
C-A-AA--------CA--GG Bacteria(100);Proteobacteria(100);Gammaproteobacteria(100);Pseudomonadales(100);Pseudomonadaceae(100);Pseudomonas(86);unclassified(86);unclassified(86);unclassified(86);

Otu0113 0 0 0 0 0 0 0 0 0 0 0 0 0 0 0 0 0 0 0 0 0 0 0 0 0 0 0 0
0 0 0 0 0 0 0 0 0 0 0 0 0 0 12 0 0 0 0 0 0 0 0 0 0 0 0 0 0
0 0 0 0 0 0 0 0 0 0 0 0 0 0 0 0 0 0 0 0 0 0 0 0 80 0 0 0 0
56 0 0 113 39 0 18 0 0 31 0 0 0 0 0 0 0 0 20 0 0 0 0 0 0 0 0
M02255_77_000000000-ADUT3_1_1105_19552_18674 T----AC---GA-AG-GGG--------GCT-A-G------C--G--T---T--GT-T-CGG-AA------TC-A--C-T--GG-GC-------GT--A-----AA-GC-GC-AC-------G-TA-G-G-C-G------------G--A-TC-T-T-T-----
------AA----G-T-C-A--------G-A-G--G--TG--A-AA-TC--C-C-AA-G-G-----CT-C-AA------C-C-T-T-G-G-A--A-C----T-G--C-C---T--T-------T--GA-T-A---C-------T--G-G--GG--A-T-C----T-C-G-A-G-T--C---C-GG-----GA-G-A--------G-G-T-G---AG-T------GG--A--ACT----
G-C-G-A-GT--GT-A-G-AG-GT--G---A--A-A---TT-C-GT-AG--AT-A-TT--C-G--C-A-A-G-A-AC-A-CC---AG--T--G--GC-GAA-G--G-C--G-------G--C-T-C-A---CTG--G--CC-C-G--------------------G-T---A-C-T--GA--CG-C-T-G--A-GG--T-G-CG-A--AA-G-C---G-TG--GG-
G--AG-C-A-AA--------CA--GG Bacteria(100);Proteobacteria(100);Alphaproteobacteria(100);Rhizobiales(100);unclassified(100);unclassified(100);unclassified(100);unclassified(100);unclassified(100);

Otu0114 0 0 0 0 0 0 0 0 0 0 0 0 0 3 0 0 0 4 0 0 1 4 2 0 0 0 0 0 0
0 0 0 0 0 0 0 0 0 0 0 0 0 0 0 0 0 0 20 0 0 0 5 0 0 1 0 3 0
0 0 0 11 0 1 0 0 0 0 0 0 0 1 0 0 0 0 0 0 0 0 0 0 0 0 0 0 0
0 0 0 0 0 0 1 2 2 0 0 2 1 0 1 0 0 0 0 0 0 0 0 0 0 0 0
M02255_76_000000000-ADUNE_1_1115_9993_22072 G----AC---AG-AA-TAA--------GCA-C-C------G--G--C---T--AA---C-T-CT------GT-G--C-C--AG-CA-------GC--C-----GC-G--GT-AA-------T-AC-A-G-A-G------------G--T-GT-G-C-A----------AG---
-C-G-T-T--------A-A-T--C--GG--A-AA-TC--C-C-CG-G-G-----CT-C-AA------C-C-T-G-G-G-A--A-C----T-G--C-A---T--C-------C--AA-A-A---C-------T--G-G--CA--A-G-C----T-A-G-A-G-T--A---T-GG-----TA-G-A--------G-G-G-T---AG-T------GG--A--ATT----T-C-C-T-GT-
-GT-A-G-CG-GT--G---A--A-A---TG-C-GT-AG--AT-A-TA--G-G--A-A-G-G-A-AC-A-CC---AG--T--G--GC-GAA-G--G-C--G-------A--C-T-A-C---CTG--G--AC-T-G--------------------A-T---A-C-T--GA--CA-C-T-G--A-GG--T-G-CG-A--AA-G-C---G-TG--GG-G--AG-C-A-
AA--------CA--GG Bacteria(100);Proteobacteria(100);Gammaproteobacteria(100);unclassified(100);unclassified(100);unclassified(100);unclassified(100);unclassified(100);unclassified(100);

Otu0115 0 0 0 0 0 58 0 0 0 0 0 0 0 0 0 0 0 0 4 4 18 3 0 0 8 16 0 42
66 0 0 0 0 0 0 0 0 81 14 151 41 0 148 0 0 27 0 0 0 0 0 0 1 2 0 0 0
0 175 0 13 0 0 0 0 0 0 0 0 7 27 18 0 43 0 0 14 0 0 0 0 0 0 0 0 0
0 136 0 25 0 0 0 0 0 0 0 0 0 0 0 20 155 0 0 0 0 0 0 0 0 0 0
M02255_77_000000000-ADUT3_1_2115_22049_12224 T----AC---GG-AG-GGA--------GCT-A-G------C--G--T---T--GT-T-CGG-AA------TT-A--C-T--GG-GC-------GT--A-----AA-GC-GC-AC-------G-TA-G-G-C-G------------G--C-GA-T-T-C------------AA----G-T-C-A--------G-A-G--G--TG--A-AA-GC--C-C-GG-G-G-----CT-C-AA------C-C-C-C-G-G-A--A-C----T-G--C-C---T--T-------T--GA-A-A---C-------T--A-G--AT--T-G-C----T-A-G-A-A-T--C---C-TG-----GA-G-A--------G-G-T-G---AG-T------GG--A--ATT----C-C-G-A-GT--GT-A-G-AG-GT--G---A--A-A---TT-C-GT-AG--AT-A-TT--C-G--G-A-A-G-A-AC-A-CC---AG--T--G--GC-GAA-G--G-C--G-------A--C-T-C-A---CTG--G--AC-A-G--------------------G-T---A-T-T--GA--CG-C-T-G--A-GG--T-G-CG-A--AA-G-C---G-TG--GG-G--AG-C-A-AA--------CA--GG Bacteria(100);Proteobacteria(100);Alphaproteobacteria(100);Sphingomonadales(100);Sphingomonadaceae(61);unclassified(57);unclassified(57);unclassified(57);unclassified(57);

Otu0116 0 0 0 0 0 0 0 0 0 0 0 0 0 0 0 0 0 0 0 0 22 0 0 0 0 0 0 15
0 0 0 0 0 0 0 0 0 99 3 0 0 0 0 0 0 0 0 0 0 0 0 0 0 0 0 0 0
0 0 0 0 0 0 0 0 0 0 0 0 0 0 0 0 0 91 0 0 0 0 0 0 0 0 0 0 0
0 0 0 0 0 0 0 0 0 0 0 0 0 0 0 0 0 70 0 0 0 0 0 0 0 0 0
M02255_77_000000000-ADUT3_1_1113_17671_9166 T----AC---GA-AG-GGG--------GCT-A-G------C--G--T---T--GC-T-CGG-AA------TC-A--C-T--GG-GC-------GT--A-----AA-GC-GC-AC-------G-TA-G-G-C-G------------G--C-TT-T-T-T-----------AA----G-T-C-A--------G-G-G--G--TG--A-AA-TC--C-T-GG-A-G-----CT-C-AA------C-T-C-C-A-G-A--A-C----T-G--C-C---T--T-------T--GA-T-A---C-------T--G-A--GA--A-G-C----T-T-G-A-G-T--C---C-GG-----GA-G-A--------G-G-T-G---AG-T------GG--A--ACT----G-C-G-A-GT--GT-A-G-AG-GT--G---A--A-A---TT-C-GT-AG--AT-A-TT--C-G--C-A-A-G-A-AC-A-CC---AG--T--G--GC-GAA-G--G-C--G-------G--C-T-C-A---CTG--G--CC-C-G--------------------G-T---A-C-T--GA--CG-C-T-G--A-GG--T-G-CG-A--AA-G-C---G-TG--GG-G--AG-C-A-AA--------CA--GG Bacteria(100);Proteobacteria(100);Alphaproteobacteria(100);Rhizobiales(100);unclassified(100);unclassified(100);unclassified(100);unclassified(100);unclassified(100);

Otu0117 0 0 0 0 0 59 0 0 43 0 0 0 0 0 0 0 0 0 0 0 0 0 0 0 0 0 0 0
0 0 0 0 0 0 0 0 0 0 0 0 0 0 0 0 0 0 0 0 0 0 0 17 4 0 0 0
0 143 0 0 0 0 0 21 0 0 0 0 0 0 0 0 13 11 0 0 0 0 0 0 0 0 0 0 0
0 0 0 0 65 0 0 0 0 0 0 0 0 22 0 0 0 0 0 153 0 0 0 0 0 0 0
M02255_77_000000000-ADUT3_1_1115_10170_12035 T----AC---GT-AG-GGG--------GCA-A-G------C--G--T---T--AT-C-CGG-AT------TT-A--C-T--GG-GT-------GT--A-----AA-GG-GA-GC-------G-CA-G-A-C-G------------G--C-GA-A-G-C-----------AA----G-T-C-T--------G-A-A--G--TG--A-AAT-G--C-A-TG-G-G-----CT-C-AA------C-C-C-A-T-G-A--A-T----T-G--C-T---T--T-------G--GA-A-A-A---C-------T--G-T--TT--G-G-C----T-T-G-A-G-T--G---T-CG-----GA-G-G--------G-G-T-A---AG-C------GG--A--ATT----C-C-T-A-GT--GT-A-G-CG-GT--G---A--A-A---TG-C-GT-AG--AT-A-TT--A-G--G-A-G-G-A-AC-A-CC---GG--A--G--GC-GAA-G--G-C--G-------G--C-T-T-A---CTG--G--AC-G-A--------------------C-A---A-C-T--GA--CG-T-T-G--A-GG--C-T-CG-A--AG-G-C---G-TG--GG-G--AG-C-A-AA--------CA--GG Bacteria(100);Firmicutes(100);Clostridia(100);Clostridiales(100);Lachnospiraceae(100);Johnsonella(100);unclassified(100);unclassified(100);unclassified(100);

Otu0118 0 0 0 0 0 0 0 0 0 0 0 0 3 0 0 2 0 1 2 0 2 4 0 0 0 0 0 0
0 0 0 0 0 0 0 0 0 0 0 0 0 0 0 0 0 0 0 0 0 0 0 0 0 0 0 0 0
7 0 0 1 8 1 0 0 0 0 1 8 9 1 0 0 0 0 0 0 0 0 0 0 0 0 0 0 0
0 0 0 0 0 0 0 0 0 0 4 0 0 0 1 0 0 0 0 0 0 0 0 0 0 0 0
M02255_76_000000000-ADUNE_1_1108_22683_17617 C----TC---CG-TG-CCA--------GCA-G-C------C--G--C---G--GT---AAT-AC------GG-A--G-G--AT-CC-------AA--G-----CG-TT-AT-CC-------G-GA-A-T-C-A------------G--T-CA-G-A-T-----------AA----G-T-C-A--------G-T-G--G--TG--A-AA-GC--C-C-AT-C-G-----CT-C-AA------C-G-G-T-G-G-A--A-C----G-G--C-C---A--T-------T--GA-T-A---C-------T--G-T--CT--G-A-C----T-T-G-A-A-T--T---A-TT-----AG-G-A--------A-G-T-A---AC-T------AG--A--ATA----T-G-T-A-GT--GT-A-G-CG-GT--G---A--A-A---TG-C-TT-AG--AG-A-TT--A-C--A-T-G-G-A-AT-A-CC---AA--T--T--GC-GAA-G--G-C--A-------G--G-T-T-A---CTA--C--TA-A-T--------------------A-T---A-T-T--GA--CG-C-T-G--A-TG--G-A-CG-A--AA-G-C---G-TG--GG-T--AG-C-G-AA--------CA--GG Bacteria(100);Bacteroidetes(100);Flavobacteria(100);Flavobacteriales(100);Flavobacteriaceae(100);Flavobacterium(100);unclassified(100);unclassified(100);unclassified(100);

Otu0119 0 0 0 0 0 0 0 0 0 0 0 1 0 0 0 0 0 4 0 0 0 0 0 4 0 0 3 0
0 0 0 0 0 0 0 0 0 0 0 0 0 0 0 0 0 0 0 0 0 99 0 3 0 0 0 0
0 0 0 0 0 0 12 0 0 0 0 0 0 0 0 0 0 0 0 0 0 0 0 0 0 0 0 0 0
0 0 0 0 0 0 0 0 20 0 0 0 0 0 0 0 0 0 0 0 0 0 0 0 0 0 0
M02255_76_000000000-ADUNE_1_1109_22780_23901 C----GG---CT-AA-CTC--------CGT-G-C------C--A--G---C--AG-C-CGC-GG------TA-A--T-A--CG-GA-------GG--A-----TC-CA-AG-CG-------T-TA-T-C-C-G------------G--T-TT-A-A-T-----------AA----G-T-C-A--------G-T-G--G--TG--A-AA-GC--C-C-AT-C-G-----CT-C-AA------C-G-G-T-G-G-A--A-C----G-G--C-C---A--T-------T--GA-T-A---C-------T--G-T--TA--G-A-C----T-T-G-A-A-T--T---A-TT-----AG-G-A--------A-G-T-A---AC-T------AG--A--ATA----T-G-T-A-GT--GT-A-G-CG-GT--G---A--A-A---TG-C-TT-AG--AG-A-TT--A-C--A-T-G-G-A-AT-A-CC---AA--T--T--GC-GAA-G--G-C--A-------G--G-T-T-A---CTA--C--TA-A-T--------------------G-G---A-T-T--GA--CG-C-T-G--A-TG--G-A-CG-A--AA-G-C---G-TG--GG-T--AG-C-G-AA--------CA--GG Bacteria(100);Bacteroidetes(100);Flavobacteria(100);Flavobacteriales(100);Flavobacteriaceae(100);Flavobacterium(100);unclassified(100);unclassified(100);unclassified(100);

Otu0120 0 0 0 0 0 0 14 90 0 0 0 1 0 0 0 0 0 0 2 2 0 0 0 5 0 0 0 8
0 0 81 0 0 25 0 0 0 0 0 19 0 0 49 0 0 0 0 0 0 0 0 58 7 0 0 0 0
0 0 0 0 8 7 0 11 0 0 0 0 0 0 0 1 0 30 0 49 14 0 1 0 0 0 0 54 0
12 60 163 9 0 0 6 0 0 0 9 0 4 0 0 57 0 0 38 20 0 79 0 0 0 0 0
M02255_77_000000000-ADUT3_1_2109_13889_11952 T----AC---GA-AG-GGG--------GCT-A-G------C--G--T---T--GC-T-CGG-AA------TT-A--C-T--GG-GC-------GT--A-----AA-GG-GA-GC-------G-TA-G-G-C-G------------G--A-CA-T-T-T----
-------AA----G-T-C-A--------G-G-G--G--TG--A-AA-TC--C-C-AG-A-G-----CT-C-AA------C-T-C-T-G-G-A--A-C----T-G--C-C---T--T-------T--GA-T-A---C-------T--G-G--GT--G-T-C----T-T-G-A-G-T--G---T-GA-----GA-G-A--------G-G-T-A---TG-T------GG--A--ACT----C-
C-G-A-GT--GT-A-G-AG-GT--G---A--A-A---TT-C-GT-AG--AT-A-TT--C-G--G-A-A-G-A-AC-A-CC---AG--T--G--GC-GAA-G--G-C--G-------A--C-A-T-A---CTG--G--CT-C-A--------------------T-T---A-C-T--GA--CG-C-T-G--A-GG--C-T-CG-A--AA-G-C---G-TG--GG-G-
-AG-C-A-AA--------CA--GG Bacteria(100);Proteobacteria(100);Alphaproteobacteria(100);Caulobacterales(100);Caulobacteraceae(100);Brevundimonas(100);unclassified(100);unclassified(100);unclassified(100);

Otu0121 0 71 0 0 0 5 0 0 27 0 0 0 0 0 0 0 0 0 4 1 16 5 27 3 0 4 1 0
0 2 3 0 0 48 0 1 0 46 2 31 0 70 0 0 0 0 0 34 70 0 2 0 15 0 0 27 126
5 0 0 4 0 0 0 0 0 4 2 3 0 10 88 0 1 86 0 65 0 0 2 0 0 0 0 0 250
0 17 0 0 83 17 0 0 0 0 0 0 3 0 0 27 0 28 5 0 0 33 0 0 0 0 39
M02255_77_000000000-ADUT3_1_2111_10084_17965 T----AC---GT-AG-GGT--------GCG-A-G------C--G--T---T--GT-C-CGG-AA------TT-A--T-T--GG-GC-------GT--A-----AA-GG-GC-TC-------G-TA-G-G-C-G------------G--T-TT-G-T-C------
-----GC----G-T-C-G--------G-G-A--G--TG--A-AA-AC--C-A-GG-T-G-----CT-T-AA------C-A-C-C-T-G-G--C-T----T-G--C-T---T--T-------C--GA-T-A---C-------G--G-G--CA--G-A-C----T-A-G-A-G-G--T---A-TG-----CA-G-G--------G-G-A-G---AA-C------GG--A--ATT----C-
C-T-G-GT--GT-A-G-CG-GT--G---A--A-A---TG-C-GC-AG--AT-A-TC--A-G--G-A-G-G-A-AC-A-CC---GG--T--G--GC-GAA-G--G-C--G-------G--T-T-C-T---CTG--G--GC-A-T--------------------G-A---C-C-T--GA--CG-C-T-G--A-GG--A-G-CG-A--AA-G-T---G-TG--GG-
G--AG-C-G-AA--------CA--GG Bacteria(100);Actinobacteria(100);Actinobacteria(100);Actinobacteridae(100);Actinomycetales(100);Propionibacterineae(100);Nocardioidaceae(100);Nocardioides(93);unclassified(93);

Otu0122 0 0 0 0 0 0 0 0 0 0 0 0 0 0 0 0 0 0 0 0 0 0 0 0 0 0 0 0
0 0 0 0 0 19 0 42 0 0 0 0 0 0 0 0 0 0 0 0 0 0 0 61 0 0 0 0 0
0 0 0 0 0 0 0 0 0 0 0 0 0 0 0 0 26 63 0 0 0 0 4 0 0 0 0 0 0
13 0 37 0 54 0 0 0 0 0 0 0 0 0 0 0 0 0 0 0 0 0 0 0 0 0 0
M02255_77_000000000-ADUT3_1_2103_20499_11907 T----AC---GA-AG-GGG--------GCT-A-G------C--G--T---T--GC-T-CGG-AA------TC-A--C-T--GG-GC-------GT--A-----AA-GC-GC-AC-------G-TA-G-G-C-G------------G--A-CT-C-T-T-----
------AA----G-T-C-G--------G-G-G--G--TG--A-AA-TC--C-T-GG-A-G-----CT-C-AA------C-T-C-C-A-G-A--A-C----T-G--C-C---T--T-------C--GA-T-A---C-------T--G-G--GA--G-T-CT---T-C-G-A-G-T--T---C-GG-----GA-G-A--------G-G-T-G---AG-T------GG--A--ACT----
G-C-G-A-GT--GT-A-G-AG-GT--G---A--A-A---TT-C-GT-AG--AT-A-TT--C-G--C-A-A-G-A-AC-A-CC---AG--T--G--GC-GAA-G--G-C--G-------G--C-T-C-A---CTG--G--CC-C-G--------------------A-T---A-C-T--GA--CG-C-T-G--A-GG--T-G-CG-A--AA-G-C---G-TG--GG-
G--AG-C-A-AA--------CA--GG Bacteria(100);Proteobacteria(100);Alphaproteobacteria(100);Rhizobiales(100);unclassified(100);unclassified(100);unclassified(100);unclassified(100);unclassified(100);

Otu0123 0 0 0 0 1 3 0 0 0 0 1 7 10 3 5 4 9 2 0 1 6 5 0 2 0 0 1 0
0 0 0 1 0 0 0 0 0 0 0 0 1 0 0 4 0 0 25 1 0 1 34 0 7 20 1 18 0
1 0 5 12 0 1 12 1 0 15 3 0 3 9 0 0 0 0 0 0 0 0 0 0 0 0 0 0 0
0 0 0 0 0 0 6 18 7 0 11 0 5 0 8 0 0 0 0 0 0 0 0 0 0 0 0
M02255_77_000000000-ADUT3_1_1112_21908_11269 T----AC---AG-AG-GGT--------GCA-A-G------C--G--T---T--AA-T-CGG-AA------TT-A--C-T--GG-GC-------GT--A-----AA-GC-GC-GC-------G-TA-G-G-T-G------------G--T-TT-G-T-T-----
------AA----G-T-T-G--------A-A-T--G--TG--A-AA-TC--C-C-CG-G-G-----CT-C-AA------C-C-T-G-G-G-A--A-C----T-G--C-A---T--C-------C--AA-A-A---C-------T--G-G--CA--A-G-C----T-A-G-A-G-T--A---T-GG-----TA-G-A--------G-G-G-T---AG-T------GG--A--ATT----T-
C-C-T-GT--GT-A-G-CG-GT--G---A--A-A---TG-C-GT-AG--AT-A-TA--G-G--A-A-G-G-A-AC-A-CC---AG--T--G--GC-GAA-G--G-C--G-------A--C-T-A-C---CTG--G--AC-T-G--------------------A-T---A-C-T--GA--CA-C-T-G--A-GG--T-G-CG-A--AA-G-C---G-TG--GG-
G--AG-C-A-AA--------CA--GG Bacteria(100);Proteobacteria(100);Gammaproteobacteria(100);Pseudomonadales(100);Pseudomonadaceae(100);Pseudomonas(97);unclassified(97);unclassified(97);unclassified(97);

Otu0124 0 3 0 0 0 0 0 0 1 0 0 0 0 9 1 1 6 0 0 1 0 0 115 86 0 0 0 0
0 0 1 0 0 0 10 7 0 0 3 0 1 0 1 1 1 0 0 7 0 0 0 0 0 0 0 0 0
0 0 1 0 0 1 1 0 0 0 1 0 0 0 0 0 0 0 12 5 0 0 0 16 11 70 0 77 47
34 35 14 0 36 8 0 0 0 3 0 1 1 1 0 0 0 0 1 0 0 0 0 0 0 0 0
M02255_76_000000000-ADUNE_1_2115_26871_8312 T----A----AG-CA-CCG--------GCT-A-A------C-----T---C--TG-T-GCC-AG------CA-G--C-C--GC-G--------GT--A-----AT-AC-AG-AG-------G-GT-G---C-G------------G--C-TT-G-T-T-----------AA----
G-T-C-G--------G-A-T--G--TG--A-AA-TC--C-C-CG-A-G-----CT-T-AA------C-T-T-G-G-G-A--A-T----T-G--C-A---T--T-------C--GA-T-A---C-------T--G-G--GA--A-G-C----T-A-G-A-G-T--A---T-GG-----GA-G-A--------G-G-A-T---GG-T------AG--A--ATT----C-C-A-G-GT-
-GT-A-G-CG-GT--G---A--A-A---TG-C-GT-AG--AG-A-TC--T-G--G-A-G-G-A-AT-A-CC---GA--T--G--GC-GAA-G--G-C--A-------G--C-C-A-T---CTG--G--CC-T-A--------------------A-T---A-C-T--GA--CG-C-T-G--A-GG--T-A-CG-A--AA-G-C---A-TG--GG-G--AG-C-A-
AA--------CA--GG Bacteria(100);Proteobacteria(100);Gammaproteobacteria(100);Pseudomonadales(100);Moraxellaceae(100);Acinetobacter(100);unclassified(100);unclassified(100);unclassified(100);

Otu0125 0 0 0 0 18 0 0 0 0 0 0 0 0 0 0 0 0 0 6 0 0 4 0 0 0 0 0 0
0 0 0 0 0 0 0 0 0 0 0 0 0 0 21 0 14 0 0 0 92 0 0 0 0 0 0 0 0
0 0 0 0 0 0 0 0 0 0 0 0 0 0 0 0 20 0 0 0 0 0 0 0 0 0 0 0 27
0 0 56 0 0 0 0 0 0 0 0 0 0 0 0 0 0 0 0 0 0 50 0 0 0 0 0
M02255_77_000000000-ADUT3_1_1116_5303_18489 T----AC---GT-AG-GTG--------GCA-A-G------C--G--T---T--GT-C-CGG-AT------TT-A--C-T--GG-GT-------GT--A-----AA-GG-GC-GT-------G-TA-G-G-C-G------------G--A-GA-T-G-C-----------
AA----G-T-T-G--------G-G-A--G--TG--A-AA-TC--C-C-GG-G-G-----CT-C-AA------C-C-C-C-G-G-A--A-C----T-G--C-T---T--T-------C--AA-A-A---C-------T--G-T--AT--C-C-C----T-T-G-A-G-T--G---T-CG-----GA-G-A--------G-G-C-A---AG-C------GG--A--ATT----C-C-T-
A-GT--GT-A-G-CG-GT--G---A--A-A---TG-C-GT-AG--AT-A-TT--A-G--G-A-G-G-A-AC-A-CC---AG--T--G--GC-GAA-G--G-C--G-------G--C-T-T-G---CTG--G--AC-G-A--------------------T-A---A-C-T--GA--CG-C-T-G--A-GG--C-G-CG-A--AA-G-C---G-TG--GG-G--
AG-C-A-AA--------CA--GG Bacteria(100);Firmicutes(88);Clostridia(70);Clostridiales(70);unclassified(63);unclassified(63);unclassified(63);unclassified(63);unclassified(63);

Otu0126 0 0 0 0 0 0 0 0 0 0 0 0 0 0 0 0 0 0 0 19 59 7 0 7 8 0 4 0
12 0 3 0 0 0 0 0 0 212 9 0 0 0 0 0 0 0 0 0 0 10 0 0 0 0 0 0
0 0 0 0 0 0 0 0 0 0 0 0 0 0 181 53 0 0 0 0 0 0 0 0 1 0 0 0 0
140 83 0 0 0 25 0 0 0 0 0 0 0 0 0 0 0 0 0 51 0 0 0 0 0 0 0
M02255_77_000000000-ADUT3_1_1105_7393_16762 T----AC---GG-AG-GAT--------GCA-A-G------C--G--T---T--AT-C-CGG-AA------TG-A--T-T--GG-GC-------GT--A-----AA-GC-GT-CC-------G-CA-G-G-T-G------------G--C-AT-A-T-C-----------AA-
---G-T-T-T--------G-C-G--G--TT--A-AA-GG--T-T-CT-G-G-----CT-C-AA------C-C-A-G-A-G-A--C-A----G-G--C-C---G--T-------G--AA-A-A---C-------T--G-A--TA--A-G-C----T-A-G-A-G-T--A---T-GG-----TA-G-G--------G-G-C-A---GA-G------GG--A--ATT----C-C-C-A-
GT--GT-A-G-CG-GT--G---A--A-A---TG-C-GT-AG--AG-A-TT--G-G--G-A-A-G-A-AC-A-CC---GG--T--G--GC-GAA-A--G-C--G-------C--T-C-T-G---CTA--G--GC-C-A--------------------A-A---A-C-T--GA--CA-C-T-G--A-GG--G-A-CG-A--AA-G-C---T-AG--GG-G--AG-
C-G-AA--------TG--GG Bacteria(100);Cyanobacteria(100);unclassified(100);unclassified(100);unclassified(100);unclassified(100);unclassified(100);unclassified(100);unclassified(100);

Otu0127 0 0 0 92 0 31 0 0 0 63 0 0 0 0 0 0 2 0 0 0 14 1 0 0 0 0 0 0
0 0 0 0 0 0 18 0 0 0 0 0 0 0 90 30 0 0 0 0 0 0 0 0 1 0 0 0
0 0 12 0 0 0 0 0 0 0 6 0 0 0 71 119 0 51 0 0 76 0 4 0 0 0 0 0 0
45 30 0 25 0 0 0 0 0 0 0 0 0 0 0 0 0 35 32 23 1 0 49 0 11 0 179
M02255_77_000000000-ADUT3_1_1112_7010_18942 T----AC---GT-AG-GGG--------GCA-A-G------C--G--T---T--AT-C-CGG-AT------TT-A--C-T--GG-GT-------GT--A-----AA-GG-GA-GC-------G-CA-G-G-C-G------------G--T-TT-T-G-C-----------AA-
---G-T-T-G--------A-G-A--G--TG--G-AA-GC--A-G-GG-G-G-----CT-C-AA------C-C-C-C-C-T-G--A-C----T-G--C-T---C--C-------C--AA-A-A---C-------T--G-T--AA--A-A-C----T-T-G-A-G-T--A---T-GG-----GA-G-A--------G-G-C-A---GG-C------GG--A--ATT----C-C-T-A-
GT--GT-A-G-CG-GT--G---A--A-A---TG-C-TT-AG--AT-A-TT--A-G--G-A-A-G-A-AC-A-CC---GG--T--G--GC-GAA-G--G-C--G-------G--C-C-T-G---CTG--G--AC-C-A--------------------A-A---A-C-T--GA--CG-C-T-G--A-GG--C-T-CG-A--AA-G-C---G-TG--GG-T--AG-C-
A-AA--------CA--GG Bacteria(100);Firmicutes(100);Clostridia(100);Clostridiales(100);Lachnospiraceae(100);Catonella(100);unclassified(100);unclassified(100);unclassified(100);

Otu0128 0 0 0 0 0 0 0 0 0 18 0 0 0 0 0 0 3 7 0 0 0 0 27 0 0 0 0 11
0 0 73 0 0 0 0 0 0 0 0 0 0 49 53 0 0 0 0 99 0 0 0 0 13 12 0 0 0
75 0 2 0 0 0 0 0 0 0 0 0 0 0 0 0 0 7 0 0 0 0 0 0 0 0 0 0 0
0 0 0 0 129 0 0 0 0 180 0 0 0 0 0 34 0 55 0 0 0 0 0 0 7 0 0
M02255_77_000000000-ADUT3_1_1114_13261_10593 T----AC---GA-AG-GGG--------GCT-A-G------C--G--T---T--GC-T-CGG-AA------TC-A--C-T--GG-GC-------GT--A-----AA-GG-GC-GC-------G-TA-G-G-C-G------------G--C-CA-T-T-C----
-------AA----G-T-C-G--------G-G-G--G--TG--A-AA-GC--C-T-GT-G-G-----CT-C-AA------C-C-A-C-A-G-A--A-T----T-G--C-C---T--T-------C--GA-T-A---C-------T--G-T--TT--G-G-C----T-T-G-A-G-A--C---C-GG-----AA-G-A--------G-G-T-A---AG-T------GG--A--ACT----
G-C-G-A-GT--GT-A-G-AG-GT--G---A--A-A---TT-C-GT-AG--AT-A-TT--C-G--C-A-A-G-A-AC-A-CC---AG--T--G--GC-GAA-G--G-C--G-------G--C-T-T-A---CTG--G--TC-C-G--------------------G-T---T-C-T--GA--CG-C-T-G--A-GG--C-G-CG-A--AA-G-C---G-TG--GG-
G--AG-C-A-AA--------CA--GG Bacteria(100);Proteobacteria(100);Alphaproteobacteria(100);Rhizobiales(100);unclassified(100);unclassified(100);unclassified(100);unclassified(100);unclassified(100);

Otu0129 0 0 0 0 0 1 0 0 0 0 0 0 5 0 0 0 2 0 0 10 39 4 35 13 0 0 0 1
3 4 133 245 0 0 0 31 0 198 85 189 21 0 0 109 458 1 3 0 0 0 133 0 3 0 27 0
0 139 4 7 0 2 0 14 0 8 20 67 8 0 112 10 0 0 398 1377 580 0 1 130 0 0 671 0 178
98 119 0 36 439 73 19 49 0 629 0 510 26 0 1 34 116 0 0 0 0 1 132 0 0 0 0
M02255_77_000000000-ADUT3_1_1101_5978_19960 T----AC---GT-AA-GGG--------GCG-A-G------C--G--T---T--GT-C-CGG-AA------TT-A--T-T--GG-GC-------GT--A-----AA-GA-GT-GC-------G-TA-G-G-C-G------------G--C-AA-A-T-T-----------
AA----G-T-C-A--------G-A-T--G--TG--A-AA-AC--T-A-AG-G-G-----CT-C-AAC-----C-C-A-T-A-G-A--T------T-G--C-A---T--C-------T--GA-A-A---C-------T--G-A--TA--T-G-C----T-T-G-A-G-T--C---A-AG-----GA-G-A--------G-G-A-A---AG-T------GG--A--ATT----C-C-T-
A-GT--GT-A-G-CG-GT--G---G--A-A---TG-C-GT-AG--AT-A-TT--A-G--G-A-G-G-A-AT-A-CC---GG--T--G--GC-GAA-G--G-C--G-------A--C-T-T-T---CTG--G--AC-T-T--------------------G-A---A-C-T--GA--CG-C-T-G--A-GG--C-A-CG-A--AA-G-C---G-TG--GG-G--
AG-C-A-AA--------CA--GG Bacteria(100);Firmicutes(100);Clostridia(100);unclassified(99);unclassified(99);unclassified(99);unclassified(99);unclassified(99);unclassified(99);

Otu0130 0 0 0 0 0 0 0 0 0 0 0 7 0 0 0 2 0 6 3 4 0 1 0 3 0 0 0 0
0 0 0 0 0 0 0 0 0 0 0 0 0 0 0 0 0 0 0 0 0 0 21 0 0 0 0 0 0
0 0 0 1 0 0 15 0 0 0 0 0 0 0 0 0 0 0 0 0 0 0 0 0 0 0 0 0 0
0 0 0 0 0 0 0 0 0 0 10 0 0 0 0 0 0 0 0 0 0 0 0 0 0 0 0
M02255_77_000000000-ADUT3_1_1109_28352_11553 T----GC---CA-GC-AGC--------CGC-G-G------T--A--A---T--AC-G-GAG-GA------TC-C--A-A--GC-GT-------TA--T-----CC-GG-AA-TC-------A-TA-G-G-C-G-------------G--T-TT-A-G-T------
-----AA----G-T-C-A--------G-T-G--G--TG--A-AA-GC--C-C-AT-C-G-----CT-C-AA------C-G-G-T-G-G-A--A-C----G-G--C-C---A--T-------T--GA-T-A---C-------T--G-C--TA--G-A-C----T-T-G-A-A-T--T---A-TT-----AG-G-A--------A-G-T-A---AC-T------AG--A--ATA----T-
G-T-A-GT--GT-A-G-CG-GT--G---A--A-A---TG-C-TT-AG--AG-A-TT--A-C--A-T-G-G-A-AT-A-CC---AA--T--T--GC-GAA-G--G-C--A-------G--G-T-T-A---CTA--C--TA-A-T--------------------G-G---A-T-T--GA--CG-C-T-G--A-TG--G-A-CG-A--AA-G-C---G-TG--GG-T--
AG-C-G-AA--------CA--GG Bacteria(100);Bacteroidetes(100);Flavobacteria(100);Flavobacteriales(100);Flavobacteriaceae(100);Flavobacterium(100);unclassified(100);unclassified(100);unclassified(100);

Otu0131 0 0 0 0 0 0 0 0 0 0 0 0 0 0 0 0 0 0 0 0 0 0 24 1 0 0 0 0
0 0 0 0 0 0 0 0 96 0 0 0 0 0 0 0 0 0 0 0 117 0 0 0 0 0 0 0 0
0 0 0 0 0 0 0 0 0 0 0 0 0 0 231 0 0 0 0 0 0 0 4 77 0 0 0 0 0
0 0 58 0 0 0 0 0 0 0 0 0 0 0 0 32 0 0 0 0 0 0 0 0 9 0 0
M02255_77_000000000-ADUT3_1_1117_5045_19079 T----AC---GA-AG-GGG--------GCG-A-G------C--G--T---T--GC-T-CGG-AA------TG-A--C-T--GG-GC-------GT--A-----AA-GG-GC-GC-------G-TA-G-G-C-G-------------G--A-CC-T-C-G-----------
CA----G-T-C-G--------G-G-C--G--TG--A-AA-TC--C-C-CG-G-G-----CT-C-AA------C-C-C-G-G-G-A--C-G----T-G--C-G---C--C-------C--GA-G-A---C-------G--C-G--GG--G-T-C----T-G-G-A-G-T--G---G-GG-----GA-G-A--------G-G-G-T---CG-C------GG--A--ATT----C-C-
C-A-GT--GT-A-G-AG-GT--G---A--A-A---TT-C-GT-AG--AT-A-TT--G-G--G-A-A-G-A-AC-A-CC---GG--T--G--GC-GAA-G--G-C--G-------G--C-G-A-C---CTG--G--CC-C-T--------------------T-G---A-C-T--GA--CG-C-T-G--A-GG--C-G-CG-A--CA-G-C---G-TG--GG-G--
AG-C-A-AA--------CA--GG Bacteria(100);Proteobacteria(100);Alphaproteobacteria(100);Rhodospirillales(100);unclassified(100);unclassified(100);unclassified(100);unclassified(100);unclassified(100);

Otu0132 0 0 1 30 34 0 0 0 32 0 0 0 0 0 0 0 0 0 4 0 6 0 0 9 0 0 0 0
0 0 0 0 20 0 0 0 0 0 0 1 0 8 8 0 0 0 0 0 0 0 0 20 0 29 0 0 0
0 0 0 0 0 0 0 0 0 0 0 0 1 0 0 0 21 9 0 0 1 0 17 0 0 0 0 0 14
0 6 0 0 0 0 0 0 0 0 0 0 0 0 0 0 0 3 0 0 0 9 0 0 0 0 0
M02255_77_000000000-ADUT3_1_1111_5974_7595 T----AC---GT-AG-GGT--------GCA-A-G------C--G--T---T--GT-C-CGG-AA------TT-A--T-T--GG-GC-------GT--A-----AA-GA-GC-TC-------G-TA-G-G-C-G-------------G--T-CT-G-T-C-----------GC-
---G-T-C-G--------G-C-T--G--TG--A-AA-AC--T-C-GG-G-G-----CT-C-AA------C-C-C-C-G-A-G--C-C----T-G--C-A---G--T-------C--GA-T-A---C-------G--G-G--CA--G-A-C----T-A-G-A-G-T--G---C-TG-----TA-G-G--------G-G-A-G---AC-T------GG--A--ATT----C-C-T-G-
GT--GT-A-G-CG-GT--G---A--A-A---TG-C-GC-AG--AT-A-TC--A-G--G-A-G-G-A-AC-A-CC---GG--T--G--GC-GAA-G--G-C--G-------G--G-T-C-T---CTG--G--GC-A-G--------------------T-A---A-C-T--GA--CG-C-T-G--A-GG--A-G-CG-A--AA-G-C---G-TG--GG-G--AG-
C-A-AA--------CA--GG Bacteria(100);Actinobacteria(100);Actinobacteria(100);Actinobacteridae(100);Actinomycetales(100);unclassified(100);unclassified(100);unclassified(100);unclassified(100);

Otu0133 0 0 0 0 0 0 0 0 0 0 0 0 0 0 0 0 0 0 0 0 0 0 0 0 0 0 0 0
2 0 0 0 0 0 0 0 0 0 0 0 0 0 0 0 0 0 0 0 0 0 0 0 0 0 0 0 0
0 0 14 0 0 0 0 0 0 0 0 0 0 0 165 206 0 0 0 0 111 0 0 0 0 0 0 0 56
0 0 0 17 72 28 0 1 0 0 0 0 0 0 0 0 0 0 0 0 0 0 0 0 0 0 0
M02255_77_000000000-ADUT3_1_1105_7777_22365 T----AC---GT-AG-GGG--------GCA-A-G------C--G--T---T--AT-C-CGG-AA------TT-A--C-T--GG-GC-------GT--A-----AA-GG-GT-GC-------G-TA-G-G-C-G-------------G--C-TA-G-T-T-----------
AA----G-T-C-G--------A-G-G--T--TA--A-AA-GG--C-A-GT-A-G-----CT-C-AA------C-T-A-C-T-G-T--T------G-G--G-C---C--T-------T--GA-A-A---C-------T--A-A--TT--A-G-C----T-T-G-A-G-T--A---T-AG-----GA-G-A--------G-G-A-A---AG-T------GG--A--ATT----C-C-C-
G-GT--GT-A-G-CG-GT--G---A--A-A---TG-C-GT-AG--AT-A-TC--G-G--G-A-G-G-A-AT-A-CC---GG--T--G--GC-GAA-G--G-C--G-------A--C-T-T-T---CTG--G--AC-T-A--------------------T-A---A-C-T--GA--CG-C-T-G--A-GG--C-A-CG-A--AA-G-C---G-TG--GG-T--
AG-C-A-AA--------CA--GG Bacteria(100);Firmicutes(100);Clostridia(100);Clostridiales(100);Peptostreptococcaceae(100);Incertae_Sedis(100);unclassified(100);unclassified(100);unclassified(100);

Otu0134 0 0 0 0 0 29 0 0 0 16 20 0 0 8 11 0 0 0 0 0 0 1 50 0 161 86 1 20
0 5 0 1 0 0 0 0 0 14 8 0 0 50 0 0 0 31 0 0 8 0 0 7 0 0 0 0 7
0 0 0 0 0 0 0 0 0 0 0 0 0 0 0 0 0 0 0 0 0 5 19 0 0 4 0 0 0
26 0 0 0 1 0 0 0 0 0 0 0 0 0 0 0 0 0 0 15 13 0 0 0 6 0 0
M02255_77_000000000-ADUT3_1_1105_11189_4306 C----TA---AC-TC-CGT--------GCC-A-G------C-----A---G--CC-G-CGG-TA------AT-A--C-G--GA-GG-------GTG-C-----GA-GCGTT-AA-------T-CG-G-A-A-T-------------T--A-CT-G-T-T-----------AA-
---G-C-A-A--------G-A-T--G--TG--A-AA-GC--C-C-GG-G-G-----CT-C-AA------C-C-T-C-G-G-A--A-C----A-G--C-A---T--T-------T--TG-A-A---C-------T--G-G--CA--G-A-C----T-A-G-A-G-T--C---T-TG-----TA-G-A--------G-G-G-G---GG-T------AG--A--ATT----T-C-A-G-
GT--GT-A-G-CG-GT--G---A--A-A---TG-C-GT-AG--AG-A-TC--T-G--A-A-G-G-A-AT-A-CC---GG--T--G--GC-GAA-G--G-C--G-------G--C-C-C-C---CTG--G--AC-A-A--------------------A-G---A-C-T--GA--CG-C-T-C--A-GA--T-G-CG-A--AA-G-C---G-TG--GG-G--AG-
C-A-AA--------CA--GG Bacteria(100);Proteobacteria(100);Gammaproteobacteria(100);Vibrionales(100);Vibrionaceae(100);Photobacterium(99);unclassified(99);unclassified(99);unclassified(99);

Otu0135 0 0 0 0 0 71 0 0 0 0 0 0 0 0 0 0 0 0 0 0 12 4 0 0 0 0 0 3
0 27 3 0 0 82 40 67 0 69 31 0 0 0 54 0 0 0 0 0 0 0 0 0 0 0 0 0
0 0 0 0 0 0 0 0 0 0 0 0 0 0 0 0 0 253 0 0 0 0 0 0 0 0 0 0
0 0 0 0 0 0 0 0 0 0 0 0 0 0 0 13 0 0 0 37 0 0 0 0 0 0 0
M02255_77_000000000-ADUT3_1_2112_15249_18248 T----AC---GA-AG-GGG--------GCT-A-G------C--G--T---T--GT-T-CGG-AA------TT-A--C-T--GG-GC-------GT--A-----AA-GC-GC-AC-------G-TA-G-G-C-G------------G--A-TT-T-G-T-----
------AA----G-T-C-A--------G-G-G--G--TG--A-AA-TC--C-C-GG-G-G-----CT-C-AA------C-C-T-C-G-G-A--A-C----T-G--C-C---T--T-------T--GA-T-A---C-------T--G-C--AA--G-T-C----T-C-G-A-G-T--C---C-GG-----AA-G-A--------G-G-T-G---AG-T------GG--A--ATT----C-
C-T-A-GT--GT-A-G-AG-GT--G---A--A-A---TT-C-GT-AG--AT-A-TT--A-G--G-A-A-G-A-AC-A-CC---AG--T--G--GC-GAA-G--G-C--G-------G--C-T-C-A---CTG--G--TC-C-G--------------------G-T---A-C-T--GA--CG-C-T-G--A-GG--T-G-CG-A--AA-G-C---G-TG--GG-G-
-AG-C-A-AA--------CA--GG Bacteria(100);Proteobacteria(100);Alphaproteobacteria(100);Rhizobiales(100);Rhodobiaceae(78);Rhodobium(78);unclassified(78);unclassified(78);unclassified(78);

Otu0136 0 0 0 0 108 0 21 2 0 0 0 0 0 0 0 0 0 0 0 0 0 0 81 3 0 6 4 0
0 0 0 135 0 0 0 20 108 0 0 0 0 0 134 356 33 0 0 2 0 0 0 32 105 0 0 0 714
0 0 0 0 0 0 0 0 0 6 0 0 0 0 0 0 0 0 0 0 0 0 0 0 56 0 0 0 19
0 0 0 178 46 0 0 0 0 0 5 43 0 0 0 0 0 0 0 0 0 45 0 1 55 0 0
M02255_77_000000000-ADUT3_1_2105_24365_12548 T----AC---GA-AG-GGG--------GCT-A-G------C--G--T---T--GT-T-CGG-AA------TT-A--C-T--GG-GC-------GT--A-----AA-GC-GC-AC-------G-TA-G-G-C-G------------G--A-TA-T-T-T-----
------AA----G-T-C-A--------G-G-G--G--TG--A-AA-TC--C-C-GC-A-G-----CT-C-AA------C-T-G-C-G-G-A--A-C----T-G--C-C---T--T-------T--GA-T-A---C-------T--G-G--GT--A-T-C----T-T-G-A-G-T--A---T-GG-----AA-G-A--------G-G-T-A---AG-T------GG--A--ATT----C-
C-G-A-GT--GT-A-G-AG-GT--G---A--A-A---TT-C-GT-AG--AT-A-TT--C-G--G-A-G-G-A-AC-A-CC---AG--T--G--GC-GAA-G--G-C--G-------G--C-T-T-A---CTG--G--TC-C-A--------------------T-T---A-C-T--GA--CG-C-T-G--A-GG--T-G-CG-A--AA-G-C---G-TG--GG-G-
-AG-C-A-AA--------CA--GG Bacteria(100);Proteobacteria(100);Alphaproteobacteria(100);Rhizobiales(100);unclassified(100);unclassified(100);unclassified(100);unclassified(100);unclassified(100);

Otu0137 0 0 0 0 0 0 0 0 0 0 318 0 0 0 0 18 0 0 0 0 0 1 0 0 0 0 0 0
0 0 0 0 0 0 0 0 0 0 0 1 0 0 0 0 0 0 3 0 0 1 0 0 0 0 0 0 0
0 1 0 0 0 0 1 0 0 0 0 0 0 0 0 0 0 0 0 0 0 0 0 0 0 0 0 0 0
0 0 0 0 0 0 0 0 1 0 0 0 0 0 0 0 0 0 0 0 0 0 0 0 0 0 0
M02255_76_000000000-ADUNE_1_2104_7077_17345 .....AC----T-AC-GT---------GCC-A-G------C--A--G---C--CG-C-GGT-AA------TA-C--G-T--AG-GT-------GG--C-----AA-GC-G--TT-------G-TC-C-G---G------------A--T-CT-C-T-T----------AA----G-
T-C-T--------G-A-T--G--TG--A-AA-GC--C-C-CC-G-G-----CT-C-AA------C-C-G-G-G-G-A--G------G-GT-C-A---T--T-------G--GA-A-A---C-------T--G-G--GG--G-A-C----T-T-G-A-G-G--A---C-AG-----AA-G-A--------G-G-A-G---AG-T------GG--A--ATT----C-C-A-A-GT--
GT-A-G-CG-GT--G---A--A-A---TG-C-GT-AG--AT-A-TT--T-G--G-A-G-G-A-AC-A-CC---AG--T--G--GC-GAA-G--G-C--G-------G--C-T-C-T---CTG--G--TC-T-G--------------------T-T---A-C-T--GA--CG-C-T-G--A-GG--C-G-CG-A--AA-G-C---G-TG--GG-G--AG-C-A-
AA--------CA--GG Bacteria(100);Firmicutes(100);Bacilli(100);Bacillales(65);unclassified(65);unclassified(65);unclassified(65);unclassified(65);unclassified(65);

Otu0138 0 0 9 11 0 10 0 8 7 0 0 0 0 0 2 0 0 0 0 0 0 0 0 1 0 0 1 3
0 0 0 7 5 8 11 0 4 9 2 0 0 0 6 8 2 2 0 3 18 0 0 7 0 1 0 2 14
0 71 2 1 3 0 0 5 0 3 0 0 3 0 0 0 1 0 3 6 0 1 0 13 0 0 2 4 23
9 24 26 19 34 13 0 0 0 1 0 1 0 3 0 0 1 14 0 16 17 9 6 0 5 0 36
M02255_76_000000000-ADUNE_1_1105_21904_6884 .....AC----T-AC-GT---------GCC-A-G------C--A--G---C--CG-C-GGT-AA------TA-C--G-T--AG-GT-------GG--C-----AA-GC-G--TT-------G----T-C-C-G------------G--A-TC-G-G-T----------CA----G-
T-C-T--------G-T-C--T--TA--A-AA-GT--T-C-GG-G-G-----CT-T-AA------C-C-C-C-G-T-G--A------T-G--G-G---A--T-------G--GA-A-A---C-------T--G-C--CA--A-T-C----T-A-G-A-G-T--A---T-CG-----GA-G-A--------G-G-A-A---AG-T------GG--A--ATT----C-C-T-A-GT--
GT-A-G-CG-GT--G---A--A-A---TG-C-GT-AG--AT-A-TT--A-G--G-A-A-G-A-AC-A-CC---AG--T--G--GC-GAA-G--G-C--G-------A--C-T-T-T---CTG--G--AC-G-A--------------------A-A---A-C-T--GA--CG-C-T-G--A-GG--C-G-CG-A--AA-G-C---C-AG--GG-G--AG-C-G-
AA--------CG--GG Bacteria(100);Firmicutes(100);Clostridia(100);Clostridiales(100);Veillonellaceae(100);Veillonella(100);unclassified(100);unclassified(100);unclassified(100);

Otu0139 0 0 0 0 0 0 0 0 0 0 0 0 0 0 0 0 0 0 0 0 0 0 0 0 0 0 0 1
0 0 0 2 0 0 0 0 0 4 7 1 3 0 0 45 20 40 0 0 0 0 0 0 0 0 0 0 0
0 0 0 0 0 0 0 0 0 0 0 0 0 0 48 59 0 0 0 0 0 0 0 0 0 0 0 15 0
5 53 32 0 0 0 0 0 0 0 0 0 0 0 0 0 0 0 0 7 0 0 0 0 0 0 0
M02255_77_000000000-ADUT3_1_1106_23356_17953 T----AC---GT-AG-GGG--------GCA-A-G------C--G--T---T--AT-C-CGG-AT------TT-A--C-T--GG-GT-------GT--A-----AA-GG-GA-GC-------G-TA-G-A-C-G------------G--C-AT-A-T-A-----
------AA----G-T-C-T--------G-A-A--G--TG--A-AA-AC--C-C-GC-A-G-----CT-C-AA------C-T-G-C-G-G-A--G-T----T-G--C-T---T--T-------G--GA-A-A---C-------T--T-A--TA--A-G-C----T-G-G-A-G-T--G---T-CG-----GA-G-G--------G-G-T-A---AG-C------GG--A--ATT----C-
C-C-A-GT--GT-A-G-CG-GT--G---A--A-A---TG-C-GT-AG--AT-A-TT--G-G--G-A-G-G-A-AC-A-CC---GG--A--G--GC-GAA-G--G-C--G-------G--C-T-T-A---CTG--G--AA-G-A--------------------T-A---A-C-T--GA--CG-T-T-G--A-GG--C-T-CG-A--AG-G-C---G-TG--GG-
T--AG-C-A-AA--------CA--GG Bacteria(100);Firmicutes(100);Clostridia(95);Clostridiales(95);Lachnospiraceae(79);unclassified(79);unclassified(79);unclassified(79);unclassified(79);

Otu0140 0 0 0 96 0 0 0 0 0 0 0 0 0 7 0 0 0 0 0 0 11 6 0 7 0 0 3 0
0 0 0 0 0 0 0 0 0 0 0 0 0 58 0 0 0 0 0 0 109 0 0 0 20 0 0 0 0
0 0 0 0 0 0 0 0 0 0 0 0 0 0 0 4 22 0 0 0 0 0 0 0 123 0 0 18 92
15 0 0 108 0 0 0 0 0 0 0 0 0 64 0 0 0 0 0 31 0 0 0 0 0 0 0
M02255_77_000000000-ADUT3_1_1111_22214_19411 T----AC---GA-AG-GGG--------GCT-A-G------C--G--T---T--GT-T-CGG-AA------TC-A--C-T--GG-GC-------GT--A-----AA-GG-GT-GC-------G-TA-G-G-C-G-------------G--A-TT-C-T-T-----
------AA----G-T-C-A--------G-G-G--G--TG--A-AA-TC--C-C-GA-G-G-----CT-C-AA------C-C-T-C-G-G-A--A-C----T-G--C-C---T--T-------T--GA-T-A---C-------T--G-G--GG--A-T-C----T-T-G-A-G-T--C---C-GG-----AA-G-A--------G-G-T-G---AG-T------GG--A--ACT----
G-C-G-A-GT--GT-A-G-AG-GT--G---A--A-A---TT-C-GT-AG--AT-A-TT--C-G--C-A-A-G-A-AC-A-CC---GG--T--G--GC-GAA-G--G-C--G-------G--C-T-C-A---CTG--G--TC-C-G--------------------G-A---A-C-T--GA--CG-C-T-G--A-GG--C-A-CG-A--CA-G-C---G-TG--
GG-G--AG-C-A-AA--------CA--GG Bacteria(100);Proteobacteria(100);Alphaproteobacteria(100);Rhizobiales(100);unclassified(100);unclassified(100);unclassified(100);unclassified(100);unclassified(100);

Otu0141 0 0 0 2 13 0 0 21 9 1 0 1 0 4 1 0 0 0 0 0 0 0 6 2 4 7 1 0
1 61 15 55 2 95 5 10 14 4 13 16 4 27 25 52 9 7 1 18 30 69 0 19 8 0 0 0 7
3 4 1 1 2 0 0 0 0 0 0 0 2 0 8 11 0 1 17 0 13 3 3 7 24 0 0 606 0
25 39 32 8 2 5 7 0 0 0 1 2 0 4 0 8 10 16 0 0 0 0 5 0 0 0 7
M02255_76_000000000-ADUNE_1_2106_20793_13793 TC---GC---AG-AA-TAA--------GCA-C-C------G--G--C---T--AACT-CTG-TG------CC-AG-C-A-GCC-GC-------GG--T-----AA-TA-CA-GA-------G-GG-T-G-C-G-------------A--G-CT-G-T-T--
---------AA----G-T-C-G--------G-A-T--G--TG--A-AA-TC--C-C-TG-A-G-----CT-T-AA------C-T-T-A-G-G-A--A-T----T-G--C-A---T--T-------C--GA-T-A---C-------T--G-G--GA--A-G-C----T-A-G-A-G-T--A---T-GG-----GA-G-A--------G-G-A-T---GG-T------AG--A--ATT----
C-C-A-G-GT--GT-A-G-CG-GT--G---A--A-A---TG-C-GT-AG--AG-A-TC--T-G--G-A-G-G-A-AT-A-CC---GA--T--G--GC-GAA-G--G-C--A-------G--C-C-A-T---CTG--G--CC-T-A--------------------A-T---A-C-T--GA--CG-C-T-G--A-GG--T-A-CG-A--AA-G-C---A-TG--GG-
G--AG-C-A-AA--------CA--GG Bacteria(100);Proteobacteria(100);Gammaproteobacteria(100);Pseudomonadales(100);Moraxellaceae(100);Acinetobacter(100);unclassified(100);unclassified(100);unclassified(100);

Otu0142 0 0 689 0 0 37 189 228 0 0 0 0 0 0 4 0 0 0 7 0 18 0 0 0 0 0 5 0
0 8 0 92 0 64 0 172 0 79 30 4 0 0 40 0 0 198 0 0 0 3 0 0 9 3 0 0 0
41 94 15 5 0 0 0 0 0 10 8 24 0 9 312 706 7 17 29 419 44 8 0 0 0 0 0 67 190
49 0 36 87 248 207 6 32 4 1283 26 645 0 34 9 0 0 0 7 46 81 128 170 0 18 0 0
M02255_77_000000000-ADUT3_1_2105_23030_22631 T----AC---GT-AT-GGG--------GCG-A-G------C--G--T---T--GT-C-CGG-AA------TT-A--T-T--GG-GC-------GT--A-----AA-GG-GT-AC-------G-TA-G-G-C-G-------------G--T-CT-A-T-T------
-----AA----G-T-C-A--------G-G-T--G--TG--A-AA-GC--G-T-GA-G-G-----CT-T-AA------C-C-T-C-A-T-T--A------A-G--C-A---C--T-------T--GA-A-A---C-------T--G-A--TA--G-A-C----T-T-G-A-G-T--G---A-AG-----GA-G-A--------G-G-A-A---AG-T------GG--A--ATT----C-
C-T-A-GT--GT-A-G-CG-GT--G---A--A-A---TG-C-GT-AG--AT-A-TT--A-G--G-A-G-G-A-AT-A-CC---GG--T--G--GC-GAA-G--G-C--G-------A--C-T-T-T---CTG--G--AC-T-T--------------------T-T---A-C-T--GA--CG-C-T-C--A-GG--T-A-CG-A--AA-G-C---G-TG--GG-G--
AG-C-A-AA--------CA--GG Bacteria(100);Firmicutes(100);Clostridia(100);Clostridiales(100);Family_XI_Incertae_Sedis(100);Parvimonas(100);unclassified(100);unclassified(100);unclassified(100);

Otu0143 0 0 0 0 66 0 0 0 0 0 0 0 0 0 0 0 0 0 0 0 4 0 0 0 0 0 0 0
0 0 0 1 0 0 36 0 0 43 0 0 34 56 0 0 0 0 0 0 0 0 0 0 0 0 0 0
0 0 0 0 8 0 0 0 0 0 0 0 0 14 0 0 0 0 0 0 0 0 0 0 0 0 0 31
0 0 0 0 0 34 0 0 0 22 0 0 0 0 0 0 0 0 0 0 0 0 0 18 0 0
M02255_77_000000000-ADUT3_1_2116_19208_15764 T----AC---GT-AG-GTC--------GCA-A-G------C--G--T---T--GT-C-CGG-AT------TT-A--C-T--GG-GC-------GT--A-----AA-GA-GC-GC-------G-CA-G-G-C-G-------------G--C-TG-G-T-C-----
------AA----G-T-C-C--------G-A-T--G--TG--A-AA-TC--T-C-CC-G-G-----CT-C-AA------C-T-G-G-G-A-A--A-C----T-G--C-A---T--C-------G--GA-A-A---C-------T--G-A--TC--G-G-C----T-T-G-A-A-G--G---T-GG-----GA-G-A--------G-G-G-T---AG-T------GG--A--ATT----
C-C-C-G-GT--GT-A-G-TG-GT--G---A--A-A---TG-C-GT-AG--AT-A-TC--G-G--G-A-G-G-A-AC-A-CC---AG--T--G--GC-GAA-G--G-C--G-------A--C-T-A-C---CTG--G--CC-C-A--------------------T-T---C-T-T--GA--CG-C-T-G--A-GG--C-G-CG-A--AA-G-C---T-AG--GG-
G--AG-C-G-AA--------CG--GG Bacteria(100);unclassified(100);unclassified(100);unclassified(100);unclassified(100);unclassified(100);unclassified(100);unclassified(100);unclassified(100);

Otu0144 0 0 0 91 0 0 0 22 0 0 0 0 0 21 0 0 0 0 0 79 0 0 59 10 0 1 3 28
0 1 4 0 0 0 0 0 0 0 60 41 45 0 0 0 0 0 0 46 0 0 8 0 16 0 0 0 0
0 0 2 0 0 0 7 0 0 0 0 4 0 0 0 59 29 174 0 126 0 6 0 79 0 0 0 0 355
0 0 0 178 0 0 0 5 0 0 0 93 0 0 0 0 0 0 0 0 0 0 0 0 6 0 0
M02255_77_000000000-ADUT3_1_1102_13249_18506 T----AC---GG-AG-GGG--------GCT-A-G------C--G--T---T--GT-T-CGG-AA------TT-A--C-T--GG-GC-------GT--A-----AA-GC-GC-AC-------G-TA-G-G-C-G-------------G--A-CC-G-G-A----
-------AA----G-T-C-A--------G-A-G--G--TG--A-AA-TC--C-C-AG-G-G-----CT-C-AA------C-C-T-T-G-G-A--A-C----T-G--C-C---T--T-------T--GA-A-A---C-------T--A-T--CG--G-T-C----T-G-G-A-G-T--T---C-GA-----GA-G-A--------G-G-T-G---AG-T------GG--A--ATT----
C-C-G-A-GT--GT-A-G-AG-GT--G---A--A-A---TT-C-GT-AG--AT-A-TT--C-G--G-A-G-G-A-AC-A-CC---AG--T--G--GC-GAA-G--G-C--G-------G--C-T-C-A---CTG--G--CT-C-G--------------------A-T---A-C-T--GA--CG-C-T-G--A-GG--T-G-CG-A--AA-G-C---G-TG--GG-
G--AG-C-A-AA--------CA--GG Bacteria(100);Proteobacteria(100);Alphaproteobacteria(100);Rhodobacterales(100);Rhodobacteraceae(100);unclassified(100);unclassified(100);unclassified(100);unclassified(100);

Otu0145 0 0 0 0 0 0 0 0 0 0 1 35 1 6 7 0 0 22 3 31 0 7 0 0 0 4 0 0
9 0 0 0 0 0 0 0 1 0 0 0 0 0 0 0 0 0 0 0 0 0 0 0 0 0 0 0 0
4 0 2 0 0 0 13 0 0 0 12 0 0 0 0 0 0 6 0 0 0 0 0 0 0 0 0 0 0
0 0 0 0 0 10 0 0 0 0 3 0 0 1 1 0 0 0 0 0 0 0 0 0 0 0 0
M02255_77_000000000-ADUT3_1_1104_14187_13141 C----TG---TG-CC-AGC--------AGC-C-G------C--G--G---T--AA-TACAG-AG------GG-T--G-C--AA-GC-------GT--T-----AA-TC-GG-AT-------T-TA-G-G-T-G------------G--C-CA-A-T-T-----
------AA----G-T-C-A--------A-A-T--G--TG--A-AA-TC--C-C-CG-A-G-----CT-T-AA------C-T-T-G-G-G-A--A-T----T-G--C-A---T--T-------C--GA-T-A---C-------T--G-G--TT--G-G-C----T-A-G-A-G-T--A---T-GG-----GA-G-A--------G-G-A-T---GG-T------AG--A--ATT----C-
C-A-G-GT--GT-A-G-CG-GT--G---A--A-A---TG-C-GT-AG--AG-A-TC--T-G--G-A-G-G-A-AT-A-CC---GA--T--G--GC-GAA-G--G-C--A-------G--C-C-A-T---CTG--G--CC-T-A--------------------A-T---A-C-T--GA--CA-C-T-G--A-GG--T-A-CG-A--AA-G-C---A-TG--GG-G-
-AG-C-A-AA--------CA--GG Bacteria(100);Proteobacteria(100);Gammaproteobacteria(100);Pseudomonadales(100);Moraxellaceae(100);Acinetobacter(100);unclassified(100);unclassified(100);unclassified(100);

Otu0146 0 0 0 130 0 0 0 0 0 0 0 0 0 0 0 0 0 0 7 0 0 0 0 0 0 0 0 1
0 0 1 111 0 0 0 0 0 0 0 48 0 0 0 44 83 0 0 0 0 0 1 31 0 0 0 0 0
0 0 0 19 0 0 0 0 0 0 0 1 0 1 77 226 0 0 153 114 132 0 0 0 0 0 0 0 2
57 0 0 0 0 35 5 22 0 137 0 52 0 0 0 56 0 37 0 33 483 0 59 0 11 0 13
M02255_77_000000000-ADUT3_1_1101_7605_10091 T----AC---GT-AG-GGG--------GCA-A-G------C--G--T---T--AT-C-CGG-AA------TT-A--T-T--GG-GC-------GT--A-----AA-GA-GT-GC-------G-TA-G-G-T-G------------G--T-TA-C-C-T----------AA-
---G-C-G-C--------A-A-G--G--TT--T-AA-TT--T-A-GA-G-G-----CT-C-AA------C-C-T-C-T-A-C--T------T-G--C-C---T--T-------G--CG-A-A---C-------T--G-G--GC--T-A-C----T-T-G-A-G-T--G---C-AG-----GA-G-G--------G-G-A-A---AG-C------GG--A--ATT----C-C-T-A-
GT--GT-A-G-CG-GT--G---A--A-A---TG-C-GT-AG--AT-A-TT--A-G--G-A-G-G-A-AC-A-CC---AG--C--G--GC-GAA-G--G-C--G-------G--C-T-T-T---CTG--G--AC-T-G--------------------T-A---A-C-T--GA--CA-C-T-G--A-GG--C-A-CG-A--AA-G-C---G-TG--GG-T--AG-C-
A-AA--------CA--GG Bacteria(100);Firmicutes(100);Clostridia(100);Clostridiales(100);Family_XIII_Incertae_Sedis(100);Mogibacterium(100);unclassified(100);unclassified(100);unclassified(100);

Otu0147 0 0 0 0 0 0 0 42 0 0 0 0 0 0 0 0 0 0 0 0 1 1 0 0 0 0 4 0
5 0 28 14 223 9 274 0 0 0 367 3 0 39 0 0 0 0 13 0 0 0 33 0 58 0 0 0 0
0 0 0 0 0 0 0 0 0 0 0 0 0 9 0 0 0 0 0 0 0 0 0 0 0 0 0 0 0
0 0 0 38 0 0 44 0 0 0 0 0 6 0 0 163 0 0 0 0 0 0 0 0 22 0 0
M02255_77_000000000-ADUT3_1_1106_24316_16982 T----AC---GA-AG-GGG--------GCT-A-G------C--G--T---T--GC-T-CGG-AA------TC-A--C-T--GG-GC-------GT--A-----AA-GG-GT-GC-------G-TA-G-G-C-G------------G--G-TT-T-T-T-----
------AA----G-T-C-A--------G-A-G--G--TG--A-AA-TC--C-T-GG-A-G-----CT-C-AA------C-T-C-C-A-G-A--A-C----T-G--C-C---T--T-------T--GA-T-A---C-------T--G-A--AA--G-T-C----T-T-G-A-G-T--A---T-GG-----GA-G-A--------G-G-T-G---AG-T------GG--A--ACT----G-
C-G-A-GT--GT-A-G-AG-GT--G---A--A-A---TT-C-GT-AG--AT-A-TT--C-G--C-A-A-G-A-AC-A-CC---AG--T--G--GC-GAA-G--G-C--G-------G--C-T-C-A---CTG--G--CC-C-A--------------------T-A---A-C-T--GA--CG-C-T-G--A-GG--C-A-CG-A--AA-G-C---G-TG--GG-G-
-AG-C-A-AA--------CA--GG Bacteria(100);Proteobacteria(100);Alphaproteobacteria(100);Rhizobiales(100);Bradyrhizobiaceae(100);unclassified(100);unclassified(100);unclassified(100);unclassified(100);

Otu0148 0 0 0 0 0 32 0 0 0 0 0 0 0 0 0 0 0 0 0 0 0 1 0 0 0 0 0 0
0 0 0 0 0 0 0 63 24 20 28 0 16 0 0 0 0 0 0 0 0 0 0 0 0 0 0 0 0
0 0 0 0 0 0 0 0 0 0 0 0 0 0 0 0 0 18 0 0 0 0 0 0 0 0 0 0 0
0 0 23 0 0 0 0 0 0 0 0 0 0 0 0 14 0 0 0 0 0 99 0 0 0 0 0
M02255_77_000000000-ADUT3_1_1109_16667_5531 C----AC---GT-AG-GCA--------CCA-A-G------C--G--T---T--GT-C-CGG-AT------TT-A--T-T--GG-GC-------GT--A-----AA-GA-GC-TC-------G-TA-G-G-C-G------------G--T-TC-G-G-T----------AA--
--G-T-C-G--------G-G-T--G--TG--A-AA-AC--T-C-TG-G-G-----CT-C-AA------C-C-C-A-G-A-G--C------C-G-CC-A---C--C-------C--GA-T-A---C-------T--G-C--TG-TG-A-C----T-C-G-A-G-T--G---C-GG-----TA-G-G--------G-G-A-G---CG-G------GG--A--ATT----C-C-T-G-
GT--GT-A-G-CG-GT--G---A--A-A---TG-C-GC-AG--AT-A-TC--A-G--G-A-G-G-A-AC-A-CC---AG--T--G--GC-GAA-G--G-C--G-------C--C-G-C-T---CTG--G--GC-C-G--------------------T-G---A-C-T--GA--CG-C-T-G--A-GG--A-G-CG-A--AA-G-C---G-TG--GG-T--AG-
C-A-AA--------CA--GG Bacteria(100);Actinobacteria(100);Actinobacteria(100);Acidimicrobidae(100);Acidimicrobiales(100);Acidimicrobineae(100);Acidimicrobiaceae(100);uncultured(75);unclassified(75);

Otu0149 335 0 0 0 0 0 0 0 0 0 0 0 0 0 0 0 0 1 0 0 0 5 0 0 1 0 3 2
0 0 0 0 0 0 0 0 0 0 0 0 0 0 0 0 0 0 0 0 0 0 0 0 19 0 0 0 0
0 0 0 0 0 0 0 0 0 0 0 0 0 0 0 0 0 0 0 0 0 0 0 0 0 0 0 0 0
0 0 0 70 0 0 37 0 0 0 0 0 0 0 0 0 0 0 0 0 0 11 0 0 0 0 0
M02255_77_000000000-ADUT3_1_1111_4054_8793 T----AC---GA-GG-GGG--------GCA-A-G------C--G--T---T--GT-T-CGG-AA------TT-A--T-T--GG-GC-------GT--A-----AA-GG-GC-GC-------G-TA-G-G-C-G------------G--T-TT-G-G-C----------
AA----G-T-T-T--------G-G-T--G--TG--A-AA-TC--T-T-CG-G-G-----CT-C-AA------C-T-C-G-A-A-G--T-C----T-G--C-A---C--C-------G--AA-A-A---C-------T--G-C--CG--G-G-C----T-T-G-A-G-T--A---T-GG-----GA-G-A--------G-G-T-G---AG-T------GG--A--ATT----T-C-C-
G-GT--GT-A-G-CG-GT--G---A--A-A---TG-C-GT-AG--AT-A-TC--G-G--A-A-G-G-A-AC-A-CC---TG--T--G--GC-GAA-A--G-C--G-------G--C-T-C-A---CTG--G--AC-C-A--------------------T-T---A-C-T--GA--CG-C-T-G--A-GG--C-G-CG-A--AA-G-C---T-AG--GG-G--
AG-C-A-AA--------CA--GG Bacteria(100);Acidobacteria(100);Acidobacteria(100);Acidobacteriales(100);Acidobacteriaceae(100);unclassified(96);unclassified(96);unclassified(96);unclassified(96);

Otu0150 0 0 0 24 0 0 68 0 0 0 0 0 0 81 73 0 0 0 0 0 7 0 0 0 0 9 9 11
0 0 9 0 0 71 0 1 1 69 50 4 1 0 168 109 34 307 0 0 0 0 0 29 60 0 0 2 0
0 0 29 1 22 11 5 0 25 9 0 0 0 0 0 0 0 19 0 0 360 0 0 0 3 0 0 0 0
17 0 77 70 206 1 7 0 0 0 0 0 0 0 0 0 0 0 303 45 0 0 0 0 0 0 21
M02255_77_000000000-ADUT3_1_1107_24322_20884 T----AC---GG-AG-GGA--------GCT-A-G------C--G--T---T--GT-T-CGG-AA------TT-A--C-T--GG-GC-------GT--A-----AA-GC-GC-AC-------G-TA-G-G-C-G------------G--T-TA-T-T-C-----
------AA----G-T-C-A--------G-A-G--G--TG--A-AA-GC--C-C-GG-G-G-----CT-C-AA------C-T-C-C-G-G-A--A-C----T-G--C-C---T--T-------T--GA-A-A---C-------T--A-G--AT--A-A-C----T-T-G-A-A-T--C---C-AG-----GA-G-A--------G-G-T-G---AG-T------GG--A--ATT----C-
C-G-A-GT--GT-A-G-AG-GT--G---A--A-A---TT-C-GT-AG--AT-A-TT--C-G--G-A-A-G-A-AC-A-CC---AG--T--G--GC-GAA-G--G-C--G-------G--C-T-C-A---CTG--G--AC-T-G--------------------G-T---A-T-T--GA--CG-C-T-G--A-GG--T-G-CG-A--AA-G-C---G-TG--GG-
G--AG-C-A-AA--------CA--GG Bacteria(100);Proteobacteria(100);Alphaproteobacteria(100);Sphingomonadales(100);Sphingomonadaceae(100);Sphingobium(80);unclassified(80);unclassified(80);unclassified(80);

Otu0151 0 0 0 0 0 0 0 0 0 0 0 9 0 1 0 13 4 0 22 4 0 0 3 0 0 0 5 3
0 0 0 0 0 0 0 6 0 0 6 0 0 0 0 0 0 0 0 0 0 0 35 0 9 0 0 0 0
0 0 0 54 0 0 0 0 0 0 0 0 0 0 0 0 0 15 0 0 0 0 0 0 0 0 0 0 8
0 0 2 0 0 0 1 0 14 0 7 0 1 0 0 54 6 0 0 0 0 0 0 0 0 0 1
M02255_76_000000000-ADUNE_1_1117_22857_24299 T----AA---GC-AC-CGG--------CTA-A-C------T--A--C---G--TG-C-CAG-CA------GC-C--G-C--GG-TA-------AT--A-----CG-TA-GG-GT-------G-CA-A-G-C-G------------G--T-TA-T-A-T------
-----CG----G-A-C-A--------G-A-T--G--TG--A-AA-TC--C-C-CG-G-G-----CT-C-AA------C-C-T-G-G-G-A--A-C----T-G--C-A---T--T-------T--GT-G-A---C-------T--G-T--AT--A-G-C----T-A-G-A-G-T--A---C-GG-----TA-G-A--------G-G-G-G---GA-T------GG--A--ATT----C-
C-G-C-GT--GT-A-G-CA-GT--G---A--A-A---TG-C-GT-AG--AT-A-TG--C-G--G-A-G-G-A-AC-A-CC---GA--T--G--GC-GAA-G--G-C--A-------A--T-C-C-C---CTG--G--AC-C-T--------------------G-T---A-C-T--GA--CG-C-T-C--A-TG--C-A-CG-A--AA-G-C---G-TG--GG-G-
-AG-C-A-AA--------CA--GG Bacteria(100);Proteobacteria(100);Betaproteobacteria(100);Burkholderiales(100);Comamonadaceae(100);unclassified(100);unclassified(100);unclassified(100);unclassified(100);

Otu0152 0 0 0 0 0 0 0 0 0 0 0 0 0 0 0 0 1 0 0 0 0 0 0 0 0 0 0 0
0 0 0 1 0 0 0 0 27 6 0 0 0 1 0 0 1 0 0 0 0 1 0 0 0 0 0 0 0
7 0 0 1 0 0 0 0 0 0 0 0 0 0 0 0 2 0 2 7 7 0 0 0 0 64 65 0 6
2 0 0 0 1 4 0 0 0 10 1 31 1 0 0 0 0 0 0 0 0 0 0 0 0 0 0
M02255_76_000000000-ADUNE_1_2107_25676_9785 C----GG---CT-AA-TTC--------CGT-G-C------C--A--G---C--AG-C-CGC-GG------TA-A--T-A--CG-GA-------AG--G-----TC-CG-G--GC-------G-TT-A-T-C-C------------G--G-AG-A-T-T-----------AT---
-T-C-G-T--------G-T-T--G--TG--A-AA-TG--T-A-GA-C-G-----CT-C-AA------C-G-T-C-T-G-C--A-C----T-G--C-A---G--C-------G--CG-A-A---C-------T--G-G--TT--T-C-C----T-T-G-A-G-T--A---C-GC-----AC-A-A--------A-G-T-G---GG-C------GG--A--ATT----C-G-T-G-GT--
GT-A-G-CG-GT--G---A--A-A---TG-C-TT-AG--AT-A-TC--A-C--G-A-A-G-A-AC-T-CC---GA--T--T--GC-GAA-G--G-C--A-------G--C-T-C-A---CTG--G--AG-C-G--------------------C-A---A-C-T--GA--CG-C-T-G--A-AG--C-T-CG-A--AA-G-T---G-CG--GG-T--AT-C-G-
AA--------CA--GG Bacteria(100);Bacteroidetes(100);Bacteroidia(100);Bacteroidales(100);Prevotellaceae(100);Prevotella(83);unclassified(83);unclassified(83);unclassified(83);

Otu0153 0 0 0 0 0 0 0 0 0 0 0 0 0 0 0 0 1 1 9 0 0 0 0 0 0 0 2 0
9 0 0 0 0 0 0 0 0 0 0 0 0 0 0 0 0 0 0 0 0 0 0 0 0 0 0 0 0
0 0 0 0 0 0 23 0 0 0 0 0 0 0 0 0 0 3 0 0 0 0 0 0 0 0 0 0 0
0 0 0 0 0 0 1 0 8 0 4 0 1 0 0 0 0 0 0 0 0 0 0 0 0 0 0
M02255_77_000000000-ADUT3_1_2116_24802_16598 T----AC---GG-AG-GAT--------CCA-A-G------C--G--T---T--AT-C-CGG-AA------TC-A--T-T--GG-GT-------TT--A-----AA-GG-GT-CC-------G-TA-G-G-C-G------------G--T-TT-A-G-T------
-----AA----G-T-C-A--------G-T-G--G--TG--A-AA-GC--C-C-AT-C-G-----CT-C-AA------C-G-G-T-G-G-A--A-C----G-G--C-C---A--T-------T--GA-T-A---C-------T--G-C--TA--A-A-C----T-T-G-A-A-T--T---A-TT-----AG-G-A--------A-G-T-A---AC-T------AG--A--ATA----T-
G-T-A-GT--GT-A-G-CG-GT--G---A--A-A---TG-C-TT-AG--AG-A-TT--A-C--A-T-G-G-A-AT-A-CC---AA--T--T--GC-GAA-G--G-C--A-------G--G-T-T-A---CTA--C--TA-A-T--------------------G-G---A-T-T--GA--CG-C-T-G--A-TG--G-A-CG-A--AA-G-C---G-TG--GG-T--
AG-C-G-AA--------CA--GG Bacteria(100);Bacteroidetes(100);Flavobacteria(100);Flavobacteriales(100);Flavobacteriaceae(100);Flavobacterium(100);unclassified(100);unclassified(100);unclassified(100);

Otu0154 0 0 60 0 0 0 0 0 0 0 0 0 0 0 0 0 0 0 2 0 0 0 0 0 0 0 0 0
0 0 0 0 0 0 0 1 0 0 0 0 0 0 0 0 0 0 0 249 526 0 0 0 0 0 0 0 0
0 0 1 0 0 0 0 0 0 6 0 0 25 20 23 19 0 0 0 0 0 0 0 0 0 0 0 0 0
547 0 0 0 0 0 11 0 0 29 0 0 0 0 0 71 0 0 19 0 0 82 0 0 0 0 0
M02255_77_000000000-ADUT3_1_1104_5195_17397 T----AC---GT-AA-GGA--------CCG-A-G------C--G--T---T--GT-C-CGG-AA------TC-A--T-T--GG-GC-------GT--A-----AA-GG-GT-AC-------G-TA-G-G-C-G------------G--G-TA-T-T-T-----------AA-
---G-T-T-A--------G-A-A--G--TC--A-AA-GG--C-T-AT-A-G-----CT-T-AA------C-T-G-T-A-G-T--A------A-G--C-T---T--C-------T--AA-A-A---C-------T--A-G--AT--A-T-C----T-T-G-A-G-A--A---A-TG-----GA-A-G--------G-G-A-A---AG-T------GG--A--ATT----C-C-T-A-GT-
-GT-A-G-CG-GT--G---G--A-A---TG-C-GC-AG--AT-A-TT--A-G--G-A-A-G-A-AT-A-CC---GG--T--G--GC-GAA-G--G-C--G-------A--C-T-T-T---CTG--G--CC-A-T--------------------T-T---T-C-T--GA--CG-C-T-G--A-GG--T-A-CG-A--AA-G-C---G-TG--GG-T--AG-C-A-
AA--------CA--GG Bacteria(100);Firmicutes(100);Clostridia(100);Clostridiales(100);Family_XI_Incertae_Sedis(100);Anaerococcus(100);unclassified(100);unclassified(100);unclassified(100);

Otu0155 0 20 22 3 0 0 0 0 0 0 0 0 0 0 0 0 0 0 0 0 0 0 0 0 0 0 0 0
0 0 0 0 0 0 17 0 0 1 0 0 0 0 0 0 0 0 0 0 0 0 0 0 0 0 0 4 7
0 0 2 0 0 0 0 0 0 0 0 0 0 0 0 0 6 0 0 8 0 0 0 0 0 0 0 0 2
12 0 0 11 23 0 0 0 0 0 0 0 0 6 0 13 0 13 0 7 0 0 8 0 0 0 18
M02255_76_000000000-ADUNE_1_2110_18689_13556 C----GG---CT-AA-TTC--------CGT-G-C------C--A--G---C--AG-C-CGC-GG------TA-A--T-A--CG-GA-------AG--G-----TC-CA-GG-CG-------T-TA-T-C-C-G------------G--ATTT-G-T-T------
-----AA----G-T-C-A--------G-C-G--G--TT--A-AA-GG--G-T-GT-G-G-----CT-C-AA------C-C-A-T-A-C----A-T----T-G--C-C---G--T-------T--GA-A-A---C-------T--G-G--CG--A-T-C----T-T-G-A-G-T--G---C-AG-----AC-A-G--------G-G-A-T---GC-C------GG--A--ATT----C-
G-T-G-GT--GT-A-G-CG-GT--G---A--A-A---TG-C-TT-AG--AT-A-TC--A-C--G-A-A-G-A-AC-T-CC---GA--T--C--GC-GAA-G--G-C--A-------G--G-T-G-T---CCG--G--GC-T-G--------------------C-A---A-C-T--GA--CG-C-T-G--A-GG--C-T-CG-A--AA-G-T---G-TG--GG-T-
-AT-C-A-AA--------CA--GG Bacteria(100);Bacteroidetes(100);unclassified(100);unclassified(100);unclassified(100);unclassified(100);unclassified(100);unclassified(100);unclassified(100);

Otu0156 0 0 0 300 0 17 30 11 26 0 0 0 0 0 0 0 0 0 0 0 6 0 0 9 0 0 0 13
3 0 0 44 0 0 60 10 0 21 1 18 0 0 0 0 0 0 0 146 0 0 28 40 7 0 0 0 114
1 355 0 0 2 0 0 0 0 0 0 0 0 0 244 360 28 0 90 0 0 5 0 0 47 0 0 0 0
36 0 0 0 0 0 1 0 0 0 0 0 0 32 0 0 0 0 0 43 71 43 9 0 0 1 81
M02255_77_000000000-ADUT3_1_1102_5427_11006 T----AC---GT-AG-GAT--------CCG-A-G------C--G--T---T--AT-C-CGG-AG------TG-A--C-T--GG-GC-------GT--A-----AA-GA-GTTGC-------G-TA-G-G-C-G------------G--T-TT-G-T-T----------AA-
---G-T-G-A--------A-T-A--G--TG--A-AA-TC--T-G-GT-G-G-----CT-C-AA------C-C-A-T-ACA-G--G-C----T-A--T-T---A--T-------T--CA-A-A---C-------T--G-G--CA--A-A-C----T-C-G-A-G-A--G---T-GG-----TA-G-A--------G-G-T-C---AC-T------GG--A--ATT----T-C-T-T-
GT--GT-A-G-GA-GT--G---A--A-A---TC-C-GT-AG--AT-A-TA--A-G--A-A-G-G-A-AC-A-CC---GA--T--G--GC-GTA-G--G-C--A-------G--G-T-G-A---CTG--G--AC-C-A--------------------T-T---T-C-T--GA--CG-C-T-A--A-GG--C-A-CG-A--AA-G-C---G-TG--GG-G--AG-
C-G-AA--------CC--GG Bacteria(100);Candidate_division_TM7(100);unclassified(100);unclassified(100);unclassified(100);unclassified(100);unclassified(100);unclassified(100);unclassified(100);

Otu0157 0 0 0 0 0 0 0 0 0 0 0 0 5 0 2 0 0 2 0 0 0 0 0 0 0 0 0 0
0 0 0 0 0 0 0 0 0 0 0 0 0 0 0 0 0 0 0 0 0 0 0 0 0 2 4 2 0
1 0 0 0 2 0 0 2 0 2 0 0 0 5 0 0 0 0 0 0 0 0 0 0 0 0 0 0 0
0 0 0 0 0 0 0 1 0 0 1 0 0 0 1 0 0 0 0 0 0 0 0 0 0 0 0
M02255_76_000000000-ADUNE_1_1116_21055_23480 T----AC---CG-AC-AGA--------ATA-A-G------C-----A---C--CG-G-CTA-AC------TC-T--G-T--GC-CA-------GC--AG----CC-GC-GG-TA-------A-TACA-G-A-G------------G--G-TA-G-T-C-----
------AA----GCG-T-T--------A-A-T--C--GG--A-AA-TC--C-C-CG-G-G-----CT-C-AA------C-C-T-G-G-G-A--A-C----T-G--C-A---T--T-------C--AA-A-A---C-------T--G-A--CT--G-A-C----T-A-G-A-G-T--A---T-GG-----TA-G-A--------G-G-G-T---GG-T------GG--A--ATT----T-
C-C-T-GT--GT-A-G-CG-GT--G---A--A-A---TG-C-GT-AG--AT-A-TA--G-G--A-A-G-G-A-AC-A-CC---AG--T--G--GC-GAA-G--G-C--G-------A--C-C-A-C---CTG--G--AC-T-G--------------------A-T---A-C-T--GA--CA-C-T-G--A-GG--T-G-CG-A--AA-G-C---G-TG--GG-
G--AG-C-A-AA--------CA--GG Bacteria(100);Proteobacteria(100);Gammaproteobacteria(100);unclassified(100);unclassified(100);unclassified(100);unclassified(100);unclassified(100);unclassified(100);

Otu0158 0 0 0 61 0 0 0 0 0 0 0 0 0 0 0 0 0 0 0 0 0 0 0 0 0 0 0 0
0 0 0 48 0 0 0 0 0 0 0 37 0 0 0 0 0 0 0 0 0 0 3 23 0 0 0 0 0
12 0 0 0 0 0 0 0 2 0 0 0 0 0 0 0 0 5 0 0 0 0 0 27 0 0 0 0 16
0 0 0 0 0 27 0 0 0 0 0 0 0 0 0 43 41 0 0 0 0 37 0 0 0 0 0
M02255_77_000000000-ADUT3_1_1104_7160_16507 T----AC---GT-AG-GGG--------TCG-A-G------C--G--T---T--GT-C-CGG-AG------TT-A--C-T--GG-GC-------GT--A-----AA-GC-GC-GT-------G-CA-G-G-T-G------------G--C-TC-A-T-T----------AC-
---G-C-C-C--------G-G-C--G--TG--A-AA-GC--C-C-CC-G-G-----CT-C-AA------C-C-G-G-G-G-A--G-G----G-T--C-G---T--C-------G--GG-G-A---C-------G--G-A--TG--A-G-C----T-T-G-A-G-G--G---T-CG-----CA-G-G--------G-G-C-T---GG-T------GG--A--ATT----C-C-C-
G-GT--GT-A-G-TG-GT--G---A--A-A---TG-C-GT-AG--AG-A-TC--G-G--G-A-G-G-A-AC-A-CC---CG--T--G--GC-G-A-A-GG-C--G-------G--C-C-A-G---CTG--G--GC-G-A--------------------C-A---C-C-T--GA--CA-C-T-G--A-GA--C-G-CG-A--AG-G-C---G-TG--GG-G--
AG-C-G-AA--------CG--GG Bacteria(100);Chloroflexi(100);Thermomicrobia(100);JG30-KF-CM45(100);unclassified(100);unclassified(100);unclassified(100);unclassified(100);unclassified(100);

Otu0159 0 0 0 0 0 0 0 0 0 0 0 0 9 0 1 1 0 8 0 0 2 1 0 0 0 0 0 0
0 0 0 0 0 0 0 0 0 0 0 0 0 0 0 0 0 1 0 0 0 0 0 0 10 29 12 0
1 1 5 3 2 0 2 13 0 11 13 4 0 8 0 0 0 0 0 0 0 0 0 0 0 0 0 0 0
0 0 0 0 0 0 2 2 0 0 4 0 6 0 0 2 0 0 0 0 0 0 0 0 0 0 0
M02255_77_000000000-ADUT3_1_2117_19777_12277 C----TG---TG-CC-AGC--------AGC-C-G------C--G--G---T--AA-TACAG-AG------GG-T--G-C--AA-GC-------GT--T-----AA-TC-GG-AA-------TTACTGGG-T-G------------G--T-TT-G-T-T---
--------AA----G-T-T-G--------G-A-T--G--TG--A-AA-TC--C-C-CG-G-G-----CT-C-AA------C-C-T-G-G-G-A--A-C----T-G--C-A---T--T-------C--AA-A-A---C-------T--G-A--CT--G-A-C----T-A-G-A-G-T--A---T-GG-----TA-G-A--------G-G-G-T---GG-T------GG--A--ATT----
T-C-C-T-GT--GT-A-G-CG-GT--G---A--A-A---TG-C-GT-AG--AT-A-TA--G-G--A-A-G-G-A-AC-A-CC---AG--T--G--GC-GAA-G--G-C--G-------A--C-C-A-C---CTG--G--AC-T-G--------------------A-T---A-C-T--GA--CA-C-T-G--A-GG--T-G-CG-A--AA-G-C---G-TG--GG-
G--AG-C-A-AA--------CA--GG Bacteria(100);Proteobacteria(100);Gammaproteobacteria(100);Pseudomonadales(100);Pseudomonadaceae(100);Pseudomonas(99);unclassified(99);unclassified(99);unclassified(99);

Otu0160 0 0 0 36 0 0 0 0 34 0 0 0 0 0 0 0 0 0 0 4 0 0 0 0 0 0 0 0
0 0 0 0 0 81 0 0 0 0 0 0 0 0 0 0 0 0 0 0 0 0 0 28 0 0 0
0 0 0 0 0 0 0 0 0 0 0 0 0 0 0 0 0 0 0 7 0 0 0 0 0 0 0
0 0 0 48 0 0 0 0 0 0 3 0 0 0 0 0 0 0 0 0 0 0 0 0 0 0
M02255_77_000000000-ADUT3_1_1113_15921_5263 T----AC---GA-AG-GGG--------GCT-A-G------C--G--T---T--GC-T-CGG-AA------TT-A--C-T--GG-GC-------GT--A-----AA-GG-GC-GC-------G-TA-G-G-C-G------------G--C-TT-G-G-T----------
TA----G-T-C-A--------G-G-C--G--TG--A-AA-TT--C-C-CG-G-G-----CT-T-AA------C-C-T-G-G-G-G--A-C----T-G--C-G---C--T-------T--GA-T-A---C-------T--G-C--TG--G-G-C----T-T-G-A-G-G--G---T-GG-----AA-G-A--------G-G-G-T---CG-T------GG--A--ATT----C-C-C-
A-GT--GT-A-G-AG-GT--G---A--A-A---TT-C-GT-AG--AT-A-TT--G-G--G-A-A-G-A-AC-A-CC---GG--T--G--GC-GAA-G--G-C--G-------G--C-G-A-C---CTG--G--TC-C-A--------------------T-T---A-C-T--GA--CG-C-T-G--A-GG--C-G-CG-A--CA-G-C---G-TG--GG-G--
AG-C-A-AA--------CA--GG Bacteria(100);Proteobacteria(100);Alphaproteobacteria(100);Rhodospirillales(100);Acetobacteraceae(100);unclassified(100);unclassified(100);unclassified(100);unclassified(100);

Otu0161 0 0 0 2 6 5 1 0 0 11 22 19 2 2 2 7 1 1 1 3 0 8 5 17 2 4 2 6
24 0 0 0 0 0 0 0 0 0 1 0 0 0 0 0 23 1 0 2 1 0 0 0 0 0 0 0
0 0 0 0 0 0 0 0 0 0 0 0 0 0 0 0 0 0 1 0 0 38 21 0 0 0 0 1 0
14 0 0 0 1 5 24 7 10 14 4 0 0 24 0 10 0 3 37 0 0 9 19 71 9 0 0
M02255_77_000000000-ADUT3_1_1108_10666_19330 T----AC---AG-AG-GGT--------GCA-A-G------C--G--T---T--AA-T-CGG-AA------TT-A--C-T--GG-GC-------GT--A-----AA-GG-GA-GC-------G-TA-G-G-T-G------------G--C-TC-T-A-T-----
------AA----G-T-C-A--------G-A-T--G--TG--A-AA-TC--C-C-CG-G-G-----CT-T-AA------C-C-T-G-G-G-A--A-C----T-G--C-A---T--C-------T--GA-A-A---C-------T--G-T--AG--A-G-C----T-A-G-A-G-T--A---T-GT-----GA-G-A--------G-G-A-A---GG-T------AG--A--ATT----C-
C-A-G-GT--GT-A-G-CG-GT--G---A--A-A---TG-C-GT-AG--AG-A-TC--T-G--G-A-G-G-A-AT-A-CC---GA--T--G--GC-GAA-G--G-C--A-------G--C-C-T-T---CTG--G--CA-T-A--------------------A-T---A-C-T--GA--CA-C-T-G--A-GG--C-T-CG-A--AA-G-C---G-TG--GG-T-
-AG-C-A-AA--------CA--GG Bacteria(100);Proteobacteria(100);Gammaproteobacteria(100);Pseudomonadales(100);Moraxellaceae(100);Psychrobacter(100);unclassified(100);unclassified(100);unclassified(100);

Otu0162 0 12 0 0 199 0 7 1 0 0 0 0 0 2 0 0 0 0 0 2 4 2 8 0 0 0 0 0
1 0 0 0 0 0 0 0 0 15 2 0 0 69 0 16 0 0 0 0 0 0 0 0 0 0 0 0
0 0 0 5 0 0 0 0 0 0 8 0 0 0 5 0 34 0 0 0 28 0 0 0 0 0 0 0 0
0 0 0 3 0 0 0 0 0 0 0 23 2 11 0 121 0 9 27 0 0 0 0 0 9 234 27
M02255_76_000000000-ADUNE_1_1106_27842_12513 .....AC----T-AC-GT---------GCC-A-G------C--A--G---C--CG-C-GGT-AA------TA-C--G-T--AG-GT-------GG--C-----AA----GC-G--------T-TA-T-C-C-G------------G--T-AA-A-A-T----------
AA----G-T-C-T--------G-A-T--G--TG--A-AA-GC--C-C-CC-G-G-----CT-C-AA------C-C-G-G-G-G-A--G------G-GT-C-A---T--T-------G--GA-A-A---C-------T--G-T--TT--T-A-C----T-T-G-A-G-T--A---C-AG-----AA-G-A--------G-G-A-G---AG-T------GG--A--ATT----C-C-A-
T-GT--GT-A-G-CG-GT--G---A--A-A---TG-C-GC-AG--AG-A-TA--T-G--G-A-G-G-A-AC-A-CC---AG--T--G--GC-GAA-G--G-C--G-------G--C-T-C-T---CTG--G--TC-T-G--------------------T-A---A-C-T--GA--CG-C-T-G--A-GG--T-G-CG-A--AA-G-C---G-TG--GG-G--
AT-C-A-AA--------CA--GG Bacteria(100);Firmicutes(100);Bacilli(100);Bacillales(100);Staphylococcaceae(100);unclassified(100);unclassified(100);unclassified(100);unclassified(100);

Otu0163 0 0 0 0 0 0 0 0 0 0 0 0 0 0 0 0 0 0 0 0 0 0 1 0 0 10 0 934
31 0 3 0 0 1 0 0 0 5 17 0 0 0 0 1 0 67 0 162 326 0 0 0 0 0 0 0
0 0 0 0 0 0 0 0 0 0 0 0 0 0 0 0 1 0 0 0 0 9 47 0 0 0 0 0 0
0 0 0 0 0 0 0 0 0 0 0 0 0 0 0 0 0 0 0 0 0 0 0 0 199 0 0
M02255_77_000000000-ADUT3_1_2111_12397_17424 T----AC---GT-AT-GTC--------GCA-A-G------C--G--T---T--AT-C-CGG-AA------TT-A--T-T--GG-GC-------GT--A-----AA-GC-GA-GT-------C-TA-G-G-C-G------------G--C-TT-G-T-T-------
----AA----G-T-C-A--------G-A-T--G--TG--A-AA-AT--G-C-GG-G-G-----CT-C-AA------C-T-C-C-G-T-A--T------T-G--C-G---T--T-------T--GA-T-A---C-------T--G-G--CA--G-G-C----T-A-G-A-G-T--A---C-TG-----GA-G-A--------G-G-T-G---GG-C------GG--A--ACT----A-
C-A-A-GT--GT-A-G-AG-GT--G---A--A-A---TT-C-GT-AG--AT-A-TT--T-G--T-A-G-G-A-AT-G-CC---GA--T--A--GT-GAA-G--A-C--A-------G--C-T-C-A---CTG--G--AC-A-G--------------------A-T---A-C-T--GA--CG-C-T-A--A-AG--C-T-CG-A--AA-G-C---G-TG--GG-G--
AG-C-A-AA--------CA--GG Bacteria(100);Fusobacteria(100);Fusobacteria(100);Fusobacteriales(100);Fusobacteriaceae(100);Psychrilyobacter(100);unclassified(100);unclassified(100);unclassified(100);

Otu0164 0 0 0 0 0 0 0 0 0 0 0 0 0 0 0 0 0 0 0 0 0 0 0 0 0 2 0 0
0 0 0 0 0 0 0 0 0 0 0 0 83 0 0 0 0 0 0 0 0 0 0 0 0 0 0 0
0 0 0 0 0 0 0 0 0 0 0 0 0 0 0 0 98 62 0 0 0 0 0 0 0 0 0 0 0
0 136 0 0 114 0 0 0 0 0 0 0 0 0 0 0 0 0 0 0 0 0 0 0 0 0 0
M02255_77_000000000-ADUT3_1_1105_24566_11451 T----AC---GG-AG-GGG--------GCT-A-G------C--G--T---T--GT-T-CGG-AA------AT-A--C-T--GG-GC-------GT--A-----AA-GC-GA-AC-------G-TA-G-G-C-G------------G--C-TT-A-G-C----
-------AA----G-T-C-A--------G-G-G--G--TG--A-AA-TC--C-C-GG-A-G-----CT-C-AA------C-T-C-C-G-G-A--A-C----T-G--C-C---C--T-------T--GA-A-A---C-------T--G-C--TT--C-G-C----T-T-G-A-A-T--C---C-TG-----GA-G-A--------G-G-C-G---AG-T------GG--A--ATT----C-
C-G-A-GT--GT-A-G-AG-GT--G---A--A-A---TT-C-GT-AG--AT-A-TT--C-G--G-A-A-G-A-AC-A-CC---AG--T--G--GC-GAA-G--G-C--G-------A--C-T-C-G---CTG--G--AC-A-G--------------------G-T---A-T-T--GA--CG-C-T-G--A-GG--T-T-CG-A--AA-G-C---G-TG--GG-G-
-AG-C-A-AA--------CA--GG Bacteria(100);Proteobacteria(100);Alphaproteobacteria(100);Sphingomonadales(73);Erythrobacteraceae(67);unclassified;unclassified;unclassified;unclassified;

Otu0165 36 0 0 0 0 0 12 0 0 0 0 0 0 0 0 0 0 0 0 0 0 0 0 2 0 0 0 0
0 0 20 0 0 10 0 0 0 0 13 0 0 0 0 0 0 0 0 0 0 0 0 0 0 0 0 0
0 0 6 0 0 0 0 0 0 0 0 0 0 0 343 172 0 18 0 14 0 1 0 0 21 0 0 0 17
17 14 27 16 10 25 2 2 0 1 0 0 0 15 0 0 0 0 8 13 0 0 10 0 0 0 19

M02255_76_000000000-ADUNE_1_1104_13666_22326 C----TA---AC-TC-CGT--------GCC-A-G------C-----A---G--CC-G-CGG-TA------AT-A--C-G--GA-GG-------GT--------GC-GA-GC--G-------T-TA-A-T-C-G------------G--A-AT-T-T-T----------AA----G-T-G-A--------G-G-T--G--TG--A-AA-TC--C-C-CG-G-G-----CT-T-AA------C-C-T-G-G-G-A--A-T----T-G--C-A---T--T-------T--CA-G-A---C-------T--G-G--GG--G-T-C----T-A-G-A-G-T--A---C-TT-----TA-G-G--------G-A-G-G---GG-T------AG--A--ATT----C-C-A-C-GT--GT-A-G-CG-GT--G---A--A-A---TG-C-GT-AG--AG-A-TG--T-G--G-A-G-G-A-AT-A-CC---GA--A--G--GC-GAA-G--G-C--A-------G--C-C-C-C---TTG--G--GA-A-T--------------------G-T---A-C-T--GA--CG-C-T-C--A-TG--T-G-CG-A--AA-G-C---G-TG--GG-G--AG-C-A-AA--------CA--GG Bacteria(100);Proteobacteria(100);Gammaproteobacteria(100);Pasteurellales(100);Pasteurellaceae(100);unclassified(99);unclassified(99);unclassified(99);unclassified(99);

Otu0166 0 0 0 0 0 0 0 0 73 49 0 0 0 0 0 0 0 0 0 0 1 0 7 0 0 11 0 0
0 0 1 0 0 0 0 0 0 0 11 0 0 0 0 41 0 0 0 0 0 0 0 29 0 0 0 0 0
0 0 0 0 0 0 0 0 0 0 0 0 0 0 15 0 0 21 0 0 0 0 0 113 0 0 0 0 0
0 0 0 0 0 0 3 0 0 0 0 0 0 0 0 31 80 0 0 0 0 0 0 0 0 0 0

M02255_77_000000000-ADUT3_1_1101_24465_15879 T----AC---GT-AG-GGA--------CCA-A-G------C--G--T---T--GT-T-CGG-AT------TT-A--C-T--GG-GC-------GT--A-----AA-GG-GC-GC-------G-TA-G-G-C-G------------G--C-GT-G-A-C-----------AA----G-T-C-A--------G-T-T--G--TG--A-AA-TC--T-C-CG-A-G-----CT-T-AA------C-T-C-G-G-A-A--C-G----G-T--C-A---A--C-------T--GA-T-A---C-------T--G-T--TG--T-G-C----T-A-G-A-G-T--G---C-AG-----AA-G-G--------G-G-C-A---AT-C------GG--A--ATT----C-T-T-G-GT--GT-A-G-CG-GT--G---A--A-A---TG-C-GT-AG--AT-A-TC--A-A--G-A-G-G-A-AC-A-CC---TG--A--G--GT-GAA-G--A-C--G-------G--G-T-T-G---CTG--G--GC-T-G--------------------A-C---A-C-T--GA--CG-C-T-G--A-GG--C-G-CG-A--AA-G-C---T-AG--GG-T--AG-C-A-AA--------CG--GG Bacteria(100);Acidobacteria(100);Acidobacteria(100);Acidobacteriales(100);Acidobacteriaceae(100);Candidatus_Chloroacidobacterium(100);unclassified(100);unclassified(100);unclassified(100);

Otu0167 0 0 0 0 0 0 0 0 47 0 0 0 0 0 0 0 0 0 0 0 0 0 0 0 0 20 97 110
33 0 0 0 0 0 0 0 0 0 0 0 0 0 0 0 0 0 0 0 0 0 0 0 0 0 0 0
0 0 0 0 0 0 3 0 0 0 0 0 0 0 0 0 0 0 0 0 0 0 64 0 0 0 0 0 0
0 0 0 0 0 0 248 9 14 0 0 0 0 0 0 0 0 0 0 0 0 44 0 0 0 0

M02255_77_000000000-ADUT3_1_1101_20996_23785 T----AC---GG-AG-GGT--------GCA-A-G------C--G--T---T--AC-T-CGG-AA------TC-A--C-T--GG-GC-------GT--A-----AA-GA-GC-GT-------G-TA-G-G-C-G------------G--G-TT-G-A-T-----------AA----G-T-T-C--------G-A-A--G--TG--A-AA-TC--C-T-AT-A-G-----CT-T-AA------C-T-A-T-A-G-A--A-C----T-G--C-T---T--T-------G--AA-A-A---C-------T--G-T--TA--A-C-C----T-A-G-A-A-T--A---T-GG-----GA-G-A--------G-G-T-A---GA-T------GG--A--ATT----T-C-T-G-GT--GT-A-G-GG-GT--A---A--A-A---TC-C-GT-AG--AG-A-TC--A-G--A-A-G-G-A-AT-A-CC---GA--T--T--GC-GAA-G--G-C--G-------A--T-C-T-A---CTG--G--AA-C-A--------------------T-T---A-T-T--GA--CG-C-T-G--A-GA--C-G-CG-A--AA-G-C---G-TG--GG-T--AG-C-A-AA--------CG--GG Bacteria(100);Proteobacteria(100);Epsilonproteobacteria(100);Campylobacterales(100);Campylobacteraceae(100);Arcobacter(100);unclassified(100);unclassified(100);unclassified(100);

Otu0168 0 0 0 0 60 0 0 0 0 0 0 0 0 0 0 0 0 0 0 0 0 0 0 0 0 1 0 0
0 0 0 0 0 0 0 0 0 0 0 0 0 0 0 0 0 0 0 0 20 0 0 0 0 0 0 0
0 0 0 0 0 0 0 0 0 0 0 0 0 0 0 0 176 0 0 0 0 0 0 0 0 0 0 0 0
5 0 0 2 0 0 4 0 0 0 0 0 0 0 0 0 0 0 0 0 0 0 0 0 0 0 0

M02255_76_000000000-ADUNE_1_1103_7274_13391 T----AA---GC-AC-CGG--------CTA-A-C------T--A--C---G--TG-C-CAG-CA------GC-C--G-C--GG-TA-------AT--A-----CG-TA-GG-GT-------G-CA-A-G-C-G------------T--T-AA-TCG-G-----------AA----G-A-C-A--------G-A-T--G--TG--A-AA-TC--C-C-CG-G-G-----CT-C-AA------C-C-T-G-G-G-A--A-C----T-G--C-A---T--T-------T--GT-G-A---C-------T--G-C--AT--T-G-C----T-G-G-A-G-T--G---C-GG-----CA-G-A--------G-G-G-G---GA-T------GG--A--ATT----C-C-G-C-GT--GT-A-G-CA-GT--G---A--A-A---TG-C-GT-AG--AT-A-TG--C-G--G-A-G-G-A-AC-A-CC---GA--T--G--GC-GAA-G--G-C--A-------A--T-C-C-C---CTG--G--GC-C-T--------------------G-C---A-C-T--GA--CG-C-T-C--A-TG--C-A-CG-A--AA-G-C---G-TG--GG-G--AG-C-A-AA--------CA--GG Bacteria(100);Proteobacteria(100);Betaproteobacteria(100);Burkholderiales(100);Comamonadaceae(100);unclassified(100);unclassified(100);unclassified(100);unclassified(100);

Otu0169 100 0 0 0 0 0 36 1 0 0 0 0 0 0 0 0 0 0 0 0 2 0 0 0 0 0 0
14 0 0 0 0 0 0 0 0 0 0 0 0 0 0 0 0 0 0 0 3 0 0 0 0 0 22 0
0 0 0 0 0 0 0 27 0 0 3 0 0 0 0 0 90 0 0 0 0 12 0 0 0 0 0 0 0
0 0 0 0 0 0 0 0 0 0 1 0 0 0 0 14 0 0 0 0 0 0 0 0 0 0 74

M02255_77_000000000-ADUT3_1_2114_17280_13300 T----AC---GT-AG-GGG--------GCT-A-G------C--G--T---T--AT-C-CGG-AT------TT-A--C-T--GG-GC-------GT--A-----AA-GG-GT-GC-------G-TA-G-G-T-G------------G--T-TT-C-T-T-----------AA----G-T-C-A--------G-G-A--G--TG--A-AA-GG--C-T-AC-G-G-----CT-C-AA------C-C-G-T-A-G-T--A------A-G--C-T---C--T-------T--GA-A-A---C-------T--G-G--GA--A-A-C----T-T-G-A-G-T--G---C-AG-----GA-G-A--------G-G-A-A---AG-T------GG--A--ATT----C-C-T-A-GT--GT-A-G-CG-GT--G---A--A-A---TG-C-GT-AG--AT-A-TT--A-G--G-A-G-G-A-AC-A-CC---AG--T--A--GC-GAA-G--G-C--G-------G--C-T-T-T---CTG--G--AC-T-G--------------------T-A---A-C-T--GA--CA-C-T-G--A-GG--C-A-CG-A--AA-G-C---G-TG--GG-G--AG-C-A-AA--------CA--GG Bacteria(100);Firmicutes(100);Clostridia(100);Clostridiales(100);Peptostreptococcaceae(100);Incertae_Sedis(100);unclassified(100);unclassified(100);unclassified(100);

Otu0170 0 0 0 46 0 0 0 0 0 0 0 0 0 0 0 0 0 0 0 1 6 0 0 0 0 4 0 0
0 1 0 0 0 0 0 0 0 34 0 0 91 0 0 0 0 0 0 0 0 0 0 29 0 19 0 0 0
0 0 0 0 0 0 0 0 0 0 0 0 3 0 9 0 0 0 0 0 0 0 0 0 0 0 0 0 0
0 0 0 0 39 0 0 0 0 0 0 0 0 0 0 0 0 0 0 0 0 0 0 0 0 0 0
M02255_77_000000000-ADUT3_1_1101_26653_16641 T----AC---GT-AG-GTG--------GCA-A-G------C--G--T---T--GT-C-CGG-AT------TT-A--C-T--GG-GC-------GT--A-----AA-GC-GA-AC-------G-CA-G-G-C-G------------G--A-CT-G-T-T-----
------AA----G-T-A-G--------A-A-A--G--TG--A-AA-GG--T-C-GG-A-G-----CT-C-AA------C-T-C-C-A-A-C--A-T----T-G--C-T---T--T-------C--TA-T-A---C-------T--G-G--CA--G-T-C----T-T-G-A-G-T--A---G-CG-----GA-G-A--------G-G-A-A---GG-G------GG--A--ACG----
A-C-A-C-GT--GT-A-G-CG-GT--G---A--A-A---TG-C-GT-TG--AT-A-TG--T-G--T-C-G-G-A-AC-A-CC---CA--T--G--GC-GAA-A--G-C--A-------C--C-C-T-T---CTG--G--AC-G-C--------------------A-A---A-C-T--GA--CG-C-T-C--A-GG--T-T-CG-A--AA-G-C---C-AA--GG-
T--AG-C-G-AA--------CA--GG Bacteria(100);unclassified(100);unclassified(100);unclassified(100);unclassified(100);unclassified(100);unclassified(100);unclassified(100);unclassified(100);

Otu0171 0 0 0 0 0 0 0 0 0 0 0 0 2 1 0 0 3 0 0 0 0 0 0 0 0 0 0 0
0 0 0 0 0 0 0 0 0 0 0 0 0 0 0 0 0 0 3 0 0 0 0 0 0 0 0 0 0
0 1 0 0 0 0 0 5 0 8 0 1 0 0 0 0 0 0 0 0 0 0 0 0 0 0 0 0 0
0 0 0 0 0 0 0 0 3 0 4 0 5 0 0 2 0 0 0 0 0 0 0 0 0 0 0
M02255_76_000000000-ADUNE_1_2115_10176_21100 T----AA---GC-AC-CGG--------CTA-A-C------T--C--T---G--TG-C-CAG-CA------GC-C--G-C--GG-TA-------AT--A-----CA-GA-GG-GT-------G-CA-A-G-C-G------------T--TAAT-C-G-G-----
------AA----G-T-T-G--------G-A-T--G--TG--A-AA-TC--C-C-CG-G-G-----CT-C-AA------C-C-T-G-G-G-A--A-C----T-G--C-A---T--T-------C--AA-A-A---C-------T--G-T--CG--A-G-C----T-A-G-A-G-T--A---T-GG-----TA-G-A--------G-G-G-T---GG-T------GG--A--ATT----T-
C-C-T-GT--GT-A-G-CG-GT--G---A--A-A---TG-C-GT-AG--AT-A-TA--G-G--A-A-G-G-A-AC-A-CC---AG--T--G--GC-GAA-G--G-C--G-------A--C-C-A-C---CTG--G--AC-T-G--------------------A-T---A-C-T--GA--CA-C-T-G--A-GG--T-G-CG-A--AA-G-C---G-TG--GG-
G--AG-C-A-AA--------CA--GG Bacteria(100);Proteobacteria(100);Gammaproteobacteria(100);Pseudomonadales(87);Pseudomonadaceae(87);unclassified(87);unclassified(87);unclassified(87);unclassified(87);

Otu0172 0 0 0 72 108 35 1 58 66 105 0 0 0 0 0 0 1 0 0 0 5 0 0 0 0 0 0 10
1 0 42 0 0 137 24 48 0 0 0 0 54 0 95 0 0 0 0 5 0 0 7 0 6 0 0 0 0
0 482 0 0 0 0 0 46 0 0 0 0 0 3 0 0 19 0 0 0 0 1 0 35 49 0 0 31 76
0 204 98 98 0 0 0 0 0 112 0 0 0 0 0 0 0 0 0 116 115 63 73 2 0 0 0
M02255_77_000000000-ADUT3_1_1103_23192_17625 T----AC---GT-AT-GTC--------GCA-A-G------C--G--T---T--AT-C-CGG-AA------TT-A--T-T--GG-GC-------AT--A-----AA-GG-GC-AT-------C-TA-G-G-C-G------------G--C-CA-G-A-T------
-----AA----G-T-C-T--------G-G-G--G--TG--A-AA-AC--T-T-GC-G-G-----CT-C-AA------C-C-G-C-A-A-G--C-C----T-G--C-C---C--T-------G--GA-A-A---C-------T--A-T--GC--G-G-C----T-A-G-A-G-T--A---C-TG-----GA-G-A--------G-G-T-G---GA-C------GG--A--ACT----G-
C-A-C-GA--GT-A-G-AG-GT--G---A--A-A---TT-C-GT-AG--AT-A-TG--T-G--C-A-G-G-A-AT-G-CC---GA--T--G--AT-GAA-G--A-T--A-------G--T-T-C-A---CTG--G--AC-G-G--------------------T-A---A-C-T--GA--CG-C-T-G--A-AG--T-G-CG-A--AA-G-C---T-AG--GG-G-
-AG-C-A-AA--------CA--GG Bacteria(100);Fusobacteria(100);Fusobacteria(100);Fusobacteriales(100);Leptotrichiaceae(100);Leptotrichia(100);unclassified(100);unclassified(100);unclassified(100);

Otu0173 0 777 625 0 0 0 131 41 70 0 0 0 0 0 0 0 2 0 4 0 0 0 0 0 0 0 0 0
0 0 0 48 0 0 0 0 0 0 0 48 0 0 0 0 0 0 0 0 0 0 0 0 0 0 0 0 0
0 0 0 0 0 0 0 0 0 0 0 0 0 0 0 0 24 30 0 0 0 0 0 0 0 0 0 0 0
0 0 0 0 0 0 0 0 0 0 0 0 0 0 0 0 0 0 0 0 0 0 0 9 183 0 0
M02255_77_000000000-ADUT3_1_1101_7841_14070 T----AC---GT-AT-GGA--------GCA-A-G------C--G--T---T--AT-C-CGG-AT------TT-A--C-T--GG-GT-------GT--A-----AA-GG-GA-GT-------G-TA-G-G-T-G------------G--C-CA-T-G-C-----------AA-
---G-T-C-A--------G-A-A--G--TG--A-AA-AT--C-C-GG-G-G-----CT-C-AA------C-C-C-C-G-G-A--A-C----T-G--C-T---T--T-------T--GA-A-A---C-------T--G-T--AA--G-G-C----T-A-G-A-G-T--G---C-AG-----GA-G-G--------G-G-T-G---AG-T------GG--A--ATT----C-C-T-A-
GT--GT-A-G-CG-GT--G---A--A-A---TG-C-GT-AG--AT-A-TT--A-G--G-A-G-G-A-AC-A-CC---AG--T--G--GC-GAA-G--G-C--G-------G--C-T-C-A---CTG--G--AC-T-G--------------------T-A---A-C-T--GA--CA-C-T-G--A-GG--C-T-CG-A--AA-G-C---G-TG--GG-G--AG-
C-A-AA--------CA--GG Bacteria(100);Firmicutes(100);Clostridia(100);Clostridiales(100);Lachnospiraceae(100);unclassified(100);unclassified(100);unclassified(100);unclassified(100);

Otu0174 293 0 0 182 0 0 42 1 0 0 0 0 0 0 0 0 0 0 0 0 0 0 0 0 0 5 0 0
0 0 0 0 0 0 0 0 0 0 0 0 0 0 0 44 116 0 0 0 0 0 0 82 16 0 0 0 0
0 0 0 0 0 0 0 0 0 0 22 30 0 20 0 0 0 0 0 74 0 0 0 0 0 0 0 0 0
0 0 0 0 0 0 5 4 0 97 9 0 0 0 2 0 0 0 0 0 0 0 0 0 0 0 0
M02255_76_000000000-ADUNE_1_1107_28496_12182 T----AC---GT-AG-GGG--------GCG-A-G------C--G--T---T--GT-C-CGG-AA------TC-A--C-T--GG-GC-------GT--A-----AA-GG-GT-TC-------G-CA-G-G-C-G------------G--A-TA-A-A-C----
-------AA----G-T-C-A--------A-G-A--G--TA--A-AA-GG--C-A-TG-G-G-----CT-T-AA------C-C-C-A-T-G-T--A------A-G--C-T---A--T-------T--GA-A-A---C-------T--G-T--TT--A-T-C----T-T-G-A-G-A--T---G-TG-----GA-G-A--------G-G-T-A---AG-T------GG--A--ATT----A-
C-T-A-GT--GT-A-G-CG-GT--G---A--A-A---TG-C-GT-AG--AT-A-TT--A-G--T-A-G-G-A-AT-A-CC---AG--T--A--GC-GAA-G--G-C--G-------A--C-T-T-A---CTG--G--AC-A-C--------------------A-A---A-C-T--GA--CG-C-T-G--A-GG--A-A-CG-A--AA-G-C---G-TG--GG-T-
-AG-C-A-AA--------CA--GG Bacteria(100);Firmicutes(100);Clostridia(100);Clostridiales(100);Family_XI_Incertae_Sedis(100);Peptoniphilus(52);unclassified(52);unclassified(52);unclassified(52);

Otu0175 0 200 214 224 0 0 176 338 177 0 0 0 0 0 0 4 0 0 0 0 0 0 0 5 0 6 4 2
4 0 0 1 0 0 0 81 144 0 0 221 0 0 95 0 0 0 0 58 0 0 0 83 30 0 0 0 0
0 0 0 0 0 0 0 0 0 0 0 0 10 0 17 0 16 152 0 0 0 2 0 0 0 0 0 0 0
0 0 121 10 0 0 0 0 1 0 0 0 0 0 0 0 0 0 18 0 0 0 0 24 322 691 0

M02255_77_000000000-ADUT3_1_1104_21315_13830 T----AC---GT-AG-GTG--------GCG-A-G------C--G--T---T--GT-C-CGG-AT------TT-A--C-T--GG-GC-------GT--A-----AA-GG-GA-GC-------G-TA-G-G-C-G------------G--A-CT-T-T-T-----
------AA----G-T-G-A--------G-A-T--G--TG--A-AA-TA--C-C-CG-G-G-----CT-C-AA------C-T-T-G-G-G-T--G-C----T-G--C-A---T--T-------T--CA-A-A---C-------T--G-G--AA--G-T-C----T-A-G-A-G-T--G---C-AG-----GA-G-A--------G-G-A-G---AA-T------GG--A--ATT----
C-C-T-A-GT--GT-A-G-CG-GT--G---A--A-A---TG-C-GT-AG--AG-A-TT--A-G--G-A-A-G-A-AC-A-CC---AG--T--G--GC-GAA-G--G-C--G-------A--T-T-C-T---CTG--G--AC-T-G--------------------T-A---A-C-T--GA--CG-C-T-G--A-GG--C-T-CG-A--AA-G-C---G-TG--GG-
G--AG-C-A-AA--------CA--GG Bacteria(100);Firmicutes(100);Clostridia(100);Clostridiales(100);Clostridiaceae(100);unclassified(68);unclassified(68);unclassified(68);unclassified(68);

Otu0176 0 0 0 0 0 1 0 0 36 0 0 2 0 10 0 0 3 0 9 0 0 0 69 8 8 0 0 0
0 0 0 0 0 0 0 18 0 0 1 18 502 69 4 0 0 0 0 24 0 0 115 253 450 17 30 0 0
41 6 0 6 15 0 0 27 0 2 0 6 27 0 0 39 19 0 0 0 0 13 14 0 220 0 0 0 0
0 0 1 0 0 0 0 0 0 0 0 0 0 0 0 34 88 0 6 0 0 0 0 2 4 20 0

M02255_77_000000000-ADUT3_1_2102_5819_10302 T----AC---GG-GG-GGG--------GCA-A-G------T--G--T---T--AT-T-CGG-AA------TG-A--C-T--GG-GC-------GT--A-----AA-GG-GC-AC-------G-TA-G-G-C-G------------G--T-GA-A-A-A-----------
GG----G-T-G-G--------A-A-A--G--TG--A-AA-GT--C-G-CC-A-A-----AA-C--A------C-T-G-G-C-G-G--G-G----T-G--C-T---T--T-------C--TT-G-A---C-------C--A-T--TT--C-A-C----T-T-G-A-G-T--G---A-GA-----TA-G-G--------G-G-A-G---AG-T------GG--A--ATT----T-C-G-
T-GT--GG-A-G-GG-AT--C---A--A-A---TC-C-TA-AG--AT-A-TA--C-G--A-A-G-G-A-AC-G-CC---AA--C--C--GC-GAA-G--G-C--A-------G--C-T-C-T---CTG--G--GT-C-C--------------------C-C---A-C-T--GA--CG-C-T-G--G-GG--T-G-CG-A--AA-G-C---A-TG--GG-G--AG-
C-A-AA--------CA--GG Bacteria(100);unclassified(100);unclassified(100);unclassified(100);unclassified(100);unclassified(100);unclassified(100);unclassified(100);unclassified(100);

Otu0177 0 0 0 0 0 0 0 0 0 0 0 0 0 0 0 0 0 0 0 0 0 0 0 12 0 6 0 0
0 0 71 0 0 0 0 19 0 0 0 0 0 0 0 0 0 0 0 0 0 0 0 7 0 0 0 0 0
0 0 0 0 0 0 0 0 0 0 0 3 0 0 0 0 0 0 0 0 7 0 0 0 0 0 0 0 0
0 0 0 0 0 0 0 0 0 0 0 0 0 0 0 0 0 0 0 0 0 0 0 4 0 0 0

M02255_77_000000000-ADUT3_1_2119_19041_14313 T----AC---GG-AG-GGG--------GCT-A-G------C--G--T---T--GT-T-CGG-AA------TT-A--C-T--GG-GC-------GT--A-----AA-GC-GC-AC-------G-TA-G-G-C-G------------G--A-TT-G-G-A----
-------AA----G-T-T-G--------G-G-G--G--TG--A-AA-TC--C-C-GG-G-G-----CT-C-AA------C-C-C-C-G-G-A--A-C----T-G--C-C---T--T-------C--AA-A-A---C-------T--C-C--CA--G-T-C----T-G-G-A-G-G--T---C-GA-----GA-G-A--------G-G-T-G---AG-T------GG--A--ATT----
C-C-G-A-GT--GT-A-G-AG-GT--G---A--A-A---TT-C-GT-AG--AT-A-TT--C-G--G-A-G-G-A-AC-A-CC---AG--T--G--GC-GAA-G--G-C--G-------G--C-T-C-A---CTG--G--CT-C-G--------------------A-T---A-C-T--GA--CG-C-T-G--A-GG--T-G-CG-A--AA-G-C---G-TG--GG-
G--AG-C-A-AA--------CA--GG Bacteria(100);Proteobacteria(100);Alphaproteobacteria(100);Rhodobacterales(100);Rhodobacteraceae(100);unclassified(100);unclassified(100);unclassified(100);unclassified(100);

Otu0178 11 20 0 0 32 0 6 31 2 0 0 0 0 0 0 0 2 0 0 0 0 3 9 0 0 0 1 3
0 0 5 60 0 0 0 0 0 0 9 1 53 17 46 3 0 0 0 0 9 0 0 15 5 178 0 0 0
0 0 0 0 0 0 3 5 0 0 0 0 0 0 11 26 0 30 0 0 0 0 0 0 4 0 0 0 0
1 9 4 64 0 0 0 0 0 9 0 0 0 0 0 0 17 5 12 7 26 81 5 0 9 0 19

M02255_77_000000000-ADUT3_1_1104_6512_11181 T----AC---GT-AG-GGT--------GCA-A-G------C--G--T---T--GT-C-CGG-AA------TT-A--T-T--GG-GC-------GT--A-----AA-GA-GC-TC-------G-TA-G-G-C-G------------G--T-TT-G-T-T-----------GC--
--G-T-C-G--------G-C-T--G--TG--A-AA-AC--C-T-GA-G-G-----CT-C-AA------C-C-T-C-A-G-G--C-C----T-G--C-A---G--T-------C--GA-T-A---C-------G--A-G--CA--G-A-C----T-A-G-A-G-T--G---T-TG-----CA-G-G--------G-G-A-G---AC-T------GG--A--ATT----C-C-T-G-
GT--GT-A-G-CG-GT--G---A--A-A---TG-C-GC-AG--AT-A-TC--A-G--G-A-G-G-A-AC-A-CC---GG--T--G--GC-GAA-G--G-C--G-------G--G-T-C-T---CTG--G--GC-A-A--------------------C-A---A-C-T--GA--CG-C-T-G--A-GG--A-G-CG-A--AA-G-C---G-TG--GG-G--AG-
C-G-AA--------CA--GG Bacteria(100);Actinobacteria(100);Actinobacteria(100);Actinobacteridae(100);Actinomycetales(100);Frankineae(100);Geodermatophilaceae(100);Blastococcus(58);unclassified(58);

Otu0179 0 0 0 0 0 0 0 0 0 0 0 2 0 0 0 0 4 1 0 0 0 0 0 0 0 0 0 0
0 0 0 0 0 0 0 0 0 0 0 0 0 0 0 0 0 0 3 0 0 0 0 0 0 0 0 2 0
0 0 0 1 0 0 0 2 0 8 1 0 0 0 0 0 0 0 0 0 0 0 0 0 0 0 0 0 0
0 0 0 0 0 1 2 0 0 0 0 0 1 0 0 0 0 0 0 0 0 0 0 0 0 0 0

M02255_76_000000000-ADUNE_1_1115_5766_8094 T----AC---CG-AC-AGA--------ATA-A-G------C-----A---C--CG-G-CTA-AC------TC-T--G-T--GC-CA-------GC--AG----CC-GC-GG-TA-------A-TACA-G-A-G------------G--T-GT-G-T-C-----------AA-
---GCG-T-T--------A-A-T--C--GG--A-AA-TC--C-C-CG-G-G-----CT-C-AA------C-C-T-G-G-G-A--A-C----T-G--C-A---T--C-------C--AA-A-A---C-------T--G-G--CA--A-G-C----T-A-G-A-G-T--A---T-GG-----TA-G-A--------G-G-G-T---AG-T------GG--A--ATT----T-C-C-T-
GT--GT-A-G-CG-GT--G---A--A-A---TG-C-GT-AG--AT-A-TA--G-G--A-A-G-G-A-AC-A-CC---AG--T--G--GC-GAA-G--G-C--G-------A--C-T-A-C---CTG--G--AC-T-G--------------------A-T---A-C-T--GA--CA-C-T-G--A-GG--T-G-CG-A--AA-G-C---G-TG--GG-G--AG-
C-A-AA--------CA--GG Bacteria(100);Proteobacteria(100);Gammaproteobacteria(100);unclassified(90);unclassified(90);unclassified(90);unclassified(90);unclassified(90);unclassified(90);

Otu0180 0 0 0 0 0 0 0 0 0 0 0 1 7 0 8 0 10 0 0 0 12 4 0 0 0 0 0 0
0 0 0 0 0 0 0 0 0 0 0 0 0 0 0 0 0 0 8 0 0 0 10 0 0 0 0 5 0
0 0 0 5 1 1 2 2 3 4 0 1 4 1 0 0 0 0 0 0 0 0 0 0 0 0 0 0 0
0 0 0 0 0 2 1 0 0 0 0 0 0 0 0 0 0 0 0 0 0 0 0 0 0 0 0
M02255_76_000000000-ADUNE_1_2101_26528_17478 G----AC---AG-AA-TAA--------GCA-C-C------G--G--C---T--AA---C-T-CT------GT-G--C-C--AG-CA-------GC--C-----GC-GG-TA-AT-------A-CA-G-A-G-G------------G--T-TG-T-C-A-------
----AG----C-G-T-T--------A-A-T--C--GG--A-AA-TC--C-C-CG-G-G-----CT-C-AA------C-C-T-G-G-G-A--A-C----T-G--C-A---T--C-------C--AA-A-A---C-------T--G-G--CA--A-G-C----T-A-G-A-G-T--A---T-GG-----TA-G-A--------G-G-G-T---AG-T------GG--A--ATT----T-
C-C-T-GT--GT-A-G-CG-GT--G---A--A-A---TG-C-GT-AG--AT-A-TA--G-G--A-A-G-G-A-AC-A-CC---AG--T--G--GC-GAA-G--G-C--G-------A--C-T-A-C---CTG--G--AC-T-G--------------------A-T---A-C-T--GA--CA-C-T-G--A-GG--T-G-CG-A--AA-G-C---G-TG--GG-
G--AG-C-A-AA--------CA--GG Bacteria(100);Proteobacteria(100);Gammaproteobacteria(100);unclassified(100);unclassified(100);unclassified(100);unclassified(100);unclassified(100);unclassified(100);

Otu0181 215 0 0 0 227 43 0 0 0 0 0 0 0 0 0 0 0 0 0 0 25 1 0 0 5 5 0 0
0 0 45 1 0 27 0 464 308 45 74 26 0 113 198 116 1 26 0 52 0 0 8 41 194 0 0 0 0
0 26 39 0 12 22 0 0 21 7 0 0 0 0 14 48 190 34 0 0 0 0 11 0 474 0 0 0 30
0 48 0 4 0 96 0 0 0 0 2 0 0 144 0 48 28 43 0 39 0 0 0 0 0 0 39
M02255_77_000000000-ADUT3_1_1103_7434_8208 T----AC---GG-AG-GGA--------GCT-A-G------C--G--T---T--AT-T-CGG-AA------TT-A--C-T--GG-GC-------GT--A-----AA-GC-GC-AC-------G-TA-G-G-C-G------------G--C-TT-T-G-T-----------AA-
---G-T-A-A--------G-A-G--G--TG--A-AA-GC--C-C-AG-A-G-----CT-C-AA------C-T-C-T-G-G-A--A-T----T-G--C-C---T--T-------T--TA-G-A---C-------T--G-C--AT--C-G-C----T-T-G-A-A-T--C---A-TG-----GA-G-A--------G-G-T-C---AG-T------GG--A--ATT----C-C-G-A-
GT--GT-A-G-AG-GT--G---A--A-A---TT-C-GT-AG--AT-A-TT--C-G--G-A-A-G-A-AC-A-CC---AG--T--G--GC-GAA-G--G-C--G-------G--C-T-G-A---CTG--G--AC-A-T--------------------G-T---A-T-T--GA--CG-C-T-G--A-GG--T-G-CG-A--AA-G-C---G-TG--GG-G--AG-
C-A-AA--------CA--GG Bacteria(100);Proteobacteria(100);Alphaproteobacteria(100);Sphingomonadales(100);Sphingomonadaceae(94);Sphingomonas(94);unclassified(94);unclassified(94);unclassified(94);

Otu0182 0 0 0 0 4 0 0 0 0 3 0 0 0 0 0 0 0 0 0 0 0 0 6 0 0 0 0 0
0 3 0 10 0 0 0 0 0 0 0 0 0 2 0 0 0 0 0 0 10 0 0 0 0 0 0 27
2 0 0 0 0 0 0 0 0 0 0 0 0 0 0 0 6 0 0 1 0 0 0 1 1 39 0 0 0
0 0 0 0 0 0 0 0 0 0 0 0 0 0 0 0 0 0 0 0 0 0 0 0 0 9 0
M02255_76_000000000-ADUNE_1_1115_5985_17887 T----AA---GC-AC-CGG--------CTA-A-C------T--A--C---G--TG-C-CAG-CA------GC-C--G-C--GG-TA-------AT--A-----CG-TA-GG-GT-------G-CG-A-G-C-G------------G--T-TA-TCG-G-----------
AA----G-A-C-A--------G-A-T--G--TG--A-AA-TC--C-C-CG-G-G-----CT-C-AA------C-C-T-G-G-G-A--A-C----T-G--C-A---T--T-------T--GT-G-A---C-------T--G-C--AT--G-G-C----T-A-G-A-G-T--A---C-GG-----TA-G-A--------G-G-G-G---GA-T------GG--A--ATT----C-C-G-
C-GT--GT-A-G-CA-GT--G---A--A-A---TG-C-GT-AG--AT-A-TG--C-G--G-A-G-G-A-AC-A-CC---GA--T--G--GC-GAA-G--G-C--A-------A--T-C-C-C---CTG--G--AC-C-T--------------------G-T---A-C-T--GA--CG-C-T-C--A-TG--C-A-CG-A--AA-G-C---G-TG--GG-G--AG-
C-A-AA--------CA--GG Bacteria(100);Proteobacteria(100);Betaproteobacteria(100);Burkholderiales(100);Comamonadaceae(100);unclassified(100);unclassified(100);unclassified(100);unclassified(100);

Otu0183 0 11 355 0 0 0 161 10 225 0 0 0 0 0 0 0 0 0 0 0 0 1 0 0 0 4 0
0 0 0 1 0 0 0 0 0 0 0 189 0 49 0 39 4 0 0 0 0 0 0 0 0 0 0 0 0
0 0 0 0 0 0 3 0 0 0 0 2 0 0 0 0 49 50 0 0 0 0 0 0 0 318 716 0 0
0 0 0 0 0 0 1 0 0 25 0 352 0 0 0 0 0 0 0 0 0 0 0 0 88 0 0
M02255_77_000000000-ADUT3_1_2109_17769_19714 T----AC---GT-AT-GGT--------GCA-A-G------C--G--T---T--AT-C-CGG-AT------TT-A--C-T--GG-GT-------GT--A-----AA-GG-GA-GC-------G-TA-G-A-C-G------------G--A-GT-G-G-C-----
------AA----G-T-C-T--------G-A-T--G--TG--A-AA-AC--C-C-GG-G-G-----CT-C-AA------C-C-C-C-G-G-G--A-C----T-G--C-A---T--T-------G--GA-A-A---C-------T--G-T--CA--A-T-C----T-A-G-A-G-T--A---C-CG-----GA-G-A--------G-G-T-A---AG-C------GG--A--ATT----C-
C-T-A-GT--GT-A-G-CG-GT--G---A--A-A---TG-C-GT-AG--AT-A-TT--A-G--G-A-G-G-A-AC-A-CC---AG--T--G--GC-GAA-G--G-C--G-------G--C-T-T-A---CTG--G--AC-G-G--------------------T-A---A-C-T--GA--CG-T-T-G--A-GG--C-T-CG-A--AA-G-C---G-TG--GG-
G--AG-C-A-AA--------CA--GG Bacteria(100);Firmicutes(100);Clostridia(100);Clostridiales(100);Lachnospiraceae(100);unclassified(69);unclassified(69);unclassified(69);unclassified(69);

Otu0184 0 0 0 0 0 0 0 0 0 0 0 0 0 0 0 0 0 0 0 0 0 0 0 0 0 0 0 0
0 0 0 0 0 0 0 47 0 0 0 0 0 82 0 0 0 0 0 0 0 0 3 0 0 0 0 17 0
0 0 0 0 0 2 0 0 0 0 0 0 0 0 0 0 0 31 0 0 0 0 0 0 0 0 0 11 0
0 0 0 0 0 0 0 0 0 0 0 0 1 0 0 0 0 0 0 0 0 0 0 0 0 0 0
M02255_77_000000000-ADUT3_1_1101_24662_14467 T----AC---GA-AG-GGG--------GCT-A-G------C--G--T---T--GT-T-CGG-AA------TT-A--C-T--GG-GC-------GT--A-----AA-GC-GC-AC-------G-TA-G-G-C-G------------G--A-TT-G-T-T-----
------AA----G-T-T-A--------G-G-G--G--TG--A-AA-TC--C-C-AG-G-G-----CT-C-AA------C-C-C-T-G-G-A--A-C----T-G--C-C---T--T-------T--AA-T-A---C-------T--G-G--CA--A-T-C----T-C-G-A-G-T--C---C-GA-----GA-G-A--------G-G-T-G---AG-T------GG--A--ATT----C-
C-G-A-GT--GT-A-G-AG-GT--G---A--A-A---TT-C-GT-AG--AT-A-TT--C-G--G-A-G-G-A-AC-A-CC---AG--T--G--GC-GAA-G--G-C--G-------G--C-T-C-A---CTG--G--CT-C-G--------------------G-T---A-C-T--GA--CG-C-T-G--A-GG--T-G-CG-A--AA-G-C---G-TG--GG-
G--AG-C-A-AA--------CA--GG Bacteria(100);Proteobacteria(100);Alphaproteobacteria(100);Rhizobiales(100);Phyllobacteriaceae(100);Mesorhizobium(54);unclassified(54);unclassified(54);unclassified(54);

Otu0185 0 0 0 0 0 0 34 0 0 0 0 0 0 0 0 0 0 0 0 0 0 0 0 0 0 0 0 0
0 0 0 0 0 0 0 0 0 0 0 0 0 0 0 0 0 0 0 0 0 0 0 0 0 0 0 0 0
0 0 3 0 0 0 0 0 0 0 0 0 0 0 154 202 0 0 0 0 0 0 3 0 0 0 0 0 0
0 0 0 0 0 0 0 0 0 0 0 0 0 0 0 0 0 0 0 0 0 0 0 0 0 0 0
M02255_77_000000000-ADUT3_1_1107_9624_21555 T----AC---GT-AG-GGG--------GCA-A-G------C--G--T---T--AT-C-CGG-AA------TT-A--C-T--GG-GC-------GT--A-----AA-GG-GT-GC-------G-TA-G-G-C-G------------G--C-TT-G-T-T----------AA-
---G-T-C-A--------A-G-G--T--TA--A-AA-GG--C-A-GC-A-G-----CT-C-AA------C-T-G-C-T-G-T--T------C-G--G-C---C--T-------T--GA-A-A---C-------T--A-A--TA--A-G-C----T-T-G-A-G-T--A---T-AG-----GA-G-A--------G-G-A-A---AG-T------GG--A--ATT----C-C-C-A-
GT--GT-A-G-CG-GT--G---A--A-A---TG-C-GT-AG--AT-A-TT--G-G--G-A-G-G-A-AT-A-CC---GG--T--G--GC-GAA-G--G-C--G-------A--C-T-T-T---CTG--G--AC-T-A--------------------A-A---A-C-T--GA--CG-C-T-G--A-GG--C-A-CG-A--AA-G-T---G-TG--GG-T--AG-C-
A-AA--------CA--GG Bacteria(100);Firmicutes(100);Clostridia(100);Clostridiales(100);unclassified(81);unclassified(81);unclassified(81);unclassified(81);unclassified(81);

Otu0186 897 0 0 0 0 65 0 0 0 0 0 0 0 0 0 0 0 0 0 0 0 0 0 0 0 0 0 0
1 0 0 0 0 0 0 0 0 0 0 0 0 0 0 0 0 0 0 0 0 0 0 0 0 0 0 0 0
0 0 0 0 0 0 0 0 0 0 0 0 0 0 13 0 0 12 0 0 0 0 0 0 0 0 0 0 0
0 0 0 136 0 0 23 0 0 0 0 0 0 0 0 0 0 0 0 0 0 0 0 0 0 0 0
M02255_77_000000000-ADUT3_1_1104_11355_9377 T----AC---GG-AG-GGG--------GCT-A-G------C--G--T---T--GT-T-CGG-AA------TT-A--C-T--GG-GC-------GT--A-----AA-GC-GC-AC-------G-TA-G-G-C-G------------G--C-TT-T-G-T----------AA-
---G-T-T-A--------G-A-G--G--TG--A-AA-GC--C-T-GG-G-G-----CT-C-AA------C-T-C-C-A-G-A--A-T----T-G--C-C---T--T-------T--AA-G-A---C-------T--G-C--AT--C-G-C----T-T-G-A-A-T--C---G-CG-----GA-G-A--------G-G-T-G---AG-T------GG--A--ATT----C-C-G-A-
GT--GT-A-G-AG-GT--G---A--A-A---TT-C-GT-AG--AT-A-TT--C-G--G-A-A-G-A-AC-A-CC---AG--T--G--GC-GAA-G--G-C--G-------G--C-T-C-A---CTG--G--AC-G-C--------------------G-T---A-T-T--GA--CG-C-T-G--A-GG--T-G-CG-A--AA-G-C---G-TG--GG-G--AG-
C-A-AA--------CA--GG Bacteria(100);Proteobacteria(100);Alphaproteobacteria(100);Sphingomonadales(100);Sphingomonadaceae(100);Sphingomonas(100);unclassified(100);unclassified(100);unclassified(100);

Otu0187 0 0 0 0 0 0 0 0 0 0 0 0 0 0 10 0 0 0 0 0 23 0 32 0 0 0 0 0
15 1 0 0 0 0 0 0 0 36 0 0 0 0 0 0 0 0 0 0 1 0 0 0 0 0 0 0 0
0 0 0 0 0 0 0 0 0 0 0 0 0 0 0 0 0 10 0 0 0 0 0 0 0 0 0 0 0
0 0 0 0 0 0 0 4 0 0 0 39 0 0 0 0 153 0 0 0 0 0 0 1 0 2394 0
M02255_77_000000000-ADUT3_1_1105_18333_9839 T----AC---GA-AG-GGG--------GCT-A-G------C--G--T---T--GT-T-CGG-AA------TT-A--C-T--GG-GC-------GT--A-----AA-GC-GC-AC-------G-TA-G-G-C-G------------G--A-TA-T-T-T----------AA-
---G-T-C-A--------G-G-G--G--TG--A-AA-TC--C-C-GG-G-G-----CT-C-AA------C-C-C-C-G-G-A--A-C----T-G--C-C---T--T-------T--GA-T-A---C-------T--G-G--GT--A-T-C----T-T-G-A-G-T--G---T-GG-----TA-G-A--------G-G-T-G---AG-T------GG--A--ATT----G-C-G-A-
GT--GT-A-G-AG-GT--G---A--A-A---TT-C-GT-AG--AT-A-TT--C-G--C-A-G-G-A-AC-A-CC---AG--T--G--GC-GAA-G--G-C--G-------G--C-T-C-A---CTG--G--AC-C-A--------------------C-A---A-C-T--GA--CG-C-T-G--A-GG--T-G-CG-A--AA-G-C---G-TG--GG-G--AG-
C-A-AA--------CA--GG Bacteria(100);Proteobacteria(100);Alphaproteobacteria(100);Rhizobiales(100);unclassified(100);unclassified(100);unclassified(100);unclassified(100);unclassified(100);

Otu0188 0 0 0 0 0 0 22 1 0 0 0 0 0 0 0 0 0 0 0 0 0 0 0 0 0 0 0 0
0 0 0 0 0 0 0 0 0 0 0 0 0 75 0 0 0 0 0 43 0 0 0 0 0 0 0 0 0
0 0 0 0 0 0 0 0 0 0 0 0 0 0 0 0 0 0 0 0 0 8 0 0 0 0 0 0 0
0 0 0 19 0 0 39 0 0 0 0 0 0 0 0 0 0 0 0 0 0 0 0 0 3 0 0
M02255_77_000000000-ADUT3_1_1117_23919_3278 T----AC---GG-AG-GGA--------GCT-A-G------C--G--T---T--GT-T-CGG-AA------TT-A--C-T--GG-GC-------GT--A-----AA-GC-GC-AC-------G-TA-G-G-C-G------------G--C-TA-C-T-C----------AA-
---G-T-C-A--------G-A-G--G--TG--A-AA-GC--C-C-GG-G-G-----CT-C-AA------C-C-C-C-G-G-A--A-C----T-G--C-C---T--T-------T--GA-A-A---C-------T--A-G--GT--G-G-C----T-A-G-A-A-T--C---T-TG-----GA-G-A--------G-G-C-G---AG-T------GG--A--ATT----C-C-G-A-
GT--GT-A-G-AG-GT--G---A--A-A---TT-C-GT-AG--AT-A-TT--C-G--G-A-A-G-A-AC-A-CC---AG--T--G--GC-GAA-G--G-C--G-------A--C-T-C-G---CTG--G--AC-A-A--------------------G-T---A-T-T--GA--CG-C-T-G--A-GG--T-G-CG-A--AA-G-C---G-TG--GG-G--AG-
C-A-AA--------CA--GG Bacteria(100);Proteobacteria(100);Alphaproteobacteria(100);Sphingomonadales(100);Sphingomonadaceae(100);unclassified(100);unclassified(100);unclassified(100);unclassified(100);

Otu0189 0 0 0 38 0 0 0 0 0 0 0 0 0 0 0 0 0 0 0 4 0 0 65 7 0 0 0 0
0 0 2 0 0 0 0 0 0 7 30 0 0 0 149 432 0 162 6 0 0 0 0 0 37 14 0 0 0
332 31 93 25 36 41 77 27 16 25 45 0 31 32 82 69 124 0 72 177 17 0 0 0 0 0 0 0 0
0 0 0 0 0 0 4 0 0 0 0 0 0 0 0 0 86 0 0 0 0 0 0 0 0 0 0
M02255_77_000000000-ADUT3_1_2111_19376_15802 T----AC---GA-AG-GGG--------GCT-A-G------C--G--T---T--GC-T-CGG-AA------TT-A--C-T--GG-GC-------GT--A-----AA-GG-GC-GC-------G-TA-G-G-C-G------------G--A-CA-G-T-T----
-------AA----G-T-T-G--------G-G-G--G--TG--A-AA-GC--C-C-GG-G-G-----CT-C-AA------C-C-T-C-G-G-A--A-T----T-G--C-C---T--T-------C--AA-T-A---C-------T--G-G--CT--G-T-C----T-T-G-A-G-T--A---C-GG-----GA-G-A--------G-G-T-G---AG-T------GG--A--ACT----
C-C-G-A-GT--GT-A-G-AG-GT--G---A--A-A---TT-C-GT-AG--AT-A-TT--C-G--G-A-A-G-A-AC-A-CC---AG--T--G--GC-GAA-G--G-C--G-------A--C-T-C-A---CTG--G--CC-C-G--------------------T-T---A-C-T--GA--CG-C-T-G--A-GG--C-G-CG-A--AA-G-C---G-TG--GG-
G--AG-C-A-AA--------CA--GG Bacteria(100);Proteobacteria(100);Alphaproteobacteria(100);Caulobacterales(100);Caulobacteraceae(100);uncultured(100);unclassified(100);unclassified(100);unclassified(100);

Otu0190 0 0 0 0 0 0 0 0 0 0 0 0 0 0 0 0 0 0 0 0 0 0 0 0 0 0 0 0
0 0 0 0 0 0 0 0 0 0 0 0 0 0 0 0 0 0 0 0 0 0 0 0 0 0 0 0 0
0 0 0 0 0 0 0 0 0 0 0 0 0 0 0 0 0 0 0 0 0 0 0 0 0 0 0 340 16
0 0 0 0 0 0 0 0 0 0 0 0 0 0 0 0 0 0 0 0 0 0 0 0 0 0 0
M02255_77_000000000-ADUT3_1_1106_23191_9858 C----GA---CT-AA-CTA--------TGT-G-C------C--A--G---C--AG-T-CGC-GG------TA-A--T-A--CA-TA-------G---G-----TT-GC-AA-GC-------GTTA-T-C-C-G------------G--A-TT-A-A-T-----------AA---
-G-T-C-T--------G-G-T--G--TT--A-AA-GG--C-A-AC-T-G-----CT-T-AA------C-G-G-T-T-G-T--A------T-G--C-A---T--T-------G--GA-A-A---C-------T--A-T--TA--G-T-C----T-A-G-A-G-T--G---T-GG-----TA-G-G--------G-A-G-T---TC-T------GG--A--ACT----C-C-A-T-GT--
GG-A-G-CG-GT--G---G--A-A---TG-C-GT-AG--AT-A-TA--T-G--G-A-A-G-A-AC-A-CC---AG--T--G--GC-GAA-G--G-C--G-------A--G-A-A-C---TTA--G--GC-C-A--------------------T-T---A-C-T--GA--CG-C-T-T--A-GG--C-T-TG-A--AA-G-T---G-TG--GG-G--AG-C-A-
AA--------TA--GG Bacteria(100);Firmicutes(98);unclassified(74);unclassified(74);unclassified(74);unclassified(74);unclassified(74);unclassified(74);unclassified(74);

Otu0191 0 89 0 0 0 0 0 0 0 0 0 0 4 0 0 0 0 0 0 0 0 0 0 0 0 0 0 0
0 0 0 68 0 0 0 0 119 0 0 0 0 0 0 0 0 0 0 1039 0 0 0 0 0 0 0 0 0
58 0 0 0 0 0 0 0 0 0 0 0 0 0 0 0 0 0 0 0 8 0 0 0 0 0 0 0 0
0 0 13 0 33 0 0 0 0 0 0 0 0 0 0 0 0 0 0 0 0 0 9 6 0 0 0
M02255_77_000000000-ADUT3_1_1103_11048_7963 T----AC---GT-AG-GGG--------GCA-A-G------C--G--T---T--AT-C-CGG-AT------TT-A--C-T--GG-GT-------GT--A-----AA-GG-GA-GC-------G-TA-G-A-C-G------------G--T-TC-T-G-C-----------AA-
---G-T-C-T--------G-G-A--G--TG--A-AA-GC--C-C-GG-G-G-----CT-C-AA------C-C-C-C-G-G-G--A-C----T-G--C-T---T--T-------G--GA-A-A---C-------T--G-T--GG--G-A-C----T-A-G-A-G-T--G---C-AG-----GA-G-A--------G-G-T-A---AG-T------GG--A--ATT----C-C-T-A-
GT--GT-A-G-CG-GT--G---A--A-A---TG-C-GT-AG--AT-A-TT--A-G--G-A-G-G-A-AC-A-CC---AG--T--G--GC-GAA-G--G-C--G-------G--C-T-T-A---CTG--G--AC-T-G--------------------T-A---A-C-T--GA--CG-T-T-G--A-GG--C-T-CG-A--AA-G-C---G-TG--GG-G--AG-
C-A-AA--------CA--GG Bacteria(100);Firmicutes(100);Clostridia(100);Clostridiales(100);Lachnospiraceae(100);Incertae_Sedis(95);unclassified(95);unclassified(95);unclassified(95);

Otu0192 7 0 0 10 0 0 0 13 3 3 0 0 0 0 1 0 0 0 0 0 3 0 0 0 0 0 1 0
0 11 3 9 4 0 0 0 15 3 4 0 1 0 10 16 14 2 0 0 6 0 0 5 0 2 0 0 3
0 1 0 1 0 0 0 0 0 0 0 0 0 0 0 0 0 0 2 2 0 1 0 4 0 5 11 0 2
0 0 4 0 0 0 0 0 0 0 0 6 0 5 0 0 11 0 0 4 1 3 4 0 4 8 3
M02255_77_000000000-ADUT3_1_1119_2603_15380 .....AC----T-AC-GT---------GCC-A-G------C--A--G---C--CG-C-GGT-AA------TA-C--G-T--AG-GT-------GG--C-----AA-GC-GT-TA-------G----G-C-G-G------------A--A-TT-A-T-T-----------AA----
G-T-C-T--------G-A-T--G--TG--A-AA-GC--C-C-AC-G-G-----CT-C-AA------C-C-G-T-G-G-A--G------G-GT-C-A---T--T-------G--GA-A-A---C-------T--G-G--AA--A-A-C----T-T-G-A-G-T--G---C-AG-----AA-G-A--------G-G-A-A---AG-T------GG--A--ATT----C-C-A-T-
GT--GT-A-G-CG-GT--G---A--A-A---TG-C-GC-AG--AG-A-TA--T-G--G-A-G-G-A-AC-A-CC---AG--T--G--GC-GAA-G--G-C--G-------A--C-T-T-T---CTG--G--TC-T-G--------------------T-A---A-C-T--GA--CG-C-T-G--A-TG--T-G-CG-A--AA-G-C---G-TG--GG-G--AT-C-
A-AA--------CA--GG Bacteria(100);Firmicutes(100);Bacilli(100);Bacillales(100);Staphylococcaceae(91);Staphylococcus(84);unclassified(84);unclassified(84);unclassified(84);

Otu0193 0 0 117 29 0 0 18 0 0 0 0 0 0 0 0 0 0 3 0 0 0 0 0 0 0 0 2 0
0 0 0 1 0 0 0 39 0 0 32 0 0 0 199 48 0 0 0 0 19 0 0 14 12 0 0 1 0
0 0 0 0 0 0 0 0 0 0 0 0 0 0 0 0 0 0 0 0 0 0 0 0 52 0 0 0 0
579 15 0 0 0 29 0 0 0 0 0 0 0 21 0 0 0 0 0 0 0 0 0 0 0 0 0
M02255_77_000000000-ADUT3_1_1117_18010_20262 T----AC---GT-AG-GGT--------ACG-A-G------C--G--T---T--GT-C-CGG-AA------TT-A--T-T--GG-GC-------GT--A-----AA-GA-GC-TC-------G-TA-G-G-T-G------------G--T-TG-G-T-C------
-----AC----G-T-C-T--------G-C-T--G--TG--G-AA-AC--G-C-AA-C-G-----CT-T-AA------C-G-T-T-G-C-G--C-G----T-G--C-A---G--T-------G--GG-T-A---C-------G--G-G--CT--G-A-C----T-A-G-A-G-T--G---C-AG-----TA-G-G--------G-G-A-G---TC-T------GG--A--ATT----C-
C-T-G-GT--GT-A-G-CG-GT--G---A--A-A---TG-C-GC-AG--AT-A-TC--A-G--G-A-G-G-A-AC-A-CC---GG--T--G--GC-GAA-G--G-C--G-------G--G-A-C-T---CTG--G--GC-T-G--------------------T-A---A-C-T--GA--CA-C-T-G--A-GG--A-G-CG-A--AA-G-C---A-TG--GG-
G--AG-C-G-AA--------CA--GG Bacteria(100);Actinobacteria(100);Actinobacteria(100);Actinobacteridae(100);Actinomycetales(100);Micrococcineae(100);Brevibacteriaceae(100);Brevibacterium(100);unclassified(100);

Otu0194 0 0 0 0 0 0 0 0 0 0 0 0 0 0 3 0 0 0 0 0 0 0 9 0 0 0 0 0
0 1 0 0 0 0 0 2 34 0 0 0 11 6 2 0 0 23 0 2 0 0 0 2 6 0 0 0 0
3 5 1 0 0 0 0 0 1 3 0 0 0 2 5 0 17 0 0 26 0 0 0 0 0 108 116 0 0
0 0 0 0 7 0 0 0 0 6 0 7 0 0 0 0 0 0 0 0 0 0 0 0 0 0 3
M02255_77_000000000-ADUT3_1_2116_22005_15107 T----AC---GT-AG-GGC--------GCA-A-G------C--G--T---T--AT-C-CGG-AA------TT-A--T-T--GG-GC-------GT--A-----AA-GA-GC-TC-------G-TA-G-G-C-G------------G--T-TT-G-T-C------
-----GC----G-T-C-T--------G-C-T--G--TG--A-AA-TC--C-C-GA-G-G-----CT-C-AA------C-C-T-C-G-G-G--C-C----T-G--C-A---G--T-------G--GG-T-A---C-------G--G-G--CA--G-A-C----T-A-G-A-G-T--G---C-GG-----TA-G-G--------G-G-A-G---AT-T------GG--A--ATT----C-
C-T-G-GT--GT-A-G-CG-GT--G---G--A-A---TG-C-GC-AG--AT-A-TC--A-G--G-A-G-G-A-AC-A-CC---GA--T--G--GC-GAA-G--G-C--A-------G--A-T-C-T---CTG--G--GC-C-G--------------------T-A---A-C-T--GA--CG-C-T-G--A-GG--A-G-CG-A--AA-G-G---G-TG--GG-
G--AG-C-A-AA--------CA--GG Bacteria(100);Actinobacteria(100);Actinobacteria(100);Actinobacteridae(100);Actinomycetales(100);Micrococcineae(100);Microbacteriaceae(100);unclassified(81);unclassified(81);

Otu0195 0 0 0 0 0 0 0 0 0 0 0 0 0 0 0 0 0 0 0 0 0 0 0 0 0 0 5 2
0 0 0 0 0 0 0 0 0 0 0 0 0 0 0 0 0 0 0 0 0 0 0 0 10 0 0 0 0
0 0 0 0 0 0 0 0 0 0 0 0 11 0 0 0 13 0 0 0 0 0 0 0 51 0 0 0 0
0 0 0 85 0 0 25 0 0 0 0 0 0 0 0 0 0 0 0 0 0 0 0 0 0 0 0
M02255_77_000000000-ADUT3_1_1101_22087_11579 T----AC---GG-AG-GGG--------GCT-A-G------C--G--T---T--GT-T-CGG-AA------TT-A--C-T--GG-GC-------GT--A-----AA-GC-GC-AC-------G-TA-G-G-C-G------------G--C-TT-G-G-T-----
-------AA----G-T-T-A--------G-A-G--G--TG--A-AA-GC--C-C-GG-G-G-----CT-C-AA------C-C-C-C-G-G-A--A-T----T-G--C-C---T--T-------T--AA-G-A---C-------T--G-C--CG--A-G-C----T-A-G-T-A-G--G---T-GA-----GA-G-A--------G-G-T-G---AG-T------GG--A--ATT----
C-C-G-A-GT--GT-A-G-AG-GT--G---A--A-A---TT-C-GT-AG--AT-A-TT--C-G--G-A-A-G-A-AC-A-CC---AG--T--G--GC-GAA-G--G-C--G-------G--C-T-C-A---CTG--G--CT-C-A--------------------T-T---C-T-A--GA--CG-C-T-G--A-GG--T-G-CG-A--AA-G-C---G-TG--GG-
G--AG-C-A-AA--------CA--GG Bacteria(100);Proteobacteria(100);Alphaproteobacteria(100);unclassified(98);unclassified(98);unclassified(98);unclassified(98);unclassified(98);unclassified(98);

Otu0196 0 0 72 0 0 0 127 73 363 0 0 0 0 0 0 0 0 0 0 0 0 0 0 10 0 0 0 0
0 0 0 0 0 95 0 0 0 0 0 237 0 0 107 0 0 1 0 0 0 0 0 0 0 0 0 0 0
0 0 0 0 0 0 0 0 0 3 0 0 6 0 0 5 0 91 0 0 0 0 0 0 0 0 0 0 0
0 0 0 0 0 0 0 0 0 0 0 0 0 0 0 0 0 0 0 0 0 0 0 8 102 0 0
M02255_77_000000000-ADUT3_1_1103_13768_17099 A----AC---GT-AG-GGT--------GCA-A-G------C--G--T---T--GT-C-CGG-AA------TT-A--C-T--GG-GT-------GT--A-----AA-GG-GA-GC-------G-CA-G-G-C-G------------G--A-CC-G-G-C----
-------AA----G-T-T-G--------G-A-A--G--TG--A-AA-AC--T-A-TG-G-G-----CT-C-AA------C-C-C-A-T-A-A--A-T----T-G--C-T---T--T-------C--AA-A-A---C-------T--G-C--TG--G-C-C----T-T-G-A-G-T--A---G-TG-----CA-G-A--------G-G-T-A---GG-T------GG--A--ATT----C-
C-C-G-GT--GT-A-G-CG-GT--G---G--A-A---TG-C-GT-AG--AT-A-TC--G-G--G-A-G-G-A-AC-A-CC---AG--T--G--GC-GAA-G--G-C--G-------A--C-C-T-A---CTG--G--GC-A-C--------------------C-A---A-C-T--GA--CG-C-T-G--A-GG--C-T-CG-A--AA-G-C---A-TG--GG-
T--AG-C-A-AA--------CA--GG Bacteria(100);Firmicutes(100);Clostridia(100);Clostridiales(100);Ruminococcaceae(100);Subdoligranulum(100);unclassified(100);unclassified(100);unclassified(100);

Otu0197 0 0 0 0 0 0 0 0 0 0 0 0 0 0 9 0 0 0 0 0 0 0 0 0 0 0 0 42
0 0 0 0 0 0 0 0 162 0 0 59 0 0 0 0 0 0 0 0 0 0 0 0 0 0 0 0 0
0 0 0 0 0 0 0 0 0 0 0 0 0 0 0 0 0 0 0 0 9 0 0 0 0 0 0 0 0
30 0 0 0 0 0 0 0 0 28 0 0 0 33 0 0 0 0 0 0 0 0 0 0 0 0 0
M02255_77_000000000-ADUT3_1_2112_16988_15998 T----AC---GA-AG-GGG--------GCT-A-G------C--G--T---T--GT-T-CGG-AA------TT-A--C-T--GG-GC-------GT--A-----AA-GC-GC-AC-------G-TA-G-G-C-G------------G--A-TC-G-T-T-----
-------AA----G-T-G-A--------G-G-G--G--TG--A-AA-TC--C-C-AG-G-G-----CT-C-AA------C-C-C-T-G-G-A--A-C----T-G--C-C---T--C-------T--CA-T-A---C-------T--G-G--CG--A-T-C----T-C-G-A-G-T--T---C-GA-----GA-G-A--------G-G-T-G---AG-T------GG--A--ATT----C-
C-G-A-GT--GT-A-G-AG-GT--G---A--A-A---TT-C-GT-AG--AT-A-TT--C-G--G-A-G-G-A-AC-A-CC---AG--T--G--GC-GAA-G--G-C--G-------G--C-T-C-A---CTG--G--CT-C-G--------------------A-C---A-C-T--GA--CG-C-T-G--A-GG--T-G-CG-A--AA-G-C---G-TG--GG-
G--AG-C-A-AA--------CA--GG Bacteria(100);Proteobacteria(100);Alphaproteobacteria(100);Rhizobiales(100);Phyllobacteriaceae(61);unclassified(61);unclassified(61);unclassified(61);unclassified(61);

Otu0198 0 0 0 0 0 0 0 0 0 0 0 0 4 0 1 0 1 1 0 0 0 0 0 0 0 0 0 0
0 0 0 0 0 0 0 0 0 0 0 0 0 0 0 0 0 0 0 0 0 0 0 0 0 0 6 2 0
2 0 1 0 0 0 0 2 1 0 1 0 0 3 0 0 0 0 0 0 0 0 0 0 0 0 0 0 0
0 0 0 0 0 0 2 0 0 0 1 0 0 0 0 0 0 0 0 0 0 0 0 0 0 0 0
M02255_77_000000000-ADUT3_1_1108_19955_10279 C----TG---TG-CC-AGC--------AGC-C-G------C--G--G---T--AA-TACAG-AG------GG-T--G-C--AA-GC-------GT--T-----AA-TC-GG-AA-------T-TA-C-T-G-G------------G--T-TT-G-T-T-----
-------AA----G-T-T-G--------G-A-T--G--TG--A-AA-TC--C-C-CG-G-G-----CT-C-AA------C-C-T-G-G-G-A--A-C----T-G--C-A---T--T-------C--AA-A-A---C-------T--G-A--CT--G-A-C----T-A-G-A-G-T--A---T-GG-----TA-G-A--------G-G-G-T---GG-T------GG--A--ATT----T-
C-C-T-GT--GT-A-G-CG-GT--G---A--A-A---TG-C-GT-AG--AT-A-TA--G-G--A-A-G-G-A-AC-A-CC---AG--T--G--GC-GAA-G--G-C--G-------A--C-C-A-C---CTG--G--AC-T-A--------------------A-T---A-C-T--GA--CA-C-T-G--A-GG--T-G-CG-A--AA-G-C---G-TG--GG-
G--AG-C-A-AA--------CA--GG Bacteria(100);Proteobacteria(100);Gammaproteobacteria(100);Pseudomonadales(100);Pseudomonadaceae(100);Pseudomonas(100);unclassified(100);unclassified(100);unclassified(100);

Otu0199 0 5 0 13 0 5 26 0 0 0 0 0 1 0 0 0 0 0 0 4 0 0 0 0 0 0 0 0
0 0 6 9 10 8 0 34 0 11 10 1 17 0 1 13 0 7 0 5 6 0 0 16 0 0 0 3 0
0 0 0 0 0 0 0 0 0 0 0 0 1 14 0 2 0 1 0 0 0 0 0 0 0 0 0 0 0
0 0 0 0 0 0 0 0 0 0 0 0 0 16 0 0 6 0 0 7 14 2 8 0 7 0 0
M02255_77_000000000-ADUT3_1_1110_6829_16585 T----AC---GT-AG-GGT--------GCG-A-G------C--G--T---T--GT-C-CGG-AA------TT-A--C-T--GG-GC-------GT--A-----AA-GA-GC-TC-------G-TA-G-G-T-G------------G--T-TT-G-T-C-----------GC-
---G-T-C-G--------T-T-T--G--TG--T-AA-GT--C-C-AC-G-G-----CT-T-AA------C-T-G-T-G-G-G--A-T----G-G--C-A---G--G-------C--GA-T-A---C-------G--G-G--CAT-A-A-C----T-T-G-A-G-T--G---C-TG-----TA-G-G--------G-G-A-G---AC-T------GG--A--ATT----C-C-T-G-
GT--GT-A-G-CG-GT--G---A--A-A---TG-C-GC-AG--AT-A-TC--A-G--G-A-G-G-A-AC-A-CC---GA--T--G--GC-GAA-G--G-C--A-------G--G-T-C-T---CTG--G--GC-A-G--------------------T-A---A-C-T--GA--CG-C-T-G--A-GG--A-G-CG-A--AA-G-C---A-TG--GG-T--AG-
C-G-AA--------CA--GG Bacteria(100);Actinobacteria(100);Actinobacteria(100);Actinobacteridae(100);Actinomycetales(100);Corynebacterineae(100);Corynebacteriaceae(100);Corynebacterium(100);unclassified(100);

Otu0200 0 0 0 0 0 0 0 0 0 0 0 0 0 0 0 0 0 0 0 0 0 0 0 0 2 0 0 0
0 0 0 4 0 0 0 0 0 0 0 2 0 0 0 7 0 0 0 0 0 0 0 0 0 0 0 0
0 0 0 0 0 0 2 0 0 0 0 0 0 0 0 0 0 0 0 3 0 1 0 4 6 0 0 18 23
19 3 14 0 5 0 0 1 0 13 0 0 0 0 0 0 0 0 0 2 0 0 0 0 0 0 0
M02255_76_000000000-ADUNE_1_1112_19861_6338 TC---GC---AG-AA-TAA--------GCA-C-C------G--G--C---T--AACT-CTG-TG------CC-AG-C-A-GCC-GC-------GG--T-----AA-TA-CA-GA-------G-GG-T-G-C-G------------A--G-CA-G-T-T----------
GA----G-T-C-G--------G-A-T--G--TG--A-AA-TC--C-C-CG-A-G-----CT-T-AA------C-T-T-G-G-G-A--A-T----T-G--C-A---T--T-------C--GA-T-A---C-------T--G-G--TT--A-G-C----T-A-G-A-G-T--G---T-GG-----GA-G-A--------G-G-A-T---GG-T------AG--A--ATT----C-C-A-
G-GT--GT-A-G-CG-GT--G---A--A-A---TG-C-GT-AG--AG-A-TC--T-G--G-A-G-G-A-AT-A-CC---GA--T--G--GC-GAA-G--G-C--A-------G--C-C-A-T---CTG--G--CC-T-A--------------------A-C---A-C-T--GA--CG-C-T-G--A-GG--T-A-CG-A--AA-G-C---A-TG--GG-G--
AG-C-A-AA--------CA--GG Bacteria(100);Proteobacteria(100);Gammaproteobacteria(100);Pseudomonadales(100);Moraxellaceae(100);Acinetobacter(79);unclassified(79);unclassified(79);unclassified(79);

Otu0201 0 0 74 0 0 0 0 0 0 0 0 0 0 0 0 0 0 0 0 0 0 0 0 0 0 0 0 0
0 0 0 0 0 0 0 0 0 0 0 37 0 0 0 0 0 0 0 0 0 0 0 6 0 0 0 0
0 0 0 0 0 0 0 0 0 0 4 0 0 0 7 0 12 0 0 0 0 0 0 0 0 0 0 0 0
0 0 0 1034 30 0 3 0 0 0 0 0 0 0 0 0 0 0 0 0 0 0 0 2 0 0 0
M02255_77_000000000-ADUT3_1_1110_20610_12237 T----AC---GG-AG-GGG--------GCT-A-G------C--G--T---T--GT-T-CGG-AA------TT-A--C-T--GG-GC-------GT--A-----AA-GC-GC-AC-------G-TA-G-G-C-G------------G--C-TT-T-G-T-----
------AA----G-T-T-A--------G-A-G--G--TG--A-AA-GC--C-C-GG-G-G-----CT-C-AA------C-C-C-C-G-G-A--A-T----T-G--C-C---T--T-------T--AA-G-A---C-------T--G-C--AG--A-G-C----T-A-G-C-A-G--G---T-GA-----GA-G-A--------G-G-T-A---AG-T------GG--A--ATT----
C-C-G-A-GT--GT-A-G-AG-GT--G---A--A-A---TT-C-GT-AG--AT-A-TT--C-G--G-A-A-G-A-AC-A-CC---AG--T--G--GC-GAA-G--G-C--G-------G--C-T-T-A---CTG--G--CT-C-A--------------------T-A---C-T-G--GA--CG-C-T-G--A-GG--T-G-CG-A--AA-G-C---G-TG--GG-
G--AG-C-A-AA--------CA--GG Bacteria(100);Proteobacteria(100);Alphaproteobacteria(100);Sphingomonadales(100);Sphingomonadaceae(88);Sphingomonas(87);unclassified(87);unclassified(87);unclassified(87);

Otu0202 82 0 0 34 0 26 22 0 0 0 0 0 0 1 4 0 0 0 24 7 46 10 14 0 0 38 27 47
53 483 280 384 153 169 125 178 253 219 158 189 89 208 148 621 56 53 13 297 603 0 4 115 15 4 0 55 393
0 0 0 0 0 0 3 0 1 0 0 0 3 2 9 6 100 22 0 0 55 0 0 0 4 0 0 21 1
16 0 238 0 0 0 0 0 0 0 0 0 0 24 0 0 0 0 0 0 0 26 0 3 1 147 0
M02255_77_000000000-ADUT3_1_1112_14068_17113 T----AC---GT-AG-GGT--------GCG-A-G------C--G--T---T--GT-C-CGG-AA------TT-A--T-T--GG-GC-------GT--A-----AA-GA-GC-TT-------G-TA-G-G-C-G------------G--T-TT-G-T-C------
-----GC----G-T-C-T--------G-C-T--G--TG--A-AA-GA--C-C-GG-G-G-----CT-T-AA------C-T-C-C-G-G-T--T-C----T-G--C-A---G--T-------G--GG-T-A---C-------G--G-G--CA--G-A-C----T-A-G-A-G-T--G---T-GG-----TA-G-G--------G-G-A-G---AC-T------GG--A--ATT----C-
C-T-G-GT--GT-A-G-CG-GT--G---A--A-A---TG-C-GC-AG--AT-A-TC--A-G--G-A-G-G-A-AC-A-CC---GA--T--G--GC-GAA-G--G-C--A-------G--G-T-C-T---CTG--G--GC-C-A--------------------T-T---A-C-T--GA--CG-C-T-G--A-GA--A-G-CG-A--AA-G-C---A-TG--GG-
G--AG-C-G-AA--------CA--GG Bacteria(100);Actinobacteria(100);Actinobacteria(100);Actinobacteridae(100);Actinomycetales(100);Micrococcineae(100);unclassified(100);unclassified(100);unclassified(100);

Otu0203 0 0 0 0 0 0 0 0 0 0 0 0 0 0 0 0 0 0 0 2 2 1 0 0 0 4 0 15
0 0 0 0 0 0 0 0 0 15 52 1 30 22 0 0 0 0 0 0 0 0 0 0 0 0 0 0
0 0 0 0 0 0 0 0 0 0 0 0 0 0 0 0 0 0 0 0 0 0 0 0 0 0 0 0
0 0 0 8 34 0 0 0 0 0 0 0 0 0 0 0 69 0 0 0 0 0 0 0 5 0 0
M02255_77_000000000-ADUT3_1_1117_10790_19901 T----AC---GT-AG-GGA--------CCA-A-G------C--G--T---T--GT-T-CGG-AT------TT-A--C-T--GG-GC-------GT--A-----AA-GG-GC-GC-------G-TA-G-G-C-G------------G--C-GT-G-G-T-----
------AA----G-T-C-A--------C-T-T--G--TG--A-AA-TC--T-C-TG-A-G-----CT-T-AA------C-T-C-A-G-A-A--C-G----G-C--C-A---A--G-------T--GA-T-A---C-------T--G-C--TG--T-G-C----T-A-G-A-G-T--G---C-AG-----AA-G-G--------G-G-C-A---AT-C------GG--A--ATT----C-
T-T-G-GT--GT-A-G-CG-GT--G---A--A-A---TG-C-GT-AG--AT-A-TC--A-A--G-A-G-G-A-AC-A-CC---TG--A--G--GT-GAA-G--A-C--G-------G--G-T-T-G---CTG--G--GC-T-G--------------------A-C---A-C-T--GA--CG-C-T-G--A-GG--C-G-CG-A--AA-G-C---C-AG--GG-
G--AG-C-A-AA--------CG--GG Bacteria(100);Acidobacteria(100);Acidobacteria(100);Acidobacteriales(100);Acidobacteriaceae(100);Candidatus_Chloroacidobacterium(100);unclassified(100);unclassified(100);unclassified(100);

Otu0204 0 0 0 0 0 22 15 0 0 0 0 0 0 0 0 0 0 1 0 0 0 0 0 0 0 0 0 0
0 0 3 0 0 0 0 23 0 0 57 3 0 0 72 70 0 0 0 0 0 0 0 0 0 0 34 0
0 0 0 0 0 0 0 0 0 0 0 0 3 0 122 131 4 0 105 0 0 0 0 0 0 0 0 0 70
14 117 0 6 49 0 0 0 0 17 0 0 9 49 0 0 0 0 0 0 0 0 0 0 8 0 0
M02255_77_000000000-ADUT3_1_1107_15299_16050 T----AC---GT-AG-GGG--------GCA-A-G------C--G--T---T--AT-C-CGG-AT------TT-A--C-T--GG-GT-------GT--A-----AA-GG-GA-GC-------G-CA-G-A-C-G------------G--C-AA-T-G-C----
-------AA----G-T-C-T--------G-A-A--G--TG--A-AA-GG--C-G-TG-G-G-----CT-C-AA------C-C-C-A-T-G-A--A-C----T-G--C-T---T--T-------G--GA-A-A---C-------T--G-T--AT--A-G-C----T-T-G-A-G-T--G---T-CG-----GA-G-G--------G-G-T-A---AG-C------GG--A--ATT----
C-C-T-A-GT--GT-A-G-CG-GT--G---A--A-A---TG-C-GT-AG--AT-A-TT--A-G--G-A-G-G-A-AC-A-CC---GG--A--G--GC-GAA-G--G-C--G-------G--C-T-T-A---CTG--G--AC-G-A--------------------C-A---A-C-T--GA--CG-T-T-G--A-GG--C-T-CG-A--AG-G-C---G-TG--
GG-G--AG-C-A-AA--------CA--GG Bacteria(100);Firmicutes(100);Clostridia(100);Clostridiales(100);Lachnospiraceae(100);Johnsonella(100);unclassified(100);unclassified(100);unclassified(100);

Otu0205 0 0 0 0 1 0 0 0 0 0 0 11 0 0 0 0 0 0 5 0 0 0 0 17 0 1 4 6
0 0 0 0 0 0 0 0 0 0 0 0 0 0 0 0 0 0 0 0 0 0 0 0 0 0 0 0 0
0 0 0 0 0 0 0 0 0 0 0 0 0 0 0 0 0 0 0 0 0 0 4 0 0 0 0 0 0
0 0 0 0 0 0 0 0 0 0 0 0 0 0 0 0 0 0 0 0 0 0 0 0 0 0 0
M02255_77_000000000-ADUT3_1_2109_29199_9415 T----AC---GG-AG-GAT--------CCA-A-G------C--G--T---T--AT-C-CGG-AA------TC-A--T-T--GG-G--------TT-----------G-G---C-----------G-G-C-----------------C-C--T-G-T----------AA----G-T-C-
A--------G-C-G--G--TG--A-AA-TC--T-C-CC-G-G-----CT-C-AA------C-C-G-G-G-A-A--A-T----G-G--C-C---G--T-------T--GA-T-A---C-------T--G-C--AG--G-G-C----T-T-G-A-A-T--T---A-GT-----AG-G-A--------A-G-T-A---AC-T------AG--A--ATA----T-G-T-A-GT--GT-A-
G-CG-GT--G---A--A-A---TG-C-TT-AG--AG-A-TT--A-C--A-T-G-G-A-AT-A-CC---AA--T--T--GC-GAA-G--G-C--A-------G--G-T-T-A---CTA--C--TA-C-T--------------------A-T---A-T-T--GA--CG-C-T-G--A-GG--G-A-CG-A--AA-G-C---G-TG--GG-T--AG-C-G-AA--------
CA--GG Bacteria(100);Bacteroidetes(100);Flavobacteria(100);Flavobacteriales(100);Flavobacteriaceae(100);unclassified(100);unclassified(100);unclassified(100);unclassified(100);

Otu0206 0 0 0 0 0 0 0 0 0 0 0 0 0 3 0 0 6 0 0 0 0 0 0 2 0 0 0 0
0 0 0 0 0 0 0 0 0 0 0 0 0 0 0 0 0 0 6 0 0 0 0 0 0 0 0 0 0
0 0 0 0 0 0 0 0 0 6 0 0 0 0 0 0 0 0 0 0 0 0 0 0 0 0 0 0 0
0 0 0 0 0 0 0 0 0 0 0 0 2 0 0 0 0 0 0 0 0 0 0 0 0 0 0
M02255_76_000000000-ADUNE_1_2110_13319_8982 T----AC---CG-AC-AGA--------ATA-A-G------C-----A---C--CG-G-CTA-AC------TC-T--G-T--GC-CA-------GC--AG----CC-GC-GG-TA-------A-TACA-G-A-G------------G--G-TT-G-C-A----------
AG----C-G-T-T--------A-A-T--C--GG--A-AA-TC--C-C-CG-G-G-----CT-C-AA------C-C-T-G-G-G-A--A-C----T-G--C-A---T--C-------C--AA-A-A---C-------T--G-G--CA--A-G-C----T-A-G-A-G-T--A---T-GG-----TA-G-A--------G-G-G-T---AG-T------GG--A--ATT----T-C-C-
T-GT--GT-A-G-CG-GT--G---A--A-A---TG-C-GT-AG--AT-A-TA--G-G--A-A-G-G-A-AC-A-CC---AG--T--G--GC-GAA-G--G-C--G-------A--C-T-A-C---CTG--G--AC-T-G--------------------A-T---A-C-T--GA--CA-C-T-G--A-GG--T-G-CG-A--AA-G-C---G-TG--GG-G--
AG-C-A-AA--------CA--GG Bacteria(100);Proteobacteria(100);Gammaproteobacteria(100);unclassified(100);unclassified(100);unclassified(100);unclassified(100);unclassified(100);

Otu0207 0 559 700 0 0 0 1520 464 2854 0 0 0 0 0 0 1 2 0 0 0 18 0 0 4 0 0 0 0
0 0 7 52 0 0 0 0 0 0 91 599 0 0 0 111 25 0 0 0 0 0 0 17 0 8 0 0 0
0 0 0 0 0 0 0 0 0 0 0 0 5 0 41 25 46 91 0 0 0 0 0 0 89 0 0 0 330
0 1 0 15 0 0 30 0 0 0 0 141 0 1 0 38 79 0 0 0 0 0 11 20 1111 1 0
M02255_77_000000000-ADUT3_1_1101_20883_14129 T----AC---GT-AG-GGA--------GCA-A-G------C--G--T---T--GT-C-CGG-AT------TT-A--C-T--GG-GT-------GT--A-----AA-GG-GT-GC-------G-TA-G-G-C-G------------G--C-TT-T-G-C-----
------AA----G-T-C-A--------G-A-T--G--TG--A-AA-TC--T-A-TG-G-G-----CT-C-AA------C-C-C-A-T-A-A--A-C----T-G--C-A---T--T-------T--GA-A-A---C-------T--G-T--AG--A-G-C----T-T-G-A-G-T--G---A-AG-----TA-G-A--------G-G-C-A---GG-C------GG--A--ATT----C-
C-C-C-GT--GT-A-G-CG-GT--G---A--A-A---TG-C-GT-AG--AG-A-TG--G-G--G-A-G-G-A-AC-A-CC---AG--T--G--GC-GAA-G--G-C--G-------G--C-C-T-G---CTG--G--GC-T-T--------------------T-A---A-C-T--GA--CG-C-T-G--A-GG--C-A-CG-A--AA-G-C---G-TG--GG-
T--AG-C-A-AA--------CA--GG Bacteria(100);Firmicutes(100);Clostridia(100);Clostridiales(100);Ruminococcaceae(100);unclassified(100);unclassified(100);unclassified(100);unclassified(100);

Otu0208 0 0 0 0 0 0 0 0 0 0 0 0 0 0 0 0 0 0 0 0 0 2 0 0 0 4 0 15
0 0 0 0 0 0 0 0 0 17 0 0 0 0 0 0 0 0 0 0 0 0 0 0 0 2 0 0 0
0 0 0 0 0 0 0 0 0 0 0 0 0 0 0 0 0 101 0 0 57 0 0 0 54 0 0 0 0
0 0 0 0 0 0 0 0 0 0 0 0 0 0 0 96 0 0 0 0 0 0 0 0 0 0 0
M02255_77_000000000-ADUT3_1_1114_9865_16668 T----AC---GA-AG-GGG--------GCT-A-G------C--G--T---T--GT-T-CGG-AT------TT-A--C-T--GG-GC-------GT--A-----AA-GC-GC-AC-------G-TA-G-G-C-G------------G--A-TC-G-T-T----------AA-
---G-T-C-G--------G-G-G--G--TG--A-AA-TC--C-T-GG-A-G-----CT-C-AA------C-T-C-C-A-G-A--A-C----T-G--C-C---T--T-------C--GA-T-A---C-------T--G-G--CG--A-T-C----T-C-G-A-G-T--C---C-GG-----AA-G-A--------G-G-T-G---AG-T------GG--A--ACT----C-C-T-A-
GT--GT-A-G-AG-GT--G---G--A-A---TT-C-GT-AG--AT-A-TT--A-G--G-A-A-G-A-AC-A-CC---AG--T--G--GC-GAA-G--G-C--G-------G--C-T-C-A---CTG--G--TC-C-G--------------------G-T---A-C-T--GA--CG-C-T-G--A-GG--T-G-CG-A--AA-G-C---G-TG--GG-G--AG-
C-A-AA--------CA--GG Bacteria(100);Proteobacteria(100);Alphaproteobacteria(100);Rhizobiales(100);Hyphomicrobiaceae(100);Devosia(92);unclassified(92);unclassified(92);unclassified(92);

Otu0209 0 415 0 0 0 24 204 32 476 0 0 0 0 0 0 1 0 0 0 4 0 4 8 1 0 0 0 0
0 930 25 141 0 0 0 0 0 0 0 320 1 0 0 0 0 0 0 6 0 0 0 0 0 0 0 0 0
0 0 0 0 0 0 0 0 0 0 8 0 1 0 8 1 0 83 0 0 0 0 0 0 0 0 0 0 0
16 0 0 15 0 0 11 0 0 0 0 58 3 36 0 0 0 0 0 0 0 0 0 12 166 0 0
M02255_76_000000000-ADUNE_1_2111_10458_13871 T----AC---GT-AG-GGG--------GCA-A-G------C--G--T---T--AT-C-CGG-AT------TT-A--C-T--GG-GT-------GT--A-----AA-GG-GA-GC-------G-TA-G-A-C-G------------G--C-AC-G-G-C----
-------AA----G-C-C-A--------G-A-T--G--TG--A-AA-GC--C-C-GG-G-G-----CT-C-AA------C-C-C-C-G-G-G--A-C----T-G--C-A---T--T-------T--GG-A-A---C-------T--G-C--TG--A-G-C----T-A-G-A-G-T--G---T-CG-----GA-G-A--------G-G-C-A---AG-T------GG--A--ATT----
C-C-T-A-GT--GT-A-G-CG-GT--G---A--A-A---TG-C-GT-AG--AT-A-TT--A-G--G-A-G-G-A-AC-A-CC---AG--T--G--GC-GAA-G--G-C--G-------G--C-T-T-G---CTG--G--AC-G-A--------------------T-G---A-C-T--GA--CG-T-T-G--A-GG--C-T-CG-A--AA-G-C---G-TG--GG-
G--AG-C-A-AA--------CA--GG Bacteria(100);Firmicutes(100);Clostridia(100);Clostridiales(100);Lachnospiraceae(100);Incertae_Sedis(99);unclassified(99);unclassified(99);unclassified(99);

Otu0210 0 0 0 0 0 0 0 0 0 0 0 0 0 0 0 0 0 0 0 0 0 0 0 8 0 0 0 0
0 0 0 0 0 0 0 0 0 0 0 0 0 0 0 0 0 0 0 0 0 0 0 71 0 0 0 0 0
0 0 0 0 0 0 0 0 0 0 0 0 4 0 30 0 0 18 0 0 0 0 0 0 0 0 0 0 0
0 0 0 0 0 0 0 0 0 0 0 0 0 0 0 0 0 0 0 0 0 0 0 0 0 0 0
M02255_76_000000000-ADUNE_1_1116_10609_14909 T----AC---GA-AG-GGG--------GCT-A-G------C--G--T---T--GC-T-CGG-AA------TT-A--C-T--GG-GC-------GT--A-----AA-GG-GC-GC-------G-TA-G-G-C-G------------G--A-CA-G-T-T----
-------TA----G-T-C-A--------G-A-G--G--TG--A-AA-GC--C-C-AG-G-G-----CT-C-AA------C-C-T-T-G-G-A--A-T----T-G--C-C---T--T-------T--GA-T-A---C-------T--G-G--CT--G-T-C----T-T-G-A-G-T--A---C-GG-----GA-G-A--------G-G-T-G---TG-T------GG--A--ACT----C-
C-G-A-GT--GT-A-G-AG-GT--G---A--A-A---TT-C-GT-AG--AT-A-TT--C-G--G-A-A-G-A-AC-A-CC---AG--T--G--GC-GAA-G--G-C--G-------A--C-A-C-A---CTG--G--CC-C-G--------------------T-T---A-C-T--GA--CG-C-T-G--A-GG--C-G-CG-A--AA-G-C---G-TG--GG-
G--AG-C-A-AA--------CA--GG Bacteria(100);Proteobacteria(100);Alphaproteobacteria(100);Caulobacterales(100);Caulobacteraceae(100);unclassified(78);unclassified(78);unclassified(78);unclassified(78);

Otu0211 0 0 0 150 0 0 0 0 0 0 0 0 0 0 0 0 0 0 0 0 0 1 0 0 0 0 0 0
65 0 0 0 0 29 0 0 0 0 0 0 0 0 0 0 0 0 0 42 0 0 0 0 0 121 0 0 0
0 0 0 0 0 0 0 0 0 0 0 0 5 0 8 0 0 820 0 0 0 0 4 0 0 0 0 0 0
0 0 0 54 0 0 0 7 0 0 0 0 0 28 0 42 0 64 0 0 0 0 26 0 0 0 0
M02255_77_000000000-ADUT3_1_1114_11060_14599 T----AC---GG-AG-GGG--------GTT-A-G------C--G--T---T--GT-T-CGG-AA------TG-A--C-T--GG-GC-------GT--A-----AA-GC-GC-GC-------G-TA-G-G-C-G------------G--A-TC-G-C-A----
-------AA----G-T-T-G--------G-G-G--G--TG--A-AA-GC--C-C-AG-G-G-----CT-C-AA------C-C-C-T-G-G-A--A-T----T-G--C-C---T--T-------C--AA-G-A---C-------T--C-G--CG--G-T-C----T-G-G-A-G-T--T---C-GA-----GA-G-A--------G-G-T-G---GG-T------GG--A--ATT----
C-C-G-A-GT--GT-A-G-AG-GT--G---A--A-A---TT-C-GT-AG--AT-A-TT--C-G--G-A-G-G-A-AC-A-CC---AG--T--G--GC-GAA-G--G-C--G-------G--C-C-C-A---CTG--G--CT-C-G--------------------A-C---A-C-T--GA--CG-C-T-G--A-GG--C-G-CG-A--AA-G-C---G-CG--
GG-G--AG-C-A-AA--------CA--GG Bacteria(100);Proteobacteria(100);Alphaproteobacteria(100);unclassified(100);unclassified(100);unclassified(100);unclassified(100);unclassified(100);unclassified(100);

Otu0212 0 241 112 0 0 0 50 2 451 0 0 0 0 0 0 0 0 0 0 0 0 0 0 0 0 0 0 0
0 0 0 0 0 0 0 0 0 0 32 17 0 0 0 0 0 0 0 0 0 0 0 0 0 0 0 0 0
0 0 0 0 0 0 0 0 0 0 0 0 0 0 0 0 21 0 0 0 0 0 0 0 0 0 0 0 72
0 0 0 0 0 0 0 0 0 0 0 0 0 0 0 0 0 0 0 0 0 0 0 0 40 0 0
M02255_77_000000000-ADUT3_1_1104_22816_9870 T----AC---GT-AG-GTG--------GCA-A-G------C--G--T---T--GT-C-CGG-AT------TT-A--C-T--GG-GT-------GT--A-----AA-GG-GC-GT-------G-TA-G---CCG------------G--G-AA-G-G-C----------
AA----G-T-C-A--------G-A-T--G--TG--A-AA-TC--C-A-CG-G-G-----CT-T-AA------C-T-C-G-T-G-A--A-C----T-G--C-A---T--T-------T--GA-A-A---C-------T--A-C--TT--T-T-C----T-T-G-A-G-T--A---T-CG-----GA-G-A--------G-G-C-A---AT-C------GG--A--ATT----C-C-T-A-
GT--GT-A-G-CG-GT--G---A--A-A---TG-C-GT-AG--AT-A-TT--A-G--G-A-G-G-A-AC-A-CC---AG--T--G--GC-GAA-G--G-C--G-------G--A-T-T-G---CTG--G--AC-G-A--------------------C-A---A-C-T--GA--CG-G-T-G--A-GG--C-G-CG-A--AA-G-C---G-TG--GG-G--AG-
C-A-AA--------CA--GG Bacteria(100);Firmicutes(100);Clostridia(100);Clostridiales(100);Ruminococcaceae(100);unclassified(100);unclassified(100);unclassified(100);unclassified(100);

Otu0213 0 0 0 0 0 0 0 0 0 0 0 0 0 0 0 0 0 0 0 0 5 0 0 0 0 0 0 0
0 0 16 0 0 0 0 19 0 44 0 12 0 0 0 2 0 16 0 0 0 0 0 5 0 0 0 1 19
0 0 0 0 0 0 0 0 0 0 0 0 0 0 0 0 0 0 0 0 0 0 0 0 0 0 0 0 0
9 0 25 5 0 0 0 0 1 0 0 0 0 0 0 0 0 467 0 0 0 0 0 0 0 0 0
M02255_77_000000000-ADUT3_1_2117_5846_7650 T----AC---GT-AG-GGC--------GCG-A-G------C--G--T---T--GT-C-CGG-AA------TT-A--T-T--GG-GC-------GT--A-----AA-GA-GC-TC-------G-TA-G-G-C-G------------G--C-TT-G-T-C-----------AC-
---G-T-C-G--------G-T-T--G--TG--A-AA-GC--C-C-GG-G-G-----CT-T-AA------C-C-C-C-G-G-G--T-C----T-G--C-A---G--T-------C--GA-T-A---C-------G--G-G--CA--G-G-C----T-A-G-A-G-T--T---C-GG-----TA-G-G--------G-G-A-G---AT-C------GG--A--ATT----C-C-T-G-
GT--GT-A-G-CG-GT--G---A--A-A---TG-C-GC-AG--AT-A-TC--A-G--G-A-G-G-A-AC-A-CC---GG--T--G--GC-GAA-G--G-C--G-------G--A-T-C-T---CTG--G--GC-C-G--------------------A-T---A-C-T--GA--CG-C-T-G--A-GG--A-G-CG-A--AA-G-C---G-TG--GG-G--AG-
C-G-AA--------CA--GG Bacteria(100);Actinobacteria(100);Actinobacteria(100);Actinobacteridae(100);Actinomycetales(100);Streptomycineae(99);Streptomycetaceae(99);Streptomyces(96);unclassified(96);

Otu0214 0 0 0 0 0 0 0 0 0 0 0 0 0 0 0 0 0 0 0 0 0 0 0 8 0 2 10 0
4 0 0 0 0 0 0 0 0 0 0 0 0 0 0 0 0 0 0 0 0 0 0 0 0 0 0 0 0
0 0 0 0 0 0 0 0 0 0 0 0 0 0 0 0 0 0 0 0 0 1 7 0 0 0 0 0 0
0 0 0 0 0 0 0 0 2 0 0 0 0 0 0 0 0 0 0 0 1 0 0 0 0 0 0
M02255_76_000000000-ADUNE_1_2114_11216_10046 C----TA---AC-TA-CGT--------GCC-A-G------C--A--G---C--CG-C-GGT-AA------TA-C--G-T--AG-GG-------CG--C-----AA-GC-G--TT-------A-TC-C-G---G------------A--T-TT-G-T-T--------
---GG----G-T-C-T--------G-C-C--G--TG--A-AA-GT--C-C-GG-G-G-----CT-C-AA------C-T-C-C-G-G-A--T-C----T-G--C-G---G--T-------G--GG-T-A---C-------G--G-G--CA--G-A-C----T-A-G-A-G-T--G---A-TG-----TA-G-G--------G-G-A-G---AC-T------GG--A--ATT----C-
C-T-G-GT--GT-A-G-CG-GT--G---A--A-A---TG-C-GC-AG--AT-A-TC--A-G--G-A-G-G-A-AC-A-CC---GA--T--G--GC-GAA-G--G-C--A-------G--G-T-C-T---CTG--G--GC-A-T--------------------T-A---A-C-T--GA--CG-C-T-G--A-GG--A-G-CG-A--AA-G-C---A-TG--GG-
G--AG-C-G-AA--------CA--GG Bacteria(100);Actinobacteria(100);Actinobacteria(100);Actinobacteridae(100);Actinomycetales(100);Micrococcineae(100);Micrococcaceae(100);unclassified(100);unclassified(100);

Otu0215 0 0 0 0 0 0 0 0 0 0 0 0 0 0 0 0 0 0 0 0 0 0 0 1 19 0 0 1
0 0 0 0 0 0 0 0 0 0 0 0 0 0 0 0 0 0 0 0 0 0 0 0 0 0 0 0
0 0 0 0 0 0 0 0 0 0 0 0 0 0 0 0 0 0 0 0 0 3 1 0 0 0 0 0 0
0 0 0 0 0 0 0 0 0 1 5 0 1 0 0 0 0 0 0 0 0 0 0 0 0 0 0
M02255_76_000000000-ADUNE_1_2110_24099_19451 T----AA---GC-AC-CGG--------CTA-A-C------T--A--C---G--TG-C-CAG-CA------GC-C--G-C--GG-TA-------AT--A-----CG-TA-GG-GT-------G-CA-A-G-C-G------------T--T-AA-TCG-G-----
------AA----G-A-C-A--------G-A-T--G--TG--A-AA-TC--C-C-CG-G-G-----CT-C-AA------C-C-T-G-G-G-A--A-T----T-G--C-A---T--C-------A--GT-G-A---C-------T--G-C--AT--A-G-C----T-T-G-A-G-T--G---C-GG-----CA-G-A--------G-G-G-G---GA-T------GG--A--ATT----
C-C-G-C-GT--GT-A-G-CA-GT--G---A--A-A---TG-C-GT-AG--AT-A-TG--C-G--G-A-G-G-A-AC-A-CC---GA--T--G--GC-GAAG---G-C--A-------A--T-C-C-C---CTG--G--GC-C-T--------------------G-C---A-C-T--GA--CG-C-T-C--A-TG--C-A-CG-A--AA-G-C---G-TG--GG-
G--AG-C-A-AA--------CA--GG Bacteria(100);Proteobacteria(100);Betaproteobacteria(100);Burkholderiales(100);Comamonadaceae(100);unclassified(100);unclassified(100);unclassified(100);unclassified(100);

Otu0216 0 0 0 0 0 132 17 307 39 76 0 0 0 1 0 0 0 0 0 0 1 0 18 4 0 0 0 0
0 0 655 370 658 1013 1002 82 91 0 87 0 0 37 0 0 0 0 0 0 0 0 0 0 0 0 0 0
0 0 0 0 0 0 0 0 0 0 0 0 0 0 0 0 0 90 0 0 0 0 0 0 0 0 0 0 60
86 115 81 0 0 0 0 0 0 145 7 0 8 0 0 13 117 28 268 0 0 0 0 0 0 0 0
M02255_76_000000000-ADUNE_1_1119_9629_8915 G----AC---GT-AG-GAT--------GCG-A-G------C--G--T---T--GT-C-CGG-AT------TT-A--T-T--GG-GC-------GT--A-----AA-GA-GT-TC---------------------------------------G-T-T----------AA----G-T-
T-T--------G-A-T--G--TT--A-AA-GA--T-C-GG-G-G-----CT-C-AA------C-C-T-C-G-G-G--A-C----A-G--C-A---T--T-------G--AA-T-A---C-------T--G-G--CA--G-A-C----T-T-G-A-G-T--A---T-GG-----TA-G-A--------G-G-C-T---AG-T------GG--A--ATT----C-C-C-A-GT--GT-
A-G-CG-GT--G---A--A-A---TG-C-GT-AG--AT-A-TT--G-G--G-A-A-G-A-AC-A-CC---GG--T--G--GC-GTA-G--G-C--G-------A--C-T-A-G---CTG--G--GC-C-A--------------------T-A---A-C-T--GA--CG-C-T-G--A-GG--A-A-CG-A--AA-G-C---C-AG--GG-G--AG-C-G-AA--
------AG--GG Bacteria(100);Cyanobacteria(100);MLE1-12(100);unclassified(100);unclassified(100);unclassified(100);unclassified(100);unclassified(100);unclassified(100);

Otu0217 0 0 11 0 0 0 0 0 0 0 0 0 0 0 0 0 0 0 0 0 0 0 0 3 0 0 0 0
0 0 0 25 0 0 1 0 0 0 0 0 19 0 0 0 0 0 0 0 0 0 0 9 0 0 0 0
0 0 0 0 0 0 0 0 0 0 0 0 0 0 0 0 0 0 39 0 0 1 0 0 0 0 0 0 0
0 0 0 0 28 0 0 0 0 0 0 0 0 1 0 0 0 0 0 0 0 0 0 0 0 0 10
M02255_76_000000000-ADUNE_1_2106_21048_13264 T----AA---GC-AC-CGG--------CTA-A-C------T--A--C---G--TG-C-CAG-CA------GC-C--G-C--GG-TA-------AT--A-----CG-TA-GG-GT-------G-CA-A-G-C-G------------T-TAATC-G-G-A----
-------AA----G-A-A-A--------G-A-T--G--TG--A-AA-TC--C-C-AG-G-G-----CT-C-AA------C-C-T-T-G-G-A--A-C----T-G--C-A---T--T-------T--TT-A-A---C-------T--A-C--CG--A-A-C----T-A-G-A-G-T--A---T-GT-----CA-G-A--------G-G-G-G---GG-T------GG--A--ATT----C-
C-A-C-GT--GT-A-G-CA-GT--G---A--A-A---TG-C-GT-AG--AT-A-TG--T-G--G-A-G-G-A-AC-A-CC---GA--T--G--GC-GAA-G--G-C--A-------G--C-C-C-C---CTG--G--GA-T-A--------------------A-T---A-C-T--GA--CG-C-T-C--A-TG--C-A-CG-A--AA-G-C---G-TG--GG-G-
-AG-C-A-AA--------CA--GG Bacteria(100);Proteobacteria(100);Betaproteobacteria(100);Burkholderiales(100);Alcaligenaceae(100);Alcaligenes(57);unclassified(57);unclassified(57);unclassified(57);

Otu0218 0 0 0 0 0 0 0 0 0 0 0 0 0 0 0 0 0 0 0 0 0 0 0 0 0 0 0 0
0 0 0 0 0 0 0 0 0 0 0 0 0 16 0 0 0 0 0 0 0 0 0 0 0 0 0 0
0 0 0 0 0 0 0 18 0 5 0 0 0 0 16 0 0 0 0 0 0 0 0 0 148 0 0 0 0
0 117 0 0 126 0 0 0 0 0 0 0 0 0 0 0 0 0 0 11 0 0 0 0 0 0 0
M02255_77_000000000-ADUT3_1_2111_10156_14895 T----AC---GT-AT-GTC--------GCG-A-G------C--G--T---T--AT-C-CGG-AA------TT-A--T-T--GG-GC-------AT--A-----AA-GG-GC-AT-------C-TA-G-G-C-G------------G--C-CG-G-G-T------
-----AA----G-T-C-C--------G-G-G--G--TG--A-AA-AC--T-G-CT-G-G-----CT-C-AA------C-C-A-G-C-A-G--C-C----T-G--C-C---T--T-------G--GA-A-A---C-------T--A-C--CC--G-G-C----T-T-G-A-G-T--G---C-CG-----GA-G-A--------G-G-T-G---GA-C------GG--A--ACT----G-
C-A-C-GA--GT-A-G-AG-GT--G---A--A-A---TT-C-GT-AG--AT-A-TG--T-G--C-A-G-G-A-AT-G-CC---GA--T--G--AT-GAA-G--A-T--A-------G--T-T-C-A---CTG--G--AC-G-G--------------------C-A---A-C-T--GA--CG-C-T-G--A-AG--T-G-CG-A--AA-G-C---T-AG--GG-G-
-AG-C-G-AA--------CA--GG Bacteria(100);Fusobacteria(100);Fusobacteria(100);Fusobacteriales(100);Leptotrichiaceae(100);Leptotrichia(100);unclassified(100);unclassified(100);unclassified(100);

Otu0219 0 0 0 0 0 0 0 0 0 0 0 0 0 0 0 0 0 0 1 0 0 0 0 0 0 0 0 0
0 0 0 0 0 0 18 0 0 0 0 0 26 0 0 0 0 0 0 0 0 27 0 0 0 0 0 8 0
0 0 0 0 0 0 0 0 0 0 0 0 0 0 0 0 3 14 24 0 0 0 0 0 68 0 0 0 0
3 5 12 3 0 8 0 0 0 11 0 0 0 17 0 30 7 0 0 0 0 0 4 0 0 0 0
M02255_76_000000000-ADUNE_1_1108_26828_7690 .....AC----T-AC-GT---------GCC-A-G------C--A--G---C--CG-C-GGT-AA------TA-C--G-T--AG-GT-------GG--C-----AA-GC-G--TT-------G----T-C-C-G------------G--A-AG-A-A-T----------AA----G-
T-C-T--------G-A-T--G--TG--A-AA-GC--C-C-TC-G-G-----CT-T-AA------C-C-G-A-G-G-A--A-C----T-G--C-A---T--C-------G--GA-A-A---C-------T--G-T--TT--T-T-C----T-T-G-A-G-T--G---C-AG-----AA-G-A--------G-G-A-G---AG-T------GG--A--ACT----C-C-A-T-GT--
GT-A-G-CG-GT--G---G--A-A---TG-C-GT-AG--AT-A-TA--T-G--G-A-A-G-A-AC-A-CC---AG--T--G--GC-GAA-G--G-C--G-------G--C-T-C-T---CTG--G--TC-T-G--------------------C-A---A-C-T--GA--CG-C-T-G--A-GG--C-T-CG-A--AA-G-C---A-TG--GG-T--AG-C-G-
AA--------CA--GG Bacteria(100);Firmicutes(100);Bacilli(100);Lactobacillales(100);Lactobacillaceae(92);unclassified(72);unclassified(72);unclassified(72);unclassified(72);

Otu0220 0 0 0 0 0 0 0 0 0 0 0 0 0 0 0 0 0 0 0 0 0 3 0 0 0 0 9 0
0 0 0 0 0 6 0 0 0 0 0 28 0 0 0 0 0 0 0 0 0 0 0 130 0 1 0 0 0
0 0 0 0 0 0 0 0 0 0 0 0 0 0 0 0 0 0 0 0 0 3 0 0 0 0 0 0 0
0 0 0 0 0 0 0 0 0 0 0 0 0 0 0 0 0 0 0 0 0 0 0 0 0 0 0
M02255_76_000000000-ADUNE_1_1112_27852_12092 T----AC---GT-AG-GGG--------GCG-A-G------C--G--T---T--GT-C-CGG-AT------TT-A--T-T--GG-GC-------GT--A-----AA-GA-GC-TC-------G-TA-------G------------G--C-TT-G-G-C--------
---AA----G-T-C-G--------G-G-T--G--TG--A-AA-CC--T-C-CA-G-G-----CT-C-AA------C-C-T-G-G-A-G--A------C-G-CC-A---C--T-------C--GA-T-A---C-------T--G-C--TA-TG-G-C----T-A-G-A-G-T--C---C-GG-----TA-G-G--------G-G-A-C---CA-C------GG--A--ATT----C-
C-T-G-GT--GT-A-G-CG-GT--G---A--A-A---TG-C-GC-AG--AT-A-TC--A-G--G-A-G-G-A-AC-A-CC---GG--T--G--GC-GAA-G--G-C--G-------G--T-G-G-T---CTG--G--GC-C-G--------------------G-C---A-C-T--GA--CG-C-T-G--A-GG--A-G-CG-A--AA-G-C---G-TG--GG-
T--AG-C-A-AA--------CA--GG Bacteria(100);Actinobacteria(100);Actinobacteria(100);unclassified(54);unclassified(54);unclassified(54);unclassified(54);unclassified(54);unclassified(54);

Otu0221 0 0 0 0 0 0 0 0 0 0 0 0 0 0 0 0 0 0 0 0 0 0 0 0 12 1 0 0
1 0 0 0 0 0 0 37 33 0 9 2 0 0 0 0 12 73 0 0 0 0 0 0 0 0 0 0 0
37 0 0 0 0 0 0 0 1 0 0 0 0 0 51 11 19 0 141 18 0 0 0 0 54 0 640 0 176
0 44 122 369 337 0 17 0 0 0 0 26 0 118 0 0 5 0 0 0 0 0 0 0 0 0 0
M02255_77_000000000-ADUT3_1_1110_5520_10047 T----AC---GT-AG-GGT--------GCA-A-G------C--G--T---T--AT-C-CGG-AA------TT-A--T-T--GG-GC-------GT--A-----AA-GG-GC-TC-------G-TA-G-G-C-G------------G--T-TC-G-T-C-----------GC-
---G-T-C-C--------G-G-T--G--TG--A-AA-GT--C-C-AT-C-G-----CT-T-AA------C-G-G-T-G-G-A--T-C----C-G--C-G---C--C-------G--GG-T-A---C-------G--G-G--CG--G-G-C----T-T-G-A-G-T--G---C-GG-----TA-G-G--------G-G-A-G---AC-T------GG--A--ATT----C-C-C-G-
GT--GT-A-A-CG-GT--G---G--A-A---TG-T-GT-AG--AT-A-TC--G-G--G-A-A-G-A-AC-A-CC---AA--T--G--GC-GAA-G--G-C--A-------G--G-T-C-T---CTG--G--GC-C-G--------------------T-T---A-C-T--GA--CG-C-T-G--A-GG--A-G-CG-A--AA-G-C---G-TG--GG-G--AG-
C-G-AA--------CA--GG Bacteria(100);Actinobacteria(100);Actinobacteria(100);Actinobacteridae(100);Bifidobacteriales(100);Bifidobacteriaceae(100);Bifidobacterium(100);unclassified(100);unclassified(100);

Otu0222 0 0 0 0 0 0 0 0 0 0 0 0 0 0 0 0 0 0 0 0 0 0 0 0 0 0 0 0
0 0 0 0 0 0 0 0 0 0 0 0 0 0 0 0 0 0 0 0 0 0 0 0 0 0 0 0 0
0 0 0 0 0 0 0 0 0 0 0 0 0 0 0 0 0 0 0 0 0 0 0 0 0 0 0 0 0
0 0 0 84 0 0 14 0 0 0 0 0 0 0 0 0 0 0 0 0 0 0 0 0 0 0 0
M02255_77_000000000-ADUT3_1_1103_12435_10329 T----AC---GA-AG-GGG--------GCT-A-G------C--G--T---T--GC-T-CGG-AA------TG-A--C-T--GG-GC-------GT--A-----AA-GG-GC-GC-------G-TA-G-G-C-G------------G--C-GG-C-C-A---
--------AA----G-T-C-A--------G-A-C--G--TG--A-AA-TT--C-C-TG-G-G-----CT-C-AA------C-C-T-G-G-G-G--G-C----T-G--C-G---T--T-------T--GA-T-A---C-------T--T-G--GT--T-G-C----T-T-G-A-G-T--G---G-GG-----AA-G-A--------G-G-G-T---CG-T------GG--A--ATT----
C-C-C-A-GT--GT-A-G-AG-GT--G---A--A-A---TT-C-GT-AG--AT-A-TT--G-G--G-A-A-G-A-AC-A-CC---GG--T--G--GC-GAA-G--G-C--G-------G--C-G-A-C---CTG--G--TC-C-T--------------------T-G---A-C-T--GA--CG-C-T-G--A-GG--C-G-CG-A--AA-G-C---G-TG--
GG-G--AG-C-A-AA--------CA--GG Bacteria(100);Proteobacteria(100);Alphaproteobacteria(100);Rhodospirillales(100);Acetobacteraceae(100);unclassified(100);unclassified(100);unclassified(100);unclassified(100);

Otu0223 0 0 0 0 15 32 2 0 0 22 48 0 0 11 11 0 0 0 0 0 0 0 84 0 200 133 2 15
0 2 0 0 0 0 0 0 17 11 0 0 0 63 0 1 14 59 3 7 14 7 0 12 0 0 1 0 5
0 0 0 0 0 0 0 0 0 0 0 0 0 0 0 0 0 0 0 0 0 14 22 19 0 0 0 0 0
38 0 0 0 0 0 0 0 0 0 0 0 0 0 0 12 0 1 0 17 53 0 2 0 15 0 0
M02255_76_000000000-ADUNE_1_1103_22532_20444 C----TA---AC-TC-CGT--------GCC-A-G------C-----A---G--CC-G-CGG-TA------AT-A--C-G--GA-GG-------GT--------GC-GA-GC-GT---------TA-A-T-C-G------------G--A-AT-G-T-T--------
---AA----G-C-A-A--------G-A-T--G--TG--A-AA-GC--C-C-GG-G-G-----CT-C-AA------C-C-T-C-G-G-A--A-C----A-G--C-A---T--T-------T--TG-A-A---C-------T--G-G--CA--G-A-C----T-A-G-A-G-T--C---T-TG-----TA-G-A--------G-G-G-G---GG-T------AG--A--ATT----T-C-
A-G-GT--GT-A-G-CG-GT--G---A--A-A---TG-C-GT-AG--AG-A-TC--T-G--A-A-G-G-A-AT-A-CC---GG--T--G--GC-GAA-G--G-C--G-------G--C-C-C-C---CTG--G--AC-A-A--------------------A-G---A-C-T--GA--CG-C-T-C--A-GA--T-G-CG-A--AA-G-C---G-TG--GG-G--
AG-C-A-AA--------CA--GG Bacteria(100);Proteobacteria(100);Gammaproteobacteria(100);Vibrionales(100);Vibrionaceae(100);Photobacterium(100);unclassified(100);unclassified(100);unclassified(100);

Otu0224 0 0 0 0 0 0 0 0 0 0 0 0 0 0 0 17 0 5 0 0 0 0 0 0 0 0 0 0
0 0 0 0 0 0 0 0 0 0 0 0 3 0 0 0 0 0 9 0 0 0 0 0 0 0 0 0 0
0 0 1 0 0 0 0 0 0 0 0 0 0 0 0 0 1 0 0 0 0 0 0 0 0 0 0 0 0
0 0 0 0 1 0 0 0 0 0 0 0 0 0 0 0 0 0 0 0 0 0 0 0 0 0 0
M02255_77_000000000-ADUT3_1_1105_13312_5384 .....AC----T-AC-GT---------GCC-A-G------C--A--G---C--CG-C-GGT-AA------TA-C--G-T--AG-GG-------GG--C-----AA-GC-GT-TG-------T-CC-G-G---A------------A--T--T-C-T-T-----------AA----G-
T-C-T--------G-A-T--G--TG--A-AA-GC--C-C-CC-G-G-----CT-C-AA------C-C-G-G-G-G-A--G------G-GT-C-A---T--T-------G--GA-A-A---C-------T--G-G--GA--G-A-C----T-T-G-A-G-G--A---C-AG-----AA-G-A--------G-G-A-G---AG-T------GG--A--ATT----C-C-A-A-GT--
GT-A-G-CG-GT--G---A--A-A---TG-C-GT-AG--AT-A-TT--T-G--G-A-G-G-A-AC-A-CC---AG--T--G--GC-GAA-G--G-C--G-------G--C-T-C-T---CTG--G--TC-T-G--------------------T-T---A-C-T--GA--CG-C-T-G--A-GG--C-G-CG-A--AA-G-C---G-TG--GG-G--AG-C-A-
AA--------CA--GG Bacteria(100);Firmicutes(100);Bacilli(100);Bacillales(100);unclassified(100);unclassified(100);unclassified(100);unclassified(100);unclassified(100);

Otu0225 0 0 0 0 0 0 0 0 0 0 0 0 0 0 0 0 0 0 0 0 0 0 5 0 0 0 0 6
0 0 0 0 0 0 0 0 0 0 25 0 0 0 0 0 0 0 0 0 0 0 0 0 14 0 0 0 0
0 27 4 0 0 0 0 0 4 0 0 0 6 0 0 10 16 3 0 0 0 0 0 0 28 0 0 0 21
0 0 0 0 0 0 0 0 0 0 0 0 0 0 0 0 0 0 0 0 0 0 0 0 0 0 0
M02255_77_000000000-ADUT3_1_1112_11485_8143 T----AC---GA-AG-GGG--------GCT-A-G------C--G--T---T--GT-T-CGG-AT------TT-A--C-T--GG-GC-------GT--A-----AA-GC-GC-AC-------G-TA-G-G-C-G------------G--T-TT-G-T-T----------AA-
---G-T-G-A--------G-A-G--G--TG--A-AA-TC--C-C-AG-A-G-----CT-C-AA------C-T-C-T-G-G-A--A-C----T-G--C-C---T--T-------T--CA-T-A---C-------T--G-G--CA--A-G-C----T-A-G-A-G-T--C---C-GG-----AA-G-A--------G-G-T-A---AG-T------GG--A--ACT----C-C-T-A-
GT--GT-A-G-AG-GT--G---G--A-A---TT-C-GT-AG--AT-A-TT--A-G--G-A-A-G-A-AC-A-CC---AG--T--G--GC-GAA-G--G-C--G-------G--C-T-T-A---CTG--G--TC-C-G--------------------G-A---A-C-T--GA--CG-C-T-G--A-GG--T-G-CG-A--AA-G-C---G-TG--GG-G--AG-
C-A-AA--------CA--GG Bacteria(100);Proteobacteria(100);Alphaproteobacteria(100);Rhizobiales(100);unclassified(85);unclassified(85);unclassified(85);unclassified(85);unclassified(85);

Otu0226 0 209 0 0 0 0 0 0 65 0 0 0 0 0 0 0 0 0 0 0 0 4 0 0 0 0 0 0
0 0 0 0 0 0 0 0 0 0 0 106 0 0 0 0 0 0 0 0 0 0 0 0 0 1 0 0
0 0 0 0 0 0 0 0 0 0 0 0 0 0 20 0 0 0 0 0 0 0 0 0 0 0 0 0 0
18 0 0 3 0 0 2 0 0 0 0 0 0 0 0 0 0 0 0 0 0 0 0 0 1 0 0
M02255_77_000000000-ADUT3_1_2117_25636_14286 T----AC---GT-AG-GGG--------GCA-A-G------C--G--T---T--AT-C-CGG-AT------TT-A--C-T--GG-GT-------GT--A-----AA-GG-GA-GC-------G-TA-G-A-C-G------------G--T-TA-A-G-C-----
------AA----G-T-C-A--------G-A-A--G--TG--A-AA-GG--C-T-GG-G-G-----CT-C-AA------C-C-C-C-G-G-G--A-C----T-G--C-T---T--T-------T--GA-A-A---C-------T--G-T--TT--A-A-C----T-A-G-A-G-T--G---C-TG-----GA-G-A--------G-G-T-A---AG-C------GG--A--ATT----C-
C-T-A-GT--GT-A-G-CG-GT--G---A--A-A---TG-C-GT-AG--AT-A-TT--A-G--G-A-G-G-A-AC-A-CC---AG--T--G--GC-GAA-G--G-C--G-------G--C-T-T-A---CTG--G--AC-A-G--------------------T-A---A-C-T--GA--CG-T-T-G--A-GG--C-T-CG-A--AA-G-C---G-TG--GG-G-
-AG-C-A-AA--------CA--GG Bacteria(100);Firmicutes(100);Clostridia(100);Clostridiales(100);Lachnospiraceae(100);unclassified(100);unclassified(100);unclassified(100);unclassified(100);

Otu0227 0 0 0 0 0 0 0 0 0 0 0 0 0 0 0 0 0 0 0 0 0 0 0 0 0 0 0 0
0 0 0 0 0 0 0 0 0 0 0 0 0 0 0 0 0 0 0 0 0 0 53 0 8 2 0 0 0
0 0 0 0 0 0 0 0 0 0 0 0 0 0 0 0 0 0 0 0 0 0 0 0 0 0 0 0 0
0 0 0 0 0 0 0 0 17 0 0 0 0 0 0 0 0 0 0 0 0 0 0 0 0 0 0
M02255_76_000000000-ADUNE_1_2119_17678_6995 C----GG---CT-AA-CTC--------CGT-T-C------C--A--G---C--AG-C-CGC-GG------TA-A--T-A--CG-GA-------GG--A-----TC-CA-AG-CG-------T-TA-T-C-C-G------------GA-T-TT-A-T-T----------AA--
--G-T-C-A--------G-G-G--G--TG--A-AA-GA--C-G-GT-G-G-----CT-C-AA------C-C-A-T-C-G-C--A-G----T-G--C-C---C--T-------T--GA-T-A---C-------T--G-A--TG--A-G-C----T-T-G-A-A-T--A---C-AC-----TA-G-A--------G-G-T-A---GG-C------GG--A--ATG----T-G-A-C-
AA--GT-A-G-CG-GT--G---A--A-A---TG-C-AT-AG--AT-A-TG--T-C--A-C-A-G-A-AC-A-CC---GA--T--T--GC-GAA-G--G-C--A-------G--C-T-T-A---CTA--T--GG-T-G--------------------T-T---A-T-T--GA--CG-C-T-G--A-GG--C-A-CG-A--AA-G-C---G-TG--GG-G--AT-C-
A-AA--------CA--GG Bacteria(100);Bacteroidetes(100);Sphingobacteria(100);Sphingobacteriales(100);Sphingobacteriaceae(100);Sphingobacteriaceae(100);Pedobacter(100);unclassified(100);unclassified(100);

Otu0228 0 14 0 0 5 0 0 0 0 0 0 0 0 0 0 0 0 0 0 0 0 0 0 0 0 0 0 0
0 0 5 0 0 0 0 0 0 0 0 0 0 0 0 0 0 0 0 0 0 0 0 0 0 0 0 0 0
0 0 0 0 0 0 0 0 0 0 0 0 0 0 0 0 0 0 0 10 0 0 0 0 0 0 0 17 0
0 0 0 0 0 8 0 3 0 0 0 0 2 0 0 0 0 0 0 0 0 0 0 0 0 0 0
M02255_77_000000000-ADUT3_1_2111_22027_15100 T----AC---GT-AG-GGT--------GCG-A-G------C--G--T---T--GT-C-CGG-AA------TT-A--T-T--GG-GC-------GT--A-----AA-GA-GC-TT-------G-TA-G-G-C-G------------G--T-TT-G-T-C------
-----GC----G-T-C-T--------G-C-T--G--TG--A-AA-AT--T-C-AG-G-G-----CT-T-AA------C-C-C-T-G-G-A--C-G----T-G--C-A---G--T-------G--GG-T-A---C-------G--G-G--CA--G-A-C----T-A-G-A-G-T--A---T-GG-----TA-G-G--------G-G-A-G---AC-T------GG--A--ATT----C-
C-T-G-GT--GT-A-G-CG-GT--G---G--A-A---TG-C-GC-AG--AT-A-TC--A-G--G-A-G-G-A-AC-A-CC---GA--T--G--GC-GAA-G--G-C--A-------G--G-T-C-T---CTG--G--GC-C-A--------------------T-T---A-C-T--GA--CG-C-T-G--A-GA--A-G-CG-A--AA-G-C---A-TG--GG-
G--AG-C-G-AA--------CA--GG Bacteria(100);Actinobacteria(100);Actinobacteria(100);Actinobacteridae(100);Actinomycetales(100);Micrococcineae(100);unclassified(100);unclassified(100);unclassified(100);

Otu0229 51 247 330 0 0 0 351 109 210 0 0 0 0 0 0 0 2 0 0 0 0 0 2 0 0 0 7 0
4 0 0 0 0 8 0 0 7 0 37 184 0 0 47 0 3 0 0 0 0 0 0 0 1 4 0 0 1
0 0 0 3 0 0 0 0 0 0 4 0 0 1 9 3 0 60 0 0 0 0 0 0 0 0 0 0 28
0 0 0 13 0 0 0 0 0 35 0 82 0 0 0 27 0 0 0 0 0 0 0 4 252 0 0
M02255_77_000000000-ADUT3_1_1111_15986_15272 T----AC---GT-AG-GGG--------GCA-A-G------C--G--T---T--AT-C-CGG-AT------TT-A--C-T--GG-GT-------GT--A-----AA-GG-GA-GC-------G-TA-G-A-C-G------------G--T-GT-G-G-C----
-------AA----G-T-C-T--------G-A-T--G--TG--A-AA-GG--C-A-TG-G-G-----CT-C-AA------C-C-T-G-T-G-G--A-C----T-G--C-A---T--T-------G--GA-A-A---C-------T--G-T--CA--T-A-C----T-T-G-A-G-T--G---C-CG-----GA-G-G--------G-G-T-A---AG-C------GG--A--ATT----
C-C-T-A-GT--GT-A-G-CG-GT--G---A--A-A---TG-C-GT-AG--AT-A-TT--A-G--G-A-G-G-A-AC-A-CC---AG--T--G--GC-GAA-G--G-C--G-------G--C-T-T-A---CTG--G--AC-G-G--------------------T-A---A-C-T--GA--CG-T-T-G--A-GG--C-T-CG-A--AA-G-C---G-TG--GG-
G--AG-C-A-AA--------CA--GG Bacteria(100);Firmicutes(100);Clostridia(100);Clostridiales(100);Lachnospiraceae(100);Blautia(100);unclassified(100);unclassified(100);unclassified(100);

Otu0230 7 0 0 0 0 0 0 5 0 0 0 0 0 0 0 0 0 0 0 0 1 0 0 0 0 0 0 0
0 6 0 1 3 0 0 0 0 0 11 0 0 1 2 12 8 0 0 0 0 6 2 0 0 0 0 1 0
0 0 0 1 0 0 0 0 0 0 0 0 0 0 0 0 0 0 0 1 0 0 0 0 0 0 0 0 0
0 0 0 0 0 0 0 0 0 0 0 5 0 2 0 0 0 0 0 2 0 4 0 0 0 0 0
M02255_77_000000000-ADUT3_1_2113_22738_22937 .....AC----T-AC-GT---------GCC-A-G------C--A--G---C--CG-C-GGT-AA------TA-C--G-T--AG-GT-------GG--C-----AA----GC-G--------T-TA-T-C-C-G-------------G--T-TT-A-T-T-----------
AA----G-T-C-T--------G-A-T--G--TG--A-AA-GC--C-C-AC-G-G-----CT-C-AA------C-C-G-T-G-G-A--G------G-GT-C-A---T--T-------G--GA-A-A---C-------T--G-G--AA--A-A-C----T-T-G-A-G-T--G---C-AG-----AA-G-A--------G-G-A-A---AG-T------GG--A--ATT----C-C-
A-T-GT--GT-A-G-CG-GT--G---A--A-A---TG-C-GC-AG--AG-A-TA--T-G--G-A-G-G-A-AC-A-CC---AG--T--G--GC-GAA-G--G-C--G-------A--C-T-T-T---CTG--G--TC-T-G--------------------T-A---A-C-T--GA--CG-C-T-G--A-TG--T-G-CG-A--AA-G-C---G-TG--GG-G--
AT-C-A-AA--------CA--GG Bacteria(100);Firmicutes(100);Bacilli(100);Bacillales(100);Staphylococcaceae(100);Staphylococcus(91);unclassified(91);unclassified(91);unclassified(91);

Otu0231 0 0 0 0 0 0 0 0 0 0 0 0 0 0 0 0 0 0 0 0 0 0 0 0 0 0 0 0
0 0 0 0 0 0 0 0 0 0 0 0 0 36 0 0 0 0 0 0 0 0 0 0 0 0 0 0 0
0 0 0 0 0 0 0 0 0 0 0 0 0 0 0 0 0 0 0 0 0 0 0 56 0 49 46 0
11 0 0 0 0 0 0 0 0 0 0 20 2 0 0 0 0 0 0 0 0 0 0 0 0 0 0
M02255_77_000000000-ADUT3_1_1117_12140_13977 T----AC---GT-AG-GGG--------GCA-A-G------C--G--T---T--AT-C-CGG-AT------TT-A--C-T--GG-GT-------GT--A-----AA-GG-GA-GC-------G-TA-G-A-C-G-------------G--A-GC-A-G-C----
-------AA----G-T-C-T--------G-A-T--G--TG--A-AA-GG--C-A-GG-G-G-----CT-C-AA------C-C-C-C-T-G-G--A-C----T-G--C-A---T--T-------G--GA-A-A---C-------T--G-T--TG--A-T-C----T-T-G-A-G-T--A---C-CG-----GA-G-G--------G-G-T-A---AG-C------GG--A--ATT----
C-C-T-A-GT--GT-A-G-CG-GT--G---A--A-A---TG-C-GT-AG--AT-A-TT--A-G--G-A-G-G-A-AC-A-CC---AG--T--G--GC-GAA-G--G-C--G-------G--C-T-T-A---CTG--G--AC-G-G--------------------T-A---A-C-T--GA--CG-T-T-G--A-GG--C-T-CG-A--AA-G-C---G-TG--GG-
G--AG-C-A-AA--------CA--GG Bacteria(100);Firmicutes(100);Clostridia(100);Clostridiales(100);Lachnospiraceae(100);unclassified(63);unclassified(63);unclassified(63);unclassified(63);

Otu0232 0 0 0 0 0 0 0 0 0 0 0 18 0 0 0 0 0 0 6 0 0 0 0 0 6 0 0 0
0 0 0 0 0 0 0 0 0 0 0 0 0 0 0 0 0 0 0 0 2 0 0 0 0 0 0 0 0
0 0 0 0 0 0 0 0 0 0 0 0 0 0 0 0 0 0 0 0 1 19 0 0 0 0 0 0 0
0 0 0 0 0 0 0 0 29 0 3 0 1 0 0 0 0 0 0 0 0 0 0 1 0 0 0
M02255_76_000000000-ADUNE_1_2115_18309_12961 C----GG---CT-AA-CTC--------CGT-G-C------C--A--G---C--AG-C-CGC-GG------TA-A--T-A--CG-GA-------GG--G-----TG-CA-AG-CG-------T-TA-T-C-C-G-------------G--A-CC-G-A-T-----
------AA----G-T-C-A--------G-T-G--G--TG--A-AA-TC--T-C-AT-A-G-----CT-T-AA------C-T-A-T-G-A-A--A-C----T-G--C-C---A--T-------T--GA-T-A---C-------T--G-T--CG--G-T-C----T-T-G-A-G-T--A---A-AT-----TA-G-A--------G-G-T-A---GC-T------GG--A--ATA----A-
G-T-A-GT--GT-A-G-CG-GT--G---A--A-A---TG-C-AT-AG--AT-A-TT--A-C--T-T-A-G-A-AC-A-CC---AA--T--T--GC-GAA-G--G-C--A-------G--G-T-T-A---CCA--T--GA-T-T--------------------T-A---A-C-T--GA--CG-C-T-G--A-GG--G-A-CG-A--AA-G-C---G-TG--GG-T--
AG-C-G-AA--------CA--GG Bacteria(100);Bacteroidetes(100);Flavobacteria(100);Flavobacteriales(100);Flavobacteriaceae(100);unclassified(92);unclassified(92);unclassified(92);unclassified(92);

Otu0233 0 0 0 11 2 2 0 0 0 0 0 0 0 0 0 0 0 0 0 0 1 0 0 0 0 0 0 0
0 4 5 0 5 0 0 0 1 4 0 0 0 3 6 1 0 0 0 0 4 0 0 0 1 0 0 0 0
0 0 0 0 0 0 0 0 0 0 0 0 0 0 0 0 0 2 0 0 0 1 0 0 0 0 0 3 7
0 0 4 0 9 0 0 0 0 0 0 0 0 0 0 0 0 7 2 11 11 3 0 0 0 0 8
M02255_77_000000000-ADUT3_1_2117_25801_13176 A----AG---CC-AC-G-G--------CTA-A-C------T--A--C---G--TG-C-CAG-CA------GC-C--G-C--GG-TA-------AT--A-----CG-TA-GG-TG-------G-CA-A-G-C-G-------------T--TGTC-C-G-G-----
------AT----G-T-A-T--------T-A-T--G--TG--A-AA-GC--C-C-CC-G-G-----CT-C-AA------C-C-G-G-G-G-A--G-G----G-T--C-A---T--T-------G--GA-A-A---C-------T--G-G--GG--A-A-C----T-T-G-A-G-T--G---C-AG-----AA-G-A--------G-G-A-G---AG-T------GG--A--ATT----
C-C-A-T-GT--GT-A-G-CG-GT--G---A--A-A---TG-C-GT-AG--AT-A-TA--T-G--G-A-G-G-A-AC-A-CC---AG--T--G--GC-GAA-G--G-C--G-------A--C-T-C-T---CTG--G--TC-T-G--------------------T-A---A-C-T--GA--CG-C-T-G--A-GG--C-T-CG-A--AA-G-C---G-TG--GG-
T--AG-C-A-AA--------CA--GG Bacteria(100);Firmicutes(100);Bacilli(100);Lactobacillales(90);unclassified(90);unclassified(90);unclassified(90);unclassified(90);unclassified(90);

Otu0234 0 0 0 0 0 0 0 0 0 0 0 0 0 0 0 0 0 0 0 0 0 0 0 0 0 0 0 0
0 0 0 0 0 0 0 19 0 0 0 0 0 41 0 0 0 0 0 0 0 0 0 0 0 7 0 0 0
0 0 0 0 0 0 0 0 0 0 0 0 0 0 111 13 0 0 0 0 0 0 0 0 0 0 0 0 0
0 0 0 0 0 0 0 0 0 0 0 0 0 0 0 0 0 0 0 41 0 0 0 0 0 0 0
M02255_77_000000000-ADUT3_1_1102_11073_5526 T----AC---GT-AG-GTG--------GCA-A-G------C--G--T---T--GT-C-CGG-AT------TT-A--C-T--GG-GC-------GT--A-----AA-GG-GA-GC-------G-TA-G-G-T-G-------------G--A-TA-T-T-T-----------AA-
---G-T-G-G--------G-A-T--G--TG--A-AA-TA--C-T-CG-G-G-----CT-T-AA------C-C-T-G-G-G-T--G-C----T-G--C-A---T--T-------C--CA-A-A---C-------T--G-G--AT--A-T-C----T-A-G-A-G-T--G---C-AG-----GA-G-A--------G-G-A-A---AG-T------AG--A--ATT----C-C-T-A-
GT--GT-A-G-CG-GT--G---A--A-A---TG-C-GT-AG--AG-A-TT--A-G--G-A-A-G-A-AT-A-CC---AG--T--G--GC-GAA-G--G-C--G-------A--C-T-T-T---CTG--G--AC-T-G--------------------T-A---A-C-T--GA--CA-C-T-G--A-GG--C-T-CG-A--AA-G-C---G-TG--GG-G--AG-C-
A-AA--------CA--GG Bacteria(100);Firmicutes(100);Clostridia(100);Clostridiales(100);Clostridiaceae(100);Clostridium(100);unclassified(100);unclassified(100);unclassified(100);

Otu0235 0 0 0 0 2 0 0 18 7 26 1 0 0 1 0 0 0 0 0 0 0 0 0 0 3 0 0 1
0 0 0 0 0 0 0 0 0 1 0 0 0 0 0 0 0 0 0 1 3 0 0 11 0 0 0 0 0
1 0 0 0 0 0 0 0 0 0 0 1 0 0 0 0 1 0 11 1 0 0 0 0 0 0 17 22 15
0 0 0 15 0 0 0 0 0 0 0 0 0 18 0 0 0 0 0 1 0 0 0 0 3 0 0
M02255_77_000000000-ADUT3_1_1110_27753_10806 T----AC---GT-AG-GGT--------GCG-A-G------C--G--T---T--GT-C-CGG-AA------TT-A--C-T--GG-GC-------GT--A-----AA-GA-GC-TC-------G-TA-G-G-T-G------------G--T-TT-G-T-C------
-----GC----G-T-C-G--------T-T-T--G--TG--T-AA-TA--C-C-GC-A-G-----CT-T-AA------C-T-G-C-G-G-G--G-T----T-G--C-A---G--G-------C--GA-T-A---C-------G--G-G--CAT-A-A-C----T-T-G-A-G-T--G---C-TG-----TA-G-G--------G-G-A-G---AC-T------GG--A--ATT----C-
C-T-G-GT--GT-A-G-CG-GT--G---G--A-A---TG-C-GC-AG--AT-A-TC--A-G--G-A-G-G-A-AC-A-CC---GA--T--G--GC-GAA-G--G-C--A-------G--G-T-C-T---CTG--G--GC-A-G--------------------T-A---A-C-T--GA--CG-C-T-G--A-GG--A-G-CG-A--AA-G-C---A-TG--GG-
T--AG-C-G-AA--------CA--GG Bacteria(100);Actinobacteria(100);Actinobacteria(100);Actinobacteridae(100);Actinomycetales(100);Corynebacterineae(100);Corynebacteriaceae(100);Corynebacterium(100);unclassified(100);

Otu0236 0 123 174 0 0 0 254 104 446 0 0 0 0 1 0 0 0 0 0 0 0 0 1 0 0 0 0 0
0 1 4 4 0 0 0 0 0 5 42 24 0 0 0 0 0 112 0 0 66 0 0 0 0 0 0 0 0
0 0 0 0 0 0 0 0 0 0 0 0 0 0 0 0 0 0 0 0 0 0 0 0 0 773 0 0 0
24 0 0 0 0 62 4 0 0 0 0 120 3 0 1 0 0 0 0 0 0 0 0 0 74 0 0
M02255_77_000000000-ADUT3_1_1108_19776_12429 T----AC---GT-AT-GGA--------GCA-A-G------C--G--T---T--AT-C-CGG-AT------TT-A--C-T--GG-GT-------GT--A-----AA-GG-GT-GC-------G-TA-G-G-T-G------------G--C-AG-T-G-C-----
------AA----G-T-C-A--------G-A-T--G--TG--A-AA-GG--C-C-GG-G-G-----CT-C-AA------C-C-C-C-G-G-A--G-C----T-G--C-A---T--T-------T--GA-A-A---C-------T--G-C--TC--G-G-C----T-A-G-A-G-T--A---C-AG-----GA-G-A--------G-G-C-A---GG-C------GG--A--ATT----
C-C-T-A-GT--GT-A-G-CG-GT--G---A--A-A---TG-C-GT-AG--AT-A-TT--A-G--G-A-G-G-A-AC-A-CC---AG--T--G--GC-GAA-G--G-C--G-------G--C-C-T-G---CTG--G--AC-T-G--------------------T-T---A-C-T--GA--CA-C-T-G--A-GG--C-A-CG-A--AA-G-C---G-TG--GG-
G--AG-C-A-AA--------CA--GG Bacteria(100);Firmicutes(100);Clostridia(100);Clostridiales(100);Lachnospiraceae(100);Incertae_Sedis(89);unclassified(89);unclassified(89);unclassified(89);

Otu0237 0 0 0 0 0 0 0 0 0 0 0 0 0 0 0 0 0 0 0 0 0 0 0 0 0 0 0 0
0 0 0 0 0 37 0 90 0 0 95 0 0 0 0 0 0 0 0 0 0 0 0 0 0 0 0 0 0
0 0 0 0 0 0 0 0 0 0 0 0 0 0 0 0 0 0 0 0 0 0 0 0 0 0 0 0 0
0 0 0 0 0 0 0 0 0 0 0 0 0 0 0 0 0 0 0 0 0 0 0 0 0 0 0
M02255_77_000000000-ADUT3_1_1104_14897_20585 T----AC---GA-AG-GGG--------GCA-A-G------C--G--T---T--GT-T-CGG-AA------TT-A--C-T--GG-GC-------GT--A-----AA-GG-GC-GC-------G-TA-G-G-C-G------------G--C-TT-A-T-C-----
------AA----G-T-C-A--------G-G-C--G--TG--A-AA-TT--C-C-CG-G-G-----CT-C-AA------C-C-T-G-G-G-G--G-C----T-G--C-G---C--T-------T--GA-T-A---C-------T--G-A--TG--A-G-C----T-T-G-A-A-T--G---C-GG-----GA-G-A--------G-G-A-T---AG-T------GG--A--ATT----
C-C-C-A-GT--GT-A-G-AG-GT--G---A--A-A---TT-C-GT-AG--AT-A-TT--G-G--G-A-A-G-A-AC-A-CC---AG--T--G--GC-GAA-G--G-C--G-------G--C-T-A-T---CTG--G--CC-C-G--------------------T-A---A-T-T--GA--CG-C-T-G--A-GG--C-G-CG-A--AA-G-C---G-TG--GG-
G--AG-C-A-AA--------CA--GG Bacteria(100);Proteobacteria(100);Alphaproteobacteria(100);Rhodospirillales(100);unclassified(100);unclassified(100);unclassified(100);unclassified(100);unclassified(100);

Otu0238 0 0 0 0 0 0 0 0 0 0 0 0 0 0 0 0 0 0 0 0 0 0 0 0 0 0 0 0
0 0 0 0 0 0 0 0 0 0 0 0 0 0 41 0 0 0 0 0 0 0 0 0 0 0 0 0 0
0 0 0 0 0 0 0 0 0 0 0 0 0 0 0 0 0 0 0 0 0 0 5 0 0 0 0 0 0
13 0 0 0 0 0 0 0 0 0 0 0 0 0 0 0 0 0 0 0 0 101 0 0 0 0 0
M02255_77_000000000-ADUT3_1_1101_13777_20000 T----AC---GT-AT-GGT--------GCA-A-G------C--G--T---T--AT-C-CGG-AT------TT-A--C-T--GG-GT-------GT--A-----AA-GG-GA-GC-------G-TA-G-A-C-G------------G--T-TG-T-G-T------
-----AA----G-T-C-T--------G-A-T--G--TG--A-AA-AC--C-C-GG-G-G-----CT-C-AA------C-C-C-C-G-G-G--A-C----T-G--C-A---T--T-------G--GA-A-A---C-------T--A-T--GC--A-G-C----T-A-G-A-G-T--G---T-CG-----GA-G-A--------G-G-T-A---AG-C------GG--A--ATT----C-
C-T-A-GT--GT-A-G-CG-GT--G---A--A-A---TG-C-GT-AG--AT-A-TT--A-G--G-A-G-G-A-AC-A-CC---AG--T--G--GC-GAA-G--G-C--G-------G--C-T-T-A---CTG--G--AC-G-A--------------------T-A---A-C-T--GA--CG-T-T-G--A-GG--C-T-CG-A--AA-G-C---G-TG--GG-G-
-AG-C-A-AA--------CA--GG Bacteria(100);Firmicutes(100);Clostridia(100);Clostridiales(100);Lachnospiraceae(100);Incertae_Sedis(89);unclassified(89);unclassified(89);unclassified(89);

Otu0239 7 1 0 0 0 0 0 0 0 0 0 0 0 4 0 0 0 0 0 0 4 0 0 0 0 1 0 0
0 0 0 49 0 0 0 0 0 0 1 5 0 13 0 5 0 0 0 196 0 0 0 4 1 0 0 0 0
0 0 2 0 5 0 4 3 0 0 0 0 0 0 0 0 0 0 0 24 0 0 1 23 0 0 65 0 18
0 9 18 0 0 0 0 0 0 0 0 0 0 0 0 0 0 0 0 0 0 0 0 0 0 0 0
M02255_76_000000000-ADUNE_1_1109_29208_15845 C----GG---CT-AA-CTC--------CGT-G-C------C--A--G---C--AG-C-CGC-GG------TA-A--T-A--CG-GA-------GG--G-----TG-CA-AG-CG-------T-TA-T-C-C-G------------G--A-CT-T-A-T------
-----TA----G-T-C-A--------G-T-G--G--TG--A-AA-GC--C-T-GT-C-G-----CT-T-AA------C-G-A-T-A-G-A--A-C----T-G--C-C---A--T-------T--GA-T-A---C-------T--G-T--AA--G-T-C----T-T-G-A-G-T--A---T-AT-----TT-G-A--------G-G-T-A---GC-T------GG--A--ATA----A-G-
T-A-GT--GT-A-G-CG-GT--G---A--A-A---TG-C-AT-AG--AT-A-TT--A-C--T-T-A-G-A-AC-A-CC---AA--T--T--GC-GAA-G--G-C--A-------G--G-T-T-A---CCA--A--GA-T-A--------------------T-A---A-C-T--GA--CG-C-T-G--A-GG--G-A-CG-A--AA-G-C---G-TG--GG-G--
AG-C-G-AA--------CA--GG Bacteria(100);Bacteroidetes(100);Flavobacteria(100);Flavobacteriales(100);Flavobacteriaceae(100);Cloacibacterium(100);unclassified(100);unclassified(100);unclassified(100);

Otu0240 0 0 80 0 0 0 51 0 0 0 0 0 0 0 0 0 0 0 0 1 5 0 0 4 0 0 0 0
0 0 0 0 0 0 0 0 0 26 0 26 102 0 25 0 22 0 0 0 0 15 0 0 0 0 0 0 0
0 0 0 0 0 0 0 0 0 0 0 6 0 0 0 0 0 0 0 0 0 0 0 0 0 0 0 0
16 0 0 0 0 0 4 0 0 34 0 0 0 14 0 0 0 0 0 0 0 0 0 0 0 0 0
M02255_77_000000000-ADUT3_1_1116_21872_12643 T----AC---GG-GG-GGT--------GCA-A-G------C--G--T---T--AC-T-CGG-AA------TC-A--C-T--GG-GC-------GT--A-----AA-GG-AC-GC-------G-TA-G-G-C-G------------G--A-TT-A-T-C-----
------AA----G-T-C-T--------T-T-T--G--TG--A-AA-TC--T-A-GT-G-G-----CT-T-AA------C-C-A-C-T-A-A--A-C----T-G--C-T---T--A-------G--GA-A-A---C-------T--G-A--TA--G-T-C----T-A-G-A-G-T--A---G-GG-----GA-G-A--------G-G-T-A---GA-T------GG--A--ATT----C-
T-T-G-GT--GT-A-G-GG-GT--A---A--A-A---TC-C-GT-AG--AG-A-TC--A-A--G-A-A-G-A-AT-A-CC---CA--T--T--GC-GAA-A--G-C--G-------A--T-C-T-G---CTG--G--AA-C-C--------------------T-A---A-C-T--GA--CG-C-T-G--A-GG--C-G-TG-A--AA-G-C---G-TG--GG-T--
AG-C-A-AA--------CA--GG Bacteria(100);Proteobacteria(100);Epsilonproteobacteria(100);Campylobacterales(100);Campylobacteraceae(100);Campylobacter(100);unclassified(100);unclassified(100);unclassified(100);

Otu0241 0 0 0 37 0 0 46 0 21 0 0 5 0 0 0 0 0 0 0 0 0 0 0 0 0 0 0 0
0 111 0 0 0 32 44 0 12 0 0 52 28 0 140 125 0 0 0 0 0 0 6 0 0 6 0 0 0
0 0 0 0 0 0 0 0 0 0 0 0 0 0 0 5 0 0 0 0 0 0 0 0 100 0 0 0 0
0 0 0 10 122 0 0 0 0 0 0 0 0 0 0 0 0 2 0 56 121 42 0 0 13 0 144
M02255_77_000000000-ADUT3_1_1103_8004_13852 T----AC---GT-AG-GGT--------GCA-A-G------C--A--T---T--AT-C-CGG-AG------TG-A--C-T--GG-GC-------GT--A-----AA-GA-GTTGC-------G-TA-G-G-C-G------------G--T-TT-A-A-T-----------
AA----G-T-G-A--------A-T-A--G--TG--A-AA-CC--T-G-GT-G-G-----CT-C-AA------C-C-A-TAC-A-G--A-C----T-A--T-T---A--T-------T--CA-A-A---C-------T--G-T--TA--A-A-C----T-C-G-A-G-A--G---T-GG-----TA-G-A--------G-G-T-A---AC-T------GG--A--ATT----T-C-T-
A-GT--GT-A-G-GA-GT--G---A--A-A---TC-C-GT-AG--AT-A-TT--A-G--A-A-G-G-A-AC-A-CC---AA--T--G--GC-GTA-G--G-C--A-------G--G-T-T-A---CTG--G--AC-C-A--------------------T-T---T-C-T--GA--CG-C-T-A--A-GG--C-A-CG-A--AA-G-C---G-TG--GG-G--AG-
C-G-AA--------CT--GG Bacteria(100);Candidate_division_TM7(100);unclassified(100);unclassified(100);unclassified(100);unclassified(100);unclassified(100);unclassified(100);

Otu0242 0 0 0 4 0 11 0 0 0 9 12 0 0 0 0 0 0 0 0 3 0 0 25 0 36 35 0 4
0 0 0 0 1 0 0 0 6 0 0 1 0 22 0 0 5 5 0 0 5 0 0 1 0 0 0 0 8
0 0 0 0 0 0 0 0 0 0 0 0 0 0 0 0 0 0 0 0 0 6 0 1 0 0 0 0
0 0 0 0 0 0 0 0 0 0 0 0 0 0 0 0 0 0 0 0 3 0 0 0 0 0 0
M02255_76_000000000-ADUNE_1_2101_13394_2931 G----AA---GC-AC-CGG--------CTA-A-C------T--C--C---G--TG-C-CAG-CA------GC-C--G-C--GG-TA-------AT--A-----CG-GA-GG-GT-------G-CG-A-G-C-T------------G--T--T-A-A-T-----------C---
-G-G-A-A--------G-A-T--G--TG--A-AA-GC--C-C-GG-G-G-----CT-C-AA------C-C-T-C-G-G-A--A-C----A-G--C-A---T--T-------T--TG-A-A---C-------T--G-G--CA--G-A-C----T-A-G-A-G-T--C---T-TG-----TA-G-A--------G-G-G-G---GG-T------AG--A--ATT----T-C-A-G-
GT--GT-A-G-CG-GT--G---A--A-A---TG-C-GT-AG--AG-A-TC--T-G--A-A-G-G-A-AT-A-CC---GG--T--G--GC-GAA-G--G-C--G-------G--C-C-C-C---CTG--G--AC-A-A--------------------A-G---A-C-T--GA--CG-C-T-C--A-GA--T-G-CG-A--AA-G-C---G-TG--GG-G--AG-
C-A-AA--------CA--GG Bacteria(100);Proteobacteria(100);Gammaproteobacteria(100);Vibrionales(100);Vibrionaceae(100);unclassified(100);unclassified(100);unclassified(100);unclassified(100);

Otu0243 0 0 0 0 0 0 0 0 0 0 0 3 0 0 0 1 0 3 4 0 0 0 0 0 0 0 0 0
0 0 0 0 0 0 0 0 0 0 0 0 0 0 0 0 0 0 0 0 0 0 0 0 0 0 1 0 0
0 1 0 0 0 0 12 0 0 0 0 0 0 0 0 0 0 0 0 0 0 0 0 0 0 0 0 0 0
0 0 0 0 0 0 0 0 0 0 0 0 0 0 1 0 0 0 0 0 0 0 0 0 0 0 0
M02255_77_000000000-ADUT3_1_2112_6049_4268 G----TG---CC-AG-CAG--------CCG-C-G---------G--T---A--AT-A-CGG-AG------GA-T--C-C--AA-GC-------GT--T-----AT-CC-GG-AA-------T-CA-G-G-C-G------------G--T-TT-A-G-T-----------AA--
--G-T-C-A--------G-T-G--G--TG--A-AA-GC--C-C-AT-C-G-----CT-C-AA------C-G-G-T-G-G-A--A-C----G-G--A-C---A--T-------T--GA-T-A---C-------T--G-C--TA--A-A-C----T-T-G-A-A-T--T---A-TT-----AG-G-A--------A-G-T-A---AC-T------AG--A--ATA----T-G-T-A-GT-
-GT-A-G-CG-GT--G---A--A-A---TG-C-TT-AG--AG-A-TT--A-C--A-T-G-G-A-AT-A-CC---AA--T--T--GC-GAA-G--G-C--A-------G--G-T-T-A---CTA--C--TA-A-T--------------------G-G---A-T-T--GA--CG-C-T-G--A-TG--G-A-CG-A--AA-G-C---G-TG--GG-T--AG-C-G-
AA--------CA--GG Bacteria(100);Bacteroidetes(100);Flavobacteria(100);Flavobacteriales(100);Flavobacteriaceae(100);Flavobacterium(100);unclassified(100);unclassified(100);unclassified(100);

Otu0244 17 0 0 0 0 0 0 0 0 0 0 0 0 0 0 0 0 0 0 0 0 0 0 0 0 0 0 0
9 0 55 0 0 0 0 0 0 0 0 0 0 0 0 0 0 0 9 0 0 0 0 0 0 0 0 0 0
0 52 0 8 0 0 5 0 10 0 0 0 0 0 0 0 0 0 0 13 5 0 0 0 0 0 0 0 0
0 0 0 0 0 0 33 0 32 0 0 0 0 0 0 0 0 18 0 0 0 0 7 0 0 0 0
M02255_76_000000000-ADUNE_1_1104_11640_9725 .....AC----T-AC-GT---------GCC-A-G------C--A--G---C--CG-C-GGT-AA------TA-C--G-T--AG-GT-------GG--C-----AA-GC-G--TT-------G----T-C-C-G------------G--T-TT-T-T-T-----------AA----G-
T-C-T--------G-A-T--G--TG--A-AA-GC--C-C-TC-G-G-----CT-C-AA------C-C-G-A-G-G-A--A------G-GT-C-A---T--T-------G--GA-A-A---C-------T--G-G--AG--A-A-C----T-T-G-A-G-T--G---C-AG-----AA-G-A--------G-G-A-G---AG-T------GG--A--ATT----C-C-A-T-GT--
GT-A-G-CG-GT--G---A--A-A---TG-C-GT-AG--AT-A-TA--T-G--G-A-G-G-A-AC-A-CC---AG--T--G--GC-GAA-G--G-C--G-------A--C-T-C-T---CTG--G--TC-T-G--------------------T-A---A-C-T--GA--CG-C-T-G--A-GG--C-T-CG-A--AA-G-C---G-TG--GG-G--AG-C-A-
AA--------CA--GG Bacteria(100);Firmicutes(100);Bacilli(100);Lactobacillales(100);unclassified(66);unclassified(66);unclassified(66);unclassified(66);unclassified(66);

Otu0245 46 0 1 0 0 0 0 0 0 0 0 0 0 0 0 0 0 0 0 0 3 0 0 0 0 0 0 0
0 0 40 57 0 0 8 0 0 19 14 0 0 0 0 106 19 16 0 0 0 0 0 0 0 0 1 0 0
0 0 0 0 0 0 0 0 0 0 0 0 0 0 120 254 0 4 0 0 0 0 0 0 102 1 0 0 0
37 0 50 0 0 42 7 1 1 13 0 0 0 0 0 50 0 34 12 126 168 157 46 0 33 0 81
M02255_76_000000000-ADUNE_1_1104_23866_19435 C----GG---CT-AA-TTC--------CGT-G-C------C--A--G---C--AG-C-CGC-GG------TA-A--T-A--CG-GA-------AG--G-----TC-CA-GG-CG-------T-TA-T-C-C-G------------G--ATTT-A-T-T------
-----AA----G-T-C-A--------G-T-G--G--TG--A-AA-GA--C-G-GT-G-G-----CT-C-AA------C-C-A-T-C-G----T-T----A-G--C-C---A--T-------T--GA-A-A---C-------T--G-G--TA--G-T-C----T-T-G-A-G-T--G---C-AG-----AC-A-G--------G-G-A-T---GC-T------GG--A--ACT----C-
G-T-G-GT--GT-A-G-CG-GT--G---A--A-A---TG-C-TT-AG--AT-A-TC--A-C--G-A-T-G-A-AC-T-CC---GA--T--C--GC-GAA-G--G-C--A-------G--G-T-G-T---CCG--G--GC-T-G--------------------C-A---A-C-T--GA--CG-C-T-G--A-GG--C-T-CG-A--AA-G-T---G-TG--GG-T--
AT-C-A-AA--------CA--GG Bacteria(100);Bacteroidetes(100);Bacteroidia(99);Bacteroidales(99);Prevotellaceae(99);Prevotella(99);unclassified(99);unclassified(99);unclassified(99);

Otu0246 0 61 0 0 0 0 0 0 99 0 0 0 0 0 0 0 0 0 0 0 0 0 0 0 0 0 0 0
0 0 0 0 0 0 0 0 0 0 0 0 0 0 0 0 0 0 0 0 0 0 0 0 0 0 0 0 0
0 0 0 0 0 0 0 0 0 0 0 0 0 0 0 0 45 0 0 0 0 0 0 0 0 0 0 0 0
0 0 0 0 0 0 0 0 0 0 0 0 0 0 0 0 0 0 0 0 0 0 0 0 76 0 0
M02255_77_000000000-ADUT3_1_1110_23676_14768 T----AC---GT-AG-GGG--------GCA-A-G------C--G--T---T--AT-C-CGG-AT------TT-A--C-T--GG-GT-------GT--A-----AA-GG-GA-GC-------G-TA-G-A-C-G------------G--C-GA-T-G-C----
-------AA----G-T-C-T--------G-A-A--G--TG--A-AA-TA--C-C-CG-G-G-----CT-C-AA------C-C-T-G-G-G-A--A-C----T-G--C-T---T--T-------G--GA-A-A---C-------T--G-T--AT--G-G-C----T-A-G-A-G-T--G---C-TG-----GA-G-A--------G-G-T-A---AG-C------GG--A--ATT----
C-C-T-A-GT--GT-A-G-CG-GT--G---A--A-A---TG-C-GT-AG--AT-A-TT--A-G--G-A-A-G-A-AC-A-CC---AG--T--G--GC-GAA-G--G-C--G-------G--C-T-T-A---CTG--G--AC-A-G--------------------T-A---A-C-T--GA--CG-T-T-C--A-GG--C-T-CG-A--AA-G-C---G-TG--GG-
G--AG-C-A-AA--------CA--GG Bacteria(100);Firmicutes(100);Clostridia(100);Clostridiales(100);Lachnospiraceae(96);unclassified(96);unclassified(96);unclassified(96);unclassified(96);

Otu0247 0 0 0 0 0 0 62 7 0 0 0 0 0 0 0 0 0 0 0 3 11 0 0 16 0 0 0 0
0 0 0 0 0 0 0 0 0 18 0 0 0 0 0 0 0 0 0 0 0 0 0 0 0 0 0 0 0
0 0 0 0 0 0 0 0 0 0 0 0 0 0 0 17 0 0 0 0 0 0 0 0 0 0 0 0 0
0 0 0 0 0 0 0 0 0 0 0 0 0 0 0 0 0 0 0 0 0 0 0 1 39 0 0
M02255_77_000000000-ADUT3_1_1108_11047_12661 T----AC---GT-AG-GTG--------GCA-A-G------C--G--T---T--GT-C-CGG-AT------TT-A--C-T--GG-GT-------GT--A-----AA-GG-GC-GT-------G-TA-G-G-C-G------------G--A-GA-T-G-C-----
------AA----G-T-C-A--------G-A-T--G--TG--A-AA-TC--C-C-CG-G-G-----CT-T-AA------C-C-C-G-G-G-A--A-C----T-G--C-A---T--T-------T--GA-A-A---C-------T--G-T--AT--C-C-C----T-T-G-A-G-T--A---T-CG-----GA-G-A--------G-G-C-A---GG-C------GG--A--ATT----C-
C-T-A-GT--GT-A-G-CG-GT--G---A--A-A---TG-C-GT-AG--AT-A-TT--A-G--G-A-G-G-A-AC-A-CC---AG--T--G--GC-GAA-G--G-C--G-------G--C-C-T-G---CTG--G--AC-G-A--------------------C-A---A-C-T--GA--CG-C-T-G--A-GG--C-G-CG-A--AA-G-C---G-TG--GG-
G--AG-C-A-AA--------CA--GG Bacteria(100);Firmicutes(100);Clostridia(100);Clostridiales(100);Ruminococcaceae(100);unclassified(100);unclassified(100);unclassified(100);unclassified(100);

Otu0248 0 0 0 0 0 0 0 0 0 0 0 4 0 0 0 0 0 0 0 0 0 0 21 23 0 6 0 0
0 0 0 0 0 0 70 18 0 0 0 0 0 0 186 0 0 0 0 0 0 0 0 0 20 0 0 0 0
0 0 0 16 0 0 0 0 0 0 0 0 0 0 1 0 0 0 0 0 0 0 0 0 0 0 0 0 0
0 0 0 0 0 0 0 0 0 0 0 0 0 0 0 0 0 0 0 0 0 0 0 0 0 0 0
M02255_77_000000000-ADUT3_1_1117_18274_9028 T----AC---GG-AG-GGA--------GCT-A-G------C--G--T---T--GT-T-CGG-AA------TT-A--C-T--GG-GC-------GT--A-----AA-GC-GC-GC-------G-TA-G-G-C-G------------G--T-TT-T-T-T-----------AA-
---G-T-C-A--------G-A-G--G--TG--A-AA-GC--C-C-GG-G-G-----CT-C-AA------C-C-C-C-G-G-A--A-T----T-G--C-C---T--T-------T--GA-A-A---C-------T--G-G--AA--A-A-C----T-T-G-A-A-T--C---T-TG-----GA-G-A--------G-G-T-C---AG-T------GG--A--ATT----C-C-G-A-
GT--GT-A-G-AG-GT--G---A--A-A---TT-C-GT-AG--AT-A-TT--C-G--G-A-A-G-A-AC-A-CC---AG--T--G--GC-GAA-G--G-C--G-------A--C-T-G-A---CTG--G--AC-A-A--------------------G-T---A-T-T--GA--CG-C-T-G--A-GG--T-G-CG-A--AA-G-C---G-TG--GG-G--AG-
C-A-AA--------CA--GG Bacteria(100);Proteobacteria(100);Alphaproteobacteria(100);Sphingomonadales(100);Sphingomonadaceae(100);Sphingopyxis(100);unclassified(100);unclassified(100);unclassified(100);

Otu0249 0 806 1233 0 0 0 549 87 455 0 0 1 0 12 0 0 0 0 0 0 0 0 29 0 0 0 0 0
0 0 0 0 41 72 0 0 0 0 0 417 0 0 0 54 0 0 0 0 0 0 0 0 0 4 0 0 0
0 0 0 0 0 0 0 0 0 0 0 0 4 0 0 18 55 60 0 1 0 0 1 0 0 0 0 0 0
0 0 0 0 0 0 12 0 0 0 0 25 0 0 1 0 0 0 0 0 0 70 0 21 734 0 32
M02255_77_000000000-ADUT3_1_1101_25893_18534 T----AC---GT-AG-GGA--------GCG-A-G------C--G--T---T--GT-C-CGG-AA------TT-A--C-T--GG-GT-------GT--A-----AA-GG-GA-GC-------G-TA-G-G-C-G------------G--G-AT-C-G-C----
-------AA----G-T-C-A--------G-A-T--G--TG--A-AA-AC--T-A-TG-G-G-----CT-T-AA------C-C-C-A-T-A-A--A-C----T-G--C-A---T--T-------T--GA-A-A---C-------T--G-T--GG--T-T-C----T-T-G-A-G-T--G---A-AG-----TA-G-A--------G-G-T-A---AG-C------GG--A--ATT----C-
C-T-A-GT--GT-A-G-CG-GT--G---A--A-A---TG-C-GT-AG--AT-A-TT--A-G--G-A-G-G-A-AC-A-TC---AG--T--G--GC-GAA-G--G-C--G-------G--C-T-T-A---CTG--G--GC-T-T--------------------T-A---A-C-T--GA--CG-C-T-G--A-GG--C-T-CG-A--AA-G-C---G-TG--GG-G-
-AG-C-A-AA--------CA--GG Bacteria(100);Firmicutes(100);Clostridia(100);Clostridiales(100);Ruminococcaceae(100);unclassified(100);unclassified(100);unclassified(100);unclassified(100);

Otu0250 0 0 30 0 0 0 38 0 0 0 0 0 0 0 0 0 0 0 0 0 0 0 0 0 0 0 0 0
0 0 0 0 0 0 0 0 0 0 0 44 0 0 0 0 0 0 0 0 0 4 0 0 19 0 0 0 0
0 0 0 0 0 0 0 0 0 0 0 0 0 0 10 0 15 0 0 0 0 0 0 0 0 0 0 0 0
0 56 0 0 0 0 0 0 0 0 0 0 0 0 0 0 0 0 0 0 0 0 0 14 20 0 0
M02255_77_000000000-ADUT3_1_1119_9660_6144 T----AC---GT-AG-GGG--------GCA-A-G------C--G--T---T--AT-C-CGG-AT------TT-A--C-T--GG-GT-------GT--A-----AA-GG-GA-GC-------G-TA-G-G-C-G------------G--C-GG-A-G-C----------
AA----G-T-C-A--------G-A-A--G--TG--A-AA-GC--C-C-GG-G-G-----CT-C-AA------C-C-C-C-G-G-G--A-C----G-G--C-T---T--T-------T--GA-A-A---C-------T--G-C--CC--T-G-C----T-T-G-A-T-T--T---C-AG-----GA-G-A--------G-G-T-A---AG-C------GG--A--ATT----C-C-T-
A-GT--GT-A-G-CG-GT--G---A--A-A---TG-C-GT-AG--AT-A-TT--A-G--G-A-G-G-A-AC-A-CC---AG--T--G--GC-GAA-G--G-C--G-------G--C-T-T-A---CTG--G--AC-T-G--------------------A-C---A-A-T--GA--CG-C-T-G--A-GG--C-T-CG-A--AA-G-C---G-TG--GG-G--
AG-C-A-AA--------CA--GG Bacteria(100);Firmicutes(100);Clostridia(100);Clostridiales(100);Lachnospiraceae(100);Coprococcus(100);unclassified(100);unclassified(100);unclassified(100);

Otu0251 0 0 0 0 0 0 0 0 0 0 0 13 0 1 1 1 0 4 11 5 0 5 0 0 0 0 0 0
7 0 0 0 0 0 0 0 0 0 0 0 0 0 0 0 1 0 0 0 0 0 0 0 0 0 0 0 0
0 0 4 0 0 1 1 0 2 3 18 0 4 0 0 0 0 1 0 0 0 0 0 0 0 0 0 0 0
0 11 2 0 0 5 0 4 0 0 0 0 1 0 0 0 0 0 0 0 0 0 0 1 0 1 0
M02255_77_000000000-ADUT3_1_1116_8081_8931 T----AC---AG-AG-GGT--------GCA-A-G------C--G--T---T--AA-T-CGG-AT------TT-A--C-T--GG-GC-------GT--A-----AA-GC-GT-GC-------G-TA-G-G-T-G------------G--C-CA-A-T-T-----------AA-
---G-T-C-A--------A-A-T--G--TG--A-AA-TC--C-C-CG-A-G-----CT-T-AA------C-T-T-G-G-G-A--A-T----T-G--C-A---T--T-------C--GA-T-A---C-------T--G-G--TT--G-G-C----T-A-G-A-G-T--A---T-GG-----GA-G-A--------G-G-A-T---GG-T------AG--A--ATT----C-C-A-G-
GT--GT-A-G-CG-GT--G---A--A-A---TG-C-GT-AG--AG-A-TC--T-G--G-A-G-G-A-AT-A-CC---GA--T--G--GC-GAA-G--G-C--A-------G--C-C-A-T---CTG--G--CC-T-A--------------------A-T---A-C-T--GA--CA-C-T-G--A-GG--T-A-CG-A--AA-G-C---A-TG--GG-G--AG-C-
A-AA--------CA--GG Bacteria(100);Proteobacteria(100);Gammaproteobacteria(100);Pseudomonadales(100);Moraxellaceae(100);Acinetobacter(100);unclassified(100);unclassified(100);unclassified(100);

Otu0252 0 0 0 0 0 0 0 0 0 0 0 0 0 0 0 0 0 0 0 0 0 0 0 0 0 0 0 0
0 0 0 0 0 0 0 0 0 0 0 0 24 0 0 0 0 0 34 0 0 0 0 0 0 0 0 0 0
0 0 0 0 0 8 0 0 0 0 0 0 0 0 0 0 0 0 0 0 0 0 0 0 0 0 0 0 0
0 0 0 0 0 0 0 2 0 0 0 0 1 0 0 0 0 0 0 0 0 0 0 0 20 0 0
M02255_77_000000000-ADUT3_1_1106_12439_14042 T----AC---GT-AG-GGC--------GCG-A-G------C--G--T---T--GT-C-CGG-AA------TT-A--T-T--GG-GC-------GT--A-----AA-GA-GC-TT-------G-TA-G-G-C-G------------G--T-TT-G-T-C------
-----GC----G-T-C-T--------G-C-T--G--TG--A-AA-GC--C-C-GG-G-G-----CT-T-AA------C-C-C-C-G-G-G--T-T----T-G--C-A---G--T-------G--GG-T-A---C-------G--G-G--CA--G-A-C----T-A-G-A-G-T--G---C-AG-----TA-G-G--------G-G-A-G---AC-T------GG--A--ATT----
C-C-T-G-GT--GT-A-G-CG-GT--G---A--A-A---TG-C-GC-AG--AT-A-TC--A-G--G-A-G-G-A-AC-A-CC---AA--T--G--GC-GAA-G--G-C--A-------G--G-T-C-T---CTG--G--GC-T-G--------------------T-A---A-C-T--GA--CG-C-T-G--A-GA--A-G-CG-A--AA-G-C---A-TG--
GG-G--AG-C-A-AA--------CA--GG Bacteria(100);Actinobacteria(100);Actinobacteria(100);Actinobacteridae(100);Actinomycetales(100);Micrococcineae(100);Micrococcaceae(100);unclassified(53);unclassified(53);

Otu0253 0 0 0 0 0 0 0 0 0 0 0 0 0 0 0 0 0 0 0 0 0 0 0 0 0 0 0 6
0 0 0 0 0 0 0 48 122 0 0 0 0 0 115 47 0 0 0 0 0 0 0 0 4 3 0 0 0
0 0 0 0 0 0 0 0 0 0 0 0 3 0 0 0 0 0 0 0 0 0 0 0 0 0 0 0 0
29 0 0 0 0 0 0 0 0 0 0 0 0 0 0 0 0 0 0 0 46 0 0 0 0 0 45
M02255_77_000000000-ADUT3_1_1109_4042_13523 T----AC---GA-AG-GGG--------GCT-A-G------C--G--T---T--GT-T-CGG-AA------TT-A--C-T--GG-GC-------GT--A-----AA-GC-GC-AC-------G-TA-G-G-C-G------------G--A-CA-T-T-T-----------AA-
---G-T-C-A--------G-G-G--G--TG--A-AA-TC--C-C-GG-G-G-----CT-C-AA------C-C-C-C-G-G-A--A-C----G-G--C-C---T--T-------T--GA-T-A---C-------T--G-G--GT--G-T-C----T-T-G-A-G-T--T---C-GG-----AA-G-A--------G-G-T-G---AG-T------GG--A--ATT----G-C-G-A-
GT--GT-A-G-AG-GT--G---A--A-A---TT-C-GT-AG--AT-A-TT--C-G--C-A-G-G-A-AC-A-CC---AG--T--G--GC-GAA-G--G-C--G-------G--C-T-C-A---CTG--G--TC-C-G--------------------A-T---A-C-T--GA--CG-C-T-G--A-GG--T-G-CG-A--AA-G-C---G-TG--GG-G--AG-C-
A-AA--------CA--GG Bacteria(100);Proteobacteria(100);Alphaproteobacteria(100);Rhizobiales(100);unclassified(100);unclassified(100);unclassified(100);unclassified(100);unclassified(100);

Otu0254 0 0 0 21 0 0 50 15 0 0 0 0 0 0 0 0 0 0 0 0 0 0 0 1 0 0 0 0
0 0 42 0 0 0 41 41 0 0 0 0 0 0 74 0 0 0 0 7 0 0 0 0 0 0 1 0 0
0 0 0 0 0 0 0 0 16 0 0 0 0 0 38 6 0 0 41 0 0 0 0 0 101 0 0 0 0
57 0 0 0 50 0 0 3 0 0 1 0 0 33 0 0 0 0 0 0 283 0 0 0 34 0 823
M02255_77_000000000-ADUT3_1_1112_23321_11471 T----AC---GG-AG-GGT--------GCA-A-G------C--G--T---T--AC-T-CGG-AA------TC-A--C-T--GG-GC-------GT--A-----AA-GG-AC-GC-------G-TA-G-G-C-G------------G--A-TT-A-T-C-----
------AA----G-T-C-T--------C-T-T--G--TG--A-AA-TC--C-T-AT-G-G-----CT-T-AA------C-C-A-T-A-G-A--A-C----T-G--C-T---T--G-------G--GA-A-A---C-------T--G-A--TA--A-T-C----T-A-G-A-G-T--G---A-GG-----GA-G-A--------G-G-C-A---GA-T------GG--A--ATT----G-
G-T-G-GT--GT-A-G-GG-GT--A---A--A-A---TC-C-GT-AG--AG-A-TC--A-C--C-A-G-G-A-AT-A-CC---CA--T--T--GC-GAA-G--G-C--G-------A--T-C-T-G---CTG--G--AA-C-T--------------------C-A---A-C-T--GA--CG-C-T-A--A-TG--C-G-TG-A--AA-G-C---G-TG--GG-G--
AG-C-A-AA--------CA--GG Bacteria(100);Proteobacteria(100);Epsilonproteobacteria(100);Campylobacterales(100);Campylobacteraceae(100);Campylobacter(100);unclassified(100);unclassified(100);unclassified(100);

Otu0255 0 0 0 0 0 0 0 0 0 0 0 0 0 0 0 0 0 0 0 0 3 0 0 0 0 0 0 0
0 1 2 0 0 0 0 0 0 22 16 329 86 0 303 0 0 0 3 1 0 153 1 160 0 132 0 22 0
0 0 0 0 0 0 0 0 0 0 0 0 22 1 0 0 0 0 0 0 0 0 0 0 0 0 0 16 18
0 0 0 0 0 0 0 0 0 0 0 0 0 0 0 0 0 0 0 0 0 0 0 0 0 0 0
M02255_76_000000000-ADUNE_1_1105_28559_13389 .....AC----T-AC-GT---------GCC-A-G------C--A--G---C--CG-C-GGT-AA------TA-C--G-T--AG-GT-------GG--C-----AA-GC-G--TT-------G-TC-C-G---G-------------A--T-CC-T-T-T-----------
AA----G-T-C-T--------G-A-T--G--TG--A-AA-GC--C-C-AC-G-G-----CT-C-AA------C-C-G-T-G-G-A--A------G-GT-C-A---T--T-------G--GA-A-A---C-------T--G-G--GG--G-A-C----T-T-G-A-G-T--G---C-AG-----AA-G-A--------G-G-A-A---AG-T------GG--A--ATT----C-C-
A-T-GT--GT-A-G-CG-GT--G---A--A-A---TG-C-GT-AG--AT-A-TA--T-G--G-A-G-G-A-AC-A-CC---AG--T--G--GC-GAA-G--G-C--G-------A--C-T-T-T---CTG--G--TC-T-G--------------------T-A---A-C-T--GA--CG-C-T-G--A-GG--C-T-CG-A--AA-G-C---G-TG--GG-G--
AG-C-G-AA--------CA--GG Bacteria(100);Firmicutes(100);Bacilli(100);unclassified(100);unclassified(100);unclassified(100);unclassified(100);unclassified(100);unclassified(100);

Otu0256 0 0 0 0 0 0 0 0 0 0 0 0 0 0 0 0 0 0 0 0 0 0 87 0 0 0 0 0
0 0 0 0 0 0 39 0 0 0 0 0 0 0 0 0 0 0 0 0 0 6 0 0 0 0 0 0
0 0 0 0 5 0 0 0 0 0 0 0 0 0 0 0 0 115 0 0 0 0 0 0 0 0 0 0
0 0 0 0 0 0 0 0 0 0 0 0 0 0 0 0 0 0 0 0 0 0 0 0 0 100 0
M02255_77_000000000-ADUT3_1_1113_17099_12527 T----AC---GG-AG-GGA--------GCT-A-G------C--G--T---T--GT-T-CGG-AA------TT-A--C-T--GG-GC-------GT--A-----AA-GC-GC-AC-------G-TA-G-G-C-G-------------G--C-TT-T-T-T------
-----AA----G-T-C-A--------G-G-G--G--TG--A-AA-TC--C-C-GG-G-G-----CT-C-AA------C-C-C-C-G-G-A--A-C----T-G--C-C---C--T-------T--GA-A-A---C-------T--G-G--GA--A-G-C----T-A-G-A-A-T--C---T-TG-----GA-G-A--------G-G-C-G---AG-T------GG--A--ATT----C-
C-G-A-GT--GT-A-G-AG-GT--G---A--A-A---TT-C-GT-AG--AT-A-TT--C-G--G-A-A-G-A-AC-A-CC---AG--T--G--GC-GAA-G--G-C--G-------A--C-T-C-G---CTG--G--AC-A-A--------------------G-T---A-T-T--GA--CG-C-T-G--A-GG--T-G-CG-A--AA-G-C---G-TG--GG-
G--AG-C-A-AA--------CA--GG Bacteria(100);Proteobacteria(100);Alphaproteobacteria(100);Sphingomonadales(100);Erythrobacteraceae(100);unclassified(59);unclassified(59);unclassified(59);unclassified(59);

Otu0257 0 0 0 0 111 0 0 0 0 0 0 0 0 0 5 0 0 0 0 0 0 0 11 0 0 0 13 0
0 0 0 0 0 0 0 0 0 0 31 0 0 0 0 0 0 0 0 0 0 0 0 16 0 0 0 0
0 0 0 0 6 0 0 0 0 0 0 0 0 0 0 12 29 0 0 0 0 0 0 46 0 0 0 0
5 0 0 0 0 0 0 0 0 0 0 0 0 0 0 16 0 0 0 0 0 0 0 0 0 0 0
M02255_77_000000000-ADUT3_1_1103_15670_14784 T----AC---GA-AG-GGG--------GCT-A-G------C--G--T---T--GC-T-CGG-AA------TT-A--C-T--GG-GC-------GT--A-----AA-GG-GC-GC-------G-TA-G-G-C-G-------------G--C-TT-A-G-T----
-------TA----G-T-C-A--------G-G-C--G--TG--A-AA-TT--C-C-TG-G-G-----CT-C-AA------C-C-T-G-G-G-G--A-C----T-G--C-G---C--T-------T--GA-T-A---C-------G--G-C--TA--G-G-C----T-A-G-A-G-G--A---T-GG-----AA-G-A--------G-G-G-T---CG-C------GG--A--ATT----
C-C-C-A-GT--GT-A-G-AG-GT--G---A--A-A---TT-C-GT-AG--AT-A-TT--G-G--G-A-A-G-A-AC-A-CC---GG--T--G--GC-GAA-G--G-C--G-------G--C-G-A-C---CTG--G--TC-C-A--------------------T-T---A-C-T--GA--CG-C-T-G--A-GG--C-G-CG-A--CA-G-C---G-TG--GG-
G--AG-C-A-AA--------CA--GG Bacteria(100);Proteobacteria(100);Alphaproteobacteria(100);Rhodospirillales(100);Acetobacteraceae(100);unclassified(100);unclassified(100);unclassified(100);unclassified(100);

Otu0258 0 0 0 0 0 0 0 0 0 0 0 0 0 0 0 0 0 0 0 0 0 0 0 0 5 0 0 0
0 0 0 0 0 0 0 0 0 0 7 0 0 0 0 4 0 0 0 0 0 0 0 0 0 0 0 0
0 0 0 0 0 0 0 0 0 0 0 0 0 0 0 0 0 13 0 0 0 0 0 0 0 0 0 0
18 0 0 0 0 0 0 0 0 0 0 0 0 0 0 0 0 0 16 0 0 0 0 0 0 0 0
M02255_77_000000000-ADUT3_1_1116_9370_20183 T----AC---GT-AG-GGC--------GCA-A-G------C--G--T---T--GT-C-CGG-AT------TT-A--T-T--GG-GC-------GT--A-----AA-GA-GC-TC-------G-TA-G-G-C-G-------------G--T-TT-G-T-C-----------GC--
--G-T-C-T--------G-C-T--G--TG--A-AA-AC--C-C-GA-G-G-----CT-C-AA------C-C-T-C-G-G-G--C-C----T-G--C-A---G--T-------G--GG-T-A---C-------G--G-G--CA--G-A-C----T-A-G-A-G-T--G---C-GG-----TA-G-G--------G-G-A-G---AC-T------GG--A--ATT----C-C-T-G-
GT--GT-A-G-CG-GT--G---G--A-A---TG-C-GC-AG--AT-A-TC--A-G--G-A-G-G-A-AC-A-CC---GA--T--G--GC-GAA-G--G-C--A-------G--G-T-C-T---CTG--G--GC-C-G--------------------C-A---A-C-T--GA--CG-C-T-G--A-GG--A-G-CG-A--AA-G-C---A-TG--GG-G--AG-
C-G-AA--------CA--GG Bacteria(100);Actinobacteria(100);Actinobacteria(100);Actinobacteridae(100);Actinomycetales(100);Micrococcineae(100);unclassified(93);unclassified(93);unclassified(93);

Otu0259 0 0 3 0 0 0 0 0 0 0 0 0 0 0 0 0 0 0 0 0 0 0 0 0 0 0 0 0
0 0 0 0 0 0 0 0 17 0 0 0 0 0 0 0 0 0 0 0 0 16 2 4 0 2 0 0 0
0 0 0 0 0 0 0 0 0 0 0 0 0 0 0 0 0 0 0 0 9 1 0 0 0 0 0 0 0
14 0 0 1 0 0 0 0 0 0 0 11 0 0 0 0 0 0 0 0 0 0 0 0 0 0 0
M02255_76_000000000-ADUNE_1_2105_12223_20003 .....AC----T-AC-GT---------GCC-A-G------C--A--G---C--CG-C-GGT-AA------TA-C--G-T--AG-GT-------GG--C-----AA-GC----G--------T-TA-T-C-C-G-------------G--T-TG-C-T-T-----------
AG----G-T-C-T--------G-A-T--G--TG--A-AA-GC--C-T-TC-G-G-----CT-T-AA------C-C-G-A-A-G-A--A-G----T-G--C-A---T--C-------G--GA-A-A---C-------C--G-G--GC--A-A-C----T-T-G-A-G-T--G---C-AG-----AA-G-A--------G-G-A-C---AG-T------GG--A--ACT----C-C-
A-T-GT--GT-A-G-CG-GT--G---G--A-A---TG-C-GT-AG--AT-A-TA--T-G--G-A-A-G-A-AC-A-CC---AG--T--G--GC-GAA-G--G-C--G-------G--C-T-G-T---CTG--G--TC-T-G--------------------C-A---A-C-T--GA--CG-C-T-G--A-GG--C-T-CG-A--AA-G-C---A-TG--GG-T--
AG-C-G-AA--------CA--GG Bacteria(100);Firmicutes(100);Bacilli(100);Lactobacillales(100);Lactobacillaceae(100);unclassified(100);unclassified(100);unclassified(100);unclassified(100);

Otu0260 0 0 0 14 0 0 3 0 0 0 0 0 0 0 0 0 0 0 0 0 1 0 0 1 0 0 0 0
0 0 2 0 0 0 0 0 0 0 9 0 2 0 79 0 29 0 0 0 0 7 0 0 0 3 0 0 0
34 0 0 0 0 0 2 0 0 0 3 0 4 13 13 23 0 0 0 0 0 0 1 0 6 0 0 12 0
271 0 0 0 0 21 3 0 0 0 6 76 0 0 0 17 19 13 0 0 0 0 0 0 10 0 0
M02255_76_000000000-ADUNE_1_2115_23954_5823 .....AC----T-AC-GT---------GCC-A-G------C--A--G---C--CG-C-GGT-AA------TA-C--G-T--AG-GT-------GG--C-----AA-GC-G--TT-------G----T-C-C-G------------G--A-TT-C-T-T-----------AA----G-T-C-T--------G-A-T--G--TG--A-AA-GC--C-T-TC-G-G-----CT-C-AA------C-C-G-A-A-G-A--A-G----T-G--C-A---T--C-------G--GA-A-A---C-------T--G-G--GA--A-A-C----T-T-G-A-G-T--G---C-AG-----AA-G-A--------G-G-A-C---AG-T------GG--A--ACT----C-C-A-T-GT--GT-A-G-CG-GT--G---A--A-A---TG-C-GT-AG--AT-A-TA--T-G--G-A-A-G-A-AC-A-CC---AG--T--G--GC-GAA-G--G-C--G-------G--C-T-G-T---CTG--G--TC-T-G--------------------T-A---A-C-T--GA--CG-C-T-G--A-GG--C-T-CG-A--AA-G-C---A-TG--GG-T--AG-C-A-AA--------CA--GG Bacteria(100);Firmicutes(100);Bacilli(100);Lactobacillales(100);Lactobacillaceae(100);unclassified(100);unclassified(100);unclassified(100);unclassified(100);

Otu0261 753 0 0 0 0 0 0 0 0 0 0 0 0 0 0 0 0 1 0 0 0 0 0 0 0 0 0 2
0 0 0 0 0 0 0 0 0 0 0 0 0 0 0 0 0 0 0 0 92 0 0 0 0 0 0 0
0 0 0 0 0 0 1 0 0 4 5 0 0 0 0 0 0 0 0 0 81 0 1 0 0 0 0 0 0
0 0 0 0 19 21 0 0 0 20 2 0 6 0 0 0 0 0 0 0 0 0 0 0 0 0 0
M02255_76_000000000-ADUNE_1_1101_28569_13911 .....AC----T-AC-GT---------GCC-A-G------C--A--G---C--CG-C-GGT-AA------TA-C--G-T--AG-GT-------GG--C-----AA-GC-G--TT-------G-TC-C-G---G-------------A--T-CT-T-T-T-----------AA----G-T-C-T--------G-A-T--G--TG--A-AA-GC--C-T-TC-G-G-----CT-T-AA------C-C-G-G-A-G-T--A-G----T-G--C-A---T--T-------G--GA-A-A---C-------T--G-G--AA--G-A-C----T-T-G-A-G-T--G---C-AG-----AA-G-A--------G-G-A-G---AG-T------GG--A--ACT----C-C-A-T-GT--GT-A-G-CG-GT--G---A--A-A---TG-C-GT-AG--AT-A-TA--T-G--G-A-A-G-A-AC-A-CC---AG--T--G--GC-GAA-A--G-C--G-------G--C-T-C-T---CTG--G--TC-T-G--------------------T-A---A-C-T--GA--CG-C-T-G--A-GG--T-T-CG-A--AA-G-C---G-TG--GG-T--AG-C-A-AA--------CA--GG Bacteria(100);Firmicutes(100);Bacilli(100);Lactobacillales(51);unclassified;unclassified;unclassified;unclassified;unclassified;

Otu0262 0 0 0 0 0 1 0 0 0 0 0 0 0 0 0 0 0 0 0 0 0 0 0 0 0 0 0 0
0 0 0 2 0 0 0 0 0 30 30 2 0 0 0 0 0 230 0 0 0 0 0 0 0 7 0 0 0
0 0 0 0 0 0 0 0 0 0 9 3 5 0 0 0 0 23 207 69 1 0 0 0 0 0 0 0 0
0 0 0 0 0 0 0 0 0 0 0 0 0 0 0 0 0 0 0 0 0 0 0 0 0 0 0
M02255_77_000000000-ADUT3_1_1105_9629_13218 G----AC---GA-AG-GGG--------GCT-A-G------C--G--T---T--GT-T-CGG-AA------TT-A--C-T--GG-GC-------GT--A-----AA-GC-GC-GT-------G-TA-G-G-C-G------------G--T-TG-T-T-C-----------AA----G-T-C-G--------G-G-T--G--TG--A-AA-GC--C-T-TG-A-G-----CT-C-AA------C-T-C-A-A-G-A--A-A----T-G--C-A---C--T-------C--GG-T-A---C-------T--G-G--AT--G-A-C----T-A-G-A-G-G--A---C-CG-----GA-G-A--------G-G-A-T---AG-T------GG--A--ATT----C-C-C-A-GT--GT-A-G-TG-GT--G---A--A-A---TA-C-GT-AG--AG-A-TT--G-G--G-A-A-G-A-AC-A-CC---AG--T--G--GC-GAA-G--G-C--G-------G--C-T-A-T---CTG--G--AC-G-G--------------------T-T---T-C-T--GA--CG-C-T-A--A-GA--C-G-CG-A--AA-G-C---G-TG--GG-G--AG-C-A-AA--------CA--GG Bacteria(100);Proteobacteria(100);Alphaproteobacteria(100);Rhodospirillales(100);wr0007(100);unclassified(100);unclassified(100);unclassified(100);unclassified(100);

Otu0263 0 0 0 0 0 0 0 0 0 0 0 0 0 2 0 0 4 0 0 0 0 0 0 0 0 0 0 0
0 0 0 6 0 41 0 54 1 0 17 0 52 0 0 2 7 0 0 26 17 0 2 0 12 0 104 3 0
0 0 2 0 0 0 0 0 0 0 0 0 0 0 0 0 0 10 0 0 0 7 0 0 0 42 267 16 0
0 0 0 0 0 0 0 0 0 0 0 0 0 0 0 0 0 0 0 0 0 0 0 0 0 0 0
M02255_77_000000000-ADUT3_1_1110_22746_10224 T----AC---GT-AG-GGT--------GCG-A-G------C--G--T---T--GT-C-CGG-AA------TT-A--C-T--GG-GC-------GT--A-----AA-GA-GT-TC-------G-TA-G-G-C-G------------G--T-TT-G-T-C-----------GC----G-T-C-G--------T-G-T--G--TG--A-AA-TC--C-C-GC-A-G-----CT-C-AA------C-T-G-C-G-G-G--C-T----T-G--C-A---G--G-------C--GA-T-A---C-------G--G-G--CA--G-A-C----T-T-G-A-G-T--A---C-TG-----CA-G-G--------G-G-A-G---AC-T------GG--A--ATT----C-C-T-G-GT--GT-A-G-CG-GT--G---A--A-A---TG-C-GC-AG--AT-A-TC--A-G--G-A-G-G-A-AC-A-CC---GG--T--G--GC-GAA-G--G-C--G-------G--G-T-C-T---CTG--G--GC-A-G--------------------T-A---A-C-T--GA--CG-C-T-G--A-GG--A-A-CG-A--AA-G-C---G-TG--GG-T--AG-C-G-AA--------CA--GG Bacteria(100);Actinobacteria(100);Actinobacteria(100);Actinobacteridae(100);Actinomycetales(100);Corynebacterineae(100);Nocardiaceae(99);Rhodococcus(99);unclassified(99);

Otu0264 0 0 0 0 1 0 0 0 0 0 0 0 0 0 0 0 0 0 0 0 0 0 0 0 0 2 0 0
0 6 1 0 0 0 0 2 0 0 0 0 0 1 0 5 0 0 0 1 0 5 0 0 0 0 0 0 0
0 0 0 0 0 0 0 0 0 0 0 0 0 0 1 2 0 0 0 0 2 0 1 0 0 0 0 31 5
0 0 0 1 0 0 0 0 0 0 0 1 0 0 0 0 0 0 0 0 0 0 0 0 0 0 0
M02255_77_000000000-ADUT3_1_2114_7771_7549 C----TA---AC-TC-TGT--------GCC-A-G------C-----A---G--CC-G-CGG-TA------AT-A--C-A--GA-GG-------GT--------GC-GA-GC-G--------T-TA-A-T-C-G------------G--C-TT-T-T-T-----------AA----G-T-C-G--------G-A-T--G--TG--A-AA-TC--C-C-TG-A-G-----CT-T-AA------C-T-T-A-G-G-A--A-T----T-G--C-A---T--T-------C--GA-T-A---C-------T--G-G--GA--A-G-C----T-A-G-A-G-T--A---T-GG-----GA-G-A--------G-G-A-T---GG-T------AG--A--ATT----C-C-A-G-GT--GT-A-G-CG-GT--G---A--A-A---TG-C-GT-AG--AG-A-TC--T-G--G-A-G-G-A-AT-A-CC---GA--T--G--GC-GAA-G--G-C--A-------G--C-C-A-T---CTG--G--CC-T-A--------------------A-T---A-C-T--GA--CG-C-T-G--A-GG--T-A-CG-A--AA-G-C---A-TG--GG-G--AG-C-A-AA--------CA--GG Bacteria(100);Proteobacteria(100);Gammaproteobacteria(100);Pseudomonadales(100);Moraxellaceae(100);Acinetobacter(100);unclassified(100);unclassified(100);unclassified(100);

Otu0265 121 0 0 0 0 0 0 0 0 0 0 3 0 0 0 0 0 0 0 0 6 0 0 16 0 1 0 2
17 30 127 0 52 21 44 11 10 23 0 46 54 0 192 17 0 0 0 57 117 132 0 27 27 1 0 0 114
0 19 3 0 0 0 0 0 0 0 0 10 2 0 243 141 28 19 0 60 40 3 8 0 14 0 0 16 0
7 0 12 7 162 0 11 3 0 16 0 0 0 14 0 21 0 167 0 10 0 0 56 1 8 0 15
M02255_76_000000000-ADUNE_1_1102_22157_12134 .....AC----T-AC-GT---------GCC-A-G------C--A--G---C--CG-C-GGT-AA------TA-C--G-T--AG-GT-------GG--C-----AA-GC-G--TT-------G-TC-C-G---G------------A--T-CT-T-T-T----------
AA----G-T-C-T--------G-A-T--G--TG--A-AA-GC--C-C-TC-G-G-----CT-C-AA------C-C-G-A-G-G-A--A------G-GT-C-A---T--T-------G--GA-A-A---C-------T--G-G--GA--G-A-C----T-T-G-A-G-T--G---C-AG-----AA-G-A--------G-G-A-G---AG-T------GG--A--ATT----C-C-
A-T-GT--GT-A-G-CG-GT--G---A--A-A---TG-C-GT-AG--AT-A-TA--T-G--G-A-G-G-A-AC-A-CC---AG--T--G--GC-GAA-G--G-C--G-------A--C-T-C-T---CTG--G--TC-T-G--------------------T-A---A-C-T--GA--CG-C-T-G--A-GG--C-T-CG-A--AA-G-C---G-TG--GG-G--
AG-C-A-AA--------CA--GG Bacteria(100);Firmicutes(100);Bacilli(100);unclassified(69);unclassified(69);unclassified(69);unclassified(69);unclassified(69);unclassified(69);

Otu0266 0 0 0 0 0 0 22 0 0 0 0 0 0 0 0 0 0 0 0 0 0 0 0 0 0 0 0 0
0 0 0 0 0 0 0 0 0 0 0 0 0 0 0 0 0 0 0 0 0 0 0 0 0 0 0 0 0
0 0 0 0 0 0 0 0 0 0 0 0 0 0 102 132 0 0 0 0 0 0 0 102 0 0 0 0 0
0 0 103 0 0 0 0 0 0 0 0 0 0 0 0 0 0 0 0 0 0 0 0 0 0 0 0
M02255_77_000000000-ADUT3_1_1105_8175_14299 T----AC---GT-AG-GAT--------CCG-A-G------C--G--T---T--AT-C-CGG-AG------TG-A--C-T--GG-GC-------GT--A-----AA-GA-GTTGC-------G-TA-G-G-C-G------------G--T-TG-T-A-T----------
AA----G-T-G-A--------A-T-A--G--TG--A-AA-TC--T-G-GT-G-G-----CT-C-AA------C-C-A-T-ACA-G--G-C----T-A--T-T---A--T-------T--CA-A-A---C-------T--G-T--AC--A-A-C----T-C-G-A-G-A--G---T-GG-----TA-G-A--------G-G-T-C---AC-T------GG--A--ATT----T-C-T-
T-GT--GT-A-G-GA-GT--G---A--A-A---TC-C-GT-AG--AT-A-TA--A-G--A-A-G-G-A-AC-A-CC---GA--T--G--GC-GTA-G--G-C--A-------G--G-T-G-A---CTG--G--AC-C-A--------------------T-T---T-C-T--GA--CG-C-T-A--A-GG--C-A-CG-A--AA-G-C---G-TG--GG-G--AG-
C-G-AA--------CC--GG Bacteria(100);Candidate_division_TM7(100);unclassified(100);unclassified(100);unclassified(100);unclassified(100);unclassified(100);unclassified(100);unclassified(100);

Otu0267 0 0 0 0 0 0 0 0 58 0 0 0 0 0 0 0 0 0 0 0 0 0 0 0 0 0 0 0
0 0 0 0 0 0 0 0 0 0 0 0 0 0 0 0 0 0 0 0 0 0 0 0 0 0 0 8 0
0 0 0 0 0 0 3 0 2 0 0 0 0 0 0 0 0 20 0 0 0 0 0 0 0 0 0 0 0
36 0 173 0 0 0 0 0 0 0 0 0 0 0 0 43 0 0 0 0 0 167 0 0 16 0 0
M02255_77_000000000-ADUT3_1_2111_18220_15969 T----AC---GA-AG-GGG--------GCT-A-G------C--G--T---T--GC-T-CGG-AA------TC-A--C-T--GG-GC-------GT--A-----AA-GG-GC-GC-------G-TA-G-G-C-G------------G--C-CG-A-T-C----
-------AA----G-T-C-A--------G-G-G--G--TG--A-AA-GC--C-T-GT-G-G-----CT-C-AA------C-C-A-C-A-G-A--A-T----T-G--C-C---T--T-------T--GA-T-A---C-------T--G-T--TT--G-G-C----T-C-G-A-G-A--C---C-GG-----AA-G-A--------G-G-T-T---GG-T------GG--A--ACT----
G-C-G-A-GT--GT-A-G-AG-GT--G---A--A-A---TT-C-GT-AG--AT-A-TT--C-G--C-A-A-G-A-AC-A-CC---AG--T--G--GC-GAA-G--G-C--G-------G--C-C-A-A---CTG--G--TC-C-G--------------------G-T---T-C-T--GA--CG-C-T-G--A-GG--C-G-CG-A--AA-G-C---G-TG--GG-
G--AG-C-A-AA--------CA--GG Bacteria(100);Proteobacteria(100);Alphaproteobacteria(100);Rhizobiales(100);unclassified(100);unclassified(100);unclassified(100);unclassified(100);unclassified(100);

Otu0268 0 0 0 0 0 0 0 0 0 0 0 0 0 0 0 0 0 0 0 7 12 0 0 0 0 0 0 0
0 0 0 0 0 0 0 0 0 47 4 0 0 0 0 0 0 0 0 0 0 0 0 0 0 0 0 0 0
0 32 516 35 30 0 3 0 0 5 63 0 0 0 0 12 0 0 358 75 0 0 0 0 0 0 0 13 0
0 0 0 0 0 0 0 0 0 0 0 0 0 0 0 0 0 0 0 0 0 0 0 0 0 0 0
M02255_77_000000000-ADUT3_1_1119_28631_13003 T----AC---GA-AG-GGG--------GCT-A-G------C--G--T---T--GT-T-CGG-AA------TC-A--C-T--GG-GC-------GT--A-----AA-GC-GC-AC-------G-TA-G-G-C-G------------G--A-CC-A-T-T-----
------AA----G-T-C-A--------G-G-G--G--TG--A-AA-GC--C-T-GG-A-G-----CT-C-AA------C-T-C-C-A-G-A--A-C----T-G--C-C---T--T-------T--GA-T-A---C-------T--G-A--TG--G-T-C----T-C-G-A-G-T--T---C-GG-----AA-G-A--------G-G-T-T---GG-T------GG--A--ACT----G-
C-G-A-GT--GT-A-G-AG-GT--G---A--A-A---TT-C-GT-AG--AT-A-TT--C-G--C-A-A-G-A-AC-A-CC---AG--T--G--GC-GAA-G--G-C--G-------G--C-C-A-A---CTG--G--TC-C-G--------------------A-T---A-C-T--GA--CG-C-T-G--A-GG--T-G-CG-A--AA-G-C---G-TG--GG-G-
-AG-C-A-AA--------CA--GG Bacteria(100);Proteobacteria(100);Alphaproteobacteria(100);Rhizobiales(100);Hyphomicrobiaceae(100);unclassified(94);unclassified(94);unclassified(94);unclassified(94);

Otu0269 0 0 0 0 0 234 0 0 0 0 0 0 0 0 0 0 0 0 0 0 0 0 0 0 0 0 0 0
0 0 0 0 0 0 0 0 0 0 0 0 0 0 0 0 0 0 0 0 0 0 0 0 0 0 0 0 0
0 0 0 0 0 0 0 0 0 0 0 0 0 0 0 0 0 0 46 0 0 0 0 0 0 0 0 0 0
0 0 51 0 0 0 0 0 0 0 0 0 0 0 0 0 0 0 0 0 0 0 0 0 0 0 0
M02255_77_000000000-ADUT3_1_1114_22211_10961 T----AC---GT-AG-GGG--------GCA-A-G------C--G--T---T--GT-C-CGG-AA------TG-A--T-T--GG-GC-------GT--A-----AA-GG-GC-GC-------G-TA-G-G-C-G------------G--C-TA-T-T-T----
-------AA----G-T-C-T--------G-G-A--G--TG--A-AA-GT--C-C-TG-C-T-----TT-C-AA------G-G-T-G-G-G-A--A-T----T-G--C-T---T--T-------G--GA-T-A---C-------T--G-G--AT--A-G-C----T-T-G-A-G-T--G---C-AG-----GA-G-A--------G-G-T-A---AG-T------GG--A--ATT----
C-C-C-A-GT--GT-A-G-CG-GT--G---A--A-A---TG-C-GT-AG--AG-A-TT--G-G--G-A-G-G-A-AC-A-CC---AG--T--G--GC-GAA-G--G-C--G-------A--C-T-T-A---CTG--G--AC-T-G--------------------T-A---A-C-T--GA--CG-C-T-G--A-GG--C-G-CG-A--AA-G-T---G-TG--
GG-G--AG-C-A-AA--------CA--GG Bacteria(100);Firmicutes(100);Clostridia(100);Clostridiales(100);Lachnospiraceae(71);uncultured(71);unclassified(71);unclassified(71);unclassified(71);

Otu0270 0 98 105 6 0 0 124 5 294 0 0 0 0 0 1 0 0 0 0 0 0 0 3 0 0 0 0 0
0 0 0 2 0 0 0 0 0 0 0 68 0 0 2 0 0 0 0 0 0 0 0 0 0 0 0 0
0 0 0 0 0 0 0 0 0 0 0 0 0 0 0 0 0 0 0 0 0 0 0 0 0 0 0 0
0 0 0 0 0 0 0 0 0 0 0 0 0 0 0 0 0 0 0 0 0 0 0 0 57 0 0
M02255_76_000000000-ADUNE_1_1106_26377_14763 T----CG---GC-TA-ACT--------CCG-T-G------C--C--A---G--CA-G-CCG-CG------GT-A--A-T--AC-GG-------AG--G-----AT-CC-GA-GC-------GTTA-T-C-C-G------------G--ATTT-A-T-T-----
------AA----G-T-C-A--------G-T-T--G--TG--A-AA-GT--T-T-GC-G-G-----CT-C-AA------C-C-G-T-A-A-A--A-T----T-G--C-A---G--T-------T--GA-T-A---C-------T--G-G--GT--G-T-C----T-T-G-A-G-T--A---C-AG-----TA-G-A--------G-G-C-A---GG-C------GG--A--ATT----C-
G-T-G-GT--GT-A-G-CG-GT--G---A--A-A---TG-C-TT-AG--AT-A-TC--A-C--G-A-A-G-A-AC-T-CC---GA--T--T--GC-GAA-G--G-C--A-------G--C-T-T-G---CTG--G--AC-T-G--------------------T-A---A-C-T--GA--CG-C-T-G--A-TG--C-T-CG-A--AA-G-T---G-TG--GG-T--
AT-C-A-AA--------CA--GG Bacteria(100);Bacteroidetes(100);Bacteroidia(100);Bacteroidales(100);Bacteroidaceae(100);Bacteroides(100);unclassified(100);unclassified(100);unclassified(100);

Otu0271 0 0 0 0 0 0 0 0 0 0 0 0 0 0 0 0 0 0 0 0 0 0 0 0 0 0 0 0
0 0 0 0 0 0 0 49 17 0 0 0 0 0 0 0 0 0 0 0 0 0 32 0 0 0 0 0
0 0 0 0 0 0 0 0 0 0 0 0 0 0 0 0 0 0 0 0 0 0 0 0 0 0 0 0
0 0 0 0 0 0 0 0 0 0 0 0 0 0 0 0 0 0 0 0 0 0 0 0 0 0 0
M02255_77_000000000-ADUT3_1_1104_15415_3015 G----AC---AG-AG-GCC--------ACG-A-G------C--G--T---T--AG-G-CGG-AA------TC-A--C-T--GG-GC-------TT--A-----AA-GC-GT-GT-------G-TA-G-G-C-G------------G--A-CC-G-T-T-----------
AA----G-T-GCC--------C-G-C--G--TG--A-AA-TC--C-C-CC-G-G-----CT-C-AA------C-C-G-G-G-G-A--A-C----G-G--C-C---G--G-------G--CA-T-A---C-------T--G-G--CG--G-T-C----T-T-G-A-G-C--G---A-TC-----CA-G-G--------G-G-T-C---AT-C------GG--A--ACA----A-A-
C-G-GT--GG-A-G-CG-GT--G---A--A-A---TG-C-GT-AG--AT-A-TC--G-T--T-T-G-G-A-AC-G-CC---GA--C--G--GT-GAA-A--A-C--A-------G--A-T-G-A---CTG--G--GG-A-T--------------------C-C---G-C-T--GA--CG-C-T-G--A-GA--C-A-CG-A--AA-G-C---C-AG--GG-G--
AG-C-A-AA--------CG--GG Bacteria(100);Planctomycetes(100);unclassified(80);unclassified(80);unclassified(80);unclassified(80);unclassified(80);unclassified(80);unclassified(80);

Otu0272 0 0 0 0 0 0 0 0 0 0 0 0 0 11 0 0 0 0 0 0 0 0 73 0 0 0 0 31
0 0 47 0 0 1 0 0 0 0 0 0 0 0 0 0 0 0 0 0 0 0 0 0 0 0 0 0
0 0 0 0 0 0 0 0 0 0 0 0 0 0 0 0 0 0 0 0 0 0 0 0 0 0 0 0
35 0 0 0 0 0 0 0 0 0 0 0 0 0 0 0 0 0 0 0 0 0 0 0 0 0 0
M02255_77_000000000-ADUT3_1_1108_21912_12979 T----AC---GG-AG-GGA--------GCT-A-G------C--G--T---T--GT-T-CGG-AA------TT-A--C-T--GG-GC-------GT--A-----AA-GC-GC-AC-------G-TA-G-G-C-G------------G--C-GA-T-T-T-----
------AA----G-T-C-A--------G-A-G--G--TG--A-AA-GC--C-C-GG-G-G-----CT-C-AA------C-C-C-C-G-G-A--A-C----T-G--C-C---T--T-------T--GA-G-A---C-------T--G-G--AT--T-G-C----T-A-G-A-A-T--C---T-TG-----GA-G-A--------G-G-C-G---GG-T------GG--A--ATT----
C-C-G-A-GT--GT-A-G-AG-GT--G---A--A-A---TT-C-GT-AG--AT-A-TT--C-G--G-A-A-G-A-AC-A-CC---AG--T--G--GC-GAA-G--G-C--G-------G--C-C-C-G---CTG--G--AC-A-A--------------------G-T---A-T-T--GA--CG-C-T-G--A-GG--T-G-CG-A--AA-G-C---G-TG--
GG-G--AG-C-A-AA--------CA--GG Bacteria(100);Proteobacteria(100);Alphaproteobacteria(100);Sphingomonadales(100);Sphingomonadaceae(100);unclassified(58);unclassified(58);unclassified(58);unclassified(58);

Otu0273 0 0 0 0 0 0 25 0 0 5 0 0 0 0 0 0 0 0 0 0 0 0 0 0 0 0 0 0
0 0 1 0 0 0 0 0 0 0 0 0 0 0 0 2 0 0 0 0 0 0 4 0 0 0 0 0
0 0 0 0 0 0 0 0 0 0 0 0 0 0 11 0 0 0 0 0 0 0 0 11 0 0 0 0
1 0 6 0 0 0 0 0 0 0 0 0 0 0 0 0 0 0 9 0 0 0 0 0 0 30
M02255_76_000000000-ADUNE_1_2104_10888_10766 T----CG---GC-TA-ACT--------CCG-T-GC-----C--A--G---C--AG-C-CGC-GG------TA-A--T-A--CG-GA-------GG--A-----TG-CG-AG-CG-------T-TA-T-T-C-G------------G--A-CTCA-T-T-----
------AA----G-T-C-A--------G-G-G--G--TG--A-AA-GG--T-T-TC-A-G-----CT-T-AA------C-T-G-A-G-A-A--A-T----T-G--C-C---T--T-------T--GA-T-A---C-------T--G-G--TA--G-T-C----T-T-G-A-A-T--A---T-CT-----GT-G-A--------A-G-T-T---CT-T------GG--A--ATG----T-
G-T-A-GT--GT-A-G-CG-GT--G---A--A-A---TG-C-TT-AG--AT-A-TT--A-C--A-C-A-G-A-AC-A-CC---GA--T--T--GC-GAA-G--G-C--A-------G--G-G-G-A---CTA--A--CA-G-A--------------------C-A---A-T-T--GA--CG-C-T-G--A-GA--G-A-CG-A--AA-G-C---G-TG--GG-G-
-AG-C-G-AA--------CA--GG Bacteria(100);Bacteroidetes(100);Flavobacteria(100);Flavobacteriales(100);Flavobacteriaceae(100);Capnocytophaga(100);unclassified(100);unclassified(100);unclassified(100);

Otu0274 0 40 49 128 0 0 549 25 191 0 0 0 5 1 0 0 0 0 0 0 0 0 0 0 0 0 0 0
0 0 0 39 0 22 0 0 0 0 0 130 0 0 0 0 0 0 0 32 0 0 0 0 0 0 0 0
0 0 0 0 0 0 0 0 0 0 0 0 0 0 0 0 13 57 0 0 0 0 0 0 0 0 0 0
0 63 0 0 0 0 12 0 0 0 0 0 0 0 0 0 0 0 0 0 0 21 0 9 212 0 0
M02255_77_000000000-ADUT3_1_1104_24464_2591 T----AC---GT-AG-GGG--------GCT-A-G------C--G--T---T--AT-C-CGG-AA------TT-A--C-T--GG-GC-------GT--A-----AA-GG-GT-GC-------G-TA-G-G-C-G------------G--T-CT-T-T-C-----------AA-
---G-T-C-A--------G-A-A--G--TG--A-AA-GG--C-T-AC-G-G-----CT-C-AA------C-C-G-T-A-G-T--A------A-G--C-T---T--T-------T--GA-A-A---C-------T--G-T--AA--G-A-C----T-T-G-A-G-T--G---C-AG-----GA-G-A--------G-G-A-G---AG-T------GG--A--ATT----C-C-T-A-
GT--GT-A-G-CG-GT--G---A--A-A---TG-C-GT-AG--AT-A-TT--A-G--G-A-G-G-A-AC-A-CC---AG--T--T--GC-GAA-G--G-C--G-------G--C-T-C-T---CTG--G--AC-T-G--------------------T-A---A-C-T--GA--CG-C-T-G--A-GG--C-A-CG-A--AA-G-C---G-TG--GG-G--AG-C-
A-AA--------CA--GG Bacteria(100);Firmicutes(100);Clostridia(100);Clostridiales(100);Peptostreptococcaceae(100);Incertae_Sedis(96);unclassified(96);unclassified(96);unclassified(96);

Otu0275 155 0 0 0 0 0 0 0 0 0 0 0 0 0 0 0 0 0 0 0 0 0 0 0 0 0 0 0
0 0 0 0 0 0 0 0 0 0 0 0 0 0 0 0 0 0 0 0 0 0 0 0 0 0 0 0
0 0 0 0 0 0 0 0 0 0 0 0 0 0 28 0 0 24 0 0 0 0 0 0 0 0 0 0
0 0 0 69 0 0 0 0 0 0 0 0 0 0 0 0 0 0 0 0 0 0 0 0 0 0 0
M02255_77_000000000-ADUT3_1_1108_5590_9779 T----AC---GA-AG-GGG--------ACT-A-G------C--G--T---T--GT-T-CGG-AA------TC-A--C-T--GG-GC-------GT--A-----AA-GC-GC-AC-------G-TA-G-G-C-G-------------G--A-TT-T-G-T-----------AA----G-T-C-A--------G-G-G--G--TG--A-AA-TC--C-C-GG-G-G-----CT-C-AA------C-C-T-C-G-G-A--A-C----T-G--C-C---T--T-------T--GA-T-A---C-------T--G-C--AA--G-T-C----T-T-G-A-G-T--C---C-GA-----TA-G-A--------G-G-T-G---GG-T------GG--A--ATT----C-C-T-A-GT--GT-A-G-AG-GT--G---A--A-A---TT-C-GT-AG--AT-A-TT--A-G--G-A-A-G-A-AC-A-CC---GG--T--G--GC-GAA-G--G-C--G-------G--C-C-C-A---CTG--G--AT-C-G--------------------G-T---A-C-T--GA--CG-C-T-G--A-GG--T-G-CG-A--AA-G-C---G-TG--GG-G--AG-C-A-AA--------CA--GG Bacteria(100);Proteobacteria(100);Alphaproteobacteria(100);Rhizobiales(100);Hyphomicrobiaceae(100);Hyphomicrobium(100);unclassified(100);unclassified(100);unclassified(100);

Otu0276 0 0 0 0 0 0 0 0 0 0 0 0 0 0 0 0 0 0 0 0 0 4 0 0 0 3 0 0
0 0 0 0 0 0 0 27 0 0 0 0 0 0 0 0 0 0 0 0 0 0 0 26 21 0 0 0 0
0 0 0 0 0 0 0 0 0 0 0 0 0 0 13 0 0 20 0 0 0 4 0 0 0 0 1 0 0
0 0 0 0 46 0 0 0 0 0 0 0 4 1 0 0 0 0 0 0 0 0 0 0 0 0 0
M02255_77_000000000-ADUT3_1_1107_7993_15723 T----AC---GT-AG-GCA--------GCG-A-G------C--G--T---T--GT-T-CGG-AG------TT-A--C-T--GG-GC-------GT--A-----AA-GA-GT-GC-------G-TA-G-G-C-G-------------G--T-TT-T-C-T-----------AA----G-T-T-T--------G-G-T--G--TG--A-AA-TC--T-C-CC-G-G-----CT-T-AA------C-T-G-G-G-A-G--G-G----T-G--C-G---C--C-------G--AA-G-A---C-------T--G-G--AA--G-G-C----T-T-G-A-G-T--A---T-GG-----GA-G-A--------G-G-T-A---AG-C------GG--A--ATT----C-C-T-G-GT--GT-A-G-CG-GT--G---A--A-A---TG-C-GT-AG--AT-A-TC--A-G--G-A-G-G-A-AC-A-CC---TG--C--G--GT-GTA-G--A-C--G-------G--C-T-T-A---CTG--G--AC-C-A--------------------T-A---A-C-T--GA--CG-C-T-G--A-GG--C-A-CG-A--AA-G-C---G-TG--GG-T--AG-C-A-AA--------CA--GG Bacteria(100);Acidobacteria(100);Acidobacteria(100);Acidobacteriales(100);Acidobacteriaceae(100);unclassified(100);unclassified(100);unclassified(100);unclassified(100);

Otu0277 0 0 0 0 0 0 0 0 0 0 0 0 0 0 0 0 0 0 0 0 0 0 0 0 0 0 0 2
0 0 0 0 0 0 0 0 0 0 0 0 0 0 0 0 0 0 0 0 0 0 0 0 0 0 0 0
0 0 11 0 0 10 9 0 0 1 13 0 0 1 0 0 0 0 0 27 0 0 0 0 0 0 0 0
33 0 0 23 0 0 0 0 0 13 0 0 0 0 0 0 0 0 0 0 0 0 0 0 0 0 0
M02255_77_000000000-ADUT3_1_1115_14772_17958 T----AC---GA-AG-GGG--------GCT-A-G------C--G--T---T--GT-T-CGG-AA------TT-A--C-T--GG-GC-------GT--A-----AA-GC-GC-AC-------G-TA-G-G-C-G-------------G--A-CA-T-T-T-----------AA----G-T-C-A--------G-G-G--G--TG--A-AA-TC--C-C-AG-A-G-----CT-C-AA------C-T-C-T-G-G-A--A-C----T-G--C-C---T--T-------T--GA-T-A---C-------T--G-G--GT--G-T-C----T-T-G-A-G-T--G---T-GG-----AA-G-A--------G-G-T-C---AG-T------GG--A--ATT----G-C-G-A-GT--GT-A-G-AG-GT--G---A--A-A---TT-C-GT-AG--AT-A-TT--C-G--C-A-G-G-A-AC-A-CC---AG--T--G--GC-GAA-G--G-C--G-------G--C-T-G-A---CTG--G--TC-C-A--------------------C-A---A-C-T--GA--CG-C-T-G--A-GG--T-G-CG-A--AA-G-C---G-TG--GG-G--AG-C-A-AA--------CA--GG Bacteria(100);Proteobacteria(100);Alphaproteobacteria(100);Rhizobiales(100);unclassified(100);unclassified(100);unclassified(100);unclassified(100);unclassified(100);

Otu0278 0 0 0 0 0 0 0 0 0 0 0 0 0 0 0 0 7 1 0 0 0 0 0 0 0 0 0 0
0 0 0 0 0 0 0 0 0 0 0 0 0 0 0 0 0 0 2 0 0 0 0 0 0 0 0 0 0
0 0 0 0 0 0 1 0 0 0 0 0 0 0 0 0 0 0 0 0 0 0 0 0 0 0 0 0
0 0 0 0 1 0 0 0 0 0 0 0 0 0 1 0 0 0 0 0 0 0 0 0 0 0 0
M02255_76_000000000-ADUNE_1_1101_24070_4723 C----TG---TG-CC-AGC--------AGC-C-G------C--G--G---T--AA-TACAG-AG------GG-T--G-C--AA-GC-------GT--T-----AA-TC-G--GA-------A-TT-A-C-T-G-------------G--T-TT-G-T-T-----------AA----G-T-T-G--------A-A-T--G--TG--A-AA-TC--C-C-CG-G-G-----CT-C-AA------C-C-T-G-G-G-A--A-C----T-G--C-A---T--C-------C--AA-A-A---C-------T--G-G--CA--A-G-C----T-A-G-A-G-T--A---T-GG-----TA-G-A--------G-G-G-T---AG-T------GG--A--ATT----T-C-C-T-GT--GT-A-G-CG-GT--G---A--A-A---TG-C-GT-AG--AT-A-TA--G-G--A-A-G-G-A-AC-A-CC---AG--T--G--GC-GAA-G--G-C--G-------A--C-T-A-C---CTG--G--AC-T-G--------------------A-T---A-C-T--GA--CA-C-T-G--A-GG--T-G-CG-A--AA-G-C---G-TG--GG-G--AG-C-A-AA--------CA--GG Bacteria(100);Proteobacteria(100);Gammaproteobacteria(100);Pseudomonadales(100);Pseudomonadaceae(100);Pseudomonas(54);unclassified(54);unclassified(54);unclassified(54);

Otu0279 0 0 81 0 0 0 0 0 0 0 0 0 0 0 0 0 0 0 0 0 0 0 0 0 0 0 0 0
0 0 0 0 0 0 0 0 0 0 0 0 0 0 0 0 0 0 0 0 114 0 0 0 0 0 0 0 0
0 25 0 0 0 0 0 0 0 0 0 0 0 0 0 0 0 0 0 0 0 0 0 0 0 0 0 0
0 0 0 0 0 0 0 0 0 0 0 0 0 0 0 0 0 0 0 0 0 28 22 0 0 0 0
M02255_77_000000000-ADUT3_1_1105_16266_18366 T----AC---GT-AT-GGA--------GCA-A-G------C--G--T---T--AT-C-CGG-AT------TT-A--C-T--GG-GT-------GT--A-----AA-GG-GA-GC-------G-TA-G-G-C-G-------------G--C-AG-A-G-C-----------AA----G-T-C-T--------G-A-T--G--TG--A-AA-GC--C-C-GG-G-G-----CT-C-AA------C-C-C-C-G-G-T--A-G----T-G--C-A---T--T-------G--GA-A-A---C-------T--G-T--TC--G-G-C----T-C-G-A-G-T--G---T-CG-----GA-G-G--------G-G-T-A---AG-C------GG--A--ATT----C-C-T-G-GT--GT-A-G-CG-GT--G---A--A-A---TG-C-GT-AG--AT-A-TC--A-G--G-A-A-G-A-AC-A-CC---GG--A--G--GC-GAA-G--G-C--G-------G--C-T-T-A---CTG--G--AC-G-A--------------------T-T---A-C-T--GA--CG-C-T-G--A-GG--C-T-CG-A--AA-G-C---G-TG--GG-G--AG-C-G-AA--------CA--GG Bacteria(100);Firmicutes(100);Clostridia(100);Clostridiales(100);Lachnospiraceae(100);Shuttleworthia(68);unclassified(68);unclassified(68);unclassified(68);

Otu0280 0 0 0 0 0 0 0 0 0 0 0 0 0 0 0 0 0 0 0 0 0 0 0 0 0 0 0 0
0 0 0 0 0 0 0 0 0 0 0 0 0 0 0 56 0 0 0 64 0 0 0 0 0 0 0 0 130
0 0 2 0 0 0 0 0 0 0 0 0 0 0 86 51 0 0 0 5 41 0 0 112 0 0 0 0 0
0 0 0 0 19 0 0 0 0 0 0 0 0 0 0 10 0 0 0 0 0 0 0 1 0 0 0
M02255_77_000000000-ADUT3_1_1101_29238_16133 T----AC---GT-AG-GGT--------GCA-A-G------C--A--T---T--AT-C-CGG-AG------TG-A--C-T--GG-GC-------GT--A-----AA-GA-GTTGC-------G-TA-G-G-C-G------------G--T-TT-A-A-T-----
------AA----G-T-G-A--------A-T-A--G--TG--A-AA-CC--T-G-GT-G-G-----CT-C-AA------C-C-A-T-ACA-G--A-C----T-A--T-T---A--T-------T--CA-A-A---C-------T--G-T--TA--A-A-C----T-C-G-A-G-A--A---T-GG-----TA-G-A--------G-G-T-A---AC-T------GG--A--ATT----T-
C-T-T-GT--GT-A-G-GA-GT--G---A--A-A---TC-C-GT-AG--AT-A-TA--A-G--A-A-G-G-A-AC-A-CC---AA--T--G--GC-GTA-G--G-C--A-------G--G-T-T-A---CTG--G--GC-C-A--------------------T-T---T-C-T--GA--CG-C-T-A--A-GG--C-A-CG-A--AA-G-C---G-TG--GG-G--
AG-C-G-AA--------CC--GG Bacteria(100);Candidate_division_TM7(100);unclassified(100);unclassified(100);unclassified(100);unclassified(100);unclassified(100);unclassified(100);unclassified(100);

Otu0281 0 0 0 0 0 0 0 0 0 0 0 0 0 0 0 0 0 0 0 0 0 0 0 0 3 0 0 0
0 0 0 0 0 31 0 0 0 0 0 0 0 0 0 0 0 0 0 0 0 0 0 26 0 0 0 0 0
0 0 0 0 0 0 0 0 0 0 0 0 0 0 0 0 0 20 0 0 1 0 0 0 0 0 0 29 0
0 0 0 0 0 0 0 0 0 0 0 0 0 0 0 0 0 24 0 0 0 0 0 0 0 0 0
M02255_77_000000000-ADUT3_1_1102_12123_12775 G----AC---GA-AG-GGG--------GCT-A-G------C--G--T---T--GT-T-CGG-AA------TT-A--C-T--GG-GC-------GT--A-----AA-GC-GA-GT-------G-TA-G-G-T-G------------G--T-TA-T-C-C-----
------AA----G-T-T-G--------G-A-T--G--TG--A-AA-GC--C-T-TG-A-G-----CT-C-AA------C-T-C-A-A-G-A--A-A----T-G--C-A---T--T-------C--AG-G-A---C-------T--G-G--AT--G-A-C----T-A-G-A-G-G--A---C-CG-----GA-G-A--------G-G-A-T---AG-T------GG--A--ATT----
C-C-C-A-GT--GT-A-G-TG-GT--G---A--A-A---TA-C-GT-AG--AG-A-TT--G-G--G-A-A-G-A-AC-A-CC---AG--T--G--GC-GAA-G--G-C--G-------G--C-T-A-T---CTG--G--AC-G-G--------------------T-T---T-C-T--GA--CA-C-T-A--A-GA--C-T-CG-A--AA-G-C---G-TG--GG-
G--AG-C-A-AA--------CA--GG Bacteria(100);Proteobacteria(100);Alphaproteobacteria(100);Rhodospirillales(100);wr0007(100);unclassified(100);unclassified(100);unclassified(100);unclassified(100);

Otu0282 0 0 0 0 0 0 0 40 0 0 0 0 0 0 0 0 0 0 0 0 0 0 20 0 0 0 0 0
0 0 0 0 0 0 0 0 0 0 0 0 0 0 26 0 0 0 0 0 0 0 0 0 0 0 0 0 0
0 50 0 0 0 0 0 0 0 0 0 0 0 0 0 0 0 0 0 0 0 7 0 0 0 0 0 0 0
0 0 0 0 0 22 0 0 0 0 0 0 0 0 0 0 0 0 0 0 0 0 0 0 0 0 0
M02255_77_000000000-ADUT3_1_1108_12649_13205 T----AC---GA-AG-GGG--------GCT-A-G------C--G--T---T--GT-T-CGG-AA------TT-A--C-T--GG-GC-------GT--A-----AA-GC-GC-AC-------G-TA-G-G-C-G------------G--A-CA-T-T-T-----
------AA----G-T-C-A--------G-G-G--G--TG--A-AA-TC--C-C-GG-G-G-----CT-C-AA------C-C-T-C-G-G-A--A-C----T-G--C-C---T--T-------T--GA-T-A---C-------T--G-G--GT--G-T-C----T-T-G-A-G-T--A---T-GG-----AA-G-A--------G-G-T-A---AG-T------GG--A--ATT----G-
C-G-A-GT--GT-A-G-AG-GT--G---A--A-A---TT-C-GT-AG--AT-A-TT--C-G--C-A-G-G-A-AC-A-CC---AG--T--G--GC-GAA-G--G-C--G-------G--C-T-T-A---CTG--G--TC-C-A--------------------T-T---A-C-T--GA--CG-C-T-G--A-GG--T-G-CG-A--AA-G-C---G-TG--GG-G-
-AG-C-A-AA--------CA--GG Bacteria(100);Proteobacteria(100);Alphaproteobacteria(100);Rhizobiales(100);unclassified(59);unclassified(59);unclassified(59);unclassified(59);unclassified(59);

Otu0283 0 0 0 0 0 0 0 0 0 0 0 0 0 0 0 0 2 0 0 0 0 0 0 0 0 4 0 0
0 0 1 0 0 0 0 0 0 0 21 34 0 24 0 0 0 0 0 1 84 0 0 0 0 13 0 25 16
28 0 0 0 0 0 0 0 0 0 0 0 8 0 0 0 0 0 0 0 0 4 0 0 0 0 0 0 0
0 0 0 0 50 0 0 0 0 0 0 0 0 0 0 0 0 0 0 0 0 0 0 0 0 0 0
M02255_77_000000000-ADUT3_1_1118_20844_16689 T----AC---GT-AG-GGT--------GCG-A-G------C--G--T---T--GT-C-CGG-AA------TT-A--T-T--GG-GC-------GT--A-----AA-GA-GC-TT-------G-TA-G-G-C-G------------G--T-TT-G-T-C------
-----GC----G-T-C-T--------G-C-C--G--TG--A-AA-AC--T-C-AG-G-G-----CT-T-AA------C-T-C-T-G-G-G--C-G----T-G--C-G---G--T-------G--GG-T-A---C-------G--G-G--CA--G-G-C----T-A-G-A-G-T--G---T-GG-----TA-G-G--------G-G-A-G---AC-T------GG--A--ATT----
C-C-T-G-GT--GT-A-G-CG-GT--G---A--A-A---TG-C-GC-AG--AT-A-TC--A-G--G-A-G-G-A-AC-A-CC---GA--T--G--GC-GAA-G--G-C--A-------G--G-T-C-T---CTG--G--GC-C-A--------------------T-A---A-C-T--GA--CG-C-T-G--A-GA--A-G-CG-A--AA-G-C---A-TG--
GG-G--AG-C-G-AA--------CA--GG Bacteria(100);Actinobacteria(100);Actinobacteria(100);Actinobacteridae(100);Actinomycetales(100);Micrococcineae(100);Dermatophilaceae(82);unclassified(82);unclassified(82);

Otu0284 0 0 0 0 0 2 0 0 0 0 0 2 0 1 1 0 0 0 0 0 0 2 0 0 0 0 0 1
0 0 0 0 0 0 0 0 0 0 0 0 0 0 0 1 3 0 0 0 0 0 0 0 0 0 0 0 0
0 0 0 0 0 0 0 0 0 0 0 0 0 0 0 0 0 0 0 0 0 3 0 4 0 0 0 0 0
0 0 0 0 0 0 0 0 0 0 0 0 0 0 1 0 0 0 0 0 0 0 0 1 0 0 0
M02255_76_000000000-ADUNE_1_2101_26731_17477 T----A----AG-CA-CCG--------GCT-A-A------C-----T---C--TG-T-GCC-AG------CA-G--C-C--GC-GG-------TA--A-----TA-CA-GA-GG-------G-T--G-C-A-A------------G--C--G-T---T----------
-AA----G-T-C-G--------G-A-A--G--TG--A-AA-TC--C-C-CG-G-G-----CT-T-AA------C-C-T-G-G-G-A--A-C----T-G--C-A---T--C-------T--GA-A-A---C-------T--G-T--AG--A-G-C----T-A-G-A-G-T--A---T-GT-----GA-G-A--------G-G-A-A---GG-T------AG--A--ATT----C-C-
A-G-GT--GT-A-G-CG-GT--G---A--A-A---TG-C-GT-AG--AG-A-TC--T-G--G-A-G-G-A-AT-A-CC---GA--T--G--GC-GAA-G--G-C--A-------G--C-C-T-T---CTG--G--CA-T-A--------------------A-T---A-C-T--GA--CA-C-T-G--A-GG--C-T-CG-A--AA-G-C---G-TG--GG-T--
AG-C-A-AA--------CA--GG Bacteria(100);Proteobacteria(100);Gammaproteobacteria(100);Pseudomonadales(73);Moraxellaceae(73);unclassified(73);unclassified(73);unclassified(73);unclassified(73);

Otu0285 0 109 0 0 0 0 24 0 173 0 0 0 0 0 0 0 0 0 0 0 0 0 0 0 0 0 0 0
0 0 0 46 0 0 0 0 0 0 0 26 0 0 0 0 0 0 0 0 0 0 0 0 0 0 0 0
0 0 0 0 0 0 0 0 0 0 0 5 0 0 0 0 0 0 0 0 0 0 1 0 0 466 0 0 23
0 0 0 0 0 0 0 0 0 56 4 71 0 0 0 0 0 0 0 0 0 0 0 0 43 0 10
M02255_77_000000000-ADUT3_1_1102_21530_19483 T----AC---GT-AG-GGG--------GCA-A-G------C--G--T---T--AT-C-CGG-AT------TT-A--C-T--GG-GT-------GT--A-----AA-GG-GA-GC-------G-CA-G-A-C-G------------G--C-AC-T-G-C-----
------AA----G-T-C-T--------G-A-A--G--TG--A-AA-GC--C-C-GG-G-G-----CT-C-AA------C-C-C-C-G-G-G--A-C----T-G--C-T---T--T-------G--GA-A-A---C-------T--G-T--AG--A-G-C----T-A-G-A-G-T--G---C-TG-----GA-G-A--------G-G-C-A---AG-C------GG--A--ATT----
C-C-T-A-GT--GT-A-G-CG-GT--G---A--A-A---TG-C-GT-AG--AT-A-TT--A-G--G-A-A-G-A-AC-A-CC---AG--T--G--GC-GAA-G--G-C--G-------G--C-T-T-G---CTG--G--AC-A-G--------------------T-A---A-C-T--GA--CG-T-T-C--A-GG--C-T-CG-A--AA-G-C---G-TG--GG-
G--AG-C-A-AA--------CA--GG Bacteria(100);Firmicutes(100);Clostridia(100);Clostridiales(100);Lachnospiraceae(100);unclassified(100);unclassified(100);unclassified(100);unclassified(100);

Otu0286 0 0 0 0 0 42 0 0 0 0 0 0 0 0 0 0 0 0 0 0 0 0 0 0 0 0 0 0
0 0 0 0 0 0 0 0 0 0 0 76 0 0 0 0 0 0 0 0 0 0 0 0 6 0 0 0
0 0 0 0 0 0 0 0 0 0 0 0 0 0 0 0 0 0 0 0 0 18 0 0 0 0 0 0
0 0 0 0 0 0 0 0 0 0 1 0 0 27 0 0 0 0 0 0 0 0 5 0 0 0
M02255_77_000000000-ADUT3_1_1107_14616_14346 T----AC---GT-AT-GGG--------GCA-A-G------C--G--T---T--GT-C-CGG-AA------TT-A--T-T--GG-GC-------GT--A-----AA-GG-GT-AC-------G-TA-G-G-C-G------------G--T-TT-A-A-T------
-----AA----G-T-C-C--------T-A-A--T--TA--A-AA-GG--C-T-AT-G-G-----CT-C-AA------C-C-A-T-A-G-T--A------T-G--G-T---T--A-------G--GA-A-A---C-------T--G-T--TG--A-A-C----T-T-G-A-G-T--A---G-AT-----GA-G-G--------G-G-A-A---AG-T------GG--A--ATT----C-C-
A-T-GT--GT-A-G-CG-GT--G---A--A-A---TG-C-GT-AG--AT-A-TA--T-G--G-A-G-G-A-AC-A-CC---AA--T--G--GC-GAA-G--G-C--A-------A--C-T-T-T---CTG--G--AA-T-C--------------------A-A---A-C-T--GA--CG-C-T-G--A-GG--T-A-CG-A--AG-G-C---G-TG--GG-G--
AG-C-A-AA--------CA--GG Bacteria(100);Firmicutes(100);Clostridia(100);Clostridiales(100);Family_XI_Incertae_Sedis(100);Helcococcus(100);unclassified(100);unclassified(100);unclassified(100);

Otu0287 123 0 0 99 0 0 0 0 0 0 0 0 0 0 0 0 0 0 0 0 0 0 0 0 10 0 0 0
0 0 0 0 0 0 0 0 0 0 0 1 0 0 0 0 0 0 0 0 0 0 0 12 0 0 0 0
0 0 0 0 0 0 0 0 0 0 0 0 0 0 0 0 0 0 22 0 0 0 0 0 0 0 51 32
0 0 0 23 0 0 0 0 0 0 0 0 0 0 0 10 0 0 0 0 0 44 0 0 0 0 18
M02255_77_000000000-ADUT3_1_1106_22050_14795 T----AC---GG-AG-GGG--------GCT-A-G------C--G--T---T--GT-T-CGG-AA------TT-A--C-T--GG-GC-------GT--A-----AA-GC-GT-AC-------G-TA-G-G-C-G------------G--C-TT-T-G-T-----
------AA----G-T-T-A--------G-A-G--G--TG--A-AA-GC--C-C-AG-A-G-----CT-C-AA------C-T-C-T-G-G-A--A-T----T-G--C-C---T--T-------T--AA-G-A---C-------T--G-C--AT--C-G-C----T-T-G-A-A-T--C---G-TG-----GA-G-A--------G-G-T-T---AG-T------GG--A--ATT----C-
C-G-A-GT--GT-A-G-AG-GT--G---A--A-A---TT-C-GT-AG--AT-A-TT--C-G--G-A-A-G-A-AC-A-CC---AG--T--G--GC-GAA-G--G-C--G-------A--C-T-A-A---CTG--G--AC-A-C--------------------G-T---A-T-T--GA--CG-C-T-G--A-GG--T-A-CG-A--AA-G-C---G-TG--GG-G-
-AG-C-A-AA--------CA--GG Bacteria(100);Proteobacteria(100);Alphaproteobacteria(100);Sphingomonadales(100);Sphingomonadaceae(100);Sphingomonas(100);unclassified(100);unclassified(100);unclassified(100);

Otu0288 324 0 0 0 0 0 145 4 0 65 0 0 0 0 0 0 0 0 0 0 0 0 0 0 0 0 0 8
0 1 0 0 162 0 0 0 0 0 17 0 0 0 63 31 1 92 0 54 1 0 0 0 0 0 0 0
0 62 0 0 0 0 0 9 0 1 0 0 0 0 303 381 0 0 0 0 0 0 7 0 97 0 0 9 157
0 68 0 0 0 0 0 8 0 0 0 0 0 38 0 0 0 0 0 94 0 94 0 0 28 0 48
M02255_77_000000000-ADUT3_1_1105_25247_19024 T----AC---GG-AG-GGT--------GCA-A-G------C--G--T---T--AC-T-CGG-AA------TC-A--C-T--GG-GC-------GT--A-----AA-GG-AC-GC-------G-TA-G-G-C-G------------G--A-TT-A-T-C-----
------AA----G-T-C-T--------C-T-T--G--TG--A-AA-TC--T-A-AC-G-G-----CT-T-AA------C-C-G-T-T-A-A--A-C----T-G--C-T---T--G-------G--GA-A-A---C-------T--G-A--TA--A-T-C----T-A-G-A-G-T--A---A-GG-----GA-G-A--------G-G-C-A---GA-T------GG--A--ATT----C-
T-T-G-GT--GT-A-G-GG-GT--A---A--A-A---TC-C-GT-AG--AT-A-TC--A-A--G-A-A-G-A-AT-A-CC---TA--T--T--GC-GAA-G--G-C--G-------A--T-C-T-G---CTG--G--AA-C-T--------------------T-A---A-C-T--GA--CG-C-T-A--A-TG--C-G-TG-A--AA-G-C---G-TG--GG-G--
AG-C-A-AA--------CA--GG Bacteria(100);Proteobacteria(100);Epsilonproteobacteria(100);Campylobacterales(100);Campylobacteraceae(100);Campylobacter(100);unclassified(100);unclassified(100);unclassified(100);

Otu0289 0 0 0 0 0 0 0 0 0 0 0 0 0 0 0 0 0 0 0 0 0 0 0 0 0 0 0 0
0 0 0 0 0 0 0 0 0 0 0 0 0 0 0 5 0 0 0 0 0 0 0 0 0 0 0 0
17 0 0 0 0 0 0 0 0 0 0 12 0 0 0 0 0 0 0 230 100 0 0 0 0 0 0 0 0
0 0 0 0 0 66 0 8 0 37 0 240 0 0 0 0 0 0 0 0 0 0 0 0 0 0
M02255_77_000000000-ADUT3_1_1110_12696_4879 T----AC---GT-AG-GGG--------GCA-A-G------C--G--T---T--AT-C-CGG-AT------TT-A--C-T--GG-GT-------GT--A-----AA-GG-GA-GC-------G-CA-G-A-C-G------------G--C-TA-T-G-C-----------
AA----G-T-C-T--------G-A-A--G--TG--A-AA-GC--C-C-GG-G-G-----CT-C-AA------C-C-C-C-G-G-G--A-C----T-G--C-T---T--T-------G--GA-A-A---C-------T--G-T--GC--A-G-C----T-A-G-A-G-T--G---T-CG-----GA-G-A--------G-G-T-A---AG-C------AG--A--ATT----C-C-C-
A-GT--GT-A-G-CG-GT--G---A--A-A---TG-C-GT-AG--AT-A-TT--G-G--G-A-A-G-A-AT-A-CC---GG--T--G--GC-GAA-G--G-C--G-------G--C-T-T-A---CTG--G--AC-G-A--------------------T-A---A-C-T--GA--CG-T-T-C--A-GG--C-T-CG-A--AG-G-C---G-TG--GG-G--
AG-C-A-AA--------CA--GG Bacteria(100);Firmicutes(100);Clostridia(100);Clostridiales(100);Lachnospiraceae(100);unclassified(100);unclassified(100);unclassified(100);unclassified(100);

Otu0290 0 0 0 0 0 1 0 0 0 0 0 4 0 0 1 0 0 1 0 0 0 0 0 1 0 6 0 1
4 0 0 0 0 0 0 0 0 0 0 0 0 0 0 0 0 0 0 0 0 0 0 0 0 0 0 0
0 0 0 0 0 0 0 0 0 0 0 0 0 0 0 0 0 0 0 0 3 18 0 0 0 0 0 0
9 0 0 0 0 0 1 0 0 0 0 0 0 0 0 0 0 0 0 0 0 0 0 1 0 0 0

M02255_76_000000000-ADUNE_1_1108_13257_9642 T----AA---CT-AC-GT---------GCC-A-G------C--A--G---C--CG-C-GGT-AA------TA-C--G-T--AG-AT-------GG--C-----AA-GC-G--TT-------G-TC-C-G---G------------A--T-TC-T-T-T-----------AA----G-T-C-T--------G-A-T--G--TG--A-AA-GA--C-C-CC-A-G-----CT-C-AA------C-T-G-G-G-G-A--A------G-GT-C-A---T--T-------G--GA-A-A---C-------T--G-G--AG--A-A-C----T-T-G-A-G-T--G---C-AG-----AA-G-A--------G-G-A-G---AG-T------GG--A--ATT----C-C-A-T-GT--GT-A-G-CG-GT--G---A--A-A---TG-C-GT-AG--AT-A-TA--T-G--G-A-G-G-A-AC-A-CC---AG--T--G--GC-GAA-G--G-C--G-------A--C-T-C-T---CTG--G--TC-T-G--------------------T-A---A-C-T--GA--CG-C-T-G--A-GG--C-T-CG-A--AA-G-C---G-TG--GG-G--AG-C-A-AA--------CA--GG Bacteria(100);Firmicutes(100);Bacilli(100);Lactobacillales(100);Carnobacteriaceae(100);Carnobacterium(71);unclassified(71);unclassified(71);unclassified(71);

Otu0291 0 0 0 0 0 0 87 71 201 0 0 0 0 0 0 0 0 0 0 2 16 0 0 0 0 0 0 0
0 3 0 0 0 0 0 0 0 36 2 141 0 0 0 0 0 0 0 0 0 0 0 0 0 0 0 0
0 0 0 0 0 0 0 0 0 0 0 0 0 0 0 4 0 0 0 0 0 0 0 0 0 0 0 0
0 0 0 0 0 0 0 0 0 0 0 0 0 0 0 0 0 0 0 0 0 0 0 0 32 0 0

M02255_77_000000000-ADUT3_1_1104_13440_15137 T----AC---GT-AG-GTG--------GCA-A-G------C--G--T---T--GT-C-CGG-AT------TT-A--C-T--GG-GT-------GT--A-----AA-GG-GC-GT-------G-CA-G-C-C-G------------G--G-TC-T-G-C-----------AA----G-T-C-A--------G-A-T--G--TG--A-AA-TC--C-A-TG-G-G-----CT-C-AA------C-C-C-A-T-G-A--A-C----T-G--C-A---T--T-------T--GA-A-A---C-------T--G-T--AG--A-T-C----T-T-G-A-G-T--G---T-CG-----GA-G-G--------G-G-C-A---AT-C------GG--A--ATT----C-C-T-A-GT--GT-A-G-CG-GT--G---A--A-A---TG-C-GT-AG--AT-A-TT--A-G--G-A-G-G-A-AC-A-CC---AG--T--G--GC-GAA-G--G-C--G-------G--A-T-T-G---CTG--G--AC-G-A--------------------T-A---A-C-T--GA--CG-G-T-G--A-GG--C-G-CG-A--AA-G-T---G-TG--GG-G--AG-C-A-AA--------CA--GG Bacteria(100);Firmicutes(100);Clostridia(100);Clostridiales(100);Ruminococcaceae(100);unclassified(100);unclassified(100);unclassified(100);unclassified(100);

Otu0292 0 0 0 0 0 0 0 0 0 0 0 0 0 0 0 0 0 0 0 0 0 0 0 0 0 0 0 0
0 0 0 0 0 0 0 0 0 0 0 0 0 0 11 0 0 0 0 0 0 0 0 0 0 0 0 0
25 65 7 29 62 24 143 21 10 16 65 27 7 4 0 0 130 0 314 266 92 0 0 0 0 0 0 0
0 0 0 0 0 0 0 0 0 0 0 0 0 0 0 0 0 0 0 0 0 0 0 0 0 0 0

M02255_77_000000000-ADUT3_1_1118_21701_7058 G----AC---GT-AG-GAT--------GCA-A-G------C--G--T---T--GT-C-CGG-AT------TT-A--T-T--GG-GC-------GT--A-----AA-GA-GT-TC-------G-TA-G-G-C-G------------G--T-TT-G-T-T-----------AA----G-T-C-T--------G-A-T--G--TT--A-AA-GA--T-C-GG-G-G-----CT-C-AA------C-C-T-C-G-G-G--A-G----T-G--C-A---T--T-------G--GA-T-A---C-------T--G-G--CA--G-A-C----T-G-G-A-G-T--G---T-GG-----TA-G-A--------G-G-T-T---AG-T------GG--A--ATT----C-C-C-A-GT--GT-A-G-CG-GT--G---A--A-A---TG-C-GT-AG--AT-A-TT--G-G--G-A-A-G-A-AC-A-CC---AG--T--G--GC-GTA-G--G-C--G-------A--C-T-A-A---CTG--G--GC-C-A--------------------T-A---A-C-T--GA--CG-C-T-G--A-GG--A-A-CG-A--AA-G-C---C-AG--GG-G--AG-C-G-AA--------TG--GG Bacteria(100);Cyanobacteria(100);MLE1-12(100);unclassified(100);unclassified(100);unclassified(100);unclassified(100);unclassified(100);unclassified(100);

Otu0293 0 0 0 0 0 0 0 0 0 0 0 0 0 0 0 0 0 0 0 0 0 0 0 0 0 0 0 0
0 0 0 0 162 0 0 0 0 0 0 82 0 0 0 0 70 0 0 0 0 0 0 71 0 0 0 0
0 88 0 0 0 0 0 0 0 0 0 15 0 0 35 0 36 0 109 137 0 0 8 0 0 0 0 0
0 0 0 0 101 0 15 13 0 11 5 60 0 0 0 0 0 0 0 0 0 0 0 0 0 0 0

M02255_77_000000000-ADUT3_1_2103_21101_18648 T----AC---GT-AG-GGG--------GCA-A-G------C--G--T---T--AT-C-CGG-AA------TT-A--T-T--GG-GC-------GT--A-----AA-GC-GT-GC-------G-TA-G-G-T-G------------G--T-AA-T-T-T-----------AA----G-C-G-C--------A-A-G--G--TT--T-AA-AC--T-A-AC-G-G-----CT-C-AA------C-C-G-T-T-A-A--A------T-G--C-C---A--T-------G--CG-A-A---C-------T--G-G--GT--T-A-C----T-T-G-A-G-T--G---C-TG-----GA-G-G--------G-G-A-A---AG-C------GG--A--ATT----C-C-T-A-GT--GT-A-G-CG-GT--G---A--A-A---TG-C-GT-AG--AT-A-TT--A-G--G-A-G-G-A-AC-A-CC---GG--C--G--GC-GAA-G--G-C--G-------G--C-T-T-T---CTG--G--AC-A-G--------------------C-A---A-C-T--GA--CA-C-T-G--A-GG--C-A-CG-A--AA-G-T---G-TG--GG-T--AG-C-A-AA--------CA--GG Bacteria(100);Firmicutes(100);Clostridia(100);Clostridiales(100);Family_XIII_Incertae_Sedis(73);unclassified(73);unclassified(73);unclassified(73);unclassified(73);

Otu0294 0 0 0 0 0 0 0 0 0 0 0 0 0 0 0 0 0 0 0 0 0 5 0 0 0 0 0 0
0 0 4 0 0 0 0 0 0 0 0 0 0 0 51 0 0 0 0 0 0 0 0 27 0 0 0 0
0 0 0 0 0 0 0 0 0 0 0 0 0 0 0 0 8 12 0 0 0 0 0 0 0 0 0 0
0 0 0 0 0 0 0 0 0 0 0 0 0 0 0 0 0 0 0 0 0 0 0 0 0 0 0

M02255_77_000000000-ADUT3_1_2103_23938_11749 T----AC---GT-AG-GGT--------GCG-A-G------C--G--T---T--GT-C-CGG-AA------TT-A--T-T--GG-GC-------GT--A-----AA-GG-GC-TC-------G-TA-G-G-C-G------------G--T-TT-G-T-C-----------GC----G-T-C-G--------G-G-A--G--TG--A-AA-AC--A-T-CC-A-G-----CT-T-AA------C-T-G-G-G-T-G--C-T----T-G--C-T---T--T-------C--GA-T-A---C-------G--G-G--CA--G-A-C----T-T-G-A-G-G--C---A-TG-----CA-G-G--------G-G-A-G---AA-T------GG--A--ATT----C-C-T-G-GT--GT-A-G-CG-GT--G---A--A-A---TG-C-GC-AG--AT-A-TC--A-G--G-A-G-G-A-AC-A-CC---GG--T--G--GC-GAA-G--G-C--G-------G--T-T-C-T---CTG--G--GC-A-T--------------------G-T---C-C-T--GA--CG-C-T-G--A-GG--A-G-CG-A--AA-G-T---G-TG--GG-G--AG-C-G-AA--------CA--GG Bacteria(100);Actinobacteria(100);Actinobacteria(100);Actinobacteridae(100);Actinomycetales(100);Propionibacterineae(100);Nocardioidaceae(100);Nocardioides(96);unclassified(96);

Otu0295 0 0 0 0 0 0 0 0 0 0 0 0 0 0 0 0 0 0 0 0 0 0 0 0 0 0 0 0
0 0 0 0 4 0 0 22 0 0 0 0 6 14 0 0 0 0 0 0 1 0 0 0 0 0 136 5 0
0 0 0 0 0 0 0 0 0 0 0 4 0 0 2 0 0 0 0 0 0 0 0 0 0 0 0 0 0
0 13 0 1 0 0 0 0 0 0 0 4 0 0 0 0 0 0 0 0 0 0 0 0 0 0 0
M02255_76_000000000-ADUNE_1_2115_27985_19871 C----GG---CT-AA-CTC--------CGT-G-C------C--A--G---C--AG-C-CGC-GG------TA-A--T-A--CG-GA-------GG--G-----TG-CA-AG-CG-------T-TA-T-C-C-G------------G--A-TT-T-G-T-----------AA----G-T-C-A--------G-T-G--G--TG--A-AA-TC--T-C-AC-A-G-----CT-T-AA------C-T-G-T-G-A-A--A-C----T-G--C-C---A--T-------T--GA-T-A---C-------T--G-C--AA--G-T-C----T-T-G-A-G-T--G---T-TG-----TT-G-A--------A-G-T-A---GC-T------GG--A--ATA----A-G-T-A-GT--GT-A-G-CG-GT--G---A--A-A---TG-C-AT-AG--AT-A-TT--A-C--T-T-A-G-A-AC-A-CC---AA--T--T--GC-GAA-G--G-C--A-------G--G-T-T-A---CTA--A--GC-A-A--------------------C-A---A-C-T--GA--CG-C-T-G--A-TG--G-A-CG-A--AA-G-C---G-TG--GG-G--AG-C-G-AA--------CA--GG Bacteria(100);Bacteroidetes(100);Flavobacteria(100);Flavobacteriales(100);Flavobacteriaceae(100);Chryseobacterium(100);unclassified(100);unclassified(100);unclassified(100);

Otu0296 0 0 0 0 0 0 0 0 0 0 0 0 0 0 0 0 0 0 3 0 0 0 0 0 0 0 0 0
0 0 0 0 0 0 0 0 0 0 0 0 0 0 0 0 27 0 0 0 0 0 0 0 0 0 0 0 0
0 0 0 0 0 0 0 0 0 0 0 0 0 0 0 0 0 0 0 0 0 0 0 0 0 0 0 0 0
0 0 103 0 0 0 0 0 0 0 0 0 0 0 0 26 0 0 0 0 0 0 0 0 0 0 0
M02255_77_000000000-ADUT3_1_1115_18751_2190 T----AC---GA-AG-GGG--------GCT-A-G------C--G--T---T--GC-T-CGG-AA------TC-A--C-T--GG-GC-------GT--A-----AA-GC-GC-AC-------G-TA-G-G-C-G------------G--G-TT-G-T-T----------AA----G-T-C-A--------G-A-G--G--TG--A-AA-TC--C-T-GG-A-G-----CT-C-AA------C-T-C-C-A-G-A--A-C----T-G--C-C---T--T-------T--GA-T-A---C-------T--G-G--CG--A-C-C----T-T-G-A-G-T--T---C-GA-----GA-G-A--------G-G-T-T---GG-T------GG--A--ACT----G-C-G-A-GT--GT-A-G-AG-GT--G---A--A-A---TT-C-GT-AG--AT-A-TT--C-G--C-A-A-G-A-AC-A-CC---AG--T--G--GC-GAA-G--G-C--G-------G--C-C-A-A---CTG--G--CT-C-G--------------------A-T---A-C-T--GA--CG-C-T-G--A-GG--T-G-CG-A--AA-G-C---G-TG--GG-G--AG-C-A-AA--------CA--GG Bacteria(100);Proteobacteria(100);Alphaproteobacteria(100);Rhizobiales(100);Hyphomicrobiaceae(100);unclassified(100);unclassified(100);unclassified(100);unclassified(100);

Otu0297 0 0 0 0 0 0 244 17 366 0 0 0 0 0 0 0 0 0 0 0 0 0 0 0 0 0 0 0
0 0 0 20 0 0 0 0 0 0 0 43 0 0 0 0 37 0 0 0 0 0 0 58 0 0 0 0 0
0 0 0 0 0 0 1 0 0 0 0 0 0 0 34 2 10 10 0 0 0 2 0 0 0 0 0 0 0
0 0 0 0 0 0 0 0 0 0 0 36 0 0 0 0 0 0 0 0 0 0 1 3 129 0 32
M02255_77_000000000-ADUT3_1_1101_28028_13412 T----AC---GT-AT-GGT--------GCA-A-G------C--G--T---T--AT-C-CGG-AT------TT-A--C-T--GG-GT-------GT--A-----AA-GG-GA-GC-------G-TA-G-A-C-G------------G--C-TG-T-G-T-----------AA----G-T-C-T--------G-A-A--G--TG--A-AA-GC--C-C-GG-G-G-----CT-C-AA------C-C-C-C-G-G-G--A-C----T-G--C-T---T--T-------G--GA-A-A---C-------T--A-T--GC--A-G-C----T-A-G-A-G-T--G---T-CG-----GA-G-A--------G-G-T-A---AG-T------GG--A--ATT----C-C-C-A-GT--GT-A-G-CG-GT--G---A--A-A---TG-C-GT-AG--AT-A-TT--G-G--G-A-G-G-A-AC-A-CC---AG--T--G--GC-GAA-G--G-C--G-------G--C-T-T-A---CTG--G--AC-G-A--------------------T-G---A-C-T--GA--CG-T-T-G--A-GG--C-T-CG-A--AA-G-C---G-TG--GG-G--AG-C-A-AA--------CA--GG Bacteria(100);Firmicutes(100);Clostridia(100);Clostridiales(100);Lachnospiraceae(100);Incertae_Sedis(99);unclassified(99);unclassified(99);unclassified(99);

Otu0298 0 0 0 26 0 0 0 0 0 0 0 0 0 0 0 0 0 0 0 0 0 0 0 0 0 0 0 0
0 0 0 0 0 15 0 0 0 0 0 0 0 0 0 0 22 0 0 0 0 0 0 0 0 0 0 0 0
0 0 0 0 0 0 0 0 0 0 0 0 0 0 0 0 0 22 0 0 0 0 0 0 0 0 0 0 0
0 0 0 0 0 0 0 0 0 0 0 0 0 0 0 0 0 27 0 0 0 0 0 0 0 0 0
M02255_77_000000000-ADUT3_1_1109_6148_14451 T----AC---GT-AG-GGT--------GCG-A-G------C--G--T---T--GT-C-CGG-AA------TT-A--T-T--GG-GC-------GT--A-----AA-GG-GC-TC-------G-TA-G-G-C-G------------G--T-TT-G-T-C-----------GC----G-T-C-G--------G-G-A--G--TG--A-AA-AC--C-C-AG-G-G-----CT-T-AA------C-T-C-T-G-G-G--C-T----T-G--C-T---T--T-------C--GA-T-A---C-------G--G-G--CA--G-A-C----T-A-G-A-G-G--T---A-TG-----CA-G-G--------G-G-A-G---AA-C------GG--A--ATT----C-C-T-G-GT--GT-A-G-CG-GT--G---A--A-A---TG-C-GC-AG--AT-A-TC--A-G--G-A-G-G-A-AC-A-CC---GG--T--G--GC-GAA-G--G-C--G-------G--T-T-C-T---CTG--G--GC-A-T--------------------T-A---C-C-T--GA--CG-C-T-G--A-GG--A-G-CG-A--AA-G-T---G-TG--GG-G--AG-C-G-AA--------CA--GG Bacteria(100);Actinobacteria(100);Actinobacteria(100);Actinobacteridae(100);Actinomycetales(100);Propionibacterineae(100);Nocardioidaceae(100);Nocardioides(100);unclassified(100);

Otu0299 0 0 0 0 0 0 0 0 0 0 0 0 0 1 0 0 0 0 2 0 0 0 1 7 0 0 0 0
2 0 0 0 1 0 0 0 0 0 0 0 0 0 0 0 0 1 0 0 0 0 0 0 0 0 0 0 0
0 0 0 0 0 0 0 0 0 0 0 0 0 0 0 0 0 0 0 0 0 3 0 0 0 0 0 0 0
0 0 0 0 0 0 1 0 0 0 1 0 0 1 0 2 0 0 2 0 0 0 2 12 0 0 0
M02255_77_000000000-ADUT3_1_1101_13167_21767 C----TG---TG-CC-AGC--------AGC-C-G------C--G--G---T--AA-TACAG-AG------GG-T--G-C--AA-GC-------GT--T-----AA-TC-GG-AA-------T-TA-G-G-T-G------------G--C-TC-T-A-T-----------AA----G-T-C-A--------G-A-T--G--TG--A-AA-TC--C-C-CG-G-G-----CT-T-AA------C-C-T-G-G-G-A--A-C----T-G--C-A---T--C-------T--GA-A-A---C-------T--G-T--AG--A-G-C----T-A-G-A-G-T--A---T-GT-----GA-G-A--------G-G-A-A---GG-T------AG--A--ATT----C-C-A-G-GT--GT-A-G-CG-GT--G---A--A-A---TG-C-GT-AG--AG-A-TC--T-G--G-A-G-G-A-AT-A-CC---GA--T--G--GC-GAA-G--G-C--A-------G--C-C-T-T---CTG--G--CA-T-A--------------------A-T---A-C-T--GA--CA-C-T-G--A-GG--C-T-CG-A--AA-G-C---G-TG--GG-T--AG-C-A-AA--------CA--GG Bacteria(100);Proteobacteria(100);Gammaproteobacteria(100);Pseudomonadales(100);Moraxellaceae(100);Psychrobacter(100);unclassified(100);unclassified(100);unclassified(100);

Otu0300 0 0 0 0 0 0 0 0 0 0 0 0 0 0 0 0 0 0 0 0 0 0 0 0 0 0 0 0
3 0 0 0 0 0 0 0 0 0 0 0 0 0 0 0 0 0 0 0 0 0 0 0 0 12 0 0 0
0 0 0 0 0 0 0 0 0 0 0 0 0 0 19 0 0 0 0 0 0 0 0 0 0 0 0 0 0
0 0 0 0 0 0 0 0 0 0 0 0 0 0 0 21 0 0 0 0 0 0 0 0 0 0 0 0
M02255_77_000000000-ADUT3_1_1110_16850_12127 T----AC---GT-AG-GTG--------GCA-A-G------C--G--T---T--GT-C-CGG-AT------TT-A--C-T--GG-GT-------GT--A-----AA-GG-GT-GC-------G-CA-G-G-C-G------------G--A-CC-T-T-T------
-----AA----G-T-A-G--------A-A-A--G--TG--A-AA-GG--T-T-GG-A-G-----CT-C-AA------C-T-C-C-G-A-C--A-C----T-G--C-T---T--C-------C--TA-T-A---C-------T--G-G--GG--G-T-C----T-T-G-A-G-T--T---T-CG-----GA-G-G--------G-G-G-A---AG-C------GG--A--ACG----A-
C-A-C-GT--GT-A-G-CG-GT--G---A--A-A---TG-C-GT-TG--AT-A-TG--T-G--T-C-G-G-A-AC-A-CC---AA--T--G--GC-GAA-A--G-C--A-------G--C-T-T-C---CTG--G--AC-G-A--------------------A-T---A-C-T--GA--CG-C-T-C--A-GG--C-A-CG-A--AA-G-C---C-AA--GG-T--
AG-C-A-AA--------CA--GG Bacteria(100);unclassified(100);unclassified(100);unclassified(100);unclassified(100);unclassified(100);unclassified(100);unclassified(100);unclassified(100);

Otu0301 0 0 0 0 0 0 0 0 0 0 0 0 0 0 0 0 0 0 0 0 0 0 0 0 0 0 0 0
0 0 0 0 0 0 0 73 0 0 0 0 0 0 0 0 0 0 0 0 0 0 0 0 0 0 0 0 0
0 0 0 0 0 0 0 0 0 0 0 0 0 0 0 0 0 0 0 0 0 0 1 0 0 0 0 0 0
0 0 0 0 0 0 0 0 0 0 0 0 0 0 0 0 0 0 0 0 0 0 0 0 0 418 0
M02255_77_000000000-ADUT3_1_1116_9154_19407 C----AC---GT-AG-GGG--------GCA-A-G------C--G--T---T--GT-C-CGG-AT------TT-A--T-T--GG-GC-------GT--A-----AA-GA-GC-TC-------G-TA-G-G-C-G------------G--T-TT-C-G-T-----------AA-
---G-T-C-G--------G-G-T--G--TG--A-AA-AC--T-C-TA-G-G-----CT-C-AA------C-C-T-G-G-A-G--A------C-G-CC-A---C--C-------C--GA-T-A---C-------T--G-C--GA-TG-A-C----T-A-G-A-G-T--C---C-GG-----TA-G-G--------G-G-A-G---TG-T------GG--A--ATT----C-C-T-G-
GT--GT-A-G-CG-GT--G---A--A-A---TG-C-GC-AG--AT-A-TC--A-G--G-A-G-G-A-AC-A-CC---AA--C--G--GC-GAA-G--G-C--A-------G--C-A-C-T---CTG--G--GC-C-G--------------------G-A---A-C-T--GA--CG-C-T-G--A-GG--A-G-CG-A--AA-G-C---G-TG--GG-T--AG-
C-G-AA--------CA--GG Bacteria(100);Actinobacteria(100);Actinobacteria(100);Acidimicrobidae(100);Acidimicrobiales(100);Acidimicrobineae(100);unclassified(86);unclassified(86);unclassified(86);

Otu0302 0 168 120 0 0 8 327 36 432 0 0 0 3 0 0 0 0 0 0 0 0 0 22 0 0 0 0 0
0 0 0 55 0 0 0 0 19 0 0 179 33 0 24 0 0 0 0 0 0 0 3 0 0 0 0 0 0
0 0 0 3 0 0 0 0 0 0 0 0 6 0 0 0 0 13 0 0 0 5 0 0 0 0 0 0 0
0 0 0 0 0 23 7 0 0 29 0 54 5 0 1 0 0 0 0 0 0 0 14 8 184 0 0
M02255_77_000000000-ADUT3_1_1108_10443_14249 T----AC---GT-AG-GGG--------GCA-A-G------C--G--T---T--AT-C-CGG-AT------TT-A--C-T--GG-GT-------GT--A-----AA-GG-GA-GC-------G-TA-G-A-C-G------------G--A-TT-A-G-C-----
------AA----G-T-C-T--------G-A-T--G--TG--A-AA-GG--C-A-GG-G-G-----CT-C-AA------C-C-C-C-T-G-G--A-C----T-G--C-A---T--T-------G--GA-A-A---C-------T--G-C--CA--G-T-C----T-T-G-A-G-T--G---C-CG-----GA-G-A--------G-G-T-A---AG-C------GG--A--ATT----
C-C-T-A-GT--GT-A-G-CG-GT--G---A--A-A---TG-C-GT-AG--AT-A-TT--A-G--G-A-G-G-A-AC-A-CC---AG--T--G--GC-GAA-G--G-C--G-------G--C-T-T-A---CTG--G--AC-G-G--------------------C-A---A-C-T--GA--CG-T-T-G--A-GG--C-T-CG-A--AA-G-C---G-TG--GG-
G--AG-C-A-AA--------CA--GG Bacteria(100);Firmicutes(100);Clostridia(100);Clostridiales(100);Lachnospiraceae(100);Blautia(93);unclassified(93);unclassified(93);unclassified(93);

Otu0303 0 0 0 0 0 0 0 0 0 0 0 0 0 0 0 0 0 0 0 0 12 1 0 0 0 0 0 0
0 0 0 0 0 48 0 0 0 29 2 60 0 0 0 0 0 0 0 0 0 0 0 0 0 0 0 0 0
0 0 0 0 0 0 0 0 0 0 0 0 0 0 0 0 0 20 0 0 0 0 0 0 0 0 0 0 0
0 0 0 0 0 0 0 0 0 0 0 0 0 0 0 0 0 0 0 0 0 0 0 0 0 0 0
M02255_77_000000000-ADUT3_1_1106_16218_10891 T----AC---GA-AG-GGG--------GCT-A-G------C--G--T---T--GT-T-CGG-AA------TC-A--C-T--GG-GC-------GT--A-----AA-GC-GC-AC-------G-TA-G-G-C-G------------G--A-TG-T-G-C----
-------CA----G-T-C-A--------G-G-G--G--TG--A-AA-TC--C-C-GG-A-G-----CT-C-AA------C-T-T-C-G-G-A--A-C----T-G--C-C---T--C-------T--GA-T-A---C-------A--G-C--AC--G-T-C----T-T-G-A-G-T--C---C-GG-----AA-G-A--------G-G-T-G---GG-T------GG--A--ATT----
C-C-T-A-GT--GT-A-G-AG-GT--G---A--A-A---TT-C-GT-AG--AT-A-TT--A-G--G-A-A-G-A-AC-A-CC---GG--T--G--GC-GAA-G--G-C--G-------G--C-C-C-A---CTG--G--TC-C-G--------------------G-T---A-C-T--GA--CG-C-T-G--A-GG--T-G-CG-A--AA-G-C---G-TG--GG-
G--AG-C-A-AA--------CA--GG Bacteria(100);Proteobacteria(100);Alphaproteobacteria(100);Rhizobiales(100);Hyphomicrobiaceae(100);unclassified(62);unclassified(62);unclassified(62);unclassified(62);

Otu0304 0 0 0 30 0 0 0 0 0 0 0 0 0 0 0 0 0 0 0 0 0 0 0 0 0 2 0 4
0 0 0 5 0 0 0 0 0 1 0 0 0 0 0 2 0 0 0 0 0 0 0 8 0 0 5 0 0
0 0 0 0 0 0 0 0 0 0 0 0 0 0 0 0 0 1 0 0 0 0 1 0 0 0 0 0 0
140 22 0 0 0 0 0 0 0 0 0 0 0 0 0 0 0 0 0 6 0 0 4 0 0 0 0
M02255_77_000000000-ADUT3_1_2109_24722_9350 T----AA---AT-AC-GTG--------CC--A-G------C--A--G---C--CG-C-GGT-AA------TA-C--G-T--AT-GT-------CC--C-----GA-GC-GT-TA-------T-CC-G-----G------------A--T-TT-A-T-T-----------AA----
G-T-C-T--------G-A-T--G--TG--A-AA-GC--C-C-GG-A-G-----CT-C-AA------C-T-C-C-G-G-A--A-A----G-G--C-A---T--T-------G--GA-A-A---C-------T--G-G--TT--A-A-C----T-T-G-A-G-T--G---C-AG-----TA-G-A--------G-G-T-A---AG-T------GG--A--ACT----C-C-A-T-GT-
-GT-A-G-CG-GT--G---G--A-A---TG-C-GT-AG--AT-A-TA--T-G--G-A-A-G-A-AC-A-CC---AG--C--G--GC-GAA-G--G-C--G-------G--C-T-T-A---CTG--G--AC-T-G--------------------T-A---A-C-T--GA--CG-T-T-G--A-GG--C-T-CG-A--AA-G-T---G-TG--GG-T--AG-C-A-
AA--------CA--GG Bacteria(100);Firmicutes(100);Bacilli(100);Lactobacillales(100);Leuconostocaceae(100);Leuconostoc(100);unclassified(100);unclassified(100);unclassified(100);

Otu0305 0 0 0 0 0 0 0 0 0 0 0 0 0 0 0 0 0 0 0 0 0 0 0 0 0 0 0 0
0 0 0 0 0 0 0 0 0 0 0 0 0 0 0 0 0 0 0 0 0 0 0 0 0 0 0 0 0
0 0 0 0 0 0 0 0 0 0 0 0 0 0 0 0 20 25 0 0 0 0 0 0 0 0 0 0 0
0 0 0 0 0 0 0 0 0 0 0 0 0 0 0 0 0 0 0 0 0 0 0 0 0 0 11
M02255_76_000000000-ADUNE_1_1102_23645_24647 C----CG---GC-TA-ACT--------TCG-T-G------C--C--A---G--CA-G-CCG-CG------GT-A--A-T--AC-GA-------AG--G-----GT-GC-AA-GC-------G-TT-GTT-C-G------------G--A-TC-A-G-C-----
------AA----G-T-C-A--------G-A-T--G--TG--A-AA-TC--T-C-GA-A-G-----CT-C-AA------C-T-T-C-G-A-A--A-C----T-G--C-G---T--C-------T--GA-A-A---C-------T--G-C--TA--G-T-C----T-A-G-A-A-T--G---T-CG-----GA-G-G--------G-G-G-C---AG-G------GG--A--ATT----T-
C-A-C-GT--GT-A-G-GG-GT--A---A--A-A---TC-C-GT-AG--AG-A-TG--T-G--A-A-G-G-A-AC-A-CC---GG--A--G--GC-GAA-G--G-C--G-------C--C-T-G-C---CTG--G--AC-G-A--------------------C-T---A-T-T--GA--CG-C-T-G--A-GG--C-G-CG-A--AA-G-C---G-TG--GG-
G--AG-C-A-AA--------CA--GG Bacteria(100);Proteobacteria(100);Deltaproteobacteria(100);Bdellovibrionales(100);Bacteriovoraceae(100);Peredibacter(100);unclassified(100);unclassified(100);unclassified(100);

Otu0306 0 2 0 0 0 0 0 0 0 1 0 4 2 0 0 0 0 4 2 0 0 0 0 0 0 0 0 0
0 0 0 0 0 0 0 0 0 0 0 0 0 0 0 0 4 0 0 0 0 0 0 0 0 0 0 0 0
0 0 0 0 0 0 0 0 0 0 0 0 0 0 0 0 0 0 0 0 0 7 0 0 0 0 0 0 0
0 0 0 0 0 2 15 0 0 0 0 0 0 0 0 5 1 0 0 0 0 0 0 0 0 0 0
M02255_77_000000000-ADUT3_1_1115_19704_2920 C----TA---AC-TC-TGT--------GCC-A-G------C-----A---G--CC-G-CGG-TA------AT-A--C-A--GA-GG-------GTG-C-----AA-GC-GT-TA-------A-TC-G-GAATT------------A--C-TT-G-A-T-----------AA-
---G-T-C-A--------G-A-T--G--TG--A-AA-TC--C-C-CG-G-G-----CT-T-AA------C-C-T-G-G-G-A--A-C----T-G--C-A---T--C-------T--GA-A-A---C-------T--G-T--TA--G-G-C----T-A-G-A-G-T--A---G-GT-----GA-G-A--------G-G-G-A---AG-T------AG--A--ATT----T-C-A-G-
GT--GT-A-G-CG-GT--G---A--A-A---TG-C-GT-AG--AG-A-TC--T-G--A-A-G-G-A-AT-A-CC---GA--T--G--GC-GAA-G--G-C--A-------G--C-T-T-C---CTG--G--CA-T-C--------------------A-T---A-C-T--GA--CA-C-T-G--A-GG--C-T-CG-A--AA-G-C---G-TG--GG-T--AG-C-
A-AA--------CA--GG Bacteria(100);Proteobacteria(100);Gammaproteobacteria(100);Pseudomonadales(100);Moraxellaceae(100);Psychrobacter(92);unclassified(92);unclassified(92);unclassified(92);

Otu0307 0 0 0 0 0 0 0 0 0 0 0 0 0 0 0 0 0 0 0 0 0 0 0 0 0 0 0 0
0 0 0 0 0 54 0 0 0 0 0 0 0 0 0 0 0 0 0 0 0 0 0 0 0 0 0 0 0
0 0 0 0 0 0 0 0 0 0 0 0 0 0 0 0 0 0 0 0 0 0 0 0 0 0 0 0 0
0 0 0 0 0 0 0 0 0 0 0 0 0 0 0 0 0 0 45 0 0 0 17 0 0 0 0
M02255_77_000000000-ADUT3_1_1102_7651_10536 G----AC---GA-AG-GGG--------GCT-A-G------C--G--T---T--GT-T-CGG-AA------TT-A--C-T--GG-GC-------GT--A-----AA-GC-GA-GT-------G-TA-G-G-T-G------------G--T-TG-T-C-G----------
TA----G-T-C-A--------G-G-C--G--TG--A-AA-GC--C-T-TG-A-G-----CT-C-AA------C-T-C-A-A-G-A--A-A----T-G--C-G---C--T-------T--GA-T-A---C-------T--G-G--GC--A-G-C----T-A-G-A-G-G--A---C-CG-----GA-G-A--------G-G-A-T---AG-T------GG--A--ATT----C-C-C-
A-GT--GT-A-G-TG-GT--G---A--A-A---TA-C-GT-AG--AG-A-TT--G-G--G-A-A-G-A-AC-A-CC---AG--T--G--GC-GAA-G--G-C--G-------G--C-T-A-T---CTG--G--AC-G-G--------------------T-T---T-C-T--GA--CA-C-T-A--A-GA--C-T-CG-A--AA-G-C---G-TG--GG-G--AG-
C-A-AA--------CA--GG Bacteria(100);Proteobacteria(100);Alphaproteobacteria(100);Rhodospirillales(100);wr0007(100);unclassified(100);unclassified(100);unclassified(100);unclassified(100);

Otu0308 0 57 0 0 0 0 0 0 0 0 0 0 0 0 0 0 0 0 0 0 0 0 0 0 0 0 0 0
0 0 0 0 0 0 0 0 0 0 0 0 0 0 0 0 0 0 0 0 0 0 0 0 0 0 0 0 173
0 0 0 0 0 0 0 0 0 0 0 0 0 0 0 0 0 0 0 0 0 0 0 0 0 0 0 0 0
0 0 0 0 0 0 0 0 0 23 0 0 0 0 0 0 0 0 0 0 0 0 0 0 0 0 0
M02255_77_000000000-ADUT3_1_1105_9047_8011 T----AC---GT-AG-GGG--------GCA-A-G------C--G--T---T--GT-C-CGG-AT------TC-A--T-T--GG-GC-------GT--A-----AA-GA-GC-TC-------G-TA-G-G-C-G------------G--C-TT-G-G-C-----------AA-
---G-T-C-G--------G-G-T--G--TG--A-AA-AC--T-T-CA-G-G-----CT-C-AA------C-C-T-G-G-A-G--C------G-G-CC-A---C--T-------C--GA-T-A---C-------T--G-C--CA-TG-G-C----T-A-G-A-G-T--C---C-GG-----TA-G-G--------G-G-A-C---CA-C------GG--A--ATT----C-C-T-G-
GT--GT-A-G-CG-GT--G---A--A-A---TG-C-GC-AG--AT-A-TC--A-G--G-A-G-G-A-AC-A-CC---GG--T--G--GC-GAA-G--G-C--G-------G--T-G-G-T---CTG--G--GC-C-G--------------------G-A---A-C-T--GA--CG-C-T-G--A-GG--A-G-CG-A--AA-G-C---G-TG--GG-G--AG-
C-G-AA--------CA--GG Bacteria(100);Actinobacteria(100);Actinobacteria(100);Acidimicrobidae(73);Acidimicrobiales(73);Acidimicrobineae(73);unclassified(73);unclassified(73);unclassified(73);

Otu0309 0 0 0 0 0 0 0 0 0 0 0 0 0 0 0 0 0 0 0 0 0 0 16 0 0 0 0 0
0 0 0 0 0 0 0 0 0 0 0 0 0 0 61 0 0 0 0 0 0 0 0 0 19 0 0 0 0
0 0 0 0 0 0 0 0 0 0 0 0 0 0 0 0 0 43 0 0 0 0 0 0 0 0 0 0 0
0 0 0 0 0 0 0 0 0 0 0 0 0 0 0 0 0 0 0 0 0 0 0 0 0 0 0
M02255_77_000000000-ADUT3_1_1104_8050_6830 T----AC---GA-AG-GGG--------GCT-A-G------C--G--T---T--GC-T-CGG-AA------TC-A--C-T--GG-GC-------GT--A-----AA-GG-GC-GC-------G-TA-G-G-C-G------------G--C-CA-T-T-C----------
AA----G-T-C-G--------G-A-G--G--TG--A-AA-GC--C-C-AG-G-G-----CT-C-AA------C-C-C-T-G-G-A--A-T----T-G--C-C---T--T-------C--GA-T-A---C-------T--G-T--TT--G-G-C----T-A-G-A-G-A--C---C-GG-----AA-G-A--------G-G-T-A---AG-T------GG--A--ACT----G-C-
G-A-GT--GT-A-G-AG-GT--G---A--A-A---TT-C-GT-AG--AT-A-TT--C-G--C-A-A-G-A-AC-A-CC---AG--T--G--GC-GAA-G--G-C--G-------G--C-T-T-A---CTG--G--TC-C-G--------------------G-T---T-C-T--GA--CG-C-T-G--A-GG--C-G-CG-A--AA-G-C---G-TG--GG-G--
AG-C-A-AA--------CA--GG Bacteria(100);Proteobacteria(100);Alphaproteobacteria(100);unclassified(89);unclassified(89);unclassified(89);unclassified(89);unclassified(89);unclassified(89);

Otu0310 0 0 0 0 0 0 0 0 0 0 0 0 0 0 0 0 0 0 0 0 0 0 0 0 1 0 0 8
0 0 0 0 0 0 0 0 0 0 0 0 0 0 0 0 0 0 0 0 0 0 0 0 23 0 0 0 0
0 0 0 0 0 0 0 0 0 0 0 0 0 0 0 0 0 0 0 0 0 0 6 0 0 0 0 0 0
0 0 0 0 0 30 0 0 0 0 0 0 0 0 0 0 0 0 0 0 0 0 0 0 0 0 0
M02255_77_000000000-ADUT3_1_1104_9764_15253 T----AC---GA-AG-GGG--------GCT-A-G------C--G--T---T--GT-T-CGG-AT------TT-A--C-T--GG-GC-------GT--A-----AA-GC-GC-AC-------G-TA-G-G-C-G------------G--A-TT-G-G-T-----------CA-
---G-T-T-A--------G-A-G--G--TG--A-AA-TC--C-C-GA-A-G-----CT-C-AA------C-T-T-C-G-G-A--A-C----T-G--C-C---T--T-------T--AA-T-A---C-------T--G-C--CA--A-T-C----T-A-G-A-G-T--C---C-GG-----AA-G-A--------G-G-T-G---AG-T------GG--A--ACT----C-C-T-A-
GT--GT-A-G-AG-GT--G---G--A-A---TT-C-GT-AG--AT-A-TT--A-G--G-A-A-G-A-AC-A-CC---AG--T--G--GC-GAA-G--G-C--G-------G--C-T-C-A---CTG--G--TC-C-G--------------------G-T---A-C-T--GA--CG-C-T-G--A-GG--T-G-CG-A--AA-G-C---G-TG--GG-G--AG-
C-A-AA--------CA--GG Bacteria(100);Proteobacteria(100);Alphaproteobacteria(100);Rhizobiales(100);unclassified(100);unclassified(100);unclassified(100);unclassified(100);unclassified(100);

Otu0311 0 1 0 0 0 1 0 0 0 0 0 0 1 0 0 0 0 0 0 0 2 0 0 0 0 0 0 0
0 0 0 2 0 0 1 0 1 0 0 0 0 0 1 1 0 0 0 0 0 0 0 0 1 0 0 1 0
0 0 0 0 0 0 0 0 0 0 0 0 0 0 0 0 1 0 1 0 0 0 0 1 0 0 0 0 6
0 0 0 0 5 0 0 0 0 0 0 0 0 0 0 0 0 0 0 0 4 0 0 0 0 0 4
M02255_76_000000000-ADUNE_1_1115_19734_3075 .....CG---GT-AT-CTT--------ACC-A-G------A--A--A---G--GG-A-CGG--C------TA-A--C-T--AC-GT-------G---C-----CA-GC-AG-CC-------G-CG-G-T-A-A------------T--A-CG-T-A-G-----------GT----
C-C-C-G--------A-G-C--G--TT--ATCC-GG--A-T-GT-G-G-----CT-T-AA------C-C-A-T-A-G-T--A------C-G--C-T---T--T-------G--GA-A-A---C-------T--G-T--TT--A-A-C----T-T-G-A-G-T--G---C-AA-----GA-G-G--------G-G-A-G---AG-T------GG--A--ATT----C-C-A-T-GT--
GT-A-G-CG-GT--G---A--A-A---TG-C-GT-AG--AT-A-TA--T-G--G-A-G-G-A-AC-A-CC---GG--T--G--GC-GAA-A--G-C--G-------G--C-T-C-T---CTG--G--CT-T-G--------------------T-A---A-C-T--GA--CG-C-T-G--A-GG--C-T-CG-A--AA-G-C---G-TG--GG-G--AG-C-A-
AA--------CA--GG Bacteria(100);unclassified(100);unclassified(100);unclassified(100);unclassified(100);unclassified(100);unclassified(100);unclassified(100);unclassified(100);

Otu0312 0 89 0 0 0 0 64 89 0 0 0 0 0 0 0 1 0 0 0 0 0 0 0 0 0 0 3 1
0 0 0 0 0 0 0 0 0 0 0 30 0 0 0 0 0 0 0 0 0 0 0 0 0 0 0 0 0
0 0 0 0 0 0 0 0 0 0 0 0 0 0 0 0 0 0 0 0 0 0 0 0 16 0 0 0 0
0 0 0 0 0 0 0 0 0 0 0 0 0 0 0 0 0 0 0 0 0 0 0 3 22 0 0
M02255_77_000000000-ADUT3_1_1108_7010_16507 T----AC---GT-AG-GGG--------GCA-A-G------C--G--T---T--AT-C-CGG-AT------TT-A--C-T--GG-GT-------GT--A-----AA-GG-GA-GC-------G-TA-G-A-C-G------------G--C-GA-G-G-C-----------
AA----G-T-C-T--------G-A-T--G--TG--A-AA-AC--C-C-GG-G-G-----CT-C--A------A-C-C-CCG-T-G--A-C----T-G--C-A---T--T-------G--GA-A-A---C-------T--G-T--TT--T-G-C----T-T-G-A-G-T--G---C-CG-----GA-G-A----------GGT-A---AG-C------GG--A--ATT----C-C-T-
A-GT--GT-A-G-CG-GT--G---A--A-A---TG-C-GT-AG--AT-A-TT--A-G--G-A-G-G-A-AC-A-CC---AG--T--G--GC-GAA-G--G-C--G-------G--C-T-T-A---CTG--G--AC-G-G--------------------C-A---A-C-T--GA--CG-T-T-G--A-GG--C-T-CG-A--AA-G-C---G-TG--GG-G--
AG-C-A-AA--------CA--GG Bacteria(100);Firmicutes(100);Clostridia(100);Clostridiales(100);Lachnospiraceae(100);Incertae_Sedis(90);unclassified(90);unclassified(90);unclassified(90);

Otu0313 0 0 0 0 0 0 0 0 0 0 0 0 0 0 0 0 0 0 0 0 0 0 0 0 1 0 0 0
0 28 0 10 0 0 0 0 0 0 4 0 1 0 0 76 30 28 0 0 0 0 0 0 0 0 0 0 0
0 0 0 0 0 0 0 0 0 0 0 0 0 0 0 0 0 0 0 0 0 0 0 0 0 0 0 0 0
18 0 0 0 0 0 0 0 0 0 0 0 0 0 0 0 0 0 0 0 0 0 0 0 0 0 0
M02255_77_000000000-ADUT3_1_1113_26462_12353 T----AC---GT-AG-GGT--------GCG-A-G------C--G--T---T--GT-C-CGG-AA------TT-A--C-T--GG-GC-------GT--A-----AA-GA-GC-TC-------G-TA-G-G-C-G------------G--T-TT-G-T-C------
-----GC----G-T-C-G--------T-C-T--G--TG--A-AA-TC--C-T-GC-A-A-----CT-C-AA------T-T-G-C-A-G-G--C-G----T-G--C-A---G--G-------C--GA-T-A---C-------G--G-G--CA--G-A-C----T-T-G-A-G-T--A---C-TA-----CA-G-G--------G-G-A-G---AC-T------GG--A--ATT----C-
C-T-G-GT--GT-A-G-CG-GT--G---A--A-A---TG-C-GC-AG--AT-A-TC--A-G--G-A-G-G-A-AC-A-CC---GG--T--G--GC-GAA-G--G-C--G-------G--G-T-C-T---CTG--G--GT-A-G--------------------T-A---A-C-T--GA--CG-C-T-G--A-GG--A-G-CG-A--AA-G-C---G-TG--GG-
G--AG-C-G-AA--------CA--GG Bacteria(100);Actinobacteria(100);Actinobacteria(100);Actinobacteridae(100);Actinomycetales(100);Corynebacterineae(100);Nocardiaceae(91);Gordonia(91);unclassified(91);

Otu0314 0 0 0 0 0 0 0 0 0 29 0 0 0 0 0 0 0 0 0 0 0 0 0 0 0 0 0 0
0 0 0 0 0 0 0 0 0 0 0 0 0 9 0 0 19 0 0 39 0 0 0 0 0 0 0 0 0
0 0 0 0 0 0 0 0 0 0 0 0 0 0 0 0 0 0 0 0 0 0 0 0 0 0 0 0 0
0 0 0 0 0 0 0 0 0 0 0 0 0 0 0 0 0 0 0 0 0 0 0 0 6 0 0
M02255_77_000000000-ADUT3_1_1111_12857_16709 G----AC---GG-GG-GGG--------GCA-A-G------T--G--T---T--CT-T-CGG-AA------TG-A--C-T--GG-GC-------GT--A-----AA-GG-GC-AC-------G-TA-G-G-C-G------------G--T-GA-A-T-C---
--------GG----G-T-T-G--------A-A-A--G--TG--A-AA-G-----T-CG-C-C-----AA-A-AA------G-T-G-G-C-G-G--A-A----T-G--C-T---C--T-------C--GA-A-A---C-------C--A-A--TT--C-A-C----T-T-G-A-G-T--G---A-GA-----CA-G-A--------G-G-A-G---AG-T------GG--A--ATT----
T-C-G-T-GT--GT-A-G-GG-GT--G---A--A-A---TC-C-GT-AA--AT-A-TA--C-G--A-A-G-G-A-AC-G-CC---AA--A--A--GC-GAA-G--G-C--A-------G--C-T-C-T---CTG--G--GT-C-C--------------------C-T---A-C-C--GA--CG-C-T-G--G-GG--T-G-CG-A--AA-G-C---A-TG--GG-
G--AG-C-G-AA--------CA--GG Bacteria(100);unclassified(100);unclassified(100);unclassified(100);unclassified(100);unclassified(100);unclassified(100);unclassified(100);unclassified(100);

Otu0315 0 0 0 0 0 0 27 0 94 0 0 0 0 0 0 0 0 0 0 0 0 0 0 0 0 0 0 0
0 0 0 0 0 0 0 0 0 0 0 0 0 0 0 0 0 0 0 0 0 0 0 0 0 0 0 0 0
0 0 0 0 0 0 0 0 0 0 0 0 0 0 0 0 0 0 0 0 0 0 0 0 0 0 0 0 0
0 0 0 0 0 0 0 0 0 0 0 0 0 0 0 0 0 0 0 0 0 0 0 0 28 0 9
M02255_77_000000000-ADUT3_1_2107_16119_16504 T----AC---GT-AG-GGG--------GCA-A-G------C--G--T---T--AT-C-CGG-AT------TT-A--C-T--GG-GT-------GT--A-----AA-GG-GA-GC-------G-TA-G-A-C-G------------G--C-AC-A-G-C----
-------AA----G-T-C-T--------G-A-A--G--TG--A-AA-TC--C-C-CG-G-G-----CT-C-AA------C-C-C-G-G-G-A--A-C----T-G--C-T---T--T-------G--GA-A-A---C-------T--G-T--TG--G-G-C----T-G-G-A-G-T--G---C-TG-----GA-G-A--------G-G-C-A---AG-C------GG--A--ATT----
C-C-T-A-GT--GT-A-G-CG-GT--G---A--A-A---TG-C-GT-AG--AT-A-TT--A-G--G-A-G-G-A-AC-A-CC---AG--T--G--GC-GAA-G--G-C--G-------G--C-T-T-G---CTG--G--AC-A-G--------------------T-A---A-C-T--GA--CG-T-T-G--A-GG--C-T-CG-A--AA-G-C---G-TG--GG-
G--AG-C-A-AA--------CA--GG Bacteria(100);Firmicutes(100);Clostridia(100);Clostridiales(100);Lachnospiraceae(66);unclassified(66);unclassified(66);unclassified(66);unclassified(66);

Otu0316 0 0 0 0 0 0 0 0 0 0 0 0 0 0 0 4 0 0 0 1 0 6 0 0 0 0 1 0
0 0 0 0 0 0 0 0 0 0 0 0 0 0 0 0 0 0 0 0 0 0 0 0 0 0 0 0 0
0 0 0 0 0 0 0 0 0 0 4 0 12 4 0 0 0 1 0 0 0 0 0 0 0 0 0 0 0
0 0 0 0 0 0 0 0 0 0 3 0 1 0 0 0 0 0 0 0 0 0 0 0 0 0 0
M02255_76_000000000-ADUNE_1_1105_5786_19038 C----TA---AC-TC-CGT--------GCC-A-G------C--A--G---C--CG-C-GGT-AA------TA-C--G-G--AG-GA-------TC--C-----AA-GC-GT--T-------A-TC-C-G-G-A------------A--T-CA-G-A-T----------AA---
-G-T-C-A--------G-T-G--G--TG--A-AA-GC--C-C-AT-C-G-----CT-C-AA------C-G-G-T-G-G-A--A-C----G-G--C-C---A--T-------T--GA-T-A---C-------T--G-T--CT--G-A-C----T-T-G-A-A-T--T---A-TT-----AG-G-A--------A-G-T-A---AC-T------AG--A--ATA----T-G-T-A-GT--
GT-A-G-CG-GT--G---A--A-A---TG-C-TT-AG--AG-A-TT--A-C--A-T-G-G-A-AT-A-CC---AA--T--T--GC-GAA-G--G-C--A-------G--G-T-T-A---CTA--C--TA-A-T--------------------A-T---A-T-T--GA--CG-C-T-G--A-TG--G-A-CG-A--AA-G-C---G-TG--GG-T--AG-C-G-AA-
-------CA--GG Bacteria(100);Bacteroidetes(100);Flavobacteria(100);Flavobacteriales(100);Flavobacteriaceae(100);Flavobacterium(100);unclassified(100);unclassified(100);unclassified(100);

Otu0317 0 199 0 0 0 0 0 0 0 0 0 0 0 0 0 0 0 0 0 0 0 0 0 0 0 0 0 0
0 0 0 0 0 0 0 0 0 0 0 26 0 0 0 0 0 0 0 0 0 0 0 0 0 0 0 0 0
0 0 0 0 0 0 0 0 0 0 0 0 0 0 0 0 0 18 0 0 0 0 0 0 61 0 0 0 0
0 0 0 0 0 0 0 0 0 0 0 0 0 0 0 0 0 0 0 0 0 0 0 0 70 0 0
M02255_77_000000000-ADUT3_1_1110_17804_19278 T----AC---GT-AT-GGT--------GCA-A-G------C--G--T---T--AT-C-CGG-AT------TT-A--C-T--GG-GT-------GT--A-----AA-GG-GA-GC-------G-CA-G-G-C-G------------G--A-AG-G-C-T-----
------AA----G-T-C-T--------G-A-T--G--TG--A-AA-GC--C-C-GG-G-G-----CT-C-AA------C-C-C-C-G-G-T--A-C----T-G--C-A---T--T-------G--GA-A-A---C-------T--G-G--TC--A-T-C----T-A-G-A-G-T--G---T-CG-----GA-G-G--------G-G-T-A---AG-T------GG--A--ATT----C-
C-T-A-GT--GT-A-G-CG-GT--G---A--A-A---TG-C-GT-AG--AT-A-TT--A-G--G-A-G-G-A-AC-A-CC---AG--T--G--GC-GAA-G--G-C--G-------G--C-T-T-A---CTG--G--AC-G-A--------------------T-A---A-C-T--GA--CG-C-T-G--A-GG--C-T-CG-A--AA-G-C---G-TG--GG-
G--AG-C-A-AA--------CA--GG Bacteria(100);Firmicutes(100);Clostridia(100);Clostridiales(100);Lachnospiraceae(100);Roseburia(100);unclassified(100);unclassified(100);unclassified(100);

Otu0318 0 0 0 92 0 114 24 107 0 0 0 0 0 0 0 0 0 0 0 0 0 0 0 0 0 0 0 5
0 0 0 0 0 0 0 0 0 0 0 0 0 0 119 0 0 0 0 0 0 7 0 0 0 0 0 0 0
0 0 0 0 0 0 0 0 0 1 0 0 1 0 0 3 0 0 0 0 0 15 0 0 0 0 0 14 0
0 0 0 14 0 0 0 0 0 10 0 0 1 0 0 0 0 29 0 55 0 0 16 0 0 0 187
M02255_76_000000000-ADUNE_1_1101_23285_18090 T----AC---GT-AG-GGG--------GCA-A-G------C--G--T---T--AT-C-CGG-AT------TT-A--C-T--GG-GT-------GT--A-----AA-GG-GA-GC-------G-C----A-------------------------G-------------
A----G-T-C-T--------G-A-A--G--TG--A-AA-TC--C-C-GA-G-G-----CT-T-AA------C-C-A-C-G-G-G--A-C----T-G--C-T---T--T-------G--GA-A-A---C-------T--G-T--GC--G-A-C----T-T-G-A-G-T--A---T-CG-----GA-G-G--------G-G-C-A---GG-C------GG--A--ATT----C-C-T-
A-GT--GT-A-G-CG-GT--G---A--A-A---TG-C-GT-AG--AT-A-TT--A-G--G-A-A-G-A-AC-A-CC---GG--T--G--GC-GAA-G--G-C--G-------G--C-C-T-G---CTG--G--AC-G-A--------------------A-A---A-C-T--GA--CG-T-T-G--A-GG--C-T-CG-A--AG-G-C---G-TG--GG-G--
AG-C-A-AA--------CA--GG Bacteria(100);Firmicutes(100);Clostridia(100);Clostridiales(100);Lachnospiraceae(100);Moryella(100);unclassified(100);unclassified(100);unclassified(100);

Otu0319 0 0 0 0 0 0 0 0 0 0 0 0 0 0 0 0 0 0 0 0 0 0 0 0 0 0 0 8
0 0 0 0 0 0 0 0 0 0 0 0 0 24 0 32 0 0 0 0 0 0 0 0 18 0 0 0 0
0 0 0 0 0 0 0 0 0 0 0 0 0 0 0 0 0 0 0 1 0 0 3 0 0 0 0 235 1
0 17 0 0 0 0 3 0 0 0 0 0 0 0 0 47 0 0 0 0 0 0 0 0 0 0 0
M02255_77_000000000-ADUT3_1_1101_25329_3851 T----AC---GA-AG-GGG--------GCT-A-G------C--G--T---T--GC-T-CGG-AA------TT-A--C-T--GG-GC-------GT--A-----AA-GG-GA-GC-------G-TA-G-G-C-G------------G--A-TA-G-TTT-----------
A----G-T-C-A--------G-A-G--G--TG--A-AA-GC--C-C-AG-G-G-----CT-C-AA------C-C-T-T-G-G-A--A-T----T-G--C-C---T--T-------T--GA-T-A---C-------T--G-G--CT--A-T-C----T-T-G-A-G-T--A---T-GG-----AA-G-A--------G-G-T-A---TG-T------GG--A--ACT----C-C-G-A-
GT--GT-A-G-AG-GT--G---A--A-A---TT-C-GT-AG--AT-A-TT--C-G--G-A-A-G-A-AC-A-CC---AG--T--G--GC-GAA-G--G-C--G-------A--C-A-T-A---CTG--G--TC-C-A--------------------T-T---A-C-T--GA--CG-C-T-G--A-GG--C-T-CG-A--AA-G-C---G-TG--GG-G--AG-C-
A-AA--------CA--GG Bacteria(100);Proteobacteria(100);Alphaproteobacteria(100);Caulobacterales(100);Caulobacteraceae(100);unclassified(84);unclassified(84);unclassified(84);unclassified(84);

Otu0320 0 0 0 0 0 0 0 0 0 0 0 0 0 0 0 0 0 0 0 0 0 0 0 0 0 0 0 0
0 0 0 0 0 0 0 0 0 0 0 0 0 15 0 0 0 0 0 0 0 0 0 0 0 0 0 0 0
0 0 0 0 0 0 0 0 7 0 0 0 0 0 0 0 0 0 0 0 0 0 0 0 0 0 0 0 0
0 0 0 26 0 0 0 0 0 0 0 0 0 0 0 0 0 0 0 0 0 0 0 0 0 0 0
M02255_77_000000000-ADUT3_1_1107_22063_14190 T----AC---GA-GG-GGG--------GCA-A-G------C--G--T---T--GT-T-CGG-AA------TT-A--T-T--GG-GC-------GT--A-----AA-GG-GT-GC-------G-TA-G-G-C-G------------G--T-TT-G-G-C----
-------AA----G-T-C-T--------T-A-T--G--TG--A-AA-TC--T-T-CG-G-G-----CT-C-AA------C-C-C-G-A-A-G--T-C----T-G--C-A---T--G-------A--GA-A-A---C-------T--G-C--CG--G-G-C----T-T-G-A-G-T--A---T-GG-----GA-G-A--------G-G-T-G---AG-T------GG--A--ATT----
T-C-C-G-GT--GT-A-G-CG-GT--G---A--A-A---TG-C-GT-AG--AT-A-TC--G-G--A-A-G-G-A-AC-A-CC---TG--T--G--GC-GAA-A--G-C--G-------G--C-T-C-A---CTG--G--AC-C-A--------------------T-A---A-C-T--GA--CG-C-T-G--A-GG--C-A-CG-A--AA-G-C---T-AG--GG-
G--AG-C-A-AA--------CA--GG Bacteria(100);Acidobacteria(100);Acidobacteria(100);Acidobacteriales(100);Acidobacteriaceae(100);unclassified(69);unclassified(69);unclassified(69);unclassified(69);

Otu0321 0 0 0 0 0 0 0 0 0 0 0 0 0 8 0 0 0 0 0 0 0 0 0 0 0 0 0 2
0 0 0 0 0 0 0 1 0 0 0 20 0 0 0 0 0 0 0 0 0 0 0 0 0 0 7 0 0
0 0 0 0 0 0 0 0 0 0 0 0 0 0 0 0 0 0 0 0 0 0 0 0 0 0 0 0 0
0 0 0 0 0 0 0 0 0 0 0 0 0 0 0 0 0 0 0 0 0 0 0 0 0 0 0
M02255_77_000000000-ADUT3_1_1107_18324_15189 T----AC---GA-AG-GGG--------GCT-A-G------C--G--T---T--GT-T-CGG-AA------TC-A--C-T--GG-GC-------GT--A-----AA-GC-GC-AC-------G-TA-G-G-C-G------------G--A-TT-G-T-T-----
------AA----G-T-C-G--------G-G-G--G--TG--A-AA-TC--C-T-GA-G-G-----CT-C-AA------C-C-T-C-A-G-A--A-C----T-G--C-C---T--T-------C--GA-T-A---C-------T--G-G--CG--A-T-C----T-T-G-A-G-T--C---C-GG-----AA-G-A--------G-G-T-T---GG-T------GG--A--ACA----G-
C-T-A-GT--GT-A-G-AG-GT--G---A--A-A---TT-C-GT-AG--AT-A-TT--A-G--C-T-A-G-A-AC-A-CC---AG--T--G--GC-GAA-G--G-C--G-------G--C-C-A-A---CTG--G--TC-C-G--------------------G-T---A-C-T--GA--CG-C-T-G--A-GG--T-G-CG-A--AA-G-C---G-TG--GG-G-
-AG-C-A-AA--------CA--GG Bacteria(100);Proteobacteria(100);Alphaproteobacteria(100);Rhizobiales(100);Xanthobacteraceae(100);Labrys(100);unclassified(100);unclassified(100);unclassified(100);

Otu0322 0 0 0 0 0 0 0 0 0 0 0 0 0 0 0 0 0 0 0 0 0 0 0 0 0 0 0 0
0 0 0 0 0 0 0 0 0 0 0 0 0 0 0 0 0 0 0 0 0 0 0 0 0 0 0 0 0
0 0 0 0 0 0 0 0 0 0 0 0 0 0 0 0 38 0 0 0 0 0 0 0 0 0 686 0 0
0 0 0 0 0 0 0 0 0 0 0 0 0 0 0 28 0 0 0 0 0 0 0 0 0 0 29
M02255_77_000000000-ADUT3_1_1106_11798_14333 T----AC---GA-AG-GGG--------GCT-A-G------C--G--T---T--GC-T-CGG-AA------TC-A--C-T--GG-GC-------GT--A-----AA-GG-GC-GC-------G-TA-G-G-C-G------------G--C-GC-C-G-C----
-------AA----G-T-C-G--------G-G-C--G--TG--A-AA-TC--C-C-CG-G-G-----CT-C-AA------C-C-C-G-G-G-G--G-C----T-G--C-G---C--C-------C--GA-G-A---C-------T--G-C--GG--T-G-C----T-C-G-A-G-G--G---C-GG-----GA-G-A--------G-G-C-T---CG-C------GG--A--ATT---
-C-C-C-A-GT--GT-A-G-AG-GT--G---A--A-A---TT-C-GT-AG--AT-A-TT--G-G--G-A-A-G-A-AC-A-CC---GG--T--G--GC-GAA-G--G-C--G-------G--C-G-A-G---CTG--G--CC-C-G--------------------T-T---T-C-T--GA--CG-C-T-G--A-GG--C-G-CG-A--CA-G-C---G-TG--
GG-G--AG-C-A-AA--------CA--GG Bacteria(100);Proteobacteria(100);Alphaproteobacteria(100);Rhodospirillales(100);unclassified(100);unclassified(100);unclassified(100);unclassified(100);unclassified(100);

Otu0323 0 0 0 0 0 0 0 0 0 0 0 0 0 0 0 0 0 0 0 0 0 0 0 0 0 0 0 0
0 0 0 0 0 0 0 0 0 0 0 0 0 0 0 0 0 0 0 0 0 0 0 0 0 0 0 0 0
0 0 0 0 0 0 0 0 0 0 0 0 0 0 24 0 0 0 0 0 0 0 0 0 0 0 0 0 0
0 0 0 0 0 0 0 0 0 0 21 0 0 28 0 0 0 0 0 0 0 0 0 0 0 0 0
M02255_77_000000000-ADUT3_1_1108_28435_13655 A----AC---GT-AG-GTC--------ACG-A-G------C--G--T---T--GT-C-CGG-AA------TT-A--C-T--GG-GT-------GT--A-----AA-GG-GA-GC-------G-CA-G-G-C-G------------G--G-TA-T-G-C----
-------AA----G-T-T-G--------G-G-A--G--TG--A-AA-TA--C-A-TG-G-G-----CT-C-AA------C-C-C-A-T-G-A--A-C----T-G--C-T---C--T-------C--AA-A-A---C-------T--G-T--GT--A-T-C----T-T-G-A-G-T--A---G-TG-----CA-G-A--------G-G-T-A---GG-C------GG--A--ATT----C-
C-C-G-GT--GT-A-G-CG-GT--G---G--A-A---TG-C-GT-AG--AT-A-TC--G-G--G-A-G-G-A-AC-A-CC---AG--T--G--GC-GAA-G--G-C--G-------G--C-C-T-A---CTG--G--GC-A-C--------------------C-A---A-C-T--GA--CG-C-T-G--A-GG--C-T-CG-A--AA-G-T---G-TG--GG-
T--AG-C-A-AA--------CA--GG Bacteria(100);Firmicutes(100);Clostridia(100);Clostridiales(100);Ruminococcaceae(100);Faecalibacterium(100);unclassified(100);unclassified(100);unclassified(100);

Otu0324 0 0 0 0 0 0 0 0 0 0 0 0 0 0 0 0 0 0 0 0 0 0 0 0 0 0 0 0
0 0 0 0 0 0 0 338 168 0 0 0 0 0 0 0 1 0 0 0 1 0 0 0 0 0 0 0 0
0 0 0 0 0 0 0 0 0 0 0 0 0 0 0 0 8 0 0 0 0 0 0 0 0 0 0 0 0
0 0 0 0 0 0 0 0 0 0 0 0 0 0 0 0 0 0 0 0 0 0 0 0 0 0 0
M02255_76_000000000-ADUNE_1_1105_11508_8805 T----AC---GT-AG-GGT--------GCG-A-G------C--G--T---T--AT-C-CGG-AA------TC-A--T-T--GG-GC-------GT--A-----AA-GA-GC-TC-------G-TA-------G------------G--T-TC-A-G-C----------AA----
G-T-C-G--------G-C-T--G--TG--A-AA-GT--C-C-GA-G-G-----CT-C-AA------C-C-T-C-G-G-A--A------T-T-GC-A---G--T-------C--GA-T-A---C-------T--G-C--TG-TG-A-C----T-C-G-A-G-T--C---C-GG-----TA-G-A--------G-G-A-G---AA-T------GG--A--ATT----C-T-C-G-GT-
-GT-A-G-CG-GT--G---A--A-A---TG-C-GC-AG--AT-A-TC--G-A--G-A-G-G-A-AC-A-CC---AG--T--A--GC-GAA-G--G-C--G-------G--T-T-C-T---CTG--G--GC-C-G--------------------G-C---A-C-T--GA--CG-C-T-G--A-GG--A-G-CG-A--AA-G-C---G-TG--GG-G--AG-C-A-
AA--------CA--GG Bacteria(100);unclassified(100);unclassified(100);unclassified(100);unclassified(100);unclassified(100);unclassified(100);unclassified(100);unclassified(100);

Otu0325 0 0 0 0 0 0 0 0 0 0 0 0 0 0 0 0 0 0 0 3 4 2 0 0 0 0 0 3
0 0 1 0 0 39 0 0 0 62 2 0 0 0 0 0 0 0 0 0 0 0 0 0 0 0 0 0
0 0 0 0 0 0 0 0 0 0 0 0 0 0 0 0 0 0 0 0 0 0 0 0 0 0 0 0
0 0 0 0 0 0 0 0 0 0 0 0 0 0 0 0 0 0 0 0 0 0 0 0 0 0 0

M02255_77_000000000-ADUT3_1_2104_6870_17085 T----AC---GG-AG-GGG--------GCT-A-G------C--G--T---T--GT-T-CGG-AA------TT-A--C-T--GG-GC-------GT--A-----AA-GC-GC-AC-------G-TA-G-G-C-G------------G--A-CC-A-T-C----------
AA----G-T-T-G--------G-G-G--G--TG--A-AA-TC--C-C-GG-G-G-----CT-C-AA------C-C-C-C-G-G-A--A-C----T-G--C-C---T--C-------C--AA-A-A---C-------T--G-C--TG--G-T-C----T-G-G-A-G-G--T---C-GA-----GA-G-A--------G-G-T-G---AG-T------GG--A--ATT----C-C-
G-A-GT--GT-A-G-AG-GT--G---A--A-A---TT-C-GT-AG--AT-A-TT--C-G--G-A-G-G-A-AC-A-CC---AG--T--G--GC-GAA-G--G-C--G-------G--C-T-C-A---CTG--G--CT-C-G--------------------A-T---A-C-T--GA--CG-C-T-G--A-GG--T-G-CG-A--AA-G-C---G-TG--GG-G--
AG-C-A-AA--------CA--GG Bacteria(100);Proteobacteria(100);Alphaproteobacteria(100);unclassified(60);unclassified(60);unclassified(60);unclassified(60);unclassified(60);unclassified(60);

Otu0326 0 58 132 0 0 0 80 101 63 0 0 0 0 0 0 0 0 0 0 0 0 0 0 0 0 0 0 0
1 0 0 0 0 0 0 0 0 0 0 332 0 0 0 0 21 0 0 0 0 0 0 54 0 0 0 0 0
0 0 6 0 0 0 0 0 0 0 0 0 0 0 0 2 0 0 0 0 0 0 0 0 6 0 0 0 0
0 0 0 0 0 0 0 0 0 0 0 43 0 0 0 0 0 0 0 0 0 0 0 4 62 0 0

M02255_77_000000000-ADUT3_1_1105_16676_21127 T----AC---GT-AT-GGT--------GCA-A-G------C--G--T---T--AT-C-CGG-AT------TT-A--C-T--GG-GT-------GT--A-----AA-GG-GA-GC-------G-TA-G-A-C-G------------G--A-TG-G-G-C-----
------AA----G-T-C-T--------G-A-A--G--TG--A-AA-GC--C-C-GG-G-G-----CT-C-AA------C-C-C-C-G-G-G--A-C----T-G--C-T---T--T-------G--GA-A-A---C-------T--G-T--TC--A-T-C----T-G-G-A-G-T--G---C-TG-----GA-G-A--------G-G-T-A---AG-T------GG--A--ATT----C-
C-T-A-GT--GT-A-G-CG-GT--G---A--A-A---TG-C-GT-AG--AT-A-TT--A-G--G-A-G-G-A-AC-A-CC---AG--T--G--GC-GAA-G--G-C--G-------G--C-T-T-A---CTG--G--AC-A-G--------------------T-A---A-C-T--GA--CG-T-T-G--A-GG--C-T-CG-A--AA-G-C---G-TG--GG-G-
-AG-C-A-AA--------CA--GG Bacteria(100);Firmicutes(100);Clostridia(100);Clostridiales(100);Lachnospiraceae(100);Incertae_Sedis(98);unclassified(98);unclassified(98);unclassified(98);

Otu0327 0 0 0 36 0 0 0 0 0 0 0 0 0 0 0 0 0 0 0 0 0 0 0 3 0 0 0 1
0 0 3 0 0 0 0 26 0 0 30 0 38 0 0 0 0 0 0 0 0 0 0 0 0 0 0 0 0
0 0 0 7 0 0 0 0 0 3 0 0 0 0 0 0 0 0 0 0 0 0 0 0 0 0 0 0 0
0 0 0 0 0 0 0 0 0 0 0 0 0 0 0 0 0 0 0 0 0 18 0 0 0 0 0

M02255_77_000000000-ADUT3_1_1117_4386_19431 T----AC---GT-AG-GGT--------CCG-A-G------C--G--T---T--GT-C-CGG-AA------TT-A--T-T--GG-GC-------GT--A-----AA-GG-GC-TC-------G-TA-G-G-C-G------------G--T-TT-G-T-C----------GC--
--G-T-C-G--------G-G-A--G--TG--A-AA-AC--C-A-TG-A-G-----CT-T-AA------C-T-C-A-T-G-G--C-T----T-G--C-T---T--C-------C--GA-T-A---C-------G--G-G--CA--G-A-C----T-A-G-A-G-G--T---A-TG-----CA-G-G--------G-G-A-G---AA-T------GG--A--ATT----C-C-T-G-
GT--GT-A-G-CG-GT--G---A--A-A---TG-C-GC-AG--AT-A-TC--A-G--G-A-G-G-A-AC-A-CC---GG--T--G--GC-GAA-G--G-C--G-------G--T-T-C-T---CTG--G--GC-A-T--------------------T-A---C-C-T--GA--CG-C-T-G--A-GG--A-G-CG-A--AA-G-T---G-TG--GG-G--AG-
C-G-AA--------CA--GG Bacteria(100);Actinobacteria(100);Actinobacteria(100);Actinobacteridae(100);Actinomycetales(100);Propionibacterineae(100);Nocardioidaceae(100);Nocardioides(100);unclassified(100);

Otu0328 0 0 0 0 0 42 53 3 0 81 0 0 0 0 0 0 0 0 0 2 16 0 0 0 0 0 0 0
0 1 0 0 0 0 0 0 0 37 2 40 0 0 0 0 0 0 1 0 0 0 0 0 0 0 0 0 0
0 0 6 0 0 0 0 7 0 0 5 8 0 0 19 0 0 0 0 197 64 8 0 0 0 0 0 0 0
0 0 68 22 0 0 15 0 0 28 0 13 7 0 0 0 0 0 0 0 0 0 0 0 37 0 0

M02255_77_000000000-ADUT3_1_1107_25179_6365 T----AC---GT-AT-GGG--------GCG-A-G------C--G--T---T--GT-C-CGG-AA------TT-A--T-T--GG-GC-------GT--A-----AA-GG-GT-AC-------G-CA-G-G-C-G------------G--T-TT-A-C-C----------AA-
---G-T-T-G--------G-A-T--G--TG--A-AA-TC--T-T-GT-A-G-----CT-C-AA------C-T-A-C-A-AAC--G------T-G--C-A---T--C-------C--AA-A-A---C-------T--G-G--CT--A-A-C----T-T-G-A-G-T--T---A-AG-----GA-G-A--------G-G-T-A---AG-T------GG--A--ATT----C-C-T-G-
GT--GT-A-G-CG-GT--G---G--A-A---TG-C-GT-AG--AT-A-TC--A-G--G-A-G-G-A-AT-A-CC---GG--T--G--GC-GAA-G--G-C--G-------A--C-T-T-A---CTG--G--AC-T-T--------------------A-T---A-C-T--GA--CG-C-T-G--A-GG--T-A-CG-A--AA-G-C---G-TG--GG-G--AG-
C-A-AA--------CA--GG Bacteria(100);Firmicutes(100);Clostridia(100);Clostridiales(100);Family_XI_Incertae_Sedis(100);unclassified(100);unclassified(100);unclassified(100);unclassified(100);

Otu0329 0 0 0 0 0 0 0 0 0 0 0 0 0 0 0 0 0 0 0 0 0 0 0 0 0 0 0 1
0 0 0 0 0 0 0 0 0 0 10 0 0 0 41 0 0 0 0 0 0 0 0 0 0 3 0 8 0
0 0 0 0 0 0 0 0 0 0 0 0 0 0 8 10 3 0 0 0 0 3 0 0 0 0 0 0 0
0 0 0 14 0 0 0 0 0 0 0 0 0 0 0 0 0 0 0 0 0 0 7 0 0 0 0

M02255_76_000000000-ADUNE_1_2115_26283_20276 C----GG---CT-AA-ATA--------CGT-G-C------C--A--G---C--AG-C-C-G-CG------GT-A--A-T--AC-GT-------AT--G-----TT-CC-AA-GC-------GTTA-T-C-C-G------------G--T-TA-T-T-T--------
---AA----G-T-C-T--------G-A-A--G--TG--A-AA-GC--C-C-TC-A-G-----CT-C-AA------C-T-G-A-G-G-A--A-G----G-G--C-T---T--T-------G--GA-A-A---C-------T--G-G--AT--A-A-C----T-T-G-A-G-T--G---C-AG-----TA-G-A--------G-G-A-A---AG-T------GG--A--ACT----C-
C-A-T-GT--GT-A-G-CG-GT--G---A--A-A---TG-C-GT-AG--AT-A-TA--T-G--G-A-A-G-A-AC-A-CC---GG--T--G--GC-GAA-A--G-C--G-------G--C-T-T-T---CTG--G--AC-T-G--------------------T-A---A-C-T--GA--CG-T-T-G--A-GG--C-T-CG-A--AA-G-T---G-TG--GG-T--
AG-C-A-AA--------CA--GG Bacteria(100);Firmicutes(100);Bacilli(100);Lactobacillales(100);Leuconostocaceae(100);Weissella(94);unclassified(94);unclassified(94);unclassified(94);

Otu0330 0 0 0 0 0 0 0 0 0 0 0 0 0 0 0 0 0 0 0 0 0 0 0 0 0 0 0 0
0 0 1 0 0 1 0 71 0 0 23 0 0 0 0 0 0 0 0 0 0 0 0 0 0 0 0 0
0 0 0 0 0 0 0 0 0 0 0 0 0 0 0 0 23 10 0 0 0 0 0 0 0 0 0 0
0 0 0 0 0 0 0 0 0 0 0 0 7 8 0 0 0 0 0 0 0 0 0 0 0 0 0
M02255_77_000000000-ADUT3_1_1111_5399_15873 G----AC---GG-AG-GGT--------GCT-A-G------C--G--T---T--GT-T-CGG-AA------TC-A--C-T--GG-GC-------GT--A-----AA-GG-GC-GC-------G-TA-G-G-C-G------------G--C-TG-T-G-G----------
CA----G-T-C-G--------G-G-T--G--TG--A-AA-GC--C-C-GG-A-G-----CT-C-AA------C-T-C-C-G-G-A--A-T----G-G--C-A---C--T-------C--GA-G-A---C-------G--C-C--AC--G-G-C----T-G-G-A-G-G--C---C-GG-----AA-G-A--------G-G-A-G---GG-T------GG--A--ATA----C-C-
C-A-GT--GT-A-G-AG-GT--G---A--A-A---TT-C-GT-AG--AT-A-TT--G-G--G-T-G-G-A-AC-A-CC---GG--T--G--GC-GAA-A--G-C--G-------G--C-T-C-T---CTG--G--TC-C-G--------------------G-A---C-C-T--GA--CG-C-T-G--A-GG--C-G-CG-A--AA-G-C---G-T-G-GG-G--
AG-C-A-AA--------CA--GG Bacteria(100);Proteobacteria(100);Alphaproteobacteria(100);unclassified(71);unclassified(71);unclassified(71);unclassified(71);unclassified(71);unclassified(71);

Otu0331 0 0 0 0 0 0 0 0 0 0 0 0 0 0 0 0 0 0 1 9 0 10 0 0 0 0 0 0
1 0 0 0 0 0 0 0 0 0 0 0 0 0 0 0 0 0 0 0 0 0 0 0 0 0 0 0
0 0 0 2 0 0 0 0 0 0 0 0 6 0 0 0 0 0 0 0 0 0 0 0 0 0 0 0
0 0 0 0 0 0 0 0 0 0 0 0 0 0 4 0 0 0 0 0 0 0 0 0 0 0 0
M02255_76_000000000-ADUNE_1_1117_16171_9739 C----TC---GC-AG-AAT--------AAG-CACC-----G--G--C---T--AACTCTGT-GC------CA-G--C-A-GCC-GC-------GG--T-----AA-TA-CA-GA-------G-GG-T-G-C-A------------A--G-CT-G-T-T----------
AA----G-T-C-G--------G-A-T--G--TG--A-AA-TC--C-C-TG-A-G-----CT-T-AA------C-T-T-A-G-G-A--A-T----T-G--C-A---T--A-------C--GA-T-A---C-------T--G-G--GA--G-A-C----T-A-G-A-G-T--A---T-GG-----GA-G-A--------G-G-A-T---GG-T------AG--A--ATT----C-C-A-
G-GT--GT-A-G-CG-GT--G---A--A-A---TG-C-GT-AG--AG-A-TC--T-G--G-A-G-G-A-AT-A-CC---GA--T--G--GC-GAA-G--G-C--A-------G--C-C-A-T---CTG--G--CC-T-A--------------------A-T---A-C-T--GA--CA-C-T-G--A-GG--T-A-CG-A--AA-G-C---A-TG--GG-G--
AG-C-A-AA--------CA--GG Bacteria(100);Proteobacteria(100);Gammaproteobacteria(100);unclassified(79);unclassified(79);unclassified(79);unclassified(79);unclassified(79);unclassified(79);

Otu0332 0 0 0 157 0 0 0 0 0 0 0 1 0 0 0 0 0 0 5 0 0 0 0 5 0 1 2 0
0 0 1 0 0 0 0 0 0 0 5 0 2 183 0 0 45 46 0 57 0 0 0 0 0 95 0 0 0
0 48 0 0 0 0 0 0 0 0 0 0 0 15 42 0 0 119 0 0 0 0 0 0 0 0 0 0
0 0 0 26 0 0 0 0 0 0 0 0 0 0 0 31 0 0 10 0 0 0 0 0 0 0 0
M02255_76_000000000-ADUNE_1_2104_25616_14147 T----AC---GA-AG-GGG--------GCT-A-G------C--G--T---T--GC-T-CGG-AA------TT-A--C-T--GG-GC-------GT--A-----AA-GG-GC-GC-------G-TA-G-G-C-G------------G--T-CA-T-T-T-----
------TA----G-T-C-A--------G-G-C--G--TG--A-AA-TT--C-C-CG-G-G-----CT-T-AA------C-C-T-G-G-G-G--A-C----T-G--C-G---C--T-------T--GA-T-A---C-------T--G-G--GT--G-A-C----T-A-G-A-G-G--G---T-GG-----AA-G-A--------G-G-G-T---CG-T------GG--A--ATT----
C-C-C-A-GT--GT-A-G-AG-GT--G---A--A-A---TT-C-GT-AG--AT-A-TT--G-G--G-A-A-G-A-AC-A-CC---GG--T--G--GC-GAA-G--G-C--G-------G--C-G-A-C---CTG--G--TC-C-A--------------------T-T---A-C-T--GA--CG-C-T-G--A-GG--C-G-CG-A--CA-G-C---G-TG--GG-
G--AG-C-A-AA--------CA--GG Bacteria(100);Proteobacteria(100);Alphaproteobacteria(100);Rhodospirillales(100);Acetobacteraceae(100);unclassified(100);unclassified(100);unclassified(100);unclassified(100);

Otu0333 0 0 0 0 0 0 0 0 0 0 0 0 0 0 0 0 0 0 0 0 0 0 0 0 0 0 0 0
0 0 0 0 0 0 0 0 0 0 0 0 0 0 0 14 0 0 0 0 0 0 0 12 0 0 0 0 0
0 0 0 0 0 0 0 0 0 0 0 0 0 0 0 0 0 0 0 0 0 0 0 0 0 0 0 0
0 37 0 0 0 0 0 0 0 0 0 0 0 0 0 0 0 0 0 0 0 0 0 0 0 0 0
M02255_77_000000000-ADUT3_1_1109_18228_18719 T----AC---GT-AG-GGT--------GCG-A-G------C--G--T---T--GT-C-CGG-AA------TT-A--T-T--GG-GC-------GT--A-----AA-GA-GC-TC-------G-TA-G-G-C-G------------G--T-TT-G-T-C------
-----GC----G-T-C-G--------G-C-C--G--TG--A-AA-AC--T-T-GG-G-G-----CT-T-AA------C-T-C-C-A-A-G--C-T----T-G--C-G---G--T-------C--GA-T-A---C-------G--G-G--CA--G-A-C----T-T-G-A-G-T--T---C-GG-----CA-G-G--------G-G-A-G---AC-T------GG--A--ATT----C-
C-T-G-GT--GT-A-G-CG-GT--G---A--A-A---TG-C-GC-AG--AT-A-TC--A-G--G-A-G-G-A-AC-A-CC---GG--T--G--GC-GAA-G--G-C--G-------G--G-T-C-T---CTG--G--GC-C-G--------------------A-C---A-C-T--GA--CG-C-T-G--A-GG--A-G-CG-A--AA-G-C---G-TG--GG-
G--AG-C-G-AA--------CA--GG Bacteria(100);Actinobacteria(100);Actinobacteria(100);Actinobacteridae(100);Actinomycetales(100);Pseudonocardineae(59);unclassified;unclassified;unclassified;

Otu0334 0 0 0 0 0 0 0 0 0 0 0 0 0 0 0 0 0 0 0 0 0 0 0 0 0 0 0 0
0 0 0 0 0 0 0 0 0 1 1 0 0 0 0 0 0 13 0 0 0 0 0 0 0 0 0 0
0 0 0 0 0 0 0 0 0 0 0 0 0 0 0 0 0 0 0 0 0 0 0 28 0 0 0 0 0
0 0 0 0 0 0 0 0 0 0 0 0 0 0 0 0 0 0 0 0 0 0 0 0 0 0 0
M02255_76_000000000-ADUNE_1_1103_21469_4931 C----CG---GC-TA-ACT--------CCG-T-G------C--C--A---G--CA-G-CCG-CG------GT-A--A-T--AC-GG-------AG--G-----GT-GC-AA-GC-------G-TT-GTC-C-G------------G--C-CA-T-T-T----------AA-
---T-T-C-T--------G-G-G--G--TG--A-AA-GC--C-C-GT-T-G-----CT-C-AA------C-A-A-C-G-G-A--A-C----T-G--C-C---C--T-------G--GA-A-A---C-------T--G-G--AT--G-G-C----T-T-G-A-G-T--A---C-AG-----AC-G-A--------G-G-G-T---GG-C------GG--A--ATG----G-A-T-G-
CT--GT-A-G-CG-GT--G---A--A-A---TG-C-AT-AG--AT-A-GC--A-T--C-C-A-G-A-AC-C-CC---GA--T--T--GC-GAA-G--G-C--A-------G--C-T-G-C---CTA--G--AC-T-G--------------------T-A---A-C-T--GA--CG-C-T-G--A-GG--C-A-CG-A--AA-G-C---G-TG--GG-G--AG-C-
G-AA--------CA--GG Bacteria(100);Bacteroidetes(100);Sphingobacteria(100);Sphingobacteriales(100);Cytophagaceae(100);Hymenobacter(100);unclassified(100);unclassified(100);unclassified(100);

Otu0335 0 0 0 24 0 0 0 0 0 0 0 0 0 0 0 0 0 0 0 0 1 0 0 0 0 0 0 0
0 0 0 29 0 0 0 0 0 5 0 0 0 0 0 0 0 0 0 0 0 0 0 0 0 82 0 0 0
0 0 0 0 0 0 0 0 0 0 0 0 0 0 0 0 0 0 0 0 0 0 0 0 0 0 0 0 0
0 0 0 1 0 4 0 0 0 0 0 0 0 3 0 20 0 0 0 0 0 0 3 1 0 0 0
M02255_77_000000000-ADUT3_1_2103_29097_16057 T----AC---GT-AG-GGT--------CCG-A-G------C--G--T---T--GT-C-CGG-AA------TT-A--T-T--GG-GC-------GT--A-----AA-GG-GC-TT-------G-TA-G-G-C-G------------G--T-CC-G-T-C------
-----GC----G-T-C-A--------G-G-A--G--TG--A-AA-AC--T-C-GG-G-G-----CT-T-AA------C-C-C-C-G-A-G--C-C----T-G--C-T---T--T-------T--GA-T-A---C-------G--G-G--CG--G-A-C----T-A-G-A-G-G--G---A-TG-----CA-G-G--------G-G-A-G---AA-C------GG--A--ATT----
C-C-T-G-GT--GG-A-G-CG-GT--G---G--A-A---TG-C-GC-AG--AT-A-TC--A-G--G-A-G-G-A-AC-A-CC---GG--T--G--GC-GAA-G--G-C--G-------G--T-T-C-T---CTG--G--GC-A-T--------------------T-A---C-C-T--GA--CG-C-T-G--A-GA--A-G-CG-A--AA-G-C---G-TG--
GG-G--AG-C-A-AA--------CA--GG Bacteria(100);Actinobacteria(100);Actinobacteria(100);Actinobacteridae(100);Actinomycetales(100);Propionibacterineae(100);Propionibacteriaceae(100);Friedmanniella(100);unclassified(100);

Otu0336 0 0 0 276 0 0 117 4 0 105 0 0 0 0 0 0 0 0 0 0 0 0 0 0 0 0 0 0
0 0 0 0 0 0 0 0 0 0 0 40 0 0 0 0 37 0 0 0 0 0 0 0 0 0 0 0 0
0 0 0 0 0 0 0 0 0 8 31 6 0 0 0 0 0 0 134 67 0 0 0 0 0 0 0 0 0
0 0 0 0 24 0 0 0 0 0 0 0 0 0 0 0 0 0 0 0 0 0 6 0 25 0 93
M02255_77_000000000-ADUT3_1_1107_26589_12742 T----AC---GT-AT-GGT--------GCG-A-G------C--G--T---T--GT-C-CGG-AA------TT-A--T-T--GG-GC-------GT--A-----AA-GG-GT-AC-------G-TA-G-G-C-G------------G--T-TA-A-A-C-----
------AA----G-T-C-A--------G-A-T--G--TA--A-AA-AC--T-T-GA-G-G-----CT-C-AA------C-C-T-C-A-T-G--T------A-G--C-A---T--T-------T--GA-A-A---C-------T--G-T--AT--A-A-C----T-T-G-A-G-T--A---G-TG-----GA-G-A--------G-G-A-A---AG-T------GG--A--ATT----C-
C-T-A-GT--GT-A-G-CG-GT--G---A--A-A---TG-C-GT-AG--AT-A-TT--A-G--G-A-G-G-A-AT-A-CC---AG--T--A--GC-GAA-G--G-C--G-------A--C-T-T-T---CTG--G--AC-A-C--------------------A-A---A-C-T--GA--CG-C-T-G--A-GG--T-A-CG-A--AA-G-C---G-TG--GG-G-
-AG-C-A-AA--------CA--GG Bacteria(100);Firmicutes(100);Clostridia(100);Clostridiales(100);Family_XI_Incertae_Sedis(76);unclassified(76);unclassified(76);unclassified(76);unclassified(76);

Otu0337 0 0 0 0 0 0 0 0 0 0 0 0 1 0 0 4 0 0 0 0 0 0 0 0 0 11 1 0
0 0 1 0 0 0 0 0 0 0 7 3 0 0 0 0 0 0 2 0 0 0 0 0 0 0 0 28 1
0 0 2 0 0 0 0 0 39 0 0 0 0 0 0 0 0 0 0 0 0 0 0 0 0 0 0 0 0
0 0 0 0 0 1 0 0 0 0 1 0 0 0 0 0 0 0 0 0 0 0 0 0 0 0 0
M02255_77_000000000-ADUT3_1_2116_24487_13915 G----AA---GC-AC-CGG--------CTA-A-C------T--C--C---G--TG-C-CAG-CA------GC-C--G-C--GG-TA-------AT--A-----CG-GA-GG-GT-------GCAA-GCGTT-A------------A--T-CG-GAA-T-
----------TA----C-T-T-A--------G-A-T--G--TG--A-AA-GC--C-C-CG-G-G-----CT-C-AA------C-C-T-G-G-G-A--A-T----T-G--C-A---T--T-------T--AA-A-A---C-------T--G-T--CC--A-G-C----T-A-G-A-G-T--C---T-TG-----TA-G-A--------G-G-G-G---GG-T------AG--A--ATT----
C-C-A-G-GT--GT-A-G-CG-GT--G---A--A-A---TG-C-GT-AG--AG-A-TC--T-G--G-A-G-G-A-AT-A-CC---GG--T--G--GC-GAA-G--G-C--G-------G--C-C-C-C---CTG--G--AC-A-A--------------------A-G---A-C-T--GA--CG-C-T-C--A-GG--T-G-CG-A--AA-G-C---G-TG--
GG-G--AG-C-A-AA--------CA--GG Bacteria(100);Proteobacteria(100);Gammaproteobacteria(100);unclassified(100);unclassified(100);unclassified(100);unclassified(100);unclassified(100);unclassified(100);

Otu0338 0 0 285 0 0 0 438 160 609 0 0 0 0 0 0 0 0 0 0 0 0 0 0 0 0 0 6 0
0 0 0 0 72 0 0 0 0 0 0 341 1 11 0 0 0 0 0 36 0 0 0 0 0 0 0 0 0
0 0 0 0 0 0 0 0 0 0 0 0 1 0 0 7 20 110 0 0 0 0 0 0 0 0 0 0 0
0 0 0 22 0 0 35 0 0 0 0 221 0 65 0 0 0 0 0 0 0 0 0 7 243 777 0
M02255_77_000000000-ADUT3_1_1101_22941_15729 A----AC---GT-AG-GGT--------GCA-A-G------C--G--T---T--GT-C-CGG-AA------TT-A--C-T--GG-GT-------GT--A-----AA-GG-GA-GC-------G-CA-G-G-C-G------------G--G-AA-G-A-C---
--------AA----G-T-T-G--------G-A-A--G--TG--A-AA-AC--C-A-TG-G-G-----CT-C-AA------C-C-C-A-T-G-A--A-T----T-G--C-T---T--T-------C--AA-A-A---C-------T--G-T--TT--T-T-C----T-T-G-A-G-T--A---G-TG-----CA-G-A--------G-G-T-A---GA-T------GG--A--ATT----C-
C-C-G-GT--GT-A-G-CG-GT--G---G--A-A---TG-C-GT-AG--AT-A-TC--G-G--G-A-G-G-A-AC-A-CC---AG--T--G--GC-GAA-G--G-C--G-------G--T-C-T-A---CTG--G--GC-A-C--------------------C-A---A-C-T--GA--CG-C-T-G--A-GG--C-T-CG-A--AA-G-C---A-TG--GG-
T--AG-C-A-AA--------CA--GG Bacteria(100);Firmicutes(100);Clostridia(100);Clostridiales(100);Ruminococcaceae(100);Subdoligranulum(100);unclassified(100);unclassified(100);unclassified(100);

Otu0339 0 0 0 0 0 0 0 0 0 0 0 0 0 0 0 0 0 1 0 0 0 0 0 0 0 0 0 0
0 0 0 0 0 0 0 0 0 0 0 0 0 0 0 0 0 0 0 0 0 0 0 0 0 0 5 4 0
0 0 0 1 0 0 0 0 0 0 0 0 0 0 0 0 0 0 0 0 0 0 0 0 0 0 0 0 0
0 0 0 0 0 0 1 0 0 0 2 0 1 0 0 0 0 0 0 0 0 0 0 0 0 0 0
M02255_76_000000000-ADUNE_1_2110_7767_22329 T----AC---CG-AC-AGA--------ATA-A-G------C-----A---C--CG-G-CTA-AC------TC-T--G-T--GC-CA-------GC--AG----CC-GC-GG-TA-------A-TA-C-A-G-A------------G--G-GT-G-C-A-----------AG-
---C-G-T-T--------A-A-T--C--GG--A-AA-TC--C-C-CG-G-G-----CT-C-AA------C-C-T-G-G-G-A--A-C----T-G--C-A---T--T-------C--AA-A-A---C-------T--G-A--CT--G-A-C----T-A-G-A-G-T--A---T-GG-----TA-G-A--------G-G-G-T---GG-T------GG--A--ATT----T-C-C-T-
GT--GT-A-G-CG-GT--G---A--A-A---TG-C-GT-AG--AT-A-TA--G-G--A-A-G-G-A-AC-A-CC---AG--T--G--GC-GAA-G--G-C--G-------A--C-C-A-C---CTG--G--AC-T-A--------------------A-T---A-C-T--GA--CA-C-T-G--A-GG--T-G-CG-A--AA-G-C---G-TG--GG-G--AG-
C-A-AA--------CA--GG Bacteria(100);Proteobacteria(100);Gammaproteobacteria(100);unclassified(100);unclassified(100);unclassified(100);unclassified(100);unclassified(100);unclassified(100);

Otu0340 0 0 58 0 0 0 0 0 129 0 0 0 0 0 0 0 0 0 0 0 0 0 0 0 0 0 0 0
0 0 0 0 0 0 0 0 0 0 0 0 0 0 0 96 0 0 0 0 0 0 0 0 0 0 0 0 0
0 0 26 0 0 0 0 0 0 0 0 0 0 0 0 19 0 0 0 0 0 0 0 0 0 0 0 0 0
0 0 0 0 0 0 0 14 0 56 0 0 0 0 0 0 0 0 0 0 0 0 13 0 0 0 0
M02255_77_000000000-ADUT3_1_1105_7056_3043 T----AC---GT-AG-GGG--------GCG-A-G------C--G--T---T--AT-C-CGG-AA------TT-A--T-T--GG-GC-------GT--A-----AA-GA-GT-GC-------G-TA-G-G-T-G------------G--C-AC-C-T-T-----------AA-
---G-C-G-C--------A-G-G--G--TT--T-AA-GG--C-A-AT-G-G-----CT-C-AA------C-C-A-T-T-G-T--T------C-G--C-C---T--T-------G--CG-A-A---C-------T--G-G--GG--T-G-C----T-T-G-A-G-T--G---C-AG-----GA-G-G--------G-G-A-A---AG-T------GG--A--ATT----C-C-T-A-
GT--GT-A-G-CG-GT--G---A--A-A---TG-C-GT-AG--AT-A-TT--A-G--G-A-G-G-A-AC-A-CC---AG--T--G--GC-GAA-G--G-C--G-------A--C-T-T-T---CTG--G--AC-T-G--------------------T-T---A-C-T--GA--CA-C-T-G--A-GG--C-A-CG-A--AA-G-C---G-TG--GG-G--AG-C-
A-AA--------CA--GG Bacteria(100);Firmicutes(100);Clostridia(100);Clostridiales(100);Family_XIII_Incertae_Sedis(100);Eubacterium(100);unclassified(100);unclassified(100);unclassified(100);

Otu0341 0 0 0 0 0 0 0 0 0 0 0 0 0 0 0 0 0 0 0 0 1 0 0 0 0 3 0 1
0 0 1 0 0 25 0 0 0 0 62 0 0 0 0 39 0 0 0 0 0 0 0 0 18 0 0 54 0
0 0 0 0 0 0 0 7 0 0 0 0 0 0 72 474 0 0 0 27 0 0 0 0 0 0 0 0 0
0 0 0 6 0 0 4 0 0 35 0 0 7 0 1 25 0 0 0 0 0 0 20 0 15 0 0
M02255_77_000000000-ADUT3_1_2118_13956_20608 T----AC---GT-AG-GGG--------GCA-A-G------C--G--T---T--AT-C-CGG-AA------TA-A--C-T--GG-GC-------GT--A-----AA-GG-GT-GC-------G-CA-G-G-T-G------------G--T-TT-A-A-C-----
------AA----G-T-T-A--------G-T-G--G--TG--A-AA-GG--C-A-TA-G-G-----CT-C-AA------C-C-A-A-T-G-T--A------A-G--C-C---A--T-------T--AA-A-A---C-------T--G-T--TT--A-A-C----T-T-G-A-G-T--G---C-AG-----GA-G-A--------G-G-A-A---AG-T------GG--A--ATT----C-
C-T-A-GT--GT-A-G-CG-GT--G---A--A-A---TG-C-GT-AG--AT-A-TT--A-G--G-A-G-G-A-AT-A-CC---AG--T--G--GC-GAA-G--G-C--G-------A--C-T-T-T---CTG--G--AC-T-G--------------------C-A---A-C-T--GA--CA-C-T-G--A-GG--C-A-CG-A--AA-G-C---G-TG--GG-G-
-AG-C-A-AA--------CA--GG Bacteria(100);Firmicutes(100);Clostridia(100);Clostridiales(100);Peptostreptococcaceae(100);Filifactor(100);unclassified(100);unclassified(100);unclassified(100);

Otu0342 0 0 0 0 0 0 0 0 0 0 0 0 0 0 0 0 0 0 0 0 0 0 0 0 0 0 0 0
0 0 0 0 0 0 0 83 0 0 0 0 0 0 0 0 0 0 0 0 0 0 0 0 0 0 0 0 0
0 0 0 0 0 0 0 0 0 0 0 0 0 0 0 0 0 0 0 0 0 0 0 0 0 0 0 0 0
0 0 0 0 0 0 0 0 0 0 0 0 0 0 0 0 0 0 0 0 0 0 0 0 0 0 0
M02255_77_000000000-ADUT3_1_1106_17177_14272 T----AC---GT-AG-GTG--------GCA-A-G------C--G--T---T--GT-C-CGG-AT------TT-A--T-T--GG-GC-------GT--A-----AA-GG-GC-GC-------G-CA-G-G-T-G------------G--T-TC-G-T-T------
-----AA----G-T-C-C--------G-A-G--G--TG--A-AA-TC--T-C-CC-G-G-----CT-C-AA------C-T-G-G-G-A-G--G------G-GT-C-T---T--C-------G--GA-T-A---C-------T--G-G--CG--G-A-C----T-T-G-A-G-G--A---G-AG-----CA-G-A--------G-G-T-G---AG-C------GG--A--ATT----
C-T-C-G-GT--GT-A-G-TG-GT--G---A--A-A---TG-C-GT-AG--AT-A-TC--G-A--G-A-G-G-A-AC-A-CC---AG--C--G--GC-GAA-G--G-C--G-------G--C-T-C-A---CTG--G--GC-T-C--------------------C-A---C-C-T--GA--CA-C-T-G--A-GG--C-G-CG-A--AA-G-C---G-TG--
GG-G--AG-C-A-AA--------CC--GG Bacteria(100);unclassified(100);unclassified(100);unclassified(100);unclassified(100);unclassified(100);unclassified(100);unclassified(100);unclassified(100);

Otu0343 0 0 0 0 0 0 0 0 0 3 0 0 0 0 0 0 0 0 0 0 0 0 9 0 0 0 0 0
0 0 0 0 0 0 20 0 0 0 0 0 0 7 0 0 7 0 0 0 0 0 1 0 0 0 0 13 0
9 0 0 0 0 0 5 0 0 0 4 0 0 0 0 0 15 0 0 0 0 0 0 0 0 0 0 8 0
0 9 0 2 0 0 1 0 0 0 0 0 0 0 0 0 24 10 0 0 0 0 0 0 0 0 0
M02255_76_000000000-ADUNE_1_1118_23203_12189 .....AC----T-AC-GT---------GCC-A-G------C--A--G---C--CG-C-GGT-AA------TA-C--G-T--AG-GT-------GG--C-----AA-GC-G--TT-------G----T-C-C-G------------G--A-TC-G-A-T----------
AA----G-T-C-T--------G-A-T--G--TG--A-AA-GC--C-T-TC-G-G-----CT-C-AA------C-C-G-G-A-G-A--A-T----T-G--C-A---T--C-------A--GA-A-A---C-------T--G-T--CG--A-G-C----T-T-G-A-G-T--A---C-AG-----AA-G-A--------G-G-A-G---AG-T------GG--A--ACT----C-C-A-
T-GT--GT-A-G-CG-GT--G---A--A-A---TG-C-GT-AG--AT-A-TA--T-G--G-A-A-G-A-AC-A-CC---GG--T--G--GC-GAA-G--G-C--G-------G--C-T-C-T---CTG--G--TC-T-G--------------------T-T---A-C-T--GA--CG-C-T-G--A-GG--C-T-CG-A--AA-G-C---A-TG--GG-T--AG-
C-G-AA--------CA--GG Bacteria(100);Firmicutes(100);Bacilli(100);Lactobacillales(100);unclassified(100);unclassified(100);unclassified(100);unclassified(100);unclassified(100);

Otu0344 0 0 0 0 0 0 0 0 0 0 0 0 0 0 0 0 0 3 0 4 0 16 0 0 0 0 0 1
1 0 0 0 0 0 0 0 0 0 0 0 0 0 0 0 0 0 0 0 0 0 0 0 0 0 0 0 0
1 0 0 0 0 0 0 0 0 0 0 0 0 0 0 0 0 0 0 0 0 0 0 0 0 0 0 0 0
0 0 0 0 0 0 0 0 6 0 0 0 0 0 0 0 0 0 0 0 0 0 0 0 0 0 0
M02255_76_000000000-ADUNE_1_2114_27851_10656 TC---GC---AG-AA-TAA--------GCA-CCGG-----C-----T---A--AC-T-CTG-TG------CC-A--G-C--AG-CC-------GC--G-----GT-AA-TA-CA-------G-AG-G-G-T-GC-----------A--A-GC-G-T-T---
--------AA----G-T-C-G--------G-A-T--G--TG--A-AA-TC--C-C-TG-A-G-----CT-T-AA------C-T-T-A-G-G-A--A-T----T-G--C-A---T--A-------C--GA-T-A---C-------T--G-G--GA--G-A-C----T-A-G-A-G-T--A---T-GG-----GA-G-A--------G-G-A-T---GG-T------AG--A--ATT----
C-C-A-G-GT--GT-A-G-CG-GT--G---A--A-A---TG-C-GT-AG--AG-A-TC--T-G--G-A-G-G-A-AT-A-CC---GA--T--G--GC-GAA-G--G-C--A-------G--C-C-A-T---CTG--G--CC-T-A--------------------A-T---A-C-T--GA--CA-C-T-G--A-GG--T-A-CG-A--AA-G-C---A-TG--GG-
G--AG-C-A-AA--------CA--GG Bacteria(100);Proteobacteria(100);Gammaproteobacteria(100);Pseudomonadales(100);Moraxellaceae(100);Acinetobacter(100);unclassified(100);unclassified(100);unclassified(100);

Otu0345 0 0 0 0 0 0 0 0 0 0 0 0 0 0 0 0 0 0 0 0 0 0 0 0 0 0 0 0
0 0 0 0 98 0 0 0 0 0 0 0 0 0 0 0 0 0 0 0 0 0 0 0 0 0 0 0 0
0 0 0 0 0 0 5 0 0 0 0 0 0 0 0 0 13 0 0 0 0 0 0 0 0 0 0 0 0
0 0 0 0 0 0 0 0 0 0 0 0 0 0 0 0 0 0 0 0 0 0 0 0 0 0 0
M02255_77_000000000-ADUT3_1_2105_9748_9756 T----AC---GT-AG-GGG--------GCG-A-G------C--G--T---T--GT-C-CGG-AA------TC-A--T-T--GG-GC-------GT--A-----AA-GG-GT-TC-------G-TA-G-G-C-G------------G--A-CT-G-A-T----------
AA----G-T-C-A--------G-A-T--G--TG--A-AA-AG--A-T-GT-G-G-----CT-T-AA------C-C-A-T-G-T-T--A------A-G--C-A---T--T-------T--GA-A-A---C-------T--G-T--CA--G-T-C----T-T-G-A-G-T--G---A-AG-----GA-G-A--------G-G-T-A---AG-T------GG--A--ATT----C-C-T-
A-GT--GT-A-G-CG-GT--G---A--A-A---TG-C-GT-AG--AT-A-TT--A-G--G-A-G-G-A-AT-A-CC---AG--T--A--GC-GAA-G--G-C--G-------A--C-T-T-A---CTG--G--CC-T-T--------------------T-A---A-C-T--GA--CG-C-T-G--A-GG--A-A-CG-A--AA-G-C---G-TG--GG-T--AG-
C-A-AA--------CA--GG Bacteria(100);Firmicutes(100);Clostridia(100);Clostridiales(100);unclassified(85);unclassified(85);unclassified(85);unclassified(85);unclassified(85);

Otu0346 0 0 0 0 0 0 0 0 0 0 0 0 0 0 0 0 0 0 0 3 10 0 0 0 0 0 0 0
0 0 0 0 0 0 0 0 0 24 4 0 0 0 34 0 0 0 0 0 0 0 0 0 0 0 0 0 0
0 0 0 0 0 0 0 0 0 0 0 0 0 0 0 0 0 22 0 0 0 0 0 0 0 0 0 0 0
0 0 0 0 0 0 0 0 0 0 0 0 0 0 0 0 0 0 0 0 0 0 0 0 0 0 0
M02255_77_000000000-ADUT3_1_1108_27465_19245 C----AC---GT-AG-GGG--------GCA-A-G------C--G--T---T--GT-C-CGG-AT------TT-A--T-T--GG-GC-------GT--A-----AA-GA-GC-TC-------G-TA-G-G-T-G------------G--T-TC-G-G-T-----
------AA----G-T-C-G--------G-G-T--G--TG--A-AA-AC--T-C-TG-G-G-----CT-C-AA------C-T-C-A-G-A-G--A------C-G-CC-A---C--C-------C--GA-T-A---C-------T--G-C--TG-TG-A-C----T-T-G-A-G-T--C---T-GG-----TA-G-G--------G-G-A-G---TG-T------GG--A--ATT----
C-C-C-A-GT--GT-A-G-CG-GT--G---A--A-A---TG-C-GC-AG--AT-A-TT--G-G--G-A-G-G-A-AC-A-CC---AG--T--G--GC-GAA-G--G-C--G-------G--C-A-C-T---CTG--G--GC-C-A--------------------G-T---A-C-T--GA--CA-C-T-G--A-GG--A-G-CG-A--AA-G-C---G-TG--
GG-T--AG-C-G-AA--------CA--GG Bacteria(100);Actinobacteria(65);Actinobacteria(65);Acidimicrobidae(65);Acidimicrobiales(65);Acidimicrobineae(65);unclassified;unclassified;unclassified;

Otu0347 0 0 0 0 0 0 20 0 0 0 0 0 0 0 0 0 0 0 0 0 0 0 0 0 0 0 0 0
0 0 0 0 0 0 0 0 0 0 0 0 0 0 0 21 0 0 0 0 0 0 0 0 0 0 0 0 0
0 0 0 0 0 0 0 0 0 0 0 0 0 0 0 9 0 0 0 0 0 0 0 0 0 0 0 0 0
9 0 0 0 0 0 0 0 0 0 0 0 0 0 0 0 0 0 0 0 0 16 0 0 0 0 0
M02255_77_000000000-ADUT3_1_1117_6943_18178 T----AC---GT-AG-GGG--------TCG-A-G------C--G--T---T--GT-C-CGG-AG------TT-A--C-T--GG-GC-------GT--A-----AA-GC-GC-AT-------G-CA-G-G-C-G------------G--C-TC-A-T-T----------AC-
---G-C-A-T--------G-G-C--G--TG--A-AA-GC--C-C-CC-G-G-----CT-T-AA------C-C-G-G-G-G-A--G-G----G-T--C-G---T--C-------G--TG-A-A---C-------G--G-G--TG--A-G-C----T-T-G-A-G-G--G---T-AT-----CA-G-G--------G-G-C-T---GG-T------AG--A--ATT----C-C-C-G-
GT--GT-A-G-TG-GT--G---A--A-A---TG-C-GT-AG--AT-A-TC--G-G--G-A-G-G-A-AT-A-CC---TG--T--G--GC-GAA-G--G-C--G-------G--C-C-A-G---CTG--G--GA-T-A--------------------C-A---C-C-T--GA--CG-C-T-G--A-GA--T-G-CG-A--AG-G-C---G-TG--GG-G--AG-
C-G-AA--------CG--GG Bacteria(100);Chloroflexi(100);Thermomicrobia(100);JG30-KF-CM45(100);unclassified(100);unclassified(100);unclassified(100);unclassified(100);unclassified(100);

Otu0348 0 0 0 0 0 0 0 0 0 0 0 0 0 0 0 0 0 0 0 1 3 6 0 0 0 0 0 0
0 2 5 0 0 0 0 0 0 31 1 0 0 0 0 0 0 0 0 0 0 0 0 0 0 0 0 0 0
0 0 0 0 0 8 0 0 0 4 0 0 0 0 0 0 0 0 0 30 0 0 1 0 0 0 0 0 0
124 0 0 0 0 771 0 83 3 0 0 418 0 0 2 0 0 0 0 0 0 0 0 0 0 0 0
M02255_77_000000000-ADUT3_1_1113_8236_20090 T----AC---GT-AT-GTC--------GCA-A-G------C--G--T---T--AT-C-CGG-AT------TT-A--T-T--GG-GC-------GT--A-----AA-GC-GC-GT-------C-TA-G-G-C-G------------G--T-CT-G-G-T----------AA---
-G-T-C-T--------G-A-T--G--TG--A-AA-AT--G-C-GG-G-G-----CT-C-AA------C-T-C-C-G-T-A--T------T-G--C-G---T--T-------G--GA-A-A---C-------T--G-C--CA--G-A-C----T-A-G-A-G-T--A---C-TG-----GA-G-A--------G-G-T-G---GG-C------GG--A--ACT----A-C-A-A-GT-
-GT-A-G-AG-GT--G---A--A-A---TT-C-GT-AG--AT-A-TT--T-G--T-A-G-G-A-AT-G-CC---GA--T--A--GA-GAA-G--T-C--A-------G--C-T-C-A---CTG--G--AC-A-G--------------------A-T---A-C-T--GA--CG-C-T-G--A-AG--C-G-CG-A--AA-G-C---A-TG--GG-G--AG-C-A-
AA--------CA--GG Bacteria(100);Fusobacteria(100);Fusobacteria(100);Fusobacteriales(100);Fusobacteriaceae(100);unclassified(100);unclassified(100);unclassified(100);unclassified(100);

Otu0349 0 0 0 0 0 0 0 0 0 0 0 0 0 0 0 0 0 0 0 0 0 0 0 0 0 0 0 0
0 0 0 0 0 0 0 0 0 0 0 0 0 0 0 0 0 0 0 0 0 0 0 0 0 0 0 0 0
0 0 0 0 0 0 0 0 0 0 0 0 0 0 0 0 0 26 0 0 0 0 0 0 0 0 0 0 0
9 0 0 0 0 0 0 0 0 0 0 0 0 0 0 16 0 0 0 0 0 0 0 0 0 0 0
M02255_77_000000000-ADUT3_1_1115_6185_11159 T----AC---GT-AG-GCA--------GCG-A-G------C--G--T---T--GT-T-CGG-AG------TT-A--C-T--GG-GC-------GT--A-----AA-GG-GT-GT-------G-TA-G-G-C-G------------G--T-TG-T-T-T----------AA-
---G-T-T-T--------G-G-T--G--TG--A-AA-TC--T-C-CC-G-G-----CT-C-AA------C-T-G-G-G-A-G--G-G----T-G--C-G---C--C-------G--AA-T-A---C-------T--G-A--AT--G-A-C----T-A-G-A-G-T--G---C-GG-----GA-G-A--------G-G-A-G---AG-T------GG--A--ATT----C-C-T-G-
GT--GT-A-G-CG-GT--G---A--A-A---TG-C-GT-AG--AT-A-TC--A-G--G-A-G-G-A-AC-A-CC---GG--T--G--GT-GTA-G--A-C--G-------G--C-T-C-T---CTG--G--AC-C-G--------------------T-A---A-C-T--GA--CG-C-T-G--A-GA--C-A-CG-A--AA-G-C---G-TG--GG-T--AG-C-
A-AA--------CA--GG Bacteria(100);Acidobacteria(100);Acidobacteria(100);Acidobacteriales(100);Acidobacteriaceae(100);unclassified(69);unclassified(69);unclassified(69);unclassified(69);

Otu0350 0 0 1 0 0 0 0 0 0 21 1 7 0 0 0 0 0 0 0 0 0 0 1 0 0 2 2 0
22 0 0 0 0 0 0 0 0 0 0 0 0 0 0 0 4 0 0 0 0 0 0 0 0 0 0 0 0
0 0 0 0 0 0 0 0 0 0 0 0 0 0 0 0 0 0 0 0 0 4 1 0 0 0 0 0 0
3 0 0 0 0 0 3 0 2 9 1 0 0 1 0 0 0 0 9 0 1 3 4 18 0 0 0
M02255_77_000000000-ADUT3_1_2107_27764_19886 T----AC---AG-AG-GGT--------GCA-A-G------C--G--T---T--AA-T-CGG-AA------TT-A-----------C--------T-----------G--G--GC-------G-TA-G-G-T-G-------------G--C-TT-G-A-T-----------
AA----G-T-C-A--------G-A-T--G--TG--A-AA-TC--C-C-CG-G-G-----CT-T-AA------C-C-T-G-G-G-A--A-C----T-G--C-A---T--C-------T--GA-T-A---C-------T--G-T--TA--G-G-C----T-A-G-A-A-T--A---G-GT-----GA-G-A--------G-G-A-A---GG-T------AG--A--ATT----C-C-A-
G-GT--GT-A-G-CG-GT--G---A--A-A---TG-C-GT-AG--AG-A-TC--T-G--G-A-G-G-A-AT-A-CC---GA--T--G--GC-GAA-G--G-C--A-------G--C-C-T-T---CTG--G--CA-T-C--------------------A-T---A-T-T--GA--CA-C-T-G--A-GG--T-T-CG-A--AA-G-C---G-TG--GG-T--AG-
C-A-AA--------CA--GG Bacteria(100);Proteobacteria(100);Gammaproteobacteria(100);Pseudomonadales(100);Moraxellaceae(100);Psychrobacter(100);unclassified(100);unclassified(100);unclassified(100);

Otu0351 0 0 0 0 0 0 0 0 0 0 0 0 0 0 0 0 0 0 0 0 0 0 0 0 0 0 0 0
0 0 5 0 0 0 0 0 0 0 139 0 0 0 0 0 0 0 0 0 0 0 0 0 14 0 0 0 0
0 0 0 0 0 0 0 0 0 0 0 0 0 0 0 0 0 0 0 0 0 0 0 0 0 0 0 0 0
0 0 0 0 0 0 0 0 0 0 0 0 0 0 0 0 0 0 0 0 0 0 0 0 0 0 0
M02255_77_000000000-ADUT3_1_1114_22460_19846 T----AC---GG-AG-GGG--------GCT-A-G------C--G--T---T--GT-T-CGG-AA------TT-A--C-T--GG-GC-------GT--A-----AA-GG-GC-AC-------G-TA-G-G-C-G------------G--G-CT-G-T-T----
-------AA----G-T-C-G--------G-G-G--G--TG--A-AA-TC--C-T-GA-G-G-----CT-C-AA------C-C-T-C-A-G-A--A-T----T-G--C-C---T--T-------C--GA-T-A---C-------T--G-G--CG--G-T-C----T-T-G-A-G-T--G---C-GG-----GA-G-A--------G-G-T-G---AG-T------GG--A--ATT----
C-C-C-A-GT--GT-A-G-AG-GT--G---A--A-A---TT-C-GT-AG--AT-A-TT--G-G--G-A-A-G-A-AC-A-CC---AG--T--G--GC-GAA-G--G-C--G-------G--C-T-C-A---CTG--G--CC-C-G--------------------C-A---A-C-T--GA--CG-C-T-G--A-GG--T-G-CG-A--AA-G-C---G-TG--GG-
G--AG-C-A-AA--------CA--GG Bacteria(100);Proteobacteria(100);Alphaproteobacteria(100);unclassified(100);unclassified(100);unclassified(100);unclassified(100);unclassified(100);unclassified(100);

Otu0352 0 0 0 0 0 0 0 0 0 0 0 0 0 0 0 0 0 0 0 0 0 0 0 0 0 0 0 0
0 0 0 0 0 0 0 0 0 0 0 0 0 0 0 0 0 0 0 0 0 0 0 0 0 0 0 0 0
0 0 0 0 0 0 0 0 0 0 0 0 0 0 93 61 0 0 0 0 0 0 0 0 0 0 0 0 0
0 0 0 0 0 0 0 0 0 0 0 0 0 0 0 0 0 0 0 0 0 0 0 0 0 0 0
M02255_77_000000000-ADUT3_1_1104_7574_20611 T----AA---GC-AC-CGG--------CTA-A-C------T--A--C---G--TG-C-CAG-CA------GC-C--G-C--GG-TA-------AT--A-----CG-TA-GG-GT-------G-CG-A-G-C-G------------T--TAAT-C-G-G-----------
AAT---T-A-C-T--------G-A-T--G--TG--A-AA-TC--C-C-CG-G-G-----CT-C-AA------C-C-T-G-G-G-A--A-C----T-G--C-A---T--T-------G--GT-G-A---C-------T--G-C--GA--A-A-C----T-G-G-A-G-T--G---C-GG-----CA-G-A--------G-G-G-A---GA-T------GG--A--ATT----C-C-
G-C-GT--GT-A-G-CA-GT--G---A--A-A---TG-C-GT-AG--AT-A-TG--C-G--G-A-G-G-A-AC-A-CC---GA--T--G--GC-GAA-G--G-C--A-------G--T-C-T-C---CTG--G--GC-C-T--------------------G-C---A-C-T--GA--CG-C-T-C--A-TG--C-A-CG-A--AA-G-C---G-TG--GG-G--
AG-C-A-AA--------CA--GG Bacteria(100);Proteobacteria(100);Betaproteobacteria(100);Burkholderiales(81);unclassified(81);unclassified(81);unclassified(81);unclassified(81);unclassified(81);

Otu0353 59 0 29 0 0 0 0 0 0 0 0 0 0 0 0 0 0 0 2 0 0 0 0 0 0 0 0 0
0 0 1 0 0 15 0 0 0 0 16 13 0 0 0 0 0 0 0 13 0 0 0 0 0 0 0 0 0
0 0 0 0 0 1 0 0 0 0 0 0 0 0 0 0 0 27 0 0 0 0 0 0 0 0 0 20 0
22 0 0 0 0 0 0 0 0 0 0 0 1 45 0 37 0 85 0 0 0 54 5 0 17 0 0
M02255_77_000000000-ADUT3_1_2118_18518_8232 T----AC---GT-AG-GGT--------GCG-A-G------C--G--T---T--GT-C-CGG-AA------TT-A--C-T--GG-GC-------GT--A-----AA-GA-GC-TC-------G-TA-G-G-C-G------------G--T-TT-G-T-C-----------AC-
---G-T-C-G--------T-C-T--G--TG--A-AA-TC--C-T-CC-A-G-----CT-T-AA------C-T-G-G-G-G-G--C-G----T-G--C-A---G--G-------C--GA-T-A---C-------G--G-G--CA--G-A-C----T-T-G-A-G-T--A---C-TA-----CA-G-G--------G-G-A-G---AC-T------GG--A--ATT----C-C-T-G-
GT--GT-A-G-CG-GT--G---A--A-A---TG-C-GC-AG--AT-A-TC--A-G--G-A-G-G-A-AC-A-CC---GG--T--G--GC-GAA-G--G-C--G-------G--G-T-C-T---CTG--G--GT-A-G--------------------T-A---A-C-T--GA--CG-C-T-G--A-GG--A-G-CG-A--AA-G-C---A-TG--GG-T--AG-
C-G-AA--------CA--GG Bacteria(100);Actinobacteria(100);Actinobacteria(100);Actinobacteridae(100);Actinomycetales(100);Corynebacterineae(100);Dietziaceae(89);Dietzia(89);unclassified(89);

Otu0354 155 0 0 0 0 0 0 0 0 0 0 0 0 0 0 0 0 0 0 0 0 0 0 0 0 0 0 0
1 0 0 0 0 0 0 0 0 0 0 0 0 14 0 0 0 0 0 0 0 0 0 0 0 0 0 0 0
0 0 0 0 0 0 0 0 0 0 0 0 0 0 0 0 0 0 0 0 0 0 0 0 0 0 0 0 0
0 121 0 0 0 0 0 0 0 0 0 0 0 0 0 0 0 0 0 0 0 0 0 0 0 0 0
M02255_76_000000000-ADUNE_1_1107_9731_10210 T----AC---GT-AG-GGA--------CCA-A-G------C--G--T---T--GT-T-CGG-AT------TT-A--C-T--GG-GC-------GT--A-----AA---------------G----G-G-C-----------------G--C-G-G-T-----------AA----G-T-
C-A--------C-T-T--G--TG--A-AA-TC--T-C-TG-A-G-----CT-T-AA------C-T-C-A-G-A-A--C-G----G-C--C-A---A--G-------T--GA-T-A---C-------T--G-C--AG--T-G-C----T-A-G-A-G-T--G---T-GG-----AA-G-G--------G-G-C-A---AT-C------GG--A--ATT----C-T-T-G-GT--GT-
A-G-CG-GT--G---A--A-A---TG-C-GT-AG--AT-A-TC--A-A--G-A-G-G-A-AC-A-CC---TG--A--G--GT-GAA-G--A-C--G-------G--G-T-T-G---CTG--G--GC-C-A--------------------A-C---A-C-T--GA--CG-C-T-G--A-GG--C-G-CG-A--AA-G-C---C-AG--GG-G--AG-C-A-AA--
------CG--GG Bacteria(100);Acidobacteria(100);Acidobacteria(100);Acidobacteriales(100);Acidobacteriaceae(100);Candidatus_Chloroacidobacterium(100);unclassified(100);unclassified(100);unclassified(100);

Otu0355 0 0 0 0 0 0 0 0 0 0 0 0 0 0 0 0 0 0 0 0 0 0 0 0 0 0 0 1
0 0 0 0 0 0 0 64 0 0 0 0 28 0 0 0 67 0 29 0 0 51 0 0 10 0 0 0 0
101 0 14 0 0 0 0 0 0 0 23 24 0 2 15 0 0 0 270 847 365 0 0 0 0 0 0 1 84
1 0 11 0 0 170 0 0 0 298 0 202 7 0 9 0 0 114 0 0 0 0 93 0 0 0 0
M02255_77_000000000-ADUT3_1_2118_25557_9143 T----AC---GT-AG-GGG--------GCT-A-G------C--G--T---T--GT-C-CGG-AA------TC-A--C-T--GG-GC-------GT--A-----AA-GG-GT-TC-------G-CA-G-G-C-G------------G--C-AA-T-G-C----------
AA----G-T-C-A--------G-A-T--G--TA--A-AA-GG--C-A-AA-G-G-----CT-C-AA------C-C-T-T-T-G-T--A------A-G--C-A---T--C-------T--GA-A-A---C-------T--G-T--AT--A-G-C----T-T-G-A-G-A--A---G-TG-----TA-G-A--------G-G-C-A---AG-T------GG--A--ATT----T-T-T-
A-GT--GT-A-G-CG-GT--G---A--A-A---TG-C-GT-AG--AT-A-TT--A-A--A-A-A-G-A-AT-A-CC---GG--T--G--GC-GAA-G--G-C--G-------A--C-T-T-G---CTG--G--GC-A-C--------------------A-A---T-C-T--GA--CG-C-T-G--A-GG--A-A-CG-A--AA-G-C---G-TG--GG-G--
AG-C-A-AA--------CA--GG Bacteria(100);Firmicutes(100);Clostridia(100);Clostridiales(100);Family_XI_Incertae_Sedis(100);Peptoniphilus(100);unclassified(100);unclassified(100);unclassified(100);

Otu0356 0 0 0 0 0 0 0 0 0 0 0 0 0 0 0 0 0 0 0 1 2 0 0 0 18 0 0 16
0 0 0 0 0 0 0 0 0 5 0 0 0 0 0 0 0 0 0 0 0 0 0 0 0 0 0 0 0
0 0 0 0 0 0 0 0 0 0 0 0 0 0 0 0 0 0 0 0 0 0 0 0 0 0 0 0 0
0 0 0 0 0 0 0 0 0 28 0 0 0 0 0 0 0 0 0 0 0 0 0 0 0 0 0
M02255_77_000000000-ADUT3_1_2103_27258_15484 T----AC---GG-AG-GGG--------GCT-A-G------C--G--T---T--GT-T-CGG-AA------TT-A--C-T--GG-GC-------GT--A-----AA-GC-GC-AC-------G-TA-G-G-C-G------------G--A-CT-G-G-A----
-------AA----G-T-T-G--------G-G-G--G--TG--A-AA-TC--C-C-GG-G-G-----CT-C-AA------C-C-T-C-G-G-A--A-C----T-G--C-C---T--T-------C--AA-A-A---C-------T--A-T--CA--G-T-C----T-G-G-A-G-T--T---C-GG-----GA-G-A--------G-G-T-G---AG-T------GG--A--ATT----
G-C-G-A-GT--GT-A-G-AG-GT--G---A--A-A---TT-C-GT-AG--AT-A-TT--C-G--C-A-G-G-A-AC-A-CC---AG--T--G--GC-GAA-G--G-C--G-------G--C-T-C-A---CTG--G--CT-C-G--------------------A-T---A-C-T--GA--CG-C-T-G--A-GG--T-G-CG-A--AA-G-C---G-TG--GG-
G--AG-C-A-AA--------CA--GG Bacteria(100);Proteobacteria(100);Alphaproteobacteria(100);Rhodobacterales(100);Rhodobacteraceae(100);unclassified(100);unclassified(100);unclassified(100);unclassified(100);

Otu0357 0 0 25 0 0 0 19 93 115 0 0 0 0 0 0 0 0 0 0 0 0 0 0 0 0 0 0 0
0 0 0 0 0 0 28 0 0 0 0 0 0 0 0 0 0 0 0 0 0 0 0 0 0 0 0 0 0
0 0 0 0 0 0 0 0 0 0 0 0 0 0 0 0 0 0 0 0 0 0 0 0 0 0 0 0 0
0 0 0 0 0 0 0 0 0 0 0 0 0 0 0 0 0 0 0 0 0 0 0 61 0 0
M02255_77_000000000-ADUT3_1_1114_4849_7739 T----AC---GT-AG-GGG--------GCG-A-G------C--G--T---T--GT-C-CGG-AA------TG-A--T-T--GG-GC-------GT--A-----AA-GG-GC-GC-------G-TA-G-G-C-G------------G--C-CT-G-C-T----------
AA----G-T-C-T--------G-A-A--G--TG--A-AA-GT--C-C-TG-C-T-----TT-C-AA------G-G-T-G-G-G-A--A-G----T-G--C-T---T--T-------G--GA-T-A---C-------T--G-G--TG--G-G-C----T-G-G-A-G-T--G---C-AG-----GA-G-A--------G-G-A-A---AG-C------GG--A--ATT----A-C-
C-G-GT--GT-A-G-CG-GT--G---A--A-A---TG-C-GT-AG--AG-A-TC--G-G--T-A-G-G-A-AC-A-CC---AG--T--G--GC-GAA-G--G-C--G-------G--C-T-T-T---CTG--G--AC-T-G--------------------A-A---A-C-T--GA--CG-C-T-G--A-GG--C-G-CG-A--AA-G-C---G-TG--GG-G--
AG-C-A-AA--------CA--GG Bacteria(100);unclassified(100);unclassified(100);unclassified(100);unclassified(100);unclassified(100);unclassified(100);unclassified(100);unclassified(100);

Otu0358 0 0 0 0 0 0 0 0 0 0 1 0 0 0 0 0 0 0 0 0 0 0 0 0 0 0 0 7
0 0 0 0 0 0 0 428 79 0 42 131 0 0 0 1 0 0 0 0 0 0 0 0 0 0 0 0 0
0 0 0 0 0 0 0 0 0 0 0 0 0 0 0 0 0 0 0 0 0 0 0 0 0 0 0 0 0
0 0 0 0 0 0 0 0 0 0 0 0 0 0 0 0 0 0 38 0 0 0 0 0 0 0 0
M02255_77_000000000-ADUT3_1_1117_13282_11517 T----AC---GT-AG-GGG--------GCG-A-G------C--G--T---T--GT-C-CGG-AA------TC-A--T-T--GG-GC-------GT--A-----AA-GA-GC-TC-------G-TA-G-G-C-G------------G--T-TC-A-G-T-----
------AA----G-T-C-G--------G-C-T--G--TG--A-AA-TT--C-C-GA-G-G-----CT-C-AA------C-C-T-C-G-G-A--A------C-T-GC-A---G--T-------C--GA-A-A---C-------T--A-T--TG-TG-A-C----T-C-G-A-G-T--C---C-GG-----TA-G-A--------G-G-A-G---AA-T------GG--A--ATT----C-
C-C-G-GT--GT-A-G-CG-GT--G---A--A-A---TG-C-GC-AG--AT-A-TC--G-G--G-A-G-G-A-AC-A-CC---AG--T--A--GC-GAA-G--G-C--G-------G--T-T-C-T---CTG--G--GC-C-G--------------------G-T---A-C-T--GA--CG-C-T-G--A-GG--A-G-CG-A--AA-G-C---G-TG--GG-
G--AG-C-A-AA--------CA--GG Bacteria(100);unclassified(100);unclassified(100);unclassified(100);unclassified(100);unclassified(100);unclassified(100);unclassified(100);unclassified(100);

Otu0359 0 0 0 0 0 0 0 0 0 0 0 0 0 0 0 0 0 0 0 0 0 0 0 0 0 0 0 0
0 0 0 1 0 0 0 0 0 0 66 3 0 0 0 0 0 0 0 0 0 0 0 0 24 0 0 0 0
0 0 0 0 0 0 0 0 0 0 0 0 0 0 0 0 0 0 0 0 0 0 0 0 0 0 0 0 0
0 0 0 0 0 0 0 0 0 0 0 0 0 0 0 0 0 0 0 0 0 0 0 0 0 0 0
M02255_77_000000000-ADUT3_1_2106_28961_11219 T----AC---GA-AG-GGG--------GCT-A-G------C--G--T---T--GT-T-CGG-AA------TC-A--C-T--GG-GC-------GT--A-----AA-GC-GC-AC-------G-TA-G-G-C-G------------G--A-CT-T-T-T-----
------AA----G-T-C-A--------G-A-G--G--TG--A-AA-TC--C-C-AA-G-G-----CT-C-AA------C-C-T-T-G-G-A--A-C----T-G--C-C---T--T-------T--GA-T-A---C-------T--G-G--GA--G-T-C----T-T-G-A-G-T--T---C-GG-----GA-G-A--------G-G-T-G---AG-T------GG--A--ACT----G-
C-G-A-GT--GT-A-G-AG-GT--G---A--A-A---TT-C-GT-AG--AT-A-TT--C-G--C-A-A-G-A-AC-A-CC---AG--T--G--GC-GAA-G--G-C--G-------G--C-T-C-A---CTG--G--CC-C-G--------------------A-T---A-C-T--GA--CG-C-T-G--A-GG--T-G-CG-A--AA-G-C---G-TG--GG-G-
-AG-C-A-AA--------CA--GG Bacteria(100);Proteobacteria(100);Alphaproteobacteria(100);Rhizobiales(100);unclassified(100);unclassified(100);unclassified(100);unclassified(100);unclassified(100);

Otu0360 0 0 0 0 0 0 0 0 0 0 0 0 0 0 0 0 0 0 0 0 0 0 0 0 0 0 0 13
0 0 0 0 0 0 0 0 0 0 0 0 0 0 0 0 0 0 0 0 0 0 0 0 0 0 0 0
0 0 0 0 0 0 0 0 0 0 0 0 0 0 0 0 0 19 0 0 0 0 0 0 0 0 0 0 0
0 0 0 0 0 0 0 0 0 0 0 0 0 0 0 34 0 0 0 0 0 0 0 0 0 0 0
M02255_77_000000000-ADUT3_1_1110_20245_17613 T----AC---GG-AG-GGA--------GCT-A-G------C--G--T---T--GT-T-CGG-AA------TT-A--C-T--GG-GC-------GT--A-----AA-GC-GC-AC-------G-TA-G-G-C-G------------G--C-TA-T-T-C-----
------AA----G-T-C-G--------G-G-G--G--TG--A-AA-GC--C-C-GG-G-G-----CT-C-AA------C-C-C-C-G-G-A--A-C----T-G--C-C---C--T-------C--GA-A-A---C-------T--A-G--GT--A-G-C----T-A-G-A-A-T--C---T-TG-----GA-G-A--------G-G-T-C---AG-T------GG--A--ATT----
C-C-G-A-GT--GT-A-G-AG-GT--G---A--A-A---TT-C-GT-AG--AT-A-TT--C-G--G-A-A-G-A-AC-A-CC---AG--T--G--GC-GAA-G--G-C--G-------A--C-T-G-A---CTG--G--AC-A-A--------------------G-T---A-T-T--GA--CG-C-T-G--A-GG--T-G-CG-A--AA-G-C---G-TG--
GG-G--AG-C-A-AA--------CA--GG Bacteria(100);Proteobacteria(100);Alphaproteobacteria(100);Sphingomonadales(100);unclassified(100);unclassified(100);unclassified(100);unclassified(100);unclassified(100);

Otu0361 0 0 0 0 0 0 0 0 0 0 0 0 0 0 0 1 0 0 0 2 0 0 0 0 0 0 0 0
0 0 0 0 0 0 0 0 0 0 0 0 0 0 0 0 0 0 0 0 0 0 0 0 46 0 0 0
0 0 0 0 0 0 0 0 0 0 0 0 0 0 0 0 0 13 0 0 0 0 2 0 0 0 0 0 0
0 0 0 0 0 0 0 0 0 0 0 0 0 8 0 0 0 0 0 0 10 0 0 0 0 0 0
M02255_77_000000000-ADUT3_1_2114_25602_13003 T----AC---GT-AG-GGT--------GCA-A-G------C--G--T---T--GT-C-CGG-AA------TT-A--T-T--GG-GC-------GT--A-----AA-GA-GC-TC-------G-TA-G-G-C-G-------------G--T-TC-G-T-C------
-----GC----G-T-C-G--------G-C-T--G--TG--A-AA-AC--C-T-GA-G-G-----CT-C-AA------C-C-T-C-A-G-G--C-C----T-G--C-A---G--T-------C--GA-T-A---C-------G--G-G--CG--G-A-C----T-T-G-A-G-T--A---C-TG-----CA-G-G--------G-G-A-G---AC-T------GG--A--ATT----C-
C-T-G-GT--GT-A-G-CG-GT--G---A--A-A---TG-C-GC-AG--AT-A-TC--A-G--G-A-G-G-A-AC-A-CC---GG--T--G--GC-GAA-G--G-C--G-------G--G-T-C-T---CTG--G--GC-A-G--------------------T-A---A-C-T--GA--CG-C-T-G--A-GG--A-G-CG-A--AA-G-C---G-TG--GG-
G--AG-C-G-AA--------CA--GG Bacteria(100);Actinobacteria(100);Actinobacteria(100);Actinobacteridae(100);Actinomycetales(100);Frankineae(91);Geodermatophilaceae(91);unclassified(91);unclassified(91);

Otu0362 0 0 0 0 0 0 0 0 0 0 0 0 0 0 0 0 0 0 0 0 0 0 0 12 0 5 0 4
0 0 0 0 0 0 0 0 0 0 0 0 0 0 0 0 0 0 0 0 0 0 0 0 0 0 0 0
0 41 5 0 0 0 0 0 0 0 0 0 0 0 0 1 0 0 0 0 0 0 0 0 0 0 0 0 0
24 0 0 51 60 0 2 0 0 0 0 0 0 0 0 0 0 0 0 0 0 0 0 0 0 0 0
M02255_77_000000000-ADUT3_1_1111_8278_14323 T----AC---GT-AT-GTC--------GCG-A-G------C--G--T---T--AT-C-CGG-AA------TT-A--T-T--GG-GC-------AT--A-----AA-GG-GC-AT-------C-TA-G-G-C-G------------G--A-TA-T-A-C-----------AA--
--G-T-C-A--------G-G-G--G--TG--A-AA-AC--T-T-AG-G-G-----CT-C-AA------C-T-C-A-A-A-G--C-T----T-G--C-C---T--T-------T--GA-A-A---C-------T--G-T--AT--A-T-C----T-A-G-A-G-T--G---C-TG-----GA-G-A--------G-G-T-G---GA-C------GG--A--ACT----A-C-A-C-
GA--GT-A-G-AG-GT--G---A--A-A---TT-C-GT-AG--AT-A-TG--T-G--T-A-G-G-A-AT-G-CC---GA--T--G--AT-GAA-G--A-T--A-------G--T-C-C-A---CTG--G--AC-A-G--------------------C-A---A-C-T--GA--CG-C-T-G--A-AG--T-G-CG-A--AA-G-C---T-AG--GG-G--AG-C-
A-AA--------CA--GG Bacteria(100);Fusobacteria(100);Fusobacteria(100);Fusobacteriales(100);Leptotrichiaceae(100);Leptotrichia(100);unclassified(100);unclassified(100);unclassified(100);

Otu0363 4 22 21 0 0 0 13 0 30 0 0 0 0 0 0 0 0 0 0 0 0 0 0 0 0 0 0 0
0 0 0 0 0 0 4 0 0 0 0 20 0 0 0 0 0 0 0 0 0 0 0 0 0 0 0 0
0 0 0 0 0 0 0 0 0 0 0 0 0 0 0 0 0 0 0 0 0 0 0 0 0 0 0 0 0
0 0 0 0 0 0 0 0 0 0 0 0 0 0 0 0 0 0 0 0 0 0 6 0 20 0 0
M02255_76_000000000-ADUNE_1_2105_19266_8185 T----CG---GC-TA-ACT--------CCG-T-G------C--C--A---G--CA-G-CCG-CG------GT-A--A-T--AC-GG-------AG--G-----AT-CC-GA-GC-------GTTA-T-C-C-G------------G--ATTT-G-T-T-----------AA-
---G-T-C-A--------G-T-T--G--TG--A-AA-GT--T-T-GC-G-G-----CT-C-AA------C-C-G-T-A-A-A--A-T----T-G--C-A---G--T-------T--GA-T-A---C-------T--G-G--CG--A-C-C----T-T-G-A-G-T--G---C-AA-----CA-G-A--------G-G-T-A---GG-C------GG--A--ATT----C-G-T-G-
GT--GT-A-G-CG-GT--G---A--A-A---TG-C-TT-AG--AT-A-TC--A-C--G-A-A-G-A-AC-T-CC---GA--T--T--GC-GAA-G--G-C--A-------G--C-T-T-A---CTG--G--AT-T-G--------------------T-A---A-C-T--GA--CG-C-T-G--A-TG--C-T-CG-A--AA-G-T---G-TG--GG-T--AT-C-A-
AA--------CA--GG Bacteria(100);Bacteroidetes(100);Bacteroidia(100);Bacteroidales(100);Bacteroidaceae(83);Bacteroides(83);unclassified(83);unclassified(83);unclassified(83);

Otu0364 0 0 0 0 0 0 0 0 0 0 0 0 0 0 0 0 0 0 0 0 0 4 0 0 0 0 0 0
0 0 0 0 0 0 0 9 0 0 0 21 0 0 0 0 0 0 0 11 0 0 0 6 0 0 0 0 0
0 0 0 0 0 0 0 0 0 0 0 0 0 0 0 0 0 23 0 0 0 0 0 0 0 0 0 0 0
1 0 0 0 0 0 0 0 0 0 0 0 0 0 0 0 31 0 0 0 0 0 0 0 0 0 0
M02255_77_000000000-ADUT3_1_1115_20054_12914 T----AC---GT-AG-GGT--------GCG-A-G------C--G--T---T--GT-C-CGG-AA------TT-A--T-T--GG-GC-------GT--A-----AA-GA-GC-TC-------G-TA-G-G-C-G-------------G--T-CT-G-T-C------
-----GC----G-T-C-G--------G-T-C--G--TG--A-AA-AC--C-T-GC-A-G-----CT-T-AA------C-T-G-T-G-G-G--C-T----T-G--C-G---G--T-------C--GA-T-A---C-------G--G-G--CAT-G-A-C----T-G-G-A-G-T--T---C-GG-----CA-G-G--------G-G-A-G---AC-T------GG--A--ATT----
C-C-T-G-GT--GT-A-G-CG-GT--G---A--A-A---TG-C-GC-AG--AT-A-TC--A-G--G-A-G-G-A-AC-A-CC---GG--T--G--GC-GAA-G--G-C--G-------G--G-T-C-T---CTG--G--GC-C-G--------------------A-T---A-C-T--GA--CG-C-T-G--A-GG--A-G-CG-A--AA-G-C---G-TG--
GG-G--AG-C-G-AA--------CA--GG Bacteria(100);Actinobacteria(100);Actinobacteria(100);Actinobacteridae(100);Actinomycetales(100);Pseudonocardineae(52);Pseudonocardiaceae(52);Pseudonocardia(52);unclassified(52);

Otu0365 0 0 0 0 0 0 0 0 0 0 0 0 0 0 0 0 0 0 0 0 0 0 0 0 0 0 0 0
0 0 0 0 0 0 0 0 0 0 0 18 0 0 0 0 0 0 0 0 0 0 0 0 0 0 0 0 0
0 0 0 0 0 0 0 0 0 0 0 0 0 0 17 35 0 0 0 37 0 0 0 0 0 0 0 24 0
0 0 0 0 0 0 0 0 0 0 0 0 4 0 0 0 0 0 0 0 0 0 0 0 0 0 0
M02255_77_000000000-ADUT3_1_2114_5807_16046 T----AC---GT-AG-GGG--------GCA-A-G------C--G--T---T--GT-C-CGG-AA------TC-A--T-T--GG-GC-------GT--A-----AA-GA-GT-AC-------G-TA-G-G-C-G------------G--T-CT-G-A-T-----------AA-
---G-C-A-C--------A-A-G--G--TT--T-AA-GG--C-A-AC-A-G-----CT-C-AA------C-T-G-T-T-G-T--T------C-G--C-C---T--T-------G--TG-A-A---C-------T--G-T--TA--G-A-C----T-T-G-A-G-T--G---C-GG-----GA-G-A--------G-G-A-A---AG-C------GG--A--ATT----C-C-T-G-
GT--GT-A-G-CG-GT--G---A--A-A---TG-C-GT-AG--AT-A-TC--A-G--G-A-G-G-A-AT-A-CC---GG--T--G--GC-GAA-G--G-C--G-------G--C-T-T-T---CTG--G--AC-C-G--------------------T-A---A-C-T--GA--CG-C-T-G--A-GG--T-A-CG-A--AA-G-C---G-TG--GG-G--AG-
C-A-AA--------CA--GG Bacteria(100);Firmicutes(52);Clostridia(52);Clostridiales(52);unclassified(52);unclassified(52);unclassified(52);unclassified(52);unclassified(52);

Otu0366 0 0 0 0 0 0 0 0 0 0 0 0 0 0 0 0 0 0 0 0 0 0 0 0 0 0 0 0
0 0 0 0 59 0 0 42 0 0 0 0 0 43 0 0 0 0 0 0 0 0 0 0 0 0 0 0 0
0 0 0 0 0 0 0 0 0 0 4 0 0 1 11 0 0 0 0 0 0 4 0 33 0 0 0 0 0
0 0 0 0 0 0 0 0 2 0 0 0 0 0 0 18 0 0 0 0 0 0 0 0 0 0 0
M02255_76_000000000-ADUNE_1_1116_24883_4572 T----AC---GT-AG-GGC--------GCA-A-G------C--G--T---T--GT-C-CGG-AA------TT-A--T-T--GG-GC-------GT--------AA------------------A---G---G------------G--C-TT-G-T-C----------GC----G-T-
C-T--------G-C-C--G--TG--A-AA-AC--T-C-GA-G-G-----CT-T-AA------C-C-T-T-G-G-G--C-G----T-G--C-G---G--T-------G--GG-T-A---C-------G--G-G--CA--G-G-C----T-A-G-A-G-T--G---C-GG-----TA-G-G--------G-G-A-G---AC-T------GG--A--ATT----C-C-T-G-GT--
GT-A-G-CG-GT--G---A--A-A---TG-C-GC-AG--AT-A-TC--A-G--G-A-G-G-A-AC-A-CC---GA--T--G--GC-GAA-G--G-C--A-------G--G-T-C-T---CTG--G--GC-C-G--------------------T-T---A-C-T--GA--CA-C-T-G--A-GA--A-G-CG-A--AA-G-C---A-TG--GG-G--AG-C-G-
AA--------CA--GG Bacteria(100);Actinobacteria(100);Actinobacteria(100);Actinobacteridae(100);Actinomycetales(100);Micrococcineae(100);Dermabacteraceae(60);unclassified;unclassified;

Otu0367 0 3 0 1 0 0 3 0 0 0 0 0 0 0 0 0 0 0 0 0 0 0 0 0 0 0 0 0
0 0 0 0 2 0 0 0 0 0 0 0 0 0 0 0 0 0 0 0 0 0 0 0 0 0 0 0 0
0 0 0 0 0 0 0 0 0 0 0 0 0 0 2 0 0 0 0 0 0 0 0 0 0 0 0 2 6
0 0 13 0 0 0 0 2 0 0 0 0 0 0 0 0 0 4 0 1 2 0 0 0 2 0 19
M02255_76_000000000-ADUNE_1_2106_9941_18425 G----AA---GC-AC-CGG--------CTA-A-C------T--C--C---G--TG-C-CAG-CA------GC-C--G-C--GG-TA-------AT--A-----CG-GA-GG-GT-------G-CG-A-G-C-G------------T--T-AATC-G-G----------
AA----G-T-G-A--------G-G-T--G--TG--A-AA-GC--C-C-CG-G-G-----CT-T-AA------C-C-T-G-G-G-A--A-T----T-G--C-A---T--T-------T--CA-T-A---C-------T--G-G--GT--C-G-C----T-A-G-A-G-T--A---C-TT-----TA-G-G--------G-A-G-G---GG-T------AG--A--ATT----C-C-A-
C-GT--GT-A-G-CG-GT--G---A--A-A---TG-C-GT-AG--AG-A-TG--T-G--G-A-G-G-A-AT-A-CC---GA--A--G--GC-GAA-G--G-C--A-------G--C-C-C-C---TTG--G--GA-A-T--------------------G-T---A-C-T--GA--CG-C-T-C--A-TG--T-G-CG-A--AA-G-C---G-TG--GG-G--
AG-C-A-AA--------CA--GG Bacteria(100);Proteobacteria(100);Gammaproteobacteria(100);Pasteurellales(100);Pasteurellaceae(100);unclassified(100);unclassified(100);unclassified(100);unclassified(100);

Otu0368 0 0 0 0 0 0 81 9 175 0 0 0 0 0 0 0 0 0 0 0 0 0 0 0 0 0 0 0
0 0 0 0 0 0 0 0 0 0 0 0 0 0 0 48 0 0 0 0 0 0 0 0 0 0 0 0 0
0 0 0 0 0 0 0 0 0 0 0 0 0 0 0 0 0 0 0 0 0 0 0 0 0 0 0 0 0
0 0 0 0 0 0 0 0 0 0 0 0 0 0 0 0 0 0 0 0 0 0 0 0 21 0 0
M02255_77_000000000-ADUT3_1_1103_13193_8254 T----AC---GT-AG-GTG--------GCA-A-G------C--G--T---T--GT-C-CGG-AT------TT-A--C-T--GG-GT-------GT--A-----AA-GG-GC-GT-------G-TA-G-G-C-G------------G--A-GA-A-G-C----------
AA----G-T-C-A--------G-A-A--G--TG--A-AA-TC--C-A-TG-G-G-----CT-T-AA------C-C-C-A-T-G-A--A-C----T-G--C-T---T--T-------T--GA-A-A---C-------T--G-T--TT--C-C-C----T-T-G-A-G-T--A---T-CG-----GA-G-A--------G-G-C-A---GG-C------GG--A--ATT----C-C-T-
A-GT--GT-A-G-CG-GT--G---A--A-A---TG-C-GT-AG--AT-A-TT--A-G--G-A-G-G-A-AC-A-CC---AG--T--G--GC-GAA-G--G-C--G-------G--C-C-T-G---CTG--G--AC-G-A--------------------C-A---A-C-T--GA--CG-C-T-G--A-GG--C-G-CG-A--AA-G-C---G-TG--GG-G--
AG-C-A-AA--------CA--GG Bacteria(100);Firmicutes(100);Clostridia(100);Clostridiales(100);unclassified(100);unclassified(100);unclassified(100);unclassified(100);unclassified(100);

Otu0369 0 0 0 0 0 0 1 0 0 0 0 0 0 0 0 0 0 0 0 0 0 0 0 0 0 0 0 0
0 0 0 0 0 0 0 0 0 0 0 0 0 0 0 0 0 0 0 0 0 0 0 0 0 0 0 0 15
0 0 0 0 0 0 0 0 0 0 0 0 0 0 0 0 0 0 0 5 0 0 0 0 0 0 0 0 0
0 0 0 0 0 0 0 0 0 0 0 0 0 0 0 0 0 0 0 3 3 36 0 0 0 0 2
M02255_76_000000000-ADUNE_1_2104_16888_9901 C----GG---CT-AA-TTC--------CGT-G-C------C--A--G---C--AG-C-CGC-GG------TA-A--T-A--CG-GA-------AG--G-----TC-CA-GG-CG-------T-TA-T-C-C-G------------GA-T-TT-G-T-T-----------AA--
--G-C-G-T--------G-T-T--G--TG--A-AA-TT--T-A-GA-T-G-----CT-C-AA------C-A-T-T-T-A-A--C-T----T-G--C-A---G--C-------G--CG-A-A---C-------T--G-T--CA--G-A-C----T-T-G-A-G-T--A---C-AC-----GC-A-A--------C-G-T-A---TG-C------GG--A--ATT----C-A-T-G-GT--
GT-A-G-CG-GT--G---A--A-A---TG-C-TT-AG--AT-A-TC--A-T--G-A-A-G-A-AC-T-CC---GA--T--T--GC-GAA-G--G-C--A-------G--C-A-T-A---CGG--G--AG-T-G--------------------T-A---A-C-T--GA--CG-C-T-T--A-AG--C-T-CG-A--AG-G-T---G-CG--GG-T--AT-C-G-
AA--------CA--GG Bacteria(100);Bacteroidetes(100);Bacteroidia(100);Bacteroidales(100);Prevotellaceae(100);Prevotella(100);unclassified(100);unclassified(100);unclassified(100);

Otu0370 0 0 0 0 0 19 345 330 0 0 0 7 0 5 0 0 0 0 0 0 0 0 0 0 0 0 0 0
0 0 1 0 3 7 0 29 0 0 0 122 609 0 0 0 149 0 0 0 102 17 0 22 26 0 0 38 0
0 0 12 0 9 15 0 6 0 4 15 39 3 19 19 9 0 26 138 564 212 19 5 0 0 0 0 52 0
3 0 0 0 119 109 10 22 0 280 0 574 0 35 2 0 0 0 0 0 0 37 59 0 34 0 0
M02255_77_000000000-ADUT3_1_1115_11861_2639 T----AC---GT-AT-GGA--------GCG-A-G------C--G--T---T--GT-C-CGG-AA------TT-A--T-T--GG-GC-------GT--A-----AA-GG-GT-GC-------G-CA-G-G-C-G------------G--C-TT-T-A-C-----------AA-
---G-T-T-G--------G-A-T--G--TG--A-AA-TA--T-T-GT-G-G-----CT-C-AA------C-C-A-C-A-AAC--G------T-G--C-A---T--C-------C--AA-A-A---C-------T--G-C--AA--A-G-C----T-T-G-A-G-T--T---A-AG-----GA-G-A--------G-G-T-A---AG-T------GG--A--ATT----C-C-T-G-
GT--GT-A-G-CG-GT--G---G--A-A---TG-C-GT-AG--AT-A-TC--A-G--G-A-G-G-A-AT-A-CC---GG--T--G--GC-GAA-G--G-C--G-------A--C-T-T-A---CTG--G--AC-T-T--------------------A-A---A-C-T--GA--CG-C-T-C--A-GG--C-A-CG-A--AA-G-C---G-TG--GG-G--AG-
C-A-AA--------CA--GG Bacteria(100);Firmicutes(100);Clostridia(100);Clostridiales(100);Family_XI_Incertae_Sedis(100);Finegoldia(100);unclassified(100);unclassified(100);unclassified(100);

Otu0371 83 0 0 0 0 0 0 0 0 0 0 0 0 0 0 0 0 0 0 0 0 0 0 0 0 0 0 0
0 0 0 0 0 0 0 0 0 0 0 0 0 0 0 0 0 0 0 0 0 0 0 30 0 0 0 0 0
0 0 0 0 0 0 0 0 0 0 0 0 0 0 0 0 0 0 0 0 0 0 0 0 0 0 0 0 0
0 0 0 0 0 0 0 0 0 0 0 0 0 0 0 0 0 0 0 0 0 0 0 0 0 0 0
M02255_77_000000000-ADUT3_1_2107_23800_14180 T----AC---GT-AG-GGT--------CCG-A-G------C--G--T---T--GT-C-CGG-AA------TT-A--T-T--GG-GC-------GT--A-----AA-GG-GC-TC-------G-TA-G-G-C-G------------G--T-TC-G-T-C------
-----GC----G-T-C-G--------G-G-A--G--TG--A-AA-AC--T-C-GG-A-G-----CT-C-AA------C-T-C-C-G-A-G--C-C----T-G--C-T---T--C-------C--GA-T-A---C-------G--G-G--CA--G-A-C----T-A-G-A-G-G--T---A-GG-----CA-G-G--------G-G-A-G---AG-C------GG--A--ACT----
C-C-T-G-GT--GT-A-G-CG-GT--G---G--A-A---TG-C-GC-AG--AT-A-TC--A-G--G-A-A-G-A-AC-A-CC---GG--T--G--GC-GAA-G--G-C--G-------G--C-T-C-T---CTG--G--GC-C-T--------------------T-A---C-C-T--GA--CG-C-T-G--A-GG--A-G-CG-A--AA-G-C---G-TG--
GG-T--AG-C-G-AA--------CA--GG Bacteria(100);Actinobacteria(100);Actinobacteria(100);Actinobacteridae(100);Actinomycetales(100);Propionibacterineae(100);Nocardioidaceae(100);Kribbella(100);unclassified(100);

Otu0372 0 0 0 0 0 1 0 0 0 0 0 0 3 1 0 1 0 1 1 0 0 0 0 0 0 0 0 0
0 0 0 0 0 0 0 0 0 0 0 0 0 0 0 0 0 0 0 0 0 0 0 0 0 8 4 3 0
0 0 0 0 1 0 1 0 0 0 0 0 0 0 0 0 0 0 0 0 0 0 0 0 0 0 0 0 0
0 0 0 0 0 0 0 1 0 0 0 0 0 0 0 0 0 0 0 0 0 0 0 0 0 0 0
M02255_76_000000000-ADUNE_1_2106_7591_16212 C----TG---TG-CC-AGC--------AGC-C-G------C--G--G---T--AA-TACAG-AG------GG-T--G-C--AA-GC-------GT--T-----AA-TC-G--GA-------A-TT-A-C-T-G------------G--T-TT-G-T-T-----------AA--
--G-T-T-G--------G-A-T--G--TG--A-AA-TC--C-C-CG-G-G-----CT-C-AA------C-C-T-G-G-G-A--A-C----T-G--C-A---T--T-------C--AA-A-A---C-------T--G-A--CT--G-A-C----T-A-G-A-G-T--A---T-GG-----TA-G-A--------G-G-G-T---GG-T------GG--A--ATT----T-C-C-T-
GT--GT-A-G-CG-GT--G---A--A-A---TG-C-GT-AG--AT-A-TA--G-G--A-A-G-G-A-AC-A-CC---AG--T--G--GC-GAA-G--G-C--G-------A--C-C-A-C---CTG--G--AC-T-G--------------------A-T---A-C-T--GA--CA-C-T-G--A-GG--T-G-CG-A--AA-G-C---G-TG--GG-G--AG-
C-A-AA--------CA--GG Bacteria(100);Proteobacteria(100);Gammaproteobacteria(100);Pseudomonadales(100);Pseudomonadaceae(100);Pseudomonas(100);unclassified(100);unclassified(100);unclassified(100);

Otu0373 0 0 0 0 0 0 0 0 0 0 0 0 0 0 0 0 0 0 0 0 0 0 0 0 0 0 0 0
0 0 0 0 0 0 0 0 0 0 0 0 0 0 0 0 0 0 0 0 0 0 0 0 0 0 5 0 0
0 0 0 0 0 0 2 0 0 3 0 0 0 0 0 0 0 0 0 62 0 0 21 0 0 0 0 0 0
0 0 43 0 0 0 4 0 0 0 0 0 0 0 0 0 0 0 0 0 0 0 0 0 0 0 0
M02255_77_000000000-ADUT3_1_1107_23659_4803 T----AC---GA-AG-GGG--------GCT-A-G------C--G--T---T--GC-T-CGG-AA------TC-A--C-T--GG-GC-------GT--A-----AA-GG-GC-GC-------G-TA-G-G-C-G------------G--A-CG-T-T-T-----------
AA----G-T-C-G--------G-G-G--G--TG--A-AA-GC--C-C-GG-G-G-----CT-C-AA------C-C-C-C-G-G-A--A-T----T-G--C-C---T--T-------C--GA-T-A---C-------T--G-G--AC--G-T-C----T-C-G-A-G-A--C---C-GG-----AA-G-A--------G-G-T-A---AG-C------GG--A--ACT----G-C-
G-A-GT--GT-A-G-AG-GT--G---A--A-A---TT-C-GT-AG--AT-A-TT--C-G--C-A-A-G-A-AC-A-CC---AG--T--G--GC-GAA-G--G-C--G-------G--C-T-T-A---CTG--G--TC-C-G--------------------G-T---T-C-T--GA--CG-C-T-G--A-GG--C-G-CG-A--AA-G-C---G-TG--GG-G--
AG-C-A-AA--------CA--GG Bacteria(100);Proteobacteria(100);Alphaproteobacteria(100);Rhizobiales(100);Beijerinckiaceae(67);Chelatococcus(67);unclassified(67);unclassified(67);unclassified(67);

Otu0374 0 0 0 0 4 10 0 0 2 6 27 0 0 1 3 0 0 0 0 1 0 0 61 0 113 88 0 2
1 0 0 0 0 0 0 0 0 1 2 0 0 47 0 0 0 16 0 1 3 0 1 2 0 0 0 0 3
0 0 0 0 0 0 0 0 0 0 0 0 0 0 0 0 0 0 0 0 0 10 7 1 0 2 0 0 0
14 0 0 0 0 0 0 0 0 0 0 0 0 0 0 1 0 1 0 0 0 3 5 0 1 0 1 0 0
M02255_77_000000000-ADUT3_1_1101_19760_5940 T----AC---GG-AG-GGT--------GCG-A-G------C--G--T---T--AA-T-CGG-AA------TT-A--C-T--GG-GC-------GT--A-----AA-GC-GC-AT-------G-CA-G-G-C-G------------G--T-CT-G-T-T-----------AA-
---G-C-A-A--------G-A-T--G--TG--A-AA-GC--C-C-GG-G-G-----CT-C-AA------C-C-T-C-G-G-A--A-C----A-G--C-A---T--T-------T--TG-A-A---C-------T--G-G--CA--G-A-C----T-A-G-A-G-T--C---T-TG-----TA-G-A--------G-G-G-G---GG-T------AG--A--ATT----T-C-A-G-
GT--GT-A-G-CG-GT--G---A--A-A---TG-C-GT-AG--AG-A-TC--T-G--A-A-G-G-A-AT-A-CC---GG--T--G--GC-GAA-G--G-C--G-------G--C-C-C-C---CTG--G--AC-A-A--------------------A-G---A-C-T--GA--CG-C-T-C--A-GA--T-G-CG-A--AA-G-C---G-TG--GG-G--AG-
C-A-AA--------CA--GG Bacteria(100);Proteobacteria(100);Gammaproteobacteria(100);Vibrionales(100);Vibrionaceae(100);Photobacterium(100);unclassified(100);unclassified(100);unclassified(100);

Otu0375 0 0 0 0 0 0 0 0 0 0 0 0 0 0 0 0 0 0 3 0 0 0 0 0 0 0 0 0
0 0 0 0 0 0 0 0 0 0 0 0 0 0 0 0 0 0 0 0 0 0 0 0 0 0 0 0 0
0 104 7 0 0 0 0 0 0 0 51 0 0 0 0 0 0 0 0 0 0 0 0 0 0 0 0 0 0
0 0 0 0 0 0 0 0 0 0 0 0 0 0 0 0 0 0 0 0 0 0 0 0 0 0 0
M02255_77_000000000-ADUT3_1_1110_17525_15058 T----AC---GA-AG-GGG--------GCT-A-G------C--G--T---T--GT-T-CGG-AA------TC-A--C-T--GG-GC-------GT--A-----AA-GC-GC-AC-------G-TA-G-G-C-G------------G--A-CT-T-T-T-----
------AA----G-T-C-A--------G-G-G--G--TG--A-AA-TC--C-C-GG-G-G-----CT-C-AA------C-C-T-C-G-G-A--A-C----T-G--C-C---T--T-------T--GA-T-A---C-------T--G-G--AA--G-T-C----T-G-G-A-G-T--C---C-GA-----GA-G-A--------G-G-T-G---AG-T------GG--A--ACT----
C-C-G-A-GT--GT-A-G-AG-GT--G---A--A-A---TT-C-GT-AG--AT-A-TT--C-G--G-A-A-G-A-AC-A-CC---AG--T--G--GC-GAA-G--G-C--G-------G--C-T-C-A---CTG--G--CT-C-G--------------------G-T---A-C-T--GA--CG-C-T-G--A-GG--T-G-CG-A--AA-G-C---G-TG--GG-
G--AG-C-A-AA--------CA--GG Bacteria(100);Proteobacteria(100);Alphaproteobacteria(100);Rhodobacterales(74);Rhodobacteraceae(74);unclassified(74);unclassified(74);unclassified(74);unclassified(74);

Otu0376 0 0 0 0 0 0 0 0 0 0 0 0 0 0 0 0 0 0 0 0 0 0 0 0 0 0 3 0
4 6 0 0 0 0 0 0 0 0 0 0 0 0 0 0 0 0 0 0 0 0 0 0 0 0 0 0 0
0 0 0 0 0 0 0 0 0 0 0 0 0 0 0 0 0 0 0 0 0 1 23 0 0 0 0 0 0
0 0 0 0 0 0 58 0 0 0 0 0 0 0 0 0 0 0 0 0 0 0 0 0 0 0 0
M02255_76_000000000-ADUNE_1_1107_8418_16265 T----AC---GG-AG-GGT--------GCA-A-G------C--G--T---T--AC-T-CGG-AA------TC-A--C-T--GG-GC-------GT---------A--A----A--------G--A---G-C-G------------G--G-TA-G-G-T-----------AA----G-
T-C-A--------G-A-A--G--TG--A-AA-TC--C-A-AT-A-G-----CT-C-AA------C-T-A-T-T-G-A--A-C----T-G--C-T---T--T-------T--GA-A-A---C-------T--G-A--TT--A-C-C----T-A-G-A-A-T--A---T-GG-----GA-G-A--------G-G-T-A---GA-T------GG--A--ATT----T-C-T-G-GT--GT-
A-G-GG-GT--A---A--A-A---TC-C-GT-AG--AG-A-TC--A-G--A-A-G-G-A-AT-A-CC---GA--T--T--GC-GAA-G--G-C--G-------A--T-C-T-A---CTG--G--AA-C-A--------------------T-T---A-T-T--GA--CG-C-T-G--A-GA--C-G-CG-A--AA-G-C---G-TG--GG-G--AG-C-A-AA---
-----CA--GG Bacteria(100);Proteobacteria(100);Epsilonproteobacteria(100);Campylobacterales(100);Campylobacteraceae(100);Arcobacter(100);unclassified(100);unclassified(100);unclassified(100);

Otu0377 0 0 0 0 0 0 12 0 0 0 0 0 0 0 0 0 0 0 0 0 0 0 0 0 0 0 0 0
0 0 0 0 0 0 16 2 0 0 0 0 0 3 0 0 0 0 0 0 0 0 4 0 0 0 0 0 0
0 0 0 0 0 0 0 0 0 0 0 0 0 0 23 39 0 0 0 0 0 0 0 0 0 0 0 0 0
0 1 1 0 0 0 0 0 0 6 0 0 0 0 0 0 0 0 0 0 7 0 0 0 0 7 17
M02255_76_000000000-ADUNE_1_1107_10448_23341 C----GG---CT-AA-TTC--------CGT-G-C------C--A--G---C--AG-C-CGC-GG------TA-A--T-A--CG-GA-------AG--G-----TC-CG-GG-CG-------T-TA-T-C-C-G------------GA-T-CT-G-T-T-----
------AA----G-C-G-T--------G-T-T--G--TG--A-AA-TT--T-A-GG-T-G-----CT-C-AA------C-A-T-T-T-A-A--C-T----T-G--C-A---G--C-------G--CG-A-A---C-------T--G-T--CA--G-A-C----T-T-G-A-G-T--A---C-AC-----GC-A-G--------C-G-C-A---GG-C------GG--A--ATT----C-
A-T-G-GT--GT-A-G-CG-GT--G---A--A-A---TG-C-TT-AG--AT-A-TC--A-T--G-A-G-G-A-AC-T-CC---GA--T--C--GC-GAA-G--G-C--A-------G--C-C-T-G---CGG--G--AG-T-G--------------------T-T---A-C-T--GA--CG-C-T-T--A-AG--C-T-CG-A--AG-G-T---G-CG--GG-T--
AT-C-G-AA--------CA--GG Bacteria(100);Bacteroidetes(100);Bacteroidia(100);Bacteroidales(100);Prevotellaceae(100);unclassified(100);unclassified(100);unclassified(100);unclassified(100);

Otu0378 0 0 0 0 0 0 0 0 0 0 0 0 0 0 0 0 0 0 0 0 9 0 0 0 0 0 0 0
0 0 0 0 0 0 0 0 0 0 0 0 0 0 0 0 0 0 0 0 0 0 0 51 0 0 0 0 0
0 0 0 0 0 0 0 0 0 0 0 0 0 0 0 0 0 0 0 0 0 0 0 0 0 0 0 0 0
19 0 0 0 0 0 4 0 0 0 0 0 0 0 0 0 0 0 0 0 0 0 0 0 0 0 0
M02255_77_000000000-ADUT3_1_1116_10664_15258 T----AC---GA-AG-GGG--------GCT-A-G------C--G--T---T--GT-T-CGG-AA------TT-A--C-T--GG-GC-------GT--A-----AA-GC-GC-AC-------G-TA-G-G-C-G------------G--A-TA-T-T-T-----
------AA----G-T-T-A--------G-G-G--G--TG--A-AA-TC--C-C-AG-G-G-----CT-C-AA------C-C-C-T-G-G-A--A-C----T-G--C-C---T--C-------T--AA-T-A---C-------T--G-G--GT--A-T-C----T-A-G-A-G-T--C---C-GA-----GA-G-A--------G-G-T-G---AG-T------GG--A--ATT----C-
C-G-A-GT--GT-A-G-AG-GT--G---A--A-A---TT-C-GT-AG--AT-A-TT--C-G--G-A-G-G-A-AC-A-CC---AG--T--G--GC-GAA-G--G-C--G-------G--C-T-C-A---CTG--G--CT-C-G--------------------G-T---A-C-T--GA--CG-C-T-G--A-GG--T-G-CG-A--AA-G-C---G-TG--GG-
G--AG-C-A-AA--------CA--GG Bacteria(100);Proteobacteria(100);Alphaproteobacteria(100);Rhizobiales(100);unclassified(73);unclassified(73);unclassified(73);unclassified(73);unclassified(73);

Otu0379 0 222 0 0 0 0 125 130 416 0 0 0 0 0 0 1 0 0 0 0 0 0 0 0 0 0 0 0
0 0 0 0 0 0 0 0 0 0 0 73 0 0 0 0 0 0 0 0 0 0 0 0 0 0 0 0 0
0 0 0 0 0 0 0 0 0 0 0 0 0 0 0 0 0 0 0 0 0 0 0 0 0 0 0 0 0
0 0 0 0 0 0 0 0 0 0 0 0 0 0 0 0 0 0 0 0 0 0 0 0 57 0 0
M02255_77_000000000-ADUT3_1_1106_24579_10771 T----AC---GT-AG-GTG--------GCG-A-G------C--G--T---T--AT-C-CGG-AT------TT-A--C-T--GG-GT-------GT--A-----AA-GG-GC-GC-------G-TA-G-G-C-G------------G--G-AA-T-G-C----
-------AA----G-T-C-A--------G-A-T--G--TG--A-AA-TC--C-A-GG-G-G-----CT-T-AA------C-C-C-T-T-G-A--A-C----T-G--C-A---T--T-------T--GA-A-A---C-------T--G-T--AT--T-T-C----T-T-G-A-G-T--G---T-CG-----GA-G-A--------G-G-T-T---GA-C------GG--A--ATT----C-
C-T-A-GT--GT-A-G-CG-GT--G---A--A-A---TG-C-GT-AG--AT-A-TT--A-G--G-A-G-G-A-AC-A-CC---AG--T--G--GC-GAA-G--G-C--G-------G--T-C-A-A---CTG--G--AC-G-A--------------------T-A---A-C-T--GA--CG-C-T-G--A-GG--C-G-CG-A--AA-G-C---G-TG--GG-
G--AG-C-A-AA--------CA--GG Bacteria(100);Firmicutes(85);Clostridia(85);Clostridiales(85);unclassified(85);unclassified(85);unclassified(85);unclassified(85);unclassified(85);

Otu0380 0 0 0 0 0 0 0 0 0 0 0 0 0 0 0 0 0 0 0 0 0 0 0 0 0 0 0 0
0 0 2 0 0 0 0 5 5 0 20 0 0 0 0 0 0 0 0 0 0 0 0 0 0 0 0 0
0 0 0 0 0 0 0 0 0 0 0 0 0 0 0 0 0 155 0 0 0 0 0 0 0 0 0 0
0 0 0 0 0 0 0 0 0 0 0 0 0 0 0 0 0 0 0 0 0 0 0 0 0 0 0
M02255_77_000000000-ADUT3_1_1112_26546_11964 T----AC---GT-AG-GGT--------CCG-A-G------C--G--T---T--AT-C-CGG-AA------TC-A--T-T--GG-GC-------GT--A-----AA-GA-GC-TC-------G-TA-G-G-C-G-------------G--T-TC-A-G-C------
-----AA----G-T-C-G--------G-C-T--G--TG--A-AA-GT--C-C-GG-G-G-----CT-C-AA------C-C-C-C-G-G-A--A-T----T-G--C-A---G--T-------C--GA-T-A---CT------G--C-T--GT--G-A-C----T-C-G-A-G-T--C---C-GG-----TA-G-A--------G-G-A-G---AA-T------GG--A--ATT----C-
C-C-G-GT--GT-A-G-CG-GT--G---A--A-A---TG-C-GC-AG--AT-A-TC--G-G--G-A-G-G-A-AC-A-CC---AG--T--A--GC-GAA-G--G-C--G-------G--T-T-C-T---CTG--G--GC-C-G--------------------G-C---A-C-T--GA--CG-C-T-G--A-GG--A-G-CG-A--AA-G-C---G-TG--GG-
G--AG-C-A-AA--------CA--GG Bacteria(100);unclassified(100);unclassified(100);unclassified(100);unclassified(100);unclassified(100);unclassified(100);unclassified(100);unclassified(100);

Otu0381 0 0 0 0 0 1 0 0 0 15 0 0 0 0 0 0 0 0 0 0 0 0 1 0 0 0 0 0
0 0 0 1 0 0 0 0 0 4 16 5 0 0 0 0 0 90 0 0 0 0 0 0 0 0 0 0
0 0 0 0 0 0 0 0 0 0 0 0 0 0 17 0 0 0 0 0 0 0 0 0 54 0 0 0 0
0 0 0 0 0 0 0 0 0 0 0 0 0 0 0 0 0 0 0 0 0 0 0 0 0 0 0
M02255_77_000000000-ADUT3_1_1114_16862_9934 T----AC---GG-AG-GGG--------GCT-A-G------C--G--T---T--GT-T-CGG-AA------TT-A--C-T--GG-GC-------GT--A-----AA-GC-GC-AC-------G-TA-G-G-C-G-------------G--C-TT-T-T-T-----------AA-
---G-T-C-A--------G-A-G--G--TG--A-AA-GC--C-C-GG-G-G-----CT-C-AA------C-C-C-C-G-G-A--A-T----T-G--C-C---T--T-------T--GA-G-A---C-------T--G-G--GA--G-G-C----T-T-G-A-T-C--G---T-CG-----GA-G-A--------G-G-T-G---AG-T------GG--A--ATT----C-C-G-A-
GT--GT-A-G-AG-GT--G---A--A-A---TT-C-GT-AG--AT-A-TT--C-G--G-A-A-G-A-AC-A-CC---AG--T--G--GC-GAA-G--G-C--G-------G--C-T-C-A---CTG--G--AC-G-A--------------------C-T---G-A-T--GA--CG-C-T-G--A-GG--T-G-CG-A--AA-G-C---G-TG--GG-G--AG-
C-A-AA--------CA--GG Bacteria(100);Proteobacteria(100);Alphaproteobacteria(100);Sphingomonadales(100);Sphingomonadaceae(58);unclassified(58);unclassified(58);unclassified(58);unclassified(58);

Otu0382 0 0 0 0 0 0 0 0 0 0 0 10 0 2 0 0 0 0 69 0 1 0 0 1 0 0 0 0
0 0 0 0 0 0 0 0 0 0 0 0 0 0 0 0 0 0 0 0 0 0 0 0 0 0 0 0
0 0 0 0 0 0 0 0 0 0 0 0 0 0 0 0 0 0 0 0 0 0 0 6 0 34 1 0
1 0 0 0 0 0 0 0 0 0 0 0 0 0 0 0 0 0 0 0 9 0 0 0 0 0 0
M02255_76_000000000-ADUNE_1_1101_12330_25075 C----GG---CT-AA-CTC--------CGT-G-C------C--A--G---C--AG-C-CGC-GG------TA-A--T-A--CG-GA-------GG--G-----TG-CA-AG-CG-------T-TA-T-C-C-G-------------G--A-TC-T-G-T------
-----AA----G-T-C-A--------G-T-G--G--TG--A-AA-TC--T-C-AT-A-G-----CT-T-AA------C-T-A-T-G-A-A--A-C----T-G--C-C---A--T-------T--GA-T-A---C-------T--G-C--AG--G-T-C----T-T-G-A-G-T--A---A-AG-----TA-G-A--------A-G-T-G---GC-T------GG--A--ATA----A-
G-T-A-GT--GT-A-G-CG-GT--G---A--A-A---TG-C-AT-AG--AT-A-TT--A-C--T-T-A-G-A-AC-A-CC---AA--T--T--GC-GAA-G--G-C--A-------G--G-T-C-A---CTA--T--GT-T-T--------------------T-A---A-C-T--GA--CG-C-T-G--A-TG--G-A-CG-A--AA-G-C---G-TG--GG-G--
AG-C-G-AA--------CA--GG Bacteria(100);Bacteroidetes(100);Flavobacteria(100);Flavobacteriales(100);Flavobacteriaceae(100);Chryseobacterium(57);unclassified(57);unclassified(57);unclassified(57);

Otu0383 0 0 0 0 0 0 0 0 0 0 0 0 0 0 0 0 0 0 0 0 0 0 0 4 0 0 0 1
0 0 0 0 0 0 0 33 0 0 0 0 0 0 0 0 0 0 0 0 0 0 0 29 0 0 0 0 0
0 0 0 0 0 0 0 0 0 0 0 0 0 0 0 2 0 32 0 66 0 0 0 0 0 0 0 0
0 0 0 0 0 0 0 0 0 0 0 0 0 0 0 24 0 0 0 0 0 0 0 0 0 0 0
M02255_77_000000000-ADUT3_1_1115_8397_22026 T----AC---GG-AG-GGG--------GCT-A-G------C--G--T---T--GT-T-CGG-AA------TT-A--C-T--GG-GC-------GT--A-----AA-GC-GC-GC-------G-TA-G-G-C-G-------------G--C-TT-A-G-C-----------
AA----G-T-T-A--------G-A-G--G--TG--A-AA-GC--C-C-GA-A-G-----CT-C-AA------C-T-T-C-G-G-A--A-T----T-G--C-C---T--T-------T--AA-A-A---C-------T--G-C--AA--G-G-C----T-A-G-A-A-T--T---G-TG-----GA-G-A--------G-G-T-G---AG-T------GG--A--ATT----C-C-G-
A-GT--GT-A-G-AG-GT--G---A--A-A---TT-C-GT-AG--AT-A-TT--C-G--G-A-A-G-A-AC-A-CC---AG--T--G--GC-GAA-G--G-C--G-------A--C-T-C-A---CTG--G--AC-A-C--------------------A-T---A-T-T--GA--CG-C-T-G--A-GG--T-G-CG-A--AA-G-C---G-TG--GG-G--AG-
C-A-AA--------CA--GG Bacteria(100);Proteobacteria(100);Alphaproteobacteria(100);unclassified(100);unclassified(100);unclassified(100);unclassified(100);unclassified(100);unclassified(100);

Otu0384 0 0 0 0 0 0 0 0 0 0 0 0 0 0 0 0 0 0 0 0 0 0 0 0 0 0 0 0
0 0 0 0 0 0 0 0 0 0 0 0 0 0 0 0 0 0 0 0 0 30 11 0 5 0 0 0 0
0 0 0 0 0 0 27 0 0 0 0 0 0 0 0 0 0 0 0 0 0 0 0 0 0 0 40 0
0 0 0 0 0 0 0 0 0 0 4 0 0 0 0 0 0 0 0 0 0 0 0 0 0 0 0
M02255_76_000000000-ADUNE_1_1105_27495_12741 T----CG---GC-TA-ACT--------CCG-T-GC-----C--A--G---C--AG-C-CGC-GG------TA-A--T-A--CG-GA-------GG--A-----TC-CA-AG-CG-------T-TA-T-C-C-G-------------G--A-TT-TAT-T-----
------AA----G-T-C-A--------G-G-G--G--TG--A-AA-GA--C-G-GT-G-G-----CT-C-AA------C-C-A-T-C-G-C--A-G----T-G--C-C---C--T-------T--GA-T-A---C-------T--G-A--AG--A-G-C----T-T-G-A-A-T--G---A-AC-----TA-G-A--------G-G-T-A---GG-C------GG--A--ATG----
T-G-A-C-AA--GT-A-G-CG-GT--G---A--A-A---TG-C-AT-AG--AT-A-TG--T-C--A-C-A-G-A-AC-A-CC---GA--T--T--GC-GAA-G--G-C--A-------G--C-T-T-A---CTA--T--GG-T-T--------------------A-A---A-T-T--GA--CG-C-T-G--A-GG--C-A-CG-A--AA-G-C---G-TG--GG-
G--AT-C-A-AA--------CA--GG Bacteria(100);Bacteroidetes(100);Sphingobacteria(100);Sphingobacteriales(100);Sphingobacteriaceae(100);Sphingobacteriaceae(100);Pedobacter(100);unclassified(100);unclassified(100);

Otu0385 0 22 0 0 0 0 3 34 26 0 0 0 0 0 0 0 0 0 0 0 0 0 0 0 0 0 0 0
0 0 0 0 0 0 0 0 0 0 0 0 0 0 0 0 0 0 0 0 0 4 0 0 0 0 0 0 0
0 0 0 0 0 0 0 0 0 0 0 0 0 0 0 0 0 0 0 0 23 0 0 0 0 0 0 0 0
0 0 0 0 0 0 2 0 0 0 0 23 0 0 0 0 0 0 0 0 0 0 0 0 0 0 0
M02255_76_000000000-ADUNE_1_2107_13226_22260 T----CG---GC-TA-ACT--------CCG-T-G------C--C--A---G--CA-G-CCG-CG------GT-A--A-T--AC-GG-------AG--G-----AT-CC-GA-GC-------GTTA-T-C-C-G-------------G--ATTT-G-T-T-----
------AA----G-T-C-A--------G-T-T--G--TG--A-AA-GT--T-T-GC-G-G-----CT-C-AA------C-C-G-T-A-A-A--A-T----T-G--C-A---G--T-------T--GA-A-A---C-------T--G-G--CA--G-T-C----T-T-G-A-G-T--A---C-AG-----TA-G-A--------G-G-T-G---GG-C------GG--A--ATT----C-
G-T-G-GT--GT-A-G-CG-GT--G---A--A-A---TG-C-TT-AG--AT-A-TC--A-C--G-A-A-G-A-AC-T-CC---GA--T--T--GC-GAA-G--G-C--A-------G--C-T-C-A---CTA--G--AC-T-G---------------------T-T---A-C-T--GA--CA-C-T-G--A-TG--C-T-CG-A--AA-G-T---G-TG--GG-T--
AT-C-A-AA--------CA--GG Bacteria(100);Bacteroidetes(100);Bacteroidia(100);Bacteroidales(100);Bacteroidaceae(100);Bacteroides(100);unclassified(100);unclassified(100);unclassified(100);

Otu0386 0 10 0 0 0 0 0 0 10 15 0 0 0 14 8 1 10 3 0 0 0 0 221 184 1 1 0 4
0 0 6 0 0 6 17 10 0 0 13 0 5 0 7 0 6 0 0 12 0 5 0 0 0 1 0 0 0
0 0 1 0 7 0 1 1 0 1 3 0 0 0 2 2 0 0 27 4 0 11 3 29 25 139 0 134 105
47 119 21 0 69 20 0 3 0 14 0 3 0 11 0 0 0 2 4 7 0 0 0 0 0 0 0
M02255_76_000000000-ADUNE_1_2105_12237_13844 T----A----AG-CA-CCG--------GCT-A-A------C-----T---C--TG-T-GCC-AG------CA-G--C-C--GC-GG-------TA--A-----TA-CA-GA-GG-------G-TG-C---G-A-------------G--C-TT-G-T-T---------
--AA----G-T-C-G--------G-A-T--G--TG--A-AA-TC--C-C-CG-A-G-----CT-T-AA------C-T-T-G-G-G-A--A-T----T-G--C-A---T--T-------C--GA-T-A---C-------T--G-G--GA--A-G-C----T-A-G-A-G-T--A---T-GG-----GA-G-A--------G-G-A-T---GG-T------AG--A--ATT----C-C-
A-G-GT--GT-A-G-CG-GT--G---A--A-A---TG-C-GT-AG--AG-A-TC--T-G--G-A-G-G-A-AT-A-CC---GA--T--G--GC-GAA-G--G-C--A-------G--C-C-A-T---CTG--G--CC-T-A---------------------A-T---A-C-T--GA--CG-C-T-G--A-GG--T-A-CG-A--AA-G-C---A-TG--GG-G--
AG-C-A-AA--------CA--GG Bacteria(100);Proteobacteria(100);Gammaproteobacteria(100);Pseudomonadales(100);Moraxellaceae(100);Acinetobacter(100);unclassified(100);unclassified(100);unclassified(100);

Otu0387 0 0 0 0 0 0 0 0 0 0 0 0 0 0 0 0 0 0 0 0 0 0 0 0 0 0 0 0
0 0 2 0 0 0 0 0 0 0 19 14 0 0 0 0 13 0 0 0 0 0 0 0 0 0 0 0 0
0 0 0 0 0 0 0 0 0 0 0 0 0 0 0 0 0 0 0 29 0 0 0 0 0 0 0 0 0
0 0 0 0 0 0 0 0 0 0 0 0 0 0 0 0 0 0 0 0 0 0 0 0 0 0 0
M02255_77_000000000-ADUT3_1_1112_8371_16097 C----AC---GT-AG-GGG--------GCG-A-G------C--G--T---T--GT-C-CGG-AA------TT-A--T-T--GG-GC-------GT--A-----AA-GA-GC-TC-------G-TA-G-G-C-G-------------G--C-TC-A-G-T-----------
AA----G-T-C-G--------G-G-T--G--TG--A-AA-CC--C-T-CA-G-G-----CT-C-AA------C-C-T-G-G-G-GA--------C-G-CC-A---C--C-------C--GA-T-A---C-------T--G-C--TG-TG-G-C----T-A-G-A-G-T--C---C-AG-----TA-G-G--------G-G-A-G---TG-T------GG--A--ATT----C-C-
C-G-GT--GT-A-G-CG-GT--G---A--A-A---TG-C-GC-AG--AT-A-TC--G-G--G-A-G-G-A-AC-A-CC---AG--C--G--GC-GAA-G--G-C--G-------G--C-A-C-T---CTG--G--GC-T-G---------------------G-T---A-C-T--GA--CG-C-T-G--A-GG--A-G-CG-A--AA-G-C---G-TG--GG-T--
AG-C-A-AA--------CA--GG Bacteria(100);Actinobacteria(100);Actinobacteria(100);Acidimicrobidae(100);Acidimicrobiales(100);Acidimicrobineae(100);unclassified(100);unclassified(100);unclassified(100);

Otu0388 0 0 0 0 0 0 0 0 0 0 0 0 0 0 0 0 0 0 0 0 0 0 0 0 0 0 0 0
0 0 28 0 0 0 0 0 0 0 0 0 54 0 45 0 0 0 0 0 0 0 0 0 0 6 0 0 0
0 0 0 0 0 0 0 0 0 0 0 0 0 0 0 14 0 0 0 36 0 0 0 0 0 0 0 0 0
21 0 0 0 0 0 7 4 0 0 0 0 0 0 0 0 0 0 0 0 0 0 0 0 0 0 41
M02255_77_000000000-ADUT3_1_1119_25738_16614 T----AC---AT-AT-GGG--------GCG-A-G------C--G--T---T--AT-C-CGG-AT------TT-A--T-T--GG-GT-------GT--A-----AA-GG-GT-GC-------G-TA-G-G-C-G-------------G--G-AA-A-A-T-----
------AA----G-T-C-A--------G-TAT--G--TG--A-AA-TC--C-C-TC-G-G-----CT-C-AA------C-T-G-A-G-G-A--A-C----T-G--C-A---A--C-------T--GA-A-A---C-------T--A-T--TT--T-T-C----T-T-G-A-G-T--G---T-TG-----AA-G-G--------G-G-A-A---AG-T------GG--A--ATT----C-
C-T-A-GT--GT-A-G-CG-GT--G---A--A-A---TG-C-GT-AG--AG-A-TT--A-G--G-A-G-G-A-AC-A-CC---AG--T--G--GC-GAA-G--G-C--G-------A--C-T-T-T---CTG--G--GC-A-A---------------------C-A---A-C-T--GA--CG-C-T-G--A-GG--C-A-CG-A--AA-G-T---G-TG--GG-
G--AG-C-A-AA--------CA--GG Bacteria(100);Firmicutes(97);Clostridia(97);Clostridiales(97);unclassified(97);unclassified(97);unclassified(97);unclassified(97);unclassified(97);

Otu0389 0 0 0 0 0 0 0 0 0 0 0 0 0 0 0 0 0 0 0 2 8 0 0 0 0 0 0 0
0 0 1 0 0 0 0 0 0 25 6 0 0 0 0 0 0 17 0 1 0 0 0 0 0 0 0 0 0
0 0 0 7 0 0 0 0 0 0 0 0 0 0 0 0 0 0 0 0 0 0 0 0 0 0 0 0 0
0 0 0 0 0 0 0 0 0 0 0 0 0 0 0 0 0 0 0 0 0 0 0 0 0 0 0
M02255_77_000000000-ADUT3_1_1119_14984_17865 G----AC---GT-AG-GGT--------GCA-A-G------C--G--T---T--GT-C-CGG-AT------TT-A--C-T--GG-GC-------GT--A-----AA-GC-GC-GC-------G-TA-G-G-C-G-------------G--C-TC-G-T-T-----
------AA----G-T-G-A--------G-A-A--G--TG--A-AA-GC--T---CT-GGG-----CT-C-AA------C-C-C-A-G-A-A--A-C----C-G--C-T---T--T-------T--CA-T-A---C-------T--G-G--CG--A-G-C----T-G-G-A-G-T--G---A-TG-----CA-G-A--------G-G-T-G---GA-T------AG--A--ATT----C-
C-C-G-GT--GT-A-G-CG-GT--G---A--A-A---TG-C-GT-TT--AT-A-TC--G-G--G-A-G-G-A-AT-A-CC---CG--T--G--GC-GAA-G--G-C--G-------G--T-C-C-A---CTG--G--GC-A-T---------------------C-A---A-C-T--GA--CG-C-T-G--A-GG--C-G-CG-A--AA-G-C---G-TG--GG-T-
-AG-C-A-AA--------CA--GG Bacteria(100);unclassified(100);unclassified(100);unclassified(100);unclassified(100);unclassified(100);unclassified(100);unclassified(100);unclassified(100);

Otu0390 0 0 0 0 0 0 0 0 0 0 0 0 0 0 1 0 0 0 0 0 0 0 0 0 0 0 0 0
0 0 0 0 0 0 0 0 0 0 0 0 34 0 77 0 0 0 0 0 0 0 0 0 0 0 0 0
0 0 0 0 0 0 0 0 0 0 0 0 0 0 6 0 0 0 0 0 0 0 0 0 0 0 0 0
0 0 81 0 0 0 0 0 0 0 0 0 5 31 1 0 0 0 0 0 0 0 0 0 0 0 0
M02255_77_000000000-ADUT3_1_1119_21401_9624 T----AC---GT-AG-GTG--------GCG-A-G------C--G--T---T--AT-C-CGG-AA------TT-A--C-T--GG-GT-------GT--A-----AA-GG-GC-GT-------G-TA-G-G-C-G------------G--C-AC-T-G-T-----------AA-
---G-T-C-A--------G-A-T--G--TG--A-AA-TC--T-C-CC-G-G-----CT-C-AA------C-C-G-G-G-A-G--C-G----T-G--C-A---T--C-------T--GA-T-A---C-------T--G-C--AG--T-A-C----T-T-G-A-G-T--G---A-TA-----GA-G-G--------G-G-A-A---AG-C------GG--A--ATT----C-C-T-A-
GT--GT-A-G-CG-GT--A---A--A-A---TG-C-GT-AG--AT-A-TT--A-G--G-A-G-G-A-AC-A-CC---AG--T--G--GC-GAA-G--G-C--G-------G--C-T-T-T---CTG--G--CT-A-T--------------------T-A---A-C-T--GA--CG-C-T-G--A-GG--C-G-CG-A--AA-G-C---G-TG--GG-G--AG-C-
A-AA--------CA--GG Bacteria(100);Firmicutes(100);Clostridia(100);Clostridiales(100);Ruminococcaceae(100);Fastidiosipila(92);unclassified(92);unclassified(92);unclassified(92);

Otu0391 0 0 0 0 0 0 0 0 0 0 0 0 0 0 0 0 0 0 0 0 0 0 0 0 0 0 0 0
0 0 0 0 114 0 0 0 0 0 0 0 0 0 0 0 0 0 0 0 0 0 0 0 0 0 0 0
0 0 0 0 0 0 0 0 0 0 0 0 0 0 0 0 0 0 0 0 0 0 0 0 0 0 0 0
22 0 0 0 0 0 0 4 0 0 0 0 0 0 0 0 0 0 0 0 0 0 0 0 0 0 0
M02255_77_000000000-ADUT3_1_2113_10509_17251 T----AC---GT-AG-GGG--------GCA-A-G------C--G--T---T--AT-C-CGG-AT------TT-A--C-T--GG-GT-------GT--A-----AA-TG-GA-GC-------G-CA-G-A-C-G------------G--C-TT-G-G-C-----
------AA----G-T-C-T--------G-A-A--G--TG--A-AA-GC--C-C-GG-G-G-----CT-T-AA------C-C-C-C-G-G-G--A-C----T-G--C-T---T--T-------G--GA-A-A---C-------T--G-T--GA--A-G-C----T-A-G-A-G-T--G---T-CG-----GA-G-A--------G-G-T-A---AG-T------GG--A--ATT----
C-C-C-A-GT--GT-A-G-CG-GT--G---A--A-A---TG-C-GT-AG--AT-A-TT--G-G--G-A-G-G-A-AC-A-CC---GG--T--G--GC-GAA-G--G-C--G-------G--C-T-T-A---CTG--G--AC-G-A--------------------T-A---A-C-T--GA--CG-T-T-G--A-GG--C-T-CG-A--AG-G-C---G-TG--
GG-G--AG-C-A-AA--------CA--GG Bacteria(100);Firmicutes(100);Clostridia(100);Clostridiales(100);Lachnospiraceae(100);unclassified(98);unclassified(98);unclassified(98);unclassified(98);

Otu0392 0 87 0 0 0 0 65 0 44 0 0 0 0 0 0 0 0 0 0 0 0 0 0 0 0 0 0 0
0 0 0 0 0 0 0 0 0 0 0 0 0 0 0 0 0 0 0 0 0 0 0 0 0 0 0 0
0 0 0 0 0 0 0 0 0 0 0 0 0 0 0 0 0 0 0 0 0 0 0 0 0 0 0 0
0 0 0 0 0 0 0 0 0 0 0 0 0 0 0 0 0 0 0 0 0 0 0 4 13 135 0
M02255_77_000000000-ADUT3_1_1103_8204_10843 T----AC---GT-AG-GGG--------GCA-A-G------C--G--T---T--AT-C-CGG-AT------TT-A--C-T--GG-GT-------GT--A-----AA-GG-GA-GC-------G-CA-G-G-C-G------------G--T-GC-G-G-C----------
AA----G-T-C-A--------G-A-T--G--TG--A-AA-AC--C-C-GG-G-G-----CT-C-AA------C-C-C-C-G-G-G--A-C----T-G--C-A---T--T-------T--GA-A-A---C-------T--G-T--CG--G-A-C----T-A-G-A-G-T--G---C-CG-----GA-G-A--------G-G-T-A---AG-T------GG--A--ATT----C-C-T-
A-GT--GT-A-G-CG-GT--G---A--A-A---TG-C-GT-AG--AT-A-TT--A-G--G-A-G-G-A-AC-A-CC---AG--T--G--GC-GAA-G--G-C--G-------G--C-T-T-A---CTG--G--AC-G-G--------------------T-A---A-C-T--GA--CG-C-T-G--A-GG--C-T-CG-A--AA-G-C---G-TG--GG-G--
AG-C-A-AA--------CA--GG Bacteria(100);Firmicutes(100);Clostridia(100);Clostridiales(100);Lachnospiraceae(100);unclassified(100);unclassified(100);unclassified(100);unclassified(100);

Otu0393 0 0 0 0 0 0 0 0 0 0 0 0 0 0 0 0 0 0 0 0 0 0 0 0 0 0 0 0
0 0 0 0 0 0 0 0 0 0 0 0 0 0 0 0 0 0 0 0 0 0 0 0 0 0 0 0
0 0 0 0 0 0 0 0 0 0 0 0 0 0 28 0 0 0 0 0 0 0 0 0 0 0 0 0
0 0 0 0 0 0 0 0 0 0 0 0 0 0 0 0 0 0 42 0 0 0 0 0 0 0 0
M02255_77_000000000-ADUT3_1_1109_28563_12093 T----AC---AG-AG-GCC--------ACG-A-G------C--G--T---T--AG-G-CGG-AA------TC-A--C-T--GG-GC-------TT--A-----AA-GC-GT-GT-------G-TA-G-G-T-G------------G--A-TG-A-G-T-----
------AA----G-T-A-C--------C-T-T--G--TG--A-AA-TC--C-C-AC-G-G-----CT-C-AA------C-C-G-T-G-G-A--A-C----T-G--C-T---T--G-------G--TA-T-A---C-------T--G-C--TT--G-T-C----T-T-G-A-G-G--C---A-CT-----CA-G-G--------G-G-C-G---AG-C------GG--A--ACA----G-
A-T-G-GT--GG-A-G-CG-GT--G---A--A-A---TG-C-GT-AG--AT-A-TC--A-T--C-T-G-G-A-AC-G-CC---AA--T--G--GT-GAA-G--A-C--A-------G--C-T-C-G---CTG--G--GG-G-T--------------------G-T---A-C-T--GA--CA-C-T-G--A-GA--C-A-CG-A--AA-G-C---C-AG--GG-
G--AG-C-A-AA--------CG--GG Bacteria(100);Planctomycetes(100);Phycisphaerae(100);WD2101(100);unclassified(100);unclassified(100);unclassified(100);unclassified(100);unclassified(100);

Otu0394 0 0 0 0 0 15 0 0 0 0 0 0 0 0 0 0 0 0 0 0 0 0 0 0 0 0 0 0
0 0 0 0 0 0 0 0 0 0 0 0 0 0 0 0 0 0 0 0 0 0 0 1 0 0 0 0 0
0 0 0 0 0 0 0 0 0 0 0 3 0 0 11 0 0 0 0 38 30 0 0 0 0 0 0 4 0
0 0 0 0 7 31 0 4 0 88 0 102 0 0 3 0 0 0 0 0 0 0 0 0 0 0 0
M02255_76_000000000-ADUNE_1_1103_5413_15978 C----GG---CT-AA-TTC--------CGT-G-C------C--A--G---C--AG-C-CGC-GG------TA-A--T-A--CG-GA-------AG--G-----TC-CA-GG-CG-------T-TA-T-C-C-G------------GA-T-TT-A-T-T-----------AA--
--G-C-G-T--------G-T-T--G--TG--A-AA-TG--T-A-GA-T-G-----CT-C-AA------C-A-T-C-T-G-A--A-T----T-G--C-A---G--C-------G--CG-A-A---C-------T--G-G--AA--G-A-C----T-T-G-A-G-T--G---C-AC-----AC-A-A--------C-G-T-A---GG-C------GG--A--ATT----C-A-T-G-
GT--GT-A-G-CG-GT--G---A--A-A---TG-C-TT-AG--AT-A-TT--A-T--G-A-A-G-A-AC-T-CC---GA--T--T--GC-GAA-G--G-C--A-------G--C-T-T-A---CGG--G--AG-T-G--------------------T-A---A-C-T--GA--CG-C-T-T--A-AG--C-T-CG-A--AG-G-T---G-CG--GG-T--AT-C-
G-AA--------CA--GG Bacteria(100);Bacteroidetes(100);Bacteroidia(100);Bacteroidales(100);Prevotellaceae(100);unclassified(100);unclassified(100);unclassified(100);unclassified(100);

Otu0395 0 0 0 0 0 0 0 0 0 0 0 0 0 0 0 0 0 0 0 0 0 0 0 0 0 0 0 0
0 0 0 0 0 0 0 0 0 0 0 1 0 1 0 0 0 0 1 0 0 2 17 0 3 2 0 0 0
0 0 0 0 0 5 0 0 8 0 0 0 0 0 0 0 0 0 0 0 0 0 0 0 0 0 0 0 0
0 0 0 0 0 0 0 0 0 0 0 0 0 0 0 0 0 0 0 0 0 0 0 0 0 0 0 0
M02255_76_000000000-ADUNE_1_2107_23064_20238 C----TA---AC-TC-CGT--------GCC-A-G------C-----A---G--CC-G-CGG-TA------AT-A--C-G--GA-GG-------GT--G-----CA--A-GC--G-------T-TA-A-T-C-G------------G--T-TT-G-T-T---------
--AA----G-T-C-A--------G-A-T--G--TG--A-AA-TC--C-C-CG-C-G-----CT-T-AA------C-G-T-G-G-G-A--A-C----T-G--C-A---T--T-------T--GA-A-A---C-------T--G-G--CA--A-G-C----T-A-G-A-G-T--C---T-TG-----TA-G-A--------G-G-G-G---GG-T------AG--A--ATT----C-C-
A-G-GT--GT-A-G-CG-GT--G---A--A-A---TG-C-GT-AG--AG-A-TC--T-G--G-A-G-G-A-AT-A-CC---GG--T--G--GC-GAA-G--G-C--G-------G--C-C-C-C---CTG--G--AC-A-A--------------------A-G---A-C-T--GA--CG-C-T-C--A-GG--T-G-CG-A--AA-G-C---G-TG--GG-G--
AG-C-A-AA--------CA--GG Bacteria(100);Proteobacteria(100);Gammaproteobacteria(100);Enterobacteriales(100);Enterobacteriaceae(100);unclassified(85);unclassified(85);unclassified(85);unclassified(85);

Otu0396 0 0 0 0 0 0 0 0 0 0 0 0 0 0 0 0 2 0 0 2 0 0 1 0 0 0 0 0
0 0 0 0 0 0 0 0 0 0 0 0 0 0 0 0 0 0 0 0 0 0 0 1 0 0 0 0 0
0 0 0 4 0 0 1 0 0 3 0 0 0 0 0 0 0 0 0 0 0 0 0 0 0 0 0 0 0
0 0 0 0 0 0 0 1 0 0 0 0 0 0 0 0 0 0 0 0 0 0 0 0 0 0 0 0
M02255_77_000000000-ADUT3_1_2105_28097_13615 G----CC---AG-CA-GCC--------GCG-G-T------A--A--T---A--CA-G-AGG-GT------GC-A--A-G--CG-TT-------AA--T-----CG-GA-AT-TA-------C-TA-G-G-T-G------------G--T-TT-G-T-T------
-----AA----G-T-T-G--------A-A-T--G--TG--A-AA-TC--C-C-CG-G-G-----CT-C-AA------C-C-T-G-G-G-A--A-C----T-G--C-A---T--C-------C--AA-A-A---C-------T--G-G--CA--A-G-C----T-A-G-A-G-T--A---T-GG-----TA-G-A--------G-G-G-T---AG-T------GG--A--ATT----T-
C-C-T-GT--GT-A-G-CG-GT--G---A--A-A---TG-C-GT-AG--AT-A-TA--G-G--A-A-G-G-A-AC-A-CC---AG--T--G--GC-GAA-G--G-C--G-------A--C-T-A-C---CTG--G--AC-T-G--------------------A-T---A-C-T--GA--CA-C-T-G--A-GG--T-G-CG-A--AA-G-C---G-TG--GG-
G--AG-C-A-AA--------CA--GG Bacteria(100);Proteobacteria(100);Gammaproteobacteria(100);Pseudomonadales(61);Pseudomonadaceae(61);unclassified(61);unclassified(61);unclassified(61);unclassified(61);

Otu0397 0 0 0 35 0 0 0 0 0 0 0 0 0 0 0 0 0 0 0 0 0 0 0 0 0 0 0 0
0 0 0 0 0 0 0 0 0 0 0 0 0 0 11 0 0 0 0 0 0 0 0 0 0 0 0 0 0
0 0 0 0 0 0 0 0 0 0 0 0 0 0 0 0 0 0 0 0 0 0 0 0 0 0 0 0 0
0 0 0 0 0 0 0 0 0 0 0 0 0 0 0 0 0 0 0 0 0 0 0 0 0 0 0 0
M02255_77_000000000-ADUT3_1_1115_24356_10819 T----AC---GT-AG-GGT--------GCA-A-G------C--G--T---T--GT-C-CGG-AA------TT-A--T-T--GG-GC-------GT--A-----AA-GA-GC-TC-------G-TA-G-G-C-G------------G--T-CT-G-T-C------
-----GC----G-T-C-T--------G-C-T--G--TG--A-AA-AC--T-C-GG-G-G-----CT-T-AA------C-C-C-C-G-G-G--C-C----T-G--C-A---G--T-------G--GG-T-A---C-------G--G-G--CA--G-A-C----T-A-G-A-G-T--G---C-GG-----TA-G-G--------G-G-A-G---AC-T------GG--A--ATT----
C-C-T-G-GT--GT-A-G-CG-GT--G---A--A-A---TG-C-GC-AG--AT-A-TC--A-G--G-A-G-G-A-AC-A-CC---GG--T--G--GC-GAA-G--G-C--G-------G--G-T-C-T---CTG--G--GC-C-G--------------------T-A---A-C-T--GA--CG-C-T-G--A-GG--A-G-CG-A--AA-G-C---A-TG--
GG-T--AG-C-G-AA--------CA--GG Bacteria(100);Actinobacteria(100);Actinobacteria(100);Actinobacteridae(100);Actinomycetales(100);unclassified(57);unclassified(57);unclassified(57);unclassified(57);

Otu0398 0 0 0 0 0 8 0 0 0 0 0 0 0 0 0 0 0 0 0 0 0 0 0 0 0 0 0 0
0 0 0 0 0 0 0 12 0 0 13 0 0 0 0 0 0 0 0 0 0 0 0 0 13 0 0 0 0
0 0 0 0 0 0 0 0 0 0 0 0 0 0 0 0 5 0 0 0 0 0 0 0 0 0 0 0 0
0 0 20 0 0 0 0 0 0 0 0 0 0 0 0 0 0 0 0 0 0 0 0 0 0 0 0 0
M02255_77_000000000-ADUT3_1_1119_8440_14873 T----AC---GT-AG-GGT--------GCA-A-G------C--G--T---T--GT-C-CGG-AA------TT-A--T-T--GG-GC-------GT--A-----AA-GA-GC-TC-------G-TA-G-G-C-G------------G--T-CT-G-T-C-----------GC-
---G-T-C-T--------G-C-T--G--TG--A-AA-AC--G-T-GG-G-G-----CT-C-AA------C-C-C-C-G-C-G--C-G----T-G--C-A---G--T-------G--GG-T-A---C-------G--G-G--CA--G-A-C----T-G-G-A-G-T--G---C-AG-----TA-G-G--------G-G-A-G---AC-T------GG--A--ATT----C-C-T-G-
GT--GT-A-G-CG-GT--G---A--A-A---TG-C-GC-AG--AT-A-TC--A-G--G-A-G-G-A-AC-A-CC---GG--T--G--GC-GAA-G--G-C--G-------G--G-T-C-T---CTG--G--GC-T-G--------------------T-T---A-C-T--GA--CG-C-T-G--A-GG--A-G-CG-A--AA-G-C---G-TG--GG-G--AG-
C-G-AA--------CA--GG Bacteria(100);Actinobacteria(100);Actinobacteria(100);Actinobacteridae(100);Actinomycetales(100);unclassified(100);unclassified(100);unclassified(100);unclassified(100);

Otu0399 0 0 0 0 0 0 0 0 0 0 0 0 0 0 0 0 0 0 0 0 0 0 0 0 23 16 0 4
0 0 0 0 0 0 0 0 0 0 0 0 0 0 0 0 0 0 0 0 0 0 0 0 0 0 0 0 0
0 0 0 0 0 0 0 0 0 0 0 0 0 0 0 0 0 0 0 0 0 0 0 3 0 0 0 0 0
0 0 0 0 0 0 0 0 0 0 0 0 0 0 0 2 0 0 0 0 0 0 0 0 0 0 0 0
M02255_76_000000000-ADUNE_1_2115_2702_18231 G----AA---GC-AC-CG---------GCT-A-A------C-----T---C--CG-T--GC-CA------GC-A--G-C--CG-CG-------GT--A-----AT-AC-GG-AG-------G-GT-G-C-G-A------------G--C-GT-T-A-A-----------TC---
-G-G-A-A--------G-C-T--G--TG--A-AA-GC--C-C-GG-G-G-----CT-C-AA------C-C-T-C-G-G-A--A-C----A-G--C-A---T--T-------T--TG-A-A---C-------T--G-G--CA--G-A-C----T-A-G-A-G-T--C---T-TG-----TA-G-A--------G-G-G-G---GG-T------AG--A--ATT----T-C-A-G-
GT--GT-A-G-CG-GT--G---A--A-A---TG-C-GT-AG--AG-A-TC--T-G--A-A-G-G-A-AT-A-CC---GG--T--G--GC-GAA-G--G-C--G-------G--C-C-C-C---CTG--G--AC-A-A--------------------A-G---A-C-T--GA--CG-C-T-C--A-GA--T-G-CG-A--AA-G-C---G-TG--GG-G--AG-
C-A-AA--------CA--GG Bacteria(100);Proteobacteria(100);Gammaproteobacteria(100);Vibrionales(100);Vibrionaceae(100);unclassified(100);unclassified(100);unclassified(100);unclassified(100);

Otu0400 0 0 0 0 0 0 0 0 0 0 0 0 0 0 0 0 0 0 0 0 0 0 0 0 0 0 0 0
0 0 0 126 0 0 0 0 0 0 0 25 0 0 0 0 0 0 0 0 0 0 0 0 0 0 0 0
0 18 0 0 16 0 0 0 0 3 8 5 0 0 95 96 0 0 0 82 0 0 6 0 0 0 0 0 0
7 60 0 14 75 55 0 4 0 92 0 80 0 0 0 0 16 0 0 0 0 0 7 0 0 0 0
M02255_77_000000000-ADUT3_1_1118_16994_14457 T----AC---GT-AG-GGG--------GCG-A-G------C--G--T---T--AT-C-CGG-AA------TT-A--C-T--GG-GC-------GT--A-----AA-GA-GT-AT-------G-TA-G-G-T-G------------G--T-TA-A-G-T-----
------AA----G-C-G-T--------A-G-G--G--TT--T-AA-GG--C-G-AC-A-G-----CC-C-AA------C-T-G-T-C-G-T--A------T-G--C-C---C--C-------G--CG-A-A---C-------T--G-T--TT--A-A-C----T-T-G-A-G-T--A---C-AG-----GA-G-G--------G-G-A-A---GG-C------GG--A--ATT----C-
C-T-A-GT--GT-A-G-CG-GT--G---A--A-A---TG-C-GT-AG--AT-A-TT--A-G--G-A-G-G-A-AC-A-CC---AG--T--G--GC-GAA-G--G-C--G-------G--C-C-T-T---CTG--G--AC-T-G--------------------T-A---A-C-T--GA--CA-C-T-G--A-GA--T-A-CG-A--AA-G-C---G-TG--GG-T--
AG-C-A-AA--------CA--GG Bacteria(100);Firmicutes(100);Clostridia(100);Clostridiales(100);Family_XIII_Incertae_Sedis(100);Eubacterium(100);unclassified(100);unclassified(100);unclassified(100);

Otu0401 0 0 0 0 0 0 0 0 0 0 1 0 1 0 0 0 0 0 0 0 0 0 0 0 0 0 0 0
0 0 0 0 0 0 0 0 0 0 0 0 0 0 0 0 0 0 0 0 0 0 0 0 0 0 0 0 0
0 0 0 0 0 0 0 0 0 0 0 0 0 0 0 0 0 0 0 0 0 0 0 0 0 0 0 0 0
0 0 0 0 0 0 5 0 7 0 0 0 0 0 0 0 0 0 0 0 0 0 0 0 0 0 0
M02255_76_000000000-ADUNE_1_1115_24627_16381 G----AA---GA-AG-GAC--------CGG-C-T------A--A--C---T--CC-G-TGC-CA------GC-A--G-C--CG-CG-------GT--A-----AT-AC-G--GA-------G-GG-T-C-C-G------------A--G-TC-G-T-T-----
------AA----T-C-G-A--------G-A-A--G--TG--A-AA-GC--C-C-CG-G-G-----CT-C-AA------C-C-T-G-G-G-A--A-T----T-G--C-A---T--T-------T--CG-A-A---C-------T--G-G--CG--A-A-C----T-A-G-A-G-T--C---T-TG-----TA-G-A--------G-G-G-G---GG-T------AG--A--ATT----
C-C-A-G-GT--GT-A-G-CG-GT--G---A--A-A---TG-C-GT-AG--AG-A-TC--T-G--G-A-G-G-A-AT-A-CC---GG--T--G--GC-GAA-G--G-C--G-------G--C-C-C-C---CTG--G--AC-A-A--------------------A-G---A-C-T--GA--CG-C-T-C--A-GG--C-A-CG-A--AA-G-C---G-TG--
GG-G--AG-C-A-AA--------CA--GG Bacteria(100);Proteobacteria(100);Gammaproteobacteria(100);unclassified(100);unclassified(100);unclassified(100);unclassified(100);unclassified(100);unclassified(100);

Otu0402 0 0 0 0 0 0 0 0 0 0 0 0 0 0 0 0 0 0 0 0 0 0 0 0 10 0 0 0
0 0 4 1 0 0 0 0 0 0 48 0 0 0 0 0 0 0 0 0 0 0 0 0 0 0 0 0 0
0 0 0 0 0 0 0 0 0 0 0 0 0 0 0 0 0 27 0 0 0 0 0 0 0 0 0 0 0
0 0 0 0 0 0 0 0 0 55 0 0 0 0 0 0 0 0 0 0 0 0 0 0 0 0 0
M02255_77_000000000-ADUT3_1_1116_15617_21338 T----AC---GA-AG-GGG--------GCT-A-G------C--G--T---T--GC-T-CGG-AA------TC-A--C-T--GG-GC-------GT--A-----AA-GC-GC-AC-------G-TA-G-G-C-G------------G--A-TC-T-T-T-----
------AA----G-T-C-A--------G-G-G--G--TG--A-AA-TC--C-T-GG-A-G-----CT-C-AA------C-T-C-C-A-G-A--A-C----T-G--C-C---T--T-------T--GA-T-A---C-------T--G-A--GG--G-T-C----T-C-G-A-G-T--C---C-GG-----GA-G-A--------G-G-T-G---AG-T------GG--A--ACT----
G-C-G-A-GT--GT-A-G-AG-GT--G---A--A-A---TT-C-GT-AG--AT-A-TT--C-G--C-A-A-G-A-AC-A-CC---AG--T--G--GC-GAA-G--G-C--G-------G--C-T-C-A---CTG--G--CC-C-G--------------------G-T---A-C-T--GA--CG-C-T-G--A-GG--T-G-CG-A--AA-G-C---G-TG--GG-
G--AG-C-A-AA--------CA--GG Bacteria(100);Proteobacteria(100);Alphaproteobacteria(100);Rhizobiales(100);unclassified(100);unclassified(100);unclassified(100);unclassified(100);unclassified(100);

Otu0403 0 0 0 0 0 0 0 0 0 0 1 0 1 0 0 0 0 0 0 0 0 0 0 0 0 0 1 1
0 0 0 0 0 0 0 0 0 1 0 0 0 0 0 0 0 0 0 0 0 0 0 0 0 0 0 0 0
0 0 0 0 0 0 0 0 0 0 0 0 0 0 0 0 0 0 0 0 0 0 0 0 0 0 0 0 0
0 0 0 0 0 0 8 0 3 0 0 0 0 0 0 0 0 0 0 0 0 0 0 0 0 0 0
M02255_76_000000000-ADUNE_1_1103_21381_22091 T----AC---GG-AG-GGT--------CCG-A-G------C--G--T---T--AA-T-CGG-AA------TT-A--C-T--GG-GC-------GT--A-----AA-GC-GT-GC-------G-CA-G-G-C-G------------G--T-TT-G-T-T-----
------AA----G-C-G-A--------G-A-T--G--TG--A-AA-GC--C-C-CG-G-G-----CT-C-AA------C-C-T-G-G-G-A--A-T----T-G--C-A---T--T-------T--CG-A-A---C-------T--G-G--CG--A-A-C----T-A-G-A-G-T--C---T-TG-----TA-G-A--------G-G-G-G---GG-T------AG--A--ATT----
C-C-A-G-GT--GT-A-G-CG-GT--G---A--A-A---TG-C-GT-AG--AG-A-TC--T-G--G-A-G-G-A-AT-A-CC---GG--T--G--GC-GAA-G--G-C--G-------G--C-C-C-C---CTG--G--AC-A-A--------------------A-G---A-C-T--GA--CG-C-T-C--A-GG--C-A-CG-A--AA-G-C---G-TG--
GG-G--AG-C-A-AA--------CA--GG Bacteria(100);Proteobacteria(100);Gammaproteobacteria(100);Alteromonadales(100);Shewanellaceae(63);Shewanella(63);unclassified(63);unclassified(63);unclassified(63);

Otu0404 0 0 0 0 0 0 0 0 0 0 0 0 0 0 0 0 0 0 0 0 0 1 0 0 0 0 0 1
0 0 0 0 0 0 0 0 0 0 0 0 0 0 0 54 0 0 0 0 0 0 0 14 0 0 0 0 0
3 27 0 0 0 0 0 0 0 0 0 0 0 0 198 234 0 6 0 0 0 2 0 0 17 0 0 6 0
21 66 0 0 19 0 1 2 0 13 0 5 0 0 0 10 0 0 4 46 114 13 0 0 0 0 0
M02255_77_000000000-ADUT3_1_2114_20307_7494 C----A----GC-A--GCC--------GCG-G-T------A--A--T---A--CG-G-AGG-AT------GC-G--A-G--CG-TT-------AT--C-----CG-GA-TT-TA-------T-TG-G-G-T-T------------G--T-TC-G-G-T-----------AA---
-G-T-C-A--------G-C-G--G--TG--A-AA-CC--T-G-AG-C-G-----CT-C-AA------C-G-T-T-C-A-G--C-C----T-G--C-C---G--T-------T--GA-A-A---C-------T--G-C--CG--G-G-C----T-T-G-A-G-T--T---C-AG-----TG-G-C--------G-G-C-A---GG-C------GG--A--ATT----C-G-T-G-
GT--GT-A-G-CG-GT--G---A--A-A---TG-C-AT-AG--AT-A-TC--A-C--G-A-G-G-A-AC-T-CC---GA--T--T--GC-GAA-G--G-C--A-------G--C-T-T-G---CCA--T--AC-T-G--------------------C-G---A-C-T--GA--CA-C-T-G--A-AG--C-A-CG-A--AG-G-C---G-TG--GG-T--AT-C-
A-AA--------CA--GG Bacteria(100);Bacteroidetes(100);Bacteroidia(100);Bacteroidales(100);Porphyromonadaceae(100);Porphyromonas(100);unclassified(100);unclassified(100);unclassified(100);

Otu0405 0 0 0 0 0 0 0 0 0 0 0 0 0 0 0 0 0 0 0 0 0 0 0 0 0 0 0 0
0 0 0 0 0 0 0 0 0 0 1 0 0 0 0 0 0 0 0 0 0 0 23 0 1 0 0 0 0
0 0 1 0 0 0 1 0 13 0 0 0 0 0 0 0 1 0 0 0 0 0 0 0 0 0 0 0 0
0 0 0 0 0 0 0 0 0 0 0 0 0 0 0 0 0 0 0 0 0 0 0 0 0 0 0
M02255_76_000000000-ADUNE_1_1101_24259_19175 T----AC---GG-AG-GGT--------GCA-A-G------C--G--T---T--AA-T-CGG-AA------TT-A--C-T--GG-GC-------GT--A-----AA-GC-GC-AC-------G-CA-G-G-C-G------------G--T-TT-G-T-T-----
------AA----G-T-C-A--------G-A-T--G--TG--A-AA-TC--C-C-CG-A-G-----CT-T-AA------C-T-T-G-G-G-A--A-C----T-G--C-A---T--T-------T--GA-A-A---C-------T--G-G--CA--A-G-C----T-A-G-A-G-T--C---T-TG-----TA-G-A--------G-G-G-G---GG-T------AG--A--ATT----C-
C-A-G-GT--GT-A-G-CG-GT--G---A--A-A---TG-C-GT-AG--AG-A-TC--T-G--G-A-G-G-A-AT-A-CC---GG--T--G--GC-GAA-G--G-C--G-------G--C-C-C-C---CTG--G--AC-A-A--------------------A-G---A-C-T--GA--CG-C-T-C--A-GG--T-G-CG-A--AA-G-C---G-TG--GG-
G--AG-C-A-AA--------CA--GG Bacteria(100);Proteobacteria(100);Gammaproteobacteria(100);Enterobacteriales(100);Enterobacteriaceae(100);unclassified(100);unclassified(100);unclassified(100);unclassified(100);

Otu0406 0 0 0 0 0 0 0 0 0 0 0 0 0 0 0 0 0 0 0 0 0 0 0 0 16 2 0 0
0 0 0 53 142 0 0 0 0 0 0 23 0 0 0 0 0 0 0 0 0 0 0 0 0 0 0 0 0
0 0 0 0 0 0 0 0 0 0 0 0 0 0 0 0 0 0 0 0 0 0 0 0 0 0 0 0 0
0 0 0 0 0 0 0 0 0 52 0 0 0 0 0 148 22 30 21 35 0 0 0 0 0 0 0
M02255_77_000000000-ADUT3_1_1116_24657_15174 G----AC---GT-AG-GAT--------GCA-A-G------C--G--T---T--GT-C-CGG-AT------TC-A--T-T--GG-GC-------GT--A-----AA-GA-GT-TC-------G-TA-G-G-C-G------------G--T-TT-G-T-T------
-----AA----G-T-C-T--------G-A-T--G--TT--A-AA-GA--T-C-GG-G-G-----CC-C-AA------C-C-C-T-G-A-G--A-G----T-G--C-A---T--T-------G--GA-T-A---C-------T--G-G--CA--G-A-C----T-G-G-A-G-T--G---C-AG-----TA-G-A--------G-G-C-A---AG-T------GG--A--ATT----C-
C-C-A-GT--GT-A-G-CG-GT--G---A--A-A---TG-C-GT-AG--AT-A-TT--G-G--G-A-A-G-A-AC-A-CC---GG--T--G--GC-GCA-G--G-C--G-------A--C-T-T-G---CTG--G--GC-T-G--------------------T-A---A-C-T--GA--CG-C-T-G--A-GG--A-A-CG-A--AA-G-C---T-AG--GG-
G--AG-C-A-AA--------TG--GG Bacteria(100);Cyanobacteria(94);MLE1-12(94);unclassified(94);unclassified(94);unclassified(94);unclassified(94);unclassified(94);unclassified(94);

Otu0407 0 0 0 0 0 0 0 0 0 0 0 0 0 0 0 0 0 0 0 0 0 0 0 0 0 0 0 0
0 0 0 0 0 0 0 0 0 0 0 0 0 0 0 0 0 0 0 0 0 0 0 0 0 0 0 0 0
0 64 0 0 0 0 0 0 0 0 9 1 0 0 0 0 0 0 0 65 0 0 1 0 0 0 0 0 0
104 0 0 0 0 60 0 6 0 37 0 0 0 0 0 0 0 0 0 0 0 0 0 0 0 0 0
M02255_77_000000000-ADUT3_1_1110_26441_20626 T----AC---GT-AG-GGG--------GCA-A-G------C--G--T---T--AT-C-CGG-AA------TT-A--C-T--GG-GC-------GT--A-----AA-GG-GT-GC-------G-TA-G-G-C-G------------G--T-AA-T-A-T-----
------AA----G-T-T-G--------G-A-G--T--TT--T-AA-AC--C-A-TG-G-G-----CT-C-AA------C-C-C-A-T-G-T--C------A-G--G-C---T--C-------C--AA-A-A---C-------T--G-T--AT--A-A-C----T-T-G-A-G-T--A---C-TG-----GA-G-A--------G-G-A-A---AG-T------GG--A--ATT----C-
C-T-A-GT--GT-A-G-CG-GT--G---A--A-A---TG-C-GT-AG--AT-A-TT--A-G--G-A-G-G-A-AT-A-CC---GG--T--G--GC-GAA-G--G-C--G-------A--C-T-T-T---CTG--G--AC-A-G--------------------T-A---A-C-T--GA--CG-C-T-G--A-GG--C-A-CG-A--AA-G-C---G-TG--GG-T-
-AG-C-A-AA--------CA--GG Bacteria(100);Firmicutes(100);Clostridia(100);Clostridiales(100);unclassified(100);unclassified(100);unclassified(100);unclassified(100);unclassified(100);

Otu0408 0 0 0 0 0 0 0 0 0 0 0 0 0 0 0 0 0 0 0 0 0 0 0 0 0 0 0 8
0 0 0 0 0 0 0 0 0 0 0 0 0 0 0 0 0 0 0 0 0 0 0 0 0 0 0 0 0
0 0 0 0 0 0 0 0 0 0 4 0 0 0 0 0 0 0 152 0 0 0 0 0 0 0 0 0 0
0 0 0 62 0 0 0 0 0 0 0 0 0 1 0 0 0 0 0 0 0 0 0 0 0 0 0
M02255_77_000000000-ADUT3_1_1113_4654_10613 T----AC---GT-AT-GTC--------GCA-A-G------C--G--T---T--AT-C-CGG-AT------TT-A--T-T--GG-GC-------GT--A-----AA-GC-GC-GT-------C-TA-G-G-T-G------------G--T-TT-G-A-T-----------AA---
-G-T-C-T--------G-A-T--G--TG--A-AA-AT--G-C-GG-A-G-----CT-C-AA------C-T-C-C-G-T-A--T------T-G--C-G---T--T-------G--GA-A-A---C-------T--G-C--CA--A-A-C----T-A-G-A-G-T--A---T-CG-----GA-G-A--------G-G-T-G---GG-C------GG--A--ACT----A-C-A-A-GT-
-GT-A-G-AG-GT--G---A--A-A---TT-C-GT-AG--AT-A-TT--T-G--T-A-G-G-A-AT-G-CC---GA--T--A--GA-GAA-G--T-C--A-------G--C-T-C-A---CTG--G--AC-G-A--------------------A-T---A-C-T--GA--CA-C-T-G--A-AG--C-G-CG-A--AA-G-C---A-TG--GG-G--AG-C-A-
AA--------CA--GG Bacteria(100);Fusobacteria(100);Fusobacteria(100);Fusobacteriales(100);Fusobacteriaceae(100);unclassified(100);unclassified(100);unclassified(100);unclassified(100);

Otu0409 0 0 0 0 0 0 0 0 0 0 0 0 0 0 0 0 0 0 0 0 0 0 9 0 0 0 0 0
0 0 0 0 0 0 0 0 0 0 0 0 0 31 0 0 0 0 0 0 0 0 0 0 0 0 0 0 0
0 0 0 0 0 0 0 0 0 0 0 0 0 0 0 0 0 24 0 0 0 0 0 0 0 0 0 0 0
0 0 0 0 0 0 0 0 0 0 0 0 0 0 0 0 0 0 0 0 0 0 0 1 0 0 0
M02255_77_000000000-ADUT3_1_1119_5514_18045 T----AC---GA-AG-GGG--------GCT-A-G------C--G--T---T--GC-T-CGG-AA------TT-A--C-T--GG-GC-------GT--A-----AA-GC-GC-AC-------G-TA-G-G-C-G------------G--G-TC-G-A-T----------
AA----G-T-C-G--------G-G-G--G--TG--A-AA-TC--C-T-GG-A-G-----CT-C-AA------C-T-C-C-A-G-A--A-C----T-G--C-C---T--C-------C--GA-A-A---C-------T--G-T--TG--A-T-C----T-T-G-A-G-T--T---C-GG-----GA-G-A--------G-G-T-G---AG-T------GG--A--ACT----G-C-G-
A-GT--GT-A-G-AG-GT--G---A--A-A---TT-C-GT-AG--AT-A-TT--C-G--C-A-A-G-A-AC-A-CC---AG--T--G--GC-GAA-G--G-C--G-------G--C-T-C-A---CTG--G--CC-C-G--------------------A-T---A-C-T--GA--CG-C-T-G--A-GG--T-G-CG-A--AA-G-C---G-TG--GG-G--AG-
C-A-AA--------CA--GG Bacteria(100);Proteobacteria(100);Alphaproteobacteria(100);Rhizobiales(100);unclassified(100);unclassified(100);unclassified(100);unclassified(100);unclassified(100);

Otu0410 0 0 0 0 0 0 1 6 0 0 0 0 0 0 0 0 0 0 0 0 0 0 0 0 0 0 0 2
0 2 1 22 0 23 4 8 0 0 3 5 0 9 0 12 0 0 1 3 0 21 0 0 0 0 0 0 0
1 0 0 0 0 0 0 0 1 0 0 0 0 0 2 2 0 0 0 0 7 1 2 0 7 0 0 203 0
0 0 0 0 0 0 0 0 0 0 0 0 0 1 0 0 1 0 0 0 0 0 0 0 0 0 0
M02255_76_000000000-ADUNE_1_1104_6490_20794 TC---GC---AG-AA-TAA--------GCA-C-C------G--G--C---T--AACT-CTG-TG------CC-A--G-C--AG-CC-------GC--G-----GT-AA-TA-CA-------G-AG-G-G-T-GC------------G--A-GC-G-T-T-----------
AA----G-T-C-G--------G-A-T--G--TG--A-AA-TC--C-C-TG-A-G-----CT-T-AA------C-T-T-A-G-G-A--A-T----T-G--C-A---T--T-------C--GA-T-A---C-------T--G-G--GA--A-G-C----T-A-G-A-G-T--A---T-GG-----GA-G-A--------G-G-A-T---GG-T------AG--A--ATT----C-C-A-
G-GT--GT-A-G-CG-GT--G---A--A-A---TG-C-GT-AG--AG-A-TC--T-G--G-A-G-G-A-AT-A-CC---GA--T--G--GC-GAA-G--G-C--A-------G--C-C-A-T---CTG--G--CC-T-A--------------------A-T---A-C-T--GA--CG-C-T-G--A-GG--T-A-CG-A--AA-G-C---A-TG--GG-G--
AG-C-A-AA--------CA--GG Bacteria(100);Proteobacteria(100);Gammaproteobacteria(100);Pseudomonadales(100);Moraxellaceae(100);Acinetobacter(100);unclassified(100);unclassified(100);unclassified(100);

Otu0411 0 0 0 0 0 0 0 0 0 0 0 0 0 0 0 0 0 0 0 0 0 0 0 0 0 0 0 0
0 0 0 0 0 0 0 0 0 0 0 0 0 0 0 0 0 0 0 0 0 0 0 0 0 0 0 0 0
0 0 0 0 0 0 0 0 0 0 0 0 0 0 0 0 0 0 0 0 0 0 0 0 0 0 0 0 0
0 0 0 0 221 0 0 0 0 0 0 0 0 0 0 0 0 0 0 0 0 0 0 0 0 0 0
M02255_76_000000000-ADUNE_1_1112_28738_14746 T----AC---GA-AG-GGG--------GCT-A-G------C--G--T---T--GC-T-CGG-AA------TG-A--C-T--GG-GC-------GT--A-----AA-GG-GC-GC-------G-TA-------------------------GG-----C----------
-----------C----------G-G-C--G--TG--A-AA-GC--C-C-TG-G-G-----CT-C-AA------C-C-C-G-G-G-G--A-C----G-G--C-G---C--T-------G--GA-T-A---C-------G--G-C--GC--G-G-C----T-C-G-A-G-G--G---C-GG-----AA-G-A--------G-G-C-T---CG-C------GG--A--ATT----C-C-C-
A-GT--GT-A-G-AG-GT--G---A--A-A---TT-C-GT-AG--AT-A-TT--G-G--G-A-A-G-A-AC-A-CC---GG--T--G--GC-GAA-G--G-C--G-------G--C-G-A-G---CTG--G--TC-C-G--------------------C-A---C-C-T--GA--CG-C-T-G--A-GG--C-G-CG-A--CA-G-C---G-TG--GG-G--
AG-C-A-AA--------CA--GG Bacteria(100);Proteobacteria(100);Alphaproteobacteria(100);Rhodospirillales(100);unclassified(100);unclassified(100);unclassified(100);unclassified(100);unclassified(100);

Otu0412 0 0 0 0 0 0 0 0 0 0 0 25 0 0 0 0 0 0 0 0 0 0 0 0 0 0 0 0
0 0 0 0 0 0 0 0 0 0 0 0 0 0 0 0 0 0 0 0 0 0 0 0 0 0 0 0 0
0 0 0 0 0 0 0 0 0 0 0 0 0 0 0 0 0 0 0 0 0 0 0 0 0 0 0 0 0
0 0 0 0 0 0 0 0 0 0 0 0 0 0 0 0 0 0 0 0 0 0 0 0 0 0 0
M02255_77_000000000-ADUT3_1_2107_27875_13369 T----AC---GT-AG-GGG--------GCA-A-G------C--G--T---T--AT-C-CGG-AA------TT-A--C-T--GG-GC-------GT--A-----AA-GG-GT-GC-------G-TA-G-G-T-G------------G--C-CT-G-A-T-----
------AA----G-T-C-A--------G-G-G--G--TC--A-AA-GG--C-A-AC-G-G-----CT-C-AA------C-C-G-T-T-G-T--A------A-G--C-C---C--C-------T--GA-A-A---C-------T--A-T--CG--G-G-C----T-T-G-A-G-T--T---C-AG-----GA-G-A--------G-G-A-A---AG-C------GG--A--ATT----C-
C-A-A-GT--GT-A-G-CG-GT--G---A--A-A---TG-C-GT-AG--AT-A-TT--T-G--G-A-G-G-A-AC-A-CC---AG--T--G--GC-GAA-G--G-C--G-------G--C-T-T-T---CTG--G--AC-T-G--------------------A-T---A-C-T--GA--CA-C-T-G--A-GG--C-A-CG-A--AA-G-C---G-TG--GG-G-
-AG-C-A-AA--------CA--GG Bacteria(100);unclassified(100);unclassified(100);unclassified(100);unclassified(100);unclassified(100);unclassified(100);unclassified(100);unclassified(100);

Otu0413 0 0 0 0 0 0 0 0 0 0 0 0 2 0 0 0 0 0 0 0 0 1 0 0 0 0 0 0
0 0 0 0 0 0 0 0 0 2 7 0 0 0 0 0 0 44 0 505 1 0 0 0 0 0 0 0 0
0 0 0 0 0 0 0 0 0 4 0 0 0 0 0 0 12 0 0 0 0 0 0 0 0 0 0 0 0
0 0 0 0 0 0 0 0 0 0 0 0 0 0 0 0 0 0 0 0 0 0 0 0 0 0 0
M02255_76_000000000-ADUNE_1_1102_23275_24267 G----TG---CC-AG-CAG--------CCG-C-G---------G--T---A--AT-A-CGG-AG------GA-T--C-C--GA-GC-------GT--T-----AT-CC-G--G--------A-TT-T-A-T-T------------G--A-CG-T-A-T---------
--AA----G-T-C-A--------G-C-T--G--TG--A-AA-GT--T-T-AC-G-G-----CT-C-AA------C-C-G-T-G-A-A--A-T----T-G--C-A---G--T-------T--GA-T-A---C-------T--G-T--AT--G-T-C----T-T-G-A-G-T--G---T-AC-----AA-G-A--------G-G-T-G---GG-C------GG--A--ATT----C-G-T-
G-GT--GT-A-G-CG-GT--G---A--A-A---TG-C-TT-AG--AT-A-TC--A-C--G-A-A-G-A-AC-T-CC---AA--T--T--GC-GAA-G--G-C--A-------G--C-T-C-A---CTG--G--GG-T-A--------------------C-A---A-C-T--GA--CA-C-T-G--A-GG--C-T-CG-A--AA-G-T---G-TG--GG-T--AT-
C-A-AA--------CA--GG Bacteria(100);Bacteroidetes(100);unclassified(92);unclassified(92);unclassified(92);unclassified(92);unclassified(92);unclassified(92);unclassified(92);

Otu0414 0 0 0 0 0 0 0 0 0 0 0 0 0 0 0 0 0 0 0 4 10 0 0 0 0 0 0 0
0 6 0 0 0 0 0 0 0 63 0 0 0 0 0 0 0 0 0 0 0 0 0 12 0 0 0 0 0
0 0 0 0 0 0 0 0 0 0 0 0 0 0 0 0 0 0 0 0 0 0 0 0 0 0 0 0 0
0 0 0 0 107 0 0 0 0 0 0 0 0 0 0 0 0 0 0 0 0 0 0 0 0 0 0
M02255_77_000000000-ADUT3_1_1101_24841_18703 G----AC---GG-AG-GAT--------GCA-A-G------T--G--T---T--AT-C-CGG-AA------TC-A--C-T--GG-GC-------GT--A-----AA-GC-GT-CT-------G-TA-G-G-T-G------------G--T-TT-A-A-A-----
------AA----G-T-C-T--------G-C-T--G--TT--A-AA-AG--T-T-GA-A-G-----CT-T-AA------C-T-T-C-A-A-A--G-A----T-G--C-C---G--T-------A--GA-A-A---C-------T--T-T--TA--A-A-C----T-C-G-A-G-G--A---T-GG-----TA-G-G--------G-G-T-A---GA-G------GG--A--ATT----T-
C-C-A-GT--GG-A-G-CG-GT--G---A--A-A---TG-C-GT-AG--AT-C-TT--G-G--A-A-A-G-A-CC-A-CC---AA--T--G--GC-GAA-G--G-C--A-------C--T-C-T-A---CTG--G--GC-C-A--------------------T-T---A-C-T--GA--CA-C-T-A--A-GA--G-A-CG-A--AA-G-C---T-AG--GG-G-
-AG-C-A-AA--------TG--GG Bacteria(100);Cyanobacteria(100);unclassified(100);unclassified(100);unclassified(100);unclassified(100);unclassified(100);unclassified(100);unclassified(100);

Otu0415 56 0 0 0 0 0 0 0 0 0 0 0 0 0 2 0 0 0 0 5 13 0 0 0 5 0 4 0
0 3 0 0 0 0 0 0 0 38 5 0 0 0 58 0 0 27 0 91 60 0 0 93 0 0 0 1 0
0 0 0 0 0 0 0 0 0 0 0 0 0 0 0 0 0 0 0 0 0 0 0 0 0 0 0 0 0
0 51 0 0 0 0 0 0 0 0 0 1 0 0 0 0 0 0 0 0 0 0 0 3 44 0 0

M02255_77_000000000-ADUT3_1_1103_18099_13233 T----AC---GT-AG-GGT--------CCG-A-G------C--G--T---T--GT-C-CGG-AA------TT-A--T-T--GG-GC-------GT--A-----AA-GG-GC-TC-------G-TA-G-G-C-G------------G--T-CT-G-T-C-----------GC----G-T-C-G--------G-G-A--G--TG--A-AA-AC--C-A-GG-T-G-----CT-T-AA------C-A-C-C-T-G-G--C-C----T-G--C-T---T--T-------C--GA-T-A---C-------G--G-G--CA--G-A-C----T-A-G-A-G-G--T---A-CT-----CA-G-G--------G-G-A-G---AA-T------GG--A--ATT----C-C-T-G-GT--GT-A-G-CG-GT--G---A--A-A---TG-C-GC-AG--AT-A-TC--A-G--G-A-G-G-A-AC-A-CC---GG--T--G--GC-GAA-G--G-C--G-------G--T-T-C-T---CTG--G--GA-G-T-------------------A-T---C-C-T--GA--CG-C-T-G--A-GG--A-G-CG-A--AA-G-T---G-TG--GG-G--AG-C-G-AA--------CA--GG Bacteria(100);Actinobacteria(100);Actinobacteria(100);Actinobacteridae(100);Actinomycetales(100);Propionibacterineae(100);Nocardioidaceae(100);Nocardioides(100);unclassified(100);

Otu0416 80 0 0 0 0 0 0 0 0 0 0 0 0 0 0 0 0 0 0 0 0 0 0 0 0 0 0 0
0 0 0 0 0 0 0 0 0 0 15 0 0 0 0 0 0 0 0 0 0 0 0 0 0 0 0 0 0
0 0 0 0 0 0 0 0 0 0 0 0 0 0 0 0 0 0 0 0 0 0 0 0 0 0 0 0 0
0 0 0 0 291 0 0 0 0 0 0 0 0 0 0 13 0 0 0 0 0 0 0 0 0 0 0

M02255_76_000000000-ADUNE_1_1113_18163_19262 C----GG---CT-AA-CTC--------CGT-G-C------C--A--G---C--AG-C-CGC-GG------TA-A--T-A--CG-GA-------GG--A-----TC-CA-AG-CG-------T-TA-T-C-C-G------------GA-T-TT-A-T-T-----------AA----G-T-C-A--------G-G-G--G--TG--A-AA-GA--C-G-GT-G-G-----CT-C-AA------C-C-A-T-C-G-C--A-G----T-G--C-C---T--T-------T--GA-T-A---C-------T--G-A--TG--A-G-C----T-T-G-A-A-T--A---C-AC-----TT-G-A--------G-G-T-A---GG-C------GG--A--ATG----T-G-A-C-AA--GT-A-G-CG-GT--G---A--A-A---TG-C-AT-AG--AT-A-TG--T-C--A-C-A-G-A-AC-A-CC---AA--T--G--GC-GAA-G--G-C--A-------G--C-T-T-A---CTA--A--GG-T-G-------------------C-A---A-T-T--GA--CG-C-T-G--A-GG--C-A-CG-A--AA-G-C---G-TG--GG-G--AT-C-A-AA--------CA--GG Bacteria(100);Bacteroidetes(100);Sphingobacteria(100);Sphingobacteriales(100);Sphingobacteriaceae(100);Sphingobacteriaceae(100);unclassified(100);unclassified(100);unclassified(100);

Otu0417 0 0 0 4 0 0 0 0 0 0 0 0 0 0 0 0 0 0 0 0 1 0 0 0 0 1 1 6
0 0 0 0 0 0 0 0 0 0 2 0 1 0 2 0 0 0 0 0 0 0 0 0 0 0 0 1 0
0 0 0 0 0 0 0 0 0 0 1 0 2 0 1 0 0 1 2 0 0 0 0 0 0 0 0 0 5
0 7 0 8 10 0 0 2 0 5 0 0 0 1 0 0 0 0 0 4 16 0 3 0 0 0 8

M02255_77_000000000-ADUT3_1_2103_19733_17383 .....AC----T-A--CGT--------GCC-A-G------C--A--G---C--CG-C-GGT-AA------TA-C--G-T--AG-GG-------TG--C-----GA-GCGTT-AA-------T-CG-G-A-C-G------------G--T-TA-C-T-T-----------AA----G-C-A-G--------G-A-T--G--TG--A-AA-TC--C-C-CG-G-G-----CT-C-AA------C-C-T-G-G-G-A--A-C----T-G--C-G---T--T-------C--TG-A-A---C-------T--G-G--GT--G-A-C----T-A-G-A-G-T--G---T-GT-----CA-G-A--------G-G-G-A---GG-T------AG--A--ATT----C-C-A-C-GT--GT-A-G-CA-GT--G---A--A-A---TG-C-GT-AG--AG-A-TG--T-G--G-A-G-G-A-AT-A-CC---GA--T--G--GC-GAA-G--G-C--A-------G--C-C-T-C---CTG--G--GA-T-A-------------------A-C---A-C-T--GA--CG-T-T-C--A-TG--C-T-CG-A--AA-G-C---G-TG--GG-T--AG-C-A-AA--------CA--GG Bacteria(100);Proteobacteria(100);Betaproteobacteria(100);Neisseriales(100);Neisseriaceae(100);Neisseria(87);unclassified(87);unclassified(87);unclassified(87);

Otu0418 0 0 0 0 0 0 0 0 0 0 0 0 0 0 0 0 0 0 0 0 11 0 0 0 0 0 0 0
0 0 0 0 0 0 0 0 0 0 0 0 0 0 0 0 11 0 0 0 0 0 0 0 0 0 0 0 0
29 0 0 0 0 0 0 0 0 0 1 9 0 0 0 0 0 0 0 70 0 0 0 0 0 0 0 0 0
0 0 0 0 0 0 0 0 0 88 0 0 0 0 0 0 0 0 0 0 0 0 0 0 0 0 0

M02255_77_000000000-ADUT3_1_1111_15162_5982 T----AC---GT-AT-GGG--------GCA-A-G------C--G--T---T--AT-C-CGG-AA------TT-A--T-T--GG-GC-------GT--A-----AA-GA-GT-GC-------G-TA-G-G-T-G------------G--T-GG-C-T-T-----------AA----G-C-G-C--------A-G-G--G--TT--T-AA-GG--C-A-AT-G-G-----CT-T-AA------C-T-A-T-T-G-T--T------C-G--C-C---T--T-------G--CG-A-A---C-------T--G-G--GT--C-A-C----T-T-G-A-G-T--A---C-AG-----GA-G-A--------G-G-A-A---AG-C------GG--A--ATT----C-C-T-A-GT--GT-A-G-CG-GT--G---A--A-A---TG-C-AT-AG--AT-A-TT--A-G--G-A-G-G-A-AC-A-CC---GG--T--G--GC-GAA-G--G-C--G-------G--C-T-T-T---CTG--G--AC-T-G-------------------C-A---A-C-T--GA--CA-C-T-G--A-GG--C-A-CG-A--AA-G-C---G-TG--GG-T--AG-C-A-AA--------CA--GG Bacteria(100);Firmicutes(100);Clostridia(100);Clostridiales(100);Family_XIII_Incertae_Sedis(100);unclassified(100);unclassified(100);unclassified(100);unclassified(100);

Otu0419 0 0 0 0 0 0 30 2 20 0 0 0 0 0 0 0 0 0 0 0 0 0 0 0 0 0 0 0
0 1 0 0 0 0 0 0 0 0 0 71 0 0 0 0 0 0 0 0 0 0 0 0 0 0 0 0 0
0 0 0 0 0 0 0 0 0 0 0 2 0 0 0 0 0 0 0 24 10 0 0 0 0 1231 1192 0 11
17 0 0 0 0 0 0 0 0 0 0 0 0 0 0 0 0 0 0 0 0 0 0 0 78 0 0

M02255_77_000000000-ADUT3_1_1105_13591_18343 T----AC---GT-AG-GGG--------GCA-A-G------C--G--T---T--AT-C-CGG-AT------TT-A--C-T--GG-GT-------GT--A-----AA-GG-GA-GC-------G-TA-G-A-C-G------------G--C-GA-G-A-C-----------AA----G-T-C-T--------G-A-A--G--TG--A-AA-GC--C-C-GG-G-G-----CT-C-AA------C-C-C-C-G-G-G--A-C----T-G--C-T---T--T-------G--GA-A-A---C-------T--G-C--CT--T-G-C----T-A-G-A-G-T--G---C-TG-----GA-G-A--------G-G-T-A---AG-T------GG--A--ATT----C-C-T-A-GT--GT-A-G-CG-GT--G---A--A-A---TG-C-GT-AG--AT-A-TT--A-G--G-A-G-G-A-AC-A-CC---AG--T--G--GC-GAA-G--G-C--G-------G--C-T-T-A---CTG--G--AC-A-G-------------------T-A---A-C-T--GA--CG-T-T-G--A-GG--C-T-CG-A--AA-G-C---G-TG--GG-G--AG-C-A-AA--------CA--GG Bacteria(100);Firmicutes(100);Clostridia(100);Clostridiales(100);Lachnospiraceae(100);unclassified(100);unclassified(100);unclassified(100);unclassified(100);

Otu0420 0 0 0 0 0 0 0 0 0 0 0 0 3 0 0 0 0 5 0 0 0 4 0 0 0 0 1 0
0 0 0 0 0 0 0 0 0 0 0 0 0 0 0 0 0 0 0 0 0 0 0 0 0 0 0 0 0
2 0 0 0 0 0 0 0 0 0 6 0 0 0 0 0 0 0 0 0 0 0 0 0 0 0 0 0 0
0 0 0 0 0 0 0 0 0 0 4 0 0 0 0 0 0 0 0 0 0 0 0 0 0 0 0
M02255_77_000000000-ADUT3_1_2114_17186_22552 C----A----GC-A--GCC--------GCG-G-T------A--A--T---A--CG-G-AGG-AT------CC-A--A-G--CG-TT-------AT--C-----CG-GA-AT-CA-------G-TA-G-G-C-G------------G--T-CA-G-A-T-------
----AA----G-T-C-A--------G-T-G--G--TG--A-AA-GC--C-C-AT-C-G-----CT-C-AA------C-G-G-T-G-G-A--A-C----G-G--C-C---A--T-------T--GA-T-A---C-------T--G-T--CT--G-A-C----T-T-G-A-A-T--T---A-TT-----AG-G-A--------A-G-T-A---AC-T------AG--A--ATA----T-G-
T-A-GT--GT-A-G-CG-GT--G---A--A-A---TG-C-TT-AG--AG-A-TT--A-C--A-T-G-G-A-AT-A-CC---AA--T--T--GC-GAA-G--G-C--A-------G--G-T-T-A---CTA--C--TA-A-T--------------------A-T---A-T-T--GA--CG-C-T-G--A-TG--G-A-CG-A--AA-G-C---G-TG--GG-T--
AG-C-G-AA--------CA--GG Bacteria(100);Bacteroidetes(100);Flavobacteria(100);Flavobacteriales(100);Flavobacteriaceae(100);Flavobacterium(100);unclassified(100);unclassified(100);unclassified(100);

Otu0421 0 0 0 0 0 0 0 0 0 0 0 0 0 0 0 0 0 0 0 0 0 0 0 0 0 0 0 0
0 0 0 0 0 0 0 0 0 0 0 0 0 0 0 0 0 0 0 0 0 0 0 0 13 0 0 0 0
0 0 0 0 0 0 0 0 0 0 0 0 0 0 0 0 0 16 0 0 0 0 0 0 0 0 0 0 86
0 0 0 0 0 0 0 0 0 0 0 0 0 0 0 0 0 0 0 0 0 0 0 0 0 0 0
M02255_77_000000000-ADUT3_1_1113_12906_7535 T----AC---GG-AG-GGG--------ACT-A-G------C--G--T---T--GT-T-CGG-AA------TT-A--C-T--GG-GC-------GT--A-----AA-GC-GC-AC-------G-TA-G-G-C-G------------G--A-TC-G-G-A----------
CA----G-T-C-A--------G-A-G--G--TG--A-AA-TC--C-C-GG-G-G-----CT-C-AA------C-C-C-C-G-G-A--A-C----T-G--C-C---T--T-------T--GA-A-A---C-------T--C-C--CG--G-T-C----T-T-G-A-G-T--T---C-GA-----GA-G-A--------G-G-T-G---AG-T------GG--A--ATT----C-C-G-
A-GT--GT-A-G-AG-GT--G---A--A-A---TT-C-GT-AG--AT-A-TT--C-G--G-A-G-G-A-AC-A-CC---AG--T--G--GC-GAA-G--G-C--G-------G--C-T-C-A---CTG--G--CT-C-G--------------------A-T---A-C-T--GA--CG-C-T-G--A-GG--T-G-CG-A--AA-G-C---G-TG--GG-G--
AG-C-A-AA--------CA--GG Bacteria(100);Proteobacteria(100);Alphaproteobacteria(100);Rhodobacterales(100);Rhodobacteraceae(100);unclassified(100);unclassified(100);unclassified(100);unclassified(100);

Otu0422 0 0 0 0 0 0 0 0 0 0 0 0 2 1 0 0 2 0 0 0 0 6 0 0 0 0 0 0
0 0 0 0 0 0 0 0 0 0 0 0 0 0 0 0 0 0 0 0 0 0 0 0 0 0 0 0 0
0 0 0 0 9 0 0 0 0 0 0 4 9 1 0 0 0 0 0 0 0 0 0 0 0 0 0 0 0
0 0 0 0 0 0 0 0 0 0 0 0 0 0 1 0 0 0 0 0 0 0 0 0 0 0 0
M02255_77_000000000-ADUT3_1_2113_12656_16234 G----TG---CC-AG-CAG--------CCG-C-G---------G--T---A--AT-A-CGGAGGA-----TC-C--A-A--GC-GT-------TA--T-----CC-GG-AA-TC-------A-TT-G-G-C-G------------G--T-CA-G-A-T-----
------AA----G-T-C-A--------G-T-G--G--TG--A-AA-GC--C-C-AT-C-G-----CT-C-AA------C-G-G-T-G-G-A--A-C----G-G--C-C---A--T-------T--GA-T-A---C-------T--G-T--CT--G-A-C----T-T-G-A-A-T--T---A-TT-----AG-G-A--------A-G-T-A---AC-T------AG--A--ATA----T-
G-T-A-GT--GT-A-G-CG-GT--G---A--A-A---TG-C-TT-AG--AG-A-TT--A-C--A-T-G-G-A-AT-A-CC---AA--T--T--GC-GAA-G--G-C--A-------G--G-T-T-A---CTA--C--TA-A-T--------------------A-T---A-T-T--GA--CG-C-T-G--A-TG--G-A-CG-A--AA-G-C---G-TG--GG-T--
AG-C-G-AA--------CA--GG Bacteria(100);Bacteroidetes(100);Flavobacteria(100);Flavobacteriales(100);Flavobacteriaceae(100);Flavobacterium(100);unclassified(100);unclassified(100);unclassified(100);

Otu0423 8 32 16 0 0 0 10 1 17 0 0 0 0 0 0 0 0 0 0 0 0 0 0 0 0 0 0 0
0 0 0 0 0 0 0 0 0 0 0 13 0 0 0 0 0 0 0 0 0 0 0 0 0 0 0 0 0
0 0 0 0 0 0 0 0 0 0 0 0 0 0 0 0 0 0 0 0 0 0 0 0 0 0 0 0 0
0 0 0 0 0 0 0 0 0 0 0 0 0 0 0 0 0 0 0 0 0 0 10 1 11 0 0
M02255_77_000000000-ADUT3_1_2118_20472_22319 G----TG---CC-AG-CAG--------CCG-C-G---------G--T---A--AT-A-CGG-AG------GA-T--C-C--GA-GC-------GT--T-----AT-CC-GG-AT-------T-TA-G-G-T-G------------G--G-TT-G-T-T------
-----AA----G-T-C-A--------G-T-T--G--TG--A-AA-GT--T-T-GC-G-G-----CT-C-AA------C-C-G-T-A-A-A--A-T----T-G--C-A---G--T-------T--GA-T-A---C-------T--G-G--CG--A-C-C----T-T-G-A-G-T--G---C-AA-----CA-G-A--------G-G-T-A---GG-C------GG--A--ATT----C-
G-T-G-GT--GT-A-G-CG-GT--G---A--A-A---TG-C-TT-AG--AT-A-TC--A-C--G-A-A-G-A-AC-T-CC---GA--T--T--GC-GAA-G--G-C--A-------G--C-T-T-A---CTG--G--AT-T-G--------------------T-A---A-C-T--GA--CG-C-T-G--A-TG--C-T-CG-A--AA-G-T---G-TG--GG-T--
AT-C-A-AA--------CA--GG Bacteria(100);Bacteroidetes(100);Bacteroidia(100);Bacteroidales(100);Bacteroidaceae(100);Bacteroides(100);unclassified(100);unclassified(100);unclassified(100);

Otu0424 0 0 0 44 0 0 0 0 0 71 0 0 0 0 0 0 0 0 0 0 0 0 0 0 0 0 0 0
0 0 0 0 0 0 0 0 0 0 0 0 0 0 0 32 0 0 2 0 21 0 0 0 0 0 0 0 0
0 0 0 0 10 0 13 14 5 0 0 0 0 0 0 0 12 0 0 0 0 0 0 0 0 0 0 150 0
154 0 35 0 34 0 0 0 0 0 10 0 15 0 0 0 0 0 0 0 0 0 0 0 0 0 0
M02255_77_000000000-ADUT3_1_2113_27378_7746 T----AC---GG-GG-GGG--------GCA-A-G------T--G--T---T--CT-T-CGG-AA------TG-A--C-T--AG-GC-------GT--A-----AA-GG-GC-AC-------T-AC-G-G-C-A------------G--T-GA-A-T-C----------
GG----G-T-T-G--------C-A-A--G--T---T-CA--G--G-T-CG-C-C-----AA-A-AA------C-C-G-G-C-G-G--A-A----T-G--C-T---C--T-------C--GA-A-A---C-------C--A-A--TT--C-A-C----T-T-G-A-G-T--G---A-GA-----CA-G-A--------G-G-A-G---AG-T------TT--C--ATT----T-C-G-T-
GT--GT-A-G-GG-GT--T---A--A-A---TC-C-GT-AG--AT-C-TA--C-G--A-A-G-G-A-AG-G-CC---AA--A--A--GC-GAA-G--G-C--A-------G--C-T-C-T---TTG--G--GT-C-C--------------------C-T---A-C-T--GA--CG-C-T-G--A-GG--T-G-CG-A--AA-G-C---A-TG--GG-G--AG-C-
A-AA--------CT--GG Bacteria(100);unclassified(100);unclassified(100);unclassified(100);unclassified(100);unclassified(100);unclassified(100);unclassified(100);unclassified(100);

Otu0425 0 0 4 0 0 0 0 0 0 0 0 0 0 0 0 0 0 0 0 0 0 0 0 0 0 0 0 0
0 23 0 0 5 0 0 0 0 0 0 0 0 0 0 0 0 0 0 0 50 0 0 0 0 0 0 0 0
0 0 0 0 0 0 0 0 1 0 0 0 0 0 18 5 0 0 9 0 0 0 0 0 0 0 0 0 0
10 0 0 0 0 0 0 0 0 0 0 0 0 0 0 2 0 0 0 0 0 0 0 0 0 0 0
M02255_76_000000000-ADUNE_1_1111_12884_7143 T----AC---GT-AG-GGG--------GCG-A-G------C--G--T---T--AT-C-CGG-AT------TC-A--T-T--GG-GC-------GT--------AA------------------A---G-C-G------------G--C-CT-G-T-T----------AG----G-T-
C-G--------G-G-A--G--TT--A-AA-GC--C-G-GG-G-G-----CT-C-AA------C-C-C-C-C-G-T--C------C-G--C-T---C--C-------C--GA-T-A---C-------C--G-G--CG--G-G-C----T-T-G-A-G-T--C---T-GG-----TA-G-G--------G-G-A-A---GG-C------GG--A--ATT----C-C-C-A-GT--GT-
A-G-CG-GT--G---G--A-A---TG-C-GC-AG--AT-A-TT--G-G--G-A-A-G-A-AC-A-CC---GG--T--G--GC-GAA-G--G-C--G-------G--C-C-T-T---CTG--G--GC-C-A--------------------C-G---A-C-T--GA--CG-C-T-G--A-GG--C-G-CG-A--AA-G-C---T-AG--GG-G--AG-C-G-AA--
------CA--GG Bacteria(100);Actinobacteria(100);Actinobacteria(100);Coriobacteridae(100);Coriobacteriales(100);Coriobacterineae(100);Coriobacteriaceae(100);Olsenella(100);unclassified(100);

Otu0426 0 0 0 82 0 0 0 0 0 0 0 0 5 0 0 0 0 0 0 0 0 0 0 0 5 0 0 0
0 0 0 0 0 0 0 0 0 0 0 0 0 0 0 0 0 0 0 0 0 0 0 0 0 71 0 0 0
0 0 0 0 0 0 0 0 0 0 4 0 0 0 23 0 0 0 0 0 0 0 0 0 0 0 0 0 0
0 0 0 0 6 0 0 0 0 0 0 0 0 0 0 0 0 0 0 0 0 0 0 0 0 0 0
M02255_77_000000000-ADUT3_1_1102_9695_19904 T----AC---GG-AG-GGT--------GCA-A-G------C--G--T---T--AT-C-CGG-AA------TC-A--C-T--GG-GC-------GT--A-----AA-GG-GC-GC-------G-TA-G-G-C-G------------G--T-TT-G-T-T----------AA-
---G-T-C-C--------G-A-T--G--TT--A-AA-GA--C-C-GA-G-G-----CT-C-AA------C-C-T-C-G-A-C--A-C----G-G--C-G---T--T-------G--GA-T-A---C-------T--G-G--CA--A-G-C----T-G-G-A-C-G--G---T-TG-----GA-G-A--------G-G-A-A---GG-T------AG--A--ATT----A-C-C-A-
GT--GT-A-G-CG-GT--G---G--A-A---TG-C-GT-AG--AT-A-CT--G-G--T-A-G-G-A-AT-A-CC---CA--T--T--GC-GAA-G--G-C--A-------G--C-C-T-T---CTG--G--AC-A-A--------------------C-A---C-G-T--GA--CG-C-T-G--A-GG--C-G-CG-A--AA-G-T---G-TG--GG-G--AG-C-
A-AA--------CC--GG Bacteria(100);Deinococcus-Thermus(100);Deinococcales(100);Trueperaceae(100);Truepera(100);unclassified(100);unclassified(100);unclassified(100);unclassified(100);

Otu0427 0 0 0 0 0 0 0 0 0 0 0 0 0 0 0 0 0 0 0 0 3 0 0 0 0 0 0 0
0 1 0 0 0 0 0 19 0 20 0 0 0 0 0 0 0 0 0 0 0 0 0 0 0 0 0 0 0
0 0 0 0 0 0 0 0 0 0 0 0 0 0 0 0 0 0 0 0 0 0 0 0 0 0 0 0 0
0 0 0 0 0 0 0 0 0 0 0 0 0 0 0 10 0 0 0 0 0 0 0 0 0 0 0
M02255_77_000000000-ADUT3_1_1118_8844_18387 T----AC---GT-AG-GGG--------CCT-A-G------C--G--T---T--GT-C-CGG-AT------TT-A--T-T--GG-GC-------GT--A-----AA-GA-GC-TC-------G-TA-G-G-C-G------------G--T-TC-G-G-T----------AA--
--G-T-C-A--------G-G-T--G--TG--A-AA-TC--T-C-CA-C-G-----CT-C-AA------C-G-T-G-G-A-G--G------C-G-CCAC---C--T---------GA-A-A---C-------T--G-C--CG-TG-A-C----T-C-G-A-G-T--C---C-GG-----TA-G-G--------G-G--AG---TG-T------GG--A--ATTC---C-C---G-
GT--GT-A-G-CG-GT--G---A--A-A---TG-C-GC-AG--AT-A-TC--G-G--G-A-G-G-A-AC-A-CC---AG--T--G--GC-GAA-G--G-C--G-------G--C-A-C-T---CTGG----GC-C-G--------------------G-T---A-C-T--GA--CG-C-T-G--A-GG--A-G-CG-A--AA-G-C---G-TG--GG-G--AG-
C-G-AA--------CA--GG Bacteria(100);Actinobacteria(82);Actinobacteria(82);unclassified(82);unclassified(82);unclassified(82);unclassified(82);unclassified(82);unclassified(82);

Otu0428 0 0 0 0 0 0 0 0 0 0 0 0 0 0 0 0 0 0 0 0 0 0 0 0 0 6 0 0
0 0 0 0 0 0 0 0 0 0 0 0 0 0 0 0 0 0 0 0 0 0 0 0 0 0 0 0 0
0 0 0 0 0 0 0 0 0 0 0 0 0 0 0 0 10 0 0 0 0 0 0 0 0 0 0 0 0
0 0 0 0 37 0 0 0 0 0 0 0 0 0 0 0 0 36 0 0 0 0 0 0 0 0 0
M02255_77_000000000-ADUT3_1_2103_20969_23951 C----AC---GT-AG-GGG--------GCA-A-G------C--G--T---T--GT-C-CGG-AT------TT-A--T-T--GG-GC-------GT--A-----AA-GA-GC-TC-------G-TA-G-G-C-G------------G--C-TG-A-G-T-----
------AA----G-T-C-G--------G-A-T--G--TT--A-AA-TC--C-C-CA-G-G-----CT-C-AA------C-C-T-G-G-G-A---------C-A-CC-A---C--C-------C--GA-T-A---C-------T--G-C--TC-TG-G-C----T-A-G-A-G-T--C---C-GG-----TA-G-G--------G-G-A-G---CG-T------GG--A--ATT----C-
C-T-G-GT--GT-A-G-CG-GT--G---A--A-A---TG-C-GC-AG--AT-A-TC--A-G--G-A-G-G-A-AC-A-CC---AG--C--G--GC-GAA-G--G-C--G-------G--C-G-C-T---CTG--G--GC-C-G--------------------G-A---A-C-T--GA--CG-C-T-G--A-GG--A-G-CG-A--AA-G-C---G-TG--GG-
T--AG-C-A-AA--------CA--GG Bacteria(100);Actinobacteria(100);Actinobacteria(100);Acidimicrobidae(100);Acidimicrobiales(100);Acidimicrobineae(100);unclassified(100);unclassified(100);unclassified(100);

Otu0429 112 0 0 0 0 0 0 0 0 0 0 0 0 0 0 0 0 0 0 0 0 0 0 0 0 0 0 0
0 0 66 0 0 0 0 0 0 0 0 0 0 0 0 65 19 0 0 0 0 0 0 0 0 0 0 0 0
0 0 0 0 0 0 0 0 0 0 0 0 0 0 0 18 0 23 0 43 0 9 0 0 0 0 0 56 0
18 0 29 0 0 0 0 0 0 0 0 0 4 0 0 0 0 21 0 0 0 0 0 0 0 0 0
M02255_77_000000000-ADUT3_1_2113_20776_15051 T----AC---GG-AG-GGG--------GCT-A-G------C--G--T---T--GT-T-CGG-AA------TT-A--C-T--GG-GC-------GT--A-----AA-GC-GC-AC-------G-TA-G-G-C-G------------G--A-TC-A-G-T-----
------CA----G-T-C-A--------G-G-G--G--TG--A-AA-TC--C-C-GG-G-G-----CT-C-AA------C-C-C-C-G-G-A--A-C----T-G--C-C---T--T-------T--GA-T-A---C-------T--G-C--TG--G-T-C----T-G-G-A-G-G--T---C-GA-----GA-G-A--------G-G-C-G---AG-T------GG--A--ATT----
C-C-G-A-GT--GT-A-G-AG-GT--G---A--A-A---TT-C-GT-AG--AT-A-TT--C-G--G-A-G-G-A-AC-A-CC---AG--T--G--GC-GAA-G--G-C--G-------G--C-T-C-G---CTG--G--CT-C-G--------------------A-T---A-C-T--GA--CG-C-T-G--A-GG--T-G-CG-A--AA-G-C---G-TG--GG-
G--AG-C-A-AA--------CA--GG Bacteria(100);Proteobacteria(100);Alphaproteobacteria(100);unclassified(100);unclassified(100);unclassified(100);unclassified(100);unclassified(100);unclassified(100);

Otu0430 0 0 0 0 0 0 0 0 91 0 0 0 0 0 0 0 0 0 0 0 0 0 0 0 0 0 0 0
0 0 0 0 0 0 0 0 0 0 0 0 0 0 0 30 0 0 0 0 0 0 0 0 0 0 0 0 0
0 0 0 0 0 0 0 0 0 0 0 0 0 0 0 0 11 0 0 0 0 0 0 0 0 0 0 0 0
0 0 0 0 0 0 0 0 0 0 0 24 0 0 0 0 0 0 0 0 0 0 0 0 0 0 0

M02255_77_000000000-ADUT3_1_1103_16943_14427 T----AC---GT-AG-GGG--------GCA-A-G------C--G--T---T--AT-C-CGG-AT------TT-A--C-T--GG-GT-------GT--A-----AA-GG-GA-GC-------G-TA-G-A-C-G------------G--C-TT-T-G-C-----
------AA----G-T-C-T--------G-A-C--G--TG--A-AA-CT--C-C-GG-G-G-----CT-C-AA------C-T-C-C-G-G-A--A-C----T-G--C-G---T--T-------G--GA-A-A---C-------T--G-T--AA--G-G-C----T-T-G-A-G-T--G---C-CG-----GA-G-A--------G-G-T-A---AG-C------GG--A--ATT----
C-C-T-A-GT--GT-A-G-CG-GT--G---A--A-A---TG-C-GT-AG--AT-A-TT--A-G--G-A-G-G-A-AC-A-CC---AG--T--G--GC-GAA-G--G-C--G-------G--C-T-T-A---CTG--G--AC-G-G--------------------C-A---A-C-T--GA--CG-T-T-G--A-GG--C-T-CG-A--AA-G-C---G-TG--GG-
G--AG-C-A-AA--------CA--GG Bacteria(100);Firmicutes(100);Clostridia(100);Clostridiales(100);Lachnospiraceae(100);unclassified(100);unclassified(100);unclassified(100);unclassified(100);

Otu0431 0 0 0 0 0 0 5 0 0 0 0 0 0 0 0 0 0 0 0 0 0 0 0 0 0 0 0 0
0 1 0 0 0 0 0 0 0 0 0 0 0 0 0 0 0 0 0 0 8 0 0 0 0 0 0 0 0
0 0 0 0 0 0 0 0 0 0 0 0 0 0 1 0 0 0 0 0 0 0 0 0 0 0 0 0 3
1 0 0 0 0 0 0 0 0 0 0 0 0 3 0 0 0 0 0 0 0 0 0 0 0 27 0

M02255_77_000000000-ADUT3_1_1106_22784_9502 T----AC---GT-AG-GGT--------GCG-A-G------C--G--T---T--GT-C-CGG-AT------TT-A--C-T--GG-GC-------GT--A-----AA-GG-GC-TC-------G-TA-G-G-T-G------------G--T-GT-G-T-C-----------GC-
---G-T-C-G--------G-C-T--G--TG--T-AA-TC--C-A-GG-G-G-----CT-T-AA------C-T-T-T-T-G-G--T-T----G-G--C-A---G--G-------C--GA-T-A---C-------G--G-G--CAT-T-G-C----T-T-G-A-G-T--G---C-TG-----TA-G-G--------G-G-A-G---AC-T------GG--A--ATT----C-C-T-G-
GT--GT-A-G-CG-GT--G---A--A-A---TG-C-GC-AG--AT-A-TC--A-G--G-A-G-G-A-AC-A-CC---GA--T--G--GC-GAA-G--G-C--A-------G--G-T-C-T---CTG--G--GC-A-G--------------------T-T---A-C-T--GA--CG-C-T-G--A-GG--A-G-CG-A--GA-G-C---A-TG--GG-T--AG-
C-G-AA--------CA--GG Bacteria(100);Actinobacteria(100);Actinobacteria(100);Actinobacteridae(100);Actinomycetales(100);Corynebacterineae(100);Corynebacteriaceae(100);Corynebacterium(100);unclassified(100);

Otu0432 2 0 0 13 0 9 0 5 0 2 0 0 0 0 0 0 0 0 0 0 0 0 0 0 0 0 0 1
0 63 0 0 0 0 0 0 7 0 0 0 0 1 13 0 0 0 0 0 17 0 0 3 0 1 0 0 0
0 10 4 0 0 0 0 0 0 0 1 0 0 0 0 0 0 0 1 0 0 0 0 1 5 0 0 8 6
0 2 0 2 1 0 0 0 0 12 0 0 0 0 0 1 0 0 0 10 0 0 0 1 0 9 0

M02255_77_000000000-ADUT3_1_1112_26196_19801 C----A----GC-A--GCC--------GCG-G-T------A--A--T---A--CG-T-AGG-GC------GC-G--A-G--CG-TT-------GT--C-----CG-GA-AT-TA-------T-TG-G-G-C-G------------G--C-TG-G-T-C-------
----GC----G-T-C-T--------G-T-C--G--TG--A-AA-TC--C-T-CT-G-G-----CT-T-AA------C-T-G-G-G-G-G--C-T----T-G--C-G---G--T-------G--GG-T-A---C-------G--G-G--CC--G-G-C----T-T-G-A-G-T--G---C-GG-----TA-G-G--------G-G-A-G---AC-T------GG--A--ACT----C-
C-T-G-GT--GT-A-G-CG-GT--G---G--A-A---TG-C-GC-AG--AT-A-TC--A-G--G-A-A-G-A-AC-A-CC---GG--T--G--GC-GAA-G--G-C--G-------G--G-T-C-T---CTG--G--GC-C-G--------------------T-T---A-C-T--GA--CG-C-T-G--A-GG--A-G-CG-A--AA-G-C---G-TG--GG-
G--AG-C-G-AA--------CA--GG Bacteria(100);Actinobacteria(100);Actinobacteria(100);Actinobacteridae(100);Actinomycetales(100);Actinomycineae(100);Actinomycetaceae(100);Actinomyces(100);unclassified(100);

Otu0433 0 146 0 0 0 0 0 0 0 0 0 0 0 0 0 0 0 0 0 0 0 0 0 0 0 0 0 0
0 0 0 0 0 0 0 0 0 0 0 0 0 0 0 0 33 0 0 0 0 0 0 0 0 0 0 0 0
0 0 0 0 0 0 0 0 0 0 0 0 0 0 0 0 0 0 0 0 0 0 0 0 0 0 0 0 0
0 0 0 0 0 0 0 0 0 0 0 0 0 0 0 0 0 0 17 0 0 45 0 0 0 0 0

M02255_77_000000000-ADUT3_1_1102_12331_20121 T----AC---GT-AG-GTT--------GCA-A-G------C--G--T---T--GT-C-CGG-AA------TT-A--C-T--GG-GT-------GT--A-----AA-GG-GC-GT-------G-TA-G-G-C-G------------G--A-GA-T-G-C-----
------AA----G-T-T-G--------G-G-A--G--TG--A-AA-TC--C-A-TG-G-G-----CT-C-AA------C-C-C-A-T-G-A--A-C----T-G--C-T---T--T-------C--AA-A-A---C-------T--G-C--AT--C-C-C----T-T-G-A-G-T--A---T-CG-----GA-G-A--------G-G-C-A---AG-C------GG--A--ATT----C-
C-T-A-GT--GT-A-G-CG-GT--G---A--A-A---TG-C-GT-AG--AT-A-TT--A-G--G-A-G-G-A-AC-A-CC---AG--T--G--GC-GAA-G--G-C--G-------G--C-T-T-G---CTG--G--AC-G-A--------------------C-A---A-C-T--GA--CG-C-T-G--A-GG--C-G-CG-A--AA-G-C---G-TG--GG-
G--AG-C-A-AA--------CA--GG Bacteria(100);Firmicutes(100);Clostridia(100);Clostridiales(100);unclassified(80);unclassified(80);unclassified(80);unclassified(80);unclassified(80);

Otu0434 0 0 0 0 0 0 0 0 0 0 0 0 0 0 0 0 0 0 0 0 0 0 0 101 0 0 3 0
0 0 0 0 0 0 0 0 0 0 0 0 65 0 43 0 85 0 0 0 0 0 0 0 18 35 0 0 0
0 0 0 0 0 0 0 0 0 0 0 0 11 0 0 5 0 0 0 0 0 0 4 296 0 0 0 0 0
0 0 0 0 0 0 0 0 0 0 0 0 0 0 0 0 0 0 0 0 0 0 0 0 0 0 0

M02255_77_000000000-ADUT3_1_2112_29021_12512 T----AC---GG-AG-GGG--------GCT-A-G------C--G--T---T--GT-T-CGG-AA------TT-A--C-T--GG-GC-------GT--A-----AA-GC-GC-AC-------G-TA-G-G-C-G------------G--C-CG-T-T-T-----
------AA----G-T-T-A--------G-A-G--G--TG--A-AA-GC--C-C-GG-G-G-----CT-C-AA------C-C-C-C-G-G-A--A-T----T-G--C-C---T--T-------T--GA-T-A---C-------T--G-G--AT--G-G-C----T-T-G-A-A-T--C---C-AG-----GA-G-A--------G-G-T-G---AG-T------GG--A--ATT----C-
C-G-A-GT--GT-A-G-AG-GT--G---A--A-A---TT-C-GT-AG--AT-A-TT--C-G--G-A-A-G-A-AC-A-CC---AG--T--G--GC-GAA-G--G-C--G-------G--C-T-C-A---CTG--G--AC-T-G--------------------G-T---A-T-T--GA--CG-C-T-G--A-GG--T-G-CG-A--AA-G-C---G-TG--GG-
G--AG-C-A-AA--------CA--GG Bacteria(100);Proteobacteria(100);Alphaproteobacteria(100);Sphingomonadales(100);unclassified(100);unclassified(100);unclassified(100);unclassified(100);unclassified(100);

Otu0435 237 0 0 0 0 0 0 0 0 0 0 0 0 0 0 0 0 0 0 0 0 0 0 0 0 0 0 0
31 0 0 0 0 0 0 115 0 0 0 0 0 0 0 0 0 0 0 0 0 0 0 0 0 0 0 0 0
0 0 0 0 0 0 0 0 0 0 0 0 0 0 0 0 0 0 0 0 0 0 0 0 0 0 0 0 58
0 0 0 176 0 0 0 0 0 0 0 0 0 0 0 0 0 0 0 0 0 0 0 0 0 0 0
M02255_77_000000000-ADUT3_1_1118_25646_21427 T----AC---GG-AG-GGG--------GCT-A-G------C--G--T---T--GT-T-CGG-AA------TT-A--C-T--GG-GC-------GT--A-----AA-GC-GT-AC-------G-TA-G-G-C-G------------G--C-TT-T-G-T-----
------AA----G-T-T-A--------G-A-G--G--TG--A-AA-GC--C-T-GG-T-G-----CT-C-AA------C-A-C-C-A-G-A--A-C----T-G--C-C---T--T-------T--AA-G-A---C-------T--G-C--AT--C-G-C----T-T-G-A-A-C--G---T-CG-----GA-G-A--------G-G-T-G---AG-T------GG--A--ATT----C-
C-G-A-GT--GT-A-G-AG-GT--G---A--A-A---TT-C-GT-AG--AT-A-TT--C-G--G-A-A-G-A-AC-A-CC---AG--T--G--GC-GAA-G--G-C--G-------G--C-T-C-A---CTG--G--AC-G-A--------------------C-T---G-T-T--GA--CG-C-T-G--A-GG--T-A-CG-A--AA-G-C---G-TG--GG-
G--AG-C-A-AA--------CA--GG Bacteria(100);Proteobacteria(100);Alphaproteobacteria(100);Sphingomonadales(100);Sphingomonadaceae(100);Sphingomonas(100);unclassified(95);unclassified(95);unclassified(95);

Otu0436 211 0 0 0 0 0 0 0 0 0 0 0 0 0 0 0 0 0 0 0 0 0 0 0 0 0 0 0
0 0 0 0 0 0 0 0 0 0 0 0 0 0 0 0 0 0 0 0 0 0 0 0 0 0 0 0 0
0 0 0 0 0 0 0 0 0 0 0 0 0 0 0 0 0 0 0 0 0 0 0 0 0 0 0 0 0
0 0 0 0 0 0 0 0 0 0 0 0 3 0 0 0 0 0 0 0 0 0 0 0 0 0 0
M02255_77_000000000-ADUT3_1_1107_8580_15783 T----AC---GT-AG-GGG--------GCA-A-G------C--G--T---T--AT-C-CGG-AA------TT-A--T-T--GG-GC-------GT--A-----AA-GG-GT-AC-------G-TA-G-G-T-G------------G--T-TA-C-C-C----------AA-
---G-C-A-C--------G-A-G--G--TA--T-AA-GG--C-A-AT-G-G-----CT-T-AA------C-C-A-T-T-G-T--T------C-G--C-C---T--T-------G--TG-A-A---C-------T--G-G--GC--T-A-C----T-T-G-A-G-T--G---C-AG-----GA-G-A--------G-G-A-A---AG-C------GG--A--ATT----C-C-T-A-
GT--GT-A-G-CG-GT--G---A--A-A---TG-C-GT-AG--AT-A-TT--A-G--G-A-G-G-A-AC-A-CC---AG--T--G--GC-GAA-G--G-C--G-------G--C-T-T-T---CTG--G--AC-T-G--------------------T-A---A-C-T--GA--CA-C-T-G--A-GG--T-A-CG-A--AA-G-C---G-TG--GG-G--AG-C-
A-AA--------CA--GG Bacteria(100);Firmicutes(100);Clostridia(100);Clostridiales(100);Family_XIII_Incertae_Sedis(100);unclassified(100);unclassified(100);unclassified(100);unclassified(100);

Otu0437 0 0 8 0 1 0 2 0 0 0 0 0 0 0 0 0 0 0 0 0 0 0 0 1 0 0 0 1
0 1 4 2 0 16 2 0 0 3 35 2 4 0 0 68 0 0 0 0 0 0 0 2 0 2 0 0 0
0 0 5 1 0 0 1 0 1 0 0 0 0 0 5 4 16 0 10 4 0 1 0 1 1 0 1 10 2
4 4 0 14 57 0 2 2 0 7 0 0 0 1 0 1 0 2 0 0 0 0 0 0 0 1 2
M02255_77_000000000-ADUT3_1_2118_28875_14859 T----AC---GT-AG-GTC--------CCG-A-G------C--G--T---T--AT-C-CGG-AT------TT-A--T-T--GG-GC-------GT--A-----AA-GC-GA-GC-------G-CA-G-G-C-G------------G--T-TA-G-A-T------
-----AA----G-T-C-T--------G-A-A--G--TT--A-AA-GG--C-T-GT-G-G-----CT-T-AA------C-C-A-T-A-G-T--A------C-G--C-T---T--T-------G--GA-A-A---C-------T--G-T--TT--A-A-C----T-T-G-A-G-T--G---C-AA-----GA-G-G--------G-G-A-G---AG-T------GG--A--ATT----C-C-
A-T-GT--GT-A-G-CG-GT--G---A--A-A---TG-C-GT-AG--AT-A-TA--T-G--G-A-G-G-A-AC-A-CC---GG--T--G--GC-GAA-A--G-C--G-------G--C-T-C-T---CTG--G--CT-T-G--------------------T-A---A-C-T--GA--CG-C-T-G--A-GG--C-T-CG-A--AA-G-C---G-TG--GG-G--
AG-C-A-AA--------CA--GG Bacteria(100);Firmicutes(100);Bacilli(100);Lactobacillales(100);Streptococcaceae(100);Streptococcus(100);unclassified(100);unclassified(100);unclassified(100);

Otu0438 0 0 8 3 0 0 0 11 0 3 0 0 0 0 0 0 0 0 0 0 0 0 0 0 0 0 0 0
0 0 0 21 0 0 0 0 0 0 0 0 0 0 0 0 0 0 0 0 0 0 0 0 0 0 0 0 0
0 16 0 0 0 0 0 0 0 0 0 0 0 0 0 0 0 0 0 0 0 0 0 0 4 0 0 0 0
0 0 0 0 0 0 0 0 0 0 0 0 0 0 0 0 0 0 0 0 0 0 0 0 0 0 0
M02255_76_000000000-ADUNE_1_2106_15712_14830 C----CG---GC-TA-ATT--------CCG-T-G------C--C--A---G--CA-G-CCG-CG------GT-A--A-T--AC-GG-------AA--G-----GT-CC-GG-GC-------G-TT-A-T-C-C------------G--G-GG-A-T-T------
-----TA----T-T-G-T--------G-T-T--G--TG--A-AA-TG--T-A-GG-C-G-----CC-C-AA------C-G-T-C-T-G-A--C-T----T-G--C-A---G--C-------G--CA-T-A---C-------T--G-G--TT--C-C-C----T-T-G-A-G-T--A---C-GC-----GC-A-A--------C-G-C-C---GG-C------GG--A--ATT----C-G-
T-C-GT--GT-A-G-CG-GT--G---A--A-A---TG-C-TT-AG--AT-A-TG--A-C--G-A-A-G-A-AC-C-CC---GA--T--T--GC-GAA-G--G-C--A-------G--C-C-G-G---CGG--G--AG-C-G--------------------C-A---A-C-T--GA--CG-C-T-G--A-AG--C-T-CG-A--AG-G-T---G-CG--GG-T--
AT-C-G-AA--------CA--GG Bacteria(100);Bacteroidetes(100);Bacteroidia(100);Bacteroidales(100);Prevotellaceae(100);Prevotella(100);unclassified(100);unclassified(100);unclassified(100);

Otu0439 0 0 0 47 0 0 0 0 0 0 0 0 0 0 0 0 0 0 0 0 0 0 0 0 0 0 0 0
0 0 0 0 0 0 0 10 0 0 57 0 0 0 69 0 0 0 0 0 0 0 0 0 0 3 0 0 0
0 0 127 0 14 1 0 0 0 3 2 0 0 0 0 0 0 31 0 0 0 0 0 0 0 0 0 0 0
0 0 0 0 0 0 0 0 0 0 0 0 0 0 0 0 0 0 0 0 0 0 0 0 0 0 0
M02255_77_000000000-ADUT3_1_2103_25761_7088 T----AC---GA-AG-GGG--------GCT-A-G------C--G--T---T--GC-T-CGG-AA------TC-A--C-T--GG-GC-------GT--A-----AA-GG-GC-GC-------G-TA-G-G-C-G------------G--A-CT-T-T-T----------
AA----G-T-C-G--------G-G-G--G--TG--A-AA-GC--C-C-AG-G-G-----CT-C-AA------C-C-C-T-G-G-A--A-T----T-G--C-C---T--T-------C--GA-T-A---C-------T--G-A--GA--G-T-C----T-T-G-A-G-T--T---C-GG-----AA-G-A--------G-G-T-T---GG-T------GG--A--ACT----G-C-
G-A-GT--GT-A-G-AG-GT--G---A--A-A---TT-C-GT-AG--AT-A-TT--C-G--C-A-A-G-A-AC-A-CC---AG--T--G--GC-GAA-G--G-C--G-------G--C-C-A-A---CTG--G--TC-C-G--------------------A-T---A-C-T--GA--CG-C-T-G--A-GG--C-G-CG-A--AA-G-C---G-TG--GG-G--
AG-C-A-AA--------CA--GG Bacteria(100);Proteobacteria(100);Alphaproteobacteria(100);Rhizobiales(100);Bradyrhizobiaceae(100);Bosea(100);unclassified(100);unclassified(100);unclassified(100);

Otu0440 0 0 0 0 0 0 0 0 0 0 0 0 0 0 0 0 0 0 0 0 0 0 0 0 0 0 0 0
0 0 0 0 0 0 0 0 0 0 0 0 0 0 0 0 0 0 0 0 0 0 0 0 0 0 0 4 8
0 0 0 0 0 0 0 0 0 0 0 0 0 0 0 0 0 0 0 0 0 0 0 0 0 0 0 0 24
0 0 0 0 16 0 0 0 0 0 0 0 0 0 0 0 0 0 0 0 0 0 0 0 0 0 0 0
M02255_77_000000000-ADUT3_1_1105_7662_21624 C----A----GC-A--GCC--------GCG-G-T------A--A--T---A--CG-T-AGG-GC------GC-A--A-G--CG-TT-------GT--C-----CG-GA-AT-TA-------T-TG-G-G-C-G-------------G--C-TG-G-T-C-----------GC----G-T-C-T--------G-T-C--G--TG--A-AA-AC--T-T-CC-G-G-----CT-C-AA------C-C-G-G-G-G-G--C-T----T-G--C-G---G--T-------G--GG-T-A---C-------G--G-G--CC--G-G-C----T-A-G-A-G-T--G---C-GG-----TA-G-G--------G-G-T-A---AC-T------GG--A--ACT----C-C-T-G-GT--GT-A-G-CG-GT--G---G--A-A---TG-C-GC-AG--AT-A-TC--A-G--G-A-A-G-A-AC-A-CC---GA--T--G--GC-GAA-G--G-C--A-------G--G-T-T-A---CTG--G--GC-C-G---------------------T-T---A-C-T--GA--CG-C-T-G--A-GG--A-G-CG-A--AA-G-C---G-TG--GG-G--AG-C-G-AA--------CA--GG Bacteria(100);Actinobacteria(100);Actinobacteria(100);Actinobacteridae(100);Actinomycetales(100);unclassified(100);unclassified(100);unclassified(100);unclassified(100);

Otu0441 0 0 0 0 0 0 0 0 0 0 0 0 0 0 0 26 0 0 3 0 0 1 0 0 0 0 1 0
2 0 0 0 0 0 0 0 0 0 0 1 0 0 0 0 0 0 1 0 1 0 0 0 1 0 0 0 0
0 0 1 0 0 0 1 0 0 0 0 0 0 0 0 0 0 0 0 0 0 0 0 0 0 0 0 0 0
0 0 0 0 0 0 0 0 0 0 0 0 0 0 0 0 0 0 0 0 0 0 0 0 0 0 0 0
M02255_76_000000000-ADUNE_1_1102_13908_5115 A----AG---CC-AC-G-G--------CTA-A-C------T--A--C---G--TG-C-CAG-CA------GC-C--G-C--GG-TA-------AT--A-----CG-TA-GG-TG-------GCAA-GCG-T-G-------------G--T-CT-C-G-G-----------AA----G-T-C-T--------G-A-T--G--TG--A-AA-GC--C-C-CC-G-G-----CT-C-AA------C-C-G-G-G-G-A--G------G-GT-C-A---T--T-------G--GA-A-A---C-------T--G-G--GA--G-A-C----T-T-G-A-G-G--A---C-AG-----AA-G-A--------G-G-A-G---AG-T------GG--A--ATT----C-C-A-A-GT--GT-A-G-CG-GT--G---A--A-A---TG-C-GT-AG--AT-A-TT--T-G--G-A-G-G-A-AC-A-CC---AG--T--G--GC-GAA-G--G-C--G-------G--C-T-C-T---CTG--G--TC-T-G---------------------T-T---A-C-T--GA--CG-C-T-G--A-GG--C-G-CG-A--AA-G-C---G-TG--GG-G--AG-C-A-AA--------CA--GG Bacteria(100);Firmicutes(100);Bacilli(100);Bacillales(100);unclassified(100);unclassified(100);unclassified(100);unclassified(100);unclassified(100);

Otu0442 0 0 0 0 0 0 0 0 0 0 0 3 0 0 0 0 0 0 0 0 0 0 0 0 0 2 6 0
0 0 0 0 0 0 0 0 0 0 0 0 0 0 0 0 0 0 0 0 0 0 0 0 0 0 0 0 0
0 0 0 0 0 0 0 0 0 0 0 0 0 0 0 0 0 0 0 0 0 0 5 0 0 0 0 0 0
0 0 0 0 0 0 0 0 3 0 0 0 0 0 0 0 0 0 0 0 0 0 0 0 0 0 0 0
M02255_77_000000000-ADUT3_1_2108_9215_14340 T----AC---GT-AG-GGT--------GCA-A-G------C--G--T---T--GT-C-CGG-AA------TT-A--T-T--GG-GC-------GT--A-----AA-GA-GC-TC-------G-TA-G-G-C-G-------------G--C-TT-G-T-C-----------GC----G-T-C-T--------G-C-T--G--TG--A-AA-AC--C-C-GA-G-G-----CT-C-AA------C-C-T-C-G-G-G--C-T----T-G--C-A---G--T-------G--GG-T-A---C-------G--G-G--CA--G-G-C----T-A-G-A-G-T--G---T-GG-----TA-G-G--------G-G-A-G---AT-T------GG--A--ATT----C-C-T-G-GT--GT-A-G-CG-GT--G---G--A-A---TG-C-GC-AG--AT-A-TC--A-G--G-A-G-G-A-AC-A-CC---GA--T--G--GC-GAA-G--G-C--A-------G--A-T-C-T---CTG--G--GC-C-A---------------------C-T---A-C-T--GA--CG-C-T-G--A-GG--A-G-CG-A--AA-G-C---A-TG--GG-G--AG-C-G-AA--------CA--GG Bacteria(100);Actinobacteria(100);Actinobacteria(100);Actinobacteridae(100);Actinomycetales(100);Micrococcineae(100);unclassified(74);unclassified(74);unclassified(74);

Otu0443 0 0 0 0 0 0 0 0 0 0 0 0 0 0 0 0 0 0 0 0 0 0 0 0 0 0 0 0
0 0 0 0 0 0 0 0 0 0 0 0 0 0 0 33 0 0 0 0 0 0 0 0 0 0 0 0 0
0 0 0 0 0 0 0 0 0 0 0 0 0 0 0 0 0 31 0 0 0 0 0 0 0 0 0 0 0
0 0 0 0 0 0 0 0 0 0 0 0 0 0 0 0 0 0 0 0 0 69 0 0 0 0 0 0
M02255_77_000000000-ADUT3_1_1118_12861_12177 T----AC---GT-AT-GTC--------GCA-A-G------C--G--T---T--AT-C-CGG-AA------TT-A--T-T--GG-GC-------TT--A-----AA-GG-GC-AT-------C-TA-G-G-C-G-------------G--T-TT-T-T-C-----------AA----G-T-C-A--------G-G-G--G--TG--A-AA-AC--T-T-AT-G-G-----CT-C-AA------C-T-A-T-A-A-G--C-T----T-G--C-C---T--T-------T--GA-A-A---C-------T--G-A--AA--A-A-C----T-A-G-A-G-T--A---C-TG-----GA-A-A--------G-G-T-G---GG-T------GG--A--ACT----A-C-A-C-GA--GT-A-G-AG-GT--G---A--A-A---TT-C-GT-AG--AT-A-TG--T-G--T-A-G-G-A-AT-G-CC---GA--T--G--AT-GAA-G--A-T--A-------A--C-T-C-A---CTG--G--AC-A-G---------------------A-A---A-C-T--GA--CG-C-T-G--A-AG--T-G-CG-A--AA-G-C---T-AG--GG-G--AG-C-A-AA--------CA--GG Bacteria(100);Fusobacteria(100);Fusobacteria(100);Fusobacteriales(100);Leptotrichiaceae(100);unclassified(100);unclassified(100);unclassified(100);unclassified(100);

Otu0444 0 0 0 0 0 0 0 0 0 0 0 0 0 0 0 0 0 0 0 0 0 0 0 0 0 0 0 0
0 0 0 0 0 0 0 0 0 0 0 0 0 0 0 0 0 0 0 0 0 0 0 0 8 0 0 0 0
0 0 0 0 0 0 0 0 0 0 0 0 0 0 0 0 0 21 0 0 0 0 0 0 0 0 0 0 0
0 0 0 0 0 0 0 0 0 0 0 0 0 0 0 0 0 0 0 0 0 0 0 0 0 0 0 0
M02255_77_000000000-ADUT3_1_1115_13639_23646 T----AC---GT-AG-GGT--------GCA-A-G------C--G--T---T--GT-C-CGG-AA------TT-A--T-T--GG-GC-------GT--A-----AA-GA-GC-TC-------G-TA-G-G-C-G-------------G--C-TT-G-T-C-----------GC----G-T-C-T--------G-C-T--G--TG--A-AA-TC--C-T-GA-G-G-----CT-C-AA------C-C-T-T-G-G-G--C-G----T-G--C-A---G--T-------G--GG-T-A---C-------G--G-G--CA--G-G-C----T-A-G-A-G-T--G---C-GG-----TA-G-G--------G-G-A-G---AT-T------GG--A--ATT----C-C-T-G-GT--GT-A-G-CG-GT--G---G--A-A---TG-C-GC-AG--AT-A-TC--A-G--G-A-G-G-A-AC-A-CC---GA--T--G--GC-GAA-G--G-C--A-------G--A-T-C-T---CTG--G--GC-C-G---------------------T-A---A-C-T--GA--CG-C-T-G--A-GG--A-G-CG-A--AA-G-C---A-TG--GG-G--AG-C-G-AA--------CA--GG Bacteria(100);Actinobacteria(100);Actinobacteria(100);Actinobacteridae(100);Actinomycetales(100);Micrococcineae(100);unclassified(73);unclassified(73);unclassified(73);

Otu0445 0 0 0 0 0 0 0 0 0 0 0 0 0 0 0 0 0 0 0 0 0 0 0 0 0 0 0 0
0 0 0 45 0 0 19 0 0 0 0 0 0 0 0 0 0 0 0 0 0 0 0 0 0 0 0 0 0
0 0 0 0 0 0 0 0 0 0 0 0 0 0 0 0 0 0 0 0 0 0 0 0 0 0 0 0 0
0 0 0 0 0 0 0 0 0 0 0 0 0 0 0 0 0 0 0 0 0 0 0 0 0 0 0
M02255_77_000000000-ADUT3_1_2101_16239_10232 T----AC---AG-AG-GGG--------GCA-A-G------C--G--T---T--GT-C-CGG-AT------TG-A--C-T--GG-GC-------GT--A-----AA-GC-GC-AC-------G-TA-G-G-T-G------------G--T-TG-G-T-T----
-------AC----G-T-G-G--------T-G-T--G--TG--A-AA-GC--T-C-AG-G-G-----CT-T-AA------C-C-C-T-G-A-T--A-G----G-C--C-A---T--G-------C--CA-A-A---C-------G--A-A--TC--G-A-C----T-C-G-A-G-G--CT--G-AG-----CA-G-A--------G-G-C-G---GG-T------GG--A--ATT----
G-C-C-G-GT--GT-A-G-TG-GT--G---A--A-A---TG-C-GT-AG--AG-A-TC--G-G--C-A-G-G-A-AC-A-CC---AA--G--G--GG-GAA-C--C-C--A-------G--C-C-C-G---CTG--G--GC-C-A--------------------G-T---C-C-T--GA--CA-C-T-G--A-GG--T-G-CG-A--CA-G-C---G-TG--
GG-G--AG-C-G-AA--------CA--GG Bacteria(100);unclassified(100);unclassified(100);unclassified(100);unclassified(100);unclassified(100);unclassified(100);unclassified(100);unclassified(100);

Otu0446 0 0 0 0 0 0 0 0 0 0 0 0 0 0 0 0 0 0 0 0 0 0 0 0 0 0 0 0
0 0 0 0 0 0 1 0 7 0 2 0 0 0 0 0 0 0 0 1 15 0 0 0 0 0 0 0 35
0 0 0 0 0 0 0 0 0 0 0 0 0 0 0 0 0 0 0 0 0 0 0 0 0 0 0 0 0
0 0 0 0 0 0 0 0 0 0 0 0 0 0 0 0 0 0 0 0 0 0 0 0 0 0 0
M02255_76_000000000-ADUNE_1_1106_27084_21255 C----TA---AC-TC-CGT--------GCC-A-G------C-----A---G--CC-G-CGG-TA------AT-A--C-G--GA-GG-------GT--G-----CA--A-GC--G-------T-TA-A-T-C-G------------G--T-TT-G-T-T---------
--AA----G-T-C-A--------G-A-T--G--TG--A-AA-TC--C-C-CG-G-G-----CT-C-AA------C-C-T-G-G-G-A--A-C----T-G--C-A---T--C-------T--GA-T-A---C-------T--G-G--CA--A-G-C----T-T-G-A-G-T--C---T-CG-----TA-G-A--------G-G-G-G---GG-T------AG--A--ATT----C-C-
A-G-GT--GT-A-G-CG-GT--G---A--A-A---TG-C-GT-AG--AG-A-TC--T-G--G-A-G-G-A-AT-A-CC---GG--T--G--GC-GAA-G--G-C--G-------G--C-C-C-C---CTG--G--AC-G-A--------------------A-G---A-C-T--GA--CG-C-T-C--A-GG--T-G-CG-A--AA-G-C---G-TG--GG-G--
AG-C-A-AA--------CA--GG Bacteria(100);Proteobacteria(100);Gammaproteobacteria(100);Enterobacteriales(100);Enterobacteriaceae(100);Enteric_Bacteria_cluster(100);unclassified(100);unclassified(100);unclassified(100);

Otu0447 0 0 0 0 0 0 0 0 0 0 0 0 0 0 0 0 0 0 0 0 0 0 0 0 0 0 0 0
0 0 0 0 0 0 0 0 79 0 0 0 0 0 0 0 0 0 0 0 0 0 0 0 0 0 0 0 0
0 0 0 0 0 0 0 0 0 0 0 0 0 0 0 0 0 17 0 0 0 0 0 0 0 0 0 0 0
0 0 0 0 0 0 0 0 0 0 0 0 0 0 0 0 0 0 0 0 0 0 0 0 0 0 0
M02255_77_000000000-ADUT3_1_2102_14017_12727 T----AC---GA-AG-GGG--------GCT-A-G------C--G--T---T--GT-T-CGG-AA------TC-A--C-T--GG-GC-------GT--A-----AA-GC-GC-AC-------G-TA-G-G-C-G------------G--A-TT-T-G-C-----
------CA----G-T-C-A--------G-G-G--G--TG--A-AA-TC--C-C-GG-G-G-----CT-C-AA------C-C-T-C-G-G-A--A-C----T-G--C-C---T--T-------T--GA-T-A---C-------A--G-C--AA--G-T-C----T-A-G-A-G-T--C---C-GG-----AA-G-A--------G-G-T-G---AG-T------GG--A--ATT----C-
C-T-A-GT--GT-A-G-AG-GT--G---A--A-A---TT-C-GT-AG--AT-A-TT--A-G--G-A-G-G-A-AC-A-CC---AG--T--G--GC-GAA-G--G-C--G-------G--C-T-C-A---CTG--G--TC-C-G--------------------G-C---A-C-T--GA--CG-C-T-G--A-GG--T-G-CG-A--AA-G-C---G-TG--GG-
G--AG-C-A-AA--------CA--GG Bacteria(100);Proteobacteria(100);Alphaproteobacteria(100);Rhizobiales(100);unclassified(100);unclassified(100);unclassified(100);unclassified(100);unclassified(100);

Otu0448 0 0 0 0 0 0 0 0 0 0 0 0 0 0 0 0 0 0 0 0 0 0 0 0 0 0 0 0
0 0 0 0 0 0 0 0 0 0 3 0 0 0 0 0 15 0 0 0 4 0 0 0 0 0 0 0 0
0 0 0 0 0 0 0 0 0 0 0 0 0 0 0 0 0 0 0 0 0 0 0 0 0 0 0 0 0
0 0 0 0 0 0 0 0 0 0 0 0 0 0 0 0 0 0 0 0 0 0 0 0 0 0 0
M02255_76_000000000-ADUNE_1_1101_14237_10572 T----AA---CT-AC-GT---------GCC-A-G------C--A--G---C--CG-C-GGT-AA------TA-C--G-T--AG-GT-------GG--C-----AA-GC-G--TT-------G-TC-C-G---G------------A--T-GA-C-T-T---------
--AA----G-T-C-T--------G-A-T--G--TG--A-AA-GC--C-C-AC-G-G-----CT-C-AA------C-C-G-T-G-G-A--G------G-GT-C-A---T--T-------G--GA-A-A---C-------T--G-G--GT--C-A-C----T-T-G-A-G-T--A---C-AG-----AA-G-A--------G-G-A-A---AG-C------GG--A--ATT----C-C-
A-T-GT--GT-A-G-CG-GT--G---A--A-A---TG-C-GT-AG--AT-A-TA--T-G--G-A-G-G-A-AC-A-CC---AG--T--G--GC-GAA-A--G-C--G-------G--C-T-T-T---CTG--G--TC-T-G--------------------A-A---A-C-T--GA--CG-C-T-G--A-GG--C-T-CG-A--AA-G-C---G-TG--GG-G--
AG-C-A-AA--------CA--GG Bacteria(100);Firmicutes(100);Bacilli(100);Lactobacillales(64);unclassified(64);unclassified(64);unclassified(64);unclassified(64);unclassified(64);

Otu0449 0 0 0 0 0 0 0 0 0 0 0 0 0 0 0 0 0 0 0 0 0 0 0 0 0 0 0 0
0 0 0 0 0 0 0 0 0 0 0 0 0 0 0 135 0 0 0 0 0 0 0 0 0 0 0 0 0
0 0 0 0 0 0 0 0 0 0 0 0 0 0 0 0 0 0 69 0 0 0 0 37 0 0 0 0 0
0 0 0 0 0 0 0 0 0 0 0 0 0 0 0 0 0 0 0 0 0 0 0 0 0 0 0
M02255_77_000000000-ADUT3_1_1115_20036_18788 T----AC---GG-AG-GGA--------GCT-A-G------C--G--T---T--GT-T-CGG-AA------TT-A--C-T--GG-GC-------GT--A-----AA-GA-GT-AC-------G-TA-G-G-C-G------------G--T-GA-T-T-C-----
------AA----G-T-C-A--------G-A-G--G--TG--A-AA-GC--C-T-GG-A-G-----CT-C-AA------C-T-C-C-A-G-A--A-C----T-G--C-C---T--T-------T--GA-A-A---C-------T--A-G--AT--C-G-C----T-A-G-A-A-T--T---A-TG-----GA-G-A--------G-G-T-T---AG-T------GG--A--ATT----C-
C-G-A-GT--GT-A-G-AG-GT--G---A--A-A---TT-C-GT-AG--AT-A-TT--C-G--G-A-A-G-A-AC-A-CC---AG--T--G--GC-GAA-G--G-C--G-------A--C-T-A-A---CTG--G--AC-A-T--------------------A-T---A-T-T--GA--CG-C-T-G--A-GG--T-A-CG-A--AA-G-C---G-TG--GG-G-
-AG-C-A-AA--------CA--GG Bacteria(100);Proteobacteria(100);Alphaproteobacteria(100);Sphingomonadales(100);unclassified(100);unclassified(100);unclassified(100);unclassified(100);unclassified(100);

Otu0450 0 0 0 0 0 0 0 0 0 0 1 2 4 1 1 0 0 1 2 0 0 1 0 0 0 0 0 0
0 0 0 0 0 0 0 0 0 0 0 0 0 0 0 1 7 0 0 0 0 0 0 0 0 0 0 0 0
0 0 0 0 0 0 0 0 0 0 0 0 0 0 0 0 0 0 0 0 0 1 0 0 0 0 0 0 0
0 0 0 0 0 0 8 0 0 0 0 0 0 0 1 4 0 0 1 0 0 0 0 16 0 0 0
M02255_76_000000000-ADUNE_1_2114_5933_17408 T----AC---AG-AG-GGT--------GCA-A-G------C--G--T---T--AA-T-CGG-AA------TT-A--C-T--GG-GC-------GT--A-----AA-GC-GA-GC-------G-TA-G-G-T-G------------G--C-TT-G-A-T-----------AA-
---G-T-C-A--------G-A-T--G--TG--A-AA-TC--C-C-CG-G-G-----CT-T-AA------C-C-T-G-G-G-A--A-C----T-G--C-A---T--C-------T--GA-A-A---C-------T--G-T--TA--G-G-C----T-A-G-A-G-T--A---G-GT-----GA-G-A--------G-G-G-A---AG-T------AG--A--ATT----T-C-A-G-
GT--GT-A-G-CG-GT--G---A--A-A---TG-C-GT-AG--AG-A-TC--T-G--A-A-G-G-A-AT-A-CC---GA--T--G--GC-GAA-G--G-C--A-------G--C-T-T-C---CTG--G--CA-T-C--------------------A-T---A-C-T--GA--CA-C-T-G--A-GG--C-T-CG-A--AA-G-C---G-TG--GG-T--AG-C-
A-AA--------CA--GG Bacteria(100);Proteobacteria(100);Gammaproteobacteria(100);Pseudomonadales(100);Moraxellaceae(100);Psychrobacter(100);unclassified(100);unclassified(100);unclassified(100);

Otu0451 0 0 0 0 0 0 0 0 0 0 0 0 0 0 0 0 0 0 0 0 0 0 0 0 0 0 0 0
0 0 7 0 0 0 103 0 0 10 18 0 0 0 0 68 0 187 0 0 0 0 0 0 0 0 1 1 0
0 0 0 0 0 0 0 0 0 0 0 0 0 0 40 0 0 0 0 0 0 0 0 0 0 0 0 0 0
38 14 0 0 0 0 0 0 0 61 0 0 0 0 0 0 0 18 0 0 0 0 0 0 0 0 0
M02255_77_000000000-ADUT3_1_1101_13217_23345 T----AC---GT-AG-GTC--------TCA-A-G------C--G--T---T--AT-C-CGG-AT------TT-A--C-T--GG-GC-------GT--A-----AA-GT-GT-CC-------G-TA-G-T-C-T-------------G--A-AT-T-G-T--------
---AA----G-T-C-T--------G-T-T--T--TC--A-AA-TC--C-T-AC-G-A-----CT-C-AA------T-C-G-T-A-G-A--A-A----G-G--G-A---A--T-------G--GA-T-A---C-------T--G-C--AA--T-T-C----T-G-G-A-A-G--T---A-TC-----TA-G-G--------G-G-T-T---AG-T------GG--A--ATT----T-C-C-
G-GT--GG-A-G-CG-GT--G---A--A-A---TG-C-GT-TG--AT-A-TC--G-G--A-A-G-G-A-AC-G-CC---GA--A--A--GC-GAA-A--G-C--A-------G--C-T-A-A---CTA--T--AG-A-A--------------------T-A---C-T-T--GA--CG-A-T-G--A-GG--G-A-CG-A--CA-G-T---T-TG--GG-G--
AG-C-A-AA--------CA--GG Bacteria(100);unclassified(100);unclassified(100);unclassified(100);unclassified(100);unclassified(100);unclassified(100);unclassified(100);unclassified(100);

Otu0452 5 0 2 6 0 0 5 8 0 4 0 0 0 0 0 0 0 0 0 2 0 0 0 0 0 0 0 0
0 8 11 8 5 8 10 4 13 4 7 0 0 3 7 15 20 0 0 0 7 0 2 1 0 0 0 2 0
0 3 0 0 0 0 0 0 0 0 0 0 0 0 0 1 3 3 0 3 5 0 0 5 0 5 0 0 0
3 0 0 0 0 5 0 0 0 7 0 7 2 0 0 0 12 0 0 4 0 3 0 0 0 8 0
M02255_77_000000000-ADUT3_1_2116_15911_18969 .....AC----T-AC-GT---------GCC-A-G------C--A--G---C--CG-C-GGT-AA------TA-C--G-T--AG-GT-------GG--C-----AA-GC-G--TT-------A-TC-C-G---G------------A--A-TT-A-T-T----------
AA----G-T-C-T--------G-A-T--G--TG--A-AA-GC--C-C-AC-G-G-----CT-C-AA------C-C-G-T-G-G-A--G------G-GT-C-A---T--T-------G--GA-A-A---C-------T--G-G--GA--A-A-C----T-T-G-A-G-T--A---C-AG-----AA-G-A--------G-G-A-A---AG-T------GG--A--ATT----C-C-
A-T-GT--GT-A-G-CG-GT--G---A--A-A---TG-C-GC-AG--AG-A-TA--T-G--G-A-G-G-A-AC-A-CC---AG--T--G--GC-GAA-G--G-C--G-------A--C-T-T-T---CTG--G--TC-T-G--------------------T-A---A-C-T--GA--CG-C-T-G--A-TG--T-G-CG-A--AA-G-C---G-TG--GG-G--
AT-C-A-AA--------CA--GG Bacteria(100);Firmicutes(100);Bacilli(100);Bacillales(100);Staphylococcaceae(100);Staphylococcus(92);unclassified(92);unclassified(92);unclassified(92);

Otu0453 0 0 0 0 0 0 0 0 0 0 0 0 0 0 0 0 0 0 0 0 0 0 0 0 0 0 0 0
0 0 0 0 0 0 0 0 39 0 0 8 0 13 0 0 21 0 0 0 0 0 0 0 0 0 0 0 0
0 0 0 0 0 0 0 0 0 0 0 0 0 0 0 0 0 0 0 0 0 0 0 0 0 0 0 0 52
0 0 7 0 76 0 0 3 0 0 0 0 0 0 0 0 0 0 0 0 0 9 0 0 0 0 0
M02255_77_000000000-ADUT3_1_1110_23174_11741 T----AC---GT-AG-GGT--------GCG-A-G------C--G--T---T--GT-C-AGG-AA------TT-A--C-T--GG-GC-------GT--A-----AA-GA-GC-TC-------G-TA-G-G-T-G------------G--T-TT-G-T-C------
-----GC----G-T-C-G--------T-T-T--G--TG--G-AA-TA--C-C-GG-G-G-----CT-T-AA------C-T-T-C-G-G-G--G-T----T-G--C-A---T--A-------C--GA-T-A---C-------G--G-G--CAT-A-A-C----T-T-G-A-G-T--G---C-TG-----TA-G-G--------G-G-A-G---AC-T------GG--A--ATT----C-
C-T-G-GT--GT-A-G-CG-GT--G---A--A-A---TG-C-GC-AG--AT-A-TC--A-G--G-A-G-G-A-AC-A-CC---GA--T--G--GC-GAA-G--G-C--A-------G--G-T-C-T---CTG--G--GC-A-G--------------------T-A---A-C-T--GA--CG-C-T-G--A-GG--A-G-CG-A--AA-G-C---A-TG--GG-
G--AG-C-G-AA--------CA--GG Bacteria(100);Actinobacteria(100);Actinobacteria(100);Actinobacteridae(100);Actinomycetales(100);Corynebacterineae(100);Corynebacteriaceae(100);Corynebacterium(100);unclassified(100);

Otu0454 0 0 0 0 0 0 0 0 0 0 0 0 0 0 0 0 0 0 0 0 0 0 0 0 0 0 0 0
0 0 0 0 2 0 0 0 0 0 0 0 0 0 0 0 0 0 0 0 0 0 0 0 0 0 0 0 0
0 0 0 0 0 0 0 0 0 0 0 0 0 0 0 0 0 0 0 26 0 0 0 0 0 0 0 0 0
0 0 0 0 0 5 0 1 0 22 0 0 0 0 0 0 0 0 0 0 0 0 0 0 0 0 0
M02255_77_000000000-ADUT3_1_2105_23540_13838 C----A----GC-A--GCC--------GCG---G------T--A--A---T--AC-G-GGG-GA------TA-C--G-A--GC-GT------TAT--C-----CG-GA-AT-TA-------T-TA-G-G-T-G------------G--C-TA-T-T-T--------
---AA----G-T-C-A--------G-G-G--G--TG--A-AA-AG--C-A-TG-A-G-----CT-C-AA------C-T-C-A-T-G-T--C-T----T-G--C-C---T--T-------T--GA-A-A---C-------T--G-G--AC--A-G-C----T-A-G-A-G-A--A---G-AG-----AC-G-A--------G-G-T-A---TG-T------GG--A--ATG----T-
G-T-G-GT--GT-A-G-CG-GT--G---A--A-A---TG-C-AT-AG--AT-A-TC--A-C--A-C-A-G-A-AC-T-CC---GA--T--T--GC-GCA-G--G-C--A-------G--C-G-T-A---CCA--G--AC-T-T--------------------A-T---T-C-T--GA--CA-C-T-G--A-AG--C-A-CG-A--AA-G-C---G-TG--GG-T--
AT-C-A-AA--------CA--GG Bacteria(100);Bacteroidetes(100);unclassified(100);unclassified(100);unclassified(100);unclassified(100);unclassified(100);unclassified(100);unclassified(100);

Otu0455 0 0 0 0 0 0 0 0 0 0 0 0 0 0 0 0 0 0 0 0 0 0 0 0 0 0 0 0
0 0 0 0 0 0 0 0 0 0 0 18 0 0 0 0 0 0 0 0 0 0 0 0 0 0 0 0 0
0 0 0 0 0 0 0 0 0 0 0 0 0 0 0 8 0 0 109 0 0 0 0 0 0 0 0 0 0
0 0 0 25 0 0 0 0 0 0 0 0 0 0 0 7 0 0 0 0 0 0 0 0 0 0 0
M02255_77_000000000-ADUT3_1_1104_20942_13462 T----AC---GG-AG-GGG--------GCT-A-G------C--G--T---T--GT-T-CGG-AA------TT-A--C-T--GG-GC-------GT--A-----AA-GC-GC-GC-------G-TA-G-G-C-G------------G--C-TT-T-G-T-----
------AA----G-T-T-A--------G-A-G--G--TG--A-AA-GC--C-C-GG-A-G-----CT-C-AA------C-T-T-C-G-G-A--A-T----T-G--C-C---T--T-------T--AA-G-A---C-------T--G-C--AT--C-G-C----T-T-G-A-A-C--A---C-GG-----GA-G-A--------G-G-T-G---AG-T------GG--A--ATT----C-
C-G-A-GT--GT-A-G-AG-GT--G---A--A-A---TT-C-GT-AG--AT-A-TT--C-G--G-A-A-G-A-AC-A-CC---AG--T--G--GC-GAA-G--G-C--G-------A--C-T-C-A---CTG--G--AC-C-G--------------------T-T---G-T-T--GA--CG-C-T-G--A-GG--T-G-CG-A--AA-G-C---G-TG--GG-G-
-AG-C-A-AA--------CA--GG Bacteria(100);Proteobacteria(100);Alphaproteobacteria(100);Sphingomonadales(100);unclassified(100);unclassified(100);unclassified(100);unclassified(100);unclassified(100);

Otu0456 4 0 1 11 0 2 13 0 0 0 0 0 0 0 0 0 0 0 0 0 0 0 0 0 0 0 0 0
0 3 1 2 13 5 23 5 0 8 3 3 0 0 0 0 0 0 0 0 1 0 0 1 0 0 0 2 0
1 0 0 0 0 0 0 0 0 0 0 0 0 0 12 14 0 6 0 2 3 0 0 1 2 0 0 23 17
4 12 14 19 17 3 0 0 0 0 0 0 0 3 0 0 0 5 0 21 25 0 0 0 0 0 33
M02255_77_000000000-ADUT3_1_1116_12817_2941 C----GG---CT-A--ACT--------ACG-T-G------C--C--A---G--CA-G-CCG-CG------GT-A--A-T--AC-GT-------AG--G-----GT-GC-GA-GC-------GTTA-A-T-C-G------------GAAT-TA-C-T-T-----------
AA----G-C-A-G--------G-A-T--G--TG--A-AA-TC--C-C-CG-G-G-----CT-C-AA------C-C-T-G-G-G-A--A-C----T-G--C-G---T--T-------C--TG-A-A---C-------T--G-G--GT--A-G-C----T-A-G-A-G-T--A---T-GT-----CA-G-A--------G-G-G-G---GG-T------AG--A--ATT----C-C-A-
C-GT--GT-A-G-CA-GT--G---A--A-A---TG-C-GT-AG--AG-A-TG--T-G--G-A-G-G-A-AT-A-CC---GA--T--G--GC-GAA-G--G-C--A-------G--C-C-C-C---CTG--G--GA-T-A--------------------A-T---A-C-T--GA--CG-T-T-C--A-TG--C-T-CG-A--AA-G-C---G-TG--GG-T--AG-
C-A-AA--------CA--GG Bacteria(100);Proteobacteria(100);Betaproteobacteria(100);Neisseriales(100);Neisseriaceae(100);unclassified(100);unclassified(100);unclassified(100);unclassified(100);

Otu0457 0 0 0 0 0 0 0 0 0 0 0 0 0 0 0 0 0 0 0 0 0 0 0 0 0 0 0 0
0 0 0 0 0 0 0 0 0 0 0 0 0 0 0 0 0 0 0 0 0 0 0 0 7 0 0 0 0
0 0 0 0 0 0 0 0 0 0 0 0 0 0 0 0 0 0 0 0 0 1 0 0 0 0 0 0 0
0 0 13 0 0 0 0 0 0 0 0 0 0 0 0 0 0 0 0 0 0 0 0 0 0 0 0
M02255_77_000000000-ADUT3_1_2108_26013_22224 G----AC---GT-AG-GGC--------GCG-A-G------C--G--T---T--GT-C-CGG-AT------TT-A--T-T--GG-GC-------GT--A-----AA-GA-GC-TC-------G-TA-G-G-C-G------------G--C-TT-G-T-T-----
------GC----G-T-C-A--------G-C-T--G--TG--A-AA-GC--C-C-AT-G-G-----CT-T-AA------C-T-G-T-G-G-G--T-T----G-G--C-A---G--C-------T--GA-T-A---C-------G--G-G--CA--G-G-C----T-A-G-A-G-T--T---C-GG-----TA-G-G--------G-G-A-G---AC-T------GG--A--ATT----
C-C-T-G-GT--GT-A-G-CG-GT--G---A--A-A---TG-C-GC-AG--AT-A-TC--A-G--G-A-G-G-A-AC-A-CC---GG--T--G--GC-GAA-G--G-C--G-------G--G-T-C-T---CTG--G--GC-C-G--------------------A-T---A-C-T--GA--CG-C-T-G--A-GG--A-G-CG-A--AA-G-C---G-TG--
GG-G--AG-C-G-AA--------CA--GG Bacteria(100);Actinobacteria(100);Actinobacteria(100);Actinobacteridae(100);Actinomycetales(100);Micromonosporineae(67);Micromonosporaceae(67);unclassified(67);unclassified(67);

Otu0458 0 0 0 0 0 0 0 0 0 0 0 0 0 0 0 0 0 0 0 0 0 0 0 0 0 0 0 0
0 0 0 0 0 74 0 52 0 0 0 0 0 0 0 0 0 0 0 0 0 0 0 0 0 0 0 0 0
0 0 0 0 0 0 0 0 0 0 0 0 0 0 0 0 0 0 0 0 0 0 0 0 0 0 0 0 0
0 0 0 0 0 0 0 0 0 0 0 0 0 0 0 0 0 0 0 0 0 0 0 0 0 0 0
M02255_77_000000000-ADUT3_1_1112_11043_19460 T----AC---GT-AG-GGT--------GCG-A-G------C--G--T---T--AT-C-CGG-AA------TC-A--T-T--GG-GC-------GT--A-----AA-GA-GC-TC-------G-TA-G-G-C-G------------G--T-TC-A-A-C-----
------AA----G-T-C-G--------G-C-T--G--TG--A-AA-GT--C-C-GA-G-G-----CT-C-AA------C-C-T-C-G-G-A--A-T----T-G--C-A---G--T-------C--GA-T-A---CT------G--T-T--GT--G-A-C----T-C-G-A-G-T--C---C-GG-----TA-G-A--------G-G-A-G---AA-C------GG--A--ATT----
C-C-C-G-GT--GT-A-G-CG-GT--G---G--A-A---TG-C-GC-AG--AT-A-TC--G-G--G-A-G-G-A-AC-A-CC---AG--T--A--GC-GAA-G--G-C--G-------G--T-T-C-T---CTG--G--GC-C-G--------------------G-C---A-C-T--GA--CG-C-T-G--A-GG--A-G-CG-A--AA-G-C---G-TG--
GG-G--AG-C-A-AA--------CA--GG Bacteria(100);unclassified(100);unclassified(100);unclassified(100);unclassified(100);unclassified(100);unclassified(100);unclassified(100);unclassified(100);

Otu0459 0 0 0 0 0 0 0 0 0 0 0 0 0 0 0 0 0 0 0 0 0 0 0 0 0 0 0 0
0 0 0 0 0 0 0 0 0 0 0 0 0 0 0 0 0 0 0 0 0 0 0 0 0 0 0 0 0
0 0 0 0 0 0 0 0 0 0 0 0 0 0 0 0 0 37 0 0 0 0 0 0 0 0 0 0 0
0 0 0 0 0 0 0 0 0 0 0 0 0 0 0 0 0 0 0 0 0 0 0 0 0 0 0
M02255_77_000000000-ADUT3_1_1116_13987_21727 T----AC---GA-AG-GGG--------GCT-A-G------C--G--T---T--GC-T-CGG-AA------TC-A--C-T--GG-GC-------GT--A-----AA-GC-GC-AC-------G-TA-G-G-T-G------------G--G-TT-T-C-T-----
------AA----G-T-C-A--------G-G-G--G--TG--A-AA-TC--C-T-GG-A-G-----CT-C-AA------C-T-C-C-A-G-A--A-C----T-G--C-C---T--T-------T--GA-T-A---C-------T--G-G--AA--A-T-C----T-TGG-A-G-T--C---C-GG-----GA-G-A--------G-G-T-G---GG-T------GG--A--ACT----
G-C-G-A-GT--GT-A-G-AG-GT--G---A--A-A---TT-C-GT-AG--AT-A-TT--C-G--C-A-A-G-A-AC-A-CC---AG--T--G--GC-GAA-G--G-C--G-------G--C-T-C-A---CTG--G--CC-C-G--------------------G-T---A-C-T--GA--CA-C-T-G--A-GG--T-G-CG-A--AA-G-C---G-TG--GG-
G--AG-C-A-AA--------CA--GG Bacteria(100);Proteobacteria(100);Alphaproteobacteria(100);Rhizobiales(100);unclassified(100);unclassified(100);unclassified(100);unclassified(100);unclassified(100);

Otu0460 0 0 0 46 0 0 0 0 0 0 0 0 0 0 0 0 0 0 0 0 0 0 0 0 0 0 0 0
0 0 0 0 0 0 0 0 0 0 0 0 0 0 0 0 0 0 0 0 0 0 0 0 0 0 0 0 0
0 0 0 0 0 0 0 0 0 0 0 0 0 0 0 0 0 0 0 0 0 8 0 0 0 0 0 0 0
0 0 0 0 0 0 0 0 0 0 0 0 0 0 0 0 0 0 0 0 0 0 0 0 0 0 0

M02255_77_000000000-ADUT3_1_1108_7467_8696 T----AC---GA-AG-GGG--------GCT-A-G------C--G--T---T--GC-T-CGG-AA------TG-A--C-T--GG-GC-------GT--A-----AA-GG-GC-GC-------G-TA-G-G-C-G------------G--G-CG-G-C-A----------
CA----G-T-C-G--------G-G-C--G--TG--A-AA-TT--C-C-TG-G-G-----CT-T-AA------C-C-T-G-G-G-G--G-C----T-G--C-G---T--C-------C--GA-G-A---C-------G--T-G--TT--G-C-C----T-A-G-A-G-T--G---G-GG-----AA-G-A--------G-G-G-T---CG-T------GG--A--ATT----C-C-
C-A-GT--GT-A-G-AG-GT--G---A--A-A---TT-C-GT-AG--AT-A-TT--G-G--G-A-A-G-A-AC-A-CC---GG--T--G--GC-GAA-G--G-C--G-------G--C-G-A-C---CTG--G--TC-C-T--------------------T-G---A-C-T--GA--CG-C-T-G--A-GG--C-G-CG-A--AA-G-C---G-TG--GG-G--
AG-C-A-AA--------CA--GG Bacteria(100);Proteobacteria(100);Alphaproteobacteria(100);Rhodospirillales(100);Acetobacteraceae(100);unclassified(100);unclassified(100);unclassified(100);unclassified(100);

Otu0461 0 0 0 0 0 0 0 0 0 0 0 0 0 0 0 0 0 0 0 0 0 0 0 0 0 0 0 0
0 0 0 0 0 0 0 0 0 0 0 0 0 0 0 0 0 0 0 0 0 0 0 0 0 0 0 0 0
0 0 0 0 0 0 0 0 0 0 0 0 0 0 0 0 0 0 32 0 0 5 0 0 0 0 0 0 0
0 0 0 0 0 0 0 0 0 0 0 0 0 0 0 0 0 0 0 0 0 0 0 0 0 0 0

M02255_77_000000000-ADUT3_1_2117_7960_11495 T----AC---GT-AG-GTG--------GCG-A-G------C--G--T---T--GT-C-CGG-AA------TT-A--C-T--GG-GC-------GT--A-----AA-GG-GT-GC-------G-TA-G-G-C-G------------G--C-CT-G-G-T----------
AA----G-T-T-G--------A-G-C--G--TG--A-AA-TT--T-T-TG-G-G-----CT-C-AA------C-C-C-A-A-A-G--G-G----A-G--C-G---C--T-------C--AA-T-A---C-------T--G-C--CG--G-G-C----T-T-G-A-G-T--G---C-GG-----GA-G-A--------G-G-A-C---GG-C------GG--A--ATT----C-C-
C-G-GT--GT-A-G-CG-GT--G---A--A-A---TG-C-GT-AG--AT-A-TC--G-G--G-A-G-G-A-AC-A-CC---AG--T--G--GC-GAA-G--G-C--G-------G--C-C-G-T---CTG--G--AC-C-G--------------------T-A---A-C-T--GA--CG-C-T-G--A-GG--C-A-CG-A--AA-G-C---G-TG--GG-G--
AG-C-A-AA--------CA--GG Bacteria(100);Firmicutes(100);Clostridia(100);Thermoanaerobacterales(100);Family_III_Incertae_Sedis(100);Caldicellulosiruptor(100);unclassified(100);unclassified(100);unclassified(100);

Otu0462 0 0 12 0 0 0 0 0 0 0 0 0 0 0 0 0 0 0 0 0 0 0 0 0 0 0 0 0
0 0 0 0 0 35 0 46 0 0 0 0 0 0 0 0 0 0 0 0 0 0 0 0 0 0 0 11 0
0 0 0 0 0 0 0 0 0 0 0 0 0 0 0 0 0 0 0 0 0 0 0 0 0 0 0 0 0
0 0 0 0 0 0 0 0 0 0 0 0 0 0 0 29 0 0 0 0 0 0 0 0 6 0 0

M02255_77_000000000-ADUT3_1_1104_25237_8885 T----AC---GT-AG-GGA--------CCA-A-G------C--G--T---T--GT-T-CGG-AT------TT-A--C-T--GG-GC-------GT--A-----AA-GG-GA-GC-------G-TA-G-G-C-G------------G--C-AA-T-T-C----------AA-
---G-T-C-A--------G-T-T--G--TG--A-AA-TC--T-C-CG-A-G-----CT-T-AA------C-T-C-G-G-A-A--C-G----G-T--C-A---A--C-------T--GA-T-A---C-------T--G-C--TT--T-G-C----T-A-G-A-G-T--A---C-AG-----AA-G-G--------G-G-C-A---AT-C------GG--A--ATT----C-T-T-G-
GT--GT-A-G-CG-GT--G---A--A-A---TG-C-GT-AG--AT-A-TC--A-A--G-A-G-G-A-AC-A-CC---TG--A--G--GT-GAA-G--A-C--G-------G--G-T-T-G---CTG--G--GC-T-G--------------------A-T---A-C-T--GA--CG-C-T-G--A-GG--C-T-CG-A--AA-G-C---T-AG--GG-G--AG-
C-A-AA--------CG--GG Bacteria(100);Acidobacteria(100);Acidobacteria(100);Acidobacteriales(100);Acidobacteriaceae(100);Candidatus_Chloroacidobacterium(100);unclassified(100);unclassified(100);unclassified(100);

Otu0463 0 0 0 0 0 0 0 0 0 0 0 0 0 0 0 0 0 0 0 0 0 0 0 0 0 0 0 0
0 0 0 0 0 0 0 0 0 0 0 0 0 0 0 0 0 0 0 52 0 0 0 0 0 0 0 0 0
0 0 0 0 0 0 0 0 0 0 0 0 0 0 0 0 0 0 0 0 0 12 0 0 0 0 0 0 0
0 0 0 0 0 0 0 0 0 0 0 0 0 79 0 0 0 0 0 0 0 0 0 0 0 0 0

M02255_77_000000000-ADUT3_1_1103_27507_16166 G----AC---GG-GG-GGG--------GCT-A-G------T--G--T---T--AT-T-CGG-AA------TG-A--C-T--AG-GC-------GT--A-----AA-GG-GC-AC-------G-TA-G-G-C-G------------G--T-GA-A-T-C----
-------CG----G-T-T-G--------A-A-A--G--TG--A-AA-GT--C-G-CC-A-G-----CT-C-AA------C-T-G-G-C-G-G--A-A----T-G--C-T---T--T-------C--AA-A-A---C-------C--A-A--TT--C-A-C----T-T-G-A-G-T--A---A-GA-----TA-G-A--------G-G-A-T---AG-T------GG--A--ATT----T-
C-G-T-GT--GT-A-G-AG-AT--A---A--A-A---TT-C-AC-AG--AT-A-TA--C-G--A-A-G-G-A-AC-A-CC---AA--A--A--GC-GAA-G--G-C--A-------G--C-T-T-T---CTG--G--GT-C-T--------------------C-T---A-C-T--GA--CG-C-T-G--A-GG--T-G-CG-A--AA-G-C---A-TA--GG-G--
AG-C-A-AA--------CA--GG Bacteria(100);unclassified(100);unclassified(100);unclassified(100);unclassified(100);unclassified(100);unclassified(100);unclassified(100);unclassified(100);

Otu0464 0 0 0 0 0 0 0 0 0 0 0 0 0 0 0 0 0 0 0 0 0 0 0 1 0 0 0 0
1 0 0 1 0 0 0 0 0 0 10 0 0 0 0 69 14 38 0 0 0 0 0 0 0 0 0 0 0
0 52 0 0 8 0 0 0 0 0 0 2 0 0 0 0 0 0 0 0 0 3 1 40 0 0 0 27 0
6 24 28 0 0 0 1 0 0 11 0 0 2 0 0 0 0 0 0 0 0 0 0 0 1 0 0

M02255_77_000000000-ADUT3_1_2117_21509_9922 .....AC----T-A--CGT--------GCC-A-G------C--A--G---C--CG-C-GGT-AA------TA-C--G-T--AG-GG-------TG--C-----GA-GC-GT-TA-------A-TC-G-G---A------------A--T-TA-T-T-T----------AA----
G-C-C-A--------G-A-T--G--TG--A-AA-TC--C-C-CG-A-G-----CC-T-AA------C-T-T-G-G-G-A--G-G----T-G--C-A---T--A-------T--GG-A-A---C-------T--G-G--GT--A-G-C----T-A-G-A-G-T--G---T-GT-----CA-G-A--------G-G-G-A---GG-T------AG--A--ACT----C-C-A-C-
GT--GT-A-G-CA-GT--G---A--A-A---TG-C-GT-AG--AG-A-TG--T-G--G-A-A-G-A-AT-A-CC---GA--T--G--GC-GAA-G--G-C--A-------G--C-C-T-C---CTG--G--GA-T-A--------------------A-C---A-C-T--GA--CG-T-T-G--A-GG--C-T-CG-A--AA-G-C---G-TG--GG-G--AG-
C-A-AA--------CA--GG Bacteria(100);Proteobacteria(100);Betaproteobacteria(100);unclassified(100);unclassified(100);unclassified(100);unclassified(100);unclassified(100);unclassified(100);

Otu0465 0 0 0 0 0 0 0 0 0 0 0 0 0 0 0 0 0 0 0 0 0 0 0 0 0 0 0 0
0 0 0 0 0 0 0 0 0 0 0 0 0 0 0 15 0 0 0 0 0 0 0 0 0 0 0 0
0 0 0 0 0 0 0 0 0 0 0 0 0 0 0 0 0 0 0 16 0 0 0 0 0 0 0 0
0 0 0 0 0 0 0 0 0 0 0 0 0 0 0 0 10 0 0 0 0 0 0 0 0 0 0
M02255_77_000000000-ADUT3_1_1104_15206_13861 T----AC---GT-AG-GGG--------CCG-A-G------C--G--T---T--GT-C-CGG-AG------TT-A--T-T--GG-GC-------GT--A-----AA-GC-GC-GC-------G-CA-G-G-C-G-------------G--C-TT-G-G-C----
-------GC----G-C-C-C--------G-A-C--G--TG--A-AA-GC--C-C-CC-G-G-----CT-T-AA------C-C-G-G-G-G-A--G-A----G-T--C-G---T--C-------G--GG-G-A---C-------G--G-C--CG--G-G-C----T-T-G-A-G-G--G---C-GG-----GA-G-A--------G-G-G-T---GG-T------GG--A--ATT--
----CCC-G-GT--GT-A-G-TG-GT--G---A--A-A---TG-C-GT-AG--AG-A-TC--G-G--G-A-G-G-A-AC-A-CC---TG--T--G--GC-GAA-G--G-C--G-------G--C-C-A-C---CTG--G--GC-C-G--------------------A-C---C-C-T--GA--CG-C-T-G--A-GG--C-G-CG-A--AG-G-C---G-TG--
GG-G--AG-C-G-AA--------CG--GG Bacteria(100);Chloroflexi(100);Thermomicrobia(100);JG30-KF-CM45(100);unclassified(100);unclassified(100);unclassified(100);unclassified(100);unclassified(100);

Otu0466 0 0 0 0 0 0 0 0 0 0 0 0 0 0 0 0 0 0 0 0 0 0 0 0 0 0 0 0
0 0 0 0 0 0 0 0 0 0 0 33 0 0 0 0 0 0 0 0 0 0 0 0 16 0 0 0 0
0 0 0 0 0 0 0 0 0 0 0 0 3 0 31 0 0 0 80 0 125 0 1 0 0 0 0 0 0
0 0 0 0 0 0 0 0 0 91 0 73 0 0 1 0 0 0 0 0 0 0 0 0 0 0 0
M02255_77_000000000-ADUT3_1_1101_21610_23233 T----AC---AT-AT-GGG--------GCG-A-G------C--G--T---T--GT-C-CGG-AT------TT-A--T-T--GG-GT-------GT--A-----AA-GG-GT-GC-------G-TA-G-G-C-G-------------G--G-AA-A-G-T-----
------AA----G-T-T-A--------G-T-T--G--TG--A-AA-TC--C-C-AC-G-G-----CT-T-AA------C-C-G-T-G-G-A--C-T----T-G--C-A---A--C-------T--AA-A-A---C-------T--G-T--TT--T-T-C----T-G-G-A-G-T--A---T-CG-----GA-G-A--------G-G-A-A---AG-C------GG--A--ATT----T-
C-T-A-GT--GT-A-G-CG-GT--G---A--A-A---TG-C-GT-AG--AT-A-TT--A-G--A-A-A-G-A-AC-A-CC---AA--A--G--GC-GAA-G--G-C--A-------G--C-T-T-T---CTG--G--AC-G-A--------------------T-A---A-C-T--GA--CG-C-T-G--A-GG--C-A-CG-A--AA-G-C---G-TG--GG-T-
-AG-C-A-AA--------CA--GG Bacteria(100);unclassified(100);unclassified(100);unclassified(100);unclassified(100);unclassified(100);unclassified(100);unclassified(100);unclassified(100);

Otu0467 0 0 0 136 0 0 0 0 0 0 0 0 0 0 0 0 0 0 0 0 0 0 0 0 0 0 0 1
0 0 0 0 0 0 0 0 0 0 0 0 0 0 0 0 0 0 0 0 0 0 0 0 0 0 0 0
0 0 0 0 0 0 0 0 0 0 0 0 0 0 0 0 0 0 0 0 0 0 0 0 0 0 0 0
0 0 0 0 0 0 0 0 0 0 0 0 0 0 0 0 0 0 0 0 0 0 0 0 0 0 0
M02255_77_000000000-ADUT3_1_1109_26458_5437 T----AC---GT-AT-GGG--------GCA-A-G------C--G--T---T--GT-C-CGG-AA------TT-A--T-T--GG-GC-------GT--A-----AA-GG-GT-AC-------G-TA-G-G-T-G-------------G--T-TA-T-T-T-----------AA--
--G-T-C-T--------G-A-A--T--TA--A-AA-GG--C-T-AT-G-G-----CT-C-AA------C-C-A-T-A-G-T--A------A-G--G-T---T--C-------A--GA-T-A---C-------T--G-G--AT--A-A-C----T-T-G-A-G-T--A---G-AT-----GA-G-G--------G-G-A-A---AA-T------GG--A--ATT----C-C-A-T-GT-
-GT-A-G-CG-GT--G---A--A-A---TG-C-GT-AG--AT-A-TA--T-G--G-A-G-G-A-AC-A-CC---AA--T--G--GC-GAA-G--G-C--A-------A--T-T-T-T---CTG--G--AA-T-C--------------------T-A---A-C-T--GA--CA-C-T-G--A-GG--T-A-CG-A--AG-G-C---G-TG--GG-T--AG-C-A-
AA--------CA--GG Bacteria(100);Firmicutes(100);Clostridia(100);Clostridiales(100);Family_XI_Incertae_Sedis(100);Helcococcus(100);unclassified(100);unclassified(100);unclassified(100);

Otu0468 0 0 0 0 0 0 0 0 0 0 0 0 0 0 0 0 0 0 0 0 0 0 0 0 0 0 0 0
0 0 0 0 0 0 96 0 0 0 0 0 0 0 0 0 0 0 0 0 0 0 0 0 0 0 0 0
0 0 0 0 0 0 0 0 0 0 0 0 0 0 0 0 0 24 0 0 0 0 0 0 0 0 0 0
0 0 0 0 0 0 0 0 0 0 0 0 0 0 0 0 0 0 0 0 0 0 0 0 0 0 0
M02255_77_000000000-ADUT3_1_1115_12808_17728 T----AC---GT-AG-GGG--------GCG-A-G------C--G--T---T--GT-C-CGG-AT------TC-A--T-T--GG-GC-------GT--A-----AA-GA-GC-TC-------G-TA-G-G-C-G-------------G--C-TT-G-G-C-----
------AA----G-C-C-G--------G-G-T--G--TG--A-AA-AC--T-C-CA-G-G-----CT-C-AA------C-C-T-G-G-A-G--A------C-G-CC-A---C--C-------C--GG-G-A---C-------T--G-C--TA-TG-G-C----T-A-G-A-G-T--C---C-GG-----TA-G-G--------G-G-A-T---CA-C------GG--A--ATT----
C-C-T-G-GT--GT-A-G-CG-GT--G---A--A-A---TG-C-GC-AG--AT-A-TC--A-G--G-A-G-G-A-AC-A-CC---GG--T--G--GC-GAA-G--G-C--G-------G--T-G-A-T---CTG--G--GC-C-G--------------------G-C---A-C-T--GA--CG-C-T-G--A-GG--A-G-CG-A--AA-G-C---G-TG--
GG-T--AG-C-A-AA--------CA--GG Bacteria(100);Actinobacteria(100);Actinobacteria(100);unclassified(100);unclassified(100);unclassified(100);unclassified(100);unclassified(100);unclassified(100);

Otu0469 0 0 0 0 0 0 0 0 0 0 0 0 0 0 0 0 0 0 0 0 0 0 13 0 0 0 0 0
0 0 0 0 0 0 0 0 0 0 0 0 0 0 0 43 0 0 0 0 0 0 0 0 0 0 0 0
0 0 0 0 0 0 0 0 0 0 0 0 0 0 0 0 0 0 0 0 0 0 0 0 0 0 0 0
0 0 0 0 0 0 0 0 0 0 0 0 0 0 0 0 0 0 0 0 0 0 0 0 0 0 0
M02255_77_000000000-ADUT3_1_1101_18980_24149 G----AC---GA-GG-GAT--------CCT-A-G------C--G--T---T--GT-T-CGG-AA------TT-A--T-T--GG-GC-------GT--A-----AA-GC-GG-GT-------G-TA-G-G-T-G-------------G--C-TA-T-G-T-----
------AA----G-T-C-A--------G-G-T--G--TG--A-AA-GC--C-T-GG-G-G-----CT-C-AA------C-C-C-C-A-G-A--A-G----T-G--C-A---T--C-------T--GA-T-A---C-------T--G-C--GT--A-G-C----T-T-G-A-G-T--G---G-TA-----GA-G-A--------G-G-A-T---AG-T------AG--A--ATT----C-
T-T-G-GT--GT-A-G-TG-GT--G---A--A-A---TA-C-GT-AG--AT-A-TC--A-A--G-A-G-G-A-AT-A-CC---GG--T--G--GC-GAA-G--G-C--G-------G--C-T-A-T---CTG--G--CT-A-C--------------------A-C---A-C-T--GA--CA-C-T-C--A-GA--C-C-CG-A--AA-G-T---G-CG--GG-G--
AT-C-A-AA--------CA--GG Bacteria(100);Proteobacteria(100);unclassified(77);unclassified(77);unclassified(77);unclassified(77);unclassified(77);unclassified(77);unclassified(77);

Otu0470 0 0 0 0 0 0 0 0 0 0 0 0 0 0 0 0 0 0 0 0 0 0 0 0 0 0 0 0
0 0 0 0 0 0 0 0 0 0 0 0 0 0 0 14 0 0 0 0 0 0 0 0 11 4 0 0 0
0 0 0 0 0 0 0 0 0 0 0 0 0 0 0 0 0 21 0 0 0 0 0 0 0 0 0 25 24
0 0 0 0 0 0 0 0 0 0 0 0 0 0 0 0 0 0 0 0 0 0 0 0 0 0 0
M02255_76_000000000-ADUNE_1_1104_23670_7426 C----CG---GC-TA-ACT--------CCG-T-G------C--C--A---G--CA-G-CCG-CG------GT-A--A-T--AC-GG-------AG--G-----GT-GC-GA-GC-------G-TT-GTC-C-G-------------G--A-TT-TAT-T-----------AA----G-T-C-C--------G-G-G--G--TG--A-AA-GC--C-C-GT-T-G-----CT-C-AA------C-A-A-C-G-G-A--A-C----T-G--C-C---C--T-------G--GA-T-A---C-------T--G-G--AA--G-G-C----T-T-G-A-G-T--A---C-AG-----AC-G-A--------G-G-T-T---GG-C------GG--A--ATG----G-A-C-G-GA--GT-A-G-CG-GT--G---A--A-A---TG-C-AT-AG--AT-A-CC--G-T--C-C-A-G-A-AC-C-CC---GA--T--T--GC-GAA-G--G-C--A-------G--C-T-G-A---CTA--G--GC-T-G---------------------T-T---A-C-T--GA--CG-C-T-G--A-GG--C-A-CG-A--AA-G-C---G-TG--GG-G--AG-C-G-AA--------CA--GG Bacteria(100);Bacteroidetes(100);Sphingobacteria(100);Sphingobacteriales(100);unclassified(79);unclassified(79);unclassified(79);unclassified(79);unclassified(79);

Otu0471 0 0 0 0 0 0 0 0 0 0 0 0 0 0 0 0 0 0 0 0 0 0 0 0 0 0 0 0
0 0 0 0 0 0 0 34 0 0 0 0 0 0 0 0 0 0 0 0 0 0 0 0 0 0 0 0 0
0 0 0 0 0 0 0 0 0 0 0 0 0 0 0 0 0 26 0 0 0 0 0 0 0 0 0 0 0
0 0 0 0 0 0 0 0 0 0 0 0 0 0 0 0 0 0 0 0 0 0 0 0 0 0 0
M02255_77_000000000-ADUT3_1_1106_26658_13913 T----AC---GA-AG-GGG--------GCT-A-G------C--G--T---T--GT-T-CGG-AA------TT-A--C-T--GG-GC-------GT--A-----AA-GC-GT-AT-------G-CA-G-G-C-G-------------G--A-TC-G-T-T-----------AA----G-T-C-G--------G-G-G--G--TG--A-AA-TC--C-C-GA-G-G-----CT-C-AA------C-C-T-C-G-G-A--A-C----T-G--C-C---T--T-------C--GA-T-A---C-------T--G-A--CG--A-T-C----T-T-G-A-G-T--C---C-GG-----GA-G-A--------G-G-T-G---AG-T------GG--A--ACT----C-C-G-A-GT--GT-A-G-AG-GT--G---A--A-A---TT-C-GT-AG--AT-A-TT--C-G--G-A-A-G-A-AC-A-CC---AG--T--G--GC-GAA-G--G-C--G-------G--C-T-C-A---CTG--G--CC-C-G---------------------G-C---A-C-T--GA--CG-C-T-G--A-GA--T-A-CG-A--AA-G-C---G-TG--GG-G--AG-C-A-AA--------CA--GG Bacteria(100);Proteobacteria(100);Alphaproteobacteria(100);unclassified(100);unclassified(100);unclassified(100);unclassified(100);unclassified(100);unclassified(100);

Otu0472 0 0 0 0 0 0 0 0 0 0 0 0 0 0 0 0 0 0 0 0 0 0 0 0 0 0 0 0
0 0 0 0 0 0 0 61 0 0 0 0 0 0 0 0 58 0 0 0 0 0 0 0 0 0 0 0 0
0 0 0 0 0 0 0 0 0 0 0 0 0 0 0 0 0 0 0 0 0 0 0 0 0 0 0 0 0
0 0 0 0 0 0 0 0 0 0 0 0 0 0 0 0 0 0 0 0 0 0 0 0 0 0 0
M02255_77_000000000-ADUT3_1_1109_25525_16039 T----AC---GT-AG-GTG--------GCG-A-G------C--G--T---T--GT-C-CGG-AT------TT-A--C-T--GG-GC-------GT--A-----AA-GA-GC-GC-------G-CA-G-G-C-G-------------G--T-CA-A-T-C-----------AA----G-T-C-G--------A-G-T--G--TG--A-AA-GC--C-C-CC-G-G-----CT-C-AA------C-T-G-G-G-G-A--G-G----G-T--C-A---C--T-------C--GA-T-A---C-------T--G-C--TT--G-A-C----T-C-G-A-A-G--G---C-GG-----GA-G-A--------G-G-G-A---AG-T------GG--A--ATT----C-C-C-G-GT--GT-A-G-TG-GT--G---A--A-A---TG-C-GT-AG--AT-A-TC--G-G--G-A-G-G-A-AC-A-CC---AG--T--G--GC-GAA-G--G-C--G-------A--C-T-T-C---CTG--G--CC-C-G---------------------T-T---C-T-T--GA--CG-C-T-G--A-GG--C-G-CG-A--AA-G-C---T-AG--GG-G--AG-C-A-AA--------CG--GG Bacteria(100);Chloroflexi(100);KD4-96(100);unclassified(100);unclassified(100);unclassified(100);unclassified(100);unclassified(100);unclassified(100);

Otu0473 0 0 0 10 0 5 0 6 0 35 0 0 0 0 0 0 0 0 0 0 0 1 0 0 0 0 0 0
0 0 22 0 83 0 96 0 0 0 14 40 0 35 0 0 0 0 0 0 0 0 0 24 0 5 0 0 124
21 0 15 0 4 0 0 0 0 0 0 0 3 0 10 11 0 3 0 4 6 0 0 0 56 0 0 89 17
13 84 0 30 46 46 12 1 0 50 0 0 0 22 0 14 0 117 0 0 183 0 0 1 0 0 12
M02255_77_000000000-ADUT3_1_1108_6786_13362 T----AC---GT-AG-GGG--------GCA-A-G------C--G--T---T--AT-C-CGG-AT------TT-A--C-T--GG-GT-------GT--A-----AA-GG-GA-GC-------G-TA-G-A-C-G-------------G--A-AT-G-G-C-----------AA----G-T-C-T--------G-A-A--G--TG--A-AA-AC--C-C-CG-G-G-----CT-C-AA------C-C-T-G-G-G-G--A-C----T-G--C-T---T--T-------G--GA-A-A---C-------T--G-T--TG--T-T-C----T-A-G-A-G-T--G---T-TG-----GA-G-A--------G-G-T-A---AG-T------GG--A--ATT----C-C-T-G-GT--GT-A-G-CG-GT--G---A--A-A---TG-C-GT-AG--AT-A-TC--A-G--G-A-A-G-A-AC-A-CC---GG--A--G--GC-GAA-G--G-C--G-------G--C-T-T-A---CTG--G--AC-A-A---------------------T-A---A-C-T--GA--CG-T-T-G--A-GG--C-T-CG-A--AA-G-C---G-TG--GG-G--AT-C-A-AA--------CA--GG Bacteria(100);Firmicutes(100);Clostridia(100);Clostridiales(100);Lachnospiraceae(100);Oribacterium(100);unclassified(100);unclassified(100);unclassified(100);

Otu0474 0 0 0 0 0 0 0 0 0 0 0 0 0 0 0 0 0 0 0 0 0 0 0 0 0 0 0 0
0 0 0 0 0 0 0 0 0 0 0 0 0 0 0 0 0 0 0 0 0 0 0 0 0 0 0 0 0
0 0 0 0 0 0 0 0 0 0 0 0 0 0 0 0 0 45 0 0 0 0 0 0 0 0 0 0 0
0 0 0 0 0 0 0 0 0 0 0 0 0 0 0 0 0 0 0 0 0 0 0 0 0 0 0
M02255_77_000000000-ADUT3_1_1108_2209_11142 T----AC---GT-AG-GGG--------CCT-A-G------C--G--T---T--GT-C-CGG-AT------TT-A--T-T--GG-GC-------GT--A-----AA-GA-GC-TC-------G-TA-G-G-C-G-------------G--T-TC-G-G-C-----------AA----G-T-C-G--------G-A-T--G--TG--A-AA-TC--T-C-CA-C-G-----CT-T-AA------C-G-T-G-G-A-G--G-G----G-C--C-A---T--T-------C--GA-A-A---C-------T--G-C-CGT--G-A-C----T-C-G-A-G-T--C---C-GG-----TA-G-G--------G-G-A-G---TG-T------GG--A--ATT----C-C-T-G-GT--GT-A-G-CG-GT--G---A--A-A---TG-C-GC-AG--AT-A-TC--A-G--G-A-G-G-A-AC-A-CC---AG--T--A--GC-GAA-G--G-C--G-------G--C-A-C-T---CTG--G--GC-C-G---------------------G-T---A-C-T--GA--CG-C-T-G--A-GG--A-G-CG-A--AA-G-C---G-TG--GG-G--AG-C-G-AA--------CA--GG Bacteria(100);Actinobacteria(100);Actinobacteria(100);unclassified(100);unclassified(100);unclassified(100);unclassified(100);unclassified(100);unclassified(100);

Otu0475 0 0 0 0 0 0 11 0 0 0 0 0 0 0 0 0 0 0 0 0 0 0 0 0 0 0 0 0
0 0 35 0 0 0 28 0 0 0 0 0 0 0 0 0 0 0 0 0 0 0 0 0 0 0 0 0
0 0 0 0 0 0 0 0 0 0 0 0 0 0 0 0 0 0 0 0 0 0 0 0 0 0 0 0
0 0 0 0 0 0 0 0 0 0 0 0 0 0 0 0 0 0 0 0 0 0 0 0 0 0 0

M02255_77_000000000-ADUT3_1_1119_8263_4200 T----AC---GT-AG-GGG--------GCA-A-G------C--G--T---T--AT-C-CGG-AT------TT-A--C-T--GG-GT-------GT--A-----AA-GG-GA-GC-------A-TA-G-A-C-G------------G--A-GT-G-G-C-----------AA----G-T-C-T--------G-A-A--G--TG--A-AA-AC--C-C-TG-G-G-----CT-T-AA------C-C-T-G-G-G-G--A-C----T-G--C-T---T--T-------G--GA-A-A---C-------T--G-T--TA--A-T-C----T-A-G-A-G-T--G---T-TG-----GA-G-A--------G-G-T-A---AG-T------GG--A--ATT----C-C-T-G-GT--GT-A-G-CG-GT--G---A--A-A---TG-C-GT-AG--AT-A-TC--A-G--G-A-A-G-A-AC-A-CC---GG--A--G--GC-GAA-G--G-C--G-------G--C-T-T-A---CTG--G--AC-A-A--------------------T-A---A-C-T--GA--CG-T-T-G--A-GG--C-T-CG-A--AA-G-C---G-TG--GG-G--AT-C-A-AA--------CA--GG Bacteria(100);Firmicutes(100);Clostridia(100);Clostridiales(100);Lachnospiraceae(100);Oribacterium(100);unclassified(100);unclassified(100);unclassified(100);

Otu0476 5 0 0 0 0 0 0 0 0 0 0 0 0 0 0 0 0 0 0 0 0 0 0 0 0 0 0 0
0 2 0 0 0 0 0 0 3 0 0 0 0 0 0 0 0 1 1 0 6 0 0 0 1 0 0 1 10
0 0 0 0 0 0 0 0 0 0 0 0 0 0 0 0 0 0 0 0 0 0 0 0 0 0 0 0
0 0 0 0 0 0 0 0 0 0 0 0 0 0 0 0 0 0 0 0 0 0 0 1 0 0 0

M02255_77_000000000-ADUT3_1_2115_7866_8684 T----AC---GG-AG-GGT--------GCA-A-G------C--G--T---T--AA-T-CGG-AA------TT-A--C-T--GG-GC-------GT--A-----AA-GC-GC-AC-------G-CA-G-G-C-G------------G--T-TT-G-T-T-----------AA----G-T-C-A--------G-A-T--G--TG--A-AA-TC--C-C-CG-G-G-----CT-C-AA------C-C-T-G-G-G-A--A-C----T-G--C-A---T--C-------T--GA-T-A---C-------T--G-G--CA--A-G-C----T-T-G-A-G-T--C---T-CG-----TA-G-A--------G-G-G-G---GG-T------AG--A--ATT----C-C-A-G-GT--GT-A-G-CG-GT--G---A--A-A---TG-C-GT-AG--AG-A-TC--T-G--G-A-G-G-A-AT-A-CC---GG--T--G--GC-GAA-G--G-C--G-------G--C-C-C-C---CTG--G--AC-G-A--------------------A-G---A-C-T--GA--CG-C-T-C--A-GG--T-G-CG-A--AA-G-C---G-TG--GG-G--AG-C-A-AA--------CA--GG Bacteria(100);Proteobacteria(100);Gammaproteobacteria(100);Enterobacteriales(100);Enterobacteriaceae(100);Enteric_Bacteria_cluster(100);unclassified(100);unclassified(100);unclassified(100);

Otu0477 0 0 0 0 0 0 0 0 0 0 0 0 0 0 0 0 0 0 0 0 0 0 0 0 0 0 0 0
0 0 0 0 0 0 0 0 0 0 0 0 0 0 0 0 0 0 0 0 0 0 0 0 0 0 0 0
0 0 0 0 0 0 0 17 0 0 0 0 0 0 0 0 0 24 0 0 0 0 0 0 0 0 0 0
0 0 0 0 0 0 0 0 0 0 0 0 0 0 0 0 0 0 0 0 0 0 0 0 0 0 0

M02255_77_000000000-ADUT3_1_2108_17175_20302 T----AC---GT-AG-GAT--------GCA-A-G------C--G--T---T--GT-C-CGG-AT------TT-A--T-T--GG-GC-------GT--A-----AA-GC-GA-AC-------G-TA-G-G-C-G------------G--T-TT-C-T-T-----------AA----G-T-T-T--------G-G-T--C--TT--A-AA-GA--C-T-GG-G-G-----CT-C-AA------C-C-T-C-A-G-G--A------G-TG-G-A---C--T-------G--AA-T-A---C-------T--G-G--GA--G-A-C----T-A-G-A-G-G--A---T-GT-----TA-G-A--------G-G-A-A---AG-T------GG--A--ATT----C-C-A-A-GT--GT-A-G-CG-GT--G---A--A-A---TG-C-GT-AG--AT-A-TT--T-G--G-A-G-G-A-AC-A-CC---AG--T--G--GC-GAA-A--G-C--G-------G--C-T-T-T---CTG--G--GA-C-A--------------------T-T---C-C-T--GA--CG-C-T-G--A-GG--T-T-CG-A--AA-G-C---T-CG--GG-G--AG-C-A-AA--------CA--GG Bacteria(100);unclassified(100);unclassified(100);unclassified(100);unclassified(100);unclassified(100);unclassified(100);unclassified(100);unclassified(100);

Otu0478 0 0 0 0 0 0 0 0 0 0 0 0 0 0 0 0 0 0 0 0 0 0 0 0 0 0 0 0
0 0 0 0 0 0 0 0 0 0 0 0 0 0 0 0 0 0 0 0 0 0 0 0 0 0 0 0
0 31 30 0 20 7 0 0 1 0 0 0 0 0 0 0 8 17 0 0 0 0 0 0 0 0 0 0
0 0 0 0 0 0 0 0 0 0 0 0 0 0 0 0 0 0 0 0 0 0 0 0 0 0 0

M02255_77_000000000-ADUT3_1_1114_11225_5802 T----AC---GG-AG-GGA--------GCT-A-G------C--G--T---T--GT-T-CGG-AA------TT-A--C-T--GG-GC-------GT--A-----AA-GC-GC-AC-------G-TA-G-G-C-G------------G--C-TT-T-T-T-----------AA----G-T-C-A--------G-G-G--G--TG--A-AA-TC--C-C-GG-G-G-----CT-C-AA------C-C-C-C-G-G-A--A-C----T-G--C-C---T--T-------T--GA-A-A---C-------T--G-C--AG--A-G-C----T-A-G-A-A-T--C---C-TG-----GA-G-A--------G-G-T-G---AG-T------GG--A--ATT----C-C-G-A-GT--GT-A-G-AG-GT--G---A--A-A---TT-C-GT-AG--AT-A-TT--C-G--G-A-A-G-A-AC-A-CC---AG--T--G--GC-GAA-G--G-C--G-------A--C-T-C-A---CTG--G--AC-A-G--------------------G-T---A-T-T--GA--CG-C-T-G--A-GG--T-G-CG-A--AA-G-C---G-TG--GG-G--AG-C-A-AA--------CA--GG Bacteria(100);Proteobacteria(100);Alphaproteobacteria(100);Sphingomonadales(100);Erythrobacteraceae(100);Erythrobacter(86);unclassified(86);unclassified(86);unclassified(86);

Otu0479 0 0 0 0 0 0 0 0 0 0 0 0 0 0 0 0 0 0 0 0 0 0 0 0 0 0 0 0
0 0 0 0 105 0 0 0 0 0 34 0 0 0 0 0 0 0 0 0 0 0 0 0 0 0 0 0
0 0 0 0 0 0 0 0 0 0 0 9 3 0 27 9 0 0 78 150 70 0 2 0 0 0 0 0
21 0 0 0 107 0 0 16 0 148 0 33 8 0 0 0 0 0 0 0 0 0 0 0 0 0 0

M02255_77_000000000-ADUT3_1_2118_26358_6301 T----AC---AT-AT-GGG--------GCG-A-G------C--G--T---T--AT-C-CGG-AT------TT-A--T-T--GG-GT-------GT--A-----AA-GG-GT-TC-------G-TA-G-G-C-G------------G--G-AA-A-G-T-----------AA----G-TCA-G--------G-A-T--G--TG--A-AA-TC--C-C-TC-G-G-----CT-C-AA------C-C-G-A-G-G-A--A-C----T-G--C-G---A--T-------T--GA-A-A---C-------T--A-T--TT--T-T-C----T-T-G-A-G-T--A---C-TG-----GA-G-G--------G-G-A-A---AG-T------GG--A--ATT----C-T-T-A-GT--GT-A-G-CG-GT--G---A--A-A---TG-C-GT-AG--AT-A-TT--A-G--G-A-A-G-A-AC-A-CC---AG--T--G--GC-GAA-G--G-C--G-------A--C-T-T-T---CTG--G--AC-A-G--------------------T-A---A-C-T--GA--CG-C-T-G--A-GG--A-A-CG-A--AA-G-T---G-TG--GG-G--AG-C-A-AA--------CA--GG Bacteria(100);unclassified(100);unclassified(100);unclassified(100);unclassified(100);unclassified(100);unclassified(100);unclassified(100);unclassified(100);

Otu0480 0 0 0 0 0 0 0 0 0 0 0 0 0 0 0 0 0 0 0 0 0 0 0 0 0 0 1 0
0 0 0 0 0 0 31 0 0 0 0 0 0 0 0 0 0 0 0 0 0 0 0 0 0 0 0 0
0 0 0 0 0 0 0 0 0 0 0 0 0 0 0 0 0 22 0 0 0 0 0 0 0 0 0 0
0 0 0 0 0 0 0 0 0 0 0 0 0 0 0 0 0 0 0 0 0 0 0 0 0 0 0
M02255_77_000000000-ADUT3_1_1117_12614_13891 C----AC---GT-AG-GGG--------GCG-A-G------C--G--T---T--GT-C-CGG-AT------TT-A--T-T--GG-GC-------GT--A-----AA-GA-GC-TC-------G-TA-G-G-C-G------------G--T-TT-G-G-C-----
------AA----G-T-C-G--------G-G-T--G--TG--A-AA-GC--T-C-GA-G-G-----CT-C-AA------C-C-T-C-G-A-G--A------C-G-CC-A---C--T-------C--GA-T-A---C-------T--G-C--CA-TG-A-C----T-G-G-A-G-T--C---C-GG-----TA-G-A--------G-G-A-G---TG-T------GG--A--ATT----
C-C-T-G-GT--GT-A-G-CG-GT--G---A--A-A---TG-C-GC-AG--AT-A-TC--A-G--G-A-G-G-A-AC-A-CC---AA--C--G--GC-GAA-G--G-C--A-------G--C-A-C-T---CTG--G--GC-C-G--------------------G-T---A-C-T--GA--CG-C-T-G--A-GG--A-G-CG-A--AA-G-C---G-TG--
GG-G--AG-C-A-AA--------CA--GG Bacteria(100);Actinobacteria(100);Actinobacteria(100);Acidimicrobidae(100);Acidimicrobiales(100);Acidimicrobineae(100);unclassified(100);unclassified(100);unclassified(100);

Otu0481 0 0 0 2 0 0 0 0 0 0 0 0 0 0 0 0 0 0 0 0 0 0 0 0 0 0 0 0
0 0 0 0 0 0 0 0 0 0 0 0 0 0 0 0 0 0 0 0 0 0 0 0 0 0 0 0
0 0 0 0 0 0 0 0 0 0 0 0 0 0 0 0 0 0 0 0 0 0 0 0 0 0 0 0
0 0 0 0 0 0 0 0 0 0 0 0 0 0 0 0 1 0 0 6 9 0 0 0 0 0 13
M02255_76_000000000-ADUNE_1_2107_13385_22829 C----GG---CT-AA-TTC--------CGT-G-C------C--A--G---C--AG-C-CGC-GG------TA-A--T-A--CG-GA-------AG--G-----TC-CG-G--GC-------G-TT-A-T-C-C------------G--G-AG-A-T-T-------
----TA----T-T-G-T--------G-T-T--G--TG--A-AA-TG--T-A-GA-C-G-----CT-C-AA------C-G-T-C-T-G-A--C-T----T-G--C-A---G--C-------G--CA-T-A---C-------T--G-G--TT--T-C-C----T-T-G-A-G-T--A---C-GC-----AC-A-A--------C-G-T-T---GG-C------GG--A--ATT----C-G-
T-C-GT--GT-A-G-CG-GT--G---A--A-A---TG-C-TT-AG--AT-A-TG--A-C--G-A-A-G-A-AC-T-CC---GA--T--T--GC-GAA-G--G-C--A-------G--C-T-G-A---CGG--G--AG-C-G--------------------C-A---A-C-T--GA--CG-C-T-T--A-AG--C-T-CG-A--AG-G-T---G-CG--GG-T--
AT-C-G-AA--------CA--GG Bacteria(100);Bacteroidetes(100);Bacteroidia(100);Bacteroidales(100);Prevotellaceae(100);Prevotella(100);unclassified(100);unclassified(100);unclassified(100);

Otu0482 0 0 0 0 0 0 0 0 0 0 48 1 0 0 0 0 0 0 0 0 0 0 0 0 0 0 0 0
0 0 0 0 0 0 0 0 0 0 0 0 0 0 0 0 0 1 0 0 0 0 0 0 0 0 0 0
0 0 0 0 0 0 0 0 0 0 0 0 0 0 0 0 1 0 0 0 0 1 1 0 0 0 0 0
0 0 0 0 0 1 0 0 1 1 0 0 0 0 0 0 0 0 1 0 0 0 0 3 0 0 0
M02255_77_000000000-ADUT3_1_1106_13712_20111 T----AC---GT-AG-GTG--------GCA-A-G------C--G--T---T--GT-C-CGG-AT------TT-A----T--------------T-------------G--G--------G-CA-G-G-C-G------------G--T-TC-T-T-T-----------AA---
-G-T-C-T--------G-A-T--G--TG--A-AA-GC--C-C-CC-G-G-----CT-C-AA------C-C-G-G-G-G-A--G------G-GT-C-A---T--T-------G--GA-A-A---C-------T--G-G--AG--A-A-C----T-T-G-A-G-T--G---C-AG-----AA-G-A--------G-G-A-G---AG-T------GG--A--ATT----C-C-A-C-
GT--GT-A-G-CG-GT--G---A--A-A---TG-C-GT-AG--AT-A-TG--T-G--G-A-G-G-A-AC-A-CC---AG--T--G--GC-GAA-G--G-C--G-------A--C-T-C-T---CTG--G--TC-T-G--------------------T-A---A-C-T--GA--CG-C-T-G--A-GG--C-T-CG-A--AA-G-C---G-TG--GG-G--AG-C-
A-AA--------CA--GG Bacteria(100);Firmicutes(100);Bacilli(100);Lactobacillales(67);Carnobacteriaceae(67);Carnobacterium(67);unclassified(67);unclassified(67);unclassified(67);

Otu0483 0 0 0 0 0 0 0 0 0 0 0 0 0 0 0 0 0 0 0 0 0 0 0 0 0 0 0 0
0 0 0 0 0 0 0 0 0 0 0 0 0 0 0 0 0 0 0 0 0 0 0 0 0 0 0 0
0 0 0 0 0 0 0 0 0 0 0 0 0 0 0 0 0 57 0 0 0 0 0 0 0 0 0 0
0 0 0 0 0 0 0 0 0 0 0 0 0 0 0 0 0 0 0 0 0 0 0 0 0 0 0
M02255_77_000000000-ADUT3_1_2106_13885_17767 T----AC---GA-AG-GGG--------GCT-A-G------C--G--T---T--GT-T-CGG-AA------TT-A--C-T--GG-GC-------GT--A-----AA-GC-GC-GC-------G-TA-G-G-C-G------------G--A-TC-G-C-T-----
------AA----G-T-C-A--------G-G-G--G--TG--A-AA-TG--C-C-GA-G-G-----CT-C-AA------C-C-T-C-G-G-A--A-C----T-G--C-C---T--T-------T--GA-T-A---C-------T--G-A--CG--A-T-C----T-G-G-A-G-A--T---C-GG-----GA-G-A--------G-G-T-T---GG-T------GG--A--ATT----
C-C-G-A-GT--GT-A-G-AG-GT--G---A--A-A---TT-C-GT-AG--AT-A-TT--C-G--G-A-G-G-A-AC-A-CC---AG--A--A--GC-GAA-G--G-C--G-------G--C-C-A-A---CTG--G--CC-C-G--------------------A-T---A-C-T--GA--CG-C-T-G--A-GG--C-G-CG-A--AA-G-C---T-TG--
GG-G--AG-C-A-AA--------CA--GG Bacteria(100);Proteobacteria(100);Alphaproteobacteria(100);unclassified(100);unclassified(100);unclassified(100);unclassified(100);unclassified(100);unclassified(100);

Otu0484 0 0 0 0 0 0 0 0 0 0 0 0 0 0 0 0 0 0 0 0 1 0 0 0 0 0 0 0
0 0 0 0 0 0 0 0 0 9 2 0 0 0 0 0 0 0 0 0 0 0 0 0 0 0 0 0
0 0 0 0 0 0 0 0 0 0 0 0 0 0 0 0 0 22 0 0 0 0 0 0 0 0 0 0
0 0 0 0 0 0 0 0 0 0 0 0 0 0 0 0 0 0 0 0 0 0 0 0 0 0 0
M02255_77_000000000-ADUT3_1_2107_22392_16702 T----AC---GA-AG-GGG--------GCT-A-G------C--G--T---T--GC-T-CGG-AA------TC-A--C-T--GG-GC-------GT--A-----AA-GC-GC-AC-------G-TA-G-G-T-G------------G--G-TC-A-T-T-----
------TA----G-T-C-A--------G-G-G--G--TG--A-AA-TC--C-T-GG-A-G-----CT-C-AA------C-T-C-C-A-G-A--A-C----T-G--C-C---T--T-------T--GA-T-A---C-------T--G-A--TG--A-T-C----T-TCG-A-G-T--C---C-GG-----GA-G-A--------G-G-T-G---GG-T------GG--A--ACT----
G-C-G-A-GT--GT-A-G-AG-GT--G---A--A-A---TT-C-GT-AG--AT-A-TT--C-G--C-A-A-G-A-AC-A-CC---AG--T--G--GC-GAA-G--G-C--G-------G--C-T-C-A---CTG--G--CC-C-G--------------------G-T---A-C-T--GA--CA-C-T-G--A-GG--T-G-CG-A--AA-G-C---G-TG--GG-
G--AG-C-A-AA--------CA--GG Bacteria(100);Proteobacteria(100);Alphaproteobacteria(100);Rhizobiales(100);unclassified(100);unclassified(100);unclassified(100);unclassified(100);unclassified(100);

Otu0485 0 0 0 0 0 0 0 0 0 0 0 0 0 0 0 0 0 0 0 0 0 0 0 0 0 0 0 0
0 0 0 0 0 97 0 0 0 0 0 0 0 0 0 0 0 0 0 0 0 0 0 0 0 0 0 0
0 0 0 0 0 0 0 0 0 0 0 0 0 0 0 0 0 17 0 0 0 0 0 0 0 0 0 0
0 0 0 0 0 0 0 0 0 0 0 0 0 0 0 0 0 0 0 0 0 0 0 0 0 0 0
M02255_77_000000000-ADUT3_1_1102_7680_5311 T----AC---GA-AG-GGG--------GCT-A-G------C--G--T---T--GT-T-CGG-AA------TT-A--C-T--GG-GC-------GT--A-----AA-GC-GC-AC-------G-TA-G-G-C-G------------G--A-TT-G-T-T-----------AA-
---G-T-C-A--------G-G-G--G--TG--A-AA-TC--C-C-AG-A-G-----CT-C-AA------C-T-C-T-G-G-A--A-C----T-G--C-C---T--T-------T--GA-T-A---C-------T--G-G--CA--A-T-C----T-A-G-A-G-T--C---C-GG-----AA-G-A--------G-G-T-T---AG-T------GG--A--ATT----C-C-C-A-
GT--GT-A-G-AG-GT--G---A--A-A---TT-C-GT-AG--AT-A-TT--G-G--G-A-A-G-A-AC-A-CC---AG--T--G--GC-GAA-G--G-C--G-------G--C-T-A-A---CTG--G--TC-C-G--------------------G-T---A-C-T--GA--CG-C-T-G--A-GG--T-G-CG-A--AA-G-C---G-TG--GG-G--AG-
C-A-AA--------CA--GG Bacteria(100);Proteobacteria(100);Alphaproteobacteria(100);Rhizobiales(100);unclassified(100);unclassified(100);unclassified(100);unclassified(100);unclassified(100);

Otu0486 0 0 0 0 0 0 0 0 0 0 0 0 0 0 0 0 0 0 0 0 0 0 0 0 0 0 0 0
0 0 0 0 0 0 0 0 0 0 0 0 0 0 0 0 0 0 0 0 0 0 0 0 0 0 0 0
0 0 0 0 181 0 0 0 0 0 0 0 0 0 0 0 0 0 0 0 0 0 0 0 0 0 0 0
0 0 0 0 0 0 0 0 0 0 0 0 0 0 0 0 0 0 0 0 0 0 0 0 0 0 0
M02255_77_000000000-ADUT3_1_2114_16297_10187 C----AA---AC-TC-CGT--------GCC-A-G------C-----A---G--CC-G-CGG-TA------AT-A--C-G--GA-GG-------GT--------GC-GA-GC-GT---------TA-G-T-C-G------------G----AT-T-G-C--------
---AA----G-C-A-T--------T-T-T--G--TG--A-AA-GA--A-C-AA-A-G-----CT-T-AA--------C-T-T-T-G-T--A-G----A-G--C-A---A--A-------A--TG-G-A---C-------G--G-C--AT--C-A-C----T-A-G-A-G-T--C---T-CT-----GA-G-A--------G-G-G-G---GG-T------AG--A--ATT----T-C-
A-A-GT--GT-A-A-CG-TT--G---A--A-A---TG-T-TT-AG--AT-A-TT--T-G--A-A-G-G-A-AT-A-CC---AG--T--G--GC-GAA-G--G-C--G-------G--C-C-C-C---CTG--G--CAGA-T--------------------G-T---A-C-T--GA--CG-C-T-C--A-GA--C-G-CG-A--AG-G-C---G-CG--GG-G--
AG-C-A-AA--------CA--GG Bacteria(100);unclassified(100);unclassified(100);unclassified(100);unclassified(100);unclassified(100);unclassified(100);unclassified(100);unclassified(100);

Otu0487 0 0 0 0 0 0 0 0 0 0 0 0 0 0 0 0 0 0 0 0 0 0 0 0 0 1 0 0
0 0 0 0 13 0 0 0 0 0 0 0 18 0 8 0 0 0 0 0 0 0 1 14 0 4 0 0 0
6 0 0 0 0 0 0 0 0 0 0 0 0 0 0 0 0 0 0 0 0 0 0 0 0 0 0 0
0 0 0 0 0 0 0 0 0 0 0 0 0 0 0 0 0 0 0 0 0 0 0 0 0 0 0
M02255_77_000000000-ADUT3_1_1102_20083_17199 T----AC---GT-AG-GGT--------GCA-A-G------C--G--T---T--GT-C-CGG-AA------TT-A--C-T--GG-GC-------GT--A-----AA-GA-GC-TC-------G-TA-G-G-C-G------------G--T-TT-G-T-C------
-----GC----G-T-C-G--------T-C-T--G--TG--A-AA-AC--C-A-GC-A-G-----CT-C-AA------C-T-G-T-T-G-G--C-T----T-G--C-A---G--G-------C--GA-T-A---C-------G--G-G--CA--G-A-C----T-T-G-A-G-T--A---T-TT-----CA-G-G--------G-G-A-G---AC-T------GG--A--ATT----C-
C-T-G-GT--GT-A-G-CG-GT--G---A--A-A---TG-C-GC-AG--AT-A-TC--A-G--G-A-G-G-A-AC-A-CC---GG--T--G--GC-GAA-G--G-C--G-------G--G-T-C-T---CTG--T--GA-A-A--------------------T-A---A-C-T--GA--CG-C-T-G--A-GG--A-G-CG-A--AA-G-C---G-TG--GG-
T--AG-C-G-AA--------CA--GG Bacteria(100);Actinobacteria(100);Actinobacteria(100);Actinobacteridae(100);Actinomycetales(100);Corynebacterineae(100);Nocardiaceae(100);Rhodococcus(100);unclassified(100);

Otu0488 0 0 0 0 0 0 0 0 0 0 0 0 0 0 0 0 0 0 0 0 0 0 0 0 0 0 0 0
0 0 0 0 0 0 0 0 0 0 0 30 0 0 0 13 0 0 0 0 0 0 0 0 0 0 0 0
0 0 0 0 1 0 0 0 0 0 0 0 0 0 18 30 0 0 0 0 0 0 0 0 0 0 0 0 20
0 0 0 0 0 0 2 0 0 0 0 0 0 56 0 0 0 0 0 0 0 0 0 0 0 0 0
M02255_76_000000000-ADUNE_1_1107_22969_20776 A----AC----T-A--TGT--------GCC-A-G------C--A--G---C--CG-C-GGT-AA------AA-C--A-T--AG-GG-------GG--C-----AA----GC-G--------T-TG-T-C-C-G------------G--T-AA-A-T-T----------
-AA----G-T-C-A--------G-G-T--G--TG--A-AA-GT--T-C-GG-G-G-----CT-C-AA------C-C-C-C-G-T-G--A-T----T-G--C-A---C--C-------T--GA-T-A---C-------T--G-A--TA--A-A-C----T-A-G-A-G-T--G---T-TG-----GA-G-A--------G-G-T-A---AG-T------GG--A--ATT----C-C-T-
A-GT--GT-A-G-CG-GT--G---G--A-A---TG-C-GT-AG--AT-A-TT--A-G--G-A-G-G-A-AC-A-CC---AG--T--G--GC-GAA-G--G-C--G-------A--C-T-T-A---CTG--G--TC-A-A--------------------T-A---A-C-T--GA--CG-C-T-G--A-GG--C-G-CG-A--AA-G-C---G-TG--GG-G--
AG-C-A-AA--------CA--GG Bacteria(100);Firmicutes(100);Clostridia(100);Clostridiales(100);Peptococcaceae(100);Peptococcus(100);unclassified(100);unclassified(100);unclassified(100);

Otu0489 0 0 0 0 0 2 0 0 0 0 0 0 0 0 0 0 0 0 0 0 0 0 0 0 0 0 0 0
0 0 0 0 0 0 0 0 1 1 0 0 0 0 0 0 0 0 0 0 0 0 0 0 0 0 0 0
0 0 3 0 0 0 0 0 0 0 0 0 0 0 0 0 22 0 0 0 0 0 0 0 0 0 0 0
0 3 0 0 0 0 0 0 0 3 0 0 0 0 0 0 0 0 0 0 0 3 0 0 0 3 0
M02255_77_000000000-ADUT3_1_2119_22358_24230 C----GG---CT-AA-CT---------ACG-T-G------C--C--A---G--CA-G-CCG-CG------GT-A--A-T--AC-GT-------AG--G-----TC-CC-GA-GC-------G-TA-G-G-C-G------------G--T-TA-G-A-T------
-----AA----G-T-C-T--------G-A-A--G--TT--A-AA-GG--C-T-GT-G-G-----CT-T-AA------C-C-A-T-A-G-T--A------C-G--C-T---T--T-------G--GA-A-A---C-------T--G-T--TT--A-A-C----T-T-G-A-G-T--G---C-AA-----GA-G-G--------G-G-A-G---AG-T------GG--A--ATT----C-C-
A-T-GT--GT-A-G-CG-GT--G---A--A-A---TG-C-GT-AG--AT-A-TA--T-G--G-A-G-G-A-AC-A-CC---GG--T--G--GC-GAA-A--G-C--G-------G--C-T-C-T---CTG--G--CT-T-G--------------------T-A---A-C-T--GA--CG-C-T-G--A-GG--C-T-CG-A--AA-G-C---G-TG--GG-G--
AG-C-A-AA--------CA--GG Bacteria(100);Firmicutes(100);Bacilli(100);Lactobacillales(100);Streptococcaceae(100);Streptococcus(100);unclassified(100);unclassified(100);unclassified(100);

Otu0490 0 0 0 0 0 0 0 74 0 0 0 0 0 0 0 0 0 0 0 0 0 0 0 0 0 0 0 0
0 0 0 0 0 0 0 0 0 0 0 0 0 0 0 0 0 0 0 0 0 0 0 0 0 0 0 0
0 0 0 0 0 0 0 0 0 0 0 0 0 0 0 0 0 0 0 0 0 0 0 0 0 0 0 0
0 0 0 0 0 0 0 0 0 0 0 0 0 0 0 0 0 0 0 0 0 0 0 0 12 0 0
M02255_77_000000000-ADUT3_1_1117_7420_17403 T----AC---GT-AG-GGG--------GCA-A-G------C--G--T---T--AT-C-CGG-AA------TT-A--T-T--GG-GC-------GT--A-----AA-GA-GT-GC-------G-TA-G-G-T-G------------G--T-TA-T-G-T-----------AA-
---G-C-G-T--------G-A-G--G--TT--T-AA-GG--C-A-GT-G-G-----CT-T-AA------C-C-A-C-T-G-T--T------C-G--C-C---T--T-------G--CG-A-A---C-------T--G-C--AT--A-A-C----T-T-G-A-G-T--G---C-AG-----GA-G-G--------G-G-A-A---AG-C------GG--A--ATT----C-C-T-A-
GT--GT-A-G-CG-GT--G---A--A-A---TG-C-GT-AG--AT-A-TT--A-G--G-A-G-G-A-AC-A-CC---AG--C--G--GC-GAA-G--G-C--G-------G--C-T-T-T---CTG--G--AC-T-G--------------------C-A---A-C-T--GA--CA-C-T-G--A-GG--C-A-CG-A--AA-G-C---G-TG--GG-G--AG-
C-A-AA--------CA--GG Bacteria(100);Firmicutes(100);Clostridia(100);Clostridiales(100);Family_XIII_Incertae_Sedis(100);unclassified(100);unclassified(100);unclassified(100);unclassified(100);

Otu0491 0 0 0 21 0 0 0 0 0 0 0 0 0 0 0 0 0 0 0 0 0 0 0 0 0 0 0 0
0 0 0 0 0 0 0 0 0 0 0 0 0 9 0 0 0 0 0 0 0 0 0 0 0 0 0 0
0 0 0 0 0 0 0 0 0 0 0 0 0 0 0 0 0 0 0 0 0 0 0 0 0 0 0 0
0 0 0 0 0 0 0 0 0 0 0 0 0 0 0 0 0 0 0 0 0 0 0 0 0 0 0
M02255_77_000000000-ADUT3_1_2114_18396_16354 T----AC---GT-AG-GGT--------CCG-A-G------C--G--T---T--GT-C-CGG-AG------TT-A--C-T--GG-GC-------GT--A-----AA-GC-GC-GC-------G-CA-G-G-C-G------------G--T-CC-C-G-C-----
------GC----G-C-C-C--------G-G-C--G--TG--A-AA-GC--C-C-CC-G-G-----CT-C-AA------C-C-G-G-G-G-A--G-G----G-T--C-G---T--C-------G--GG-G-A---C-------G--G-C--GG--G-A-C----T-T-G-A-G-G--G---C-GG-----TA-G-G--------G-G-C-C---GG-T------GG--A--ATC---
-C-C-T-G-GT--GT-A-G-TG-GT--G---A--A-A---TG-C-GT-AG--AG-A-TC--A-G--G-G-G-G-A-AC-A-CC---CG--T--G--GC-GAA-G--G-C--G-------G--C-C-G-G---CTG--G--GC-C-G--------------------A-C---C-C-T--GA--CG-C-T-G--A-GG--C-G-CG-A--AG-G-C---G-TG--
GG-G--AG-C-G-AA--------CG--GG Bacteria(100);Chloroflexi(100);Thermomicrobia(100);JG30-KF-CM45(100);unclassified(100);unclassified(100);unclassified(100);unclassified(100);unclassified(100);

Otu0492 0 0 28 0 0 0 28 0 24 0 0 0 0 0 0 0 0 0 0 0 0 0 0 0 0 0 0 0
0 0 0 0 0 0 0 0 0 0 0 0 0 0 0 0 0 0 0 0 0 0 0 0 0 0 0 0
0 0 0 0 0 0 0 0 0 0 0 0 0 0 0 0 0 0 0 0 0 0 0 0 0 0 0 0
0 0 0 0 0 0 0 0 0 0 0 0 0 0 0 0 0 0 0 0 0 0 0 0 0 0 0
M02255_77_000000000-ADUT3_1_1102_8467_14181 T----AC---GT-AG-GGG--------GCA-A-G------C--G--T---T--AT-C-CGG-AT------TT-A--C-T--GG-GT-------GT--A-----AA-GG-GA-GC-------G-TA-G-A-C-G------------G--C-AA-G-G-C-----------
AA----G-T-C-T--------G-A-T--G--TG--A-AA-GG--C-T-GG-G-G-----CT-C-AA------C-C-C-C-G-G-G--A-C----T-G--C-A---T--T-------G--GA-A-A---C-------T--G-T--CC--T-G-C----T-G-G-A-G-T--G---C-CG-----GA-G-A--------G-G-T-A---AG-C------GG--A--ATT----C-C-T-
A-GT--GT-A-G-CG-GT--G---A--A-A---TG-C-GT-AG--AT-A-TT--A-G--G-A-G-G-A-AC-A-CC---AG--T--G--GC-GAA-G--G-C--G-------G--C-T-T-A---CTG--G--AC-G-G--------------------T-A---A-C-T--GA--CG-T-T-G--A-GG--C-T-CG-A--AA-G-C---G-TG--GG-G--
AG-C-A-AA--------CA--GG Bacteria(100);Firmicutes(100);Clostridia(100);Clostridiales(100);Lachnospiraceae(100);unclassified(100);unclassified(100);unclassified(100);unclassified(100);

Otu0493 0 0 0 0 0 0 0 0 0 0 0 0 0 0 0 0 0 0 0 0 0 0 0 0 0 0 0 0
0 186 0 0 0 0 0 0 0 0 0 0 0 0 91 0 0 0 0 0 0 0 0 0 0 0 0 0
0 0 0 0 0 0 0 0 0 0 0 0 0 0 0 0 0 0 0 0 0 0 0 0 0 0 0 0
0 0 0 0 0 0 0 0 0 0 0 0 0 0 0 0 0 0 0 0 0 0 0 0 0 0 0
M02255_77_000000000-ADUT3_1_1102_20784_11021 T----AC---GT-AG-GTG--------GCG-A-G------C--G--T---T--GT-C-CGG-AT------TT-A--C-T--GG-GC-------GT--A-----AA-GG-AT-GC-------G-TA-G-G-C-G------------G--A-TA-T-T-T------
-----AA----G-T-G-G--------G-A-T--G--TG--A-AA-TA--C-C-CG-G-G-----CT-T-AA------C-C-T-G-G-G-T--G-C----T-G--C-A---T--T-------C--CA-A-A---C-------T--G-G--AT--A-T-C----T-A-G-A-G-T--G---C-AG-----GA-G-A--------G-G-A-A---AG-C------GG--A--ATT----C-
C-T-A-GT--GT-A-G-CG-GT--G---A--A-A---TG-C-GT-AG--AG-A-TT--A-G--G-A-A-G-A-AC-A-CC---AG--T--G--GC-GAA-G--G-C--G-------G--C-T-T-T---CTG--G--AC-T-G--------------------T-A---A-C-T--GA--CG-C-T-G--A-GG--C-A-TG-A--AA-G-C---G-TG--GG-G-
-AG-C-A-AA--------CA--GG Bacteria(100);Firmicutes(100);Clostridia(100);Clostridiales(100);Clostridiaceae(100);Clostridium(100);unclassified(100);unclassified(100);unclassified(100);

Otu0494 0 0 0 0 0 0 0 0 0 0 0 0 0 0 0 0 0 0 0 0 0 0 0 0 0 0 0 0
0 1 0 0 0 0 0 0 0 13 0 0 0 0 0 0 0 0 0 0 0 0 0 26 0 0 0 0
0 0 0 0 0 0 0 0 0 0 0 0 0 0 0 0 0 0 0 0 0 0 0 0 0 0 0 0
0 0 0 0 0 0 0 0 0 0 0 0 0 0 0 0 0 0 0 0 0 0 0 0 0 0 0
M02255_77_000000000-ADUT3_1_1107_17108_13279 G----AC---AG-AG-GTG--------CCA-A-G------C--G--T---T--AG-G-CGG-AA------TC-A--C-T--GG-GC-------TT--A-----AA-GC-GT-GT-------G-TA-G-G-C-G------------G--A-TT-G-G-T----
-------AA----G-T-A-C--------C-T-T--G--TG--A-AA-TC--C-C-AC-A-G-----CT-C-AA------C-T-G-T-G-G-A--A-C----T-G--C-T---C--G-------G--TA-T-A---C-------T--G-C--TT--A-T-C----T-T-G-A-G-C--C---A-CT-----TA-G-G--------G-G-C-T---GT-C------GG--A--ACA----A-
A-C-G-GT--GG-A-G-CG-GT--G---A--A-A---TG-C-GT-AG--AT-A-TC--G-T--T-T-G-G-A-AC-G-CC---AA--A--G--GT-GAA-A--A-C--A-------G--A-C-A-G---CTG--G--GA-G-T--------------------G-T---G-C-T--GA--CG-C-T-G--A-GA--C-A-CG-A--AA-G-C---C-AG--GG-
G--AG-C-G-AA--------CG--GG Bacteria(100);Planctomycetes(100);Phycisphaerae(100);WD2101(100);unclassified(100);unclassified(100);unclassified(100);unclassified(100);unclassified(100);

Otu0495 1 1 56 0 0 0 82 4 268 0 0 0 0 0 0 0 0 0 0 0 0 0 0 0 0 0 0 0
0 0 0 0 0 0 0 0 0 0 0 191 0 0 0 28 0 0 0 0 0 0 0 0 0 0 0 0 0
0 0 0 0 0 0 0 0 0 1 0 0 0 0 0 0 0 21 0 0 0 0 0 0 0 0 0 0 0
0 0 0 0 0 0 0 0 0 0 0 0 0 0 0 0 0 0 1 0 0 0 0 9 249 0 0
M02255_77_000000000-ADUT3_1_1107_18303_16516 T----AC---GT-AT-GGA--------GCA-A-G------C--G--T---T--AT-C-CGG-AT------TT-A--C-T--GG-GT-------GT--A-----AA-GG-GA-GT-------G-TA-G-G-T-G-------------G--T-AT-C-A-C-----------AA----G-T-C-A--------G-A-A--G--TG--A-AA-GC--C-C-GG-G-G-----CT-C-AA------C-C-C-C-G-G-G--A-C----T-G--C-T---T--T-------T--GA-A-A---C-------T--G-T--GG--A-A-C----T-G-G-A-G-T--G---C-AG-----GA-G-A--------G-G-T-A---AG-T------GG--A--ATT----C-C-T-A-GT--GT-A-G-CG-GT--G---A--A-A---TG-C-GT-AG--AT-A-TT--A-G--G-A-G-G-A-AC-A-CC---AG--T--G--GC-GAA-G--G-C--G-------G--C-T-T-A---CTG--G--AC-T-G---------------------T-A---A-C-T--GA--CA-C-T-G--A-GG--C-T-CG-A--AA-G-C---G-TG--GG-G--AG-C-A-AA--------CA--GG Bacteria(100);Firmicutes(100);Clostridia(100);Clostridiales(100);Lachnospiraceae(100);unclassified(100);unclassified(100);unclassified(100);unclassified(100);

Otu0496 0 0 0 0 0 0 0 0 0 0 0 0 0 0 0 0 0 0 0 0 0 1 0 0 0 0 0 0
0 0 0 0 0 0 0 0 0 0 6 0 0 0 0 0 0 0 0 0 0 0 0 0 0 0 0 0 0
0 0 0 11 0 0 0 0 0 0 0 0 0 0 7 0 0 0 0 0 0 0 0 0 0 0 0 0 0
0 0 0 0 0 0 0 0 0 0 0 0 0 0 1 0 0 0 0 0 0 0 0 0 0 0 0
M02255_77_000000000-ADUT3_1_1119_2714_12249 T----AC---GA-AG-GGG--------GCT-A-G------C--G--T---T--GT-T-CGG-AA------TT-A--C-T--GG-GC-------GT--A-----AA-GC-GC-GC-------G-TA-G-G-C-G-------------G--T-GG-G-C-A-----------TA----G-T-C-A--------G-G-G--G--TG--A-AA-TC--C-C-GG-G-G-----CT-C-AA------C-C-T-C-G-G-A--A-T----T-G--C-C---T--T-------T--GA-T-A---C-------T--G-G--CA--C-G-C----T-T-G-A-A-T--C---C-GT-----GA-G-A--------G-G-G-T---GG-T------GG--A--ATG----G-C-C-A-GT--GT-A-G-AG-GT--G---A--A-A---TT-C-GT-AG--AT-A-TT--G-G--C-C-G-G-A-AC-A-CC---AG--T--G--GC-GAA-G--G-C--G-------G--C-C-A-C---CTG--G--CG-C-G---------------------G-T---A-T-T--GA--CG-C-T-G--A-GG--C-G-CG-A--AA-G-C---G-TG--GG-G--AG-C-A-AA--------CA--GG Bacteria(100);Proteobacteria(100);Alphaproteobacteria(100);Rhodospirillales(100);Rhodospirillaceae(100);Caenispirillum(100);unclassified(100);unclassified(100);unclassified(100);

Otu0497 0 0 0 0 0 0 0 0 0 0 0 0 0 0 0 0 0 0 0 0 0 0 0 0 0 0 0 0
0 0 4 0 0 0 0 0 0 0 21 0 0 0 0 0 0 0 0 0 110 0 0 0 0 0 0 0 0
0 0 0 0 0 0 0 0 0 0 0 0 0 0 0 0 0 0 0 0 0 0 0 0 0 0 0 0 0
0 0 0 0 0 0 0 0 0 0 0 0 0 0 0 0 0 0 0 0 0 0 0 0 0 0 0
M02255_76_000000000-ADUNE_1_2101_19631_14325 T----AC---GT-AG-GGG--------CCG-A-G------C--G--T---T--GT-C-CGG-AG------TT-A--T-T--GG-GC-------GT--A-----AA-GC-GC-GC-------G-CA---------------------------G-T---C------------A----G-C-C-C--------G-A-C--G--TG--A-AA-GC--C-C-CC-G-G-----CT-T-AA------C-C-G-G-G-G-A--G-G----G-T--C-G---T--G-------G--GG-G-A---C-------G--G-C--AC--C-G-C----T-T-G-A-G-G--G---C-GG-----GA-G-A--------G-G-T-C---GG-T------GG--A--ATT----C-C-C-G-GT--GT-A-G-TG-GT--G---A--A-A---TG-C-GT-AG--AG-A-TC--G-G--G-A-G-G-A-AC-A-CC---TG--T--G--GC-GAA-G--G-C--G-------G--C-C-G-A---CTG--G--GC-C-G---------------------A-C---C-C-T--GA--CG-C-T-G--A-GG--C-G-CG-A--AG-G-C---G-TG--GG-G--AG-C-G-AA--------CG--GG Bacteria(100);Chloroflexi(100);Thermomicrobia(100);JG30-KF-CM45(100);unclassified(100);unclassified(100);unclassified(100);unclassified(100);unclassified(100);

Otu0498 0 0 0 0 0 0 0 0 0 0 0 0 3 0 0 0 0 0 0 0 0 0 0 0 0 0 0 0
0 0 0 0 0 0 0 0 0 0 4 0 0 0 0 0 0 29 0 303 0 0 0 0 0 0 0 0 0
0 0 0 0 0 0 0 0 0 0 0 0 0 0 0 0 7 0 0 0 0 0 0 0 0 0 0 0 0
0 0 0 0 0 0 0 0 0 0 0 0 0 0 0 0 0 0 0 0 0 0 0 0 0 0 0
M02255_77_000000000-ADUT3_1_1108_25031_12624 T----AC---GG-AG-GAT--------CCG-A-G------C--G--T---T--AT-C-CGG-AT------TT-A--T-T--GG-G--------TT--------------GA--C--------------G-T------------------A--------T-----------AA----G-T-C-A--------G-C-T--G--TG--A-AA-GT--T-T-AC-G-G-----CT-C-AA------C-C-G-T-G-A-A--A-T----T-G--C-A---G--T-------T--GA-T-A---C-------T--G-T--AT--G-T-C----T-T-G-A-G-T--G---T-AC-----AA-G-A--------G-G-T-G---GG-C------GG--A--ATT----C-G-T-G-GT--GT-A-G-CG-GT--G---A--A-A---TG-C-TT-AG--AT-A-TC--A-C--G-A-A-G-A-AC-T-CC---AA--T--T--GC-GAA-G--G-C--A-------G--C-T-C-A---CTG--G--GG-T-A---------------------C-A---A-C-T--GA--CA-C-T-G--A-GG--C-T-CG-A--AA-G-T---G-TG--GG-T--AT-C-A-AA--------CA--GG Bacteria(100);Bacteroidetes(100);unclassified(72);unclassified(72);unclassified(72);unclassified(72);unclassified(72);unclassified(72);unclassified(72);

Otu0499 0 0 0 0 0 0 0 0 0 0 0 0 0 0 0 0 0 0 0 0 0 0 4 0 0 0 0 0
0 0 0 0 0 0 0 10 0 0 0 0 1 0 0 0 0 0 0 0 0 0 0 0 0 0 0 0 0
0 0 0 0 3 0 0 0 0 0 0 0 0 0 0 0 0 0 1 0 0 0 0 34 8 0 6 3 17
18 0 0 0 0 0 0 0 0 3 0 0 0 0 0 0 0 0 0 1 0 0 0 0 0 0 0
M02255_76_000000000-ADUNE_1_2106_27416_18708 T----AA---GC-AC-CG---------GCT-A-A------C-----T---T--CG-T--GC-CA------GC-A--G-C--CG-CG-------GT--A-----AT-AC-GA-AG-------G-GT-G-C-A-A-------------G-----C-G-T-T-----------AA----G-T-C-G--------G-A-A--G--TG--A-AA-GC--C-C-CG-G-G-----CT-C-AA------C-C-T-G-G-G-A--A-C----T-G--C-A---T--C-------C--AA-A-A---C-------T--G-G--CG--A-G-C----T-A-G-A-G-T--A---T-GG-----CA-G-A--------G-G-G-T---GG-T------GG--A--ATT----T-C-C-T-GT--GT-A-G-CG-GT--G---A--A-A---TG-C-GT-AG--AT-A-TA--G-G--A-A-G-G-A-AC-A-CC---AG--T--G--GC-GAA-G--G-C--G-------A--C-C-A-C---CTG--G--GC-T-A---------------------A-T---A-C-T--GA--CA-C-T-G--A-GG--T-G-CG-A--AA-G-C---G-TG--GG-G--AG-C-A-AA--------CA--GG Bacteria(100);Proteobacteria(100);Gammaproteobacteria(100);Pseudomonadales(100);Pseudomonadaceae(100);unclassified(100);unclassified(100);unclassified(100);unclassified(100);

Otu0500 0 0 0 0 0 0 0 0 0 0 0 0 0 0 0 0 0 0 0 0 0 0 0 0 0 0 0 0
0 0 0 0 0 0 0 0 0 0 0 0 0 0 0 0 0 0 0 0 0 0 0 28 0 0 0 0 0
0 0 0 0 0 0 0 0 0 0 0 0 0 0 0 0 0 0 0 0 0 0 0 0 0 0 0 0 0
0 0 0 0 0 0 0 0 0 0 0 0 0 0 0 0 0 0 0 0 0 0 0 0 0 0 0
M02255_77_000000000-ADUT3_1_1105_21363_15295 T----AC---GT-AG-GAT--------GCA-A-G------C--G--T---T--GT-C-CGG-AA------TT-A--T-T--GG-GC-------GT--A-----AA-GA-GC-GC-------G-CA-G-G-C-G------------G--T-CG-A-T-C-----
------AA----G-T-C-A--------G-A-T--G--TG--A-AA-TA--G-T-CC-G-G-----CT-T-AA------C-C-G-G-A-A-G--G------G-GT-C-A---T--T-------T--GA-A-A---C-------T--G-G--TC--G-A-C----T-A-G-A-G-T--A---C-AG-----CA-G-A--------G-G-A-T---AG-C------GG--A--ATT----
C-C-C-G-GT--GT-A-G-TG-GT--G---A--A-A---TG-C-GT-AG--AT-A-TC--G-G--G-A-G-G-A-AC-A-CC---AG--T--G--GC-GAA-G--G-C--G-------G--C-T-A-T---CTG--G--GC-T-G--------------------A-A---A-C-T--GA--CG-C-T-G--A-GG--C-G-CG-A--AG-G-C---T-AG--
GG-G--AG-C-A-AA--------CA--GG Bacteria(100);unclassified(100);unclassified(100);unclassified(100);unclassified(100);unclassified(100);unclassified(100);unclassified(100);unclassified(100);

Otu0501 0 0 0 0 0 0 0 0 0 0 0 0 0 0 0 0 0 0 0 0 0 0 0 0 0 0 0 0
0 0 0 0 0 0 0 0 0 0 0 3 0 0 0 0 0 0 0 0 0 0 0 0 0 0 0 0 0
0 0 0 0 0 6 0 0 0 0 0 0 0 0 0 0 0 0 0 59 0 0 0 0 0 0 0 0 0
0 0 0 0 44 0 0 0 0 17 0 0 0 0 1 0 0 0 0 0 0 0 0 0 0 0 0
M02255_76_000000000-ADUNE_1_1106_23666_12767 T----AC---GT-AG-GGA--------GCA-A-G------C--G--T---T--GT-C-CGG-AA------TC-A--C-T--GG-GC-------GT--A-----AA-GG-GT-TC-------G-CA-G-G-C--------------------AA-T-G-C-------
----AA----G-T-C-T--------C-G-T--G--TG--A-AA-GG--C-A-AG-G-G-----CT-C-AA------C-C-C-T-T-G-T--A------A-G--C-A---C--A-------A--GA-A-A---C-------T--G-C--AT--A-G-C----T-T-G-A-G-T--A---G-TG-----GA-G-A--------G-G-C-A---AG-T------GG--A--ATT----C-C-
T-A-GT--GT-A-G-CG-GT--G---A--A-A---TG-C-GT-AG--AT-A-TT--A-G--G-A-G-G-A-AT-A-CC---GG--T--G--GC-GAA-G--G-C--G-------A--C-T-T-G---CTG--G--AC-A-C--------------------A-A---A-C-T--GA--CG-C-T-G--A-GG--A-A-CG-A--AA-G-C---G-TG--GG-G--
AG-C-A-AA--------CA--GG Bacteria(100);Firmicutes(100);Clostridia(100);Clostridiales(100);unclassified(100);unclassified(100);unclassified(100);unclassified(100);unclassified(100);

Otu0502 0 0 0 0 0 0 0 0 0 0 0 0 0 0 0 0 0 0 0 0 0 0 0 0 0 0 0 0
0 0 0 0 0 0 0 0 0 0 0 0 0 0 0 0 0 0 0 0 0 0 0 0 0 0 0 0 0
27 0 0 101 4 19 0 20 0 5 0 4 9 0 15 9 0 0 0 0 0 0 0 0 0 0 0 0 0
0 0 0 0 0 0 0 0 0 0 0 0 0 0 0 0 0 0 0 0 0 0 0 0 0 0 0
M02255_77_000000000-ADUT3_1_2110_7383_14236 T----AC---GG-AG-GAT--------GCA-A-G------C--G--T---T--AT-C-CGG-AA------TG-A--T-T--GG-GC-------GT--A-----AA-GC-GT-CC-------G-CA-G-G-T-G------------G--C-TT-T-G-C-----------AA-
---G-T-C-T--------G-C-T--G--TC--A-AA-GC--T-C-AG-G-G-----CT-T-AA------C-C-C-T-G-A-A--T-C----G-G--C-G---G--T-------G--GA-A-A---C-------T--G-A--AA--A-G-C----T-A-G-A-G-T--G---C-GG-----TA-G-G--------G-G-T-A---GA-G------GG--A--ATT----C-C-C-A-
GT--GT-A-G-CG-GT--G---A--A-A---TG-C-GT-AG--AG-A-TT--G-G--G-A-A-G-A-AC-G-CC---AG--T--G--GC-GAA-A--G-C--G-------C--T-C-T-G---CTA--G--AC-C-G--------------------C-A---A-C-T--GA--CA-C-T-C--A-TG--G-A-CG-A--AA-G-C---T-AG--GG-G--AG-
C-G-AA--------TG--GG Bacteria(100);Cyanobacteria(100);unclassified(100);unclassified(100);unclassified(100);unclassified(100);unclassified(100);unclassified(100);unclassified(100);

Otu0503 0 0 0 0 0 0 0 0 0 0 0 0 0 0 0 0 0 0 0 0 0 0 0 0 0 0 0 0
0 0 0 0 0 0 0 0 0 0 47 0 0 0 0 0 0 0 0 0 0 0 0 0 0 0 0 0 0
0 0 0 0 0 0 0 0 0 0 0 0 0 0 0 0 0 0 0 0 0 0 0 0 0 0 0 0 0
0 0 0 0 0 0 0 0 0 0 0 0 0 0 0 0 0 0 0 0 0 0 0 0 0 0 0
M02255_77_000000000-ADUT3_1_1119_23444_24047 T----AC---GT-AG-GTG--------GCA-A-G------C--G--T---T--GT-C-CGG-AT------TT-A--T-T--GG-GC-------GT--A-----AA-GG-GC-GC-------G-CA-G-G-C-G------------G--T-CC-G-T-T-----
------AA----G-T-C-T--------G-A-G--G--TG--A-AA-TC--T-C-CT-G-G-----CT-C-AA------C-T-G-G-G-A-G--G------G-GT-C-T---T--C-------A--GA-T-A---C-------T--G-G--CG--G-A-C----T-T-G-A-G-G--A---G-TG-----CA-G-A--------G-G-T-G---AG-T------GG--A--ATT----
C-T-C-G-GT--GT-A-G-TG-GT--G---A--A-A---TG-C-GT-AG--AT-A-TC--G-A--G-A-G-G-A-AC-A-CC---AG--T--G--GC-GAA-G--G-C--G-------G--C-T-C-A---CTG--G--GC-A-C--------------------C-A---C-C-T--GA--CG-C-T-G--A-GG--C-G-CG-A--AA-G-C---G-TG--
GG-G--AG-C-G-AA--------CC--GG Bacteria(100);unclassified(100);unclassified(100);unclassified(100);unclassified(100);unclassified(100);unclassified(100);unclassified(100);unclassified(100);

Otu0504 0 0 0 0 0 0 0 0 0 0 0 0 0 0 0 0 0 0 0 0 0 0 0 0 0 0 0 0
0 0 0 0 0 0 0 0 0 0 0 0 0 0 0 0 0 0 0 0 0 0 0 0 0 0 0 0 0
0 0 0 0 0 0 2 0 0 0 0 0 0 0 79 158 0 0 0 0 0 0 0 0 0 0 0 52 0
0 0 0 0 0 0 0 0 0 0 0 0 0 0 0 0 0 0 0 0 0 0 0 0 0 0 0
M02255_77_000000000-ADUT3_1_2119_27577_10255 T----AC---GT-AG-GGG--------GCA-A-G------C--G--T---T--AT-C-CGG-AA------TT-A--T-T--GG-GC-------GT--A-----AA-GA-GT-GC-------G-TA-G-G-T-G------------G--T-CT-A-C-T-----
------AA----G-C-G-C--------G-A-G--G--TG--A-AA-GG--C-A-AT-G-G-----CT-C-AA------C-C-A-T-T-G-T--T------A-G--C-C---T--T-------G--CG-A-A---C-------T--G-G--CA--G-A-C----T-T-G-A-G-T--G---C-AA-----GA-G-A--------G-G-A-A---AG-C------GG--A--ATT----
C-C-T-A-GT--GT-A-G-CG-GT--G---A--A-A---TG-C-GC-AG--AT-A-TT--A-G--G-A-G-G-A-AC-A-CC---GG--C--G--GC-GAA-G--G-C--G-------G--C-T-T-T---CTG--G--AT-T-G--------------------T-A---A-C-T--GA--CA-C-T-G--A-GG--C-A-CG-A--AA-G-C---G-TG--GG-
T--AG-C-A-AA--------CA--GG Bacteria(100);Firmicutes(100);Clostridia(100);Clostridiales(100);Family_XIII_Incertae_Sedis(100);unclassified(100);unclassified(100);unclassified(100);unclassified(100);

Otu0505 0 0 0 0 0 0 0 0 0 0 0 0 0 0 0 0 0 0 0 0 0 0 0 0 0 0 0 0
0 0 0 0 0 0 0 0 0 0 0 0 0 0 101 0 0 0 0 0 0 0 7 0 0 0 0 0 0
0 0 0 0 0 0 0 0 0 0 0 0 0 0 28 89 0 0 0 0 0 0 0 0 0 0 0 0 38
0 0 0 0 0 0 0 0 0 0 0 0 0 0 0 0 0 0 0 0 0 0 0 0 0 0 0
M02255_77_000000000-ADUT3_1_1114_5334_14061 T----AC---GT-AG-GGG--------GCA-A-G------C--G--T---T--AT-C-CGG-AT------TT-A--C-T--GG-GT-------GT--A-----AA-GG-GA-GC-------G-TA-G-A-C-G-------------G--C-GA-T-G-T-----------AA----G-T-C-T--------G-A-A--G--TG--A-AA-GC--C-C-AC-G-G-----CT-C-AA------C-T-G-T-G-G-G--A-C----T-G--C-T---T--T-------G--GA-A-A---C-------T--A-T--GT--A-G-C----T-A-G-A-G-T--A---T-CG-----GA-G-G--------G-G-C-A---AG-C------GG--A--ATT----C-C-T-A-GT--GT-A-G-CG-GT--G---A--A-A---TG-C-GT-AG--AT-A-TT--A-G--G-A-G-G-A-AC-A-CC---GG--A--G--GC-GAA-G--G-C--G-------G--C-T-T-G---CTG--G--AC-G-A--------------------A-G---A-C-T--GA--CG-T-T-G--A-GG--C-T-CG-A--AG-G-C---G-TG--GG-G--AG-C-A-AA--------CA--GG Bacteria(100);Firmicutes(100);Clostridia(100);Clostridiales(100);Lachnospiraceae(100);unclassified(100);unclassified(100);unclassified(100);unclassified(100);

Otu0506 0 0 0 0 0 0 1 0 0 0 0 0 1 0 0 0 0 0 0 0 0 0 0 0 0 0 0 0
2 0 0 0 0 0 0 0 0 0 0 0 0 0 0 0 0 0 0 0 0 0 0 0 0 0 0 0 0
0 0 0 0 0 0 0 0 0 0 0 0 0 0 11 11 0 0 0 0 0 0 0 0 0 0 0 4 0
25 0 0 0 0 0 0 0 0 0 0 0 0 0 0 0 0 0 0 0 0 0 0 0 0 0 0
M02255_77_000000000-ADUT3_1_1112_6135_9646 T----AC---GT-AG-GGT--------GCG-A-G------C--G--T---T--GT-C-CGG-AT------TT-A--T-T--GG-GC-------GT--A-----AA-GA-GC-TT-------G-TA-G-G-C-G-------------G--T-TT-G-T-C-----------GC----G-T-C-G--------G-G-A--G--TG--A-AA-AC--T-T-GG-G-G-----CT-T-AA------C-C-C-T-G-A-G--C-T----T-G--C-T---T--T-------C--GA-T-A---C-------G--G-G--CT--G-A-C----T-T-G-A-G-G--G---A-GG-----TA-G-G--------G-G-A-G---AA-T------GG--A--ATT----C-C-T-G-GT--GG-A-G-CG-GT--G---G--A-A---TG-C-GC-AG--AT-A-TC--A-G--G-A-G-G-A-AC-A-CC---GG--T--G--GC-GAA-G--G-C--G-------G--T-T-C-T---CTG--G--AC-C-T--------------------T-T---C-C-T--GA--CG-C-T-G--A-GA--A-G-CG-A--AA-G-C---G-TG--GG-G--AG-C-G-AA--------CA--GG Bacteria(100);Actinobacteria(100);Actinobacteria(100);Actinobacteridae(100);Actinomycetales(100);Propionibacterineae(100);Propionibacteriaceae(100);unclassified(100);unclassified(100);

Otu0507 0 0 0 0 0 0 0 0 0 0 0 0 0 0 0 0 0 0 0 0 0 0 0 0 0 0 0 0
0 0 0 0 0 0 0 0 0 0 0 0 0 0 0 0 0 0 0 0 0 0 0 0 0 0 0 0 0
221 0 31 0 41 19 89 32 16 28 65 10 13 21 21 7 6 0 0 308 201 0 0 0 0 0 0 35 33
0 0 0 0 0 0 0 0 0 0 0 0 0 0 0 13 84 0 18 0 0 0 0 0 0 0 0
M02255_77_000000000-ADUT3_1_1105_22193_11153 G----AC---GT-AG-GAT--------GCA-A-G------C--G--T---T--GT-C-CGG-AT------TT-A--T-T--GG-GC-------GT--A-----AA-GA-GT-TC-------G-TA-G-G-T-G-------------G--T-TT-G-T-T-----------AA----G-T-T-T--------G-G-T--G--TT--A-AA-GA--T-T-GG-G-G-----CT-C-AA------C-C-C-T-G-A-A--A-C----T-G--C-A---C--T-------G--AA-T-A---C-------T--G-G--CA--G-A-C----T-C-G-A-G-T--G---T-GG-----TA-G-A--------G-G-C-T---AG-T------GG--A--ATT----C-C-C-A-GT--GT-A-G-CG-GT--G---A--A-A---TG-C-GT-AG--AT-A-TT--G-G--G-A-A-G-A-AC-A-CC---GG--T--G--GC-GTA-G--G-C--G-------A--C-T-A-G---CTG--G--GC-C-A--------------------T-A---A-C-T--GA--CA-C-T-G--A-GG--A-A-CG-A--AA-G-C---C-AG--GG-G--AG-C-G-AA--------TG--GG Bacteria(100);Cyanobacteria(100);MLE1-12(100);unclassified(100);unclassified(100);unclassified(100);unclassified(100);unclassified(100);unclassified(100);

Otu0508 0 0 0 0 0 0 0 0 0 0 0 0 0 0 0 0 0 0 0 0 0 0 0 0 0 0 0 0
0 0 0 0 0 0 0 0 0 0 0 0 0 0 0 0 0 0 0 0 0 0 0 0 0 0 0 0 0
0 0 0 0 9 0 0 0 4 0 0 0 0 0 0 0 0 0 0 0 0 0 0 0 0 0 0 0 0
0 0 0 0 0 0 0 0 0 0 0 0 0 0 0 0 0 0 0 0 0 0 0 0 0 0 0
M02255_77_000000000-ADUT3_1_1108_6665_16169 T----AC---GT-AG-GTG--------GCG-A-G------C--G--T---T--GT-C-CGG-AA------TT-A--T-T--GG-GC-------GT--A-----AA-GG-GT-GC-------G-TA-G-G-C-G-------------G--T-TT-T-T-T-----------AA----G-T-G-G--------G-A-T--G--TG--A-AA-TC--C-A-TG-G-G-----CT-T-AA------C-C-C-A-T-G-A--A-C----T-G--C-A---T--T-------C--CA-A-A---C-------T--G-G--AA--A-G-C----T-T-G-A-G-T--G---C-AG-----GA-G-A--------G-G-A-A---AG-C------GG--A--ATT----C-C-C-A-GT--GT-A-G-CG-GT--G---A--A-A---TG-C-GT-AG--AT-A-TT--G-G--G-A-G-G-A-AC-A-CC---AG--T--G--GC-GAA-G--G-C--G-------G--C-T-T-T---CTG--G--AC-T-G--------------------T-A---A-C-T--GA--CG-C-T-G--A-GG--C-A-CG-A--AA-G-C---G-TG--GG-G--AG-C-A-AA--------CA--GG Bacteria(100);unclassified(100);unclassified(100);unclassified(100);unclassified(100);unclassified(100);unclassified(100);unclassified(100);unclassified(100);

Otu0509 0 0 0 0 0 0 0 0 0 0 0 0 0 0 0 0 0 0 0 0 0 0 0 0 0 0 0 0
0 0 0 0 0 0 0 0 0 0 86 0 0 0 0 0 0 0 0 0 0 0 0 0 0 0 0 0 0
0 0 0 0 0 0 0 0 0 0 0 0 0 0 0 0 0 0 0 0 0 0 0 0 0 0 0 0 0
0 0 0 0 0 0 0 0 0 0 0 0 0 0 0 0 0 0 0 0 0 0 0 0 0 0 0
M02255_77_000000000-ADUT3_1_1108_9978_9218 T----AC---GA-AG-GGG--------GCT-A-G------C--G--T---T--GT-T-CGG-AA------TC-A--C-T--GG-GC-------GT--A-----AA-GC-GC-AC-------G-TA-G-G-C-G-------------G--A-TC-G-T-T-----------AA----G-T-C-G--------G-G-G--G--TG--A-AA-TC--C-C-AG-G-G-----CT-C-AA------C-C-C-T-G-G-A--A-C----T-G--C-C---T--T-------C--GA-T-A---C-------T--G-G--CG--ATC-T----T-C-G-A-G-T--C---C-GG-----GA-G-A--------G-G-T-G---AG-T------GG--A--ACT----C-C-G-A-GT--GT-A-G-AG-GT--G---A--A-A---TT-C-GT-AG--AT-A-TT--C-G--G-A-A-G-A-AC-A-CC---AG--T--G--GC-GAA-G--G-C--G-------G--C-T-C-A---CTG--G--CC-C-G--------------------G-T---A-C-T--GA--CG-C-T-G--A-GG--T-G-CG-A--AA-G-C---G-TG--GG-G--AG-C-A-AA--------CA--GG Bacteria(100);Proteobacteria(100);Alphaproteobacteria(100);Rhizobiales(100);unclassified(100);unclassified(100);unclassified(100);unclassified(100);unclassified(100);

Otu0510 0 0 0 0 0 0 0 0 0 0 0 0 0 0 0 0 0 0 0 0 0 0 0 0 0 0 0 0
0 0 0 0 0 0 0 0 0 0 0 0 0 0 18 0 0 0 0 0 0 0 0 0 0 0 0 0 0
0 0 0 0 0 0 0 0 4 0 0 0 0 0 0 0 0 0 0 0 0 0 0 0 0 0 0 0 0
0 0 0 0 0 0 0 0 0 0 0 0 0 0 0 0 0 0 0 0 0 0 0 0 0 0 0 0

M02255_77_000000000-ADUT3_1_2114_9484_17319 G----AC---GT-AG-GGT--------CCA-A-G------C--G--T---T--GT-C-CGG-AT------TT-A--C-T--GG-GC-------GT--A-----AA-GA-GC-GC-------G-TA-G-G-C-G------------G--C-TC-G-T-T-----------AA----G-T-G-T--------G-A-A--G--TG--A-AA-TC--C-C-CA-GGG-----CT-C-AA------C-C-C-G-G-A-A--A-C----T-G--C-T---T--T-------A--CA-T-A---C-------T--G-G--CG--A-G-C----T-G-G-A-G-T--G---A-TG-----AA-G-A--------G-G-T-T---TG-T------GG--A--ATT----C-C-T-G-GT--GT-A-G-CG-GT--G---A--A-A---TG-C-GT-TG--AT-A-TC--A-G--G-A-G-G-A-AC-A-CC---CA--T--G--GC-GAA-G--G-C--A-------G--C-A-A-A---CTG--G--TC-A-T--------------------C-A---A-C-T--GA--CG-C-T-G--A-GG--C-G-CG-A--AA-G-C---G-TG--GG-T--AG-C-A-AA--------CA--GG Bacteria(100);unclassified(100);unclassified(100);unclassified(100);unclassified(100);unclassified(100);unclassified(100);unclassified(100);unclassified(100);

Otu0511 0 0 0 0 0 0 0 0 0 0 0 0 0 0 0 0 0 0 0 0 0 0 0 0 0 0 0 0
0 0 0 0 0 0 0 0 0 0 0 0 0 0 0 0 0 0 0 0 0 0 0 0 0 0 0 17 0
0 0 0 0 0 0 0 0 16 0 0 0 0 0 0 0 0 0 0 0 0 0 0 0 0 0 0 0 0
0 0 0 0 0 1 0 0 0 0 0 0 0 0 0 0 0 0 0 0 0 0 0 0 0 0 0 0

M02255_76_000000000-ADUNE_1_2119_21695_14039 TC---GC---AG-AA-GAA--------GCA-C-C------G--G--C---T--AA---C-T-CC------GT-G--C-C--AG-CA-------GC--C-----GC-G--GT-AA-------TAC--G-G-A-G-------------G--T-TG-G-A-A-----------AA----G-T-T-A--------G-A-T--G--TG--A-AA-GC--C-C-CG-G-G-----CT-C-AA------C-C-T-G-G-G-A--A-T----T-G--C-A---T--T-------T--AA-A-A---C-------T--G-T--CC--A-G-C----T-A-G-A-G-T--C---T-TG-----TA-G-A--------G-G-G-G---GG-T------AG--A--ATT----C-C-A-G-GT--GT-A-G-CG-GT--G---A--A-A---TG-C-GT-AG--AG-A-TC--T-G--G-A-G-G-A-AT-A-CC---GG--T--G--GC-GAA-G--G-C--G-------G--C-C-C-C---CTG--G--AC-A-A--------------------A-G---A-C-T--GA--CG-C-T-C--A-GG--T-G-CG-A--AA-G-C---G-TG--GG-G--AG-C-A-AA--------CA--GG Bacteria(100);Proteobacteria(100);Gammaproteobacteria(100);unclassified(100);unclassified(100);unclassified(100);unclassified(100);unclassified(100);unclassified(100);

Otu0512 0 0 0 0 0 0 0 0 0 0 0 0 0 0 0 0 0 0 0 0 0 0 0 0 0 0 0 0
0 0 0 0 0 0 0 68 0 0 55 0 0 0 0 0 0 0 0 0 0 0 0 0 0 0 0 0 0
0 0 0 0 0 0 0 0 0 0 0 0 0 0 0 0 0 0 0 0 0 0 0 0 0 0 0 0 0
0 0 0 0 0 0 0 0 0 0 0 0 0 0 0 0 0 0 0 0 0 0 0 0 0 0 0 0

M02255_77_000000000-ADUT3_1_1102_9784_15449 T----AC---GG-AG-GGG--------GCT-A-G------C--G--T---T--GT-T-CGG-AA------TT-A--C-T--GG-GC-------GT--A-----AA-GC-GG-GT-------G-TA-G-G-C-G------------G--A-TC-T-C-T-----------AA----G-T-C-G--------G-G-G--G--TG--A-AA-GC--C-C-TG-G-G-----CT-C-AA------C-C-C-A-G-G-A--A-C----T-G--C-C---T--T-------C--GA-T-A---C-------T--G-G--AG--A-T-C----T-C-G-A-G-T--C---T-GG-----GA-G-A--------G-G-T-G---AG-C------GG--A--ATT----C-C-G-A-GT--GT-A-G-AG-GT--G---A--A-A---TT-C-GT-AG--AT-A-TT--C-G--G-A-G-G-A-AC-A-CC---AG--A--G--GC-GAA-G--G-C--G-------G--C-T-C-A---CTG--G--AC-C-A--------------------G-T---A-C-T--GA--CG-C-T-G--A-GA--C-C-CG-A--AA-G-C---G-TG--GG-G--AG-C-A-AA--------CA--GG Bacteria(100);Proteobacteria(100);unclassified(100);unclassified(100);unclassified(100);unclassified(100);unclassified(100);unclassified(100);unclassified(100);

Otu0513 0 0 19 0 0 0 43 3 0 0 0 0 0 0 0 0 0 0 0 0 0 0 0 0 0 0 0 0
0 0 0 0 0 0 0 0 0 0 0 0 0 0 0 0 0 0 0 0 0 0 0 0 0 0 0 0 0
0 0 0 0 0 0 0 0 0 0 0 0 0 0 0 0 0 0 0 0 0 0 0 0 0 0 0 0 0
0 0 0 0 0 21 0 0 0 0 0 0 0 0 0 0 0 0 0 0 40 0 0 0 0 0 54

M02255_77_000000000-ADUT3_1_1116_27511_16037 T----AC---GT-AG-GAT--------CCG-A-G------C--A--T---T--AT-C-CGG-AG------TG-A--C-T--GG-GT-------GT--A-----AA-GA-GTTGC-------G-TA-G-G-T-G------------G--C-AA-G-G-T-----------AA----G-T-A-G--------A-T-A--G--TG--A-AA-TC--T--GGT-G-G-----CT-C-AA------C-C-A-T-TCA-G--A-C----T-A--T-T---A--T-------C--TA-A-A---C-------T--A-T--CT--A-G-C----T-C-G-A-G-A--C---T-GT-----TA-T-G--------G-G-T-A---AC-T------GG--A--ATT----T-C-T-A-GT--GT-A-G-GA-GT--G---A--A-A---TC-C-GT-AG--AT-A-TT--A-G--A-A-G-GA--AC-A-CC---GA--T--A--GC-GTA-G--G-C--A-------G--G-T-T-A---CTG--G--GA-C-A--------------------G-T---T-C-T--GA--CA-C-T-A--A-GG--C-A-CG-A--AA-G-C---G-TA--GG-G--AG-C-A-AA--------CG--GG Bacteria(100);Candidate_division_TM7(100);unclassified(100);unclassified(100);unclassified(100);unclassified(100);unclassified(100);unclassified(100);unclassified(100);

Otu0514 0 0 0 0 0 0 0 0 0 0 0 0 0 0 0 0 0 0 0 0 0 0 0 0 0 0 0 0
0 0 0 0 0 0 0 0 0 0 0 0 0 0 0 0 0 0 0 0 110 0 0 0 0 0 0 0 119
0 0 0 0 0 0 0 0 0 0 0 0 0 0 0 0 0 0 0 0 0 0 0 0 0 0 0 0 0
0 0 0 0 0 30 0 0 0 0 0 0 3 0 0 0 0 0 0 0 0 0 0 0 0 0 0 0

M02255_77_000000000-ADUT3_1_1108_25182_17694 T----AC---GG-AG-GGG--------GCT-A-G------C--G--T---T--GT-T-CGG-AA------TT-A--C-T--GG-GC-------GT--A-----AA-GC-GC-AC-------G-TA-G-G-C-G------------G--A-TA-G-T-T-----------AA----G-T-C-G--------G-G-G--G--TG--A-AA-TC--C-C-GG-G-G-----CT-C-AA------C-C-C-C-G-G-A--A-C----T-G--C-C---T--T-------C--GA-T-A---C-------T--G-G--CT--G-T-C----T-A-G-A-G-A--T---C-GA-----GA-G-A--------G-G-T-G---AG-T------GG--A--ATT----C-C-G-A-GT--GT-A-G-AG-GT--G---A--A-A---TT-C-GT-AG--AT-A-TT--C-G--G-A-G-G-A-AC-A-CC---AG--T--G--GC-GAA-G--G-C--G-------G--C-T-C-A---CTG--G--CT-C-G--------------------A-T---A-C-T--GA--CG-C-T-G--A-GG--T-G-CG-A--AA-G-C---G-TG--GG-G--AG-C-A-AA--------CA--GG Bacteria(100);Proteobacteria(100);Alphaproteobacteria(100);Rhodobacterales(100);Rhodobacteraceae(100);unclassified(100);unclassified(100);unclassified(100);unclassified(100);

Otu0515 0 0 0 0 0 0 0 0 0 0 0 0 0 0 0 0 0 0 0 0 0 0 0 0 0 0 0 0
0 0 0 0 0 0 0 0 0 0 0 0 0 0 0 0 12 0 0 0 0 0 0 0 0 7 0 0 0
0 0 0 0 0 0 0 0 0 0 0 0 0 0 0 0 0 0 0 0 0 0 0 0 0 0 0 0 0
0 0 0 0 0 0 0 0 0 0 0 0 0 0 0 0 0 0 0 0 0 0 0 0 0 0 0
M02255_77_000000000-ADUT3_1_1111_4340_13965 T----AC---GG-AG-GGG--------GCT-A-G------C--G--T---T--GT-T-CGG-AA------TT-A--C-T--GG-GC-------GT--A-----AA-GC-GC-AC-------G-TA-G-G-C-G------------G--A-TA-G-T-T-----------
AA----G-T-T-A--------G-A-G--G--TG--A-AA-GC--C-C-AG-G-G-----CT-C-AA------C-C-C-T-G-G-A--A-C----T-G--C-C---T--T-------T--AA-T-A---C-------T--G-G--CT--G-T-C----T-A-G-A-G-A--T---C-GA-----GA-G-A--------G-G-T-G---AG-T------GG--A--ATT----C-C-G-
A-GT--GT-A-G-AG-GT--G---A--A-A---TT-C-GT-AG--AT-A-TT--C-G--G-A-G-G-A-AC-A-CC---AG--T--G--GC-GAA-G--G-C--G-------G--C-T-C-A---CTG--G--CT-C-G--------------------A-T---A-C-T--GA--CG-C-T-G--A-GG--T-G-CG-A--AA-G-C---G-TG--GG-G--
AG-C-A-AA--------CA--GG Bacteria(100);Proteobacteria(100);Alphaproteobacteria(100);unclassified(64);unclassified(64);unclassified(64);unclassified(64);unclassified(64);unclassified(64);

Otu0516 0 0 0 0 0 0 0 0 0 0 0 0 0 0 0 0 0 0 0 0 0 2 0 0 0 0 0 0
0 0 0 0 0 0 0 0 0 0 0 0 0 0 0 0 0 0 0 0 0 0 0 0 47 0 0 0
0 0 0 0 0 0 0 0 0 0 0 0 0 0 0 0 0 0 0 0 0 0 0 0 0 0 0 0 0
0 0 0 0 0 0 0 0 0 0 0 0 0 0 0 0 0 0 0 0 0 0 0 0 0 0 0
M02255_77_000000000-ADUT3_1_1118_4536_14012 T----AC---GA-AG-GGG--------GCT-A-G------C--G--T---T--GC-T-CGG-AA------TT-A--C-T--GG-GC-------GT--A-----AA-GC-GC-GC-------G-TA-G-G-C-G------------G--A-TC-T-T-T-----------AA-
---G-T-C-A--------G-G-G--G--TG--A-AA-TC--C-C-GG-G-G-----CT-C-AA------C-C-C-C-G-G-A--A-C----T-G--C-C---T--T-------T--GA-T-A---C-------T--G-G--GG--A-T-C----T-T-G-A-G-T--T---C-GA-----GA-G-A--------G-G-T-C---GG-T------GG--A--ACT----G-C-G-A-
GT--GT-A-G-AG-GT--G---A--A-A---TT-C-GT-AG--AT-A-TT--C-G--C-A-A-G-A-AC-A-CC---AG--T--G--GC-GAA-G--G-C--G-------G--C-C-G-A---CTG--G--CT-C-G--------------------A-C---A-C-T--GA--CG-C-T-G--A-GG--C-G-CG-A--AA-G-C---G-TG--GG-G--AG-
C-A-AA--------CA--GG Bacteria(100);Proteobacteria(100);Alphaproteobacteria(100);Rhizobiales(88);unclassified(88);unclassified(88);unclassified(88);unclassified(88);unclassified(88);

Otu0517 0 0 0 0 0 0 0 0 0 0 0 0 0 1 0 0 0 0 0 0 0 0 0 0 0 0 0 0
0 0 0 0 0 0 0 0 0 0 0 0 0 0 0 0 0 0 0 0 0 0 0 0 10 0 0 0
0 0 0 0 0 0 0 0 0 0 0 0 0 0 0 0 0 0 0 0 0 0 0 0 0 0 0 0 0
0 0 21 0 0 0 0 0 0 0 0 0 0 0 0 0 0 0 0 0 0 0 0 0 0 0 0
M02255_77_000000000-ADUT3_1_2114_21976_16697 T----AC---GT-AG-GGT--------GCGAG--------C--G--T---T--GT-C-CGG-AT------TT-A--T-T--GG-GC-------GT--A-----AA-GA-GC-TC-------G-TA-G-G-T-G------------G--T-TT-G-T-T------
-----GC----G-T-C-G--------G-G-A--G--TG--A-AA-AC--A-C-GG-G-G-----CT-T-AA------C-T-T-C-G-T-G--C-T----T-G--C-T---T--T-------C--GA-T-A---C-------G--G-G--CA--G-A-C----T-T-G-A-G-G--A---A-GG-----TA-G-G--------G-G-A-G---AA-C------GG--A--ATT----C-
C-T-G-GT--GG-A-G-CG-GT--G---G--A-A---TG-C-GC-AG--AT-A-TC--A-G--G-A-G-G-A-AC-A-CC---GG--T--G--GC-GAA-G--G-C--G-------G--T-T-C-T---CTG--G--AC-C-T--------------------T-T---C-C-T--GA--CG-C-T-G--A-GG--A-G-CG-A--AA-G-C---G-TG--GG-
G--AG-C-A-AA--------CA--GG Bacteria(100);Actinobacteria(100);Actinobacteria(100);Actinobacteridae(100);Actinomycetales(100);unclassified(100);unclassified(100);unclassified(100);unclassified(100);

Otu0518 0 100 0 0 0 0 77 5 31 0 0 0 0 0 0 0 0 0 0 0 0 0 0 0 0 0 0 0
0 0 0 0 0 0 0 0 0 0 0 22 0 0 0 0 0 0 0 0 0 0 0 0 0 0 0 0 0
0 0 0 0 0 0 0 0 0 0 0 0 0 0 0 0 0 0 0 0 0 0 0 0 0 0 0 0 0
0 0 0 0 0 0 0 0 0 0 0 0 0 0 0 0 0 0 0 0 0 0 0 1 11 0 0
M02255_77_000000000-ADUT3_1_2108_20327_11889 T----AC---GT-AG-GGG--------GCA-A-G------C--G--T---T--AT-C-CGG-AT------TT-A--C-T--GG-GT-------GT--A-----AA-GG-GA-GC-------G-TA-G-A-C-G------------G--A-AC-A-G-C----
-------AA----G-T-C-T--------G-A-T--G--TG--A-AA-AC--C-C-GG-G-G-----CT-C-AA------C-C-C-C-G-G-G--A-C----T-G--C-A---T--T-------G--GA-A-A---C-------T--G-T--TG--A-T-C----T-A-G-A-G-T--G---T-CG-----GA-G-A--------G-G-T-A---AG-T------GG--A--ATT----
C-C-T-A-GT--GT-A-G-CG-GT--G---A--A-A---TG-C-GT-AG--AT-A-TT--A-G--G-A-G-G-A-AC-A-CC---AG--T--G--GC-GAA-G--G-C--G-------G--C-T-T-A---CTG--G--AC-G-A--------------------T-G---A-C-T--GA--CG-T-T-G--A-GG--C-T-CG-A--AA-G-C---G-TG--GG-
G--AG-C-A-AA--------CA--GG Bacteria(100);Firmicutes(100);Clostridia(100);Clostridiales(100);Lachnospiraceae(100);unclassified(100);unclassified(100);unclassified(100);unclassified(100);

Otu0519 0 0 0 0 0 2 1 0 0 0 0 0 1 0 0 0 0 0 0 0 0 0 0 0 0 1 0 0
0 0 0 0 0 0 0 0 0 0 0 0 0 0 0 0 3 0 0 0 0 0 0 0 0 0 0 0 0
0 0 0 0 0 0 0 0 0 0 0 0 0 0 0 0 0 0 0 0 0 4 0 0 0 0 0 0 0
1 0 0 0 0 0 0 0 0 0 0 0 0 0 0 0 1 0 0 0 0 0 0 0 0 0 0
M02255_77_000000000-ADUT3_1_1113_16935_4619 C----TA---AC-TC-TGT--------GCC-A-G------C--A--G---C--CG-C-GGT-AA------TA-C--A-G--AG-GG-------GG--C-----AA-GC-GT-TA-------A-TC-G-GAATT------------A--C-TC-T-A-T-----------AA-
---G-T-C-A--------G-A-T--G--TG--A-AA-TC--C-C-CG-G-G-----CT-T-AA------C-C-T-G-G-G-A--A-C----T-G--C-A---T--C-------T--GA-A-A---C-------T--G-T--AG--A-G-C----T-A-G-A-G-T--A---T-GT-----GA-G-A--------G-G-A-A---GG-T------AG--A--ATT----C-C-A-G-
GT--GT-A-G-CG-GT--G---A--A-A---TG-C-GT-AG--AG-A-TC--T-G--G-A-G-G-A-AT-A-CC---GA--T--G--GC-GAA-G--G-C--A-------G--C-C-T-T---CTG--G--CA-T-A--------------------A-T---A-C-T--GA--CA-C-T-G--A-GG--C-T-CG-A--AA-G-C---G-TG--GG-T--AG-C-
A-AA--------CA--GG Bacteria(100);Proteobacteria(100);Gammaproteobacteria(100);Pseudomonadales(100);Moraxellaceae(100);Psychrobacter(65);unclassified(65);unclassified(65);unclassified(65);

Otu0520 0 0 0 0 0 0 49 0 226 0 0 0 0 0 0 0 0 0 0 0 0 0 0 0 0 0 0 0
0 0 0 1 0 0 0 0 0 0 0 0 0 0 0 0 0 0 0 0 0 0 0 0 0 0 0 0
0 0 0 0 0 0 0 0 0 0 0 0 0 0 0 0 0 0 0 0 0 0 0 0 0 0 0 0
0 0 0 0 0 0 0 0 0 0 0 0 0 0 0 0 0 0 0 0 0 0 0 0 0 0 0

M02255_77_000000000-ADUT3_1_1106_10451_19332 T----AC---GT-AG-GGG--------GCG-A-G------C--G--T---T--AT-C-CGG-AA------TT-A--C-T--GG-GT-------GT--A-----AA-GG-GT-GC-------G-TA-G-G-C-G------------G--T-AC-G-G-C----
-------AA----G-T-T-T--------G-A-T--G--TG--A-AA-CC--C-G-CG-G-G-----CT-T-AA------C-C-C-G-A-G-G--C-A----T-G--C-A---T--C-------G--AA-A-A---C-------T--A-C--TG--A-A-C----T-A-G-A-G-T--G---C-AG-----GA-G-A--------G-G-A-A---AG-C------GG--A--ATT----
C-C-T-A-GT--GT-A-G-CG-GT--G---A--A-A---TG-C-GT-AG--AT-A-TT--A-G--G-A-A-G-A-AC-A-CC---AG--T--G--GC-GAA-G--G-C--G-------G--C-T-T-T---CTG--G--AC-T-G--------------------C-A---A-C-T--GA--CG-C-T-G--A-GG--C-A-CG-A--AA-G-C---G-TG--GG-
G--AG-C-A-AA--------CA--GG Bacteria(100);unclassified(82);unclassified(82);unclassified(82);unclassified(82);unclassified(82);unclassified(82);unclassified(82);unclassified(82);

Otu0521 0 84 76 0 0 0 223 59 837 0 0 0 0 0 0 0 0 0 0 0 0 0 0 0 0 4 0 0
0 0 0 59 0 0 0 0 0 0 0 69 0 0 0 0 0 0 0 0 0 0 0 0 0 0 0 0
27 0 0 0 0 0 0 1 0 0 0 0 0 0 0 3 0 66 0 0 0 0 4 0 0 23 0 0 0
0 0 0 0 0 0 2 0 0 0 0 57 0 57 0 0 0 0 0 0 0 0 0 5 133 0 0

M02255_77_000000000-ADUT3_1_1111_15155_14051 T----AC---GT-AG-GGG--------GCG-A-G------C--G--T---T--AT-C-CGG-AT------TT-A--C-T--GG-GT-------GT--A-----AA-GG-GA-GT-------G-TA-G-A-C-G------------G--C-GT-A-T-C-----
------AA----G-T-C-T--------G-A-T--G--TG--A-AA-GG--C-A-GG-G-G-----CT-T-AA------C-C-C-C-T-G-G--A-C----T-G--C-A---T--T-------G--GA-A-A---C-------T--G-G--TA--T-G-C----T-T-G-A-G-T--G---C-CG-----GA-G-G--------G-G-T-A---AG-C------GG--A--ATT----
C-C-T-A-GT--GT-A-G-CG-GT--G---A--A-A---TG-C-GT-AG--AT-A-TT--A-G--G-A-G-G-A-AC-A-CC---AG--T--G--GC-GAA-G--G-C--G-------G--C-T-T-A---CTG--G--AC-G-G--------------------T-A---A-C-T--GA--CG-T-T-G--A-GG--C-T-CG-A--AA-G-C---G-TG--GG-
G--AG-C-A-AA--------CA--GG Bacteria(100);Firmicutes(100);Clostridia(100);Clostridiales(100);Lachnospiraceae(100);Blautia(99);unclassified(99);unclassified(99);unclassified(99);

Otu0522 0 0 0 314 0 0 0 0 0 0 0 0 0 0 0 0 0 0 0 0 0 0 0 0 0 0 0 0
0 0 0 0 0 0 0 0 0 0 0 0 0 0 0 0 0 0 0 0 0 0 0 0 0 0 19 0
0 0 0 0 0 0 0 0 0 0 0 0 0 0 0 0 0 0 0 0 0 0 0 0 0 0 0 0
0 0 0 0 0 0 0 0 0 0 0 0 0 0 0 0 0 0 0 0 0 0 0 0 0 0 0

M02255_77_000000000-ADUT3_1_1115_7678_20352 T----AC---GG-AG-GGG--------GTT-A-G------C--G--T---T--GT-T-CGG-AA------TG-A--C-T--GG-GC-------GT--A-----AA-GC-GC-GC-------G-TA-G-G-C-G------------G--A-TC-A-G-C-----------
AA----G-T-C-A--------G-G-G--G--TG--A-AA-TC--C-C-GG-G-G-----CT-C-AA------C-C-C-C-G-G-C--A-C----T-G--C-C---T--T-------T--GA-G-A---C-------T--G-T--TG--G-T-C----T-G-G-A-G-T--T---C-GA-----GA-G-A--------G-G-T-G---GG-T------GG--A--ATT----C-C-G-
A-GT--GT-A-G-AG-GT--G---A--A-A---TT-C-GT-AG--AT-A-TT--C-G--G-A-G-G-A-AC-A-CC---AG--T--G--GC-GAA-G--G-C--G-------G--C-C-C-A---CTG--G--CT-C-G--------------------A-C---A-C-T--GA--CG-C-T-G--A-GG--C-G-CG-A--AA-G-C---G-CG--GG-G--
AG-C-A-AA--------CA--GG Bacteria(100);Proteobacteria(100);Alphaproteobacteria(100);Rhodobacterales(95);Rhodobacteraceae(95);unclassified(95);unclassified(95);unclassified(95);unclassified(95);

Otu0523 0 0 0 0 0 0 16 1 40 0 0 0 0 0 0 0 0 0 0 0 0 0 7 0 0 0 0 0
0 0 0 0 0 0 0 0 0 0 0 41 0 0 0 0 0 0 0 0 0 0 0 0 0 0 0 0
0 0 0 0 0 0 0 0 0 0 0 0 0 0 0 6 0 23 0 0 0 0 0 0 0 0 0 0 203
0 0 0 0 0 0 0 0 0 0 0 46 0 0 0 0 0 0 0 0 0 0 0 15 153 0 0

M02255_77_000000000-ADUT3_1_1116_15727_17296 T----AC---GT-AT-GGT--------GCA-A-G------C--G--T---T--AT-C-CGG-AT------TT-A--C-T--GG-GT-------GT--A-----AA-GG-GA-GC-------G-TA-G-G-T-G------------G--C-AA-G-G-C-----
------AA----G-C-C-A--------G-A-A--G--TG--A-AA-AC--C-C-GG-G-G-----CT-C-AA------C-C-G-C-G-G-G--A-T----T-G--C-T---T--T-------T--GG-A-A---C-------T--G-C--CA--T-G-C----T-A-G-A-G-T--G---C-AG-----GA-G-G--------G-G-T-G---AG-C------GG--A--ATT----
C-C-T-A-GT--GT-A-G-CG-GT--G---A--A-A---TG-C-GT-AG--AT-A-TT--A-G--G-A-G-G-A-AC-A-CC---AG--A--G--GC-GAA-G--G-C--G-------G--C-T-C-A---CTG--G--AC-T-G--------------------T-A---A-C-T--GA--CA-C-T-G--A-GG--C-T-CG-A--AA-G-C---G-TG--GG-
G--AG-C-A-AA--------CA--GG Bacteria(100);Firmicutes(100);Clostridia(100);Clostridiales(100);Lachnospiraceae(100);unclassified(100);unclassified(100);unclassified(100);unclassified(100);

Otu0524 0 0 0 0 0 0 0 0 0 0 0 0 0 1 0 0 0 0 0 0 0 0 0 0 0 0 0 0
0 0 0 0 0 0 0 0 0 0 0 29 0 0 0 0 0 0 0 0 0 0 0 0 0 0 0 0
0 0 0 0 0 0 0 0 0 0 0 0 0 4 5 0 0 0 0 0 0 0 0 0 0 0 0 0
0 0 0 0 0 0 0 0 0 0 0 86 0 0 0 9 0 0 0 0 0 0 0 0 0 0 0

M02255_77_000000000-ADUT3_1_2110_19549_21157 T----AC---GT-AG-GGG--------GCA-A-G------C--G--T---T--AT-C-CGG-AA------TT-A--T-T--GG-GC-------GT--A-----AA-GA-GT-GC-------G-TA-G-G-T-G------------GT---TA-T-C-T------
-----AA----G-C-G-T--------A-A-G--G--TT--T-AA-GC--G-T-GC-A-G-----CT-C-AA------C-T-G-C-A-T-C--T------C-G--C-C---T--T-------G--CG-A-A---C-------T--G-G--AC--T-A-C----T-T-G-A-G-T--G---C-AG-----GA-G-G--------G-G-A-A---AG-C------GG--A--ATT----C-
C-T-A-GT--GT-A-G-CG-GT--G---A--A-A---TG-C-GT-AG--AT-A-TT--A-G--G-A-G-G-A-AC-G-CC---AG--C--G--GC-GAA-G--G-C--G-------G--C-T-T-T---CTG--G--AC-T-G--------------------T-A---A-C-T--GA--CA-C-T-G--A-GG--C-A-CG-A--AA-G-C---G-TG--GG-G-
-AG-C-A-AA--------CA--GG Bacteria(100);Firmicutes(100);Clostridia(100);Clostridiales(100);Family_XIII_Incertae_Sedis(100);unclassified(100);unclassified(100);unclassified(100);unclassified(100);

Otu0525 0 0 0 0 0 0 0 0 0 0 0 0 8 0 0 0 0 0 0 0 0 0 0 0 0 0 0 0
0 0 0 0 0 0 0 0 0 0 0 0 14 0 0 0 0 0 0 586 0 0 0 0 0 0 0 0 0
0 0 0 0 0 0 0 0 0 0 0 0 0 0 0 0 0 0 0 0 0 0 0 16 0 0 0 0 0
35 0 0 0 0 0 0 0 0 0 0 0 0 0 0 0 0 0 0 0 0 0 0 0 0 0 0
M02255_77_000000000-ADUT3_1_1106_19317_18027 T----AC---GT-AG-GTG--------GCG-A-G------C--G--T---T--GT-C-CGG-AA------TT-A--T-T--GG-GC-------GT--A-----AA-GG-AT-GC-------G-TA-G-G-T-G------------G--A-TA-T-T-T------
-----AA----G-T-G-G--------G-A-T--G--TG--A-AA-TC--C-C-CG-G-G-----CT-C-AA------C-C-C-G-G-G-A--A-C----T-G--C-A---T--T-------C--CA-T-A---C-------T--G-G--AT--G-T-C----T-A-G-A-G-T--G---C-AG-----GA-G-A--------G-G-A-A---AG--C-----GG--A--ATT----C-
C-T-A-GT--GT-A-G-CG-GT--G---A--A-A---TG-C-GT-AG--AT-A-TT--A-G--G-A-G-G-A-AC-A-CC---AG--T--G--GC-GAA-G--G-C--G-------G--C-T-T-T---CTG--G--AC-T-G--------------------T-A---A-C-T--GA--CA-C-T-G--A-GG--C-A-TG-A--AA-G-C---G-TG--GG-T-
-AG-C-A-AA--------CA--GG Bacteria(100);Firmicutes(100);Clostridia(100);Clostridiales(100);Clostridiaceae(98);Clostridium(98);unclassified(98);unclassified(98);unclassified(98);

Otu0526 0 0 0 0 0 0 0 0 0 0 0 0 0 0 0 0 0 0 0 0 0 0 0 0 0 0 0 0
0 0 0 0 0 0 0 0 0 17 0 0 0 0 0 0 0 0 0 0 0 0 0 0 12 0 0 0 0
0 0 0 0 0 0 0 0 0 0 0 0 0 0 0 0 0 0 0 0 0 0 0 0 0 0 0 0 0
0 0 0 0 0 0 0 0 0 0 0 0 0 0 0 0 0 0 0 0 0 0 0 0 0 0 0
M02255_77_000000000-ADUT3_1_2102_20632_17000 T----AC---GA-AG-GGG--------GCT-A-G------C--G--T---T--GC-T-CGG-AA------TC-A--C-T--GG-GC-------GT--A-----AA-GG-GC-GC-------G-TA-G-G-C-G------------G--C-GG-A-C-T----
-------TA----G-T-C-G--------G-G-C--G--TG--A-AA-TC--C-C-CG-G-G-----CT-C-AA------C-C-C-G-G-G-G--G-C----T-G--C-G---C--C-------C--GA-T-A---C-------G--G-G--TC--C-G-C----T-G-G-A-G-G--A---C-GG-----GA-G-A--------G-G-C-T---CG-C------GG--A--ATT----
C-C-C-A-GT--GT-A-G-AG-GT--G---A--A-A---TG-C-GT-AG--AT-A-TT--G-G--G-A-A-G-A-AC-A-CC---GG--T--G--GC-GAA-G--G-C--G-------G--C-G-A-G---CTG--G--CC-C-G--------------------T-T---T-C-T--GA--CG-C-T-G--A-GG--C-G-CG-A--CA-G-C---G-TG--
GG-G--AG-C-A-AA--------CA--GG Bacteria(100);Proteobacteria(100);Alphaproteobacteria(100);Rhodospirillales(100);unclassified(100);unclassified(100);unclassified(100);unclassified(100);unclassified(100);

Otu0527 0 0 0 37 0 0 0 0 0 0 0 0 0 0 0 0 0 0 0 0 0 0 0 0 0 0 0 0
0 0 0 0 0 0 0 0 0 0 0 0 0 0 0 0 0 0 0 0 0 0 0 0 0 0 0 0 62
0 0 0 0 0 0 0 0 0 0 0 0 0 0 0 0 0 0 0 0 0 0 0 0 0 0 0 0 0
0 0 0 0 0 0 0 0 0 0 0 0 0 0 0 0 0 0 0 0 0 0 0 0 0 0 0
M02255_77_000000000-ADUT3_1_1106_9021_22356 T----AC---GT-AG-GGT--------GCG-A-G------C--G--T---T--GT-C-CGG-AA------TT-A--T-T--GG-GC-------GT--A-----AA-GG-GC-TT-------G-TA-G-G-C-G------------G--T-TC-G-T-C----------AC-
---G-T-C-G--------A-A-A--G--TG--A-AA-AC--T-C-AG-G-G-----CT-T-AA------C-C-C-T-G-A-G--C-C----T-G--C-T---T--T-------C--GA-T-A---C-------G--G-G--TG--A-A-C----T-A-G-A-G-G--A---A-TG-----CA-G-G--------G-G-A-G---AA-C------GG--A--ATT----C-C-T-G-
GT--GG-A-G-CG-GT--G---G--A-A---TG-C-GC-AG--AT-A-TC--A-G--G-A-G-G-A-AC-A-CC---GG--T--G--GC-GAA-G--G-C--G-------G--T-T-C-T---CTG--G--GC-A-T--------------------T-A---C-C-T--GA--CG-C-T-G--A-GA--A-G-CG-A--AA-G-C---G-TG--GG-G--AG-
C-A-AA--------CA--GG Bacteria(100);Actinobacteria(100);Actinobacteria(100);Actinobacteridae(100);Actinomycetales(100);Propionibacterineae(100);Propionibacteriaceae(100);unclassified(100);unclassified(100);

Otu0528 123 37 11 28 74 12 37 8 18 10 0 3 0 0 0 0 0 0 0 0 16 4 0 0 0 0 3 5
0 0 0 25 18 23 15 64 60 49 47 5 8 26 25 54 97 0 0 0 14 0 0 18 2 0 0 5 0
0 36 0 1 0 0 1 0 0 0 0 0 0 1 5 4 16 8 0 2 0 0 0 4 8 0 0 0 0
0 0 0 0 2 3 0 0 0 6 0 4 0 16 0 5 0 0 4 71 30 66 19 2 17 10 0
M02255_77_000000000-ADUT3_1_1112_14569_18307 T----AC---GT-AG-GGT--------GCG-A-G------C--G--T---T--GT-C-CGG-AT------TT-A--T-T--GG-GC-------GT--A-----AA-GG-GC-TC-------G-TA-G-G-C-G------------G--T-TG-A-T-C------
-----GC----G-T-C-G--------G-A-A--G--TG--G-AA-AC--T-C-GA-T-G-----CT-T-AA------C-G-T-T-G-A-G--C-G----T-G--C-T---T--T-------C--GA-T-A---C-------G--G-G--TT--G-A-C----T-T-G-A-G-G--A---A-GG-----TA-G-G--------G-G-A-G---AA-T------GG--A--ATT----C-
C-T-G-GT--GG-A-G-CG-GT--G---G--A-A---TG-C-GC-AG--AT-A-TC--A-G--G-A-G-G-A-AC-A-CC---AG--T--G--GC-GAA-G--G-C--G-------G--T-T-C-T---CTG--G--AC-C-T--------------------T-T---C-C-T--GA--CG-C-T-G--A-GG--A-G-CG-A--AA-G-C---G-TG--GG-
G--AG-C-G-AA--------CA--GG Bacteria(100);Actinobacteria(100);Actinobacteria(100);Actinobacteridae(100);Actinomycetales(100);Propionibacterineae(100);Propionibacteriaceae(100);Propionibacterium(100);unclassified(100);

Otu0529 233 0 0 0 0 0 0 0 0 0 0 0 0 0 0 0 0 0 0 0 0 0 0 0 0 0 0 0
0 0 0 0 0 0 0 0 0 0 0 0 0 0 0 0 0 0 0 0 0 0 0 0 0 15 0 0 0
0 0 0 0 0 0 0 0 0 0 0 0 0 0 0 0 0 0 0 0 0 0 0 0 0 0 0 0 0
0 0 0 0 0 0 0 0 0 0 0 0 0 0 0 0 0 0 0 0 0 0 0 0 0 0 0
M02255_77_000000000-ADUT3_1_1105_14410_17807 T----AC---GG-AG-GGG--------GCT-A-G------C--G--T---T--GT-T-CGG-AA------TT-A--C-T--GG-GC-------GT--A-----AA-GC-GC-AC-------G-TA-G-G-C-G------------G--C-TT-T-G-T-----
------AA----G-T-T-A--------G-A-G--G--TG--A-AA-GC--C-T-GG-A-G-----CT-C-AA------C-T-C-C-A-G-A--A-T----T-G--C-C---T--T-------T--GA-T-A---C-------T--G-C--AT--G-G-C----T-T-G-A-A-T--C---C-GG-----GA-G-A--------G-G-T-G---AG-T------GG--A--ATT----C-
C-G-A-GT--GT-A-G-AG-GT--G---A--A-A---TT-C-GT-AG--AT-A-TT--C-G--G-A-A-G-A-AC-A-CC---AG--T--G--GC-GAA-G--G-C--G-------G--C-T-C-A---CTG--G--AC-C-G--------------------G-C---A-T-T--GA--CG-C-T-G--A-GG--T-G-CG-A--AA-G-C---G-TG--GG-
G--AG-C-A-AA--------CA--GG Bacteria(100);Proteobacteria(100);Alphaproteobacteria(100);Sphingomonadales(100);Sphingomonadaceae(94);unclassified(94);unclassified(94);unclassified(94);unclassified(94);

Otu0530 0 0 0 0 0 0 0 0 0 0 0 0 0 0 0 0 0 0 0 0 0 0 0 0 0 0 0 0
0 0 0 0 0 0 0 0 0 0 0 0 0 0 0 90 0 0 0 0 0 0 0 0 0 0 0 0 0
26 136 0 0 0 0 0 0 1 0 0 0 0 0 0 0 0 0 0 0 0 0 0 0 0 0 0 0 0
0 0 0 0 0 0 0 0 0 0 0 0 0 0 0 0 0 0 0 0 0 0 0 0 0 0 0 0
M02255_77_000000000-ADUT3_1_2116_10727_22179 T----AC---GG-AG-GGT--------GCA-A-G------C--G--T---T--AC-T-CGG-AA------TC-A--C-T--GG-GC-------GT--A-----AA-GG-AC-GC-------G-TA-G-G-C-G------------G--A-TT-A-T-C-----
------AA----G-T-C-T--------C-T-T--G--TG--A-AA-TC--T-A-GT-G-G-----CT-T-AA------C-C-A-C-T-A-A--A-C----T-G--C-T---T--G-------G--GA-A-A---C-------T--G-A--TA--A-T-C----T-A-G-A-G-T--A---A-GG-----GA-G-A--------G-G-C-A---GA-T------GG--A--ATT----C-
T-T-G-GT--GT-A-G-GG-GT--A---A--A-A---TC-C-GT-AG--AG-A-TC--A-A--G-A-A-G-A-AT-A-CT---TA--T--T--GC-GAA-G--G-C--G-------A--T-C-T-G---CTA--G--AA-C-T--------------------T-A---A-C-T--GA--CG-C-T-A--A-TG--C-G-TG-A--AA-G-C---G-TG--GG-G--
AG-C-A-AA--------CA--GG Bacteria(100);Proteobacteria(100);Epsilonproteobacteria(100);Campylobacterales(100);Campylobacteraceae(100);Campylobacter(100);unclassified(100);unclassified(100);unclassified(100);

Otu0531 0 0 0 0 0 0 0 0 0 0 0 0 0 0 0 0 0 0 0 0 0 0 0 0 0 0 0 0
0 0 0 0 0 0 0 0 0 0 0 0 0 0 0 10 0 0 0 0 0 0 0 0 0 0 0 0 0
0 38 0 0 0 0 0 0 0 0 0 0 0 0 0 0 0 0 0 0 0 0 0 0 0 0 0 0 0
0 0 0 0 0 0 0 0 0 0 0 0 0 0 0 0 0 0 0 0 0 0 0 0 0 0 0 0
M02255_77_000000000-ADUT3_1_1102_12426_18927 T----AC---GT-AG-GAT--------GCA-A-G------C--G--T---T--AT-C-CGG-AT------TT-A--C-T--GG-GT-------GT--A-----AA-GG-GT-GC-------G-TA-G-G-C-G------------G--A-CT-T-T-C------
-----AA----G-T-T-G--------G-A-T--G--TG--A-AA-AC--C-C-GG-G-G-----CT-T-AA------C-C-C-C-G-T-G--A-T----T-G--C-A---T--C-------C--AA-A-A---C-------T--G-T--AG--G-T-C----T-T-G-A-T-T--G---G-AG-----TA-G-A--------G-G-T-A---AG-T------GG--A--ATT----C-
C-G-A-GT--GT-A-G-CG-GT--G---A--A-A---TG-C-GC-AG--AT-A-TT--C-G--G-A-A-G-A-AC-A-CC---GG--T--T--GC-G-AAG--G-C--G-------A--C-T-T-A---CTG--G--GC-T-C--------------------T-A---A-A-T--GA--CG-C-T-G--A-GG--C-A-CG-A--AA-G-T---G-TG--GG-G-
-AG-C-A-AA--------CA--GG Bacteria(100);unclassified(100);unclassified(100);unclassified(100);unclassified(100);unclassified(100);unclassified(100);unclassified(100);unclassified(100);

Otu0532 0 0 0 0 0 0 0 0 0 0 0 0 0 0 0 0 0 0 0 0 0 0 0 0 0 0 0 0
0 0 0 0 0 0 0 0 0 0 0 0 0 0 0 0 0 0 0 0 0 0 0 0 0 0 0 0 0
0 74 0 0 0 0 0 0 0 0 0 0 0 0 0 0 0 0 0 0 0 0 0 0 0 0 0 0 0
0 0 0 0 0 0 0 0 0 0 0 0 0 0 0 0 0 0 0 0 0 0 0 0 0 0 0 0
M02255_77_000000000-ADUT3_1_1107_21841_17750 T----AC---GT-AT-GGG--------GCG-A-G------C--G--T---T--AT-C-CGG-AA------TT-A--T-T--GG-GC-------GT--A-----AA-GA-GT-AC-------G-TA-G-G-T-G------------G--T-TT-C-T-T------
-----AA----G-C-G-C--------A-G-G--G--TT--T-AA-GG--C-T-AC-G-G-----CT-T-AA------C-C-G-T-A-G-T--T------T-G--C-C---T--T-------G--TG-A-A---C-------T--G-G--GA--G-A-C----T-T-G-A-G-T--G---C-AG-----GA-G-A--------G-G-A-A---AG-C------GG--A--ATT----C-
C-T-A-GT--GT-A-G-CG-GT--G---A--A-A---TG-C-GT-AG--AT-A-TT--A-G--G-A-G-G-A-AC-A-CC---GG--T--G--GC-GAA-G--G-C--G-------G--C-T-T-T---CTG--G--AC-T-G--------------------T-A---A-C-T--GA--CA-C-T-G--A-GG--T-A-CG-A--AA-G-C---G-TG--GG-G-
-AG-C-A-AA--------CA--GG Bacteria(100);Firmicutes(100);Clostridia(100);Clostridiales(100);unclassified(100);unclassified(100);unclassified(100);unclassified(100);unclassified(100);

Otu0533 0 0 34 0 0 0 0 0 51 0 0 0 0 0 0 0 0 0 0 0 0 0 0 0 0 0 0 0
0 0 0 1 0 0 0 0 0 0 0 27 0 0 0 0 0 0 0 0 0 0 0 0 0 0 0 0 0
0 14 0 0 0 0 0 0 0 0 0 0 0 0 0 0 0 0 0 0 0 0 0 0 0 0 0 0 58
0 0 0 0 0 0 0 0 0 0 0 0 0 0 0 0 0 0 0 0 0 0 0 0 7 0 0
M02255_77_000000000-ADUT3_1_1111_28683_16096 T----AC---GT-AG-GTG--------GCA-A-G------C--G--T---T--GT-C-CGG-AA------TT-A--C-T--GG-GT-------GT--A-----AA-GG-GA-GC-------G-TA-G-G-C-G------------G--G-GA-G-G-C---
--------AA----G-T-T-G--------A-A-T--G--TC--T-AA-AC--T-A-TC-G-G-----CT-C-AA------C-T-G-A-T-A-G----T----C-G--C-G---T--T-------C--AA-A-A---C-------T--G-C--CA--C-T-C----T-T-G-A-G-T--G---C-AG-----TA-G-A--------G-G-T-A---GG-C------GG--A--ATT----C-
C-T-A-GT--GT-A-G-CG-GT--G---A--A-A---TG-C-GT-AG--AT-A-TT--A-G--G-A-G-G-A-AC-A-CC---AG--T--G--GC-GAA-G--G-C--G-------G--C-C-T-A---CTG--G--GC-T-G--------------------T-A---A-C-T--GA--CG-C-T-G--A-GG--C-T-CG-A--AA-G-C---G-TG--GG-T-
-AG-C-A-AA--------CA--GG Bacteria(100);Firmicutes(100);Clostridia(100);Clostridiales(100);unclassified(63);unclassified(63);unclassified(63);unclassified(63);unclassified(63);

Otu0534 0 0 0 0 0 0 0 0 0 0 0 0 0 0 0 0 0 0 0 0 0 0 0 0 0 1 0 0
0 0 0 0 0 0 0 0 17 0 0 0 0 0 0 0 0 0 0 0 0 0 0 0 0 0 0 0 0
0 20 0 0 0 0 0 0 0 0 0 0 0 0 20 0 0 0 0 0 0 0 0 0 0 0 0 0 0
0 0 0 0 0 0 0 19 0 0 0 0 0 0 0 0 0 0 0 0 0 0 0 0 0 0 0 0
M02255_77_000000000-ADUT3_1_1117_6754_11993 T----AC---GT-AG-GGA--------GCA-A-G------C--G--T---T--AT-C-CGG-AT------TT-A--C-T--GG-GT-------GT--A-----AA-GG-GC-GT-------G-TA-G-G-C-G------------G--G-AG-T-G-C----------
AA----G-T-C-A--------G-A-T--G--TG--A-AA-AC--T-A-TG-G-G-----CT-C-AA------C-C-C-A-T-A-G--C-C----T-G--C-A---T--T-------T--GA-A-A---C-------T--G-T--AC--T-T-C----T-T-G-A-G-T--A---C-TG-----GA-G-A--------G-G-C-A---GG-C------GG--A--ATT----C-C-G-
T-GT--GT-A-G-CG-GT--G---A--A-A---TG-C-GT-AG--AT-A-TA--C-G--G-A-G-G-A-AC-A-CC---AG--T--G--GC-GAA-G--G-C--G-------G--C-C-T-G---CTG--G--AC-A-G--------------------C-A---A-C-T--GA--CG-C-T-G--A-GG--C-G-CG-A--AA-G-C---G-TG--GG-G--
AG-C-A-AA--------CA--GG Bacteria(100);Firmicutes(100);Clostridia(100);Clostridiales(100);Ruminococcaceae(100);Incertae_Sedis(100);unclassified(100);unclassified(100);unclassified(100);

Otu0535 0 0 0 0 0 0 0 0 0 0 0 0 0 0 0 0 0 0 0 0 0 0 0 0 0 0 0 0
2 0 0 0 0 1 0 0 0 0 0 0 0 27 20 0 0 0 0 0 0 0 0 0 8 0 0 0 0
0 0 0 0 0 0 0 0 0 0 0 0 0 0 0 0 0 0 0 0 0 0 0 0 0 0 0 0 0
0 0 0 0 0 0 0 0 0 0 0 0 0 0 0 0 0 0 0 0 0 0 0 0 0 0 0
M02255_77_000000000-ADUT3_1_1103_6810_21124 T----AC---GT-AG-GGG--------GCA-A-G------C--G--T---T--AT-C-CGG-AT------TT-A--C-T--GG-GT-------GT--A-----AA-GG-GA-GC-------G-TA-G-A-C-G------------G--A-CG-G-G-C----------
AA----G-T-C-T--------G-A-A--G--TG--A-AA-GG--C-A-GG-G-G-----CT-C-AA------C-C-C-C-T-G-G--A-C----T-G--C-T---T--T-------G--GA-A-A---C-------T--G-T--CC--A-T-C----T-A-G-A-G-T--G---C-CG-----GA-G-A--------G-G-T-A---AG-C------GG--A--ATT----C-C-T-
A-GT--GT-A-G-CG-GT--G---A--A-A---TG-C-GT-AG--AT-A-TT--A-G--G-A-G-G-A-AC-A-CC---AG--T--G--GC-GAA-G--G-C--G-------G--C-T-T-A---CTG--G--AC-G-G--------------------T-A---A-C-T--GA--AG-T-T-G--A-GG--C-T-CG-A--AA-G-C---G-TG--GG-G--
AG-C-A-AA--------CA--GG Bacteria(100);Firmicutes(100);Clostridia(100);Clostridiales(100);Lachnospiraceae(100);unclassified(100);unclassified(100);unclassified(100);unclassified(100);

Otu0536 0 0 0 0 0 0 0 0 0 0 0 0 0 0 0 0 0 0 0 0 0 0 0 0 0 0 0 0
0 0 0 0 0 0 0 0 0 0 38 0 0 0 0 0 0 0 0 0 0 0 0 0 0 0 0 0 0
0 19 0 0 0 0 0 0 0 0 0 0 0 0 0 0 0 0 0 0 0 0 0 0 0 0 0 0 0
0 0 0 0 0 0 0 0 0 0 0 0 0 0 0 0 0 0 0 0 0 0 0 0 0 0 0
M02255_77_000000000-ADUT3_1_2112_16879_10346 T----AC---GG-AG-GGG--------GCT-A-G------C--G--T---T--GT-T-CGG-AA------TT-A--C-T--GG-GC-------GT--A-----AA-GC-GC-AC-------G-TA-G-G-C-G------------G--C-TT-G-A-A-----
------AA----G-T-T-G--------G-G-G--G--TG--A-AA-GC--C-C-GG-A-G-----CT-C-AA------C-T-C-C-G-G-A--A-T----T-G--C-C---T--T-------C--AA-A-A---C-------T--C-T--CA--A-G-C----T-G-G-A-G-T--T---C-GG-----AA-G-A--------G-G-A-G---AG-T------GG--A--ATT----
C-C-C-A-GT--GT-A-G-AG-GT--G---A--A-A---TT-C-GT-AG--AT-A-TT--G-G--G-A-A-G-A-AC-A-CC---AG--T--G--GC-GAA-G--G-C--G-------G--C-T-C-T---CTG--G--TC-C-G--------------------A-T---A-C-T--GA--CG-C-T-G--A-GG--T-G-CG-A--AA-G-C---G-TG--GG-
G--AG-C-A-AA--------CA--GG Bacteria(100);Proteobacteria(100);Alphaproteobacteria(100);unclassified(100);unclassified(100);unclassified(100);unclassified(100);unclassified(100);unclassified(100);

Otu0537 0 0 0 0 0 0 0 0 0 0 0 0 0 0 0 0 0 0 0 0 0 0 0 0 0 0 0 0
0 0 0 0 0 0 0 0 0 0 0 24 0 0 0 0 0 0 0 0 0 0 0 0 17 0 0 0 0
0 0 0 0 0 0 0 0 0 0 0 0 0 0 0 0 0 0 0 0 0 0 0 0 0 0 0 0 0
0 0 0 0 0 0 0 0 0 0 0 0 0 0 0 0 0 0 0 0 0 0 0 0 0 0 0
M02255_77_000000000-ADUT3_1_1103_18659_19897 T----AC---GT-AG-GAC--------CCA-A-G------C--G--T---T--AT-C-CGG-AG------TG-A--C-T--GG-GC-------GT--A-----AA-GA-GTTGC-------G-TA-G-G-C-G------------G--C-TT-A-A-T-----
------AA----G-T-G-G--------A-T-A--G--TG--A-AA-CC--T-G-GT-G-G-----CT-C-AA------C-C-A-TAC-A-G--A-C----T-A--T-T---A--T-------C--CA-A-A---C-------T--G-T--TA--A-G-C----T-C-G-A-G-A--A---T-GG-----TA-G-A--------G-G-T-G---GC-T------GG--A--ATT----T-
C-T-A-GT--GT-A-G-GA-GT--G---A--A-A---TC-C-GT-AG--AT-A-TT--A-G--A-A-G-G-A-AC-A-CC---GA--T--G--GC-GTA-G--G-C--A-------G--G-C-C-A---CTG--G--AC-C-A--------------------T-T---T-C-T--GA--CG-C-T-A--A-GG--C-A-CG-A--AA-G-C---G-TG--GG-G-
-AG-C-G-AA--------CC--GG Bacteria(100);Candidate_division_TM7(100);unclassified(100);unclassified(100);unclassified(100);unclassified(100);unclassified(100);unclassified(100);unclassified(100);

Otu0538 0 94 0 0 0 0 32 1 0 0 0 0 0 0 0 0 0 0 0 0 0 0 0 0 0 0 0 0
0 0 0 0 0 0 0 0 0 0 0 81 0 0 0 0 0 0 0 0 0 0 0 0 0 0 0 0 0
0 0 0 0 0 0 0 0 0 0 0 0 0 0 0 0 0 0 0 0 0 0 0 0 0 0 0 0 0
0 0 0 0 0 0 0 0 0 0 0 0 0 0 0 0 0 0 0 0 0 0 0 0 0 0 0
M02255_77_000000000-ADUT3_1_1104_25878_15560 T----AC---GT-AT-GGT--------GCA-A-G------C--G--T---T--AT-C-CGG-AT------TT-A--C-T--GG-GT-------GT--A-----AA-GG-GA-GC-------G-CA-G-G-C-G------------G--T-TT-G-G-C-----
------AA----G-T-C-T--------G-A-T--G--TG--A-AA-AT--C-C-GG-G-G-----CT-C-AA------C-T-C-C-G-G-A--A-C----T-G--C-A---T--T-------G--GA-A-A---C-------T--G-T--CA--G-A-C----T-A-G-A-G-T--G---T-CG-----GA-G-A--------G-G-T-A---AG-T------GG--A--ATT----C-
C-T-A-GT--GT-A-G-CG-GT--G---A--A-A---TG-C-GT-AG--AT-A-TT--A-G--G-A-G-G-A-AC-A-CC---AG--T--G--GC-GAA-G--G-C--G-------G--C-T-T-A---CTG--G--AC-G-A--------------------T-A---A-C-T--GA--CG-C-T-G--A-GG--C-T-CG-A--AA-G-C---G-TG--GG-
G--AG-C-A-AA--------CA--GG Bacteria(100);Firmicutes(100);Clostridia(100);Clostridiales(100);Lachnospiraceae(100);unclassified(100);unclassified(100);unclassified(100);unclassified(100);

Otu0539 0 0 0 0 0 0 0 0 0 0 0 0 0 0 0 0 0 0 0 0 0 0 0 0 0 0 0 0
0 0 0 0 0 0 0 0 0 0 0 0 0 0 0 0 0 0 0 0 0 0 0 0 0 0 0 0 0
0 6 0 0 0 0 0 0 0 0 0 0 0 0 15 20 0 0 0 0 0 0 0 0 0 0 0 0 0
0 0 0 0 6 18 0 0 0 31 0 5 0 0 0 0 0 0 0 0 0 0 0 0 0 0 0
M02255_77_000000000-ADUT3_1_2112_14015_9545 T----AC---GT-GC-CAG--------CAGCC-G------CG-G--T---A--AT-A-CGT-AG------GT-G--G-C--GA-GC------GTT--AT----CC-GGAAT-TA-------T-TG-G-G-C-G------------G--T-CT-G-T-T----------
AA----G-T-T-C--------A-T-G--G--TC--A-AA-TT--G-T-GG-G-G-----CT-C-AA------C-C-C-C-A-T-C--G------C-G--C-C---A--T-------G--GA-T-A---C-------T--G-G--CA--G-A-C----T-A-G-A-G-T--A---T-TG-----GA-G-A--------G-G-C-A---AG-T------GG--A--ATT----C-C-A-
T-GT--GT-A-G-CG-GT--A---A--A-A---TG-C-GT-AG--AT-A-TA--T-G--G-A-G-G-A-AC-A-CC---GG--T--G--GC-GAA-G--G-C--G-------A--C-T-T-G---CTA--G--CC-A-A--------------------A-G---A-C-T--GA--CG-C-T-G--A-GG--C-A-CG-A--AA-G-C---G-TG--GG-G--
AG-C-A-AA--------TA--GG Bacteria(100);Firmicutes(100);Erysipelotrichi(100);Erysipelotrichales(100);Erysipelotrichaceae(100);Bulleidia(100);unclassified(100);unclassified(100);unclassified(100);

Otu0540 0 0 0 0 0 0 0 0 0 0 0 0 0 0 0 0 0 0 0 0 0 0 0 9 0 0 0 0
0 0 0 0 0 0 0 0 0 0 0 0 0 0 0 0 0 0 0 0 0 0 0 0 15 0 0 0 0
0 0 0 0 0 0 0 0 0 0 0 0 0 0 0 0 0 0 0 0 0 0 0 0 0 0 0 0 0
0 0 0 0 0 0 0 0 0 0 0 0 0 0 0 0 0 0 0 0 0 0 0 0 0 0 0
M02255_77_000000000-ADUT3_1_1101_21294_12063 T----AC---GA-AG-GGG--------GCT-A-G------C--G--T---T--GT-T-CGG-AA------TT-A--C-T--GG-GC-------GT--A-----AA-GC-GC-AC-------G-TA-G-G-C-G------------G--A-CA-T-T-T-----
------AA----G-T-C-A--------G-G-G--G--TG--A-AA-TC--C-C-GG-G-G-----CT-C-AA------C-C-C-C-G-G-A--A-C----T-G--C-C---T--T-------T--GA-T-A---C-------T--G-G--GT--G-T-C----T-T-G-A-G-T--G---T-GG-----AA-G-A--------G-G-T-C---GG-T------GG--A--ATT----
G-C-G-A-GT--GT-A-G-AG-GT--G---A--A-A---TT-C-GT-AG--AT-A-TT--C-G--C-A-G-G-A-AC-A-CC---AG--T--G--GC-GAA-G--G-C--G-------G--C-C-G-A---CTG--G--TC-C-A--------------------T-T---A-C-T--GA--CG-C-T-G--A-GG--T-G-CG-A--AA-G-C---G-TG--GG-
G--AG-C-A-AA--------CA--GG Bacteria(100);Proteobacteria(100);Alphaproteobacteria(100);Rhizobiales(100);unclassified(100);unclassified(100);unclassified(100);unclassified(100);unclassified(100);

Otu0541 0 0 0 0 0 0 0 0 0 0 0 0 0 0 0 0 0 0 0 0 0 0 0 0 0 0 0 0
0 0 0 0 0 0 0 0 0 0 0 0 0 0 0 0 0 0 0 0 0 0 0 0 0 0 0 0 0
0 8 0 0 0 0 0 0 0 0 0 0 0 0 0 0 0 0 0 0 0 0 0 0 0 0 0 0 19
0 0 0 0 0 0 0 0 0 0 0 12 0 0 0 0 0 0 0 0 0 0 0 0 0 0 0
M02255_76_000000000-ADUNE_1_1103_18938_18238 C----GG---CT-AA-CT---------ACG-T-G------C--C--A---G--CA-G-CCG-CG------GT-A--A-T--AC-GT-------AG--G-----TC-AC-AA-GC-------G-TT-GTC-C-G-------------G--A-TT-G-T-T-------
----AA----G-T-C-T--------G-A-T--G--TG--A-AA-GC--C-C-AC-G-G-----CT-C-AA------C-C-G-T-G-G-A--A------C-GG-C-A---T--T-------G--GA-A-A---C-------T--G-G--CA--A-A-C----T-T-G-A-G-T--A---T-AG-----AA-G-A--------G-G-A-A---AG-T------GG--A--ATT----C-
C-A-T-GT--GT-A-G-CG-GT--G---A--A-A---TG-C-GT-AG--AT-A-TA--T-G--G-A-G-G-A-AC-A-CC---AG--T--G--GC-GAA-G--G-C--G-------A--C-T-T-T---CTG--G--TC-T-A--------------------T-A---A-C-T--GA--CG-C-T-G--A-GG--C-T-CG-A--AA-G-C---G-TG--GG-G--
AG-C-G-AA--------CA--GG Bacteria(100);Firmicutes(100);Bacilli(100);Lactobacillales(100);Aerococcaceae(100);Facklamia(100);unclassified(100);unclassified(100);unclassified(100);

Otu0542 0 0 0 0 0 0 0 0 0 0 0 0 0 0 0 0 0 0 0 0 0 0 0 0 0 0 0 0
0 0 0 0 0 53 0 75 0 0 0 0 0 0 0 0 0 0 0 0 0 0 0 0 0 0 0 0 0
0 0 0 0 0 0 0 0 0 0 0 0 0 0 0 0 0 0 0 0 0 0 0 0 0 0 0 0 0
0 0 0 0 0 0 0 0 0 0 0 0 0 0 0 0 0 0 0 0 0 0 0 0 0 0 0
M02255_77_000000000-ADUT3_1_1111_14882_9688 T----AC---GA-AG-GGG--------GCT-A-G------C--G--T---T--GT-T-CGG-AA------TC-A--C-T--GG-GC-------GT--A-----AA-GA-GC-AC-------G-TA-G-G-C-G------------G--A-CT-T-G-T-----------TA-
---G-T-C-A--------G-G-G--G--TG--A-AA-TC--C-C-GG-G-G-----CT-C-AA------C-C-C-C-G-G-A--A-C----T-G--C-C---C--T-------T--GA-A-A---C-------T--G-C--AA--G-T-C----T-T-G-A-G-T--C---C-GA-----GA-G-A--------G-G-T-G---AG-T------GG--A--ATT----C-C-T-A-
GT--GT-A-G-AG-GT--G---A--A-A---TT-C-GT-AG--AT-A-TT--A-G--G-A-A-G-A-AC-A-CC---AG--T--G--GC-GAA-G--G-C--G-------G--C-T-C-A---CTG--G--CT-C-G--------------------G-T---A-C-T--GA--CG-C-T-G--A-GG--T-G-CG-A--AA-G-C---G-TG--GG-G--AG-
C-A-AA--------CA--GG Bacteria(100);Proteobacteria(100);Alphaproteobacteria(100);unclassified(100);unclassified(100);unclassified(100);unclassified(100);unclassified(100);unclassified(100);

Otu0543 0 0 0 0 0 0 0 0 0 0 0 0 0 0 0 0 0 0 0 0 0 0 0 0 0 0 0 0
0 0 0 0 0 0 0 20 0 0 9 14 0 0 0 0 0 0 0 0 0 0 0 0 0 0 0 0 0
0 0 0 0 0 0 0 0 0 0 0 0 0 0 0 0 0 0 0 0 0 0 0 0 0 0 0 0 0
0 0 0 0 0 0 0 0 0 0 0 0 0 0 0 0 0 0 0 0 0 0 0 0 0 0 0
M02255_77_000000000-ADUT3_1_2111_24375_7657 T----AC---GG-GG-GGG--------GCA-A-G------C--G--T---T--GT-T-CGG-AA------TT-A--C-T--GG-GC-------GT--A-----AA-GG-GT-GC-------G-TA-G-G-C-G------------G--C-TC-T-C-T-----------
AA----G-T-C-G--------G-A-T--G--TG--A-AA-AC--T-C-TG-G-G-----CT-C-AA------C-C-C-A-G-A-G--C-C----T-G--C-A---T--C-------T--GA-A-A---C-------T--G-G--AG--G-G-C----T-A-G-G-G-T--T---C-TG-----GA-G-G--------G-G-A-A---AG-C------GG--A--ATT----C-C-
T-G-GT--GT-A-G-CG-GT--G---A--A-A---TG-C-GT-AG--AT-A-TC--A-G--G-A-G-G-A-AC-A-CC---GG--T--G--GC-GAA-G--G-C--G-------G--C-T-T-T---CTG--G--AC-A-G--------------------A-T---A-C-C--GA--CG-C-T-G--A-GG--C-A-CG-A--AA-G-C---T-AG--GG-G--
AG-C-A-AA--------CA--GG Bacteria(100);unclassified(54);unclassified(54);unclassified(54);unclassified(54);unclassified(54);unclassified(54);unclassified(54);unclassified(54);

Otu0544 0 0 0 0 0 0 0 0 0 0 0 0 0 0 0 0 0 0 0 0 0 0 0 0 0 0 0 0
0 0 0 73 0 0 0 0 0 0 0 0 0 0 0 95 0 0 0 0 0 0 0 1 0 0 0 0 0
0 0 0 0 0 0 0 0 0 0 8 4 0 0 0 0 0 0 116 0 0 0 0 0 0 0 0 0 0
0 0 0 0 60 0 2 0 0 51 0 0 0 0 0 0 0 0 0 0 0 227 0 0 0 0 0
M02255_77_000000000-ADUT3_1_1105_12267_14903 T----AC---GT-AG-GTG--------GCA-A-G------C--G--T---T--AT-C-CGG-AT------TT-A--C-T--GG-GT-------GT--A-----AA-GG-GC-GT-------G-TA-G-G-C-G------------G--A-TT-C-T-C------
-----AA----G-T-T-G--------G-A-T--G--TG--A-AA-CC--C-C-TT-G-G-----CT-A-AA------C-T-A-A-G-G-G--C-T----T-G--C-A---T--T-------C--AA-A-A---C-------T--G-A--GG--A-C-C----T-T-G-A-G-T--A---T-CA-----GA-G-G--------G-G-A-A---AG-T------GG--A--ATT----C-
C-T-G-GT--GG-A-G-CG-GT--A---A--A-A---TG-C-GT-AG--AG-A-TC--A-G--G-A-G-G-A-AC-A-CC---GG--T--G--GC-GAA-G--G-C--G-------A--C-T-T-T---CTG--G--CT-G-A--------------------T-A---A-C-T--GA--CG-C-T-G--A-GG--C-G-CG-A--AA-G-C---G-TG--GG-
G--AG-C-A-AA--------CA--GG Bacteria(100);Firmicutes(100);Clostridia(100);Clostridiales(100);Ruminococcaceae(100);Fastidiosipila(100);unclassified(100);unclassified(100);unclassified(100);

Otu0545 0 0 0 0 0 0 0 0 0 0 0 0 0 0 0 0 0 0 0 0 0 0 0 0 0 0 7 0
0 0 0 0 0 0 0 0 0 0 0 0 0 0 0 0 0 0 0 0 0 0 0 0 0 0 0 0
0 0 0 0 0 0 0 0 0 0 0 0 0 0 0 14 0 0 0 0 0 0 0 0 0 0 0 0
0 0 0 0 0 0 0 0 0 0 0 0 0 0 0 0 0 0 0 0 0 0 0 0 0 0 0
M02255_77_000000000-ADUT3_1_1104_7397_7653 T----AC---GG-AG-AGG--------GCT-A-G------C--G--T---T--AT-T-CGG-AA------TT-A--T-T--GG-GC-------GT--A-----AA-GG-GC-GC-------G-TA-G-G-C-G------------G--A-TT-A-G-T-----------
AA----G-T-T-A--------A-A-A--G--TG--A-AA-TC--C-C-AA-G-G-----CT-C-AA------C-C-T-T-G-G-A--A-T----T-G--C-T---T--T-------T--AA-A-A---C-------T--G-C--TA--A-T-C----T-A-G-A-G-A--T---T-GA-----AA-G-A--------G-G-A-T---AG-A------GG--A--ATT----C-C-T-
A-GT--GT-A-G-AG-GT--G---A--A-A---TT-C-GT-AA--AT-A-TT--A-G--G-A-G-G-A-AC-A-CC---AG--T--G--GC-GAA-G--G-C--G-------T--C-T-A-T---CTG--G--TT-C-A--------------------A-A---T-C-T--GA--CG-C-T-G--A-GG--C-G-CG-A--AG-G-C---G-TG--GG-G--AG-
C-A-AA--------CA--GG Bacteria(100);Proteobacteria(100);Alphaproteobacteria(100);Rickettsiales(100);Anaplasmataceae(100);Wolbachia(100);unclassified(100);unclassified(100);unclassified(100);

Otu0546 0 0 0 0 0 0 0 0 0 0 0 0 0 0 0 0 0 0 0 0 0 0 28 0 0 0 0 0
0 0 0 0 0 0 0 82 0 0 0 0 0 0 0 0 0 0 0 0 0 0 0 0 0 0 0 0
0 0 0 0 0 0 0 0 0 0 0 0 0 0 0 13 0 0 0 0 0 0 0 0 0 0 0 0
0 0 0 0 0 0 0 0 0 0 0 0 0 0 0 0 0 0 0 0 0 0 0 0 0 0 0
M02255_77_000000000-ADUT3_1_1112_13640_14634 T----AC---GG-AG-GGG--------GCT-A-G------C--G--T---T--GT-T-CGG-AA------TC-A--C-T--GG-GC-------GT--A-----AA-GC-GC-AC-------G-TA-G-G-C-G------------G--C-GA-T-C-T-----
------AA----G-T-C-A--------G-A-G--G--TG--A-AA-GC--C-C-GG-G-G-----CT-C-AA------C-C-C-C-G-G-A--A-T----T-G--C-C---T--T-------T--GA-G-A---C-------T--G-G--AT--T-G-C----T-T-G-A-A-C--G---T-CG-----GA-G-A--------G-G-T-G---GG-T------GG--A--ATT----
C-C-G-A-GT--GT-A-G-AG-GT--G---A--A-A---TT-C-GT-AG--AT-A-TT--C-G--G-A-A-G-A-AC-A-CC---AG--T--G--GC-GAA-G--G-C--G-------G--C-C-C-A---CTG--G--AC-G-A--------------------C-T---G-T-T--GA--CG-C-T-G--A-GG--T-G-CG-A--AA-G-C---G-TG--
GG-G--AG-C-A-AA--------CA--GG Bacteria(100);Proteobacteria(100);Alphaproteobacteria(100);Sphingomonadales(100);Sphingomonadaceae(100);Sphingobium(90);unclassified(90);unclassified(90);unclassified(90);

Otu0547 0 0 0 0 0 0 0 0 0 0 0 0 0 0 0 0 0 0 0 0 0 0 0 0 0 0 0 0
0 0 0 0 0 0 0 87 0 0 0 0 0 0 0 0 0 0 0 0 0 0 0 0 0 0 0 0
0 0 0 0 0 0 0 0 0 0 0 0 0 0 0 0 0 0 0 0 0 0 0 0 0 0 0 0
0 0 0 0 0 0 0 0 0 0 0 0 0 0 0 0 0 0 0 0 0 0 0 0 0 0 0
M02255_77_000000000-ADUT3_1_2111_24631_9113 C----AC---GT-AG-GCA--------CCA-A-G------C--G--T---T--GT-C-CGG-AT------TT-A--T-T--GG-GC-------GT--A-----AA-GA-GC-TT-------G-TA-G-G-C-G------------G--T-TC-G-G-C-----------AA--
--G-T-C-G--------G-G-T--G--TG--A-AA-AC--T-C-TG-G-G-----CT-C-AA------C-T-C-A-G-A-G--A------T-G-CC-A---C--C-------C--GA-T-A---C-------T--G-C--TG-TG-A-C----T-A-G-A-G-T--G---C-AG-----TA-G-G--------G-G-A-G---CG-G------GG--A--ATT----C-C-T-G-
GT--GT-A-G-CG-GT--G---A--A-A---TG-C-GC-AG--AT-A-TC--A-G--G-A-G-G-A-AC-A-CC---AG--T--G--GC-GAA-G--G-C--G-------C--C-G-C-T---CTG--G--GC-T-G--------------------T-A---A-C-T--GA--CG-C-T-G--A-GA--A-G-CG-A--AA-G-C---G-TG--GG-T--AG-
C-A-AA--------CA--GG Bacteria(100);Actinobacteria(100);Actinobacteria(100);Acidimicrobidae(100);Acidimicrobiales(100);Acidimicrobineae(100);Acidimicrobiaceae(100);unclassified(100);unclassified(100);

Otu0548 0 0 0 0 0 0 0 0 0 0 0 0 0 0 0 0 0 0 0 0 0 0 0 0 0 0 0 0
0 0 0 0 0 0 0 0 104 0 0 0 0 0 0 0 0 0 0 0 0 0 0 0 0 0 0 0
0 0 0 0 0 0 0 0 0 0 0 0 0 0 0 0 23 0 0 0 0 0 0 0 0 0 0 0
0 0 0 0 0 0 0 5 0 0 0 0 0 0 0 0 0 0 0 0 0 0 0 0 0 0 0
M02255_77_000000000-ADUT3_1_1104_28969_16166 T----AC---GG-AG-GGG--------GCT-A-G------C--G--T---T--GT-T-CGG-AA------TT-A--C-T--GG-GC-------GT--A-----AA-GC-GC-AC-------G-TA-G-G-C-G------------G--T-TT-T-G-C-----
------AA----G-T-C-A--------G-A-G--G--TG--A-AA-GC--C-C-GG-A-G-----CT-C-AA------C-T-C-C-G-G-A--A-T----T-G--C-C---T--T-------T--GA-G-A---C-------T--G-C--AT--C-A-C----T-T-G-A-A-C--G---T-CG-----GA-G-A--------G-G-T-G---AG-T------GG--A--ATT----C-
C-G-A-GT--GT-A-G-AG-GT--G---A--A-A---TT-C-GT-AG--AT-A-TT--C-G--G-A-A-G-A-AC-A-CC---AG--T--G--GC-GAA-G--G-C--G-------G--C-T-C-A---CTG--G--AC-G-A--------------------C-T---G-T-T--GA--CG-C-T-G--A-GG--T-G-CG-A--AA-G-C---G-TG--GG-
G--AG-C-A-AA--------CA--GG Bacteria(100);Proteobacteria(100);Alphaproteobacteria(100);Sphingomonadales(100);Sphingomonadaceae(100);Sphingomonas(100);unclassified(100);unclassified(100);unclassified(100);

Otu0549 0 0 0 0 0 0 0 0 0 0 0 0 0 0 0 0 0 0 0 0 0 0 0 0 0 0 0 0
0 0 0 0 0 0 0 0 0 0 0 0 0 0 0 0 0 0 0 0 0 0 0 12 0 0 0 0
0 0 0 0 0 0 0 0 0 0 0 0 0 0 0 0 25 0 0 0 0 0 0 0 0 0 0 0
0 0 0 0 0 0 0 0 0 0 0 0 0 0 0 0 0 0 0 0 0 0 0 0 0 0 0
M02255_77_000000000-ADUT3_1_1106_16871_21111 T----AC---GT-AG-GTG--------GCA-A-G------C--G--T---T--AT-C-CGG-AT------TT-A--C-T--GG-GT-------GT--A-----AA-GG-GC-GT-------G-TA-G-G-C-G------------G--G-TC-T-G-C-----
------AA----G-T-C-A--------G-A-T--G--TG--A-AA-TC--T-C-CG-G-G-----CT-T-AA------C-T-C-G-G-A-A--G-T----G-T--C-A---T--C-------T--GA-A-A---C-------T--A-C--GG--A-T-C----T-T-G-A-G-T--A---C-TG-----GA-G-A--------G-G-A-T---AG-T------GG--A--ATT----C-
C-T-A-GT--GT-A-G-CG-GT--A---A--A-A---TG-C-GT-AG--AT-A-TT--A-G--G-A-G-G-A-AC-A-CC---AG--T--G--GC-GAA-G--G-C--G-------G--C-T-A-T---CTG--G--AC-A-G--------------------T-A---A-C-T--GA--CG-C-T-G--A-GG--C-G-CG-A--AA-G-C---G-TG--GG-
G--AG-C-A-AA--------CA--GG Bacteria(100);Firmicutes(100);Clostridia(100);Clostridiales(100);Ruminococcaceae(100);unclassified(68);unclassified(68);unclassified(68);unclassified(68);

Otu0550 0 0 0 0 0 0 0 0 0 0 0 0 0 0 0 0 0 0 0 0 0 0 0 0 0 0 0 0
0 0 0 0 0 0 0 0 0 0 0 20 0 0 0 0 0 0 0 0 0 0 0 0 0 0 0 0 0
0 0 0 0 0 0 0 0 0 0 0 0 0 0 0 0 30 0 0 0 0 0 0 0 0 0 0 0 0
0 0 0 0 0 0 0 0 0 0 0 0 0 0 0 0 0 0 0 0 0 0 0 0 0 0 0 0
M02255_77_000000000-ADUT3_1_1108_12388_19105 T----AC---GT-AG-GCA--------GCG-A-G------C--G--T---T--GT-T-CGG-AG------TT-A--C-T--GG-GC-------GT--A-----AA-GC-GT-GC-------G-TA-G-G-C-G------------G--T-GT-C-T-T-----
------AA----G-T-T-T--------G-T-T--G--TG--A-AA-TC--T-C-CC-G-G-----CT-T-AA------C-C-G-G-G-A-G--G-G----T-G--C-G---G--C-------G--AA-T-A---C-------T--G-A--GA--T-G-C-----TG-G-A-G-T--G---C-GG-----GA-G-A--------G-G-T-A---AG-C------GG--A--ATT----
C-C-C-G-GT--GT-A-G-CG-GT--G---A--A-A---TG-C-GT-AG--AT-A-TC--G-G--G-A-G-G-A-AC-A-CC---TG--A--G--AT-GTA-G--A-T--G-------G--C-T-T-A---CTG--G--AC-C-G--------------------T-A---A-C-T--GA--CG-C-T-G--A-GG--C-A-CG-A--AA-G-C---G-TG--GG-
T--AG-C-A-AA--------CA--GG Bacteria(100);unclassified(100);unclassified(100);unclassified(100);unclassified(100);unclassified(100);unclassified(100);unclassified(100);unclassified(100);

Otu0551 0 0 0 0 0 0 0 0 0 0 0 0 0 0 0 0 0 0 0 0 0 0 0 0 0 0 0 0
0 0 0 0 0 0 0 0 0 0 0 0 0 0 0 0 0 0 0 0 0 0 0 0 0 0 0 0 0
0 0 0 0 0 0 0 0 0 0 0 0 0 0 0 0 34 0 0 0 0 0 0 0 0 0 0 0 0
0 0 0 0 0 0 0 0 0 0 0 0 0 0 0 0 0 0 0 0 0 0 0 0 0 0 0 0
M02255_77_000000000-ADUT3_1_1119_7295_18738 T----AC---GT-AG-GGA--------GCA-A-G------C--G--T---T--GT-C-CGG-AT------TT-A--T-T--GG-GC-------GT--A-----AA-GA-GC-TC-------G-TA-G-G-C-G------------G--T-TA-A-G-A-----------AA-
---G-T-C-A--------G-G-T--G--TG--A-AA-CC--T-C-CA-G-G-----CT-C-AA------C-C-T-G-G-A-G--A-C----G-C--C-A---C--T-------T--GA-T-A---CT------T--C-T--TT--G-A-C----T-T-G-A-G-T--T---C-GG-----TA-G-G--------G-G-A-T---CA-C------GG--A--ATT----C-C-T-G-
GT--GT-A-G-CG-GT--G---A--A-A---TG-C-GC-AG--AT-A-TC--A-G--G-A-G-G-A-AC-A-CC---GA--T--G--GC-GAA-G--G-C--A-------G--T-G-A-T---CTG--G--GC-C-G--------------------A-T---A-C-T--GA--CG-C-T-G--A-GG--A-G-CG-A--AA-G-C---G-TG--GG-G--AG-
C-G-AA--------CA--GG Bacteria(100);Actinobacteria(100);Actinobacteria(100);unclassified(100);unclassified(100);unclassified(100);unclassified(100);unclassified(100);unclassified(100);

Otu0552 0 0 0 0 0 0 0 0 97 0 0 0 0 0 0 0 0 0 0 0 0 0 0 0 0 0 0 0
0 0 0 0 0 0 0 0 0 0 0 0 0 0 0 0 0 0 0 219 0 0 0 0 0 0 0 0 0
0 0 0 0 0 0 0 0 0 0 0 0 0 0 0 0 0 0 0 0 0 0 0 0 0 0 0 0 0
0 0 0 0 0 0 0 0 0 0 0 0 0 0 0 0 0 0 0 0 0 0 0 0 0 0 0 0
M02255_77_000000000-ADUT3_1_1101_14338_16919 T----AC---GT-AG-GTG--------GCA-A-G------C--G--T---T--AT-C-CGG-AT------TT-A--C-T--GG-GT-------GT--A-----AA-GG-GC-GA-------G-TA-G-G-C-G------------G--G-AC-T-G-C-----
------AA----G-T-C-A--------G-A-T--G--TG--A-AA-AC--T-A-TG-G-G-----CT-T-AA------C-C-C-A-T-A-G--A-C----T-G--C-A---T--T-------T--GA-A-A---C-------T--G-T--GG--T-T-C----T-T-G-A-G-T--G---A-TG-----GA-G-A--------G-G-C-A---GG-C------GG--A--ATT----C-
C-C-G-GT--GT-A-G-CG-GT--G---G--A-A---TG-C-GT-AG--AT-A-TC--G-G--G-A-G-G-A-AC-A-CC---AG--T--G--GC-GAA-G--G-C--G-------G--C-C-T-G---CTG--G--AC-A-T--------------------T-A---A-C-T--GA--CG-C-T-G--A-GG--C-G-CG-A--AA-G-T---G-TG--GG-
G--AG-C-A-AA--------CA--GG Bacteria(100);Firmicutes(100);Clostridia(100);Clostridiales(100);Ruminococcaceae(100);unclassified(100);unclassified(100);unclassified(100);unclassified(100);

Otu0553 0 0 0 0 0 0 0 0 0 0 0 0 0 0 0 0 0 0 3 0 0 0 0 0 0 0 0 0
0 0 0 0 0 0 0 0 0 0 0 0 0 0 0 0 0 0 0 0 0 0 0 0 17 0 0 0 0
0 0 0 0 0 0 0 0 0 0 0 0 0 0 0 0 17 0 0 0 0 0 0 0 0 0 0 0 0
0 0 0 0 0 0 0 0 0 0 0 0 0 0 0 0 0 0 0 0 0 0 0 0 0 0 0 0
M02255_77_000000000-ADUT3_1_2115_27363_12144 G----AC---GG-AG-GGT--------GCT-A-G------C--G--T---T--GT-T-CGG-AA------TC-A--C-T--GG-GC-------GT--A-----AA-GG-GC-GC-------G-TA-G-G-C-G------------G--C-TT-T-G-G----
-------CA----G-T-C-G--------GTT-T--G--TG--A-AA-GC--C-C-GG-G-G-----CT-C-AA------C-C-C-C-G-G-A--G-G----T-G--C-G---G--C-------C--GA-A-A---C-------A--C-C--AA--G-G-C----T-G-G-A-G-G--C---C-GG-----AA-G-A--------G-G-A-G---GG-T------GG--A--ATA-
---C-C-C-A-GT--GT-A-G-AG-GT--G---A--A-A---TT-C-GT-AG--AT-A-TT--G-G--G-T-G-G-A-AC-A-CC---GG--T--G--GC-GAA-A--G-C--G-------G--C-T-C-T---CTG--G--TC-C-G--------------------G-T---C-C-T--GA--CG-C-T-G--A-GG--C-G-CG-A--AA-G-C---G-T-G-
GG-G--AG-C-A-AA--------CA--GG Bacteria(100);Proteobacteria(100);Alphaproteobacteria(100);Rhodospirillales(100);unclassified(100);unclassified(100);unclassified(100);unclassified(100);unclassified(100);

Otu0554 0 0 0 0 0 0 0 0 44 0 0 0 0 0 0 0 0 0 0 0 0 0 0 0 0 0 0 0
0 0 0 0 0 0 0 0 0 0 0 0 0 0 0 0 0 0 0 0 0 0 0 0 0 0 0 0 0
0 0 0 0 0 0 0 0 0 0 0 0 0 0 0 0 25 0 0 0 0 0 0 0 0 0 0 0 0
0 0 0 0 0 0 0 0 0 0 0 0 0 0 0 0 0 0 0 0 0 0 0 0 0 0 0 0
M02255_77_000000000-ADUT3_1_1104_15766_13966 G----AC---AG-AG-GGT--------GCT-A-G------C--G--T---T--GT-T-CGG-AA------TT-A--C-T--GG-GC-------GT--A-----AA-GG-GC-GC-------G-TA-G-G-C-G------------G--C-TC-G-T-T-----
------TA----G-T-C-G--------G-G-T--G--TG--A-AA-TC--C-C-AA-G-G-----CT-C-AA------C-C-T-T-G-G-A--A-C----T-G--C-A---T--T-------C--GA-T-A---C-------T--G-G--CG--G-G-C----T-A-G-A-G-A--C---C-GG-----AA-G-A--------G-G-T-G---GG-T------GG--A--ATA----
C-C-C-A-GT--GT-A-G-AG-GT--G---A--A-A---TT-C-GT-AG--AT-A-TT--G-G--G-T-G-G-A-AC-A-CC---GG--T--G--GC-GAA-G--G-C--G-------G--C-T-C-A---CTG--G--TC-C-G--------------------G-T---T-C-T--GA--CG-C-T-G--A-GG--C-G-CG-A--AA-G-C---G-TG--GG-
G--AG-C-A-AA--------CA--GG Bacteria(100);Proteobacteria(100);Alphaproteobacteria(100);unclassified(100);unclassified(100);unclassified(100);unclassified(100);unclassified(100);unclassified(100);

Otu0555 0 0 81 0 0 0 305 14 154 0 0 0 0 0 0 0 0 0 0 0 0 0 0 0 0 0 0 0
0 0 124 0 0 0 0 0 0 0 0 301 0 0 0 0 0 0 0 0 0 0 0 0 0 0 0 0
0 0 0 0 0 0 0 0 0 0 0 0 0 0 0 11 0 0 0 0 0 0 0 0 0 0 0 0
0 0 0 0 0 0 0 0 0 0 0 0 0 0 0 0 0 0 0 0 0 0 0 0 0 0
M02255_76_000000000-ADUNE_1_1107_26528_13445 T----AC---GT-AT-GGT--------GCA-A-G------C--G--T---T--AT-C-CGG-AT------TT-A--C-T--GG-GT-------GT--A-----AA-GG-GT-GC-------G-TA-------G------------G-----A-----C----------
AA----G-T-C-T--------G-A-A--G--TG--A-AA-AT--C-C-GG-G-G-----CT-T-AA------C-C-C-C-G-G-A--A-C----T-G--C-T---T--T-------G--GA-A-A---C-------T--G-C--CT--G-A-C----T-A-G-A-G-T--A---C-AG-----GA-G-A--------G-G-T-A---AG-T------GG--A--ATT----C-C-T-
A-GT--GT-A-G-CG-GT--G---A--A-A---TG-C-GT-AG--AT-A-TT--A-G--G-A-G-G-A-AC-A-CC---AG--T--G--GC-GAA-G--G-C--G-------A--C-T-T-A---CTG--G--AC-T-G--------------------C-T---A-C-T--GA--CA-C-T-G--A-GG--C-A-CG-A--AA-G-C---G-TG--GG-G--
AG-C-A-AA--------CA--GG Bacteria(100);Firmicutes(100);Clostridia(100);Clostridiales(100);Lachnospiraceae(100);Coprococcus(100);unclassified(100);unclassified(100);unclassified(100);

Otu0556 0 0 0 0 0 0 0 0 0 0 0 0 0 0 0 0 0 0 0 0 0 0 0 0 0 0 0 0
0 0 0 0 0 0 0 0 0 0 0 3 0 0 0 0 4 0 0 0 0 0 0 3 0 0 0 0 0
0 0 0 0 0 0 0 0 0 0 0 0 0 0 0 0 0 0 0 15 9 0 0 0 0 0 0 0 0
0 0 0 0 2 11 0 0 0 17 0 22 0 0 0 0 0 0 0 0 0 0 0 0 0 0
M02255_77_000000000-ADUT3_1_1104_6514_17424 C----A----GC-A--GCC--------GCG-G-T------A--A--T---A--CG-G-AGG-AT------GC-G--A-G--CG-TT-------AT--C-----CG-GA-AT-TA-------T-TG-G-G-T-G------------G--C-AA-G-G-G----------AA--
--G-T-C-A--------G-G-G--G--TG--A-AA-AG--C-T-GT-A-G-----CT-C-AA------C-T-A-T-G-G-T--C-T----T-G--C-C---T--T-------T--GA-A-A---C-------T--C-T--CT--A-G-C----T-A-G-A-G-T--G---T-AC-----TG-G-A--------G-G-T-A---CG-T------GG--A--ACG----T-G-T-G-GT-
-GT-A-G-CG-GT--G---A--A-A---TG-C-AT-AG--AT-A-TC--A-C--A-C-A-G-A-AC-T-CC---GA--T--T--GC-GCA-G--G-C--A-------G--C-G-T-A---CTA--C--AT-T-A--------------------C-A---A-C-T--GA--CA-C-T-G--A-AG--C-A-CG-A--AA-G-C---G-TG--GG-T--AT-C-C-
AA--------CA--GG Bacteria(100);Bacteroidetes(100);Bacteroidia(100);Bacteroidales(100);Porphyromonadaceae(100);Porphyromonas(100);unclassified(100);unclassified(100);unclassified(100);

Otu0557 0 0 0 0 0 0 0 0 0 0 0 0 0 0 0 0 0 0 0 0 0 0 0 0 9 0 0 0
0 0 0 0 0 0 0 0 0 0 0 0 0 0 0 0 0 0 0 0 0 0 0 0 0 0 0 0
0 0 0 0 0 0 0 0 0 0 0 0 0 0 0 0 25 26 0 0 0 0 0 0 0 0 0 0 0
0 0 0 0 0 0 0 0 0 0 0 0 0 0 0 0 0 0 0 0 0 0 0 0 0 0
M02255_77_000000000-ADUT3_1_1102_9193_11590 T----AC---GT-AG-GTG--------GCA-A-G------C--G--T---T--GT-C-CGG-AT------TT-A--C-T--GG-GC-------GT--A-----AA-GA-GC-GC-------G-CA-G-G-C-G------------G--T-CG-A-G-T----------
AA----G-T-C-G--------A-A-T--G--TG--A-AA-GC--C-C-CC-G-G-----CT-C-AA------C-T-G-G-G-G-A--G-G----G-T--C-A---T--T-------C--GA-T-A---C-------T--G-T--TC--G-A-C----T-C-G-A-A-G--G---C-AG-----GA-G-A--------G-A-G-A---AG-T------GG--A--ATT----C-C-
C-G-GT--GT-A-G-TG-GT--G---A--A-A---TG-C-GT-AG--AT-A-TC--G-G--G-A-G-G-A-AC-A-CC---AG--T--G--GC-GAA-G--G-C--G-------A--C-T-T-C---TTG--G--CC-T-G--------------------T-T---C-T-T--GA--CG-C-T-G--A-GG--C-G-CG-A--AA-G-C---T-GG--GG-G--
AG-C-A-AA--------CG--GG Bacteria(100);unclassified(100);unclassified(100);unclassified(100);unclassified(100);unclassified(100);unclassified(100);unclassified(100);unclassified(100);

Otu0558 0 0 0 0 0 0 0 0 0 0 0 0 0 0 0 0 0 0 0 0 0 0 0 0 0 0 4 0
0 0 0 0 0 0 0 0 0 0 0 0 0 0 0 0 0 0 3 0 0 0 0 0 0 0 0 0
0 0 0 0 0 0 0 0 0 0 0 0 0 0 0 0 0 0 0 0 0 0 0 0 0 0 0 0 0
0 0 0 0 0 0 0 0 0 0 0 0 0 0 0 0 0 0 0 0 0 0 0 0 0 0
M02255_76_000000000-ADUNE_1_2112_6703_12887 T----AC---CT-AC-AG---------AAG-A-A------G--G--A---C--CG-G-C-T-AA------CT-C--C-G--TG-CC-------AG--C-----AG-CC-GC-GG-------T-AA-T-A-C-G------------G--A-GG-G-TCC----------GA--
--G-C-GTT--------A-A-T--C--GG--A-AA-GC--C-C-CG-G-G-----CT-C-AA------C-C-T-G-G-G-G--A-T----T-G--C-A---T--T-------T--CG-A-A---C-------T--G-G--CG--A-A-C----T-A-G-A-G-T--C---T-TG-----TA-G-A--------G-G-G-G---GG-T------AG--A--ATT----C-C-A-G-
GT--GT-A-G-CG-GT--G---A--A-A---TG-C-GT-AG--AG-A-TC--T-G--G-A-G-G-A-AT-A-CC---GG--T--G--GC-GAA-G--G-C--G-------G--C-C-C-C---CTG--G--AC-A-A--------------------A-G---A-C-T--GA--CG-C-T-C--A-GG--C-A-CG-A--AA-G-C---G-TG--GG-G--AG-
C-A-AA--------CA--GG Bacteria(100);Proteobacteria(100);Gammaproteobacteria(100);unclassified(100);unclassified(100);unclassified(100);unclassified(100);unclassified(100);unclassified(100);

Otu0559 0 0 0 0 0 0 0 0 0 0 0 0 0 0 0 0 0 0 8 0 16 0 190 0 2 4 0 0
11 225 0 45 49 0 22 0 0 0 117 0 157 0 0 0 0 0 0 0 1 0 0 0 0 0 0 0 0
0 0 0 0 0 0 0 0 0 0 0 0 0 0 0 0 0 0 106 0 0 8 0 0 0 0 0 21 0
11 90 168 24 39 0 6 0 0 168 0 0 26 0 0 25 0 0 18 57 0 0 1 0 0 0 0
M02255_77_000000000-ADUT3_1_1110_5505_16120 T----AC---GA-AG-GGG--------GCT-A-G------C--G--T---T--GC-T-CGG-AA------TC-A--C-T--GG-GC-------GT--A-----AA-GG-GC-GC-------G-TA-G-G-C-G------------G--C-TT-A-T-C----------
AA----G-T-C-G--------G-G-G--G--TG--A-AA-GC--C-C-GT-G-G-----CT-C-AA------C-C-A-C-G-G-A--A-T----G-G--C-C---T--T-------C--GA-T-A---C-------T--G-G--TA--G-G-C----T-T-G-A-G-A--C---C-GG-----AA-G-A--------G-G-A-C---AG-C------GG--A--ACT----G-C-
G-A-GT--GT-A-G-AG-GT--G---A--A-A---TT-C-GT-AG--AT-A-TT--C-G--C-A-A-G-A-AC-A-CC---AG--T--G--GC-GAA-G--G-C--G-------G--C-T-G-T---CTG--G--TC-C-G--------------------G-T---T-C-T--GA--CG-C-T-G--A-GG--C-G-CG-A--AA-G-C---G-TG--GG-G--
AG-C-A-AA--------CA--GG Bacteria(100);Proteobacteria(100);Alphaproteobacteria(100);Rhizobiales(100);Methylobacteriaceae(100);Methylobacterium(100);unclassified(100);unclassified(100);unclassified(100);

Otu0560 0 0 0 0 0 0 0 0 0 0 0 0 0 0 0 0 0 0 0 0 0 0 0 0 0 0 0 0
0 0 0 0 0 0 0 0 0 0 0 0 0 0 0 0 0 0 0 0 0 8 0 0 0 0 0 0 0
0 0 0 0 0 0 0 0 0 0 0 0 0 0 0 0 0 22 0 0 0 0 0 0 0 0 0 0 0
0 0 0 0 0 0 0 0 0 0 0 0 0 0 0 0 0 0 0 0 0 0 0 0 0 0 0
M02255_76_000000000-ADUNE_1_1102_17594_14796 T----AC---GT-AG-GGT--------GCG-A-G------C--G--T---T--GT-C-CGG-AA------TT-A--T-T--GG-GC-------GT---------A--A---------------A---G---G------------G--C-TT-G-T-C-----------
GC----G-T-C-T--------G-C-T--G--TG--A-AA-TC--C-C-GG-G-G-----CT-T-AA------C-C-C-C-G-G-G--C-T----T-G--C-A---G--T-------G--GG-T-A---C-------G--G-G--CA--G-G-C----T-A-G-A-G-T--G---T-GG-----TA-G-G--------G-G-A-G---AC-T------GG--A--ATT----C-C-
T-G-GT--GT-A-G-CG-GT--G---G--A-A---TG-C-GC-AG--AT-A-TC--A-G--G-A-G-G-A-AC-A-CC---GA--T--G--GC-GAA-G--G-C--A-------G--G-T-C-T---CTG--G--GC-C-A--------------------T-T---A-C-T--GA--CG-C-T-G--A-GA--A-G-CG-A--AA-G-C---G-TG--GG-G--
AG-C-G-AA--------CA--GG Bacteria(100);Actinobacteria(100);Actinobacteria(100);Actinobacteridae(100);Actinomycetales(100);Micrococcineae(100);unclassified(100);unclassified(100);unclassified(100);

Otu0561 0 0 0 0 0 0 0 0 0 0 0 0 0 0 0 0 0 0 0 0 0 0 0 0 0 0 0 0
0 1 0 0 0 0 0 96 0 0 28 1 0 0 0 0 0 0 0 0 0 0 0 0 0 0 0 0 0
0 0 0 0 0 0 0 0 0 0 0 0 0 0 0 0 0 0 0 0 0 0 0 0 0 0 0 0 0
0 0 0 0 0 0 0 0 0 0 0 0 0 0 0 0 0 0 0 0 0 0 0 0 0 0 0
M02255_77_000000000-ADUT3_1_1109_17321_19549 T----AC---GT-AG-GTG--------GCA-A-G------C--G--T---T--GT-C-CGG-AT------TT-A--C-T--GG-GC-------GT--A-----AA-GA-GC-GC-------G-CA-G-G-C-G------------G--C-TG-G-G-C----
-------AA----G-T-C-C--------G-A-T--G--TG--A-AA-GC--T-T-CC-G-G-----CT-T-AA------C-T-G-G-A-A-A--A-T----T-G--C-A---T--C-------G--GA-A-A---C-------T--G-C--TC--G-G-C----T-T-G-A-A-G--G---T-GG-----GA-G-A--------G-G-G-T---AG-C------GG--A--ATT----
C-C-C-G-GT--GT-A-G-TG-GT--G---A--A-A---TG-C-GT-AG--AT-A-TC--G-G--G-A-G-G-A-AC-A-CC---AG--T--G--GC-GAA-G--G-C--G-------G--C-T-A-C---CTG--G--CC-C-A--------------------C-T---C-T-T--GA--CG-C-T-G--A-GG--C-G-CG-A--AA-G-C---T-AG--GG-
G--AG-C-A-AA--------CG--GG Bacteria(100);unclassified(100);unclassified(100);unclassified(100);unclassified(100);unclassified(100);unclassified(100);unclassified(100);unclassified(100);

Otu0562 0 0 0 52 0 0 0 0 0 0 0 0 0 0 0 0 0 0 0 0 0 0 0 0 0 0 0 0
0 0 0 0 0 0 0 0 0 0 30 0 0 0 0 0 0 0 0 0 0 0 0 0 0 0 0 0 0
0 0 0 0 0 0 0 0 0 0 0 0 0 0 0 0 0 40 0 0 0 0 0 0 0 0 0 0 0
0 0 0 0 0 0 0 0 0 0 0 0 0 0 0 0 0 0 0 0 0 0 0 0 0 0 0
M02255_77_000000000-ADUT3_1_1103_16324_15260 T----AC---GG-AG-GGG--------GCT-A-G------C--G--T---T--GT-T-CGG-AA------TT-A--C-T--GG-GC-------GT--A-----AA-GG-GC-GC-------G-TA-G-G-C-G------------G--T-GC-G-G-T----
-------AA----G-T-T-A--------G-G-C--G--TG--A-AA-GG--C-C-CG-G-G-----CT-C-AA------C-C-C-G-G-G-A--A-C----T-G--C-G---C--T-------T--AA-A-A---C-------T--G-C--CG--C-G-C----TCT-G-A-G-T--C---C-GA-----GA-G-A--------G-G-A-T---AG-C------GG--A--ATT----
C-C-C-A-GT--GT-A-G-AG-GT--G---A--A-A---TT-C-GT-AG--AT-A-TT--G-G--G-A-A-G-A-AC-A-CC---AG--T--G--GC-GAA-G--G-C--G-------G--C-T-A-T---CTG--G--CT-C-G--------------------G-T---A-C-T--GA--CG-C-T-G--A-GG--C-G-CG-A--AA-G-C---G-TG--GG-
G--AG-C-A-AA--------CA--GG Bacteria(100);Proteobacteria(100);Alphaproteobacteria(100);unclassified(100);unclassified(100);unclassified(100);unclassified(100);unclassified(100);unclassified(100);

Otu0563 0 0 0 0 0 0 0 0 0 0 0 0 0 0 0 0 0 0 0 0 0 0 0 0 0 0 0 6
0 0 0 0 0 0 0 0 0 0 0 0 0 0 0 0 0 0 0 0 0 0 0 0 0 0 0 0 0
0 0 0 0 0 0 0 0 0 0 0 0 0 0 0 0 0 21 0 0 0 0 0 0 73 0 0 0 0 0
0 0 0 0 0 0 0 0 0 0 0 0 0 0 0 0 0 0 0 0 0 0 0 0 0 0 0
M02255_77_000000000-ADUT3_1_1104_21083_13214 T----AC---GA-AG-GGG--------ACT-A-G------C--G--T---T--GT-T-CGG-AT------TT-A--C-T--GG-GC-------GT--A-----AA-GC-GC-GC-------G-TA-G-G-C-G------------G--A-TA-T-T-T------
-----AA----G-T-C-A--------G-G-G--G--TG--A-AA-TC--C-C-AG-G-G-----CT-C-AA------C-C-C-T-G-G-A--A-C----T-G--C-C---T--T-------T--GA-T-A---C-------T--G-G--AT--G-T-C----T-G-G-A-G-A--T---C-GG-----GA-G-A--------G-G-T-G---AG-T------GG--A--ATT----C-
C-G-A-GT--GT-A-G-AG-GT--G---A--A-A---TT-C-GT-AG--AT-A-TT--C-G--G-A-G-G-A-AC-A-CC---AG--A--A--GC-GAA-G--G-C--G-------G--C-T-C-A---CTG--G--CC-C-G--------------------A-T---A-C-T--GA--CG-C-T-G--A-GG--C-G-CG-A--AA-G-C---G-TG--GG-
G--AG-C-A-AA--------CA--GG Bacteria(100);Proteobacteria(100);Alphaproteobacteria(100);unclassified(100);unclassified(100);unclassified(100);unclassified(100);unclassified(100);unclassified(100);

Otu0564 0 0 0 0 0 0 0 0 0 0 0 0 0 0 0 0 0 0 0 0 0 0 0 0 0 0 0 0
0 0 0 0 0 0 0 0 0 0 0 0 0 0 0 95 0 0 0 0 0 0 0 0 0 0 0 0 0
0 0 0 0 0 0 0 0 0 0 0 0 0 0 0 0 0 0 0 0 0 0 0 0 0 0 0 0 0
0 0 0 0 0 0 0 0 0 0 0 0 0 0 0 0 0 0 0 0 0 23 0 0 0 0 0
M02255_77_000000000-ADUT3_1_1116_10195_14906 T----AC---GT-AG-GAT--------CCG-A-G------C--A--T---T--AT-C-CGG-AG------TG-A--C-T--GG-GT-------GT--A-----AA-GA-GTTGC-------G-TA-G-G-T-G------------G--C-AT-A-G-T-----
------AA----G-T-A-G--------A-T-A--G--TG--A-AA-TC--T--GGT-G-G-----CT-C-AA------C-C-A-T-TCA-G--A-C----T-A--T-T---A--T-------C--TA-A-A---C-------T--A-C--TA--A-G-C----T-C-G-A-G-A--T---A-GT-----CA-T-G--------G-G-T-A---AC-T------GG--A--ATT----T-
C-T-A-GT--GT-A-G-GA-GT--G---A--A-A---TC-C-GT-AG--AT-A-TT--A-G--A-A-G-GA--AC-A-CC---GA--T--A--GC-GTA-G--G-C--A-------G--G-T-T-A---CTG--G--GA-C-T--------------------A-T---T-C-T--GA--CA-C-T-A--A-GG--C-A-CG-A--AA-G-C---G-TA--GG-G--
AG-C-A-AA--------CG--GG Bacteria(100);Candidate_division_TM7(100);unclassified(100);unclassified(100);unclassified(100);unclassified(100);unclassified(100);unclassified(100);unclassified(100);

Otu0565 0 0 0 0 0 0 0 0 0 0 0 0 0 0 0 0 0 0 0 0 0 0 0 0 0 0 0 0
0 0 2 0 0 0 0 29 0 0 32 0 0 0 0 0 0 0 0 0 0 0 0 0 0 0 0 0
0 0 0 0 0 0 0 0 0 0 0 0 0 0 0 0 0 0 0 0 0 0 0 0 0 0 0 0
0 0 0 0 0 0 0 0 0 0 0 0 0 0 0 0 0 0 0 0 0 0 0 0 0 0 0
M02255_77_000000000-ADUT3_1_1105_26738_15862 T----AC---GT-AG-GTG--------GCG-A-G------C--G--T---T--GT-C-CGG-AT------TT-A--C-T--GG-GC-------GT--A-----AA-GG-GC-GC-------G-CA-G-G-C-G------------G--T-CT-G-T-T-----
------TA----G-T-C-T--------A-G-G--G--TG--A-AA-GC--T-C-CC-G-G-----CT-C-AA------C-T-G-G-G-A-G--A-G----G-T--C-C---T--T-------G--GA-T-A---C-------T--G-G--CA--G-A-C----T-C-G-A-G-G--G---A-TG-----CA-G-A--------G-G-A-A---AG-T------GG--A--ATT----
C-C-C-G-GT--GT-A-G-TG-GT--G---A--T-A---TG-C-GT-AG--AT-A-TC--G-G--G-A-G-G-A-AC-A-CC---AG--T--G--GC-GAA-G--G-C--G-------G--C-T-T-T---CTG--G--GC-A-T--------------------T-A---C-C-T--GA--CG-C-T-G--A-GG--C-G-CG-A--AA-G-C---G-TG--GG-
G--AG-C-G-AA--------CC--GG Bacteria(100);Chloroflexi(100);S085(54);unclassified(54);unclassified(54);unclassified(54);unclassified(54);unclassified(54);unclassified(54);

Otu0566 0 0 0 0 0 0 0 0 0 0 0 0 0 0 0 0 0 0 0 0 0 0 0 0 0 0 0 0
0 0 0 0 0 0 0 0 0 0 0 0 0 0 0 0 0 0 0 0 0 0 0 43 0 0 0 0
0 0 0 0 0 0 0 0 0 0 0 0 0 0 0 0 0 15 0 0 0 0 0 0 0 0 0 0
0 0 0 0 0 0 0 0 0 0 0 0 0 0 0 0 0 0 0 0 0 0 0 0 0 0 0
M02255_77_000000000-ADUT3_1_1107_12603_12830 T----AC---GT-AG-GGG--------GCA-A-G------C--G--T---T--GT-C-CGG-AT------TT-A--T-T--GG-GC-------GT--A-----AA-GA-GC-TC-------G-TA-G-G-C-G------------G--T-TC-G-A-C-----
------AA----G-T-C-G--------A-A-T--G--TG--A-AA-TC--T-C-CA-C-G-----CT-C-AA------C-G-T-G-G-A-G--G------G-G--TCA---T--T-------C--GA-A-A---C-------T--G-T--CG-TG-A-C----T-T-G-A-G-T--A---C-GG-----TA-G-G--------G-G--AG---TG-T------GG--A--ATTC---
C-C---G-GT--GT-A-G-CG-GT--G---A--A-A---TG-C-GC-AG--AG-A-TC--G-G--G-A-G-G-A-AC-A-CC---CG--T--A--GC-GAA-G--G-C--G-------G--C-A-C-T---CTGG----GC-C-G--------------------A-T---A-C-T--GA--CG-C-T-G--A-GG--A-G-CG-A--AA-G-C---G-TG--
GG-G--AG-C-A-AA--------CA--GG Bacteria(100);Actinobacteria(100);Actinobacteria(100);Acidimicrobidae(100);Acidimicrobiales(100);Acidimicrobineae(100);uncultured(100);unclassified(100);unclassified(100);

Otu0567 0 0 0 0 0 0 0 0 0 0 0 0 0 0 0 0 0 0 0 0 0 0 0 0 5 0 0 0
0 0 0 0 0 0 0 0 0 0 0 0 0 0 0 11 0 0 0 0 0 0 0 0 0 0 0 0
0 0 0 0 0 0 0 0 0 0 0 0 0 0 0 0 0 0 0 0 0 0 0 0 0 0 0 0
0 0 0 0 0 0 0 0 0 0 0 0 0 0 0 0 0 0 0 0 0 0 0 0 0 0 0
M02255_77_000000000-ADUT3_1_1117_21479_17832 G----AC---AG-AG-GCC--------ACG-A-G------C--G--T---T--AG-G-CGG-AA------TC-A--C-T--GG-GC-------TT--A-----AA-GC-GT-GT-------G-TA-G-G-C-G------------G--A-CC-C-G-T----
-------AA----G-T-G-T--------C-T-T--G--TG--A-AA-TC--C-C-AC-G-G-----CT-C-AA------C-C-G-T-G-G-A--A-C----T-G--C-T---G--G-------G--CA-T-A---C-------T--G-C--GG--G-T-C----T-T-G-A-G-C--C---G-GC-----CA-G-G--------G-G-T-C---AC-C------GG--A--ACA----
A-G-C-G-GT--GG-A-G-CG-GT--G---A--A-A---TG-C-GT-AG--AT-A-TC--G-C--T-T-G-G-A-AC-G-CC---GA--C--G--GT-GAA-A--A-C--A-------G--G-T-G-A---CTG--G--GG-C-C--------------------G-C---G-C-T--GA--CG-C-T-G--A-GA--C-A-CG-A--AA-G-C---C-AG--
GG-G--AG-C-G-AA--------CG--GG Bacteria(100);Planctomycetes(100);Phycisphaerae(100);WD2101(100);unclassified(100);unclassified(100);unclassified(100);unclassified(100);unclassified(100);

Otu0568 0 0 0 0 0 0 0 0 0 0 0 0 0 0 0 0 0 0 0 0 0 0 0 0 0 0 0 0
0 0 0 0 0 0 0 88 0 0 0 1 0 0 0 0 0 0 0 0 0 0 0 0 0 0 0 0
0 0 0 0 0 0 0 0 0 0 0 0 0 0 0 0 0 0 0 0 0 0 0 0 0 0 0 0
0 0 0 0 0 0 0 0 0 0 0 0 0 0 0 0 0 0 0 0 0 0 0 0 0 0 0
M02255_77_000000000-ADUT3_1_1103_5176_13178 T----AC---GT-AG-GGG--------GCA-A-G------C--G--T---T--GT-C-CGG-AT------TT-A--T-T--GG-GC-------GT--A-----AA-GG-GT-GC-------G-CA-G-G-C-G------------G--T-CT-A-T-T-----------AA-
---G-T-C-A--------G-A-T--G--TA--A-AA-TA--C-C-GA-G-G-----CT-C-AA------C-C-T-C-G-G-G--G------A-G--C-A---A--C-------T--GA-A-A---C-------T--T-T--TA--G-A-C----T-T-G-A-G-T--T---A-AG-----GA-G-A--------G-G-G-A---AG-T------GG--A--ATT----C-C-T-A-
GT--GT-A-G-CG-GT--G---A--A-A---TG-C-GT-AG--AT-A-TT--A-G--G-A-A-G-A-AT-A-CC---GG--T--G--GC-GAA-G--G-C--G-------A--C-T-T-T---CTG--G--AC-T-T--------------------A-A---A-C-T--GA--CG-C-T-G--A-GG--C-A-CG-A--AA-G-T---G-TG--GG-G--AG-C-
A-AA--------CA--GG Bacteria(100);Firmicutes(68);Clostridia(68);Clostridiales(68);unclassified(68);unclassified(68);unclassified(68);unclassified(68);unclassified(68);

Otu0569 0 0 0 0 0 0 0 0 0 0 0 0 0 0 0 0 0 0 0 0 0 0 0 0 0 0 0 0
0 0 4 0 0 0 0 0 0 8 8 11 0 0 0 0 0 85 0 0 0 0 0 0 0 0 0 0
0 0 0 0 0 0 0 0 0 0 0 0 0 0 0 0 0 22 0 0 0 0 0 0 0 0 0 0
0 0 0 0 0 0 0 0 0 0 0 0 0 0 0 0 0 0 0 0 0 0 0 0 0 0 0
M02255_77_000000000-ADUT3_1_1111_19889_12178 T----AC---GA-AG-GGG--------GCT-A-G------C--G--T---T--GT-T-CGG-AT------TT-A--C-T--GG-GC-------GT--A-----AA-GC-GC-GT-------G-TA-G-G-C-G------------G--G-CT-G-G-A----
-------AA----G-T-T-G--------G-G-G--G--TG--A-AA-TC--C-C-GA-G-G-----CT-C-AA------C-C-T-C-G-G-A--A-C----T-G--C-C---T--C-------C--AA-A-A---C-------T--T-C--CA--G-C-C----T-G-G-A-C-T--C---A-AG-----TA-G-A--------G-G-C-A---AG-T------GG--A--ATT----
G-C-G-A-GT--GT-A-G-AG-GT--G---A--A-A---TT-C-GT-AG--AT-A-TT--C-G--C-A-G-G-A-AC-A-CC---AG--T--G--GC-GAA-G--G-C--G-------A--C-T-T-G---CTG--G--AC-T-T--------------------G-T---A-G-T--GA--CG-C-T-G--A-GA--C-G-CG-A--AA-G-C---G-TG--GG-
G--AG-C-A-AA--------CA--GG Bacteria(100);Proteobacteria(100);unclassified(85);unclassified(85);unclassified(85);unclassified(85);unclassified(85);unclassified(85);unclassified(85);

Otu0570 0 0 0 0 0 0 0 0 0 0 0 0 0 0 0 0 0 0 0 0 0 0 0 0 0 0 0 0
0 0 0 0 0 0 0 0 0 0 0 0 0 0 0 29 0 0 0 0 0 0 0 0 0 0 0 0 0
0 1 5 0 0 0 0 0 0 0 0 0 0 0 248 112 3 0 0 0 0 0 0 0 0 0 0 0 0
0 0 0 0 0 0 0 0 0 0 0 0 0 0 0 0 0 0 0 0 0 0 0 0 0 0 0 0

M02255_76_000000000-ADUNE_1_1106_20145_6284 T----AC---GT-AG-GGG--------GCG-A-G------C--G--T---T--GT-C-CGG-AA------TT-A--C-T--GG-GC-------GT--A-----AA-GG-GC-AC-------G-CA-G-G-C-T------------G--T-GC-T-T-C-----------AA----G-T-C-A--------G-C-T--G--TA--A-AA-GG--A-T-GC-G-G-----CT-T-AA------C-C-G-T-G-T-T--A------T-G--C-G---G--C-------T--GA-G-A---C-------T--G-A--GG--T-G-C----T-G-G-A-G-T--A---C-CG-----GA-G-A--------G-G-C-A---AG-T------GG--A--ATT----C-C-C-A-GT--GT-A-G-CG-GT--G---A--A-A---TG-C-GT-AG--AT-A-TT--G-G--G-A-A-G-A-AC-A-TC---GG--T--G--GC-GAA-G--G-C--G-------A--C-T-T-G---CTG--G--AC-G-G--------------------T-A---A-C-T--GA--CG-C-T-G--A-GG--T-G-CG-A--AA-G-C---C-AG--GG-T--AG-C-G-AA--------CG--GG Bacteria(100);Synergistetes(100);Synergistia(100);Synergistales(100);Synergistaceae(100);uncultured(100);unclassified(100);unclassified(100);unclassified(100);

Otu0571 0 0 0 0 0 0 0 0 0 0 0 0 0 0 0 0 0 0 0 0 0 0 0 0 0 0 0 0
0 0 0 0 0 0 0 0 0 0 0 0 0 0 0 0 0 0 0 0 122 0 0 0 0 0 0 0 0
0 0 0 0 0 0 0 0 0 0 0 0 0 0 0 0 0 0 0 0 0 0 0 0 0 0 0 0 0
0 0 0 0 0 0 0 0 0 0 0 0 0 0 0 0 0 0 0 0 0 0 0 0 0 0 0 0

M02255_76_000000000-ADUNE_1_2119_10318_8447 C----TA---AC-TC-CGT--------GCC-A-G------C--A--G---C--CG-C-GGT-AA------TA-C--G-G--AG-GG-------TC--C-----GA-GC-GT-TA-------T-C--C-G---G------------A--T-CA-A-T-T-----------AA----G-T-C-A--------G-A-G--G--TG--A-AA-TC--C-C-AT-A-G-----CT-C-AA------C-T-A-T-G-G-A--A-C----T-G--C-C---T--T-------T--GA-T-A---C-------T--G-G--TT--G-A-C----T-T-G-A-G-T--C---A-TA-----TG-G-A--------A-G-T-A---GA-T------AG--A--ATG----T-G-T-A-GT--GT-A-G-CG-GT--G---A--A-A---TG-C-AT-AG--AT-A-TT--A-C--A-C-A-G-A-AT-A-CC---GA--T--T--GC-GAA-G--G-C--A-------G--T-C-T-A---CTA--C--GT-A-T--------------------G-T---A-C-T--GA--CG-C-T-G--A-GG--G-A-CG-A--AA-G-C---G-TG--GG-G--AG-C-G-AA--------CA--GG Bacteria(100);Bacteroidetes(100);Flavobacteria(100);Flavobacteriales(100);Flavobacteriaceae(100);Polaribacter(100);unclassified(100);unclassified(100);unclassified(100);

Otu0572 0 0 0 0 0 0 0 0 0 0 0 0 0 0 0 0 0 0 0 0 0 0 0 0 0 0 0 0
0 0 0 0 0 0 0 0 0 0 0 0 0 0 44 0 0 0 0 0 0 0 0 35 0 0 0 0 0
0 0 0 0 0 0 0 0 0 0 0 0 0 0 0 0 0 0 0 0 0 0 0 0 0 0 0 0 0
0 0 0 0 0 0 0 0 0 0 0 0 0 0 0 0 0 0 0 0 0 0 0 0 0 0 0 0

M02255_77_000000000-ADUT3_1_2112_25087_4149 T----AC---GT-AG-GGG--------GCG-A-G------C--G--T---T--GT-C-CGG-AT------TT-A--T-T--GG-GC-------GT--A-----AA-GA-GC-TC-------G-TA-G-G-C-G------------G--T-TT-G-G-C-----------AA----G-T-C-G--------G-G-T--G--TG--A-AA-AC--T-C-CG-G-G-----CT-C-AA------C-T-C-G-G-A-G--A------C-G-CC-A---C--T-------C--GA-T-A---C-------T--G-C--CA-TG-A-C----T-C-G-A-G-T--C---C-GG-----TA-G-G--------G-G-A-G---TG-C------GG--A--ATT----C-C-T-G-GT--GT-A-G-CG-GT--G---A--A-A---TG-C-GC-AG--AT-A-TC--A-G--G-A-G-G-A-AC-A-CC---GG--T--G--GC-GAA-G--G-C--G-------G--C-A-C-T---CTG--G--GC-C-G--------------------G-T---A-C-T--GA--CG-C-T-G--A-GG--A-G-CG-A--AA-G-C---G-TG--GG-G--AG-C-G-AA--------CA--GG Bacteria(100);Actinobacteria(100);Actinobacteria(100);unclassified(100);unclassified(100);unclassified(100);unclassified(100);unclassified(100);unclassified(100);

Otu0573 0 0 0 0 0 0 0 0 0 0 0 0 0 0 0 0 0 0 0 0 0 0 0 0 0 0 0 0
0 0 0 0 0 0 0 14 0 0 0 0 0 15 0 2 0 0 0 0 0 0 0 0 0 0 0 0 0
0 0 0 0 0 0 0 0 0 0 0 1 0 0 0 0 0 0 0 0 70 0 0 0 0 0 0 0 0
0 0 0 0 0 0 0 0 0 0 0 0 0 0 0 0 0 0 0 0 0 0 0 0 0 0 0 0

M02255_77_000000000-ADUT3_1_1105_18560_23449 T----AC---GT-AG-GGG--------GCA-A-G------C--G--T---T--GT-C-CGG-AA------TG-A--T-T--GG-GC-------GT--A-----AA-GG-GC-TC-------G-TA-G-G-T-G------------G--C-AT-G-T-C-----------AA----G-T-C-T--------G-A-A--G--TG--A-AA-GT--C-C-TA-C-T-----TT-T-AA------G-G-T-G-G-G-A--A-T----T-G--C-T---T--T-------G--GA-A-A---C-------T--G-G--CA--A-G-C----T-T-G-A-G-T--A---T-AG-----GA-G-A--------G-G-T-T---AG-T------GG--A--ATT----C-C-T-A-GT--GT-A-G-CG-GT--G---A--A-A---TG-C-GT-AG--AG-A-TT--A-G--G-A-G-G-A-AC-A-TC---AG--T--G--GC-GAA-G--G-C--G-------A--C-T-A-A---CTG--G--AC-T-A--------------------A-A---A-C-T--GA--CA-C-T-G--A-GG--A-G-CG-A--AA-G-T---G-TG--GG-T--AG-C-A-AA--------CA--GG Bacteria(100);unclassified(100);unclassified(100);unclassified(100);unclassified(100);unclassified(100);unclassified(100);unclassified(100);unclassified(100);

Otu0574 0 0 0 0 0 0 0 0 0 0 0 0 0 0 0 0 0 0 0 0 0 0 0 0 0 0 0 0
0 0 0 0 0 0 0 0 0 0 0 97 0 0 0 18 0 0 0 0 0 0 0 0 0 0 0 0 0
0 0 0 0 0 0 0 0 0 0 0 2 0 5 0 0 0 0 135 195 0 0 0 0 0 0 0 0 0
0 0 0 0 0 0 0 0 2 114 0 121 0 0 5 0 0 0 0 0 0 0 0 0 0 0 0 0

M02255_77_000000000-ADUT3_1_2119_17560_17371 T----AC---GT-AT-GGA--------CCG-A-G------C--G--T---T--AT-C-CGG-AA------TC-A--T-T--GG-GC-------GT--A-----AA-GG-GT-AC-------G-TA-G-G-C-G------------G--T-TA-A-A-T-----------AA----G-T-T-A--------G-A-A--G--TG--A-AA-TC--C-C-AT-A-G-----CT-C-AA------C-T-A-T-A-G-T--A------A-G--C-T---T--C-------T--AA-A-A---C-------T--G-T--AT--A-A-C----T-T-G-A-G-A--A---A-TG-----AA-A-G--------G-G-A-A---AG-T------GG--A--ATT----C-C-T-A-GT--GT-A-G-CG-GT--G---A--A-A---TG-C-GC-AG--AT-A-TT--A-G--G-A-G-G-A-AT-A-CC---GG--T--G--GC-GAA-G--G-C--G-------A--C-T-T-T---CTG--G--TC-A-T--------------------A-T---T-C-T--GA--CG-C-T-G--A-GG--T-A-CG-A--AA-G-C---G-TG--GG-T--AG-C-A-AA--------CA--GG Bacteria(100);Firmicutes(100);Clostridia(100);Clostridiales(100);Family_XI_Incertae_Sedis(100);Anaerococcus(100);unclassified(100);unclassified(100);unclassified(100);

Otu0575 11 0 0 0 0 0 0 0 0 0 0 0 0 0 0 0 0 0 0 0 0 0 0 0 0 0 0 0
0 0 0 0 0 0 0 0 0 0 0 0 0 0 20 0 0 0 0 0 0 0 0 0 0 0 0 0 0
0 0 0 0 0 0 0 0 0 0 0 0 0 0 0 0 0 0 0 0 0 0 0 0 0 0 0 7 0
0 0 0 0 0 0 0 0 0 8 0 0 0 0 0 0 0 0 0 0 0 0 0 0 0 0 0
M02255_76_000000000-ADUNE_1_1106_16485_19660 C----GG---CT-AA-CT---------ACG-T-G------C--C--A---G--CA-G-CCG-CG------GT-A--A-T--AC-GT-------AG--G-----TG-GC-AA-GC-------G-TT-G-T-C-G------------G--T-GA-T-T-T-----------AA----G-T-C-T--------G-A-T--G--TG--A-AA-GC--C-C-CC-G-G-----CT-C-AA------C-C-G-G-G-G-A--G------G-GT-C-A---T--T-------G--GA-A-A---C-------T--G-G--AT--C-A-C----T-T-G-A-G-T--G---C-AG-----AA-G-A--------G-G-A-G---AG-T------GG--A--ATT----C-C-A-T-GT--GT-A-G-CG-GT--G---A--A-A---TG-C-GT-AG--AT-A-TA--T-G--G-A-G-G-A-AC-A-CC---AG--T--G--GC-GAA-G--G-C--G-------G--C-T-C-T---CTG--G--TC-T-G--------------------T-A---A-C-T--GA--CG-C-T-G--A-GG--C-T-CG-A--AA-G-C---G-TG--GG-T--AG-C-A-AA--------CA--GG Bacteria(100);Firmicutes(100);Bacilli(100);Lactobacillales(100);unclassified(61);unclassified(61);unclassified(61);unclassified(61);unclassified(61);

Otu0576 0 0 0 0 0 0 0 0 0 0 0 0 0 0 0 0 0 0 0 0 0 0 0 0 0 0 0 0
0 0 6 0 0 0 0 0 0 0 102 0 0 0 0 0 0 0 0 0 0 0 0 0 0 0 0 0 0
0 0 0 0 0 0 0 0 0 0 0 0 5 0 0 0 0 0 0 0 0 0 0 0 0 0 0 0 0
0 0 0 0 0 0 0 0 0 0 0 0 0 0 0 0 0 0 0 0 0 0 0 0 0 0 0
M02255_77_000000000-ADUT3_1_2101_20853_22567 T----AC---GA-AG-GGG--------GCT-A-G------C--G--T---T--GT-T-CGG-AA------TT-A--C-T--GG-GC-------GT--A-----AA-GC-GC-AC-------G-TA-G-G-C-G------------G--A-CT-T-G-T-----------CA----G-T-C-A--------G-G-G--G--TG--A-AA-TC--C-C-GA-G-G-----CT-T-AA------C-C-T-C-G-G-A--A-C----T-G--C-C---T--T-------T--GA-T-A---C-------T--G-C--AA--G-T-C----T-T-G-A-G-G--A---T-GA-----GA-G-A--------G-G-T-A---AG-T------GG--A--ACT----G-C-G-A-GT--GT-A-G-AG-GT--G---A--A-A---TT-C-GT-AG--AT-A-TT--C-G--C-A-A-G-A-AC-A-CC---AG--T--G--GC-GAA-G--G-C--G-------G--C-T-T-A---CTG--G--CT-C-A--------------------T-T---T-C-T--GA--CG-C-T-G--A-GG--T-G-CG-A--AA-G-C---G-TG--GG-G--AG-C-A-AA--------CA--GG Bacteria(100);Proteobacteria(100);Alphaproteobacteria(100);unclassified(100);unclassified(100);unclassified(100);unclassified(100);unclassified(100);

Otu0577 0 0 0 0 0 0 0 0 0 0 0 0 0 0 0 0 0 0 0 0 0 0 0 0 0 0 0 0
0 0 0 0 0 0 0 0 0 0 0 0 0 0 58 0 0 0 0 0 0 0 0 0 0 0 0 0 0
0 0 0 0 0 0 0 0 0 0 0 0 6 0 0 0 0 0 0 0 0 0 0 0 0 0 0 0 0
0 0 0 0 0 0 0 0 0 0 0 0 0 0 0 0 0 0 0 0 0 0 0 0 0 0 0
M02255_77_000000000-ADUT3_1_1107_21296_23386 T----AC---GA-AG-GGG--------GCT-A-G------C--G--T---T--GC-T-CGG-AA------TT-A--C-T--GG-GC-------GT--A-----AA-GG-GC-GC-------G-TA-G-G-C-G------------G--C-GC-A-T-T-----------TA----G-T-C-A--------G-G-C--G--TG--A-AA-TT--C-C-TG-G-G-----CT-T-AA------C-C-T-G-G-G-G--A-C----T-G--C-G---C--T-------T--GA-T-A---C-------G--G-G--TG--T-G-C----T-T-G-A-G-G--A---C-GG-----AA-G-A--------G-G-C-T---CG-C------GG--A--ATT----C-C-C-A-GT--GT-A-G-AG-GT--G---A--A-A---TT-C-GT-AG--AT-A-TT--G-G--G-A-A-G-A-AC-A-CC---GG--T--G--GC-GAA-G--G-C--G-------G--C-G-A-G---CTG--G--TC-C-G--------------------T-A---T-C-T--GA--CG-C-T-G--A-GG--C-G-CG-A--CA-G-C---G-TG--GG-G--AG-C-A-AA--------CA--GG Bacteria(100);Proteobacteria(100);Alphaproteobacteria(100);Rhodospirillales(100);Acetobacteraceae(100);unclassified(100);unclassified(100);unclassified(100);unclassified(100);

Otu0578 0 0 0 0 0 0 0 0 0 0 0 0 0 0 0 0 0 0 0 0 0 0 0 0 0 0 0 0
0 0 0 0 0 0 0 0 0 4 0 0 0 0 0 1 0 14 0 0 0 0 0 0 0 0 0 0 0
0 0 0 0 0 0 0 0 0 0 0 0 0 0 0 0 0 0 0 0 0 0 0 0 0 0 0 0 0
7 3 0 0 0 0 0 0 0 3 0 0 0 0 0 0 0 0 0 0 0 0 0 0 0 0 0
M02255_76_000000000-ADUNE_1_1119_3185_14127 T----AC---GT-AG-GTC--------TCA-A-G------C--G--T---T--AT-C-CGG-AT------TT-A--C-T--GG-GC-------GT--A-----AA----GT----------G--A-T-C-C--------------G-------T--------------AA----G-T-C-T--------G-T-T--T--TC--A-AA-TC--C-T-AC-G-A-----CT-C-AA------T-C-G-T-A-G-A--A-A----G-G--G-A---A--T-------G--GA-T-A---C-------T--G-C--AG--A-T-C----T-A-G-A-A-G--T---A-TC-----TA-G-G--------G-G-T-T---AG-T------GG--A--ATT----T-C-C-G-GT--GG-A-G-CG-GT--G---A--A-A---TG-C-GT-TG--AT-A-TC--G-G--A-A-G-G-A-AC-G-CC---GA--A--A--GC-GAA-A--G-C--A-------G--C-T-A-A---CTA--T--AG-A-A--------------------T-A---C-T-T--GA--CG-A-T-G--A-GG--G-A-CG-A--CA-G-T---T-TG--GG-G--AG-C-A-AA--------CA--GG Bacteria(100);unclassified(100);unclassified(100);unclassified(100);unclassified(100);unclassified(100);unclassified(100);unclassified(100);unclassified(100);

Otu0579 0 0 0 0 0 0 0 0 0 0 0 0 1 0 0 0 0 0 0 0 0 0 0 0 0 0 1 0
0 0 0 0 0 0 0 0 0 0 0 0 0 0 0 0 0 0 0 0 0 0 0 0 0 0 0 0 0
0 0 0 0 0 0 0 0 0 0 0 0 4 0 0 0 0 0 0 0 0 0 0 0 0 0 0 0 0
0 0 0 0 0 0 0 0 0 0 0 0 1 0 0 0 0 0 0 0 0 0 0 0 0 0 0
M02255_77_000000000-ADUT3_1_2107_6535_21828 G----TG---CC-AG-CAG--------CCG-C-G------G--T--A---A--GA-C-GGAGGA------TC-C--A-A--GC-GT-------TA--T-----CC-GG-AA-TC-------A-TA-G-G---G------------G--T-CA-G-A-T-----------AA----G-T-C-A--------G-T-G--G--TG--A-AA-GC--C-C-AT-C-G-----CT-C-AA------C-G-G-T-G-G-A--A-C----G-G--C-C---A--T-------T--GA-T-A---C-------T--G-T--CT--G-A-C----T-T-G-A-A-T--T---A-TT-----AG-G-A--------A-G-T-A---AC-T------AG--A--ATA----T-G-T-A-GT--GT-A-G-CG-GT--G---A--A-A---TG-C-TT-AG--AG-A-TT--A-C--A-T-G-G-A-AT-A-CC---AA--T--T--GC-GAA-G--G-C--A-------G--G-T-T-A---CTA--C--TA-A-T--------------------A-T---A-T-T--GA--CG-C-T-G--A-TG--G-A-CG-A--AA-G-C---G-TG--GG-T--AG-C-G-AA--------CA--GG Bacteria(100);Bacteroidetes(100);Flavobacteria(100);Flavobacteriales(100);Flavobacteriaceae(100);Flavobacterium(100);unclassified(100);unclassified(100);unclassified(100);

Otu0580 0 0 0 0 0 0 0 0 0 0 0 0 0 0 0 0 0 0 0 0 0 0 3 0 0 0 0 0
0 0 0 0 0 0 0 0 0 1 0 4 0 0 0 0 0 0 0 2 0 0 0 0 0 0 0 0 16
0 0 0 0 0 0 0 0 0 0 0 0 0 0 0 0 1 0 0 0 0 0 0 0 0 0 0 0 0
0 0 0 0 0 0 0 0 0 0 0 0 0 0 0 0 0 2 0 0 0 1 0 0 0 23 0
M02255_77_000000000-ADUT3_1_1117_10057_20499 T----AC---GT-AG-GGT--------GCA-A-G------C--G--T---T--GT-C-CGG-AA------TT-A--T-T--GG-GC-------GT--A-----AA-GA-GC-TC-------G-TA-G-G-C-G------------G--T-TC-G-T-C------
-----GC----G-T-C-G--------G-C-T--G--TG--A-AA-AC--C-C-GG-A-G-----CT-C-AA------C-T-C-C-G-G-G--C-C----T-G--C-A---G--T-------C--GA-T-A---C-------G--G-G--CG--G-A-C----T-T-G-A-G-T--G---T-TG-----CA-G-G--------G-G-A-G---AC-T------GG--A--ATT----C-
C-T-G-GT--GT-A-G-CG-GT--G---A--A-A---TG-C-GC-AG--AT-A-TC--A-G--G-A-G-G-A-AC-A-CC---GG--T--G--GC-GAA-G--G-C--G-------G--G-T-C-T---CTG--G--GC-A-A--------------------C-T---A-C-T--GA--CG-C-T-G--A-GG--A-G-CG-A--AA-G-C---G-TG--GG-
G--AG-C-G-AA--------CA--GG Bacteria(100);Actinobacteria(100);Actinobacteria(100);Actinobacteridae(100);Actinomycetales(100);Frankineae(100);Geodermatophilaceae(100);Geodermatophilus(100);unclassified(100);

Otu0581 0 0 0 0 0 0 0 0 0 0 0 0 0 45 6 0 0 0 0 6 9 0 0 0 0 24 0 29
21 0 0 0 0 0 0 0 0 0 0 0 0 0 157 0 47 0 0 0 0 0 0 0 0 0 0 0 0
0 7 0 0 0 0 0 0 0 0 0 0 2 5 0 0 0 20 0 0 0 0 0 0 0 0 0 0 0
0 0 0 72 34 0 0 0 0 0 0 0 0 20 0 0 69 0 73 31 0 0 0 3 25 65 0
M02255_77_000000000-ADUT3_1_2117_12112_7260 T----AC---GG-AG-GGG--------GCT-A-G------C--G--T---T--GT-T-CGG-AA------TT-A--C-T--GG-GC-------GT--A-----AA-GC-GC-AC-------G-TA-G-G-C-G------------G--C-TT-T-G-T-----------AA-
---G-T-C-A--------G-G-G--G--TG--A-AA-GC--C-T-GG-A-G-----CT-C-AA------C-T-C-C-A-G-A--A-C----T-G--C-C---T--T-------T--GA-G-A---C-------T--G-C--AG--C-G-C----T-T-G-A-A-T--C---C-GG-----GA-G-A--------G-G-T-A---AG-T------GG--A--ATT----C-C-G-A-
GT--GT-A-G-AG-GT--G---A--A-A---TT-C-GT-AG--AT-A-TT--C-G--G-A-A-G-A-AC-A-CC---AG--T--G--GC-GAA-G--G-C--G-------G--C-T-T-A---CTG--G--AC-C-G--------------------G-G---A-T-T--GA--CG-C-T-G--A-GG--T-G-CG-A--AA-G-C---G-TG--GG-G--AG-
C-A-AA--------CA--GG Bacteria(100);Proteobacteria(100);Alphaproteobacteria(100);Sphingomonadales(100);unclassified(100);unclassified(100);unclassified(100);unclassified(100);unclassified(100);

Otu0582 0 0 0 0 0 0 0 0 0 0 0 0 0 2 0 0 0 0 0 0 0 0 0 0 0 0 0 0
0 0 0 0 0 0 0 0 0 4 3 1 18 0 10 2 0 26 0 0 0 44 1 10 8 0 0 0 10
0 0 0 0 0 0 0 0 0 0 0 0 0 0 0 5 1 0 0 0 0 0 0 0 0 0 0 0 0
0 0 0 0 0 0 0 0 0 0 0 0 0 0 0 0 0 0 0 0 0 0 0 0 0 0 0
M02255_76_000000000-ADUNE_1_1118_11936_24395 C----A----GC-A--GCC--------GCG-G-T------A--A--T---A--CG-G-AGG-GT------GC-A--A-G--CG-TT-------AC--C-----CG-GA-AT-CA-------C-TG-G-G-C-G------------G--A-CA-C-T-T-------
----AA----G-T-C-T--------G-G-T--T--TT--A-AA-GA--C-C-GG-G-G-----CT-C-AA------C-C-C-C-G-G-G--C-A----T-G--G-A---C--T-------G--GG-T-A---C-------T--G-G--GT--G-T-C----T-G-G-A-C-C--T---C-TG-----GA-G-A--------G-A-G-A---AC-T------GG--A--ATT----C-
C-T-G-GT--GT-A-G-CG-GT--G---G--A-A---TG-C-GT-AG--AT-A-CC--A-G--G-A-G-G-A-AC-A-CC---AA--T--G--GC-GAA-G--G-C--A-------G--G-T-T-C---TTG--G--AC-A-G--------------------A-A---G-G-T--GA--CG-C-T-G--A-GG--C-G-CG-A--AA-G-T---G-TG--GG-
G--AG-C-G-AA--------CC--GG Bacteria(100);Deinococcus-Thermus(100);Deinococcales(100);Deinococcaceae(100);Deinococcus(100);unclassified(100);unclassified(100);unclassified(100);unclassified(100);

Otu0583 0 0 0 0 0 0 0 0 0 0 0 0 0 0 0 0 0 0 0 23 0 15 0 0 0 0 0 0
1 0 0 0 0 0 0 0 0 0 0 0 0 0 0 0 0 0 0 0 0 0 0 0 0 0 0 0 0
0 0 0 0 0 0 0 0 0 0 0 0 16 5 1 0 0 0 0 0 0 0 0 0 0 0 0 0 0
0 0 0 0 0 0 0 0 0 0 0 0 0 0 2 0 0 0 0 0 0 0 0 0 0 0 0
M02255_76_000000000-ADUNE_1_2111_17527_23784 C----TC---GC-AG-AAT--------AAG-CACC-----G--G--C---T--AACT-CTG-TG------CC-A--G-C--AG-CC-------GC--G-----GT-AA-TA-CA-------G-AG-G-G-T-G------------C--A-AG-T-G-T---
--------TA----A-T-C-G--------G-A-T--G--TG--A-AA-TC--C-C-TG-A-G-----CT-T-AA------C-T-T-A-G-G-A--A-T----T-G--C-A---T--A-------C--GA-T-A---C-------T--G-G--GA--G-A-C----T-A-G-A-G-T--A---T-GG-----GA-G-A--------G-G-A-T---GG-T------AG--A--ATT----
C-C-A-G-GT--GT-A-G-CG-GT--G---A--A-A---TG-C-GT-AG--AG-A-TC--T-G--G-A-G-G-A-AT-A-CC---GA--T--G--GC-GAA-G--G-C--A-------G--C-C-A-T---CTG--G--CC-T-A--------------------A-T---A-C-T--GA--CA-C-T-G--A-GG--T-A-CG-A--AA-G-C---A-TG--GG-
G--AG-C-A-AA--------CA--GG Bacteria(100);Proteobacteria(100);Gammaproteobacteria(100);Pseudomonadales(100);Moraxellaceae(100);Acinetobacter(100);unclassified(100);unclassified(100);unclassified(100);

Otu0584 0 0 0 0 0 0 0 0 0 0 0 0 0 0 0 0 0 0 0 0 0 0 0 0 0 0 0 0
0 0 0 0 0 0 0 42 0 0 0 0 0 0 0 0 0 0 0 0 0 0 0 0 0 0 0 0 0
0 0 0 0 0 0 0 0 0 0 0 0 0 0 0 0 1 0 0 0 0 0 0 0 0 0 0 0 0
0 0 0 0 0 0 0 0 0 0 0 0 0 0 0 0 0 0 0 0 0 0 0 0 0 0 0
M02255_76_000000000-ADUNE_1_1109_15746_16540 T----AC---GT-GC-CAGC-------AGC-C-G------CG-G--T---A--AT-A-CGT-AG------GT-G--G-C--AA-GC-------GT--T-----GT-CC-G--GA-------T-TT-A-C-T-G------------G--G-CG-T-T-A-----
------AA----GAG-C-G--------C-G-C--A--GG--A-AA-GC--C-C-CC-G-G-----CT-C-AA------C-T-G-G-G-G-A--G-G----G-T--C-A---C--G-------C--GA-T-A---C-------T--G-A--TC--G-A-C----T-C-G-A-A-G--G---C-AG-----GA-G-A--------G-G-G-T---AG-T------GG--A--ATT---
-C-C-C-G-GT--GT-A-G-TG-GT--G---A--A-A---TG-C-GT-AG--AT-A-TC--G-G--G-A-G-G-A-AC-A-CC---AG--T--G--GC-GAA-G--G-C--G-------A--C-T-A-C---CTG--G--CC-T-GT-------------------T-T---C-T-T--GA--CG-C-T-G--A-GG--C-G-CG-A--AA-G-C---T-AG--
GG-G--AG-C-A-AA--------CG--GG Bacteria(100);Chloroflexi(100);KD4-96(100);unclassified(100);unclassified(100);unclassified(100);unclassified(100);unclassified(100);unclassified(100);

Otu0585 0 0 0 0 0 0 0 0 0 0 0 0 0 0 0 0 0 0 1 0 0 0 0 0 0 0 0 0
0 0 0 0 0 0 0 0 0 0 0 0 0 0 0 0 0 0 0 0 0 0 0 0 1 0 0 0
0 12 0 0 0 0 0 0 0 0 0 0 0 0 0 0 0 0 0 7 0 0 0 3 24 0 0 0 0
3 0 0 3 0 0 0 0 0 2 0 0 0 0 0 0 0 0 0 0 0 2 0 0 0 0 0
M02255_76_000000000-ADUNE_1_1106_18796_11884 .....AC----T-AC-GT---------GCC-A-G------C--A--G---C--CG-C-GGT-AA------TA-C--G-T--AG-GG-------GG--C-----AA-GC-G--TT-------G-TC-C-G---G------------A--T-CA-A-T-T----------
AA----G-T-C-T--------G-A-T--G--TG--A-AA-GC--C-C-CC-G-G-----CT-C-AA------C-C-G-G-G-G-A--G------G-GT-C-A---T--T-------G--GA-A-A---C-------T--G-G--TT--G-A-C----T-T-G-A-G-T--G---C-AG-----AA-G-A--------G-G-A-G---AG-T------GG--A--ATT----C-C-
A-T-GT--GT-A-G-CG-GT--G---A--A-A---TG-C-GT-AG--AT-A-TA--T-G--G-A-G-G-A-AC-A-CC---AG--T--G--GC-GAA-G--G-C--G-------A--C-T-C-T---CTG--G--TC-T-G--------------------T-A---A-C-T--GA--CG-C-T-G--A-GG--C-T-CG-A--AA-G-C---G-TG--GG-T--
AG-C-A-AA--------CA--GG Bacteria(100);Firmicutes(100);Bacilli(100);Lactobacillales(100);unclassified(100);unclassified(100);unclassified(100);unclassified(100);unclassified(100);

Otu0586 0 0 0 0 0 0 0 0 0 0 0 0 0 0 0 0 0 0 0 0 0 0 0 0 0 0 0 0
0 0 0 0 0 0 0 0 0 0 0 0 0 0 0 0 0 0 0 0 0 0 0 0 0 0 13 0
0 0 0 0 0 0 0 0 0 0 0 0 0 0 210 74 0 0 0 0 0 0 0 0 0 0 0 42 0
0 0 0 0 0 0 0 0 0 0 0 0 0 0 0 0 0 0 0 0 0 0 0 0 0 0 0
M02255_77_000000000-ADUT3_1_1105_17115_13889 T----AC---GT-AG-GGT--------GCG-A-G------C--A--T---T--AT-C-CGG-AG------TG-A--C-T--GG-GC-------GT--A-----AA-GA-GTTGC-------G-TA-G-G-C-G------------G--T-AA-G-T-C----
-------AA----G-T-G-A--------A-T-A--G--TG--A-AA-CC--T-G-GT-G-G-----CT-C-AA------C-C-A-T-ACA-G--A-C----T-A--T-T---A--T-------T--CA-A-A---C-------T--G-G--CT--T-A-C----T-C-G-A-G-A--G---T-GG-----TA-G-A--------G-G-T-C---AC-T------GG--A--ATT----T-
C-T-T-GT--GT-A-G-GA-GT--G---A--A-A---TC-C-GT-AG--AT-A-TA--A-G--A-A-G-G-A-AC-A-CC---GA--T--G--GC-GTA-G--G-C--A-------G--G-T-G-A---CTG--G--AC-C-A--------------------T-T---T-C-T--GA--CG-C-T-A--A-GG--C-A-CG-A--AA-G-C---G-TG--GG-G-
-AG-C-G-AA--------CC--GG Bacteria(100);Candidate_division_TM7(100);unclassified(100);unclassified(100);unclassified(100);unclassified(100);unclassified(100);unclassified(100);unclassified(100);

Otu0587 0 0 0 0 0 0 0 0 0 0 0 0 0 0 0 0 0 0 0 0 0 0 0 0 0 0 0 0
0 0 0 0 0 0 0 0 0 0 0 0 9 26 0 0 0 0 0 0 0 0 0 0 0 0 0 0 63
0 0 0 0 0 0 0 0 0 0 0 0 0 0 19 0 0 0 0 0 0 0 0 0 0 0 0 0 0
0 0 0 0 0 0 0 0 0 0 0 0 0 0 0 0 0 0 0 0 0 0 0 0 0 0 0
M02255_77_000000000-ADUT3_1_1107_5007_12715 T----AC---GT-AG-GGT--------GCG-A-G------C--G--T---T--GT-C-CGG-AA------TT-A--T-T--GG-GC-------GT--A-----AA-GA-GC-TC-------G-TA-G-G-C-G------------G--T-TC-G-T-C----------GC-
---G-T-C-G--------A-A-T--G--TG--A-AA-AC--C-C-GG-G-G-----CT-C-AA------C-T-C-C-G-G-G--C-C----T-G--C-A---T--T-------C--GA-T-A---C-------G--G-G--CG--G-A-C----T-A-G-A-G-T--T---C-GG-----TA-G-G--------G-G-A-G---AC-T------GG--A--ATT----C-C-T-G-
GT--GT-A-G-CG-GT--G---A--A-A---TG-C-GC-AG--AT-A-TC--A-G--G-A-G-G-A-AC-A-CC---GG--T--G--GC-GAA-G--G-C--G-------G--G-T-C-T---CTG--G--GC-C-G--------------------A-T---A-C-T--GA--CG-C-T-G--A-GG--A-G-CG-A--AA-G-C---G-TG--GG-G--AG-
C-G-AA--------CA--GG Bacteria(100);Actinobacteria(100);Actinobacteria(100);Actinobacteridae(100);Actinomycetales(100);unclassified(100);unclassified(100);unclassified(100);unclassified(100);

Otu0588 0 0 0 0 0 0 0 0 0 0 0 0 0 0 0 0 0 0 0 0 0 0 0 0 0 0 0 0
0 0 0 0 0 0 0 0 0 0 0 0 0 0 0 0 0 0 0 0 0 0 0 0 0 0 0 0
0 0 0 0 0 0 0 0 0 0 0 0 0 0 21 0 0 34 0 0 0 0 0 0 0 0 0 0 0
0 0 0 0 0 0 0 0 0 0 0 0 0 0 0 0 0 0 0 0 0 0 0 0 0 0 0
M02255_77_000000000-ADUT3_1_1107_25011_14873 T----AC---GT-AG-GGG--------GCA-A-G------C--G--T---T--GT-C-CGG-AT------TT-A--T-T--GG-GC-------GT--A-----AA-GA-GC-TC-------G-TA-G-G-C-G------------G--T-TT-G-G-T-----
------AA----G-T-C-A--------G-G-T--G--TG--A-AA-TC--T-C-CA-C-G-----CT-C-AA------C-G-T-G-G-A-G--G------G-G-CCAC---C--T----------GA-T-A---C-------T--G-C--CA-TG-A-C----T-G-G-A-G-T--C---C-GG-----TA-G-A--------G-G--AG---TG-T------GG--A--ATTC---
C-C---G-GT--GT-A-G-CG-GT--G---A--A-A---TG-C-GC-AG--AT-A-TC--G-G--G-A-G-G-A-AC-A-CC---CG--T--A--GC-GAA-G--G-C--G-------G--C-A-C-T---CTGG----GC-C-G--------------------G-T---A-C-T--GA--CG-C-T-G--A-GG--A-G-CG-A--AA-G-C---G-TG--
GG-G--AG-C-A-AA--------CA--GG Bacteria(100);Actinobacteria(100);Actinobacteria(100);Acidimicrobidae(100);Acidimicrobiales(100);Acidimicrobineae(100);unclassified(100);unclassified(100);unclassified(100);

Otu0589 0 0 0 0 0 0 0 0 0 0 0 0 0 0 0 0 0 0 0 0 0 0 0 0 0 6 0 8
65 0 0 0 0 0 0 0 0 0 0 0 1 0 0 0 0 0 0 0 0 0 0 0 0 0 0 0
0 0 0 0 0 0 0 0 0 0 0 0 0 0 39 0 0 0 0 0 0 0 0 0 0 0 0 0 0
0 0 0 0 0 0 0 0 0 0 0 0 0 0 0 0 0 0 0 0 0 0 0 0 0 0 0
M02255_77_000000000-ADUT3_1_1108_15962_10076 T----AC---GG-AG-GGG--------GCT-A-G------C--G--T---T--GT-T-CGG-AA------TT-A--C-T--GG-GC-------GT--A-----AA-GC-GC-AC-------G-TA-G-G-C-G------------G--C-TA-T-T-C-----
------AA----G-T-C-A--------G-A-G--G--TG--A-AA-GC--C-C-GG-G-G-----CT-C-AA------C-C-C-C-G-G-A--A-T----T-G--C-C---T--T-------T--GA-A-A---C-------T--A-G--AT--A-G-C----T-T-G-A-A-T--C---A-CG-----GA-G-A--------G-G-T-G---GG-T------GG--A--ATT----
C-C-G-A-GT--GT-A-G-AG-GT--G---A--A-A---TT-C-GT-AG--AT-A-TT--C-G--G-A-A-G-A-AC-A-CC---AG--T--G--GC-GAA-G--G-C--G-------G--C-C-C-A---CTG--G--AC-G-T--------------------G-T---A-T-T--GA--CG-C-T-G--A-GG--T-G-CG-A--AA-G-C---G-TG--GG-
G--AG-C-A-AA--------CA--GG Bacteria(100);Proteobacteria(100);Alphaproteobacteria(100);Sphingomonadales(100);Sphingomonadaceae(100);unclassified(68);unclassified(68);unclassified(68);unclassified(68);

Otu0590 0 0 0 0 0 0 0 0 0 0 0 0 0 0 0 0 0 0 0 0 0 0 0 0 0 0 0 0
0 0 0 0 0 0 0 73 64 0 0 0 0 0 0 0 0 0 0 0 0 0 0 0 0 0 0 0
0 0 0 0 0 0 0 0 0 0 0 0 0 0 0 0 0 0 0 0 0 0 0 0 0 0 0 0
0 0 0 0 0 0 0 0 0 0 0 0 0 0 0 0 0 0 0 0 0 0 0 0 0 0 0
M02255_77_000000000-ADUT3_1_1119_5513_12373 T----AC---GT-AG-GGG--------GCA-A-G------C--G--T---T--GT-C-CGG-AA------TT-A--T-T--GG-GC-------GT--A-----AA-GA-GC-TC-------G-TA-G-G-C-G------------G--C-TC-A-G-C-----------AA----G-T-C-G--------G-C-T--G--TG--A-AA-TC--C-C-GA-G-G-----CT-T-AA------C-C-T-C-G-G-A--A-C----T-G--C-A---G--T-------C--GA-T-A---C-------T--G-C--TGT-G-G-C----T-A-G-A-G-T--C---T-GG-----TA-G-A--------G-G-A-G---AA-T------GG--A--ATT----C-C-C-G-GT--GT-A-G-CG-GT--G---A--A-A---TG-C-GC-AG--AT-A-TC--G-G--G-A-G-G-A-AC-A-CC---AG--T--A--GC-GAA-G--G-C--G-------G--T-T-C-T---CTG--G--GC-C-A--------------------G-T---A-C-T--GA--CG-C-T-G--A-GG--A-G-CG-A--AA-G-C---G-TG--GG-G--AG-C-A-AA--------CA--GG Bacteria(100);unclassified(100);unclassified(100);unclassified(100);unclassified(100);unclassified(100);unclassified(100);unclassified(100);unclassified(100);

Otu0591 0 0 0 0 0 0 0 0 0 0 0 0 0 0 0 0 0 0 0 0 0 0 1 0 0 0 0 0
0 0 0 0 0 0 0 0 0 0 0 0 0 0 0 0 0 0 0 18 0 0 4 0 0 0 0 0 80
0 0 0 0 0 0 0 0 0 0 0 0 0 0 0 0 0 0 0 0 0 0 0 0 0 0 0 0
4 0 0 0 0 17 0 0 0 0 3 0 0 0 0 37 0 0 0 0 0 0 0 0 0 0 0
M02255_77_000000000-ADUT3_1_1115_15462_7256 T----AC---GT-AG-GGC--------GCA-A-G------C--G--T---T--GT-C-CGG-AA------TT-A--T-T--GG-GC-------GT--A-----AA-GA-GC-TC-------G-TA-G-G-C-G------------G--C-TT-G-T-C-----------GC----G-T-C-T--------G-C-G--G--TG--A-AA-GC--C-C-GG-G-G-----CT-T-AA------C-T-C-C-G-G-G--T-G----T-G--C-A---G--T-------G--GG-T-A---C-------G--G-G--CA--G-G-C----T-A-G-A-G-T--G---C-AG-----TA-G-G--------G-G-T-A---AC-T------GG--A--ATT----C-C-T-G-GT--GT-A-G-CG-GT--G---A--A-A---TG-C-GC-AG--AT-A-TC--A-G--G-A-G-G-A-AC-A-CC---GA--T--G--GC-GAA-G--G-C--A-------G--G-T-T-A---CTG--G--GC-T-G--------------------T-T---A-C-T--GA--CG-C-T-G--A-GG--A-G-CG-A--AA-G-C---A-TG--GG-G--AG-C-G-AA--------CA--GG Bacteria(100);Actinobacteria(100);Actinobacteria(100);Actinobacteridae(100);Actinomycetales(100);Micrococcineae(100);Micrococcaceae(100);Kocuria(99);unclassified(99);

Otu0592 0 0 0 0 0 0 0 0 0 0 0 0 0 0 0 0 0 0 0 0 0 0 0 4 0 0 0 0
0 0 0 0 0 0 0 0 0 0 0 0 0 0 0 0 0 0 0 51 0 0 0 0 0 0 0 0 0
0 0 0 0 0 0 0 0 0 0 0 0 0 0 0 0 0 0 0 0 0 0 7 0 0 0 0 0 0
0 0 0 0 0 0 0 0 0 0 0 0 0 0 0 0 0 0 0 0 0 0 0 0 0 0 0
M02255_77_000000000-ADUT3_1_1110_25175_18753 T----AC---GT-AG-GAT--------CCA-A-G------C--G--T---T--AT-C-CGG-AG------TG-A--C-T--GG-GC-------GT--A-----AA-GA-GTTGC-------G-TA-G-G-T-G------------G--T-TA-T-A-T-----------AA----G-T-G-A--------A-T-A--G--TG--A-AA-TC--T-G-GC-G-G-----CT-C-AA------C-C-G-T-ACA-G--G-C----T-A--T-T---A--T-------T--CA-A-A---C-------T--G-T--AT--A-A-C----T-C-G-A-G-A--A---T-GG-----TA-G-A--------G-G-T-A---AC-T------GG--A--ATT----T-C-T-T-GT--GT-A-G-GA-GT--G---A--A-A---TC-C-GT-AG--AT-A-TA--A-G--A-A-G-G-A-AC-A-CC---AA--T--G--GC-GTA-G--G-C--A-------G--G-T-T-A---CTG--G--AC-C-A--------------------T-T---T-C-T--GA--CA-C-T-G--A-GG--C-A-CG-A--AA-G-C---G-TG--GG-G--AG-C-G-AA--------CC--GG Bacteria(100);Candidate_division_TM7(100);unclassified(100);unclassified(100);unclassified(100);unclassified(100);unclassified(100);unclassified(100);unclassified(100);

Otu0593 0 0 0 0 0 0 0 0 0 0 0 0 0 0 0 0 0 0 0 0 0 0 0 0 0 0 0 0
0 0 0 0 0 0 0 0 0 0 0 0 0 0 0 0 0 0 0 0 0 0 0 0 0 0 0 0
0 0 0 0 0 0 0 0 0 0 0 0 0 0 16 14 0 0 0 0 0 0 0 0 0 0 0 0
0 0 0 0 0 0 0 0 0 2 0 0 0 0 0 0 0 0 0 0 0 0 0 0 0 0 0
M02255_76_000000000-ADUNE_1_2106_9517_19446 T----CG---GC-TA-ACT--------CCG-T-GC-----C--A--G---C--AG-C-CGC-GG------TA-A--T-A--CG-GA-------GG--A-----TT-CG-AG-CG-------T-TA-T-C-C-G------------G--A-CT-C-T-T----------ACA----G-T-C-A--------G-C-G--G--TG--A-AA-TG--T-C-TG-G-G-----CT-T-AC------C-C-T-A-G-G-A--A-T----T-G--C-C---G--T-------T--GA-T-A---C-------T--G-G--GA--G-T-C----T-T-G-G-A-T--A---A-GC-----AT-A-A--------G-G-A-C---GG-C------GG--A--ATG----C-G-T-A-GT--GT-A-G-CG-GT--G---A--A-A---TG-C-TT-AG--AT-A-TT--A-C--G-T-A-G-A-AG-A-CC---AA--T--T--GC-GAA-G--G-C--A-------G--C-T-G-T---CTG--G--GT-C-T--------------------T-T---A-T-C--GA--CG-C-T-C--A-AG--C-A-CG-A--AA-G-C---G-TG--GG-G--AG-C-A-AA--------CA--GG Bacteria(100);unclassified(100);unclassified(100);unclassified(100);unclassified(100);unclassified(100);unclassified(100);unclassified(100);unclassified(100);

Otu0594 0 0 0 0 0 0 0 0 0 0 0 0 0 0 0 0 0 0 0 0 0 0 0 0 0 0 0 0
0 0 0 0 0 0 0 0 0 0 0 0 0 0 0 0 0 0 0 0 0 0 0 0 0 0 0 0
0 0 0 0 0 0 0 0 0 0 0 0 0 0 27 28 0 0 0 0 0 0 0 0 6 0 0 1 0
0 0 0 0 0 0 0 0 0 3 0 0 0 0 0 0 0 0 0 0 0 0 0 0 0 0 0
M02255_77_000000000-ADUT3_1_1118_4551_18475 T----AA---GC-AC-CGG--------CTA-A-C------T--A--C---G--TG-C-CAG-CA------GC-C--G-C--GG-TA-------AT--A-----CG-TA-GG-GT-------G-CA-A-G-C-G------------T--TAAT-C-G-G-----------AAT---T-A-C-T--------G-A-T--G--TG--A-AA-TC--C-C-CG-G-G-----CT-T-AA------C-C-T-G-G-G-A--A-C----T-G--C-A---T--T-------G--GT-G-A---C-------T--G-C--AA--G-G-C----T-A-G-A-G-T--G---T-GT-----CA-G-A--------G-G-G-A---GG-T------GG--A--ATT----C-C-G-C-AT--GT-A-G-CA-GT--G---A--A-A---TG-C-GT-AG--AT-A-TG--C-G--G-A-G-G-A-AC-A-CC---GA--T--G--GC-GAA-G--G-C--A-------G--C-C-T-C---CTG--G--GA-T-A--------------------A-C---A-C-T--GA--CG-C-T-C--A-TG--C-A-CG-A--AA-G-C---G-TG--GG-G--AG-C-A-AA--------CA--GG Bacteria(100);Proteobacteria(100);Betaproteobacteria(100);Burkholderiales(100);Alcaligenaceae(54);unclassified(54);unclassified(54);unclassified(54);unclassified(54);

Otu0595 0 0 0 0 0 0 0 0 0 0 0 0 0 0 0 0 0 0 0 0 0 0 0 0 0 0 0 0
0 0 0 0 0 0 0 0 0 0 0 0 0 0 0 0 0 0 0 137 0 0 0 0 0 0 0 0 0
0 0 0 0 0 0 0 0 0 0 0 0 0 0 0 0 0 0 0 0 0 0 0 0 0 0 0 0 0
0 0 0 0 0 0 0 0 0 0 0 0 0 0 0 0 0 0 0 0 0 0 0 0 0 0 0
M02255_77_000000000-ADUT3_1_1110_17124_12915 G----AC---GT-AG-GAT--------GCA-A-G------C--G--T---T--GT-C-CGG-AA------TC-A--T-T--GG-GC-------GT--A-----AA-GC-GT-TC-------G-TA-G-G-C-G------------G--C-AT-G-T-C-----
------AA----G-T-C-T--------G-A-T--G--TT--A-AA-GC--C-C-GG-A-G-----CT-C-AA------C-T-C-C-G-G-T--T--C---G-G--C-A---T--T-------G--GA-T-A---C-------T--G-G--CA--A-G-C----T-A-G-A-G-T--G---C-AA-----TA-G-A--------G-G-T-A---GG-G------GG--A--ATT----C-
C-T-A-GT--GT-A-G-CG-GT--G---A--A-A---TG-C-GT-AG--AT-A-TT--A-G--G-A-G-G-A-AC-A-CC---GG--T--G--GC-GAA-A--G-C--G-------C--C-C-T-A---CTG--G--GT-T-G--------------------T-A---A-C-T--GA--CG-C-T-G--A-GG--A-A-CG-A--AA-G-C---C-AG--GG-T-
-AG-C-A-AA--------TG--GG Bacteria(100);unclassified(100);unclassified(100);unclassified(100);unclassified(100);unclassified(100);unclassified(100);unclassified(100);unclassified(100);

Otu0596 0 0 0 0 0 0 0 0 0 0 0 0 0 0 0 0 0 0 0 0 0 0 0 0 0 0 0 0
0 0 0 0 0 0 0 0 0 0 0 0 0 0 0 0 0 0 0 0 0 0 0 0 0 0 0 0 0
0 0 0 0 0 0 0 25 0 0 0 0 0 0 6 14 0 0 0 0 0 0 0 0 0 0 0 0 0
17 15 0 0 10 0 0 0 0 0 0 0 0 0 0 0 0 0 0 0 0 0 0 0 0 0 0
M02255_76_000000000-ADUNE_1_1108_17572_2975 C----GG---CT-AA-CT---------ACG-T-G------C--C--A---G--CA-G-CCG-CG------GT-A--A-T--AC-GT-------AG--G-----TG-GC-GA-GC-------G-TT-GTC-C-G------------G--A-CA-T-A-T-----------AA-
---G-T-C-C--------A-T-C--T--TA--A-AA-GT--G-C-GG-G-G-----CT-C-AA------C-C-C-C-G-T-G--A------G-G--G-G---A--T-------G--GA-A-A---C-------T--G-T--AT--G-C-C----T-T-G-A-G-T--G---C-AG-----GA-G-A--------G-G-A-A---AG-C------GG--A--ATT----C-C-C-A-
GT--GT-A-G-CG-GT--G---A--A-A---TG-C-GT-AG--AT-A-TT--G-G--G-A-G-G-A-AC-A-CC---AG--T--G--GC-GAA-G--G-C--G-------G--C-T-T-T---CTG--G--AC-T-G--------------------C-A---A-C-T--GA--CG-C-T-G--A-GG--C-T-CG-A--AA-G-C---C-AG--GG-G--AG-
C-G-AA--------CG--GG Bacteria(100);Firmicutes(100);Clostridia(100);Clostridiales(100);Veillonellaceae(100);Selenomonas(100);unclassified(100);unclassified(100);unclassified(100);

Otu0597 0 0 0 0 0 0 0 0 0 0 0 0 0 0 0 0 0 0 1 0 0 0 0 0 0 0 0 0
0 1 0 0 0 0 0 0 0 0 0 0 0 0 2 0 0 0 0 0 0 0 0 0 0 0 0 0 0
0 0 0 0 0 0 0 1 0 0 0 0 0 0 0 1 0 0 0 3 0 0 0 0 2 0 0 3 0
0 1 0 0 6 7 0 0 0 0 0 13 0 0 0 0 0 0 0 0 0 0 0 0 0 0 0
M02255_76_000000000-ADUNE_1_2111_5790_10049 T----AC---GT-AG-GGG--------GCT-A-G------C--G--T---T--AT-C-CGG-AT------TT-A--C-T--GG-GC-------GT--A-----AA---------------G-T--G-G-T--------------G-----C-G-T------------AG----G-T-
C-G--------G-T-G--G--TT--A-AA-GG--C-T-AC-G-G-----CT-C-AA------C-C-G-T-A-G-T--A------A-G--C-C---G--C-------C--GA-A-A---C-------T--G-G--AG--G-A-C----T-T-G-A-G-T--G---C-AG-----GA-G-A--------G-G-A-A---AG-T------GG--A--ATT----C-C-C-A-GT--GT-
A-G-CG-GT--G---A--A-A---TG-C-GT-AG--AT-A-TT--G-G--G-A-G-G-A-AC-A-CC---AG--T--A--GC-GAA-G--G-C--G-------G--C-T-T-T---CTG--G--AC-T-G--------------------C-A---A-C-T--GA--CA-C-T-G--A-GG--C-A-CG-A--AA-G-C---G-TG--GG-T--AG-C-A-AA---
-----CA--GG Bacteria(100);Firmicutes(100);Clostridia(100);Clostridiales(100);Peptostreptococcaceae(100);Peptostreptococcus(100);unclassified(100);unclassified(100);unclassified(100);

Otu0598 0 0 0 0 0 0 0 0 0 0 0 0 0 0 0 0 0 0 0 0 0 0 0 0 0 0 0 0
0 0 0 0 0 0 0 0 0 0 0 0 0 0 0 0 0 0 0 0 0 0 0 0 0 0 0 0 0
0 0 0 0 0 0 0 0 0 0 0 0 0 0 0 0 0 0 0 0 0 0 0 0 0 0 0 0 0
16 0 0 0 0 21 0 0 0 0 0 0 0 0 0 0 0 0 0 0 0 0 0 0 0 0 0
M02255_77_000000000-ADUT3_1_2114_25434_15603 T----AC---GT-AG-GGG--------GCA-A-G------C--G--T---T--AT-C-CGG-AA------TT-A--T-T--GG-GC-------GT--A-----AA-GC-GT-GC-------G-TA-G-G-T-G------------G--T-TA-C-C-T------
-----AA----G-C-G-C--------A-A-G--G--TT--T-AA-TT--T-A-GC-A-G-----CT-C-AA------C-T-G-C-T-A-C--A------T-G--C-C---T--T-------G--CG-A-A---C-------T--G-G--GC--T-A-C----T-T-G-A-G-T--G---C-AG-----GA-G-G--------G-G-A-A---AG-C------GG--A--ATT----C-
C-T-A-GT--GT-A-G-CG-GT--G---A--A-A---TG-C-GT-AG--AT-A-TT--A-G--G-A-G-G-A-AC-A-CC---GG--C--G--GC-GAA-G--G-C--G-------G--C-T-T-T---CTG--G--AC-T-G--------------------T-A---A-C-T--GA--CA-C-T-G--A-GG--C-A-CG-A--AA-G-C---G-TG--GG-G-
-AG-C-A-AA--------CA--GG Bacteria(100);Firmicutes(100);Clostridia(100);Clostridiales(100);Family_XIII_Incertae_Sedis(100);Mogibacterium(100);unclassified(100);unclassified(100);unclassified(100);

Otu0599 0 137 324 0 0 0 71 9 316 0 0 0 0 0 0 0 0 0 0 0 0 0 0 0 0 0 0 0
0 0 0 0 0 0 0 0 0 0 0 113 0 0 0 0 0 0 0 0 0 0 0 0 0 0 0 0 0
0 0 0 0 0 0 0 0 0 0 0 0 0 0 0 0 0 0 0 0 0 0 0 0 0 0 0 0 0
0 0 0 0 0 0 29 0 0 0 0 43 0 0 3 0 0 30 0 0 0 0 0 2 126 0 0
M02255_77_000000000-ADUT3_1_2109_18256_17276 A----AC---GT-AG-GTC--------ACA-A-G------C--G--T---T--GT-C-CGG-AA------TT-A--C-T--GG-GT-------GT--A-----AA-GG-GA-GC-------G-CA-G-G-C-G------------G--G-CG-A-T-C----
-------AA----G-T-T-G--------G-A-A--G--TG--A-AA-TC--C-A-TG-G-G-----CT-C-AA------C-C-C-A-T-G-A--A-C----T-G--C-T---T--T-------C--AA-A-A---C-------T--G-A--TT--G-T-C----T-T-G-A-G-T--A---G-TG-----CA-G-A--------G-G-T-A---GG-C------GG--A--ATT----C-
C-C-G-GT--GT-A-G-CG-GT--G---G--A-A---TG-C-GT-AG--AT-A-TC--G-G--G-A-G-G-A-AC-A-CC---AG--T--G--GC-GAA-G--G-C--G-------G--C-C-T-A---CTG--G--GC-A-C--------------------C-A---A-C-T--GA--CG-C-T-G--A-GG--C-T-CG-A--AA-G-T---G-TG--GG-
T--AG-C-A-AA--------CA--GG Bacteria(100);Firmicutes(100);Clostridia(100);Clostridiales(100);Ruminococcaceae(100);Faecalibacterium(100);unclassified(100);unclassified(100);unclassified(100);

Otu0600 0 0 0 0 0 0 0 0 0 0 0 0 0 0 0 0 0 0 0 0 0 0 13 0 0 0 0 0
33 0 0 0 0 0 0 0 0 0 0 0 0 0 0 0 0 0 0 0 0 0 0 0 0 0 0 0
0 0 0 0 0 0 0 0 0 0 0 0 0 0 0 0 0 0 0 0 36 0 0 0 0 0 0 0
0 0 0 0 0 0 0 0 0 0 0 0 0 0 0 0 0 0 0 0 0 0 0 0 0 0 0
M02255_77_000000000-ADUT3_1_1103_7219_8491 T----AC---GG-AG-GAT--------GCA-A-G------C--G--T---T--AT-C-CGG-AA------TT-A--T-T--GG-GC-------GT--A-----AA-GC-GT-CC-------G-CA-G-G-T-G------------G--C-AA-T-T-C-----------AA-
---G-T-C-T--------G-C-T--G--TT--A-AA-GG--T-T-CT-G-G-----CT-C-AA------C-C-A-G-A-G-A--C-A----G-G--C-A---G--T-------G--GA-A-A---C-------T--G-A--AT--A-G-C----T-A-G-A-G-T--G---C-GG-----TA-G-G--------G-G-C-A---GA-G------GG--A--ATT----C-C-C-A-
GT--GT-A-G-CG-GT--G---A--A-A---TG-C-GT-AG--AG-A-TT--G-G--G-A-A-G-A-AC-A-CC---GG--T--G--GC-GAA-A--G-C--G-------C--T-C-T-G---CTA--G--GC-C-G--------------------T-A---A-C-T--GA--CA-C-T-C--A-TG--G-A-CG-A--AA-G-C---T-AG--GG-G--AG-
C-G-AA--------TG--GG Bacteria(100);Cyanobacteria(100);unclassified(100);unclassified(100);unclassified(100);unclassified(100);unclassified(100);unclassified(100);unclassified(100);

Otu0601 0 0 0 0 0 0 0 0 22 0 0 0 0 0 0 0 0 0 0 0 7 0 0 0 0 0 0 0
0 0 0 0 0 26 0 60 0 18 10 0 0 0 0 0 0 0 0 0 0 0 0 0 0 0 0 0
0 0 0 0 0 0 0 0 0 0 0 0 0 0 0 0 0 65 0 0 0 0 0 0 0 0 0 0
0 0 0 0 0 0 0 0 0 0 0 0 0 0 0 0 0 0 0 0 0 0 0 0 0 0 0
M02255_77_000000000-ADUT3_1_1105_25942_10977 C----AC---GT-AG-GCA--------CCA-A-G------C--G--T---T--GT-C-CGG-AT------TT-A--T-T--GG-GC-------GT--A-----AA-GA-GC-TC-------G-TA-G-G-C-G------------G--T-TC-A-G-T------
-----AA----G-T-C-G--------G-G-T--G--TG--A-AA-AC--T-C-TG-G-G-----CT-C-AA------C-C-C-A-G-A-G--A------C-G-CC-A---C--C-------C--GA-T-A---C-------T--G-C--TG-TG-A-C----T-T-G-A-G-T--A---C-GG-----TA-G-G--------G-G-A-G---TG-G------GG--A--ATT----
C-C-T-G-GT--GT-A-G-CG-GT--G---A--A-A---TG-C-GC-AG--AT-A-TC--A-G--G-A-G-G-A-AC-A-CC---AG--T--G--GC-GAA-G--G-C--G-------C--C-A-C-T---CTG--G--GC-C-G--------------------T-A---A-C-T--GA--CG-C-T-G--A-GG--A-G-CG-A--AA-G-C---G-TG--
GG-T--AG-C-A-AA--------CA--GG Bacteria(100);Actinobacteria(100);Actinobacteria(100);Acidimicrobidae(100);Acidimicrobiales(100);Acidimicrobineae(100);Acidimicrobiaceae(100);unclassified(100);unclassified(100);

Otu0602 0 0 0 0 0 0 0 0 0 0 0 0 0 0 0 0 0 0 0 0 0 0 0 0 4 3 0 9
0 0 0 0 0 0 0 0 0 0 0 0 0 0 0 0 0 0 0 0 0 0 0 0 0 0 0 0
0 0 0 0 0 0 0 0 0 0 0 0 0 0 0 0 0 0 0 0 0 10 0 0 0 0 0 0
0 0 0 0 0 0 0 0 0 0 0 0 0 0 0 0 0 0 0 0 0 0 0 0 0 0 0
M02255_76_000000000-ADUNE_1_1106_15060_17325 T----AC---GG-AG-GGG--------GCT-A-G------C--G--T---T--AT-T-CGG-AA------TT-A--C-T--GG-GC-------GT--A-----AA-GC-GC-AC-------G-TA-G-G-C-G------------G--G-TC-A-G-C----
-------AA----G-T-T-G--------G-G-G--G--TG--A-AA-TC--C-C-GG-G-G-----CT-C-AA------C-C-C-C-G-G-A--A-C----G-G--C-C---C--T-------C--AA-A-A---C-------T--G-T--TG--G-T-C----T-T-G-A-G-T--T---C-GA-----GA-G-A--------G-G-T-G---AG-T------GG--A--ATT----
C-C-G-A-GT--GT-A-G-AG-GT--G---A--A-A---TT-C-GT-AG--AT-A-TT--C-G--G-A-G-G-A-AC-A-CC---AG--T--G--GC-GAA-G--G-C--G-------G--C-T-C-A---CTG--G--CT-C-G--------------------A-T---A-C-T--GA--CG-C-T-G--A-GG--T-G-CG-A--AA-G-C---G-TG--GG-
G--AG-C-A-AA--------CA--GG Bacteria(100);Proteobacteria(100);Alphaproteobacteria(100);Rhodobacterales(100);Rhodobacteraceae(100);unclassified(100);unclassified(100);unclassified(100);unclassified(100);

Otu0603 0 0 0 0 0 0 0 0 0 0 0 0 0 0 0 0 0 0 0 0 0 0 0 0 0 0 0 0
0 0 0 0 0 19 35 46 0 0 0 44 0 0 0 0 0 0 0 0 0 0 0 0 0 0 0 0
0 0 0 0 0 0 0 0 0 0 0 0 0 0 0 0 0 0 0 0 0 0 0 0 0 0 0 0
19 0 0 0 0 0 0 0 0 0 0 0 0 0 0 0 0 0 0 0 0 0 0 0 0 0 0
M02255_77_000000000-ADUT3_1_2101_12360_13769 T----AC---GT-AG-GGT--------GCG-A-G------C--G--T---T--GT-C-CGG-AA------TT-A--T-T--GG-GC-------GT--A-----AA-GG-GC-TC-------G-TA-G-G-C-G------------G--T-TA-G-T-C-----
------GC----G-T-C-G--------G-G-A--G--TG--A-AA-AC--T-C-GG-A-G-----CT-T-AA------C-T-C-C-G-A-G--C-T----T-G--C-T---T--C-------T--GA-T-A---C-------G--G-G--CT--G-A-C----T-A-G-A-G-G--T---A-TG-----CA-G-G--------G-G-A-G---AA-T------GG--A--ATT----
C-C-T-G-GT--GT-A-G-CG-GT--G---A--A-A---TG-C-GC-AG--AT-A-TC--A-G--G-A-G-G-A-AC-A-CC---GG--T--G--GC-GAA-G--G-C--G-------G--T-T-C-T---CTG--G--GC-A-T--------------------T-A---C-C-T--GA--CG-C-T-G--A-GG--A-G-CG-A--AA-G-T---G-TG--
GG-G--AG-C-G-AA--------CA--GG Bacteria(100);Actinobacteria(100);Actinobacteria(100);Actinobacteridae(100);Actinomycetales(100);Propionibacterineae(100);Nocardioidaceae(100);unclassified(89);unclassified(89);

Otu0604 0 0 3 1 8 1 0 0 3 0 0 0 0 0 0 0 0 0 0 0 1 0 0 0 0 0 0 0
12 8 0 0 1 0 0 0 0 0 0 0 0 0 0 0 0 7 0 0 0 4 0 0 0 0 0 8
0 0 0 0 0 0 0 0 0 0 0 0 0 0 2 0 0 0 6 2 0 0 0 1 0 9 0 0 0
0 0 0 0 0 0 0 0 0 0 0 0 0 0 0 0 0 0 1 0 5 1 0 0 0 12 0
M02255_76_000000000-ADUNE_1_2101_23595_12818 T----AA---GC-AC-CGG--------CTA-A-C------T--A--C---G--TG-C-CAG-CA------GC-C--G-C--GG-TA-------AT--A-----CG-TA-GG-GT-------G-CA-A-G-C-GT-----------T--A-AT-C-G-G-----
------AA----G-A-C-A--------G-A-T--G--TG--A-AA-TC--C-C-CG-G-G-----CT-C-AA------C-C-T-G-G-G-A--A-C----T-G--C-A---T--T-------T--GT-G-A---C-------T--G-C--AT--G-G-C----T-A-G-A-G-T--G---C-GG-----CA-G-A--------G-G-G-G---GA-T------GG--A--ATT----
C-C-G-C-GT--GT-A-G-CA-GT--G---A--A-A---TG-C-GT-AG--AT-A-TG--C-G--G-A-G-G-A-AC-A-CC---GA--T--G--GC-GAA-G--G-C--A-------A--T-C-C-C---CTG--G--GC-C-T--------------------G-C---A-C-T--GA--CG-C-T-C--A-TG--C-A-CG-A--AA-G-C---G-TG--GG-
G--AG-C-A-AA--------CA--GG Bacteria(100);Proteobacteria(100);Betaproteobacteria(100);Burkholderiales(100);Comamonadaceae(100);unclassified(100);unclassified(100);unclassified(100);unclassified(100);

Otu0605 0 0 0 0 0 0 0 0 0 0 0 0 0 0 0 0 0 0 0 0 0 0 0 0 0 0 0 0
0 0 0 0 0 0 0 0 22 0 0 0 0 0 0 0 0 0 0 0 0 0 0 0 0 0 0 0
0 0 0 0 0 0 0 0 0 0 0 0 0 0 0 0 0 0 0 0 0 0 0 0 0 0 0 0
14 0 0 0 0 0 0 0 0 0 0 0 0 0 0 0 0 0 0 0 0 0 0 0 0 0 0
M02255_77_000000000-ADUT3_1_1111_23892_16904 T----AC---GT-AG-GGA--------GCG-A-G------C--G--T---T--GT-C-CGG-AT------TT-A--C-T--GG-GT-------GT--A-----AA-GG-GT-GC-------G-TA-G-G-C-G------------G--A-TT-G-G-C----
-------AA----G-T-C-A--------G-A-A--G--TG--A-AA-TC--C-A-TG-G-G-----CT-T-AA------C-C-C-A-T-G-A--A-C----T-G--C-T---T--T-------T--GA-A-A---C-------T--G-T--TA--G-T-C----T-T-G-A-G-T--G---A-AG-----TA-G-A--------G-G-T-A---GG-C------GG--A--ATT----C-
C-C-G-GT--GT-A-G-CG-GT--G---A--A-A---TG-C-GT-AG--AG-A-TC--G-G--G-A-G-G-A-AC-A-CC---AG--T--G--GC-GAA-G--G-C--G-------G--C-C-T-A---CTG--G--GC-T-T--------------------T-A---A-C-T--GA--CG-C-T-G--A-GG--C-A-CG-A--AA-G-T---G-TG--GG-
T--AG-C-A-AA--------CA--GG Bacteria(100);Firmicutes(62);Clostridia(62);Clostridiales(62);unclassified(62);unclassified(62);unclassified(62);unclassified(62);unclassified(62);

Otu0606 0 0 0 0 1 0 0 0 0 0 0 0 0 0 0 0 0 0 0 0 0 0 0 0 0 1 0 0
0 0 0 0 0 0 0 0 0 0 0 0 0 0 0 0 1 0 0 0 0 0 0 0 0 0 0 0
0 0 0 0 0 0 0 0 0 0 0 0 0 0 0 0 0 0 0 0 0 0 0 0 0 0 0 0
2 0 0 0 0 0 0 0 3 0 0 0 1 2 0 0 0 0 0 0 0 4 1 4 0 0 0
M02255_77_000000000-ADUT3_1_2103_29305_10373 C----TG---TG-CC-AGC--------AGC-C-G------C--G--G---T--AA-TACAG-AG------GG-T--G-C--AA-GC-------GT--T-----AATCGGAATTA-------C-TG-G-G-T-G------------G--C-TT-G-A-T--
---------AA----G-T-C-A--------G-A-T--G--TG--A-AA-TC--C-C-CG-G-G-----CT-T-AA------C-C-T-G-G-G-A--A-C----T-G--C-A---T--C-------T--GA-T-A---C-------T--G-T--TA--G-G-C----T-A-G-A-A-T--A---G-GT-----GA-G-A--------G-G-A-A---GG-T------AG--A--ATT----
C-C-A-G-GT--GT-A-G-CG-GT--G---A--A-A---TG-C-GT-AG--AG-A-TC--T-G--G-A-G-G-A-AT-A-CC---GA--T--G--GC-GAA-G--G-C--A-------G--C-C-T-T---CTG--G--CA-T-C--------------------A-T---A-T-T--GA--CA-C-T-G--A-GG--T-T-CG-A--AA-G-C---G-TG--GG-
T--AG-C-A-AA--------CA--GG Bacteria(100);Proteobacteria(100);Gammaproteobacteria(100);Pseudomonadales(100);Moraxellaceae(100);Psychrobacter(100);unclassified(100);unclassified(100);unclassified(100);

Otu0607 0 0 0 0 0 0 0 0 0 0 0 0 0 0 0 0 0 0 0 0 0 0 0 0 0 0 0 0
0 0 0 0 0 0 0 1 0 0 0 0 0 0 0 0 0 0 0 0 0 0 66 0 0 0 0 0
0 0 0 0 0 0 0 0 0 0 0 0 0 0 0 0 0 0 0 0 0 0 0 0 0 0 0 0
0 0 0 0 0 0 0 0 0 0 0 0 0 0 0 12 0 0 0 0 0 0 0 0 0 0 0
M02255_77_000000000-ADUT3_1_1116_7460_13336 T----AC---GT-AG-GAC--------CCA-A-G------C--G--T---T--AT-C-CGG-AG------TG-A--C-T--GG-GC-------GT--A-----AA-GA-GTTGC-------G-TA-G-G-C-G------------G--C-TC-G-T-T----------
AA----G-C-G-A--------G-T-A--G--TG--A-AA-TC--G-T-GT-G-G-----CT-C-AA------C-C-A-T-AC--G--G-C----T-A--T-T---A--C-------T--CG-A-A---C-------T--G-G--CG--A-G-C----T-C-G-A-G-A--A---T-GG-----TA-G-A--------G-G-T-A---GC-T------GG--A--ATT----T-C-T-
A-GT--GT-A-G-GA-GT--G---A--A-A---TC-C-GT-AG--AT-A-TT--A-G--A-A-G-G-A-AC-A-CC---AA--T--G--GC-GTA-G--G-C--A-------G--G-C-T-A---CTG--G--AC-C-A--------------------T-T---T-C-T--GA--CG-C-T-A--A-GG--C-A-CG-A--AA-G-C---G-TG--GG-G--AG-
C-G-AA--------CC--GG Bacteria(100);Candidate_division_TM7(100);unclassified(100);unclassified(100);unclassified(100);unclassified(100);unclassified(100);unclassified(100);unclassified(100);

Otu0608 0 0 0 0 0 0 0 0 0 0 0 0 0 0 0 0 0 0 0 0 0 0 0 13 0 0 0 0
0 0 0 0 0 0 0 0 0 0 0 141 0 0 0 0 0 0 0 0 0 0 0 0 0 0 0 0
0 0 0 0 0 0 0 0 0 0 0 0 0 0 0 15 0 0 0 0 0 0 0 0 0 0 0 0
0 0 0 0 0 0 4 0 0 0 0 14 0 0 0 0 0 0 0 0 0 0 0 0 0 0 0
M02255_77_000000000-ADUT3_1_1110_27725_18600 T----AC---GT-AG-GTG--------GCA-A-G------C--G--T---T--GT-C-CGG-AA------TT-A--C-T--GG-GT-------GT--A-----AA-GG-GA-GC-------G-TA-G-G-C-G------------G--G-AT-G-C-C----
-------AA----G-T-C-A--------G-C-T--G--TG--A-AA-AC--T-A-TG-G-G-----CT-T-AA------C-C-C-G-T-A-G--A-C----T-G--C-A---G--T-------T--GA-A-A---C-------T--G-G--TA--T-T-C----T-T-G-A-G-T--G---A-AG-----TA-G-A--------G-G-T-T---GG-C------GG--A--ATT----C-
C-G-A-GT--GT-A-G-CG-GT--G---A--A-A---TG-C-GT-AG--AT-A-TT--C-G--G-A-G-G-A-AC-A-CC---GG--T--G--GC-GAA-G--G-C--G-------G--C-C-A-A---CTG--G--GC-T-T--------------------T-A---A-C-T--GA--CG-C-T-G--A-GG--C-T-CG-A--AA-G-T---G-TG--GG-
G--AG-C-A-AA--------CA--GG Bacteria(100);Firmicutes(100);unclassified(91);unclassified(91);unclassified(91);unclassified(91);unclassified(91);unclassified(91);unclassified(91);

Otu0609 0 0 0 0 0 0 0 0 0 0 0 0 0 0 0 0 0 0 0 0 0 0 0 0 0 0 0 0
0 0 0 0 0 0 0 0 0 0 0 0 0 0 0 19 0 0 0 0 0 0 0 0 0 0 0 0
0 0 0 0 0 0 0 0 0 0 0 0 0 0 0 0 0 0 0 0 0 0 0 0 0 0 0 0
15 0 0 0 0 15 0 0 0 25 0 0 0 0 0 0 0 0 0 0 0 0 0 0 0 0 0
M02255_77_000000000-ADUT3_1_1115_22438_8104 T----AC---GG-AG-GGT--------GCT-A-G------C--G--T---T--GT-T-CGG-AA------TT-A--C-T--GG-GC-------GT--A-----AA-GG-GC-GC-------G-TA-G-G-C-G------------G--C-CG-T-T-T-----------GC-
---G-T-C-G--------G-A-T--G--TG--A-AA-GC--C-C-GG-G-G-----CT-C-AA------C-C-T-C-G-G-A--A-C----G-G--C-A---T--C-------C--GA-T-A---C-------G--G-G--GC--G-G-C----T-G-G-A-G-A--C---C-GG-----GA-G-A--------G-G-A-G---GG-T------GG--A--ATA----G-C-C-
A-GT--GT-A-G-AG-GT--G---A--A-A---TT-C-GT-AG--AT-A-TT--G-G--C-T-G-G-A-AC-A-CC---GG--T--G--GC-GAA-A--G-C--G-------G--C-T-C-T---CTG--G--CC-C-G--------------------G-A---T-C-T--GA--CG-C-T-G--A-GG--C-G-CG-A--AA-G-C---G-T-G-GG-G--
AG-C-A-AA--------CA--GG Bacteria(100);Proteobacteria(100);Alphaproteobacteria(100);unclassified(100);unclassified(100);unclassified(100);unclassified(100);unclassified(100);unclassified(100);

Otu0610 0 0 0 0 0 0 0 0 0 0 0 0 0 0 0 0 0 0 0 2 14 0 0 0 0 0 0 1
0 1 0 0 0 0 0 0 0 26 0 0 0 0 0 0 0 0 0 0 0 0 0 0 0 0 0 0
0 0 0 0 0 0 0 0 0 0 0 0 0 0 0 0 0 0 0 0 0 0 0 0 0 0 0 0
0 0 0 0 0 0 0 0 0 0 0 0 0 0 0 11 0 0 0 0 0 0 0 0 0 0 0

M02255_76_000000000-ADUNE_1_1109_16403_8442 .....AC----T-AC-GTG---------CC-A-G------C--A--G---C--CG-C-GGT-AA------TA-C--G-T--AG-GT-------GA--C-----AA-GC-G--TT-------G-TC-C-G---G------------A--T-CT-T-T-T----------AA----G-T-C-T--------G-A-T--G--TG--A-AA-GC--C-C-AC-G-G-----CT-C-AA------C-C-G-T-G-G-A--T------G-GT-C-A---T--T-------G--GA-A-A---C-------T--G-G--AA--G-A-C----T-T-G-A-A-T--G---C-AG-----AA-G-A--------G-G-A-A---AG-T------GG--A--ATT----C-C-A-T-GT--GT-A-G-CG-GT--G---A--A-A---TG-C-GT-AG--AT-A-TA--T-G--G-A-G-G-A-AC-A-CC---AG--T--G--GC-GAA-G--G-C--G-------A--C-T-T-T---CTG--G--TC-T-G--------------------T-A---A-T-T--GA--CG-C-T-G--A-GG--C-T-CG-A--AA-G-C---G-TG--GG-G--AG-C-G-AA--------CA--GG Bacteria(100);Firmicutes(100);Bacilli(100);Lactobacillales(100);Carnobacteriaceae(100);unclassified(79);unclassified(79);unclassified(79);unclassified(79);

Otu0611 0 0 0 0 0 0 0 0 0 0 0 0 0 0 0 0 0 0 0 1 0 0 0 0 0 0 0 28
0 0 1 0 0 0 0 0 0 28 1 0 0 0 0 0 0 0 0 0 0 0 0 0 0 0 0 0
0 0 0 0 0 0 0 0 0 0 0 0 0 0 0 0 0 0 0 0 0 0 0 0 0 0 0 0
0 0 0 0 0 0 0 0 0 0 0 0 0 0 0 0 0 0 0 0 0 0 0 0 0 0 0

M02255_77_000000000-ADUT3_1_1115_10312_10472 T----AC---GA-AG-GGG--------GCT-A-G------C--G--T---T--GC-T-CGG-AA------TC-A--C-T--GG-GC-------GT--A-----AA-GG-GC-GC-------G-TA-G-G-C-G------------G--C-CC-T-G-T-----------TG----G-T-C-G--------G-G-C--G--TG--A-AA-TC--C-C-CG-G-G-----CT-C-AA------C-C-C-G-G-G-G--G-C----T-G--C-G---C--C-------C--GA-A-A---C-------G--G-C--GG--G-G-C----T-G-G-A-G-G--C---A-AG-----GA-G-A--------G-G-C-T---CG-C------GG--A--ATT----C-C-C-A-GT--GT-A-G-AG-GT--G---A--A-A---TT-C-GT-AG--AT-A-TT--G-G--G-A-A-G-A-AC-A-CC---GG--T--G--GC-GAA-G--G-C--G-------G--C-G-A-G---CTG--G--CC-T-T--------------------G-A---A-C-T--GA--CG-C-T-G--A-GG--C-G-CG-A--CA-G-C---G-TG--GG-G--AG-C-A-AA--------CA--GG Bacteria(100);Proteobacteria(100);Alphaproteobacteria(100);Rhodospirillales(100);unclassified(100);unclassified(100);unclassified(100);unclassified(100);unclassified(100);

Otu0612 0 0 0 0 0 0 0 0 0 0 0 0 0 0 0 0 0 0 0 2 0 0 0 0 0 0 0 0
0 1 0 0 0 0 0 0 0 4 1 0 0 0 0 0 0 0 0 0 0 0 0 0 0 0 0 0
0 0 0 0 0 0 0 0 0 0 0 0 0 0 0 0 0 0 0 0 0 0 0 0 0 0 0 0
0 24 0 0 0 0 0 0 0 0 0 0 0 0 0 0 0 0 0 4 0 0 0 0 0 0 29

M02255_77_000000000-ADUT3_1_1103_18593_16365 T----AC---GT-AG-GAT--------CCG-A-G------C--G--T---T--AT-C-CGG-AG------TG-A--C-T--GG-GC-------GT--A-----AA-GA-GTTGC-------G-TA-G-G-T-G------------G--T-TT-A-A-T-----------AA----G-C-G-A--------A-T-A--G--TG--A-AA-TC--T-G-GT-G-G-----CT-C-AA------C-C-A-T-TCA-G--A-C----T-A--T-T---A--T-------T--CG-A-A---C-------T--G-T--TA--A-A-C----T-C-G-A-G-A--G---C-AG-----AA-G-A--------G-G-T-A---GC-T------GG--A--ATT----T-C-T-A-GT--GT-A-G-GA-GT--G---A--A-A---TC-C-GT-AG--AT-A-TT--A-G--A-A-G-G-A-AC-A-CC---GA--T--G--GC-GTA-G--G-C--A-------G--G-C-T-A---CTG--G--GC-T-G--------------------T-T---T-C-T--GA--CA-C-T-G--A-GG--C-A-CG-A--AA-G-C---G-TG--GG-G--AG-C-G-AA--------CC--GG Bacteria(100);Candidate_division_TM7(100);unclassified(100);unclassified(100);unclassified(100);unclassified(100);unclassified(100);unclassified(100);unclassified(100);

Otu0613 0 309 108 0 0 0 225 4 117 0 0 0 0 0 1 0 0 0 0 0 0 0 0 0 0 0 0 0
0 0 0 0 0 0 0 0 0 0 0 0 0 0 0 0 0 0 0 0 0 0 0 0 5 0 0 0
0 0 0 0 0 0 0 0 0 0 0 0 0 0 0 0 0 0 0 0 0 10 1 0 0 0 0 0
0 0 0 0 0 0 0 0 0 0 0 0 0 0 0 0 0 0 0 0 0 0 0 50 0 0 0

M02255_77_000000000-ADUT3_1_1101_19843_7140 T----AC---GT-AG-GGA--------GCA-A-G------C--G--T---T--AT-C-CGG-AT------TT-A--C-T--GG-GT-------GT--A-----AA-GG-GC-GC-------G-CA-G-G-C-G------------G--G-CC-G-G-C----------AA----G-T-T-G--------G-G-A--G--TG--A-AA-TC--C-C-GG-G-G-----CT-T-AA------C-C-C-C-G-G-A--A-C----T-G--C-T---T--T-------C--AA-A-A---C-------T--G-C--TG--G-T-C----T-T-G-A-G-T--G---A-TG-----GA-G-A--------G-G-C-A---GG-C------GG--A--ATT----C-C-G-T-GT--GT-A-G-CG-GT--G---A--A-A---TG-C-GT-AG--AT-A-TA--C-G--G-A-G-G-A-AC-A-CC---AG--T--G--GC-GAA-G--G-C--G-------G--C-C-T-G---CTG--G--AC-A-T--------------------T-A---A-C-T--GA--CG-C-T-G--A-GG--C-G-CG-A--AA-G-C---G-TG--GG-G--AG-C-A-AA--------CA--GG Bacteria(100);Firmicutes(100);Clostridia(100);Clostridiales(100);Ruminococcaceae(100);Incertae_Sedis(100);unclassified(100);unclassified(100);unclassified(100);

Otu0614 0 0 0 0 0 0 0 0 0 0 0 0 0 0 0 0 0 0 3 0 0 0 0 0 0 18 0 35
0 0 0 0 0 0 0 0 0 0 0 0 0 0 0 0 0 0 0 86 0 0 0 0 0 0 0 0
0 272 0 0 0 0 0 0 0 0 0 0 0 0 0 0 0 0 0 0 0 0 0 0 0 0 0 0
0 0 0 15 0 0 0 0 0 0 0 31 3 0 0 0 0 0 0 0 0 0 0 0 0 0 0

M02255_77_000000000-ADUT3_1_1103_17751_9172 T----AC---GT-AT-GTC--------GCG-A-G------C--G--T---T--AT-C-CGG-AA------TT-A--T-T--GG-GC-------AT--A-----AA-GG-GC-AT-------C-TA-G-G-C-G------------G--C-CC-T-G-T-----------AA----G-T-C-T--------A-G-G--G--TG--A-AA-AC--C-T-GC-G-G-----CT-C-AA------C-C-G-C-A-G-G--C-C----T-G--C-C---C--C-------G--GA-A-A---C-------T--A-C--AG--G-G-C----T-A-G-A-G-T--G---C-TG-----GA-G-A--------G-G-T-G---GA-C------GG--A--ACT----G-C-A-C-GA--GT-A-G-AG-GT--G---A--A-A---TT-C-GT-AG--AT-A-TG--T-G--C-A-G-G-A-AT-G-CC---GA--T--G--AT-GAA-G--A-T--A-------G--T-T-C-A---CTG--G--AC-G-G--------------------C-A---A-C-T--GA--CG-C-T-G--A-AG--T-G-CG-A--AA-G-C---T-AG--GG-G--AG-C-A-AA--------CA--GG Bacteria(100);Fusobacteria(100);Fusobacteria(100);Fusobacteriales(100);Leptotrichiaceae(100);Leptotrichia(100);unclassified(100);unclassified(100);unclassified(100);

Otu0615 0 0 0 0 96 0 0 0 0 0 0 0 0 0 0 0 0 0 0 0 0 0 0 0 0 0 0 0
0 0 0 0 0 0 0 0 0 0 0 0 0 0 0 0 0 0 0 0 0 0 0 0 0 0 0 0 0
0 0 0 0 0 0 0 0 0 0 0 0 0 0 0 0 0 0 0 0 0 0 0 0 15 0 0 0 0
0 0 0 0 0 0 0 0 0 0 0 0 0 0 0 0 0 0 29 0 0 0 0 0 0 0 0
M02255_77_000000000-ADUT3_1_1101_2986_10392 T----AC---GA-GT-GCC--------TCA-A-G------C--G--T---T--AT-C-CGG-AA------TC-A--T-T--GG-GC-------GT--A-----AA-GG-TT-GT-------G-TA-G-G-T-G------------G--T-TA-T-A-T-----------TA---
-G-T-C-T--------T-T-T--G--TT--A-AA-TT--C-T-TC-G-G-----CT-T-AA------C-C-G-G-G-G-G--C-A----T-G--C-A---A--A-------G--GA-A-A---C-------G--G-T--AT--G-AC-----T-A-G-A-G-G--A---T-GC-----GA-G-G--------G-G-T-C---TG-C------GG--A--ACT----C-A-T-A-GT-
-GT-A-G-CG-GT--G---A--A-A---TG-C-GT-TG--AT-A-TT--A-T--G-G-G-G-A-AC-A-CC---AA--A--A--GC-GAA-G--G-C--A-------G--C-A-G-A---CTG--G--AG-C-A--------------------C-T---C-C-T--GA--CA-C-T-G--A-AA--C-A-AG-A--AA-G-C---G-TG--GG-T--CG-C-G-
AA--------TG--GG Bacteria(100);unclassified(100);unclassified(100);unclassified(100);unclassified(100);unclassified(100);unclassified(100);unclassified(100);unclassified(100);

Otu0616 0 200 0 0 0 0 2 0 0 0 0 0 0 0 0 0 0 0 0 0 0 0 0 0 0 0 0 0
0 0 0 0 0 0 0 0 0 0 0 0 0 0 0 0 0 0 0 0 0 0 0 0 0 4 0 0 0
0 0 0 0 0 0 0 0 0 0 0 0 0 0 0 0 0 0 0 0 0 0 0 0 0 0 0 0 0
0 0 0 0 0 0 0 0 0 0 0 0 0 0 0 0 0 0 0 0 0 0 0 0 0 0 0
M02255_77_000000000-ADUT3_1_1105_20390_9750 T----AC---GT-AG-GGG--------GCA-A-G------C--G--T---T--AT-C-CGG-AA------TT-A--T-T--GG-GC-------GT--A-----AA-GA-GT-AC-------G-TA-G-G-T-G------------G--T-TT-T-C-T-----------AA--
--G-C-A-C--------G-G-G--G--TT--T-AA-GG--C-A-AT-G-G-----CT-T-AA------C-C-A-T-T-G-T--T------C-G--C-C---T--T-------G--TG-A-A---C-------T--G-G--AA--G-A-C----T-T-G-A-G-T--G---C-AG-----GA-G-A--------G-G-A-A---AG-C------GG--A--ATT----C-C-T-A-
GT--GT-A-G-CG-GT--G---A--A-A---TG-C-GT-AG--AT-A-TT--A-G--G-A-G-G-A-AC-A-CC---AG--T--G--GC-GAA-G--G-C--G-------G--C-T-T-T---CTG--G--AC-T-G--------------------T-A---A-C-T--GA--CA-C-T-G--A-GG--T-A-CG-A--AA-G-C---G-TG--GG-G--AG-C-
A-AA--------CA--GG Bacteria(100);Firmicutes(100);Clostridia(100);Clostridiales(100);Family_XIII_Incertae_Sedis(100);unclassified(100);unclassified(100);unclassified(100);unclassified(100);

Otu0617 0 0 0 0 0 0 0 0 0 0 0 0 0 0 0 0 0 0 0 0 0 0 8 0 0 0 0 0
0 0 0 0 0 0 0 0 0 0 0 0 0 0 0 0 0 0 0 0 0 0 0 0 0 0 0 0 0
0 0 0 0 0 0 0 0 0 0 0 0 0 0 0 0 0 0 0 0 0 0 0 0 0 0 0 0 0
0 0 0 0 0 16 0 0 0 0 0 0 0 0 0 0 0 0 0 0 0 0 0 0 0 0 0
M02255_77_000000000-ADUT3_1_2108_27630_7402 T----AC---GT-AG-GGT--------GCA-A-G------C--G--T---T--GT-C-CGG-AT------TT-A--T-T--GG-GC-------GT--A-----AA-GA-GC-TC-------G-TA-G-G-C-G------------G--T-TT-G-T-C-----------GC--
--G-T-C-G--------A-C-T--G--TG--A-AA-GC--C-C-GG-G-G-----CT-C-AA------C-T-C-C-G-G-G--T-C----T-G--C-A---G--T-------C--GA-T-A---C-------G--G-G--CA--G-A-C----T-A-G-A-G-T--T---C-GG-----TA-G-G--------G-G-A-G---AC-T------GG--A--ATT----C-C-T-G-
GT--GT-A-G-CG-GT--G---A--A-A---TG-C-GC-AG--AT-A-TC--A-G--G-A-G-G-A-AC-A-CC---GG--T--G--GC-GAA-G--G-C--G-------G--G-T-C-T---CTG--G--GC-C-G--------------------A-T---A-C-T--GA--CG-C-T-G--A-GG--A-G-CG-A--AA-G-C---G-TG--GG-G--AG-
C-A-AA--------CA--GG Bacteria(100);Actinobacteria(100);Actinobacteria(100);Actinobacteridae(100);Actinomycetales(100);unclassified(67);unclassified(67);unclassified(67);unclassified(67);

Otu0618 0 8 0 0 0 0 0 0 13 0 0 0 0 0 0 0 0 0 0 0 0 0 0 0 0 0 0 0
0 0 0 0 0 0 0 0 0 0 0 0 0 0 0 0 0 0 0 0 0 0 0 0 0 0 0 0 0
0 0 0 0 0 0 0 0 0 0 0 0 0 0 0 0 0 0 0 0 0 0 0 0 0 0 0 0 0
0 0 0 0 0 0 3 0 0 0 0 13 0 0 0 0 0 0 0 0 0 0 0 0 0 0 0
M02255_77_000000000-ADUT3_1_2115_6016_15235 G----TG---CC-AG-CAG--------CCG-C-G---------G--T---A--AT-A-CGG-AG------GA-T--C-C--GA-GC-------GT--T-----AT-CC-GG-AT-------T-TA-G-G-T-G------------G--G-TT-G-T-T-----------AA--
--G-T-C-A--------G-T-T--G--TG--A-AA-GT--T-T-GC-G-G-----CT-C-AA------C-C-G-T-A-A-A--A-T----T-G--C-A---G--T-------T--GA-A-A---C-------T--G-G--CA--G-T-C----T-T-G-A-G-T--A---C-AG-----TA-G-A--------G-G-T-G---GG-C------GG--A--ATT----C-G-T-G-
GT--GT-A-G-CG-GT--G---A--A-A---TG-C-TT-AG--AT-A-TC--A-C--G-A-A-G-A-AC-T-CC---GA--T--T--GC-GAA-G--G-C--A-------G--C-T-C-A---CTA--G--AC-T-G--------------------T-T---A-C-T--GA--CA-C-T-G--A-TG--C-T-CG-A--AA-G-T---G-TG--GG-T--AT-C-A-
AA--------CA--GG Bacteria(100);Bacteroidetes(100);Bacteroidia(100);Bacteroidales(100);Bacteroidaceae(100);Bacteroides(100);unclassified(100);unclassified(100);unclassified(100);

Otu0619 0 0 0 0 0 59 0 0 0 0 0 0 0 0 0 0 0 0 0 0 0 0 49 0 0 0 0 0
0 0 0 0 0 0 0 0 0 0 0 0 0 0 0 0 0 0 0 0 0 0 0 0 0 0 0 0 0
0 0 0 0 0 0 0 0 0 0 0 0 0 0 0 0 0 0 0 0 0 0 0 0 0 0 0 0 0
0 0 0 0 0 0 0 0 0 0 0 0 0 0 0 0 0 0 0 0 0 0 0 0 0 0 0
M02255_77_000000000-ADUT3_1_2119_18293_22835 T----AC---GA-AG-GGG--------GCT-A-G------C--G--T---T--GC-T-CGG-AA------TT-A--C-T--GG-GC-------GT--A-----AA-GG-GA-GC-------G-TA-G-G-C-G------------G--G-TT-A-T-C----
-------AA----G-T-T-G--------G-A-G--G--TG--A-AA-GC--C-C-AG-G-G-----CT-C-AA------C-C-T-T-G-G-A--A-T----T-G--C-C---T--T-------C--AA-A-A---C-------T--G-A--TA--G-C-C----T-A-G-A-G-T--A---T-GA-----TA-G-A--------G-G-T-A---AG-T------GG--A--ACT----
C-C-G-A-GT--GT-A-G-AG-GT--G---A--A-A---TT-C-GT-AG--AT-A-TT--C-G--G-A-A-G-A-AC-A-CC---AG--T--G--GC-GAA-G--G-C--G-------A--C-T-T-A---CTG--G--AT-C-A--------------------T-G---A-C-T--GA--CG-C-T-G--A-GG--C-T-CG-A--AA-G-C---G-TG--GG-
G--AG-C-A-AA--------CA--GG Bacteria(100);Proteobacteria(100);Alphaproteobacteria(100);Caulobacterales(100);Caulobacteraceae(100);unclassified(55);unclassified(55);unclassified(55);unclassified(55);

Otu0620 0 0 0 0 0 0 0 0 0 0 0 0 0 0 0 0 0 0 0 0 0 0 0 2 0 0 0 0
0 0 0 0 0 0 0 0 0 0 0 0 0 0 0 0 0 0 0 0 0 0 0 0 0 0 0 0 0
0 0 0 0 0 0 0 0 0 0 0 0 0 0 0 0 0 0 0 0 0 0 0 0 0 0 0 0 18
0 0 0 0 0 0 0 0 0 0 0 0 0 0 0 0 0 0 0 0 0 0 0 0 0 0 0
M02255_77_000000000-ADUT3_1_1117_25241_18621 G----AC---GA-AC-CGT--------GCG-A-A------C--G--T---T--AT-T-CGG-AA------TT-A--C-T--GG-GC-------TT--A-----AA-GC-GC-GT-------G-TA-G-G-C-G------------G--G-CC-G-G-C-----
------AC----G-T-C-C--------GAC-G--C--TG--A-AA-TC--C-C-CC-G-G-----CT-C-AA------C-C-G-G-G-G-A--A-G----T-G--G-C---G--G-------G--GA-T-A---C-------G--A-C--CG--G-C-C----T-C-G-A-G-G--G---G-GG-----TA-G-G--------G-G-G-A---CC-T------GG--A--ACT---
-T-C-C-G-GT--GG-A-G-CG-GT--G---A--A-A---TG-C-GT-TG--AG-A-TC--G-G--A-A-G-G-A-AC-G-CC---CG--T--G--GC-GAA-A--G-C--G-------A--G-G-T-C---CTG--G--AC-C-C--------------------T-T---T-C-T--GA--CG-C-T-G--A-GA--C-G-CG-A--AA-G-C---C-AG--
GG-G--AG-C-G-AA--------CG--GG Bacteria(100);Planctomycetes(100);Planctomycetacia(100);Planctomycetales(100);Planctomycetaceae(100);Singulisphaera(100);unclassified(100);unclassified(100);unclassified(100);

Otu0621 0 0 0 0 0 0 0 0 0 0 0 0 0 0 0 0 0 0 0 0 0 0 0 0 0 0 0 0
0 0 0 0 0 0 0 0 0 0 0 0 0 0 0 0 0 0 0 0 0 0 0 0 0 0 0 0 0
0 0 0 0 0 0 0 0 0 0 0 0 0 0 0 0 0 0 0 0 0 0 0 0 0 0 0 0 0
0 0 0 0 0 0 0 0 0 0 0 0 0 0 0 46 0 0 0 0 0 0 0 0 0 0 0
M02255_76_000000000-ADUNE_1_1119_7158_8330 .....AC----T-AC-GTG---------CC-A-G------C--A--G---C--CG-C-GGT-AA------TA-C--G-T--AG-GT-------GA--C-----AA-GC-G--TT-------G----T-C-C-G------------G--T-CT-T-T-T----------AA----G-
T-C-T--------G-A-T--G--TG--A-AA-GC--C-C-AC-G-G-----CT-C-AA------C-C-G-T-G-G-A--T------G-GT-C-A---T--T-------G--GA-A-A---C-------T--G-G--AG--G-A-C----T-T-G-A-A-T--A---C-AG-----AA-G-A--------G-G-T-A---AG-T------GG--A--ATT----C-C-A-T-GT--
GT-A-G-CG-GT--G---A--A-A---TG-C-GT-AG--AT-A-TA--T-G--G-A-G-G-A-AC-A-CC---AG--T--G--GC-GAA-G--G-C--G-------A--C-T-T-A---CTG--G--TC-T-G--------------------T-T---A-T-T--GA--CG-C-T-G--A-GG--C-T-CG-A--AA-G-C---G-TG--GG-G--AG-C-G-
AA--------CA--GG Bacteria(100);Firmicutes(100);Bacilli(100);Lactobacillales(100);Carnobacteriaceae(100);unclassified(100);unclassified(100);unclassified(100);unclassified(100);

Otu0622 0 0 0 0 0 0 0 0 0 0 0 0 0 0 0 0 0 0 0 0 0 0 0 0 0 0 0 0
0 0 0 0 0 0 0 0 0 0 0 0 0 0 0 0 0 0 0 0 0 0 0 0 0 0 0 0 0
0 0 0 0 0 0 0 0 0 0 0 0 0 0 0 0 0 0 0 0 0 0 0 0 0 0 0 0 0
0 0 0 0 0 0 6 0 0 0 0 0 0 0 0 0 0 0 0 0 0 174 0 0 0 0 0
M02255_77_000000000-ADUT3_1_1102_16413_22482 T----AC---GA-AG-GGG--------GCT-A-G------C--G--T---T--GC-T-CGG-AA------TT-A--C-T--GG-GC-------GT--A-----AA-GG-GC-GC-------G-TA-G-G-C-G------------G--G-TC-G-T-T----
-------AA----G-T-T-A--------G-A-G--G--TG--A-AA-GC--C-C-AG-G-G-----CT-C-AA------C-C-C-T-G-G-A--A-T----T-G--C-C---T--T-------T--GA-T-A---C-------T--G-G--CG--A-C-C----T-T-G-A-G-T--A---T-GG-----CA-G-A--------G-G-T-G---TG-T------GG--A--ACT----
C-C-G-A-GT--GT-A-G-AG-GT--G---A--A-A---TT-C-GT-AG--AT-A-TT--C-G--G-A-A-G-A-AC-A-CC---AG--T--G--GC-GAA-G--G-C--G-------A--C-A-C-A---CTG--G--GC-C-A--------------------T-T---A-C-T--GA--CG-C-T-G--A-GG--C-G-CG-A--AA-G-C---G-TG--GG-
G--AG-C-A-AA--------CA--GG Bacteria(100);Proteobacteria(100);Alphaproteobacteria(100);Caulobacterales(100);Caulobacteraceae(100);unclassified(100);unclassified(100);unclassified(100);unclassified(100);

Otu0623 0 0 0 0 0 0 0 0 0 0 0 0 0 0 0 0 0 0 0 0 0 0 0 0 13 0 0 0
0 0 0 0 0 0 1 0 0 0 0 0 0 0 0 0 0 0 0 0 0 0 0 0 0 0 0 0 0
0 0 0 0 0 0 0 0 0 0 0 0 0 0 0 0 0 0 0 0 0 0 0 0 0 0 0 0 0
2 0 0 0 0 0 0 0 0 0 0 0 0 0 0 0 0 0 0 0 2 0 0 0 0 0 0
M02255_77_000000000-ADUT3_1_2118_23697_6859 C----CG---TG-CC-AGC--------AGC-C-G------C--G--G---T--AA-TACGG-AG------GG-T--G-C--GA-GC-------GT--T-----AA-TCGGAATT-------ACTG-G-G-C-G------------G--T-CT-G-T-T----------
AA----G-C-A-A--------G-A-T--G--TG--A-AA-GC--C-C-GG-G-G-----CT-C-AA------C-C-T-C-G-G-A--A-C----A-G--C-A---T--T-------T--TG-A-A---C-------T--G-G--CA--G-A-C----T-A-G-A-G-T--C---T-TG-----TA-G-A--------G-G-G-G---GG-T------AG--A--ATT----T-C-A-
G-GT--GT-A-G-CG-GT--G---A--A-A---TG-C-GT-AG--AG-A-TC--T-G--A-A-G-G-A-AT-A-CC---GG--T--G--GC-GAA-G--G-C--G-------G--C-C-C-C---CTG--G--AC-A-A--------------------A-G---A-C-T--GA--CG-C-T-C--A-GA--T-G-CG-A--AA-G-C---G-TG--GG-G--
AG-C-A-AA--------CA--GG Bacteria(100);Proteobacteria(100);Gammaproteobacteria(100);Vibrionales(100);Vibrionaceae(100);Photobacterium(100);unclassified(100);unclassified(100);unclassified(100);

Otu0624 0 0 0 0 0 0 0 0 0 0 0 0 0 0 0 0 0 0 0 0 0 0 0 0 0 0 0 0
0 0 0 0 0 0 0 0 0 0 0 0 0 0 0 0 0 0 0 0 0 0 0 0 0 0 0 0 0
0 0 0 0 0 0 0 0 0 0 0 0 0 0 0 0 0 0 0 0 0 0 0 0 0 0 0 0 11
0 0 0 0 0 0 0 0 0 0 0 0 0 0 0 0 0 0 0 0 0 0 0 0 0 0 10
M02255_76_000000000-ADUNE_1_1106_23524_22293 .....AC----T-AC-GT---------GCC-A-G------C--A--G---C--CG-C-GGT-AA------TA-C--G-T--AG-GT-------GG--C-----AA--G-CG-TT-------G----T-C-C-G------------G--A-TT-C-T-T----------
AA----G-T-C-T--------G-A-T--G--TG--A-AA-GC--C-C-AC-G-G-----CT-T-AA------C-C-G-T-G-G-A--A-G----T-G--C-A---T--T-------G--GA-A-A---C-------T--G-G--GG--A-A-C----T-T-G-A-G-T--A---C-AG-----AA-G-A--------G-G-A-A---AG-T------GG--A--ACT----C-C-
A-T-GT--GT-A-G-CG-GT--G---G--A-A---TG-C-GT-AG--AT-A-TA--T-G--G-A-A-G-A-AC-A-CC---AG--T--G--GC-GAA-G--G-C--G-------A--C-T-T-T---CTG--G--TC-T-G--------------------T-C---A-C-T--GA--CG-C-T-G--A-GG--C-C-CG-A--AA-G-C---G-TG--GG-T--
AG-C-A-AA--------CA--GG Bacteria(100);Firmicutes(100);Bacilli(100);Lactobacillales(100);Aerococcaceae(100);Aerococcus(100);unclassified(100);unclassified(100);unclassified(100);

Otu0625 0 0 0 0 0 0 0 0 0 0 0 0 0 0 0 0 0 0 0 0 0 0 0 0 0 0 0 0
0 0 0 0 0 0 0 0 0 0 0 0 0 0 0 0 0 0 0 0 0 0 0 0 0 0 0 0 0
0 0 0 0 0 0 0 0 0 0 0 0 0 0 0 0 0 0 0 0 0 0 0 0 0 0 0 0 0
0 0 0 0 0 14 0 0 0 0 0 0 0 0 0 0 0 0 0 0 112 0 0 0 0 0 0
M02255_77_000000000-ADUT3_1_1114_22034_7545 T----AC---GA-AG-GGG--------GCT-A-G------C--G--T---T--GC-T-CGG-AA------TG-A--C-T--GG-GC-------GT--A-----AA-GG-GC-GC-------G-TA-G-G-C-G------------G--A-TT-G-G-T----------
CA----G-T-C-G--------G-G-C--G--TG--A-AA-TT--C-C-TG-G-G-----CT-C-AA------C-C-T-G-G-G-G--A-C----T-G--C-G---T--T-------C--GA-G-A---C-------G--G-C--TG--G-T-C----T-A-G-A-G-T--G---G-GG-----AA-G-A--------G-G-G-T---CG-T------GG--A--ATT----C-C-
C-A-GT--GT-A-G-AG-GT--G---A--A-A---TT-C-GT-AG--AT-A-TT--G-G--G-A-A-G-A-AC-A-CC---GG--T--G--GC-GAA-G--G-C--G-------G--C-G-A-C---CTG--G--TC-C-T--------------------T-G---A-C-T--GA--CG-C-T-G--A-GG--C-G-CG-A--AA-G-C---G-TG--GG-G--
AG-C-A-AA--------CA--GG Bacteria(100);Proteobacteria(100);Alphaproteobacteria(100);Rhodospirillales(100);Acetobacteraceae(100);unclassified(100);unclassified(100);unclassified(100);unclassified(100);

Otu0626 0 0 0 0 0 0 0 0 0 0 0 0 0 0 0 0 0 0 0 0 0 0 0 0 0 0 0 0
1 0 0 0 0 0 0 29 0 0 10 0 0 0 0 0 0 0 0 0 0 0 0 0 0 0 0 0 0
0 0 0 0 0 0 0 0 0 0 0 0 0 0 0 0 0 11 0 0 0 0 0 0 0 0 0 0 0
0 0 0 0 0 0 0 0 0 0 0 0 0 0 0 0 0 0 0 0 0 0 0 0 4 0 0
M02255_77_000000000-ADUT3_1_1113_10399_7307 C----AC---GT-AG-GGG--------GCA-A-G------C--G--T---T--GT-C-CGG-AT------TT-A--T-T--GG-GC-------GT--A-----AA-GA-GC-TC-------G-TA-G-G-T-G------------G--T-TC-A-G-T-----------AA-
---G-T-C-G--------G-G-T--G--TG--A-AA-CC--T-C-CA-G-G-----CT-C-AA------C-C-T-G-GAG-A---------C-G-CC-A---C--C-------C--GA-T-A---C-------T--G-C--TG-TG-A-C----T-A-G-A-G-T--C---C-AG-----TA-G-G--------G-G-A-G---CG-T------GG--A--ATT----C-C-T-G-
GT--GT-A-G-CG-GT--G---A--A-A---TG-C-GC-AG--AT-A-TC--A-G--G-A-G-G-A-AC-A-CC---AG--T--G--GC-GAA-G--G-C--G-------G--C-G-C-T---CTG--G--GC-T-G--------------------G-A---A-C-T--GA--CA-C-T-G--A-GG--A-G-CG-A--AA-G-C---G-TG--GG-G--AG-
C-G-AA--------CA--GG Bacteria(100);Actinobacteria(100);Actinobacteria(100);Acidimicrobidae(100);Acidimicrobiales(100);Acidimicrobineae(100);unclassified(100);unclassified(100);unclassified(100);

Otu0627 0 0 0 0 0 0 0 0 0 0 0 0 0 0 0 0 0 0 0 0 0 0 0 0 0 0 0 0
0 0 0 0 0 0 0 0 0 0 0 0 0 0 0 0 0 0 0 0 0 18 0 0 0 0 0 0 0
0 0 0 0 0 0 0 0 0 0 0 0 0 0 0 0 0 0 0 0 0 0 0 0 0 0 0 0 12
0 0 0 0 0 0 0 0 0 0 0 0 0 0 0 0 0 0 0 0 0 0 0 0 0 0 0
M02255_76_000000000-ADUNE_1_2112_26976_5287 C----GG---CT-AA-CT---------ACG-T-G------C--C--A---G--CA-G-CCG-CG------GT-A--A-T--AC-GT-------AG--G-----TG-GC-GA-GC-------G-TT-G-T-C-G------------G--G-AA-A-G-T-----------AA-
---G-T-C-A--------G-T-C--T--TA--A-AA-GT--G-C-GG-G-G-----CT-C-AA------C-C-C-C-G-T-G--A------G-G--G-G---A--T-------T--GA-A-A---C-------T--A-C--TT--T-T-C----T-T-G-A-G-T--G---C-AG-----GA-G-A--------G-G-A-A---AG-C------GG--A--ATT----C-C-T-A-
GT--GT-A-G-CG-GT--G---A--A-A---TG-C-GT-AG--AT-A-TT--A-G--G-A-G-G-A-AC-A-CC---AG--T--G--GC-GAA-G--G-C--G-------G--C-T-T-T---CTG--G--AC-T-G--------------------T-A---A-C-T--GA--CA-C-T-G--A-GG--C-T-CG-A--AA-G-C---C-AG--GG-G--AG-C-
G-AA--------CG--GG Bacteria(100);Firmicutes(100);Clostridia(100);Clostridiales(100);Veillonellaceae(100);unclassified(100);unclassified(100);unclassified(100);unclassified(100);

Otu0628 0 0 0 0 0 0 0 0 0 0 0 0 0 0 0 0 0 0 0 0 0 0 0 0 0 0 0 0
0 0 0 0 0 0 0 0 0 0 0 0 0 0 0 0 0 0 0 0 0 0 0 0 0 0 0 0 0
0 0 0 0 0 0 0 0 0 0 0 0 0 0 0 0 0 0 0 0 0 0 0 0 0 0 0 0 0
0 89 0 35 0 0 0 0 0 0 0 0 0 0 0 0 0 0 0 0 0 0 0 0 0 0 0
M02255_77_000000000-ADUT3_1_1114_23985_15860 T----AC---GT-AG-GGG--------GCA-A-G------C--G--T---T--GT-C-CGG-AA------TA-A--T-T--GG-GC-------GT--A-----AA-GG-GC-GC-------G-TA-G-G-C-G------------G--C-TC-G-G-T----
-------AA----G-T-C-T--------G-G-A--G--TG--A-AA-GT--C-C-TG-C-T-----TT-T-AA------G-G-T-G-G-G-A--A-T----T-G--C-T---T--T-------G--GA-T-A---C-------T--G-T--CG--G-G-C----T-T-G-A-G-T--G---C-AG-----GA-G-A--------G-G-T-T---AG-T------GG--A--ATT----
C-C-C-A-GT--GT-A-G-CG-GT--G---A--A-A---TG-C-GT-AG--AG-A-TT--G-G--G-A-G-G-A-AC-A-CC---AG--T--G--GC-GAA-G--G-C--G-------A--C-T-A-A---CTG--G--AC-T-G--------------------T-A---A-C-T--GA--CG-C-T-G--A-GG--C-G-CG-A--AA-G-T---G-TG--
GG-G--AG-C-A-AA--------CA--GG Bacteria(100);Firmicutes(100);Clostridia(100);Clostridiales(100);Lachnospiraceae(100);uncultured(100);unclassified(100);unclassified(100);unclassified(100);

Otu0629 0 0 0 0 0 0 0 0 0 0 0 0 0 0 0 0 0 0 0 0 0 0 0 0 0 0 0 0
0 0 0 0 0 1 0 0 0 0 0 0 0 0 0 0 0 0 0 0 0 0 0 0 0 0 0 1 278
0 0 0 0 0 0 0 0 0 0 0 0 0 0 0 0 0 0 0 0 0 0 0 0 0 0 0 0 0
0 0 0 0 0 0 8 0 0 0 0 0 0 42 0 0 0 0 0 0 0 0 0 0 0 0 0
M02255_77_000000000-ADUT3_1_2104_15367_20207 T----AC---GT-AT-GTG--------GCA-A-G------C--G--T---T--GT-C-CGG-AA------TT-A--T-T--GG-GC-------GT--A-----AA-GG-GT-AC-------G-TA-G-G-C-G------------G--T-TA-T-T-T------
-----AA----G-T-C-T--------A-A-A--T--TA--A-AA-GG--C-A-TT-G-G-----CT-C-AA------C-C-A-A-T-G-T--A------A-G--G-T---T--T-------A--GA-T-A---C-------T--G-G--AT--A-A-C----T-T-G-A-G-T--A---G-AT-----GA-G-G--------G-G-A-A---AG-T------GG--A--ATT----C-C-
A-T-GT--GT-A-G-CG-GT--G---A--A-A---TG-C-GT-AG--AT-A-TA--T-G--G-A-G-G-A-AC-A-CC---AA--T--G--GC-GAA-G--G-C--A-------A--C-T-T-T---CTG--G--AA-T-C--------------------A-A---A-C-T--GA--CG-C-T-G--A-GG--T-A-CG-A--AG-G-C---G-TG--GG-G--
AG-C-A-AA--------CA--GG Bacteria(100);Firmicutes(100);Clostridia(100);Clostridiales(100);Family_XI_Incertae_Sedis(100);Helcococcus(100);unclassified(100);unclassified(100);unclassified(100);

Otu0630 0 0 0 0 0 0 0 0 0 0 0 0 0 0 0 0 0 0 1 5 0 2 0 0 0 0 0 0
0 0 0 0 0 0 0 0 0 0 0 0 0 0 0 0 0 0 0 0 0 0 0 0 0 0 0 0 0
0 0 0 0 0 0 0 0 0 0 0 0 3 0 0 0 0 0 0 0 0 0 0 0 0 0 0 0 0
0 0 0 0 0 0 0 0 0 0 0 0 0 0 1 0 0 0 0 0 0 0 0 0 0 0 0
M02255_76_000000000-ADUNE_1_2107_17385_14870 C----TC---GC-AG-AAT--------AAG-CACC-----G--G--C---T--AACTCTGT-GC------CA-G--C-A-GCC-GCG------GT--A-----AT-AC-AG-AG-------G-GT-G-C-A-G------------G--T-CT-G-T-T-
----------AA----G-T-C-G--------G-A-T--G--TG--A-AA-TC--C-C-TG-A-G-----CT-T-AA------C-T-T-A-G-G-A--A-T----T-G--C-A---T--A-------C--GA-T-A---C-------T--G-G--GA--G-A-C----T-A-G-A-G-T--A---T-GG-----GA-G-A--------G-G-A-T---GG-T------AG--A--ATT---
-C-C-A-G-GT--GT-A-G-CG-GT--G---A--A-A---TG-C-GT-AG--AG-A-TC--T-G--G-A-G-G-A-AT-A-CC---GA--T--G--GC-GAA-G--G-C--A-------G--C-C-A-T---CTG--G--CC-T-A--------------------A-T---A-C-T--GA--CA-C-T-G--A-GG--T-A-CG-A--AA-G-C---A-TG--
GG-G--AG-C-A-AA--------CA--GG Bacteria(100);Proteobacteria(100);Gammaproteobacteria(100);Pseudomonadales(100);Moraxellaceae(100);Acinetobacter(59);unclassified(59);unclassified(59);unclassified(59);

Otu0631 0 0 0 0 0 0 0 0 0 0 0 0 0 0 0 0 0 0 0 0 0 0 0 0 0 0 0 0
0 0 0 0 0 0 0 0 0 0 0 0 0 0 0 0 0 0 0 0 0 0 0 0 0 0 0 0 0
0 7 0 0 0 0 0 0 0 0 0 0 0 0 0 0 0 0 0 0 0 0 0 0 0 0 0 0 0
68 0 0 0 0 0 0 0 0 0 0 0 0 0 0 0 0 0 0 0 0 0 0 0 0 0 0
M02255_77_000000000-ADUT3_1_1101_15626_10296 T----AC---GT-AG-GGT--------CCG-A-G------C--G--T---T--GT-C-CGG-AA------TT-A--T-T--GG-GC-------GT--A-----AA-GG-GC-TC-------G-TA-G-G-C-G------------G--T-TT-G-T-C------
-----GC----G-T-C-G--------G-G-A--G--TG--A-AA-AC--T-C-AG-G-G-----CT-T-AA------C-T-C-T-G-A-G--C-G----T-G--C-T---T--T-------C--GA-T-A---C-------G--G-G--CA--G-A-C----T-T-G-A-G-G--G---A-TG-----CA-G-G--------G-G-A-G---AA-T------GG--A--ATT----C-
C-T-G-GT--GT-A-G-CG-GT--G---G--A-A---TG-C-GC-AG--AT-A-TC--A-G--G-A-G-G-A-AC-A-CC---GG--T--G--GC-GAA-G--G-C--G-------G--T-T-C-T---CTG--G--GC-A-T--------------------T-T---C-C-T--GA--CG-C-T-G--A-GG--A-G-CG-A--AA-G-C---A-TG--GG-
G--AG-C-G-AA--------CA--GG Bacteria(100);Actinobacteria(100);Actinobacteria(100);Actinobacteridae(100);Actinomycetales(100);Propionibacterineae(100);unclassified(91);unclassified(91);unclassified(91);

Otu0632 0 0 0 0 0 0 0 0 0 0 0 0 0 0 0 0 0 0 0 0 0 0 0 0 0 0 0 0
0 0 0 0 0 0 0 0 0 0 0 0 0 0 0 0 0 0 0 0 0 0 0 0 0 0 0 0 0
0 0 0 0 0 0 0 0 0 0 0 0 0 0 0 0 0 18 0 0 0 0 0 0 0 0 0 0 0
0 0 0 0 0 0 4 0 0 0 0 0 0 0 0 0 0 0 0 0 0 0 0 0 0 0 0
M02255_77_000000000-ADUT3_1_1111_27549_18897 T----AC---GT-AG-GCA--------GCG-A-G------C--G--T---T--GT-T-CGG-AG------TT-A--C-T--GG-GC-------GT--A-----AA-GA-GT-GC-------G-TA-G-G-C-G------------G--T-GG-C-C-C----
-------AA----G-T-C-T--------G-G-T--G--TG--A-AA-TC--T-C-CC-G-G-----CT-C-AA------C-C-G-G-G-A-G--G-G----T-G--C-G---C--C-------G--GA-A-A---C-------T--G-G--GT--T-G-C----T-T-G-A-G-T--A---C-TG-----GA-G-A--------G-G-C-A---AG-C------GG--A--ATT----
C-C-T-G-GT--GT-A-G-CG-GT--G---A--A-A---TG-C-GT-AG--AT-A-TC--A-G--G-A-G-G-A-AC-A-CC---CG--T--G--GT-GTA-G--A-C--G-------G--C-T-T-G---CTG--G--AC-A-G--------------------T-C---A-C-T--GA--CG-C-T-G--A-GG--C-A-CG-A--AA-G-C---G-TG--GG-
G--AG-C-A-AA--------CA--GG Bacteria(100);unclassified(100);unclassified(100);unclassified(100);unclassified(100);unclassified(100);unclassified(100);unclassified(100);unclassified(100);

Otu0633 0 0 0 0 0 0 0 0 0 0 0 0 0 0 0 0 0 0 0 0 0 0 0 0 0 0 0 0
0 0 0 0 0 0 0 0 0 0 0 0 0 0 0 0 46 0 0 0 0 0 0 0 0 0 0 0 0
0 0 0 0 0 0 0 0 0 0 0 0 0 0 0 0 0 0 0 0 0 0 0 0 0 0 0 0 0
316 0 0 0 0 0 0 0 0 0 0 0 0 0 0 0 0 0 0 0 0 0 0 0 0 0 0
M02255_77_000000000-ADUT3_1_1105_6715_19018 T----AC---GG-AG-GAT--------GCA-A-G------C--G--T---T--AT-C-CGG-AA------TG-A--T-T--GG-GC-------GT--A-----AA-GC-GT-CC-------G-CA-G-G-T-G------------G--C-AA-T-G-T-----------AA-
---G-T-C-T--------G-C-T--G--TT--A-AA-GA--G-T-CT-A-G-----CT-C-AA------C-T-A-G-A-T-A--A-G----A-G--C-A---G--T-------G--GA-A-A---C-------T--A-C--AT--A-G-C----T-A-G-A-G-T--A---C-GT-----TC-G-G--------G-G-C-A---GA-G------GG--A--ATT----C-C-T-G-
GT--GT-A-G-CG-GT--G---A--A-A---TG-C-GT-AG--AG-A-TC--A-G--G-A-A-G-A-AC-A-CC---AG--T--G--GC-GAA-G--G-C--G-------C--T-C-T-G---CTA--G--GC-C-G--------------------T-A---A-C-T--GA--CA-C-T-G--A-GG--G-A-CG-A--AA-G-C---T-AG--GG-G--AG-
C-G-AA--------TG--GG Bacteria(100);Cyanobacteria(100);SubsectionIV(100);Nostoc(100);unclassified(100);unclassified(100);unclassified(100);unclassified(100);unclassified(100);

Otu0634 0 0 0 0 0 0 0 0 0 0 5 0 0 0 0 0 0 0 0 0 0 0 4 0 4 0 0 0
0 0 0 0 0 0 0 0 0 0 0 0 0 0 0 0 0 0 0 0 0 0 0 0 0 0 0 0 0
0 0 0 0 0 0 0 0 0 0 0 0 0 0 0 0 0 0 0 0 0 0 0 0 0 0 0 0 0
0 0 0 0 0 0 0 0 0 0 0 0 0 0 0 0 0 0 0 0 0 0 0 0 0 0 0
M02255_76_000000000-ADUNE_1_2112_15473_20468 A----AC---AG-AA-GAA--------GCA-C-C------G--G--C---T--AACT-CCG-TG------CC-A--G-C--AG-CC-------GC--G-----GT-AA-TA-CG-------G-AG-G-G-T-GC-----------G--A-GC-G-T-T---
--------AA----G-C-A-A--------G-A-T--G--TG--A-AA-GC--C-C-GG-G-G-----CT-C-AA------C-C-T-C-G-G-A--A-C----A-G--C-A---T--T-------G--TG-A-A---C-------T--G-G--CA--G-A-C----T-A-G-A-G-T--C---T-TG-----TA-G-A--------G-G-G-G---GG-T------AG--A--ATT---
-T-C-A-G-GT--GT-A-G-CG-GT--G---A--A-A---TG-C-GT-AG--AG-A-TC--T-G--A-A-G-G-A-AT-A-CC---GG--T--G--GC-GAA-G--G-C--G-------G--C-C-C-C---CTG--G--AC-A-A--------------------A-G---A-C-T--GA--CG-C-T-C--A-GA--T-G-CG-A--AA-G-C---G-TG--
GG-G--AG-C-A-AA--------CA--GG Bacteria(100);Proteobacteria(100);Gammaproteobacteria(100);Vibrionales(100);Vibrionaceae(100);unclassified(100);unclassified(100);unclassified(100);unclassified(100);

Otu0635 0 0 0 0 0 0 0 0 0 0 0 0 5 0 0 0 0 0 0 3 7 0 0 0 0 0 0 0
0 1 0 0 0 0 0 0 0 35 0 0 0 0 0 0 0 0 0 0 0 0 0 0 0 0 0 0
0 0 0 0 0 0 0 0 0 0 0 0 0 0 0 0 0 0 0 0 0 0 0 0 0 0 0 0
0 0 0 0 0 0 0 0 0 0 0 0 0 0 0 0 0 0 0 0 0 0 0 0 0 0 0
M02255_77_000000000-ADUT3_1_1119_2882_9488 T----AC---GA-AG-GGG--------GCT-A-G------C--G--T---T--GT-T-CGG-AA------TC-A--C-T--GG-GC-------GT--A-----AA-GC-GT-AC-------G-TA-G-G-C-G------------G--A-TC-G-T-T-----------AA-
---G-T-C-A--------G-G-G--G--TG--A-AA-TC--C-C-GA-G-G-----CT-C-AA------C-C-T-C-G-G-A--A-C----T-G--C-C---T--T-------T--GA-T-A---C-------T--G-G--CG--A-T-C----T-T-G-A-G-T--C---C-GG-----GA-G-A--------G-G-T-G---AG-T------GG--A--ACT----C-C-T-A-
GT--GT-A-G-AG-GT--G---A--A-A---TT-C-GT-AG--AT-A-TT--A-G--G-A-A-G-A-AC-A-CC---AG--T--G--GC-GAA-G--G-C--G-------G--C-T-C-A---CTG--G--CC-C-G--------------------G-A---A-C-T--GA--CG-C-T-G--A-GG--T-A-CG-A--AA-G-C---G-TG--GG-G--AG-
C-A-AA--------CA--GG Bacteria(100);Proteobacteria(100);Alphaproteobacteria(100);Rhizobiales(100);unclassified(91);unclassified(91);unclassified(91);unclassified(91);unclassified(91);

Otu0636 0 0 0 0 0 0 0 0 0 0 0 0 0 0 0 0 0 0 0 0 0 0 0 0 0 0 0 0
0 0 0 0 0 0 0 0 0 0 0 0 0 0 0 0 0 0 0 0 0 0 0 0 0 0 0 0
0 0 0 0 0 0 0 0 0 0 0 0 0 0 0 0 0 40 0 0 0 0 0 0 0 0 0 0
0 0 24 0 0 0 6 0 0 0 0 0 0 0 0 0 0 0 0 0 0 0 0 0 0 0 0
M02255_77_000000000-ADUT3_1_1114_16288_13793 T----AC---GG-AG-GGG--------GCT-A-G------C--G--T---T--GT-T-CGG-AA------TT-A--C-T--GG-GC-------GT--A-----AA-GC-GC-AC-------G-TA-G-G-C-G------------G--C-TT-T-A-C-----
------AA----G-T-C-A--------G-A-G--G--TG--A-AA-GC--C-C-GG-A-G-----CT-C-AA------C-T-C-C-G-G-A--A-T----T-G--C-C---T--T-------T--GA-G-A---C-------T--G-T--AT--C-G-C----T-T-G-A-A-C--A---C-GG-----GA-G-A--------G-G-T-G---AG-T------GG--A--ATT----C-
C-G-A-GT--GT-A-G-AG-GT--G---A--A-A---TT-C-GT-AG--AT-A-TT--C-G--G-A-A-G-A-AC-A-CC---AG--T--G--GC-GAA-G--G-C--G-------G--C-T-C-A---CTG--G--AC-C-G--------------------T-T---G-T-T--GA--CG-C-T-G--A-GG--T-G-CG-A--AA-G-C---G-TG--GG-G-
-AG-C-A-AA--------CA--GG Bacteria(100);Proteobacteria(100);Alphaproteobacteria(100);unclassified(92);unclassified(92);unclassified(92);unclassified(92);unclassified(92);unclassified(92);

Otu0637 0 0 0 0 0 0 0 0 0 0 0 0 0 0 0 0 0 0 0 0 0 0 0 0 0 0 0 0
0 0 0 0 0 0 0 0 0 0 0 0 0 0 0 0 0 0 0 0 0 0 0 0 0 0 0 0
0 0 0 0 0 0 0 0 0 0 0 0 0 0 0 0 0 0 0 0 0 0 4 0 0 0 0 0 0
0 0 0 0 0 0 0 0 0 0 0 0 0 0 0 16 0 0 0 0 0 0 0 0 0 0 0
M02255_77_000000000-ADUT3_1_1108_19390_22270 T----AC---GA-AG-GGG--------GCT-A-G------C--G--T---T--GC-T-CGG-AA------TG-A--C-T--GG-GC-------GT--A-----AA-GG-GC-GC-------G-TA-G-G-C-G------------G--C-AC-G-G-A---
--------CA----G-T-C-A--------G-G-C--G--TG--A-AA-TT--C-C-TG-G-T-----CT-T-AA------C-C-T-G-G-G-G--G-C----T-G--C-G---T--T-------T--GA-T-A---C-------G--T-T--CG--A-G-C----T-A-G-A-G-G--A---T-GG-----AA-G-A--------G-G-C-T---CG-T------GG--A--ATT----
C-C-C-A-GT--GT-A-G-AG-GT--G---A--A-A---TT-C-GT-AG--AT-A-TT--G-G--G-A-A-G-A-AC-A-CC---GG--T--G--GC-GAA-G--G-C--G-------G--C-G-A-G---CTG--G--TC-C-A--------------------T-T---A-C-T--GA--CG-C-T-G--A-GG--C-G-CG-A--AA-G-C---G-TG--
GG-G--AG-C-A-AA--------CA--GG Bacteria(100);Proteobacteria(100);Alphaproteobacteria(100);Rhodospirillales(100);Acetobacteraceae(100);unclassified(100);unclassified(100);unclassified(100);unclassified(100);

Otu0638 0 0 0 0 0 0 0 0 0 0 0 0 0 2 0 0 0 0 0 2 10 0 2 0 0 0 0 2
0 8 6 26 0 0 0 12 0 20 8 13 4 18 0 3 3 0 0 41 22 0 0 46 0 0 0 0 1
1 0 0 0 0 0 0 0 0 0 0 0 0 0 0 0 0 0 0 0 0 1 0 0 0 0 10 0
5 0 0 10 0 0 1 0 0 1 0 0 2 11 0 0 0 0 0 0 0 0 0 0 0 0 0
M02255_77_000000000-ADUT3_1_2102_15172_9536 G----AC---GG-GG-GGG--------GCA-A-G------T--G--T---T--AT-T-CGG-AA------TG-A--C-T--AG-GC-------GT--A-----AA-GG-GC-AC-------G-TA-G-G-C-G------------G----TG-A-A-T-----------
CT----G-C-A-G--------G-G-T--T--GG-AAGGGAAA--G-T-CG-C-C-----AA-C-AA------T-T-G-G-C-G-G--G-G----T-G--C-T---T--T-------C--GG-A-A---C-------C--A-A--TT--C-A-C----T-T-G-A-G-T--G---A-GA-----TA-G-G--------G-G-A-G---AG-T------GG--A--ATT----T-
C-G-T-GT--GT-A-G-GG-AT--C---A--A-A---TC-C-GG-AG--AT-C-TA--C-G--A-A-G-G-A-AC-G-CC---AA--A--A--GC-GAA-G--G-C--A-------G--C-T-C-T---CTG--G--GT-C-T--------------------A-C---A-C-T--GA--CG-C-T-G--G-GG--T-G-CG-A--AA-G-C---A-TG--GG-G-
-AG-C-A-AA--------CA--GG Bacteria(100);unclassified(100);unclassified(100);unclassified(100);unclassified(100);unclassified(100);unclassified(100);unclassified(100);unclassified(100);

Otu0639 0 0 0 0 0 0 0 0 0 0 0 0 0 0 0 0 0 0 0 0 0 0 0 0 0 0 12 0
0 0 0 0 0 0 0 0 0 0 0 0 0 0 0 0 0 0 0 0 0 0 0 0 0 0 0 0
0 0 0 0 0 0 0 0 0 0 0 0 0 0 0 0 0 0 0 0 0 0 0 0 0 0 0 0
0 0 46 0 0 0 0 0 0 0 0 0 0 0 0 0 0 0 0 0 0 0 0 0 0 0 0
M02255_77_000000000-ADUT3_1_1112_19727_7325 T----AC---GA-AG-GGG--------GCT-A-G------C--G--T---T--GC-T-CGG-AA------TG-A--C-T--GG-GC-------GT--A-----AA-GG-GC-GC-------G-TA-G-G-C-G------------G--A-TC-A-C-G-----------
CA----G-T-C-A--------G-A-T--G--TG--A-AA-TT--C-C-TG-G-G-----CT-T-AA------C-C-T-G-G-G-G--G-C----T-G--C-A---T--T-------T--GA-G-A---C-------G--T-G--TG--G-T-C----T-A-G-A-G-T--G---T-GA-----AA-G-A--------G-G-G-T---CG-T------GG--A--ATT----C-C-C-
A-GT--GT-A-G-AG-GT--G---A--A-A---TT-C-GT-AG--AT-A-TT--G-G--G-A-A-G-A-AC-A-CC---GG--T--G--GC-GAA-G--G-C--G-------G--C-G-A-C---CTG--G--TT-C-A--------------------T-A---A-C-T--GA--CG-C-T-G--A-GG--C-G-CG-A--AA-G-C---G-TG--GG-G--
AG-C-A-AA--------CA--GG Bacteria(100);Proteobacteria(100);Alphaproteobacteria(100);Rhodospirillales(100);Acetobacteraceae(100);unclassified(100);unclassified(100);unclassified(100);unclassified(100);

Otu0640 0 0 0 0 0 0 0 0 76 0 0 0 0 0 0 0 0 0 0 0 0 0 0 0 0 0 0 0
0 0 0 0 0 0 0 0 0 0 0 0 0 0 0 0 0 0 0 0 0 0 0 0 0 0 0 0 0
0 0 0 0 0 0 0 0 0 0 0 0 0 0 0 0 0 0 0 0 0 0 0 0 0 0 0 0 0
0 0 138 0 0 0 0 0 0 0 0 0 0 0 0 0 0 0 0 0 0 0 0 0 0 0 0
M02255_77_000000000-ADUT3_1_1112_4712_19514 T----AC---GT-AG-GGG--------GCA-A-G------C--G--T---T--AT-C-CGG-AT------TT-A--C-T--GG-GT-------GT--A-----AA-GG-GA-GT-------G-TA-G-G-T-G------------G--T-CT-G-G-C----------AA-
---G-T-C-A--------G-A-T--G--TG--A-AA-GC--C-C-GG-G-G-----CT-C-AA------C-T-C-C-G-G-G--A-C----T-G--C-A---T--T-------T--GA-A-A---C-------T--G-T--CA--G-A-C----T-A-G-A-G-T--G---C-AG-----GA-G-A--------G-G-A-A---AG-T------GG--A--ATT----C-C-T-A-
GT--GT-A-G-CG-GT--G---A--A-A---TG-C-GT-AG--AT-A-TT--A-G--G-A-A-G-A-AC-A-CC---AG--T--G--GC-GAA-G--G-C--G-------G--C-T-T-T---CTG--G--AC-T-G--------------------T-A---A-C-T--GA--CA-C-T-G--A-GG--C-T-CG-A--AA-G-C---G-TG--GG-G--AG-C-
A-AA--------CA--GG Bacteria(100);Firmicutes(100);Clostridia(100);Clostridiales(100);Lachnospiraceae(100);unclassified(100);unclassified(100);unclassified(100);unclassified(100);

Otu0641 0 0 0 0 0 0 0 0 0 0 0 0 0 0 0 0 0 0 0 0 0 0 0 0 0 0 0 0
0 0 0 0 0 0 0 0 0 0 0 0 0 0 0 0 0 0 0 0 0 0 0 0 0 0 0 0 27
0 0 0 0 0 0 0 0 0 0 0 0 0 0 0 0 0 0 0 0 0 0 0 0 0 0 0 0 18
0 0 57 0 0 0 0 0 0 0 0 0 0 0 0 0 0 0 0 0 0 0 0 0 0 0 0
M02255_77_000000000-ADUT3_1_1113_18640_16642 G----AC---GT-AG-GGC--------GCG-A-G------C--G--T---T--GT-C-CGG-AT------TT-A--T-T--GG-GC-------GT--A-----AA-GA-GC-TC-------G-TA-G-G-C-G------------G--C-TC-G-T-T-----
------GC----G-T-C-G--------G-C-T--G--TG--A-AA-AC--C-C-GC-A-G-----CT-C-AA------C-T-G-T-G-G-G--C-T----T-G--C-A---G--C-------C--GA-T-A---C-------G--G-G--CG--G-G-C----T-G-G-A-G-T--T---C-GG-----TA-G-G--------G-G-A-G---AC-T------GG--A--ATT----
C-C-T-G-GT--GT-A-G-CG-GT--G---A--A-A---TG-C-GC-AG--AT-A-TC--A-G--G-A-G-G-A-AC-A-CC---GG--T--G--GC-GAA-G--G-C--G-------G--G-T-C-T---CTG--G--GC-C-G--------------------A-T---A-C-T--GA--CG-C-T-G--A-GG--A-G-CG-A--AA-G-C---G-TG--
GG-G--AG-C-G-AA--------CA--GG Bacteria(100);Actinobacteria(100);Actinobacteria(100);Actinobacteridae(100);Actinomycetales(100);Micromonosporineae(100);Micromonosporaceae(100);unclassified(56);unclassified(56);

Otu0642 0 97 1882 0 0 0 107 6 258 0 0 0 0 0 0 0 0 1 0 0 0 0 0 0 0 0 0 0
0 0 0 0 0 0 0 0 0 0 0 0 0 0 0 0 0 0 0 0 0 0 0 0 0 0 0 0 0
0 0 0 0 0 0 0 0 0 0 0 0 3 0 0 2 15 39 0 0 0 0 0 0 0 0 0 0 0
0 0 0 0 0 0 0 0 0 0 0 0 0 0 0 0 0 0 0 0 0 0 0 7 99 0 0
M02255_77_000000000-ADUT3_1_2111_7601_16394 T----AC---GT-AG-GGA--------GCG-A-G------C--G--T---T--GT-C-CGG-AA------TT-A--C-T--GG-GT-------GT--A-----AA-GG-GA-GC-------G-TA-G-G-C-G------------G--G-AT-G-G-C----------
AA----G-T-C-A--------G-A-T--G--TG--A-AA-AC--T-A-TG-G-G-----CT-C-AA------C-C-C-A-T-A-G--A-C----T-G--C-A---T--T-------T--GA-A-A---C-------T--G-T--TG--T-G-C----T-T-G-A-G-T--G---A-GG-----TA-G-A--------G-G-T-A---AG-C------GG--A--ATT----C-C-T-
G-GT--GT-A-G-CG-GT--G---A--A-A---TG-C-GT-AG--AG-A-TC--A-G--G-A-G-G-A-AC-A-TC---GG--T--G--GC-GAA-G--G-C--G-------G--C-T-T-A---CTG--G--GC-C-T--------------------T-T---A-C-T--GA--CG-C-T-G--A-GG--C-T-CG-A--AA-G-C---G-TG--GG-G--
AG-C-A-AA--------CA--GG Bacteria(100);Firmicutes(100);Clostridia(100);Clostridiales(100);Ruminococcaceae(100);Ruminococcus(100);unclassified(100);unclassified(100);unclassified(100);

Otu0643 0 0 0 0 0 0 0 0 0 0 0 0 0 0 0 0 0 0 0 0 0 0 0 0 0 0 0 0
0 0 0 0 0 0 0 0 0 0 0 0 0 0 18 0 0 0 0 0 0 0 0 0 0 0 0 0 0
0 0 0 0 0 0 0 0 0 0 0 0 0 0 0 0 0 0 0 0 0 0 0 0 0 0 0 0 0
0 0 12 0 0 0 0 0 0 0 0 0 0 0 0 0 0 0 0 0 0 0 0 0 0 0 0
M02255_77_000000000-ADUT3_1_2116_18700_16534 C----TC---CG-TG-CCA--------GCAGCCG------CG-G--T---A--AT-A-CGG-AG------GG-T--G-C--AA-GC-------GT--T-----AT-CC-GG-AT-------T-TA-G-C-T-G------------G--G-TC-T-G-T----
-------AA----G-T-C-A--------G-T-G--G--TG--A-AA-TC--T-C-CG-A-G-----CT-T-AA------C-T-C-G-G-A-A--A-C----T-G--C-C---A--T-------T--GA-T-A---C-------T--A-T--AG--A-T-C----T-T-G-A-A-T--T---A-TC-----TG-G-A--------G-G-T-A---AG-C------GG--A--ATA----T-
G-T-C-AT--GT-A-G-CG-GT--G---A--A-A---TG-C-TT-AG--AT-A-TG--A-C--A-T-A-G-A-AC-A-CC---AA--T--T--GC-GAA-G--G-C--A-------G--C-T-T-A---CTA--C--GG-A-T--------------------C-G---A-T-T--GA--CA-C-T-G--A-GG--C-A-CG-A--AA-G-C---G-TG--GG-G--
AT-C-A-AA--------CA--GG Bacteria(100);Bacteroidetes(100);Sphingobacteria(100);Sphingobacteriales(100);Chitinophagaceae(100);unclassified(100);unclassified(100);unclassified(100);unclassified(100);

Otu0644 0 0 0 0 0 0 0 0 0 0 0 0 0 0 0 0 0 0 0 0 0 0 0 0 0 0 0 0
0 0 0 0 0 0 0 0 0 0 0 0 0 0 0 0 0 0 0 0 0 0 0 0 0 0 0 0 0
0 0 0 0 0 0 0 0 0 0 0 0 0 0 0 0 0 0 0 0 0 0 0 0 0 0 0 0 0
0 0 90 0 0 0 0 0 0 0 0 0 0 0 0 0 0 0 0 0 0 0 0 0 0 0 0
M02255_76_000000000-ADUNE_1_2115_21736_8913 C----AC---GT-AG-GGG--------GCG-A-G------C--G--T---T--GT-C-CGG-AT------TT-A--T-T--GG-GC-------GT--A-----AA-GA-GC-TC-------G-TA-------G------------G----TC-G-G-------------A------
-----------------T--G--TG--A-AA-AC--T-C-CA-G-G-----CT-C-AA------C-T-T-G-G-A-G--A------C-G-CC-A---T--T-------C--GA-T-A---C-------T--G-C--GA-TG-A-C----T-T-G-A-G-T--C---C-GG-----TA-G-G--------G-G-A-G---TG-T------GG--A--ATT----C-C-T-G-GT--GT-
A-G-CG-GT--G---A--A-A---TG-C-GC-AG--AT-A-TC--A-G--G-A-G-G-A-AC-A-CC---GG--C--G--GC-GAA-G--G-C--G-------G--C-A-C-T---CTG--G--GC-C-G--------------------G-T---A-C-T--GA--CG-C-T-G--A-GG--A-G-CG-A--AA-G-C---G-TG--GG-T--AG-C-A-AA-
-------CA--GG Bacteria(100);Actinobacteria(100);Actinobacteria(100);unclassified(100);unclassified(100);unclassified(100);unclassified(100);unclassified(100);unclassified(100);

Otu0645 0 338 60 0 0 1 313 14 481 0 0 0 0 0 0 0 0 0 1 0 0 1 0 0 0 1 0 0
0 0 5 67 0 0 0 0 0 5 16 60 0 0 0 0 0 105 0 1 0 0 0 0 0 0 0 0 0
0 0 0 0 0 0 0 0 0 0 0 3 0 0 0 4 33 27 0 0 0 0 0 0 0 1 0 0 0
0 0 0 0 0 0 11 0 0 35 0 189 0 0 1 0 0 0 0 0 0 0 0 0 148 161 0
M02255_77_000000000-ADUT3_1_2113_24365_8959 T----AC---GT-AG-GGG--------GCA-A-G------C--G--T---T--AT-C-CGG-AT------TT-A--C-T--GG-GT-------GT--A-----AA-GG-GA-GC-------G-TA-G-A-C-G------------G--C-AA-G-G-C----------
AA----G-T-C-T--------G-A-T--G--TG--A-A-AGC--C-C-AG-G-G-----CT-T-AA------C-C-C-T-G-G-G--A-C----T-G--C-A---T--T-------G--GA-A-A---C-------T--G-T--CT--G-G-C----T-C-G-A-G-T--G---C-CG-----GA-G-A--------G-G-T-A---AG-C------GG--A--ATT----C-C-T-
A-GT--GT-A-G-CG-GT--G---A--A-A---TG-C-GT-AG--AT-A-TT--AG---G-A-A-G-A-AC-A-CC---AG--T--G--GC-GAA-G--G-C--G-------G--C-T-TA----CTG--G--AC-G-G--------------------TA----A-C-T--GA--CG-T-T-G--A-GG--C-T-CG-A--AA-G-C---G-TG--GG-G--
AG-C-A-AA--------CA--GG Bacteria(100);Firmicutes(100);Clostridia(100);Clostridiales(100);Lachnospiraceae(100);Incertae_Sedis(100);unclassified(100);unclassified(100);unclassified(100);

Otu0646 0 0 0 0 0 0 0 0 0 0 0 0 0 0 0 0 0 0 0 0 0 0 0 0 0 0 0 0
0 0 0 0 0 0 0 0 0 0 0 0 0 0 0 0 0 0 0 0 0 0 0 0 0 0 0 0 0
0 0 0 0 0 0 0 0 0 0 0 0 0 0 0 0 0 0 0 0 0 0 0 0 0 0 0 0 0
0 0 63 0 0 0 0 0 0 0 0 0 0 0 0 0 0 0 0 0 0 0 0 0 0 0 0
M02255_76_000000000-ADUNE_1_1110_23710_13030 .....AC----T-AC-GT---------GCC-A-G------C--A--G---C--CG-C-GGT-AA------TA-C--G-T--AG-GT-------GG--C-----AA-GC-G--TT-------G----T-C-C-G------------G--T-CT-T-T-T----------
AA----G-T-C-T--------G-A-T--G--TG--A-AA-GC--C-C-CC-G-G-----CT-T-AA------C-C-G-G-G-G-A--G------G-GT-C-A---T--T-------G--GA-A-A---C-------T--G-G--AA--G-A-C----T-G-G-A-G-T--G---C-AG-----AA-G-A--------G-G-A-G---AG-T------GG--A--ATT----C-C-
A-C-GT--GT-A-G-CG-GT--G---A--A-A---TG-C-GT-AG--AT-A-TG--T-G--G-A-G-G-A-AC-A-CC---AG--T--G--GC-GAA-G--G-C--G-------A--C-T-C-T---CTG--G--TC-T-G--------------------T-A---A-C-T--GA--CG-C-T-G--A-GG--C-G-CG-A--AA-G-C---G-TG--GG-G--
AG-C-A-AA--------CA--GG Bacteria(100);Firmicutes(100);Bacilli(100);Bacillales(100);Listeriaceae(100);Listeria(100);unclassified(100);unclassified(100);unclassified(100);

Otu0647 0 0 0 0 0 0 0 0 0 0 0 0 0 0 0 0 0 0 0 0 0 0 0 12 0 0 3 0
61 0 0 0 0 0 0 0 0 0 0 0 0 0 0 0 0 0 0 0 0 0 0 0 0 0 0 0 0
0 0 0 0 0 0 0 0 0 0 0 0 0 0 0 0 0 0 0 0 0 0 0 0 0 0 0 0 0
0 0 0 0 0 0 0 0 0 0 0 0 0 0 0 0 139 0 0 0 0 0 0 0 0 0 0
M02255_77_000000000-ADUT3_1_1115_11278_14352 T----AC---GG-AG-GGG--------GCT-A-G------C--G--T---T--GT-T-CGG-AA------TT-A--C-T--GG-GC-------GT--A-----AA-GC-GC-AC-------G-TA-G-G-C-G------------G--C-TT-T-G-T-----
------AA----G-T-T-A--------G-A-G--G--TG--A-AA-GC--C-T-GG-A-G-----CT-T-AA------C-T-C-C-A-G-A--A-T----T-G--C-C---T--T-------T--AA-G-A---C-------T--G-C--AT--C-G-C----T-T-G-A-A-T--C---C-GA-----GA-G-A--------G-G-T-G---AG-T------GG--A--ATT----C-
C-G-A-GT--GT-A-G-AG-GT--G---A--A-A---TT-C-GT-AG--AT-A-TT--C-G--G-A-A-G-A-AC-A-CC---AG--T--G--GC-GAA-G--G-C--G-------G--C-T-C-A---CTG--G--CT-C-G--------------------G-T---A-T-T--GA--CG-C-T-G--A-GG--T-G-CG-A--AA-G-C---G-TG--GG-G-
-AG-C-A-AA--------CA--GG Bacteria(100);Proteobacteria(100);Alphaproteobacteria(100);Sphingomonadales(100);unclassified(95);unclassified(95);unclassified(95);unclassified(95);unclassified(95);

Otu0648 0 0 0 0 0 0 0 0 0 0 0 3 0 0 0 0 0 0 0 0 8 0 0 0 0 0 0 2
0 0 0 0 0 0 0 0 0 0 0 0 0 0 0 0 0 0 0 0 0 0 0 0 0 0 0 0 0
0 0 16 0 0 0 0 46 0 0 0 0 0 0 0 0 0 0 0 0 0 0 0 13 0 0 0 0 0
0 0 0 0 0 0 0 0 0 6 0 0 0 0 0 0 0 0 0 0 0 0 0 0 0 0 0
M02255_77_000000000-ADUT3_1_2115_19867_16474 T----AC---GT-AG-GGG--------GCA-A-G------C--G--T---T--AT-C-CGG-AT------TT-A--C-T--GG-GT-------GT--A-----AA-GG-GA-GC-------G-CA-G-A-C-G------------G--C-TC-T-G-C-----
------AA----G-T-C-T--------G-A-A--G--TG--A-AA-GC--C-C-GC-G-G-----CT-T-AA------C-C-G-C-G-G-G--A------C-TG-C-T---T--T-------G--GA-A-A---C-------T--G-C--AA--G-G-C----T-T-G-A-G-T--A---T-CG-----GA-G-G--------G-G-C-A---GG-T------GG--A--ATT----
C-C-T-A-GT--GT-A-G-CG-GT--G---A--A-A---TG-C-GT-AG--AT-A-TT--A-G--G-A-A-G-A-AC-A-CC---GG--T--G--GC-GAA-G--G-C--G-------G--C-C-T-G---CTG--G--AC-G-A--------------------A-A---A-C-T--GA--CG-T-T-G--A-GG--C-T-CG-A--AG-G-C---G-TG--
GG-G--AG-C-A-AA--------CA--GG Bacteria(100);Firmicutes(100);Clostridia(100);Clostridiales(100);Lachnospiraceae(100);Moryella(100);unclassified(100);unclassified(100);unclassified(100);

Otu0649 0 0 0 0 0 0 0 0 0 0 0 0 0 0 0 0 0 0 0 0 0 0 0 0 0 0 0 15
0 0 4 0 0 0 0 0 0 0 68 11 0 0 0 0 0 0 0 0 0 0 0 0 0 0 0 0 0
0 0 0 0 0 0 0 0 0 0 0 0 0 0 0 0 0 0 0 0 0 0 0 0 0 0 0 0 0
0 0 0 20 0 0 0 0 0 0 0 0 0 0 0 0 0 0 0 0 0 0 0 0 0 0 0
M02255_77_000000000-ADUT3_1_1119_23851_9475 T----AC---GA-AG-GGG--------GCT-A-G------C--G--T---T--GT-T-CGG-AA------TT-A--C-T--GG-GC-------GT--A-----AA-GC-GC-AC-------G-TA-G-G-C-G------------G--A-TA-T-T-T----------AA-
---G-T-C-A--------G-G-G--G--TG--A-AA-TC--C-C-GG-G-G-----CT-C-AA------C-C-C-C-G-G-A--A-C----T-G--C-C---T--T-------T--GA-T-A---C-------T--G-G--GT--A-T-C----T-A-G-A-G-T--A---T-GG-----TA-G-A--------G-G-T-A---AG-T------GG--A--ATT----C-C-G-A-
GT--GT-A-G-AG-GT--G---A--A-A---TT-C-GT-AG--AT-A-TT--C-G--G-A-G-G-A-AC-A-CC---AG--T--G--GC-GAA-G--G-C--G-------G--C-T-T-A---CTG--G--AC-C-A--------------------T-T---A-C-T--GA--CG-C-T-G--A-GG--T-G-CG-A--AA-G-C---G-TG--GG-G--AG-C-
A-AA--------CA--GG Bacteria(100);Proteobacteria(100);Alphaproteobacteria(100);Rhizobiales(100);unclassified(100);unclassified(100);unclassified(100);unclassified(100);unclassified(100);

Otu0650 0 0 0 0 0 0 0 0 0 0 0 0 0 0 7 0 0 0 0 0 0 0 0 0 0 0 3 6
11 0 0 0 0 0 0 0 0 0 0 0 0 0 0 0 0 0 0 0 0 0 0 0 0 0 0 0
0 0 0 0 0 0 0 0 0 0 0 0 0 0 0 0 14 0 0 0 0 0 0 0 0 0 0 0
0 0 0 0 0 0 0 0 0 0 0 0 0 0 0 0 0 0 0 0 0 0 0 0 0 0 0
M02255_77_000000000-ADUT3_1_2105_10814_8506 T----AC---GA-GG-GAT--------CCT-A-G------C--G--T---T--GT-T-CGG-AA------TC-A--T-T--GG-GC-------GT--A-----AA-GG-GT-GT-------G-TA-G-G-C-G------------G--C-CT-T-G-T----------AA----G-T-C-A--------G-T-T--G--TG--A-AA-GC--C-C-CG-G-G-----CT-C-AA------C-C-C-G-G-G-A--A-G----T-G--C-T---T--C-------T--GA-T-A---C-------T--G-C--TT--G-G-C----T-T-G-A-G-T--A---T-TG-----GA-T-A--------G-G-G-G---AG-T------GG--A--ATT----C-C-A-G-GT--GT-A-G-TG-GT--G---A--A-A---TA-C-GT-AG--AT-A-TC--T-G--G-A-G-G-A-AC-A-CC---GG--C--G--GC-GAA-G--G-C--G-------G--C-T-C-A---CTG--G--CC-A-T--------------------A-T---A-C-T--GA--CG-C-T-G--A-AA--C-A-CG-A--AA-G-C---G-TG--GG-T--AG-C-A-AA--------CA--GG Bacteria(100);unclassified(100);unclassified(100);unclassified(100);unclassified(100);unclassified(100);unclassified(100);unclassified(100);unclassified(100);

Otu0651 0 0 0 0 0 0 0 0 0 0 0 0 0 0 0 0 0 0 0 0 0 0 0 0 0 0 0 0
0 0 0 0 0 0 0 0 0 0 0 0 0 0 55 0 0 0 0 0 0 0 0 0 0 0 0 0
0 0 0 0 0 0 0 0 0 0 0 0 0 0 0 0 0 0 0 0 0 0 0 0 0 0 0 0
0 0 0 34 0 0 0 0 0 0 0 0 0 0 0 0 0 0 0 0 0 0 0 0 0 0 0
M02255_77_000000000-ADUT3_1_1104_17470_22577 T----AC---GA-GG-GGG--------GCA-A-G------C--G--T---T--GT-T-CGG-AA------TT-A--T-T--GG-GC-------GT--A-----AA-GG-GT-GC-------G-TA-G-G-C-G------------G--T-TT-G-A-T-----------AA----G-T-T-T--------G-A-T--G--TG--A-AA-TC--T-A-TG-G-G-----CT-C-AA------C-C-C-A-T-A-G--T-C----T-G--C-A---T--C-------G--AA-A-A---C-------T--G-T--CG--G-G-C----T-T-G-A-G-T--A---T-GG-----GA-G-A--------G-G-T-G---AG-T------GG--A--ATT----T-C-C-G-GT--GT-A-G-CG-GT--G---A--A-A---TG-C-GT-AG--AT-A-TC--G-G--A-A-G-G-A-AC-A-CC---TG--T--G--GC-GAA-A--G-C--G-------G--C-T-C-A---CTG--G--AC-C-A--------------------T-T---A-C-T--GA--CG-C-T-G--A-TG--C-A-CG-A--AA-G-C---T-AG--GG-G--AG-C-A-AA--------CA--GG Bacteria(100);Acidobacteria(100);Acidobacteria(100);Acidobacteriales(100);Acidobacteriaceae(100);Edaphobacter(62);unclassified(62);unclassified(62);unclassified(62);

Otu0652 0 0 0 0 0 0 0 0 0 0 0 0 0 0 0 0 0 0 0 0 6 0 0 0 0 0 0 0
0 1 1 0 0 0 0 0 0 16 0 0 0 0 0 0 0 0 0 0 0 0 0 0 0 0 0 0
0 0 0 0 0 0 0 0 0 0 0 0 0 0 0 0 0 0 0 0 0 0 0 0 0 0 0 0
0 0 0 0 0 0 0 0 0 0 0 0 0 0 0 0 0 0 0 0 0 0 0 0 0 0 0
M02255_77_000000000-ADUT3_1_2106_23184_12869 T----AC---GT-AG-GGG--------GCG-A-G------C--G--T---T--AT-C-CGG-AT------TT-A--C-T--GG-GC-------GT--A-----AA-GC-GC-GC-------T-AA-G-G-C-G------------G--C-CG-G-C-T-----------AA----G-T-T-G--------G-T-G--G--TG--A-AA-TT--T-C-GG-G-G-----CT-C-AA------C-C-C-C-G-A-A--A-C----T-G--C-C---T--C-------C--AA-T-A---C-------T--G-G--TC--G-G-C----T-C-G-A-G-T--G---C-GG-----GA-G-A--------G-G-A-A---GG-T------GG--A--ATT----C-C-C-G-GT--GT-A-G-TA-GT--G---A--A-A---TG-C-GT-AG--AT-A-TC--G-G--G-A-G-G-A-AC-A-CC---AG--T--G--GC-GAA-G--G-C--G-------G--C-C-T-T---CTG--G--CC-C-G--------------------C-A---A-C-T--GA--CG-C-T-G--T-AG--C-G-CG-A--AA-G-C---G-TG--GG-G--AG-C-G-AA--------CG--GG Bacteria(100);unclassified(100);unclassified(100);unclassified(100);unclassified(100);unclassified(100);unclassified(100);unclassified(100);unclassified(100);

Otu0653 0 0 0 0 0 0 0 0 0 0 0 0 0 0 0 0 0 0 0 0 0 0 0 0 0 0 0 0
0 0 0 0 0 0 90 0 0 0 0 0 0 0 0 0 0 0 0 0 0 0 0 0 0 0 0 0
0 0 0 0 0 0 0 0 0 0 0 0 0 0 0 0 0 0 0 0 0 0 0 0 0 0 0 0
0 0 0 2 0 0 0 0 0 0 0 0 0 0 0 0 130 0 0 0 0 0 0 0 0 0 0
M02255_77_000000000-ADUT3_1_1106_7690_8296 T----AC---GT-AG-GTG--------GCA-A-G------C--G--T---T--GT-C-CGG-AT------TT-A--C-T--GG-GC-------GT--A-----AA-GG-AT-GC-------G-TA-G-G-C-G------------G--A-CA-T-T-T----------AA----G-T-C-A--------G-A-T--G--TG--A-AA-TA--C-C-CG-A-G-----CT-T-AA------C-T-T-G-G-G-T--G-C----T-G--C-A---T--T-------T--GA-A-A---C-------T--G-G--GT--G-T-C----T-A-G-A-G-T--G---C-AG-----GA-G-A--------G-G-T-A---AG-T------GG--A--ATT----C-C-T-A-GT--GT-A-G-CG-GT--G---A--A-A---TG-C-GT-AG--AG-A-TT--A-G--G-A-A-G-A-AC-A-CC---AG--T--G--GC-GAA-G--G-C--G-------A--C-T-T-A---CTG--G--AC-T-G--------------------T-A---A-C-T--GA--CG-C-T-G--A-GG--C-A-TG-A--AA-G-C---G-TG--GG-G--AG-C-A-AA--------CA--GG Bacteria(100);Firmicutes(100);Clostridia(100);Clostridiales(100);Clostridiaceae(100);Clostridium(100);unclassified(100);unclassified(100);unclassified(100);

Otu0654 0 162 21 42 0 0 2 0 0 0 0 0 0 0 0 0 0 0 0 0 0 0 0 0 0 0 0 0
0 0 0 0 0 0 0 0 0 0 0 0 0 0 0 0 0 0 0 0 0 0 0 0 0 0 0 0
0 0 0 0 0 0 0 0 0 4 0 0 0 0 0 0 0 0 0 0 0 0 0 0 0 0 0 30
0 0 0 0 0 0 0 0 0 0 0 0 0 0 0 0 0 0 0 123 0 0 0 0 0 0 0
M02255_77_000000000-ADUT3_1_1110_19970_16645 G----AC---GG-GG-GGG--------GCA-A-G------T--G--T---T--CT-T-CGG-AA------TG-G--C-T--GG-GC-------GT--A-----AA-GG-GC-AC-------G-TA-G-G-C-G------------G--T-GA-A-T-C-----------GG----G-T-T-T--------C-A-A--G--TT--C-AA-----G-T-CG-C-C-----AA-A-AA------G-A-G-G-C-G-G--A-A----T-G--C-T---C--T-------C--GA-A-A---C-------C--A-A--TT--C-A-C----T-T-G-A-G-T--G---A-GA-----CA-G-A--------G-G-A-G---AG-T------TT--C--ATT----T-C-G-T-GT--GT-A-G-GG-GT--G---A--A-A---TC-C-TC-AT--AT-C-TA--C-G--A-A-G-G-A-AG-G-CC---AA--A--T-GC-GAA-G--G-C--A-------G--C-T-C-T---CTG--G--GT-C-C--------------------C-T---A-C-C--GA--CG-C-T-G--G-GG--T-G-CG-A--AA-G-C---A-TG--GG-G--AG-C-G-AA--------CG--GG Bacteria(100);unclassified(100);unclassified(100);unclassified(100);unclassified(100);unclassified(100);unclassified(100);unclassified(100);unclassified(100);

Otu0655 0 0 0 0 0 0 0 0 0 0 0 0 0 0 0 0 0 0 0 0 0 0 0 0 0 0 3 0
0 0 0 0 0 0 0 0 0 0 0 0 0 0 0 0 0 0 0 0 0 0 0 0 0 0 0 0
0 0 0 0 0 0 0 0 0 0 0 0 0 0 0 0 0 0 0 0 0 0 0 0 0 0 0 0
0 0 0 35 0 0 0 0 0 0 0 0 0 0 0 0 0 0 0 0 0 0 0 0 0 0 0
M02255_77_000000000-ADUT3_1_1109_26111_10660 T----AC---GA-AG-GGG--------GCT-A-G------C--G--T---T--GC-T-CGG-AA------TG-A--C-T--GG-GC-------GT--A-----AA-GG-GC-GC-------G-TA-G-G-C-G-------------G--T-TT-G-T-A----
-------CA----G-T-C-A--------G-A-T--G--TG--A-AA-TT--C-C-TG-G-G-----CT-C-AA------C-C-T-G-G-G-G--A-C----T-G--C-A---T--T-------T--GA-T-A---C-------G--T-A--CG--G-A-C----T-T-G-A-G-T--G---G-AG-----AA-G-A--------G-G-G-T---CG-T------GG--A--ATT----
T-C-C-A-GT--GT-A-G-AG-GT--G---A--A-A---TT-C-GT-AG--AT-A-TT--G-G--A-A-A-G-A-AC-A-CC---GG--T--G--GC-GAA-G--G-C--G-------G--C-G-A-C---CTG--G--TC-T-T--------------------T-A---A-C-T--GA--CG-C-T-G--A-GG--C-G-CG-A--AA-G-C---G-TG--GG-
G--AG-C-A-AA--------CA--GG Bacteria(100);Proteobacteria(100);Alphaproteobacteria(100);Rhodospirillales(100);Acetobacteraceae(100);unclassified(100);unclassified(100);unclassified(100);unclassified(100);

Otu0656 0 0 0 0 0 0 0 0 0 0 0 0 0 0 0 0 0 0 0 0 0 0 0 0 0 0 0 0
0 0 0 0 0 0 0 0 0 0 25 0 0 0 0 0 0 0 0 0 0 0 0 0 0 0 0 0
0 0 0 0 0 0 0 0 0 0 0 0 0 0 0 0 0 0 0 0 0 0 0 0 0 0 0 0
0 0 0 22 0 0 0 0 0 0 0 0 0 0 0 0 0 0 0 0 0 0 0 0 0 0 0
M02255_77_000000000-ADUT3_1_1110_16333_11606 T----AC---GG-AG-GAT--------GCA-A-G------C--G--T---T--GT-C-CGG-AA------TC-A--T-T--GG-GC-------GT--A-----AA-GG-GT-CC-------T-GA-G-G-T-G-------------G--T-TT-G-C-T-----
------TA----G-T-C-C--------A-G-T--G--TG--A-AA-GT--T-C-AG-G-G-----CT-T-AC------C-C-T-T-G-A-A--A-A----T-G--C-G---G--T-------G--GA-A-A---C-------T--A-G--CA--G-G-C----T-A-G-A-G-T--A---C-GG-----TG-G-G--------G-G-C-A---GA-G------GG--A--ATT----
C-C-C-G-AT--GT-A-G-CG-GT--G---A--A-A---TG-C-AT-AG--AT-A-TC--G-G--G-A-A-G-A-AC-A-CC---GA--T--G--GC-GAA-G--G-C--A-------C--T-C-T-G---CTG--G--GC-C-G--------------------A-A---A-C-T--GA--CA-C-T-G--A-AA--G-A-CG-A--AA-G-C---C-AG--GG-
G--AG-C-G-AA--------TG--GG Bacteria(100);unclassified(100);unclassified(100);unclassified(100);unclassified(100);unclassified(100);unclassified(100);unclassified(100);unclassified(100);

Otu0657 0 229 19 0 0 0 49 0 224 0 0 0 0 0 4 0 0 0 0 0 0 0 0 0 0 0 0 0
1 0 32 0 0 0 0 0 0 0 59 0 0 0 0 0 0 0 0 0 0 0 0 0 0 0 0 0
13 0 0 0 0 0 0 0 0 1 0 0 0 0 0 0 4 0 0 1 0 0 0 0 173 149 0 0
10 8 0 0 0 0 0 0 0 11 0 0 0 0 0 0 0 0 0 0 0 0 0 9 39 0 0
M02255_76_000000000-ADUNE_1_1118_8884_21219 G----TG---CC-AG-CAG--------CCG-C-G---------G--T---A--AT-A-CGG-AG------GA-T-C-C--GA-GC-------GT--T-----AT-CC-G--GA-------T-TT-A-T-T-G-------------G--G-TT-G-T-T-----------AA----
G-T-C-A--------G-T-T--G--TG--A-AA-GT--T-T-GC-G-G-----CT-C-AA------C-C-G-T-A-A-A--A-T----T-G--C-A---A--T-------T--GA-T-A---C-------T--G-G--CA--G-T-C----T-T-G-A-G-T--A---C-AG-----TT-G-A--------G-G-T-A---GG-C------GG--A--ATT----C-G-T-G-GT--
GT-A-G-CG-GT--G---A--A-A---TG-C-TT-AG--AT-A-TC--A-C--G-A-A-G-A-AC-T-CC---GA--T--T--GC-GAA-G--G-C--A-------G--C-T-T-A---CTA--A--CC-T-G--------------------T-A---A-C-T--GA--CA-T-T-G--A-TG--C-T-CG-A--AA-G-T---G-TG--GG-T--AT-C-A-AA--
------CA--GG Bacteria(100);Bacteroidetes(100);Bacteroidia(100);Bacteroidales(100);Bacteroidaceae(100);Bacteroides(100);unclassified(100);unclassified(100);unclassified(100);

Otu0658 0 0 0 0 0 0 0 0 0 0 0 0 0 0 0 0 0 0 0 0 0 0 0 0 11 0 0 16
0 0 0 0 0 0 0 0 0 0 0 0 0 0 0 0 0 0 0 0 0 0 0 0 0 0 0 0
0 0 0 0 0 0 0 0 0 0 0 0 0 0 0 0 0 0 0 0 0 0 0 0 0 0 0 0
0 0 0 0 0 0 0 0 0 0 0 0 0 0 0 0 0 0 0 0 0 0 0 0 0 0 0
M02255_77_000000000-ADUT3_1_2101_24056_6986 T----AC---GT-AG-GGA--------CCA-A-G------C--G--T---T--GT-T-CGG-AT------TT-A--C-T--GG-GC-------GT--A-----AA-GG-GC-GC-------G-TA-G-G-C-G-------------G--C-AA-T-T-C-----------AA-
---G-T-C-A--------G-C-T--G--TG--A-AA-TC--T-C-TG-G-G-----CT-T-AA------C-C-C-A-G-A-A--C-G----G-C--C-A---G--C-------T--GA-T-A---C-------T--G-C--TT--T-G-C----T-A-G-A-G-T--G---C-AG-----AA-G-G--------G-G-C-A---AT-C------GG--A--ATT----C-T-T-G-
GT--GT-A-G-CG-GT--G---A--A-A---TG-C-GT-AG--AT-A-TC--A-A--G-A-G-G-A-AC-A-CC---TG--A--G--GT-GAA-G--A-C--G-------G--G-T-T-G---CTG--G--GC-T-G--------------------A-C---A-C-T--GA--CG-C-T-G--A-GG--C-G-CG-A--AA-G-C---C-AG--GG-G--AG-
C-A-AA--------CG--GG Bacteria(100);Acidobacteria(100);Acidobacteria(100);Acidobacteriales(100);Acidobacteriaceae(100);Candidatus_Chloroacidobacterium(100);unclassified(100);unclassified(100);unclassified(100);

Otu0659 0 0 0 0 0 0 0 0 0 0 0 0 0 0 0 0 0 0 0 0 0 0 0 0 0 0 0 0
0 0 0 0 0 0 0 0 0 0 0 0 0 0 0 0 0 0 0 0 0 0 0 0 0 0 0 0
0 0 0 0 0 0 0 0 0 0 0 0 0 0 0 0 0 0 0 0 0 0 0 0 0 0 0 0
0 0 0 0 0 0 0 0 0 0 0 0 0 0 0 0 0 33 0 0 0 0 0 0 0 0 0
M02255_76_000000000-ADUNE_1_2111_5430_9102 T----AC---AG-AG-GCC--------CCA-A-G------C--G--T---T--AT-C-CGG-AA------TC-A--C-T--GG-GC-------GT--A-----AA----G--G--------G-T------C---------------G-------T-G-T-----------TA----G-T-
C-C--------T-T-C--G--TG--A-AAGAT--C-T-TC-G-G-----CT-T-AA------C-A-G-A-G-G-A--G-A----C-G--C-G---G--A-------G--GA-A-A---C-------G--G-C--AC--G-A-C----TC--G-A-G-G--T---T-GC-----GA-G-A--------G-G-T-A---TA-C------GG--A--ACT----C-A-T-A-GT--
GT-A-G-GG-GT--G---A--A-A---TC-C-GT-TG--AT-A-TT--A-T--G-G-G-G-A-AC-A-CC---AA--A--T--GC-GAA-G--G-C--A-------G--T-A-T-A---CTG--G--CG-C-A--------------------C-A---C-C-T--GA--CG-C-T-G--A-GA--C-A-CG-A--AA-G-C---G-TG--GG-A--AT-C-G-
AA--------CG--GG Bacteria(100);unclassified(100);unclassified(100);unclassified(100);unclassified(100);unclassified(100);unclassified(100);unclassified(100);unclassified(100);

Otu0660 0 0 0 0 0 0 0 0 0 0 0 0 0 0 0 0 0 0 0 0 0 0 0 0 0 0 0 0
0 0 0 0 0 0 0 0 0 0 0 0 0 0 0 0 0 0 0 0 0 0 0 0 0 0 0 0 0
0 0 0 0 0 0 0 0 0 0 0 0 0 0 0 0 0 0 0 0 0 0 0 0 0 0 0 0 0
0 132 0 0 0 0 0 0 0 0 0 0 0 0 0 0 0 0 0 0 0 0 0 0 0 0 0 0
M02255_77_000000000-ADUT3_1_2112_17732_15896 T----AC---GG-AG-GGG--------GCT-A-G------C--G--T---T--AT-T-CGG-AA------TT-A--C-T--GG-GC-------GT--A-----AA-GC-GC-AC-------G-TA-G-G-C-G------------G--A-CC-A-G-A----
-------AA----G-T-T-A--------G-G-G--G--TG--A-AA-TC--C-C-GG-G-G-----CT-C-AA------C-C-T-C-G-G-A--A-C----T-G--C-C---T--C-------T--AA-A-A---C-------G--C-C--TG--G-T-C----T-T-G-A-G-G--T---C-GA-----GA-G-A--------G-G-T-G---AG-T------GG--A--ATT----
C-C-G-A-GT--GT-A-G-AG-GT--G---A--A-A---TT-C-GT-AG--AT-A-TT--C-G--G-A-G-G-A-AC-A-CC---AG--T--G--GC-GAA-G--G-C--G-------G--C-T-C-A---CTG--G--CT-C-G--------------------A-T---A-C-T--GA--CG-C-T-G--A-GG--T-G-CG-A--AA-G-C---G-TG--GG-
G--AG-C-A-AA--------CA--GG Bacteria(100);Proteobacteria(100);Alphaproteobacteria(100);unclassified(100);unclassified(100);unclassified(100);unclassified(100);unclassified(100);unclassified(100);

Otu0661 0 117 0 0 0 0 0 0 0 0 0 0 0 0 0 0 0 0 0 0 16 0 0 0 0 1 0 0
0 0 0 0 0 0 77 0 0 0 0 0 0 0 0 0 0 0 0 0 0 0 0 0 16 0 0 0 0
0 0 0 0 0 0 0 0 0 0 0 0 0 0 0 0 0 0 0 0 0 0 0 0 0 0 0 0 0
0 0 0 0 0 0 2 0 0 0 0 0 0 0 0 54 148 0 0 0 0 0 17 0 0 0 0 0
M02255_77_000000000-ADUT3_1_1105_11619_18899 T----AC---GA-AG-GGG--------GCT-A-G------C--G--T---T--GC-T-CGG-AA------TG-A--C-T--GG-GC-------GT--A-----AA-GG-GC-GC-------G-TA-G-G-C-G------------G--T-AT-G-G-A---
--------CA----G-T-C-A--------G-G-T--G--TG--A-AA-TT--C-C-CG-G-G-----CT-T-AA------C-C-T-G-G-G-G--A-C----T-G--C-A---T--T-------T--GA-T-A---C-------G--T-T--CA--A-A-C----T-A-G-A-G-T--G---T-GG-----AA-G-A--------G-G-G-T---CG-T------GG--A--ATT----
C-C-C-A-GT--GT-A-G-AG-GT--G---A--A-A---TT-C-GT-AG--AT-A-TT--G-G--G-A-A-G-A-AC-A-CC---GG--T--G--GC-GAA-G--G-C--G-------G--C-G-A-C---CTG--G--TC-C-A--------------------T-T---A-C-T--GA--CG-C-T-G--A-GG--C-G-CG-A--AA-G-C---G-TG--GG-
G--AG-C-A-AA--------CA--GG Bacteria(100);Proteobacteria(100);Alphaproteobacteria(100);Rhodospirillales(100);Acetobacteraceae(100);unclassified(100);unclassified(100);unclassified(100);unclassified(100);

Otu0662 0 0 0 0 0 0 15 0 0 0 0 0 1 0 0 0 0 0 0 3 5 0 0 0 0 0 0 0
0 0 48 0 0 0 0 0 7 14 0 0 0 8 0 0 0 0 0 0 0 0 0 18 6 0 0 0 0
0 0 0 0 0 0 0 0 0 0 0 0 0 0 0 0 0 0 0 0 0 0 0 70 0 0 0 0 0
0 80 0 0 0 30 1 0 0 7 0 0 2 0 0 0 0 0 0 0 0 0 0 0 0 0 0 0
M02255_77_000000000-ADUT3_1_2117_22324_16347 T----AC---GT-AG-GGC--------GCA-A-G------C--G--T---T--GT-C-CGG-AA------TT-A--T-T--GG-GC-------GT--A-----AA-GA-GC-TC-------G-TA-G-G-C-G------------G--T-TT-G-T-C------
-----GC----G-T-C-T--------G-C-T--G--TG--A-AA-TC--C-C-GA-G-G-----CT-C-AA------C-C-T-C-G-G-G--C-T----T-G--C-A---G--T-------G--GG-T-A---C-------G--G-G--CA--G-A-C----T-A-G-A-G-T--G---C-GG-----TA-G-G--------G-G-A-G---AC-T------GG--A--ACT----C-
C-T-G-GT--GT-A-G-CG-GT--G---G--A-A---TG-C-GC-AG--AT-A-TC--A-G--G-A-A-G-A-AC-A-CC---GA--T--G--GC-GAA-G--G-C--A-------G--G-T-C-T---CTG--G--GC-C-G--------------------T-A---A-C-T--GA--CG-C-T-G--A-GG--A-G-CG-A--AA-G-C---A-TG--GG-
G--AG-C-G-AA--------CA--GG Bacteria(100);Actinobacteria(100);Actinobacteria(100);Actinobacteridae(100);Actinomycetales(100);Micrococcineae(100);Microbacteriaceae(89);unclassified(89);unclassified(89);

Otu0663 0 0 0 0 0 0 0 0 0 0 0 0 0 0 0 0 0 0 0 0 0 0 0 0 0 0 0 0
0 0 0 0 0 0 0 0 0 0 0 0 0 0 0 0 0 0 0 0 0 0 0 0 0 0 0 0 0
0 0 0 0 0 0 0 0 0 0 0 0 0 0 0 0 0 0 0 0 0 0 0 0 0 0 0 0 0
0 0 0 0 0 37 0 0 0 0 0 0 0 0 0 0 0 0 0 0 0 0 0 0 0 0 0 0
M02255_77_000000000-ADUT3_1_1105_22468_9812 T----AC---GT-AG-GGA--------GCG-A-G------C--G--T---T--AT-C-CGG-AA------TT-A--C-T--GG-GC-------GT--A-----AA-GG-GC-GC-------G-TA-G-G-C-G------------G--T-GA-C-T-C-----------
AA----G-T-C-G--------T-T-C--G--TT--A-AA-TA--T-T-GA-G-G-----CT-T-AA------C-C-T-C-A-A-G--G------A-G--C-G---G--C-------C--GA-A-A---C-------T--G-A--GA--C-A-C----T-G-G-A-G-A--C---A-CA-----GA-G-A--------G-G-C-A---AA-C------AG--A--ATT----C-C-T-
G-GT--GT-A-G-CG-GT--G---A--A-A---TG-C-GT-AG--AT-A-TC--A-G--G-A-A-G-A-AT-A-CC---GG--T--G--GC-GAA-G--G-C--G-------G--T-T-T-G---CTG--G--AT-G-T--------------------G-A---T-C-T--GA--CG-C-T-G--A-AG--C-G-CG-A--AA-G-C---C-GG--GG-G--
AG-C-G-AA--------CG--GG Bacteria(100);unclassified(100);unclassified(100);unclassified(100);unclassified(100);unclassified(100);unclassified(100);unclassified(100);unclassified(100);

Otu0664 0 0 0 0 0 0 0 0 0 0 0 0 0 0 0 0 0 0 0 0 0 0 0 0 0 0 0 0
0 0 0 0 0 0 0 0 0 0 0 0 0 0 0 0 0 0 0 0 0 0 0 0 0 0 0 0 0
0 0 0 0 0 0 0 0 0 0 0 0 0 0 0 0 0 0 0 0 0 0 0 0 0 0 0 0 0
0 0 0 0 0 0 0 0 0 0 0 0 0 0 0 22 0 0 0 0 0 0 0 0 0 0 0 0
M02255_77_000000000-ADUT3_1_2113_27026_10338 T----AC---AG-AG-GCC--------CCA-A-G------C--G--T---T--AT-C-CGG-AA------TC-A--C-T--GG-GC-------GT--A-----AA-GG-GT-GC-------G-TA-G-G-C-G------------G--T-CG-T-G-T-----
------TA----G-T-C-T--------T-T-C--G--TG--A-AAGAT--C-T-TC-G-G-----CT-T-AA------C-C-G-A-G-G-A--G-A----C-G--C-G---A--G-------G--GA-A-A---C-------G--G-C--AC--G-A-C----T-T-G-A-G-G--T---T-GC-----AA-G-A--------G-G-T-A---TA-C------GG--A--ACT----
C-A-T-A-GT--GT-A-G-GG-GT--G---A--A-A---TC-C-GT-TG--AT-A-TT--A-T--G-G-G-G-A-AC-A-CC---AA--A--T--GC-GAA-G--G-C--A-------G--T-A-T-A---CTG--G--TG-C-A--------------------C-A---C-C-T--GA--CG-C-T-G--A-GG--C-A-CG-A--AA-G-C---G-TG--GG-
A--AT-C-G-AA--------CG--GG Bacteria(100);unclassified(100);unclassified(100);unclassified(100);unclassified(100);unclassified(100);unclassified(100);unclassified(100);unclassified(100);

Otu0665 0 0 0 0 0 0 0 0 0 0 0 0 0 14 0 0 0 0 0 0 0 0 15 0 0 0 0 0
7 0 0 0 0 0 0 0 0 0 0 0 0 0 0 0 0 0 0 0 0 0 0 0 0 0 0 0
0 0 0 0 0 0 0 0 0 0 0 0 0 0 0 0 0 0 0 0 0 0 0 0 0 0 0 0
0 0 0 0 21 0 0 0 0 0 0 0 0 0 0 0 0 0 0 0 0 0 0 0 0 0 0

M02255_77_000000000-ADUT3_1_1111_7146_13032 T----AC---GA-AG-GGG--------GCT-A-G------C--G--T---T--GT-T-CGG-AA------TT-A--C-T--GG-GC-------GT--A-----AA-GG-GC-GC-------G-TA-G-G-C-G------------G--C-CT-G-T-T----------AA----G-T-T-G--------G-A-A--G--TG--A-AA-GC--C-C-CG-G-G-----CT-C-AA------C-C-T-G-G-G-A--A-T----T-G--C-T---T--T-------C--GA-T-A---C-------T--G-G--CA--G-G-C----T-T-G-A-G-T--T---C-CG-----GA-G-A--------G-G-A-T---GG-T------GG--A--ATT----C-C-C-A-GT--GT-A-G-AG-GT--G---A--A-A---TT-C-GT-AG--AT-A-TT--G-G--G-A-A-G-A-AC-A-CC---GG--T--G--GC-GAA-G--G-C--G-------G--C-C-A-T---CTG--G--AC-G-G--------------------A-C---A-C-T--GA--CG-C-T-G--A-GG--C-G-CG-A--AA-G-C---G-TG--GG-G--AG-C-A-AA--------CA--GG Bacteria(100);Proteobacteria(100);Alphaproteobacteria(100);Rhodospirillales(100);Rhodospirillaceae(100);Azospirillum(100);unclassified(100);unclassified(100);unclassified(100);

Otu0666 0 52 0 144 1 0 0 0 0 0 0 0 0 0 0 0 0 0 2 0 0 0 0 0 0 1 0 0
0 0 0 0 0 0 0 0 1 0 0 0 0 3 0 0 0 0 0 1 0 0 0 3 6 2 0 0 0
6 0 0 0 0 0 0 0 0 0 0 0 2 0 1 50 10 0 0 0 0 0 1 0 0 0 0 0 0
0 0 5 0 41 0 0 0 0 0 0 0 3 0 0 2 0 0 18 0 0 0 0 0 0 0 21

M02255_76_000000000-ADUNE_1_1112_6284_9083 C----A----GC-A--GCC--------GCG-G-T------A--A--T---A--CG-T-AGG-GC------GC-A--A-G--CG-TT-------AT--C-----CG-GA-AT-TA-------T-TG-G-G-C-G------------G--T-TT-G-T-C----------GC----G-T-C-T--------G-C-C--G--TG--A-AA-GT--C-C-GG-G-G-----CT-T-AA------C-G-C-C-G-G-A--T-C----T-G--C-G---G--T-------G--GG-T-A---C-------G--G-G--CA--G-A-C----T-A-G-A-G-T--G---C-AG-----TA-G-G--------G-G-A-G---AC-T------GG--A--ATT----C-C-T-G-GT--GT-A-G-CG-GT--G---A--A-A---TG-C-GC-AG--AT-A-TC--A-G--G-A-G-G-A-AC-A-CC---GA--T--G--GC-GAA-G--G-C--A-------G--G-T-C-T---CTG--G--GC-T-G--------------------T-A---A-C-T--GA--CG-C-T-G--A-GG--A-G-CG-A--AA-G-C---A-TG--GG-G--AG-C-G-AA--------CA--GG Bacteria(100);Actinobacteria(100);Actinobacteria(100);Actinobacteridae(100);Actinomycetales(100);Micrococcineae(100);Micrococcaceae(100);unclassified(100);unclassified(100);

Otu0667 0 0 0 0 0 0 0 0 0 0 0 0 0 0 9 0 0 0 0 0 0 0 36 0 0 0 6 11
0 0 0 0 0 0 0 0 0 0 0 0 0 54 39 0 0 0 0 0 0 0 0 0 0 0 0 0
0 0 0 0 0 0 0 0 0 0 0 0 0 0 0 0 65 0 0 0 0 0 0 0 0 0 0 0
0 1 0 0 0 0 0 0 0 0 0 0 0 0 0 8 165 0 52 0 102 121 0 0 0 0 0

M02255_77_000000000-ADUT3_1_1102_17940_16164 T----AC---GA-AG-GGG--------GCT-A-G------C--G--T---T--GT-T-CGG-AA------TC-A--C-T--GG-GC-------GT--A-----AA-GC-GC-AC-------G-TA-G-G-C-G------------G--A-CT-T-T-T-----------AA----G-T-C-G--------G-A-G--G--TG--A-AA-TC--C-C-AA-G-G-----CT-C-AA------C-C-T-T-G-G-A--A-C----T-G--C-C---C--C-------C--GA-T-A---C-------T--G-G--GA--G-T-C----T-T-G-A-G-T--A---T-GG-----TA-G-A--------G-G-T-T---AG-T------GG--A--ACT----G-C-G-A-GT--GT-A-G-AG-GT--G---A--A-A---TT-C-GT-AG--AT-A-TT--C-G--C-A-A-G-A-AC-A-CC---AG--T--G--GC-GAA-G--G-C--G-------G--C-T-A-A---CTG--G--AC-C-A--------------------T-T---A-C-T--GA--CG-C-T-G--A-GG--T-G-CG-A--AA-G-C---G-TG--GG-G--AG-C-A-AA--------CA--GG Bacteria(100);Proteobacteria(100);Alphaproteobacteria(100);Rhizobiales(100);unclassified(100);unclassified(100);unclassified(100);unclassified(100);unclassified(100);

Otu0668 0 0 0 0 0 0 0 0 0 0 0 0 0 0 0 0 0 0 0 0 0 0 0 0 0 0 0 3
2 0 0 0 0 0 0 0 0 0 0 0 0 0 0 0 0 0 0 0 0 0 0 0 0 0 0 0
0 0 0 0 0 0 0 17 0 0 0 0 0 0 0 0 0 0 0 0 0 0 0 0 0 0 0 0
0 0 0 45 0 5 0 0 0 0 0 17 0 0 0 0 0 0 0 0 0 0 0 0 0 0 0

M02255_77_000000000-ADUT3_1_1110_6026_19815 T----AC---GT-AG-GGG--------GCA-A-G------C--G--T---T--AT-C-CGG-AT------TT-A--C-T--GG-GT-------GT--A-----AA-GG-GA-GC-------G-TA-G-A-C-G------------G--A-AG-A-G-C----------AA----G-T-C-T--------G-A-T--G--TG--A-AA-GG--C-T-GG-G-G-----CT-T-AA------C-C-C-C-A-G-G--A-C----T-G--C-A---T--T-------G--GA-A-A---C-------T--G-T--TT--T-T-C----T-A-G-A-G-T--G---C-CG-----GA-G-A--------G-G-T-A---AG-C------GG--A--ATT----C-C-T-A-GT--GT-A-G-CG-GT--G---A--A-A---TG-C-GT-AG--AT-A-TT--A-G--G-A-G-G-A-AC-A-CC---AG--T--G--GC-GAA-G--G-C--G-------G--C-T-T-A---CTG--G--AC-G-G--------------------T-A---A-C-T--GA--CG-T-T-G--A-GG--C-T-CG-A--AA-G-C---G-TG--GG-G--AG-C-A-AA--------CA--GG Bacteria(100);Firmicutes(100);Clostridia(100);Clostridiales(100);Lachnospiraceae(100);Blautia(100);unclassified(100);unclassified(100);unclassified(100);

Otu0669 0 0 0 0 0 0 0 0 0 0 0 0 0 0 0 0 0 0 0 0 0 0 0 0 0 0 0 21
0 0 0 0 0 0 0 0 0 0 0 0 0 0 0 0 0 0 0 0 0 0 0 0 0 0 0 0
0 0 0 0 0 0 0 0 0 0 0 0 0 0 0 0 0 0 0 0 0 0 0 0 0 0 0 0
0 0 0 0 0 0 0 0 0 0 0 0 0 0 0 0 0 0 0 0 0 0 0 0 0 0 0

M02255_77_000000000-ADUT3_1_1117_20207_24273 T----AC---GG-AG-GGG--------GCT-A-G------C--G--T---T--GT-T-CGG-AA------TT-A--C-T--GG-GC-------GT--A-----AA-GC-GC-AC-------G-TA-G-G-C-G------------G--A-TG-C-T-C-----------AA----G-T-C-G--------G-G-G--G--TG--A-AA-TC--C-C-GG-G-G-----CT-C-AA------C-C-C-C-G-G-A--A-C----T-G--C-C---T--C-------C--GA-T-A---C-------T--G-G--GT--G-T-C----T-G-G-A-G-G--A---T-GG-----GA-G-A--------G-G-C-G---AG-T------GG--A--ATT----C-C-G-A-GT--GT-A-G-AG-GT--G---A--A-A---TT-C-GT-AG--AT-A-TT--C-G--G-A-G-G-A-AC-A-CC---AG--T--G--GC-GAA-G--G-C--G-------G--C-T-C-G---CTG--G--CC-C-A--------------------T-T---A-C-T--GA--CG-C-T-G--A-GG--T-G-CG-A--AA-G-C---G-TG--GG-G--AG-C-A-AA--------CA--GG Bacteria(100);Proteobacteria(100);Alphaproteobacteria(100);unclassified(100);unclassified(100);unclassified(100);unclassified(100);unclassified(100);unclassified(100);

Otu0670 0 0 0 0 0 0 0 0 0 0 0 0 0 0 0 0 0 0 0 0 0 0 8 0 0 0 0 0
0 0 0 0 0 0 0 0 0 0 0 0 0 0 0 0 0 0 0 0 0 0 0 0 26 0 0 0 0
0 0 0 0 0 0 0 0 0 0 0 0 0 0 0 0 0 0 0 0 0 0 0 0 0 0 0 0 0
0 0 0 0 0 0 0 0 0 0 0 0 0 0 0 0 0 0 0 0 0 0 0 0 0 0 0
M02255_77_000000000-ADUT3_1_1114_21048_20290 T----AC---GT-AG-GGC--------GCA-A-G------C--G--T---T--AT-C-CGG-AA------TT-A--T-T--GG-GC-------GT--A-----AA-GA-GC-TC-------G-TA-G-G-C-G------------G--T-TT-G-T-C------
-----GC----G-T-C-T--------G-C-T--G--TG--A-AA-GA--C-C-GG-G-G-----CT-C-AA------C-T-C-C-G-G-T--T-C----T-G--C-A---G--T-------G--GG-T-A---C-------G--G-G--CA--G-A-C----T-A-G-A-G-T--G---C-AG-----TA-G-G--------G-G-A-G---AC-T------GG--A--ATT----C-
C-T-G-GT--GT-A-G-CG-GT--G---A--A-A---TG-C-GC-AG--AT-A-TC--A-G--G-A-G-G-A-AC-A-CC---GA--T--G--GC-GAA-G--G-C--A-------G--G-T-C-T---CTG--G--GC-T-G--------------------T-A---A-C-T--GA--CG-C-T-G--A-GG--A-G-CG-A--AA-G-C---A-TG--GG-
G--AG-C-G-AA--------CA--GG Bacteria(100);Actinobacteria(100);Actinobacteria(100);Actinobacteridae(100);Actinomycetales(100);Micrococcineae(100);Micrococcaceae(100);Arthrobacter(77);unclassified(77);

Otu0671 0 0 0 0 0 0 0 0 0 0 0 0 0 0 0 0 0 0 0 0 0 0 0 0 0 0 0 0
0 0 0 0 0 0 0 0 0 0 0 0 0 0 0 0 0 0 0 0 0 0 0 22 0 0 0 0 0
0 0 0 0 0 0 0 0 0 0 0 0 0 0 0 0 0 0 0 0 0 0 0 0 0 0 0 0 0
0 0 0 0 0 0 0 0 0 0 0 0 0 0 0 0 0 40 0 0 0 0 0 0 0 0 0
M02255_77_000000000-ADUT3_1_2103_13707_17756 T----AC---GT-AG-GGG--------GCA-A-G------C--G--T---T--AT-C-CGG-AT------TT-A--C-T--GG-GT-------GT--A-----AA-GG-GA-GC-------G-TA-G-A-C-G------------G--T-TG-T-G-C-----
------AA----G-T-C-T--------G-A-A--G--TG--A-AA-GC--C-C-GG-G-G-----CT-C-AA------C-C-C-C-G-G-G--A-C----T-G--C-T---T--T-------G--GA-A-A---C-------T--G-T--AT--A-A-C----T-G-G-A-G-T--G---C-AG-----GA-G-A--------G-G-T-A---AG-C------GG--A--ATT----
C-C-T-A-GT--GT-A-G-CG-GT--G---A--A-A---TG-C-GT-AG--AT-A-TT--A-G--G-A-G-G-A-AC-A-CC---AG--T--G--GC-GAA-G--G-C--G-------G--C-T-T-A---CTG--G--AC-T-G--------------------T-A---A-C-T--GA--CG-T-T-G--A-GG--C-T-CG-A--AA-G-C---G-TG--GG-
G--AG-C-A-AA--------CA--GG Bacteria(100);Firmicutes(100);Clostridia(100);Clostridiales(100);Lachnospiraceae(100);unclassified(100);unclassified(100);unclassified(100);unclassified(100);

Otu0672 0 0 0 0 0 0 0 0 0 0 0 0 0 0 0 0 0 0 0 0 0 0 0 0 0 0 0 0
0 0 0 0 0 0 0 0 0 0 0 0 0 0 0 0 0 0 0 0 0 0 0 0 0 0 0 0 0
0 0 0 0 0 0 0 0 0 0 0 0 0 0 0 0 0 25 0 0 0 0 0 0 0 0 0 0 0
0 0 141 0 0 0 0 0 0 0 0 0 0 0 0 0 0 0 0 0 0 0 0 0 0 0 0
M02255_77_000000000-ADUT3_1_1103_3688_13633 T----AC---GA-AG-GGG--------GCT-A-G------C--G--T---T--GT-T-CGG-AA------TT-A--C-T--GG-GC-------GT--A-----AA-GC-GC-AC-------G-TA-G-G-C-G------------G--A-TT-G-G-T-----------CA-
---G-T-T-A--------G-G-G--G--TG--A-AA-TC--C-C-AG-G-G-----CT-C-AA------C-C-C-T-G-G-A--A-C----T-G--C-C---T--T-------T--AA-T-A---C-------T--G-C--CG--G-T-C----T-T-G-A-G-T--T---C-GA-----GA-G-A--------G-G-T-G---AG-T------GG--A--ATT----C-C-G-A-
GT--GT-A-G-AG-GT--G---A--A-A---TT-C-GT-AG--AT-A-TT--C-G--G-A-G-G-A-AC-A-CC---AG--T--G--GC-GAA-G--G-C--G-------G--C-T-C-A---CTG--G--CT-C-G--------------------A-T---A-C-T--GA--CG-C-T-G--A-GG--T-G-CG-A--AA-G-C---G-TG--GG-G--AG-
C-A-AA--------CA--GG Bacteria(100);Proteobacteria(100);Alphaproteobacteria(100);unclassified(100);unclassified(100);unclassified(100);unclassified(100);unclassified(100);unclassified(100);

Otu0673 0 0 0 0 0 0 0 0 0 0 0 0 0 0 0 0 0 0 0 0 0 0 0 0 0 0 0 0
7 0 0 0 0 0 0 0 0 0 0 0 0 0 0 0 0 0 0 0 0 0 0 0 0 0 0 0 0
0 0 0 0 0 0 0 0 0 0 0 0 0 0 0 0 0 0 0 0 0 0 0 0 0 0 0 0 0
0 0 75 0 0 0 0 0 0 0 0 0 0 0 0 0 0 0 0 0 0 0 0 0 0 0 0
M02255_77_000000000-ADUT3_1_1103_7230_18288 T----AC---GA-AG-GGG--------GCT-A-G------C--G--T---T--GT-T-CGG-AA------TC-A--C-T--GG-GC-------GT--A-----AA-GC-GC-AC-------G-TA-G-G-C-G------------G--A-TC-T-T-T-----------AA-
---G-T-C-A--------G-G-G--G--TG--A-AA-TC--C-C-GA-G-G-----CT-C-AA------C-C-T-C-G-G-A--A-C----T-G--C-C---T--T-------T--GA-T-A---C-------T--G-G--GG--A-T-C----T-C-G-A-G-T--C---C-GG-----AA-G-A--------G-G-T-A---AG-T------GG--A--ACT----G-C-G-A-
GT--GT-A-G-AG-GT--G---A--A-A---TT-C-GT-AG--AT-A-TT--C-G--C-A-A-G-A-AC-A-CC---AG--T--G--GC-GAA-G--G-C--G-------G--C-T-T-A---CTG--G--TC-C-G--------------------G-A---A-C-T--GA--CG-C-T-G--A-GG--T-G-CG-A--AA-G-C---G-TG--GG-G--AG-C-
A-AA--------CA--GG Bacteria(100);Proteobacteria(100);Alphaproteobacteria(100);Rhizobiales(100);unclassified(100);unclassified(100);unclassified(100);unclassified(100);unclassified(100);

Otu0674 0 0 0 0 0 0 0 0 0 0 0 0 0 0 0 0 0 0 0 0 0 0 0 0 0 0 0 5
0 0 0 0 0 0 0 0 0 0 0 0 0 0 0 85 0 0 0 0 0 0 4 0 0 0 0 0 0
0 1 0 0 0 0 0 0 0 0 0 0 0 0 179 98 0 0 0 0 0 0 0 0 0 0 0 0 0
0 0 0 0 0 0 0 0 0 0 0 0 0 0 0 0 0 0 0 0 0 0 0 0 0 0 0
M02255_77_000000000-ADUT3_1_1107_17248_8320 T----AC---GT-AG-GGG--------GCA-A-G------C--G--T---T--AT-C-CGG-AA------TT-A--T-T--GG-GC-------GT--A-----AA-GA-GT-AC-------G-TA-G-G-T-G------------G--C-TA-T-A-T-----------AA-
---G-C-G-C--------A-T-G--G--TG--A-AA-GG--C-A-GA-G-G-----CT-T-AA------C-C-A-C-T-G-T--T------A-G--C-C---T--T-------G--CG-A-A---C-------T--G-T--AT--A-G-C----T-T-G-A-G-T--G---C-AG-----GA-G-A--------G-G-G-A---AG-C------GG--A--ATT----C-C-T-A-
GT--GT-A-G-CG-GT--G---A--A-A---TG-C-GT-AG--AT-A-TT--A-G--G-A-G-G-A-AC-A-CC---AG--T--G--GC-GAA-G--G-C--G-------G--C-T-T-T---CTG--G--AC-T-G--------------------T-A---A-C-T--GA--CA-C-T-G--A-GG--T-A-CG-A--AA-G-C---G-TG--GG-G--AG-C-
A-AA--------CA--GG Bacteria(100);Firmicutes(100);Clostridia(100);Clostridiales(100);Family_XIII_Incertae_Sedis(100);uncultured(100);unclassified(100);unclassified(100);unclassified(100);

Otu0675 0 95 17 31 0 0 0 6 0 0 0 0 0 0 0 0 0 0 0 0 0 0 0 0 0 0 0 0
0 0 0 0 0 0 0 0 0 0 0 0 0 0 0 0 0 0 0 0 0 0 0 0 0 0 0 0 0
0 0 0 0 0 0 0 0 0 0 0 0 0 0 0 0 0 0 0 0 0 0 0 0 0 0 0 0 37
0 0 0 0 0 0 4 0 0 0 0 0 0 0 0 0 0 0 0 70 0 0 0 0 0 0 0
M02255_77_000000000-ADUT3_1_1112_11715_11048 G----AC---GG-GG-GGG--------GCA-A-G------T--G--T---T--CT-T-CGG-AA------TG-A--C-T--GG-GC-------GT--A-----AA-GG-GC-AC-------G-TA-G-G-C-G------------G--G-GA-A-T-C---
--------GG----G-T-T-T--------C-A-A--G--TT--C-AA-G---T-C-GC-C-A-----AA-A-AG------A-G-G-C-G-G-A--A------T-G--C-T---C--T-------C--GA-A-A---C-------C--A-A--TT--C-A-C----T-T-G-A-G-T--G---A-GA-----CA-G-A--------G-G-A-G---AG-T------TT--C--ATT----T-
C-G-T-GT--GT-A-G-GG-GT--G---A--A-A---TC-C-TC-AT--AT-C-TA--C-G--A-A-G-G-A-AG-G-CC---AA--A--T--GC-GAA-G--G-C--A-------G--C-T-C-T---CTG--G--GT-C-C--------------------C-T---A-C-C--GA--CG-C-T-G--G-GG--T-G-CG-A--AA-G-C---A-TG--GG-G-
-AG-C-G-AA--------CG--GG Bacteria(100);unclassified(100);unclassified(100);unclassified(100);unclassified(100);unclassified(100);unclassified(100);unclassified(100);unclassified(100);

Otu0676 0 0 0 0 0 0 0 0 0 0 0 0 0 0 0 0 0 0 0 0 1 0 0 0 0 1 0 0
0 0 0 0 0 0 1 0 0 5 1 0 0 0 6 0 0 8 0 0 0 22 1 0 6 0 0 0 2
0 0 0 0 0 0 0 0 0 0 0 0 0 0 0 0 1 0 0 0 0 0 0 0 0 0 0 0 0
0 0 0 0 0 0 0 0 0 0 0 0 0 0 0 0 0 0 0 0 0 0 0 0 0 0 0
M02255_76_000000000-ADUNE_1_2106_15826_24272 T----AC---GG-AG-GGT--------GCA-A-G------C--G--T---T--AC-C-CGG-AA------TC-A-----------C--------T-----------GG-GC----------G-T------------------------A-CA-C-T-T-----------AA----
G-T-C-T--------G-G-T--T--TT--A-AA-GA--C-C-GG-G-G-----CT-C-AA------C-C-C-C-G-G-G--C-A----T-G--G-A---C--T-------G--GG-T-A---C-------T--G-G--GT--G-T-C----T-G-G-A-C-C--T---C-TG-----GA-G-A--------G-A-G-A---AC-T------GG--A--ATT----C-C-T-G-GT-
-GT-A-G-CG-GT--G---G--A-A---TG-C-GT-AG--AT-A-CC--A-G--G-A-G-G-A-AC-A-CC---AA--T--G--GC-GAA-G--G-C--A-------G--G-T-T-C---TTG--G--AC-A-G--------------------A-A---G-G-T--GA--CG-C-T-G--A-GG--C-G-CG-A--AA-G-T---G-TG--GG-G--AG-C-
G-AA--------CC--GG Bacteria(100);Deinococcus-Thermus(100);Deinococcales(100);Deinococcaceae(100);Deinococcus(100);unclassified(100);unclassified(100);unclassified(100);unclassified(100);

Otu0677 0 0 0 0 0 0 0 0 0 0 0 0 0 0 0 0 0 0 0 0 0 0 0 0 0 0 0 0
0 0 0 0 0 0 0 0 0 0 0 0 0 0 24 0 0 0 0 0 0 0 0 0 0 0 0 0 0
0 0 0 0 0 0 0 0 0 0 0 0 0 0 0 0 0 0 0 0 0 0 0 0 0 0 0 0 0
0 0 0 42 0 0 6 0 0 0 0 0 0 0 0 0 0 0 0 0 0 0 0 0 0 0 0
M02255_77_000000000-ADUT3_1_1102_13280_16706 T----AC---GA-AG-GGG--------GCT-A-G------C--G--T---T--GC-T-CGG-AA------TG-A--C-T--GG-GC-------GT--A-----AA-GG-GC-GC-------G-TA-G-G-C-G------------G--T-TT-G-T-A----
-------CA----G-T-C-A--------G-A-T--G--TG--A-AA-TT--C-C-TG-G-G-----CT-T-AA------C-C-T-G-G-G-G--G-C----T-G--C-A---T--T-------T--GA-T-A---C-------G--T-G--CG--A-G-C----T-T-G-A-G-T--G---T-GA-----AA-G-A--------G-G-G-T---CG-T------GG--A--ATT----
C-C-C-A-GT--GT-A-G-AG-GT--G---A--A-A---TT-C-GT-AG--AT-A-TT--G-G--G-A-A-G-A-AC-A-CC---GG--T--G--GC-GAA-G--G-C--G-------G--C-G-A-C---CTG--G--TT-C-A--------------------T-T---A-C-T--GA--CG-C-T-G--A-GG--C-G-CG-A--AA-G-C---G-TG--GG-
G--AG-C-A-AA--------CA--GG Bacteria(100);Proteobacteria(100);Alphaproteobacteria(100);Rhodospirillales(100);Acetobacteraceae(100);uncultured(77);unclassified(77);unclassified(77);unclassified(77);

Otu0678 0 0 0 0 0 0 0 0 0 0 0 0 0 0 0 0 1 0 0 0 0 0 5 4 0 0 0 0
0 0 0 0 0 0 0 0 0 0 0 0 0 0 0 0 0 0 0 0 0 0 0 0 0 0 0 0 0
0 0 0 0 0 0 0 0 0 0 0 0 0 0 0 0 0 0 0 0 0 0 0 0 0 0 0 0 0
2 0 0 0 0 0 0 0 0 0 0 0 0 0 0 0 0 0 0 0 0 0 0 0 0 0 0
M02255_76_000000000-ADUNE_1_2112_6585_8141 C----TA---AC-TC-TGT--------GCC-A-G------C-----A---G--CC-G-CGG-TA------AT-A--C-A--GA-GG-------GT--------GC-GA-GC-GT-------T-AA-T---C-G------------G--C-TT-T-T-T-----------AA----
G-T-C-G--------G-A-T--G--TG--A-AA-GC--C-C-CG-A-G-----CT-T-AA------C-T-T-G-G-G-A--A-T----T-G--C-A---T--T-------C--GA-T-A---C-------T--G-G--GA--A-G-C----T-A-G-A-G-T--A---T-GG-----GA-G-A--------G-G-A-T---GG-T------AG--A--ATT----C-C-A-G-GT-
-GT-A-G-CG-GT--G---A--A-A---TG-C-GT-AG--AG-A-TC--T-G--G-A-G-G-A-AT-A-CC---GA--T--G--GC-GAA-G--G-C--A-------G--C-C-A-T---CTG--G--CC-T-A--------------------A-T---A-C-T--GA--CG-C-T-G--A-GG--T-A-CG-A--AA-G-C---A-TG--GG-G--AG-C-A-
AA--------CA--GG Bacteria(100);Proteobacteria(100);Gammaproteobacteria(100);Pseudomonadales(100);Moraxellaceae(100);Acinetobacter(100);unclassified(100);unclassified(100);unclassified(100);

Otu0679 0 0 0 0 0 0 0 0 0 0 0 0 0 0 0 0 0 0 0 0 0 0 0 3 0 0 0 1
0 0 0 0 0 0 0 0 19 0 0 0 0 0 0 0 0 0 0 0 0 0 0 0 0 0 0 0 0
0 0 0 0 0 0 0 0 0 0 0 0 0 0 0 0 0 0 0 0 0 0 0 0 0 0 0 6 0
0 0 0 0 0 0 0 0 0 0 0 0 0 0 0 0 0 0 0 0 0 0 0 0 0 0 0
M02255_76_000000000-ADUNE_1_2105_8866_13878 .....AC----T-AC-GT---------GCC-A-G------C--A--G---C--CG-C-GGT-AA------TA-C--G-T--AG-GT-------GG--C-----AA-GC-G--TT-------G----T-C-C-G------------G--C-TT-C-T-T-----------AA----G-
T-C-T--------G-A-T--G--TG--A-AA-TC--T-T-GC-G-G-----CT-C-AA------C-C-G-C-A-A-G--C-G----G-T--C-A---T--T-------G--GA-A-A---C-------T--G-G--GA--G-G-C----T-T-G-A-G-T--G---C-AG-----AA-G-A--------G-G-A-G---AG-T------GG--A--ATT----C-C-A-C-GT--
GT-A-G-CG-GT--G---A--A-A---TG-C-GT-AG--AG-A-TG--T-G--G-A-G-G-A-AC-A-CC---AG--T--G--GC-GAA-G--G-C--G-------G--C-T-C-T---CTG--G--TC-T-G--------------------T-A---A-C-T--GA--CG-C-T-G--A-GG--C-G-CG-A--AA-G-C---G-TG--GG-G--AG-C-A-
AA--------CA--GG Bacteria(100);Firmicutes(100);Bacilli(100);Bacillales(100);unclassified(100);unclassified(100);unclassified(100);unclassified(100);unclassified(100);

Otu0680 0 0 4 3 0 0 0 0 0 0 0 0 0 0 0 0 0 0 0 0 0 0 0 0 0 0 0 0
0 0 0 0 15 0 0 4 0 5 0 0 0 0 0 0 0 0 0 0 0 0 0 0 0 0 0 0 0
0 0 0 0 0 0 0 0 0 0 0 0 0 0 0 0 0 0 0 0 0 0 0 0 0 0 0 10 7
15 0 0 1 0 0 0 1 0 0 0 0 0 0 0 0 1 0 0 0 0 0 1 0 0 0 0
M02255_76_000000000-ADUNE_1_1104_17091_17735 T----A----AG-CA-CCG--------GCT-A-A------C-----T---C--TG-T-GCC-AG------CA-G--C-C--GC-G--------GT--A-----AT-AC-AG-AG-------G-GT-G-C-G-A------------G--C-TA-G-T-T--------
---GA----A-T-C-G--------G-A-T--G--TG--A-AA-TC--C-C-CG-A-G-----CT-T-AA------C-T-T-G-G-G-A--A-T----T-G--C-A---T--T-------C--GA-T-A---C-------T--G-G--TT--A-G-C----T-A-G-A-G-T--G---T-GG-----GA-G-A--------G-G-A-T---GG-T------AG--A--ATT----C-C-
A-G-GT--GT-A-G-CG-GT--G---A--A-A---TG-C-GT-AG--AG-A-TC--T-G--G-A-G-G-A-AT-A-CC---GA--T--G--GC-GAA-G--G-C--A-------G--C-C-A-T---CTG--G--CC-T-A--------------------A-C---A-C-T--GA--CG-C-T-G--A-GG--T-A-CG-A--AA-G-C---A-TG--GG-G--
AG-C-A-AA--------CA--GG Bacteria(100);Proteobacteria(100);Gammaproteobacteria(100);Pseudomonadales(100);Moraxellaceae(100);Acinetobacter(100);unclassified(100);unclassified(100);unclassified(100);

Otu0681 0 0 0 0 0 0 0 0 0 0 0 0 0 0 0 0 0 0 0 0 0 0 0 0 0 0 1 0
0 0 0 0 0 0 0 0 0 0 0 0 0 0 0 0 0 0 0 0 0 0 0 0 0 0 0 0 0
0 0 0 0 0 0 0 0 0 0 0 0 0 0 0 0 0 0 0 0 0 1 0 0 0 0 0 0 0
0 0 0 0 0 0 1 0 0 0 0 0 0 0 0 0 0 0 0 0 0 0 0 3 0 0 0
M02255_76_000000000-ADUNE_1_2119_3546_9455 T----GC---AG-AA-TAA--------GCA-C-C------G--G--C---T--AACT-CTG-TG------CC-A--G-C--AG-CC-------GC--G-----GT-AA-TA-CA-------G-AG-G-G-T-G------------C--A-AG-C-G-T-----------
TA----A-T-C-G--------G-A-A--G--GG--A-AA-TC--C-C-CG-G-G-----CT-T-AA------C-C-T-G-G-G-A--A-C----T-G--C-A---T--C-------T--GA-A-A---C-------T--G-T--AG--A-G-C----T-A-G-A-G-T--A---T-GT-----GA-G-A--------G-G-A-A---GG-T------AG--A--ATT----C-C-A-
G-GT--GT-A-G-CG-GT--G---A--A-A---TG-C-GT-AG--AG-A-TC--T-G--G-A-G-G-A-AT-A-CC---GA--T--G--GC-GAA-G--G-C--A-------G--C-C-T-T---CTG--G--CA-T-A--------------------A-T---A-C-T--GA--CA-C-T-G--A-GG--C-T-CG-A--AA-G-C---G-TG--GG-T--AG-
C-A-AA--------CA--GG Bacteria(100);Proteobacteria(100);Gammaproteobacteria(100);Pseudomonadales(100);Moraxellaceae(100);unclassified(100);unclassified(100);unclassified(100);unclassified(100);

Otu0682 0 0 0 0 0 0 0 0 0 0 0 0 0 0 0 0 0 0 0 0 0 0 0 0 0 0 0 0
0 0 0 0 0 0 0 0 0 0 0 0 0 0 0 0 0 0 0 0 0 0 0 0 0 0 0 0 0
0 0 0 0 0 0 0 0 0 0 0 0 0 0 0 0 0 0 0 0 0 0 0 0 0 0 0 0 0
0 0 0 0 0 0 0 0 0 0 0 0 0 0 0 0 0 0 0 0 0 0 0 6 0 0 0
M02255_77_000000000-ADUT3_1_2114_27135_5500 C----TC---TG-TG-CCA--------GC--A-G------C--C--G---C--GG-T-AAT-AC------AG-A--G-G--GT-GC-------AA--G-----CG-TT-AA-TC-------G-GA-A-G-T-G------------A--C-TC-T-A-T-----------AA---
-G-T-C-A--------G-A-T--G--TG--A-AA-TC--C-C-CG-G-G-----CT-T-AA------C-C-T-G-G-G-A--A-C----T-G--C-A---T--C-------T--GA-A-A---C-------T--G-T--AG--A-G-C----T-A-G-A-G-T--A---T-GT-----GA-G-A--------G-G-A-A---GG-T------AG--A--ATT----C-C-A-G-
GT--GT-A-G-CG-GT--G---A--A-A---TG-C-GT-AG--AG-A-TC--T-G--G-A-G-G-A-AT-A-CC---GA--T--G--GC-GAA-G--G-C--A-------G--C-C-T-T---CTG--G--CA-T-A--------------------A-T---A-C-T--GA--CA-C-T-G--A-GG--C-T-CG-A--AA-G-C---G-TG--GG-T--AG-C-
A-AA--------CA--GG Bacteria(100);Proteobacteria(100);Gammaproteobacteria(100);Pseudomonadales(100);Moraxellaceae(100);Psychrobacter(100);unclassified(100);unclassified(100);unclassified(100);

Otu0683 0 0 0 0 0 0 0 0 0 0 0 0 0 0 0 0 0 0 0 0 0 0 0 0 0 0 0 0
0 0 0 0 0 0 0 0 0 0 0 0 0 4 0 0 0 0 0 0 4 0 0 0 0 0 0 0 0
0 0 0 0 0 0 0 0 0 0 0 0 0 0 0 0 0 0 0 0 0 0 0 0 0 0 0 0 0
0 0 0 3 0 0 0 0 0 0 0 0 0 8 0 0 0 0 0 0 0 0 0 0 0 0 0
M02255_76_000000000-ADUNE_1_1117_20979_23622 T----AC---GA-AG-GGG--------GCT-A-G------C--G--T---T--GT-T-CGG-AA------TC-A--C-T--GG-GC-------GT--A-----AA-G--G----------G-T----G-C--------------G----------T----------
AG----G-T-C-A--------G-G-G--G--TG--A-AA-TC--C-C-GA-G-G-----CT-C-AA------C-C-T-C-G-G-A--A-C----T-G--C-C---T--T-------T--GA-T-A---C-------T--G-G--GG--T-C-C----T-T-G-A-G-T--C---C-GG-----AA-G-A--------G-G-T-G---AG-T------GG--A--ACT----G-C-
G-A-GT--GT-A-G-AG-GT--G---A--A-A---TT-C-GT-AG--AT-A-TT--C-G--C-A-A-G-A-AC-A-CC---GG--T--G--GC-GAA-G--G-C--G-------G--C-T-C-A---CTG--G--TC-C-G--------------------G-A---A-C-T--GA--CG-C-T-G--A-GG--C-A-CG-A--CA-G-C---G-TG--GG-G--
AG-C-A-AA--------CA--GG Bacteria(100);Proteobacteria(100);Alphaproteobacteria(100);Rhizobiales(100);unclassified(100);unclassified(100);unclassified(100);unclassified(100);unclassified(100);

Otu0684 0 0 0 0 0 0 0 0 0 0 0 0 0 0 0 0 0 0 0 0 0 0 0 0 0 0 0 0
0 0 0 0 0 0 0 0 0 0 0 0 0 0 0 0 0 0 0 0 0 0 0 0 1 0 0 0 0
0 0 0 0 0 0 0 0 0 0 1 0 1 0 0 0 0 0 0 0 0 0 0 0 0 0 0 0 0
0 0 0 0 0 0 0 0 0 0 3 0 3 0 0 0 0 0 0 0 0 0 0 0 0 0 0
M02255_76_000000000-ADUNE_1_1118_5008_4743 G----AC---AG-AA-TAA--------GCA-C-C------G--G--C---T--AA---C-T-CT------GT-G--C-C--AG-CA-------GC--C-----GC-GG-TA-AT-------A-CA-G-A-G-G------------T--G-TG-C-A-A-----------GC---
-G-T-T-G--------A-A-T--C--GG--A-AA-TC--C-C-CG-G-G-----CT-C-AA------C-C-T-G-G-G-A--A-C----T-G--C-A---T--C-------C--AA-A-A---C-------T--G-G--CA--A-G-C----T-A-G-A-G-T--A---T-GG-----TA-G-A--------G-G-G-T---GG-T------GG--A--ATT----T-C-C-T-GT-
-GT-A-G-CG-GT--G---A--A-A---TG-C-GT-AG--AT-A-TA--G-G--A-A-G-G-A-AC-A-CC---AG--T--G--GC-GAA-G--G-C--G-------A--C-C-A-C---CTG--G--AC-T-G--------------------A-T---A-C-T--GA--CA-C-T-G--A-GG--T-G-CG-A--AA-G-C---G-TG--GG-G--AG-C-A-
AA--------CA--GG Bacteria(100);Proteobacteria(100);Gammaproteobacteria(100);unclassified(100);unclassified(100);unclassified(100);unclassified(100);unclassified(100);unclassified(100);

Otu0685 192 0 0 0 0 0 0 0 0 0 0 0 0 0 0 0 0 0 0 0 0 0 0 0 0 0 0 0
0 0 0 0 0 0 0 0 0 0 0 0 0 0 0 0 0 0 0 0 0 0 0 0 0 0 0 0 0
0 0 0 0 0 0 0 0 0 0 0 0 0 0 0 0 0 0 0 0 0 0 0 0 0 0 0 0 0
0 0 0 0 0 0 0 0 0 0 0 0 0 0 0 0 0 0 0 0 0 0 0 0 0 0 0 0
M02255_77_000000000-ADUT3_1_1101_3293_9693 T----AC---GT-AG-GGA--------GCA-A-G------C--G--T---T--GT-T-CGG-AT------TT-A--C-T--GG-GC-------GT--A-----AA-GG-GC-GC-------G-TA-G-G-C-G------------G--C-GC-G-G-T----------
AA----G-T-C-A--------C-T-T--G--TG--A-AA-TC--T-C-TG-A-G-----CT-T-AA------C-T-C-A-G-A-A--C-G----G-C--C-A---A--G-------T--GA-T-A---C-------T--G-C--AG--T-G-C----T-A-G-A-G-T--G---C-AG-----AA-A-G--------G-G-C-A---AT-C------GG--A--ATT----C-T-T-
G-GT--GT-A-G-CG-GT--G---A--A-A---TG-C-GT-AG--AT-A-TC--A-A--G-A-G-G-A-AC-A-CC---TG--A--G--GC-GAA-G--G-C--G-------G--G-T-T-G---CTA--G--GC-T-G--------------------A-C---A-C-T--GA--CG-C-T-G--AGTG--C-G-CG-A--AA-G-C---C-AG--GG-G--
AG-C-A-AA--------CG--GG Bacteria(100);Acidobacteria(100);Acidobacteria(100);Acidobacteriales(100);Acidobacteriaceae(100);Candidatus_Chloroacidobacterium(100);unclassified(100);unclassified(100);unclassified(100);

Otu0686 0 0 65 0 0 0 0 0 0 0 0 0 0 0 0 0 0 0 0 0 0 0 0 3 0 0 0 0
0 0 0 0 0 0 0 0 0 0 0 0 0 0 0 0 0 0 0 0 0 0 0 0 0 0 0 0 0
0 0 0 0 0 0 0 0 0 0 0 0 0 0 0 0 11 0 0 0 0 0 0 0 0 0 0 0 0
0 0 0 0 21 0 0 0 0 0 0 0 0 0 0 0 18 0 0 0 0 0 0 0 18 0 0 0
M02255_77_000000000-ADUT3_1_1119_20309_10142 T----AC---GT-AG-GGG--------GCA-A-G------C--G--T---T--AT-C-CGG-AT------TT-A--C-T--GG-GT-------GT--A-----AA-GG-GA-GC-------G-TA-G-A-C-G------------G--T-GT-T-G-C-----
------AA----G-T-C-T--------G-A-T--G--TG--A-AA-GG--C-G-GG-G-G-----CT-C-AA------C-C-C-C-T-G-G--A-C----T-G--C-A---T--T-------G--GA-A-A---C-------T--G-T--GA--T-A-C----T-C-G-A-G-T--G---C-CG-----GA-G-A--------G-G-T-A---AG-C------GG--A--ATT----
C-C-T-A-GT--GT-A-G-CG-GT--G---A--A-A---TG-C-GT-AG--AT-A-TT--A-G--G-A-G-G-A-AC-A-CC---AG--T--G--GC-GAA-G--G-C--G-------G--C-T-T-A---CTG--G--AC-G-G--------------------T-A---A-C-T--GA--CG-T-T-G--A-GG--C-T-CG-A--AA-G-C---G-TG--GG-
G--AG-C-A-AA--------CA--GG Bacteria(100);Firmicutes(100);Clostridia(100);Clostridiales(100);Lachnospiraceae(100);unclassified(100);unclassified(100);unclassified(100);unclassified(100);

Otu0687 0 0 0 0 0 0 0 0 0 0 0 0 0 0 0 0 0 0 0 0 0 0 0 0 0 0 0 0
0 0 0 0 0 0 0 0 0 0 0 0 0 0 0 0 0 0 0 0 0 0 0 0 0 0 0 0 0
0 0 0 0 0 0 0 0 0 0 0 0 0 0 0 0 0 0 0 0 0 0 0 0 0 91 0 0 0
0 13 0 0 0 0 0 0 0 0 0 0 0 0 0 0 0 0 0 0 0 0 0 0 0 0 0 0
M02255_76_000000000-ADUNE_1_1107_16973_4964 T----CG---GC-TA-ACT--------ACG-T-G------C--C--A---G--CA-G-CCG-CG------GT-A--A-T--AC-GG-------AG--G-----AT-GC-GA-GC-------GTTA-T-C-C-G------------G--G-TT-T-T-T----------AA-
---G-T-C-A--------G-C-T--G--TG--A-AA-GT--T-T-GG-G-G-----CT-C-AA------C-C-T-T-A-A-A--A-T----T-G--C-A---G--T-------T--GA-T-A---C-------T--G-G--AG--A-C-C----T-T-G-A-G-T--G---C-AG-----TT-G-A--------G-G-C-A---GG-C------GG--A--ATT----C-G-T-G-
GT--GT-A-G-CG-GT--G---A--A-A---TG-C-TT-AG--AT-A-TC--A-C--G-A-A-G-A-AC-T-CC---GA--T--T--GC-GAA-G--G-C--A-------G--C-T-T-G---CTA--A--AG-T-G--------------------T-A---A-C-T--GA--CG-T-T-C--A-TG--C-T-CG-A--AA-G-T---G-TG--GG-T--AT-C-A-
AA--------CA--GG Bacteria(100);Bacteroidetes(100);Bacteroidia(100);Bacteroidales(100);Bacteroidaceae(100);Bacteroides(100);unclassified(100);unclassified(100);unclassified(100);

Otu0688 0 0 0 0 0 0 0 0 0 0 0 0 0 0 0 0 0 0 0 0 0 0 0 0 0 0 0 0
1 0 0 0 0 0 0 11 0 0 0 11 0 0 0 0 0 0 0 0 0 0 0 0 0 0 0 0 0
0 25 0 0 0 0 0 0 0 0 0 0 0 0 0 0 0 0 0 0 0 0 0 0 0 0 0 0 0
0 0 0 0 0 0 0 0 0 0 0 0 5 0 0 0 0 0 0 0 0 0 0 0 0 0 0 0
M02255_76_000000000-ADUNE_1_2112_9170_9454 C----TC---CG-TG-CCA--------GCAGCCG------CG-G--T---A--AT-A-CGG-AG------GG-T--G-C--AA-GC-------GT--T-----GT-CC-G--GA-------T-TT-A-T-T-G------------G--C-TT-G-T-T----------AA-
---G-T-C-C--------G-G-G--G--TG--A-AA-GC--C-C-AC-A-G-----CT-C-AA------C-T-G-T-G-G-A--A-C----T-G--C-C---C--T-------G--GA-T-A---C-------T--G-G--CG--A-G-C----T-T-G-A-G-T--C---C-AG-----AC-G-A--------G-G-T-T---GG-C------GG--A--ATG----G-A-A-G-
CT--GT-A-G-CG-GT--G---A--A-A---TG-C-AT-AG--AT-A-GC--T-T--C-C-A-G-A-AC-C-CC---GA--T--T--GC-GTA-G--G-C--A-------G--C-T-G-A---CTA--G--GC-T-G--------------------G-T---A-C-T--GA--CA-C-T-G--A-GG--C-A-CG-A--AA-G-C---G-TG--GG-G--AG-C-
G-AA--------CA--GG Bacteria(100);Bacteroidetes(100);Sphingobacteria(100);Sphingobacteriales(100);Cytophagaceae(100);Hymenobacter(100);unclassified(100);unclassified(100);unclassified(100);

Otu0689 0 0 0 0 0 0 0 0 0 0 0 0 0 1 0 0 0 0 0 0 0 0 0 0 0 0 0 0
0 0 0 0 0 0 0 0 0 0 0 0 0 0 0 0 0 0 0 0 0 0 0 0 0 0 0 0 0
0 0 0 0 0 0 0 0 0 0 0 0 0 0 0 0 0 0 0 0 0 0 0 0 0 0 0 0 0
0 0 0 0 0 0 7 0 0 0 0 0 0 0 0 0 1 0 0 0 1 0 0 3 0 0 0 0
M02255_77_000000000-ADUT3_1_2114_25470_3820 C----TG---TG-CC-AGC--------AGC-C-G------C--G--G---T--AA-TACAG-AG------GG-T--G-C--AA-GC-------GT--T-----AATCGGAATTA-------C-TA-G-G-T-G------------G--C-TT-G-A-T----------
AA----G-T-C-A--------G-A-T--G--TG--A-AC-TC--C-C-CG-G-G-----CT-T-AA------C-C-T-G-G-G-A--A-C----T-G--C-A---T--C-------T--GA-A-A---C-------T--G-T--TA--G-G-C----T-A-G-A-G-T--A---G-GT-----GA-G-A--------G-G-G-A---AG-T------AG--A--ATT----T-C-A-
G-GT--GT-A-G-CG-GT--G---A--A-A---TG-C-GT-AG--AG-A-TC--T-G--A-A-G-G-A-AT-A-CC---GA--T--G--GC-GAA-G--G-C--A-------G--C-T-T-C---CTG--G--CA-T-C--------------------A-T---A-C-T--GA--CA-C-T-G--A-GG--C-T-CG-A--AA-G-C---G-TG--GG-T--AG-
C-A-AA--------CA--GG Bacteria(100);Proteobacteria(100);Gammaproteobacteria(100);Pseudomonadales(100);Moraxellaceae(100);Psychrobacter(100);unclassified(100);unclassified(100);unclassified(100);

Otu0690 0 0 0 0 0 0 0 0 0 0 0 0 0 0 0 0 0 0 0 0 0 0 0 0 0 0 0 0
0 0 0 0 0 0 0 0 0 0 0 0 0 0 0 0 0 0 0 0 0 0 0 0 0 0 0 0 0
0 0 0 0 0 0 0 0 0 0 0 0 0 0 0 0 0 0 0 0 0 0 0 0 0 43 51 0 0
0 0 0 0 0 0 0 0 0 0 0 0 0 0 0 0 0 0 0 0 0 0 0 0 0 0 0
M02255_77_000000000-ADUT3_1_2106_3192_11017 T----AC---GG-AG-GGT--------GCA-A-G------C--G--T---T--AT-C-CGG-AT------TC-A--C-T--GG-G--------TT---------A----G--GC-------G----G-A-C-------------------TT---G-T-----------AA----G-T-
C-C--------G-T-G--G--TG--A-AA-TC--T-C-CG-A-G-----CT-T-AA------C-T-C-G-G-A-A--A-C----T-G--C-C---A--T-------G--GA-T-A---C-------T--A-T--AG--G-T-C----T-T-G-A-A-T--G---T-TG-----TG-G-A--------G-G-T-T---AG-C------GG--A--ATA----G-T-T-C-AT--GT-
A-G-CG-GT--G---A--A-A---TG-C-TT-AG--AT-A-TG--A-C--C-T-A-G-A-AC-A-CC---GA--T--T--GC-GAA-G--G-C--A-------G--C-T-G-G---CTA--C--AC-A-A--------------------C-G---A-T-T--GA--CG-C-T-G--A-GG--C-A-CG-A--AA-G-C---G-TG--GG-G--AT-C-A-AA----
----CA--GG Bacteria(100);Bacteroidetes(100);Sphingobacteria(100);Sphingobacteriales(100);Chitinophagaceae(100);unclassified(100);unclassified(100);unclassified(100);unclassified(100);

Otu0691 0 0 0 0 0 0 0 0 0 0 0 0 0 0 0 0 0 0 0 0 0 0 0 0 0 0 0 0
0 0 0 0 0 0 0 0 0 0 0 0 0 0 23 0 0 0 0 0 0 0 0 0 0 0 0 0 0
0 0 0 0 0 0 0 0 0 0 0 0 0 0 0 0 0 0 0 0 0 0 0 0 0 0 0 0 0
0 0 0 0 0 0 0 0 0 0 0 0 0 0 0 0 0 0 0 0 0 0 0 6 0 0 0
M02255_77_000000000-ADUT3_1_1113_3639_17456 T----AC---GT-AG-GGT--------GCA-A-G------C--G--T---T--GT-C-CGG-AA------TT-A--T-T--GG-GC-------GT--A-----AA-GA-GC-TC-------G-TA-G-G-C-G------------G--T-CT-G-T-C-----------GC-
---G-T-C-T--------G-G-T--G--TG--A-AA-AT--C-C-AG-G-G-----CT-T-AA------C-C-C-T-G-G-A--C-C----T-G--C-A---T--T-------G--GG-T-A---C-------G--G-G--CA--G-A-C----T-A-G-A-G-T--G---C-GG-----TA-G-G--------G-G-A-G---AT-C------GG--A--ATT----C-C-T-G-
GT--GT-A-G-CG-GT--G---A--A-A---TG-C-GC-AG--AT-A-TC--A-G--G-A-G-G-A-AC-A-CC---GG--T--G--GC-GAA-G--G-C--G-------G--A-T-C-T---CTG--G--GC-C-G--------------------T-A---A-C-T--GA--CG-C-T-G--A-GG--A-G-CG-A--AA-G-C---A-TG--GG-G--AG-
C-G-AA--------CA--GG Bacteria(100);Actinobacteria(100);Actinobacteria(100);Actinobacteridae(100);Actinomycetales(100);unclassified(100);unclassified(100);unclassified(100);unclassified(100);

Otu0692 0 0 0 0 0 0 0 0 0 42 0 0 0 0 0 0 0 0 0 0 0 0 0 2 0 0 0 0
0 0 0 0 0 0 21 0 0 0 0 18 0 0 0 0 0 0 0 0 0 0 0 0 0 0 0 0 0
0 0 0 0 0 0 0 0 0 0 0 0 0 0 0 0 0 0 0 0 0 0 0 0 0 0 0 0 0
0 0 0 0 0 0 0 0 0 0 0 0 0 0 0 0 0 0 0 0 0 0 0 0 0 0 0
M02255_77_000000000-ADUT3_1_1111_11898_20548 T----AC---GT-AG-GGT--------GCG-A-G------C--G--T---T--GT-C-CGG-AA------TT-A--C-T--GG-GC-------GT--A-----AA-GA-GC-TC-------G-TA-G-G-C-G------------G--T-TT-G-T-C------
-----GC----G-T-C-G--------T-T-C--G--TG--A-AA-AC--T-T-GG-G-G-----CT-T-AA------C-T-C-C-A-A-G--C-G----T-G--C-G---G--G-------C--GA-T-A---C-------G--G-G--CA--G-A-C----T-T-G-A-G-T--A---C-TA-----CA-G-G--------G-G-A-G---AC-T------GG--A--ATT----C-
C-T-G-GT--GT-A-G-CG-GT--G---A--A-A---TG-C-GC-AG--AT-A-TC--A-G--G-A-G-G-A-AC-A-CC---GG--T--G--GC-GAA-G--G-C--G-------G--G-T-C-T---CTG--G--GT-A-G--------------------T-A---A-C-T--GA--CG-C-T-G--A-GG--A-G-CG-A--AA-G-C---G-TG--GG-
T--AG-C-G-AA--------CA--GG Bacteria(100);Actinobacteria(100);Actinobacteria(100);Actinobacteridae(100);Actinomycetales(100);Corynebacterineae(76);unclassified(76);unclassified(76);unclassified(76);

Otu0693 0 0 0 0 0 0 0 0 0 0 0 0 0 0 1 0 0 0 0 0 0 0 0 7 0 1 0 0
6 0 0 0 0 0 0 0 0 0 0 0 1 0 0 0 0 0 0 0 0 0 0 0 0 0 0 0 0
0 0 0 0 0 0 0 0 0 0 0 0 0 0 0 0 0 0 0 0 0 0 0 0 0 0 0 0 0
0 0 0 0 0 0 0 0 0 0 0 0 0 0 0 0 0 0 0 0 0 1 0 3 0 0 0
M02255_76_000000000-ADUNE_1_2114_21873_23625 T----GC---AG-AA-TAA--------GCA-C-C------G--G--C---T--AACT-CTG-TG------CC-A--G-C--AG-CC-------GC--G-----GT-AA-TA-CA-------G-AG-G-G-T-GC-----------A--A-GC-G-T-T----
-------AA----G-T-C-G--------G-A-A--G--TG--A-AA-TC--C-C-CG-G-G-----CT-C-AA------C-C-T-G-G-G-A--A-C----T-G--C-A---T--C-------T--GA-T-A---C-------T--G-T--TG--A-G-C----T-A-G-A-G-T--A---T-GT-----GA-G-A--------G-G-A-A---GG-T------AG--A--ATT----
C-C-A-G-GT--GG-A-G-CG-GT--G---A--A-A---TG-C-GT-AG--AG-A-TC--T-G--G-A-G-G-A-AT-A-CC---GA--T--G--GC-GAA-G--G-C--A-------G--C-C-T-T---CTG--G--CA-T-A--------------------A-T---A-C-T--GA--CA-C-T-G--A-GG--C-T-CG-A--AA-G-C---G-TG--GG-
T--AG-C-A-AA--------CA--GG Bacteria(100);Proteobacteria(100);Gammaproteobacteria(100);Pseudomonadales(85);Moraxellaceae(85);unclassified(85);unclassified(85);unclassified(85);unclassified(85);

Otu0694 0 0 0 0 0 0 0 0 0 0 0 0 0 0 0 0 0 0 0 0 0 0 0 0 0 0 0 0
0 0 0 0 0 0 0 0 0 0 0 0 0 0 0 0 0 0 0 0 0 0 0 0 0 0 0 0 0
0 0 0 0 0 0 0 0 0 4 0 0 0 0 0 0 0 0 0 0 0 0 0 0 0 0 0 0 0
0 0 0 0 0 0 0 0 0 0 1 0 1 0 0 0 0 0 0 0 0 0 0 0 0 0 0
M02255_76_000000000-ADUNE_1_1113_8994_21177 G----AC---AG-AA-TAA--------G-C-A-C------CG-G--C---T--AACT-CTG-TG------CC-A--G-C--AG-CC-------GC--G-----GT-AA-TA-CA-------G-AG-G-G-T-G------------A--A-GC-G-T-T-----------
AA----G-T-C-G--------G-A-A--G--TG--A-AA-TC--C-C-CG-G-G-----CT-C-AA------C-C-T-G-G-G-A--A-C----T-G--C-A---T--T-------C--AA-A-A---C-------T--G-T--CG--A-G-C----T-A-G-A-G-T--A---T-GG-----TA-G-A--------G-G-G-T---GG-T------GG--A--ATT----T-C-C-
T-GT--GT-A-G-CG-GT--G---A--A-A---TG-C-GT-AG--AT-A-TA--G-G--A-A-G-G-A-AC-A-CC---AG--T--G--GC-GAA-G--G-C--G-------A--C-C-A-C---CTG--G--AC-T-A--------------------A-T---A-C-T--GA--CA-C-T-G--A-GG--T-G-CG-A--AA-G-C---G-TG--GG-G--
AG-C-A-AA--------CA--GG Bacteria(100);Proteobacteria(100);Gammaproteobacteria(100);unclassified(100);unclassified(100);unclassified(100);unclassified(100);unclassified(100);unclassified(100);

Otu0695 0 0 0 0 0 0 0 0 0 0 0 0 0 0 0 0 0 0 5 0 0 0 0 0 0 0 0 0
0 0 0 0 0 0 0 0 0 0 0 0 0 0 0 0 0 0 0 0 0 0 0 0 0 0 0 0 0
0 0 0 0 0 0 0 0 0 0 0 0 0 0 0 0 0 0 0 0 0 0 0 0 0 0 0 0 0
0 0 0 0 0 0 0 0 0 0 0 0 0 0 0 74 0 0 0 0 0 0 0 10 0 0 0
M02255_77_000000000-ADUT3_1_2104_17621_13932 T----AC---GG-AG-GGA--------GCT-A-G------C--G--T---T--GT-T-CGG-AA------TT-A--C-T--GG-GC-------GT--A-----AA-GC-GT-AC-------G-TA-G-G-C-G------------G--T-TA-T-T-C------
-----AA----G-T-C-A--------G-A-G--G--TG--A-AA-GC--C-C-GG-G-G-----CT-C-AA------C-C-C-C-G-G-A--A-C----G-G--C-C---T--T-------T--GA-A-A---C-------T--A-G--AT--A-G-C----T-A-G-A-G-T--C---T-TG-----GA-G-A--------G-G-T-T---AG-T------GG--A--ATT----C-
C-G-A-GT--GT-A-G-AG-GT--G---A--A-A---TT-C-GT-AG--AT-A-TT--C-G--G-A-A-G-A-AC-A-CC---AG--T--G--GC-GAA-G--G-C--G-------A--C-T-A-A---CTG--G--AC-A-A--------------------G-T---A-C-T--GA--CG-C-T-G--A-GG--T-A-CG-A--AA-G-C---G-TG--GG-
G--AG-C-A-AA--------CA--GG Bacteria(100);Proteobacteria(100);Alphaproteobacteria(100);Sphingomonadales(100);Sphingomonadaceae(100);Novosphingobium(100);unclassified(100);unclassified(100);unclassified(100);

Otu0696 0 0 0 0 0 0 0 0 0 0 0 0 0 0 0 0 0 0 0 0 0 0 0 16 0 0 0 0
0 0 0 0 0 0 0 0 0 0 0 0 0 0 0 0 0 0 0 0 0 0 0 0 0 0 0 0 0
0 0 0 0 0 0 0 0 0 0 0 0 0 0 0 0 0 0 0 0 0 0 0 0 0 0 0 0 0
0 0 0 0 0 0 0 0 0 0 0 0 0 0 0 0 0 0 0 0 0 0 0 5 0 0 0
M02255_77_000000000-ADUT3_1_2101_23598_7201 T----AC---GG-AG-GGG--------GCT-A-G------C--G--T---T--GT-T-CGG-AA------TT-A--C-T--GG-GC-------GT--A-----AA-GC-GC-AC-------G-TA-G-G-C-G------------G--A-CT-G-G-A----------
AA----G-T-T-A--------G-A-G--G--TG--A-AA-TC--C-C-GG-G-G-----CT-C-AA------C-C-C-C-G-G-A--A-C----T-G--C-C---T--T-------T--AA-A-A---C-------T--C-T--CA--G-T-C----T-T-G-A-G-T--T---C-GA-----GA-G-A--------G-G-T-G---AG-T------GG--A--ATT----C-C-G-
A-GT--GT-A-G-AG-GT--G---A--A-A---TT-C-GT-AG--AT-A-TT--C-G--G-A-G-G-A-AC-A-CC---AG--T--G--GC-GAA-G--G-C--G-------G--C-T-C-A---CTG--G--CT-C-G--------------------A-T---A-C-T--GA--CG-C-T-G--A-GG--T-G-CG-A--AA-G-C---G-TG--GG-G--
AG-C-A-AA--------CA--GG Bacteria(100);Proteobacteria(100);Alphaproteobacteria(100);Rhodobacterales(77);Rhodobacteraceae(77);unclassified(77);unclassified(77);unclassified(77);unclassified(77);

Otu0697 0 0 0 0 0 0 0 0 0 0 0 0 0 0 0 0 0 0 0 0 0 0 0 0 0 6 0 0
0 0 0 0 0 0 0 0 0 0 0 0 0 0 0 0 0 0 0 0 0 0 0 0 0 0 0 0 0
0 0 0 0 0 0 0 0 0 0 0 0 0 0 0 0 0 0 0 0 0 0 0 112 0 0 0 0 0
0 0 0 0 0 0 0 0 0 0 0 0 0 0 0 0 0 0 0 0 0 0 0 0 0 0 0
M02255_77_000000000-ADUT3_1_1101_17052_22306 T----AC---GA-AG-GGG--------GCT-A-G------C--G--T---T--GT-T-CGG-AA------TT-A--C-T--GG-GC-------GT--A-----AA-GC-GC-AC-------G-TA-G-G-C-G------------G--G-TG-G-T-T----
-------AA----G-T-T-A--------G-G-G--G--TG--A-AA-TC--C-C-GG-A-G-----CT-C-AA------C-T-C-C-G-G-A--A-C----T-G--C-C---T--T-------T--GA-T-A---C-------T--G-G--CC--A-T-C----T-A-G-A-G-A--T---C-GG-----AA-G-A--------G-G-T-G---AG-T------GG--A--ATT----
C-C-C-A-GT--GT-A-G-AG-GT--G---A--A-A---TT-C-GT-AG--AT-A-TT--G-G--G-A-A-G-A-AC-A-CC---GG--T--G--GC-GAA-G--G-C--G-------G--C-T-C-A---CTG--G--TC-C-G--------------------A-T---A-C-T--GA--CG-C-T-G--A-GG--T-G-CG-A--AA-G-C---G-TG--GG-
G--AG-C-A-AA--------CA--GG Bacteria(100);Proteobacteria(100);Alphaproteobacteria(100);Rhizobiales(100);unclassified(100);unclassified(100);unclassified(100);unclassified(100);unclassified(100);

Otu0698 0 0 0 0 0 0 0 0 0 0 0 0 0 0 0 0 0 0 0 0 0 0 13 12 1 0 0 6
2 0 0 0 0 0 0 0 0 0 0 0 0 0 0 0 0 0 0 14 14 0 0 0 0 0 0 0 0
0 0 0 0 0 0 0 0 0 0 0 0 0 0 0 0 0 0 0 0 0 92 18 0 0 0 0 0 0
0 0 0 0 0 0 0 0 0 0 0 0 0 0 0 0 0 0 0 0 0 0 0 0 17 0 0
M02255_76_000000000-ADUNE_1_1116_14770_13985 G----AA---GC-AC-CGG--------CTA-A-C------T--C--C---G--TG-C-CAG-CA------GC-C--G-C--GG-TA-------AT--A-----CG-GA-GG-GT-------G-CG-A-G-C-G------------G--T-TA-A-TGT----
-------AA----G-T-C-A--------G-A-T--G--TG--A-AA-TC--C-C-AG-G-G-----CT-C-AA------C-C-T-T-G-G-A--A-C----T-G--C-A---T--T-------T--GA-A-A---C-------T--G-G--TT--A-A-C----T-A-G-A-G-T--T---T-TG-----TA-G-A--------G-G-G-T---GG-T------AG--A--ATT----T-
C-A-G-GT--GT-A-G-CG-GT--G---A--A-A---TG-C-GT-AG--AG-A-TC--T-G--A-A-G-G-A-AT-A-CC---AG--T--G--GC-GAA-G--G-C--G-------G--C-C-A-C---CTG--G--AC-A-A--------------------A-G---A-C-T--GA--CG-C-T-G--A-GG--C-G-CG-A--AG-G-C---G-TG--GG-
G--AG-C-G-AA--------CG--GG Bacteria(100);Proteobacteria(100);Gammaproteobacteria(100);Alteromonadales(100);Psychromonadaceae(100);Psychromonas(100);unclassified(100);unclassified(100);unclassified(100);

Otu0699 0 0 0 0 0 2 0 0 0 0 0 1 0 0 0 0 1 0 0 0 0 0 0 5 0 0 0 0
1 0 0 0 0 0 0 0 0 0 0 0 0 0 0 0 0 0 0 0 0 0 0 0 0 0 0 0 0
0 0 0 0 0 0 0 0 0 0 0 0 0 0 0 0 0 0 0 0 0 1 4 0 0 0 0 0 0
0 0 0 0 0 0 0 0 5 0 0 0 0 0 0 0 0 0 3 0 0 0 3 0 1 0 0
M02255_76_000000000-ADUNE_1_1115_28438_17513 T----AA---GC-AC-CGG--------CTA-A-C------T--C--T---G--TG-C-CAG-CA------GC-C--G-C--GG-TA-------AT--A-----CA-GA-GG-GT-------G-CA-A-G-C-GT-----------T--A-AT-C-G-G-----
------AA----G-T-C-A--------G-A-T--G--TG--A-AA-TC--C-C-CG-G-G-----CT-C-AA------C-C-T-G-G-G-A--A-C----T-G--C-A---T--C-------T--GA-T-A---C-------T--G-T--TG--A-G-C----T-A-G-A-G-T--A---T-GT-----GA-G-A--------G-G-A-A---GG-T------AG--A--ATT----C-
C-A-G-GT--GT-A-G-CG-GT--G---A--A-A---TG-C-GT-AG--AG-A-TC--T-G--G-A-G-G-A-AT-A-CC---GA--T--G--GC-GAA-G--G-C--A-------G--C-C-T-T---CTG--G--CA-T-A--------------------A-T---A-C-T--GA--CA-C-T-G--A-GG--C-T-CG-A--AA-G-C---G-TG--GG-T-
-AG-C-A-AA--------CA--GG Bacteria(100);Proteobacteria(100);Gammaproteobacteria(100);Pseudomonadales(100);Moraxellaceae(100);unclassified(100);unclassified(100);unclassified(100);unclassified(100);

Otu0700 0 0 0 0 0 0 0 0 0 0 0 0 0 0 0 0 0 0 0 0 0 0 0 0 0 0 0 12
0 0 0 1 0 0 0 0 0 0 29 1 0 0 0 0 0 0 0 0 0 0 0 0 0 0 0 0
0 0 0 0 0 0 0 0 0 0 0 0 0 0 0 0 0 0 0 0 0 0 0 0 0 0 0 0
0 0 0 0 0 0 0 0 0 0 0 0 0 0 0 0 0 0 0 0 0 0 0 0 0 0 0
M02255_77_000000000-ADUT3_1_1109_14054_20139 G----AC---GG-AG-GGT--------GCT-A-G------C--G--T---T--GT-T-CGG-AA------TG-A--C-T--GG-GC-------GT--A-----AA-GG-GC-GC-------G-TA-G-G-C-G-------------G--C-CG-C-T-T----
-------GC----G-T-C-G--------G-A-T--G--TG--A-AA-GC--C-C-GG-G-G-----CT-C-AA------C-C-C-C-G-G-A--A-C----T-G--C-A---T--C-------C--GA-T-A---C-------G--G-G--GC--G-G-C----T-C-G-A-G-A--C---C-GA-----GA-G-A--------G-G-T-G---AG-T------GG--A--ATA---
-C-C-C-A-GT--GT-A-G-AG-GT--G---A--A-A---TT-C-GT-AG--AT-A-TT--G-G--G-T-G-G-A-AC-A-CC---GG--T--G--GC-GAA-A--G-C--G-------G--C-T-C-A---CTG--G--CT-C-G---------------------G-T---T-C-T--GA--CG-C-T-G--A-GG--C-G-CG-A--AA-G-C---G-T-G-
GG-G--AG-C-A-AA--------CA--GG Bacteria(100);Proteobacteria(100);Alphaproteobacteria(100);unclassified(100);unclassified(100);unclassified(100);unclassified(100);unclassified(100);unclassified(100);

Otu0701 0 0 0 0 0 0 0 0 0 0 0 0 1 0 0 0 0 0 0 1 0 0 0 2 0 0 0 0
0 0 0 0 0 0 0 0 0 0 0 0 0 0 0 0 0 0 0 0 0 0 0 0 0 0 0 0
0 0 0 0 0 0 0 0 0 0 0 0 0 0 0 0 0 0 0 0 4 0 0 0 0 0 0 0
3 0 0 0 0 0 0 0 0 3 0 0 1 3 0 0 0 0 0 0 1 0 6 15 1 0 0
M02255_76_000000000-ADUNE_1_2112_4534_7687 T----GC---AG-AA-TAA--------GCA-C-C------G--G--C---T--AA---C-T-CT------GT-G--C-C--AG-CA-------GC--C-----GC-GG-TA-AT-------A-CA---G-A-G-------------G--G-TG-C-A-A-----------GC---
-G-T-T-A--------G-A-T--C--GG--A-AA-TC--C-C-CG-G-G-----CT-T-AA------C-C-T-G-G-G-A--A-C----T-G--C-A---T--C-------T--GA-A-A---C-------T--G-T--AG--A-G-C----T-A-G-A-G-T--A---T-GT-----GA-G-A--------G-G-A-A---GG-T------AG--A--ATT----C-C-A-G-
GT--GT-A-G-CG-GT--G---A--A-A---TG-C-GT-AG--AG-A-TC--T-G--G-A-G-G-A-AT-A-CC---GA--T--G--GC-GAA-G--G-C--A-------G--C-C-T-T---CTG--G--CA-T-A---------------------A-T---A-C-T--GA--CA-C-T-G--A-GG--C-T-CG-A--AA-G-C---G-TG--GG-T--AG-C-
A-AA--------CA--GG Bacteria(100);Proteobacteria(100);Gammaproteobacteria(100);Pseudomonadales(100);Moraxellaceae(100);unclassified(100);unclassified(100);unclassified(100);unclassified(100);

Otu0702 0 0 0 0 0 0 1 29 0 0 0 0 0 0 0 0 0 0 0 0 0 0 0 0 0 0 0 0
0 0 0 0 0 0 0 0 0 0 0 0 0 0 0 0 0 0 0 0 0 0 0 0 0 0 0 0
0 0 0 0 0 0 0 0 0 0 0 0 0 0 0 0 0 0 0 0 8 0 0 0 0 0 0 0
0 0 0 0 0 0 0 0 0 0 0 0 0 0 0 0 0 0 0 0 0 0 0 0 0 0 0
M02255_77_000000000-ADUT3_1_1116_9980_13623 T----AC---GT-AG-GGA--------GCA-A-G------C--G--T---T--AT-C-CGG-AT------TT-A--T-T--GG-GT-------GT--A-----AA-GG-GT-GC-------G-TA-G-A-C-G-------------G--G-AA-G-G-T-----------
AA----G-T-T-A--------G-T-T--G--TG--A-AA-TC--C-C-TC-G-G-----CT-C-AA------C-T-G-A-G-G-A--A-C----T-G--C-G---A--C-------T--AA-A-A---C-------T--G-C--TT--T-T-C----T-T-G-A-G-T--G---C-TG-----GA-G-A--------G-G-A-A---AG-T------GG--A--ATT----C-C-T-
A-GT--GT-A-G-CG-GT--G---A--A-A---TG-C-GT-AG--AT-A-TT--A-G--G-A-G-G-A-AC-A-CC---AG--T--G--GC-GAA-G--G-C--G-------A--C-T-T-T---CTG--G--AC-A-G---------------------T-A---A-C-T--GA--CG-T-T-G--A-GG--C-A-CG-A--AA-G-T---G-TG--GG-G--
AG-C-A-AA--------CA--GG Bacteria(100);Firmicutes(100);Clostridia(100);Clostridiales(100);Ruminococcaceae(100);uncultured(100);unclassified(100);unclassified(100);unclassified(100);

Otu0703 0 0 0 0 0 0 0 0 0 0 0 0 0 0 0 0 0 0 0 2 2 0 0 0 0 0 0 0
0 0 0 0 0 0 0 0 0 20 0 0 0 20 0 0 0 0 0 0 140 0 0 0 0 0 0 0
0 0 0 0 0 0 0 0 0 0 0 0 0 0 0 0 0 0 0 0 0 0 0 0 0 0 0 0
18 0 0 0 0 0 0 0 0 0 0 0 3 0 0 0 0 0 0 0 0 0 0 0 0 0 0
M02255_77_000000000-ADUT3_1_2116_25388_18113 T----AC---GT-AG-GGT--------ACT-A-G------C--G--T---T--GT-C-CGG-AA------TT-A--T-T--GG-GC-------GT--A-----AA-GA-GC-TC-------G-TA-G-G-T-G-------------G--T-TT-G-T-C-------
----GC----G-T-C-T--------G-C-T--G--TG--G-AA-AG--G-T-GC-C-G-----CT-T-AA------C-G-G-T-G-C-G--C-G----T-G--C-A---G--T-------G--GG-T-A---C-------G--G-G--CG--G-A-C----T-A-G-A-G-T--G---C-AG-----TA-G-G--------G-G-A-G---TC-T------GG--A--ATT----C-
C-T-G-GT--GT-A-G-CG-GT--G---A--A-A---TG-C-GC-AG--AT-A-TC--A-G--G-A-G-G-A-AC-A-CC---GG--T--G--GC-GAA-G--G-C--G-------G--G-A-C-T---CTG--G--GC-T-G---------------------T-T---A-C-T--GA--CG-C-T-G--A-GG--A-G-CG-A--AA-G-C---A-TG--GG-
G--AG-C-G-AA--------CA--GG Bacteria(100);Actinobacteria(100);Actinobacteria(100);Actinobacteridae(100);Actinomycetales(100);Micrococcineae(100);unclassified(100);unclassified(100);unclassified(100);

Otu0704 0 0 0 0 0 0 0 0 0 2 0 0 0 0 0 0 0 0 0 0 0 0 0 0 0 0 0 0
0 0 0 4 0 0 0 0 0 0 0 0 6 0 11 0 0 0 0 0 0 0 0 0 1 0 0 0 9
0 0 0 0 5 0 0 0 0 0 0 0 0 0 0 0 0 0 0 0 3 0 0 0 0 0 0 4 0
0 0 3 0 0 0 0 0 0 0 0 0 0 0 0 0 0 0 0 0 0 0 0 0 0 0 0
M02255_76_000000000-ADUNE_1_2106_26449_5125 G----AA---GC-AC-CG---------GCT-A-A------C-----T---C--CG-T--GC-CA------GC-A--G-C--CG-CG-------GT--A-----AT-AC-GG-A--------G-GG-T-G-C-A-------------A--G-CT-G-T-T-----------AA----
G-T-C-G--------G-A-A--G--TG--A-AA-TC--C-C-CG-G-G-----CT-T-AA------C-C-T-G-G-G-A--A-C----T-G--C-A---T--T-------T--GA-A-A---C-------T--G-G--CA--G-G-C----T-T-G-A-G-T--C---T-TG-----TA-G-A--------G-G-G-G---GG-T------AG--A--ATT----C-C-A-G-GT-
-GT-A-G-CG-GT--G---A--A-A---TG-C-GT-AG--AG-A-TC--T-G--G-A-G-G-A-AT-A-CC---GG--T--G--GC-GAA-G--G-C--G-------G--C-C-C-C---CTG--G--AC-A-A---------------------A-G---A-C-T--GA--CG-C-T-C--A-GG--T-G-CG-A--AA-G-C---G-TG--GG-G--AG-C-A-
AA--------CA--GG Bacteria(100);Proteobacteria(100);Gammaproteobacteria(100);Enterobacteriales(100);Enterobacteriaceae(100);Enteric_Bacteria_cluster(100);unclassified(100);unclassified(100);unclassified(100);

Otu0705 0 0 0 0 0 0 0 0 0 0 0 0 0 0 0 0 0 0 0 0 0 0 0 0 0 0 0 0
0 0 0 0 0 0 0 0 0 0 0 0 0 38 79 30 0 0 0 0 0 0 0 0 31 9 0 0 0
0 0 0 0 0 0 0 0 0 0 0 0 0 0 0 0 0 0 0 0 0 0 0 0 0 0 0 0 0
0 0 0 0 0 0 0 0 0 0 0 0 0 24 0 0 0 25 0 2 0 0 0 0 0 0 0
M02255_77_000000000-ADUT3_1_1114_10222_13542 G----AC---GG-AG-GGG--------GCT-A-G------C--G--T---T--GT-T-CGG-AA------TG-A--C-T--GG-GC-------GT--A-----AA-GG-GC-GC-------G-TA-G-G-C-G------------G--T-TT-T-T-T----
-------AA----G-T-G-A--------G-G-C--G--TG--A-AA-GC--C-C-TG-G-G-----CT-T-AA------C-C-C-A-G-G-A--G-G----T-G--C-G---T--T-------T--CA-T-A---C-------T--G-G--AA--G-A-C----T-T-G-A-G-T--A---C-GA-----GA-G-A--------G-G-A-A---AG-C------GG--A--ATT----
C-C-T-A-GT--GT-A-G-AG-GT--G---A--A-A---TT-C-GT-AG--AT-A-TT--A-G--G-A-A-G-A-AC-A-CC---AG--T--G--GC-GAA-G--G-C--G-------G--C-T-T-T---CTG--G--CT-C-G--------------------T-A---A-C-T--GA--CG-C-T-G--A-GG--C-G-CG-A--AA-G-C---G-TG--GG-
G--AG-C-A-AA--------CA--GG Bacteria(100);Proteobacteria(79);unclassified(79);unclassified(79);unclassified(79);unclassified(79);unclassified(79);unclassified(79);unclassified(79);

Otu0706 0 0 0 0 0 0 0 0 0 0 0 0 0 0 0 0 0 0 0 0 0 0 0 0 0 0 0 0
0 0 0 0 0 0 0 0 0 0 0 0 0 0 0 0 0 0 0 0 0 0 0 0 0 0 0 0 0
0 0 0 0 0 0 0 0 0 0 0 0 0 0 0 0 0 0 0 0 0 0 0 0 0 0 0 0 52
0 0 0 0 0 0 0 0 0 0 0 0 0 0 0 0 0 0 0 0 0 0 0 0 0 0 0
M02255_77_000000000-ADUT3_1_1106_4216_6191 T----AC---GT-AT-GGT--------GCA-A-G------C--G--T---T--AT-C-CGG-AT------TT-A--C-T--GG-GT-------GT--A-----AA-GG-GT-GC-------G-TA-G-G-T-G------------G--T-AT-G-G-C-----------AA-
---G-T-C-A--------G-A-A--G--TG--A-AA-GG--C-T-GG-G-G-----CT-C-AA------C-C-C-C-G-G-G--A-C----T-G--C-T---T--T-------T--GA-A-A---C-------T--G-T--CA--A-A-C----T-A-G-A-G-T--A---C-AG-----GA-G-A--------G-G-A-A---AG-C------GG--A--ATT----C-C-T-A-
GT--GT-A-G-CG-GT--G---A--A-A---TG-C-GT-AG--AT-A-TT--A-G--G-A-G-G-A-AC-A-CC---AG--T--G--GC-GAA-G--G-C--G-------G--C-T-T-T---CTG--G--AC-T-G--------------------A-A---A-C-T--GA--CA-C-T-G--A-GG--C-A-CG-A--AA-G-C---G-TG--GG-G--AG-
C-A-AA--------CA--GG Bacteria(100);Firmicutes(100);Clostridia(100);Clostridiales(100);Lachnospiraceae(100);unclassified(100);unclassified(100);unclassified(100);unclassified(100);

Otu0707 0 0 0 0 0 0 0 0 0 0 0 0 0 0 0 0 0 0 0 0 0 0 0 0 0 0 0 0
0 0 1 22 0 0 0 0 0 0 0 0 0 0 0 0 0 9 0 0 0 0 0 0 0 0 0 0 0
0 0 0 0 0 0 0 0 0 0 0 0 0 0 0 0 0 0 0 0 0 0 0 0 0 0 0 0 0
0 0 0 0 0 0 0 0 0 0 0 0 0 0 0 0 0 0 0 0 0 0 0 0 0 0 0
M02255_77_000000000-ADUT3_1_1111_18620_14640 G----AC---GT-AG-GGC--------GCG-A-G------C--G--T---T--GT-C-CGG-AT------TT-A--T-T--GG-GC-------GT--A-----AA-GA-GC-TC-------G-TA-G-G-C-G------------G--C-TT-G-T-C-----
------GC----G-T-C-G--------G-C-T--G--TG--A-AA-TC--C-C-GT-G-G-----CT-C-AA------C-T-G-C-G-G-G--C-T----T-G--C-A---G--C-------C--GA-T-A---C-------G--G-G--CA--G-G-C----T-A-G-A-G-T--T---C-GG-----TA-G-G--------G-G-A-G---AC-T------GG--A--ATT----
C-C-T-G-GT--GT-A-G-CG-GT--G---A--A-A---TG-C-GC-AG--AT-A-TC--A-G--G-A-G-G-A-AC-A-CC---GG--T--G--GC-GAA-G--G-C--G-------G--G-T-C-T---CTG--G--GC-C-G--------------------A-T---A-C-T--GA--CG-C-T-G--A-GG--A-G-CG-A--AA-G-C---G-TG--
GG-G--AG-C-G-AA--------CA--GG Bacteria(100);Actinobacteria(100);Actinobacteria(100);Actinobacteridae(100);Actinomycetales(100);Micromonosporineae(100);Micromonosporaceae(100);unclassified(69);unclassified(69);

Otu0708 0 0 0 0 0 0 0 0 0 0 0 4 0 0 1 0 0 4 0 0 0 0 0 0 0 0 0 0
0 0 0 0 0 0 0 0 0 0 0 0 0 0 0 0 0 0 0 0 0 0 0 0 0 0 0 0 0
0 0 0 0 0 0 0 0 0 0 0 0 0 0 0 0 0 0 0 0 0 0 0 0 0 0 0 0 0
0 0 0 0 0 2 0 0 0 0 0 0 0 0 0 0 0 0 0 0 0 0 0 0 0 0 0
M02255_77_000000000-ADUT3_1_1108_19997_5721 C----TA---AC-TC-TGT--------GCC-A-G------C-----A---G--CC-G-CGG-TA------AT-A--C-A--GA-GG-------GTG-C-----AA-GC-GT-TA-------A-TC-G-GAT-G------------G--C-CA-A-T-T-----------AA-
---G-T-C-A--------A-A-T--G--TG--A-AA-TC--C-C-CG-A-G-----CT-T-AA------C-T-T-G-G-G-A--A-T----T-G--C-A---T--T-------C--GA-T-A---C-------T--G-G--TT--G-G-C----T-A-G-A-G-T--A---T-GG-----GA-G-A--------G-G-A-T---GG-T------AG--A--ATT----C-C-A-G-
GT--GT-A-G-CG-GT--G---A--A-A---TG-C-GT-AG--AG-A-TC--T-G--G-A-G-G-A-AT-A-CC---GA--T--G--GC-GAA-G--G-C--A-------G--C-C-A-T---CTG--G--CC-T-A--------------------A-T---A-C-T--GA--CA-C-T-G--A-GG--T-A-CG-A--AA-G-C---A-TG--GG-G--AG-C-
A-AA--------CA--GG Bacteria(100);Proteobacteria(100);Gammaproteobacteria(100);Pseudomonadales(100);Moraxellaceae(100);Acinetobacter(100);unclassified(100);unclassified(100);unclassified(100);

Otu0709 0 0 0 0 0 0 0 0 0 0 0 0 0 0 0 0 0 0 0 0 0 0 0 0 0 0 0 0
0 0 0 0 0 0 0 0 0 0 0 0 0 0 0 0 0 0 0 0 0 0 0 0 12 0 0 0 0
0 0 0 0 0 0 0 0 0 0 0 0 0 0 0 0 0 0 0 0 0 0 0 0 0 0 0 0 0
0 0 0 0 0 0 0 0 0 0 0 0 4 0 0 0 0 0 0 0 0 0 0 0 0 0 0
M02255_77_000000000-ADUT3_1_2115_24363_5982 T----AC---GA-AG-GGG--------GCT-A-G------C--G--T---T--GC-T-CGG-AA------TG-A--C-T--GG-GC-------GT--A-----AA-GG-GC-GC-------G-TA-G-G-C-G------------G--T-TT-A-C-A-----------
CA----G-T-C-A--------G-G-T--G--TG--A-AA-TT--C-C-CG-G-G-----CT-T-AA------C-C-T-G-G-G-G--G-C----T-G--C-A---T--T-------T--GA-T-A---C-------G--T-G--TA--G-A-C----T-A-G-A-G-T--G---T-GA-----GA-G-A--------G-G-G-T---TG-T------GG--A--ATT----C-T-C-
A-GT--GT-A-G-AG-GT--G---A--A-A---TT-C-GT-AG--AT-A-TT--G-G--G-A-A-G-A-AC-A-CC---GG--T--G--GC-GAA-G--G-C--G-------G--C-A-A-C---CTG--G--CT-C-A--------------------T-C---A-C-T--GA--CG-C-T-G--A-GG--C-G-CG-A--AA-G-C---G-TG--GG-G--
AG-C-A-AA--------CA--GG Bacteria(100);Proteobacteria(100);Alphaproteobacteria(100);Rhodospirillales(100);Acetobacteraceae(100);unclassified(100);unclassified(100);unclassified(100);unclassified(100);

Otu0710 0 0 0 0 0 0 0 0 0 0 16 0 0 0 0 3 0 0 0 0 0 0 0 0 0 0 0 0
0 0 0 0 0 0 0 0 0 0 0 0 0 0 0 0 0 0 0 0 0 0 0 0 0 0 0 0
0 0 0 0 0 0 0 0 0 0 0 0 0 0 0 0 0 0 0 0 0 0 0 0 0 0 0 0
0 0 0 0 0 0 0 0 0 0 0 0 0 0 0 0 0 0 0 0 0 0 0 0 0 0 0
M02255_76_000000000-ADUNE_1_1117_16642_9063 C----GG---CT-AA-CTC--------TGT-G-C------C--A--G---C--AG-C-CGC-GG------TA-A--T-A--CG-TA-------G---G-----TG-GC-AA-GC-------G-TT-GTC-C-G-------------G--T-TT-T-T-T-----------AA---
-G-T-C-T--------G-A-T--G--TG--A-AA-GC--C-C-CC-G-G-----CT-C-AA------C-C-G-G-G-G-A--G------G-GT-C-A---T--T-------G--GA-A-A---C-------T--G-G--AG--A-A-C----T-T-G-A-G-T--G---C-AG-----AA-G-A--------G-G-A-G---AG-T------GG--A--ATT----C-C-A-C-
GT--GT-A-G-CG-GT--G---A--A-A---TG-C-GT-AG--AT-A-TG--T-G--G-A-G-G-A-AC-A-CC---AG--T--G--GC-GAA-G--G-C--G-------A--C-T-C-T---CTG--G--TC-T-G--------------------T-A---A-C-T--GA--CG-C-T-G--A-GG--C-T-CG-A--AA-G-C---G-TG--GG-G--AG-C-
A-AA--------CA--GG Bacteria(100);Firmicutes(100);Bacilli(100);unclassified(100);unclassified(100);unclassified(100);unclassified(100);unclassified(100);unclassified(100);

Otu0711 0 0 0 0 0 0 0 0 0 0 0 0 0 1 0 18 0 0 1 0 0 1 0 0 0 0 0 0
0 0 0 0 0 0 0 0 0 0 0 0 0 0 0 0 0 0 0 0 0 0 0 0 0 0 0 1
0 0 0 0 0 0 0 0 0 0 0 0 0 0 0 0 0 0 0 0 0 0 0 0 0 0 0 0
0 0 0 0 0 0 0 0 0 0 0 0 0 0 0 0 0 0 0 0 0 0 0 0 0 0 0
M02255_77_000000000-ADUT3_1_1110_27116_9784 A----AG---CC-AC-G-G--------CTA-A-C------T--A--C---G--TG-C-CAG-CA------GC-C--G-C--GG-TA-------AT--A-----CG-TA-GG-TG-------G-CA-A-G-C-G-------------G--T--T-G-T-C-----------CG---
-G-A-C-T--------G-A-T--G--TG--A-AA-GC--C-C-CC-G-G-----CT-C-AA------C-C-G-G-G-G-A--G------G-GT-C-A---T--T-------G--GA-A-A---C-------T--G-G--GA--G-A-C----T-T-G-A-G-G--A---C-AG-----AA-G-A--------G-G-A-G---AG-T------GG--A--ATT----C-C-A-A-
GT--GT-A-G-CG-GT--G---A--A-A---TG-C-GT-AG--AT-A-TT--T-G--G-A-G-G-A-AC-A-CC---AG--T--G--GC-GAA-G--G-C--G-------G--C-T-C-T---CTG--G--TC-T-G--------------------T-T---A-C-T--GA--CG-C-T-G--A-GG--C-G-CG-A--AA-G-C---G-TG--GG-G--AG-C-
A-AA--------CA--GG Bacteria(100);Firmicutes(100);Bacilli(100);Bacillales(100);unclassified(100);unclassified(100);unclassified(100);unclassified(100);unclassified(100);

Otu0712 0 0 0 0 0 0 0 0 0 0 55 1 0 0 0 0 0 0 0 0 0 0 1 0 0 0 0 0
0 0 1 0 0 0 0 0 0 0 1 0 0 0 0 0 0 0 0 0 0 0 0 0 0 0 0 0
0 0 0 0 0 0 0 0 5 0 0 0 0 0 1 0 0 0 0 0 0 0 0 0 0 0 0 0
0 0 0 0 0 0 0 1 1 0 0 0 0 0 0 0 0 0 3 0 0 0 0 0 0 0 0
M02255_76_000000000-ADUNE_1_2101_25339_5746 A----AG---CC-AC-G-G--------CTA-A-C------T--A--C---G--TG-C-CAG-CA------GC-C--G-C--GG-TA-------AT--A-----CG-TA-GG-TG-------G-CA-A-G-C-G-------------T--TGTC-C-G-G-----------AA-
---G-T-C-T--------G-A-T--G--TG--A-AA-GC--C-C-CC-G-G-----CT-C-AA------C-C-G-G-G-G-A--G------G-GT-C-A---T--T-------G--GA-A-A---C-------T--G-G--AG--A-A-C----T-T-G-A-G-T--G---C-AG-----AA-G-A--------G-G-A-G---AG-T------GG--A--ATT----C-C-A-C-
GT--GT-A-G-CG-GT--G---A--A-A---TG-C-GT-AG--AT-A-TG--T-G--G-A-G-G-A-AC-A-CC---AG--T--G--GC-GAA-G--G-C--G-------A--C-T-C-T---CTG--G--TC-T-G--------------------T-A---A-C-T--GA--CG-C-T-G--A-GG--C-T-CG-A--AA-G-C---G-TG--GG-G--AG-C-
A-AA--------CA--GG Bacteria(100);Firmicutes(100);Bacilli(100);unclassified(100);unclassified(100);unclassified(100);unclassified(100);unclassified(100);unclassified(100);

Otu0713 0 0 0 0 0 0 0 0 0 0 0 0 0 0 0 0 0 0 0 0 0 0 0 0 0 0 0 0
0 0 0 85 0 0 0 0 0 0 0 0 0 0 0 0 0 0 0 0 0 0 0 0 0 0 0 0
0 0 0 0 0 0 0 0 0 0 0 0 0 0 0 0 0 0 0 0 0 0 0 0 0 0 0 0
0 0 0 0 0 0 0 0 0 0 0 0 0 0 0 0 0 0 0 0 0 0 0 0 0 0 0
M02255_77_000000000-ADUT3_1_2105_14360_10388 G----AC---GG-AG-GGC--------GCG-A-G------C--G--T---T--AT-T-CGT-TT------TG-A--T-T--GG-GT-------GT--A-----AA-GG-GT-AT-------G-TA-G-G-C-G-------------GCCC-CT-A-G-T----
-------TT----T-T-T-G--------G-C-T--A--AA--A-AA-GC--G-G-AG-T-G-----TT-C-CT------A-T-G-C-T-A-T--A-T----G-G--C-C---T--T-------T--GA-A-A-----------A--A-G--GG--G-G-C----T-T-G-T-G-T--T---G-AT-----GA-T-A--------G-G-T-C---GG-G------GC--A--AGT----T-
C-T-T-AT--GT-A-G-GG-GT--A---G--A-A---TC-C-CA-CC--AT-A-TG--A-G--A-A-C-G-A-AC-G-AC---AGC-T--G--GC-GAA-G--G-C--G-------C--C-C-T-T---CCC--G---C-T-T--------------------C-A---A-C-A--GA--CG-C-T-A--A-GG--T-A-CG-G--AA-G-C---T-TA--GG-T--
AG-C-A-AA--------CG--GG Bacteria(100);unclassified(100);unclassified(100);unclassified(100);unclassified(100);unclassified(100);unclassified(100);unclassified(100);unclassified(100);

Otu0714 0 0 0 0 0 0 0 0 0 0 44 5 0 0 1 1 0 0 0 0 0 3 0 0 0 0 0 0
0 0 0 0 0 0 0 0 0 0 0 0 0 0 0 0 0 1 0 0 0 0 0 0 0 0 0 0
0 0 0 0 0 0 0 0 3 0 1 0 0 0 0 0 0 0 0 0 0 0 0 0 0 0 0 0
0 0 0 0 0 0 0 0 0 0 0 0 0 0 0 0 0 0 0 0 0 0 0 0 0 0 0
M02255_76_000000000-ADUNE_1_2106_27273_12167 .....AC----T-AC-GTG--------CCA-G-C------A--G--C---T--G--C-GGT-AA------TA-C--G-T--AG-GT-------GG--C-----AA-GC-G--TT-------G----T-C-C-G-------------G--A-TC-T-T-T-----------
AA----G-T-C-T--------G-A-T--G--TG--A-AA-GC--C-C-CC-G-G-----CT-C-AA------C-C-G-G-G-G-A--G------G-GT-C-A---T--T-------G--GA-A-A---C-------T--G-G--AG--A-A-C----T-T-G-A-G-T--G---C-AG-----AA-G-A--------G-G-A-G---AG-T------GG--A--ATT----C-C-
A-C-GT--GT-A-G-CG-GT--G---A--A-A---TG-C-GT-AG--AT-A-TG--T-G--G-A-G-G-A-AC-A-CC---AG--T--G--GC-GAA-G--G-C--G-------A--C-T-C-T---CTG--G--TC-T-G--------------------T-A---A-C-T--GA--CG-C-T-G--A-GG--C-T-CG-A--AA-G-C---G-TG--GG-G--
AG-C-A-AA--------CA--GG Bacteria(100);Firmicutes(100);Bacilli(100);unclassified(100);unclassified(100);unclassified(100);unclassified(100);unclassified(100);unclassified(100);

Otu0715 0 0 0 0 0 0 22 0 0 0 0 0 0 0 0 0 0 0 0 0 0 0 0 0 0 0 0 0
0 0 0 0 0 0 0 0 0 0 0 0 0 0 0 0 0 0 0 0 0 0 0 0 0 0 0 0 0
0 0 0 0 0 0 0 0 0 0 0 0 0 0 0 0 0 0 0 0 0 0 0 0 0 0 0 0 0
0 0 0 0 0 0 0 8 0 0 0 0 0 0 0 0 0 0 0 0 0 0 0 0 0 0 0
M02255_77_000000000-ADUT3_1_1107_3904_10302 T----AC---GG-AG-GGA--------GCT-A-G------C--G--T---T--GT-T-CGG-AA------TT-A--C-T--GG-GC-------GT--A-----AA-GC-GT-AC-------G-TA-G-G-C-G------------G--T-TA-T-T-C----------AA-
---G-T-C-A--------G-A-G--G--TG--A-AA-GC--C-T-GG-A-G-----CT-C-AA------C-T-C-C-A-G-A--A-C----T-G--C-C---T--T-------T--GA-A-A---C-------T--A-G--AT--A-G-C----T-A-G-A-A-T--C---T-TG-----GA-G-A--------G-G-T-G---AG-T------GG--A--ATT----C-C-G-A-
GT--GT-A-G-AG-GT--G---A--A-A---TT-C-GT-AG--AT-A-TT--C-G--G-A-A-G-A-AC-A-CC---AG--T--G--GC-GAA-G--G-C--G-------A--C-T-C-A---CTG--G--AC-A-A--------------------G-T---A-T-T--GA--CG-C-T-G--A-GG--T-A-CG-A--AA-G-C---G-TG--GG-G--AG-C-
A-AA--------CA--GG Bacteria(100);Proteobacteria(100);Alphaproteobacteria(100);Sphingomonadales(100);unclassified(100);unclassified(100);unclassified(100);unclassified(100);unclassified(100);

Otu0716 0 0 0 0 0 0 0 0 0 0 0 0 0 0 0 0 0 0 0 0 0 0 0 0 0 0 0 0
0 0 0 0 0 0 0 0 0 0 0 0 0 0 0 0 0 0 0 0 0 0 0 0 0 0 0 0 0
0 0 0 0 0 0 0 0 0 0 0 0 0 0 0 0 0 0 0 0 0 0 0 0 0 0 0 0 0
0 0 0 0 0 0 0 0 0 0 0 0 0 0 0 0 0 0 0 0 0 131 6 0 5 0 0
M02255_76_000000000-ADUNE_1_2104_4629_10581 .....AC----T-AC-GT---------GCC-A-G------C--A--G---C--CG-C-GGT-AA------TA-C--G-T--AG-GT-------GG--C-----AA-GC-G--TT-------G----T-C-C-G------------G--T-CC-G-T-T----------AA----G-
T-C-T--------G-A-T--G--TG--A-AA-GC--C-C-CC-G-G-----CT-C-AA------C-C-G-G-G-G-A--G------G-GT-C-A---T--T-------G--GA-A-A---C-------T--G-G--CG--A-A-C----T-T-G-A-G-T--G---C-AG-----AA-G-A--------G-G-A-G---AG-T------GG--A--ATT----C-C-A-T-GT--
GT-A-G-CG-GT--G---A--A-A---TG-C-GT-AG--AT-A-TA--T-G--G-A-G-G-A-AC-A-CC---AG--T--G--GC-GAA-G--G-C--G-------G--C-T-C-T---CTG--G--TC-T-G--------------------T-A---A-C-T--GA--CG-C-T-G--A-GG--C-T-CG-A--AA-G-C---G-TG--GG-T--AG-C-A-
AA--------CA--GG Bacteria(100);Firmicutes(100);Bacilli(100);unclassified(85);unclassified(85);unclassified(85);unclassified(85);unclassified(85);unclassified(85);

Otu0717 0 0 0 0 0 0 0 0 0 0 0 0 0 1 0 0 0 0 0 0 2 0 0 0 3 2 0 0
0 0 0 0 0 0 0 0 0 0 1 1 0 0 0 0 1 0 0 0 1 0 0 0 0 0 0 0 0
0 0 0 0 0 0 0 0 0 0 0 0 0 0 0 0 0 0 0 0 0 0 1 0 0 0 0 0 0
3 0 0 0 0 0 0 0 0 0 0 0 0 0 0 0 0 0 0 0 0 0 0 0 0 0 0
M02255_76_000000000-ADUNE_1_2114_21549_9896 G----AA---GA-AG-CAC--------CGG-C-T------A--A--C---T--CC-G-TGC-CA------GC-A--G-C--CG-CG-------GT--A-----AT-AC-GG-AG-------G-GT-G-C-G-A------------G--C-GT-T-A-A----------
TC----G-G-A-A--------G-A-T--G--TG--A-AA-GC--C-C-GG-G-G-----CT-C-AA------C-C-T-C-G-G-A--A-C----A-G--C-A---T--T-------T--TG-A-A---C-------T--G-G--CA--G-A-C----T-A-G-A-G-T--C---T-TG-----TA-G-A--------G-G-G-G---GG-T------AG--A--ATT----T-C-A-
G-GT--GT-A-G-CG-GT--G---A--A-A---TG-C-GT-AG--AG-A-TC--T-G--A-A-G-G-A-AT-A-CC---GG--T--G--GC-GAA-G--G-C--G-------G--C-C-C-C---CTG--G--AC-A-A--------------------A-G---A-C-T--GA--CG-C-T-C--A-GA--T-G-CG-A--AA-G-C---G-TG--GG-G--
AG-C-A-AA--------CA--GG Bacteria(100);Proteobacteria(100);Gammaproteobacteria(100);Vibrionales(100);Vibrionaceae(100);unclassified(100);unclassified(100);unclassified(100);unclassified(100);

Otu0718 0 0 0 0 0 0 0 0 0 0 0 0 0 0 0 0 0 0 0 4 0 0 5 0 0 0 0 0
0 0 0 0 0 0 0 0 0 0 0 0 0 0 3 0 0 0 0 0 0 0 0 0 0 0 0 0 0
0 0 0 0 0 0 0 0 0 0 0 0 0 0 0 0 0 0 0 0 0 0 0 0 0 0 0 0 24
0 0 17 0 0 0 0 0 0 0 0 0 0 0 0 0 0 0 0 0 0 0 0 0 0 0 0
M02255_77_000000000-ADUT3_1_1108_24720_4276 T----AC---GT-AG-GGG--------TCG-A-G------C--G--T---T--GT-C-CGG-AG------TT-A--C-T--GG-GC-------GT--A-----AA-GC-GC-GC-------G-CA-G-G-T-G------------G--C-TC-A-T-T----------AC-
---G-C-C-C--------G-G-C--G--TG--A-AA-GC--C-C-CC-G-G-----CT-C-AA------C-C-G-G-G-G-A--A-G----G-T--C-G---T--C-------G--GG-T-A---C-------G--G-G--TG--A-G-C----T-T-G-A-G-G--G---T-AT-----CA-G-G--------G-G-C-A---GG-T------AG--A--ATT----C-C-C-G-
GT--GT-A-G-TG-GT--G---A--A-A---TG-C-GT-AG--AG-A-TC--G-G--G-A-G-G-A-AT-A-CC---CG--T--G--GC-G-A-A-GG-C--G-------G--C-C-T-G---CTG--G--GA-T-A--------------------C-A---C-C-T--GA--CA-C-T-G--A-GG--C-G-CG-A--AG-G-C---G-TG--GG-G--AG-
C-G-AA--------CG--GG Bacteria(100);Chloroflexi(100);Thermomicrobia(100);JG30-KF-CM45(100);unclassified(100);unclassified(100);unclassified(100);unclassified(100);unclassified(100);

Otu0719 0 0 0 0 0 0 0 0 0 0 0 0 0 0 0 0 0 0 0 0 0 0 0 0 0 0 0 0
0 0 0 0 0 0 0 0 0 0 0 0 0 0 0 0 0 0 0 0 0 0 0 0 0 0 0 0 0
0 0 0 0 0 0 0 0 0 0 0 0 0 0 24 2 0 0 0 0 0 0 0 0 0 0 0 0 0
0 0 0 0 0 0 0 0 0 0 0 0 0 0 0 0 0 0 0 0 0 0 69 0 0 0 0
M02255_76_000000000-ADUNE_1_1102_8376_20695 T----AC---GT-AG-GTG--------GCA-A-G------C--G--T---T--GT-C-CGG-AT------TT-A--C-T--GG-GC-------GT--A-----AA-GG-GA-GC-------G-TA-G-G-C-G------------G--A-C--T-TTT----------AA-
---G-T-G-G--------G-A-T--G--TG--A-AA-TA--C-C-CG-G-G-----CT-T-AA------C-C-T-G-G-G-T--G-C----T-G--C-A---T--T-------C--CA-A-A---C-------T--G-G--AA--G-T-C----T-A-G-A-G-T--A---C-AG-----GA-G-G--------G-G-A-A---AG-T------GG--A--ATT----C-C-T-A-
GT--GT-A-G-CG-GT--G---A--A-A---TG-C-GT-AG--AG-A-TT--A-G--G-A-A-G---AA-CACC---AG--T--G--GC-GAA-G--G-C--G-------G--C-T-T-T---CTG--G--AC-T-G--------------------T-A---A-C-T--GA--CG-C-T-G--A-GG--C-T-CG-A--AA-G-C---G-TG--GG-G--AG-
C-A-AA--------CA--GG Bacteria(100);Firmicutes(100);Clostridia(100);Clostridiales(100);Clostridiaceae(100);Clostridium(73);unclassified(73);unclassified(73);unclassified(73);

Otu0720 0 35 0 0 0 0 14 0 9 0 0 0 0 0 0 0 0 0 0 0 0 0 0 0 0 0 0 0
0 0 0 0 0 0 0 0 0 0 0 0 0 0 0 0 0 0 0 0 0 0 0 0 0 0 0 0
0 0 0 0 0 0 0 0 0 0 0 0 0 0 0 0 0 0 0 0 0 3 0 0 0 0 0 0
0 0 0 0 0 0 0 0 0 0 0 0 0 0 0 0 0 0 0 0 0 0 0 0 0 0 0
M02255_76_000000000-ADUNE_1_1117_8307_6013 T----AC---GT-AG-GGA--------GCA-A-G------C--G--T---T--AT-C-CGG-AT------TT-A--C-T--GG-GT-------GT--A-----AA----G--G--------G-C----G-C-----------------G--C----------------AA----G-T-
T-G--------G-A-A--G--TG--A-AA-TC--T-A-TG-G-G-----CT-T-AA------C-C-C-A-T-A-A--A-C----T-G--C-T---T--T-------C--AA-A-A---C-------T--G-C--TG--G-T-C----T-T-G-A-G-T--G---A-TG-----GA-G-A--------G-G-C-A---GG-C------GG--A--ATT----C-C-G-T-GT--GT-
A-G-CG-GT--G---A--A-A---TG-C-GT-AG--AT-A-TA--C-G--G-A-G-G-A-AC-A-CC---AG--T--G--GC-GAA-G--G-C--G-------G--C-C-T-G---CTG--G--AC-A-T--------------------T-A---A-C-T--GA--CG-C-T-G--A-GG--C-G-CG-A--AA-G-C---G-TG--GG-G--AG-C-A-AA--
------CA--GG Bacteria(100);Firmicutes(100);Clostridia(100);Clostridiales(100);Ruminococcaceae(100);Incertae_Sedis(100);unclassified(100);unclassified(100);unclassified(100);

Otu0721 0 0 0 0 0 0 1 20 0 0 0 0 0 0 0 0 0 0 0 0 0 0 0 0 0 0 0 0
0 0 0 0 0 0 0 0 0 0 0 0 0 0 0 0 0 0 0 0 0 0 0 0 2 0 13 0
0 0 0 0 0 0 0 0 0 0 0 0 0 0 0 0 0 2 0 0 0 0 0 0 0 0 27 0
0 0 0 13 0 0 0 0 0 0 0 0 0 0 0 0 17 0 0 0 0 0 0 0 0 0 0
M02255_76_000000000-ADUNE_1_1102_9679_13523 T----GG---CT-A--ACT--------ACG-T-G------C--C--A---G--CA-G-CCG-CG------GT-A--A-T--AC-GT-------AG--A-----TG-GC-AA-GC-------G-TT-GTC-C-G-------------G--T-TC-T-T-T-----------AA---
-G-T-C-T--------G-A-T--G--TG--A-AA-GC--C-C-CT-G-G-----CT-T-AA------C-C-A-G-G-G-A--G------G-GT-C-A---T--T-------G--GA-A-A---C-------T--G-G--AG--A-A-C----T-T-G-A-G-T--G---C-AG-----AA-G-A--------G-G-A-A---AG-T------GG--A--ATT----C-C-A-T-
GT--GT-A-G-CG-GT--G---A--A-A---TG-C-GT-AG--AT-A-TA--T-G--G-A-G-G-A-AC-A-CC---AG--T--G--GC-GAA-G--G-C--G-------A--C-T-T-T---CTG--G--TC-T-G--------------------T-A---A-C-T--GA--CG-C-T-G--A-GG--C-T-CG-A--AA-G-C---A-TG--GG-G--AG-C-
A-AA--------CA--GG Bacteria(100);Firmicutes(100);Bacilli(100);Lactobacillales(100);Carnobacteriaceae(100);Desemzia(100);unclassified(100);unclassified(100);unclassified(100);

Otu0722 0 0 0 0 0 12 0 0 0 0 0 0 0 0 0 0 0 0 0 0 0 0 0 0 0 0 0 25
10 0 0 0 0 0 0 0 0 0 67 24 0 0 0 0 18 0 0 0 0 0 0 0 0 16 0 0 0
0 0 0 0 0 0 0 0 0 0 0 0 0 0 3 0 5 0 0 0 0 0 0 0 0 0 0 60
25 0 0 0 0 0 0 0 0 0 0 0 0 0 0 0 0 0 0 0 0 0 0 0 0 0 0
M02255_77_000000000-ADUT3_1_1118_20060_18242 T----AC---GG-AG-GGG--------GTT-A-G------C--G--T---T--GT-T-CGG-AA------TG-A--C-T--GG-GC-------GT--A-----AA-GC-GC-GC-------G-TA-G-G-C-G-------------G--G-CT-G-G-A---
--------AA----G-T-C-A--------G-A-G--G--TG--A-AA-GC--C-C-GG-G-G-----CT-C-AA------C-C-C-C-G-G-A--A-T----G-G--C-C---T--T-------T--GA-A-A---C-------T--C-C--CA--G-C-C----T-T-G-A-G-T--T---C-GA-----GA-G-A--------G-G-T-G---AG-T------GG--A--ATT----
C-C-G-A-GT--GT-A-G-AG-GT--G---A--A-A---TT-C-GT-AG--AT-A-TT--C-G--G-A-G-G-A-AC-A-CC---AG--T--G--GC-GAA-G--G-C--G-------G--C-T-C-A---CTG--G--CT-C-G--------------------A-C---A-C-T--GA--CG-C-T-G--A-GG--T-G-CG-A--AA-G-C---G-TG--GG-
G--AG-C-A-AA--------CA--GG Bacteria(100);Proteobacteria(100);Alphaproteobacteria(100);Rhodobacterales(100);Rhodobacteraceae(100);unclassified(100);unclassified(100);unclassified(100);unclassified(100);

Otu0723 0 0 0 0 0 0 0 0 0 0 0 0 0 0 0 0 0 0 0 0 0 0 0 0 0 0 0 0
0 0 0 0 0 0 0 0 0 0 0 0 0 0 0 0 0 0 0 0 0 8 0 0 0 0 0 0
0 0 0 0 0 0 0 0 0 0 0 0 0 0 0 0 0 0 0 0 0 0 0 0 0 0 0 0
0 0 0 0 0 0 0 0 0 0 0 0 0 0 0 0 0 0 0 0 0 10 0 0 0 0 0
M02255_77_000000000-ADUT3_1_2115_26550_11434 T----AC---GT-AG-GGG--------CCG-A-G------C--G--T---T--GT-C-CGG-AG------TT-A--C-T--GG-GC-------GT--A-----AA-GC-GT-GC-------G-CA-G-G-C-G-------------G--C-GC-G-T-T----
-------CT----G-C-C-C--------G-G-C--G--TG--A-AA-GC--C-C-CC-G-G-----CT-C-AA------C-C-G-G-G-G-A--A-C----G-T--C-G---T--C-------G--GG-G-A---C-------G--G-G--CG--C-G-C----T-T-G-A-G-G--G---C-GG-----CA-G-G--------G-G-C-T---GG-T------GG--A--ACT---
---CCC-G-GT--GT-A-G-TG-GT--G---A--A-A---TG-C-GT-AG--AG-A-TC--G-G--G-A-A-G-A-AC-A-CC---TG--T--G--GC-GAA-G--G-C--G-------G--C-C-A-G---CTG--G--GC-C-G--------------------G-C---C-C-T--GA--CG-C-T-G--A-GG--C-A-CG-A--AG-G-C---G-TG--
GG-G--AG-C-G-AA--------CG--GG Bacteria(100);Chloroflexi(100);Thermomicrobia(100);JG30-KF-CM45(100);unclassified(100);unclassified(100);unclassified(100);unclassified(100);unclassified(100);

Otu0724 0 0 0 0 0 0 0 0 0 0 0 0 0 0 0 0 0 0 0 0 0 0 0 0 0 0 0 0
0 0 0 0 0 0 0 0 0 0 0 0 0 0 0 0 0 0 0 0 0 0 0 0 0 0 0 0
0 0 0 0 0 0 0 0 0 0 0 0 0 0 0 0 0 0 0 0 0 0 0 0 0 0 0 0
0 46 0 0 0 0 0 0 0 0 0 0 0 0 0 0 0 0 0 0 0 38 0 0 0 0 0
M02255_77_000000000-ADUT3_1_2113_20797_20817 T----AC---GA-AG-GGG--------GCT-A-G------C--G--T---T--GT-T-CGG-AA------TT-A--C-T--GG-GC-------GT--A-----AA-GC-GC-AC-------G-TA-G-G-C-G-------------G--A-TT-G-G-T-----
------CA----G-T-T-A--------G-G-G--G--TG--A-AA-TC--C-C-GG-A-G-----CT-C-AA------C-T-C-C-G-G-A--A-C----T-G--C-C---T--T-------T--AA-T-A---C-------T--G-C--CA--G-T-C----T-A-G-A-G-A--C---C-GA-----GA-G-A--------G-G-T-A---AG-T------GG--A--ACT----C-
C-T-A-GT--GT-A-G-AG-GT--G---G--A-A---TT-C-GT-AG--AT-A-TT--A-G--G-A-A-G-A-AC-A-CC---AG--T--G--GC-GAA-G--G-C--G-------G--C-T-T-A---CTG--G--CT-C-G--------------------G-A---A-C-T--GA--CG-C-T-G--A-GG--T-G-CG-A--AA-G-C---G-TG--GG-
G--AG-C-A-AA--------CA--GG Bacteria(100);Proteobacteria(100);Alphaproteobacteria(100);Rhizobiales(100);Hyphomicrobiaceae(100);Devosia(100);unclassified(100);unclassified(100);unclassified(100);

Otu0725 0 0 0 0 0 0 0 0 0 0 0 0 0 0 0 0 0 0 0 0 0 0 0 0 0 0 0 0
0 0 0 0 0 0 0 0 0 0 0 0 0 0 0 0 0 0 0 0 0 0 0 0 0 0 0 0
0 0 0 0 0 0 0 0 0 0 0 0 0 0 0 0 0 35 0 0 0 0 0 0 0 0 0 0
0 0 0 0 0 0 0 4 0 0 0 0 0 0 0 0 0 0 0 0 0 0 0 0 0 0 0
M02255_77_000000000-ADUT3_1_2114_17653_20849 T----AC---GT-AG-GGG--------GCG-A-G------C--G--T---T--GT-C-CGT-AT------TT-A--T-T--GG-GC-------GT--A-----AA-GG-GT-TC-------G-CA-G-G-T-G------------G--C-CT-G-T-T------
-----AA----G-T-C-A--------G-A-T--G--TG--A-AA-GA--T-C-AC-A-G-----CT-C-AA------C-T-G-T-G-G-T--A------A-G--C-A---T--T-------T--GA-A-A---C-------T--G-A--AA--G-G-C----T-T-G-A-G-T--T---A-AG-----GA-G-A--------G-G-A-A---AG-T------GG--A--ATT----C-
C-T-A-GT--GT-A-G-CG-GT--G---A--A-A---TG-C-GT-AG--AT-A-TT--A-G--G-A-G-G-A-AT-A-CC---AG--T--G--GC-GAA-G--G-C--G-------A--C-T-T-T---CTG--G--AC-T-T--------------------A-T---A-C-T--GA--CA-C-T-G--A-GG--A-A-CG-A--AA-G-C---G-TG--GG-G--
AG-C-A-AA--------CA--GG Bacteria(100);Firmicutes(100);Clostridia(100);Clostridiales(100);unclassified(90);unclassified(90);unclassified(90);unclassified(90);unclassified(90);

Otu0726 0 0 0 0 0 0 0 0 0 0 0 0 0 0 0 0 0 0 0 0 0 0 0 0 0 0 0 0
0 0 0 0 0 0 0 0 0 0 0 0 0 0 0 0 0 0 0 0 0 0 0 0 0 0 0 0
0 0 0 0 0 0 0 0 0 0 0 0 0 0 0 0 0 0 0 0 0 0 0 0 0 0 30 0
0 0 0 0 0 0 0 0 0 0 0 0 0 0 0 0 0 0 0 0 0 0 0 0 0 0 0
M02255_77_000000000-ADUT3_1_2103_17387_17374 T----AC---GT-AG-GGG--------GCA-A-G------C--G--T---T--AT-C-CGG-AT------TT-A--C-T--GG-GT-------GT--A-----AA-GG-GA-GC-------G-CA-G-G-C-G------------G--C-GA-G-G-C----
-------AA----G-T-C-T--------G-A-T--G--TG--A-AA-GC--C-C-GG-G-G-----CT-C-AA------C-C-C-C-G-G-G--A-C----T-G--C-A---T--T-------G--GA-A-A---C-------T--G-T--CT--T-G-C----T-T-G-A-G-T--G---T-CG-----GA-G-A--------G-G-T-A---AG-T------GG--A--ATT----
C-C-T-A-GT--GT-A-G-CG-GT--G---A--A-A---TG-C-GT-AG--AT-A-TT--A-G--G-A-G-G-A-AC-A-CC---AG--T--G--GC-GAA-G--G-C--G-------G--C-T-T-A---CTG--G--AC-G-A--------------------C-A---A-C-T--GA--CG-C-T-G--A-GG--C-T-CG-A--AA-G-C---G-TG--GG-
G--AG-C-A-AA--------CA--GG Bacteria(100);Firmicutes(100);Clostridia(100);Clostridiales(100);Lachnospiraceae(100);unclassified(100);unclassified(100);unclassified(100);unclassified(100);

Otu0727 0 0 0 0 0 0 0 0 0 0 0 0 0 0 0 0 0 0 0 0 0 0 0 0 0 0 0 0
0 0 0 0 0 0 0 0 0 0 0 0 0 0 0 0 0 0 0 0 0 0 0 0 0 0 0 0
17 0 0 0 0 0 0 0 0 0 0 0 0 0 0 0 0 0 0 0 0 0 0 0 0 0 60 1
0 0 0 0 0 0 0 0 0 0 0 0 0 0 0 0 0 0 0 0 0 0 0 0 0 0 0
M02255_77_000000000-ADUT3_1_1101_12180_17483 T----AC---GA-AG-GGG--------GCT-A-G------C--G--T---T--GT-T-CGG-AA------TT-A--C-T--GG-GC-------GT--A-----AA-GC-GC-AC-------G-CA-G-G-C-G------------G--T-CC-G-A-T-----
------TA----G-T-C-A--------G-A-A--G--TG--A-AA-GC--C-C-CG-G-G-----CT-C-AA------C-C-T-G-G-G-A--A-C----T-G--C-T---T--T-------T--GA-T-A---C-------T--G-T--CG--G-G-C----T-T-G-A-A-T--C---A-CG-----GA-G-A--------G-G-G-T---AG-T------GG--A--ATT----C-
C-G-A-GT--GT-A-G-AG-GT--G---A--A-A---TT-C-GT-AG--AT-A-TT--C-G--G-A-A-G-A-AC-A-CC---AG--T--G--GC-GAA-G--G-C--G-------A--C-T-A-C---CTG--G--CC-G-T--------------------C-G---A-T-T--GA--CG-C-T-C--A-TG--T-G-CG-A--AA-G-C---G-TG--GG-G-
-AG-C-A-AA--------CA--GG Bacteria(100);Proteobacteria(100);Alphaproteobacteria(100);Rhodospirillales(100);Rhodospirillaceae(100);Magnetospirillum(100);unclassified(100);unclassified(100);unclassified(100);

Otu0728 0 0 0 0 0 0 0 0 0 0 0 0 0 0 0 0 0 0 0 0 0 0 0 0 0 0 0 0
0 0 0 0 0 0 0 0 0 0 0 0 0 0 0 0 0 0 0 0 0 0 0 0 0 0 0 0
0 0 0 0 0 0 0 0 0 0 0 0 0 0 0 0 0 16 0 0 0 0 0 0 0 0 0 0
0 0 0 0 0 0 0 0 0 0 0 0 0 0 0 19 0 0 0 0 0 0 0 0 0 0 0
M02255_76_000000000-ADUNE_1_1106_20003_9249 C----TA---AC-TA-CGT--------GCC-A-G------C--A--G---C--CG-C-GGT-AA------TA-C--G-T--AG-GG-------GG--C-----AA-GC-G--TT-------G-TC-C-G---G------------A--T-TA-G-G-T-----------AG---
-G-T-C-C--------G-T-T--G--TG--A-AA-AC--T-C-GA-G-G-----CT-C-AA------C-C-T-C-G-A-G--A-C----G-C--C-G---A--T-------G--GA-A-A---C-------C--A-T--CT--G-A-C----T-A-G-A-G-T--C---C-GG-----AA-G-A--------G-G-A-G---AG-T------GG--A--ATT----C-C-T-G-
GT--GT-A-G-CG-GT--G---A--A-A---TG-C-GC-AG--AT-A-TC--A-G--G-A-A-G-A-AC-A-CC---CG--T--G--GC-TAA-G--G-C--G-------G--C-T-C-T---CTA--G--TA-C-G--------------------T-G---A-C-T--GA--CG-C-T-G--A-GA--C-G-CG-A--AA-G-C---G-TG--GG-G--AG-C-
G-AA--------CA--GG Bacteria(100);Actinobacteria(100);Actinobacteria(100);unclassified(55);unclassified(55);unclassified(55);unclassified(55);unclassified(55);unclassified(55);

Otu0729 0 0 0 0 0 0 0 0 0 0 0 0 0 0 0 0 0 0 0 0 0 0 0 0 0 0 0 0
0 0 0 0 0 0 0 0 0 0 22 0 0 0 0 0 0 0 0 0 0 0 0 0 0 0 0 0
0 0 0 0 0 0 0 0 0 0 0 0 0 0 0 0 0 0 0 0 0 0 0 0 0 0 0 41
0 0 0 0 0 0 0 0 0 0 0 0 0 0 0 0 0 0 0 0 0 0 0 0 0 0 0
M02255_77_000000000-ADUT3_1_1105_18686_9378 C----AC---GT-AG-GCA--------CCA-A-G------C--G--T---T--GT-C-CGG-AT------TT-A--T-T--GG-GC-------GT--A-----AA-GA-GC-TC-------G-TA-G-G-C-G------------G--T-TC-G-G-T-----------AA--
--G-T-C-G--------G-G-T--G--TG--A-AA-AC--T-C-TG-G-G-----CT-C-AA------C-C-C-A-G-A-G--C------C-G-CC-A---C--C-------C--GA-T-A---C-------T--G-C--TG-TG-A-C----T-C-G-A-G-T--G---T-GG-----TA-G-G--------G-G-A-G---TG-G------GG--A--ATT----C-C-T-G-
GT--GT-A-G-CG-GT--G---A--A-A---TG-C-GC-AG--AT-A-TC--A-G--G-A-G-G-A-AC-A-CC---AG--T--G--GC-GAA-G--G-C--G-------C--C-A-C-T---CTG--G--GC-C-A--------------------T-T---A-C-T--GA--CG-C-T-G--A-GG--A-G-CG-A--AA-G-C---G-TG--GG-T--AG-
C-A-AA--------CA--GG Bacteria(100);Actinobacteria(100);Actinobacteria(100);Acidimicrobidae(100);Acidimicrobiales(100);Acidimicrobineae(100);Acidimicrobiaceae(100);unclassified(100);unclassified(100);

Otu0730 0 73 150 0 0 0 77 11 59 0 0 0 0 0 0 0 0 0 0 0 0 0 0 0 0 0 0 0
0 0 0 0 0 7 0 0 0 0 0 49 0 0 0 3 0 0 0 0 0 0 0 0 0 1 0 0 0
0 0 0 0 0 0 0 0 0 0 0 0 0 0 0 0 3 9 0 0 0 0 0 0 0 0 0 0 0
0 0 0 0 0 0 2 0 0 0 0 1 0 0 0 0 0 0 0 0 0 13 0 0 95 0 0
M02255_76_000000000-ADUNE_1_2112_11984_23285 T----AC---GT-AG-GGA--------GCG-A-G------C--G--T---T--GT-C-CGG-AA------TT-A--C-T--GG-GT-------GT--A-----AA----G--G--------G--A---G-C-----------------G----T--------------
AA----G-T-C-A--------G-A-T--G--TG--A-AA-AC--T-A-TG-G-G-----CT-T-AA------C-C-C-A-T-A-A--A-C----T-G--C-A---T--T-------T--GA-A-A---C-------T--G-T--GG--T-T-C----T-T-G-A-G-T--G---A-AG-----TA-G-A--------G-G-T-A---AG-C------GG--A--ATT----C-C-T-
A-GT--GT-A-G-CG-GT--G---A--A-A---TG-C-GT-AG--AT-A-TT--A-G--G-A-G-G-A-AC-A-TC---AG--T--G--GC-GAA-G--G-C--G-------G--C-T-T-A---CTG--G--GC-T-T--------------------T-A---A-C-T--GA--CG-C-T-G--A-GG--C-T-CG-A--AA-G-C---G-TG--GG-G--AG-
C-A-AA--------CA--GG Bacteria(100);Firmicutes(100);Clostridia(100);Clostridiales(100);Ruminococcaceae(100);unclassified(70);unclassified(70);unclassified(70);unclassified(70);

Otu0731 0 0 0 0 0 0 0 0 0 0 0 0 0 0 0 0 3 0 0 0 0 0 0 0 0 0 0 0
0 0 0 0 0 0 0 0 0 0 0 0 0 0 0 0 0 0 0 0 0 0 0 0 0 0 0 0 0
0 0 0 0 0 0 0 0 0 0 0 0 0 0 0 0 0 0 0 0 0 0 0 0 9 0 0 0 0
0 0 0 0 0 0 0 0 0 0 0 0 0 0 0 0 0 0 0 0 0 0 7 0 0 0 0
M02255_77_000000000-ADUT3_1_1115_11387_16571 T----AC---GT-AG-GGT--------GCA-A-G------C--G--T---T--GT-C-CGG-AA------TT-A--T-T--GG-GC-------GT--A-----AA-GA-GC-TC-------G-TA-G-G-C-G------------G--T-GT-G-T-C-----
------GC----G-T-C-T--------G-C-T--G--TG--A-AA-AC--T-C-AG-G-G-----CT-C-AA------C-T-C-T-G-A-G--C-T----T-G--C-A---G--T-------G--GG-T-A---C-------G--G-G--CA--C-A-C----T-A-G-A-G-T--G---C-TG-----TA-G-G--------G-G-A-G---AC-T------GG--A--ATT----C-
C-T-G-GT--GT-A-G-CG-GT--G---A--A-A---TG-C-GC-AG--AT-A-TC--A-G--G-A-G-G-A-AC-A-CC---GG--T--G--GC-GAA-G--G-C--G-------G--G-T-C-T---CTG--G--GC-A-G--------------------T-T---A-C-T--GA--CG-C-T-G--A-GG--A-G-CG-A--AA-G-C---A-TG--GG-
G--AG-C-G-AA--------CA--GG Bacteria(100);Actinobacteria(100);Actinobacteria(100);Actinobacteridae(100);Actinomycetales(100);Kineosporiineae(100);Kineosporiaceae(100);Kineococcus(100);unclassified(100);

Otu0732 0 0 0 0 0 0 0 0 0 0 0 0 0 0 0 0 0 0 0 0 0 0 0 0 0 0 0 0
0 0 0 0 0 0 0 0 0 0 0 0 0 0 0 0 0 0 0 0 0 0 0 0 0 0 0 0 0
0 0 0 0 0 0 0 0 0 0 0 0 0 0 32 0 0 0 0 0 0 0 0 0 0 0 0 0 0
0 0 0 0 0 0 0 0 0 0 0 0 0 6 0 0 0 0 0 0 0 0 0 0 0 0 0
M02255_76_000000000-ADUNE_1_1119_23344_14748 C----TA---AC-TC-TGT--------GCC-A-G------C-----A---G--CC-G-CGG-TA------AT-A--C-A--GA-GG-------GT--------GC-TA-GC-G--------T-TA-A-T-C-G------------G--A-TT-G-T-T---------
--AA----G-T-C-G--------G-T-T--G--TG--A-AA-GC--C-C-CG-G-G-----CT-C-AA------C-C-T-G-G-G-A--A-T----T-G--C-A---G--T-------C--GA-T-A---C-------T--G-G--CA--G-A-C----T-A-G-A-G-A--A---C-GG-----TA-G-A--------G-G-G-A---GG-C------GG--A--ACT----C-
C-A-G-GT--GT-A-G-CG-GT--G---A--A-A---TG-C-GT-AG--AT-A-TC--T-G--G-A-A-G-A-AC-A-CC---GA--T--G--GC-GAA-G--G-C--A-------A--C-C-T-C---CTG--G--GC-C-T--------------------G-T---T-C-T--GA--CG-C-T-G--A-GG--C-A-CG-A--AA-G-C---G-TG--GG-T-
-AG-C-A-AA--------CA--GG Bacteria(100);Proteobacteria(100);Gammaproteobacteria(100);Xanthomonadales(100);Sinobacteraceae(100);Nevskia(100);unclassified(100);unclassified(100);unclassified(100);

Otu0733 0 0 0 0 0 0 0 0 0 0 0 0 0 0 0 0 0 0 0 0 0 0 0 0 0 0 0 0
0 0 0 0 0 0 0 0 0 0 0 0 0 0 0 0 0 0 0 0 0 0 0 0 0 0 0 0 0
0 0 0 0 0 0 0 0 0 0 0 0 0 0 0 0 7 0 0 0 0 0 0 0 0 0 190 0 0
0 0 0 0 0 0 0 0 0 0 0 0 0 0 0 0 0 0 0 0 0 0 0 0 0 0 10
M02255_76_000000000-ADUNE_1_1114_24283_9653 T----AC---GA-AG-GGG--------GCT-A-G------C--G--T---T--GC-T-CGG-AA------TC-A--C-T--GG-GC-------GT--A-----AA-G--G--GC-------------G-C-----------------C-G------T-----------AG----G-T-
C-G--------G-G-C--G--TG--A-AA-TC--C-C-CG-G-G-----CT-C-AA------C-C-C-G-G-G-G--G-C----T-G--C-G---C--C-------C--GA-G-A---C-------T--G-C--GG--T-G-C----T-C-G-A-G-G--G---C-GG-----GA-G-A--------G-G-C-T---CG-C------GG--A--ATT----C-C-C-A-GT--
GT-A-G-AG-GT--G---A--A-A---TT-C-GT-AG--AT-A-TT--G-G--G-A-A-G-A-AC-A-CC---GG--T--G--GC-GAA-G--G-C--G-------G--C-G-A-G---CTG--G--CC-C-G--------------------T-T---T-C-T--GA--CG-C-T-G--A-GG--C-G-CG-A--CA-G-C---G-TA--GG-G--AG-C-A-
AA--------CA--GG Bacteria(100);Proteobacteria(100);Alphaproteobacteria(100);Rhodospirillales(100);unclassified(100);unclassified(100);unclassified(100);unclassified(100);unclassified(100);

Otu0734 0 0 101 0 0 0 9 0 169 0 0 0 0 0 0 0 0 0 0 0 0 0 0 0 0 0 0 0
0 0 0 0 0 0 0 0 0 0 0 0 0 0 0 0 0 0 0 0 0 0 0 0 0 0 0 0 0
0 0 0 0 0 0 0 0 0 0 0 0 0 0 0 0 0 0 0 0 0 0 0 0 0 0 0 0 0
0 0 0 0 0 0 0 0 0 0 0 0 0 0 0 0 0 0 0 0 0 0 0 0 0 0 0
M02255_76_000000000-ADUNE_1_2106_24242_13935 T----AC---GT-AG-GTG--------GCA-A-G------C--G--T---T--GT-C-CGG-AT------TT-A--C-T--GG-GT-------GT--A-----AA-GG-GC----------G-T--G---C--------------------------------------AA-
---G-T-C-A--------G-A-T--G--TG--A-AA-TC--T-C-AG-G-G-----CT-T-AA------C-C-C-T-G-A-A--A-C----T-G--C-A---T--T-------T--GA-A-A---C-------T--G-T--AT--G-T-C----T-T-G-A-G-T--G---C-CG-----GA-G-A--------G-G-T-A---AT-C------GG--A--ATT----C-C-T-T-GT-
-GT-A-G-CG-GT--G---A--A-A---TG-C-GT-AG--AT-A-TA--A-G--G-A-A-G-A-AC-A-CC---AG--T--G--GC-GAA-G--G-C--G-------G--A-T-T-A---CTG--G--AC-G-G--------------------T-A---A-C-T--GA--CG-G-T-G--A-GG--C-G-CG-A--AA-G-C---G-TG--GG-G--AG-C-
G-AA--------CA--GG Bacteria(100);Firmicutes(100);Clostridia(100);Clostridiales(100);Ruminococcaceae(100);unclassified(100);unclassified(100);unclassified(100);unclassified(100);

Otu0735 0 0 0 0 0 0 0 0 0 0 0 0 0 0 0 0 0 0 0 0 0 0 0 0 0 0 0 0
0 0 0 0 0 0 0 0 0 0 0 0 0 0 0 0 0 0 0 0 0 0 0 0 0 0 0 0 0
0 0 0 0 0 0 0 0 0 0 0 0 0 0 0 4 0 0 0 0 0 0 0 0 84 0 0 0 47
0 0 0 0 0 0 0 0 0 0 0 0 0 0 0 0 0 0 0 58 0 0 0 0 0 0 0
M02255_77_000000000-ADUT3_1_1105_12733_11765 T----AC---GT-AG-GGA--------GCG-A-G------C--G--T---T--AT-C-CGG-AT------TT-A--T-T--GG-GT-------GT--A-----AA-GG-GT-GC-------G-TA-G-A-C-G------------G--A-AA-T-A-C-----
------AA----G-T-T-A--------G-T-T--G--TG--A-AA-TA--C-C-TC-G-G-----CT-T-AA------C-T-G-G-G-G-A--A-C----T-G--C-A---A--C-------T--AA-A-A---C-------T--A-T--AT--T-T-C----T-T-G-A-G-T--A---T-CG-----GA-G-G--------G-G-T-T---TG-T------GG--A--ATT----C-T-
T-A-GT--GT-A-G-CG-GT--G---A--A-A---TG-C-GT-AG--AT-A-TT--A-G--G-A-A-G-A-AC-A-CC---GG--T--G--GC-GAA-G--G-C--G-------A--C-A-A-A---CTG--G--AC-G-A--------------------T-A---A-C-T--GA--CG-T-T-G--A-GG--C-A-CG-A--AA-G-T---G-TG--GG-G--
AG-C-A-AA--------CA--GG Bacteria(100);Firmicutes(100);Clostridia(100);Clostridiales(100);Ruminococcaceae(100);uncultured(100);unclassified(100);unclassified(100);unclassified(100);

Otu0736 0 0 0 0 0 0 0 0 0 0 0 0 0 0 0 0 0 0 2 0 0 0 0 0 0 0 0 0
0 0 3 0 0 0 32 0 0 2 2 0 0 21 100 0 87 53 0 0 59 0 0 0 0 0 0 4 99
0 0 0 0 0 0 0 0 0 0 0 0 0 0 0 0 0 0 0 0 0 0 0 0 0 0 0 32 0
4 0 0 0 0 0 0 0 0 4 0 0 4 0 0 0 0 0 11 0 0 29 5 0 0 0 0
M02255_76_000000000-ADUNE_1_1118_17072_9809 .....AC----T-AC-GT---------GCC-A-G------C--A--G---C--CG-C-GGT-AA------TA-C--G-T--AG-GT-------GG--C-----AA-GC-G--TT-------G----T-C-C-G------------G--A-AT-G-A-T-----------AA----G-
T-C-T--------G-A-T--G--TG--A-AA-GC--C-C-AC-G-G-----CT-C-AA------C-C-G-T-G-G-A--A-C----T-G--C-A---T--C-------G--GA-A-A---C-------T--G-T--CA--T-T-C----T-T-G-A-G-T--G---C-AG-----AA-G-A--------G-G-A-G---AG-T------GG--A--ACT----C-C-A-T-GT--
GT-A-G-CG-GT--G---G--A-A---TG-C-GT-AG--AT-A-TA--T-G--G-A-A-G-A-AC-A-CC---AG--T--G--GC-GAA-G--G-C--G-------G--C-T-C-T---CTG--G--TC-T-G--------------------C-A---A-C-T--GA--CG-C-T-G--A-GG--C-T-CG-A--AA-G-C---A-TG--GG-T--AG-C-G-
AA--------CA--GG Bacteria(100);Firmicutes(100);Bacilli(100);Lactobacillales(100);unclassified(57);unclassified(57);unclassified(57);unclassified(57);unclassified(57);

Otu0737 0 0 0 0 0 0 0 0 0 0 0 0 0 0 0 0 0 0 3 0 0 0 0 0 0 0 0 0
0 2 0 0 0 0 0 0 0 9 19 1 0 0 0 0 0 104 0 1 0 0 0 0 0 0 0 1 0
0 0 0 0 0 0 0 0 0 0 0 0 0 0 0 0 0 0 0 0 0 0 0 0 0 0 0 0 0
0 0 0 0 0 0 0 0 0 0 0 0 0 0 0 0 0 0 0 0 0 0 0 0 0 0 0
M02255_77_000000000-ADUT3_1_1110_14104_17809 T----AC---GG-AG-GAG--------GCA-A-G------C--G--T---T--AT-C-CGG-AA------TT-A--T-T--GG-GC-------GT--A-----AA-GA-GT-CC-------G-TA-G-G-T-G------------G--G-TA-A-C-C-----
------AA----G-T-C-A--------G-T-T--G--TT--A-AA-TA--G-T-GA-A-G-----CT-T-AA------C-T-T-C-A-T-A--G-A----G-G--C-G---A--C-------T--GA-A-A---C-------T--G-G--TT--A-T-C----T-A-G-A-G-T--G---T-GG-----TA-G-G--------G-G-T-A---GA-G------GG--A--ATT----T-
C-C-A-GT--GT-A-G-CG-GT--G---A--A-A---TG-C-GT-AG--AT-A-TT--G-G--A-A-A-G-A-AC-A-CC---GG--T--G--GC-GAA-A--G-C--G-------C--T-C-T-A---CTG--G--GC-C-T--------------------G-C---A-C-T--GA--CA-C-T-G--A-GG--G-A-CG-A--AA-G-C---T-AG--GG-G-
-AG-C-G-AA--------AG--GG Bacteria(100);Cyanobacteria(100);unclassified(98);unclassified(98);unclassified(98);unclassified(98);unclassified(98);unclassified(98);unclassified(98);

Otu0738 0 0 0 0 0 0 0 0 0 0 0 0 0 0 0 0 0 0 0 0 0 0 1 0 0 0 0 0
0 0 119 0 0 0 0 0 0 0 51 10 0 0 153 0 0 0 0 0 49 0 0 0 0 0 0 49 0
0 0 0 0 0 0 0 0 0 1 0 0 0 2 0 0 236 0 0 0 0 0 0 0 0 0 0 0 0
0 0 0 0 0 0 0 0 0 0 0 0 0 0 0 0 0 0 11 51 0 0 0 0 0 0 58
M02255_76_000000000-ADUNE_1_1102_18715_10107 T----AC---GA-AG-GGG--------GCT-A-G------C--G--T---T--GC-T-CGG-AA------TT-A--C-T--GG-GC-------GT--A-----AA-GG-GC-GC-------G-TA------------------------GG-C-C-C---------
--AA----G-T-C-A--------G-G-C--G--TG--A-AA-TT--C-C-TG-G-G-----CT-C-AA------C-C-T-G-G-G-G--A-C----T-G--C-G---C--T-------T--GA-T-A---C-------T--G-G--GT--T-G-C----T-T-G-A-G-G--A---T-GG-----AA-G-A--------G-G-C-T---CG-T------GG--A--ATT----C-C-
C-A-GT--GT-A-G-AG-GT--G---A--A-A---TT-C-GT-AG--AT-A-TT--G-G--G-A-A-G-A-AC-A-CC---GG--T--G--GC-GAA-G--G-C--G-------G--C-G-A-G---CTG--G--TC-C-A--------------------T-T---A-C-T--GA--CG-C-T-G--A-GG--C-G-CG-A--CA-G-C---G-TG--GG-G--
AG-C-A-AA--------CA--GG Bacteria(100);Proteobacteria(100);Alphaproteobacteria(100);Rhodospirillales(100);Acetobacteraceae(100);Roseomonas(100);unclassified(100);unclassified(100);unclassified(100);

Otu0739 0 0 0 0 0 0 0 0 0 0 0 0 0 0 0 0 0 0 0 0 0 0 0 0 0 0 0 0
0 0 0 0 0 0 0 0 0 0 0 60 0 0 0 0 0 0 0 0 0 0 0 0 0 0 0 0 0
0 0 0 0 0 0 0 0 0 0 0 0 0 0 0 0 0 0 0 0 0 0 0 0 0 0 0 0 0
0 0 0 0 0 0 0 0 0 0 0 0 0 0 0 0 0 0 0 0 0 0 0 0 0 0 0
M02255_77_000000000-ADUT3_1_1108_11929_7997 T----AC---GT-AG-GGG--------GCA-A-G------C--G--T---T--GT-C-CGG-AA------TG-A--T-T--GG-GC-------GT--A-----AA-GG-GC-GC-------G-TA-G-G-C-G------------G--C-TT-G-G-T-----------
AA----G-T-T-T--------G-A-A--G--TG--A-AA-GT--C-C-TT-C-T-----TT-T-AA------G-G-A-G-G-G-A--A-C----T-G--C-T---T--T-------G--AA-A-A---C-------T--G-T--CG--G-G-C----T-T-G-A-G-T--G---C-AG-----GA-G-A--------G-G-T-A---AG-C------GG--A--ATT----C-C-C-
G-GT--GT-A-G-CG-GT--G---A--A-A---TG-C-GT-AG--AG-A-TC--G-G--G-A-G-G-A-AC-A-CC---AG--T--G--GC-GAA-G--G-C--G-------G--C-T-T-A---CTG--G--AC-T-G--------------------T-T---A-C-T--GA--CG-C-T-G--A-GG--C-G-CG-A--AA-G-T---G-TG--GG-G--
AG-C-A-AA--------CA--GG Bacteria(100);unclassified(100);unclassified(100);unclassified(100);unclassified(100);unclassified(100);unclassified(100);unclassified(100);unclassified(100);

Otu0740 0 0 0 0 0 0 0 0 0 0 0 0 0 0 0 0 0 0 0 0 0 0 0 0 0 0 0 0
0 0 0 0 0 0 0 0 0 0 0 20 0 0 0 0 0 0 0 0 0 0 0 0 0 0 0 0
0 0 0 0 0 0 0 0 0 0 0 0 0 0 0 0 0 0 0 0 0 0 0 0 0 0 0 0
0 0 0 0 0 0 0 0 0 0 0 0 0 0 0 0 0 0 0 0 0 0 0 0 0 0 0
M02255_77_000000000-ADUT3_1_2108_16554_9942 T----AC---GA-AG-GGG--------GCT-A-G------C--G--T---T--GC-T-CGG-AA------TG-A--C-T--GG-GC-------GT--A-----AA-GG-GC-GC-------G-TA-G-G-C-G------------G--A-TG-G-C-T----------
TT----G-T-C-G--------G-G-C--G--TG--A-AA-TT--C-C-AG-G-G-----CT-T-AA------C-C-T-T-G-G-G--G-C----T-G--C-G---T--T-------C--GA-G-A---C-------G--G-G--TT--G-T-C----T-T-G-A-G-T--T---C-GG-----AA-G-A--------G-G-G-T---CG-T------GG--A--ATT----C-C-C-
A-GT--GT-A-G-AG-GT--G---A--A-A---TT-C-GT-AG--AT-A-TT--G-G--G-A-A-G-A-AC-A-CC---GG--T--G--GC-GAA-G--G-C--G-------G--C-G-A-C---CTG--G--TC-C-G--------------------A-G---A-C-T--GA--CG-C-T-G--A-GG--C-G-CG-A--AA-G-C---G-TG--GG-G--
AG-C-A-AA--------CA--GG Bacteria(100);Proteobacteria(100);Alphaproteobacteria(100);Rhodospirillales(100);Acetobacteraceae(100);unclassified(100);unclassified(100);unclassified(100);unclassified(100);

Otu0741 0 0 0 0 0 0 0 0 0 0 0 0 0 0 0 0 0 0 0 0 0 0 18 0 0 0 0 0
0 0 0 0 0 0 0 0 0 0 0 0 0 0 0 0 0 0 0 0 0 0 0 0 0 0 0 0
0 0 0 0 0 0 0 0 0 0 0 0 0 0 0 0 0 0 0 0 0 0 0 0 0 0 0 0
0 0 0 0 0 0 0 0 0 0 0 0 0 0 0 0 0 0 0 0 0 0 0 0 0 0 0
M02255_77_000000000-ADUT3_1_2115_18474_18035 G----AC---GG-AG-GAG--------GCT-A-G------C--G--T---T--GT-T-CGG-AA------TT-A--C-T--GG-GC-------GT--A-----AA-GC-GT-GC-------G-CA-G-G-T-T------------G--T-GA-G-T-T-----
------CA----G-T-T-G--------G-A-T--G--TG--A-AA-GC--C-C-GG-G-G-----CT-T-AA------C-C-T-C-G-G-A--T-G----T-G--C-A---T--C-------C--AA-T-A---C-------T--G-G--CT--C-G-C----T-G-G-A-G-G--T---T-GG-----AA-G-A--------G-G-A-G---AG-T------GG--A--ATT----
C-C-C-A-GT--GT-A-G-AG-GT--G---A--A-A---TT-C-GT-AG--AT-A-TT--G-G--G-A-A-G-A-AC-A-CC---AG--T--G--GC-GAA-G--G-C--G-------G--C-T-C-T---CTG--G--TC-C-A--------------------T-A---C-C-T--GA--CA-C-T-C--A-TG--C-A-CG-A--AA-G-C---G-TG--GG-
G--AG-C-A-AA--------CA--GG Bacteria(100);Proteobacteria(100);Alphaproteobacteria(100);MNG3(100);unclassified(100);unclassified(100);unclassified(100);unclassified(100);unclassified(100);

Otu0742 0 0 0 0 0 0 0 0 0 0 0 0 0 0 0 0 0 0 0 0 0 0 0 0 0 0 0 0
0 0 0 0 0 0 0 0 0 0 0 16 0 0 0 0 0 0 0 0 0 0 0 0 0 0 0 0
0 0 0 0 0 0 0 0 0 0 0 0 0 0 0 0 0 0 0 0 0 0 0 0 0 0 0 0
0 0 0 0 0 0 0 0 0 0 0 0 0 0 0 0 0 0 0 0 0 0 0 0 21 0 0
M02255_77_000000000-ADUT3_1_1108_19712_10930 T----AC---GT-AG-GTG--------GCA-A-G------C--G--T---T--GT-C-CGG-AT------TT-A--C-T--GG-GT-------GT--A-----AA-GG-GC-GT-------G-CA-G-C-C-G------------G--G-CC-G-G-C-----
------AA----G-T-C-A--------G-A-T--G--TG--A-AA-TC--C-A-CG-G-G-----CT-T-AA------C-C-C-G-T-G-A--A-C----T-G--C-A---T--T-------T--GA-A-A---C-------T--G-T--TG--G-T-C----T-T-G-A-G-T--A---T-CG-----GA-G-A--------G-G-T-A---AT-C------GG--A--ATT----C-
C-T-T-GT--GT-A-G-CG-GT--G---A--A-A---TG-C-GT-AG--AT-A-TA--A-G--G-A-A-G-A-AC-A-CC---AG--T--G--GC-GAA-G--G-C--G-------G--A-T-T-A---CTG--G--AC-G-A--------------------C-A---A-C-T--GA--CG-G-T-G--A-GG--C-G-CG-A--AA-G-C---G-TG--GG-
G--AG-C-A-AA--------CA--GG Bacteria(100);Firmicutes(100);Clostridia(100);Clostridiales(100);Ruminococcaceae(100);unclassified(100);unclassified(100);unclassified(100);unclassified(100);

Otu0743 0 0 0 0 0 0 0 0 0 0 0 0 0 0 0 0 0 0 0 0 0 0 0 0 0 0 0 0
0 0 0 0 0 0 0 0 0 0 0 17 0 0 0 0 0 0 0 0 0 0 0 0 0 0 0 0
0 0 0 0 0 0 0 0 0 0 0 0 0 0 0 0 0 0 0 0 0 0 0 0 0 0 0 0
0 0 0 0 0 0 0 0 0 0 0 0 0 0 0 0 0 0 0 0 0 0 0 0 0 0 0
M02255_77_000000000-ADUT3_1_1105_16395_10343 T----AC---GT-AG-GGC--------GCG-A-G------C--G--T---T--GT-C-CGG-AA------TT-A--T-T--GG-GC-------GT--A-----AA-GA-GC-TC-------G-TA-G-G-C-G------------G--C-TG-G-T-C-----
------GC----G-T-C-T--------G-C-C--G--TG--A-AA-G-G-C-C-TG-G-G-----CT-T-AA------C-T-C-G-G-G-T--T-T----T-G--C-G---G--T-------G--GA-T-A---C-------G--G-G--CC--G-G-C----T-A-G-A-G-G--T---A-GG-----TA-G-G--------G-G-A-G---AA-C------GG--A--ATT----
C-C-T-G-GT--GT-A-G-CG-GT--G---A--A-A---TG-C-GC-AG--AT-A-TC--A-G--G-A-G-G-A-AC-A-CC---GG--T--G--GC-GAA-G--G-C--G-------G--T-T-C-T---CTG--G--GC-C-T--------------------A-T---C-C-T--GA--CG-C-T-G--A-GG--A-G-CG-A--AA-G-C---G-TG--
GG-G--AG-C-G-AA--------CA--GG Bacteria(100);Actinobacteria(100);Actinobacteria(100);Actinobacteridae(100);Actinomycetales(100);Streptosporangineae(100);unclassified(100);unclassified(100);unclassified(100);

Otu0744 0 0 0 0 0 0 0 0 0 0 0 0 0 0 0 0 0 0 0 0 0 4 0 0 0 0 0 0
0 0 0 0 0 0 0 0 0 0 0 0 0 0 0 0 0 0 0 0 0 0 0 0 0 0 0 0
0 0 0 0 0 0 0 0 0 0 0 0 0 0 0 0 0 0 0 0 0 0 0 0 0 0 0 0
0 0 0 0 0 0 0 0 0 0 0 0 0 0 0 0 0 0 0 0 0 0 0 0 0 0 0
M02255_76_000000000-ADUNE_1_2112_7258_22015 TC---GC---AG-AA-TAA--------GCA-CCGG-----C--T--A---A--CT-C-TGT-GC------CA-G--C-A-GCC-GC-------GG--T-----AA-TA-CA-GA-------G-GG-T-G-C-G------------A--G-CG-T-T-T----------
AA----G-T-C-G--------G-A-T--G--TG--A-AA-TC--C-C-TG-A-G-----CT-T-AA------C-T-T-A-G-G-A--A-T----T-G--C-A---T--T-------C--GA-T-A---C-------T--G-G--GA--G-A-C----T-A-G-A-G-T--A---T-GG-----GA-G-A--------G-G-A-T---GG-T------AG--A--ATT----C-C-A-
G-GT--GT-A-G-CG-GT--G---A--A-A---TG-C-GT-AG--AG-A-TC--T-G--G-A-G-G-A-AT-A-CC---GA--T--G--GC-GAA-G--G-C--A-------G--C-C-A-T---CTG--G--CC-T-A--------------------A-T---A-C-T--GA--CA-C-T-G--A-GG--C-A-CG-A--AA-G-C---A-TG--GG-G--
AG-C-A-AA--------CA--GG Bacteria(100);Proteobacteria(100);Gammaproteobacteria(100);unclassified(100);unclassified(100);unclassified(100);unclassified(100);unclassified(100);unclassified(100);

Otu0745 0 0 0 0 0 0 0 0 0 0 0 0 0 0 0 0 0 0 0 0 0 4 0 0 0 0 0 0
0 0 0 0 0 0 0 0 0 0 0 0 0 0 0 0 0 0 0 0 0 0 0 0 0 0 0 0 0
0 0 0 0 0 0 0 0 0 0 0 0 0 0 0 0 0 0 0 0 0 0 0 0 0 0 0 0 0
0 0 0 0 0 0 0 0 0 0 0 0 0 0 0 29 0 0 0 0 0 0 0 0 0 0 0
M02255_77_000000000-ADUT3_1_2103_9602_10556 T----AC---GG-AG-GGT--------GCA-A-G------C--G--T---T--AA-T-CGG-AA------TA-A--C-T--GG-GC-------GT--A-----AA-GG-GC-AT-------G-CA-G-G-C-G------------G--T-TA-T-A-C----------
AA----G-T-A-G--------G-A-T--G--TG--A-AA-GC--C-T-GG-G-G-----CT-C-AA------C-C-T-C-A-G-A--A-C----T-G--C-A---T--T-------C--TA-A-A---C-------T--G-T--GT--G-A-C----T-A-G-A-G-T--A---T-TG-----CA-G-G--------G-G-G-A---GA-C------GG--A--ATT----T-C-A-
G-GT--GT-A-G-CG-GT--G---G--A-A---TG-C-GT-AG--AT-A-TC--T-G--A-A-A-G-A-AC-A-CC---AA--A--G--GC-GAA-G--G-C--A-------G--T-C-T-C---CTG--G--GC-A-A--------------------A-T---A-C-T--GA--CG-C-T-C--A-TA--T-G-CG-A--AA-G-C---G-TG--GG-T--AG-
C-A-AA--------CA--GG Bacteria(100);Proteobacteria(100);Gammaproteobacteria(100);Aeromonadales(100);Succinivibrionaceae(100);Succinivibrio(100);unclassified(100);unclassified(100);unclassified(100);

Otu0746 0 0 0 0 0 0 0 0 0 0 0 0 0 0 0 0 0 0 0 0 0 0 0 0 0 0 0 0
0 0 0 0 0 0 0 0 0 0 0 14 0 0 0 0 0 0 0 0 0 0 0 0 0 0 0 0 0
0 0 0 0 0 0 0 0 0 0 0 0 0 0 0 0 109 0 0 0 0 0 0 0 0 0 0 0 0
0 0 0 0 0 0 0 0 0 0 0 0 0 0 0 0 0 0 0 0 0 0 0 0 0 0 0
M02255_77_000000000-ADUT3_1_1105_17467_16662 T----AC---GA-AG-GGG--------GCT-A-G------C--G--T---T--GT-T-CGG-AA------TT-A--C-T--GG-GC-------GT--A-----AA-GC-GC-AC-------G-TA-G-G-C-G------------G--A-TC-A-T-T-----
------TA----G-T-C-A--------G-G-G--G--TG--A-AA-TC--C-C-GG-G-G-----CT-C-AA------C-C-T-C-G-G-A--A-C----T-G--C-C---T--T-------T--GA-T-A---C-------T--G-A--TG--G-T-C----T-C-G-A-G-T--C---C-GA-----TA-G-A--------G-G-T-G---AG-T------GG--A--ATT----C-
C-T-A-GT--GT-A-G-AG-GT--G---A--A-A---TT-C-GT-AG--AT-A-TT--A-G--G-A-A-G-A-AC-A-CC---AG--T--G--GC-GAA-G--G-C--G-------G--C-T-C-A---CTG--G--AT-C-G--------------------G-T---A-C-T--GA--CG-C-T-G--A-GG--T-G-CG-A--AA-G-C---G-TG--GG-G-
-AG-C-A-AA--------CA--GG Bacteria(100);Proteobacteria(100);Alphaproteobacteria(100);Rhizobiales(100);Hyphomicrobiaceae(100);unclassified(100);unclassified(100);unclassified(100);unclassified(100);

Otu0747 0 0 0 0 0 0 0 0 0 0 0 0 0 0 0 0 0 0 0 0 0 0 0 0 0 0 0 0
0 0 0 0 0 0 0 0 0 0 0 7 0 0 0 0 0 0 0 0 0 0 0 0 0 0 0 0 0
0 0 0 0 0 0 0 0 0 0 0 0 0 0 0 0 0 0 0 0 0 0 0 0 0 0 0 0 0
0 0 0 0 12 0 0 0 0 0 0 0 0 0 0 21 0 0 0 0 0 0 0 0 0 0 0
M02255_77_000000000-ADUT3_1_2112_21036_22412 T----AC---GT-AG-GGT--------GCG-A-G------C--G--T---T--GT-C-CGG-AA------TT-A--T-T--GG-GC-------GT--A-----AA-GA-GC-TT-------G-TA-G-G-C-G------------G--T-TT-G-T-T------
-----GC----G-T-C-A--------G-A-A--G--TG--A-AA-AC--T-C-AG-G-G-----CT-T-AA------C-C-C-T-G-A-G--C-C----T-G--C-T---T--C-------T--GA-T-A---C-------G--G-G--CA--G-A-C----T-A-G-A-G-G--A---A-GT-----TA-G-G--------G-G-A-G---AA-C------GG--A--ATT----C-
C-T-G-GT--GG-A-G-CG-GT--G---G--A-A---TG-C-GC-AG--AT-A-TC--A-G--G-A-G-G-A-AC-A-CC---GG--T--G--GC-GAA-G--G-C--G-------G--T-T-C-T---CTG--G--GA-C-T--------------------T-T---C-C-T--GA--CG-C-T-G--A-GA--A-G-CG-A--AA-G-C---G-TG--GG-
G--AG-C-A-AA--------CA--GG Bacteria(100);Actinobacteria(100);Actinobacteria(100);Actinobacteridae(100);Actinomycetales(100);Propionibacterineae(100);Propionibacteriaceae(100);unclassified(100);unclassified(100);

Otu0748 0 0 0 0 0 0 0 0 0 0 0 0 0 0 0 0 0 0 0 0 0 0 0 0 0 0 0 0
0 0 0 0 0 0 0 0 0 0 0 1 0 0 0 0 0 0 0 0 0 0 0 0 0 0 0 0 0
0 0 0 0 0 0 0 0 0 0 0 0 0 0 19 0 0 0 0 0 0 0 0 0 0 0 0 0 0
0 0 0 0 0 0 0 0 0 0 0 0 0 0 0 0 0 0 0 0 0 0 0 0 0 0 0
M02255_77_000000000-ADUT3_1_2104_25730_7401 T----AC---AG-AG-GGT--------GCA-A-G------C--G--T---T--AA-T-CGG-AA------TT-A--C-T--GG-GC-------GT--A-----AA-GC-GC-GC-------G-TA-G-G-T-G------------G--T-TC-G-T-T-----------AA-
---G-T-T-G--------G-A-T--G--TG--A-AA-GC--C-C-CG-G-G-----CT-C-AA------C-C-T-G-G-G-A--A-C----T-G--C-A---T--C-------C--AA-A-A---C-------T--G-G--CG--A-G-C----T-A-G-A-G-T--A---C-GG-----TA-G-A--------G-G-G-T---GG-T------GG--A--ATT----T-C-C-T-
GT--GT-A-G-CG-GT--G---A--A-A---TG-C-GT-AG--AT-A-TA--G-G--A-A-G-G-A-AC-A-CC---AG--T--G--GC-GAA-G--G-C--G-------A--C-C-A-C---CTG--G--AC-T-G--------------------A-T---A-C-T--GA--CA-C-T-G--A-GG--T-G-CG-A--AA-G-C---G-TG--GG-G--AG-
C-A-AA--------CA--GG Bacteria(100);Proteobacteria(100);Gammaproteobacteria(100);Pseudomonadales(100);Pseudomonadaceae(100);Pseudomonas(100);unclassified(100);unclassified(100);unclassified(100);

Otu0749 0 0 0 0 0 0 0 0 0 0 0 0 0 0 0 0 0 0 0 0 0 0 0 0 0 0 0 0
0 1 0 0 0 0 0 0 0 9 1 0 0 0 0 0 0 0 0 0 0 0 0 0 0 0 0 0 0
0 0 0 0 0 0 0 0 0 0 0 0 0 0 0 0 0 0 0 0 0 0 0 0 0 0 0 0 0
0 0 0 0 0 0 0 0 0 0 0 0 0 0 0 0 0 0 0 0 0 0 0 0 0 0 0
M02255_76_000000000-ADUNE_1_2119_25705_17841 A----CG---GCTAA-CTA--------CGT-G-C------C--A--G---C--AG-C-CGC-GG------TA-A--T-A--CG-TA-------GG--T-----GG-CG-AG-CG-------T-TA-T-C-C-G------------G--A-AT-G-A-T-----
------AA----G-T-C-T--------G-G-A--G--TG--A-AA-GG--C-T-AC-A-G-----CT-C-AA------C-T-G-T-A-G-T--A------T-G--C-T---C--T-------G--GA-A-A---C-------T--G-T--CA--A-G-C----T-A-G-A-G-T--G---C-AG-----AA-G-A--------G-G-G-C---AA-T------GG--A--ACT----C-
C-A-T-GT--GT-A-G-CG-GT--A---A--A-A---TG-C-GT-AG--AT-A-TA--T-G--G-A-A-G-A-AC-A-CC---GG--T--G--GC-GAA-G--G-C--G-------G--T-T-G-C---CTG--G--TC-T-G--------------------A-A---A-C-T--GA--CG-C-T-G--A-AG--T-A-CG-A--AA-G-C---G-TG--GG-G-
-AG-C-A-AA--------TA--GG Bacteria(100);unclassified(100);unclassified(100);unclassified(100);unclassified(100);unclassified(100);unclassified(100);unclassified(100);unclassified(100);

Otu0750 0 0 0 0 0 0 0 0 0 0 0 0 0 0 0 0 0 0 0 0 0 0 0 0 0 0 0 0
0 0 0 0 0 0 0 0 0 0 0 0 19 0 0 0 0 0 0 0 0 0 0 0 0 0 0 0
0 0 0 0 0 0 0 0 0 0 0 0 0 0 0 0 0 0 0 0 0 0 0 0 0 0 0 0
0 0 0 0 0 0 0 0 0 0 0 0 0 0 0 0 0 0 0 0 0 0 0 0 0 0 0
M02255_76_000000000-ADUNE_1_1113_25735_20100 C----TA---AC-TC-TGT--------GCC-A-G------C-----A---G--CC-G-CGG-TA------AT-A--C-A--GA-GG-------GT--G-----CA--A-GC-G--------T-TG-T-T-C-G------------G--ATCC-T-G-C--------
---AA----G-T-C-A--------G-A-A--G--TG--A-AA-TC--T-C-AT-T-G-----CT-C-AA------C-G-A-T-G-A-G--G-C----T-G--C-T---T--T-------T--GA-T-A---C-------T--G-C--GG--A-T-C----T-G-G-A-G-A--C---C-GG-----TA-G-A--------G-G-C-T---GG-T------AG--A--ATT----G-C-
A-G-GT--GT-A-G-CG-GT--G---G--A-A---TG-C-GT-AG--AG-A-TC--T-G--C-A-A-G-A-AT-A-CC---CG--T--G--GC-GAA-G--G-C--G-------G--C-C-A-G---CTG--G--GC-C-G--------------------G-T---T-C-T--GA--CG-C-T-G--A-GG--C-G-CG-A--AA-G-C---G-TG--GG-G--
AG-C-A-AA--------CA--GG Bacteria(100);unclassified(100);unclassified(100);unclassified(100);unclassified(100);unclassified(100);unclassified(100);unclassified(100);unclassified(100);

Otu0751 0 0 0 0 0 0 0 0 0 0 0 0 0 0 0 0 0 0 0 0 0 0 1 0 2 0 0 0
0 0 0 0 0 0 0 2 0 0 0 0 0 0 0 0 0 0 0 0 0 0 0 0 0 39 0 0
0 0 0 0 0 0 0 0 0 0 0 0 0 0 0 0 0 0 0 0 0 0 0 0 0 0 0 0
0 0 0 0 0 0 0 0 0 0 0 0 0 0 0 0 0 0 0 0 0 0 0 0 0 0 0
M02255_77_000000000-ADUT3_1_2108_22084_23328 T----AC---GA-AG-GGG--------GCT-A-G------C--G--T---T--GT-T-CGG-AT------TT-A--C-T--GG-GC-------GT--A-----AA-GC-GC-AC-------G-TA-G-G-C-G------------G--A-CT-A-T-T-----
------AA----G-T-C-A--------G-G-G--G--TG--A-AA-TC--C-C-GG-G-G-----CT-C-AA------C-C-C-C-G-G-A--A-C----T-G--C-C---T--T-------T--GA-T-A---C-------T--G-G--TA--G-T-C----T-T-G-A-G-T--T---C-GA-----GA-G-A--------G-G-T-G---AG-T------GG--A--ATT----C-
C-G-A-GT--GT-A-G-AG-GT--G---A--A-A---TT-C-GT-AG--AT-A-TT--C-G--G-A-G-G-A-AC-A-CC---AG--T--G--GC-GAA-G--G-C--G-------A--C-C-A-C---CTG--G--AC-T-A--------------------A-T---A-C-T--GA--CA-C-T-G--A-GG--T-G-CG-A--AA-G-C---G-TG--GG-G-
-AG-C-A-AA--------CA--GG Bacteria(100);Proteobacteria(100);Alphaproteobacteria(100);Rhizobiales(100);unclassified(100);unclassified(100);unclassified(100);unclassified(100);unclassified(100);

Otu0752 0 0 0 0 0 0 0 0 0 0 0 0 0 0 0 0 0 0 0 0 0 0 19 0 0 0 0 0
0 0 0 0 0 0 0 0 0 0 0 0 0 0 0 0 0 0 0 0 0 0 0 0 0 0 0 0
0 0 0 0 0 0 0 0 0 0 0 0 0 0 0 0 0 0 0 0 0 0 0 0 0 0 0 0
0 0 0 0 0 0 0 0 0 0 0 0 0 0 0 0 0 0 0 0 0 0 0 0 0 0 0
M02255_77_000000000-ADUT3_1_2119_20634_13659 T----AC---GA-AG-GGA--------GCG-A-G------C--G--T---T--GT-T-CGG-AA------TC-A--C-T--GG-GC-------TT--A-----AA-GC-GT-GT-------G-TA-G-G-T-G------------G--T-CA-T-G-T-----
------AA----G-T-A-A--------G-A-A--G--TG--A-AA-GC--C-C-CG-G-G-----CT-T-AA------C-C-T-G-G-G-A--A-T----T-G--C-T---T--T-------T--TA-G-A---C-------T--G-C--AT--G-G-C----T-G-G-A-G-T--T---C-TG-----GA-G-A--------G-G-T-T---AG-G------AG--A--ATT----C-
C-G-A-GT--GT-A-G-AG-GT--G---A--A-A---TT-C-GC-AG--AT-A-TT--C-G--G-A-G-G-A-AT-A-CC---AG--T--G--GC-GAA-G--G-C--G-------C--C-T-A-A---CTG--G--AC-A-G--------------------A-T---A-C-T--GA--CA-C-T-G--A-AA--C-A-CG-A--AA-G-C---G-TG--GG-T-
-AG-C-A-AA--------CA--GG Bacteria(100);Proteobacteria(100);unclassified(100);unclassified(100);unclassified(100);unclassified(100);unclassified(100);unclassified(100);unclassified(100);

Otu0753 0 0 0 0 0 0 0 0 0 0 0 0 0 0 0 0 0 0 0 0 0 0 0 0 0 0 0 0
0 0 0 0 0 0 0 0 0 0 0 45 0 0 0 0 0 0 0 0 0 0 0 0 0 0 0 0
0 0 0 0 0 0 0 0 0 0 0 0 0 0 0 0 0 0 0 0 0 0 0 0 0 0 0 24
0 0 0 0 0 0 0 0 0 0 0 0 0 0 0 0 0 0 0 0 0 0 0 0 0 0 0
M02255_77_000000000-ADUT3_1_1107_19114_20543 T----AC---GT-AG-GTG--------GCA-A-G------C--G--T---T--GT-C-CGG-AA------TT-A--C-T--GG-GT-------GT--A-----AA-GG-GA-GT-------G-TA-G-G-C-G------------G--G-AT-A-T-C-----
------AA----G-T-C-A--------G-A-A--G--TG--A-AA-AT--T-A-CG-G-G-----CT-C-AA------C-T-C-G-T-A-A--C-C----T-G--C-T---T--T-------T--GA-A-A---C-------T--G-A--CA--T-T-C----T-T-G-A-G-T--G---A-AG-----TA-G-A--------G-G-C-A---AG-C------GG--A--ATT----C-
C-T-A-GT--GT-A-G-CG-GT--G---A--A-A---TG-C-GT-AG--AT-A-TT--A-G--G-A-G-G-A-AC-A-CC---AG--T--G--GC-GAA-G--G-C--G-------G--C-T-T-G---CTG--G--GC-T-T--------------------T-T---A-C-T--GA--CG-C-T-G--A-GG--C-T-CG-A--AA-G-C---G-TG--GG-G-
-AG-C-A-AA--------CA--GG Bacteria(100);Firmicutes(100);Clostridia(100);Clostridiales(100);unclassified(100);unclassified(100);unclassified(100);unclassified(100);unclassified(100);

Otu0754 0 0 0 0 0 0 0 0 0 0 0 0 0 0 0 0 0 0 0 0 0 0 0 0 0 0 0 0
0 0 0 0 0 0 0 0 0 0 0 35 0 0 0 0 0 0 0 0 0 0 0 0 0 0 0 0
0 0 0 0 0 0 0 0 0 0 0 0 0 0 0 0 0 0 0 0 0 0 0 0 0 0 0 0
0 0 0 0 0 0 0 0 0 0 0 0 0 0 0 0 0 0 0 0 0 0 0 0 0 0 0
M02255_77_000000000-ADUT3_1_1116_14690_15130 T----AC---GT-AG-GAC--------CCA-A-G------C--G--T---T--AT-C-CGG-AG------TG-A--C-T--GG-GC-------GT--A-----AA-GA-GTTGC-------G-TA-G-G-T-G------------G--T-TT-G-T-T-----
------AA----G-C-G-A--------A-T-G--G--TG--A-AA-TC--T-G-GC-G-G-----CT-C-AA------C-C-G-T-ACA-G--A-C----T-A--T-T---A--T-------T--CG-A-A---C-------T--G-G--CA--A-A-C----T-C-G-A-G-A--A---C-AG-----CA-G-A--------G-G-T-A---AC-T------GG--A--ATT----T-
C-T-G-AA--GT-A-G-GG-GT--A---A--T-A---TC-C-GT-AG--AT-A-TC--A-G--A-A-G-G-A-AC-A-CC---AA--T--G--GC-GTA-G--G-C--A-------G--G-T-T-A---CTG--G--GC-T-G--------------------T-T---T-C-T--GA--CA-C-T-G--A-GG--C-A-CG-A--AA-G-C---G-TG--GG-G--
AG-C-G-AA--------CC--GG Bacteria(100);Candidate_division_TM7(100);unclassified(100);unclassified(100);unclassified(100);unclassified(100);unclassified(100);unclassified(100);unclassified(100);

Otu0755 0 0 0 0 0 0 0 0 0 0 0 0 0 0 0 0 0 0 0 0 0 0 0 0 0 0 0 0
0 0 0 0 0 0 0 0 0 0 0 57 0 0 0 0 0 0 0 0 0 0 0 0 0 0 0 0
0 0 0 0 0 0 0 0 0 0 0 0 0 0 0 0 0 0 0 0 0 0 0 0 0 0 0 0
0 0 0 0 0 0 0 0 0 0 0 0 0 0 0 0 0 0 0 0 0 0 0 0 0 0 0
M02255_77_000000000-ADUT3_1_1117_7675_23689 G----AC---GT-AG-GAG--------GCG-A-G------C--G--T---T--AT-C-CGG-AT------TC-A--C-T--GG-GC-------GT--A-----AA-GC-GC-AT-------G-TA-G-G-C-G------------G--T-TT-G-T-T-----------AA----G-T-C-C--------G-G-T--G--TT--A-AA-GC--C-C-CT-G-G-----CT-C-AA------C-T-G-G-G-G-G--A-G----G-T--C-A---T--T-------G--GA-G-A---C-------T--A-A--CA--G-A-C----T-T-G-A-G-G--G---C-AA-----CA-G-A--------G-G-C-A---CG-T------GG--A--ACT----C-C-C-G-GT--GT-A-G-CG-GT--G---G--A-A---TG-C-GT-AG--AG-A-TC--G-G--G-A-A-G-A-AC-A-CC---AG--T--G--GC-GAA-G--G-C--G-------A--C-G-T-G---CTG--G--GT-T-G--------------------C-A---A-C-T--GA--CG-C-T-G--A-GA--T-G-CG-A--AA-G-T---G-TG--GG-G--AG-C-A-AA--------CG--GG Bacteria(100);unclassified(100);unclassified(100);unclassified(100);unclassified(100);unclassified(100);unclassified(100);unclassified(100);unclassified(100);

Otu0756 0 0 0 0 0 0 0 0 0 0 0 0 0 0 0 0 0 0 0 0 0 0 0 0 0 0 0 0
0 3 0 0 0 0 0 0 0 16 3 0 0 0 0 0 0 0 0 0 0 0 0 0 0 0 0 0
0 0 0 0 0 0 0 0 0 0 0 0 0 0 0 0 0 0 0 0 0 0 0 0 0 0 0 0
0 0 0 0 0 0 0 0 0 0 0 0 0 0 0 0 0 0 0 0 0 0 0 0 0 0 0
M02255_77_000000000-ADUT3_1_2111_23617_16176 T----AC---AG-AG-GGC--------TCG-A-G------C--G--T---T--AA-T-CGG-AA------TC-A--C-T--GG-GC-------TT--A-----AA-GC-GT-GC-------G-TA-G-G-C-G------------G--A-TT-T-G-A-----------AG----G-C-G-C--------T-T-T--G--TG--A-AA-TC--C-C-TC-G-G-----CT-C-AA------C-C-G-A-G-G-A--A-C----T-G--C-T---T--G-------G--CG-A-A---C-------C--A-C--AA--A-T-C----T-T-G-A-G-G--C---T-GG-----TA-G-A--------G-G-T-G---GT-C------GG--A--ACA----A-T-A-G-GT--GG-A-G-CG-GT--G---A--A-A---TG-C-GT-AG--AT-A-TC--T-A--T-T-G-G-A-AC-A-CC---GA--A--G--GT-GAA-G--A-C--A-------G--G-C-C-A---CTG--G--GC-C-A--------------------G-T---C-C-T--GA--CG-C-T-G--A-GG--C-A-CG-A--AA-G-C---G-TG--GG-T--AG-C-A-AA--------CG--GG Bacteria(100);Planctomycetes(100);Phycisphaerae(100);Phycisphaerales(100);Phycisphaeraceae(100);CL500-3(100);unclassified(100);unclassified(100);unclassified(100);

Otu0757 0 0 0 0 0 0 0 0 0 0 0 0 0 0 0 0 0 0 0 0 0 0 9 0 0 0 0 0
0 0 0 0 0 0 0 0 0 0 0 0 0 0 0 0 0 0 0 0 0 0 0 0 0 0 0 0
0 0 0 0 0 0 0 0 0 0 0 0 0 0 0 0 0 6 0 0 0 0 0 0 0 0 0 0
0 0 0 0 0 0 0 0 0 0 0 0 0 0 0 0 0 0 0 0 0 0 0 0 0 0 0
M02255_76_000000000-ADUNE_1_1112_17353_22182 .....AC----T-AC-GT---------GCC-A-G------C--A--G---C--CG-C-GGT-AA------TA-C--G-T--AG-GT-------GG--C-----AA-GC-G--TT-------G----T-C-C-G------------G--A-CG-A-T-T----------AA----G-T-C-T--------G-A-T--G--TG--A-AA-TC--C-C-AC-G-G-----CT-T-AA------C-C-G-T-G-G-A--A------G-GT-C-A---T--T-------G--GA-A-A---C-------T--G-A--TT--G-G-C----T-T-G-A-G-T--G---C-AG-----AA-G-A--------G-G-A-G---AG-T------GG--A--ATT----C-C-A-C-GT--GT-A-G-CG-GT--G---A--A-A---TG-C-GT-AG--AT-A-TG--T-G--G-A-G-G-A-AC-A-CC---AG--T--G--GC-GAA-G--G-C--G-------G--C-T-C-T---CTG--G--TC-T-G--------------------C-A---A-C-T--GA--CG-C-T-G--A-GG--C-G-CG-A--AA-G-C---G-TG--GA-T--AG-C-A-AA--------CA--GG Bacteria(100);Firmicutes(100);unclassified(100);unclassified(100);unclassified(100);unclassified(100);unclassified(100);unclassified(100);unclassified(100);

Otu0758 0 0 0 0 0 0 0 0 0 0 0 0 0 0 0 0 0 0 0 0 0 0 0 0 0 0 0 0
0 0 0 0 0 0 0 0 0 0 0 23 0 0 0 0 0 0 0 0 0 0 0 0 0 0 0 0
0 0 0 0 0 0 0 0 0 0 0 0 0 0 0 0 0 0 0 0 0 0 0 0 0 0 0 0
0 0 0 0 0 0 0 0 0 0 0 0 0 0 0 0 0 0 0 0 0 0 0 0 0 0 0
M02255_77_000000000-ADUT3_1_1116_8450_20867 T----AC---GG-AG-GGG--------GCT-A-G------C--G--T---T--GT-T-CGG-AA------TT-A--C-T--GG-GC-------GT--A-----AA-GC-GC-AC-------G-TA-G-G-C-G------------G--C-TT-T-G-T-----------AA----G-T-T-A--------G-A-G--G--TG--A-AA-GC--C-C-GG-G-G-----CT-C-AA------C-T-C-C-G-G-A--A-T----T-G--C-C---T--T-------T--AA-G-A---C-------T--G-C--AT--C-G-C----T-A-G-A-A-C--A---C-GG-----GA-G-A--------G-G-T-A---AG-C------GG--A--ATT----C-C-G-A-GT--GT-A-G-AG-GT--G---A--A-A---TT-C-GT-AG--AT-A-TT--C-G--G-A-A-G-A-AC-A-CC---AG--T--G--GC-GAA-G--G-C--G-------G--C-T-T-A---CTG--G--AC-C-G--------------------T-T---G-T-T--GA--CG-C-T-G--A-GG--T-G-CG-A--AA-G-C---G-TG--GG-G--AG-C-A-AA--------CA--GG Bacteria(100);Proteobacteria(100);Alphaproteobacteria(100);Sphingomonadales(100);unclassified(100);unclassified(100);unclassified(100);unclassified(100);unclassified(100);

Otu0759 0 0 0 0 0 0 0 0 0 0 0 0 0 0 0 0 0 0 0 0 0 0 1 0 0 0 0 0
0 0 0 0 0 0 0 0 0 0 0 0 0 0 0 0 0 0 0 0 113 0 0 0 0 0 0 0
0 0 0 0 0 0 0 0 0 0 0 0 0 0 0 0 0 0 0 0 0 0 0 0 0 0 0 0
0 0 0 0 0 0 0 0 0 0 0 0 0 0 0 0 0 0 0 0 0 0 0 0 0 0 0
M02255_77_000000000-ADUT3_1_2108_15109_21181 T----AC---GT-AG-GGG--------GCG-A-G------C--G--T---T--AT-C-CGG-AA------TT-A--T-T--GG-GC-------GT--A-----AA-GA-GT-AC-------G-TA-G-G-C-G------------G--T-TT-T-T-T-----------AA----G-C-G-A--------G-G-G--G--TA--T-AA-GG--C-A-GC-G-G-----CT-T-AA------C-T-G-C-T-G-T--T------G-G--C-C---C--C-------T--CG-A-A---C-------T--G-G--AG--G-A-C----T-T-G-A-G-T--G---T-CG-----GA-G-A--------G-G-A-A---AG-C------GG--A--ATT----C-C-T-A-GT--GT-A-G-CG-GT--G---A--A-A---TG-C-GT-AG--AG-A-TT--A-G--G-A-G-G-A-AC-A-CC---AG--T--G--GC-GAA-G--G-C--G-------G--C-T-T-T---CTG--G--AC-G-A--------------------C-A---A-C-T--GA--CG-C-T-G--A-GG--T-A-CG-A--AA-G-C---G-TG--GG-G--AG-C-A-AA--------CA--GG Bacteria(100);Firmicutes(100);unclassified(100);unclassified(100);unclassified(100);unclassified(100);unclassified(100);unclassified(100);unclassified(100);

Otu0760 0 0 0 0 0 0 0 0 0 0 0 0 0 1 0 0 0 0 0 0 0 0 0 0 0 0 0 0
0 0 0 97 0 0 0 0 0 0 0 0 0 0 0 0 0 0 0 0 0 0 0 0 0 0 0 0 0
0 0 0 0 0 0 0 0 0 0 0 0 0 0 0 0 0 0 0 0 0 0 0 0 0 0 0 0 0
0 0 0 0 0 0 0 0 0 0 0 0 0 0 0 0 0 0 0 0 0 0 0 0 0 0 0
M02255_77_000000000-ADUT3_1_2109_6445_15208 T----AC---GT-AG-GTG--------GCA-A-G------C--G--T---T--GT-C-CGG-AT------TT-A--C-T--GG-GT-------GT--A-----AA-GG-GC-GT-------G-TA-G-G-C-G------------G--G-AC-T-G-C----------AA-
---G-T-C-T--------G-A-T--G--TG--T-AA-TC--T-G-GT-G-G-----CT-C-AA------C-C-A-C-C-A-A--A-C----T-G--C-A---T--T-------G--GA-A-A---C-------T--G-T--AG--T-T-C----T-T-G-A-G-T--A---T-CG-----GA-G-A--------G-G-C-A---GG-C------GG--A--ATT----C-C-T-A-
GT--GT-A-G-CG-GT--G---A--A-A---TG-C-GT-AG--AT-A-TT--A-G--G-A-A-G-A-AC-A-CC---AG--T--G--GC-GAA-G--G-C--G-------G--C-C-T-G---CTG--G--AC-G-A--------------------C-A---A-C-T--GA--CG-C-T-G--A-GG--C-G-CG-A--AA-G-C---G-TG--GG-G--AG-
C-A-AA--------CA--GG Bacteria(100);Firmicutes(100);Clostridia(100);Clostridiales(100);unclassified(100);unclassified(100);unclassified(100);unclassified(100);unclassified(100);

Otu0761 0 0 0 0 0 0 0 0 0 0 0 0 0 0 0 0 0 0 0 0 0 0 24 0 0 0 0 0
0 0 0 0 0 0 0 0 0 0 0 0 0 0 0 0 0 0 0 0 0 0 0 0 0 0 0 0 0
0 0 0 0 0 0 0 0 0 0 0 0 0 0 0 0 0 0 0 0 0 0 0 0 0 0 0 0 0
0 0 0 0 0 0 0 0 0 0 0 0 0 0 0 0 0 0 0 0 0 0 0 0 0 0 0
M02255_77_000000000-ADUT3_1_1102_6528_19327 T----AC---AG-AG-GGA--------GCT-A-G------C--G--T---T--GT-T-CGG-AA------TC-A--C-T--GG-GC-------GT--A-----AA-GC-GC-GC-------G-TA-G-G-T-G------------G--C-TT-A-G-T----------AA-
---G-T-T-A--------G-G-A--G--TG--A-AA-TC--C-T-GG-G-G-----CT-T-AA------C-C-T-C-A-G-A--A-T----T-G--C-T---C--T-------T--AA-G-A---C-------T--G-C--TA--A-G-C----T-A-G-A-G-G--T---A-GG-----CA-G-A--------G-G-A-T---GG-T------GG--A--ATT----C-T-T-A-
GT--GT-A-G-CG-GT--G---A--A-A---TG-C-GT-AG--AT-A-TT--A-A--G-A-G-G-A-AC-A-CC---AG--T--G--GC-GAA-A--G-C--G-------G--C-C-A-T---CTG--G--GC-C-T--------------------A-T---T-C-T--GA--CA-C-T-A--A-AG--C-G-CG-A--AA-G-C---G-TG--GG-G--AG-C-
A-AA--------CA--GG Bacteria(100);unclassified(100);unclassified(100);unclassified(100);unclassified(100);unclassified(100);unclassified(100);unclassified(100);unclassified(100);

Otu0762 0 0 0 0 0 0 0 0 0 0 0 0 0 0 0 0 0 0 0 0 0 0 0 0 0 0 0 0
0 0 0 0 0 0 0 0 0 0 0 57 0 0 0 0 0 0 0 0 0 0 0 0 0 0 0 0 0
0 0 0 0 0 0 0 0 0 0 0 0 0 0 0 0 0 0 0 0 0 0 0 0 0 0 0 0 0
0 0 0 0 0 0 0 0 0 0 0 0 0 0 0 0 0 0 0 0 0 0 0 0 0 0 0
M02255_77_000000000-ADUT3_1_1118_17544_10868 T----AC---GT-AG-GGA--------GCA-A-G------C--G--T---T--AT-C-CGG-AT------TT-A--C-T--GG-GT-------GT--A-----AA-GG-GC-GT-------G-TA-G-G-C-G------------G--G-AT-T-G-C-----
------AA----G-T-C-A--------G-A-T--G--TG--A-AA-AC--T-C-GG-A-G-----CT-C-AA------C-T-C-C-G-A-G--C-C----T-G--C-A---T--T-------T--GA-A-A---C-------T--G-T--AG--T-T-C----T-T-G-A-G-T--G---G-TG-----GA-G-A--------G-G-C-A---AA-T------GG--A--ATT----C-
C-T-A-GT--GT-A-G-CG-GT--G---A--A-A---TG-C-GT-AG--AT-A-TT--A-G--G-A-A-G-A-AC-A-CC---AG--T--G--GC-GAA-G--G-C--G-------A--T-T-T-G---CTG--G--AC-A-C--------------------T-A---A-C-T--GA--CG-C-T-G--A-GG--C-G-CG-A--AA-G-C---G-TG--GG-G-
-AG-C-A-AA--------CA--GG Bacteria(100);unclassified(100);unclassified(100);unclassified(100);unclassified(100);unclassified(100);unclassified(100);unclassified(100);unclassified(100);

Otu0763 0 0 0 0 0 0 0 0 0 0 0 0 0 0 0 0 0 0 0 0 0 0 24 0 0 0 0 0
0 0 0 0 0 0 0 0 0 0 0 0 0 0 0 0 0 0 0 0 0 0 0 0 0 0 0 0 0
0 0 0 0 0 0 0 0 0 0 0 0 0 0 0 0 0 0 0 0 0 0 0 0 0 0 0 0 0
0 0 0 0 0 0 0 0 0 0 0 0 0 0 0 0 0 0 0 0 0 0 0 0 0 0 0
M02255_77_000000000-ADUT3_1_1112_22480_12476 T----AC---GT-AG-GGT--------CCA-A-G------C--G--T---T--AT-C-CGG-AA------TC-A--C-T--GG-GC-------GT--A-----AA-GC-GT-GC-------G-TA-G-G-T-G------------G--A-TT-G-T-T------
-----AA----G-T-G-C--------G-A-T--G--CG--A-AA-TC--C-T-GT-G-G-----CT-C-AA------C-C-A-T-A-T-G--G-AC---T-G--T-A---T--C-------G--CA-T-A---C-------T--G-G--CT--A-T-C----T-A-G-A-G-G--A---T-AT-----GA-G-A--------G-G-C-A---AG-T------GG--A--ATT----A-
C-T-G-GT--GT-A-G-CA-GT--G---A--A-A---TG-C-GT-AG--AT-A-TC--A-G--T-A-G-G-A-AC-A-CC---AA--T--G--GC-GAA-G--G-C--A-------G--C-T-T-G---CTG--G--CA-T-A--------------------T-T---T-C-T--GA--CG-C-T-A--A-GG--C-A-CG-A--AA-G-C---G-TG--GG-G--
AG-C-G-AA--------CA--GG Bacteria(100);WCHB1-60(100);unclassified(100);unclassified(100);unclassified(100);unclassified(100);unclassified(100);unclassified(100);unclassified(100);

Otu0764 0 0 0 0 0 0 0 0 0 0 0 0 0 0 0 0 0 0 0 0 0 1 0 0 0 0 0 0
0 0 0 0 0 0 0 0 0 0 0 0 0 0 0 0 0 0 0 0 0 0 0 0 0 0 0 0 0
0 0 0 0 0 0 0 0 0 0 0 0 1 0 0 0 0 0 0 0 0 0 0 0 0 0 0 0 0
0 0 0 0 0 0 0 0 0 0 0 0 0 0 5 0 0 0 0 0 0 0 0 0 0 0 0
M02255_76_000000000-ADUNE_1_2106_26423_8154 T----AC--GAG-AA-GAA--------ACA-C-T------G--G--T---T--CG-T--GA-AC------CA-G--C-T--TG-AC-------G---G-----TA-TC-GT-AA-------G-AA-T-A-A-G------------G--A-TC-G-G-C----------TA--
--A-C-T-C--------C-G-T--G--CC--A-GC-AG--C-C-GC-G-G-----TA-A-TA------C-G-G-A-G-G-A--T-C----C-A--A-G---C--G-------T--TA-T-C---C-------G--G-A--AT--C-A-T----T-G-G-G-T-T--T---A-AA-----GG-G-T--------C-C-G-T---AG-G------CG--G--TCA----G-A-T-A-
AG--TC-A-G-TG-GT--G---A--A-A---TG-C-GT-AG--AT-A-TA--G-G--A-A-G-G-A-AC-A-CC---AG--T--G--GC-GAA-G--G-C--G-------A--C-T-A-C---CTG--G--AC-T-G--------------------A-T---A-C-T--GA--CA-C-T-G--A-GG--T-G-CG-A--AA-G-C---G-TG--GG-G--AG-
C-A-AA--------CA--GG Bacteria(100);unclassified(100);unclassified(100);unclassified(100);unclassified(100);unclassified(100);unclassified(100);unclassified(100);unclassified(100);

Otu0765 0 0 0 0 0 0 0 0 0 0 0 0 0 0 0 0 0 0 0 0 0 0 19 0 0 0 0 0
0 0 0 0 0 0 0 0 0 0 0 0 0 0 0 0 0 0 0 0 0 0 0 0 0 0 0 0 0
0 0 0 0 0 0 0 0 0 0 0 0 0 0 0 0 0 0 0 0 0 0 0 0 0 0 0 0 0
0 0 0 0 0 0 0 0 0 0 0 0 0 0 0 0 0 0 0 0 0 0 0 0 0 0 0 0
M02255_77_000000000-ADUT3_1_1112_12071_11125 T----AC---GG-AG-GAT--------CCA-A-G------C--G--T---T--AT-C-CGG-AA------TT-A--C-T--GG-GC-------GT--A-----AA-GA-GTTGC-------G-TA-G-G-C-G------------G--C-AA-A-G-T----
-------AA----G-C-A-G--------G-T-A--G--TG--A-AA-GC--G-T-GT-G-G-----CT-C-AA------C-C-A-T-A-C-T--C-A----C-A--T-T---A--T-------C--TG-A-A---C-------T--G-C--TT--A-G-C----T-A-G-A-G-G--A---T-GA-----GA-G-A--------G-G-T-C---AC-T------GG--A--ATT----C-
C-T-T-GT--GT-A-G-GA-GT--G---A--A-A---TC-C-GT-AG--AT-A-TA--A-G--G-A-G-G-A-AC-A-CC---GA--T--G--GC-GTA-G--G-C--A-------G--G-T-G-A---CTG--G--CT-C-A--------------------T-T---C-C-T--GA--CG-C-T-C--A-GG--C-A-CG-A--AA-G-C---G-TG--GG-G-
-AG-C-A-AA--------CG--GG Bacteria(100);Candidate_division_TM7(100);unclassified(100);unclassified(100);unclassified(100);unclassified(100);unclassified(100);unclassified(100);unclassified(100);

Otu0766 0 0 0 0 0 0 0 0 0 0 0 0 0 0 0 0 0 0 0 0 0 0 1 0 0 0 0 6
1 0 0 0 0 0 0 0 0 0 0 0 0 0 0 0 0 0 0 0 0 0 0 0 0 0 0 1 0
0 0 0 0 0 0 0 0 0 0 0 0 0 0 0 0 0 0 0 0 0 0 0 0 0 0 0 0 0
0 0 0 0 0 0 0 0 0 0 0 0 0 0 0 0 0 0 0 0 0 0 0 0 0 0 0 0
M02255_76_000000000-ADUNE_1_2101_16763_16254 TG---AC---AG-AA-GAA--------GCA-C-C------G--G--C---T--AACT-CCG-TG------CC-A--G-C--AG-CC-------GC--G-----GT-AA-TA-CG-------G-AG-G-G-T-GC------------G--A-GC-G-T-T--
---------AA----T-C-G-A--------G-A-A--G--TG--A-AA-GC--C-C-CG-G-G-----CT-C-AA------C-C-T-G-G-G-A--A-C----T-G--C-A---T--T-------T--CG-A-A---C-------T--G-G--CA--A-A-C----T-A-G-A-G-T--G---T-GA-----TA-G-A--------G-G-G-T---GG-T------AG--A--ATT---
-T-C-A-G-GT--GT-A-G-CG-GT--G---A--A-A---TG-C-GT-AG--AG-A-TC--T-G--A-A-G-G-A-AT-A-CC---GA--T--G--GC-GAA-G--G-C--A-------G--C-C-A-C---CTG--G--GT-C-A--------------------A-C---A-C-T--GA--CG-C-T-C--A-TG--T-A-CG-A--AA-G-C---G-TG--
GG-G--AG-C-A-AA--------CG--GG Bacteria(100);Proteobacteria(100);Gammaproteobacteria(100);Alteromonadales(100);unclassified(100);unclassified(100);unclassified(100);unclassified(100);unclassified(100);

Otu0767 0 0 0 0 0 0 0 0 0 0 0 0 0 0 0 0 0 0 0 0 0 0 0 0 0 0 0 0
0 0 0 0 0 0 0 0 0 0 0 0 0 0 0 0 0 0 0 0 0 0 0 0 0 0 0 0 0
0 0 0 0 0 0 0 0 0 0 0 0 0 0 0 0 0 0 0 0 0 0 0 0 0 0 0 0 0
0 0 0 0 0 0 0 0 2 0 0 0 1 0 0 0 0 0 0 0 0 0 0 0 0 0 0 0
M02255_77_000000000-ADUT3_1_2109_8979_20201 T----AA---GC-AC-CGG--------CTA-A-C------T--A--C---G--TG-C-CAG-CA------GC-C--G-C--GG-TA-------AT--A-----CG-TA-GG-GT-------G-CA-A-G-C-G------------T--T-AA-T-C-G-----------GA-
-----A-T-T--------A-C-T--G--TG--A-AA-TC--C-C-CG-G-G-----CT-C-AA------C-C-T-G-G-G-A--A-C----T-G--C-A---T--T-------T--GT-G-A---C-------T--G-T--AT--A-G-C----T-A-G-A-G-T--A---C-GG-----TA-G-A--------G-G-G-G---GA-T------GG--A--ATT----C-C-G-C-
GT--GT-A-G-CA-GT--G---A--A-A---TG-C-GT-AG--AT-A-TG--C-G--G-A-G-G-A-AC-A-CC---GA--T--G--GC-GAA-G--G-C--A-------A--T-C-C-C---CTG--G--AC-C-T--------------------G-T---A-C-T--GA--CG-C-T-C--A-TG--C-A-CG-A--AA-G-C---G-TG--GG-G--AG-C-
A-AA--------CA--GG Bacteria(100);Proteobacteria(100);Betaproteobacteria(100);Burkholderiales(100);Comamonadaceae(100);unclassified(100);unclassified(100);unclassified(100);unclassified(100);

Otu0768 0 0 0 0 0 0 0 0 0 0 0 0 0 0 0 0 0 0 0 0 0 0 0 0 0 0 0 0
0 0 0 0 0 0 0 0 0 0 0 24 0 0 0 0 0 0 0 0 0 0 0 0 0 0 0 0 0
0 0 0 0 0 0 0 0 0 0 0 0 0 0 0 0 0 0 0 0 0 0 0 0 0 0 0 0 0
0 0 0 0 0 0 0 0 0 0 0 0 0 0 0 0 0 0 0 0 0 0 0 0 0 0 0 0
M02255_77_000000000-ADUT3_1_1112_3979_11475 T----AC---GT-AG-GGG--------GCG-A-G------C--G--T---T--AT-C-CGG-AA------TT-A--T-T--GG-GC-------GT--A-----AA-GA-GT-GC-------G-TA-G-G-T-G------------G--T-AA-C-T-T-----------AA-
---G-C-G-C--------G-G-G--G--TT--T-AA-GG--C-A-AT-G-G-----CT-T-AA------C-C-G-T-T-G-T--T------C-G--C-C---C--C-------G--CG-A-A---C-------T--G-G--GA--T-A-C----T-T-G-A-G-T--G---C-AG-----GA-G-A--------G-G-A-A---AG-C------GG--A--ATT----C-C-T-A-
GT--GT-A-G-CG-GT--G---A--A-A---TG-C-GT-AG--AT-A-TT--A-G--G-A-G-G-A-AC-A-CC---AG--T--G--GC-GAA-G--G-C--G-------G--C-T-T-T---CTG--G--AC-T-G--------------------A-A---A-C-T--GA--CA-C-T-G--A-GG--C-A-CG-A--AA-G-T---G-TG--GG-G--AG-
C-A-AA--------CA--GG Bacteria(100);Firmicutes(100);Clostridia(100);Clostridiales(100);Family_XIII_Incertae_Sedis(100);unclassified(100);unclassified(100);unclassified(100);unclassified(100);

Otu0769 0 0 0 0 0 0 0 0 0 0 0 0 0 0 0 0 0 0 0 0 0 0 14 0 0 0 0 0
0 0 0 0 0 0 0 0 0 0 0 0 0 0 0 0 0 0 0 0 0 0 0 0 0 0 0 0 0
0 0 0 0 0 0 0 0 0 0 0 0 0 0 0 0 0 0 0 0 0 0 0 0 0 0 0 0 0
0 0 0 0 0 0 0 0 0 0 0 0 0 0 0 0 0 0 0 0 0 0 0 0 0 0 0 0
M02255_77_000000000-ADUT3_1_1102_20374_8981 T----AC---GT-AG-GAT--------CCA-A-G------C--G--T---T--AT-C-CGG-AG------TG-A--C-T--GG-GC-------GT--A-----AA-GA-GTTGC-------G-TA-G-G-T-G------------G--T-TT-G-T-T-----------AA-
---G-T-A-G--------A-T-A--G--TG--A-AA-TC--T-G-GC-G-G-----CT-C-AA------C-C-G-T-ACA-G--G-C----T-A--T-T---A--T-------C--TA-A-A---C-------T--G-A--CA--A-A-C----T-C-G-A-G-A--A---T-GG-----TA-G-A--------G-G-T-A---AC-T------GG--A--ATT----T-C-T-T-
GT--GT-A-G-GA-GT--G---A--A-A---TC-C-GT-AG--AT-A-TA--A-G--A-A-G-G-A-AC-A-CC---AA--T--G--GC-GTA-G--G-C--A-------G--G-T-T-A---CTG--G--AC-C-A--------------------T-T---T-C-T--GA--CA-C-T-G--A-GG--C-A-CG-A--AA-G-C---G-TG--GG-G--AG-C-
G-AA--------CC--GG Bacteria(100);Candidate_division_TM7(100);unclassified(100);unclassified(100);unclassified(100);unclassified(100);unclassified(100);unclassified(100);unclassified(100);

Otu0770 0 0 0 0 0 0 0 0 0 0 0 0 0 0 0 0 0 0 0 0 0 0 0 0 0 0 0 0
0 0 0 0 0 0 0 0 0 0 0 34 0 0 0 0 0 0 0 0 0 0 0 0 0 0 0 0 0
0 0 0 0 0 0 0 0 0 0 0 0 0 0 0 0 0 0 0 0 0 0 0 0 0 0 0 0 0
0 0 0 0 0 0 0 0 0 0 0 0 0 0 0 0 0 0 0 0 0 0 0 0 0 0 0
M02255_77_000000000-ADUT3_1_1111_23758_5762 T----AC---GT-AG-GTG--------GCG-A-G------C--G--T---T--AT-C-CGG-AT------TT-A--C-T--GG-GC-------GT--A-----AA-GC-GC-GC-------G-CA-G-G-C-G------------G--C-TG-T-T-C-----------AA----G-T-C-C--------G-A-T--G--TG--A-AA-TC--T-T-CC-G-G-----CT-T-AA------C-T-G-G-A-A-A--A-C----G-G--C-A---T--C-------G--GA-T-A---C-------T--G-G--AC--G-G-C----T-G-G-A-A-G--G---T-GG-----GA-G-A--------G-G-G-T---AG-C------GG--A--ATT----C-C-C-G-GT--GT-A-G-TG-GT--G---A--A-A---TG-C-GT-AG--AT-A-TC--G-G--G-A-G-G-A-AC-A-CC---AG--T--G--GC-GAA-A--G-C--G-------G--C-T-A-C---CTG--G--CC-C-A--------------------C-T---C-T-T--GA--CG-C-T-G--A-GG--C-G-CG-A--AA-G-C---T-AG--GG-G--AG-C-A-AA--------CG--GG Bacteria(100);unclassified(100);unclassified(100);unclassified(100);unclassified(100);unclassified(100);unclassified(100);unclassified(100);unclassified(100);

Otu0771 0 0 0 0 0 0 0 0 0 0 0 0 0 0 0 0 0 0 0 0 0 0 0 0 0 0 0 0
0 0 0 0 0 0 0 0 0 0 0 35 0 0 0 0 0 0 0 0 0 0 0 0 0 0 0 0 0
0 0 0 0 0 0 0 0 0 0 0 0 0 0 0 0 0 0 0 0 0 0 0 0 0 0 0 0 0
0 0 0 0 0 0 0 0 0 0 0 0 0 0 0 0 0 0 0 0 0 0 0 0 0 0 0
M02255_77_000000000-ADUT3_1_2111_6066_10438 T----AC---GT-AG-GGG--------GCG-A-G------C--G--T---T--GT-C-CGG-AA------TT-A--C-T--GG-GC-------GT--A-----AA-GG-GT-GC-------G-TA-G-G-C-G------------G--C-CA-T-T-T-----------AA----G-T-C-C--------T-G-T--G--TG--A-AA-GG--C-C-CC-G-G-----CT-T-AA------C-C-G-G-G-G-A--G------G-GC-C-A---T--G-------G--GA-A-A---C-------T--G-G--AT--G-G-C----T-T-G-A-G-T--G---C-CG-----GA-G-A--------G-G-G-T---GG-C------GG--A--ATT----C-C-C-G-GT--GT-A-G-CG-GT--G---A--A-A---TG-C-GT-AG--AT-A-TC--G-G--G-A-G-G-A-AC-A-CC---AG--T--G--GC-GAA-G--G-C--G-------G--C-C-A-C---CTG--G--AC-G-G--------------------T-A---A-C-T--GA--CG-C-T-G--A-GG--C-A-CG-A--CA-G-C---T-GG--GG-G--AG-C-A-AA--------CA--GG Bacteria(100);unclassified(100);unclassified(100);unclassified(100);unclassified(100);unclassified(100);unclassified(100);unclassified(100);unclassified(100);

Otu0772 0 0 0 0 0 0 0 0 0 0 0 0 0 0 0 0 0 0 0 0 0 0 0 0 0 0 0 0
0 0 0 0 0 0 0 0 0 0 0 21 0 0 0 0 0 0 0 0 0 0 0 0 0 0 0 0 0
0 0 0 0 0 0 0 0 0 0 0 0 0 0 0 0 0 0 0 0 0 0 0 0 0 0 0 0 0
0 0 0 0 0 0 0 0 0 0 0 0 0 0 0 0 0 0 0 0 0 0 0 0 0 0 0
M02255_77_000000000-ADUT3_1_1115_25201_8853 T----AC---GG-AG-GGG--------GCT-A-G------C--G--T---T--GT-T-CGG-AA------TT-A--C-T--GG-GC-------GT--A-----AA-GC-GC-GC-------G-TA-G-G-C-G------------G--A-TC-T-G-C-----------AA----G-T-C-A--------G-G-G--G--TG--A-AA-TC--C-C-GA-G-G-----CT-C-AA------C-C-T-C-G-G-A--A-C----T-G--C-C---T--T-------T--GA-T-A---C-------T--G-C--GG--A-T-C----T-T-G-A-G-T--C---C-GA-----GA-G-A--------G-G-T-G---AG-T------GG--A--ATT----C-C-T-A-GT--GT-A-G-AG-GT--G---A--A-A---TT-C-GT-AG--AT-A-TT--A-G--G-A-A-G-A-AC-A-CC---AG--T--G--GC-GAA-G--G-C--G-------G--C-T-C-A---CTG--G--CT-C-G--------------------G-T---A-C-T--GA--CG-C-T-G--A-GG--C-G-CG-A--AA-G-C---G-TG--GG-G--AG-C-A-AA--------CA--GG Bacteria(100);Proteobacteria(100);Alphaproteobacteria(100);unclassified(100);unclassified(100);unclassified(100);unclassified(100);unclassified(100);unclassified(100);

Otu0773 0 0 0 0 0 0 0 0 0 0 0 0 0 0 0 0 0 0 0 0 0 0 12 0 0 0 0 0
0 0 0 0 0 0 0 0 0 0 0 0 0 0 0 0 0 0 0 0 0 0 0 0 0 0 0 0 0
0 0 0 0 0 0 0 0 0 0 0 0 0 0 0 0 0 0 0 0 0 0 0 0 0 0 0 0 0
0 0 0 0 0 0 0 0 0 0 0 0 0 0 0 0 0 0 0 0 0 0 0 0 0 0 0
M02255_77_000000000-ADUT3_1_1106_7183_20466 T----AC---AG-AG-ACC--------TCA-A-G------C--G--T---T--AT-C-CGG-AT------TT-A--T-T--GG-GC-------GT--A-----AA-GG-GT-CC-------G-CA-G-G-T-G------------G--C-TG-T-G-T-----------AA----G-T-C-A--------T-G-C--G--TC--A-AA-TC--T-C-TG-G-G-----CT-C-AA------C-C-C-A-G-A-A--A-C----C-G--C-G---T--G-------T--GA-T-A---C-------T--G-C--GC--A-G-C----T-A-G-A-G-G--C---T-GG-----GA-G-A--------G-G-T-G---CA-C------GG--A--ATT----A-C-T-G-GT--GT-A-G-CG-GT--A---A--A-A---TG-C-GT-TA--AT-A-TC--A-G--T-A-A-G-A-AC-A-CC---AA--A--G--GC-GAA-G--G-C--A-------G--T-G-C-A---CTG--G--AA-C-A--------------------G-T---C-C-T--GA--CA-C-T-C--A-GG--G-A-CG-A--AA-G-C---G-TG--GG-G--AG-C-G-AA--------AG--GG Bacteria(100);unclassified(100);unclassified(100);unclassified(100);unclassified(100);unclassified(100);unclassified(100);unclassified(100);unclassified(100);

Otu0774 0 0 0 0 0 0 0 0 0 0 0 0 0 0 0 0 0 0 0 0 0 0 21 0 0 0 0 0
0 0 0 0 0 0 0 0 0 0 0 0 0 0 0 0 0 0 0 0 0 0 0 0 0 0 0 0 0
0 0 0 0 0 0 0 0 0 0 0 0 0 0 0 0 0 0 0 0 0 0 0 0 0 0 0 0 0
0 0 0 0 0 0 0 0 0 0 0 0 0 0 0 0 0 0 0 0 0 0 0 0 0 0 0
M02255_77_000000000-ADUT3_1_1109_5524_12893 T----AC---AG-AG-GTC--------TCA-A-G------C--G--T---T--GT-T-CGG-AT------TC-A--T-T--GG-GC-------GT--A-----AA-GG-GT-GC-------G-TA-G-G-T-G------------G--T-CA-T-G-T-----------AA----G-T-C-T--------A-A-T--G--TG--A-AA-TC--T-C-GG-G-G-----CT-T-AA------C-C-C-C-G-A-A--A-T----T-G--C-A---T--C-------G--GA-T-A---C-------T--G-C--GT--G-A-C----T-A-G-A-G-G--A---T-TG-----TA-G-A--------G-G-A-G---AT-T------GG--A--ATT----C-A-C-G-GT--GT-A-G-CA-GT--G---A--A-A---TG-C-GT-AG--AT-A-TC--G-T--G-A-G-G-A-AG-A-CC---AG--T--T--GC-GAA-G--G-C--G-------A--A-T-C-T---CTG--G--GC-A-A--------------------T-T---C-C-T--GA--CA-C-T-G--A-GG--C-A-CG-A--AG-G-C---C-AG--GG-G--AG-C-A-AA--------CG--GG Bacteria(100);Verrucomicrobia(100);Spartobacteria(100);unclassified(100);unclassified(100);unclassified(100);unclassified(100);unclassified(100);unclassified(100);

Otu0775 0 0 0 0 0 0 0 0 0 0 0 0 0 0 0 0 0 0 0 0 0 0 0 0 0 0 0 0
0 0 0 0 0 0 0 0 0 0 0 25 0 0 0 0 0 0 0 0 0 0 0 0 0 0 0 0
0 0 0 0 0 0 0 0 0 0 0 0 0 0 0 0 0 0 0 0 0 0 0 0 0 0 0 0
0 0 0 0 0 0 0 0 0 0 0 0 0 0 0 0 0 0 0 0 0 0 0 0 0 0 0
M02255_77_000000000-ADUT3_1_2101_20932_16178 G----AC---GG-AG-GGT--------GCT-A-G------C--G--T---T--GT-T-CGG-AA------TC-A--C-T--GG-GC-------GT--A-----AA-GG-GC-GC-------G-TA-G-G-C-G------------G--C-CA-A-G-G---
--------AA----G-T-C-G--------G-A-T--G--TG--A-AA-GC--C-C-GG-G-G-----CT-C-AA------C-C-T-C-G-G-A--G-G----T-G--C-A---T--C-------C--GA-G-A---C-------T--C-T--TT--G-G-C----T-G-G-A-G-G--C---C-GG-----AA-G-A--------G-G-A-G---GG-T------GG--A--ATA--
--C-C-C-A-GT--GT-A-G-AG-GT--G---A--A-A---TT-C-GT-AG--AT-A-TT--G-G--G-T-G-G-A-AC-A-CC---GG--T--G--GC-GAA-A--G-C--G-------G--C-T-C-T---CTG--G--TC-C-G--------------------G-A---C-C-T--GA--CG-C-T-G--A-GG--C-G-CG-A--AA-G-C---G-T-G-
GG-G--AG-C-A-AA--------CA--GG Bacteria(100);Proteobacteria(100);Alphaproteobacteria(100);unclassified(100);unclassified(100);unclassified(100);unclassified(100);unclassified(100);unclassified(100);

Otu0776 0 0 0 0 0 0 0 0 0 0 0 0 0 0 0 0 0 0 0 0 0 0 6 0 0 0 0 0
0 0 0 0 0 0 0 0 0 0 0 0 0 0 0 0 0 0 0 0 0 0 0 0 0 0 0 0
4 0 0 0 0 0 0 0 0 6 1 0 2 0 0 0 0 0 0 0 0 0 0 0 0 0 0 0
0 0 0 0 0 0 0 0 0 0 0 0 0 0 0 0 0 0 0 0 0 0 0 0 0 0 0
M02255_77_000000000-ADUT3_1_2114_4055_16501 T----AC---GT-AG-GGT--------CCG-A-G------C--G--T---T--GT-C-CGG-AA------TT-A--T-T--GG-GC-------GT--A-----AA-GG-GC-TC-------G-TA-G-G-C-G------------G--T-CT-G-T-T-----------GC--
--G-T-C-G--------G-G-A--G--TG--A-AA-AC--C-C-AG-G-G-----CT-T-AA------C-C-C-T-G-G-G--C-C----T-G--C-T---T--T-------C--GA-T-A---C-------G--G-G--CA--G-A-C----T-A-G-A-G-G--C---A-TT-----CA-G-G--------G-G-A-G---AA-C------GG--A--ATT----C-C-T-G-
GT--GT-A-G-CG-GT--G---A--A-A---TG-C-GC-AG--AT-A-TC--A-G--G-A-G-G-A-AC-A-CC---GG--T--G--GC-GAA-G--G-C--G-------G--T-T-C-T---CTG--G--GA-A-T--------------------G-T---T-C-T--GA--CG-C-T-G--A-GG--A-G-CG-A--AA-G-T---G-TG--GG-G--AG-
C-G-AA--------CA--GG Bacteria(100);Actinobacteria(100);Actinobacteria(100);Actinobacteridae(100);Actinomycetales(100);Propionibacterineae(100);Nocardioidaceae(100);Nocardioides(100);unclassified(100);

Otu0777 0 0 0 0 0 0 0 0 0 0 0 0 0 0 0 0 0 0 0 0 0 0 8 0 0 0 0 0
0 0 0 0 0 0 0 0 0 0 0 0 0 0 0 0 0 0 0 0 0 0 0 0 0 0 0 0
0 0 0 0 0 0 0 0 0 0 0 0 0 0 0 0 0 0 0 0 0 0 0 0 0 0 0 0
0 0 0 0 0 0 0 0 0 0 0 0 0 0 0 0 0 0 0 0 0 0 0 0 0 0 0
M02255_77_000000000-ADUT3_1_1110_17222_7181 T----AC---GG-AG-GGG--------GTT-A-G------C--G--T---T--GT-T-CGG-AA------TT-A--C-T--GG-GC-------GT--A-----AA-GC-GC-AC-------G-TA-G-G-C-G------------G--A-TC-A-A-T-----------AA-
---G-T-T-A--------G-A-G--G--TG--A-AA-TC--C-C-GG-G-G-----CT-C-AA------C-C-C-C-G-G-A--A-C----T-G--C-C---T--T-------T--AA-T-A---C-------T--G-T--TG--A-T-C----T-A-G-A-G-T--T---C-GA-----GA-G-A--------G-G-T-A---AG-T------GG--A--ATT----C-C-G-A-
GT--GT-A-G-AG-GT--G---A--A-A---TT-C-GT-AG--AT-A-TT--C-G--G-A-G-G-A-AC-A-CC---AG--T--G--GC-GAA-G--G-C--G-------G--C-T-T-A---CTG--G--CT-C-G--------------------A-T---A-C-T--GA--CG-C-T-G--A-GG--T-G-CG-A--AA-G-T---G-TG--GG-G--AG-C-
A-AA--------CA--GG Bacteria(100);Proteobacteria(100);Alphaproteobacteria(100);Rhodobacterales(100);Rhodobacteraceae(100);unclassified(100);unclassified(100);unclassified(100);unclassified(100);

Otu0778 0 0 0 0 0 0 0 0 0 0 0 0 0 0 0 0 0 0 0 0 0 0 0 0 0 0 0 0
0 0 0 0 0 0 0 0 0 0 0 37 0 0 123 0 0 0 0 0 0 0 0 0 0 0 0 0
0 0 0 0 0 0 0 0 0 0 0 0 0 0 0 0 0 0 0 0 0 0 0 0 0 0 0 0
0 0 0 0 0 0 0 0 0 0 0 0 0 0 0 0 0 0 0 0 0 0 0 0 0 0 0
M02255_77_000000000-ADUT3_1_1108_2297_12960 T----AC---GT-AG-GCA--------GCG-A-G------C--G--T---T--GT-T-CGG-AG------TT-A--C-T--GG-GC-------GT--A-----AA-GC-GT-GC-------G-TA-G-G-C-G------------G--T-GC-C-A-T-----------AA-
---G-T-C-T--------G-T-T--G--TG--A-AA-TC--T-C-CC-G-G-----CT-C-AA------C-T-G-G-G-A-G--G-G----T-G--C-G---G--T-------G--GA-A-A---C-------T--G-T--GG--T-G-C----T-G-G-A-G-T--G---T-GG-----GA-G-A--------G-G-T-A---GG-C------GG--A--ATT----C-C-T-G-
GT--GT-A-G-CG-GT--G---A--A-A---TG-C-GT-AG--AT-A-TC--A-G--G-A-G-G-A-AC-A-CC---TG--T--G--GT-GTA-G--A-C--G-------G--C-T-T-A---CTG--G--AC-C-A--------------------T-G---A-C-T--GA--CG-C-T-G--A-GG--C-A-CG-A--AA-G-C---G-TG--GG-G--AG-C-
A-AA--------CA--GG Bacteria(100);unclassified(100);unclassified(100);unclassified(100);unclassified(100);unclassified(100);unclassified(100);unclassified(100);unclassified(100);

Otu0779 0 0 0 0 0 0 0 0 0 0 0 0 0 0 0 0 0 0 0 0 0 0 0 0 0 0 0 0
0 0 0 0 0 0 0 0 0 0 0 17 0 0 0 0 0 0 0 0 0 0 0 0 0 0 0 0
0 0 0 0 0 0 0 0 0 0 0 0 0 0 0 0 0 0 0 0 0 0 0 0 0 0 0 0
0 0 0 0 0 0 0 0 0 0 0 0 0 0 0 0 0 0 0 0 0 0 0 0 0 0 0
M02255_77_000000000-ADUT3_1_1116_4262_13251 T----AC---GG-AG-GAT--------CCA-A-G------C--G--T---T--AT-C-CGG-AA------TT-A--C-T--GG-GC-------GT--A-----AA-GA-GTTGC-------G-TA-G-G-T-G------------G--C-AT-T-G-T-----------AA-
---G-T-T-T--------G-T-A--G--TG--A-AA-GC--G-T-GG-G-G-----CT-C-AA------C-C-C-C-A-T-A--T-C----C-A--T-T---A--T-------G--AA-A-A---C-------T--G-C--AA--A-G-C----T-A-G-A-G-G--A---C-AA-----GA-G-A--------G-G-T-T---AT-T------GG--A--ATT----C-C-T-A-
GT--GT-A-G-GA-GT--G---A--A-A---TC-C-GT-AG--AT-A-TT--A-G--G-A-G-G-A-AC-A-CC---GA--T--G--GC-GTA-G--G-C--A-------G--A-T-A-A---CTG--G--CT-T-G--------------------T-T---C-C-T--GA--CA-C-T-A--A-GG--C-A-CG-A--AA-G-C---A-TG--GG-T--AG-C-
A-AA--------CG--GG Bacteria(100);unclassified(100);unclassified(100);unclassified(100);unclassified(100);unclassified(100);unclassified(100);unclassified(100);unclassified(100);

Otu0780 0 0 0 0 0 0 0 0 0 0 0 0 0 0 0 0 0 0 0 0 0 0 0 0 0 0 0 0
0 0 0 0 0 0 0 0 0 0 0 26 0 0 0 0 0 0 0 0 0 0 0 0 0 0 0 0
0 0 0 0 0 0 0 0 0 0 0 0 0 0 0 0 0 21 0 0 0 0 0 0 0 0 0 0
0 0 0 0 0 0 0 0 0 0 0 0 0 0 0 0 0 0 0 0 0 0 0 0 0 0 0
M02255_76_000000000-ADUNE_1_1111_8402_15885 C----TA---AC-TC-TGT--------GCC-A-G------C-----A---G--CC-G-CGG-TA------AT-A--C-A--GA-GA-------CT--G-----CA--A-GC-GT-------T-AT-T-C-G-G------------A--T-TG-G-G-T----------GT----
G-T-C-A--------G-A-T--G--TG--A-AA-TC--C-C-GA-G-G-----CT-C-AA------C-C-T-C-G-G-A--A-C----T-G--C-G---T--C-------T--GA-A-A---C-------T--A-C--TC--G-A-C----T-A-G-A-G-T--A---C-TG-----GA-G-A--------G-G-G-T---AA-C------GG--A--ATT----C-A-C-G-GT--
GT-A-G-CA-GT--G---A--A-A---TG-C-GT-AG--AT-A-TC--G-T--G-A-G-G-A-AC-A-CC---AG--A--G--GC-GAA-G--G-C--G-------G--T-T-A-C---CTG--G--AC-A-G--------------------T-T---A-C-T--GA--CG-C-T-C--A-GG--C-A-CG-A--AA-G-C---A-TG--GG-G--AG-C-A-
AA--------AG--GG Bacteria(100);Verrucomicrobia(100);Opitutae(100);Opitutales(100);Opitutaceae(100);Opitutus(100);unclassified(100);unclassified(100);unclassified(100);

Otu0781 0 0 0 0 0 0 0 0 0 0 0 0 0 0 0 0 0 0 0 0 0 0 0 0 0 0 0 0
0 0 0 0 0 0 0 0 0 0 0 19 0 0 0 0 0 0 0 0 0 0 0 0 0 0 0 0
0 0 0 0 0 0 0 0 0 0 0 0 0 0 0 0 0 0 0 0 0 0 0 0 0 0 0 0
0 0 0 0 0 0 0 0 0 0 0 0 0 0 0 0 0 0 0 0 0 0 0 0 0 0 0
M02255_77_000000000-ADUT3_1_1110_26460_11965 T----AC---GT-AG-GGA--------GCA-A-G------C--G--T---T--GT-C-CGG-AA------TT-A--C-T--GG-GT-------GT--A-----AA-GG-GT-GC-------G-TA-G-G-C-G------------G--C-CC-T-G-C-----
------AA----G-T-C-A--------G-G-C--G--TG--A-AA-AC--C-A-TG-G-G-----CT-C-AA------C-C-C-G-T-G-G--A-C----T-G--C-G---T--T-------T--GA-A-A---C-------T--G-T--GG--G-G-C----T-T-G-A-G-T--G---A-AG-----TA-G-A--------G-G-T-A---GG-C------GG--A--ATT----
C-C-C-G-GT--GT-A-G-CG-GT--G---A--A-A---TG-C-GT-AG--AG-A-TC--G-G--G-A-G-G-A-AC-A-CC---AG--T--G--GC-GAA-G--G-C--G-------G--C-C-T-A---CTG--G--GC-T-T--------------------T-A---A-C-T--GA--CG-C-T-G--A-GG--C-A-CG-A--AA-G-C---A-TG--
GG-T--AG-C-A-AA--------CA--GG Bacteria(100);unclassified(100);unclassified(100);unclassified(100);unclassified(100);unclassified(100);unclassified(100);unclassified(100);unclassified(100);

Otu0782 0 0 0 0 0 0 0 0 0 0 0 0 0 0 0 0 0 0 0 0 0 0 0 0 0 0 0 0
0 0 0 0 0 0 0 0 0 0 0 0 0 0 0 0 0 0 0 0 0 0 0 0 0 0 3 0
0 0 0 0 0 0 0 0 0 0 0 0 0 0 0 3 0 0 0 3 0 0 0 0 0 0 0 0
0 0 0 0 0 7 0 0 0 0 0 0 0 0 0 0 0 0 0 0 0 0 0 0 0 0 0
M02255_76_000000000-ADUNE_1_2112_13852_20996 C----GG---CT-AA-TTC--------CGT-G-C------C--A--G---C--AG-C-CGC-GG------TA-A--T-A--CG-GA-------AG--G-----TC-CA-GG-CG-------T-TA-T-C-C-G------------GA-T-TG-G-T-T-----
------AA----G-C-G-T--------G-T-T--G--TG--A-AA-TG--T-A-GA-T-G-----CT-C-AA------C-A-T-C-T-G-A--C-T----T-G--C-A---G--C-------G--CG-A-A---C-------T--G-G--CT--G-A-C----T-T-G-A-G-T--A---C-AC-----AC-A-A--------C-G-T-A---GG-C------GG--A--ATT----C-
A-T-G-GT--GT-A-G-CG-GT--G---A--A-A---TG-C-TT-AG--AT-A-TC--A-T--G-A-A-G-A-AC-T-CC---GA--T--T--GC-GAA-G--G-C--A-------G--C-T-T-A---CGG--G--AG-T-G--------------------T-T---A-C-T--GA--CG-C-T-T--A-AG--C-T-CG-A--AG-G-T---G-CG--GG-T--
AT-C-G-AA--------CA--GG Bacteria(100);Bacteroidetes(100);Bacteroidia(100);Bacteroidales(100);Prevotellaceae(100);unclassified(100);unclassified(100);unclassified(100);unclassified(100);

Otu0783 0 0 0 0 0 0 0 0 0 0 0 0 0 0 0 0 0 0 0 0 0 6 0 0 0 0 0 0
0 0 0 0 0 0 0 0 0 0 0 0 0 0 0 0 0 0 0 0 0 0 0 0 0 0 0 0
0 0 0 0 0 0 0 0 0 0 0 0 0 0 0 0 0 0 0 0 0 0 0 0 0 0 0 0
0 0 0 0 0 0 0 0 0 0 0 0 0 0 0 0 0 0 0 0 0 0 0 0 0 0 0
M02255_77_000000000-ADUT3_1_2102_18731_14552 G----AC---GG-AG-GAT--------CCA-A-G------C--G--T---T--AT-C-CGG-AG------TG-A--C-T--GG-GC-------GT--A-----AA-GA-GTTGC-------G-TA-G-G-C-G------------G--T-TT-G-T-T----
-------AA----G-T-A-G--------A-T-A--G--TG--A-AA-TC--A-A-GT-G-G-----CT-C-AA------C-C-A-T-T-T-G--G-C----T-A--T-T---A--T-------C--TA-A-A---C-------T--G-G--CA--A-A-C----T-T-G-A-C-A--A---T-GA-----GA-G-A--------G-G-T-A---GC-T------GG--A--ATT----C-
C-T-A-GT--GT-A-G-GA-GT--G---A--A-A---TC-C-GT-AG--AT-A-TT--A-G--G-A-G-G-A-AC-A-CC---AA--T--G--GC-GTA-G--G-C--A-------G--G-C-T-A---CTG--G--CT-C-A--------------------T-T---T-G-T--GA--CG-C-T-G--A-GG--C-A-CG-A--AA-G-C---G-TG--GG-G-
-AG-C-G-AA--------CC--GG Bacteria(100);unclassified(100);unclassified(100);unclassified(100);unclassified(100);unclassified(100);unclassified(100);unclassified(100);unclassified(100);

Otu0784 0 0 0 0 0 0 0 0 0 0 0 0 0 0 0 0 0 0 0 0 0 1 0 0 0 16 23 11
76 0 3 1 0 0 0 0 0 1 96 1 0 0 0 0 0 0 0 0 0 0 16 0 3 0 0 0
0 0 0 0 0 0 0 0 0 0 0 0 0 0 0 0 0 0 0 0 0 0 0 0 0 0 0 0
0 0 0 0 0 0 0 0 0 0 0 0 0 0 0 0 0 10 0 0 0 0 0 0 0 0 0
M02255_77_000000000-ADUT3_1_1114_9402_6030 G----AC---GA-GG-GAT--------CCT-A-G------C--G--T---T--GT-T-CGG-AA------TC-A--T-T--GG-GC-------GT--A-----AA-GC-GC-AT-------G-TA-G-G-C-T------------G--C-CA-T-T-C----------AA--
--G-T-C-A--------G-T-T--G--TG--A-AA-GC--C-C-CG-G-G-----CT-C-AA------C-C-C-G-G-G-A--A-G----T-G--C-T---T--C-------T--GA-T-A---C-------T--G-T--TT--G-G-C----T-T-G-A-G-T--A---C-TA-----GA-G-A--------G-G-A-T---AG-T------AG--A--ATT----C-C-A-G-
GT--GT-A-G-TG-GT--G---A--A-A---TA-C-GT-AG--AT-A-TC--T-G--G-A-G-G-A-AT-A-CC---GG--T--G--GC-GAA-G--G-C--G-------G--C-T-A-T---CTG--G--CT-A-T--------------------G-T---A-C-T--GA--CG-C-T-G--A-GA--T-G-CG-A--AA-G-C---G-TG--GG-G--AG-C-
A-AA--------CA--GG Bacteria(100);Proteobacteria(100);unclassified(100);unclassified(100);unclassified(100);unclassified(100);unclassified(100);unclassified(100);unclassified(100);

Otu0785 0 0 0 0 0 0 0 0 0 0 0 0 0 0 0 0 0 0 0 0 0 0 0 0 0 0 0 0
0 0 0 0 0 0 0 0 0 0 0 0 0 49 0 0 0 0 0 0 0 0 0 0 0 0 0 0
0 0 0 0 0 0 0 0 0 4 0 0 0 0 0 0 0 0 0 0 0 0 0 0 0 0 0 0
0 0 0 0 0 0 0 0 0 0 0 0 0 0 0 0 0 14 0 0 0 0 0 0 0 0 0
M02255_77_000000000-ADUT3_1_1116_23193_14043 G----AC---GG-AG-GAT--------GCA-A-G------C--G--T---T--AT-C-CGG-AA------TG-A--T-T--GG-GC-------GT--A-----AA-GC-GT-CT-------G-TA-G-G-T-G------------G--T-TT-A-T-T------
-----AA----G-T-C-T--------G-C-T--G--TT--A-AA-GC--A-C-AG-G-G-----CT-C-AA------C-C-C-T-G-T-A--T-A----G-G--C-G---G--T-------A--GA-A-A---C-------T--G-G--TA--G-G-C----T-T-G-A-G-T--A---T-GG-----TA-G-G--------G-G-T-A---GA-G------GG--A--ATT----C-
C-C-G-AT--GT-A-G-TG-GT--G---A--A-A---TG-C-GT-TG--AT-A-TC--G-G--G-A-A-G-A-AC-A-CC---AA--A--G--GC-GAA-A--G-C--A-------C--T-C-T-G---CTG--G--GC-C-A--------------------A-T---A-C-T--GA--CA-C-T-G--A-GA--G-A-CG-A--AA-G-C---T-AG--GG-G-
-AG-C-A-AA--------TG--GG Bacteria(100);Cyanobacteria(100);unclassified(100);unclassified(100);unclassified(100);unclassified(100);unclassified(100);unclassified(100);unclassified(100);

Otu0786 0 0 0 0 0 0 0 0 0 0 0 0 0 0 0 0 0 0 0 0 0 0 2 1 0 0 0 0
0 0 0 0 0 0 0 0 0 0 0 0 0 0 7 0 0 0 0 0 0 0 0 0 0 0 0 0
0 0 0 0 0 0 3 0 0 0 0 0 0 0 0 0 0 0 0 0 0 0 0 0 0 0 0 0
7 0 0 0 0 0 0 0 0 0 0 0 0 0 0 0 0 0 0 0 0 0 0 0 0 0 0
M02255_76_000000000-ADUNE_1_1113_16414_22687 C----GG---CT-AA-CTC--------CGT-G-C------C--A--G---C--AG-C-CGC-GG------TA-A--T-A--CG-GA-------GG--G-----TG-CA-AG-CG-------T-TA-T-C-C-G------------G--A-TT-A-A-T------
-----CA----G-T-C-A--------G-T-G--G--TG--A-AA-TC--T-C-AT-A-G-----CT-T-AA------C-T-A-T-G-A-A--A-C----T-G--C-C---A--T-------T--GA-T-A---C-------T--G-T--TA--G-T-C----T-T-G-A-G-T--G---A-TG-----TT-G-A--------A-G-T-T---GC-T------GG--A--ATG----T-G-
T-A-GT--GT-A-G-CG-GT--G---A--A-A---TG-C-TT-AG--AT-A-TT--A-C--G-C-A-G-A-AC-A-CC---AA--T--T--GC-GAA-G--G-C--A-------G--G-T-G-A---CTA--A--AC-A-T--------------------T-A---A-C-T--GA--CG-C-T-G--A-TG--G-A-CG-A--AA-G-C---G-TG--GG-G--
AG-C-G-AA--------CA--GG Bacteria(100);Bacteroidetes(100);Flavobacteria(100);Flavobacteriales(100);Flavobacteriaceae(100);Wautersiella(100);unclassified(100);unclassified(100);unclassified(100);

Otu0787 0 0 0 0 0 0 0 0 0 0 0 0 0 0 0 0 0 0 0 0 0 0 24 0 0 0 0 0
0 0 0 0 0 0 0 0 0 0 0 0 0 0 0 0 0 0 0 0 0 0 0 0 0 0 0 0
0 0 0 0 0 0 0 0 0 0 0 0 0 0 0 0 0 0 0 0 0 0 0 0 0 0 0 0
0 0 0 0 0 0 0 0 0 0 0 0 0 0 0 0 0 0 0 0 0 0 0 0 0 0 0
M02255_77_000000000-ADUT3_1_1119_14071_16889 C----AC---GT-AG-GGG--------CCA-A-G------C--G--T---T--GT-C-CGG-AT------TT-A--T-T--GG-GC-------GT--A-----AA-GA-GC-TC-------G-TA-G-G-C-G------------G--T-TC-A-G-T------
-----CA----G-T-C-G--------G-G-T--G--TG--A-AA-AC--T-C-TG-G-G-----CT-C-AA------C-T-C-G-G-A-G--A------C-G-CC-A---C--C-------C--GA-T-A---C-------T--G-C--TG-TG-A-C----T-T-G-A-G-T--C---C-GG-----TA-G-G--------G-G-A-G---CA-G------GG--A--ATT----C-
C-A-G-GT--GT-A-G-CG-GT--G---A--A-A---TG-C-GC-AG--AT-A-TC--T-G--G-A-G-G-A-AC-A-CC---GG--T--G--GC-GAA-G--G-C--G-------C--T-G-C-T---CTG--G--GC-C-G--------------------G-T---A-C-T--GA--CG-C-T-G--A-GG--A-G-CG-A--AA-G-C---G-TG--GG-
T--AG-C-A-AA--------CA--GG Bacteria(100);Actinobacteria(100);Actinobacteria(100);Acidimicrobidae(100);Acidimicrobiales(100);Acidimicrobineae(100);unclassified(100);unclassified(100);unclassified(100);

Otu0788 0 0 0 0 0 0 0 0 0 0 0 0 0 0 0 0 0 0 0 0 0 0 0 0 0 0 0 0
0 0 0 0 0 0 0 0 0 0 0 0 0 0 0 0 0 0 71 0 0 0 0 0 0 0 0 0
0 0 0 0 0 0 0 0 0 0 0 0 0 0 0 0 0 0 0 0 0 0 0 0 0 0 0 0
0 0 0 0 0 0 0 0 0 0 0 0 0 0 0 0 0 0 0 0 0 0 0 0 0 0 0
M02255_77_000000000-ADUT3_1_1107_5376_11481 T----AC---GT-AG-GGG--------GCA-A-G------C--G--T---T--AT-C-CGG-AA------TT-A--C-T--GG-GC-------GT--A-----AA-GG-GT-GC-------G-TA-G-G-C-G------------G--T-TT-A-T-C-----------AA-
---G-T-C-A--------G-G-G--G--TG--A-AA-GG--C-T-AC-G-G-----CT-C-AA------C-C-G-T-A-G-T--G------A-G--C-C---T--C-------T--GA-A-A---C-------T--G-G--TG--A-A-C----T-T-G-A-G-T--G---T-AG-----GA-G-A--------G-G-A-A---AG-T------GG--A--ATT----C-C-C-A-
GT--GT-A-G-CG-GT--G---A--A-A---TG-C-GT-AG--AT-A-TT--G-G--G-A-G-G-A-AC-A-CC---AG--T--G--GC-GAA-G--G-C--G-------A--C-T-T-T---CTG--G--AC-T-A--------------------C-A---A-C-T--GA--CG-C-T-G--A-GG--C-A-CG-A--AA-G-C---G-TG--GG-G--AG-
C-A-AA--------CA--GG Bacteria(100);Firmicutes(100);Clostridia(100);Clostridiales(100);Peptostreptococcaceae(100);Incertae_Sedis(100);unclassified(100);unclassified(100);unclassified(100);

Otu0789 0 0 0 0 0 0 0 0 0 0 0 0 0 0 0 0 0 0 0 0 0 0 0 0 0 0 0 0
0 0 0 0 0 0 0 0 0 0 0 49 0 0 0 0 0 0 0 0 0 0 0 0 0 0 0 0
0 0 0 0 0 0 0 0 0 0 0 0 0 0 0 0 0 0 0 0 0 0 0 0 0 0 0 0
0 0 0 0 0 0 0 0 0 0 0 0 0 0 0 0 0 0 0 0 0 0 0 0 0 0 0
M02255_77_000000000-ADUT3_1_1103_8189_15102 T----AC---GA-AG-GGG--------GCA-A-G------C--G--T---T--GT-T-CGG-AA------TT-A--C-T--GG-GC-------GT--A-----AA-GG-GC-GC-------G-CA-G-G-C-G------------G--C-CT-G-T-C-----------
AA----G-T-C-A--------G-A-T--G--TG--A-AA-GC--C-C-CG-G-G-----CT-C-AA------C-C-T-G-G-G-A--A-T----A-G--C-A---T--T-------T--GG-G-A---C-------T--G-G--CA--G-G-C----T-A-G-A-G-T--T---C-GG-----GA-G-A--------G-G-A-G---AG-T------GG--A--ATT----C-C-
C-A-GT--GT-A-G-AG-GT--G---A--A-A---TT-C-GT-AG--AT-A-TT--G-G--G-A-A-G-A-AC-A-CC---GG--T--G--GC-GAA-G--G-C--G-------G--C-T-C-T---CTG--G--AC-C-G--------------------A-T---A-C-T--GA--CG-C-T-G--A-GG--C-G-CG-A--AA-G-C---G-TG--GG-G--
AG-C-A-AA--------CA--GG Bacteria(100);Proteobacteria(100);Alphaproteobacteria(100);Rhodospirillales(100);unclassified(100);unclassified(100);unclassified(100);unclassified(100);unclassified(100);

Otu0790 0 0 0 0 0 0 0 0 0 0 0 0 0 0 0 0 0 0 0 0 0 0 0 0 0 0 0 0
0 0 0 0 0 0 0 0 0 0 0 20 0 0 0 0 0 0 0 0 0 0 0 0 0 0 0 0
0 0 0 0 0 0 0 0 0 0 0 0 0 0 0 0 0 17 0 0 0 0 0 0 0 0 0 0
0 0 0 0 0 0 0 0 0 0 0 0 0 0 0 0 0 0 0 0 0 0 0 0 0 0 0
M02255_77_000000000-ADUT3_1_1109_24534_17859 T----AC---GA-AC-TGT--------GCG-A-A------C--G--T---T--AT-T-CGG-AA------TC-A--C-T--GG-GC-------TT--A-----CA-GG-GT-GC-------G-TA-G-G-C-G------------G--C-CT-T-C-C-------
----AA----G-T-C-A--------G-G-T--G--TG--A-AA-TC--C-C-AC-A-G-----CT-C-AA------C-T-G-T-G-G-A--A-C----A-G--C-G---C--T-------T--GA-A-A---C-------T--G-G--TT--G-G-C----T-T-G-A-G-T--C---A-GC-----TA-G-A--------G-G-T-A---CA-C------GG--A--ACT----T-C-
C-G-GT--GG-A-G-CG-GT--G---A--A-A---TG-T-GT-TG--AT-A-TC--G-G--A-A-G-G-A-AC-A-CC---AG--A--G--GC-GAA-A--G-C--G-------G--T-G-T-A---CTG--G--AG-C-T--------------------G-G---T-C-T--GA--CG-C-T-G--A-GG--C-A-CG-A--AA-G-C---C-AG--GG-G--
AG-C-G-AA--------CG--GG Bacteria(100);Planctomycetes(100);Planctomycetacia(100);Planctomycetales(100);Planctomycetaceae(100);Planctomyces(100);unclassified(100);unclassified(100);unclassified(100);

Otu0791 0 0 0 0 0 0 0 0 0 0 0 0 0 0 0 0 0 0 0 0 0 0 1 0 0 0 0 0
0 0 0 0 0 0 0 0 0 0 0 0 0 0 0 0 0 0 0 0 169 0 0 0 0 0 0 0
0 0 0 0 0 0 0 0 0 0 0 0 0 0 0 0 0 0 0 0 0 0 0 0 0 0 0 0
0 0 0 0 0 0 0 0 0 0 0 0 0 0 0 0 0 0 0 0 0 0 0 0 0 0 0
M02255_77_000000000-ADUT3_1_2101_21204_12706 C----AC---GT-AG-GGG--------GCA-A-G------C--G--T---T--GT-C-CGG-AT------TT-A--T-T--GG-GC-------GT--A-----AA-GA-GC-TC-------G-TA-G-G-T-G------------G--C-TG-A-G-T-----
------AA----G-T-C-G--------G-A-T--G--TC--A-AA-TC--C-C-CA-G-G-----CT-C-AA------C-C-T-G-G-G-A---------T-C-CC-A---T--C-------C--GA-T-A---C-------T--G-C--TC-TG-G-C----T-A-G-A-G-T--C---C-GG-----TA-G-G--------G-G-A-G---CG-T------GG--A--ATT----C-
C-T-G-GT--GT-A-G-CG-GT--G---A--A-A---TG-C-GC-AG--AT-A-TC--A-G--G-A-G-G-A-AC-A-CC---AG--C--G--GC-GAA-G--G-C--G-------G--C-G-C-T---CTG--G--GC-C-G--------------------G-T---A-C-T--GA--CA-C-T-G--A-GG--A-G-CG-A--AA-G-C---G-TG--GG-
T--AG-C-A-AA--------CA--GG Bacteria(100);Actinobacteria(100);Actinobacteria(100);Acidimicrobidae(100);Acidimicrobiales(100);Acidimicrobineae(100);unclassified(100);unclassified(100);unclassified(100);

Otu0792 0 0 0 0 0 0 0 0 0 0 0 0 0 0 0 0 0 0 0 0 0 0 14 0 0 0 0 0
0 0 0 0 0 0 0 0 0 0 0 0 0 0 0 0 0 0 0 0 0 0 0 0 0 0 0 0
0 0 0 0 0 0 0 0 0 0 0 0 0 0 0 0 0 0 0 0 0 0 0 0 0 0 0 0
0 0 0 0 0 0 0 0 0 0 0 0 0 0 0 0 0 0 0 0 0 0 0 0 0 0 0
M02255_77_000000000-ADUT3_1_2101_12142_19871 T----AC---GA-AG-GGG--------GCT-A-G------C--G--T---T--GT-T-CGG-AA------TT-A--C-T--GG-GC-------GT--A-----AA-GC-GC-GC-------G-CA-G-G-C-G------------G--T-TC-G-A-T-----
------AA----G-T-T-A--------G-G-G--G--TG--A-AA-TC--C-C-CG-G-G-----CT-C-AA------C-C-C-G-G-G-A--A-T----G-G--C-C---T--T-------T--AA-G-A---C-------T--G-T--CG--G-G-C----T-A-G-A-G-G--A---C-GA-----GA-G-A--------G-G-T-G---AG-T------GG--A--ATT----
C-C-C-A-GT--GT-A-G-AG-GT--G---A--A-A---TT-C-GT-AG--AT-A-TT--G-G--G-A-A-G-A-AC-A-CC---GG--T--G--GC-GAA-G--G-C--G-------G--C-T-C-A---CTG--G--CT-C-G--------------------T-T---T-C-T--GA--CG-C-T-G--A-GG--C-G-CG-A--AA-G-C---G-TG--GG-
G--AG-C-A-AA--------CA--GG Bacteria(100);Proteobacteria(100);Alphaproteobacteria(100);Rhizobiales(100);Methylocystaceae(100);uncultured(100);unclassified(100);unclassified(100);unclassified(100);

Otu0793 0 0 0 0 0 0 0 0 0 0 0 0 0 0 0 0 0 0 0 0 0 0 0 0 0 0 0 0
0 0 0 0 0 0 0 0 0 0 0 0 12 18 34 0 0 0 0 0 0 0 0 5 307 0 0 0
0 0 0 0 0 0 0 0 0 0 0 0 0 0 0 2 0 0 0 0 0 0 0 0 0 0 0 0
0 0 0 0 0 0 0 0 0 0 0 0 0 22 0 0 0 0 0 0 0 1 0 0 0 0 0
M02255_77_000000000-ADUT3_1_1102_25590_8401 T----AC---GT-AG-GGT--------GCG-A-G------C--G--T---T--GT-C-CGG-AA------TT-A--T-T--GG-GC-------GT--A-----AA-GA-GC-TT-------G-TA-G-G-T-G------------G--T-TT-G-T-C-----------GC--
--G-T-C-T--------G-C-G--G--TG--A-AA-TC--C-C-AA-C-G-----CT-T-AA------C-G-T-T-G-G-G--C-T----T-G--C-C---G--T-------G--GG-T-A---C-------G--G-G--CA--G-A-C----T-A-G-A-G-T--G---T-GG-----TA-G-G--------G-G-A-G---AC-T------GG--A--ATT----C-C-T-G-
GT--GT-A-G-CG-GT--G---A--A-A---TG-C-GC-AG--AT-A-TC--A-G--G-A-G-G-A-AC-A-CC---GA--T--G--GC-GAA-G--G-C--A-------G--G-T-C-T---CTG--G--GC-C-A--------------------T-A---A-C-T--GA--CA-C-T-G--A-GA--A-G-CG-A--AA-G-C---A-TG--GG-G--AG-
C-G-AA--------CA--GG Bacteria(100);Actinobacteria(100);Actinobacteria(100);Actinobacteridae(100);Actinomycetales(100);Micrococcineae(100);unclassified(100);unclassified(100);unclassified(100);

Otu0794 0 0 0 0 0 0 0 0 0 0 0 0 0 0 0 0 0 0 0 0 0 0 0 0 0 0 0 0
0 0 0 0 0 0 0 0 0 0 0 0 0 0 52 0 0 0 0 0 0 0 0 0 0 0 0 0
0 0 0 0 0 0 0 0 0 0 0 0 0 0 0 0 0 0 0 0 0 0 0 0 0 0 0 0
0 0 0 0 0 0 0 0 0 0 0 0 0 0 0 0 0 0 0 0 0 0 0 0 0 0 0
M02255_77_000000000-ADUT3_1_1112_10361_13372 T----AC---GA-AG-GGG--------GCG-A-G------C--G--T---T--GT-T-CGG-AA------TT-A--T-T--GG-GC-------GT--A-----AA-GG-GA-GC-------G-TA-G-G-C-G------------G--T-TC-G-A-T----
-------AA----G-T-C-A--------C-T-T--G--TG--A-AA-TC--T-C-TG-G-G-----CT-C-AA------C-T-C-A-G-A-G--T-C----T-G--C-A---A--G-------C--GA-A-A---C-------T--G-T--CG--G-G-C----T-T-G-A-G-T--A---T-GG-----GA-G-A--------G-G-T-G---AG-T------GG--A--ATT----
C-C-T-G-GT--GT-A-G-CG-GT--G---A--A-A---TG-C-GT-AG--AT-A-TC--A-G--G-A-G-G-A-AC-A-CC---TG--T--G--GC-GAA-A--G-C--G-------G--C-T-C-A---CTG--G--AC-C-A--------------------T-T---A-C-T--GA--CG-C-T-G--A-TG--C-T-CG-A--AA-G-C---T-AG--GG-
G--AG-C-A-AA--------CA--GG Bacteria(100);Acidobacteria(100);Acidobacteria(100);Acidobacteriales(100);Acidobacteriaceae(100);unclassified(100);unclassified(100);unclassified(100);unclassified(100);

Otu0795 0 0 0 0 0 0 0 0 0 0 0 0 0 0 0 0 0 0 0 0 0 0 0 0 0 0 0 0
0 0 0 0 0 0 0 0 0 0 0 0 0 0 0 66 0 0 0 0 0 0 0 0 1 0 0 0 0
0 0 0 0 0 0 0 0 0 0 0 0 0 0 0 0 0 0 0 0 0 0 0 0 0 0 0 0 0
0 0 0 0 0 0 0 0 0 0 0 0 0 0 0 0 0 0 0 0 0 0 0 0 0 0 0 0
M02255_77_000000000-ADUT3_1_1102_15282_3459 T----AC---GT-AG-GGA--------GCA-A-G------C--G--T---T--GT-C-CGG-AT------TT-A--T-T--GG-GC-------GT--A-----AA-GG-GT-GC-------G-TA-G-G-C-G------------G--C-CT-G-A-T-----------AA----G-T-C-A--------G-A-T--G--TG--A-AA-GG--C-G-GA-G-G-----CT-C-AA------C-C-T-C-C-G-T--T------A-G--C-A---T--C-------T--GA-A-A---C-------T--G-T--CA--G-G-C----T-T-G-A-G-T--G---A-AG-----GA-G-A--------G-G-A-A---AG-T------GG--A--ATT----C-C-T-A-GT--GT-A-G-CG-GT--G---A--A-A---TG-C-GT-AG--AT-A-TT--A-G--G-A-G-G-A-AT-A-CC---GG--T--G--GC-GAA-G--G-C--G-------A--C-T-T-T---CTG--G--AC-T-T--------------------A-A---A-C-T--GA--CG-C-T-G--A-GG--C-A-CG-A--AA-G-T---G-TG--GG-G--AG-C-A-AA--------CA--GG Bacteria(100);Firmicutes(100);Clostridia(100);Clostridiales(100);Family_XI_Incertae_Sedis(100);Finegoldia(100);unclassified(100);unclassified(100);unclassified(100);

Otu0796 0 0 0 0 0 0 0 0 0 0 0 0 0 0 0 0 0 0 0 0 0 0 0 0 0 0 0 0
0 0 0 0 0 0 0 0 0 0 0 0 0 0 0 87 0 0 0 0 0 0 0 0 0 0 0 0 0
0 0 0 0 0 0 0 0 0 0 0 0 0 0 0 0 0 0 0 0 0 0 0 0 0 0 0 0 0
0 0 0 0 0 0 0 0 0 0 0 0 0 0 0 0 0 0 0 0 0 0 0 0 0 0 0 0
M02255_77_000000000-ADUT3_1_1101_17178_12006 T----AC---GA-AG-GGG--------GCT-A-G------C--G--T---T--GC-T-CGG-AA------TG-A--C-T--GG-GC-------GT--A-----AA-GG-GC-GT-------G-TA-G-G-C-G------------G--T-TT-G-C-A-----------CA----G-T-T-A--------G-A-T--G--TG--A-AA-TC--C-C-CG-G-G-----CT-T-AA------C-C-T-G-G-G-A--G-C----T-G--C-A---T--T-------T--AA-T-A---C-------G--T-G--CA--G-A-C----T-A-G-A-G-T--G---T-GA-----GA-G-A--------G-G-G-T---TG-T------GG--A--ATT----C-C-C-A-GT--GT-A-G-AG-GT--G---A--A-A---TT-C-GT-AG--AT-A-TT--G-G--G-A-A-G-A-AC-A-CC---GG--T--G--GC-GAA-G--G-C--G-------G--C-A-A-C---CTG--G--CT-C-A--------------------T-G---A-C-T--GA--CG-C-T-G--A-GG--C-G-CG-A--AA-G-C---G-TG--GG-G--AG-C-A-AA--------CA--GG Bacteria(100);Proteobacteria(100);Alphaproteobacteria(100);Rhodospirillales(100);Acetobacteraceae(100);Acetobacter(100);unclassified(100);unclassified(100);unclassified(100);

Otu0797 0 0 0 0 0 0 0 0 0 0 0 0 0 0 0 0 2 0 0 0 0 0 0 0 0 0 0 0
0 0 0 0 0 0 0 0 0 0 0 0 0 0 0 0 0 0 0 0 0 0 0 0 0 0 0 0 0
0 0 0 0 0 0 0 0 0 0 0 0 0 0 0 0 0 0 0 0 0 0 0 0 0 0 0 0 0
0 0 0 0 0 0 0 0 0 0 0 0 0 0 0 0 0 0 0 0 0 0 0 0 0 0 0 0
M02255_77_000000000-ADUT3_1_1113_2732_15178 T----AC---GT-AT-GTT--------GCG-A-G------C--G--T---T--AT-C-CGG-AA------TT-A--T-T--GG-GC-------TT--A-----AA-GG-GC-AT-------C-TA-G-G-C-G------------G--T-AA-G-A-C-----------AA----G-T-T-G--------A-A-G--G--TG--A-AA-AC--C-T-GT-G-G-----CT-C-AA------C-C-A-T-A-G-G--C-T----T-G--C-C---T--A-------C--AA-A-A---C-------T--G-T--TG--A-A-C----T-A-G-A-G-T--A---C-TG-----GA-A-A--------G-G-T-G---GG-T------GG--A--ACT----A-C-A-C-GA--GT-A-G-AG-GT--G---A--A-A---TT-C-GT-AG--AT-A-TG--T-G--T-A-G-G-A-AT-G-CC---GA--T--G--AT-GAA-G--A-T--A-------A--C-T-C-A---CTG--G--AC-A-G--------------------A-A---A-C-T--GA--CG-C-T-G--A-AG--T-G-CG-A--AA-G-C---T-AG--GG-G--AG-C-A-AA--------CA--GG Bacteria(100);Fusobacteria(100);Fusobacteria(100);Fusobacteriales(100);Leptotrichiaceae(100);Sneathia(100);unclassified(100);unclassified(100);unclassified(100);

Otu0798 0 0 0 0 0 0 0 0 0 0 0 0 0 0 0 0 3 0 0 0 0 0 0 0 0 0 0 0
0 0 0 0 0 0 0 0 0 0 0 0 0 0 0 0 0 0 0 0 0 0 0 0 0 0 0 0 0
0 0 0 0 0 0 0 0 0 0 0 0 0 0 0 0 0 0 0 0 0 0 0 0 0 0 0 0 0
0 0 0 0 0 0 0 0 0 0 0 0 0 0 0 0 0 0 0 0 0 0 0 0 0 0 0 0
M02255_77_000000000-ADUT3_1_2117_23107_8474 T----AC---GT-AG-GCA--------GCA-A-G------C--G--T---T--GT-T-CGG-AA------TT-A--C-T--GG-GC-------GT--A-----AC-GA-GT-GT-------G-TA-G-G-C-G------------G--T-GA-A-C-T-----------AA----G-T-T-C--------G-G-T--G--TG--A-AA-TC--T-C-CC-G-G-----CT-C-AA------C-T-G-G-G-A-G--G-G----T-G--C-G---C--C-------G--GA-A-A---C-------T--G-G--TT--T-G-C----T-G-G-A-G-C--G---T-GG-----GA-G-A--------G-G-A-A---AG-C------GG--A--ATT----C-C-T-G-GT--GT-A-G-CG-GT--G---A--A-A---TG-C-GT-AG--AT-A-TC--A-G--G-A-G-G-A-AC-A-CC---TG--T--G--GT-GTA-G--A-C--A-------G--C-T-T-T---CTG--G--AC-C-A--------------------T-A---G-C-T--GA--CG-C-T-G--A-GA--C-A-CG-A--AA-G-C---G-TG--GG-T--AG-C-A-AA--------CA--GG Bacteria(100);unclassified(100);unclassified(100);unclassified(100);unclassified(100);unclassified(100);unclassified(100);unclassified(100);unclassified(100);

Otu0799 0 0 0 0 0 0 0 0 0 0 0 0 0 0 0 0 1 0 0 0 0 0 0 0 0 0 0 0
0 0 0 0 0 0 0 0 0 0 0 0 0 0 0 0 0 0 0 0 0 0 0 0 0 0 0 0 0
0 0 0 1 0 0 0 0 0 0 0 0 0 0 0 0 0 0 0 0 0 0 0 0 0 0 0 0 0
0 0 0 0 0 0 0 0 0 0 0 0 0 0 0 0 0 0 0 0 0 0 0 0 0 0 0 0
M02255_76_000000000-ADUNE_1_1113_4513_20301 G----AC---AG-AA-TAA--------GCA-C-C------G--G--C---T--AA---C-T-CT------GT-G--C-C--AG-CA-------GC--C-----GC-GG-TA-AT-------A-CA-G-A-T-G------------G--T-TT-G-T-C-----------AA----GCG-T-T--------A-A-T--C--GG--A-AA-TC--C-C-CG-G-G-----CT-C-AA------C-C-T-G-G-G-A--A-C----T-G--C-A---T--C-------C--AA-A-A---C-------T--G-G--CA--A-G-C----T-A-G-A-G-T--A---T-GG-----TA-G-A--------G-G-G-T---AG-T------GG--A--ATT----T-C-C-T-GT--GT-A-G-CG-GT--G---A--A-A---TG-C-GT-AG--AT-A-TA--G-G--A-A-G-G-A-AC-A-CC---AG--T--G--GC-GAA-G--G-C--G-------A--C-T-A-C---CTG--G--AC-T-G--------------------A-T---A-C-T--GA--CA-C-T-G--A-GG--T-G-CG-A--AA-G-C---G-TG--GG-G--AG-C-A-AA--------CA--GG Bacteria(100);Proteobacteria(100);Gammaproteobacteria(100);unclassified(100);unclassified(100);unclassified(100);unclassified(100);unclassified(100);unclassified(100);

Otu0800 0 0 0 0 0 0 0 0 0 0 0 0 0 0 0 0 0 0 38 0 0 0 0 0 0 0 0 0
0 0 0 0 0 0 0 0 0 0 0 0 0 0 0 0 0 0 0 0 0 0 0 0 0 0 0 0 0
0 0 0 0 0 0 0 0 0 0 0 0 0 0 0 0 0 0 0 0 0 0 0 0 0 0 0 0 0
0 0 0 0 0 0 0 0 0 0 0 0 0 0 0 0 0 0 0 0 0 0 0 0 0 0 0 0
M02255_76_000000000-ADUNE_1_2119_19617_13887 C----GG---CT-AA-CTC--------CGT-G-C------C--A--G---C--AG-C-CGC-GG------TA-A--T-A--CG-GA-------GG--G-----TG-CA-AG-CG-------T-TA-T-C-C-G------------G--A-CT-G-A-T-----
------CA----G-T-C-A--------G-T-G--G--TG--A-AA-TC--C-T-AT-C-G-----CT-T-AA------C-G-A-T-A-G-A--A-C----T-G--C-C---A--T-------T--GA-T-A---C-------T--G-T--TA--G-T-C----T-T-G-A-A-T--T---A-GT-----TT-G-A--------A-G-T-G---GC-T------GG--A--ATG----T-G-
T-A-GT--GT-A-G-CG-GT--G---A--A-A---TG-C-TT-AG--AT-A-TT--A-C--G-C-A-G-A-AC-A-CC---AA--T--T--GC-GAA-G--G-C--A-------G--G-T-C-A---CTA--A--GT-C-T--------------------A-T---A-T-T--GA--CG-C-T-G--A-TG--G-A-CG-A--AA-G-C---G-TG--GG-G--
AG-C-G-AA--------CA--GG Bacteria(100);Bacteroidetes(100);Flavobacteria(100);Flavobacteriales(100);Flavobacteriaceae(100);unclassified(100);unclassified(100);unclassified(100);unclassified(100);

Otu0801 0 0 0 0 0 0 0 0 0 0 0 0 0 0 0 0 0 0 1 0 0 0 0 0 0 0 0 0
0 0 0 0 0 0 0 0 0 0 0 0 0 0 0 0 0 0 0 0 0 0 0 0 0 0 0 0 0
0 0 0 0 0 0 0 0 0 0 0 0 0 0 0 0 0 0 0 0 0 0 0 0 0 0 0 0 0
0 0 0 23 0 0 0 0 0 0 0 0 0 0 0 0 0 0 0 0 0 0 0 0 0 0 0 0
M02255_77_000000000-ADUT3_1_1110_10330_20197 T----AC---GT-AT-GGT--------GCA-A-G------C--G--T---T--AT-C-CGG-AT------TT-A--C-T--GG-GT-------GT--A-----AA-GG-GA-GC-------G-TA-G-A-C-G------------G--A-CT-T-G-C------
-----AA----G-T-C-T--------G-A-A--G--TG--A-AA-GC--C-C-GG-G-G-----CT-C-AA------C-C-C-C-G-G-G--A-C----T-G--C-T---T--T-------G--GA-A-A---C-------T--G-T--AG--G-T-C----T-A-G-A-G-T--G---C-TG-----GA-G-A--------G-G-T-A---AG-T------GG--A--ATT----C-
C-T-A-GT--GT-A-G-CG-GT--G---A--A-A---TG-C-GT-AG--AT-A-TT--A-G--G-A-G-G-A-AC-A-CC---AG--T--G--GC-GAA-G--G-C--G-------G--C-T-T-A---CTG--G--AC-A-G--------------------T-A---A-C-T--GA--CG-T-T-G--A-GG--C-T-CG-A--AA-G-C---G-TG--GG-G-
-AG-C-A-AA--------CA--GG Bacteria(100);Firmicutes(100);Clostridia(100);Clostridiales(100);Lachnospiraceae(100);unclassified(100);unclassified(100);unclassified(100);unclassified(100);

Otu0802 0 0 0 0 0 0 0 0 0 0 0 0 0 0 0 0 0 0 1 0 0 0 0 0 0 0 0 0
0 0 0 0 0 0 0 0 0 0 0 0 0 0 0 0 0 0 0 0 0 0 0 0 0 0 0 0 0
0 0 0 0 0 0 0 0 0 0 0 0 0 0 15 0 0 0 0 0 0 0 0 0 0 0 0 0 0
0 0 0 0 0 44 0 0 0 0 0 0 0 0 0 0 0 0 0 0 0 0 0 0 0 0 0 0
M02255_77_000000000-ADUT3_1_2109_11343_9850 T----AC---GT-AG-GGG--------ACG-A-G------C--G--T---T--GT-C-CGG-AA------TC-A--C-T--GG-GC-------GT--A-----AA-GG-GC-GC-------G-TA-G-G-C-G------------G--T-TT-T-A-T----------AA-
---G-T-C-A--------G-A-T--G--TG--A-AA-GG--T-A-CC-G-G-----CT-C-AA------C-C-G-G-T-G-A--C-G----T-G--C-A---T--T-------T--GA-A-A---C-------T--G-T--AA--G-A-C----T-T-G-A-G-T--A---C-TG-----AA-G-A--------G-G-C-A---AG-C------GG--A--ATT----C-C-T-A-
GT--GT-A-G-CG-GT--G---A--A-A---TG-C-GT-AG--AT-A-TT--A-G--G-A-A-G-A-AC-A-CC---GG--T--G--GC-GAA-G--G-C--G-------G--C-T-T-G---CTG--G--GC-A-G--------------------A-T---A-C-T--GA--CG-C-T-G--A-GG--T-G-CG-A--AA-G-C---G-TG--GG-G--AG-
C-G-AA--------CA--GG Bacteria(100);Firmicutes(100);Clostridia(100);Clostridiales(100);Eubacteriaceae(100);Eubacterium(100);unclassified(100);unclassified(100);unclassified(100);

Otu0803 0 0 0 0 0 0 0 0 0 0 0 0 0 0 0 0 0 0 0 0 0 0 0 0 0 0 0 0
0 0 0 0 0 0 0 0 0 0 0 0 0 0 23 0 0 0 0 0 0 0 0 0 0 2 0 0 0
0 0 0 0 0 0 0 0 0 0 0 0 0 0 0 0 0 0 0 0 0 0 0 0 0 0 0 0 0
0 0 0 0 0 0 0 0 0 0 0 0 0 0 0 0 0 0 0 0 0 0 0 0 0 0 0 0
M02255_77_000000000-ADUT3_1_2119_22764_18095 G----AC---AG-GA-GAT--------GCA-A-G------T--G--T---T--AT-C-CGG-AA------TG-A--T-T--GG-GC-------GT--A-----AA-GG-GT-CT-------G-TA-G-G-T-G------------G--T-TT-G-A-A-----
------AA----G-T-C-T--------G-T-T--G--TT--A-AA-TA--C-T-AG-G-G-----CT-T-AA------C-C-C-T-G-G-T--C-A----G-G--C-A---A--T-------A--GA-A-A---C-------T--A-T--TA--G-A-C----T-A-G-A-G-T--A---C-GG-----TA-G-A--------G-G-C-A---GA-G------GG--A--ATT----T-
T-C-G-GT--GG-A-G-TG-GT--G---A--A-A---TG-C-GT-AG--AT-C-TC--G-A--A-A-A-G-A-AC-A-CC---AA--T--G--GC-GAA-G--G-C--A-------C--T-C-T-G---CTG--G--GC-C-G--------------------A-T---A-C-T--GA--CA-T-T-G--A-GA--G-A-CG-A--AA-G-C---T-AG--GG-G-
-AG-C-A-AA--------AG--GG Bacteria(100);Cyanobacteria(100);unclassified(100);unclassified(100);unclassified(100);unclassified(100);unclassified(100);unclassified(100);unclassified(100);

Otu0804 0 0 0 0 0 0 0 0 0 0 0 0 0 0 0 0 0 0 0 0 0 0 0 0 0 0 0 0
0 0 0 0 0 0 0 0 0 0 0 0 0 0 33 0 0 0 0 0 0 0 0 0 0 0 0 0 0
0 0 0 0 0 0 0 0 0 0 0 0 0 0 0 0 0 0 0 0 0 0 0 0 0 0 0 0 0
0 0 0 0 0 0 0 0 0 0 0 0 0 0 0 0 0 0 0 0 0 0 0 0 0 0 0 0
M02255_77_000000000-ADUT3_1_2113_9565_19962 G----AC---GG-AG-GGG--------GCT-A-G------C--G--T---T--GT-T-CGG-AA------TT-A--C-T--GG-GC-------GT--A-----AA-GA-GT-GC-------G-TA-G-G-T-G------------G--T-TT-A-G-T-----------
AA----G-T-T-G--------G-A-A--G--TG--A-AA-GC--C-C-GG-G-G-----CT-T-AA------C-C-T-C-G-G-A--A-T----T-G--C-T---T--T-------C--AA-A-A---C-------T--A-C--TA--A-T-C----T-A-G-A-G-T--G---T-AG-----TA-G-G--------G-G-A-T---GA-T------GG--A--ATT----C-C-T-
A-GT--GT-A-G-AG-GT--G---A--A-A---TT-C-TT-AG--AT-A-TT--A-G--G-A-G-G-A-AC-A-CC---GG--T--G--GC-GAA-G--G-C--G-------G--T-C-A-T---CTG--G--GC-T-A--------------------C-A---A-C-T--GA--CG-C-T-G--A-TG--C-A-CG-A--AA-G-C---G-TG--GG-G--AG-
C-A-AA--------CA--GG Bacteria(100);Proteobacteria(100);Alphaproteobacteria(100);Rickettsiales(100);Rickettsiaceae(100);Rickettsia(100);unclassified(100);unclassified(100);unclassified(100);

Otu0805 0 0 0 0 0 0 0 0 0 0 0 0 0 0 0 0 0 0 5 0 0 0 0 0 0 0 0 0
0 0 0 0 0 0 0 0 0 0 0 0 0 0 0 0 0 0 0 0 0 0 0 0 0 0 0 0 0
0 0 0 0 0 0 0 0 0 0 0 0 0 0 0 0 0 0 0 0 0 0 0 0 0 0 0 0 0
0 0 0 0 0 0 0 0 0 0 0 0 0 0 0 0 0 0 0 0 0 0 0 0 0 0 0

M02255_77_000000000-ADUT3_1_2112_17525_9790 T----AC---GT-AG-GGG--------GCA-A-G------C--G--T---T--AT-C-CGG-AA------TC-A--T-T--GG-GC-------GT--A-----AA-GC-GT-GC-------G-TA-G-G-C-G------------G--T-TT-G-A-A----------
GG----G-T-C-A--------G-A-G--G--TG--A-AA-GG--T-C-GG-G-G-----CT-T-AA------C-C-C-C-G-G-T--T------A-G--C-C---T--T-------T--GA-A-A---C-------C--G-G--CA--G-A-C----T-T-G-A-G-T--A---C-AG-----GA-G-G--------G-G-A-A---AG-T------GG--A--ATT----T-C-T-
A-GT--GT-A-G-CG-GT--G---G--A-A---TG-C-GT-AG--AT-A-TT--A-G--A-A-G-G-A-AC-A-CC---AG--T--G--GC-GAA-G--G-C--G-------A--C-T-T-T---CTG--G--AC-T-G--------------------T-A---A-C-T--GA--CG-C-T-G--A-GG--C-A-CG-A--AA-G-C---G-TG--GG-T--AG-
C-G-AA--------CA--GG Bacteria(100);Firmicutes(100);Clostridia(100);Clostridiales(100);unclassified(100);unclassified(100);unclassified(100);unclassified(100);unclassified(100);

Otu0806 0 0 0 0 0 0 0 0 0 0 0 0 0 0 0 0 0 0 0 0 0 0 0 0 0 0 0 0
0 0 0 0 0 0 0 0 0 0 0 0 0 0 55 0 0 0 0 0 0 0 0 0 0 0 0 0 0
0 0 0 0 0 0 0 0 0 0 0 0 0 0 0 0 0 0 0 0 0 0 0 0 0 0 0 0 0
0 0 0 0 0 0 0 0 0 0 0 0 0 0 0 0 0 0 0 0 0 0 0 0 0 0 0

M02255_77_000000000-ADUT3_1_1117_4791_12506 T----AC---GT-AG-GTG--------GCG-A-G------C--G--T---T--GT-C-CGG-AT------TT-A--C-T--GG-GT-------GT--A-----AA-GG-GC-GT-------G-TA-G-G-C-G------------G--G-CA-G-T-C----------
AA----G-T-C-A--------G-A-T--G--TG--A-AA-TA--C-C-GG-G-G-----CT-T-AA------C-C-C-C-G-G-A--G-C----T-G--C-A---T--T-------T--GA-A-A---C-------T--G-A--CA--G-T-C----T-T-G-A-G-T--G---C-CG-----GA-G-A--------G-G-A-A---AG-C------GG--A--ATT----C-C-T-
A-GT--GT-A-G-CG-GT--G---A--A-A---TG-C-GT-AG--AT-A-TT--A-G--G-A-G-G-A-AC-A-CC---AG--T--G--GC-GAA-G--G-C--G-------G--C-T-T-T---CTG--G--AC-G-G--------------------T-A---A-C-T--GA--CG-C-T-G--A-GG--C-G-CG-A--AA-G-C---A-TG--GG-G--
AG-C-A-AA--------CA--GG Bacteria(100);Firmicutes(100);Clostridia(100);Clostridiales(100);Ruminococcaceae(100);unclassified(100);unclassified(100);unclassified(100);unclassified(100);

Otu0807 0 0 0 0 0 0 0 0 0 0 0 0 0 0 0 0 1 0 0 0 0 0 0 0 0 0 0 0
0 0 1 0 0 0 0 0 0 0 0 0 0 0 0 0 0 0 0 0 0 5 0 0 0 0 0 0 0
0 5 0 0 0 0 0 0 1 0 0 0 0 0 0 0 0 0 0 0 0 0 0 0 0 0 0 0 0
0 0 0 0 0 0 0 0 0 2 0 10 0 0 0 0 0 0 0 0 0 0 0 0 0 0 0

M02255_76_000000000-ADUNE_1_1115_7306_18567 .....AC----T-AC-GTG---------CC-A-G------C--A--G---C--CG-C-GGT-AA------TA-C--G-T--AG-GT-------GA--C-----AA-GC-G--TT-------G-TC-C-G---G---------------T-CT-G-T-T-----------TA----G-
T-C-T--------A-A-T--G--TG--A-AA-GC--C-C-AC-G-G-----CT-T-AA------C-C-G-T-G-G-A--A------C-GG-C-A---T--T-------G--GA-A-A---C-------T--G-A--CA--G-A-C----T-T-G-A-A-T--G---T-AG-----AA-G-A--------G-G-A-A---AA-T------GG--A--ATT----C-C-A-A-GT--
GT-A-G-CG-GT--G---G--A-A---TG-C-GT-AG--AT-A-TT--T-G--G-A-G-G-A-AC-A-CC---AG--T--G--GC-GAA-G--G-C--G-------A--T-T-T-T---CTG--G--TC-T-A--------------------A-C---A-T-T--GA--CG-C-T-G--A-GG--C-T-CG-A--AA-G-C---G-TG--GG-G--AG-C-G-
AA--------CA--GG Bacteria(100);Firmicutes(100);Bacilli(100);Lactobacillales(100);Carnobacteriaceae(100);Dolosigranulum(100);unclassified(100);unclassified(100);unclassified(100);

Otu0808 0 0 0 0 0 0 0 0 0 0 0 0 0 0 0 0 0 0 2 3 0 0 0 0 0 0 0 0
0 0 0 0 0 0 0 0 0 0 0 0 0 0 0 0 0 0 0 0 0 0 0 0 0 0 0 0 0
0 0 0 0 0 0 0 0 0 0 0 0 0 0 0 0 0 0 0 0 0 0 0 0 0 0 0 0 0
0 0 0 0 0 0 0 1 0 0 0 0 0 0 0 0 0 0 0 0 0 0 0 0 0 0 0

M02255_76_000000000-ADUNE_1_2107_15756_15592 C----TA---AC-TC-TGT--------GC--CAGC-----A--G--C---C--GC-G-GTA-AT------AC-A--G-A--GG-GT-------G---C-----AA-GC-GT-----------TA-A-T-C-G------------G--T-CT-T-T-T----------
-AA----G-T-C-G--------G-A-T--G--TG--A-AA-TC--C-C-TG-A-G-----CT-T-AA------C-T-T-A-G-G-A--A-T----T-G--C-A---T--A-------C--GA-T-A---C-------T--G-G--GA--G-A-C----T-A-G-A-G-T--A---T-GG-----GA-G-A--------G-G-A-T---GG-T------AG--A--ATT----C-C-
A-G-GT--GT-A-G-CG-GT--G---A--A-A---TG-C-GT-AG--AG-A-TC--T-G--G-A-G-G-A-AT-A-CC---GA--T--G--GC-GAA-G--G-C--A-------G--C-C-A-T---CTG--G--CC-T-A--------------------A-T---A-C-T--GA--CA-C-T-G--A-GG--T-A-CG-A--AA-G-C---A-TG--GG-G--
AG-C-A-AA--------CA--GG Bacteria(100);Proteobacteria(100);Gammaproteobacteria(100);Pseudomonadales(100);Moraxellaceae(100);Acinetobacter(100);unclassified(100);unclassified(100);unclassified(100);

Otu0809 0 0 0 0 0 0 0 0 0 0 0 0 0 0 0 0 0 0 0 0 0 0 0 0 0 0 0 0
0 0 0 0 0 0 0 0 0 0 0 0 0 0 62 0 0 0 0 0 0 0 0 0 0 0 0 0 0
0 0 0 0 0 0 0 0 0 0 0 0 0 0 0 0 0 0 0 0 0 0 0 0 0 0 0 0 0
0 0 0 0 0 0 0 0 0 0 0 0 0 0 0 0 0 0 0 0 0 0 0 0 0 0 0

M02255_77_000000000-ADUT3_1_1112_9911_5900 T----AC---GA-AG-GGG--------GCG-A-G------C--G--T---T--GT-T-CGG-AT------TT-A--C-T--GG-GC-------GT--A-----AA-GG-GC-GC-------G-TA-G-G-C-G------------G--C-CC-T-C-T-----------TT-
---G-T-C-G--------C-G-T--G--TG--A-AA-GC--C-C-CG-G-G-----CT-C-AA------C-C-T-G-G-G-A--A-T----T-G--C-G---C--G-------C--GA-A-A---C-------G--G-G--GG--G-G-C----T-T-G-A-G-T--T---C-GG-----GA-G-A--------G-G-A-G---CG-T------GG--A--ATT----C-C-C-A-
GT--GT-A-G-AG-GT--G---A--A-A---TT-C-GT-AG--AT-A-TT--G-G--G-A-A-G-A-AC-A-CC---GG--T--G--GC-GAA-G--G-C--G-------G--C-G-C-T---CTG--G--AC-C-G--------------------A-G---A-C-T--GA--CG-C-T-G--A-GG--C-G-CG-A--AA-G-C---G-TG--GG-G--AG-
C-A-AA--------CA--GG Bacteria(100);Proteobacteria(100);Alphaproteobacteria(100);Rhodospirillales(100);unclassified(100);unclassified(100);unclassified(100);unclassified(100);unclassified(100);

Otu0810 0 0 0 0 0 0 0 0 0 0 0 0 0 0 0 0 0 0 3 0 0 0 0 0 0 0 0 0
0 0 0 0 0 0 0 0 0 0 0 0 0 0 0 0 0 0 0 0 0 0 0 0 0 0 0 0 0
0 0 0 0 0 0 0 0 0 0 0 0 0 0 0 0 0 0 0 0 0 0 0 0 0 0 0 0 0
0 0 0 0 0 0 0 0 0 0 0 0 0 0 0 0 0 0 0 0 0 0 0 0 0 0 0
M02255_77_000000000-ADUT3_1_1106_13520_18930 T----AC---GT-AG-GGT--------GCA-A-G------C--G--T---T--GT-C-CGG-AA------TT-A--T-T--GG-GC-------GT--A-----AA-GA-GC-TT-------G-TA-G-G-C-G------------G--T-TT-G-T-C-----------GC----G-T-C-G--------G-A-T--G--TG--A-AA-AC--T-C-AG-G-G-----CT-T-AA------C-C-C-T-G-A-G--C-G----T-G--C-T---T--T-------C--GA-T-A---C-------G--G-G--CA--G-A-C----T-A-G-A-G-G--A---A-GT-----TA-G-G--------G-G-A-G---AA-C------GG--A--ATT----C-C-T-G-GT--GG-A-G-CG-GT--G---G--A-A---TG-C-GC-AG--AT-A-TC--A-G--G-A-G-G-A-AC-A-CC---AG--T--G--GC-GAA--G-G-C--G-------G--T-T-C-T---CTG--G--GA-C-T--------------------T-T---C-C-T--GA--CG-C-T-G--A-GA--A-G-CG-A--AA-G-C---A-TG--GG-G--AT-C-A-AA--------CA--GG Bacteria(100);Actinobacteria(100);Actinobacteria(100);Actinobacteridae(100);Actinomycetales(100);Propionibacterineae(100);unclassified(100);unclassified(100);unclassified(100);

Otu0811 0 0 0 0 0 0 0 0 0 0 0 0 0 0 0 0 0 0 3 0 0 0 0 0 0 0 0 0
0 0 0 0 0 0 0 0 0 0 0 0 0 0 0 0 0 0 0 0 0 0 0 0 0 0 0 0 0
0 0 0 0 0 0 0 0 0 0 0 0 0 0 0 0 0 0 0 0 0 0 0 0 0 0 0 0 0
0 0 0 0 0 0 0 0 0 0 0 0 0 0 0 0 0 0 0 0 0 0 0 0 0 0 0
M02255_77_000000000-ADUT3_1_2109_14360_22340 T----AC---GG-AG-GGT--------GCA-A-G------C--G--T---T--AT-C-CGG-AT------TT-A--T-T--GG-G--------GT---------A--G-G---C-------G----G-G-C--------------------T--T-G-T-----------AA----G-T-C-A--------G-T-G--G--TG--A-AA-TC--T-C-AT-A-G-----CT-T-AA------C-T-A-T-G-A-A--A-C----T-G--C-C---A--T-------T--GA-T-A---C-------T--G-C--AG--G-T-C----T-T-G-A-G-T--A---A-AG-----TA-G-A--------A-G-T-G---GC-T------GG--A--ATA----A-G-T-A-GT--GT-A-G-CG-GT--G---A--A-A---TG-C-AT-AG--AT-A-TT--A-C--T-T-A-G-A-AC-A-CC---AA--T--T--GC-GAA-G--G-C--A-------G--G-T-C-A---CTA--T--GT-T-T--------------------T-A---A-C-T--GA--CG-C-T-G--A-TG--G-A-CG-A--AA-G-C---G-TG--GG-G--AG-C-G-AA--------CA--GG Bacteria(100);Bacteroidetes(100);Flavobacteria(100);Flavobacteriales(100);Flavobacteriaceae(100);unclassified(100);unclassified(100);unclassified(100);unclassified(100);

Otu0812 0 0 0 0 0 0 0 0 0 0 0 0 0 0 0 0 0 0 0 0 0 0 0 0 0 0 0 0
0 0 0 0 0 0 0 0 0 0 0 0 0 0 72 0 0 0 0 0 0 0 0 0 0 0 0 0 0
0 0 0 0 0 0 0 0 0 0 0 0 0 0 0 0 0 0 0 0 0 0 0 0 0 0 0 0 0
0 0 0 0 0 0 0 0 0 0 0 0 0 0 0 0 0 0 0 0 0 0 0 0 0 0 0
M02255_77_000000000-ADUT3_1_1110_11128_13227 T----AC---GG-AG-GGT--------GCG-A-G------C--G--T---T--AT-C-CGG-AA------TC-A--C-T--GG-GC-------GT--A-----AA-GG-GC-GC-------G-TA-G-G-C-G------------G--T-TT-G-T-C-----------AA----G-T-C-C--------G-A-T--G--TT--A-AA-GC--G-T-GA-G-G-----CT-C-AA------C-C-T-C-A-T-C--A-C----G-G--C-G---T--T-------G--GA-T-A---C-------T--A-G--CA--A-G-C----T-T-G-A-C-G--G---T-TG-----GA-G-A--------G-G-A-A---GG-T------AG--A--ATT----A-C-C-A-GT--GT-A-G-CG-GT--G---G--A-A---TG-C-GT-AG--AT-A-CT--G-G--T-A-G-G-A-AT-A-CC---CA--T--T--GC-GAA-G--G-C--A-------G--C-C-T-T---CTG--G--AC-A-A--------------------C-A---C-G-T--GA--CG-C-T-G--A-GG--C-G-CG-A--AA-G-T---G-TG--GG-G--AG-C-A-AA--------CC--GG Bacteria(100);Deinococcus-Thermus(100);Deinococcales(100);Trueperaceae(100);Truepera(100);unclassified(100);unclassified(100);unclassified(100);unclassified(100);

Otu0813 0 0 0 0 0 0 0 0 0 0 0 0 0 0 0 0 0 0 3 0 0 0 11 0 0 0 0 0
0 0 0 0 0 0 0 0 0 0 0 0 0 0 0 0 0 0 0 0 0 0 0 0 0 0 0 0 0
0 0 0 0 0 0 0 0 0 0 0 0 0 0 0 0 0 54 0 0 0 0 0 0 0 0 0 0 0
0 0 0 0 0 0 0 0 0 0 0 0 0 0 0 0 0 0 0 0 0 0 0 0 0 0 0
M02255_77_000000000-ADUT3_1_2108_15775_10231 T----AC---GG-AG-GGG--------GCT-A-G------C--G--T---T--GT-T-CGG-AA------TT-A--C-T--GG-GC-------GT--A-----AA-GC-GC-GC-------G-TA-G-G-C-G------------G--T-TC-T-T-C-----------AA----G-T-C-A--------G-A-G--G--TG--A-AA-GC--C-C-GG-T-G-----CT-C-AA------C-A-C-C-G-G-A--A-T----T-G--C-C---T--T-------T--GA-A-A---C-------T--A-G--GG--A-A-C----T-T-G-A-A-T--C---C-AG-----GA-G-A--------G-G-T-G---AG-T------GG--A--ATT----C-C-G-A-GT--GT-A-G-AG-GT--G---A--A-A---TT-C-GT-AG--AT-A-TT--C-G--G-A-A-G-A-AC-A-CC---AG--T--G--GC-GAA-G--G-C--G-------G--C-T-C-A---CTG--G--AC-T-G--------------------G-T---A-T-T--GA--CG-C-T-G--A-GG--T-G-CG-A--AA-G-C---G-TG--GG-G--AG-C-A-AA--------CA--GG Bacteria(100);Proteobacteria(100);Alphaproteobacteria(100);Sphingomonadales(100);unclassified(100);unclassified(100);unclassified(100);unclassified(100);unclassified(100);

Otu0814 0 0 0 0 0 0 0 0 0 0 0 0 0 0 0 0 0 0 0 0 0 0 0 0 0 0 0 0
0 0 0 0 0 0 0 0 0 0 0 0 0 0 44 0 0 0 0 0 0 0 0 0 0 0 0 0 0
0 0 0 0 0 0 0 0 0 0 0 0 0 0 0 0 0 0 0 0 0 0 0 0 0 0 0 0 0
0 0 0 0 0 0 0 0 0 0 0 0 0 0 0 0 0 0 0 0 0 0 0 0 0 0 0
M02255_77_000000000-ADUT3_1_1110_10823_6024 T----AC---AG-GG-GGC--------GCG-A-G------C--G--T---T--GC-T-CGG-AA------TC-A--C-T--GG-GC-------GT--A-----AA-GC-GT-AC-------G-CA-G-G-C-G------------G--A-CC-G-T-T-----------AA----G-T-C-G--------G-G-T--G--TG--A-AA-TC--C-C-AG-G-G-----CT-C-AA------C-C-C-T-G-G-A--C-G----T-G--C-A---T--T-------C--GA-T-A---C-------T--G-G--CG--G-T-C----T-C-G-A-G-T--A---C-GG-----GA-G-A--------G-G-T-G---AG-T------GG--A--ATT----C-C-T-G-GT--GT-A-G-AG-GT--G---A--A-A---TT-C-GT-AG--AT-A-TC--A-G--G-A-G-G-A-AC-A-CC---GG--T--G--GC-GAA-A--G-C--G-------G--C-T-C-A---CTG--G--CC-C-G--------------------A-T---A-C-T--GA--CG-C-T-C--A-TG--T-A-CG-A--AA-G-C---G-TG--GG-G--AG-C-A-AA--------CA--GG Bacteria(100);unclassified(100);unclassified(100);unclassified(100);unclassified(100);unclassified(100);unclassified(100);unclassified(100);unclassified(100);

Otu0815 0 0 0 0 0 0 0 0 0 0 0 0 0 0 0 0 0 0 0 3 0 0 0 0 0 0 0 0
0 0 1 0 0 0 0 0 0 17 1 0 0 0 0 0 0 0 0 0 0 0 0 0 0 0 0 0
0 0 0 0 0 0 0 0 0 0 0 0 0 0 0 0 0 0 0 0 0 0 0 0 0 0 0 0
0 0 15 0 0 0 0 0 0 0 0 0 0 0 0 0 0 0 0 0 0 0 0 0 0 0 0
M02255_77_000000000-ADUT3_1_1108_8539_15257 G----AC---GG--G-GGG--------GCA-A-G------T--G--T---T--CT-T-CGG-AA------TG-A--C-T--AG-GT-------GT--A-----AA-GG-GC-AC-------G-TA-G-G-C-G------------G--T-GA-A-T-C----------
GG----G-T-T-G--------A-A-A--G--CG--A-AA-----G-T-CG-C-C-----AA-A-AA------T-T-G-G-T-G-G--A-A----T-G--C-T---C--T-------C--GA-A-A---C-------C--A-A--TT--C-A-C----T-T-G-A-G-T--G---A-GA-----CA-G-A--------G-G-A-G---AG-T------GG--A--ATT----T-C-G-
T-GTG-GC-A-T-GG-GT--G---A--A-A---TC-C-TA-AG--AT-C-TA--C-G--A-A-G-G-A-AA-G-CC---AA--A--A--GC-GAA-G--G-C--A-------G--C-T-C-T---CTG--G--GT-C-C--------------------C-T---A-C-C--GA--CG-C-T-G--G-AG--T-G-CG-A--AA-G-C---A-TG--GG-G--
AG-C-G-AA--------CG--GG Bacteria(100);unclassified(100);unclassified(100);unclassified(100);unclassified(100);unclassified(100);unclassified(100);unclassified(100);unclassified(100);

Otu0816 0 0 0 0 0 0 0 0 0 0 0 0 0 0 0 0 0 0 0 0 0 0 0 0 0 0 0 0
0 0 0 0 0 0 0 0 0 0 0 0 0 0 28 0 0 0 0 0 0 0 0 0 0 0 0 0
0 0 0 0 0 0 0 0 0 0 0 0 0 0 0 0 0 0 0 0 0 0 0 0 0 0 0 0
0 0 0 0 0 0 0 0 0 0 0 0 0 0 0 0 0 0 0 0 0 0 0 0 0 0 0
M02255_77_000000000-ADUT3_1_1109_25304_16706 T----AC---GG-AG-GGG--------GCA-A-G------C--G--T---T--GT-T-CGG-AA------TT-A--C-T--GG-GC-------GT--A-----AA-GG-GC-GC-------G-TA-G-G-C-G------------G--A-AC-A-T-T----
--------AA----G-T-T-A--------G-G-G--G--TG--A-AA-GG--C-C-TG-G-G-----CT-C-AA------C-C-T-G-G-G-A--A-C----G-G--C-C---T--C-------T--AA-G-A---C-------T--G-A--TG--A-T-C----T-G-G-A-G-T--C---C-GG-----AA-G-A--------G-G-A-T---GG-C------GG--A--ATT----
C-C-C-A-GT--GT-A-G-AG-GT--G---A--A-A---TT-C-GT-AG--AT-A-TT--G-G--G-A-A-G-A-AC-A-CC---GG--T--G--GC-GAA-G--G-C--G-------G--C-C-A-T---CTG--G--TC-C-G--------------------G-C---A-C-T--GA--CG-C-T-G--A-GG--C-G-CG-A--AA-G-CG----TG--
GG-G--AG-C-A-AA--------CA--GG Bacteria(100);Proteobacteria(100);unclassified(100);unclassified(100);unclassified(100);unclassified(100);unclassified(100);unclassified(100);unclassified(100);

Otu0817 0 0 0 0 0 0 0 0 0 0 0 0 0 0 0 0 0 0 0 2 0 0 0 0 0 0 0 0
0 0 1 0 0 0 0 0 0 20 0 0 0 0 0 0 0 0 0 0 0 0 0 0 0 0 0 0
0 0 0 0 0 0 0 0 0 0 0 0 0 0 0 0 0 5 0 0 0 0 0 0 0 0 0 0
0 0 0 0 0 0 0 0 0 0 0 0 0 0 0 0 0 0 0 0 0 0 0 0 0 0 0
M02255_77_000000000-ADUT3_1_1111_5162_14501 T----AC---GT-AG-GGG--------TCG-A-G------C--G--T---T--GT-C-CGG-AG------TT-A--C-T--GG-GC-------GT--A-----AA-GC-GC-AT-------G-CA-G-G-C-G------------G--C-TC-A-T-T----------AC-
---G-C-A-T--------G-G-C--G--TG--A-AA-GC--C-C-CC-G-G-----CT-C-AA------C-G-G-G-G-G-A--G-G----G-T--C-G---T--C-------G--TG-A-A---C-------G--G-G--TG--A-G-C----T-T-G-A-G-G--G---T-AT-----CA-G-G--------G-G-C-T---GG-T------GG--A--ATT----C-C-C-G-
GT--GT-A-G-TG-GT--G---A--A-A---TG-C-GT-AG--AG-A-TC--G-G--G-A-G-G-A-AC-A-CC---TG--T----GGC-GAA-G--G-C--G-------G--C-C-A-G---CTG--G--GA-T-A--------------------C-A---C-C-T--GA--CG-C-T-G--A-GA--T-G-CG-A--AG-G-C---GTGG--G--G--AG-
C-G-AA--------CG--GG Bacteria(100);Chloroflexi(100);Thermomicrobia(100);JG30-KF-CM45(100);unclassified(100);unclassified(100);unclassified(100);unclassified(100);unclassified(100);

Otu0818 0 0 0 0 0 0 0 0 0 0 0 0 0 0 0 0 0 0 0 0 0 0 0 0 0 0 0 0
0 0 0 0 0 0 0 0 0 0 0 0 0 0 114 0 0 0 0 0 0 0 0 0 0 0 0 0
0 0 0 0 0 0 0 0 0 0 0 0 0 0 0 0 0 0 0 0 0 0 0 0 0 0 0 0
0 0 0 0 0 0 0 0 0 0 0 0 0 0 0 0 0 0 0 0 0 0 0 0 0 0 0
M02255_77_000000000-ADUT3_1_1109_13260_6368 T----AC---GG-AG-GGG--------GCT-A-G------C--G--T---T--GT-T-CGG-AA------TT-A--C-T--GG-GC-------GT--A-----AA-GC-GC-AC-------G-TA-G-G-C-G------------G--T-TG-C-T-C----------AA-
---G-T-C-A--------G-A-G--G--TG--A-AA-GC--C-T-GG-A-G-----CT-C-AA------C-T-C-C-A-G-A--A-C----T-G--C-C---T--T-------T--GA-A-A---C-------T--A-G--GC--A-A-C----T-T-G-A-A-C--G---T-CG-----GA-G-A--------G-G-T-G---AG-T------GG--A--ATT----C-C-G-A-
GT--GT-A-G-AG-GT--G---A--A-A---TT-C-GT-AG--AT-A-TT--C-G--G-A-A-G-A-AC-A-CC---AG--T--G--GC-GAA-G--G-C--G-------G--C-T-C-A---CTG--G--AC-G-A--------------------C-T---G-T-T--GA--CG-C-T-G--A-GG--T-G-CG-A--AA-G-C---G-TG--GG-G--AG-
C-A-AA--------CA--GG Bacteria(100);Proteobacteria(100);Alphaproteobacteria(100);Sphingomonadales(100);unclassified(100);unclassified(100);unclassified(100);unclassified(100);unclassified(100);

Otu0819 0 0 0 0 0 0 0 0 0 0 0 0 0 0 0 0 0 0 0 3 0 0 0 0 0 0 0 0
0 4 0 0 0 0 0 0 0 28 0 0 0 0 0 0 0 0 0 0 0 0 0 0 0 0 0 0
0 0 0 0 0 0 0 0 0 0 0 0 0 0 0 0 0 0 0 0 0 0 0 0 0 0 0 0
0 0 0 0 0 0 0 0 0 0 0 0 0 0 0 0 0 0 0 0 0 0 0 0 0 0 0
M02255_77_000000000-ADUT3_1_1119_12401_20327 T----AC---GA-AG-GGG--------GCT-A-G------C--G--T---T--GC-T-CGG-AA------TT-A--C-T--GG-GC-------GT--A-----AA-GG-GC-GC-------G-TA-G-G-C-G------------G--A-TA-T-T-T-----
------AA----G-T-C-G--------G-G-G--G--TG--A-AA-GC--C-C-AG-G-G-----CT-C-AA------C-C-C-T-G-G-A--A-C----A-G--C-C---T--T-------C--GA-T-A---C-------T--G-G--AT--A-T-C----T-T-G-A-G-T--A---C-GG-----GA-G-A--------G-G-T-G---TG-T------GG--A--ACT----
C-C-G-A-GT--GT-A-G-AG-GT--G---A--A-A---TT-C-GT-AG--AT-A-TT--C-G--G-A-A-G-A-AC-A-CC---AG--T--G--GC-GAA-G--G-C--G-------A--C-A-C-A---CTG--G--CC-C-G--------------------T-T---A-C-T--GA--CG-C-T-G--A-GG--C-G-CG-A--AA-G-C---G-TG--GG-
G--AG-C-A-AA--------CA--GG Bacteria(100);Proteobacteria(100);Alphaproteobacteria(100);Caulobacterales(100);Caulobacteraceae(100);unclassified(100);unclassified(100);unclassified(100);unclassified(100);

Otu0820 0 0 0 0 0 0 0 0 0 0 0 0 0 0 0 0 1 0 0 0 0 0 0 0 0 0 0 0
0 0 0 0 0 0 0 0 0 0 0 0 0 0 0 0 0 0 0 0 0 0 0 0 0 0 0 0
0 0 0 1 0 0 0 0 0 1 0 0 0 0 0 0 0 0 0 0 0 0 0 0 0 0 0 0
0 0 0 0 0 0 0 0 0 0 0 0 0 0 0 0 0 0 0 0 0 0 0 0 0 0 0

M02255_76_000000000-ADUNE_1_2101_12372_24548 T----AC---CG-AC-AGA--------ATA-A-G------C-----A---C--CG-G-CTA-AC------TC-T--G-T--GC-CA-------GC--AG----CC-GC-GG-TA-------A-TACA-G-A-G-------------G--T-GT-G-C-A-----
-----AGC----G-T-T-G--------A-A-T--C--GG--A-AA-TC--C-C-CG-G-G-----CT-C-AA------C-C-T-G-G-G-A--A-C----T-G--C-A---T--C-------C--AA-A-A---C-------T--G-G--CA--A-G-C----T-A-G-A-G-T--A---T-GG-----TA-G-A--------G-G-G-T---AG-T------GG--A--ATT----
T-C-C-T-GT--GT-A-G-CG-GT--G---A--A-A---TG-C-GT-AG--AT-A-TA--G-G--A-A-G-G-A-AC-A-CC---AG--T--G--GC-GAA-G--G-C--G-------A--C-T-A-C---CTG--G--AC-T-G--------------------A-T---A-C-T--GA--CA-C-T-G--A-GG--T-G-CG-A--AA-G-C---G-TG--GG-
G--AG-C-A-AA--------CA--GG Bacteria(100);Proteobacteria(100);Gammaproteobacteria(100);unclassified(100);unclassified(100);unclassified(100);unclassified(100);unclassified(100);unclassified(100);

Otu0821 0 0 0 0 0 0 0 0 0 0 0 0 0 0 0 0 0 0 0 0 0 0 0 0 0 0 0 0
0 0 3 0 0 0 0 0 0 1 28 0 0 0 0 0 0 0 0 0 0 0 0 0 0 0 0 0
0 0 0 0 0 0 0 0 0 0 0 0 0 0 0 0 0 0 0 0 0 0 0 0 0 0 0 0
0 0 0 0 0 0 0 0 0 0 0 0 0 0 0 0 0 0 0 0 0 0 0 0 0 0 0

M02255_77_000000000-ADUT3_1_2119_19219_10339 T----AC---GT-AG-GTG--------TCA-A-G------C--G--T---T--GT-C-CGG-AT------TT-A--T-T--GG-GC-------GT--A-----AA-GC-GC-CC-------G-CA-G-G-C-G-------------G--C-TT-G-G-C------
-----AA----G-T-T-C--------G-A-G--G--TG--A-AA-TC--T-C-CC-G-G-----CT-C-AA------C-T-G-G-G-A-G--G-C----T-G--C-C---T--C-------G--AA-A-A---C-------T--G-C--TA--G-G-C----T-T-G-A-G-G--C---A-GG-----GA-G-A--------G-G-G-A---TG-T------GG--A--ATT----C-
C-C-G-GT--GT-A-G-TG-GT--G---A--A-A---TG-C-GT-AG--AT-A-TC--G-G--G-A-G-G-A-AC-A-CC---AG--T--G--GC-GAA-G--G-C--G-------G--C-A-T-C---CTG--GC-CC---T--------------------G-T---C-C-T--GA--CG-C-T-C--A-GG--G-G-CG-A--AA-G-C---G-TG--GG-
G--AG-C-A-AA---------CC-GG Bacteria(100);Chloroflexi(100);TK10(100);unclassified(100);unclassified(100);unclassified(100);unclassified(100);unclassified(100);unclassified(100);

Otu0822 0 0 0 0 0 0 0 0 0 0 0 0 0 1 0 0 0 0 0 0 0 0 0 0 0 8 0 0
0 0 0 0 0 0 0 0 0 0 0 0 0 0 0 0 0 0 0 0 0 0 0 0 0 0 0 0
0 0 0 0 0 0 0 0 0 0 0 0 0 0 0 0 0 7 0 0 0 0 0 0 0 0 0 0
0 0 0 0 0 0 0 0 0 0 0 0 0 0 0 0 0 0 0 0 0 0 0 0 0 0 0

M02255_76_000000000-ADUNE_1_1110_27402_11160 C----A----GC-A--GCC--------GCG-G-T------A--A--T---A--CG-G-AGG-GT------GC-A--A-G--CG-TT-------AC--C-----CG-GA-AT-CA-------C-TG-G-G-C-G-------------G--C-CT-G-C-C------
-----AA----G-T-C-T--------G-G-T--T--TT--A-AA-GC--C-T-GC-G-G-----CT-C-AA------C-C-G-C-A-G-A--G-A----T-G--G-A---C--T-------G--GA-G-A---C-------T--G-G--TA--G-G-C----T-A-G-A-C-C--T---C-TG-----GA-G-A--------G-A-G-A---AC-T------GG--A--ATT----C-
C-T-G-GT--GT-A-G-CG-GT--G---G--A-A---TG-C-GT-AG--AT-A-CC--A-G--G-A-G-G-A-AC-A-CC---GA--T--G--GC-GAA-G--G-C--A-------G--G-T-T-C---TTG--G--AC-A-G--------------------A-A---G-G-T--GA--CG-C-T-G--A-GG--C-G-CG-A--AA-G-T---G-TG--GG-
G--AG-C-A-AA--------CC--GG Bacteria(100);Deinococcus-Thermus(100);Deinococcales(100);Deinococcaceae(100);Deinococcus(100);unclassified(100);unclassified(100);unclassified(100);unclassified(100);

Otu0823 0 0 0 0 0 0 0 0 0 0 0 0 0 1 0 0 0 0 0 0 0 0 0 0 0 0 0 0
0 0 58 0 0 0 0 0 0 0 0 24 0 0 0 0 0 0 0 0 0 0 0 0 0 0 0 0
0 0 0 0 0 0 0 0 0 0 0 0 0 0 0 0 0 0 0 0 0 0 0 0 0 0 0 0
0 0 0 0 0 0 0 0 0 0 0 0 0 0 0 0 0 0 0 0 0 0 0 0 0 0 0

M02255_77_000000000-ADUT3_1_1101_11727_12548 T----AC---GT-AG-GGG--------GCA-A-G------C--G--T---T--AT-C-CGG-AT------TT-A--C-T--GG-GT-------GT--A-----AA-GG-GA-GC-------G-CA-G-G-C-G-------------G--T-CT-G-T-C-----
------AA----G-T-C-T--------G-A-T--G--TG--A-AA-TC--C-C-GG-G-G-----CT-C-AA------C-C-C-T-G-G-A--A-C----T-G--C-A---T--T-------G--GA-A-A---C-------T--G-T--CA--G-A-C----T-A-G-A-G-T--G---C-CG-----GA-G-A--------G-G-T-A---AG-T------GG--A--ATT----C-
C-T-A-GT--GT-A-G-CG-GT--G---A--A-A---TG-C-GT-AG--AT-A-TT--A-G--G-A-G-G-A-AC-A-CC---AG--T--G--GC-GAA-G--G-C--G-------G--C-T-T-A---CTG--G--AC-G-G--------------------T-A---A-C-T--GA--CG-C-T-G--A-GG--C-T-CG-A--AA-G-C---G-TG--GG-
G--AG-C-A-AA--------CA--GG Bacteria(100);unclassified(100);unclassified(100);unclassified(100);unclassified(100);unclassified(100);unclassified(100);unclassified(100);unclassified(100);

Otu0824 0 0 0 0 0 0 0 0 0 0 0 0 0 0 0 0 0 0 0 0 0 0 0 0 0 0 0 0
0 0 0 0 0 0 0 0 0 0 0 0 0 0 0 12 0 0 0 0 0 0 0 0 0 0 0 0
0 0 0 0 0 0 0 0 0 0 0 0 0 0 0 0 0 0 0 0 0 0 0 0 0 0 0 0
0 0 0 0 0 0 0 0 0 0 0 0 0 0 0 0 0 0 0 0 0 0 0 0 0 0 0

M02255_77_000000000-ADUT3_1_1107_19093_13126 T----AC---GT-AG-GGA--------GCA-A-G------C--G--T---T--AT-C-CGG-AT------TT-A--T-T--GG-GT-------GT--A-----AA-GG-GT-GC-------G-TA-G-A-C-G-------------G--G-AA-A-G-C-----
------AA----G-T-T-A--------G-T-T--G--TG--A-AA-TC--C-C-TC-G-G-----CT-T-AA------C-T-G-A-G-G-A--A-C----T-A--C-A---A--C-------T--AA-A-A---C-------T--A-C--TT--T-T-C----T-T-G-A-G-T--G---T-TG-----GA-G-A--------G-G-A-A---AG-T------GG--A--ATT----C-C-
T-A-GT--GT-A-G-CG-GT--G---A--A-A---TG-C-GT-AG--AT-A-TT--A-G--G-A-G-G-A-AC-A-CC---GG--T--G--GC-GAA-G--G-C--G-------A--C-T-T-T---CTG--G--AC-A-A--------------------T-A---A-C-T--GA--CG-T-T-G--A-GG--C-A-CG-A--AA-G-T---G-TG--GG-G--
AG-C-A-AA--------CA--GG Bacteria(100);Firmicutes(100);Clostridia(100);Clostridiales(100);Ruminococcaceae(100);uncultured(100);unclassified(100);unclassified(100);unclassified(100);

Otu0825 0 0 0 0 0 0 0 0 0 0 0 0 0 0 8 0 0 0 0 4 20 5 0 0 0 0 0 0
0 0 4 0 0 0 0 0 0 62 0 0 8 0 66 0 19 0 0 0 0 0 0 0 9 0 0 0 0
0 116 0 0 0 6 3 40 0 15 49 67 0 8 89 0 0 0 0 673 84 0 0 0 0 0 0 0 55
26 0 0 56 86 243 5 24 0 404 6 283 0 0 0 0 0 0 0 0 0 34 0 1 0 0 0
M02255_77_000000000-ADUT3_1_1105_11025_10148 T----AC---GT-AG-GGG--------GCT-A-G------C--G--T---T--GT-C-CGG-AA------TC-A--C-T--GG-GC-------GT--A-----AA-GG-GT-TC-------G-CA-G-G-C-G------------G--A-AT-A-A-C-----
------AA----G-T-C-A--------G-A-T--G--TG--A-AA-GG--C-A-TG-G-G-----CT-C-AA------C-C-C-A-T-G-T--A------A-G--C-A---T--T-------T--GA-A-A---C-------T--G-T--AA--T-T-C----T-T-G-A-G-A--A---G-TG-----GA-G-A--------G-G-T-A---AG-T------GG--A--ATT----C-
C-T-A-GT--GT-A-G-CG-GT--G---A--A-A---TG-C-GT-AG--AT-A-TT--A-G--G-A-G-G-A-AT-A-CC---TG--T--G--GC-GAA-G--G-C--G-------A--C-T-T-A---CTG--G--AC-A-C--------------------A-A---ATC-T--GA--CG-C-T-G--A-GG--A-A-CG-A--AA-G-C---G-TG--GG-
T--AG-C-A-AA--------CA--GG Bacteria(100);Firmicutes(100);Clostridia(100);Clostridiales(100);Family_XI_Incertae_Sedis(100);unclassified(100);unclassified(100);unclassified(100);unclassified(100);

Otu0826 0 0 0 0 0 0 0 0 0 0 0 0 0 0 0 0 0 0 0 0 0 0 0 0 0 0 0 0
0 0 0 0 0 0 0 0 0 0 0 0 0 0 0 22 0 0 0 0 0 0 0 0 0 0 0 0 0
5 18 0 0 0 0 0 0 0 0 0 0 0 0 145 149 0 0 0 0 0 0 0 0 7 0 0 7 0
15 35 0 0 20 0 0 0 0 7 0 4 0 0 0 0 0 0 0 46 86 12 0 0 0 0 0
M02255_76_000000000-ADUNE_1_2107_21260_21189 C----TA---AC-TC-CGT--------GCC-A-G------C--A--G---C--CG-C-GGT-AA------TA-C--G-G--AG-GA-------TG--C-----GA-GC-GT-TA-------T-CC-G-GATTT------------A--T-TC-G-G-T-----
------AA----G-T-C-A--------G-C-G--G--TG--A-AA-CC--T-G-AG-C-G-----CT-C-AA------C-G-T-T-C-A-G--C-C----T-G--C-C---G--T-------T--GA-A-A---C-------T--G-C--CG--G-G-C----T-T-G-A-G-T--T---C-AG-----TG-G-C--------G-G-C-A---GG-C------GG--A--ATT----C-
G-T-G-GT--GT-A-G-CG-GT--G---A--A-A---TG-C-AT-AG--AT-A-TC--A-C--G-A-G-G-A-AC-T-CC---GA--T--T--GC-GAA-G--G-C--A-------G--C-T-T-G---CCA--T--AC-T-G--------------------C-G---A-C-T--GA--CA-C-T-G--A-AG--C-A-CG-A--AG-G-C---G-TG--GG-T--
AT-C-A-AA--------CA--GG Bacteria(100);Bacteroidetes(100);Bacteroidia(100);Bacteroidales(100);Porphyromonadaceae(100);Porphyromonas(100);unclassified(100);unclassified(100);unclassified(100);

Otu0827 0 0 0 0 0 0 0 0 0 0 0 0 0 0 0 0 0 0 0 0 0 0 0 0 0 0 0 0
0 0 0 0 0 0 0 0 0 0 0 0 0 0 0 3 0 0 0 0 0 0 0 0 0 0 0 0 0
0 0 0 0 0 0 0 0 0 0 0 0 0 0 14 16 0 0 0 0 0 0 0 0 2 0 0 2 0
2 0 0 0 0 0 0 0 0 3 0 0 0 0 0 0 0 0 0 5 13 1 0 0 0 0 0
M02255_77_000000000-ADUT3_1_2114_11604_11352 C----GT---GC-CA-GCA--------GCC-G-C------G--G--T---A--AT-A-CGG-AG------GA-T--G-C--GA-GC-------GT--T-----AT-CC-GG-AT-------T-TA-T-T-G-G------------G--T-TC-G-G-T------
-----AA----G-T-C-A--------G-C-G--G--TG--A-AA-CC--T-G-AG-C-G-----CT-C-AA------C-G-T-T-C-A-G--C-C----T-G--C-C---G--T-------T--GA-A-A---C-------T--G-C--CG--G-G-C----T-T-G-A-G-T--T---C-AG-----TG-G-C--------G-G-C-A---GG-C------GG--A--ATT----C-
G-T-G-GT--GT-A-G-CG-GT--G---A--A-A---TG-C-AT-AG--AT-A-TC--A-C--G-A-G-G-A-AC-T-CC---GA--T--T--GC-GAA-G--G-C--A-------G--C-T-T-G---CCA--T--AC-T-G--------------------C-G---A-C-T--GA--CA-C-T-G--A-AG--C-A-CG-A--AG-G-C---G-TG--GG-T--
AT-C-A-AA--------CA--GG Bacteria(100);Bacteroidetes(100);Bacteroidia(100);Bacteroidales(100);Porphyromonadaceae(100);Porphyromonas(100);unclassified(100);unclassified(100);unclassified(100);

Otu0828 0 0 0 0 0 0 0 0 0 0 0 0 0 0 3 0 0 0 0 0 0 0 0 0 0 9 14 9
13 0 0 0 0 0 0 0 0 0 0 0 0 0 0 0 0 0 0 0 0 0 0 0 0 0 0 0 0
0 0 0 0 0 0 0 0 0 0 0 0 0 0 0 0 0 0 0 0 0 0 0 0 0 0 0 0 0
20 0 0 0 0 0 0 0 0 0 0 0 0 0 0 0 0 0 51 21 0 0 0 0 0 0 0
M02255_77_000000000-ADUT3_1_1115_3356_9371 G----AC---GA-GG-GGT--------CCA-A-G------C--G--T---T--GT-T-CGG-AA------TC-A--T-T--GG-GC-------GT--A-----AA-GC-GG-GT-------G-TA-G-G-T-G------------G--C-TT-T-G-T-----------AA-
---G-T-C-A--------G-G-A--G--TG--A-AA-TC--C-C-CG-A-G-----CT-T-AA------C-T-C-G-G-G-A--A-G----T-G--C-T---T--T-------T--GA-A-A---C-------T--G-C--AA--A-G-C----T-T-G-A-A-T--G---T-GG-----GA-G-A--------G-G-A-T---CG-T------GG--A--ATT----C-C-A-G-
GT--GT-A-G-TG-GT--G---A--A-A---TA-C-GT-AG--AT-A-TC--T-G--G-A-G-G-A-AC-A-CC---GG--T--G--GC-GAA-G--G-C--G-------G--C-G-A-T---CTG--G--CC-C-A--------------------A-C---A-T-T--GA--CA-C-T-G--A-GA--C-C-CG-A--AA-G-C---G-TG--GG-G--AT-C-
A-AA--------CA--GG Bacteria(100);unclassified(100);unclassified(100);unclassified(100);unclassified(100);unclassified(100);unclassified(100);unclassified(100);unclassified(100);

Otu0829 0 0 0 0 0 0 0 0 0 0 0 0 0 0 0 0 0 0 0 0 0 0 0 0 0 0 0 0
0 0 0 0 0 0 0 0 0 0 0 0 0 0 0 1 0 0 0 0 0 0 0 0 0 0 0 0 0
0 0 0 0 0 0 0 0 0 0 0 10 1 0 8 0 0 0 0 0 0 0 0 0 0 0 0 0 0
78 0 0 0 0 0 0 11 0 22 0 65 2 0 0 0 0 0 0 0 0 0 0 0 0 0 0
M02255_77_000000000-ADUT3_1_1108_28195_10148 T----AC---GT-AG-GGG--------GCA-A-G------C--G--T---T--AT-C-CGG-AT------TT-A--C-T--GG-GT-------GT--A-----AA-GG-GA-GC-------G-TA-G-A-C-G------------G--C-AT-G-G-C----
-------AA----G-C-C-A--------G-A-T--G--TG--A-AA-GC--C-C-GG-G-G-----CT-C-AA------C-C-C-C-G-G-G--A-C----T-G--C-A---T--T-------T--GG-A-A---C-------T--G-T--CA--G-G-C----T-A-G-A-G-T--G---T-CG-----GA-G-A--------G-G-A-A---AG-C------GG--A--ATT----
C-C-T-A-GT--GT-A-G-CG-GT--G---A--A-A---TG-C-GT-AG--AT-A-TT--A-G--G-A-G-G-A-AC-A-CC---AG--T--G--GC-GAA-G--G-C--G-------G--C-T-T-T---CTG--G--AC-G-A--------------------T-G---A-C-T--GA--CG-T-T-G--A-GG--C-T-CG-A--AA-G-C---G-TG--GG-
G--AG-C-A-AA--------CA--GG Bacteria(100);Firmicutes(100);Clostridia(100);Clostridiales(100);Lachnospiraceae(100);Incertae_Sedis(100);unclassified(100);unclassified(100);unclassified(100);

Otu0830 0 0 0 0 0 0 0 0 0 0 0 0 0 0 0 4 0 0 0 0 0 0 1 0 0 0 0 0
0 0 0 0 0 0 0 0 0 0 0 0 0 0 0 0 0 0 0 0 0 0 0 0 0 0 0 0 0
0 0 0 0 0 0 0 0 0 0 0 0 0 0 0 0 0 0 0 0 0 0 0 0 0 0 0 0 0
0 0 0 0 0 0 0 0 0 0 0 0 0 0 0 0 0 0 0 0 0 0 0 0 0 0 0
M02255_76_000000000-ADUNE_1_1109_14064_10115 T----AT---CT-AA-CCA--------GAA-A-G------C--C--A---C--G--G-C-T-AA------CT-A--C-G--TG-CC-------AG--C-----AG-CC-GC-GG-------T-AA-T-A-C-GT------------A--G-GT-G-G-C-------
----AA----GCG-T-T--------G-T-C--C--GG--A-AA-GC--C-C-CC-G-G-----CT-C-AA------C-C-G-G-G-A--G------G-GG-C-A---T--T-------G--GA-A-A---C-------T--G-G--GA--G-A-C----T-T-G-A-G-G--A---C-AG-----AA-G-A--------G-G-A-G---AG-T------GG--A--ATT----
C-C-A-A-GT--GT-A-G-CG-GT--G---A--A-A---TG-C-GT-AG--AT-A-TT--T-G--G-A-G-G-A-AC-A-CC---AG--T--G--GC-GAA-G--G-C--G-------G--C-T-C-T---CTG--G--TC-T-G--------------------T-T---A-C-T--GA--CG-C-T-G--A-GG--C-G-CG-A--AA-G-C---G-TG--GG-
G--AG-C-A-AA--------CA--GG Bacteria(100);Firmicutes(100);Bacilli(100);Bacillales(100);unclassified(100);unclassified(100);unclassified(100);unclassified(100);unclassified(100);

Otu0831 0 0 0 0 0 0 0 0 0 0 0 0 0 0 0 0 0 0 0 0 0 0 0 0 0 0 0 0
0 0 0 0 0 0 0 0 0 0 0 0 0 0 0 42 0 0 0 0 0 0 0 0 0 0 0 0 0
0 0 0 0 0 0 0 0 0 0 0 0 0 0 0 0 0 0 0 0 0 0 0 0 0 0 0 0 0
0 0 0 0 0 0 0 0 0 0 0 0 0 0 0 0 0 0 0 0 0 0 0 0 0 0 0
M02255_77_000000000-ADUT3_1_2101_23042_11302 T----AC---GT-AG-GAT--------CCG-A-G------C--G--T---T--AT-C-CGG-AG------TG-A--C-T--GG-GC-------GT--A-----AA-GA-GTTGC-------G-TA-G-G-T-G------------G--T-TA-G-T-A----
-------AA----G-C-G-A--------A-T-A--G--TG--A-AA-TC--T-G-GC-G-G-----CT-C-AA------C-C-G-T-ACA-G--G-C----T-A--T-T---A--T-------T--CG-A-A---C-------T--C-A--CT--A-A-C----T-C-G-A-G-A--A---T-GG-----TA-G-A--------G-G-T-T---AC-T------GG--A--ATT----T-
C-T-T-GT--GT-A-G-GA-GT--G---A--A-A---TC-C-GT-AG--AT-A-TA--A-G--A-A-G-G-A-AC-A-CC---AA--T--G--GC-GTA-G--G-C--A-------G--G-T-A-A---CTG--G--AC-C-A--------------------T-T---T-C-T--GA--CA-C-T-A--A-GG--C-A-CG-A--AA-G-C---G-TG--GG-G--
AG-C-G-AA--------CC--GG Bacteria(100);Candidate_division_TM7(100);unclassified(100);unclassified(100);unclassified(100);unclassified(100);unclassified(100);unclassified(100);unclassified(100);

Otu0832 0 0 0 0 0 0 0 0 0 0 0 0 0 0 0 1 0 0 1 0 0 0 0 0 0 0 0 0
0 0 0 0 0 0 0 0 0 0 0 0 0 0 0 0 0 0 0 0 0 0 0 0 0 0 0 0 0
0 0 0 0 0 0 0 0 0 0 0 0 0 0 0 0 0 0 0 0 0 0 0 0 0 0 0 0 0
0 0 0 0 0 0 0 0 0 0 0 0 0 0 11 0 0 0 0 0 0 0 0 0 0 0 0
M02255_76_000000000-ADUNE_1_2112_19484_8441 C----TA---AC-TC-TGT--------GCC-A-G------C-----A---G--CC-G-CGG-TA------AT-A--C-A--GA-GG-------GT--G-----CA--A-GC-G--------T-TA-A-T-C-G------------G--A-TT-T-G-T----------AA----
G-T-C-T--------G-A-T--G--TG--A-AA-TC--C-C-CG-G-G-----CT-C-AA------C-C-T-G-G-G-A--A-T----T-G--C-A---T--T-------G--GA-G-A---C-------T--G-C--AA--G-G-C----T-A-G-A-A-T--C---T-GG-----CA-G-A--------G-G-G-G---GG-T------AG--A--ATT----C-C-A-C-GT-
-GT-A-G-CA-GT--G---A--A-A---TG-C-GT-AG--AT-A-TG--T-G--G-A-G-G-A-AC-A-CC---GA--T--G--GC-GAA-G--G-C--A-------G--C-C-C-C---CTG--G--GT-C-A--------------------A-G---A-T-T--GA--CG-C-T-C--A-TG--C-A-CG-A--AA-G-C---G-TG--GG-G--AG-C-A-
AA--------CA--GG Bacteria(100);Proteobacteria(100);Betaproteobacteria(100);Burkholderiales(100);Oxalobacteraceae(100);Janthinobacterium(100);unclassified(100);unclassified(100);unclassified(100);

Otu0833 0 0 0 0 0 0 0 0 0 0 0 0 0 0 0 0 0 0 0 3 6 0 0 0 0 0 0 0
0 2 0 0 0 0 0 0 0 40 2 0 0 0 0 0 0 0 0 0 0 0 0 0 0 0 0 0 0
0 0 0 0 0 0 0 0 0 0 0 0 0 0 0 0 0 0 0 0 0 0 0 0 0 0 0 0 0
0 0 0 0 0 0 0 0 0 0 0 0 0 0 0 0 0 0 0 0 0 0 0 0 0 0 0
M02255_77_000000000-ADUT3_1_2119_23057_9922 T----AC---GG-AG-GAG--------GCA-A-G------C--G--T---T--AT-C-CGG-AA------TT-A--T-T--GG-GC-------GT--A-----AA-GC-GT-CC-------G-TA-G-G-T-G------------G--C-TG-C-T-C----------AA-
---G-T-C-T--------G-C-G--G--TC--A-AA-GA--C-C-GG-G-G-----CT-T-AA------C-C-C-T-G-G-A--A-A----G-G--C-C---G--T-------G--GA-A-A---C-------T--G-A--GC--A-G-C----T-A-G-A-G-T--G---C-AG-----TA-G-G--------G-G-T-A---GA-G------GG--A--ATT----C-C-C-G-
GT--GT-A-G-CG-GT--G---A--A-A---TG-C-GT-AG--AG-C-TC--G-G--G-A-A-G-A-AC-A-TC---GG--T--G--GC-GAA-A--G-C--G-------C--T-C-T-A---CTG--G--AC-T-G--------------------C-A---A-C-T--GA--CG-C-T-G--A-GG--G-A-CG-A--AA-G-C---T-AG--GG-G--AG-
C-G-AA--------TG--GG Bacteria(100);Cyanobacteria(100);SubsectionIII(100);unclassified(100);unclassified(100);unclassified(100);unclassified(100);unclassified(100);unclassified(100);

Otu0834 0 0 0 0 0 0 0 0 0 0 0 0 0 0 0 0 9 0 0 0 0 0 0 12 0 0 0 0
0 0 0 0 0 0 0 0 0 0 0 0 0 0 0 0 0 0 0 0 0 0 0 0 0 0 0 0 0
0 299 0 0 0 0 0 0 0 8 0 0 0 17 0 0 0 13 0 0 87 12 14 0 0 0 0 0 0
0 0 0 0 0 0 0 0 0 0 2 62 5 0 0 0 0 0 0 0 0 0 0 0 0 1 0
M02255_77_000000000-ADUT3_1_1112_4415_10453 T----AC---GT-AT-GTC--------GCA-A-G------C--G--T---T--AT-C-CGG-AA------TT-A--T-T--GG-GC-------TT--A-----AA-GG-GC-AT-------C-TA-G-G-C-G------------G--T-AT-A-A-C----------AA---
-G-T-T-G--------A-A-G--G--TG--A-AA-AG--C-T-GT-A-G-----CT-C-AA------C-T-A-T-A-G-T--C-T----T-G--C-C---T--A-------C--AA-A-A---C-------T--G-T--AA--T-A-C----T-A-G-A-G-T--A---C-TG-----GA-A-A--------G-G-T-G---GG-T------GG--A--ACT----A-C-A-C-GA-
-GT-A-G-AG-GT--G---A--A-A---TT-C-GT-AG--AT-A-TG--T-G--T-A-G-G-A-AT-G-CC---GA--T--G--AT-GAA-G--A-T--A-------A--C-T-C-A---CTG--G--AC-A-G--------------------A-A---A-C-T--GA--CG-C-T-G--A-AG--T-G-CG-A--AA-G-C---T-AG--GG-G--AG-C-A-
AA--------CA--GG Bacteria(100);Fusobacteria(100);Fusobacteria(100);Fusobacteriales(100);Leptotrichiaceae(100);unclassified(100);unclassified(100);unclassified(100);unclassified(100);

Otu0835 0 0 0 0 0 0 0 0 0 0 0 0 0 0 0 0 10 0 0 0 0 0 0 0 0 0 0 0
0 0 0 0 0 0 0 0 0 0 0 0 0 0 0 0 0 0 0 0 0 0 0 0 0 0 0 0 0
0 0 0 0 0 0 0 0 0 0 0 0 0 0 0 0 0 0 0 0 0 0 0 0 0 0 0 0 0
0 0 0 0 0 0 0 0 0 0 0 0 0 0 0 0 0 0 0 0 0 0 0 0 0 0 0 0
M02255_77_000000000-ADUT3_1_2102_25699_4028 T----AC---GA-AG-GGG--------GCT-A-G------C--G--T---T--GC-T-CGG-AA------TG-A--C-T--GG-GC-------GT--A-----AA-GG-GC-GC-------G-TA-G-G-C-G------------G--T-TT-G-C-C----------
AA----G-T-T-A--------G-A-T--G--TG--A-AA-TT--C-C-TG-G-G-----CT-T-AA------C-C-T-G-G-G-G--G-C----T-G--C-A---T--T-------T--GA-T-A---C-------T--G-G--CA--G-G-C----T-T-G-A-G-T--A---T-GG-----AA-G-A--------G-G-G-T---CG-T------GG--A--ATT----C-C-C-
A-GT--GT-A-G-AG-GT--G---A--A-A---TT-C-GT-AG--AT-A-TT--G-G--G-A-A-G-A-AC-A-CC---GG--T--G--GC-GAA-G--G-C--G-------G--C-G-A-C---CTG--G--TC-C-A--------------------T-A---A-C-T--GA--CG-C-T-G--A-GG--C-G-CG-A--AA-G-C---G-TG--GG-G--
AG-C-A-AA--------CA--GG Bacteria(100);Proteobacteria(100);Alphaproteobacteria(100);Rhodospirillales(100);Acetobacteraceae(100);unclassified(100);unclassified(100);unclassified(100);unclassified(100);

Otu0836 0 0 0 0 0 0 0 0 0 0 0 0 0 0 0 0 0 0 0 0 0 0 0 0 0 0 0 0
0 0 0 0 0 0 0 0 0 0 0 0 0 0 0 33 0 0 0 0 0 0 0 0 0 0 0 0 0
0 0 0 0 0 0 0 0 0 0 0 0 0 0 0 0 0 0 0 16 0 0 0 0 0 0 0 0 0
0 0 0 0 0 0 0 0 0 0 0 0 0 0 0 19 0 0 0 0 0 0 0 0 0 0 0 0
M02255_77_000000000-ADUT3_1_1114_20016_19866 T----AC---GT-AG-GGT--------GCG-A-G------C--G--T---T--GT-C-CGG-AA------TT-A--T-T--GG-GC-------GT--A-----AA-GA-GC-TC-------G-TA-G-G-C-G------------G--T-CT-G-T-C------
-----GC----G-T-C-G--------A-A-T--G--TG--A-AA-AC--C-C-GG-G-G-----CT-C-AA------C-T-C-C-G-G-G--C-T----T-G--C-A---T--T-------C--GA-T-A---C-------G--G-G--CA--G-G-C----T-A-G-A-G-T--T---C-GG-----TA-G-G--------G-G-A-G---TC-T------GG--A--ATT----C-
C-T-G-GT--GT-A-G-CG-GT--G---A--A-A---TG-C-GC-AG--AT-A-TC--A-G--G-A-G-G-A-AC-A-CC---GG--T--G--GC-GAA-G--G-C--G-------G--G-A-C-T---CTG--G--GC-C-G--------------------A-T---A-C-T--GA--CG-C-T-G--A-GG--A-G-CG-A--AA-G-C---G-TG--GG-
G--AG-C-G-AA--------CA--GG Bacteria(100);Actinobacteria(100);Actinobacteria(100);Actinobacteridae(100);Actinomycetales(100);Frankineae(100);unclassified(100);unclassified(100);unclassified(100);

Otu0837 0 0 0 0 0 0 0 0 0 0 0 0 0 0 0 0 0 0 0 0 0 0 0 0 0 0 0 0
0 0 0 0 0 0 0 0 0 0 0 0 0 0 0 19 0 0 0 0 0 0 0 0 0 0 0 0 0
0 0 0 0 0 0 0 0 0 0 0 0 0 0 0 0 0 0 0 0 0 0 0 0 0 0 0 0 0
0 0 0 0 0 0 0 0 0 0 0 0 0 0 0 0 0 0 0 0 0 0 0 0 0 0 0 0
M02255_77_000000000-ADUT3_1_1113_25546_15628 T----AC---GT-AG-GGG--------TCG-A-G------C--G--T---T--GT-C-CGG-AG------TT-A--C-T--GG-GC-------GT--A-----AA-GC-GT-GC-------G-CA-G-G-C-G------------G--C-GG-T-C-T----
-------GC----G-C-C-C--------G-G-C--G--TG--A-AA-GC--C-C-CC-G-G-----CT-C-AA------C-C-G-G-G-G-A--G-G----G-T--C-G---T--C-------G--GG-G-A---C-------G--G-G--AC--C-G-C----T-T-G-A-G-G--G---T-AT-----CA-G-G--------G-G-C-T---GG-T------GG--A--ATT---
-C-C-C-G-GT--GT-A-G-TG-GT--G---A--A-A---TG-C-GT-AG--AG-A-TC--G-G--G-A-G-G-A-AC-A-CC---CG--T--G--GC-GAA-G--G-C--G-------G--C-C-A-G---CTG--G--GA-T-A--------------------C-A---C-C-T--GA--CG-C-T-G--A-GG--C-A-CG-A--AG-G-C---G-TG--
GG-G--AG-C-G-AA--------CG--GG Bacteria(100);Chloroflexi(100);Thermomicrobia(100);JG30-KF-CM45(100);unclassified(100);unclassified(100);unclassified(100);unclassified(100);unclassified(100);

Otu0838 0 0 0 0 0 0 0 0 0 0 0 0 0 0 0 0 0 0 0 0 0 0 0 0 0 0 0 0
0 0 0 0 0 0 0 0 0 0 0 0 0 0 0 22 0 0 0 0 0 0 0 0 0 0 0 0 0
0 0 0 0 0 0 0 0 0 0 0 0 0 0 0 0 0 0 0 0 0 0 0 0 0 0 0 0 0
0 0 0 0 0 0 0 0 0 0 0 0 0 0 0 0 0 0 0 0 0 0 0 0 0 0 0 0
M02255_77_000000000-ADUT3_1_1113_5925_6452 T----AC---GA-AG-GGG--------GCT-A-G------C--G--T---T--GC-T-CGG-AA------TG-A--C-T--GG-GC-------GT--A-----AA-GG-GC-GC-------G-TA-G-G-C-G------------G--T-TT-G-G-A----------
CA----G-T-C-A--------G-A-T--G--TG--A-AA-TT--C-C-CG-G-G-----CT-T-AA------C-C-T-G-G-G-G--G-C----T-G--C-A---T--T-------T--GA-G-A---C-------G--T-C--CA--G-G-C----T-T-G-A-G-T--G---T-GA-----GA-G-A--------G-G-G-T---TG-T------GG--A--ATT----C-C-C-
A-GT--GT-A-G-AG-GT--G---A--A-A---TT-C-GT-AG--AT-A-TT--G-G--G-A-A-G-A-AC-A-CC---GG--T--G--GC-GAA-G--G-C--G-------G--C-A-A-C---CTG--G--CT-C-A--------------------T-G---A-C-T--GA--CG-C-T-G--A-GG--C-G-CG-A--AA-G-C---G-TG--GG-G--
AG-C-A-AA--------CA--GG Bacteria(100);Proteobacteria(100);Alphaproteobacteria(100);Rhodospirillales(100);Acetobacteraceae(100);unclassified(100);unclassified(100);unclassified(100);unclassified(100);

Otu0839 0 0 0 0 0 0 0 0 0 0 0 0 0 0 0 0 0 0 0 0 0 0 0 0 0 0 0 0
0 0 0 0 0 0 0 0 0 0 0 0 0 0 0 61 0 0 0 0 0 0 0 0 0 0 0 0 0
0 0 0 0 0 0 0 0 0 0 0 0 0 0 0 0 0 0 0 0 0 0 0 0 0 0 0 0 0
0 0 0 0 0 0 0 0 0 0 0 0 0 0 0 0 0 0 0 0 0 0 0 0 0 0 0 0
M02255_77_000000000-ADUT3_1_1110_26846_14999 T----AC---GA-AG-GGG--------GCT-A-G------C--G--T---T--AC-T-CGG-AA------TG-A--C-T--GG-GC-------GT--A-----AA-GG-GC-GC-------G-TA-G-G-C-G------------G--T-TT-G-T-A----
-------CA----G-T-C-A--------G-G-C--G--TG--A-AA-TT--C-C-TG-G-G-----CT-T-AA------C-C-T-G-G-G-G--G-C----T-G--C-G---T--T-------T--GA-T-A---C-------G--T-G--CA--G-A-C----T-A-G-A-G-T--G---G-GG-----AA-G-A--------G-G-G-T---CG-T------GG--A--ATT----
C-C-C-A-GT--GT-A-G-AG-GT--G---A--A-A---TT-C-GT-AG--AT-A-TT--G-G--G-A-A-G-A-AC-A-CC---GG--T--G--GC-GAA-G--G-C--G-------G--C-G-A-C---CTG--G--TC-C-T--------------------T-G---A-C-T--GA--CG-C-T-G--A-GG--C-G-CG-A--AA-G-C---G-TG--
GG-G--AG-C-A-AA--------CA--GG Bacteria(100);Proteobacteria(100);Alphaproteobacteria(100);Rhodospirillales(100);Acetobacteraceae(100);unclassified(100);unclassified(100);unclassified(100);unclassified(100);

Otu0840 0 0 0 0 0 0 0 0 0 0 0 0 0 0 0 0 0 0 0 0 0 0 0 0 0 0 0 0
0 0 0 0 0 0 0 0 0 0 0 0 0 0 0 24 0 0 0 0 0 0 0 0 0 0 0 0 0
0 0 0 0 0 0 0 0 0 0 0 0 0 0 0 0 0 0 0 0 0 0 0 0 0 0 0 0 0
0 0 0 0 0 0 0 0 0 0 0 0 0 0 0 0 0 0 0 0 0 0 0 0 0 0 0
M02255_77_000000000-ADUT3_1_1110_12416_11681 T----AC---GT-AG-GGG--------ACA-A-G------C--G--T---T--GT-C-CGG-AA------TG-A--C-T--GG-GC-------GT--A-----AA-GG-GC-GC-------G-TA-G-G-C-G------------G--T-CT-A-T-T-----
------AA----G-T-C-T--------G-A-T--G--TG--A-AA-GG--T-A-CC-G-G-----CT-C-AA------C-C-G-G-T-G-A--A-G----T-G--C-A---T--T-------G--GA-A-A---C-------T--G-G--TA--G-A-C----T-T-G-A-G-T--A---T-TG-----GA-G-A--------G-G-C-A---AG-T------GG--A--ATT----
C-C-T-A-GT--GT-A-G-CG-GT--G---A--A-A---TG-C-GT-AG--AT-A-TT--A-G--G-A-G-G-A-AC-A-CC---AG--T--G--GC-GAA-G--G-C--G-------G--C-T-T-G---CTG--G--AC-A-A--------------------A-T---A-C-T--GA--CG-C-T-G--A-GG--T-G-CG-A--AA-G-C---G-TG--
GG-G--AG-C-G-AA--------CA--GG Bacteria(100);Firmicutes(100);Clostridia(100);Clostridiales(100);Eubacteriaceae(100);Eubacterium(100);unclassified(100);unclassified(100);unclassified(100);

Otu0841 0 0 0 0 0 0 0 0 0 0 0 0 0 0 0 0 2 0 0 0 0 0 0 0 0 0 0 0
0 0 0 0 0 0 0 0 0 0 0 0 0 0 0 0 0 0 0 0 0 0 0 0 0 0 0 0 0
0 0 0 0 0 0 0 0 0 0 0 0 0 0 0 0 0 0 0 0 0 0 0 0 0 0 0 0 0
0 0 0 0 0 0 0 0 0 0 0 0 0 0 0 0 0 0 0 0 0 0 0 0 0 0 0
M02255_76_000000000-ADUNE_1_1110_24335_9534 C----GG---CT-AA-TTC--------CGT-G-C------C--A--G---C--AG-C-CGC-GG------TA-A--T-A--CG-GA-------AG--G-----TC-CA-G--GC-------G-TT-A-T-C-C------------G--G-GG-A-T-T-----------TA---
-G-T-G-T--------G-T-T--G--TG--A-AA-TG--T-A-GA-C-G-----CT-C-AA------C-G-T-C-T-G-A--C-T----T-G--C-A---G--C-------G--CA-T-A---C-------T--G-G--TT--C-C-C----T-T-G-A-G-T--A---C-GC-----AC-A-A--------C-G-T-C---GG-C------GG--A--ATT----C-G-T-C-GT--
GT-A-G-CG-GT--G---A--A-A---TG-C-TT-AG--AT-A-TG--A-C--G-A-A-G-A-AC-T-CC---AA--T--T--GC-GAA-G--G-C--A-------G--C-T-G-A---CGG--G--GG-C-G--------------------C-A---A-C-T--GA--CG-C-T-T--A-AG--C-T-CG-A--AG-G-T---G-CG--GG-T--AT-C-G-
AA--------CA--GG Bacteria(100);Bacteroidetes(100);Bacteroidia(100);Bacteroidales(100);Prevotellaceae(100);Prevotella(100);unclassified(100);unclassified(100);unclassified(100);

Otu0842 0 0 0 0 0 0 0 0 0 0 0 0 0 0 0 0 0 0 0 0 0 0 0 0 0 0 0 0
0 0 0 0 0 0 0 0 0 0 0 0 0 0 0 0 0 0 0 0 0 0 0 0 0 0 0 0 0
0 0 0 0 0 0 0 0 0 0 0 0 0 0 0 0 0 0 0 0 0 0 0 0 0 0 681 0 0
0 0 0 0 0 0 0 0 0 38 1 0 0 0 0 0 0 0 0 0 0 0 0 0 0 0 0
M02255_77_000000000-ADUT3_1_1105_23085_17063 T----AC---GT-AG-GGG--------GCA-A-G------C--G--T---T--GT-C-CGG-AA------TC-A--C-T--GG-GC-------GT--A-----AA-GG-GT-TC-------G-CA-G-G-C-G------------G--A-AA-T-G-C----
-------AA----G-T-C-A--------G-T-T--G--TG--A-AA-GG--C-A-GA-G-G-----CT-C-AA------C-C-T-C-T-G-T--A------A-G--C-A---A--T-------T--GA-A-A---C-------T--G-T--AT--T-T-C----T-T-G-A-G-A--T---G-TG-----GA-G-A--------G-G-C-A---AG-T------GG--A--ATT----C-
C-T-A-GT--GT-A-G-CG-GT--G---A--A-A---TG-C-GT-AG--AT-A-TT--A-G--G-A-A-G-A-AT-A-CC---GG--T--G--GC-GAA-G--G-C--G-------A--C-T-T-G---CTG--G--AC-A-C--------------------A-A---A-C-T--GA--CG-C-T-G--A-GG--A-A-CG-A--AA-G-C---G-TG--GG-T-
-AG-C-A-AA--------CA--GG Bacteria(100);Firmicutes(100);Clostridia(100);Clostridiales(100);Family_XI_Incertae_Sedis(100);Peptoniphilus(100);unclassified(100);unclassified(100);unclassified(100);

Otu0843 0 0 0 0 0 0 0 0 0 0 0 0 0 0 0 0 0 0 0 0 0 0 0 0 0 0 0 0
0 0 0 0 0 0 0 0 0 0 0 0 0 0 0 51 0 0 0 0 0 0 0 0 0 0 0 0 0
0 0 0 0 0 0 0 0 0 0 0 0 0 0 0 0 0 0 0 0 0 0 0 0 0 0 0 0 0
0 0 0 0 0 0 0 0 0 0 0 0 0 0 0 0 0 0 0 0 0 0 0 0 0 0 0
M02255_77_000000000-ADUT3_1_1104_22614_17422 T----AC---GT-AG-GTG--------GCG-A-G------C--G--T---T--GT-C-CGG-AT------TT-A--C-T--GG-GC-------GT--A-----AA-GG-GA-GC-------G-TA-G-G-T-G------------G--T-C--T-TTT------
-----AA----G-T-G-G--------G-A-T--G--TG--A-AA-TC--C-C-TG-G-G-----CT-T-AA------C-C-C-A-G-G-A--A-T----T-G--C-A---T--T-------C--CA-T-A---C-------T--G-G--GA--G-A-C----T-A-G-A-G-T--G---C-AG-----GA-G-A--------G-G-A-A---AG-T------GG--A--ATT----C-
C-A-A-GT--GT-A-G-CG-GT--G---A--A-A---TG-C-GT-AG--AG-A-TT--T-G--G-A-G-G---AA-CACC---AG--T--G--GC-GAA-G--G-C--G-------A--C-T-T-T---CTG--G--AC-T-G--------------------T-A---A-C-T--GA--CA-C-T-G--A-GG--C-T-CG-A--GA-G-C---G-TG--GG-G-
-AG-C-A-AA--------CA--GG Bacteria(100);Firmicutes(100);unclassified(100);unclassified(100);unclassified(100);unclassified(100);unclassified(100);unclassified(100);unclassified(100);

Otu0844 0 0 0 0 0 0 0 0 0 0 0 0 0 0 0 0 0 0 0 0 0 0 0 0 0 0 0 0
0 0 0 0 0 0 0 0 0 0 0 0 0 0 0 19 0 0 0 0 0 0 0 0 0 0 0 0 0
0 0 0 0 0 0 0 0 0 0 0 0 0 0 0 0 0 0 0 0 0 0 0 0 0 0 0 0 0
0 0 0 0 0 0 0 0 0 0 0 0 0 0 0 0 0 0 0 0 0 0 0 0 0 0 0
M02255_77_000000000-ADUT3_1_1102_6810_21460 T----AC---GT-AG-GGT--------CCG-A-G------C--G--T---T--GT-C-CGG-AT------TT-A--T-T--GG-GC-------GT--A-----AA-GA-GC-TC-------G-TA-G-G-C-G------------G--C-TC-A-G-T-----------AA--
--G-T-C-A--------G-A-T--G--TG--A-AA-TC--T-C-AG-G-G-----CT-C-AA------C-C-C-G-G-A-A--A------C-T-GC-A---T--C-------T--GA-T-A---C-------T--G-C--TG-TG-G-C----T-T-G-A-G-T--C---T-GG-----TA-G-G--------G-G-A-A---CA-C------GG--A--ATA----C-C-T-G-
GT--GT-A-G-CG-GT--G---A--A-A---TG-C-GC-AG--AT-A-TC--A-G--G-T-G-G-A-AC-A-CC---GA--T--G--GC-GAA-G--G-C--A-------G--T-G-T-T---CTG--G--GC-C-A--------------------G-T---A-C-T--GA--CG-C-T-G--A-GG--A-G-CG-A--AA-G-C---G-TG--GG-G--AG-
C-A-AA--------CA--GG Bacteria(100);unclassified(100);unclassified(100);unclassified(100);unclassified(100);unclassified(100);unclassified(100);unclassified(100);unclassified(100);

Otu0845 0 0 0 0 0 0 0 0 0 0 0 0 0 0 0 0 0 0 0 0 6 0 0 0 0 0 0 0
0 2 0 0 0 0 0 0 0 17 1 0 0 0 0 0 0 0 0 0 0 0 0 0 0 0 0 0
0 0 0 0 0 0 0 0 0 0 0 0 0 0 0 0 0 0 0 0 0 0 0 0 0 0 0 0
0 0 0 0 0 0 0 0 0 0 0 0 0 0 0 0 0 0 0 0 0 0 0 0 0 0 0
M02255_77_000000000-ADUT3_1_2117_9410_16626 T----AC---GA-AG-GGG--------GCT-A-G------C--G--T---T--GT-T-CGG-AA------TT-A--C-T--GG-GC-------GT--A-----AA-GC-GC-AC-------G-TA-G-G-C-G------------G--G-TT-T-G-T-----------AA-
---G-T-A-A--------G-G-G--G--TG--A-AA-TC--C-C-AG-G-G-----CT-C-AA------C-C-C-T-G-G-A--A-C----T-G--C-C---T--C-------T--TA-G-A---C-------T--G-C--AA--G-C-C----T-G-G-A-G-G--T---C-AG-----GA-G-A--------G-G-C-G---AG-T------GG--A--ATT----C-C-G-A-
GT--GT-A-G-AG-GT--G---A--A-A---TT-C-GT-AG--AT-A-TT--C-G--G-A-G-G-A-AC-A-CC---AG--T--G--GC-GAA-G--G-C--G-------A--C-T-C-G---CTG--G--AC-T-G--------------------A-T---A-C-T--GA--CG-C-T-G--A-GG--T-G-CG-A--AA-G-C---G-TG--GG-G--AG-
C-A-AA--------CA--GG Bacteria(100);Proteobacteria(100);Alphaproteobacteria(100);unclassified(100);unclassified(100);unclassified(100);unclassified(100);unclassified(100);unclassified(100);

Otu0846 0 0 0 0 0 0 0 0 0 0 0 0 0 0 0 0 0 0 0 0 0 0 0 0 0 0 0 0
0 0 0 0 0 0 0 0 0 0 0 0 0 18 0 0 0 0 0 0 0 0 0 0 0 0 0 0
0 0 0 0 0 0 0 0 0 0 0 0 0 0 0 0 0 0 0 0 0 0 0 0 0 0 0 0
0 0 0 0 0 0 0 0 0 0 0 0 0 0 0 0 0 0 0 0 0 0 0 0 0 0 0
M02255_77_000000000-ADUT3_1_1115_13208_20472 T----AC---GT-AT-GGG--------GCA-A-G------C--G--T---T--GT-C-CGG-AA------TT-A--T-T--GG-GC-------GT--A-----AA-GG-GT-AC-------G-TA-G-G-C-G------------G--T-TA-A-T-T------
-----AA----G-T-C-T--------G-A-A--T--TA--A-AA-GG--C-T-GT-G-G-----CT-C-AA------C-C-A-T-G-G-T--A------A-G--G-T---T--T-------A--GA-T-A---C-------T--G-G--TT--A-A-C----T-T-G-A-G-T--A---G-AT-----GA-G-G--------G-G-A-A---AG-T------GG--A--ATT----C-
C-A-T-GT--GT-A-G-CG-GT--G---A--A-A---TG-C-GT-AG--AT-A-TA--T-G--G-A-G-G-A-AC-A-CC---TG--T--G--GC-GAA-G--G-C--G-------A--C-T-T-T---CTA--G--AA-T-C--------------------T-A---A-C-T--GA--CG-C-T-G--A-GG--T-A-CG-A--AG-G-C---G-TG--GG-G-
-AG-C-A-AA--------CA--GG Bacteria(100);Firmicutes(100);Clostridia(100);Clostridiales(100);Family_XI_Incertae_Sedis(100);Helcococcus(100);unclassified(100);unclassified(100);unclassified(100);

Otu0847 0 0 0 0 0 0 0 0 0 0 0 0 0 0 0 0 0 0 0 0 4 0 0 0 0 0 0 0
0 4 0 0 0 0 0 0 0 18 1 0 0 0 0 0 0 0 0 0 0 0 0 0 0 0 0 0
0 0 0 0 0 0 0 0 0 0 0 0 0 0 0 0 0 9 0 0 0 0 0 0 0 0 0 0
0 0 0 0 0 0 0 0 0 0 0 0 0 0 0 0 0 0 0 0 0 0 0 0 0 0 0
M02255_76_000000000-ADUNE_1_2110_15964_8041 C----CG---GC-TA-ACT--------CCG-T-G------C--C--A---G--CA-G-CCG-CG------GT-A--A-T--AC-GG-------AG--G-----GT-GC-AA-GC-------G-TT-GTT-C-G------------G--T-TT-C-T-T-----------AA--
--G-T-C-T--------G-A-T--G--TG--A-AA-GC--C-C-TG-G-G-----CT-C-AA------C-C-C-A-G-G-A--A-G----T-G--C-A---T--T-------G--GA-T-A---C-------T--G-G--GA--G-A-C----T-T-G-A-A-T--A---C-GG-----GA-G-A--------G-G-G-T---AG-T------GG--A--ATT----C-C-T-A-
GT--GT-A-G-GA-GT--G---A--A-A---TC-C-GT-AG--AT-A-TT--A-G--G-A-G-G-A-AC-A-CC---GG--T--G--GC-GAA-G--G-C--G-------G--C-T-A-C---CTG--G--AC-C-G--------------------A-T---A-T-T--GA--CG-C-T-G--A-GA--C-G-CG-A--AA-G-C---G-TG--GG-G--AG-
C-A-AA--------CA--GG Bacteria(100);Proteobacteria(100);Deltaproteobacteria(100);Desulfuromonadales(100);Geobacteraceae(100);Geobacter(100);unclassified(100);unclassified(100);unclassified(100);

Otu0848 0 0 0 0 0 0 0 0 0 0 0 0 0 0 0 0 0 0 0 0 0 0 0 0 0 0 0 0
0 0 0 0 0 0 0 0 0 0 0 0 0 18 0 0 0 0 0 0 0 0 0 0 0 0 0 0
0 0 0 0 0 0 0 0 0 0 0 0 0 0 0 0 0 0 0 0 0 0 0 0 0 0 0 0
0 0 0 0 0 0 0 0 0 0 0 0 0 0 0 0 0 0 0 0 0 0 0 0 0 0 0
M02255_77_000000000-ADUT3_1_1113_19557_19577 T----AC---GT-AG-GTG--------GCA-A-G------C--G--T---T--AT-C-CGG-AT------TT-A--C-T--GG-GT-------GT--A-----AA-GG-GC-GT-------G-TA-G-A-C-G------------G--G-TT-T-G-A-----
------AA----G-T-C-A--------G-G-C--G--TG--A-AA-TA--C-C-GC-A-G-----CT-T-AA------C-T-G-C-G-G-G--G-C----T-G--C-G---C--T-------T--GA-A-A---C-------T--T-C--AA--A-T-C----T-T-G-A-G-T--G---C-TG-----GA-G-A--------G-G-A-A---AG-C------GG--A--ATT----C-
C-T-A-GT--GT-A-G-CG-GT--G---A--A-A---TG-C-GT-AG--AT-A-TT--A-G--G-A-G-G-A-AC-A-CC---AG--T--G--GC-GAA-G--G-C--G-------G--C-T-T-T---CTG--G--AC-A-G--------------------T-A---A-C-T--GA--CG-T-T-G--A-GG--C-G-CG-A--AA-G-T---G-TG--GG-G-
-AG-C-A-AA--------CA--GG Bacteria(100);Firmicutes(100);Clostridia(100);Clostridiales(100);unclassified(100);unclassified(100);unclassified(100);unclassified(100);unclassified(100);

Otu0849 0 0 0 0 0 0 0 0 0 0 0 0 0 0 0 0 0 0 0 0 0 0 0 0 0 0 0 0
0 0 0 0 0 0 0 0 0 0 0 0 0 19 0 0 0 0 0 0 0 0 0 0 0 0 0 0
0 0 0 0 0 0 0 0 0 0 0 0 0 0 0 0 0 0 0 0 0 0 0 0 0 0 0 0
0 0 0 0 0 0 0 0 0 0 0 0 0 0 0 0 0 0 0 0 0 0 0 0 0 0 0
M02255_77_000000000-ADUT3_1_1112_8165_7019 T----AC---GT-AG-GGG--------GCA-A-G------C--G--T---T--AT-C-CGG-AT------TT-A--C-T--GG-GT-------GT--A-----AA-GG-GA-GC-------G-TA-G-A-C-G------------G--T-TA-A-T-C-----------AA-
---G-T-C-A--------G-A-T--G--TG--A-AA-GC--C-C-GG-G-G-----CT-T-AA------C-C-G-C-G-G-G--A-C----T-G--C-A---T--T-------T--GA-A-A---C-------T--G-G--AG--A-A-C----T-G-G-A-G-T--G---T-CG-----GA-G-A--------G-G-T-A---AG-C------GG--A--ATT----C-C-T-A-
GT--GT-A-G-CG-GT--G---A--A-A---TG-C-GT-AG--AT-A-TT--A-G--G-A-G-G-A-AC-A-CC---AG--T--G--GC-GAA-G--G-C--G-------G--C-T-T-A---CTG--G--AC-G-A--------------------T-G---A-C-T--GA--CG-T-T-G--A-GG--C-T-CG-A--AA-G-C---G-TG--GG-G--AG-
C-A-AA--------CA--GG Bacteria(100);Firmicutes(100);Clostridia(100);Clostridiales(100);Lachnospiraceae(100);unclassified(100);unclassified(100);unclassified(100);unclassified(100);

Otu0850 0 0 0 0 0 0 0 0 0 0 0 0 0 0 0 0 0 0 0 0 6 0 0 0 0 0 0 0
0 0 0 0 0 0 0 0 0 20 0 44 0 0 0 0 89 0 0 0 0 0 0 0 0 0 0 0
34 45 0 0 0 1 0 0 0 0 1 26 0 0 59 0 0 0 0 0 0 0 0 0 0 0 0 0
0 0 0 0 160 0 0 23 0 173 0 4 0 0 0 0 0 0 0 0 0 0 0 0 0 0 0
M02255_76_000000000-ADUNE_1_2111_24374_12348 T----AC---GT-AG-GGG--------GCG-A-A------C--G--T---T--AT-C-CGG-AA------TG-A--T-T--GG-GC-------GT--A-----AA-GG-GT-AC-------G-TA-G-G-C-G------------G--C-GC-T-T-T-----
------AA----G-T-C-A--------G-G-T--G--TG--A-AA-GG--C-A-TG-G-G-----CT-C-AA------C-C-C-A-T-G-T--A------A-G--C-A---C--T-------T--GA-A-A---C-------T--G-G--AG--C-G-C----T-T-G-A-G-G--G---A-AG-----GA-G-A--------G-G-A-A---AG-T------GG--A--ATT----
C-C-T-A-GT--GT-A-G-CG-GT--G---A--A-A---TG-C-GT-AG--AT-A-TT--A-G--G-A-G-G-A-AT-A-CC---GG--T--G--GC-GAA-G--G-C--G-------A--C-T-T-T---CTG--G--AC-T-T--------------------T-T---T-C-T--GA--CG-C-T-G--A-GG--T-A-CG-A--AA-G-C---G-TG--GG-
G--AG-C-A-AA--------CA--GG Bacteria(100);Firmicutes(100);Clostridia(100);Clostridiales(100);Family_XI_Incertae_Sedis(100);uncultured(100);unclassified(100);unclassified(100);unclassified(100);

Otu0851 0 0 0 0 0 0 0 0 0 0 0 0 0 0 0 0 0 0 0 0 0 0 0 0 0 0 0 0
0 0 0 0 0 0 0 0 0 0 0 0 0 42 0 0 0 0 0 0 0 0 0 0 0 0 0 0
0 0 0 0 0 0 0 0 0 0 0 0 0 0 0 0 0 0 0 0 0 0 0 0 0 0 0 0
0 0 0 0 0 0 0 0 0 0 0 0 0 0 0 0 0 0 0 0 0 0 0 0 0 0 0
M02255_77_000000000-ADUT3_1_1110_24589_5811 T----AC---AG-AG-GTC--------TCA-A-G------C--G--T---T--GT-T-CGG-AT------TT-A--C-T--GG-GC-------GT--A-----AA-GG-GT-GC-------G-TA-G-G-C-G------------G--C-GG-C-G-T-----------AA-
---G-T-C-G--------A-G-T--G--TG--A-AA-TC--T-T-CC-G-G-----CT-C-AA------C-C-G-G-A-A-A--A-T----T-G--C-A---C--T-------T--GA-T-A---C-------T--G-T--GT--C-G-C----T-A-G-A-G-G--A---C-TG-----TA-G-A--------G-G-A-G---AC-T------GG--A--ATT----C-A-C-G-
GT--GT-A-G-CA-GT--G---A--A-A---TG-C-GT-AG--AT-A-TC--G-T--G-A-G-G-A-AG-A-CC---GG--T--G--GC-GAA-A--G-C--G-------A--G-T-C-T---CTG--G--GC-A-G--------------------T-T---C-C-T--GA--CG-C-T-G--A-GG--C-A-CG-A--AG-G-C---T-AG--GG-G--AG-
C-A-AA--------CG--GG Bacteria(100);Verrucomicrobia(100);Spartobacteria(100);unclassified(100);unclassified(100);unclassified(100);unclassified(100);unclassified(100);unclassified(100);

Otu0852 0 0 0 0 0 0 0 0 0 0 0 0 0 0 0 0 0 0 0 0 13 1 0 0 0 0 0 0
0 0 0 1 0 0 0 0 0 23 3 0 0 0 0 0 0 0 0 0 0 0 0 0 0 0 0 0
0 0 0 0 0 0 0 0 0 0 0 0 0 0 0 0 0 0 0 0 0 0 0 0 0 0 0 0
0 0 0 0 0 0 0 0 0 0 0 0 0 0 0 0 0 0 0 0 0 0 0 0 0 0 0
M02255_77_000000000-ADUT3_1_1107_26294_10594 T----AC---GT-AG-GGG--------GCA-A-G------C--G--T---T--AT-C-CGG-AT------TT-A--C-T--GG-GT-------GT--A-----AA-GG-GA-GC-------G-TA-G-G-T-G------------G--C-TC-G-A-T-----
------AA----G-T-C-A--------G-A-A--G--TG--A-AA-GC--C-C-AA-G-G-----CT-C-AA------C-C-T-T-G-G-G--A-C----T-G--C-T---T--T-------T--GA-A-A---C-------T--A-T--CG--G-G-C----T-A-G-A-G-T--G---C-AG-----GA-G-A--------G-G-T-A---AG-T------GG--A--ATT----C-
C-T-A-GT--GT-A-G-CG-GT--G---A--A-A---TG-C-GT-AG--AT-A-TT--A-G--G-A-G-G-A-AC-A-CC---AG--T--G--GC-GAA-G--G-C--G-------A--C-T-T-A---CTG--G--AC-T-G--------------------T-A---A-C-T--GA--CA-C-T-G--A-GG--C-T-CG-A--AA-G-C---G-TG--GG-G-
-AG-C-A-AA--------CA--GG Bacteria(100);Firmicutes(100);Clostridia(100);Clostridiales(100);Lachnospiraceae(100);unclassified(100);unclassified(100);unclassified(100);unclassified(100);

Otu0853 0 0 0 0 0 0 0 0 0 0 0 0 0 0 0 0 0 0 0 0 0 0 0 0 0 0 0 0
0 0 0 0 0 0 0 0 0 0 0 0 0 31 0 0 0 0 0 0 0 0 0 0 0 0 0 0
0 0 0 0 0 0 0 0 0 0 0 0 0 0 0 0 0 0 0 0 0 0 0 0 0 0 0 0
0 0 0 0 0 0 0 0 0 0 0 0 0 0 0 0 0 0 0 0 0 0 0 0 0 0 0
M02255_77_000000000-ADUT3_1_1108_7814_4546 T----AC---GT-AG-GTG--------GCA-A-G------C--G--T---T--GT-C-CGG-AA------TT-A--C-T--GG-GT-------GT--A-----AA-GG-GA-GC-------G-CA-G-G-C-G------------G--G-AG-A-T-C-----------
AA----G-T-C-A--------G-A-C--G--TG--A-AA-AC--T-A-TG-G-G-----CT-C-AA------C-C-C-A-T-A-A--C-G----T-G--C-G---T--T-------T--GA-A-A---C-------T--G-T--TC--T-T-C----T-T-G-A-G-T--G---A-AG-----TA-G-A--------G-G-T-A---GG-C------GG--A--ATT----C-C-G-
A-GT--GT-A-G-CG-GT--G---A--A-A---TG-C-GT-AG--AT-A-TT--C-G--G-A-G-G-A-AC-A-CC---AG--T--G--GC-GAA-G--G-C--G-------G--C-C-T-A---CTG--G--GC-T-T--------------------T-T---A-C-T--GA--CG-C-T-G--A-GG--C-T-CG-A--AA-G-T---G-TG--GG-G--AG-
C-A-AA--------CA--GG Bacteria(100);unclassified(100);unclassified(100);unclassified(100);unclassified(100);unclassified(100);unclassified(100);unclassified(100);unclassified(100);

Otu0854 0 0 0 0 0 0 0 0 0 0 0 0 0 0 0 0 0 0 0 0 9 0 0 0 0 0 0 0
0 0 0 0 0 0 0 0 0 0 0 0 0 0 0 0 0 0 0 0 0 0 0 0 0 0 0 0
0 0 0 0 0 0 0 0 0 0 0 0 0 0 0 0 0 0 0 0 0 0 0 0 0 0 0 0
0 0 0 0 0 0 0 0 0 0 0 0 0 0 0 0 0 0 0 0 0 0 0 0 0 0 0
M02255_77_000000000-ADUT3_1_1111_15672_11362 T----AC---GT-AG-ATG--------GCG-A-G------C--G--T---T--GT-C-CGG-AA------TT-A--C-T--GG-GT-------GT--A-----AA-GG-GA-GT-------G-TA-G-G-C-G------------G--G-AT-C-A-T-----
------AA----G-T-T-G--------C-G-T--G--TG--A-AA-TG--C-A-GG-G-G-----CT-C-AA------C-C-C-C-T-G-A--A-C----T-G--C-G---C--G-------C--AA-A-A---C-------T--G-T--GG--T-T-C----T-T-G-A-G-T--G---A-AG-----TA-G-A--------G-G-C-A---GG-C------GG--A--ATT----
C-C-C-G-GT--GT-A-G-CG-GT--G---G--A-A---TG-C-GT-AGA-T--A-TC--G-G--G-A-G-G-A-AC-A-CC---AG--T--G--GC-GAA-G--G-C--G-------G--C-C-T-G---CTG--G--GC-T-T--------------------T-T---A-C-T--GA--CG-C-T-G--A-GG--C-T-CG-A--AA-G-C---A-TG--GG-
G--AG-C-A-AA--------CA--GG Bacteria(100);unclassified(100);unclassified(100);unclassified(100);unclassified(100);unclassified(100);unclassified(100);unclassified(100);unclassified(100);

Otu0855 0 0 0 0 0 0 0 0 0 0 0 0 0 0 0 0 0 0 0 0 0 0 0 0 0 0 0 0
0 0 0 0 0 0 0 0 0 0 0 0 0 30 0 0 0 0 0 0 0 0 0 0 0 0 0 0 0
0 0 0 0 0 0 0 0 0 0 0 0 0 0 0 0 0 0 0 0 0 0 0 0 0 0 0 0 0
0 0 0 0 0 0 0 0 0 0 0 0 0 0 0 0 0 0 0 0 0 0 0 0 0 0 0 0
M02255_77_000000000-ADUT3_1_1105_17302_23191 T----AC---GT-AG-GGG--------GCA-A-G------C--G--T---T--GT-C-CGG-AA------TG-A--T-T--GG-GC-------GT--A-----AA-GG-GC-GC-------G-TA-G-G-C-G------------G--C-CC-G-T-T----
-------AA----G-T-C-T--------G-G-A--G--TG--A-AA-GT--C-C-TG-C-T-----TT-T-AA------G-G-T-G-G-G-A--A-T----T-G--C-T---T--T-------G--GA-T-A---C-------T--G-A--TG--G-G-C----T-T-G-A-G-T--G---C-AG-----GA-G-A--------G-G-T-T---AT-C------GG--A--ATT----C-
C-C-G-GT--GT-A-G-CG-GT--G---A--A-A---TG-C-GT-AG--AG-A-TC--G-G--G-A-G-G-A-AC-A-CC---AG--T--G--GC-GAA-G--G-C--G-------G--G-T-A-A---CTG--G--AC-T-G--------------------C-A---A-C-T--GA--CG-C-T-G--A-GG--C-G-CG-A--AA-G-T---G-TG--GG-
G--AG-C-A-AA--------CA--GG Bacteria(100);Firmicutes(100);unclassified(100);unclassified(100);unclassified(100);unclassified(100);unclassified(100);unclassified(100);unclassified(100);

Otu0856 0 0 0 0 0 0 0 0 0 0 0 0 0 0 0 0 0 0 0 0 3 0 0 0 0 0 0 5
0 0 0 0 0 0 0 0 0 0 0 0 0 0 0 0 0 0 0 0 0 0 0 0 0 0 0 0 0
0 0 0 0 0 0 0 0 0 0 0 0 0 0 0 0 0 0 0 0 0 0 0 0 0 0 0 0 0
0 0 0 0 0 0 0 0 0 0 0 0 0 0 0 0 0 0 0 0 0 0 0 0 0 0 0 0
M02255_77_000000000-ADUT3_1_1119_26354_17042 T----AC---GA-AG-GGG--------GCT-A-G------C--G--T---T--GT-T-CGG-AT------TT-A--C-T--GG-GC-------GT--A-----AA-GC-GC-AC-------G-TA-G-G-C-G------------G--A-TT-G-T-T-----
------AA----G-T-C-G--------G-G-G--G--TG--A-AA-TC--C-T-GG-A-G-----CT-C-AA------C-T-C-C-A-G-A--A-T----T-G--C-C---T--T-------C--GA-T-A---C-------T--G-G--CT--T-T-C----T-A-G-A-G-G--C---T-GG-----AA-G-A--------G-G-T-T---AG-T------GG--A--ATT----C-
C-C-A-GT--GT-A-G-AG-GT--G---A--A-A---TT-C-GT-AG--AT-A-TT--G-G--G-A-A-G-A-AC-A-CC---AG--T--G--GC-GAA-G--G-C--G-------G--C-T-A-A---CTG--G--TC-C-A--------------------G-A---T-C-T--GA--CG-C-T-G--A-GG--T-G-CG-A--AA-G-C---G-TG--GG-
G--AG-C-A-AA--------CA--GG Bacteria(100);Proteobacteria(100);Alphaproteobacteria(100);Rhizobiales(100);unclassified(100);unclassified(100);unclassified(100);unclassified(100);unclassified(100);

Otu0857 0 0 0 0 0 0 0 0 0 0 0 0 0 0 0 0 0 0 0 0 0 0 0 0 0 0 0 0
0 0 0 0 0 0 0 0 0 0 0 0 0 63 0 0 0 0 0 0 0 0 0 0 0 0 0 0 0
0 0 0 0 0 0 0 0 0 0 0 0 0 0 0 0 0 0 0 0 0 0 0 0 0 0 0 0 0
0 0 0 0 0 0 0 0 0 0 0 0 0 0 0 0 0 0 0 0 0 0 0 0 0 0 0 0
M02255_77_000000000-ADUT3_1_1105_25479_18455 T----AC---GTA-G-GGG--------GCG-A-G------C--G--T---T--GT-C-CGG-AT------TT-A--C-T--GG-GC-------GT--A-----AA-GG-GT-GA-------G-TA-G-G-C-G------------G--T-TA-T-A-T-----
------AT----G-T-C-A--------G-A-T--G--TA--A-AA-GG--C-T-AT-G-G-----CT-T-AA------C-C-A-T-A-G-T--T------A-G--C-A---T--T-------T--GA-A-A---C-------T--G-T--AT--G-A-C----T-T-G-A-G-T--G---C-AG-----GA-G-A--------G-G-T-A---AG-T------GG--A--ATT----C-
C-T-A-GT--GT-A-G-CG-GT--G---A--A-A---TG-C-GT-AG--AT-A-TT--A-G--G-A-G-G-A-AC-A-CC---AG--T--G--GC-GAA-G--G-C--G-------A--C-T-T-A---CTG--G--AC-T-G--------------------T-A---A-C-T--GA--CG-C-T-G--A-GT--C-A-CG-A--AA-G-C---G-TG--GG-T--
AG-C-A-AA--------CA--GG Bacteria(100);Firmicutes(100);Clostridia(100);Clostridiales(100);Family_XI_Incertae_Sedis(100);Sedimentibacter(100);unclassified(100);unclassified(100);unclassified(100);

Otu0858 0 0 0 0 0 0 0 0 0 0 0 0 0 0 0 0 0 0 0 0 0 0 0 0 0 0 0 0
0 0 0 0 0 0 0 0 0 0 0 0 0 9 0 0 0 0 0 0 0 0 0 0 0 0 0 0 0
0 0 0 0 0 0 0 0 0 0 0 0 0 0 0 0 0 0 0 0 0 0 0 0 0 0 0 0 0
0 0 0 0 0 0 0 0 0 0 0 0 0 0 0 0 0 0 0 0 0 0 0 0 0 0 0 0
M02255_77_000000000-ADUT3_1_2111_7392_15478 T----AC---GT-AG-GGG--------GCA-A-G------C--G--T---T--GT-C-CGG-AA------TC-A--T-T--GG-GC-------GT--A-----AA-GA-GC-GT-------G-TA-G-G-C-G------------G--T-CC-G-G-T-----------
AA----G-T-C-G--------G-C-T--G--TG--A-AA-GT--C-C-AG-G-G-----CT-C-AA------C-T-C-T-G-G-G--A------T-G-CC-G---G--T-------C--GA-T-A---C-------T--G-C--CG--G-A-C----T-A-G-A-G-T--T---C-GG-----AA-G-A--------G-G-C-G---AG-T------GG--A--ATT----C-C-
C-G-GT--GT-A-G-CG-GT--G---A--A-A---TG-C-GC-AG--AT-A-TC--G-G--G-A-G-G-A-AC-A-CC---TA--T--G--GC-GAA-G--G-C--A-------G--C-T-C-G---CTG--G--GA-C-G--------------------T-T---A-C-T--GA--CG-C-T-G--A-GA--C-G-CG-A--AA-G-C---G-TG--GG-G--
AG-C-A-AA--------CA--GG Bacteria(100);Actinobacteria(100);Actinobacteria(100);unclassified(100);unclassified(100);unclassified(100);unclassified(100);unclassified(100);unclassified(100);

Otu0859 0 0 0 0 0 0 0 0 0 0 0 0 0 0 0 0 0 0 0 0 0 0 0 0 0 0 0 0
0 0 0 0 0 0 0 0 0 0 0 0 8 0 0 12 7 0 0 0 0 15 0 0 0 0 0 0 0
0 0 0 0 0 0 0 0 0 0 0 0 0 0 0 0 0 0 0 0 0 0 0 0 0 0 0 0 0
0 0 0 0 20 0 0 0 0 0 0 0 0 0 0 0 0 0 0 0 0 0 0 0 0 0 0 0
M02255_76_000000000-ADUNE_1_1110_20838_21457 T----AC---GTGCC-AGCA-------GCC-G-C------G--G--T---A--AT-A-CGT-AG------GG-C--G-C--GA-GC-------GT--T-----GT-CC-GG-AT-------T-TA---T-T-G------------G--G-TC-G-T-C-----
------GC----G-T-C-T--------G-T-C--G--TG--A-AA-GC--C-A-GC-A-G-----CT-T-AA------C-T-G-T-T-G-G--T-C----T-G--C-G---G--T-------G--GG-T-A---C-------G--G-G--CG--G-G-C----T-T-G-A-G-T--G---C-GG-----TA-G-G--------G-G-T-G---AC-T------GG--A--ATT----
C-C-T-G-GT--GT-A-G-CG-GT--G---G--A-A---TG-C-GC-AG--AT-A-TC--A-G--G-A-G-G-A-AC-A-CC---GA--T--G--GC-GAA-G--G-C--A-------G--G-T-C-A---CTG--G--GC-C-G--------------------T-T---A-C-T--GA--CA-C-T-G--A-GG--A-G-CG-A--AA-G-C---G-TG--
GG-G--AG-C-G-AA--------CA--GG Bacteria(100);Actinobacteria(100);Actinobacteria(100);Actinobacteridae(100);Actinomycetales(100);Actinomycineae(100);Actinomycetaceae(100);Mobiluncus(100);unclassified(100);

Otu0860 0 0 0 0 0 0 0 0 0 0 0 0 0 0 0 0 0 0 0 0 0 0 0 0 0 0 0 0
0 0 0 0 0 0 0 0 0 0 0 0 1 0 0 0 0 0 0 0 0 0 0 0 0 0 0 0
0 0 0 0 0 0 0 0 0 0 0 0 0 0 0 0 0 0 0 0 0 0 1 0 0 0 0 21 0
0 0 0 0 0 0 0 0 0 0 0 0 0 1 0 0 0 0 0 0 0 0 0 0 0 0 0
M02255_76_000000000-ADUNE_1_2112_27195_16751 TC---GC---AG-AA-TAA--------GCA-C-C------G--G--C---T--AACT-CTG-TG------CC-A--G-C--AG-CC-------GC--G-----GT-AA-TA-CA-------G-AG-G-G-T-G------------C--G-AG-C-G-T---
--------TA----A-T-C-G--------G-A-T--G--TG--A-AA-TC--C-C-TG-A-G-----CT-T-AA------C-T-T-A-G-G-A--A-T----T-G--C-A---T--T-------C--GA-T-A---C-------T--G-G--GA--A-G-C----T-A-G-A-G-T--A---T-GG-----GA-G-A--------G-G-A-T---GG-T------AG--A--ATT----
C-C-A-G-GT--GT-A-G-CG-GT--G---A--A-A---TG-C-GT-AG--AG-A-TC--T-G--G-A-G-G-A-AT-A-CC---GA--T--G--GC-GAA-G--G-C--A-------G--C-C-A-T---CTG--G--CC-T-A--------------------A-T---A-C-T--GA--CG-C-T-G--A-GG--T-A-CG-A--AA-G-C---A-TG--GG-
G--AG-C-A-AA--------CA--GG Bacteria(100);Proteobacteria(100);Gammaproteobacteria(100);Pseudomonadales(100);Moraxellaceae(100);Acinetobacter(100);unclassified(100);unclassified(100);unclassified(100);

Otu0861 0 0 0 0 0 0 0 0 0 0 0 0 0 0 0 0 0 0 0 0 3 0 0 0 0 0 0 0
0 0 0 0 0 0 0 0 0 16 3 0 0 0 0 0 0 0 0 0 0 0 0 0 0 0 0 0
0 0 0 0 0 0 0 0 0 0 0 0 0 0 0 0 0 0 0 0 0 0 0 0 0 0 0 0
0 0 0 0 18 0 0 0 0 0 0 0 0 0 0 0 0 0 0 0 0 0 0 0 0 0 0
M02255_76_000000000-ADUNE_1_2110_21079_10138 C----GG---CT-AA-ATA--------CGT-G-C------C--A--G---C--AG-C-C-G-CG------GT-A--A-T--AC-GT-------AT--G-----TT-CC-AA-GC-------GTTA-T-C-C-G------------G--T-TA-T-T-T--------
---AA----G-T-C-C--------G-A-A--G--TG--A-AA-GC--C-C-AC-A-G-----CT-T-AA------C-T-G-T-G-G-A--A-G----T-G--C-T---T--T-------G--GA-A-A---C-------T--G-G--AT--A-A-C----T-T-G-A-G-T--G---C-AG-----TA-G-A--------G-G-A-G---AG-T------GG--A--ACT----C-C-
A-T-GT--GT-A-G-CG-GT--G---A--A-A---TG-C-GT-AG--AT-A-TA--T-G--G-A-A-G-A-AC-A-CC---AG--T--G--GC-GAA-G--G-C--G-------G--C-T-C-T---CTG--G--AC-T-G--------------------T-A---A-C-T--GA--CG-T-T-G--A-GG--C-T-CG-A--AA-G-T---G-TG--GG-T--
AG-C-A-AA--------CA--GG Bacteria(100);Firmicutes(100);Bacilli(100);Lactobacillales(100);Leuconostocaceae(100);unclassified(100);unclassified(100);unclassified(100);unclassified(100);

Otu0862 0 0 0 0 0 0 0 0 0 0 0 0 0 0 0 0 0 0 0 0 7 0 0 0 0 0 0 0
0 0 0 0 0 0 0 0 0 0 0 0 0 0 0 0 0 0 0 0 0 0 0 0 0 0 0 0
0 0 0 0 0 0 0 0 0 0 0 0 0 0 0 0 0 0 0 0 0 0 0 0 0 0 0 0
0 0 0 0 0 0 0 0 0 0 0 0 0 0 0 0 0 0 0 0 0 0 0 0 0 0 0
M02255_77_000000000-ADUT3_1_1103_6104_20140 T----AC---GG-AG-GGG--------GCT-A-G------C--G--T---T--GT-T-CGG-AA------TT-A--C-T--GG-GC-------GT--A-----AA-GA-GT-AC-------G-TA-G-G-C-G------------G--T-TT-T-T-T----------AA-
---G-T-C-A--------G-A-G--G--TG--A-AA-TC--C-C-GG-A-G-----CT-C-AA------C-T-C-C-G-G-A--A-C----T-G--C-C---T--T-------T--GA-A-A---C-------T--G-G--AA--A-G-C----T-A-G-A-A-T--C---A-TG-----GA-G-A--------G-G-T-T---AG-T------GG--A--ATT----C-C-G-A-
GT--GT-A-G-AG-GT--G---A--A-A---TT-C-GT-AG--AT-A-TT--C-G--G-A-A-G-A-AC-A-CC---AG--T--G--GC-GAA-G--G-C--G-------A--C-T-G-A---CTG--G--AC-A-T--------------------G-T---A-T-T--GA--CG-C-T-G--A-GG--T-A-CG-A--AA-G-C---G-TG--GG-G--AG-C-
A-AA--------CA--GG Bacteria(100);Proteobacteria(100);Alphaproteobacteria(100);Sphingomonadales(100);GOBB3-C201(100);unclassified(100);unclassified(100);unclassified(100);unclassified(100);

Otu0863 0 0 0 0 0 0 0 0 0 0 0 0 0 0 0 0 0 0 0 0 6 0 0 0 0 0 0 0
0 0 0 0 0 0 0 0 0 0 0 0 0 0 0 0 0 0 0 0 0 0 0 0 0 0 0 0
0 0 0 0 0 0 0 0 0 0 0 0 0 0 0 0 0 0 0 0 0 0 0 0 0 0 0 0
0 0 0 0 0 0 0 0 0 0 0 0 0 0 0 0 0 0 0 0 0 0 0 0 0 0 0
M02255_77_000000000-ADUT3_1_2112_14429_13204 T----AC---GG-AG-GGT--------GCA-A-G------C--G--T---T--AC-T-CGG-AA------TC-A--C-T--GG-GC-------GT--A-----AA-GG-AC-GC-------G-TA-G-G-C-G------------G--A-TT-A-T-C-----
------AA----G-T-C-T--------T-T-T--G--TG--A-AA-TC--T-A-AT-G-G-----CT-C-AA------C-C-A-T-T-A-A--A-C----T-G--C-T---T--G-------A--GA-A-A---C-------T--G-A--TA--A-T-C----T-A-G-A-G-T--G---A-GG-----GA-G-A--------G-G-C-A---GA-T------GG--A--ATT----G-
G-T-G-GT--GT-A-G-GG-GT--A---A--A-A---TC-C-GT-AG--AG-A-TC--A-C--C-A-G-G-A-AT-A-CC---CA--T--T--GC-GAA-G--G-C--G-------A--T-C-T-G---CTG--G--AA-C-T--------------------T-A---A-C-T--GA--CG-C-T-A--A-TG--C-G-TG-A--AA-G-C---G-TG--GG-G--
AG-C-A-AA--------CA--GG Bacteria(100);Proteobacteria(100);Epsilonproteobacteria(100);Campylobacterales(100);Campylobacteraceae(100);Campylobacter(100);unclassified(100);unclassified(100);unclassified(100);

Otu0864 0 0 0 0 0 0 0 0 0 0 0 0 0 0 0 0 0 0 0 0 0 0 0 0 0 0 0 0
0 0 0 0 0 0 0 0 0 0 0 0 23 0 0 0 0 0 0 0 0 0 0 0 0 0 0 0
0 0 0 0 0 0 0 0 0 0 0 0 0 0 0 0 0 0 0 0 0 0 0 0 0 0 0 0
0 0 0 0 0 0 0 0 0 0 0 0 0 0 0 0 0 0 0 0 0 0 0 0 0 0 0
M02255_77_000000000-ADUT3_1_1109_25994_12864 T----AC---GT-AG-GGT--------GCA-A-G------C--G--T---T--GT-C-CGG-AA------TT-A--T-T--GG-GC-------GT--A-----AA-GA-GC-TC-------G-TA-G-G-T-G------------G--C-TT-G-T-T------
-----GC----G-T-C-G--------C-C-T--G--TG--A-AA-GC--C-C-GG-G-G-----CT-T-AA------C-T-C-C-G-G-G--T-C----T-G--C-A---G--G-------C--GA-T-A---C-------G--G-G--CA--G-G-C----T-T-G-A-G-G--G---C-AG-----CA-G-G--------G-G-A-G---AC-T------GG--A--ATT----
C-C-T-G-GT--GT-A-G-CG-GT--G---A--A-A---TG-C-GC-AG--AT-A-TC--A-G--G-A-G-G-A-AC-A-CC---GG--T--G--GC-GAA-G--G-C--G-------G--G-T-C-T---CTG--G--GC-T-G--------------------T-A---C-C-T--GA--CA-C-T-G--A-GG--A-G-CG-A--AA-G-C---G-TG--
GG-G--AG-C-A-AA--------CA--GG Bacteria(100);Actinobacteria(100);Actinobacteria(100);Actinobacteridae(100);Actinomycetales(100);unclassified(100);unclassified(100);unclassified(100);unclassified(100);

Otu0865 0 0 0 0 0 0 0 0 0 0 0 0 0 0 0 0 0 0 0 0 0 0 0 0 0 0 0 0
0 0 0 0 0 0 0 0 0 0 0 0 33 0 0 0 0 0 0 0 0 0 0 0 0 0 0 0 0
0 0 0 0 0 0 0 0 0 0 0 0 0 0 0 0 0 0 0 0 0 0 0 0 0 0 0 0 0
0 0 0 0 0 0 0 0 0 0 0 0 0 0 0 0 0 0 0 0 0 0 0 0 0 0 0 0
M02255_77_000000000-ADUT3_1_2102_28047_6699 T----AC---GG-AG-GAT--------GCA-A-G------C--G--T---T--AT-C-CGG-AC------TG-A--T-T--GG-GT-------GT--A-----AA-GC-AT-CT-------G-TA-G-G-C-G------------G--T-TT-T-G-G----------AA-
---G-T-C-T--------A-C-G--G--TT--A-AA-TC--C-C-AG-A-G-----CT-T-AA------C-T-T-T-G-GTT--C-A----G-C--C-G---T--A-------G--AG-T-A---C-------T--T-C--AA--A-G-C----T-T-G-A-G-T--A---T-GG-----TA-G-G--------G-G-T-A---GA-G------GG--A--ACT----T-C-T-C-
GT--GG-A-G-CG-GT--G---A--A-A---TG-C-AC-AG--AG-A-TG--A-G--A-A-G-G-A-AC-A-CC---AG--T--G--GC-GAA-A--G-C--G-------C--T-C-T-A---CTG--G--GC-C-A--------------------T-G---A-C-T--GA--CG-C-T-G--AGAG----A-TG-A--CA-G-C---T-GA--GG-G--AG-
C-G-AA--------TA--GG Bacteria(100);unclassified(100);unclassified(100);unclassified(100);unclassified(100);unclassified(100);unclassified(100);unclassified(100);unclassified(100);

Otu0866 0 0 0 0 0 0 0 0 0 0 0 0 0 0 0 0 0 0 0 0 10 0 0 0 0 0 0 0
0 0 0 0 0 0 0 0 0 0 0 0 0 0 0 0 0 0 0 0 0 0 0 0 0 0 0 0 0
0 0 0 0 0 0 0 0 0 0 0 0 0 0 0 0 0 0 0 0 0 0 0 0 0 0 0 0 0
0 0 0 0 0 0 0 0 0 0 0 0 0 0 0 0 0 0 0 0 0 0 0 0 0 0 0 0
M02255_77_000000000-ADUT3_1_1109_13514_8790 T----AC---GA-AG-GGG--------GCT-A-G------C--G--T---T--GC-T-CGG-AA------TG-A--C-T--GG-GC-------GT--A-----AA-GG-GC-GC-------G-TA-G-G-C-G------------G--T-TT-A-T-G----------
CA----G-T-C-A--------G-A-T--G--TG--A-AA-TC--C-C-CG-G-G-----CT-T-AA------C-C-T-G-G-G-A--A-C----T-G--C-A---T--T-------T--GA-G-A---C-------G--C-A--TA--G-A-C----T-A-G-A-G-G--T---C-GA-----GA-G-A--------G-G-G-T---TG-T------GG--A--ATT----C-C-C-
A-GT--GT-A-G-AG-GT--G---A--A-A---TT-C-GT-AG--AT-A-TT--G-G--G-A-A-G-A-AC-A-CC---GG--T--G--GC-GAA-G--G-C--G-------G--C-A-A-C---CTG--G--CT-C-G--------------------A-T---A-C-T--GA--CG-C-T-G--A-GG--C-G-CG-A--AA-G-C---G-TG--GG-G--
AG-C-A-AA--------CA--GG Bacteria(100);Proteobacteria(100);Alphaproteobacteria(100);Rhodospirillales(100);Acetobacteraceae(100);Gluconobacter(100);unclassified(100);unclassified(100);unclassified(100);

Otu0867 0 0 0 0 0 0 0 0 0 0 0 0 0 0 0 0 0 0 0 0 1 0 0 0 0 0 0 0
0 0 0 0 0 0 0 0 0 0 20 0 0 0 0 0 0 0 0 0 0 0 0 0 0 0 0 0 0
0 0 0 0 0 0 0 0 0 0 0 0 0 0 0 0 0 0 0 0 0 0 0 0 0 0 0 0 0
0 0 0 0 0 0 0 0 0 0 0 0 0 0 0 0 0 0 0 0 0 0 0 0 0 0 0 0
M02255_77_000000000-ADUT3_1_1112_7152_22009 T----AC---GA-AG-GGT--------GCT-A-G------C--G--T---T--GT-T-CGG-AA------TC-A--C-T--GG-GC-------GT--A-----AA-GC-GC-AC-------G-TA-G-G-C-G------------G--T-CC-G-CAC----------
CA----G-T-C-A--------G-G-C--G--TG--A-AA-GC--C-C-CA-G-G-----CT-C-AA------C-C-T-G-G-G-A--A-T----T-G--C-G---C--T-------T--GA-T-A---C-------T--A-G--CG--G-G-C----T-A-G-A-G-G--C---C-GG-----GA-G-A--------G-G-G-T---AG-T------GG--A--ATT----C-C-
C-A-GT--GT-A-G-AG-GT--G---A--A-A---TT-C-GT-AG--AT-A-TT--G-G--G-A-A-G-A-AC-A-CC---AG--T--G--GC-GAA-G--G-C--G-------G--C-T-A-C---CTG--G--AC-C-G--------------------G-T---T-C-T--GA--CG-C-T-G--A-GG--T-G-CG-A--AA-G-C---G-TG--GG-G--
AG-C-A-AA--------CA--GG Bacteria(100);Proteobacteria(100);Alphaproteobacteria(100);Rhodospirillales(100);unclassified(100);unclassified(100);unclassified(100);unclassified(100);unclassified(100);

Otu0868 0 0 0 0 0 0 0 0 0 0 0 0 0 0 0 0 0 0 0 0 0 0 0 0 0 0 0 0
0 0 0 0 0 0 0 0 0 0 0 0 30 0 0 0 0 0 0 0 0 0 0 0 0 0 0 0 0
0 0 0 0 0 0 0 0 0 0 0 0 0 0 0 0 0 0 0 0 0 0 0 0 0 0 0 0 0
0 0 0 0 0 0 0 0 0 0 0 0 0 0 0 0 0 0 0 0 0 0 0 0 0 0 0 0
M02255_77_000000000-ADUT3_1_1118_23401_20327 T----AC---GG-AG-GGG--------GCA-A-G------C--G--T---T--GT-T-CGG-AA------TT-A--C-T--GG-GC-------GT--A-----AA-GG-GC-GC-------G-TA-G-G-C-G------------G----TA-T-G-T-----
-----CGC----G-T-C-A--------G-G-T--G--TG--A-AA-GC--C-C-CG-G-G-----CT-C-AA------C-C-T-G-G-G-A--T-G----T-G--C-A---C--T-------T--GA-T-A---C-------G--G-G--CA--T-G-C----T-T-G-A-G-T--T---C-GA-----GA-G-A--------G-G-T-G---AG-T------GG--A--ATT----
C-C-C-A-GT--GT-A-G-AG-GT--G---A--A-A---TT-C-GT-AG--AT-A-TT--G-G--G-A-A-G-A-AC-A-CC---AG--C--G--GC-GAA-G--G-C--G-------G--C-T-C-A---CTG--G--CT-C-G--------------------A-A---A-C-T--GA--CG-C-T-G--A-GG--C-G-CG-A--AA-G-C---G-TG--
GG-G--AG-C-A-AA--------CA--GG Bacteria(100);unclassified(100);unclassified(100);unclassified(100);unclassified(100);unclassified(100);unclassified(100);unclassified(100);unclassified(100);

Otu0869 0 0 0 0 0 0 0 0 0 0 0 0 0 0 0 0 0 0 0 0 0 0 0 0 0 0 0 0
0 0 0 0 0 0 0 0 0 0 0 0 18 0 0 0 0 0 0 0 0 0 0 0 0 0 0 0 0
0 0 8 0 0 0 0 0 0 0 0 0 0 0 0 0 0 0 0 0 0 0 0 0 0 0 0 0 0
0 0 0 0 0 0 0 0 0 0 0 0 0 0 0 0 0 0 0 0 0 0 0 0 0 0 0 0
M02255_77_000000000-ADUT3_1_1118_14342_16961 T----AC---GT-AG-GGA--------GCG-A-G------C--G--T---T--GT-C-CGG-AA------TT-A--C-T--GG-GC-------GT--A-----AA-GG-GC-GC-------G-TA-G-G-T-G------------G--T-CC-A-T-T-----
------AA----G-T-T-A--------C-T-G--G--TG--A-AA-GT--T-C-GG-G-G-----CT-T-AA------C-C-C-C-G-T-G--AT-----T-G--C-C---T--G-------T--AA-T-A---C-------T--G-G--TG--G-A-C----T-T-G-A-G-T--G---C-AG-----GA-G-A--------G-G-G-G---AG-T------GG--A--ATT----C-
C-C-A-GT--GT-A-G-CG-GT--G---A--A-A---TG-C-GT-AG--AT-A-TT--G-G--G-A-G-G-A-AC-A-CC---AG--T--G--GC-GAA-G--G-C--G-------G--C-T-C-T---CTG--G--AC-T-G--------------------T-A---A-C-T--GA--CA-C-T-G--A-GG--C-G-CG-A--AA-G-C---G-TG--GG-
G--AG-C-A-AA--------CA--GG Bacteria(100);Firmicutes(100);Clostridia(100);Clostridiales(100);unclassified(100);unclassified(100);unclassified(100);unclassified(100);unclassified(100);

Otu0870 0 0 0 0 0 0 0 0 0 0 0 0 0 0 0 0 0 0 0 0 0 0 0 0 0 0 0 0
0 0 0 0 0 0 0 0 0 0 0 0 0 0 0 0 0 0 0 0 0 0 0 0 0 0 0 0 0
0 0 0 0 1 0 0 0 0 0 0 0 0 0 0 0 0 0 0 0 0 0 0 9 0 0 0 0 0
0 0 0 0 0 0 0 0 0 0 0 0 0 0 0 0 0 0 0 0 0 0 0 0 0 0 0 0
M02255_77_000000000-ADUT3_1_1117_25030_13456 G----AC---GG-GG-GGG--------GCA-A-G------T--G--T---T--CT-T-CGG-AA------TG-A--C-T--GG-GC-------GT--A-----AA-GG-GC-AC-------G-TA-G-G-C-G------------G--T-GA-A-T-C---
--------GG----G-T-T-G--------A-A-A--G--TG--A-AA-G---T-C-G--C-C-----AA-A-AA------C-T-G-G-G-G-G--A-A----T-G--C-T---C--T-------C--GA-A-A---C-------C--A-A--TT--C-A-C----T-T-G-A-G-T--G---A-GA-----CA-G-A--------G-G-A-G---AG-T------GG--A--ATT----
T-C-G-T-GT--GT-A-G-GG-GT--G---A--A-A---TC-C-GG-AG--AT-C-TA--C-G--A-A-G-G-A-AC-G-CC---AA--A--A--GC-GAA-G--G-C--A-------G--C-T-C-T---CTG--G--GT-C-C--------------------C-T---A-C-C--GA--CG-C-T-G--G-AG--T-G-CG-A--AA-G-C---A-TG--GG-
G--AG-C-G-AA--------CG--GG Bacteria(100);unclassified(100);unclassified(100);unclassified(100);unclassified(100);unclassified(100);unclassified(100);unclassified(100);unclassified(100);

Otu0871 0 0 0 0 0 0 0 0 0 0 0 0 0 0 0 0 0 0 0 0 0 0 0 0 0 0 0 0
0 0 0 0 0 0 0 0 0 0 0 0 0 0 60 0 0 0 0 0 0 0 0 0 0 0 0 0 0
0 0 0 0 0 0 0 0 0 0 0 0 0 0 0 0 0 0 0 0 0 0 0 0 0 0 0 0 0
0 0 0 0 0 0 0 0 0 0 0 0 0 0 0 0 0 0 0 0 0 0 0 0 0 0 0 0
M02255_77_000000000-ADUT3_1_1101_14138_3640 T----AC---GT-AG-GGG--------GCA-A-G------C--G--T---T--AT-C-CGG-AA------TC-A--T-T--GG-GC-------GT--A-----AA-GG-GT-GC-------G-TA-G-G-C-T------------G--C-CC-T-A-T----------AG-
---G-T-C-A--------G-A-G--G--TC--A-AA-GG--C-T-AC-G-G-----CT-C-AA------C-C-G-T-A-G-T--A------A-G--C-C---T--T-------T--GA-A-A---C-------T--G-T--AG--G-G-C----T-T-G-A-G-T--G---C-AG-----GA-G-A--------G-G-A-G---AG-T------GG--A--ATT----C-C-T-A-
GT--GT-A-G-CG-GT--G---A--A-A---TG-C-GT-AG--AT-A-TT--A-G--G-A-G-G-A-AC-A-CC---AG--T--G--GC-GAA-G--G-C--G-------A--C-T-C-T---CTG--G--AC-T-G--------------------C-A---A-C-T--GA--CG-C-T-G--A-GG--C-A-CG-A--AA-G-C---G-TG--GG-T--AG-C-
G-AA--------CA--GG Bacteria(100);Firmicutes(100);Clostridia(100);Clostridiales(100);unclassified(100);unclassified(100);unclassified(100);unclassified(100);unclassified(100);

Otu0872 0 0 0 0 0 0 0 0 0 0 0 0 0 0 0 0 0 0 0 2 6 0 0 0 0 0 0 0
0 0 0 0 0 0 0 0 0 12 0 0 0 0 0 0 0 0 0 0 0 0 0 0 0 0 0 0 0
0 0 0 0 0 0 0 0 0 0 0 0 0 0 0 0 0 0 0 0 0 0 0 0 0 0 0 0 0
0 0 0 0 0 0 0 0 0 0 0 0 0 0 0 0 0 0 0 0 0 0 0 0 0 0 0 0
M02255_77_000000000-ADUT3_1_2118_4938_8350 T----AC---GG-AG-GAT--------CCA-A-G------C--G--T---T--AT-C-CGG-AA------TT-A--C-T--GG-GC-------GT--A-----AA-GA-GTTGC-------G-TA-G-G-T-G------------G--C-AT-A-G-T----------
AA----G-T-A-A--------G-T-A--G--TG--A-AA-GC--C-T-GG-G-G-----CT-C-AA------C-C-C-T-T-T-A--A-A----C-A--T-T---A--T-------T--TA-A-A---C-------T--G-C--TA--A-G-C----T-A-G-A-G-G--G---C-GA-----GA-G-A--------G-G-T-T---AC-T------AG--A--ATT----C-C-T-
T-GT--GT-A-G-GA-GT--G---A--A-A---TC-C-GT-AG--AT-A-TA--A-G--G-A-G-G-A-AT-A-CC---GA--T--G--GC-GTA-G--G-C--A-------G--G-T-A-A---CTG--G--CT-C-G--------------------T-C---C-C-T--GA--CA-C-T-A--A-GG--C-A-CG-A--AA-G-C---G-TG--GG-T--AG-
C-A-AA--------CG--GG Bacteria(100);Candidate_division_TM7(100);unclassified(100);unclassified(100);unclassified(100);unclassified(100);unclassified(100);unclassified(100);unclassified(100);

Otu0873 0 0 0 0 0 0 0 0 0 0 0 0 0 0 0 0 0 0 0 0 0 0 0 0 0 0 0 0
0 0 0 0 0 0 0 0 0 0 0 0 0 0 31 0 0 0 0 0 0 0 0 0 0 0 0 0 0
0 0 0 0 0 0 0 0 0 0 0 0 0 0 0 0 0 0 0 0 0 0 0 0 0 0 0 0 0
0 0 0 0 0 0 0 0 0 0 0 0 0 0 0 0 0 0 0 0 0 0 0 0 0 0 0 0
M02255_77_000000000-ADUT3_1_1108_12315_24609 T----AC---GT-AG-GGC--------GCA-A-G------C--G--T---T--GT-C-CGG-AA------TT-A--T-T--GG-GC-------GT--A-----AA-GA-GC-TC-------G-TA-G-G-C-G------------G--T-CT-G-T-C------
-----AC----G-T-C-T--------G-C-T--G--TG--A-AA-AC--C-C-GA-G-G-----CT-C-AA------C-C-T-C-G-G-G--C-T----T-G--C-A---G--T-------G--GG-T-A---C-------G--G-G--CA--G-A-C----T-A-G-A-G-T--G---C-GG-----TA-G-G--------G-G-A-G---AC-T------GG--A--ATT----C-
C-T-G-GT--GT-A-G-CG-GT--A---A--A-A---TG-C-AC-AG--AT-A-TC--A-G--G-A-G-G-A-AC-A-CC---GA--T--G--GC-GAA-G--G-C--A-------G--G-T-C-T---CTG--G--GC-C-G--------------------C-A---A-C-T--GA--CG-C-T-G--A-GG--A-G-CG-A--AA-G-C---G-TG--GG-
T--AG-C-A-AA--------CA--GG Bacteria(100);Actinobacteria(100);Actinobacteria(100);Actinobacteridae(100);Actinomycetales(100);Micrococcineae(100);unclassified(100);unclassified(100);unclassified(100);

Otu0874 0 0 0 0 0 0 0 0 0 0 0 0 0 0 0 0 0 0 0 0 0 0 0 0 0 0 0 0
0 0 0 0 0 0 0 0 0 0 0 0 0 0 46 0 0 0 0 0 0 0 0 0 0 0 0 0 0
0 0 0 0 0 0 0 0 0 0 0 0 0 0 0 0 0 0 0 0 0 0 0 0 0 0 0 0 0
0 0 0 0 0 0 0 0 0 0 0 0 0 0 0 0 0 0 0 0 0 0 0 0 0 0 0 0
M02255_77_000000000-ADUT3_1_1105_17420_11769 C----AC---GT-AG-GCA--------CCA-A-G------C--G--T---T--GT-C-CGG-AT------TT-A--T-T--GG-GC-------GT--A-----AA-GA-GC-TC-------G-TA-G-G-C-G------------G--T-TC-G-A-T------
-----AA----G-T-C-G--------G-G-T--G--TG--A-AA-AC--T-T-TG-G-G-----CT-C-AA------C-C-C-A-A-A-G--C------G-T-GC-A---T--T-------C--GA-T-A---C-------T--G-T--TG-TG-A-C----T-A-G-A-G-T--T---C-GG-----TA-G-G--------G-G-A-G---TG-G------GG--A--ATT----C-
C-T-A-GT--GT-A-G-CG-GT--G---A--A-A---TG-C-GC-AG--AT-A-TT--A-G--G-A-G-G-A-AC-A-CC---GG--T--G--GC-GAA-G--G-C--G-------C--C-A-C-T---CTG--G--GC-C-G--------------------A-A---A-C-T--GA--CG-C-T-G--A-GG--A-G-CG-A--AA-G-C---G-TG--GG-
T--AG-C-A-AA--------CA--GG Bacteria(100);Actinobacteria(100);Actinobacteria(100);Acidimicrobidae(100);Acidimicrobiales(100);Acidimicrobineae(100);unclassified(100);unclassified(100);unclassified(100);

Otu0875 0 0 0 0 0 0 0 0 0 0 0 0 0 0 0 0 0 0 0 0 0 0 0 0 0 0 0 0
0 0 0 0 0 0 0 0 0 0 0 0 0 0 64 0 0 0 0 0 0 0 0 0 0 0 0 0
0 0 0 0 0 0 0 0 0 0 0 0 0 0 0 0 0 0 0 0 0 0 0 0 0 0 0 0
0 0 0 0 0 0 0 0 0 0 0 0 0 0 0 0 0 0 0 0 0 0 0 0 0 0 0
M02255_77_000000000-ADUT3_1_1105_7977_9509 G----AC---AG-AG-GGT--------GCT-A-G------C--G--T---T--GT-T-CGG-AA------TT-A--C-T--GG-GC-------GT--A-----AA-GG-GC-GC-------G-TA-G-G-C-G------------G--C-CA-T-G-T----------
AA----G-T-C-G--------G-A-T--G--TG--A-AA-TC--C-C-GG-G-G-----CT-C-AA------C-C-C-C-G-G-A--A-C----T-G--C-A---T--T-------C--GA-T-A---C-------T--G-T--GT--G-G-C----T-A-G-A-G-A--C---C-GG-----AA-G-A--------G-G-T-G---GG-T------GG--A--ATA----C-C-
C-A-GT--GT-A-G-AG-GT--G---A--A-A---TT-C-GT-AG--AT-A-TT--G-G--G-T-G-G-A-AC-A-CC---GG--T--G--GC-GAA-G--G-C--G-------G--C-T-C-A---CTG--G--TC-C-G--------------------G-T---T-C-T--GA--CG-C-T-G--A-GG--C-G-CG-A--AA-G-C---G-T-G-GG-G--
AG-C-A-AA--------CA--GG Bacteria(100);Proteobacteria(100);Alphaproteobacteria(100);unclassified(100);unclassified(100);unclassified(100);unclassified(100);unclassified(100);unclassified(100);

Otu0876 0 0 0 0 0 0 0 0 0 0 0 0 0 0 0 0 0 0 0 0 0 0 0 0 0 0 0 0
0 0 0 0 0 0 0 0 0 0 0 0 0 0 84 0 0 0 0 0 0 0 0 0 0 0 0 0
0 0 0 0 0 0 0 0 0 0 0 0 0 0 0 0 0 0 0 0 0 0 0 0 0 0 0 0
0 0 0 0 0 0 0 0 0 0 0 0 0 0 0 0 0 0 0 0 0 0 0 0 0 0 0
M02255_77_000000000-ADUT3_1_1105_21288_9159 T----AC---GT-AG-GGG--------GCT-A-G------C--G--T---T--AT-C-CGG-AA------TT-A--T-T--GG-GC-------GT--A-----AA-GG-GT-GC-------G-TA-G-G-C-G------------G--T-CC-T-A-C----------AA-
---G-T-C-A--------G-A-G--G--TG--A-AA-GG--C-T-AC-G-G-----CT-C-AA------C-C-G-T-A-G-T--A------A-G--C-C---T--T-------T--GA-A-A---C-------T--G-T--AG--G-A-C----T-T-G-A-G-T--G---C-AG-----GA-G-A--------G-G-A-G---AG-T------GG--A--ATT----C-C-C-A-
GT--GT-A-G-CG-GT--G---A--A-A---TG-C-GT-AG--AT-A-TT--G-G--G-A-G-G-A-AC-A-CC---AG--T--T--GC-GAA-G--G-C--G-------G--C-T-C-T---CTG--G--AC-T-G--------------------T-A---A-C-T--GA--CG-C-T-G--A-GG--C-A-CG-A--AA-G-C---G-TG--GG-G--AG-
C-G-AA--------CA--GG Bacteria(100);Firmicutes(100);Clostridia(100);Clostridiales(100);Peptostreptococcaceae(100);Sporacetigenium(100);unclassified(100);unclassified(100);unclassified(100);

Otu0877 0 0 0 0 0 0 0 0 0 0 0 0 0 0 0 0 0 0 0 3 9 0 0 0 0 0 0 0
0 0 0 0 0 0 0 0 0 30 1 0 0 0 0 0 0 0 0 0 0 0 0 0 0 0 0 0
0 0 0 0 0 0 0 0 0 0 0 0 0 0 0 0 0 0 0 0 0 0 0 0 0 0 0 0
0 0 0 0 0 0 0 0 0 0 0 0 0 0 0 0 0 0 0 0 0 0 0 0 0 0 0
M02255_77_000000000-ADUT3_1_1110_19519_10174 T----AC---GT-AG-GTG--------GCA-A-G------C--G--T---T--GT-C-CGG-AT------TT-A--C-T--GG-GC-------GT--A-----AA-GG-GC-GT-------G-CA-G-G-C-G------------G--T-TT-G-T-T------
-----AA----G-T-C-T--------A-G-G--G--TG--A-AA-GC--T-C-CC-G-G-----CT-C-AA------C-T-G-G-G-A-G--A-G----G-T--C-C---T--T-------G--GA-T-A---C-------T--G-G--CA--G-A-C----T-T-G-A-G-G--A---G-AG-----TA-G-A--------G-G-A-G---AG-T------GG--A--ATT----
C-C-C-G-GT--GT-A-G-TG-GT--G---A--T-A---TG-C-GT-AG--AT-A-TC--G-G--G-A-G-G-A-AC-A-CC---AG--T--G--GC-GAA-G--G-C--G-------G--C-T-C-T---CTG--G--GC-T-C--------------------T-A---C-C-T--GA--CG-C-T-G--A-GA--C-G-CG-A--AA-G-C---G-TG--GG-
G--AG-C-G-AA--------CC--GG Bacteria(100);Chloroflexi(100);S085(100);unclassified(100);unclassified(100);unclassified(100);unclassified(100);unclassified(100);unclassified(100);

Otu0878 0 0 0 0 0 0 0 0 0 0 0 0 0 0 0 0 0 0 0 0 0 0 0 0 0 0 0 0
0 0 0 0 0 0 0 0 0 0 0 0 0 0 112 0 0 0 0 0 0 0 0 0 0 0 0 0
0 0 0 0 0 0 0 0 0 0 0 0 0 0 0 0 0 0 0 0 0 0 0 0 0 0 0 0
0 0 0 0 0 0 0 0 0 0 0 0 0 0 0 0 0 0 0 0 0 0 0 0 0 0 0
M02255_77_000000000-ADUT3_1_1102_24387_21281 T----AC---GA-AG-GGG--------GCA-A-G------C--G--T---T--GT-T-CGG-AT------TT-A--C-T--GG-GC-------GT--A-----AA-GG-GC-GC-------G-CA-G-G-C-G------------G--C-TC-T-T-C-----
------AA----G-T-C-A--------G-A-T--G--TG--A-AA-GC--C-C-CG-G-G-----CT-C-AA------C-C-T-G-G-G-A--A-C----T-G--C-A---T--T-------T--GA-G-A---C-------T--G-A--AG--A-A-C----T-C-G-A-G-T--T---C-GG-----GA-G-A--------G-G-A-T---GG-C------GG--A--ATT----
C-C-C-A-GT--GT-A-G-AG-GT--G---A--A-A---TT-C-GT-AG--AT-A-TT--G-G--G-A-A-G-A-AC-A-CC---GG--T--G--GC-GAA-G--G-C--G-------G--C-C-A-T---CTG--G--AC-C-G--------------------A-G---A-C-T--GA--CG-C-T-G--A-GG--C-G-CG-A--AA-G-C---G-TG--
GG-G--AG-C-A-AA--------CA--GG Bacteria(100);Proteobacteria(100);Alphaproteobacteria(100);Rhodospirillales(100);unclassified(100);unclassified(100);unclassified(100);unclassified(100);unclassified(100);

Otu0879 0 0 0 0 0 0 0 0 0 0 0 0 0 0 0 0 0 0 0 0 0 0 0 0 0 0 0 0
0 0 0 0 0 0 0 0 0 0 0 0 0 0 53 0 0 0 0 0 0 0 0 0 0 0 0 0
0 0 0 0 0 0 0 0 0 0 0 0 0 0 0 0 0 0 0 0 0 0 0 0 0 0 0 0
0 0 0 0 0 0 0 0 0 0 0 0 0 0 0 0 0 0 0 0 0 0 0 0 0 0 0
M02255_77_000000000-ADUT3_1_1102_13661_17734 C----AC---GT-AG-GCA--------CCA-A-G------C--G--T---T--GT-C-CGG-AT------TT-A--T-T--GG-GC-------GT--A-----AA-GA-GC-TC-------G-TA-G-G-C-G------------G--T-TC-G-G-T------
-----AA----G-T-C-G--------G-G-T--G--TG--A-AA-AC--C-T-TG-G-G-----CT-C-AA------C-C-C-A-A-G-G--A------C-T-GC-A---C--T-------C--GA-T-A---C-------T--G-C--TG-TG-A-C----T-T-G-A-G-T--C---C-GG-----TA-G-G--------G-G-A-G---TG-G------GG--A--ATT----C-
C-T-G-GT--GT-A-G-CG-GT--G---G--A-A---TG-C-GC-AG--AT-A-TC--A-G--G-A-G-G-A-AC-A-CC---GG--T--G--GC-GAA-G--G-C--G-------C--C-A-C-T---CTG--G--GC-C-G--------------------G-T---A-C-T--GA--CG-C-T-G--A-GG--A-G-CG-A--AA-G-C---G-TG--GG-
G--AG-C-G-AA--------CA--GG Bacteria(100);Actinobacteria(100);Actinobacteria(100);Acidimicrobidae(100);Acidimicrobiales(100);Acidimicrobineae(100);unclassified(100);unclassified(100);unclassified(100);

Otu0880 0 0 0 0 0 0 0 0 0 0 0 0 0 0 0 0 0 0 0 1 0 0 0 0 0 0 0 0
0 0 0 0 0 0 0 0 0 12 0 0 0 0 0 0 0 0 0 0 0 0 0 0 0 0 0 0 0
0 0 0 0 0 0 0 0 0 0 0 0 0 0 0 0 0 0 0 0 0 0 0 0 0 0 0 0 0
0 0 0 0 0 0 0 0 0 0 0 0 0 0 0 0 0 0 0 0 0 0 0 0 0 0 0
M02255_77_000000000-ADUT3_1_2109_23326_6892 T----AC---GT-AG-GGT--------GCG-A-G------C--G--T---T--GT-C-CGG-AT------TT-A--T-T--GG-GC-------GT--A-----AA-GA-GC-TT-------G-TA-G-G-C-G------------G--T-TT-G-T-C----------AC--
--G-T-C-G--------A-A-A--G--TG--A-AA-AC--C-T-GG-G-C-----CT-T-AA------T-C-C-C-G-G-G--C-T----T-G--C-T---T--T-------C--GA-T-A---C-------G--G-G--CT--G-A-C----T-G-G-A-G-G--A---A-GG-----TA-G-G--------G-G-A-G---AA-C------GG--A--ATT----C-C-T-G-
GT--GG-A-G-CG-GT--G---G--A-A---TG-C-GC-AG--AT-A-TC--A-G--G-A-G-G-A-AC-A-CC---GG--T--G--GC-GAA-G--G-C--G-------G--T-T-C-T---CTG--G--AC-C-T--------------------T-T---C-C-T--GA--CG-C-T-G--A-GA--A-G-CG-A--AA-G-C---G-TG--GG-G--AG-
C-A-AA--------CA--GG Bacteria(100);Actinobacteria(100);Actinobacteria(100);Actinobacteridae(100);Actinomycetales(100);Propionibacterineae(100);Propionibacteriaceae(100);unclassified(100);unclassified(100);

Otu0881 0 0 0 0 0 0 0 0 0 0 0 0 0 0 0 0 0 0 0 1 0 0 0 0 0 0 0 0
0 0 1 0 0 0 0 0 0 7 0 8 0 0 0 0 0 0 0 0 0 0 0 48 0 0 0 0 0
0 0 0 0 0 0 0 10 0 0 0 12 0 0 0 0 0 0 0 23 234 73 0 0 0 0 667 0 0 306
41 0 0 0 0 21 1 18 0 171 5 662 3 28 4 0 34 0 22 0 0 0 0 0 0 0 0
M02255_77_000000000-ADUT3_1_1110_17783_15269 T----AC---GG-AG-GGT--------GCA-A-G------C--G--T---T--AA-T-CGG-AA------TA-A--C-T--GG-GC-------GT--A-----AA-GG-GC-AT-------G-CA-G-G-C-G------------G--T-TC-A-T-C-----
------AA----G-T-A-G--------G-A-T--G--TG--A-AA-TC--C-C-CG-G-G-----CT-C-AA------C-C-T-G-G-G-A--A-C----A-G--C-A---T--A-------C--TA-A-A---C-------T--G-G--TG--G-A-C----T-A-G-A-G-T--A---T-TG-----CA-G-G--------G-G-G-A---GA-C------GG--A--ATT----
C-C-A-G-GT--GT-A-G-CG-GT--G---G--A-A---TG-C-GT-AG--AT-A-TC--T-G--G-A-A-G-A-AC-A-CC---AA--A--G--GC-GAA-G--G-C--A-------G--T-C-T-C---CTG--G--GC-A-A--------------------A-T---A-C-T--GA--CG-C-T-C--A-TA--T-G-CG-A--AA-G-C---G-TG--GG-
T--AG-C-A-AA--------CA--GG Bacteria(100);Proteobacteria(100);Gammaproteobacteria(100);Aeromonadales(100);Succinivibrionaceae(100);Succinivibrio(100);unclassified(100);unclassified(100);unclassified(100);

Otu0882 0 0 0 0 0 0 0 0 0 0 0 0 0 0 0 0 0 0 0 1 0 0 0 0 0 0 0 0
0 0 0 0 0 0 0 0 0 0 0 0 0 0 0 0 0 0 0 0 1 7 0 0 0 0 0 0 0
0 0 0 0 0 0 0 0 0 0 0 0 0 0 0 0 0 0 0 0 0 0 0 0 0 0 0 0 0
0 0 0 0 0 0 0 0 0 0 0 0 0 0 0 0 0 0 0 0 0 0 0 0 0 0 0
M02255_76_000000000-ADUNE_1_2107_6475_4912 C----GT---GC-CA-GCA--------GCC-G-C------G--G--T---A--AT-A-CGT-AT------GG-A--G-C--GA-GC------GTT--G-----TC-CG-GA-AT-------T-AT-T-G-C-G------------G--C-GT-A-A-A----------
GG----G-TAC-G--------C-A-G--G--TT--A-AA-GA--T-C-GG-G-G-----CT-C-AA------C-C-C-C-G-T-A--A------A-G--C-A---T--T-------G--GA-A-A---C-------T--G-A--TA--A-A-C----T-T-G-A-G-T--A---G-TG-----GA-G-A--------G-G-A-A---AG-T------GG--A--ATT----C-C-
T-A-GT--GT-A-G-TG-GT--G---A--A-A---TA-C-GT-AG--AT-A-TT--A-G--G-A-G-G-A-AT-A-CC---AG--T--A--GC-GAA-G--G-C--G-------A--C-T-T-T---CTG--G--AC-A-C--------------------A-A---A-C-T--GA--CG-C-T-G--A-GG--T-A-CG-A--AA-G-C---G-TG--GG-G--
AG-C-A-AA--------CA--GG Bacteria(100);Firmicutes(100);Clostridia(100);Clostridiales(100);Family_XI_Incertae_Sedis(100);Finegoldia(100);unclassified(100);unclassified(100);unclassified(100);

Otu0883 0 0 0 0 0 0 0 0 0 0 0 0 0 0 0 0 0 0 0 0 0 0 0 0 0 0 0 0
0 0 0 0 0 0 0 0 0 0 0 0 0 0 30 69 37 0 0 0 0 0 0 0 0 0 0 0 0
0 0 0 0 0 0 0 0 0 0 0 0 0 0 0 0 0 0 0 0 0 0 0 0 0 0 0 0 0
0 0 0 0 0 0 0 0 0 0 0 48 0 12 0 0 0 0 0 0 0 0 0 0 0 0 0
M02255_77_000000000-ADUT3_1_1101_9878_23086 T----AC---GT-AG-GGG--------GCT-A-G------C--G--T---T--AT-C-CGG-AT------TT-A--C-T--GG-GC-------GT--A-----AA-GG-GT-GC-------G-TA-G-G-C-G------------G--T-CT-T-T-C----------AA--
--G-T-C-A--------G-G-A--G--TT--A-AA-GG--C-T-AC-G-G-----CT-C-AA------C-C-G-T-A-G-T--A------A-G--C-T---C--C-------T--GA-T-A---C-------T--G-T--CT--G-A-C----T-T-G-A-G-T--G---C-AG-----GA-G-A--------G-G-A-A---AG-C------GG--A--ATT----C-C-C-A-
GT--GT-A-G-CG-GT--G---A--A-A---TG-C-GT-AG--AT-A-TT--G-G--G-A-G-G-A-AC-A-CC---AG--T--A--GC-GAA-G--G-C--G-------G--C-T-T-T---CTG--G--AC-T-G--------------------T-A---A-C-T--GA--CG-C-T-G--A-GG--C-A-CG-A--AA-G-C---G-TG--GG-G--AG-
C-A-AA--------CA--GG Bacteria(100);Firmicutes(100);Clostridia(100);Clostridiales(100);Peptostreptococcaceae(100);unclassified(100);unclassified(100);unclassified(100);unclassified(100);

Otu0884 0 0 0 0 0 0 0 0 0 0 0 0 0 0 0 0 0 0 0 0 0 0 0 0 0 0 0 0
0 0 0 0 0 0 0 0 0 0 0 0 35 0 0 0 0 0 0 0 0 0 0 0 0 0 0 0 0
0 0 0 0 0 0 0 0 0 0 0 0 0 0 0 0 0 0 0 0 0 0 0 0 0 0 0 0 0
0 0 0 0 0 0 0 0 0 0 0 0 0 0 0 0 0 0 0 0 0 0 0 0 5 0 0
M02255_77_000000000-ADUT3_1_1110_10390_16595 T----AC---GT-AG-GGA--------GCA-A-G------C--G--T---T--GT-T-CGG-AT------TT-A--C-T--GG-GC-------GT--A-----AA-GG-GC-GC-------G-TA-G-G-C-G------------G--C-GT-G-T-T-----
------AA----G-T-C-A--------G-C-T--G--TG--A-AA-TC--T-C-CA-A-G-----CT-C-AA------C-T-T-G-G-A-A--C-G----G-C--C-A---G--C-------T--GA-T-A---C-------T--G-A--TG--T-G-C----T-A-G-A-G-T--G---C-AG-----AA-G-G--------G-G-C-A---AT-C------GG--A--ATT----C-
T-T-G-GT--GT-A-G-CG-GT--G---A--A-A---TG-C-GT-AG--AT-A-TC--A-A--G-A-G-G-A-AC-A-CC---TG--A--G--GT-GAA-G--A-C--G-------G--G-T-T-G---CTG--G--GC-T-G--------------------A-C---A-C-T--GA--CG-C-T-G--A-GG--C-G-CG-A--AA-G-C---C-AG--GG-
G--AG-C-A-AA--------CG--GG Bacteria(100);Acidobacteria(100);Acidobacteria(100);Acidobacteriales(100);Acidobacteriaceae(100);Candidatus_Chloroacidobacterium(100);unclassified(100);unclassified(100);unclassified(100);

Otu0885 0 0 0 0 0 0 0 0 0 0 0 0 0 0 0 0 0 0 0 0 0 0 0 0 0 0 0 0
0 0 0 0 0 0 0 0 0 0 0 0 0 0 17 0 0 0 0 0 0 0 0 0 0 0 0 0
0 0 0 0 0 0 0 0 0 0 0 0 0 0 0 0 0 0 0 0 0 0 0 0 0 0 0 0
0 0 0 0 0 0 0 0 0 0 0 0 0 0 0 0 0 0 0 0 0 0 0 0 0 0 0
M02255_76_000000000-ADUNE_1_1113_17346_25056 C----TA---AC-TC-CGT--------GCC-A-G------C-----A---G--CC-G-CGG-TA------AT-A--C-G--GA-GG-------GT--G-----CA--A-GC--G-------T-TA-A-T-C-G------------G--A-TT-G-G-T--------
---CA----G-T-C-A--------G-A-T--G--TG--A-AA-GC--C-C-TC-G-G-----CT-T-AA------C-C-G-G-G-G-A--A-C----T-G--C-A---T--T-------T--GA-T-A---C-------T--G-C--CA--G-G-C----T-T-G-A-G-T--G---T-CG-----GA-G-A--------G-G-G-T---GG-C------GG--A--ATT----C-C-
A-G-GT--GT-A-G-GA-GT--G---A--A-A---TC-C-GT-AG--AT-A-TC--T-G--G-A-G-G-A-AC-A-CC---AG--T--G--GC-GAA-G--G-C--G-------G--C-C-A-C---CTG--G--AC-G-A--------------------C-A---A-C-T--GA--CG-C-T-G--A-GG--A-G-CG-A--AA-G-C---G-TG--GG-T--
AG-C-A-AA--------CA--GG Bacteria(100);Proteobacteria(100);Deltaproteobacteria(100);Desulfovibrionales(100);Desulfovibrionaceae(100);unclassified(100);unclassified(100);unclassified(100);unclassified(100);

Otu0886 0 0 0 0 0 0 0 0 0 0 0 0 0 0 0 0 0 0 0 0 0 0 0 0 0 0 0 0
0 0 0 0 0 0 0 0 0 0 0 0 0 0 0 0 5 5 0 0 0 0 0 0 0 0 0 0
0 0 0 0 0 0 0 0 0 0 0 0 0 0 0 0 0 0 0 0 0 0 0 0 0 0 0 0
0 0 0 0 0 0 0 0 0 0 0 0 0 0 0 0 0 0 0 0 0 0 0 0 0 0 0
M02255_76_000000000-ADUNE_1_1106_15785_17092 C----ACG--GC-TA-ACT--------ACG-T-G------C--C--A---G--CA-G-CCG-CG------GT-A--A-T--AC-GT-------AG--G-----TG-GC-AA-GC-------G-TT-G-T-C-C------------G--G-AT-G-A-T-----
------AA----G-T-C-T--------G-A-T--G--TG--A-AA-GC--C-C-AC-G-G-----CT-C-AA------C-C-G-T-G-G-A--A-C----T-G--C-A---T--C-------G--GA-A-A---C-------T--G-T--CA--T-T-C----T-T-G-A-G-T--G---C-AG-----AA-G-A--------G-G-A-G---AG-T------GG--A--ATT----C-
C-A-T-GT--GT-A-G-CG-GT--G---G--A-A---TG-C-GT-AG--AT-A-TA--T-G--G-A-A-G-A-AC-A-CC---AG--T--G--GC-GAA-G--G-C--G-------G--C-T-C-T---CTG--G--TC-T-G--------------------C-A---A-C-T--GA--CG-C-T-G--A-GG--C-T-CG-A--AA-G-C---A-TG--GG-T--
AG-C-G-AA--------CA--GG Bacteria(100);Firmicutes(100);Bacilli(100);Lactobacillales(100);unclassified(100);unclassified(100);unclassified(100);unclassified(100);unclassified(100);

Otu0887 0 0 0 0 0 0 0 0 0 0 0 0 0 0 0 0 0 0 0 0 0 0 0 0 0 0 0 0
0 0 0 0 0 0 0 0 0 0 0 0 0 2 0 0 0 0 0 0 0 0 0 0 0 0 0 0
0 0 0 0 0 0 0 0 0 0 0 0 0 2 0 0 0 0 0 0 0 0 0 0 0 0 0 6
0 0 0 0 0 0 0 0 0 0 0 0 0 0 0 0 0 0 0 0 0 0 0 0 0 0 0
M02255_76_000000000-ADUNE_1_2112_28395_18425 T----CG---GC-TA-ACT--------CCG-T-GC-----C--A--G---C--AG-C-CGC-GG------TA-A--T-A--CG-GA-------GG--A-----TC-CG-AG-CG-------T-TA-T-C-C-G------------G--C-AC-T-T-T------
-----AA----G-T-C-A--------G-G-G--G--TG--A-AA-GA--C-G-GC-A-G-----CT-C-AA------C-T-G-T-C-G-C--A-G----T-G--C-C---C--T-------T--GA-T-A---C-------T--G-A--AG--T-G-C----T-T-G-A-A-T--G---C-GG-----TT-G-A--------A-G-A-C---GG-C------GG--A--ATG----A-
G-A-C-AA--GT-A-G-CG-GT--G---A--A-A---TG-C-AT-AG--AT-A-TG--T-C--T-C-A-G-A-AC-A-CC---GA--T--T--GC-GAA-G--G-C--A-------G--C-T-G-T---CTA--A--GC-C-G--------------------T-T---A-T-T--GA--CG-C-T-G--A-TG--C-A-CG-A--AA-G-C---G-TG--GG-G--
AT-C-G-AA--------CA--GG Bacteria(100);Bacteroidetes(100);Sphingobacteria(100);Sphingobacteriales(100);Sphingobacteriaceae(100);Sphingobacteriaceae(100);Sphingobacterium(100);unclassified(100);unclassified(100);

Otu0888 0 0 0 0 0 0 0 0 0 0 0 0 0 0 0 0 0 0 0 1 10 0 0 0 11 0 0 0
0 2 0 0 0 0 0 0 0 27 0 0 0 0 0 0 0 0 0 0 0 0 0 0 0 0 0 0
0 0 0 0 0 0 0 0 0 0 0 0 0 0 0 0 0 0 0 0 0 0 0 0 50 0 0 0
0 0 0 0 17 0 0 0 0 0 0 0 0 0 0 0 0 0 0 0 0 0 0 0 0 0 41
M02255_77_000000000-ADUT3_1_1117_22532_19561 G----AC---GG-AG-GAG--------GCT-A-G------C--G--T---T--GT-T-CGG-AA------TT-A--C-T--GG-GC-------GT--A-----AA-GC-GC-GC-------G-CA-G-G-C-G------------G--C-TT-A-G-C----
-------AA----G-T-C-A--------G-G-G--G--TG--A-AA-GC--C-C-GG-G-G-----CT-C-AA------C-C-T-C-G-G-A--A-C----T-G--C-C---T--T-------T--GA-G-A---C-------T--G-C--TA--G-G-C----T-C-G-A-G-A--C---C-GA-----GA-G-A--------G-G-A-G---AG-T------GG--A--ATT----
C-C-C-A-GT--GT-A-G-AG-GT--G---A--A-A---TT-C-GT-AG--AT-A-TT--G-G--G-A-A-G-A-AC-A-CC---AG--T--G--GC-GAA-G--G-C--G-------G--C-T-C-T---CTG--G--CT-C-G--------------------G-T---A-C-T--GA--CG-C-T-G--A-GG--C-G-CG-A--AA-G-C---G-TG--GG-
G--AG-C-A-AA--------CA--GG Bacteria(100);Proteobacteria(100);Alphaproteobacteria(100);unclassified(100);unclassified(100);unclassified(100);unclassified(100);unclassified(100);unclassified(100);

Otu0889 0 0 0 0 0 0 0 0 0 0 0 0 0 0 0 0 0 0 0 1 0 0 0 4 0 0 5 0
0 0 0 0 0 0 0 0 0 0 0 0 0 0 0 0 0 0 0 0 0 0 0 0 0 0 0 0
0 0 0 0 0 0 0 0 0 0 0 0 0 0 0 0 0 0 0 0 0 0 0 0 0 0 0 0
0 0 0 0 0 0 0 0 0 0 0 0 0 0 0 0 0 0 0 0 0 0 0 0 0 0 0
M02255_76_000000000-ADUNE_1_2115_6530_20338 C----GG---CT-AA-CTC--------CGT-G-C------C--A--G---C--AG-C-CGC-GG------TA-A--T-A--CG-GA-------GG--A-----TC-CA-AG-CG-------T-TA-T-C-C-G------------GA-C-CC-T-G-T-----------AA-
---G-T-C-A--------G-C-G--G--TG--A-AA-TC--T-C-CC-G-G-----CT-C-AA------C-C-G-G-G-A-A--A-T----G-G--C-C---G--T-------T--GA-T-A---C-------T--G-C--AG--G-G-C----T-T-G-A-A-T--T---A-GT-----AG-G-A--------A-G-T-A---AC-T------AG--A--ATA----T-G-T-A-
GT--GT-A-G-CG-GT--G---A--A-A---TG-C-TT-AG--AG-A-TT--A-C--A-T-G-G-A-AT-A-CC---AA--T--T--GC-GAA-G--G-C--A-------G--G-T-T-A---CTA--C--TA-C-T--------------------A-T---A-T-T--GA--CG-C-T-G--A-GG--G-A-CG-A--AA-G-C---G-TG--GG-T--AG-C-
G-AA--------CA--GG Bacteria(100);Bacteroidetes(100);Flavobacteria(100);Flavobacteriales(100);Flavobacteriaceae(100);unclassified(100);unclassified(100);unclassified(100);unclassified(100);

Otu0890 0 0 0 0 0 0 0 0 0 0 0 0 0 0 0 0 0 0 0 0 0 0 0 0 0 0 0 0
0 0 0 0 0 0 0 0 0 0 0 0 0 1 0 0 0 0 0 0 0 0 0 0 0 0 0 0
0 0 0 0 0 0 0 0 0 0 0 0 0 0 0 0 0 0 0 0 0 0 0 0 0 0 0 0
0 0 0 0 18 0 0 0 0 23 0 0 0 0 0 0 0 0 0 0 0 0 0 0 0 0 0
M02255_77_000000000-ADUT3_1_1104_23462_12033 T----AC---GA-AG-GGA--------CCT-A-G------C--G--T---A--GT-T-CGG-AA------TT-A--C-T--GG-GC-------GT--A-----AA-GC-GC-GC-------G-TA-G-G-C-C------------G--T-TG-A-G-T-----
------TA----G-T-T-A--------A-T-T--G--TG--A-AA-TC--C-C-AA-A-G-----CT-T-AA------C-T-T-T-G-G-A--A-C----T-G--C-A---A--T-------T--AA-A-A---C-------T--G-C--TC--G-A-C----T-A-G-A-G-T--T---T-GA-----TA-G-A--------G-G-A-A---AG-C------GG--A--ATA----C-
A-T-A-GT--GT-A-G-AG-GT--G---A--A-A---TT-C-GT-AG--AT-A-TT--A-T--G-T-A-G-A-AC-A-CC---AG--T--T--GC-GAA-G--G-C--G-------G--C-T-T-T---CTG--G--AT-C-A--------------------A-C---A-C-T--GA--CG-C-T-G--A-GG--C-G-CG-A--AA-G-T---A-TG--GG-T--
AG-C-A-AA--------GA--GG Bacteria(100);Proteobacteria(100);Alphaproteobacteria(100);SAR11(100);unclassified(100);unclassified(100);unclassified(100);unclassified(100);unclassified(100);

Otu0891 0 0 0 0 0 0 0 0 0 0 0 0 0 0 0 0 0 0 0 0 2 0 0 0 0 0 0 0
0 0 0 0 0 0 0 0 0 10 0 0 0 0 0 0 0 0 0 0 0 0 0 0 0 0 0 0
0 0 0 0 0 0 0 0 0 0 0 0 0 0 0 0 0 0 0 0 0 0 0 0 0 0 0 0
0 0 0 0 0 0 0 0 0 0 0 0 0 0 0 0 0 0 0 0 0 0 0 0 0 0 0
M02255_76_000000000-ADUNE_1_1102_10766_11218 T----AC---GT-AG-GGG--------CCT-A-G------C--G--T---T--GT-C-CGG-AT------TT-A--T-T--GG-GC-------GT--A-----AA-GA-GC-TC-------G----G-T-A-G------------G----TC----------------
-A----------G--------G---T--G--TG--A-AA-TC--T-C-CA-C-G-----CT-C-AA------C-G-T-G-G-A-G--G------C-G-CCAC---C--T----------GA-A-A---C-------T--G-C--CG-TG-A-C----T-C-G-A-G-T--C---C-GG-----TA-G-G--------G-G--AG---TG-T------GG--A--ATTC---C-C---G-
GT--GT-A-G-CG-GT--G---A--A-A---TG-C-GC-AG--AT-A-TC--G-G--G-A-G-G-A-AC-A-CC---AG--T--G--GC-GAA-G--G-C--G-------G--C-A-C-T---CTGG----GC-C-G--------------------G-T---A-C-T--GA--CG-C-T-G--A-GG--A-G-CG-A--AA-G-C---G-TG--GG-G--AG-
C-G-AA--------CA--GG Bacteria(100);unclassified(100);unclassified(100);unclassified(100);unclassified(100);unclassified(100);unclassified(100);unclassified(100);unclassified(100);

Otu0892 0 0 0 0 0 0 0 0 0 0 0 0 0 0 0 0 0 0 0 0 0 0 0 0 0 0 0 0
0 0 0 0 0 0 0 0 0 0 0 0 0 0 0 0 0 0 0 0 0 0 0 0 0 0 0 0
0 0 0 0 0 0 0 0 0 0 0 0 0 0 0 0 0 84 0 0 0 0 0 0 0 0 0 0
0 0 0 0 0 0 0 0 0 0 0 0 0 0 0 0 0 0 0 0 0 11 0 0 0 0 0
M02255_76_000000000-ADUNE_1_1111_16534_18622 T----AA---GC-AC-CG---------GCT-A-A------C--T--A---C--GT-G-CCAGCA------GC-C--G-C--GG-TA-------AT--A-----CG-TA-GG-GT-------G-CA-A-G-C-G------------G--T-TA-T-A-T------
-----CG----G-A-C-A--------G-A-T--G--TG--A-AA-TC--C-C-CG-G-G-----CT-C-AA------C-C-T-G-G-G-A--A-C----T-G--C-A---T--T-------T--GT-G-A---C-------T--G-T--AT--A-G-C----T-A-G-A-G-T--A---C-GG-----CA-G-A--------G-G-G-G---GA-T------GG--A--ATT----C-
C-G-C-GT--GT-A-G-CA-GT--G---A--A-A---TG-C-GT-AG--AT-A-TG--C-G--G-A-G-G-A-AC-A-CC---GA--T--G--GC-GAA-G--G-C--A-------A--T-C-C-C---CTG--G--GC-C-T--------------------G-T---A-C-T--GA--CG-C-T-C--A-TG--C-A-CG-A--AA-G-C---G-TG--GG-G-
-AG-C-A-AA--------CA--GG Bacteria(100);Proteobacteria(100);Betaproteobacteria(100);Burkholderiales(100);Comamonadaceae(100);unclassified(100);unclassified(100);unclassified(100);unclassified(100);

Otu0893 0 0 0 0 0 0 0 0 0 0 0 0 0 0 0 0 0 0 0 0 0 0 0 0 0 0 0 0
0 0 0 0 0 0 0 0 0 0 0 0 0 29 0 0 0 0 0 0 0 0 0 0 0 0 0 0
0 0 0 0 0 0 0 0 0 0 0 0 0 0 0 0 0 0 0 0 0 0 0 0 0 0 0 0
0 0 0 0 0 0 0 0 0 0 0 0 0 0 0 0 0 0 0 0 0 0 0 0 0 0 0
M02255_77_000000000-ADUT3_1_1104_19700_16577 G----AC---GG-AG-GAT--------GCG-A-G------T--G--T---T--AT-C-CGG-AA------TC-A--C-T--GG-GC-------GT--A-----AA-GC-GT-CT-------G-TA-G-G-T-G------------G--T-TT-A-A-A-----
------AA----G-T-C-T--------A-C-T--G--TT--A-AA-GC--T-T-GA-A-G-----CT-T-AA------C-T-T-C-A-A-A--A-A----T-G--C-C---G--T-------A--GA-A-A---C-------T--T-T--TA--A-A-C----T-T-G-A-G-T--G---T-GG-----TA-G-G--------G-G-T-A---GA-G------GG--A--ATT----T-
C-C-A-GT--GG-A-G-CG-GT--G---A--A-A---TG-C-GT-AG--AT-C-TT--G-G--A-A-A-G-A-CC-A-CC---AA--T--G--GC-GAA-G--G-C--A-------C--T-C-T-A---CTG--G--GC-C-A--------------------T-T---A-C-T--GA--CA-C-T-A--A-GA--G-A-CG-A--AA-G-C---T-AG--GG-G-
-AG-C-A-AA--------TG--GG Bacteria(100);Cyanobacteria(100);unclassified(100);unclassified(100);unclassified(100);unclassified(100);unclassified(100);unclassified(100);unclassified(100);

Otu0894 0 0 0 0 0 0 0 0 0 0 0 0 0 0 0 0 0 0 0 0 6 0 0 0 0 0 0 0
0 0 0 0 0 0 0 0 0 26 0 0 0 0 0 0 0 0 0 0 0 0 0 0 0 0 0 0
0 0 0 0 0 0 0 0 0 0 0 0 0 0 0 0 0 0 0 0 0 0 0 0 0 0 0 0
0 0 0 0 0 0 0 0 0 0 0 0 0 0 0 0 0 0 0 0 0 0 0 0 0 0 0
M02255_76_000000000-ADUNE_1_1108_24659_4216 T----AA---CT-A--CGT--------GCC-A-G------C--A--G---C--CG-C-GGT-AA------TA-C--G-T--AG-GG-------GG--C-----AA-GC-GT-TG-------T-CC-G-G---A------------C--T--T-A-T-T----------AA----
G-T-C-T--------G-A-T--G--TG--A-AA-GC--C-C-AC-G-G-----CT-C-AA------C-C-G-T-G-G-A--G------G-GC-C-A---T--T-------G--GA-A-A---C-------T--G-G--TA--G-G-C----T-T-G-A-G-T--G---C-AG-----GA-G-A--------G-G-A-G---AG-C------GG--A--ATT----C-C-C-G-
GT--GT-A-G-CG-GT--G---A--A-A---TG-C-GT-AG--AT-A-TC--G-G--G-A-G-G-A-AC-A-CC---CG--T--G--GC-GAA-G--G-C--G-------G--C-T-C-T---CTG--G--CC-T-G--------------------T-A---A-C-T--GA--CG-C-T-G--A-GG--C-G-CG-A--AA-G-C---G-TG--GG-G--AG-
C-A-AA--------CA--GG Bacteria(100);Firmicutes(100);Bacilli(100);Bacillales(100);Paenibacillaceae(100);Ammoniphilus(100);unclassified(100);unclassified(100);unclassified(100);

Otu0895 0 0 0 0 0 0 0 0 0 0 0 0 0 0 0 0 0 0 0 0 0 0 0 0 0 0 0 0
0 0 0 0 0 0 0 0 0 0 0 0 0 2 0 0 0 0 0 0 0 0 0 13 0 0 0 0 0
0 0 0 0 0 0 0 0 0 0 0 0 0 0 0 0 0 0 0 0 0 0 0 0 0 0 0 0 0
0 0 0 0 0 0 0 0 0 0 0 0 0 0 0 0 0 0 0 0 0 0 0 0 0 0 0
M02255_77_000000000-ADUT3_1_2111_22576_22368 T----AC---GT-AG-GGT--------CCG-A-G------C--G--T---T--GT-C-CGG-AA------TT-A--C-T--GG-GC-------GT--A-----AA-GA-GC-TT-------G-TA-G-G-C-G------------G--T-TC-G-T-C-----------GC----G-T-C-G--------G-T-T--G--TG--A-AA-AC--T-T-GG-G-G-----CT-C-AA------C-C-T-T-A-A-G--C-T----T-G--C-A---G--G-------C--GA-T-A---C-------G--G-G--CG--G-A-C----T-A-G-A-G-T--A---C-TT-----CA-G-G--------G-G-A-G---AC-T------GG--A--ATT----C-C-T-G-GT--GT-A-G-CG-GT--G---A--A-A---TG-C-GC-AG--AT-A-TC--A-G--G-A-G-G-A-AC-A-CC---GG--T--G--GC-GAA-G--G-C--G-------G--G-T-C-T---CTG--G--GA-A-G--------------------T-A---A-C-T--GA--CG-C-T-G--A-GA--A-G-CG-A--AA-G-C---A-TG--GG-T--AG-C-A-AA--------CA--GG Bacteria(100);Actinobacteria(100);Actinobacteria(100);Actinobacteridae(100);Actinomycetales(100);Corynebacterineae(100);Nocardiaceae(100);Nocardia(100);unclassified(100);

Otu0896 0 0 0 0 0 0 0 0 0 0 0 0 0 0 0 0 0 0 0 0 0 0 0 0 0 0 0 0
0 0 0 0 0 0 0 6 9 0 0 0 0 0 0 0 0 0 0 0 0 0 0 0 0 0 0 0 0
0 0 0 0 0 0 0 0 0 0 0 0 0 0 0 0 0 0 0 0 0 0 0 0 0 0 0 0 0
0 0 0 0 0 0 0 0 0 0 0 0 0 0 0 0 0 0 0 0 0 0 0 0 0 0 0
M02255_76_000000000-ADUNE_1_1102_6846_16750 C----GA---GA-GA-AGA--------AGC-C-C------C--G--G---C--TA-A-CTC-CG------TG-C--C-A-GCA-GC-------CG--C-----GG-TA-AT-AC-------G-GG-G-G-G-G------------G--C-AA-G-CGT-----------TG----T-T-C-G--------G-A-C--G--TG--A-AA-TC--C-C-TC-G-G-----CT-T-AA------C-C-G-G-G-G-A--A-C----T-G--C-G---T--C-------C--GA-T-A---C-------T--G-G--AT--G-G-C----T-T-G-A-G-T--T---T-GG-----GA-G-A--------G-G-G-A---TG-C------GG--A--ATT----C-C-A-G-GT--GT-A-G-CG-GT--G---A--A-A---TG-C-GT-AG--AT-A-TC--T-G--G-A-G-G-A-AC-A-CCG--G---T--G--GC-GAA-G--G-C--G-------G--C-A-T-C---CTG--G--AC-C-A--------------------A-C---A-C-T--GA--CG-C-T-G--A-GG--A-G-CG-A--AA-G-C---C-AG--GG-G--AG-C-A-AA--------CG--GG Bacteria(100);Acidobacteria(100);unclassified(100);unclassified(100);unclassified(100);unclassified(100);unclassified(100);unclassified(100);unclassified(100);

Otu0897 0 0 0 0 0 0 0 0 0 0 0 0 0 0 0 0 0 0 0 0 0 0 0 0 0 0 0 0
0 0 0 0 0 0 0 0 0 0 0 0 35 0 0 0 0 0 0 0 0 0 0 0 0 0 0 0 0
0 0 0 0 0 0 0 0 0 0 0 0 0 0 0 0 0 0 0 0 0 0 0 0 0 0 0 0 0
0 0 0 0 0 0 0 0 0 0 0 0 0 0 0 0 0 0 0 0 0 0 0 0 0 0 0
M02255_77_000000000-ADUT3_1_1117_3846_12018 T----AC---GT-AG-GTG--------GCG-A-G------C--G--T---T--AT-C-CGG-AT------TT-A--C-T--GG-GC-------GT--A-----AA-GG-GA-GC-------G-TA-G-G-C-G------------G--A-TG-A-T-T-----------AA----G-T-G-G--------G-A-T--G--TG--A-AA-TA--C-C-CG-G-G-----CT-C-AA------C-T-T-G-G-G-T--G-C----T-G--C-A---T--T-------C--CA-A-A---C-------T--G-G--TT--A-T-C----T-A-G-A-G-T--G---C-AG-----GA-G-A--------G-G-A-G---AG-T------GG--A--ATT----C-C-T-A-GT--GT-A-G-CG-GT--G---A--A-A---TG-C-GT-AG--AG-A-TT--A-G--G-A-A-G-A-AC-A-CC---AG--T--G--GC-GAA-G--G-C--G-------A--C-T-C-T---CTG--G--AC-T-G--------------------T-A---A-C-T--GA--CG-C-T-G--A-GG--C-T-CG-A--AA-G-C---G-TG--GG-G--AG-C-A-AA--------CA--GG Bacteria(100);Firmicutes(100);Clostridia(100);Clostridiales(100);Clostridiaceae(100);unclassified(100);unclassified(100);unclassified(100);unclassified(100);

Otu0898 0 0 0 0 0 0 0 0 0 0 0 0 0 0 0 0 0 0 0 0 0 0 0 0 0 0 0 0
0 0 0 0 0 0 0 50 0 0 0 0 0 0 0 0 0 0 0 0 0 0 0 0 0 0 0 0 0
0 0 0 0 0 0 0 0 0 0 0 0 0 0 0 0 0 0 0 0 0 0 0 0 0 0 0 0 0
0 0 0 0 0 0 0 0 0 0 0 0 0 0 0 0 0 0 0 0 0 0 0 0 0 0 0
M02255_77_000000000-ADUT3_1_1101_16227_10788 C----AC---GT-AG-GAG--------GCG-A-G------C--G--T---T--AT-C-CGG-AT------TC-A--C-T--GG-GC-------GT--A-----AA-GC-GC-GT-------G-CA-G-G-C-G------------G--T-TT-G-G-T-----------AA----G-T-T-G--------G-A-T--G--TG--A-AA-GC--T-C-CC-G-G-----CT-C-AA------C-T-G-G-G-A-G--A-G----G-T--C-G---T--T-------C--AA-T-A---C-------T--A-C--CG--A-A-C----T-T-G-A-G-A--G---T-AG-----AA-G-A--------G-G-A-A---GA-T------GG--A--ATT----C-C-C-G-GT--GT-A-G-TG-GT--G---A--A-A---TG-C-GT-AG--AT-A-TC--G-G--G-A-G-G-A-AC-A-CC---AG--T--G--GC-GAA-G--G-C--G-------A--T-C-T-T---CTG--G--TC-T-A--------------------T-T---T-C-T--GA--CG-C-T-C--A-GA--C-G-CG-A--CA-G-C---T-AG--GG-T--AG-T-A-AA--------CG--GG Bacteria(100);Chloroflexi(100);Anaerolineae(100);Anaerolineales(100);Anaerolineaceae(100);uncultured(100);unclassified(100);unclassified(100);unclassified(100);

Otu0899 0 0 0 0 0 0 0 0 0 0 0 0 0 0 0 0 0 0 0 0 0 0 0 0 0 0 0 0
0 0 0 0 0 0 0 29 0 0 0 0 0 0 0 0 0 0 0 0 0 0 0 0 0 0 0 0 0
0 0 0 0 0 0 0 0 0 0 0 0 0 0 0 0 0 0 0 0 0 0 0 0 0 0 0 0 3
0 0 0 0 0 0 0 0 0 0 0 0 0 0 0 0 0 1 0 0 0 0 0 0 0 0 0
M02255_76_000000000-ADUNE_1_2107_8229_20926 T----AC---GT-AG-GTG--------GCA-A-G------C--G--T---T--GT-C-CGG-AT------TT-A--T-T--GG-GC-------GT--A-----AA-GC-GA-GC-------G-CA-G-G-C-G------------G--T-TC-C-T-T-----------AA----G-T-C-T--------G-A-T--G--TG--A-AA-GC--C-C-CC-G-G-----CT-C-AA------C-C-G-G-G-G-A--G-G----G-T--C-A---T--T-------G--GA-A-A---C-------T--G-G--GG--A-A-C----T-T-G-A-G-T--G---C-AG-----AA-G-A--------G-G-A-G---AG-T------GG--A--ATT----C-C-A-T-GT--GT-A-G-CG-GT--G---A--A-A---TG-C-GT-AG--AT-A-TA--T-G--G-A-G-G-A-AC-A-CC---AG--T--G--GC-GAA-G--G-C--G-------A--C-T-C-T---CTG--G--TC-T-G--------------------T-A---A-C-T--GA--CG-C-T-G--A-GG--C-T-CG-A--AA-G-C---G-TG--GG-T--AG-C-A-AA--------CA--GG Bacteria(100);Firmicutes(100);Bacilli(100);Lactobacillales(100);unclassified(100);unclassified(100);unclassified(100);unclassified(100);unclassified(100);

Otu0900 0 0 0 0 0 0 0 0 0 0 0 0 0 0 0 0 0 0 0 0 0 0 0 0 0 0 0 0
0 0 0 0 0 0 0 20 0 0 0 19 0 0 0 0 0 0 0 0 0 0 0 0 1 0 0 0 0
0 0 0 0 0 0 0 0 0 0 0 0 0 0 0 0 0 0 0 0 0 0 0 0 0 0 0 0 0
0 0 0 0 0 0 0 0 0 0 0 0 0 0 0 0 0 0 0 0 0 0 0 0 0 0 0
M02255_76_000000000-ADUNE_1_2101_15709_7300 G----AA---GC-CC-CGG--------CTA-A-C------T--C--C---G--TG-C-CAG-CA------GC-C--G-C--GG-TA-------AT--A-----CG-GG-GG-GG-------G-CG-A-G-C-G------------T--T-AT-TCG-G----------
AA----G-T-C-A--------T-G-G--G--TG--A-AA-TC--C-C-TC-G-G-----CT-C-AA------C-C-G-A-G-G-A--A-T----T-G--C-C---C--T-------T--GA-A-A---C-------T--G-C--GT--G-G-C----T-T-G-A-G-G--C---C-GG-----GA-G-G--------G-G-G-C---AG-T------GG--A--ATT----C-C-C-
A-GT--GT-A-G-CG-GT--G---A--A-A---TG-C-GT-AG--AT-A-TT--G-G--G-A-G-G-A-AC-A-CC---AG--T--G--GC-GAA-G--G-C--G-------G--C-T-G-C---CTG--G--AC-C-G--------------------G-T---T-C-T--GA--CG-C-T-G--A-TG--C-G-CG-A--AA-G-C---G-TG--GG-T--
AG-C-A-AA--------CA--GG Bacteria(100);unclassified(100);unclassified(100);unclassified(100);unclassified(100);unclassified(100);unclassified(100);unclassified(100);unclassified(100);

Otu0901 0 0 0 0 0 0 0 0 0 0 0 0 0 0 0 0 0 0 0 0 0 0 0 0 0 0 0 0
0 0 0 0 0 0 0 121 0 0 0 0 0 0 0 0 0 0 0 0 0 0 0 0 0 0 0 0 0
0 0 0 0 0 0 0 0 0 0 0 0 0 0 0 0 0 0 0 0 0 0 0 0 0 0 0 0 0
0 0 0 0 0 0 0 0 0 0 0 0 0 0 0 0 0 0 0 0 0 0 0 0 0 0 0
M02255_76_000000000-ADUNE_1_1118_26136_15453 G----AC---GT-AG-GAG--------GCG-A-G------C--G--T---T--AT-C-CGG-AT------TC-A--C-T--GG-GC-------GT--A-----AA-GC-GC-AT-------G-TA-G-G-C-G------------G--T-TG-T-C-T-----
------AA----G-T-C-C--------G-C-T--G--TT--A-AA-GC--C-C-CC-G-G-----CT-C-AA------C-T-G-G-G-G-G--A-G----G-T--C-A---G--T-------G--GA-G-A---C-------T--A-G--TC--G-A-C----T-T-G-A-G-G--G---C-AA-----CA-G-A--------G-G-C-A---CG-T------GG--A--ACT----
C-C-C-G-GT--GT-A-G-CG-GT--G---G--A-A---TG-C-GT-AG--AG-A-TC--G-G--G-A-A-G-A-AC-A-CC---AG--T--G--GC-GAA-G--G-C--G-------A--C-G-T-G---CTG--G--GT-T-G--------------------C-G---A-C-T--GA--CG-C-T-G--A-GA--T-G-CG-A--AA-G-T---G-TG--
GG-G--AG-C-A-AA--------CG--GG Bacteria(100);unclassified(100);unclassified(100);unclassified(100);unclassified(100);unclassified(100);unclassified(100);unclassified(100);unclassified(100);

Otu0902 0 0 0 0 0 0 0 0 0 0 0 0 0 0 0 0 0 0 0 0 0 0 0 0 0 0 0 0
0 0 0 0 0 0 0 26 0 0 0 0 0 0 0 0 0 0 0 0 0 0 0 0 0 0 0 0 0
0 0 0 0 0 0 0 0 0 0 0 0 0 0 0 0 0 0 0 0 0 0 0 0 0 0 0 0 0
0 0 0 0 0 0 0 0 0 0 0 0 0 0 0 0 0 0 0 0 0 0 0 0 0 0 0
M02255_76_000000000-ADUNE_1_1107_15597_25022 G----CA---AG-CA-CCG--------GCT-A-A------C--T--C---T--GTGC-CAG-CA------GC-C--G-C--GG-TA-------AG--A-----CA-GA-GG-GT-------G-CA-A-A-C-GTT-----------G--T-TC-G-G-A--
---------AT----G-T-C-G--------G-A-T--G--TG--A-AA-GC--C-C-TG-G-G-----CT-C-AA------C-C-C-A-G-G-A--A-G----T-G--C-A---T--T-------C--GA-A-A---C-------T--C-C--CG--A-G-C----T-T-G-A-G-T--T---C-CG-----GA-G-A--------G-G-A-A---GG-C------GG--A--ATT---
-C-T-C-G-GT--GT-A-G-AG-GT--G---A--A-A---TT-C-GT-AG--AT-A-TC--G-A--G-A-G-G-A-AC-A-CC---GG--T--G--GC-GAA-G--G-C--G-------G--C-C-T-T---CTG--G--AC-G-G--------------------T-G---A-C-T--GA--CG-C-T-G--A-GA--C-G-CG-A--AA-G-C---G-TG--
GG-G--AG-C-A-AA--------CA--GG Bacteria(100);Proteobacteria(100);Deltaproteobacteria(100);Myxococcales(100);Sorangiineae(100);Polyangiaceae(100);unclassified(100);unclassified(100);unclassified(100);

Otu0903 0 0 0 0 0 0 0 0 0 0 0 0 0 0 0 0 0 0 0 0 0 0 0 0 0 0 0 0
0 0 0 0 0 0 0 31 0 0 0 0 0 0 0 0 0 0 0 0 0 0 0 0 0 0 0 0 0
0 0 0 0 0 0 0 0 0 0 0 0 0 0 0 0 0 0 0 0 0 0 0 0 0 0 0 0 0
0 0 0 0 0 0 0 0 0 0 0 0 0 0 0 0 0 0 0 0 0 0 0 0 0 0 0
M02255_76_000000000-ADUNE_1_1106_8092_16029 C----GG---CT-AA-CTT--------CGT-G-C------C--A--G---C--AG-C-CGC-GG------TA-A--G-A--CG-AA-------GG--T-----GG-CA-AC-CG-------T-TG-T-T-C-G------------G--A-TA-C-G-T-----------AA-
---G-T-C-G--------G-G-T--G--TG--A-AA-TC--C-C-TC-G-G-----CC-T-AA------C-C-G-A-G-G-A--A-C----T-G--C-G---C--C-------C--GA-T-A---C-------T--G-C--GC--G-T-C----T-T-G-A-G-G--A---C-AG-----GA-G-G--------G-G-A-G---CG-T------GG--A--ATT----C-C-T-G-
GT--GG-A-G-CG-GT--G---A--A-A---TG-C-GT-TG--AT-A-TC--A-G--G-A-G-G-A-AC-A-CC---GG--C--G--GC-GAA-G--G-C--G-------A--C-G-C-T---CTG--G--TC-T-G--------------------T-T---T-C-T--GA--CG-C-T-G--A-GG--T-G-CG-A--AA-G-C---C-AG--GG-G--AG-
C-A-AA--------CG--GG Bacteria(100);unclassified(100);unclassified(100);unclassified(100);unclassified(100);unclassified(100);unclassified(100);unclassified(100);unclassified(100);

Otu0904 0 0 0 0 0 0 0 0 0 0 0 0 0 0 0 0 0 0 0 0 0 0 0 0 0 0 0 0
2 0 0 0 0 0 0 0 0 0 0 0 0 0 0 0 0 0 0 0 0 0 0 0 0 0 0 0 0
0 0 0 0 0 0 0 0 0 0 0 0 0 0 0 0 0 0 0 0 0 0 0 0 0 0 0 0 71
0 0 0 0 0 0 0 0 0 0 0 0 0 0 0 0 0 0 0 0 0 0 0 0 0 0 0
M02255_77_000000000-ADUT3_1_1111_6732_11243 T----AC---GT-AG-GGG--------GCA-A-G------C--G--T---T--AT-C-CGG-AA------TT-A--C-T--GG-GC-------GT--A-----AA-GG-GT-GC-------G-TA-G-G-C-G------------G--C-TT-A-G-T-----------
AA----G-T-C-A--------A-G-A--T--TT--A-AA-GG--C-A-GC-A-G-----CT-C-AA------C-T-G-C-T-G-T--T------C-G--G-T---C--T-------T--GA-A-A---C-------T--A-T--TG--A-G-C----T-T-G-A-G-T--A---T-AG-----GA-G-A--------G-G-A-A---AG-T------GG--A--ATT----C-C-C-
A-GT--GT-A-G-CG-GT--G---A--A-A---TG-C-GT-AG--AT-A-TT--G-G--G-A-G-G-A-AT-A-CC---GG--T--G--GC-GAA-G--G-C--G-------A--C-T-T-T---CTG--G--AC-T-A--------------------T-A---A-C-T--GA--CG-C-T-G--A-GG--C-A-CG-A--AA-G-C---G-TG--GG-T--AG-
C-A-AA--------CA--GG Bacteria(100);unclassified(100);unclassified(100);unclassified(100);unclassified(100);unclassified(100);unclassified(100);unclassified(100);unclassified(100);

Otu0905 0 0 0 0 0 0 0 0 0 0 0 0 0 0 0 0 0 0 0 0 0 0 0 0 0 0 0 0
0 0 0 0 0 0 0 41 0 0 0 0 0 0 0 0 0 0 0 0 0 0 0 0 0 0 0 0 0
0 0 0 0 0 0 0 0 0 0 0 0 0 0 0 0 0 0 0 0 0 0 0 0 0 0 0 0 0
0 0 0 0 0 0 0 0 0 0 0 0 0 0 0 0 0 0 0 0 0 0 0 0 0 0 0
M02255_76_000000000-ADUNE_1_1105_26108_17627 C----GG---CT-AA-TTC--------CGT-G-C------C--A--G---C--AG-C-CGC-GG------TA-A--TAC--GG-AA-------GG--G-----GC-AA-GC-GT---------TG-T-T-C-G------------G--T-CG-C-G-T-----
------AT----G-T-C-T--------G-C-T--G--TG--A-AA-GC--A-C-AC-G-G-----CT-T-AA------C-T-G-T-G-T-G----A----A-GT-C-G---G--T-------A--GA-A-A---C-------T--G-C--GA--G-A-C----T-T-G-A-G-G--C---T-GG-----GA-G-A--------G-G-T-T---GG-T------GG--A--ATT----
C-C-C-T-GT--GT-A-G-CG-GT--G---A--A-A---TG-C-GT-AG--AT-A-TA--G-G--G-G-A-G-A-AC-A-CT---CG--T--G--GC-GAA-G--G-C--G-------G--C-C-A-G---CTG--G--AC-C-A--------------------G-C---C-C-T--GA--CG-C-T-G--A-GG--C-G-CG-A--CA-G-C---G-TG--
GG-G--AG-C-A-AA--------CA--GG Bacteria(100);unclassified(100);unclassified(100);unclassified(100);unclassified(100);unclassified(100);unclassified(100);unclassified(100);unclassified(100);

Otu0906 0 0 0 0 0 0 0 0 0 0 0 0 0 0 0 0 0 0 0 0 0 0 0 0 0 0 0 0
0 0 0 0 0 0 0 163 0 0 0 0 0 0 0 0 0 0 0 0 0 0 0 0 0 0 0 0 0
0 0 0 0 0 0 0 0 0 0 0 0 0 0 0 0 0 0 0 0 0 0 0 0 0 0 0 0 0
0 0 0 0 0 0 0 0 0 0 0 0 0 0 0 0 0 0 0 0 0 0 0 0 0 0 0
M02255_76_000000000-ADUNE_1_1104_21714_16964 T----AC---GT-AG-GCG--------GCA-A-G------C--G--T---T--AC-C-CGG-AT------TT-A--T-T--GG-GC-------GT--A-----AA-GC-GT-GC-------G-CA-G-G-C-G------------G--T-TT-T-G-A------
-----AA----G-T-C-A--------T-C-A--T--TG--A-AA-TC--T-T-GG-G-G-----CT-C-AA------C-C-C-C-A-A-G--C-C----T-G--G-T---G--G-------T--GA-T-A---C-------T--T-C--AA--G-A-C----T-A-G-G-T-T--G---T-TT-----GA-G-G--------G-G-G-T---AC-C------GG--A--ACA----G-
T-T-G-GT--GG-A-G-CA-GT--G---A--A-A---TG-C-GT-TG--AT-A-TC--A-A--C-T-G-G-A-AC-A-CC---AA--A--G--GC-GAA-G--G-C--A-------G--G-T-A-C---CTG--G--CA-A-A--------------------C-T---A-A-C--GA--CG-C-T-G--T-TG--C-A-CG-A--AA-G-C---G-TG--GG-G-
-AG-C-A-AA--------AC--GG Bacteria(100);unclassified(100);unclassified(100);unclassified(100);unclassified(100);unclassified(100);unclassified(100);unclassified(100);unclassified(100);

Otu0907 0 0 0 0 0 0 0 0 0 0 0 0 0 0 0 0 0 0 0 0 0 0 0 0 0 0 0 0
1 0 0 0 0 0 0 2 0 0 0 0 0 0 0 0 0 0 0 0 0 0 0 0 0 0 20 0 0
0 0 0 0 0 0 0 0 0 0 0 0 0 0 0 0 0 0 0 0 0 0 0 0 0 0 0 0 0
0 0 0 0 0 0 0 0 0 0 0 0 0 0 0 0 0 0 0 0 0 0 0 0 0 0 0
M02255_76_000000000-ADUNE_1_2111_25601_17402 C----TA---AC-TC-CGT--------GCC-A-G------C--A--G---C--CG-C-GGT-AA------TA-C--G-G--AG-GG-------TG--C-----AA-GC-GT-TA-------T-CC-G-GAT-T------------T--A-TT-T-G-T------
-----AA----G-T-C-A--------G-T-G--G--TG--A-AA-TC--T-C-AC-A-G-----CT-T-AA------C-T-G-T-G-A-A--A-C----T-G--C-C---A--T-------T--GA-T-A---C-------T--G-C--AA--G-T-C----T-T-G-A-G-T--G---T-TG-----TT-G-A--------A-G-T-A---GC-T------GG--A--ATA----A-G-
T-A-GT--GT-A-G-CG-GT--G---A--A-A---TG-C-AT-AG--AT-A-TT--A-C--T-T-A-G-A-AC-A-CC---AA--T--T--GC-GAA-G--G-C--A-------G--G-T-T-A---CTA--A--GC-A-A--------------------C-A---A-C-T--GA--CG-C-T-G--A-TG--G-A-CG-A--AA-G-C---G-TG--GG-G--
AG-C-G-AA--------CA--GG Bacteria(100);Bacteroidetes(100);Flavobacteria(100);Flavobacteriales(100);Flavobacteriaceae(100);Chryseobacterium(100);unclassified(100);unclassified(100);unclassified(100);

Otu0908 0 0 0 0 0 0 0 0 0 0 0 0 0 0 0 0 0 0 0 0 0 0 0 0 0 0 0 0
0 0 0 0 0 0 0 17 0 0 0 0 0 0 0 0 0 0 0 0 0 0 0 0 0 0 0 0 0
0 0 0 0 0 0 0 0 0 0 0 0 0 0 0 0 0 0 0 0 0 0 0 0 0 0 0 0 0
0 0 0 0 0 0 0 0 0 0 0 0 0 0 0 0 0 0 0 0 0 0 0 0 0 0 0
M02255_76_000000000-ADUNE_1_1103_23005_13506 C----CG---GC-TA-ACT--------TCG-T-G------C--C--A---G--CA-G-CCG-CG------GT-A--A-T--AC-GA-------AG--G-----GT-GC-AA-AC-------G-TT-GCT-C-G------------G--C-AT-T-G-C-----
------AA----G-T-C-G--------G-A-T--G--TG--A-AA-TC--C-C-TC-G-G-----CT-T-AA------C-C-A-A-G-G-A--A-G----T-G--C-A---T--C-------C--GA-A-A---C-------T--G-C--CG--T-G-C----T-T-G-A-G-T--A---C-TT-----AA-G-A--------G-G-A-T---CG-C------GG--A--ATT----C-
C-C-G-GT--GT-A-G-AG-GT--G---A--A-A---TT-C-GT-AG--AT-A-TC--G-G--G-A-G-G-A-AC-A-CC---AG--T--G--GC-GAA-G--G-C--G-------G--C-G-A-T---CTG--G--GA-A-G--------------------A-T---A-C-T--GA--CG-C-T-G--A-GG--T-G-CG-A--AA-G-C---G-TG--GG-
G--AG-C-A-AA--------CA--GG Bacteria(100);Proteobacteria(100);Deltaproteobacteria(100);Myxococcales(100);Nannocystineae(100);Haliangiaceae(100);Haliangium(100);unclassified(100);unclassified(100);

Otu0909 0 0 0 0 0 0 0 0 0 0 0 0 0 0 0 0 0 0 0 0 0 0 0 0 0 0 0 0
0 0 0 0 0 0 0 20 0 0 0 0 0 0 0 0 0 0 0 0 0 0 0 0 0 0 0 0 0
0 0 0 0 0 0 0 0 0 0 0 0 0 0 0 0 0 0 0 0 0 0 0 0 0 0 0 0 0
0 0 0 0 0 0 0 0 0 0 0 0 0 0 0 0 0 0 0 0 0 0 0 0 0 0 0
M02255_77_000000000-ADUT3_1_1101_23575_14385 A----AC---GT-AG-GAG--------GCG-A-G------C--G--T---T--AT-C-CGG-AG------TT-A--C-T--GG-GC-------GT--A-----AA-GC-GT-GC-------G-TA-G-G-C-G------------G--G-TC-G-G-T----
-------AA----G-T-G-G--------G-T-C--G--TG--A-AA-GC--G-C-CG-G-G-----CT-A-AA------C-C-G-G-G-C-G--A-G----G-A--C-G---A--A-------C--TA-G-A---C-------T--G-C--CG--G-T-C----T-A-G-A-G-G--C---G-GA-----TA-G-A--------G-G-G-G---TG-C------GG--A--ATT--
--C-C-G-G-GT--GT-A-G-CG-GT--G---A--A-A---TG-T-GT-AG--AG-A-TC--C-G--G-A-G-G-A-AC-A-CC---AG--T--G--GT-GCA-A--A-C--G-------G--C-G-C-C---CTG--G--GT-C-C--------------------G-A---A-C-T--GA--CG-C-T-G--A-GG--C-A-CG-A--AA-G-C---G-TG--
GG-G--AG-C-G-AA--------CG--GG Bacteria(100);Chloroflexi(100);Anaerolineae(100);Anaerolineales(100);Anaerolineaceae(100);uncultured(100);unclassified(100);unclassified(100);unclassified(100);

Otu0910 0 0 0 0 0 0 0 0 0 0 0 0 0 0 0 0 0 0 0 0 0 0 0 0 0 0 0 0
0 181 0 0 0 0 0 0 0 0 0 0 0 0 0 0 0 0 0 0 0 0 0 0 0 0 0 0
0 0 0 0 0 0 0 0 0 0 0 0 0 0 0 0 0 0 0 0 0 0 0 0 0 0 0 0
0 0 0 0 0 0 0 3 0 32 0 0 0 0 0 0 0 0 0 0 0 0 0 0 0 0 0
M02255_77_000000000-ADUT3_1_1101_18351_12947 T----AC---GT-AG-GTG--------GCA-A-G------C--G--T---T--AT-C-CGG-AT------TT-A--C-T--GG-GT-------GT--A-----AA-GG-GC-GT-------G-TA-G-G-C-G------------G--G-AT-C-G-C-----
------AA----G-T-C-A--------G-A-T--G--TG--A-AA-AC--T-G-GA-G-G-----CT-C-AA------C-C-T-C-C-A-G--C-C----T-G--C-A---T--T-------T--GA-A-A---C-------T--G-T--GG--T-T-C----T-T-G-A-G-T--A---C-TG-----GA-G-A--------G-G-C-A---GA-C------GG--A--ATT----C-
C-T-A-GT--GT-A-G-CG-GT--G---A--A-A---TG-C-GT-AG--AT-A-TT--A-G--G-A-G-G-A-AC-A-CC---AG--T--G--GC-GAA-G--G-C--G-------G--T-C-T-G---CTG--G--AC-A-G--------------------C-A---A-C-T--GA--CG-C-T-G--A-GG--C-G-CG-A--AA-G-C---G-TG--GG-
G--AG-C-A-AA--------CA--GG Bacteria(100);Firmicutes(100);Clostridia(100);Clostridiales(100);unclassified(100);unclassified(100);unclassified(100);unclassified(100);unclassified(100);

Otu0911 0 0 0 0 0 0 0 0 0 0 0 0 0 0 0 0 0 0 0 0 0 0 0 0 0 0 0 0
0 0 0 0 0 0 0 36 0 0 0 0 0 0 0 0 0 0 0 0 0 0 0 0 0 0 0 0
0 0 0 0 0 0 0 0 0 0 0 0 0 0 0 0 0 5 0 0 0 0 0 0 0 0 0 0
0 0 0 0 0 0 0 0 0 0 0 0 0 0 0 0 0 0 0 0 0 0 0 0 0 0 0
M02255_76_000000000-ADUNE_1_1102_20855_12687 G----AC---GT-AG-GAT--------CCG-A-G------C--G--T---T--GT-C-CGG-AG------TT-A--C-T--GG-GC-------GT--A-----AA-------GC-------G-C----G-C----------------------G---C-----------G--
---G---C----------G------G--TG--A-AA-GC--C-C-CC-C-G-----CT-C-AA------C-G-G-G-G-G-A--G-G----G-T--C-C---G--T-------C--GA-G-A---T-------G--G-C--GT--G-T-C----T-G-G-A-G-G--C---A-GG-----GA-G-A--------G-G-C-C---AG-T------GG--A--ATT----C-C-G-G-
GT--GT-A-G-TG-GT--G---A--A-A---TG-C-GT-AG--AG-A-TC--C-G--G-A-G-G-A-AC-A-CC---AG--T--G--GC-GAA-G--G-C--G-------G--C-T-G-G---CTG--G--AC-C-T--------------------G-A---C-C-T--GA--CG-C-T-GA-G--G--C-G-CG-A--AG-G-C---G-TG--GG-G--AG-
C-G-AA--------CG--GG Bacteria(100);Chloroflexi(100);unclassified(100);unclassified(100);unclassified(100);unclassified(100);unclassified(100);unclassified(100);unclassified(100);

Otu0912 0 0 0 0 0 0 0 0 0 0 0 0 0 0 0 0 0 0 0 0 0 0 0 0 0 0 0 0
0 0 0 0 0 0 0 25 0 0 0 0 0 0 0 0 0 0 0 0 0 0 0 0 0 0 0 0
0 0 0 0 0 0 0 0 0 0 0 0 0 0 0 0 0 0 0 0 0 0 0 0 0 0 0 0
0 0 0 0 0 0 0 0 0 0 0 0 0 0 0 0 0 0 0 0 0 0 0 0 0 0 0
M02255_76_000000000-ADUNE_1_1101_24422_23499 T----AA---CT-A--CGT--------GCC-A-G------C--A--G---C--CG-C-GGT-AA------TA-C--G-T--AG-GG-------GG--C-----AA-GC-G--TT-------G----T-C-C-G------------G--A-CT-T-T-T----------
-AA----G-T-C-T--------G-A-T--G--TG--A-AA-GC--C-C-AC-G-G-----CT-T-AA------C-C-G-T-G-G-A--G------G-GT-C-A---T--T-------G--GA-A-A---C-------T--G-G--AG--G-A-C----T-T-G-A-G-T--A---C-AG-----AA-G-A--------G-G-A-G---AG-T------GG--A--ATT----C-C-
A-C-GT--GT-A-G-CG-GT--G---A--A-A---TG-C-GT-AG--AG-A-TG--T-G--G-A-G-G-A-AC-A-CC---AG--T--G--GC-GAA-G--G-C--G-------A--C-T-C-T---CTG--G--TC-T-G--------------------T-A---A-C-T--GA--CG-C-T-G--A-GG--T-G-CG-A--AA-G-C---G-TG--GG-G--
AG-C-G-AA--------CA--GG Bacteria(100);Firmicutes(100);Bacilli(100);Bacillales(100);Bacillaceae(100);unclassified(100);unclassified(100);unclassified(100);unclassified(100);

Otu0913 0 0 0 0 0 0 0 0 0 0 0 0 0 0 0 0 0 0 0 0 0 0 0 0 0 0 0 0
0 12 5 7 0 0 0 0 0 0 0 0 0 0 0 0 0 0 0 0 0 0 0 0 0 0 0 0
0 0 0 0 0 0 0 0 0 0 0 0 0 0 0 0 0 0 0 0 0 0 0 0 0 0 0 0
0 0 0 0 0 0 0 0 0 0 0 0 0 0 0 0 0 0 0 0 0 0 0 0 0 0 0
M02255_77_000000000-ADUT3_1_1113_20927_7747 C----AA---AC-TC-TGT--------GCC-A-G------C-----A---G--CC-G-CGG-TA------AT-A--C-A--GA-GG-------GT--G-----CA--A-GC-GT---------TG-T-T-C-G------------G--A-AT-T-G-A-----------CA----
G-T-C-A--------C-G-G--G--TG--A-AA-TC--C-C-CA-A-G-----CT-T-AA------C-T-T-G-G-G-A--A-C----T-G--C-C---T--G-------T--GA-G-A---C-------C--T-C--AG--T-A-C----T-A-G-A-G-T--G---T-GA-----GA-G-G--------G-G-A-T---AG-T------GG--A--ATA----C-C-C-A-GT-
-GT-A-G-CG-GT--G---A--A-A---TG-C-GT-AG--AG-A-TT--G-G--G-T-G-G-A-AC-A-CC---GG--T--G--GC-GAA-G--G-C--G-------G--C-T-A-T---CTG--G--CT-C-A--------------------C-A---A-C-T--GA--CG-A-T-C--A-GG--C-A-CG-A--AA-G-C---G-TG--GG-G--AG-C-A-
AA--------CA--GG Bacteria(100);unclassified(100);unclassified(100);unclassified(100);unclassified(100);unclassified(100);unclassified(100);unclassified(100);unclassified(100);

Otu0914 0 0 0 0 0 0 0 0 0 0 0 0 0 0 0 0 0 0 0 0 0 0 0 0 0 0 0 0
0 47 0 0 0 0 0 0 0 0 0 0 0 0 0 0 0 0 0 0 0 0 0 0 0 0 0 0
0 0 0 0 0 0 0 0 0 0 0 0 0 0 0 0 0 0 0 0 0 0 0 0 0 0 0 0
0 0 0 0 0 0 0 0 0 0 0 0 0 0 0 0 0 0 0 0 0 0 0 0 0 0 0
M02255_77_000000000-ADUT3_1_2106_26558_12956 C----GT---GC-CA-GCA--------GCC-G-C------G--G--T---A--AT-A-CGG-AG------GA-T--G-C--GA-GC-------GT--T-----AT-CC-GG-AT-------T-TA-G-G-T-G------------G--G-TT-G-T-T-----
------AA----G-T-C-A--------G-C-G--G--TG--A-AA-TG--T-T-TA-A-G-----CT-C-AA------C-T-T-A-G-A-C--A-C----T-G--C-C---G--T-------T--GA-A-A---C-------T--G-G--CG--A-G-C----T-T-G-A-G-T--G---C-AA-----AG-G-A--------G-G-T-A---GG-C------GG--A--ATT----C-
G-T-G-GT--GT-A-G-CG-GT--G---A--A-A---TG-C-AT-AG--AT-A-TC--A-C--G-A-G-G-A-AC-T-CC---GA--T--T--GC-GTA-G--G-C--A-------G--C-T-T-A---CTA--T--CA-T-G--------------------C-A---A-C-T--GA--CA-C-T-G--A-AG--C-A-CG-A--AA-G-C---G-TG--GG-T--
AT-C-A-AA--------CA--GG Bacteria(100);Bacteroidetes(100);Bacteroidia(100);Bacteroidales(100);unclassified(100);unclassified(100);unclassified(100);unclassified(100);unclassified(100);

Otu0915 0 0 0 0 0 0 0 0 0 0 0 0 0 0 0 0 0 0 0 0 0 0 0 0 0 0 0 0
0 0 0 0 0 0 53 0 0 0 0 0 0 0 0 0 0 0 0 0 0 0 0 0 0 0 0 0 0
0 0 0 0 0 0 0 0 0 0 0 0 0 0 0 0 0 0 0 0 0 0 0 0 0 0 0 0 0
0 0 0 0 0 0 0 0 0 0 0 0 0 0 0 0 0 0 0 0 0 0 0 0 0 0 0
M02255_77_000000000-ADUT3_1_1114_5051_17539 T----AC---GA-AG-GGG--------GCT-A-G------C--G--T---T--GC-T-CGG-AA------TC-A--C-T--GG-GC-------GT--A-----AA-GG-GC-GC-------G-TA-G-G-C-G------------G--A-TG-A-T-T----------
AA----G-T-C-G--------G-A-G--G--TG--A-AA-GC--C-C-AG-G-G-----CT-C-AA------C-C-C-T-G-G-A--A-T----T-G--C-C---T--T-------C--GA-T-A---C-------T--G-A--TC--G-T-C----T-C-G-A-G-A--A---T-GG-----AA-G-A--------G-G-T-A---AG-T------GG--A--ATA----C-C-G-
A-GT--GT-A-G-AG-GT--G---A--A-A---TT-C-GT-AG--AT-A-TT--C-G--G-T-G-G-A-AC-A-CC---AG--T--G--GC-GAA-G--G-C--G-------A--C-T-T-A---CTG--G--TC-C-A--------------------T-T---T-C-T--GA--CG-C-T-G--A-GG--C-G-CG-A--AA-G-C---G-TG--GG-G--AT-
C-A-AA--------CA--GG Bacteria(100);Proteobacteria(100);Alphaproteobacteria(100);unclassified(100);unclassified(100);unclassified(100);unclassified(100);unclassified(100);unclassified(100);

Otu0916 0 0 0 0 0 0 0 0 0 0 0 0 0 0 0 0 0 0 0 0 0 0 0 0 0 0 0 0
0 0 0 0 0 0 21 0 0 0 0 0 0 0 0 0 0 0 0 0 0 0 0 0 0 0 0 0 0
0 0 0 0 0 0 0 0 0 0 0 0 0 0 0 0 0 0 0 0 0 0 0 0 0 0 0 0 0
0 0 0 0 0 0 0 0 0 0 0 0 0 0 0 0 0 0 0 0 0 0 0 0 0 0 0
M02255_77_000000000-ADUT3_1_1112_6996_10726 T----AC---GT-AG-GGG--------GCA-A-G------C--G--T---T--GT-C-CGG-AT------TT-A--T-T--GG-GC-------GT--A-----AA-GA-GC-TC-------G-TA-G-G-C-G------------G--T-TT-G-G-T-----------AA-
---G-T-C-G--------G-A-T--G--TG--A-AA-TC--T-C-CA-G-G-----CT-C-AA------C-T-T-G-G-A-G--G------G-G-TC-A---T--T-------C--GA-T-A---C-------T--G-C--CA-TG-A-C----T-A-G-A-G-T--T---T-GG-----TA-G-G--------G-G-A-G---TG-T------GG--A--ATT----T-C-T-G-
GT--GG-A-G-CG-GT--G---A--A-A---TG-C-GC-AG--AT-A-TC--A-G--A-A-G-G-A-AC-A-CC---CG--T--A--GC-GAA-G--G-C--G-------G--C-A-C-T---CTG--G--GC-C-A--------------------T-A---A-C-T--GA--CG-C-T-G--A-GG--A-G-CG-A--AA-G-C---G-TG--GG-G--AG-
C-G-AA--------CA--GG Bacteria(100);Actinobacteria(100);Actinobacteria(100);unclassified(100);unclassified(100);unclassified(100);unclassified(100);unclassified(100);unclassified(100);

Otu0917 0 0 0 0 0 0 0 0 0 0 0 0 0 0 0 0 0 0 0 0 0 0 0 0 0 0 0 0
0 0 0 0 0 0 34 0 0 0 0 0 0 0 0 0 0 0 0 0 0 0 0 0 0 0 0 0 0
0 0 0 0 0 0 0 0 0 0 0 0 0 0 0 0 0 0 0 0 0 0 0 0 0 0 0 0 0
0 0 0 0 0 0 0 0 0 0 0 0 0 0 0 0 0 0 0 0 0 0 0 0 0 0 0
M02255_77_000000000-ADUT3_1_1103_6476_20314 T----AC---GT-AG-GGG--------GCG-A-G------C--G--T---T--GT-C-CGG-AT------TT-A--T-T--GG-GC-------GT--A-----AA-GA-GC-TC-------G-TA-G-G-C-G------------G--C-TT-A-G-C-----------AA-
---G-T-C-A--------G-G-T--G--TG--A-AA-AC--T-C-CA-G-G-----CT-C-AA------C-C-T-G-GAG-A---------C-G-CC-A---C--T-------T--GA-G-A---C-------T--G-C--TA-TG-G-C----T-A-G-A-G-T--C---C-GG-----TA-G-G--------G-G-A-C---CA-C------GG--A--ATT----C-C-T-G-
GT--GT-A-G-CG-GT--G---A--A-A---TG-C-GC-AG--AT-A-TC--A-G--G-A-G-G-A-AC-A-CC---AG--T--G--GC-GAA-G--G-C--G-------G--T-G-G-T---CTG--G--GC-C-G--------------------G-A---A-C-T--GA--CG-C-T-G--A-GG--A-G-CG-A--AA-G-C---G-TG--GG-T--AG-
C-A-AA--------CA--GG Bacteria(100);Actinobacteria(100);Actinobacteria(100);unclassified(100);unclassified(100);unclassified(100);unclassified(100);unclassified(100);unclassified(100);

Otu0918 0 0 0 0 0 0 0 0 0 0 0 0 0 0 0 0 0 0 0 0 0 0 0 0 0 0 0 0
0 0 0 0 0 0 20 0 0 0 0 0 0 0 0 0 0 0 0 0 0 0 0 0 0 0 0 0 0
0 0 0 0 0 0 0 0 0 0 0 0 0 0 0 0 0 0 0 0 0 0 0 0 0 0 0 0 0
0 0 0 0 0 0 0 0 0 0 0 0 0 0 0 0 0 0 0 0 0 0 0 0 0 0 0
M02255_77_000000000-ADUT3_1_2106_19145_10695 G----AC---GA-GG-GAT--------CCT-A-G------C--G--T---T--GC-T-CGG-AA------TC-A--T-T--GG-GC-------GT--A-----AA-GC-GG-GT-------G-TA-G-G-G-G------------G--C-TT-G-A-T----
-------AA----G-T-C-A--------G-G-T--G--TG--A-AA-TC--C-C-AG-G-G-----CT-C-AA------C-C-C-T-G-G-A--A-G----T-G--C-A---T--T-------T--GA-T-A---C-------T--G-T--TG--A-G-C----T-T-G-A-A-T--G---T-GG-----GA-G-A--------G-G-C-T---AC-T------AG--A--ATT----C-
C-T-G-GT--GT-A-G-TG-GT--G---A--A-A---TA-C-GT-AG--AT-A-TC--A-G--G-A-G-G-A-AT-T-CC---GG--A--G--GC-GAA-G--G-C--G-------G--G-T-A-G---CTG--G--CC-C-A--------------------A-C---A-T-T--GA--CC-C-T-G--A-GA--C-C-CG-A--AA-G-C---G-TG--GG-
G--AT-C-A-AA--------CA--GG Bacteria(100);Proteobacteria(100);Deltaproteobacteria(100);Bdellovibrionales(100);Bdellovibrionaceae(100);Bdellovibrio(100);unclassified(100);unclassified(100);unclassified(100);

Otu0919 0 0 0 0 0 0 0 0 0 0 0 0 0 0 0 0 0 0 0 0 0 0 0 0 0 0 0 0
0 1 2 0 0 0 1 0 2 0 0 0 1 3 0 0 2 3 0 0 0 0 0 0 0 0 0 0 0
0 0 0 0 0 0 0 0 0 0 0 0 0 0 0 0 0 0 0 0 0 0 1 0 0 0 0 0 0
0 1 0 0 1 0 0 0 0 0 0 0 0 0 0 0 0 0 0 0 2 0 0 0 0 0 0
M02255_76_000000000-ADUNE_1_2115_19484_17688 T----AC---GTGCC-AGC--------AGC-C-G------C--G--G---T-GAT-A-CGT-AG------GG-T--G-C--GA-GC------GTT--G-----TC-CG-GA-TT-------T-AT-T-G-G-G------------C--G-TA-A-A-G---
--------GG----C-T-C-G--------G-A-A--G--TG--T-AA-TC--T-T-GG-G-G-----CT-T-AA------C-C-C-T-G-A-G--C-G----T-G--C-T---T--T-------C--GA-T-A---C-------G--G-G--TT--G-A-C----T-T-G-A-G-G--A---A-GG-----TA-G-G--------G-G-A-G---AA-T------GG--A--ATT----
C-C-T-G-GT--GG-A-G-CG-GT--G---G--A-A---TG-C-GC-AG--AT-A-TC--A-G--G-A-G-G-A-AC-A-CC---AG--T--G--GC-GAA-G--G-C--G-------G--T-T-C-T---CTG--G--GC-C-T--------------------T-T---C-C-T--GA--CG-C-T-G--A-GG--A-G-CG-A--AA-G-C---G-TG--
GG-G--AG-C-G-AA--------CA--GG Bacteria(100);Actinobacteria(100);Actinobacteria(100);Actinobacteridae(100);Actinomycetales(100);Propionibacterineae(100);Propionibacteriaceae(100);Propionibacterium(100);unclassified(100);

Otu0920 0 0 0 0 0 0 0 0 0 0 0 0 0 0 0 0 0 0 0 0 0 0 0 0 0 0 0 0
0 1 4 0 0 0 0 0 0 0 79 0 0 0 0 0 0 0 0 0 0 0 0 0 0 0 0 0 0
0 0 0 0 0 0 0 0 0 0 0 0 0 0 0 0 0 0 0 0 0 0 0 0 0 0 0 0 0
0 0 0 0 0 0 0 0 0 0 0 0 0 0 0 0 0 0 0 0 0 0 0 0 0 0 0
M02255_77_000000000-ADUT3_1_1115_6901_19873 T----AC---GA-AG-GGG--------GCT-A-G------C--G--T---T--GT-T-CGG-AA------TT-A--C-T--GG-GC-------GT--A-----AA-GC-GC-AC-------G-TA-G-G-C-G------------G--A-CC-C-G-T-----------
AA----G-T-C-A--------G-A-G--G--TG--A-AA-GC--C-T-GG-A-G-----CT-T-AA------C-T-C-C-A-G-A--A-C----T-G--C-C---T--T-------T--GA-T-A---C-------T--G-C--GG--G-T-C----T-T-G-A-G-G--A---T-GG-----GA-G-A--------G-G-T-G---AG-T------GG--A--ACT----G-C-
G-A-GT--GT-A-G-AG-GT--G---A--A-A---TT-C-GT-AG--AT-A-TT--C-G--C-A-A-G-A-AC-A-CC---AG--T--G--GC-GAA-G--G-C--G-------G--C-T-C-A---CTG--G--CC-C-A--------------------T-T---A-C-T--GA--CG-C-T-G--A-GG--T-G-CG-A--AA-G-C---G-TG--GG-G--
AG-C-A-AA--------CA--GG Bacteria(100);Proteobacteria(100);Alphaproteobacteria(100);Rhizobiales(100);unclassified(100);unclassified(100);unclassified(100);unclassified(100);unclassified(100);

Otu0921 0 0 0 0 0 0 0 0 0 0 0 0 0 0 0 0 0 0 0 0 0 0 0 0 0 0 0 0
0 0 0 0 0 0 0 62 0 0 0 0 0 0 0 0 0 0 0 0 0 0 0 0 0 14 0 0 0
0 0 0 0 0 0 0 0 0 0 0 0 0 0 0 0 0 0 0 0 0 0 0 0 0 0 0 0 0
0 0 0 0 0 0 0 0 0 0 0 0 0 0 0 0 0 0 0 0 0 0 0 0 0 0 0
M02255_77_000000000-ADUT3_1_1109_8036_4281 G----AC---GG-AG-GAT--------GCA-A-G------C--G--T---T--AT-C-CGG-AA------TG-A--T-T--GG-GC-------GT--A-----AA-GC-GT-CT-------G-TA-G-G-T-G------------G--A-TT-G-T-A-----------AA-
---G-T-C-C--------T-C-T--G--TT--A-AA-GA--T-C-GG-G-G-----CT-T-AA------C-C-C-A-G-T-T--C-A----A-G--C-A---G--T-------G--GA-A-A---C-------T--T-A--TA--A-T-C----T-A-G-A-G-T--A---C-GG-----TA-G-G--------G-G-C-A---GA-G------GG--A--ATT----C-C-C-G-
GT--GT-A-G-CG-GT--G---A--A-A---TG-C-GT-AG--AT-A-TC--G-G--G-A-A-G-A-AC-A-CC---GA--C--A--GC-GAA-A--G-C--A-------C--T-C-T-G---CTG--G--GC-C-G--------------------A-A---A-C-T--GA--CA-C-T-A--A-GA--G-A-CG-A--AA-G-C---T-AG--GG-G--AG-
C-A-AA--------AA--GG Bacteria(100);Cyanobacteria(100);unclassified(100);unclassified(100);unclassified(100);unclassified(100);unclassified(100);unclassified(100);unclassified(100);

Otu0922 0 0 0 0 0 0 0 0 0 0 0 0 0 0 0 0 0 0 0 0 0 0 0 0 0 0 0 2
0 0 0 0 0 0 0 0 0 0 0 0 0 0 0 0 0 0 0 0 0 0 0 0 0 2 0 0 0
0 0 0 0 0 0 0 0 0 0 0 0 0 0 0 0 0 0 0 0 0 0 0 0 0 0 0 0 0
0 0 0 0 0 0 0 0 0 0 0 0 0 0 0 0 0 0 0 0 0 0 0 0 0 0 0
M02255_77_000000000-ADUT3_1_2101_10529_11214 T----AC---GT-AG-GTG--------TCA-A-G------C--G--T---T--GT-C-CGG-AT------TT-A--T-T--GG-GC-------GT--A-----AA-GC-GC-CC-------G-CA-G-G-C-G------------G--C-TT-G-C-C------
-----AA----G-T-T-C--------G-A-T--G--TG--A-AA-GC--T-C-CC-G-G-----CT-T-AA------C-T-G-G-G-A-G--G-G----T-G--C-A---T--C-------G--AA-A-A---C-------T--G-G--CA--G-G-C----T-T-G-A-G-G--C---A-GG-----GA-G-A--------G-G-G-A---TG-T------GG--A--ATT----
C-C-A-G-GT--GT-A-G-TG-GT--G---A--A-A---TG-C-GT-AG--AT-A-TC--T-G--G-A-G-G-A-AC-A-CC---AG--T--G--GC-GAA-G--G-C--G-------G--C-A-T-C---CTG--GC-CC---T--------------------G-T---C-C-T--GA--CG-C-T-C--A-GG--G-G-CG-A--AA-G-C---G-TG--GG-
G--AG-C-A-AA---------CC-GG Bacteria(100);Chloroflexi(100);unclassified(100);unclassified(100);unclassified(100);unclassified(100);unclassified(100);unclassified(100);unclassified(100);

Otu0923 0 0 0 0 0 0 0 0 0 0 0 0 0 0 0 0 0 0 0 0 0 0 0 0 0 0 0 2
0 0 0 0 0 0 0 0 0 0 0 0 0 0 0 0 0 0 0 0 0 0 0 0 0 0 0 0 0
0 0 0 0 0 0 0 0 0 0 0 0 0 0 0 0 0 8 0 0 0 0 0 0 0 0 0 0 0
0 0 0 0 0 0 0 0 0 0 0 0 0 0 0 0 0 0 0 0 0 0 0 0 0 0 0
M02255_77_000000000-ADUT3_1_2118_24183_16844 T----AC---GG-AG-GAT--------CCA-A-G------C--G--T---T--AT-T-CGG-AG------TG-A--C-T--GG-GC-------GT--A-----AA-GA-GTTGC-------G-TA-G-G-G-G------------G--T-TT-G-T-T----
-------AA----G-C-A-A--------A-T-A--G--TG--A-AA-TC--T-T-GT-G-G-----CT-C-AA------C-C-A-T-G-A-G--G-C----T-A--T-T---A--T-------T--TG-A-A---C-------T--G-G--CA--G-A-C----T-T-G-A-G-A--A---T-AT-----GA-G-A--------G-G-T-A---GA-T------GG--A--ATT----C-
C-C-A-GT--GT-A-G-GA-GT--G---A--A-A---TC-C-GT-AG--AT-A-TT--G-G--G-A-G-G-A-AC-A-CC---AA--T--G--GC-GTA-G--G-C--A-------G--T-C-T-A---CTG--G--CA-T-A--------------------T-T---T-C-T--GA--CC-C-T-G--A-GG--C-A-CG-A--AA-G-C---G-TG--GG-G--
AG-C-G-AA--------CC--GG Bacteria(100);unclassified(100);unclassified(100);unclassified(100);unclassified(100);unclassified(100);unclassified(100);unclassified(100);unclassified(100);

Otu0924 0 0 0 0 0 0 0 0 0 0 0 0 0 0 0 0 0 0 0 0 0 0 0 0 0 0 0 0
0 0 0 0 0 0 0 26 0 0 0 0 0 0 0 0 0 0 0 0 0 0 0 0 0 0 0 0 0
0 0 0 0 0 0 0 0 0 0 0 0 0 0 0 0 0 0 0 0 0 0 0 0 0 0 0 0 0
0 0 0 0 0 0 0 0 0 0 0 0 0 0 0 0 0 0 0 0 0 0 0 0 0 0 0
M02255_77_000000000-ADUT3_1_1115_21615_13288 T----AC---AG-AG-ACC--------TCA-A-G------C--G--T---T--GT-C-CGG-AT------TT-A--T-T--GG-GC-------GT--A-----AA-GA-GT-GC-------G-TA-G-G-T-G------------G--T-TA-G-T-T------
-----AA----G-T-C-A--------A-T-G--G--TC--A-AA-TC--T-C-CC-C-G-----CT-T-AA------C-G-G-G-G-A-A--A-C----T-G--C-T---G--T-------T--GA-A-A---C-------T--G-G--CT--G-A-C----T-T-G-A-G-T--C---T-GG-----AA-G-A--------G-G-C-A---AA-C------GG--A--ATT----G-
C-T-G-GT--GT-A-G-GG-GT--A---A--A-A---TC-C-GT-TG--AT-A-TC--A-G--C-A-G-G-A-AC-A-CC---AA--A--T--GC-GAA-A--G-C--A-------G--T-T-T-G---CTG--G--GA-C-A--------------------G-T---A-C-T--GA--CA-C-T-G--T-TG--C-A-CG-A--AA-G-C---G-TG--GG-G--
AT-C-A-AA--------GA--GG Bacteria(100);unclassified(100);unclassified(100);unclassified(100);unclassified(100);unclassified(100);unclassified(100);unclassified(100);unclassified(100);

Otu0925 0 0 0 0 0 0 0 0 0 0 0 0 0 0 0 0 0 0 0 0 0 0 0 0 0 0 0 3
0 0 0 0 0 0 0 0 0 0 0 0 0 0 0 0 0 0 0 0 0 0 0 0 0 0 0 0 0
0 0 0 0 0 0 0 0 0 0 0 0 0 0 0 0 0 0 0 0 0 0 2 0 0 0 0 0 0
0 0 0 0 0 0 0 0 2 0 0 0 0 0 0 0 0 0 0 0 0 0 0 0 0 0 0

M02255_76_000000000-ADUNE_1_2107_18840_10733 C----GG---CT-AA-CTA--------CGT-G-C------C--A--G---C--AG-C-CGC-GG------TA-A--T-A--CG-TA-------GG--G-----CG-CA-AG-CG-------T-TA-T-C-C-G------------G--T-TT-G-T-T------
-----GG----G-T-C-T--------G-C-C--G--TG--A-AA-GT--C-C-GG-G-G-----CT-C-AA------C-C-C-C-G-G-A--T-C----T-G--C-G---G--T-------G--GG-T-A---C-------G--G-G--CA--G-A-C----T-A-G-A-G-T--G---A-TG-----TA-G-G--------G-G-A-G---AC-T------GG--A--ATT----
C-C-T-G-GT--GT-A-G-CG-GT--G---A--A-A---TG-C-GC-AG--AT-A-TC--A-G--G-A-G-G-A-AC-A-CC---GA--T--G--GC-GAA-G--G-C--A-------G--G-T-C-T---CTG--G--GC-A-T--------------------T-A---A-C-T--GA--CG-C-T-G--A-GG--A-G-CG-A--AA-G-C---A-TG--
GG-G--AG-C-G-AA--------CA--GG Bacteria(100);Actinobacteria(100);Actinobacteria(100);Actinobacteridae(100);Actinomycetales(100);Micrococcineae(100);Micrococcaceae(100);unclassified(100);unclassified(100);

Otu0926 0 0 0 0 0 0 0 0 0 0 0 0 0 0 0 0 0 0 0 0 0 0 0 0 0 0 0 0
0 0 0 0 0 0 0 28 0 0 0 0 0 0 0 0 0 0 0 0 0 0 0 0 0 0 0 0 0
0 0 0 0 0 0 0 0 0 0 0 0 0 0 0 0 0 0 0 0 0 0 0 0 0 0 0 0 0
0 0 0 0 0 0 0 0 0 0 0 0 0 0 0 0 0 0 0 0 0 0 0 0 0 0 0

M02255_77_000000000-ADUT3_1_1114_4395_14222 T----AC---GT-AG-GTG--------GCA-A-G------C--G--T---T--GT-C-CGG-AT------TT-A--C-T--GG-GC-------GT--A-----AA-GG-GC-GT-------G-CA-G-G-C-G------------G--T-TC-G-T-T-----------AA-
---G-T-T-C--------G-A-G--G--TG--A-AA-GC--T-C-CC-G-G-----CT-C-AA------C-T-G-G-G-A-G--A-G----G-T--C-C---C--C-------G--GA-T-A---C-------T--G-G--CG--G-A-C----T-T-G-A-G-G--G---A-GG-----CA-G-A--------G-G-A-A---AG-T------GG--A--ATT----C-C-C-G-
GT--GT-A-G-TG-GT--G---A--T-A---TG-C-GT-AG--AT-A-TC--G-G--G-A-G-G-A-AC-A-CC---AG--T--G--GC-GAA-G--G-C--G-------G--C-T-T-T---CTG--G--GC-C-T--------------------T-A---C-C-T--GA--CG-C-T-G--A-GA--C-G-CG-A--AA-G-C---G-TG--GG-G--AG-C-
G-AA--------CC--GG Bacteria(100);Chloroflexi(100);S085(100);unclassified(100);unclassified(100);unclassified(100);unclassified(100);unclassified(100);unclassified(100);

Otu0927 0 0 0 0 0 0 0 0 0 0 0 0 0 0 0 0 0 0 0 0 0 0 0 0 0 0 0 0
0 0 0 0 0 0 0 21 0 0 0 0 0 0 0 0 0 0 0 0 0 0 0 0 0 0 0 0 0
0 0 0 0 0 0 0 0 0 0 0 0 0 0 0 0 0 0 0 0 0 0 0 0 0 0 0 0 0
0 0 0 0 0 0 0 0 0 0 0 0 0 0 0 0 0 0 0 0 0 0 0 0 0 0 0

M02255_77_000000000-ADUT3_1_1114_9520_11847 T----AC---GA-AT-GGG--------GCA-A-G------C--G--T---T--GT-T-CGG-AA------TA-A--C-T--GG-GC-------TT--A-----AA-GC-GT-GC-------G-TA-G-G-C-G------------G--C-CA-A-G-C-----------
AA----G-T-C-A--------G-A-A--G--TG--A-AA-TC--C-C-CG-G-G-----CT-T-AA------C-C-C-G-G-G-A--A-T----T-G--C-T---T--T-------T--GA-A-A---C-------T--G-C--AT--G-G-C----T-A-G-A-A-T--C---C-GG-----TA-G-A--------G-G-T-C---GG-C------AG--A--ATT----C-C-C-
A-GT--GT-A-G-AG-GT--G---A--A-A---TT-C-GC-AG--AT-A-TT--G-G--G-A-G-G-A-AT-A-CC---AG--T--G--GC-GTA-G--G-C--G-------G--C-C-G-A---CTG--G--AC-C-G--------------------G-T---T-T-T--GA--CG-C-T-G--A-GG--T-A-CG-A--AA-G-C---G-TG--GG-G--
AG-C-A-AA--------CA--GG Bacteria(100);Proteobacteria(100);unclassified(100);unclassified(100);unclassified(100);unclassified(100);unclassified(100);unclassified(100);unclassified(100);

Otu0928 0 0 0 0 0 0 0 0 0 0 0 0 0 0 0 0 0 0 0 0 0 0 0 0 0 0 0 3
0 0 0 0 0 0 0 53 0 0 0 0 0 0 0 0 0 0 0 0 0 0 0 0 0 0 0 0 0
0 0 0 0 0 0 0 0 0 0 0 0 0 0 0 0 0 0 0 0 0 0 0 0 0 0 0 0 0
0 0 0 0 0 0 0 0 0 0 0 0 0 0 0 0 0 0 0 0 0 0 0 0 0 0 0

M02255_77_000000000-ADUT3_1_1108_19702_8745 T----AC---GT-AG-GGG--------GCA-A-G------C--G--T---T--GT-C-CGG-AA------TT-A--T-T--GG-GC-------GT--A-----AA-GA-GC-TC-------G-TA-G-G-C-G------------G--C-TC-A-G-C-----------AA-
---G-T-C-G--------G-C-T--G--TG--A-AA-TC--C-C-GA-G-G-----CT-C-AA------C-C-T-C-G-G-A--A-C----T-G--C-A---G--T-------C--GA-T-A---CT------G--C-T--GT--G-G-C----T-A-G-A-G-T--C---T-GG-----TA-G-A--------G-G-A-G---AA-T------GG--A--ATT----C-C-C-G-
GT--GT-A-G-CG-GT--G---A--A-A---TG-C-GC-AG--AT-A-TC--G-G--G-A-G-G-A-AC-A-CC---AG--T--A--GC-GAA-G--G-C--G-------G--T-T-C-T---CTG--G--GC-C-A--------------------G-T---A-C-T--GA--CG-C-T-G--A-GG--A-G-CG-A--AA-G-C---G-TG--GG-G--AG-
C-A-AA--------CA--GG Bacteria(100);unclassified(100);unclassified(100);unclassified(100);unclassified(100);unclassified(100);unclassified(100);unclassified(100);unclassified(100);

Otu0929 0 0 0 0 0 0 0 0 0 0 0 0 0 0 0 0 0 0 0 0 0 0 0 0 0 0 0 0
0 0 0 0 0 0 109 0 0 0 0 0 0 0 0 0 0 0 0 0 0 0 6 0 0 0 0 0 0
0 0 0 0 0 0 0 0 0 0 0 0 0 0 44 4 0 0 0 0 0 0 0 0 0 0 0 0 0
0 0 0 0 0 0 0 0 0 0 0 0 0 0 0 0 0 0 0 0 0 0 0 0 0 0 0

M02255_77_000000000-ADUT3_1_2106_17287_14532 T----AC---GT-AT-GTC--------GCG-A-G------C--G--T---T--AT-C-CGG-AA------TT-A--T-T--GG-GC-------AT--A-----AA-GG-GC-AT-------C-TA-G-G-C-G------------G--C-AC-G-A-C------
-----AA----G-T-C-A--------G-G-G--G--TG--A-AA-AC--T-T-GC-G-G-----CT-C-AA------C-T-G-C-A-A-G--C-T----T-G--C-C---T--T-------T--GA-A-A---C-------T--G-T--AG--T-G-C----T-A-G-A-G-T--A---T-TG-----GA-A-A--------G-G-T-G---GG-C------GG--A--ACT----A-
C-A-C-GA--GT-A-G-AG-GT--G---A--A-A---TT-C-GT-AG--AT-A-TG--T-G--T-A-G-G-A-AT-G-CC---GA--T--G--AT-GAA-G--A-T--A-------G--C-T-C-A---CTG--G--AC-G-A--------------------T-A---A-C-T--GA--CG-C-T-G--A-AG--T-G-CG-A--AA-G-C---T-AG--GG-G-
-AG-C-G-AA--------CA--GG Bacteria(100);Fusobacteria(100);Fusobacteria(100);Fusobacteriales(100);Leptotrichiaceae(100);Leptotrichia(100);unclassified(100);unclassified(100);unclassified(100);

Otu0930 0 0 0 0 0 0 0 0 0 0 0 0 0 0 0 0 0 0 0 0 0 0 0 0 0 0 0 0
0 0 0 0 0 0 0 18 0 0 0 0 0 0 0 0 0 0 0 0 0 0 0 0 0 0 0 0 0
0 0 0 0 0 0 0 0 0 0 0 0 0 0 0 0 0 0 0 0 0 0 0 0 0 0 0 0 0
0 0 0 0 0 0 0 0 0 0 0 0 0 0 0 0 0 0 0 0 0 0 0 0 0 0 0
M02255_77_000000000-ADUT3_1_1109_13693_20753 C----AC---GT-AG-GGA--------CCA-A-G------C--G--T---T--GT-C-CGG-AT------TC-A--T-T--GG-GC-------GT--A-----AA-GA-GC-TC-------G-TA-G-G-C-G------------G--T-TC-G-A-T------
-----AA----G-T-C-G--------G-G-T--G--TG--A-AA-CC--T-C-CA-G-G-----CT-C-AA------C-C-T-G-GAG-A---------C-G-CC-A---C--C-------C--GA-T-A---C-------T--G-T--CG-TG-A-C----T-A-G-A-G-T--C---C-AG-----TA-G-G--------G-G-A-G---CG-T------GG--A--ATT----C-
C-T-G-GT--GT-A-G-CG-GT--G---A--A-A---TG-C-GC-AG--AT-A-TC--A-G--G-A-G-G-A-AC-A-CC---AG--C--G--GC-GAA-G--G-C--G-------G--C-G-C-T---CTG--G--GC-T-G--------------------G-A---A-C-T--GA--CG-C-T-G--A-GG--A-G-CG-A--AA-G-C---G-TG--GG-
G--AG-C-G-AA--------CA--GG Bacteria(100);Actinobacteria(100);Actinobacteria(100);Acidimicrobidae(100);Acidimicrobiales(100);Acidimicrobineae(100);unclassified(100);unclassified(100);unclassified(100);

Otu0931 0 0 0 0 0 0 0 0 0 0 0 0 0 0 0 0 0 0 0 0 0 0 0 0 0 0 0 6
0 0 0 0 0 0 0 0 0 0 0 0 0 0 0 0 0 0 0 0 0 0 0 0 0 0 0 0 0
0 0 0 0 0 0 0 0 0 0 0 0 0 0 0 0 0 0 0 0 0 0 0 0 0 0 0 0 0
0 0 0 0 0 0 0 0 0 0 0 0 0 0 0 0 0 0 0 0 0 0 0 0 0 0 0
M02255_77_000000000-ADUT3_1_1114_10619_20633 G----AC---GT-AG-GAT--------GCA-A-G------C--G--T---T--GT-C-CGG-AA------TT-A--T-T--GG-GC-------GT--A-----AA-GA-GT-TC-------G-TA-G-G-C-G------------G--T-TT-G-T-T------
-----AA----G-T-C-T--------G-G-T--G--TT--A-AA-GA--C-G-GG-A-G-----CT-C-AA------C-T-C-C-C-G-C--A--T---G-G--C-A---T--C-------G--GA-T-A---C-------T--G-G--CA--G-G-C----T-A-G-A-G-T--G---T-GG-----TA-G-A--------G-G-T-C---TC-G------GG--A--ATT----C-
C-A-G-GT--GT-A-G-CG-GT--G---A--A-A---TG-C-GT-AG--AT-A-TC--T-G--G-A-G-G-A-AC-A-CC---GG--T--G--GC-GAA-A--G-C--G-------C--G-A-G-A---CTG--G--GC-C-A--------------------T-A---A-C-T--GA--CG-C-T-G--A-GG--A-A-CG-A--AA-G-C---C-AG--GG-
G--AG-C-A-AA--------AG--GG Bacteria(100);unclassified(100);unclassified(100);unclassified(100);unclassified(100);unclassified(100);unclassified(100);unclassified(100);unclassified(100);

Otu0932 0 0 0 0 0 0 0 0 0 0 0 0 0 0 0 0 0 0 0 0 0 0 0 0 0 0 0 6
0 0 0 0 0 0 0 0 0 0 0 0 0 0 0 0 0 0 0 0 0 0 0 0 0 0 0 0 0
0 0 0 0 0 0 0 0 0 0 0 0 0 0 0 0 0 0 0 0 0 0 0 0 0 0 0 0 0
0 0 0 0 0 0 0 0 0 0 0 0 0 0 0 0 0 0 0 0 0 0 0 0 0 0 0
M02255_77_000000000-ADUT3_1_2104_7652_4899 T----AC---GA-AG-GGG--------GCT-A-G------C--G--T---T--GT-T-CGG-AA------TT-A--C-T--GG-GC-------GT--A-----AA-GC-GC-GC-------G-CA-G-G-C-G------------G--T-CC-G-T-C----------
AA----G-T-C-A--------G-G-G--G--TG--A-AA-GC--C-C-AG-A-G-----CT-C-AA------C-T-C-T-G-G-A--A-C----T-G--C-C---T--T-------T--GA-G-A---C-------T--G-T--AG--G-A-C----T-T-G-G-G-T--A---C-GG-----GA-G-A--------G-G-T-G---AG-T------GG--A--ATT----C-C-
G-A-GT--GT-A-G-AG-GT--G---A--A-A---TT-C-GT-AG--AT-A-TT--C-G--G-A-A-G-A-AC-A-CC---AG--T--G--GC-GAA-G--G-C--G-------G--C-T-C-A---CTG--G--CC-C-G--------------------T-T---A-C-C--GA--CG-C-T-C--A-GG--C-G-CG-A--CA-G-C---G-TG--GG-G--
AG-C-A-AA--------CA--GG Bacteria(100);Proteobacteria(100);Alphaproteobacteria(100);unclassified(100);unclassified(100);unclassified(100);unclassified(100);unclassified(100);unclassified(100);

Otu0933 0 0 0 0 0 0 0 0 0 0 0 0 0 0 0 0 0 0 0 0 0 0 0 0 0 0 0 0
0 0 0 0 0 0 0 63 0 0 0 0 0 0 0 0 0 0 0 0 0 0 0 0 0 0 0 0 0
0 0 0 0 0 0 0 0 0 0 0 0 0 0 0 0 0 0 0 0 0 0 0 0 0 0 0 0 0
0 0 0 0 0 0 0 0 0 0 0 0 0 0 0 0 0 0 0 0 0 0 0 0 0 0 0
M02255_77_000000000-ADUT3_1_1109_7369_18530 T----AC---GA-AG-GGG--------GCT-A-G------C--G--T---T--GT-T-CGG-AT------TT-A--C-T--GG-GC-------GT--A-----AA-GC-GC-GC-------G-TA-G-G-C-G------------G--G-TC-G-T-T----------AA-
---G-T-C-G--------G-G-G--G--TG--A-AA-TC--C-C-GA-G-G-----CT-C-AA------C-C-T-C-G-G-A--A-C----T-G--C-C---T--T-------C--GA-T-A---C-------T--G-G--CG--A-C-C----T-A-G-A-G-T--C---C-GG-----GA-G-A--------G-G-T-G---TA-T------GG--A--ACT----C-C-T-A-
GT--GT-A-G-AG-GT--G---G--A-A---TT-C-GT-AG--AT-A-TT--A-G--G-A-A-G-A-AC-A-CC---GG--T--G--GC-GAA-G--G-C--G-------A--T-A-C-A---CTG--G--CC-C-G--------------------G-T---A-C-T--GA--CG-C-T-G--A-GG--C-G-CG-A--AA-G-C---G-TG--GG-G--AG-
C-A-AA--------CA--GG Bacteria(100);Proteobacteria(100);Alphaproteobacteria(100);Rhizobiales(100);unclassified(100);unclassified(100);unclassified(100);unclassified(100);unclassified(100);

Otu0934 0 0 0 0 0 0 0 0 0 0 0 0 0 0 0 0 0 0 0 0 0 0 0 0 0 0 0 0
0 0 0 0 0 0 0 0 0 0 45 0 0 0 0 0 0 0 0 0 0 0 0 0 0 0 0 0 0
0 0 0 0 0 0 0 0 0 0 0 0 0 0 0 0 0 0 0 0 0 0 0 0 0 0 0 0 0
0 0 0 0 0 0 0 0 0 0 0 0 0 0 0 0 0 0 0 0 0 0 0 0 0 0 0
M02255_77_000000000-ADUT3_1_1103_8784_15915 T----AC---AG-AG-GCC--------CCA-A-G------C--A--T---T--AT-C-CGG-AA------TC-A--C-T--GG-GC-------GT--A-----AA-GG-GT-GT-------G-TA-G-G-C-G------------G--T-TA-T-G-T----------TA--
--G-T-C-T--------T-T-T--G--TG--A-AAGGT--C-T-TG-G-G-----CT-T-AA------C-C-C-A-G-G-A--A-C----C-G--C-A---G--G-------G--GA-A-A---C-------G--G-C--AT--A-A-C----TTA-G-A-G-G--A---T-GT-----GA-G-A--------G-G-T-A---AA-G------GG--A--ACT----C-A-T-G-
GT--GT-A-G-GG-GT--G---A--A-A---TC-C-GT-TG--AT-A-TC--A-T--G-G-G-G-A-AC-A-CC---AA--A--T--GC-GAA-G--G-C--A-------C--T-T-T-A---CTG--G--CA-C-A--------------------C-T---C-C-T--GA--CG-C-T-G--A-GA--C-A-CG-A--AA-G-C---G-TG--GG-A--AT-C-
G-AA--------CG--GG Bacteria(100);Candidate_division_OD1(100);unclassified(100);unclassified(100);unclassified(100);unclassified(100);unclassified(100);unclassified(100);unclassified(100);

Otu0935 0 0 0 0 0 0 0 0 0 0 0 0 0 0 0 0 0 0 0 0 0 0 0 0 0 0 0 2
0 0 0 0 0 0 0 0 0 3 0 0 0 0 0 0 0 0 0 0 0 0 0 0 0 0 0 0 0
0 0 0 0 0 0 0 0 0 0 0 0 0 0 0 0 0 0 0 0 0 0 0 0 0 0 0 0 0
0 0 0 0 0 0 0 0 0 0 0 0 0 0 0 0 0 0 0 0 0 0 0 0 0 0 0
M02255_76_000000000-ADUNE_1_1111_24863_23515 T----AC---GA-AG-GGG--------GCT-A-G------C--G--T---T--GC-T-CGG-AA------TC-A--C-T--GG-GC-------GT--A-----AA-G--G--GC-------------G-C-----------------T-G------T----------
AG----G-T-C-G--------G-G-C--G--TG--A-AA-TC--C-C-CG-G-G-----CT-C-AA------C-C-C-G-G-G-G--G-C----T-G--C-G---C--C-------C--GA-A-A---C-------G--G-C--GG--G-G-C----T-G-G-A-G-G--C---A-AG-----GA-G-A--------G-G-C-T---CG-C------GG--A--ATT----C-C-
C-A-GT--GT-A-G-AG-GT--G---A--A-A---TT-C-GT-AG--AT-A-TT--G-G--G-A-A-G-A-AC-A-CC---GG--T--G--GC-GAA-G--G-C--G-------G--C-G-A-G---CTG--G--CC-T-T--------------------G-A---A-C-T--GA--CG-C-T-G--A-GG--C-G-CG-A--CA-G-C---G-TG--GG-G--
AG-C-A-AA--------CA--GG Bacteria(100);Proteobacteria(100);Alphaproteobacteria(100);Rhodospirillales(100);unclassified(100);unclassified(100);unclassified(100);unclassified(100);unclassified(100);

Otu0936 0 0 0 0 0 0 0 0 0 0 0 0 0 0 0 0 0 0 0 0 0 0 0 0 0 0 0 0
0 0 0 0 0 0 0 85 0 0 0 0 0 0 0 0 0 0 0 0 0 0 0 0 0 0 0 0 0
0 0 0 0 0 0 0 0 0 0 0 0 0 0 0 0 0 0 0 0 0 0 0 0 0 0 0 0 0
0 0 0 0 0 0 0 0 0 0 0 0 0 0 0 0 0 0 0 0 0 0 0 0 0 0 0
M02255_77_000000000-ADUT3_1_1107_18767_9698 T----AC---GT-AG-GTG--------GCA-A-G------C--G--T---T--AT-C-CGG-AA------TT-A--C-T--GG-GC-------GT--A-----AA-GC-GC-AC-------G-CA-G-G-C-G------------G--T-CT-G-T-T-----------AA-
---G-T-T-T--------G-A-C--G--TG--A-AA-GC--T-C-CC-G-G-----CT-T-AA------C-T-G-G-G-A-G--A-G----G-T--C-G---T--T-------G--AA-G-A---C-------T--G-G--CA--G-A-C----T-T-G-A-G-G--C---A-AT-----GA-G-A--------G-G-G-G---TG-T------AG--A--ATT----C-C-A-G-
GT--GT-A-G-TG-GT--G---G--A-A---TG-C-GT-AG--AT-A-TC--T-G--G-A-G-G-A-AT-A-CC---AG--T--G--GC-GAA-A--G-C--G-------G--C-A-C-C---CTA--G--CG-T-T--------------------G-T---T-C-T--GA--CG-C-T-C--A-TG--T-G-CG-A--AA-G-C---G-TG--GG-G--AG-C-
G-AA--------CG--GG Bacteria(100);Chloroflexi(100);Caldilineae(100);Caldilineales(100);Caldilineaceae(100);Caldilinea(100);unclassified(100);unclassified(100);unclassified(100);

Otu0937 0 0 0 0 0 0 0 0 0 0 0 0 0 0 0 0 0 0 0 0 0 0 0 0 0 0 0 0
0 0 0 0 0 0 0 108 0 0 23 27 0 0 0 0 0 0 0 0 0 0 0 0 0 0 0 0 0
0 0 0 0 0 0 0 0 0 0 0 0 0 0 0 0 0 0 0 0 0 0 0 0 0 0 0 0 0
0 0 0 0 0 0 0 0 0 0 0 0 0 0 0 0 0 0 0 0 0 0 0 0 0 0 0
M02255_77_000000000-ADUT3_1_1105_21113_20753 G----AC---GG-AG-GGT--------GCT-A-G------C--G--T---T--GT-T-CGG-AA------TC-A--C-T--GG-GC-------GT--A-----AA-GG-GC-GC-------G-TA-G-G-C-G------------G--C-CC-A-T-G----
-------CA----G-T-C-G--------G-T-TC-G--TG--A-AA-GC--C-C-GG-G-G-----CT-C-AA------C-C-C-C-G-G-A--G-G----T-G--C-G---G--C-------C--GA-A-A---C-------A--C-A--TG--G-G-C----T-G-G-A-G-G--C---C-GG-----AA-G-A--------G-G-A-G---GG-T------GG--A--ATA-
---C-C-C-A-GT--GT-A-G-AG-GT--G---A--A-A---TT-C-GT-AG--AT-A-TT--G-G--G-T-G-G-A-AC-A-CC---GG--T--G--GC-GAA-A--G-C--G-------G--C-T-C-T---CTG--G--TC-C-G--------------------G-T---C-C-T--GA--CG-C-T-G--A-GG--C-G-CG-A--AA-G-C---G-T-G-
GG-G--AG-C-A-AA--------CA--GG Bacteria(100);Proteobacteria(100);Alphaproteobacteria(100);Rhodospirillales(100);unclassified(100);unclassified(100);unclassified(100);unclassified(100);unclassified(100);

Otu0938 0 0 0 0 0 0 0 0 0 0 0 0 0 0 0 0 0 0 0 0 0 0 0 0 0 0 0 0
0 0 0 0 0 0 0 60 0 0 0 0 0 0 0 0 0 0 0 0 0 0 0 0 0 0 0 0 0
0 0 0 0 0 0 0 0 0 0 0 0 0 0 0 0 0 0 0 0 0 0 0 0 0 0 0 0 0
0 0 0 0 0 0 0 0 0 0 0 0 0 0 0 0 0 0 0 0 0 0 0 0 0 0 0
M02255_77_000000000-ADUT3_1_1104_6490_8922 T----AC---GT-AG-GGC--------GCA-A-G------C--G--T---T--GT-C-CGG-AA------TT-A--T-T--GG-GC-------GT--A-----AA-GA-GC-TC-------G-TA-G-G-T-G------------G--C-TG-G-T-C-----------GC-
---G-T-C-T--------G-C-C--G--TG--A-AA-GC--C-C-GC-A-G-----CT-T-AA------C-T-G-C-G-G-G--T-C----T-G--C-G---G--T-------G--GA-T-A---C-------G--G-G--CC--G-G-C----T-A-G-A-G-G--T---A-GG-----TA-G-G--------G-G-C-A---AG-T------GG--A--ATT----C-C-T-G-
GT--GT-A-G-CG-GT--G---A--A-A---TG-C-GC-AG--AT-A-TC--A-G--G-A-G-G-A-AC-A-CC---GG--T--G--GC-GAA-G--G-C--G-------G--C-T-T-G---CTG--G--GC-C-T--------------------T-A---C-C-T--GA--CG-C-T-G--A-GG--A-G-CG-A--AA-G-C---G-TG--GG-G--AG-
C-G-AA--------CA--GG Bacteria(100);Actinobacteria(100);Actinobacteria(100);Actinobacteridae(100);Actinomycetales(100);Streptosporangineae(100);Streptosporangiaceae(100);Nonomuraea(100);unclassified(100);

Otu0939 0 0 0 0 0 0 0 0 0 0 0 0 0 0 0 0 0 0 0 0 0 0 0 0 0 0 0 0
0 0 0 0 0 0 0 62 0 0 0 0 0 0 0 0 0 0 0 0 0 0 0 0 0 0 0 0 0
0 0 0 0 0 0 0 0 0 0 0 0 0 0 0 0 0 0 0 0 0 0 0 0 0 0 0 0 0
0 0 0 0 0 0 0 0 0 0 0 0 0 0 0 0 0 0 0 0 0 0 0 0 0 0 0
M02255_77_000000000-ADUT3_1_1103_14243_25140 T----AC---GT-AG-GTG--------GCA-A-G------C--G--T---T--GT-C-CGG-AT------TT-A--C-T--GG-GC-------GT--A-----AA-GC-GC-GT-------G-TA-G-G-T-G------------G--T-TT-G-G-A-----
------AA----G-T-C-C--------G-G-G--G--TG--A-AA-TC--T-T-GC-G-G-----CT-C-AA------C-T-G-C-A-A-G--A-G----G-T--C-C---T--T-------G--GA-T-A---C-------T--T-C--CA--G-A-C----T-T-G-A-G-G--G---A-GG-----CA-G-A--------G-G-A-A---GG-T------GG--A--ATT----
C-C-C-G-GT--GT-A-G-TG-GT--G---A--A-A---TG-C-GT-AG--AT-A-TC--G-G--G-A-G-G-A-AC-A-CC---AG--T--G--GC-GAA-G--G-C--G-------G--C-C-T-T---CTG--G--GC-C-T--------------------T-T---C-C-T--GA--CG-C-T-G--A-GG--C-G-CG-A--AA-G-C---G-TG--GG-
T--AG-C-A-AA--------CC--GG Bacteria(100);unclassified(100);unclassified(100);unclassified(100);unclassified(100);unclassified(100);unclassified(100);unclassified(100);unclassified(100);

Otu0940 0 0 0 0 0 0 0 0 0 0 0 0 0 0 0 0 0 0 0 0 0 0 0 0 0 0 0 0
0 0 0 0 0 0 0 36 0 0 0 0 0 0 0 0 0 0 0 0 0 0 0 0 0 0 0 0 0
0 0 0 0 0 0 0 0 0 0 0 0 0 0 0 0 0 0 0 0 0 0 0 0 0 0 0 0 0
0 0 0 0 0 0 0 0 0 0 0 0 0 0 0 0 0 0 0 0 0 0 0 0 0 0 0

M02255_77_000000000-ADUT3_1_1103_9123_13953 G----AC---AG-AG-GTG--------CCA-A-G------C--G--T---T--AG-G-CGG-AA------TC-A--C-T--GG-GC-------TT--A-----AA-GC-GT-GT-------G-TA-G-G-C-G------------G--A-CG-G-C-T----------AA----G-T-G-T--------C-T-T--G--TG--A-AA-TC--C-C-AC-A-G-----CT-T-AA------C-T-G-T-G-G-A--A-C----T-G--C-T---C--G-------A--CA-T-A---C-------T--G-G--TC--G-T-C----T-T-G-A-G-C--A---A-CC-----TA-G-G--------G-G-C-G---AG-C------GG--A--ACA----A-G-T-G-GT--GG-A-G-CG-GT--G---A--A-A---TG-C-GT-AG--AT-A-TC--A-C--T-T-G-G-A-AC-G-CC---AA--T--G--GT-GAA-A--A-C--A-------G--C-T-C-G---CTG--G--GG-G-T--------------------T-T---G-C-T--GA--CG-C-T-G--A-GA--C-A-CG-A--AA-G-C---C-AG--GG-G--AG-C-A-AA--------CG--GG Bacteria(100);Planctomycetes(100);Phycisphaerae(100);WD2101(100);unclassified(100);unclassified(100);unclassified(100);unclassified(100);unclassified(100);

Otu0941 0 0 0 0 0 0 0 0 0 0 0 0 0 0 0 0 0 0 0 0 0 0 0 0 0 0 0 0
2 0 0 0 0 0 0 0 0 0 0 0 0 0 0 0 0 0 0 0 0 0 0 0 0 0 0 0 0
0 0 0 0 0 0 0 0 0 0 0 0 0 0 0 0 0 0 0 0 0 0 0 0 0 0 0 0 0
0 0 0 0 0 0 0 0 0 0 0 0 0 0 0 0 0 0 0 0 0 0 0 1 0 0 0

M02255_76_000000000-ADUNE_1_2115_2556_16708 T----GC---AG-AA-TAA--------GCA-C-C------G--G--C---T--AA---C-T-CT------GT-G--C-C--AG-CA-------GC--C-----GC-GG-TA-AT-------A-CA-G-A-G-G------------G--T-GC-T-A-A----------GC----G-T-T-A--------G-A-T--G--GG--A-AA-TC--C-C-CG-G-G-----CT-T-AA------C-C-T-G-G-G-A--A-C----T-G--C-A---T--C-------T--GA-A-A---C-------T--G-T--AG--A-G-C----T-A-G-A-G-T--A---T-GT-----GA-G-A--------G-G-A-A---GG-T------AG--A--ATT----C-C-A-G-GT--GT-A-G-CG-GT--G---A--A-A---TG-C-GT-AG--AG-A-TC--T-G--G-A-G-G-A-AT-A-CC---GA--T--G--GC-GAA-G--G-C--A-------G--C-C-T-T---CTG--G--CA-T-A--------------------A-T---A-C-T--GA--CA-C-T-G--A-GG--C-T-CG-A--AA-G-C---G-TG--GG-T--AG-C-A-AA--------CA--GG Bacteria(100);Proteobacteria(100);Gammaproteobacteria(100);Pseudomonadales(100);Moraxellaceae(100);unclassified(100);unclassified(100);unclassified(100);unclassified(100);

Otu0942 0 0 0 0 0 0 0 0 0 0 0 0 0 0 0 0 0 0 0 0 0 0 0 0 0 0 0 0
27 0 0 0 0 0 0 0 0 0 0 0 0 0 0 0 0 0 0 0 0 0 0 0 0 0 0 0 0
0 0 0 0 0 0 0 0 0 0 0 0 0 0 0 0 0 0 0 0 0 0 0 0 0 0 0 0 0
6 0 0 0 0 0 0 8 0 0 0 0 0 34 0 0 0 0 0 0 0 0 0 0 0 0 0

M02255_77_000000000-ADUT3_1_1104_17099_16574 T----AC---GT-AG-GGG--------GCA-A-G------C--G--T---T--AT-C-CGG-AT------TT-A--C-T--GG-GT-------GT--A-----AA-GG-GA-GC-------G-TA-G-A-C-G------------G--C-AG-T-G-C-----------AA----G-T-C-T--------G-A-A--G--TG--A-AA-GC--C-C-GG-G-G-----CT-C-AA------C-C-C-C-G-G-G--A-C----T-G--C-T---T--T-------G--GA-A-A---C-------T--G-T--GC--A-G-C----T-A-G-A-G-T--G---T-CG-----GA-G-A--------G-G-C-A---AG-C------GG--A--ATT----C-C-T-A-GT--GT-A-G-CG-GT--G---A--A-A---TG-C-GT-AG--AT-A-TT--A-G--G-A-G-G-A-AC-A-CC---AG--T--G--GC-GAA-G--G-C--G-------G--C-T-T-G---CTG--G--AC-G-A--------------------T-G---A-C-T--GA--CG-T-T-G--A-GG--C-T-CG-A--AA-G-C---G-TG--GG-G--AG-C-A-AA--------CA--GG Bacteria(100);Firmicutes(100);Clostridia(100);Clostridiales(100);Lachnospiraceae(100);unclassified(100);unclassified(100);unclassified(100);unclassified(100);

Otu0943 0 0 0 0 0 0 0 0 0 0 0 0 0 0 0 0 0 0 0 0 0 0 0 0 0 0 0 0
0 0 0 0 0 0 0 50 0 0 0 0 0 0 0 0 0 0 0 0 0 0 0 0 0 0 0 0 0
0 0 0 0 0 0 0 0 0 0 0 0 0 0 0 0 0 0 0 0 0 0 0 0 0 0 0 0 0
0 0 0 0 0 0 0 0 0 0 0 0 0 0 0 0 0 0 0 0 0 0 0 0 0 0 0

M02255_77_000000000-ADUT3_1_1102_7831_12093 T----AC---GA-AG-GGG--------GCT-A-G------C--G--T---T--GT-T-CGG-AT------TT-A--C-T--GG-GC-------GT--A-----AA-GC-GC-GC-------G-TA-G-G-C-G------------G--G-CA-T-T-T----------AA----G-T-C-A--------G-G-G--G--TG--A-AA-TC--C-C-AG-G-G-----CT-C-AA------C-C-C-T-G-G-A--A-C----G-G--C-C---T--T-------T--GA-T-A---C-------T--G-G--AT--G-C-C----T-G-G-A-G-A--T---C-GG-----GA-G-A--------G-G-T-T---GG-T------GG--A--ATT----C-C-G-A-GT--GT-A-G-AG-GT--G---A--A-A---TT-C-GT-AG--AT-A-TT--C-G--G-A-G-G-A-AC-A-CC---AG--A--A--GC-GAA-G--G-C--G-------G--C-C-A-A---CTG--G--CC-C-G--------------------A-T---A-C-T--GA--CG-C-T-G--A-GG--C-G-CG-A--AA-G-C---G-TG--GG-G--AG-C-A-AA--------CA--GG Bacteria(100);Proteobacteria(100);Alphaproteobacteria(100);unclassified(100);unclassified(100);unclassified(100);unclassified(100);unclassified(100);unclassified(100);

Otu0944 0 0 0 0 0 0 0 0 0 0 0 0 0 0 0 0 0 0 0 0 0 0 0 0 0 0 0 0
0 0 0 0 0 0 0 145 0 0 0 0 0 0 0 0 0 0 0 0 0 0 0 0 0 0 0 0 0
0 0 0 0 0 0 0 0 0 0 0 0 0 0 0 0 0 0 0 0 0 0 0 0 0 0 0 0 0
0 0 0 0 0 0 0 0 0 0 0 0 0 0 0 0 0 0 0 0 0 0 0 0 0 0 0

M02255_77_000000000-ADUT3_1_1102_7130_8250 T----AC---GG-AG-GGG--------ACT-A-G------C--G--T---T--GT-T-CGG-AA------TT-A--C-T--GG-GC-------GT--A-----AA-GC-GC-GC-------G-TA-G-G-C-G------------G--A-TT-T-G-C----------AA----G-T-C-G--------G-G-G--G--TG--A-AA-GC--C-C-GA-G-G-----CT-C-AA------C-C-T-C-G-G-A--A-C----T-G--C-C---T--T-------C--GA-T-A---C-------T--G-C--AA--A-T-C----T-C-G-A-G-T--C---C-GA-----GA-G-A--------G-G-T-G---AG-T------GG--A--ATT----C-C-T-A-GT--GT-A-G-AG-GT--G---A--A-A---TT-C-GT-AG--AT-A-TT--A-G--G-A-A-G-A-AC-A-CC---AG--T--G--GC-GAA-G--G-C--G-------G--C-T-C-A---CTG--G--CT-C-G--------------------G-T---A-C-T--GA--CG-C-T-G--A-GG--C-G-CG-A--AA-G-C---G-TG--GG-G--AG-C-A-AA--------CA--GG Bacteria(100);Proteobacteria(100);Alphaproteobacteria(100);Rhizobiales(100);Rhodobiaceae(100);Rhodobium(100);unclassified(100);unclassified(100);unclassified(100);

Otu0945 0 0 0 0 0 0 0 0 0 0 0 0 0 0 0 0 0 0 0 0 0 0 0 0 0 0 0 0
0 0 0 0 0 0 0 76 0 0 0 0 0 0 0 0 0 0 0 0 0 0 0 0 0 0 1 0 0
0 0 0 0 0 0 0 0 0 0 0 0 0 0 0 0 0 0 0 0 0 0 0 0 0 0 0 0 0
0 0 0 0 0 0 0 0 0 0 0 0 0 0 0 0 0 0 0 0 0 0 0 0 0 0 0 0

M02255_77_000000000-ADUT3_1_1102_7876_3975 T----GC---GT-AG-GGG--------CCA-A-A------C--G--T---T--AT-C-CGG-AA------TT-A--C-T--GG-GC-------GT--A-----AA-GA-GAAGC-------G-TA-G-G-C-G------------T--C-TC-G-A-T-----------AA----G-T-C-T--------C-A-T--G--TG--A-AA-GC--C-C-GA-G-G-----CT-C-AA------C-C-T-C-G-G-A--A-A----C-G--C-G---T--G-------A--GA-A-A---C-------T--A-T--CA--A-G-A----T-T-G-A-G-T--C---A-GA-----TA-G-G--------G-G-A-A---AG-C------GG--A--ATT----C-C-T-G-GT--GT-A-G-CG-GT--G---A--A-A---TG-C-GT-TG--AT-A-TC--A-G--G-A-G-G-A-AC-A-CC---AA--T--G--GC-GAA-G--G-C--A-------G--C-T-T-T---CTG--G--AT-C-T--------------------A-G---A-C-T--GA--CG-C-T-A--A-GG--C-T-CG-A--AA-G-C---G-TG--GG-T--AG-C-A-AA--------AC--GG Bacteria(100);unclassified(100);unclassified(100);unclassified(100);unclassified(100);unclassified(100);unclassified(100);unclassified(100);unclassified(100);

Otu0946 0 0 0 0 0 0 0 0 0 0 0 0 0 0 0 0 0 0 0 0 0 0 0 0 0 0 0 0
0 0 0 0 0 26 0 0 0 0 0 0 0 0 0 0 0 0 0 0 0 0 0 0 0 0 0 0 0
0 0 0 0 0 0 0 0 0 0 0 0 0 0 0 0 0 0 0 0 0 0 0 0 0 0 0 0 0
0 0 0 0 0 0 0 0 0 0 0 0 0 0 0 0 0 0 0 0 0 0 0 0 0 0 0 0

M02255_76_000000000-ADUNE_1_1106_20563_20441 G----AC---GG-AG-GGT--------GCT-A-G------C--G--T---T--GT-T-CGG-AA------TC-A--C-T--GG-GC-------GT--A-----AA-GG-GC-GC-------G-TA-G-G-C---------------C--A-GG-C-A-A-----------GT------C-G-G--------C-T-T--G--TG--A-AA-GC--C-C-GG-G-G-----CT-C-AA------C-C-C-C-G-G-A--G-G----T-G--C-G---G--C-------C--GG-G-A---C-------T--G-C--TT--G-G-C----T-G-G-A-G-G--C---C-GG-----AA-G-A--------G-G-A-G---GG-T------GG--A--ATA----C-C-C-A-GT--GT-A-G-AG-GT--G---A--A-A---TT-C-GT-AG--AT-A-TT--G-G--G-T-G-G-A-AC-A-CC---GG--T--G--GC-GAA-A--G-C--G-------G--C-T-C-T---CTG--G--TC-C-G--------------------G-T---C-C-T--GA--CG-C-T-G--A-GG--C-G-CG-A--AA-G-C---G-T-G-GG-G--AG-C-A-AA--------CA--GG Bacteria(100);Proteobacteria(100);Alphaproteobacteria(100);Rhodospirillales(100);unclassified(100);unclassified(100);unclassified(100);unclassified(100);unclassified(100);

Otu0947 0 0 0 0 0 0 0 0 0 0 0 0 0 0 0 0 0 0 0 0 0 0 0 0 0 0 0 0
0 0 0 0 0 41 31 9 0 0 0 0 0 0 0 0 0 0 0 0 0 0 0 0 0 0 0 0 0
0 0 0 0 0 0 0 0 0 0 0 0 0 0 0 0 0 0 0 0 0 0 0 0 0 0 0 0 0
0 0 0 0 0 20 0 0 0 51 0 0 0 22 0 58 51 57 40 0 0 0 0 0 0 0 0

M02255_77_000000000-ADUT3_1_1109_13471_7650 G----AC---GA-AC-CGT--------GCG-A-A------C--G--T---T--GT-T-CGG-AA------TC-A--C-T--GG-GC-------TT--A-----AA-GG-GC-GC-------G-TA-G-G-C-G------------G--G-CT-G-T-C-----------AA----G-T-C-C--------G-G-G--G--TG--A-AA-TC--C-T-CC-C-G-----CT-C-AA------C-G-G-G-A-G-A--A-C----G-G--C-C---T--C-------G--GA-T-A---C-------T--G-A--CG--A-T-C----T-C-G-A-G-G--G---G-GA-----TA-G-G--------G-G-C-A---CG-C------GG--A--ACT----G-T-G-G-GT--GG-A-G-CG-GT--G---A--A-A---TG-C-GT-TG--AT-A-TC--C-A--T-A-G-G-A-AC-T-CC---GG--T--G--GC-GAA-G--G-C--G-------G--C-G-T-G---CTG--G--AT-C-C--------------------C-T---T-C-T--GA--CG-C-T-G--A-GG--C-G-CG-A--AA-G-C---C-AG--GG-G--AG-C-A-AA--------CG--GG Bacteria(100);unclassified(100);unclassified(100);unclassified(100);unclassified(100);unclassified(100);unclassified(100);unclassified(100);unclassified(100);

Otu0948 0 0 0 0 0 0 0 0 0 0 0 0 0 0 0 0 0 0 0 0 0 0 0 0 0 0 0 0
0 0 0 0 0 9 7 0 0 0 0 0 0 0 0 0 0 0 0 0 0 0 0 0 0 0 0 0 0
0 0 0 0 0 0 0 0 0 0 0 0 0 0 0 0 0 0 0 0 0 0 0 0 0 0 0 0 0
0 0 0 0 0 0 0 0 0 0 0 0 0 0 0 0 0 0 0 0 0 0 0 0 0 0 0 0

M02255_77_000000000-ADUT3_1_1108_21009_20260 T----AC---AG-AG-GCC--------CCA-A-G------C--A--T---T--AT-C-CGG-AA------TC-A--C-T--GG-GC-------GT--A-----AA-GG-GT-GT-------G-CA-G-G-C-G------------G--T-TT-T-G-T-----------TA----G-T-C-C--------T-T-C--G--TA--A-AAGCT--T-T-CG-G-A-----CT-C-AA------T-C-C-G-G-A-A--A-A----T-G--C-G---G--T-------G--GA-A-A---C-------G--G-C--AA--A-A-C----TTC-G-A-G-G--A---C-GC-----GA-G-A--------G-G-T-A---GA-G------GG--A--ACT----C-A-T-G-GT--GT-A-G-GG-GT--G---A--A-A---TC-C-GT-TG--AT-A-TC--A-T--G-G-G-G-A-AC-A-CC---AA--A--T--GC-GAA-G--G-C--A-------C--T-C-T-A---CTG--G--CG-C-G--------------------C-T---C-C-T--GA--CG-C-T-C--A-TA--C-A-CG-A--AA-G-C---G-TG--GG-A--AT-C-G-AA--------CG--GG Bacteria(100);unclassified(100);unclassified(100);unclassified(100);unclassified(100);unclassified(100);unclassified(100);unclassified(100);unclassified(100);

Otu0949 0 0 0 0 0 0 0 0 0 0 0 0 0 0 0 0 0 0 0 0 0 0 0 0 0 0 0 0
0 0 0 0 0 28 0 0 0 0 0 0 0 0 0 0 0 0 0 89 0 0 0 0 0 0 0 0 0
0 0 0 0 0 0 0 0 0 0 0 0 0 0 0 0 0 0 0 0 0 0 0 0 0 0 0 0 0
0 0 0 0 0 0 0 0 0 0 0 0 0 0 0 0 0 0 0 0 0 0 0 0 0 0 0 0

M02255_77_000000000-ADUT3_1_1105_23899_16401 T----AC---GT-AG-GGG--------GCA-A-G------C--G--T---T--AT-C-CGG-AA------TT-A--C-T--GG-GT-------GT--A-----AA-GG-GA-GA-------G-TA-G-G-C-G------------G--C-AT-G-G-T-----------AA----G-T-T-A--------G-A-T--G--TG--A-AA-GC--C-C-GA-G-G-----CT-T-AA------C-C-T-C-G-G-G--A-T----T-G--C-A---T--T-------T--AA-A-A---C-------T--A-T--CA--A-G-C----T-A-G-A-G-T--A---C-AG-----GA-G-A--------G-G-T-A---AG-T------GG--A--ATT----C-C-T-A-GT--GT-A-G-CG-GT--G---A--A-A---TG-C-GT-AG--AT-A-TT--A-G--G-A-A-G-A-AC-A-CC---AG--T--G--GC-GAA-G--G-C--G-------A--C-T-T-A---CTG--G--AC-T-G--------------------A-A---A-C-T--GA--CG-C-T-G--A-GG--C-T-CG-A--AA-G-C---G-TG--GG-G--AG-C-G-AA--------CA--GG Bacteria(100);Firmicutes(100);Clostridia(100);Clostridiales(100);Lachnospiraceae(100);unclassified(100);unclassified(100);unclassified(100);unclassified(100);

Otu0950 0 0 0 0 0 0 0 0 0 0 0 0 0 0 0 0 0 0 0 0 0 0 0 0 0 0 0 0
0 0 1 2 0 0 0 0 0 0 75 0 0 0 0 0 0 0 0 0 0 0 0 0 0 0 0 0 0
0 0 0 0 0 0 0 0 0 0 0 0 0 0 0 0 0 0 0 0 0 0 0 0 0 0 0 0 0
0 0 0 0 0 0 0 0 0 0 0 0 0 0 0 0 0 0 0 0 0 0 0 0 0 0 0 0
M02255_77_000000000-ADUT3_1_2113_21773_11991 T----AC---GG-AG-GGG--------GCT-A-G------C--G--T---T--GT-T-CGG-AA------TT-A--C-T--GG-GC-------GT--A-----AA-GG-GC-GT-------G-TA-G-G-C-G------------G--T-TT-G-C-C-----
------GA----G-T-C-A--------G-A-T--G--TG--A-AA-GC--C-C-CG-G-G-----CT-T-AA------C-C-T-G-G-G-A--A-C----T-G--C-A---T--T-------T--GA-G-A---C-------T--G-G--CA--G-G-C----T-A-G-A-G-G--T---T-GG-----AA-G-A--------G-G-A-G---AG-T------GG--A--ATT----
C-C-C-A-GT--GT-A-G-AG-GT--G---A--A-A---TT-C-GT-AG--AT-A-TT--G-G--G-A-A-G-A-AC-A-CC---AG--T--G--GC-GAA-G--G-C--G-------G--C-T-C-T---CTG--G--TC-C-A--------------------C-A---C-C-T--GA--CG-C-T-G--A-GG--C-G-CG-A--AA-G-C---G-TG--GG-
G--AG-C-A-AA--------CA--GG Bacteria(100);Proteobacteria(100);unclassified(100);unclassified(100);unclassified(100);unclassified(100);unclassified(100);unclassified(100);unclassified(100);

Otu0951 0 0 0 0 0 0 0 0 0 0 0 0 0 0 0 0 0 0 0 0 0 0 0 0 0 0 0 0
0 0 1 0 0 0 0 0 0 9 1 0 0 0 0 0 0 0 0 0 0 0 0 0 0 0 0 0 0
0 0 0 0 0 0 0 0 0 0 0 0 0 0 0 0 0 0 0 0 0 0 0 0 0 0 0 0 0
0 0 0 0 0 0 0 0 0 0 0 0 0 0 0 0 0 0 0 0 0 0 0 0 0 0 0 0
M02255_77_000000000-ADUT3_1_2113_15047_19035 T----AC---GT-AG-GGC--------GCA-A-G------C--G--T---T--GT-C-CGG-AA------TT-A--T-T--GG-GC-------GT--A-----AA-GA-GC-TC-------G-TA-G-G-C-G------------G--T-TT-G-T-C------
-----GC----G-T-C-T--------G-C-C--G--TG--A-AA-AC--C-T-AG-T-G-----CT-T-AA------C-A-C-T-G-G-G--C-G----T-G--C-G---G--T-------G--GG-T-A---C-------G--G-G--CA--G-G-C----T-A-G-A-G-T--G---T-GG-----TA-G-G--------G-G-A-G---AC-T------GG--A--ATT----
C-C-T-G-GT--GT-A-G-CG-GT--G---G--A-A---TG-C-GC-AG--AT-A-TC--A-G--G-A-G-G-A-AC-A-CC---GA--T--G--GC-GAA-G--G-C--A-------G--G-T-C-T---CTG--G--GC-C-A--------------------T-T---A-C-T--GA--CG-C-T-G--A-GG--A-G-CG-A--AA-G-C---A-TG--
GG-T--AG-C-G-AA--------CA--GG Bacteria(100);Actinobacteria(100);Actinobacteria(100);Actinobacteridae(100);Actinomycetales(100);Micrococcineae(100);unclassified(100);unclassified(100);unclassified(100);

Otu0952 0 0 0 0 0 0 0 0 0 0 0 0 0 0 0 0 0 0 0 0 0 0 0 0 0 0 0 0
0 0 1 0 0 0 0 0 0 0 36 0 0 0 0 0 0 0 0 0 0 0 0 0 0 0 0 0 0
0 0 0 0 0 0 0 0 0 0 0 0 0 0 0 0 0 0 0 0 0 0 0 0 0 0 0 0 0
0 0 0 0 0 0 0 0 0 0 0 0 0 0 0 0 0 0 0 0 0 0 0 0 0 0 0 0
M02255_77_000000000-ADUT3_1_2118_10573_17407 T----AC---GT-AG-GAG--------GCA-A-G------C--G--T---T--AT-C-CGG-AT------TA-A--C-T--GG-GC-------GT--A-----AA-GC-GT-GC-------G-TA-G-G-C-G------------G--T-TG-T-C-T-----
------AA----G-T-C-G--------C-C-T--T--TT--A-AA-GC--C-T-GG-G-G-----CT-T-AA------C-C-C-C-A-G-A--T-T----T-G--G-A---G--G-------T--GA-T-A---C-------T--G-G--AT--T-A-C----T-T-G-AGG-A--T---C-GT-----TC-G-G--------G-G-T-A---TC--------GG--A--ACA----G-
T-T-G-GT--GT-A-G-CG-GT--G---A--A-A---TG-C-GT-TG--AT-A-TC--A-A--C-T-G-G-A-AC-A-CC---GA--A--G--GC-GAA-G--G-C--A-------G--A-T-A-C---CTG--G--AA-T-T--------------------T-T---C-C-T--GA--CG-C-T-G--A-GG--T-A-CG-A--AA-G-C---T-AG--GG-T--
AG-C-G-AA--------AG--GG Bacteria(100);unclassified(100);unclassified(100);unclassified(100);unclassified(100);unclassified(100);unclassified(100);unclassified(100);unclassified(100);

Otu0953 0 0 0 0 0 0 0 0 0 0 0 0 0 0 0 0 0 0 0 0 0 0 0 0 0 0 0 0
0 0 0 26 0 0 0 0 0 0 0 0 0 0 0 0 0 0 0 0 0 0 0 0 0 0 0 0 0
0 0 0 0 0 0 0 0 0 0 0 0 0 0 0 0 0 0 0 0 0 0 0 0 0 0 0 0 0
0 0 0 0 0 0 0 0 0 0 0 0 0 0 0 0 0 0 0 0 0 0 0 0 0 0 0 0
M02255_77_000000000-ADUT3_1_1103_7402_7106 T----AC---GT-AG-GTG--------GCA-A-G------C--G--T---T--GT-C-CGG-AT------TT-A--C-T--GG-GT-------GT--A-----AA-GG-GC-GT-------G-TA-G-G-C-G------------G--G-AT-T-G-C-----------AA-
---G-T-C-A--------G-G-C--G--TG--A-AA-AC--C-A-GG-G-G-----CT-C-AA------C-C-T-C-T-G-G--C-C----T-G--C-G---T--T-------T--GA-A-A---C-------T--G-T--AG--T-T-C----T-T-G-A-G-T--A---C-TG-----GA-G-A--------G-G-T-T---GA-C------GG--A--ATT----C-C-T-A-
GT--GT-A-G-CG-GT--G---A--A-A---TG-C-GT-AG--AT-A-TT--A-G--G-A-G-G-A-AC-A-CC---AG--T--G--GC-GAA-G--G-C--G-------G--T-C-A-A---CTG--G--AC-A-G--------------------C-A---A-C-T--GA--CG-C-T-G--A-GG--C-G-CG-A--AA-G-C---G-TG--GG-G--AG-
C-A-AA--------CA--GG Bacteria(100);Firmicutes(100);Clostridia(100);Clostridiales(100);unclassified(100);unclassified(100);unclassified(100);unclassified(100);unclassified(100);

Otu0954 0 0 0 0 0 0 0 0 0 0 0 0 0 0 0 0 0 0 0 0 0 0 0 0 0 0 0 0
0 0 0 130 87 169 262 0 0 0 0 34 0 0 0 0 0 0 0 0 0 0 0 0 0 0 0 0 0
0 0 0 0 0 0 0 0 0 0 0 0 0 0 0 0 0 0 0 0 0 0 0 0 0 0 0 0 0
0 0 0 0 0 0 0 0 0 0 0 0 0 0 0 0 0 0 0 0 0 0 0 0 0 0 0 0
M02255_77_000000000-ADUT3_1_1108_10557_11837 T----AC---GT-AG-GCA--------GCA-A-G------C--G--T---T--GT-T-CGG-AA------TT-A--C-T--GG-GC-------GT--A-----AA-GA-GT-TC-------G-TA-G-G-C-G------------G--T-GC-G-G-C-----
------AA----G-T-T-C--------G-G-T--G--TG--A-AA-TC--T-C-CC-G-G-----CT-C-AA------C-T-G-G-G-A-G--G-G----T-G--C-G---C--T-------A--AA-T-A---C-------T--G-C--CG--T-G-C----T-C-G-A-G-T--A---T-AG-----GA-G-A--------G-G-T-A---AG-C------GG--A--ATT----C-
C-T-G-GT--GT-A-G-CG-GT--G---A--A-A---TG-C-GT-AG--AT-A-TC--A-G--G-A-G-G-A-AC-A-CC---TG--T--G--GT-GTA-G--A-C--G-------G--C-T-T-A---CTG--G--AC-T-A--------------------T-A---A-C-T--GA--CG-C-T-G--A-GG--A-A-CG-A--AA-G-C---G-TG--GG-T-
-AG-C-A-AA--------CA--GG Bacteria(100);unclassified(100);unclassified(100);unclassified(100);unclassified(100);unclassified(100);unclassified(100);unclassified(100);unclassified(100);

Otu0955 0 0 0 0 0 0 0 0 0 0 0 0 0 0 0 0 0 0 0 0 0 0 0 0 0 0 0 0
0 0 0 33 0 0 0 0 0 0 0 0 0 0 0 0 0 0 0 0 0 0 0 0 0 0 0 0 0
0 0 0 0 0 0 0 0 0 0 0 0 0 0 0 0 0 0 0 0 0 0 0 0 0 0 0 0 0
0 0 0 0 0 0 0 0 0 0 0 0 0 0 0 0 0 0 0 0 0 0 0 0 0 0 0 0
M02255_77_000000000-ADUT3_1_1109_5320_16278 T----AC---GT-AG-GGT--------CCG-A-G------C--G--T---T--GT-C-CGG-AG------TT-A--C-T--GG-GC-------GT--A-----AA-GC-GC-GC-------G-CA-G-G-C-G------------G--C-CG-T-T-C----------AA-
---G-T-C-T--------G-G-C--G--TG--A-AA-GC--C-C-CC-G-G-----CT-C-AA------C-C-G-G-G-G-A--G-G----G-T--C-G---T--C-------G--GA-G-A---C-------T--G-G--GC--G-G-C----T-T-G-A-G-A--G---T-GG-----CA-G-G--------G-G-C-T---GG-T------GG--A--ATT------CCC-
G-GT--GT-A-G-TG-GT--G---A--A-A---TG-C-GT-AG--AG-A-TC--G-G--G-A-G-G-A-AC-A-CC---CG--T--G--GC-GAA-G--G-C--G-------G--C-C-A-G---CTG--G--GC-C-A--------------------C-C---T-C-T--GA--CG-C-T-G--A-GG--C-G-CG-A--AG-G-C---G-TG--GG-G--
AG-C-G-AA--------CG--GG Bacteria(100);Chloroflexi(100);Thermomicrobia(100);JG30-KF-CM45(100);unclassified(100);unclassified(100);unclassified(100);unclassified(100);unclassified(100);

Otu0956 0 0 0 0 0 0 0 0 0 0 0 0 0 0 0 0 0 0 0 0 0 0 0 0 0 0 0 0
0 0 0 0 0 85 0 0 0 0 0 0 0 0 0 0 0 0 0 0 0 0 0 0 0 0 0 0 0
0 0 0 0 0 0 0 0 0 0 0 0 0 0 0 0 0 0 0 0 0 0 0 0 0 0 0 0 0
0 0 0 0 0 0 0 0 0 0 0 0 0 0 0 0 0 0 0 0 0 0 0 0 0 0 0 0
M02255_77_000000000-ADUT3_1_1102_16737_8378 G----AC---GA-AG-GGC--------GCG-G-G------T--G--T---T--AT-T-CGT-GT------TG-A--T-T--GG-GT-------GT--A-----AA-GG-GT-AG-------G-TA-G-G-C-G------------G--A-TT-C-T-A----------TT-
---T-T-T-T--------T-T-G--G--AC--A-AA-AG--CGC-GG-A-G-----TG-T-TC------C-T-A-C-GCG-A--A-G----T-T--C-T---T--T-------T--AT-T-A---A-------A--T-G---A--G-T-C----T-T-G-T-G-T--T---A-AG-----GG-G-A--------G-G-T-T---AA-G------AC--A--ATT----CTG-T-G-
AG--TT-A-G-GA-GT--A---G--A-A---TC-C-TT-AT--AG-C-CA--C-G--G-A-G-G-AATG-T-CG---AT--C--G--GC-GAA-G--G-C---------A--C-T-T-T---TCC--A--AC-T-T--------------------T-A---A-C-A--GA--CG-C-T-G--A-GC--T-A-CG-G--AA-G-C---T-TA--GG-G--AG-C-
A-AA--------CA--GG Bacteria(100);unclassified(100);unclassified(100);unclassified(100);unclassified(100);unclassified(100);unclassified(100);unclassified(100);unclassified(100);

Otu0957 0 0 0 0 0 0 0 0 0 0 0 0 0 0 0 0 0 0 0 0 0 0 0 0 0 0 0 0
0 0 0 33 0 0 0 0 0 0 0 0 0 0 0 0 0 0 0 0 0 0 0 0 0 0 0 0 0
0 0 0 0 0 0 0 0 0 0 0 0 0 0 0 0 0 0 0 0 0 0 0 0 0 0 0 0 0
0 0 0 0 0 0 0 0 0 0 0 0 0 0 0 0 0 0 0 0 0 0 0 0 0 0 0 0
M02255_77_000000000-ADUT3_1_1112_16452_19966 T----AC---GT-AG-GGG--------GCA-A-G------C--G--T---T--GT-C-CGG-AT------TT-A--T-T--GG-GC-------GT--A-----AA-GC-GC-TC-------G-CA-G-G-T-G------------G--C-TC-G-T-C------
-----GC----G-T-C-G--------T-G-C--G--T--GA-AA-GA--C-C-CC-G-G-----CT-C-AA------C-T-G-G-G-G-A--G------G-GT-C-G---T--G-------C--GA-T-A---C-------G--G-G--CG--A-G-C----T-G-G-A-G-G--C---C-AG-----TA-G-G--------G-G-G-A---TG-C------GG--A--ATT----
C-C-G-G-GT--GT-A-G-TG-GT--G---G--A-A---TG-C-GT-AG--AG-A-TC--C-G--G-A-G-G-A-AC-A-CC---AG--C--G--GC-GAA-G--G-C--G-------G--C-A-T-C---CTG--G--AC-T-G--------------------G-T---C-C-T--GA--CA-C-T-C--A-GG--A-G-CG-A--AA-G-C---G-TG--
GG-G--AG-C-G-AT--------CC--GG Bacteria(100);unclassified(100);unclassified(100);unclassified(100);unclassified(100);unclassified(100);unclassified(100);unclassified(100);unclassified(100);

Otu0958 0 0 0 0 0 0 0 0 0 0 0 0 0 0 0 0 0 0 0 0 0 0 0 0 0 0 0 0
0 0 0 0 0 92 0 0 0 0 0 0 0 0 0 0 0 0 0 0 0 0 0 0 0 0 0 0 0
0 0 0 0 0 0 0 0 0 0 0 0 0 0 0 0 0 0 0 0 0 0 0 0 0 0 0 0 0
0 0 0 0 0 0 0 0 0 0 0 0 0 0 0 0 0 0 0 0 0 0 0 0 0 0 0 0
M02255_77_000000000-ADUT3_1_1101_24443_15950 T----AC---GA-AG-GGG--------GCG-A-G------C--G--T---T--GT-T-CGG-AT------TT-A--C-T--GG-GC-------GT--A-----AA-GG-GC-GC-------G-CA-G-G-C-G------------G--C-GA-C-A-T----
-------TT----G-T-C-G--------G-G-C--G--TG--A-AA-GC--C-C-CG-G-G-----CT-T-AA------C-C-T-G-G-G-A--A-C----T-G--C-G---T--T-------T--GA-T-A---C-------G--G-T--GT--G-G-C----T-A-G-A-G-T--T---C-GG-----GA-G-A--------G-G-A-G---AG-T------GG--A--ATT----
C-C-C-A-GT--GT-A-G-AG-GT--G---A--A-A---TT-C-GT-AG--AT-A-TT--G-G--G-A-A-G-A-AC-A-CC---GG--T--G--GC-GAA-G--G-C--G-------G--C-T-C-T---CTG--G--AC-C-G--------------------A-G---A-C-T--GA--CG-C-T-G--A-GG--C-G-CG-A--AA-G-C---G-TG--
GG-G--AG-C-A-AA--------CA--GG Bacteria(100);Proteobacteria(100);Alphaproteobacteria(100);Rhodospirillales(100);unclassified(100);unclassified(100);unclassified(100);unclassified(100);unclassified(100);

Otu0959 0 0 0 0 0 0 0 0 0 0 0 0 0 0 0 0 0 0 0 0 0 0 0 0 0 0 0 0
0 0 1 0 0 0 0 0 0 0 0 28 0 0 0 0 0 0 0 0 0 0 0 0 0 0 0 0 0
0 0 0 0 0 0 0 0 0 0 0 0 0 0 0 0 0 0 0 0 0 0 0 0 0 0 0 0 0
0 0 0 0 0 0 0 0 0 0 0 0 0 0 0 0 0 0 0 0 0 0 0 0 0 0 0 0
M02255_77_000000000-ADUT3_1_2109_19306_13180 T----AC---GT-AG-GGG--------GCA-A-G------C--G--T---T--AT-C-CGG-AT------TT-A--C-T--GG-GT-------GT--A-----AA-GG-GA-GC-------G-TA-G-A-C-G------------G--C-GA-A-G-C----
-------AA----G-T-C-T--------G-A-A--G--TG--A-AA-GC--C-C-GG-G-G-----CT-C-AA------C-C-G-C-G-G-G--A-C----T-G--C-T---T--T-------G--GA-A-A---C-------T--G-T--TT--T-G-C----T-G-G-A-G-T--G---C-TG-----GA-G-A--------G-G-C-A---GG-C------GG--A--ATT----
C-C-T-A-GT--GT-A-G-CG-GT--G---A--A-A---TG-C-GT-AG--AT-A-TT--A-G--G-A-G-G-A-AC-A-CC---AG--T--G--GC-GAA-G--G-C--G-------G--C-C-T-G---CTG--G--AC-A-G--------------------T-A---A-C-T--GA--CG-T-T-G--A-GG--C-T-CG-A--AA-G-C---G-TG--GG-
G--AG-C-A-AA--------CA--GG Bacteria(100);Firmicutes(100);Clostridia(100);Clostridiales(100);Lachnospiraceae(100);unclassified(100);unclassified(100);unclassified(100);unclassified(100);

Otu0960 0 0 0 0 0 0 0 0 0 0 0 0 0 0 0 0 0 0 0 0 0 0 0 0 0 0 0 0
0 0 0 5 0 2 0 0 0 0 6 0 0 0 0 0 0 0 0 0 0 0 0 2 0 0 0 0 0
0 0 0 0 0 0 0 0 0 0 0 0 0 0 0 0 0 0 10 0 0 0 0 0 0 0 0 0 0
0 0 16 2 0 0 0 0 0 0 0 0 0 0 0 0 0 0 0 0 0 0 0 0 3 0 16
M02255_77_000000000-ADUT3_1_2111_4486_11348 G----AC---GG-GG-GGG--------GCA-A-G------T--G--T---T--CT-T-CGG-AA------TG-A--C-T--GG-GC-------GT--A-----AA-GG-GC-AC-------G-TA-G-G-C-G------------G--T-GA-A-T-C----------
GG----G-T-T-G--------A-A-A--G--TG--A-AA-G---T-C-GC-C-A-----AA-A-AC------T-G-G-C-G-G-A--A------T-G--C-T---C--T-------C--GA-A-A---C-------C--A-A--TT--C-A-C----T-T-G-A-G-T--G---A-GA-----CA-G-A--------G-G-A-G---AG-T------GG--A--ATT----T-C-G-
T-GT--GT-A-G-GG-GT--G---A--A-A---TC-C-GG-AG--AT-C-TA--C-G--A-A-G-G-A-AC-G-CC---AA--A--A--GC-GAA-G--G-C--A-------G--C-T-C-T---CTG--G--GT-C-C--------------------C-T---A-C-C--GA--CG-C-T-G--G-AG--T-G-CG-A--AA-G-C---A-TG--GG-G--
AG-C-G-AA--------CG--GG Bacteria(100);unclassified(100);unclassified(100);unclassified(100);unclassified(100);unclassified(100);unclassified(100);unclassified(100);unclassified(100);

Otu0961 0 0 0 0 0 0 0 0 0 0 0 0 0 0 0 0 0 0 0 0 0 0 0 0 0 0 0 0
0 0 0 0 0 57 0 0 0 0 0 0 0 0 0 0 0 0 0 0 0 0 0 0 0 0 0 0 0
0 0 0 0 0 0 0 0 0 0 0 0 0 0 0 0 0 0 0 0 0 0 0 0 0 0 0 0 0
0 0 0 0 0 0 0 0 0 0 0 0 0 0 0 0 0 0 0 0 0 0 0 0 0 0 0
M02255_76_000000000-ADUNE_1_1106_5513_18760 C----CG---GC-TA-ACT--------ACG-T-G------C--C--A---G--CA-G-CTG-CG------GT-A--A-T--AC-GT-------AG--G-----GT-GC-AA-GCA------T-TA-A-T-C-G------------G--A-AT-T-G-T----------AA-
---G-T-C-A--------G-A-T--G--TG--A-AA-TC--C-C-GG-A-G-----CT-T-AA------C-T-C-C-G-G-A--A-C----G-G--C-A---T--T-------T--GA-T-A---C-------T--G-C--TT--T-T-C----T-A-G-A-G-G--A---T-AG-----AC-G-G--------A-G-A-A---AA-C------GG--A--ATT----C-C-A-C-
AA--GT-A-G-CG-GT--G---A--A-A---TG-C-GT-AG--AT-A-TG--T-G--G-A-A-G-A-AC-A-CC---TG--T--G--GC-GAA-A--G-C--G-------G--T-T-T-T---CTA--G--TT-T-A--------------------T-T---C-C-T--GA--CG-C-T-G--A-TG--C-G-CG-A--AA-G-C---T-AG--GG-G--AG-C-
A-AA--------CA--GG Bacteria(100);Chlamydiae(100);Chlamydiae(100);Chlamydiales(100);unclassified(100);unclassified(100);unclassified(100);unclassified(100);unclassified(100);

Otu0962 0 0 0 0 0 0 0 0 0 0 0 0 0 0 0 0 0 0 0 0 0 0 0 0 0 0 0 0
0 0 0 1 0 0 0 0 0 0 62 0 0 0 0 0 0 0 0 0 0 0 0 0 0 0 0 0 0
0 0 0 0 0 0 0 0 0 0 0 0 0 0 0 0 0 0 0 0 0 0 0 0 0 0 0 0 0
0 0 0 0 0 0 0 0 0 0 0 0 0 0 0 0 0 0 0 0 0 0 0 0 0 0 0
M02255_77_000000000-ADUT3_1_1101_20012_10694 T----AC---GG-AG-GGG--------GCT-A-G------C--G--T---T--GT-T-CGG-AA------TT-A--C-T--GG-GC-------GT--A-----AA-GC-GC-AC-------G-TA-G-G-C-G------------G--G-CT-G-T-T-----
------AA----G-T-C-A--------G-T-G--G--TG--A-AA-TC--C-C-GG-G-G-----CT-C-AA------C-C-T-C-G-G-A--A-T----T-G--C-C---T--T-------T--GA-T-A---C-------T--G-G--CA--G-C-C----T-T-G-A-G-T--C---C-GG-----GA-G-A--------G-G-T-G---AG-T------GG--A--ATT----C-
C-G-A-GT--GT-A-G-AG-GT--G---A--A-A---TT-C-GT-AG--AT-A-TT--C-G--G-A-G-G-A-AC-A-CC---AG--T--G--GC-GAA-G--G-C--G-------G--C-T-C-A---CTG--G--CC-C-G--------------------G-T---A-C-T--GA--CG-C-T-G--A-GG--T-G-CG-A--AA-G-C---G-TG--GG-
G--AG-C-A-AA--------CA--GG Bacteria(100);Proteobacteria(100);Alphaproteobacteria(100);unclassified(100);unclassified(100);unclassified(100);unclassified(100);unclassified(100);unclassified(100);

Otu0963 0 0 0 0 0 0 0 0 0 0 0 0 0 0 0 0 0 0 0 0 0 0 0 0 0 0 0 0
0 0 0 1 0 0 0 0 0 0 22 0 0 0 0 0 0 0 0 0 0 0 0 0 0 0 0 0 0
0 0 0 0 0 0 0 0 0 0 0 0 0 0 0 0 0 0 0 0 0 0 0 0 0 0 0 0 0
0 0 0 0 0 0 0 0 0 0 0 0 0 0 0 0 0 0 0 0 0 0 0 0 0 0 0
M02255_77_000000000-ADUT3_1_1107_18329_22326 A----AC---GT-AG-GAG--------GCA-A-G------C--G--T---T--AT-C-CGG-AG------TT-A--C-T--GG-GC-------GT--A-----AA-GC-GT-GC-------G-TA-G-G-C-G------------G--A-TG-G-G-T----
-------AA----G-T-G-G--------G-T-G--G--TG--A-AA-GC--G-C-TC-G-G-----CT-A-AA------C-C-G-G-G-C-G--A-G----T-G--C-C---A--T-------T--CA-G-A---C-------T--G-T--CC--A-T-C----T-A-G-A-G-G--C---G-GA-----TA-G-A--------G-G-G-G---TG-C------GG--A--ATT----
C-C-G-G-GT--GT-A-G-CG-GT--G---A--A-A---TG-T-GT-AG--AG-A-TC--C-G--G-A-G-G-A-AC-A-CC---AG--T--G--GT-GCA-A--A-C--G-------G--C-G-C-C---CTG--G--GT-C-C--------------------G-A---A-C-T--GA--CG-C-T-G--A-GG--C-A-CG-A--AA-G-C---G-TG--
GG-G--AG-C-G-AA--------CG--GG Bacteria(100);Chloroflexi(100);Anaerolineae(100);Anaerolineales(100);Anaerolineaceae(100);uncultured(100);unclassified(100);unclassified(100);unclassified(100);

Otu0964 0 0 0 0 0 0 0 0 0 0 0 0 0 0 0 0 0 0 0 0 0 0 0 0 0 0 0 0
0 0 0 0 0 29 0 0 0 0 0 0 1 0 0 0 0 0 0 0 0 0 0 0 0 0 0 0 0
0 0 0 0 0 0 0 0 0 0 0 0 0 0 0 0 0 0 0 0 0 0 0 0 0 0 0 0 0
0 0 0 0 0 10 0 0 0 10 0 0 0 9 0 15 16 11 10 0 0 0 0 0 0 0 0
M02255_76_000000000-ADUNE_1_1102_14807_8973 G----TT---CG-TG-CCA--------GCA-G-C------C--G--C---G--GT-A-AGACGA------AC-C--G-T--GC-GA-------AC--G-----TT-GT-TCGGA-------A-TC-A-C-T-G------------G--G-CT-G-T-A----------
AA----G-G-G-C--------G-G-G--G--TG--A-AA-TC--C-T-CC-C-G-----CT-C-AA------C-G-G-G-A-G-A--A-C----G-G--C-C---T--C-------G--GA-T-A---C-------T--G-A--CG--A-T-C----T-C-G-A-G-G--G---G-GA-----TA-G-G--------G-G-C-A---CG-C------GG--A--ACT----G-T-
G-G-GT--GG-A-G-CG-GT--G---A--A-A---TG-C-GT-TG--AT-A-TC--C-A--T-A-G-G-A-AC-T-CC---GG--T--G--GC-GAA-G--G-C--G-------G--C-G-T-G---CTG--G--AT-C-C--------------------C-T---T-C-T--GA--CG-C-T-G--A-GG--C-G-CG-A--AA-G-C---C-AG--GG-G--
AG-C-A-AA--------CG--GG Bacteria(100);unclassified(100);unclassified(100);unclassified(100);unclassified(100);unclassified(100);unclassified(100);unclassified(100);unclassified(100);

Otu0965 0 0 0 0 0 0 0 0 0 0 0 0 0 0 0 0 0 0 0 0 0 0 0 0 0 0 0 0
0 0 0 4 0 0 0 0 0 0 79 0 0 0 0 0 0 0 0 0 0 0 0 0 0 0 0 0
0 0 0 0 0 0 0 0 0 0 0 0 0 0 0 0 0 0 0 0 0 0 0 0 0 0 0 0
0 0 0 0 0 0 0 0 0 0 0 0 0 0 0 0 0 0 0 0 0 0 0 0 0 0 0
M02255_77_000000000-ADUT3_1_1117_25795_4194 T----AC---GG-AG-GGG--------GCT-A-G------C--G--T---T--GT-T-CGG-AA------TT-A--C-T--GG-GC-------GT--A-----AA-GC-GC-GC-------G-TA-G-G-C-G-------------G--A-CC-T-G-C-----------
AA----G-T-C-G--------G-G-G--G--TG--A-AA-GC--C-C-GA-G-G-----CT-C-AA------C-C-T-C-G-G-A--A-C----T-G--C-C---T--T-------C--GA-T-A---C-------T--G-C--GG--G-T-C----T-C-G-A-G-T--C---C-GG-----GA-G-A--------G-G-T-G---AG-T------GG--A--ATT----C-C-
T-A-GT--GT-A-G-AG-GT--G---A--A-A---TT-C-GT-AG--AT-A-TT--A-G--G-A-A-G-A-AC-A-CC---AG--T--G--GC-GAA-G--G-C--G-------G--C-T-C-A---CTG--G--CC-C-G--------------------G-T---A-C-T--GA--CG-C-T-G--A-GG--C-G-CG-A--AA-G-C---G-TG--GG-G--
AG-C-A-AA--------CA--GG Bacteria(100);Proteobacteria(100);Alphaproteobacteria(100);Rhizobiales(100);Rhodobiaceae(100);Rhodobium(100);unclassified(100);unclassified(100);unclassified(100);

Otu0966 0 0 0 0 0 0 0 0 0 0 0 0 0 0 0 0 0 0 0 0 0 0 0 0 0 0 0 0
0 0 0 9 0 0 0 0 0 0 0 0 0 0 0 0 0 0 0 0 0 0 0 0 0 0 0 0
0 0 0 0 0 0 0 0 0 0 0 0 0 0 0 0 0 0 0 5 0 0 0 0 0 0 0 0
0 0 0 8 0 0 0 0 0 0 0 0 0 0 0 0 0 0 0 0 0 0 0 0 0 0 0
M02255_76_000000000-ADUNE_1_2111_6601_12928 C----GG---CT-AA-TTC--------CGT-G-C------C--A--G---C--AG-C-CGC-GG------TA-A--T-A--CG-GA-------AG--G-----TC-CG-GG-CG-------T-TA-T-C-C-G-------------GA-T-CT-A-T-T-----------AA-
---G-C-G-T--------G-T-T--G--TG--A-AA-TT--T-A-CC-G-G-----CT-C-AA------C-C-G-G-T-G-G--C-T----T-G--C-A---G--C-------G--CG-A-A---C-------T--G-G--TC--G-A-C----T-T-G-A-G-T--A---T-GC-----AG-G-A--------A-G-T-A---GG-C------GG--A--ATT----C-A-T-G-
GT--GT-A-G-CG-GT--G---A--A-A---TG-C-TT-AG--AT-A-TC--A-T--G-A-C-G-A-AC-T-CC---GA--T--T--GC-GCA-G--G-C--A-------G--C-T-T-A---CTG--T--AG-C-A--------------------T-A---A-C-T--GA--CG-C-T-G--A-TG--C-T-CG-A--AA-G-T---G-CG--GG-T--AT-C-A-
AA--------CA--GG Bacteria(100);Bacteroidetes(100);Bacteroidia(100);Bacteroidales(100);Prevotellaceae(100);Prevotella(100);unclassified(100);unclassified(100);unclassified(100);

Otu0967 0 0 0 0 0 0 0 0 0 0 0 0 0 0 0 0 0 0 0 0 0 0 0 0 0 0 0 0
0 0 0 1 0 0 0 0 0 0 0 1 1 0 0 0 0 21 0 0 0 0 0 0 0 0 0 0
0 0 0 0 0 0 0 0 0 0 0 0 0 0 0 0 0 0 0 0 0 0 0 0 0 0 0 0
0 0 0 0 0 0 0 0 0 0 0 0 0 0 0 0 0 0 0 0 0 0 0 0 0 0 0
M02255_77_000000000-ADUT3_1_1108_10465_18961 G----AC---GT-AG-GAT--------GCA-A-G------C--G--T---T--GT-C-CGG-AT------TT-A--T-T--GG-GC-------GT--A-----AA-GA-GT-TC-------G-TA-G-G-T-G-------------G--T-TT-G-T-T------
-----AA----G-T-C-T--------G-A-T--G--TT--A-AA-GA--C-T-GA-G-G-----CT-C-AA------C-C-T-C-G-G-G--A-G----T-G--C-A---T--T-------G--GA-T-A---C-------T--G-G--CA--G-A-C----T-A-G-A-G-T--A---T-GG-----TA-G-A--------G-G-C-A---AG-C------GG--A--ATT----C-
C-C-A-GT--GT-A-G-CG-GT--G---A--A-A---TG-C-GT-AG--AT-A-TT--G-G--G-A-A-G-A-AC-A-CC---GG--T--G--GC-GTA-G--G-C--G-------G--C-T-T-G---CTG--G--GC-C-A--------------------T-A---A-C-T--GA--CA-C-T-G--A-GG--A-A-CG-A--AA-G-C---C-AG--GG-
G--AG-C-G-AA--------TG--GG Bacteria(100);Cyanobacteria(100);MLE1-12(100);unclassified(100);unclassified(100);unclassified(100);unclassified(100);unclassified(100);unclassified(100);

Otu0968 0 0 0 0 0 0 0 0 0 0 0 0 0 0 0 0 0 0 0 0 0 0 0 0 0 0 0 0
0 0 0 0 25 0 0 0 0 0 0 0 0 0 0 0 0 0 0 0 0 0 0 0 0 0 0 0
0 0 0 0 0 0 0 0 0 0 0 0 0 0 0 0 0 0 0 0 0 0 0 0 0 0 0 0
0 0 0 0 0 0 0 0 0 0 0 0 0 0 0 0 0 0 0 0 0 0 0 0 0 0 0
M02255_76_000000000-ADUNE_1_1109_19496_9232 C----TA---AC-TC-CGT--------GCC-A-G------C-----A---G--CC-G-CGG-TA------AT-A--C-G--GA-GG-------GT--G-----CA--A-GC--G-------T-TA-T-T-C-G-------------G--C-AA-T-T-T-----------AA----
G-T-C-A--------G-A-T--G--TG--A-AA-GC--C-C-CG-G-G-----CT-T-AA------C-C-T-G-G-G-A--A-C----T-G--C-A---T--T-------T--GA-A-A---C-------T--G-G--AT--A-G-C----T-A-G-A-G-T--A---T-GT-----TA-G-A--------G-G-A-A---GG-C------GG--A--ATT----T-C-C-A-GT--
GT-A-G-CA-GT--G---A--A-A---TG-C-GT-AG--AT-A-TT--G-G--A-A-G-G-A-AC-A-CC---GA--T--G--GC-GAA-G--G-C--A-------G--C-C-T-T---CTG--G--GG-C-A--------------------A-T---A-C-T--GA--CG-C-T-C--A-TG--T-G-CG-A--AA-G-C---G-TG--GG-T--AG-C-A-
AA--------CA--GG Bacteria(100);Proteobacteria(100);Gammaproteobacteria(100);Cardiobacteriales(100);Cardiobacteriaceae(100);unclassified(100);unclassified(100);unclassified(100);unclassified(100);

Otu0969 0 0 0 0 0 0 0 0 0 0 0 0 0 0 0 0 0 0 0 0 0 0 0 0 0 0 0 0
0 0 0 0 130 0 0 0 0 0 0 0 0 0 0 0 0 0 0 0 0 0 0 0 0 0 0 0
0 0 0 0 0 0 0 0 0 0 0 0 0 0 0 0 0 0 0 0 0 0 0 0 0 0 0 0
0 0 0 0 0 0 0 0 0 0 0 0 0 0 0 0 0 0 0 0 0 0 0 0 0 0 0
M02255_77_000000000-ADUT3_1_1102_10338_10670 T----AC---GG-AG-GGG--------GCT-A-G------C--G--T---T--GT-T-CGG-AA------TT-A--C-T--GG-GC-------GT--A-----AA-GC-GC-AC-------G-TA-G-G-C-G-------------G--C-TT-T-G-T-----
------AA----G-T-C-A--------G-A-G--G--TG--A-AA-GC--C-C-AG-G-G-----CT-C-AA------C-C-T-T-G-G-A--A-C----T-G--C-C---T--T-------T--GA-G-A---C-------T--G-C--AG--A-G-C----T-T-G-A-A-T--C---A-CG-----GA-G-A--------G-G-T-G---GG-T------GG--A--ATT----
C-C-G-A-GT--GT-A-G-AG-GT--G---A--A-A---TT-C-GT-AG--AT-A-TT--C-G--G-A-A-G-A-AC-A-CC---AG--T--G--GC-GAA-G--G-C--G-------G--C-C-C-A---CTG--G--AC-G-T--------------------G-T---A-T-T--GA--CG-C-T-G--A-GG--T-G-CG-A--AA-G-C---G-TG--GG-
G--AG-C-A-AA--------CA--GG Bacteria(100);Proteobacteria(100);Alphaproteobacteria(100);unclassified(100);unclassified(100);unclassified(100);unclassified(100);unclassified(100);unclassified(100);

Otu0970 0 0 0 0 0 0 0 0 0 0 0 0 0 0 0 0 0 0 0 0 0 0 0 0 0 0 0 0
0 0 0 0 60 0 0 0 0 0 0 0 0 0 0 0 0 0 0 0 0 0 0 0 0 0 0 0
0 0 0 0 0 0 0 0 0 0 0 0 0 0 0 0 0 0 0 0 0 0 0 0 0 0 0 0
0 0 0 0 0 0 0 0 0 0 0 0 0 0 0 0 0 0 0 0 0 0 0 0 0 0 0
M02255_77_000000000-ADUT3_1_1106_28560_8182 G----AC---GA-GG-GGT--------CCT-A-G------C--G--T---T--GT-T-CGG-AA------TT-A--C-T--GG-GC-------GT--A-----AA-GC-GC-AT-------G-TA-G-G-T-T-------------G--T-TT-G-A-C-----------AA----G-T-C-A--------G-G-T--G--TG--A-AA-GC--C-T-CG-G-G-----CT-T-AA------C-C-C-G-A-G-A--A-G----T-G--C-A---T--T-------T--GA-T-A---C-------T--G-T--CA--A-T-C----T-T-G-A-A-T--A---T-GG-----GA-G-A--------G-G-G-T---AG-T------AG--A--ATT----C-C-T-G-GT--GT-A-G-TG-GT--G---A--A-A---TA-C-GT-AG--AT-A-TC--A-G--G-A-G-G-A-AT-A-CC---GG--A--G--GC-GAA-G--G-C--G-------G--C-T-A-C---CTG--G--CC-T-A--------------------A-T---A-T-T--GA--CA-C-T-G--A-GA--T-G-CG-A--AA-G-C---G-TG--GG-G--AT-C-A-AA--------CA--GG Bacteria(100);unclassified(100);unclassified(100);unclassified(100);unclassified(100);unclassified(100);unclassified(100);unclassified(100);unclassified(100);

Otu0971 0 0 0 0 0 0 0 0 0 0 0 0 0 0 0 0 0 0 0 0 0 0 0 0 0 0 0 0
0 0 1 0 0 0 0 0 0 0 29 0 0 0 0 0 0 0 0 0 0 0 0 0 0 0 0 0
0 0 0 0 0 0 0 0 0 0 0 0 0 0 0 0 0 0 0 0 0 0 0 0 0 0 0 0
0 0 0 0 0 0 0 0 0 0 0 0 0 0 0 0 0 0 0 0 0 0 0 0 0 0 0
M02255_77_000000000-ADUT3_1_1102_18616_11835 T----AC---GT-AG-GTG--------GCA-A-G------C--G--T---T--GT-C-CGG-AT------TT-A--C-T--GG-GT-------GT--A-----AA-GG-GC-GT-------G-TA-G-G-C-G-------------G--A-TA-A-A-T-----------AA----G-T-T-G--------G-G-T--G--TG--A-AA-TAC-C---AC-G-G-----CT-C-AA------C-C-G-T-G-G-G--G-G----T-G--C-A---T--T-------C--AA-A-A---C-------T--A-T--TT--A-C-C----T-T-G-A-G-T--G---C-TG-----GA-G-A--------G-G-A-A---AG-C------GG--A--ATT----C-C-T-A-GT--GT-A-G-CG-GT--G---A--A-A---TG-C-GT-AG--AT-A-TT--A-G--G-A-G-G-A-AC-A-CC---AG--T--G--GC-GAA-G--G-C--G-------G--C-T-T-T---CTG--G--AC-A-G--------------------T-A---A-C-T--GA--CG-C-T-G--A-GG--C-G-CG-A--AA-G-C---G-TG--GG-G--AG-C-A-AA--------CA--GG Bacteria(100);unclassified(100);unclassified(100);unclassified(100);unclassified(100);unclassified(100);unclassified(100);unclassified(100);unclassified(100);

Otu0972 0 0 0 0 0 0 0 0 0 0 0 0 0 0 0 0 0 0 0 0 0 0 0 0 0 0 0 0
0 0 16 0 20 16 25 0 0 0 0 0 0 0 0 0 0 0 0 0 0 0 0 0 0 0 0 0
0 0 0 0 0 0 0 0 0 0 0 0 0 0 0 0 0 0 0 0 0 0 0 0 0 0 0 0
0 0 0 0 0 0 0 0 0 0 0 0 0 0 0 0 0 0 0 0 0 0 0 0 7 0 0
M02255_77_000000000-ADUT3_1_1107_24657_20368 G----AC---GT-AG-GAT--------GCA-A-G------C--G--T---T--GT-C-CGG-AT------TC-A--T-T--GG-GC-------GT--A-----AA-GA-GT-TC-------G-TA-G-G-T-G-------------G--T-TT-G-T-T-----------AA----G-T-T-T--------G-G-T--G--TT--A-AA-GA--A-C-GG-G-G-----CC-T-AA------C-C-C-C-G-G-T--A-G----T-G--C-A---C--T-------G--AA-T-A---C-------T--G-G--CA--G-A-C----T-T-G-A-G-T--G---C-GG-----TA-G-A--------G-G-T-A---AG-T------GG--A--ATT----C-C-A-A-GT--GT-A-G-CG-GT--G---A--A-A---TG-C-AT-AG--AT-A-TT--T-G--G-A-A-G-A-AC-A-CC---GG--T--G--GC-GTA-A--G-C--G-------A--C-T-T-A---CTG--G--GC-C-G--------------------T-A---A-C-T--GA--CA-C-T-G--A-GG--A-A-CG-A--AA-G-C---C-GG--GG-T--AG-C-G-AA--------TG--GG Bacteria(100);unclassified(100);unclassified(100);unclassified(100);unclassified(100);unclassified(100);unclassified(100);unclassified(100);unclassified(100);

Otu0973 0 0 0 0 0 0 0 0 0 0 0 0 0 0 0 0 0 0 0 0 0 0 0 0 0 0 0 0
0 0 0 0 0 0 37 0 0 0 0 0 0 0 0 0 0 0 0 0 0 0 0 0 0 0 0 0
0 0 0 0 0 0 0 0 0 0 0 0 0 0 0 0 0 0 0 0 0 0 0 0 0 0 0 0
0 0 0 0 0 0 0 0 0 0 0 0 0 0 0 0 0 0 0 0 0 0 0 0 0 0 0
M02255_77_000000000-ADUT3_1_1107_18083_16279 T----AC---GT-AG-GTG--------GCA-A-G------C--G--T---T--GT-C-CGG-AT------TT-A--T-T--GG-GC-------GT--A-----AA-GC-GC-GC-------G-CA-G-G-T-G-------------G----TTCG-T-T-----------GC----G-T-C-C--------G-A-G--G--TG--A-AA-TC--T-C-CC-G-G-----CT-C-AA------C-T-G-G-G-A-G--G------G-GT-C-T---T--C-------G--GA-T-A---C-------G--G-G--CG--G-A-C----T-T-G-A-G-G--A---G-AG-----CA-G-A--------G-G-T-A---GG-C------GG--A--ATT----C-T-G-G-GT--GT-A-G-TG-GT--G---A--A-A---TG-C-GT-AG--AT-A-TC--C-A--G-A-G-G-A-AC-A-CC---AG--T--G--GC-GAA-G--G-C--G-------G--C-C-T-A---CTG--G--GC-T-C--------------------C-A---C-C-T--GA--CA-C-T-G--A-GG--C-G-CG-A--AA-G-C---G-TG--GG-G--AG-C-G-AA--------CC--GG Bacteria(100);unclassified(100);unclassified(100);unclassified(100);unclassified(100);unclassified(100);unclassified(100);unclassified(100);unclassified(100);

Otu0974 0 0 0 0 0 0 0 0 0 0 0 0 0 0 0 0 0 0 0 0 0 0 0 0 0 0 0 0
0 0 0 0 0 0 64 4 0 0 0 3 0 0 0 0 0 0 0 0 0 0 0 0 0 0 0 0
0 0 0 0 0 0 0 0 0 0 0 0 0 0 0 0 0 0 0 0 0 0 0 0 0 0 0 0
0 0 0 0 0 0 0 0 0 0 0 0 0 0 0 0 0 0 0 0 0 0 0 0 0 0 0
M02255_77_000000000-ADUT3_1_1106_10326_9875 G----AC---GG-GG-GGG--------GCA-A-G------T--G--T---T--AT-T-CGG-AA------TG-A--C-T--AG-GC-------GT--A-----AA-GG-GC-AC-------G-TA-G-G-C-G-------------G--T-GA-A-T-C-----------GG----G-T-T-G--------G-A-A--G--TG--A-AA-----G-T-CG-C-C-----AA-C-AA------T-T-G-G-C-G-G--G-G----T-G--C-T---T--T-------C--GG-A-A---C-------C--A-A--TT--C-A-C----T-T-G-A-G-T--G---A-GA-----TA-G-G--------G-G-A-G---AG-T------GG--A--ATT----T-C-G-T-GT--GT-A-G-GG-AT--C---A--A-A---TC-C-GG-AG--AT-C-TA--C-G--A-A-G-G-A-AC-G-CC---AA--A--A--GC-GAA-G--G-C--A-------G--C-T-C-T---CTG--G--GT-C-T--------------------A-C---A-C-T--GA--CG-C-T-G--G-GG--T-G-CG-A--AA-G-C---A-TG--GG-G--AG-C-A-AA--------CA--GG Bacteria(100);unclassified(100);unclassified(100);unclassified(100);unclassified(100);unclassified(100);unclassified(100);unclassified(100);unclassified(100);

Otu0975 0 0 0 0 0 0 0 0 0 0 0 0 0 0 0 0 0 0 0 0 0 0 0 0 0 0 0 0
0 0 0 0 0 0 0 0 0 0 33 0 0 0 0 0 0 0 0 0 0 0 0 0 0 0 0 0 0
0 0 0 0 0 0 0 0 0 0 0 0 0 0 0 0 0 0 0 0 0 0 0 0 0 0 0 0 0
0 0 0 0 0 0 0 0 0 0 0 0 0 0 0 0 0 0 0 0 0 0 0 0 0 0 0
M02255_77_000000000-ADUT3_1_1119_12795_18069 T----AC---GG-AG-GGG--------GCT-A-G------C--G--T---T--GT-T-CGG-AG------TT-A--C-T--GG-GC-------GT--A-----AA-GG-GC-GC-------G-TA-G-G-C-G------------G--C-TG-T-G-C----
-------AA----G-T-C-A--------G-G-T--G--TG--A-AA-GG--C-C-CG-G-G-----CT-C-AA------C-C-C-G-G-G-G--A-C----G-G--C-G---C--T-------T--GC-G-A---C-------T--G-C--AC--G-G-CT---T-G-G-A-G-T--C---C-GG-----GA-G-A--------G-G-A-T---GG-T------GG--A--ATT--
--C-C-C-A-GT--GT-A-G-AG-GT--G---A--A-A---TT-C-GT-AG--AT-A-TT--G-G--G-A-A-G-A-AC-A-CC---GG--T--G--GC-GAA-G--G-C--G-------G--C-C-A-T---CTG--G--AC-C-G--------------------G-T---A-C-T--GA--CG-C-T-G--A-GG--C-G-CG-A--AA-G-C---GTGG--
GG----AG-C-A-AA--------CA--GG Bacteria(100);Proteobacteria(100);Alphaproteobacteria(100);Rhodospirillales(100);unclassified(100);unclassified(100);unclassified(100);unclassified(100);unclassified(100);

Otu0976 0 0 0 0 0 0 0 0 0 0 0 0 0 0 0 0 0 0 0 0 0 0 0 0 0 0 0 0
0 0 0 0 0 0 57 0 0 0 0 0 0 0 0 0 0 0 0 0 0 0 0 0 0 0 0 0 0
0 0 0 0 0 0 0 0 0 0 0 0 0 0 0 0 0 0 0 0 0 0 0 0 0 0 0 0 0
0 0 0 0 0 0 0 0 0 0 0 0 0 0 0 0 0 0 0 0 0 0 0 0 0 0 0
M02255_77_000000000-ADUT3_1_1102_6631_12227 T----AC---GG-AG-GAT--------CCA-A-G------C--G--T---T--AT-C-CGG-AA------TT-A--C-T--GG-GC-------GT--A-----AA-GA-GTTGC-------G-TA-G-G-T-G------------G--C-AT-A-G-T-----------
AA----G-T-A-G--------A-T-A--G--TG--A-AA-GC--G-T-GT-G-G-----CT-C-AA------C-C-A-T-A-C-A--T-A----C-A--T-T---A--T-------C--TA-A-A---C-------T--G-C--TA--A-G-C----T-A-G-A-A-G--A---T-GA-----GA-G-A--------G-G-T-A---GA-T------GG--A--ATT----C-C-C-
T-GT--GT-A-G-GA-GT--G---A--A-A---TC-C-GT-AG--AT-A-TA--G-G--G-A-G-G-A-AC-A-CC---GA--T--G--GC-GTA-G--G-C--A-------G--T-C-T-A---CTG--G--CT-C-A--------------------T-T---C-T-T--GA--CA-C-T-A--A-GG--C-A-CG-A--AA-G-C---G-TG--GG-T--AG-
C-A-AA--------CG--GG Bacteria(100);unclassified(100);unclassified(100);unclassified(100);unclassified(100);unclassified(100);unclassified(100);unclassified(100);unclassified(100);

Otu0977 0 0 0 0 0 0 0 0 0 0 0 0 0 0 0 0 0 0 0 0 0 0 0 0 0 0 0 0
0 0 0 0 0 0 17 0 0 0 0 0 0 0 0 0 0 0 0 0 0 0 0 0 0 0 0 0 0
0 0 0 0 0 0 0 0 0 0 0 0 0 0 0 0 0 0 0 0 0 0 0 0 0 0 0 0 0
0 0 0 0 0 0 0 0 0 0 0 0 0 0 0 0 0 0 0 0 0 0 0 0 0 0 0
M02255_77_000000000-ADUT3_1_1102_12098_10428 G----AT---AG-AG-AAT--------GCA-A-G------T--G--T---T--AT-C-CAG-AA------TG-A--T-T--GG-GT-------GT--A-----AA-GC-AT-CT-------G-TA-G-G-T-G------------G--C-TT-T-T-T-------
----AA----G-T-C-C--------G-T-T--G--TC--A-AA-TC--C-C-AG-G-G-----CT-C-AA------C-C-C-T-G-G-A--C-A----G-G--C-G---G--T-------G--GA-A-A---C-------T--A-C--CA--A-G-C----T-G-G-A-G-T--A---T-GG-----TA-G-G--------G-A-C-A---GA-G------GG--A--ATT----T-
T-C-G-AT--AA-A-G-CG-AT--G---A--A-A---TG-C-GT-AG--AG-A-TC--G-A--A-A-A-G-A-AC-A-CC---AA--C--G--AT-GAA-A--G-C--A-------C--T---T-G---CTA--G--GT-C-G--------------------A-C---A-C-T--GA--CA-C-T-G--A-GA--G-A-TG-A--AA-G-C---T-AG--AG-G--
AG-C-G-AA--------TG--GG Bacteria(100);unclassified(100);unclassified(100);unclassified(100);unclassified(100);unclassified(100);unclassified(100);unclassified(100);unclassified(100);

Otu0978 0 0 0 0 0 0 0 0 0 0 0 0 0 0 0 0 0 0 0 0 0 0 0 0 0 0 0 0
0 0 1 0 0 0 0 0 0 0 6 0 0 0 0 0 0 21 0 0 0 0 0 0 0 0 0 0 0
0 0 0 0 0 0 0 0 0 0 0 0 0 0 0 0 0 0 0 0 0 0 0 0 0 0 0 0 0
0 0 0 0 0 0 0 0 0 0 0 0 0 0 0 0 0 0 0 0 0 0 0 0 0 0 0
M02255_77_000000000-ADUT3_1_2102_9073_15933 T----AC---GT-AG-GGG--------GCA-A-G------C--G--T---T--AT-C-CGG-AT------TT-A--C-T--GG-GT-------GT--A-----AA-GG-GA-GC-------G-TA-G-A-C-G------------G--C-AT-G-G-C-----------
AA----G-T-C-T--------G-A-T--G--TG--A-AA-AC--T-T-GG-G-G-----CT-C-AA------C-C-C-C-A-A-G--A-C----T-G--C-A---T--T-------G--GA-A-A---C-------T--G-T--TA--A-G-C----T-A-G-A-G-T--G---C-AG-----GA-G-A--------G-G-T-A---AG-C------GG--A--ATT----C-C-T-
A-GT--GT-A-G-CG-GT--G---A--A-A---TG-C-GT-AG--AT-A-TT--A-G--G-A-G-G-A-AC-A-CC---AG--T--G--GC-GAA-G--G-C--G-------G--C-T-T-A---CTG--G--AC-T-G--------------------T-A---A-C-T--GA--CG-T-T-G--A-GG--C-T-CG-A--AA-G-C---G-TG--GG-G--
AG-C-A-AA--------CA--GG Bacteria(100);Firmicutes(100);Clostridia(100);Clostridiales(100);Lachnospiraceae(100);Incertae_Sedis(100);unclassified(100);unclassified(100);unclassified(100);

Otu0979 0 0 0 0 0 0 0 0 0 0 0 0 0 0 0 0 0 0 0 0 0 0 0 0 0 0 0 0
0 0 0 0 0 0 0 0 22 0 0 0 0 0 0 0 0 0 0 0 0 0 0 0 0 0 0 0 0
0 0 0 0 0 0 0 0 0 0 0 0 0 0 0 0 0 0 0 0 0 0 0 0 0 0 0 0 0
0 0 0 0 0 0 0 0 0 0 0 0 0 0 0 0 0 0 0 0 0 0 0 0 0 0 0
M02255_76_000000000-ADUNE_1_1108_11237_24940 C----GG---CT-AA-CTC--------CGT-G-C------C--A--G---C--AG-C-CGC-GG------TA-A--T-A--CG-GA-------GG--G-----TG-CA-AG-CG-------T-TA-T-C-C-G------------G--A-TTCT-G-T-----
------AA----G-T-C-A--------G-T-G--G--TG--A-AA-TC--T-C-CG-G-G-----CT-T-AA------C-C-C-G-G-A-A--A-C----T-G--C-C---G--T-------T--GA-T-A---C-------T--A-T--AG--A-T-C----T-T-G-A-A-T--G---T-TG-----TG-G-A--------G-G-T-T---AG-C------GG--A--ATA----T-
G-T-C-AT--GT-A-G-CG-GT--G---A--A-A---TG-C-AT-AG--AT-A-TG--A-C--A-T-A-G-A-AC-A-CC---AA--T--T--GC-GAA-G--G-C--A-------G--C-T-G-G---CTA--C--AC-A-A--------------------A-T---A-T-T--GA--CG-C-T-G--A-GG--C-A-CG-A--AA-G-C---G-TG--GG-G--
AT-C-A-AA--------CA--GG Bacteria(100);Bacteroidetes(100);Sphingobacteria(100);Sphingobacteriales(100);Chitinophagaceae(100);Chitinophaga(100);unclassified(100);unclassified(100);unclassified(100);

Otu0980 0 0 0 0 0 0 0 0 0 0 0 0 0 0 0 0 0 0 0 0 0 0 0 0 0 0 0 0
0 0 0 0 0 0 70 0 0 0 0 0 0 0 0 0 0 0 0 0 0 0 0 0 0 0 0 0 0
0 0 0 0 0 0 0 0 0 0 0 0 0 0 0 0 0 0 0 0 0 0 0 0 0 0 0 0 0
0 0 0 0 0 0 0 0 0 0 0 0 0 0 0 0 0 0 0 0 0 0 0 0 0 0 0 0
M02255_77_000000000-ADUT3_1_1101_6792_3894 T----AC---GT-AG-GGG--------GCG-A-G------C--G--T---T--AT-C-CGG-AA------TT-A--T-T--GG-GC-------GT--A-----AA-GC-GC-GC-------G-CA-G-G-C-G------------G--T-CA-T-T-T-----------AA-
---G-T-C-A--------G-G-T--G--TG--A-AA-GG--C-T-GC-G-G-----CT-C-AA------C-C-G-C-A-G-A--G------C-GG-C-A---C--C-------T--GA-A-A---C-------T--G-G--AT--G-A-C----T-T-G-A-G-T--G---C-AG-----GA-G-A--------G-G-A-G---AG-C------GG--A--ATT----C-C-C-G-
GT--GT-A-G-CG-GT--G---G--A-A---TG-C-GT-AG--AG-A-TC--G-G--G-A-G-G-A-AC-A-CC---AG--T--G--GC-GAA-G--G-C--G-------G--C-T-C-T---CTG--G--CC-T-G--------------------T-T---T-C-T--GA--CG-C-T-G--A-GG--C-G-CG-A--AA-G-C---G-TG--GG-G--AG-
C-A-AA--------CA--GG Bacteria(100);Firmicutes(100);Bacilli(100);Bacillales(100);Thermoactinomycetaceae(100);Laceyella(100);unclassified(100);unclassified(100);unclassified(100);

Otu0981 0 0 0 0 0 0 0 0 0 0 0 0 0 0 0 0 0 0 0 0 0 0 0 0 0 0 0 0
0 0 0 0 0 0 25 0 0 0 0 0 0 0 0 0 0 0 0 0 0 0 0 0 0 0 0 0 0
0 0 0 0 0 0 0 0 0 0 0 0 0 0 0 0 0 0 0 0 0 0 0 0 0 0 0 0 0
0 0 0 0 0 0 0 0 0 0 0 0 0 0 0 0 0 0 0 0 0 0 0 0 0 0 0 0
M02255_76_000000000-ADUNE_1_1116_24723_21577 C----CG---GC-TA-ACT--------TCG-T-G------C--C--A---G--CA-G-CCG-CG------GT-A--A-T--AC-GA-------AG--G-----GT-CC-AA-GC-------G-TT-GTT-C-G------------G--A-TC-A-G-C-----
------AA----G-T-C-A--------G-A-T--G--TG--A-AA-TC--T-C-GA-A-G-----CT-C-AA------C-T-T-C-G-A-A--A-C----T-G--C-G---T--C-------T--GA-A-A---C-------T--G-C--TA--G-T-C----T-A-G-A-A-T--G---T-CG-----GA-G-G--------G-G-A-T---AG-G------GG--A--ATT----T-
C-A-C-GT--GT-A-G-GG-GT--A---A--A-A---TC-C-GT-AG--AG-A-TG--T-G--A-A-G-G-A-AC-A-CC---GG--A--G--GC-GAA-G--G-C--G-------C--C-T-A-T---CTG--G--AC-G-A--------------------C-T---A-T-T--GA--CG-C-T-G--A-GG--C-T-CG-A--AA-G-C---G-TG--GG-
G--AG-C-A-AA--------CA--GG Bacteria(100);Proteobacteria(100);Deltaproteobacteria(100);Bdellovibrionales(100);Bacteriovoraceae(100);Peredibacter(100);unclassified(100);unclassified(100);unclassified(100);

Otu0982 0 0 0 0 0 0 0 0 0 0 0 0 0 0 0 0 0 0 0 0 0 0 0 0 0 0 0 0
0 0 0 0 0 0 0 0 0 0 0 0 0 0 0 0 0 0 0 0 0 0 0 1 0 0 0 0 0
0 0 0 0 0 0 0 0 0 0 0 0 0 0 0 0 0 0 0 0 0 0 0 0 0 0 0 0 0
0 8 0 0 0 0 0 0 0 0 0 0 0 3 0 0 0 0 0 0 0 0 0 0 0 0 0 0
M02255_76_000000000-ADUNE_1_2110_12608_23643 C----GG---CT-AA-TTC--------CGT-G-C------C--A--G---C--AG-C-CGC-GG------TA-A--T-A--CG-GA-------AG--G-----TC-CA-GG-CG-------T-TA-T-C-C-G------------G--A-TT-T-A-T-------
----TA----G-C-G-T--------G-C-T--G--TG--A-AA-TA--T-A-GT-G-G-----CT-C-AA------C-C-T-C-T-A-T--C-G----T-G--C-A---G--C-------G--CG-A-A---C-------T--G-T--CG--A-A-C----T-T-G-A-G-T--G---C-GT-----AG-T-A--------G-G-T-A---GG-C------GG--A--ATT----C-G-
T-G-GT--GT-A-G-CG-GT--G---A--A-A---TG-C-TT-AG--AT-A-TC--A-C--G-A-A-G-A-AC-T-CC---GA--T--T--GC-GAA-G--G-C--A-------G--C-T-T-A---CCG--T--AA-C-G--------------------T-T---A-C-T--GA--CG-C-T-T--A-AG--C-A-CG-A--AG-G-T---G-CG--GG-T--AT-
C-G-AA--------CA--GG Bacteria(100);Bacteroidetes(100);Bacteroidia(100);Bacteroidales(100);unclassified(100);unclassified(100);unclassified(100);unclassified(100);unclassified(100);

Otu0983 0 0 0 0 0 0 0 0 0 0 0 0 0 0 0 0 0 0 0 0 0 0 0 0 0 0 0 7
0 0 0 0 0 0 0 0 0 0 0 0 0 0 0 0 0 0 0 0 0 0 0 0 0 0 0 0 0
0 0 0 0 0 0 0 0 0 0 0 0 0 0 0 0 0 0 0 0 0 0 0 0 0 0 0 0 0
0 0 0 0 0 0 0 0 0 0 0 0 0 0 0 0 0 0 0 0 0 0 0 0 0 0 0 0
M02255_77_000000000-ADUT3_1_1115_13081_19849 T----AC---GG-AG-GGG--------GCT-A-G------C--G--T---T--GT-T-CGG-AA------TT-A--C-T--GG-GC-------GT--A-----AA-GG-GC-GC-------G-TA-G-G-C-G------------G--T-TT-A-C-C-----
------CA----G-T-C-A--------G-G-C--G--TG--A-AA-GG--C-C-TG-G-G-----CT-C-AA------C-C-C-G-G-G-A--A-C----T-G--C-G---C--T-------T--GA-T-A---C-------T--G-G--TA--G-A-C----T-C-G-A-G-T--C---C-GG-----GA-G-A--------G-G-A-T---GG-C------GG--A--ATT----
C-C-C-A-GT--GT-A-G-AG-GT--G---A--A-A---TG-C-GT-AG--AT-A-TT--G-G--G-A-A-G-A-AC-A-CC---GG--T--G--GC-GAA-G--G-C--G-------G--C-C-A-T---CTG--G--AC-C-G--------------------G-C---A-C-T--GA--CG-C-T-G--A-GG--C-G-CG-A--AA-G-C---GTGG--
GG----AG-C-A-AA--------CA--GG Bacteria(100);Proteobacteria(100);Alphaproteobacteria(100);Rhodospirillales(100);unclassified(100);unclassified(100);unclassified(100);unclassified(100);unclassified(100);

Otu0984 0 0 0 0 0 0 0 0 0 0 0 0 0 0 0 0 0 0 0 0 0 0 0 0 0 0 0 0
0 0 2 0 0 0 10 0 0 0 11 0 0 0 0 0 0 0 0 0 0 0 0 0 0 0 0 0 0
0 0 0 0 0 0 0 0 0 0 0 0 0 0 0 0 0 0 0 0 0 0 0 0 0 0 0 0 0
0 0 0 0 0 0 0 0 0 0 0 0 0 0 0 0 0 0 0 0 0 0 0 0 0 0 0 0
M02255_77_000000000-ADUT3_1_2117_12121_14681 T----ACG--GG--G-GGG--------GCA-A-G------C--G--T---T--GT-T-CGG-AA------TT-A--C-T--GG-GC-------GT--A-----AA-GG-GC-GC-------G-TA-G-G-C-G------------G--T-TG-G-A-T----
-------AA----G-T-C-A--------G-C-T--G--TG--A-AA-AC--C-A-CG-G-G-----CT-T-AA------C-T-C-G-T-G-G--C-C----T-G--C-A---G--T-------T--GA-G-A---C-------T--G-T--CT--G-G-C----T-T-G-A-G-T--G---T-GG-----GA-G-A--------G-G-A-G---AG-T------GG--A--ATT----
C-C-T-G-GT--GT-A-G-CG-GT--G---A--A-A---TG-C-GT-AG--AT-A-TC--A-G--G-A-G-G-A-AC-A-CC---GG--T--G--GC-GAA-G--G-C--G-------G--C-T-C-T---CTG--G--AC-C-A--------------------C-A---A-C-T--GA--CG-C-T-G--A-GG--C-G-CG-A--AA-G-C---T-AG--
GG-G--AG-C-A-AA--------CA--GG Bacteria(100);unclassified(100);unclassified(100);unclassified(100);unclassified(100);unclassified(100);unclassified(100);unclassified(100);unclassified(100);

Otu0985 0 0 0 0 0 0 0 0 0 0 0 0 0 0 0 0 0 0 0 0 0 0 0 0 0 0 0 0
0 0 1 0 0 0 0 0 0 0 28 0 0 0 0 0 0 0 0 0 0 0 0 0 0 0 0 0
0 0 0 0 0 0 0 0 0 0 0 0 0 0 0 0 0 0 0 0 0 0 0 0 0 0 0 0
0 0 0 0 0 0 0 0 0 0 0 0 0 0 0 0 0 0 0 0 0 0 0 0 0 0
M02255_77_000000000-ADUT3_1_1111_10354_21173 G----AC---AG-AG-GTG--------CCA-A-G------C--G--T---T--AG-G-CGG-AA------TC-A--C-T--GG-GC-------TT--A-----AA-GC-GT-GT-------G-TA-G-G-C-G------------G--A-CT-T-C-T-----
------AA----G-T-A-C--------C-T-T--G--TG--A-AA-TC--C-C-AC-G-G-----CT-C-AA------C-C-G-T-G-G-A--A-C----T-G--C-T---C--G-------G--TA-T-A---C-------T--G-G--TT--G-T-C----T-T-G-A-G-C--C---A-CT-----TA-G-G--------G-G-C-T---AC-C------GG--A--ACA----A-
A-T-G-GT--GG-A-G-CG-GT--G---A--A-A---TG-C-GT-AG--AT-A-TC--A-T--T-T-G-G-A-AC-G-CC---AA--T--G--GT-GAA-A--A-C--A-------G--G-T-A-G---CTG--G--GA-G-T--------------------G-T---G-C-T--GA--CG-C-T-G--A-GA--C-A-CG-A--AA-G-C---C-AG--GG-
G--AG-C-G-AA--------CG--GG Bacteria(100);Planctomycetes(100);Phycisphaerae(100);WD2101(100);unclassified(100);unclassified(100);unclassified(100);unclassified(100);unclassified(100);

Otu0986 0 0 0 0 0 0 0 0 0 0 0 0 0 0 0 0 0 0 0 0 0 0 0 0 0 0 0 0
0 0 0 0 0 8 0 0 8 0 0 0 0 0 0 0 0 0 0 0 0 0 0 0 0 0 0 0
0 0 0 0 0 0 0 0 0 0 0 0 0 0 0 0 0 0 0 0 0 0 0 0 0 0 0 0
0 0 0 0 0 0 0 0 0 0 0 0 0 0 0 0 0 0 0 0 0 0 0 0 0 0
M02255_76_000000000-ADUNE_1_1116_21605_9074 T----CC---AG-AA-GAA--------GCC-C-C------G--G--C---T--AA-C-TAC-GT------GC-C--A-G--CA-GC-------CG--C-----GG-TA-AT-AC-------G-TA-G-G-G-G------------G--C-AA-GCG-T-----------
TG----T-C-C-G--------GAT-T--G--TG--A-AA-AC--T-G-GA-G-G-----CT-C-AA------C-C-T-T-C-A-G--A-G----G-T--C-G---A--C-------C--GA-A-A---C-------C--A-T--CT--G-G-C----T-A-G-A-G-T--C---C-GG-----GA-G-A--------G-G-A-G---AG-T------GG--A--ATT----C-C-
T-G-GT--GT-A-G-CG-GT--G---A--A-A---TG-C-GC-AG--AT-A-TC--A-G--G-A-A-G-A-AC-A-CC---CG--T--G--GC-GAA-A--G-C--G-------G--C-T-C-T---CTG--G--AA-C-G--------------------G-T---A-C-T--GA--CG-C-T-G--A-GA--C-G-CG-A--AA-G-C---G-TG--GG-G--
AG-C-G-AA--------CA--GG Bacteria(100);Actinobacteria(100);Actinobacteria(100);Rubrobacteridae(100);AKIW543(100);unclassified(100);unclassified(100);unclassified(100);unclassified(100);

Otu0987 0 0 0 0 0 0 0 0 0 0 0 0 0 0 0 0 0 0 0 0 0 0 0 0 0 0 0 0
0 0 0 0 0 0 0 0 0 0 0 0 0 0 0 0 0 0 0 0 0 0 0 0 0 0 0 0
0 0 0 0 0 0 0 0 0 0 0 0 0 0 0 0 0 0 0 0 0 0 0 0 0 0 9 0
0 0 0 0 0 0 0 0 0 0 0 0 1 2 0 0 0 0 0 0 0 0 0 0 0 0
M02255_76_000000000-ADUNE_1_1111_13896_7608 T----A----AG-CA-CCG--------GCT-A-A------C-----T---C--TG-T-GCC-AG------CA-G--C-C--GC-G--------GT--A-----AT-AC--A-GA-------G-GG-T-G-C-G------------A--G-CT-G-T-T-----------AA----
G-T-C-G--------G-A-T--G--TG--A-AA-TC--C-C-TG-A-G-----CT-T-AA------C-T-T-A-G-G-A--A-T----T-G--C-A---T--T-------C--GA-T-A---C-------T--G-G--GA--A-G-C----T-A-G-A-G-T--A---T-GG-----GA-G-A--------G-G-A-T---GG-T------AG--A--ATT----C-C-A-G-GT--
GT-A-G-CG-GT--G---A--A-A---TG-C-GT-AG--AG-A-TC--T-G--G-A-G-G-A-AT-A-CC---GA--T--G--GC-GAA-G--G-C--A-------G--C-C-A-T---CTG--G--CC-T-A--------------------A-T---A-C-T--GA--CG-C-T-G--A-GG--T-A-CG-A--AA-G-C---A-TG--GG-G--AG-C-A-
AA--------CA--GG Bacteria(100);Proteobacteria(100);Gammaproteobacteria(100);Pseudomonadales(100);Moraxellaceae(100);Acinetobacter(100);unclassified(100);unclassified(100);unclassified(100);

Otu0988 0 0 0 0 0 0 0 0 0 0 0 0 0 0 0 0 0 0 0 0 0 0 0 0 0 0 0 0
0 0 0 0 0 17 0 0 0 0 0 0 0 0 0 0 0 0 0 0 0 0 0 0 0 0 0 0
0 0 0 0 0 0 0 0 0 0 0 0 0 0 0 0 0 0 0 0 0 0 0 0 0 0 0 0
0 0 0 24 0 0 0 0 0 0 0 0 0 32 0 0 0 0 0 0 0 0 0 0 0 0
M02255_77_000000000-ADUT3_1_1117_11204_14635 T----AC---GT-AG-GGA--------CCA-A-G------C--G--T---T--GT-T-CGG-AT------TT-A--C-T--GG-GC-------GT--A-----AA-GG-GC-GC-------G-TA-G-G-C-G------------G--C-AT-G-A-C-----
------AA----G-T-C-A--------C-T-T--G--TG--A-AA-TC--T-C-CG-G-G-----CT-T-AA------C-T-C-G-G-A-A--C-G----G-C--C-A---A--G-------T--GA-T-A---C-------T--G-T--CG--C-G-C----T-A-G-A-G-T--G---C-AG-----AA-G-G--------G-G-C-A---AT-C------GG--A--ATT----C-
T-T-G-GT--GT-A-G-CG-GT--G---A--A-A---TG-C-GT-AG--AT-A-TC--A-A--G-A-G-G-A-AC-A-CC---TG--A--G--GT-GAA-G--A-C--G-------G--G-T-T-G---CTG--G--GC-T-G--------------------A-C---A-C-T--GA--CG-C-T-G--A-GG--C-G-CG-A--AA-G-C---T-AG--GG-
T--AG-C-A-AA--------CG--GG Bacteria(100);Acidobacteria(100);Acidobacteria(100);Acidobacteriales(100);Acidobacteriaceae(100);Candidatus_Chloroacidobacterium(100);unclassified(100);unclassified(100);unclassified(100);

Otu0989 0 0 0 0 0 0 0 0 0 0 0 0 0 0 0 0 0 0 0 0 0 0 0 0 0 0 0 0
0 0 0 0 0 21 0 0 0 0 0 0 0 0 0 0 0 0 0 0 0 0 0 0 0 0 0 0
0 0 0 0 0 0 0 0 0 0 0 0 0 0 0 0 0 0 0 0 0 0 0 0 0 0 0 0
0 0 0 0 0 0 0 0 0 0 0 0 0 0 0 0 0 0 0 0 0 0 0 0 0 0
M02255_77_000000000-ADUT3_1_2116_8294_6558 T----AC---GT-AG-GTG--------GCA-A-G------C--G--T---T--GT-C-CGG-AT------TT-A--C-T--GG-GC-------GT--A-----AA-GA-GC-GC-------G-CA-G-G-C-G------------G--T-CG-G-A-T-----------CA-
---G-T-C-G--------A-A-T--G--TG--A-AA-GC--C-C-CC-G-G-----CT-C-AA------C-T-G-G-G-G-A--G-G----G-T--C-A---T--T-------C--GA-T-A---C-------T--G-T--CC--G-A-C----T-C-G-A-A-G--G---C-AG-----GA-G-A--------G-G-G-A---AG-C------GG--A--ATT----C-C-C-G-
GT--GT-A-G-TG-GT--G---A--A-A---TG-C-GT-AG--AT-A-TC--G-G--G-A-G-G-A-AC-A-CC---AG--T--G--GC-GAA-G--G-C--G-------G--C-T-T-T---CTG--G--CC-T-G--------------------T-T---C-T-T--GA--CG-C-T-G--A-GG--C-G-CG-A--AA-G-C---T-AG--GG-G--AG-C-
G-AA--------CG--GG Bacteria(100);Chloroflexi(100);KD4-96(100);unclassified(100);unclassified(100);unclassified(100);unclassified(100);unclassified(100);unclassified(100);

Otu0990 0 0 0 0 0 0 0 0 0 0 0 0 0 0 0 0 0 0 0 0 0 0 0 0 0 0 0 0
0 0 0 0 0 31 0 0 0 0 0 0 0 0 0 0 0 0 0 0 0 0 0 0 0 0 0 0 0
0 0 0 0 0 0 0 0 0 0 0 0 0 0 0 0 0 0 0 0 0 0 0 0 0 0 0 0 0
0 0 0 0 0 0 0 0 0 0 0 0 0 0 0 0 0 0 0 0 0 0 0 0 0 0 0 0
M02255_77_000000000-ADUT3_1_2113_26940_9890 T----AC---GT-AG-GAC--------CCA-A-G------C--G--T---T--AT-C-CGG-AG------TG-A--C-T--GG-GT-------GT--A-----AA-GA-GTTGC-------G-TA-G-G-T-G------------G--C-TC-G-T-T-----------AA-
---G-T-A-G--------A-T-A--G--TG--A-AA-TC--G-T-GT-G-G-----CT-C-AA------C-C-A-T-TAC-G--G-C----T-A--T-T---A--T-------C--TA-A-A---C-------T--G-G--CG--G-G-C----T-C-G-A-G-A--A---T-GG-----TA-G-A--------G-G-T-A---GC-T------GG--A--ATT----T-C-T-A-
GT--GT-A-G-GA-GT--G---A--A-A---TC-C-GT-AG--AT-A-TT--A-G--A-A-G-G-A-AC-A-CC---AA--T--G--GC-GTA-G--G-C--A-------G--G-C-T-A---CTG--G--AC-C-A--------------------T-T---T-C-T--GA--CA-C-T-G--A-GG--C-A-CG-A--AA-G-C---G-TG--GG-G--AG-C-
G-AA--------CC--GG Bacteria(100);Candidate_division_TM7(100);unclassified(100);unclassified(100);unclassified(100);unclassified(100);unclassified(100);unclassified(100);unclassified(100);

Otu0991 0 0 0 0 0 0 0 0 0 0 0 0 0 0 0 0 0 0 0 0 0 0 0 0 0 0 0 0
0 0 4 0 0 0 0 0 0 0 54 1 0 0 0 0 0 0 0 0 0 0 0 0 0 0 0 0 0
0 0 0 0 0 0 0 0 0 0 0 0 0 0 0 0 0 0 0 0 0 0 0 0 0 0 0 0 0
0 0 0 0 0 0 0 0 0 0 0 0 0 0 0 0 0 0 0 0 0 0 0 0 0 0 0 0
M02255_76_000000000-ADUNE_1_1118_14683_6626 .....AC----T-A--CGT--------GCC-A-G------C--A--G---C--CG-C-GGT-AA------TA-C--G-T--AG-GG-------GG--C-----AA----GC-G--------T-TG-T-C-C-G------------G--A-TA-G-T-T-----------AA----G-
T-C-C--------G-G-T--G--TG--A-AA-GA--T-C-AG-G-G-----CT-C-AA------C-C-C-T-G-A-G--A-G----T-G--C-A---T--C-------G--GA-A-A---C-------T--G-G--TT--A-T-C----T-T-G-A-G-G--A---C-AG-----GA-G-A--------G-G-A-A---AG-T------GG--A--ATT----C-C-T-A-GT--
GT-A-G-CG-GT--G---A--A-A---TG-C-GT-AG--AT-A-TT--A-G--G-A-G-G-A-AC-A-CC---AG--T--G--GC-GAA-G--G-C--G-------A--C-T-T-T---CTG--G--AC-T-G--------------------T-A---A-C-T--GA--CG-C-T-G--A-GG--C-G-CG-A--AA-G-C---G-TG--GG-G--AG-C-A-
AA--------CA--GG Bacteria(100);Firmicutes(100);Clostridia(100);Clostridiales(100);Peptococcaceae(100);Desulfosporosinus(100);unclassified(100);unclassified(100);unclassified(100);

Otu0992 0 0 0 0 0 0 0 0 0 0 0 0 0 0 0 0 0 0 0 0 0 0 0 0 0 0 0 0
0 0 0 0 0 23 0 0 0 0 0 0 0 0 0 0 0 0 0 0 0 0 0 0 0 0 0 0 0
0 0 0 0 0 0 0 0 0 0 0 0 0 0 0 0 0 0 0 0 0 0 0 0 0 0 0 0 0
0 0 0 0 0 0 0 0 0 0 0 0 0 0 0 0 0 0 0 0 0 0 0 0 0 0 0 0
M02255_77_000000000-ADUT3_1_1116_23965_23976 T----AC---AG-AG-ACC--------TCA-A-G------C--G--T---T--AT-C-CGG-AT------TT-A--T-T--GG-GC-------GT--A-----AA-GC-GT-GT-------G-TA-G-G-C-G------------G--T-CT-G-A-T-------
----TA----G-T-C-A--------G-A-T--G--TT--A-AA-TA--T-C-AG-G-G-----CT-C-AA------C-C-C-T-G-A-A--C-C----A-G--C-A---T--T-------T--GA-A-A---C-------G--G-T--CA--G-A-C----T-A-G-A-G-A--A---T-GT-----GA-G-A--------G-A-T-C---AG-T------GG--A--ATT----T-A-
T-G-GT--GT-A-G-TA-GT--G---A--A-A---TG-C-GT-TG--AT-A-TC--A-T--A-A-A-G-A-AC-A-CC---CA--A--G--GC-GAA-G--G-C--A-------A--C-T-G-A---TTG--G--CA-C-A--------------------T-T---T-C-T--GA--CG-C-T-G--A-GA--C-A-CG-A--AA-G-C---G-TG--GG-G--
AG-C-G-AA--------TG--GG Bacteria(100);unclassified(100);unclassified(100);unclassified(100);unclassified(100);unclassified(100);unclassified(100);unclassified(100);unclassified(100);

Otu0993 0 0 0 0 0 0 0 0 0 0 0 0 0 0 0 0 0 0 0 0 0 0 0 0 0 0 0 0
0 0 0 0 0 49 0 0 0 0 0 0 0 0 0 0 0 0 0 0 0 0 0 0 0 0 0 0 0
0 0 0 0 0 0 0 0 0 0 0 0 0 0 0 0 0 0 0 0 0 0 0 0 0 0 0 0 0
0 0 0 0 0 0 0 0 0 0 0 0 0 0 0 0 0 0 0 0 0 0 0 0 0 0 0 0
M02255_77_000000000-ADUT3_1_1113_12620_3061 G----AC---GG-AG-GGT--------GCT-A-G------C--G--T---T--GT-T-CGG-AA------TC-A--C-T--GG-GC-------GT--A-----AA-GG-GC-GC-------G-TA-G-G-C-G------------G--C-CA-G-G-C-----------
AA----G-T-C-G--------GCT-T--G--TG--A-AA-GC--C-C-GG-G-G-----CT-C-AA------C-C-C-C-G-G-A--G-G----T-G--C-G---G--C-------C--GG-G-A---C-------T--G-C--TT--G-G-C----T-G-G-A-G-G--C---C-GG-----AA-G-A--------G-G-A-G---GG-T------GG--A--ATA----C-
C-C-A-GT--GT-A-G-AG-GT--G---A--A-A---TT-C-GT-AG--AT-A-TT--G-G--G-T-G-G-A-AC-A-CC---GG--T--G--GC-GAA-A--G-C--G-------G--C-T-C-T---CTG--G--TC-C-G--------------------G-T---C-C-T--GA--CG-C-T-G--A-GG--C-G-CG-A--AA-G-C---G-T-G-GG-G-
-AG-C-A-AA--------CA--GG Bacteria(100);Proteobacteria(100);Alphaproteobacteria(100);Rhodospirillales(100);unclassified(100);unclassified(100);unclassified(100);unclassified(100);unclassified(100);

Otu0994 0 0 0 0 0 0 0 0 0 0 0 0 0 0 0 0 0 0 0 0 0 0 0 0 0 0 0 0
0 0 0 0 0 37 0 0 0 0 0 41 0 0 0 0 0 0 0 0 0 0 0 0 0 0 0 0 0
0 0 0 0 0 0 0 0 0 0 0 0 0 0 0 0 0 0 0 0 0 0 0 0 0 0 0 0 0
0 0 0 0 0 0 0 0 0 0 0 0 0 0 0 0 0 0 0 0 0 0 0 0 0 0 0 0
M02255_77_000000000-ADUT3_1_1110_10171_15421 A----AC---GT-AG-GGG--------GCG-A-G------C--G--T---T--GT-C-CGG-AA------TC-A--C-T--GG-GC-------GT--A-----AA-GG-GT-GC-------G-TA-G-G-C-G------------G--C-CG-C-T-T----
-------AA----G-T-C-C--------T-G-T--G--TG--A-AA-GG--C-C-CC-G-G-----CT-C-AA------C-C-G-G-G-G-A--AG-----G-C--C-A---T--G-------G--GA-A-A---C-------T--G-A--GC--G-G-C----T-T-G-A-G-T--G---C-TG-----GA-G-A--------G-G-G-T---TG-C------GG--A--ATT----
C-C-C-G-GT--GT-A-G-CG-GT--G---A--A-A---TG-C-GT-AG--AT-A-TC--G-G--G-A-G-G-A-AC-A-CC---AG--T--G--GC-GAA-G--G-C--G-------G--C-A-A-C---CTG--G--AC-A-G--------------------T-A---A-C-T--GA--CG-C-T-G--A-GG--C-A-CG-A--CA-G-C---T-GG--
GG-G--AG-C-A-AA--------CA--GG Bacteria(100);unclassified(100);unclassified(100);unclassified(100);unclassified(100);unclassified(100);unclassified(100);unclassified(100);unclassified(100);

Otu0995 0 0 0 0 0 0 0 0 0 0 0 0 0 0 0 0 0 0 0 0 0 0 0 0 0 0 0 0
0 0 0 0 0 0 0 0 0 0 0 0 0 0 0 17 0 0 0 0 0 0 0 0 0 0 0 0 0
0 0 0 0 0 0 0 0 0 0 0 0 0 0 0 0 0 0 0 0 0 0 0 0 0 0 0 0 0
0 0 0 0 0 0 0 0 0 0 0 0 0 0 0 0 0 0 0 0 0 0 0 0 0 0 0
M02255_77_000000000-ADUT3_1_1111_14200_12363 T----AC---GT-AG-GGT--------GCA-A-G------C--G--T---T--GT-C-CGG-AA------TT-A--T-T--GG-GC-------GT--A-----AA-GA-GC-TC-------G-TA-G-G-C-G------------G--T-TT-G-T-C-----------GC----G-T-C-G--------G-A-T--G--TG--A-AA-AC--C-C-AA-G-G-----CT-C-AA------C-C-T-T-G-G-G--C-C----T-G--C-A---T--T-------C--GA-T-A---C-------G--G-G--CA--G-A-C----T-A-G-A-G-T--T---C-GG-----TA-G-G--------G-G-A-G---AC-T------GG--A--ATT----C-C-T-G-GT--GT-A-G-CG-GT--G---A--A-A---TG-C-GC-AG--AT-A-TC--A-G--G-A-G-G-A-AC-A-CC---GG--T--G--GC-GAA-G--G-C--G-------G--G-T-C-T---CTG--G--GC-C-G--------------------A-T---A-C-T--GA--CG-C-T-G--A-GG--A-G-CG-A--AA-G-C---G-TG--GG-G--AG-C-G-AA--------CA--GG Bacteria(100);Actinobacteria(100);Actinobacteria(100);Actinobacteridae(100);Actinomycetales(100);unclassified(100);unclassified(100);unclassified(100);unclassified(100);

Otu0996 0 0 0 0 0 0 0 0 0 0 0 0 0 0 0 0 0 0 0 0 0 0 0 0 0 0 0 0
0 0 0 0 0 0 0 0 0 0 0 23 0 0 0 0 0 0 0 0 0 0 0 0 0 0 0 0 0
0 0 0 0 0 0 0 0 0 0 0 0 0 0 0 0 0 0 0 0 0 0 0 0 0 0 0 0 0
0 0 0 0 0 0 0 0 0 0 0 0 0 0 0 0 0 0 0 0 0 0 0 0 0 0 0
M02255_76_000000000-ADUNE_1_1103_4841_10400 C----TA---AC-TC-CGT--------GCC-A-G------C-----A---G--CC-G-CGG-TA------AT-A--C-G--GA-GG-------GT--G-----CA-AG-CG-TT-------A-TC-C-G-G-A------------T--T-CA-C-T-T-----------AA----G-T-C-A--------G-T-G--G--TG--A-AA-TC--T-C-CG-G-G-----CT-C-AA--------C-C-C-GGA-A--A-C----T-G--C-C---A--T-------T--GA-T-A---C-------T--A-T--TT--A-T-C----T-T-G-A-A-T--T---C-TG-----TT-G-A--------G-G-T-G---GG-C------GG--A--ATA----C-A-T-C-AT--GT-A-G-CG-GT--G---A--A-A---TG-C-TT-AG--AT-A-TG--A-T--G-T-A-G-A-AC-A-CC---GA--T--T--GC-GAA-G--G-C--A-------G--C-T-C-A---CTA--A--GC-A-G--------------------C-G---A-T-T--GA--CG-C-T-G--A-GG--C-A-CG-A--AA-G-C---G-TG--GG-G--AT-C-A-AA--------CA--GG Bacteria(100);Bacteroidetes(100);Sphingobacteria(100);Sphingobacteriales(100);Chitinophagaceae(100);uncultured(100);unclassified(100);unclassified(100);unclassified(100);

Otu0997 0 0 0 0 0 0 0 0 0 0 0 0 0 0 0 0 0 0 0 0 0 0 0 0 0 0 0 0
0 0 0 0 0 0 0 0 0 0 0 4 0 0 0 0 0 0 0 0 0 0 0 1 0 0 0 0 0
0 0 0 0 0 0 0 0 0 0 0 0 0 0 0 0 1 0 0 4 0 0 0 0 0 0 0 0 0
0 0 0 0 3 0 0 0 0 0 1 0 0 0 1 0 0 0 0 0 0 0 0 0 0 0 0
M02255_76_000000000-ADUNE_1_1102_24296_16857 T----AC---GT-AG-GGG--------GCA-A-G------C--G--T---T--AT-C-CGG-AA------TT-A--T-T--GG-GC-------GT--------AA--A----GC-------G-T--G-G-T-A------------A--T-T------T-----------AA----G-C-G-C--------A-A-G--G--TT--T-AA-AC--T-A-AC-G-G-----CT-C-AA------C-C-G-T-T-A-A--A------T-G--C-C---A--T-------G--CG-A-A---C-------T--G-G--GT--T-A-C----T-T-G-A-G-T--G---C-TG-----GA-G-G--------G-G-A-A---AG-C------GG--A--ATT----C-C-T-A-GT--GT-A-G-CG-GT--G---A--A-A---TG-C-GT-AG--AT-A-TT--A-G--G-A-G-G-A-AC-A-CC---GG--C--G--GC-GAA-G--G-C--G-------G--C-T-T-T---CTG--G--AC-A-G--------------------C-A---A-C-T--GA--CA-C-T-G--A-GG--C-A-CG-A--AA-G-T---G-TG--GG-T--AG-C-A-AA--------CA--GG Bacteria(100);Firmicutes(100);Clostridia(100);Clostridiales(100);unclassified(100);unclassified(100);unclassified(100);unclassified(100);unclassified(100);

Otu0998 0 0 0 0 0 0 0 0 0 0 0 0 0 0 0 0 0 0 0 0 0 0 0 0 2 0 0 0
0 0 0 0 0 0 0 0 0 0 0 0 0 0 0 0 0 0 0 0 0 0 0 0 0 0 0 0 0
0 0 0 0 0 0 0 0 0 0 0 0 0 0 0 0 0 0 0 0 0 0 0 0 0 0 0 0 0
0 0 0 679 0 0 0 0 0 0 0 0 0 0 0 0 0 0 0 0 0 0 0 0 0 0 0
M02255_77_000000000-ADUT3_1_2113_18291_6674 T----AC---GA-AG-GGG--------GCT-A-G------C--G--T---T--GT-T-CGG-AA------TT-A--C-T--GG-GC-------GT--A-----AA-GC-GC-AC-------G-CA-G-G-C-G------------G--C-TC-G-A-T-----------AA----A-T-T-A--------G-A-A--G--TG--A-AA-GC--C-T-TG-G-G-----CT-T-AA------C-C-T-G-A-G-A--A-T----T-G--C-T---T--T-------T--AA-G-A---C-------T--G-T--CG--A-G-C----T-A-G-A-A-T--C---C-AG-----AA-G-A--------G-G-G-T---AG-T------GG--A--ATT----C-C-G-A-GT--GT-A-G-AG-GT--G---A--A-A---TT-C-GT-AG--AT-A-TT--C-G--G-A-A-G-A-AC-A-CC---AG--T--G--GC-GAA-G--G-C--G-------A--C-T-A-C---CTG--G--TC-T-G--------------------G-C---A-T-T--GA--CG-C-T-C--A-TG--T-G-CG-A--AA-G-C---G-TG--GG-G--AG-C-A-AA--------CA--GG Bacteria(100);Proteobacteria(100);Alphaproteobacteria(100);Rhodospirillales(100);Rhodospirillaceae(100);unclassified(100);unclassified(100);unclassified(100);unclassified(100);

Otu0999 0 0 0 0 0 0 0 0 0 0 0 0 0 0 0 0 0 0 0 0 0 0 0 0 0 0 0 0
0 0 0 0 0 0 0 0 0 0 5 0 0 0 0 8 0 29 0 0 0 0 0 0 0 0 0 0 0
0 0 0 0 0 0 0 0 0 0 0 0 0 0 0 0 0 0 0 0 0 0 0 0 0 0 0 0 0
0 0 0 0 0 0 0 0 0 0 0 0 0 0 0 0 0 0 0 0 0 0 0 0 0 0 0
M02255_76_000000000-ADUNE_1_1119_3026_12801 .....AC----T-AC-GT---------GCC-A-G------C--A--G---C--CG-C-GGT-AA------TA-C--G-T--AG-GT-------GG--C-----AA-GC-G--TT-------G----T-C-C-G------------G--T-TT-T-T-T-----------AA----G-T-C-T--------G-A-T--G--TG--A-AA-GC--C-T-TC-G-G-----CT-T-AA------C-C-G-G-A-G-A--T-G----T-G--C-A---T--T-------G--GA-A-A---C-------T--G-G--AA--G-A-C----T-T-G-A-G-T--G---C-AG-----AA-G-A--------G-G-A-G---AG-T------GG--A--ACT----C-C-A-T-GT--GT-A-G-CG-GT--G---A--A-A---TG-C-GT-AG--AT-A-TA--T-G--G-A-A-G-A-AC-A-CC---AG--T--G--GC-GAA-A--G-C--G-------G--C-T-C-T---CTG--G--TC-T-G--------------------T-A---A-C-T--GA--CG-C-T-G--A-GG--T-T-CG-A--AA-G-C---A-TG--GG-T--AG-C-G-AA--------CA--GG Bacteria(100);Firmicutes(100);Bacilli(100);Lactobacillales(100);Lactobacillaceae(100);Lactobacillus(100);unclassified(100);unclassified(100);unclassified(100);

Otu1000 0 0 0 0 0 0 0 0 0 0 0 0 0 0 0 0 0 0 0 0 0 0 0 0 0 0 0 0
0 0 0 0 0 0 0 0 0 0 5 0 0 0 0 7 0 25 0 0 0 0 0 0 0 0 0 0 0
0 0 0 0 0 0 0 0 0 0 0 0 0 0 0 0 0 0 0 0 0 0 0 0 0 0 0 0 0
0 0 0 0 0 0 0 0 0 0 0 0 0 0 0 0 0 0 0 0 0 0 0 0 0 0 0
M02255_77_000000000-ADUT3_1_1105_6963_14042 G----AC---GT-AG-GGT--------CCG-A-G------C--G--T---T--GT-C-CGG-AT------TT-A--C-T--GG-GC-------GT--A-----AA-GA-GC-GC-------G-TA-G-G-C-G------------G--C-CC-G-T-T-----------AA----G-T-G-A--------G-G-A--G--TG--A-AA-GC--T-C-C--GGG-----CT-C-AA------C-C-C-G-G-A-A--A-T----C-G--C-T---T--T-------T--CA-T-A---C-------T--G-G--CG--G-G-C----T-A-G-A-G-G--G---T-GG-----CA-G-A--------G-G-C-T---TG-T------GG--A--ATT----C-C-T-G-GT--GT-A-G-CG-GT--G---A--A-A---TG-C-GT-AG--AT-A-TC--A-G--G-A-G-G-A-AC-A-CC---CA--T--G--GC-GAA-G--G-C--A-------G--C-A-A-G---CTG--G--GC-C-G--------------------C-A---T-C-T--GA--CG-C-T-G--A-GG--C-G-CG-A--AA-G-C---G-TG--GG-T--AG-C-A-AA--------CA--GG Bacteria(100);unclassified(100);unclassified(100);unclassified(100);unclassified(100);unclassified(100);unclassified(100);unclassified(100);unclassified(100);

Otu1001 0 0 0 0 0 0 0 0 0 0 0 0 0 0 0 0 0 0 0 0 0 0 0 0 6 0 0 0
0 0 0 0 0 0 0 0 0 0 0 0 0 0 0 0 0 0 0 0 0 0 0 0 0 0 0 0 0
0 0 0 0 0 0 0 0 0 0 0 0 0 0 0 0 0 0 21 0 0 0 0 0 0 0 0 0 0
0 0 1 0 0 72 0 0 0 31 0 58 0 0 0 0 0 0 0 0 0 0 0 0 0 0 0
M02255_77_000000000-ADUT3_1_2114_20544_15063 T----AC---GT-AG-GGG--------ACA-A-G------C--G--T---T--AT-C-CGG-AA------TT-A--C-T--GG-GC-------GT--A-----AA-GG-GT-GC-------G-CA-G-G-T-G------------G--T-CA-G-A-T-----------AA----G-T-T-A--------G-A-G--G--TT--A-AA-AG--C-A-GA-G-G-----CT-C-AA------C-C-A-C-T-G-T--C------A-G--C-C---T--T-------T--AA-A-A---C-------T--A-T--CT--G-A-C----T-T-G-A-G-T--G---C-AG-----GA-G-A--------G-G-A-A---AG-A------GG--A--ATT----C-C-C-G-GT--GT-A-G-CG-GT--G---A--A-A---TG-C-GT-AG--AT-A-TC--G-G--G-A-G-G-A-AT-A-CC---GG--T--G--GC-GAA-G--G-C--G-------C--C-T-T-T---CTG--G--AC-T-G--------------------T-A---A-C-T--GA--CA-C-T-G--A-GG--C-A-CG-A--AA-G-C---G-TG--GG-G--AG-C-A-AA--------CA--GG Bacteria(100);unclassified(100);unclassified(100);unclassified(100);unclassified(100);unclassified(100);unclassified(100);unclassified(100);unclassified(100);

Otu1002 0 0 0 0 0 0 0 0 0 0 0 0 0 0 0 0 0 0 0 0 0 0 0 0 0 0 0 0
0 0 0 0 0 0 0 0 0 0 4 0 0 0 0 0 0 0 0 0 0 0 0 0 0 0 0 0 0
0 0 0 0 0 0 0 0 7 0 0 0 0 0 0 0 0 0 0 0 0 0 0 0 0 0 0 0 0
0 0 0 0 0 0 0 0 0 0 0 0 0 0 0 0 0 0 0 0 0 0 0 0 0 0 0
M02255_77_000000000-ADUT3_1_2115_12166_10948 T----AC---GG-AG-GGT--------GCA-A-G------C--G--T---T--AA-T-CGG-AA------TT-A--C-T--GG-GC-------GT--A-----AA-GC-GC-AC-------G-CA-G-G-C-G------------G--T-TG-G-A-T-----------AA----G-T-T-A--------G-A-T--G--TG--A-AA-GC--C-C-CG-G-G-----CT-C-AA------C-C-T-G-G-G-A--A-T----T-G--C-A---T--T-------T--AA-A-A---C-------T--G-T--CC--A-G-C----T-A-G-A-G-T--C---T-TG-----TA-G-A--------G-G-G-G---GG-T------AG--A--ATT----C-C-A-G-GT--GT-A-G-CG-GT--G---A--A-A---TG-C-GT-AG--AG-A-TC--T-G--G-A-G-G-A-AT-A-CC---GG--T--G--GC-GAA-G--G-C--G-------G--C-C-C-C---CTG--G--AC-A-A--------------------A-G---A-C-T--GA--CG-C-T-C--A-GG--T-G-CG-A--AA-G-C---G-TG--GG-G--AG-C-A-AA--------CA--GG Bacteria(100);Proteobacteria(100);Gammaproteobacteria(100);Aeromonadales(100);Aeromonadaceae(100);Aeromonas(100);unclassified(100);unclassified(100);unclassified(100);

Otu1003 0 0 0 0 0 0 0 0 0 0 0 0 0 0 0 0 0 0 0 0 0 0 0 0 0 0 0 0
0 0 0 0 0 0 0 0 0 0 2 0 0 0 0 0 0 0 0 0 0 0 0 0 0 0 0 0 0
0 9 0 0 0 0 0 0 0 0 0 0 0 0 0 0 0 0 0 0 0 0 0 0 0 0 0 0 0
0 0 0 0 0 0 0 0 0 0 0 0 0 0 0 0 0 0 0 0 0 0 0 0 0 0 0
M02255_76_000000000-ADUNE_1_2106_15640_12702 C----GG---CT-AA-TTC--------CGT-G-C------C--A--G---C--AG-C-CGC-GG------TA-A--T-A--CG-GA-------AG--G-----TC-CT-G--GC-------G-TT-A-T-C-C------------G--G-AC-T-T-T-----------AT----G-C-G-T--------G-T-T--G--TG--A-AA-TG--T-A-CC-G-G-----CT-C-AA------C-C-G-G-T-A-A--C-G----T-G--C-A---G--C-------G--CG-A-A---C-------T--G-G--GG--T-G-C----T-T-G-A-G-T--A---C-GA-----AG-A-G--------G-G-A-A---GG-C------GG--A--ACT----C-G-T-G-GT--GT-A-G-CG-GT--G---A--A-A---TG-C-TT-AG--AT-A-TC--A-C--G-A-G-G-A-AC-T-CC---GA--T--C--GC-GAA-G--G-C--A-------G--C-T-T-T---CCG--T--TT-C-G--------------------G-A---A-C-T--GA--CG-C-T-G--A-GG--C-T-CG-A--AA-G-T---G-CG--GG-T--AT-C-G-AA--------CA--GG Bacteria(100);Bacteroidetes(100);Bacteroidia(100);Bacteroidales(100);Prevotellaceae(100);Prevotella(100);unclassified(100);unclassified(100);unclassified(100);

Otu1004 0 0 0 0 0 0 0 0 0 0 0 0 0 0 0 0 0 0 0 0 0 0 0 0 0 0 0 0
0 0 0 0 0 0 0 0 0 0 30 0 0 0 0 0 0 0 0 0 0 0 0 0 0 0 0 0 0
0 0 0 0 0 0 0 0 0 0 0 0 0 0 0 0 0 0 0 0 0 0 0 0 0 0 0 0 0
0 0 0 0 0 0 0 0 0 0 0 0 0 0 0 0 0 0 0 0 0 0 0 0 0 0 0
M02255_77_000000000-ADUT3_1_1118_13603_17747 G----AC---GA-GG-GAT--------CCT-A-G------C--G--T---T--GT-T-CGG-AA------TC-A--T-T--GG-GC-------GT--A-----AA-GC-GT-AT-------G-TA-G-G-T-G------------G--C-TA-T-G-T-----------AA----G-T-C-A--------G-G-T--G--TG--A-AA-TC--C-C-TG-G-G-----CT-C-AA------C-C-C-A-G-G-A--C-G----T-G--C-A---T--T-------T--GA-A-A---C-------T--G-C--GT--A-G-C----T-T-G-A-A-T--A---T-GG-----GA-G-A--------G-G-G-T---AG-T------GG--A--ATT----C-C-T-G-GT--GT-A-G-TG-GT--G---A--A-A---TA-C-GT-AG--AT-A-TC--A-G--G-A-G-G-A-AC-A-CC---GG--T--G--GC-GAA-G--G-C--G-------G--C-T-A-C---CTG--G--CC-C-A--------------------A-T---A-T-T--GA--CA-C-T-G--A-GA--T-A-CG-A--AA-G-C---G-TG--GG-G--AT-C-A-AA--------CA--GG Bacteria(100);Proteobacteria(100);unclassified(100);unclassified(100);unclassified(100);unclassified(100);unclassified(100);unclassified(100);unclassified(100);

Otu1005 0 0 0 0 0 0 0 0 0 0 0 0 0 0 0 0 0 0 0 0 0 0 0 0 4 0 0 0
0 0 0 0 0 0 0 0 0 0 0 0 0 0 0 0 0 0 0 0 0 0 0 0 0 0 0 0
0 0 0 0 0 0 0 0 0 0 0 0 0 0 0 0 0 0 0 0 0 0 0 0 0 0 0 0
0 0 0 0 0 0 0 0 0 0 0 0 0 0 0 0 0 0 0 0 0 0 0 0 0 0 0
M02255_77_000000000-ADUT3_1_2112_18070_8917 T----AC---GT-AG-GTT--------GCG-A-G------C--G--T---T--GT-C-CGG-AT------TT-A--C-T--GG-GT-------GT--A-----AA-GC-GC-GC-------G-CA-G-G-C-G-------------G--T-CT-C-G-A-----------AA-
---G-T-C-T--------A-C-G--G--TG--A-AA-GC--T-C-CC-G-------GC-C-CA------A-C-T-G-G-G-A--G-A----G-G--C-C---C--G-------C--GG-A-A---A-------C--T-T--CGA-G-A-C----T-T-G-A-AGG------C-GG-----GA-G-A--------G-GCA-A---A--T------GG--A--ATT----T-C-C-G-
GT--GT-A-G-CG-GT--A---A--A-A---TG-C-GT-AG--AT-A-CC--G-G--A-A-G-G-A-AC-A-CC---AG--T--G--GC-GAA-G--G-C--G-------A--T-T-T-G---CTG--G--CC-C-G--------------------C-C---C-T-T--GA--CG-C-T-C--A-GG--C-G-CG-A--AA-G-C---T-AG--GG-G--AG-C-
G-AC--------CG--GG Bacteria(100);unclassified(100);unclassified(100);unclassified(100);unclassified(100);unclassified(100);unclassified(100);unclassified(100);unclassified(100);

Otu1006 0 0 0 0 0 0 0 0 0 0 0 0 0 0 0 0 0 0 0 0 0 0 0 0 3 0 0 0
0 0 0 0 0 0 0 0 0 0 0 0 0 0 0 0 0 0 0 0 0 0 0 10 0 0 0 0 0
0 0 0 0 0 0 0 0 0 0 0 0 0 0 0 0 0 0 0 0 0 0 0 0 0 0 0 0
0 0 0 0 0 0 0 0 0 0 0 0 0 0 0 0 0 0 0 0 0 0 0 0 0 0 0
M02255_77_000000000-ADUT3_1_2108_23824_13018 T----AC---GT-AG-GGT--------GCA-A-G------C--G--T---T--GT-C-CGG-AA------TT-A--T-T--GG-GC-------GT--A-----AA-GA-GC-TC-------G-TA-G-G-C-G-------------G--T-CT-G-T-C------
-----GC----G-T-C-G--------G-A-C--G--TG--A-AA-AT--C-C-AG-G-G-----CT-C-AA------C-C-C-T-G-G-A--C-C----T-G--C-G---T--T-------C--GA-T-A---C-------G--G-G--CA--G-A-C----T-A-G-A-G-T--T---C-GG-----TA-G-G--------G-G-A-G---AC-T------GG--A--ATT----C-
C-T-G-GT--GT-A-G-CG-GT--G---A--A-A---TG-C-GC-AG--AT-A-TC--A-G--G-A-G-G-A-AC-A-CC---GG--T--G--GC-GAA-G--G-C--G-------G--G-T-C-T---CTG--G--GC-C-G--------------------A-A---A-C-T--GA--CG-C-T-G--A-GG--A-G-CG-A--AA-G-C---G-TG--GG-
G--AG-C-A-AA--------CA--GG Bacteria(100);Actinobacteria(100);Actinobacteria(100);Actinobacteridae(100);Actinomycetales(100);unclassified(100);unclassified(100);unclassified(100);unclassified(100);

Otu1007 0 0 0 0 0 0 0 0 0 0 0 0 0 0 0 0 0 0 0 0 0 0 0 0 0 0 0 0
0 0 0 0 0 0 0 0 0 0 21 0 0 0 0 0 0 0 0 0 0 0 0 0 0 0 0 0
0 0 0 0 0 0 0 0 0 0 0 0 0 0 0 0 0 0 0 0 0 0 0 0 0 0 0 0
0 0 0 0 0 0 0 0 0 0 0 0 0 0 0 0 0 0 0 0 0 0 0 0 0 0 0
M02255_77_000000000-ADUT3_1_1110_17680_5998 T----AC---GA-AG-GGG--------GCT-A-G------C--G--T---T--GT-T-CGG-AA------TC-A--C-T--GG-GC-------GT--A-----AA-GC-GC-AC-------G-TA-G-G-C-G-------------G--A-CA-T-T-T-----------AA-
---G-T-C-A--------G-G-G--G--TG--A-AA-GC--C-T-GG-A-G-----CT-C-AA------C-T-C-C-A-G-A--A-C----T-G--C-C---C--T-------T--GA-T-A---C-------T--G-G--GT--G-T-C----T-C-G-A-G-T--C---C-GG-----AA-G-A--------G-G-T-A---AG-T------GG--A--ACT----G-C-G-A-
GT--GT-A-G-AG-GT--G---A--A-A---TT-C-GT-AG--AT-A-TT--C-G--C-A-A-G-A-AC-A-CC---AG--T--G--GC-GAA-G--G-C--G-------G--C-T-T-A---CTG--G--TC-C-G--------------------G-T---A-C-T--GA--CG-C-T-G--A-GG--T-G-CG-A--AA-G-C---G-TG--GG-G--AG-C-
A-AA--------CA--GG Bacteria(100);Proteobacteria(100);Alphaproteobacteria(100);Rhizobiales(100);Hyphomicrobiaceae(100);Angulomicrobium(100);unclassified(100);unclassified(100);unclassified(100);

Otu1008 0 0 0 0 0 0 0 0 0 0 0 0 0 0 0 0 0 0 0 0 0 0 0 0 6 0 0 0
0 0 0 0 0 0 0 0 0 0 0 0 0 0 0 0 0 0 0 0 0 0 0 0 0 0 0 0
0 0 0 0 0 0 0 0 0 0 0 0 0 0 0 0 0 0 0 0 0 0 0 0 0 0 0 0
0 0 0 0 0 0 0 0 0 0 0 0 0 0 0 0 0 15 0 0 0 0 0 0 0 0 0
M02255_77_000000000-ADUT3_1_1111_20978_16249 T----AC---GT-AG-GTG--------GCA-A-G------C--G--T---T--GT-C-CGG-AA------TT-A--C-T--GG-GT-------GT--A-----AA-GG-GT-GC-------G-CA-G-G-C-G-------------G--G-AT-G-G-C----
-------AA----G-T-T-G--------G-A-T--G--TG--A-AA-AC--T-C-CG-G-G-----CT-C-AA------C-C-C-G-G-A-G--C-C----T-G--C-A---T--T-------C--AA-A-A---C-------T--G-C--TG--T-T-C----T-T-G-A-G-T--G---A-AG-----TA-G-A--------G-G-C-A---GG-C------GG--A--ATT----
C-C-C-G-GT--GT-A-G-CG-GT--G---A--A-A---TG-C-GT-AGA-T--A-TC--G-G--G-A-G-G-A-AC-A-CC---AG--T--G--GC-GAA-G--G-C--G-------G--C-C-T-G---CTG--G--GC-T-T--------------------T-T---A-C-T--GA--CG-C-T-G--A-GG--C-A-CG-A--AA-G-C---A-TG--GG-
T--AG-C-A-AA--------CA--GG Bacteria(100);Firmicutes(100);Clostridia(100);Clostridiales(100);Ruminococcaceae(100);Ethanoligenens(100);unclassified(100);unclassified(100);unclassified(100);

Otu1009 0 0 0 0 0 0 0 0 0 0 0 0 0 0 0 0 0 0 0 0 0 0 0 0 0 6 0 0
0 0 0 0 0 0 0 0 0 0 0 0 0 0 0 0 0 0 0 0 0 0 0 0 0 0 0 0
0 0 0 0 0 0 0 0 0 0 0 0 0 0 0 0 0 0 0 0 0 0 0 0 0 0 0 0
0 0 17 0 0 0 0 0 0 0 0 0 0 0 0 0 0 0 0 0 0 0 0 0 0 0 0
M02255_76_000000000-ADUNE_1_2107_24759_9643 C----GT---GC-CA-GCA--------GCC-G-C------G--G--T---A--AT-A-CGG-AG------GG-T--G-C--AA-GC-------GT--T-----AC-CC-G--GA-------A-T--C-A-C-G-------------G--G-AG-C-G-T-----------AA--
--G-T-C-T--------G-G-T--T--TT--A-AA-GA--C-T-GA-G-G-----CT-C-AA------C-C-T-C-A-G-G--A-G----T-G--G-A---C--T-------G--GA-T-A---C-------T--G-C--GA--T-C-C----T-T-G-A-C-C--T---C-TG-----GA-G-A--------G-G-C-A---AC-C------GG--A--ATT----C-C-T-G-GT-
-GT-A-G-CG-GT--G---G--A-A---TG-C-GT-AG--AT-A-CC--A-G--G-A-G-G-A-AC-A-CC---AA--T--G--GC-GAA-G--G-C--A-------G--G-T-T-G---CTG--G--AC-A-G--------------------A-A---G-G-T--GA--CG-C-T-G--A-GG--C-G-CG-A--AA-G-T---G-TG--GG-G--AG-C-
A-AA--------CC--GG Bacteria(100);Deinococcus-Thermus(100);Deinococcales(100);Deinococcaceae(100);Deinococcus(100);unclassified(100);unclassified(100);unclassified(100);unclassified(100);

Otu1010 0 0 0 0 0 0 0 0 0 0 0 0 0 0 0 0 0 0 0 0 0 0 0 0 0 5 0 0
0 0 0 0 0 0 0 0 0 0 0 0 0 0 0 0 0 0 0 0 0 0 0 0 0 0 0 0 0
0 0 0 0 0 0 0 0 0 0 0 0 0 0 0 0 0 0 0 0 0 0 0 0 0 0 0 0 0
0 0 0 0 0 0 0 0 0 0 0 0 0 0 0 0 0 0 0 0 0 0 0 0 0 0 0
M02255_77_000000000-ADUT3_1_2106_20511_19618 T----AC---GT-AG-GTG--------GCG-A-G------C--G--T---T--GT-C-CGG-AA------TG-A--C-T--GG-GC-------GT--A-----AA-GG-GC-GT-------G-TA-G-G-T-G------------G--A-CT-G-A-T----
-------CA----G-T-C-G--------G-A-T--G--GG--A-AA-GC--C-C-CA-G-G-----CT-T-AA------C-C-T-G-G-G-A--A-C----C-G--C-A---T--C-------C--GA-T-A---C-------G--G-T--CA--G-T-C----T-T-G-A-G-T--G---C-GG-----GA-G-A--------G-G-G-C---AG-T------GG--A--ATT----
C-C-C-A-GT--GT-A-G-CG-GT--G---A--A-A---TG-C-GT-AG--AG-A-TT--G-G--G-A-G-G-A-AC-A-CC---AG--T--G--GC-GAA-G--G-C--G-------G--C-T-G-C---CTG--G--AC-C-G--------------------T-A---A-C-T--GA--CA-C-T-G--A-GG--C-G-CG-A--AA-G-C---G-TG--
GG-G--AG-C-A-AA--------CA--GG Bacteria(100);unclassified(100);unclassified(100);unclassified(100);unclassified(100);unclassified(100);unclassified(100);unclassified(100);unclassified(100);

Otu1011 0 0 0 0 0 0 0 0 0 0 0 0 0 0 0 0 0 0 0 0 0 0 0 0 0 5 0 0
0 0 0 0 0 0 0 0 0 0 0 0 0 0 0 0 0 0 0 0 0 0 0 0 0 0 0 0 0
0 0 0 0 0 0 0 0 0 0 0 0 0 0 0 0 0 0 0 0 0 0 0 0 0 0 0 0 0
0 0 0 0 0 0 0 0 0 0 0 0 0 0 0 0 0 0 0 0 0 0 0 0 0 0 0
M02255_77_000000000-ADUT3_1_2112_17512_13554 T----AC---GA-AG-GGG--------GCT-A-G------C--G--T---T--GC-T-CGG-AT------TT-A--C-T--GG-GC-------GT--A-----AA-GG-GC-GC-------G-TA-G-G-C-G------------G--A-TG-A-C-C----
-------AA----G-T-T-G--------G-G-G--G--TG--A-AA-TC--C-C-GG-G-G-----CT-C-AA------C-C-T-C-G-G-A--A-C----T-G--C-C---T--C-------C--AA-A-A---C-------T--G-G--TG--A-T-C----T-T-G-A-G-T--A---T-GA-----GA-G-A--------G-G-T-G---TG-T------GG--A--ACT----
C-C-G-A-GT--GT-A-G-AG-GT--G---A--A-A---TT-C-GT-AG--AT-A-TT--C-G--G-A-A-G-A-AC-A-CC---AG--T--G--GC-GAAG---G-C--G-------A--C-A-C-A---CTG--G--CT-C-A--------------------T-T---A-C-T--GA--CG-C-T-G--A-GG--C-G-CG-A--AA-G-C---G-TG--GG-
G--AG-C-A-AA--------CA--GG Bacteria(100);Proteobacteria(100);Alphaproteobacteria(100);unclassified(100);unclassified(100);unclassified(100);unclassified(100);unclassified(100);unclassified(100);

Otu1012 0 0 0 0 0 0 0 0 0 0 0 0 0 0 0 0 0 0 0 0 0 0 0 0 0 2 0 0
0 0 0 0 0 0 0 0 0 0 0 0 0 0 0 0 0 0 1 0 0 0 0 0 0 0 0 0 0
0 0 0 0 0 0 0 0 2 0 0 0 0 0 0 0 0 0 0 0 0 0 0 0 0 0 0 0 0
0 0 0 0 0 0 0 0 0 0 0 0 0 0 0 0 0 0 0 0 0 0 0 0 0 0 0
M02255_77_000000000-ADUT3_1_2115_17938_15504 A----AG---CA-CC-GGC--------TAA-C-T------C--C--G---T--GC-C-AGC--A------GC-C--G-C--GG-TA-------AT--A-----CG-GA-GG-GT-------G-CA-A-G-C-G------------T-TAATCGG-A-T---
--------TA----C-T-T-A--------G-A-T--G--TG--A-AA-GC--C-C-CG-G-G-----CT-C-AA------C-C-T-G-G-G-A--A-T----T-G--C-A---T--T-------T--AA-A-A---C-------T--G-T--CC--A-G-C----T-A-G-A-G-T--C---T-TG-----TA-G-A--------G-G-G-G---GG-T------AG--A--ATT----
C-C-A-G-GT--GT-A-G-CG-GT--G---A--A-A---TG-C-GT-AG--AG-A-TC--T-G--G-A-G-G-A-AT-A-CC---GG--T--G--GC-GAA-G--G-C--G-------G--C-C-C-C---CTG--G--AC-A-A--------------------A-G---A-C-T--GA--CG-C-T-C--A-GG--T-G-CG-A--AA-G-C---G-TG--
GG-G--AG-C-A-AA--------CA--GG Bacteria(100);Proteobacteria(100);Gammaproteobacteria(100);unclassified(100);unclassified(100);unclassified(100);unclassified(100);unclassified(100);unclassified(100);

Otu1013 0 0 0 0 0 0 0 0 0 0 0 0 0 0 0 0 0 0 0 0 0 0 0 0 0 2 0 10
0 0 0 0 0 0 0 0 0 0 0 0 0 0 0 0 0 0 0 0 0 0 0 0 0 0 0 0 0
0 0 0 0 0 0 0 0 0 0 0 0 0 0 0 0 0 0 0 0 0 0 0 0 0 0 0 0 0
0 0 0 0 0 0 0 0 0 0 0 0 0 0 0 0 0 0 0 0 0 0 0 0 0 0 0
M02255_76_000000000-ADUNE_1_2115_4070_18462 G----AA---GC-AC-CGG--------CTA-A-C------T--T--C---G--TG-C-CAG-CA------GC-C--G-C--GG-TA-------AT--A-----CG-AG-GG-GT-------G-CA-A-G-C-G------------G--TTAATC-G-G-----------
AA----G-T-T-G--------G-A-T--G--TG--A-AA-GC--C-C-CG-G-G-----CT-C-AA------C-C-T-G-G-G-A--A-T----T-G--C-A---T--T-------C--AA-A-A---C-------T--G-G--TT--G-A-C----T-A-G-A-G-T--A---T-GT-----GA-G-A--------G-G-G-G---GG-T------AG--A--ATT----C-C-A-
A-GT--GT-A-G-CG-GT--G---A--A-A---TG-C-GT-AG--AG-A-TT--T-G--G-A-G-G-A-AT-A-CC---AG--T--G--GC-GAA-G--G-C--G-------G--C-C-C-C---CTG--G--CA-C-A--------------------A-T---A-C-T--GA--CG-C-T-G--A-GG--T-A-CG-A--AA-G-C---G-TG--GG-G--
AG-C-A-AA--------CA--GG Bacteria(100);Proteobacteria(100);Gammaproteobacteria(100);Chromatiales(100);Chromatiaceae(100);unclassified(100);unclassified(100);unclassified(100);unclassified(100);

Otu1014 0 0 0 0 0 0 0 0 0 0 0 0 0 0 0 0 0 0 0 0 0 0 0 0 0 0 0 0
0 0 0 0 0 0 0 0 0 0 28 0 0 0 0 0 0 0 0 0 0 0 0 0 0 0 0 0 0
0 0 0 0 0 0 0 0 0 0 0 0 0 0 0 0 0 0 0 0 0 0 0 0 0 0 0 0 0
0 0 0 0 0 0 0 0 0 0 0 0 0 0 0 0 0 0 0 0 0 0 0 0 0 0 0
M02255_77_000000000-ADUT3_1_1105_17835_21152 T----AC---GT-AG-GGG--------GCG-A-G------C--G--T---T--GT-C-CGG-AA------TT-A--C-T--GG-GC-------GT--A-----AA-GG-GT-AC-------G-TA-G-G-C-G------------G--C-TA-G-G-T----
-------AA----G-T-C-A--------G-G-T--G--TG--A-AA-TC--C-A-TG-G-G-----CT-C-AA------C-C-C-A-T-G-T--A------A-G--C-A---C--T-------T--GA-A-A---C-------T--G-C--TT--A-G-C----T-T-G-A-G-T--T---A-AG-----GA-G-A--------G-G-A-A---AG-C------GG--A--ATT----C-
C-T-G-GT--GT-A-G-CG-GT--G---A--A-A---TG-C-GT-AG--AT-A-TC--A-G--G-A-G-G-A-AC-A-CC---AG--T--G--GC-GAA-G--G-C--G-------G--C-T-T-T---CTG--G--CC-T-T--------------------G-G---A-C-T--GA--CG-C-T-G--A-GG--T-A-CG-A--AA-G-C---G-TG--GG-G-
-AG-C-A-AA--------CA--GG Bacteria(100);Firmicutes(100);Clostridia(100);Clostridiales(100);unclassified(100);unclassified(100);unclassified(100);unclassified(100);unclassified(100);

Otu1015 0 0 0 0 0 0 0 0 0 0 0 0 0 0 0 0 0 0 0 0 0 0 0 0 0 2 0 2
0 0 0 0 0 0 0 0 0 0 0 0 0 0 0 0 0 0 0 0 0 0 0 0 0 0 0 0 0
0 0 0 0 0 0 0 0 0 0 0 0 0 0 0 0 0 0 0 0 0 0 0 0 0 0 0 0 0
0 0 0 0 0 0 0 0 0 0 0 0 0 0 0 0 0 0 0 0 0 0 0 0 0 0 0
M02255_77_000000000-ADUT3_1_1115_5308_18657 T----AC---GA-AG-GGA--------GCT-A-G------C--G--T---T--GT-T-CGG-AA------TT-A--C-T--GG-GC-------GT--A-----AA-GG-GC-GC-------G-TA-G-G-CGG----------------C-TT-T-G-T-----------AA----G-T-C-A--------G-A-T--G--TG--A-AA-TC--C-C-CG-G-G-----CT-T-AA------C-C-T-G-G-G-A--A-C----T-G--C-A---T--T-------T--GA-T-A---C-------T--G-C--AA--G-G-C----T-A-G-A-G-A--T---T-GA-----GA-G-A--------G-G-T-A---AG-T------GG--A--ATTA-----T-G-A-GT--GT-A-G-AG-GT--G---A--A-A---TT-C-GT-AG--AT-A-TT--C-A--T-A-A-G-A-AC-A-TC---AG--T--G--GC-GAA-G--G-C--G-------G--C-T-T-A---CTG--G--CT-C-A---------------------A-T---A-C-T--GA--CG-C-T-A--A-GG--C-G-CG-A--AA-G-C---G-TG--GG-G--AG-C-A-AA--------CA--GG Bacteria(100);Proteobacteria(100);unclassified(100);unclassified(100);unclassified(100);unclassified(100);unclassified(100);unclassified(100);unclassified(100);

Otu1016 0 0 0 0 0 0 0 0 0 0 0 0 0 0 0 0 0 0 0 0 0 0 0 0 0 1 0 0
0 0 0 0 0 0 0 0 0 0 0 0 0 0 38 0 0 0 0 0 0 0 0 0 0 0 0 0 0
0 0 0 0 0 0 0 0 0 0 0 0 0 0 0 0 0 0 0 0 0 0 0 0 0 0 0 0 0
0 0 0 0 0 0 0 0 0 0 0 0 0 0 0 0 76 0 0 0 0 0 0 0 0 0 0
M02255_77_000000000-ADUT3_1_1115_10045_20545 T----AC---GA-AG-GGG--------GCT-A-G------C--G--T---T--GT-T-CGG-AA------TT-A--C-T--GG-GC-------GT--A-----AA-GC-GC-AC-------G-TA-G-G-C-G-------------G--A-TT-A-G-C-----------AA----G-T-C-A--------G-G-G--G--TG--A-AA-TC--C-C-GG-G-G-----CT-C-AA------C-C-T-C-G-G-A--A-C----T-G--C-C---T--T-------T--GA-T-A---C-------T--G-C--TA--G-T-C----T-A-G-A-G-T--C---C-GG-----AA-G-A--------G-G-T-G---GG-T------GG--A--ATT----C-C-T-A-GT--GT-A-G-AG-GT--G---A--A-A---TT-C-GC-AG--AT-A-TT--A-G--G-A-A-G-A-AC-A-CC---AG--T--G--GC-GAA-G--G-C--G-------G--C-C-C-A---CTG--G--TC-C-G---------------------G-A---A-C-T--GA--CG-C-T-G--A-GG--T-G-CG-A--AA-G-C---G-TG--GG-G--AG-C-A-AA--------CA--GG Bacteria(100);Proteobacteria(100);Alphaproteobacteria(100);Rhizobiales(100);unclassified(100);unclassified(100);unclassified(100);unclassified(100);unclassified(100);

Otu1017 0 0 0 0 0 0 0 0 0 0 0 0 0 0 0 0 0 0 0 0 0 0 0 0 0 0 0 0
0 0 0 0 0 0 0 0 0 0 31 1 0 0 0 0 0 0 0 0 0 0 0 0 0 0 0 0 0
0 0 0 0 0 0 0 0 0 0 0 0 0 0 0 0 0 0 0 0 0 0 0 0 0 0 0 0 0
0 0 0 0 0 0 0 0 0 0 0 0 0 0 0 0 0 0 0 0 0 0 0 0 0 0 0
M02255_77_000000000-ADUT3_1_1104_3043_17971 T----AC---GT-AG-GTG--------GCA-A-G------C--G--T---T--GT-C-CGG-AT------TT-A--C-T--GG-GC-------GT--A-----AA-GG-GC-GT-------G-CA-G-G-C-G-------------G--T-TT-G-C-T-----------AA----G-T-C-T--------A-G-G--G--TG--A-AA-GC--T-C-CC-G-G-----CT-C-AA------C-T-G-G-G-A-G--A-G----G-T--C-C---T--T-------G--GA-T-A---C-------T--G-G--CG--A-A-C----T-T-G-A-G-G--G---A-TG-----CA-G-A--------G-G-A-A---AG-T------GG--A--ATT----C-C-C-G-GT--GT-A-G-TG-GT--G---A--T-A---TG-C-GT-AG--AT-A-TC--G-G--G-A-G-G-A-AC-A-CC---AG--T--G--GC-GAA-G--G-C--G-------G--C-T-T-T---CTG--G--GC-A-T---------------------T-T---C-C-T--GA--CG-C-T-G--A-GA--C-G-CG-A--AA-G-C---G-TG--GG-G--AG-C-G-AA--------CC--GG Bacteria(100);Chloroflexi(100);unclassified(100);unclassified(100);unclassified(100);unclassified(100);unclassified(100);unclassified(100);unclassified(100);

Otu1018 0 0 0 0 0 0 0 0 0 0 0 0 0 0 0 0 0 0 0 0 0 0 0 0 0 7 0 0
0 0 0 0 0 0 0 0 0 0 0 0 0 0 0 0 0 0 0 0 0 0 0 0 0 0 0 0 0
0 0 0 0 0 0 0 0 0 0 0 0 0 0 0 0 0 0 0 0 0 0 0 0 0 0 0 0 0
0 0 0 0 0 0 0 0 0 0 0 0 0 0 0 0 0 0 0 0 0 0 0 0 0 0 0
M02255_77_000000000-ADUT3_1_1117_8620_17358 T----AC---GG-AG-GGG--------GCT-A-G------C--G--T---T--GT-T-CGG-AA------TT-A--C-T--GG-GC-------GT--A-----AA-GC-GC-AC-------G-TA-G-G-C-G-------------G--C-TT-T-A-C-----------AA----G-T-C-A--------G-A-G--G--TG--A-AA-GC--C-C-GG-A-G-----CT-C-AA------C-T-C-C-G-G-A--A-T----T-G--C-C---T--T-------T--GA-G-A---C-------T--G-T--AT--C-G-C----T-T-G-A-A-T--C---A-TG-----GA-G-A--------G-G-C-G---GG-T------GG--A--ATT----C-C-G-A-GT--GT-A-G-AG-GT--G---A--A-A---TT-C-GT-AG--AT-A-TT--C-G--G-A-A-G-A-AC-A-CC---AG--T--G--GC-GAA-G--G-C--G-------G--C-C-C-G---CTG--G--AC-A-T---------------------G-T---A-T-T--GA--CG-C-T-G--A-GG--T-G-CG-A--AA-G-C---G-TG--GG-G--AG-C-A-AA--------CA--GG Bacteria(100);Proteobacteria(100);Alphaproteobacteria(100);Sphingomonadales(100);Sphingomonadaceae(100);unclassified(100);unclassified(100);unclassified(100);unclassified(100);

Otu1019 0 0 0 0 0 0 0 0 0 0 0 0 0 0 0 0 0 0 0 0 0 0 0 0 0 0 0 0
0 0 0 0 0 0 0 0 0 0 8 0 0 0 0 0 0 0 0 0 0 0 0 0 0 0 0 1 0
0 0 0 0 0 0 0 0 0 0 0 0 0 0 0 0 0 0 0 0 0 0 0 0 0 0 0 0 0
0 0 0 0 0 0 0 0 0 0 0 0 0 0 0 0 0 0 0 0 0 0 17 34 0 0 0 0 0
M02255_77_000000000-ADUT3_1_1102_2995_14389 G----AC---GT-AG-GGC--------GCG-A-G------C--G--T---T--GT-C-CGG-AT------TT-A--T-T--GG-GC-------GT--A-----AA-GA-GC-TC-------G-TA-G-G-C-G-------------G--C-TT-G-T-C-----------GC----G-T-C-G--------A-A-T--G--TG--A-AA-TC--T-C-AG-G-G-----CT-C-AA------C-T-C-T-G-A-C--A-T----T-G--C-A---T--T-------C--GA-T-A---C-------G--G-G--CA--G-G-C----T-A-G-A-G-T--T---C-GG-----TA-G-G--------G-G-A-G---AC-T------GG--A--ATT----C-C-T-G-GT--GT-A-G-CG-GT--G---A--A-A---TG-C-GC-AG--AT-A-TC--A-G--G-A-G-G-A-AC-A-CC---GG--T--G--GC-GAA-G--G-C--G-------G--G-T-C-T---CTG--G--GC-C-G---------------------A-T---A-C-T--GA--CG-C-T-G--A-GG--A-G-CG-A--AA-G-C---G-TG--GG-G--AG-C-G-AA--------CA--GG Bacteria(100);Actinobacteria(100);Actinobacteria(100);Actinobacteridae(100);Actinomycetales(100);Micromonosporineae(100);Micromonosporaceae(100);Actinoplanes(100);unclassified(100);

Otu1020 0 0 0 0 0 0 0 0 0 0 0 0 0 0 0 0 0 0 0 0 0 0 0 0 0 0 0 0
0 0 0 0 0 0 0 0 0 0 0 50 0 0 0 0 0 0 0 0 0 0 0 0 0 0 0 0 0
0 0 0 0 0 0 0 0 0 0 0 0 0 0 0 0 0 0 0 0 0 0 0 0 0 0 0 0 0
0 0 0 0 0 0 0 0 0 0 0 0 0 0 0 0 0 0 0 0 0 0 0 0 0 0 0
M02255_77_000000000-ADUT3_1_1103_6886_12447 T----AC---GT-AG-GGG--------GCG-A-G------C--G--T---T--GT-C-CGG-AA------TT-A--C-T--GG-GC-------GT--A-----AA-GG-GT-GC-------G-TA-G-G-C-G------------G--C-TT-A-G-T----------
AA----G-T-C-A--------G-G-T--G--TG--A-AA-GG--C-A-TG-G-G-----CT-C-AA------C---C-C-A-T-G--T-A----A-G--C-A---C--T-------T--GA-A-A---C-------T--G-C--TT--A-G-C----T-T-G-A-G-T--T---A-AG-----GA-G-A--------G-G-G-A---AG-C------GG--A--ATT----C-C-T-
G-GT--GT-A-G-CG-GT--G---A--A-A---TG-C-GT-AG--AT-A-TC--A-G--G-A-G-G-A-AC-A-CC---AG--T--G--GC-GAA-G--G-C--G-------G--C-T-T-C---CTG--G--CC-T-T--------------------G-G---A-C-T--GA--CG-C-T-G--A-GG--C-A-CG-A--AA-G-C---G-TG--GG-G--
AG-C-A-AA--------CA--GG Bacteria(100);Firmicutes(100);Clostridia(100);Clostridiales(100);unclassified(100);unclassified(100);unclassified(100);unclassified(100);unclassified(100);

Otu1021 0 0 0 0 0 0 0 0 0 0 0 0 0 0 0 0 0 0 0 0 0 0 0 27 0 0 0 0
0 0 0 0 0 0 0 0 0 0 0 0 0 0 0 0 0 0 0 0 0 0 0 0 0 0 0 0 0
0 0 0 0 0 0 0 0 0 0 0 0 0 0 0 0 0 0 0 0 0 0 0 0 0 0 0 0 0
0 0 0 0 0 0 0 0 0 0 0 0 0 0 0 0 0 0 0 0 0 0 0 0 0 0 0
M02255_77_000000000-ADUT3_1_1106_5860_16419 T----AC---GG-AG-GGG--------GCT-A-G------C--G--T---T--GT-T-CGG-AA------TT-A--C-T--GG-GC-------GT--A-----AA-GC-GC-GC-------G-TA-G-G-C-G------------G--C-CA-T-T-T-----------AA-
---G-T-C-A--------G-A-G--G--TG--A-AA-GC--C-C-GG-T-G-----CT-C-AA------C-A-T-C-G-G-A--A-T----A-G--C-C---T--T-------T--GA-G-A---C-------T--G-G--GT--G-G-C----T-A-G-A-A-T--C---C-AG-----GA-G-A--------G-G-T-G---AG-T------GG--A--ATT----C-C-G-A-
GT--GT-A-G-AG-GT--G---A--A-A---TT-C-GT-AG--AT-A-TT--C-G--G-A-A-G-A-AC-A-CC---AG--T--G--GC-GAA-G--G-C--G-------G--C-T-C-A---CTG--G--AC-T-G--------------------G-T---A-T-T--GA--CG-C-T-G--A-GG--T-G-CG-A--AA-G-C---G-TG--GG-G--AG-
C-A-AA--------CA--GG Bacteria(100);Proteobacteria(100);Alphaproteobacteria(100);unclassified(100);unclassified(100);unclassified(100);unclassified(100);unclassified(100);unclassified(100);

Otu1022 0 0 0 0 0 0 0 0 0 0 0 0 0 0 0 0 0 0 0 0 0 0 0 18 0 0 0 0
0 0 0 0 0 0 0 0 0 0 0 0 0 0 0 0 0 0 0 0 0 0 0 0 0 0 0 0 0
0 0 0 0 0 0 0 0 0 0 0 0 0 0 0 0 0 0 0 0 0 0 0 0 0 0 0 0 0
0 0 0 0 0 0 0 0 0 0 0 0 0 0 0 0 0 0 0 0 0 0 0 0 0 0 0
M02255_77_000000000-ADUT3_1_1112_19263_11399 T----AC---GT-AG-GGG--------CCA-A-G------C--G--T---T--GT-C-CGG-AG------TT-A--C-T--GG-GC-------GT--A-----AA-GC-GT-GC-------G-CA-G-G-C-G------------G--C-TC-G-T-T-----
------GC----G-C-C-C--------G-A-C--G--TG--A-AA-GC--C-C-CC-G-G-----CT-C-AA------C-C-G-G-G-G-A--G-G----G-T--C-G---T--C-------G--GG-G-A---C-------G--G-G--CG--A-G-C----T-T-G-A-G-G--G---T-AT-----CA-G-G--------G-G-C-T---GG-T------GG--A--ACT---
-C-C-C-G-GT--GT-A-G-TG-GT--G---A--A-A---TG-C-GT-AG--AG-A-TC--G-G--G-A-A-G-A-AC-A-CC---CG--T--G--GC-GAA-G--G-C--G-------G--C-C-A-G---CTG--G--GA-T-A--------------------C-A---C-C-T--GA--CG-C-T-G--A-GG--C-A-CG-A--AG-G-C---G-TG--
GG-G--AG-C-G-AA--------CG--GG Bacteria(100);Chloroflexi(100);Thermomicrobia(100);JG30-KF-CM45(100);unclassified(100);unclassified(100);unclassified(100);unclassified(100);unclassified(100);

Otu1023 0 0 0 0 0 0 0 0 0 0 0 0 0 0 0 0 0 0 0 0 0 0 0 6 0 0 0 0
0 0 0 0 0 0 0 0 0 0 0 0 0 0 0 0 0 0 0 0 0 0 0 0 0 0 0 0 0
0 0 0 0 0 0 0 0 0 0 0 5 0 0 0 0 0 0 0 22 0 0 0 0 0 0 0 0 23
32 0 0 0 86 0 7 7 0 155 0 91 12 0 3 0 0 0 0 0 0 0 0 0 0 0 0
M02255_77_000000000-ADUT3_1_1115_15344_15735 T----AC---GT-AG-GGG--------GCG-A-G------C--G--T---T--GT-T-CGG-AA------TT-A--T-T--GG-GC-------GT--A-----AA-GG-GT-AC-------G-TA-G-G-C-G------------G--T-TT-G-T-T-----
------AA----G-T-T-T--------G-G-C--G--TT--A-AA-TC--A-C-GG-G-G-----CT-C-AA------C-C-C-C-G-T-T--C------A-G--C-G---T--T-------G--AA-A-A---C-------T--G-G--CA--A-A-C----T-T-G-A-G-T--A---G-TA-----GA-G-G--------G-G-A-C---AG-T------GG--A--ATT----C-
C-T-A-GT--GT-A-G-CG-GT--G---A--A-A---TG-C-GT-AG--AG-A-TT--A-G--G-A-A-G-A-AT-A-CC---GG--T--G--GC-GAA-G--G-C--G-------A--C-T-G-T---CTG--G--AT-A-C--------------------A-T---A-C-T--GA--CG-C-T-C--A-GG--T-A-CG-A--AA-G-C---G-TG--GG-G-
-AG-C-A-AA--------CA--GG Bacteria(100);unclassified(100);unclassified(100);unclassified(100);unclassified(100);unclassified(100);unclassified(100);unclassified(100);unclassified(100);

Otu1024 0 0 0 0 0 0 0 0 0 0 0 0 0 0 0 0 0 0 0 0 0 0 0 0 0 0 0 0
0 0 0 0 0 0 0 0 0 0 0 76 0 0 0 0 0 0 0 0 0 0 0 0 0 0 0 0 0
0 0 0 0 0 0 0 0 0 0 0 0 0 0 0 0 0 0 0 0 0 0 0 0 0 0 0 0 0
0 0 0 0 0 0 0 0 0 0 0 0 0 0 0 0 0 0 0 0 0 0 0 0 0 0 0
M02255_77_000000000-ADUT3_1_1105_7682_11232 T----AC---GT-AG-GAC--------CCA-A-G------C--G--T---T--AT-C-CGG-AG------TG-A--C-T--GG-GC-------GT--A-----AA-GA-GTTGC-------G-TA-G-G-C-G------------G--C-TT-A-T-T-----------AA-
---G-T-G-G--------A-T-A--G--TG--A-AA-CC--T-G-GT-G-G-----CT-C-AA------C-C-A-T-ACA-G--A-C----T-A--T-T---A--T-------C--CA-A-A---C-------T--G-A--TA--A-G-C----T-T-G-A-G-A--A---T-GG-----TA-G-A--------G-G-T-G---AT-T------GG--A--ATT----T-C-T-A-
GT--GT-A-G-GG-GT--A---A--T-A---TC-C-GT-AG--AT-A-TT--A-G--A-A-G-G-A-AC-A-CC---AA--T--G--GC-GTA-G--G-C--A-------G--A-T-C-A---CTG--G--AC-C-A--------------------T-T---T-C-T--GA--CG-C-T-G--A-GG--C-A-CG-A--AA-G-C---G-TG--GG-G--AG-C-
G-AA--------CC--GG Bacteria(100);Candidate_division_TM7(100);unclassified(100);unclassified(100);unclassified(100);unclassified(100);unclassified(100);unclassified(100);unclassified(100);

Otu1025 0 0 0 0 0 0 0 0 0 0 0 0 0 0 0 0 0 0 0 0 0 0 0 2 0 0 0 0
0 0 0 0 0 0 0 0 0 0 0 0 0 0 0 3 0 0 0 0 0 0 0 0 0 0 0 0 0
0 0 0 0 0 0 0 0 0 0 0 0 0 0 0 0 0 0 0 0 0 5 0 0 0 0 0 0 0
0 0 0 0 0 0 0 0 0 0 0 0 0 0 0 0 0 0 0 0 0 0 0 0 0 0 0
M02255_77_000000000-ADUT3_1_2101_14199_12521 T----AC---GT-AG-GTG--------GCG-A-G------C--G--T---T--GT-C-CGG-AT------TT-A--C-T--GG-GC-------GT--A-----AA-GG-GA-GC-------G-TA-G-G-C-G------------G--A-TT-C-T-T-----
------AA----G-T-G-G--------G-A-T--G--TG--A-AA-TA--C-C-CG-G-G-----CT-T-AA------C-C-T-G-G-G-T--G-C----T-G--C-A---T--T-------C--CA-A-A---C-------T--G-G--GA--A-T-C----T-A-G-A-G-T--G---C-AG-----GA-G-G--------G-G-A-G---AG-T------GG--A--ATT----
C-C-T-A-GT--GT-A-G-CG-GT--G---A--A-A---TG-C-GT-AG--AG-A-TT--A-G--G-A-A-G-A-AC-A-CC---AG--T--G--GC-GAA-G--G-C--G-------A--C-T-C-T---CTG--G--AC-T-G--------------------T-A---A-C-T--GA--CG-C-T-G--A-GG--C-T-CG-A--AA-G-C---G-TG--GG-
G--AG-C-A-AA--------CA--GG Bacteria(100);Firmicutes(100);Clostridia(100);Clostridiales(100);Clostridiaceae(100);Clostridium(100);unclassified(100);unclassified(100);unclassified(100);

Otu1026 0 0 0 0 0 0 0 0 0 0 0 0 0 0 0 0 0 0 0 0 0 0 0 0 0 0 0 0
0 0 0 0 0 0 0 0 0 0 0 81 0 0 0 0 0 0 0 0 0 0 0 0 0 0 0 0 0
0 0 0 0 0 0 0 0 0 0 0 0 0 0 0 0 0 0 0 0 0 0 0 0 0 0 0 0 0
0 0 0 0 0 0 0 0 0 0 0 0 0 0 2 0 0 0 0 0 0 0 0 0 0 0 0
M02255_77_000000000-ADUT3_1_1105_4953_10215 T----AC---GT-AG-GGA--------GCG-A-G------C--G--T---T--GT-C-CGG-AA------TG-A--C-T--GG-GT-------GT--A-----AA-GG-GA-GC-------G-TA-G-G-C-G------------G--G-AT-G-G-C----------
AA----G-T-C-A--------G-A-T--G--TG--A-AA-CC--T-G-AG-G-G-----CT-C-AA------C-C-T-T-C-A-G--A-C----T-G--C-A---T--T-------T--GA-A-A---C-------T--G-C--TG--T-T-C----T-T-G-A-G-T--G---A-AG-----TA-G-A--------G-G-T-A---AG-C------GG--A--ATT----C-C-T-
G-GT--GT-A-G-CG-GT--G---A--A-A---TG-C-GT-AG--AG-A-TC--A-G--G-A-G-G-A-AC-A-TC---GG--T--G--GC-GAA-G--G-C--G-------G--C-T-T-A---CTG--G--GC-T-T--------------------T-T---A-C-T--GA--CG-C-T-G--A-GG--C-T-CG-A--AA-G-C---G-TG--GG-G--
AG-C-A-AA--------CA--GG Bacteria(100);Firmicutes(100);Clostridia(100);Clostridiales(100);Ruminococcaceae(100);Ruminococcus(100);unclassified(100);unclassified(100);unclassified(100);

Otu1027 0 0 0 0 0 0 0 0 0 0 0 0 0 0 0 0 0 0 0 0 0 0 0 0 0 0 0 0
0 0 0 0 0 0 0 0 0 0 0 40 0 0 0 0 0 0 0 0 0 0 0 0 0 0 0 0 0
0 0 0 0 0 0 0 0 0 0 0 0 0 0 0 0 0 0 0 0 0 0 0 0 0 0 0 0 0
0 0 0 0 0 0 0 0 0 0 0 0 0 0 0 0 0 0 0 0 0 0 0 0 0 0 0
M02255_77_000000000-ADUT3_1_1105_12927_9652 G----AC---GA-AC-CGT--------GCT-A-A------C--G--T---T--GT-T-CGG-AA------TC-A--C-T--GG-GC-------TT--A-----AA-GG-GC-GC-------G-TA-G-G-C-G------------G--T-TT-A-C-C----------AA--
--G-T-C-G--------G-G-G--G--TG--A-AA-TC--T-T-TC-G-G-----CT-C-AA------C-C-G-G-A-A-A--A-G----G-G--C-C---T--T-------C--GA-T-A---C-------T--G-G--TA--A-A-C----T-A-G-A-G-C--G---A-GG-----TA-G-G--------G-G-C-A---GA-T------GG--A--ACT----T-C-C-G-
GT--GG-A-G-CG-GT--G---A--A-A---TG-T-GT-TG--AT-A-TC--G-G--A-A-G-G-A-AC-G-CC---GG--T--G--GC-GAA-A--G-C--G-------A--T-C-T-G---CTG--G--AC-C-T--------------------C-T---T-C-T--GA--CG-C-T-G--A-GG--C-G-CG-A--AA-G-C---C-AG--GG-G--AG-
C-A-AA--------CG--GG Bacteria(100);unclassified(100);unclassified(100);unclassified(100);unclassified(100);unclassified(100);unclassified(100);unclassified(100);unclassified(100);

Otu1028 0 0 0 0 0 0 0 0 0 0 0 0 0 0 0 0 0 0 0 0 0 0 0 9 0 0 0 0
0 0 0 0 0 0 0 0 0 0 0 0 0 0 0 0 0 0 0 0 0 0 0 0 0 0 0 0 0
0 0 0 0 0 0 0 0 0 0 0 0 0 0 0 0 0 0 0 0 0 0 0 0 0 0 0 0 0
0 0 0 0 0 0 0 0 0 0 0 0 0 0 0 0 0 0 0 0 0 0 0 0 0 0 0
M02255_76_000000000-ADUNE_1_1113_26671_10200 C----CG---GC-TA-ACT--------CCG-T-G------C--C--A---G--CA-G-CCG-CG------GT-A--A-T--AC-GG-------AG--G-----GT-CC-AA-GC-------GTTA-A-T-C-G------------G--A-TA-A-T-T-----
------AA----G-T-C-A--------G-A-T--G--TG--A-AA-TC--C-C-TG-G-G-----CT-C-AA------C-C-T-A-G-G-A--A-C----T-G--C-A---T--T-------T--GA-T-A---C-------T--G-G--TT--A-T-C----T-A-G-A-G-T--A---T-GG-----TA-G-A--------G-G-T-G---AG-T------GG--A--ATT----T-
C-A-G-GT--GT-A-G-CG-GT--G---A--A-A---TG-C-GT-AG--AG-A-TC--T-G--A-A-G-G-A-AC-A-TC---AG--T--G--GC-GAA-G--G-C--G-------A--C-T-C-A---CTG--G--GC-C-A--------------------T-T---A-C-T--GA--CG-C-T-G--A-GG--T-G-CG-A--AA-G-C---G-TG--GG-T-
-AG-C-A-AA--------CA--GG Bacteria(100);Proteobacteria(100);Gammaproteobacteria(100);unclassified(100);unclassified(100);unclassified(100);unclassified(100);unclassified(100);unclassified(100);

Otu1029 0 0 0 0 0 0 0 0 0 0 0 0 0 0 0 0 0 0 0 0 0 0 0 0 0 0 0 0
0 0 0 0 0 0 0 0 0 0 0 46 18 0 0 0 0 0 0 0 0 0 0 0 0 0 0 0 0
0 0 0 0 0 0 0 0 0 0 0 0 0 0 81 78 0 0 0 0 0 0 0 0 124 0 0 0 0
0 0 0 30 0 0 0 0 0 50 0 0 0 0 0 0 0 0 0 0 0 0 0 0 0 0 0
M02255_77_000000000-ADUT3_1_1104_11791_8280 T----AC---GT-AG-GTC--------CCG-A-G------C--G--T---T--AT-C-CGG-AG------TG-A--C-T--GG-GC-------GT--A-----AA-GA-GTTGC-------G-TA-G-G-C-G------------G--T-CA-A-G-T----------
TA----G-C-G-A--------G-C-G--A--TG--A-AA-AC--T-A-TC-G-G-----CT-C-AA------C-C-G-A-T-A-G--C-T----T-G--T-T---G--T-------T--CG-A-A---C-------T--G-C--TT--G-A-C----T-C-G-A-G-A--G---T-AT-----CA-G-A--------G-G-T-C---GC-T------GG--A--ATT----C-C-T-
A-GT--GT-A-G-CA-GT--G---A--A-A---TG-C-GT-AG--AT-A-TT--A-G--G-A-A-G-A-AC-A-CC---GA--T--G--GC-GTA-G--G-C--A-------G--G-C-G-A---CTG--G--GA-T-A--------------------T-T---T-C-T--GA--CG-C-T-A--A-GG--C-A-CG-A--AA-G-C---G-TG--GG-T--AG-
C-G-AA--------CC--GG Bacteria(100);Candidate_division_TM7(100);unclassified(100);unclassified(100);unclassified(100);unclassified(100);unclassified(100);unclassified(100);unclassified(100);

Otu1030 0 0 0 0 0 0 0 0 0 0 0 0 0 0 0 0 0 0 0 0 0 0 0 1 0 0 3 0
0 0 0 0 0 0 0 0 0 0 0 0 0 0 0 0 0 0 0 0 0 0 0 0 0 0 0 0 0
0 0 0 0 0 0 0 0 0 0 0 0 0 0 0 0 0 0 0 0 0 0 0 0 0 0 0 0 0
0 0 0 0 0 0 0 0 0 0 0 0 0 0 0 0 0 0 0 0 0 0 0 0 0 0 0
M02255_76_000000000-ADUNE_1_2110_28238_7836 C----GG---CT-AA-CTC--------CGT-G-C------C--A--G---C--AG-C-CGC-GG------TA-A--T-A--CG-GA-------GG--G-----TC-CA-AG-CG-------T-TA-T-C-C-G-------------G--C-CT-T-A-T-----------AA--
--G-T-C-A--------G-T-G--G--TG--A-AA-TC--T-C-CT-A-G-----CT-C-AA------C-T-A-G-G-A-A--A-C----T-G--C-C---A--T-------T--GA-T-A---C-------T--G-T--AG--G-G-C----T-T-G-A-A-T--T---T-TT-----GT-G-A--------A-G-T-A---AC-T------AG--A--ATA----T-G-T-A-GT--
GT-A-G-CG-GT--G---A--A-A---TG-C-TT-AG--AT-A-TT--A-C--A-T-G-G-A-AT-A-CC---AA--T--T--GC-GAA-G--G-C--A-------G--G-T-T-A---CTA--A--CA-A-A--------------------C-G---A-T-T--GA--CG-C-T-G--A-TG--G-A-CG-A--AA-G-C---G-TG--GG-G--AG-C-G-
AA--------CA--GG Bacteria(100);Bacteroidetes(100);Flavobacteria(100);Flavobacteriales(100);Flavobacteriaceae(100);unclassified(100);unclassified(100);unclassified(100);unclassified(100);

Otu1031 0 0 0 0 0 0 0 0 0 0 0 0 0 0 0 0 0 0 0 0 0 0 0 0 0 0 0 0
0 0 0 0 0 0 0 0 0 0 0 30 0 0 0 0 0 0 0 0 0 0 0 0 0 0 0 0 0
0 0 0 0 0 0 0 0 0 0 0 0 0 0 0 0 0 0 0 0 0 0 0 0 0 0 0 0 0
0 0 0 0 0 0 0 0 0 0 0 0 0 0 0 0 0 0 0 0 0 0 0 0 0 0 0
M02255_77_000000000-ADUT3_1_1103_19656_19942 A----AC---GT-AG-GTG--------GCA-A-G------C--G--T---T--GT-C-CGG-AA------TT-A--C-T--GG-GT-------GT--A-----AA-GG-GA-GC-------G-CA-G-G-C-G-------------G--A-TT-T-G-C----
-------AA----G-T-T-G--------G-A-A--G--TG--A-AA-CT--C-A-TG-G-G-----CT-C-AA------C-C-C-A-T-G-A--A-C----T-G--C-T---T--T-------C--AA-A-A---C-------T--G-C--AG--A-T-C----T-T-G-A-G-T--G---G-TG-----TA-G-A--------G-G-T-A---GG-C------GG--A--ATT----C-
C-C-G-GT--GT-A-G-CG-GT--G---G--A-A---TG-C-GT-AG--AT-A-TC--G-G--G-A-G-G-A-AC-A-CC---AG--T--G--GC-GAA-G--G-C--G-------G--C-C-T-A---CTG--G--GC-A-C--------------------T-A---A-C-T--GA--CG-C-T-G--A-GG--C-T-CG-A--AA-G-C---A-TG--GG-
T--AG-C-A-AA--------CA--GG Bacteria(100);Firmicutes(100);Clostridia(100);Clostridiales(100);Ruminococcaceae(100);unclassified(100);unclassified(100);unclassified(100);unclassified(100);

Otu1032 0 0 0 0 0 0 0 0 0 0 0 0 0 0 0 0 0 0 0 0 0 0 0 0 0 0 0 0
0 0 0 0 0 0 0 0 0 0 21 0 0 0 0 0 0 0 0 0 0 0 0 0 0 0 0 0 0
0 0 0 0 0 0 0 0 0 0 0 0 0 0 0 0 0 0 0 0 0 0 0 0 0 0 0 0 0
0 0 0 0 0 0 0 0 0 0 0 0 0 0 0 0 0 0 0 0 0 0 0 0 0 0 0
M02255_76_000000000-ADUNE_1_1108_18387_10675 T----AC---CA-GC-ACC--------CCG-A-G------T--G--G---T--CG-G-GAC-GA------TT-A--T-T--GG-GC-------CT--A-----AA-GC-AT-CC-------G-TA-G-C-C-G-------------G--T-CC-T-G-C------
-----AA----G-T-C-C--------T-C-C--G--TT--A-AA-TC--C-A-CC-C-G-----CT-C-AA------C-G-G-T-T-G-G--G-C----C-G--C-G---G--G-------G--GA-T-A---C-------T--A-C--AG--G-G-C----T-A-G-G-A-G--G---C-GG-----GA-G-A--------G-G-C-A---AG-C------GG--T--ACT----C-
G-A-T-GG--GT-A-G-GG-GT--A---A--A-A---TC-C-AC-TG--AT-C-CA--T-T--G-A-A-G-A-CC-A-CC---AG--T--G--GC-GAA-G--G-C--G-------G--C-T-T-G---CCA--G--AA-C-G--------------------C-G---C-T-C--GA--CG-G-T-G--A-GG--G-A-TG-A--AA-G-C---T-GG--GG-
G--AG-C-A-AA--------CC--GG Bacteria(100);unclassified(100);unclassified(100);unclassified(100);unclassified(100);unclassified(100);unclassified(100);unclassified(100);unclassified(100);

Otu1033 0 0 0 0 0 0 0 0 0 0 0 0 0 0 0 0 0 0 0 0 0 0 0 0 0 0 0 0
0 0 0 0 0 0 0 0 0 0 0 27 0 0 0 0 0 0 0 0 0 0 0 0 0 0 0 0 0
0 0 0 0 0 0 0 0 0 0 0 0 0 0 0 0 0 0 0 0 0 0 0 0 0 0 0 0 0
0 0 0 0 0 0 0 0 0 0 0 0 0 0 0 0 0 0 0 0 0 0 0 0 0 0 0
M02255_77_000000000-ADUT3_1_1102_17943_22346 T----AC---GG-AG-GAT--------GCA-A-G------C--G--T---T--AT-C-CGG-AA------TT-A--T-T--GG-GC-------GT--A-----AA-GC-GT-CC-------G-CA-G-G-T-G-------------G--C-AT-T-T-C------
-----AA----G-T-C-T--------G-C-T--G--TC--A-AA-GA--C-C-GG-G-G-----CT-T-AA------C-C-C-T-G-G-A--A-A----G-G--C-A---G--T-------G--GA-A-A---C-------T--G-A--AA--C-G-C----T-A-G-A-G-T--G---T-CG-----TA-G-G--------G-G-T-A---GA-G------GG--A--ATT----
C-C-T-G-GT--GT-A-G-CG-GT--G---A--A-A---TG-C-GT-AG--AG-A-TC--A-G--G-A-A-G-A-AC-A-TC---GG--T--G--GC-GAA-A--G-C--G-------C--T-C-T-A---CTG--G--AC-G-A--------------------C-A---A-C-T--GA--CA-C-T-C--A-GG--G-A-CG-A--AA-G-C---T-AG--
GG-G--AG-C-G-AA--------TG--GG Bacteria(100);Cyanobacteria(100);SubsectionIII(100);unclassified(100);unclassified(100);unclassified(100);unclassified(100);unclassified(100);unclassified(100);

Otu1034 0 0 0 0 0 0 0 0 0 0 0 0 0 0 0 0 0 0 0 0 0 0 0 2 0 0 0 0
0 0 0 0 0 0 0 0 0 0 0 0 0 0 0 0 0 0 0 0 0 0 0 0 0 0 0 0 0
0 0 0 0 0 0 0 0 0 0 0 0 0 0 0 0 0 0 0 0 0 0 0 0 0 0 0 0 0
0 0 0 0 0 0 3 0 0 0 0 0 0 0 0 0 0 0 0 0 0 0 0 0 0 0 0
M02255_77_000000000-ADUT3_1_2107_7832_20233 T----AC---GA-AG-GGG--------GCG-A-G------C--G--T---T--GT-T-CGG-AA------TT-A--C-T--GG-GC-------GT--A-----AA-GG-GT-GT-------G-TA-G-G-C-G-------------G--T-TA-A-G-T-----------
AA----G-A-T-A--------G-T-G--G--TG--A-AA-TG--C-C-GG-G-G-----CT-C-AA------C-C-T-C-G-G-A--A-T----T-G--C-C---A--T-------T--AT-G-A---C-------T--A-T--TT--A-G-C----T-A-G-A-A-T--G---A-TG-----CA-G-A--------G-G-A-T---AG-C------GG--A--ATA----C-C-C-
A-GT--GT-A-G-AG-GT--G---A--A-A---TT-C-GT-AG--AT-A-TT--G-G--G-T-A-G-A-AC-A-CC---AG--A--G--GC-GAA-G--G-C--G-------G--C-T-A-T---CTG--G--GC-A-T--------------------T-G---A-T-T--GA--CG-C-T-G--A-GG--C-A-CG-A--AA-G-C---A-TG--GG-G--AT-
C-A-AA--------CA--GG Bacteria(100);Proteobacteria(100);Alphaproteobacteria(100);unclassified(100);unclassified(100);unclassified(100);unclassified(100);unclassified(100);unclassified(100);

Otu1035 0 0 0 0 0 0 0 0 0 0 0 0 0 0 0 0 0 0 0 0 0 0 0 2 0 0 0 0
0 0 0 3 0 0 0 0 0 0 0 7 0 0 0 2 9 0 0 0 0 0 0 0 0 0 0 1 0
0 5 0 0 0 0 0 0 0 0 2 0 0 0 0 0 0 0 6 39 16 0 0 0 0 0 22 0 0
0 5 0 0 16 2 0 0 0 17 0 21 0 0 0 0 0 0 0 0 0 0 4 0 0 0 0
M02255_77_000000000-ADUT3_1_2110_11611_21365 T----AC---GT-AA-GGG--------GCG-A-G------C--G--T---T--GT-C-CGG-AA------TT-A--T-T--GG-GC-------GT--A-----AA-GA-GT-GC-------G-TA-G-G-C-G------------G--C-AA-A-T-T-----
------AA----G-T-C-A--------G-A-T--G--TG--A-AA-AC--T-A-AG-G-G-----CT-C-AA------C-C-C-A-T-A-G--A-T----T-G--C-A---T--C-------T--GA-A-A---C-------T--G-A--TA--T-G-C----T-T-G-A-G-T--C---A-AG-----GA-G-A--------G-G-A-A---AG-T------GG--A--ATT----C-
C-T-A-GT--GT-A-G-CG-GT--G---G--A-A---TG-C-GT-AG--AT-A-TT--A-G--G-A-G-G-A-AT-G-CC---GG--T--G--GC-GAA-G--G-C--G-------A--C-T-T-T---CTG--G--AC-T-T--------------------G-A---A-C-T--GA--CG-C-T-G--A-GG--C-A-CG-A--AA-G-C---G-TG--GG-G-
-AG-C-A-AA--------CA--GG Bacteria(100);unclassified(100);unclassified(100);unclassified(100);unclassified(100);unclassified(100);unclassified(100);unclassified(100);unclassified(100);

Otu1036 0 0 0 0 0 0 0 0 0 0 0 0 0 0 0 0 0 0 0 0 0 0 0 0 0 0 0 0
0 0 0 0 0 0 0 0 0 0 0 32 0 0 0 0 0 0 0 0 0 0 0 0 0 0 0 0 0
0 0 0 0 0 0 0 0 0 0 0 0 0 0 0 0 0 0 0 0 0 0 0 0 0 0 0 0 0
0 0 0 0 0 0 0 0 0 0 0 0 0 0 0 0 0 0 0 0 0 0 0 0 0 0 0
M02255_77_000000000-ADUT3_1_1102_20964_21329 T----AC---GT-AG-GGC--------GCA-A-G------C--G--T---T--GT-C-CGG-AA------TT-A--T-T--GG-GC-------GT--A-----AA-GA-GC-TC-------G-TA-G-G-C-G------------G--T-TT-A-T-C------
-----AC----G-T-C-G--------G-C-T--G--TG--A-AA-TC--C-C-GA-G-G-----CT-T-AA------C-C-T-C-G-G-G--C-C----T-G--C-A---G--T-------C--GA-T-A---C-------G--G-G--TT--G-A-C----T-A-G-A-G-T--G---A-AG-----CA-G-G--------G-G-A-G---AC-T------GG--A--ATT----C-
C-T-G-GT--GT-A-G-CG-GT--G---A--A-A---TG-C-GC-AG--AT-A-TC--A-G--G-A-G-G-A-AC-A-CC---GG--T--G--GC-GAA-G--G-C--G-------G--G-T-C-T---CTG--G--GC-T-T--------------------T-A---A-C-T--GA--CG-C-T-G--A-GG--A-G-CG-A--AA-G-C---G-TG--GG-
T--AG-C-A-AA--------CA--GG Bacteria(100);Actinobacteria(100);Actinobacteria(100);Actinobacteridae(100);Actinomycetales(100);unclassified(100);unclassified(100);unclassified(100);unclassified(100);

Otu1037 0 0 0 0 0 0 0 0 0 0 0 0 0 0 0 0 0 0 0 0 0 0 0 0 7 0 0 0
0 0 0 0 0 0 0 0 0 0 0 0 0 0 0 0 0 0 0 0 0 0 0 0 0 0 0 0 0
0 0 0 0 0 0 0 0 0 0 0 0 0 0 0 0 0 0 0 0 0 0 0 0 0 0 0 0 0
0 0 0 0 0 0 0 0 0 0 0 0 0 0 0 1 0 10 0 0 0 0 0 0 0 0 0
M02255_76_000000000-ADUNE_1_1110_28552_13713 T----AC---GT-AG-GTG--------GCA-A-G------C--G--T---T--GT-C-CGG-AA------TT-A--C-T--GG-GT-------GT--A-----AA----G--G--------G--A-----------------------T-GC---G-C----------
AA----G-T-T-G--------G-A-T--G--TG--A-AA-AC--T-C-CG-G-G-----CT-C-AA------C-C-C-G-G-A-G--C-C----T-G--C-A---T--T-------C--AA-A-A---C-------T--G-C--TG--T-T-C----T-T-G-A-G-T--G---A-AG-----TA-G-A--------G-G-C-A---GG-C------GG--A--ATT----C-C-C-
G-GT--GT-A-G-CG-GT--G---A--A-A---TG-C-GT-AGA-T--A-TC--G-G--G-A-G-G-A-AC-A-CC---AG--T--G--GC-GAA-G--G-C--G-------G--C-C-T-G---CTG--G--GC-T-T--------------------T-T---A-C-T--GA--CG-C-T-G--A-GG--C-A-CG-A--AA-G-C---A-TG--GG-T--
AG-C-A-AA--------CA--GG Bacteria(100);Firmicutes(100);Clostridia(100);Clostridiales(100);Ruminococcaceae(100);Ethanoligenens(100);unclassified(100);unclassified(100);unclassified(100);

Otu1038 0 0 0 0 0 0 0 0 0 0 0 0 0 0 0 0 0 0 0 0 0 0 0 0 14 0 0 0
0 0 0 0 0 0 0 0 0 0 0 0 0 0 0 0 0 0 0 0 0 0 0 0 0 0 0 0 0
0 0 0 0 0 0 0 0 0 0 0 0 0 0 0 0 0 0 0 0 0 0 0 0 0 0 0 0 0
0 0 0 0 0 0 0 0 0 0 0 0 0 0 0 0 0 0 0 0 0 0 0 0 0 0 0
M02255_77_000000000-ADUT3_1_1102_9137_9211 G----AC---GG-AG-GGT--------GCT-A-G------C--G--T---T--GT-T-CGG-AA------TT-A--C-T--GG-GC-------GT--A-----AA-GG-GC-GC-------G-TA-G-G-C-G------------G--C-TC-T-T-T-----------CA-
---G-T-C-G--------G-A-T--G--TG--A-AA-GC--C-C-GG-G-G-----CT-C-AA------C-C-C-C-G-G-A--A-C----G-G--C-A---T--C-------C--GA-G-A---C-------G--G-G--AG--G-G-C----T-C-G-A-G-A--C---C-GG-----GA-G-A--------G-G-A-G---GG-T------GG--A--ATA----G-C-C-
A-GT--GT-A-G-AG-GT--G---A--A-A---TT-C-GT-AG--AT-A-TT--G-G--C-T-G-G-A-AC-A-CC---GG--T--G--GC-GAA-A--G-C--G-------G--C-T-C-T---CTG--G--CC-C-G--------------------G-T---T-C-T--GA--CG-C-T-G--A-GG--C-G-CG-A--AA-G-C---G-T-G-GG-G--AG-
C-A-AA--------CA--GG Bacteria(100);Proteobacteria(100);Alphaproteobacteria(100);Rhodospirillales(100);Candidatus_Alysiosphaera(100);unclassified(100);unclassified(100);unclassified(100);unclassified(100);

Otu1039 0 0 0 0 0 0 0 0 0 0 0 0 0 0 0 0 0 0 0 0 0 0 0 0 10 0 0 0
0 0 0 0 0 0 0 0 0 0 0 0 0 0 0 0 0 0 0 0 0 0 0 0 0 0 0 0 0
0 0 0 0 0 0 0 0 0 0 0 0 0 0 0 0 0 0 0 0 0 0 0 0 0 0 0 0 0
0 0 0 0 0 0 0 0 0 0 0 0 0 0 0 0 0 0 0 0 0 0 0 0 0 0 0
M02255_77_000000000-ADUT3_1_1107_5276_18246 T----AC---GT-AG-GTG--------GCA-A-G------C--G--T---T--GT-C-CGG-AA------TT-A--T-T--GG-GC-------GT--A-----AA-GG-GC-GC-------G-TA-G-G-C-G------------G--C-CT-T-C-T-----------AA-
---G-T-C-A--------G-A-T--G--TG--A-AA-TC--C-C-TG-G-G-----CT-T-AA------C-C-T-G-G-G-T--A-G----T-G--C-A---T--T-------T--GA-A-A---C-------T--G-G--AG--G-G-C----T-T-G-A-G-T--G---C-AG-----GA-G-A--------G-G-T-A---AG-T------GG--A--ATT----C-C-T-A-
GT--GT-A-G-CG-GT--G---A--A-A---TG-C-GT-AG---ATA-TT--A-G--G-A-G-G-A-AC-A-CC---AG--T--G--GC-GAA-G--G-C--G-------A--C-T-T-A---CTG--G--AC-T-G--------------------T-A---A-C-T--GA--CG-C-T-G--A-GG--C-G-CG-A--AA-G-C---G-TG--GG-G--AG-
C-A-AA--------CA--GG Bacteria(100);Firmicutes(100);Clostridia(100);Clostridiales(100);unclassified(100);unclassified(100);unclassified(100);unclassified(100);unclassified(100);

Otu1040 0 0 0 0 0 0 0 0 0 0 0 0 0 0 0 0 0 0 0 0 0 0 0 0 5 0 0 0
0 0 0 0 0 0 0 0 0 0 0 0 0 0 0 0 0 0 0 0 0 0 0 0 0 0 0 0 0
0 0 0 0 0 0 0 0 0 0 0 0 0 0 0 0 0 0 0 0 0 0 0 0 0 0 0 0 0
0 0 0 0 0 0 0 0 0 0 0 0 0 0 0 0 0 0 0 0 0 0 0 0 0 0 0
M02255_77_000000000-ADUT3_1_2117_20533_9161 T----AC---GT-AG-GGG--------GCG-A-G------C--G--T---T--GT-C-CGA-AG------TT-A--C-T--GG-GC-------GT--A-----AA-GA-GC-GC-------G-TA-G-G-C-G------------G--G-TT-C-T-T----------
AA----G-T-G-A--------G-G-G--G--TG--A-AA-GG--T-TCAG-G-G-----CT-C-AA------C-C-C-G-G-A-C--A-C----T-G--C-C---T--T-------T--CA-T-A---C-------T--G-G--GA--A-C-C----T-T-G-A-G-T--G---C-GG-----GA-G-A--------G-G-C-G---AG-T------GG--A--ATG----G-C-
T-G-GT--GT-A-G-CG-GT--G---A--A-A---TG-C-GT-AG--AT-A-TC--A-G--T-C-G-G-A-AC-A-CC---CA--T--G--GC-GAA-G--G-C--A-------G--C-T-C-G---CTG--G--CC-T-G--------------------C-A---A-C-T--GA--CG-C-T-G--A-GG--C-G-CG-A--AA-G-C---G-TG--GG-G--
AG-C-A-AA--------CA--GG Bacteria(100);unclassified(100);unclassified(100);unclassified(100);unclassified(100);unclassified(100);unclassified(100);unclassified(100);unclassified(100);

Otu1041 0 0 0 0 0 0 0 0 0 0 0 0 0 0 0 0 0 0 0 0 0 0 0 0 0 0 0 0
0 0 0 0 0 0 0 0 0 0 0 30 0 0 0 0 0 0 0 0 0 0 0 0 0 0 0 0 0
0 0 0 0 0 0 0 0 0 0 0 0 0 0 0 0 0 0 0 0 0 0 0 0 0 0 0 0 0
0 0 0 0 0 0 0 0 0 0 0 0 0 0 0 0 0 0 0 0 0 0 0 0 0 0 0
M02255_77_000000000-ADUT3_1_1101_4213_10105 T----AC---GA-AG-GGG--------GCT-A-G------C--G--T---T--GT-T-CGG-AA------TT-A--C-T--GG-GC-------GT--A-----AA-GC-GC-AC-------G-TA-G-G-C-G------------G--A-TT-G-T-T-----------AA-
---G-T-G-A--------G-G-G--G--TG--A-AA-TC--C-C-AG-G-G-----CT-C-AA------C-C-C-T-G-G-A--A-C----T-G--C-C---T--C-------T--CA-T-A---C-------T--G-G--CA--A-T-C----T-T-G-A-G-T--A---T-GG-----AA-G-A--------G-G-T-G---AG-T------GG--A--ATT----C-C-G-A-
GT--GT-A-G-AG-GT--G---A--A-A---TT-C-GT-AG--AT-A-TT--C-G--G-A-G-G-A-AC-A-CC---AG--T--G--GC-GAA-G--G-C--G-------G--C-T-C-A---CTG--G--TC-C-A--------------------T-T---A-C-T--GA--CG-C-T-G--A-GG--T-G-CG-A--AA-G-C---G-TG--GG-G--AG-C-
A-AA--------CA--GG Bacteria(100);Proteobacteria(100);Alphaproteobacteria(100);Rhizobiales(100);unclassified(100);unclassified(100);unclassified(100);unclassified(100);unclassified(100);

Otu1042 0 0 0 0 0 0 0 0 0 0 0 0 0 0 0 0 0 0 0 0 0 0 0 0 8 0 6 0
0 0 0 0 0 0 0 0 0 0 0 0 0 0 0 0 0 0 0 0 0 0 0 0 0 0 0 0 0
0 0 0 0 0 0 0 0 0 0 0 0 0 0 0 14 0 8 0 0 0 0 0 0 0 0 0 0 0
0 0 0 115 0 0 0 0 0 0 0 0 0 0 0 0 0 0 0 0 0 0 0 0 9 0 0
M02255_77_000000000-ADUT3_1_2118_14877_11874 T----AC---GG-AG-GGG--------GCT-A-G------C--G--T---T--GT-T-CGG-AA------AT-A--C-T--GG-GC-------GT--A-----AA-GC-GA-AC-------G-TA-G-G-C-G------------G--C-TT-G-G-C----
-------AA----G-T-C-A--------G-G-G--G--TG--A-AA-TC--C-C-AG-T-G-----CT-C-AA------C-A-C-T-G-G-A--A-C----T-G--C-C---T--T-------T--GA-A-A---C-------T--G-C--CA--G-G-C----T-T-G-A-A-T--C---C-TG-----GA-G-A--------G-G-C-G---AG-T------GG--A--ATT----
C-C-G-A-GT--GT-A-G-AG-GT--G---A--A-A---TT-C-GT-AG--AT-A-TT--C-G--G-A-A-G-A-AC-A-CC---AG--T--G--GC-GAA-G--G-C--G-------A--C-T-C-G---CTG--G--AC-A-G--------------------G-T---A-T-T--GA--CG-C-T-G--A-GG--T-T-CG-A--AA-G-C---G-TG--GG-
G--AG-C-A-AA--------CA--GG Bacteria(100);Proteobacteria(100);Alphaproteobacteria(100);Sphingomonadales(100);unclassified(100);unclassified(100);unclassified(100);unclassified(100);unclassified(100);

Otu1043 0 0 0 0 0 0 0 0 0 0 0 0 0 0 0 0 0 0 0 0 0 0 0 0 0 0 0 0
0 0 0 0 0 0 0 0 0 0 0 18 0 0 0 0 0 0 0 0 0 0 0 0 0 0 0 0 0
0 0 0 0 0 0 0 0 0 0 0 0 0 0 0 0 0 0 0 0 0 0 0 0 0 0 0 0 0
0 0 0 0 0 0 0 0 0 0 0 0 0 0 0 0 0 0 0 0 0 0 0 0 0 0 0
M02255_76_000000000-ADUNE_1_1105_19166_19700 T----A----AC-TC-TGT--------GCC-A-G------C-----A---G--CC-G-CGG-TA------AG-A--C-A--GA-GG-------GT--G-----CA--A-AC-GT---------TG-C-T-C-G------------G--G-AC-T-T-T---------
--AA----G-T-C-G--------G-G-T--G--TG--A-AA-TC--C-C-TG-G-G-----CT-C-AA------C-C-T-A-G-G-A--A-C----T-G--C-A---T--C-------C--GA-A-A---C-------T--G-A--GG--T-T-C----T-G-G-A-G-T--A---G-TG-----GA-G-A--------G-G-A-A---AG-C------GG--A--ATT----C-C-
G-G-GT--GT-A-G-AG-GT--G---A--A-A---TT-C-GT-AG--AT-A-TC--C-G--G-A-G-G-A-AC-A-CC---AG--T--G--GC-GAA-G--G-C--G-------G--C-T-T-T---CTG--G--AC-A-C--------------------T-A---A-C-T--GA--CG-C-T-G--A-GA--T-G-CG-A--AA-G-C---G-TG--GG-G--
AG-C-A-AA--------CA--GG Bacteria(100);Proteobacteria(100);unclassified(100);unclassified(100);unclassified(100);unclassified(100);unclassified(100);unclassified(100);unclassified(100);

Otu1044 0 0 0 0 0 0 0 0 0 0 0 0 0 0 0 0 0 0 0 0 0 0 0 0 0 0 0 0
0 0 0 0 0 0 0 0 0 0 0 0 0 0 0 0 0 0 0 0 0 0 0 0 0 0 0 0 0
0 0 0 0 0 0 0 0 0 0 0 0 0 0 0 0 0 0 0 75 0 0 0 0 0 0 0 0 0
0 0 0 0 0 0 0 0 0 0 0 0 0 0 0 0 0 0 0 0 0 0 0 0 0 0 0
M02255_77_000000000-ADUT3_1_1119_18609_17999 T----AC---GA-AG-GGG--------GCT-A-G------C--G--T---T--GT-T-CGG-AA------TT-A--C-T--GG-GC-------GT--A-----AA-GC-GC-AC-------G-TA-G-G-C-G------------G--A-TT-G-T-T-----
------AA----G-T-G-A--------G-G-G--G--TG--A-AA-TC--C-C-AG-G-G-----CT-C-AA------C-C-C-T-G-G-A--A-C----T-G--C-C---T--T-------T--CA-T-A---C-------T--G-G--CA--A-T-C----T-A-G-A-G-T--C---C-AG-----AA-G-A--------G-G-T-G---AG-T------GG--A--ATT----C-
C-G-A-GT--GT-A-G-AG-GT--G---A--A-A---TT-C-GT-AG--AT-A-TT--C-G--G-A-G-G-A-AC-A-CC---AG--T--G--GC-GAA-G--G-C--G-------G--C-T-C-A---CTG--G--TC-T-G--------------------G-A---A-C-T--GA--CG-C-T-G--A-GG--T-G-CG-A--AA-G-C---G-TG--GG-
G--AG-C-A-AA--------CA--GG Bacteria(100);Proteobacteria(100);Alphaproteobacteria(100);Rhizobiales(100);unclassified(100);unclassified(100);unclassified(100);unclassified(100);unclassified(100);

Otu1045 0 0 0 0 0 0 0 0 0 0 0 0 0 0 0 0 0 0 0 0 0 0 0 0 0 0 0 36
0 0 0 0 0 0 0 0 0 0 0 0 0 0 0 0 0 0 0 0 0 0 0 0 0 0 0 0 0
0 0 0 0 0 0 0 0 0 0 0 0 0 0 0 0 0 0 0 0 0 0 0 0 0 0 0 0 0
0 0 0 0 0 0 0 0 0 0 0 0 0 0 0 0 0 0 0 0 0 0 0 0 0 0 0
M02255_77_000000000-ADUT3_1_1105_22292_24909 T----AC---GT-AG-GGA--------CCA-A-G------C--G--T---T--GT-T-CGG-AT------TT-A--C-T--GG-GC-------GT--A-----AA-GG-GT-GC-------G-TA-G-G-C-G------------G--C-GT-G-T-C-----
------AA----G-T-C-A--------G-T-T--G--TG--A-AA-TC--T-T-AA-G-G-----CT-T-AA------C-C-T-T-A-A-G--C-G----G-T--C-A---A--C-------T--GA-T-A---C-------T--G-T--CA--T-G-C----T-A-G-A-G-T--G---C-AG-----AA-G-G--------G-G-C-A---AT-C------GG--A--ATT----C-
T-T-G-GT--GT-A-G-CG-GT--G---A--A-A---TG-C-GT-AG--AT-A-TC--A-A--G-A-G-G-A-AC-A-CC---TG--A--G--GT-GAA-G--A-C--G-------G--G-T-T-G---CTG--G--GC-T-G--------------------A-C---A-C-T--GA--CG-C-T-G--A-GG--C-A-CG-A--AA-G-C---T-AG--GG-T-
-AG-C-A-AA--------CG--GG Bacteria(100);Acidobacteria(100);Acidobacteria(100);Acidobacteriales(100);Acidobacteriaceae(100);Candidatus_Chloroacidobacterium(100);unclassified(100);unclassified(100);unclassified(100);

Otu1046 0 0 0 0 0 0 0 0 0 0 0 0 0 0 0 0 0 0 0 0 0 0 0 0 0 0 0 0
0 0 0 0 0 0 0 0 15 0 0 0 0 0 0 0 0 0 0 0 0 0 0 0 0 0 0 0 0
0 0 0 0 0 0 0 0 0 0 0 0 0 0 0 0 0 0 0 0 0 0 0 0 0 0 0 0 0
0 0 0 0 0 0 0 0 0 0 0 0 0 0 0 0 0 0 0 0 0 0 0 0 0 0 0
M02255_77_000000000-ADUT3_1_1108_13484_19049 C----AC---GT-AG-GCA--------CCA-A-G------C--G--T---T--GT-C-CGG-AT------TT-A--T-T--GG-GC-------GT--A-----AA-GA-GC-TC-------G-TA-G-G-C-G------------G--T-TC-A-G-T------
-----AA----G-T-C-G--------G-G-T--G--TG--A-AA-AC--T-C-TG-G-G-----CT-T-AA------C-C-C-A-G-A-G--A------C-G-CC-A---C--T-------C--GA-T-A---C-------T--A-T--TG-TG-A-C----T-A-G-A-G-T--T---C-GG-----TA-G-G--------G-G-A-G---CG-G------GG--A--ATT----C-
C-T-A-GT--GT-A-G-CG-GT--G---A--A-A---TG-C-GC-AG--AT-A-TT--A-G--G-A-G-G-A-AC-A-CC---GG--T--G--GC-GAA-G--G-C--G-------C--C-G-C-T---CTG--G--GC-C-G--------------------A-T---A-C-T--GA--CG-C-T-G--A-GG--A-G-CG-A--AA-G-C---G-TG--GG-
T--AG-C-A-AA--------CA--GG Bacteria(100);Actinobacteria(100);Actinobacteria(100);Acidimicrobidae(100);Acidimicrobiales(100);Acidimicrobineae(100);Acidimicrobiaceae(100);uncultured(100);unclassified(100);

Otu1047 0 0 0 0 0 0 0 0 0 0 0 0 0 0 0 0 0 0 0 0 0 0 0 0 0 0 0 0
0 0 0 0 0 0 0 0 31 0 0 0 0 0 0 0 0 0 0 0 0 0 0 0 0 0 0 0 0
0 0 0 0 0 0 0 0 0 0 0 0 0 0 0 0 0 0 0 0 0 0 0 0 0 0 0 0 0
0 0 0 0 0 0 0 0 0 0 0 0 0 0 0 0 0 0 0 0 0 0 0 0 0 0 0
M02255_77_000000000-ADUT3_1_1105_6064_15731 T----AC---GA-AG-GGG--------GCT-A-G------C--G--T---T--GT-T-CGG-AA------TC-A--C-T--GG-GC-------GT--A-----AA-GC-GC-GC-------G-TA-G-G-C-G------------G--A-TA-T-G-T-----------
AA----G-T-C-A--------G-A-G--G--TG--A-AA-GC--C-C-AG-G-G-----CC-T-AA------C-C-T-T-G-G-A--A-C----T-G--C-C---T--T-------T--GA-A-A---C-------T--A-C--AT--A-T-C----T-A-G-A-G-A--C---T-CG-----GA-G-G--------G-G-A-T---AG-C------GG--A--ATT----C-C-A-
A-AT--GT-A-G-GG-GT--G---A--A-A---TC-C-GT-AG--AT-A-TT--T-G--G-A-G-G-A-AC-A-CC---GG--T--G--GC-GAA-G--G-C--G-------G--C-T-A-T---CTG--G--AC-G-A--------------------G-T---A-C-T--GA--CG-C-T-G--A-GG--T-G-CG-A--AA-G-C---G-TG--GG-G--
AG-C-A-AA--------CA--GG Bacteria(100);Proteobacteria(100);unclassified(100);unclassified(100);unclassified(100);unclassified(100);unclassified(100);unclassified(100);unclassified(100);

Otu1048 0 0 0 0 0 0 0 0 0 0 0 0 0 0 0 0 0 0 0 0 0 0 0 0 0 0 0 12
0 0 0 0 0 0 0 0 0 0 0 0 0 0 0 0 0 0 0 0 0 0 0 0 0 0 0 0 0
0 0 0 0 0 0 0 0 0 0 0 0 0 0 0 0 0 0 0 0 0 0 0 0 0 0 0 0 0
0 0 0 0 0 0 0 0 0 0 0 0 0 0 0 0 0 0 0 0 0 0 0 0 0 0 0
M02255_77_000000000-ADUT3_1_1106_8261_10458 T----AC---GA-AG-GGG--------GCT-A-G------C--G--T---T--GT-T-CGG-AA------TC-A--C-T--GG-GC-------GT--A-----AA-GC-GC-AC-------G-TA-G-G-C-G------------G--A-CT-T-T-C-----------AA-
---G-T-C-A--------G-G-G--G--TG--A-AA-TC--C-C-GG-G-G-----CT-C-AA------C-C-T-C-G-G-A--A-C----T-G--C-C---T--T-------T--GA-T-A---C-------T--G-T--TG--G-T-C----T-C-G-A-G-T--C---C-GA-----TA-G-A--------G-G-T-G---GG-T------GG--A--ATT----C-C-T-A-
GT--GT-A-G-AG-GT--G---A--A-A---TT-C-GT-AG--AT-A-TT--A-G--G-A-A-G-A-AC-A-CC---GG--T--G--GC-GAA-G--G-C--G-------G--C-C-C-A---CTG--G--AT-C-G--------------------G-T---A-C-T--GA--CG-C-T-G--A-GG--T-G-CG-A--AA-G-C---G-TG--GG-G--AG-
C-A-AA--------CA--GG Bacteria(100);Proteobacteria(100);Alphaproteobacteria(100);Rhizobiales(100);Hyphomicrobiaceae(100);unclassified(100);unclassified(100);unclassified(100);unclassified(100);

Otu1049 0 0 0 0 0 0 0 0 0 0 0 0 0 0 0 0 0 0 0 0 0 0 0 0 0 0 0 0
0 0 0 0 0 0 0 0 39 0 0 0 0 0 0 0 0 0 0 0 0 0 0 0 0 0 0 0 0
0 0 0 0 0 0 0 0 0 0 0 0 0 0 0 0 0 0 0 0 0 0 0 0 0 0 0 0 0
0 0 0 0 0 0 0 0 0 0 0 0 0 0 0 0 0 0 0 0 0 0 0 0 0 0 0
M02255_77_000000000-ADUT3_1_1101_27602_13163 T----AC---GA-AG-GGG--------GCT-A-G------C--G--T---T--GC-T-CGG-AA------TT-A--C-T--GG-GC-------GT--A-----AA-GG-GC-GC-------G-TA-G-G-C-G------------G--C-TC-T-G-T----
-------TA----G-T-C-A--------G-G-C--G--TG--A-AA-GC--C-C-TG-G-G-----CT-C-AA------C-C-T-G-G-G-G--A-C----T-G--C-G---C--T-------T--GA-G-A---C-------G--G-C--AG--G-G-C----T-T-G-A-G-G--G---C-AG-----AA-G-A--------G-G-C-T---CG-C------GG--A--ATT----
C-C-C-A-GT--GT-A-G-AG-GT--G---A--A-A---TT-C-GT-AG--AT-A-TT--G-G--G-A-A-G-A-AC-A-CC---GG--T--G--GC-GAA-G--G-C--G-------G--C-G-A-G---CTG--G--TC-T-G--------------------C-A---C-C-T--GA--CG-C-T-G--A-GG--C-G-CG-A--TA-G-C---G-TG--
GG-G--AG-C-A-AA--------CA--GG Bacteria(100);Proteobacteria(100);Alphaproteobacteria(100);Rhodospirillales(100);Acetobacteraceae(100);unclassified(100);unclassified(100);unclassified(100);unclassified(100);

Otu1050 0 0 0 0 0 0 0 0 0 0 0 0 0 0 0 0 0 0 0 0 0 0 0 0 0 0 0 0
0 0 0 0 0 0 0 0 12 0 0 0 0 0 0 0 0 0 0 0 0 0 0 0 0 0 0 0 0
0 0 0 0 0 0 0 0 0 0 0 0 0 0 0 0 0 0 0 0 0 0 0 0 0 0 0 0 0
0 0 0 0 0 0 0 0 0 0 0 0 0 0 0 0 0 0 0 0 0 0 0 0 0 0 0 0
M02255_76_000000000-ADUNE_1_1119_26109_9337 C----TA---AC-TC-CGT--------GCC-A-G------C-----A---G--CC-G-CGG-TA------AT-A--C-G--GG-GG-------GG--G-----CA-AG-CG-TT-------G-T----T-C-G------------G--A-CA-A-C-T----------AA---
-G-T-C-A--------G-A-C--G--TG--A-AA-TC--C-C-CA-G-G-----CT-C-AA------C-C-T-G-G-G-A--A-C----T-G--C-G---T--C-------T--GA-T-A---C-------T--G-G--AT--G-G-C----T-T-G-A-A-T--T---C-GG-----GA-G-A--------G-G-G-A---TG-C------AG--A--ATT----C-C-A-G-
GT--GT-A-G-CG-GT--G---A--A-A---TG-C-GT-AG--AT-A-TC--T-G--G-A-G-G-A-AT-A-CC---GG--T--G--GC-GAA-G--G-C--G-------G--C-A-T-C---CTG--G--AC-C-G--------------------A-C---A-T-T--GA--CG-C-T-G--A-GG--A-G-CG-A--AA-G-C---T-AG--GG-G--AG-
C-A-AA--------CG--GG Bacteria(100);Acidobacteria(100);Acidobacteria(100);Acidobacteriales(100);Acidobacteriaceae(100);uncultured(100);unclassified(100);unclassified(100);unclassified(100);

Otu1051 0 0 0 0 0 0 0 0 0 0 0 0 0 0 0 0 0 0 0 0 0 0 0 0 0 0 0 30
0 0 0 0 0 0 0 0 0 5 9 0 0 0 0 0 51 50 0 0 0 0 0 0 0 0 0 0 0
0 0 0 0 0 0 0 0 0 0 0 0 0 0 0 0 0 0 0 0 23 0 0 0 0 0 0 0 0
0 0 0 0 0 0 0 0 0 71 0 0 0 0 0 0 0 0 0 0 0 0 0 0 0 0 0 0
M02255_77_000000000-ADUT3_1_1107_11172_15391 T----AC---GT-AT-GTC--------GCA-A-G------C--G--T---T--AT-C-CGG-AT------TT-A--T-T--GG-GC-------GT--A-----AA-GC-GC-GT-------C-TA-G-G-C-G------------G--G-AA-A-G-C------
-----AA----G-T-C-T--------G-A-T--G--TG--A-AA-AT--G-C-GG-G-G-----CT-C-AA------C-T-C-C-G-T-A--T------T-G--C-G---T--T-------G--GA-A-A---C-------T--A-C--TT--T-T-C----T-A-G-A-G-T--A---T-CA-----GA-G-A--------G-G-T-G---GG-C------GG--A--ACT----A-C-
A-A-GT--GT-A-G-AG-GT--G---A--A-A---TT-C-GT-AG--AT-A-TT--T-G--T-A-G-G-A-AT-G-CC---GA--T--G--GA-GAA-G--T-C--A-------G--C-T-C-A---CTG--G--AT-G-A--------------------A-T---A-C-T--GA--CG-C-T-A--A-AG--C-G-CG-A--AA-G-C---G-TG--GG-G--
AG-C-A-AA--------CA--GG Bacteria(100);Fusobacteria(100);Fusobacteria(100);Fusobacteriales(100);Fusobacteriaceae(100);unclassified(100);unclassified(100);unclassified(100);unclassified(100);

Otu1052 0 0 0 0 0 0 0 0 0 0 0 0 0 0 0 0 0 0 0 0 0 0 0 0 0 0 0 15
0 0 0 0 0 0 0 0 0 0 0 0 0 0 0 0 0 0 0 0 0 0 0 0 0 0 0 0 0
0 0 0 0 0 0 0 0 0 0 0 0 0 0 0 0 0 0 0 0 0 0 0 0 0 0 0 0 0
0 0 0 0 0 0 0 0 0 0 0 0 0 0 0 0 0 0 0 0 0 0 0 0 0 0 0 0
M02255_77_000000000-ADUT3_1_1107_7750_22606 T----AC---GA-GT-GCC--------CCA-A-G------C--G--T---T--AT-C-CGG-AA------TT-A--T-T--GG-GC-------GT--A-----AA-GG-GT-GT-------G-TA-G-G-T-G------------G--T-TT-C-G-T----------TA--
--G-T-C-T--------T-C-T--G--TC--A-AA-TT--C-T-TC-G-G-----CT-T-AA------C-C-G-G-G-G-G--C-A----T-G--C-A---G--G-------G--GA-A-A---C-------G--G-C--GG--A-AC-----T-A-G-A-G-G--A---T-GC-----GA-G-A--------G-G-T-A---AA-T------GG--A--ACT----C-A-T-G-
GA--GT-A-G-GG-GT--G---A--A-A---TC-C-GT-TG--AT-A-TC--A-T--G-G-G-G-A-AC-A-CC---AA--A--A--GC-GAA-G--G-C--A-------A--T-T-T-A---CTG--G--CG-C-A--------------------C-T---C-C-T--GA--CA-C-T-G--A-GA--C-A-CG-A--AA-G-C---G-TA--GG-T--AG-C-
G-AA--------TG--GG Bacteria(100);unclassified(100);unclassified(100);unclassified(100);unclassified(100);unclassified(100);unclassified(100);unclassified(100);unclassified(100);

Otu1053 0 0 0 0 0 0 0 0 0 0 0 0 0 0 0 0 0 0 0 0 0 0 0 0 0 0 0 0
0 0 0 0 0 0 0 17 0 0 0 0 0 0 0 0 0 0 0 0 0 0 0 0 0 0 0 0 0
0 0 0 0 0 0 0 0 0 0 0 0 0 0 0 0 0 0 0 0 0 0 0 0 0 0 0 0 0
0 0 0 0 0 0 0 0 0 0 0 0 0 0 0 0 0 0 0 0 0 0 0 0 0 0 0 0
M02255_77_000000000-ADUT3_1_1110_16453_19855 C----AC---GT-AG-GGG--------GCA-A-G------C--G--T---T--GT-C-CGG-AT------TT-A--T-T--GG-GC-------GT--A-----AA-GC-GC-TC-------G-TA-G-G-C-G------------G--C-TC-A-G-T-----
------AA----G-T-C-G--------G-G-T--G--TG--A-AA-TC--T-C-TG-G-G-----CT-C-AAC-----C-C-A-G-A-G-A---------A-G-CC-G---C--C-------C--GA-T-A---C-------T--G-C--TG-TG-G-C----T-A-G-A-G-T--C---C-GG-----TA-G-G--------G-G-A-G---CG-T------GG--A--ATT----
C-C-C-G-GT--GT-A-G-CG-GT--G---A--A-A---TG-C-GC-AG--AT-A-TC--G-G--G-A-G-G-A-AC-A-CC---AG--C--G--GC-GAA-G--G-C--G-------G--C-G-C-T---CTG--G--GC-C-G--------------------G-A---A-C-T--GA--CG-C-T-G--A-GG--A-G-CG-A--AA-G-C---G-TG--
GG-G--AG-C-A-AA--------CA--GG Bacteria(100);Actinobacteria(100);Actinobacteria(100);Acidimicrobidae(100);Acidimicrobiales(100);Acidimicrobineae(100);unclassified(100);unclassified(100);unclassified(100);

Otu1054 0 0 0 0 0 0 0 0 0 0 0 0 0 0 0 0 0 0 0 0 0 0 0 0 0 0 0 0
0 0 0 0 0 0 0 3 0 0 0 0 0 0 0 0 0 0 0 0 0 0 0 0 0 0 13 1 0
0 0 0 0 0 0 0 0 0 0 0 0 0 0 0 0 0 0 0 0 0 0 0 0 0 0 0 0 0
0 0 0 0 0 0 0 0 0 0 0 0 0 0 0 0 0 0 0 0 0 0 0 0 0 0 0 0
M02255_77_000000000-ADUT3_1_1108_13375_16637 T----AC---GG-AG-GGT--------GCA-A-G------C--G--T---T--AT-C-CGG-AT------TT-A------------------TT----------GG-G----------G-TA-G-G-C-T------------G--A-TT-T-G-T----------AA--
--G-T-C-A--------G-T-G--G--TG--A-AA-TC--T-C-AC-A-G-----CT-T-AA------C-T-G-T-G-A-A--A-C----T-G--C-C---A--T-------T--GA-T-A---C-------T--G-C--AA--G-T-C----T-T-G-A-G-T--G---T-TG-----TT-G-A--------A-G-T-A---GC-T------GG--A--ATA----A-G-T-A-GT--
GT-A-G-CG-GT--G---A--A-A---TG-C-AT-AG--AT-A-TT--A-C--T-T-A-G-A-AC-A-CC---AA--T--T--GC-GAA-G--G-C--A-------G--G-T-T-A---CTA--A--GC-A-A--------------------C-A---A-C-T--GA--CG-C-T-G--A-TG--G-A-CG-A--AA-G-C---G-TG--GG-G--AG-C-G-
AA--------CA--GG Bacteria(100);Bacteroidetes(100);Flavobacteria(100);Flavobacteriales(100);Flavobacteriaceae(100);Chryseobacterium(100);unclassified(100);unclassified(100);unclassified(100);

Otu1055 0 0 0 0 0 0 0 0 0 0 0 0 0 0 0 0 0 0 0 0 0 0 0 0 0 0 0 11
0 0 0 0 0 0 0 0 0 0 0 0 0 0 0 0 0 0 0 0 0 0 0 0 0 0 0 0 0
0 0 0 0 0 0 0 0 0 0 0 0 0 0 0 0 0 0 0 0 0 0 0 0 0 0 0 0 0
0 0 0 0 0 0 0 0 0 0 0 0 0 0 0 0 0 0 0 0 0 0 0 0 0 0 0
M02255_77_000000000-ADUT3_1_1101_14667_11060 T----AC---GG-AG-GGT--------CCA-A-G------C--G--T---T--GT-C-CGG-AA------TC-A--C----------------T-----------G-G--GT-------G-TA-G-G-C-G------------G--A-TT-T-G-T----------
AA----G-T-C-A--------G-A-G--G--TG--A-AA-GC--C-C-AC-G-G-----CT-T-AA------C-C-G-T-G-G-A--A-C----T-G--C-C---T--T-------T--GA-T-A---C-------T--G-C--AA--G-T-C----T-T-G-A-G-T--C---C-TG-----GA-G-G--------G-G-C-T---GG-C------GG--A--ATT----C-G-T-
G-GT--GT-A-G-CG-GT--G---A--A-A---TG-C-GT-AG--AT-A-TC--A-C--G-A-G-G-A-AC-A-CC---GG--A--G--GT-GTA-G--A-C--G-------G--C-C-A-G---CTG--G--AC-A-G--------------------G-T---A-C-T--GA--CG-C-T-G--A-GG--C-A-CG-A--AA-G-C---G-TG--GG-G--
AG-C-G-AA--------CA--GG Bacteria(100);unclassified(100);unclassified(100);unclassified(100);unclassified(100);unclassified(100);unclassified(100);unclassified(100);unclassified(100);

Otu1056 0 0 0 0 0 0 0 0 0 0 0 0 0 0 0 0 0 0 0 0 0 0 0 0 0 0 0 7
0 0 0 0 0 0 0 0 0 0 0 0 0 0 0 0 0 0 0 0 0 0 0 0 0 0 0 0 0
0 0 0 0 0 0 0 0 0 0 0 0 0 0 0 0 0 0 0 0 0 0 0 0 0 0 0 0 0
0 0 0 0 0 0 0 0 0 0 0 0 0 0 0 0 0 0 0 0 0 0 0 0 0 0 0
M02255_77_000000000-ADUT3_1_1108_6276_3454 T----AC---GT-AG-GGA--------GCA-A-G------C--G--T---T--GT-C-CGG-AT------TT-A--T-T--GG-GC-------GT--A-----AA-GA-GC-TC-------G-TA-G-G-C-G------------G--C-TT-A-G-C-----------AA-
---G-T-C-A--------G-G-T--G--TG--A-AA-AC--T-C-CA-G-G-----CT-C-AA------C-C-T-G-G-A-G--A------C-G-CC-A---C--T-------T--GA-T-A---C-------T--G-C--TA-TG-G-C----T-T-G-A-G-T--T---T-GG-----TA-G-G--------G-G-T-A---AG-C------GG--A--ATT----C-C-T-G-
GT--GT-A-G-CG-GT--G---A--A-A---TG-C-GC-AG--AT-A-TC--A-G--G-A-G-G-A-AC-A-CC---GA--T--G--GC-GAA-G--G-C--A-------G--C-T-T-A---CTG--G--GC-C-A--------------------A-T---A-C-T--GA--CG-C-T-G--A-GG--A-G-CG-A--AA-G-C---G-TG--GG-G--AG-
C-G-AA--------CA--GG Bacteria(100);Actinobacteria(100);Actinobacteria(100);unclassified(100);unclassified(100);unclassified(100);unclassified(100);unclassified(100);unclassified(100);

Otu1057 0 0 0 0 0 0 0 0 0 0 0 0 0 0 0 0 0 0 0 0 0 0 0 0 0 0 0 0
0 0 0 0 0 0 0 0 27 0 0 0 0 0 0 0 0 0 0 0 0 0 0 0 0 0 0 0 0
0 0 0 0 0 0 0 0 0 0 0 0 0 0 0 0 0 0 0 0 0 0 0 0 0 0 0 0 0
0 0 0 0 0 0 0 0 0 0 0 0 0 0 0 0 0 0 0 0 0 0 0 0 0 0 0
M02255_77_000000000-ADUT3_1_1109_6081_8861 T----AC---GT-AG-GTG--------GCA-A-G------C--G--T---T--GT-C-CGG-AA------TT-A--T-T--GG-GC-------GT--A-----AA-GC-GC-GC-------G-CA-G-G-C-G------------G--T-TC-T-T-T-----------AA--
--G-T-T-G--------G-G-T--G--TT--A-AA-GG--C-A-GC-G-G-----CT-C-AA------C-C-G-C-T-G-T--T------C-G--C-A---C--C-------C--AA-A-A---C-------T--G-G--GG--A-A-C----T-T-G-A-G-T--G---C-AG-----GA-G-A--------G-G-A-T---AG-T------GG--A--ATT----C-C-C-G-
GT--GT-A-G-CG-GT--G---G--A-A---TG-C-GT-AG--AG-A-TC--G-G--G-A-G-G-A-AC-A-CC---AG--T--G--GC-GAA-G--G-C--G-------A--C-T-G-T---CTG--G--CC-T-G--------------------C-A---A-C-T--GA--CG-C-T-G--A-GG--C-G-CG-A--AA-G-C---G-TG--GG-G--AG-
C-A-AA--------CA--GG Bacteria(100);Firmicutes(100);unclassified(100);unclassified(100);unclassified(100);unclassified(100);unclassified(100);unclassified(100);unclassified(100);

Otu1058 0 0 0 0 0 0 0 0 0 0 0 0 0 0 0 0 0 0 0 0 0 0 0 0 0 0 0 0
0 0 0 0 0 0 0 38 0 0 0 0 0 0 0 0 0 0 0 0 0 0 0 0 0 0 0 0 0
0 0 0 0 0 0 0 0 0 0 0 0 0 0 0 0 0 0 0 0 0 0 0 0 0 0 0 0 0
0 0 0 0 0 0 0 0 0 0 0 0 0 0 0 0 0 0 0 0 0 0 0 0 0 0 0
M02255_76_000000000-ADUNE_1_1111_20928_23726 T----AC---GG-AA-GGC--------CCG-A-G------C--G--T---T--AA-T-CGG-AA------TT-A--C-T--GG-GC-------TT--A-----AA-GC-GT-GC-------G-TA-G-G-C-G------------G--A-TC-G-A-A-----
------GG----G-T-G-T--------C-T-T--G--TG--A-AA-TC--C-C-TC-G-G-----CT-C-AA------C-C-G-G-G-G-A--A-C----T-G--C-A---G--G-------G--CA-A-A---C-------C--G-C--CG--A-T-C----T-T-G-A-G-G--C---A-GG-----TA-G-G--------G-G-T-C---GA-T------GG--A--ACT----
G-T-C-G-GT--GG-A-G-CG-GT--G---A--A-A---TG-C-GT-AG--AT-A-TC--G-A--C-A-G-G-A-AC-G-CC---GG--T--G--GC-GAA-G--G-C--G-------A--T-C-G-A---CTG--G--GC-C-T--------------------G-T---C-C-T--GA--CG-C-T-C--A-GG--C-A-CG-A--AA-G-C---G-TG--
GG-G--AG-C-A-AA--------CA--GG Bacteria(100);unclassified(100);unclassified(100);unclassified(100);unclassified(100);unclassified(100);unclassified(100);unclassified(100);unclassified(100);

Otu1059 0 0 0 0 0 0 0 0 0 0 0 0 0 0 0 0 0 0 0 0 0 0 0 0 0 0 0 0
0 0 0 0 0 0 0 26 0 0 0 0 0 0 0 0 0 0 0 0 0 0 0 0 0 0 0 0 0
0 0 0 0 0 0 0 0 0 0 0 0 0 0 0 0 0 0 0 0 0 0 0 0 0 0 0 0 0
0 0 0 0 0 0 0 0 0 0 0 0 0 0 0 0 0 0 0 0 0 0 0 0 0 0 0
M02255_77_000000000-ADUT3_1_2118_18981_10228 G----AC---GA-AC-CGA--------GCG-A-A------C--G--T---T--GT-T-CGG-AA------TC-A--C-T--GG-GC-------TT--A-----AA-GG-GC-GC-------G-TA-G-G-C-G------------G--G-CT-G-T-C-----
------AA----G-T-C-T--------G-G-G--G--TG--A-AA-TC--C-C-AC-G-G-----CT-C-AA------C-C-G-T-G-G-A--A-C----T-G--C-C---T--T-------A--GA-T-A---C-------T--G-A--CG--G-C-C----T-C-G-A-G-G--G---A-GG-----TA-G-G--------G-G-C-G---AG-C------GG--A--ACT----
G-T-G-A-GT--GG-A-G-CG-GT--G---A--A-A---TG-C-GT-TG--AT-A-TT--C-A--C-A-G-G-A-AC-T-CC---GG--T--G--GC-GAA-G--G-C--G-------G--C-T-C-G---CTG--G--AC-C-T--------------------T-T---T-C-T--GA--CG-C-T-G--A-GG--C-G-CG-A--AA-G-C---T-AG--GG-
G--AG-C-A-AA--------CG--GG Bacteria(100);unclassified(100);unclassified(100);unclassified(100);unclassified(100);unclassified(100);unclassified(100);unclassified(100);unclassified(100);

Otu1060 0 0 0 0 0 0 0 0 0 0 0 0 0 0 0 0 0 0 0 0 0 0 0 0 0 0 0 0
0 0 0 0 0 0 0 15 0 0 0 0 0 0 0 0 0 0 0 0 0 0 0 0 0 0 0 0 0
0 0 0 0 0 0 0 0 0 0 0 0 0 0 0 0 0 0 0 0 0 0 0 0 0 0 0 0 0
0 0 0 0 0 0 0 0 0 0 0 0 0 0 0 0 0 0 0 0 0 0 0 0 0 0 0
M02255_77_000000000-ADUT3_1_2108_21480_11212 C----AC---GT-AG-GGT--------CCG-A-G------C--G--T---T--GT-C-CGG-AA------TT-A--C-T--GG-GC-------GT--A-----AA-GC-GC-GC-------G-CA-G-G-C-G------------G--T-CC-A-T-T-----
------CA----G-T-C-G--------C-T-G--G--TG--A-AA-GC--C-C-CA-C-G-----CT-C-AA------C-G-T-G-G-G-A--A-G----G-T--C-C---G--G-------C--GA-G-A---C-------G--G-G--TG--G-A-C----T-G-G-A-G-G--C---A-GG-----GA-G-A--------G-G-T-C---GG-T------GG--A--ATT---
-C-C-G-G-GT--GT-A-G-TG-GT--G---A--A-A---TG-C-GT-AG--AG-A-TC--C-G--G-A-G-G-A-AC-A-CC---AG--A--G--GC-GAA-G--G-C--G-------G--C-C-G-A---CTG--G--AC-C-T--------------------G-A---C-C-T--GA--CG-C-T-G--A-GG--C-G-CG-A--AG-G-C---G-TG--
GG-G--AG-C-G-AA--------CG--GG Bacteria(100);unclassified(100);unclassified(100);unclassified(100);unclassified(100);unclassified(100);unclassified(100);unclassified(100);unclassified(100);

Otu1061 0 0 0 0 0 0 0 0 0 0 0 0 0 0 0 0 0 0 0 0 0 0 0 0 0 0 0 2
0 0 0 0 0 0 0 0 0 0 0 0 0 0 0 0 0 0 0 0 0 0 2 0 0 0 0 0 0
0 0 0 0 0 0 0 0 0 0 0 0 0 0 0 0 0 0 0 0 0 0 0 0 0 0 0 0 0
0 0 0 30 21 0 0 0 0 0 0 0 0 0 0 0 0 0 0 0 0 0 0 0 0 0 0
M02255_76_000000000-ADUNE_1_2112_20876_15478 C----CG---GC-TA-ACT--------TCG-T-G------C--C--A---G--CA-G-CCG-CG------GT-A--A-T--AC-GA-------AG--G-----GT-GC-AA-GC-------GTTA-A-T-C-G------------G--A-GA-G-T-T-----
------AA----G-T-C-T--------G-C-T--G--TG--A-AA-TC--C-C-CG-G-G-----CT-C-AA------C-C-T-G-G-G-A--A-T----G-G--C-A---G--T-------G--GA-T-A---C-------T--G-G--CT--C-G-C----T-A-G-A-G-T--G---T-GT-----CA-G-A--------G-G-A-T---GG-T------GG--A--ATT----C-
C-C-G-GT--GT-A-G-CG-GT--G---A--A-A---TG-C-GT-AG--AG-A-TC--G-G--G-A-G-G-A-AC-A-TC---AG--T--G--GC-GAA-G--G-C--G-------G--C-C-A-T---CTG--G--GA-C-A--------------------A-C---A-C-T--GA--CG-C-T-G--A-GG--C-A-CG-A--AA-G-C---G-TG--GG-
G--AG-C-A-AA--------CA--GG Bacteria(100);Proteobacteria(100);Gammaproteobacteria(100);Xanthomonadales(100);Xanthomonadaceae(100);unclassified(100);unclassified(100);unclassified(100);unclassified(100);

Otu1062 0 0 0 0 0 0 0 0 0 0 0 0 0 0 0 0 0 0 0 0 0 0 0 0 0 0 0 0
0 0 0 0 0 0 0 61 0 0 0 0 0 0 0 0 0 0 0 0 0 0 0 0 0 0 0 0 0
0 0 0 0 0 0 0 0 0 0 0 0 0 0 0 0 0 13 0 0 0 0 0 0 0 0 0 0 0
0 0 0 0 0 0 0 0 0 0 0 0 0 0 0 0 0 0 0 0 0 0 0 0 0 0 0
M02255_77_000000000-ADUT3_1_2107_17364_14687 G----AC---GT-AG-GAT--------CCG-A-G------C--G--T---T--GT-C-CGG-AG------TT-A--C-T--GG-GC-------GT--A-----AA-GC-GC-GC-------G-CA-G-G-C-G------------G--A-CA-C-G-C----
-------GC----A-T-C-G--------G-C-G--G--TG--A-AA-GC--C-C-CC-C-G-----CT-C-AA------C-G-G-G-G-G-A--G-G----G-T--C-C---G--T-------C--GA-G-A---T-------G--G-C--GT--G-T-C----T-G-G-A-G-G--C---A-GG-----GA-G-A--------G-G-C-C---AG-T------GG--A--ATT---
-C-C-G-G-GT--GT-A-G-TG-GT--G---A--A-A---TG-C-GT-AG--AG-A-TC--C-G--G-A-G-G-A-AC-A-CC---AG--T--G--GC-GAA-G--G-C--G-------G--C-T-G-G---CTG--G--AC-C-T--------------------G-A---C-C-T--GA--CG-C-T-GA-G--G--C-G-CG-A--AG-G-C---G-TG--
GG-G--AG-C-G-AA--------CG--GG Bacteria(100);Chloroflexi(100);unclassified(100);unclassified(100);unclassified(100);unclassified(100);unclassified(100);unclassified(100);unclassified(100);

Otu1063 0 0 0 0 0 0 0 0 0 0 0 0 0 0 0 0 0 0 0 0 0 0 0 0 0 0 0 2
0 0 0 0 0 0 0 0 0 0 0 0 0 0 0 0 0 0 0 0 0 0 0 0 0 0 0 0 0
0 0 0 0 0 0 0 0 0 0 0 0 0 0 0 0 0 0 0 0 0 0 0 0 0 0 0 0 0
0 0 0 0 0 0 0 0 0 0 0 0 0 0 0 0 0 56 0 0 0 0 0 0 0 0 0
M02255_77_000000000-ADUT3_1_1114_22384_18854 T----AC---GA-AG-GGG--------GCT-A-G------C--G--T---T--GT-T-CGG-AA------TT-A--C-T--GG-GC-------GT--A-----AA-GC-GC-AC-------G-TA-G-G-C-G------------G--G-TT-G-T-T-----
------AA----G-T-C-A--------G-G-G--G--TG--A-AA-TC--C-C-GG-A-G-----CT-C-AA------C-T-C-C-G-G-A--A-C----T-G--C-C---T--T-------T--GA-T-A---C-------T--G-G--CA--A-T-C----T-A-G-A-G-A--C---T-GG-----AA-G-A--------G-G-T-T---AG-T------GG--A--ATT----C-
C-C-A-GT--GT-A-G-AG-GT--G---A--A-A---TT-C-GT-AG--AT-A-TT--G-G--G-A-A-G-A-AC-A-CC---AG--T--G--GC-GAA-G--G-C--G-------G--C-T-A-A---CTG--G--TC-C-A--------------------G-T---A-C-T--GA--CG-C-T-G--A-GG--T-G-CG-A--AA-G-C---G-TG--GG-
G--AG-C-A-AA--------CA--GG Bacteria(100);Proteobacteria(100);Alphaproteobacteria(100);Rhizobiales(100);unclassified(100);unclassified(100);unclassified(100);unclassified(100);unclassified(100);

Otu1064 0 0 0 0 0 0 0 0 0 0 0 0 0 0 0 0 0 0 0 0 0 0 0 0 0 0 0 0
0 0 0 0 0 0 0 29 0 0 0 0 0 0 0 0 0 0 0 0 0 0 0 0 0 0 0 0 0
0 0 0 0 0 0 0 0 0 0 0 0 0 0 0 0 0 0 0 0 0 0 0 0 0 0 0 0 0
0 0 0 0 0 0 0 0 0 0 0 0 0 0 0 0 0 0 0 0 0 0 0 0 0 0 0
M02255_77_000000000-ADUT3_1_2104_11116_14423 T----AC---GA-AG-GGG--------GCT-A-G------C--G--T---T--GT-T-CGG-AT------TT-A--C-T--GG-GC-------GT--A-----AA-GC-GC-GT-------G-TA-G-G-C-G------------G--G-TC-G-T-T-----
------AA----G-T-T-G--------G-G-G--G--TG--A-AA-TC--C-C-GA-G-G-----CT-C-AA------C-C-T-C-G-G-A--A-C----T-G--C-C---T--T-------C--AA-A-A---C-------T--G-G--CG--A-C-C----T-G-G-A-C-T--C---A-AG-----CA-G-A--------G-G-C-A---AG-T------GG--A--ATT----
G-C-G-A-GT--GT-A-G-AG-GT--G---A--A-A---TT-C-GT-AG--AT-A-TT--C-G--C-A-G-G-A-AC-A-CC---AG--T--G--GC-GAA-G--G-C--G-------A--C-T-T-G---CTG--G--GC-T-T--------------------G-T---A-G-T--GA--CG-C-T-G--A-GA--C-G-CG-A--AA-G-C---G-TG--GG-
G--AG-C-A-AA--------CA--GG Bacteria(100);Proteobacteria(100);Alphaproteobacteria(100);unclassified(100);unclassified(100);unclassified(100);unclassified(100);unclassified(100);unclassified(100);

Otu1065 0 0 0 0 0 0 0 0 0 0 0 0 0 0 0 0 0 0 0 0 0 0 0 0 0 0 0 13
0 0 0 0 0 0 0 0 0 0 0 0 0 0 0 0 0 0 0 0 0 0 0 0 0 0 0 0
0 0 0 0 0 0 0 0 0 0 0 0 0 0 0 0 0 0 0 0 0 0 0 0 0 0 0 0
0 0 0 0 0 0 0 0 0 0 0 0 0 0 0 0 0 0 0 0 0 0 0 0 0 0 0
M02255_77_000000000-ADUT3_1_2106_18670_10312 T----AC---AG-AG-GTG--------CCG-A-G------C--G--T---T--AG-G-CGG-AA------TC-A--C-T--GG-GC-------TT--A-----AA-GC-GT-GT-------G-TA-G-G-C-G------------G--A-GG-C-C-T----
-------AA----G-T-A-C--------C-T-T--G--TG--A-AA-TC--C-C-AC-A-G-----CT-C-AA------C-T-G-T-G-G-A--A-C----T-G--C-T---T--G-------G--TA-T-A---C-------T--G-G--GT--C-T-C----T-T-G-A-G-C--C---A-CT-----TA-G-G--------G-G-C-A---AT-C------GG--A--ACA----A-
A-C-G-GT--GG-A-G-CG-GT--G---A--A-A---TG-C-GT-AG--AT-A-TC--G-T--T-T-G-G-A-AC-G-CC---AA--T--G--GT-GAA-A--A-C--A-------G--A-T-T-G---CTG--G--GA-G-T--------------------G-T---G-C-T--GA--CG-C-T-G--A-GA--C-A-CG-A--AA-G-C---C-AG--GG-
G--AG-C-G-AA--------CG--GG Bacteria(100);Planctomycetes(100);Phycisphaerae(100);WD2101(100);unclassified(100);unclassified(100);unclassified(100);unclassified(100);unclassified(100);

Otu1066 0 0 0 0 0 0 0 0 0 0 0 0 0 0 0 0 0 0 0 0 0 0 0 0 0 0 0 0
0 0 0 0 0 0 0 27 0 0 0 0 0 0 0 0 0 0 0 0 0 0 0 0 0 0 0 0
0 0 0 0 0 0 0 0 0 0 0 0 0 0 0 0 0 0 0 0 0 0 0 0 0 0 0 0
0 0 0 0 0 0 0 0 0 0 0 0 0 0 0 0 0 0 0 0 0 0 0 0 0 0 0
M02255_77_000000000-ADUT3_1_1119_14708_19047 A----AC---GT-AG-GAG--------GCA-A-G------C--G--T---T--AT-C-CGG-AT------TC-A--C-T--GG-GC-------GT--A-----AA-GC-GC-GT-------G-CA-G-G-C-G------------G--T-GC-G-G-T----
-------AA----G-T-T-G--------G-G-T--G--TG--A-AA-TC--T-C-CC-G-G-----CT-C-AA------C-T-G-G-G-A-G--A-G----G-T--C-A---T--T-------C--AA-T-A---C-------T--A-C--CG--T-A-C----T-T-G-A-G-A--G---C-AG-----AA-G-A--------G-G-A-A---GA-T------GG--A--ATT----
C-C-C-G-GT--GT-A-G-TG-GT--G---A--A-A---TG-C-GT-AG--AT-A-TC--G-G--G-A-G-G-A-AC-A-CC---AG--T--G--GC-GAA-G--G-C--G-------A--T-C-T-T---CTG--G--TC-T-G--------------------T-T---T-C-T--GA--CG-C-T-C--A-GA--C-G-CG-A--CA-G-C---T-AG--GG-
T--AG-T-A-AA--------CG--GG Bacteria(100);Chloroflexi(100);Anaerolineae(100);Anaerolineales(100);Anaerolineaceae(100);uncultured(100);unclassified(100);unclassified(100);unclassified(100);

Otu1067 0 0 0 0 0 0 0 0 0 0 0 0 0 0 0 0 0 0 0 0 0 0 0 0 0 0 0 9
0 0 0 0 0 0 0 0 0 0 0 0 0 0 0 0 0 0 0 0 0 0 0 0 0 0 0 0
0 0 0 0 0 0 0 0 0 0 0 0 0 0 0 0 0 0 0 0 0 0 0 0 0 0 0 0
0 0 0 0 0 0 0 0 0 0 0 0 0 0 0 0 0 0 0 0 0 0 0 0 0 0 0
M02255_77_000000000-ADUT3_1_1110_6818_14666 T----AC---GA-AG-GGA--------GCT-A-G------C--G--T---T--GT-T-CGG-AA------TT-A--C-T--GG-GC-------GT--A-----AA-GG-GC-GC-------G-TA-G-G-CGG----------------C-CT-T-G-T-----------AA-
---G-T-C-A--------G-A-T--G--TG--A-AA-GC--C-C-CG-G-G-----CT-T-AA------C-C-T-G-G-G-A--A-C----T-G--C-A---T--T-------T--GA-T-A---C-------T--G-C--AA--G-G-C----T-A-G-A-G-A--T---C-GA-----GA-G-A--------G-G-A-A---AG-T------GG--A--ATTA-----C-G-A-
GT--GT-A-G-AA-GT--G---A--A-A---TT-C-GT-AG--AT-A-TT--C-G--T-A-G-G-A-AC-A-CC---AG--T--G--GC-GAA-G--G-C--G-------A--C-T-T-T---CTG--G--CT-C-G--------------------A-T---A-C-T--GA--CG-C-T-A--A-AG--C-G-CG-A--AA-G-C---G-TG--GG-G--AG-C-
A-AA--------CA--GG Bacteria(100);Proteobacteria(100);Alphaproteobacteria(100);Rickettsiales(100);Candidatus_Odyssella(100);unclassified(100);unclassified(100);unclassified(100);unclassified(100);

Otu1068 0 0 0 0 0 0 0 0 0 0 0 0 0 0 0 0 0 0 0 0 0 0 0 0 0 0 0 0
0 0 0 0 0 0 0 28 0 0 0 0 0 0 0 0 0 0 0 0 0 0 0 0 0 0 0 0
0 0 0 0 0 0 0 0 0 0 0 0 0 0 0 0 0 0 0 0 0 0 0 0 0 0 0 0
0 0 0 0 0 0 0 0 0 0 0 0 0 0 0 0 0 0 0 0 0 0 0 0 0 0 0
M02255_77_000000000-ADUT3_1_1117_17029_8980 T----AC---GT-AG-GTG--------GCA-A-G------C--G--T---T--GT-C-CGG-AT------TT-A--C-T--GG-GC-------GT--A-----AA-GG-GC-GT-------G-CA-G-G-C-G------------G--T-TT-G-T-C-----------TA-
---G-T-C-T--------A-G-G--G--TG--A-AA-GC--C-C-CC-G-G-----CT-T-AA------C-T-G-G-G-G-G--A-G----G-T--C-C---T--T-------G--GA-T-A---C-------T--G-G--CA--A-A-C----T-T-G-A-G-G--G---A-GG-----CA-G-A--------G-G-A-A---AG-T------GG--A--ATT----C-C-C-G-
GT--GT-A-G-TG-GT--G---A--T-A---TG-C-GT-AG--AT-A-TC--G-G--G-A-G-G-A-AC-A-CC---AG--T--G--GC-GAA-G--G-C--G-------G--C-T-T-T---CTG--G--GC-C-T--------------------T-A---C-C-T--GA--CG-C-T-G--A-GA--C-G-CG-A--AA-G-C---G-TG--GG-G--AG-C-
G-AA--------CC--GG Bacteria(100);unclassified(100);unclassified(100);unclassified(100);unclassified(100);unclassified(100);unclassified(100);unclassified(100);unclassified(100);

Otu1069 0 0 0 0 0 0 0 0 0 0 0 0 0 0 0 0 0 0 0 0 0 0 0 0 0 0 1 0
0 0 0 0 0 0 0 0 0 0 0 0 0 0 0 0 0 0 1 0 0 0 0 0 0 0 0 0
0 0 0 0 0 0 0 0 0 0 0 0 0 0 0 0 0 0 0 0 0 0 0 0 0 0 0 0
0 0 0 0 0 0 0 0 0 0 0 0 0 0 0 0 0 0 0 0 0 0 0 0 0 0 0
M02255_76_000000000-ADUNE_1_2119_28522_18282 A----AA---GC-CC-CGG--------CTA-A-C------T--A--C---G--TG-C-CAG-CA------GC-C--G-C--GG-TA-------AT--A-----CG-TA-GG-TG-------G-CA-A-G-C-G------------T--T-GTCC-G-G----
-------AA----G-T-C-T--------G-A-T--G--TG--A-AA-GC--C-C-CC-G-G-----CT-C-AA------C-C-G-G-G-G-A--G------G-GG-C-A---T--T-------G--GA-A-A---C-------T--G-G--GA--G-A-C----T-T-G-A-G-G--A---C-AG-----AA-G-A--------G-G-A-G---AG-T------GG--A--ATT---
-C-C-A-A-GT--GT-A-G-CG-GT--G---A--A-A---TG-C-GT-AG--AT-A-TT--T-G--G-A-G-G-A-AC-A-CC---AG--T--G--GC-GAA-G--G-C--G-------G--C-T-C-T---CTG--G--TC-T-G--------------------T-T---A-C-T--GA--CG-C-T-G--A-GG--C-G-CG-A--AA-G-C---G-TG--
GG-G--AG-C-A-AA--------CA--GG Bacteria(100);Firmicutes(100);Bacilli(100);Bacillales(100);unclassified(100);unclassified(100);unclassified(100);unclassified(100);unclassified(100);

Otu1070 0 0 0 0 0 0 0 0 0 0 0 0 0 0 0 0 0 0 0 0 0 0 0 0 0 0 0 0
0 0 0 0 0 0 0 0 0 0 51 75 0 0 0 0 0 0 0 0 0 0 0 0 0 0 0 0
0 0 0 0 0 0 0 0 0 0 0 0 0 0 0 0 0 0 0 0 0 0 0 0 0 0 0 0
0 0 0 0 0 0 0 0 0 0 0 0 0 0 0 0 0 0 0 0 0 0 0 0 0 0 0
M02255_76_000000000-ADUNE_1_1104_15368_21290 T----AC---GG-AG-GGT--------GCA-A-G------C--G--T---T--AC-C-CGG-AT------TT-A--C-T--GG-GT-------GT--A-----AA-GG-GC-GT-------G-TA-G-G-C-G------------G--T-CT-C-T-C-----
------AA----G-T-C-C--------G-A-T--G--CT--A-AA-GA--C-C-AA-G-G-----CT-C-AA------C-C-T-C-G-G-G--A-G----T-G--C-G---T--T-------G--GA-T-A---C-------T--G-T--GA--G-G-C----T-A-G-A-C-G--G---T-CG-----GA-G-A--------G-G-G-T---AG-C------GG--A--ATT----
T-C-C-G-GA--GT-A-G-CG-GT--G---A--A-A---TG-C-GC-AG--AT-A-CC--G-G--A-A-G-G-A-AC-G-CC---AA--T--A--GC-GAA-A--G-C--A-------G--C-T-A-C---CTG--G--AC-G-A--------------------T-T---T-G-T--GA--CG-C-T-G--A-GG--C-G-CG-A--AA-G-C---G-TG--
GG-G--AG-C-A-AA--------CC--GG Bacteria(100);Deinococcus-Thermus(100);Thermales(100);Thermaceae(100);Meiothermus(100);unclassified(100);unclassified(100);unclassified(100);unclassified(100);

Otu1071 0 0 0 0 0 0 0 0 0 0 0 0 0 0 0 0 0 0 0 0 0 0 0 0 0 8 0 0
0 0 0 0 0 0 0 0 0 0 0 0 0 0 0 0 0 0 0 0 0 0 0 0 0 0 0 0
0 0 0 0 0 0 0 0 0 0 0 0 0 0 0 0 0 0 0 0 0 0 0 0 0 0 0 0
0 0 0 0 0 0 0 0 0 0 0 0 0 0 0 0 0 0 0 0 0 0 0 0 0 0 0
M02255_77_000000000-ADUT3_1_1103_13736_12274 T----AC---GA-AG-GGG--------GCT-A-G------C--G--T---T--GT-T-CGG-AT------TT-A--C-T--GG-GC-------GT--A-----AA-GC-GC-GC-------G-TA-G-G-C-G------------G--A-TA-A-A-T-----
------TA----G-T-T-A--------G-A-G--G--TG--A-AA-TC--C-C-AG-G-G-----CT-C-AA------C-C-C-T-G-G-A--A-C----T-G--C-C---T--T-------T--AA-T-A---C-------T--G-T--TT--A-T-C----T-A-G-A-G-T--A---T-GA-----AA-G-A--------G-G-T-G---AG-T------GG--A--ATT----C-
C-G-A-GT--GT-A-G-AG-GT--G---A--A-A---TT-C-GT-AG--AT-A-TT--C-G--G-A-G-G-A-AC-A-TC---AG--T--G--GC-GAA-G--G-C--G-------G--C-T-C-A---CTG--G--TT-C-A--------------------T-T---A-C-T--GA--CG-C-T-G--A-GG--T-G-CG-A--AA-G-C---G-TG--GG-G-
-AG-C-A-AA--------CA--GG Bacteria(100);Proteobacteria(100);Alphaproteobacteria(100);unclassified(100);unclassified(100);unclassified(100);unclassified(100);unclassified(100);unclassified(100);

Otu1072 0 0 0 0 0 0 0 0 0 0 0 0 0 0 0 0 0 0 0 0 0 0 0 0 0 2 1 0
0 0 0 0 0 0 0 0 0 0 0 0 0 0 0 0 0 0 0 0 0 0 0 0 0 0 0 0
0 0 0 0 0 0 0 0 0 0 0 0 0 0 0 0 0 0 0 0 0 0 0 0 0 0 0 0
0 0 0 0 0 0 0 0 0 0 0 0 0 0 0 0 0 0 0 0 0 0 0 0 0 0 0
M02255_76_000000000-ADUNE_1_2114_26021_18835 T----GG---CT-AA-CTA--------CGT-T-C------C--A--G---C--AG-C-CGC-GG------TA-A--T-A--CG-TA-------G---A-----TG-GC-AA-GC-------G-TT-GTC-C-G------------G--A-TC-T-T-T-------
----AA----G-T-C-T--------G-A-T--G--TG--A-AA-GA--C-C-CC-A-G-----CT-C-AA------C-T-G-G-G-G-A--A------G-GT-C-A---T--T-------G--GA-A-A---C-------T--G-G--AG--A-A-C----T-T-G-A-G-T--G---C-AG-----AA-G-A--------G-G-A-G---AG-T------GG--A--ATT----C-
C-A-T-GT--GT-A-G-CG-GT--G---A--A-A---TG-C-GT-AG--AT-A-TA--T-G--G-A-G-G-A-AC-A-CC---AG--T--G--GC-GAA-G--G-C--G-------A--C-T-C-T---CTG--G--TC-T-G--------------------T-A---A-C-T--GA--CG-C-T-G--A-GG--C-T-CG-A--AA-G-C---G-TG--GG-G-
-AG-C-A-AA--------CA--GG Bacteria(100);Firmicutes(100);Bacilli(100);Lactobacillales(100);Carnobacteriaceae(100);unclassified(100);unclassified(100);unclassified(100);unclassified(100);

Otu1073 0 0 0 0 0 0 0 0 0 0 0 0 0 0 0 0 0 0 0 0 0 0 0 0 0 2 0 0
0 0 0 0 0 0 0 0 0 0 0 0 0 0 0 0 0 0 0 0 0 0 0 0 0 0 0 0
0 0 0 0 0 0 0 0 0 0 0 0 0 0 0 0 0 0 0 0 0 0 0 0 0 0 0 0
0 0 0 0 0 0 0 0 0 0 0 0 0 0 0 9 0 0 0 0 0 0 0 0 0 0 0
M02255_77_000000000-ADUT3_1_1106_17027_16655 T----AC---GT-AG-GGG--------GCA-A-G------C--G--T---T--AT-C-CGG-AT------TC-A--T-T--GG-GC-------GT--A-----AA-GC-GC-GC-------G-TA-G-G-C-G------------G--T-CT-G-T-T-----
------AG----G-T-C-A--------G-G-A--G--TT--A-AA-TC--T-G-GG-G-G-----CT-C-AA------C-C-C-C-T-A-T--C------C-G--C-T---C--C-------T--GA-T-A---C-------C--G-G--CA--G-G-C----T-T-G-A-G-T--C---T-GG-----TA-G-G--------G-G-A-A---GA-T------GG--A--ATT----C-
C-A-A-GT--GT-A-G-CG-GT--G---A--A-A---TG-C-GC-AG--AT-A-TT--T-G--G-A-A-G-A-AC-A-CC---GG--T--G--GC-GAA-G--G-C--G-------G--T-C-T-T---CTG--G--GC-C-A--------------------T-G---A-C-T--GA--CG-C-T-G--A-GG--C-G-CG-A--AA-G-C---T-AG--GG-
G--AG-C-G-AA--------CA--GG Bacteria(100);Actinobacteria(100);Actinobacteria(100);Coriobacteridae(100);Coriobacteriales(100);Coriobacterineae(100);Coriobacteriaceae(100);unclassified(100);unclassified(100);

Otu1074 0 0 0 0 0 0 0 0 0 0 0 0 0 0 0 0 0 0 0 0 0 0 0 0 0 0 0 0
0 0 0 0 0 0 0 0 0 1 0 0 0 0 0 25 2 8 0 0 0 0 0 0 0 0 1 0
0 9 0 0 0 0 0 0 0 0 0 0 0 0 0 0 0 0 0 0 0 0 8 0 0 0 8 2
0 6 10 0 0 0 0 0 0 3 0 0 0 0 0 0 0 0 0 0 0 0 0 0 0 0 0
M02255_77_000000000-ADUT3_1_1113_19220_17537 C----GG---CT-A--ACT--------ACG-T-G------C--C--A---G--CA-G-CCG-CG------GT-A--A-T--AC-GT-------AG--G-----GT-GC-GA-GC-------GTTA-A-T-C-G------------GA-T-TA-T-T-T------
-----AA----G-C-C-A--------G-A-T--G--TG--A-AA-TA--C-C-CG-A-G-----CC-T-AA------C-T-T-G-G-G-A--G-G----T-G--C-A---T--A-------T--GG-A-A---C-------T--G-G--GT--A-G-C----T-A-G-A-G-T--G---T-GT-----CA-G-A--------G-G-G-A---GG-T------AG--A--ACT----C-
C-A-C-GT--GT-A-G-CA-GT--G---A--A-A---TG-C-GT-AG--AG-A-TG--T-G--G-A-A-G-A-AT-A-CC---GA--T--G--GC-GAA-G--G-C--A-------G--C-C-T-C---CTG--G--GA-T-A--------------------A-C---A-C-T--GA--CG-T-T-G--A-GG--C-T-CG-A--AA-G-C---G-TG--GG-
G--AG-C-A-AA--------CA--GG Bacteria(100);Proteobacteria(100);Betaproteobacteria(100);unclassified(100);unclassified(100);unclassified(100);unclassified(100);unclassified(100);unclassified(100);

Otu1075 0 0 0 0 0 0 0 0 0 0 0 0 0 0 0 0 0 0 0 0 0 0 0 0 0 4 0 0
0 0 0 0 0 0 0 0 0 0 0 0 0 0 0 0 0 0 0 0 0 0 0 0 0 0 0 0 0
0 0 0 0 0 0 0 0 0 0 0 0 0 0 0 0 0 0 0 0 0 0 0 0 0 0 0 0 0
0 0 0 61 0 0 0 0 0 0 0 0 0 0 0 0 0 0 0 0 0 0 0 0 0 0 0
M02255_77_000000000-ADUT3_1_1111_17220_10208 T----AC---GA-AG-GGG--------GCT-A-G------C--G--T---T--GT-T-CGG-AT------TT-A--C-T--GG-GC-------GT--A-----AA-GC-GC-AC-------G-TA-G-G-C-G------------G--A-AA-T-T-T-----
------AA----G-T-C-A--------G-G-G--G--TG--A-AA-TC--C-C-GA-G-G-----CT-C-AA------C-C-T-C-G-G-A--A-C----T-G--C-C---T--T-------T--GA-T-A---C-------T--G-G--GT--T-T-C----T-T-G-A-G-T--C---C-GG-----AA-G-A--------G-G-T-G---AG-T------GG--A--ACT----G-
C-G-A-GT--GT-A-G-AG-GT--G---A--A-A---TT-C-GT-AG--AT-A-TT--C-G--C-A-A-G-A-AC-A-CC---AG--T--G--GC-GAA-G--G-C--G-------G--C-T-C-A---CTG--G--TC-C-G--------------------G-A---A-C-T--GA--CG-C-T-G--A-GG--T-G-CG-A--AA-G-C---G-TG--GG-
G--AG-C-A-AA--------CA--GG Bacteria(100);Proteobacteria(100);Alphaproteobacteria(100);Rhizobiales(100);unclassified(100);unclassified(100);unclassified(100);unclassified(100);unclassified(100);

Otu1076 0 0 0 0 0 0 0 0 0 0 0 0 0 0 0 0 0 0 0 0 0 0 0 0 0 0 6 0
0 0 0 0 0 0 0 0 0 0 0 0 0 0 0 0 0 0 0 0 0 0 0 0 4 0 0 0 0
0 0 0 0 0 0 0 0 0 0 0 0 0 0 0 0 0 0 0 0 0 0 0 0 0 0 0 0 0
0 0 0 0 0 0 0 0 0 0 0 0 0 0 0 0 0 0 0 0 0 0 0 0 0 0 0
M02255_77_000000000-ADUT3_1_1101_9280_11182 T----AC---GT-AG-GAT--------GCA-A-G------C--G--T---T--GT-C-CGG-AA------TT-A--T-T--GG-GC-------GT--A-----AA-GC-GA-AC-------G-TA-G-G-C-G------------G--T-TA-C-T-T-----------AA--
--G-T-T-T--------G-G-T--C--TT--A-AA-GA--C-C-GG-G-G-----CT-C-AA------C-T-C-C-G-G-G--A------A-TG-G-A---C--T-------G--AA-T-A---C-------T--G-G--GT--G-A-C----T-A-G-A-G-G--A---T-GT-----CA-G-A--------G-G-A-A---AG-T------GG--A--ATT----C-C-A-A-
GT--GT-A-G-CG-GT--G---A--A-A---TG-C-GT-AG--AT-A-TT--T-G--G-A-G-G-A-AC-A-CC---AG--T--G--GC-GAA-A--G-C--G-------G--C-T-T-T---CTG--G--GA-C-A--------------------T-T---C-C-T--GA--CG-C-T-G--A-GG--T-T-CG-A--AA-G-C---T-CG--GG-G--AG-C-
A-AA--------CA--GG Bacteria(100);unclassified(100);unclassified(100);unclassified(100);unclassified(100);unclassified(100);unclassified(100);unclassified(100);unclassified(100);

Otu1077 0 0 0 0 0 0 0 0 0 0 0 0 0 0 0 0 0 0 0 0 0 0 0 0 0 0 4 0
0 0 0 0 0 0 0 0 0 0 0 0 0 0 0 0 0 0 0 0 0 0 0 0 0 0 0 0 0
0 0 0 0 0 0 0 0 0 0 0 0 0 0 0 0 0 0 0 0 0 0 0 0 0 0 0 0 0
0 0 0 0 0 0 0 0 0 0 0 0 0 0 0 0 0 0 0 0 0 0 0 0 0 0 0
M02255_77_000000000-ADUT3_1_1105_28471_10798 G----AC---GA-GG-GAC--------CCT-A-G------C--G--T---T--GT-T-CGG-AA------TC-A--T-T--GG-GC-------GT--A-----AA-GC-GG-AG-------G-TA-G-G-T-G------------G--C-TT-G-G-T----
-------AA----G-T-C-G--------A-G-T--G--TG--A-AA-GC--C-C-AG-G-G-----CT-C-AA------C-C-C-T-G-G-A--A-G----T-G--C-A---T--T-------C--GA-A-A---C-------T--G-C--TG--A-G-C----T-T-G-A-G-T--G---C-TA-----GA-G-A--------G-G-T-T---AC-T------AG--A--ATG----
C-C-T-G-GT--GT-A-G-TG-GT--G---A--A-A---TA-C-GT-AG--AT-A-TC--A-G--G-C-G-G-A-AT-A-CC---GG--A--G--GC-GAA-G--G-C--G-------G--G-T-A-A---CTG--G--CT-A-G--------------------A-C---A-C-T--GA--CA-C-T-C--A-AT--T-C-CG-A--AA-G-C---G-TG--GG-
G--AT-C-A-AA--------CA--GG Bacteria(100);Proteobacteria(100);Deltaproteobacteria(100);Bdellovibrionales(100);Bdellovibrionaceae(100);Bdellovibrio(100);unclassified(100);unclassified(100);unclassified(100);

Otu1078 0 0 0 0 0 0 0 0 0 0 0 0 0 0 0 0 0 0 0 0 0 0 0 0 0 0 25 0
3 0 0 0 0 0 0 0 0 0 0 0 0 0 0 0 0 0 0 0 0 0 0 0 0 0 0 0 0
0 0 0 0 0 0 0 0 0 0 0 0 0 0 0 0 0 0 0 0 0 0 0 0 0 0 0 0 0
0 0 0 32 0 0 0 0 0 0 0 0 0 0 0 0 0 0 0 0 0 0 0 0 0 0 0
M02255_77_000000000-ADUT3_1_1110_16451_24536 T----AC---GG-AG-GGG--------GCT-A-G------C--G--T---T--GT-T-CGG-AA------TT-A--C-T--GG-GC-------GT--A-----AA-GC-GC-AC-------G-TA-G-G-C-T------------G--C-TT-T-G-T------
-----AA----G-T-C-A--------G-A-G--G--TG--A-AA-GC--C-C-GG-G-G-----CT-C-AA------C-C-C-C-G-G-A--T-G----T-G--C-C---T--T-------T--GA-G-A---C-------T--G-C--AG--A-G-C----T-A-G-A-A-T--C---G-CG-----GA-G-A--------G-G-T-G---AG-T------GG--A--ATT----
C-C-G-A-GT--GT-A-G-AG-GT--G---A--A-A---TT-C-GT-AG--AT-A-TT--C-G--G-A-A-G-A-AC-A-CC---AG--T--G--GC-GAA-G--G-C--G-------G--C-T-C-A---CTG--G--AC-G-C--------------------G-T---A-T-T--GA--CG-C-T-G--A-GG--T-G-CG-A--AA-G-C---G-TG--GG-
G--AG-C-A-AA--------CA--GG Bacteria(100);Proteobacteria(100);Alphaproteobacteria(100);Sphingomonadales(100);unclassified(100);unclassified(100);unclassified(100);unclassified(100);unclassified(100);

Otu1079 0 0 0 0 0 0 0 0 0 0 0 0 0 0 0 0 0 0 0 0 0 0 0 0 0 0 0 0
0 0 0 0 0 0 0 0 0 0 0 0 0 0 0 0 0 0 0 0 0 0 0 0 0 0 0 24 0
0 0 0 0 0 0 0 0 0 0 0 0 0 0 0 0 0 0 0 0 0 0 0 0 0 0 0 0 0
0 0 0 0 0 0 0 0 0 0 0 0 0 0 0 0 0 0 0 0 0 0 0 0 0 0 0
M02255_77_000000000-ADUT3_1_1114_9658_17890 T----AC---GA-AG-GGG--------GCT-A-G------C--G--T---T--GC-T-CGG-AA------TT-A--C-T--GG-GC-------GT--A-----AA-GG-GA-GC-------G-TA-G-G-C-G------------G--G-TC-G-T-C-----------
AA----G-T-T-G--------G-G-G--G--TG--A-AA-GC--C-C-AG-G-G-----CT-C-AA------C-C-C-T-G-G-A--A-C----T-G--C-C---T--T-------C--AA-A-A---C-------T--G-G--CG--A-C-C----T-A-G-A-G-T--A---T-GG-----TA-G-A--------G-G-T-G---TG-T------GG--A--ACT----C-C-G-
A-GT--GT-A-G-AG-GT--G---A--A-A---TT-C-GT-AG--AT-A-TT--C-G--G-A-A-G-A-AC-A-CC---AG--T--G--GC-GAA-G--G-C--G-------A--C-A-C-A---CTG--G--GC-C-A--------------------T-T---A-C-T--GA--CG-C-T-G--A-GG--C-T-CG-A--AA-G-C---G-TG--GG-G--AG-
C-A-AA--------CA--GG Bacteria(100);Proteobacteria(100);Alphaproteobacteria(100);Caulobacterales(100);Caulobacteraceae(100);unclassified(100);unclassified(100);unclassified(100);unclassified(100);

Otu1080 0 0 0 0 0 0 0 0 0 0 0 0 0 0 0 0 0 0 0 0 0 0 0 0 0 0 4 0
0 0 0 0 0 0 0 0 0 0 0 0 0 0 0 0 0 0 0 0 0 0 0 0 0 0 0 0 0
0 0 0 0 0 0 0 0 0 0 0 0 0 0 0 0 0 0 0 0 0 0 0 0 0 0 0 0 0
0 0 0 0 0 0 0 0 0 0 0 0 0 0 0 0 0 0 0 0 0 0 0 0 0 0 0
M02255_76_000000000-ADUNE_1_2119_12805_17690 G----AC---GA-AC-CGT--------GCG-A-A------C--G--T---T--AT-T-CGG-AA------TC-A--C-T--GG-GC-------TT--A-----AA----G--G--------G-TA----------------------------G-A-T-----------AA-
---G-T-C-G--------A-G-G--G--TG--A-AA-GC--C-T-CT-A-G-----CT-T-AA------C-T-A-G-A-G-A--A-G----T-G--C-C---C--T-------C--GA-A-A---C-------T--A-T--TT--G-T-C----T-T-G-A-G-A--G---A-GA-----TA-G-G--------G-G-C-A---TA-C------GG--A--ACT----T-C-C-G-
GT--GG-A-G-CG-GT--G---A--A-A---TG-C-GT-AG--AG-A-TC--G-G--A-A-G-G-A-AC-G-CC---GG--T--G--GC-GAA-A--G-C--G-------G--T-A-T-G---CTG--G--AT-C-T--------------------T-T---T-C-T--GA--CG-C-T-G--A-GG--C-G-CG-A--AA-G-C---T-AG--GG-G--AG-
C-G-AA--------CG--GG Bacteria(100);Planctomycetes(100);Planctomycetacia(100);Planctomycetales(100);Planctomycetaceae(100);uncultured(100);unclassified(100);unclassified(100);unclassified(100);

Otu1081 0 0 0 0 0 0 0 0 0 0 0 0 0 0 0 0 0 0 0 0 0 0 0 0 0 0 0 0
0 0 0 0 0 0 0 0 0 0 0 25 0 0 0 0 0 0 0 0 0 0 0 0 0 0 0 0 0
0 0 0 0 0 0 0 0 0 0 0 0 0 0 0 0 0 0 0 0 0 0 0 0 0 0 0 0 0
0 0 0 0 0 0 0 0 0 0 0 0 0 0 0 0 0 0 0 0 0 0 0 0 0 0 0
M02255_77_000000000-ADUT3_1_1107_10442_12377 G----AC---GG-GG-GGT--------CCA-A-G------C--G--T---T--GT-T-CGG-AA------TC-A--T-T--GG-GC-------GT--A-----AA-GC-GG-GC-------G-TA-G-G-T-G------------G--C-CT-T-G-C----
-------AA----G-T-C-A--------G-G-A--G--TG--A-AA-GC--C-C-TG-G-G-----CT-C-AA------C-C-C-A-G-G-A--A-G----T-G--C-T---T--T-------T--GA-A-A---C-------T--G-C--AA--G-G-C----T-T-G-A-A-T--G---T-GG-----GA-G-A--------G-G-G-T---AG-T------AG--A--ATT----
C-C-T-G-GT--GT-A-G-TG-GT--G---A--A-A---TA-C-GT-AG--AT-A-TC--A-G--G-A-G-G-A-AT-A-CC---GG--T--G--GC-GAA-G--G-C--G-------G--C-T-A-C---CTG--G--CC-C-A--------------------A-C---A-T-T--GA--CA-C-T-G--A-GG--C-C-CA-A--AA-G-C---G-TG--GG-
G--AT-C-A-AA--------CA--GG Bacteria(100);unclassified(100);unclassified(100);unclassified(100);unclassified(100);unclassified(100);unclassified(100);unclassified(100);unclassified(100);

Otu1082 0 0 0 0 0 0 0 0 0 0 0 0 0 0 0 0 0 0 0 0 0 0 0 0 0 0 6 1
0 0 0 0 0 0 0 0 0 0 0 0 0 0 0 0 0 0 0 0 0 0 0 0 0 0 0 0 0
0 0 0 0 0 0 0 0 0 0 0 0 0 0 0 0 0 0 0 0 0 0 0 0 0 0 0 0 0
0 0 0 0 0 0 0 0 0 0 0 0 0 0 0 0 0 0 0 0 0 0 0 0 0 0 0
M02255_76_000000000-ADUNE_1_1115_25054_20841 G----AA---TA-AG-CAC--------CGG-C-T------A--A--C---T--CT-G-TGC-CA------GC-A--G-C--CG-CG-------GT--A-----AT-AC-AG-AG-------G-GT-G-C-G-A------------G--C-TT-G-T-T------
-----AA----G-T-C-G--------G-A-T--G--TG--A-AA-GC--C-C-TG-A-G-----CT-T-AA------C-T-T-A-G-G-A--A-T----T-G--C-A---T--T-------C--GA-T-A---C-------T--G-G--TA--A-G-C----T-A-G-A-G-T--A---T-GG-----GA-G-A--------G-G-A-A---GG-T------AG--A--ATT----C-
C-A-G-GT--GT-A-G-CG-GT--G---A--A-A---TG-C-GT-AG--AT-A-TC--T-G--G-A-G-G-A-AT-A-CC---GA--T--G--GC-GAA-G--G-C--A-------G--C-C-T-T---CTG--G--CC-T-A--------------------A-T---A-C-T--GA--CG-C-T-G--A-GG--T-G-CG-A--AA-G-C---A-TG--GG-G-
-AG-C-A-AA--------CA--GG Bacteria(100);Proteobacteria(100);Gammaproteobacteria(100);unclassified(100);unclassified(100);unclassified(100);unclassified(100);unclassified(100);unclassified(100);

Otu1083 0 0 0 0 0 0 0 0 0 0 0 0 0 0 0 0 0 0 0 0 0 0 0 0 0 0 0 0
0 0 0 0 0 0 0 0 0 15 2 0 0 0 0 0 0 0 0 0 0 0 0 0 0 0 0 0 0
0 0 0 0 0 0 0 0 0 0 0 0 0 0 0 0 0 0 0 0 0 0 0 0 0 0 0 0 0
0 0 0 0 0 0 0 0 0 0 0 0 0 0 0 0 0 0 0 0 0 0 0 0 0 0 0
M02255_77_000000000-ADUT3_1_2102_9746_9272 T----AC---GG-AG-GAT--------CCA-A-G------C--G--T---T--AT-C-CGG-AA------TT-A--C-T--GG-GC-------GT--A-----AA-GA-GTTGC-------G-TA-G-G-T-G------------G--C-AT-A-G-T-----------AT-
---G-T-C-G--------G-T-A--G--TG--A-AA-GC--G-T-GT-G-G-----CT-C-AA------C-C-A-T-A-T-A--C-A----C-A--T-T---A--T-------C--GA-A-A---C-------T--G-C--TA--A-G-C----T-A-G-A-G-G--A---C-GA-----GA-G-A--------G-G-T-T---AT-T------GG--A--ATT----C-C-C-A-
GT--GT-A-G-GA-GT--G---A--A-A---TC-C-GT-AG--AT-A-TT--G-G--G-A-G-G-A-AC-A-CC---GA--T--G--GC-GTA-G--G-C--A-------G--A-T-A-A---CTG--G--CT-C-G--------------------T-T---C-C-T--GA--CA-C-T-A--A-GG--C-A-CG-A--AA-G-C---G-TG--GG-G--AG-C-
A-AA--------CG--GG Bacteria(100);Candidate_division_TM7(100);unclassified(100);unclassified(100);unclassified(100);unclassified(100);unclassified(100);unclassified(100);unclassified(100);

Otu1084 0 0 0 0 0 0 0 0 0 0 0 0 0 0 0 0 0 0 0 0 0 0 0 0 0 0 0 0
0 0 0 0 0 0 0 0 0 2 0 0 0 0 0 0 0 0 0 0 0 0 0 0 0 0 0 0 0
0 0 0 0 0 0 0 0 0 0 0 0 0 0 0 0 0 0 0 0 0 0 0 0 0 0 10 0 0
1 0 4 0 1 0 0 0 0 0 0 0 0 0 0 1 0 0 0 0 0 0 0 0 0 0 0
M02255_77_000000000-ADUT3_1_1101_7129_20279 T----AA---GC-AC-CGG--------CTA-A-C------T--A--C---G--TG-C-CAG-CA------GC-C--G-C--GG-TA-------AT--A-----CG-TA-GG-GT-------G-CA-A-G-C-G------------T--TAAT-C-G-G-----------
AAT---T-A-C-T--------G-A-G--G--TG--A-AA-TC--C-C-CG-G-G-----CT-C-AA------C-C-T-G-G-G-A--A-C----T-G--C-C---T--T-------T--GT-G-A---C-------T--G-C--AA--G-G-C----T-G-G-A-G-T--G---C-GG-----CA-G-A--------G-G-G-G---GA-T------GG--A--ATT----C-C-
G-C-GT--GT-A-G-CA-GT--G---A--A-A---TG-C-GT-AG--AT-A-TG--C-G--G-A-G-G-A-AC-A-CC---GA--T--G--GC-GAA-G--G-C--A-------A--T-C-C-C---CTG--G--GC-C-T--------------------G-C---A-C-T--GA--CG-C-T-C--A-TG--C-A-CG-A--AA-G-C---G-TG--GG-G--
AG-C-A-AA--------CA--GG Bacteria(100);Proteobacteria(100);Betaproteobacteria(100);Burkholderiales(100);Comamonadaceae(100);unclassified(100);unclassified(100);unclassified(100);unclassified(100);

Otu1085 0 0 0 0 0 0 0 0 0 0 0 0 0 0 0 0 0 0 0 0 0 0 0 0 0 0 0 0
0 0 0 0 0 0 0 0 1 0 0 1 0 0 0 0 0 0 0 0 0 0 0 0 0 0 0 0 0
0 0 0 0 0 0 0 0 0 0 0 0 0 0 0 2 0 0 1 0 0 0 0 0 1 0 0 4 7
1 0 2 0 9 0 0 0 0 0 0 0 1 0 0 0 0 0 0 0 7 0 0 0 0 0 9

M02255_76_000000000-ADUNE_1_2104_23423_20247 T----AA---GC-AC-CGG--------CTA-A-C------T--A--C---G--TG-C-CAG-CA------GC-C--G-C--GG-TA-------AT--A-----CG-TA-GG-GT-------G-CG-A-G-C-G------------T--T-AATC-G-G----
-------AA----G-C-A-G--------G-A-T--G--TG--A-AA-TC--C-C-CG-G-G-----CT-C-AA------C-C-T-G-G-G-A--A-C----T-G--C-G---T--T-------C--TG-A-A---C-------T--G-G--GT--G-A-C----T-A-G-A-G-T--G---T-GT-----CA-G-A--------G-G-G-A---GG-T------AG--A--ATT----
C-C-A-C-GT--GT-A-G-CA-GT--G---A--A-A---TG-C-GT-AG--AG-A-TG--T-G--G-A-G-G-A-AT-A-CC---GA--T--G--GC-GAA-G--G-C--A-------G--C-C-T-C---CTG--G--GA-T-A--------------------A-C---A-C-T--GA--CG-T-T-C--A-TG--C-T-CG-A--AA-G-C---G-TG--GG-
T--AG-C-A-AA--------CA--GG Bacteria(100);Proteobacteria(100);Betaproteobacteria(100);Neisseriales(100);Neisseriaceae(100);unclassified(100);unclassified(100);unclassified(100);unclassified(100);

Otu1086 0 0 0 0 0 0 0 0 0 0 0 0 0 0 0 0 0 0 0 0 0 0 0 0 0 0 5 0
0 0 0 0 0 0 0 0 0 0 0 0 0 0 0 0 0 0 0 0 0 0 0 0 0 0 0 0 0
0 0 0 0 0 0 0 0 0 0 0 0 0 0 0 0 0 0 0 0 0 0 0 0 0 0 0 0 0
0 0 0 0 0 0 0 0 0 0 0 0 0 0 0 0 0 0 0 0 0 0 0 0 0 0 0

M02255_77_000000000-ADUT3_1_2118_18943_22183 T----AC---GT-AG-GGA--------GCA-A-G------C--G--T---T--GT-T-CGG-AT------TT-A--C-T--GG-GC-------GT--A-----AA-GG-GC-GC-------G-TA-G-G-C-G------------G--C-GC-A-G-T----
-------AA----G-T-T-A--------C-T-T--G--TG--A-AA-TC--T-C-TG-A-G-----CT-T-AA------C-T-C-A-G-A-A--C-G----G-C--C-A---A--G-------T--AA-T-A---C-------T--G-C--AG--T-G-C----T-A-G-A-G-T--G---C-AG-----AA-A-G--------G-G-C-A---AT-C------GG--A--ATT----C-
T-T-G-GT--GT-A-G-CG-GT--G---A--A-A---TG-C-GT-AG--AT-A-TC--A-A--G-A-G-G-A-AC-A-CC---TG--A--G--GC-GAA-G--G-C--G-------G--G-T-T-G---CTT--G--TC-T-G--------------------A-C---A-C-T--GA--CG-C-T-G--A-GG--C-G-CG-A--AA-G-C---C-AG--GG-
G--AG-C-A-AA--------CG--GG Bacteria(100);Acidobacteria(100);Acidobacteria(100);Acidobacteriales(100);Acidobacteriaceae(100);Candidatus_Chloroacidobacterium(100);unclassified(100);unclassified(100);unclassified(100);

Otu1087 0 0 0 0 0 0 0 0 0 0 0 0 0 0 0 0 0 0 0 0 0 0 0 0 0 0 0 0
0 0 0 0 0 0 0 0 15 0 0 0 0 0 0 0 0 0 0 0 0 0 0 0 0 0 0 0 0
0 0 0 0 0 0 0 0 0 0 0 0 0 0 0 0 0 0 0 0 0 0 0 0 0 0 0 0 0
0 0 0 0 0 0 0 0 0 0 0 0 0 0 0 0 0 0 0 0 0 0 0 0 0 0 0

M02255_77_000000000-ADUT3_1_1116_26109_19119 G----AC---AG-GG-GGC--------CCG-A-G------C--G--T---T--AG-T-CGG-AA------TT-A--C-T--GG-GC-------TT--A-----AA-GG-GT-GC-------G-TA-G-G-C-G------------G--G-CC-C-G-T----
-------AA----G-T-G-T--------C-G-C--G--TG--A-AA-GC--C-C-AT-C-G-----CT-C-AA------C-G-A-T-G-G-A--A-C----C-G--C-G---C--G-------G--CA-A-A---C-------T--G-C--GG--G-T-C----T-C-G-A-G-G--A---A-GG-----TA-G-A--------G-G-C-G---GG-T------GG--A--ACG---
-C-T-T-G-GT--GG-A-G-CG-GT--G---G--A-A---TG-C-GT-AG--AT-A-TC--A-A--G-C-G-G-A-AC-G-CC---GA--A--G--GT-GAA-G--A-C--A-------G--C-C-C-G---CTG--G--GC-C-T--------------------T-T---C-C-T--GA--CG-C-T-G--A-GG--C-A-CG-A--AA-G-C---G-TG--
GG-G--AG-C-A-AA--------CA--GG Bacteria(100);unclassified(100);unclassified(100);unclassified(100);unclassified(100);unclassified(100);unclassified(100);unclassified(100);unclassified(100);

Otu1088 0 0 0 0 0 0 0 0 0 0 0 0 0 0 0 0 0 0 0 0 0 0 0 0 0 0 0 2
0 0 0 0 0 0 0 0 0 0 1 0 0 0 0 0 0 6 0 0 0 0 0 0 0 0 0 0 0
0 0 0 0 0 0 0 0 0 0 0 0 0 0 0 0 0 0 0 0 0 0 0 0 0 0 0 0 0
0 0 0 0 0 0 0 0 0 4 0 0 0 0 0 0 0 0 0 0 0 0 0 0 0 0 0

M02255_76_000000000-ADUNE_1_2107_24733_8688 C----GT---GC-CA-GCA--------GCC-G-C------G--G--T---A--AT-A-CGT-AT------GT-C--G-C--AA-GC-------GT--T-----AT-CC-GG-AT-------TTATTGGG-C-G------------G--G-AA-A-G-C-----------
GC----G-T-C-T--------G-A-T--G--TG--A-AA-AT--G-C-GG-G-G-----CT-C-AA------C-T-C-C-G-T-A--T------T-G--C-G---T--T-------G--GA-A-A---C-------T--A-C--TT--T-T-C----T-A-G-A-G-T--A---T-CA-----GA-G-A--------G-G-T-G---GG-C------GG--A--ACT----A-C-A-
A-GT--GT-A-G-AG-GT--G---A--A-A---TT-C-GT-AG--AT-A-TT--T-G--T-A-G-G-A-AT-G-CC---GA--T--G--GA-GAA-G--T-C--A-------G--C-T-C-A---CTG--G--AT-G-A--------------------A-T---A-C-T--GA--CG-C-T-A--A-AG--C-G-CG-A--AA-G-C---G-TG--GG-G--AG-
C-A-AA--------CA--GG Bacteria(100);Fusobacteria(100);Fusobacteria(100);Fusobacteriales(100);Fusobacteriaceae(100);unclassified(100);unclassified(100);unclassified(100);unclassified(100);

Otu1089 0 0 0 0 0 0 0 0 0 0 0 0 0 0 0 0 0 0 0 0 0 0 0 0 0 0 0 0
0 0 0 0 0 0 0 0 8 0 0 22 0 0 0 0 0 0 0 0 0 0 0 0 0 0 0 0 0
0 0 0 0 0 0 0 0 0 0 0 0 0 0 0 0 0 0 0 0 0 0 0 0 0 0 0 0 0
0 0 0 0 0 0 0 0 0 0 0 0 0 0 0 0 0 0 0 0 0 0 0 0 0 0 0

M02255_77_000000000-ADUT3_1_1112_12362_6967 T----AC---GT-AG-GAA--------GCA-A-G------C--G--T---T--GC-C-CGG-AA------TT-A--C-T--GG-GC-------GT--A-----AA-GA-GC-TC-------G-TA-G-G-C-G------------G--G-GG-C-G-T-----------
GC----G-T-C-C--------G-G-G--A--AG--A-AA-TC--T-C-AA-G-G-----CT-C-AAC-----C-T-T-G-G-G-G--C------T-A--T-C---T--C-------G--GA-T-A---C-------G--G-C--GC--T-T-C----T-T-G-A-G-G--G---T-GA-----GA-G-A--------G-G-A-A---AG-T------AG--A--ATT----C-C-C-
G-GT--GT-A-G-CG-GT--G---A--A-A---TG-C-GT-AG--AT-A-TC--G-G--G-A-G-G-A-AT-A-CC---AG--C--G--GC-GAA-G--G-C--G-------A--C-T-T-T---CTG--G--CT-C-A--------------------T-T---C-C-T--GA--CG-C-T-G--A-GG--A-G-CG-A--AA-G-C---G-TG--GG-T--AG-
C-G-AA--------CG--GG Bacteria(100);unclassified(100);unclassified(100);unclassified(100);unclassified(100);unclassified(100);unclassified(100);unclassified(100);unclassified(100);

Otu1090 0 0 0 0 0 0 0 0 0 0 0 0 0 0 0 0 0 0 0 0 0 0 0 0 0 0 0 10
0 0 0 0 0 0 0 0 0 0 0 0 0 0 0 0 0 0 0 0 0 0 0 0 0 0 0 0 0
0 0 0 0 0 0 0 0 0 0 0 0 0 0 0 0 0 0 0 0 0 0 0 0 0 0 0 0 0
0 0 0 0 0 0 0 0 0 0 0 0 0 0 0 0 0 0 0 0 0 0 0 0 0 0 0
M02255_77_000000000-ADUT3_1_1104_20747_22488 T----AC---GA-AG-GGG--------GCT-A-G------C--G--T---T--GC-T-CGG-AA------TC-A--C-T--GG-GC-------GT--A-----AA-GC-GC-AC-------G-TA-G-G-C-G------------G--C-TT-A-T-T-----
------AA----G-T-C-G--------G-G-G--G--TG--A-AA-TC--C-T-GG-A-G-----CT-C-AA------C-T-C-C-A-G-A--A-C----T-G--C-C---T--T-------C--GA-T-A---C-------T--G-A--TG--A-G-C----T-T-G-A-G-T--C---C-GG-----GA-G-A--------G-G-T-G---AG-T------GG--A--ACT----C-
C-G-A-GT--GT-A-G-AG-GT--G---A--A-A---TT-C-GT-AG--AT-A-TT--C-G--G-A-A-G-A-AC-A-CC---AG--T--G--GC-GAA-G--G-C--G-------G--C-T-C-A---CTG--G--CC-C-G--------------------G-T---A-C-T--GA--CG-C-T-G--A-GG--T-G-CG-A--AA-G-C---G-TG--GG-
G--AG-C-A-AA--------CA--GG Bacteria(100);Proteobacteria(100);Alphaproteobacteria(100);Rhizobiales(100);unclassified(100);unclassified(100);unclassified(100);unclassified(100);unclassified(100);

Otu1091 0 0 0 0 0 0 0 0 0 0 0 0 0 0 0 0 0 0 0 0 0 0 0 0 0 0 0 38
0 0 0 0 0 0 0 0 0 0 0 0 0 0 0 0 0 0 0 0 0 0 0 0 0 0 0 0 0
0 0 0 0 0 0 0 0 0 0 0 0 0 0 0 0 0 0 0 0 0 0 0 0 0 0 0 0 0
0 0 0 0 0 0 0 0 0 0 0 0 0 0 0 0 0 0 0 0 0 0 0 0 0 0 0
M02255_77_000000000-ADUT3_1_1105_16181_19288 T----AC---GG-AG-GGT--------GCA-A-G------C--G--T---T--AC-T-CGG-AA------TC-A--C-T--GG-GC-------GT--A-----AA-GA-GC-GT-------G-TA-G-G-C-G------------G--A-TA-G-A-T----
-------AA----G-T-T-C--------G-A-A--G--TG--A-AA-TC--C-A-AT-G-G-----CT-C-AA------C-C-A-T-T-G-A--A-C----T-G--C-T---T--T-------G--AA-A-A---C-------T--G-T--TT--A-T-C----T-A-G-A-A-T--A---T-GG-----GA-G-A--------G-G-T-A---GA-T------GG--A--ATT----T-
C-T-G-GT--GT-A-G-GG-GT--A---A--A-A---TC-C-GT-AG--AG-A-TC--A-G--A-A-G-G-A-AT-A-CC---GA--T--T--GC-GAA-G--G-C--G-------A--T-C-T-A---CTG--G--AA-C-A--------------------T-T---A-T-T--GA--CG-C-T-G--A-GA--C-G-CG-A--AA-G-C---G-TG--GG-G-
-AG-C-A-AA--------CA--GG Bacteria(100);Proteobacteria(100);Epsilonproteobacteria(100);Campylobacterales(100);Campylobacteraceae(100);Arcobacter(100);unclassified(100);unclassified(100);unclassified(100);

Otu1092 0 0 0 0 0 0 0 0 0 0 0 0 0 0 0 0 0 0 0 0 0 0 0 0 0 0 0 20
0 0 0 0 0 0 0 0 0 0 0 0 0 0 0 0 0 0 0 0 0 0 0 0 0 0 0 0 0
0 0 0 0 0 0 0 0 0 0 0 0 0 0 0 0 0 0 0 0 0 0 0 0 0 0 0 0 0
0 0 0 0 0 0 0 0 0 0 0 0 0 0 0 0 0 0 0 0 0 0 0 0 0 0 0
M02255_77_000000000-ADUT3_1_1105_15107_22950 T----AC---GT-AG-GGA--------GCA-A-G------C--G--T---T--GT-T-CGG-AT------TT-A--C-T--GG-GC-------GT--A-----AA-GG-GC-GC-------G-TA-G-G-C-G------------G--C-AG-A-T-C-----
------AA----G-T-C-A--------G-C-T--G--TG--A-AA-TC--T-C-CG-A-G-----CT-T-AA------C-T-C-G-G-A-A--C-G----G-T--C-A---G--C-------T--GA-T-A---C-------T--G-T--TC--T-G-C----T-A-G-A-G-T--G---C-AG-----AA-A-G--------G-G-C-A---AT-C------GG--A--ATT----C-
T-T-G-GT--GT-A-G-CG-GT--G---A--A-A---TG-C-GT-AG--AT-A-TC--A-A--G-A-G-G-A-AC-A-CC---TG--A--G--GC-GAA-G--G-C--G-------G--G-T-T-G---CTA--G--GC-T-G--------------------A-C---A-C-T--GA--CG-C-T-G--AG-G--C-G-CG-A--AA-G-C---C-AG--GG-
G--AG-C-A-AA--------CG--GG Bacteria(100);Acidobacteria(100);Acidobacteria(100);Acidobacteriales(100);Acidobacteriaceae(100);Candidatus_Chloroacidobacterium(100);unclassified(100);unclassified(100);unclassified(100);

Otu1093 0 0 0 0 0 0 0 0 0 0 0 0 0 0 0 0 0 0 0 0 0 0 0 0 0 0 0 0
0 0 0 0 0 0 0 0 0 0 0 0 0 0 0 0 0 0 0 0 0 0 0 0 0 0 0 0 0
0 0 0 0 0 0 0 0 0 0 0 0 0 0 0 0 0 0 0 0 0 0 0 0 0 0 0 0 0
0 0 18 0 0 0 0 0 0 0 0 0 0 0 0 0 0 0 0 0 0 0 0 0 0 0 0
M02255_77_000000000-ADUT3_1_1119_20760_18170 T----AC---AG-GG-GCC--------TCG-A-G------C--G--T---T--AA-T-CGG-AA------TC-A--C-T--GG-GC-------TT--A-----AA-GG-GT-GC-------G-CA-G-G-C-G------------G--T-CT-G-G-A-----
------AA----G-T-G-T--------C-C-T--G--TG--A-AA-TC--C-C-AC-G-G-----CT-C-AA------C-C-G-T-G-G-A--A-T----T-G--C-G---G--G-------G--CA-A-A---C-------T--T-C--CA--G-A-C----T-T-G-A-G-G--A---A-GG-----TA-G-A--------G-G-C-T---GT-T------GG--A--ACG----
A-T-A-G-GT--GG-A-G-CG-GT--G---G--A-A---TG-C-GT-AG--AG-A-TC--T-A--T-C-G-G-A-AC-G-CC---GA--A--G--GT-GAA-G--A-C--A-------G--A-C-A-G---CTG--G--GC-C-T--------------------T-T---C-C-T--GA--CG-C-T-C--A-TG--C-A-CG-A--AA-G-C---G-TG--GG-
G--AG-C-A-AA--------CC--GG Bacteria(100);unclassified(100);unclassified(100);unclassified(100);unclassified(100);unclassified(100);unclassified(100);unclassified(100);unclassified(100);

Otu1094 0 0 0 0 0 0 0 0 0 0 0 0 0 0 0 0 0 0 0 0 0 0 0 0 0 0 0 0
0 0 0 0 0 0 0 0 0 0 0 0 0 0 0 0 0 0 0 0 0 0 0 0 0 0 0 0 0
0 0 0 0 0 0 0 0 0 0 0 0 0 0 0 0 0 0 0 0 0 0 0 0 0 0 0 0 0
0 0 13 0 0 0 0 0 0 0 0 0 0 0 0 0 0 0 0 0 0 0 0 0 0 0 0
M02255_76_000000000-ADUNE_1_2107_28680_16898 T----CG---GC-TA-ACT--------CCG-T-GC-----C--A--G---C--AG-C-CGC-GG------TA-A--T-A--CG-GA-------GG--A-----TC-CA-AG-CG-------T-TA-T-C-C-G------------G--C-CT-A-T-T------
-----AA----G-T-C-A--------G-G-G--G--TG--A-AA-GA--C-G-GT-A-G-----CT-C-AA------C-T-A-T-C-G-C--A-G----T-G--C-C---C--T-------T--GA-T-A---C-------T--G-A--TG--G-G-C----T-T-G-A-A-T--G---G-AC-----TA-G-A--------G-G-T-A---GG-C------GG--A--ATG----T-
G-A-C-AA--GT-A-G-CG-GT--G---A--A-A---TG-C-AT-AG--AT-A-TG--T-C--A-C-A-G-A-AC-A-CC---GA--T--T--GC-GAA-G--G-C--A-------G--C-T-T-A---CTA--T--GG-T-C--------------------T-T---A-T-T--GA--CG-C-T-G--A-GG--C-A-CG-A--AA-G-C---G-TG--GG-G--
AT-C-A-AA--------CA--GG Bacteria(100);Bacteroidetes(100);Sphingobacteria(100);Sphingobacteriales(100);Sphingobacteriaceae(100);Sphingobacteriaceae(100);unclassified(100);unclassified(100);unclassified(100);

Otu1095 0 0 0 0 0 0 0 0 0 0 0 0 0 0 0 0 0 0 0 0 0 0 0 0 0 0 0 0
0 0 0 0 0 0 0 0 0 0 0 0 0 0 0 0 0 0 0 0 0 0 0 0 0 0 0 0 0
0 0 0 0 0 0 0 0 0 0 0 0 0 0 0 0 0 0 0 0 0 0 0 0 0 0 0 0 0
0 0 46 0 0 0 0 0 0 0 0 0 0 0 0 0 0 0 0 0 0 0 0 0 0 0 0 0
M02255_77_000000000-ADUT3_1_1103_27340_11493 T----AC---GT-AT-GGG--------GCA-A-G------C--G--T---T--AT-C-CGG-AA------TT-A--T-T--GG-GC-------GT--A-----AA-GA-GT-GC-------G-TA-G-G-T-G-------------G--T-TA-C-C-T------
-----AA----G-C-G-C--------A-A-G--G--TT--T-AA-TC--T-T-GA-G-G-----CT-C-AA------C-C-T-C-A-T-T--C------T-G--C-C---T--T-------G--CG-A-A---C-------T--G-G--GC--T-A-C----T-T-G-A-G-T--G---C-AG-----GA-G-G--------G-G-A-A---AG-C------GG--A--ATT----C-C-
C-A-GT--GT-A-G-CG-GT--G---A--A-A---TG-C-GT-AG--AT-A-TT--G-G--G-A-A-G-A-AC-A-CC---GG--T--G--GC-GAA-G--G-C--G-------G--C-T-T-T---CTG--G--AC-T-G--------------------T-A---A-C-T--GA--CA-C-T-G--A-GG--C-A-CG-A--AA-G-C---G-TG--GG-G--
AG-C-A-AA--------CA--GG Bacteria(100);Firmicutes(100);Clostridia(100);Clostridiales(100);Family_XIII_Incertae_Sedis(100);unclassified(100);unclassified(100);unclassified(100);unclassified(100);

Otu1096 0 0 0 0 0 0 0 0 0 0 0 0 0 0 0 0 0 0 0 0 0 0 0 0 0 0 0 0
0 0 0 0 0 0 0 0 0 0 0 0 0 0 0 0 0 0 0 0 0 0 0 0 0 0 0 0 0
0 0 0 0 0 0 0 0 0 0 0 0 0 0 0 0 0 0 0 0 0 0 0 0 0 0 0 0 0
0 0 40 0 0 0 0 0 0 0 0 0 0 0 0 0 0 0 0 0 0 0 0 0 0 0 0 0
M02255_77_000000000-ADUT3_1_1104_18218_13698 T----AC---GT-AG-GGT--------CCA-A-G------C--G--T---T--GT-C-CGG-AT------TT-A--T-T--GG-GC-------GT--A-----AA-GA-GC-TC-------G-TA-G-G-C-G------------G--T-TC-C-G-T------
-----AA----G-T-C-G--------G-A-T--G--TG--A-AA-AC--T-C-AG-G-G-----CT-C-AA------C-C-C-G-G-A-G--A------C-G-CC-A---T--C-------T--GA-T-A---C-------T--G-C--GG-TG-A-C----T-G-G-A-G-T--C---T-GG-----TA-G-G--------G-G-A-T---CA-C------GG--A--ATA----
C-C-T-G-GT--GT-A-G-CG-GT--G---A--A-A---TG-C-GC-AG--AT-A-TC--A-G--G-T-G-G-A-AC-A-CC---GA--T--G--GC-GAA-G--G-C--A-------G--T-G-A-T---CTG--G--GC-C-A--------------------G-T---A-C-T--GA--CG-C-T-G--A-GG--A-G-CG-A--AA-G-C---G-TG--
GG-G--AG-C-A-AA--------CA--GG Bacteria(100);unclassified(100);unclassified(100);unclassified(100);unclassified(100);unclassified(100);unclassified(100);unclassified(100);unclassified(100);

Otu1097 0 0 0 0 0 0 0 0 0 0 0 0 0 0 0 0 0 0 0 0 0 0 0 0 0 0 0 0
0 0 0 0 0 0 0 0 0 0 0 0 0 0 0 0 0 0 0 0 0 0 0 0 0 0 0 0 0
0 0 0 0 0 0 0 0 0 0 0 0 0 0 0 0 0 0 0 0 0 0 0 0 0 0 0 0 0
0 0 153 0 0 0 0 0 0 0 0 0 0 0 0 0 0 0 0 0 0 0 0 0 0 0 0 0
M02255_77_000000000-ADUT3_1_1106_9089_22388 T----AC---GT-AG-GTG--------GCA-A-G------C--G--T---T--GT-C-CGG-AT------TT-A--C-T--GG-GC-------GT--A-----AA-GC-GC-GC-------G-CA-G-G-C-G------------G--A-AG-G-T-T-----------
AA----G-T-A-G--------G-A-A--G--TG--A-AA-GG--T-T-GG-A-G-----CT-C-AA------C-T-C-C-A-A-C--A-C----T-G--C-T---C--C-------C--TA-T-A---C-------T--G-G--CC--C-T-C----T-T-G-A-G-T--C---C-CG-----GA-G-A--------G-G-A-A---AG-C------GG--A--ACG----A-C-A-
C-GT--GG-A-G-CG-GT--G---A--A-A---TG-C-GT-TG--AT-A-TG--T-G--T-C-G-G-A-AC-A-CC---AA--T--G--GC-GAA-G--G-C--A-------G--C-T-T-T---CTG--G--AC-G-G--------------------G-A---A-C-T--GA--CG-C-T-C--A-GG--C-G-CG-A--AA-G-C---C-GA--GG-T--AG-
C-G-AA--------CA--GG Bacteria(100);unclassified(100);unclassified(100);unclassified(100);unclassified(100);unclassified(100);unclassified(100);unclassified(100);unclassified(100);

Otu1098 0 0 0 0 0 0 0 0 0 0 0 0 0 0 0 0 0 0 0 0 0 0 0 0 0 0 0 0
0 0 0 0 0 0 0 0 0 0 0 0 0 0 0 0 0 0 0 0 0 0 0 0 0 0 0 0 0
0 0 0 0 0 0 0 0 0 0 0 0 0 0 0 0 0 0 0 0 0 0 0 0 0 0 0 0 0
0 0 29 0 0 0 0 0 0 0 0 0 0 0 0 0 0 0 0 0 0 0 0 0 0 0 0 0
M02255_77_000000000-ADUT3_1_1107_11076_7786 T----AC---GT-AG-GGT--------GCA-A-G------C--A--T---T--AT-C-CGG-AG------TG-A--C-T--GG-GC-------GT--A-----AA-GA-GTTGC-------G-TA-G-G-T-G------------G--T-TA-G-T-A-----------
AA----G-T-G-A--------A-T-A--G--TG--A-AA-TC--T-G-GC-G-G-----CT-C-AA------C-C-G-T-ACA-G--A-C----T-A--T-T---A--T-------T--CA-A-A---C-------T--C-A--CT--A-A-C----T-C-G-A-G-A--A---T-GG-----TA-G-A--------G-G-T-A---AC-T------GG--A--ATT----T-C-T-
T-GT--GT-A-G-GA-GT--G---A--A-A---TC-C-GT-AG--AT-A-TA--A-G--A-A-G-G-A-AC-A-CC---AA--T--G--GC-GTA-G--G-C--A-------G--G-T-T-A---CTG--G--AC-C-A--------------------T-T---T-C-T--GA--CA-C-T-G--A-GG--C-A-CG-A--AA-G-C---G-TG--GG-G--AG-
C-G-AA--------CC--GG Bacteria(100);Candidate_division_TM7(100);unclassified(100);unclassified(100);unclassified(100);unclassified(100);unclassified(100);unclassified(100);unclassified(100);

Otu1099 0 0 0 0 0 0 0 0 0 0 0 0 0 0 0 0 0 0 0 0 0 0 0 0 0 0 0 0
0 0 0 0 0 0 0 0 0 0 0 0 0 0 0 0 0 0 0 0 0 0 0 0 0 0 0 0 0
0 0 0 0 0 0 0 0 0 0 0 0 0 0 0 0 0 0 0 0 0 0 0 0 0 0 0 0 0
0 0 61 0 0 0 0 0 0 0 0 0 0 0 0 0 0 0 0 0 0 0 0 0 0 0 0 0
M02255_77_000000000-ADUT3_1_1109_15257_10855 T----AC---AG-AG-GGG--------GCA-A-G------C--G--T---T--AT-C-CGG-AT------TG-A--C-T--GG-GC-------GT--A-----AA-GC-GT-GT-------G-TA-G-G-C-G------------G--T-AG-C-G-C----
-------AG----G-T-G-G--------C-G-C--G--GT--A-CA-GC--T-C-CC-G-G-----CT-T-AA------C-T-G-G-G-A-A--G-C----G-C--C-G---C--A-------C--CA-A-A---C-------C--G-C--GC--G-A-C----T-C-G-A-G-C--C---G-TG-----CA-G-A--------G-G-T-C---GG-T------GG--A--ATT----
G-C-C-C-GT--GT-A-G-CA-GT--G---A--A-A---TG-C-GT-AG--AG-A-TG--G-G--C-A-G-G-A-AC-A-CC---AA--G--G--GC-GCA-A--G-C--A-------G--C-C-G-A---CTG--G--GC-A-C--------------------G-A---G-C-T--GA--CG-C-T-G--A-GG--C-A-CG-A--CA-G-C---G-TG--
GG-G--AG-C-A-AA--------CA--GG Bacteria(100);unclassified(100);unclassified(100);unclassified(100);unclassified(100);unclassified(100);unclassified(100);unclassified(100);unclassified(100);

Otu1100 0 0 0 0 0 0 0 0 0 0 0 0 0 0 0 0 0 0 0 0 0 0 0 0 0 0 0 0
0 0 0 0 0 0 0 0 0 0 0 0 0 0 0 0 0 0 0 0 0 0 0 0 0 0 0 0 0
0 0 0 0 0 0 0 0 0 0 0 0 0 0 0 0 0 0 0 0 0 0 0 0 0 0 0 0 0
0 0 65 0 0 0 0 0 0 0 0 0 0 0 0 0 0 0 0 0 0 0 0 0 0 0 0

M02255_77_000000000-ADUT3_1_1112_6190_5961 T----AC---GT-AG-GTG--------GCA-A-G------C--G--T---T--GT-C-CGG-AA------TT-A--C-T--GG-GT-------GT--A-----AA-GG-GA-GC-------G-CA-G-G-C-G------------G--G-AC-A-G-C----------
AA----G-T-T-G--------G-A-T--G--TG--A-AA-TA--C-C-GA-G-G-----CT-T-AA------C-C-T-C-G-G-G--G-C----T-G--C-A---T--C-------C--AA-A-A---C-------T--G-T--TG--T-T-C----T-T-G-A-G-T--G---A-AG-----TA-G-A--------G-G-T-T---GG-C------GG--A--ATT----C-C-G-
A-GT--GT-A-G-CG-GT--G---A--A-A---TG-C-GT-AG--AT-A-TT--C-G--G-A-G-G-A-AC-A-CC---AG--T--G--GC-GAA-G--G-C--G-------G--C-C-A-A---CTG--G--GC-T-T--------------------T-A---A-C-T--GA--CG-C-T-G--A-GG--C-T-CG-A--AA-G-C---A-TG--GG-G--
AG-C-A-AA--------CA--GG Bacteria(100);Firmicutes(100);Clostridia(100);Clostridiales(100);unclassified(100);unclassified(100);unclassified(100);unclassified(100);unclassified(100);

Otu1101 0 0 0 0 0 0 0 0 0 0 0 0 0 0 0 0 0 0 0 0 0 0 0 0 0 0 0 0
0 0 0 0 0 0 0 0 0 0 0 0 0 0 0 0 0 0 0 0 0 0 0 0 0 0 0 0 0
0 0 0 0 0 0 0 0 0 0 0 0 0 0 0 0 0 0 0 0 0 0 0 0 0 0 0 0 0
0 0 50 0 0 0 0 0 0 0 0 0 0 0 0 0 0 0 0 0 0 0 0 0 0 0 0

M02255_77_000000000-ADUT3_1_1113_14217_6885 G----AC---AG-AG-GTC--------CCA-A-G------C--G--T---T--AG-G-CGG-AA------TC-A--C-T--GG-GC-------TT--A-----AA-GC-GT-GT-------G-TA-G-G-C-G------------G--G-TC-G-T-T-----------AA-
---G-T-G-T--------C-T-T--G--TG--A-AA-TC--C-C-AT-C-G-----CT-C-AC------C-G-A-T-G-G-A--A-C----T-G--C-T---G--G-------A--CA-T-A---C-------T--G-G--CG--A-T-C----T-T-G-A-G-C--C---A-CT-----TA-G-G--------G-G-C-A---AC-C------GG--A--ACA----A-A-C-G-
GT--GG-A-G-CG-GT--G---A--A-A---TG-C-GT-AG--AT-A-TC--G-T--T-T-G-G-A-AC-G-CC---AA--T--G--GT-GAA-A--A-C--A-------G--G-T-T-G---CTG--G--GA-G-T--------------------G-T---G-C-T--GA--CG-C-T-G--A-GA--C-A-CG-A--AA-G-C---C-AG--GG-G--AG-
C-A-AA--------CG--GG Bacteria(100);Planctomycetes(100);Phycisphaerae(100);WD2101(100);unclassified(100);unclassified(100);unclassified(100);unclassified(100);unclassified(100);

Otu1102 0 0 0 0 0 0 0 0 0 0 0 0 0 0 0 0 0 0 0 0 0 0 0 0 0 0 0 0
0 0 0 0 0 0 0 0 0 0 0 0 0 0 0 0 0 0 0 0 0 0 0 0 0 0 0 0 0
0 0 0 0 0 0 0 0 0 0 0 0 0 0 0 0 0 0 0 0 0 0 0 0 0 0 0 0 0
0 0 20 0 0 0 0 0 0 0 0 0 0 0 0 0 0 0 0 0 0 0 0 0 0 0 0

M02255_76_000000000-ADUNE_1_1106_15196_7023 .....AC----T-AC-GT---------GCC-A-G------C--A--G---C--CG-C-GGT-AA------TA-C--G-T--AG-GT-------GG--C-----AA-GC-G--TT-------G----T-C-C-G------------G--T-TT-C-T-T----------AA----G-
T-C-T--------G-A-T--G--TG--A-AA-GC--C-C-AC-G-G-----CT-T-AA------C-C-G-T-G-G-A--G------G-GT-C-A---T--T-------G--GA-A-A---C-------T--G-G--GA--G-A-C----T-T-G-A-G-T--G---C-AG-----AA-G-A--------G-G-A-A---AG-T------GG--A--ATT----C-C-A-A-GT--
GT-A-G-CG-GT--G---A--A-A---TG-C-GT-AG--AG-A-TT--T-G--G-A-G-G-A-AC-A-CC---AG--T--G--GC-GAA-G--G-C--G-------A--C-T-T-T---CTG--G--TC-T-G--------------------C-A---A-C-T--GA--CG-C-T-G--A-GG--C-G-CG-A--AA-G-C---A-TG--GG-G--AG-C-A-
AA--------CA--GG Bacteria(100);Firmicutes(100);Bacilli(100);Bacillales(100);Planococcaceae(100);Kurthia(100);Kurthia(100);unnamed(100);unclassified(100);

Otu1103 0 0 0 0 0 0 0 0 0 0 0 0 0 0 0 0 0 0 0 0 0 0 0 0 0 0 0 0
0 0 0 0 0 0 0 0 0 0 0 0 0 0 0 0 0 0 0 0 0 0 0 0 0 0 0 0 0
0 0 0 0 0 0 0 0 0 0 0 0 0 0 0 0 0 0 0 0 0 0 0 0 0 0 0 0 0
0 0 18 0 0 0 0 0 0 0 0 0 0 0 0 0 0 0 0 0 0 0 0 0 0 0 0

M02255_77_000000000-ADUT3_1_2117_12128_15278 G----AC---GG-AG-GGT--------GCT-A-G------C--G--T---T--GT-T-CGG-AA------TT-A--C-T--GG-GC-------GT--A-----AA-GG-GC-GC-------G-TA-G-G-C-G------------G--C-TT-C-T-T-----
------GC----G-T-C-G--------G-A-T--G--TG--A-AA-GC--C-C-GG-G-G-----CT-C-AA------C-C-T-C-G-G-A--A-C----G-G--C-A---T--C-------C--GA-T-A---C-------G--G-G--GG--A-G-C----T-G-G-A-G-A--C---C-GG-----GA-G-A--------G-G-A-G---GG-T------GG--A--ATA---
-G-C-C-A-GT--GT-A-G-AG-GT--G---A--A-A---TT-C-GT-AG--AT-A-TT--G-G--C-T-G-G-A-AC-A-CC---GG--T--G--GC-GAA-A--G-C--G-------G--C-T-C-T---CTG--G--CC-C-G--------------------G-T---T-C-T--GA--CG-C-T-G--A-GG--C-G-CG-A--AA-G-C---G-T-G-
GG-G--AG-C-A-AA--------CA--GG Bacteria(100);Proteobacteria(100);Alphaproteobacteria(100);unclassified(100);unclassified(100);unclassified(100);unclassified(100);unclassified(100);unclassified(100);

Otu1104 0 0 0 0 0 0 0 0 0 0 0 0 0 0 0 0 0 0 0 0 0 0 0 0 0 0 0 0
0 0 0 0 0 0 0 0 0 0 0 0 0 0 0 0 0 0 0 0 0 0 0 0 0 0 0 0 0
0 0 0 0 0 0 0 0 0 0 0 0 0 0 0 0 0 0 0 0 0 0 0 0 0 0 0 0 0
0 0 21 0 0 0 0 0 0 0 0 0 0 0 0 0 0 0 0 0 0 0 0 0 0 0 0

M02255_76_000000000-ADUNE_1_1101_8274_14502 G----GC---TA-AT-TTC--------GTG-C-C------A--G--C---A-GCC-G-CGG-TA------AT-A--C-G--AA-AG-------GT--C-----CA--A-GC-G--------T-TA-C-T-C-G------------G--A-CT-T-A-T----------AT----
G-T-C-G--------A-T-T--G--TG--A-AA-GC--C-C-CA-G-G-----CT-C-AA------C-C-T-G-G-G-A--A-C----C-G--C-A---G--T-------C--GA-A-A---C-------T--G-T--GA--G-A-C----T-T-G-A-A-T--G---T-TG-----GA-G-A--------G-G-G-T---GG-G------GG--A--ATA----C-C-T-A-
GT--TG-A-G-AA-GT--G---A--A-A---TT-C-GT-AG--AG-A-TT--A-G--G-T-G-G-A-AC-A-CC---GG--A--G--GC-GAA-G--G-C--G-------C--C-T-A-C---CTG--G--CC-A-A--------------------A-C---A-T-T--GA--CG-C-T-A--A-GG--C-G-CG-A--AA-G-C---G-TG--GG-G--AG-
C-A-AA--------CA--GG Bacteria(100);unclassified(100);unclassified(100);unclassified(100);unclassified(100);unclassified(100);unclassified(100);unclassified(100);unclassified(100);

Otu1105 0 0 0 0 0 0 0 0 0 0 0 0 0 0 0 0 0 0 0 0 0 0 0 0 0 0 0 0
0 0 0 0 0 0 0 0 0 0 0 0 0 0 0 0 0 0 0 0 0 0 0 0 0 0 0 0 0
0 0 0 0 0 0 0 0 0 0 0 0 0 0 0 0 0 0 0 0 0 0 0 0 0 0 0 0 0
0 0 0 19 0 0 0 0 0 0 0 0 0 0 0 0 0 0 0 0 0 0 0 0 0 0 0
M02255_76_000000000-ADUNE_1_1112_23464_24737 C----GG---CT-AA-CTC--------CGT-G-C------C--A--G---C--AG-C-CGC-GG------TA-A--T-A--CG-GA-------GG--A-----TC-CA-AG-CG-------T-TA-T-C-C-G------------GA-T-TT-A-T-T------
-----AA----G-T-C-A--------G-G-G--G--TG--A-AA-AC--T-T-GC-G-G-----CT-C-AA------C-C-G-T-A-A-G--C-T----T-G--C-C---T--T-------T--GA-T-A---C-------T--G-A--GG--A-G-C----T-T-G-A-A-T--A---C-AC-----TT-G-A--------G-G-T-A---GG-C------GG--A--ATG----T-
G-A-C-AA--GT-A-G-CG-GT--G---A--A-A---TG-C-AT-AG--AT-A-TG--T-C--A-C-A-G-A-AC-A-CC---AA--T--T--GC-GAA-G--G-C--A-------G--C-T-T-A---CTA--A--AG-T-G--------------------T-G---A-T-T--GA--CG-C-T-G--A-GG--C-A-CG-A--AA-G-C---G-TG--GG-G-
-AT-C-A-AA--------CA--GG Bacteria(100);Bacteroidetes(100);Sphingobacteria(100);Sphingobacteriales(100);Sphingobacteriaceae(100);unclassified(100);unclassified(100);unclassified(100);unclassified(100);

Otu1106 0 0 0 0 0 0 0 0 0 0 0 0 0 0 0 0 0 0 0 0 0 0 0 0 0 0 0 0
0 0 0 0 0 0 0 0 0 0 0 0 0 0 0 0 0 0 0 0 0 0 0 0 0 0 0 0 0
0 0 0 0 0 0 0 0 0 0 0 0 0 0 0 0 0 0 0 0 0 0 0 0 0 0 0 0 0
0 0 0 15 0 0 0 0 0 0 0 0 0 0 0 0 0 0 0 0 0 0 0 0 0 0 0
M02255_76_000000000-ADUNE_1_1113_18848_12640 T----AA---GC-AC-CGG--------CTA-A-C------T--A--C---G--TG-C-CAG-CA------GC-C--G-C--GG-TA-------AT--A-----CG-TA-GG-GT-------G-CA-A-G-C-G------------T--T-AA-TCG-G-----
------AA----G-A-C-C--------G-A-T--G--TG--A-AA-TC--C-C-CG-G-G-----CT-C-AA------C-C-T-G-G-G-A--A-C----T-G--C-A---T--T-------G--GT-G-A---C-------T--G-C--AT--C-G-C----T-T-G-A-G-T--A---T-GG-----CA-G-A--------G-G-G-G---GG-T------AG--A--ATT----C-
C-A-C-GT--GT-A-G-CA-GT--G---A--A-A---TG-C-GT-AG--AG-A-TG--T-G--G-A-G-G-A-AT-A-CC---GA--T--G--GC-GAA-G--G-C--A-------G--C-C-C-C---CTG--G--GT-C-A--------------------A-T---A-C-T--GA--CG-C-T-C--A-TG--C-A-CG-A--AA-G-C---G-TG--GG-G-
-AG-C-A-AA--------CA--GG Bacteria(100);Proteobacteria(100);Betaproteobacteria(100);unclassified(100);unclassified(100);unclassified(100);unclassified(100);unclassified(100);unclassified(100);

Otu1107 0 0 0 0 0 0 0 0 0 0 0 0 0 0 0 0 0 0 0 0 0 0 0 0 0 0 0 0
0 0 0 0 0 0 0 0 0 0 0 0 0 0 0 0 0 0 0 0 0 0 0 0 0 0 0 0 0
0 0 0 0 0 0 0 0 0 0 0 0 0 0 0 0 0 0 0 0 0 0 0 0 0 0 0 0 0
0 0 0 26 0 0 0 0 0 0 0 0 0 0 0 0 0 0 0 0 0 0 0 0 0 0 0
M02255_77_000000000-ADUT3_1_1104_5280_16508 T----AC---GA-GG-GGG--------GCG-A-G------C--G--T---T--GT-T-CGG-AA------TT-A--T-T--GG-GC-------GT--A-----AA-GG-GT-GC-------G-TA-G-G-C-G------------G--T-TT-G-G-T-----------
AA----G-T-C-T--------G-A-T--G--TG--A-AA-TC--T-A-TG-G-G-----CT-C-AA------C-T-C-A-T-A-G--T-C----T-G--C-A---T--C-------G--GA-A-A---C-------T--G-C--CG--G-G-C----T-T-G-A-G-T--A---T-GG-----GA-G-A--------G-G-T-G---AG-T------GG--A--ATT----C-C-T-
G-GT--GT-A-G-CG-GT--G---A--A-A---TG-C-GT-AG--AT-A-TC--A-G--G-A-G-G-A-AC-A-CC---TG--T--G--GC-GAA-A--G-C--G-------G--C-T-C-A---CTG--G--AC-C-A--------------------T-A---A-C-T--GA--CG-C-T-G--A-TG--C-A-CG-A--AA-G-C---T-AG--GG-G--
AG-C-A-AA--------CA--GG Bacteria(100);Acidobacteria(100);Acidobacteria(100);Acidobacteriales(100);Acidobacteriaceae(100);Acidobacterium(100);unclassified(100);unclassified(100);unclassified(100);

Otu1108 0 0 0 0 0 0 0 0 0 0 0 0 0 0 0 0 0 0 0 0 0 0 0 0 0 0 0 0
0 0 0 0 0 0 0 0 0 0 0 0 0 0 0 0 0 0 0 0 0 0 0 0 0 0 0 0 0
0 0 0 0 0 0 0 0 0 0 0 0 0 0 0 0 0 0 0 0 0 0 0 0 0 0 0 0 0
0 0 0 27 0 0 1 0 0 0 0 0 0 0 0 0 0 0 0 0 0 0 0 0 0 0 0
M02255_77_000000000-ADUT3_1_1108_7485_4275 T----AC---GT-AG-GGG--------GCA-A-G------C--G--T---T--GT-T-CGG-AT------TT-A--C-T--GG-GC-------GT--A-----AA-GG-GC-GC-------G-TA-G-G-C-G------------G--C-GT-G-A-C-----------
AA----G-T-C-A--------G-T-T--G--TG--A-AA-TC--T-C-TG-A-G-----CT-T-AA------C-T-C-A-G-A-A--C-G----G-T--C-A---A--C-------T--GA-T-A---C-------T--G-T--TG--T-G-C----T-A-G-A-G-T--G---C-AG-----AA-G-G--------G-G-C-A---AC-C------GG--A--ATT----C-T-T-
G-GT--GT-A-G-CG-GT--G---A--A-A---TG-C-GT-AG--AT-A-TC--A-A--G-A-G-G-A-AC-A-CC---TG--A--G--GT-GAA-G--A-C--G-------G--G-T-T-G---CTG--G--GC-T-G--------------------A-C---A-C-T--GA--CG-C-T-G--A-GG--C-G-CG-A--AA-G-C---T-AG--GG-T--
AG-C-A-AA--------CG--GG Bacteria(100);Acidobacteria(100);Acidobacteria(100);Acidobacteriales(100);Acidobacteriaceae(100);Candidatus_Chloroacidobacterium(100);unclassified(100);unclassified(100);unclassified(100);

Otu1109 0 0 0 0 0 0 0 0 0 0 0 0 0 0 0 0 0 0 0 0 0 0 0 0 0 0 0 0
0 0 0 0 0 0 0 0 0 0 0 0 0 0 0 0 0 0 0 0 0 0 0 0 0 0 0 0 0
0 0 0 0 0 0 0 0 0 0 0 0 0 0 0 0 0 0 0 0 0 0 0 0 0 0 0 0 0
0 0 0 33 0 0 0 0 0 0 0 0 0 0 0 0 0 0 0 0 0 0 0 0 0 0 0
M02255_77_000000000-ADUT3_1_1110_5363_16680 T----AC---AG-AG-GTG--------CCA-A-G------C--G--T---T--AG-G-CGG-AA------TC-A--C-T--GG-GC-------TT--A-----AA-GC-GC-GT-------G-TA-G-G-T-G------------G--A-CG-G-G-T-----------
AA----G-T-A-T--------C-T-T--G--TG--A-AA-TC--C-C-AC-G-G-----CT-C-AA------C-C-G-T-G-G-A--A-C----T-G--C-T---G--G-------A--TA-T-A---C-------T--G-C--TT--G-T-C----T-T-G-A-G-G--C---A-CT-----TA-G-G--------G-G-C-A---GC-T------GG--A--ACA----A-A-T-
G-GT--GG-A-G-CG-GT--G---A--A-A---TG-C-GT-AG--AT-A-TC--A-T--T-T-G-G-A-AC-G-CC---AA--A--G--GC-GAA-A--G-C--A-------G--G-C-T-G---CTG--G--GA-G-T--------------------G-A---T-C-T--GA--CA-C-T-G--A-GA--C-G-CG-A--AA-G-C---C-AG--GT-G--
AG-C-A-AA--------CG--GG Bacteria(100);Planctomycetes(100);Phycisphaerae(100);unclassified(100);unclassified(100);unclassified(100);unclassified(100);unclassified(100);unclassified(100);

Otu1110 0 0 0 0 0 0 0 0 0 0 0 0 0 0 0 0 0 0 0 0 0 0 0 0 0 0 0 0
0 0 0 0 0 0 0 0 0 0 0 0 0 0 0 0 0 0 0 0 0 0 0 0 0 0 0 0 0
0 0 0 0 0 0 0 0 0 0 0 0 0 0 0 0 0 0 0 0 0 0 0 0 0 0 0 0 0
0 88 0 0 0 0 0 0 0 0 0 0 0 0 0 0 0 0 0 0 0 0 0 0 0 0 0
M02255_77_000000000-ADUT3_1_1119_7036_21703 T----AC---GA-AG-GGG--------GCT-A-G------C--G--T---T--GC-T-CGG-AA------TC-A--C-T--GG-GC-------GT--A-----AA-GG-GC-GC-------G-TA-G-G-C-G------------G--A-CA-T-T-T----------
AA----G-T-C-G--------G-G-G--G--TG--A-AA-GC--C-C-AG-G-G-----CT-C-AA------C-C-C-T-G-G-A--A-T----T-G--C-C---T--C-------C--GA-T-A---C-------T--G-G--GT--G-T-C----T-C-G-A-G-A--C---C-GG-----AA-G-A--------G-G-T-G---AG-T------GG--A--ACT----G-C-
G-A-GT--GT-A-G-AG-GT--G---A--A-A---TT-C-GT-AG--AT-A-TT--C-G--C-A-A-G-A-AC-A-CC---AG--T--G--GC-GAA-G--G-C--G-------G--C-T-C-A---CTG--G--TC-C-G--------------------G-T---T-C-T--GA--CG-C-T-G--A-GG--C-G-CG-A--AA-G-C---G-TG--GG-G--
AG-C-A-AA--------CA--GG Bacteria(100);Proteobacteria(100);Alphaproteobacteria(100);Rhizobiales(100);unclassified(100);unclassified(100);unclassified(100);unclassified(100);unclassified(100);

Otu1111 0 0 0 0 0 0 0 0 0 0 0 0 0 0 0 0 0 0 0 0 0 0 0 0 0 0 0 0
0 0 0 0 0 0 0 0 0 0 0 0 0 0 0 0 0 0 0 0 0 0 0 0 0 0 0 0 0
0 0 0 0 0 0 0 0 0 0 0 0 0 0 0 0 0 0 0 0 0 0 0 0 0 0 0 0 0
0 30 0 0 0 0 0 0 0 0 0 0 0 0 0 0 0 0 0 0 0 0 0 0 0 0 0
M02255_76_000000000-ADUNE_1_1106_8121_6593 G----TG---CC-AG-CAG--------CCG-C-G---------G--T---A--AG-A-CGT-AG------GG-C--G-C--GA-GC-------GT--T-----GT-CC-GG-AT-------T-TA-T-T-G-G------------G--C-TC-G-T-C----------GC--
--G-T-C-T--------G-C-C--G--TG--A-AA-GA--C-C-AT-G-G-----CT-T-AA------C-T-G-T-G-G-T--T-T----T-G--C-G---G--T-------G--GA-T-A---C-------G--G-G--CG--G-G-C----T-T-G-A-G-G--C---C-AG-----TA-G-G--------G-G-A-G---AT-C------GG--A--ATT----C-T-A-C-
GT--GT-A-G-CG-GT--G---A--A-A---TG-C-GC-AG--AT-A-TG--T-G--G-A-G-G-A-AC-A-CC---GG--T--G--GC-GAA-G--G-C--G-------G--A-T-C-T---CTG--G--GC-T-G--------------------G-T---T-C-T--GA--CG-C-T-G--A-GG--A-G-CG-A--AA-G-C---A-TG--GG-G--AG-
C-G-AA--------CA--GG Bacteria(100);Actinobacteria(100);Actinobacteria(100);Actinobacteridae(100);Actinomycetales(100);Glycomycineae(100);Glycomycetaceae(100);Glycomyces(100);Glycomyces(100);

Otu1112 0 0 0 0 0 0 0 0 0 0 0 0 0 0 0 0 0 0 0 0 0 0 0 0 0 0 0 0
0 0 0 0 0 0 0 0 0 0 0 0 0 0 0 0 0 0 0 0 0 0 0 0 0 0 0 0 0
0 0 0 0 0 0 0 0 0 0 0 0 0 0 0 0 0 0 0 0 0 0 0 0 0 0 0 0 0
0 23 0 0 0 0 0 0 0 0 0 0 0 0 0 0 0 0 0 0 0 0 0 0 0 0 0
M02255_77_000000000-ADUT3_1_1101_18348_11528 T----AC---GT-AG-GTG--------GCG-A-G------C--G--T---T--AT-C-CGG-AT------TT-A--C-T--GG-GT-------GT--A-----AA-GG-GT-GT-------G-TA-G-G-C-G------------G--G-AC-T-G-C-----
------AA----G-T-C-A--------G-A-T--G--TG--A-AA-AC--T-A-TG-G-G-----CT-T-AA------C-T-C-A-T-A-G--C-C----T-G--C-A---T--T-------T--GA-A-A---C-------T--G-T--GG--T-T-C----T-T-G-A-G-G--G---T-CG-----GA-G-G--------G-G-T-T---AC-T------GG--A--ATT----C-
C-C-G-GT--GT-A-G-CG-GT--G---A--A-A---TG-C-GT-AG--AT-A-TC--G-G--G-A-G-G-A-AC-A-CC---GG--T--G--GC-GAA-G--G-C--G-------A--G-T-A-A---CTG--G--AC-G-A--------------------T-A---C-C-T--GA--CG-C-T-G--A-GA--C-A-CG-A--AA-G-T---G-TG--GG-
G--AG-C-A-AA--------CA--GG Bacteria(100);unclassified(100);unclassified(100);unclassified(100);unclassified(100);unclassified(100);unclassified(100);unclassified(100);unclassified(100);

Otu1113 0 0 0 0 0 0 0 0 0 0 0 0 0 0 0 0 0 0 0 0 0 0 0 0 0 0 0 0
0 0 0 0 0 0 0 0 0 0 0 0 0 0 0 0 0 0 0 0 0 0 0 0 0 0 0 0 0
0 0 0 0 0 0 0 0 0 0 0 0 0 0 0 0 0 0 0 0 0 0 0 0 0 0 0 0 0
0 41 0 0 0 0 0 0 0 0 0 0 0 0 0 0 0 0 0 0 0 0 0 0 0 0 0
M02255_77_000000000-ADUT3_1_1109_17253_13215 T----AC---GA-AG-GGG--------GCT-A-G------C--G--T---T--GC-T-CGG-AA------TG-A--C-T--GG-GC-------GT--A-----AA-GG-GC-GC-------G-TA-G-G-C-G------------G--T-TT-G-T-A----
-------CA----G-T-C-A--------G-G-T--G--TG--A-AA-TT--C-C-CG-G-G-----CT-C-AA------C-C-T-G-G-G-G--G-C----T-G--C-A---C--T-------T--GA-T-A---C-------G--T-A--TA--G-A-C----T-T-G-A-G-T--G---A-GG-----AA-G-A--------G-G-G-T---CG-T------GG--A--ATT----
C-C-C-A-GT--GT-A-G-AG-GT--G---A--A-A---TT-C-GT-AG--AT-A-TT--G-G--G-A-A-G-A-AC-A-CC---GG--T--G--GC-GAA-G--G-C--G-------G--C-G-A-C---CTG--G--TC-C-T--------------------T-T---A-C-T--GA--CG-C-T-G--A-GG--C-G-CG-A--AA-G-C---G-TG--GG-
G--AG-C-A-AA--------CA--GG Bacteria(100);Proteobacteria(100);Alphaproteobacteria(100);Rhodospirillales(100);Acetobacteraceae(100);unclassified(100);unclassified(100);unclassified(100);unclassified(100);

Otu1114 0 0 0 0 0 0 0 0 0 0 0 0 0 0 0 0 0 0 0 0 0 0 0 0 0 0 0 0
0 0 0 0 0 0 0 0 0 0 0 0 0 0 0 0 0 0 0 0 0 0 0 0 0 0 0 0 0
0 0 0 0 0 0 0 0 0 0 0 0 0 0 0 0 0 0 0 0 0 0 0 0 0 0 0 0 0
0 28 0 0 0 0 0 0 0 0 0 0 0 0 0 0 0 0 0 0 0 0 0 0 0 0 0
M02255_77_000000000-ADUT3_1_1110_26740_10092 T----AC---GT-AG-GGT--------GCG-A-G------C--G--T---T--GC-C-CGG-AT------TT-A--T-T--GG-GC-------GT--A-----AA-GG-GC-GC-------G-TA-G-G-C-G------------G--C-CT-G-G-C----
-------GC----G-T-C-G--------G-C-T--G--TG--A-AA-TC--T-C-CC-G-G-----CT-C-AA------C-T-G-G-G-A-G--G-G----G-A--C-G---G--C-------C--GA-T-A---C-------G--G-C--TG--G-G-C----T-T-G-A-G-C--G---A-GG-----TA-G-G--------G-G-A-G---AG-T------GG--A--ATT---
-C-C-C-G-GT--GT-A-G-TG-GT--G---G--A-A---TG-C-GT-AG--AT-A-TC--G-G--G-A-G-G-A-AC-A-CC---AG--T--G--GC-GAA-G--G-C--G-------G--C-T-C-T---CTG--G--AC-C-T--------------------C-T---G-C-T--GA--CG-C-T-G--A-GG--C-G-CGA---AA-G-C---G-TG--
GG-G--AG-C-G-AT--------C---CG Bacteria(100);unclassified(100);unclassified(100);unclassified(100);unclassified(100);unclassified(100);unclassified(100);unclassified(100);unclassified(100);

Otu1115 0 0 0 0 0 0 0 0 0 0 0 0 0 0 0 0 0 0 0 0 0 0 0 0 0 0 0 0
0 0 0 0 0 0 0 0 0 0 0 0 0 0 0 0 0 0 0 0 0 0 0 0 0 0 0 0
0 0 0 0 0 0 0 0 0 0 0 0 0 0 0 0 0 0 0 0 0 0 0 0 0 0 0 0
0 58 0 0 0 0 0 0 0 0 0 0 0 0 0 0 0 0 0 0 0 0 0 0 0 0 0
M02255_77_000000000-ADUT3_1_1112_21952_10668 T----AC---GT-AG-GGT--------GCA-A-G------C--G--T---T--AT-C-CGG-AA------TT-A--T-T--GG-GC-------GT--A-----AA-GA-GC-TC-------G-TA-G-G-C-G------------G--T-TT-G-T-C------
-----AC----G-C-C-T--------G-G-T--G--TG--A-AA-GT--C-C-AT-C-G-----CT-T-AA------C-G-G-T-G-G-A--T-C----T-G--C-G---C--C-------G--GG-T-A---C-------G--G-G--CA--G-G-C----T-A-G-A-G-T--G---C-AG-----TA-G-G--------G-G-A-G---AT-T------GG--A--ATT----C-
C-C-G-GT--GT-A-A-CG-GT--G---G--A-A---TG-T-GT-AG--AT-A-TC--G-G--G-A-A-G-A-AC-A-CC---AA--T--G--GC-GAA-G--G-C--A-------G--A-T-C-T---CTG--G--GC-T-G--------------------T-T---A-C-T--GA--CG-C-T-G--A-GG--A-G-CG-A--AA-G-C---A-TG--GG-
G--AG-C-G-AA--------CA--GG Bacteria(100);Actinobacteria(100);Actinobacteria(100);Actinobacteridae(100);unclassified(100);unclassified(100);unclassified(100);unclassified(100);unclassified(100);

Otu1116 0 0 0 0 0 0 0 0 0 0 0 0 0 0 0 0 0 0 0 0 0 0 0 0 0 0 0 0
0 0 0 0 0 0 0 0 0 0 0 0 0 0 0 0 0 0 0 0 0 0 0 0 0 0 0 0
0 0 0 0 0 0 0 0 0 0 0 0 0 0 0 0 0 0 0 0 0 0 0 0 0 0 0 0
0 60 0 0 0 0 0 0 0 0 0 0 0 0 0 0 0 0 0 0 0 0 0 0 0 0 0
M02255_77_000000000-ADUT3_1_1113_7414_4044 T----AC---GT-AG-GGT--------CCA-A-G------C--G--T---T--GT-C-CGG-AT------TT-A--T-T--GG-GC-------GT--A-----AA-GA-GC-TC-------G-TA-G-G-C-G------------G--T-TC-G-G-T-----------AA--
--G-T-C-G--------G-G-T--G--TG--A-AA-AC--C-C-CA-G-G-----CT-C-AA------C-C-T-G-G-G-G--A------C-G-CC-A---C--T-------C--GA-T-A---C-------T--G-C--TG-TG-A-C----T-T-G-A-G-T--T---C-GG-----TA-G-G--------G-G-A-G---CA-C------GG--A--ATT----C-C-T-G-
GT--GT-A-G-CG-GT--G---A--A-A---TG-C-GC-AG--AT-A-TC--A-G--G-A-G-G-A-AC-A-CC---GG--T--G--GC-GAA-G--G-C--G-------G--T-G-C-T---CTG--G--GC-C-G--------------------A-A---A-C-T--GA--CG-C-T-G--A-GG--A-G-CG-A--AA-G-C---G-TG--GG-T--AG-
C-A-AA--------CA--GG Bacteria(100);Actinobacteria(100);Actinobacteria(100);Acidimicrobidae(100);Acidimicrobiales(100);Acidimicrobineae(100);unclassified(100);unclassified(100);unclassified(100);

Otu1117 0 0 0 0 0 0 0 0 0 0 0 0 0 0 0 0 0 0 0 0 0 0 0 0 0 0 0 0
0 0 0 0 0 0 0 0 0 0 0 0 0 0 0 0 0 0 0 0 0 0 0 0 0 0 0 0
0 0 0 0 0 0 0 0 0 0 0 0 0 0 0 0 0 0 0 0 0 0 0 0 0 0 0 0
0 36 0 0 0 0 0 0 0 0 0 0 0 0 0 0 0 0 0 0 0 0 0 0 0 0 0
M02255_77_000000000-ADUT3_1_1115_14242_17429 G----AC---GT-AG-GGA--------GCG-A-G------C--G--T---T--GT-C-CGG-AT------TC-A--T-T--GG-GC-------GT--A-----AA-GC-GC-GC-------G-TA-G-G-C-G------------G--T-TT-G-G-C----
-------AA----G-T-C-G--------G-A-T--G--TG--A-AA-TC--C-C-GA-G-G-----CT-C-AA------C-C-T-C-G-G-G--A-C----T-G--C-A---T--C-------C--GA-T-A---C-------T--G-C--CC--G-A-C----T-A-G-A-G-G------TAGG-----TA-G-G--------G-G-A-G---AT-C------GG--A--ATT----
C-C-T-G-GT--GT-A-G-CG-GT--G---A--A-A---TG-C-GC-AG--AT-A-TC--A-G--G-A-G-G-A-AC-A-CC---GG--T--G--GC-GAA-G--G-C--G-------GATC-T-------CTG--G--GC-C-T--------------------T-A---C-C-T--GA--CG-C-T-G--A-GG--C-G-CG-A--AA-G-C---T-AG--
GG-G--AG-C-G-AA--------CA--GG Bacteria(100);Actinobacteria(100);Actinobacteria(100);unclassified(100);unclassified(100);unclassified(100);unclassified(100);unclassified(100);unclassified(100);

Otu1118 0 0 0 0 0 0 0 0 0 0 0 0 0 0 0 0 0 0 0 0 0 0 0 0 0 0 0 0
0 0 0 0 0 0 0 0 0 0 0 0 0 0 0 0 0 0 0 0 0 0 0 0 0 0 0 0
0 0 0 0 0 0 0 0 0 0 0 0 0 0 0 0 0 0 0 0 0 0 0 0 0 0 0 0
0 19 0 0 0 0 0 0 0 0 0 0 0 0 0 0 0 0 0 0 0 0 0 0 0 0 0
M02255_77_000000000-ADUT3_1_1115_11401_19305 T----AC---GA-AG-GGG--------GCT-A-G------C--G--T---T--GC-T-CGG-AA------TT-A--C-T--GG-GC-------GT--A-----AA-GG-GC-GC-------G-TA-G-G-C-G------------G--C-GC-T-T-C----
-------AA----G-T-C-A--------G-G-C--G--TG--A-AA-TT--C-C-TG-G-G-----CT-T-AA------C-C-T-G-G-G-G--A-C----T-G--C-G---C--T-------T--GA-G-A---C-------T--G-T--GG--T-G-C----T-A-G-A-G-G--T---C-AG-----AA-G-A--------G-G-G-T---CG-T------GG--A--ATT----
C-C-C-A-GT--GT-A-G-AG-GT--G---A--A-A---TT-C-GT-AG--AT-A-TT--G-G--G-A-A-G-A-AC-A-CC---GG--T--G--GC-GAA-G--G-C--G-------G--C-G-A-C---CTG--G--TC-T-G--------------------A-T---A-C-T--GA--CG-C-T-G--A-GG--C-G-CG-A--CA-G-C---G-TG--
GG-G--AG-C-A-AA--------CA--GG Bacteria(100);Proteobacteria(100);Alphaproteobacteria(100);Rhodospirillales(100);Acetobacteraceae(100);unclassified(100);unclassified(100);unclassified(100);unclassified(100);

Otu1119 0 0 0 0 0 0 0 0 0 0 0 0 0 0 0 0 0 0 0 0 0 0 0 0 0 0 0 0
0 0 0 0 0 0 0 0 0 0 0 0 0 0 0 0 0 0 0 0 0 0 0 0 0 0 0 0
0 0 0 0 0 0 0 0 0 0 0 0 0 0 0 0 0 0 0 0 0 0 0 0 0 0 0 0
0 0 0 29 0 0 0 0 0 0 0 0 0 0 0 0 0 0 0 0 0 0 0 0 0 0 0
M02255_77_000000000-ADUT3_1_2102_18172_9200 T----AC---GT-AG-GGG--------GCA-A-G------C--G--T---T--AT-C-CGG-AT------TT-A--C-T--GG-GT-------GT--A-----AA-GG-GA-GC-------G-TA-G-G-C-G------------G--C-AG-G-G-C-----------
AA----G-T-C-A--------G-A-A--G--TG--A-AA-GC--C-C-GG-G-G-----CT-C-AA------C-C-C-C-G-G-G--A-C----T-G--C-T---T--T-------T--GA-A-A---C-------T--G-T--CT--T-G-C----T-A-G-A-G-T--A---T-CG-----GA-G-A--------G-G-T-A---AG-T------GG--A--ATT----C-C-T-
A-GT--GT-A-G-CG-GT--G---A--A-A---TG-C-GT-AG--AT-A-TT--A-G--G-A-G-G-A-AC-A-CC---AG--T--G--GC-GAA-G--G-C--G-------A--C-T-T-A---CTG--G--AC-G-A--------------------C-A---A-C-T--GA--CG-C-T-G--A-GG--C-T-CG-A--AA-G-C---G-TG--GG-G--
AG-C-A-AA--------CA--GG Bacteria(100);Firmicutes(100);Clostridia(100);Clostridiales(100);Lachnospiraceae(100);unclassified(100);unclassified(100);unclassified(100);unclassified(100);

Otu1120 0 0 0 0 0 0 0 0 0 0 0 0 0 0 0 0 0 0 0 0 0 0 0 0 0 0 0 0
0 0 0 0 0 0 0 0 0 0 0 0 0 0 0 0 0 0 0 0 0 0 0 0 0 0 0 0 0
0 0 0 0 0 0 0 0 0 0 0 0 0 0 0 0 0 0 0 0 0 0 0 0 0 0 0 0 0
0 26 0 0 0 0 0 0 0 0 0 0 0 0 0 0 0 0 0 0 0 0 0 0 0 0 0
M02255_76_000000000-ADUNE_1_1105_15937_6118 .....AC----T-AC-GTG---------CC-A-G------C--A--G---C--CG-C-GGT-AA------TA-C--G-T--AG-GT-------GA--C-----AA-GC-G--TT-------G----T-C-C-G------------G--T-TC-G-G-T----------AA----G-
T-C-T--------G-A-T--G--TG--A-AA-GC--C-C-AC-G-G-----CT-C-AA------C-C-G-T-G-G-A--A-C----G-G--C-A---T--T-------G--GA-A-A---C-------T--G-G--CG--G-A-C----T-T-G-A-A-T--G---T-AG-----CA-G-A--------G-G-A-A---AG-T------GG--A--ATT----C-C-A-T-GT--
GT-A-G-CG-GT--G---G--A-A---TG-C-GT-AG--AT-A-TA--T-G--G-A-G-G-A-AC-A-CC---AG--T--G--GC-GAA-A--G-C--G-------A--C-T-T-T---CTG--G--GC-T-A--------------------T-G---A-T-T--GA--CG-C-T-G--A-GG--C-T-CG-A--AA-G-C---G-TG--GG-G--AG-C-G-
AA--------CA--GG Bacteria(100);Firmicutes(100);Bacilli(100);Lactobacillales(100);Carnobacteriaceae(100);Alloiococcus(100);unclassified(100);unclassified(100);unclassified(100);

Otu1121 0 0 0 0 0 0 0 0 0 0 0 0 0 0 0 0 0 0 0 0 0 0 0 0 0 0 0 0
0 0 0 0 0 0 0 0 0 0 0 0 0 0 0 0 0 0 0 0 0 0 0 0 0 0 0 0 0
0 0 0 0 0 0 0 0 0 0 0 0 0 0 0 0 0 0 0 0 0 0 0 0 0 0 0 0 0
0 16 0 0 0 0 0 0 0 0 0 0 0 0 0 0 0 0 0 0 0 0 0 0 0 0 0
M02255_77_000000000-ADUT3_1_1105_9137_14584 C----AC---GT-AG-GGG--------GCA-A-G------C--G--T---T--GT-C-CGG-AT------TT-A--C-T--GG-GC-------GT--A-----AA-GA-GC-TC-------G-TA-G-G-C-G------------G--T-TC-G-G-C----------AA-
---G-T-C-G--------G-G-T--G--TG--A-CA-GC--T-C-CA-G-G-----CT-C-AA------C-C-T-G-GAG-A---------G-G-CC-A---C--T-------C--GA-T-A---C-------T--G-C--TG-TG-A-C----T-A-G-A-G-T--C---C-GG-----TA-G-G--------G-G-A-G---CG-T------GG--A--ATT----C-C-C-G-
GT--GT-A-G-CG-GT--G---A--A-A---TG-C-GC-AG--AT-A-TC--G-G--G-A-G-G-A-AC-A-CC---AG--C--G--GC-GAA-G--G-C--G-------G--C-G-C-T---CTG--G--GC-C-G--------------------G-A---A-C-T--GA--CG-C-T-G--A-GG--A-G-CG-A--AA-G-C---G-TG--GG-T--AG-
C-A-AA--------CA--GG Bacteria(100);Actinobacteria(100);Actinobacteria(100);Acidimicrobidae(100);Acidimicrobiales(100);Acidimicrobineae(100);unclassified(100);unclassified(100);unclassified(100);

Otu1122 0 0 0 0 0 0 0 0 0 0 0 0 0 0 0 0 0 0 0 0 0 0 0 0 0 0 0 0
0 0 0 0 0 0 0 0 0 0 0 0 0 0 0 0 0 0 0 0 0 0 0 0 0 0 0 0 0
0 0 0 0 0 0 0 0 0 0 0 0 0 0 0 0 0 0 0 0 0 0 0 0 0 0 0 0 0
0 15 0 0 0 0 0 0 0 0 0 0 0 0 0 0 0 0 0 0 0 0 0 0 0 0 0
M02255_77_000000000-ADUT3_1_2103_24611_15304 T----AC---AG-AG-GGG--------GCA-A-G------C--G--T---T--GT-C-CGG-AA------TT-A--C-T--GG-GC-------GT--A-----AA-GC-GC-GC-------G-CA-G-G-C-G------------G--T-CG-G-C-G---
--------AT----G-T-G-C--------C-G-T--G--TG--A-CA-GG--C-G-GG-G-G-----CT-C-AA------C-C-C-C-C-G-AT-T------G-G--C-A---G--G-------G--TA-G-A---C-------A--C-G--CC--A-A-C----T-T-G-A-G-G--C---A-GG-----GA-G-A--------G-G-G-A---TG-T------GG--A--ATG--
--C-C-C-G-GT--GG-A-G-CG-GT--G---A--A-A---TG-C-GT-AG--AG-A-TC--G-G--G-C-G-G-A-AC-A-CC---AG--C--G--GC-GAA-G--G-C--G-------G--C-A-T-C---CTG--G--CC-C-T--------------------G-A---C-C-T--GA--CG-C-T-G--A-GG--C-G-CG-A--GA-G-C---G-TG--
GG-G--AG-C-G-AA--------CT--GG Bacteria(100);unclassified(100);unclassified(100);unclassified(100);unclassified(100);unclassified(100);unclassified(100);unclassified(100);unclassified(100);

Otu1123 0 0 0 0 0 0 0 0 0 0 0 0 0 0 0 0 0 0 0 0 0 0 0 0 0 0 0 0
0 0 0 0 0 0 0 0 0 0 0 0 0 0 0 0 0 0 0 0 0 0 0 0 0 0 0 0 0
0 0 0 0 0 0 0 0 0 0 0 0 0 0 0 0 0 0 0 0 0 0 0 0 0 0 0 0 0
0 9 0 0 0 0 0 0 0 0 0 0 0 0 0 0 0 0 0 0 0 0 0 0 0 0 0
M02255_77_000000000-ADUT3_1_1109_13946_18673 T----AC---GT-AG-GGG--------GCA-A-G------C--G--T---T--GT-C-CGG-AT------TT-A--T-T--GG-GC-------GT--A-----AA-GC-GC-GC-------G-CA-G-G-C-G------------G--C-TT-G-G-T-----
------AA----G-T-C-G--------G-C-C--G--TG--A-AA-TC--T-C-CA-G-G-----CT-C-AA------C-T-T-G-G-A-G--G------G-GT-C-G---G--G-------C--GA-G-A---C-------T--G-C--CA--G-G-C----T-T-G-A-G-G--T---G-GG-----TA-G-A--------G-G-G-C---AG-T------GG--A--ATT----
C-C-G-G-GT--GT-A-G-TG-GT--G---G--A-A---TG-C-GT-AG--AG-A-TC--C-G--G-A-G-G-A-AC-A-CC---AG--T--G--GC-GAA-G--G-C--G-------G--C-T-G-C---CTG--G--GC-C-T--------------------G-G---C-C-T--GA--CG-C-T-G--A-GG--C-G-CG-A--AA-G-C---G-TG--
GG-G--AG-C-A-AT--------CC--GG Bacteria(100);unclassified(100);unclassified(100);unclassified(100);unclassified(100);unclassified(100);unclassified(100);unclassified(100);unclassified(100);

Otu1124 0 0 0 0 0 0 0 0 0 0 0 0 0 0 0 0 0 0 0 0 0 0 0 0 0 0 0 0
0 0 0 0 0 0 0 0 0 0 0 0 0 0 0 0 0 0 0 0 0 0 0 0 0 0 0 0 0
0 0 0 0 0 0 0 0 0 0 0 0 0 0 0 0 0 0 0 0 0 0 0 0 0 0 0 0 0
0 0 49 0 0 0 0 0 0 0 0 0 0 0 0 0 0 0 0 0 0 0 0 0 0 0 0
M02255_76_000000000-ADUNE_1_1101_12429_13298 C----TC---CG-TG-CCA--------GCAGCCG------CG-G--T---A--AT-A-CGG-AG------GG-T--G-C--AA-GC-------GT--T-----GT-CC-G--GA-------T-TT-A-T-T-G------------G--C-CT-T-G-T-----
------AA----G-T-C-C--------G-T-G--G--TG--A-AA-GC--C-T-CC-T-G-----CT-C-AA------C-A-G-G-A-G-A--A-C----T-G--C-C---A--T-------G--GA-T-A---C-------T--G-C--CG--G-G-C----T-T-G-A-G-T--G---T-AG-----AC-G-A--------G-G-G-T---GG-C------GG--A--ATA----
G-A-G-G-CT--GT-A-G-CG-GT--G---A--A-A---TG-C-AT-AG--AT-A-GC--C-T--C-T-A-G-A-AC-C-CC---GA--T--T--GC-GTA-G--G-C--A-------G--C-T-G-C---CTA--G--AC-T-A--------------------C-A---A-C-T--GA--CA-C-T-G--A-GG--C-A-CG-A--AA-G-C---G-TG--GG-
G--AG-C-G-AA--------CA--GG Bacteria(100);Bacteroidetes(100);Sphingobacteria(100);Sphingobacteriales(100);unclassified(100);unclassified(100);unclassified(100);unclassified(100);unclassified(100);

Otu1125 0 0 0 0 0 0 0 0 0 0 0 0 0 0 0 0 0 0 0 0 0 0 0 0 0 0 0 0
0 0 0 0 0 0 0 0 0 0 0 0 0 0 0 0 0 0 0 0 0 0 0 0 0 0 0 0 0
0 0 0 0 0 0 0 0 0 0 0 0 0 0 0 0 0 0 0 0 0 0 0 0 0 0 0 0 0
0 0 13 0 0 0 0 0 0 0 0 0 0 0 0 0 0 0 0 0 0 0 0 0 0 0 0 0
M02255_76_000000000-ADUNE_1_1103_15699_9353 .....AC----T-A--CGT--------GCC-A-G------C--A--G---C--CG-C-GGT-AA------TA-C--G-T--AG-GG-------GG--C-----AA-GC-G--TT-------G----T-C-C-G------------G--A-TT-C-G-T----------CA----G-
T-C-C--------G-A-T--G--TG--A-CA-GG--C-C-AC-G-G-----CT-C-AA------C-C-G-T-G-G-A--A------G-GC-C-A---T--T-------G--GA-A-A---C-------T--G-C--GA--A-A-C----T-T-G-A-G-G--A---C-AG-----AA-G-A--------G-G-A-G---AG-T------GG--A--ATT----C-C-A-C-GT--
GT-A-G-CG-GT--G---A--A-A---TG-C-GT-AG--AT-A-TG--T-G--G-A-G-G-A-AC-A-CC---AG--T--G--GC-GAA-G--G-C--G-------A--C-T-C-T---CTG--G--TC-T-G--------------------T-A---C-C-T--GA--CG-C-T-G--A-GG--T-G-CG-A--AA-G-C---A-TG--GG-G--AG-C-A-
AA--------CA--GG Bacteria(100);Firmicutes(100);Bacilli(100);Bacillales(100);unclassified(100);unclassified(100);unclassified(100);unclassified(100);unclassified(100);

Otu1126 0 0 0 0 0 0 0 0 0 0 0 0 0 0 0 0 0 0 0 0 0 0 0 0 0 0 0 0
0 0 0 0 0 0 0 0 0 0 0 0 0 0 0 0 0 0 0 0 0 0 0 0 0 0 0 0 0
0 0 0 0 0 0 0 0 0 0 0 0 0 0 0 0 0 0 0 0 0 0 0 0 0 0 0 0 0
0 0 20 0 0 0 0 0 0 0 0 0 0 0 0 0 0 0 0 0 0 0 0 0 0 0 0 0
M02255_76_000000000-ADUNE_1_1104_17697_8025 T----A----AC-TC-CGT--------GCC-A-G------C-----A---G--CC-G-CGG-TA------AT-A--C-G--GA-GG-------GT--G-----CA--A-GC-G--------T-TG-T-T-C-G------------G--A-TT-G-A-T----------AA----
G-T-C-A--------G-A-T--G--TG--A-AA-GC--C-C-AC-G-G-----CT-T-AA------C-C-G-T-G-G-A--A-G----T-G--C-A---T--T-------T--GA-T-A---C-------T--G-T--CA--G-A-C----T-T-G-A-G-T--A---C-CA-----GA-G-G--------G-G-A-A---AG-T------GG--A--ATT----C-C-C-G-GT--
GT-A-G-AG-GT--G---A--A-A---TT-C-GT-AG--AT-A-TC--G-G--G-A-G-G-A-AT-A-CC---GG--T--G--GC-GAA-G--G-C--G-------A--C-T-T-T---CTG--G--CT-G-G--------------------A-T---A-C-T--GA--CG-C-T-G--A-GG--C-G-CG-A--AA-G-C---G-TG--GG-G--AG-C-A-
AA--------CA--GG Bacteria(100);Proteobacteria(100);Deltaproteobacteria(100);Desulfobacterales(100);Desulfobulbaceae(100);Desulfobulbus(100);unclassified(100);unclassified(100);unclassified(100);

Otu1127 0 0 0 0 0 0 0 0 0 0 0 0 0 0 0 0 0 0 0 0 0 0 0 0 0 0 0 0
0 0 0 0 0 0 0 0 0 0 0 0 0 0 0 0 0 0 0 0 0 0 0 0 0 0 0 0 0
0 0 0 0 0 0 0 0 0 0 0 0 0 0 0 0 0 0 0 0 0 0 0 0 0 0 0 0 0
0 0 0 0 0 0 20 0 0 0 0 0 0 0 0 0 0 0 0 0 0 0 0 0 0 0 0 0
M02255_77_000000000-ADUT3_1_1102_27155_20332 T----AC---GG-AG-GGG--------GCT-A-G------C--G--T---T--GT-T-CGG-AA------TT-A--C-T--GG-GC-------GT--A-----AA-GC-GC-AC-------G-TA-G-G-C-G------------G--C-TG-T-T-C-----
------AA----G-T-C-A--------G-A-G--G--TG--A-AA-GC--C-C-GG-G-G-----CT-C-AA------C-C-C-C-G-G-A--A-T----A-G--C-C---T--T-------T--GA-G-A---C-------T--G-G--AT--A-G-C----T-T-G-A-A-T--C---T-TG-----GA-G-A--------G-G-C-G---AG-T------GG--A--ATT----
C-C-G-A-GT--GT-A-G-AG-GT--G---A--A-A---TT-C-GT-AG--AT-A-TT--C-G--G-A-A-G-A-AC-A-CC---AG--T--G--GC-GAA-G--G-C--G-------G--C-T-C-G---CTG--G--AC-A-A--------------------G-T---A-T-T--GA--CG-C-T-G--A-GG--T-G-CG-A--AA-G-C---G-TG--
GG-G--AG-C-A-AA--------CA--GG Bacteria(100);Proteobacteria(100);Alphaproteobacteria(100);Sphingomonadales(100);Sphingomonadaceae(100);Sphingobium(100);unclassified(100);unclassified(100);unclassified(100);

Otu1128 0 0 0 0 0 0 0 0 0 0 0 0 0 0 0 0 0 0 0 0 0 0 0 0 0 0 0 0
0 0 0 0 0 0 0 0 0 0 0 0 0 0 0 0 0 0 0 0 0 0 0 0 0 0 0 0 0
0 0 0 0 0 0 0 0 0 0 0 0 0 0 0 0 0 0 0 0 0 0 0 0 0 0 0 0 0
0 0 0 0 32 0 0 0 0 0 0 0 0 0 0 0 0 0 0 0 0 0 0 0 0 0 0 0
M02255_77_000000000-ADUT3_1_2109_23997_11059 T----AC---AG-AG-GTC--------TCA-A-G------C--G--T---T--GT-T-CGG-AT------TC-A--T-T--GG-GC-------GT--A-----AA-GG-GT-GC-------G-CA-G-G-C-T------------G--T-GG-T-G-T------
-----AA----G-T-C-G--------A-G-T--G--TG--A-AA-TC--T-C-GG-G-G-----CT-C-AA------C-C-C-C-G-A-A--A-T----T-G--C-A---C--T-------C--GA-T-A---C-------T--G-C--AT--C-A-C----T-A-G-A-G-G--A---C-TG-----TA-G-A--------G-G-A-G---AG-T------GG--A--ATT----C-
A-C-G-GT--GT-A-G-CA-GT--G---A--A-A---TG-C-GT-AG--AT-A-TC--G-T--G-A-G-G-A-AG-A-CC---AG--T--T--GC-GAA-A--G-C--G-------A--C-T-C-T---CTG--G--GC-A-G--------------------T-T---C-C-T--GA--CG-C-T-C--A-TG--C-A-CG-A--AG-G-C---C-AG--GG-G--
AG-C-A-AA--------CG--GG Bacteria(100);Verrucomicrobia(100);Spartobacteria(100);unclassified(100);unclassified(100);unclassified(100);unclassified(100);unclassified(100);unclassified(100);

Otu1129 0 0 0 0 0 0 0 0 0 0 0 0 0 0 0 0 0 0 0 0 0 0 0 0 0 0 0 0
0 0 0 0 0 0 0 0 0 0 0 0 0 0 0 0 0 0 0 0 0 0 0 0 0 0 0 0 0
0 0 0 0 0 0 0 0 0 0 0 0 0 0 0 0 0 0 0 0 0 0 0 0 0 0 0 0 0
0 0 0 0 0 21 0 0 0 0 0 0 0 0 0 0 0 0 0 0 0 0 0 0 0 0 0 0
M02255_77_000000000-ADUT3_1_2103_14663_12054 T----AC---GA-AG-GGA--------GCA-A-G------C--G--T---T--GC-T-CGG-AT------TT-A--T-T--GG-GC-------GT--A-----AA-GG-GT-GT-------G-TA-G-G-T-G------------G--T-TT-G-G-T-----
------AA----G-T-T-A--------G-G-T--G--TG--A-AA-GC--C-C-AA-A-G-----CT-C-AA------C-T-T-T-G-G-A--A-T----T-G--C-A---C--T-------T--AA-A-A---C-------T--A-C--TA--G-A-C----T-T-G-A-A-G--A---T-CG-----GA-G-A--------G-G-T-A---AG-C------AG--A--ATT----C-
C-C-A-GT--GT-A-G-AG-GT--G---A--A-A---TT-C-GT-AG--AT-A-TT--G-G--G-A-A-G-A-AT-A-CC---GA--T--G--GC-GTA-G--G-C--A-------G--C-T-T-A---CTA--G--TC-G-A--------------------A-T---C-T-T--GA--CA-C-T-G--A-GG--C-A-CG-A--AA-G-C---G-TG--GG-G--
AG-C-A-AA--------CA--GG Bacteria(100);unclassified(100);unclassified(100);unclassified(100);unclassified(100);unclassified(100);unclassified(100);unclassified(100);unclassified(100);

Otu1130 0 0 0 0 0 0 0 0 0 0 0 0 0 0 0 0 0 0 0 0 0 0 0 0 0 0 0 0
0 0 0 0 0 0 0 0 0 0 0 0 0 0 0 0 0 0 0 0 0 0 0 0 0 0 0 0 0
0 0 0 0 0 0 0 0 0 0 0 0 0 0 0 0 0 0 0 0 0 0 0 0 0 0 0 0 0
0 0 0 0 0 45 0 0 0 0 0 0 0 0 0 0 0 0 0 0 0 0 0 0 0 0 0

M02255_77_000000000-ADUT3_1_1101_26799_17371 T----AC---GA-GG-GAT--------CCT-A-G------C--G--T---T--GT-T-CGG-AA------TT-A--T-T--GG-GC-------GT--A-----AA-GC-GT-GT-------G-TA-G-G-C-G------------G--C-TT-T-G-C------
-----AA----G-T-C-A--------G-T-T--G--TG--A-AA-GC--C-C-CG-G-G-----CT-C-AA------C-C-C-G-G-G-A--A-G----T-G--C-T---T--C-------T--GA-T-A---C-------T--G-C--TA--A-G-C----T-T-G-A-G-T--A---C-TG-----GA-G-A--------G-G-A-T---AG-T------GG--A--ATT----C-
C-A-G-GT--GT-A-G-TG-GT--G---A--A-A---TA-C-GT-AG--AT-A-TC--T-G--G-A-G-G-A-AC-A-CC---GG--T--G--GC-GAA-G--G-C--G-------G--C-T-A-T---CTG--G--CC-A-T--------------------G-T---A-C-T--GA--CG-C-T-G--A-GA--C-A-CG-A--AA-G-C---G-TG--GG-G-
-AG-C-A-AA--------CA--GG Bacteria(100);Proteobacteria(100);unclassified(100);unclassified(100);unclassified(100);unclassified(100);unclassified(100);unclassified(100);unclassified(100);

Otu1131 0 0 0 0 0 0 0 0 0 0 0 0 0 0 0 0 0 0 0 0 0 0 0 0 0 0 0 0
0 0 0 0 0 0 0 0 0 0 0 0 0 0 0 0 0 0 0 0 0 0 0 0 0 0 0 0 0
0 0 0 0 0 0 0 0 0 0 0 0 0 0 0 0 0 0 0 0 0 0 0 0 0 0 0 0 0
0 0 0 0 0 22 0 0 0 0 0 0 0 0 0 0 0 0 0 0 0 0 0 0 0 0 0

M02255_77_000000000-ADUT3_1_1104_15379_15781 T----AC---GT-AG-GGA--------GCG-A-G------C--G--T---T--AT-C-CGG-AT------TC-A--T-T--GG-GC-------GT--A-----AA-GC-GC-GC-------G-TA-G-G-C-G------------G--C-CC-G-T-C-----
------AA----G-C-G-G--------G-G-T--T--TC--A-AA-TC--C-A-GG-G-G-----CT-C-AA------C-C-T-C-T-G-G--C------C-G--G-A---C--C-------C--CG-A-A---C-------T--G-G--CG--G-G-C----T-C-G-A-G-T--G---C-GG-----TA-G-A--------G-G-A-A---GG-T------GG--A--ATT----
C-C-C-A-GT--GT-A-G-CG-GT--G---A--A-A---TG-C-GC-AG--AT-A-TT--G-G--G-A-A-G-A-AC-A-CC---GA--T--G--GC-GAA-G--G-C--A-------G--C-C-T-T---CTG--G--GC-C-G--------------------C-C---A-C-T--GA--CG-C-T-G--A-GG--C-G-CG-A--AA-G-C---T-AG--
GG-G--AG-C-G-AA--------CA--GG Bacteria(100);Actinobacteria(100);Actinobacteria(100);Coriobacteridae(100);Coriobacteriales(100);Coriobacterineae(100);Coriobacteriaceae(100);unclassified(100);unclassified(100);

Otu1132 0 0 0 0 0 0 0 0 0 0 0 0 0 0 0 0 0 0 0 0 0 0 0 0 0 0 0 0
0 0 0 0 0 0 0 0 0 0 0 0 0 0 0 0 0 0 0 0 0 0 0 0 0 0 0 0 0
0 0 0 0 0 0 0 0 0 0 0 0 0 0 0 0 0 0 0 0 0 0 0 0 0 0 0 0 0
0 0 0 0 0 20 0 0 0 0 0 0 0 0 0 0 0 0 0 0 0 0 0 0 0 0 0

M02255_77_000000000-ADUT3_1_1115_9114_17629 T----AC---GT-AG-GGT--------CCA-A-G------C--G--T---T--GT-C-CGG-AT------TT-A--T-T--GG-GC-------GT--A-----AA-GA-GC-TC-------G-TA-G-G-C-G------------G--C-TC-A-G-T-----------AA--
--G-T-C-G--------G-A-T--G--TG--A-AA-AC--T-C-CA-G-G-----CT-C-AA------C-C-T-G-G-A-G--A------C-G-CC-A---T--T-------C--GA-T-A---C-------T--G-C--TG-TG-G-C----T-T-G-A-G-T--C---T-GG-----TA-G-G--------G-G-A-G---CA-C------GG--A--ATT----C-C-T-G-
GT--GT-A-G-CG-GT--G---A--A-A---TG-C-GC-AG--AT-A-TC--A-G--G-A-G-G-A-AC-A-CC---GG--T--G--GC-GAA-G--G-C--G-------G--T-G-C-T---CTG--G--GC-C-A--------------------G-T---A-C-T--GA--CG-C-T-G--A-GG--A-G-CG-A--AA-G-C---G-TG--GG-G--AG-
C-A-AA--------CA--GG Bacteria(100);Actinobacteria(100);Actinobacteria(100);unclassified(100);unclassified(100);unclassified(100);unclassified(100);unclassified(100);unclassified(100);

Otu1133 0 0 0 0 0 0 0 0 0 0 0 0 0 0 0 0 0 0 0 0 0 0 0 0 0 0 0 0
0 0 0 0 0 0 0 0 0 0 0 0 0 0 0 0 0 0 0 0 0 0 0 0 0 0 0 0 0
0 0 0 0 0 0 0 0 0 0 0 0 0 0 0 0 0 0 0 0 0 0 0 0 0 0 0 0 0
0 0 0 0 0 5 0 0 0 15 0 0 0 0 0 0 0 0 0 0 0 0 0 0 0 0 0

M02255_77_000000000-ADUT3_1_2109_24060_18276 T----AC---GT-AG-GGG--------GCA-A-G------C--G--T---T--AT-C-CGG-AT------TT-A--C-T--GG-GT-------GT--A-----AA-GG-GA-GC-------G-TA-G-A-C-G------------G--C-AG-T-G-C----
-------AA----G-T-C-T--------G-A-A--G--TG--A-AA-TG--C-A-TG-G-G-----CT-C-AA------C-T-C-A-T-G-A--A-C----T-G--C-T---T--T-------G--GA-A-A---C-------T--G-T--AC--A-G-C----T-A-G-A-G-T--A---C-CG-----GA-G-G--------G-G-T-A---AG-C------GG--A--ATT----
C-C-T-G-GT--GT-A-G-CG-GT--G---A--A-A---TG-C-GT-AG--AT-A-TC--A-G--G-A-G-G-A-AC-A-CC---GG--T--G--GC-GAA-G--G-C--G-------G--C-T-T-A---CTG--G--AC-G-G--------------------A-A---A-C-T--GA--CG-T-T-A--A-GG--C-T-CG-A--AA-G-C---G-TG--
GG-G--AG-C-A-AA--------CA--GG Bacteria(100);Firmicutes(100);Clostridia(100);Clostridiales(100);Lachnospiraceae(100);unclassified(100);unclassified(100);unclassified(100);unclassified(100);

Otu1134 0 0 0 0 0 0 0 0 0 0 0 0 0 0 0 0 0 0 0 0 0 0 0 0 0 0 0 0
0 0 0 0 0 0 0 0 0 0 0 0 0 0 0 0 0 0 0 0 0 0 0 0 0 0 0 0 0
0 0 0 0 0 0 0 0 0 0 0 0 0 0 0 0 0 0 0 0 0 0 0 0 0 0 0 0 0
0 0 0 0 0 0 4 0 0 0 0 0 0 0 0 0 0 0 0 0 0 0 0 0 0 0 0

M02255_76_000000000-ADUNE_1_1105_12564_18328 C----GG---CT-AA-CTC--------CGT-G-C------C--A--G---C--AG-C-CGC-GG------TA-A--T-A--CG-GA-------GG--A-----TC-CG-AG-CG-------T-TA-T-C-C-G------------GA-C-CT-A-T-T------
-----AA----G-T-C-A--------G-G-G--G--TG--A-AA-GA--C-G-GC-A-G-----CT-C-AA------C-T-G-T-C-G-C--A-G----T-G--C-C---T--T-------T--GA-T-A---C-------T--G-A--TG--G-G-C----T-T-G-A-A-T--G---C-AG-----CT-G-A--------G-G-T-A---GG-C------GG--A--ATG----T-
G-A-C-AA--GT-A-G-CG-GT--G---A--A-A---TG-C-AT-AG--AT-A-TG--T-C--A-C-A-G-A-AC-A-CC---GA--T--T--GC-GAA-G--G-C--A-------G--C-T-T-A---CTA--A--AG-T-G--------------------C-G---A-T-T--GA--CG-C-T-G--A-GG--C-A-CG-A--AA-G-C---G-TG--GG-G-
-AT-C-A-AA--------CA--GG Bacteria(100);Bacteroidetes(100);Sphingobacteria(100);Sphingobacteriales(100);Sphingobacteriaceae(100);Sphingobacteriaceae(100);unclassified(100);unclassified(100);unclassified(100);

Otu1135 0 0 0 0 0 0 0 0 0 0 0 0 0 0 0 0 0 0 0 0 0 0 0 0 0 0 0 0
0 0 0 0 0 0 0 0 0 0 0 0 0 0 0 0 0 0 0 0 0 0 0 0 0 0 0 0 0
0 0 0 0 0 0 0 0 0 0 0 0 0 0 0 0 0 0 0 0 0 0 0 0 0 0 0 0 0
0 0 0 0 0 0 8 0 0 0 0 0 0 0 0 0 0 0 0 0 0 0 0 0 0 0 0
M02255_77_000000000-ADUT3_1_1102_6707_10670 T----AC---GA-GG-GGG--------GCA-A-G------C--G--T---T--GT-T-CGG-AA------TT-A--T-T--GG-GC-------GT--A-----AA-GG-GT-GC-------G-TA-G-G-C-G------------G--T-TT-G-A-T-----------
AA----G-T-C-A--------T-C-T--G--TG--A-AA-TC--T-C-TG-G-G-----CT-C-AA------C-C-C-A-G-A-G--T-C----T-G--C-A---G--G-------C--GA-A-A---C-------T--G-T--CG--G-G-C----T-T-G-A-G-T--A---T-GG-----GA-G-A--------G-G-T-G---AG-T------GG--A--ATT----T-C-C-
G-GT--GT-A-G-CG-GT--G---A--A-A---TG-C-GT-AG--AT-A-TC--G-G--A-A-G-G-A-AC-A-CC---TG--T--G--GC-GAA-A--G-C--G-------G--C-T-C-A---CTG--G--AC-C-A--------------------T-A---A-C-T--GA--CG-C-T-G--A-TG--C-A-CG-A--AA-G-C---T-AG--GG-G--
AG-C-A-AA--------CA--GG Bacteria(100);Acidobacteria(100);Acidobacteria(100);Acidobacteriales(100);Acidobacteriaceae(100);Edaphobacter(100);unclassified(100);unclassified(100);unclassified(100);

Otu1136 0 0 0 0 0 0 0 0 0 0 0 0 0 0 0 0 0 0 0 0 0 0 0 0 0 0 0 0
0 0 0 0 0 0 0 0 0 0 0 0 0 0 0 0 0 0 0 0 0 0 0 0 0 0 0 0 0
0 0 0 0 0 0 0 0 0 0 0 0 0 0 0 0 0 0 0 0 0 0 0 0 0 0 0 0 0
0 0 0 0 103 0 0 0 0 0 0 0 0 0 0 0 0 0 0 0 0 0 0 0 0 0 0
M02255_77_000000000-ADUT3_1_1107_16510_19043 T----AC---GT-AG-GAT--------GCA-A-G------C--G--T---T--GT-C-CGG-AT------TT-A--T-T--GG-GC-------GT--A-----AA-GC-GA-AC-------G-TA-G-G-C-G------------G--C-TT-C-T-T------
-----AA----G-T-T-T--------G-G-T--C--TT--A-AA-GA--C-C-GG-G-G-----CT-C-AA------C-C-C-C-G-G-G--A-A----T-G--G-A---C--T-------G--AA-T-A---C-------T--G-G--GA--G-G-C----T-A-G-A-G-G--A---T-GT-----CA-G-A--------G-G-A-A---AG-T------GG--A--ATT----C-
C-T-A-GT--GT-A-G-CG-GT--G---A--A-A---TG-C-GT-AG--AT-A-TT--A-G--G-A-G-G-A-AC-A-CC---AG--T--G--GC-GAA-A--G-C--G-------A--C-T-T-T---CTG--G--GA-C-A--------------------T-T---T-C-T--GA--CG-C-T-G--A-GG--T-T-CG-A--AA-G-C---T-CG--GG-G--
AG-C-A-AA--------CA--GG Bacteria(100);unclassified(100);unclassified(100);unclassified(100);unclassified(100);unclassified(100);unclassified(100);unclassified(100);unclassified(100);

Otu1137 0 0 0 0 0 0 0 0 0 0 0 0 0 0 0 0 0 0 0 0 0 0 0 0 0 0 0 0
0 0 0 0 0 0 0 0 0 0 0 0 0 0 0 0 0 0 0 0 0 0 0 0 0 0 0 0 0
0 0 0 0 0 0 0 0 0 0 0 0 0 0 0 0 0 0 0 0 0 0 0 0 0 0 0 0 0
0 0 0 0 0 0 103 0 0 0 0 0 0 0 0 0 0 0 0 0 0 0 0 0 0 0 0
M02255_77_000000000-ADUT3_1_1103_18170_10056 T----AC---GG-AG-GGA--------GCG-A-G------C--G--T---T--GT-T-CGG-AA------TT-A--C-T--GG-GC-------GT--A-----AA-GC-GT-AT-------G-TA-G-G-C-G------------G--T-TT-T-G-T-----
------AA----G-A-C-A--------G-A-T--G--TG--A-AA-TC--C-C-AG-A-G-----CT-T-AA------C-T-T-T-G-G-A--A-C----T-G--C-A---T--T-------T--GT-G-A---C-------T--G-C--AT--G-A-C----T-C-G-A-G-T--A---T-TG-----GA-G-A--------G-G-T-T---GG-C------GG--A--ATT----C-
C-T-A-GT--GT-A-G-CA-GT--G---A--A-A---TG-C-GC-AG--AT-A-TT--A-G--G-A-G-G-A-AC-A-CC---GA--T--G--GC-GTA-A--G-C--A-------G--C-C-A-A---CTG--G--AC-A-A--------------------T-T---A-C-T--GA--CG-C-T-G--A-GA--T-A-CG-A--AA-G-T---A-TG--GG-G-
-AG-C-A-AA--------CA--GG Bacteria(100);unclassified(100);unclassified(100);unclassified(100);unclassified(100);unclassified(100);unclassified(100);unclassified(100);unclassified(100);

Otu1138 0 0 0 0 0 0 0 0 0 0 0 0 0 0 0 0 0 0 0 0 0 0 0 0 0 0 0 0
0 0 0 0 0 0 0 0 0 0 0 0 0 0 0 0 0 0 0 0 0 0 0 0 0 0 0 0 0
0 0 0 0 0 0 0 0 0 0 0 0 0 0 0 0 0 0 0 0 0 0 0 0 0 0 0 0 0
0 0 0 0 0 0 4 0 0 0 0 0 0 0 0 0 0 0 0 0 0 0 0 0 0 0 0
M02255_77_000000000-ADUT3_1_1109_8906_23133 T----AC---GT-AG-GGG--------GCG-A-G------C--G--T---T--GT-C-CGG-AA------TG-A--T-T--GG-GC-------GT--A-----AA-GG-GC-GC-------G-TA-G-G-C-G------------G--C-TT-A-T-C-----------
AA----G-T-C-T--------G-G-A--G--TG--G-AA-GT--C-C-TA-T-T-----TT-C-AA------G-G-T-A-G-G-A--A-T----T-G--C-T---T--T-------G--GA-A-A---C-------T--G-G--TA--A-G-C----T-T-G-A-G-T--G---C-AG-----GA-G-A--------G-G-T-C---AT-C------GG--A--ATT----C-C-C-
G-GT--GT-A-G-CG-GT--G---A--A-A---TG-C-GT-AG--AG-A-TC--G-G--G-A-G-G-A-AC-A-CC---AG--T--G--GC-GAA-G--G-C--G-------G--G-T-G-A---CTG--G--AC-T-G--------------------C-A---A-C-T--GA--CG-C-T-G--A-GG--C-G-CG-A--AA-G-T---G-TG--GG-G--
AG-C-A-AA--------CA--GG Bacteria(100);Firmicutes(100);unclassified(100);unclassified(100);unclassified(100);unclassified(100);unclassified(100);unclassified(100);unclassified(100);

Otu1139 0 0 0 0 0 0 0 0 0 0 0 0 0 0 0 0 0 0 0 0 0 0 0 0 0 0 0 0
0 0 0 0 0 0 0 0 0 0 0 0 0 0 0 0 0 0 0 0 0 0 0 0 0 0 0 0 0
0 0 0 0 0 0 0 0 0 0 0 0 0 0 0 0 0 0 0 0 0 0 0 0 0 0 0 0 0
0 0 0 0 0 0 15 0 0 0 0 0 0 0 0 0 0 0 0 0 0 0 0 0 0 0 0
M02255_77_000000000-ADUT3_1_1110_24127_15812 T----AC---GG-AG-GGA--------GCT-A-G------C--G--T---T--GT-T-CGG-AA------TT-A--C-T--GG-GC-------GT--A-----AA-GC-GC-AC-------G-TA-G-G-C-G------------G--T-CA-G-C-C-----
------AA----G-T-C-A--------G-A-G--G--TG--A-AA-GC--C-C-GG-A-G-----CT-C-AA------C-T-T-C-G-G-A--A-C----T-G--C-C---T--T-------T--GA-A-A---C-------T--A-T--CT--G-A-C----T-A-G-A-A-T--C---T-TG-----GA-G-A--------G-G-C-G---GG-T------GG--A--ATT----C-
C-G-A-GT--GT-A-G-AG-GT--G---A--A-A---TT-C-GT-AG--AT-A-TT--C-G--G-A-A-G-A-AC-A-CC---AG--T--G--GC-GAA-G--G-C--G-------G--C-C-C-G---CTG--G--AC-A-A--------------------G-T---A-T-T--GA--CG-C-T-G--A-GG--T-G-CG-A--AA-G-C---G-TG--GG-
G--AG-C-A-AA--------CA--GG Bacteria(100);Proteobacteria(100);Alphaproteobacteria(100);Sphingomonadales(100);Sphingomonadaceae(100);Novosphingobium(100);unclassified(100);unclassified(100);unclassified(100);

Otu1140 0 0 0 0 0 0 0 0 0 0 0 0 0 0 0 0 0 0 0 0 0 0 0 0 0 0 0 0
0 0 0 0 0 0 0 0 0 0 0 0 0 0 0 0 0 0 0 0 0 0 0 0 0 0 0 0 0
0 0 0 0 0 0 0 0 0 0 0 0 0 0 0 0 0 0 0 0 0 0 0 0 0 0 0 0 0
0 0 0 0 0 0 8 0 0 0 0 0 0 0 0 0 0 0 0 0 0 0 0 0 0 0 0
M02255_77_000000000-ADUT3_1_1113_15469_8018 T----AC---GA-AG-GGG--------GCT-A-G------C--G--T---T--GC-T-CGG-AA------TG-A--C-T--GG-GC-------GT--A-----AA-GG-GC-GC-------G-TA-G-G-C-G------------G--C-AT-A-T-A----------
CA----G-T-C-A--------G-A-A--G--TG--A-AA-TT--C-C-TG-G-G-----CT-C-AA------C-C-T-G-G-G-G--A-C----T-G--C-T---T--T-------T--GA-T-A---C-------G--T-G--TA--A-G-C----T-A-G-A-G-T--G---G-AG-----AA-G-A--------G-G-G-T---TG-T------GG--A--ATT----T-C-C-
A-GT--GT-A-G-AG-GT--G---A--A-A---TT-C-GT-AG--AT-A-TT--G-G--A-A-A-G-A-AC-A-CC---GG--T--G--GC-GAA-G--G-C--G-------G--C-A-A-C---CTG--G--TC-T-T--------------------C-T---A-C-T--GA--CG-C-T-G--A-GG--C-G-CG-A--AA-G-C---G-TG--GG-G--
AG-C-A-AA--------CA--GG Bacteria(100);Proteobacteria(100);Alphaproteobacteria(100);Rhodospirillales(100);Acetobacteraceae(100);Acidocella(100);unclassified(100);unclassified(100);unclassified(100);

Otu1141 0 0 0 0 0 0 0 0 0 0 0 0 0 0 0 0 0 0 0 0 0 0 0 0 0 0 0 0
0 0 0 0 0 0 0 0 0 0 0 0 0 0 0 0 0 0 0 0 0 0 0 0 0 0 0 0 0
0 0 0 0 0 0 0 0 0 0 0 0 0 0 0 0 0 0 0 0 0 0 0 0 0 0 0 0 0
0 0 0 0 0 0 8 0 0 0 0 0 0 0 0 0 0 0 0 0 0 0 0 0 0 0 0
M02255_77_000000000-ADUT3_1_2103_24132_19995 T----AC---AG-AG-GGT--------GCA-A-G------C--G--T---T--GT-T-CGG-AA------TT-A--C-T--GG-GC----------------------G--GC---------------------------------T-TA-T---T----------AA----
G-T-C-A--------G-A-T--G--TG--A-AA-TC--C-C-TT-G-G-----CT-C-AA------C-T-G-A-G-G-A--A-C----T-G--C-A---T--C-------T--GA-T-A---C-------T--G-G--TA--A-G-C----T-T-G-A-G-T--G---T-CG-----GA-G-G--------G-G-A-T---GG-T------AG--A--ATT----C-C-T-G-GT--
GT-A-G-AG-GT--G---A--A-A---TT-C-GT-AG--AT-A-TC--A-G--G-A-G-G-A-AT-A-CC---TG--T--G--GC-GAA-G--G-C--G-------A--C-C-A-T---CTG--G--AA-G-A--------------------C-A---A-C-T--GA--CG-C-T-G--A-GA--C-T-CG-A--AA-G-C---G-TG--GG-T--AG-C-A-
AA--------CA--GG Bacteria(100);Proteobacteria(100);unclassified(100);unclassified(100);unclassified(100);unclassified(100);unclassified(100);unclassified(100);unclassified(100);

Otu1142 0 0 0 0 0 0 0 0 0 0 0 0 0 0 0 0 0 0 0 0 0 0 0 0 0 0 0 0
0 0 0 0 0 0 0 0 0 0 0 0 0 0 0 0 0 0 0 0 0 0 0 0 0 0 0 0 0
0 0 0 0 0 0 0 0 0 0 0 0 0 0 0 0 0 0 0 0 0 0 0 0 0 0 0 0 0
0 0 0 0 0 0 6 0 0 0 0 0 0 0 0 10 0 0 0 0 0 0 0 0 0 0 0
M02255_77_000000000-ADUT3_1_2108_23146_16905 T----AC---GA-AG-GGT--------GCT-A-G------C--G--T---T--GT-T-CGG-AA------TA-A--C-T--GG-GC-------GT--A-----AA-GG-GC-GT-------G-TA-G-G-C-G------------G--T-TT-T-A-T-----
------GC----G-T-C-A--------G-A-T--G--TG--A-AA-GT--C-C-TG-G-G-----CT-C-AA------C-C-T-G-G-G-G--A-C----G-G--C-A---T--T-------T--GA-T-A---C-------G--G-T--AA--G-A-C----T-A-G-A-G-G--T---T-AT-----GA-G-A--------G-G-A-A---GG-C------GG--A--ATT----
G-T-G-C-GT--GT-A-G-GA-GT--G---A--A-A---TC-C-GT-AG--AG-A-TG--C-A--C-A-G-G-A-AC-A-CC---AG--T--T--GC-GAA-G--G-C--G-------G--C-C-T-T---CTG--G--TA-T-T--------------------A-A---C-C-T--GA--CG-C-T-A--A-GG--C-G-CG-A--AA-G-C---G-TG--GG-
G--AG-C-A-AA--------CA--GG Bacteria(100);unclassified(100);unclassified(100);unclassified(100);unclassified(100);unclassified(100);unclassified(100);unclassified(100);unclassified(100);

Otu1143 0 0 0 0 0 0 0 0 0 0 0 0 0 0 0 0 0 0 0 0 0 0 0 0 0 0 0 0
0 0 0 0 0 0 0 0 0 0 0 0 0 0 0 0 0 0 0 0 0 0 0 0 0 0 0 0 0
0 0 0 0 0 0 0 0 0 0 0 0 0 0 0 0 0 0 0 0 0 0 0 0 0 0 0 0 0
0 0 0 0 0 0 6 0 0 0 0 0 0 0 0 0 0 0 0 0 0 0 0 0 0 0 0
M02255_77_000000000-ADUT3_1_2119_23290_19130 T----AC---AG-AG-GTC--------TCA-A-G------C--G--T---T--GT-T-CGG-AT------TC-A--T-T--GG-GC-------GT--A-----AA-GG-GT-GC-------G-TA-G-G-T-G------------G--C-TT-G-G-T------
-----AA----G-T-T-G--------A-A-G--G--TG--A-AA-TC--T-C-GG-G-G-----CT-T-AA------C-T-C-C-G-A-A--A-C----T-G--C-C---T--T-------C--AA-T-A---C-------T--G-C--TG--A-G-C----T-C-G-A-G-G--A---T-TG-----TA-G-A--------G-G-A-G---AA-T------GG--A--ATT----C-
A-C-G-GT--GT-A-G-CA-GT--G---A--A-A---TG-C-GT-AG--AT-A-TC--G-T--G-A-G-G-A-AG-A-CC---AG--T--G--GC-GAA-G--G-C--G-------A--T-T-C-T---CTG--G--GC-A-A--------------------T-T---C-C-T--GA--CA-C-T-G--A-GG--C-A-CG-A--AG-G-C---C-AG--GG-G-
-AG-C-A-AA--------CG--GG Bacteria(100);Verrucomicrobia(100);Spartobacteria(100);unclassified(100);unclassified(100);unclassified(100);unclassified(100);unclassified(100);unclassified(100);

Otu1144 0 0 0 0 0 0 0 0 0 0 0 0 0 0 0 0 0 0 0 0 0 0 0 0 0 0 0 0
0 0 0 0 0 0 0 0 0 0 0 0 0 0 0 0 0 0 0 0 0 0 0 0 0 0 0 0 0
0 0 0 0 0 0 0 0 0 0 0 0 0 0 0 0 0 0 0 0 0 0 0 0 0 0 0 0 0
10 0 0 0 0 0 0 0 0 0 0 0 0 0 0 0 0 0 0 0 0 0 0 0 0 0 0
M02255_76_000000000-ADUNE_1_1113_12144_2859 C----GG---CT-AA-ATA--------CGT-G-C------C--A--G---C--AG-C-CGC-GG------TA-A--T-A--CG-G--------AT--G-----TC-CC-GA-GC-------GTTA-T-C-C-G------------G--T-TG-G-T-T----------AA--
--G-T-C-T--------G-A-T--G--TG--A-AA-GC--C-C-GG-A-G-----CT-C-AA------C-T-C-C-G-G-A--A-A----G-G--C-A---T--T-------G--GA-A-A---C-------T--G-G--TT--A-A-C----T-T-G-A-G-T--G---C-AG-----TA-G-A--------G-G-T-A---AG-T------GG--A--ACT----C-C-A-T-
GT--GT-A-G-CG-GT--G---G--A-A---TG-C-GT-AG--AT-A-TA--T-G--G-A-A-G-A-AC-A-CC---AG--C--G--GC-GAA-G--G-C--G-------G--C-T-T-A---CTG--G--AC-T-G--------------------T-A---A-C-T--GA--CG-T-T-G--A-GG--C-T-CG-A--AA-G-T---G-TG--GG-T--AG-C-
A-AA--------CA--GG Bacteria(100);Firmicutes(100);Bacilli(100);Lactobacillales(100);Leuconostocaceae(100);Leuconostoc(100);unclassified(100);unclassified(100);unclassified(100);

Otu1145 0 0 0 0 0 0 0 0 0 0 0 0 0 0 0 0 0 0 0 0 0 0 0 0 0 0 0 0
0 0 0 0 0 0 0 0 0 0 0 0 0 0 0 0 0 0 0 0 0 0 0 0 0 0 0 0 0
0 0 0 0 0 0 0 0 0 0 0 0 0 0 0 0 0 0 0 0 0 0 0 0 0 0 0 0 0
0 0 0 0 26 0 0 0 0 0 0 0 0 0 0 0 0 0 0 0 0 0 0 0 0 0 0
M02255_77_000000000-ADUT3_1_1106_23820_5584 T----AC---GG-AG-GAT--------CCA-A-G------C--G--T---T--AT-C-CGG-AT------TT-A-------------------TT--------------G--G--------G-TA-G-G-C-G-------------G--C-AT-G-C-T-----------AA----G-T-C-
A--------G-G-G--G--TG--A-AA-TA--C-G-GT-G-G-----CT-C-AA------C-C-A-T-C-G-C--A-G----T-G--C-C---T--T-------T--GA-T-A---C-------T--G-G--CG--T-G-C----T-T-G-A-A-T--G---T-AC-----AT-G-A--------G-G-T-A---GG-C------GG--A--ATG----A-G-A-C-AA--GT-A-
G-CG-GT--G---A--A-A---TG-C-AT-AG--AT-A-TG--T-C--T-C-A-G-A-AC-T-CC---GA--T--T--GC-GAA-G--G-C--A-------G--C-T-T-A---CTA--A--GG-T-A--------------------T-G---A-T-T--GA--CG-C-T-G--A-GG--C-A-CG-A--AA-G-C---G-TG--GG-G--AT-C-A-AA--------
CA--GG Bacteria(100);Bacteroidetes(100);Sphingobacteria(100);Sphingobacteriales(100);Sphingobacteriaceae(100);Sphingobacteriaceae(100);unclassified(100);unclassified(100);unclassified(100);

Otu1146 0 0 0 0 0 0 0 0 0 0 0 0 0 0 0 0 0 0 0 0 0 0 0 0 0 0 0 0
0 0 0 0 0 0 0 0 0 0 0 0 0 0 0 0 0 0 0 0 0 0 0 0 0 0 0 0 0
0 0 0 0 0 0 0 0 0 0 0 0 0 0 0 0 0 0 0 0 0 0 0 0 0 0 0 0 0
0 0 0 0 76 0 0 0 0 0 0 0 0 0 0 0 0 0 0 0 0 0 0 0 0 0 0
M02255_77_000000000-ADUT3_1_1102_12657_16690 T----AC---GT-AG-GGA--------GCG-A-G------C--G--T---T--AT-C-CGG-AA------TT-A--T-T--GG-GT-------GT--A-----AA-GG-GT-GC-------G-TA-G-A-C-G------------G--G-AA-G-A-T----
-------AA----G-T-T-A--------G-T-T--G--TG--A-AA-TC--C-C-TC-G-G-----CT-T-AA------C-T-G-A-G-G-A--A-C----T-G--C-A---A--C-------T--AA-A-A---C-------T--G-T--TT--T-T-C----T-T-G-A-G-T--G---C-AG-----GA-G-A--------G-G-T-A---AG-T------GG--A--ATT----C-
C-T-A-GT--GT-A-G-CG-GT--G---A--A-A---TG-C-GT-AG--AT-A-TT--A-G--G-A-G-G-A-AC-A-CC---AG--T--G--GC-GAA-G--G-C--G-------A--C-T-T-A---CTG--G--AC-T-G--------------------T-A---A-C-T--GA--CG-T-T-G--A-GG--C-A-CG-A--AA-G-T---G-TG--GG-G-
-AG-C-A-AA--------CA--GG Bacteria(100);Firmicutes(100);Clostridia(100);Clostridiales(100);Ruminococcaceae(100);uncultured(100);unclassified(100);unclassified(100);unclassified(100);

Otu1147 0 0 0 0 0 0 0 0 0 0 0 0 0 0 0 0 0 0 0 0 0 0 0 0 0 0 0 0
0 0 0 0 0 0 0 0 0 0 0 0 0 0 0 0 0 0 0 0 0 0 0 0 0 0 0 0 0
0 0 0 0 0 0 0 0 0 0 0 0 0 0 0 0 0 0 0 0 0 0 0 0 0 0 0 0 0
0 0 0 0 43 0 0 0 0 0 0 0 0 0 0 0 0 0 0 0 0 0 0 0 0 0 0
M02255_77_000000000-ADUT3_1_1102_18303_12106 T----AC---GA-AG-GGG--------GCT-A-G------C--G--T---T--GC-T-CGG-AA------TT-A--C-T--GG-GC-------GT--A-----AA-GG-GC-GC-------G-TA-G-G-C-G------------G--C-GC-T-C-C----
-------AA----G-T-T-A--------G-G-C--G--TG--A-AA-TT--C-C-CG-G-G-----CT-C-AA------C-C-T-G-G-G-G--A-C----T-G--C-G---C--T-------T--AA-G-A---C-------T--G-G--AG--T-G-C----T-T-G-A-G-G--A---T-GG-----AA-G-A--------G-G-G-T---TG-T------GG--A--ATT----
C-C-C-A-GT--GT-A-G-AG-GT--G---A--A-A---TT-C-GT-AG--AT-A-TT--G-G--G-A-A-G-A-AC-A-CC---GG--T--G--GC-GAA-G--G-C--G-------G--C-A-A-C---CTG--G--TC-C-A--------------------T-T---A-C-T--GA--CG-C-T-G--A-GG--C-G-CG-A--TA-G-C---G-TG--GG-
G--AG-C-A-AA--------CA--GG Bacteria(100);Proteobacteria(100);Alphaproteobacteria(100);Rhodospirillales(100);Acetobacteraceae(100);unclassified(100);unclassified(100);unclassified(100);unclassified(100);

Otu1148 0 0 0 0 0 0 0 0 0 0 0 0 0 0 0 0 0 0 0 0 0 0 0 0 0 0 0 0
0 0 0 0 0 0 0 0 0 0 0 0 0 0 0 0 0 0 0 0 0 0 0 0 0 0 0 0 0
0 0 0 0 0 0 0 0 0 0 0 0 0 0 0 0 0 0 0 0 0 0 0 0 0 0 0 0 0
0 0 0 3 12 0 0 0 0 0 0 0 0 0 0 0 0 0 0 0 0 0 0 0 0 0 0
M02255_77_000000000-ADUT3_1_1114_12003_8461 T----AC---GG-AG-GAT--------GCA-A-G------C--G--T---T--AT-C-CGG-AA------TG-A--T-T--GG-GC-------GT--A-----AA-GC-GT-TT-------T-AA-G-G-T-G------------G--T-TT-G-G-T-----------AA-
---G-T-C-T--------A-C-T--G--TT--A-AA-TA--T-C-AG-G-G-----CT-T-AA------C-C-C-T-G-A-A--C-A----G-G--C-A---G--T-------G--GA-A-A---C-------T--A-G--CA--A-G-C----T-T-G-A-G-T--A---C-GG-----TA-G-G--------G-G-C-A---AA-G------GG--A--ATT----C-C-C-G-
GT--GT-A-G-CG-GT--G---A--A-A---TG-C-GT-AG--AT-A-TC--G-G--G-A-A-G-A-AC-A-CC---AA--A--G--GC-GAA-A--G-C--A-------C--T-T-T-G---CTG--G--GC-C-G--------------------A-A---A-C-T--GA--CA-C-T-G--A-TA--G-A-CG-A--AA-G-C---T-AG--GG-G--AG-
C-A-AA--------TG--GG Bacteria(100);Cyanobacteria(100);unclassified(100);unclassified(100);unclassified(100);unclassified(100);unclassified(100);unclassified(100);unclassified(100);

Otu1149 0 0 0 0 0 0 0 0 0 0 0 0 0 0 0 0 0 0 0 0 0 0 0 0 0 0 0 0
0 0 0 0 0 0 0 0 0 0 0 0 0 0 0 0 0 0 0 0 0 0 0 0 0 0 0 0 0
0 0 0 0 0 0 0 0 0 0 0 0 0 0 0 0 0 0 0 0 0 0 0 0 0 0 0 0 0
0 0 0 20 0 0 0 0 0 0 0 0 0 0 0 0 0 0 0 0 0 0 0 0 0 0 0
M02255_77_000000000-ADUT3_1_1103_5112_5410 T----AC---GT-AG-GAC--------CCA-A-G------C--G--T---T--AT-C-CGG-AG------TG-A--C-T--GG-GC-------GT--A-----AA-GA-GTTGC-------G-TA-G-G-C-G------------G--A-CA-A-G-T-----------
AA----G-C-G-A--------A-T-A--G--TG--A-AA-TC--T-G-GT-G-G-----CT-C-AA------C-C-A-T--CA-A--C-A----C-A--T-T---G--T-------T--CG-A-A---C-------T--G-C--TC--G-T-C----T-C-G-A-G-A--A---T-GG-----TA-G-A--------G-G-T-A---CT-T------GG--A--ATT----T-C-T-T-
GT--GT-A-G-GA-GT--G---A--A-A---TC-C-GT-AG--AT-A-TA--A-G--A-A-G-G-A-AC-A-CC---AA--T--G--GC-GTA-G--G-C--A-------G--A-G-T-A---CTG--G--AC-C-A--------------------T-T---T-C-T--GA--CG-C-T-G--A-GG--C-A-CG-A--AA-G-C---G-TG--GG-G--AG-C-
G-AA--------CC--GG Bacteria(100);Candidate_division_TM7(100);unclassified(100);unclassified(100);unclassified(100);unclassified(100);unclassified(100);unclassified(100);unclassified(100);

Otu1150 0 0 0 0 0 0 0 0 0 0 0 0 0 0 0 0 0 0 0 0 0 0 0 0 0 0 0 0
0 0 0 0 0 0 0 0 0 0 0 0 0 0 0 0 0 0 0 0 0 0 0 0 0 0 0 0 0
0 0 0 0 0 0 0 0 0 0 0 0 0 0 0 0 0 0 0 0 0 0 0 0 0 0 0 0 0
0 0 0 17 0 0 0 0 0 0 0 0 0 0 0 0 0 0 0 0 0 0 0 0 0 0 0
M02255_77_000000000-ADUT3_1_2101_12668_14938 G----AC---AG-AG-GTG--------CCA-A-G------C--G--T---T--AG-G-CGG-AA------TC-A--C-T--GG-GC-------TT--A-----AA-GC-GT-GT-------G-TA-G-G-C-G-------------G--A-CG-G-G-T-----------AA----G-T-A-C--------C-T-T--G--TG--A-AA-TC--C-C-AC-G-G-----CT-C-AA------C-C-G-T-G-G-A--A-C----T-G--C-T---C--G-------G--TA-T-A---C-------T--G-C--CC--G-T-C----T-T-G-A-G-C--C---A-CT-----CA-G-G--------G-G-T-C---GT-C------GG--A--ACA----A-G-C-G-GT--GG-A-G-CG-GT--G---A--A-A---TG-C-GT-AG--AT-A-TC--G-C--T-T-G-G-A-AC-G-CC---AA--A--G--GT-GAA-A--A-C--A-------G--A-C-G-A---CTG--G--GG-G-T--------------------G-T---G-C-T--GA--CG-C-T-G--A-GA--C-A-CG-A--AA-G-C---C-AG--GG-G--AG-C-G-AA--------CG--GG Bacteria(100);Planctomycetes(100);Phycisphaerae(100);WD2101(100);unclassified(100);unclassified(100);unclassified(100);unclassified(100);unclassified(100);

Otu1151 0 0 0 0 0 0 0 0 0 0 0 0 0 0 0 0 0 0 0 0 0 0 0 0 0 0 0 0
0 0 0 0 0 0 0 0 0 0 0 0 0 0 0 0 0 0 0 0 0 0 0 0 0 0 0 0 0
0 0 0 0 0 0 0 0 0 0 0 0 0 0 0 0 0 0 0 0 0 0 0 0 0 0 0 0 0
0 0 0 15 0 0 0 0 0 0 0 0 0 0 0 0 0 0 0 0 0 0 0 0 0 0 0
M02255_77_000000000-ADUT3_1_1113_13949_15325 T----AC---GA-AG-GGG--------GCT-A-G------C--G--T---T--GC-T-CGG-AA------TG-A--C-T--GG-GC-------GT--A-----AA-GG-GC-GC-------G-TA-G-G-C-T-------------G--T-TT-G-T-A-----------CA----G-T-C-G--------G-G-T--G--TG--A-AA-TT--C-C-TG-G-G-----CT-T-AA------C-C-T-G-G-G-G--G-C----T-G--C-A---C--C-------C--GA-T-A---C-------G--T-G--CA--G-G-C----T-T-G-A-G-T--G---C-GG-----AA-G-A--------G-G-G-T---CG-T------GG--A--ATT----C-C-C-A-GT--GT-A-G-AG-GT--G---A--A-A---TT-C-GT-AG--AT-A-TT--G-G--G-A-A-G-A-AC-A-CC---GG--T--G--GC-GAA-G--G-C--G-------G--C-G-A-C---CTG--G--TC-C-G--------------------T-G---A-C-T--GA--CG-C-T-G--A-GG--C-G-CG-A--AA-G-C---G-TG--GG-G--AG-C-A-AA--------CA--GG Bacteria(100);Proteobacteria(100);Alphaproteobacteria(100);Rhodospirillales(100);Acetobacteraceae(100);unclassified(100);unclassified(100);unclassified(100);unclassified(100);

Otu1152 0 0 0 0 0 0 0 0 0 0 0 0 0 0 0 0 0 0 0 0 0 0 0 0 0 0 0 0
0 0 0 0 0 0 0 0 0 0 0 0 0 0 0 0 0 0 0 0 0 0 0 0 0 0 0 0 0
0 0 0 0 0 0 0 0 0 0 0 0 0 0 0 0 0 0 0 0 0 0 0 0 0 0 0 0 0
0 0 0 24 0 0 0 0 0 0 0 0 0 0 0 0 0 0 0 0 0 0 0 0 0 0 0
M02255_76_000000000-ADUNE_1_2115_13229_21279 C----CA---AC-TA-CGT--------GCC-A-G------C--A--G---C--CG-C-GGT-AA------TA-C--G-T--AG-G--------GT--C-----CG-AG-CG-TT-------G-TC-C-G---G-------------A--A-TT-G-T-T-----------GC----G-T-C-G--------G-G-A--G--TG--A-AA-AC--C-C-AG-G-G-----CT-T-AA------C-T-C-T-G-G-G--C-T----G-G--C-T---T--T-------C--GA-T-A---C-------G--G-G--CA--G-A-C----T-A-G-A-G-G--C---A-TT-----CA-G-G--------G-G-A-G---AA-C------GG--A--ATT----C-C-T-G-GT--GT-A-G-CG-GT--G---A--A-A---TG-C-GC-AG--AT-A-TC--A-G--G-A-G-G-A-AC-A-CC---GG--T--G--GC-GAA-G--G-C--G-------G--T-T-C-T---CTG--G--GA-A-T--------------------G-T---T-C-T--GA--CG-C-T-G--A-GG--A-G-CG-A--AA-G-T---G-TG--GG-G--AG-C-G-AA--------CA--GG Bacteria(100);Actinobacteria(100);Actinobacteria(100);Actinobacteridae(100);Actinomycetales(100);Propionibacterineae(100);Nocardioidaceae(100);unclassified(100);unclassified(100);

Otu1153 0 0 0 0 0 0 0 0 0 0 0 0 0 0 0 0 0 0 0 0 0 0 0 0 0 0 0 0
0 0 0 0 0 0 0 0 0 0 0 0 0 0 0 0 0 0 0 0 0 0 0 0 0 0 0 0 0
0 0 0 0 0 0 0 0 0 0 0 0 0 0 0 0 0 0 0 0 0 0 0 0 0 0 0 0 0
0 0 0 61 0 0 0 0 0 0 0 0 0 0 0 0 0 0 0 0 0 0 0 0 0 0 0
M02255_77_000000000-ADUT3_1_2117_29612_14400 T----AC---GT-AG-GGT--------CCG-A-G------C--G--T---T--GT-C-CGG-AA------TT-A--T-T--GG-GC-------GT--A-----AA-GG-GC-TC-------G-TA-G-G-C-G-------------G--T-TT-G-T-T-----------GC----G-T-C-G--------G-G-A--G--TG--A-AA-TC--C-C-CC-A-G-----CT-T-AA------C-T-G-G-G-G-G--T-C----T-G--C-T---T--T-------C--GA-T-A---C-------G--G-G--CA--G-A-C----T-A-G-A-G-G--C---A-TT-----CA-G-G--------G-G-A-G---AA-C------GG--A--ATT----C-C-T-G-GT--GT-A-G-CG-GT--G---A--A-A---TG-C-GC-AG--AT-A-TC--A-G--G-A-G-G-A-AC-A-CC---GG--T--G--GC-GAA-G--G-C--G-------G--T-T-C-T---CTG--G--GA-A-T--------------------G-T---T-C-T--GA--CG-C-T-G--A-GG--A-G-CG-A--AA-G-T---G-TG--GG-G--AG-C-G-AA--------CA--GG Bacteria(100);Actinobacteria(100);Actinobacteria(100);Actinobacteridae(100);Actinomycetales(100);Propionibacterineae(100);Nocardioidaceae(100);unclassified(100);unclassified(100);

Otu1154 0 0 0 0 0 0 0 0 0 0 0 0 0 0 0 0 0 0 0 0 0 0 0 0 0 0 0 0
0 0 0 0 0 0 0 0 0 0 0 0 0 0 0 0 0 0 0 0 0 0 0 0 0 0 0 0 0
0 0 0 0 0 0 0 0 0 0 0 0 0 0 0 0 0 0 0 0 0 0 0 0 0 0 0 0 0
0 0 0 57 0 0 0 0 0 0 0 0 0 0 0 0 0 0 0 0 0 0 0 0 0 0 0
M02255_77_000000000-ADUT3_1_2112_2773_7920 T----AC---AG-AC-GGT--------GCG-A-G------C--G--T---T--AT-T-CAG-CA------TA-A--A-T--GG-GT-------GT--A-----AA-GG-GT-AT-------G-TA-G-G-T-G-------------G--T-TT-A-GTTT----------TA----A-G-C-T--------A-A-A--T--AG--A-AA-TG--C-T-AG-G-C-----GT-A-AA------T-C-T-T-A-G-C--A-G----A-T--A-T---T--T-------T--AT-T-A---C-------T--T-A--CTA-G-A-C----T-T-G-A-G-T--A---T-AA-----TT-G-G--------T-G-T-A---AA-A------GG--A--ATT----T-C-T-A-CA-GTT-A-G-TG-GT--T---A--A-A---TA-C-GA-GG--AG-A-GT--A-G--A-A-G-G-A-AC-G-CC---AA--A--G--GT-GAA-G--A-C--A-------T--T-T-T-T---CAA--A--AT---T--------------------A-T---A-C-T--GA--CA-C-T-G--A-GG--T-A-CG-G--AG-G-T---T-TG--GG-G--AG-C-A-AA--------TC--GG Bacteria(100);unclassified(100);unclassified(100);unclassified(100);unclassified(100);unclassified(100);unclassified(100);unclassified(100);unclassified(100);

Otu1155 0 0 0 0 0 0 0 0 0 0 0 0 0 0 0 0 0 0 0 0 0 0 0 0 0 0 0 0
0 0 0 0 0 0 0 0 0 0 0 0 0 0 0 0 0 0 0 0 0 0 0 0 0 0 0 0 0
0 0 0 0 0 0 0 0 0 0 0 0 0 0 0 0 0 0 0 0 0 0 0 0 0 0 0 0 0
0 0 0 27 0 0 0 0 0 0 0 0 0 0 0 0 0 0 0 0 0 0 0 0 0 0 0
M02255_77_000000000-ADUT3_1_2109_23226_11179 T----AC---GA-AG-GGG--------GCT-A-G------C--G--T---T--GC-T-CGG-AA------TG-A--C-T--GG-GC-------GT--A-----AA-GG-GC-GC-------G-TA-G-G-C-G------------G--T-TT-G-T-A----
-------CA----G-T-C-G--------G-A-T--G--TG--A-AA-TT--C-C-TG-G-G-----CT-T-AA------C-C-T-G-G-G-G--G-C----T-G--C-A---T--T-------C--GA-T-A---C-------G--T-G--CA--G-G-C----T-T-G-A-G-T--G---T-GG-----AA-G-A--------G-G-G-T---TG-T------GG--A--ATT----
C-C-C-A-GT--GT-A-G-AG-GT--G---A--A-A---TT-C-GT-AG--AT-A-TT--G-G--G-A-A-G-A-AC-A-CC---GG--T--G--GC-GAA-G--G-C--G-------G--C-A-A-C---CTG--G--TC-C-A--------------------T-G---A-C-T--GA--CG-C-T-G--A-TG--C-G-CG-A--AA-G-C---G-TG--GG-
G--AG-C-A-AA--------CA--GG Bacteria(100);Proteobacteria(100);Alphaproteobacteria(100);Rhodospirillales(100);Acetobacteraceae(100);unclassified(100);unclassified(100);unclassified(100);unclassified(100);

Otu1156 0 0 0 0 0 0 0 0 0 0 0 0 0 0 0 0 0 0 0 0 0 0 0 0 0 0 0 0
0 0 0 0 0 0 0 0 0 0 0 0 0 0 0 0 0 0 0 0 0 0 0 0 0 0 0 0 0
0 0 0 0 0 0 0 0 0 0 0 0 0 0 0 0 0 0 0 0 0 0 0 0 0 0 0 0 0
0 0 0 21 0 0 0 0 0 0 0 0 0 0 0 0 0 0 0 0 0 0 0 0 0 0 0
M02255_77_000000000-ADUT3_1_2106_18147_12497 T----AC---GT-AG-GGG--------CCG-A-G------C--G--T---T--GT-C-CGG-AA------TT-A--C-T--GG-GC-------GT--A-----AA-GC-GC-GC-------G-CA-G-G-C-G------------G--C-TG-A-T-C-----
------AA----G-C-C-T--------G-A-C--G--TG--A-AA-GC--C-C-CC-G-G-----CT-C-AA------C-C-G-G-G-G-A--G-G----G-T--C-G---T--C-------G--GG-G-A---C-------T--G-A--TT--G-G-C----T-T-G-A-G-G--G---C-GG-----TA-G-G--------G-G-C-C---GG-T------GG--A--ATT----
C-C-C-G-GT--GT-A-G-TG-GT--G---A--A-A---TG-C-GT-AG--AG-A-TC--G-G--G-A-G-G-A-AC-A-CC---AG--T--G--GC-GAA-G--G-C--G-------G--C-C-G-G---CTG--G--GC-C-G--------------------A-C---C-C-T--GA--CG-C-T-G--A-GG--C-G-CG-A--AG-G-C---G-TG--
GG-G--AG-C-G-AA--------CG--GG Bacteria(100);unclassified(100);unclassified(100);unclassified(100);unclassified(100);unclassified(100);unclassified(100);unclassified(100);unclassified(100);

Otu1157 0 0 0 0 0 0 0 0 0 0 0 0 0 0 0 0 0 0 0 0 0 0 0 0 0 0 0 0
0 0 0 0 0 0 0 0 0 0 0 0 0 0 0 0 0 0 0 0 0 0 0 0 0 0 0 0 0
0 0 0 0 0 0 0 0 0 0 0 0 0 0 0 0 0 0 0 0 0 0 0 0 0 0 0 0 0
0 0 0 20 0 0 0 0 0 0 0 0 0 0 0 0 0 0 0 0 0 0 0 0 0 0 0
M02255_77_000000000-ADUT3_1_2102_6079_19923 T----AC---GT-AT-GTC--------GCG-A-G------C--G--T---T--AT-C-CGG-AA------TT-A--T-T--GG-GC-------AT--A-----AA-GG-GC-AT-------C-TA-G-G-C-G------------G--C-CA-G-A-T-----------AA--
--G-T-C-C--------G-A-G--G--TG--A-AA-AC--T-G-CC-G-G-----CT-C-AA------C-C-A-G-C-A-G--C-T----T-G--C-C---T--T-------G--GA-A-A---C-------T--A-C--CT--G-G-C----T-A-G-A-G-T--A---T-TG-----GA-G-A--------G-G-T-G---GA-C------GG--A--ACT----A-C-A-C-
GA--GT-A-G-AG-GT--G---A--A-A---TT-C-GT-AG--AT-A-TG--T-G--T-A-G-G-A-AT-G-CC---GA--T--G--AT-GAA-G--A-T--A-------G--T-T-C-A---CTG--G--AT-G-A--------------------T-A---A-C-T--GA--CG-C-T-G--A-AG--T-G-CG-A--AA-G-C---T-AG--GG-G--AG-C-
G-AA--------CA--GG Bacteria(100);Fusobacteria(100);Fusobacteria(100);Fusobacteriales(100);Leptotrichiaceae(100);Leptotrichia(100);unclassified(100);unclassified(100);unclassified(100);

Otu1158 0 0 0 0 0 0 0 0 0 0 0 0 0 0 0 0 0 0 0 0 0 0 0 0 0 0 0 0
0 0 0 0 0 0 0 0 0 0 0 0 0 0 0 0 0 0 0 0 0 0 0 0 0 0 0 0 0
0 0 0 0 0 0 0 0 0 0 0 0 0 0 0 0 0 0 0 0 0 0 0 0 0 0 0 0 0
0 0 0 43 0 0 0 0 0 0 0 0 0 0 0 0 0 0 0 0 0 0 0 0 0 0 0
M02255_77_000000000-ADUT3_1_2102_19156_18206 T----AC---GA-AG-GGG--------GCT-A-G------C--G--T---T--GC-T-CGG-AA------TG-A--C-T--GG-GC-------GT--A-----AA-GG-GC-GC-------G-TA-G-G-C-G------------G--C-GC-G-T-A---
--------CA----G-T-C-A--------G-A-A--G--TG--A-AA-TT--C-C-TG-G-G-----CT-C-AA------C-C-T-G-G-G-G--A-C----T-G--C-T---T--T-------T--GA-T-A---C-------G--T-G--CG--T-G-C----T-T-G-A-G-T--G---A-GG-----AA-G-A--------G-G-G-T---TG-T------GG--A--ATT----
T-C-C-A-GT--GT-A-G-AG-GT--G---A--A-A---TT-C-GT-AG--AT-A-TT--G-G--A-A-A-G-A-AC-A-CC---GG--T--G--GC-GAA-G--G-C--G-------G--C-A-A-C---CTG--G--TC-C-T--------------------T-T---A-C-T--GA--CG-C-T-G--A-GG--C-G-CG-A--AA-G-C---G-TG--GG-
G--AG-C-A-AA--------CA--GG Bacteria(100);Proteobacteria(100);Alphaproteobacteria(100);Rhodospirillales(100);Acetobacteraceae(100);Acidocella(100);unclassified(100);unclassified(100);unclassified(100);

Otu1159 0 0 0 0 0 0 0 0 0 0 0 0 0 0 0 0 0 0 0 0 0 0 0 0 0 0 0 0
0 0 0 0 0 0 0 0 0 0 0 0 0 0 0 0 0 0 0 0 0 0 0 0 0 0 0 0 0
0 0 0 0 0 0 0 0 0 0 0 0 0 0 0 0 0 0 0 0 0 0 0 0 0 0 0 0 0
0 0 0 20 0 0 0 0 0 0 0 0 0 0 0 0 0 0 0 0 0 0 0 0 0 0 0
M02255_77_000000000-ADUT3_1_2102_23380_13288 T----AC---GG-AG-GGG--------GCT-A-G------C--G--T---T--GT-T-CGG-AA------TT-A--C-T--GG-GC-------GT--A-----AA-GC-GC-AC-------G-TA-G-G-C-G------------G--C-TT-C-C-T-----
------AA----G-T-C-A--------G-A-G--G--TG--A-AA-GC--C-C-GG-A-G-----CT-C-AA------C-T-C-C-G-G-A--A-T----A-G--C-C---T--T-------T--GA-G-A---C-------T--G-G--GT--C-G-C----T-T-G-A-A-T--C---C-AG-----GA-G-A--------G-G-T-G---AG-T------GG--A--ATT----
C-C-G-A-GT--GT-A-G-AG-GT--G---A--A-A---TT-C-GT-AG--AT-A-TT--C-G--G-A-A-G-A-AC-A-CC---AG--T--G--GC-GAA-G--G-C--G-------G--C-T-C-A---CTG--G--AC-T-G--------------------G-T---A-T-T--GA--CG-C-T-G--A-GG--T-G-CG-A--AA-G-C---G-TG--GG-
G--AG-C-A-AA--------CA--GG Bacteria(100);Proteobacteria(100);Alphaproteobacteria(100);Sphingomonadales(100);Sphingomonadaceae(100);unclassified(100);unclassified(100);unclassified(100);unclassified(100);

Otu1160 0 0 0 0 0 0 0 0 0 0 0 0 0 0 0 0 0 0 0 0 0 0 0 0 0 0 0 0
0 0 0 0 0 0 0 0 0 0 0 0 0 0 0 0 0 0 0 0 0 0 0 0 0 0 0 0 0
0 0 0 0 0 0 0 0 0 0 0 0 0 0 0 0 0 0 0 0 0 0 0 0 11 0 0 0 0
0 0 0 0 0 0 0 0 0 0 0 0 0 0 0 0 0 0 0 0 0 0 0 0 0 0 0

M02255_77_000000000-ADUT3_1_1115_21119_17035 T----AC---GA-AG-GGG--------GCT-A-G------C--G--T---T--GC-T-CGG-AA------TG-A--C-T--GG-GC-------GT--A-----AA-GG-GC-GC-------G-TA-G-G-C-G-------------G--C-TC-A-T-T----
-------TT----G-T-C-A--------G-G-C--G--TG--A-AA-GT--C-C-TG-G-G-----CT-C-AA------C-C-T-G-G-G-G--A-C----G-G--C-G---C--T-------T--GG-G-A---C-------G--G-G--TG--G-G-C----T-T-G-A-G-T--G---G-GG-----AA-G-A--------G-G-G-T---CG-T------GG--A--ATT---
-C-C-C-A-GT--GT-A-G-AG-GT--G---A--A-A---TT-C-GT-AG--AT-A-TT--G-G--G-A-A-G-A-AC-A-CC---GG--T--G--GC-GAA-G--G-C--G-------G--C-G-A-C---CTG--G--TC-CTT--------------------T-T---A-C-T--GA--CG-C-T-G--A-GG--C-G-CG-A--GA-G-C---G-TG--
GG-G--AG-C-A-AA--------CA--GG Bacteria(100);Proteobacteria(100);Alphaproteobacteria(100);Rhodospirillales(100);Acetobacteraceae(100);unclassified(100);unclassified(100);unclassified(100);unclassified(100);

Otu1161 0 0 0 0 0 0 0 0 0 0 0 0 0 0 0 0 0 0 0 0 0 0 0 0 0 0 0 0
0 0 0 0 0 0 0 0 0 0 0 0 0 0 0 0 0 0 0 0 0 0 0 0 0 0 0 0 0
0 0 0 0 0 0 0 0 0 0 0 0 0 0 0 0 0 0 0 0 0 0 0 44 0 0 0 0 0
0 0 0 0 0 0 0 0 0 0 0 0 0 0 0 0 0 0 0 0 0 0 0 0 0 0 0

M02255_77_000000000-ADUT3_1_2118_20326_21662 T----AC---GT-AG-GGG--------GCG-A-G------C--G--T---T--GT-C-CGG-AA------TT-A--C-T--GG-GC-------GT--A-----AA-GG-GT-GC-------G-TA-G-G-C-G-------------G--C-CT-T-G-T----
-------AA----G-T-C-A--------G-A-T--G--TG--A-AA-TA--C-C-AT-G-G-----CT-T-AA------C-T-G-T-G-G-G--G-G----T-G--C-A---T--T-------T--GA-A-A---C-------T--A-T--AG--G-G-C----T-T-G-A-G-T--A---C-AG-----GA-G-A--------G-G-A-A---AG-T------GG--A--ATT----
C-C-T-A-GT--GT-A-G-CG-GT--G---A--A-A---TG-C-GT-AG--AG-A-TT--A-G--G-A-G-G-A-AC-A-CC---AG--T--G--GC-GAA-G--G-C--G-------A--C-T-T-T---CTG--G--AC-T-G--------------------T-A---A-C-T--GA--CG-C-T-G--A-GG--C-A-CG-A--AA-G-C---G-TG--GG-
T--AG-C-G-AA--------CA--GG Bacteria(100);Firmicutes(100);Clostridia(100);Clostridiales(100);unclassified(100);unclassified(100);unclassified(100);unclassified(100);unclassified(100);

Otu1162 0 0 0 0 0 0 0 0 0 0 0 0 0 0 0 0 0 0 0 0 0 0 0 0 0 0 0 0
0 0 0 0 0 0 0 0 0 0 0 0 0 0 0 0 0 0 0 0 0 0 0 0 0 0 0 0 0
0 0 0 0 0 0 0 0 0 0 0 0 0 0 0 0 0 0 0 0 0 0 0 8 0 0 0 0 0
0 0 0 0 0 0 0 0 0 0 0 0 0 0 0 0 0 0 0 0 0 0 0 0 0 0 0

M02255_76_000000000-ADUNE_1_1103_14176_9331 C----TA---AC-TC-CGT--------GCC-A-G------C--A--G---C--CG-C-GGT-AA------TA-C--G-G--AG-GG-------TC--C-----AA-GC-GT-TA-------T-CC-G-G---A-------------A--T-C--A-T-T-----------AA----
G-T-C-A--------G-T-G--G--TG--A-AA-TC--T-C-TT-G-G-----CT-T-AA------C-C-A-A-G-A-A--A-T----T-G--C-C---A--T-------T--GA-A-A---C-------T--A-C--TA--G-T-C----T-T-G-A-A-T--T---C-GG-----TC-G-A--------A-G-T-G---GG-C------GG--A--ATG----T-G-T-C-AT--
GT-A-G-CG-GT--G---A--A-A---TG-C-AT-AG--AT-A-TG--A-C--A-C-A-G-A-AC-A-CC---GA--T--A--GC-GAA-G--G-C--A-------G--C-T-C-A---CTA--G--GC-C-T--------------------G-A---A-T-T--GA--CG-C-T-C--A-GG--G-A-CG-A--AA-G-C---G-TG--GG-G--AG-C-G-
AA--------CA--GG Bacteria(100);Bacteroidetes(100);Flavobacteria(100);Flavobacteriales(100);Cryomorphaceae(100);unclassified(100);unclassified(100);unclassified(100);unclassified(100);

Otu1163 0 0 0 0 0 0 0 0 0 0 0 0 0 0 0 0 0 0 0 0 0 0 0 0 0 0 0 0
0 0 0 0 0 0 0 0 0 0 0 0 0 0 0 0 0 0 0 0 0 0 0 0 0 0 0 0 0
0 0 0 0 0 0 0 0 0 0 0 0 0 0 0 0 0 0 0 0 0 0 0 1 0 0 0 0 0
1 0 0 0 0 0 0 0 0 0 0 0 0 0 0 15 0 0 0 0 0 0 0 0 0 0 0

M02255_76_000000000-ADUNE_1_1108_14998_24345 T----AA---GC-AC-CGG--------CTA-A-C------T--A--C---G--TG-C-CAG-CA------GC-C--G-C--GG-TA-------AT--A-----CG-TA-GG-GT-------G-CA-A-G-C-G-------------T--TAAT-C-G-G-----
------AA----G-C-C-A--------G-A-T--G--TG--A-AA-TC--C-C-CA-A-G-----CT-T-AA------C-T-T-G-G-G-A--C-G----T-G--C-A---T--T-------T--GG-A-A---C-------T--G-G--GT--G-A-C----T-A-G-A-G-T--G---T-GT-----CA-G-A--------G-G-G-A---GG-T------AG--A--ATT----C-
C-A-C-AT--GT-A-G-CG-GT--G---G--A-A---TG-C-GT-AG--AG-A-TG--T-G--G-A-G-G-A-AT-A-CC---GA--T--G--GC-GAA-G--G-C--A-------G--C-C-T-C---CTG--G--GA-T-A--------------------A-C---A-C-T--GA--CG-T-T-G--A-GG--C-T-CG-A--AA-G-C---G-TG--GG-
G--AG-C-A-AA--------CA--GG Bacteria(100);Proteobacteria(100);unclassified(100);unclassified(100);unclassified(100);unclassified(100);unclassified(100);unclassified(100);unclassified(100);

Otu1164 0 0 0 0 0 0 0 0 0 0 0 0 0 0 0 0 0 0 0 0 0 0 0 0 0 0 0 0
0 0 0 0 0 0 0 0 0 0 0 0 0 0 0 0 0 0 0 0 0 0 0 0 0 0 0 0 0
0 0 0 0 0 0 0 0 0 0 0 0 0 0 0 0 0 0 0 0 0 0 0 0 13 0 0 0 0
0 0 0 0 0 0 0 0 0 0 0 0 0 0 0 0 0 0 0 0 0 0 0 0 0 0 0

M02255_76_000000000-ADUNE_1_1110_24028_7088 C----GG---CT-AA-CTC--------CGT-G-C------C--A--G---C--AG-C-CGC-GG------TA-A--T-A--CG-GA-------GG--A-----TC-CG-AG-CG-------T-TA-T-C-C-G-------------GA-T-TT-A-T-T-----------AA--
--G-T-C-A--------G-G-G--G--TG--A-AA-GA--C-G-GT-A-G-----CT-A-AA------C-T-A-T-C-G-C--A-G----T-G--C-C---T--T-------T--GA-T-A---C-------T--G-A--AG--A-G-C----T-T-G-A-A-T--A---C-AC-----TT-G-A--------G-G-T-A---GG-C------GG--A--ATG----T-G-A-C-
AA--GT-A-G-CG-GT--G---A--A-A---TG-C-AT-AG--AT-A-TG--T-C--A-C-A-G-A-AC-A-CC---AA--T--T--GC-GAA-G--G-C--A-------G--C-T-T-A---CTA--A--AG-T-G--------------------T-G---A-T-T--GA--CG-C-T-G--A-GG--C-A-CG-A--AA-G-C---G-TG--GG-G--AT-C-
A-AA--------CA--GG Bacteria(100);Bacteroidetes(100);Sphingobacteria(100);Sphingobacteriales(100);Sphingobacteriaceae(100);Sphingobacteriaceae(100);unclassified(100);unclassified(100);unclassified(100);

Otu1165 0 0 0 0 0 0 0 0 0 0 0 0 0 0 0 0 0 0 0 0 0 0 0 0 0 0 0 0
0 0 0 0 0 0 0 0 0 0 0 0 0 0 0 0 0 0 0 0 0 0 0 0 0 0 0 0 0
0 0 0 0 0 0 0 0 0 0 0 0 0 0 0 0 0 0 0 0 0 0 0 0 32 0 0 0 0
0 0 0 0 0 0 0 0 0 0 0 0 0 0 0 0 0 0 0 0 0 0 0 0 0 0 0

M02255_76_000000000-ADUNE_1_1113_24540_18763 C----GG---CT-AA-CTC--------CGT-G-C------C--A--G---C--AG-C-CGC-GG------TA-A--T-A--CG-GA-------GG--A-----TC-CA-AG-CG-------T-TA-T-C-C-G------------G--A-TT-TAT-T-----------AA----G-T-C-A--------G-G-G--G--TG--A-AA-GA--C-G-GT-G-G-----CT-C-AA------C-C-A-T-C-G-C--A-G----T-G--C-C---C--T-------T--GA-T-A---C-------T--G-A--AG--A-G-C----T-T-G-A-A-T--G---G-AC-----TA-G-A--------G-G-T-A---GG-C------GG--A--ATG----T-G-A-C-AA--GT-A-G-CG-GT--G---A--A-A---TG-C-AT-AG--AT-A-TG--T-C--A-C-A-G-A-AC-A-CC---GA--T--T--GC-GAA-G--G-C--A-------G--C-T-T-A---CTA--T--GG-T-T--------------------T-T---A-T-T--GA--CG-C-T-G--A-GG--C-A-CG-A--AA-G-C---G-TG--GG-G--AT-C-A-AA--------CA--GG Bacteria(100);Bacteroidetes(100);Sphingobacteria(100);Sphingobacteriales(100);Sphingobacteriaceae(100);Sphingobacteriaceae(100);Pedobacter(100);unclassified(100);unclassified(100);

Otu1166 0 0 0 0 0 0 0 0 0 0 0 0 0 0 0 0 0 0 0 0 0 0 0 0 0 0 0 0
0 0 0 0 0 0 0 0 0 0 0 0 0 0 0 0 0 0 0 0 0 0 0 0 0 0 0 0 0
0 0 0 0 0 0 0 0 0 0 0 0 0 0 0 0 0 0 0 0 0 0 0 0 35 0 0 0 0
0 0 0 0 0 0 0 0 0 0 0 0 0 0 0 0 0 0 0 0 0 0 0 0 0 0 0

M02255_77_000000000-ADUT3_1_1101_24464_5674 T----AC---GT-AG-GGG--------CCG-A-G------C--G--T---T--GT-C-CGG-AG------TT-A--T-T--GG-GC-------GT--A-----AA-GC-GC-GC-------G-CA-G-G-C-G------------G--C-TT-C-A-C-----------GC----G-C-C-C--------G-A-C--G--TG--A-AA-GC--C-C-CC-G-G-----CT-T-AA------C-C-G-G-G-G-A--G-A----G-T--C-G---T--T-------G--GG-G-A---C-------G--G-T--GG--A-G-C----T-T-G-A-G-G--G---C-GG-----GA-G-A--------G-G-T-C---GG-T------GG--A--ATT----C-C-C-G-GT--GT-A-G-TG-GT--G---A--A-A---TG-C-GT-AG--AG-A-TC--G-G--G-A-G-G-A-AC-A-CC---TG--T--G--GC-GAA-G--G-C--G-------G--C-C-G-A---CTG--G--GC-C-G--------------------A-C---C-C-T--GA--CG-C-T-G--A-GG--C-G-CG-A--AG-G-C---G-TG--GG-G--AG-C-G-AA--------CG--GG Bacteria(100);Chloroflexi(100);Thermomicrobia(100);unclassified(100);unclassified(100);unclassified(100);unclassified(100);unclassified(100);unclassified(100);

Otu1167 0 0 0 0 0 0 0 0 0 0 0 0 0 0 0 0 0 0 0 0 0 0 0 0 0 0 0 0
0 0 0 0 0 0 0 0 0 0 0 0 0 0 0 0 0 0 0 0 0 0 0 0 0 0 0 0 0
0 0 0 0 0 0 0 0 0 0 0 0 0 0 0 0 0 0 0 0 0 0 0 0 48 0 0 0 0
0 0 0 0 0 0 0 0 0 0 0 0 0 0 0 0 0 0 0 0 0 0 0 0 0 0 0

M02255_77_000000000-ADUT3_1_1106_6265_5567 T----AC---GT-AG-GGG--------GCG-A-G------C--G--T---T--GT-C-CGG-AA------TT-A--C-T--GG-GT-------GT--A-----AA-GG-GC-GT-------G-TA-G-G-C-G------------G--G-TA-A-G-C-----------AA----G-T-T-A--------T-C-G--G--TA--A-AA-AC--C-C-AG-G-G-----CT-C-AA------C-C-T-T-G-G-G--C-C----T-G--C-C---G--A-------T--AA-A-A---C-------T--G-C--AT--A-C-C----T-T-G-A-G-T--G---C-AG-----GA-G-A--------G-G-G-G---AG-T------GG--A--ATT----C-C-C-G-GA--GT-A-G-CG-GT--G---A--A-A---TG-C-GT-AG--AT-A-CC--G-G--G-A-G-G-A-AC-A-CC---GG--T--G--GC-GAA-G--G-C--G-------G--C-T-C-C---CTG--G--CC-T-G--------------------T-A---A-C-T--GA--CG-C-T-G--A-GG--C-G-CG-A--AA-G-C---T-GG--GG-G--AG-C-G-AA--------CG--GG Bacteria(100);Firmicutes(100);Clostridia(100);unclassified(100);unclassified(100);unclassified(100);unclassified(100);unclassified(100);unclassified(100);

Otu1168 0 0 0 0 0 0 0 0 0 0 0 0 0 0 0 0 0 0 0 0 0 0 0 0 0 0 0 0
0 0 0 0 0 0 0 0 0 0 0 0 0 0 0 0 0 0 0 0 0 0 0 0 0 0 0 0 0
0 0 0 0 0 0 0 0 0 0 0 0 0 0 0 0 0 0 0 0 0 0 0 0 25 0 0 0 0
0 0 0 0 0 0 0 0 0 0 0 0 0 0 0 0 0 0 0 0 0 0 0 0 0 0 0

M02255_77_000000000-ADUT3_1_1115_19830_9736 T----AC---GA-AG-GGG--------GCT-A-G------C--G--T---T--GT-T-CGG-AA------TT-A--C-T--GG-GC-------GT--A-----AA-GC-GC-GC-------G-TA-G-G-C-G------------G--A-TT-T-G-C-----------CA----G-T-C-A--------G-G-G--G--TG--A-AA-TG--C-C-AG-G-G-----CT-T-AA------C-C-T-T-G-G-A--A-C----T-G--C-C---T--T-------T--GA-T-A---C-------T--G-C--AA--G-T-C----T-G-G-A-G-T--A---C-GG-----GA-G-A--------G-G-T-G---AG-T------GG--A--ATT----C-C-G-A-GT--GT-A-G-AG-GT--G---A--A-A---TT-C-GT-AG--AT-A-TT--C-G--G-A-G-G-A-AC-A-CC---AG--A--A--GC-GAA-G--G-C--G-------G--C-T-C-A---CTG--G--CC-C-G--------------------A-T---A-C-T--GA--CG-C-T-G--A-GG--C-G-CG-A--AA-G-C---G-TG--GG-G--AG-C-A-AA--------CA--GG Bacteria(100);Proteobacteria(100);Alphaproteobacteria(100);unclassified(100);unclassified(100);unclassified(100);unclassified(100);unclassified(100);unclassified(100);

Otu1169 0 0 0 0 0 0 0 0 0 0 0 0 0 0 0 0 0 0 0 0 0 0 0 0 0 0 0 0
0 0 0 0 0 0 0 0 0 0 0 0 0 0 0 0 0 0 0 0 0 0 0 0 0 0 0 0 0
0 0 0 0 0 0 0 0 0 0 0 0 0 0 0 0 0 0 0 0 0 0 0 17 0 0 0 0 0
0 0 0 0 0 0 0 0 0 0 0 0 0 0 0 0 0 0 0 0 0 0 0 0 0 0 0

M02255_77_000000000-ADUT3_1_2111_9923_6958 T----AC---GG-AG-GGA--------GCT-A-G------C--G--T---T--GT-T-CGG-AA------TT-A--C-T--GG-GC-------GT--A-----AA-GC-GC-AC-------G-TA-G-G-C-G------------G--C-TA-T-T-T-----------AA----G-T-C-A--------G-G-G--G--TG--A-AA-TC--C-C-GG-G-G-----CT-C-AA------C-C-C-C-G-G-A--A-C----T-G--C-C---T--C-------T--GA-A-A---C-------T--G-G--AT--A-G-C----T-A-G-A-A-T--C---T-TG-----GA-G-A--------G-G-T-C---AG-T------GG--A--ATT----C-C-G-A-GT--GT-A-G-AG-GT--G---A--A-A---TT-C-GT-AG--AT-A-TT--C-G--G-A-A-G-A-AC-A-CC---AG--T--G--GC-GAA-G--G-C--G-------A--C-T-G-A---CTG--G--AC-A-A--------------------G-T---A-T-T--GA--CG-C-T-G--A-GG--T-G-CG-A--AA-G-C---G-TG--GG-G--AG-C-A-AA--------CA--GG Bacteria(100);Proteobacteria(100);Alphaproteobacteria(100);Sphingomonadales(100);unclassified(100);unclassified(100);unclassified(100);unclassified(100);unclassified(100);

Otu1170 0 0 0 0 0 0 0 0 0 0 0 0 0 0 0 0 0 0 0 0 0 0 0 0 0 0 0 0
0 0 0 0 0 0 0 0 0 0 0 0 0 0 0 0 0 0 0 0 0 0 0 0 0 0 0 0 0
0 0 0 0 0 0 0 0 0 0 0 0 0 0 0 0 0 0 0 0 0 0 0 0 13 0 0 0 0
0 0 0 0 0 0 0 0 0 0 0 0 0 0 0 0 0 0 0 0 0 0 0 0 0 0 0 0
M02255_76_000000000-ADUNE_1_1106_28249_15233 G----TT---CG-TG-CCA--------GCA-G-C------C--G--C---G--GT-A-AGACGA------AC-C--G-T--GC-GA-------AC--G-----TT-GT-TCGGA-------A-TC-A-C-T-G------------G--G-CT-G-T-A----
-------AA----G-T-C-T--------G-G-G--G--TG--A-AA-TG--C-C-AC-G-G-----CT-C-AA------C-C-G-T-G-G-A--A-C----T-G--C-C---T--C-------A--GA-T-A---C-------T--G-A--CG--G-C-C----T-C-G-A-G-G--A---A-GG-----TA-G-G--------G-G-C-A---TG-C------GG--A--ACT----
G-T-A-G-GT--GG-A-G-CG-GT--G---A--A-A---TG-C-GT-TG--AT-A-TC--T-A--C-A-G-G-A-AC-T-CC---GG--T--G--GC-GAA-A--G-C--G-------G--C-G-T-G---CTG--G--AC-C-T--------------------T-T---T-C-T--GA--CG-C-T-G--A-GG--C-G-CG-A--AA-G-C---T-AG--GG-
G--AG-C-A-AA--------CG--GG Bacteria(100);Planctomycetes(100);unclassified(100);unclassified(100);unclassified(100);unclassified(100);unclassified(100);unclassified(100);unclassified(100);

Otu1171 0 0 0 0 0 0 0 0 0 0 0 0 0 0 0 0 0 0 0 0 0 0 0 0 0 0 0 0
0 0 0 0 0 0 0 0 0 0 0 0 0 0 0 0 0 0 0 0 0 0 0 0 0 0 0 0 0
0 0 0 0 0 0 0 0 0 0 0 0 0 0 0 0 0 0 0 0 0 0 0 0 0 0 0 0 0
2 4 18 0 0 2 0 0 0 1 0 0 0 0 0 0 0 0 0 0 0 0 0 0 0 0 0 0
M02255_76_000000000-ADUNE_1_1119_17386_19831 T----AC---AG-AA-GAA--------GCA-C-C------G--G--C---T--AACTCCGT-GC------CA-G--C-A-GCC-GC-------GG--T-----AA-TA-CG-GG-------G-GG-T-G-C-G------------G--A-GC-G-T-T--
---------AA----G-T-C-G--------G-A-A--G--TG--A-AA-GC--C-C-CG-G-G-----CT-T-AA------C-C-T-G-G-G-A--A-T----T-G--C-A---T--T-------T--CA-T-A---C-------T--G-G--GT--C-G-C----T-A-G-A-G-T--A---C-TT-----TA-G-G--------G-A-G-G---GG-T------AG--A--ATT----
C-C-A-C-GT--GT-A-G-CG-GT--G---A--A-A---TG-C-GT-AG--AG-A-TG--T-G--G-A-G-G-A-AT-A-CC---GA--A--G--GC-GAA-G--G-C--A-------G--C-C-C-C---TTG--G--GA-A-T--------------------G-T---A-C-T--GA--CG-C-T-C--A-TG--T-G-CG-A--AA-G-C---G-TG--
GG-G--AG-C-A-AA--------CA--GG Bacteria(100);Proteobacteria(100);Gammaproteobacteria(100);Pasteurellales(100);Pasteurellaceae(100);unclassified(100);unclassified(100);unclassified(100);unclassified(100);

Otu1172 0 0 0 0 0 0 0 0 0 0 0 0 0 0 0 0 0 0 0 0 0 0 0 0 0 0 0 0
0 0 0 0 0 0 0 0 0 0 0 0 0 0 0 0 0 0 0 0 0 0 0 0 0 0 0 0 0
0 0 0 0 0 0 0 0 0 0 0 0 0 0 0 0 0 0 0 0 0 0 0 0 6 0 125 0 0
0 0 0 0 0 0 0 0 0 0 0 0 0 0 0 0 0 0 0 0 0 0 0 0 0 0 0 0
M02255_76_000000000-ADUNE_1_1108_21661_18014 T----AA---CT-AC-GTG--------CCA-G-C------A--G--C---C--GC-G-GTA-AT------AC----G-T--AG-GT-------CC--C-----GA-GC-GT--T-------G-C--G---C-G------------A--A-TG-G-G-T---------
--AA----G-C-G-G--------T-T-C--G--TC--A-AA-GC--C-C-AC-C-G-----CT-C-AA------C-G-G-T-G-G-A--A-A----G-G--C-G---T--A-------C--CG-A-A---C-------T--G-C--CC--A-T-C----T-T-G-A-G-G--C---A-CG-----CT-C-A--------G-G-C-A---GG-C------GG--A--ATT----C-C-
C-G-GT--GT-A-G-CG-GT--G---A--A-A---TG-C-GT-AG--AT-A-TC--G-G--G-A-G-G-A-AC-A-CC---GA--A--G--GC-GAA-G--G-C--A-------G--C-C-T-G---CTG--G--GG-C-T--------------------G-T---T-C-T--GA--CG-G-T-C--A-GG--G-A-CG-A--AA-G-C---T-GG--GG-G--
AG-C-A-AA--------CC--GG Bacteria(100);Aquificae(100);Aquificae(100);Aquificales(100);Aquificaceae(100);Hydrogenobacter(100);unclassified(100);unclassified(100);unclassified(100);

Otu1173 0 0 0 0 0 0 0 0 0 0 0 0 0 0 0 0 0 0 0 0 0 0 0 0 0 0 0 0
0 0 0 0 0 0 0 0 0 0 0 0 0 0 0 0 0 0 0 0 0 0 0 0 0 0 0 0 0
0 0 0 0 0 0 0 0 0 0 0 0 0 0 0 0 0 0 0 0 0 0 0 0 0 40 47 0 0
0 0 0 0 0 0 0 0 0 0 0 0 0 0 0 0 0 0 0 0 0 0 0 0 0 0 0 0
M02255_76_000000000-ADUNE_1_1107_23557_14483 C----GG---CT-AA-CTC--------CGT-G-C------C--A--G---C--AG-C-CGC-GG------TA-A--T-A--CG-GA-------GG--G-----TG-CA-AG-CG-------T-TA-T-C-C-G------------G--A-CT-T-G-T------
-----AA----G-T-C-C--------G-T-G--G--TG--A-AA-TC--T-C-CG-A-G-----CT-T-AA------C-T-C-G-G-A-A--A-C----T-G--C-C---A--T-------G--GA-T-A---C-------T--A-T--AG--G-T-C----T-T-G-A-A-T--G---T-TG-----TG-G-A--------G-G-T-T---AG-C------GG--A--ATA----G-
T-T-C-AT--GT-A-G-CG-GT--G---A--A-A---TG-C-TT-AG--AT-A-TG--A-C--C-T-A-G-A-AC-A-CC---GA--T--T--GC-GAA-G--G-C--A-------G--C-T-G-G---CTA--C--AC-A-A--------------------C-G---A-T-T--GA--CG-C-T-G--A-GG--C-A-CG-A--AA-G-C---G-TG--GG-G--
AT-C-A-AA--------CA--GG Bacteria(100);Bacteroidetes(100);Sphingobacteria(100);Sphingobacteriales(100);Chitinophagaceae(100);unclassified(100);unclassified(100);unclassified(100);unclassified(100);

Otu1174 0 0 0 0 0 0 0 0 0 0 0 0 0 0 0 0 0 0 0 0 0 0 0 0 0 0 0 0
0 0 0 0 0 0 0 0 0 0 0 0 0 0 0 0 0 0 0 0 0 0 0 0 0 0 0 0 0
0 0 0 0 0 0 0 0 0 0 0 0 0 0 0 0 0 0 0 0 0 0 0 0 0 102 134 0 0
0 0 0 0 0 0 0 0 0 0 0 0 0 0 0 0 0 0 0 0 0 0 0 0 0 0 0 0
M02255_77_000000000-ADUT3_1_2117_25395_23028 T----AC---GG-AG-GGT--------GCA-A-G------C--G--T---T--AT-C-CGG-AT------TC-A--C----------------T-----------G-G--G--------G-TA-G-G-C-G------------G--A-CT-T-G-T-----------AA-
---G-T-C-C--------G-T-G--G--TG--A-AA-TC--T-C-CG-A-G-----CT-T-AA------C-T-C-G-G-A-A--A-C----T-G--C-C---A--T-------G--GA-T-A---C-------T--A-T--AG--G-T-C----T-T-G-A-A-T--G---T-TG-----TG-G-A--------G-G-T-T---AG-C------GG--A--ATA----G-T-T-C-
AT--GT-A-G-CG-GT--G---A--A-A---TG-C-TT-AG--AT-A-TG--A-C--C-T-A-G-A-AC-A-CC---GA--T--T--GC-GAA-G--G-C--A-------G--C-T-G-G---CTA--C--AC-A-A--------------------C-G---A-T-T--GA--CG-C-T-G--A-GG--C-A-CG-A--AA-G-C---G-TG--GG-G--AT-C-
A-AA--------CA--GG Bacteria(100);Bacteroidetes(100);Sphingobacteria(100);Sphingobacteriales(100);Chitinophagaceae(100);unclassified(100);unclassified(100);unclassified(100);unclassified(100);

Otu1175 0 0 0 0 0 0 0 0 0 0 0 0 0 0 0 0 0 0 0 0 0 0 0 0 0 0 0 0
0 0 0 0 0 0 0 0 0 0 0 0 0 0 0 0 0 0 0 0 0 0 0 0 0 0 0 0 0
0 0 0 0 0 0 0 0 0 0 0 0 0 0 0 0 0 0 0 0 0 0 0 0 0 12 11 0 0
0 0 0 0 0 0 0 0 0 0 0 0 0 0 0 0 0 0 0 0 0 0 0 0 0 0 0
M02255_76_000000000-ADUNE_1_1103_6870_15631 .....AC---TC-CG-TG---------CCA-G-C------A--G--C---C--GC-G-GTA-AT------AC-G--G-A--GG-GT-------GC--A-----AG-CG-TT-AT-------C-CG-G-A-T-G-------------G--A-CT-T-G-T-----------AA----
G-T-C-C--------G-T-G--G--TG--A-AA-TC--T-C-CG-A-G-----CT-T-AA------C-T-C-G-G-A-A--A-C----T-G--C-C---A--T-------G--GA-T-A---C-------T--A-T--AG--G-T-C----T-T-G-A-A-T--G---T-TG-----TG-G-A--------G-G-T-T---AG-C------GG--A--ATA----G-T-T-C-AT--
GT-A-G-CG-GT--G---A--A-A---TG-C-TT-AG--AT-A-TG--A-C--C-T-A-G-A-AC-A-CC---GA--T--T--GC-GAA-G--G-C--A-------G--C-T-G-G---CTA--C--AC-A-A--------------------C-G---A-T-T--GA--CG-C-T-G--A-GG--C-A-CG-A--AA-G-C---G-TG--GG-G--AT-C-A-
AA--------CA--GG Bacteria(100);Bacteroidetes(100);Sphingobacteria(100);Sphingobacteriales(100);Chitinophagaceae(100);unclassified(100);unclassified(100);unclassified(100);unclassified(100);

Otu1176 0 0 0 0 0 0 0 0 0 0 0 0 0 0 0 0 0 0 0 0 0 0 0 0 0 0 0 0
0 0 0 0 0 0 0 0 0 0 0 0 0 0 0 0 0 0 0 0 0 0 0 0 0 0 0 0 0
0 0 0 0 0 0 0 0 0 0 0 0 0 0 0 0 0 0 0 0 0 0 0 0 0 14 0 0 0
0 0 0 0 0 0 0 0 0 0 0 0 0 0 0 0 0 0 0 0 0 0 0 0 0 0 0
M02255_77_000000000-ADUT3_1_2112_15558_24875 G----AC---GA-AC-CGT--------GCG-A-A------C--G--T---T--GT-T-CGG-AA------TC-A--C-T--GG-GC-------TT--A-----AA-GG-GC-GC-------------G-C-T------------G----C------C-----------
AA----G-T-C-T--------G-G-G--G--TG--A-AA-TC--C-T-CC-G-G-----CT-C-AA------C-C-G-G-A-G-A--A-C----G-G--C-C---T--C-------G--GA-T-A---C-------T--G-G--CG--G-T-C----T-C-G-A-G-G--G---A-GG-----TA-G-G--------G-G-C-G---TG-C------GG--A--ACT----A-A-
G-G-GT--GG-A-G-CG-GT--G---A--A-A---TG-C-GT-TG--AT-A-TC--C-T--T-A-G-G-A-AC-T-CC---GG--T--G--GC-GAA-G--G-C--G-------G--C-A-C-G---CTG--G--AC-C-T--------------------C-T---T-C-T--GA--CG-C-T-G--A-GG--C-G-CG-A--AA-G-C---C-AG--GG-G--
AG-C-A-AA--------CG--GG Bacteria(100);unclassified(100);unclassified(100);unclassified(100);unclassified(100);unclassified(100);unclassified(100);unclassified(100);unclassified(100);

Otu1177 0 0 0 0 0 0 0 0 0 0 0 0 0 0 0 0 0 0 0 0 0 0 0 0 0 0 0 0
0 0 0 0 0 0 0 0 0 0 0 0 0 0 0 0 0 0 0 0 0 0 0 0 0 0 0 0 0
0 0 0 0 0 0 0 0 0 0 0 0 0 0 0 0 0 0 0 0 0 0 7 0 0 0 0 0 0
0 0 0 0 0 0 0 0 0 0 0 0 0 0 0 0 0 0 0 0 0 0 0 0 0 0 0
M02255_77_000000000-ADUT3_1_1107_10657_15376 T----AC---GA-AG-GGG--------GCT-A-G------C--G--T---T--GT-T-CGG-AT------TT-A--C-T--GG-GC-------GT--A-----AA-GC-GC-AC-------G-TA-G-G-C-G------------G--A-TT-G-G-T-----
------CA----G-T-T-A--------G-G-G--G--TG--A-AA-TC--C-C-AG-G-G-----CT-T-AA------C-C-C-T-G-G-A--A-C----T-G--C-C---T--T-------T--AA-T-A---C-------T--G-C--CA--G-T-C----T-T-G-A-G-T--A---T-GG-----AA-G-A--------G-G-T-G---AG-T------GG--A--ATT----G-
C-G-A-GT--GT-A-G-AG-GT--G---A--A-A---TT-C-GT-AG--AT-A-TT--C-G--C-A-G-G-A-AC-A-CC---AG--T--G--GC-GAA-G--G-C--G-------G--C-T-C-A---CTG--G--TC-C-A--------------------T-T---A-C-T--GA--CG-C-T-G--A-GG--T-G-CG-A--AA-G-C---G-TG--GG-G-
-AG-C-A-AA--------CA--GG Bacteria(100);Proteobacteria(100);Alphaproteobacteria(100);Rhizobiales(100);unclassified(100);unclassified(100);unclassified(100);unclassified(100);unclassified(100);

Otu1178 0 0 0 0 0 0 0 0 0 0 0 0 0 0 0 0 0 0 0 0 0 0 0 0 0 0 0 0
0 0 0 0 0 0 0 0 0 0 0 0 0 0 0 0 0 0 0 0 0 0 0 0 0 0 0 0 0
0 0 0 0 0 0 0 0 0 0 0 0 0 0 0 0 0 0 0 0 0 4 0 0 0 0 0 0 0
0 0 0 0 0 0 0 0 0 0 0 0 0 0 0 0 0 0 0 0 0 0 0 0 0 0 0
M02255_77_000000000-ADUT3_1_1117_14677_23567 T----AC---GA-AG-GGG--------GCT-A-G------C--G--T---T--GC-T-CGG-AA------TC-A--C-T--GG-GC-------GT--A-----AA-GG-GC-GC-------G-TA-G-G-C-G------------G--A-TC-T-T-T-----
------AA----G-T-C-G--------G-G-G--G--TG--A-AA-GC--C-C-AG-G-G-----CT-C-AA------C-C-C-T-G-G-A--A-T----T-G--C-C---T--T-------C--GA-T-A---C-------T--G-A--AG--A-T-C----T-T-G-A-G-A--C---C-GG-----AA-G-A--------G-G-T-T---AG-T------GG--A--ACG----
G-C-G-A-GT--GT-A-G-AG-GT--G---A--A-A---TT-C-GT-AG--AT-A-TT--C-G--C-A-A-G-A-AC-A-CC---AG--T--G--GC-GAA-G--G-C--G-------G--C-T-A-A---CTG--G--TC-C-G--------------------G-T---T-C-T--GA--CG-C-T-G--A-GG--C-G-CG-A--AA-G-C---G-TG--GG-
G--AG-C-A-AA--------CA--GG Bacteria(100);Proteobacteria(100);Alphaproteobacteria(100);Rhizobiales(100);unclassified(100);unclassified(100);unclassified(100);unclassified(100);unclassified(100);

Otu1179 0 0 0 0 0 0 0 0 0 0 0 0 0 0 0 0 0 0 0 0 0 0 0 0 0 0 0 0
0 0 0 0 0 0 0 0 0 0 0 0 0 0 0 0 0 0 0 0 0 0 0 0 0 0 0 0 0
0 0 0 0 0 0 0 0 0 0 0 0 0 0 0 0 0 0 0 0 0 3 0 0 0 0 0 0 0
0 0 0 0 0 0 0 0 0 0 0 0 0 0 0 0 0 0 0 0 0 0 0 0 0 0 0
M02255_76_000000000-ADUNE_1_2110_24194_2355 T----AC---GT--G-CCA--------GCA-G-C------C--G--C---G--GT-A-ATA-CG------TA-G--G-G--GGCAA-------GC--G-----TT-AT-CC-GG-------A-TT-T-A-C-G------------G--T-GG-T-G-T-----------AA-
---G-T-C-A--------G-A-T--G--TG--A-AA-GC--C-C-GG-G-G-----CT-C-AA------C-C-C-C-G-G-G--A-C----T-G--C-A---T--T-------T--GA-A-A---C-------T--A-T--GT--T-G-C----T-G-G-A-G-T--G---C-AG-----GA-G-A--------G-G-T-A---AG-T------GG--A--ATT----C-C-T-A-
GT--GT-A-G-CG-GT--G---A--A-A---TG-C-GT-AG--AT-A-TT--A-G--G-A-G-G-A-AC-A-CC---AG--T--G--GC-GAA-G--G-C--G-------G--C-T-T-A---CTG--G--AC-T-G--------------------T-A---A-C-T--GA--CG-T-T-G--A-GG--C-T-CG-A--AA-G-C---G-TG--GG-G--AG-
C-A-AA--------CA--GG Bacteria(100);Firmicutes(100);Clostridia(100);Clostridiales(100);Lachnospiraceae(100);unclassified(100);unclassified(100);unclassified(100);unclassified(100);

Otu1180 0 0 0 0 0 0 0 0 0 0 0 0 0 0 0 0 0 0 0 0 0 0 0 0 0 0 0 0
0 0 0 0 0 0 0 0 0 0 0 0 0 0 0 0 0 0 0 0 0 0 0 0 0 0 0 0 0
0 0 0 0 0 0 0 0 0 0 0 0 0 0 0 0 0 0 0 0 0 14 0 0 0 0 0 0 0
0 0 0 0 0 0 0 0 0 0 0 0 0 0 0 0 0 0 0 0 0 0 0 0 0 0 0
M02255_77_000000000-ADUT3_1_1117_22199_12525 T----AC---GG-AG-GGG--------ACT-A-G------C--G--T---T--GT-T-CGG-AA------TT-A--C-T--GG-GC-------GT--A-----AA-GC-GT-GC-------G-TA-G-G-C-G------------G--A-TC-A-G-C-----
------AA----G-T-T-G--------G-G-G--G--TG--A-AA-TC--C-C-GG-G-G-----CT-C-AA------C-C-C-C-G-G-A--A-C----T-G--C-C---T--C-------C--AA-A-A---C-------T--G-T--TG--G-T-C----T-A-G-A-G-T--T---C-GA-----GA-G-A--------G-G-T-G---AG-T------GG--A--ATT----C-
C-G-A-GT--GT-A-G-AG-GT--G---A--A-A---TT-C-GT-AG--AT-A-TT--C-G--G-A-G-G-A-AC-A-TC---AG--T--G--GC-GAA-G--G-C--G-------G--C-T-C-A---CTG--G--CT-C-G--------------------A-T---A-C-T--GA--CG-C-T-G--A-GG--T-A-CG-A--AA-G-T---G-TG--GG-G-
-AG-C-A-AA--------CA--GG Bacteria(100);Proteobacteria(100);Alphaproteobacteria(100);Rhodobacterales(100);Rhodobacteraceae(100);unclassified(100);unclassified(100);unclassified(100);unclassified(100);

Otu1181 0 0 0 0 0 0 0 0 0 0 0 0 0 0 0 0 0 0 0 0 0 0 0 0 0 0 0 0
0 0 0 0 0 0 0 0 0 0 0 0 0 0 0 0 0 0 0 0 0 0 0 0 0 0 0 0 0
0 0 0 0 0 0 0 0 0 0 0 0 0 0 0 0 0 0 0 0 0 3 0 0 0 0 0 0 0
0 0 0 0 0 0 0 0 0 0 0 0 0 0 0 0 0 0 0 0 0 0 0 0 0 0 0
M02255_77_000000000-ADUT3_1_1117_7141_14690 T----AC---GA-AG-GGG--------GCG-A-G------C--G--T---T--GT-T-CGG-AA------TT-A--T-T--GG-GC-------GT--A-----AA-GG-GC-GC-------G-TA-G-G-C-G-------------G--T-GC-G-G-T-----------
AA----G-A-G-T--------G-T-T--G--TG--A-AA-TC--C-T-CG-G-G-----CT-C-AA------C-T-C-G-A-G-G--C-C----T-G--C-A---A--C-------G--CT-G-A---C-------T--G-C--CG--T-G-C----T-G-G-A-G-T--G---T-GG-----GA-G-A--------G-G-T-G---CG-T------GG--A--ATT----C-C-C-
G-GT--GT-A-G-CG-GT--G---A--A-A---TG-C-GT-AG--AT-A-TC--G-G--G-A-G-G-A-AC-A-CC---TG--T--G--GC-GAA-A--G-C--G-------G--C-G-C-A---CTG--G--AC-C-A--------------------C-A---A-C-T--GA--CG-C-T-G--A-GG--C-G-CG-A--AA-G-C---T-AG--GG-G--
AG-C-A-AA--------CA--GG Bacteria(100);Acidobacteria(100);Acidobacteria(100);Acidobacteriales(100);Acidobacteriaceae(100);unclassified(100);unclassified(100);unclassified(100);unclassified(100);

Otu1182 0 0 0 0 0 0 0 0 0 0 0 0 0 0 0 0 0 0 0 0 0 0 0 0 0 0 0 0
0 0 0 0 0 0 0 0 0 0 0 0 0 0 0 0 0 0 0 0 0 0 0 0 0 0 0 0 0
0 0 0 0 0 0 0 0 0 0 0 0 0 0 0 0 0 0 0 0 0 1 0 0 0 0 0 0 0
0 0 0 0 0 0 0 0 0 0 0 0 0 0 0 1 0 0 0 0 0 0 0 3 0 0 0
M02255_77_000000000-ADUT3_1_1105_10873_2274 C----TG---TG-CC-AGC--------AGC-C-G------C--G--G---T--AA-TACAG-AG------GG-G--G-C--AA-GC------GTT--A----ATC-GG-AATTA-------C-TA-G-G-T-G-------------G--C-TC-T-A-T-----------
AA----G-T-C-A--------G-A-T--G--TG--A-AA-TC--C-C-CG-G-G-----CT-T-AA------C-C-T-G-G-G-A--A-C----T-G--C-A---T--C-------T--GA-A-A---C-------T--G-T--AG--A-G-C----T-A-G-A-G-T--A---T-GT-----GA-G-A--------G-G-A-A---GG-T------AG--A--ATT----C-C-A-
G-GT--GT-A-G-CG-GT--G---A--A-A---TG-C-GT-AG--AG-A-TC--T-G--G-A-G-G-A-AT-A-CC---GA--T--G--GC-GAA-G--G-C--A-------G--C-C-T-T---CTG--G--CA-T-A--------------------A-T---A-C-T--GA--CA-C-T-G--A-GG--C-T-CG-A--AA-G-C---G-TG--GG-T--AG-
C-A-AA--------CA--GG Bacteria(100);Proteobacteria(100);Gammaproteobacteria(100);Pseudomonadales(100);Moraxellaceae(100);Psychrobacter(100);unclassified(100);unclassified(100);unclassified(100);

Otu1183 0 0 0 0 0 0 0 0 0 0 0 0 0 0 0 0 0 0 0 0 0 0 0 0 0 0 0 0
0 0 0 0 0 0 0 0 0 0 0 0 0 0 0 0 0 0 0 0 0 0 0 0 0 0 0 0 0
0 0 0 0 0 0 0 0 0 0 0 0 0 0 0 0 0 0 0 0 0 0 14 0 0 0 0 0 0
0 0 0 0 0 0 0 0 0 0 0 0 0 0 0 0 0 0 0 0 0 0 0 0 0 0 0
M02255_77_000000000-ADUT3_1_1104_17918_13894 T----AC---GT-AG-GTG--------GCA-A-G------C--G--T---T--GT-C-CGG-AT------TT-A--C-T--GG-GC-------GT--A-----AA-GA-AT-GC-------G-TA-G-G-C-G-------------G--A-TA-T-T-T------
-----AA----G-T-G-A--------G-A-T--G--TG--A-AA-TT--C-C-CG-G-G-----CT-T-AA------C-T-T-G-G-G-A--G-C----T-G--C-A---T--T-------T--CA-A-A---C-------T--G-G--GT--A-T-C----T-A-G-A-G-T--G---C-GG-----GA-G-A--------G-G-A-A---AG-T------GG--A--ATT----C-
C-T-A-GT--GT-A-G-CG-GT--G---A--A-A---TG-C-GT-AG--AG-A-TT--A-G--G-A-A-G-A-AC-A-CC---AG--T--G--GC-GAA-G--G-C--G-------A--C-T-T-T---CTG--G--AC-C-G--------------------T-A---A-C-T--GA--CG-C-T-G--A-GG--C-A-TG-A--AA-G-C---G-TG--GG-G-
-AG-C-A-AA--------CA--GG Bacteria(100);Firmicutes(100);Clostridia(100);Clostridiales(100);Clostridiaceae(100);Clostridium(100);unclassified(100);unclassified(100);unclassified(100);

Otu1184 0 0 0 0 0 0 0 0 0 0 0 0 0 0 0 0 0 0 0 0 0 0 0 0 0 0 0 0
0 0 0 0 0 0 0 0 0 0 0 0 0 0 0 0 0 0 0 0 0 0 0 0 0 0 0 0 0
0 0 0 0 0 0 0 0 0 0 0 0 0 0 0 0 0 0 0 0 0 0 4 0 0 0 0 0 0
0 0 0 0 0 0 0 0 0 0 0 0 0 0 0 0 0 0 0 0 0 0 0 0 0 0 0
M02255_77_000000000-ADUT3_1_1102_6580_11943 T----AC---GT-AG-GGG--------GCA-A-G------C--G--T---T--GT-C-CGG-AA------TT-A--T-T--GG-GC-------GT--A-----AA-GA-GC-TC-------G-TA-G-G-C-G------------G--T-CC-A-T-T-----------AA-
---G-T-C-A--------G-A-T--G--TG--A-AA-TC--T-C-AG-G-G-----CT-C-AA------C-C-C-T-G-A-G--C-C----T-G--C-A---T--C-------T--GA-T-A---C-------T--G-G--TG--G-A-C----T-T-G-A-G-G--C---A-GG-----TA-G-G--------G-G-A-A---GG-T------GG--A--ATT----C-C-T-G-
GT--GT-A-G-CG-GT--G---A--A-A---TG-C-GC-AG--AT-A-TC--A-G--G-A-G-G-A-AC-A-CC---AG--T--G--GC-GAA-G--G-C--G-------G--C-C-T-T---CTG--G--GC-C-T--------------------G-T---T-C-T--GA--CG-C-T-G--A-GG--A-G-CG-A--AA-G-C---G-TG--GG-G--AG-
C-G-AA--------CA--GG Bacteria(100);Actinobacteria(100);Actinobacteria(100);unclassified(100);unclassified(100);unclassified(100);unclassified(100);unclassified(100);unclassified(100);

Otu1185 0 0 0 0 0 0 0 0 0 0 0 0 0 0 0 0 0 0 0 0 0 0 0 0 0 0 0 0
0 0 0 0 0 0 0 0 0 0 0 0 0 0 0 0 0 0 0 0 0 0 0 0 0 0 0 0 0
0 0 0 0 0 0 0 0 0 0 0 0 0 0 0 0 0 0 0 0 0 0 4 0 0 0 0 0 0
0 0 0 0 0 0 0 0 0 0 0 0 0 0 0 0 0 0 0 0 0 0 0 0 0 0 0
M02255_77_000000000-ADUT3_1_1105_16581_22349 T----AC---GT-AG-GGT--------GCG-A-G------C--G--T---T--GT-C-CGG-AA------TT-A--C-T--GG-GC-------GT--A-----AA-GG-GC-TC-------G-TA-G-G-T-G------------G--T-TT-G-T-C------
-----GC----G-T-C-G--------T-C-T--G--TG--A-AA-TT--C-T-GG-G-G-----CT-T-AA------C-T-C-C-G-G-G--C-G----T-G--C-A---G--G-------C--GA-T-A---C-------G--G-G--CAT-A-A-C----T-T-G-A-G-T--G---C-TG-----TA-G-G--------G-G-T-A---AC-T------GG--A--ATT----C-
C-T-G-GT--GT-A-G-CG-GT--G---A--A-A---TG-C-GC-AG--AT-A-TC--A-G--G-A-G-G-A-AC-A-CC---GA--T--G--GC-GAA-G--G-C--A-------G--G-T-T-A---CTG--G--GC-A-G--------------------T-T---A-C-T--GA--CG-C-T-G--A-GG--A-G-CG-A--AA-G-C---A-TG--GG-
T--AG-C-G-AA--------CA--GG Bacteria(100);Actinobacteria(100);Actinobacteria(100);Actinobacteridae(100);Actinomycetales(100);Corynebacterineae(100);Corynebacteriaceae(100);Corynebacterium(100);unclassified(100);

Otu1186 0 0 0 0 0 0 0 0 0 0 0 0 0 0 0 0 0 0 0 0 0 0 0 0 0 0 0 0
0 0 0 0 0 0 0 0 0 0 0 0 0 0 0 0 0 0 0 0 0 0 0 0 0 0 0 0 0
0 0 0 0 0 0 0 0 0 0 0 0 0 0 0 0 0 0 0 0 0 0 0 0 0 0 37 0 0
0 0 0 0 0 0 0 0 0 0 0 0 0 0 0 0 0 0 0 0 0 0 0 0 0 0 0
M02255_76_000000000-ADUNE_1_1119_10110_23425 C----GG---CT-AA-CT---------ACG-T-G------C--C--A---G--CA-G-CCG-CG------GT-A--A-T--AC-GT-------AG--G-----TG-GC-GA-GC-------G-TT-G-T-C-C------------G--G-AA-G-G-T------
-----AA----G-T-C-T--------A-T-C--T--TA--A-AA-GT--G-C-GG-G-G-----CT-C-AA------C-C-C-C-G-T-G--A------G-G--G-G---A--T-------G--GA-A-A---C-------T--A-T--CT--T-T-C----T-T-G-A-G-T--G---C-AG-----GA-G-A--------G-G-A-A---AG-T------GG--A--ATT----C-
C-T-A-GT--GT-A-G-CG-GT--G---A--A-A---TG-C-GT-AG--AT-A-TT--A-G--G-A-G-G-A-AC-A-CC---AG--T--G--GC-GAA-G--G-C--G-------G--C-T-T-T---CTG--G--AC-T-G--------------------T-A---A-C-T--GA--CG-C-T-G--A-GG--C-T-CG-A--AA-G-C---G-TG--GG-G-
-AG-C-G-AA--------CA--GG Bacteria(100);Firmicutes(100);Clostridia(100);Clostridiales(100);Veillonellaceae(100);Mitsuokella(100);unclassified(100);unclassified(100);unclassified(100);

Otu1187 0 0 0 0 0 0 0 0 0 0 0 0 0 0 0 0 0 0 0 0 0 0 0 0 0 0 0 0
0 0 0 0 0 0 0 0 0 0 0 0 0 0 0 0 0 0 0 0 0 0 0 0 0 0 0 0 0
0 0 0 0 0 0 0 0 0 0 0 0 0 0 0 0 0 0 0 0 0 0 5 0 0 0 0 0 0
0 0 0 0 0 0 0 0 0 0 0 0 0 0 0 0 0 0 0 0 0 0 0 0 0 0 0
M02255_77_000000000-ADUT3_1_1115_15726_8254 T----AC---GG-AG-GGG--------GCT-A-G------C--G--T---T--GT-T-CGG-AA------TT-A--C-T--GG-GC-------GT--A-----AA-GC-GC-AC-------G-TA-G-G-C-G------------G--A-CT-G-A-A-----------
AA----G-T-T-G--------G-A-G--G--TG--A-AA-TC--C-T-GG-G-G-----CT-C-AA------C-C-C-C-A-G-A--A-C----G-G--C-C---T--T-------C--AA-A-A---C-------T--A-T--CA--G-T-C----T-G-G-A-G-T--T---C-GA-----GA-G-A--------G-G-T-G---AG-T------GG--A--ATT----C-C-G-
A-GT--GT-A-G-AG-GT--G---A--A-A---TT-C-GT-AG--AT-A-TT--C-G--G-A-G-G-A-AC-A-CC---AG--T--G--GC-GAA-G--G-C--G-------G--C-T-C-A---CTG--G--CT-C-G--------------------A-T---A-C-T--GA--CG-C-T-G--A-GG--T-G-CG-A--AA-G-C---G-TG--GG-G--
AG-C-A-AA--------CA--GG Bacteria(100);Proteobacteria(100);Alphaproteobacteria(100);Rhodobacterales(100);Rhodobacteraceae(100);unclassified(100);unclassified(100);unclassified(100);unclassified(100);

Otu1188 0 0 0 0 0 0 0 0 0 0 0 0 0 0 0 0 0 0 0 0 0 0 0 0 0 0 0 0
0 0 0 0 0 0 0 0 0 0 0 0 0 0 0 0 0 0 0 0 0 0 0 0 0 0 0 0 0
0 0 0 0 0 0 0 0 0 0 0 0 0 0 0 0 0 0 0 0 0 0 1 0 0 0 0 0 0
0 0 0 14 0 0 0 2 0 0 0 0 0 0 0 0 89 0 0 0 0 0 0 0 0 0 0
M02255_77_000000000-ADUT3_1_2114_9419_15477 T----AC---GT-AG-GGC--------GCA-A-G------C--G--T---T--AT-C-CGG-AA------TT-A--T-T--GG-GC-------GT--A-----AA-GA-GC-TT-------G-TA-G-G-C-G------------G--T-TC-G-T-C-----------GC--
--G-T-C-T--------G-G-T--G--TG--A-AA-GC--C-C-AT-C-G-----CT-T-AA------C-G-G-T-G-G-G--T-C----T-G--C-G---C--C-------G--GG-T-A---C-------G--G-G--CG--G-G-C----T-A-G-A-G-T--G---C-AG-----TA-G-G--------G-G-A-G---AC-T------GG--A--ATT----C-C-C-G-
GT--GT-A-A-CG-GT--G---G--A-A---TG-T-GT-AG--AT-A-TC--G-G--G-A-A-G-A-AC-A-CC---AA--T--G--GC-GAA-G--G-C--A-------G--G-T-C-T---CTG--G--GC-T-G--------------------T-T---A-C-T--GA--CG-C-T-G--A-GA--A-G-CG-A--AA-G-C---G-TG--GG-G--AG-
C-G-AA--------CA--GG Bacteria(100);Actinobacteria(100);Actinobacteria(100);Actinobacteridae(100);Bifidobacteriales(100);Bifidobacteriaceae(100);Gardnerella(100);unclassified(100);unclassified(100);

Otu1189 0 0 0 0 0 0 0 0 0 0 0 0 0 0 0 0 0 0 0 0 0 0 0 0 0 0 0 0
0 0 0 0 0 0 0 0 0 0 0 0 0 0 0 0 0 0 0 0 0 0 0 0 0 0 0 0 0
0 0 0 0 0 0 0 0 0 0 0 0 0 0 0 0 0 0 0 0 0 0 0 8 0 0 0 0 0
0 0 0 0 0 0 0 0 0 0 0 0 0 0 0 0 0 0 0 0 0 0 0 0 0 0 0
M02255_76_000000000-ADUNE_1_1113_27055_13219 C----GG---CT-A--ACT--------ACG-T-G------C--C--A---G--CA-G-CCG-CG------GT-A--A-A--AC-GT-------AG--G-----GG-GC-GA-GC-------G-TT-GTC-C-G------------G--A-CC-T-T-T-----
------AA----G-T-C-T--------G-A-T--G--TG--A-AA-TT--C-C-GC-G-G-----CT-C-AA------C-C-G-C-G-G-G--T-G----T-G--C-A---T--T-------G--GA-A-A---C-------T--G-G--GG--G-A-C----T-T-G-A-G-T--G---C-GG-----AA-G-A--------G-G-G-G---AG-C------GG--A--ATT----
C-C-C-G-GT--GT-A-G-CG-GT--G---A--A-A---TG-C-GT-AG--AG-A-TC--G-G--G-A-G-G-A-AC-A-CC---AG--T--G--GC-GAA-G--G-C--G-------G--C-T-C-T---CTG--G--TC-C-T--------------------C-A---A-C-T--GA--CG-C-T-G--A-GG--C-G-CG-A--AA-G-C---G-TG--
GG-G--AG-C-G-AA--------CA--GG Bacteria(100);Firmicutes(100);unclassified(100);unclassified(100);unclassified(100);unclassified(100);unclassified(100);unclassified(100);unclassified(100);

Otu1190 0 0 0 0 0 0 0 0 0 0 0 0 0 0 0 0 0 0 0 0 0 0 0 0 0 0 0 0
0 0 0 0 0 0 0 0 0 0 0 0 0 0 0 0 0 0 0 0 0 0 0 0 0 0 0 0 0
0 0 0 0 0 0 0 0 0 0 0 0 0 0 0 0 0 0 0 0 0 0 0 19 0 0 0 0 0
0 0 0 0 0 0 0 0 0 0 0 0 0 0 0 0 0 0 0 0 0 0 0 0 0 0 0 0

M02255_76_000000000-ADUNE_1_1117_14636_11354 .....AC----T-A--CGT--------GCC-A-G------C--A--G---C--CG-C-GGT-AA------TA-C--G-T--AG-GG-------GG--C-----AA-GC-GT------------TA-T-C-C-G-------------G--A-AT-A-A-T-----------AA----G-T-C-T--------A-A-G--G--TT--A-AA-GT--G-C-GG-G-G-----CT-C-AA------C-C-C-C-G-T-G--A------T-G--C-C---T--T-------G--GA-A-A---C-------T--G-T--TA--A-G-C----T-T-G-A-G-T--A---T-GG-----TA-G-A--------G-G-C-A---AG-T------GG--A--ATT----T-C-A-T-GT--GT-A-G-CG-GT--A---A--A-A---TG-C-GT-AA--AT-A-TA--T-G--A-A-G-G-A-AC-A-CC---AG--T--G--GC-GAA-G--G-C--G-------G--C-T-T-G---CTG--G--GC-C-A---------------------T-T---A-C-T--GA--CG-C-T-G--A-GG--C-A-CG-A--AA-G-C---G-TG--GG-G--AG-C-A-AA--------CA--GG Bacteria(100);Firmicutes(100);Mollicutes(100);unclassified(100);unclassified(100);unclassified(100);unclassified(100);unclassified(100);unclassified(100);

Otu1191 0 0 0 0 0 0 0 0 0 0 0 0 0 0 0 0 0 0 0 0 0 0 0 0 0 0 0 0
0 0 0 0 0 0 0 0 0 0 0 0 0 0 0 0 0 0 0 0 0 0 0 0 0 0 0 0 0
0 0 0 0 0 0 0 0 0 0 0 0 0 0 0 0 0 0 0 0 0 0 0 52 0 0 0 0 0
0 0 0 0 0 0 0 0 0 0 0 0 0 0 0 0 0 0 0 0 0 0 0 0 0 0 0 0

M02255_77_000000000-ADUT3_1_1102_24563_4474 T----AC---GT-AG-GTG--------GCA-A-G------C--G--T---T--AT-C-CGG-AA------TT-A--T-T--GG-GC-------GT--A-----AA-GG-GA-GC-------G-TA-G-G-C-G-------------G--A-TA-T-T-T-----------AA----G-T-G-G--------G-A-T--G--TG--A-AA-TA--C-T-TG-G-G-----CT-C-AA------C-C-C-G-A-G-T--G-C----T-G--C-A---T--T-------C--CA-A-A---C-------T--G-G--AT--A-T-C----T-A-G-A-G-T--G---C-AG-----GA-G-A--------G-G-A-G---AG-T------GG--A--ATT----C-C-T-A-GT--GT-A-G-CG-GT--G---A--A-A---TG-C-GT-AG--AG-A-TT--A-G--G-A-A-G-A-AC-A-CC---AG--T--G--GC-GAA-G--G-C--G-------A--C-T-C-T---CTG--G--AC-T-G---------------------T-A---A-C-T--GA--CG-C-T-G--A-GG--C-T-CG-A--AA-G-C---G-TG--GG-G--AG-C-G-AA--------CA--GG Bacteria(100);Firmicutes(100);Clostridia(100);Clostridiales(100);Clostridiaceae(100);unclassified(100);unclassified(100);unclassified(100);unclassified(100);

Otu1192 0 0 0 0 0 0 0 0 0 0 0 0 0 0 0 0 0 0 0 0 0 0 0 0 0 0 0 0
0 0 0 0 0 0 0 0 0 0 0 0 0 0 0 0 0 0 0 0 0 0 0 0 0 0 0 0 0
0 0 0 0 0 0 0 0 0 0 0 0 0 0 0 0 0 0 0 0 0 0 0 41 0 0 0 0 0
0 0 0 0 0 0 0 0 0 0 0 0 0 0 0 0 0 0 0 0 0 0 0 0 0 0 0 0

M02255_77_000000000-ADUT3_1_1113_26250_22061 T----AC---GG-AG-GGG--------GCT-A-G------C--G--T---T--GT-T-CGG-AA------TT-A--C-T--GG-GC-------GT--A-----AA-GC-GC-AC-------G-TA-G-G-C-G-------------G--C-TT-T-T-T-----------AA----G-T-C-A--------G-A-G--G--TG--A-AA-GC--C-C-GG-A-G-----CT-C-AA------C-T-C-C-G-G-A--A-T----T-G--C-C---T--T-------T--GA-G-A---C-------T--G-G--AA--G-G-C----T-T-G-A-A-T--C---A-CG-----GA-G-A--------G-G-T-G---GG-T------GG--A--ATT----C-C-G-A-GT--GT-A-G-AG-GT--G---A--A-A---TT-C-GT-AG--AT-A-TT--C-G--G-A-A-G-A-AC-A-CC---AG--T--G--GC-GAA-G--G-C--G-------G--C-C-C-A---CTG--G--AC-G-T---------------------G-T---A-T-T--GA--CG-C-T-G--A-GG--T-G-CG-A--AA-G-C---G-TG--GG-G--AG-C-A-AA--------CA--GG Bacteria(100);Proteobacteria(100);Alphaproteobacteria(100);Sphingomonadales(100);Sphingomonadaceae(100);unclassified(100);unclassified(100);unclassified(100);unclassified(100);

Otu1193 0 0 0 0 0 0 0 0 0 0 0 0 0 0 0 0 0 0 0 0 0 0 0 0 0 0 0 0
0 0 0 0 0 0 0 0 0 0 0 0 0 0 0 0 0 0 0 0 0 0 0 0 0 0 0 0 0
0 0 0 0 0 0 0 0 0 0 0 0 0 0 0 0 0 0 0 0 0 0 0 78 0 0 0 0 0
0 0 0 0 0 0 0 0 0 0 0 0 0 0 0 0 0 0 0 0 0 0 0 0 0 0 0 0

M02255_77_000000000-ADUT3_1_2108_17403_9516 T----AC---GA-AG-GGG--------GCT-A-G------C--G--T---T--GC-T-CGG-AA------TT-A--C-T--GG-GC-------GT--A-----AA-GG-GA-GC-------G-TA-G-G-C-G-------------G--A-CA-G-T-T-----------TA----T-T-C-A--------G-A-G--G--TG--A-AA-GC--C-C-AG-G-G-----CT-C-AA------C-C-T-T-G-G-A--A-T----T-G--C-C---T--T-------T--GA-T-A---C-------T--G-A--TT--G-T-C----T-T-G-A-G-T--A---T-GG-----GA-G-A--------G-G-T-T---AG-C------GG--A--ACT----C-C-G-A-GT--GT-A-G-AG-GT--G---A--A-A---TT-C-GT-AG--AT-A-TT--C-G--G-A-A-G-A-AC-A-CC---AG--T--G--GC-GAA-G--G-C--G-------G--C-T-A-A---CTG--G--CC-C-A---------------------T-T---A-C-T--GA--CG-C-T-G--A-GG--C-T-CG-A--AA-G-C---G-TG--GG-G--AG-C-A-AA--------CA--GG Bacteria(100);Proteobacteria(100);Alphaproteobacteria(100);Caulobacterales(100);Caulobacteraceae(100);unclassified(100);unclassified(100);unclassified(100);unclassified(100);

Otu1194 0 0 0 0 0 0 0 0 0 0 0 0 0 0 0 0 0 0 0 0 0 0 0 0 0 0 0 0
0 0 0 0 0 0 0 0 0 0 0 0 0 0 0 0 0 0 0 0 0 0 0 0 0 0 0 0 0
0 0 0 0 0 0 0 0 0 0 0 0 0 0 0 0 0 0 0 0 0 0 0 0 0 0 0 0 0
33 0 0 0 0 0 0 0 0 0 0 0 0 0 0 0 0 0 0 0 0 0 0 0 0 0 0 0

M02255_77_000000000-ADUT3_1_1114_23677_9552 T----AC---GG-AG-GAT--------GCA-A-G------C--G--T---T--AT-C-CGG-AA------TT-A--T-T--GG-GC-------GT--A-----AA-GC-GT-CC-------G-TA-G-G-T-G-------------G--C-AC-T-T-C-----------AA----G-T-C-T--------T-T-T--G--TC--A-AA-TC--G-T-GC-A-G-----CT-T-AA------C-T-G-C-A-T-A--C------G-GG-C-G---G--A-------G--GA-A-A---C-------T--G-G--AG--C-G-C----T-A-G-A-G-T--G---C-GA-----TA-G-G--------G-G-T-C---AC-A------GG--A--ATT----C-C-C-A-GT--GT-A-G-CG-GT--G---A--A-A---TG-C-GT-AG--AT-A-TT--G-G--G-A-A-G-A-AC-A-CC---AG--C--G--GC-GAA-A--G-C--G-------T--G-T-G-A---CTG--G--GT-C-T---------------------G-C---A-C-T--GA--CA-C-T-G--A-GG--G-A-CG-A--AA-G-C---T-AG--GG-G--AG-C-G-AA--------AG--GG Bacteria(100);Cyanobacteria(100);SubsectionIII(100);unclassified(100);unclassified(100);unclassified(100);unclassified(100);unclassified(100);unclassified(100);

Otu1195 0 0 0 0 0 0 0 0 0 0 0 0 0 0 0 0 0 0 0 0 0 0 0 0 0 0 0 0
0 0 0 0 0 0 0 0 0 0 0 0 0 0 0 0 0 0 0 0 0 0 0 0 0 0 0 0 0
0 0 0 0 0 0 0 0 0 0 0 0 0 0 0 0 0 0 0 0 0 0 0 0 0 0 0 0 25
0 0 0 0 0 0 0 0 0 0 0 0 0 0 0 0 0 0 0 0 0 0 0 0 0 0 0 0
M02255_76_000000000-ADUNE_1_1118_8404_15380 T----CG---GC-TA-ACT--------CCG-T-GC-----C--A--G---C--AG-C-CGC-GG------TA-A--T-A--CG-GA-------GG--A-----TC-CG-AG-CG-------T-TA-T-C-C-G------------GA-T-CT-A-T-T-----------AA----G-T-C-A--------G-G-G--G--TG--A-AA-TA--C-G-GT-G-G-----CT-C-AA------C-C-A-T-C-G-C--A-G----T-G--C-C---T--T-------T--GA-T-A---C-------T--G-A--TG--G-G-C----T-T-G-A-A-T--C---C-AT-----TT-G-A--------A-G-T-G---GG-C------GG--A--ATA----A-G-A-C-AA--GT-A-G-CG-GT--G---A--A-A---TG-C-AT-AG--AT-A-TG--T-C--T-T-A-G-A-AC-T-CC---GA--T--T--GC-GAA-G--G-C--A-------G--C-T-C-A---CTA--A--GC-T-G--------------------G-T---A-T-T--GA--CG-C-T-G--A-TG--C-A-CG-A--AA-G-C---G-TG--GG-G--AT-C-G-AA--------CA--GG Bacteria(100);Bacteroidetes(100);Sphingobacteria(100);Sphingobacteriales(100);Sphingobacteriaceae(100);Sphingobacteriaceae(100);Sphingobacterium(100);unclassified(100);unclassified(100);

Otu1196 0 0 0 0 0 0 0 0 0 0 0 0 0 0 0 0 0 0 0 0 0 0 0 0 0 0 0 0
0 0 0 0 0 0 0 0 0 0 0 0 0 0 0 0 0 0 0 0 0 0 0 0 0 0 0 0 0
0 0 0 0 0 0 0 0 0 0 0 0 0 0 0 0 0 0 0 0 0 0 0 0 0 0 0 0 10
0 0 0 0 0 0 0 0 0 0 0 0 0 0 0 0 0 0 0 0 0 0 0 0 0 0 0 0
M02255_76_000000000-ADUNE_1_2101_14276_7513 C----TA---AC-TC-TGT--------GCC-A-G------C-----A---G--CC-G-CGG-TA------AT-A--C-A--GA-GG-------GT--------GC-TA-GC-GT---------TG-T-T-C-G-------------G--A-CA-G-G-C-----------AA----G-T-C-G--------G-G-T--G--TG--A-AA-GC--C-C-TC-A-G-----CT-T-AA------C-T-G-A-G-G-A--A-G----T-G--C-G---C--C-------C--GA-A-A---C-------T--A-C--TT--G-G-C----T-T-G-A-G-T--A---C-CG-----GA-G-A--------G-G-G-T---GG-C------GG--A--ATT----C-C-C-G-GT--GT-A-G-AG-GT--G---A--A-A---TT-C-GT-AG--AT-A-TC--G-G--G-A-G-G-A-AC-A-CC---AG--T--G--GC-GAA-G--G-C--G-------G--C-C-A-C---CTG--G--AC-G-G--------------------C-T---A-C-T--GA--CG-C-T-G--A-GA--C-G-CG-A--AA-G-C---G-TG--GG-G--AG-C-A-AA--------CA--GG Bacteria(100);Proteobacteria(100);Deltaproteobacteria(100);Myxococcales(100);unclassified(100);unclassified(100);unclassified(100);unclassified(100);unclassified(100);

Otu1197 0 0 0 0 0 0 0 0 0 0 0 0 0 0 0 0 0 0 0 0 0 0 0 0 0 0 0 0
0 0 0 0 0 0 0 0 0 0 0 0 0 0 0 0 0 0 0 0 0 0 0 0 0 0 0 0 0
0 0 0 0 0 0 0 0 0 0 0 0 0 0 0 0 0 0 0 0 0 0 0 0 0 0 0 0 11
0 0 0 0 0 0 0 0 0 0 0 0 0 0 0 0 0 0 0 0 0 0 0 0 0 0 0 0
M02255_76_000000000-ADUNE_1_1113_19559_21734 C----GG---CT-AA-CTC--------CGT-G-C------C--A--G---C--AG-C-CGC-GG------TA-A--T-A--CG-GA-------GG--A-----TC-CA-AG-CG-------T-TA-T-C-CGG-------------G--C-TT-A-T-T-----------AA----G-T-C-A--------G-G-G--G--TG--A-AA-GA--C-G-GT-A-G-----CT-T-AA------C-T-A-T-C-G-C--A-G----T-G--C-C---T--T-------T--GA-T-A---C-------T--G-A--TG--A-G-C----T-T-G-A-A-T--A---C-AG-----TT-G-A--------A-G-T-G---GG-C------GG--A--ATG----T-G-A-C-AA--GT-A-G-CG-GT--G---A--A-A---TG-C-AT-AG--AT-A-TG--T-C--A-C-A-G-A-AC-A-CC---GA--T--T--GC-GAA-G--G-C--A-------G--C-T-C-A---CTA--A--AG-T-G--------------------T-G---A-T-T--GA--CA-C-T-G--A-GG--C-A-CG-A--AA-G-C---G-TG--GG-G--AT-C-A-AA--------CA--GG Bacteria(100);Bacteroidetes(100);Sphingobacteria(100);Sphingobacteriales(100);Sphingobacteriaceae(100);unclassified(100);unclassified(100);unclassified(100);unclassified(100);

Otu1198 0 0 0 0 0 0 0 0 0 0 0 0 0 0 0 0 0 0 0 0 0 0 0 0 0 0 0 0
0 0 0 0 0 0 0 0 0 0 0 0 0 0 0 0 0 0 0 0 0 0 0 0 0 0 0 0 0
0 0 0 0 0 0 0 0 0 0 0 0 0 0 0 0 0 0 0 0 0 0 0 0 0 0 0 0 0
13 0 0 0 0 0 0 0 0 0 0 0 0 0 0 0 0 0 0 0 0 0 0 0 0 0 0 0
M02255_76_000000000-ADUNE_1_1116_3461_15781 C----GG---CT-AA-ATA--------CGT-G-C------C--A--G---C--AG-C-C-G-CG------GT-A--A-T--AC-GT-------AT--G-----TC-CC-AA-GC-------GTTA-T-C-C-G-------------G--T-TA-T-T-T-----------AA----G-T-C-T--------G-A-T--G--TG--A-AA-GC--C-T-AC-G-G-----CT-C-AA------C-C-G-T-A-G-A--A-T----T-G--C-A---T--C-------G--GA-A-A---C-------T--G-G--AT--G-A-C----T-T-G-A-G-T--G---C-AG-----TA-G-A--------G-G-A-G---AG-T------GG--A--ACT----C-C-A-T-GT--GT-A-G-CG-GT--G---A--A-A---TG-C-GT-AG--AT-A-TA--T-G--G-A-A-G-A-AC-A-CC---AG--A--G--GC-GAA-G--G-C--G-------G--C-T-C-T---CTG--G--AC-T-G--------------------T-A---A-C-T--GA--CG-C-T-G--A-GG--C-T-CG-A--AA-G-T---G-TG--GG-T--AG-C-A-AA--------CA--GG Bacteria(100);Firmicutes(100);Bacilli(100);unclassified(100);unclassified(100);unclassified(100);unclassified(100);unclassified(100);unclassified(100);

Otu1199 0 0 0 0 0 0 0 0 0 0 0 0 0 0 0 0 0 0 0 0 0 0 0 0 0 0 0 0
0 0 0 0 0 0 0 0 0 0 0 0 0 0 0 0 0 0 0 0 0 0 0 0 0 0 0 0 0
0 0 0 0 0 0 0 0 0 0 0 0 0 0 0 0 0 0 0 0 0 0 0 0 0 0 0 0 0
29 0 0 0 0 0 0 0 0 0 0 0 0 0 0 0 0 0 0 0 0 0 0 0 0 0 0 0
M02255_77_000000000-ADUT3_1_1105_8243_10993 T----AC---GG-AG-GGG--------GCT-A-G------C--G--T---T--GT-T-CGG-AA------TT-A--C-T--GG-GC-------GT--A-----AA-GC-GC-GC-------G-TA-G-G-C-G-------------G--C-TT-G-G-T-----------AA----G-T-T-A--------G-A-G--G--TG--A-AA-GC--C-C-GG-G-G-----CT-C-AA------C-C-C-C-G-G-A--A-T----A-G--C-C---T--T-------T--AA-G-A---C-------T--G-C--TT--C-G-C----T-T-G-A-A-C--G---T-CG-----GA-G-A--------G-G-T-G---AG-T------GG--A--ATT----C-C-G-A-GT--GT-A-G-AG-GT--G---A--A-A---TT-C-GT-AG--AT-A-TT--C-G--G-A-A-G-A-AC-A-CC---AG--T--G--GC-GAA-G--G-C--G-------G--C-T-C-A---CTG--G--AC-G-A--------------------C-T---G-T-T--GA--CG-C-T-G--A-GG--T-G-CG-A--AA-G-C---G-TG--GG-G--AG-C-A-AA--------CA--GG Bacteria(100);Proteobacteria(100);Alphaproteobacteria(100);Sphingomonadales(100);Sphingomonadaceae(100);Sphingomonas(100);Sphingomonadaceae(100);Sphingomonas(100);unclassified(100);

Otu1200 0 0 0 0 0 0 0 0 0 0 0 0 0 0 0 0 0 0 0 0 0 0 0 0 0 0 0 0
0 0 0 0 0 0 0 0 0 0 0 0 0 0 0 0 0 0 0 0 0 0 0 0 0 0 0 0 0
0 0 0 0 0 0 0 0 0 0 0 0 0 0 0 0 0 0 0 0 0 0 0 0 0 0 0 0 0
46 0 0 0 0 0 0 0 0 0 0 0 0 0 0 0 0 0 0 0 0 0 0 0 0 0 0
M02255_77_000000000-ADUT3_1_1107_15285_13755 T----AC---GT-AG-GTG--------GCA-A-G------C--G--T---T--GT-C-CGG-AT------TT-A--C-T--GG-GC-------GT--A-----AA-GG-AT-GC-------G-TA-G-G-C-G------------G--A-TA-C-T-T------
-----AA----G-T-C-A--------G-A-T--G--TG--A-AA-AT--C-C-CG-A-G-----CT-T-AA------C-T-T-G-G-G-A--A-C----T-G--C-A---T--T-------T--GA-A-A---C-------T--G-G--GT--A-T-C----T-A-G-A-G-T--G---T-GG-----GA-G-A--------G-G-A-A---AG-T------GG--A--ATT----C-
C-T-A-GT--GT-A-G-CG-GT--G---A--A-A---TG-C-GT-AG--AG-A-TT--A-G--G-A-A-G-A-AC-A-CC---AG--T--G--GC-GAA-G--G-C--G-------A--C-T-T-T---CTG--G--AC-C-A--------------------T-A---A-C-T--GA--CG-C-T-G--A-GG--C-A-TG-A--AA-G-C---G-TG--GG-G-
-AG-C-A-AA--------CA--GG Bacteria(100);Firmicutes(100);Clostridia(100);Clostridiales(100);Clostridiaceae(100);Clostridium(100);unclassified(100);unclassified(100);unclassified(100);

Otu1201 0 0 0 0 0 0 0 0 0 0 0 0 0 0 0 0 0 0 0 0 0 0 0 0 0 0 0 0
0 0 0 0 0 0 0 0 0 0 0 0 0 0 0 0 0 0 0 0 0 0 0 0 0 0 0 0 0
0 0 0 0 0 0 0 0 0 0 0 0 0 0 0 0 0 0 0 0 0 0 0 0 0 0 0 0 0
22 0 0 0 0 0 0 0 0 0 0 0 0 0 0 0 0 0 0 0 0 0 0 0 0 0 0
M02255_77_000000000-ADUT3_1_1107_12664_15591 G----AC---GG-AG-GAG--------GCA-A-G------C--G--T---T--AT-C-CGG-AA------TT-A--T-T--GG-GC-------GT--A-----AA-GC-GT-CC-------G-CA-G-G-C-G------------G--C-TT-T-T-T-----
------AA----G-T-C-T--------G-T-T--G--TT--A-AA-GC--C-C-AC-A-G-----CT-C-AA------C-T-G-T-G-G-A--T-C----G-G--C-A---A--T-------G--GA-A-A---C-------T--G-G--GA--G-G-C----T-A-G-A-G-T--G---T-GG-----TA-G-G--------G-G-T-A---GA-G------GG--A--ATT----
C-C-C-G-GT--GT-A-G-CG-GT--G---A--A-A---TG-C-GT-AG--AT-A-TC--G-G--G-A-A-G-A-AC-A-CC---AG--T--G--GC-GAA-G--G-C--G-------C--T-C-T-A---CTG--G--GC-C-A--------------------C-A---A-C-T--GA--CG-C-T-G--A-GG--G-A-CG-A--AA-G-C---T-AG--
GG-G--AG-C-G-AA--------AG--GG Bacteria(100);Cyanobacteria(100);SubsectionIII(100);unclassified(100);unclassified(100);unclassified(100);unclassified(100);unclassified(100);unclassified(100);

Otu1202 0 0 0 0 0 0 0 0 0 0 0 0 0 0 0 0 0 0 0 0 0 0 0 0 0 0 0 0
0 0 0 0 0 0 0 0 0 0 0 0 0 0 0 0 0 0 0 0 0 0 0 0 0 0 0 0 0
0 0 0 0 0 0 0 0 0 0 0 0 0 0 0 0 0 0 0 0 0 0 0 0 0 0 0 0 0
28 0 0 0 0 0 0 0 0 0 0 0 0 0 0 0 0 0 0 0 0 0 0 0 0 0 0
M02255_77_000000000-ADUT3_1_1111_14194_13027 T----AC---GT-AG-GCA--------GCG-A-G------C--G--T---T--GT-T-CGG-AG------TT-A--C-T--GG-GC-------GT--A-----AA-GC-GT-GC-------G-TA-G-G-C-G------------G--T-GG-T-C-C-----
------AA----G-T-C-T--------G-G-T--G--TG--A-AA-TC--T-C-CC-G-G-----CT-C-AA------C-T-G-G-G-A-G--G-G----T-G--C-G---C--C-------G--GA-A-A---C-------T--G-G--GC--C-G-C----T-G-G-A-G-T--A---C-GG-----GA-G-A--------G-G-C-A---AG-C------GG--A--ATT----
C-C-A-G-GT--GT-A-G-CG-GT--G---A--A-A---TG-C-GT-AG--AT-A-TC--T-G--G-A-G-G-A-AC-A-CC---CG--T--G--GT-GCA-G--A-C--G-------G--C-T-T-G---CTG--G--AC-A-G--------------------T-G---A-C-T--GA--CG-C-T-G--A-GG--C-A-CG-A--AA-G-C---G-TG--
GG-T--AG-C-A-AA--------CA--GG Bacteria(100);unclassified(100);unclassified(100);unclassified(100);unclassified(100);unclassified(100);unclassified(100);unclassified(100);unclassified(100);

Otu1203 0 0 0 0 0 0 0 0 0 0 0 0 0 0 0 0 0 0 0 0 0 0 0 0 0 0 0 0
0 0 0 0 0 0 0 0 0 0 0 0 0 0 0 0 0 0 0 0 0 0 0 0 0 0 0 0 0
0 0 0 0 0 0 0 0 0 0 0 0 0 0 0 0 0 0 0 0 0 0 0 0 0 0 0 0 12
0 0 0 0 0 0 0 0 0 0 0 0 0 0 0 0 0 0 0 0 0 0 0 0 0 0 0
M02255_77_000000000-ADUT3_1_2114_24120_16997 T----AC---GT-AG-GGT--------CCG-A-G------C--G--T---T--GT-C-CGG-AA------TT-A--T-T--GG-GC-------GT--A-----AA-GA-GC-TC-------G-TA-G-G-C-G------------G--T-TC-G-T-C------
-----GC----G-T-C-T--------G-C-C--G--TG--A-AA-TC--C-T-CA-G-G-----CT-C-AA------C-C-T-G-G-G-G--C-G----T-G--C-G---G--T-------G--GG-T-A---C-------G--G-G--CG--G-A-C----T-A-G-A-G-T--G---C-GG-----TA-G-G--------G-G-A-G---AA-T------GG--A--ACT----
C-C-T-G-GT--GT-A-G-CG-GT--G---G--A-A---TG-C-GC-AG--AT-A-TC--A-G--G-A-A-G-A-AC-A-CC---GA--T--G--GC-GAA-G--G-C--A-------G--T-T-C-T---CTG--G--GC-C-G--------------------T-A---A-C-T--GA--CG-C-T-G--A-GG--A-G-CG-A--AA-G-C---G-TG--
GG-G--AG-C-G-AA--------CA--GG Bacteria(100);Actinobacteria(100);Actinobacteria(100);Actinobacteridae(100);Actinomycetales(100);unclassified(100);unclassified(100);unclassified(100);unclassified(100);

Otu1204 0 0 0 0 0 0 0 0 0 0 0 0 0 0 0 0 0 0 0 0 0 0 0 0 0 0 0 0
0 0 0 0 0 0 0 0 0 0 0 0 0 0 0 0 0 0 0 0 0 0 0 0 0 0 0 0 0
0 0 0 0 0 0 0 0 0 0 0 0 0 0 0 0 0 0 0 0 0 0 0 0 0 0 0 0 0
23 0 0 0 0 0 0 0 0 0 0 0 0 0 0 0 0 0 0 0 0 0 0 0 0 0 0
M02255_77_000000000-ADUT3_1_1119_13604_5411 T----AC---GT-AG-GGG--------GCA-A-G------C--G--T---T--AT-C-CGG-AT------TT-A--C-T--GG-GT-------GT--A-----AA-GG-GA-GC-------G-TA-G-A-C-G------------G--T-TT-A-G-C-----------AA-
---G-T-C-T--------G-A-A--G--TG--A-AA-TC--C-C-GG-G-G-----CT-T-AA------C-C-C-C-G-G-A--G-C----T-G--C-T---T--T-------G--GA-A-A---C-------T--G-T--TA--G-A-C----T-T-G-A-G-T--G---T-CG-----GA-G-A--------G-G-T-A---AG-T------AG--A--ATT----C-C-T-A-
GT--GT-A-G-CG-GT--G---A--A-A---TG-C-GT-AG--AT-A-TT--A-G--G-A-G-G-A-AT-A-CC---AG--T--G--GC-GAA-G--G-C--G-------A--C-T-T-A---CTG--G--AC-G-A--------------------T-A---A-C-T--GA--CG-T-T-G--A-GG--C-T-CG-A--AA-G-C---G-TG--GG-G--AG-
C-A-AA--------CA--GG Bacteria(100);Firmicutes(100);Clostridia(100);Clostridiales(100);Lachnospiraceae(100);unclassified(100);unclassified(100);unclassified(100);unclassified(100);

Otu1205 0 0 0 0 0 0 0 0 0 0 0 0 0 0 0 0 0 0 0 0 0 0 0 0 0 0 0 0
0 0 0 0 0 0 0 0 0 0 0 0 0 0 0 0 0 0 0 0 0 0 0 0 0 0 0 0 0
0 0 0 0 0 0 0 0 0 0 0 0 0 0 0 0 0 0 0 0 0 0 0 0 0 0 0 0 0
9 0 0 0 0 0 0 0 0 0 0 0 0 0 0 0 0 0 0 0 0 0 0 0 0 0 0 0
M02255_76_000000000-ADUNE_1_2112_15726_18412 C----GG---CT-AA-TTT--------CGT-G-C------C--A--G---C--AG-C-CGC-GG------TA-A--T-A--CG-AA-------AG--G-----TC-CA-AG-CG-------T-TA-C-T-C-G------------G--A-TT-T-G-T-------
----AT----G-T-C-G--------A-T-T--G--TG--A-AA-GC--C-C-CG-G-G-----CT-C-AA------C-C-T-G-G-G-A--A-C----T-G--C-A---G--T-------C--GA-A-A---C-------T--G-C--AA--A-G-C----T-T-G-A-A-T--G---T-TG-----GA-G-A--------G-G-T-T---GG-G------GG--A--ATA----C-
C-T-A-GT--GG-A-G-AA-GT--G---A--A-A---TT-C-GT-AG--AG-A-TT--A-G--G-T-G-G-A-AC-A-CC---AG--A--G--GC-GAA-G--G-C--G-------C--C-T-G-A---CTG--G--CC-A-A--------------------A-C---A-T-T--GA--CG-C-T-G--A-GG--C-G-CG-A--AA-G-C---G-TG--GG-
T--AG-C-A-AA--------CA--GG Bacteria(100);unclassified(100);unclassified(100);unclassified(100);unclassified(100);unclassified(100);unclassified(100);unclassified(100);unclassified(100);

Otu1206 0 0 0 0 0 0 0 0 0 0 0 0 0 0 0 0 0 0 0 0 0 0 0 0 0 0 0 0
0 0 0 0 0 0 0 0 0 0 0 0 0 0 0 0 0 0 0 0 0 0 0 0 0 0 0 0 0
0 0 0 0 0 0 0 0 0 0 0 0 0 0 0 0 0 0 0 0 0 0 0 0 0 0 0 0 0
11 0 0 0 0 0 0 0 0 0 0 0 0 0 0 0 0 0 0 0 0 0 0 0 0 0 0 0
M02255_77_000000000-ADUT3_1_1110_14598_19444 A----AC---GT-AG-GAG--------GCG-A-G------C--G--T---T--AT-C-CGG-AT------TT-A--C-T--GG-GT-------GT--A-----AA-GC-GC-GT-------G-CA-G-G-C-G------------G--T-TT-A-T-T------
-----AA----G-T-T-G--------G-G-T--G--TG--A-AA-GC--T-C-CT-G-G-----CT-C-AA------C-T-A-G-G-A-G--A-G----G-T--C-G---C--T-------C--AA-G-A---C-------T--G-A--TA--G-A-C----T-C-G-A-G-A--G---C-AG-----TA-G-G--------G-G-A-A---GG-T------GG--A--ATT----
C-C-G-G-GA--GT-A-G-TG-GT--G---A--A-A---TG-C-GT-AG--AT-A-TC--C-G--G-A-G-G-A-AC-A-CC---AG--T--G--GC-GAA-G--G-C--G-------G--C-C-T-T---CTA--G--AC-T-G--------------------T-A---T-C-T--GA--CG-C-T-C--A-GA--C-G-CG-A--AA-G-C---T-AG--GG-
T--AG-C-A-AA--------CG--GG Bacteria(100);Chloroflexi(100);Anaerolineae(100);Anaerolineales(100);Anaerolineaceae(100);unclassified(100);unclassified(100);unclassified(100);unclassified(100);

Otu1207 0 0 0 0 0 0 0 0 0 0 0 0 0 0 0 0 0 0 0 0 0 0 0 0 0 0 0 0
0 0 0 0 0 0 0 0 0 0 0 0 0 0 0 0 0 0 0 0 0 0 0 0 0 0 0 0 0
0 0 0 0 0 0 0 0 0 0 0 0 0 0 0 0 0 0 0 0 0 0 0 0 0 0 0 0 0
13 0 0 0 0 0 0 0 0 0 0 0 0 0 0 0 0 0 0 0 0 0 0 0 0 0 0 0
M02255_77_000000000-ADUT3_1_2108_16615_18854 T----AC---GG-AG-GGG--------GCT-A-G------C--G--T---T--GT-T-CGG-AA------TT-A--C-T--GG-GC-------GT--A-----AA-GC-GC-GC-------G-TA-G-G-C-G------------G--A-CC-G-T-T----
-------AA----G-T-C-G--------G-G-G--G--TG--A-AA-TC--C-C-GG-G-G-----CT-C-AA------C-C-C-C-G-G-A--A-C----G-G--C-C---T--T-------C--GA-T-A---C-------T--G-G--CG--G-T-C----T-G-G-A-G-G--T---C-GA-----GA-G-A--------G-G-T-G---GG-T------GG--A--ATT----
C-C-G-A-GT--GT-A-G-AG-GT--G---A--A-A---TT-C-GT-AG--AT-A-TT--C-G--G-A-G-G-A-AC-A-CC---GG--T--G--GC-GAA-G--G-C--G-------G--C-C-C-A---CTG--G--CT-C-G--------------------A-T---T-C-T--GA--CG-C-T-G--A-GG--C-G-CG-A--AA-G-C---G-TG--GG-
G--AG-C-A-AA--------CA--GG Bacteria(100);Proteobacteria(100);Alphaproteobacteria(100);unclassified(100);unclassified(100);unclassified(100);unclassified(100);unclassified(100);unclassified(100);

Otu1208 0 0 0 0 0 0 0 0 0 0 0 0 0 0 0 0 0 0 0 0 0 0 0 0 0 0 0 0
0 0 0 0 0 0 0 0 0 0 0 0 0 0 0 0 0 0 0 0 0 0 0 0 0 0 0 0 0
0 0 0 0 0 0 0 0 0 0 0 0 0 0 0 0 0 0 0 0 0 0 0 0 0 0 0 0 0
11 0 0 0 0 0 0 0 0 0 0 0 0 0 0 0 0 0 0 0 0 0 0 0 0 0 0 0
M02255_77_000000000-ADUT3_1_1109_8570_17849 T----AC---GT-AG-GGC--------GCA-A-G------C--G--T---T--GT-C-CGG-AA------TT-A--T-T--GG-GC-------GT--A-----AA-GA-GC-TC-------G-TA-G-G-C-G------------G--C-TT-G-T-C-----------GC-
---G-T-C-T--------G-C-C--G--TG--A-AA-GT--C-C-GG-G-G-----CT-T-AA------C-T-C-C-G-G-A--T-C----T-G--C-G---G--T-------G--GG-T-A---C-------G--G-G--CA--G-G-C----T-A-G-A-G-T--G---C-GG-----TA-G-G--------G-G-T-G---AC-T------GG--A--ATT----C-C-T-G-
GT--GT-A-G-CG-GT--G---G--A-A---TG-C-GC-AG--AT-A-TC--A-G--G-A-G-G-A-AC-A-CC---GA--T--G--GC-GAA-G--G-C--A-------G--G-T-C-A---CTG--G--GC-C-G--------------------C-A---A-C-T--GA--CG-C-T-G--A-GG--A-G-CG-A--AA-G-C---A-TG--GG-G--AG-
C-G-AA--------CA--GG Bacteria(100);Actinobacteria(100);Actinobacteria(100);Actinobacteridae(100);Actinomycetales(100);Micrococcineae(100);unclassified(100);unclassified(100);unclassified(100);

Otu1209 0 0 0 0 0 0 0 0 0 0 0 0 0 0 0 0 0 0 0 0 0 0 0 0 0 0 0 0
0 0 0 0 0 0 0 0 0 0 0 0 0 0 0 0 0 0 0 0 0 0 0 0 0 0 0 0 0
0 0 0 0 0 0 0 0 0 0 0 0 0 0 0 0 0 0 0 0 0 0 0 0 0 0 0 0 0
21 0 0 0 0 0 0 0 0 0 0 0 0 0 0 0 0 0 0 0 0 0 0 0 0 0 0 0
M02255_77_000000000-ADUT3_1_1114_9697_12836 T----AC---GT-AG-GAC--------CCA-A-G------C--G--T---T--AT-C-CGG-AG------TG-A--C-T--GG-GC-------GT--A-----AA-GA-GTTGC-------G-TA-G-G-T-G------------G--T-TT-G-T-A-----------
AA----G-T-G-A--------A-T-A--G--TG--A-AA-TC--T-G-GC-G-G-----CT-C-AA------C-C-G-T-ACA-G--G-C----T-A--T-T---A--T-------T--CA-A-A---C-------T--C-A--CA--A-A-C----T-C-G-A-G-A--A---T-GG-----TA-G-A--------G-G-T-T---AC-T------GG--A--ATT----T-C-T-
A-GT--GT-A-G-GA-GT--G---A--A-A---TC-C-GT-AG--AT-A-TT--A-G--A-A-G-G-A-AC-A-CC---AA--T--G--GC-GTA-G--G-C--A-------G--G-T-A-A---CTG--G--AC-C-A--------------------T-T---T-C-T--GA--CA-C-T-A--A-GG--C-A-CG-A--AA-G-C---G-TG--GG-G--AG-
C-G-AA--------CC--GG Bacteria(100);Candidate_division_TM7(100);unclassified(100);unclassified(100);unclassified(100);unclassified(100);unclassified(100);unclassified(100);unclassified(100);

Otu1210 0 0 0 0 0 0 0 0 0 0 0 0 0 0 0 0 0 0 0 0 0 0 0 0 0 0 0 0
0 0 0 0 0 0 0 0 0 0 0 0 0 0 0 0 0 0 0 0 0 0 0 0 0 0 0 0 0
0 0 0 0 0 0 0 0 0 0 0 0 0 0 0 0 0 0 0 0 0 0 0 0 0 0 0 0 0
11 0 0 0 0 0 0 0 0 0 0 0 0 0 0 0 0 0 0 0 0 0 0 0 0 0 0 0
M02255_77_000000000-ADUT3_1_2106_16800_19909 C----GG---CT-AA-AT---------ACGTG-C------C--A--G---C--AG-C-C-G-CG------GT-A--A-T--AC-GT-------AT--G-----TC-CC-GAGCG-------T-TA-T-A-C-G-------------G--T-TA-A-T-T-------
----AA----G-T-C-T--------G-A-T--G--TG--A-AA-GC--C-C-GG-A-G-----CT-C-AA------C-T-C-C-G-G-A--A-A----G-G--C-A---T--T-------G--GA-A-A---C-------T--G-G--TT--A-A-C----T-T-G-A-G-T--G---C-AG-----TA-G-A--------G-G-T-A---AG-T------GG--A--ACT----C-
C-A-T-GT--GT-A-G-CG-GT--G---G--A-A---TG-C-GT-AG--AT-A-TA--T-G--G-A-A-G-A-AC-A-CC---AG--C--G--GC-GAA-G--G-C--G-------G--C-T-T-A---CTG--G--AC-T-G--------------------T-A---A-C-T--GA--CG-T-T-G--A-GG--C-T-CG-A--AA-G-T---G-TG--GG-T-
-AG-C-A-AA--------CA--GG Bacteria(100);Firmicutes(100);Bacilli(100);Lactobacillales(100);Leuconostocaceae(100);Leuconostoc(100);unclassified(100);unclassified(100);unclassified(100);

Otu1211 0 0 0 0 0 0 0 0 0 0 0 0 0 0 0 0 0 0 0 0 0 0 0 0 0 0 0 0
0 0 0 0 0 0 0 0 0 0 0 0 0 0 0 0 0 0 0 0 0 0 0 0 0 0 0 0 0
0 0 0 0 0 0 0 0 0 0 0 0 0 0 0 0 0 0 0 0 0 0 0 0 0 0 0 0 0
0 0 0 0 0 0 7 0 0 0 0 0 0 0 0 0 0 0 0 0 0 0 0 0 0 0 0 0
M02255_77_000000000-ADUT3_1_1117_7739_4845 T----AC---AG-AC-GGT--------GCG-G-G------C--G--T---T--AT-T-CAG-CT------TA-A--A-T--GG-GT-------GT--A-----AA-GG-GT-AT-------G-TA-G-G-T-G-------------G--C-CA-G-T-C-----------CT--
--G-A-T-A--------A-G-T--A--GA--A-AT-TC--T-G-GG-G-T-----CA-T-AA------C-A-G-T-A-GCA--G-A----T-A--C-T---T--T-------G--TA-A-A---C-------A--GTT--CA--A-G-C----TCC-G-A-G-T--A---T-AG-----AC-G-G--------G-G-T-A---TG-G------GT--A--ATT----T-C-T-T-
CA--GT-T-A-GTGTT--T---A--T-A---GA-C-CG-AG--AT-A-GA--A-G--A-A-G-G-A-GC-A-TC---AT--T--G--GT-GAA-G--A-C--G-------T--C-A-T-T---CTA--G---T-T-T--------------------A-T---A-C-T--GA--CA-C-T-G--A-GG--T-A-CG-G--AG-G-T---T-AG--GG-G--AG-C-
A-AA--------TC--GG Bacteria(100);unclassified(100);unclassified(100);unclassified(100);unclassified(100);unclassified(100);unclassified(100);unclassified(100);unclassified(100);

Otu1212 0 0 0 0 0 0 0 0 0 0 0 0 0 0 0 0 0 0 0 0 0 0 0 0 0 0 0 0
0 0 0 0 0 0 0 0 0 0 0 0 0 0 0 0 0 0 0 0 0 0 0 0 0 0 0 0 0
0 0 0 0 0 0 0 0 0 0 0 0 0 0 0 0 0 0 0 0 0 0 0 0 0 0 0 0 11
0 0 0 0 0 0 0 0 0 0 0 0 0 0 0 0 0 0 0 0 0 0 0 0 0 0 0 0
M02255_77_000000000-ADUT3_1_1111_7555_23813 T----AC---GG-AG-AGG--------GCT-A-G------C--G--T---T--AT-T-CGG-AA------TT-A--T-T--GG-GC-------GT--A-----AA-GG-GC-GC-------G-TA-G-G-C-T-------------G--G-TT-A-A-T-----------AA-
---G-T-T-A--------A-A-A--G--TG--A-AA-TC--C-C-GA-G-G-----CT-T-AA------C-C-T-T-G-G-A--A-T----T-G--C-T---T--T-------T--AA-A-A---C-------T--A-T--TA--A-T-C----T-A-G-A-G-A--T---T-GA-----AA-G-A--------G-G-A-T---AG-A------GG--A--ATT----C-C-T-G-AT-
-GT-A-G-AG-GT--A---A--A-A---TT-C-GT-AA--AT-A-TT--A-G--G-A-G-G-A-AC-A-CC---AG--T--G--GC-GAA-G--G-C--G-------T--C-T-A-T---CTG--G--TT-C-A--------------------A-A---T-C-T--GA--CG-C-T-G--A-AG--C-G-CG-A--AG-G-C---G-TG--GG-G--AG-C-A-
AA--------CA--GG Bacteria(100);Proteobacteria(100);Alphaproteobacteria(100);Rickettsiales(100);Anaplasmataceae(100);Wolbachia(100);unclassified(100);unclassified(100);unclassified(100);

Otu1213 0 0 0 0 0 0 0 0 0 0 0 0 0 0 0 0 0 0 0 0 0 0 0 0 0 0 0 0
0 0 0 0 0 0 0 0 0 0 0 0 0 0 0 0 0 0 0 0 0 0 0 0 0 0 0 0 0
0 0 0 0 0 0 0 0 0 0 0 0 0 0 0 0 0 0 0 0 0 0 0 0 0 0 0 0 27
0 51 0 0 0 0 0 0 0 0 0 0 0 0 0 0 0 0 0 0 0 0 0 0 0 0 0 0
M02255_77_000000000-ADUT3_1_1108_9007_20157 T----AC---GA-AG-GGG--------GCT-A-G------C--G--T---T--GC-T-CGG-AA------TG-A--C-T--GG-GC-------GT--A-----AA-GG-GC-GC-------G-TA-G-G-C-G-------------G--A-AT-G-C-T-----------
TT----G-T-C-G--------G-G-C--G--TG--A-AA-TT--C-C-AG-G-G-----CT-T-AA------C-C-T-T-G-G-G--A-C----T-G--C-G---T--T-------C--GA-G-A---C-------G--G-G--TA--T-T-C----T-A-G-A-G-T--G---G-AG-----AA-G-A--------G-G-G-T---CG-T------GG--A--ATT----C-C-C-
A-GT--GT-A-G-AG-GT--G---A--A-A---TT-C-GT-AG--AT-A-TT--G-G--G-A-A-G-A-AC-A-CC---GG--T--G--GC-GAA-G--G-C--G-------G--C-G-A-C---CTG--G--TC-T-T--------------------T-T---A-C-T--GA--CG-C-T-G--A-GG--C-G-CG-A--AA-G-C---G-TG--GG-G--
AG-C-A-AA--------CA--GG Bacteria(100);Proteobacteria(100);Alphaproteobacteria(100);Rhodospirillales(100);Acetobacteraceae(100);Acidiphilium(100);unclassified(100);unclassified(100);unclassified(100);

Otu1214 0 0 0 0 0 0 0 0 0 0 0 0 0 0 0 0 0 0 0 0 0 0 0 0 0 0 0 0
0 0 0 0 0 0 0 0 0 0 0 0 0 0 0 0 0 0 0 0 0 0 0 0 0 0 0 0 0
0 0 0 0 0 0 0 0 0 0 0 0 0 0 0 0 0 0 0 0 0 0 0 0 0 0 0 0 40
0 0 0 0 0 0 0 0 0 0 0 0 0 0 0 0 0 0 0 0 0 0 0 0 0 0 0 0
M02255_77_000000000-ADUT3_1_1106_11926_16646 G----AC---GG-AG-GGG--------GCT-A-G------C--G--T---T--GT-T-CGG-AA------TG-A--C-T--GG-GC-------GT--A-----AA-GG-GC-GC-------G-TA-G-G-C-G-------------G--T-TT-T-T-T----
-------AA----G-T-T-G--------G-G-C--G--TG--A-AA-GC--C-C-CA-G-G-----CT-T-AA------C-C-T-G-G-G-A--A-C----T-G--C-G---C--T-------T--GA-T-A---C-------T--G-A--AA--G-A-C----T-A-G-A-G-G--A---C-GA-----GA-G-A--------G-G-A-A---AG-T------GG--A--ATT----
C-C-G-A-GT--GT-A-G-AG-GT--G---A--A-A---TT-C-GT-AG--AT-A-TT--C-G--G-A-G-G-A-AC-A-TC---AG--A--G--GC-GAA-G--G-C--G-------G--C-T-T-T---CTG--G--CT-C-G--------------------T-A---T-C-T--GA--CG-C-T-G--A-GG--C-G-CG-A--AA-G-C---G-TG--GG-
G--AG-C-A-AA--------CA--GG Bacteria(100);unclassified(100);unclassified(100);unclassified(100);unclassified(100);unclassified(100);unclassified(100);unclassified(100);unclassified(100);

Otu1215 0 0 0 0 0 0 0 0 0 0 0 0 0 0 0 0 0 0 0 0 0 0 0 0 0 0 0 0
0 0 0 0 0 0 0 0 0 0 0 0 0 0 0 0 0 0 0 0 0 0 0 0 0 0 0 0 0
0 0 0 0 0 0 0 0 0 0 0 0 0 0 0 0 0 0 0 0 0 0 0 0 0 0 0 0 60
0 0 0 0 0 0 0 0 0 0 0 0 0 0 0 0 0 0 0 0 0 0 0 0 0 0 0
M02255_77_000000000-ADUT3_1_1105_17063_22789 T----AC---GT-AG-GGA--------GCA-A-G------C--G--T---T--AT-C-CGG-AA------TT-A--C-T--GG-GT-------GT--A-----AA-GG-GA-GT-------G-TA-G-G-C-G------------G--G-AT-T-A-C-----
------AA----G-T-C-A--------G-A-T--G--TG--A-AA-AC--T-G-TG-G-G-----CT-C-AA------C-T-C-A-C-A-G--A-T----T-G--C-A---T--T-------T--GA-A-A---C-------T--A-T--AG--T-T-C----T-T-G-A-G-T--G---A-AG-----TA-G-A--------G-G-T-A---AG-T------GG--A--ATT----C-
C-T-A-GT--GT-A-G-CG-GT--G---A--A-A---TG-C-GT-AG--AT-A-TT--A-G--G-A-G-G-A-AC-A-TC---AG--T--G--GC-GAA-G--G-C--G-------A--C-T-T-A---CTG--G--GC-T-T--------------------T-A---A-C-T--GA--CG-C-T-G--A-GG--C-T-CG-A--AA-G-C---G-TG--GG-G-
-AG-C-A-AA--------CA--GG Bacteria(100);Firmicutes(100);Clostridia(100);Clostridiales(100);unclassified(100);unclassified(100);unclassified(100);unclassified(100);unclassified(100);

Otu1216 0 0 0 0 0 0 0 0 0 0 0 0 0 0 0 0 0 0 0 0 0 0 0 0 0 0 0 0
0 0 0 0 0 0 0 0 0 0 0 0 0 0 0 0 0 0 0 0 0 0 0 0 0 0 0 0 0
0 0 0 0 0 0 0 0 0 0 0 0 0 0 0 0 0 0 0 0 0 0 0 0 0 0 0 0 50
0 0 0 0 0 0 0 0 0 0 0 0 0 0 0 0 0 0 0 0 0 0 0 0 0 0 0
M02255_77_000000000-ADUT3_1_1103_15167_16095 T----AC---GT-AG-GTG--------GCG-A-G------C--G--T---T--AT-C-CGG-AA------TT-A--C-T--GG-GT-------GT--A-----AA-GG-GT-GT-------G-TA-G-G-C-G------------G--G-GA-A-G-C----
-------AA----G-T-C-A--------G-A-T--G--TG--A-AA-AC--C-A-AA-G-G-----CT-C-AA------C-C-T-T-T-G-G--C-T----T-G--C-A---T--T-------T--GA-A-A---C-------T--G-T--TT--T-T-C----T-T-G-A-G-A--G---T-GG-----GA-G-A--------G-G-T-A---AA-C------GG--A--ATT----C-
C-T-A-GT--GT-A-G-TA-GT--G---A--A-A---TG-C-GT-AG--AT-A-TT--A-G--G-A-G-G-A-AC-A-CC---GG--T--G--GC-GAA-G--G-C--G-------G--T-T-T-A---CTG--G--AC-C-A--------------------C-A---A-C-T--GA--CG-C-T-G--A-GA--C-A-CG-A--AA-G-C---G-TG--GG-G-
-AG-C-A-AA--------CA--GG Bacteria(100);unclassified(100);unclassified(100);unclassified(100);unclassified(100);unclassified(100);unclassified(100);unclassified(100);unclassified(100);

Otu1217 0 0 0 0 0 0 0 0 0 0 0 0 0 0 0 0 0 0 0 0 0 0 0 0 0 0 0 0
0 0 0 0 0 0 0 0 0 0 0 0 0 0 0 0 0 0 0 0 0 0 0 0 0 0 0 0 0
0 0 0 0 0 0 0 0 0 0 0 0 0 0 0 0 0 0 0 0 0 0 0 0 0 0 0 3 0
2 0 3 0 0 0 0 0 0 0 0 0 0 0 0 0 0 0 0 0 0 0 0 0 0 0 1
M02255_76_000000000-ADUNE_1_2115_6267_9939 C----TA---AC-TC-TGT--------GCC-A-G------C-----A---G--CC-G-CGG-TA------AT-A--C-A--GA-GG-------GT--------GC-GA-GC-G--------T-TA-A-T-C-G------------G--C-TA-A-T-T-----------GA----
G-T-C-G--------G-A-T--G--TG--A-AA-TC--C-C-CG-A-G-----CT-T-AA------C-T-T-G-G-G-A--A-T----T-G--C-A---T--T-------C--GA-T-A---C-------T--G-G--TT--A-G-C----T-A-G-A-G-T--G---T-GG-----GA-G-A--------G-G-A-T---GG-T------AG--A--ATT----C-C-A-G-GT--
GT-A-G-CG-GT--G---A--A-A---TG-C-GT-AG--AG-A-TC--T-G--G-A-G-G-A-AT-A-CC---GA--T--G--GC-GAA-G--G-C--A-------G--C-C-A-T---CTG--G--CC-T-A--------------------A-C---A-C-T--GA--CG-C-T-G--A-GG--T-A-CG-A--AA-G-C---A-TG--GG-G--AG-C-A-
AA--------CA--GG Bacteria(100);Proteobacteria(100);Gammaproteobacteria(100);Pseudomonadales(100);Moraxellaceae(100);Acinetobacter(100);unclassified(100);unclassified(100);unclassified(100);

Otu1218 0 0 0 0 0 0 0 0 0 0 0 0 0 0 0 0 0 0 0 0 0 0 0 0 0 0 0 0
0 0 0 0 0 0 0 0 0 0 0 0 0 0 0 0 0 0 0 0 0 0 0 0 0 0 0 0 0
0 0 0 0 0 0 0 0 0 0 0 0 0 0 0 0 0 0 0 0 0 0 0 0 0 0 0 20 0
0 0 0 0 0 0 0 0 0 0 0 0 0 0 0 0 0 0 0 0 0 0 0 0 0 0 0
M02255_77_000000000-ADUT3_1_2104_6270_10883 T----AC---GT-AG-GGT--------GCG-A-G------C--G--T---T--AT-C-CGG-AT------TT-A----T--------------T-----------GG-G---C-------G-TA-G-G-C-G------------G--T-TC-G-T-C-----------GC----G-T-
C-C--------G-G-T--G--TG--A-AA-GT--C-C-AT-C-G-----CC-T-AA------C-G-G-T-G-G-A--T-C----T-G--C-G---C--C-------G--GG-T-A---C-------G--G-G--CG--G-G-C----T-G-G-A-G-T--G---C-GG-----TA-G-G--------G-G-A-G---AC-T------GG--A--ATT----C-C-C-G-GT--
GT-A-A-CG-GT--G---G--A-A---TG-T-GT-AG--AT-A-TC--G-G--G-A-A-G-A-AC-A-CC---AA--T--G--GC-GAA-G--G-C--A-------G--G-T-C-T---CTG--G--GC-C-G--------------------T-C---A-C-T--GA--CG-C-T-G--A-GG--A-G-CG-A--AA-G-C---G-TG--GG-G--AG-C-G-
AA--------CA--GG Bacteria(100);Actinobacteria(100);Actinobacteria(100);Actinobacteridae(100);Bifidobacteriales(100);Bifidobacteriaceae(100);Bifidobacterium(100);unclassified(100);unclassified(100);

Otu1219 0 0 0 0 0 0 0 0 0 0 0 0 0 0 0 0 0 0 0 0 0 0 0 0 0 0 0 0
0 0 0 0 0 0 0 0 0 0 0 0 0 0 0 0 0 0 0 0 0 0 0 0 0 0 0 0 0
0 0 0 0 0 0 0 0 0 0 0 0 0 0 0 0 0 0 0 0 0 0 0 0 0 0 0 13 0
0 0 0 0 0 0 0 0 0 0 0 0 0 0 0 0 0 0 0 0 0 0 0 0 0 0 0
M02255_77_000000000-ADUT3_1_1117_17225_12544 T----AC---GA-AG-GGG--------GCT-A-G------C--G--T---T--GT-T-CGG-AA------TT-A--C-T--GG-GC-------GT--A-----AA-GC-GT-GC-------G-CA-G-G-C-T------------G--C-CA-T-T-T------
-----AA----G-T-C-A--------G-G-G--G--TG--A-AA-GC--C-C-AG-A-G-----CT-C-AA------C-T-C-T-G-G-A--A-T----T-G--C-C---T--T-------T--GA-A-A---C-------T--A-T--TT--G-G-C----T-T-G-A-G-T--G---C-GG-----GA-G-A--------G-G-T-G---AG-T------GG--A--ATT----C-
C-C-A-GT--GT-A-G-AG-GT--G---A--A-A---TT-C-GT-AG--AT-A-TT--G-G--G-A-A-G-A-AC-A-CC---GG--T--G--GC-GAA-G--G-C--G-------G--C-T-C-A---CTG--G--CC-C-G--------------------T-T---T-C-T--GA--CG-C-T-C--A-TG--C-A-CG-A--TA-G-C---G-TG--GG-G--
AT-C-A-AA--------CA--GG Bacteria(100);Proteobacteria(100);unclassified(100);unclassified(100);unclassified(100);unclassified(100);unclassified(100);unclassified(100);unclassified(100);

Otu1220 0 0 0 0 0 0 0 0 0 0 0 0 0 0 0 0 0 0 0 0 0 0 0 0 0 0 0 0
0 0 0 0 0 0 0 0 0 0 0 0 0 0 0 0 0 0 0 0 0 0 0 0 0 0 0 0 0
0 0 0 0 0 0 0 0 0 0 0 0 0 0 0 0 0 0 0 0 0 0 0 0 0 0 0 22 0
0 0 0 0 0 0 0 0 0 0 0 0 0 0 0 0 0 0 0 0 0 0 0 0 0 0 0 0
M02255_76_000000000-ADUNE_1_1106_6775_11584 C----CG---GC-TA-ACT--------TCG-T-G------C--C--A---G--CA-G-CCG-CG------GT-A--A-T--AC-GA-------AG--G-----GT-GC-GA-GC-------G-TT-GTT-C-G------------G--G-AG-C-T-T-----------AA----G-T-C-A--------A-G-T--G--TG--A-AA-TC--C-C-CA-G-G-----CT-C-AA------C-C-T-G-G-G-A--C-G----T-G--C-A---C--C-------T--GA-A-A---C-------T--G-G--GC--T-T-C----T-T-G-A-A-T--C---T-TG-----GA-G-A--------G-G-G-T---GA-T------GG--A--ATT----G-C-T-G-GT--GT-A-G-GA-GT--G---A--C-A---TC-C-GT-AG--AG-A-TC--A-G--C-A-G-G-A-AC-A-CC---GG--A--G--GC-GAA-G--G-C--G-------A--T-C-A-C---CTG--G--CC-A-A--------------------A-G---A-T-T--GA--CG-C-T-G--A-GG--A-A-CG-A--AA-G-C---G-TG--GG-G--AG-C-A-AA--------CA--GG Bacteria(100);unclassified(100);unclassified(100);unclassified(100);unclassified(100);unclassified(100);unclassified(100);unclassified(100);unclassified(100);

Otu1221 0 0 0 0 0 0 0 0 0 0 0 0 0 0 0 0 0 0 0 0 0 0 0 0 0 0 0 0
0 0 0 0 0 0 0 0 0 0 0 0 0 0 0 0 0 0 0 0 0 0 0 0 0 0 0 0 0
0 0 0 0 0 0 0 0 0 0 0 0 0 0 0 0 0 0 0 0 0 0 0 0 0 0 0 52 0
0 0 0 0 0 0 0 0 0 0 0 0 0 0 0 0 0 0 0 0 0 0 0 0 0 0 0 0
M02255_77_000000000-ADUT3_1_1114_6905_11830 T----AC---GA-AG-GGG--------GCT-A-G------C--G--T---T--GC-T-CGG-AA------TC-A--C-T--GG-GC-------GT--A-----AA-GC-GC-AC-------G-TA-G-G-C-G------------G--A-TC-G-T-T-----------AA----G-T-C-A--------G-G-G--G--TG--A-AA-TC--C-T-GG-G-G-----CT-C-AA------C-C-T-C-A-G-A--A-C----T-G--C-C---C--T-------T--GA-T-A---C-------T--G-G--CG--A-T-C----T-C-G-A-G-T--T---C-GA-----GA-G-A--------G-G-T-T---GG-T------GG--A--ACT----C-C-G-A-GT--GT-A-G-AG-GT--G---A--A-A---TT-C-GT-AG--AT-A-TT--C-G--G-A-A-G-A-AC-A-CC---AG--T--G--GC-GAA-G--G-C--G-------G--C-C-A-A---CTG--G--CT-C-G--------------------A-T---A-C-T--GA--CG-C-T-G--A-GG--T-G-CG-A--AA-G-C---G-TG--GG-G--AG-C-A-AA--------CA--GG Bacteria(100);Proteobacteria(100);Alphaproteobacteria(100);Rhizobiales(100);Hyphomicrobiaceae(100);unclassified(100);unclassified(100);unclassified(100);unclassified(100);

Otu1222 0 0 0 0 0 0 0 0 0 0 0 0 0 0 0 0 0 0 0 0 0 0 0 0 0 0 0 0
0 0 0 0 0 0 0 0 0 0 0 0 0 0 0 0 0 0 0 0 0 0 0 0 0 0 0 0 0
0 0 0 0 0 0 0 0 0 0 0 0 0 0 0 0 0 0 0 0 0 0 0 0 0 0 0 10 0
0 0 0 0 0 0 0 0 0 0 0 0 0 0 0 0 0 0 0 0 0 0 0 0 0 0 0 0
M02255_77_000000000-ADUT3_1_1110_10703_2323 T----A----AG-CA-CCG--------GCT-A-A------C-----T---C--TG-T-GCC-AG------CA-G--C-C--GC-G--------GT--A-----AT-AC-AG-TA-------G-GG-T-G-C--------------G--A-GC-G-T-T-----------AA----G-T-C-G--------G-A-T--G--TG--A-CA-TC--C-C-TG-A-G-----CT-T-AA------C-T-T-A-G-G-A--A-T----T-G--C-A---T--T-------C--GA-T-A---C-------T--G-G--GA--A-G-C----T-A-G-A-G-T--A---T-GG-----GA-G-A--------G-G-A-T---GG-T------AG--A--ATT----C-C-A-G-GT--GT-A-G-CG-GT--G---A--A-A---TG-C-GT-AG--AG-A-TC--T-G--G-A-G-G-A-AT-A-CC---GA--T--G--GC-GAA-G--G-C--A-------G--C-C-A-T---CTG--G--CC-T-A--------------------A-T---A-C-T--GA--CG-C-T-G--A-GG--T-A-CG-A--AA-G-C---A-TG--GG-G--AG-C-A-AA--------CA--GG Bacteria(100);Proteobacteria(100);Gammaproteobacteria(100);Pseudomonadales(100);Moraxellaceae(100);Acinetobacter(100);unclassified(100);unclassified(100);unclassified(100);

Otu1223 0 0 0 0 0 0 0 0 0 0 0 0 0 0 0 0 0 0 0 0 0 0 0 0 0 0 0 0
0 0 0 0 0 0 0 0 0 0 0 0 0 0 0 0 0 0 0 0 0 0 0 0 0 0 0 0 0
0 0 0 0 0 0 0 0 0 0 0 0 0 0 0 0 0 0 0 0 0 0 0 0 0 0 0 27 0
0 0 0 0 0 0 0 0 0 0 0 0 0 0 0 0 0 0 0 0 0 0 0 0 0 0 0 0
M02255_77_000000000-ADUT3_1_1109_15675_18273 T----AC---GG-AA-GGG--------GCT-A-G------C--G--T---T--GT-T-CGG-AA------TT-A--C-T--GG-GC-------GT--A-----AA-GC-GC-GC-------G-TA-G-G-C-G------------G--C-TT-T-T-C-----------AA----G-T-G-A--------G-G-C--G--TG--A-AA-GT--C-C-TG-G-G-----CT-C-AA------C-C-T-G-G-G-G--A-C----G-G--C-G---C--T-------T--CA-G-A---C-------T--G-T--TT--G-G-C----T-A-G-A-G-T--C---C-GG-----AA-G-A--------G-G-G-T---GG-T------GG--A--ATT----C-C-G-A-GT--GT-A-G-AG-GT--G---A--A-A---TT-C-GT-AG--AT-A-TT--C-G--G-A-G-G-A-AC-A-CC---GG--T--G--GC-GAA-G--G-C--G-------G--C-C-A-C---CTG--G--TC-C-G--------------------G-T---A-C-T--GA--CG-C-T-C--A-AG--T-G-CG-A--AA-G-C---G-TG--GG-G--AG-C-A-AA--------CA--GG Bacteria(100);Proteobacteria(100);Alphaproteobacteria(100);Rhodospirillales(100);Rhodospirillaceae(100);Defluviicoccus(100);unclassified(100);unclassified(100);unclassified(100);

Otu1224 0 0 0 0 0 0 0 0 0 0 0 0 0 0 0 0 0 0 0 0 0 0 0 0 0 0 0 0
0 0 0 0 0 0 0 0 0 0 0 0 0 0 0 0 0 0 0 0 0 0 0 0 0 0 0 0 0
0 0 0 0 0 0 0 0 0 0 0 0 0 0 0 0 0 0 0 0 0 0 0 0 0 0 0 48 7
0 0 0 0 0 0 0 0 0 0 0 0 0 0 0 0 0 0 0 0 0 0 0 0 0 0 0 0
M02255_76_000000000-ADUNE_1_2112_16320_24833 T----AA---CT-AT-GT---------GCC-A-G------C--A--G---T--CG-C-GGT-AA------TA-C--A-T--AG-GT-------TG--C-----AA-GC-GT-TA-------T-CC-G-G---G-------------G--A-TT-A-A-T-----------AA----G-T-C-T--------G-G-T--G--TT--A-AA-GG--C-A-AC-T-G-----CT-T-AA------C-G-G-T-G-T--A------T-G--C-A---T--T-------G--GA-A-A---C-------T--A-T--TA--G-T-C----T-A-G-A-G-T--G---T-GG-----TA-G-G--------G-A-G-T---TC-T------GG--A--ACT----C-C-A-T-GT--GG-A-G-CG-GT--G---G--A-A---TG-C-GT-AG--AT-A-TA--T-G--G-A-A-G-A-AC-A-CC---AG--T--G--GC-GAA-G--G-C--G-------A--G-A-A-C---TTA--G--GC-C-A--------------------T-T---A-C-T--GA--CG-C-T-T--A-GG--C-T-TG-A--AA-G-T---G-TG--GG-G--AG-C-A-AA--------TA--GG Bacteria(100);Firmicutes(100);Mollicutes(100);unclassified(100);unclassified(100);unclassified(100);unclassified(100);unclassified(100);unclassified(100);

Otu1225 0 0 0 0 0 0 0 0 0 0 0 0 0 0 0 0 0 0 0 0 0 0 0 0 0 0 0 0
0 0 0 0 0 0 0 0 0 0 0 0 0 0 0 0 0 0 0 0 0 0 0 0 0 0 0 0 0
0 0 0 0 0 0 0 0 0 0 0 0 0 0 0 0 0 0 0 0 0 0 0 0 0 0 13 0 0
2 0 0 0 0 0 0 0 0 0 0 4 1 0 0 0 0 0 0 0 0 0 0 0 0 0 0 0
M02255_76_000000000-ADUNE_1_1106_9338_11659 T----AC---GT-AG-GGG--------GCA-A-G------C--G--T---T--AT-C-CGG-AT------TT-A--C-T--GG-GT-------GT--A-----AA----G--G--------G--A-G---C-----------------A-GT-A-G-------------A----G-T-C-T--------G-A-T--G--TG--A-AA-GG--C-G-GG-G-G-----CT-C-AA------C-C-C-C-C-G-G--A-C----T-G--C-A---T--T-------G--GA-A-A---C-------T--G-T--TG--A-T-C----T-T-G-A-G-T--A---C-CG-----GA-G-A--------G-G-T-A---AG-C------GG--A--ATT----C-C-T-A-GT--GT-A-G-CG-GT--G---A--A-A---TG-C-GT-AG--AT-A-TT--A-G--G-A-G-G-A-AC-A-CC---AG--T--G--GC-GAA-G--G-C--G-------G--C-T-T-A---CTG--G--AC-G-G--------------------T-A---A-C-T--GA--CG-T-T-G--A-GG--C-T-CG-A--AA-G-C---G-TG--GG-G--AG-C-A-AA--------CA--GG Bacteria(100);Firmicutes(100);Clostridia(100);Clostridiales(100);Lachnospiraceae(100);Blautia(100);unclassified(100);unclassified(100);unclassified(100);

Otu1226 0 0 0 0 0 0 0 0 0 0 0 0 0 0 0 0 0 0 0 0 0 0 0 0 0 0 0 0
0 0 0 0 0 0 0 0 0 0 0 0 0 0 0 0 0 0 0 0 0 0 0 0 0 0 0 0 0
0 0 0 0 0 0 0 0 0 0 0 0 0 0 0 0 0 0 0 0 0 0 0 0 0 0 520 0 0
12 0 0 40 0 0 0 0 0 0 0 0 0 0 0 0 0 0 0 0 0 0 0 0 0 0 0 0
M02255_77_000000000-ADUT3_1_1101_12680_11432 T----AC---GA-AG-GGT--------GCT-A-G------C--G--T---T--GT-T-CGG-AA------TC-A--C-T--GG-GC-------GT--A-----AA-GC-GC-GC-------G-TA-G-G-C-G-------------G--C-GT-T-T-C-----------AA----G-T-C-A--------G-G-G--G--TG--A-AA-TC--C-C-GA-G-G-----CT-C-AA------C-C-T-C-G-G-A--A-T----T-G--C-C---T--T-------T--GA-A-A---C-------T--G-A--AA--C-G-C----T-A-G-A-A-T--A---T-CG-----GA-G-G--------G-G-A-T---AG-C------GG--A--ATT----T-C-T-A-AT--GT-A-G-CG-GT--G---A--A-A---TG-C-GT-AG--AT-A-TT--A-G--A-A-G-G-A-AC-A-CC---GG--T--G--GC-GAA-G--G-C--G-------G--C-T-A-T---CTG--G--AC-G-A--------------------T-T---A-T-T--GA--CG-C-T-G--A-GG--C-G-CG-A--AA-G-C---G-TG--GG-G--AT-C-A-AA--------CA--GG Bacteria(100);unclassified(100);unclassified(100);unclassified(100);unclassified(100);unclassified(100);unclassified(100);unclassified(100);unclassified(100);

Otu1227 0 0 0 0 0 0 0 0 0 0 0 0 0 0 0 0 0 0 0 0 0 0 0 0 0 0 0 0
0 0 0 0 0 0 0 0 0 0 0 0 0 0 0 0 0 0 0 0 0 0 0 0 0 0 0 0 0
0 0 0 0 0 0 0 0 0 0 0 0 0 0 0 0 0 0 0 0 0 0 0 0 0 0 0 0 0
0 0 0 0 0 0 0 0 0 0 0 0 0 0 0 0 0 0 0 39 0 0 0 0 0 0 0 0
M02255_77_000000000-ADUT3_1_1102_10488_19348 T----AC---GG-AG-GGG--------GCT-A-G------C--G--T---T--GT-T-CGG-AA------TT-A--C-T--GG-GC-------GT--A-----AA-GC-GC-GC-------G-TA-G-G-C-G-------------G--C-TT-T-G-T-----------AA----G-T-C-A--------G-A-G--G--TG--A-AA-TC--C-C-GG-G-G-----CT-C-AA------C-T-C-C-G-G-A--A-C----T-G--C-C---T--T-------T--GA-A-A---C-------T--G-C--AT--C-G-C----T-T-G-A-A-T--G---T-CG-----GA-G-A--------G-G-T-G---AG-T------GG--A--ATT----C-C-G-A-GT--GT-A-G-AG-GT--G---A--A-A---TT-C-GT-AG--AT-A-TT--C-G--G-A-A-G-A-AC-A-CC---AG--T--G--GC-GAA-G--G-C--G-------G--C-T-C-A---CTG--G--AC-G-A--------------------C-T---A-T-T--GA--CG-C-T-G--A-GG--T-G-CG-A--AA-G-C---G-TG--GG-G--AG-C-A-AA--------CA--GG Bacteria(100);Proteobacteria(100);Alphaproteobacteria(100);unclassified(100);unclassified(100);unclassified(100);unclassified(100);unclassified(100);unclassified(100);

Otu1228 0 0 0 0 0 0 0 0 0 0 0 0 0 0 0 0 0 0 0 0 0 0 0 0 0 0 0 0
0 0 0 0 0 0 0 0 0 0 0 0 0 0 0 0 0 0 0 0 0 0 0 0 0 0 0 0 0
0 0 0 0 0 0 0 0 0 0 0 0 0 0 0 0 0 0 0 0 0 0 0 0 0 0 0 0 0
0 0 0 0 0 0 0 0 0 0 0 0 0 0 0 0 0 0 14 0 0 0 0 0 0 0 0 0
M02255_77_000000000-ADUT3_1_1110_21502_19200 T----AC---GA-AG-GGA--------GCT-A-G------C--G--T---T--GT-T-CGG-AA------TT-A--C-T--GG-GC-------GT--A-----AA-GG-GC-GC-------G-TA-G-G-C-G-------------G--C-CT-T-G-T-----------AA----G-T-C-A--------G-A-T--G--TG--A-AA-GC--C-C-CG-G-G-----CT-T-AA------C-C-T-G-G-G-A--A-C----T-G--C-A---T--T-------T--GA-T-A---C-------T--G-C--AA--G-G-C----T-A-G-A-G-A--A---T-GT-----GA-G-A--------G-G-T-A---AG-T------GG--A--ATT----A-T-G-A-GT--GT-A-G-AG-GT--G---A--A-A---TT-C-GT-AG--AT-A-TT--C-A--T-A-A-G-A-AC-A-TC---AG--T--G--GC-GAA-G--G-C--G-------G--C-T-T-A---CTG--G--CA-C-A--------------------T-T---A-C-T--GA--CG-C-T-A--A-GG--C-G-CG-A--AA-G-C---G-TG--GG-G--AG-C-A-AA--------CA--GG Bacteria(100);Proteobacteria(100);unclassified(100);unclassified(100);unclassified(100);unclassified(100);unclassified(100);unclassified(100);unclassified(100);

Otu1229 0 0 0 0 0 0 0 0 0 0 0 0 0 0 0 0 0 0 0 0 0 0 0 0 0 0 0 0
0 0 0 0 0 0 0 0 0 0 0 0 0 0 0 0 0 0 0 0 0 0 0 0 0 0 0 0 0
0 0 0 0 0 0 0 0 0 0 0 0 0 0 0 0 0 0 0 0 0 0 0 0 0 0 0 0 0
0 0 0 0 0 0 0 0 0 0 0 0 0 0 0 0 0 0 14 0 0 0 0 0 0 0 0 0
M02255_77_000000000-ADUT3_1_1116_13488_24283 T----AC---GT-AT-GGT--------GCA-A-G------C--G--T---T--AT-C-CGG-AT------TT-A--C-T--GG-GT-------GT--A-----AA-GG-GA-GC-------G-TA-G-G-C-G-------------G--T-TT-T-A-C-----------AA----G-T-C-T--------G-A-T--G--TG--A-AA-GC--C-C-GG-G-G-----CT-C-AA------C-T-C-C-G-G-T--A-T----T-G--C-A---T--T-------G--GA-A-A---C-------T--G-T--AG--A-A-C----T-A-G-A-G-T--G---T-CG-----GA-G-G--------G-G-T-A---AG-T------GG--A--ATT----C-C-T-A-GT--GT-A-G-CG-GT--G---A--A-A---TG-C-GT-AG--AT-A-TT--A-G--G-A-G-G-A-AC-A-CC---AG--T--G--GC-GAA-G--G-C--G-------G--C-T-T-A---CTG--G--AC-G-A--------------------T-T---A-C-T--GA--CG-C-T-G--A-GG--C-T-CG-A--AA-G-C---G-TG--GG-G--AG-C-A-AA--------CA--GG Bacteria(100);Firmicutes(100);Clostridia(100);Clostridiales(100);Lachnospiraceae(100);Butyrivibrio-Pseudobutyrivibrio(100);unclassified(100);unclassified(100);unclassified(100);

Otu1230 0 0 0 0 0 0 0 0 0 0 0 0 0 0 0 0 0 0 0 0 0 0 0 0 0 0 0 0
0 0 0 0 0 0 0 0 0 0 0 0 0 0 0 0 0 0 0 0 0 0 0 0 0 0 0 0 0
0 0 0 0 0 0 0 0 0 0 0 0 0 0 0 0 0 0 0 0 0 0 0 0 0 0 0 0 0
0 0 0 0 0 0 0 0 0 0 0 0 0 0 0 0 0 0 0 13 0 0 0 0 0 0 0
M02255_76_000000000-ADUNE_1_1112_24526_5228 T----AA---CT-A--CGT--------GCC-A-G------C--A--G---C--CG-C-GGT-AA------TA-C--G-T--AG-GG-------GG--C-----GA-GC-G--TT-------G-TC-C-G---G------------A--T-TC-G-G-T-----------AA----
G-T-C-C--------G-T-C--G--TG--A-AA-TC--C-T-GA-G-G-----CT-C-AA------C-C-T-T-G-G-A--C-G----T-G--C-G---A--T-------G--GA-T-A---C-------T--G-C--CG--G-G-C----T-G-G-A-G-G--A---T-GG-----TA-G-A--------G-G-C-G---AG-T------GG--A--ATT----C-C-C-G-GT-
-GT-A-G-CG-GT--G---A--A-A---TG-C-GC-AG--AT-A-TC--G-G--G-A-G-G-A-AC-A-CC---AG--T--A--GC-GAA-G--G-C--G-------G--C-T-C-G---CTG--G--GC-C-A--------------------T-T---C-C-T--GA--CG-C-T-G--A-GA--C-G-CG-A--AA-G-C---T-AG--GG-G--AG-C-G-
AA--------CA--GG Bacteria(100);Actinobacteria(100);Actinobacteria(100);Rubrobacteridae(100);Rubrobacterales(100);Rubrobacterineae(100);Rubrobacteriaceae(100);Rubrobacter(100);unclassified(100);

Otu1231 0 0 0 0 0 0 0 0 0 0 0 0 0 0 0 0 0 0 0 0 0 0 0 0 0 0 0 0
0 0 0 0 0 0 0 0 0 0 0 0 0 0 0 0 0 0 0 0 0 0 0 0 0 0 0 0 0
0 0 0 0 0 0 0 0 0 0 0 0 0 0 0 0 0 0 0 0 0 0 0 0 0 0 0 0 0
0 0 0 0 0 0 0 0 0 0 0 0 0 0 0 0 0 0 0 9 0 0 0 0 0 0 0
M02255_76_000000000-ADUNE_1_2105_18742_21319 T----AA---CT-A--CGT--------GCC-A-G------C--A--G---C--CG-C-GGT-AA------TA-C--G-T--AG-G--------G---G-----GC-GA-GC-GT---------TG-T-C-C-G------------G--T-TC-G-G-T---------
--AA----G-T-C-C--------G-T-C--G--TG--A-AA-TC--C-T-GA-G-G-----CT-C-AA------C-C-T-T-G-G-A--C-G----T-G--C-G---A--T-------G--GA-T-A---C-------T--G-C--CG--G-G-C----T-G-G-A-G-G--A---T-GG-----TA-G-A--------G-G-C-G---AG-T------GG--A--ATT----C-C-
C-G-GT--GT-A-G-CG-GT--G---A--A-A---TG-C-GC-AG--AT-A-TC--G-G--G-A-G-G-A-AC-A-CC---AG--T--A--GC-GAA-G--G-C--G-------G--C-T-C-G---CTG--G--GC-C-A--------------------T-T---C-C-T--GA--CG-C-T-G--A-GA--C-G-CG-A--AA-G-C---T-AG--GG-G--
AG-C-G-AA--------CA--GG Bacteria(100);Actinobacteria(100);Actinobacteria(100);Rubrobacteridae(100);Rubrobacterales(100);Rubrobacterineae(100);Rubrobacteriaceae(100);Rubrobacter(100);unclassified(100);

Otu1232 0 0 0 0 0 0 0 0 0 0 0 0 0 0 0 0 0 0 0 0 0 0 0 0 0 0 0 0
0 0 0 0 0 0 0 0 0 0 0 0 0 0 0 0 0 0 0 0 0 0 0 0 0 0 0 0 0
0 0 0 0 0 0 0 0 0 0 0 0 0 0 0 0 0 0 0 0 0 0 0 0 0 0 0 0 0
0 0 0 0 0 0 0 0 0 0 0 0 0 0 0 0 0 0 0 33 0 0 0 0 0 0 0
M02255_77_000000000-ADUT3_1_1101_13499_14686 T----AC---GA-AG-GGG--------GCT-A-G------C--G--T---T--GT-T-CGG-AA------TT-A--C-T--GG-GC-------GT--A-----AA-GC-GC-AC-------G-TA-G-G-C-G------------G--A-TT-G-T-T-----
------AA----G-T-T-A--------G-G-G--G--TG--A-AA-TC--C-C-GG-G-G-----CT-C-AA------C-C-T-C-G-G-A--A-C----T-G--C-C---T--T-------T--GA-T-A---C-------T--G-G--CA--A-T-C----T-T-G-A-G-T--C---C-GG-----AA-G-A--------G-G-T-G---AG-T------GG--A--ATT----C-
C-T-A-GT--GT-A-G-AG-GT--G---A--A-A---TT-C-GT-AG--AT-A-TT--A-G--G-A-A-G-A-AC-A-CC---AG--T--G--GC-GAA-G--G-C--G-------G--C-T-C-A---CTG--G--TC-C-G--------------------G-C---A-C-T--GA--CG-C-T-G--A-GG--T-G-CG-A--AA-G-C---G-TG--GG-G-
-AG-C-A-AA--------CA--GG Bacteria(100);Proteobacteria(100);Alphaproteobacteria(100);Rhizobiales(100);unclassified(100);unclassified(100);unclassified(100);unclassified(100);unclassified(100);

Otu1233 0 0 0 0 0 0 0 0 0 0 0 0 0 0 0 0 0 0 0 0 0 0 0 0 0 0 0 0
0 0 0 0 0 0 0 0 0 0 0 0 0 0 0 0 0 0 0 0 0 0 0 0 0 0 0 0 0
0 0 0 0 0 0 0 0 0 0 0 0 0 0 0 0 0 0 0 0 0 0 0 0 0 0 0 0 0
0 0 0 0 0 0 0 0 0 0 0 0 0 0 0 0 0 0 0 28 0 0 0 0 0 0 0
M02255_77_000000000-ADUT3_1_1102_18382_19663 T----AC---GT-AG-GTG--------GCG-A-G------C--G--T---T--AT-C-CGG-AA------TT-A--C-T--GG-GT-------GT--A-----AA-GG-GT-GT-------G-TA-G-G-C-G------------G--G-GA-G-G-C----
-------AA----G-T-C-A--------G-A-T--G--TG--A-AA-AC--C-A-AA-A-G-----CT-C-AA------C-T-T-T-T-G-G--C-C----T-G--C-A---T--T-------T--GA-A-A---C-------T--G-T--TT--C-T-C----T-T-G-A-G-A--G---T-GG-----GA-G-A--------G-G-T-A---AA-T------GG--A--ATT----C-
C-T-G-GT--GT-A-G-TA-GT--G---A--A-A---TG-C-GT-AG--AT-A-TC--A-G--G-A-G-G-A-AC-A-CC---GG--T--G--GC-GAA-G--G-C--G-------A--T-T-T-A---CTG--G--AC-C-A--------------------T-T---A-C-T--GA--CG-C-T-G--A-GA--C-A-CG-A--AA-G-C---G-TG--GG-G-
-AG-C-A-AA--------CA--GG Bacteria(100);unclassified(100);unclassified(100);unclassified(100);unclassified(100);unclassified(100);unclassified(100);unclassified(100);unclassified(100);

Otu1234 0 0 0 0 0 0 0 0 0 0 0 0 0 0 0 0 0 0 0 0 0 0 0 0 0 0 0 0
0 0 0 0 0 0 0 0 0 0 0 0 0 0 0 0 0 0 0 0 0 0 0 0 0 0 0 0 0
0 0 0 0 0 0 0 0 0 0 0 0 0 0 0 0 0 0 0 0 0 0 0 0 0 0 0 0 0
0 0 0 0 0 0 0 0 0 0 0 0 0 0 0 0 0 0 0 24 0 0 0 0 0 0 0
M02255_77_000000000-ADUT3_1_1119_20613_21728 T----AC---AG-AG-GGT--------GCA-A-G------C--G--T---T--GT-T-CGG-AA------TT-A--T-T--GG-GC-------GT--A-----AA-GG-GC-GC-------G-TA-G-G-C-G------------G--T-GC-G-G-T----
-------AA----G-T-C-T--------C-T-T--G--TG--A-AA-TC--T-C-CG-G-G-----CT-C-AA------C-T-C-G-G-A-G--T-C----T-G--C-A---A--G-------G--GA-A-A---C-------T--G-C--CG--T-G-C----T-T-G-A-G-T--G---T-GG-----GA-G-A--------G-G-T-G---AG-T------GG--A--ATT----
C-C-C-G-GT--GT-A-G-CG-GT--G---A--A-A---TG-C-GT-AG--AT-A-TC--G-G--G-A-G-G-A-AC-A-CC---TG--T--G--GC-GAA-A--G-C--G-------G--C-T-C-A---CTG--G--AC-C-A--------------------C-A---A-C-T--GA--CG-C-T-G--A-GG--C-G-CG-A--AA-G-C---T-AG--
GG-G--AG-C-A-AA--------CA--GG Bacteria(100);Acidobacteria(100);Acidobacteria(100);Acidobacteriales(100);Acidobacteriaceae(100);Candidatus_Koribacter(100);unclassified(100);unclassified(100);unclassified(100);

Otu1235 0 0 0 0 0 0 0 0 0 0 0 0 0 0 0 0 0 0 0 0 0 0 0 0 0 0 0 0
0 0 0 0 0 0 0 0 0 0 0 0 0 0 0 0 0 0 0 0 0 0 0 0 0 0 0 0 0
0 0 0 0 0 0 0 0 0 0 0 0 0 0 0 0 0 0 0 0 0 0 0 0 0 0 0 0 0
0 0 0 0 0 0 0 0 0 0 0 0 0 0 0 0 0 0 0 10 0 0 0 0 0 0 0
M02255_77_000000000-ADUT3_1_2106_24191_11147 T----AC---GT-AG-GTG--------GCA-A-G------C--G--T---T--GT-C-CGG-AT------TT-A--C-T--GG-GC-------GT--A-----AA-------GC---------A-G-G-C-G------------G--A-TA-C-T-T----------
AA----G-T-A-G--------G-A-A--G--TG--A-AA-GG--T-G-GG-A-G-----CT-C-AA------C-T-C-C-C-A-C--A-T----T-G--C-T---C--C-------C--TA-T-A---C-------T--G-G--GT--G-T-C----T-T-G-A-G-T--G---G-CG-----GA-G-A--------G-G-G-A---GA-T------GG--A--ACG----A-C-
A-C-GT--GT-A-G-CG-GT--G---A--A-A---TG-C-GT-TG--AT-A-TG--T-G--T-C-G-G-A-AC-A-CC---AA--T--G--GC-GAA-A--G-C--A-------A--T-C-T-C---CTG--G--AC-G-C--------------------A-A---A-C-T--GA--CG-C-T-G--A-GA--T-A-CG-A--AA-G-C---C-AA--GG-T--
AG-C-A-AA--------CA--GG Bacteria(100);unclassified(100);unclassified(100);unclassified(100);unclassified(100);unclassified(100);unclassified(100);unclassified(100);unclassified(100);

Otu1236 0 0 0 0 0 0 0 0 0 0 0 0 0 0 0 0 0 0 0 0 0 0 0 0 0 0 0 0
0 0 0 0 0 0 0 0 0 0 0 0 0 0 0 0 0 0 0 0 0 0 0 0 0 0 0 0 0
0 0 0 0 0 0 0 0 0 0 0 0 0 0 0 0 0 0 0 0 0 0 0 0 0 0 0 0 0
0 0 0 0 0 0 0 0 0 0 0 0 0 0 0 0 0 0 28 0 0 0 0 0 0 0 0
M02255_77_000000000-ADUT3_1_1119_4791_5629 T----AC---GT-AG-GGT--------GCG-A-G------C--G--T---T--GT-C-CGG-AA------TT-A--T-T--GG-GC-------GT--A-----AA-GG-GC-TT-------G-TA-G-G-C-G------------G--T-TG-G-T-C-----------GC-
---G-T-C-G--------G-A-A--G--TG--A-AA-TC--T-C-AG-G-G-----CT-T-AA------C-T-C-T-G-A-G--T-C----T-G--C-T---T--C-------T--GA-T-A---C-------G--G-G--CT--G-A-C----T-T-G-A-G-G--G---A-TG-----CA-G-G--------G-G-A-G---AA-C------GG--A--ATT----C-C-T-G-
GT--GG-A-G-CG-GT--G---G--A-A---TG-C-GC-AG--AT-A-TC--A-G--G-A-G-G-A-AC-A-CC---GG--T--G--GC-GAA-G--G-C--G-------G--T-T-C-T---CTG--G--GC-A-T--------------------T-T---C-C-T--GA--CG-C-T-G--A-GA--A-G-CG-A--AA-G-C---G-TG--GG-G--AG-
C-A-AA--------CA--GG Bacteria(100);Actinobacteria(100);Actinobacteria(100);Actinobacteridae(100);Actinomycetales(100);Propionibacterineae(100);Propionibacteriaceae(100);unclassified(100);unclassified(100);

Otu1237 0 0 0 0 0 0 0 0 0 0 0 0 0 0 0 0 0 0 0 0 0 0 0 0 0 0 0 0
0 0 0 0 0 0 0 0 0 0 0 0 0 0 0 0 0 0 0 0 0 0 0 0 0 0 0 0 0
0 0 0 0 0 0 0 0 0 0 0 0 0 0 0 0 0 0 0 0 0 0 0 0 0 0 0 0 0
0 0 0 0 0 0 0 0 0 0 0 0 0 0 0 0 0 0 0 8 0 0 0 0 0 0 0
M02255_77_000000000-ADUT3_1_1106_23506_10260 T----AC---GT-AG-GGG--------GCG-A-G------C--G--T---T--GT-C-CGG-AT------TC-A--T-T--GG-GC-------GT--A-----AA-GA-GC-TC-------G-TA-G-G-C-G------------G--T-TC-A-G-T-----
------AA----G-T-C-A--------G-G-T--G--TT--A-AA-GC--T-C-CG-A-G-----CT-C-AA------CTC-G-G-A-G-A---------G-G-CC-A---T--T-------T--GA-T-A---C-------T--G-C--TG-TG-A-C----T-A-G-A-A-T--C---C-AG-----TA-G-G--------G-G-A-G---CG-T------GG--A--ATT----C-
C-T-G-GT--GT-A-G-CG-GT--G---A--A-A---TG-C-GC-AG--AT-A-TC--A-G--G-A-G-G-A-AC-A-CC---AG--C--G--GC-GAA-G--G-C--G-------G--C-G-C-T---CTG--G--GC-T-G--------------------G-A---A-T-T--GA--CG-C-T-G--A-GG--A-G-CG-A--AA-G-T---G-TG--GG-
T--AG-C-A-AA--------CA--GG Bacteria(100);Actinobacteria(100);Actinobacteria(100);Acidimicrobidae(100);Acidimicrobiales(100);Acidimicrobineae(100);unclassified(100);unclassified(100);unclassified(100);

Otu1238 0 0 0 0 0 0 0 0 0 0 0 0 0 0 0 0 0 0 0 0 0 0 0 0 0 0 0 0
0 0 0 0 0 0 0 0 0 0 0 0 0 0 0 0 0 0 0 0 0 0 0 0 0 0 0 0 0
0 0 0 0 0 0 0 0 0 0 0 0 0 0 0 0 0 0 0 0 0 0 0 0 0 0 0 0 0
0 0 0 0 0 0 0 0 0 0 0 0 0 0 0 0 0 0 0 0 18 0 0 0 0 0 0
M02255_77_000000000-ADUT3_1_1107_18710_13462 T----AC---GT-AG-GGG--------GCG-A-G------C--G--T---T--GT-C-CGG-AT------TT-A--C-T--GG-GC-------GT--A-----AA-GC-GC-GT-------G-TA-G-G-C-G------------G--C-TG-T-T-T-----
------AA----G-T-T-G--------G-G-T--G--TG--A-AA-GC--C-C-GG-G-G-----CT-C-AA------C-C-C-C-G-G-G--A-C----G-C--C-G---C--C-------C--AA-G-A---C-------T--G-G--AT--G-G-C----T-T-G-A-G-G--A---C-GG-----AA-G-A--------G-G-G-G---CG-T------GG--A--ATT----
C-C-T-G-GT--GT-A-G-CG-GT--G---A--A-A---TG-C-GT-AG--AG-A-TC--A-G--G-A-G-G-A-AC-A-CC---CG--T--G--GC-GAA-G--G-C--G-------G--C-G-C-C---CTG--G--TC-C-G--------------------T-T---T-C-T--GA--CG-C-T-G--A-GA--C-G-CG-A--AA-G-C---G-CG--
GG-G--AG-C-G-AA--------CG--GG Bacteria(100);unclassified(100);unclassified(100);unclassified(100);unclassified(100);unclassified(100);unclassified(100);unclassified(100);unclassified(100);

Otu1239 0 0 0 0 0 0 0 0 0 0 0 0 0 0 0 0 0 0 0 0 0 0 0 0 0 0 0 0
0 0 0 0 0 0 0 0 0 0 0 0 0 0 0 0 0 0 0 0 0 0 0 0 0 0 0 0 0
0 0 0 0 0 0 0 0 0 0 0 0 0 0 0 0 0 0 0 0 0 0 0 0 0 0 0 0 0
0 0 0 0 0 0 0 0 0 0 0 0 0 0 0 0 0 0 0 0 0 16 0 0 0 0 0
M02255_76_000000000-ADUNE_1_1115_15743_12245 .....AC----T-AC-GT---------GCC-A-G------C--A--G---C--CG-C-GGT-AA------TA-C--G-T--AG-GT-------GG--C-----AA-GC-G--TT-------G-TC-C-G---G------------A--T-CT-C-T-T----------
AA----G-T-C-T--------G-A-T--G--TG--A-AA-TC--T-T-GC-G-G-----CT-T-AA------C-C-G-T-A-A-G--T------G-GT-C-A---T--T-------G--GA-A-A---C-------T--G-G--GA--G-G-C----T-T-G-A-G-T--G---C-AG-----AA-G-A--------G-G-A-G---AG-T------GG--A--ATT----C-C-A-
T-GT--GT-A-G-CG-GT--G---A--A-A---TG-C-GT-AG--AG-A-TA--T-G--G-A-G-G-A-AC-A-CC---AG--T--G--GC-GAA-G--G-C--G-------A--C-T-C-T---CTG--G--TC-T-G--------------------T-A---A-C-T--GA--CG-C-T-G--A-GG--A-G-CG-A--AA-G-C---G-TG--GG-G--
AG-C-G-AA--------CA--GG Bacteria(100);Firmicutes(100);Bacilli(100);unclassified(100);unclassified(100);unclassified(100);unclassified(100);unclassified(100);unclassified(100);

Otu1240 0 0 0 0 0 0 0 0 0 0 0 0 0 0 0 0 0 0 0 0 0 0 0 0 0 0 0 0
0 0 0 0 0 0 0 0 0 0 0 0 0 0 0 0 0 0 0 0 0 0 0 0 0 0 0 0 0
0 0 0 0 0 0 0 0 0 0 0 0 0 0 0 0 0 0 0 0 0 0 0 0 0 0 0 0 0
0 0 0 0 0 0 0 0 0 0 0 0 0 0 0 0 0 0 0 0 0 14 0 0 0 0 0
M02255_76_000000000-ADUNE_1_1117_13149_10690 C----AC---GT-AG-GGG--------GCG-A-G------C--G--T---T--GT-C-CGG-AT------TT-A--T-T--GG-GC-------GT--A-----AA-GA-GC-TC----------A-G-T-A-G------------G----TC-G-G----------
---GT----G-T-G-A--------A-A-T--T--TC----AG-GG--C-T-CA-------------AC------C-C-T-G-A-A-A---------C-G-CC-A---C--C-------C--GA-T-A---C-------T--G-C--TG-TG-G-C----T-A-G-A-G-T--C---C-GG-----TA-G-G--------G-G-A-G---CG-T------GG--A--ATT----C-C-T-G-
GT--GT-A-G-CG-GT--G---A--A-A---TG-C-GC-AG--AT-A-TC--A-G--G-A-G-G-A-AC-A-CC---AG--C--G--GC-GAA-G--G-C--G-------G--C-G-C-T---CTG--G--GC-C-G--------------------G-A---A-C-T--GA--CG-C-T-G--A-GG--A-G-CG-A--AA-G-C---A-TG--GG-T--AG-
C-G-AA--------CA--GG Bacteria(100);Actinobacteria(100);Actinobacteria(100);Acidimicrobidae(100);Acidimicrobiales(100);Acidimicrobineae(100);unclassified(100);unclassified(100);unclassified(100);

Otu1241 0 0 0 0 0 0 0 0 0 0 0 0 0 0 0 0 0 0 0 0 0 0 0 0 0 0 0 0
0 0 0 0 0 0 0 0 0 0 0 0 0 0 0 0 0 0 0 0 0 0 0 0 0 0 0 0 0
0 0 0 0 0 0 0 0 0 0 0 0 0 0 0 0 0 0 0 0 0 0 0 0 0 0 0 0 0
0 0 0 0 0 0 0 0 0 0 0 0 0 0 0 0 0 0 0 0 0 74 0 0 0 0 0
M02255_76_000000000-ADUNE_1_2105_8559_11800 T----AC---GT-AA-GGA--------CCA-A-G------C--G--T---T--GT-C-CGG-AA------TC-A--T-T--GG-GC-------GT--A-----AA-GG-GT-AC-------G-TA-G-G-C-T-------------G--C-CT-A-G-T-----------AA-
---G-T-T-A--------G-A-A--G--TG--A-AA-GC--A-T-AT-A-G-----CT-T-AA------C-T-A-T-A-T-A--A------A-G--C-T---T--T-------T--AA-A-A---C-------T--G-C--TA--G-G-C----T-T-G-A-G-A--G---A-TG-----AA-A-G--------G-G-A-A---AG-T------GG--A--ATT----C-C-T-A-
GT--GT-A-G-CG-GT--G---A--A-A---TG-C-GC-AG--AT-A-TT--A-G--G-A-A-G-A-AT-A-CC---AG--T--G--GC-GAA-G--G-C--G-------A--C-T-T-T---CTG--G--TC-A-T--------------------C-C---T-C-T--GA--CG-C-T-G--A-GG--T-A-CG-A--AA-G-C---G-TG--GG-T--AG-C-
A-AA--------CA--GG Bacteria(100);Firmicutes(100);Clostridia(100);Clostridiales(100);Family_XI_Incertae_Sedis(100);unclassified(100);unclassified(100);unclassified(100);unclassified(100);

Otu1242 0 0 0 0 0 0 0 0 0 0 0 0 0 0 0 0 0 0 0 0 0 0 0 0 0 0 0 0
0 0 0 0 0 0 0 0 0 0 0 0 0 0 0 0 0 0 0 0 0 0 0 0 0 0 0 0 0
0 0 0 0 0 0 0 0 0 0 0 0 0 0 0 0 0 0 0 0 0 0 0 0 0 0 0 0 0
0 0 0 0 0 0 0 0 0 0 0 0 0 0 0 0 0 0 0 0 0 56 0 0 0 0 0
M02255_77_000000000-ADUT3_1_1103_4972_7951 T----AC---GT-AG-GGG--------GCG-A-G------C--G--T---T--GT-C-CGG-AC------TT-A--T-T--GG-GC-------GT--A-----AA-GA-GC-AT-------G-TA-G-G-C-G-------------G--T-CC-T-G-T-----------AA-
---G-T-C-A--------G-A-T--G--TG--A-AA-TC--T-C-CG-G-G-----CT-C-AA------C-C-C-G-G-A-A--A-C----T-G--C-A---T--C-------T--GA-T-A---C-------T--G-C--AG--G-A-C----T-T-G-A-G-G--T---A-GG-----TA-G-A--------G-G-A-G---AG-T------GG--A--ATT----T-C-C-G-
GT--GT-A-G-CG-GT--G---G--A-A---TG-C-GC-AG--AT-A-TC--G-G--A-A-G-G-A-AC-A-CC---GA--T--G--GC-GAA-G--G-C--A-------G--C-T-C-T---CTG--G--GC-C-T--------------------C-A---C-C-T--GA--CG-C-T-G--A-GA--T-G-CG-A--AA-G-C---G-TG--GG-T--AG-C-
A-AA--------CA--GG Bacteria(100);unclassified(100);unclassified(100);unclassified(100);unclassified(100);unclassified(100);unclassified(100);unclassified(100);unclassified(100);

Otu1243 0 0 0 0 0 0 0 0 0 0 0 0 0 0 0 0 0 0 0 0 0 0 0 0 0 0 0 0
0 0 0 0 0 0 0 0 0 0 0 0 0 0 0 0 0 0 0 0 0 0 0 0 0 0 0 0 0
0 0 0 0 0 0 0 0 0 0 0 0 0 0 0 0 0 0 0 0 0 0 0 0 0 0 0 0 0
0 0 0 0 0 0 0 0 0 0 0 0 0 0 0 0 0 0 0 0 0 95 0 0 0 0 0
M02255_77_000000000-ADUT3_1_1105_27494_9381 T----AC---GG-AG-GGG--------GCT-A-G------C--G--T---T--GT-T-CGG-AA------TT-A--C-T--GG-GC-------GT--A-----AA-GC-GC-AC-------G-TA-G-G-C-G-------------G--A-CC-T-G-C-----------
AA----G-T-T-G--------G-G-G--G--TG--A-AA-TC--C-C-GG-G-G-----CT-C-AA------C-C-C-C-G-G-A--A-C----T-G--C-C---T--C-------C--AG-G-A---C-------T--G-C--CG--G-T-C----T-G-G-A-G-T--T---C-GG-----GA-G-A--------G-G-C-G---AG-T------GG--A--ATT----C-C-
G-A-GT--GT-A-G-AG-GT--G---A--A-A---TT-C-GT-AG--AT-A-TT--C-G--G-A-G-G-A-AC-A-CC---AG--T--G--GC-GAA-G--G-C--G-------G--C-T-C-G---CTG--G--CC-C-G--------------------A-T---A-C-T--GA--CG-C-T-G--A-GG--T-G-CG-A--AA-G-C---G-TG--GG-G--
AG-C-A-AA--------CA--GG Bacteria(100);Proteobacteria(100);Alphaproteobacteria(100);unclassified(100);unclassified(100);unclassified(100);unclassified(100);unclassified(100);unclassified(100);

Otu1244 0 0 0 0 0 0 0 0 0 0 0 0 0 0 0 0 0 0 0 0 0 0 0 0 0 0 0 0
0 0 0 0 0 0 0 0 0 0 0 0 0 0 0 0 0 0 0 0 0 0 0 0 0 0 0 0 0
0 0 0 0 0 0 0 0 0 0 0 0 0 0 0 0 0 0 0 0 0 0 0 0 0 0 0 0 0
0 0 0 0 0 0 0 0 0 0 0 0 0 0 0 0 0 0 59 0 0 0 0 0 0 0 0
M02255_77_000000000-ADUT3_1_1101_11044_8253 T----AC---GA-AG-GGA--------GCT-A-G------C--G--T---T--GT-T-CGG-AA------TT-A--C-T--GG-GC-------GT--A-----AA-GG-GC-GC-------G-TA-G-G-C-G-------------G--T-TT-G-A-C-----------
AA----G-T-T-G--------G-A-T--G--TG--A-AA-GC--C-C-AG-G-G-----CT-C-AA------C-C-C-T-G-G-A--A-T----A-G--C-A---T--T-------C--AA-G-A---C-------T--G-T--TT--T-A-C----T-T-G-A-A-T--T---C-GG-----TA-G-A--------G-G-T-G---AG-T------GG--A--ATT----C-C-C-
A-GT--GT-A-G-AG-GT--G---A--A-A---TT-C-GT-AG--AT-A-TT--G-G--G-A-A-G-A-AC-A-CC---AG--T--G--GC-GAA-G--G-C--G-------G--C-T-C-A---CTG--G--AC-C-G--------------------A-C---A-T-T--GA--CG-C-T-G--A-GG--C-G-CG-A--AA-G-C---G-TG--GG-G--
AT-C-A-AA--------CA--GG Bacteria(100);Proteobacteria(100);Alphaproteobacteria(100);unclassified(100);unclassified(100);unclassified(100);unclassified(100);unclassified(100);unclassified(100);

Otu1245 0 0 0 0 0 0 0 0 0 0 0 0 0 0 0 0 0 0 0 0 0 0 0 0 0 0 0 0
0 0 0 0 0 0 0 0 0 0 0 0 0 0 0 0 0 0 0 0 0 0 0 0 0 0 0 0 0
0 0 0 0 0 0 0 0 0 0 0 0 0 0 0 0 0 0 0 0 0 0 0 0 0 0 0 0 0
0 0 0 0 0 0 0 0 0 0 0 0 0 0 0 0 0 28 0 0 0 0 0 0 0 0 0 0
M02255_77_000000000-ADUT3_1_1110_6961_6589 T----AC---GT-AG-GAT--------GCA-A-G------C--G--T---T--AT-C-CGG-AT------TT-A--C-T--GG-GT-------GT--A-----AA-GG-GA-GC-------G-CA-G-G-C-G------------G--G-AC-T-G-C----------
AA----G-T-T-G--------G-A-T--G--TG--A-AA-TA--C-C-GT-G-G-----CT-T-AA------C-C-A-C-G-G-A--A-C----T-G--C-A---T--C-------C--AA-A-A---C-------T--G-T--AG--T-T-C----T-T-G-A-G-T--G---A-AG-----TA-G-A--------G-G-C-A---AG-C------GG--A--ATT----C-C-G-
A-GT--GT-A-G-CG-GT--G---A--A-A---TG-C-GT-AG--AT-A-TT--C-G--G-A-G-G-A-AC-A-CC---AG--T--G--GC-G-AAG--G-C--G-------G--C-T-T-G---CTG--G--GC-T-T--------------------T-A---A-C-T--GA--CG-C-T-G--A-GG--C-T-CG-A--AA-G-T---G-TG--GG-G--AG-
C-A-AA--------CA--GG Bacteria(100);Firmicutes(100);unclassified(100);unclassified(100);unclassified(100);unclassified(100);unclassified(100);unclassified(100);unclassified(100);

Otu1246 0 0 0 0 0 0 0 0 0 0 0 0 0 0 0 0 0 0 0 0 0 0 0 0 0 0 0 0
0 0 0 0 0 0 0 0 0 0 0 0 0 0 0 0 0 0 0 0 0 0 0 0 0 0 0 0 0
0 0 0 0 0 0 0 0 0 0 0 0 0 0 0 0 0 0 0 0 0 0 0 0 0 0 0 0 0
0 0 0 0 0 0 0 0 0 0 0 0 0 0 0 0 0 36 0 0 0 0 0 0 0 0 0 0
M02255_77_000000000-ADUT3_1_1114_13464_16095 T----AC---GA-GG-GGG--------GCG-A-G------C--G--T---T--GT-T-CGG-AA------TT-A--T-T--GG-GC-------GT--A-----AA-GG-GT-GC-------G-TA-G-G-C-G-------------G--T-TT-G-G-T----
-------AA----G-T-C-T--------T-A-T--G--TG--A-AA-TC--T-A-TG-G-G-----CT-C-AA------C-T-C-A-T-A-G--A-C----T-G--C-A---T--G-------A--GA-A-A---C-------T--G-C--CG--G-G-C----T-T-G-A-G-T--G---T-GG-----GA-G-A--------G-G-T-G---AG-T------GG--A--ATT----
T-C-C-G-GT--GT-A-G-CG-GT--G---A--A-A---TG-C-GT-AG--AT-A-TC--G-G--A-A-G-G-A-AC-A-CC---TG--T--G--GC-GAA-A--G-C--G-------G--C-T-C-A---CTG--G--AC-C-A---------------------C-A---A-C-T--GA--CG-C-T-G--A-TG--C-A-CG-A--AA-G-C---T-AG--GG-
G--AG-C-A-AA--------CA--GG Bacteria(100);Acidobacteria(100);Acidobacteria(100);Acidobacteriales(100);Acidobacteriaceae(100);Acidobacterium(100);unclassified(100);unclassified(100);unclassified(100);

Otu1247 0 0 0 0 0 0 0 0 0 0 0 0 0 0 0 0 0 0 0 0 0 0 0 0 0 0 0 0
0 0 0 0 0 0 0 0 0 0 0 0 0 0 0 0 0 0 0 0 0 0 0 0 0 0 0 0 0
0 0 0 0 0 0 0 0 0 0 0 0 0 0 0 0 0 0 0 0 0 0 0 0 0 0 0 0 0
0 0 0 0 0 0 0 0 0 0 0 0 0 0 0 0 0 12 0 0 0 0 0 0 0 0 0 0
M02255_77_000000000-ADUT3_1_1117_14350_8074 T----AC---GT-AG-GGG--------GCA-A-G------C--G--T---T--AT-C-CGG-AT------TT-A--C-T--GG-GT-------GT--A-----AA-GG-GA-GC-------G-TA-G-G-C-G------------G--T-TA-A-G-C----------
AA----G-T-C-A--------G-A-A--G--TG--A-AA-GC--C-C-GG-G-G-----CT-C-AA------C-C-T-C-G-G-G--A-C----T-G--C-T---T--T-------T--GA-A-A---C-------T--G-T--TT--A-A-C----T-A-G-A-T-T--G---C-AG-----GA-G-A--------G-G-T-A---AG-T------GG--A--ATT----C-C-T-
A-GT--GT-A-G-CG-GT--G---A--A-A---TG-C-GT-AG--AT-A-TT--A-G--G-A-G-G-A-AC-A-CC---AG--T--G--GC-GAA-G--G-C--G-------G--C-T-T-A---CTG--G--AC-T-G--------------------T-A---A-A-T--GA--CG-C-T-G--A-GG--C-T-CG-A--AA-G-C---G-TG--GG-G--
AG-C-A-AA--------CA--GG Bacteria(100);Firmicutes(100);Clostridia(100);Clostridiales(100);Lachnospiraceae(100);unclassified(100);unclassified(100);unclassified(100);unclassified(100);

Otu1248 0 0 0 0 0 0 0 0 0 0 0 0 0 0 0 0 0 0 0 0 0 0 0 0 0 0 0 0
0 0 0 0 0 0 0 0 0 0 0 0 0 0 0 0 0 0 0 0 0 0 0 0 0 0 0 0 0
0 0 0 0 0 0 0 0 0 0 0 0 0 0 0 0 0 0 0 0 0 0 0 0 0 0 0 0 0
0 0 0 0 0 0 0 0 0 0 0 0 0 0 0 0 0 28 0 0 0 0 0 0 0 0 0 0
M02255_77_000000000-ADUT3_1_2101_21800_11369 T----AC---GT-AG-GGT--------GCA-A-G------C--G--T---T--GT-C-CGG-AA------TT-A--T-T--GG-GC-------GT--A-----AA-GA-GC-TC-------G-TA-G-G-C-G-------------G--T-TT-G-T-C------
-----GC----G-T-C-C--------G-C-G--G--TG--A-AA-AC--C-T-GA-T-G-----CT-T-AA------C-A-T-T-G-G-G--C-T----T-G--C-C---G--T-------G--GG-T-A---C-------G--G-G--CA--G-A-C----T-A-G-A-G-T--G---T-GG-----TA-G-G--------G-G-A-G---AC-T------GG--A--ATT----C-
C-T-G-GT--GT-A-G-CG-GT--G---G--A-A---TG-C-GC-AG--AT-A-TC--A-G--G-A-G-G-A-AC-A-CC---GA--T--G--GC-GAA-G--G-C--A-------G--G-T-C-T---CTG--G--GC-C-A---------------------T-T---A-C-T--GA--CG-C-T-G--A-GG--A-G-CG-A--AA-G-C---G-TG--GG-
T--AG-C-G-AA--------CA--GG Bacteria(100);Actinobacteria(100);Actinobacteria(100);Actinobacteridae(100);Actinomycetales(100);unclassified(100);unclassified(100);unclassified(100);unclassified(100);

Otu1249 0 0 0 0 0 0 0 0 0 0 0 0 0 0 0 0 0 0 0 0 0 0 0 0 0 0 0 0
0 0 0 0 0 0 0 0 0 0 0 0 0 0 0 0 0 0 0 0 0 0 0 0 0 0 0 0 0
0 0 0 0 0 0 0 0 0 0 0 0 0 0 0 0 0 0 0 0 0 0 0 0 0 0 0 0 0
0 0 0 0 0 0 0 0 0 0 0 0 0 0 0 0 0 39 0 0 0 0 0 0 0 0 0 0
M02255_77_000000000-ADUT3_1_2106_20840_9178 T----AC---GT-AG-GGG--------GCA-A-G------C--G--T---T--AT-C-CGG-AT------TT-A--C-T--GG-GT-------GT--A-----AA-GG-GA-GC-------G-TA-G-A-C-G------------G--C-AG-G-G-C----------
AA----G-T-C-C--------G-A-T--G--TG--A-AA-AC--C-C-GG-G-G-----CT-C-AA------C-C-G-C-G-G-G--A-T----T-G--C-A---T--T-------G--GA-A-A---C-------T--G-C--TC--G-G-C----T-C-G-A-G-T--A---C-TG-----GA-G-G--------G-G-T-A---AG-C------GG--A--ATT----C-C-T-
A-GT--GT-A-G-CG-GT--G---A--A-A---TG-C-GT-AG--AT-A-TT--A-G--G-A-G-G-A-AC-A-CC---GG--C--G--GC-GAA-G--G-C--G-------G--C-T-T-A---CTG--G--AC-A-G--------------------T-A---A-C-T--GA--CG-T-T-G--A-GG--C-T-CG-A--AA-G-C---G-TG--GG-G--
AG-C-A-AA--------CA--GG Bacteria(100);Firmicutes(100);Clostridia(100);Clostridiales(100);Lachnospiraceae(100);unclassified(100);unclassified(100);unclassified(100);unclassified(100);

Otu1250 0 0 0 0 0 0 0 0 0 0 0 0 0 0 0 0 0 0 0 0 0 0 0 0 0 0 0 0
0 0 0 0 0 0 0 0 0 0 0 0 0 0 0 0 0 0 0 0 0 0 0 0 0 0 0 0 0
0 0 0 0 0 0 0 0 0 0 0 0 0 0 0 0 0 0 0 0 0 0 0 0 0 0 0 0 0
0 0 0 0 0 0 0 0 0 0 0 0 0 0 0 0 0 38 12 0 0 0 0 0 0 0 0
M02255_77_000000000-ADUT3_1_1106_21788_20393 T----AC---GA-AG-GGG--------GCT-A-G------C--G--T---T--GT-T-CGG-AA------TT-A--C-T--GG-GC-------GT--A-----AA-GG-GC-GC-------G-TA-G-G-C-G------------G--C-GC-A-T-C----
-------AA----G-T-C-A--------G-G-C--G--TG--A-AA-GC--C-C-CG-G-G-----CT-T-AA------C-C-T-G-G-G-A--A-C----T-G--C-G---T--T-------T--GA-G-A---C-------T--G-G--TG--T-G-C----T-A-G-A-G-T--T---C-GG-----GA-G-A--------G-G-T-G---AG-T------GG--A--ATT----
C-C-C-A-GT--GT-A-G-AG-GT--G---A--A-A---TT-C-GT-AG--AT-A-TT--G-G--G-A-A-G-A-AC-A-CC---GG--T--G--GC-GAA-G--G-C--G-------G--C-T-C-A---CTG--G--AC-C-G--------------------A-C---A-C-T--GA--CG-C-T-G--A-GG--C-G-CG-A--AA-G-C---G-TG--
GG-G--AG-C-A-AA--------CA--GG Bacteria(100);Proteobacteria(100);Alphaproteobacteria(100);Rhodospirillales(100);unclassified(100);unclassified(100);unclassified(100);unclassified(100);unclassified(100);

Otu1251 0 0 0 0 0 0 0 0 0 0 0 0 0 0 0 0 0 0 0 0 0 0 0 0 0 0 0 0
0 0 0 0 0 0 0 0 0 0 0 0 0 0 0 0 0 0 0 0 0 0 0 0 0 0 0 0 0
0 0 0 0 0 0 0 0 0 0 0 0 0 0 0 0 0 0 0 0 0 0 0 0 0 0 0 0 0
0 0 0 0 0 0 0 0 0 0 0 0 0 0 0 0 0 3 0 0 25 0 0 0 0 0 0
M02255_77_000000000-ADUT3_1_1109_3797_20219 T----AC---GT-AG-GGT--------GCA-A-G------C--G--T---T--GT-C-CGG-AA------TT-A--T-T--GG-GC-------GT--A-----AA-GA-GC-TC-------G-TA-G-G-C-G------------G--T-TC-G-T-C-----------GC-
---G-T-C-G--------G-C-T--G--TG--A-AA-TC--C-C-GA-G-G-----CT-C-AA------C-C-T-C-G-G-G--T-C----T-G--C-A---G--T-------C--GA-T-A---C-------G--G-G--CG--G-A-C----T-G-G-A-G-T--T---C-GG-----CA-G-G--------G-G-A-G---AC-T------GG--A--ATT----C-C-T-G-
GT--GT-A-G-CG-GT--G---A--A-A---TG-C-GC-AG--AT-A-TC--A-G--G-A-G-G-A-AC-A-CC---GG--T--G--GC-GAA-G--G-C--G-------G--G-T-C-T---CTG--G--GC-C-G--------------------A-T---A-C-T--GA--CG-C-T-G--A-GG--A-G-CG-A--AA-G-C---G-TG--GG-G--AG-
C-G-AA--------CA--GG Bacteria(100);Actinobacteria(100);Actinobacteria(100);Actinobacteridae(100);Actinomycetales(100);unclassified(100);unclassified(100);unclassified(100);unclassified(100);

Otu1252 0 0 0 0 0 0 0 0 0 0 0 0 0 0 0 0 0 0 0 0 0 0 0 0 0 0 0 0
0 0 0 0 0 0 0 0 0 0 0 0 0 0 0 0 0 0 0 0 0 0 0 0 0 0 0 0 0
0 0 0 0 0 0 0 0 0 0 0 0 0 0 0 0 0 0 0 0 0 0 0 0 0 0 0 0 0
0 0 0 0 0 0 0 0 0 0 0 0 0 0 0 0 0 26 0 0 0 0 0 0 0 0 0
M02255_77_000000000-ADUT3_1_2104_11921_9338 C----TC---CG-TG-CCA--------GCAGCCG------CG-G--T---A--AT-A-CGG-AG------GG-T--G-C--AA-GC-------GT--T-----AT-CC-GG-AT-------T-TA-G-G-T-G------------G--G-TT-G-G-T-----------
AA----G-T-C-A--------G-T-G--G--TG--A-AA-TC--T-C-CG-A-G-----CT-T-AA------C-T-C-G-G-A-A--A-C----T-G--C-C---A--T-------T--GA-T-A---C-------T--A-T--CA--A-T-C----T-T-G-A-A-T--A---T-CC-----TG-G-A--------G-G-T-G---AG-C------GG--A--ATA----T-G-T-C-
AT--GT-A-G-CG-GT--G---A--A-A---TG-C-TT-AG--AT-A-TG--A-C--A-T-A-G-A-AC-A-CC---AA--T--T--GC-GAA-G--G-C--A-------G--C-T-C-A---CTA--C--GG-G-G--------------------T-C---A-T-T--GA--CA-C-T-G--A-GG--C-A-CG-A--AA-G-C---G-TG--GG-G--AT-C-
A-AA--------CA--GG Bacteria(100);Bacteroidetes(100);Sphingobacteria(100);Sphingobacteriales(100);Chitinophagaceae(100);unclassified(100);unclassified(100);unclassified(100);unclassified(100);

Otu1253 0 0 0 0 0 0 0 0 0 0 0 0 0 0 0 0 0 0 0 0 0 0 0 0 0 0 0 0
0 0 0 0 0 0 0 0 0 0 0 0 0 0 0 0 0 0 0 0 0 0 0 0 0 0 0 0 0
0 0 0 0 0 0 0 0 0 0 0 0 0 0 0 0 0 0 0 0 0 0 0 0 0 0 0 0 0
0 0 0 0 0 0 0 0 0 0 0 0 0 0 0 0 0 0 0 0 0 32 0 0 0 0 0
M02255_77_000000000-ADUT3_1_1105_20549_15513 C----AC---GT-AG-GGG--------GCG-A-G------C--G--T---T--GT-C-CGG-AT------TT-A--T-T--GG-GC-------GT--A-----AA-GA-GC-TC-------G-TA-G-G-C-G------------G--C-TC-A-G-T-----
------AA----G-T-C-G--------G-G-T--G--TG--A-AA-TT--T-C-AG-G-G-----CT-C-AAC-----C-C-T-G-A-A-A---------C-G-CC-A---C--C-------C--GA-T-A---C-------T--G-C--TG-TG-G-C----T-A-G-A-G-T--C---C-GG-----TA-G-G--------G-G-A-G---CG-T------GG--A--ATT----
C-C-T-G-GT--GT-A-G-CG-GT--G---A--A-A---TG-C-GC-AG--AT-A-TC--A-G--G-A-G-G-A-AC-A-CC---AG--C--G--GC-GAA-G--G-C--G-------G--C-G-C-T---CTG--G--GC-C-G--------------------G-A---A-C-T--GA--CG-C-T-G--A-GG--A-G-CG-A--AA-G-C---A-TG--
GG-T--AG-C-G-AA--------CA--GG Bacteria(100);Actinobacteria(100);Actinobacteria(100);Acidimicrobidae(100);Acidimicrobiales(100);Acidimicrobineae(100);unclassified(100);unclassified(100);unclassified(100);

Otu1254 0 0 0 0 0 0 0 0 0 0 0 0 0 0 0 0 0 0 0 0 0 0 0 0 0 0 0 0
0 0 0 0 0 0 0 0 0 0 0 0 0 0 0 0 0 0 0 0 0 0 0 0 0 0 0 0 0
0 0 0 0 0 0 0 0 0 0 0 0 0 0 0 0 0 0 0 0 0 0 0 0 0 0 0 0 0
0 0 0 0 0 0 0 0 0 0 0 0 0 0 0 0 0 0 22 0 0 0 0 0 0 0 0
M02255_77_000000000-ADUT3_1_1101_13212_24079 T----AC---GT-AG-GTG--------GCA-A-G------C--G--T---T--GT-C-CGG-AT------TT-A--T-T--GG-GC-------GT--A-----AA-GC-AT-GC-------G-TA-G-G-C-G------------G--A-TA-T-T-T------
-----AA----G-T-G-G--------G-A-T--G--TG--A-AA-TC--C-C-GG-G-G-----CT-T-AA------C-C-C-C-G-G-T--G-C----T-G--C-A---T--T-------T--CA-A-A---C-------T--G-G--AT--A-T-C----T-A-G-A-G-T--G---C-AG-----GA-G-A--------G-G-A-A---AG-C------GG--A--ATT----C-
C-T-A-GT--GT-A-G-CG-GT--G---A--A-A---TG-C-GT-AG--AT-A-TT--A-G--G-A-A-G-A-AC-A-CC---AG--T--G--GC-GAA-G--G-C--G-------G--C-T-T-T---CTG--G--AC-T-G--------------------T-A---A-C-T--GA--CG-C-T-G--A-GG--C-A-TG-A--AA-G-C---G-TG--GG-T-
-AG-C-A-AA--------CA--GG Bacteria(100);Firmicutes(100);Clostridia(100);Clostridiales(100);Clostridiaceae(100);Clostridium(100);unclassified(100);unclassified(100);unclassified(100);

Otu1255 0 0 0 0 0 0 0 0 0 0 0 0 0 0 0 0 0 0 0 0 0 0 0 0 0 0 0 0
0 0 0 0 0 0 0 0 0 0 0 0 0 0 0 0 0 0 0 0 0 0 0 0 0 0 0 0 0
0 0 0 0 0 0 0 0 0 0 0 0 0 0 0 0 0 0 0 0 0 0 0 0 0 0 0 0 0
0 0 0 0 0 0 0 0 0 0 0 0 0 0 0 0 0 0 16 0 0 0 0 0 0 0 0

M02255_77_000000000-ADUT3_1_1105_11827_14973 T----AC---AT-AG-GGT--------GCA-A-G------C--G--T---T--GT-C-CGG-AA------TT-A--T-T--GG-GC-------GT--A-----AA-GA-GC-TC-------G-TA-G-G-T-G------------G--T-TC-G-T-C-----------AC----G-T-C-G--------G-A-T--G--TG--A-AA-CT--C-T-GG-G-G-----CT-T-AA------C-C-C-C-A-G-A--C-C----T-G--C-A---T--T-------C--GA-T-A---C-------G--G-G--CG--A-G-C----T-T-G-A-G-T--A---T-GG-----TA-G-G--------G-G-A-G---TC-T------GG--A--ATT----C-C-T-G-GT--GT-A-G-CG-GT--G---G--A-A---TG-C-GC-AG--AT-A-TC--A-G--G-A-G-G-A-AC-A-CC---AA--T--G--GC-GAA-G--G-C--A-------G--G-A-C-T---CTG--G--GC-C-A--------------------T-T---A-C-T--GA--CA-C-T-G--A-GG--A-G-CG-A--AA-G-C---G-TG--GG-G--AG-C-G-AA--------CA--GG Bacteria(100);Actinobacteria(100);Actinobacteria(100);Actinobacteridae(100);Actinomycetales(100);Frankineae(100);Sporichthyaceae(100);hgcI_clade(100);unclassified(100);

Otu1256 0 0 0 0 0 0 0 0 0 0 0 0 0 0 0 0 0 0 0 0 0 0 0 0 0 0 0 0
0 0 0 0 0 0 0 0 0 0 0 0 0 0 0 0 0 0 0 0 0 0 0 0 0 0 0 0 0
0 0 0 0 0 0 0 0 0 0 0 0 0 0 0 0 0 0 0 0 0 0 0 0 0 0 0 0 0
0 0 0 0 0 0 0 0 0 0 0 0 0 0 0 0 0 0 20 0 0 0 0 0 0 0 0

M02255_77_000000000-ADUT3_1_1106_16150_9689 G----AC---GT-AG-GAT--------GCG-A-G------C--G--T---T--GT-C-CGG-AT------TT-A--T-T--GG-GC-------GT--A-----AA-GA-GT-TC-------G-TA-G-G-T-G------------G--C-TT-G-T-C-----------AA----G-T-T-T--------G-A-T--G--TT--A-AA-GA--T-C-GG-G-G-----CT-C-AA------C-C-C-T-G-G-G--A-C----T-G--C-A---T--T-------G--AA-T-A---C-------T--G-G--CA--G-G-C----T-T-G-A-G-T--G---T-GG-----TA-G-A--------G-G-C-T---AG-T------GG--A--ATT----C-C-C-A-GT--GT-A-G-CG-GT--G---A--A-A---TG-C-GT-AG--AT-A-TT--G-G--G-A-A-G-A-AC-A-CC---GG--T--G--GC-GTA-G--G-C--G-------A--C-T-A-G---CTG--G--GC-C-A--------------------T-A---A-C-T--GA--CA-C-T-G--A-GG--A-A-CG-A--AA-G-C---C-AG--GG-T--AG-C-G-AA--------TG--GG Bacteria(100);Cyanobacteria(100);MLE1-12(100);unclassified(100);unclassified(100);unclassified(100);unclassified(100);unclassified(100);unclassified(100);

Otu1257 0 0 0 0 0 0 0 0 0 0 0 0 0 0 0 0 0 0 0 0 0 0 0 0 0 0 0 0
0 0 0 0 0 0 0 0 0 0 0 0 0 0 0 0 0 0 0 0 0 0 0 0 0 0 0 0 0
0 0 0 0 0 0 0 0 0 0 0 0 0 0 0 0 0 0 0 0 0 0 0 0 0 0 0 0 0
0 0 0 0 0 0 0 0 0 0 0 0 0 0 0 0 0 0 22 0 0 0 0 0 0 0 0

M02255_77_000000000-ADUT3_1_1108_6703_8097 T----AC---GT-AG-GTG--------GCA-A-G------C--G--T---T--AT-C-CGG-AT------TT-A--C-T--GG-GT-------GT--A-----AA-GG-GC-GT-------G-TA-G-G-C-G------------G--C-TT-T-G-C-----------AA----G-T-C-A--------G-A-A--G--TG--A-AA-TT--C-T-CG-A-G-----CT-C-AA------C-T-C-G-G-G-C--G-C----T-G--C-T---T--C-------T--GA-A-A---C-------T--G-T--AA--A-A-C----T-T-G-A-G-T--A---C-TG-----GA-G-G--------G-G-A-A---AG-C------GG--A--ATT----C-C-T-A-GT--GT-A-G-CG-GT--A---A--A-A---TG-C-GC-AG--AT-A-TT--A-G--G-A-G-G-A-AC-A-CC---GG--T--G--GC-GAA-G--G-C--G-------G--C-T-T-T---CTG--G--AC-A-G--------------------T-A---A-C-T--GA--CG-C-T-G--A-GG--C-G-CG-A--AA-G-C---G-TG--GG-G--AG-C-A-AA--------CA--GG Bacteria(100);Firmicutes(100);Clostridia(100);Clostridiales(100);unclassified(100);unclassified(100);unclassified(100);unclassified(100);unclassified(100);

Otu1258 0 0 0 0 0 0 0 0 0 0 0 0 0 0 0 0 0 0 0 0 0 0 0 0 0 0 0 0
0 0 0 0 0 0 0 0 0 0 0 0 0 0 0 0 0 0 0 0 0 0 0 0 0 0 0 0 0
0 0 0 0 0 0 0 0 0 0 0 0 0 0 0 0 0 0 0 0 0 0 0 0 0 0 0 0 0
0 0 0 0 0 0 0 0 0 0 0 0 0 0 0 0 0 0 31 0 0 0 0 0 0 0 0

M02255_77_000000000-ADUT3_1_1112_20779_4411 T----AC---GG-AG-GGG--------GCT-A-G------C--G--T---T--GT-T-CGG-AA------TT-A--C-T--GG-GC-------GT--A-----AA-GC-GC-AC-------G-TA-G-G-C-G------------G--C-TT-C-C-T-----------AA----G-T-C-A--------G-A-G--G--TG--A-AA-GC--C-T-GG-G-G-----CT-C-AA------C-C-C-C-A-G-A--A-C----T-G--C-C---T--T-------T--GA-G-A---C-------T--G-G--GT--C-G-C----T-T-G-A-A-T--C---C-GG-----GA-G-A--------G-G-T-G---AG-T------GG--A--ATT----C-C-G-A-GT--GT-A-G-AG-GT--G---A--A-A---TT-C-GT-AG--AT-A-TT--C-G--G-A-A-G-A-AC-A-CC---AG--T--G--GC-GAA-G--G-C--G-------G--C-T-C-A---CTG--G--AC-C-G--------------------G-C---A-T-T--GA--CG-C-T-G--A-GG--T-G-CG-A--AA-G-C---G-TG--GG-G--AG-C-A-AA--------CA--GG Bacteria(100);Proteobacteria(100);Alphaproteobacteria(100);Sphingomonadales(100);unclassified(100);unclassified(100);unclassified(100);unclassified(100);unclassified(100);

Otu1259 0 0 0 0 0 0 0 0 0 0 0 0 0 0 0 0 0 0 0 0 0 0 0 0 0 0 0 0
0 0 0 0 0 0 0 0 0 0 0 0 0 0 0 0 0 0 0 0 0 0 0 0 0 0 0 0 0
0 0 0 0 0 0 0 0 0 0 0 0 0 0 0 0 0 0 0 0 0 0 0 0 0 0 0 0 0
0 0 0 0 0 0 0 0 0 0 0 0 0 0 0 0 0 0 34 0 0 0 0 0 0 0 0

M02255_77_000000000-ADUT3_1_1115_17857_18984 T----AC---GT-AG-GGC--------GCA-A-G------C--G--T---T--GT-C-CGG-AA------TC-A--T-T--GG-GC-------GT--A-----AA-GA-GC-TC-------G-TA-G-G-C-G------------G--C-TT-G-T-C-----------AC----G-T-C-T--------G-C-T--G--TG--A-AA-TC--C-C-GG-G-G-----CT-C-AA------C-C-C-C-G-G-G--C-G----T-G--C-A---G--T-------G--GA-T-A---C-------G--G-G--CA--G-G-C----T-A-G-A-G-G--C---A-GG-----CA-G-G--------G-G-A-G---AA-C------GG--A--ATT----C-C-C-G-GT--GT-A-G-CG-GT--G---A--A-A---TG-C-GC-AG--AT-A-TC--G-G--G-A-G-G-A-AC-A-CC---GG--T--G--GC-GAA-G--G-C--G-------G--T-T-C-T---CTG--G--GC-C-T--------------------G-T---A-C-T--GA--CG-C-T-G--A-GG--A-G-CG-A--AA-G-C---G-TG--GG-G--AG-C-G-AA--------CA--GG Bacteria(100);Actinobacteria(100);Actinobacteria(100);Actinobacteridae(100);Actinomycetales(100);unclassified(100);unclassified(100);unclassified(100);unclassified(100);

Otu1260 0 0 0 0 0 0 0 0 0 0 0 0 0 0 0 0 0 0 0 0 0 0 0 0 0 0 0 0
0 0 0 0 0 0 0 0 0 0 0 0 0 0 0 0 0 0 0 0 0 0 0 0 0 0 0 0 0
0 0 0 0 0 0 0 0 0 0 0 0 0 0 0 0 0 0 0 0 0 0 0 0 0 0 0 0 0
0 0 0 0 0 0 0 0 0 0 0 0 0 0 0 0 0 0 30 0 0 0 0 0 0 0 0
M02255_77_000000000-ADUT3_1_1116_7525_23353 T----AC---GA-AG-GGG--------GCT-A-G------C--G--T---T--GT-T-CGG-AA------TT-A--C-T--GG-GC-------GT--A-----AA-GC-GC-GC-------G-CA-G-G-C-G------------G--T-TC-T-T-C-----------AA-
---G-T-C-A--------G-A-G--G--TG--A-AA-GC--C-C-AG-A-G-----CT-C-AA------C-T-C-T-G-G-A--A-T----G-G--C-C---T--T-------T--GA-G-A---C-------T--A-G--AG--A-A-C----T-A-G-G-G-T--A---C-GG-----GA-G-A--------G-G-T-G---AG-T------GG--A--ATT----C-C-G-A-
GT--GT-A-G-AG-GT--G---A--A-A---TT-C-GT-AG--AT-A-TT--C-G--G-A-A-G-A-AC-A-CC---AG--T--G--GC-GAA-G--G-C--G-------G--C-T-C-A---CTG--G--CC-C-G--------------------T-T---A-C-C--GA--CG-C-T-C--A-GG--C-G-CG-A--CA-G-C---G-TG--GG-G--AG-C-
A-AA--------CA--GG Bacteria(100);Proteobacteria(100);Alphaproteobacteria(100);unclassified(100);unclassified(100);unclassified(100);unclassified(100);unclassified(100);unclassified(100);

Otu1261 0 0 0 0 0 0 0 0 0 0 0 0 0 0 0 0 0 0 0 0 0 0 0 0 0 0 0 0
0 0 0 0 0 0 0 0 0 0 0 0 0 0 0 0 0 0 0 0 0 0 0 0 0 0 0 0 0
0 0 0 0 0 0 0 0 0 0 0 0 0 0 0 0 0 0 0 0 0 0 0 0 0 0 0 0 0
0 0 0 0 0 0 0 0 0 0 0 0 0 0 0 0 0 0 0 0 0 0 0 0 27 0 0
M02255_77_000000000-ADUT3_1_1112_24579_14818 T----AC---GG-AG-GGG--------GCT-A-G------C--G--T---T--GT-T-CGG-AA------TT-A--C-T--GG-GC-------GT--A-----AA-GC-GC-GC-------G-TA-G-G-C-G------------G--C-TT-T-G-C-----
------AA----G-T-C-A--------G-A-G--G--TG--A-AA-GC--C-C-GG-G-G-----CT-C-AA------C-C-C-C-G-G-A--A-T----T-G--C-C---T--T-------T--GA-A-A---C-------T--G-C--AT--C-G-C----T-A-G-A-A-C--G---T-CG-----GA-G-A--------G-G-T-G---AG-T------GG--A--ATT----
C-C-G-A-GT--GT-A-G-AG-GT--G---A--A-A---TT-C-GT-AG--AT-A-TT--C-G--G-A-A-G-A-AC-A-CC---AG--T--G--GC-GAA-G--G-C--G-------A--C-T-C-A---CTG--G--AC-G-A--------------------C-T---G-T-T--GA--CG-C-T-G--A-GG--T-G-CG-A--AA-G-C---G-TG--GG-
G--AG-C-A-AA--------CA--GG Bacteria(100);Proteobacteria(100);Alphaproteobacteria(100);Sphingomonadales(100);unclassified(100);unclassified(100);unclassified(100);unclassified(100);unclassified(100);

Otu1262 0 0 0 0 0 0 0 0 0 0 0 0 0 0 0 0 0 0 0 0 0 0 0 0 0 0 0 0
0 0 0 0 0 0 0 0 0 0 0 0 0 0 0 0 0 0 0 0 0 0 0 0 0 0 0 0 0
0 0 0 0 0 0 0 0 0 0 0 0 0 0 0 0 0 0 0 0 0 0 0 0 0 0 0 0 0
0 0 0 0 0 0 0 0 0 0 0 0 0 0 0 0 0 0 0 0 0 0 0 0 14 0 0
M02255_77_000000000-ADUT3_1_1101_3204_18259 T----AC---GT-AG-GGG--------GCA-A-G------C--G--T---T--AT-C-CGG-AA------TT-A--T-T--GG-GC-------GT--A-----AA-GA-GT-AC-------G-TA-G-G-C-G------------G--T-TT-C-T-T-----------AA--
--G-C-G-C--------A-G-G--G--TC--T-AA-GG--C-T-AT-G-G-----CT-C-AA------C-C-A-T-A-G-T--T------C-G--C-C---C--T-------G--TG-A-A---C-------T--G-G--GA--G-A-C----T-T-G-A-G-T--A---T-CG-----GA-G-A--------G-G-A-A---AG-C------GG--A--ATT----C-C-T-A-
GT--GT-A-G-CG-GT--G---A--A-A---TG-C-GT-AG--AT-A-TT--A-G--G-A-G-G-A-AC-A-CC---AG--T--G--GC-GAA-G--G-C--G-------G--C-T-T-T---CTG--G--AC-G-A--------------------A-T---A-C-T--GA--CG-C-T-G--A-GG--T-A-CG-A--AA-G-C---G-TG--GG-G--AG-
C-A-AA--------CA--GG Bacteria(100);Firmicutes(100);Clostridia(100);Clostridiales(100);unclassified(100);unclassified(100);unclassified(100);unclassified(100);unclassified(100);

Otu1263 0 0 0 0 0 0 0 0 0 0 0 0 0 0 0 0 0 0 0 0 0 0 0 0 0 0 0 0
0 0 0 0 0 0 0 0 0 0 0 0 0 0 0 0 0 0 0 0 0 0 0 0 0 0 0 0 0
0 0 0 0 0 0 0 0 0 0 0 0 0 0 0 0 0 0 0 0 0 0 0 0 0 0 0 0 0
0 0 0 0 0 0 0 0 0 0 0 0 0 0 0 0 0 0 0 0 0 0 0 0 18 0 0
M02255_77_000000000-ADUT3_1_1102_6524_8289 T----AC---GT-AG-GGA--------GCG-A-G------C--G--T---T--GT-C-CGG-AA------TT-A--C-T--GG-GT-------GT--A-----AA-GG-GA-GC-------G-TA-G-G-C-G------------G--G-AA-G-G-C-----------
AA----G-T-T-G--------G-A-T--G--TG--A-AA-AC--T-G-TG-G-G-----CT-C-AA------C-C-G-A-C-A-G--A-C----T-G--C-A---T--T-------C--AA-A-A---C-------T--G-T--TT--T-T-C----T-T-G-A-G-T--G---A-AG-----TA-G-A--------G-G-C-A---AG-C------GG--A--ATT----C-C-T-
A-GT--GT-A-G-CG-GT--G---A--A-A---TG-C-GT-AG--AT-A-TT--A-G--G-A-G-G-A-AC-A-CC---AG--T--G--GC-GAA-G--G-C--G-------G--C-T-T-G---CTG--G--GC-T-T--------------------T-T---A-C-T--GA--CG-C-T-G--A-GG--C-T-CG-A--AA-G-T---G-TG--GG-G--AG-
C-A-AA--------CA--GG Bacteria(100);Firmicutes(100);Clostridia(100);Clostridiales(100);unclassified(100);unclassified(100);unclassified(100);unclassified(100);unclassified(100);

Otu1264 0 0 0 0 0 0 0 0 0 0 0 0 0 0 0 0 0 0 0 0 0 0 0 0 0 0 0 0
0 0 0 0 0 0 0 0 0 0 0 0 0 0 0 0 0 0 0 0 0 0 0 0 0 0 0 0 0
0 0 0 0 0 0 0 0 0 0 0 0 0 0 0 0 0 0 0 0 0 0 0 0 0 0 0 0 0
0 0 0 0 0 0 0 0 0 0 0 0 0 0 0 0 0 0 0 0 0 0 0 0 14 0 0
M02255_77_000000000-ADUT3_1_1109_5050_14066 T----AC---GT-AG-GGG--------GCA-A-G------C--G--T---T--AT-C-CGG-AT------TT-A--C-T--GG-GT-------GT--A-----AA-GG-GA-GC-------G-TA-G-G-T-G------------G--T-TT-G-G-T-----------AA-
---G-T-C-A--------G-G-T--G--TG--A-AA-GC--C-C-GG-G-G-----CT-C-AA------C-T-C-C-G-G-G--A-T----T-G--C-A---C--T-------T--GA-A-A---C-------T--A-T--CA--A-A-C----T-A-G-A-G-T--G---C-AG-----GA-G-A--------G-G-T-A---AG-T------GG--A--ATT----C-C-T-A-
GT--GT-A-G-CG-GT--G---A--A-A---TG-C-GT-AG--AT-A-TT--A-G--G-A-G-G-A-AC-A-CC---AG--T--G--GC-GAA-G--G-C--G-------G--C-T-T-A---CTG--G--AC-T-G--------------------T-A---A-C-T--GA--CA-C-T-G--A-GG--C-T-CG-A--AA-G-C---G-TG--GG-G--AG-
C-A-AA--------CA--GG Bacteria(100);Firmicutes(100);Clostridia(100);Clostridiales(100);unclassified(100);unclassified(100);unclassified(100);unclassified(100);unclassified(100);

Otu1265 0 0 0 0 0 0 0 0 0 0 0 0 0 0 0 0 0 0 0 0 0 0 0 0 0 0 0 0

0 0 0 0 0 0 0 0 0 0 0 0 0 0 0 0 0 0 0 0 0 0 0 0 0 0 0 0 0

0 0 0 0 0 0 0 0 0 0 0 0 0 0 0 0 0 0 0 0 0 0 0 0 0 0 0 0 0

0 0 0 0 0 0 0 0 0 0 0 0 0 0 0 0 0 0 0 0 0 0 0 0 28 0 0

M02255_77_000000000-ADUT3_1_1109_16426_21261 T----AC---GA-AG-GGG--------GCT-A-G------C--G--T---T--GT-T-CGG-AT------TT-A--C-T--GG-GC-------GT--A-----AA-GC-GC-AC-------G-TA-G-G-C-G------------G--A-TC-A-T-T-----
------AA----G-T-C-A--------G-G-G--G--TG--A-AA-TC--C-C-GA-G-G-----CT-C-AA------C-C-T-C-G-G-A--A-C----T-G--C-C---T--T-------T--GA-T-A---C-------T--G-G--TG--A-T-C----T-T-G-A-G-T--C---C-GG-----GA-G-A--------G-G-T-G---AG-T------GG--A--ACT----G-
C-G-A-GT--GT-A-G-AG-GT--G---A--A-A---TT-C-GT-AG--AT-A-TT--C-G--C-A-A-G-A-AC-A-CC---AG--T--G--GC-GAA-G--G-C--G-------G--C-T-C-A---CTG--G--CC-C-G--------------------G-A---A-C-T--GA--CG-C-T-G--A-GG--T-G-CG-A--AA-G-C---G-TG--GG-
G--AG-C-A-AA--------CA--GG Bacteria(100);Proteobacteria(100);Alphaproteobacteria(100);Rhizobiales(100);unclassified(100);unclassified(100);unclassified(100);unclassified(100);unclassified(100);

Otu1266 0 0 0 0 0 0 0 0 0 0 0 0 0 0 0 0 0 0 0 0 0 0 0 0 0 0 0 0

0 0 0 0 0 0 0 0 0 0 0 0 0 0 0 0 0 0 0 0 0 0 0 0 0 0 0 0 0

0 0 0 0 0 0 0 0 0 0 0 0 0 0 0 0 0 0 0 0 0 0 0 0 0 0 0 0 0

0 0 0 0 0 0 0 0 0 0 0 0 0 0 0 0 0 0 0 0 0 0 0 0 29 0 0

M02255_77_000000000-ADUT3_1_1115_8423_10154 T----AC---GA-AG-GGG--------GCT-A-G------C--G--T---T--GC-T-CGG-AA------TC-A--T-T--GG-GC-------GT--A-----AA-GG-GC-GC-------G-TA-G-G-C-G------------G--C-CC-C-G-T-----------
TA----G-T-C-A--------G-G-C--G--TG--A-AA-CC--C-C-CG-G-G-----CT-C-AA------C-C-C-G-G-G-G--A-C----T-G--C-G---C--T-------T--GA-T-A---C-------G--G-C--GG--G-G-C----T-T-G-A-G-G--A---C-GG-----GA-G-A--------G-G-C-T---CG-C------GG--A--ATT----C-C-
C-A-GT--GT-A-G-AG-GT--G---A--A-A---TT-C-GT-AG--AT-A-TT--G-G--G-A-A-G-A-AC-A-CC---GG--T--G--GC-GAA-G--G-C--G-------G--C-G-A-G---CTG--G--CC-C-G--------------------T-T---T-C-T--GA--CG-C-T-G--A-GG--C-G-CG-A--CA-G-C---G-TG--GG-G--
AG-C-A-AA--------CA--GG Bacteria(100);Proteobacteria(100);Alphaproteobacteria(100);Rhodospirillales(100);unclassified(100);unclassified(100);unclassified(100);unclassified(100);unclassified(100);

Otu1267 0 0 0 0 0 0 0 0 0 0 0 0 0 0 0 0 0 0 0 0 0 0 0 0 0 0 0 0

0 0 0 0 0 0 0 0 0 0 0 0 0 0 0 0 0 0 0 0 0 0 0 0 0 0 0 0 0

0 0 0 0 0 0 0 0 0 0 0 0 0 0 0 0 0 0 0 0 0 0 0 0 0 0 0 0 0

0 0 0 0 0 0 0 0 0 0 0 0 0 0 0 0 0 0 0 0 0 0 0 0 14 0 0

M02255_77_000000000-ADUT3_1_2105_23119_10145 T----AC---GT-AA-GGG--------GCA-A-G------C--G--T---T--AT-C-CGG-AT------TT-A--C-T--GG-GT-------GT--A-----AA-GG-GA-GC-------G-TA-G-A-C-G------------G--T-TT-G-G-C-----
------AA----G-T-C-T--------G-A-T--G--TG--A-AA-GG--C-A-TG-G-G-----CT-C-AA------C-C-T-G-T-G-G--A-C----T-G--C-A---T--T-------G--GA-A-A---C-------T--G-T--CA--G-A-C----T-T-G-A-G-T--G---C-CG-----GA-G-A--------G-G-C-A---AG-C------GG--A--ATT----
C-C-T-A-GT--GT-A-G-CG-GT--G---A--A-A---TG-C-GT-AG--AT-A-TT--A-G--G-A-G-G-A-AC-A-CC---AG--T--G--GC-GAA-G--G-C--G-------G--CC--T-G---CTG--G--AC-G-G--------------------T-A---A-C-T--GA--CG-T-T-G--A-GG--C-T-CG-A--AA-G-C---G-TG--
GG-G--AG-C-A-AA--------CA--GG Bacteria(100);Firmicutes(100);Clostridia(100);Clostridiales(100);Lachnospiraceae(100);Blautia(100);unclassified(100);unclassified(100);unclassified(100);

Otu1268 0 0 0 0 0 0 0 0 0 0 0 0 0 0 0 0 0 0 0 0 0 0 0 0 0 0 0 0

0 0 0 0 0 0 0 0 0 0 0 0 0 0 0 0 0 0 0 0 0 0 0 0 0 0 0 0 0

0 0 0 0 0 0 0 0 0 0 0 0 0 0 0 0 0 0 0 0 0 0 0 0 0 0 0 0 0

0 0 0 0 0 0 0 0 0 0 0 0 0 0 0 0 0 0 0 0 0 0 0 0 22 0 0

M02255_77_000000000-ADUT3_1_2109_4842_19235 T----AC---GG-AG-GGT--------GCA-A-G------C--G--T---T--GT-T-CGG-AA------TT-A--C-T--GG-GC-------GT--A-----AA-GC-GT-AC-------G-TA-G-G-C-G------------G--C-GA-T-T-T-----------AA-
---G-T-C-A--------A-A-T--G--TT--A-AA-GC--C-C-GA-G-G-----CT-C-AA------C-C-T-C-G-T-G--G-A----T-G--C-C---T--T-------T--GA-T-A---C-------T--G-G--AT--T-G-C----T-A-G-A-G-G--G---T-GA-----TA-G-A--------G-G-A-A---AG-T------GG--A--ATT----C-C-G-A-
GT--GT-A-G-CG-GT--G---A--A-A---TG-C-GT-AG--AT-A-TT--C-G--G-A-G-G-A-AC-A-TC---AG--T--A--GC-GAA-G--G-C--G-------A--C-T-T-T---CTG--G--GT-C-A--------------------C-A---C-C-T--GA--CG-C-T-G--A-GG--T-A-CG-A--AA-G-C---G-TG--GG-T--AG-C-
A-AA--------CA--GG Bacteria(100);unclassified(100);unclassified(100);unclassified(100);unclassified(100);unclassified(100);unclassified(100);unclassified(100);unclassified(100);

Otu1269 0 0 0 0 0 0 0 0 0 0 0 0 0 0 0 0 0 0 0 0 0 0 0 0 0 0 0 0

0 0 0 0 0 0 0 0 0 0 0 0 0 0 0 0 0 0 0 0 0 0 0 0 0 0 0 0 0

0 0 0 0 0 0 0 0 0 0 0 0 0 0 0 0 0 0 0 0 0 0 0 0 0 0 0 0 0

0 0 0 0 0 0 0 0 0 0 0 0 0 0 0 0 0 0 0 0 0 0 0 0 13 0 0

M02255_77_000000000-ADUT3_1_2118_19974_9864 T----AC---GT-AG-GGT--------GCG-A-G------C--G--T---T--GT-C-CGG-AT------TT-A--T-T--GG-GC-------GT--A-----AA-GA-GC-TC-------G-TA-G-G-T-G------------G--T-TT-G-T-T-----------GC--
--G-T-C-G--------G-A-A--G--TG--A-AA-AC--C-T-GC-A-G-----CT-T-AA------C-T-G-T-G-G-G--C-T----T-G--C-T---T--T-------C--GA-T-A---C-------G--G-G--CA--G-A-C----T-T-G-A-G-G--A---A-GG-----TA-G-G--------G-G-A-G---AA-C------GG--A--ATT----C-C-T-G-
GT--GG-A-G-CG-GT--G---G--A-A---TG-C-GC-AG--AT-A-TC--A-G--G-A-G-G-A-AC-A-CC---GG--T--G--GC-GAA-G--G-C--G-------G--T-T-C-T---CTG--G--AC-C-T--------------------T-T---C-C-T--GA--CG-C-T-G--A-GG--A-G-CG-A--AA-G-C---G-TG--GG-G--AG-
C-G-AA--------CA--GG Bacteria(100);Actinobacteria(100);Actinobacteria(100);Actinobacteridae(100);Actinomycetales(100);unclassified(100);unclassified(100);unclassified(100);unclassified(100);

Otu1270 0 0 0 0 0 0 0 0 0 0 0 0 0 0 0 0 0 0 0 0 0 0 0 0 0 0 0 0
0 0 0 0 0 0 0 0 0 0 0 0 0 0 0 0 0 0 0 0 0 0 0 0 0 0 0 0 0
0 0 0 0 0 0 0 0 0 0 0 0 0 0 0 0 0 0 0 0 0 0 0 0 0 0 0 0 0
0 0 0 0 0 0 0 0 0 0 0 0 0 0 0 0 0 0 0 0 0 0 0 3 0 0 0
M02255_76_000000000-ADUNE_1_1119_5111_17127 T----TA---GC-TG-CAG--------AAT-A-A------G--C--A---C--CG-G-CTA-AC------TC-T--G-T--GC-CA-------GC--AG----CC-GC-GG-TA-------A-TACA-G-A-G------------G--G-TC-C-A-A----------
GC----G-T-C-A--------G-A-T--C--GG--A-AA-TC--C-C-CG-G-G-----CT-T-AA------C-C-T-G-G-G-A--A-C----T-G--C-A---T--C-------T--GA-A-A---C-------T--G-T--AG--A-G-C----T-A-G-A-G-T--A---T-GT-----GA-G-A--------G-G-A-A---GG-T------AG--A--ATT----C-C-A-
G-GT--GT-A-G-CG-GT--G---A--A-A---TG-C-GT-AG--AG-A-TC--T-G--G-A-G-G-A-AT-A-CC---GA--T--G--GC-GAA-G--G-C--A-------G--C-C-T-T---CTG--G--CA-T-A--------------------A-T---A-C-T--GA--CA-C-T-G--A-GG--C-T-CG-A--AA-G-C---G-TG--GG-T--AG-
C-A-AA--------CA--GG Bacteria(100);Proteobacteria(100);Gammaproteobacteria(100);unclassified(100);unclassified(100);unclassified(100);unclassified(100);unclassified(100);unclassified(100);

Otu1271 0 0 0 0 0 0 0 0 0 0 0 0 0 0 0 0 0 0 0 0 0 0 0 0 0 0 0 0
0 0 0 0 0 0 0 0 0 0 0 0 0 0 0 0 0 0 0 0 0 0 0 0 0 0 0 0 0
0 0 0 0 0 0 0 0 0 0 0 0 0 0 0 0 0 0 0 0 0 0 0 0 0 0 0 0 0
0 0 0 0 0 0 0 0 0 0 0 0 0 0 0 0 0 0 0 0 0 0 0 0 17 0 0
M02255_77_000000000-ADUT3_1_1107_7155_9886 T----AC---GT-AG-GTG--------GCA-A-G------C--G--T---T--AT-C-CGG-AA------TT-A--C-T--GG-GT-------GT--A-----AA-GG-GT-GT-------G-TA-G-G-C-G------------G--G-GC-G-A-C----------
AA----G-T-C-A--------G-A-T--G--TG--A-AA-AT--T-A-TG-G-G-----CT-C-AA------C-C-C-A-T-A-A--C-C----T-G--C-G---T--C-------T--GA-A-A---C-------T--G-T--TG--C-T-C----T-T-G-A-G-A--G---T-GG-----GA-G-A--------G-G-T-A---AA-T------GG--A--ATT----C-C-C-
G-GT--GT-A-G-CG-GT--G---A--A-A---TG-C-GT-AG--AT-A-TC--G-G--G-A-G-G-A-AC-A-CC---AG--T--G--GC-GAA-G--G-C--G-------A--T-T-T-A---CTG--G--AC-C-A--------------------C-A---A-C-T--GA--CG-C-T-G--A-GA--C-A-CG-A--AA-G-C---G-TG--GG-G--
AG-C-A-AA--------CA--GG Bacteria(100);unclassified(100);unclassified(100);unclassified(100);unclassified(100);unclassified(100);unclassified(100);unclassified(100);unclassified(100);

Otu1272 0 0 0 0 0 0 0 0 0 0 0 0 0 0 0 0 0 0 0 0 0 0 0 0 0 0 0 0
0 0 0 0 0 0 0 0 0 0 0 0 0 0 0 0 0 0 0 0 0 0 0 0 0 0 0 0 0
0 0 0 0 0 0 0 0 0 0 0 0 0 0 0 0 0 0 0 0 0 0 0 0 0 0 0 0 0
0 0 0 0 0 0 0 0 0 0 0 0 0 0 0 0 0 0 0 0 0 0 0 0 0 15 0
M02255_76_000000000-ADUNE_1_2114_12107_8729 .....AC----T-A--CGT--------GCC-A-G------C--A--G---C--CG-C-GGT-AA------CA-C--G-T--AG-GG-------GG--C-----AA-GC-G--TT-------G-T----C-C-G------------G--A-TT-C-G-T----------AA----G-
T-T-G--------G-G-C--G--TA--AAGA-GCT-C-G-TA-G-G-----CT-C-AA------C-C-T-G-GAG-A---------C-G-CC-A---C--C-------C--GA-T-A---C-------T--G-C--GA-TG-A-C----T-A-G-A-G-T--C---C-GG-----TA-G-G--------G-G-A-G---TG-T------GG--A--ATT----C-C-T-G-GT-
-GT-A-G-CG-GT--G---A--A-A---TG-C-GC-AG--AT-A-TC--A-G--G-A-G-G-A-AC-A-CC---AA--C--G--GC-GAA-G--G-C--A-------G--C-A-C-T---CTG--G--GC-C-G--------------------G-A---A-C-T--GA--CG-C-T-G--A-GG--A-G-CG-A--AA-G-C---G-TG--GG-T--AG-C-G-
AA--------CA--GG Bacteria(100);Actinobacteria(100);Actinobacteria(100);Acidimicrobidae(100);Acidimicrobiales(100);Acidimicrobineae(100);unclassified(100);unclassified(100);unclassified(100);

Otu1273 0 0 0 0 0 0 0 0 0 0 0 0 0 0 0 0 0 0 0 0 0 0 0 0 0 0 0 0
0 0 0 0 0 0 0 0 0 0 0 0 0 0 0 0 0 0 0 0 0 0 0 0 0 0 0 0 0
0 0 0 0 0 0 0 0 0 0 0 0 0 0 0 0 0 0 0 0 0 0 0 0 0 0 0 0 0
0 0 0 0 0 0 0 0 0 0 0 0 0 0 0 0 0 0 0 0 0 0 0 0 0 13 0
M02255_76_000000000-ADUNE_1_1115_13351_10104 A----AG---GG-AC-GGC--------TAA-C-T------A--C--G---T--GC-C-AGC-AG------CC-G--C-G-GTA-AT-------AC--G-----TA-GG-TC-CC-------G-AG-C-G-T-T------------G--T-CC-G-G-A----
-------AA----G-T-C-T--------G-G-A--G--TA--A-AA-GG--C-T-AT-G-G-----CT-C-AA------C-C-A-T-A-G-T--G------T-G--C-T---C--T-------G--GA-A-A---C-------T--G-T--CT--G-A-C----T-T-G-A-G-T--G---C-AG-----AA-G-G--------G-G-A-G---AG-T------GG--A--ATT----C-
C-A-T-GT--GT-A-G-CG-GT--G---A--A-A---TG-C-GT-AG--AT-A-TA--T-G--G-A-G-G-A-AC-A-CC---AG--T--G--GC-GAA-A--G-C--G-------G--C-T-C-T---CTG--G--TC-T-G--------------------T-C---A-C-T--GA--CG-C-T-G--A-GG--C-T-CG-A--AA-G-C---G-TG--GG-T--
AG-C-G-AA--------CA--GG Bacteria(100);Firmicutes(100);Bacilli(100);Lactobacillales(100);unclassified(100);unclassified(100);unclassified(100);unclassified(100);unclassified(100);

Otu1274 0 0 0 0 0 0 0 0 0 0 0 0 0 0 0 0 0 0 0 0 0 0 0 0 0 0 0 0
0 0 0 0 0 0 0 0 0 0 0 0 0 0 0 0 0 0 0 0 0 0 0 0 0 0 0 0 0
0 0 0 0 0 0 0 0 0 0 0 0 0 0 0 0 0 0 0 0 0 0 0 0 0 0 0 0 0
0 0 0 0 0 0 0 0 0 0 0 0 0 0 0 0 0 0 0 0 0 0 0 0 0 0 26
M02255_77_000000000-ADUT3_1_1105_21679_18263 T----AC---GA-AG-GTG--------GCG-A-A------T--G--T---T--GC-T-CGG-AT------TC-A--C-T--GG-GC-------GT--A-----AA-GC-GT-CT-------G-TA-G-G-T-G------------G--C-TT-G-T-T------
-----AA----G-T-T-A--------G-A-G--G--TG--A-AA-GC--C-C-CG-C-G-----CT-C-AA------C-G-T-G-G-G-A--A-C----T-G--C-C---T--T-------T--AA-A-A---C-------T--G-G--CG--A-G-C----T-T-G-A-G-A--G---T-AT-----CA-G-A--------G-G-T-A---GG-C------GG--A--ATA----A-
C-T-G-GT--GT-A-G-AG-GT--A---A--A-A---TT-C-AT-AG--AT-A-TC--A-G--T-T-G-G-A-AC-A-TC---CA--T--G--GC-GAA-G--G-C--A-------G--C-T-T-A---CTG--G--GA-T-A--------------------T-T---T-C-T--GA--CA-C-T-G--A-GA--G-A-CG-A--CA-G-C---G-TG--GG-G--
AG-C-A-AA--------CA--GG Bacteria(100);unclassified(100);unclassified(100);unclassified(100);unclassified(100);unclassified(100);unclassified(100);unclassified(100);unclassified(100);

Otu1275 0 0 0 0 0 0 0 0 0 0 0 0 0 0 0 0 0 0 0 0 0 0 0 0 0 0 0 0
0 0 0 0 0 0 0 0 0 0 0 0 0 0 0 0 0 0 0 0 0 0 0 0 0 0 0 0 0
0 0 0 0 0 0 0 0 0 0 0 0 0 0 0 0 0 0 0 0 0 0 0 0 0 0 0 0 0
0 0 0 0 0 0 0 0 0 0 0 0 0 0 0 0 0 0 0 0 0 0 0 0 0 0 12
M02255_77_000000000-ADUT3_1_1108_16029_12335 T----AC---GT-AG-GAC--------CCA-A-G------C--G--T---T--AT-C-CGG-AG------TG-A--C-T--GG-GC-------GT--A-----AA-GA-GTTGC-------G-TA-G-G-C-G------------G--T-CT-G-T-A----
-------AA----G-C-G-A--------A-T-A--G--TG--A-AA-GA--T-G-AT-G-G-----CT-C-AA------C-C-A-T--CA-G--A-A----C-A--T-T---G--T-------T--CG-A-A---C-------T--C-A--CA--G-A-C----T-C-G-A-G-A--A---T-GG-----TA-G-A--------G-G-T-A---GC-T------GG--A--ATT----
T-C-G-T-GT--GT-A-G-GA-GT--G---A--A-A---TC-C-GT-AG--AT-A-TA--C-G--A-A-G-G-A-AC-A-CC---AA--T--G--GC-GTA-G--G-C--A-------G--G-C-T-A---CTG--G--AC-C-A--------------------T-T---T-C-T--GA--CG-C-T-G--A-GG--C-A-CG-A--AA-G-C---G-TG--GG-
G--AG-C-G-AA--------CC--GG Bacteria(100);Candidate_division_TM7(100);unclassified(100);unclassified(100);unclassified(100);unclassified(100);unclassified(100);unclassified(100);unclassified(100);

Otu1276 0 0 0 0 0 0 0 0 0 0 0 0 0 0 0 0 0 0 0 0 0 0 0 0 0 0 0 0
0 0 0 0 0 0 0 0 0 0 0 0 0 0 0 0 0 0 0 0 0 0 0 0 0 0 0 0 0
0 0 0 0 0 0 0 0 0 0 0 0 0 0 0 0 0 0 0 0 0 0 0 0 0 0 0 0 0
0 0 0 0 0 0 0 0 0 0 0 0 0 0 0 0 0 0 0 0 0 0 0 0 0 0 16
M02255_77_000000000-ADUT3_1_2113_28220_12171 T----AC---AG-GG-GGT--------GCA-A-G------C--G--T---T--GC-T-CGG-AA------TC-A--C-T--GG-GC-------GT--A-----AA-GC-GT-AC-------G-CA-G-G-C-G------------G--A-CC-G-A-T----
-------AA----G-T-C-G--------G-G-T--G--TG--A-AA-GC--C-C-AG-G-G-----CT-C-AA------C-C-C-T-G-G-A--A-C----T-G--C-A---T--T-------C--GA-A-A---C-------T--G-T--CG--G-T-C----T-T-G-A-G-T--A---C-GG-----GA-G-A--------G-G-T-G---AG-T------GG--A--ATT----
C-T-T-G-GT--GT-A-G-AG-GT--G---A--A-A---TT-C-GT-AG--AT-A-TC--A-G--G-A-A-G-A-AC-A-CC---GG--T--G--GC-GAA-A--G-C--G-------G--C-T-C-A---CTG--G--CC-C-G--------------------A-T---A-C-T--GA--CG-C-T-C--A-TG--T-A-CG-A--AA-G-C---G-TG--GG-
G--AG-C-A-AA--------CA--GG Bacteria(100);Proteobacteria(100);unclassified(100);unclassified(100);unclassified(100);unclassified(100);unclassified(100);unclassified(100);unclassified(100);

Otu1277 0 0 0 0 0 0 0 0 0 0 0 0 0 0 0 0 0 0 0 0 0 0 0 0 0 0 0 0
0 0 0 0 0 0 0 0 0 0 0 0 0 0 0 0 0 0 0 0 0 0 0 0 0 0 0 0 0
0 0 0 0 0 0 0 0 0 0 0 0 0 0 0 0 0 0 0 0 0 0 0 0 0 0 0 0 0
0 0 0 0 0 0 0 0 0 0 0 0 0 0 0 0 0 0 0 0 0 0 0 0 0 0 25
M02255_77_000000000-ADUT3_1_1104_13451_20462 T----AC---GA-AT-GGA--------GCG-A-G------C--G--T---T--GT-T-CGG-AA------TC-A--C-T--GG-GC-------GT--A-----AA-GG-GT-GC-------G-CA-G-G-C-G------------G--T-CA-T-G-A----
-------AA----G-T-T-A--------G-A-A--G--TG--A-AA-TC--C-C-AG-G-G-----CT-T-AA------C-C-T-T-G-G-A--A-T----T-G--C-T---T--T-------T--AA-A-A---C-------T--C-C--AT--G-A-C----T-A-G-A-G-T--A---C-TG-----GA-G-A--------G-G-T-T---GG-C------GG--A--ATT----C-
C-G-A-GT--GT-A-G-AG-GT--G---A--A-A---TT-C-GC-AG--AT-A-TT--C-G--G-A-G-G-A-AC-A-CC---AA--T--G--GC-GTA-G--G-C--A-------G--C-C-A-A---CTG--G--AC-A-G--------------------T-A---A-C-T--GA--CG-C-T-C--A-TG--C-A-CG-A--AA-G-C---G-TG--GG-G-
-AT-C-A-AA--------CA--GG Bacteria(100);Proteobacteria(100);unclassified(100);unclassified(100);unclassified(100);unclassified(100);unclassified(100);unclassified(100);unclassified(100);

Otu1278 0 0 0 0 0 0 0 0 0 0 0 0 0 0 0 0 0 0 0 0 0 0 0 0 0 0 0 0
0 0 0 0 0 0 0 0 0 0 0 0 0 0 0 0 0 0 0 0 0 0 0 0 0 0 0 0 0
0 0 0 0 0 0 0 0 0 0 0 0 0 0 0 0 0 0 0 0 0 0 0 0 0 0 0 0 0
0 0 0 0 0 0 0 0 0 0 0 0 0 0 0 0 0 19 0 0 0 0 0 0 0 0 0
M02255_77_000000000-ADUT3_1_1107_11819_17641 T----AC---GG-AG-GGG--------GCT-A-G------C--G--T---T--GT-T-CGG-AA------TT-A--C-T--GG-GC-------GT--A-----AA-GC-GT-AC-------G-TA-G-G-C-G------------G--C-TT-T-T-T------
-----AA----G-T-C-A--------G-A-G--G--TG--A-AA-TC--C-C-GG-G-G-----CT-C-AA------C-T-C-C-G-G-A--A-C----T-G--C-C---T--T-------T--GA-G-A---C-------T--G-G--AT--C-G-C----T-T-G-A-A-C--G---T-CG-----GA-G-A--------G-G-T-G---AG-T------GG--A--ATT----C-
C-G-A-GT--GT-A-G-AG-GT--G---A--A-A---TT-C-GT-AG--AT-A-TT--C-G--G-A-A-G-A-AC-A-CC---AG--T--G--GC-GAA-G--G-C--G-------G--C-T-C-A---CTG--G--AC-G-A--------------------C-T---G-T-T--GA--CG-C-T-G--A-GG--T-A-CG-A--AA-G-C---G-TG--GG-
G--AG-C-A-AA--------CA--GG Bacteria(100);Proteobacteria(100);Alphaproteobacteria(100);Sphingomonadales(100);unclassified(100);unclassified(100);unclassified(100);unclassified(100);unclassified(100);

Otu1279 0 0 0 0 0 0 0 0 0 0 0 0 0 0 0 0 0 0 0 0 0 0 0 0 0 0 0 0
0 0 0 0 0 0 0 0 0 0 0 0 0 0 0 0 0 0 0 0 0 0 0 0 0 0 0 0 0
0 0 0 0 0 0 0 0 0 0 0 0 0 0 0 0 0 0 0 0 0 0 0 0 0 0 0 0 0
0 0 0 0 0 0 0 0 0 0 0 0 0 0 0 0 0 0 0 0 0 0 0 3 0 0 0
M02255_77_000000000-ADUT3_1_1105_6867_18856 T----AC---GT-AG-GGA--------GCA-A-G------C--G--T---T--AT-C-CGG-AT------TT-A--C-T--GG-GT-------GT--A-----AA-GG-GT-GC-------G-TA-G-G-C-G------------G--C-TT-G-G-T-----------AA-
---G-T-C-T--------A-A-A--G--TG--A-AA-TT--T-C-GG-G-G-----CT-C-AA------C-C-C-C-G-G-A--G-C----T-G--C-T---T--T-------G--GA-A-A---C-------T--G-C--CG--A-G-C----T-A-G-A-G-T--G---T-GG-----GA-G-A--------G-G-A-A---AG-C------GG--A--ACT----T-T-G-A-
GT--GT-A-G-CG-GT--G---A--A-A---TG-C-GT-AG--AG-A-TT--C-A--A-A-G-G-A-AC-A-CC---AG--T--A--GC-GAA-G--G-C--G-------G--C-T-T-T---CTG--G--AC-C-A--------------------T-A---A-C-T--GA--CG-C-T-G--A-GG--C-A-CG-A--AA-G-C---G-TG--GG-G--AG-
C-A-AA--------CA--GG Bacteria(100);unclassified(100);unclassified(100);unclassified(100);unclassified(100);unclassified(100);unclassified(100);unclassified(100);unclassified(100);

Otu1280 0 0 0 0 0 0 0 0 0 0 0 0 0 0 0 0 0 0 0 0 0 0 0 0 0 0 0 0
0 0 0 0 0 0 0 0 0 0 0 0 0 0 0 0 0 0 0 0 0 0 0 0 0 0 0 0 0
0 0 0 0 0 0 0 0 0 0 0 0 0 0 0 0 0 0 0 0 0 0 0 0 0 0 0 0 0
0 0 0 0 0 0 0 0 0 0 0 0 0 0 0 0 0 0 0 0 0 0 9 0 0 0 0
M02255_76_000000000-ADUNE_1_2111_14859_19479 T----A----AC-TC-CGT--------GCC-A-G------C--A--G---C--CG-C-GGT-AA------TA-C--G-G--AG-GG-------TG--C-----AA-GC-GT-TA-------T-CC-G-G---A-------------T--T-CT-T---T---------AA----G-T-C-A--------G-A-G--G--TG--A-AA-TC--C-T-GC-A-G-----CT-C-AA------C-T-G-C-A-G-A--A-C----T-G--C-C---T--T-------T--GA-T-A---C-------T--G-G--AA--A-T-C----T-T-G-A-G-T--T---T-AG-----TT-G-A--------A-G-T-G---GG-C------GG--A--ATA----T-G-A-C-AT--GT-A-G-CG-GT--G---A--A-A---TG-C-AT-AG--AT-A-TG--T-C--A-T-A-G-A-AC-A-CC---GA--T--A--GC-GAA-G--G-C--A-------G--C-T-C-A---CTA--A--AC-T-T--------------------G-A---A-C-T--GA--CA-C-T-G--A-TG--T-A-CG-A--AA-G-C---G-TG--GG-G--AG-C-G-AA--------CA--GG Bacteria(100);Bacteroidetes(100);unclassified(100);unclassified(100);unclassified(100);unclassified(100);unclassified(100);unclassified(100);unclassified(100);

Otu1281 0 0 0 0 0 0 0 0 0 0 0 0 0 0 0 0 0 0 0 0 0 0 0 0 0 0 0 0
0 0 0 0 0 0 0 0 0 0 0 0 0 0 0 0 0 0 0 0 0 0 0 0 0 0 0 0 0
0 0 0 0 0 0 0 0 0 0 0 0 0 0 0 0 0 0 0 0 0 0 0 0 0 0 0 0 0
0 0 0 0 0 0 0 0 0 0 0 0 0 0 0 0 0 0 0 0 0 0 7 0 0 0 0
M02255_77_000000000-ADUT3_1_2113_24120_11194 T----AC---GT-AG-GGT--------GCA-A-G------C--G--T---T--GT-C-CGG-AT------TT-A--T-T--GG-GC-------GT--A-----AA-GA-GC-TC-------G-TA-G-G-C-G-------------G--T-TT-G-T-C-----------AC----G-T-C-G--------G-C-T--G--TG--A-AA-AT--C-C-GG-G-G-----CT-C-AA------C-C-C-C-G-G-A--C-C----T-G--C-A---G--T-------C--GA-T-A---C-------G--G-G--CA--G-A-C----T-A-G-A-G-T--T---C-GG-----CA-G-G--------G-G-A-G---AC-T------GG--A--ATT----C-C-T-G-GT--GT-A-G-CG-GT--G---A--A-A---TG-C-GC-AG--AT-A-TC--A-G--G-A-G-G-A-AC-A-CC---GG--T--G--GC-GAA-G--G-C--G-------G--G-T-C-T---CTG--G--GC-C-G--------------------A-A---A-C-T--GA--CG-C-T-G--A-GG--A-G-CG-A--AA-G-C---G-TG--GG-G--AG-C-A-AA--------CA--GG Bacteria(100);Actinobacteria(100);Actinobacteria(100);Actinobacteridae(100);Actinomycetales(100);unclassified(100);unclassified(100);unclassified(100);unclassified(100);

Otu1282 0 0 0 0 0 0 0 0 0 0 0 0 0 0 0 0 0 0 0 0 0 0 0 0 0 0 0 0
0 0 0 0 0 0 0 0 0 0 0 0 0 0 0 0 0 0 0 0 0 0 0 0 0 0 0 0 0
0 0 0 0 0 0 0 0 0 0 0 0 0 0 0 0 0 0 0 0 0 0 0 0 0 0 0 0 0
0 0 0 0 0 0 0 0 0 0 0 0 0 0 0 0 0 0 0 0 0 0 33 0 0 0 0
M02255_77_000000000-ADUT3_1_1109_8107_19829 T----AC---GT-AG-GCA--------GCG-A-G------C--G--T---T--GT-T-CGG-AA------TT-A--C-T--GG-GC-------GT--A-----AA-GA-GT-GT-------G-TA-G-G-C-G------------G--T-GC-T-C-T-----------AA----G-T-T-C--------G-G-T--G--TG--A-AA-TC--T-C-CT-G-G-----CT-C-AA------C-T-G-G-G-A-G--G-G----T-G--C-G---C--C-------G--GA-A-A---C-------T--G-G--AA--T-G-C----T-T-G-A-G-C--G---T-GG-----GA-G-A--------G-G-A-A---AG-C------GG--A--ATT----C-C-T-G-GT--GT-A-G-CG-GT--G---A--A-A---TG-C-GT-AG--AT-A-TC--A-G--G-A-G-G-A-AC-A-CC---TG--T--G--GT-GTA-G--A-C--G-------G--C-T-T-T---CTG--G--AC-C-A--------------------T-A---G-C-T--GA--CG-C-T-G--A-GA--C-A-CG-A--AA-G-C---G-TG--GG-T--AG-C-A-AA--------CA--GG Bacteria(100);unclassified(100);unclassified(100);unclassified(100);unclassified(100);unclassified(100);unclassified(100);unclassified(100);unclassified(100);

Otu1283 0 0 0 0 0 0 0 0 0 0 0 0 0 0 0 0 0 0 0 0 0 0 0 0 0 0 0 0
0 0 0 0 0 0 0 0 0 0 0 0 0 0 0 0 0 0 0 0 0 0 0 0 0 0 0 0 0
0 0 0 0 0 0 0 0 0 0 0 0 0 0 0 0 0 0 0 0 0 0 0 0 0 0 0 0 0
0 0 0 0 0 0 0 0 0 0 0 0 0 0 0 0 0 0 0 0 0 0 7 0 0 0 0
M02255_77_000000000-ADUT3_1_1107_26687_10542 C----GG---CT-AA-CTA---------CG-T-G------C--C--A---G--CA-G-CCG-CG------GT-A--A-T--AC-GT-------AG--G-----GA-GC-GA-GC-------GTTA-T-C-C-G-------------G--A-TT-T-A-T-----------AA----G-T-C-G--------G-T-G--G--TC--T-AA-GT--C-C-GA-A-G-----CT-T-AA------C-T-T-C-G-G-T--T------C-G--C-C---A--T-------T--GA-A-A---C-------T--G-T--AG--A-G-C----T-T-G-A-G-T--A---T-GG-----AA-G-A--------G-G-T-A---AG-T------GG--A--ATT----T-C-T-A-GT--GT-A-G-CG-GT--T---A--A-A---TG-C-GT-AG--AT-A-TT--A-G--A-A-G-G-A-AC-A-CC---AG--T--G--GC-GAA-A--G-C--G-------A--C-T-T-A---CTG--G--TC-C-A--------------------C-T---A-C-T--GA--CG-C-T-G--A-GG--C-A-CG-A--AA-G-C---G-TG--GG-G--AG-C-A-AA--------TA--GG Bacteria(100);Firmicutes(100);Mollicutes(100);RF9(100);unclassified(100);unclassified(100);unclassified(100);unclassified(100);unclassified(100);

Otu1284 0 0 0 0 0 0 0 0 0 0 0 0 0 0 0 0 0 0 0 0 0 0 0 0 0 0 0 0
0 0 0 0 0 0 0 0 0 0 0 0 0 0 0 0 0 0 0 0 0 0 0 0 0 0 0 0 0
0 0 0 0 0 0 0 0 0 0 0 0 0 0 0 0 0 0 0 0 0 0 0 0 0 0 0 0 0
0 0 0 0 0 0 0 0 0 0 0 0 0 0 0 0 0 0 0 0 0 0 48 0 0 0 0
M02255_77_000000000-ADUT3_1_1102_10182_13778 G----AC---GA-GG-GAT--------CCT-A-G------C--G--T---T--GT-T-CGG-AA------TC-A--T-T--GG-GC-------GT--A-----AA-GC-GG-GT-------G-TA-G-G-T-G-------------G--C-TT-T-G-T-----------AA----G-T-C-G--------A-G-T--G--TG--A-AA-GC--C-T-GG-G-G-----CT-C-AA------C-C-C-C-A-G-A--A-G----T-G--C-A---T--T-------C--GA-T-A---C-------T--G-C--GA--A-G-C----T-T-G-A-G-T--G---C-CG-----GA-G-A--------G-G-T-T---AG-T------AG--A--ATT----G-T-T-G-GT--GT-A-G-TG-GT--G---A--A-A---TA-C-GT-AG--AT-A-TC--A-A--C-A-G-G-A-AT-A-CC---GG--A--G--GC-GAA-G--G-C--G-------G--C-T-A-A---CTG--G--CC-G-G--------------------A-C---A-C-T--GA--CA-C-T-G--A-GA--C-C-CG-A--AA-G-C---G-TG--GG-G--AT-C-A-AA--------CA--GG Bacteria(100);Proteobacteria(100);Deltaproteobacteria(100);Bdellovibrionales(100);Bdellovibrionaceae(100);Bdellovibrio(100);unclassified(100);unclassified(100);unclassified(100);

Otu1285 0 0 0 0 0 0 0 0 0 0 0 0 0 0 0 0 0 0 0 0 0 0 0 0 0 0 0 0
0 0 0 0 0 0 0 0 0 0 0 0 0 0 0 0 0 0 0 0 0 0 0 0 0 0 0 0 0
0 0 0 0 0 0 0 0 0 0 0 0 0 0 0 0 0 0 0 0 0 0 0 0 0 0 0 0 0
0 0 0 0 0 0 0 0 0 0 0 0 0 0 0 0 0 0 0 0 0 0 8 0 0 0 0
M02255_76_000000000-ADUNE_1_2111_14262_22103 GT---TC---GT-GCCAGC--------AGC-C-G------C--G--G---T--AA-G-ACG-AA------CC-G--T-G--CG-AA-------CG--T-----TA-TT-CG-GA-------A-TC-A-C-T-G------------G--C-CC-G-G-C----
-------GC----G-T-C-C--------TTC-G--C--TG--A-AA-GC--C-C-CC-G-G-----CT-C-AA------C-C-G-G-G-G-A--A-G----T-G--G-C---G--G-------G--GA-T-A---C-------G--G-C--CG--G-G-C----T-C-G-A-G-G--G---G-GG-----TA-G-G--------G-G-G-A---GC-C------GG--A--ACT-
---C-A-C-G-GT--GG-A-G-CG-GT--G---A--A-A---TG-C-GT-TG--AG-A-TC--G-T--G-A-G-G-A-AC-G-CC---CG--T--G--GC-GAA-A--G-C--G-------G--G-C-T-C---CTG--G--GC-C-C--------------------C-G---C-C-T--GA--CG-C-T-G--A-GA--C-G-CG-A--AA-G-C---C-AG--
GG-G--AG-C-G-AA--------CG--GG Bacteria(100);Planctomycetes(100);Planctomycetacia(100);Planctomycetales(100);Planctomycetaceae(100);Singulisphaera(100);unclassified(100);unclassified(100);unclassified(100);

Otu1286 0 0 0 0 0 0 0 0 0 0 0 0 0 0 0 0 0 0 0 0 0 0 0 0 0 0 0 0
0 0 0 0 0 0 0 0 0 0 0 0 0 0 0 0 0 0 0 0 0 0 0 0 0 0 0 0 0
0 0 0 0 0 0 0 0 0 0 0 0 0 0 0 0 0 0 0 0 0 0 0 0 0 0 0 0 0
0 0 0 0 0 0 0 0 0 0 0 0 0 0 0 0 0 0 0 0 0 12 0 0 0 0 0
M02255_76_000000000-ADUNE_1_2106_17927_23565 C----TA---AC-TC-CGT--------GCC-A-G------C--A--G---C--CG-C-GGT-AA------TA-C--G-G--AG-GG-------TG--C-----AA-GC-GT------------TA-T-C-C-G------------G--A-CT-T-G-T---------
--TA----G-T-C-A--------G-T-G--G--TG--A-AA-TC--T-C-AT-A-G-----CT-T-AA------C-T-A-T-G-A-A--A-C----T-G--C-C---A--T-------T--GA-T-A---C-------T--G-C--AG--G-T-C----T-T-G-A-G-T--A---A-AT-----TT-G-A--------A-G-T-G---GC-T------GG--A--ATA----A-G-T-
A-GT--GT-A-G-CG-GT--G---A--A-A---TG-C-AT-AG--AT-A-TT--A-C--T-T-A-G-A-AC-A-CC---AA--T--T--GC-GAA-G--G-C--A-------G--G-T-C-A---CTA--A--GA-T-T--------------------T-A---A-C-T--GA--CG-C-T-G--A-TG--G-A-CG-A--AA-G-C---G-TG--GG-G--AG-
C-G-AA--------CA--GG Bacteria(100);Bacteroidetes(100);Flavobacteria(100);Flavobacteriales(100);Flavobacteriaceae(100);Candidatus_Amoebinatus(100);unclassified(100);unclassified(100);unclassified(100);

Otu1287 0 0 0 0 0 0 0 0 0 0 0 0 0 0 0 0 0 0 0 0 0 0 0 0 0 0 0 0
0 0 0 0 0 0 0 0 0 0 0 0 0 0 0 0 0 0 0 0 0 0 0 0 0 0 0 0 0
0 0 0 0 0 0 0 0 0 0 0 0 0 0 0 0 0 0 0 0 0 0 0 0 0 0 0 0 0
0 0 0 0 0 0 0 0 0 0 0 0 0 0 0 0 0 0 0 0 0 0 9 0 0 0 0
M02255_76_000000000-ADUNE_1_2105_22601_15535 T----AA---CT-A--CGT--------GCC-A-G------C--A--G---C--CG-C-GGT-AA------TA-C--G-T--AG-GG-------GG--C-----AA-GC-G--TT-------G-TC-C-G---G------------A--T-TT-A-T-T---------
--AA----G-T-C-T--------G-A-T--G--TG--A-AA-GC--C-C-AC-G-G-----CT-C-AA------C-C-G-T-G-G-A--G------G-GC-C-A---T--T-------G--GA-A-A---C-------T--G-G--TA--G-G-C----T-T-G-A-G-T--G---C-AG-----GA-G-A--------G-G-A-G---AG-C------GG--A--ATT----C-
C-C-G-GT--GT-A-G-CG-GT--G---A--A-A---TG-C-GT-AG--AT-A-TC--G-G--G-A-G-G-A-AC-A-CC---CG--T--G--GC-GAA-G--G-C--G-------G--C-T-C-T---CTG--G--CC-T-G--------------------T-A---A-C-T--GA--CG-C-T-G--A-GG--C-G-CG-A--AA-G-C---G-TG--GG-
G--AG-C-A-AA--------CA--GG Bacteria(100);Firmicutes(100);Bacilli(100);Bacillales(100);Paenibacillaceae(100);Ammoniphilus(100);unclassified(100);unclassified(100);unclassified(100);

Otu1288 0 0 0 0 0 0 0 0 0 0 0 0 0 0 0 0 0 0 0 0 0 0 0 0 0 0 0 0
0 0 0 0 0 0 0 0 0 0 0 0 0 0 0 0 0 0 0 0 0 0 0 0 0 0 0 0 0
0 0 0 0 0 0 0 0 0 0 0 0 0 0 0 0 0 0 0 0 0 0 0 0 0 0 0 0 0
0 0 0 0 0 0 0 0 0 0 0 0 0 0 0 0 0 0 0 0 0 43 0 0 34 0 0
M02255_77_000000000-ADUT3_1_2104_16088_20785 T----AC---GT-AG-GGG--------GCA-A-G------C--G--T---T--AT-C-CGG-AT------TT-A--C-T--GG-GT-------GT--A-----AA-GG-GA-GC-------G-TA-G-G-T-G------------G--T-AT-G-G-C----
-------AA----G-T-C-A--------G-A-G--G--TG--A-AA-AC--C-C-AG-G-G-----CT-T-AA------C-C-T-T-G-G-G--A-T----T-G--C-C---T--T-------T--GA-A-A---C-------T--G-T--CA--G-A-C----T-A-G-A-G-T--G---C-AG-----GA-G-G--------G-G-T-A---AG-T------GG--A--ATT----
C-C-T-A-GT--GT-A-G-CG-GT--G---A--A-A---TG-C-GT-AG--AT-A-TT--A-G--G-A-G-G-A-AC-A-CC---AG--T--G--GC-GAA-G--G-C--G-------G--C-T-T-A---CTG--G--AC-T-G--------------------T-A---A-C-T--GA--CA-C-T-G--A-GG--C-T-CG-A--AA-G-C---G-TG--GG-
G--AG-C-A-AA--------CA--GG Bacteria(100);Firmicutes(100);Clostridia(100);Clostridiales(100);unclassified(100);unclassified(100);unclassified(100);unclassified(100);unclassified(100);

Otu1289 0 0 0 0 0 0 0 0 0 0 0 0 0 0 0 0 0 0 0 0 0 0 0 0 0 0 0 0
0 0 0 0 0 0 0 0 0 0 0 0 0 0 0 0 0 0 0 0 0 0 0 0 0 0 0 0 0
0 0 0 0 0 0 0 0 0 0 0 0 0 0 0 0 0 0 0 0 0 0 0 0 0 0 0 0 0
0 0 0 0 0 0 0 0 0 0 0 0 0 0 0 0 0 0 0 0 0 56 0 0 0 0 0
M02255_77_000000000-ADUT3_1_1117_10863_20771 T----AC---GT-AG-GGT--------GCG-A-G------C--G--T---T--GT-C-CGG-AA------TT-A--T-T--GG-GC-------GT--A-----AA-GA-GC-TC-------G-TA-G-G-C-G------------G--T-TT-G-T-T------
-----GC----G-C-C-T--------G-C-T--G--TG--A-AA-AC--C-T-GG-G-G-----CT-T-AA------C-T-T-T-G-G-G--A-T----T-G--C-A---G--T-------G--GG-T-A---C-------G--G-G--CA--G-A-C----T-A-G-A-G-T--G---T-GG-----TA-G-G--------G-G-T-A---AT-T------GG--A--ATT----C-
C-T-G-GT--GT-A-G-CG-GT--G---G--A-A---TG-C-GC-AG--AT-A-TC--A-G--G-A-G-G-A-AC-A-CC---GA--T--G--GC-GAA-G--G-C--A-------G--G-T-T-A---CTG--G--GC-C-A--------------------C-C---A-C-T--GA--CG-C-T-G--A-GG--A-G-CG-A--AA-G-C---G-TG--GG-
T--AG-C-G-AA--------CA--GG Bacteria(100);Actinobacteria(100);Actinobacteria(100);Actinobacteridae(100);Actinomycetales(100);unclassified(100);unclassified(100);unclassified(100);unclassified(100);

Otu1290 0 0 0 0 0 0 0 0 0 0 0 0 0 0 0 0 0 0 0 0 0 0 0 0 0 0 0 0
0 0 0 0 0 0 0 0 0 0 0 0 0 0 0 0 0 0 0 0 0 0 0 0 0 0 0 0 0
0 0 0 0 0 0 0 0 0 0 0 0 0 0 0 0 0 0 0 0 0 0 0 0 0 0 0 0 0
0 0 0 0 0 0 0 0 0 0 0 0 0 0 0 0 0 0 0 0 0 11 0 0 0 0 0
M02255_77_000000000-ADUT3_1_1115_7693_19807 C----GG---CT-AA-CT---------ACG-T-G------C--C--A---G--CA-G-CCG-CG------GT-A--A-T--AC-GT-------AG--G-----TG-GC-AA-GC-------G-TT-G-T-C-C------------G----GA-T-T-T-----------A----
T-T-C-T--------G-A-T--G--TG--A-AA-GC--C-C-CC-G-G-----CT-C-AA------C-C-G-G-G-G-A--G------G-GT-C-A---T--T-------G--GA-A-A---C-------T--G-G--CG--A-A-C----T-T-G-A-G-T--G---C-AG-----AA-G-A--------G-G-A-G---AG-T------GG--A--ATT----C-C-A-T-
GT--GT-A-G-CG-GT--G---A--A-A---TG-C-GT-AG--AT-A-TA--T-G--G-A-G-G-A-AC-A-CC---AG--T--G--GC-GAA-G--G-C--G-------G--C-T-C-T---CTG--G--TC-T-G--------------------T-A---A-C-T--GA--CG-C-T-G--A-GG--C-T-CG-A--AA-G-C---G-TG--GG-T--AG-C-
A-AA--------CA--GG Bacteria(100);Firmicutes(100);Bacilli(100);Lactobacillales(100);unclassified(100);unclassified(100);unclassified(100);unclassified(100);unclassified(100);

Otu1291 0 0 0 0 0 0 0 0 0 0 0 0 0 0 0 0 0 0 0 0 0 0 0 0 0 0 0 0
0 0 0 0 0 0 0 0 0 0 0 0 0 0 0 0 0 0 0 0 0 0 0 0 0 0 0 0 0
0 0 0 0 0 0 0 0 0 0 0 0 0 0 0 0 0 0 0 0 0 0 0 0 0 0 0 0 0
0 0 0 0 0 0 0 0 0 0 0 0 0 0 0 0 0 0 0 0 0 38 0 0 0 0 0
M02255_77_000000000-ADUT3_1_1114_4807_12334 T----AC---GT-AT-GGA--------GCA-A-G------C--G--T---T--AT-C-CGG-AT------TT-A--C-T--GG-GT-------GT--A-----AA-GG-GA-GC-------G-TA-G-A-C-G------------G--T-TA-A-G-C-----------AA-
---G-T-C-T--------G-A-A--G--TG--A-AA-GC--C-C-GG-G-G-----CC-C-AA------C-C-C-C-G-G-A--A-C----T-G--C-T---T--T-------G--GA-A-A---C-------T--G-T--TG--A-A-C----T-A-G-A-G-T--G---C-AG-----GA-G-A--------G-G-T-A---AG-T------GG--A--ATT----C-C-T-A-
GT--GT-A-G-CG-GT--G---A--A-A---TG-C-GT-AG--AT-A-TT--A-G--G-A-G-G-A-AC-A-CC---AG--T--G--GC-GAA-G--G-C--G-------G--C-T-T-A---CTG--G--AC-T-G--------------------T-A---A-C-T--GA--CG-T-T-G--A-GG--C-T-CG-A--AA-G-C---G-TG--GG-G--AG-
C-A-AA--------CA--GG Bacteria(100);Firmicutes(100);Clostridia(100);Clostridiales(100);Lachnospiraceae(100);unclassified(100);unclassified(100);unclassified(100);unclassified(100);

Otu1292 0 0 0 0 0 0 0 0 0 0 0 0 0 0 0 0 0 0 0 0 0 0 0 0 0 0 0 0
0 0 0 0 0 0 0 0 0 0 0 0 0 0 0 0 0 0 0 0 0 0 0 0 0 0 0 0 0
0 0 0 0 0 0 0 0 0 0 0 0 0 0 0 0 0 0 0 0 0 0 0 0 0 0 0 0 0
0 0 0 0 0 0 0 0 0 0 0 0 0 0 0 0 0 0 0 0 0 17 0 0 0 0 0
M02255_77_000000000-ADUT3_1_1112_23394_6639 T----AC---GT-AG-GGG--------GCA-A-G------C--G--T---T--AT-C-CGG-AA------TT-A--T-T--GG-GC-------GT--A-----AA-GC-GT-GC-------G-TA-G-G-T-G------------G--T-TC-T-T-T----------AA--
--G-C-G-C--------A-G-G--G--TT--T-AA-GG--C-A-AT-G-G-----CC-C-AA------C-C-A-T-T-G-T--T------C-G--C-C---C--T-------G--CG-A-A---C-------T--G-G--AG--A-A-C----T-T-G-A-G-T--G---C-AG-----GA-G-A--------G-G-A-A---AG-C------GG--A--ATT----C-C-T-A-
GT--GT-A-G-CG-GT--G---A--A-A---TG-C-GT-AG--AT-A-TT--A-G--G-A-G-G-A-AC-A-CC---AG--T--G--GC-GAA-G--G-C--G-------G--C-T-T-T---CTG--G--AC-T-G--------------------T-A---A-C-T--GA--CA-C-T-G--A-GG--C-A-CG-A--AA-G-C---G-TG--GG-G--AG-
C-A-AA--------CA--GG Bacteria(100);Firmicutes(100);Clostridia(100);Clostridiales(100);Family_XIII_Incertae_Sedis(100);unclassified(100);unclassified(100);unclassified(100);unclassified(100);

Otu1293 0 0 0 0 0 0 0 0 0 0 0 0 0 0 0 0 0 0 0 0 0 0 0 0 0 0 0 0
0 0 0 0 0 0 0 0 0 0 0 0 0 0 0 0 0 0 0 0 0 0 0 0 0 0 0 0 0
0 0 0 0 0 0 0 0 0 0 0 0 0 0 0 0 0 0 0 0 0 0 0 0 0 0 0 0 0
0 0 0 0 0 0 0 0 0 0 0 0 0 0 0 0 0 0 0 0 0 78 0 0 0 0 0
M02255_77_000000000-ADUT3_1_1107_24472_19389 T----AC---GG-AG-GGG--------GCT-A-G------C--G--T---T--GT-T-CGG-AA------TT-A--C-T--GG-GC-------GT--A-----AA-GC-GC-AC-------G-TA-G-G-C-G------------G--A-CC-G-A-C----
-------AA----G-T-C-G--------G-G-G--G--TG--A-AA-TC--C-C-GG-G-G-----CT-C-AA------C-C-C-C-G-G-A--A-C----T-G--C-C---T--C-------C--GA-A-A---C-------T--G-T--CG--G-T-C----T-G-G-A-G-G--A---T-GA-----GA-G-A--------G-G-C-G---AG-T------GG--A--ATT----
C-C-G-A-GT--GT-A-G-AG-GT--G---A--A-A---TT-C-GT-AG--AT-A-TT--C-G--G-A-G-G-A-AC-A-CC---AG--T--G--GC-GAA-G--G-C--G-------G--C-T-C-G---CTG--G--CT-C-A--------------------T-T---T-C-T--GA--CG-C-T-G--A-GG--T-G-CG-A--AA-G-C---G-TG--GG-
G--AG-C-A-AA--------CA--GG Bacteria(100);Proteobacteria(100);Alphaproteobacteria(100);unclassified(100);unclassified(100);unclassified(100);unclassified(100);unclassified(100);unclassified(100);

Otu1294 0 0 0 0 0 0 0 0 0 0 0 0 0 0 0 0 0 0 0 0 0 0 0 0 0 0 0 0
0 0 0 0 0 0 0 0 0 0 0 0 0 0 0 0 0 0 0 0 0 0 0 0 0 0 0 0 0
0 0 0 0 0 0 0 0 0 0 0 0 0 0 0 0 0 0 0 0 0 0 0 0 0 0 0 0 0
0 0 0 0 0 0 0 0 0 0 0 0 0 9 0 0 0 0 0 0 0 0 0 0 0 0 0
M02255_77_000000000-ADUT3_1_1117_7834_18841 T----AC---GT-AG-GTG--------GCA-A-G------C--G--T---T--GT-C-CGG-AT------TT-A--C-T--GG-GT-------GT--A-----AA-GG-GT-GC-------G-CA-G-G-C-G------------G--G-AC-T-G-C-----------
AA----G-T-T-G--------G-A-T--G--TG--A-AA-AT--C-C-GA-G-G-----CT-C-AA------C-C-T-C-G-G-A--A-C----T-G--C-A---T--C-------C--AA-A-A---C-------T--G-T--GG--T-T-C----T-T-G-A-G-T--G---G-AG-----CA-G-A--------G-G-T-A---AG-C------GG--A--ATT----C-C-G-
A-GT--GT-A-G-CG-GT--G---A--A-A---TG-C-GT-AG--AT-A-TT--C-G--G-A-G-G-A-AC-A-CC---AG--T--G--GC-G-AAG--G-C--G-------G--C-T-T-A---CTG--G--GC-T-C--------------------T-A---A-C-T--GA--CG-C-T-G--A-GG--C-A-CG-A--AA-G-T---G-TG--GG-T--AG-
C-A-AA--------CA--GG Bacteria(100);unclassified(100);unclassified(100);unclassified(100);unclassified(100);unclassified(100);unclassified(100);unclassified(100);unclassified(100);

Otu1295 0 0 0 0 0 0 0 0 0 0 0 0 0 0 0 0 0 0 0 0 0 0 0 0 0 0 0 0
0 0 0 0 0 0 0 0 0 0 0 0 0 0 0 0 0 0 0 0 0 0 0 0 0 0 0 0
0 0 0 0 0 0 0 0 0 0 0 0 0 0 0 0 0 0 0 0 0 0 0 0 0 0 0 0
0 0 0 0 0 0 0 0 0 0 0 0 7 0 0 0 0 0 0 0 0 0 0 0 0 0 0

M02255_77_000000000-ADUT3_1_2113_21336_9373 T----AC---GT-AG-GCA--------GCG-A-G------C--G--T---T--GT-T-CGG-AG------TT-A--C-T--GG-GC-------GT--A-----AA-GC-GT-GC-------G-TA-G-G-C-G------------G--T-TG-T-C-T----------AA----G-T-T-T--------G-G-T--G--TG--A-AA-TC--T-C-CC-G-G-----CT-T-AA------C-T-G-G-G-A-G--G-G----T-G--C-G---C--C-------G--AA-A-A---C-------T--G-G--GT--G-A-C-----TG-G-A-G-T--G---T-CG-----GA-G-A--------G-G-T-A---AG-C------GG--A--ATT----C-C-T-G-GT--GT-A-G-CG-GT--G---A--A-A---TG-C-GT-AG--AG-A-TC--A-G--G-A-G-G-A-AC-A-CC---TG--T--G--GT-GTA-G--A-C--A-------G--C-T-T-A---CTG--G--AC-G-A--------------------T-A---A-C-T--GA--CG-C-T-G--A-GG--C-A-CG-A--AA-G-C---G-TG--GG-T--AG-C-A-AA--------CA--GG Bacteria(100);unclassified(100);unclassified(100);unclassified(100);unclassified(100);unclassified(100);unclassified(100);unclassified(100);unclassified(100);

Otu1296 0 0 0 0 0 0 0 0 0 0 0 0 0 0 0 0 0 0 0 0 0 0 0 0 0 0 0 0
0 0 0 0 0 0 0 0 0 0 0 0 0 0 0 0 0 0 0 0 0 0 0 0 0 0 0 0
0 0 0 0 0 0 0 0 0 0 0 0 0 0 0 0 0 0 0 0 0 0 0 0 0 0 0 0
0 0 0 0 0 0 0 0 0 0 0 0 4 0 0 0 0 0 0 0 0 0 0 0 0 0 0

M02255_76_000000000-ADUNE_1_1103_20720_18691 T----AC---GT-AA-GGT--------CCG-A-G------C--G--T---T--GT-C-CGG-AA------TC-A--T-T--GG-GC-------GT--A-----AA-GG-GT-AC-------G-TA-G-G-C-G------------G--C-CA-A-A-C-----------AA----G-T-T-A--------G-A-A--G--TG--A-AA-TC--C-T-AT-A-G-----CT-C-AA------C-T-A-T-A-G-T--A------A-G--C-T---T--T-------T--AA-A-A---C-------T--A-T--TT--G-G-C----T-T-G-A-G-A--T---A-TG-----GA-A-G--------G-G-A-A---AG-T------GG--A--ATT----C-C-T-A-GT--GT-A-G-CG-GT--G---A--A-A---TG-C-GC-AG--AT-A-TT--A-G--G-A-G-G-A-AT-A-CC---GG--T--G--GC-GAA-G--G-C--G-------A--C-T-T-T---CTG--G--CC-A-T--------------------G-A---T-C-T--GA--CG-C-T-G--A-GG--T-A-CG-A--AA-G-C---G-TG--GG-T--AG-C-A-AA--------CA--GG Bacteria(100);Firmicutes(100);Clostridia(100);Clostridiales(100);Family_XI_Incertae_Sedis(100);Anaerococcus(100);unclassified(100);unclassified(100);unclassified(100);

Otu1297 0 0 0 0 0 0 0 0 0 0 0 0 0 0 0 0 0 0 0 0 0 0 0 0 0 0 0 0
0 0 0 0 0 0 0 0 0 0 0 0 0 0 0 0 0 0 0 0 0 0 0 0 0 0 0 0
0 0 0 0 0 0 0 0 0 0 0 0 0 0 0 0 0 0 0 0 0 0 0 0 0 0 0 0
0 0 0 0 0 0 0 0 0 0 0 0 1 0 1 0 0 0 0 0 0 0 0 0 0 0 0

M02255_76_000000000-ADUNE_1_1101_16679_4786 T----AA---GC-AC-CG---------GCT-A-A------C---------T--AC-G-TGC-CA------GC-A--G-C--CG-CG-------GT--A-----AT-AC-GT--A-------G----G-G-T-G------------C--A-AG-C-G-T----------TA----A-T-C-G--------G-A-T--G--TG--A-AA-TC--C-C-CG-G-G-----CT-C-AA------C-C-T-G-G-G-A--A-T----T-G--C-A---T--T-------G--GA-G-A---C-------T--G-C--AA--G-G-C----T-A-G-A-A-T--C---T-GG-----CA-G-A--------G-G-G-G---GG-T------AG--A--ATT----C-C-A-C-GT--GT-A-G-CA-GT--G---A--A-A---TG-C-GT-AG--AT-A-TG--T-G--G-A-G-G-A-AC-A-CC---GA--T--G--GC-GAA-G--G-C--A-------G--C-C-C-C---CTG--G--GT-C-A--------------------A-G---A-T-T--GA--CG-C-T-C--A-TG--C-A-CG-A--AA-G-C---G-TG--GG-G--AG-C-A-AA--------CA--GG Bacteria(100);Proteobacteria(100);Betaproteobacteria(100);Burkholderiales(100);Oxalobacteraceae(100);Janthinobacterium(100);unclassified(100);unclassified(100);unclassified(100);

Otu1298 0 0 0 0 0 0 0 0 0 0 0 0 0 0 0 0 0 0 0 0 0 0 0 0 0 0 0 0
0 0 0 0 0 0 0 0 0 0 0 0 0 0 0 0 0 0 0 0 0 0 0 0 0 0 0 0
0 0 0 0 0 0 0 0 0 0 0 0 0 0 0 0 0 0 0 0 0 0 0 0 0 0 0 0
0 0 0 0 0 0 0 0 0 0 0 0 0 18 0 0 0 0 0 0 0 0 0 0 0 0 0

M02255_76_000000000-ADUNE_1_1108_9926_9559 .....AC----T-AC-GT---------GCC-A-G------C--A--G---C--CG-C-GGT-AA------TA-C--G-T--AG-GT-------GG--C-----AA-GC-G--TT-------G----T-C-C-G------------G--T-CT-T-T-T----------AA----G-T-C-T--------G-A-T--G--TG--A-AA-TA--T-C-GG-G-G-----CT-C-AA------C-C-C-C-G-A-G--G------G-GT-C-A---T--T-------G--GA-A-A---C-------T--G-G--GA--G-A-C----T-T-G-A-G-T--A---C-AG-----AA-G-A--------G-G-A-G---AG-T------GG--A--ATT----C-C-A-C-GT--GT-A-G-CG-GT--G---A--A-A---TG-C-GT-AG--AT-A-TG--T-G--G-A-G-G-A-AC-A-CC---AG--T--G--GC-GAA-G--G-C--G-------A--C-T-C-T---CTG--G--TC-T-G--------------------T-A---A-C-T--GA--CG-C-T-G--A-GG--C-G-CG-A--AA-G-C---G-TG--GG-G--AG-C-A-AA--------CA--GG Bacteria(100);Firmicutes(100);Bacilli(100);Bacillales(100);unclassified(100);unclassified(100);unclassified(100);unclassified(100);unclassified(100);

Otu1299 0 0 0 0 0 0 0 0 0 0 0 0 0 0 0 0 0 0 0 0 0 0 0 0 0 0 0 0
0 0 0 0 0 0 0 0 0 0 0 0 0 0 0 0 0 0 0 0 0 0 0 0 0 0 0 0
0 0 0 0 0 0 0 0 0 0 0 0 0 0 0 0 0 0 0 0 0 0 0 0 0 0 0 0
0 0 0 0 0 0 0 0 0 0 0 0 0 8 0 0 0 0 0 0 0 0 0 0 0 0 0

M02255_76_000000000-ADUNE_1_2110_20926_23895 T----AC---GG-AG-GGA--------GCT-A-G------C--G--T---T--GT-T-CGG-AA------AT-A--C-T--GG-GC-------GT--A-----AA-GC-G----------G-CA---T-C-------------G----------T----------AG----G-T-C-A--------G-G-G--G--TG--A-AA-TC--C-C-GG-G-G-----CT-C-AA------C-C-C-C-G-G-A--A-C----T-G--C-C---C--T-------T--GA-A-A---C-------T--G-C--AG--T-G-C----T-A-G-A-A-T--C---T-TG-----GA-G-A--------G-G-C-G---AG-T------GG--A--ATT----C-C-G-A-GT--GT-A-G-AG-GT--G---A--A-A---TT-C-GT-AG--AT-A-TT--C-G--G-A-A-G-A-AC-A-CC---AG--T--G--GC-GAA-G--G-C--G-------A--C-T-C-G---CTG--G--AC-A-A--------------------G-T---A-T-T--GA--CG-C-T-G--A-GG--T-G-CG-A--AA-G-C---G-TG--GG-G--AG-C-A-AA--------CA--GG Bacteria(100);Proteobacteria(100);Alphaproteobacteria(100);Sphingomonadales(100);Erythrobacteraceae(100);unclassified(100);unclassified(100);unclassified(100);unclassified(100);

Otu1300 0 0 0 0 0 0 0 0 0 0 0 0 0 0 0 0 0 0 0 0 0 0 0 0 0 0 0 0
0 0 0 0 0 0 0 0 0 0 0 0 0 0 0 0 0 0 0 0 0 0 0 0 0 0 0 0 0
0 0 0 0 0 0 0 0 0 0 0 0 0 0 0 0 0 0 0 0 0 0 0 0 0 0 0 0 0
0 0 0 0 0 0 0 0 0 0 0 0 0 55 0 0 0 0 0 0 0 0 0 0 0 0 0
M02255_77_000000000-ADUT3_1_1101_14447_23173 T----AC---GA-AT-GGG--------GCA-A-G------C--G--T---T--GT-T-CGG-AA------TC-A--C-T--GG-GC-------GT--A-----AA-GC-GT-GT-------G-TA-G-G-C-G------------G--T-TT-A-T-C------
-----AA----G-T-C-A--------G-A-A--G--TG--A-AA-GC--C-C-AG-G-G-----CT-T-AA------C-C-C-T-G-G-A--A-T----T-G--C-T---T--T-------T--GA-A-A---C-------T--G-A--TA--G-G-C----T-A-G-A-G-T--A---C-TG-----GA-G-A--------G-G-T-T---AG-C------AG--A--ATT----C-
C-A-A-GT--GT-A-G-AG-GT--G---A--A-A---TT-C-GC-AG--AT-A-TT--T-G--G-A-G-G-A-AT-A-CC---AG--T--G--GC-GCA-A--G-C--G-------G--C-T-A-A---CTG--G--AC-A-G--------------------T-A---A-C-T--GA--CG-C-T-G--A-GA--C-A-CG-A--AA-G-C---G-TG--GG-G-
-AT-C-A-AA--------CA--GG Bacteria(100);Proteobacteria(100);Alphaproteobacteria(100);DB1-14(100);unclassified(100);unclassified(100);unclassified(100);unclassified(100);unclassified(100);

Otu1301 0 0 0 0 0 0 0 0 0 0 0 0 0 0 0 0 0 0 0 0 0 0 0 0 0 0 0 0
0 0 0 0 0 0 0 0 0 0 0 0 0 0 0 0 0 0 0 0 0 0 0 0 0 0 0 0 0
0 0 0 0 0 0 0 0 0 0 0 0 0 0 0 0 0 0 0 0 0 0 0 0 0 0 0 0 0
0 0 0 0 0 0 0 0 0 0 0 0 0 26 0 0 0 0 0 0 0 0 0 0 0 0 0
M02255_77_000000000-ADUT3_1_1110_18231_23580 T----AC---GT-AG-GTG--------GCA-A-G------C--G--T---T--AT-C-CGG-AT------TT-A--C-T--GG-GT-------GT--A-----AA-GG-GC-GT-------G-TA-G-G-C-G------------G--G-AA-A-G-C-----
------AA----G-T-C-A--------G-A-T--G--TG--A-AA-AC--T-G-TG-G-G-----CT-C-AA------C-C-C-A-C-A-G--C-C----T-G--C-A---T--T-------T--GA-A-A---C-------T--G-T--TT--T-T-C----T-T-G-A-G-T--A---C-TG-----GA-G-A--------G-G-C-A---GA-T------GG--A--ATT----C-
C-T-A-GT--GT-A-G-CG-GT--G---A--A-A---TG-C-GT-AG--AT-A-TT--A-G--G-A-G-G-A-AC-A-CC---AG--T--G--GC-GAA-G--G-C--G-------A--T-C-T-G---CTG--G--AC-A-G--------------------C-A---A-C-T--GA--CG-C-T-G--A-GG--C-G-CG-A--AA-G-C---G-TG--GG-
G--AG-C-A-AA--------CA--GG Bacteria(100);unclassified(100);unclassified(100);unclassified(100);unclassified(100);unclassified(100);unclassified(100);unclassified(100);unclassified(100);

Otu1302 0 0 0 0 0 0 0 0 0 0 0 0 0 0 0 0 0 0 0 0 0 0 0 0 0 0 0 0
0 0 0 0 0 0 0 0 0 0 0 0 0 0 0 0 0 0 0 0 0 0 0 0 0 0 0 0 0
0 0 0 0 0 0 0 0 0 0 0 0 0 0 0 0 0 0 0 0 0 0 0 0 0 0 0 0 0
0 0 0 0 0 0 0 0 0 0 0 0 0 25 0 0 0 0 0 0 0 0 0 0 0 0 0
M02255_77_000000000-ADUT3_1_1113_11589_14477 T----AC---GG-AG-GGG--------GCT-A-G------C--G--T---T--GT-T-CGG-AA------TT-A--C-T--GG-GC-------GT--A-----AA-GC-GC-AC-------G-TA-G-G-C-G------------G--C-TA-T-C-C-----
------AA----G-T-T-G--------G-G-G--G--TG--A-AA-TC--C-C-TG-G-G-----CT-T-AA------C-C-C-A-G-G-A--A-C----T-G--C-C---T--T-------C--AA-A-A---C-------T--G-G--AT--G-G-C----T-A-G-A-G-T--C---C-GA-----GA-G-A--------G-G-T-G---AG-T------GG--A--ATT----
T-C-C-A-GT--GT-A-G-AG-GT--G---A--A-A---TT-C-GT-AG--AT-A-TT--G-G--A-A-A-G-A-AC-A-CC---AG--T--G--GC-GAA-G--G-C--G-------G--C-T-C-A---CTG--G--CT-C-G--------------------G-T---A-C-T--GA--CG-C-T-G--A-GG--T-G-CG-A--CA-G-C---G-TG--GG-
G--AG-C-A-AA--------CA--GG Bacteria(100);Proteobacteria(100);Alphaproteobacteria(100);Rhizobiales(100);Rhodobiaceae(100);Parvibaculum(100);unclassified(100);unclassified(100);unclassified(100);

Otu1303 0 0 0 0 0 0 0 0 0 0 0 0 0 0 0 0 0 0 0 0 0 0 0 0 0 0 0 0
0 0 0 0 0 0 0 0 0 0 0 0 0 0 0 0 0 0 0 0 0 0 0 0 0 0 0 0 0
0 0 0 0 0 0 0 0 0 0 0 0 0 0 0 0 0 0 0 0 0 0 0 0 0 0 0 0 0
0 0 0 0 0 0 0 0 0 0 0 25 0 0 0 0 0 0 0 0 0 0 0 0 0 0 0
M02255_77_000000000-ADUT3_1_1117_11605_11861 T----AC---GT-AG-GGG--------GCG-A-G------C--G--T---T--AT-C-CGG-AA------TC-A--T-T--GG-GC-------GT--A-----AA-GC-GC-GC-------G-CA-G-G-C-G------------G--G-CT-T-T-C-----
------AA----G-C-G-G--------C-GGC--G--TC--G-AA-GC--C-G-GG-G-G-----CT-C-AA------C-C-C-C-C-G-G--A------A-G--C-G---C--C-------C--CG-A-A---C-------T--G-G--AA--G-C-C----T-C-G-G-A-T--G---C-GG-----CA-G-G--------G-G-G-A---GG-C------GG--A--ATT---
-C-C-C-G-GT--GT-A-G-CG-GT--G---A--A-A---TG-C-GC-AG--AT-A-TC--G-G--G-A-A-G-A-AC-A-CC---GA--C--G--GC-GAA-G--G-C--A-------G--C-C-T-C---CTG--G--GC-C-G--------------------G-C---A-T-C--GA--CG-C-T-G--A-GG--C-G-CG-A--AA-G-C---T-GG--
GG-G--AG-C-G-AA--------CA--GG Bacteria(100);Actinobacteria(100);Actinobacteria(100);Coriobacteridae(100);Coriobacteriales(100);Coriobacterineae(100);Coriobacteriaceae(100);Slackia(100);unclassified(100);

Otu1304 0 0 0 0 0 0 0 0 0 0 0 0 0 0 0 0 0 0 0 0 0 0 0 0 0 0 0 0
0 0 0 0 0 0 0 0 0 0 0 0 0 0 0 0 0 0 0 0 0 0 0 0 0 0 0 0 0
0 0 0 0 0 0 0 0 0 0 0 0 0 0 0 0 0 0 0 0 0 0 0 0 0 0 0 0 0
0 0 0 0 0 0 0 0 0 0 0 0 0 10 0 0 0 0 0 0 0 0 0 0 0 0 0
M02255_77_000000000-ADUT3_1_1118_25169_10978 T----AC---GA-AG-GGG--------GCT-A-G------C--G--T---T--GC-T-CGG-AA------TG-A--C-T--GG-GC-------GT--A-----AA-GG-GC-GC-------G-TA-G-G-C-G------------G--A-TG-G-G-A---
--------CA----G-T-C-A--------G-A-T--G--TG--A-AA-TT--C-C-CG-G-G-----CT-T-AA------C-C-T-G-G-G-G--G-C----T-G--C-A---T--T-------T--GA-T-A---C-------G--T-G--TT--G-T-C----T-A-G-A-G-T--T---C-GG-----AA-G-A--------G-G-G-T---CG-T------GG--A--ATT----
C-C-C-A-GT--GT-A-G-AG-GT--G---A--A-A---TT-C-GT-AG--AT-A-TT--G-G--G-A-A-G-A-AC-A-CC---GG--T--G--GC-GAA-G--G-C--G-------G--C-G-A-C---CTG--G--TC-C-T--------------------G-G---A-C-T--GA--CG-C-T-G--A-GG--C-G-CG-A--AA-G-C---G-TG--
GG-G--AG-C-A-AA--------CA--GG Bacteria(100);Proteobacteria(100);Alphaproteobacteria(100);Rhodospirillales(100);Acetobacteraceae(100);unclassified(100);unclassified(100);unclassified(100);unclassified(100);

Otu1305 0 0 0 0 0 0 0 0 0 0 0 0 0 0 0 0 0 0 0 0 0 0 0 0 0 0 0 0
0 0 0 0 0 0 0 0 0 0 0 0 0 0 0 0 0 0 0 0 0 0 0 0 0 0 0 0 0
0 0 0 0 0 0 0 0 0 0 0 0 0 0 0 0 0 0 0 0 0 0 0 0 0 0 0 0 0
0 0 0 0 0 0 0 0 0 0 0 0 0 34 0 0 0 0 0 0 0 0 0 0 0 0 0
M02255_77_000000000-ADUT3_1_1106_5009_4856 G----AC---GT-AG-GGA--------GCA-A-G------C--G--T---T--GT-C-CGG-AT------TT-A--T-T--GG-GC-------GT--A-----AA-GG-GC-TC-------C-TA-G-G-T-G------------G--T-TC-A-G-T-----------AA-
---G-T-C-G--------A-C-T--G--TG--A-AA-AT--C-C-AA-G-G-----CT-C-AA------C-C-T-T-G-G-G--A------C-G-CC-A---G--T-------C--GA-T-A---C-------T--G-C--TG-TG-A-C----T-T-G-A-G-T--T---C-GG-----TA-G-A--------G-G-A-G---TG-T------GG--A--ATT----T-C-T-G-
GT--GT-A-G-CG-GT--G---A--A-A---TG-C-GC-AG--AT-A-TC--A-G--A-A-G-G-A-AC-A-CC---AA--C--G--GC-GAA-G--G-C--A-------G--C-A-C-T---CTG--G--GC-C-G--------------------A-T---A-C-T--GA--CA-C-T-G--A-AG--A-G-CG-A--AA-G-C---G-TG--GG-G--AG-
C-A-AA--------CA--GG Bacteria(100);Actinobacteria(100);Actinobacteria(100);unclassified(100);unclassified(100);unclassified(100);unclassified(100);unclassified(100);unclassified(100);

Otu1306 0 0 0 0 0 0 0 0 0 0 0 0 0 0 0 0 0 0 0 0 0 0 0 0 0 0 0 0
0 0 0 0 0 0 0 0 0 0 0 0 0 0 0 0 0 0 0 0 0 0 0 0 0 0 0 0 0
0 0 0 0 0 0 0 0 0 0 0 0 0 0 0 0 0 0 0 0 0 0 0 0 0 0 0 0 0
0 0 0 0 0 0 0 0 0 0 0 0 0 15 0 0 0 0 0 0 0 0 0 0 0 0 0
M02255_77_000000000-ADUT3_1_1112_5686_12538 T----AC---GG-AG-GGT--------GCA-A-G------C--G--T---T--GT-T-CGG-AA------TC-A--T-T--GG-GC-------GT--A-----AA-GC-GC-GC-------G-CA-G-G-C-G------------G--T-CT-G-T-C-----------AA-
---G-T-C-T--------G-A-T--G--TG--A-AA-TC--C-C-GG-G-G-----CT-C-AA------C-C-C-C-G-G-G--C-G----T-G--C-A---T--T-------G--GA-A-A---C-------T--G-A--TG--G-A-C----T-G-G-A-G-T--T---T-GG-----GA-G-A--------G-G-C-T---AG-T------GG--A--ATT----C-C-A-G-
GT--GT-A-G-GG-GT--G---A--A-A---TC-C-GT-AG--AG-A-TC--T-G--G-A-G-G-A-AC-A-CC---GG--T--G--GC-GAA-G--G-C--G-------A--C-T-G-G---CTG--G--AC-C-A--------------------A-T---A-C-T--GA--CG-C-T-G--A-GG--C-G-CG-A--AA-G-C---G-TG--GG-G--AG-
C-A-AA--------CA--GG Bacteria(100);Proteobacteria(100);unclassified(100);unclassified(100);unclassified(100);unclassified(100);unclassified(100);unclassified(100);unclassified(100);

Otu1307 0 0 0 0 0 0 0 0 0 0 0 0 0 0 0 0 0 0 0 0 0 0 0 0 0 0 0 0
0 0 0 0 0 0 0 0 0 0 0 0 0 0 0 0 0 0 0 0 0 0 0 0 0 0 0 0 0
0 0 0 0 0 0 0 0 0 0 0 0 0 0 0 0 0 0 0 0 0 0 0 0 0 0 0 0 0
0 0 0 0 0 0 0 0 0 0 0 0 0 6 0 0 0 0 0 0 0 0 0 0 0 0 0
M02255_77_000000000-ADUT3_1_2117_14869_20931 T----AC---GT-AG-GGG--------CCG-A-G------C--G--T---T--GT-C-CGG-AA------TT-A--C-T--GG-GC-------GT--A-----AA-GC-GC-GC-------G-CA-G-G-C-G------------G--C-TC-G-T-C-----
------AA----G-C-C-T--------G-A-C--G--TG--A-AA-GC--C-C-CC-G-G-----CT-C-AA------C-C-G-G-G-G-A--G-G----G-T--C-G---T--C-------G--GG-G-A---C-------T--G-G--CG--A-G-C----T-T-G-A-G-G--T---C-GG-----CA-G-G--------G-G-C-T---GG-T------GG--A--ATT----
C-C-C--GGT--GT-A-G-TG-GT--G---A--A-A---TG-C-GT-AG--AG-A-TC--G-G--G-A-G-G-A-AC-A-CC---CG--T--G--GC-GAA-G--G-C---------GG--C-C-A-G---CTG--G--GC-C-G--------------------G-A---C-C-T--GA--CG-C-T-G----AGG-C-G-CG----AA-GGC---G-T---
GGGG--AG-C-G-AA--------CG--GG Bacteria(100);Chloroflexi(100);Thermomicrobia(100);JG30-KF-CM45(100);unclassified(100);unclassified(100);unclassified(100);unclassified(100);unclassified(100);

Otu1308 0 0 0 0 0 0 0 0 0 0 0 0 0 0 0 0 0 0 0 0 0 0 0 0 0 0 0 0
0 0 0 0 0 0 0 0 0 0 0 0 0 0 0 0 0 0 0 0 0 0 0 0 0 0 0 0 0
0 0 0 0 0 0 0 0 0 0 0 0 0 0 0 0 0 0 0 0 0 0 0 0 0 0 0 0 0
0 0 0 0 0 0 0 0 0 0 0 0 0 1 0 0 0 0 0 0 0 59 0 0 0 0 0
M02255_77_000000000-ADUT3_1_1105_24500_11047 T----AC---GT-AG-GGG--------GCG-A-G------C--G--T---T--GT-C-CGG-AA------TG-A--C-T--GG-GC-------GT--A-----AA-GG-GC-GT-------G-TA-G-G-C-G------------G--C-CT-G-T-T----
-------AA----G-T-T-A--------G-G-A--G--TG--A-AA-GT--C-C-CG-C-T-----TT-C-AA------G-G-T-G-G-G-A--A-T----T-G--C-T---T--T-------T--AA-T-A---C-------T--G-A--TG--G-G-C----T-T-G-A-G-T--G---C-AG-----AA-G-A--------G-G-T-A---AG-T------GG--A--ATT----
C-C-C-A-GT--GT-A-G-CG-GT--G---A--A-A---TG-C-GT-AG--AG-A-TT--G-G--G-A-G-G-A-AC-A-CC---AG--T--G--GC-GAA-G--G-C--G-------A--C-T-T-A---CTG--G--GC-T-G--------------------T-A---A-C-T--GA--CG-C-T-G--A-GG--C-G-CG-A--AA-G-C---G-TG--
GG-G--AG-C-G-AA--------CA--GG Bacteria(100);Firmicutes(100);Clostridia(100);Clostridiales(100);unclassified(100);unclassified(100);unclassified(100);unclassified(100);unclassified(100);

Otu1309 0 0 0 0 0 0 0 0 0 0 0 0 0 0 0 0 0 0 0 0 0 0 0 0 0 0 0 0
0 0 0 0 0 0 0 0 0 0 0 0 0 0 0 0 0 0 0 0 0 0 0 0 0 0 0 0 0
0 0 0 0 0 0 0 0 0 0 0 0 0 0 0 0 0 0 0 0 0 0 0 0 0 0 0 0 0
0 0 0 0 0 0 0 0 0 0 0 0 0 0 0 34 0 0 0 0 0 0 0 0 0 0 0
M02255_76_000000000-ADUNE_1_1107_20727_20880 T----AC---GG-AG-GGG--------GTT-A-G------C--G--T---T--GT-T-CGG-AA------TG-A--C-T--GG-GC-------GT--A-----AA-GC-GC-GC-------G-TA-G-G-C-G------------G--G-CT-G-G-A---
--------AA----G-T-C-A--------G-A-G--G--TG--A-AA-GC--C-C-GG-G-G-----CT-C-AA------C-C-C-C-G-G-A--A-C----T-G--C-C---T--T-------T--GA-G-A---C-------T--T-C--CA--G-C-C----T-C-G-A-G-T--T---C-GA-----GA-G-A--------G-G-T-G---GG-T------GG--A--ATT----
C-C-G-A-GT--GT-A-G-AG-GT--G---A--A-A---TT-C-GT-AG--AT-A-TT--C-G--G-A-G-G-A-AC-A-CC---AG--T--G--GC-GAA-G--G-C--G-------G--C-C-C-A---CTG--G--CT-C-G--------------------A-C---A-C-T--GA--CG-C-T-G--A-GG--C-G-CG-A--AA-G-C---G-CG--
GG-G--AG-C-A-AA--------CA--GG Bacteria(100);Proteobacteria(100);Alphaproteobacteria(100);unclassified(100);unclassified(100);unclassified(100);unclassified(100);unclassified(100);unclassified(100);

Otu1310 0 0 0 0 0 0 0 0 0 0 0 0 0 0 0 0 0 0 0 0 0 0 0 0 0 0 0 0
0 0 0 0 0 0 0 0 0 0 0 0 0 0 0 0 0 0 0 0 0 0 0 0 0 0 0 0 0
0 0 0 0 0 0 0 0 0 0 0 0 0 0 0 0 0 0 0 0 0 0 0 0 0 0 0 0 0
0 0 0 0 0 0 0 0 0 0 0 0 0 0 0 11 0 0 0 0 0 0 0 0 0 0 0 0
M02255_76_000000000-ADUNE_1_1115_22116_23019 GT---TC---GT-GCCAGC--------AGC-C-G------C--G--G---T--AA-G-ACG-AA------CC-G--T-G--CG-AA-------CG--T-----TA-TT-CG-GA-------A-TC-A-C-T-G------------G--G-CC-G-G-A----
-------AC----G-T-C-C--------GTA-G--C--TG--A-AA-TC--C-C-CC-G-G-----CT-C-AA------C-C-G-G-G-G-A--A-G----T-G--G-C---G--T-------G--GA-A-A---C-------G--A-C--CG--G-C-C----T-G-G-A-G-T--G---G-AG-----GA-G-G--------G-G-G-A---TC-C------GG--A--ACT--
--C-C-C-G-GT--GG-A-G-CG-GT--G---A--A-A---TG-C-GT-TG--AG-A-TC--G-G--G-A-G-G-A-AC-G-CC---GG--C--G--GC-GAA-A--G-C--G-------G--G-A-T-C---CTG--G--AC-T-T--------------------C-G---A-C-T--GA--CG-C-T-G--A-GG--C-G-CG-A--AA-G-C---C-AG--
GG-G--AG-C-G-AA--------CG--GG Bacteria(100);Planctomycetes(100);Planctomycetacia(100);Planctomycetales(100);Planctomycetaceae(100);unclassified(100);unclassified(100);unclassified(100);unclassified(100);

Otu1311 0 0 0 0 0 0 0 0 0 0 0 0 0 0 0 0 0 0 0 0 0 0 0 0 0 0 0 0
0 0 0 0 0 0 0 0 0 0 0 0 0 0 0 0 0 0 0 0 0 0 0 0 0 0 0 0 0
0 0 0 0 0 0 0 0 0 0 0 0 0 0 0 0 0 0 0 0 0 0 0 0 0 0 0 0 0
0 0 0 0 0 0 0 0 0 25 0 0 0 0 0 0 0 0 0 0 0 0 0 0 0 0 0 0
M02255_77_000000000-ADUT3_1_1108_5255_13667 T----AC---GT-AG-GGG--------GCA-A-G------C--G--T---T--AT-C-CGG-AA------TT-A--C-T--GG-GC-------GT--A-----AA-GG-GT-GC-------G-TA-G-G-C-G------------G--T-TT-T-A-C-----------AA-
---G-T-T-G--------A-A-G--T--TT--T-AA-AC--T-A-TG-G-G-----CT-T-AA------C-C-C-A-T-A-T--C------A-G--G-C---T--T-------C--AA-A-A---C-------T--G-T--AA--G-A-C----T-T-G-A-G-T--G---C-AG-----GA-G-A--------G-G-A-A---AG-T------GG--A--ATT----C-C-C-G-GT-
-GT-A-G-CG-GT--G---A--A-A---TG-C-GT-AG--AT-A-TC--G-G--G-A-G-G-A-AT-A-CC---GG--T--G--GC-GAA-G--G-C--G-------A--C-T-T-T---CTG--G--AC-T-G--------------------T-A---A-C-T--GA--CG-C-T-G--A-GG--C-A-CG-A--AA-G-C---G-TG--GG-T--AG-C-A-
AA--------CA--GG Bacteria(100);unclassified(100);unclassified(100);unclassified(100);unclassified(100);unclassified(100);unclassified(100);unclassified(100);unclassified(100);

Otu1312 0 0 0 0 0 0 0 0 0 0 0 0 0 0 0 0 0 0 0 0 0 0 0 0 0 0 0 0
0 0 0 0 0 0 0 0 0 0 0 0 0 0 0 0 0 0 0 0 0 0 0 0 0 0 0 0 0
0 0 0 0 0 0 0 0 0 0 0 0 0 0 0 0 0 0 0 0 0 0 0 0 0 0 0 0 0
0 0 0 0 0 0 5 0 0 0 0 0 0 0 0 0 0 0 0 0 0 0 0 0 0 0 0 0
M02255_76_000000000-ADUNE_1_2110_3033_12308 C----TA---AC-TC-TGT--------GCC-A-G------C-----A---G--CC-G-CGG-TA------AT-A--C-A--GA-GG-------GT--G-----CA-AG-CG-TT-------G-TT-C-G---G------------A--A-TT-A-C-T-----------AA----
G-T-C-A--------G-A-T--G--TG--A-AA-TC--C-C-TT-G-G-----CT-C-AA------C-T-G-A-G-G-A--A-C----T-G--C-A---T--C-------T--GA-T-A---C-------T--G-G--TA--A-G-C----T-T-G-A-G-T--G---T-CG-----GA-G-G--------G-G-A-T---GG-T------AG--A--ATT----C-C-T-G-GT--
GT-A-G-AG-GT--G---A--A-A---TT-C-GT-AG--AT-A-TC--A-G--G-A-G-G-A-AT-A-CC---TG--T--G--GC-GAA-G--G-C--G-------A--C-C-A-T---CTG--G--AA-G-A--------------------C-A---A-C-T--GA--CG-C-T-G--A-GA--C-T-CG-A--AA-G-C---G-TG--GG-T--AG-C-A-
AA--------CA--GG Bacteria(100);Proteobacteria(100);unclassified(100);unclassified(100);unclassified(100);unclassified(100);unclassified(100);unclassified(100);unclassified(100);

Otu1313 0 0 0 0 0 0 0 0 0 0 0 0 0 0 0 0 0 0 0 0 0 0 0 0 0 0 0 0
0 0 0 0 0 0 0 0 0 0 0 0 0 0 0 0 0 0 0 0 0 0 0 0 0 0 0 0 0
0 0 0 0 0 0 0 0 0 0 0 0 0 0 0 0 0 0 0 0 0 0 0 0 0 0 0 0 0
0 0 0 0 0 0 0 4 0 0 0 0 0 0 0 0 0 0 0 0 0 0 0 0 0 0 0 0
M02255_77_000000000-ADUT3_1_1109_21078_19365 T----AC---GT-AG-GTG--------GCA-A-G------C--G--T---T--AT-C-CGG-AT------TT-A--C-T--GG-GT-------GT--A-----AA-GG-GT-GT-------G-TA-G-G-C-G------------G--G-AA-A-G-C-----
------AA----G-T-C-A--------G-A-T--G--TG--A-AA-TT--T-A-CG-G-G-----CT-C-AA------C-T-C-G-T-G-A--A-C----T-G--C-G---T--C-------T--GA-A-A---C-------T--G-T--TT--T-T-C----T-T-G-A-G-G--G---T-CG-----GA-G-A--------G-G-T-A---AC-T------GG--A--ATT----C-
C-T-G-GT--GT-A-G-CG-GT--G---A--A-A---TG-C-GT-AG--AT-A-TC--A-G--G-A-G-G-A-AC-A-CC---GG--T--G--GC-GAA-G--G-C--G-------A--G-T-T-A---CTG--G--AC-G-A--------------------T-T---A-C-T--GA--CG-C-T-G--A-GA--C-A-CG-A--AA-G-T---G-TG--GG-
G--AG-C-A-AA--------CA--GG Bacteria(100);unclassified(100);unclassified(100);unclassified(100);unclassified(100);unclassified(100);unclassified(100);unclassified(100);unclassified(100);

Otu1314 0 0 0 0 0 0 0 0 0 0 0 0 0 0 0 0 0 0 0 0 0 0 0 0 0 0 0 0
0 0 0 0 0 0 0 0 0 0 0 0 0 0 0 0 0 0 0 0 0 0 0 0 0 0 0 0 0
0 0 0 0 0 0 0 0 0 0 0 0 0 0 0 0 0 0 0 0 0 0 0 0 0 0 0 0 0
0 0 0 0 0 0 0 10 0 0 0 0 0 0 0 0 0 0 0 0 0 0 0 0 0 0 0 0
M02255_77_000000000-ADUT3_1_2101_26815_16754 T----AC---GT-AT-GGG--------GCA-A-G------C--G--T---T--AT-C-CGG-AT------TT-A--C-T--GG-GT-------GT--A-----AA-GG-GA-GC-------G-TA-G-A-C-G------------G--T-AC-G-G-C-----
------AA----G-C-C-C--------G-A-T--G--TG--A-AA-AC--C-C-GC-A-G-----CT-C-AA------C-T-G-C-G-G-G--A-T----T-G--C-A---T--T-------A--GG-A-A---C-------T--G-T--CG--A-A-C----T-A-G-A-G-T--G---T-CG-----GA-G-A--------G-G-T-A---AG-T------GG--A--ATT----
C-C-T-A-GT--GT-A-G-CG-GT--G---A--A-A---TG-C-GT-AG--AT-A-TT--A-G--G-A-G-G-A-AC-A-CC---AG--T--G--GC-GAA-G--G-C--G-------G--C-T-T-A---CTG--G--AC-G-A--------------------T-G---A-C-T--GA--CG-T-T-G--A-GG--C-T-CG-A--AA-G-C---G-TG--GG-
G--AG-C-A-AA--------CA--GG Bacteria(100);Firmicutes(100);Clostridia(100);Clostridiales(100);Lachnospiraceae(100);unclassified(100);unclassified(100);unclassified(100);unclassified(100);

Otu1315 0 0 0 0 0 0 0 0 0 0 0 0 0 0 0 0 0 0 0 0 0 0 0 0 0 0 0 0
0 0 0 0 0 0 0 0 0 0 0 0 0 0 0 0 0 0 0 0 0 0 0 0 0 0 0 0 0
0 0 0 0 0 0 0 0 0 0 0 0 0 0 0 0 0 0 0 0 0 0 0 0 0 0 0 0 0
0 0 0 0 0 0 0 8 0 0 0 0 0 0 0 0 0 0 0 0 0 0 0 0 0 0 0
M02255_77_000000000-ADUT3_1_2113_14103_14321 T----AC---GG-AG-GGT--------GCG-A-G------C--G--T---T--AT-C-CGG-AA------TT-A--C-T--GG-G---------T---------A----G--GC-----------G-G-C-------------------TT-T-G-T-----------AA--
--G-T-C-A--------G-G-A--G--TG--A-AA-GT--T-T-GC-G-G-----CT-C-AA------C-C-G-T-A-A-A--A-T----T-G--C-T---T--C-------T--GA-T-A---C-------T--G-C--AT--A-G-C----T-A-G-A-A-T--C---A-GG-----AT-G-A--------G-G-T-C---AG-C------GG--A--ATG----T-G-G-C-
AT--GT-A-G-CG-GT--G---A--A-A---TG-C-AT-AG--AT-A-TG--C-C--A-T-A-G-A-AC-A-CC---AA--T--T--GC-GAA-G--G-C--A-------G--C-T-G-G---CTA--G--AC-C-T--------------------G-T---A-T-T--GA--CG-C-T-G--A-GG--C-A-CG-A--AA-G-C---G-TG--GG-G--AG-C-
G-AA--------CA--GG Bacteria(100);Bacteroidetes(100);unclassified(100);unclassified(100);unclassified(100);unclassified(100);unclassified(100);unclassified(100);unclassified(100);

Otu1316 0 0 0 0 0 0 0 0 0 0 0 0 0 0 0 0 0 0 0 0 0 0 0 0 0 0 0 0
0 0 0 0 0 0 0 0 0 0 0 0 0 0 0 0 0 0 0 0 0 0 0 0 0 0 0 0 0
0 0 0 0 0 0 0 0 0 0 0 0 0 0 0 0 0 0 0 0 0 0 0 0 0 0 0 0 0
0 0 0 0 0 0 0 13 0 0 0 0 0 0 0 0 0 0 0 0 0 0 0 0 0 0 0
M02255_77_000000000-ADUT3_1_2116_18198_7987 G----AC---GG-AG-GGG--------GCT-A-G------C--G--T---T--GT-T-CGG-AA------TT-A--C-T--GG-GC-------GT--A-----AA-GG-GC-GC-------G-TA-G-G-C-G-------------G--A-TT-A-G-T-----------
AA----G-T-T-G--------G-G-A--G--TG--A-AA-TC--C-C-GG-G-G-----CT-T-AA------C-C-T-C-G-G-A--A-C----T-G--C-T---T--T-------C--AA-A-A---C-------T--A-C--TA--G-T-C----T-T-G-A-G-T--G---A-AG-----TA-G-A--------G-G-A-T---GA-T------GG--A--ATT----T-C-T-
A-GT--GT-A-G-AG-GT--G---A--A-A---TT-C-TT-AG--AT-A-TT--A-G--A-A-G-G-A-AC-A-CC---GG--T--G--GC-GAA-G--G-C--G-------G--T-C-A-T---CTG--G--AC-T-T--------------------C-A---A-C-T--GA--CG-C-T-G--A-GG--C-G-CG-A--AA-G-C---G-TG--GG-G--AG-
C-A-AA--------CA--GG Bacteria(100);Proteobacteria(100);Alphaproteobacteria(100);unclassified(100);unclassified(100);unclassified(100);unclassified(100);unclassified(100);unclassified(100);

Otu1317 0 0 0 0 0 0 0 0 0 0 0 0 0 0 0 0 0 0 0 0 0 0 0 0 0 0 0 0
0 0 0 0 0 0 0 0 0 0 0 0 0 0 0 0 0 0 0 0 0 0 0 0 0 0 0 0 0
0 0 0 0 0 0 0 0 0 0 0 0 0 0 0 0 0 0 0 0 0 0 0 0 0 0 0 0 0
0 0 0 0 0 0 0 6 0 0 0 0 0 0 0 0 0 0 0 0 0 0 0 0 0 0 0
M02255_77_000000000-ADUT3_1_2101_17891_20822 T----AC---GG-AG-GAT--------GCA-A-G------C--G--T---T--AT-C-CGG-AA------TG-A--T-T--GG-GC-------GT--A-----AA-GC-GT-CC-------G-CA-G-G-T-G-------------G--C-AT-T-T-C-----
------AA----G-T-C-T--------G-C-G--G--TC--A-AA-GG--T-C-GA-G-G-----CT-C-AA------C-C-T-C-G-A-A--C-A----G-G--C-C---G--T-------G--GA-A-A---C-------T--G-A--AA--A-G-C----T-A-G-A-G-T--G---T-GG-----TA-G-G--------G-G-C-A---GA-G------GG--A--ATT----
C-C-C-A-GT--GT-A-G-CG-GT--G---A--A-A---TG-C-GT-AG--AG-A-TT--G-G--G-A-A-G-A-AC-A-CC---GG--T--G--GC-GAA-A--G-C--G-------C--T-C-T-G---CTA--G--GC-C-A--------------------A-G---A-C-T--GA--CA-C-T-G--A-GG--G-A-CG-A--AA-G-C---T-AG--
GG-G--AG-C-G-AA--------TG--GG Bacteria(100);Cyanobacteria(100);unclassified(100);unclassified(100);unclassified(100);unclassified(100);unclassified(100);unclassified(100);unclassified(100);

Otu1318 0 0 0 0 0 0 0 0 0 0 0 0 0 0 0 0 0 0 0 0 0 0 0 0 0 0 0 0
0 0 0 0 0 0 0 0 0 0 0 0 0 0 0 0 0 0 0 0 0 0 0 0 0 0 0 0 0
0 0 0 0 0 0 0 0 0 0 0 0 0 0 0 0 0 0 0 0 0 0 0 0 0 0 0 0 0
0 0 0 0 0 0 0 0 2 0 1 0 0 0 0 0 0 0 0 0 0 0 0 0 0 0 0
M02255_76_000000000-ADUNE_1_2110_9582_8051 .....AC----T-A--CGT--------GCC-A-G------C-----A---G--CC-G-CGG-TA------AT-A--C-G--TA-GG-------GT--G-----CA--A-GC-GT-------T-CA---T-C-G-------------G--T-TT-T-G-T-----------AA----G-
T-C-T--------G-A-T--G--TG--A-AA-TC--C-C-CG-G-G-----CT-C-AA------C-C-T-G-G-G-A--A-T----T-G--C-A---T--T-------G--GA-G-A---C-------T--G-C--AA--G-G-C----T-A-G-A-A-T--C---T-GG-----CA-G-A--------G-G-G-G---GG-T------AG--A--ATT----C-C-A-C-GT--
GT-A-G-CA-GT--G---A--A-A---TG-C-GT-AG--AT-A-TG--T-G--G-A-G-G-A-AC-A-CC---GA--T--G--GC-GAA-G--G-C--A-------G--C-C-C-C---CTG--G--GT-C-A--------------------A-G---A-T-T--GA--CG-C-T-C--A-TG--C-A-CG-A--AA-G-C---G-TG--GG-G--AG-C-A-
AA--------CA--GG Bacteria(100);Proteobacteria(100);Betaproteobacteria(100);Burkholderiales(100);Oxalobacteraceae(100);Janthinobacterium(100);unclassified(100);unclassified(100);unclassified(100);

Otu1319 0 0 0 0 0 0 0 0 0 0 0 0 0 0 0 0 0 0 0 0 0 0 0 0 0 0 0 0
0 0 0 0 0 0 0 0 0 0 0 0 0 0 0 0 0 0 0 0 0 0 0 0 0 0 0 0 0
0 0 0 0 0 0 0 0 0 0 0 0 0 0 0 0 0 0 0 0 0 0 0 0 0 0 0 0 0
0 0 0 0 0 0 0 0 0 44 0 0 0 0 0 0 0 0 0 0 0 0 0 0 0 0 0
M02255_77_000000000-ADUT3_1_1105_13652_4052 T----AC---GT-AG-GAT--------CCG-A-G------C--G--T---T--AT-C-CGG-AG------TG-A--C-T--GG-GC-------GT--A-----AA-GA-GTTGC-------G-TA-G-G-C-G-------------G--T-TA-G-T-A-----------
AA----G-C-G-A--------A-T-A--G--TG--A-AA-CC--T-G-GT-G-G-----CT-C-AA------C-C-A-T--CA-A--C-C----C-A--T-T---A--T-------T--CG-A-A---C-------T--C-A--CT--A-A-C----T-C-G-A-G-A--G---T-AG-----TA-G-A--------G-G-T-A---CC-T------GG--A--ATT----C-C-C-T-
GT--GT-A-G-CG-GT--G---A--A-A---TG-C-GT-AG--AT-A-TA--G-G--G-A-G-G-A-AC-A-CC---AA--T--G--GC-GTA-G--G-C--A-------G--G-G-T-A---CTG--G--AC-T-A--------------------T-T---T-C-T--GA--CG-C-T-G--A-GG--C-A-CG-A--AA-G-C---G-TG--GG-G--AG-
C-G-AA--------CC--GG Bacteria(100);Candidate_division_TM7(100);unclassified(100);unclassified(100);unclassified(100);unclassified(100);unclassified(100);unclassified(100);unclassified(100);

Otu1320 0 0 0 0 0 0 0 0 0 0 0 0 0 0 0 0 0 0 0 0 0 0 0 0 0 0 0 0
0 0 0 0 0 0 0 0 0 0 0 0 0 0 0 0 0 0 0 0 0 0 0 0 0 0 0 0 0
0 0 0 0 0 0 0 0 0 0 0 0 0 0 0 0 0 0 0 0 0 0 0 0 0 0 0 0 0
0 0 0 0 0 0 0 0 0 0 0 0 0 0 0 15 0 0 0 0 0 0 0 0 0 0 0 0
M02255_76_000000000-ADUNE_1_2114_20743_11527 .....AC----T-A--CGT--------GCC-A-G------C--A--G---C--CG-C-GGT-AA------TA-C--G-T--AG-GG-------GG--C-----AA-GC-G--TT-------G-T----C-C-G------------G--T-TT-C-T-T-----------AA----G-T-C-T--------G-A-T--G--TG--A-AA-TC--T-T-GC-G-G-----CT-C-AA------C-C-G-C-A-A-G--C------G-GT-C-A---T--T-------G--GA-A-A---C-------T--G-G--GG--A-A-C----T-T-G-A-G-T--G---C-AG-----AA-G-A--------G-G-A-G---AG-T------GG--A--ATT----C-C-A-C-GT--GT-A-G-CG-GT--G---A--A-A---TG-C-GT-AG--AG-A-TG--T-G--G-A-G-G-A-AC-A-CC---AG--T--G--GC-GAA-G--G-C--G-------A--C-T-C-T---CTG--G--TC-T-G--------------------T-A---A-C-T--GA--CG-C-T-G--A-GG--T-G-CG-A--AA-G-C---G-TG--GG-T--AG-C-G-AA--------CA--GG Bacteria(100);Firmicutes(100);Bacilli(100);Bacillales(100);Bacillaceae(100);unclassified(100);unclassified(100);unclassified(100);unclassified(100);

Otu1321 0 0 0 0 0 0 0 0 0 0 0 0 0 0 0 0 0 0 0 0 0 0 0 0 0 0 0 0
0 0 0 0 0 0 0 0 0 0 0 0 0 0 0 0 0 0 0 0 0 0 0 0 0 0 0 0 0
0 0 0 0 0 0 0 0 0 0 0 0 0 0 0 0 0 0 0 0 0 0 0 0 0 0 0 0 0
0 0 0 0 0 0 0 0 0 25 0 0 0 0 0 0 0 0 0 0 0 0 0 0 0 0 0 0
M02255_77_000000000-ADUT3_1_1102_22875_6970 T----AC---GT-AG-GGT--------CCA-A-G------C--G--T---T--GT-C-CGG-AT------TT-A--T-T--GG-GC-------GT--A-----AA-GA-GC-TC-------G-TA-G-G-T-G-------------G--T-TC-G-G-T-----------AA----G-T-C-G--------G-A-T--G--TG--A-AA-AC--T-C-GG-G-G-----CT-C-AA------C-C-C-C-G-A-G--A------C-G-CC-A---T--C-------C--GA-T-A---C-------T--G-C--TG-TG-A-C----T-T-G-A-G-T--C---T-GG-----TA-G-G--------G-G-A-G---AT-C------GG--A--ATT----C-C-T-G-GT--GT-A-G-CG-GT--G---A--A-A---TG-C-GC-AG--AT-A-TC--A-G--G-A-G-G-A-AC-A-CC---GG--T--G--GC-GAA-G--G-C--G-------G--A-T-C-T---CTG--G--GC-C-A--------------------G-T---A-C-T--GA--CA-C-T-G--A-GG--A-G-CG-A--AA-G-C---G-TG--GG-T--AG-C-A-AA--------CA--GG Bacteria(100);Actinobacteria(100);Actinobacteria(100);unclassified(100);unclassified(100);unclassified(100);unclassified(100);unclassified(100);unclassified(100);

Otu1322 0 0 0 0 0 0 0 0 0 0 0 0 0 0 0 0 0 0 0 0 0 0 0 0 0 0 0 0
0 0 0 0 0 0 0 0 0 0 0 0 0 0 0 0 0 0 0 0 0 0 0 0 0 0 0 0 0
0 0 0 0 0 0 0 0 0 0 0 0 0 0 0 0 0 0 0 0 0 0 0 0 0 0 0 0 0
0 0 0 0 0 0 0 0 0 0 3 0 0 0 0 0 0 0 0 0 0 0 0 0 0 0 0 0
M02255_76_000000000-ADUNE_1_2111_8688_15542 A----AC---GA-GG-AAG--------CCA-C-G------G--C--T---A--AC-T-ACG-TG------CC-A--G-C--AG-CC-------GC--G-----GT-AA-TA--C-------G-TA-G-G-C-G-------------G--C-AAGCGT-T-----------GT----C-C-G-G--------A-C-T--T--TA--A-AA-GT--G-C-GG-G-G-----CT-C-AA------C-C-C-C-G-T-G--A------G-G--G-G---G--T-------C--CG-A-A---C-------T--G-A--CT--T-T-C----T-T-G-A-G-T--G---C-AG-----GA-G-A--------G-G-G-A---AG-C------GG--A--ATT----C-C-T-A-GT--GT-A-G-CG-GT--G---A--A-A---TG-C-GT-AG--AT-A-TT--A-G--G-A-A-G-A-AC-A-CC---AG--T--G--GC-GAA-G--G-C--G-------G--C-T-T-T---CTG--G--AC-T-G--------------------T-A---A-C-T--GA--CG-C-T-G--A-GG--C-T-CG-A--AA-G-C---T-AG--GG-T--AG-C-G-AA--------CG--GG Bacteria(100);Firmicutes(100);Clostridia(100);Clostridiales(100);Veillonellaceae(100);Megamonas(100);unclassified(100);unclassified(100);unclassified(100);

Otu1323 0 0 0 0 0 0 0 0 0 0 0 0 0 0 0 0 0 0 0 0 0 0 0 0 0 0 0 0
0 0 0 0 0 0 0 0 0 0 0 0 0 0 0 0 0 0 0 0 0 0 0 0 0 0 0 0 0
0 0 0 0 0 0 0 0 0 0 0 0 0 0 0 0 0 0 0 0 0 0 0 0 0 0 0 0 0
0 0 0 0 0 0 0 0 0 0 6 0 0 0 0 0 0 0 0 0 0 0 0 0 0 0 0 0
M02255_77_000000000-ADUT3_1_1108_17632_14207 T----AC---GA-AG-GGG--------GCG-A-G------C--G--T---T--AT-T-CGG-AA------TC-A--C-T--GG-GC-------GT--A-----AA-GA-GT-GC-------G-TAGG---C-G-------------G--T-TA-T-A-A-----------AA----G-T-T-A--------GAA----G--TG--A-AA-GC--C-C-AG-G-G-----CT-C-AA------C-C-T-T-G-G-A--A-T----T-G--C-T---T--T-------T--AA-T-A---C-------T--G-T--AT--A-A-C----T-C-G-A-A-T--T---C-GG-----GA-G-A--------G-G-A-T---AG-C------GG--A--ATT----G-T-C-A-GT--GT-A-G-CA-GT--G---A--A-A---TG-C-GT-AG--AT-A-TT--G-A--C-A-G-G-A-AC-A-CC---GG--T--G--GC-GTA-A--G-C--G-------G--C-T-A-T---CTG--G--AC-C-G--------------------A-C---A-T-T--GA--CG-C-T-G--A-GG--C-A-CG-A--AA-G-C---G-TG--GG-G--AT-C-A-AA--------CA--GG Bacteria(100);unclassified(100);unclassified(100);unclassified(100);unclassified(100);unclassified(100);unclassified(100);unclassified(100);unclassified(100);

Otu1324 0 0 0 0 0 0 0 0 0 0 0 0 0 0 0 0 0 0 0 0 0 0 0 0 0 0 0 0
0 0 0 0 0 0 0 0 0 0 0 0 0 0 0 0 0 0 0 0 0 0 0 0 0 0 0 0 0
0 0 0 0 0 0 0 0 0 0 0 0 0 0 0 0 0 0 0 0 0 0 0 0 0 0 0 0 0
0 0 0 0 0 0 0 0 0 0 7 0 0 0 0 0 0 0 0 0 0 0 0 0 0 0 0 0
M02255_77_000000000-ADUT3_1_2112_3903_16509 G----AC---AG-AA-GAT--------GCA-A-G------C--A--T---T--AT-C-CAG-AA------TG-A--T-T--GG-GC-------AT--A-----AA-GC-AT-CT-------G-TA-G-G-T-G-------------G--C-TT-T-T-T-----------AA----G-T-T-C--------A-T-C--G--TC--A-TA-TC--C-C-AG-G-G-----CC-C-AA------C-C-C-T-A-G-A--C-A----G-G--C-A---A--T-------G--GA-A-A---C-------T--A-C--CA--A-G-C----T-G-G-A-G-T--A---T-AG-----TA-G-T--------G-A-T-A---GA-G------GA--A--ATT----T-T-C-G-AT--GG-A-G-CG-CT--G---A--A-A---TG-C-GT-AG--AG-A-TC--A-A--A-A-A-G-A-AC-A-CC---AA--C--G--GC-AAA-A--G-C--A-------C--T-C-T-G---CTA--G--GC-T-G--------------------A-C---A-C-T--AA--CA-C-T-G--A-GA--G-A-TA-A--AA-T-C---T-AG--GA-G--AG-T-G-AA--------TG--GG Bacteria(100);unclassified(100);unclassified(100);unclassified(100);unclassified(100);unclassified(100);unclassified(100);unclassified(100);unclassified(100);

Otu1325 0 0 0 0 0 0 0 0 0 0 0 0 0 0 0 0 0 0 0 0 0 0 0 0 0 0 0 0
0 0 0 0 0 0 0 0 0 0 0 0 0 0 0 0 0 0 0 0 0 0 0 0 0 0 0 0 0
0 0 0 0 0 0 0 0 0 0 0 0 0 0 0 0 0 0 0 0 0 0 0 0 0 0 0 0 0
0 0 0 0 0 0 0 0 0 0 4 0 0 0 0 0 0 0 0 0 0 0 0 0 0 0 0
M02255_77_000000000-ADUT3_1_1110_6583_6697 T----AC---GA-AG-GGG--------GCT-A-G------C--G--T---T--GC-T-CGG-AA------TG-A--C-T--GG-GC-------GT--A-----AA-GG-GC-GC-------G-TA-G-G-C-G------------G--C-GA-T-A-T----------
CA----G-T-C-A--------G-A-T--G--TG--A-AA-TT--C-C-TG-G-G-----CT-T-AA------C-C-T-G-G-G-G--G-C----T-G--C-A---T--T-------T--GA-T-A---C-------G--G-T--AT--T-G-C----T-T-G-A-G-T--A---G-GG-----AA-G-A--------G-G-G-T---CG-T------GG--A--ATT----C-C-C-
A-GT--GT-A-G-AG-GT--G---A--A-A---TT-C-GT-AG--AT-A-TT--G-G--G-A-A-G-A-AC-A-CC---GG--T--G--GC-GAA-G--G-C--G-------G--C-G-A-C---CTG--G--TC-C-T--------------------T-T---A-C-T--GA--CG-C-T-G--A-GG--C-G-CG-A--AA-G-C---G-TG--GG-G--
AG-C-A-AA--------CA--GG Bacteria(100);Proteobacteria(100);Alphaproteobacteria(100);Rhodospirillales(100);Acetobacteraceae(100);unclassified(100);unclassified(100);unclassified(100);unclassified(100);

Otu1326 0 0 0 0 0 0 0 0 0 0 0 0 0 0 0 0 0 0 0 0 0 0 0 0 0 0 0 0
0 0 0 0 0 0 0 0 0 0 0 0 0 0 0 0 0 0 0 0 0 0 0 0 0 0 0 0 0
0 0 0 0 0 0 0 0 0 0 0 0 0 0 0 0 0 0 0 0 0 0 0 0 0 0 0 0 0
0 0 0 0 0 0 0 0 0 0 8 0 0 0 0 0 0 0 0 0 0 0 0 0 0 0 0
M02255_77_000000000-ADUT3_1_2113_26458_7201 T----AC---GT-AG-GGA--------GCA-A-G------C--G--T---T--AT-C-CGG-AT------TT-A--T-T--GG-GT-------GT--A-----AA-GG-GT-GC-------G-TA-G-A-C-G------------G--G-AG-A-G-C----------
AA----G-T-T-G--------G-T-T--G--TG--A-AA-TC--C-C-TC-G-G-----CT-T-AA------C-T-G-A-G-G-A--A-C----T-G--C-A---A--C-------C--AA-A-A---C-------T--A-T--TC--T-T-C----T-T-G-A-G-T--G---C-TG-----GA-G-A--------G-G-A-A---AG-T------GG--A--ATT----C-C-T-
A-GT--GT-A-G-CG-GT--G---A--A-A---TG-C-GT-AG--AT-A-TT--A-G--G-A-G-G-A-AC-A-CC---AG--T--G--GC-GAA-G--G-C--G-------A--C-T-T-T---CTG--G--AC-A-G--------------------T-A---A-C-T--GA--CG-T-T-G--A-GG--C-A-CG-A--AA-G-T---G-TG--GG-G--
AG-C-A-AA--------CA--GG Bacteria(100);Firmicutes(100);Clostridia(100);Clostridiales(100);Ruminococcaceae(100);uncultured(100);unclassified(100);unclassified(100);unclassified(100);

Otu1327 0 0 0 0 0 0 0 0 0 0 0 0 0 0 0 0 0 0 0 0 0 0 0 0 0 0 0 0
0 0 0 0 0 0 0 0 0 0 0 0 0 0 0 0 0 0 0 0 0 0 0 0 0 0 0 0 0
0 0 0 0 0 0 0 0 0 0 0 0 0 0 0 0 0 0 0 0 0 0 0 0 0 0 0 0 0
0 0 0 0 0 0 0 0 0 0 6 0 0 0 0 0 0 0 0 0 0 0 0 0 0 0 0
M02255_77_000000000-ADUT3_1_2119_24891_5946 T----AC---GA-AG-GGG--------GCT-A-G------C--G--T---T--GC-T-CGG-AA------TC-A--C-T--GG-GC-------GT--A-----AA-GG-GC-GC-------G-TA-G-G-C-G------------G--C-CC-C-G-T----------
TG----G-T-C-G--------G-G-C--G--TG--A-AA-TC--C-C-CG-G-G-----CT-C-AA------C-C-C-G-G-G-G--G-C----T-G--C-G---C--C-------C--GA-G-A---C-------G--G-C--GG--G-G-C----T-G-G-A-G-G--A---C-GG-----GA-G-A--------G-G-C-T---CG-C------GG--A--ATT----C-C-
C-A-GT--GT-A-G-AG-GT--G---A--A-A---TT-C-GT-AG--AT-A-TT--G-G--G-A-A-G-A-AC-A-CC---GG--T--G--GC-GAA-G--G-C--G-------G--C-G-A-G---CTG--G--CC-C-G--------------------T-T---T-C-T--GA--CG-C-T-G--A-GG--C-G-CG-A--CA-G-C---G-TG--GG-G--
AG-C-A-AA--------CA--GG Bacteria(100);Proteobacteria(100);Alphaproteobacteria(100);Rhodospirillales(100);unclassified(100);unclassified(100);unclassified(100);unclassified(100);unclassified(100);

Otu1328 0 0 0 0 0 0 0 0 0 0 0 0 0 0 0 0 0 0 0 0 0 0 0 0 0 0 0 0
0 0 0 0 0 0 0 0 0 0 0 0 0 0 0 0 0 0 0 0 0 0 0 0 0 0 0 0 0
0 0 0 0 0 0 0 0 0 0 0 0 0 0 0 0 0 0 0 0 0 0 0 0 0 0 0 0 0
0 0 0 0 0 0 0 0 0 0 0 0 0 0 0 0 58 0 0 0 0 0 0 0 0 0 0
M02255_77_000000000-ADUT3_1_1103_17636_11519 C----AC---GT-AG-GGG--------GCG-A-G------C--G--T---T--GT-C-CGG-AT------TT-A--C-T--GG-GC-------GT--A-----AA-GA-GC-GC-------G-TA-G-G-T-G------------G--T-TA-G-G-T----
-------AA----G-T-C-A--------G-G-T--G--TG--A-AA-GG--C-C-AG-G-G-----CT-C-AA------C-C-CTG-G-G-A--T------T-G--C-A---C--T-------T--GA-A-A---C-------T--G-T--TT--A-A-C----T-T-G-A-G-G--G---T-AG-----GA-G-A--------G-G-T-A---AG-T------GG--A--ATT----
C-C-T-G-GT--GT-A-G-CG-GT--G---A--A-A---TG-C-GT-AG--AT-A-TC--A-G--G-A-G-G-A-AC-A-CC---AG--T--G--GC-GAA-A--G-C--G-------A--C-T-T-A---CTG--G--CC-T-A--------------------G-C---C-C-T--GA--CA-C-T-G--A-GG--C-G-CG-A--AA-G-C---G-TG--GG-
G--AG-C-G-AA--------CA--GG Bacteria(100);unclassified(100);unclassified(100);unclassified(100);unclassified(100);unclassified(100);unclassified(100);unclassified(100);unclassified(100);

Otu1329 0 0 0 0 0 0 0 0 0 0 0 0 0 0 0 0 0 0 0 0 0 0 0 0 0 0 0 0
0 0 0 0 0 0 0 0 0 0 0 0 0 0 0 0 0 0 0 0 0 0 0 0 0 0 0 0 0
0 0 0 0 0 0 0 0 0 0 0 0 0 0 0 0 0 0 0 0 0 0 0 0 0 0 0 0 0
0 0 0 0 0 0 0 0 0 0 0 0 0 0 0 15 0 0 0 0 0 0 0 0 0 0 0
M02255_76_000000000-ADUNE_1_1106_25844_8758 T----AA---CT-A--CGT--------GCC-A-G------C--A--G---C--CG-C-GGT-AA------TA-C--G-T--AG-GG-------GG--C-----AA-GC-G--TT-------G-T----C-C-G------------G--T-TC-T-T-T----------AA----
G-T-C-T--------G-A-T--G--TG--A-AA-TC--T-T-GC-G-G-----CT-C-AA------C-C-G-C-A-A-G--C------G-GT-C-A---T--T-------G--GA-A-A---C-------T--G-G--AG--A-A-C----T-T-G-A-G-G--A---C-AG-----AA-G-A--------G-G-A-G---AG-T------GG--A--ATT----C-C-A-C-GT-
-GT-A-G-CG-GT--G---A--A-A---TG-C-GT-AG--AT-A-TG--T-G--G-A-G-G-A-AC-A-CC---AG--T--G--GC-GAA-G--G-C--G-------A--C-T-C-T---CTG--G--TC-T-G--------------------T-A---A-C-T--GA--CG-C-T-G--A-GG--T-G-CG-A--AA-G-C---G-TG--GG-T--AG-C-G-
AA--------CA--GG Bacteria(100);Firmicutes(100);Bacilli(100);Bacillales(100);Bacillaceae(100);unclassified(100);unclassified(100);unclassified(100);unclassified(100);

Otu1330 0 0 0 0 0 0 0 0 0 0 0 0 0 0 0 0 0 0 0 0 0 0 0 0 0 0 0 0
0 0 0 0 0 0 0 0 0 0 0 0 0 0 0 0 0 0 0 0 0 0 0 0 0 0 0 0 0
0 0 0 0 0 0 0 0 0 0 0 0 0 0 0 0 0 0 0 0 0 0 0 0 0 0 0 0 0
0 0 0 0 0 0 0 0 0 0 0 0 0 0 0 12 0 0 0 0 0 0 0 0 0 0 0
M02255_77_000000000-ADUT3_1_2101_18503_17455 A----AC---GT-AG-GTT--------CCG-A-G------C--G--T---T--AT-C-CGG-AT------TT-A--C-T--GG-GC-------GT--A-----AA-GC-GC-GT-------T-CA-G-G-C-G------------G--C-TG-G-T-C------
-----AA----G-T-C-G--------G-G-C--A--TG--A-AA-GC--T-C-TC-G-G-----CT-C-AA------C-C-G-G-G-A-G--A-G----G-C--T-G---T--C-------C--GA-T-A---C-------T--G-A--TC--G-G-C----T-T-G-A-G-G--G---C-AG-----TA-G-A--------G-G-G-T---GG-T------GG--A--ATT----
C-C-G-G-GT--GT-A-G-TG-GT--G---A--A-A---TG-C-GT-AG--AT-A-TC--C-G--G-A-G-G-A-AC-A-CC---AG--T--G--GC-GAA-A--G-C--G-------G--C-C-A-C---CTG--G--AC-T-G--------------------TA----C-C-T--GA--CG-C-T-C--A-AA--C-G-CG-A--AA-G-C---T-AG--GG-
G--AG-C-G-AA--------CG--GG Bacteria(100);Chloroflexi(100);Anaerolineae(100);Anaerolineales(100);Anaerolineaceae(100);unclassified(100);unclassified(100);unclassified(100);unclassified(100);

Otu1331 0 0 0 0 0 0 0 0 0 0 0 0 0 0 0 0 0 0 0 0 0 0 0 0 0 0 0 0
0 0 0 0 0 0 0 0 0 0 0 0 0 0 0 0 0 0 0 0 0 0 0 0 0 0 0 0 0
0 0 0 0 0 0 0 0 0 0 0 0 0 0 0 0 0 0 0 0 0 0 0 0 0 0 0 0 0
0 0 0 0 0 0 0 0 0 0 0 0 0 0 0 12 0 0 0 0 0 0 0 0 0 0 0
M02255_77_000000000-ADUT3_1_2104_18725_9767 T----AC---GT-AG-GGT--------CCG-A-G------C--G--T---T--GT-C-CGG-AG------TT-A--C-T--GG-GC-------GT--A-----AA-GC-GC-GC-------G-CA-G-G-C-G------------G--T-TC-G-A-T-----------AA-
---G-A-C-T--------G-G-C--G--TG--A-AA-GC--C-C-CC-G-G-----CT-C-AA------C-C-G-G-G-G-A--G-G----G-T--C-G---T--T-------G--GT-G-A---C-------T--G-T--CG--G-A-C----T-T-G-A-A-G--A---C-AG-----CA-G-G--------G-G-C-A---GG-T------GG--A--ATT----C-C-C--
GGT--GT-A-G-TG-GT--G---A--A-A---TG-C-GT-AG--AG-A-TC--G-G--G-A-G-G-A-AC-A-CC---CG--T--G--GC-GAA-G--G-C---------GG--C-C-T-G---CTG--G--GC-T-G--------------------G-T---C-T-T--GA--CG-C-T-G----AGG-C-G-CG----AA-GGC---G-T---GGGG--
AG-C-G-AA--------CG--GG Bacteria(100);Chloroflexi(100);Thermomicrobia(100);JG30-KF-CM45(100);unclassified(100);unclassified(100);unclassified(100);unclassified(100);unclassified(100);

Otu1332 0 0 0 0 0 0 0 0 0 0 0 0 0 0 0 0 0 0 0 0 0 0 0 0 0 0 0 0
0 0 0 0 0 0 0 0 0 0 0 0 0 0 0 0 0 0 0 0 0 0 0 0 0 0 0 0 0
0 0 0 0 0 0 0 0 0 0 0 0 0 0 0 0 0 0 0 0 0 0 0 0 0 0 0 0 0
0 0 0 0 0 0 0 0 0 0 0 0 0 0 0 10 0 0 0 0 0 0 0 0 0 0 0
M02255_77_000000000-ADUT3_1_1111_8533_13564 T----AC---GT-AG-GGG--------GCG-A-G------C--G--T---T--GT-C-CGG-AA------TG-A--C-T--GG-GC-------GT--A-----AA-GG-GC-GT-------G-TA-G-G-C-G------------G--C-CG-A-C-T-----------
AA----G-T-C-T--------G-A-T--G--TG--A-AA-TA--C-C-CG-C-T-----TT-T-AA------G-G-T-G-G-G-T--C-G----T-G--C-A---T--T-------G--GA-A-A---C-------T--G-G--AT--G-G-C----T-A-G-A-G-T--G---C-GG-----AA-G-A--------G-G-T-A---AG-T------GG--A--ATT----C-C-C-
A-GT--GT-A-G-CG-GT--G---A--A-A---TG-C-GT-AG--AG-A-TT--G-G--G-A-G-G-A-AC-A-CC---AG--T--G--GC-GAA-G--G-C--G-------A--C-T-T-A---CTG--G--GC-C-G--------------------C-A---A-C-T--GA--CG-C-T-G--A-GG--C-G-CG-A--AA-G-C---G-TG--GG-G--
AG-C-A-AA--------CA--GG Bacteria(100);Firmicutes(100);unclassified(100);unclassified(100);unclassified(100);unclassified(100);unclassified(100);unclassified(100);unclassified(100);

Otu1333 0 0 0 0 0 0 0 0 0 0 0 0 0 0 0 0 0 0 0 0 0 0 0 0 0 0 0 0
0 0 0 0 0 0 0 0 0 0 0 0 0 0 0 0 0 0 0 0 0 0 0 0 0 0 0 0 0
0 0 0 0 0 0 0 0 0 0 0 0 0 0 0 0 0 0 0 0 0 0 0 0 0 0 0 0 0
0 0 0 0 0 0 0 0 0 0 0 0 0 0 0 0 47 0 0 0 0 0 0 0 0 0 0
M02255_77_000000000-ADUT3_1_1101_27369_11056 T----AC---GG-AG-GGA--------GCT-A-G------C--G--T---T--GT-T-CGG-AA------TT-A--C-T--GG-GC-------GT--A-----AA-GC-GC-GC-------G-TA-G-G-C-G------------G--T-TT-T-T-C------
-----AA----G-T-C-A--------G-G-G--G--TG--A-AA-GC--C-C-GG-A-G-----CT-C-AA------C-T-C-C-G-G-A--A-C----T-G--C-C---T--C-------T--GA-A-A---C-------T--G-G--AT--G-A-C----T-T-G-A-G-T--C---T-TG-----GA-G-A--------G-G-T-C---AG-T------GG--A--ATT----C-
C-G-A-GT--GT-A-G-AG-GT--G---A--A-A---TT-C-GT-AG--AT-A-TT--C-G--G-A-A-G-A-AC-A-CC---AG--T--G--GC-GAA-G--G-C--G-------A--C-T-G-A---CTG--G--AC-A-A--------------------G-T---A-C-T--GA--CG-C-T-G--A-GG--T-G-CG-A--AA-G-C---G-TG--GG-
G--AG-C-A-AA--------CA--GG Bacteria(100);Proteobacteria(100);Alphaproteobacteria(100);Sphingomonadales(100);unclassified(100);unclassified(100);unclassified(100);unclassified(100);unclassified(100);

Otu1334 0 0 0 0 0 0 0 0 0 0 0 0 0 0 0 0 0 0 0 0 0 0 0 0 0 0 0 0
0 0 0 0 0 0 0 0 0 0 0 0 0 0 0 0 0 0 0 0 0 0 0 0 0 0 0 0 0
0 0 0 0 0 0 0 0 0 0 0 0 0 0 0 0 0 0 0 0 0 0 0 0 0 0 0 0 0
0 0 0 0 0 0 0 0 0 0 0 0 0 0 0 0 46 0 0 0 0 0 0 0 0 0 0
M02255_77_000000000-ADUT3_1_1101_26441_16658 G----AC---GT-AG-GAT--------GCA-A-G------C--G--T---T--GT-C-CGG-AT------TT-A--T-T--GG-GC-------GT--A-----AA-GA-GT-TC-------G-TA-G-G-C-G------------G--T-TC-G-T-T------
-----AA----G-T-C-T--------G-A-T--G--TT--A-AA-GA--T-C-GG-G-G-----CT-C-AA------C-C-C-T-G-G-G--A-G----T-G--C-A---T--T-------G--GA-T-A---C-------T--G-G--CG--G-A-C----T-G-G-A-G-T--G---C-GG-----TA-G-A--------G-G-C-T---AG-T------GG--A--ATT----C-
C-C-A-GT--GT-A-G-CG-GT--G---A--A-A---TG-C-GT-AG--AT-A-TT--G-G--G-A-A-G-A-AC-A-CC---AG--T--G--GC-GTA-G--G-C--G-------A--C-T-A-G---CTG--G--GC-C-G--------------------T-A---A-C-T--GA--CG-C-T-G--A-GG--A-A-CG-A--AA-G-C---C-AG--GG-
G--AG-C-G-AA--------TG--GG Bacteria(100);Cyanobacteria(100);MLE1-12(100);unclassified(100);unclassified(100);unclassified(100);unclassified(100);unclassified(100);unclassified(100);

Otu1335 0 0 0 0 0 0 0 0 0 0 0 0 0 0 0 0 0 0 0 0 0 0 0 0 0 0 0 0
0 0 0 0 0 0 0 0 0 0 0 0 0 0 0 0 0 0 0 0 0 0 0 0 0 0 0 0 0
0 0 0 0 0 0 0 0 0 0 0 0 0 0 0 0 0 0 0 0 0 0 0 0 0 0 0 0 0
0 0 0 0 0 0 0 0 0 0 0 0 0 0 0 0 161 0 0 0 0 0 0 0 0 0 0
M02255_77_000000000-ADUT3_1_1101_26201_20236 T----AC---GG-AG-GGG--------GCT-A-G------C--G--T---T--GT-T-CGG-AA------TT-A--C-T--GG-GC-------GT--A-----AA-GG-GT-GC-------G-TA-G-G-C-G------------G--C-TG-T-T-C-----
------AA----G-T-C-A--------G-A-C--G--TG--A-AA-GG--C-C-TG-G-G-----CT-C-AA------C-C-T-G-G-G-A--A-C----T-G--C-G---T--T-------T--GA-G-A---C-------T--G-G--AC--G-G-C----T-T-G-A-G-T--C---C-GG-----GA-G-A--------G-G-T-G---AG-C------GG--A--ATT----
C-C-C-A-GT--GT-A-G-AG-GT--G---A--A-A---TT-C-GT-AG--AT-A-TT--G-G--G-A-A-G-A-AC-A-CC---GG--T--G--GC-GAA-G--G-C--G-------G--C-T-C-A---CTG--G--AC-C-G--------------------G-T---A-C-T--GA--CG-C-T-G--A-GG--C-A-CG-A--AA-G-C---G-TG--
GG-G--AG-C-A-AA--------CA--GG Bacteria(100);Proteobacteria(100);Alphaproteobacteria(100);unclassified(100);unclassified(100);unclassified(100);unclassified(100);unclassified(100);unclassified(100);

Otu1336 0 0 0 0 0 0 0 0 0 0 0 0 0 0 0 0 0 0 0 0 0 0 0 0 0 0 0 0
0 0 0 0 0 0 0 0 0 0 0 0 0 0 0 0 0 0 0 0 0 0 0 0 0 0 0 0 0
0 0 0 0 0 0 0 0 0 0 0 0 0 0 0 0 0 0 0 0 0 0 0 0 0 0 0 0 0
0 0 0 0 0 0 0 0 0 0 0 0 0 0 0 0 60 0 0 0 0 0 0 0 0 0 0
M02255_77_000000000-ADUT3_1_1102_27452_16424 T----AC---GA-AG-GGG--------GCT-A-G------C--G--T---T--GT-T-CGG-AA------TT-A--C-T--GG-GC-------GT--A-----AA-GC-GC-GT-------G-TA-G-G-C-G------------G--G-CT-T-T-T-----
------AA----G-T-C-A--------G-G-G--G--TG--A-AA-TG--C-C-AA-G-G-----CT-C-AA------C-C-T-T-G-G-A--A-C----T-G--C-C---T--T-------T--GA-T-A---C-------T--G-G--AA--G-T-C----T-T-G-A-G-T--C---C-GG-----GA-G-A--------G-G-T-G---AG-T------GG--A--ACT----
G-C-G-A-GT--GT-A-G-AG-GT--G---A--A-A---TT-C-GT-AG--AT-A-TT--C-G--C-A-A-G-A-AC-A-CC---AG--T--G--GC-GAA-G--G-C--G-------G--C-T-C-A---CTG--G--CC-C-G--------------------G-A---A-C-T--GA--CG-C-T-G--A-GA--C-G-CG-A--AA-G-C---G-TG--
GG-G--AG-C-A-AA--------CA--GG Bacteria(100);Proteobacteria(100);Alphaproteobacteria(100);Rhizobiales(100);unclassified(100);unclassified(100);unclassified(100);unclassified(100);unclassified(100);

Otu1337 0 0 0 0 0 0 0 0 0 0 0 0 0 0 0 0 0 0 0 0 0 0 0 0 0 0 0 0
0 0 0 0 0 0 0 0 0 0 0 0 0 0 0 0 0 0 0 0 0 0 0 0 0 0 0 0 0
0 0 0 0 0 0 0 0 0 0 0 0 0 0 0 0 0 0 0 0 0 0 0 0 0 0 0 0 0
0 0 0 0 0 0 0 0 0 0 0 0 0 0 0 28 0 0 0 0 0 0 0 0 0 0 0
M02255_77_000000000-ADUT3_1_2101_18250_10192 T----AC---GT-AA-GGG--------GCG-A-G------C--G--T---T--GT-C-CGG-AA------TT-A--T-T--GG-GC-------GT--A-----AA-GG-GT-TC-------G-CA-G-G-C-G------------G--C-CT-A-T-T-----
------AA----G-T-C-A--------G-A-T--G--TT--A-AA-TC--T-C-AT-G-G-----CC-T-AA------C-C-A-T-G-A-T--A------A-G--C-A---T--T-------T--GA-A-A---C-------T--G-A--AA--G-G-C----T-T-G-A-G-T--T---A-AG-----GA-G-A--------G-G-A-A---AG-T------GG--A--ATT----C-
C-T-A-GT--GT-A-G-CG-GT--G---A--A-A---TG-C-GT-AG--AT-A-TT--A-G--G-A-G-G-A-AT-A-CC---AG--T--G--GC-GAA-G--G-C--G-------A--C-T-T-T---CTG--G--AC-T-T--------------------A-T---A-C-T--GA--CG-C-T-G--A-GG--A-A-CG-A--AA-G-C---A-TG--GG-T--
AG-C-A-AA--------CA--GG Bacteria(100);Firmicutes(100);Clostridia(100);Clostridiales(100);unclassified(100);unclassified(100);unclassified(100);unclassified(100);unclassified(100);

Otu1338 0 0 0 0 0 0 0 0 0 0 0 0 0 0 0 0 0 0 0 0 0 0 0 0 0 0 0 0
0 0 0 0 0 0 0 0 0 0 0 0 0 0 0 0 0 0 0 0 0 0 0 0 0 0 0 0 0
0 0 0 0 0 0 0 0 0 0 0 0 0 0 0 0 0 0 0 0 0 0 0 0 0 0 0 0 0
0 0 0 0 0 0 0 0 0 0 0 0 0 0 0 0 41 0 0 0 0 0 0 0 0 0 0
M02255_77_000000000-ADUT3_1_1103_19570_18641 G----AC---AG-AG-GTG--------CCG-A-G------C--G--T---T--AG-G-CGG-AA------TC-A--C-T--GG-GC-------TT--A-----AA-GC-GT-GT-------G-TA-G-G-C-G------------G--A-CC-G-T-T----
-------AA----G-T-A-C--------C-T-T--G--TG--A-AA-TC--C-C-AC-G-G-----CT-C-AA------C-C-G-T-G-G-A--A-C----G-G--C-T---G--G-------G--TA-T-A---C-------T--G-G--CG--G-T-C----T-T-G-A-G-C--G---G-CC-----TA-G-G--------G-G-C-A---GA-C------GG--A--ACA----
A-G-T-G-GT--GG-A-G-CG-GT--G---A--A-A---TG-C-GT-AG--AT-A-TC--A-C--T-T-G-G-A-AC-G-CC---GA--C--G--GT-GAA-A--A-C--A-------G--T-C-T-G---CTG--G--GG-G-C--------------------C-T---G-C-T--GA--CG-C-T-G--A-GA--C-A-CG-A--AA-G-C---C-AG--
GG-G--AG-C-G-AA--------CG--GG Bacteria(100);Planctomycetes(100);Phycisphaerae(100);WD2101(100);unclassified(100);unclassified(100);unclassified(100);unclassified(100);unclassified(100);

Otu1339 0 0 0 0 0 0 0 0 0 0 0 0 0 0 0 0 0 0 0 0 0 0 0 0 0 0 0 0
0 0 0 0 0 0 0 0 0 0 0 0 0 0 0 0 0 0 0 0 0 0 0 0 0 0 0 0 0
0 0 0 0 0 0 0 0 0 0 0 0 0 0 0 0 0 0 0 0 0 0 0 0 0 0 0 0 0
0 0 0 0 0 0 0 0 0 0 0 0 0 0 0 0 22 0 0 0 0 0 0 0 0 0 0
M02255_77_000000000-ADUT3_1_1113_8332_10673 T----AC---AG-AG-GCC--------CCA-A-G------C--G--T---T--AG-G-CGG-AA------TC-A--C-T--GG-GC-------TT--A-----AA-GC-GT-GC-------G-TA-G-G-C-G------------G--C-CC-C-T-C-----------AA-
---G-T-G-C--------T-T-T--G--TG--A-AA-TC--C-C-CC-G-G-----CT-C-AA------C-C-G-G-G-G-A--A-C----T-G--C-T---T--G-------G--CA-T-A---C-------T--G-G--GG--G-G-C----T-T-G-A-A-T--C---A-TC-----TA-G-G--------G-G-C-A---GC-T------GG--A--ACT----G-A-C-G-
GT--GG-A-G-CG-GT--G---A--A-A---TG-C-GT-AG--AT-A-TC--G-T--C-A-G-G-A-AC-G-CC---GA--C--G--GC-GAA-A--G-C--A-------G--G-C-T-G---CTG--G--AG-A-T--------------------G-T---A-T-T--GA--CG-C-T-G--A-GG--C-A-CG-A--AA-G-C---C-AG--GG-G--AG-
C-G-AA--------CG--GG Bacteria(100);unclassified(100);unclassified(100);unclassified(100);unclassified(100);unclassified(100);unclassified(100);unclassified(100);unclassified(100);

Otu1340 0 0 0 0 0 0 0 0 0 0 0 0 0 0 0 0 0 0 0 0 0 0 0 0 0 0 0 0
0 0 0 0 0 0 0 0 0 0 0 0 0 0 0 0 0 0 0 0 0 0 0 0 0 0 0 0
0 0 0 0 0 0 0 0 0 0 0 0 0 0 0 0 0 0 0 0 0 0 0 0 0 0 0 0
0 0 0 0 0 0 0 0 0 0 0 0 0 0 0 0 37 0 0 0 0 0 0 0 0 0 0
M02255_77_000000000-ADUT3_1_1106_13113_22549 T----AC---GT-AG-GGG--------GCG-A-G------C--G--T---T--GT-C-CGG-AA------TT-A--C-T--GG-GC-------GT--A-----AA-GC-GC-GC-------G-TA-G-G-T-G------------G--T-TT-T-C-T-----
------AA----G-A-C-C--------G-A-T--G--TG--A-AA-TC--T-C-CG-C-G-----CT-T-AC------C-G-T-G-G-A-G--A-G----G-T--C-G---T--T-------G--GT-G-A---C-------T--G-G--GA--G-A-C----T-T-G-A-G-T--T---T-GG-----CA-G-A--------G-G-T-G---AG-T------GG--A--ATT----
C-C-C-G-GT--GG-A-G-CG-GT--G---A--A-A---TG-C-GT-AG--AG-A-TC--G-G--G-A-G-G-A-AC-A-CC---AG--T--G--GC-GAA-G--G-C--G-------G--C-T-C-A---CTG--G--GC-C-A--------------------T-G---A-C-T--GA--CG-C-T-G--A-GG--C-G-CG-A--AA-G-C---G-TG--
GG-G--AG-C-G-AA--------CC--GG Bacteria(100);unclassified(100);unclassified(100);unclassified(100);unclassified(100);unclassified(100);unclassified(100);unclassified(100);unclassified(100);

Otu1341 0 0 0 0 0 0 0 0 0 0 0 0 0 0 0 0 0 0 0 0 0 0 0 0 0 0 0 0
0 0 0 0 0 0 0 0 0 0 0 0 0 0 0 0 0 0 0 0 0 0 0 0 0 0 0 0
0 0 0 0 0 0 0 0 0 0 0 0 0 0 0 0 0 0 0 0 0 0 0 0 0 0 0 0
0 0 0 0 0 0 0 0 0 0 0 0 0 0 0 0 20 0 0 0 0 0 0 0 0 0 0
M02255_76_000000000-ADUNE_1_1107_15193_10923 T----AC---GG-AG-GGT--------GCA-A-G------C--G--T---T--AC-C-CGG-AA------TC-A-----------C--------T-----------GG-GC----------G------G---G------------G-----A-C-T-T-----------AA----
G-T-C-T--------G-G-C--T--TT--A-AA-GA--C-A-GC-G-G-----CT-C-AA------C-C-G-C-T-G-G--A-A----T-G--G-G---C--T-------G--GG-T-A---C-------T--G-G--GT--T-T-C----T-T-G-A-C-C--T---C-TG-----GA-G-A--------G-G-A-A---GC-T------GG--A--ATT----C-C-T-G-GT--
GT-A-G-CG-GT--G---G--A-A---TG-C-GT-AG--AT-A-CC--A-G--G-A-G-G-A-AC-A-CC---AA--T--G--GC-GAA-G--G-C--A-------G--G-C-T-T---CTG--G--AC-A-G--------------------A-A---G-G-T--GA--CG-C-T-G--A-GG--C-G-CG-A--AA-G-T---G-TG--GG-G--AG-C-G-
AA--------CC--GG Bacteria(100);unclassified(100);unclassified(100);unclassified(100);unclassified(100);unclassified(100);unclassified(100);unclassified(100);unclassified(100);

Otu1342 0 0 0 0 0 0 0 0 0 0 0 0 0 0 0 0 0 0 0 0 0 0 0 0 0 0 0 0
0 0 0 0 0 0 0 0 0 0 0 0 0 0 0 0 0 0 0 0 0 0 0 0 0 0 0 0
0 0 0 0 0 0 0 0 0 0 0 0 0 0 0 0 0 0 0 0 0 0 0 0 0 0 0 0
0 0 0 0 0 0 0 0 0 0 0 0 0 0 0 0 0 56 0 0 0 0 0 0 0 0 0
M02255_77_000000000-ADUT3_1_1101_3831_13729 T----AC---GA-AG-GGG--------GCT-A-G------C--G--T---T--GC-T-CGG-AA------TG-A--C-T--GG-GC-------GT--A-----AA-GG-GC-GC-------G-TA-G-G-C-G------------G--T-TT-G-A-A----------
CA----G-T-T-G--------G-G-C--G--TG--A-AA-TT--C-C-AG-G-G-----CT-T-AA------C-C-T-T-G-G-G--A-C----T-G--C-G---T--T-------C--AA-T-A---C-------G--T-T--TA--G-A-C----T-T-G-A-G-T--T---C-GG-----AA-G-A--------G-G-G-T---CG-T------GG--A--ATT----C-C-C-
A-GT--GT-A-G-AG-GT--G---A--A-A---TT-C-GT-AG--AT-A-TT--G-G--G-A-A-G-A-AC-A-CC---GG--T--G--GC-GAA-G--G-C--G-------G--C-G-A-C---CTG--G--TC-C-G--------------------A-A---A-C-T--GA--CG-C-T-G--A-GG--C-G-CG-A--AA-G-C---G-TG--GG-G--
AG-C-A-AA--------CA--GG Bacteria(100);Proteobacteria(100);Alphaproteobacteria(100);Rhodospirillales(100);Acetobacteraceae(100);unclassified(100);unclassified(100);unclassified(100);unclassified(100);

Otu1343 0 0 0 0 0 0 0 0 0 0 0 0 0 0 0 0 0 0 0 0 0 0 0 0 0 0 0 0
0 0 0 0 0 0 0 0 0 0 0 0 0 0 0 0 0 0 0 0 0 0 0 0 0 0 0 0
0 0 0 0 0 0 0 0 0 0 0 0 0 0 0 0 0 0 0 0 0 0 0 0 0 0 0 0
0 0 0 0 0 0 0 0 0 0 0 0 0 0 0 0 0 30 0 0 0 0 0 0 0 0 0
M02255_77_000000000-ADUT3_1_1103_10669_14002 T----AC---GA-GG-GGG--------GCA-A-G------C--G--T---T--GT-T-CGG-AA------TT-A--T-T--GG-GC-------GT--A-----AA-GG-GC-GC-------G-TA-G-G-C-G------------G--T-GC-G-G-T----
-------AA----G-T-C-A--------C-C-T--G--TG--A-AA-TC--T-C-CC-G-G-----CT-C-AA------C-T-G-G-G-A-G--C-C----T-G--C-A---G--G-------C--GA-T-A---C-------T--G-C--CG--T-G-C----T-T-G-A-G-G--G---T-GG-----GA-G-A--------G-G-T-G---CG-T------GG--A--ATT----
C-C-C-G-GT--GT-A-G-CG-GT--G---A--A-A---TG-C-GT-AG--AT-A-TC--G-G--G-A-G-G-A-AC-A-CC---TG--T--G--GC-GAA-A--G-C--G-------G--C-G-C-A---CTG--G--AC-C-A--------------------C-T---T-C-T--GA--CG-C-T-G--A-GG--C-G-CG-A--AA-G-C---T-AG--
GG-G--AG-C-A-AA--------CA--GG Bacteria(100);unclassified(100);unclassified(100);unclassified(100);unclassified(100);unclassified(100);unclassified(100);unclassified(100);unclassified(100);

Otu1344 0 0 0 0 0 0 0 0 0 0 0 0 0 0 0 0 0 0 0 0 0 0 0 0 0 0 0 0
0 0 0 0 0 0 0 0 0 0 0 0 0 0 0 0 0 0 0 0 0 0 0 0 0 0 0 0
0 0 0 0 0 0 0 0 0 0 0 0 0 0 0 0 0 0 0 0 0 0 0 0 0 0 0 0
0 0 0 0 0 0 0 0 0 0 0 0 0 0 0 0 0 25 0 0 0 0 0 0 0 0 0
M02255_77_000000000-ADUT3_1_1104_6691_12808 T----AC---GG-AG-GGT--------GCA-A-G------C--G--T---T--GT-C-CGG-AT------TT-A--T-T--GG-G---------T---------A----G--GC-----------G-G-C-----------------C-CT---G-T-----------AA----G-T-C-
A--------G-T-A--T--TA--A-AA-GA--C-G-GT-A-G-----CT-T-AA------C-T-A-T-C-G-G--G-C----T-G--G-T---A--T-------T--GA-T-A---C-------T--G-C--AG--G-G-C----T-T-G-A-G-T--A---G-AG-----AC-G-A--------G-G-T-A---GG-C------GG--A--ATT----G-A-T-A-AT--GT-A-
G-CG-GT--G---A--A-A---TG-C-AT-AG--AT-A-TT--A-T--C-A-G-G-A-AC-T-CC---GA--T--T--GC-GAA-G--G-C--A-------G--C-T-T-A---CTA--G--GC-T-C--------------------A-A---A-C-T--GA--CG-C-T-G--A-GG--C-A-CG-A--AA-G-T---G-CG--GG-G--AG-C-A-AA-------
-CA--GG Bacteria(100);Bacteroidetes(100);unclassified(100);unclassified(100);unclassified(100);unclassified(100);unclassified(100);unclassified(100);unclassified(100);

Otu1345 0 0 0 0 0 0 0 0 0 0 0 0 0 0 0 0 0 0 0 0 0 0 0 0 0 0 0 0
0 0 0 0 0 0 0 0 0 0 0 0 0 0 0 0 0 0 0 0 0 0 0 0 0 0 0 0
0 0 0 0 0 0 0 0 0 0 0 0 0 0 0 0 0 0 0 0 0 3 0 0 0 0 0 0
0 0 0 0 0 0 0 0 0 0 0 0 0 0 0 0 0 0 0 0 0 0 0 0 0 0 0
M02255_76_000000000-ADUNE_1_1105_27429_5980 C----CG---GC-TA-ACT--------CCG-T-G------C--C--A---G--CA-G-CCG-CG------GT-A--A-T--AC-GG-------AG--G-----GT-GC-AA-AC-------G-TT-GCT-C-G------------G--A-CC-A-T-C-----------AA-
---G-T-C-G--------G-A-T--G--TG--A-AA-TC--C-C-TC-G-G-----CT-T-AA------C-C-G-A-G-G-A--C-G----T-G--C-A---T--T-------C--GA-A-A---C-------T--G-C--TG--G-C-C----T-T-G-A-G-T--A---C-GA-----CA-G-A--------G-G-A-T---CG-C------GG--A--ATT----C-C-C-G-
GT--GT-A-G-AG-GT--G---A--A-A---TT-C-GT-AG--AT-A-TC--G-G--G-A-G-G-A-AC-A-CC---AG--T--G--GC-GAA-G--G-C--G-------G--C-G-A-T---CTG--G--GT-C-G--------------------A-T---A-C-T--GA--CG-C-T-G--A-GG--T-G-CG-A--AA-G-C---G-TG--GG-G--AG-
C-A-AA--------CA--GG Bacteria(100);Proteobacteria(100);Deltaproteobacteria(100);unclassified(100);unclassified(100);unclassified(100);unclassified(100);unclassified(100);unclassified(100);

Otu1346 0 0 0 0 0 0 0 0 0 0 0 0 0 0 0 0 0 0 0 0 0 0 0 0 0 0 0 0
0 0 0 0 0 0 0 0 0 0 0 0 0 0 0 0 0 0 0 0 0 0 0 0 0 0 0 0
0 0 0 0 0 0 0 0 0 0 0 0 0 0 0 0 0 0 0 0 0 0 0 0 0 0 0 0
0 0 0 0 0 0 0 0 0 0 0 0 0 0 0 12 0 0 0 0 0 0 0 0 0 0 0
M02255_77_000000000-ADUT3_1_1108_7045_17060 G----AC---AG-AG-GTC--------CCG-A-G------C--G--T---T--AG-G-CGG-AA------TC-A--C-T--GG-GC-------TT--A-----AA-GC-GT-GT-------G-TA-G-G-C-G------------G--A-TG-C-C-T----------
AA----G-T-A-C--------C-T-T--G--TG--A-AA-TC--C-C-AC-G-G-----CT-C-AC------C-C-G-T-G-G-A--A-C----T-G--C-T---T--G-------G--TA-T-A---C-------T--G-G--GT--G-T-C----T-T-G-A-G-C--C---A-CT-----TA-G-G--------G-G-C-T---AC-C------GG--A--ACA----A-A-T-
G-GT--GG-A-G-CG-GT--G---A--A-A---TG-C-GT-AG--AT-A-TC--A-T--T-T-G-G-A-AC-G-CC---AA--T--G--GT-GAA-A--A-C--A-------G--G-T-A-G---CTG--G--GA-G-T--------------------G-T---G-C-T--GA--CG-C-T-G--A-GA--C-A-CG-A--AA-G-C---C-AG--GG-G--
AG-C-A-AA--------CG--GG Bacteria(100);Planctomycetes(100);Phycisphaerae(100);WD2101(100);unclassified(100);unclassified(100);unclassified(100);unclassified(100);unclassified(100);

Otu1347 0 0 0 0 0 0 0 0 0 0 0 0 0 0 0 0 0 0 0 0 0 0 0 0 0 0 0 0
0 0 0 0 0 0 0 0 0 0 0 0 0 0 0 0 0 0 0 0 0 0 0 0 0 0 0 0
0 0 0 0 0 0 0 0 0 0 0 0 0 0 0 0 0 0 0 0 0 0 0 0 0 0 0 0
0 0 0 0 0 0 0 0 0 0 0 0 0 0 0 22 0 0 0 0 0 0 0 0 0 0 0
M02255_77_000000000-ADUT3_1_1106_15327_19254 T----AC---GA-AG-GGG--------GCT-A-G------C--G--T---T--GC-T-CGG-AA------TG-A--C-T--GG-GC-------GT--A-----AA-GG-GC-GC-------G-TA-G-G-C-G------------G--C-TC-A-T-A----
-------CA----G-T-C-A--------G-G-C--G--TG--A-AA-TT--C-C-TG-G-G-----CT-C-AA------C-C-T-G-G-G-G--A-C----T-G--C-G---C--T-------T--GA-G-A---C-------G--T-G--TG--G-G-C----T-T-G-A-G-T--G---G-GG-----AA-G-A--------G-G-G-T---CG-T------GG--A--ATT----
C-C-C-A-GT--GT-A-G-AG-GT--G---A--A-A---TT-C-GT-AG--AT-A-TT--G-G--G-A-A-G-A-AC-A-CC---GG--T--G--GC-GAA-G--G-C--G-------G--C-G-A-C---CTG--G--TC-C-T--------------------T-G---A-C-T--GA--CG-C-T-G--A-GG--C-G-CG-A--AA-G-C---G-TG--
GG-G--AG-C-A-AA--------CA--GG Bacteria(100);Proteobacteria(100);Alphaproteobacteria(100);Rhodospirillales(100);Acetobacteraceae(100);unclassified(100);unclassified(100);unclassified(100);unclassified(100);

Otu1348 0 0 0 0 0 0 0 0 0 0 0 0 0 0 0 0 0 0 0 0 0 0 0 0 0 0 0 0
0 0 0 0 0 0 0 0 0 0 0 0 0 0 0 0 0 0 0 0 0 0 0 0 0 0 0 0
0 0 0 0 0 0 0 0 0 0 0 0 0 0 0 0 0 0 0 0 0 0 0 0 0 0 0 0
0 0 0 0 0 0 0 0 0 0 0 0 0 0 0 8 0 6 23 8 0 0 0 0 0 0 0
M02255_76_000000000-ADUNE_1_2112_19346_21029 G----AC---GT-AG-GAT--------GCA-A-G------C--G--T---T--GT-C-CGG-AT------TT-A--T-T--GG-GC-------GT--A-----AA-G--G----------G-T------T-C------------G-----T---T------------AA-
---G-T-C-T--------G-A-T--G--TT--A-AA-GA--C-T-GG-G-G-----CT-C-AA------C-C-C-T-G-G-G--A-G----T-G--C-A---T--T-------G--GA-T-A---C-------T--G-G--CG--G-A-C----T-G-G-A-G-T--G---C-AG-----TA-G-A--------G-G-C-A---AG-C------GG--A--ATT----C-C-A-G-
GT--GT-A-G-CG-GT--G---A--A-A---TG-C-GT-AG--AT-A-TC--T-G--G-A-A-G-A-AC-A-CC---GG--T--G--GC-GCA-A--G-C--G-------G--C-T-T-G---CTG--G--GC-T-G--------------------T-A---A-C-T--GA--CG-C-T-G--A-GG--A-A-CG-A--AA-G-C---T-AG--GG-T--AG-C-
G-AA--------TG--GG Bacteria(100);unclassified(100);unclassified(100);unclassified(100);unclassified(100);unclassified(100);unclassified(100);unclassified(100);unclassified(100);

Otu1349 0 0 0 0 0 0 0 0 0 0 0 0 0 0 0 0 0 0 0 0 0 0 0 0 0 0 0 0
0 0 0 0 0 0 0 0 0 0 0 0 0 0 0 0 0 0 0 0 0 0 0 0 0 0 0 0
0 0 0 0 0 0 0 0 0 0 0 0 0 0 0 0 0 0 0 0 0 0 0 0 0 0 0 0
0 0 0 0 0 0 0 0 0 0 0 0 0 0 0 20 0 0 0 0 0 0 0 0 0 0 0
M02255_76_000000000-ADUNE_1_1101_15042_22107 .....AC----T-AC-GT---------GCC-A-G------C--A--G---C--CG-C-GGT-AA------TA-C--G-T--AG-GT-------GG--C-----AA----GC-G--------T-TA-T-C-C-G------------G--A-AT-G-A-T----------
AA----G-T-C-T--------G-A-T--G--TG--A-AA-TC--C-C-CC-G-G-----CT-C-AA------C-C-G-G-G-G-A--G------G-GT-C-A---T--T-------G--GA-A-A---C-------T--G-T--CA--T-G-C----T-T-G-A-G-T--G---C-AG-----AA-G-A--------G-G-A-G---AG-T------GG--A--ATT----C-C-A-
T-GT--GT-A-G-CG-GT--G---A--A-A---TG-C-GC-AG--AG-A-TA--T-G--G-A-G-G-A-AC-A-CC---AG--T--G--GC-GAA-G--G-C--G-------G--C-T-C-T---CTG--G--TC-T-G--------------------T-A---A-C-T--GA--CG-C-T-G--A-TG--T-G-CG-A--AA-G-C---G-TG--GG-G--AT-
C-A-AA--------CA--GG Bacteria(100);Firmicutes(100);Bacilli(100);Bacillales(100);Staphylococcaceae(100);unclassified(100);unclassified(100);unclassified(100);unclassified(100);

Otu1350 0 0 0 0 0 0 0 0 0 0 0 0 0 0 0 0 0 0 0 0 0 0 0 0 0 0 0 0
0 0 0 0 0 0 0 0 0 0 0 0 0 0 0 0 0 0 0 0 0 0 0 0 0 0 0 0 0
0 0 0 0 0 0 0 0 0 0 0 0 0 0 0 0 0 0 0 0 0 0 0 0 0 0 0 0 0
0 0 0 0 0 0 0 0 0 0 0 0 0 0 0 11 0 0 0 0 0 0 0 0 0 0 0
M02255_77_000000000-ADUT3_1_2118_14114_21404 C----GT---GC-CA-GCA--------GCC-G-C------G--G--T---A--AT-A-CGG-AG------GG-T--G-C--GA-GC-------GT--T-----GT-CC-GG-AT-------T-TA-G-G-T-G------------G--G-TT-G-A-T-----
------AA----G-T-C-A--------G-T-G--G--TG--A-AA-TA--C-A-GC-C-G-----CT-C-AA------C-G-G-T-T-G-A--G-G----T-G--C-C---A--T-------T--GA-T-A---C-------T--G-T--CA--A-G-C----T-T-G-A-A-A--G---A-AT-----TG-G-A--------G-G-C-T---GC-C------GG--A--ATG----G-
A-T-G-GT--GT-A-G-CG-GT--G---A--A-A---TG-C-AT-AG--AT-A-TC--A-T--C-C-A-G-A-AC-A-CC---GA--T--T--GC-GAA-G--G-C--A-------G--G-T-G-G---CTA--C--GA-T-T--------------------G-A---T-T-T--GA--CA-C-T-G--A-GG--C-A-CG-A--AA-G-C---A-TG--GG-G--
AG-C-A-AA--------CA--GG Bacteria(100);Bacteroidetes(100);Sphingobacteria(100);Sphingobacteriales(100);Cytophagaceae(100);Dyadobacter(100);unclassified(100);unclassified(100);unclassified(100);

Otu1351 0 0 0 0 0 0 0 0 0 0 0 0 0 0 0 0 0 0 0 0 0 0 0 0 0 0 0 0
0 0 0 0 0 0 0 0 0 0 0 0 0 0 0 0 0 0 0 0 0 0 0 0 0 0 0 0 0
0 0 0 0 0 0 0 0 0 0 0 0 0 0 0 0 0 0 0 0 0 0 0 0 0 0 0 0 0
0 0 0 0 0 0 0 0 0 0 0 0 0 0 0 11 0 0 0 0 0 0 0 0 0 0 0
M02255_77_000000000-ADUT3_1_2112_24531_16378 T----AC---GA-AG-GGG--------GCT-A-G------C--G--T---T--GC-T-CGG-AA------TT-A--C-T--GG-GC-------GT--A-----AA-GG-GC-GC-------G-TA-G-G-C-G------------G--C-CT-G-C-T----
-------TA----G-T-C-A--------G-A-C--G--TG--A-AA-TT--C-C-CG-G-G-----CT-T-AA------C-C-T-G-G-G-G--G-C----T-G--C-G---T--T-------T--GA-G-A---C-------G--G-G--CG--G-G-C----T-T-G-A-G-G--A---T-GG-----AA-G-A--------G-G-C-T---CG-T------GG--A--ATT----
C-C-C-A-GT--GT-A-G-AG-GT--G---A--A-A---TT-C-GT-AG--AT-A-TT--G-G--G-A-A-G-A-AC-A-CC---GG--T--G--GC-GAA-G--G-C--G-------G--C-G-A-G---CTG--G--TC-C-A--------------------T-T---A-C-T--GA--CG-C-T-G--A-GG--C-G-CG-A--CA-G-C---G-TG--
GG-G--AG-C-A-AA--------CA--GG Bacteria(100);Proteobacteria(100);Alphaproteobacteria(100);Rhodospirillales(100);Acetobacteraceae(100);unclassified(100);unclassified(100);unclassified(100);unclassified(100);

Otu1352 0 0 0 0 0 0 0 0 0 0 0 0 0 0 0 0 0 0 0 0 0 0 0 0 0 0 0 0
0 0 0 0 0 0 0 0 0 0 0 0 0 0 0 0 0 0 0 0 0 0 0 0 0 0 0 0 0
0 0 0 0 0 0 0 0 0 0 0 0 0 0 0 0 0 0 0 0 0 0 0 0 0 0 0 0 0
0 0 0 0 0 0 0 0 0 0 0 0 0 0 0 26 0 0 0 0 0 0 0 0 0 0 0
M02255_77_000000000-ADUT3_1_2107_24298_17196 T----AC---GT-AG-GGG--------GCG-A-G------C--G--T---T--GT-C-CGG-AA------TT-A--T-T--GG-GC-------GT--A-----AA-GG-GC-GC-------G-TA-G-A-C-G------------G--C-CG-A-G-T----
-------AA----G-T-T-A--------C-T-G--G--TG--A-AA-GC--C-C-AT-C-T-----TT-T-AA------G-G-A-T-G-G-A--A-C----T-G--C-C---T--G-------T--AA-T-A---C-------T--G-C--TT--G-G-C----T-T-G-A-G-T--G---C-AG-----GA-G-A--------G-G-G-A---AG-C------GG--A--ATT----C-
C-T-A-GT--GT-A-G-CG-GT--G---A--A-A---TG-C-GT-AG--AT-A-TT--A-G--G-A-G-G-A-AC-A-CC---AG--T--G--GC-GAA-G--G-C--G-------G--C-T-T-T---CTG--G--AC-T-G--------------------T-A---A-C-T--GA--CG-T-T-G--A-GG--C-G-CG-A--AA-G-C---G-TG--GG-G-
-AG-C-A-AA--------CA--GG Bacteria(100);unclassified(100);unclassified(100);unclassified(100);unclassified(100);unclassified(100);unclassified(100);unclassified(100);unclassified(100);

Otu1353 0 0 0 0 0 0 0 0 0 0 0 0 0 0 0 0 0 0 0 0 0 0 0 0 0 0 0 0
0 0 0 0 0 0 0 0 0 0 0 0 0 0 0 0 0 0 0 0 0 0 0 0 0 0 0 0 0
0 0 0 0 0 0 0 0 0 0 0 0 0 0 0 0 0 0 0 0 0 0 0 0 0 0 0 0 0
0 0 0 0 0 0 0 0 0 0 0 0 0 0 0 14 0 0 0 0 0 0 0 0 0 0 0
M02255_77_000000000-ADUT3_1_2106_6146_14735 T----AC---GA-AG-GGG--------GCT-A-G------C--G--T---T--GC-T-CGG-AA------TT-A--C-T--GG-GC-------GT--A-----AA-GG-GC-GC-------G-TA-G-G-C-G------------G--C-CT-G-C-T-----------
TA----G-T-C-T--------G-G-C--G--TG--A-AA-TG--C-C-TG-G-G-----CT-C-AA------C-C-T-G-G-G-G--A-C----T-G--C-G---C--T-------G--GA-T-A---C-------G--G-G--TG--G-G-C----T-T-G-A-G-G--G---C-AG-----GA-G-A--------G-G-C-T---CG-C------GG--A--ATT----C-C-
C-A-GT--GT-A-G-AG-GT--G---A--A-A---TT-C-GT-AG--AT-A-TT--G-G--G-A-A-G-A-AC-A-CC---GG--T--G--GC-GAA-G--G-C--G-------G--C-G-A-G---CTG--G--CC-T-G--------------------T-G---A-C-T--GA--CG-C-T-G--A-GG--C-G-CG-A--CA-G-C---G-TG--GG-G--
AG-C-A-AA--------CA--GG Bacteria(100);Proteobacteria(100);Alphaproteobacteria(100);Rhodospirillales(100);Acetobacteraceae(100);unclassified(100);unclassified(100);unclassified(100);unclassified(100);

Otu1354 0 0 0 0 0 0 0 0 0 0 0 0 0 0 0 0 0 0 0 0 0 0 0 0 0 0 0 0
0 0 0 0 0 0 0 0 0 0 0 0 0 0 0 0 0 0 0 0 0 0 0 0 0 0 0 0 0
0 0 0 0 0 0 0 0 0 0 0 0 0 0 0 0 0 0 0 0 0 0 0 0 0 0 0 0 0
0 0 0 0 0 0 0 0 0 0 0 0 0 0 0 14 0 0 0 0 0 0 0 0 0 0 0
M02255_77_000000000-ADUT3_1_1118_12659_14848 T----AC---AG-AG-GTG--------CCG-A-G------C--G--T---T--AG-G-CGG-AA------TC-A--C-T--GG-GC-------TT--A-----AA-GC-GC-GT-------G-TA-G-G-C-G------------G--G-TC-G-T-T-----
------AA----G-T-GTC--------C-G-C--G--TG--A-AA-TC--C-C-AC-G-G-----CT-C-AA------C-C-G-T-G-G-A--A-C----G-G--C-C---G--G-------G--CA-T-A---C-------T--G-G--CG--A-C-C----T-T-G-A-G-G--C---A-TT-----CA-G-G--------G-G-C-G---AG-C------GG--A--ACA----
A-A-C-G-GT--GG-A-G-CG-GT--G---A--A-A---TG-C-GT-AG--AT-A-TC--G-T--T-T-G-G-A-AC-G-CC---GA--C--G--GT-GAA-A--A-C--A-------G--C-T-C-G---CTG--G--GA-A-T--------------------G-T---C-C-T--GA--CG-C-T-G--A-GA--C-G-CG-A--AA-G-C---C-AG--
GG-G--AG-C-G-AA--------CG--GG Bacteria(100);unclassified(100);unclassified(100);unclassified(100);unclassified(100);unclassified(100);unclassified(100);unclassified(100);unclassified(100);

Otu1355 0 0 0 0 0 0 0 0 0 0 0 0 0 0 0 0 0 0 0 0 0 0 0 0 0 0 0 0
0 0 0 0 0 0 0 0 0 0 0 0 0 0 0 0 0 0 0 0 0 0 0 0 0 0 0 0 0
0 0 0 0 0 0 0 0 0 0 0 0 0 0 0 0 0 0 0 0 0 0 0 0 0 0 0 0 0
0 0 0 0 0 0 0 0 0 0 0 0 0 0 0 29 0 0 0 0 0 0 0 0 0 0 0
M02255_77_000000000-ADUT3_1_1113_21868_14502 T----AC---AG-AG-GTT--------ACA-A-G------C--A--T---T--GT-C-CGG-AA------TC-A--C-T--GG-GC-------GT--A-----AA-GC-GTTTC-------G-CA-G-G-T-G------------T--T-TT-T-C-T-----------AA----G-T-T-A--------T-G-T--T--TG--A-AA-GA--C-C-GG-G-G-----CT-C-AA------C-C-T-C-G-G-G--A-A----C-G--G-G---C--G-------T--AA-T-A---C-------T--G-G--AA--A-A-A----T-T-G-A-A-G--G---G-TA-----TC-G-G--------G-G-C-T---AT-T------GG--A--ACT----G-T-C-G-GT--GT-A-G-GG-GT--G---A--A-A---TC-C-GT-TG--AT-A-TC--G-A--C-A-G-G-A-AC-T-CC---GA--G--G--GC-GTA-G--G-C--A-------G--A-T-A-G---CTA--G--GT-A-C--------------------C-T---C-T-T--GA--CA-C-T-C--A-TG--A-A-CG-A--AA-G-C---T-AG--GG-G--AG-C-G-AA--------AG--GG Bacteria(100);unclassified(100);unclassified(100);unclassified(100);unclassified(100);unclassified(100);unclassified(100);unclassified(100);unclassified(100);

Otu1356 0 0 0 0 0 0 0 0 0 0 0 0 0 0 0 0 0 0 0 0 0 0 0 0 0 0 0 0
0 0 0 0 0 0 0 0 0 0 0 0 0 0 0 0 0 0 0 0 0 0 0 0 0 0 0 0 0
0 0 0 0 0 0 0 0 0 0 0 0 0 0 0 0 0 0 0 0 0 0 0 0 0 0 0 0 0
0 0 0 0 0 0 0 0 0 0 0 0 0 0 0 16 0 0 0 0 0 0 0 0 0 0 0
M02255_77_000000000-ADUT3_1_1110_19076_21094 T----AC---GG-AG-GGG--------GCT-A-G------C--G--T---T--GT-T-CGG-AA------TC-A--C-T--GG-GC-------GT--A-----AA-GC-GC-GC-------G-TA-G-G-C-G------------G--C-TT-G-C-C-----------AA----G-T-C-A--------G-G-G--G--TG--A-AA-GC--C-C-GC-G-G-----CT-C-AA------C-C-G-C-G-G-A--A-C----T-G--C-C---C--T-------T--GA-G-A---C-------T--G-G--CT--C-G-C----T-C-G-A-A-C--A---C-GG-----GA-G-A--------G-G-C-G---GG-T------GG--A--ATT----C-C-G-A-GT--GT-A-G-AG-GT--G---A--A-A---TT-C-GT-AG--AT-A-TT--C-G--G-A-A-G-A-AC-A-CC---GG--T--G--GC-GAA-G--G-C--G-------G--C-C-C-G---CTG--G--AC-C-G--------------------T-T---G-T-T--GA--CG-C-T-G--A-GG--C-G-CG-A--AA-G-C---G-TG--GG-G--AG-C-A-AA--------CA--GG Bacteria(100);unclassified(100);unclassified(100);unclassified(100);unclassified(100);unclassified(100);unclassified(100);unclassified(100);unclassified(100);

Otu1357 0 0 0 0 0 0 0 0 0 0 0 0 0 0 0 0 0 0 0 0 0 0 0 0 0 0 0 0
0 0 0 0 0 0 0 0 0 0 0 0 0 0 0 0 0 0 0 0 0 0 0 0 0 0 0 0 0
0 0 0 0 0 0 0 0 0 0 0 0 0 0 0 0 0 0 0 0 0 0 0 0 0 0 0 0 0
0 0 0 0 0 0 0 0 0 0 0 0 0 0 0 40 0 0 0 0 0 0 0 0 0 0 0
M02255_77_000000000-ADUT3_1_1109_12832_18343 G----AC---GG-AG-GGT--------GCT-A-G------C--G--T---T--GT-T-CGG-AA------TC-A--C-T--GG-GC-------GT--A-----AA-GG-GC-GC-------G-TA-G-G-C-G------------G--C-CT-G-T-T-----------AA----G-T-C-G--------G-A-T--G--TG--A-AA-GC--C-C-GG-G-G-----CT-C-AA------C-C-C-C-G-G-A--A-C----T-G--C-A---T--C-------C--GA-G-A---C-------T--G-G--CA--G-G-C----T-T-G-A-G-A--C---C-GG-----GA-G-A--------G-G-A-G---GG-T------GG--A--ATA----T-C-C-A-GT--GT-A-G-AG-GT--G---A--A-A---TT-C-GT-AG--AT-A-TT--G-G--A-T-G-G-A-AC-A-CC---GG--T--G--GC-GAA-A--G-C--G-------G--C-T-C-T---CTG--G--CC-C-G--------------------G-A---T-C-T--GA--CG-C-T-G--A-GG--C-G-CG-A--AA-G-C---G-T-G-GG-G--AG-C-A-AA--------CA--GG Bacteria(100);Proteobacteria(100);Alphaproteobacteria(100);Rhodospirillales(100);unclassified(100);unclassified(100);unclassified(100);unclassified(100);unclassified(100);

Otu1358 0 0 0 0 0 0 0 0 0 0 0 0 0 0 0 0 0 0 0 0 0 0 0 0 0 0 0 0
0 0 0 0 0 0 0 0 0 0 0 0 0 0 0 0 0 0 0 0 0 0 0 0 0 0 0 0 0
0 0 0 0 0 0 0 0 0 0 0 0 0 0 0 0 0 0 0 0 0 0 0 0 0 0 0 0 0
0 0 0 0 0 0 0 0 0 0 0 0 0 0 0 15 0 0 0 0 0 0 0 0 0 0 0
M02255_77_000000000-ADUT3_1_1104_7008_21476 T----AC---GT-AG-GGG--------GCG-A-G------C--G--T---T--GT-C-CGG-AA------TG-A--T-T--GG-GC-------GT--A-----AA-GG-GC-GC-------G-TA-G-G-C-G------------G--C-CA-T-T-T-----------AA----G-T-C-T--------G-G-A--G--TG--A-AA-GT--C-C-TG-T-T-----TT-T-AA------G-A-T-G-G-G-A--A-C----T-G--C-T---T--T-------G--GA-T-A---C-------T--G-G--AT--G-G-C----T-T-G-A-G-T--G---C-AG-----GA-G-A--------G-G-T-T---AT-C------GG--A--ATT----C-C-C-G-GT--GT-A-G-CG-GT--G---A--A-A---TG-C-GT-AG--AG-A-TC--G-G--G-A-G-G-A-AC-A-CC---AG--T--G--GC-GAA-G--G-C--G-------G--G-T-A-A---CTG--G--AC-T-G--------------------C-A---A-C-T--GA--CG-C-T-G--A-GG--C-G-CG-A--AA-G-T---G-TG--GG-G--AG-C-A-AA--------CA--GG Bacteria(100);Firmicutes(100);unclassified(100);unclassified(100);unclassified(100);unclassified(100);unclassified(100);unclassified(100);unclassified(100);

Otu1359 0 0 0 0 0 0 0 0 0 0 0 0 0 0 0 0 0 0 0 0 0 0 0 0 0 0 0 0
0 0 0 0 0 0 0 0 0 0 0 0 0 0 0 0 0 0 0 0 0 0 0 0 0 0 0 0 0
0 0 0 0 0 0 0 0 0 0 0 0 0 0 0 0 0 0 0 0 0 0 0 0 0 0 0 0 0
0 0 0 0 0 0 0 0 0 0 0 0 0 0 0 35 0 0 0 0 0 0 0 0 0 0 0
M02255_77_000000000-ADUT3_1_1101_7909_21025 T----AC---GA-AG-GGG--------GCT-A-G------C--G--T---T--GC-T-CGG-AT------TT-A--C-T--GG-GC-------GT--A-----AA-GG-GA-GC-------G-TA-G-G-C-G------------G--G-AC-G-A-C-----------AA----G-T-T-G--------G-G-G--G--TG--A-AA-GC--C-C-GG-G-G-----CT-C-AA------C-C-T-C-G-G-A--A-T----T-G--C-C---T--T-------C--AA-G-A---C-------T--G-T--CG--A-C-C----T-G-G-A-G-T--T---T-GG-----GA-G-A--------G-G-T-G---AG-T------GG--A--ACT----C-C-G-A-GT--GT-A-G-AG-GT--G---A--A-A---TT-C-GT-AG--AT-A-TT--C-G--G-A-A-G-A-AC-A-CC---AG--T--G--GC-GAA-G--G-C--G-------A--C-T-C-A---CTG--G--CC-C-A--------------------A-T---A-C-T--GA--CG-C-T-G--A-GG--C-T-CG-A--AA-G-C---G-TG--GG-G--AG-C-A-AA--------CA--GG Bacteria(100);Proteobacteria(100);Alphaproteobacteria(100);unclassified(100);unclassified(100);unclassified(100);unclassified(100);unclassified(100);unclassified(100);

Otu1360 0 0 0 0 0 0 0 0 0 0 0 0 0 0 0 0 0 0 0 0 0 0 0 0 0 0 0 0
0 0 0 0 0 0 0 0 0 0 0 0 0 0 0 0 0 0 0 0 0 0 0 0 0 0 0 0
0 0 0 0 0 0 0 0 0 0 0 0 0 0 0 0 0 0 0 0 0 0 0 0 0 0 0 0
0 0 0 0 0 0 0 0 0 0 0 0 0 0 0 53 0 34 160 58 0 0 0 0 0 0 0
M02255_77_000000000-ADUT3_1_1101_7903_17884 G----AC---GT-AG-GAT--------GCA-A-G------C--G--T---T--GT-C-CGG-AT------TT-A--T-T--GG-GC-------GT--A-----AA-GA-GT-TC-------G-TA-G-G-C-G------------G--T-TC-G-T-T----------AA--
--G-T-C-T--------G-A-T--G--TT--A-AA-GA--C-T-GG-G-G-----CT-C-AA------C-C-C-T-G-G-G--A-G----T-G--C-A---T--T-------G--GA-T-A---C-------T--G-G--CG--G-A-C----T-G-G-A-G-T--G---C-AG-----TA-G-A--------G-G-C-A---AG-C------GG--A--ATT----C-C-A-G-
GT--GT-A-G-CG-GT--G---A--A-A---TG-C-GT-AG--AT-A-TC--T-G--G-A-A-G-A-AC-A-CC---GG--T--G--GC-GCA-A--G-C--G-------G--C-T-T-G---CTG--G--GC-T-G--------------------T-A---A-C-T--GA--CG-C-T-G--A-GG--A-A-CG-A--AA-G-C---T-AG--GG-T--AG-C-
G-AA--------TG--GG Bacteria(100);unclassified(100);unclassified(100);unclassified(100);unclassified(100);unclassified(100);unclassified(100);unclassified(100);unclassified(100);

Otu1361 0 0 0 0 0 0 0 0 0 0 0 0 0 0 0 0 0 0 0 0 0 0 0 0 0 0 0 0
0 0 0 0 0 0 0 0 0 0 0 0 0 0 0 0 0 0 0 0 0 0 0 8 0 0 0 0
0 0 0 0 0 0 0 0 0 0 0 0 0 0 0 0 0 0 0 0 0 0 0 0 0 0 0 0
0 0 0 0 0 0 0 0 0 0 0 0 0 0 0 0 0 0 0 0 0 0 0 0 0 0 0
M02255_77_000000000-ADUT3_1_2107_22321_19415 T----AC---GT-AT-GGG--------GCG-A-G------C--G--T---T--GT-C-CGG-AA------TC-A--T-T--GG-GC-------GT--A-----AA-GG-GT-TC-------G-TA-G-G-C-G------------G--T-TT-A-T-T------
-----AA----G-T-C-G--------A-G-T--G--TG--A-AA-GG--T-T-GG-G-G-----CT-C-AA------C-C-C-C-A-T-A--A------A-G--C-A---C--T-------C--GA-A-A---C-------T--G-A--TA--A-A-C----T-T-G-A-G-A--T---G-TG-----GA-G-A--------G-G-A-A---AG-T------GG--A--ATT----C-
C-T-A-GT--GT-A-G-CG-GT--G---A--C-A---TG-C-GT-AG--AT-A-TT--A-G--G-A-G-G-A-AT-A-CC---AG--T--A--GC-GAA-G--G-C--G-------A--C-T-T-T---CTG--G--AC-A-C--------------------A-A---A-C-T--GA--CG-C-T-A--A-GG--A-A-CG-A--AA-G-C---G-TG--GG-G-
-AG-C-A-AA--------CA--GG Bacteria(100);Firmicutes(100);Clostridia(100);Clostridiales(100);unclassified(100);unclassified(100);unclassified(100);unclassified(100);unclassified(100);

Otu1362 0 0 0 0 0 0 0 0 0 0 0 0 0 0 0 0 0 0 0 0 0 0 0 0 0 0 0 0
0 0 0 0 0 0 0 0 0 0 0 0 0 0 0 0 0 0 0 0 0 0 0 0 0 0 0 0
0 0 0 0 0 0 0 0 0 0 0 0 0 0 0 0 0 0 0 0 0 0 0 14 0 0 0 0
0 0 0 1 0 0 0 0 0 0 0 0 0 0 0 0 0 0 0 0 0 0 0 0 0 0 0
M02255_77_000000000-ADUT3_1_1105_5313_18244 T----AC---GT-AG-GGT--------GCA-A-G------C--G--T---T--GT-C-CGG-AA------TT-A--T-T--GG-GC-------GT--A-----AA-GA-GC-TC-------G-TA-G-G-C-G------------G--T-CT-G-T-C----------GC-
---G-T-C-T--------G-C-T--G--TG--A-AA-AC--T-T-CG-G-G-----CT-C-AA------C-C-C-G-A-A-G--C-T----G-G--C-A---G--T-------G--GG-T-A---C-------G--G-G--CG--G-A-C----T-G-G-A-G-T--G---C-AG-----TA-G-G--------G-G-A-G---AC-T------GG--A--ATT----C-C-T-G-
GT--GT-A-G-CG-GT--G---A--A-A---TG-C-GC-AG--AT-A-TC--A-G--G-A-G-G-A-AC-A-CC---GG--T--G--GC-GAA-G--G-C--G-------G--G-T-C-T---CTG--G--GC-T-G--------------------T-T---A-C-T--GA--CG-C-T-G--A-GG--A-G-CG-A--AA-G-C---G-TG--GG-G--AG-
C-G-AA--------CA--GG Bacteria(100);Actinobacteria(100);Actinobacteria(100);Actinobacteridae(100);Actinomycetales(100);unclassified(100);unclassified(100);unclassified(100);unclassified(100);

Otu1363 0 0 0 0 0 0 0 0 0 0 0 0 0 0 0 0 0 0 0 0 0 0 0 0 0 0 0 0
0 0 0 0 0 0 0 0 0 0 0 0 0 0 0 0 0 0 0 0 0 0 0 7 0 0 0 0
0 0 0 0 0 0 0 0 0 0 0 0 0 0 0 0 0 0 0 0 0 0 0 0 0 0 0 0
0 0 0 0 0 0 0 0 0 0 0 0 0 0 0 0 0 0 0 0 0 0 0 0 0 0 0
M02255_77_000000000-ADUT3_1_1111_23055_9937 T----AC---GT-AG-GGG--------GCA-A-G------C--G--T---T--AT-C-CGG-AT------TT-A--C-T--GG-GT-------GT--A-----AA-GG-GA-GC-------G-TA-G-A-C-G------------G--C-GA-G-A-C----------
AA----G-T-C-T--------G-A-A--G--TG--A-AA-GC--C-C-GG-G-G-----CT-C-AA------C-C-C-C-GCG-G--A-C----T-G--C-T---T--T-------G--GA-A-A---C-------T--G-C--CT--T-G-C----T-G-G-A-G-T--G---T-CG-----GA-G-A--------G-G-T-A---AG-T------GG--A--ATT----C-C-
T-A-GT--GT-A-G-CG-GT--G---A--A-A---TG-C-GT-AG--AT-A-TT--A-G--G-A-G-G-A-AC-A-CC---AG--T--G--GC-GAA-G--G-C--G-------G--C-T-T-A---CTG--G--AC-G-A--------------------T-A---A-C-T--GA--CG-T-T-G--A-GG--C-T-CG-A--AA-G-C---G-TG--GG-G--
AG-C-A-AA--------CA--GG Bacteria(100);Firmicutes(100);Clostridia(100);Clostridiales(100);Lachnospiraceae(100);unclassified(100);unclassified(100);unclassified(100);unclassified(100);

Otu1364 0 0 0 0 0 0 0 0 0 0 0 0 0 0 0 0 0 0 0 0 0 0 0 0 0 0 0 0
0 0 0 0 0 0 0 0 0 0 0 0 0 0 0 0 0 0 0 0 0 0 0 5 0 0 0 0
0 0 0 0 0 0 0 0 0 0 0 0 0 0 0 0 0 7 0 0 0 0 0 0 0 0 0 0
0 0 0 0 0 0 0 0 0 0 0 0 0 0 0 0 0 0 0 0 0 0 0 0 0 0 0
M02255_77_000000000-ADUT3_1_1108_4384_9296 T----AC---AG-AG-GTG--------CCG-A-G------C--G--T---T--AG-G-CGG-AA------TC-A--C-T--GG-GC-------TT--A-----AA-GC-GC-GT-------G-TA-G-G-C-G------------G--G-TC-G-T-T----------
AA----G-TGT-C--------C-G-C--G--TG--A-AA-TC--C-C-AC-G-G-----CT-C-AA------C-C-G-T-G-G-A--A-C----T-G--C-C---G--G-------G--CA-T-A---C-------T--G-G--CG--A-C-C----T-T-G-A-G-G--C---A-CT-----TA-G-G--------G-G-C-G---AG-C------GG--A--ACA----A-A-
T-G-GT--GG-A-G-CG-GT--G---A--A-A---TG-C-GT-AG--AT-A-TC--A-T--T-T-G-G-A-AC-G-CC---GA--C--G--GT-GAA-A--A-C--A-------G--C-T-C-G---CTG--G--GA-G-T--------------------G-T---C-C-T--GA--CG-C-T-G--A-GA--C-G-CG-A--AA-G-C---C-AG--GG-G--
AG-C-G-AA--------CG--GG Bacteria(100);Planctomycetes(100);Phycisphaerae(100);WD2101(100);unclassified(100);unclassified(100);unclassified(100);unclassified(100);unclassified(100);

Otu1365 0 0 0 0 0 0 0 0 0 0 0 0 0 0 0 0 0 0 0 0 0 0 0 0 0 0 0 0
0 0 0 0 0 0 0 0 0 0 0 0 0 0 0 0 0 0 0 0 0 0 0 0 8 0 0 0 0
0 0 0 0 0 0 0 0 0 0 0 0 0 0 0 0 0 0 0 0 0 0 0 0 0 0 0 0 0
0 0 0 0 0 0 0 0 0 0 0 0 0 0 0 0 0 0 0 0 0 0 0 0 0 0 0

M02255_77_000000000-ADUT3_1_1112_28223_11342 T----AC---GT-AG-GTG--------GCA-A-G------C--G--T---T--GT-C-CGG-AT------TT-A--C-T--GG-GT-------GT--A-----AA-GG-GC-GT-------G-TA-G-G-C-G------------G--A-GA-T-G-C-----
------AA----G-T-C-A--------G-G-A--G--TG--A-AA-TC--T-A-GG-G-G-----CT-C-AA------C-C-C-C-T-A-A--A-C----T-G--C-T---T--T-------T--GA-A-A---C-------T--G-T--AT--C-C-C----T-T-G-A-G-T--A---T-CG-----GA-G-A--------G-G-C-A---GG-C------GG--A--ATT----C-
C-T-A-GT--GT-A-G-CG-GT--G---A--A-A---TG-C-GT-AG--AT-A-TT--A-G--G-A-G-G-A-AC-A-CC---AG--T--G--GC-GAA-G--G-C--G-------G--C-C-T-G---CTG--G--AC-G-A--------------------C-A---A-C-T--GA--CG-C-T-G--A-GG--C-G-CG-A--AA-G-C---G-TG--GG-
G--AG-C-A-AA--------CA--GG Bacteria(100);Firmicutes(100);Clostridia(100);Clostridiales(100);Ruminococcaceae(100);unclassified(100);unclassified(100);unclassified(100);unclassified(100);

Otu1366 0 0 0 0 0 0 0 0 0 0 0 0 0 0 0 0 0 0 0 0 0 0 0 0 0 0 0 0
0 0 0 0 0 0 0 0 0 0 0 0 0 0 0 0 0 0 0 0 0 0 0 0 11 0 0 0 0
0 0 0 0 0 0 0 0 0 0 0 0 0 0 0 0 0 0 0 0 0 0 0 0 0 0 0 0 0
0 0 0 0 0 0 0 0 0 0 0 0 0 0 0 0 0 0 0 0 0 0 0 0 0 0 0

M02255_77_000000000-ADUT3_1_1114_19063_12573 T----AC---GG-AG-GGA--------GCT-A-G------C--G--T---T--GT-T-CGG-AA------TT-A--C-T--GG-GC-------GT--A-----AA-GC-GT-AC-------G-TA-G-G-C-G------------G--T-GA-C-T-C-----
------AA----G-T-C-A--------G-A-G--G--TG--A-AA-GC--C-T-GG-A-G-----CT-C-AA------C-T-C-C-A-G-A--A-C----T-G--C-C---T--T-------T--GA-A-A---C-------T--A-G--GT--C-G-C----T-T-G-A-A-T--C---A-CG-----GA-G-A--------G-G-T-G---AG-T------GG--A--ATT----C-
C-G-A-GT--GT-A-G-AG-GT--G---A--A-A---TT-C-GT-AG--AT-A-TT--C-G--G-A-A-G-A-AC-A-CC---AG--T--G--GC-GAA-G--G-C--G-------G--C-T-C-A---CTG--G--AC-G-T--------------------G-T---A-T-T--GA--CG-C-T-G--A-GG--T-A-CG-A--AA-G-C---G-TG--GG-G-
-AG-C-A-AA--------CA--GG Bacteria(100);Proteobacteria(100);Alphaproteobacteria(100);Sphingomonadales(100);unclassified(100);unclassified(100);unclassified(100);unclassified(100);unclassified(100);

Otu1367 0 0 0 0 0 0 0 0 0 0 0 0 0 0 0 0 0 0 0 0 0 0 0 0 0 0 0 0
0 0 0 0 0 0 0 0 0 0 0 0 0 0 0 0 0 0 0 0 0 0 0 0 7 0 0 0 0
0 0 0 0 0 0 0 0 0 0 0 0 0 0 0 0 0 0 0 0 0 0 0 0 0 0 0 0 0
0 0 0 0 0 0 0 0 0 0 0 0 0 0 0 0 0 0 0 0 0 0 0 0 0 0 0

M02255_77_000000000-ADUT3_1_2105_20572_11539 G----AC---AG-AG-GGG--------GCA-A-G------C--G--T---T--GT-C-CGG-AG------TT-A--C-T--GG-GC-------GT--A-----AA-GC-GT-CC-------G-CA-G-G-C-G------------G--T-CG-G-G-T---
--------AA----G-T-G-G--------T-G-T--G--TG--A-AA-GC--G-C-GC-A-G-----CT-C-AA------C-T-G-C-G-C-G--A-G----G-C--C-A---T--G-------C--CA-G-A---C-------T--G-C--TC--A-A-C----T-C-G-A-G-T--C---C-GG-----TA-G-A--------G-G-T-T---CG-T------CG--A--ATT----
G-C-C-G-GT--GT-A-G-TG-GT--G---A--A-A---TG-C-GT-AG--AG-A-TC--G-G--C-A-G-G-A-AG-A-CC---AA--G--G--GT-GCA-G--A-C--A-------G--C-G-A-A---CTG--G--GA-C-G--------------------G-T---A-C-T--GA--CG-C-TGA----AG--G-A-CG-A--AA-G-C---G-TG--
GG-G--AG-C-G-AA--------CC--GG Bacteria(100);unclassified(100);unclassified(100);unclassified(100);unclassified(100);unclassified(100);unclassified(100);unclassified(100);unclassified(100);

Otu1368 0 0 0 0 0 0 0 0 0 0 0 0 0 0 0 0 0 0 0 0 0 0 0 0 0 0 0 0
0 0 0 0 0 0 0 0 0 0 0 0 0 0 0 0 0 0 0 0 0 0 0 0 9 0 0 0 0
0 0 0 0 0 0 0 0 0 0 0 0 0 0 0 0 0 0 0 0 0 0 0 0 0 0 0 0 0
0 0 0 0 0 0 0 0 0 0 0 0 0 0 0 0 0 0 0 0 0 0 0 0 0 0 0

M02255_77_000000000-ADUT3_1_2107_27019_10397 T----AC---GA-GG-GGG--------GCA-A-G------C--G--T---T--GT-T-CGG-AA------TT-A--T-T--GG-GC-------GT--A-----AA-GG-GT-GC-------G-TA-G-G-C-G------------G--T-TC-A-G-C----
-------AA----G-T-C-T--------C-G-T--G--TG--A-AA-TC--T-T-CG-G-G-----CT-C-AA------C-C-C-G-A-A-G--T-C----T-G--C-A---C--G-------G--GA-A-A---C-------T--A-C--TG--G-G-C----T-T-G-A-G-T--A---T-GG-----GA-G-A--------G-G-T-G---AG-T------GG--A--ATT----
T-C-C-G-GT--GT-A-G-CG-GT--G---A--A-A---TG-C-GT-AG--AT-A-TC--G-G--A-A-G-G-A-AC-A-CC---TG--T--G--GC-GAA-A--G-C--G-------G--C-T-C-A---CTG--G--AC-C-A--------------------T-A---A-C-T--GA--CG-C-T-G--A-TG--C-A-CG-A--AA-G-C---T-AG--GG-
G--AG-C-A-AA--------CA--GG Bacteria(100);Acidobacteria(100);Acidobacteria(100);Acidobacteriales(100);Acidobacteriaceae(100);unclassified(100);unclassified(100);unclassified(100);unclassified(100);

Otu1369 0 0 0 0 0 0 0 0 0 0 0 0 0 0 0 0 0 0 0 0 0 0 0 0 0 0 0 0
0 0 0 0 0 0 0 0 0 0 0 0 0 0 0 0 0 0 0 0 0 0 0 0 7 0 0 0 0
0 0 0 0 0 0 0 0 0 0 0 0 0 0 0 0 0 0 0 0 0 0 0 0 0 0 0 0 0
0 0 0 0 0 0 0 0 0 0 0 0 0 0 0 0 0 0 0 0 0 0 0 0 0 0 0

M02255_77_000000000-ADUT3_1_2111_8821_18161 T----AC---AG-AG-GAT--------GCA-A-G------C--G--T---T--AT-C-CGG-AA------TG-A--T-T--GG-GC-------GT--A-----AA-GC-GT-TT-------G-TA-G-G-T-G------------G--T-TT-A-T-T-----------AA--
--G-T-C-T--------T-C-T--G--GC--A-AA-GC--T-C-AG-G-G-----CT-T-AA------C-C-C-T-G-T-T--T-A----G-G--C-A---G--T-------A--GA-A-A---C-------T--A-A--TA--G-G-C----T-A-G-A-G-T--A---C-GG-----TA-G-G--------G-G-C-A---GA-G------GG--A--ATT----C-C-C-G-
GT--GT-A-G-CG-GT--G---A--A-A---TG-C-GC-AG--AT-A-TC--G-G--G-A-A-G-A-AC-A-CC---GA--T--G--GC-GAA-A--G-C--A-------C--T-C-T-G---CTG--G--GC-C-G--------------------A-A---A-C-T--GA--CA-C-T-G--A-GA--G-A-CG-A--AA-G-C---T-AG--GG-G--AG-
C-G-AA--------TG--GG Bacteria(100);Cyanobacteria(100);unclassified(100);unclassified(100);unclassified(100);unclassified(100);unclassified(100);unclassified(100);unclassified(100);

Otu1370 0 0 0 0 0 0 0 0 0 0 0 0 0 0 0 0 0 0 0 0 0 0 0 0 0 0 0 0
0 0 0 0 0 0 0 0 0 0 0 0 0 0 0 0 0 0 0 0 0 0 0 0 15 0 0 0 0
0 0 0 0 0 0 0 0 0 0 0 0 0 0 0 0 0 0 0 0 0 0 0 0 0 0 0 0 0
0 0 0 0 0 0 0 0 0 0 0 0 0 0 0 0 0 0 0 0 0 0 0 0 0 0 0
M02255_77_000000000-ADUT3_1_2115_13643_11130 T----AC---GA-AG-GGG--------GCT-A-G------C--G--T---T--GC-T-CGG-AA------TT-A--C-T--GG-GC-------GT--A-----AA-GG-GC-GC-------G-TA-G-G-C-G------------G--A-CG-T-T-T----
-------AA----G-T-C-A--------G-A-G--G--TA--A-AA-GC--C-C-AG-G-G-----CT-C-AA------C-C-T-T-G-G-A--A-C----T-G--C-C---T--T-------T--GA-T-A---C-------T--G-G--AC--G-T-C----T-T-G-A-G-T--A---C-GG-----GA-G-A--------G-G-T-G---TG-T------GG--A--ACT----
C-C-G-A-GT--GT-A-G-AG-GT--G---A--A-A---TT-C-GT-AG--AT-A-TT--C-G--G-A-A-G-A-AC-A-CC---AG--T--G--GC-GAA-G--G-C--G-------A--C-A-C-A---CTG--G--CC-C-G--------------------T-T---A-C-T--GA--CG-C-T-G--A-GG--C-G-CG-A--AA-G-C---G-TG--GG-
G--AG-C-A-AA--------CA--GG Bacteria(100);Proteobacteria(100);Alphaproteobacteria(100);Caulobacterales(100);Caulobacteraceae(100);unclassified(100);unclassified(100);unclassified(100);unclassified(100);

Otu1371 0 0 0 0 0 0 0 0 0 0 0 0 0 0 0 0 0 0 0 0 0 0 0 0 0 0 0 0
0 0 0 0 0 0 0 0 0 0 0 0 0 0 0 0 0 0 0 0 0 0 0 0 9 0 0 0 0
0 0 0 0 0 0 0 0 0 0 0 0 0 0 0 0 0 0 0 0 0 0 0 0 0 0 0 0 0
0 0 0 0 0 0 0 0 0 0 0 0 0 0 0 0 0 0 0 0 0 0 0 0 0 0 0
M02255_77_000000000-ADUT3_1_1113_5384_15115 T----AC---GT-AG-GGT--------GCG-A-G------C--G--T---T--GT-C-CGG-AT------TT-A--T-T--GG-GC-------GT--A-----AA-GA-GC-TT-------G-TA-G-G-C-G------------G--T-TT-G-T-T----------GC--
--G-T-C-G--------G-A-A--G--TG--A-AA-AC--T-C-AG-G-G-----CT-T-AA------C-T-C-T-G-A-G--C-C----T-G--C-T---T--T-------C--GA-T-A---C-------G--G-G--CA--G-A-C----T-T-G-A-G-G--A---A-GG-----TA-G-G--------G-G-A-G---AA-T------GG--A--ATT----C-C-T-G-
GT--GA-A-G-CG-GT--G---G--A-A---TG-C-GT-AG--AT-A-TC--A-G--G-A-G-G-A-AC-A-CC---GG--T--G--GC-GAA-G--G-C--G-------G--T-T-C-T---CTG--G--AC-C-T--------------------T-T---C-C-T--GA--CG-C-T-G--A-GA--A-G-CG-A--AA-G-C---G-TG--GG-G--AG-
C-A-AA--------CA--GG Bacteria(100);Actinobacteria(100);Actinobacteria(100);Actinobacteridae(100);Actinomycetales(100);Propionibacterineae(100);Propionibacteriaceae(100);unclassified(100);unclassified(100);

Otu1372 0 0 0 0 0 0 0 0 0 0 0 0 0 0 0 0 0 0 0 0 0 0 0 0 0 0 0 0
0 0 0 0 0 0 0 0 0 0 0 0 0 0 0 0 0 0 0 0 0 0 0 0 0 11 0 0 0
0 0 0 0 0 0 0 0 0 0 0 0 0 0 0 0 0 0 0 0 0 0 0 0 0 0 0 0 0
0 0 0 0 0 0 0 0 0 0 0 0 0 0 0 0 0 0 0 0 0 0 0 0 0 0 0
M02255_77_000000000-ADUT3_1_2101_26246_12116 G----AT---AG-AG-GAT--------GCA-A-G------C--A--T---T--AT-C-TGG-AA------TG-A--T-T--GG-GC-------GT--A-----AA-GC-GT-CT-------G-TA-G-G-T-G------------G--C-TT-T-T-C------
-----AA----G-T-C-C--------G-C-T--G--TC--A-AA-TC--C-C-CG-G-G-----CT-C-AA------C-C-C-T-G-G-A--C-A----G-G--C-G---G--T-------G--GA-A-A---C-------T--A-A--CA--A-G-A----T-G-G-A-G-T--A---C-GG-----TA-G-G--------G-G-C-A---GA-G------GG--A--ATT----T-
G-C-G-GT--GG-A-G-GG-GT--G---A--A-A---TG-C-GT-TG--AG-A-TC--G-G--A-A-A-T-A-AC-A-CC---AA--C--G--GC-GAA-A--G-C--A-------C--T-C-T-G---CTG--G--GC-C-G--------------------A-C---A-T-T--GA--CA-T-T-G--A-GA--G-A-CG-A--AA-G-C---T-AG--GG-
G--AG-A-A-AA--------TG--GG Bacteria(100);Cyanobacteria(100);unclassified(100);unclassified(100);unclassified(100);unclassified(100);unclassified(100);unclassified(100);unclassified(100);

Otu1373 0 0 0 0 0 0 0 0 0 0 0 0 0 0 0 0 0 0 0 0 0 0 0 0 0 0 0 0
0 0 0 0 0 0 0 0 0 0 0 0 0 0 0 0 0 0 0 0 0 0 0 0 0 6 0 0 0
0 0 0 0 0 0 0 0 0 0 0 0 0 0 0 0 0 0 0 0 0 0 0 0 0 0 0 0 0
0 0 0 0 0 0 0 0 0 0 0 0 0 0 0 0 0 0 0 0 0 0 0 0 0 0 0
M02255_77_000000000-ADUT3_1_2113_7028_8880 T----AC---GT-AG-GAT--------CCG-A-G------C--G--T---T--AT-C-CGG-AG------TG-A--C-T--GG-GC-------GT--A-----AA-GA-GTTGC-------G-TA-G-G-C-G------------G--A-CG-T-G-T----------
AA----G-C-G-A--------A-T-A--G--TG--A-AA-TC--G-A-GT-G-G-----CT-C-AA------C-C-A-TCTTC-G--G-C----T-A--T-T---A--T-------T--CG-A-A---C-------T--G-T--AC--G-T-C----T-C-G-A-G-A--G---T-AG-----TA-G-A--------G-G-T-A---CC-T------GG--A--ATT----C-C-C-
T-GT--GT-A-G-CG-GT--G---A--A-A---TG-C-GT-AG--AT-A-TA--G-G--G-A-G-G-A-AC-A-CC---AA--T--G--GC-GTA-G--G-C--A-------G--G-G-T-A---CTA--G--AC-T-A--------------------T-T---T-C-T--GA--CG-C-T-G--A-GG--C-A-CG-A--AA-G-C---G-TG--GG-G--
AG-C-G-AA--------CC--GG Bacteria(100);unclassified(100);unclassified(100);unclassified(100);unclassified(100);unclassified(100);unclassified(100);unclassified(100);unclassified(100);

Otu1374 0 0 0 0 0 0 0 0 0 0 0 0 0 0 0 0 0 0 0 0 0 0 0 0 0 0 0 0
0 0 0 0 0 0 0 0 0 0 0 0 0 0 0 0 0 0 0 0 0 0 0 0 0 8 0 0 0
0 0 0 0 0 0 0 0 0 0 0 0 0 0 0 0 0 0 0 0 0 0 0 0 0 0 0 0 0
0 0 0 0 0 0 0 0 0 0 0 0 0 0 0 0 0 0 0 0 0 0 0 0 0 0 0
M02255_77_000000000-ADUT3_1_1110_15347_5235 T----AC---GA-AG-GGG--------GCT-A-G------C--G--T---T--GC-T-CGG-AA------TT-A--C-T--GG-GC-------GT--A-----AA-GG-GA-GC-------G-TA-G-G-C-G------------G--G-TT-A-T-C----------
AA----G-T-T-G--------G-A-G--G--TG--A-AA-GC--C-C-AG-G-G-----CT-C-AA------C-C-T-T-G-G-A--A-T----T-G--C-C---T--T-------C--AA-A-A---C-------T--G-A--TA--A-C-C----T-A-G-A-G-G--A---T-AG-----AA-G-A--------G-G-T-A---AG-T------GG--A--ACT----C-C-G-
A-GT--GT-A-G-AG-GT--G---A--A-A---TT-C-GT-AG--AT-A-TT--C-G--G-A-A-G-A-AC-A-CC---AG--T--G--GC-GAA-G--G-C--G-------A--C-T-T-A---CTG--G--TC-T-A--------------------T-T---A-C-T--GA--CG-C-T-G--A-GG--C-T-CG-A--AA-G-C---G-TG--GG-G--AG-
C-A-AA--------CA--GG Bacteria(100);Proteobacteria(100);Alphaproteobacteria(100);Caulobacterales(100);Caulobacteraceae(100);Asticcacaulis(100);unclassified(100);unclassified(100);unclassified(100);

Otu1375 0 0 0 0 0 0 0 0 0 0 0 0 0 0 0 0 0 0 0 0 0 0 0 0 0 0 0 0
0 0 0 0 0 0 0 0 0 0 0 0 0 0 0 0 0 0 0 0 0 0 0 0 0 0 6 0 0
0 0 0 0 0 0 0 0 0 0 0 0 0 0 0 0 0 0 0 0 0 0 0 0 0 0 0 0 0
0 0 0 0 0 0 0 0 0 0 0 0 0 0 0 0 0 0 0 0 0 0 0 0 0 0 0
M02255_76_000000000-ADUNE_1_1101_7090_23734 C----GG---CT-AA-CTC--------TGT---G------C--C--A---G--CA-G-CCG-CG------GT-A--A-T--AC-GA-------AG--G-----GG-GC-TA-GC-------G-TT-GTT-C-G------------G--A-CT-A-T-T-----------AA--
--G-T-C-A--------G-G-G--G--TG--A-AA-TC--C-C-GG-G-G-----CT-C-AA------C-C-C-C-G-G-A--A-C----T-G--C-C---T--T-------T--GA-T-A---C-------T--G-G--TA--G-T-C----T-T-G-A-G-T--T---C-GA-----GA-G-A--------G-G-T-G---AG-T------GG--A--ATT----C-C-G-A-
GT--GT-A-G-AG-GT--G---A--A-A---TT-C-GT-AG--AT-A-TT--C-G--G-A-G-G-A-AC-A-CC---AG--T--G--GC-GAA-G--G-C--G-------G--C-T-C-A---CTG--G--CT-C-G--------------------A-T---A-C-T--GA--CG-C-T-G--A-GG--T-G-CG-A--AA-G-C---G-TG--GG-G--AG-
C-A-AA--------CA--GG Bacteria(100);Proteobacteria(100);Alphaproteobacteria(100);Rhizobiales(100);unclassified(100);unclassified(100);unclassified(100);unclassified(100);unclassified(100);

Otu1376 0 0 0 0 0 0 0 0 0 0 0 0 0 0 0 0 0 0 0 0 0 0 0 0 0 0 0 0
0 0 0 0 0 0 0 0 0 0 0 0 0 0 0 0 0 0 0 0 0 0 0 0 0 0 13 0 0
0 0 0 0 0 0 0 0 0 0 0 0 0 0 0 0 0 0 0 0 0 0 0 0 0 0 0 0 0
0 0 0 0 0 0 0 0 0 0 0 0 0 0 0 0 0 0 0 0 0 0 0 0 0 0 0
M02255_76_000000000-ADUNE_1_1108_8709_11797 C----TC---CG-TG-CCA--------GCA-G-C------C--G--C---G--GT---AAT-AC------GG-A--G-G--GT-GC-------AA--G-----CG-TT-AT-CC-------G-GA---T-C-T------------G--A-TT-T-G-T-----------AA----
G-T-C-A--------G-T-G--G--TG--A-AA-TC--T-C-AC-A-G-----CT-T-AA------C-T-G-T-G-A-A--A-C----T-G--C-C---A--T-------T--GA-T-A---C-------T--G-C--AA--G-T-C----T-T-G-A-G-T--G---T-TG-----TT-G-A--------A-G-T-A---GC-T------GG--A--ATA----A-G-T-A-GT--
GT-A-G-CG-GT--G---A--A-A---TG-C-AT-AG--AT-A-TT--A-C--T-T-A-G-A-AC-A-CC---AA--T--T--GC-GAA-G--G-C--A-------G--G-T-T-A---CTA--A--GC-A-A--------------------C-A---A-C-T--GA--CG-C-T-G--A-TG--G-A-CG-A--AA-G-C---G-TG--GG-G--AG-C-G-
AA--------CA--GG Bacteria(100);Bacteroidetes(100);Flavobacteria(100);Flavobacteriales(100);Flavobacteriaceae(100);Chryseobacterium(100);unclassified(100);unclassified(100);unclassified(100);

Otu1377 0 0 0 0 0 0 0 0 0 0 0 0 0 0 0 0 0 0 0 0 0 0 0 0 0 0 0 0
0 0 0 0 0 0 0 0 0 0 0 0 0 0 0 0 0 0 0 0 0 0 0 0 0 0 4 0 0
0 0 0 0 0 0 0 0 0 0 0 0 0 0 0 0 0 0 0 0 0 0 0 0 0 0 0 0 0
0 0 0 0 0 0 0 0 0 0 0 0 0 0 0 0 0 0 0 0 0 0 0 0 0 0 0
M02255_76_000000000-ADUNE_1_2101_8761_13276 C----CG---GC-TA-ACT--------TCG-T-G------C--C--A---G--CA-G-CCG-CG------GT-A--A-T--AC-GA-------AG--G-----GG-GC-TA-GC-------G-TT-GTT-C-G------------G--A-CT-A-T-T-----------AA-
---G-T-C-A--------G-G-G--G--TG--A-AA-TC--C-C-GG-G-G-----CT-C-AA------C-C-C-C-G-G-A--A-C----T-G--C-C---T--T-------T--GA-T-A---C-------T--G-G--TA--G-T-C----T-T-G-A-G-T--T---C-GA-----GA-G-A--------G-G-T-G---AG-T------GG--A--ATT----C-C-G-A-
GT--GT-A-G-AG-GT--G---A--A-A---TT-C-GT-AG--AT-A-TT--C-G--G-A-G-G-A-AC-A-CC---AG--T--G--GC-GAA-G--G-C--G-------G--C-T-C-A---CTG--G--CT-C-G--------------------A-T---A-C-T--GA--CG-C-T-G--A-GG--T-G-CG-A--AA-G-C---G-TG--GG-G--AG-
C-A-AA--------CA--GG Bacteria(100);Proteobacteria(100);Alphaproteobacteria(100);Rhizobiales(100);Phyllobacteriaceae(100);unclassified(100);unclassified(100);unclassified(100);unclassified(100);

Otu1378 0 0 0 0 0 0 0 0 0 0 0 0 0 0 0 0 0 0 0 0 0 0 0 0 0 0 0 0
0 0 0 0 0 0 0 0 0 0 0 0 0 0 0 0 0 0 0 0 0 0 0 0 8 0 0 0 0
0 0 0 0 0 0 0 0 0 0 0 0 0 0 0 0 0 0 0 0 0 0 0 0 0 0 0 0 0
0 0 0 0 0 0 0 0 0 0 0 0 0 0 0 0 0 0 0 0 0 0 0 0 0 0 0
M02255_77_000000000-ADUT3_1_1102_7694_8196 T----AC---AG-AG-GGG--------GCA-A-G------C--G--T---T--GT-C-CGG-AT------TT-A--C-T--GG-GC-------GT--A-----CA-GC-GC-GC-------G-CA-G-G-C-G------------G--A-CC-G-C-G-----------
AC----G-T-G-C--------C-G-T--G--TG--A-CA-AG--C-C-AC-C-G-----CT-T-AC------C-G-G-T-G-G-A--G-G----G-C--C-A---T--G-------G--TA-C-A---C-------G--A-G--CG--G-T-C----T-G-G-A-G-G--C---A-GG-----GA-G-A--------G-G-G-A---TG-T------GG--A--ATG----C-C-
C-G-GT--GG-A-G-CG-GT--G---A--A-A---TG-C-GT-AG--AG-A-TC--G-G--G-C-G-G-A-AC-A-CC---AG--C--G--GC-GAA-G--G-C--G-------G--C-A-T-C---CTG--G--TC-C-T--------------------G-T---A-C-T--GA--CG-C-T-G--A-GG--C-G-CG-A--GA-G-C---G-TG--GG-G--
AG-C-A-AA--------CT--GG Bacteria(100);unclassified(100);unclassified(100);unclassified(100);unclassified(100);unclassified(100);unclassified(100);unclassified(100);unclassified(100);

Otu1379 0 0 0 0 0 0 0 0 0 0 0 0 0 0 0 0 0 0 0 0 0 0 0 0 0 0 0 0
0 0 0 0 0 0 0 0 0 0 0 0 0 0 0 0 0 0 0 0 0 0 0 0 14 0 0 0 0
0 0 0 0 0 0 0 0 0 0 0 0 0 0 0 0 0 0 0 0 0 0 0 0 0 0 0 0 0
0 0 0 0 0 0 0 0 0 0 0 0 0 0 0 0 0 0 0 0 0 0 0 0 0 0 0
M02255_77_000000000-ADUT3_1_1101_15610_15008 T----AC---GA-AG-GGG--------GCT-A-G------C--G--T---T--AC-T-CGG-AA------TG-A--C-T--GG-GC-------GT--A-----AA-GG-GC-GC-------G-TA-G-G-C-G------------G--T-TT-G-T-T----
-------TA----G-T-T-A--------G-G-C--G--TG--A-AA-TT--C-C-TG-G-G-----CT-C-AA------C-C-T-G-G-G-G--G-C----T-G--C-G---C--T-------T--AA-T-A---C-------A--G-G--CA--G-A-C----T-A-G-A-G-T--G---T-GA-----AA-G-A--------G-G-G-T---CG-T------GG--A--ATT----
T-C-C-A-GT--GT-A-G-AG-GT--G---A--A-A---TT-C-GT-AG--AT-A-TT--G-G--A-A-A-G-A-AC-A-CC---GG--T--G--GC-GAA-G--G-C--G-------G--C-G-A-C---CTG--G--TT-C-A--------------------T-T---A-C-T--GA--CG-C-T-G--A-GG--C-G-CG-A--AA-G-C---G-TG--GG-
G--AG-C-A-AA--------CA--GG Bacteria(100);Proteobacteria(100);Alphaproteobacteria(100);Rhodospirillales(100);Acetobacteraceae(100);unclassified(100);unclassified(100);unclassified(100);unclassified(100);

Otu1380 0 0 0 0 0 0 0 0 0 0 0 0 0 0 0 0 0 0 0 0 0 0 0 0 0 0 0 0
0 0 0 0 0 0 0 0 0 0 0 0 0 0 0 0 0 0 0 0 0 0 0 0 69 0 0 0 0
0 0 0 0 0 0 0 0 0 0 0 0 0 0 0 0 0 0 0 0 0 0 0 0 0 0 0 0 0
0 0 0 0 0 0 0 0 0 0 0 0 0 0 0 0 0 0 0 0 0 0 0 0 0 0 0
M02255_77_000000000-ADUT3_1_1104_10079_6749 T----AC---GA-AG-GGG--------GCT-A-G------C--G--T---T--AC-T-CGG-AA------TT-A--C-T--GG-GC-------GT--A-----AA-GG-GC-GC-------G-TA-G-G-C-G------------G--C-GC-A-C-C----------
AA----G-T-T-A--------G-G-C--G--TG--A-AA-GT--C-C-TG-G-G-----CT-C-AA------C-C-T-G-G-G-A--A-C----T-G--C-G---C--T-------T--GA-T-A---C-------T--G-G--TG--T-G-C----T-T-G-A-G-G--A---T-GG-----AA-G-A--------G-G-G-T---TG-T------GG--A--ATT----C-C-C-
A-GT--GT-A-G-AG-GT--G---A--A-A---TT-C-GT-AG--AT-A-TT--G-G--G-A-A-G-A-AC-A-CC---GG--T--G--GC-GAA-G--G-C--G-------G--C-A-A-C---CTG--G--TC-C-A--------------------T-T---A-C-T--GA--CG-C-T-G--A-GG--C-G-CG-A--CA-G-C---G-TG--GG-G--
AG-C-A-AA--------CA--GG Bacteria(100);Proteobacteria(100);Alphaproteobacteria(100);Rhodospirillales(100);Acetobacteraceae(100);unclassified(100);unclassified(100);unclassified(100);unclassified(100);

Otu1381 0 0 0 0 0 0 0 0 0 0 0 0 0 0 0 0 0 0 0 0 0 0 0 0 0 0 0 0
0 0 0 0 0 0 0 0 0 0 0 0 0 0 0 0 0 0 0 0 0 0 0 0 7 0 0 0 0
0 0 0 0 0 0 0 0 0 0 0 0 0 0 0 0 0 0 0 0 0 0 0 0 0 0 0 0 0
0 0 0 0 0 0 0 0 0 0 0 0 0 0 0 0 0 0 0 0 0 0 0 0 0 0 0
M02255_77_000000000-ADUT3_1_1111_22479_10578 T----AC---GT-AG-GGT--------GCA-A-G------C--A--T---T--AT-C-CGG-AG------TG-A--C-T--GG-GC-------GT--A-----AA-GA-GTTGC-------G-TA-G-G-C-G------------G--T-TA-G-T-A----
-------AA----G-C-G-A--------A-T-A--G--TG--A-AA-TC--T-G-GT-G-G-----CT-C-AA------C-C-A-T-ACA-G--A-C----T-A--T-T---A--T-------T--CG-A-A---C-------T--C-A--CT--A-A-C----T-C-G-A-G-A--A---T-GG-----TA-G-A--------G-G-T-A---AC-T------GG--A--ATT----T-
C-T-A-GT--GT-A-G-GA-GT--G---A--A-A---TC-C-GT-AG--AT-A-TT--A-G--A-A-G-G-A-AC-A-CC---AA--T--G--GC-GTA-G--G-C--A-------G--G-T-T-A---CTG--G--AC-C-A--------------------T-T---T-C-T--GA--CG-C-T-A--A-GG--C-A-CG-A--AA-G-C---G-TG--GG-G--
AG-C-G-AA--------CC--GG Bacteria(100);Candidate_division_TM7(100);unclassified(100);unclassified(100);unclassified(100);unclassified(100);unclassified(100);unclassified(100);unclassified(100);

Otu1382 0 0 0 0 0 0 0 0 0 0 0 0 0 0 0 0 0 0 0 0 0 0 0 0 0 0 0 0
0 0 0 0 0 0 0 0 0 0 0 0 0 0 0 0 0 0 0 0 0 0 0 0 28 0 0 0 0
0 0 0 0 0 0 0 0 0 0 0 0 0 0 0 0 0 0 0 0 0 0 0 0 0 0 0 0 0
0 0 0 0 0 0 0 0 0 0 0 0 0 0 0 0 0 0 0 0 0 0 0 0 0 0 0
M02255_77_000000000-ADUT3_1_2101_20143_9713 G----AC---GG-AG-GGG--------GCT-A-G------C--G--T---T--GT-T-CGG-AA------TG-A--C-T--GG-GC-------GT--A-----AA-GC-GC-GC-------G-TA-G-G-C-G------------G--T-TT-G-G-T----------
AA----G-T-T-G--------G-A-T--G--TG--A-AA-GC--C-C-GG-G-G-----CT-C-AA------C-C-C-C-G-G-A--T-G----T-G--C-G---T--T-------C--AA-A-A---C-------T--G-C--TA--G-A-C----T-T-G-A-G-A--C---C-GG-----AA-G-G--------G-G-A-G---AG-C------GG--A--ATT----C-C-
C-A-GT--GT-A-G-AG-GT--G---A--A-A---TT-C-GT-AG--AT-A-TT--G-G--G-A-A-G-A-AC-A-CC---GG--T--G--GC-GAA-G--G-C--G-------G--C-T-C-T---CTG--G--TC-C-G--------------------G-T---T-C-T--GA--CG-C-T-G--A-GG--C-G-CG-A--AA-G-C---G-TG--GG-G--
AG-C-A-AA--------CA--GG Bacteria(100);Proteobacteria(100);unclassified(100);unclassified(100);unclassified(100);unclassified(100);unclassified(100);unclassified(100);unclassified(100);

Otu1383 0 0 0 0 0 0 0 0 0 0 0 0 0 0 0 0 0 0 0 0 0 0 0 0 0 0 0 0
0 0 0 0 0 0 0 0 0 0 0 0 0 0 0 0 0 0 0 0 0 0 0 0 7 0 0 0 0
0 0 0 0 0 0 0 0 0 0 0 0 0 0 0 0 0 0 0 0 0 0 0 0 0 0 0 0 0
0 0 0 0 0 0 0 0 0 0 0 0 0 0 0 0 0 0 0 0 0 0 0 0 0 0 0
M02255_77_000000000-ADUT3_1_2105_27610_19560 G----AC---AG-AG-GAT--------GTA-A-G------C--A--T---T--AT-C-TGG-AA------TG-A--T-T--GG-GA-------AT--A-----AA-AC-GT-CT-------G-TA-G-G-T-G------------G--C-TT-T-T-C------
-----AA----G-T-C-T--------G-T-C--G--TC--A-AA-CC--C-C-AG-G-G-----CT-C-AA------C-C-C-T-G-G-A--T-A----G-G--T-G---G--T-------G--GA-A-A---C-------T--A-C--CA--A-G-C----T-G-G-A-G-T--A---C-GG-----TA-G-G--------G-G-C-A---GA-G------GG--A--ATT----T-
T-C-G-AT--GG-A-G-TG-GT--G---A--A-A---TG-C-GT-TG--AG-A-TC--G-G--A-A-A-G-A-AC-A-CC---AA--C--G--GC-AAA-A--G-C--A-------C--T-C-T-G---CTG--G--GC-C-G--------------------A-C---A-C-T--AA--CA-C-T-G--A-GA--G-A-TG-A--AA-G-C---T-AG--GG-G-
-AG-C-A-AA--------TG--GG Bacteria(100);Cyanobacteria(100);unclassified(100);unclassified(100);unclassified(100);unclassified(100);unclassified(100);unclassified(100);unclassified(100);

Otu1384 0 0 0 0 0 0 0 0 0 0 0 0 0 0 0 0 0 0 0 0 0 0 0 0 0 0 0 0
0 0 0 0 0 0 0 0 0 0 0 0 0 0 0 0 0 0 0 0 0 0 0 0 10 0 0 0 0
0 0 0 0 0 0 0 0 0 0 0 0 0 0 0 0 0 0 0 0 0 0 0 0 0 0 0 0 0
0 0 0 0 0 0 0 0 0 0 0 0 0 0 0 0 0 0 0 0 0 0 0 0 0 0 0
M02255_77_000000000-ADUT3_1_2106_26844_18278 T----AC---GA-AG-GGG--------GCG-A-G------C--G--T---T--GT-T-CGG-AT------TT-A--C-T--GG-GC-------GT--A-----AA-GG-GC-GC-------G-CA-G-G-C-G------------G--C-GG-A-C-C---
--------AA----G-T-C-A--------G-G-C--G--TG--A-AA-GC--C-C-CG-G-G-----CT-T-AA------C-C-T-G-G-G-A--A-C----T-G--C-G---T--C-------T--GG-G-A---C-------T--G-G--TA--T-G-C----T-A-G-A-G-T--T---C-GG-----GA-G-A--------G-G-A-G---AG-T------GG--A--ATT---
-C-C-C-A-GT--GT-A-G-AG-GT--G---A--A-A---TT-C-GT-AG--AT-A-TT--G-G--G-A-A-G-A-AC-A-CC---GG--T--G--GC-GAA-G--G-C--G-------G--C-T-C-T---CTG--G--AC-C-G--------------------A-G---A-C-T--GA--CG-C-T-G--A-GG--C-G-CG-A--AA-G-C---G-TG--
GG-G--AG-C-A-AA--------CA--GG Bacteria(100);Proteobacteria(100);Alphaproteobacteria(100);Rhodospirillales(100);unclassified(100);unclassified(100);unclassified(100);unclassified(100);unclassified(100);

Otu1385 0 0 0 0 0 0 0 0 0 0 0 0 0 0 0 0 0 0 0 0 0 0 0 0 0 0 0 0
0 0 0 0 0 0 0 0 0 0 0 0 0 0 0 0 0 0 0 0 0 0 0 0 17 0 0 0 0
0 0 0 0 0 0 0 0 0 0 0 0 0 0 0 0 0 0 0 0 0 0 0 0 0 0 0 0 0
0 0 0 0 0 0 0 0 0 0 0 0 0 0 0 0 0 0 0 0 0 0 0 0 0 0 0
M02255_77_000000000-ADUT3_1_2114_26802_13459 T----AC---GA-AG-GGG--------GCT-A-G------C--G--T---T--GT-T-CGG-AA------TT-A--C-T--GG-GC-------GT--A-----AA-GC-GC-GT-------G-TA-G-G-C-G------------G--G-CT-T-T-C-----
------AA----G-T-C-A--------G-G-G--G--TG--A-AA-TC--C-C-GG-G-G-----CT-C-AA------C-C-T-C-G-G-A--A-C----T-G--C-C---T--T-------T--GA-T-A---C-------T--G-T--TA--G-C-C----T-C-G-A-G-T--C---C-GG-----AA-G-A--------G-G-T-T---GG-T------GG--A--ATT----C-
C-G-A-GT--GT-A-G-AG-GT--G---A--A-A---TT-C-GT-AG--AT-A-TT--C-G--G-A-G-G-A-AC-A-CC---AG--A--G--GC-GAA-G--G-C--G-------G--C-C-A-A---CTG--G--TC-C-G--------------------G-T---A-C-T--GA--CG-C-T-G--A-GA--C-G-CG-A--AA-G-C---G-TG--GG-
G--AG-C-A-AA--------CA--GG Bacteria(100);Proteobacteria(100);Alphaproteobacteria(100);unclassified(100);unclassified(100);unclassified(100);unclassified(100);unclassified(100);unclassified(100);

Otu1386 0 0 0 0 0 0 0 0 0 0 0 0 0 0 0 0 0 0 0 0 0 0 0 0 0 0 0 0
0 0 0 0 0 0 0 0 0 0 0 0 0 0 0 0 0 0 0 0 0 0 0 0 7 0 0 0 0
0 0 0 0 0 0 0 0 0 0 0 0 0 0 0 0 0 0 0 0 0 0 0 0 0 0 0 0 0
0 0 0 0 0 0 0 0 0 0 0 0 0 0 0 0 0 0 0 0 0 0 0 0 0 0 0
M02255_77_000000000-ADUT3_1_2117_16606_15169 T----AC---GT-AT-GGT--------GCA-A-G------C--G--T---T--AT-C-CGG-AT------TT-A--C-T--GG-GT-------GT--A-----AA-GG-GT-GC-------G-TA-G-G-T-G------------G--T-AT-G-G-T------
-----AA----G-T-C-A--------G-A-A--G--TG--A-AA-GC--A-C-GG-G-G-----CT-C-AA------C-C-C-C-G-T-G--A-C----T-G--C-T---T--T-------T--GA-A-A---C-------T--A-T--CA--A-A-C----T-A-G-A-G-T--C---C-AG-----GA-G-A--------G-G-T-A---AG-C------GG--A--ATT----C-
C-T-A-GT--GT-A-G-CG-GT--G---A--A-A---TG-C-GT-AG--AT-A-TT--A-G--G-A-G-G-A-AC-A-CC---AG--T--G--GC-GAA-A--G-C--G-------G--C-T-T-A---CTG--G--AC-T-G--------------------G-T---A-C-T--GA--CA-C-T-G--A-GG--C-A-CG-A--AA-G-C---G-TG--GG-G-
-AG-C-A-AA--------CA--GG Bacteria(100);Firmicutes(100);Clostridia(100);Clostridiales(100);Lachnospiraceae(100);unclassified(100);unclassified(100);unclassified(100);unclassified(100);

Otu1387 0 0 0 0 0 0 0 0 0 0 0 0 0 0 0 0 0 0 0 0 0 0 0 0 0 0 0 0
0 0 0 0 0 0 0 0 0 0 0 0 0 0 0 0 0 0 0 0 0 0 0 0 0 0 1 0 0
0 0 0 0 0 0 0 0 0 0 0 0 0 0 0 0 0 0 0 0 0 0 0 0 0 0 0 0 0
0 0 0 388 0 0 2 0 0 0 0 0 0 0 0 0 185 0 62 0 0 24 0 0 0 0 0
M02255_77_000000000-ADUT3_1_1116_22401_21236 T----AC---GG-AG-GGA--------GCT-A-G------C--G--T---T--GT-T-CGG-AA------TT-A--C-T--GG-GC-------GT--A-----AA-GC-GC-AC-------G-TA-G-G-C-G------------G--T-AC-A-T-C-----
------AA----G-T-C-A--------G-G-G--G--TG--A-AA-GC--C-C-GG-A-G-----CT-C-AA------C-T-C-C-G-G-A--A-C----T-G--C-C---C--T-------T--GA-A-A---C-------T--A-G--TG--T-A-C----T-A-G-A-A-T--C---T-TG-----GA-G-A--------G-G-C-G---GG-T------GG--A--ATT----
C-C-G-A-GT--GT-A-G-AG-GT--G---A--A-A---TT-C-GT-AG--AT-A-TT--C-G--G-A-A-G-A-AC-A-CC---AG--T--G--GC-GAA-G--G-C--G-------G--C-C-C-G---CTG--G--AC-A-A--------------------G-T---A-T-T--GA--CG-C-T-G--A-GG--T-G-CG-A--AA-G-C---G-TG--
GG-G--AG-C-A-AA--------CA--GG Bacteria(100);Proteobacteria(100);Alphaproteobacteria(100);Sphingomonadales(100);unclassified(100);unclassified(100);unclassified(100);unclassified(100);unclassified(100);

Otu1388 0 0 0 0 0 0 0 0 0 0 0 0 0 0 0 0 0 0 0 0 0 0 0 0 0 0 0 0
0 0 0 0 0 0 0 0 0 0 0 0 0 0 0 0 0 0 0 0 0 0 0 0 9 0 0 0 0
0 0 0 0 0 0 0 0 0 0 0 0 0 0 0 0 0 0 0 0 0 0 0 0 0 0 0 0 0
0 0 0 0 0 0 0 0 0 0 0 0 0 0 0 0 0 0 0 0 0 0 0 0 0 0 0
M02255_77_000000000-ADUT3_1_1103_12505_20808 T----AC---GT-AG-GAC--------CCA-A-G------C--G--T---T--AT-C-CGG-AG------TG-A--C-T--GG-GC-------GT--A-----AA-GA-GTTGC-------G-TA-G-G-T-G------------G--C-TT-G-T-A-----
------AA----G-T-G-A--------A-T-A--G--TG--A-AA-TC--T-G-GG-G-G-----CT-C-AA------C-C-T-C-ACA-G--G-C----T-A--T-T---A--T-------T--CA-A-A---C-------T--C-A--CA--A-G-C----T-C-G-A-G-A--A---T-GA-----TA-G-A--------G-G-T-A---AC-T------GG--A--ATT----T-
C-T-G-AA--GT-A-G-GG-GT--A---A--T-A---TC-C-GT-AG--AT-A-TC--A-G--A-A-G-G-A-AC-A-CC---AA--T--G--GC-GTA-G--G-C--A-------G--G-T-T-A---CTG--G--AT-C-A--------------------T-T---T-C-T--GA--CA-C-T-G--A-GG--C-A-CG-A--AA-G-C---G-TG--GG-G--
AG-C-G-AA--------CC--GG Bacteria(100);Candidate_division_TM7(100);unclassified(100);unclassified(100);unclassified(100);unclassified(100);unclassified(100);unclassified(100);unclassified(100);

Otu1389 0 0 0 0 0 0 0 0 0 0 0 0 0 0 0 0 0 0 0 0 0 0 0 0 0 0 0 0
0 0 0 0 0 0 0 0 0 0 0 0 0 0 0 0 0 0 0 0 0 0 0 0 20 0 0 0 0
0 0 0 0 0 0 0 0 0 0 0 0 0 0 0 0 0 0 0 0 0 0 0 0 0 0 0 0 0
0 0 0 0 0 0 0 0 0 0 0 0 0 0 0 0 0 0 0 0 0 0 0 0 0 0 0
M02255_77_000000000-ADUT3_1_1106_11100_16782 T----AC---GA-AG-GGG--------GCT-A-G------C--G--T---T--GC-T-CGG-AA------TG-A--C-T--GG-GC-------GT--A-----AA-GG-GC-GC-------G-TA-G-G-C-G------------G--G-TG-G-T-A---
--------CA----G-T-C-A--------G-A-T--G--TG--A-AA-TT--C-C-TG-G-G-----CT-C-AA------C-C-T-G-G-G-G--G-C----T-G--C-A---T--T-------T--GA-T-A---C-------G--T-G--CT--G-T-C----T-T-G-A-G-T--T---C-GG-----AA-G-A--------G-G-G-T---CG-T------GG--A--ATT----
C-C-C-A-GT--GT-A-G-AG-GT--G---A--A-A---TT-C-GT-AG--AT-A-TT--G-G--G-A-A-G-A-AC-A-CC---GG--T--G--GC-GAA-G--G-C--G-------G--C-G-A-C---CTG--G--TC-C-G--------------------A-A---A-C-T--GA--CG-C-T-G--A-GG--C-G-CG-A--AA-G-C---G-TG--
GG-G--AG-C-A-AA--------CA--GG Bacteria(100);Proteobacteria(100);Alphaproteobacteria(100);Rhodospirillales(100);Acetobacteraceae(100);unclassified(100);unclassified(100);unclassified(100);unclassified(100);

Otu1390 0 0 0 0 0 0 0 0 0 0 0 0 0 0 0 0 0 0 0 0 0 0 0 0 0 0 0 0
0 0 0 0 0 0 0 0 0 0 0 0 0 0 0 0 0 0 0 0 0 0 0 0 8 0 0 0 0
0 0 0 0 0 0 0 0 0 0 0 0 0 0 0 0 0 0 0 0 0 0 0 0 0 0 0 0
0 0 0 0 0 0 0 0 0 0 0 0 0 0 0 0 0 0 0 0 0 0 0 0 0 0 0
M02255_77_000000000-ADUT3_1_1107_22063_15733 T----AC---GA-AG-GGG--------GCT-A-G------C--G--T---T--GC-T-CGG-AA------TT-A--C-T--GG-GC-------GT--A-----AA-GG-GC-GC-------G-TA-G-G-C-G------------G--C-CT-T-G-T----
-------TA----G-T-C-A--------G-G-C--G--TG--A-AA-AT--C-C-CG-G-G-----CT-T-AA------C-C-T-G-G-G-G--A-C----T-G--C-G---C--T-------T--GA-T-A---C-------G--G-C--AG--G-G-C----T-T-G-A-G-G--A---T-GG-----AA-G-A--------G-G-C-T---CG-T------GG--A--ATT----
C-C-C-A-GT--GT-A-G-AG-GT--G---A--A-A---TT-C-GT-AG--AT-A-TT--G-G--G-A-A-G-A-AC-A-CC---GG--T--G--GC-GAA-G--G-C--G-------G--C-G-A-G---CTG--G--TC-C-A--------------------T-T---A-C-T--GA--CG-C-T-G--A-GG--C-G-CG-A--CA-G-C---G-TG--
GG-G--AG-C-A-AA--------CA--GG Bacteria(100);Proteobacteria(100);Alphaproteobacteria(100);Rhodospirillales(100);Acetobacteraceae(100);unclassified(100);unclassified(100);unclassified(100);unclassified(100);

Otu1391 0 0 0 0 0 0 0 0 0 0 0 0 0 0 0 0 0 0 0 0 0 0 0 0 0 0 0 0
0 0 0 0 0 0 0 0 0 0 0 0 0 0 0 0 0 0 0 0 0 0 0 0 15 0 0 0 0
0 0 0 0 0 0 0 0 0 0 0 0 0 0 0 0 0 0 0 0 0 0 0 0 0 0 0 0
0 0 0 0 0 0 0 0 0 0 0 0 0 0 0 0 0 0 0 0 0 0 0 0 0 0 0
M02255_77_000000000-ADUT3_1_1113_24976_5924 T----AC---GA-AG-GGG--------GCT-A-G------C--G--T---T--GT-T-CGG-AA------TT-A--C-T--GG-GC-------GT--A-----AA-GC-GC-AC-------G-TA-G-G-C-T------------G--A-TC-G-T-T-----------AA-
---G-T-G-A--------G-G-G--G--TG--A-AA-TC--C-C-GA-G-G-----CT-C-AA------C-C-T-C-G-G-A--A-C----T-G--C-C---C--T-------T--CA-T-A---C-------T--G-G--CG--A-T-C----T-C-G-A-G-T--C---C-GG-----AA-G-A--------G-G-T-T---AG-T------GG--A--ATT----C-C-C-A-
GT--GT-A-G-AG-GT--G---A--A-A---TT-C-GT-AG--AT-A-TT--G-G--G-A-A-G-A-AC-A-CC---AG--T--G--GC-GAA-G--G-C--G-------G--C-T-A-A---CTG--G--TC-C-G--------------------G-T---A-C-T--GA--CG-C-T-G--A-GG--T-G-CG-A--AA-G-C---G-TG--GG-G--AG-
C-A-AA--------CA--GG Bacteria(100);Proteobacteria(100);Alphaproteobacteria(100);Rhizobiales(100);unclassified(100);unclassified(100);unclassified(100);unclassified(100);unclassified(100);

Otu1392 0 0 0 0 0 0 0 0 0 0 0 0 0 0 0 0 0 0 0 0 0 0 0 0 0 0 0 0
0 0 0 0 0 0 0 0 0 0 0 0 0 0 0 0 0 0 0 0 0 0 0 0 15 0 0 0 0
0 0 0 0 0 0 0 0 0 0 0 0 0 0 0 0 0 0 0 0 0 0 0 0 0 0 0 0
0 0 0 0 0 0 0 0 0 0 0 0 0 0 0 0 0 0 0 0 0 0 0 0 0 0 0
M02255_77_000000000-ADUT3_1_1113_6206_7782 T----AC---GT-AG-GGT--------GCA-A-G------C--A--T---T--AT-C-CGG-AA------TT-A--T-T--GG-GC-------GT--A-----AA-GA-GT-TT-------G-TA-G-G-C-G------------G--T-AA-A-T-T-----------AA--
--G-T-A-G--------G-A-G--G--TG--A-AA-TC--T-C-TG-G-G-----CC-C-AA------C-C-C-A-G-A-A--C-C----C-G--T-T---T--C-------C--TA-A-A---C-------T--G-G--TT--T-A-C----T-A-G-A-G-G--T---A-TG-----TA-G-A--------G-G-T-A---AG-T------GG--A--ATT----T-C-C-G-GT-
-GT-A-G-GG-GT--A---A--A-A---TC-C-GT-TG--AG-A-TC--G-G--A-A-G-G-A-AC-G-CC---AA--A--A--GC-GAA-G--G-C--A-------G--C-T-T-A---CTG--G--AC-A-T----------------------A---C-C-T--GA--CG-C-T-G--A-GA--A-A-CG-A--AA-G-C---G-TG--GG-G--AG-C-A-
AA--------CA--GG Bacteria(100);unclassified(100);unclassified(100);unclassified(100);unclassified(100);unclassified(100);unclassified(100);unclassified(100);unclassified(100);

Otu1393 0 0 0 0 0 0 0 0 0 0 0 0 0 0 0 0 0 0 0 0 0 0 0 0 0 0 0 0
0 0 0 0 0 0 0 0 0 0 0 0 0 0 0 0 0 0 0 0 0 0 0 0 16 0 0 0 0
0 0 0 0 0 0 0 0 0 0 0 0 0 0 0 0 0 0 0 0 0 0 0 0 0 0 0 0
0 0 0 0 0 0 0 0 0 0 0 0 0 0 0 0 0 0 0 0 0 0 0 0 0 0 0
M02255_77_000000000-ADUT3_1_2105_26397_18519 T----AC---AG-AG-GCC--------CCA-A-G------C--G--T---T--AG-G-CGG-AA------TC-A--C-T--GG-GC-------TT--A-----AA-GC-GT-GT-------G-TA-G-G-C-G------------G--G-AT-G-G-T-----
------AA----G-T-A-C--------T-T-T--G--TG--A-AA-TC--C-C-AC-G-G-----CT-C-AA------C-T-G-T-G-G-A--A-T----T-G--C-T---T--A-------G--TA-T-A---C-------T--G-C--CG--T-T-C----T-T-G-A-G-T--C---A-TT-----TA-G-G--------G-G-C-A---GC-C------GG--A--ACA----G-A-
T-G-GT--GG-A-G-CG-GT--G---A--A-A---TG-C-GT-AG--AT-A-TC--A-T--C-T-G-G-A-AC-G-CC---AA--T--G--GT-GCA-G--A-C--A-------G--G-C-T-G---CTG--G--GA-A-T--------------------G-A---A-C-T--GA--CG-C-T-G--A-GG--C-A-CG-A--AA-G-C---T-AG--GG-G--
AG-C-G-AA--------CG--GG Bacteria(100);unclassified(100);unclassified(100);unclassified(100);unclassified(100);unclassified(100);unclassified(100);unclassified(100);unclassified(100);

Otu1394 0 0 0 0 0 0 0 0 0 0 0 0 0 0 0 0 0 0 0 0 0 0 0 0 0 0 0 0
0 0 0 0 0 0 0 0 0 0 0 0 0 0 0 0 0 0 0 0 0 0 0 0 13 0 0 0 0
0 0 0 0 0 0 0 0 0 0 0 0 0 0 0 0 0 0 0 0 0 0 0 0 0 0 0 0
0 0 0 0 0 0 0 0 0 0 0 0 0 0 0 0 0 0 0 0 0 0 0 0 0 0 0
M02255_77_000000000-ADUT3_1_2106_22646_14571 T----AC---GT-AG-GTG--------GCA-A-G------C--G--T---T--GT-C-CGG-AA------TT-A--C-T--GG-GC-------GT--A-----AA-GG-AT-GC-------G-TA-G-G-C-G------------G--A-TG-C-T-T-----
------AA----G-T-C-A--------G-A-T--G--TG--A-AA-AT--C-C-CG-G-G-----CT-T-AA------C-T-T-G-G-G-A--A-C----T-G--C-A---T--T-------T--GA-A-A---C-------T--G-G--GC--A-T-C----T-A-G-A-G-T--G---C-AG-----GA-G-A--------G-G-A-G---AG-T------GG--A--ATT----
C-C-T-A-GT--GT-A-G-CG-GT--G---A--A-A---TG-C-GT-AG--AG-A-TT--A-G--G-A-A-G-A-AC-A-CC---AG--T--G--GC-GAA-G--G-C--G-------G--C-T-C-T---CTG--G--AC-T-G--------------------T-A---A-C-T--GA--CG-C-T-G--A-GG--C-A-TG-A--AA-G-C---G-TG--GG-
G--AG-C-A-AA--------CA--GG Bacteria(100);Firmicutes(100);Clostridia(100);Clostridiales(100);Clostridiaceae(100);Clostridium(100);unclassified(100);unclassified(100);unclassified(100);

Otu1395 0 0 0 0 0 0 0 0 0 0 0 0 0 0 0 0 0 0 0 0 0 0 0 0 0 0 0 0
0 0 0 0 0 0 0 0 0 0 0 0 0 0 0 0 0 0 0 0 0 0 0 0 0 0 0 0 0
0 3 0 0 0 2 0 0 0 0 0 0 0 0 0 0 0 0 0 0 0 0 0 0 0 0 0 0 0
0 0 0 0 0 0 0 0 0 0 0 0 0 0 0 0 0 0 0 0 0 0 0 0 0 0 0
M02255_77_000000000-ADUT3_1_1107_22152_21720 T----AC---GT-AG-GTC--------CCG-A-G------C--G--T---T--GT-C-CGG-AT------TT-A--T-T--GG-GC-------GT--A-----AA-GC-GA-GC-------G-CA-G-G-T-G------------G--T-TT-A-A-T-----
-----AA----G-T-C-T--------G-A-T--G--TA--A-AA-GG--C-A-GT-G-G-----CT-C-AA------C-C-A-T-T-G-T--G------T-G--C-A---T--T-------G--GA-A-A---C-------T--G-T--TA--G-A-C----T-T-G-A-G-T--G---C-AG-----TA-G-A--------G-G-A-G---AG-T------GG--A--ATT----C-
C-A-T-GT--GT-A-G-CG-GT--G---A--A-A---TG-C-GT-AG--AT-A-TA--T-G--G-A-G-G-A-AC-A-CC---GG--T--G--GC-GAA-A--G-C--G-------G--C-T-C-T---CTG--G--AC-T-G--------------------T-A---A-C-T--GA--CA-C-T-G--A-GG--C-T-CG-A--AA-G-C---G-TG--GG-T-
-AG-C-A-AA--------CA--GG Bacteria(100);Firmicutes(100);Bacilli(100);Lactobacillales(100);Streptococcaceae(100);Lactococcus(100);unclassified(100);unclassified(100);unclassified(100);

Otu1396 0 0 0 0 0 0 0 0 0 0 0 0 0 0 0 0 0 0 0 0 0 0 0 0 0 0 0 0
0 0 0 0 0 0 0 0 0 0 0 0 0 0 0 0 0 0 0 0 0 0 0 0 0 0 0 0 0
0 29 0 0 0 36 0 0 0 0 0 0 0 0 0 0 0 0 0 0 0 0 0 0 0 0 0 0 0
0 0 0 0 0 0 0 0 0 0 0 0 0 0 0 0 0 0 0 0 0 0 0 0 0 0 0
M02255_77_000000000-ADUT3_1_1105_12228_7051 T----AC---GA-AG-GGG--------GCT-A-G------C--G--T---T--GT-T-CGG-AT------TT-A--C-T--GG-GC-------GT--A-----AA-GC-GC-AC-------G-TA-G-G-C-G------------G--G-CT-A-A-T-----------AA-
---G-T-C-A--------G-G-G--G--TG--A-AA-TC--C-C-GG-G-G-----CT-C-AA------C-C-C-C-G-G-A--A-C----T-G--C-C---T--T-------T--GA-T-A---C-------T--G-T--TA--G-T-C----T-T-G-A-G-T--A---T-GG-----AA-G-A--------G-G-T-G---AG-T------GG--A--ATT----C-C-G-A-
GT--GT-A-G-AG-GT--G---A--A-A---TT-C-GT-AG--AT-A-TT--C-G--G-A-G-G-A-AC-A-CC---AG--T--G--GC-GAA-G--G-C--G-------G--C-T-C-A---CTG--G--TC-C-A--------------------T-T---A-C-T--GA--CG-C-T-G--A-GG--T-G-CG-A--AA-G-C---G-TG--GG-G--AG-C-
A-AA--------CA--GG Bacteria(100);Proteobacteria(100);Alphaproteobacteria(100);Rhizobiales(100);Brucellaceae(100);Ochrobactrum(100);unclassified(100);unclassified(100);unclassified(100);

Otu1397 0 0 0 0 0 0 0 0 0 0 0 0 0 0 0 0 0 0 0 0 0 0 0 0 0 0 0 0
0 0 0 0 0 0 0 0 0 0 0 0 0 0 0 0 0 0 0 0 0 0 0 0 0 0 0 0 0
0 24 0 0 0 0 0 0 0 0 0 0 0 0 24 10 17 0 0 0 0 0 0 0 0 0 0 0 0
0 0 0 0 0 0 0 0 0 0 0 0 0 0 0 0 0 0 0 0 0 0 0 0 0 0 0
M02255_77_000000000-ADUT3_1_1106_2141_13736 T----AC---GT-AG-GGG--------GCA-A-G------C--G--T---T--AT-C-CGG-AT------TT-A--C-T--GG-GT-------GT--A-----AA-GG-GA-GC-------G-TA-G-A-C-G------------G--C-GA-A-G-C----------
AA----G-T-C-T--------G-A-A--G--TG--A-AA-AT--C-C-GG-G-G-----CT-C-AA------C-T-C-C-G-G-A--A-G----T-G--C-T---T--T-------G--GA-A-A---C-------T--G-T--TT--T-G-A----T-A-G-A-G-T--G---T-CG-----GA-G-A--------G-G-T-A---AG-T------GG--A--ATT----C-C-T-
G-GT--GT-A-G-CG-GT--G---A--A-A---TG-C-GT-AG--AT-A-TC--A-G--G-A-A-G-A-AC-A-CC---GG--A--G--GC-GAA-G--G-C--G-------G--C-T-T-A---CTG--G--AC-G-A--------------------T-A---A-C-T--GA--CG-T-T-G--A-GG--C-T-CG-A--AA-G-C---G-TG--GG-A--
AG-C-A-AA--------CA--GG Bacteria(100);Firmicutes(100);Clostridia(100);Clostridiales(100);Lachnospiraceae(100);Oribacterium(100);unclassified(100);unclassified(100);unclassified(100);

Otu1398 0 0 0 0 0 0 0 0 0 0 0 0 0 0 0 0 0 0 0 0 0 0 0 0 0 0 0 0
0 0 0 0 0 0 0 0 0 0 0 0 0 0 0 0 0 0 0 0 0 0 0 0 0 0 0 0 0
0 57 0 0 0 0 0 0 0 0 0 0 0 0 0 0 0 0 0 0 0 0 0 0 0 0 0 0 0
0 0 0 0 0 0 0 0 0 0 0 0 0 0 0 0 0 0 0 0 0 0 0 0 0 0 0
M02255_77_000000000-ADUT3_1_1106_14357_19836 T----AC---GT-AT-GTC--------GCA-A-G------C--G--T---T--AT-C-CGG-AA------TT-A--T-T--GG-GC-------AT--A-----AA-GG-GC-AT-------C-TA-G-G-C-G------------G--C-CC-T-G-T-------
----AA----G-T-C-T--------A-G-G--G--TG--A-AA-AC--C-T-GC-G-G-----CT-C-AA------C-C-G-C-A-G-G--C-C----T-G--C-C---C--C-------G--GA-A-A---C-------T--A-T--AG--G-G-C----T-G-G-A-G-T--A---C-TG-----GA-G-A--------G-G-T-G---GA-C------GG--A--ACT----A-
C-A-C-GA--GT-A-G-AG-GT--G---A--A-A---TT-C-GT-AG--AT-A-TG--T-G--T-A-G-G-A-AT-G-CC---GA--T--G--AT-GAA-G--A-T--A-------G--T-T-C-A---CTG--G--AC-G-G--------------------T-A---A-C-T--GA--CG-C-T-G--A-AG--T-G-CG-A--AA-G-C---T-GG--GG-G-
-AG-C-A-AA--------CA--GG Bacteria(100);Fusobacteria(100);Fusobacteria(100);Fusobacteriales(100);Leptotrichiaceae(100);Leptotrichia(100);unclassified(100);unclassified(100);unclassified(100);

Otu1399 0 0 0 0 0 0 0 0 0 0 0 0 0 0 0 0 0 0 0 0 0 0 0 0 0 0 0 0
0 0 0 0 0 0 0 0 0 0 0 0 0 0 0 0 0 0 0 0 0 0 0 0 0 0 0 0 0
0 8 0 0 0 0 0 0 0 0 0 0 0 0 0 0 0 0 0 0 0 0 0 0 0 0 0 0 0
0 0 0 0 0 0 0 0 0 0 0 0 0 0 0 0 0 0 0 0 0 0 0 0 0 0 0
M02255_77_000000000-ADUT3_1_1110_16336_10523 T----AC---GT-AG-GGG--------GCA-A-G------C--G--T---T--GT-C-CGG-AA------TG-A--C-T--GG-GC-------GT--A-----AA-GG-GT-GT-------G-TA-G-G-C-G------------G--G-CT-C-G-C----
-------AA----G-T-T-G--------G-A-T--G--TG--T-AA-TA--C-CCAG-A-G-----CT-T-AA------C-T-C-G-G-G-T--G-C----T-G--C-A---T--C-------T--GA-A-A---C-------T--A-C--GA--G-T-C----T-T-G-A-G-T--G---T-CG-----GA-G-A--------G-G-T-A---AG-T------GG--A--ATT----
C-C-T-A-GT--GT-A-G-CG-GT--G---G--A-A---TG-C-GT-AG--AT-A-TT--A-G--G-A-G-G-A-AC-A-TC---AG--T--G--GC-GAA-G--G-C--G-------A--C-T-T-A---CTG--G--AC-G-A--------------------T-A---A-C-T--GA--CG-C-T-G--A-GG--C-A-CG-A--AA-G-C---G-TG--GG-
G--AG-C-A-AA--------CA--GG Bacteria(100);unclassified(100);unclassified(100);unclassified(100);unclassified(100);unclassified(100);unclassified(100);unclassified(100);unclassified(100);

Otu1400 0 0 0 0 0 0 0 0 0 0 0 0 0 0 0 0 0 0 0 0 0 0 0 0 0 0 0 0
0 0 0 0 0 0 0 0 0 0 0 0 0 0 0 0 0 0 0 0 0 0 0 0 0 0 0 0 0
0 23 0 0 0 0 0 0 0 0 0 0 0 0 0 22 0 0 0 0 0 0 0 0 0 0 0 0 0
0 0 0 0 0 0 0 0 0 0 0 0 0 0 0 0 0 0 0 0 0 0 0 0 0 0 0 0
M02255_77_000000000-ADUT3_1_1111_13317_21543 T----AC---GT-AG-GGG--------GCG-A-G------C--G--T---T--GT-C-CGG-AA------TG-A--C-T--GG-GC-------GT--A-----AA-GG-GT-GC-------G-TA-G-G-T-G------------G--T-TT-A-A-T----
-------AA----G-T-T-A--------G-A-A--G--TG--T-AA-TA--CCC-AG-G-G-----CT-T-AA------C-T-C-G-G-G-T--G-C----T-G--C-T---T--C-------T--AA-A-A---C-------T--G-T--TA--G-G-C----T-T-G-A-G-T--G---T-AG-----TA-G-A--------G-G-A-A---AG-C------GG--A--ATT----
C-A-A-T-GT--GT-A-G-CG-GT--G---G--A-A---TG-C-GT-AG--AT-A-TA--T-T--G-A-A-G-A-AC-A-TC---AG--T--G--GC-GAA-G--G-C--G-------G--C-T-T-T---CTG--G--AC-T-A--------------------T-A---A-C-T--GA--CA-C-T-G--A-GG--C-A-CG-A--AA-G-C---G-TG--GG-
G--AG-C-A-AA--------CA--GG Bacteria(100);Firmicutes(100);Clostridia(100);Clostridiales(100);Lachnospiraceae(100);uncultured(100);unclassified(100);unclassified(100);unclassified(100);

Otu1401 0 0 0 0 0 0 0 0 0 0 0 0 0 0 0 0 0 0 0 0 0 0 0 0 0 0 0 0
0 0 0 0 0 0 0 0 0 0 0 0 0 0 0 0 0 0 0 0 0 0 0 0 0 0 0 0 0
0 72 0 0 0 0 0 0 0 0 0 0 0 0 0 0 0 0 0 0 0 0 0 0 0 0 0 0 0
0 0 0 0 0 0 0 0 0 0 0 0 0 0 0 0 0 0 0 0 0 0 0 0 0 0 0 0
M02255_77_000000000-ADUT3_1_1112_13415_13065 T----AC---GA-AG-GGG--------GCT-A-G------C--G--T---T--GC-T-CGG-AA------TG-A--C-T--GG-GC-------GT--A-----AA-GG-GC-GC-------G-TA-G-G-C-G------------G--A-TG-G-T-T----
-------CA----G-T-C-A--------G-A-C--G--TG--A-AA-TT--C-C-TG-G-G-----CT-T-AA------C-C-T-G-G-G-G--G-C----T-G--C-G---T--T-------T--GA-G-A---C-------G--G-T--TC--A-T-C----T-A-G-A-G-T--T---T-GG-----AA-G-A--------G-G-G-T---CG-T------GG--A--ATT----
C-C-C-A-GT--GT-A-G-AG-GT--G---A--A-A---TT-C-GT-AG--AT-A-TT--G-G--G-A-A-G-A-AC-A-CC---GG--T--G--GC-GAA-G--G-C--G-------G--C-G-A-C---CTG--G--TC-C-T--------------------T-G---A-C-T--GA--CG-C-T-G--A-GG--C-G-CG-A--AA-G-C---G-TG--
GG-G--AG-C-A-AA--------CA--GG Bacteria(100);Proteobacteria(100);Alphaproteobacteria(100);Rhodospirillales(100);Acetobacteraceae(100);unclassified(100);unclassified(100);unclassified(100);unclassified(100);

Otu1402 0 0 0 0 0 0 0 0 0 0 0 0 0 0 0 0 0 0 0 0 0 0 0 0 0 0 0 0
0 0 0 0 0 0 0 0 0 0 0 0 0 0 0 0 0 0 0 0 0 0 0 0 0 0 0 0 0
0 38 159 0 83 20 0 0 65 24 0 0 0 0 0 0 166 0 0 0 0 0 0 0 0 0 0 0 0
0 0 0 0 0 0 0 0 0 0 0 0 0 0 0 0 0 0 0 0 0 0 0 0 0 0 0 0
M02255_77_000000000-ADUT3_1_1119_22138_19486 T----AC---GT-AG-GTG--------GCG-A-G------C--G--T---T--GT-C-CGG-AA------TT-A--T-T--GG-GC-------GT--A-----AA-GG-GT-GC-------G-TA-G-G-C-G------------G--C-TC-T-T-T------
-----AA----G-T-G-G--------G-A-T--G--TG--A-AA-TC--C-C-TG-G-G-----CT-C-AA------C-C-C-A-G-G-A--A-C----T-G--C-A---T--T-------C--CA-A-A---C-------T--G-G--AG--A-G-C----T-A-G-A-G-T--G---C-AG-----GA-G-A--------G-G-A-A---AG-C------GG--A--ATT----
C-C-C-A-GT--GT-A-G-CG-GT--G---A--A-A---TG-C-GT-AG--AG-A-TT--G-G--G-A-G-G-A-AC-A-CC---AG--T--G--GC-GAA-G--G-C--G-------G--C-T-T-T---CTG--G--AC-T-G--------------------T-A---A-C-T--GA--CG-C-T-G--A-GG--C-A-CG-A--AA-G-C---G-TG--
GG-G--AG-C-A-AA--------CA--GG Bacteria(100);Firmicutes(100);Clostridia(100);Clostridiales(100);Clostridiaceae(100);Caloramator(100);unclassified(100);unclassified(100);unclassified(100);

Otu1403 0 0 0 0 0 0 0 0 0 0 0 0 0 0 0 0 0 0 0 0 0 0 0 0 0 0 0 0
0 0 0 0 0 0 0 0 0 0 0 0 0 0 0 0 0 0 0 0 0 0 0 0 0 0 0 0 0
0 24 10 0 10 0 0 0 10 0 0 0 0 0 0 0 36 0 0 0 0 0 0 0 0 0 0 0 0
0 0 0 0 0 0 0 0 0 0 0 0 0 0 0 0 0 0 0 0 0 0 0 0 0 0 0 0
M02255_77_000000000-ADUT3_1_1102_16371_11042 T----AC---GT-AG-GGG--------GCG-A-G------C--G--T---T--GT-C-CGG-AA------TT-A--C-T--GG-GC-------GT--A-----AA-GA-GC-AC-------G-TA-G-G-C-G------------G--C-TA-T-A-A----
-------AA----G-T-C-A--------G-A-T--G--TG--A-AA-AA--C-C-TG-G-G-----CT-C-AA------C-C-G-A-G-G-G--T-A----T-G--C-A---T--C-------T--GA-A-A---C-------T--A-A--AT--A-G-C----T-T-G-A-G-T--C---A-AG-----GA-G-A--------G-G-A-G---AG-C------GG--A--ATT----
C-C-T-G-GT--GT-A-G-CG-GT--G---A--A-A---TG-C-GT-AG--AG-A-TC--A-G--G-A-A-G-A-AT-A-CC---AG--T--G--GC-GAA-A--G-C--G-------G--C-T-C-T---CTG--G--AC-T-T--------------------G-A---A-C-T--GA--CG-C-T-G--A-GG--T-G-CG-A--AA-G-C---G-TG--GG-
G--AG-C-A-AA--------CA--GG Bacteria(100);Firmicutes(100);Clostridia(100);Thermoanaerobacterales(100);Family_III_Incertae_Sedis(100);Thermoanaerobacter(100);unclassified(100);unclassified(100);unclassified(100);

Otu1404 0 0 0 0 0 0 0 0 0 0 0 0 0 0 0 0 0 0 0 0 0 0 0 0 0 0 0 0
0 0 0 0 0 0 0 0 0 0 0 0 0 0 0 0 0 0 0 0 0 0 0 0 0 0 0 0 0
4 0 0 0 0 0 0 4 0 0 0 0 0 0 0 0 0 0 0 5 7 0 0 0 0 0 0 0 0
0 0 0 0 0 0 0 0 0 0 0 0 0 0 0 0 0 0 0 0 0 0 0 0 0 0 0 0
M02255_76_000000000-ADUNE_1_2114_28128_18151 T----AC---GA-AG-GGG--------GCT-A-G------C--G--T---T--GC-T-CGG-AA------TT-A--C-T--GG-GC-------GT--A-----AA----G--G--------G--A--------------------G----C--G-T-T-----------
AG----G-T-T-A--------G-A-G--G--TG--A-AA-GC--C-C-AG-G-G-----CT-C-AA------C-C-T-T-G-G-A--A-T----T-G--C-C---T--T-------T--GA-T-A---C-------T--G-G--CA--G-T-C----T-T-G-A-G-T--A---C-GG-----AA-G-A--------G-G-T-A---TG-T------GG--A--ACT----C-C-G-
A-GT--GT-A-G-AG-GT--G---A--A-A---TT-C-GT-AG--AT-A-TT--C-G--G-A-A-G-A-AC-A-CC---AG--T--G--GC-GAA-G--G-C--G-------A--C-A-T-A---CTG--G--TC-C-G--------------------T-T---A-C-T--GA--CG-C-T-G--A-GG--C-T-CG-A--AA-G-C---G-TG--GG-G--AG-
C-A-AA--------CA--GG Bacteria(100);Proteobacteria(100);Alphaproteobacteria(100);Caulobacterales(100);Caulobacteraceae(100);Caulobacter(100);unclassified(100);unclassified(100);unclassified(100);

Otu1405 0 0 0 0 0 0 0 0 0 0 0 0 0 0 0 0 0 0 0 0 0 0 0 0 0 0 0 0
0 0 0 0 0 0 0 0 0 0 0 0 0 0 0 0 0 0 0 0 0 0 0 0 0 0 0 0 0
0 8 0 0 0 0 0 0 0 0 0 0 0 0 0 0 0 0 0 0 0 0 0 0 0 0 0 0 0
0 0 0 0 0 0 0 0 0 0 0 0 0 0 0 0 0 0 0 0 0 0 0 0 0 0 0
M02255_76_000000000-ADUNE_1_2105_9336_13064 C----GG---CT-AA-CTT--------CGT-G-C------C--A--G---C--AG-C-CGC-GG------TA-A--T-A--CG-AA-------GG--A-----TC-CA-AG-CG-------T-TA-T-C-C-G------------GA-T-TT-A-T-T-----------AA----G-T-C-A--------G-A-G--G--TG--A-AA-GC--C-T-GG-A-G-----CT-T-AA------C-T-C-C-A-G-A--A-C----T-G--C-C---T--T-------T--GA-T-A---C-------T--G-A--TG--A-G-C----T-T-G-A-A-T--A---T-AG-----TT-G-A--------G-G-T-A---GG-C------GG--A--ATG----T-G-T-C-AT--GT-A-G-CG-GT--G---A--A-A---TG-C-TT-AG--AT-A-TG--A-C--A-C-A-G-A-AC-A-CC---GA--T--T--GC-GAA-G--G-C--A-------G--C-T-T-G---CTA--A--AC-T-A--------------------T-A---A-T-T--GA--CG-C-T-G--A-GG--C-A-CT-A--AA-G-C---G-TG--GG-G--AT-C-A-AA--------CA--GG Bacteria(100);Bacteroidetes(100);Sphingobacteria(100);Sphingobacteriales(100);unclassified(100);unclassified(100);unclassified(100);unclassified(100);unclassified(100);

Otu1406 0 0 0 0 0 0 0 0 0 0 0 0 0 0 0 0 0 0 0 0 0 0 0 0 0 0 0 0
0 0 0 0 0 0 0 0 0 0 0 0 0 0 0 0 0 0 0 0 0 0 0 0 0 0 0 0 0
0 1 0 0 2 0 0 1 0 0 0 0 0 0 0 0 0 0 0 9 1 0 0 0 0 0 0 0 0
0 0 0 0 0 0 0 0 0 0 0 0 0 0 0 0 0 0 0 0 0 0 0 0 0 0 0
M02255_76_000000000-ADUNE_1_2104_10973_9965 G----AC---GT-AG-GAT--------GCA-A-G------C--G--T---T--GT-C-CGG-AT------TT-A--T-T--GG-GC-------GT--A-----AA-G--G----------G-T------T-C------------G-----T---T------------AA----G-T-C-T--------G-A-T--G--TT--A-AA-GA--T-C-GG-G-G-----CT-C-AA------C-C-T-C-G-G-G--A-G----T-G--C-A---T--T-------G--GA-T-A---C-------T--G-G--CA--G-A-C----T-G-G-A-G-T--G---T-GG-----TA-G-A--------G-G-T-T---AG-T------GG--A--ATT----C-C-C-A-GT--GT-A-G-CG-GT--G---A--A-A---TG-C-GT-AG--AT-A-TT--G-G--G-A-A-G-A-AC-A-CC---AG--T--G--GC-GTA-G--G-C--G-------A--C-T-A-A---CTG--G--GC-C-A--------------------T-A---A-C-T--GA--CG-C-T-G--A-GG--A-A-CG-A--AA-G-C---C-AG--GG-G--AG-C-G-AA--------TG--GG Bacteria(100);Cyanobacteria(100);MLE1-12(100);unclassified(100);unclassified(100);unclassified(100);unclassified(100);unclassified(100);unclassified(100);

Otu1407 0 0 0 0 0 0 0 0 0 0 0 0 0 0 0 0 0 0 0 0 0 0 0 0 0 0 0 0
0 0 0 0 0 0 0 0 0 0 0 0 0 0 0 0 0 0 0 0 0 0 0 0 0 0 0 0 0
0 6 0 1 0 0 0 0 0 0 0 0 0 0 0 0 0 0 0 0 0 0 3 0 0 0 0 0 0
0 0 0 0 0 0 0 0 0 0 0 0 0 0 0 0 0 0 0 0 0 0 0 0 0 0 0
M02255_76_000000000-ADUNE_1_1108_20360_19990 .....AC----T-AC-GT---------GCC-A-G------C--A--G---C--CG-C-GGT-AA------TA-C--G-T--AG-GT-------GG--C-----AA-GC-G--TT-------G----T-C-C-G------------G--T-TT-C-T-T----------AA----G-T-C-T--------G-A-T--G--TG--A-AA-GC--C-C-CC-G-G-----CT-C-AA------C-C-G-G-G-G-A--G------G-GT-C-A---T--T-------G--GA-A-A---C-------T--G-G--GG--A-A-C----T-T-G-A-G-T--G---C-AG-----AA-G-A--------G-G-A-G---AG-T------GG--A--ATT----C-C-A-C-GT--GT-A-G-CG-GT--G---A--A-A---TG-C-GT-AG--AG-A-TG--T-G--G-A-G-G-A-AC-A-CC---AG--T--G--GC-GAA-G--G-C--G-------A--C-T-C-T---CTG--G--TC-T-G--------------------T-A---A-C-T--GA--CG-C-T-G--A-GG--A-G-CG-A--AA-G-C---G-TG--GG-G--AG-C-G-AA--------CA--GG Bacteria(100);Firmicutes(100);Bacilli(100);unclassified(100);unclassified(100);unclassified(100);unclassified(100);unclassified(100);unclassified(100);

Otu1408 0 0 0 0 0 0 0 0 0 0 0 0 0 0 0 0 0 0 0 0 0 0 0 0 0 0 0 0
0 0 0 0 0 0 0 0 0 0 0 0 0 0 0 0 0 0 0 0 0 0 0 0 0 0 0 0 0
0 0 61 0 37 5 0 0 14 10 1 0 0 0 0 0 75 0 0 0 0 0 0 0 0 0 0 0 0
0 0 0 0 0 0 0 0 0 0 0 0 0 0 0 0 0 0 0 0 0 0 0 0 0 0 0
M02255_77_000000000-ADUT3_1_1102_9741_16744 T----AC---GT-AG-GTG--------GCG-A-G------C--G--T---T--GT-C-CGG-AA------TT-A--T-T--GG-GC-------GT--A-----AA-GG-GT-GC-------G-TA-G-G-C-G------------G--T-AG-T-T-T-----------AA----G-T-G-G--------G-G-C--G--TG--A-AA-TC--C-C-CG-G-G-----CT-T-AA------C-C-C-G-G-G-A--G-C----T-G--C-G---C--T-------C--CA-A-A---C-------T--G-G--AC--T-A-C----T-C-G-A-G-T--G---C-AG-----GA-G-A--------G-G-A-A---AG-C------GG--A--ATT----C-C-C-A-GT--GT-A-G-CG-GT--G---A--A-A---TG-C-GT-AG--AG-A-TT--G-G--G-A-G-G-A-AC-A-CC---AG--T--G--GC-GAA-G--G-C--G-------G--C-T-T-T---CTG--G--AC-T-G--------------------T-A---A-C-T--GA--CG-C-T-G--A-GG--C-A-CG-A--AA-G-C---G-TG--GG-G--AG-C-A-AA--------CA--GG Bacteria(100);unclassified(100);unclassified(100);unclassified(100);unclassified(100);unclassified(100);unclassified(100);unclassified(100);unclassified(100);

Otu1409 0 0 0 0 0 0 0 0 0 0 0 0 0 0 0 0 0 0 0 0 0 0 0 0 0 0 0 0
0 0 0 0 0 0 0 0 0 0 0 0 0 0 0 0 0 0 0 0 0 0 0 0 0 0 0 0 0
0 0 16 0 0 0 0 2 2 1 0 0 0 0 0 0 2 0 0 0 0 0 0 0 0 0 0 0 0
0 0 0 0 0 0 0 0 0 0 0 0 0 0 0 0 0 0 0 0 0 0 0 0 0 0 0
M02255_77_000000000-ADUT3_1_1119_19729_8193 T----AC---GT-AG-GGT--------CCA-A-G------C--G--T---T--AA-T-CGG-AA------TT-A--C-T--GG-GC-------GT--A-----AA-GC-GT-GC-------G-CA-G-G-C-G------------G--T-TG-T-G-C-----------AA----G-A-C-C--------G-A-T--G--TG--A-AA-TC--C-C-CG-A-G-----CT-T-AA------C-T-T-G-G-G-A--A-T----T-G--C-A---T--T-------G--GT-G-A---C-------T--G-C--AC--G-G-C----T-A-G-A-G-T--G---T-GT-----CA-G-A--------G-G-G-G---GG-T------AG--A--ATT----C-C-A-C-GT--GT-A-G-CA-GT--G---A--A-A---TG-C-GT-AG--AG-A-TG--T-G--G-A-G-G-A-AT-A-CC---GA--T--G--GC-GAA-G--G-C--A-------G--C-C-C-C---CTG--G--GA-T-A--------------------A-C---A-C-T--GA--CG-C-T-C--A-TG--C-A-CG-A--AA-G-C---G-TG--GG-G--AG-C-A-AA--------CA--GG Bacteria(100);Proteobacteria(100);Betaproteobacteria(100);Burkholderiales(100);Burkholderiaceae(100);Ralstonia(100);unclassified(100);unclassified(100);unclassified(100);

Otu1410 0 0 0 0 0 0 0 0 0 0 0 0 0 0 0 0 0 0 0 0 0 0 0 0 0 0 0 0
0 0 0 0 0 0 0 0 0 0 0 0 0 0 0 0 0 0 0 0 0 0 0 0 0 0 0 0 0
0 0 15 0 0 0 0 0 0 0 0 0 0 0 0 0 0 0 0 0 0 0 0 0 0 0 0 0 0
0 0 0 0 0 0 0 0 0 0 0 0 0 0 0 0 0 0 0 0 0 0 0 0 0 0 0 0
M02255_77_000000000-ADUT3_1_1110_28706_16264 T----AC---GT-AG-GGG--------GCA-A-G------C--G--T---T--GT-C-CGG-AA------TT-A--T-T--GG-GC-------GT--A-----AA-GC-GC-GC-------G-CA-G-G-C-G------------G--T-TA-A-T-T-----
------AA----G-T-C-A--------G-G-T--G--TG--A-AA-GG--C-T-AC-G-G-----CT-C-AA------C-C-G-T-A-G-A--G------T-TG-C-A---T--T-------T--GA-A-A---C-------T--G-G--TT--A-A-C----T-T-G-A-G-T--G---C-AG-----GA-G-A--------G-G-T-A---AG-T------GG--A--ATT----C-
C-C-G-GT--GT-A-G-CG-GT--G---G--A-A---TG-C-GT-AG--AG-A-TC--G-G--G-A-G-G-A-AC-A-CC---AG--T--G--GC-GAA-G--G-C--G-------G--C-T-T-A---CTG--G--CC-T-G--------------------C-A---A-C-T--GA--CG-C-T-G--A-GG--C-G-CG-A--AA-G-C---G-TG--GG-
G--AG-C-A-AA--------CA--GG Bacteria(100);Firmicutes(100);unclassified(100);unclassified(100);unclassified(100);unclassified(100);unclassified(100);unclassified(100);unclassified(100);

Otu1411 0 0 0 0 0 0 0 0 0 0 0 0 0 0 0 0 0 0 0 0 0 0 0 0 0 0 0 0
0 0 0 0 0 0 0 0 0 0 0 0 0 0 0 0 0 0 0 0 0 0 0 0 0 0 0 0 0
0 0 6 2 0 0 0 0 0 0 4 0 0 0 0 0 0 0 0 0 0 0 0 0 0 0 0 0 0
0 0 0 0 0 0 0 0 0 0 0 0 0 0 0 0 0 0 0 0 0 0 0 0 0 0 0 0
M02255_76_000000000-ADUNE_1_2114_22876_15582 T----AA---GG-AC-CGG--------CTA-A-C------T--A--C---G--TG-C-CAG-CA------GC-C--G-C--GG-TA-------AT--A-----CG-TA-GG-GT-------C-CA-A-G-C-G------------T--T-AA-TCG-G-----
------AA----G-A-C-C--------G-A-T--G--TG--A-AA-TC--C-C-CG-A-G-----CT-T-AA------C-T-T-G-G-G-A--A-T----T-G--C-A---T--T-------G--GT-G-A---C-------T--G-C--AC--G-G-C----T-A-G-A-G-T--G---T-GT-----CA-G-A--------G-G-G-G---GG-T------AG--A--ATT----C-
C-A-C-GT--GT-A-G-CA-GT--G---A--A-A---TG-C-GT-AG--AG-A-TG--T-G--G-A-G-G-A-AT-A-CC---GA--T--G--GC-GAA-G--G-C--A-------G--C-C-C-C---CTG--G--GA-T-A--------------------A-C---A-C-T--GA--CG-C-T-C--A-TG--C-A-CG-A--AA-G-C---G-TG--GG-
G--AG-C-A-AA--------CA--GG Bacteria(100);Proteobacteria(100);Betaproteobacteria(100);Burkholderiales(100);unclassified(100);unclassified(100);unclassified(100);unclassified(100);unclassified(100);

Otu1412 0 0 0 0 0 0 0 0 0 0 0 0 0 0 0 0 0 0 0 0 0 0 0 0 0 0 0 0
0 0 0 0 0 0 0 0 0 0 0 0 0 0 0 0 0 0 0 0 0 0 0 0 0 0 0 0 0
0 0 0 0 0 0 0 0 0 0 0 0 0 0 0 0 0 0 0 0 0 0 0 0 0 0 0 0 0
29 0 0 0 0 0 0 0 0 0 0 0 0 0 0 0 0 0 0 0 0 0 0 0 0 0 0 0
M02255_77_000000000-ADUT3_1_1104_21040_19221 T----AC---GT-AG-GTG--------GCA-A-G------C--G--T---T--GT-C-CGG-AT------TT-A--C-T--GG-GT-------GT--A-----AA-GG-GG-GC-------G-CA-G-G-C-G------------G--G-AA-G-C-T----
-------AA----G-T-T-G--------G-A-T--G--TG--A-AA-TA--C-C-GG-G-G-----CT-C-AA------C-T-C-C-G-G-A--G-C----T-G--C-A---T--C-------C--AA-A-A---C-------T--G-G--TT--T-T-C----T-T-G-A-G-T--G---G-AG-----TA-G-A--------G-G-C-A---AG-C------GG--A--ATT----
C-C-G-A-GT--GT-A-G-CG-GT--G---A--A-A---TG-C-GT-AG--AT-A-TT--C-G--G-A-G-G-A-AC-A-CC---AG--T--G--GC-GAA-G--G-C--G-------G--C-T-T-G---CTG--G--GC-T-C--------------------T-A---A-C-T--GA--CG-C-T-G--A-GG--C-C-CG-A--AA-G-C---G-TG--GG-
G--AG-C-A-AA--------CA--GG Bacteria(100);unclassified(100);unclassified(100);unclassified(100);unclassified(100);unclassified(100);unclassified(100);unclassified(100);unclassified(100);

Otu1413 0 0 0 0 0 0 0 0 0 0 0 0 0 0 0 0 0 0 0 0 0 0 0 0 0 0 0 0
0 0 0 0 0 0 0 0 0 0 0 0 0 0 0 0 0 0 0 0 0 0 0 0 0 0 0 0 0
9 0 0 0 0 0 0 0 0 0 0 0 0 0 0 0 0 0 0 0 0 0 0 0 0 0 8 0 0
0 0 0 0 0 0 0 0 0 0 0 0 0 0 0 0 0 0 0 0 0 0 0 0 0 0 0 0
M02255_76_000000000-ADUNE_1_1102_14127_12584 G----AA---TA-AG-CAC--------CGG-C-T------A--A--C---T--AC-G-TGC-CA------GC-A--G-C--CG-CG-------GT--A-----AT-AC-GT--A-------G-GG-T-G-C-G------------A--G-CT-G-T-T------
-----AA----G-T-C-G--------G-A-A--G--TG--A-AA-TC--C-C-CG-G-G-----CT-C-AA------C-C-T-G-G-G-A--A-C----T-G--C-A---T--T-------T--GT-G-A---C-------T--G-G--CA--G-G-C----T-A-G-A-G-T--A---T-GG-----CA-G-A--------G-G-G-G---GG-T------AG--A--ATT----C-
C-A-C-GT--GT-A-G-CA-GT--G---A--A-A---TG-C-GT-AG--AG-A-TG--T-G--G-A-G-G-A-AT-A-CC---GA--T--G--GC-GAA-G--G-C--A-------G--C-C-C-C---CTG--G--GC-C-A--------------------A-T---A-C-T--GA--CG-C-T-C--A-TG--C-A-CG-A--AA-G-C---G-TG--GG-G-
-AG-C-A-AA--------CA--GG Bacteria(100);Proteobacteria(100);Betaproteobacteria(100);unclassified(100);unclassified(100);unclassified(100);unclassified(100);unclassified(100);unclassified(100);

Otu1414 0 0 0 0 0 0 0 0 0 0 0 0 0 0 0 0 0 0 0 0 0 0 0 0 0 0 0 0
0 0 0 0 0 0 0 0 0 0 0 0 0 0 0 0 0 0 0 0 0 0 0 0 0 0 0 0 0
6 0 0 0 0 0 0 0 0 0 0 0 0 0 0 0 0 0 0 14 0 0 0 0 0 0 0 0 0
5 0 0 0 0 0 0 0 0 0 0 5 0 0 0 0 0 0 0 0 0 0 0 0 0 0 0 0
M02255_77_000000000-ADUT3_1_1109_12351_10624 T----AC---GT-AG-GGG--------GCG-A-G------C--G--T---T--AT-C-CGG-AT------TC-A--T-T--GG-GC-------GT--A-----AA-GC-GC-GC-------G-TA-G-G-C-G------------G--C-CC-G-G-C----
-------AG----G-C-A-G--------G-G-G--G--TC--A-AA-TG--G-C-GG-G-G-----CT-C-AA------C-C-C-C-G-T-C--C------C-G--C-C---C--C-------C--TG-A-A---C-------C--G-C--CG--G-G-C----T-C-G-G-G-T--C---C-GG-----TA-G-G--------G-G-A-G---GG-T------GG--A--ACA----
C-C-C-G-GT--GT-A-G-CG-GT--G---G--A-A---TG-C-GC-AG--AT-A-TC--G-G--G-T-G-G-A-AC-A-CC---GG--T--G--GC-GAA-G--GC---G-------G--C-C-C-T---CTG--G--GC-C-G--------------------A-G---A-C-C--GA--CG-C-T-G--A-GG--C-G-CG-A--AA-G-C---T-GG--
GG-G--AG-C-G-AA--------CA--GG Bacteria(100);Actinobacteria(100);Actinobacteria(100);Coriobacteridae(100);Coriobacteriales(100);Coriobacterineae(100);Coriobacteriaceae(100);Collinsella(100);unclassified(100);

Otu1415 0 0 0 0 0 0 0 0 0 0 0 0 0 0 0 0 0 0 0 0 0 0 0 0 0 0 0 0
0 0 0 0 0 0 0 0 0 0 0 0 0 0 0 0 0 0 0 0 0 0 0 0 0 0 0 0 0
23 0 0 0 0 0 0 0 0 0 0 0 0 0 0 0 0 0 0 0 0 0 0 0 0 0 0 0 0
0 0 0 0 0 0 0 0 0 0 0 0 0 0 0 0 0 0 0 0 0 0 0 0 0 0 0 0
M02255_77_000000000-ADUT3_1_1118_15907_22717 T----AC---GG-AG-GGA--------GCT-A-G------C--G--T---T--GT-T-CGG-AA------TT-A--C-T--GG-GC-------GT--A-----AA-GC-GT-AC-------G-TA-G-G-C-G------------G--C-TT-T-G-T-----
------AA----G-T-C-A--------G-A-G--G--TG--A-AA-GC--C-C-AC-G-A-----CT-C-AA------T-C-G-T-G-G-A--A-C----T-G--C-C---T--T-------T--GA-G-A---C-------T--G-C--TT--C-G-C----T-T-G-A-A-G--A---T-GG-----GA-G-A--------G-G-T-T---AG-T------GG--A--ATT----C-
C-G-A-GT--GT-A-G-AG-GT--G---A--A-A---TT-C-GT-AG--AT-A-TT--C-G--G-A-A-G-A-AC-A-CC---AG--T--G--GC-GAA-G--G-C--G-------G--C-T-A-A---CTG--G--AC-C-A--------------------T-T---C-T-T--GA--CG-C-T-G--A-GG--T-A-CG-A--AA-G-C---G-TG--GG-G-
-AG-C-A-AA--------CA--GG Bacteria(100);unclassified(100);unclassified(100);unclassified(100);unclassified(100);unclassified(100);unclassified(100);unclassified(100);unclassified(100);

Otu1416 0 0 0 0 0 0 0 0 0 0 0 0 0 0 0 0 0 0 0 0 0 0 0 0 0 0 0 0
0 0 0 0 0 0 0 0 0 0 0 0 0 0 0 0 0 0 0 0 0 0 0 0 0 0 0 0 0
34 0 0 0 0 0 0 0 0 0 0 0 0 0 0 0 0 0 0 0 0 0 0 0 0 0 0 0 0
0 0 0 0 0 0 0 0 0 0 0 0 0 0 0 0 0 0 0 0 0 0 0 0 0 0 0 0
M02255_77_000000000-ADUT3_1_1117_7589_17799 T----AC---GG-AG-GAT--------GCA-A-G------C--G--T---T--AT-C-CGG-AA------TG-A--T-T--GG-GC-------GT--A-----AA-GC-GT-CC-------G-CA-G-G-T-G------------G--C-AA-T-G-T-----------AA-
---G-T-C-T--------G-C-T--G--TC--A-AA-GC--G-T-CT-G-G-----CT-C-AA------C-C-A-G-A-T-A--A-A----G-G--C-A---G--T-------G--GA-A-A---C-------T--A-C--AT--A-G-C----T-A-G-A-G-T--A---A-GG-----TA-G-G--------G-G-T-A---GA-G------GG--A--ATT----C-C-T-G-
GT--GT-A-G-CG-GT--G---A--A-A---TG-C-GT-AG--AG-A-TC--A-G--G-A-A-G-A-AC-A-CC---GG--T--G--GC-GAA-A--G-C--G-------C--T-C-T-G---CTG--G--AC-C-T--------------------T-A---A-C-T--GA--CA-C-T-G--A-GG--G-A-CG-A--AA-G-C---T-AG--GG-G--AG-
C-G-AA--------TG--GG Bacteria(100);Cyanobacteria(100);unclassified(100);unclassified(100);unclassified(100);unclassified(100);unclassified(100);unclassified(100);unclassified(100);

Otu1417 0 0 0 0 0 0 0 0 0 0 0 0 0 0 0 0 0 0 0 0 0 0 0 0 0 0 0 0
0 0 0 0 0 0 0 0 0 0 0 0 0 0 0 0 0 0 0 0 0 0 0 0 0 0 0 0 0
5 48 0 120 0 2 0 7 5 0 0 0 0 0 0 9 17 0 0 0 0 0 0 0 0 0 0 0 0
0 0 0 0 0 0 0 0 0 0 0 0 0 0 0 0 0 0 0 0 0 0 0 0 0 0 0 0
M02255_77_000000000-ADUT3_1_1111_22116_19660 T----AC---GG-AG-GAT--------GCA-A-G------C--G--T---T--AT-C-CGG-AA------TG-A--T-T--GG-GC-------GT--A-----AA-GC-GT-CC-------G-CA-G-G-T-G------------G--C-TT-A-T-C-----
------AA----G-T-C-G--------A-T-T--G--TC--A-AA-GG--T-C-CT-G-G-----CT-C-AA------C-T-G-G-G-A-A--C-A----G-G--C-A---G--T-------G--GA-A-A---C-------T--G-A--AA--A-G-C----T-A-G-A-G-T--G---C-GG-----TA-G-G--------G-G-T-A---GA-G------GG--A--ATT----
C-C-C-G-GT--GT-A-G-CG-GT--G---A--A-A---TG-C-GT-AG--AG-A-TC--G-G--G-A-A-G-A-AC-A-CC---AG--T--G--GC-GAA-A--G-C--G-------C--T-C-T-A---CTA--G--GC-C-T--------------------G-C---A-C-T--GA--CA-C-T-C--A-TG--G-A-CG-A--AA-G-C---T-AG--GG-
G--AG-C-G-AA--------TG--GG Bacteria(100);Cyanobacteria(100);SubsectionII(100);SubgroupII(100);Chroococcidiopsis(100);unclassified(100);unclassified(100);unclassified(100);unclassified(100);

Otu1418 0 0 0 0 0 0 0 0 0 0 0 0 0 0 0 0 0 0 0 0 0 0 0 0 0 0 0 0
0 0 0 0 0 0 0 0 0 0 0 0 0 0 0 0 0 0 0 0 0 0 0 0 0 0 0 0 0
10 0 0 0 0 0 0 0 0 0 0 0 0 0 0 0 0 0 0 0 0 0 0 0 0 0 0 0 0
0 0 0 0 0 0 0 0 0 0 0 0 0 0 0 0 0 0 0 0 0 0 0 0 0 0 0 0
M02255_77_000000000-ADUT3_1_1110_17261_4206 T----AC---AG-AG-GGG--------GCA-A-G------C--G--T---T--GT-C-CGG-AC------TT-A--C-T--GG-GC-------GT--A-----CA-GC-GC-GC-------G-CA-G-G-C-G------------G--C-CG-T-G-T-----------
AT----G-T-G-C--------T-G-G--G--TG--A-CA-TC--C-T-CC-C-G-----CT-C-AA------C-G-G-G-A-G-G--C-G----G-C--C-C---C--G-------G--TA-G-A---C-------A--G-C--AG--G-G-C----T-G-G-A-G-G--C---A-GG-----GA-G-A--------G-G-G-A---TG-T------GG--A--ATG----C-C-
C-G-GT--GG-A-G-CG-GT--G---A--A-A---TG-C-GT-AG--AG-A-TC--G-G--G-C-G-G-A-AC-A-CC---AG--C--G--GC-GAA-G--G-C--G-------G--C-A-T-C---CTG--G--CC-C-T--------------------G-A---C-C-T--GA--CG-C-T-G--A-GG--C-G-CG-A--TA-G-C---G-TG--GG-G--
AG-C-A-AA--------CG--GG Bacteria(100);unclassified(100);unclassified(100);unclassified(100);unclassified(100);unclassified(100);unclassified(100);unclassified(100);unclassified(100);

Otu1419 0 0 0 0 0 0 0 0 0 0 0 0 0 0 0 0 0 0 0 0 0 0 0 0 0 0 0 0
0 0 0 0 0 0 0 0 0 0 0 0 0 0 0 0 0 0 0 0 0 0 0 0 0 0 0 0 213
0 0 0 0 0 0 0 0 0 0 0 0 0 0 0 0 0 0 0 0 0 0 0 0 0 0 0 0 0
0 0 0 0 0 0 0 0 0 0 0 0 0 0 0 0 0 0 0 0 0 0 0 0 0 0 0 0
M02255_77_000000000-ADUT3_1_1115_26670_17085 G----AC---AG-AG-GCC--------TCA-A-G------C--G--T---T--AG-G-CGG-AA------TC-A--C-T--GG-GC-------TT--A-----AA-GC-GT-GT-------G-TA-G-G-C-G------------G--A-TC-G-T-T-----
------AA----G-T-G-T--------C-T-T--G--TG--A-AA-TC--C-C-AC-G-G-----CT-C-AA------C-C-G-T-G-G-A--A-C----G-G--C-T---G--G-------G--CA-T-A---C-------T--G-G--CG--A-T-C----T-T-G-A-G-C--A---G-CC-----TA-G-G--------G-G-C-G---AC-C------GG--A--ACA----
A-A-C-G-GT--GG-A-G-CG-GT--G---A--A-A---TG-C-GT-AG--AT-A-TC--G-T--T-T-G-G-A-AC-G-CC---AA--T--G--GT-GAA-A--A-C--A-------G--G-T-C-G---CTG--G--GG-G-C--------------------T-C---G-C-T--GA--CG-C-T-G--A-GA--C-A-CG-A--AA-G-C---C-AG--
GG-G--AG-C-A-AA--------CG--GG Bacteria(100);Planctomycetes(100);Phycisphaerae(100);WD2101(100);unclassified(100);unclassified(100);unclassified(100);unclassified(100);unclassified(100);

Otu1420 0 0 0 0 0 0 0 0 0 0 0 0 0 0 0 0 0 0 0 0 0 0 0 0 0 0 0 0
0 0 0 0 0 0 0 0 0 0 0 0 0 0 0 0 0 0 0 0 0 0 0 0 0 0 0 0 19
0 0 0 0 0 0 0 0 0 0 0 0 0 0 0 0 0 0 0 0 0 0 0 0 0 0 0 0 0
0 0 0 0 0 0 0 0 0 0 0 0 0 0 0 0 0 0 0 0 0 0 0 0 0 0 0
M02255_77_000000000-ADUT3_1_1109_13524_17850 G----AC---AG-AG-GCC--------CCA-A-G------C--G--T---T--AG-G-CGG-AA------TC-A--C-T--GG-GC-------TT--A-----AA-GC-GT-GT-------G-TA-G-G-C-G------------G--A-CG-C-C-T-----
------AA----G-T-G-T--------C-T-T--G--TG--A-AA-TC--C-C-AC-G-G-----CT-C-AC------C-C-G-T-G-G-A--A-C----T-G--C-T---G--G-------G--CA-T-A---C-------T--G-G--GC--G-T-C----T-T-G-G-G-C--C---A-AC-----TA-G-G--------G-G-C-A---GC-C------GG--A--ACA----A-
G-C-G-GT--GG-A-G-CG-GT--G---A--A-A---TG-C-GT-AG--AT-A-TC--G-C--T-T-G-G-A-AC-G-CC---GA--A--G--GT-GAA-A--A-C--A-------G--G-C-T-G---CTG--G--GG-T-T--------------------G-C---G-C-C--GA--CG-C-T-G--A-GA--C-A-CG-A--AA-G-C---C-AG--GG-
G--AG-C-A-AA--------CG--GG Bacteria(100);Planctomycetes(100);Phycisphaerae(100);WD2101(100);unclassified(100);unclassified(100);unclassified(100);unclassified(100);unclassified(100);

Otu1421 0 0 0 0 0 0 0 0 0 0 0 0 0 0 0 0 0 0 0 0 0 0 0 0 0 0 0 0
0 0 0 0 0 0 0 0 0 0 0 0 0 0 0 0 0 0 0 0 0 0 0 0 0 0 0 0 154
0 0 0 0 0 0 0 0 0 0 0 0 0 0 0 0 0 0 0 0 0 0 0 0 0 0 0 0 0
0 0 0 0 0 0 0 0 0 0 0 20 0 0 0 0 0 0 0 0 0 0 0 0 0 0 0
M02255_77_000000000-ADUT3_1_1105_7747_7291 G----AC---GT-AG-GGG--------GCG-A-G------C--G--T---T--GT-C-CGG-AA------TT-A--T-T--GG-GC-------GT--A-----AA-GG-GC-TC-------C-TA-G-G-C-G------------G--T-TC-A-G-T----------
AA----G-T-C-G--------A-C-T--G--TG--A-AA-AT--C-C-AA-G-G-----CT-C-AA------C-C-T-T-G-G-G--A------C-G-CC-A---G--T-------C--GA-T-A---C-------T--G-C--TG-TG-A-C----T-C-G-A-G-T--T---C-GG-----TA-G-A--------G-G-A-G---TG-T------GG--A--ATT----C-C-T-
G-GT--GT-A-G-CG-GT--G---A--A-A---TG-C-GC-AG--AT-A-TC--A-G--G-A-G-G-A-AC-A-CC---AA--C--G--GC-GAA-G--G-C--A-------G--C-A-C-T---CTG--G--GC-C-G--------------------A-T---A-C-T--GA--CG-C-T-G--A-AG--A-G-CG-A--AA-G-C---G-TG--GG-G--
AG-C-A-AA--------CA--GG Bacteria(100);Actinobacteria(100);Actinobacteria(100);unclassified(100);unclassified(100);unclassified(100);unclassified(100);unclassified(100);unclassified(100);

Otu1422 0 0 0 0 0 0 0 0 0 0 0 0 0 0 0 0 0 0 0 0 0 0 0 0 0 0 0 0
0 0 0 0 0 0 0 0 0 0 0 0 0 0 0 0 0 0 0 0 0 0 0 0 0 0 0 0 46
0 0 0 0 0 0 0 0 0 0 0 0 0 0 0 0 0 0 0 0 0 0 0 0 0 0 0 0 0
0 0 0 0 0 0 0 0 0 0 0 0 0 0 0 0 0 0 0 0 0 0 0 0 0 0 0
M02255_76_000000000-ADUNE_1_1103_20755_8199 C----TA---AC-TC-TGT--------GCC-A-G------C-----A---G--CC-G-CGG-TA------AT-A--C-A--GA-GG-------GT--G-----CA--A-GC-GT---------TG-T-T-C-G------------G--A-CT-G-C-T----------AA----
G-T-C-A--------T-G-T--G--TG--A-AA-TC--C-C-TC-G-G-----CT-T-AA------C-C-G-A-G-G-A--A-C-----G-A--C-G---C--A-------T--GA-A-A---C-------T--G-G--CA--A-G-C----T-A-G-A-G-T--A---C-CA-----AA-G-A--------G-G-G-G---GG-T------GG--A--ATT----C-C-C-G-
GT--GT-A-G-CG-GT--G---A--A-A---TG-C-GT-AG--AT-A-TC--G-G--G-A-G-G-A-AC-A-CC---GG--T--G--GC-GAA-G--G-C--G-------G--C-C-C-C---CTG--G--TT-G-G--------------------A-T---A-C-T--GA--CG-C-T-G--A-GA--C-G-CG-A--AA-G-C---G-TG--GG-G--AG-
C-A-AA--------CA--GG Bacteria(100);Proteobacteria(100);Deltaproteobacteria(100);GR-WP33-30(100);uncultured(100);unclassified(100);unclassified(100);unclassified(100);unclassified(100);

Otu1423 0 0 0 0 0 0 0 0 0 0 0 0 0 0 0 0 0 0 0 0 0 0 0 0 0 0 0 0
0 0 0 0 0 0 0 0 0 0 0 0 0 0 0 0 0 0 0 0 0 0 0 0 0 0 0 0 16
0 0 0 0 0 0 0 0 0 0 0 0 0 0 0 0 0 0 0 0 0 0 0 0 0 0 0 0 0
0 0 0 0 0 0 0 0 0 0 0 0 0 0 0 0 0 0 0 0 0 0 0 0 0 0 0
M02255_76_000000000-ADUNE_1_1103_24980_5045 .....AC----T-AC-GT---------GCC-A-G------C--A--G---C--CT-C-GGT-AA------TA-C--G-T--AG-GT-------GG--C-----AA-GC-G--TT-------G----T-C-C-G------------G--A-TT-C-T-T----------AA----G-
T-C-T--------G-A-T--G--TG--A-AA-TC--T-C-GG-G-G-----CT-C-AA------C-C-C-C-G-A-G--C------G-GC-C-A---T--T-------G--GA-A-A---C-------T--G-G--GG--A-G-C----T-T-G-A-G-T--G---C-AG-----AA-G-A--------G-G-A-G---AG-T------GG--A--ATT----C-C-A-C-GT--
GT-A-G-CG-GT--G---A--A-A---TG-C-GT-AG--AG-A-TG--T-G--G-A-G-G-A-AC-A-CC---AG--T--G--GC-GAA-G--G-C--G-------A--C-T-C-T---CTG--G--TC-T-G--------------------T-A---A-C-T--GA--CG-C-T-G--A-GG--C-G-CG-A--AA-G-C---G-TG--GG-G--AG-C-A-
AA--------CA--GG Bacteria(100);Firmicutes(100);Bacilli(100);unclassified(100);unclassified(100);unclassified(100);unclassified(100);unclassified(100);unclassified(100);

Otu1424 0 0 0 0 0 0 0 0 0 0 0 0 0 0 0 0 0 0 0 0 0 0 0 0 0 0 0 0
0 0 0 0 0 0 0 0 0 0 0 0 0 0 0 0 0 0 0 0 0 0 0 0 0 0 0 2 53
0 0 0 0 0 0 0 0 0 0 0 0 0 0 0 0 0 0 0 0 0 0 0 0 0 0 0 0 0
0 0 0 0 0 0 0 0 0 0 0 0 0 0 0 0 0 0 0 0 0 0 0 0 0 0 0
M02255_77_000000000-ADUT3_1_1114_25083_3995 G----AC---AG-AG-GTG--------CCA-A-G------C--G--T---T--AG-G-CGG-AA------TC-A--C-T--GG-GC-------TT--A-----AA-GC-GT-GT-------G-TA-G-G-C-G------------G--A-CC-T-C-T----------AA-
---G-T-A-C--------C-T-T--G--TG--A-AA-TC--C-C-AC-G-G-----CT-C-AA------C-C-G-T-G-G-A--A-C----T-G--C-T---C--G-------G--TA-T-A---C-------T--G-G--GG--G-T-C----T-T-G-A-G-C--C---A-CT-----TA-G-G--------G-G-T-C---GT-C------GG--A--ACA----A-G-C-G-
GT--GG-A-G-CG-GT--G---A--A-A---TG-C-GT-AG--AT-A-TC--G-C--T-T-G-G-A-AC-G-CC---AA--C--G--GT-GAA-A--A-C--A-------G--A-C-G-A---CTG--G--GA-G-T--------------------G-C---G-C-T--GA--CG-C-T-G--A-GA--C-A-CG-A--AA-G-C---C-AG--GG-G--AG-
C-G-AA--------CG--GG Bacteria(100);Planctomycetes(100);Phycisphaerae(100);WD2101(100);unclassified(100);unclassified(100);unclassified(100);unclassified(100);unclassified(100);

Otu1425 0 0 0 0 0 0 0 0 0 0 0 0 0 0 0 0 0 0 0 0 0 0 0 0 0 0 0 0
0 0 0 0 0 0 0 0 0 0 0 0 0 0 0 0 0 0 0 0 0 0 0 0 0 0 9 0
0 0 0 0 0 0 0 0 0 0 0 0 0 0 0 0 0 0 0 0 0 0 0 0 0 0 0 0
0 0 0 0 0 0 0 0 0 0 0 0 0 0 0 0 0 0 0 0 0 0 0 0 0 0 0
M02255_76_000000000-ADUNE_1_1118_13857_14464 .....AC----T-AC-GTG--------CCA-G-C------A--G--C---C--G--C-GGT-AA------TA-C--G-T--AG-GT-------GG--C-----AA-AC-G--TT-------G----T-C-C-G-------------G--T-TT-A-T-T-----------
AA----G-T-C-T--------G-A-T--G--TG--A-AAG-C--C-T-TC-G-G-----CT-T-AA------C-C-G-G-A-G-A--A-G----T-G--C-A---T--C-------G--GA-A-A---C-------T--G-A--TA--G-A-C----T-T-G-A-G-T--G---C-AG-----AA-A-A--------G-G-A-T---AG-T------GG--A--ACT----T-C-A-
T-GT--GT-A-G-CG-GT--G---A--A-A---TG-C-GT-AG--AT-A-TA--T-G--A-A-G-G-A-AC-A-CC---AG--T--G--GC-GAA-G--G-C--G-------G--C-T-A-T---CTG--G--TC-T-G--------------------T-A---A-C-T--GA--CG-C-T-G--A-GG--C-T-CG-A--AA-G-C---A-TG--GG-T--AG-
C-A-AA--------CA--GG Bacteria(100);Firmicutes(100);Bacilli(100);Lactobacillales(100);Lactobacillaceae(100);Lactobacillus(100);unclassified(100);unclassified(100);unclassified(100);

Otu1426 0 0 0 0 0 0 0 0 0 0 0 0 0 0 0 0 0 0 0 0 0 0 0 0 0 0 0 0
0 0 0 0 0 0 0 0 0 0 0 0 0 0 0 0 0 0 0 0 0 0 0 0 0 0 6 0
0 0 0 0 0 0 0 0 0 0 0 0 0 0 0 0 0 0 0 0 0 0 0 0 0 0 0 0
0 0 0 0 0 0 0 0 0 0 0 0 0 0 0 0 0 0 0 0 0 0 0 0 0 0 0
M02255_77_000000000-ADUT3_1_2103_21061_20643 T----AC---GT-AG-GTG--------GCT-A-G------C--G--T---T--GT-C-CGG-AT------TT-A--C-T--GG-GC-------GT--A-----AA-GA-GC-GC-------G-CA-G-G-C-G-------------G--T-CG-T-T-C------
-----AA----G-T-C-G--------A-G-T--G--TG--A-AA-GC--C-C-CC-G-G-----CT-C-AA------C-T-G-G-G-G-A--G-G----G-T--C-A---C--T-------C--GA-T-A---C-------T--G-A--TC--G-A-C----T-T-G-A-A-G--G---C-GG-----GA-G-A--------G-G-G-A---AG-C------GG--A--ATT----
C-C-C-G-GT--GT-A-G-TG-GT--G---A--A-A---TG-C-GT-AG--AT-A-TC--G-G--G-A-G-G-A-AC-A-CC---AG--T--G--GC-GAA-G--G-C--G-------G--C-T-T-T---CTG--G--CC-C-G--------------------T-T---C-T-T--GA--CG-C-T-G--A-GG--C-G-CG-A--AA-G-C---T-AG--GG-
T--AG-C-A-AA--------CG--GG Bacteria(100);Chloroflexi(100);KD4-96(100);unclassified(100);unclassified(100);unclassified(100);unclassified(100);unclassified(100);unclassified(100);

Otu1427 0 0 0 0 0 0 0 0 0 0 0 0 0 0 0 0 0 0 0 0 0 0 0 0 0 0 0 0
0 0 0 0 0 0 0 0 0 0 0 0 0 0 0 0 0 0 0 0 0 0 0 0 0 0 24 0
0 0 0 0 0 0 0 0 0 0 0 0 0 0 0 0 0 0 0 0 0 0 0 0 0 0 0 0
0 0 0 0 0 0 0 0 0 0 0 0 0 0 0 0 0 0 0 0 0 0 0 0 0 0 0
M02255_77_000000000-ADUT3_1_1105_5010_19515 T----AC---GT-AG-GGG--------GCG-A-G------C--G--T---T--GT-C-CGG-AG------TT-A--C-T--GG-GC-------GT--A-----AA-GC-GC-AC-------G-CA-G-G-C-G-------------G--T-GT-G-C-C-----------
AA----G-T-T-T--------G-C-A--G--TG--A-CA-GT--T-C-CC-G-G-----CT-T-AA------C-T-G-G-G-G-A--A-G----G-A--C-T---G--T-------G--AA-A-A---C-------T--G-G--TA--C-A-C----T-T-G-A-G-G--G---T-TT-----CA-G-A--------G-G-G-A---CA-T------GG--A--ATT----C-C-G-
G-GT--GG-A-G-TG-GT--G---A--A-A---TG-C-GT-AG--AG-A-TC--C-G--G-A-G-G-A-AC-A-CC---AA--T--G--GC-GAA-G--G-C--A-------G--T-G-T-C---CTG--G--GA-A-G--------------------C-T---C-C-T--GA--CG-C-T-C--A-GG--T-G-CG-A--AA-G-C---C-AG--GG-G--
AG-C-G-AA--------CG--GG Bacteria(100);unclassified(100);unclassified(100);unclassified(100);unclassified(100);unclassified(100);unclassified(100);unclassified(100);unclassified(100);

Otu1428 0 0 0 0 0 0 0 0 0 0 0 0 0 0 0 0 0 0 0 0 0 0 0 0 0 0 0 0
0 0 0 0 0 0 0 0 0 0 0 0 0 0 0 0 0 0 0 73 0 0 0 0 0 0 0 0
0 0 0 0 0 0 0 0 0 0 0 0 0 0 6 15 0 0 0 0 0 0 0 0 0 0 0 0
0 0 0 0 0 0 8 0 0 0 0 0 0 0 0 0 0 0 11 73 1 0 0 0 0 0 0
M02255_77_000000000-ADUT3_1_1106_5218_19762 T----AC---GT-AG-GGG--------GCA-A-G------C--G--T---T--AT-C-CGG-AT------TT-A--C-T--GG-GT-------GT--A-----AA-GG-GA-GC-------G-TA-G-A-C-G-------------G--A-AT-G-G-C-----------
AA----G-T-C-T--------G-A-A--G--TG--A-AA-TG--C-C-CG-G-G-----CT-C-AA------C-C-C-G-G-G-A--A-C----T-G--C-T---T--T-------G--GA-A-A---C-------T--G-T--CG--A-T-C----T-G-G-A-G-T--G---T-TG-----GA-G-A--------G-G-T-A---AG-T------GG--A--ATT----C-C-T-
G-GT--GT-A-G-CG-GT--G---A--A-A---TG-C-GT-AG--AG-A-TC--A-G--G-A-A-G-A-AC-A-CC---GG--A--G--GC-GAA-G--G-C--G-------G--C-T-T-A---CTG--G--AC-A-A--------------------A-G---A-C-T--GA--CG-T-T-G--A-GG--C-T-CG-A--AA-G-C---G-TG--GG-A--
AG-C-A-AA--------CA--GG Bacteria(100);Firmicutes(100);Clostridia(100);Clostridiales(100);Lachnospiraceae(100);Oribacterium(100);unclassified(100);unclassified(100);unclassified(100);

Otu1429 0 0 0 0 0 0 0 0 0 0 0 0 0 0 0 0 0 0 0 0 0 0 0 0 0 0 0 0
0 0 0 0 0 0 0 0 0 0 0 0 0 0 0 0 0 0 0 708 0 0 0 0 0 0 0 0
0 0 0 0 0 0 0 0 0 0 0 0 0 0 0 0 0 0 0 0 0 1 0 0 0 0 0 0
0 0 0 0 0 0 0 0 0 0 0 0 0 0 0 71 0 0 0 0 0 0 0 0 0 0 0
M02255_77_000000000-ADUT3_1_1101_7009_9824 T----AC---GT-AG-GGA--------GCG-A-G------C--G--T---T--AT-C-CGG-AT------TT-A--C-T--GG-GT-------GT--A-----AA-GG-GC-GA-------G-TA-G-G-C-G-------------G--G-AC-G-G-C-----------
AA----G-T-T-G--------G-G-A--G--TG--A-AA-TC--T-G-GG-G-G-----CT-T-AA------C-C-C-C-C-A-A--A-C----T-G--C-T---T--T-------C--AA-A-A---C-------T--G-C--TG--T-T-C----T-T-G-A-G-T--G---A-TG-----GA-G-A--------G-G-C-A---GG-C------GG--A--ATT----C-C-C-
A-GT--GT-A-G-CG-GT--G---A--A-A---TG-C-GT-AG--AT-A-TT--G-G--G-A-G-G-A-AC-A-CC---AG--T--G--GC-GAA-G--G-C--G-------G--C-C-T-G---CTG--G--AC-A-T--------------------T-A---A-C-T--GA--CG-C-T-G--A-GG--A-G-CG-A--AA-G-C---G-TG--GG-G--
AG-C-A-AA--------CA--GG Bacteria(100);Firmicutes(100);Clostridia(100);Clostridiales(100);unclassified(100);unclassified(100);unclassified(100);unclassified(100);unclassified(100);

Otu1430 0 0 0 0 0 0 0 0 0 0 0 0 0 0 0 0 0 0 0 0 0 0 0 0 0 0 0 0
0 0 0 0 0 0 0 0 0 0 0 0 0 0 0 0 0 0 0 51 0 0 0 0 0 0 0 0 0
0 0 0 0 0 0 0 0 0 0 0 0 0 0 0 0 0 0 0 0 0 0 0 0 0 0 0 0 0
0 0 0 0 0 0 0 0 0 0 0 0 0 0 0 0 0 0 0 0 0 0 0 0 0 0 0
M02255_77_000000000-ADUT3_1_1103_22319_10893 T----AC---GT-AG-GGG--------GCA-A-G------C--G--T---T--AT-C-CGG-AT------TT-A--C-T--GG-GT-------GT--A-----AA-GG-GA-GC-------G-TA-G-A-C-G------------G--C-GA-T-G-C----
-------AA----G-T-C-T--------G-G-A--G--TG--A-AA-GC--C-C-GG-G-G-----CT-C-AA------C-C-C-C-G-G-G--A-C----T-G--C-T---T--T-------G--GA-A-A---C-------T--G-T--GT--T-G-C----T-G-G-A-G-T--G---C-AG-----GA-G-A--------G-G-T-A---AG-T------GG--A--ATT----
C-C-T-A-GT--GT-A-G-CG-GT--G---A--A-A---TG-C-GT-AG--AT-A-TT--A-G--G-A-G-G-A-AC-A-CC---AG--T--G--GC-GAA-G--G-C--G-------G--C-T-T-A---CTG--G--AC-T-G--------------------T-A---A-C-T--GA--CG-T-T-G--A-GG--C-T-CG-A--AA-G-C---G-TG--GG-
G--AG-C-A-AA--------CA--GG Bacteria(100);Firmicutes(100);Clostridia(100);Clostridiales(100);Lachnospiraceae(100);Incertae_Sedis(100);unclassified(100);unclassified(100);unclassified(100);

Otu1431 0 0 0 0 0 0 0 0 0 0 0 0 0 0 0 0 0 0 0 0 0 0 0 0 0 0 0 0
0 0 0 0 0 0 0 0 0 0 0 0 0 0 0 0 0 0 0 138 0 0 0 0 0 0 0 0 0
0 0 0 0 0 0 0 0 0 0 0 0 0 0 0 0 0 0 0 0 0 0 0 0 0 0 0 0 0
0 0 0 0 0 0 0 0 0 0 0 0 0 0 0 0 0 0 0 0 0 0 0 0 0 0 0
M02255_77_000000000-ADUT3_1_1106_7208_3210 G----TG---CC-AG-CAG--------CCG-C-G---------G--T---A--AT-A-CGG-AG------GA-T--C-C--GA-GC-------GT--T-----AT-CC-GG-AT-------T-TA-G-G-T-G------------G--G-TT-G-A-T----------AA--
--G-T-C-A--------G-C-G--G--TG--A-AA-GT--T-T-GC-A-G-----CT-T-AA------C-T-G-T-A-A-A--A-A----T-G--C-C---G--T-------T--GA-A-A---C-------T--G-T--CG--G-A-C----T-T-G-A-G-T--G---T-AA-----AT-G-A--------G-G-T-A---GG-C------GG--A--ATG----C-G-T-G-
GT--GT-A-G-CG-GT--G---A--A-A---TG-C-AT-AG--AT-A-TC--A-C--G-C-A-G-A-AC-T-CC---GA--T--T--GC-GAA-G--G-C--A-------G--C-T-T-A---CTA--A--GC-T-A--------------------C-A---A-C-T--GA--CA-C-T-G--A-AG--C-A-CG-A--AA-G-C---G-TG--GG-G--AT-C-
A-AA--------CA--GG Bacteria(100);Bacteroidetes(100);Bacteroidia(100);Bacteroidales(100);Porphyromonadaceae(100);Parabacteroides(100);unclassified(100);unclassified(100);unclassified(100);

Otu1432 0 0 0 0 0 0 0 0 0 0 0 0 0 0 0 0 0 0 0 0 0 0 0 0 0 0 0 0
0 0 0 0 0 0 0 0 0 0 0 0 0 0 0 0 0 0 0 49 0 0 0 0 0 0 0 0 0
0 0 0 0 0 0 0 0 0 0 0 0 0 0 0 0 0 0 0 0 0 0 0 0 0 0 0 0 0
0 0 0 0 0 0 0 0 0 0 0 0 0 0 0 0 0 0 0 0 0 0 0 0 0 0 0
M02255_77_000000000-ADUT3_1_1106_6444_13621 T----AC---GT-AG-GGG--------ACA-A-G------C--G--T---T--GT-C-CGG-AT------TT-A--C-T--GG-GC-------GT--A-----AA-GG-GC-AC-------G-CA-G-G-C-G------------G--T-TT-T-T-T----------AA-
---G-T-C-A--------G-A-T--G--TG--A-AA-GG--T-A-CC-G-G-----CT-C-AA------C-C-G-G-T-G-A--C-A----T-G--C-A---T--T-------T--GA-A-A---C-------T--G-A--AA--G-A-C----T-T-G-A-G-T--A---T-TG-----GA-G-A--------G-G-C-A---AG-T------GG--A--ATT----C-C-T-A-
GT--GT-A-G-CG-GT--G---A--A-A---TG-C-GT-AG--AT-A-TT--A-G--G-A-G-G-A-AC-A-CC---GG--T--G--GC-GAA-G--G-C--G-------G--C-T-T-G---CTG--G--AC-A-A--------------------A-T---A-C-T--GA--CG-C-T-G--A-GG--T-G-CG-A--AA-G-C---G-TG--GG-G--AG-
C-A-AA--------CA--GG Bacteria(100);Firmicutes(100);Clostridia(100);Clostridiales(100);Eubacteriaceae(100);Acetobacterium(100);unclassified(100);unclassified(100);unclassified(100);

Otu1433 0 0 0 0 0 0 0 0 0 0 0 0 0 0 0 0 0 0 0 0 0 0 0 0 0 0 0 0
0 0 0 0 0 0 0 0 0 0 0 0 0 0 0 0 0 0 0 52 0 0 0 0 0 0 0 0 0
0 0 0 0 0 0 0 0 0 0 0 0 0 0 0 0 0 0 0 0 0 0 0 0 0 0 0 0 0
0 0 0 0 0 0 0 0 0 0 0 0 0 0 0 0 0 0 0 0 0 0 0 0 0 0 0
M02255_77_000000000-ADUT3_1_1107_22186_5761 T----AC---GT-AG-GCA--------GCG-A-G------C--G--T---T--GT-T-CGG-AA------TT-A--C-T--GG-GC-------GT--A-----AA-GA-GT-GT-------G-TA-G-G-T-G------------G--T-GT-C-G-T----------AA-
---G-T-T-T--------G-G-T--G--TG--A-AA-TC--T-C-CC-G-G-----CT-T-AA------C-T-G-G-G-A-G--G-G----T-G--C-G---C--C-------G--AA-G-A---C-------T--G-C--GA--T-G-C----T-G-G-A-G-T--A---T-GG-----GA-G-A--------G-G-A-A---AG-C------GG--A--ATT----C-C-T-G-
GT--GT-A-G-CG-GT--G---A--A-A---TG-C-GT-AG--AT-A-TC--A-G--G-A-G-G-A-AC-A-CC---TG--C--G--GT-GTA-G--A-C--G-------G--C-T-T-T---CTG--G--AC-C-A--------------------T-T---A-C-T--GA--CA-C-T-G--A-GA--C-A-CG-A--AA-G-C---G-TG--GG-T--AG-C-
A-AA--------CA--GG Bacteria(100);Acidobacteria(100);Acidobacteria(100);Acidobacteriales(100);Acidobacteriaceae(100);unclassified(100);unclassified(100);unclassified(100);unclassified(100);

Otu1434 0 0 0 0 0 0 0 0 0 0 0 0 0 0 0 0 0 0 0 0 0 0 0 0 0 0 0 0
0 0 0 0 0 0 0 0 0 0 0 0 0 0 0 0 0 0 0 35 0 0 0 0 0 0 0 0 0
0 0 0 0 0 0 0 0 0 0 0 0 0 0 0 0 0 0 0 0 0 0 0 0 0 0 0 0 0
0 0 0 0 0 0 0 0 0 0 0 0 0 0 0 0 0 0 0 0 0 0 0 0 0 0 0
M02255_77_000000000-ADUT3_1_1108_9180_19910 T----AC---GA-AG-GGG--------GCT-A-G------C--G--T---T--GC-T-CGG-AA------TC-A--C-T--GG-GC-------GT--A-----AA-GC-GC-AC-------G-TA-G-G-C-G------------G--A-TC-T-T-T----------AA-
---G-T-C-A--------G-G-G--G--TG--A-AA-TC--C-C-AA-G-G-----CT-C-AA------C-C-T-T-G-G-A--A-C----T-G--C-C---T--T-------T--GA-T-A---C-------T--G-G--GG--A-T-C----T-A-G-A-G-T--C---C-GG-----AA-G-A--------G-G-T-T---GG-T------GG--A--ACT----G-C-G-A-
GT--GT-A-G-AG-GT--G---A--A-A---TT-C-GT-AG--AT-A-TT--C-G--C-A-A-G-A-AC-A-CC---AG--T--G--GC-GAA-G--G-C--G-------G--C-C-A-A---CTG--G--TC-C-G--------------------G-T---A-C-T--GA--CG-C-T-G--A-GG--T-G-CG-A--AA-G-C---G-TG--GG-G--AG-
C-A-AA--------CA--GG Bacteria(100);Proteobacteria(100);Alphaproteobacteria(100);Rhizobiales(100);unclassified(100);unclassified(100);unclassified(100);unclassified(100);unclassified(100);

Otu1435 0 0 0 0 0 0 0 0 0 0 0 0 0 0 0 0 0 0 0 0 0 0 0 0 0 0 0 0
0 0 0 0 0 0 0 0 0 0 0 0 0 0 0 0 0 0 0 46 0 0 0 0 0 0 0 0 0
0 0 0 0 0 0 0 0 0 0 0 0 0 0 0 0 0 0 0 0 0 0 0 0 0 0 0 0 0
0 0 0 0 0 0 0 0 0 0 0 0 0 0 0 0 0 0 0 0 0 0 0 0 0 0 0 0
M02255_77_000000000-ADUT3_1_1111_19495_20151 T----AC---AG-AG-GCC--------ACA-A-G------C--G--T---T--AG-G-CGG-AA------TC-A--C-T--GG-GC-------TT--A-----AA-GC-GT-GT-------G-TA-G-G-C-G------------G--G-AT-G-G-T----
-------AA----G-T-A-C--------T-T-T--G--TG--A-AA-TC--C-C-AC-G-G-----CT-C-AA------C-C-G-T-G-G-A--A-A----T-G--C-T---T--G-------G--TA-T-A---C-------T--G-C--CG--T-T-C----T-T-G-A-A-T--C---A-CC-----TA-G-G--------G-G-C-A---GC-T------GG--A--ACT----G-
A-T-G-GT--GG-A-G-CG-GT--G---A--A-A---TG-C-AT-AG--AT-A-TC--A-T--C-A-G-G-A-AC-G-CC---AA--T--G--GT-GCA-G--A-C--A-------A--G-C-T-G---CTG--G--GG-G-T--------------------G-T---A-T-T--GA--CG-C-T-G--A-GG--C-A-CG-A--AA-G-C---T-AG--GG-
G--AG-C-A-AA--------CG--GG Bacteria(100);unclassified(100);unclassified(100);unclassified(100);unclassified(100);unclassified(100);unclassified(100);unclassified(100);unclassified(100);

Otu1436 0 0 0 0 0 0 0 0 0 0 0 0 0 0 0 0 0 0 0 0 0 0 0 0 0 0 0 0
0 0 0 0 0 0 0 0 0 0 0 0 0 0 0 0 0 0 0 36 0 0 0 0 0 0 0 0 0
0 0 0 0 0 0 0 0 0 0 0 0 0 0 0 0 0 0 0 0 0 0 0 0 0 0 0 0 0
0 0 0 0 0 0 0 0 0 0 0 0 0 0 0 0 0 0 0 0 0 0 0 0 0 0 0 0
M02255_76_000000000-ADUNE_1_2111_13746_14505 T----AC---GT-AG-GGC--------GCA-A-G------C--G--T---T--AT-C-CGG-AT------TT-A--T-T--GG-GC-------GT--------AA------------------A---G---G------------G--C-TC-G-T-C-----------GC-
---G-T-C-C--------G-G-T--G--TG--A-AA-GT--C-C-AT-C-G-----CT-T-AA------C-G-G-T-G-G-A--T-C----T-G--C-G---C--C-------G--GG-T-A---C-------G--G-G--CG--G-G-C----T-G-G-A-G-T--G---C-GG-----TA-G-G--------G-G-A-G---AC-T------GG--A--ATT----C-C-C-G-
GT--GT-A-A-CG-GT--G---G--A-A---TG-T-GT-AG--AT-A-TC--G-G--G-A-A-G-A-AC-A-CC---GA--T--G--GC-GAA-G--G-C--A-------G--G-T-C-T---CTG--G--GC-C-G--------------------T-C---A-C-T--GA--CG-C-T-G--A-GG--A-G-CG-A--AA-G-C---G-TG--GG-G--AG-
C-G-AA--------CA--GG Bacteria(100);Actinobacteria(100);Actinobacteria(100);Actinobacteridae(100);Bifidobacteriales(100);Bifidobacteriaceae(100);Bifidobacterium(100);unclassified(100);unclassified(100);

Otu1437 0 0 0 0 0 0 0 0 0 0 0 0 0 0 0 0 0 0 0 0 0 0 0 0 0 0 0 0
0 0 0 0 0 0 0 0 0 0 0 0 0 0 0 0 0 0 0 28 0 0 0 0 0 0 0 0 0
0 0 0 0 0 0 0 0 0 0 0 0 0 0 0 0 0 0 0 0 0 0 0 0 0 0 0 0 0
0 0 0 0 0 0 0 0 0 0 0 0 0 0 0 0 0 0 0 0 0 0 0 0 0 0 0 0
M02255_76_000000000-ADUNE_1_1109_17355_14788 C----CG---TG-CC-AGC--------AGC-C-G------C--G--G---T--AA-TACGG-GG------GG-T--G-C--AA-GC-------GT--T-----AC-TC-GG-AA-------T-CA-C-T-G-G------------G--A-TT-A-A-T----
-------AA----G-T-T-T--------G-A-A--G--TG--A-AA-TC--C-T-AT-A-G-----CT-T-AA------C-T-A-T-A-G-A--A-C----T-G--C-T---T--T-------G--AA-A-A---C-------T--G-T--TA--A-T-C----T-A-G-A-A-T--G---T-GG-----GA-G-A--------G-G-T-A---GA-T------GG--A--ATT----T-
C-T-G-GT--GT-A-G-GG-GT--A---A--A-A---TC-C-GT-AG--AG-A-TC--A-G--A-A-G-G-A-AT-A-CC---GA--T--T--GC-GAA-G--G-C--G-------A--T-C-T-A---CTG--G--AA-C-A--------------------T-T---A-T-T--GA--CG-C-T-G--A-GA--T-G-CG-A--AA-G-C---G-TG--GG-G-
-AG-C-A-AA--------CA--GG Bacteria(100);Proteobacteria(100);Epsilonproteobacteria(100);Campylobacterales(100);Campylobacteraceae(100);Arcobacter(100);unclassified(100);unclassified(100);unclassified(100);

Otu1438 0 0 0 0 0 0 0 0 0 0 0 0 0 0 0 0 0 0 0 0 0 0 0 0 0 0 0 0
0 0 0 0 0 0 0 0 0 0 0 0 0 0 0 0 0 0 0 28 0 0 0 0 0 0 0 0 0
0 0 0 0 0 0 0 0 0 0 0 0 0 0 0 0 0 0 0 0 0 0 0 0 0 0 0 0 0
0 0 0 0 0 0 0 0 0 0 0 0 0 0 0 0 0 0 0 0 0 0 0 0 0 0 0 0
M02255_77_000000000-ADUT3_1_1107_17174_13846 T----AC---GT-AG-GGT--------CCA-A-G------C--G--T---T--AT-C-CGG-AT------TT-A--C-T--GG-GT-------GT--A-----AA-GG-GC-GA-------G-TA-G-A-C-G------------G--T-TA-G-G-C-----
------AA----G-T-C-A--------G-A-T--G--TG--A-AA-AC--T-C-AG-G-G-----CT-C-AA------C-T-T-T-G-A-G--A-C----T-G--C-A---T--C-------T--GA-A-A---C-------T--G-T--TT--A-A-C----T-A-G-A-G-T--G---C-AG-----AA-G-A--------G-G-T-A---AG-T------GG--A--ATT----C-
C-T-A-GT--GT-A-G-CG-GT--G---A--A-A---TG-C-GT-AG--AT-A-TT--A-G--G-A-G-G-A-AC-A-CC---AG--T--G--GC-GAA-G--G-C--G-------A--C-T-T-A---CTG--G--GC-T-G--------------------T-A---A-C-T--GA--CG-T-T-G--A-GG--C-G-CG-A--AA-G-T---G-TG--GG-G-
-AG-C-A-AA--------CA--GG Bacteria(100);unclassified(100);unclassified(100);unclassified(100);unclassified(100);unclassified(100);unclassified(100);unclassified(100);unclassified(100);

Otu1439 0 0 0 0 0 0 0 0 0 0 0 0 0 0 0 0 0 0 0 0 0 0 0 0 0 0 0 0
0 0 0 0 0 0 0 0 0 0 0 0 0 0 0 0 0 0 0 1 0 0 0 0 0 0 0 4 0
0 0 0 0 0 0 0 0 0 0 0 0 0 0 0 0 0 0 0 0 0 0 0 0 0 0 0 0 0
0 0 0 0 0 0 0 0 0 0 0 0 0 0 0 0 0 0 0 0 0 0 0 0 0 0 0 0
M02255_77_000000000-ADUT3_1_1111_3505_8514 T----AC---GG-AG-GAG--------GCA-A-G------C--G--T---T--AT-C-CGG-AA------TT-A--T-T--GG-GC-------GT--A-----AA-GC-GT-CC-------G-TA-G-G-T-G------------G--C-TT-TCT-C-----------AA-
---G-T-C-T--------G-C-G--G--TT--A-AA-GA--C-T-GG-G-G-----CT-T-AA------C-T-C-C-A-G-A--A-A----G-G--C-C---G--T-------G--GA-A-A---C-------T--G-A--AA--G-G-C----T-A-G-A-G-A--G---C-CG-----TA-G-G--------G-G-T-A---GA-G------GG--A--ATT----C-C-C-G-
GT--GT-A-G-CG-GT--G---A--A-A---TG-C-GT-AG--AG-C-TC--G-G--G-A-A-G-A-AC-A-TC---GG--T--G--GC-GAA-A--G-C--G-------C--T-C-T-A---CTG--G--AC-G-G--------------------T-A---T-C-T--GA--CA-C-T-G--A-GG--G-A-CG-A--AA-G-C---T-AG--GG-G--AG-
C-G-AA--------TG--GG Bacteria(100);Cyanobacteria(100);unclassified(100);unclassified(100);unclassified(100);unclassified(100);unclassified(100);unclassified(100);unclassified(100);

Otu1440 0 0 0 0 0 0 0 0 0 0 0 0 0 0 0 0 0 0 0 0 0 0 0 0 0 0 0 0
0 0 0 0 0 0 0 0 0 0 0 0 0 0 0 0 0 0 0 1 0 0 0 0 0 3 0 0 0
0 0 0 0 0 0 0 0 0 0 0 0 0 0 0 0 0 6 0 0 0 0 0 0 0 0 0 0 0
0 0 0 0 0 0 0 0 0 0 0 0 0 0 0 0 0 0 0 0 0 0 0 0 0 0 0
M02255_76_000000000-ADUNE_1_2119_22317_21913 A----GA---AA-CC-CCG--------GCT-A-A------C--T--C---C--GTGC-CAG-CA------GC-C--G-C--GG-TA-------AT--A-----CG-GA-GG-GG-------G-TT-A-G-C-GT-----------T--G-TT-C-G-G---
--------AAT---G-A-C-T--------G-G-GC-G--TG--A-AA-GCG-CGCGTAGG-G-----CT-C-AA------C-C-C-T-G-G-A--A-T----T-G--C-C---T--T-------C--AA-G-A---C-------T--C-G--CG--G-T-C----T-G-G-A-G-T--T---C-GA-----GA-G-A--------G-G-T-G---GG-T------GG--A--
ATT----C-C-G-A-GT--GT-A-G-AG-GT--G---A--A-A---TT-C-GT-AG--AT-A-TT--C-G--G-A-G-G-A-AC-A-CC---AG--T--G--GC-GAA-G--G-C--G-------G--C-C-C-A---CTG--G--CT-C-G--------------------A-C---A-C-T--GA--CG-C-T-G--A-GG--C-G-CG-A--AA-G-C---G-
CG--GG-G--AG-C-A-AA--------CA--GG Bacteria(100);Proteobacteria(100);Alphaproteobacteria(100);unclassified(100);unclassified(100);unclassified(100);unclassified(100);unclassified(100);unclassified(100);

Otu1441 0 0 0 0 0 0 0 0 0 0 0 0 0 0 0 0 0 0 0 0 0 0 0 0 0 0 0 0
0 0 0 0 0 0 0 0 0 0 0 0 0 0 0 0 0 0 0 0 0 0 0 0 0 0 0 0 0
0 0 0 0 0 0 0 0 0 0 0 0 0 0 0 0 0 0 0 0 0 0 0 0 0 3 3 0 0
0 0 0 0 0 0 0 0 0 7 0 4 0 2 0 0 0 0 0 0 0 0 0 0 0 0 0
M02255_77_000000000-ADUT3_1_1105_13001_23478 C----GT---GC-CA-GCA--------GCC-G-C------G--G--T---A--AT-A-CGT-AT------GG-A--G-C--GA-GC-------GT--T-----GT-CC-GG-AA-------T-TA-T-T-G-G------------G--C-GT-A-A-A-----
------GG----G-TAC-G--------C-A-G--G--TT--A-AA-GA--T-C-GG-G-G-----CT-C-AA------C-C-C-C-G-T-A--A------A-G--C-A---T--T-------G--GA-A-A---C-------T--G-A--TA--A-A-C----T-T-G-A-G-T--A---G-TG-----GA-G-A--------G-G-A-A---AG-T------GG--A--ATT----
C-C-T-A-GT--GT-A-G-TG-GT--G---A--A-A---TA-C-GT-AG--AT-A-TT--A-G--G-A-G-G-A-AT-A-CC---AG--T--A--GC-GAA-G--G-C--G-------A--C-T-T-T---CTG--G--AC-A-C--------------------A-A---A-C-T--GA--CG-C-T-G--A-GG--T-A-CG-A--AA-G-C---G-TG--GG-
G--AG-C-A-AA--------CA--GG Bacteria(100);Firmicutes(100);Clostridia(100);Clostridiales(100);Family_XI_Incertae_Sedis(100);Finegoldia(100);unclassified(100);unclassified(100);unclassified(100);

Otu1442 0 0 0 0 0 0 0 0 0 0 0 0 0 0 0 0 0 0 0 0 0 0 0 0 0 0 0 0
0 0 0 0 0 0 0 0 0 0 0 0 0 0 0 0 0 0 0 0 0 41 0 0 0 0 0 0 0
0 0 0 0 0 0 0 0 0 0 0 0 0 0 0 0 0 0 0 0 0 0 0 0 0 0 0 0 0
0 0 0 0 0 0 0 0 0 0 0 0 0 0 0 0 0 0 0 0 0 0 0 0 0 0 0
M02255_76_000000000-ADUNE_1_1105_27940_11581 C----CG---GC-TA-ACT--------TCG-T-G------C--C--A---G--CA-G-CCG-CG------GT-A--A-T--AC-GA-------AG--G-----GT-CC-AA-GC-------G-TT-GTT-C-G------------G--A-TC-A-G-C-----
------AA----G-T-C-A--------G-G-T--G--TG--A-AA-TC--T-C-GG-G-G-----CT-C-AA------C-C-C-C-G-A-A--A-C----T-G--C-G---C--C-------T--GA-A-A---C-------T--G-C--TA--G-T-C----T-A-G-A-A-T--A---T-CG-----GA-G-G--------G-G-G-C---AG-G------GG--A--ATT----
T-C-A-C-GT--GT-A-G-GG-GT--A---A--A-A---TC-C-GT-AG--AG-A-TG--T-G--A-A-G-G-A-AC-A-CC---GG--G--G--GC-GAA-G--G-C--G-------C--C-T-G-C---CTG--G--AC-G-A--------------------T-T---A-T-T--GA--CG-C-T-G--A-GG--C-G-CG-A--AA-G-C---G-TG--
GG-G--AG-C-A-AA--------CA--GG Bacteria(100);Proteobacteria(100);Deltaproteobacteria(100);Bdellovibrionales(100);Bacteriovoraceae(100);Peredibacter(100);unclassified(100);unclassified(100);unclassified(100);

Otu1443 0 0 0 0 0 0 0 0 0 0 0 0 0 0 0 0 0 0 0 0 0 0 0 0 0 0 0 0
0 0 0 0 0 0 0 0 0 0 0 0 0 0 0 0 0 0 0 0 0 12 0 0 0 0 0 0 0
0 0 0 0 0 0 0 0 0 0 0 0 0 0 0 0 0 0 0 0 0 0 0 0 0 0 0 0 0
0 0 0 0 0 0 0 0 0 0 0 0 0 0 0 0 0 0 0 0 0 0 0 0 0 0 0
M02255_76_000000000-ADUNE_1_1113_25764_17739 T----AC---GA-GG-GGT--------CCT-A-G------C--G--T---T--GT-T-CGG-AA------TC-A--T-T--GG-GC-------GT--A-----AA-G--G----------G-T----G-T--------------G----------T-----------AA-
---G-T-C-A--------G-G-T--G--TG--A-AA-GC--C-C-CG-G-G-----CT-C-AA------C-C-C-G-G-G-A--A-G----T-G--C-A---T--C-------T--GA-T-A---C-------T--G-C--GT--A-G-C----T-T-G-A-G-T--A---C-CG-----AA-G-A--------G-G-A-T---AG-T------GG--A--ATT----C-C-A-G-
GT--GT-A-G-TG-GT--G---A--A-A---TA-C-GT-AG--AT-A-TC--T-G--G-A-G-G-A-AC-A-CC---GG--T--G--GC-GAA-G--G-C--G-------G--C-T-A-T---CTG--G--TC-G-A--------------------G-T---A-C-T--GA--CA-C-T-G--A-GA--C-A-CG-A--AA-G-C---A-TG--GG-G--AT-C-
A-AA--------CA--GG Bacteria(100);Proteobacteria(100);unclassified(100);unclassified(100);unclassified(100);unclassified(100);unclassified(100);unclassified(100);unclassified(100);

Otu1444 0 0 0 0 0 0 0 0 0 0 0 0 0 0 0 0 0 0 0 0 0 0 0 0 0 0 0 0
0 0 0 0 0 0 0 0 0 0 0 0 0 0 0 0 0 0 0 0 0 9 0 0 0 0 0 0 0
0 0 0 0 0 0 0 0 0 0 0 0 0 0 0 0 0 0 0 0 0 0 0 0 0 0 0 0 0
0 0 0 0 0 0 0 0 0 0 0 0 0 0 0 0 0 0 0 0 0 0 0 0 0 0 0
M02255_76_000000000-ADUNE_1_1117_13467_12684 T----AA---CT-A--CGT--------GCC-A-G------C--A--G---C--CG-C-GGT-AA------TA-C--G-T--AG-GG-------CG--C-----GA-GC-G--TT-------G-TC-C-G---G------------A--T-TT-A-T-T---------
--GG----G-C-C-T--------G-T-C--G--TG--T-AA-TC--C-T-CT-G-G-----CT-T-AA------C-T-G-G-G-G-G--T-G----T-G--C-G---G--T-------G--GG-T-A---C-------G--G-G--CA--G-G-C----T-T-G-A-G-T--G---C-GG-----TA-G-G--------G-G-A-G---AT-T------GG--A--ATT----C-C-
T-G-GT--GT-A-G-CG-GT--G---G--A-A---TG-C-GC-AG--AT-A-TC--A-G--G-A-G-G-A-AC-A-CC---GG--T--G--GC-GAA-G--G-C--G-------G--G-T-C-T---CTG--G--GC-C-G--------------------T-T---A-C-T--GA--CG-C-T-G--A-GG--A-G-CG-A--AA-G-C---G-TG--GG-G--
AG-C-G-AA--------CA--GG Bacteria(100);Actinobacteria(100);Actinobacteria(100);Actinobacteridae(100);Actinomycetales(100);unclassified(100);unclassified(100);unclassified(100);unclassified(100);

Otu1445 0 0 0 0 0 0 0 0 0 0 0 0 0 0 0 0 0 0 0 0 0 0 0 0 0 0 0 0
0 0 0 0 0 0 0 0 0 0 0 0 0 0 0 0 0 11 0 0 0 0 0 0 0 0 0 0 0
0 0 0 0 0 0 0 0 0 0 0 0 0 0 0 0 0 0 0 0 0 0 0 0 0 0 0 0 0
0 0 0 0 0 0 0 0 0 0 0 0 0 0 0 0 0 0 0 0 0 0 0 0 0 0 0
M02255_76_000000000-ADUNE_1_1111_3260_12175 T----AC---GT-AG-GGG--------GCA-A-G------C--G--T---T--AT-C-CGG-AT------TT-A--C-T--GG-GT-------GT--A-----AA--G-G--G--------G----G---C--------------G-----T-A-T-A-----------AA----G-
T-C-T--------G-A-A--G--TG--A-AA-AC--C-C-GC-A-G-----CT-C-AA------C-T-G-C-G-G-A--G-T----T-G--C-T---T--T-------G--GA-A-A---C-------T--T-A--TA--A-G-C----T-G-G-A-G-T--G---T-CG-----GA-G-G--------G-G-T-A---AG-C------GG--A--ATT----C-C-C-A-GT--
GT-A-G-CG-GT--G---A--A-A---TG-C-GT-AG--AT-A-TT--G-G--G-A-G-G-A-AC-A-CC---GG--A--G--GC-GAA-G--G-C--G-------G--C-T-T-A---CTG--G--AA-G-A--------------------T-A---A-C-T--GA--CG-T-T-G--A-GG--C-T-CG-A--AG-G-C---G-TG--GG-T--AG-C-A-
AA--------CA--GG Bacteria(100);Firmicutes(100);Clostridia(100);Clostridiales(100);unclassified(100);unclassified(100);unclassified(100);unclassified(100);unclassified(100);

Otu1446 0 0 0 0 0 0 0 0 0 0 0 0 0 0 0 0 0 0 0 0 0 0 0 0 0 0 0 0
0 0 0 0 0 0 0 0 0 0 0 0 0 0 0 0 4 0 0 0 0 0 0 0 0 0 0 0 0
0 0 0 0 0 0 0 0 0 0 0 0 0 0 0 0 0 0 0 11 0 0 0 0 0 0 0 21 0
0 0 0 0 0 5 0 0 0 0 0 0 0 0 0 36 0 0 0 0 0 0 0 0 0 0 0
M02255_76_000000000-ADUNE_1_2101_14357_8337 C----TA---AC-TC-CGT--------GCC-A-G------C--A--G---C--CG-C-GGT-AA------TA-C--G-G--AG-GG-------TG--C-----AA-GC-GT--T-------A-TC-C-G---G------------A--C-TT-A-T-T-----------AA----
G-T-C-A--------G-T-G--G--TG--A-AA-TC--T-C-AT-A-G-----CT-C-AA------C-T-A-T-G-A-A--A-C----T-G--C-C---A--T-------T--GA-T-A---C-------T--G-A--TA--G-T-C----T-T-G-A-G-T--A---T-AT-----TT-G-A--------A-G-T-A---GC-T------GG--A--ATA----A-G-T-A-GT--
GT-A-G-CG-GT--G---A--A-A---TG-C-AT-AG--AT-A-TT--A-C--T-T-A-G-A-AC-A-CC---AA--T--A--GC-GAA-G--G-C--A-------G--G-T-T-A---CTA--A--GA-T-A--------------------T-T---A-C-T--GA--CG-C-T-G--A-GG--G-A-CG-A--GA-G-C---G-TA--GG-G--AG-C-G-
AA--------CA--GG Bacteria(100);Bacteroidetes(100);Flavobacteria(100);Flavobacteriales(100);Flavobacteriaceae(100);unclassified(100);unclassified(100);unclassified(100);unclassified(100);

Otu1447 0 0 0 0 0 0 0 0 0 0 0 0 0 0 0 0 0 0 0 0 0 0 0 0 0 0 0 0
0 0 0 0 0 0 0 0 0 0 0 0 0 0 0 0 9 0 0 0 0 0 0 0 0 0 0 0 0
0 0 0 0 0 0 0 0 0 0 0 0 0 0 0 0 0 0 0 0 0 0 0 0 0 0 0 0 0
0 0 0 0 0 0 0 0 0 0 0 0 0 0 0 0 0 0 0 0 0 0 0 0 0 0 0
M02255_76_000000000-ADUNE_1_2104_23038_9753 C----GG---CT-AA-TTC--------CGT-G-C------C--A--G---C--AG-C-CGC-GG------TA-A--T-A--CG-GA-------AG--G-----TC-CA-GG-CG-------T-TA-T-C-C-G------------G--A-CT-G-T-T-----------AT--
--G-T-C-A--------G-C-G--G--TT--A-AA-GA--G-T-GT-G-G-----CT-C-AA------C-C-A-T-G-C----G-A----T-G--C-C---G--T-------T--GA-A-A---C-------T--G-G--TG--G-T-C----T-T-G-A-G-T--G---C-AC-----AC-A-G--------G-G-A-T---GC-T------GG--A--ACT----C-G-T-G-
GT--GT-A-G-CG-GT--G---A--A-A---TG-C-TT-AG--AT-A-TC--A-C--G-A-T-G-A-AC-T-CC---GA--T--C--GC-GAA-G--G-C--A-------G--G-T-G-T---CCG--G--GG-T-G--------------------T-A---A-C-T--GA--CG-C-T-G--A-GG--C-T-CG-A--AA-G-T---G-TG--GG-T--AT-C-
A-AA--------CA--GG Bacteria(100);Bacteroidetes(100);unclassified(100);unclassified(100);unclassified(100);unclassified(100);unclassified(100);unclassified(100);unclassified(100);

Otu1448 0 0 0 0 0 0 0 0 0 0 0 0 0 0 0 0 0 0 0 0 0 0 0 0 0 0 0 0
0 0 0 0 0 0 0 0 0 0 0 0 0 0 0 0 32 0 0 0 0 0 0 0 0 0 0 0 0
0 0 0 0 0 0 0 0 0 0 0 0 0 0 0 0 0 0 0 0 0 0 0 0 0 0 0 0 0
0 0 0 0 0 0 0 0 0 0 0 0 0 0 0 0 0 0 0 0 0 0 0 0 0 0 0
M02255_77_000000000-ADUT3_1_1109_9123_12671 G----AC---GG-AG-GAG--------GCT-A-G------C--G--T---T--GT-T-CTG-AA------TT-A--C-T--GG-GC-------GT--A-----AA-GC-GT-AC-------G-CA-G-G-T-G------------G--C-GA-G-A-C-----------
AA----G-T-C-G--------G-A-T--G--TG--A-AA-GC--C-C-GG-G-G-----CT-C-AA------C-C-C-C-G-G-A--T-G----T-G--C-A---T--C-------C--GA-T-A---C-------T--G-T--TT--T-G-C----T-G-G-A-G-T--C---C-GG-----AA-G-A--------G-G-A-G---AG-C------GG--A--ATT----C-C-C-
A-GT--GT-A-G-AG-GT--G---A--A-A---TT-C-GT-AG--AT-A-TT--G-G--G-A-A-G-A-AC-A-CC---GG--T--G--GC-GAA-G--G-C--G-------G--C-T-C-T---CTG--G--TC-C-G--------------------G-T---A-C-T--GA--CA-C-T-C--A-AG--T-A-CG-A--AA-G-C---G-TG--GG-G--AG-
C-A-AA--------CA--GG Bacteria(100);Proteobacteria(100);unclassified(100);unclassified(100);unclassified(100);unclassified(100);unclassified(100);unclassified(100);unclassified(100);

Otu1449 0 0 0 0 0 0 0 0 0 0 0 0 0 0 0 0 0 0 0 0 0 0 0 0 0 0 0 0
0 0 0 0 0 0 0 0 0 0 0 0 0 0 0 0 17 0 0 0 0 0 0 0 0 0 0 0 0
0 0 0 0 0 0 0 0 0 0 0 0 0 0 0 0 0 0 0 0 0 0 0 0 0 0 0 0 0
0 0 0 0 0 0 0 0 0 0 0 0 0 0 0 0 0 0 0 0 0 0 0 0 0 0 0
M02255_77_000000000-ADUT3_1_1112_12425_11233 T----AC---GA-AG-GGG--------GCT-A-G------C--G--T---T--GC-T-CGG-AA------TT-A--C-T--GG-GC-------GT--A-----AA-GG-GC-GC-------G-TA-G-G-C-G------------G--C-CA-G-A-T----
-------TA----G-T-C-A--------G-G-C--G--TG--A-AA-TT--C-C-TG-G-G-----CT-C-AA------C-C-T-G-G-G-G--A-C----T-G--C-G---C--T-------T--GA-T-A---C-------G--G-T--TT--G-G-C----T-T-G-A-G-G--G---C-AG-----GA-G-A--------G-G-C-T---CG-C------GG--A--ATT----
C-C-C-A-GT--GT-A-G-AG-GT--G---A--A-A---TT-C-GT-AG--AT-A-TT--G-G--G-A-A-G-A-AC-A-CC---GG--T--G--GC-GAA-G--G-C--G-------G--C-G-A-G---CTG--G--CC-T-G--------------------T-T---A-C-T--GA--CG-C-T-G--A-GG--C-G-CG-A--CA-G-C---G-TG--
GG-G--AG-C-A-AA--------CA--GG Bacteria(100);Proteobacteria(100);Alphaproteobacteria(100);Rhodospirillales(100);Acetobacteraceae(100);unclassified(100);unclassified(100);unclassified(100);unclassified(100);

Otu1450 0 0 0 0 0 0 0 0 0 0 0 0 0 0 0 0 0 0 0 0 0 0 0 0 0 0 0 0
0 0 0 0 0 0 0 0 0 0 0 0 0 0 0 0 29 0 0 0 0 0 0 0 0 0 0 0 0
0 0 0 0 0 0 0 0 0 0 0 0 0 0 0 0 0 0 0 0 0 0 0 0 0 0 0 0 0
0 0 0 0 0 0 0 0 0 0 0 0 0 0 0 0 0 0 0 0 0 0 0 0 0 0 0

M02255_77_000000000-ADUT3_1_1113_13666_15503 C----AC---GT-AG-GGG--------GCA-A-G------C--G--T---T--GT-C-CGG-AT------TT-A--T-T--GG-GC-------GT--A-----AA-GA-GC-TC-------G-TA-G-G-T-G-------------G--C-TC-G-G-T-----
------AA----G-T-C-G--------G-G-T--G--TG--A-AA-TT--T-C-GA-G-G-----CT-C-AA------C-C-TCG-A-G-A---------C-G-CC-A---C--C-------T--GA-T-A---C-------T--G-C--TG-TG-G-C----T-A-G-A-G-T--C---C-GG-----TA-G-G--------G-G-A-G---CG-T------GG--A--ATT----
C-C-T-G-GT--GT-A-G-CG-GT--G---A--A-A---TG-C-GC-AG--AT-A-TC--A-G--G-A-G-G-A-AC-A-CC---AG--C--G--GC-GAA-G--G-C--G-------G--C-G-C-T---CTG--G--GC-C-G---------------------G-T---A-C-T--GA--CA-C-T-G--A-GG--A-G-CG-A--AA-G-C---G-TG--
GG-G--AT-C-A-AA--------CA--GG Bacteria(100);Actinobacteria(100);Actinobacteria(100);Acidimicrobidae(100);Acidimicrobiales(100);Acidimicrobineae(100);unclassified(100);unclassified(100);unclassified(100);

Otu1451 0 0 0 0 0 0 0 0 0 0 0 0 0 0 0 0 0 0 0 0 0 0 0 0 0 0 0 0
0 0 0 0 0 0 0 0 0 0 0 0 0 0 0 0 28 0 0 0 0 0 0 0 0 0 0 0 0
0 0 0 0 0 0 0 0 0 0 0 0 0 0 0 0 0 0 0 0 0 0 0 0 0 0 0 0 0
0 0 0 0 0 0 0 0 0 0 0 0 0 0 0 0 0 0 0 0 0 0 0 0 0 0 0

M02255_77_000000000-ADUT3_1_2116_14262_23204 T----AC---GT-AG-GTG--------GCG-A-G------C--G--T---T--AT-C-CGG-AT------TT-A--C-T--GG-GT-------GT--A-----AA-GG-GC-GT-------G-TA-G-G-C-G-------------G--T-CT-T-G-T------
-----AA----G-T-C-A--------A-A-T--G--TG--A-AA-TC--T-C-CC-G-G-----CT-C-AA------C-T-G-G-G-A-G--T-G----T-G--C-A---T--C-------T--GA-A-A---C-------T--A-T--GG--G-A-C----T-T-G-A-G-T--A---C-TG-----GA-G-G--------G-G-A-A---AG-C------GG--A--ATT----C-
C-T-A-GT--GT-A-G-CG-GT--A---A--A-A---TG-C-GT-AG--AG-A-TT--A-G--G-A-G-G-A-AC-A-CC---GG--T--G--GC-GAA-G--G-C--G-------G--C-T-T-T---CTG--G--AC-A-G---------------------T-A---A-C-T--GA--CG-C-T-G--A-GG--C-G-CG-A--AA-G-C---G-TG--GG-
G--AG-A-A-AA--------CA--GG Bacteria(100);Firmicutes(100);Clostridia(100);unclassified(100);unclassified(100);unclassified(100);unclassified(100);unclassified(100);unclassified(100);

Otu1452 0 0 0 0 0 0 0 0 0 0 0 0 0 0 0 0 0 0 0 0 0 0 0 0 0 0 0 0
0 0 0 0 0 0 0 0 0 0 0 0 0 0 0 0 10 0 0 0 0 0 0 0 0 0 0 0 0
0 0 0 0 0 0 0 0 0 0 0 0 0 0 0 0 0 0 0 0 0 0 0 0 0 0 0 0 0
0 0 0 0 0 0 0 0 0 0 0 0 0 0 0 0 0 0 0 0 0 0 0 0 0 0 0

M02255_77_000000000-ADUT3_1_1105_6094_3047 T----AC---GG-GG-GGA--------GC--AAG------C--G--T---T--GT-T-CGG-AT------TT-A-----------C--------T------------G-G--GC-----------G-G-C------------------C-AC-C-G-C-----------AA----G-T-C-
G--------A-C-T--G--TG--A-AA-TC--T-C-CG-G-G-----CT-T-AA------C-C-C-G-G-A-A--A-G----G-T--C-A---G--C-------C--GA-T-A---C-------T--G-C--GG--G-G-C----T-A-G-A-G-T--G---C-AG-----AA-G-G--------G-G-C-A---AC-T------GG--A--ATT----C-T-C-G-GT--GT-
A-G-CG-GT--G---A--A-A---TG-C-GT-AG--AT-A-TC--G-A--G-A-G-G-A-AC-A-CC---TG--C--G--GC-GAA-G--G-C--G-------G--G-T-T-G---CTG--G--GC-T-G---------------------A-C---A-C-T--GA--CG-C-T-G--A-GG--C-G-CG-A--AA-G-C---T-AG--GG-G--AG-C-G-AA--
------CG--GG Bacteria(100);Acidobacteria(100);Acidobacteria(100);Acidobacteriales(100);Acidobacteriaceae(100);uncultured(100);unclassified(100);unclassified(100);unclassified(100);

Otu1453 0 0 0 0 0 0 0 0 0 0 0 0 0 0 0 0 0 0 0 0 0 0 0 0 0 0 0 0
0 0 0 0 0 0 0 0 0 0 0 0 0 0 0 0 7 0 0 0 0 0 0 0 0 0 0 0 70
0 0 0 0 0 0 0 0 0 0 0 0 0 0 0 0 0 0 0 0 0 0 0 0 0 0 0 0 0
0 0 0 0 0 0 0 0 0 0 0 0 0 0 0 0 0 0 0 0 0 0 0 0 0 0 0

M02255_77_000000000-ADUT3_1_1116_12972_14521 T----AC---AG-AG-GGG--------GCA-A-G------C--G--T---T--GT-C-CGG-AA------TT-A--C-T--GG-GC-------GT--A-----CA-GC-GC-GC-------G-CA-G-G-C-G-------------G--A-CC-G-C-C----
-------AC----G-T-G-C--------C-G-T--G--TG--A-CA-AG--C-C-AC-T-G-----CT-C-AA------C-A-G-T-G-G-A--G-G----G-C--C-A---T--G-------G--TA-C-A---C-------G--A-G--CA--G-T-C----T-G-G-A-G-G--C---A-GG-----GA-G-A--------G-G-G-A---TG-T------GG--A--ATG---
-C-C-C-G-GT--GG-A-G-CG-GT--G---A--A-A---TG-C-GT-AG--AG-A-TC--G-G--G-C-G-G-A-AC-A-CC---AG--C--G--GC-GAA-G--G-C--G-------G--C-A-T-C---CTG--G--TC-C-T---------------------G-T---T-C-T--GA--CG-C-T-G--A-GG--C-G-CG-A--GA-G-C---G-TG--
GG-G--AG-C-A-AA--------CT--GG Bacteria(100);unclassified(100);unclassified(100);unclassified(100);unclassified(100);unclassified(100);unclassified(100);unclassified(100);unclassified(100);

Otu1454 0 0 0 0 0 0 0 0 0 0 0 0 0 0 0 0 0 0 0 0 0 0 0 0 0 0 0 0
0 0 0 0 0 0 0 0 0 0 0 0 0 0 0 0 0 0 0 0 0 20 0 0 0 0 0 0 0
0 0 0 0 0 0 0 0 0 0 0 0 0 0 0 0 0 0 0 0 0 0 0 0 0 0 0 0 0
0 0 0 0 0 0 0 0 0 0 0 0 0 0 0 0 0 0 0 0 0 0 0 0 0 0 0

M02255_76_000000000-ADUNE_1_1117_7565_20411 C----AC---GT-AG-GGG--------GCA-A-G------C--G--T---T--GT-C-CGG-AT------TT-A--T-T--GG-GC-------GT--------AA--A------------G--A---------------------G--C-TC-G-G-T-----------AG----G-T-
C-A--------G-G-T--G--TG--A-AA-CC--C-T-CG-G-G-----CT-C-AA------C-C-C-G-G-G-GA--------C-G-CC-A---C--C-------T--GA-T-A---C-------T--G-C--CA-TG-G-C----T-T-G-A-G-T--C---C-GG-----TA-G-G--------G-G-A-G---CG-T------GG--A--ATT----C-C-T-G-GT--
GT-A-G-CG-GT--G---A--A-A---TG-C-GC-AG--AT-A-TC--A-G--G-A-G-G-A-AC-A-CC---AG--C--G--GC-GAA-G--G-C--G-------G--C-G-C-T---CTG--G--GC-C-G---------------------G-A---A-C-T--GA--CG-C-T-G--A-GG--A-G-CG-A--AA-G-C---G-TG--GG-G--AG-C-A-
AA--------CA--GG Bacteria(100);Actinobacteria(100);Actinobacteria(100);Acidimicrobidae(100);Acidimicrobiales(100);Acidimicrobineae(100);unclassified(100);unclassified(100);unclassified(100);

Otu1455 0 0 0 0 0 0 0 0 0 0 0 0 0 0 0 0 0 0 0 0 0 0 0 0 0 0 0 0
0 0 0 0 0 0 0 0 0 0 0 0 0 0 0 0 0 0 9 0 0 0 0 0 0 0 0 0 0
0 0 0 0 0 0 0 0 0 0 0 0 0 0 0 0 0 0 0 0 0 0 0 0 0 0 0 0 0
0 0 0 0 0 0 0 0 0 0 0 0 0 0 0 0 0 0 0 0 0 0 0 0 0 0 0
M02255_77_000000000-ADUT3_1_1110_24998_8949 T----AC---GT-AG-GCA--------GCA-A-G------C--G--T---T--GT-T-CGG-AA------TT-A--C-T--GG-GC-------GT--A-----AA-GG-GT-GC-------G-TA-G-G-C-G------------G--C-TT-T-T-T----------AA--
--G-T-T-T--------G-T-C--G--TG--A-AA-TC--T-C-CC-G-G-----CT-C-AA------C-C-G-G-G-A-G--G-G----T-G--C-G---G--C-------G--AA-T-A---C-------T--G-A--GA--A-G-C----T-A-G-A-G-T--G---T-GG-----GA-G-A--------G-G-A-A---AG-T------GG--A--ATT----C-C-T-G-
GT--GT-A-G-CG-GT--G---A--A-A---TG-C-GT-AG--AT-A-TC--A-G--G-A-G-G-A-AC-A-CC---GG--T--G--GT-GTA-A--A-C--G-------G--C-T-T-T---CTG--G--AC-C-A--------------------T-A---A-C-T--GA--CG-C-T-G--A-GG--C-A-CG-A--AA-G-C---G-TG--GG-T--AG-C-
A-AA--------CA--GG Bacteria(100);Acidobacteria(100);Acidobacteria(100);Acidobacteriales(100);Acidobacteriaceae(100);Candidatus_Solibacter(100);unclassified(100);unclassified(100);unclassified(100);

Otu1456 0 0 0 0 0 0 0 0 0 0 0 0 0 0 0 0 0 0 0 0 0 0 0 0 0 0 0 0
0 0 0 0 0 0 0 0 0 0 0 0 0 0 0 0 0 0 3 0 0 0 0 0 0 0 0 0 0
0 0 0 0 0 0 0 0 0 0 0 0 0 0 0 0 0 0 0 0 0 0 0 0 0 0 0 0 0
0 0 0 0 0 0 0 0 0 0 0 0 0 0 0 0 0 0 0 0 0 0 0 0 0 0 0
M02255_77_000000000-ADUT3_1_2102_9988_18372 T----AC---GG-AG-GAT--------GCA-A-G------C--G--T---T--AT-C-CGG-AA------TG-A--T-T--GG-GC-------GT--A-----AA-GC-GT-CC-------G-CA-G-G-T-G------------G--C-AG-T-G-T-----------
AA----G-T-C-T--------G-C-T--G--TC--A-AA-GG--T-C-AG-G-G-----CT-T-AA------C-C-C-T-G-A-A--C-A----G-G--C-A---G--T-------G--GA-A-A---C-------T--A-C--AC--A-G-A----T-A-G-A-G-T--G---C-GG-----TG-G-G--------G-G-C-A---GA-G------GG--A--ATT----C-C-
C-A-GT--GT-A-G-CG-GT--G---A--A-A---TG-C-GT-AG--AG-A-TT--G-G--G-A-A-G-A-AC-A-CC---GG--T--G--GC-GAA-A--G-C--G-------C--T-C-T-G---CTA--G--GC-C-G--------------------C-A---A-C-T--GA--CA-C-T-C--A-TG--G-A-CG-A--AA-G-C---T-AG--GG-G--
AG-C-G-AA--------TG--GG Bacteria(100);Cyanobacteria(100);unclassified(100);unclassified(100);unclassified(100);unclassified(100);unclassified(100);unclassified(100);unclassified(100);

Otu1457 0 0 0 0 0 0 0 0 0 0 0 0 0 0 0 0 0 0 0 0 0 0 0 0 0 0 0 0
0 0 0 0 0 0 0 0 0 0 0 0 0 0 0 0 0 0 4 0 0 0 0 0 0 0 0 0 0
0 0 0 0 0 0 0 0 0 0 0 0 0 0 0 0 0 0 0 0 0 0 0 0 0 0 0 0 0
0 0 0 0 0 0 0 0 0 0 0 0 0 0 0 0 0 0 0 0 0 0 0 0 0 0 0
M02255_77_000000000-ADUT3_1_2111_25678_12362 T----AC---GG-AG-GGA--------GCT-A-G------C--G--T---T--GT-T-CGG-AA------TT-A--C-T--GG-GC-------GT--A-----AA-GC-GC-AC-------G-TA-G-G-C-G------------G--C-GA-C-T-C-----
------AA----G-T-C-A--------G-A-G--G--TG--A-AA-GC--C-T-GG-A-G-----CT-C-AA------C-T-C-C-A-G-A--A-C----T-G--C-C---T--T-------T--GA-A-A---C-------T--A-G--GT--C-G-C----T-A-G-A-A-T--C---T-TG-----GA-G-A--------G-G-C-G---AG-T------GG--A--ATT----C-
C-G-A-GT--GT-A-G-AG-GT--G---A--A-A---TT-C-GT-AG--AT-A-TT--C-G--G-A-A-G-A-AC-A-CC---AG--T--G--GC-GAA-G--G-C--G-------A--C-T-C-G---CTG--G--AC-A-A--------------------G-T---A-T-T--GA--CG-C-T-G--A-GG--T-G-CG-A--AA-G-C---G-TG--GG-
G--AG-C-A-AA--------CA--GG Bacteria(100);Proteobacteria(100);Alphaproteobacteria(100);Sphingomonadales(100);unclassified(100);unclassified(100);unclassified(100);unclassified(100);unclassified(100);

Otu1458 0 0 0 0 0 0 0 0 0 0 0 0 0 0 0 0 0 0 0 0 0 0 0 0 0 0 0 0
0 0 0 0 0 0 0 0 0 0 0 0 0 0 0 0 0 0 0 468 0 0 0 0 0 0 0 0 0
0 0 0 0 0 0 0 0 0 0 0 0 0 0 0 0 0 0 0 0 0 0 0 0 0 0 0 0 0
0 0 0 0 0 0 0 0 0 0 0 0 0 0 0 0 0 0 0 0 0 0 0 0 0 0 0
M02255_76_000000000-ADUNE_1_1101_16891_16797 C----GG---CT-AA-CTA---------CG-T-G------C--C--A---G--CA-G-CCG-CG------GT-A--A-T--AC-GT-------AG--G-----GA-GC-GA-GC-------GTTA-T-C-C-G------------G--A-AA-T-A-T-----
------AA----G-T-C-T--------A-A-G--G--TT--T-AA-GC--C-C-GG-A-G-----CT-C-AA------C-T-C-C-G-G-T--T------C-G--C-C---T--T-------A--GA-A-A---C-------T--G-T--AT--T-T-C----T-T-G-A-G-T--A---T-GG-----TA-G-A--------G-G-C-A---AG-T------GG--A--ATT----T-C-
T-A-GT--GT-A-G-CG-GT--A---A--A-A---TG-C-GT-AG--AT-A-TT--A-G--A-A-G-G-A-AC-A-CC---AG--T--G--GC-GAA-G--G-C--G-------A--C-T-T-G---CTG--G--GC-C-A--------------------T-T---A-C-T--GA--CG-C-T-G--A-GA--C-A-CG-A--AA-G-C---G-TG--GG-G--
AG-C-A-AA--------TA--GG Bacteria(100);unclassified(100);unclassified(100);unclassified(100);unclassified(100);unclassified(100);unclassified(100);unclassified(100);unclassified(100);

Otu1459 0 0 0 0 0 0 0 0 0 0 0 0 0 0 0 0 0 0 0 0 0 0 0 0 0 0 0 0
0 0 0 0 0 0 0 0 0 0 0 0 0 0 0 0 0 0 0 139 0 0 0 0 0 0 0 0 0
0 0 0 0 0 0 0 0 0 0 0 0 0 0 0 0 0 0 0 0 0 0 0 0 0 0 0 0 0
0 0 0 0 0 0 0 0 0 0 0 0 0 0 0 0 0 0 0 0 0 0 0 0 0 0 0
M02255_76_000000000-ADUNE_1_1103_9305_8127 T----CG---GC-TA-ACT--------CCG-T-G------C--C--A---G--CA-G-CCG-CG------GT-A--A-T--AC-GG-------AG--G-----AT-GC-GA-GC-------GTTA-T-C-C-G------------GA-T-TT-A-T-T-----------AA-
---G-T-C-A--------G-C-G--G--TA--A-AA-TT--T-C-CG-G-G-----CT-C-AA------C-C-C-G-G---T--C-G----T-G--C-C---G--T-------T--GA-A-A---C-------T--G-G--CA--G-G-C----T-T-G-A-G-T--A---T-GG-----AT-G-A--------A-G-T-G---GG-C------GG--A--ATT----T-G-T-A-
GT--GT-A-G-CG-GT--G---A--A-A---TG-C-TT-AG--AT-A-TT--A-C--A-A-G-G-A-AC-T-CC---GA--T--T--GC-GCA-G--G-C--A-------G--C-T-C-A---CTA--A--AC-C-A--------------------T-A---A-C-T--GA--CG-C-T-G--A-AG--C-A-CG-A--AA-G-C---G-TG--GG-T--AT-C-
A-AA--------CA--GG Bacteria(100);Bacteroidetes(100);Bacteroidia(100);Bacteroidales(100);Porphyromonadaceae(100);unclassified(100);unclassified(100);unclassified(100);unclassified(100);

Otu1460 0 0 0 0 0 0 0 0 0 0 0 0 0 0 0 0 0 0 0 0 0 0 0 0 0 0 0 0
0 0 0 0 0 0 0 0 0 0 0 0 0 0 0 0 0 0 0 0 12 0 0 0 0 0 0 0 0
0 0 0 0 0 0 0 0 0 0 0 0 0 0 0 0 0 0 0 0 0 0 0 0 0 0 0 0 0
0 0 0 0 0 0 0 0 0 0 0 0 0 0 0 0 0 0 0 0 0 0 0 0 0 0 0
M02255_76_000000000-ADUNE_1_1103_17910_17046 G----AA---GC-CC-CGG--------CTA-A-C------T--A--C---G--TG-C-CAG-CA------GC-C--G-C--GG-TA-------AT--A-----CG-TA-GG-GG-------GCGA-GCG-T-T-------------G--T-CC-G-G-T---
--------AA----G-T-C-T--------G-C-C--G--TG--A-AA-AC--C-T-GA-G-G-----CT-C-AA------C-C-T-C-G-G-G--C-G----T-G--C-G---G--T-------G--GA-T-A---C-------T--G-C--CG--G-G-C----T-A-G-A-G-G--A---C-GG-----TA-G-A--------G-G-C-G---AG-T------GG--A--ATT---
-C-C-C-G-GT--GT-A-G-CG-GT--G---A--A-A---TG-C-GC-AG--AT-A-TC--G-G--G-A-G-G-A-AC-A-CC---AG--T--A--GC-GAA-G--G-C--G-------G--C-T-C-G---CTG--G--GC-C-G--------------------T-T---C-C-T--GA--CG-C-T-G--A-GA--C-G-CG-A--AA-G-C---T-AG--
GG-G--AG-C-G-AA--------CA--GG Bacteria(100);Actinobacteria(100);Actinobacteria(100);Rubrobacteridae(100);Rubrobacterales(100);Rubrobacterineae(100);Rubrobacteriaceae(100);Rubrobacter(100);unclassified(100);

Otu1461 0 0 0 0 0 0 0 0 0 0 0 0 0 0 0 0 0 0 0 0 0 0 0 0 0 0 0 0
0 0 0 0 0 0 0 0 0 0 0 0 0 0 0 0 0 0 0 40 0 0 0 0 0 0 0 0 0
0 0 0 0 0 0 0 0 0 0 0 0 0 0 0 0 0 0 0 0 0 0 0 0 0 0 0 0 0
0 0 0 0 0 0 0 0 0 0 0 0 0 0 0 0 0 0 0 0 0 0 0 0 0 0 0
M02255_76_000000000-ADUNE_1_1105_11612_23442 T----CG---GC-TA-ACT--------CCG-T-GC-----C--A--G---C--AG-C-CGC-GG------TA-A--T-A--CG-GA-------GG--A-----TC-CG-AG-CG-------T-TA-T-C-C-G-------------G--T-TT-G-A-T------
-----AA----G-T-C-A--------G-C-G--G--TG--A-AA-GT--T-T-GC-A-G-----CT-T-AA------C-T-G-T-A-A-A--A-A----T-G--C-C---G--T-------T--GA-A-A---C-------T--G-T--CG--G-A-C----T-T-G-A-G-T--G---T-AA-----AT-G-A--------G-G-T-A---GG-C------GG--A--ATG----C-
G-T-G-GT--GT-A-G-CG-GT--G---A--A-A---TG-C-AT-AG--AT-A-TC--A-C--G-C-A-G-A-AC-T-CC---GA--T--T--GC-GAA-G--G-C--A-------G--C-T-T-A---CTA--A--GC-T-A--------------------C-A---A-C-T--GA--CA-C-T-G--A-AG--C-A-CG-A--AA-G-C---G-TG--GG-G--
AT-C-A-AA--------CA--GG Bacteria(100);Bacteroidetes(100);Bacteroidia(100);Bacteroidales(100);Porphyromonadaceae(100);Parabacteroides(100);unclassified(100);unclassified(100);unclassified(100);

Otu1462 0 0 0 0 0 0 0 0 0 0 0 0 0 0 0 0 0 0 0 0 0 0 0 0 0 0 0 0
0 0 0 0 0 0 0 0 0 0 0 0 0 0 0 0 0 0 0 0 0 0 0 18 0 0 0 0 0
0 0 0 0 0 0 0 0 0 0 0 0 0 0 0 0 0 0 0 0 0 0 0 0 0 0 0 0 0
0 0 0 0 0 0 0 0 0 0 0 0 0 0 0 0 0 0 0 0 0 0 0 0 0 0 0
M02255_77_000000000-ADUT3_1_2116_13925_12900 C----AC---GT-AG-GGT--------GCA-A-G------C--G--T---T--GT-C-CGG-AA------TC-A--T-T--GG-GC-------GT--A-----AA-GA-GC-TC-------G-TA-G-G-C-G-------------G--C-TT-A-G-T-----
------AA----G-T-C-G--------G-G-T--G--TG--A-AA-CC--C-C-CA-G-G-----CT-C-AA------C-C-T-G-G-G-GA--------C-G-CC-A---C--C-------C--GA-T-A---C-------T--G-C--TA-TA-G-C----T-A-G-A-G-T--C---C-AG-----TA-G-G--------G-G-A-G---TG-C------GG--A--ATT----
C-C-C-G-GT--GT-A-G-CG-GT--G---A--A-A---TG-C-GC-AG--AT-A-TC--G-G--G-A-G-G-A-AC-A-CC---AG--C--G--GC-GAA-G--G-C--G-------G--C-A-C-T---CTG--G--GC-T-G--------------------G-T---A-C-T--GA--CG-C-T-C--A-GG--A-G-CG-A--AA-G-C---G-TG--
GG-T--AG-C-G-AA--------CA--GG Bacteria(100);Actinobacteria(100);Actinobacteria(100);Acidimicrobidae(100);Acidimicrobiales(100);Acidimicrobineae(100);unclassified(100);unclassified(100);unclassified(100);

Otu1463 0 0 0 0 0 0 0 0 0 0 0 0 0 0 0 0 0 0 0 0 0 0 0 0 0 0 0 0
0 0 0 0 0 0 0 0 0 0 0 0 0 0 0 0 0 0 0 0 0 0 0 27 0 0 0 0 0
0 0 0 0 0 0 0 0 0 0 0 0 0 0 0 0 0 0 0 0 0 0 0 0 0 0 0 0 0
0 0 0 0 0 0 0 0 0 0 0 0 0 0 0 0 0 0 0 0 0 0 0 0 0 0 0
M02255_77_000000000-ADUT3_1_1110_17089_19834 T----AC---GT-AG-GGT--------CCG-A-G------C--G--T---T--GT-C-CGG-AT------TT-A--T-T--GG-GC-------GT--A-----AA-GA-GC-TC-------G-TA-G-G-T-G-------------G--C-TC-C-G-T------
-----AA----G-T-C-G--------G-G-T--G--TG--A-AA-AC--C-C-CA-G-G-----CT-C-AA------C-C-T-G-G-G-G--A------C-G-CC-A---C--T-------C--GA-T-A---C-------T--G-C--GG-TA-G-C----T-T-G-A-G-T--C---T-GG-----TA-G-G--------G-G-A-T---CA-C------GG--A--ATT----C-
C-T-G-GT--GT-A-G-CG-GT--G---A--A-A---TG-C-GC-AG--AT-A-TC--A-G--G-A-G-G-A-AC-A-CC---AG--T--G--GC-GAA-G--G-C--G-------G--T-G-A-T---CTG--G--GC-C-A--------------------G-T---A-C-T--GA--CA-C-T-G--A-GG--A-G-CG-A--AA-G-C---G-TG--GG-
G--AG-C-G-AA--------CA--GG Bacteria(100);Actinobacteria(100);Actinobacteria(100);Acidimicrobidae(100);Acidimicrobiales(100);Acidimicrobineae(100);uncultured(100);unclassified(100);unclassified(100);

Otu1464 0 0 0 0 0 0 0 0 0 0 0 0 0 0 0 0 0 0 0 0 0 0 0 0 0 0 0 0
0 0 0 0 0 0 0 0 0 0 0 0 0 0 0 0 0 0 0 0 0 0 0 14 0 0 0 0 0
0 0 0 0 0 0 0 0 0 0 0 0 0 0 0 0 0 0 0 0 0 0 0 0 0 0 0 0 0
0 0 0 0 0 0 0 0 0 0 0 0 0 0 0 0 0 0 0 0 0 0 0 0 0 0 0
M02255_77_000000000-ADUT3_1_1111_20760_15733 T----AT---AG-AG-GAT--------GCA-A-A------C--G--T---T--AT-C-CAA-AA------TG-G--T-T--GG-GC-------GT--A-----AA-GC-GT-CT-------G-TA-G-G-T-G-------------G--C-TT-T-T-T-------
----AA----G-T-C-C--------G-C-C--G--TC--A-AA-TC--C-C-AA-G-G-----CT-C-AA------C-C-C-C-G-G-A--C-A----G-G--C-G---G--T-------G--GA-A-A---C-------T--A-C--CA--A-G-C----T-G-G-A-G-T--A---C-GG-----TA-G-G--------G-G-C-A---GA-G------GG--A--ATT----T-
C-C-G-GT--GG-A-G-CG-GT--G---A--A-A---TG-C-GT-AG--AG-A-TC--G-G--A-A-A-G-A-AC-A-CC---AA--T--G--GA-GAA-A--G-C--A-------C--T-C-T-G---CTG--G--GC-C-G--------------------A-C---A-C-T--GA--CA-C-T-G--A-GA--G-A-CA-A--AA-G-C---T-AG--GG-
G--AG-C-G-AA--------TG--GG Bacteria(100);Cyanobacteria(100);unclassified(100);unclassified(100);unclassified(100);unclassified(100);unclassified(100);unclassified(100);unclassified(100);

Otu1465 0 0 0 0 0 0 0 0 0 0 0 0 0 0 0 0 0 0 0 0 0 0 0 0 0 0 0 0
0 0 0 0 0 0 0 0 0 0 0 0 0 0 0 0 0 0 0 0 0 0 0 55 0 0 0 0 0
0 0 0 0 0 0 0 0 0 0 0 0 0 0 0 0 0 0 0 0 0 0 0 0 0 0 0 0 0
0 0 0 0 0 0 0 0 0 0 0 0 0 0 0 0 0 0 0 0 0 0 0 0 0 0 0
M02255_77_000000000-ADUT3_1_1112_5986_16812 T----AC---GA-AG-GGA--------GCT-A-G------C--G--T---T--GT-T-CGG-AA------TA-A--C-T--GG-GC-------GT--A-----AA-GG-GT-GC-------G-TA-G-G-T-G------------G--G-TG-T-G-T----------
GC----G-T-T-A--------G-A-T--G--TG--A-AA-TC--C-C-CG-G-G-----CT-T-AA------C-C-T-G-G-G-A--A-C----T-G--C-A---T--T-------T--AA-T-A---C-------G--G-C--AC--G-T-C----T-A-G-A-G-T--G---C-GA-----GA-G-G--------G-G-A-A---AG-C------GG--A--ATG----G-C-
G-A-GT--GG-A-G-CA-GT--G---A--A-A---TG-C-AT-TG--AG-A-TT--C-G--C-T-A-G-A-AC-A-CC---AG--A--G--GC-GAA-G--G-C--G-------G--C-T-T-T---CTG--G--CT-C-G--------------------C-T---A-C-T--GA--CA-C-T-A--A-GG--C-A-CG-A--AA-G-C---G-TG--GG-G--
AG-C-A-AA--------CA--GG Bacteria(100);unclassified(100);unclassified(100);unclassified(100);unclassified(100);unclassified(100);unclassified(100);unclassified(100);unclassified(100);

Otu1466 0 0 0 0 0 0 0 0 0 0 0 0 0 0 0 0 0 0 0 0 0 0 0 0 0 0 0 0
0 0 0 0 0 0 0 0 0 0 0 0 0 0 0 0 0 0 0 0 0 0 0 25 0 0 0 0 0
0 0 0 0 0 0 0 0 0 0 0 0 0 0 0 0 0 0 0 0 0 0 0 0 0 0 0 0 0
0 0 0 0 0 0 0 0 0 0 0 0 0 0 0 0 0 0 0 0 0 0 0 0 0 0 0
M02255_77_000000000-ADUT3_1_1113_18211_15294 T----AC---GT-AG-GTG--------GCA-A-G------C--G--T---T--AT-C-CGG-AT------TT-A--C-T--GG-GT-------GT--A-----AA-GG-GC-GT-------G-TA-G-G-C-G------------G--G-TC-T-G-C-----
------AA----G-T-C-G--------G-A-T--G--TG--A-AA-TT--C-C-CG-G-G-----CT-T-AA------C-T-C-G-G-G-C--G-G----G-T--C-A---T--C-------C--GA-A-A---C-------T--G-C--GG--A-T-C----T-T-G-A-G-T--A---C-TG-----GA-G-A--------G-G-A-T---AG-T------GG--A--ATT----C-
C-T-A-GT--GT-A-G-CG-GT--A---A--A-A---TG-C-GT-AG--AT-A-TT--A-G--G-A-G-G-A-AC-A-CC---AG--T--G--GC-GAA-G--G-C--G-------G--C-T-A-T---CTG--G--AC-A-G--------------------T-A---A-C-T--GA--CG-C-T-G--A-GG--C-G-CG-A--AA-G-C---G-TG--GG-
G--AG-C-A-AA--------CA--GG Bacteria(100);Firmicutes(100);Clostridia(100);Clostridiales(100);Ruminococcaceae(100);Fastidiosipila(100);unclassified(100);unclassified(100);unclassified(100);

Otu1467 0 0 0 0 0 0 0 0 0 0 0 0 0 0 0 0 0 0 0 0 0 0 0 0 0 0 0 0
0 0 0 0 0 0 0 0 0 0 0 0 0 0 0 0 0 0 0 0 0 0 0 16 0 0 0 0 0
0 0 0 0 0 0 0 0 0 0 0 0 0 0 0 0 0 0 0 0 0 0 0 0 0 0 0 0 0
0 0 0 0 0 0 0 0 0 0 0 0 0 0 0 0 0 0 0 0 0 0 0 0 0 0 0
M02255_77_000000000-ADUT3_1_1115_18850_12125 T----AC---GT-AG-GGG--------GCG-A-G------C--G--T---T--GT-C-CGG-AT------TT-A--T-T--GG-GC-------GT--A-----AA-GA-GC-TC-------G-TA-G-G-C-G------------G--C-TG-G-G-C----
-------AA----G-T-C-G--------G-G-T--G--TG--A-AA-AC--T-C-CA-G-G-----CT-C-AA------C-T-T-G-G-A-G--A------C-G-CC-A---C--T-------T--GA-T-A---C-------T--G-C--CC-TG-G-C----T-T-G-A-G-T--C---C-GG-----TA-G-G--------G-G-A-G---CG-C------GG--A--ATT----
C-C-T-G-GT--GT-A-G-CG-GT--G---A--A-A---TG-C-GC-AG--AG-A-TC--A-G--G-A-G-G-A-AC-A-CC---GG--T--G--GC-GAA-G--G-C--G-------G--C-G-C-T---CTG--G--GC-C-G--------------------G-T---A-C-T--GA--CG-C-T-G--A-GG--A-G-CG-A--AA-G-C---G-TG--
GG-G--AG-C-G-AA--------CA--GG Bacteria(100);Actinobacteria(100);Actinobacteria(100);unclassified(100);unclassified(100);unclassified(100);unclassified(100);unclassified(100);unclassified(100);

Otu1468 0 0 0 0 0 0 0 0 0 0 0 0 0 0 0 0 0 0 0 0 0 0 0 0 0 0 0 0
0 0 0 0 0 0 0 0 0 0 0 0 0 0 0 0 0 0 0 0 0 0 0 24 0 0 0 0 0
0 0 0 0 0 0 0 0 0 0 0 0 0 0 0 0 0 0 0 0 0 0 0 0 0 0 0 0 0
0 0 0 0 0 0 0 0 0 0 0 0 0 0 0 0 0 0 0 0 0 0 0 0 0 0 0
M02255_77_000000000-ADUT3_1_1116_22331_15015 T----AC---GT-AG-GGT--------GCG-A-G------C--G--T---T--GT-C-CGG-AA------TT-A--C-T--GG-GC-------GT--A-----AA-GA-GC-TT-------G-TA-G-G-C-G------------G--T-CT-G-T-C------
-----GC----G-T-C-T--------T-C-T--G--TG--A-AA-AC--T-T-GG-G-G-----CT-C-AA------C-C-T-T-A-A-G--C-T----T-G--C-A---G--G-------G--GA-T-A---C-------G--G-G--CA--G-A-C----T-A-G-A-G-T--A---C-TT-----CA-G-G--------G-G-A-G---AC-T------GG--A--ATT----C-
C-T-G-GT--GT-A-G-CG-GT--G---A--A-A---TG-C-GC-AG--AT-A-TC--A-G--G-A-G-G-A-AC-A-CC---GG--T--G--GC-GAA-G--G-C--G-------G--G-T-C-T---CTG--G--GA-A-G--------------------T-A---A-C-T--GA--CG-C-T-G--A-GA--A-G-CG-A--AA-G-C---G-TG--GG-
T--AG-C-G-AA--------CA--GG Bacteria(100);Actinobacteria(100);Actinobacteria(100);Actinobacteridae(100);Actinomycetales(100);Corynebacterineae(100);Nocardiaceae(100);Nocardia(100);unclassified(100);

Otu1469 0 0 0 0 0 0 0 0 0 0 0 0 0 0 0 0 0 0 0 0 0 0 0 0 0 0 0 0
0 0 0 0 0 0 0 0 0 0 0 0 0 0 0 0 0 0 0 0 0 0 0 27 0 0 0 0 0
0 0 0 0 0 0 0 0 0 0 0 0 0 0 0 0 0 0 0 0 0 0 0 0 0 0 0 0 0
0 0 0 0 0 0 0 0 0 0 0 0 0 0 0 0 0 0 0 0 0 0 0 0 0 0 0
M02255_77_000000000-ADUT3_1_1118_26201_13474 T----AC---GA-AG-GGG--------GCT-A-G------C--G--T---T--GT-T-CGG-AA------TT-A--C-T--GG-GC-------GT--A-----AA-GC-GC-GC-------G-TA-G-G-C-G------------G--A-TA-T-T-T-----
------AA----G-T-C-A--------G-G-G--G--TG--A-AA-TG--C-C-AG-G-G-----CT-C-AA------C-C-T-T-G-G-A--A-C----T-G--C-C---T--T-------T--GA-T-A---C-------T--G-G--GT--A-T-C----T-G-G-A-G-T--T---T-GG-----GA-G-A--------G-G-T-G---AG-T------GG--A--ATT----C-
C-G-A-GT--GT-A-G-AG-GT--G---A--A-A---TT-C-GT-AG--AT-A-TT--C-G--G-A-G-G-A-AC-A-CC---AG--A--A--GC-GAA-G--G-C--G-------G--C-T-C-A---CTG--G--CC-C-A--------------------A-T---A-C-T--GA--CG-C-T-G--A-GG--C-G-CG-A--AA-G-C---G-TG--GG-
G--AG-C-A-AA--------CA--GG Bacteria(100);Proteobacteria(100);Alphaproteobacteria(100);unclassified(100);unclassified(100);unclassified(100);unclassified(100);unclassified(100);unclassified(100);

Otu1470 0 0 0 0 0 0 0 0 0 0 0 0 0 0 0 0 0 0 0 0 0 0 0 0 0 0 0 0
0 0 0 0 0 0 0 0 0 0 0 0 0 0 0 0 0 0 0 0 0 0 0 18 0 0 0 0 0
0 0 0 0 0 0 0 0 0 0 0 0 0 0 0 0 0 0 0 0 0 0 0 0 0 0 0 0 0
0 0 0 0 0 0 0 0 0 0 0 0 0 0 0 0 0 0 0 0 0 0 0 0 0 0 0 0
M02255_77_000000000-ADUT3_1_2115_16602_13316 C----AC---GT-AG-GGG--------GCA-A-G------C--G--T---T--AC-C-CGG-AT------TT-A--C-T--GG-GC-------GT--A-----CA-GC-GC-GC-------G-TA-G-G-C-G------------G--C-TG-G-C-C-----
------AA----G-C-G-T--------G-G-T--G--TG--A-AA-TC--T-C-CG-G-G-----CT-C-AA------C-C-C-G-G-A-A--A-C----T-G--C-A---C--C-------G--CG-A-A---C-------TG-G-T--CT--G-G-C----T-A-G-A-G-T--G---C-AG-----GA-G-G--------G-G-G-T---GG-T------GG--A--ATT----
C-C-C-G-GT--GT-A-G-CG-GT--G---A--A-A---TG-C-GT-AG--AT-A-TC--G-G--G-A-G-G-A-AC-A-CC---AG--T--G--GC-GAA-G--G-C--G-------A--C-C-A-C---CTG--G--CC-T-G--------------------T-A---A-C-T--GA--CG-C-T-G--A-TG--C-G-CG-A--AA-G-C---G-TG--
GG-G--AG-C-G-AA--------CC--GG Bacteria(100);unclassified(100);unclassified(100);unclassified(100);unclassified(100);unclassified(100);unclassified(100);unclassified(100);unclassified(100);

Otu1471 0 0 0 0 0 0 0 0 0 0 0 0 0 0 0 0 0 0 0 0 0 0 0 0 0 0 0 0
0 0 0 0 0 0 0 0 0 0 0 0 0 0 0 0 0 0 0 0 0 0 0 14 0 0 0 0 0
0 0 0 0 0 0 0 0 0 0 0 0 0 0 0 0 0 0 0 0 0 0 0 0 0 0 0 0 0
0 0 0 0 0 0 0 0 0 0 0 0 0 0 0 0 0 0 0 0 0 0 0 0 0 0 0 0
M02255_77_000000000-ADUT3_1_1109_8539_17151 G----AC---GT-AG-GGC--------GCA-A-G------C--G--T---T--GT-C-CGG-AT------TT-A--T-T--GG-GC-------GT--A-----AA-GA-GC-TC-------G-TA-G-G-C-G------------G--C-TT-G-T-C-----------AC-
---G-T-C-G--------T-C-C--G--TG--A-AA-AC--T-T-CA-G-G-----CT-T-AA------C-C-T-G-G-A-G--C-G----T-G--C-G---G--T-------C--GA-T-A---C-------G--G-G--CT--G-G-C----T-A-G-A-G-G--C---A-GG-----TA-G-G--------G-G-A-G---AT-C------GG--A--ATT----C-C-T-G-
GT--GT-A-G-CG-GT--G---A--A-A---TG-C-GC-AG--AT-A-TC--A-G--G-A-G-G-A-AC-A-CC---GG--T--G--GC-GAA-G--G-C--G-------G--A-T-C-T---CTG--G--GC-C-T--------------------G-T---A-C-T--GA--CG-C-T-G--A-GG--A-G-CG-A--AA-G-C---G-TG--GG-G--AG-
C-G-AA--------CA--GG Bacteria(100);Actinobacteria(100);Actinobacteria(100);Actinobacteridae(100);Actinomycetales(100);unclassified(100);unclassified(100);unclassified(100);unclassified(100);

Otu1472 0 0 0 0 0 0 0 0 0 0 0 0 0 0 0 0 0 0 0 0 0 0 0 0 0 0 0 0
0 0 0 0 0 0 0 0 0 0 0 0 0 0 0 0 0 0 0 0 0 0 0 12 0 0 0 0 0
0 0 0 0 0 0 0 0 0 0 0 0 0 0 0 0 0 0 0 0 0 0 0 0 0 0 0 0 0
0 0 0 0 0 0 0 0 0 0 0 0 0 0 0 0 0 0 0 0 0 0 0 0 0 0 0 0
M02255_76_000000000-ADUNE_1_1117_12473_9704 C----TA---AC-TC-CGT--------GCC-A-G------C-----A---G--CC-G-CGG-TA------AT-A--C-G--GA-GG-------GT--G-----CA--A-GC-G--------T-TA-A-T-C-G------------G--A-TT-G-C-T----------CA----
G-T-C-G--------G-A-T--G--TG--A-AA-GC--C-C-CG-G-G-----CT-T-AA------C-C-T-G-G-G-A--A-T----T-G--C-A---T--T-------C--GA-T-A---C-------T--G-G--CA--G-A-C----T-A-G-A-G-T--G---T-GG-----AA-G-A--------G-G-A-A---GG-T------GG--A--ATT----C-C-A-G-
GT--GT-A-G-CG-GT--G---A--A-A---TG-C-GT-AG--AT-A-TC--T-G--G-A-G-G-A-AC-A-TC---AG--T--G--GC-GAA-G--G-C--G-------G--C-C-T-T---CTG--G--TC-C-A--------------------A-C---A-C-T--GA--CG-C-T-G--A-GG--T-G-CG-A--AA-G-C---G-TG--GG-G--AG-C-
A-AA--------CA--GG Bacteria(100);Proteobacteria(100);Gammaproteobacteria(100);unclassified(100);unclassified(100);unclassified(100);unclassified(100);unclassified(100);unclassified(100);

Otu1473 0 0 0 0 0 0 0 0 0 0 0 0 0 0 0 0 0 0 0 0 0 0 0 0 0 0 0 0
0 0 0 0 0 0 0 0 0 0 0 0 0 0 0 0 0 0 0 0 0 0 0 14 0 0 0 0 0
0 0 0 0 0 0 0 0 0 0 0 0 0 0 0 0 0 0 0 0 0 0 0 0 0 0 0 0 0
0 0 0 0 0 0 0 0 0 0 0 0 0 0 0 0 0 0 0 0 0 0 0 0 0 0 0 0
M02255_77_000000000-ADUT3_1_1108_19885_20520 T----AC---GG-AG-GGG--------GCA-A-G------C--G--T---T--GT-T-CGG-AA------TT-A--C-T--GG-GC-------GT--A-----AA-GG-GC-GC-------G-TA-G-G-C-G------------G--T-CT-G-T-C----
-------GC----G-T-C-A--------G-G-T--G--TG--A-AA-GC--C-C-CG-G-G-----CT-C-AA------C-C-T-G-G-G-A--G-G----T-G--C-A---C--T-------T--GA-T-A---C-------G--G-G--CG--G-G-C----T-G-G-A-G-T--C---C-GA-----GA-G-A--------G-G-T-G---AG-T------GG--A--ATT---
-C-C-C-A-GT--GT-A-G-AG-GT--G---A--A-A---TT-C-GT-AG--AT-A-TT--G-G--G-A-A-G-A-AC-A-CC---AG--T--G--GC-GAA-G--G-C--G-------G--C-T-C-A---CTG--G--CT-C-G--------------------G-A---A-C-T--GA--CG-C-T-G--A-GG--C-G-CG-A--AA-G-C---G-TG--
GG-G--AG-C-A-AA--------CA--GG Bacteria(100);Proteobacteria(100);Alphaproteobacteria(100);unclassified(100);unclassified(100);unclassified(100);unclassified(100);unclassified(100);unclassified(100);

Otu1474 0 0 0 0 0 0 0 0 0 0 0 0 0 0 0 0 0 0 0 0 0 0 0 0 0 0 0 0
0 0 0 0 0 0 0 0 0 0 0 0 0 0 0 0 0 0 0 0 0 0 0 12 0 0 0 0 0
0 0 0 0 0 0 0 0 0 0 0 0 0 0 0 0 0 0 0 0 0 0 0 0 0 0 0 0 0
0 0 0 0 0 0 0 0 0 0 0 0 0 0 0 0 0 0 0 0 0 0 0 0 0 0 0 0
M02255_77_000000000-ADUT3_1_2108_9597_9522 T----AC---GT-AG-GGC--------GCA-A-G------C--G--T---T--GT-C-CGG-AA------TT-A--T-T--GG-GC-------GT--A-----AA-GA-GC-TC-------G-TA-G-G-C-G------------G--T-TT-G-T-C-----------GC-
---G-T-C-T--------G-T-C--G--TG--A-AA-GC--C-C-AC-G-G-----CT-T-AA------C-C-G-T-G-G-G--T-C----T-G--C-G---G--T-------G--GA-T-A---C-------G--G-G--CA--G-A-C----T-A-G-A-G-G--C---A-GG-----TA-G-G--------G-G-A-G---AA-T------GG--A--ATT----C-C-C-G-
GT--GT-A-G-CG-GT--G---A--A-A---TG-C-GC-AG--AT-A-TC--G-G--G-A-G-G-A-AC-A-CC---GG--T--G--GC-GAA-G--G-C--G-------G--T-T-C-T---CTG--G--GC-C-T--------------------G-T---A-C-T--GA--CG-C-T-G--A-GG--A-G-CG-A--AA-G-C---G-TG--GG-G--AG-
C-G-AA--------CA--GG Bacteria(100);Actinobacteria(100);Actinobacteria(100);Actinobacteridae(100);Actinomycetales(100);Streptosporangineae(100);Thermomonosporaceae(100);Actinomadura(100);unclassified(100);

Otu1475 0 0 0 0 0 0 0 0 0 0 0 0 0 0 0 0 0 0 0 0 0 0 0 0 0 0 0 0
0 0 0 0 0 0 0 0 0 0 0 0 0 0 0 0 0 0 0 0 0 0 0 18 0 0 0 0 0
0 0 0 0 0 0 0 0 0 0 0 0 0 0 0 0 0 0 0 0 0 0 0 0 0 0 0 0 0
0 0 0 0 0 0 0 0 0 0 0 0 0 0 0 0 0 0 0 0 0 0 0 0 0 0 0 0
M02255_77_000000000-ADUT3_1_2117_5422_11496 T----AC---GT-AG-GGT--------GCG-A-G------C--G--T---T--GT-C-CGG-AA------TT-A--T-T--GG-GC-------GT--A-----AA-GA-GC-TC-------G-TA-G-G-C-G------------G--T-TT-G-T-C-----------GC-
---G-T-C-G--------T-T-C--G--TG--A-AA-AC--T-C-CA-C-G-----CT-T-AA------C-G-T-G-G-A-G--C-G----T-G--C-G---G--G-------C--GA-T-A---C-------G--G-G--CA--G-A-C----T-T-G-A-G-T--T---C-GG-----TA-G-G--------G-G-A-G---AC-T------GG--A--ATT----C-C-T-G-
GT--GT-A-G-CG-GT--G---A--A-A---TG-C-GC-AG--AT-A-TC--A-G--G-A-G-G-A-AC-A-CC---GG--T--G--GC-GAA-G--G-C--G-------G--G-T-C-T---CTG--G--GC-C-G--------------------A-T---A-C-T--GA--CG-C-T-G--A-GG--A-G-CG-A--AA-G-C---G-TG--GG-G--AG-
C-G-AA--------CA--GG Bacteria(100);Actinobacteria(100);Actinobacteria(100);Actinobacteridae(100);Actinomycetales(100);Pseudonocardineae(100);Pseudonocardiaceae(100);Amycolatopsis(100);unclassified(100);

Otu1476 0 0 0 0 0 0 0 0 0 0 0 0 0 0 0 0 0 0 0 0 0 0 0 0 0 0 0 0
0 0 0 0 0 0 0 0 0 0 0 0 0 0 0 0 0 0 0 0 0 0 0 24 0 0 0 0 0
0 0 0 0 0 0 0 0 0 0 0 0 0 0 0 0 0 0 0 0 0 0 0 0 0 0 0 0 0
0 0 0 0 0 0 0 0 0 0 0 0 0 0 0 0 0 0 0 0 0 0 0 0 0 0 0 0
M02255_77_000000000-ADUT3_1_2118_6911_11562 T----AC---GT-AG-GAC--------CCA-A-G------C--G--T---T--AT-C-CGG-AA------TT-A--C-T--GG-GC-------GT--A-----AA-GA-GT-TT-------G-TA-G-G-C-G------------G--T-TT-T-T-C-----------AA---
-G-T-C-A--------G-T-T--G--CG--A-AA-TC--C-T-TC-C-G-----CT-C-AA------C-G-G-AAT-G-G--A-C----T-G--T-G---A--T-------T--GA-A-A---C-------T--G-A--AT--T-A-C----T-A-G-A-G-G--T---T-GG-----TA-G-A--------G-G-T-G---CG-T------GG--A--ATT----T-C-T-G-GT-
-GT-A-G-GA-GT--G---A--C-A---TC-C-GT-AG--AT-A-TC--A-G--A-A-G-G-A-AC-A-CC---AA--T--G--GC-GAA-G--G-C--A-------G--C-G-C-A---CTG--G--GC-C-A--------------------T-A---C-C-T--GA--CG-C-T-G--A-GA--A-A-CG-A--AA-G-C---G-TG--GG-G--AG-C-G-
AA--------CG--GG Bacteria(100);unclassified(100);unclassified(100);unclassified(100);unclassified(100);unclassified(100);unclassified(100);unclassified(100);unclassified(100);

Otu1477 0 0 0 0 0 0 0 0 0 0 0 0 0 0 0 0 0 0 0 0 0 0 0 0 0 0 0 0
0 0 0 0 0 0 0 0 0 0 0 0 0 0 0 0 0 0 0 0 0 0 0 13 0 0 0 0 0
0 0 0 0 0 0 0 0 0 0 0 0 0 0 0 0 0 0 0 0 0 0 0 0 0 0 0 0 0
0 0 0 0 0 0 0 0 0 0 0 0 0 0 0 0 0 0 0 0 0 0 0 0 0 0 0 0
M02255_77_000000000-ADUT3_1_1105_7984_20512 T----AC---AG-AG-GCC--------CCA-A-G------C--A--T---T--AT-C-CGG-AT------TC-A--C-T--GG-GC-------GT--A-----AA-GG-GT-GC-------G-TA-G-G-C-G------------G--T-GT-G-A-T-----------TA-
---G-T-C-G--------G-G-T--G--TT--A-AA-TC--C-T-GG-G-G-----CT-C-AA------C-C-T-C-A-G-A--A-T----C-G--C-A---T--T-------C--GA-A-A---C-------G--G-T--CA--T-A-C----T-A-G-A-A-G--A---A-GT-----CA-G-A--------G-G-T-G---AA-C------AG--A--ACT----C-T-C-G-
GT--GT-A-G-GG-GT--G---A--A-A---TC-C-GT-TG--AT-A-TC--G-A--G-G-G-G-A-AT-A-CC---AA--A--T--GC-GAA-G--G-C--A-------G--T-T-C-A---CTG--G--GG-C-T--------------------T-T---C-T-T--GA--CG-C-T-G--A-GG--C-A-CG-A--AA-G-C---G-TG--GG-T--AG-C-
G-AA--------CG--GG Bacteria(100);Candidate_division_OD1(100);unclassified(100);unclassified(100);unclassified(100);unclassified(100);unclassified(100);unclassified(100);unclassified(100);

Otu1478 0 0 0 0 0 0 0 0 0 0 0 0 0 0 0 0 0 0 0 0 0 0 0 0 0 0 0 0
0 0 0 0 0 0 0 0 0 0 0 0 0 0 0 0 0 0 0 0 0 0 0 13 0 0 0 0 0
0 0 0 0 0 0 0 0 0 0 0 0 0 0 0 0 0 0 0 0 0 0 0 0 0 0 0 0 0
0 0 0 0 0 0 0 0 0 0 0 0 0 0 0 0 0 0 0 0 0 0 0 0 0 0 0 0
M02255_76_000000000-ADUNE_1_2105_13844_11716 T----AC---GT-AG-GGT--------CCG-A-G------C--G--T---T--GT-C-CGG-AA------TT-A--T-T--GG-GC-------GT--A-----AA----------------G----G-G-C--------------------TC-G-T-C-----------AC-
---G-T-C-G--------G-G-A--G--TG--A-AA-AC--T-C-GG-G-G-----CT-T-AA------C-C-C-C-G-A-G--C-C----T-G--C-T---T--C-------C--GA-T-A---C-------G--G-G--CA--G-A-C----T-A-G-A-G-G--T---A-GG-----CA-G-G--------G-G-A-G---AG-C------GG--A--ACT----C-C-T-G-
GT--GT-A-G-CG-GT--G---G--A-A---TG-C-GC-AG--AT-A-TC--A-G--G-A-A-G-A-AC-A-CC---GG--T--G--GC-GAA-G--G-C--G-------G--C-T-C-T---CTG--G--GC-C-T--------------------T-A---C-C-T--GA--CG-C-T-G--A-GG--A-G-CG-A--AA-G-C---G-TG--GG-T--AG-
C-G-AA--------CA--GG Bacteria(100);Actinobacteria(100);Actinobacteria(100);Actinobacteridae(100);Actinomycetales(100);Propionibacterineae(100);Nocardioidaceae(100);Kribbella(100);unclassified(100);

Otu1479 0 0 0 0 0 0 0 0 0 0 0 0 0 0 0 0 0 0 0 0 0 0 0 0 0 0 0 0
0 0 0 0 0 0 0 0 0 0 0 0 0 0 0 0 0 0 0 0 0 0 0 0 0 0 0 0 0
0 0 0 14 0 0 0 0 0 0 0 0 0 0 0 0 0 0 0 0 0 0 0 0 0 0 0 0 0
0 0 0 0 0 0 0 0 0 0 0 0 0 0 0 0 0 0 0 0 0 0 0 0 0 0 0 0
M02255_77_000000000-ADUT3_1_1105_12266_7061 T----AC---GA-AG-GGG--------GCT-A-G------C--G--T---T--GC-T-CGG-AA------TG-A--C-T--GG-GC-------GT--A-----AA-GG-GC-GC-------G-TA-G-G-C-G------------G--C-GT-G-C-A-----------
TA----G-T-C-A--------G-A-T--G--TG--A-AA-TT--C-C-TG-G-G-----CT-T-AA------C-C-T-G-G-G-G--G-C----T-G--C-A---T--T-------T--GA-T-A---C-------G--T-G--TG--T-G-C----T-G-G-A-G-T--G---T-GA-----AA-G-A--------G-G-G-T---CG-T------GG--A--ATT----C-C-C-
A-GT--GT-A-G-AG-GT--G---A--A-A---TT-C-GT-AG--AT-A-TT--G-G--G-A-A-G-A-AC-A-CC---GG--T--G--GC-GAA-G--G-C--G-------G--C-G-A-C---CTG--G--TT-C-A--------------------T-T---A-C-T--GA--CG-C-T-G--A-GG--C-G-CG-A--AA-G-C---G-TG--GG-G--
AG-C-A-AA--------CA--GG Bacteria(100);Proteobacteria(100);Alphaproteobacteria(100);Rhodospirillales(100);Acetobacteraceae(100);unclassified(100);unclassified(100);unclassified(100);unclassified(100);

Otu1480 0 0 0 0 0 0 0 0 0 0 0 0 0 0 0 0 0 0 0 0 0 0 0 0 0 0 0 0
0 0 0 0 0 0 0 0 0 0 0 0 0 0 0 0 0 0 0 0 0 0 0 47 0 0 0 0 0
0 0 0 0 0 0 0 0 0 0 0 0 0 0 0 0 0 0 0 0 0 0 0 0 0 0 0 0 0
0 0 0 0 0 0 0 0 0 0 0 0 0 0 0 0 0 0 0 0 0 0 0 0 0 0 0
M02255_77_000000000-ADUT3_1_1108_26633_15992 T----AC---GT-AG-GGA--------GCA-A-G------C--G--T---T--GT-C-CGG-AT------TT-A--T-T--GG-GC-------GT--A-----AA-GA-GC-TC-------G-TA-G-G-C-G------------G--T-TC-G-G-T-----
------AA----G-T-C-G--------G-G-T--G--TG--A-AA-AC--T-C-TG-G-G-----CT-C-AA------C-C-C-G-G-A-G--A------G-G-CC-A---C--T-------C--GA-T-A---C-------T--G-C--CG-TG-A-C----T-T-G-A-G-T--C---T-GG-----TA-G-G--------G-G-A-G---CA-C------GG--A--ATT----
C-C-T-G-GT--GT-A-G-CG-GT--G---A--A-A---TG-C-AC-AG--AT-A-TC--A-G--G-A-G-G-A-AC-A-CC---GG--T--G--GC-GAA-G--G-C--G-------G--T-G-C-T---CTG--G--GC-C-A--------------------G-T---A-C-T--GA--CG-C-T-G--A-GG--A-G-CG-A--AA-G-C---G-TG--
GG-G--AG-C-G-AA--------CA--GG Bacteria(100);unclassified(100);unclassified(100);unclassified(100);unclassified(100);unclassified(100);unclassified(100);unclassified(100);unclassified(100);

Otu1481 0 0 0 0 0 0 0 0 0 0 0 0 0 0 0 0 0 0 0 0 0 0 0 0 0 0 0 0
0 0 0 0 0 0 0 0 0 0 0 0 0 0 0 0 0 0 0 0 0 0 0 93 0 0 0 0 0
0 0 0 0 0 0 0 0 0 0 0 0 0 0 0 0 0 0 0 0 0 0 0 0 0 0 0 0 0
0 0 0 0 0 0 0 0 0 0 0 0 0 0 0 0 0 0 0 0 0 0 0 0 0 0 0
M02255_77_000000000-ADUT3_1_1104_12857_17142 T----AC---GG-AG-GGA--------GCT-A-G------C--G--T---T--GT-T-CGG-AA------AT-A--C-T--GG-GC-------GT--A-----AA-GC-GC-AC-------G-TA-G-G-C-G------------G--C-TA-T-C-T-----
------AA----G-T-C-A--------G-G-G--G--TG--A-AA-TC--C-C-GG-A-G-----CT-C-AA------C-T-C-C-G-G-A--A-C----T-G--C-C---T--T-------T--GA-A-A---C-------T--G-G--AT--G-G-C----T-A-G-A-A-T--C---T-TG-----GA-G-A--------G-G-C-G---AG-T------GG--A--ATT----C-
C-G-A-GT--GT-A-G-AG-GT--G---A--A-A---TT-C-GT-AG--AT-A-TT--C-G--G-A-A-G-A-AC-A-CC---AG--T--G--GC-GAA-G--G-C--G-------A--C-T-C-G---CTG--G--AC-A-A--------------------G-T---A-T-T--GA--CG-C-T-G--A-GG--T-G-CG-A--AA-G-C---G-TG--GG-
G--AG-C-A-AA--------CA--GG Bacteria(100);Proteobacteria(100);Alphaproteobacteria(100);Sphingomonadales(100);unclassified(100);unclassified(100);unclassified(100);unclassified(100);unclassified(100);

Otu1482 0 0 0 0 0 0 0 0 0 0 0 0 0 0 0 0 0 0 0 0 0 0 0 0 0 0 0 0
0 0 0 0 0 0 0 0 0 0 0 0 0 0 0 0 0 0 0 0 0 0 0 30 0 0 0 0 0
0 0 0 0 0 0 0 0 0 0 0 0 0 0 0 0 0 0 0 0 0 0 0 0 0 0 0 0 0
0 0 0 0 0 0 0 0 0 0 0 0 0 0 0 0 0 0 0 0 0 0 0 0 0 0 0
M02255_77_000000000-ADUT3_1_1103_21944_16789 T----AC---GT-AG-GGG--------GCG-A-A------C--G--T---T--AT-C-CGG-AA------TG-A--T-T--GG-GC-------GT--A-----AA-GG-GT-AC-------G-TA-G-G-C-G------------G--A-CC-T-G-T----
-------AA----G-T-C-A--------G-G-T--G--TG--A-AA-GG--C-A-TG-G-G-----CT-C-AA------C-C-C-A-T-G-T--A------A-G--C-A---C--T-------T--GA-A-A---C-------T--G-C--GG--G-C-C----T-T-G-A-G-G--G---A-AG-----GA-G-A--------G-G-T-A---AG-T------GG--A--ATT----
C-C-T-A-GT--GT-A-G-CG-GT--G---A--A-A---TG-C-GT-AG--AT-A-TT--A-G--G-A-G-G-A-AT-A-CC---GG--T--G--GC-GAA-G--G-C--G-------A--C-T-T-A---CTG--G--AC-T-T--------------------T-T---A-C-T--GA--CG-C-T-G--A-GG--T-A-CG-A--AA-G-C---G-TG--GG-
G--AG-C-A-AA--------CA--GG Bacteria(100);Firmicutes(100);Clostridia(100);Clostridiales(100);Family_XI_Incertae_Sedis(100);uncultured(100);unclassified(100);unclassified(100);unclassified(100);

Otu1483 0 0 0 0 0 0 0 0 0 0 0 0 0 0 0 0 0 0 0 0 0 0 0 0 0 0 0 0
0 0 0 0 0 0 0 0 0 0 0 0 0 0 0 0 0 0 0 0 0 0 0 34 0 0 0 0 0
0 0 0 0 0 0 0 0 0 0 0 0 0 0 0 0 0 0 0 0 0 0 0 0 0 0 0 0 0
0 0 0 0 0 0 0 0 0 0 0 0 0 0 0 0 0 0 0 0 0 0 0 0 0 0 0
M02255_77_000000000-ADUT3_1_1102_21199_18226 T----AC---GT-AT-GGA--------GCA-A-G------C--G--T---T--AT-C-CGG-AT------TT-A--C-T--GG-GT-------GT--A-----AA-GG-GA-GC-------G-TA-G-A-C-G------------G--C-AT-G-G-C-----
------AA----G-T-C-T--------G-A-T--G--TGA-A-A--AC--C-C-TG-G-G-----CT-C-AA------C-C-C-G-G-G-G--A-C----T-G--C-A---T--T-------G--GA-A-A---C-------T--G-T--TA--A-G-C----T-A-G-A-G-T--G---C-AG-----GA-G-A--------G-G-T-A---AG-T------GG--A--ATT----
C-C-T-A-GT--GT-A-G-CG-GT--G---A--A-A---TG-C-GT-AG--AT-A-TT--A-G--G-A-G-G-A-AC-A-CC---AG--T--G--GC-GAA-G--G-C--G-------G--C-T-T-A---CTG--G--AC-T-G--------------------T-A---A-C-T--GA--CG-T-T-G--A-GG--C-T-CG-A--AA-G-C---G-TG--GG-
G--AG-C-A-AA--------CA--GG Bacteria(100);Firmicutes(100);Clostridia(100);Clostridiales(100);Lachnospiraceae(100);unclassified(100);unclassified(100);unclassified(100);unclassified(100);

Otu1484 0 0 0 0 0 0 0 0 0 0 0 0 0 0 0 0 0 0 0 0 0 0 0 0 0 0 0 0
0 0 0 0 0 0 0 0 0 0 0 0 0 0 0 0 0 0 0 0 0 0 0 24 0 0 0 0 0
0 0 0 0 0 0 0 0 0 0 0 0 0 0 0 0 0 0 0 0 0 0 0 0 0 0 0 0 0
0 0 0 0 0 0 0 0 0 0 0 0 0 0 0 0 0 0 0 0 0 0 0 0 0 0 0
M02255_77_000000000-ADUT3_1_1101_11582_14431 T----AC---GT-AG-GGT--------GCG-A-G------C--G--T---T--GT-C-CGG-AA------TT-A--C-T--GG-GC-------GT--A-----AA-GA-GC-TC-------G-TA-G-G-T-G------------G--T-TT-G-T-C------
-----GC----G-T-T-G--------T-T-C--G--TG--A-AA-AC--T-A-AA-G-G-----CT-T-AA------C-C-T-T-T-G-G--C-G----T-G--C-G---G--G-------C--GA-T-A---C-------G--G-G--CA--G-A-C----T-G-G-A-G-T--A---C-TG-----CA-G-G--------G-G-A-G---AC-T------GG--A--ATT----C-
C-T-G-GT--GT-A-G-CG-GT--G---G--A-A---TG-C-GC-AG--AT-A-TC--A-G--G-A-G-G-A-AC-A-CC---GG--T--G--GC-GAA-G--G-C--G-------G--G-T-C-T---CTG--G--GC-A-G--------------------T-A---A-C-T--GA--CG-C-T-G--A-GG--A-G-CG-A--AA-G-C---G-TG--GG-
G--AG-C-G-AA--------CA--GG Bacteria(100);Actinobacteria(100);Actinobacteria(100);Actinobacteridae(100);Actinomycetales(100);Corynebacterineae(100);unclassified(100);unclassified(100);unclassified(100);

Otu1485 0 0 0 0 0 0 0 0 0 0 0 0 0 0 0 0 0 0 0 0 0 0 0 0 0 0 0 0
0 0 0 0 0 0 0 0 0 0 0 0 0 0 0 0 0 0 0 0 0 0 0 53 0 0 0 0 0
0 0 0 0 0 0 0 0 0 0 0 0 0 0 0 0 0 0 0 0 0 0 0 0 0 0 0 0 0
0 0 0 0 0 0 0 0 0 0 0 0 0 0 0 0 0 0 0 0 0 0 0 0 0 0 0
M02255_77_000000000-ADUT3_1_1101_5096_11408 T----AC---GT-AG-GGA--------GCA-A-G------C--G--T---T--GT-C-CGG-AA------TC-A--T-T--GG-GC-------GT--A-----AA-GG-GC-GC-------G-TA-G-G-C-G------------G--T-CT-G-G-T----------
AA----G-T-C-A--------A-A-T--G--TG--A-AA-TG--C-A-CA-G-G-----CT-T-AA--------C-C-T-G-T-G--A-GC---T-G--C-G---T--T-------T--GA-A-A---C-------T--G-T--CA--G-A-C----T-G-G-A-G-A--T---C-AG-----GA-G-A--------G-G-A-A---AG-T------GG--A--ATT----C-C-T-
A-GT--GT-A-G-CG-GT--G---A--A-A---TG-C-GT-AG--AT-A-TT--A-G--G-A-A-G-A-AC-A-TC---AA--T--G--GC-GAA-G--G-C--A-------G--C-T-T-T---CTG--G--CC-T-G--------------------A-A---A-C-T--GA--CG-C-T-G--A-GG--C-G-CG-A--AA-G-C---G-TG--GG-G--
AG-C-A-AA--------CA--GG Bacteria(100);unclassified(100);unclassified(100);unclassified(100);unclassified(100);unclassified(100);unclassified(100);unclassified(100);unclassified(100);

Otu1486 0 0 0 0 0 0 0 0 0 0 0 0 0 0 0 0 0 0 0 0 0 0 0 0 0 0 0 0
0 0 0 0 0 0 0 0 0 0 0 0 0 0 0 0 0 0 0 0 0 0 0 18 0 0 0 0 0
0 0 0 0 0 0 0 0 0 0 0 0 0 0 0 0 0 0 0 0 0 0 0 0 0 0 0 0 0
0 0 0 0 0 0 0 0 0 0 0 0 0 0 0 0 0 0 0 0 0 0 0 0 0 0 0
M02255_76_000000000-ADUNE_1_1102_18935_7879 T----AC---AA-AG-GAT--------GCA-A-G------T--G--T---T--AT-C-AAG-AA------TG-A--T-T--GG-GT-------GT--A-----AA-GT-GT--TT------G-TA-G-G-T-G------------A--C-TT-T-T-T----------AA---
-G-T-C-C--------G-C-C--G--TC--A-AA-TC----C------G-----------A----------C-------A----A------G--T-G---G--T-------G--GA-A-A---C-------T--A-C--CA--A-G-C----T-G-G-A-G-T--A---C-AA-----TA-G-G--------G-G-C-A---TA-G------GG--A--ATT----T-C-T-A-GT--GG-A-G-
TG-GT--G---A--A-A---TA-T-GT-AG--AG-A-TC--A-T--A-A-A-G-A-AC-A-CC---AA--T--G--AA-GAA-A--G-C--A-------C--T-T-T-G---TTG--G--GC-C-G--------------------A-C---A-T-T--GA--CA-T-T-G--A-GA--G-A-CG-A--AA-G-C---T-AG--GG-G--AG-T-G-AA--------
TG--AG Bacteria(100);unclassified(100);unclassified(100);unclassified(100);unclassified(100);unclassified(100);unclassified(100);unclassified(100);unclassified(100);

Otu1487 0 0 0 0 0 0 0 0 0 0 0 0 0 0 0 0 0 0 0 0 0 0 0 0 0 0 0 0
0 0 0 0 0 0 0 0 0 0 0 0 0 0 0 0 0 0 0 0 0 0 8 0 0 0 0 0 0
0 0 0 0 0 0 0 0 0 0 0 0 0 0 0 0 0 0 0 0 0 0 0 0 0 0 0 0 0
0 0 0 0 0 0 0 0 0 0 0 0 0 0 0 0 0 0 0 0 0 0 0 0 0 0 0
M02255_77_000000000-ADUT3_1_1109_4041_18703 G----AC---GG-AG-GGT--------GCT-A-G------C--G--T---T--GT-T-CGG-AA------TC-A--C-T--GG-GC-------GT--A-----AA-GG-GC-GC-------G-TA-G-G-C-G------------G--C-CT-T-T-G----------
GT----G-T-C-G--------G-A-T--G--TG--A-AA-GC--C-C-GG-G-G-----CT-C-AA------C-T-C-C-G-G-A--G-G----T-G--C-A---T--C-------C--GA-A-A---C-------T--C-A--GG--G-G-C----T-C-G-A-G-G--C---C-GG-----AA-G-A--------G-G-A-G---GG-T------GG--A--ATA----C-C-
C-A-GT--GT-A-G-AG-GT--G---A--A-A---TT-C-GT-AG--AT-A-TT--G-G--G-T-G-G-A-AC-A-CC---GG--T--G--GC-GAA-A--G-C--G-------G--C-T-C-T---CTG--G--TC-C-G--------------------G-T---C-C-T--GA--CG-C-T-G--A-GG--C-G-CG-A--AA-G-C---G-T-G-GG-G--
AG-C-A-AA--------CA--GG Bacteria(100);Proteobacteria(100);Alphaproteobacteria(100);Rhodospirillales(100);unclassified(100);unclassified(100);unclassified(100);unclassified(100);unclassified(100);

Otu1488 0 0 0 0 0 0 0 0 0 0 0 0 0 0 0 0 0 0 0 0 0 0 0 0 0 0 0 0
0 0 0 0 0 0 0 0 0 0 0 0 0 0 0 0 0 0 0 0 0 0 9 0 0 0 0 0 0
0 0 0 0 0 0 0 0 0 0 0 0 0 0 0 0 0 0 0 0 0 0 0 0 0 0 0 0 0
0 0 0 0 0 0 0 0 0 0 0 0 0 0 0 0 0 0 0 0 0 0 0 0 0 0 0
M02255_76_000000000-ADUNE_1_2119_27979_18851 T----CG---GC-TA-ACT--------CCG-T-G------C--C--A---G--CA-G-CCG-CG------GT-A--A-T--AC-GG-------AG--G-----AT-CC-AA-GC-------GTGA-T-C-C-G------------GA-T-TT-A-T-T-----
------AA----G-T-C-A--------G-G-G--G--TG--A-AA-GA--C-G-GT-G-G-----CT-C-AA------C-C-A-T-C-G-C--A-G----T-G--C-C---C--T-------T--GA-T-A---C-------T--G-A--TG--A-G-C----T-T-G-A-A-T--A---C-AC-----TA-G-A--------G-G-T-A---GG-C------GG--A--ATG----
T-G-A-C-AA--GT-A-G-CG-GT--G---A--A-A---TG-C-AT-AG--AT-A-TG--T-C--A-C-A-G-A-AC-A-CC---GA--T--T--GC-GAA-G--G-C--A-------G--C-T-T-A---CTA--T--GG-T-G--------------------T-T---A-T-T--GA--CG-C-T-G--A-GG--C-A-CG-A--AA-G-C---G-TG--GG-
G--AT-C-A-AA--------CA--GG Bacteria(100);Bacteroidetes(100);Sphingobacteria(100);Sphingobacteriales(100);Sphingobacteriaceae(100);Sphingobacteriaceae(100);Pedobacter(100);unclassified(100);unclassified(100);

Otu1489 0 0 0 0 0 0 0 0 0 0 0 0 0 0 0 0 0 0 0 0 0 0 0 0 0 0 0 0
0 0 0 0 0 0 0 0 0 0 0 0 0 0 0 0 0 0 0 0 0 0 1 0 0 0 0 0 0
0 0 0 0 0 0 0 0 0 0 0 0 0 0 0 0 0 0 0 0 0 0 0 0 0 0 0 0 0
0 4 28 0 0 0 0 0 0 0 0 0 0 0 0 0 0 0 0 13 0 0 0 0 0 0 0
M02255_77_000000000-ADUT3_1_2105_17535_9351 T----AC---GT-AG-GCA--------GCA-A-G------C--G--T---T--GT-T-CGG-AG------TT-A--C-T--GG-GC-------GT--A-----AA-GA-GT-GC-------G-TA-G-G-C-G------------G--T-TG-A-C-C----------AA-
---G-T-C-T--------G-T-C--G--TG--A-AA-TC--T-C-CT-G-G-----CT-T-AA------C-T-G-G-G-A-G--G-G----T-G--C-G---G--T-------G--GA-A-A---C-------T--G-G--TT--G-G-C----T-T-G-A-G-A--G---T-GG-----GA-G-A--------G-G-T-A---AG-C------GG--A--ATT----C-C-T-G-
GT--GT-A-G-CG-GT--G---A--A-A---TG-C-GT-AG--AT-A-TC--A-G--G-A-G-G-A-AC-A-CC---GG--T--G--GT-GTA-G--A-C--G-------G--C-T-T-A---CTG--G--AC-C-A--------------------C-G---A-C-T--GA--CG-C-T-G--A-GG--C-A-CG-A--AA-G-C---G-TG--GG-T--AG-
C-A-AA--------CA--GG Bacteria(100);unclassified(100);unclassified(100);unclassified(100);unclassified(100);unclassified(100);unclassified(100);unclassified(100);unclassified(100);

Otu1490 0 0 0 0 0 0 0 0 0 0 0 0 0 0 0 0 0 0 0 0 0 0 0 0 0 0 0 0
0 0 0 0 0 0 0 0 0 0 0 0 0 0 0 0 0 0 0 0 0 0 0 0 0 4 0 0 0
0 0 0 0 0 0 0 0 0 0 0 0 0 3 0 0 0 0 0 0 0 0 0 0 0 0 0 0 0
0 0 0 0 0 0 0 0 0 0 0 0 0 0 0 0 0 0 0 0 0 0 0 0 0 0 0
M02255_77_000000000-ADUT3_1_1114_7894_24450 T----AC---GTGCC-AGC--------AGC-C-G------C--G--G---T--AA-TACGT-AG------GG-T--G-C--AA-GC-------GT--T-----AATCGGAATTA-------C-TG-G-G-C-G------------G--T-TT-T-G-T----------
AA----G-T-C-T--------G-A-T--G--TG--A-AA-TC--C-C-CG-G-G-----CT-C-AA------C-C-T-G-G-G-A--A-T----T-G--C-A---T--T-------G--GA-G-A---C-------T--G-C--AA--G-G-C----T-A-G-A-A-T--C---T-GG-----CA-G-A--------G-G-G-G---GG-T------AG--A--ATT----C-C-A-
C-GT--GT-A-G-CA-GT--G---A--A-A---TG-C-GT-AG--AT-A-TG--T-G--G-A-G-G-A-AC-A-CC---GA--T--G--GC-GAA-G--G-C--A-------G--C-C-C-C---CTG--G--GT-C-A--------------------A-G---A-T-T--GA--CG-C-T-C--A-TG--C-A-CG-A--AA-G-C---G-TG--GG-G--
AG-C-A-AA--------CA--GG Bacteria(100);Proteobacteria(100);Betaproteobacteria(100);Burkholderiales(100);Oxalobacteraceae(100);Janthinobacterium(100);unclassified(100);unclassified(100);unclassified(100);

Otu1491 0 0 0 0 0 0 0 0 0 0 0 0 0 0 0 0 0 0 0 0 0 0 0 0 0 0 0 0
0 0 0 0 0 0 0 0 0 0 0 0 0 0 0 0 0 0 0 0 0 0 4 0 0 0 0 0 0
0 0 0 0 0 0 0 0 0 0 0 0 0 0 0 0 0 0 0 0 0 0 0 0 0 0 0 0 0
0 0 0 25 0 0 0 0 0 0 0 0 0 0 0 0 0 0 0 0 0 0 0 0 0 0 0
M02255_77_000000000-ADUT3_1_1119_16297_19108 T----AC---GA-AG-GGG--------GCT-A-G------C--G--T---T--GT-T-CGG-AA------TG-A--C-T--GG-GC-------GT--A-----AA-GG-GC-GC-------G-TA-G-G-C-G------------G--T-TT-G-T-C----
-------AA----G-T-T-G--------G-G-G--G--TG--A-AA-TC--C-C-GG-G-G-----CT-T-AA------C-C-T-C-G-G-A--A-T----T-G--C-C---T--T-------C--AA-G-A---C-------T--G-G--CA--G-G-C----T-G-G-A-G-T--A---T-GG-----AA-G-A--------G-G-G-T---CG-T------GG--A--ATT----
C-C-C-A-GT--GT-A-G-AG-GT--G---A--A-A---TT-C-GT-AG--AT-A-TT--G-G--G-A-A-G-A-AC-A-CC---GG--T--G--GC-GAA-G--G-C--G-------G--C-G-A-C---CTG--G--TC-C-A--------------------T-T---A-C-T--GA--CG-C-T-G--A-GG--C-G-CG-A--CA-G-C---G-TG--GG-
G--AG-C-A-AA--------CA--GG Bacteria(100);Proteobacteria(100);Alphaproteobacteria(100);Rhodospirillales(100);unclassified(100);unclassified(100);unclassified(100);unclassified(100);unclassified(100);

Otu1492 0 0 0 0 0 0 0 0 0 0 0 0 0 0 0 0 0 0 0 0 0 0 0 0 0 0 0 0
0 0 0 0 0 0 0 0 0 0 0 0 0 0 0 0 0 0 0 0 0 10 0 0 0 0 0 0 0
0 0 0 0 0 0 0 0 0 0 0 0 0 0 0 0 0 0 0 0 0 0 0 0 0 0 0 0 0
0 0 0 0 0 0 0 0 0 0 0 0 0 0 0 0 0 0 0 0 0 0 0 0 0 0 0
M02255_76_000000000-ADUNE_1_1106_25558_22587 C----TA---AC-TC-CGT--------GCC-A-G------C-----A---G--CC-G-CGG-TA------AT-A--C-G--GG-GG-------GG--G-----CA-AG-CG-TT-------G-T----T-C-G------------G--A-CA-A-C-T--------
---AA----G-T-C-A--------G-A-C--G--TG--A-AA-TC--C-C-TC-G-G-----CT-T-AA------C-C-G-G-G-G-A--A-C----T-G--C-G---T--C-------T--GA-T-A---C-------T--G-G--AT--G-G-C----T-T-G-A-G-A--T---T-GG-----GA-G-A--------G-G-G-A---TG-C------GG--A--ATT----C-C-
A-G-GT--GT-A-G-CG-GT--G---A--A-A---TG-C-GT-AG--AT-A-TC--T-G--G-A-G-G-A-AC-A-CC---GG--T--G--GC-GAA-G--G-C--G-------G--C-A-T-C---CTG--G--AC-C-A--------------------A-C---A-C-T--GA--CG-C-T-G--A-GG--A-G-CG-A--AA-G-C---C-AG--GG-G--
AG-C-A-AA--------CG--GG Bacteria(100);Acidobacteria(100);Acidobacteria(100);Acidobacteriales(100);Acidobacteriaceae(100);uncultured(100);unclassified(100);unclassified(100);unclassified(100);

Otu1493 0 0 0 0 0 0 0 0 0 0 0 0 0 0 0 0 0 0 0 0 0 0 0 0 0 0 0 0
0 0 0 0 0 0 0 0 0 0 0 0 0 0 0 0 0 0 0 0 0 7 0 0 0 0 0 0 0
0 0 0 0 0 0 0 0 0 0 0 0 0 0 0 0 0 0 0 0 0 0 0 0 0 0 0 0 0
0 0 0 0 0 0 0 0 0 0 0 0 0 0 0 0 0 0 0 0 0 0 0 0 0 0 0
M02255_77_000000000-ADUT3_1_2112_12348_16566 C----CG---GC-TA-ACT--------CCG-T-G------C--C--A---G--CA-G-CCG-CG------GT-A--A-T--AC-GG-------AG--G-----GT-GC-AA-GC-------G-TT-GTC-C-G------------G--A-TT-C-A-T-----
------AA----G-T-C-C--------G-T-G--G--TG--A-AA-GC--C-A-AC-A-G-----CT-C-AA------C-T-G-T-T-G-A--A-C----T-G--C-C---A--T-------G--GA-T-A---C-------T--G-T--GA--G-G-C----T-T-G-A-G-T--A---C-AG-----AC-G-A--------G-G-T-A---GG-C------GG--A--ATG----
G-A-T-C-GT--GT-A-G-CG-GT--G---A--A-A---TG-C-AT-AG--AT-A-CG--A-T--C-C-A-G-A-AC-T-CC---GA--T--T--GC-GAA-G--G-C--A-------G--C-T-T-A---CTA--G--GC-T-G--------------------T-A---A-C-T--GA--CG-C-T-G--A-GG--C-A-CG-A--AA-G-C---G-TG--GG-
G--AG-C-G-AA--------CA--GG Bacteria(100);Bacteroidetes(100);Sphingobacteria(100);Sphingobacteriales(100);unclassified(100);unclassified(100);unclassified(100);unclassified(100);unclassified(100);

Otu1494 0 0 0 0 0 0 0 0 0 0 0 0 0 0 0 0 0 0 0 0 0 0 0 0 0 0 0 0
0 0 0 0 0 0 0 0 0 0 0 0 0 0 0 0 0 0 0 0 0 7 0 0 0 0 0 0 0
0 0 0 0 0 0 0 0 0 0 0 0 0 0 0 0 0 0 0 0 0 0 0 0 0 0 0 0 0
0 0 0 0 0 0 0 0 0 0 0 0 0 0 0 0 0 0 0 0 0 0 0 0 0 0 0
M02255_76_000000000-ADUNE_1_1104_12629_15171 .....AC----T-A--CGT--------GCC-A-G------C--A--G---C--CG-C-GGT-AA------TA-C--G-T--AG-GG-------GG--C-----AA-GC-G--TT-------G-TC-C-G---G------------A--T-TA-T-A-C----------
AG----G-T-C-C--------G-C-T--G--TG--A-AA-AC--T-G-GA-G-G-----CT-T-AA------C-C-T-C-C-A-G--A-C----G-T--C-G---G--T-------G--GA-A-A---C-------C--G-T--AT--G-A-C----T-A-G-A-G-T--C---C-GG-----AA-G-A--------G-G-A-G---AG-T------GG--A--ATT----C-C-T-
G-GT--GT-A-G-CG-GT--G---A--A-A---TG-C-GC-AG--AT-A-TC--A-G--G-A-A-G-A-AC-A-CC---CG--T--G--GC-GAA-G--G-C--G-------G--C-T-C-T---CTG--G--GA-C-G--------------------G-T---A-C-T--GA--CG-C-T-G--A-GA--C-G-CG-A--AA-G-C---G-TG--GG-G--
AG-C-G-AA--------CA--GG Bacteria(100);Actinobacteria(100);Actinobacteria(100);Rubrobacteridae(100);AKIW543(100);unclassified(100);unclassified(100);unclassified(100);unclassified(100);

Otu1495 0 0 0 0 0 0 0 0 0 0 0 0 0 0 0 0 0 0 0 0 0 0 0 0 0 0 0 0
0 0 0 0 0 0 0 0 0 0 0 0 0 0 0 0 0 0 0 0 0 0 0 0 0 0 0 0 0
0 0 0 0 0 0 0 0 0 0 0 0 0 0 0 0 0 23 0 0 0 0 0 0 0 0 0 0 0
0 0 0 0 0 0 0 0 0 0 0 0 0 0 0 0 0 0 0 0 0 0 0 0 0 0 0

M02255_77_000000000-ADUT3_1_1119_17992_13613 T----AC---GA-AG-GGG--------GCT-A-G------C--G--T---T--GC-T-CGG-AA------TT-A--C-T--GG-GC-------GT--A-----AA-GG-GC-GC-------G-TA-G-G-C-G------------G--C-GC-C-A-G----
-------TA----G-T-C-A--------G-G-C--G--TG--A-AA-TT--C-C-TG-G-G-----CT-C-AA------C-C-T-G-G-G-G--G-C----T-G--C-G---C--T-------T--GA-T-A---C-------G--C-T--GG--T-G-C----T-A-G-A-G-G--A---C-GG-----AA-G-A--------G-G-C-T---CG-C------GG--A--ATT----
C-C-C-A-GT--GT-A-G-AG-GT--G---A--A-A---TT-C-GT-AG--AT-A-TT--G-G--G-A-A-G-A-AC-A-CC---GG--T--G--GC-GAA-G--G-C--G-------G--C-G-A-G---CTG--G--TC-C-G--------------------T-T---A-C-T--GA--CG-C-T-G--A-GG--C-G-CG-A--CA-G-C---G-TG--
GG-G--AG-C-A-AA--------CA--GG Bacteria(100);Proteobacteria(100);Alphaproteobacteria(100);Rhodospirillales(100);Acetobacteraceae(100);unclassified(100);unclassified(100);unclassified(100);unclassified(100);

Otu1496 0 0 0 0 0 0 0 0 0 0 0 0 0 0 0 0 0 0 0 0 0 0 0 0 0 0 0 0
0 0 0 0 0 0 0 0 0 0 0 0 0 0 0 0 0 0 0 0 0 0 0 0 0 0 0 0 0
0 0 0 0 0 0 0 0 0 0 0 0 0 0 0 0 0 46 0 0 0 0 0 0 0 0 0 0 0
0 0 0 0 0 0 0 0 0 0 0 0 0 0 0 0 0 0 0 0 0 0 0 0 0 0 0

M02255_77_000000000-ADUT3_1_1112_28147_8734 T----AC---GA-AG-GGG--------GCT-A-G------C--G--T---T--GT-T-CGG-AT------TT-A--C-T--GG-GC-------GT--A-----AA-GC-GC-GC-------G-TA-G-G-C-G------------G--A-TT-G-T-T-----------AA-
---G-T-C-A--------G-G-G--G--TG--A-AA-TC--C-C-AG-G-G-----CT-C-AA------C-C-C-T-G-G-A--A-C----T-G--C-C---T--T-------T--GA-T-A---C-------T--G-G--CA--A-T-C----T-G-G-A-G-A--T---C-GG-----GA-G-A--------G-G-T-T---GG-T------GG--A--ATT----C-C-G-A-
GT--GT-A-G-AG-GT--G---A--A-A---TT-C-GT-AG--AT-A-TT--C-G--G-A-G-G-A-AC-A-CC---AG--A--A--GC-GAA-G--G-C--G-------G--C-C-A-A---CTG--G--CC-C-G--------------------A-T---A-C-T--GA--CG-C-T-G--A-GG--C-G-CG-A--AA-G-C---G-TG--GG-G--AG-
C-A-AA--------CA--GG Bacteria(100);Proteobacteria(100);Alphaproteobacteria(100);unclassified(100);unclassified(100);unclassified(100);unclassified(100);unclassified(100);unclassified(100);

Otu1497 0 0 0 0 0 0 0 0 0 0 0 0 0 0 0 0 0 0 0 0 0 0 0 0 0 0 0 0
0 0 0 0 0 0 0 0 0 0 0 0 0 0 0 0 0 0 0 0 0 0 0 0 0 0 0 0 0
0 0 0 0 0 0 0 0 0 0 0 0 0 0 0 0 0 28 0 0 0 0 0 0 0 0 0 0 0
0 0 0 0 0 0 0 0 0 0 0 0 0 0 0 0 0 0 0 0 0 0 0 0 0 0 0

M02255_77_000000000-ADUT3_1_1114_8522_19061 G----AC---GT-AG-GAG--------GCG-A-G------C--G--T---T--AT-C-CGG-AT------TC-A--C-T--GG-GC-------GT--A-----AA-GC-GC-AT-------G-TA-G-G-C-G------------G--C-TG-T-G-C-----------
AA----G-T-C-G--------T-G-T--G--TT--A-AA-GC--C-C-CT-G-G-----CT-C-AA------C-T-G-G-G-G-G--A-G----G-T--C-A---T--G-------C--GA-G-A---C-------T--G-C--GC--G-G-C----T-G-G-A-G-G--G---C-AG-----TA-G-A--------G-G-C-A---CA-T------GG--A--ACT----C-C-
C-G-GT--GT-A-G-CG-GT--G---G--A-A---TG-C-GT-AG--AG-A-TC--G-G--G-A-A-G-A-AC-A-CC---CG--T--G--GC-GAA-G--G-C--G-------G--T-G-T-G---CTG--G--GC-T-G--------------------C-A---C-C-T--GA--CG-C-T-G--A-GA--T-G-CG-A--AA-G-T---G-TG--GG-G--
AG-C-A-AA--------CG--GG Bacteria(100);Chloroflexi(100);Caldilineae(100);Caldilineales(100);Caldilineaceae(100);uncultured(100);unclassified(100);unclassified(100);unclassified(100);

Otu1498 0 0 0 0 0 0 0 0 0 0 0 0 0 0 0 0 0 0 0 0 0 0 0 0 0 0 0 0
0 0 0 0 0 0 0 0 0 0 0 0 0 0 0 0 0 0 0 0 0 0 0 0 0 0 0 0 0
0 0 0 0 0 0 0 0 0 0 0 0 0 0 0 0 0 16 0 0 0 0 0 0 0 0 0 0 0
0 0 0 0 0 0 0 0 0 0 0 0 0 0 0 0 0 0 0 0 0 0 0 0 0 0 0

M02255_77_000000000-ADUT3_1_1114_25715_20690 T----AC---GT-AG-GCA--------GCA-A-G------C--G--T---T--GT-T-CGG-AG------TT-A--C-T--GG-GC-------GT--A-----AA-GA-GT-GT-------G-TA-G-G-C-G------------G--T-TT-G-C-C-----
------AA----G-T-C-T--------A-G-T--G--TG--A-AA-TC--T-C-CC-G-G-----CT-T-AA------C-T-G-G-G-A-G--G-G----T-G--C-G---C--C-------G--GA-T-A---C-------T--G-G--CA--A-G-C----T-AG--A-G-T--G---C-GG-----GA-G-A--------G-G-G-A---AG-C------GG--A--ATT----
C-C-T-G-GT--GT-A-G-CG-GT--G---A--A-A---TG-C-GT-AG--AT-A-TC--A-G--G-A-G-G-A-AC-A-CC---GG--T--G--GT-GTA-G--A-C--G-------G--C-T-T-C---CTG--G--AC-C-G--------------------C-C---A-C-T--GA--CG-C-T-G--A-GA--C-A-CG-A--AA-G-C---G-TG--GG-
T--AG-C-A-AA--------CA--GG Bacteria(100);Acidobacteria(100);Acidobacteria(100);Acidobacteriales(100);Acidobacteriaceae(100);unclassified(100);unclassified(100);unclassified(100);unclassified(100);

Otu1499 0 0 0 0 0 0 0 0 0 0 0 0 0 0 0 0 0 0 0 0 0 0 0 0 0 0 0 0
0 0 0 0 0 0 0 0 0 0 0 0 0 0 0 0 0 0 0 0 0 0 0 0 0 0 0 0 0
0 0 0 0 0 0 0 0 0 0 0 0 0 0 0 0 0 16 0 0 0 0 0 0 0 0 0 0 0
0 0 0 0 0 0 0 0 0 0 0 0 0 0 0 0 0 0 0 0 0 0 0 0 0 0 0

M02255_77_000000000-ADUT3_1_1114_12571_25138 T----AC---GA-AG-GGG--------GCT-A-G------C--G--T---T--GT-T-CGG-AA------TT-A--C-T--GG-GC-------GT--A-----AA-GC-GC-AC-------G-TA-G-G-C-G------------G--A-CT-A-T-T-----
------AA----G-T-C-A--------G-G-G--G--TG--A-AA-TC--C-T-GG-A-G-----CT-C-AA------C-T-C-C-A-G-A--A-C----T-G--C-C---T--T-------T--GA-T-A---C-------T--G-A--TG--G-T-C----T-C-G-A-G-T--C---C-GG-----AA-G-A--------G-G-T-G---AG-T------GG--A--ATT----C-
C-G-A-GT--GT-A-G-AG-GT--G---A--A-A---TT-C-GT-AG--AT-A-TT--C-G--G-A-G-G-A-AC-A-CC---AG--T--G--GC-GAA-G--G-C--G-------G--C-T-C-A---CTG--G--TC-C-G--------------------G-T---A-C-T--GA--CG-C-T-G--A-GG--T-G-CG-A--AA-G-C---G-TG--GG-
G--AG-C-A-AA--------CA--GG Bacteria(100);Proteobacteria(100);Alphaproteobacteria(100);Rhizobiales(100);unclassified(100);unclassified(100);unclassified(100);unclassified(100);unclassified(100);

Otu1500 0 0 0 0 0 0 0 0 0 0 0 0 0 0 0 0 0 0 0 0 0 0 0 0 0 0 0 0
0 0 0 0 0 0 0 0 0 0 0 0 0 0 0 0 0 0 0 0 0 0 0 0 0 0 0 0 0
0 0 0 0 0 0 0 0 0 0 0 0 0 0 0 0 0 42 0 0 0 0 0 0 0 0 0 0 0
0 0 0 0 0 0 0 0 0 0 0 0 0 0 0 0 0 0 0 0 0 0 0 0 0 0 0
M02255_77_000000000-ADUT3_1_1115_13850_10052 T----AC---GA-AG-GGG--------GCG-A-G------C--G--T---T--GT-T-CGG-AA------TT-A--C-T--GG-GC-------GT--A-----AA-GG-GC-GC-------G-TA-G-G-C-G------------G--C-GT-A-C-C----
-------AA----G-T-C-A--------G-G-C--G--TG--A-AA-GC--C-C-TG-G-G-----CT-T-AA------C-C-C-A-G-G-A--A-C----T-G--C-G---T--T-------T--GA-G-A---C-------T--G-G--TA--T-G-C----T-G-G-A-G-T--G---C-AG-----GA-G-A--------G-G-T-C---GG-C------GG--A--ATT----
C-C-C-A-GT--GT-A-G-AG-GT--G---A--A-A---TT-C-GT-AG--AG-A-TT--G-G--G-A-A-G-A-AC-A-CC---GG--T--G--GC-GTA-G--G-C--G-------G--C-C-G-A---CTG--G--AC-T-G--------------------T-A---A-C-T--GA--CG-C-T-G--A-GG--C-G-CG-A--AA-G-C---G-TG--
GG-G--AG-C-A-AA--------CA--GG Bacteria(100);Proteobacteria(100);Alphaproteobacteria(100);unclassified(100);unclassified(100);unclassified(100);unclassified(100);unclassified(100);unclassified(100);

Otu1501 0 0 0 0 0 0 0 0 0 0 0 0 0 0 0 0 0 0 0 0 0 0 0 0 0 0 0 0
0 0 0 0 0 0 0 0 0 0 0 0 0 0 0 0 0 0 0 0 0 0 0 0 0 0 0 0 0
0 0 0 0 0 0 0 0 0 0 0 0 0 0 0 0 0 19 0 0 0 0 0 0 0 0 0 0 0
0 0 0 0 0 0 0 0 0 0 0 0 0 0 0 0 0 0 0 0 0 0 0 0 0 0 0
M02255_77_000000000-ADUT3_1_1116_23223_21076 T----AC---GT-AG-GGG--------GCA-A-G------C--G--T---T--GT-C-CGG-AT------TT-A--T-T--GG-GC-------GT--A-----AA-GA-GC-TC-------G-TA-G-G-C-G------------G--T-TT-G-G-T-----
------AA----G-T-C-G--------G-G-T--G--TG--A-AA-TC--T-C-CG-T-G-----CT-C-AA------C-A-C-G-G-A-G--G------G-G-CC-A---C--C-------C--GA-T-A---C-------T--G-C--CA-TG-A-C----T-T-G-A-G-T--C---C-GG-----TA-G-A--------G-G-A-G---AG-T------GG--A--ATT----
C-C-C-G-GT--GT-A-G-CG-GT--G---A--A-A---TG-C-GC-AG--AT-A-TC--G-G--G-A-G-G-A-AC-A-CC---AG--T--A--GC-GAA-G--G-C--G-------G--C-T-C-T---CTG--G--GC-C-G--------------------G-T---A-C-T--GA--CG-C-T-G--A-GG--A-G-CG-A--AA-G-C---G-TG--
GG-G--AG-C-G-AA--------CA--GG Bacteria(100);unclassified(100);unclassified(100);unclassified(100);unclassified(100);unclassified(100);unclassified(100);unclassified(100);unclassified(100);

Otu1502 0 0 0 0 0 0 0 0 0 0 0 0 0 0 0 0 0 0 0 0 0 0 0 0 0 0 0 0
0 0 0 0 0 0 0 0 0 0 0 0 0 0 0 0 0 0 0 0 0 0 0 0 0 0 0 0 0
0 0 0 0 0 0 0 0 0 0 0 0 0 0 0 0 0 21 0 0 0 0 0 0 0 0 0 0 0
0 0 0 0 0 0 0 0 0 0 0 0 0 0 0 0 0 0 0 0 0 0 0 0 0 0 0
M02255_77_000000000-ADUT3_1_1117_14789_21871 T----AC---GT-AG-GGA--------GCG-A-G------C--G--T---T--GT-C-CGG-AT------TT-A--T-T--GG-GC-------GT--A-----AA-GA-GC-GC-------G-TA-G-G-C-G------------G--C-TT-G-A-T-----
------AA----G-C-C-G--------G-A-T--G--TG--A-AA-CC--C-C-CA-A-G-----CT-C-AA------C-T-T-G-G-G-G--C------G-G-CC-A---T--T-------C--GG-A-A---C-------T--G-T--CA-TG-G-C----T-A-G-A-G-T--C---T-GG-----TA-G-G--------G-G-A-A---CA-C------GG--A--ATT----
C-C-T-G-GT--GT-A-G-CG-GT--G---A--A-A---TG-C-TT-AG--AT-A-TC--A-G--G-A-G-G-A-AC-A-CC---AG--T--G--GC-GAA-G--G-C--G-------G--T-G-T-T---CTG--G--GC-C-A--------------------G-T---A-C-T--GA--CG-C-T-G--A-GG--C-G-CG-A--AA-G-C---G-TG--
GG-G--AG-C-G-AA--------CA--GG Bacteria(100);unclassified(100);unclassified(100);unclassified(100);unclassified(100);unclassified(100);unclassified(100);unclassified(100);unclassified(100);

Otu1503 0 0 0 0 0 0 0 0 0 0 0 0 0 0 0 0 0 0 0 0 0 0 0 0 0 0 0 0
0 0 0 0 0 0 0 0 0 0 0 0 0 0 0 0 0 0 0 0 0 0 0 0 0 0 0 0 0
0 0 0 0 0 0 0 0 0 0 0 0 0 0 0 0 0 29 0 0 0 0 0 0 0 0 0 0 0
0 0 0 0 0 0 0 0 0 0 0 0 0 0 0 0 0 0 0 0 0 0 0 0 0 0 0
M02255_77_000000000-ADUT3_1_1118_10343_19173 G----AC---GG-AG-GAG--------GCT-A-G------C--G--T---T--GT-T-CGG-AA------TT-A--C-T--GG-GC-------GT--A-----AA-GC-GC-GC-------G-CG-G-G-C-G------------G--T-CT-A-G-C----
-------AA----G-T-C-A--------G-G-G--G--TG--A-AA-GC--C-C-GA-G-G-----CT-C-AA------C-T-T-C-G-G-A--A-C----G-G--C-C---C--T-------T--GA-G-A---C-------T--G-T--TG--G-A-C----T-G-G-A-G-A--C---C-GG-----GA-G-A--------G-G-A-G---AG-T------GG--A--ATT---
-C-C-C-A-GT--GT-A-G-AG-GT--G---A--A-A---TT-C-GT-AG--AT-A-TT--G-G--G-A-A-G-A-AC-A-CC---AG--T--G--GC-GAA-G--G-C--G-------G--C-T-C-T---CTG--G--CC-C-G--------------------G-A---C-C-T--GA--CG-C-T-G--A-GG--C-G-CG-A--AA-G-C---G-TG--
GG-G--AG-C-A-AA--------CA--GG Bacteria(100);Proteobacteria(100);Alphaproteobacteria(100);unclassified(100);unclassified(100);unclassified(100);unclassified(100);unclassified(100);unclassified(100);

Otu1504 0 0 0 0 0 0 0 0 0 0 0 0 0 0 0 0 0 0 0 0 0 0 0 0 0 0 0 0
0 0 0 0 0 0 0 0 0 0 0 0 0 0 0 0 0 0 0 0 0 0 0 0 0 0 0 0 0
0 0 0 0 0 0 0 0 0 0 0 0 0 0 0 0 0 24 0 0 0 0 0 0 0 0 0 0 0
0 0 0 0 0 0 0 0 0 0 0 0 0 0 0 0 0 0 0 0 0 0 0 0 0 0 0
M02255_77_000000000-ADUT3_1_1111_7187_15590 T----AC---GA-AG-GGG--------GCT-A-G------C--G--T---T--GC-T-CGG-AA------TC-A--C-T--GG-GC-------GT--A-----AA-GG-GC-GC-------G-TA-G-G-C-G------------G--A-TC-T-T-T-----------
AA----G-T-C-A--------G-G-G--G--TG--A-AA-GC--C-T-GA-G-G-----CT-C-AA------C-C-T-C-A-G-A--A-T----T-G--C-C---T--T-------T--GA-T-A---C-------T--G-A--AG--A-T-C----T-T-G-A-G-A--C---C-GG-----AA-G-A--------G-G-T-A---AG-T------GG--A--ACT----G-C-G-
A-GT--GT-A-G-AG-GT--G---A--A-A---TT-C-GT-AG--AT-A-TT--C-G--C-A-A-G-A-AC-A-CC---AG--T--G--GC-GAA-G--G-C--G-------G--C-T-T-A---CTG--G--TC-C-G--------------------G-T---T-C-T--GA--CG-C-T-G--A-GG--C-G-CG-A--AA-G-C---G-TG--GG-G--AG-
C-A-AA--------CA--GG Bacteria(100);Proteobacteria(100);Alphaproteobacteria(100);Rhizobiales(100);unclassified(100);unclassified(100);unclassified(100);unclassified(100);unclassified(100);

Otu1505 0 0 0 0 0 0 0 0 0 0 0 0 0 0 0 0 0 0 0 0 0 0 0 0 0 0 0 0
0 0 0 0 0 0 0 0 0 0 0 0 0 0 0 0 0 0 0 0 0 0 0 0 0 0 0 0 0
0 0 0 0 0 0 0 0 0 0 0 0 0 0 0 0 0 22 0 0 0 0 0 0 0 0 0 0 0
0 0 0 0 0 0 0 0 0 0 0 0 0 0 0 0 0 0 0 0 0 0 0 0 0 0 0 0
M02255_77_000000000-ADUT3_1_1119_11468_17641 T----AC---GA-AG-GGG--------GCT-A-G------C--G--T---T--GT-T-CGG-AA------TT-A--C-T--GG-GC-------GT--A-----AA-GC-GC-GC-------G-TA-G-G-T-T------------G--T-GT-G-G-C-----
------AA----G-T-C-A--------G-T-G--G--TG--A-AA-TC--C-C-GG-A-G-----CT-T-AA------C-T-T-C-G-G-A--A-C----T-G--C-C---A--T-------T--GA-A-A---C-------T--G-C--TG--C-A-C----T-A-G-A-A-T--C---T-CA-----GA-G-G--------G-G-G-T---AG-C------GG--A--ATT----C-
C-A-A-AT--GT-A-G-GG-GT--G---A--A-A---TC-C-GT-AG--AT-A-TT--T-G--G-A-G-G-A-AC-A-CC---GG--T--G--GC-GAA-G--G-C--G-------G--C-T-A-C---CTG--G--AT-G-A--------------------G-T---A-T-T--GA--CA-C-T-G--A-GG--C-G-CG-A--AA-G-C---G-TG--GG-
G--AT-C-A-AA--------CA--GG Bacteria(100);Proteobacteria(100);Alphaproteobacteria(100);Rickettsiales(100);SM2D12(100);unclassified(100);unclassified(100);unclassified(100);unclassified(100);

Otu1506 0 0 0 0 0 0 0 0 0 0 0 0 0 0 0 0 0 0 0 0 0 0 0 0 0 0 0 0
0 0 0 0 0 0 0 0 0 0 0 0 0 0 0 0 0 0 0 0 0 0 0 0 0 0 0 0 0
0 0 0 0 0 0 0 0 0 0 0 0 0 0 0 0 0 27 0 0 0 0 0 0 0 0 0 0 0
0 0 0 0 0 0 0 0 0 0 0 0 0 0 0 0 0 0 0 0 0 0 0 0 0 0 0 0
M02255_77_000000000-ADUT3_1_2101_18236_11884 T----AC---GT-AG-GGG--------GCG-A-G------C--G--T---T--GT-C-CGG-AT------TT-A--T-T--GG-GC-------GT--A-----AA-GA-GC-TC-------G-TA-G-G-C-G------------G--T-TT-G-G-C-----
------AA----G-T-C-G--------G-A-T--G--TG--A-AA-TC--T-C-CA-G-G-----CT-C-AA------C-C-T-G-G-A-G--G-G----G-T--C-A---T--T-------C--GA-A-A---C-------T--G-C--CAT-G-A-C----T-A-G-A-G-T--T---C-GG-----TA-G-A--------G-G-A-G---TG-T------GG--A--ATT----
C-C-T-G-GT--GG-A-G-CG-GT--G---A--A-A---TG-C-GC-AG--AT-A-TC--A-G--G-A-G-G-A-AC-A-CC---CG--T--A--GC-GAA-G--G-C--G-------G--C-A-C-T---CTG--G--GC-C-G--------------------A-T---A-C-T--GA--CG-C-T-G--A-GG--A-G-CG-A--AA-G-C---G-TG--
GG-G--AG-C-G-AA--------CA--GG Bacteria(100);Actinobacteria(100);Actinobacteria(100);Acidimicrobidae(100);Acidimicrobiales(100);Acidimicrobineae(100);unclassified(100);unclassified(100);unclassified(100);

Otu1507 0 0 0 0 0 0 0 0 0 0 0 0 0 0 0 0 0 0 0 0 0 0 0 0 0 0 0 0
0 0 0 0 0 0 0 0 0 0 0 0 0 0 0 0 0 0 0 0 0 0 0 0 0 0 0 0 0
0 0 0 0 0 0 0 0 0 0 0 0 0 0 0 0 0 19 0 0 0 0 0 0 0 0 0 0 0
0 0 0 0 0 0 0 0 0 0 0 0 0 0 0 0 0 0 0 0 0 0 0 0 0 0 0 0
M02255_77_000000000-ADUT3_1_2103_28583_13361 T----AC---GT-AG-GTG--------GCA-A-G------C--G--T---T--GT-C-CGG-AT------TT-A--C-T--GG-GC-------GT--A-----AA-GA-GC-GC-------G-CA-G-G-C-G------------G--C-CG-A-G-T-----
------AA----G-T-C-G--------A-A-T--G--TG--A-AA-GC--C-C-CCGG-------CT-C-AA------C-T-G-G-G-G-A--A-C----G-T--C-A---T--T-------C--GA-T-A---C-------T--G----TTC-G-G-C----T-C-G-A-A-GA-----T-AG-----GA-G-A--------G-G-G-A---AG-C------GG--A--ATT----
T-C-A-G-GT--GT-A-G-TG-GT--G---A--A-A---TG-C-GT-AG--AT-A-TC--T-G--A-A-G-G-A-AC-A-CC---AG--T--G--GC-GAA-G--G-C--G-------G--C-T-T-T---CTG--G--CC-T-A--------------------T-T---C-T-T--GA--CG-C-T-G--A-GG--C-G-CG-A--AA-G-C---T-AG--GG-
G--AG-C-G-AA--------CG--GG Bacteria(100);unclassified(100);unclassified(100);unclassified(100);unclassified(100);unclassified(100);unclassified(100);unclassified(100);unclassified(100);

Otu1508 0 0 0 0 0 0 0 0 0 0 0 0 0 0 0 0 0 0 0 0 0 0 0 0 0 0 0 0
0 0 0 0 0 0 0 0 0 0 0 0 0 0 0 0 0 0 0 0 0 0 0 0 0 0 0 0 0
0 0 0 0 0 0 0 0 0 0 0 0 0 0 0 0 0 23 0 0 0 0 0 0 0 0 0 0 0
0 0 0 0 0 0 0 0 0 0 0 0 0 0 0 0 0 0 0 0 0 0 0 0 0 0 0 0
M02255_77_000000000-ADUT3_1_2107_9236_17761 T----AC---GG-AG-GGA--------GCT-A-G------C--G--T---T--AT-T-CGG-AA------TT-A--C-T--GG-GC-------GT--A-----AA-GC-GC-GC-------G-TA-G-G-C-G------------G--T-TT-T-T-T-----------AA--
--G-T-C-A--------G-A-G--G--TG--A-AA-GC--C-T-GG-A-G-----CT-C-AA------C-T-C-C-A-G-A--A-C----T-G--C-C---T--T-------T--GA-A-A---C-------T--G-G--AA--G-A-C----T-T-G-A-A-T--C---T-TG-----GA-G-A--------G-G-T-C---AG-T------GG--A--ATT----C-C-G-A-
GT--GT-A-G-AG-GT--G---A--A-A---TT-C-GT-AG--AT-A-TT--C-G--G-A-A-G-A-AC-A-CC---AG--T--G--GC-GAA-G--G-C--G-------A--C-T-G-A---CTG--G--AC-A-A--------------------G-T---A-T-T--GA--CG-C-T-G--A-GG--T-G-CG-A--AA-G-C---G-TG--GG-G--AG-
C-A-AA--------CA--GG Bacteria(100);Proteobacteria(100);Alphaproteobacteria(100);Sphingomonadales(100);unclassified(100);unclassified(100);unclassified(100);unclassified(100);unclassified(100);

Otu1509 0 0 0 0 0 0 0 0 0 0 0 0 0 0 0 0 0 0 0 0 0 0 0 0 0 0 0 0
0 0 0 0 0 0 0 0 0 0 0 0 0 0 0 0 0 0 0 0 0 0 0 0 0 0 0 0 0
0 0 0 0 0 0 0 0 0 0 0 0 0 0 0 0 0 15 0 0 0 0 0 0 0 0 0 0 0
0 0 0 0 0 0 0 0 0 0 0 0 0 0 0 0 0 0 0 0 0 0 0 0 0 0 0 0
M02255_77_000000000-ADUT3_1_2116_12337_19537 T----AC---GA-AG-GGG--------GCT-A-G------C--G--T---T--GT-T-CGG-AT------TT-A--C-T--GG-GC-------GT--A-----AA-GC-GC-GC-------G-TA-G-G-C-G------------G--A-TA-T-T-T-----
------AA----G-T-C-G--------G-G-G--G--TG--A-AA-TC--C-C-GG-G-G-----CT-C-AA------C-C-C-C-G-G-A--A-T----G-G--C-C---T--T-------C--GA-T-A---C-------T--G-G--AT--G-T-C----T-G-G-A-G-A--T---C-GG-----GA-G-A--------G-G-T-T---GG-T------GG--A--ATT----
C-C-G-A-GT--GT-A-G-AG-GT--G---A--A-A---TT-C-GT-AG--AT-A-TT--C-G--G-A-G-G-A-AC-A-CC---AG--A--A--GC-GAA-G--G-C--G-------G--C-C-A-A---CTG--G--CC-C-G--------------------A-T---A-C-T--GA--CG-C-T-G--A-GG--C-G-CG-A--AA-G-C---G-TG--
GG-G--AG-C-A-AA--------CA--GG Bacteria(100);Proteobacteria(100);Alphaproteobacteria(100);unclassified(100);unclassified(100);unclassified(100);unclassified(100);unclassified(100);unclassified(100);

Otu1510 0 0 0 0 0 0 0 0 0 0 0 0 0 0 0 0 0 0 0 0 0 0 0 0 0 0 0 0
0 0 0 0 0 0 0 0 0 0 0 0 0 0 0 0 0 0 0 0 0 0 0 0 0 0 0 0 0
0 0 0 0 0 0 0 0 0 0 0 0 0 0 0 0 0 20 0 0 0 0 0 0 0 0 0 0 0
0 0 0 0 0 0 0 0 0 0 0 0 0 0 0 0 0 0 0 0 0 0 0 0 0 0 0
M02255_77_000000000-ADUT3_1_2117_22755_8453 T----AC---GG-AA-GAT--------CCA-A-G------C--G--T---T--AT-C-CGG-AA------TT-A--C-T--GG-GC-------GT--A-----AA-GA-GTTGC-------G-TA-G-G-T-G------------G--C-AT-A-G-C----------
AA----G-T-A-G--------G-T-A--G--TG--A-AA-GC--G-T-TC-G-G-----CT-C-AA------C-C-G-G-A-T-A--T-C----C-A--T-T---A--C-------C--TA-A-A---C-------T--G-T--TA--A-G-C----T-A-G-A-G-G--A---T-GA-----GA-G-A--------G-G-T-T---AT-T------GG--A--ATT----C-C-C-
T-GT--GT-A-G-GA-GT--G---A--A-A---TC-C-GT-AG--AT-A-TA--G-G--G-A-G-G-A-AC-A-CC---GA--T--G--GC-GTA-G--G-C--A-------G--A-T-A-A---CTG--G--CT-C-A--------------------T-T---C-C-T--GA--CA-C-T-A--A-GG--C-A-CG-A--AA-G-C---G-TG--GG-G--AG-
C-A-AA--------CG--GG Bacteria(100);Candidate_division_TM7(100);unclassified(100);unclassified(100);unclassified(100);unclassified(100);unclassified(100);unclassified(100);unclassified(100);

Otu1511 0 0 0 0 0 0 0 0 0 0 0 0 0 0 0 0 0 0 0 0 0 0 0 0 0 0 0 0
0 0 0 0 0 0 0 0 0 0 0 0 0 0 0 0 0 0 0 0 0 0 0 0 0 0 0 0 0
0 0 0 0 0 0 0 0 0 0 0 0 0 0 0 0 0 24 0 0 0 0 0 0 0 0 0 0 0
0 0 0 0 0 0 0 0 0 0 0 0 0 0 0 0 0 0 0 0 0 0 0 0 0 0 0
M02255_77_000000000-ADUT3_1_2118_28353_17498 T----AC---GA-AG-GGG--------GCT-A-G------C--G--T---T--GT-T-CGG-AA------TT-A--C-T--GG-GC-------GT--A-----AA-GC-GT-AC-------G-TA-G-G-C-G------------G--A-TT-T-G-T-----
------AA----G-T-A-G--------G-G-G--G--TG--A-AA-TC--C-C-CA-G-G-----CT-C-AA------C-C-T-G-G-G-A--A-C----T-G--C-C---T--T-------C--TA-G-A---C-------T--G-C--AA--G-T-C----T-T-G-A-G-A--T---C-AG-----GA-G-A--------G-G-C-G---AG-T------GG--A--ATT----
C-C-G-A-GT--GT-A-G-AG-GT--G---A--A-A---TT-C-GT-AG--AT-A-TT--C-G--G-A-G-G-A-AC-A-CC---AG--T--G--GC-GAA-G--G-C--G-------A--C-T-C-G---CTG--G--AC-T-G--------------------A-T---A-C-T--GA--CG-C-T-G--A-GG--T-A-CG-A--AA-G-C---G-TG--GG-
G--AG-C-A-AA--------CA--GG Bacteria(100);Proteobacteria(100);Alphaproteobacteria(100);unclassified(100);unclassified(100);unclassified(100);unclassified(100);unclassified(100);unclassified(100);

Otu1512 0 0 0 0 0 0 0 0 0 0 0 0 0 0 0 0 0 0 0 0 0 0 0 0 0 0 0 0
0 0 0 0 0 0 0 0 0 0 0 0 0 0 0 0 0 0 0 0 0 0 0 0 0 0 0 0 0
0 0 0 0 0 0 0 0 0 0 0 0 0 0 0 0 0 26 0 0 0 0 0 0 0 0 0 0 0
0 0 0 0 0 0 0 0 0 0 0 0 0 0 0 0 0 0 0 0 0 0 0 0 0 0 0
M02255_77_000000000-ADUT3_1_1106_12928_14988 T----AC---GG-AG-GGG--------GCT-A-G------C--G--T---T--GT-T-CGG-AA------TT-A--C-T--GG-GC-------GT--A-----AA-GC-GC-AC-------G-TA-G-G-C-G------------G--A-CC-T-A-C-----
------AA----G-T-C-G--------G-G-G--G--TG--A-AA-TC--C-C-GG-G-G-----CT-C-AA------C-C-C-C-G-G-A--A-C----T-G--C-C---T--T-------C--GA-A-A---C-------T--G-T--CG--G-T-C----T-G-G-A-G-T--C---C-GA-----GA-G-A--------G-G-T-G---AG-T------GG--A--ATT----
C-C-G-A-GT--GT-A-G-AG-GT--G---A--A-A---TT-C-GT-AG--AT-A-TT--C-G--G-A-G-G-A-AC-A-CC---AG--T--G--GC-GAA-G--G-C--G-------G--C-T-C-A---CTG--G--CT-C-G--------------------G-T---A-C-T--GA--CG-C-T-G--A-GG--T-G-CG-A--AA-G-C---G-TG--GG-
G--AG-C-A-AA--------CA--GG Bacteria(100);Proteobacteria(100);Alphaproteobacteria(100);unclassified(100);unclassified(100);unclassified(100);unclassified(100);unclassified(100);unclassified(100);

Otu1513 0 0 0 0 0 0 0 0 0 0 0 0 0 0 0 0 0 0 0 0 0 0 0 0 0 0 0 0
0 0 0 0 0 0 0 0 0 0 0 0 0 0 0 0 0 0 0 0 0 0 0 0 0 0 0 0 0
0 0 0 0 0 0 0 0 0 0 0 0 0 0 0 0 0 16 0 0 0 0 0 0 0 0 0 0 0
0 0 0 0 0 0 0 0 0 0 0 0 0 0 0 0 0 0 0 0 0 0 0 0 0 0 0
M02255_76_000000000-ADUNE_1_1115_13378_8762 .....AC----T-AC-GT---------GCC-A-G------C--A--G---C--CG-C-GGT-AA------TA-C--G-T--AG-GT-------GG--C-----AA-GC----G--------T-TA-T-C-C-G------------G--A-TT-T-T-T-----------AA----G-
T-C-T--------G-A-T--G--TG--A-AA-GC--C-T-TC-G-G-----CT-T-AA------C-C-G-A-A-G-A--A-G----T-G--C-A---T--C-------G--GA-A-A---C-------T--G-G--GA--A-A-C----T-T-G-A-G-T--G---C-AG-----AA-G-A--------G-G-A-C---AG-T------GG--A--ACT----C-C-A-T-GT--
GT-A-G-CG-GT--G---A--A-A---TG-C-GT-AG--AT-A-TA--T-G--G-A-A-G-A-AC-A-CC---AG--T--G--GC-GAA-G--G-C--G-------G--C-T-G-T---CTG--G--TC-T-G--------------------T-A---A-C-T--GA--CG-C-T-G--A-GG--C-T-CG-A--AA-G-C---A-TG--GG-T--AG-C-G-
AA--------CA--GG Bacteria(100);Firmicutes(100);Bacilli(100);Lactobacillales(100);Lactobacillaceae(100);unclassified(100);unclassified(100);unclassified(100);unclassified(100);

Otu1514 0 0 0 0 0 0 0 0 0 0 0 0 0 0 0 0 0 0 0 0 0 0 0 0 0 0 0 0
0 0 0 0 0 0 0 0 0 0 0 0 0 0 0 0 0 0 0 0 0 0 0 0 0 0 0 0 0
0 0 0 0 0 0 0 0 0 0 0 0 0 0 0 0 0 19 0 0 0 0 0 0 0 0 0 0 0
0 0 0 0 0 0 0 0 0 0 0 0 0 0 0 0 0 0 0 0 0 0 0 0 0 0 0
M02255_76_000000000-ADUNE_1_2111_22102_10996 T----AC---GA-AG-GGG--------GCT-A-G------C--G--T---T--GC-T-CGG-AA------TC-A--C-T--GG-GC-------GT--A-----AA-GC-GC-AC-------G-TA-G-G---G------------T--C-AT---T-T-------
-----A----G-T-C-A--------G-G-G--G--TG--A-AA-TC--C-T-GG-A-G-----CT-C-AA------C-T-C-C-A-G-A--A-C----T-G--C-C---T--T-------T--GA-T-A---C-------T--G-A--TG--A-T-C----T-TGG-A-G-T--C---C-GG-----GA-G-A--------G-G-T-G---GG-T------GG--A--ACT----G-
C-G-A-GT--GT-A-G-AG-GT--G---A--A-A---TT-C-GT-AG--AT-A-TT--C-G--C-A-A-G-A-AC-A-CC---AG--T--G--GC-GAA-G--G-C--G-------G--C-T-C-A---CTG--G--CC-C-G--------------------G-T---A-C-T--GA--CA-C-T-G--A-GG--T-G-CG-A--AA-G-C---G-TG--GG-G-
-AG-C-A-AA--------CA--GG Bacteria(100);Proteobacteria(100);Alphaproteobacteria(100);Rhizobiales(100);unclassified(100);unclassified(100);unclassified(100);unclassified(100);unclassified(100);

Otu1515 0 0 0 0 0 0 0 0 0 0 0 0 0 0 0 0 0 0 0 0 0 0 0 0 0 0 0 0
0 0 0 0 0 0 0 0 0 0 0 0 0 0 0 0 0 0 0 0 0 0 0 0 0 0 0 0 0
0 0 0 0 0 0 0 0 0 0 0 0 0 0 0 0 0 48 0 0 0 0 0 0 0 0 0 0 0
0 0 0 0 0 0 0 0 0 0 0 0 0 0 0 0 0 0 0 0 0 0 0 0 0 0 0
M02255_77_000000000-ADUT3_1_1101_24143_8271 T----AC---GA-AG-GGG--------GCT-A-G------C--G--T---T--GT-T-CGG-AA------TT-A--C-T--GG-GC-------GT--A-----AA-GG-GC-GC-------G-TA-G-G-C-G------------G--T-CT-T-G-T-----------AA-
---G-T-C-A--------G-A-T--G--TG--A-AA-GC--C-C-CG-G-G-----CT-C-AA------C-C-C-G-G-G-A--A-T----A-G--C-A---T--T-------T--GA-T-A---C-------T--G-C--GA--G-G-C----T-T-G-A-G-T--C---C-GG-----GA-G-A--------G-G-A-T---GG-C------GG--A--ATT----C-C-C-A-
GT--GT-A-G-AG-GT--G---A--A-A---TT-C-GT-AG--AT-A-TT--G-G--G-A-A-G-A-AC-A-CC---GG--T--G--GC-GAA-G--G-C--G-------G--C-C-A-T---CTG--G--AC-C-G--------------------G-T---A-C-T--GA--CG-C-T-G--A-GG--C-G-CG-A--AA-G-C---GTGG--GG----AG-
C-A-AA--------CA--GG Bacteria(100);Proteobacteria(100);Alphaproteobacteria(100);Rhodospirillales(100);MND8(100);unclassified(100);unclassified(100);unclassified(100);unclassified(100);

Otu1516 0 0 0 0 0 0 0 0 0 0 0 0 0 0 0 0 0 0 0 0 0 0 0 0 0 0 0 0
0 0 0 0 0 0 0 0 0 0 0 0 0 0 0 0 0 0 0 0 0 0 0 0 0 0 0 0 0
0 0 0 0 0 0 0 0 0 0 0 0 0 0 0 0 0 37 0 0 0 0 0 0 0 0 0 0 0
0 0 0 0 0 0 0 0 0 0 0 0 0 0 0 0 0 0 0 0 0 0 0 0 0 0 0
M02255_77_000000000-ADUT3_1_1103_3393_8760 T----AC---GA-AG-GGG--------GCG-A-G------C--G--T---T--GT-T-CGG-AT------TT-A--C-T--GG-GC-------GT--A-----AA-GG-GC-GC-------G-CA-G-G-C-G------------G--C-TT-A-T-T-----------TT-
---G-T-C-A--------G-G-C--G--TG--A-AA-GC--C-C-CG-G-G-----CT-T-AA------C-C-T-G-G-G-A--A-C----T-G--C-G---C--T-------T--GA-G-A---C-------G--G-A--TG--G-G-C----T-A-G-A-G-T--T---C-GG-----GA-G-A--------G-G-A-G---AG-T------GG--A--ATT----C-C-C-A-
GT--GT-A-G-AG-GT--G---A--A-A---TT-C-GT-AG--AT-A-TT--G-G--G-A-A-G-A-AC-A-CC---GG--T--G--GC-GAA-G--G-C--G-------G--C-T-C-T---CTG--G--AC-C-G--------------------A-G---A-C-T--GA--CG-C-T-G--A-GG--C-G-CG-A--AA-G-C---G-TG--GG-G--AG-
C-A-AA--------CA--GG Bacteria(100);Proteobacteria(100);Alphaproteobacteria(100);Rhodospirillales(100);unclassified(100);unclassified(100);unclassified(100);unclassified(100);unclassified(100);

Otu1517 0 0 0 0 0 0 0 0 0 0 0 0 0 0 0 0 0 0 0 0 0 0 0 0 0 0 0 0
0 0 0 0 0 0 0 0 0 0 0 0 0 0 0 0 0 0 0 0 0 0 0 0 0 0 0 0 0
0 0 0 0 0 0 0 0 0 0 0 0 0 0 0 0 0 76 0 0 0 0 0 0 0 0 0 0 0
0 0 0 0 0 0 0 0 0 0 0 0 0 0 0 0 0 0 0 0 0 0 0 0 0 0 0
M02255_77_000000000-ADUT3_1_1104_13675_10283 T----AC---GG-AG-GGG--------GCT-A-G------C--G--T---T--GT-T-CGG-AA------TT-A--C-T--GG-GC-------GT--A-----AA-GC-GC-AC-------G-TA-G-G-C-G------------G--G-CT-G-G-A----
-------AA----G-T-T-G--------G-G-G--G--TG--A-AA-TC--C-C-GG-G-G-----CT-C-AA------C-C-C-C-G-G-A--A-C----T-G--C-C---C--T-------C--AA-A-A---C-------T--T-T--CA--G-T-C----T-G-G-A-G-T--C---C-GA-----GA-G-A--------G-G-T-G---AG-T------GG--A--ATT----
C-C-G-A-GT--GT-A-G-AG-GT--G---A--A-A---TT-C-GT-AG--AT-A-TT--C-G--G-A-G-G-A-AC-A-CC---AG--T--G--GC-GAA-G--G-C--G-------G--C-T-C-A---CTG--G--CT-C-G--------------------G-T---A-C-T--GA--CG-C-T-G--A-GG--T-G-CG-A--AA-G-C---G-TG--GG-
G--AG-C-A-AA--------CA--GG Bacteria(100);Proteobacteria(100);Alphaproteobacteria(100);unclassified(100);unclassified(100);unclassified(100);unclassified(100);unclassified(100);unclassified(100);

Otu1518 0 0 0 0 0 0 0 0 0 0 0 0 0 0 0 0 0 0 0 0 0 0 0 0 0 0 0 0
0 0 0 0 0 0 0 0 0 0 0 0 0 0 0 0 0 0 0 0 0 0 0 0 0 0 0 0 0
0 0 0 0 0 0 0 0 0 0 0 0 0 0 0 0 0 16 0 0 0 0 0 0 0 0 0 0 0
0 0 0 0 0 0 0 0 0 0 0 0 0 0 0 0 0 0 0 0 0 0 0 0 0 0 0
M02255_77_000000000-ADUT3_1_1105_16387_20366 T----AC---GA-AG-GGT--------GCT-A-G------C--G--T---T--GT-T-CGG-AA------TC-A--C-T--GG-GC-------GT--A-----AA-GC-GC-AC-------G-TA-G-G-C-G------------GT-C-CG-C-A-C----
-------CA----G-T-C-A--------G-G-C--G--TG--A-AA-GC--C-C-CG-G-G-----CT-C-AA------C-C-C-G-G-G-A--A-T----A-G--C-G---C--T-------T--GA-T-A---C-------T--A-G--CG--G-G-C----T-A-G-A-G-T--C---C-GG-----GA-G-A--------G-G-A-T---AG-T------GG--A--ATT----
C-C-C-A-GT--GT-A-G-AG-GT--G---A--A-A---TT-C-GT-AG--AT-A-TT--G-G--G-A-A-G-A-AC-A-CC---AG--T--G--GC-GAA-G--G-C--G-------G--C-T-A-T---CTG--G--AC-C-G--------------------G-T---A-C-T--GA--CG-C-T-G--A-GG--T-G-CG-A--AA-G-C---GTGG--
GG----AG-C-A-AA--------CA--GG Bacteria(100);Proteobacteria(100);Alphaproteobacteria(100);unclassified(100);unclassified(100);unclassified(100);unclassified(100);unclassified(100);unclassified(100);

Otu1519 0 0 0 0 0 0 0 0 0 0 0 0 0 0 0 0 0 0 0 0 0 0 0 0 0 0 0 0
0 0 0 0 0 0 0 0 0 0 0 0 0 0 0 0 0 0 0 0 0 0 0 0 0 0 0 0 0
0 0 0 0 0 0 0 0 0 0 0 0 0 0 0 0 0 23 0 0 0 0 0 0 0 0 0 0 0
0 0 0 0 0 0 0 0 0 0 0 0 0 0 0 0 0 0 0 0 0 0 0 0 0 0 0
M02255_77_000000000-ADUT3_1_1105_19008_23029 T----AC---GA-AG-GGG--------GCT-A-G------C--G--T---T--GT-T-CGG-AT------TT-A--C-T--GG-GC-------GT--A-----AA-GC-GC-GT-------G-TA-G-G-C-G------------G--A-CT-T-G-T-----
------AA----G-T-T-G--------G-G-G--G--TG--A-AA-TC--C-C-GA-G-G-----CT-C-AA------C-C-T-C-G-G-A--A-C----T-G--C-C---T--C-------C--AA-A-A---C-------T--A-C--AA--G-T-C----T-G-G-A-C-T--C---A-AG-----TA-G-A--------G-G-C-A---AG-T------GG--A--ATT----G-
C-G-A-GT--GT-A-G-AG-GT--G---A--A-A---TT-C-GT-AG--AT-A-TT--C-G--C-A-G-G-A-AC-A-CC---AG--T--G--GC-GAA-G--G-C--G-------A--C-T-T-G---CTG--G--AC-T-T--------------------G-T---A-G-T--GA--CG-C-T-G--A-GA--C-G-CG-A--AA-G-C---G-TG--GG-G-
-AG-C-A-AA--------CA--GG Bacteria(100);Proteobacteria(100);Alphaproteobacteria(100);unclassified(100);unclassified(100);unclassified(100);unclassified(100);unclassified(100);unclassified(100);

Otu1520 0 0 0 0 0 0 0 0 0 0 0 0 0 0 0 0 0 0 0 0 0 0 0 0 0 0 0 0
0 0 0 0 0 0 0 0 0 0 0 0 0 0 0 0 0 0 0 0 0 0 0 0 0 0 0 0 0
0 0 0 0 0 0 0 0 0 0 0 0 0 0 0 0 0 31 0 0 0 0 0 0 0 0 0 0 0
0 0 0 0 0 0 0 0 0 0 0 0 0 0 0 0 0 0 0 0 0 0 0 0 0 0 0

M02255_77_000000000-ADUT3_1_1106_16204_8221 T----AC---GG-AG-GAG--------GCA-A-G------C--G--T---T--GT-T-CGG-AA------TC-A--C-T--GG-GC-------TT--A-----AA-GC-GC-GT-------G-TA-G-G-C-G------------G--G-AA-G-G-C----------GG----G-T-G-T--------C-C-T--G--TG--A-AA-GC--C-C-TC-G-G-----CT-C-AA------C-C-G-G-G-G-A--A-T----T-G--C-A---G--A-------G--CA-A-A---C-------T--A-C--CT--T-T-C----T-T-G-A-G-G--C---A-GG-----TA-G-A--------G-G-C-A---AG-T------GG--A--ACT----A-T-G-G-GT--GG-A-G-CG-GT--G---A--A-A---TG-C-GT-AG--AT-A-TC--C-A--T-G-G-G-A-AC-G-CC---GG--T--G--GC-GAA-A--G-C--G-------G--C-T-T-G---CTG--G--GC-C-T--------------------G-T---C-C-T--GA--CG-C-T-G--A-GA--C-G-CG-A--AA-G-C---G-TA--GG-G--AG-C-A-AA--------CA--GG Bacteria(100);unclassified(100);unclassified(100);unclassified(100);unclassified(100);unclassified(100);unclassified(100);unclassified(100);unclassified(100);

Otu1521 0 0 0 0 0 0 0 0 0 0 0 0 0 0 0 0 0 0 0 0 0 0 0 0 0 0 0 0
0 0 0 0 0 0 0 0 0 0 0 0 0 0 0 0 0 0 0 0 0 0 0 0 0 0 0 0 0
0 0 0 0 0 0 0 0 0 0 0 0 0 0 0 0 0 15 0 0 0 0 0 0 0 0 0 0 0
0 0 0 0 0 0 0 0 0 0 0 0 0 0 0 0 0 0 0 0 0 0 0 0 0 0 0

M02255_76_000000000-ADUNE_1_1105_12990_4908 T----AC---GA-AG-GGG--------GCT-A-G------C--G--T---T--GT-T-CGG-AT------TT-A--C-T--GG-GC-------GT--A-----AA-GC-GC-GC-------G-CA-------G-------------G--C-CT-T-G-C-----------AA----G-T-C-G--------A-G-G--G--TG--A-AA-TC--C-C-AG-A-G-----CT-C-AA------C-T-C-T-G-G-A--A-T----T-G--C-C---T--T-------C--GA-G-A---C-------T--G-C--TT--G-G-C----T-TCG-A-G-T--A---C-GG-----GA-G-A--------G-G-T-G---AG-T------GG--A--ATT----C-C-C-A-GT--GT-A-G-AG-GT--G---A--A-A---TT-C-GT-AG--AT-A-TT--G-G--G-A-A-G-A-AC-A-CC---GG--T--G--GC-GAA-G--G-C--G-------G--C-T-C-A---CTG--G--CC-C-G--------------------T-T---A-C-T--GA--CG-C-T-C--A-GG--C-G-CG-A--CA-G-C---G-TG--GG-G--AT-C-A-AA--------CA--GG Bacteria(100);Proteobacteria(100);Alphaproteobacteria(100);unclassified(100);unclassified(100);unclassified(100);unclassified(100);unclassified(100);unclassified(100);

Otu1522 0 0 0 0 0 0 0 0 0 0 0 0 0 0 0 0 0 0 0 0 0 0 0 0 0 0 0 0
0 0 0 0 0 0 0 0 0 0 0 0 0 0 0 0 0 0 0 0 0 0 0 0 0 0 0 0 0
0 0 0 0 0 0 0 0 0 0 0 0 0 0 0 0 0 23 0 0 0 0 0 0 0 0 0 0 0
0 0 0 0 0 0 0 0 0 0 0 0 0 0 0 0 0 0 0 0 0 0 0 0 0 0 0

M02255_77_000000000-ADUT3_1_1107_26838_11597 T----AC---GA-AA-GGG--------GCT-A-G------C--G--T---T--GT-T-CGG-AA------TT-A--C-T--GG-GC-------GT--A-----AA-GC-GC-AC-------G-TA-G-G-C-G------------G--C-AT-T-G-T-----------AA----G-T-T-A--------G-G-G--G--TG--A-AA-TC--C-C-CG-G-G-----CT-C-AA------C-C-C-G-G-G-A--A-C----T-G--C-C---T--T-------T--AA-G-A---C-------T--G-C--AA--A-G-C----T-T-G-A-G-G--A---T-GC-----GA-G-A--------G-G-T-T---GG-C------GG--A--ATT----C-C-G-A-GT--GT-A-G-AG-GT--G---A--A-A---TT-C-GT-AG--AT-A-TT--C-G--G-A-A-G-A-AC-A-CC---AG--T--G--GC-GAA-G--G-C--G-------G--C-C-A-A---CTG--G--CG-C-A--------------------T-T---T-C-T--GA--CG-C-T-G--A-GG--T-G-CG-A--AA-G-C---G-TG--GG-G--AG-C-A-AA--------CA--GG Bacteria(100);Proteobacteria(100);Alphaproteobacteria(100);Parvularculales(100);Parvularculaceae(100);Parvularcula(100);unclassified(100);unclassified(100);unclassified(100);

Otu1523 0 0 0 0 0 0 0 0 0 0 0 0 0 0 0 0 0 0 0 0 0 0 0 0 0 0 0 0
0 0 0 0 0 0 0 0 0 0 0 0 0 0 0 0 0 0 0 0 0 0 0 0 0 0 0 0 0
0 0 0 0 0 0 0 0 0 0 0 0 0 0 0 0 0 26 0 0 0 0 0 0 0 0 0 0 0
0 0 0 0 0 0 0 0 0 0 0 0 0 0 0 0 0 0 0 0 0 0 0 0 0 0 0

M02255_77_000000000-ADUT3_1_1107_17079_17986 T----AC---GA-AG-GGG--------GCT-A-G------C--G--T---T--GT-T-CGG-AT------TT-A--C-T--GG-GC-------GT--A-----AA-GC-GC-AC-------G-TA-G-G-C-G------------G--G-TC-G-T-T-----------AA----G-T-C-A--------G-G-G--G--TG--A-AA-TC--C-C-GG-A-G-----CT-C-AA------C-T-C-C-G-G-A--A-C----T-G--C-C---T--T-------T--GA-T-A---C-------T--G-G--CG--A-C-C----T-T-G-A-G-G--C---T-GG-----AA-G-A--------G-G-T-T---AG-T------GG--A--ATT----C-C-C-A-GT--GT-A-G-AG-GT--G---A--A-A---TT-C-GT-AG--AT-A-TT--G-G--G-A-A-G-A-AC-A-CC---AG--T--G--GC-GAA-G--G-C--G-------G--C-T-A-A---CTG--G--TC-C-A--------------------G-A---T-C-T--GA--CG-C-T-G--A-GG--T-G-CG-A--AA-G-C---G-TG--GG-G--AG-C-A-AA--------CA--GG Bacteria(100);Proteobacteria(100);Alphaproteobacteria(100);Rhizobiales(100);Nordella(100);unclassified(100);unclassified(100);unclassified(100);unclassified(100);

Otu1524 0 0 0 0 0 0 0 0 0 0 0 0 0 0 0 0 0 0 0 0 0 0 0 0 0 0 0 0
0 0 0 0 0 0 0 0 0 0 0 0 0 0 0 0 0 0 0 0 0 0 0 0 0 0 0 0 0
0 0 0 0 0 0 0 0 0 0 0 0 0 0 0 0 0 21 0 0 0 0 0 0 0 0 0 0 0
0 0 0 0 0 0 0 0 0 0 0 0 0 0 0 0 0 0 0 0 0 0 0 0 0 0 0

M02255_77_000000000-ADUT3_1_1108_20091_4695 T----AC---GT-AG-GGG--------GCG-A-G------C--G--T---T--GT-C-CGA-AG------TT-A--C-T--GG-GC-------GT--A-----AA-GC-GC-GC-------G-TA-G-G-C-G------------G--T-TC-A-T-T-----------TA----G-T-T-C--------G-G-G--G--TG--A-AA-GG--T-T-CAAC-G-----CT-C-AA------C-G-T-G-A-A-C--A-G----T-G--C-C---T--T-------G--AA-T-A---C-------T--G-G--TG--G-A-C----T-T-G-A-G-T--T---C-GG-----GA-G-A--------G-G-G-C---AG-T------GG--A--ATT----C-C-T-G-GT--GT-A-G-CG-GT--G---A--A-A---TG-C-GT-AG--AT-A-TC--A-G--G-A-G-G-A-AC-A-CC---GA--T--G--GC-GAA-G--G-C--A-------G--C-T-G-C---CTG--G--CC-T-A--------------------G-A---A-C-T--GA--CG-C-T-G--A-GG--T-G-CG-A--AA-G-C---G-TG--GG-G--AG-C-G-AA--------CA--GG Bacteria(100);unclassified(100);unclassified(100);unclassified(100);unclassified(100);unclassified(100);unclassified(100);unclassified(100);unclassified(100);

Otu1525 0 0 0 0 0 0 0 0 0 0 0 0 0 0 0 0 0 0 0 0 0 0 0 0 0 0 0 0
0 0 0 0 0 0 0 0 0 0 0 0 0 0 0 0 0 0 0 0 0 0 0 0 0 0 0 0 0
0 0 0 0 0 0 0 0 0 0 0 0 0 0 0 0 0 46 0 0 0 0 0 0 0 0 0 0 0
0 0 0 0 0 0 0 0 0 0 0 0 0 0 0 0 0 0 0 0 0 0 0 0 0 0 0 0
M02255_77_000000000-ADUT3_1_1108_6366_8067 T----AC---GA-AG-GGG--------ACT-A-G------C--G--T---T--GT-T-CGG-AA------TT-A--C-T--GG-GC-------GT--A-----AA-GC-GC-GC-------G-TA-G-G-C-G------------G--A-TC-G-C-T----------AA-
---G-T-C-A--------G-G-G--G--TG--A-AA-TC--C-C-AA-G-G-----CT-C-AA------C-C-T-T-G-G-A--A-C----T-G--C-C---T--T-------T--GA-T-A---C-------T--G-A--CG--A-T-C----T-G-G-A-G-A--T---T-GG-----GA-G-A--------G-G-T-G---AG-T------GG--A--ATT----C-C-G-A-
GT--GT-A-G-AG-GT--G---A--A-A---TT-C-GT-AG--AT-A-TT--C-G--G-A-G-G-A-AC-A-CC---AG--A--A--GC-GAA-G--G-C--G-------G--C-T-C-A---CTG--G--CC-C-A--------------------A-T---A-C-T--GA--CG-C-T-G--A-GG--C-G-CG-A--AA-G-C---G-TG--GG-G--AG-
C-A-AA--------CA--GG Bacteria(100);Proteobacteria(100);Alphaproteobacteria(100);unclassified(100);unclassified(100);unclassified(100);unclassified(100);unclassified(100);unclassified(100);

Otu1526 0 0 0 0 0 0 0 0 0 0 0 0 0 0 0 0 0 0 0 0 0 0 0 0 0 0 0 0
0 0 0 0 0 0 0 0 0 0 0 0 0 0 0 0 0 0 0 0 0 0 0 0 0 0 0 0 0
0 0 0 0 0 0 0 0 0 0 0 0 0 0 0 0 0 64 0 0 0 0 0 0 0 0 0 0 0
0 0 0 0 0 0 0 0 0 0 0 0 0 0 0 0 0 0 0 0 0 0 0 0 0 0 0 0
M02255_77_000000000-ADUT3_1_1109_17464_15658 T----AC---GA-AG-GGG--------GCT-A-G------C--G--T---T--GC-T-CGG-AT------TT-A--C-T--GG-GC-------GT--A-----AA-GG-GC-GC-------G-TA-G-G-C-G------------G--A-TG-A-C-C----
-------AA----G-T-T-G--------G-G-G--G--TG--A-AA-GC--C-C-GG-G-G-----CT-C-AA------C-C-T-C-G-G-A--A-T----T-G--C-C---T--T-------C--AA-A-A---C-------T--G-G--TT--G-T-C----T-T-G-A-G-T--A---T-GG-----GA-G-A--------G-G-T-G---TG-T------GG--A--ACT----
C-C-G-A-GT--GT-A-G-AG-GT--G---A--A-A---TT-C-GT-AG--AT-A-TT--C-G--G-A-A-G-A-AC-A-CC---AG--T--G--GC-GAA-G--G-C--G-------A--C-A-C-A---CTG--G--CC-C-A--------------------T-T---A-C-T--GA--CG-C-T-G--A-GG--C-G-CG-A--AA-G-C---G-TG--GG-
G--AG-C-A-AA--------CA--GG Bacteria(100);Proteobacteria(100);Alphaproteobacteria(100);Caulobacterales(100);unclassified(100);unclassified(100);unclassified(100);unclassified(100);unclassified(100);

Otu1527 0 0 0 0 0 0 0 0 0 0 0 0 0 0 0 0 0 0 0 0 0 0 0 0 0 0 0 0
0 0 0 0 0 0 0 0 0 0 0 0 0 0 0 0 0 0 0 0 0 0 0 0 0 0 0 0 0
0 0 0 0 0 0 0 0 0 0 0 0 0 0 0 0 0 15 0 0 0 0 0 0 0 0 0 0 0
0 0 0 0 0 0 0 0 0 0 0 0 0 0 0 0 0 0 0 0 0 0 0 0 0 0 0 0
M02255_77_000000000-ADUT3_1_1110_14149_16330 T----AC---GA-AG-GGG--------GCT-A-G------C--G--T---T--GC-T-CGG-AA------TC-A--C-T--GG-GC-------GT--A-----AA-GG-GC-GC-------G-TA-G-G-C-G------------G--A-TA-C-T-T----
-------AA----G-T-C-A--------G-G-G--G--TG--A-AA-GC--C-C-GA-G-G-----CT-C-AA------C-C-T-C-G-G-A--A-C----T-G--C-C---T--T-------T--GA-T-A---C-------T--G-A--GT--A-T-C----T-T-G-A-G-A--C---C-GG-----TA-G-A--------G-G-T-A---AG-T------GG--A--ACT----
G-C-G-A-GT--GT-A-G-AG-GT--G---A--A-A---TT-C-GT-AG--AT-A-TT--C-G--C-A-A-G-A-AC-A-CC---AG--T--G--GC-GAA-G--G-C--G-------G--C-T-T-A---CTG--G--AC-C-G--------------------G-T---T-C-T--GA--CG-C-T-G--A-GG--C-G-CG-A--AA-G-C---G-TG--GG-
G--AG-C-A-AA--------CA--GG Bacteria(100);Proteobacteria(100);Alphaproteobacteria(100);Rhizobiales(100);unclassified(100);unclassified(100);unclassified(100);unclassified(100);unclassified(100);

Otu1528 0 0 0 0 0 0 0 0 0 0 0 0 0 0 0 0 0 0 0 0 0 0 0 0 0 0 0 0
0 0 0 0 0 0 0 0 0 0 0 0 0 0 0 0 0 0 0 0 0 0 0 0 0 0 0 0 0
0 0 0 0 0 0 0 0 0 0 0 0 0 0 0 0 0 15 0 0 0 0 0 0 0 0 0 0 0
0 0 0 0 0 0 0 0 0 0 0 0 0 0 0 0 0 0 0 0 0 0 0 0 0 0 0 0
M02255_77_000000000-ADUT3_1_1111_22645_11961 T----AC---GT-AG-GGG--------TCT-A-G------C--G--T---T--GT-C-CGG-AA------TC-A--T-T--GG-GC-------GT--A-----AA-GA-GC-TC-------G-TA-G-G-C-G------------G--T-TC-G-G-C-----
------AA----G-T-C-G--------G-A-T--G--TG--A-AA-TC--T-C-CA-C-G-----CT-C-AA------C-G-T-G-G-A-G--G-G----G-C--C-A---T--T-------C--GA-A-A---C-------T--G-C-CGT--G-A-C----T-G-G-A-G-T--C---C-GG-----TA-G-G--------G-G-A-G---TG-C------GG--A--ATT----
C-C-C-G-GT--GT-A-G-CG-GT--G---A--A-A---TG-C-GC-AG--AT-A-TC--G-G--G-A-G-G-A-AC-A-CC---AG--T--G--GC-GAA-G--G-C--G-------G--C-A-C-T---CTG--G--GC-C-G--------------------G-A---A-C-T--GA--CG-C-T-G--A-GG--A-G-CG-A--AA-G-C---G-TG--
GG-G--AG-C-G-AA--------CA--GG Bacteria(100);unclassified(100);unclassified(100);unclassified(100);unclassified(100);unclassified(100);unclassified(100);unclassified(100);unclassified(100);

Otu1529 0 0 0 0 0 0 0 0 0 0 0 0 0 0 0 0 0 0 0 0 0 0 0 0 0 0 0 0
0 0 0 0 0 0 0 0 0 0 0 0 0 0 0 0 0 0 0 0 0 0 0 0 0 0 0 0 0
0 0 0 0 0 0 0 0 0 0 0 0 0 0 0 0 0 0 0 80 0 0 0 0 0 0 0 0 0
0 0 0 0 0 0 0 0 0 0 0 0 0 0 0 0 0 0 0 0 0 0 0 0 0 0 0 0
M02255_77_000000000-ADUT3_1_1104_13394_20369 T----AC---GT-AG-GGG--------GCG-A-G------C--G--T---T--GT-T-CGG-AA------TT-A--T-T--GG-GC-------GT--A-----AA-GG-GT-AC-------G-TA-G-G-C-G------------G--A-TC-G-T-T-----
------AA----G-T-T-T--------G-G-C--G--TT--A-AA-TT--C-T-GG-G-G-----CT-C-AA------C-C-C-C-A-G-C--A------A-G--C-G---T--T-------G--AA-A-A---C-------T--G-A--CG--A-C-C----T-T-G-A-G-T--A---G-TA-----GA-G-G--------G-G-A-C---AG-T------GG--A--ATT----C-
C-T-A-GT--GT-A-G-CG-GT--G---A--A-A---TG-C-GT-AG--AG-A-TT--A-G--G-A-A-G-A-AT-A-CC---GG--T--G--GC-GAA-G--G-C--G-------A--C-T-G-T---CTG--G--AT-A-C--------------------A-T---A-C-T--GA--CG-C-T-G--A-GG--T-A-CG-A--AA-G-C---G-TG--GG-
G--AG-C-G-AA--------CA--GG Bacteria(100);unclassified(100);unclassified(100);unclassified(100);unclassified(100);unclassified(100);unclassified(100);unclassified(100);unclassified(100);

Otu1530 0 0 0 0 0 0 0 0 0 0 0 0 0 0 0 0 0 0 0 0 0 0 0 0 0 0 0 0
0 0 0 0 0 0 0 0 0 0 0 0 0 0 0 0 0 0 0 0 0 0 0 0 0 0 0 0 0
0 0 0 0 0 0 0 0 0 0 0 0 0 0 0 0 0 0 46 0 0 0 0 0 0 0 0 0 0
0 0 0 0 0 0 0 0 0 0 0 0 0 0 0 0 0 0 0 0 0 0 0 0 0 0 0
M02255_77_000000000-ADUT3_1_1104_10969_9432 T----AC---GT-AG-GGA--------GCA-A-G------C--G--T---T--AT-C-CGG-AT------TT-A--T-T--GG-GT-------GT--A-----AA-GG-GT-GC-------G-TA-G-A-C-G------------G--G-AA-A-T-T-----------AA----G-T-T-A--------G-T-T--G--TG--A-AA-TC--C-C-TC-G-G-----CT-T-AA------C-T-G-A-G-G-A--A-C----T-G--C-A---A--C-------T--AA-A-A---C-------T--G-G--TT--T-T-C----T-T-G-A-G-T--G---C-GG-----GA-G-A--------G-G-A-A---AG-T------GG--A--ATT----C-C-T-A-GT--GT-A-G-CG-GT--G---A--A-A---TG-C-GT-AG--AT-A-TT--A-G--G-A-G-G-A-AC-A-CC---AG--T--G--GC-GAA-G--G-C--G-------A--C-T-T-T---CTG--G--AC-C-G--------------------T-A---A-C-T--GA--CG-T-T-G--A-GG--C-A-CG-A--AA-G-T---G-TG--GG-G--AG-C-A-AA--------CA--GG Bacteria(100);Firmicutes(100);Clostridia(100);Clostridiales(100);Ruminococcaceae(100);uncultured(100);unclassified(100);unclassified(100);unclassified(100);

Otu1531 0 0 0 0 0 0 0 0 0 0 0 0 0 0 0 0 0 0 0 0 0 0 0 0 0 0 0 0
0 0 0 0 0 0 0 0 0 0 0 0 0 0 0 0 0 0 0 0 0 0 0 0 0 0 0 0 0
0 0 0 0 0 0 0 0 0 0 0 0 0 0 0 0 0 0 77 0 0 0 0 0 74 0 0 0 0
0 0 0 0 0 0 0 0 0 0 0 0 0 0 0 0 0 0 0 0 0 0 0 0 0 0 0
M02255_77_000000000-ADUT3_1_1104_18185_22607 T----AC---GT-AG-GTG--------GCA-A-G------C--G--T---T--GT-C-CGG-AA------TT-A--C-T--GG-GC-------GT--A-----AA-GG-GT-GC-------G-TA-G-G-C-G------------G--T-CC-T-T-T-----------AA----G-T-G-G--------G-A-T--G--TG--A-AA-TC--C-C-TG-G-G-----CT-T-AA------C-C-C-A-G-G-A--A-C----T-G--C-A---T--T-------C--CA-A-A---C-------T--G-G--AG--G-A-C----T-A-G-A-G-T--G---C-AG-----GA-G-A--------G-G-A-A---AG-C------GG--T--ATT----C-C-C-A-GT--GT-A-G-CG-GT--G---A--A-A---TG-C-GT-AG--AG-A-TT--G-G--G-A-G-G-A-AC-A-CC---AG--T--A--GC-GAA-G--G-C--G-------G--C-T-T-T---CTG--G--AA-T-G--------------------T-A---A-C-T--GA--CG-C-T-G--A-GG--C-A-CG-A--AA-G-C---G-TG--GG-G--AG-C-A-AA--------CA--GG Bacteria(100);Firmicutes(100);Clostridia(100);Clostridiales(100);Clostridiaceae(100);unclassified(100);unclassified(100);unclassified(100);unclassified(100);

Otu1532 0 0 0 0 0 0 0 0 0 0 0 0 0 0 0 0 0 0 0 0 0 0 0 0 0 0 0 0
0 0 0 0 0 0 0 0 0 0 0 0 0 0 0 0 0 0 0 0 0 0 0 0 0 0 0 0 0
0 0 0 0 0 0 0 0 0 0 0 0 0 0 0 0 0 0 123 0 0 0 0 0 0 0 0 0 0
0 0 0 0 0 0 0 0 0 0 0 0 0 0 0 0 0 0 0 0 0 0 0 0 0 0 0
M02255_77_000000000-ADUT3_1_1104_17845_22883 T----AC---GT-AG-GTG--------GCA-A-G------C--G--T---T--GT-C-CGG-AA------TT-A--C-T--GG-GT-------GT--A-----AA-GG-GA-GC-------G-CA-G-G-C-G------------G--G-AC-T-G-C-----------AA----G-T-T-G--------G-A-A--G--TG--A-AA-TA--C-C-GC-A-G-----CT-T-AA------C-T-G-C-G-G-A--G-C----T-G--C-T---T--C-------C--AA-A-A---C-------T--G-T--AG--T-T-C----T-T-G-A-G-T--G---G-AG-----TA-G-A--------G-G-T-A---GG-C------GG--A--ATT----C-C-G-A-GT--GT-A-G-CG-GT--G---A--A-A---TG-C-GT-AG--AT-A-TT--C-G--G-A-G-G-A-AC-A-CC---AG--T--G--GC-GAA-G--G-C--G-------G--C-C-T-A---CTG--G--GC-T-C--------------------T-T---A-C-T--GA--CG-C-T-G--A-GG--C-T-CG-A--AA-G-T---G-TG--GG-G--AG-C-A-AA--------CA--GG Bacteria(100);Firmicutes(100);Clostridia(100);Clostridiales(100);unclassified(100);unclassified(100);unclassified(100);unclassified(100);unclassified(100);

Otu1533 0 0 0 0 0 0 0 0 0 0 0 0 0 0 0 0 0 0 0 0 0 0 0 0 0 0 0 0
0 0 0 0 0 0 0 0 0 0 0 0 0 0 0 0 0 0 0 0 0 0 0 0 0 0 0 0 0
0 0 0 0 0 0 0 0 0 0 0 0 0 0 0 0 0 0 132 0 0 0 0 0 0 0 0 0 0
0 0 0 0 0 0 0 0 0 0 0 0 0 0 0 0 0 0 0 0 0 0 0 0 0 0 0
M02255_77_000000000-ADUT3_1_1105_15034_23589 T----AC---GG-AG-GGG--------GCT-A-G------C--G--T---T--GT-T-CGG-AA------TT-A--C-T--GG-GC-------GT--A-----AA-GC-GT-AC-------G-TA-G-G-C-G------------G--T-CT-T-T-T-----------AA----G-T-C-A--------G-A-G--G--TG--A-AA-GC--C-C-GG-G-G-----CT-C-AA------C-T-C-C-G-G-A--A-T----T-G--C-C---T--T-------T--GA-A-A---C-------T--G-G--AA--G-A-C----T-T-G-A-A-C--A---T-GG-----GA-G-A--------G-G-T-A---AG-T------GG--A--ATT----C-C-G-A-GT--GT-A-G-AG-GT--G---A--A-A---TT-C-GT-AG--AT-A-TT--C-G--G-A-G-G-A-AC-A-CC---AG--T--G--GC-GAA-G--G-C--G-------G--C-T-T-A---CTG--G--AC-C-A--------------------T-T---G-T-T--GA--CG-C-T-G--A-GG--T-A-CG-A--AA-G-C---G-TG--GG-G--AG-C-A-AA--------CA--GG Bacteria(100);Proteobacteria(100);Alphaproteobacteria(100);unclassified(100);unclassified(100);unclassified(100);unclassified(100);unclassified(100);unclassified(100);

Otu1534 0 0 0 0 0 0 0 0 0 0 0 0 0 0 0 0 0 0 0 0 0 0 0 0 0 0 0 0
0 0 0 0 0 0 0 0 0 0 0 0 0 0 0 0 0 0 0 0 0 0 0 0 0 0 0 0 0
0 0 0 0 0 0 0 0 0 0 0 0 0 0 0 0 0 0 56 0 0 0 0 0 0 0 0 0 0
0 0 0 0 0 0 0 0 0 0 0 0 0 0 0 0 0 0 0 0 0 0 0 0 0 0 0
M02255_77_000000000-ADUT3_1_1108_26954_19116 T----AC---GT-AG-GTG--------GCA-A-G------C--G--T---T--AT-C-CGG-AT------TT-A--C-T--GG-GT-------GT--A-----AA-GG-GC-GC-------G-TA-G-G-C-G------------G--G-GA-T-G-C-----------AA----G-T-C-A--------G-G-T--G--TG--A-AA-TC--T-A-TG-G-G-----CT-T-AA------C-C-C-A-T-A-A--A-C----T-G--C-A---T--T-------T--GA-A-A---C-------T--G-T--AT--C-T-C----T-T-G-A-G-T--G---C-TG-----GA-G-A--------G-G-T-A---GA-T------GG--A--ATT----C-C-T-T-GT--GT-A-G-CG-GT--G---A--A-A---TG-C-GT-AG--AT-A-TA--A-G--G-A-A-G-A-AC-A-CC---AG--T--G--GC-GAA-G--G-C--G-------A--T-C-T-A---CTG--G--AC-A-G--------------------T-A---A-C-T--GA--CG-C-T-G--A-GG--C-G-CG-A--AA-G-C---G-TG--GG-G--AG-C-A-AA--------CA--GG Bacteria(100);Firmicutes(100);Clostridia(100);Clostridiales(100);Ruminococcaceae(100);unclassified(100);unclassified(100);unclassified(100);unclassified(100);

Otu1535 0 0 0 0 0 0 0 0 0 0 0 0 0 0 0 0 0 0 0 0 0 0 0 0 0 0 0
0 0 0 0 0 0 0 0 0 0 0 0 0 0 0 0 0 0 0 0 0 0 0 0 0 0 0 0
0 0 0 0 0 0 0 0 0 0 0 0 0 0 0 0 0 0 36 0 0 0 0 0 0 0 0 0
0 0 0 0 0 0 0 0 0 0 0 0 0 0 0 0 0 0 0 0 0 0 0 0 0 0 0
M02255_77_000000000-ADUT3_1_1113_12972_18835 T----AC---GT-AG-GTG--------GCG-A-G------C--G--T---T--AT-C-CGG-AT------TT-A--C-T--GG-GT-------GT--A-----AA-GG-GT-GT-------G-TA-G-G-C-G------------G--G-AA-G-G-T----
-------AA----G-T-C-A--------G-A-T--G--TG--A-AA-TG--T-A-TG-G-G-----CT-C-AA------C-T-C-A-T-A-C--C-C----T-G--C-G---T--C-------T--GA-A-A---C-------T--G-C--TT--T-T-C----T-T-G-A-G-T--G---T-CG-----GA-G-A--------G-G-T-A---AG-T------GG--A--ATT----C-
C-C-G-GT--GT-A-G-CG-GT--G---A--A-A---TG-C-GT-AG--AT-A-TC--G-G--G-A-G-G-A-AC-A-CC---GG--T--G--GC-GAA-G--G-C--G-------G--C-T-T-A---CTG--G--AC-G-A--------------------C-A---A-C-T--GA--CG-C-T-G--A-GA--C-A-CG-A--AA-G-T---G-TG--GG-
G--AG-C-A-AA--------CA--GG Bacteria(100);unclassified(100);unclassified(100);unclassified(100);unclassified(100);unclassified(100);unclassified(100);unclassified(100);unclassified(100);

Otu1536 0 0 0 0 0 0 0 0 0 0 0 0 0 0 0 0 0 0 0 0 0 0 0 0 0 0 0
0 0 0 0 0 0 0 0 0 0 0 0 0 0 0 0 0 0 0 0 0 0 0 0 0 0 0 0
0 0 0 0 0 0 0 0 0 0 0 0 0 0 0 0 0 0 27 0 0 0 0 0 0 0 0 0
0 0 0 0 0 0 0 0 0 0 0 0 0 0 0 0 0 0 0 0 0 0 0 0 0 0 0
M02255_77_000000000-ADUT3_1_2103_14782_20951 T----AC---GT-AG-GGG--------GCA-A-G------C--G--T---T--AT-C-CGG-AT------TT-A--C-T--GG-GT-------GT--A-----AA-GG-GA-GC-------G-TA-G-A-C-G------------G--C-AG-T-G-C----
-------AA----G-T-C-T--------G-A-A--G--TG--A-AA-TT--T-C-GC-G-G-----CT-C-AA------C-C-G-C-G-A-A--A-C----T-G--C-T---T--T-------G--GA-A-A---C-------T--G-T--AC--A-G-C----T-A-G-A-G-T--A---C-CG-----GA-G-G--------G-G-T-A---AG-C------GG--A--ATT----
C-C-T-A-GT--GT-A-G-CG-GT--G---A--A-A---TG-C-GT-AG--AT-A-TT--A-G--G-A-A-G-A-AC-A-CC---GG--T--G--GC-GAA-G--G-C--G-------G--C-T-T-A---CTG--G--AC-G-G--------------------T-A---A-C-T--GA--CG-T-T-G--A-GG--C-T-CG-A--AA-G-C---G-TG--GG-
G--AG-C-A-AA--------CA--GG Bacteria(100);Firmicutes(100);Clostridia(100);Clostridiales(100);Lachnospiraceae(100);unclassified(100);unclassified(100);unclassified(100);unclassified(100);

Otu1537 0 0 0 0 0 0 0 0 0 0 0 0 0 0 0 0 0 0 0 0 0 0 0 0 0 0 0
0 0 0 0 0 0 0 0 0 0 0 0 0 0 0 0 0 0 0 0 0 0 0 0 0 0 0 0
0 0 0 0 0 0 0 0 0 0 0 0 0 0 0 0 0 0 46 0 0 0 0 0 0 0 0 0
0 0 0 0 0 0 0 0 0 0 0 0 0 0 0 0 0 0 0 0 0 0 0 0 0 0 0
M02255_77_000000000-ADUT3_1_1106_12441_15759 T----AC---GT-AG-GTG--------GCA-A-G------C--G--T---T--AT-C-CGG-AT------TT-A--C-T--GG-GT-------GT--A-----AA-GG-GT-GA-------G-TA-G-G-C-G------------G--G-AG-A-T-C-----
------AA----G-T-C-A--------G-A-T--G--TG--A-AA-TA--C-A-AA-G-G-----CT-T-AA------C-C-T-T-T-G-A--A-C----T-G--C-A---T--T-------T--GA-A-A---C-------T--G-T--TC--T-T-C----T-T-G-A-G-T--A---T-CG-----GA-G-A--------G-G-T-A---AT-T------GG--A--ATT----C-C-
C-A-GT--GT-A-G-CG-GT--G---A--A-A---TG-C-GT-AG--AG-A-TT--G-G--G-A-G-G-A-AC-A-CC---AG--T--G--GC-GAA-G--G-C--G-------G--A-T-T-A---CTG--G--AC-G-A--------------------T-A---A-C-T--GA--CG-C-T-G--A-GG--C-A-CG-A--AA-G-T---G-TG--GG-G--
AG-C-A-AA--------CA--GG Bacteria(100);unclassified(100);unclassified(100);unclassified(100);unclassified(100);unclassified(100);unclassified(100);unclassified(100);unclassified(100);

Otu1538 0 0 0 0 0 0 0 0 0 0 0 0 0 0 0 0 0 0 0 0 0 0 0 0 0 0 0
0 0 0 0 0 0 0 0 0 0 0 0 0 0 0 0 0 0 0 0 0 0 0 0 0 0 0 0
0 0 0 0 0 0 0 0 0 0 0 0 0 0 0 0 0 0 29 0 0 0 0 0 0 0 0 0
0 0 0 0 0 0 0 0 0 0 0 0 0 0 0 0 0 0 0 0 0 0 0 0 0 0 0
M02255_77_000000000-ADUT3_1_1103_26239_16610 T----AC---GA-AG-GGT--------GCA-A-G------C--G--T---T--GT-T-CGG-AA------TT-A--C-T--GG-GT-------GT--A-----AA-GG-GC-GT-------G-TA-G-G-C-G------------G--A-TT-G-T-C-----
------AA----G-T-T-A--------G-T-G--G--TG--A-AA-CC--C-C-TG-G-G-----CT-T-AA------C-C-T-G-G-G-A--A-C----T-G--C-C---A--T-------T--GA-T-A---C-------T--G-A--TA--G-T-C----T-T-G-A-G-T--A---T-TG-----GA-G-A--------G-G-A-T---AA-C------GG--A--ATA----T-
C-C-A-GT--GT-A-G-AG-GT--G---A--A-A---TT-C-GT-AG--AT-A-TT--G-G--A-T-A-G-A-AC-A-CC---GG--T--G--GC-GAA-G--G-C--G-------G--T-T-A-T---CTG--G--CC-G-A--------------------T-T---A-C-T--GA--CG-C-T-G--A-GG--C-G-CG-A--GA-G-C---G-TG--GG-G-
-AG-C-A-AA--------CA--GG Bacteria(100);unclassified(100);unclassified(100);unclassified(100);unclassified(100);unclassified(100);unclassified(100);unclassified(100);unclassified(100);

Otu1539 0 0 0 0 0 0 0 0 0 0 0 0 0 0 0 0 0 0 0 0 0 0 0 0 0 0 0
0 0 0 0 0 0 0 0 0 0 0 0 0 0 0 0 0 0 0 0 0 0 0 0 0 0 0 0
0 0 0 0 0 0 0 0 0 0 0 0 0 0 0 0 0 0 0 7 0 0 0 0 0 0 0 0
4 0 0 0 0 7 0 0 0 0 0 10 0 0 0 0 0 0 0 0 0 0 0 0 0 0 0
M02255_76_000000000-ADUNE_1_2114_10632_17368 C----AA---GC-CA-CGG--------CTA-A-C------T--A--C---G--TG-C-CAG-CA------GC-C--G-C--GG-TA-------AT--A-----CG-TA-GG-TG-------G-CA-A-G-C-G------------T--T-GT-C-C-G------
-----GA----G-T-C-C--------A-T-C--T--TA--A-AA-GT--G-C-GG-G-G-----CT-T-AA------C-C-C-C-G-T-G--A------T-G--G-G---A--T-------G--GA-A-A---C-------T--G-G--GA--A-G-C----T-G-G-A-G-T--A---T-CG-----GA-G-A--------G-G-A-A---AG-T------GG--A--ATT----C-
C-T-A-GT--GT-A-G-CG-GT--G---A--A-A---TG-C-GT-AG--AG-A-TT--A-G--G-A-A-G-A-AC-A-CC---GG--T--G--GC-GAA-G--G-C--G-------A--C-T-T-T---CTG--G--AC-G-A--------------------A-A---A-C-T--GA--CG-C-T-G--A-GG--C-G-CG-A--AA-G-C---G-TG--GG-
G--AG-C-A-AA--------CA--GG Bacteria(100);Firmicutes(100);Clostridia(100);Clostridiales(100);Veillonellaceae(100);Dialister(100);unclassified(100);unclassified(100);unclassified(100);

Otu1540 0 0 0 0 0 0 0 0 0 0 0 0 0 0 0 0 0 0 0 0 0 0 0 0 0 0 0 0
0 0 0 0 0 0 0 0 0 0 0 0 0 0 0 0 0 0 0 0 0 0 0 0 0 0 0 0 0
0 0 0 0 0 0 0 0 0 0 0 0 0 0 0 0 0 0 0 0 0 0 0 0 0 0 0 0 0
0 0 0 0 0 9 0 0 0 8 0 0 0 0 0 0 0 0 0 0 0 0 0 0 0 0 0
M02255_77_000000000-ADUT3_1_1102_15601_12055 T----AC---GT-AG-GGG--------GCG-A-G------C--G--T---T--AT-C-CGG-AT------TC-A--T-T--GG-GC-------GT--A-----AA-GC-GC-GC-------G-TA-G-G-C-G------------G--C-CT-G-T-T-----
------AG----G-T-C-A--------A-G-A--G--TT--A-AA-TC--C-G-GG-G-G-----CT-C-AA------C-C-C-C-C-G-T--C------C-G--C-T---C--C-------T--GA-T-A---C-------C--G-G--CA--G-G-C----T-T-G-A-G-T--C---T-GG-----TA-G-G--------G-G-A-A---GG-C------GG--A--ATT----C-
C-A-A-GT--GT-A-G-CG-GT--G---G--A-A---TG-C-GC-AG--AT-A-TT--T-G--G-A-A-G-A-AC-A-CC---GG--T--G--GC-GAA-G--G-C--G-------G--C-C-T-T---CTG--G--GC-C-A--------------------C-G---A-C-T--GA--CG-C-T-G--A-GG--C-G-CG-A--AA-G-C---T-AG--GG-
G--AG-C-A-AA--------CA--GG Bacteria(100);Actinobacteria(100);Actinobacteria(100);Coriobacteridae(100);Coriobacteriales(100);Coriobacterineae(100);Coriobacteriaceae(100);Olsenella(100);unclassified(100);

Otu1541 0 0 0 0 0 0 0 0 0 0 0 0 0 0 0 0 0 0 0 0 0 0 0 0 0 0 0 0
0 0 0 0 0 0 0 0 0 0 0 0 0 0 0 0 0 0 0 0 0 0 0 0 0 0 0 0 0
0 0 0 0 0 0 0 0 0 0 0 0 0 0 0 0 0 0 0 1 0 0 0 0 0 0 0 26 0
0 0 0 0 0 0 4 0 0 0 0 0 0 0 0 0 0 0 0 0 0 0 0 0 0 0 0
M02255_77_000000000-ADUT3_1_1104_6099_15840 T----AC---GT-AG-GGG--------GCG-A-G------C--G--T---T--AT-C-CGG-AA------TT-A--T-T--GG-GC-------GTA-------AA-GA-GT-GC-------G-TA-G-G-T-G------------G--T-TA-T-C-T-----------AA-
---G-C-G-T--------G-G-G--G--TG--A-AA-GG--C-A-GT-G-G-----CT-T-AA------C-C-A-T-T-G-T--A------A-G--C-C---T--T-------G--CG-A-A---C-------T--G-G--AT--A-G-C----T-T-G-A-G-T--G---C-AG-----GA-G-G--------G-G-A-A---AG-T------GG--A--ATT----C-C-T-A-
GT--GT-A-G-CG-GT--G---A--A-A---TG-C-GT-AG--AT-A-TT--A-G--G-A-G-G-A-AC-A-CC---GG--T--G--GC-GAA-G--G-C--G-------G--C-T-T-T---CTG--G--AC-T-G--------------------T-A---A-C-T--GA--CA-C-T-G--A-GG--C-A-CG-A--AA-G-C---G-TG--GG-T--AG-C-
A-AA--------CA--GG Bacteria(100);Firmicutes(100);Clostridia(100);Clostridiales(100);Family_XIII_Incertae_Sedis(100);unclassified(100);unclassified(100);unclassified(100);unclassified(100);

Otu1542 0 0 0 0 0 0 0 0 0 0 0 0 0 0 0 0 0 0 0 0 0 0 0 0 0 0 0 0
0 0 0 0 0 0 0 0 0 0 0 0 0 0 0 0 0 0 0 0 0 0 0 0 0 0 0 0 0
0 0 0 0 0 0 0 0 0 0 0 0 0 0 0 0 0 0 0 0 33 0 0 0 0 0 0 0 0
0 0 0 0 0 0 0 0 0 0 0 0 0 0 0 0 0 0 0 0 0 0 0 0 0 0 0
M02255_77_000000000-ADUT3_1_1102_21345_16078 T----AC---AG-AG-GGT--------GCT-A-G------C--G--T---T--GT-C-CGG-AA------TC-A--C-T--GG-GC-------GT--A-----AA-GG-GC-GC-------G-TA-G-G-C-G------------G--C-CC-G-A-T----
-------AA----G-T-A-G--------G-G-G--G--TG--A-AA-TC--T-T-GC-G-G-----CT-C-AA------C-C-G-C-A-A-G--G-C----T-G--C-C---T--T-------C--TA-A-A---C-------T--G-T--CG--G-G-C----T-G-G-A-G-C--A---C-GG-----TA-G-A--------G-G-C-A---GA-T------GG--A--ATT----
C-C-C-G-GT--GT-A-G-CG-GT--G---G--A-A---TG-C-GT-AG--AG-A-TC--G-G--G-A-A-G-A-AC-A-CC---AG--T--G--GC-GAA-G--G-C--G-------G--T-C-T-G---CTG--G--GC-CG--------------------T-T---G-C-T--GA--CG-C-T-G--A-GG--C-G-CG-A--CA-G-C---G-TG--
GG-G--AG-C-A-AA--------CA--GG Bacteria(100);unclassified(100);unclassified(100);unclassified(100);unclassified(100);unclassified(100);unclassified(100);unclassified(100);unclassified(100);

Otu1543 0 0 0 0 0 0 0 0 0 0 0 0 0 0 0 0 0 0 0 0 0 0 0 0 0 0 0 0
0 0 0 0 0 0 0 0 0 0 0 0 0 0 0 0 0 0 0 0 0 0 0 0 0 0 0 0 0
0 0 0 0 0 0 0 0 0 0 0 0 0 0 0 0 0 0 0 0 39 0 0 0 0 0 0 0 0
0 0 0 0 0 0 0 0 0 0 0 0 0 0 0 0 0 0 0 0 0 0 0 0 0 0 0
M02255_77_000000000-ADUT3_1_1105_5671_7124 G----AC---GG-AG-GAT--------GCA-A-G------C--G--T---T--AT-C-CGG-AA------TG-A--T-T--GG-GC-------GT--A-----AA-GC-GT-CC-------G-CA-G-G-T-G------------G--C-TA-T-T-C-----------AA-
---G-T-C-T--------G-C-T--G--TC--A-AA-GA--G-C-AA-G-G-----CT-C-AA------C-C-T-T-G-T-C--A-A----G-G--C-A---G--T-------G--GA-A-A---C-------T--G-A--AA--G-G-C----T-A-G-A-G-T--G---T-GG-----TA-G-G--------G-G-T-A---GA-G------GG--A--ATT----C-C-C-A-
GT--GT-A-G-CG-GT--G---A--A-A---TG-C-GT-AG--AG-A-TT--G-G--G-A-A-G-A-AC-A-CC---GG--T--G--GC-GAA-A--G-C--G-------C--T-C-T-A---CTG--G--AC-C-T--------------------G-C---A-C-T--GA--CA-C-T-G--A-GG--G-A-CG-A--AA-G-C---T-AG--GG-G--AG-
C-G-AA--------TG--GG Bacteria(100);Cyanobacteria(100);unclassified(100);unclassified(100);unclassified(100);unclassified(100);unclassified(100);unclassified(100);unclassified(100);

Otu1544 0 0 0 0 0 0 0 0 0 0 0 0 0 0 0 0 0 0 0 0 0 0 0 0 0 0 0 0
0 0 0 0 0 0 0 0 0 0 0 0 0 0 0 0 0 0 0 0 0 0 0 0 0 0 0 0 0
0 0 0 0 0 0 0 0 0 0 0 0 0 0 0 0 0 0 0 0 0 6 0 0 0 0 0 0 0
0 0 0 0 0 0 0 0 0 0 0 0 0 0 0 0 0 0 0 0 0 0 0 0 0 0 0
M02255_77_000000000-ADUT3_1_1108_12409_13968 T----AC---GG-AG-GGG--------GTT-A-G------C--G--T---T--GT-T-CGG-AA------TT-A--C-T--GG-GC-------GT--A-----AA-GC-GT-AC-------G-TA-G-G-C-G------------G--A-TC-A-G-C-----
------AA----G-T-A-T--------G-G-G--G--TG--A-AA-TC--C-C-GG-G-G-----CT-C-AA------C-C-C-C-G-G-A--A-C----T-G--C-C---T--C-------G--TA-A-A---C-------T--G-T--TG--G-T-C----T-T-G-A-G-T--T---C-GA-----GA-G-A--------G-G-T-G---AG-T------GG--A--ATT----C-
C-G-A-GT--GT-A-G-AG-GT--G---A--A-A---TT-C-GT-AG--AT-A-TT--C-G--G-A-G-G-A-AC-A-CC---AG--T--G--GC-GAA-G--G-C--G-------G--C-T-C-A---CTG--G--CT-C-G--------------------A-T---A-C-T--GA--CG-C-T-G--A-GG--T-A-CG-A--AA-G-T---G-TG--GG-G-
-AG-C-A-AA--------CA--GG Bacteria(100);Proteobacteria(100);Alphaproteobacteria(100);Rhodobacterales(100);Rhodobacteraceae(100);unclassified(100);unclassified(100);unclassified(100);unclassified(100);

Otu1545 0 0 0 0 0 0 0 0 0 0 0 0 0 0 0 0 0 0 0 0 0 0 0 0 0 0 0 0
0 0 0 0 0 0 0 0 0 0 0 0 0 0 0 0 0 0 0 0 0 0 0 0 0 0 0 0 0
0 0 0 0 0 0 0 0 0 0 0 0 0 0 0 0 0 0 0 0 0 9 0 0 0 0 0 0 0
0 0 0 0 0 0 0 0 0 0 0 0 0 0 0 0 0 0 0 0 0 0 0 0 0 0 0
M02255_77_000000000-ADUT3_1_2103_24119_6977 T----AC---AG-AG-GCC--------TCA-A-G------C--G--T---T--AT-C-CGG-AT------TT-A--T-T--GG-GC-------GT--A-----AA-GC-GT-AT-------G-TA-G-G-C-G------------G--G-TA-T-A-T-----------TA--
--G-T-C-G--------C-A-G--G--TC--A-AA-TC--T-C-CG-A-G-----CC-C-AA------C-T-C-G-G-A-A--A-C----T-G--C-T---T--G-------C--GA-A-A---C-------G--G-T--AT--A-T-C----T-A-G-A-G-G--A---T-GT-----GA-G-A--------G-A-T-G---AC-T------GG--A--ACT----C-A-T-G-
GT--GT-A-G-TA-GT--G---A--A-A---TG-C-GT-TG--AT-A-TC--A-T--G-G-G-G-A-AC-A-CC---AA--A--G--GC-GAA-G--G-C--A-------G--G-T-C-A---TTG--G--CA-C-A--------------------T-T---C-C-T--GA--CG-C-T-C--A-G-A-T-A-CG-A--AA-G-C---G-TG--GG-T--AG-C-
G-AA--------TG--GG Bacteria(100);unclassified(100);unclassified(100);unclassified(100);unclassified(100);unclassified(100);unclassified(100);unclassified(100);unclassified(100);

Otu1546 0 0 0 0 0 0 0 0 0 0 0 0 0 0 0 0 0 0 0 0 0 0 0 0 0 0 0 0
0 0 0 0 0 0 0 0 0 0 0 0 0 0 0 0 0 0 0 0 0 0 0 0 0 0 0 0 0
0 0 0 0 0 0 0 0 0 0 0 0 0 0 0 0 0 22 0 0 0 0 0 0 0 0 0 0 0
0 0 0 0 0 0 0 0 0 0 0 0 0 0 0 0 0 0 0 0 0 0 0 0 0 0 0
M02255_76_000000000-ADUNE_1_1101_7382_19940 T----A----AC-TC-TGT--------GCC-A-G------C-----A---G--CC-G-CGG-TA------AG-A--C-A--GA-GG-------GT--G-----CA--A-AC-GT---------TG-C-T-C-G------------G--A-TT-G-G-G----------AA----
G-T-C-G--------G-G-T--G--TG--A-AA-TC--C-C-TG-G-G-----CT-C-AA------C-C-T-A-G-G-A--A-C----T-G--C-A---T--T-------C--GA-A-A---C-------T--C-T--CA--A-T-C----T-A-G-A-G-T--G---A-CG-----GA-G-A--------G-G-A-A---AG-C------GG--A--ATT----C-C-C-G-GT--
GT-A-G-AG-GT--G---A--A-A---TT-C-GT-AG--AT-A-TC--G-G--G-A-G-G-A-AC-A-CC---AG--A--G--GC-GAA-G--G-C--G-------G--C-T-T-T---CTG--G--AC-G-T--------------------C-A---A-C-T--GA--CG-C-T-G--A-GG--C-G-CG-A--AA-G-C---G-TG--GG-G--AG-C-A-
AA--------CA--GG Bacteria(100);Proteobacteria(100);unclassified(100);unclassified(100);unclassified(100);unclassified(100);unclassified(100);unclassified(100);unclassified(100);

Otu1547 0 0 0 0 0 0 0 0 0 0 0 0 0 0 0 0 0 0 0 0 0 0 0 0 0 0 0 0
0 0 0 0 0 0 0 0 0 0 0 0 0 0 0 0 0 0 0 0 0 0 0 0 0 0 0 0 0
0 0 0 0 0 0 0 0 0 0 0 0 0 0 0 0 0 0 85 0 0 0 0 0 0 0 0 0 0
0 0 0 0 0 0 0 0 0 0 0 0 0 0 0 0 0 0 0 0 0 0 0 0 0 0 0
M02255_77_000000000-ADUT3_1_1103_6493_13789 T----AC---GT-AG-GGA--------CCA-A-G------C--G--T---T--GT-T-CGG-AT------TT-A--C-T--GG-GC-------GT--A-----AA-GG-GC-GC-------G-TA-G-G-C-G------------G--C-AA-T-T-C----------AA-
---G-T-C-A--------G-C-T--G--TG--A-AA-TC--T-C-CG-G-G-----CT-T-AA------C-T-C-G-G-A-A--C-G----G-T--C-A---G--C-------T--GA-T-A---C-------T--G-T--TT--T-G-C----T-A-G-A-G-T--G---C-AG-----AA-G-G--------G-G-C-A---AT-C------GG--A--ATT----C-T-C-G-
GT--GT-A-G-CG-GT--G---A--A-A---TG-C-GT-AG--AT-A-TC--G-A--G-A-G-G-A-AC-A-CC---TG--A--G--GT-GAA-G--A-C--G-------G--G-T-T-G---CTG--G--GC-T-G--------------------A-C---A-C-T--GA--CG-C-T-G--A-TG--C-G-CG-A--AA-G-C---C-AG--GG-T--AG-
C-A-AA--------CG--GG Bacteria(100);Acidobacteria(100);Acidobacteria(100);Acidobacteriales(100);Acidobacteriaceae(100);Candidatus_Chloroacidobacterium(100);unclassified(100);unclassified(100);unclassified(100);

Otu1548 0 0 0 0 0 0 0 0 0 0 0 0 0 0 0 0 0 0 0 0 0 0 0 0 0 0 0 0
0 0 0 0 0 0 0 0 0 0 0 0 0 0 0 0 0 0 0 0 0 0 0 0 0 0 0 0 0
0 0 0 0 0 0 0 0 0 0 0 0 0 0 0 0 0 0 57 0 0 0 0 0 0 0 0 0 0
10 0 0 0 0 0 0 0 0 0 0 0 0 0 0 0 0 0 0 0 0 0 0 0 0 0 0
M02255_77_000000000-ADUT3_1_1101_27410_15960 T----AC---GT-AG-GGA--------GCG-A-G------C--G--T---T--AT-C-CGG-AA------TT-A--C-T--GG-GC-------GT--A-----AA-GG-GC-GC-------G-TA-G-G-C-G------------G--A-AT-G-A-C----
-------AA----G-T-C-A--------G-CAA--G--TG--A-AA-GC--G-T-GG-G-G-----CT-C-AA------C-C-C-C-A-T-G--A------T-G--C-G---G--C-------T--GA-A-A---C-------T--G-T--TA--T-T-C----T-A-G-A-G-G--C---A-TG-----GA-G-A--------G-G-C-A---AA-C------GG--A--ATT----
C-C-C-G-GT--GT-A-G-CG-GT--G---A--A-A---TG-C-GT-AG--AT-A-TC--G-G--G-A-A-G-A-AC-A-CC---AG--T--G--GC-GAA-G--G-C--G-------G--T-T-T-G---CTG--G--CC-A-T--------------------G-A---A-C-T--GA--CG-C-T-C--ATAG--C-G-CG-A--AA-G-C---T-AG--
GG-T--AG-C-A-AA--------CG--GG Bacteria(100);Synergistetes(100);Synergistia(100);Synergistales(100);Synergistaceae(100);Jonquetella(100);unclassified(100);unclassified(100);unclassified(100);

Otu1549 0 0 0 0 0 0 0 0 0 0 0 0 0 0 0 0 0 0 0 0 0 0 0 0 0 0 0 0
0 0 0 0 0 0 0 0 0 0 0 0 0 0 0 0 0 0 0 0 0 0 0 0 0 0 0 0 0
0 0 0 0 0 0 0 0 0 0 0 0 0 0 0 0 0 0 23 0 0 0 0 0 0 0 0 0 0
0 0 0 0 0 0 0 0 0 0 0 0 0 0 0 0 0 0 0 0 0 0 0 0 0 0 0
M02255_77_000000000-ADUT3_1_1101_20031_9546 T----AC---GT-AG-GTG--------GCA-A-G------C--G--T---T--GT-C-CGG-AT------TT-A--C-T--GG-GT-------GT--A-----AA-GG-GC-GT-------G-TA-G-A-C-G------------G--G-AC-T-G-C----------AA-
---G-T-C-A--------G-A-T--G--TG--A-AA-TT--T-A-TG-G-G-----CT-C-AA------C-C-C-A-T-A-A--C-C----T-G--C-A---T--T-------T--GA-A-A---C-------T--G-T--GG--T-T-C----T-T-G-A-G-T--G---A-TG-----GA-G-A--------G-G-T-A---AG-C------GG--A--ATT----C-C-T-A-
GT--GT-A-G-CG-GT--G---A--A-A---TG-C-GT-AG--AT-A-TT--A-G--G-A-G-G-A-AC-A-CC---GG--T--G--GC-GAA-G--G-C--G-------G--C-T-T-A---CTG--G--AC-A-T--------------------T-A---A-C-T--GA--CG-T-T-G--A-GG--C-G-CG-A--AA-G-T---G-TG--GG-G--AG-
C-A-AA--------CA--GG Bacteria(100);Firmicutes(100);Clostridia(100);Clostridiales(100);unclassified(100);unclassified(100);unclassified(100);unclassified(100);unclassified(100);

Otu1550 0 0 0 0 0 0 0 0 0 0 0 0 0 0 0 0 0 0 0 0 0 0 0 0 0 0 0 0
0 0 0 0 0 0 0 0 0 0 0 0 0 0 0 0 0 0 0 0 0 0 0 0 0 0 0 0 0
0 0 0 0 0 0 0 0 0 0 0 0 0 0 0 0 0 20 0 0 0 0 0 0 0 0 0 0 0
0 0 0 0 0 0 0 0 0 0 0 0 0 63 0 287 236 83 0 0 0 0 0 0 0 0 0
M02255_77_000000000-ADUT3_1_1118_6019_18654 T----AC---GT-AG-GCA--------GCA-A-G------C--G--T---T--GT-T-CGG-AA------TT-A--C-T--GG-GC-------GT--A-----AA-GA-GC-TC-------G-TA-G-G-C-G------------G--T-TT-G-C-T----------AA--
--G-T-T-T--------G-G-T--G--TG--A-AA-TC--T-C-CC-G-G-----CT-T-AA------C-T-G-G-G-A-G--G-G----T-G--C-G---C--C-------G--AA-A-A---C-------T--G-G--CA--G-A-C----T-C-G-A-G-T--G---C-GG-----GA-A-A--------G-G-A-A---AG-C------GG--A--ATT----C-C-T-G-
GT--GT-A-G-CG-GT--G---A--A-A---TG-C-GT-AG--AT-A-TC--A-G--G-A-G-G-A-AC-A-CC---TG--T--G--GT-GTA-G--A-C--G-------G--C-T-T-T---CTG--G--AC-C-G--------------------T-A---A-C-T--GA--CG-C-T-G--A-GG--A-G-CG-A--AA-G-C---G-TG--GG-T--AG-C-
A-AA--------CA--GG Bacteria(100);unclassified(100);unclassified(100);unclassified(100);unclassified(100);unclassified(100);unclassified(100);unclassified(100);unclassified(100);

Otu1551 0 0 0 0 0 0 0 0 0 0 0 0 0 0 0 0 0 0 0 0 0 0 0 0 0 0 0 0
0 0 0 0 0 0 0 0 0 0 0 0 0 0 0 0 0 0 0 0 0 0 0 0 0 0 0 0 0
0 0 0 0 0 0 0 0 0 0 0 0 0 0 0 0 0 15 0 0 0 0 0 0 0 0 0 0 0
0 0 0 0 0 0 0 0 0 0 0 0 0 0 0 0 0 0 0 0 0 0 0 0 0 0 0
M02255_77_000000000-ADUT3_1_1118_4385_14764 G----AC---GA-AG-GGG--------GCG-A-G------C--G--T---T--GT-T-CGG-AA------TC-A--C-T--GG-GC-------GT--A-----AA-GG-GC-GC-------G-TA-G-G-C-G------------G--C-GC-A-A-T----------
CA----G-T-C-A--------G-G-C--G--TG--A-AA-GC--C-C-CG-A-G-----CT-C-AA------C-T-T-G-G-G-A--A-T----A-G--C-G---C--T-------T--GA-T-A---C-------T--A-T--TG--T-G-C----T-A-G-A-G-A--G---C-GG-----GA-G-A--------G-G-A-T---GG-T------GG--A--ATT----C-C-C-
A-GT--GT-A-G-AG-GT--G---A--A-A---TT-C-GT-AG--AT-A-TT--G-G--G-A-A-G-A-AC-A-CC---GG--T--G--GC-GAA-G--G-C--G-------G--C-C-A-T---CTG--G--AC-C-G--------------------T-T---T-C-T--GA--CG-C-T-G--A-GG--C-G-CG-A--AA-G-C---G-TG--GG-G--
AG-C-A-AA--------CA--GG Bacteria(100);Proteobacteria(100);Alphaproteobacteria(100);Rhodospirillales(100);unclassified(100);unclassified(100);unclassified(100);unclassified(100);unclassified(100);

Otu1552 0 0 0 0 0 0 0 0 0 0 0 0 0 0 0 0 0 0 0 0 0 0 0 0 0 0 0 0
0 0 0 0 0 0 0 0 0 0 0 0 0 0 0 0 0 0 0 0 0 0 0 0 0 0 0 0 0
0 0 0 0 0 0 0 0 0 0 0 0 0 0 0 0 0 19 0 0 0 0 0 0 0 0 0 0 0
0 0 0 0 0 0 0 0 0 0 0 0 0 0 0 0 0 0 0 0 0 0 0 0 0 0 0
M02255_77_000000000-ADUT3_1_1111_24050_19375 G----AC---GA-GG-GAT--------CCT-A-G------C--G--T---T--GT-T-CGG-AA------TC-A--T-T--GG-GC-------GT--A-----AA-GC-GG-GT-------G-TA-G-G-T-G------------G--G-CT-T-C-T-----
------AA----G-T-C-A--------G-A-T--G--TG--A-AA-TC--C-C-AG-G-G-----CT-C-AA------C-C-C-T-G-G-A--A-G----T-G--C-A---T--T-------T--GA-T-A---C-------T--G-G--AA--G-T-C----T-T-G-A-G-T--G---T-GG-----TA-G-A--------G-G-T-T---AC-T------GG--A--ATT----G-
T-T-G-GT--GT-A-G-TG-GT--G---A--A-A---TA-C-GT-AG--AT-A-TC--A-A--C-A-G-G-A-AT-A-CC---GG--A--G--GC-GAA-G--G-C--G-------A--G-T-A-A---CTG--G--GC-C-A--------------------A-C---A-C-T--GA--CA-C-T-G--A-GA--C-C-CG-A--AA-G-C---G-TG--GG-
G--AT-C-A-AA--------CA--GG Bacteria(100);Proteobacteria(100);Deltaproteobacteria(100);Bdellovibrionales(100);Bdellovibrionaceae(100);Bdellovibrio(100);unclassified(100);unclassified(100);unclassified(100);

Otu1553 0 0 0 0 0 0 0 0 0 0 0 0 0 0 0 0 0 0 0 0 0 0 0 0 0 0 0 0
0 0 0 0 0 0 0 0 0 0 0 0 0 0 0 0 0 0 0 0 0 0 0 0 0 0 0 0 0
0 0 0 0 0 0 0 0 0 0 0 0 0 0 0 0 0 15 0 0 0 0 0 0 0 0 0 0 0
0 0 0 0 0 0 0 0 0 0 0 0 0 0 0 0 0 0 0 0 0 0 0 0 0 0 0
M02255_77_000000000-ADUT3_1_2107_14300_14994 T----AC---GT-AG-GGA--------GCG-A-G------C--G--T---T--GT-C-CGG-AT------TT-A--C-T--GG-GT-------GT--A-----AA-GG-GT-GC-------G-TA-G-G-C-G------------G--A-AG-A-G-C----
-------AA----G-T-C-A--------G-T-A--G--TG--A-AA-TC--C-A-TG-G-G-----CT-T-AA------C-C-C-G-T-G-A--A-C----T-G--C-T---A--T-------T--GA-A-A---C-------T--G-T--TC--T-T-C----T-T-G-A-G-T--G---A-AG-----TA-G-A--------G-G-T-A---GG-C------GG--A--ATT----C-
C-C-G-GT--GT-A-G-CG-GT--G---A--A-A---TG-C-GT-AG--AG-A-TC--G-G--G-A-G-G-A-AC-A-CC---AG--T--G--GC-GAA-G--G-C--G-------G--C-C-T-A---CTG--G--GC-T-T--------------------T-A---A-C-T--GA--CG-C-T-G--A-GG--C-A-CG-A--AA-G-C---A-TG--GG-
T--AG-C-A-AA--------CA--GG Bacteria(100);unclassified(100);unclassified(100);unclassified(100);unclassified(100);unclassified(100);unclassified(100);unclassified(100);unclassified(100);

Otu1554 0 0 0 0 0 0 0 0 0 0 0 0 0 0 0 0 0 0 0 0 0 0 0 0 0 0 0 0
0 0 0 0 0 0 0 0 0 0 0 0 0 0 0 0 0 0 0 0 0 0 0 0 0 0 0 0 0
0 0 0 0 0 0 0 0 0 0 0 0 0 0 0 0 0 20 0 0 0 0 3 0 0 0 0 0 0
0 0 0 0 0 0 0 0 0 0 0 0 0 0 0 0 0 0 0 0 0 0 0 0 0 0 0
M02255_77_000000000-ADUT3_1_1119_20598_11969 C----AC---GT-AG-GGT--------GCA-A-G------C--G--T---T--GT-C-CGG-AT------TT-A--T-T--GG-GC-------GT--A-----AA-GG-GC-TC-------G-TA-G-G-C-G------------G--T-TC-G-A-C-----
------AA----G-T-C-G--------G-G-T--G--TG--A-AA-CC--T-C-CA-G-G-----CT-C-AA------C-C-T-G-G-A-G----T----C-G-CC-A---C--T-------C--GA-A-A---C-------T--G-T--TG-TG-A-C----T-A-G-A-G-T--C---C-GG-----TA-G-G--------G-G-A-G---CA-T------GG--A--ATT----
C-C-T-G-GT--GT-A-G-CG-GT--G---G--A-A---TG-C-GC-AG--AT-A-TC--A-G--G-A-G-G-A-AC-A-CC---AG--T--G--GC-GAA-G--G-C--G-------A--T-G-C-T---CTG--G--GC-C-G--------------------G-T---A-C-T--GA--CG-C-T-G--A-GG--A-G-CG-A--AA-G-C---A-TG--
GG-G--AG-C-G-AA--------CA--GG Bacteria(100);Actinobacteria(100);Actinobacteria(100);unclassified(100);unclassified(100);unclassified(100);unclassified(100);unclassified(100);unclassified(100);

Otu1555 0 0 0 0 0 0 0 0 0 0 0 0 0 0 0 0 0 0 0 0 0 0 0 0 0 0 0 0
0 0 0 0 0 0 0 0 0 0 0 0 0 0 0 0 0 0 0 0 0 0 0 0 0 0 0 0 0
0 0 0 0 0 0 0 0 0 0 0 0 0 0 0 0 0 25 0 0 0 0 0 0 0 0 0 0 0
0 0 0 0 0 0 0 0 0 0 0 0 0 0 0 0 0 0 0 0 0 0 0 0 0 0 0
M02255_77_000000000-ADUT3_1_2113_19415_19303 T----AC---AG-AG-GGT--------GCG-A-G------C--G--T---T--GT-C-CGG-AA------TC-A--C-T--GG-GC-------GT--A-----AA-GG-GC-GC-------G-TA-G-G-C-G------------G--C-CT-G-G-T----
-------AA----G-T-C-G--------G-G-G--G--TG--A-AA-CG--C-T-GC-G-G-----CT-C-AA------C-C-G-C-A-G-C--A-C----T-G--C-C---T--T-------C--GA-G-A---C-------T--G-T--CA--G-G-C----T-T-G-A-G-C--A---C-GG-----TA-G-A--------G-G-C-T---GG-T------GG--A--ATT----
C-C-C-G-GT--GT-A-G-CG-GT--G---G--A-A---TG-C-GT-AG--AG-A-TC--G-G--G-A-A-G-A-AC-A-TC---GG--T--G--GC-GAA-G--G-C--G-------G--C-C-A-G---CTG--G--GC-CG--------------------T-T---G-C-T--GA--CG-C-T-G--A-GG--C-G-CG-A--CA-G-C---G-TG--
GG-G--AG-C-A-AA--------CA--GG Bacteria(100);unclassified(100);unclassified(100);unclassified(100);unclassified(100);unclassified(100);unclassified(100);unclassified(100);unclassified(100);

Otu1556 0 0 0 0 0 0 0 0 0 0 0 0 0 0 0 0 0 0 0 0 0 0 0 0 0 0 0 0
0 0 0 0 0 0 0 0 0 0 0 0 0 0 0 0 0 0 0 0 0 0 0 0 0 0 0 0 0
0 0 0 0 0 0 0 0 0 0 0 0 0 0 0 0 0 16 0 0 0 0 0 0 0 0 0 0 0
0 0 0 0 0 0 0 0 0 0 0 0 0 0 0 0 0 0 0 0 0 0 0 0 0 0 0
M02255_77_000000000-ADUT3_1_2113_15588_17173 T----AC---GT-AG-GGG--------GCG-A-G------C--G--T---T--GT-C-CGG-AT------TT-A--T-T--GG-GC-------GT--A-----AA-GA-GC-TC-------G-TA-G-G-C-G------------G--T-TC-G-G-T-----
------AA----G-T-C-G--------G-A-T--G--TG--A-AA-TC--T-C-CA-C-G-----CT-C-AA------C-G-T-G-G-A-G--G------G-G--CCA---T--T-------C--GA-T-A---C-------T--G-C--TG-TG-A-C----T-C-G-A-G-T--C---C-GG-----TA-G-G--------G-G--AG---TG-T------GG--A--ATTC---
C-C---G-GT--GT-A-G-CG-GT--G---A--A-A---TG-C-GC-AG--AG-A-TC--G-G--G-A-G-G-A-AC-A-CC---CG--T--A--GC-GAA-G--G-C--G-------G--C-A-C-T---CTGG----GC-C-G--------------------G-T---A-C-T--GA--CG-C-T-G--A-GG--A-G-CG-A--AA-G-C---G-TG--
GG-G--AG-C-A-AA--------CA--GG Bacteria(100);Actinobacteria(100);Actinobacteria(100);Acidimicrobidae(100);Acidimicrobiales(100);Acidimicrobineae(100);uncultured(100);unclassified(100);unclassified(100);

Otu1557 0 0 0 0 0 0 0 0 0 0 0 0 0 0 0 0 0 0 0 0 0 0 0 0 0 0 0 0
0 0 0 0 0 0 0 0 0 0 0 0 0 0 0 0 0 0 0 0 0 0 0 0 0 0 0 0 0
0 0 0 0 0 0 0 0 0 0 0 0 0 0 0 0 0 26 0 0 0 0 0 0 0 0 0 0 0
0 0 0 0 0 0 0 0 0 0 0 0 0 0 0 0 0 0 0 0 0 0 0 0 0 0 0
M02255_77_000000000-ADUT3_1_1118_11490_14483 T----AC---GA-AG-GGG--------GCT-A-G------C--G--T---T--GT-T-CGG-AA------TT-A--C-T--GG-GC-------GT--A-----AA-GG-GC-GC-------G-TA-G-G-C-G------------G--C-GC-A-T-T----
-------AA----G-T-C-A--------G-G-T--G--TG--A-AA-GC--C-C-TG-G-G-----CT-C-AA------C-C-C-A-G-G-A--A-T----T-G--C-A---C--T-------T--GA-T-A---C-------T--G-A--TG--T-G-C----T-T-G-A-A-T--C---C-GG-----AA-G-A--------G-G-A-T---GA-C------GG--A--ATT----
C-C-C-A-GT--GT-A-G-AG-GT--G---A--A-A---TT-C-AT-TG--AT-A-TT--G-G--G-A-A-G-A-AC-A-CC---AG--T--G--GC-GAA-G--G-C--G-------G--T-C-A-T---CTG--G--TC-C-G--------------------G-T---A-T-T--GA--CG-C-T-G--A-GG--C-G-CG-A--AA-G-C---G-TG--GG-
G--AG-C-A-AA--------CA--GG Bacteria(100);Proteobacteria(100);Alphaproteobacteria(100);unclassified(100);unclassified(100);unclassified(100);unclassified(100);unclassified(100);unclassified(100);

Otu1558 0 0 0 0 0 0 0 0 0 0 0 0 0 0 0 0 0 0 0 0 0 0 0 0 0 0 0 0
0 0 0 0 0 0 0 0 0 0 0 0 0 0 0 0 0 0 0 0 0 0 0 0 0 0 0 0 0
0 0 0 0 0 0 0 0 0 0 0 0 0 0 0 0 0 27 0 0 0 0 0 0 0 0 0 0 0
0 0 0 0 0 0 0 0 0 0 0 0 0 0 0 0 0 0 0 0 0 0 0 0 0 0 0
M02255_77_000000000-ADUT3_1_1115_20850_23281 T----AC---GA-AG-GGG--------GCT-A-G------C--G--T---T--GT-T-CGG-AT------TT-A--C-T--GG-GC-------GT--A-----AA-GC-GC-AT-------G-TA-G-G-C-G------------G--G-TC-G-T-T-----
------AA----G-T-T-A--------G-G-G--G--TG--A-AA-TC--C-C-AA-G-G-----CT-C-AA------C-C-T-T-G-G-A--A-C----T-G--C-C---T--C-------T--AA-A-A---C-------T--G-G--CG--A-C-C----T-G-G-A-G-C--A---A-GA-----TA-G-A--------G-G-C-G---AG-T------GG--A--ATT----C-
C-G-A-GT--GT-A-G-AG-GT--G---A--A-A---TT-C-GT-AG--AT-A-TT--C-G--G-A-G-G-A-AC-A-CC---AG--T--G--GC-GAA-G--G-C--G-------A--C-T-C-G---CTG--G--AT-C-T--------------------T-T---G-C-T--GA--CG-C-T-G--A-GA--T-G-CG-A--AA-G-C---G-TG--GG-G-
-AG-C-A-AA--------CA--GG Bacteria(100);Proteobacteria(100);Alphaproteobacteria(100);unclassified(100);unclassified(100);unclassified(100);unclassified(100);unclassified(100);unclassified(100);

Otu1559 0 0 0 0 0 0 0 0 0 0 0 0 0 0 0 0 0 0 0 0 0 0 0 0 0 0 0 0
0 0 0 0 0 0 0 0 0 0 0 0 0 0 0 0 0 0 0 0 0 0 0 0 0 0 0 0 0
0 0 0 0 0 0 0 0 0 0 0 0 0 0 0 0 0 16 0 0 0 0 0 0 0 0 0 0 0
0 0 0 0 0 0 0 0 0 0 0 0 0 0 0 0 0 0 0 0 0 0 0 0 0 0 0
M02255_77_000000000-ADUT3_1_1112_22408_18041 A----AC---GT-AG-GAG--------GCG-A-G------C--G--T---T--AT-C-CGG-AT------TT-A--C-T--GG-GT-------GT--A-----AA-GC-GC-GT-------G-CA-G-G-C-G------------G--T-TC-G-C-T-----
------AA----G-T-T-G--------G-A-T--G--TG--A-AA-GC--T-C-CC-G-G-----CT-C-AA------C-T-G-G-G-A-G--A-G----G-T--C-G---T--A-------C--AA-G-A---C-------T--G-G--CG--G-A-C----T-G-G-A-G-T--G---T-GG-----TA-G-G--------G-G-A-A---GG-T------GG--A--ATT----
C-C-G-G-GT--GT-A-G-TG-GT--G---A--A-A---TG-C-GT-AG--AT-A-TC--C-G--G-A-G-G-A-AC-A-CC---AG--T--G--GC-GAA-A--G-C--G-------G--C-C-T-T---CTA--G--AC-C-A--------------------C-A---A-C-T--GA--CG-C-T-C--A-GA--C-G-CG-A--AA-G-C---T-AG--GG-
T--AG-C-A-AA--------CG--GG Bacteria(100);Chloroflexi(100);Anaerolineae(100);Anaerolineales(100);Anaerolineaceae(100);unclassified(100);unclassified(100);unclassified(100);unclassified(100);

Otu1560 0 0 0 0 0 0 0 0 0 0 0 0 0 0 0 0 0 0 0 0 0 0 0 0 0 0 0 0
0 0 0 0 0 0 0 0 0 0 0 0 0 0 0 0 0 0 0 0 0 0 0 0 0 0 0 0
0 0 0 0 0 0 0 0 0 0 0 0 0 0 0 0 0 23 0 0 0 0 0 0 0 0 0 0
0 0 0 0 0 0 0 0 0 0 0 0 0 0 0 0 0 0 0 0 0 0 0 0 0 0 0
M02255_77_000000000-ADUT3_1_1109_8394_15483 T----AC---GT-AG-GGG--------GCG-A-G------C--G--T---T--GT-C-CGG-AA------TT-A--T-T--GG-GC-------GT--A-----AA-GA-GC-TC-------G-TA-G-G-C-G------------G--C-TC-A-G-C----------
AA----G-T-C-G--------G-C-T--G--TG--A-AA-TC--C-C-GA-G-G-----CT-C-AA------C-C-T-C-G-G-A--A------C-T-GC-A---G--T-------C--GA-T-A---C-------T--G-C--TG-TG-G-C----T-A-G-A-G-T--C---T-GG-----TA-G-A--------G-G-A-G---AG-T------GG--A--ATT----C-C-C-
G-GT--GT-A-G-CG-GT--G---A--A-A---TG-C-GC-AG--AT-A-TC--G-G--G-A-G-G-A-AC-A-CC---AG--T--A--GC-GAA-G--G-C--G-------G--C-T-C-T---CTG--G--GC-C-A--------------------G-T---A-C-T--GA--CG-C-T-G--A-GG--A-G-CG-A--AA-G-C---G-TG--GG-G--
AG-C-A-AA--------CA--GG Bacteria(100);unclassified(100);unclassified(100);unclassified(100);unclassified(100);unclassified(100);unclassified(100);unclassified(100);unclassified(100);

Otu1561 0 0 0 0 0 0 0 0 0 0 0 0 0 0 0 0 0 0 0 0 0 0 0 0 0 0 0 0
0 0 0 0 0 0 0 0 0 0 0 0 0 0 0 0 0 0 0 0 0 0 0 0 0 0 0 0
0 0 0 0 0 0 0 0 0 0 0 0 0 0 0 0 0 18 0 0 0 0 0 0 0 0 0 0
0 0 0 0 0 0 0 0 0 0 0 0 0 0 0 0 0 0 0 0 0 0 0 0 0 0 0
M02255_77_000000000-ADUT3_1_1108_5627_11754 T----AC---GT-AG-GGT--------GCG-A-G------C--G--T---T--GT-C-CGG-AT------TT-A--T-T--GG-GC-------GT--A-----AA-GG-GA-GC-------G-TA-G-G-C-G------------G--C-CA-T-T-T-----------AA-
---G-T-T-T--------G-G-T--C--TT--A-AA-GA--C-T-GG-G-G-----CT-C-AA------C-C-C-C-A-G-G--A------C-TG-G-A---C--T-------G--AA-T-A---C-------T--G-G--GT--G-G-C----T-A-G-A-G-G--A---T-GT-----CA-G-A--------G-G-A-A---AG-T------GG--A--ATT----C-C-G-A-
GT--GT-A-G-CG-GT--G---A--A-A---TG-C-GT-AG--AT-A-TT--C-G--G-A-G-G-A-AC-A-CC---AG--T--G--GC-GAA-G--G-C--G-------A--C-T-T-T---CTG--G--GA-C-A--------------------T-T---C-C-T--GA--CG-C-T-G--A-GG--C-T-CG-A--AA-G-C---T-CG--GG-G--AG-C-
A-AA--------CA--GG Bacteria(100);unclassified(100);unclassified(100);unclassified(100);unclassified(100);unclassified(100);unclassified(100);unclassified(100);unclassified(100);

Otu1562 0 0 0 0 0 0 0 0 0 0 0 0 0 0 0 0 0 0 0 0 0 0 0 0 0 0 0 0
0 0 0 0 0 0 0 0 0 0 0 0 0 0 0 0 0 0 0 0 0 0 0 0 0 0 0 0
0 0 0 0 0 0 0 0 0 0 0 0 5 0 0 0 0 0 0 0 0 0 0 0 0 0 0 0
0 0 0 0 0 0 0 0 0 0 0 0 0 0 0 0 0 0 0 0 0 0 0 0 0 0 0
M02255_77_000000000-ADUT3_1_1118_25261_8734 C----TC---TG-TG-CCA--------GCA-GCCG-----C--G--G---T--AA-TACAG-AG------GG-T--G-C--AA-GC-------GG--T-----AA-TC-GG-AT-------T-TA-C-G-T-G------------G--T-CT-T-T-T----------
AA----G-T-C-G--------G-A-T--G--TG--A-AA-TC--C-C-TG-A-G-----CT-T-AA------C-T-T-A-G-G-A--A-T----T-G--C-A---T--A-------C--GA-T-A---C-------T--G-G--GA--G-A-C----T-A-G-A-G-T--A---T-GG-----GA-G-A--------G-G-A-T---GG-T------AG--A--ATT----C-C-A-
G-GT--GT-A-G-CG-GT--G---A--A-A---TG-C-GT-AG--AG-A-TC--T-G--G-A-G-G-A-AT-A-CC---GA--T--G--GC-GAA-G--G-C--A-------G--C-C-A-T---CTG--G--CC-T-A--------------------A-T---A-C-T--GA--CA-C-T-G--A-GG--T-A-CG-A--AA-G-C---A-TG--GG-G--
AG-C-A-AA--------CA--GG Bacteria(100);Proteobacteria(100);Gammaproteobacteria(100);Pseudomonadales(100);Moraxellaceae(100);Acinetobacter(100);unclassified(100);unclassified(100);unclassified(100);

Otu1563 0 0 0 0 0 0 0 0 0 0 0 0 0 0 0 0 0 0 0 0 0 0 0 0 0 0 0 0
0 0 0 0 0 0 0 0 0 0 0 0 0 0 0 0 0 0 0 0 0 0 0 0 0 0 0 0
0 0 0 0 0 0 0 0 4 0 0 0 0 0 0 0 0 0 0 0 0 0 0 0 0 0 0 0
0 0 0 0 0 0 0 0 0 0 0 0 0 0 0 0 0 0 0 0 0 0 0 0 0 0 0
M02255_77_000000000-ADUT3_1_1110_26081_6594 T----AC---GA-AG-GGA--------GCT-A-G------C--G--T---T--GT-T-CGG-AA------TC-A--C-T--GG-GC-------GT--A-----AA-GC-GC-AC-------G-TA-G-G-C-G------------G--A-TA-T-G-T-----------CA-
---G-T-C-A--------G-G-G--G--TG--A-AA-TC--C-C-GG-A-G-----CT-C-AA------C-T-T-C-G-G-A--A-C----T-G--C-C---T--T-------T--GA-T-A---C-------A--G-C--AT--A-T-C----T-C-G-A-G-T--C---C-GA-----GA-G-A--------G-G-T-G---AG-T------GG--A--ATT----C-C-T-A-
GT--GT-A-G-AG-GT--G---A--A-A---TT-C-GT-AG--AT-A-TT--A-G--G-A-A-G-A-AC-A-CC---GG--T--G--GC-GAA-G--G-C--G-------G--C-T-C-A---CTG--G--CT-C-G--------------------G-T---A-C-T--GA--CG-C-T-G--A-GG--T-G-CG-A--AA-G-C---G-TG--GG-G--AG-
C-A-AA--------CA--GG Bacteria(100);Proteobacteria(100);Alphaproteobacteria(100);Rhizobiales(100);Hyphomicrobiaceae(100);Pedomicrobium(100);unclassified(100);unclassified(100);unclassified(100);

Otu1564 0 0 0 0 0 0 0 0 0 0 0 0 0 0 0 0 0 0 0 0 0 0 0 0 0 0 0 0
0 0 0 0 0 0 0 0 0 0 0 0 0 0 0 0 0 0 0 0 0 0 0 0 0 0 0 0
0 0 0 0 0 0 0 0 1 0 0 0 0 0 0 0 0 0 0 0 0 0 2 0 0 0 0 0
0 0 0 0 0 0 9 0 0 0 0 0 0 0 0 0 0 6 0 0 0 0 0 0 0 0 0
M02255_76_000000000-ADUNE_1_1107_10556_23220 .....AC----T-AC-GT---------GCC-A-G------C--A--G---C--CG-C-GGT-AA------TA-C--G-T--AG-GT-------GG--C-----AA-GC-GT-TG-------T-CC-G-G-A-T-------------G--A-C----T-T-----------
AA----G-T-C-T--------G-A-T--G--TG--A-AA-GC--C-C-AC-G-G-----CT-T-AA------C-C-G-T-G-G-A--G------G-GT-C-A---T--T-------G--GA-A-A---C-------T--G-G--GT--C-A-C----T-T-G-A-G-T--G---C-AG-----AA-G-A--------G-G-A-A---AG-C------GG--A--ATT----C-C-
A-T-GT--GT-A-G-CG-GT--G---A--A-A---TG-C-GT-AG--AT-A-TA--T-G--G-A-G-G-A-AC-A-CC---AG--T--G--GC-GAA-G--G-C--G-------G--C-T-T-T---CTG--G--TC-T-G--------------------T-A---A-C-T--GA--CG-C-T-G--A-GG--C-T-CG-A--AA-G-C---G-TG--GG-G--
AG-C-A-AA--------CA--GG Bacteria(100);Firmicutes(100);Bacilli(100);unclassified(100);unclassified(100);unclassified(100);unclassified(100);unclassified(100);unclassified(100);

Otu1565 0 0 0 0 0 0 0 0 0 0 0 0 0 0 0 0 0 0 0 0 0 0 0 0 0 0 0 0
0 0 0 0 0 0 0 0 0 0 0 0 0 0 0 0 0 0 0 0 0 0 0 0 0 0 0 0 0
0 0 0 0 0 0 0 0 0 6 0 0 0 0 0 0 0 0 0 0 0 0 0 0 0 0 0 0 0
0 0 0 0 0 0 0 0 0 0 0 0 0 0 0 0 0 0 0 0 0 0 0 0 0 0 0
M02255_77_000000000-ADUT3_1_1112_15075_18667 T----AA---TG-AG-GGT--------GCA-A-G------C--G--T---T--AA-T-CGG-AA------TT-A--C-T--GC-GC-------GT--A-----AA-GC-GA-AC-------G-CA-G-G-C-G------------G--T-TT-G-T-T-----------AA----G-T-C-A--------G-A-T--G--TG--A-AA-TC--C-C-CG-C-G-----CT-C-AA------C-G-T-G-G-G-A--A-C----G-G--C-A---T--T-------T--GA-G-A---C-------T--G-G--CA--A-G-C----T-A-G-A-G-T--C---T-TG-----TA-G-A--------G-G-G-G---TG-T------AG--A--ATT----C-C-A-G-GT--GT-A-G-CG-GT--G---A--A-A---TG-C-GT-AG--AG-A-TC--T-G--G-A-G-G-A-AT-A-CC---GG--T--G--GC-GAA-G--G-C--G-------G--C-C-C-C---CTG--G--AC-A-A--------------------A-G---A-C-T--GA--CG-C-T-C--A-GA--T-G-CG-A--AA-G-C---G-TG--GG-G--AG-C-A-AA--------CA--GG Bacteria(100);Proteobacteria(100);Gammaproteobacteria(100);Enterobacteriales(100);Enterobacteriaceae(100);unclassified(100);unclassified(100);unclassified(100);unclassified(100);

Otu1566 0 0 0 0 0 0 0 0 0 0 0 0 0 0 0 0 0 0 0 0 0 0 0 0 0 0 0 0
0 0 0 0 0 0 0 0 0 0 0 0 0 0 0 0 0 0 0 0 0 0 0 0 0 0 0 0 0
0 0 0 0 0 0 0 0 0 4 0 0 0 0 0 0 0 0 0 0 0 0 0 0 0 0 0 0 0
0 0 0 0 0 0 0 0 0 0 0 0 0 0 0 0 0 0 0 0 0 0 0 0 0 0 0
M02255_77_000000000-ADUT3_1_2107_14368_11244 T----AC---AG-AG-GAG--------CCA-A-G------C--G--T---T--GT-C-CGG-AT------TG-A--C-T--GG-GC-------GT--A-----AA-GC-GC-AC-------G-CA-G-G-C-G------------G--A-TG-C-G-C-----------GC----G-T-G-G--------G-G-T--G--TG--A-AA-TC--T-G-GC-C-G-----CT-T-AA------C-G-G-C-C-A-G--G-C----G-C--C-A---T--C-------C--CA-T-A---C-------G--G-C--GC--G-T-C----T-G-G-A-G-T--G---A-TG-----CA-G-A--------G-G-T-C---GG-T------GG--A--ATT----G-C-C-G-GT--GT-A-G-TG-GT--G---A--A-A---TG-C-GT-AG--AG-A-TC--G-G--C-A-G-G-A-AC-A-CC---CA--G--G--GG-GAA-A--C-C--A-------G--C-C-G-A---CTG--G--GC-A-T--------------------CTA-----C-T--GA--CG-C-T-G--A-GG--T-G-CG-A--GA-G-C---G-TG--GG-G--AG-C-G-AA--------CC--GG Bacteria(100);Chloroflexi(100);Chloroflexi(100);Chloroflexales(100);Chloroflexaceae(100);Roseiflexus(100);unclassified(100);unclassified(100);unclassified(100);

Otu1567 0 0 0 0 0 0 0 0 0 0 0 0 0 0 0 0 0 0 0 0 0 0 0 0 0 0 0 0
0 0 0 0 0 0 0 0 0 0 0 0 0 0 0 0 0 0 0 0 0 0 0 0 0 0 0 0 0
0 0 0 0 0 0 0 0 0 4 0 0 0 0 0 0 0 0 0 0 0 0 0 0 0 0 0 0 0
0 0 0 0 0 0 0 0 0 0 0 0 0 0 0 0 0 0 0 0 0 0 0 0 0 0 0
M02255_77_000000000-ADUT3_1_2117_9142_13367 T----AC---GA-AG-GGG--------GCT-A-G------C--G--T---T--GT-T-CGG-AA------TT-A--C-T--GG-GC-------GT--A-----AA-GC-GC-AC-------G-TA-G-G-C-G------------G--A-CT-A-T-T-----------AA----G-T-C-A--------G-G-G--G--TG--A-AA-TC--C-C-AG-G-G-----CT-C-AA------C-C-C-T-G-G-A--A-C----T-G--C-C---T--T-------T--GA-T-A---C-------T--G-G--TA--G-T-C----T-T-G-A-G-T--A---C-GA-----GA-G-A--------G-G-T-G---AG-T------GG--A--ATT----C-C-G-A-GT--GT-A-G-AG-GT--G---A--A-A---TT-C-GT-AG--AT-A-TT--C-G--G-A-A-G-A-AC-A-CC---AG--T--G--GC-GAA-G--G-C--G-------A--C-T-C-A---CTG--G--CT-C-G--------------------T-A---A-C-T--GA--CG-C-T-G--A-GG--T-G-CG-A--AA-G-C---G-TG--GG-G--AG-C-A-AA--------CA--GG Bacteria(100);Proteobacteria(100);Alphaproteobacteria(100);unclassified(100);unclassified(100);unclassified(100);unclassified(100);unclassified(100);unclassified(100);

Otu1568 0 0 0 0 0 0 0 0 0 0 0 0 0 0 0 0 0 0 0 0 0 0 0 0 0 0 0 0
0 0 0 0 0 0 0 0 0 0 0 0 0 0 0 0 0 0 0 0 0 0 0 0 0 0 0 0 0
0 0 0 0 0 0 0 0 0 0 6 0 0 0 0 0 0 0 0 0 0 0 0 0 0 0 0 0 0
0 0 0 0 0 0 0 0 0 0 0 0 0 0 0 0 0 0 0 0 0 0 0 0 0 0 0
M02255_77_000000000-ADUT3_1_1102_26422_10091 T----AC---GA-AG-GGG--------GCT-A-G------C--G--T---T--GC-T-CGG-AA------TG-A--C-T--GG-GC-------GT--A-----AA-GG-GC-GC-------G-TA-G-G-C-G------------G--A-TG-G-T-A-----------CA----G-T-C-A--------G-A-T--G--TG--A-AA-TT--C-C-TG-G-G-----CT-C-AA------C-C-T-G-G-G-G--G-C----T-G--C-A---T--T-------T--GA-T-A---C-------G--T-G--CT--G-T-C----T-A-G-A-G-T--G---T-GG-----AA-G-A--------G-G-G-T---TG-T------GG--A--ATT----C-C-C-A-GT--GT-A-G-AG-GT--G---A--A-A---TT-C-GT-AG--AT-A-TT--G-G--G-A-A-G-A-AC-A-CC---GG--T--G--GC-GAA-G--G-C--G-------G--C-A-A-C---CTG--G--TC-C-A--------------------T-T---A-C-T--GA--CG-C-T-G--A-GG--C-G-CG-A--AA-G-C---G-TG--GG-G--AG-C-A-AA--------CA--GG Bacteria(100);Proteobacteria(100);Alphaproteobacteria(100);Rhodospirillales(100);Acetobacteraceae(100);unclassified(100);unclassified(100);unclassified(100);unclassified(100);

Otu1569 0 0 0 0 0 0 0 0 0 0 0 0 0 0 0 0 0 0 0 0 0 0 0 0 0 0 0 0
0 0 0 0 0 0 0 0 0 0 0 0 0 0 0 0 0 0 0 0 0 0 0 0 0 0 0 0 0
0 0 0 0 0 0 0 0 0 0 6 0 0 0 0 0 0 0 0 0 0 0 0 0 0 0 0 0 0
0 0 0 0 0 0 0 0 0 0 0 0 0 0 0 0 0 0 0 0 0 0 0 0 0 0 0
M02255_77_000000000-ADUT3_1_2107_6977_20335 T----AC---GT-AG-GAC--------CCA-A-G------C--G--T---T--AT-C-CGG-AG------TG-A--C-T--GG-GC-------GT--A-----AA-GA-GTTGC-------G-TA-G-G-C-G------------G--C-TT-A-G-T-----------AA----G-T-G-G--------G-T-A--G--TG--A-AA-CC--G-G-GT-G-G-----CT-C-AA------C-C-A-T-TCA-G--A-C----T-A--T-T---A--C-------C--CA-A-A---C-------T--G-C--TA--G-G-C----T-C-G-A-G-A--A---T-GG-----TA-G-A--------G-G-T-G---GC-T------GG--A--ATT----T-C-C-T-GT--GT-A-G-GA-GT--G---A--A-A---TC-C-GT-AG--AT-A-TA--G-G--A-A-G-G-A-AC-A-CC---GA--T--G--GC-GTA-G--G-C--A-------G--G-C-C-A---CTG--G--AC-C-A--------------------T-T---T-C-T--GA--CG-C-T-A--A-GG--C-A-CG-A--AA-G-C---G-TG--GG-G--AG-C-G-AA--------CC--GG Bacteria(100);Candidate_division_TM7(100);unclassified(100);unclassified(100);unclassified(100);unclassified(100);unclassified(100);unclassified(100);unclassified(100);

Otu1570 0 0 0 0 0 0 0 0 0 0 0 0 0 0 0 0 0 0 0 0 0 0 0 0 0 0 0 0
0 0 0 0 0 0 0 0 0 0 0 0 0 0 0 0 0 0 0 0 0 0 0 0 0 0 0 0
0 0 0 0 0 0 0 0 0 0 0 4 0 0 4 1 0 0 0 0 0 0 0 0 0 0 0 0
0 0 0 0 65 0 0 0 0 23 0 0 0 0 0 0 0 0 0 0 0 0 0 0 0 0 0
M02255_77_000000000-ADUT3_1_2119_20448_10437 T----AC---GT-AG-GTG--------GCA-A-G------C--G--T---T--AT-C-CGG-AA------TT-A--C-T--GG-GT-------GT--A-----AA-GG-GC-GC-------G-TA-G-G-C-G------------G--G-CG-T-G-C----
-------AA----G-T-T-A--------G-A-T--G--TG--A-AA-AC--C-T-GG-G-G-----CT-C-AA------C-T-C-C-A-G-A--C-T----T-G--C-A---T--C-------T--AA-A-A---C-------T--G-C--AC--A-T-C----T-T-G-A-G-T--G---T-CA-----GA-G-G--------G-G-A-A---AG-T------GG--A--ATT----C-
C-T-A-GT--GG-A-G-CG-GT--A---A--A-A---TG-C-GT-AG--AG-A-TT--A-G--G-A-G-G-A-AC-A-TC---GG--T--G--GC-GAA-G--G-C--G-------A--C-T-T-T---CTG--G--CT-G-A--------------------C-A---A-C-T--GA--CG-C-T-G--A-GG--C-G-CG-A--AA-G-C---G-TG--GG-
T--AG-C-G-AA--------CA--GG Bacteria(100);unclassified(100);unclassified(100);unclassified(100);unclassified(100);unclassified(100);unclassified(100);unclassified(100);unclassified(100);

Otu1571 0 0 0 0 0 0 0 0 0 0 0 0 0 0 0 0 0 0 0 0 0 0 0 0 0 0 0 0
0 0 0 0 0 0 0 0 0 0 0 0 0 0 0 0 0 0 0 0 0 0 0 0 0 0 0 0
0 0 0 0 0 0 0 0 5 0 0 0 0 0 0 0 0 0 0 90 0 0 0 0 0 0 0 0
0 0 0 0 0 0 0 0 0 0 0 0 0 0 0 0 0 0 0 0 0 0 0 0 0 0 0
M02255_77_000000000-ADUT3_1_2117_20635_12350 T----AC---GA-AG-GGG--------GCT-A-G------C--G--T---T--GT-T-CGG-AA------TT-A--C-T--GG-GC-------GT--A-----AA-GC-GC-AC-------G-TA-G-G-C-G------------G--A-TT-G-T-T-----
------AA----G-T-G-A--------G-G-G--G--TG--A-AA-TC--C-C-GG-G-G-----CT-C-AA------C-C-C-C-G-G-A--A-C----T-G--C-C---T--C-------T--CA-T-A---C-------T--G-G--CA--A-T-C----T-C-G-A-G-T--T---T-GG-----GA-G-A--------G-G-T-G---AG-T------GG--A--ATT----C-
C-G-A-GT--GT-A-G-AG-GT--G---A--A-A---TT-C-GT-AG--AT-A-TT--C-G--G-A-G-G-A-AC-A-CC---AG--T--G--GC-GAA-G--G-C--G-------G--C-T-C-A---CTG--G--CC-C-A--------------------A-A---A-C-T--GA--CG-C-T-G--A-GG--T-G-CG-A--AA-G-C---G-TG--GG-
G--AG-C-A-AA--------CA--GG Bacteria(100);Proteobacteria(100);Alphaproteobacteria(100);Rhizobiales(100);unclassified(100);unclassified(100);unclassified(100);unclassified(100);unclassified(100);

Otu1572 0 0 0 0 0 0 0 0 0 0 0 0 0 0 0 0 0 0 0 0 0 0 0 0 0 0 0 0
0 0 0 0 0 0 0 0 0 0 0 0 0 0 0 0 0 0 0 0 0 0 0 0 0 0 0 0
0 0 0 0 0 0 0 0 0 0 0 0 4 0 0 0 0 0 0 0 0 0 0 0 0 0 0 0
0 0 0 0 0 0 0 0 0 0 0 0 0 0 0 0 0 0 0 0 0 0 0 0 0 0 0
M02255_77_000000000-ADUT3_1_1113_14241_13422 T----AC---GT-AG-GGG--------GCG-A-G------C--G--T---T--GT-C-CGG-AT------TT-A--T-T--GG-GC-------GT--A-----AA-GC-GC-CC-------G-CA-G-G-C-G------------G--T-CG-C-G-T-----
------CA----G-T-T-T--------G-G-G--G--TG--A-CA-GC--T-C-CT-G-G-----CT-T-AA------C-C-G-G-G-A-G--A-G----G-A--C-C---T--C-------G--AA-G-A---C-------G--G-C--GT--G-A-C----T-T-G-A-G-G--G---C-CA-----GA-G-A--------G-G-G-A---CA-T------GG--A--ACT----
C-C-G-G-GT--GG-A-G-TG-GT--G---A--A-A---TG-C-GT-AG--AG-A-TC--C-G--G-A-A-G-A-AC-A-CC---AA--A--G--GC-GAA-G--G-C--A-------G--T-G-T-C---CTG--G--CT-G-G--------------------C-A---C-C-T--GA--CG-C-T-G--A-GG--G-G-CG-A--AA-G-C---G-AG--
GG-G--AG-C-G-AA--------CG--GG Bacteria(100);unclassified(100);unclassified(100);unclassified(100);unclassified(100);unclassified(100);unclassified(100);unclassified(100);unclassified(100);

Otu1573 0 0 0 0 0 0 0 0 0 0 0 0 0 0 0 0 0 0 0 0 0 0 0 0 0 0 0 0
0 0 0 0 0 0 0 0 0 0 0 0 0 0 0 0 0 0 0 0 0 0 0 0 0 0 0 0
0 0 0 0 0 0 0 0 0 0 0 0 4 0 0 0 0 0 0 0 0 0 0 0 0 0 0 0
0 0 0 0 0 0 0 0 0 0 0 0 0 0 0 0 0 0 0 0 0 0 0 0 0 0 0
M02255_77_000000000-ADUT3_1_1113_13472_22947 T----AC---GA-AT-GGA--------GCG-A-G------C--G--T---T--GT-T-CGG-AA------TC-A--C-T--GG-GC-------GT--A-----AA-GG-GT-GC-------G-CA-G-G-C-G------------G--T-CA-T-G-A----
-------AA----G-T-T-A--------G-A-A--G--TG--A-AA-TC--C-C-TA-G-G-----CT-C-AA------C-C-T-A-G-G-A--A-T----T-G--C-T---T--T-------T--AA-A-A---C-------T--C-C--AT--G-A-C----T-A-G-A-G-T--A---C-TG-----GA-G-A--------G-G-T-T---GG-C------GG--A--ATT----C-
C-A-A-GT--GT-A-G-CA-GT--G---A--A-A---TG-C-GT-AG--AT-A-TT--T-G--G-A-G-G-A-AC-A-CC---AA--T--G--GC-GTA-G--G-C--A-------G--C-C-A-A---CTG--G--AC-A-G--------------------T-T---A-C-T--GA--CG-C-T-C--A-TG--C-A-CG-A--AA-G-C---G-TG--GG-G--
AT-C-A-AA--------CA--GG Bacteria(100);unclassified(100);unclassified(100);unclassified(100);unclassified(100);unclassified(100);unclassified(100);unclassified(100);unclassified(100);

Otu1574 0 0 0 0 0 0 0 0 0 0 0 0 0 0 0 0 0 0 0 0 0 0 0 0 0 0 0 0
0 0 0 0 0 0 0 0 0 0 0 0 0 0 0 0 0 0 0 0 0 0 0 0 0 0 0 0
0 0 0 0 0 0 0 0 0 0 0 0 6 0 0 0 0 0 0 0 0 0 0 0 0 0 0 0
0 0 0 0 0 0 0 0 0 0 0 0 0 0 0 0 0 0 0 0 0 0 0 0 0 0 0
M02255_77_000000000-ADUT3_1_2102_17983_9694 T----AC---GT-AG-GTG--------GCA-A-G------C--G--T---T--GT-C-CGG-AT------TT-A--C-T--GG-GT-------GT--A-----AA-GG-GT-GC-------G-CA-G-G-C-G------------G--G-CC-T-C-T-----------AA-
---G-T-A-A--------G-A-A--G--TG--A-AA-GG--T-T-TG-A-G-----CT-C-AA------C-T-C-G-G-A-C--A-C----T-G--C-T---T--C-------T--TA-T-A---C-------T--G-G--GG--G-C-C----T-T-G-A-G-G--C---T-CG-----GA-G-G--------G-G-A-A---AG-C------GG--A--ACG----A-C-A-C-
GT--GT-A-G-CG-GT--G---A--A-A---TG-C-GT-TG--AT-A-TG--T-G--T-C-G-G-A-AC-A-CC---AA--T--G--GC-GAA-A--G-C--A-------G--C-T-T-T---CTG--G--AC-G-A--------------------G-T---A-C-T--GA--CG-C-T-C--A-GG--C-A-CG-A--AA-G-C---C-AA--GG-T--AG-C-
A-AA--------CA--GG Bacteria(100);unclassified(100);unclassified(100);unclassified(100);unclassified(100);unclassified(100);unclassified(100);unclassified(100);unclassified(100);

Otu1575 0 0 0 0 0 0 0 0 0 0 0 0 0 0 0 0 0 0 0 0 0 0 0 0 0 0 0 0
0 0 0 0 0 0 0 0 0 0 0 0 0 0 0 0 0 0 0 0 0 0 0 0 0 0 0 0 0
0 0 0 0 0 0 0 0 0 0 0 0 6 0 0 0 0 0 0 0 0 0 0 0 0 0 0 0 0
0 0 0 0 0 0 0 0 0 0 0 0 0 0 0 0 0 0 0 0 0 0 0 0 0 0 0
M02255_77_000000000-ADUT3_1_1101_9189_19327 T----AC---GG-AG-GAT--------GCA-A-G------C--G--T---T--AT-C-CGG-AA------TG-A--T-T--GG-GC-------GT--A-----AA-GG-GT-TC-------G-CA-G-G-T-G------------G--T-AG-T-G-A----------
AA----G-T-T-T--------G-C-T--G--TT--A-AA-AG--T-C-GG-G-G-----CT-C-AA------C-C-C-C-G-A-A--C-C----G-G--C-G---G--T-------G--AA-A-A---C-------T--A-C--AC--A-A-C----T-A-G-A-G-T--G---C-GG-----TA-G-G--------G-G-C-A---GA-G------GG--A--ATT----C-C-T-
G-GT--GT-A-G-CG-GT--G---A--A-A---TG-C-GT-AG--AG-C-TC--A-G--G-A-A-G-A-AC-A-CC---GG--T--G--GC-GAA-A--G-C--G-------C--T-C-T-G---CTA--G--AC-C-T--------------------G-C---A-C-T--GA--CA-C-T-G--A-GG--G-A-CG-A--AA-G-C---T-AG--GG-G--
AG-C-G-AA--------TG--GG Bacteria(100);Cyanobacteria(100);unclassified(100);unclassified(100);unclassified(100);unclassified(100);unclassified(100);unclassified(100);unclassified(100);

Otu1576 0 0 0 0 0 0 0 0 0 0 0 0 0 0 0 0 0 0 0 0 0 0 0 0 0 0 0 0
0 0 0 0 0 0 0 0 0 0 0 0 0 0 0 0 0 0 0 0 0 0 0 0 0 0 0 0 0
0 0 0 0 0 0 0 0 0 0 0 0 0 6 0 0 0 0 0 0 0 0 0 0 0 0 0 0 0
0 0 0 0 0 0 0 0 0 0 0 0 0 0 0 0 0 0 0 0 0 0 0 0 0 0 0
M02255_77_000000000-ADUT3_1_2103_19181_23516 T----AC---GA-AG-GGG--------GCT-A-G------C--G--T---T--GT-T-CGG-AA------TT-A--C-T--GG-GC-------GT--A-----AA-GC-GC-AC-------G-TA-G-G-C-G------------G--A-TT-G-A-T-----
------CA----G-T-C-A--------G-G-G--G--TG--A-AA-TC--C-C-AG-A-G-----CT-C-AA------C-T-C-T-G-G-A--A-C----T-G--C-C---T--T-------T--GA-T-A---C-------T--G-T--CA--A-T-C----T-A-G-A-G-T--C---C-AG-----AA-G-A--------G-G-T-G---AG-T------GG--A--ATT----C-
C-G-A-GT--GT-A-G-AG-GT--G---A--A-A---TT-C-GT-AG--AT-A-TT--C-G--G-A-G-G-A-AC-A-CC---AG--T--G--GC-GAA-G--G-C--G-------G--C-T-C-A---CTG--G--TC-T-G--------------------G-T---A-C-T--GA--CG-C-T-G--A-GG--T-G-CG-A--AA-G-C---G-TG--GG-
G--AG-C-A-AA--------CA--GG Bacteria(100);Proteobacteria(100);Alphaproteobacteria(100);Rhizobiales(100);Rhizobiaceae(100);unclassified(100);unclassified(100);unclassified(100);unclassified(100);

Otu1577 0 0 0 0 0 0 0 0 0 0 0 0 0 0 0 0 0 0 0 0 0 0 0 0 0 0 0 0
0 0 0 0 0 0 0 0 0 0 0 0 0 0 0 0 0 0 0 0 0 0 0 0 0 0 0 0 0
0 0 0 0 0 0 0 0 0 0 0 0 0 6 0 0 0 0 0 0 0 0 0 0 0 0 0 0 0
0 0 0 0 0 0 0 0 0 0 0 0 0 0 0 0 0 0 0 0 0 0 0 0 0 0 0
M02255_77_000000000-ADUT3_1_1104_26982_16185 T----AC---GT-AG-GAC--------CCA-A-G------C--G--T---T--AT-C-CGG-AG------TG-A--C-T--GG-GC-------GT--A-----AA-GA-GTTGC-------G-TA-G-G-C-G------------G--T-GA-T-T-T-----
------AA----G-C-A-G--------A-T-A--G--TG--A-AA-GC--A-T-GT-G-G-----CT-C-AA------C-C-A-T-TAT-G--T-C----T-A--T-T---A--T-------C--TG-A-A---C-------T--G-A--AT--C-A-C----T-T-G-A-G-A--A---T-GG-----TA-G-A--------G-G-T-A---GC-T------GG--A--ATT----T-
C-T-A-GT--GT-A-G-GA-GT--G---A--A-A---TC-C-GT-AG--AT-A-TT--A-G--A-A-G-G-A-AC-A-CC---AA--T--G--GC-GTA-G--G-C--A-------G--G-C-T-A---CTG--G--AC-C-A--------------------T-T---T-C-T--GA--CG-C-T-G--A-GG--C-A-CG-A--AA-G-C---G-TG--GG-G--
AG-C-G-AA--------CC--GG Bacteria(100);unclassified(100);unclassified(100);unclassified(100);unclassified(100);unclassified(100);unclassified(100);unclassified(100);unclassified(100);

Otu1578 0 0 0 0 0 0 0 0 0 0 0 0 0 0 0 0 0 0 0 0 0 0 0 0 0 0 0 0
0 0 0 0 0 0 0 0 0 0 0 0 0 0 0 0 0 0 0 0 0 0 0 0 0 0 0 0 0
0 0 0 0 0 0 0 0 0 0 0 0 0 2 0 0 0 13 0 0 0 0 0 0 0 0 0 0 0
0 0 0 0 0 0 0 0 0 0 0 0 0 0 0 0 0 0 0 0 0 0 0 0 0 0 0
M02255_77_000000000-ADUT3_1_1117_13063_16755 T----AC---GT-AG-GGG--------CCG-A-G------C--G--T---T--GT-C-CGG-AG------TT-A--C-T--GG-GC-------GT--A-----AA-GC-GT-GC-------G-CA-G-G-C-G------------G--C-GC-G-T-T----
-------CT----G-C-C-C--------G-G-C--G--TG--A-AA-GC--C-C-CC-G-G-----CT-C-AA------C-C-G-G-G-G-A--G-G----G-T--C-G---T--C-------G--GG-G-A---C-------G--G-G--CG--C-G-C----T-T-G-A-G-G--G---T-AT-----CA-G-G--------G-G-C-T---GG-T------GG--A--ATT---
---CCC-G-GT--GT-A-G-TG-GT--G---A--A-A---TG-C-GT-AG--AG-A-TC--G-G--G-A-G-G-A-AC-A-CC---TG--T--G--GC-GAA-G--G-C--G-------G--C-C-A-G---CTG--G--GA-T-A--------------------C-A---C-C-T--GA--CG-C-T-G--A-GG--C-A-CG-A--AG-G-C---G-TG--
GG-G--AG-C-G-AA--------CG--GG Bacteria(100);Chloroflexi(100);Thermomicrobia(100);JG30-KF-CM45(100);unclassified(100);unclassified(100);unclassified(100);unclassified(100);unclassified(100);

Otu1579 0 0 0 0 0 0 0 0 0 0 0 0 0 0 0 0 0 0 0 0 0 0 0 0 0 0 0 0
0 0 0 0 0 0 0 0 0 0 0 0 0 0 0 0 0 0 0 0 0 0 0 0 0 0 0 0 0
0 0 0 0 0 1 0 0 0 0 0 0 0 0 1 0 0 0 0 0 0 0 0 0 0 0 0 0 0
0 0 0 0 0 6 0 0 0 0 0 8 0 0 0 0 0 0 0 0 0 0 0 0 0 0 0
M02255_76_000000000-ADUNE_1_2106_25950_15086 C----GT---GC-CA-GCA--------GCC-G-C------G--G--T---A--AT-A-CGT-AG------GG-G--G-C--TA-GC-------GT--T-----AT-CC-G--GA-------T-TT-A-C-T-G------------G--G-CT-G-T-A-------
----AA----G-T-C-G--------G-T-G--C--GT--A-AA-GG--C-T-AC-G-G-----CT-C-AA------C-C-G-T-A-G-T--T------A-G--C-C---T--C-------C--GA-A-A---C-------T--G-G--AA--G-A-C----T-T-G-A-G-T--G---C-AG-----GA-G-A--------G-G-A-A---AG-T------GG--A--ATT----C-
C-C-A-GT--GT-A-G-CG-GT--G---A--A-A---TG-C-GT-AG--AT-A-TT--G-G--G-A-G-G-A-AC-A-CC---AG--T--A--GC-GAA-G--G-C--G-------G--C-T-T-T---CTG--G--AC-T-G--------------------C-A---A-C-T--GA--CA-C-T-G--A-GG--C-A-CG-A--AA-G-C---G-TG--GG-T-
-AG-C-A-AA--------CA--GG Bacteria(100);Firmicutes(100);Clostridia(100);Clostridiales(100);Peptostreptococcaceae(100);Peptostreptococcus(100);unclassified(100);unclassified(100);unclassified(100);

Otu1580 0 0 0 0 0 0 0 0 0 0 0 0 0 0 0 0 0 0 0 0 0 0 0 0 0 0 0 0
0 0 0 0 0 0 0 0 0 0 0 0 0 0 0 0 0 0 0 0 0 0 0 0 0 0 0 0 0
0 0 0 1 0 0 0 0 0 0 0 0 0 0 0 0 0 0 0 0 0 0 0 0 0 0 0 0 0
0 0 0 7 0 0 0 0 0 0 0 0 0 0 0 0 0 0 0 0 0 0 0 0 0 0 0 0
M02255_76_000000000-ADUNE_1_1118_29031_16527 T----AC---AG-AC-GGT--------GCG-A-G------C--G--T---T--AT-T-CAG-CA------TA-A--A-T--GG-GT-------GT--A-----AA-GG-GT-AT-------G-TA--------------------G--T-TT-----T-----------
AA----G-C-T----------A-A-A--T--AG--A-AA-TG--C-T-AG-G-C-----GT-A-AA------T-C-T-T-A-G-C--A-G----A-T--A-T---T--T-------T--AT-T-A---C-------T--T-A--CTA-G-A-C----T-T-G-A-G-T--A---T-AA-----TT-G-G--------T-G-T-A---AA-A------GG--A--ATT----T-C-T-A-
CA-GTT-A-G-TG-GT--T---A--A-A---TA-C-GA-GG--AG-A-GT--A-G--A-A-G-G-A-AC-G-CC---AA--A--G--GT-GAA-G--A-C--A-------T--T-T-T-T---CAA--A--AT---T--------------------A-T---A-C-T--GA--CA-C-T-G--A-GG--T-A-CG-G--AG-G-T---T-TG--GG-G--AG-C-
A-AA--------TC--GG Bacteria(100);unclassified(100);unclassified(100);unclassified(100);unclassified(100);unclassified(100);unclassified(100);unclassified(100);unclassified(100);

Otu1581 0 0 0 0 0 0 0 0 0 0 0 0 0 0 0 0 0 0 0 0 0 0 0 0 0 0 0 0
0 0 0 0 0 0 0 0 0 0 0 0 0 0 0 0 0 0 0 0 0 0 0 0 0 0 0 0 0
0 0 0 1 0 0 0 0 0 0 0 0 0 0 0 24 0 0 0 0 0 0 0 0 0 0 0 0 0
0 0 0 0 0 0 0 0 0 0 0 0 0 0 0 0 0 0 0 0 0 0 0 0 0 0 0 0
M02255_77_000000000-ADUT3_1_1105_22688_11482 T----AC---GT-AG-GGG--------GCG-A-G------C--G--T---T--AT-C-CGG-AT------TC-A--T-T--GG-GC-------GT--A-----AA-GC-GC-GC-------G-CA-G-G-C-G------------G--C-CC-G-C-C-----
------AA----G-C-G-G--------CCT-C--G--TC--G-AA-GC--C-G-GG-G-G-----CT-C-AA------C-C-C-C-C-G-G--A------A-G--C-G---A--C-------C--CG-A-A---C-------T--G-G--CG--G-G-C----T-C-G-A-G-T--G---G-GG-----CA-G-G--------G-G-A-G---GA-T------GG--A--ATT---
-C-C-C-G-GT--GT-A-G-CG-GT--G---A--A-A---TG-C-GC-AG--AT-A-TC--G-G--G-A-G-G-A-AC-A-CC---GA--C--G--GC-GAA-G--G-C--A-------G--C-C-C-T---CTG--G--GC-C-T--------------------T-C---A-C-T--GA--CG-C-T-G--A-GG--C-G-CG-A--AA-G-C---T-GG--
GG-G--AG-C-G-AA--------CA--GG Bacteria(100);Actinobacteria(100);Actinobacteria(100);Coriobacteridae(100);Coriobacteriales(100);Coriobacterineae(100);Coriobacteriaceae(100);Slackia(100);unclassified(100);

Otu1582 0 0 0 0 0 0 0 0 0 0 0 0 0 0 0 0 0 0 0 0 0 0 0 0 0 0 0 0
0 0 0 0 0 0 0 0 0 0 0 0 0 0 0 0 0 0 0 0 0 0 0 0 0 0 0 0 0
0 0 0 1 0 0 0 0 0 13 0 0 0 0 0 0 0 0 0 0 0 0 0 0 0 0 0 0 0
0 0 0 0 0 0 0 0 0 0 0 0 0 0 0 0 0 0 0 0 0 0 0 0 0 0 0 0
M02255_77_000000000-ADUT3_1_1119_4630_20182 T----AC---GG-AG-GGA--------GCT-A-G------C--G--T---T--GT-T-CGG-AA------TT-A--C-T--GG-GC-------GT--A-----AA-GC-GC-AC-------G-TA-G-G-C-G------------G--C-TT-T-G-T-----------AA-
---G-T-C-A--------G-A-G--G--TG--A-AA-GC--C-T-GG-A-G-----CT-C-AA------C-T-C-C-A-G-A--A-C----T-G--C-C---T--T-------T--GA-G-A---C-------T--G-C--AT--C-G-C----T-T-G-A-A-T--C---C-AG-----GA-G-A--------G-G-T-G---AG-T------GG--A--ATT----C-C-G-A-
GT--GT-A-G-AG-GT--G---A--A-A---TT-C-GT-AG--AT-A-TT--C-G--G-A-A-G-A-AC-A-CC---AG--T--G--GC-GAA-G--G-C--G-------A--C-T-A-C---CTG--G--AC-T-G--------------------A-T---A-C-T--GA--CA-C-T-G--A-GG--T-G-CG-A--AA-G-C---G-TG--GG-G--AG-C-
A-AA--------CA--GG Bacteria(100);Proteobacteria(100);Alphaproteobacteria(100);Sphingomonadales(100);unclassified(100);unclassified(100);unclassified(100);unclassified(100);unclassified(100);

Otu1583 0 0 0 0 0 0 0 0 0 0 0 0 0 0 0 0 0 0 0 0 0 0 0 0 0 0 0 0
0 0 0 0 0 0 0 0 0 0 0 0 0 0 0 0 0 0 0 0 0 0 0 0 0 0 0 0 0
0 0 0 0 9 0 0 0 0 0 0 0 0 0 0 0 0 0 0 0 0 0 0 0 0 0 0 0 0
0 0 0 0 0 0 0 0 0 0 0 0 0 0 0 0 0 0 0 0 0 0 0 0 0 0 0 0
M02255_77_000000000-ADUT3_1_1110_25431_5497 T----AC---GA-AG-GGG--------GCT-A-G------C--G--T---T--GC-T-CGG-AA------TT-A--C-T--GG-GC-------GT--A-----AA-GG-GC-GC-------G-TA-G-G-C-G------------G--C-TT-G-G-C-----------
CA----G-T-C-A--------G-G-C--G--TG--A-AA-TT--C-C-TG-G-G-----CT-T-AA------C-C-T-G-G-G-G--A-C----T-G--C-G---C--T-------T--GA-T-A---C-------T--G-C--CA--G-G-C----T-T-G-A-G-G--C---C-GA-----GA-G-A--------G-G-G-T---GG-T------GG--A--ATT----C-C-C-
A-GT--GT-A-G-AG-GT--G---A--A-A---TT-C-GT-AG--AT-A-TT--G-G--G-A-A-G-A-AC-A-CC---GG--T--G--GC-GAA-G--G-C--G-------A--C-C-A-C---CTG--G--CT-C-G---------------------G-T---A-C-T--GA--CG-C-T-G--A-GG--C-G-CG-A--CA-G-C---G-TG--GG-G--
AG-C-A-AA--------CA--GG Bacteria(100);Proteobacteria(100);Alphaproteobacteria(100);Rhodospirillales(100);Acetobacteraceae(100);unclassified(100);unclassified(100);unclassified(100);unclassified(100);

Otu1584 0 0 0 0 0 0 0 0 0 0 0 0 0 0 0 0 0 0 0 0 0 0 0 0 0 0 0 0
0 0 0 0 0 0 0 0 0 0 0 0 0 0 0 0 0 0 0 0 0 0 0 0 0 0 0 0 0
0 0 0 0 10 0 0 0 0 0 0 0 0 0 0 0 0 0 0 0 0 0 0 0 0 0 0 0 0
0 0 0 0 0 0 0 0 0 0 0 0 0 0 0 0 0 0 0 0 0 0 0 0 0 0 0 0
M02255_77_000000000-ADUT3_1_1119_17935_13113 T----AC---GT-AG-GCA--------GCA-A-G------C--G--T---T--GT-T-CGG-AG------TT-A--C-T--GG-GC-------GT--A-----AA-GG-GT-GC-------G-TA-G-G-C-G------------G--C-TG-T-T-T-----
------AA----G-T-T-T--------G-G-T--G--TG--A-AA-TC--T-C-CC-G-G-----CT-C-AA------C-C-G-G-G-A-G--G-G----T-G--C-G---C--C-------G--AA-T-A---C-------T--G-A--GT--G-G-C----T-A-G-A-G-T--G---C-GG-----GA-G-A--------G-G-A-A---AG-T------GG--A--ATT----
C-C-T-G-GT--GT-A-G-CG-GT--G---A--A-A---TG-C-GT-AG--AT-A-TC--A-G--G-A-G-G-A-AC-A-CC---GG--T--G--GT-GTA-A--A-C--G-------G--C-T-T-T---CTG--G--AC-C-G--------------------T-A---A-C-T--GA--CG-C-T-G--A-GG--C-A-CG-A--AA-G-C---G-TG--GG-
T--AG-C-A-AA--------CA--GG Bacteria(100);Acidobacteria(100);Acidobacteria(100);Acidobacteriales(100);Acidobacteriaceae(100);Candidatus_Solibacter(100);unclassified(100);unclassified(100);unclassified(100);

Otu1585 0 0 0 0 0 0 0 0 0 0 0 0 0 0 0 0 0 0 0 0 0 0 0 0 0 0 0 0
0 0 0 0 0 0 0 0 0 0 0 0 0 0 0 0 0 0 0 0 0 0 0 0 0 0 0 0 0
0 0 0 0 5 0 0 0 0 0 0 0 0 0 0 0 0 0 0 0 0 0 0 0 0 0 0 0 0
0 0 0 0 0 0 0 0 0 0 0 0 0 4 0 0 0 0 0 0 0 0 0 0 0 0 0
M02255_77_000000000-ADUT3_1_1119_22950_9139 T----AC---GT-AG-GGT--------CCG-A-G------C--G--T---T--GT-C-CGG-AA------TT-A--T-T--GG-GC-------GT--A-----AA-GG-GC-TC-------G-TA-G-G-C-G------------G--T-TT-G-T-C-----------GC--
--G-T-C-G--------G-G-A--G--TG--A-AA-AC--G-C-AG-G-C-----CT-T-AA------T-T-C-T-G-C-G--C-T----T-G--C-T---T--T-------C--GA-T-A---C-------G--G-G--CA--G-A-C----T-A-G-A-G-G--T---A-TG-----CA-G-G--------G-G-A-G---AA-C------GG--A--ATT----C-C-T-G-
GT--GT-A-G-CG-GT--G---A--A-A---TG-C-GC-AG--AT-A-TC--A-G--G-A-G-G-A-AC-A-CC---GG--T--G--GC-GAA-G--G-C--G-------G--T-T-C-T---CTG--G--GC-A-T--------------------G-T---C-C-T--GA--CG-C-T-G--A-GG--A-G-CG-A--AA-G-T---G-TG--GG-G--AG-
C-G-AA--------CA--GG Bacteria(100);Actinobacteria(100);Actinobacteria(100);Actinobacteridae(100);Actinomycetales(100);Propionibacterineae(100);Nocardioidaceae(100);unclassified(100);unclassified(100);

Otu1586 0 0 0 0 0 0 0 0 0 0 0 0 0 0 0 0 0 0 0 0 0 0 0 0 0 0 0 0
0 0 0 0 0 0 0 0 0 0 0 0 0 0 0 0 0 0 0 0 0 0 0 0 0 0 0 0 0
0 0 0 0 11 0 0 0 0 0 0 0 0 0 0 0 0 0 0 0 0 0 0 0 0 0 0 0 0
0 0 0 0 0 0 0 0 0 0 0 0 0 0 0 0 0 0 0 0 0 0 0 0 0 0 0
M02255_77_000000000-ADUT3_1_2101_24265_14931 T----AC---GA-AG-GGG--------GCT-A-G------C--G--T---T--GC-T-CGG-AA------TT-A--C-T--GG-GC-------GT--A-----AA-GG-GA-GC-------G-TA-G-G-C-G------------G--A-TC-G-T-T----
-------TA----G-T-T-G--------G-G-G--G--TG--A-AA-TC--C-C-GG-G-G-----CT-C-AA------C-C-T-C-G-G-A--A-C----T-G--C-C---T--T-------C--AA-T-A---C-------T--G-G--CG--A-T-C----T-T-G-A-G-T--A---T-GG-----GA-G-A--------G-G-T-G---AG-T------GG--A--ACT----
C-C-G-A-GT--GT-A-G-AG-GT--G---A--A-A---TT-C-GT-AG--AT-A-TT--C-G--G-A-A-G-A-AC-A-CC---AG--T--G--GC-GAA-G--G-C--G-------A--C-T-C-A---CTG--G--CC-C-A--------------------T-T---A-C-T--GA--CG-C-T-G--A-GG--C-T-CG-A--AA-G-C---G-TG--GG-
G--AG-C-A-AA--------CA--GG Bacteria(100);Proteobacteria(100);Alphaproteobacteria(100);Caulobacterales(100);Caulobacteraceae(100);unclassified(100);unclassified(100);unclassified(100);unclassified(100);

Otu1587 0 0 0 0 0 0 0 0 0 0 0 0 0 0 0 0 0 0 0 0 0 0 0 0 0 0 0 0
0 0 0 0 0 0 0 0 0 0 0 0 0 0 0 0 0 0 0 0 0 0 0 0 0 0 0 0 0
0 0 0 0 9 0 0 0 0 0 0 0 0 0 0 0 0 0 0 0 0 0 0 0 0 0 0 0 0
0 0 0 0 0 0 0 0 0 0 0 0 0 0 0 0 0 0 0 0 0 0 0 0 0 0 0
M02255_77_000000000-ADUT3_1_1108_25734_15369 T----AC---GT-AG-GTG--------GCA-A-G------C--G--T---T--GT-C-CGG-AT------TT-A--C-T--GG-GT-------GT--A-----AA-GG-GC-GT-------G-TA-G-G-C-G------------G--G-TT-A-A-C-----
------AA----G-T-C-A--------G-A-T--G--TG--A-AA-TTC-C---GG-G-G-----CT-C-AA------C-C-C-C-G-G-C--G-C----T-G--C-A---T--C-------T--GA-A-A---C-------T--G-T--TA--A-T-C----T-T-G-A-G-T--G---C-TG-----GA-G-A--------G-G-A-A---AG-C------GG--A--ATT----C-
C-T-A-GT--GT-A-G-CG-GT--G---A--A-A---TG-C-GT-AG--AT-A-TT--A-G--G-A-G-G-A-AC-A-CC---AG--T--G--GC-GAA-G--G-C--G-------G--C-T-T-T---CTG--G--AC-A-G--------------------T-A---A-C-T--GA--CG-C-T-G--A-GG--C-G-CG-A--AA-G-C---G-TG--GG-
G--AG-C-A-AA--------CA--GG Bacteria(100);Firmicutes(100);Clostridia(100);Clostridiales(100);Ruminococcaceae(100);Acetivibrio(100);unclassified(100);unclassified(100);unclassified(100);

Otu1588 0 0 0 0 0 0 0 0 0 0 0 0 0 0 0 0 0 0 0 0 0 0 0 0 0 0 0 0
0 0 0 0 0 0 0 0 0 0 0 0 0 0 0 0 0 0 0 0 0 0 0 0 0 0 0 0 0
0 0 0 0 0 0 0 0 0 0 0 0 0 0 6 8 0 0 0 0 0 0 0 0 0 0 0 0 0
0 0 0 0 0 0 0 0 0 0 0 0 0 0 0 0 0 0 0 0 0 0 0 0 0 0 0
M02255_76_000000000-ADUNE_1_1102_15025_7955 G----AG---GA-AG-CCA--------CGG-C-T------A--A--C---T--AC-G-TGC-CA------GC-A--G-C--CG-CG-------GT--A-----AT-AC-GT-AG-------G-TG-G-C-G-A------------G--C-GT-T-G-T-----------CC-
---G-G-A-T--------T-T-C--T--TA--A-AA-GT--T-C-GG-G-G-----CT-C-AA------C-C-C-C-G-T-G--A------T-G--G-G---A--A-------A--GA-A-A---C-------T--A-C--AT--G-T-C----T-T-G-A-G-T--A---C-AG-----GA-G-A--------G-G-A-A---AG-C------GG--A--ATT----C-C-C-A-
GT--GT-A-G-CG-GT--G---A--A-A---TG-C-GT-AG--AT-A-TT--G-G--G-A-G-G-A-AC-A-CC---AG--T--G--GC-GAA-G--G-C--G-------G--C-T-T-T---CTG--G--AC-T-G--------------------C-A---A-C-T--GA--CG-C-T-G--A-GG--C-T-CG-A--AA-G-C---C-AG--GG-G--AG-
C-G-AA--------CG--GG Bacteria(100);Firmicutes(100);Clostridia(100);Clostridiales(100);Veillonellaceae(100);unclassified(100);unclassified(100);unclassified(100);unclassified(100);

Otu1589 0 0 0 0 0 0 0 0 0 0 0 0 0 0 0 0 0 0 0 0 0 0 0 0 0 0 0 0
0 0 0 0 0 0 0 0 0 0 0 0 0 0 0 0 0 0 0 0 0 0 0 0 0 0 0 0 0
0 0 0 0 0 5 0 0 0 0 0 0 0 0 0 0 0 0 0 0 0 0 0 0 0 0 0 0 0
0 0 0 0 0 0 0 0 0 0 0 0 0 0 0 0 0 0 0 0 0 0 0 0 0 0 0
M02255_77_000000000-ADUT3_1_2104_3357_16010 T----AA---CT-AC-GTG--------CC--A-G------C--A--G---C--CG-C-GGT-AA------TA-C--G-T--AG-GT-------CC--C-----GA-GC-GT-TG-------T-CC-G-G-T-G------------G--T-TT-A-A-T-----------AA---
-G-T-C-T--------G-A-T--G--TA--A-AA-GG--C-A-GT-G-G-----CT-C-AA------C-C-A-T-T-G-T--G------T-G--C-A---T--T-------G--GA-A-A---C-------T--G-T--TA--G-A-C----T-G-G-A-G-T--G---C-AG-----TA-G-A--------G-G-A-G---AG-T------GG--A--ATT----C-C-A-T-GT-
-GT-A-G-CG-GT--G---A--A-A---TG-C-GT-AG--AT-A-TA--T-G--G-A-G-G-A-AC-A-CC---GG--T--G--GC-GAA-A--G-C--G-------G--C-T-C-T---CTG--G--AC-T-G--------------------T-A---A-C-T--GA--CA-C-T-G--A-GG--C-T-CG-A--AA-G-C---G-TG--GG-T--AG-C-A-
AA--------CA--GG Bacteria(100);Firmicutes(100);Bacilli(100);Lactobacillales(100);Streptococcaceae(100);Lactococcus(100);unclassified(100);unclassified(100);unclassified(100);

Otu1590 0 0 0 0 0 0 0 0 0 0 0 0 0 0 0 0 0 0 0 0 0 0 0 0 0 0 0 0
0 0 0 0 0 0 0 0 0 0 0 0 0 0 0 0 0 0 0 0 0 0 0 0 0 0 0 0
0 0 0 0 0 0 5 0 0 0 0 0 0 0 0 0 0 0 0 0 0 0 0 0 0 0 0 0
0 0 0 0 0 0 0 0 0 0 0 0 0 0 0 0 0 0 0 0 0 0 0 0 0 0 0

M02255_77_000000000-ADUT3_1_1112_13731_8674 T----AC---GG-AA-GAT--------CCA-A-G------C--G--T---T--AT-C-CGG-AA------TT-A--C-T--GG-GC-------GT--A-----AA-GA-GTTGC-------G-TA-G-G-C-G------------G--C-AT-G-A-T----------AA----G-T-C-A--------G-C-A--G--TG--A-AA-GC--G-T-GG-G-G-----CT-C-AA------C-C-C-C-A-T-A--T-A----C-A--T-T---G--T-------T--GA-A-A---C-------T--A-T--TA--A-G-C----T-A-G-A-G-G--A---T-GA-----GA-G-A--------G-G-T-T---AT-T------GG--A--ATT----C-C-C-A-GT--GT-A-G-GA-GT--G---A--A-A---TC-C-GT-AG--AT-A-TT--G-G--G-A-G-G-A-AC-A-CC---GA--T--G--GC-GTA-G--G-C--A-------G--A-T-A-A---CTG--G--CT-C-A--------------------T-T---C-C-T--GA--CG-C-T-A--A-GG--C-A-CG-A--AA-G-C---G-TG--GG-G--AG-C-A-AA--------CG--GG Bacteria(100);Candidate_division_TM7(100);unclassified(100);unclassified(100);unclassified(100);unclassified(100);unclassified(100);unclassified(100);unclassified(100);

Otu1591 0 0 0 0 0 0 0 0 0 0 0 0 0 0 0 0 0 0 0 0 0 0 0 0 0 0 0 0
0 0 0 0 0 0 0 0 0 0 0 0 0 0 0 0 0 0 0 0 0 0 0 0 0 0 0 0
0 0 0 0 0 0 7 0 0 0 0 0 0 0 0 0 1 0 0 0 0 0 0 0 0 0 0 0
0 0 0 0 0 0 0 0 0 0 0 0 0 0 0 0 0 0 0 0 0 0 0 0 0 0 0

M02255_77_000000000-ADUT3_1_1116_14278_15046 G----AC---GA-GG-GGT--------CCA-A-G------C--G--T---T--GT-T-CGG-AA------TC-A--T-T--GG-GC-------GT--A-----AA-GC-GG-GT-------G-TA-G-G-C-G------------G--C-CT-T-G-C-----------AA----G-T-C-A--------G-A-A--G--TG--A-AA-GC--C-C-TG-G-G-----CT-C-AA------C-C-C-A-G-G-A--A-G----T-G--C-T---T--T-------T--GA-A-A---C-------T--G-C--AG--G-G-C----T-T-G-A-G-T--G---T-GG-----GA-G-A--------G-G-A-T---AG-T------GG--A--ATT----C-C-A-G-GT--GT-A-G-TG-GT--G---A--A-A---TA-C-GT-AG--AG-A-TC--T-G--G-A-G-G-A-AC-A-CC---GG--T--G--GC-GAA-G--G-C--G-------G--C-T-A-T---CTG--G--CC-C-A--------------------A-C---A-C-T--GA--CG-C-T-C--A-GA--C-C-CG-A--AA-G-C---G-TG--GG-G--AT-C-A-AA--------CA--GG Bacteria(100);unclassified(100);unclassified(100);unclassified(100);unclassified(100);unclassified(100);unclassified(100);unclassified(100);unclassified(100);

Otu1592 0 0 0 0 0 0 0 0 0 0 0 0 0 0 0 0 0 0 0 0 0 0 0 0 0 0 0 0
0 0 0 0 0 0 0 0 0 0 0 0 0 0 0 0 0 0 0 0 0 0 0 0 0 0 0 0
0 0 0 0 0 0 0 5 0 0 0 0 0 0 0 0 0 0 0 0 0 0 0 0 0 0 0 0
0 0 0 0 0 0 0 0 0 0 0 0 0 0 0 0 0 0 0 0 0 0 0 0 0 0 0

M02255_77_000000000-ADUT3_1_1101_18852_13360 T----AC---GT-AG-GGT--------CCG-A-G------C--G--T---T--GT-C-CGG-AT------TT-A--T-T--GG-GC-------GT--A-----AA-GA-GC-TC-------G-TA-G-G-C-G------------G--T-TC-G-C-T-----------AA----G-T-C-G--------G-A-T--G--TG--A-AA-AC--T-C-GG-G-G-----CT-C-AA------C-C-C-C-G-A-G--A-C----G-C--C-A---T--C-------C--GA-T-A---C-------T--G-G-CGT--G-A-C----T-T-G-A-G-T--C---T-GG-----TA-G-G--------G-G-A-G---AT-C------GG--A--ATT----C-C-T-G-GT--GT-A-G-CG-GT--G---A--A-A---TG-C-GC-AG--AT-A-TC--A-G--G-A-G-G-A-AC-A-CC---GG--T--G--GC-GAA-G--G-C--G-------G--A-T-C-T---CTG--G--GC-C-A--------------------G-T---A-C-T--GA--CG-C-T-G--A-GG--A-G-CG-A--AA-G-C---G-TG--GG-G--AG-C-A-AA--------CA--GG Bacteria(100);Actinobacteria(100);Actinobacteria(100);unclassified(100);unclassified(100);unclassified(100);unclassified(100);unclassified(100);unclassified(100);

Otu1593 0 0 0 0 0 0 0 0 0 0 0 0 0 0 0 0 0 0 0 0 0 0 0 0 0 0 0 0
0 0 0 0 0 0 0 0 0 0 0 0 0 0 0 0 0 0 0 0 0 0 0 0 0 0 0 0
0 0 0 0 0 0 0 6 0 0 0 0 0 0 0 0 0 0 0 0 0 0 0 0 0 0 0 0
0 0 0 0 0 0 0 0 0 0 0 0 0 0 0 0 0 0 0 0 0 0 0 0 0 0 0

M02255_77_000000000-ADUT3_1_1113_23430_19752 T----AC---AG-AG-GCC--------CCA-A-G------C--G--T---T--AT-C-CGG-AA------TC-A--C-T--GG-GC-------GT--A-----AA-GG-GT-GT-------G-TA-G-G-C-G------------G--T-CG-T-G-T-----------TA----G-T-C-T--------T-T-C--G--TG--A-AAGCT--T-C-TG-G-G-----CT-T-AA------C-C-C-A-G-A-A--A-A----C-G--C-G---G--T-------G--GA-A-A---C-------G--G-C--AC--G-A-C----TTA-G-A-G-G--T---T-GC-----GA-G-A--------G-G-T-A---TA-C------GG--A--ACT----C-A-T-A-GT--GT-A-G-GG-GT--G---A--A-A---TC-C-TT-TG--AT-A-TT--A-T--G-G-G-G-A-AC-A-CC---AA--A--T--GC-GAA-G--G-C--A-------G--T-A-T-A---CTG--G--CG-C-A--------------------C-A---C-C-T--GA--CG-C-T-G--A-GA--C-A-CG-A--AA-G-C---G-TG--GG-A--AT-C-G-AA--------CG--GG Bacteria(100);unclassified(100);unclassified(100);unclassified(100);unclassified(100);unclassified(100);unclassified(100);unclassified(100);unclassified(100);

Otu1594 0 0 0 0 0 0 0 0 0 0 0 0 0 0 0 0 0 0 0 0 0 0 0 0 0 0 0 0
0 0 0 0 0 0 0 0 0 0 0 0 0 0 0 0 0 0 0 0 0 0 0 0 0 0 0 0
0 0 0 0 0 0 0 4 0 0 0 0 0 0 0 0 0 0 0 0 0 0 0 0 0 0 0 0
0 0 0 0 0 0 0 0 0 0 0 0 0 0 0 0 0 0 0 0 0 0 0 0 0 0 0

M02255_76_000000000-ADUNE_1_2115_9010_21278 T----AC---AG-AG-GCC--------CCA-A-G------C--G--T---T--AT-C-CGG-AA------TC-A--C-T--GG-GC-------GT--A-----AA----G--G--------G-T------C--------------G-------T-G-T----------TA----G-T-C-T--------T-T-C--G--TG--A-AAGCT--G-C-TG-G-G-----CT-T-AA------C-C-C-A-G-A-A--A-A----C-G--C-G---G--T-------G--GA-A-A---C-------G--G-C--AC--G-A-C----TTA-G-A-G-G--T---T-GC-----GA-G-A--------G-G-T-A---TA-C------GG--A--ACT----C-A-T-A-GT--GT-A-G-GG-GT--G---A--A-A---TC-C-GT-TG--AT-A-TT--A-T--G-G-G-G-A-AC-A-CC---AA--A--T--GC-GAA-G--G-C--A-------G--T-A-T-A---CTG--G--CG-C-A--------------------C-A---C-C-T--GA--CG-C-T-G--A-GA--C-A-CG-A--AA-G-C---G-TG--GG-A--AT-C-G-AA--------CG--GG Bacteria(100);unclassified(100);unclassified(100);unclassified(100);unclassified(100);unclassified(100);unclassified(100);unclassified(100);unclassified(100);

Otu1595 0 0 0 0 0 0 0 0 0 0 0 0 0 0 0 0 0 0 0 0 0 0 0 0 0 0 0 0
0 0 0 0 0 0 0 0 0 0 0 0 0 0 0 0 0 0 0 0 0 0 0 0 0 0 0 0 0
0 0 0 0 0 0 0 1 4 0 0 0 0 0 0 0 0 0 0 0 0 0 0 0 0 0 0 0 0
0 0 0 0 0 0 0 0 0 0 0 0 0 0 0 0 0 0 0 0 0 0 0 0 0 0 0
M02255_76_000000000-ADUNE_1_1119_8445_7056 C----TA---AC-TC-CGT--------GCC-A-G------C-----A---G--CC-G-CGG-TA------AT-A--C-G--GA-GG-------GT--G-----CA--A-GC-GT-------T-AA-T-C-G-G------------A--T-TT-G-T-T----------AA----
G-T-C-A--------G-A-T--G--TG--A-AA-TC--C-C-CG-A-G-----CT-T-AA------C-T-T-G-G-G-A--A-C----T-G--C-A---T--T-------T--GA-A-A---C-------T--G-G--CA--A-G-C----T-A-G-A-G-T--C---T-TG-----TA-G-A--------G-G-G-G---GG-T------AG--A--ATT----C-C-A-G-GT--
GT-A-G-CG-GT--G---A--A-A---TG-C-GT-AG--AG-A-TC--T-G--G-A-G-G-A-AT-A-CC---GG--T--G--GC-GAA-G--G-C--G-------G--C-C-C-C---CTG--G--AC-A-A--------------------A-G---A-C-T--GA--CG-C-T-C--A-GG--T-G-CG-A--AA-G-C---G-TG--GG-G--AG-C-A-
AA--------CA--GG Bacteria(100);Proteobacteria(100);Gammaproteobacteria(100);Enterobacteriales(100);Enterobacteriaceae(100);unclassified(100);unclassified(100);unclassified(100);unclassified(100);

Otu1596 0 0 0 0 0 0 0 0 0 0 0 0 0 0 0 0 0 0 0 0 0 0 0 0 0 0 0 0
0 0 0 0 0 0 0 0 0 0 0 0 0 0 0 0 0 0 0 0 0 0 0 0 0 0 0 0 0
0 0 0 0 0 0 0 0 0 0 0 0 0 0 0 0 40 0 0 0 0 0 0 0 0 0 0 0 0
0 0 0 0 0 0 0 0 0 0 0 0 0 0 0 0 0 0 0 0 0 0 0 0 0 0 0
M02255_77_000000000-ADUT3_1_1109_9078_17931 T----AC---GG-AG-GGG--------GCT-A-G------C--G--T---T--GT-T-CGG-AA------TT-A--C-T--GG-GC-------GT--A-----AA-GC-GC-AC-------G-TA-G-G-C-G------------G--A-TC-A-G-T----------
AA----G-T-T-A--------G-G-G--G--TG--A-AA-GC--C-C-AG-G-G-----CT-C-AA------C-C-C-T-G-G-A--A-C----T-G--C-C---T--T-------T--AA-T-A---C-------T--G-C--TG--G-T-C----T-G-G-A-G-T--C---C-GA-----GA-G-A--------G-G-T-G---AG-T------GG--A--ATT----C-C-G-
A-GT--GT-A-G-AG-GT--G---A--A-A---TT-C-GT-AG--AT-A-TT--C-G--G-A-G-G-A-AC-A-CC---AG--T--G--GC-GAA-G--G-C--G-------G--C-T-C-A---CTG--G--CT-C-G--------------------G-T---A-C-T--GA--CG-C-T-G--A-GG--T-G-CG-A--AA-G-C---G-TG--GG-G--
AG-C-A-AA--------CA--GG Bacteria(100);Proteobacteria(100);Alphaproteobacteria(100);unclassified(100);unclassified(100);unclassified(100);unclassified(100);unclassified(100);unclassified(100);

Otu1597 0 0 0 0 0 0 0 0 0 0 0 0 0 0 0 0 0 0 0 0 0 0 0 0 0 0 0 0
0 0 0 0 0 0 0 0 0 0 0 0 0 0 0 0 0 0 0 0 0 0 0 0 0 0 0 0 0
0 0 0 0 0 0 0 0 0 0 0 0 0 0 0 20 0 0 0 0 0 0 0 0 0 0 0 0 0
0 0 0 0 0 0 0 0 0 0 0 0 0 0 0 0 0 0 0 0 0 0 0 0 0 0 0
M02255_77_000000000-ADUT3_1_1103_20435_12803 T----AC---GT-AG-GTG--------ACA-A-G------C--G--T---T--AT-C-CGG-AT------TT-A--C-T--GG-GC-------GT--A-----AA-GG-GC-GT-------G-TA-G-G-C-G------------G--T-TT-G-G-C-----
------AA----G-T-C-A--------G-A-A--G--TG--A-AA-TT--C-C-TG-A-G-----CT-C-AA------C-T-C-G-G-G-C--G-C----T-G--C-T---T--C-------T--GA-A-A---C-------T--G-C--TA--G-A-C----T-T-G-A-G-T--G---C-TG-----GA-G-G--------G-G-A-T---AG-C------GG--A--ATT----C-
C-T-A-GT--GG-A-G-CG-GT--A---A--A-A---TG-C-GC-AG--AT-A-TT--A-G--G-A-A-G-A-AC-A-CC---GG--T--G--GC-GAA-G--G-C--G-------G--C-T-A-T---CTG--G--AC-A-G--------------------T-A---A-C-T--GA--CG-C-T-G--A-GG--C-G-CG-A--AA-G-C---G-TG--GG-
G--AG-C-A-AA--------CA--GG Bacteria(100);unclassified(100);unclassified(100);unclassified(100);unclassified(100);unclassified(100);unclassified(100);unclassified(100);unclassified(100);

Otu1598 0 0 0 0 0 0 0 0 0 0 0 0 0 0 0 0 0 0 0 0 0 0 0 0 0 0 0 0
0 0 0 0 0 0 0 0 0 0 0 0 0 0 0 0 0 0 0 0 0 0 0 0 0 0 0 0 0
0 0 0 0 0 0 0 0 0 0 0 0 0 0 0 36 0 0 0 0 0 0 0 0 0 0 0 0 0
0 0 0 0 0 0 0 0 0 0 0 0 0 0 0 0 0 0 0 0 0 0 0 0 0 0 0
M02255_77_000000000-ADUT3_1_2108_13253_10506 T----AC---GT-AG-GTG--------ACA-A-G------C--G--T---T--AT-C-CGG-AT------TT-A--C-T--GG-GT-------GT--A-----AA-GG-GC-GT-------G-TA-G-G-C-G------------G--T-TT-G-G-C------
-----AA----G-T-C-A--------G-A-A--G--TG--A-AA-TT--C-C-CG-G-G-----CT-T-AA------C-T-T-G-G-G-C--G-C----T-G--C-T---T--C-------T--GA-A-A---C-------T--G-C--TT--A-A-C----T-T-G-A-G-T--A---C-TG-----GA-G-A--------G-G-A-T---AG-T------GG--A--ATT----C-C-
T-A-GT--GT-A-G-CG-GT--A---A--A-A---TG-C-AC-AG--AT-A-TT--A-G--G-A-G-G-A-AC-A-CC---GG--T--G--GC-GAA-G--G-C--G-------G--C-T-A-T---CTG--G--AC-A-G--------------------T-A---A-C-T--GA--CG-C-T-G--A-GG--C-G-CG-A--AA-G-C---A-CG--GG-G--
AG-C-A-AA--------CA--GG Bacteria(100);Firmicutes(100);Clostridia(100);Clostridiales(100);unclassified(100);unclassified(100);unclassified(100);unclassified(100);unclassified(100);

Otu1599 0 0 0 0 0 0 0 0 0 0 0 0 0 0 0 0 0 0 0 0 0 0 0 0 0 0 0 0
0 0 0 0 0 0 0 0 0 0 0 0 0 0 0 0 0 0 0 0 0 0 0 0 0 0 0 0 0
0 0 0 0 0 0 0 0 0 0 0 0 0 0 0 4 0 0 0 0 0 0 0 0 0 0 0 0 0
0 0 0 0 0 0 0 0 0 0 0 0 0 0 0 0 0 0 0 0 0 0 0 0 0 0 48
M02255_77_000000000-ADUT3_1_1103_5672_8577 T----AC---GT-AG-GGG--------GCG-A-G------C--G--T---T--GT-C-CGG-AA------TT-A--C-T--GG-GC-------GT--A-----AA-GG-GA-GC-------G-TA-G-G-C-G------------G--T-CT-G-A-T----------
AA----G-T-T-G--------G-A-T--G--TG--A-AA-TA--C-C-CG-G-G-----CT-T-AA------C-T-T-G-G-G-G--G-G----T-G--C-A---T--C-------C--AA-T-A---C-------T--G-T--TG--G-A-C----T-A-G-A-G-T--A---C-AG-----GA-G-A--------G-G-A-A---AG-C------GG--A--ATT----C-C-T-
A-GT--GT-A-G-CG-GT--G---A--A-A---TG-C-AT-AG--AT-A-TT--A-G--G-A-G-G-A-AC-A-TC---GG--T--G--GC-GAA-G--G-C--G-------G--C-T-T-T---CTG--G--AC-T-G--------------------C-A---A-C-T--GA--CG-C-T-G--A-GG--C-T-CG-A--AA-G-C---G-TG--GG-G--
AG-C-A-AA--------CA--GG Bacteria(100);Firmicutes(100);unclassified(100);unclassified(100);unclassified(100);unclassified(100);unclassified(100);unclassified(100);unclassified(100);

Otu1600 0 0 0 0 0 0 0 0 0 0 0 0 0 0 0 0 0 0 0 0 0 0 0 0 0 0 0 0
0 0 0 0 0 0 0 0 0 0 0 0 0 0 0 0 0 0 0 0 0 0 0 0 0 0 0 0 0
0 0 0 0 0 0 0 0 0 0 0 0 0 0 0 14 0 0 0 0 0 0 0 0 0 0 0 0 0
0 0 0 0 0 0 0 0 0 0 0 0 0 0 0 0 0 0 0 0 0 0 0 0 0 0 0

M02255_77_000000000-ADUT3_1_2107_18042_19017 T----AC---GG-AG-GGG--------GCT-A-G------C--G--T---T--GT-T-CGG-AA------TG-A--C-T--GG-GC-------GT--A-----AA-GC-GC-GC-------G-TA-G-G-C-G------------G--A-TC-G-G-A---
--------CA----G-T-C-A--------G-A-G--G--TG--A-AA-GC--C-C-GG-G-G-----CT-C-AA------C-C-C-C-G-G-A--A-C----G-G--C-C---T--T-------T--GA-T-A---C-------T--T-C--CG--G-T-C----T-T-G-A-G-T--T---C-GA-----GA-G-A--------G-G-T-G---GG-T------GG--A--ATT----
C-C-G-A-GT--GT-A-G-AG-GT--G---A--A-A---TT-C-GT-AG--AT-A-TT--C-G--G-A-G-G-A-AC-A-CC---AG--T--G--GC-GAA-G--G-C--G-------G--C-C-C-A---CTG--G--CT-C-G--------------------A-C---A-C-T--GA--CG-C-T-G--A-GG--C-G-CG-A--AA-G-C---G-CG--
GG-G--AG-C-A-AA--------CA--GG Bacteria(100);Proteobacteria(100);Alphaproteobacteria(100);unclassified(100);unclassified(100);unclassified(100);unclassified(100);unclassified(100);unclassified(100);

Otu1601 0 0 0 0 0 0 0 0 0 0 0 0 0 0 0 0 0 0 0 0 0 0 0 0 0 0 0 0
0 0 0 0 0 0 0 0 0 0 0 0 0 0 0 0 0 0 0 0 0 0 0 0 0 0 0 0 0
0 0 0 0 0 0 0 0 0 0 0 0 0 0 0 2 0 0 0 0 0 0 0 0 0 0 0 0 0
0 0 0 0 0 0 0 0 0 0 0 0 0 15 0 0 0 0 0 0 0 0 0 0 0 0 0

M02255_77_000000000-ADUT3_1_1101_18231_7711 T----AC---GT-AG-GAT--------CCG-A-G------C--G--T---T--AT-C-CGG-AG------TG-A--C-T--GG-GC-------GT--A-----AA-GA-GTTGC-------G-TA-G-G-T-G------------G--T-TT-A-T-T-----------AA-
---G-T-A-G--------G-T-G--A--TG--A-AA-GC--T-G-GT-G-G-----CT-C-AA------C-C-A-T-TCA-G--A-T----T-G--T-T---A--T-------C--TA-A-A---C-------T--G-G--TA--A-A-C----T-T-G-A-G-A--G---T-AG-----CA-G-A--------G-G-T-A---AC-T------GG--A--ATT----T-C-T-A-
GT--GT-A-G-GA-GT--G---A--A-A---TC-C-GT-AG--AT-A-TT--A-G--A-A-G-G-A-AC-A-CC---AA--T--G--GC-GTA-G--G-C--A-------G--G-T-T-A---CTG--G--GC-T-A--------------------T-T---T-C-T--GA--CA-C-T-A--A-GG--C-A-CG-A--AA-G-C---G-TG--GG-G--AG-C-
G-AA--------CG--GG Bacteria(100);Candidate_division_TM7(100);unclassified(100);unclassified(100);unclassified(100);unclassified(100);unclassified(100);unclassified(100);unclassified(100);

Otu1602 0 0 0 0 0 0 0 0 0 0 0 0 0 0 0 0 0 0 0 0 0 0 0 0 0 0 0 0
0 0 0 0 0 0 0 0 0 0 0 0 0 0 0 0 0 0 0 0 0 0 0 0 0 0 0 0 0
0 0 0 0 0 0 0 0 0 0 0 0 0 0 0 0 67 0 0 0 0 0 0 0 0 0 0 0 0
0 0 0 0 0 0 0 0 0 0 0 0 0 0 0 0 0 0 0 0 0 0 0 0 0 0 0

M02255_77_000000000-ADUT3_1_1105_24063_19483 T----AC---GT-AG-GGG--------GCA-A-G------C--G--T---T--AT-C-CGG-AT------TT-A--C-T--GG-GT-------GT--A-----AA-GG-GA-GC-------G-TA-G-G-C-G------------G--C-AA-G-G-C----
-------AA----G-T-C-T--------G-A-T--G--TG--A-AA-AC--C-C-GG-G-G-----CT-C-AA------C-C-C-C-G-G-G--A-C----T-G--C-A---T--T-------G--GA-A-A---C-------T--G-C--TT--A-G-C----T-A-G-A-G-T--G---T-CG-----GA-G-A--------G-G-T-A---AG-T------GG--A--ATT----
C-C-T-A-GT--GT-A-G-CG-GT--G---A--A-A---TG-C-GT-AG--AT-A-TT--A-G--G-A-G-G-A-AC-A-CC---AG--T--G--GC-GAA-G--G-C--G-------A--C-T-T-A---CTG--G--AC-G-A--------------------T-A---A-C-T--GA--CG-C-T-G--A-GG--C-T-CG-A--AA-G-C---G-TG--GG-
G--AG-C-A-AA--------CA--GG Bacteria(100);Firmicutes(100);Clostridia(100);Clostridiales(100);Lachnospiraceae(100);unclassified(100);unclassified(100);unclassified(100);unclassified(100);

Otu1603 0 0 0 0 0 0 0 0 0 0 0 0 0 0 0 0 0 0 0 0 0 0 0 0 0 0 0 0
0 0 0 0 0 0 0 0 0 0 0 0 0 0 0 0 0 0 0 0 0 0 0 0 0 0 0 0 0
0 0 0 0 0 0 0 0 0 0 0 0 0 0 0 0 20 0 0 0 0 0 0 0 0 0 0 0 0
0 0 0 0 0 0 0 0 0 0 0 0 0 0 0 0 0 0 0 0 0 0 0 0 0 0 0

M02255_77_000000000-ADUT3_1_1107_14182_18275 T----AC---GT-AT-GGA--------GCA-A-G------C--G--T---T--AT-C-CGG-AT------TT-A--C-T--GG-GT-------GT--A-----AA-GG-GA-GC-------G-TA-G-A-C-G------------G--A-TT-T-G-C------
-----AA----G-T-C-T--------G-A-T--G--TG--A-AA-GC--C-T-GG-G-G-----CT-C-AA------C-C-C-C-G-G-A--A-C----T-G--C-A---T--T-------G--GA-A-A---C-------T--G-C--AG--A-T-C----T-T-G-A-G-T--G---C-CG-----GA-G-A--------G-G-T-A---AG-C------GG--A--ATT----C-
C-T-A-GT--GT-A-G-CG-GT--G---A--A-A---TG-C-GT-AG--AT-A-TT--A-G--G-A-G-G-A-AC-A-CC---AG--T--G--GC-GAA-G--G-C--G-------G--C-T-T-A---CTG--G--AC-G-G--------------------T-A---A-C-T--GA--CG-T-T-G--A-GG--C-T-CG-A--AA-G-C---G-TG--GG-
G--AG-C-A-AA--------CA--GG Bacteria(100);Firmicutes(100);Clostridia(100);Clostridiales(100);Lachnospiraceae(100);unclassified(100);unclassified(100);unclassified(100);unclassified(100);

Otu1604 0 0 0 0 0 0 0 0 0 0 0 0 0 0 0 0 0 0 0 0 0 0 0 0 0 0 0 0
0 0 0 0 0 0 0 0 0 0 0 0 0 0 0 0 0 0 0 0 0 0 0 0 0 0 0 0 0
0 0 0 0 0 0 0 0 0 0 0 0 0 0 0 0 42 0 0 0 0 0 0 0 0 0 0 0 0
0 0 0 0 0 0 0 0 0 0 0 0 0 0 0 0 0 0 0 0 0 0 0 0 0 0 0

M02255_77_000000000-ADUT3_1_1109_17493_16293 T----AC---GT-AG-GTC--------CCA-A-G------C--G--T---T--AT-C-CGG-AA------TT-A--C-T--GG-GC-------GT--A-----AA-GA-GT-CT-------G-TA-G-C-T-G------------G--T-CT-C-G-T-------
----AA----G-T-C-T--------A-T-T--T--TC--A-AA-TA--C-C-TT-G-G-----CT-T-AA------C-C-A-A-G-G-A--A-A----G-G--G-G---A--T-------G--GA-T-A---C-------T--A-C--GA--G-T-C----T-G-G-A-A-G--T---T-TT-----CA-G-G--------G-G-T-A---AG-T------AG--A--ATT----T-C-
C-G-GT--GG-A-G-AG-GT--G---A--A-A---TT-C-GT-TG--AT-A-TC--G-G--A-A-G-G-A-AT-G-CC---AA--A--A--GC-GAA-A--G-C--A-------G--C-T-T-A---CTA--T--GA-A-T--------------------A-T---C-T-T--GA--CA-G-T-G--A-GA--G-A-CG-A--AA-G-T---T-TG--GG-T--
AG-C-A-AA--------CG--GG Bacteria(100);unclassified(100);unclassified(100);unclassified(100);unclassified(100);unclassified(100);unclassified(100);unclassified(100);unclassified(100);

Otu1605 0 0 0 0 0 0 0 0 0 0 0 0 0 0 0 0 0 0 0 0 0 0 0 0 0 0 0 0
0 0 0 0 0 0 0 0 0 0 0 0 0 0 0 0 0 0 0 0 0 0 0 0 0 0 0 0 0
0 0 0 0 0 0 0 0 0 0 0 0 0 0 1 0 0 0 0 0 0 0 0 0 0 0 0 0 0
0 0 0 0 0 0 0 0 0 0 0 60 0 0 0 0 0 0 0 0 0 0 0 0 0 0 0
M02255_77_000000000-ADUT3_1_1111_25165_4428 T----AC---GG-AG-GGT--------GCA-A-G------C--G--T---T--AC-T-CGG-AA------TC-A--C-T--GG-GC-------GT--A-----AA-GG-GT-GC-------G-TA-G-G-T-G------------G--A-TT-A-T-C-----------AA----G-T-C-T--------C-T-T--G--TG--A-AA-TC--T-A-AT-A-G-----CT-T-AA------C-T-A-T-T-A-A--A-T----T-G--C-T---T--G-------G--GA-A-A---C-------T--G-A--TA--G-T-C----T-A-G-A-G-T--A---G-GG-----GA-G-A--------G-G-C-A---GA-T------GG--A--ACT----C-T-T-G-GT--GT-A-G-GA-GT--A---A--A-A---TC-C-GT-AG--AT-A-TC--A-A--G-A-A-G-A-AT-A-CC---TA--T--T--GC-GAA-A--G-C--G-------A--T-C-T-G---CTA--G--AA-C-C--------------------T-A---A-C-T--GA--CA-C-T-G--A-TG--C-A-CG-A--AA-G-C---G-TG--GG-G--AG-C-A-AA--------CA--GG Bacteria(100);Proteobacteria(100);Epsilonproteobacteria(100);Campylobacterales(100);Campylobacteraceae(100);Campylobacter(100);unclassified(100);unclassified(100);unclassified(100);

Otu1606 0 0 0 0 0 0 0 0 0 0 0 0 0 0 0 0 0 0 0 0 0 0 0 0 0 0 0 0
0 0 0 0 0 0 0 0 0 0 0 0 0 0 0 0 0 0 0 0 0 0 0 0 0 0 0 0 0
0 0 0 0 0 0 0 0 0 0 0 0 0 0 0 0 20 0 0 0 0 0 0 0 0 0 0 0 0
0 0 0 0 0 0 0 0 0 0 0 0 0 0 0 0 0 0 0 0 0 0 0 0 0 0 0
M02255_77_000000000-ADUT3_1_1113_18487_10182 T----AC---GT-AG-GGG--------GCA-A-G------C--G--T---T--AT-C-CGG-AT------TT-A--C-T--GG-GT-------GT--A-----AA-GG-GA-GC-------G-TA-G-A-C-G------------G--T-AT-G-G-T-----------AA----G-T-C-A--------G-A-T--G--TG--A-AA-GC--C-C-GG-A-G-----CT-C-AA------C-T-C-C-G-G-G--A-C----T-G--C-A---T--T-------T--GA-A-A---C-------T--A-T--CA--G-A-C----T-A-G-A-G-T--G---C-AG-----GA-G-A--------G-G-T-A---AG-T------GG--A--ATT----C-C-T-A-GT--GT-A-G-CG-GT--G---A--A-A---TG-C-GT-AG--AT-A-TT--A-G--G-A-G-G-A-AC-A-CC---AG--T--G--GC-GAA-G--G-C--G-------G--C-T-T-A---CTG--G--AC-T-G--------------------T-A---A-C-T--GA--CG-T-T-G--A-GG--C-T-CG-A--AA-G-C---G-TG--GG-G--AG-C-A-AA--------CA--GG Bacteria(100);Firmicutes(100);Clostridia(100);Clostridiales(100);Lachnospiraceae(100);unclassified(100);unclassified(100);unclassified(100);unclassified(100);

Otu1607 0 0 0 0 0 0 0 0 0 0 0 0 0 0 0 0 0 0 0 0 0 0 0 0 0 0 0 0
0 0 0 0 0 0 0 0 0 0 0 0 0 0 0 0 0 0 0 0 0 0 0 0 0 0 0 0 0
0 0 0 0 0 0 0 0 0 0 0 0 0 0 0 0 15 0 0 0 0 0 0 0 0 0 0 0 0
0 0 0 0 0 0 0 0 0 0 0 0 0 0 0 0 0 0 0 0 0 0 0 0 0 0 0
M02255_77_000000000-ADUT3_1_1119_18640_20364 T----AC---GT-AT-GGA--------GCA-A-G------C--G--T---T--AT-C-CGG-AT------TT-A--C-T--GG-GT-------GT--A-----AA-GG-GT-GC-------G-TA-G-G-C-G------------G--C-AT-G-G-C-----------AA----G-T-C-A--------G-A-A--G--TG--A-AA-GC--C-C-GG-G-G-----CT-C-AA------C-C-C-C-G-G-T--A-G----T-G--C-T---T--T-------T--GA-A-A---C-------T--G-C--TG--A-G-C----T-T-G-A-G-T--A---C-GG-----GA-G-A--------G-G-T-A---AG-C------GG--A--ATT----C-C-T-A-GT--GT-A-G-CG-GT--G---A--A-A---TG-C-GT-AG--AT-A-TT--A-G--G-A-G-G-A-AC-A-CC---AG--T--G--GC-GAA-G--G-C--G-------G--C-T-T-A---CTG--G--AC-C-G--------------------T-A---A-C-T--GA--CG-C-T-G--A-GG--C-A-CG-A--AA-G-C---G-TG--GG-G--AG-C-A-AA--------CA--GG Bacteria(100);Firmicutes(100);Clostridia(100);Clostridiales(100);Lachnospiraceae(100);unclassified(100);unclassified(100);unclassified(100);unclassified(100);

Otu1608 0 0 0 0 0 0 0 0 0 0 0 0 0 0 0 0 0 0 0 0 0 0 0 0 0 0 0 0
0 0 0 0 0 0 0 0 0 0 0 0 0 0 0 0 0 0 0 0 0 0 0 0 0 0 0 0 0
0 0 0 0 0 0 0 0 0 0 0 0 0 0 0 0 24 0 0 0 0 0 0 0 0 0 0 0 0
0 0 0 0 0 0 0 0 0 0 0 0 0 0 0 0 0 0 0 0 0 0 0 0 0 0 0
M02255_77_000000000-ADUT3_1_2114_21990_16470 T----AC---GT-AG-GGG--------GCA-A-G------C--G--T---T--AT-C-CGG-AT------TC-A--T-T--GG-GC-------GT--A-----AA-GA-GC-GC-------G-TA-G-G-C-G------------G--A-TG-T-T-T-----------AA----G-C-A-G--------A-A-T--C--TC--T-AA-TC--T-G-AG-G-G-----CT-C-AA------C-C-C-C-C-A-G--C------C-G--G-A---T--T-------C--TG-A-A---C-------T--G-G--AT--G-T-C----T-C-G-A-G-T--T---T-GG-----TA-G-A--------G-G-A-A---GA-T------GG--A--ATT----C-C-C-G-GT--GT-A-G-CG-GT--G---G--A-A---TG-C-GC-AG--AT-A-TC--G-G--G-A-A-G-A-AC-A-CC---GA--T--G--GC-GAA-G--G-C--A-------G--T-C-T-T---CTG--G--GC-C-A--------------------T-A---A-C-T--GA--CG-C-T-G--A-GG--C-G-CG-A--AA-G-C---T-AG--GG-G--AG-C-A-AA--------CA--GG Bacteria(100);unclassified(100);unclassified(100);unclassified(100);unclassified(100);unclassified(100);unclassified(100);unclassified(100);unclassified(100);

Otu1609 0 0 0 0 0 0 0 0 0 0 0 0 0 0 0 0 0 0 0 0 0 0 0 0 0 0 0 0
0 0 0 0 0 0 0 0 0 0 0 0 0 0 0 0 0 0 0 0 0 0 0 0 0 0 0 0 0
0 0 0 0 0 0 0 0 0 0 0 0 0 0 0 0 24 0 0 0 0 0 0 0 0 0 0 0 0
0 0 0 0 0 0 0 0 0 0 0 0 0 0 0 0 0 0 0 0 0 0 0 0 0 0 0
M02255_77_000000000-ADUT3_1_1105_10523_10873 T----AC---GT-AG-GGG--------GCA-A-G------C--G--T---T--AT-C-CGG-AT------TT-A--C-T--GG-GT-------GT--A-----AA-GG-GA-GC-------G-TA-G-G-C-G------------G--T-GA-T-G-C-----------AA----G-T-C-A--------G-A-A--G--TG--A-AA-AC--C-C-GG-G-G-----CT-C-AA------C-C-C-C-GCG-G--A-T----T-G--C-T---T--T-------T--GA-G-A---C-------T--G-T--GT--T-G-C----T-G-G-A-G-T--A---C-AG-----GA-G-G--------G-G-C-A---GG-C------GG--A--ATT----C-C-T-G-GT--GT-A-G-CG-GT--G---A--A-A---TG-C-GT-AG--AT-A-TC--A-G--G-A-G-G-A-AC-A-CC---GG--T--G--GC-GAA-G--G-C--G-------G--C-C-T-G---CTG--G--AC-T-G--------------------T-A---A-C-T--GA--CG-C-T-G--A-GG--C-T-CG-A--AG-G-C---G-TG--GG-G--AG-C-A-AA--------CA--GG Bacteria(100);Firmicutes(100);Clostridia(100);Clostridiales(100);Lachnospiraceae(100);unclassified(100);unclassified(100);unclassified(100);unclassified(100);

Otu1610 0 0 0 0 0 0 0 0 0 0 0 0 0 0 0 0 0 0 0 0 0 0 0 0 0 0 0 0
0 0 0 0 0 0 0 0 0 0 0 0 0 0 0 0 0 0 0 0 0 0 0 0 0 0 0 0
0 0 0 0 0 0 0 0 0 0 0 0 0 0 0 0 14 0 0 0 0 0 0 0 0 0 0 0
0 0 0 0 0 0 0 0 0 0 0 0 0 0 0 0 0 0 0 0 0 0 0 0 0 0 0
M02255_77_000000000-ADUT3_1_2102_18863_17666 T----AC---GT-AG-GGG--------GCA-A-G------C--G--T---T--GT-C-CGG-AA------TG-A--T-T--GG-GC-------GT--A-----AA-GG-GC-GC-------G-TA-G-G-C-G-------------G--C-TT-T-A-T-----------AA----G-T-C-T--------G-G-A--G--TG--A-AA-GT--C-C-TC-T-T-----TT-C-AA------G-G-G-A-G-G-A--A-T----T-G--C-T---T--T-------G--GA-A-A---C-------T--A-T--AG--A-G-C----T-T-G-A-G-T--G---C-AG-----GA-G-A--------G-G-T-T---AT-C------GG--A--ATT----C-C-C-G-GT--GT-A-G-CG-GT--G---A--A-A---TG-C-GT-AG--AT-A-TC--G-G--G-A-G-G-A-AC-A-TC---GG--T--G--GC-GAA-G--G-C--G-------G--G-T-A-A---CTG--G--AC-T-G---------------------C-A---A-C-T--GA--CG-C-T-G--A-GG--C-G-CG-A--AA-G-T---G-TG--GG-G--AG-C-A-AA--------CA--GG Bacteria(100);unclassified(100);unclassified(100);unclassified(100);unclassified(100);unclassified(100);unclassified(100);unclassified(100);unclassified(100);

Otu1611 0 0 0 0 0 0 0 0 0 0 0 0 0 0 0 0 0 0 0 0 0 0 0 0 0 0 0 0
0 0 0 0 0 0 0 0 0 0 0 0 0 0 0 0 0 0 0 0 0 0 0 0 0 0 0 0
0 0 0 0 0 0 0 0 0 0 0 0 0 0 0 0 5 6 0 0 0 0 0 0 0 0 0 0
0 0 0 0 0 0 0 0 0 0 0 0 0 0 0 0 0 0 0 0 0 0 0 0 0 0 0
M02255_77_000000000-ADUT3_1_2118_11161_13835 T----AC---GT-AG-GGT--------GCG-A-G------C--G--T---T--GT-C-CGG-AA------TT-A--T-T--GG-GC-------GT--A-----AA-GA-GC-TT-------G-TA-G-G-C-G-------------G--T-TT-G-T-C-----------GC----G-T-C-T--------G-C-T--G--TG--A-AA-AC--T-C-GG-G-G-----CT-T-AA------C-C-C-C-G-A-G--C-C----T-G--C-A---G--T-------G--GG-T-A---C-------G--G-G--CA--G-A-C----T-A-G-A-G-T--A---T-GG-----TA-G-G--------G-G-A-G---AC-T------GG--A--ATT----C-C-T-G-GT--GT-A-G-CG-GT--G---G--A-A---TG-C-GC-AG--AT-A-TC--A-G--G-A-G-G-A-AC-A-CC---GA--T--G--GC-GAA-G--G-C--A-------G--G-T-C-T---CTG--G--GC-C-A---------------------T-A---A-C-T--GA--CG-C-T-G--A-GA--A-G-CG-A--AA-G-C---G-TG--GG-T--AG-C-G-AA--------CA--GG Bacteria(100);Actinobacteria(100);Actinobacteria(100);Actinobacteridae(100);Actinomycetales(100);Micrococcineae(100);Intrasporangiaceae(100);Ornithinicoccus(100);unclassified(100);

Otu1612 0 0 0 0 0 0 0 0 0 0 0 0 0 0 0 0 0 0 0 0 0 0 0 0 0 0 0 0
0 0 0 0 0 0 0 0 0 0 0 0 0 0 0 0 0 0 0 0 0 0 0 0 0 0 0 0
0 0 0 0 0 0 0 0 0 0 0 0 0 0 0 0 7 0 0 0 0 0 0 0 0 0 0 0
14 0 0 0 0 0 0 0 0 0 0 0 0 0 0 0 0 0 0 0 0 0 0 0 0 0 0
M02255_77_000000000-ADUT3_1_1102_21140_10770 T----AC---GT-AG-GGT--------GCA-A-G------C--A--T---T--AT-C-CGG-AG------TG-A--C-T--GG-GC-------GT--A-----AA-GA-GTTGC-------G-TA-G-G-T-G-------------G--T-CA-G-T-A-----------AA----G-T-G-A--------A-T-A--G--TG--A-AA-TC--T-G-GT-G-G-----CT-C-AA------C-C-A-T-TCA-G--G-C----T-A--T-T---A--T-------T--CA-A-A---C-------T--C-A--CT--G-A-C----T-C-G-A-G-A--A---T-AG-----CA-G-A--------G-G-T-A---AC-T------GG--A--ATT----T-C-T-T-GT--GT-A-G-GA-GT--G---A--A-A---TC-C-GT-AG--AT-A-TA--A-G--A-A-G-G-A-AC-A-CC---AA--T--G--GC-GTA-G--G-C--A-------G--G-T-T-A---CTG--G--GC-T-A---------------------T-T---T-C-T--GA--CA-C-T-G--A-GG--C-A-CG-A--AA-G-C---G-TG--GG-G--AG-C-G-AA--------CG--GG Bacteria(100);Candidate_division_TM7(100);unclassified(100);unclassified(100);unclassified(100);unclassified(100);unclassified(100);unclassified(100);unclassified(100);

Otu1613 0 0 0 0 0 0 0 0 0 0 0 0 0 0 0 0 0 0 0 0 0 0 0 0 0 0 0 0
0 0 0 0 0 0 0 0 0 0 0 0 0 0 0 0 0 0 0 0 0 0 0 0 0 0 0 0
0 0 0 0 0 0 0 0 0 0 0 0 0 0 0 0 0 0 0 0 0 0 0 0 0 0 5 0
0 0 0 0 0 1 0 0 0 0 0 0 0 0 0 9 0 0 0 0 0 0 0 0 0 0 0
M02255_76_000000000-ADUNE_1_1112_5487_15700 C----GG---CT-AA-CTC--------CGT-G-C------C--A--G---C--AG-C-CGC-GG------TA-A--T-A--CG-GA-------GG--G-----TG-CA-AG-CG-------T-TA-T-C-C-G-------------G--G-TT-A-T-T-----------AA----G-T-C-A--------G-T-G--G--TG--A-AA-TC--T-C-AT-A-G-----CT-C-AA------C-T-A-T-G-A-A--A-C----T-G--C-C---A--T-------T--GA-T-A---C-------T--G-A--TA--G-T-C----T-T-G-A-G-T--A---T-AT-----TT-G-A--------A-G-T-A---GC-T------GG--A--ATA----A-G-T-A-GT--GT-A-G-CG-GT--G---A--A-A---TG-C-AT-AG--AT-A-TT--A-C--T-T-A-G-A-AC-A-CC---AA--T--A--GC-GAA-G--G-C--A-------G--G-T-T-A---CTA--A--GA-T-A---------------------T-T---A-C-T--GA--CG-C-T-G--A-GG--G-A-CG-A--GA-G-C---G-TA--GG-G--AG-C-G-AA--------CA--GG Bacteria(100);Bacteroidetes(100);Flavobacteria(100);Flavobacteriales(100);Flavobacteriaceae(100);unclassified(100);unclassified(100);unclassified(100);unclassified(100);

Otu1614 0 0 0 0 0 0 0 0 0 0 0 0 0 0 0 0 0 0 0 0 0 0 0 0 0 0 0 0
0 0 0 0 0 0 0 0 0 0 0 0 0 0 0 0 0 0 0 0 0 0 0 0 0 0 0 0
0 0 0 0 0 0 0 0 0 0 0 0 0 0 19 0 0 0 0 0 0 0 0 0 0 0 0 0
0 0 0 0 0 0 0 0 0 0 0 0 0 0 0 0 0 0 0 0 0 0 0 0 0 0 0
M02255_77_000000000-ADUT3_1_2113_16331_14533 T----AC---GA-AG-GGG--------GCA-A-G------C--G--T---T--GT-T-CGG-AA------TG-A--C-T--GG-GC-------GT--A-----AA-GG-GC-GC-------G-TA-G-G-C-G-------------G--T-TC-G-T-T-----------GC----G-T-C-A--------G-A-T--G--TG--A-AA-GC--C-C-CG-G-G-----CT-C-AA------C-C-T-G-G-G-A--A-C----T-G--C-A---T--T-------T--GA-T-A---C-------G--G-G--CG--A-G-C----T-T-G-A-A-T--C---C-AA-----GA-G-A--------G-G-G-T---GG-T------GG--A--ATT----C-C-C-A-GT--GT-A-G-AG-GT--G---A--A-A---TT-C-GT-AG--AT-A-TT--G-G--G-A-A-G-A-AC-A-CC---AG--T--G--GC-GAA-G--G-C--G-------G--C-C-A-C---CTG--G--CT-T-G---------------------G-T---A-T-T--GA--CG-C-T-G--A-GG--C-G-CG-A--AA-G-C---G-TG--GG-G--AG-C-A-AA--------CA--GG Bacteria(100);Proteobacteria(100);Alphaproteobacteria(100);Rhodospirillales(100);Rhodospirillaceae(100);unclassified(100);unclassified(100);unclassified(100);unclassified(100);

Otu1615 0 0 0 0 0 0 0 0 0 0 0 0 0 0 0 0 0 0 0 0 0 0 0 0 0 0 0 0
0 0 0 0 0 0 0 0 0 0 0 0 0 0 0 0 0 0 0 0 0 0 0 0 0 0 0 0
0 0 0 0 0 0 0 0 0 0 0 0 0 0 8 9 0 0 0 0 0 0 0 0 0 0 0 0
0 0 0 0 0 0 0 0 0 0 0 0 0 0 0 0 0 0 0 0 0 0 0 0 0 0 0
M02255_77_000000000-ADUT3_1_2113_25337_7595 A----CT---AC-GT-GCC--------AGC-A-G------C--C--G---C--GG-T-GAT-AC------GT-A--G-G--GC-GC-------G---A-----GC-GT-TG-TC-------C-GG-A-A-T-T------------A--T-TG-G-G-C-----------GC--
--G-T-C-T--------G-T-C--G--TG--A-AA-AC--C-C-TC-G-G-----CT-T-AA------C-C-G-G-G-G-G--C-T----T-G--C-G---G--T-------G--GG-T-A---C-------G--G-G--CC--G-G-C----T-T-G-A-G-T--G---C-GG-----CA-G-G--------G-G-A-G---AC-T------GG--A--ATT----C-C-T-G-
GT--GT-A-G-CG-GT--G---G--A-A---TG-C-GC-AG--AT-A-TC--A-G--G-A-G-G-A-AC-A-CC---GG--T--G--GC-GAA-G--G-C--G-------G--G-T-C-T---CTG--G--GC-C-G--------------------T-T---A-C-T--GA--CG-C-T-G--A-GG--A-G-CG-A--AA-G-C---G-TG--GG-G--AG-
C-G-AA--------CA--GG Bacteria(100);Actinobacteria(100);Actinobacteria(100);Actinobacteridae(100);Actinomycetales(100);unclassified(100);unclassified(100);unclassified(100);unclassified(100);

Otu1616 0 0 0 0 0 0 0 0 0 0 0 0 0 0 0 0 0 0 0 0 0 0 0 0 0 0 0 0
0 0 0 0 0 0 0 0 0 0 0 0 0 0 0 0 0 0 0 0 0 0 0 0 0 0 0 0
0 0 0 0 0 0 0 0 0 0 0 0 0 0 23 7 0 0 0 0 0 0 0 0 0 0 0 0
0 0 0 0 0 0 0 0 0 0 0 0 0 0 0 0 0 0 0 0 0 0 0 0 0 0 0
M02255_76_000000000-ADUNE_1_2119_18955_13517 T----CG---GC-TA-ACT--------CCG-T-G------C--C--A---G--CA-G-CCG-CG------GT-A--A-T--AC-GG-------AG--G-----AT-GC-GA-GC-------GTTA-T-C-C-G------------GA-T-CT-A-T-T-----
------AA----G-T-C-A--------G-T-G--G--TG--A-AA-GT--T-T-GT-C-G-----CT-C-AA------C-G-A-T-A-A-A--A-T----T-G--C-C---G--T-------T--GA-A-A---C-------T--G-G--TA--G-T-C----T-T-G-A-G-T--A---T-GG-----AT-G-A--------A-G-T-A---GG-C------GG--A--ATG----C-
G-T-G-GT--GT-A-G-CG-GT--G---A--A-A---TG-C-AT-AG--AG-A-TC--A-C--G-C-A-G-A-AC-T-CC---GA--T--T--GC-GAA-G--G-C--A-------G--C-T-T-A---CTA--A--GG-C-A--------------------T-A---A-C-T--GA--CA-C-T-G--A-AG--C-A-CG-A--AA-G-C---G-TG--GG-T--
AT-C-A-AA--------CA--GG Bacteria(100);Bacteroidetes(100);Bacteroidia(100);Bacteroidales(100);Porphyromonadaceae(100);Tannerella(100);unclassified(100);unclassified(100);unclassified(100);

Otu1617 0 0 0 0 0 0 0 0 0 0 0 0 0 0 0 0 0 0 0 0 0 0 0 0 0 0 0 0
0 0 0 0 0 0 0 0 0 0 0 0 0 0 0 0 0 0 0 0 0 0 0 0 0 0 0 0
0 0 0 0 0 0 0 0 0 0 0 0 0 0 17 0 0 0 0 0 0 0 0 0 0 0 0 0
0 0 0 0 0 0 0 0 0 0 0 0 0 0 0 0 0 0 0 0 0 0 0 0 0 0 0
M02255_77_000000000-ADUT3_1_2114_20704_17107 T----AC---GA-AG-GGG--------GCT-A-G------C--G--T---T--GC-T-CGG-AA------TC-A--C-T--GG-GC-------GT--A-----AA-GG-GC-GC-------G-TA-G-G-C-G------------G--A-TT-G-T-T----
-------AA----G-T-C-A--------G-A-G--G--TG--A-AA-GC--C-C-AG-G-G-----CT-C-AA------C-C-C-T-G-G-A--A-T----T-G--C-C---T--T-------T--GA-T-A---C-------T--G-G--CA--A-T-C----T-T-G-A-G-A--C---C-GG-----AA-G-A--------G-G-T-T---AG-T------GG--A--ACT----
G-C-G-A-GT--GT-A-G-AG-GT--G---A--A-A---TT-C-GT-AG--AT-A-TT--C-G--C-A-A-G-A-AC-A-CC---AG--T--G--GC-GAA-G--G-C--G-------G--C-T-G-A---CTG--G--TC-C-G--------------------G-T---T-C-T--GA--CG-C-T-G--A-GG--C-G-CG-A--AA-G-C---G-TG--GG-
G--AG-C-A-AA--------CA--GG Bacteria(100);Proteobacteria(100);Alphaproteobacteria(100);unclassified(100);unclassified(100);unclassified(100);unclassified(100);unclassified(100);unclassified(100);

Otu1618 0 0 0 0 0 0 0 0 0 0 0 0 0 0 0 0 0 0 0 0 0 0 0 0 0 0 0 0
0 0 0 0 0 0 0 0 0 0 0 0 0 0 0 0 0 0 0 0 0 0 0 0 0 0 0 0
0 0 0 0 0 0 0 0 0 0 0 0 0 0 20 53 0 0 0 0 0 0 0 0 0 0 0 0
0 0 0 0 0 0 0 0 0 26 0 0 0 0 0 0 0 0 0 0 0 0 0 0 0 0 0
M02255_77_000000000-ADUT3_1_1112_20582_10688 T----AC---AT-AG-GGG--------GCA-A-G------C--G--T---T--AT-C-CGG-AA------TG-A--C-T--GG-GT-------GT--A-----AA-GG-GT-GC-------G-TA-G-G-C-G------------G--T-AA-G-G-T----
-------AA----G-T-C-T--------G-A-T--G--TG--A-AA-GA--C-T-GG-G-G-----CT-C-AA------C-T-C-C-A-G-G--G-G----T-G--C-A---T--T-------G--GA-A-A---C-------T--A-T--GT--T-A-C----T-A-G-A-G-T--A---C-AG-----GA-G-A--------G-G-A-A---AG-C------GG--A--ATT----
C-C-T-A-GT--GT-A-G-CG-GT--G---A--A-A---TG-C-GT-AG--AT-A-TT--A-G--G-A-G-G-A-AC-A-CC---GG--T--G--GC-GAA-G--G-C--G-------G--C-T-T-T---CTG--G--AC-T-G--------------------A-A---A-C-T--GA--CG-C-T-G--A-GG--C-A-CG-A--AA-G-C---G-TG--GG-
G--AG-C-A-AA--------CA--GG Bacteria(100);Firmicutes(100);Clostridia(100);Clostridiales(100);unclassified(100);unclassified(100);unclassified(100);unclassified(100);unclassified(100);

Otu1619 0 0 0 0 0 0 0 0 0 0 0 0 0 0 0 0 0 0 0 0 0 0 0 0 0 0 0 0
0 0 0 0 0 0 0 0 0 0 0 0 0 0 0 0 0 0 0 0 0 0 0 0 0 0 0 0
0 0 0 0 0 0 0 0 0 0 0 0 0 0 17 17 0 0 0 0 0 0 0 0 0 0 0 1
0 0 0 0 0 0 0 0 0 0 0 0 0 0 0 0 0 0 0 0 0 0 0 0 0 0 0
M02255_76_000000000-ADUNE_1_1106_18404_10928 C----TA---AC-TC-CGT--------GCC-A-G------C--A--G---C--CG-C-GGT-AA------TA-C--G-G--AG-GA-------TG--C-----GA-GC-GT--T-------A-TC-C-G---G------------A--G-TT-A-T-T--------
---AA----G-T-C-A--------A-G-G--G--TG--A-AA-GT--T-T-GT-T-G-----CT-T-AA------C-G-A-T-A-A-A--A-T----T-G--C-C---T--T-------T--GA-T-A---C-------T--G-G--TA--G-T-C----T-T-G-A-A-T--T---T-TT-----GT-G-A--------A-G-T-T---GC-T------GG--A--ATG----T-G-T-
A-GT--GT-A-G-CG-GT--G---A--A-A---TG-C-AT-AG--AT-A-TT--A-C--A-C-A-G-A-AC-A-CC---GA--T--T--GC-GAA-G--G-C--A-------T--G-T-G-A---CTA--A--CA-A-A--------------------G-G---A-T-T--GA--CG-C-T-G--A-GA--G-A-CG-A--AA-G-C---G-TG--GG-G--AG-
C-G-AA--------CA--GG Bacteria(100);Bacteroidetes(100);Flavobacteria(100);Flavobacteriales(100);Flavobacteriaceae(100);Capnocytophaga(100);unclassified(100);unclassified(100);unclassified(100);

Otu1620 0 0 0 0 0 0 0 0 0 0 0 0 0 0 0 0 0 0 0 0 0 0 0 0 0 0 0 0
0 0 0 0 0 0 0 0 0 0 0 0 0 0 0 0 0 0 0 0 0 0 0 0 0 0 0 0 0
0 0 0 0 0 0 0 0 0 0 0 0 0 0 27 57 0 0 0 0 0 0 0 0 0 0 0 0 0
0 0 0 0 0 0 5 0 0 0 0 0 0 0 0 0 0 0 0 0 0 0 0 0 0 0 0
M02255_77_000000000-ADUT3_1_2118_27681_15192 T----AC---GT-AG-GGG--------GCA-A-G------C--G--T---T--AT-C-CGG-AT------TT-A--C-T--GG-GT-------GT--A-----AA-GG-GA-GC-------G-CA-G-G-C-G------------G--T-CT-G-G-C----
-------AA----G-T-T-G--------A-G-A--G--TG--G-AA-GC--A-G-GG-G-G-----CT-C-AA------C-C-C-C-C-T-G--A-C----T-G--C-T---C--C-------C--AA-A-A---C-------T--G-T--TG--G-A-C----T-G-G-A-G-T--A---T-GG-----GA-G-A--------G-G-C-A---GG-C------GG--A--ATT----
C-C-T-A-GT--GT-A-G-CG-GT--G---A--A-A---TG-C-TC-AG--AT-A-TT--A-G--G-A-A-G-A-AC-A-CC---GG--T--G--GC-GAA-G--G-C--G-------G--C-C-T-G---CTG--G--AC-C-A--------------------A-A---A-C-T--GA--CG-C-T-G--A-GG--C-T-CG-A--GA-G-C---G-TG--GG-
G--AG-C-G-AA--------CA--GG Bacteria(100);Firmicutes(100);Clostridia(100);Clostridiales(100);Lachnospiraceae(100);Catonella(100);unclassified(100);unclassified(100);unclassified(100);

Otu1621 0 0 0 0 0 0 0 0 0 0 0 0 0 0 0 0 0 0 0 0 0 0 0 0 0 0 0 0
0 0 0 0 0 0 0 0 0 0 0 0 0 0 0 0 0 0 0 0 0 0 0 0 0 0 0 0 0
0 0 0 0 0 0 0 0 0 0 0 0 0 0 18 0 0 0 0 0 0 0 0 0 0 0 0 0 0
0 0 0 0 0 0 0 0 0 0 0 0 0 0 0 0 0 0 0 0 0 0 0 0 0 0 0
M02255_77_000000000-ADUT3_1_1112_18973_14722 T----AC---GA-AG-GGG--------GCT-A-G------C--G--T---T--GT-T-CGG-AA------TT-A--C-T--GG-GC-------GT--A-----AA-GC-GC-GC-------G-CA-G-G-C-G------------G--C-TA-T-C-C-----
------AA-----G-T-C-A--------G-T-G--G--TG--A-AA-GC--C-C-GG-A-G-----CT-C-AA------C-T-C-C-G-G-A--A-C----T-G--C-C---A--T-------T--GA-A-A---C-------T--G-T--TT--A-G-C----T-T-G-A-G-G--A---C-GA-----GA-G-A--------G-G-T-G---AG-T------GG--A--ATT----
C-C-C-A-GT--GT-A-G-AG-GT--G---A--A-A---TT-C-GT-AG--AT-A-TT--G-G--G-A-A-G-A-AC-A-CC---GG--T--G--GC-GAA-G--G-C--G-------G--C-T-C-A---CTG--G--CT-C-G--------------------T-T---T-C-T--GA--CG-C-T-C--A-GG--C-G-CG-A--TA-G-C---G-TG--GG-
G--AT-C-A-AA--------CA--GG Bacteria(100);Proteobacteria(100);Alphaproteobacteria(100);Rhizobiales(100);Methylocystaceae(100);uncultured(100);unclassified(100);unclassified(100);unclassified(100);

Otu1622 0 0 0 0 0 0 0 0 0 0 0 0 0 0 0 0 0 0 0 0 0 0 0 0 0 0 0 0
0 0 0 0 0 0 0 0 0 0 0 0 0 0 0 0 0 0 0 0 0 0 0 0 0 0 0 0 0
0 0 0 0 0 0 0 0 0 0 0 0 0 0 90 0 0 0 0 0 0 0 0 0 0 0 0 0 0
0 0 0 0 0 0 0 0 0 0 0 0 0 0 0 0 0 0 0 0 0 0 0 0 0 0 0
M02255_77_000000000-ADUT3_1_1108_20310_16503 T----AC---GA-AG-GGG--------GCT-A-G------C--G--T---T--GC-T-CGG-AA------TG-A--C-T--GG-GC-------GT--A-----AA-GG-GC-GC-------G-TA-G-G-C-G------------G--A-CA-T-A-T----
-------TA-----G-T-C-A--------G-G-C--G--TG--A-AA-TT--C-C-CG-G-G-----CT-T-AA------C-C-T-G-G-G-G--G-C----T-G--C-G---T--T-------T--GA-T-A---C-------G--G-T--GT--G-T-C----T-T-G-A-G-T--G---G-GG-----AA-G-A--------G-G-G-T---CG-T------GG--A--ATT----
C-C-C-A-GT--GT-A-G-AG-GT--G---A--A-A---TT-C-GT-AG--AT-A-TT--G-G--G-A-A-G-A-AC-A-CC---GG--T--G--GC-GAA-G--G-C--G-------G--C-G-A-C---CTG--G--TC-C-T--------------------T-G---A-C-T--GA--CG-C-T-G--A-GG--C-G-CG-A--AA-G-C---G-TG--
GG-G--AG-C-A-AA--------CA--GG Bacteria(100);Proteobacteria(100);Alphaproteobacteria(100);Rhodospirillales(100);Acetobacteraceae(100);unclassified(100);unclassified(100);unclassified(100);unclassified(100);

Otu1623 0 0 0 0 0 0 0 0 0 0 0 0 0 0 0 0 0 0 0 0 0 0 0 0 0 0 0 0
0 0 0 0 0 0 0 0 0 0 0 0 0 0 0 0 0 0 0 0 0 0 0 0 0 0 0 0 0
0 0 0 0 0 0 0 0 0 0 0 0 0 0 36 0 0 0 0 0 0 0 0 223 0 0 0 0 0
0 0 0 0 0 0 0 0 0 0 0 0 0 0 0 0 0 0 0 0 0 0 0 0 0 0 0
M02255_77_000000000-ADUT3_1_1108_22151_12522 T----AC---GA-AG-GGG--------GCT-A-G------C--G--T---T--GC-T-CGG-AT------TT-A--C-T--GG-GC-------GT--A-----AA-GG-GA-GC-------G-TA-G-G-C-G------------G--A-AC-G-A-C----
-------AA-----G-T-C-A--------G-G-G--G--TG--A-AA-GC--C-C-GG-G-G-----CT-C-AA------C-C-T-C-G-G-A--A-T----T-G--C-C---C--T-------T--GA-G-A---C-------T--G-T--CG--A-T-C----T-A-G-A-G-T--T---T-GG-----GA-G-A--------G-G-T-G---TG-T------GG--A--ACT----
C-C-G-A-GT--GT-A-G-AG-GT--G---A--A-A---TT-C-GT-AG--AT-A-TT--C-G--G-A-A-G-A-AC-A-CC---AG--T--G--GC-GAA-G--G-C--G-------A--C-A-C-A---CTG--G--CC-C-A--------------------A-T---A-C-T--GA--CG-C-T-G--A-GG--C-T-CG-A--AA-G-C---G-TG--GG-
G--AG-C-A-AA--------CA--GG Bacteria(100);Proteobacteria(100);Alphaproteobacteria(100);unclassified(100);unclassified(100);unclassified(100);unclassified(100);unclassified(100);unclassified(100);

Otu1624 0 0 0 0 0 0 0 0 0 0 0 0 0 0 0 0 0 0 0 0 0 0 0 0 0 0 0 0
0 0 0 0 0 0 0 0 0 0 0 0 0 0 0 0 0 0 0 0 0 0 0 0 0 0 0 0 0
0 0 0 0 0 0 0 0 0 0 0 0 0 0 20 25 0 0 0 0 0 0 0 0 0 0 0 0 0
0 0 0 0 0 0 0 0 0 0 0 0 0 0 0 0 0 0 0 0 0 0 0 0 0 0 0
M02255_77_000000000-ADUT3_1_1107_12702_20784 A----AC---GT-AG-GAG--------ACG-A-G------C--G--T---T--AT-C-CGG-AT------TT-A--C-T--GG-GC-------GT--A-----AA-GA-GC-GT-------G-TA-G-G-A-G------------G--C-AA-A-A-C----
-------AA-----G-A-C-G--------G-A-T--G--TG--A-AA-GC--C-C-CC-G-G-----CT-T-AA------C-C-G-G-G-G-G--A-G----G-T--C-G---T--A-------C--GT-G-A---C-------T--G-T--AG--A-G-C----T-A-G-A-G-T--G---A-GT-----TA-G-A--------G-G-T-A---AG-C------GG--A--ACT----
C-C-G-G-GA--GT-A-G-TG-GT--G---A--A-A---TG-C-GT-AG--AT-A-TC--C-G--G-A-A-G-A-AC-A-TC---AG--A--G--GC-GAA-A--G-C--G-------G--C-T-T-A---CTG--G--GA-C-T--------------------T-A---A-C-T--GA--CA-C-T-G--A-GA--C-G-CG-A--AA-G-C---C-AG--
GG-T--AG-C-A-AA--------CG--GG Bacteria(100);Chloroflexi(100);Anaerolineae(100);Anaerolineales(100);Anaerolineaceae(100);uncultured(100);unclassified(100);unclassified(100);unclassified(100);

Otu1625 0 0 0 0 0 0 0 0 0 0 0 0 0 0 0 0 0 0 0 0 0 0 0 0 0 0 0 0
0 0 0 0 0 0 0 0 0 0 0 0 0 0 0 0 0 0 0 0 0 0 0 0 0 0 0 0 0
0 0 0 0 0 0 0 0 0 0 0 0 0 0 139 168 0 0 0 0 0 0 0 0 0 0 0 0 0
0 0 0 0 0 0 0 0 0 0 0 0 0 0 0 0 0 0 0 0 0 0 0 0 0 0 0
M02255_77_000000000-ADUT3_1_1107_17240_10394 C----A----GC-A--GCC--------GCG-G-T------G--A--T---A--CG-T-AGG-GC------GC-G--A-G--CG-TT-------GT--C-----CG-GA-AT-TA-------T-TG-G-G-C-G------------G--C-TG-G-T-C-----
-----GC----G-T-C-T--------G-T-C--G--TG--A-AA-AC--C-C-TC-G-G-----CT-T-AA------C-C-G-G-G-G-G--C-T----T-G--C-G---G--T-------G--GG-T-A---C-------G--G-G--CC--G-G-C----T-T-G-A-G-T--G---C-GG-----CA-G-G--------G-G-A-G---AC-T------GG--A--ATT----
C-C-T-G-GT--GT-A-G-CG-GT--G---G--A-A---TG-C-GC-AG--AT-A-TC--A-G--G-A-G-G-A-AC-A-CC---GG--T--G--GC-GAA-G--G-C--G-------G--G-T-C-T---CTG--G--GC-C-G--------------------T-T---A-C-T--GA--CG-C-T-G--A-GG--A-G-CG-A--AA-G-C---G-TG--
GG-G--AG-C-G-AA--------CA--GG Bacteria(100);Actinobacteria(100);Actinobacteria(100);Actinobacteridae(100);Actinomycetales(100);unclassified(100);unclassified(100);unclassified(100);unclassified(100);

Otu1626 0 0 0 0 0 0 0 0 0 0 0 0 0 0 0 0 0 0 0 0 0 0 0 0 0 0 0 0
0 0 0 0 0 0 0 0 0 0 0 0 0 0 0 0 0 0 0 0 0 0 0 0 0 0 0 0 0
0 0 0 0 0 0 0 0 0 0 0 0 0 0 84 57 0 0 0 0 0 0 0 0 0 0 0 0 0
0 0 0 0 0 0 0 0 0 0 3 0 0 0 0 0 0 0 0 0 0 0 0 0 0 0 0
M02255_77_000000000-ADUT3_1_1104_17646_14396 T----AC---GT-AG-GAT--------CCG-A-G------C--G--T---T--AT-C-CGG-AG------TG-A--C-T--GG-GC-------GT--A-----AA-GA-GTTGC-------G-TA-G-G-C-G------------G--T-TT-G-T-T-----
------AA----G-C-G-A--------A-T-A--G--TG--A-AA-TC--T-G-GT-G-G-----CT-C-AA------C-C-A-TAC-A-G--A-C----T-A--T-T---A--T-------T--CG-A-A---C-------T--G-G--CA--A-A-C----T-C-G-A-G-A--G---T-AG-----CA-G-A--------G-G-T-A---AC-T------GG--A--ATT----T-
C-T-A-GT--GT-A-G-GA-GT--G---A--A-A---TC-C-GT-AG--AT-A-TT--A-G--A-A-G-G-A-AC-A-CC---GA--T--G--GC-GTA-G--G-C--A-------G--G-T-T-A---CTG--G--GC-T-A--------------------T-T---T-C-T--GA--CG-C-T-A--A-GG--C-A-CG-A--AA-G-C---G-TG--GG-G--
AG-C-G-AA--------CC--GG Bacteria(100);Candidate_division_TM7(100);unclassified(100);unclassified(100);unclassified(100);unclassified(100);unclassified(100);unclassified(100);unclassified(100);

Otu1627 0 0 0 0 0 0 0 0 0 0 0 0 0 0 0 0 0 0 0 0 0 0 0 0 0 0 0 0
0 0 0 0 0 0 0 0 0 0 0 0 0 0 0 0 0 0 0 0 0 0 0 0 0 0 0 0 0
0 0 0 0 0 0 0 0 0 0 0 0 0 0 10 32 0 0 0 0 0 0 0 0 0 0 0 0 0
0 0 0 0 0 0 0 0 0 0 0 0 0 0 0 0 0 0 0 0 0 0 0 0 0 0 0
M02255_77_000000000-ADUT3_1_1102_18362_20766 T----AC---GT-AG-GGG--------GCA-A-G------C--G--T---T--AT-C-CGG-AT------TT-A--C-T--GG-GT-------GT--A-----AA-GG-GA-GC-------G-TA-G-A-C-G------------G--T-AC-T-G-C-----
------AA----G-T-C-T--------G-G-A--G--TG--A-AA-CC--C-G-CG-G-G-----CT-C-AA------C-C-T-G-C-G-G--C-A----T-G--C-T---T--T-------G--GA-A-A---C-------T--G-T--GG--T-A-C----T-G-G-A-G-T--G---T-CG-----GA-G-A--------G-G-T-A---AG-C------GG--A--ATT----
C-C-C-A-GT--GT-A-G-CG-GT--G---A--A-A---TG-C-GT-AG--AT-A-TT--G-G--G-A-A-G-A-AC-A-CC---TG--G--G--GC-GAA-G--G-C--G-------G--C-T-T-A---CTG--G--AC-G-A--------------------T-T---A-C-T--GA--CG-T-T-G--A-GG--C-T-CG-A--AG-G-C---G-TG--GG-
G--AG-C-A-AA--------CA--GG Bacteria(100);unclassified(100);unclassified(100);unclassified(100);unclassified(100);unclassified(100);unclassified(100);unclassified(100);unclassified(100);

Otu1628 0 0 0 0 0 0 0 0 0 0 0 0 0 0 0 0 0 0 0 0 0 0 0 0 0 0 0 0
0 0 0 0 0 0 0 0 0 0 0 0 0 0 0 0 0 0 0 0 0 0 0 0 0 0 0 0 0
0 0 0 0 0 0 0 0 0 0 0 0 0 0 22 10 0 0 0 0 0 0 0 0 0 0 0 0 0
0 0 0 0 0 0 0 0 0 0 0 0 0 0 0 0 0 0 0 0 0 0 0 0 0 0 0
M02255_76_000000000-ADUNE_1_1105_9733_24596 G----TG---CC-AG-CAG--------CCG-C-G---------G--T---A--AT-A-CGT-AG------GG-G--G-C--GA-GC-------GT--T-----GT-CC-GG-AA-------T-TA-C-T-G-G------------G--C-GT-A-A-A-----------GG-
---G-C-A-C--------G-C-T--G--TA--A-AA-GG--A-T-GC-G-G-----CT-T-AA------C-C-G-T-G-T-T--A------T-G--C-G---G--C-------T--GA-G-A---C-------T--G-A--GG--T-G-C----T-G-G-A-G-T--A---C-CG-----GA-G-A--------G-G-C-A---AG-T------GG--A--ATT----C-C-C-A-
GT--GT-A-G-CG-GT--G---A--A-A---TG-C-GT-AG--AT-A-TT--G-G--G-A-A-G-A-AC-A-TC---GG--T--G--GC-GAA-G--G-C--G-------A--C-T-T-G---CTG--G--AC-G-G--------------------T-A---A-C-T--GA--CG-C-T-G--A-GG--T-G-CG-A--AA-G-C---C-AG--GG-T--AG-
C-G-AA--------CG--GG Bacteria(100);Synergistetes(100);Synergistia(100);Synergistales(100);Synergistaceae(100);uncultured(100);unclassified(100);unclassified(100);unclassified(100);

Otu1629 0 0 0 39 0 0 0 0 0 0 0 0 0 0 0 0 0 0 0 0 0 0 0 0 0 0 0 0
0 0 0 0 0 0 0 0 0 0 0 0 0 0 0 0 0 0 0 0 0 0 0 0 0 0 0 0 0
0 0 0 0 0 0 0 0 0 0 0 0 0 0 0 0 0 0 0 0 0 0 0 0 0 0 0 0 0
0 0 0 0 0 0 0 0 0 0 0 0 0 0 0 0 0 0 0 0 0 0 0 0 0 0 0
M02255_77_000000000-ADUT3_1_1103_21753_19784 T----AC---GT-AG-GGG--------GCA-A-G------C--G--T---T--AT-C-CGG-AT------TT-A--C-T--GG-GT-------GT--A-----AA-GG-GA-GC-------G-TA-G-A-C-G------------G--C-GT-A-T-C-----
------AA----G-T-C-T--------G-A-T--G--TG--A-AA-GG--C-A-GG-G-G-----CT-C-AA------C-C-C-C-T-G-G--A-C----T-G--C-A---T--T-------G--GA-A-A---C-------T--G-G--TA--T-G-C----T-C-A-A-G-T--G---C-CG-----GA-G-A--------G-G-T-A---AG-C------GG--A--ATT----
C-C-T-A-GT--GT-A-G-CG-GT--G---A--A-A---TG-C-GT-AG--AT-A-TT--A-G--G-A-G-G-A-AC-A-CC---AG--T--G--GC-GAA-G--G-C--G-------G--C-T-T-A---CTG--G--AC-G-G--------------------T-A---A-C-T--GA--CG-T-T-G--A-GG--C-T-CG-A--AA-G-C---G-TG--GG-
G--AG-C-A-AA--------CA--GG Bacteria(100);Firmicutes(100);Clostridia(100);Clostridiales(100);Lachnospiraceae(100);unclassified(100);unclassified(100);unclassified(100);unclassified(100);

Otu1630 0 0 0 51 0 0 0 0 0 0 0 0 0 0 0 0 0 0 0 0 0 0 0 0 0 0 0 5
4 0 2 0 0 0 0 0 0 0 58 0 0 0 0 0 0 0 0 0 0 0 0 0 0 4 0 0 0
0 0 0 0 0 0 0 0 0 0 0 0 0 0 0 0 0 27 0 0 0 12 0 0 0 0 0 0 0
0 0 0 0 0 0 0 0 0 0 0 0 0 0 0 0 0 0 0 0 0 0 0 0 0 0 0

M02255_77_000000000-ADUT3_1_1105_24209_12964 T----AC---GTAGG-GGG---------CT-A-G------C--G--T---T--AT-C-CGG-AA------TT-A--C-T--GG-GC-------GT--A-----AA-GG-GT-GC-------G-TA-G-G-C-G------------G--T-CT-T-T-C------
-----AA----G-C-C-A--------G-A-A--G--TG--A-AA-GG--C-T-AC-G-G-----CT-C-AA------C-C-G-T-A-G-T--A------A-G--C-T---T--T-------T--GG-A-A---C-------T--G-T--AA--G-A-C----T-T-G-A-G-T--G---C-AG-----GA-G-A--------G-G-A-G---AG-T------GG--A--ATT----C-
C-T-A-GT--GT-A-G-CG-GT--G---A--A-A---TG-C-GT-AG--AT-A-TT--A-G--G-A-G-G-A-AC-A-CC---AG--T--A--GC-GAA-G--G-C--G-------G--C-T-C-T---CTG--G--AC-T-G--------------------T-A---A-C-T--GA--CG-C-T-G--A-GG--C-A-CG-A--AA-G-C---G-TG--GG-G-
-AG-C-A-AA--------CA--GG Bacteria(100);Firmicutes(100);Clostridia(100);Clostridiales(100);Peptostreptococcaceae(100);Incertae_Sedis(100);unclassified(100);unclassified(100);unclassified(100);

Otu1631 7 0 0 0 0 0 0 0 0 0 0 0 0 0 0 0 0 0 0 0 0 0 0 7 0 0 0 0
0 0 0 0 0 0 0 0 0 0 0 0 0 0 10 0 4 0 0 0 0 1 0 0 0 0 0 0 0
0 0 0 1 0 0 0 0 0 0 0 0 0 0 0 0 0 0 0 0 0 0 0 0 0 0 0 0 0
0 0 8 0 0 0 0 0 0 0 1 0 0 0 0 1 0 0 0 0 0 0 0 0 0 4 0

M02255_76_000000000-ADUNE_1_1104_20386_19744 C----TA---AC-TC-TGT--------GCC-A-G------C-----A---G--CC-G-CGG-TA------AT-A--C-A--GA-GG-------GT-----------GC-AA-GC-------G-TT-A-ATC-G------------G--A-TA-A-T-T---------
--AA----G-T-C-A--------A-A-T--G--TG--A-AA-TC--C-C-CG-A-G-----CT-T-AA------C-T-T-G-G-G-A--A-T----T-G--C-A---T--T-------C--GA-T-A---C-------T--G-G--TT--A-G-C----T-A-G-A-G-T--G---T-GG-----GA-G-A--------G-G-A-T---GG-T------AG--A--ATT----C-C-
A-G-GT--GT-A-G-CG-GT--G---A--A-A---TG-C-GT-AG--AG-A-TC--T-G--G-A-G-G-A-AT-A-CC---GA--T--G--GC-GAA-G--G-C--A-------G--C-C-A-T---CTG--G--CC-T-A--------------------A-C---A-C-T--GA--CG-C-T-G--A-GG--T-G-CG-A--AA-G-C---A-TG--GG-G--
AG-C-A-AA--------CA--GG Bacteria(100);Proteobacteria(100);Gammaproteobacteria(100);Pseudomonadales(100);Moraxellaceae(100);Acinetobacter(100);unclassified(100);unclassified(100);unclassified(100);

Otu1632 0 0 0 69 0 0 0 0 0 0 0 0 0 0 0 0 0 0 0 0 0 0 0 0 0 0 0 0
0 0 0 0 0 0 0 0 0 0 0 0 0 0 0 0 0 0 0 0 0 0 0 0 0 0 0 0 0
0 0 0 0 0 0 0 0 0 0 0 0 0 0 0 0 0 0 0 0 0 0 0 0 0 0 0 0 0
0 0 0 0 0 0 0 0 0 0 0 0 0 0 0 0 0 0 0 0 0 0 0 0 0 0 0

M02255_77_000000000-ADUT3_1_1104_13844_23439 T----AC---GT-AG-GGG--------GCA-A-G------C--G--T---T--AT-C-CGG-AT------TT-A--C-T--GG-GT-------GT--A-----AA-GG-GA-GC-------G-TA-G-A-C-G------------G--A-TG-T-G-C-----
------AA----G-T-C-T--------G-A-A--G--TG--A-AA-GC--C-C-GG-G-G-----CT-C-AA------C-C-C-C-G-G-G--A-C----T-G--C-T---T--T-------G--GA-A-A---C-------T--G-T--GT--A-T-C----T-A-G-A-G-T--G---C-CG-----GA-G-A--------G-G-T-A---AG-C------GG--A--ATT----
C-C-C-A-GT--GT-A-G-CG-GT--G---A--A-A---TG-C-GT-AG--AT-A-TT--G-G--G-A-G-G-A-AC-A-CC---AG--T--G--GC-GAA-G--G-C--G-------G--C-T-T-A---CTG--G--AC-G-G--------------------T-A---A-C-T--GA--CG-T-T-G--A-GG--C-T-CG-A--AA-G-C---G-TG--
GG-G--AG-C-A-AA--------CA--GG Bacteria(100);Firmicutes(100);Clostridia(100);Clostridiales(100);Lachnospiraceae(100);unclassified(100);unclassified(100);unclassified(100);unclassified(100);

Otu1633 0 0 0 0 0 0 38 0 20 0 0 0 0 0 0 0 0 0 0 0 0 0 0 0 0 0 0 0
0 0 0 0 0 0 0 0 0 0 0 17 0 0 0 0 0 0 0 0 0 0 0 0 0 0 0 0 0
0 0 0 0 0 0 0 0 0 0 0 0 0 0 0 0 0 0 0 0 0 0 0 0 0 0 0 0 0
0 0 0 0 0 0 0 0 0 0 0 0 0 0 0 0 0 0 0 0 0 0 0 0 0 0 0

M02255_77_000000000-ADUT3_1_1101_5753_16798 T----AC---GT-AG-GTG--------GCA-A-G------C--G--T---T--GT-C-CGG-AA------TT-A--C-T--GG-GT-------GT--A-----AA-GG-GA-GC-------G-TA-G-G-C-G------------G--G-CA-G-G-C----------
AA----G-T-C-A--------G-G-C--G--TG--A-AA-TA--T-A-TC-G-G-----CT-C-AA------C-C-G-G-T-A-A----C----G-G--C-G---C--T-------T--GA-A-A---C-------T--G-C--AG--G-T-C----T-T-G-A-G-T--G---A-AG-----TA-G-A--------G-G-T-T---GG-C------GG--A--ATT----C-C-T-
A-GT--GT-A-G-CG-GT--G---A--A-A---TG-C-GT-AG--AT-A-TT--A-G--G-A-G-G-A-AC-A-CC---AG--T--G--GC-GAA-G--G-C--G-------G--C-C-A-A---CTG--G--GC-T-T--------------------T-T---A-C-T--GA--CG-C-T-G--A-GG--C-T-CG-A--AA-G-T---G-TG--GG-G--
AG-C-A-AA--------CA--GG Bacteria(100);Firmicutes(100);Clostridia(100);Clostridiales(100);unclassified(100);unclassified(100);unclassified(100);unclassified(100);unclassified(100);

Otu1634 0 0 0 0 0 0 32 3 56 0 0 0 0 0 0 0 0 0 0 0 0 0 0 0 0 0 0 0
0 0 0 0 0 0 0 0 0 0 0 0 0 0 0 0 0 0 0 0 0 0 0 0 0 0 0 0 0
0 0 0 0 0 0 0 0 0 0 0 0 0 0 0 0 0 0 0 0 0 0 0 0 0 0 0 0 0
0 0 0 0 0 0 0 0 0 0 0 0 0 0 0 0 0 0 0 0 0 0 0 0 0 0 0

M02255_77_000000000-ADUT3_1_1101_19481_13943 T----AC---GT-AG-GGA--------GCG-A-G------C--G--T---T--GT-C-CGG-AA------TT-A--C-T--GG-GT-------GT--A-----AA-GG-GT-GC-------G-TA-G-G-C-G------------G--A-TA-C-G-C----
-------AA----G-T-C-A--------G-T-A--G--TG--A-AA-TG--C-A-TG-G-G-----CT-T-AA------C-C-C-A-T-G-A--C-G----T-G--C-T---A--T-------T--GA-A-A---C-------T--G-T--GT--A-T-C----T-T-G-A-G-T--G---A-AG-----TA-G-A--------G-G-C-A---GG-C------GG--A--ATT----C-
C-C-G-GT--GT-A-G-CG-GT--G---A--A-A---TG-C-GT-AG--AG-A-TC--G-G--G-A-G-G-A-AC-A-CC---AG--T--G--GC-GAA-G--G-C--G-------G--C-C-T-G---CTG--G--GC-T-T--------------------T-A---A-C-T--GA--CG-C-T-G--A-GG--C-A-CG-A--AA-G-T---G-TG--GG-
T--AG-C-A-AA--------CA--GG Bacteria(100);unclassified(100);unclassified(100);unclassified(100);unclassified(100);unclassified(100);unclassified(100);unclassified(100);unclassified(100);

Otu1635 0 0 0 0 15 0 0 0 0 0 0 0 0 0 0 0 0 0 0 0 0 0 0 0 0 0 0 0
0 0 0 0 0 0 0 0 0 0 0 0 0 0 0 0 0 0 0 0 0 0 0 0 0 0 0 0 0
0 0 0 0 0 0 0 0 0 0 0 0 0 0 0 0 0 0 0 0 0 0 0 0 0 0 0 0 0
0 0 0 0 0 0 0 0 0 0 0 0 0 0 0 0 0 0 0 0 0 0 0 0 0 0 0 0
M02255_76_000000000-ADUNE_1_1107_14196_19934 .....AC----T-AC-GT---------GCC-A-G------C--A--G---C--CG-C-GGT-AA------TA-C--G-T--AG-GT-------GG--C-----AA-GC-G--TT-------G-TC-C-G---G------------A--T-GA-C-T-T----------
AA----G-T-C-T--------G-A-T--G--TG--A-AA-GC--C-C-AC-G-G-----CT-C-AA------C-C-G-T-G-G-A--G------G-GT-C-A---T--T-------G--GA-A-A---C-------T--G-G--GT--C-A-C----T-T-G-A-G-T--A---C-AG-----AA-G-A--------G-G-A-A---AG-T------GG--A--ATT----C-C-
A-T-GT--GT-A-G-AG-GT--G---A--A-A---TT-C-GT-AG--AT-A-TA--T-G--G-A-G-G-A-AC-A-CC---AG--T--G--GC-GAA-A--G-C--G-------A--C-T-T-T---CTA--T--TC-T-G--------------------A-C---A-C-T--GA--CG-C-T-G--A-GG--C-T-CG-A--AA-G-C---G-TG--GG-T--
AG-C-A-AA--------CA--GG Bacteria(100);Firmicutes(100);Bacilli(100);Lactobacillales(100);unclassified(100);unclassified(100);unclassified(100);unclassified(100);unclassified(100);

Otu1636 0 0 0 5 0 0 0 0 0 0 0 0 0 0 0 0 0 0 0 0 0 0 0 0 0 0 0 0
0 0 0 0 0 0 0 0 0 1 1 1 0 0 6 0 0 0 0 0 0 0 0 0 0 0 0 0 0
0 0 0 0 0 0 0 0 0 0 0 0 0 0 2 0 0 0 0 0 0 0 0 0 0 0 0 0 0
0 0 0 0 0 0 0 0 0 0 0 0 0 0 0 0 0 0 0 0 0 0 0 0 0 0 0 0
M02255_77_000000000-ADUT3_1_1104_17411_19641 C----GG---CT-AA-CT---------ACG-T-G------C--C--A---G--CA-G-CCG-CG------GT-A--A-T--AC-GT-------AG--G-----TC-CC-GA-GC-------G-TT-GTC-C-G------------G--T-TT-A-T-T-------
----AA----G-T-C-T--------G-G-T--G--TA--A-AA-GG--C-A-GT-G-G-----CT-C-AA------C-C-A-T-T-G-T--A------T-G--C-A---T--T-------G--GA-A-A---C-------T--G-G--TA--G-A-C----T-T-G-A-G-T--G---C-AG-----GA-G-A--------G-G-A-G---AG-T------GG--A--ATT----C-
C-A-T-GT--GT-A-G-CG-GT--G---A--A-A---TG-C-GT-AG--AT-A-TA--T-G--G-A-G-G-A-AC-A-CC---GG--T--G--GC-GAA-A--G-C--G-------G--C-T-C-T---CTG--G--CC-T-G--------------------T-A---A-C-T--GA--CA-C-T-G--A-GG--C-T-CG-A--AA-G-C---G-TG--GG-G-
-AG-C-A-AA--------CA--GG Bacteria(100);Firmicutes(100);Bacilli(100);Lactobacillales(100);Streptococcaceae(100);Lactococcus(100);unclassified(100);unclassified(100);unclassified(100);

Otu1637 0 0 0 0 0 0 0 0 0 0 21 0 0 0 0 0 0 0 0 0 0 0 0 0 0 0 0 0
0 0 0 0 0 0 0 0 0 0 0 0 0 0 0 0 0 0 0 0 0 0 0 0 0 0 0 0 0
0 0 0 0 0 0 0 0 0 0 0 0 0 0 0 0 0 0 0 0 0 0 0 0 0 0 0 0 0
0 0 0 0 0 0 0 0 0 0 0 0 0 0 0 0 0 0 0 0 0 0 0 0 0 0 0 0
M02255_76_000000000-ADUNE_1_1119_4959_17893 C----GG---CT-AA-CT---------CCG-T-G------C--C--A---G--CA-G-CCG-CG------GT-A--A-T--AC-GT-------AG--G-----TG-GC-AA-GC-------G-TT-GTC-C-G------------G--A-TC-T-T-T----------AA--
--G-T-C-T--------G-A-T--G--TG--A-AA-GC--C-C-CC-G-G-----CT-C-AA------C-C-G-G-G-G-A--G------G-GT-C-A---T--T-------G--GA-A-A---C-------T--G-G--AG--A-A-C----T-T-G-A-G-T--G---C-AG-----AA-G-A--------G-G-A-G---AG-T------GG--A--ATT----C-C-A-C-
GT--GT-A-G-CG-GT--G---A--A-A---TG-C-GT-AG--AT-A-TG--T-G--G-A-G-G-A-AC-A-CC---AG--T--G--GC-GAA-G--G-C--G-------A--C-T-C-T---CTG--G--TC-T-G--------------------T-A---A-C-T--GA--CG-C-T-G--A-GG--C-T-CG-A--AA-G-C---G-TG--GG-G--AG-C-
A-AA--------CA--GG Bacteria(100);Firmicutes(100);Bacilli(100);unclassified(100);unclassified(100);unclassified(100);unclassified(100);unclassified(100);unclassified(100);

Otu1638 0 0 0 0 0 0 0 0 0 0 0 0 0 0 0 0 0 0 0 0 0 0 3 0 0 1 0
0 0 0 0 0 0 0 0 0 0 0 0 0 0 0 0 0 0 0 0 0 0 0 0 0 0 0 0 0
0 0 0 0 0 0 0 0 0 0 0 0 0 0 0 0 0 0 0 0 0 0 0 0 0 0 0 0 0
0 0 0 0 0 0 0 0 0 0 0 0 0 0 0 0 0 0 0 0 0 0 0 0 0 0 0 0
M02255_76_000000000-ADUNE_1_1108_14636_22495 T----AA---GC-AC-CGG--------CTA-A-C------T--C--T---G--TG-C-CAG-CA------GC-C--G-C--GG-TA-------AT--AC---AGA-GG-GT-GC-------A-AG-C-G-T-T------------A--A-TC-G-G-T----
-------AA----G-T-C-A--------G-A-T--G--TG--A-AA-TC--C-C-CG-G-G-----CT-C-AA------C-C-T-G-G-G-A--A-C----T-G--C-A---T--C-------T--GA-T-A---C-------T--G-T--TG--A-G-C----T-A-G-A-G-T--A---T-GT-----GA-G-A--------G-G-A-A---GG-T------AG--A--ATT----
C-C-A-G-GT--GT-A-G-CG-GT--G---A--A-A---TG-C-GT-AG--AG-A-TC--T-G--G-A-G-G-A-AT-A-CC---GA--T--G--GC-GAA-G--G-C--A-------G--C-C-T-T---CTG--G--CA-T-A--------------------A-T---A-C-T--GA--CA-C-T-G--A-GG--C-T-CG-A--AA-G-C---G-TG--GG-
T--AG-C-A-AA--------CA--GG Bacteria(100);Proteobacteria(100);Gammaproteobacteria(100);Pseudomonadales(100);Moraxellaceae(100);unclassified(100);unclassified(100);unclassified(100);unclassified(100);

Otu1639 0 0 0 0 0 0 0 0 59 0 0 0 0 0 0 0 0 0 0 0 0 0 0 0 0 0 0 0
0 0 0 0 0 0 0 0 0 0 0 0 0 0 0 0 0 0 0 0 0 0 0 0 0 0 0 0 0
0 0 0 0 0 0 0 0 0 0 0 0 0 0 0 0 0 0 0 0 0 0 0 0 0 0 0 0 0
0 0 0 0 0 0 0 0 0 56 0 0 0 0 0 0 0 0 0 0 0 0 0 0 0 0 0 0
M02255_76_000000000-ADUNE_1_1110_27863_13995 C----GG---CT-A--ACT--------ATG-T-G------C--C--A---G--CA-G-CCG-CG------GT-A--A-T--AC-AT-------AG--G-----AT-GC-GA-GC-------GTTA-T-C-C-G------------G--A-TT-T-A-C-------
----AA----G-T-C-T--------G-G-T--G--TG--A-AA-GG--C-A-AT-T-G-----CT-T-AA------C-G-ATT---G-T--T------T-G--C-A---T--T-------G--GA-A-A---C-------T--G-T--AA--G-T-C----T-A-G-A-G-T--A---T-AG-----TA-G-G--------G-A-G-T---TC-T------GG--A--ACT----C-C-
A-T-GT--GG-A-G-CG-GT--G---G--A-A---TG-C-GT-AG--AT-A-TA--T-G--G-A-A-G-A-AC-A-CC---AG--T--G--GC-GAA-G--G-C--G-------A--G-A-A-C---TTT--G--GC-T-A--------------------T-A---A-C-T--GA--CG-C-T-T--A-GG--C-T-CG-A--AA-G-T---G-TG--GG-G--
AG-C-A-AA--------TA--GG Bacteria(100);Firmicutes(100);Mollicutes(100);Mycoplasmatales(100);Mycoplasmataceae(100);unclassified(100);unclassified(100);unclassified(100);unclassified(100);

Otu1640 0 0 0 0 0 0 0 0 15 0 0 0 0 0 0 0 0 0 0 0 0 0 0 0 0 0 0 0
0 0 0 0 0 0 0 0 0 0 0 0 0 0 0 0 0 0 0 0 0 0 0 0 0 0 0 0
0 0 0 0 0 0 0 0 0 0 0 0 0 0 0 0 0 0 0 0 0 0 0 0 0 0 0 0
0 0 0 0 0 0 0 0 0 0 0 0 0 0 1 0 0 0 0 0 0 0 0 0 0 0 0
M02255_76_000000000-ADUNE_1_2112_20153_7675 T----AC---GT-AG-GGG--------GCA-A-G------C--G--T---T--AT-C-CGG-AT------TT-A--C-T--GG-GT-------GT--A-----AA-GG-GA-GC-----------G---C-A------------G----A--T-G-C----------AA----
G-T-C-T--------G-G-A--G--TG--A-AA-GC--C-C-GG-G-G-----CT-C-AA------C-C-C-C-G-G-G--A-C----T-G--C-T---T--T-------G--GA-A-A---C-------T--G-T--AT--G-G-C----T-A-G-A-G-T--G---C-TG-----GA-G-A--------G-G-C-A---AG-C------GG--A--ATT----C-C-T-A-GT-
-GT-A-G-CG-GT--G---A--A-A---TG-C-GT-AG--AT-A-TT--A-G--G-A-A-G-A-AC-A-CC---AG--T--G--GC-GAA-G--G-C--G-------G--C-T-T-G---CTG--G--AC-A-G--------------------T-A---A-C-T--GA--CG-T-T-C--A-GG--C-T-CG-A--AA-G-C---G-TG--GG-G--AG-C-A-
AA--------CA--GG Bacteria(100);Firmicutes(100);Clostridia(100);Clostridiales(100);Lachnospiraceae(100);unclassified(100);unclassified(100);unclassified(100);unclassified(100);

Otu1641 0 0 0 0 12 0 0 0 0 0 0 0 0 0 0 0 0 0 1 0 0 0 0 0 0 0 0 0
0 0 0 0 0 0 0 0 0 0 0 5 0 0 0 0 0 0 0 0 0 0 0 0 0 0 0 0
0 0 0 0 1 0 0 0 0 0 0 0 0 0 0 0 0 0 0 0 0 0 0 0 6 0 0 0 0 0
0 0 0 0 0 0 0 0 0 0 0 0 0 0 0 3 0 0 0 0 0 0 0 0 0 0 0
M02255_76_000000000-ADUNE_1_2105_15191_16657 T----AC---GA-AG-GGG--------GCT-A-G------C--G--T---T--GC-T-CGG-AA------TT-A--C-T--GG-GC-------GT--A-----AA--G-G--GC-----------G-G-C-G------------T--A-GG----------------
TA----G-T-C-A--------G-G-C--G--TG--A-AA-TT--C-C-TG-G-G-----CT-C-AA------C-C-T-G-G-G-G--A-C----T-G--C-G---C--T-------T--GA-T-A---C-------G--G-C--TA--G-G-C----T-A-G-A-G-G--A---T-GG-----AA-G-A--------G-G-G-T---CG-C------GG--A--ATT----C-C-
C-A-GT--GT-A-G-AG-GT--G---A--A-A---TT-C-GT-AG--AT-A-TT--G-G--G-A-A-G-A-AC-A-CC---GG--T--G--GC-GAA-G--G-C--G-------G--C-G-A-C---CTG--G--TC-C-A--------------------T-T---A-C-T--GA--CG-C-T-G--A-GG--C-G-CG-A--CA-G-C---G-TG--GG-G--
AG-C-A-AA--------CA--GG Bacteria(100);Proteobacteria(100);Alphaproteobacteria(100);Rhodospirillales(100);Acetobacteraceae(100);unclassified(100);unclassified(100);unclassified(100);unclassified(100);

Otu1642 0 0 0 0 0 0 0 0 79 0 0 0 0 0 0 0 0 0 0 0 0 0 0 0 0 0 0 0
0 0 0 0 0 0 0 0 0 0 0 0 0 0 0 0 0 0 0 0 0 0 0 0 0 0 0 0
0 0 0 0 0 0 0 0 0 0 0 0 0 0 0 0 0 0 0 0 0 0 0 0 0 0 0 0
0 0 0 0 0 0 0 0 0 0 0 0 0 0 0 0 0 0 0 0 0 0 0 0 4 0 0
M02255_77_000000000-ADUT3_1_1101_6754_3878 T----AC---GT-AG-GGA--------GCG-A-G------C--G--T---T--GT-C-CGG-AA------TT-A--C-T--GG-GT-------GT--A-----AA-GG-GA-GC-------G-TA-G-G-C-G------------G--G-AA-A-G-C----------
AA----G-T-T-G--------G-A-A--G--TG--A-AA-TG--C-A-TG-G-G-----CT-T-AA------C-C-C-A-T-G-A--G-C----T-G--C-T---T--T-------C--AA-A-A---C-------T--G-T--TT--T-T-C----T-T-G-A-G-T--G---A-AG-----TA-G-A--------G-G-C-A---GG-C------GG--A--ATT----C-C-T-
A-GT--GT-A-G-CG-GT--G---A--A-A---TG-C-GT-AG--AT-A-TT--A-G--G-A-G-G-A-AC-A-CC---AG--T--G--GC-GAA-G--G-C--G-------G--C-C-T-G---CTG--G--GC-T-T--------------------T-A---A-C-T--GA--CG-C-T-G--A-GG--C-T-CG-A--AA-G-C---G-TG--GG-T--AG-
C-A-AA--------CA--GG Bacteria(100);Firmicutes(100);Clostridia(100);Clostridiales(100);Ruminococcaceae(100);unclassified(100);unclassified(100);unclassified(100);unclassified(100);

Otu1643 0 0 0 0 0 0 0 0 0 0 0 0 0 7 0 0 0 0 0 0 0 0 0 0 0 0 0 0
0 0 0 0 1 0 0 0 0 0 0 0 0 0 0 0 0 0 0 0 0 0 0 0 0 0 0 0
0 0 0 0 0 0 0 0 0 0 0 0 0 0 0 0 0 0 0 0 0 0 0 0 0 0 0 0
0 0 0 0 0 0 0 0 0 0 0 0 0 0 0 0 0 0 0 0 0 0 0 0 0 0 0
M02255_77_000000000-ADUT3_1_2108_8463_21662 G----AC---AG-AG-GAT--------GCA-A-G------C--G--T---T--AT-C-CGA-AA------TG-A--T-T--GG-GC-------GT--A-----AA-GC-GT-CT-------G-TA-G-G-T-G------------G--A-TT-T-T-C----------AA--
--G-T-C-C--------G-C-C--G--TC--A-AA-TC--C-C-AG-G-G-----CT-C-AA------C-C-C-T-A-G-A--T-A----G-G--C-G---G--T-------G--GA-A-A---C-------T--C-C--CA--A-G-C----T-G-G-A-G-T--A---C-AG-----TA-G-G--------G-G-T-A---GA-G------GG--A--ATT----T-C-T-G-
GT--GG-A-G-TC-AT--G---A--A-A---TG-T-GT-TG--AG-A-TC--A-G--A-A-A-G-A-AC-A-CC---AA--T--G--GA-GAA-A--G-C--A-------C--T-T-T-G---CTG--G--GC-C-G--------------------A-C---A-C-T--AA--CA-C-T-G--A-GA--G-A-TG-A--AA-T-C---T-AG--GG-G--AG-C-
A-AA--------TG--GG Bacteria(100);Cyanobacteria(100);unclassified(100);unclassified(100);unclassified(100);unclassified(100);unclassified(100);unclassified(100);unclassified(100);

Otu1644 0 0 0 89 0 0 0 0 0 0 0 0 0 0 0 0 0 0 0 0 0 0 0 0 0 0 0 0
0 0 0 0 0 0 0 0 0 0 0 0 0 0 0 0 0 0 0 0 0 0 0 0 0 0 0 0
0 0 0 0 0 0 0 0 0 0 0 0 0 0 0 0 0 0 0 0 0 0 0 0 0 0 0 0
0 0 0 0 0 0 0 0 0 0 0 0 0 0 0 0 0 0 0 0 0 0 0 0 0 0 0
M02255_77_000000000-ADUT3_1_1104_11174_6477 T----AC---GG-AG-GGG--------GCT-A-G------C--G--T---T--GT-T-CGG-AA------TT-A--C-T--GG-GC-------GT--A-----AA-GC-GC-AC-------G-TA-G-G-C-G------------G--T-TA-T-T-C----------AA-
---G-T-C-A--------G-A-G--G--TG--A-AA-GC--C-C-GG-A-G-----CT-C-AA------C-T-C-C-G-G-A--A-C----T-G--C-C---T--T-------T--GA-A-A---C-------T--A-G--AT--A-A-C----T-C-G-A-A-T--C---A-TG-----GA-G-A--------G-G-C-G---AG-T------GG--A--ATT----C-C-G-A-
GT--GT-A-G-AG-GT--G---A--A-A---TT-C-GT-AG--AT-A-TT--C-G--G-A-A-G-A-AC-A-CC---AG--T--G--GC-GAA-G--G-C--G-------G--C-T-C-G---CTG--G--AC-A-T--------------------G-T---A-T-T--GA--CG-C-T-G--A-GG--T-G-CG-A--AA-G-C---G-TG--GG-G--AG-
C-A-AA--------CA--GG Bacteria(100);Proteobacteria(100);Alphaproteobacteria(100);Sphingomonadales(100);unclassified(100);unclassified(100);unclassified(100);unclassified(100);unclassified(100);

Otu1645 0 0 0 83 0 0 0 0 0 0 0 0 0 0 0 0 0 0 0 0 0 0 0 0 0 0 0 0
0 0 0 0 0 0 0 0 0 0 0 0 0 0 0 0 0 0 0 0 0 0 0 0 0 0 0 0 0
0 0 0 0 0 0 0 0 0 0 0 0 0 0 0 0 0 0 0 0 0 0 0 0 0 0 0 0 0
0 0 0 0 0 0 0 0 0 0 0 0 0 0 0 29 0 0 0 0 0 0 0 0 0 0 0 0
M02255_77_000000000-ADUT3_1_1105_6555_17898 T----AC---GT-AG-GTG--------GCA-A-G------C--G--T---T--GT-C-CGG-AT------TT-A--C-T--GG-GC-------GT--A-----AA-GC-GT-GC-------G-CA-G-G-C-G-------------G--A-CT-T-T-T-----------AA--
--G-T-A-G--------A-A-A--G--TG--A-AA-GG--T-T-GG-A-G-----CT-C-AA------C-T-C-C-G-A-C--A-C----T-G--C-T---T--C-------C--TA-T-A---C-------T--G-G--GA--G-T-C----T-T-G-A-A-T--G---C-CG-----GA-G-A--------G-G-A-A---GA-T------GG--A--ACG----A-C-A-C-
GT--GT-A-G-CG-GT--G---A--A-A---TG-C-GT-TG--AG-A-TG--T-G--T-C-G-G-A-AC-A-CC---AA--T--G--GC-GAA-A--G-C--A-------A--T-C-T-T---CTG--G--AC-G-G---------------------A-T---A-T-T--GA--CG-C-T-C--A-TG--C-A-CG-A--AA-G-C---T-AA--GG-T--AG-C-
A-AA--------CA--GG Bacteria(100);unclassified(100);unclassified(100);unclassified(100);unclassified(100);unclassified(100);unclassified(100);unclassified(100);unclassified(100);

Otu1646 0 0 0 67 0 0 0 0 0 0 0 0 0 0 0 0 0 0 0 0 0 0 0 0 0 0 0 0
0 0 0 0 0 0 0 0 0 0 0 0 0 0 0 0 0 0 0 0 0 0 0 0 0 0 0 0 0
0 0 0 0 0 0 0 0 0 0 0 0 0 0 0 0 0 0 0 0 0 0 0 0 0 0 0 0 0
0 0 0 0 0 0 0 0 0 0 0 0 0 0 0 0 0 0 0 0 0 0 0 0 0 0 0 0
M02255_77_000000000-ADUT3_1_1103_10815_19532 T----AC---GA-AG-GGG--------GCT-A-G------C--G--T---T--GC-T-CGG-AA------TT-A--C-T--GG-GC-------GT--A-----AA-GG-GC-GC-------G-TA-G-G-C-G-------------G--A-CA-G-T-T----
-------AA----G-T-T-G--------G-G-G--G--TG--A-AA-GC--C-C-GG-G-G-----CT-C-AA------C-C-T-C-G-G-A--A-T----T-G--C-C---T--T-------C--AA-T-A---C-------T--G-G--CT--G-T-C----T-T-G-A-G-T--G---T-GA-----GA-G-A--------G-G-T-G---TG-T------GG--A--ACT----
C-C-G-A-GT--GT-A-G-AG-GT--G---A--A-A---TT-C-GT-AG--AT-A-TT--C-G--G-A-A-G-A-AC-A-CC---AG--T--G--GC-GAA-G--G-C--G-------A--C-A-C-A---CTG--G--CT-C-A---------------------C-T---A-C-T--GA--CG-C-T-G--A-GG--C-G-CG-A--AA-G-C---G-TG--GG-
G--AG-C-A-AA--------CA--GG Bacteria(100);Proteobacteria(100);Alphaproteobacteria(100);Caulobacterales(100);Caulobacteraceae(100);unclassified(100);unclassified(100);unclassified(100);unclassified(100);

Otu1647 0 0 0 0 0 0 22 10 26 0 0 0 0 0 0 0 0 0 0 0 0 0 0 0 0 0 0 0
0 0 0 0 0 0 0 0 0 0 0 6 0 0 0 0 0 0 0 0 0 0 0 0 0 0 0 0 0
0 0 0 0 0 0 0 0 0 0 0 0 0 0 0 0 0 0 0 0 0 0 0 0 0 0 0 0 0
0 0 0 0 0 0 0 0 0 0 0 0 0 0 0 0 0 0 0 0 0 0 0 0 0 0 0 0
M02255_76_000000000-ADUNE_1_1104_20163_7171 T----CG---GC-TA-ACT--------CCG-T-GC-----C--A--G---C--AG-C-CGC-GG------TA-A--T-A--CG-GA-------GG--A-----TC-CG-AG-CG-------T-TA-T-C-C-G-------------G--A-TT-TAT-T-----------AA-
---G-T-C-A--------G-C-G--G--TG--A-AA-GT--C-T-GT-G-G-----CT-C-AA------C-C-A-T-A-G-A--A-T----T-G--C-C---G--T-------T--GA-A-A---C-------T--G-G--GG--G-G-C----T-T-G-A-G-T--A---T-GT-----TT-G-A--------G-G-C-A---GG-C------GG--A--ATG----C-G-T-G-
GT--GT-A-G-CG-GT--G---A--A-A---TG-C-AT-AG--AT-A-TC--A-C--G-C-A-G-A-AC-C-CC---GA--T--T--GC-GAA-G--G-C--A-------G--C-C-T-G---CCA--A--GC-C-A---------------------T-G---A-C-T--GA--CG-C-T-G--A-TG--C-A-CG-A--AA-G-C---G-TG--GG-G--AT-C-
A-AA--------CA--GG Bacteria(100);Bacteroidetes(100);Bacteroidia(100);Bacteroidales(100);Porphyromonadaceae(100);Parabacteroides(100);unclassified(100);unclassified(100);unclassified(100);

Otu1648 0 0 0 0 0 0 18 0 15 0 0 0 0 0 0 0 0 0 0 0 0 0 0 0 0 0 0 0
0 0 0 0 0 0 0 0 0 0 0 0 0 0 0 0 0 0 0 0 0 0 0 0 0 0 0 0 0
0 0 0 0 0 0 0 0 0 0 0 0 0 0 0 0 0 0 0 0 0 0 0 0 0 0 0 0 0
0 0 0 0 0 0 0 0 0 0 0 0 0 0 0 0 0 0 0 0 0 0 0 0 0 0 0 0
M02255_76_000000000-ADUNE_1_1101_18729_15257 T----AC---GT-AG-GGG--------GCA-A-G------C--G--T---T--AT-C-CGG-AT------TT-A--C-T--GG-GT-------GT--A-----AA-GG-GA-GC-------G----G---C---------------A--T-GA-T--------------
-AA----G-T-C-T--------G-A-T--G--TG--A-AA-AC--C-C-AA-G-G-----CT-C-AA------C-C-A-T-G-G-G--A-C----T-G--C-A---T--T-------G--GA-A-A---C-------T--G-T--CG--T-G-C----T-G-G-A-G-T--G---T-CG-----GA-G-A--------G-G-T-A---AG-C------GG--A--ATT----C-C-
T-A-GT--GT-A-G-CG-GT--G---A--A-A---TG-C-GT-AG--AT-A-TT--A-G--G-A-G-G-A-AC-A-CC---AG--T--G--GC-GAA-G--G-C--G-------G--C-T-T-A---CTG--G--AC-G-A---------------------T-G---A-C-T--GA--CG-C-T-G--A-GG--C-T-CG-A--AA-G-C---G-TG--GG-G--
AG-C-A-AA--------CA--GG Bacteria(100);Firmicutes(100);Clostridia(100);Clostridiales(100);Lachnospiraceae(100);unclassified(100);unclassified(100);unclassified(100);unclassified(100);

Otu1649 0 0 0 0 0 0 0 0 0 0 0 0 0 0 0 0 0 0 0 0 0 0 0 0 0 0 0 0
0 0 0 0 0 0 0 0 0 0 0 0 0 0 0 0 0 0 0 0 0 0 0 0 0 0 0 0 0
0 0 0 0 0 0 0 0 0 0 0 0 0 0 7 0 0 0 0 0 0 0 0 0 0 0 0 0 0
0 0 0 0 0 0 0 0 0 0 0 0 0 0 0 0 0 0 0 0 0 0 4 0 0 0 0 0
M02255_77_000000000-ADUT3_1_1101_3866_9648 T----AC---GT-AG-GGT--------GCG-A-G------C--G--T---T--GT-C-CGG-AA------TT-A--T-T--GG-GC-------GT--A-----AA-GA-GC-TC-------G-TA-G-G-C-G-------------G--T-CT-G-T-C-----------GC-
---G-T-C-G--------A-T-C--G--TG--A-AA-AC--T-T-GG-G-G-----CT-T-AA------C-C-C-T-G-A-G--C-T----T-G--C-G---G--T-------C--GA-T-A---C-------G--G-G--CAT-G-A-C----T-G-G-A-G-T--T---C-GG-----CA-G-G--------G-G-A-G---AC-T------GG--A--ATT----C-C-T-G-
GT--GT-A-G-CG-GT--G---A--A-A---TG-C-GC-AG--AT-A-TC--A-G--G-A-G-G-A-AC-A-CC---GG--T--G--GC-GAA-G--G-C--G-------G--G-T-C-T---CTG--G--GC-C-G---------------------A-T---A-C-T--GA--CG-C-T-G--A-GG--A-G-CG-A--AA-G-C---G-TG--GG-G--AG-
C-G-AA--------CA--GG Bacteria(100);Actinobacteria(100);Actinobacteria(100);Actinobacteridae(100);Actinomycetales(100);Pseudonocardineae(100);Pseudonocardiaceae(100);Pseudonocardia(100);unclassified(100);

Otu1650 3 0 0 0 0 0 0 0 0 0 0 0 0 0 0 0 0 0 0 0 0 0 0 0 0 1 0 0
0 0 0 0 0 0 0 0 0 0 0 0 0 0 0 0 0 0 0 0 0 0 0 0 0 0 0 0 0
0 0 0 0 0 0 0 0 0 0 0 0 0 0 0 0 0 5 0 0 0 0 0 0 0 0 0 0 0
0 5 0 0 0 0 0 0 0 0 0 0 0 0 0 0 0 0 0 0 0 0 0 0 0 0 0

M02255_77_000000000-ADUT3_1_2117_6779_9115 C----TC---CG-TG-CCA--------GCAGCCG------CG-G--T---A--AT-A-CGG-AG------GG-T--G-C--AA-GC-------GT--T-----AT-CC-G--GA-------T-TC-A-C-T-G------------G--G-TT-G-G-T----------
AA----G-T-C-A--------G-T-G--G--TG--A-AA-TC--T-C-CG-T-G-----CT-T-AA------C-A-T-G-G-A-A--A-C----T-G--C-C---A--T-------T--GA-T-A---C-------T--A-C--TG--G-T-C----T-T-G-A-A-T--T---T-TC-----TG-G-A--------G-G-T-A---AG-C------GG--A--ATA----T-G-T-C-
AT--GT-A-G-CG-GT--G---A--A-A---TG-C-TT-AG--AT-A-TG--A-C--A-T-A-G-A-AC-A-CC---AA--T--T--GC-GAA-G--G-C--A-------G--C-T-T-A---CTA--C--AG-G-G--------------------A-T---A-T-T--GA--CA-C-T-G--A-GG--C-A-CG-A--AA-G-C---G-TG--GG-G--AT-C-
A-AA--------CA--GG Bacteria(100);Bacteroidetes(100);Sphingobacteria(100);Sphingobacteriales(100);Chitinophagaceae(100);unclassified(100);unclassified(100);unclassified(100);unclassified(100);

Otu1651 0 0 0 0 0 0 0 0 22 0 0 0 0 0 0 0 0 0 0 0 0 0 0 0 0 0 0 0
0 0 0 0 0 0 0 0 0 0 0 0 0 0 0 0 0 0 0 0 0 0 0 0 0 0 0 0 0
0 0 0 0 0 0 0 0 0 0 0 0 0 0 0 0 0 0 0 0 0 0 0 0 0 0 0 0 0
0 0 0 0 0 0 0 0 0 0 0 0 0 0 0 0 0 0 0 0 0 0 0 0 0 0 0

M02255_77_000000000-ADUT3_1_1103_3735_19287 T----AC---GT-AG-GGG--------GCA-A-G------C--G--T---T--AT-C-CGG-AA------TT-A--T-T--GG-GC-------GT--A-----AA-GA-GT-GC-------G-TA-G-G-T-G------------G--T-TA-C-C-T----------AA-
---G-C-G-C--------A-G-G--G--TA--T-AA-GG--C-A-AT-G-G-----CT-C-AA------C-C-A-T-T-G-T--T------G-G--C-C---C--T-------G--CG-A-A---C-------T--G-G--GC--T-A-C----T-T-G-A-G-T--G---C-AG-----GA-G-A--------G-G-A-A---AG-C------GG--A--ATT----C-C-T-A-
GT--GT-A-G-CG-GT--G---A--A-A---TG-C-GT-AG--AT-A-TT--A-G--G-A-G-G-A-AC-A-CC---AG--T--G--GC-GAA-G--G-C--G-------G--C-T-T-T---CTG--G--AC-T-G--------------------T-A---A-C-T--GA--CA-C-T-G--A-GG--C-A-CG-A--AA-G-C---G-TG--GG-G--AG-
C-A-AA--------CA--GG Bacteria(100);Firmicutes(100);Clostridia(100);Clostridiales(100);Family_XIII_Incertae_Sedis(100);unclassified(100);unclassified(100);unclassified(100);unclassified(100);

Otu1652 0 0 0 74 0 0 0 0 0 0 0 0 0 0 0 0 0 0 0 0 0 0 0 0 0 0 0 0
0 0 0 0 0 0 0 0 0 0 0 0 0 0 0 0 0 0 0 0 0 0 0 0 0 0 0 0 0
0 0 0 0 0 0 0 0 0 0 0 0 0 0 0 0 0 0 0 0 0 0 0 0 0 0 0 0 0
0 0 0 0 0 0 0 0 0 0 0 0 0 0 0 0 0 0 0 0 0 0 0 0 0 0 0

M02255_77_000000000-ADUT3_1_1101_19494_14229 T----AC---GG-AG-GGG--------GCA-A-G------C--G--T---T--GT-T-CGG-AT------TT-A--C-T--GG-GC-------GT--A-----AA-GG-GC-GC-------G-TA-G-G-C-G------------G--T-TT-A-G-C----
-------AA----G-T-C-A--------C-T-T--G--TG--A-AA-TC--T-T-TG-G-G-----CT-T-AA------C-T-C-A-A-A-G--C-G----G-C--C-A---A--G-------T--GA-T-A---C-------T--G-C--TA--G-A-C----T-A-G-A-G-T--G---C-AG-----AA-G-G--------G-G-C-A---AT-C------GG--A--ATT----C-
T-T-G-GT--GT-A-G-CG-GT--G---A--A-A---TG-C-GT-AG--AT-A-TC--A-A--G-A-G-G-A-AC-A-CC---TG--A--G--GC-GAA-G--G-C--G-------G--A-T-T-G---CTG--G--GC-T-G--------------------A-C---A-C-T--GA--CG-C-T-G--A-GG--C-G-CG-A--AA-G-C---T-AG--GG-
T--AG-C-A-AA--------CG--GG Bacteria(100);Acidobacteria(100);Acidobacteria(100);Acidobacteriales(100);Acidobacteriaceae(100);Candidatus_Chloroacidobacterium(100);unclassified(100);unclassified(100);unclassified(100);

Otu1653 0 0 0 0 0 1 0 0 0 0 0 0 0 0 0 0 0 0 0 0 0 0 0 0 0 0 0 0
0 0 0 0 0 0 0 0 0 0 0 0 0 0 0 0 0 0 0 0 0 0 0 23 0 0 0 0 0
0 0 0 0 0 0 0 0 0 0 0 0 0 0 0 0 0 0 0 0 0 0 0 0 0 0 0 0 0
0 0 0 0 0 0 0 0 0 0 0 0 0 0 0 0 0 0 0 0 0 0 0 0 0 0 0

M02255_76_000000000-ADUNE_1_1102_11928_6867 C----TA---AC-TC-TGT--------GCC-A-G------C-----A---G--CC-G-CGG-TA------AT-A--C-A--GA-GG-------GT--G-----CA--A-GC-G--------T-TA-A-T-C-G------------G--A-AC-A-A-T----------AA----
G-T-T-T--------G-A-T--G--TG--A-AA-GC--C-C-TG-G-G-----CT-T-AA------C-C-T-G-G-G-A--A-T----G-G--C-G---T--T-------G--AA-G-A---C-------T--G-T--TG--T-T-C----T-A-G-A-G-T--T---T-GA-----CA-G-A--------G-G-G-T---CG-T------GG--A--ATT----T-C-C-G-GT--
GT-A-G-CG-GT--G---A--A-A---TG-C-GT-AG--AG-A-TC--G-G--A-A-A-G-A-AC-A-CC---AG--T--G--GC-GAA-G--G-C--G-------G--C-G-A-C---CTG--G--GT-C-T--------------------A-A---A-C-T--GA--CA-C-T-G--A-GG--T-G-CG-A--AA-G-C---G-TG--GG-G--AG-C-A-
AA--------CA--GG Bacteria(100);Proteobacteria(100);unclassified(100);unclassified(100);unclassified(100);unclassified(100);unclassified(100);unclassified(100);unclassified(100);

Otu1654 0 0 0 0 7 0 0 0 0 0 0 0 0 0 0 0 0 0 0 0 0 0 0 0 0 0 0 0
0 0 0 0 0 0 0 0 0 0 0 0 0 0 0 0 0 0 0 0 0 0 0 0 0 0 0 0 0
0 0 0 0 0 0 0 0 0 0 0 0 0 0 0 0 0 0 0 0 0 0 0 0 0 0 0 0 0
0 0 0 0 0 0 0 0 0 0 0 0 0 0 0 0 0 0 0 0 0 0 0 0 0 0 0

M02255_76_000000000-ADUNE_1_2105_15891_16502 T----AC---GT-AG-GGT--------GCA-A-G------C--G--T---T--AA-T-CGG-AA------TT-A--C-T--GG-GC-------GT--A-----AA-GC-GT-GC-------G-CA-G-G-C-G------------G--T-AA-T-G-T-----
------AA----G-A-C-A--------G-A-T--G--TG--A-AA-TC--C-C-CG-G-G-----CT-C-AA------C-C-T-G-G-G-A--A-C----T-G--C-A---T--T-------T--GT-G-A---C-------T--G-C--AT--T-G-C----T-G-G-A-G-T--G---C-GG-----CA-G-A--------G-G-G-G---GA-T------GG--A--ATT----
C-C-G-C-GT--GT-A-G-CA-GT--G---A--A-A---TG-C-GT-AG--AT-A-TG--C-G--G-A-G-G-A-AC-A-CC---GA--T--G--GC-GAA-G--G-C--A-------A--T-C-C-C---CTG--G--GC-C-T--------------------G-C---A-C-T--GA--CG-C-T-C--A-TG--C-A-CG-A--AA-G-C---G-TG--GG-
G--AG-C-A-AA--------CA--GG Bacteria(100);Proteobacteria(100);Betaproteobacteria(100);Burkholderiales(100);Comamonadaceae(100);unclassified(100);unclassified(100);unclassified(100);unclassified(100);

Otu1655 0 0 0 0 0 1 0 0 0 0 0 0 0 0 0 0 0 0 0 0 0 0 0 0 0 0 0 0
0 1 4 1 0 0 0 0 0 6 22 1 0 0 0 0 0 115 0 0 0 0 0 0 0 0 0 0 0
0 0 0 0 0 0 0 0 0 0 0 0 0 0 0 0 0 0 0 0 0 0 0 0 0 0 0 0 0
0 0 0 0 0 0 0 0 0 0 0 0 0 0 0 0 0 0 0 0 0 0 0 0 0 0 0
M02255_77_000000000-ADUT3_1_1104_4510_16214 T----AC---GA-GA-GGC--------CCG-A-G------C--G--T---T--AA-T-CGG-AA------TC-A--C-T--GG-GC-------TT--A-----AA-GG-GT-GC-------G-TA-G-G-C-G------------G--A-GC-C-G-C----------
AA----G-T-G-T--------C-C-T--G--TG--A-AA-TC--C-C-CC-G-T-----CT-C-AA------T-C-G-G-G-G-A--A-T----G-G--C-G---G--G-------G--CA-T-A---C-------T--G-C--GG--T-T-C----T-T-G-A-G-G--C---G-GG-----TA-G-G--------G-G-T-T---GT-C------GG--A--ACG----C-T-C-
G-GT--GG-A-G-CG-GT--G---A--A-A---TG-C-GT-AG--AT-A-TC--G-A--G-C-G-G-A-AC-G-CC---GA--T--G--GC-GAA-A--G-C--A-------G--A-C-A-A---CTG--G--GC-C-C--------------------G-T---C-C-T--GA--CG-C-T-G--A-GG--C-A-CG-A--AA-G-C---G-TG--GG-G--
AG-C-A-AA--------CA--GG Bacteria(100);unclassified(100);unclassified(100);unclassified(100);unclassified(100);unclassified(100);unclassified(100);unclassified(100);unclassified(100);

Otu1656 0 0 0 0 0 1 0 0 0 0 0 0 0 0 0 0 0 0 0 0 0 0 0 0 0 0 0 0
0 0 0 0 0 0 0 0 0 0 0 0 0 0 0 0 0 0 0 0 0 25 0 0 0 0 1 0 0
0 0 0 0 0 0 0 0 0 0 0 0 0 0 0 0 0 0 0 0 0 0 0 0 0 0 0 0 0
0 0 0 0 0 0 0 0 0 0 0 0 0 0 0 0 0 0 0 0 0 0 0 0 0 0 0
M02255_76_000000000-ADUNE_1_1109_21110_22627 C----GG---CT-A--ACT--------ACG-T-G------C--C--A---G--CA-G-CCG-CG------GT-A--A-T--AC-GT-------AG--G-----GT-GC-AA-GC-------GTTA-A-T-C-G------------G--A-CC-G-C-T------
-----AA----G-A-C-A--------G-A-T--G--TG--A-AA-TC--C-C-CG-G-G-----CT-T-AA------C-C-T-G-G-G-A--A-C----T-G--C-A---T--T-------T--GT-G-A---C-------T--G-G--CG--G-G-C----T-A-G-A-G-T--A---T-GG-----CA-G-A--------G-G-G-G---GG-T------AG--A--ATT----C-
C-A-C-GT--GT-A-G-CA-GT--G---A--A-A---TG-C-GT-AG--AG-A-TG--T-G--G-A-G-G-A-AT-A-CC---GA--T--G--GC-GAA-G--G-C--A-------G--C-C-C-C---CTG--G--GC-C-A--------------------A-T---A-C-T--GA--CG-C-T-C--A-TG--C-A-CG-A--AA-G-C---G-TG--GG-G-
-AG-C-A-AA--------CA--GG Bacteria(100);Proteobacteria(100);Betaproteobacteria(100);unclassified(100);unclassified(100);unclassified(100);unclassified(100);unclassified(100);unclassified(100);

Otu1657 0 0 0 67 0 0 0 0 0 0 0 0 0 0 0 0 0 0 0 0 0 0 0 0 0 0 0 0
0 0 0 0 0 0 0 37 0 0 0 0 0 0 0 0 0 0 0 0 0 0 0 0 0 0 0 0 0
0 0 0 0 0 0 0 0 0 0 0 0 0 0 0 0 0 0 0 0 0 0 0 0 0 0 0 0 0
0 0 0 0 0 0 0 0 0 0 0 0 0 0 0 0 0 0 0 0 0 0 0 0 0 0 0
M02255_77_000000000-ADUT3_1_1101_21628_12209 T----AC---GT-AG-GTG--------GCA-A-G------C--G--T---T--GT-C-CGG-AT------TT-A--C-T--GG-GC-------GT--A-----AA-GG-GC-AA-------G-CA-G-G-C-G------------G--A-CT-G-T-T-----
------AA----G-T-A-G--------G-A-A--G--TG--A-AA-GG--T-C-GG-A-G-----CT-C-AA------C-T-C-C-A-A-C--A-T----T-G--C-T---C--C-------C--TA-T-A---C-------T--G-G--CA--G-T-C----T-T-G-A-G-T--C---T-CG-----GA-G-A--------G-G-A-A---AG-C------GG--A--ACG----A-
T-A-C-GT--GT-A-G-CG-GT--G---A--A-A---TG-C-GT-TG--AT-A-TG--T-A--T-C-G-G-A-AC-A-CC---AA--T--G--GC-GAA-G--G-C--A-------G--C-T-T-T---CTG--G--AC-G-A--------------------G-A---A-C-T--GA--CG-C-T-C--A-TT--T-G-CG-A--AA-G-C---C-GA--GG-T--
AG-C-G-AA--------CA--GG Bacteria(100);Candidate_division_OP10(100);unclassified(100);unclassified(100);unclassified(100);unclassified(100);unclassified(100);unclassified(100);unclassified(100);

Otu1658 0 0 0 0 0 0 0 0 48 0 0 0 0 0 0 0 0 0 0 0 0 0 0 0 0 0 0 0
0 0 0 0 0 0 0 0 0 0 0 0 0 0 0 0 0 0 0 0 0 0 0 0 0 0 0 0 0
0 0 0 0 0 0 0 0 0 0 0 0 0 0 0 0 0 0 0 0 0 0 0 0 0 0 0 0 0
0 0 0 0 0 0 0 0 0 0 0 0 0 0 0 0 0 0 0 0 0 0 0 0 0 0 0
M02255_77_000000000-ADUT3_1_1110_9886_6363 T----AC---GT-AG-GTG--------GCA-A-G------C--G--T---T--GT-C-CGG-AT------TT-A--C-T--GG-GT-------GT--A-----AA-GG-GC-GC-------G-TA-G-G-C-G------------G--G-GA-G-A-C----------
AA----G-T-C-A--------G-A-T--G--TG--A-AA-TC--T-A-TG-G-G-----CT-T-AA------C-C-C-A-T-A-A--A-C----T-G--C-A---T--T-------T--GA-A-A---C-------T--A-T--TT--C-T-C----T-T-G-A-G-T--G---A-TG-----GA-G-A--------G-G-C-A---AG-C------GG--A--ATT----C-C-T-A-
GT--GT-A-G-CG-GT--G---A--A-A---TG-C-GT-AG--AT-A-TT--A-G--G-A-G-G-A-AC-A-CC---AG--T--G--GC-GAA-G--G-C--G-------G--C-T-T-G---CTG--G--AC-A-T--------------------T-A---A-C-T--GA--CG-C-T-G--A-GG--C-G-CG-A--AA-G-C---G-TG--GG-G--AG-
C-A-AA--------CA--GG Bacteria(100);Firmicutes(100);Clostridia(100);Clostridiales(100);Ruminococcaceae(100);unclassified(100);unclassified(100);unclassified(100);unclassified(100);

Otu1659 0 0 0 0 5 0 0 0 0 0 0 0 0 0 0 0 0 0 0 0 0 0 0 0 0 0 0 0
0 0 0 0 0 0 0 0 0 0 0 0 0 0 0 0 0 0 0 0 0 0 0 0 0 0 0 0 0
0 0 0 0 0 0 0 0 0 0 0 0 0 0 0 0 0 0 0 0 0 0 0 0 0 0 0 0 0
0 0 0 0 0 0 0 0 0 0 0 0 0 0 0 0 0 0 0 3 0 0 0 0 0 0 0
M02255_76_000000000-ADUNE_1_1101_15009_23295 G----AA---GA-AG-CAC--------CGG-C-T------A--A--C---T--CC-G-TGC-CA------GC-A--G-C--CG-CG-------GT--A-----AT-AC-GG-A--------G-GG-T-G-C-A------------A--G-CT-G-T-T------
-----AA----G-T-C-G--------G-A-A--G--TG--A-AA-TC--C-C-CG-G-G-----CT-C-AA------C-C-T-G-G-G-A--A-C----T-G--C-A---T--T-------C--GA-A-A---C-------T--G-G--CA--G-G-C----T-A-G-A-G-T--C---T-TG-----TA-G-A--------G-G-G-G---GG-T------AG--A--ATT----C-
C-A-G-GT--GT-A-G-CG-GT--G---A--A-A---TG-C-GT-AG--AG-A-TC--T-G--G-A-G-G-A-AT-A-CC---GG--T--G--GC-GAA-G--G-C--G-------G--C-C-C-C---CTG--G--AC-A-A--------------------A-G---A-C-T--GA--CG-C-T-C--A-GG--T-G-CG-A--AA-G-C---G-TG--GG-
G--AG-C-A-AA--------CA--GG Bacteria(100);Proteobacteria(100);Gammaproteobacteria(100);Enterobacteriales(100);Enterobacteriaceae(100);Enteric_Bacteria_cluster(100);unclassified(100);unclassified(100);unclassified(100);

Otu1660 0 0 0 0 0 0 0 0 22 0 0 0 0 0 0 0 0 0 0 0 0 0 0 0 0 0 0 0
0 0 0 0 0 0 0 0 0 0 0 0 0 0 0 0 0 0 0 0 0 0 0 0 0 0 0 0 0
0 0 0 0 0 0 0 0 0 0 0 0 0 0 0 0 0 0 0 0 0 0 0 0 0 0 0 0 0
0 0 0 0 0 0 0 0 0 0 0 0 0 0 0 0 0 0 0 0 0 0 0 0 0 0 0 0
M02255_77_000000000-ADUT3_1_1110_13320_18371 T----AC---GT-AG-GGG--------GCG-A-G------C--G--T---T--GT-C-CGG-AT------TT-A--T-T--GG-GC-------GT--A-----AA-GA-GC-TC-------G-TA-G-G-C-G------------G--C-TC-G-G-T-----
------AA----G-T-C-G--------G-A-T--G--TG--A-AA-TC--T-C-CA-G-G-----CT-T-AA------C-C-T-G-G-A-G--A------G-G-CC-A---T--T-------C--GA-T-A---C-------T--G-C--TG-TG-G-C----T-T-G-A-G-T--C---C-GG-----TA-G-A--------G-G-A-G---CA-T------GG--A--ATT----C-
C-C-G-GT--GT-A-G-CG-GT--G---A--A-A---TG-C-GC-AG--AT-A-TC--G-G--G-A-G-G-A-AC-A-CC---AG--T--A--GC-GAA-G--G-C--G-------G--T-G-C-T---CTG--G--GC-C-G--------------------G-T---A-C-T--GA--CG-C-T-G--A-GG--A-G-CG-A--AA-G-C---G-TG--GG-
G--AG-C-G-AA--------CA--GG Bacteria(100);Actinobacteria(100);Actinobacteria(100);Acidimicrobidae(100);Acidimicrobiales(100);Acidimicrobineae(100);unclassified(100);unclassified(100);unclassified(100);

Otu1661 0 0 0 0 0 0 0 19 0 0 0 0 0 0 0 0 0 0 0 0 0 0 0 0 0 0 0 0
0 0 0 0 0 0 0 0 0 0 0 0 0 0 0 0 0 0 0 0 0 0 0 0 0 0 0 0 0
0 0 0 0 0 0 0 0 0 0 0 0 0 0 0 0 0 0 0 0 0 0 0 0 0 0 0 0 0
0 0 0 0 0 0 0 0 0 0 0 0 0 0 0 0 0 0 0 0 0 0 0 0 0 0 0 0
M02255_77_000000000-ADUT3_1_2107_16433_9564 G----AC---AG-AG-GTG--------CCG-A-G------C--G--T---T--AG-G-CGG-AA------TC-A--C-T--GG-GC-------TT--A-----AA-GC-GT-GT-------G-TA-G-G-C-G------------G--G-CC-C-G-T-----------
AA----G-T-G-T--------C-T-T--G--TG--A-AA-TC--C-C-AC-G-G-----CT-C-AA------C-C-G-T-G-G-A--A-C----G-G--C-T---G--G-------G--CA-T-A---C-------T--G-C--GG--G-C-C----T-C-G-G-G-C--A---A-CT-----CA-G-G--------G-G-C-A---GA-C------GG--A--ACA----A-G-
T-G-GT--GG-A-G-CG-GT--G---A--A-A---TG-C-GT-AG--AT-A-TC--A-C--T-T-G-G-A-AC-G-CC---GG--C--G--GT-GAA-A--A-C--G-------G--T-C-T-G---CTG--G--GG-G-T--------------------T-T---G-C-C--GA--CG-C-T-G--A-GA--C-A-CG-A--AA-G-C---C-AG--GG-G--
AG-C-G-AA--------CG--GG Bacteria(100);Planctomycetes(100);unclassified(100);unclassified(100);unclassified(100);unclassified(100);unclassified(100);unclassified(100);unclassified(100);

Otu1662 0 0 0 0 0 0 0 0 0 0 0 0 0 0 0 0 0 0 0 0 0 0 0 0 0 0 0 0
0 0 0 0 0 0 0 0 0 0 0 0 0 0 0 0 0 0 0 0 0 0 0 0 0 0 0 0 0
0 0 0 0 0 0 0 0 0 0 0 0 0 0 0 0 0 0 0 0 0 0 4 0 0 0 0 0 0
0 0 0 0 0 0 0 0 0 0 0 0 0 0 0 0 0 0 0 0 0 0 0 0 0 0 0 0
M02255_76_000000000-ADUNE_1_1102_7462_9203 C----CG---GC-TA-ACT--------CCG-T-G------C--C--A---G--CA-G-CCG-CG------GT-A--A-T--AC-GG-------AG--G-----GT-GC-AA-GC-------G-TT-GTC-C-G------------G--A-TT-G-A-T-----------
AA----G-T-C-C--------G-T-G--G--TG--A-AA-GC--C-A-AC-A-G-----CT-C-AA------C-T-G-T-T-G-A--A-C----T-G--C-C---A--T-------G--GA-T-A---C-------T--G-T--CG--G-G-C----T-T-G-A-G-T--A---C-AG-----AC-G-A--------G-G-T-A---GG-C------GG--A--ATT----G-A-T-
G-GT--GT-A-G-CG-GT--G---A--A-A---TG-C-AT-AG--AT-A-CC--A-T--C-A-A-G-A-AC-C-CC---GA--T--T--GC-GAA-G--G-C--A-------G--C-T-T-A---CTA--G--GC-T-G--------------------T-A---A-C-T--GA--CG-C-T-G--A-GG--C-A-CG-A--AA-G-C---G-TG--GG-G--AG-
C-G-AA--------CA--GG Bacteria(100);Bacteroidetes(100);Sphingobacteria(100);Sphingobacteriales(100);unclassified(100);unclassified(100);unclassified(100);unclassified(100);unclassified(100);

Otu1663 0 0 0 0 0 0 5 57 0 0 0 0 0 0 0 0 0 0 0 0 0 0 0 0 0 0 0 0
0 0 0 0 0 0 0 0 0 0 0 0 0 0 0 0 0 0 0 0 0 0 0 0 0 0 0 0 0
0 0 0 0 0 0 0 0 0 0 0 0 0 0 0 0 0 0 0 0 0 0 0 0 0 0 0 0 0
0 0 0 0 0 0 0 0 0 0 0 0 0 0 0 0 0 0 0 0 0 0 0 0 0 0 0 0
M02255_77_000000000-ADUT3_1_1108_5059_16054 T----AC---GG-AA-GGG--------GCT-A-G------C--G--T---T--GT-T-CGG-AA------TC-A--C-T--GG-GC-------GT--A-----AA-GC-GC-GC-------G-TA-G-G-C-G------------G--C-TT-T-G-C-----------AA-
---G-T-G-C--------G-G-C--G--TG--A-AA-GT--C-C-TG-G-G-----CT-C-AA------C-C-T-G-G-G-G--A-C----G-G--C-G---C--T-------G--CG-G-A---C-------T--G-C--TT--G-G-C----T-G-G-A-G-T--C---C-GG-----AA-G-A--------G-G-G-T---GG-T------GG--A--ATT----C-C-G-A-
GT--GT-A-G-AG-GT--G---A--A-A---TT-C-GT-AG--AT-A-TT--C-G--G-A-G-G-A-AC-A-CC---GG--T--G--GC-GAA-G--G-C--G-------G--C-C-A-C---CTG--G--TC-C-G--------------------G-T---A-C-T--GA--CG-C-T-G--A-AG--C-G-CG-A--AA-G-C---G-TG--GG-G--AG-
C-A-AA--------CA--GG Bacteria(100);Proteobacteria(100);Alphaproteobacteria(100);Rhodospirillales(100);Rhodospirillaceae(100);Defluviicoccus(100);unclassified(100);unclassified(100);unclassified(100);

Otu1664 0 0 0 0 0 0 0 42 0 0 0 0 0 0 0 0 0 0 0 0 0 0 0 0 0 0 0 0
0 0 0 0 0 0 0 0 0 0 0 0 0 0 0 0 0 0 0 0 0 0 0 0 0 0 0 0 0
0 0 0 0 0 0 0 0 0 0 0 0 0 0 0 0 0 0 0 0 0 0 0 0 0 0 0 0 0
0 0 0 0 0 0 0 0 0 0 0 0 0 0 0 0 0 0 0 0 0 0 0 0 0 0 0 0
M02255_77_000000000-ADUT3_1_1115_23720_14711 T----AC---GT-AG-GGG--------GCG-A-G------C--G--T---T--GT-C-CGA-AG------TT-A--C-T--GG-GC-------GT--A-----AA-GC-GC-GC-------G-TA-G-G-C-G------------G--T-CA-T-T-T-----
------AA----G-T-T-C--------G-G-G--G--TG--A-AA-GG--T-T-CAGC-G-----CT-C-AA------C-G-C-G-A-A-C--A-G----T-G--C-C---T--T-------G--AA-T-A---C-------T--G-G--GT--G-A-C----T-T-G-A-G-T--T---C-GG-----GA-G-A--------G-G-G-C---AG-T------GG--A--ATT----
C-C-T-G-GT--GT-A-G-CG-GT--G---A--A-A---TG-C-GT-AG--AT-A-TC--A-G--G-A-G-G-A-AC-A-CC---GA--T--G--GC-GAA-G--G-C--A-------G--C-T-G-C---CTG--G--CC-T-A--------------------G-A---A-C-T--GA--CG-C-T-G--A-GG--T-G-CG-A--AA-G-C---G-TG--
GG-G--AG-C-G-AA--------CA--GG Bacteria(100);unclassified(100);unclassified(100);unclassified(100);unclassified(100);unclassified(100);unclassified(100);unclassified(100);unclassified(100);

Otu1665 0 0 0 17 0 0 0 0 0 0 0 0 0 0 0 0 0 0 0 0 2 0 0 0 0 0 0 0
0 0 0 0 0 0 0 0 0 0 3 0 0 2 20 0 1 0 0 0 0 0 0 0 1 0 0 0 0
0 0 0 0 0 0 0 0 0 0 0 0 0 0 2 6 0 0 0 0 0 0 0 0 0 0 0 0 0
0 0 0 0 2 9 0 0 0 0 0 0 0 0 0 0 0 0 0 2 0 0 0 0 0 0 0
M02255_77_000000000-ADUT3_1_1116_17839_10608 T----AA---CT-AC-GTG--------CC--A-G------C--A--G---C--CG-C-GGT-AA------TA-C--G-T--AG-GT-------CC--C-----GA-GC-G--TT-------G-TC-C-G---G------------A--T-TT-A-T-T---------
-AA----G-T-C-T--------G-G-T--G--TA--A-AA-GG--C-A-GT-G-G-----CT-C-AA------C-C-A-T-T-G-T--A------T-G--C-A---T--T-------G--GA-A-A---C-------T--G-G--TA--G-A-C----T-T-G-A-G-T--G---C-AG-----GA-G-A--------G-G-A-G---AG-T------GG--A--ATT----C-C-
A-T-GT--GT-A-G-CG-GT--G---A--A-A---TG-C-GT-AG--AT-A-TA--T-G--G-A-G-G-A-AC-A-CC---GG--T--G--GC-GAA-A--G-C--G-------G--C-T-C-T---CTG--G--CC-T-G--------------------T-A---A-C-T--GA--CA-C-T-G--A-GG--C-T-CG-A--AA-G-C---G-TG--GG-G--
AG-C-A-AA--------CA--GG Bacteria(100);Firmicutes(100);Bacilli(100);Lactobacillales(100);Streptococcaceae(100);Lactococcus(100);unclassified(100);unclassified(100);unclassified(100);

Otu1666 0 0 0 0 88 0 0 0 0 0 0 0 0 0 0 0 0 0 0 0 0 1 0 0 0 0 0 0
0 0 0 0 0 0 0 0 0 0 0 0 0 0 0 0 0 0 0 0 0 0 0 0 0 0 0 0 0
0 0 0 0 0 0 0 0 0 0 0 0 0 0 0 0 0 0 0 0 0 0 0 0 0 0 0 0 0
0 0 0 0 0 0 0 0 0 0 0 0 0 0 0 0 0 0 0 0 0 0 0 0 0 0 0
M02255_76_000000000-ADUNE_1_1102_23868_15546 C----TA---AC-TC-TGT--------GCC-A-G------C-----A---G--CC-G-CGG-TA------AT-A--C-A--GA-GG-------GT--------GC-TA-GC-G--------T-TA-A-T-C-G------------G--A-TT-G-A-T---------
--AA----G-T-C-G--------G-T-T--G--TG--A-AA-GC--C-C-CG-G-G-----CT-C-AA------C-C-T-G-G-G-A--A-T----T-G--C-G---G--C-------C--GA-T-A---C-------T--G-T--CA--G-A-C----T-A-G-A-A-A--A---C-GG-----TA-G-A--------G-G-G-A---GG-T------GG--A--ACT----C-C-
A-G-GT--GT-A-G-CG-GT--G---A--A-A---TG-C-GT-AG--AT-A-TC--T-G--G-A-A-G-A-AC-A-CC---GA--T--G--GC-GAA-G--G-C--A-------G--C-C-T-C---CTG--G--GC-C-T--------------------G-T---T-T-T--GA--CG-C-T-G--A-GG--C-A-CG-A--AA-G-C---G-TG--GG-T--
AG-C-A-AA--------CA--GG Bacteria(100);Proteobacteria(100);Gammaproteobacteria(100);Xanthomonadales(100);Sinobacteraceae(100);Nevskia(100);unclassified(100);unclassified(100);unclassified(100);

Otu1667 0 0 0 0 0 0 0 0 0 0 0 0 0 2 0 0 0 0 0 0 0 0 0 0 0 0 0 0
0 0 0 0 0 0 0 0 9 0 0 0 0 0 0 17 0 0 0 0 0 0 0 0 0 0 0 0 0
0 0 0 0 0 0 0 0 0 0 0 0 0 0 0 0 0 0 0 0 0 0 0 0 0 0 0 0 8
3 0 0 0 0 0 0 0 0 0 0 0 0 0 0 0 0 0 0 0 0 0 0 0 2 0 0
M02255_76_000000000-ADUNE_1_2106_16997_11159 A----AG---TC-AC-GGC--------TAA-C-T------A--C--G---T--GC-C-AGC-AG------CC-G--C-G--GT-AA-------TA--C-----GT-AG-GT-GA-------C-AA-GCG-T-T------------G--T-CC-G-GAT----
-------AA----G-T-C-T--------G-A-T--G--TG--A-AA-GC--C-C-AC-G-G-----CT-C-AA------C-C-G-T-G-G-A--A-C----G-G--C-A---T--T-------G--GA-A-A---C-------T--G-G--CG--G-A-C----T-T-G-A-A-T--G---T-AG-----CA-G-A--------G-G-A-A---AG-T------GG--A--ATT----
C-C-A-T-GT--GT-A-G-CG-GT--G---G--A-A---TG-C-GT-AG--AT-A-TA--T-G--G-A-G-G-A-AC-A-CC---AG--T--G--GC-GAA-A--G-C--G-------A--C-T-T-T---CTG--G--GC-T-A--------------------T-G---A-T-T--GA--CG-C-T-G--A-GG--C-T-CG-A--AA-G-C---G-TG--GG-
G--AG-C-G-AA--------CA--GG Bacteria(100);Firmicutes(100);Bacilli(100);Lactobacillales(100);Carnobacteriaceae(100);Alloiococcus(100);unclassified(100);unclassified(100);unclassified(100);

Otu1668 0 0 0 0 0 0 0 0 0 0 0 0 0 0 0 0 0 0 0 0 2 0 0 0 0 0 0 0
0 0 0 0 0 0 0 0 0 0 0 0 0 0 0 0 0 0 0 0 0 0 0 0 0 0 0 0 0
0 0 0 0 0 0 0 0 0 0 0 0 0 0 17 18 0 0 0 0 0 0 0 0 0 0 0 0 3
0 0 0 0 0 0 0 0 0 0 0 0 0 0 0 0 0 0 0 0 0 0 0 0 0 0 0
M02255_77_000000000-ADUT3_1_2114_9373_10246 C----GT---GC-CA-GCA--------GCC-G-C------G--G--T---A--AT-A-CGG-AG------GA-T--G-C--GA-GC-------GT--T-----AT-CC-GG-AT-------T-TA-G-T-T-G------------G--G-TT-G-T-T-----------AA--
--G-T-C-C--------G-C-G--G--TG--A-AA-GT--T-T-GT-C-G-----CT-T-AA------C-G-A-T-A-A-A--A-T----T-G--C-C---G--T-------T--GA-A-A---C-------T--G-G--TA--G-T-C----T-T-G-A-G-T--A---T-AG-----AT-G-A--------A-G-T-A---GG-C------GG--A--ATG----C-G-T-G-
GT--GT-A-G-CG-GT--G---A--A-A---TG-C-AT-AG--AG-A-TC--A-C--G-C-A-G-A-AC-T-CC---GA--T--T--GC-GAA-G--G-C--A-------G--C-T-T-A---CTA--A--GG-T-A--------------------T-A---A-C-T--GA--CA-C-T-G--A-AG--C-A-CG-A--AA-G-C---G-TG--GG-T--AT-C-
A-AA--------CA--GG Bacteria(100);Bacteroidetes(100);Bacteroidia(100);Bacteroidales(100);Porphyromonadaceae(100);Tannerella(100);unclassified(100);unclassified(100);unclassified(100);

Otu1669 0 0 0 0 0 0 0 33 0 0 0 0 0 0 0 0 0 0 0 0 0 0 0 0 0 0 0 0
0 0 0 0 0 0 0 0 0 0 0 0 0 0 0 0 0 0 0 0 0 0 0 0 0 0 0 0 0
0 0 0 0 0 0 0 0 0 0 0 0 0 0 0 0 0 0 0 0 0 0 0 0 0 0 0 0 0
0 0 0 0 0 0 0 0 0 0 0 0 0 0 0 0 0 0 0 0 0 0 0 0 0 0 0
M02255_77_000000000-ADUT3_1_1109_8832_19112 T----AC---GT-AG-GGA--------GCG-A-G------C--G--T---T--GT-C-CGG-AT------TT-A--C-T--GG-GT-------GT--A-----AA-GG-GT-GC-------G-TA-G-G-C-G------------G--A-TC-T-G-C-----------AA-
---G-T-C-A--------G-T-A--G--TG--A-AA-TC--C-C-AG-G-G-----CT-T-AA------C-C-C-T-G-G-A--A-C----T-G--C-T---A--T-------T--GA-A-A---C-------T--G-T--GG--G-T-C----T-T-G-A-G-T--G---A-GG-----TA-G-A--------G-G-C-A---GG-C------GG--A--ATT----C-C-C-G-
GT--GT-A-G-CG-GT--G---A--A-A---TG-C-GT-AG--AG-A-TC--G-G--G-A-G-G-A-AC-A-CC---AG--T--G--GC-GAA-G--G-C--G-------G--C-C-T-G---CTG--G--GC-C-T--------------------T-A---A-C-T--GA--CG-C-T-G--A-GG--C-A-CG-A--AA-G-C---A-TG--GG-T--AG-
C-A-AA--------CA--GG Bacteria(100);unclassified(100);unclassified(100);unclassified(100);unclassified(100);unclassified(100);unclassified(100);unclassified(100);unclassified(100);

Otu1670 0 0 0 0 0 0 0 14 0 0 0 0 0 0 0 0 0 0 0 0 2 0 0 0 0 0 0 0
1 0 0 0 0 0 0 0 0 4 0 0 0 0 0 0 0 0 0 0 0 0 0 0 0 0 0 0
0 0 0 0 0 0 0 0 0 0 0 0 0 0 0 0 0 0 0 0 0 0 0 0 0 0 0 0
0 0 0 0 0 0 0 0 0 0 0 0 0 0 0 1 0 0 0 0 0 0 0 0 0 0 0
M02255_77_000000000-ADUT3_1_1105_7413_12065 T----AC---GT-AG-GGT--------GCA-A-G------C--G--T---T--GT-C-CGG-AA------TT-A--T-T--GG-GC-------GT--A-----AA-GA-GC-TC-------G-TA-G-G-T-G-------------G--T-TC-G-T-C-----------GC--
--G-T-C-T--------G-C-T--G--TG--A-AA-GT--C-C-GA-G-G-----CT-T-AA------C-C-T-C-G-G-A--T-C----T-G--C-A---G--T-------G--GG-T-A---C-------G--G-G--CG--G-A-C----T-A-G-A-G-T--G---C-GG-----TA-G-A--------G-G-A-G---AT-G------GG--A--ATT----C-C-T-G-
GT--GT-A-G-CG-GT--G---G--A-A---TG-C-GC-AG--AT-A-TC--A-G--G-A-G-G-A-AC-A-CC---GG--T--G--GC-GAA-G--G-C--G-------C--A-T-C-T---CTG--G--GC-C-G--------------------T-A---A-C-T--GA--CG-C-T-G--A-GG--A-G-CG-A--AA-G-C---A-TG--GG-G--AG-
C-G-AA--------CA--GG Bacteria(100);Actinobacteria(100);Actinobacteria(100);Actinobacteridae(100);Actinomycetales(100);unclassified(100);unclassified(100);unclassified(100);unclassified(100);

Otu1671 0 0 0 0 0 0 16 1 43 0 0 0 0 0 0 0 0 0 0 0 0 0 0 0 0 0 0 0
0 0 0 0 0 0 0 0 0 0 0 0 0 0 0 0 0 0 0 0 0 0 0 0 0 0 0 0
0 0 0 0 0 0 0 0 0 0 0 0 0 0 0 0 0 0 0 0 0 0 0 0 0 0 0 0
0 0 0 6 0 0 1 0 0 0 0 0 0 0 0 0 0 0 0 0 0 0 0 3 54 0 0
M02255_77_000000000-ADUT3_1_1109_6560_14917 T----AC---GT-AG-GTG--------ACA-A-G------C--G--T---T--GT-C-CGG-AT------TT-A--C-T--GG-GT-------GT--A-----AA-GG-GC-GC-------G-TA-G-G-C-G------------G--A--C-T-GTC-----------AA-
---G-T-C-A--------G-T-C--G--TG--A-AA-TA--C-C-GG-G-G-----CT-T-AA------C-C-C-C-G-G-G--G-C----T-G--C-G---A--T-------T--GA-A-A---C-------T--G-A--CA--G-C-C----T-T-G-A-G-T--A---T-CG-----GA-G-A--------G-G-A-A---AG-C------GG--A--ATT----C-C-T-A-
GT--GT-A-G-CG-GT--G---A--A-A---TG-C-GT-AG--AT-A-TT--A-G--G-A-G-G-A-AC-A-CC---AG--T--G--GC-GAA-G--G-C--G-------G--C-T-T-T---CTG--G--AC-G-A--------------------C-A---A-C-T--GA--CG-C-T-G--A-GG--C-G-CG-A--AA-G-T---G-TG--GG-G--AG-
C-A-AA--------CA--GG Bacteria(100);Firmicutes(100);Clostridia(100);Clostridiales(100);unclassified(100);unclassified(100);unclassified(100);unclassified(100);unclassified(100);

Otu1672 0 0 0 0 0 0 48 4 172 0 0 0 0 0 0 0 0 0 0 0 0 0 0 0 0 0 0 0
0 0 0 0 0 0 0 0 0 0 0 0 0 0 0 0 0 0 0 0 0 0 0 0 0 0 0 0
0 0 0 0 0 0 0 0 0 0 0 0 0 0 0 2 0 0 0 0 0 0 0 0 0 0 0 0
0 0 0 0 0 0 0 0 0 0 0 0 0 0 0 0 0 0 0 0 0 0 0 2 28 0 0
M02255_77_000000000-ADUT3_1_1109_17302_14845 T----AC---GT-AT-GGA--------GCA-A-G------C--G--T---T--AT-C-CGG-AT------TT-A--T-T--GG-GC-------GT--A-----AA-GC-GC-GC-------G-TA-G-G-C-G------------G--C-TT-G-T-T------
-----AA----G-C-A-A--------G-G-T--C--TT--A-AA-TC--T-T-GG-G-G-----CT-C-AA------C-C-T-C-A-A-G--C------C-G--G-A---T--C-------T--TG-A-A---C-------T--G-G--CA--G-G-C----T-C-G-A-G-T--A---T-GG-----TA-G-A--------G-G-A-A---AG-T------GG--A--ATT----C-
C-C-A-GT--GT-A-G-CG-GT--G---G--A-A---TG-C-GC-AG--AT-A-TT--G-G--G-A-A-G-A-AC-A-CC---GA--T--G--GC-GAA-G--G-C--A-------G--C-T-T-T---CTG--G--GC-C-A--------------------T-T---A-C-T--GA--CG-C-T-G--A-GG--T-G-CG-A--AA-G-C---T-AG--GG-G-
-AG-C-A-AA--------CA--GG Bacteria(100);unclassified(100);unclassified(100);unclassified(100);unclassified(100);unclassified(100);unclassified(100);unclassified(100);unclassified(100);

Otu1673 0 0 0 0 178 0 0 0 0 0 0 0 0 0 0 0 0 0 0 0 0 0 0 0 0 0 0 0
0 0 0 0 0 0 0 0 0 0 0 0 0 0 0 0 0 0 0 0 0 0 0 0 0 0 0 0
0 0 0 0 0 0 0 0 0 0 0 0 0 0 0 0 0 0 0 0 0 0 0 0 0 0 0 0
0 0 0 0 0 0 0 0 0 0 0 0 0 0 0 0 0 0 0 0 0 0 0 0 0 0 0
M02255_77_000000000-ADUT3_1_1104_18394_10042 T----AC---GT-AG-GGA--------GCA-A-G------C--G--T---T--GT-C-CGG-AA------TT-A--C-T--GG-GT-------GT--A-----AA-GG-GA-GC-------G-TA-G-G-C-G------------G--G-AA-T-G-C----
-------AA----G-T-T-G--------A-A-T--G--TC--A-AA-TC--T-A-CC-G-G-----CT-T-AA------C-T-G-G-T-A-G----C----C-G--C-G---T--T-------C--AA-A-A---C-------T--G-C--GT--T-T-C----T-T-G-A-G-T--G---A-AG-----TA-G-A--------G-G-C-A---GG-C------GG--A--ATT----C-
C-T-A-GT--GT-A-G-CG-GT--G---A--A-A---TG-C-GT-AGA-T--A-TT--A-G--G-A-G-G-A-AC-A-CC---AG--T--G--GC-GAA-G--G-C--G-------G--C-C-T-G---CTG--G--GC-T-T--------------------T-T---A-C-T--GA--CG-C-T-G--A-GG--C-T-CG-A--AA-G-T---G-TG--GG-T--
AG-C-A-AA--------CA--GG Bacteria(100);Firmicutes(100);Clostridia(100);Clostridiales(100);Ruminococcaceae(100);unclassified(100);unclassified(100);unclassified(100);unclassified(100);

Otu1674 0 0 0 35 0 0 0 0 0 0 0 0 0 0 0 0 0 0 0 0 0 0 0 0 0 0 0 0
0 0 0 0 0 0 0 0 0 0 0 0 0 0 0 0 0 0 0 0 0 0 0 0 0 0 0 0
0 0 0 0 0 0 0 0 0 0 0 0 0 0 0 0 0 0 0 0 0 0 0 0 0 0 0 0
0 0 0 0 0 0 0 0 0 0 0 0 0 0 0 0 0 0 0 0 0 0 0 0 0 0 0
M02255_77_000000000-ADUT3_1_1111_10326_9035 T----AC---GA-AG-GGG--------GCT-A-G------C--G--T---T--GC-T-CGG-AA------TG-A--C-T--GG-GC-------GT--A-----AA-GG-GC-GC-------G-TA-G-G-C-G------------G--A-TG-T-C-T-----------
TT----G-T-C-G--------G-G-C--G--TG--A-AA-TT--C-C-AG-G-G-----CT-C-AA------C-C-T-T-G-G-G--A-C----T-G--C-G---T--T-------C--GA-G-A---C-------G--G-G--AT--G-T-C----T-A-G-A-G-T--T---T-GG-----CA-G-A--------G-G-G-T---CG-T------GG--A--ATT----C-C-C-
A-GT--GT-A-G-AG-GT--G---A--A-A---TT-C-GT-AG--AT-A-TT--G-G--G-A-A-G-A-AC-A-CC---GG--T--G--GC-GAA-G--G-C--G-------G--C-G-A-C---CTG--G--TC-C-T--------------------T-G---A-C-T--GA--CG-C-T-G--A-TG--C-G-CG-A--AA-G-C---G-TG--GG-G--
AG-C-A-AA--------CA--GG Bacteria(100);Proteobacteria(100);Alphaproteobacteria(100);Rhodospirillales(100);unclassified(100);unclassified(100);unclassified(100);unclassified(100);unclassified(100);

Otu1675 0 0 0 76 0 0 0 0 0 0 0 0 0 0 0 0 0 0 0 0 0 0 0 0 0 0 0 0
0 0 0 0 0 0 0 0 0 0 0 0 0 0 0 0 0 0 0 0 0 0 0 0 0 0 0 0 0
0 0 0 0 0 0 0 0 0 0 0 0 0 0 0 0 0 0 0 0 0 0 0 0 0 0 0 0 0
0 0 0 0 0 0 0 0 0 0 0 0 0 0 0 0 0 0 0 0 0 0 0 0 0 0 0

M02255_77_000000000-ADUT3_1_1111_11517_5654 G----AC---GG-AG-GGT--------GCT-A-G------C--G--T---T--GT-T-CGG-AA------TT-A--C-T--GG-GC-------GT--A-----AA-GG-GC-GC-------G-TA-G-G-C-G------------G--C-TC-T-T-G----------AA----G-T-C-G--------G-A-T--G--TG--A-AA-GC--C-C-GG-G-G-----CT-C-AA------C-C-T-C-G-G-A--A-C----G-G--C-A---T--C-------C--GA-T-A---C-------T--C-T--TG--G-G-C----T-T-G-A-G-A--C---C-GG-----AA-G-A--------G-G-T-G---AG-T------GG--A--ATA----C-C-C-A-GT--GT-A-G-AG-GT--G---A--A-A---TT-C-GT-AG--AT-A-TT--G-G--G-T-G-G-A-AC-A-CC---GG--T--G--GC-GAA-A--G-C--G-------G--C-T-C-A---CTG--G--TC-C-G--------------------G-T---T-C-T--GA--CG-C-T-G--A-GG--C-G-CG-A--AA-G-C---G-T-G-GG-G--AG-C-A-AA--------CA--GG Bacteria(100);Proteobacteria(100);Alphaproteobacteria(100);unclassified(100);unclassified(100);unclassified(100);unclassified(100);unclassified(100);unclassified(100);

Otu1676 0 0 0 0 168 0 0 0 0 0 0 0 0 0 0 0 0 0 0 0 0 0 0 0 0 0 0 0
0 0 0 0 0 0 0 0 0 0 0 0 0 0 0 0 0 0 0 0 0 0 0 0 0 0 0 0 0
0 0 0 0 0 0 0 0 0 0 0 0 0 0 0 0 0 0 0 0 0 0 0 0 0 0 0 0 0
0 0 0 0 0 0 0 0 0 0 0 0 0 0 0 0 0 0 0 0 0 0 0 0 0 0 0

M02255_77_000000000-ADUT3_1_1104_21820_11000 T----AC---AG-AG-GCC--------CCG-A-G------C--G--T---T--AG-T-CGG-AA------TC-A--C-T--GG-GC-------TT--A-----AA-GG-GT-GC-------G-TA-G-G-C-G------------G--A-CT-T-G-T-----------AA----G-T-G-C--------T-T-T--G--TG--A-AA-GC--C-C-CG-G-G-----CT-C-AA------C-C-C-G-G-G-A--A-C----C-G--C-A---G--G-------G--CA-T-A---C-------T--G-C--AA--G-T-C----T-T-G-A-G-G--A---A-GC-----TA-G-A--------G-G-C-G---GG-T------AG--A--ACG----A-T-C-G-GT--GG-A-G-CG-GT--G---G--A-A---TG-C-GT-AG--AG-A-TC--G-A--T-C-G-G-A-AT-G-CC---AA--G--G--GT-GAA-G--A-C--A-------A--C-C-C-G---CTG--G--GG-C-T--------------------T-T---C-C-T--GA--CG-C-T-G--A-GG--C-A-CG-A--AA-G-C---G-TG--GG-G--AT-C-A-AA--------CA--GG Bacteria(100);unclassified(100);unclassified(100);unclassified(100);unclassified(100);unclassified(100);unclassified(100);unclassified(100);unclassified(100);

Otu1677 0 0 0 0 0 0 0 0 48 0 0 0 0 0 0 0 0 0 0 0 0 0 0 0 0 0 0 0
0 0 0 0 0 0 0 0 0 0 0 0 0 0 0 0 0 0 0 0 0 0 0 0 0 0 0 0 0
0 0 0 0 0 0 0 0 0 0 0 0 0 0 0 0 0 0 0 0 0 0 0 0 0 0 0 0 0
0 0 0 0 0 0 0 0 0 0 0 0 0 0 0 0 0 0 0 0 0 0 0 0 0 0 0

M02255_77_000000000-ADUT3_1_1111_4413_8206 T----AC---GT-AG-GTG--------GCA-A-G------C--G--T---T--AT-C-CGG-AT------TT-A--C-T--GG-GT-------GT--A-----AA-GG-GC-GT-------G-TA-G-G-C-G------------G--G-AA-G-G-C----------AA----G-T-C-A--------G-A-T--G--TG--A-AA-AC--T-A-TG-G-G-----CT-C-AA------C-C-C-A-T-A-G--C-C----T-G--C-A---T--T-------T--GA-A-A---C-------T--G-T--TT--T-T-C----T-T-G-A-G-T--G---C-TG-----GA-G-A--------G-G-C-A---AT-C------GG--A--ATT----C-C-G-T-GT--GT-A-G-CG-GT--G---A--A-A---TG-C-GT-AG--AT-A-TA--C-G--G-A-G-G-A-AC-A-CC---AG--T--G--GC-GAA-G--G-C--G-------G--A-T-T-G---CTG--G--AC-A-G--------------------T-A---A-C-T--GA--CG-C-T-G--A-GG--C-G-CG-A--AA-G-C---G-TG--GG-G--AG-C-A-AA--------CA--GG Bacteria(100);Firmicutes(100);Clostridia(100);Clostridiales(100);Ruminococcaceae(100);Incertae_Sedis(100);unclassified(100);unclassified(100);unclassified(100);

Otu1678 0 0 0 0 0 0 0 0 0 0 0 0 0 7 0 0 0 0 0 0 0 0 0 0 0 0 0 0
0 0 0 0 0 0 0 0 0 0 0 0 0 0 0 0 0 0 0 0 0 0 0 0 0 0 0 0 0
0 0 0 0 0 0 0 0 0 0 0 0 0 0 0 0 0 0 0 0 0 0 0 0 0 0 0 0 0
0 0 0 0 0 0 0 0 0 0 0 0 0 0 0 0 0 0 0 0 0 0 0 0 0 0 0

M02255_77_000000000-ADUT3_1_2119_26179_21370 T----AC---GG-AG-GGG--------GCT-A-G------C--G--T---T--GT-T-CGG-AA------AT-A--C-T--GG-GC-------GT--A-----AA-GC-GT-AC-------G-TA-G-G-C-G------------G--C-AT-G-G-C-----------AA----G-T-C-A--------G-G-G--G--TG--A-AA-TC--C-C-GG-A-G-----CT-C-AA------C-T-C-C-G-G-A--A-C----T-G--C-C---C--T-------T--GA-A-A---C-------T--G-C--TT--C-G-C----T-A-G-A-A-T--C---T-TG-----GA-G-A--------G-G-T-C---AG-T------GG--A--ATT----C-C-G-A-GT--GT-A-G-AG-GT--G---A--A-A---TT-C-GT-AG--AT-A-TT--C-G--G-A-A-G-A-AC-A-CC---AG--T--G--GC-GAA-G--G-C--G-------A--C-T-G-A---CTG--G--AC-A-A--------------------G-T---A-T-T--GA--CG-C-T-G--A-GG--T-A-CG-A--AA-G-C---G-TG--GG-G--AG-C-A-AA--------CA--GG Bacteria(100);Proteobacteria(100);Alphaproteobacteria(100);Sphingomonadales(100);unclassified(100);unclassified(100);unclassified(100);unclassified(100);unclassified(100);

Otu1679 0 0 0 76 0 0 0 0 0 0 0 0 0 0 0 0 0 0 0 0 0 0 0 0 0 0 0 0
0 0 0 0 0 0 0 0 0 0 0 0 0 0 0 0 0 0 0 0 0 0 0 0 0 0 0 0 0
0 0 0 0 0 0 0 0 0 0 0 0 0 0 0 0 0 0 0 0 0 0 0 0 0 0 0 0 0
0 0 0 0 0 0 0 0 0 0 0 0 0 0 0 0 0 0 0 0 0 0 0 0 0 0 0

M02255_77_000000000-ADUT3_1_1108_7341_18101 T----AC---GA-AG-GGG--------GCT-A-G------C--G--T---T--GT-T-CGG-AA------TT-A--C-T--GG-GC-------GT--A-----AA-GC-GC-AC-------G-TA-G-G-C-G------------G--A-TA-T-T-T-----------AA----G-T-C-G--------G-G-G--G--TG--A-AA-TC--C-C-GG-G-G-----CT-C-AA------C-C-C-C-G-G-A--A-C----G-G--C-C---T--T-------C--GA-T-A---C-------T--G-G--GT--A-T-C----T-T-G-A-G-T--C---C-GG-----AA-G-A--------G-G-T-G---AG-T------GG--A--ATT----G-C-G-A-GT--GT-A-G-AG-GT--G---A--A-A---TT-C-GT-AG--AT-A-TT--C-G--C-A-G-G-A-AC-A-CC---AG--T--G--GC-GAA-G--G-C--G-------G--C-T-C-A---CTG--G--TC-C-G--------------------G-A---A-C-T--GA--CG-C-T-G--A-GG--T-G-CG-A--AA-G-C---G-TG--GG-G--AG-C-A-AA--------CA--GG Bacteria(100);Proteobacteria(100);Alphaproteobacteria(100);unclassified(100);unclassified(100);unclassified(100);unclassified(100);unclassified(100);unclassified(100);

Otu1680 0 0 0 0 0 0 24 3 58 0 0 0 0 0 0 0 0 0 0 0 0 0 0 0 0 0 0 0
0 0 0 0 0 0 0 0 0 0 0 0 0 0 0 0 0 0 0 0 0 0 0 0 0 0 0 0
0 0 0 0 0 0 0 0 0 0 0 0 0 0 0 0 0 0 0 0 0 0 0 0 0 0 0 0
0 0 0 0 0 0 0 0 0 0 0 0 0 0 0 0 0 0 0 0 0 0 0 0 0 0 0

M02255_77_000000000-ADUT3_1_1107_4089_13402 T----AC---GT-AG-GGG--------GCA-A-G------C--G--T---T--AT-C-CGG-AT------TT-A--C-T--GG-GT-------GT--A-----AA-GG-GA-GC-------G-CA-G-G-C-G------------G--C-AT-G-A-T----------
AA----G-T-C-T--------G-A-T--G--TG--A-AA-AC--C-C-AA-G-G-----CT-C-AA------C-C-A-T-G-G-G--A-C----T-G--C-A---T--T-------G--GA-A-A---C-------T--G-T--CG--T-G-C----T-G-G-A-G-T--G---T-CG-----GA-G-A--------G-G-T-A---AG-C------GG--A--ATT----C-C-T-
A-GT--GT-A-G-CG-GT--G---A--A-A---TG-C-GT-AG--AT-A-TT--A-G--G-A-G-G-A-AC-A-CC---AG--T--G--GC-GAA-G--G-C--G-------G--C-T-T-A---CTG--G--AC-G-A--------------------T-G---A-C-T--GA--CG-C-T-G--A-GG--C-T-CG-A--AA-G-C---G-TG--GG-G--
AG-C-A-AA--------CA--GG Bacteria(100);Firmicutes(100);Clostridia(100);Clostridiales(100);Lachnospiraceae(100);unclassified(100);unclassified(100);unclassified(100);unclassified(100);

Otu1681 0 0 0 0 147 0 0 0 0 0 0 0 0 0 0 0 0 0 0 0 0 0 0 0 0 0 0 0
0 0 0 0 0 0 0 0 0 0 0 0 0 0 0 0 0 0 0 0 0 0 0 0 0 0 0 0
0 0 0 0 0 0 0 0 0 0 0 0 0 0 0 0 0 0 0 0 0 0 0 0 0 0 0 0
0 0 0 0 0 0 0 0 0 0 0 0 0 0 0 0 0 0 0 0 0 0 0 0 0 0 0

M02255_77_000000000-ADUT3_1_1105_17526_14122 T----AC---GA-AG-GGT--------GCT-A-G------C--G--T---T--GT-T-CGG-AA------TC-A--C-T--GG-GC-------GT--A-----AA-GC-GC-GC-------G-TA-G-G-C-G------------G--C-TT-G-T-C-----
------AA----G-T-C-A--------G-G-G--G--TG--A-AA-TC--C-T-GA-G-G-----CT-C-AA------C-C-T-C-A-G-A--A-T----T-G--C-C---T--T-------T--GA-A-A---C-------T--A-G--CA--G-G-C----T-G-G-A-G-T--G---T-CG-----GA-G-G--------G-G-A-T---AG-C------GG--A--ATT----
C-C-T-A-AT--GT-A-G-AG-GT--G---A--A-A---TT-C-GT-AG--AT-A-TT--A-G--G-A-G-G-A-AC-A-CC---GG--T--G--GC-GAA-G--G-C--G-------G--C-T-A-T---CTG--G--AC-G-A--------------------C-A---A-C-T--GA--CG-C-T-G--A-GG--C-G-CG-A--AA-G-C---G-TG--
GG-G--AT-C-A-AA--------CA--GG Bacteria(100);unclassified(100);unclassified(100);unclassified(100);unclassified(100);unclassified(100);unclassified(100);unclassified(100);unclassified(100);

Otu1682 0 0 0 0 0 0 0 24 0 0 0 0 0 0 0 0 0 0 0 0 0 0 0 0 0 0 0 0
0 0 0 0 0 0 0 0 0 0 0 0 0 0 0 0 0 0 0 0 0 0 0 0 0 0 0 0
0 0 0 0 0 0 0 0 0 0 0 0 0 0 0 0 0 0 0 0 0 0 0 0 0 0 0 0
0 0 0 0 0 0 0 0 0 0 0 0 0 0 0 0 0 0 0 0 0 0 0 0 0 0 0

M02255_77_000000000-ADUT3_1_2114_28763_14140 G----AC---AG-AG-GTG--------CCA-A-G------C--G--T---T--AG-G-CGG-AA------TC-A--C-T--GG-GC-------TT--A-----AA-GC-GT-GT-------G-TA-G-G-C-G------------G--A-TC-G-T-T-----
------AA----G-T-A-C--------C-T-T--G--TG--A-AA-TC--C-C-AC-G-G-----CT-C-AA------C-C-G-T-G-G-A--A-C----T-G--C-T---C--G-------G--TA-T-A---C-------T--G-G--CG--G-T-C----T-T-G-A-G-C--C---A-CC-----TA-G-G--------G-G-C-A---GC-T------GG--A--ACA----A-
G-C-G-GT--GG-A-G-CG-GT--G---A--A-A---TG-C-GT-AG--AT-A-TC--G-C--T-T-G-G-A-AC-G-CC---AA--C--G--GT-GAA-A--A-C--A-------G--G-C-T-G---CTG--G--GG-G-T--------------------G-T---G-C-T--GA--CG-C-T-G--A-GA--C-A-CG-A--AA-G-C---C-AG--GG-
G--AG-C-G-AA--------CG--GG Bacteria(100);Planctomycetes(100);Phycisphaerae(100);WD2101(100);unclassified(100);unclassified(100);unclassified(100);unclassified(100);unclassified(100);

Otu1683 1 1 0 0 0 0 0 0 0 0 0 0 0 0 0 0 0 0 0 0 0 0 0 0 0 0 0 0
0 0 0 0 0 0 1 0 0 0 0 0 0 0 0 2 0 0 0 1 0 0 0 0 0 0 0 0
0 0 0 0 0 0 0 0 0 0 0 0 0 0 0 0 0 0 0 0 0 0 0 0 0 0 1 0
0 0 0 0 0 0 4 0 0 0 0 0 0 0 0 0 0 0 0 1 0 0 0 0 0 0 0

M02255_76_000000000-ADUNE_1_1108_26976_5194 T----AC---GT-AG-GTC--------CCG-A-G------C--G--T---T--GT-C-CGG-AT------TT-A--T-T--GG-GC-------GT--A-----AA-GC-GA-GC-------G-CA-G-G-C-G------------G--T-TT-G-A-T-----------AA--
--G-T-C-T--------G-A-A--G--TT--A-AA-GG--C-T-GT-G-G-----CT-C-AA------C-C-A-T-A-G-T--T------C-G--C-T---T--T-------G--GA-A-A---C-------T--G-T--CA--A-A-C----T-T-G-A-G-T--G---C-AG-----AA-G-G--------G-G-A-G---AG-T------GG--A--ATT----C-C-A-T-GT-
-GT-A-G-CG-GT--G---A--A-A---TG-C-GT-AG--AT-A-TA--T-G--G-A-G-G-A-AC-A-CC---GG--T--G--GC-GAA-A--G-C--G-------G--C-T-C-T---CTG--G--TC-T-G--------------------T-A---A-C-T--GA--CG-C-T-G--A-GG--C-T-CG-A--AA-G-C---G-TG--GG-G--AG-C-G-
AA--------CA--GG Bacteria(100);Firmicutes(100);Bacilli(100);Lactobacillales(100);Streptococcaceae(100);Streptococcus(100);unclassified(100);unclassified(100);unclassified(100);

Otu1684 15 0 0 0 0 0 0 0 0 0 0 0 0 0 0 0 0 0 0 0 0 0 0 0 0 0 0 0
0 0 0 0 0 0 0 0 0 0 0 0 0 0 0 0 0 0 0 0 0 0 0 0 0 0 0 0
0 0 0 0 0 0 0 0 0 0 0 0 0 0 0 0 0 0 0 0 0 0 0 0 0 0 0 0
0 0 0 0 0 0 0 0 0 0 0 0 0 0 0 0 0 0 0 0 0 0 0 0 0 0 0

M02255_76_000000000-ADUNE_1_1113_24400_8594 T----AC---GT-AG-GAG--------GCG-A-G------C--G--T---T--GT-C-CGG-AT------TT-A--C-T--GG-GC-------GT--A-----AA-GC-GC-GC-------G-CA-----T-G------------G----CA-A-G-T-----------AA---
----------------G-G-T--G--TG--A-AA-TC--T-C-CC-T-G-----CC-T-AA------C-G-G-G-G-A-G--G-G----T-G--C-A---C--T-------T--TA-G-A---C-------T--G-C--CA--A-G-C----T-A-G-A-G-G--A---C-GG-----TA-G-A--------G-G-G-G---GG-T------AG--A--ATT----G-C-T-G-GT--
GT-A-G-CG-GT--G---A--A-A---TG-C-GT-AG--AG-A-TC--A-G--C-A-G-G-A-AT-A-CC---CG--T--G--GC-GAA-G--G-C--G-------G--C-C-C-C---CTG--G--GC-C-G--------------------A-A---C-C-T--GA--CG-C-T-G--A-GG--C-G-CG-A--AG-G-C---T-AG--GG-G--AG-C-G-
AA--------CG--GG Bacteria(100);unclassified(100);unclassified(100);unclassified(100);unclassified(100);unclassified(100);unclassified(100);unclassified(100);unclassified(100);

Otu1685 0 0 0 38 0 0 0 0 0 0 0 0 0 0 0 0 0 0 0 0 0 0 0 0 0 0 0 0
0 0 0 0 0 0 0 0 0 0 0 0 0 0 0 0 0 0 0 0 0 0 0 0 0 0 0 0 0
0 0 0 0 0 0 0 0 0 0 0 0 0 0 0 0 0 0 0 0 0 0 0 0 0 0 0 0 0
0 0 0 0 0 0 0 0 0 0 0 0 0 0 0 0 0 0 0 0 0 0 0 0 0 0 0

M02255_77_000000000-ADUT3_1_1108_10222_4794 T----AC---GT-AG-GGG--------GCG-A-G------C--G--T---T--AT-C-CGG-AA------TT-A--T-T--GG-GC-------GT--A-----AA-GA-GT-GC-------G-TA-G-G-T-G------------G--T-TA-C-C-T----------AA-
---G-C-A-G--------G-G-G--G--TT--T-AA-GG--C-T-AT-G-G-----CT-T-AA------C-C-A-T-A-G-T--T------C-G--C-C---C--C-------C--TG-A-A---C-------T--G-G--GC--T-A-C----T-T-G-A-G-T--G---C-AG-----GA-G-A--------G-G-A-A---AG-C------GG--A--ATT----C-C-T-A-
GT--GT-A-G-CG-GT--G---A--A-A---TG-C-GT-AG--AT-A-TT--A-G--G-A-G-G-A-AC-A-CC---AG--T--G--GC-GAA-G--G-C--G-------G--C-T-T-T---CTG--G--AC-T-G--------------------T-A---A-C-T--GA--CA-C-T-G--A-GG--C-A-CG-A--AA-G-C---G-TG--GG-G--AG-
C-A-AA--------CA--GG Bacteria(100);Firmicutes(100);Clostridia(100);Clostridiales(100);unclassified(100);unclassified(100);unclassified(100);unclassified(100);unclassified(100);

Otu1686 0 0 0 0 18 0 0 46 0 0 0 0 0 0 0 0 0 0 0 0 0 0 0 0 0 0 0 0
0 0 42 0 0 0 0 0 0 0 0 0 0 0 0 0 0 0 0 0 0 0 5 0 0 0 0 0 0
0 0 0 0 0 0 0 0 0 0 7 0 0 0 0 0 0 0 0 0 0 0 0 0 0 0 0 0 0
18 0 0 0 0 0 0 0 0 30 0 0 0 0 0 0 0 0 0 0 0 0 0 0 11 0 0

M02255_77_000000000-ADUT3_1_1117_25190_8008 T----AC---GA-AG-GGG--------GCG-A-G------C--G--T---T--GT-T-CGG-AA------TC-A--C-T--GG-GC-------TT--A-----AA-GC-GT-GC-------G-TA-G-G-T-G------------G--G-CA-G-G-A----------
TA----G-T-C-A--------G-A-A--G--TG--A-AA-TC--C-C-GG-G-G-----CT-T-AA------C-C-C-C-G-G-A--A-C----T-G--C-T---T--T-------T--GA-A-A---C-------T--T-C--CT--G-T-C----T-G-G-A-A-T--T---C-GG-----TA-G-A--------G-G-C-T---GA-T------GG--A--ATA----C-C-C-
A-GT--GT-A-G-AG-GT--G---A--A-A---TT-C-GC-AG--AT-A-TT--G-G--G-T-G-G-A-AC-A-CC---AG--T--G--GC-GAA-G--G-C--G-------A--T-C-A-G---CTG--G--GC-C-G--------------------A-T---A-T-T--GA--CA-C-T-G--A-GG--C-A-CG-A--AA-G-C---G-TG--GG-T--
AG-C-A-AA--------CA--GG Bacteria(100);Proteobacteria(100);Alphaproteobacteria(100);unclassified(100);unclassified(100);unclassified(100);unclassified(100);unclassified(100);unclassified(100);

Otu1687 0 0 0 0 0 0 77 7 0 0 0 0 0 0 0 0 0 0 2 0 0 0 0 0 0 0 0 0
0 0 0 0 0 0 47 0 126 0 0 0 0 0 0 0 0 0 0 0 0 0 0 0 0 0 0 0 0
0 0 0 0 0 0 0 0 0 0 0 0 0 0 0 0 0 0 0 0 0 0 0 0 44 0 0 40 0
0 0 0 0 0 0 4 7 0 0 0 35 0 0 0 0 0 0 0 36 0 0 0 1 0 0 6

M02255_77_000000000-ADUT3_1_1104_1957_14465 T----AC---GG-AG-GGG--------GCA-A-G------C--G--T---T--AT-C-CGG-AA------TT-A--T-T--GG-GC-------GT--A-----AA-GG-GT-AC-------G-TA-G-G-T-G------------G--C-CT-T-T-T----------AA-
---G-C-G-T--------A-G-G--G--TA--T-AA-GG--C-A-AT-G-G-----CT-C-AA------C-C-A-T-T-G-T--T------C-G--C-C---C--T-------A--TG-A-A---C-------T--G-G--AA--G-G-C----T-T-G-A-G-T--G---C-AG-----GA-G-A--------G-G-A-A---AG-C------GG--A--ATT----C-C-T-A-
GT--GT-A-G-CG-GT--G---G--A-A---TG-C-AT-AG--AT-A-TT--A-G--G-A-G-G-A-AC-A-TC---AG--C--G--GC-GAA-G--G-C--G-------G--C-T-T-T---CTG--G--AC-T-G--------------------C-A---A-C-T--GA--CA-C-T-G--A-GG--T-A-CG-A--AA-G-C---G-TG--GG-G--AG-
C-A-AA--------CA--GG Bacteria(100);Firmicutes(100);Clostridia(100);Clostridiales(100);Family_XIII_Incertae_Sedis(100);Eubacterium(100);unclassified(100);unclassified(100);unclassified(100);

Otu1688 0 0 0 0 0 0 0 0 0 0 0 2 0 0 0 0 0 0 0 0 0 0 0 0 0 0 0 0
1 0 0 0 0 0 0 0 0 0 0 0 0 0 0 0 0 0 0 0 0 0 0 0 0 0 0 0 0
0 0 0 0 0 0 0 0 0 0 0 0 0 0 0 0 0 0 0 0 0 1 0 0 0 0 0 0 0
0 0 0 0 0 0 0 0 0 0 0 0 0 0 0 0 0 0 0 0 0 0 0 0 0 0 0

M02255_77_000000000-ADUT3_1_2108_22471_16737 C----TG---TG-CC-AGC--------AGC-C-G------C--G--G---T--AA-TACAG-AG------GG-T--G-C--AA-GC-------GT--T-----AA-TC-GG-AA-------T-TA-G-G-T-G------------G--C-TT-G-A-T-----
------AA----G-T-C-A--------G-A-T--G--TG--A-AA-TC--C-C-CG-G-G-----CT-T-AA------C-C-T-G-G-G-A--A-C----T-G--C-A---T--C-------T--GA-T-A---C-------T--G-T--TA--G-G-C----T-A-G-A-A-T--A---G-GT-----GA-G-A--------G-G-A-A---GG-T------AG--A--ATT----C-
C-A-G-GT--GT-A-G-CG-GT--G---A--A-A---TG-C-GT-AG--AG-A-TC--T-G--G-A-G-G-A-AT-A-CC---GA--T--G--GC-GAA-G--G-C--A-------G--C-C-T-T---CTG--G--CA-T-C--------------------A-T---A-T-T--GA--CA-C-T-G--A-GG--T-T-CG-A--AA-G-C---G-TG--GG-T--
AG-C-A-AA--------CA--GG Bacteria(100);Proteobacteria(100);Gammaproteobacteria(100);Pseudomonadales(100);Moraxellaceae(100);Psychrobacter(100);unclassified(100);unclassified(100);unclassified(100);

Otu1689 0 0 0 0 0 0 57 180 0 0 0 0 0 0 0 0 0 0 0 0 0 0 0 0 0 0 0 0
0 0 0 0 0 0 0 0 0 0 0 0 0 0 66 0 0 0 0 0 0 0 0 0 0 0 0 0 0
0 0 0 0 0 0 0 0 4 0 0 0 0 0 0 0 0 0 0 0 0 0 0 0 0 0 0 0 0
0 0 0 0 0 0 0 0 0 0 0 0 0 0 0 0 0 0 0 0 0 0 0 0 0 0 0

M02255_77_000000000-ADUT3_1_1104_22968_10622 T----AC---GT-AT-GGG--------GCA-A-G------C--G--T---T--GT-C-CGG-AA------TT-A--T-T--GG-GC-------GT--A-----AA-GG-GT-AC-------G-TA-G-G-C-G------------G--T-TA-T-T-T-----
-----AA----G-T-C-C--------T-A-A--T--TA--A-AA-GG--C-A-AT-G-G-----CT-C-AA------C-C-A-T-T-G-T--A------A-G--G-T---T--A-------G--GA-A-A---C-------T--A-G--AT--A-A-C----T-T-G-A-G-T--A---G-AT-----GA-G-G--------G-G-A-A---AG-T------GG--A--ATT----C-
C-A-T-GT--GT-A-G-CG-GT--G---A--A-A---TG-C-GT-AG--AT-A-TA--T-G--G-A-G-G-A-AC-A-CC---TG--T--G--GC-GAA-G--G-C--G-------A--C-T-T-T---CTG--G--AA-T-C--------------------A-A---A-C-T--GA--CG-C-T-G--A-GG--T-A-CG-A--AG-G-C---G-TG--GG-G-
-AG-C-A-AA--------CA--GG Bacteria(100);Firmicutes(100);Clostridia(100);Clostridiales(100);Family_XI_Incertae_Sedis(100);Helcococcus(100);unclassified(100);unclassified(100);unclassified(100);

Otu1690 2 0 0 0 0 0 0 0 0 0 0 0 0 0 0 0 0 0 0 0 0 0 0 0 0 0 0 0
0 0 0 0 0 0 2 0 0 0 0 0 0 0 0 0 0 0 0 0 0 0 0 0 0 0 0 1 0
0 0 0 0 0 0 0 0 0 0 0 0 0 0 0 0 0 0 0 0 0 0 0 0 0 0 0 0 0
0 0 0 3 0 0 0 0 0 0 0 0 0 0 0 0 0 2 0 0 5 0 0 0 0 0 6

M02255_77_000000000-ADUT3_1_1115_14942_2302 .....AC----T-A--CGT--------GCC-A-G------C--A--G---C--CG-C-GGT-AA------TA-C--G-T--AG-GG-------TG--C-----GA-GC-GT-GA-------A-TC-G---G-A------------A--T-TA-C-T-T----------AA----G-C-A-G--------G-A-T--G--TG--A-AA-TC--C-C-CG-G-G-----CT-C-AA------C-C-T-G-G-G-A--A-C----T-G--C-G---T--T-------C--TG-A-A---C-------T--G-G--GT--G-A-C----T-A-G-A-G-T--G---T-GT-----CA-G-A--------G-G-G-A---GG-T------AG--A--ATT----C-C-A-C-GT--GT-A-G-CA-GT--G---A--A-A---TG-C-GT-AG--AG-A-TG--T-G--G-A-G-G-A-AT-A-CC---GA--T--G--GC-GAA-G--G-C--A-------G--C-C-T-C---CTG--G--GA-T-A--------------------A-C---A-C-T--GA--CG-T-T-C--A-TG--C-T-CG-A--AA-G-C---G-TG--GG-T--AG-C-A-AA--------CA--GG Bacteria(100);Proteobacteria(100);Betaproteobacteria(100);Neisseriales(100);Neisseriaceae(100);Neisseria(100);unclassified(100);unclassified(100);unclassified(100);

Otu1691 0 0 0 54 0 0 0 0 0 0 0 0 0 0 0 0 0 0 3 0 0 0 0 0 0 0 0 0
0 0 0 0 0 0 0 0 0 0 0 0 0 0 0 0 0 0 0 0 0 0 0 0 0 0 0 0 0
0 0 0 0 0 0 0 0 0 0 0 0 0 0 0 0 0 0 0 0 0 0 0 0 0 0 0 0 0
0 0 0 0 0 0 0 0 0 0 0 0 0 0 0 0 0 0 0 0 0 0 0 0 0 0 0

M02255_77_000000000-ADUT3_1_1105_7395_23170 T----AC---GT-AG-GTG--------ACA-A-G------C--G--T---T--GT-C-CGG-AT------TT-A--C-T--GG-GT-------GT--A-----AA-GG-GC-GT-------G-TA-G-G-C-G------------G--G-AT-A-G-C-----------AA----G-T-C-A--------G-T-C--G--TG--A-AA-TA--C-C-GA-G-G-----CT-C-AA------C-C-T-C-G-G-G--G-C----T-G--C-G---A--T-------T--GA-A-A---C-------T--G-T--TA--T-T-C----T-T-G-A-G-T--A---T-CG-----GA-G-A--------G-G-A-A---AG-C------GG--A--ATT----C-C-C-A-GT--GT-A-G-CG-GT--G---A--A-A---TG-C-GT-AG--AT-A-TT--G-G--G-A-G-G-A-AC-A-CC---AG--T--G--GC-GAA-G--G-C--G-------G--C-T-T-T---CTG--G--AC-G-A--------------------C-A---A-C-T--GA--CG-C-T-G--A-GG--C-G-CG-A--AA-G-T---G-TG--GG-G--AG-C-A-AA--------CA--GG Bacteria(100);Firmicutes(100);Clostridia(100);Clostridiales(100);unclassified(100);unclassified(100);unclassified(100);unclassified(100);unclassified(100);

Otu1692 0 0 0 0 0 0 0 0 12 0 0 0 0 0 0 0 0 0 0 0 0 0 0 0 0 0 0 0
0 0 0 0 0 0 0 0 0 0 0 0 0 0 0 0 0 0 0 0 0 0 0 0 0 0 0 0 0
0 0 0 0 0 0 0 0 0 0 0 0 0 0 0 0 1 0 0 0 0 0 0 0 4 0 0 0 0
0 0 0 0 0 0 0 0 0 0 0 0 0 0 0 0 0 0 0 0 0 0 0 0 1 0 0

M02255_76_000000000-ADUNE_1_1113_24061_15740 T----CG---GC-TA-ACT--------CCG-T-GC-----C--A--G---C--AG-C-CGC-GG------TA-A--T-A--CG-GA-------GG--A-----TC-CG-GG-CG-------T-TA-T-C-C-G------------G--A-TG-TAT-T-----------AA----G-T-C-A--------G-T-T--G--TG--A-AA-GT--T-T-GC-G-G-----CT-C-AA------C-C-G-T-A-A-A--A-T----T-G--C-A---G--T-------T--GA-T-A---C-------T--G-G--AT--A-T-C----T-T-G-A-G-T--G---C-AG-----TT-G-A--------G-G-C-A---GG-C------GG--A--ATT----C-G-T-G-GT--GT-A-G-CG-GT--G---A--A-A---TG-C-TT-AG--AT-A-TC--A-C--G-A-A-G-A-AC-T-CC---GA--T--T--GC-GAA-G--G-C--A-------G--C-C-T-G---CTA--A--GC-T-G--------------------C-A---A-C-T--GA--CA-T-T-G--A-GG--C-T-CG-A--AA-G-T---G-TG--GG-T--AT-C-A-AA--------CA--GG Bacteria(100);Bacteroidetes(100);Bacteroidia(100);Bacteroidales(100);Bacteroidaceae(100);Bacteroides(100);unclassified(100);unclassified(100);unclassified(100);

Otu1693 0 0 0 0 0 0 0 0 0 0 0 0 4 0 0 0 0 0 0 0 0 0 0 0 0 0 0 0
0 0 0 0 0 0 22 0 0 0 0 0 0 0 0 0 0 0 0 734 0 0 0 0 0 0 0 0 1
0 0 0 0 0 0 0 0 0 0 0 0 0 0 0 0 0 0 0 0 0 0 0 0 0 0 0 0 0
0 0 0 0 0 0 0 0 0 0 0 0 0 0 0 0 0 0 0 0 0 0 0 0 0 0 0

M02255_77_000000000-ADUT3_1_2114_23450_11350 T----AC---GG-AG-GGT--------GCA-A-G------C--G--T---T--AC-T-CGG-AA------TC-A--C-T--GG-GC-------GT--A-----AA-GG-AT-GC-------G-TA-G-G-C-T------------G--T-AA-T-A-T-----------AA----G-T-C-A--------G-A-A--G--TG--A-AA-TC--C-A-AC-G-G-----CT-T-AA------C-C-G-T-T-G-A--A-C----T-G--C-T---T--T-------T--GA-A-A---C-------T--G-T--TT--T-A-C----T-A-G-A-A-T--A---T-GG-----GA-G-A--------G-G-T-A---GA-T------GG--A--ATT----G-G-T-G-GT--GT-A-G-GG-GT--A---A--A-A---TC-C-GT-AG--AT-A-TC--A-C--C-A-G-G-A-AT-A-CC---GA--T--T--GC-GAA-G--G-C--G-------A--T-C-T-A---CTG--G--AA-C-A--------------------T-T---A-T-T--GA--CG-C-T-G--A-GG--C-A-TG-A--AA-G-C---G-TG--GG-G--AG-C-A-AA--------CA--GG Bacteria(100);Proteobacteria(100);Epsilonproteobacteria(100);Campylobacterales(100);Campylobacteraceae(100);Sulfurospirillum(100);unclassified(100);unclassified(100);unclassified(100);

Otu1694 0 0 0 0 0 2 0 0 0 0 0 0 0 0 0 0 0 0 0 0 0 0 0 0 9 8 0 0
0 0 0 0 0 0 0 0 0 0 0 0 0 0 0 0 0 0 0 0 0 0 0 0 0 0 0 0 0
0 0 0 0 0 0 0 0 0 0 0 0 0 0 0 0 0 0 0 0 0 0 0 0 0 0 0 0 0
0 0 0 0 0 0 0 0 0 0 0 0 0 0 0 0 0 0 0 1 0 0 0 0 0 1 0 0

M02255_76_000000000-ADUNE_1_2114_25308_22707 G----AA---GC-AC-CGG--------CTA-A-C------T--C--C---G--TG-C-CAG-CA------GC-C--G-C--GG-TA-------AT--A-----CG-GA-GG-GT-------G-CG-A-G-C-G------------T--TAAT-C-G-G-----------AA----G-C-A-A--------G-A-T--G--TG--A-AA-GC--C-C-GG-G-G-----CT-C-AA------C-C-T-C-G-G-A--A-C----A-G--C-A---T--T-------T--TG-A-A---C-------T--G-G--CA--G-A-C----T-A-G-A-G-T--C---T-TG-----TA-G-A--------G-G-G-G---GG-T------AG--A--ATT----T-C-A-G-GT--GT-A-G-CG-GT--G---A--A-A---TG-C-GT-AG--AG-A-TC--T-G--A-A-G-G-A-AT-A-CC---GG--T--G--GC-GAA-G--G-C--G-------G--C-C-C-C---CTG--G--AC-A-A--------------------A-G---A-C-T--GA--CG-C-T-C--A-GA--T-G-CG-A--AA-G-C---G-TG--GG-G--AG-C-A-AA--------CA--GG Bacteria(100);Proteobacteria(100);Gammaproteobacteria(100);Vibrionales(100);Vibrionaceae(100);Photobacterium(100);unclassified(100);unclassified(100);unclassified(100);

Otu1695 0 0 0 0 0 0 0 0 0 1 0 0 0 0 0 0 0 0 0 0 0 0 0 0 8 0 0 0
0 0 0 0 0 0 0 0 0 0 0 0 0 0 0 0 0 0 0 0 0 0 0 0 0 0 0 0
0 0 0 0 0 0 0 0 0 0 0 0 0 0 0 0 0 0 0 0 0 0 0 0 0 0 0 0
0 0 0 0 0 0 0 0 0 0 0 0 0 0 0 0 0 0 0 1 0 0 0 0 0 0 0

M02255_76_000000000-ADUNE_1_1107_19073_2634 A----AC---AG-AA-GAA--------GCA-C-C------G--G--C---T--AACT-CCG-TG------CC-AG-C-A-GCC-GC-------GG--T-----AA-TA-CG-GA-------G-GG-T-G-C-G------------A--G-CT-G-T-T----------AA----T-C-A-G--------G-A-A--G--TG--A-AA-GC--C-C-GG-G-G-----CT-C-AA------C-C-T-C-G-G-A--A-C----A-G--C-A---T--T-------T--TG-A-A---C-------T--G-G--CA--G-A-C----T-A-G-A-G-T--C---T-TG-----TA-G-A--------G-G-G-G---GG-T------AG--A--ATT----T-C-A-G-GT--GT-A-G-CG-GT--G---A--A-A---TG-C-GT-AG--AG-A-TC--T-G--A-A-G-G-A-AT-A-CC---GG--T--G--GC-GAA-G--G-C--G-------G--C-C-C-C-C---CTG--G--AC-A-A--------------------A-G---A-C-T--GA--CG-C-T-C--A-GA--T-G-CG-A--AA-G-C---G-TG--GG-G--AG-C-A-AA--------CA--GG Bacteria(100);Proteobacteria(100);Gammaproteobacteria(100);Vibrionales(100);Vibrionaceae(100);unclassified(100);unclassified(100);unclassified(100);unclassified(100);

Otu1696 0 0 44 0 0 0 0 0 0 0 0 0 0 0 0 0 0 0 0 0 0 0 0 0 0 0 0 0
0 0 0 0 0 0 0 0 0 0 0 0 0 0 0 0 0 0 0 0 0 0 0 0 0 0 0 0
0 0 0 0 0 0 0 0 0 0 0 0 0 0 0 0 0 0 0 0 0 0 0 0 0 0 0 0
0 0 0 0 0 0 2 0 0 0 0 0 0 0 0 0 0 0 0 0 0 0 0 0 0 0 0

M02255_77_000000000-ADUT3_1_2116_29435_14777 T----AC---GT-AG-GTG--------GCA-A-G------C--G--T---T--AT-C-CGG-AT------TT-A--T-T--GG-GT-------GT--A-----AA-GG-GC-GT-------G-TA-G-G-C-G------------G--G-AT-T-G-C-----------AA----G-T-C-A--------G-A-T--G--TG--A-AA-AC--T-G-GG-G-G-----CT-C-AA------C-C-T-C-C-A-G--C-C----T-G--C-A---T--T-------T--GA-A-A---C-------T--G-T--AG--T-T-C----T-T-G-A-G-T--G---T-CG-----GA-G-A--------G-G-C-A---AT-C------GG--A--ATT----C-C-G-T-GT--GT-A-G-CG-GT--G---A--A-A---TG-C-GT-AG--AT-A-TA--C-G--G-A-G-G-A-AC-A-CC---AG--T--G--GC-GAA-G--G-C--G-------G--A-T-T-G---CTG--G--AC-G-A--------------------T-A---A-C-T--GA--CG-C-T-G--A-GG--C-G-CG-A--AA-G-C---G-TG--GG-G--AG-C-A-AA--------CA--GG Bacteria(100);Firmicutes(100);Clostridia(100);Clostridiales(100);Ruminococcaceae(100);unclassified(100);unclassified(100);unclassified(100);unclassified(100);

Otu1697 0 163 55 0 0 0 0 0 0 0 0 0 0 0 0 0 0 0 0 0 0 0 0 0 0 0 0 0
0 0 0 0 0 0 0 0 0 0 0 76 0 0 0 0 0 0 0 0 0 0 0 0 0 0 0 0
0 0 0 0 0 0 0 0 0 0 0 0 0 0 0 0 0 0 0 0 0 0 1 0 0 0 0 0
27 0 0 0 0 0 0 0 0 0 0 0 0 0 0 0 0 0 0 0 0 0 0 0 10 0 0

M02255_77_000000000-ADUT3_1_1106_8732_13626 T----AC---GT-AG-GGG--------GCA-A-G------C--G--T---T--AT-C-CGG-AT------TT-A--C-T--GG-GT-------GT--A-----AA-GG-GT-GC-------G-TA-G-G-T-G------------G--C-AA-G-G-C----------AA----G-T-C-A--------G-A-T--G--TG--A-AA-GC--C-C-GG-G-G-----CT-C-AA------C-C-C-C-G-G-T--A-C----T-G--C-A---T--T-------T--GA-A-A---C-------T--G-T--CT--A-G-C----T-A-G-A-G-T--G---C-AG-----GA-G-A--------G-G-T-A---AG-C------GG--A--ATT----C-C-T-A-GT--GT-A-G-CG-GT--G---A--A-A---TG-C-GT-AG--AT-A-TT--A-G--G-A-G-G-A-AC-A-CC---AG--T--G--GC-GAA-G--G-C--G-------G--C-T-T-A---CTG--G--AC-T-G--------------------T-A---A-C-T--GA--CA-C-T-G--A-GG--C-A-CG-A--AA-G-C---G-TG--GG-G--AG-C-A-AA--------CA--GG Bacteria(100);Firmicutes(100);Clostridia(100);Clostridiales(100);Lachnospiraceae(100);unclassified(100);unclassified(100);unclassified(100);unclassified(100);

Otu1698 0 63 0 0 0 0 0 0 0 0 0 0 0 0 0 0 0 0 0 0 0 0 0 0 0 0 0 0
0 0 0 0 0 0 0 0 0 0 0 0 0 0 0 0 0 0 0 0 0 0 0 0 0 0 0 0
0 0 0 0 0 0 0 0 0 0 0 0 0 0 0 0 0 0 0 0 0 0 0 0 0 0 0 0
0 0 0 0 0 0 0 0 0 0 0 0 0 0 0 0 0 0 0 0 0 0 0 0 0 0 0

M02255_77_000000000-ADUT3_1_1107_24542_12219 T----AC---GT-AG-GTG--------GCA-A-G------C--G--T---T--GT-C-CGG-AA------TT-A--C-T--GG-GT-------GT--A-----AA-GG-GA-GC-------G-TA-G-G-C-G------------G--G-AA-G-A-C-----------AA----G-T-T-G--------G-G-A--G--TG--A-AA-TG--T-A-TG-G-G-----CT-T-AA------C-C-C-A-TAA-A----C----T-G--C-T---T--T-------C--AA-A-A---C-------T--G-T--TT--T-T-C----T-T-G-A-G-T--G---A-AG-----TA-G-A--------G-G-C-A---GG-C------GG--A--ATT----C-C-T-A-GT--GT-A-G-CG-GT--G---A--A-A---TG-C-GT-AAA-T--A-TT--A-G--G-A-G-G-A-AC-A-CC---AG--T--G--GC-GAA-G--G-C--G-------G--C-C-T-G---CTG--G--GC-T-T--------------------T-A---A-C-T--GA--CG-C-T-G--A-GG--C-T-CG-A--AA-G-C---G-TG--GG-T--AG-C-A-AA--------CA--GG Bacteria(100);Firmicutes(100);Clostridia(100);Clostridiales(100);Ruminococcaceae(100);unclassified(100);unclassified(100);unclassified(100);unclassified(100);

Otu1699 0 0 0 0 0 0 0 0 0 5 0 0 0 0 0 0 0 0 0 0 0 0 0 0 0 0 0 2
0 0 0 0 0 0 0 0 0 0 0 0 0 0 0 0 0 0 0 0 2 0 0 0 0 0 0 0
0 0 0 0 0 0 0 0 0 0 0 0 0 0 0 0 0 0 0 0 0 0 0 0 0 0 0 0
0 0 0 0 0 0 0 0 0 0 0 0 0 0 0 0 0 0 0 0 0 0 0 0 0 0 0

M02255_76_000000000-ADUNE_1_2114_19309_22621 A----AC---AG-AA-GAA--------GCA-C-C------G--G--C---T--AACT-CCG-TG------CC-A--G-C--AG-CC-------GC--G-----GT-AA-TA-CG-------G-AG-G-G-T-GC------------G--A-GC-G-T-T-----------AA----G-T-C-G--------G-A-A--G--TG--A-AA-GC--C-C-GG-G-G-----CT-C-AA------C-C-T-C-G-G-A--A-G----G-T--C-A---T--T-------T--GA-A-A---C-------T--G-G--CA--A-A-C----T-A-G-A-G-T--A---C-TG-----TA-G-A--------G-G-G-G---GG-T------AG--A--ATT----T-C-A-G-GT--GT-A-G-CG-GT--G---A--A-A---TG-C-GT-AG--AG-A-TC--T-G--A-A-G-G-A-AT-A-CC---AG--T--G--GC-GAA-G--G-C--G-------G--C-C-C-C-C---CTG--G--AC-A-G--------------------A-T---A-C-T--GA--CA-C-T-C--A-GA--T-G-CG-A--AA-G-C---G-TG--GG-G--AG-C-A-AA--------CA--GG Bacteria(100);Proteobacteria(100);Gammaproteobacteria(100);Vibrionales(100);Vibrionaceae(100);unclassified(100);unclassified(100);unclassified(100);unclassified(100);

Otu1700 0 39 0 0 0 0 0 0 0 0 0 0 0 0 0 0 0 0 0 0 0 0 0 0 0 0 0 6
0 0 0 0 134 0 79 0 0 0 0 0 0 0 0 0 0 0 0 0 0 0 0 0 1 0 0 0 0
0 0 0 0 0 0 0 0 0 0 0 0 0 0 0 0 0 0 0 0 0 0 0 0 0 0 0 0 15
0 0 0 0 0 0 0 1 0 0 0 0 0 0 0 60 0 0 0 0 0 0 0 0 0 0 0

M02255_77_000000000-ADUT3_1_1109_13800_9467 T----AC---GA-AG-GGG--------GCT-A-G------C--G--T---T--GT-T-CGG-AA------TC-A--C-T--GG-GC-------GT--A-----AA-GC-GC-AC-------G-TA-G-G-C-G------------G--A-CC-G-T-T----------
AA----G-T-C-G--------G-A-G--G--TG--A-AA-GC--C-T-GG-G-G-----CT-C-AA------C-C-C-C-A-G-A--A-C----T-G--C-C---T--T-------C--GA-T-A---C-------T--G-G--CG--G-T-C----T-T-G-A-G-T--A---T-GG-----TA-G-A--------G-G-T-T---GG-T------GG--A--ACT----C-C-G-
A-GT--GT-A-G-AG-GT--G---A--A-A---TT-C-GT-AG--AT-A-TT--C-G--G-A-A-G-A-AC-A-CC---AG--T--G--GC-GAA-G--G-C--G-------G--C-C-A-A---CTG--G--AC-C-A--------------------T-T---A-C-T--GA--CG-C-T-G--A-GG--T-G-CG-A--AA-G-C---G-TG--GG-G--
AG-C-A-AA--------CA--GG Bacteria(100);Proteobacteria(100);Alphaproteobacteria(100);Rhizobiales(100);unclassified(100);unclassified(100);unclassified(100);unclassified(100);unclassified(100);

Otu1701 0 0 28 0 27 6 7 0 0 0 0 0 0 1 0 0 0 0 0 0 0 0 0 0 0 0 0 1
0 0 14 0 0 0 0 0 10 0 0 0 0 0 0 0 2 0 0 0 0 0 0 0 0 0 0 0 0
0 0 0 0 0 0 0 0 0 0 0 0 0 0 0 0 0 0 0 7 0 0 0 6 0 0 0 0 14
4 0 4 9 0 0 0 0 0 0 0 0 0 6 0 0 0 0 5 19 13 7 16 0 0 0 0

M02255_77_000000000-ADUT3_1_1109_23501_18373 T----AC---GT-GC-CAG--------CAGCC-G------CG-G--T---A--AT-A-CGT-AG------GT-G--G-C--GA-GC------GTT--AT----CC-GGAAT-TA-------T-TG-G-G-C-G-------------G--C-CT-G-T-T--
---------AA----G-T-A-A--------G-T-G--G--TT--A-AA-TT--G-T-TG-G-G-----CT-C-AA------C-C-C-A-A-T-C--C------A-G--C-C---A--C-------T--TA-A-A---C-------T--G-G--CA--G-G-C----T-A-G-A-G-T--A---T-TG-----GA-G-A--------G-G-C-A---AG-T------GG--A--ATT----
C-C-A-T-GT--GT-A-G-CG-GT--A---A--A-A---TG-C-GT-AG--AT-A-TA--T-G--G-A-G-G-A-AC-A-CC---AG--T--G--GC-GAA-G--G-C--G-------G--C-T-T-G---CTA--G--CC-A-A--------------------A-G---A-C-T--GA--CG-C-T-C--A-TG--C-A-CG-A--AA-G-C---G-TG--GG-
G--AG-C-A-AA--------TA--GG Bacteria(100);Firmicutes(100);Erysipelotrichi(100);Erysipelotrichales(100);Erysipelotrichaceae(100);Solobacterium(100);unclassified(100);unclassified(100);unclassified(100);

Otu1702 0 49 0 0 0 0 0 0 0 0 0 0 0 0 0 0 0 0 0 0 0 0 0 0 0 0 0 0
0 0 0 0 0 0 0 0 0 0 0 0 0 0 0 0 0 0 0 0 0 0 0 0 0 0 0 0 0
0 0 0 0 0 0 0 0 0 0 0 0 0 0 0 0 0 0 0 0 0 0 0 0 0 0 0 0 0
0 0 0 0 0 0 0 0 0 0 0 0 0 0 0 0 0 0 0 0 0 0 0 0 0 0 0

M02255_77_000000000-ADUT3_1_1111_16102_18618 T----AC---GT-AG-GGG--------GCG-A-G------C--G--T---T--GT-C-CGG-AA------TT-A--C-T--GG-GC-------GT--A-----AA-GG-GA-GC-------G-TA-G-G-C-G------------G--T-CC-T-T-T-----
------AA----G-T-T-G--------G-A-T--G--TG--A-AA-GC--C-C-CG-G-G-----CT-T-AA------C-T-T-G-G-G-A--A-G----T-G--C-A---T--C-------C--AA-T-A---C-------T--G-G--AG--G-A-CT---T-G-G-A-G-T--A---C-AG-----GA-G-A--------G-G-G-A---AG-C------GG--A--ATT----
C-C-T-A-GT--GT-A-G-CG-GT--G---A--A-A---TG-C-GT-AG--AT-A-TT--A-G--G-A-G-G-A-AC-A-CC---AG--T--G--GC-GAA-G--G-C--G-------G--C-T-T-T---CTG--G--AC-T-G--------------------A-C---A-C-T--GA--CG-C-T-G--A-GG--C-T-CG-A--AA-G-C---G-TG--GG-
G--AG-C-A-AA--------CA--GG Bacteria(100);unclassified(100);unclassified(100);unclassified(100);unclassified(100);unclassified(100);unclassified(100);unclassified(100);unclassified(100);

Otu1703 0 0 0 0 0 48 0 0 0 0 0 0 0 0 0 0 0 0 0 0 0 0 0 0 0 0 0 0
0 0 0 0 0 0 0 0 0 0 0 0 0 0 0 0 0 0 0 0 0 0 0 0 0 0 0 0 0
0 0 0 0 0 0 0 0 0 0 0 0 0 0 0 0 0 0 0 0 0 0 0 0 0 0 0 0 0
0 0 0 0 0 0 0 0 0 0 0 0 0 0 0 0 0 0 0 0 0 0 0 0 0 0 0

M02255_77_000000000-ADUT3_1_1101_10895_14827 T----AC---GA-AG-GGA--------GCT-A-G------C--G--T---T--GT-T-CGG-AT------TT-A--C-T--GG-GC-------GT--A-----AA-GA-GC-GT-------G-TA-G-G-C-G------------G--C-TT-T-G-C-----
------AA----G-T-T-G--------G-G-A--G--TG--A-AA-TC--C-C-GG-A-G-----CT-C-AA------C-T-C-C-G-G-A--A-C----T-G--C-T---T--T-------C--AA-A-A---C-------T--G-C--TT--A-G-C----T-A-G-A-G-T--A---T-GT-----AA-G-A--------G-G-G-T---GA-C------GG--A--ATT----C-
C-T-A-GT--GT-A-G-AG-GT--G---A--A-A---TT-C-AT-AG--AT-A-TT--A-G--G-A-G-G-A-AC-A-CC---AG--T--G--GC-GAA-G--G-C--G-------G--T-C-A-C---CTG--G--GA-C-A--------------------T-T---A-C-T--GA--CG-C-T-G--A-GA--C-G-CG-A--AA-G-C---G-TG--GG-G-
-AG-C-A-AA--------CA--GG Bacteria(100);unclassified(100);unclassified(100);unclassified(100);unclassified(100);unclassified(100);unclassified(100);unclassified(100);unclassified(100);

Otu1704 0 0 0 0 0 0 0 0 0 0 0 0 3 0 0 0 0 0 0 0 0 0 0 0 0 0 0 0
0 0 0 0 0 0 0 0 0 0 0 0 0 0 0 0 0 0 0 0 0 0 0 0 0 0 0 0 0
0 0 0 0 0 0 0 0 0 0 0 0 0 0 0 0 0 0 0 0 0 0 0 0 0 0 0 0 0
0 0 0 0 0 0 0 0 0 0 0 0 0 0 0 0 0 0 0 0 0 0 0 0 0 0 0

M02255_77_000000000-ADUT3_1_1112_13041_4839 C----TA---AC-TC-TGT--------GCC-A-G------C--A--G---C--CG-C-GGT-AA------TA-C--A-G--AG-GG-------GG--C-----AA-GC-GT-TA-------A-TC-G-G---A------------A--T-TA-G-T-T----------AA--
--G-T-T-G--------G-A-T--G--TG--A-AA-TC--C-C-CG-G-G-----CT-C-AA------C-C-T-G-G-G-A--A-C----T-G--C-A---T--T-------C--AA-A-A---C-------T--G-A--CT--G-A-C----T-A-G-A-G-T--A---T-GG-----TA-G-A--------G-G-G-T---GG-T------GG--A--ATT----T-C-C-T-
GT--GT-A-G-CG-GT--G---A--A-A---TG-C-GT-AG--AT-A-TA--G-G--A-A-G-G-A-AC-A-CC---AG--T--G--GC-GAA-G--G-C--G-------A--C-C-A-C---CTG--G--AC-T-G--------------------A-T---A-C-T--GA--CA-C-T-G--A-GG--T-G-CG-A--AA-G-C---G-TG--GG-G--AG-
C-A-AA--------CA--GG Bacteria(100);Proteobacteria(100);Gammaproteobacteria(100);Pseudomonadales(100);Pseudomonadaceae(100);unclassified(100);unclassified(100);unclassified(100);unclassified(100);

Otu1705 0 14 0 0 0 0 0 0 0 0 0 0 0 0 0 0 0 0 0 0 0 0 0 0 0 0 0 0
0 0 0 0 0 0 0 0 0 1 0 0 0 0 0 1 0 0 0 0 0 0 0 0 0 0 0 0
0 0 0 0 0 0 0 0 0 0 0 0 0 0 0 0 0 0 0 0 0 0 0 0 0 0 0 0
0 0 0 0 0 0 0 1 0 0 0 0 0 0 0 0 0 0 0 0 0 0 0 0 0 0 0

M02255_76_000000000-ADUNE_1_1103_15069_22243 T----A----AG-CA-CCG--------GCT-A-A------C-----T---C--TG-T-GCC-AG------CA-G--C-C--GC-G--------GT--A-----AT-AC-AG-AG-------G-GT-G---C-G------------G--A-GC-G-T-T----------AA----G-T-C-G--------G-A-T--G--TG--A-AA-TC--C-C-CG-G-G-----CT-C-AA------C-C-T-G-G-G-A--A-T----T-G--C-A---T--T-------C--GA-T-A---C-------T--G-G--CA--A-G-C----T-A-G-A-G-T--A---T-GG-----GA-G-A--------G-G-A-A---GG-T------AG--A--ATT----C-C-A-G-GT--GT-A-G-CG-GT--G---A--A-A---TG-C-GT-AG--AG-A-TC--T-G--G-A-G-G-A-AT-A-CC---GA--T--G--GC-GAA-G--G-C--A-------G--C-C-T-T---CTG--G--CC-T-A--------------------A-T---A-C-T--GA--CG-C-T-G--A-GG--T-G-CG-A--AA-G-C---A-TG--GG-G--AG-C-A-AA--------CA--GG Bacteria(100);Proteobacteria(100);Gammaproteobacteria(100);unclassified(100);unclassified(100);unclassified(100);unclassified(100);unclassified(100);unclassified(100);

Otu1706 0 6 0 0 0 0 0 0 3 0 0 0 0 0 0 0 0 0 0 0 0 0 0 0 0 0 0 0
0 0 0 1 0 0 0 0 0 0 0 0 0 0 0 0 0 0 0 0 0 0 0 0 0 0 0 0
0 0 0 0 0 0 0 0 0 0 0 0 0 0 0 0 0 0 0 0 0 0 0 0 0 0 0 0
0 0 0 0 0 0 0 0 0 4 0 0 0 0 1 0 0 0 0 0 0 0 0 0 0 0 0

M02255_76_000000000-ADUNE_1_1105_6378_9077 TC---ACG--GCTAA-CTA--------CGT-G-C------C--A--G---C--AG-C-CGC-GG------TA-A--T-A--CG-TA-------GG--T-----GG-CG-AG-CG-------T-TA-T-C-C-G------------G--A-AG-A-T-C-----------AA----G-T-C-T--------G-G-A--G--TA--A-AA-GG--T-A-TG-G-G-----CT-C-AA------C-C-C-G-T-A-C--T------G-G--C-T---C--T-------G--GA-A-A---C-------T--G-A--TC--A-G-C----T-A-G-A-G-A--A---C-AG-----AA-G-A--------G-G-A-C---GG-C------GG--A--ACT----C-C-A-T-GT--GT-A-G-CG-GT--A---A--A-A---TG-C-GT-AG--AT-A-TA--T-G--G-A-A-G-A-AC-A-CC---GG--T--G--GC-GAA-G--G-C--G-------G--C-C-G-T---CTG--G--TC-T-G--------------------G-AT--T-C-T--GA--CA-C-T-G--A-AG--C-A-CG-A--AA-G-C---G-TG--GG-G--AG-C-A-AA--------TA--GG Bacteria(100);unclassified(100);unclassified(100);unclassified(100);unclassified(100);unclassified(100);unclassified(100);unclassified(100);unclassified(100);

Otu1707 0 0 50 0 0 0 0 0 0 0 0 0 0 0 0 0 0 0 0 0 0 0 0 0 0 0 0 0
0 0 0 0 0 0 0 0 0 0 0 13 0 0 0 0 0 0 0 0 0 0 0 0 0 0 0 0
0 0 0 0 0 0 0 0 0 0 0 0 0 0 0 0 0 0 0 0 0 0 0 0 0 0 0 0
0 0 0 0 0 0 0 0 0 0 0 0 0 0 0 0 0 0 0 0 0 0 0 0 0 0 0

M02255_77_000000000-ADUT3_1_1107_5205_4398 T----AC---GT-AG-GGT--------GCA-A-G------C--G--T---T--GT-C-CGG-AA------TT-A--T-T--GG-GC-------GT--A-----AA-GA-GC-TC-------G-TA-G-G-C-G------------G--T-TT-G-T-C-----------GC----G-T-C-T--------G-G-T--G--TG--A-AA-GT--C-C-AT-C-G-----CT-T-AA------C-G-G-T-G-G-A--T-T----G-G--C-G---C--T-------G--GG-T-A---C-------G--G-G--CA--G-G-C----T-A-G-A-G-T--G---T-AG-----TA-G-G--------G-G-A-G---AC-T------GG--A--ATT----C-T-C-G-GT--GT-A-A-CG-GT--G---G--A-A---TG-T-GT-AG--AT-A-TC--G-G--G-A-A-G-A-AC-A-CC---AA--T--G--GC-GAA-G--G-C--A-------G--G-T-C-T---CTG--G--GC-T-A--------------------T-T---A-C-T--GA--CG-C-T-G--A-GG--A-G-CG-A--AA-G-C---G-TG--GG-G--AG-C-G-AA--------CA--GG Bacteria(100);Actinobacteria(100);Actinobacteria(100);Actinobacteridae(100);Bifidobacteriales(100);Bifidobacteriaceae(100);Alloscardovia(100);unclassified(100);unclassified(100);

Otu1708 0 0 27 0 0 0 0 0 0 0 0 0 0 0 0 0 0 0 0 0 0 0 0 0 0 0 0 0
0 0 0 0 0 0 0 0 0 0 0 0 0 0 0 0 0 0 0 0 0 0 0 0 0 0 0 0
0 0 0 0 0 0 0 0 0 0 0 0 0 0 0 0 0 0 0 0 0 0 0 0 0 0 0 0
0 0 0 0 0 0 0 0 0 0 0 0 0 0 0 0 0 0 0 0 0 0 0 0 0 0 0

M02255_77_000000000-ADUT3_1_2102_23786_14315 T----AC---GT-AG-GGG--------GCG-A-G------C--G--T---T--AT-C-CGG-AT------TC-A--T-T--GG-GC-------GT--A-----AA-GC-GC-GC-------G-TA-G-G-C-G------------G--C-CG-C-C-T-----------AA----G-C-G-G--------A-A-C--C--TC--T-AA-CC--C-C-GG-G-G-----CT-C-AA------C-C-T-C-G-G-G--C------C-G--G-G---T--T-------C--CG-G-A---C-------T--G-G--GC--G-G-C----T-C-G-A-G-T--G---C-GG-----TA-G-A--------G-G-C-A---GG-C------GG--A--ATT----C-C-C-G-GT--GT-A-G-CG-GT--G---G--A-A---TG-C-GC-AG--AT-A-TC--G-G--G-A-A-G-A-AC-A-CC---GA--T--G--GC-GAA-G--G-C--A-------G--C-C-T-G---CTG--G--GC-C-G--------------------C-C---A-C-T--GA--CG-C-T-G--A-GG--C-G-CG-A--AA-G-C---T-GG--GG-G--AG-C-G-AA--------CA--GG Bacteria(100);Actinobacteria(100);Actinobacteria(100);Coriobacteridae(100);Coriobacteriales(100);Coriobacterineae(100);Coriobacteriaceae(100);Adlercreutzia(100);unclassified(100);

Otu1709 0 0 106 0 0 0 165 14 182 0 0 0 0 0 0 0 0 0 0 0 0 0 0 0 0 0 0 2
0 0 0 0 0 0 0 0 0 0 0 0 0 0 0 0 0 0 0 0 0 0 0 0 0 0 0 0
0 0 0 0 0 0 0 0 0 0 0 0 0 0 4 0 0 0 0 0 0 0 0 0 0 0 0 0
0 0 0 0 0 0 0 0 0 0 0 41 0 23 0 0 0 0 0 0 0 0 24 0 56 0 0

M02255_77_000000000-ADUT3_1_1105_15933_7322 T----AC---GT-AT-GGG--------GCA-A-G------C--G--T---T--AT-C-CGG-AT------TT-A--C-T--GG-GT-------GT--A-----AA-GG-GA-GC-------G-TA-G-G-C-G------------G--C-AT-G-G-C-----------AA----G-T-C-A--------G-A-A--G--TG--A-AA-GC--C-T-GG-G-G-----CT-C-AA------C-C-C-C-G-G-A--A-T----T-G--C-T---T--T-------T--GA-A-A---C-------T--G-T--CA--G-G-C----T-A-G-A-G-T--G---T-CG-----GA-G-G--------G-G-T-A---AG-C------GG--A--ATT----C-C-T-A-GT--GT-A-G-CG-GT--G---A--A-A---TG-C-GT-AG--AT-A-TT--A-G--G-A-G-G-A-AC-A-CC---GG--T--G--GC-GAA-G--G-C--G-------G--C-T-T-A---CTG--G--AC-G-A--------------------T-T---A-C-T--GA--CG-C-T-G--A-GG--C-T-CG-A--AA-G-C---G-TG--GG-G--AG-C-A-AA--------CA--GG Bacteria(100);Firmicutes(100);Clostridia(100);Clostridiales(100);Lachnospiraceae(100);unclassified(100);unclassified(100);unclassified(100);unclassified(100);

Otu1710 0 0 24 0 0 0 0 0 0 0 0 0 0 0 0 0 0 0 0 0 0 0 0 0 0 0 0 0
0 0 0 0 0 0 0 0 0 0 0 0 0 0 0 0 0 0 0 0 0 0 0 0 0 0 0 0 0
0 0 0 0 0 0 0 0 0 0 0 0 0 0 0 0 0 0 0 0 0 0 0 0 0 0 0 0 0
0 0 0 0 0 0 0 0 0 0 0 0 0 0 0 0 0 0 0 0 0 0 0 0 0 0 0
M02255_77_000000000-ADUT3_1_1103_2317_9858 T----AC---GT-AG-GTG--------GCG-A-G------C--G--T---T--AT-C-CGG-AA------TT-A--C-T--GG-GT-------GT--A-----AA-GG-GT-GT-------G-TA-G-G-C-G------------G--G-AC-G-A-C----------
AA----G-T-C-A--------G-A-T--G--TG--A-AA-AT--T-G-CA-G-G-----CT-C-AA------C-C-T-G-G-A-A--A-G----T-G--C-A---T--T-------T--GA-A-A---C-------T--G-C--CG--T-T-C----T-T-G-A-G-A--G---T-CG-----GA-G-A--------G-G-T-A---AA-T------GG--A--ATT----C-C-C-
G-GT--GT-A-G-CG-GT--G---A--A-A---TG-C-GT-AG--AT-A-TC--G-G--G-A-G-G-A-AC-A-CC---AG--T--G--GC-GAA-G--G-C--G-------A--T-T-T-A---CTG--G--AC-G-A--------------------C-A---A-C-T--GA--CG-C-T-G--A-GA--C-A-CG-A--AA-G-C---G-TG--GG-G--
AG-C-A-AA--------CA--GG Bacteria(100);unclassified(100);unclassified(100);unclassified(100);unclassified(100);unclassified(100);unclassified(100);unclassified(100);unclassified(100);

Otu1711 0 0 0 0 0 0 0 0 0 0 4 3 0 0 1 4 0 0 10 4 0 5 0 1 0 0 3 0
0 0 0 0 0 0 0 0 0 0 0 0 0 0 0 0 0 0 6 0 0 0 39 0 21 62 2 0 0
1 2 4 6 2 14 5 0 26 18 3 1 0 19 0 0 1 4 0 0 0 0 0 0 0 0 0 0 0
0 0 0 0 0 0 2 0 37 0 17 0 1 9 41 0 0 0 0 0 0 0 0 0 0 0 0
M02255_77_000000000-ADUT3_1_2116_21351_24941 T----AC---GT-AG-GGT--------GCA-A-G------C--G--T---T--AA-T-CGG-AA------TT-A--C-T--GG-GC-------GT--A-----AA-GC-GT-GC-------G-CA-G-G-C-G------------G--T-TT-T-G-T------
-----AA----G-T-C-T--------G-A-T--G--TG--A-AA-TC--C-C-CG-G-G-----CT-C-AA------C-C-T-G-G-G-A--A-T----T-G--C-A---T--T-------G--GA-G-A---C-------T--G-C--AA--G-G-C----T-A-G-A-A-T--C---T-GG-----CA-G-A--------G-G-G-G---GG-T------AG--A--ATT----C-
C-A-C-GT--GT-A-G-CA-GT--G---A--A-A---TG-C-GT-AG--AT-A-TG--T-G--G-A-G-G-A-AC-A-CC---GA--T--G--GC-GAA-G--G-C--A-------G--C-C-C-C---CTG--G--GT-C-A--------------------A-G---A-T-T--GA--CG-C-T-C--A-TG--C-A-CG-A--AA-G-C---G-TG--GG-G-
-AG-C-A-AA--------CA--GG Bacteria(100);Proteobacteria(100);Betaproteobacteria(100);Burkholderiales(100);Oxalobacteraceae(100);Janthinobacterium(100);unclassified(100);unclassified(100);unclassified(100);

Otu1712 0 0 0 0 0 0 0 0 0 0 6 0 0 1 0 14 0 0 3 0 0 0 0 1 0 0 0 0
3 0 0 0 0 0 0 0 0 0 0 1 0 0 0 0 0 0 3 0 5 0 0 0 0 0 0 0 1
0 0 0 0 0 0 0 0 0 0 1 0 0 1 0 0 0 0 0 0 0 0 0 0 0 0 0 0 0
0 0 0 0 0 0 0 0 0 0 0 0 0 0 0 0 0 1 0 0 0 0 0 0 0 0 0
M02255_77_000000000-ADUT3_1_2109_26812_11010 T----AC---GT-AG-GTG--------GCA-A-G------C--G--T---T--GT-C-CGG-AA------TT-A--T-T--GG-GC-------GT--A-----AA-GC-GC-GC-------G-CA-G-G-C-G------------G--T-CT-C-T-T------
-----AA----G-T-C-T--------G-A-T--G--TG--A-AA-GC--C-C-CC-G-G-----CT-C-AA------C-C-G-G-G-G-A--G------G-GT-C-A---T--T-------G--GA-A-A---C-------T--G-G--GA--G-A-C----T-T-G-A-G-G--A---C-AG-----AA-G-A--------G-G-A-G---AG-T------GG--A--ATT----
C-C-A-A-GT--GT-A-G-CG-GT--G---A--A-A---TG-C-GT-AG--AT-A-TT--T-G--G-A-G-G-A-AC-A-CC---AG--T--G--GC-GAA-G--G-C--G-------G--C-T-C-T---CTG--G--TC-T-G--------------------T-T---A-C-T--GA--CG-C-T-G--A-GG--C-G-CG-A--AA-G-C---G-TG--GG-
G--AG-C-A-AA--------CA--GG Bacteria(100);Firmicutes(100);Bacilli(100);Bacillales(100);unclassified(100);unclassified(100);unclassified(100);unclassified(100);unclassified(100);

Otu1713 0 0 151 0 0 0 106 0 32 0 0 0 0 0 0 0 0 0 0 0 0 0 0 0 0 0 0 0
0 0 0 0 0 0 0 0 0 0 0 0 0 0 0 0 0 0 0 0 0 0 0 0 0 0 0 0 0
0 0 0 0 0 0 0 0 0 0 0 0 0 0 0 0 0 0 0 0 0 0 0 0 0 0 0 0 0
0 0 0 0 0 4 0 0 0 0 0 19 0 0 1 0 0 0 0 0 0 0 0 9 28 0 0
M02255_77_000000000-ADUT3_1_1101_5501_21493 T----AC---GT-AT-GGA--------GCA-A-G------C--G--T---T--AT-C-CGG-AT------TT-A--C-T--GG-GT-------GT--A-----AA-GG-GA-GC-------G-TA-G-G-C-G------------G--T-CC-T-G-C-----------AA-
---G-T-C-T--------G-A-T--G--TG--A-AA-GG--C-C-GG-G-G-----CT-C-AA------C-C-C-C-G-G-G--A-C----T-G--C-A---T--T-------G--GA-A-A---C-------T--G-T--AG--G-A-C----T-A-G-A-G-T--G---T-CG-----GA-G-G--------G-G-T-A---AG-T------GG--A--ATT----C-C-T-A-
GT--GT-A-G-CG-GT--G---A--A-A---TG-C-GT-AG--AT-A-TT--A-G--G-A-G-G-A-AC-A-CC---AG--T--G--GC-GAA-G--G-C--G-------G--C-T-T-A---CTG--G--AC-G-A--------------------C-T---A-C-T--GA--CG-C-T-G--A-GG--C-T-CG-A--AA-G-C---G-TG--GG-G--AG-
C-A-AA--------CA--GG Bacteria(100);Firmicutes(100);Clostridia(100);Clostridiales(100);Lachnospiraceae(100);unclassified(100);unclassified(100);unclassified(100);unclassified(100);

Otu1714 0 0 112 0 0 0 0 0 0 36 0 0 0 0 0 0 0 0 0 0 0 0 0 0 0 0 0 0
0 0 0 0 0 0 0 0 0 0 0 0 0 0 0 0 0 0 0 0 0 0 0 0 0 0 0 0 0
0 0 0 0 0 0 0 0 0 0 0 0 0 0 0 0 0 0 0 0 0 0 0 0 0 0 0 0 0
0 0 0 0 0 0 0 0 0 0 0 0 0 0 0 0 0 0 0 0 0 0 0 0 0 0 0
M02255_77_000000000-ADUT3_1_1101_27468_11603 T----AC---GG-AA-GGG--------GCT-A-G------C--G--T---T--GT-T-CGG-AT------TT-A--C-T--GG-GC-------GT--A-----AA-GC-GC-AT-------G-TA-G-G-C-G------------G--C-GG-A-T-C-----
------AA----G-T-G-G--------G-G-C--G--TG--A-AA-GG--C-C-TG-G-G-----CT-C-AA------C-C-T-G-G-G-A--A-C----T-G--C-G---C--T-------T--CA-G-A---C-------T--G-G--TT--C-G-C----T-C-G-A-G-T--T---C-GG-----GA-G-A--------G-G-G-T---GG-T------GG--A--ATT----
C-C-C-A-GT--GT-A-G-AG-GT--G---A--A-A---TT-C-GT-AG--AT-A-TT--G-G--G-A-G-G-A-AC-A-CC---GG--T--G--GC-GAA-G--G-C--G-------G--C-C-A-C---CTG--G--CC-C-G--------------------A-G---A-C-T--GA--CG-C-T-C--A-AG--T-G-CG-A--AA-G-C---G-TG--
GG-G--AG-C-A-AA--------CA--GG Bacteria(100);Proteobacteria(100);Alphaproteobacteria(100);Rhodospirillales(100);Rhodospirillaceae(100);unclassified(100);unclassified(100);unclassified(100);unclassified(100);

Otu1715 0 0 24 0 0 0 0 0 0 0 0 0 0 0 0 0 0 0 0 0 0 0 0 0 0 0 0 0
0 0 0 0 0 0 0 0 0 0 0 0 0 0 0 0 0 0 0 0 0 0 0 0 0 0 0 0
0 0 0 0 0 0 0 0 0 0 0 0 0 0 0 0 0 0 0 0 0 0 0 0 0 0 0 0
0 0 0 0 0 0 0 0 0 0 0 0 0 0 0 0 0 0 0 0 0 0 0 0 0 0 0

M02255_76_000000000-ADUNE_1_1111_15137_10294 .....AC----T-AC-GT---------GCC-A-G------C--A--G---C--CG-C-GGT-AA------TA-C--G-T--AG-GT-------GG--C-----AA-GC-GT-TA-------T-C------C-G------------G--A-AC-T-A-A----------
GG----G-T-C-T--------G-T-G--G--TG--A-AA-GA--T-C-GA-A-G-----CT-T-AA------C-T-T-C-G-G-T--A------A-G--C-C---A--T-------G--GA-A-A---C-------C--G-T--AG--A-G-C----T-A-G-A-G-T--G---T-GT-----GA-G-A--------G-G-A-T---CG-T------GG--A--ATT----C-C-A-
T-GT--GT-A-G-CG-GT--G---A--A-A---TG-C-GT-AG--AT-A-TA--T-G--G-A-G-G-A-AC-A-CC---AG--T--G--GC-GAA-G--G-C--G-------A--C-G-A-T---CTG--G--CG-C-A--------------------T-A---A-C-T--GA--CG-C-T-C--A-GT--C-C-CG-A--AA-G-C---G-TG--GG-G--AG-
C-A-AA--------TA--GG Bacteria(100);Firmicutes(100);Erysipelotrichi(100);Erysipelotrichales(100);Erysipelotrichaceae(100);unclassified(100);unclassified(100);unclassified(100);unclassified(100);

Otu1716 0 39 0 0 0 0 0 0 0 0 0 0 0 0 0 0 0 0 0 0 0 0 0 0 0 0 0 0
0 0 0 0 0 0 0 0 0 0 0 0 0 0 0 0 0 0 0 0 0 0 0 0 0 0 0 0
0 0 0 0 0 0 0 0 0 0 0 0 0 0 0 0 0 0 0 0 0 0 0 0 0 0 0 0
0 0 0 0 0 0 0 0 0 0 0 0 0 0 0 0 0 0 0 0 0 0 0 0 0 0 0

M02255_77_000000000-ADUT3_1_1104_5655_18482 T----AC---GT-AG-GGA--------GCG-A-G------C--G--T---T--AT-C-CGG-AT------TC-A--T-T--GG-GC-------GT--A-----AA-GA-GC-GC-------G-TA-G-G-C-G------------G--C-CT-C-T-C-----------AA-
---G-C-G-G--------G-A-T--C--TC--T-AA-TC--C-G-AG-G-G-----CT-C-AA------C-C-C-C-C-G-G--C------C-G--G-A---T--C-------C--CG-A-A---C-------T--G-G--GA--G-G-C----T-C-G-A-G-T--T---C-GG-----TA-G-A--------G-G-C-A---GG-C------GG--A--ATT----C-C-C-G-
GT--GT-A-G-CG-GT--G---G--A-A---TG-C-GC-AG--AT-A-TC--G-G--G-A-A-G-A-AC-A-CC---GA--T--G--GC-GAA-G--G-C--A-------G--C-C-T-G---CTG--G--GC-C-G--------------------C-A---A-C-T--GA--CG-C-T-G--A-GG--C-G-CG-A--AA-G-C---T-AG--GG-G--AG-
C-G-AA--------CA--GG Bacteria(100);Actinobacteria(100);Actinobacteria(100);Coriobacteridae(100);Coriobacteriales(100);Coriobacterineae(100);Coriobacteriaceae(100);Eggerthella(100);unclassified(100);

Otu1717 0 0 0 0 0 30 0 0 0 0 0 0 0 0 0 0 0 0 0 0 0 0 0 0 0 0 0 0
0 0 0 0 0 0 0 0 0 0 0 0 0 0 0 0 0 0 0 0 0 0 0 0 0 0 0 0
0 0 0 0 0 0 0 0 0 0 0 0 0 0 0 0 0 0 0 0 0 0 0 0 0 0 0 0
0 0 0 0 0 0 0 0 0 0 0 0 0 0 0 0 0 0 0 0 0 0 0 0 0 0 0

M02255_77_000000000-ADUT3_1_2116_18764_12619 G----AC---GT-AG-GAA--------GCA-A-G------C--G--T---T--GC-C-CGG-AA------TT-A--C-T--GG-GC-------GT--A-----AA-GA-GC-TC-------G-TA-G-G-C-G------------G--G-GG-C-G-T----
-------GC----G-T-C-C--------G-A-A--A--GG--A-AA-TC--T-C-AA-G-G-----CT-C-AA------C-C-T-T-G-G-G--G-C----T-T--T-T---T--C-------G--GA-T-A---C-------G--G-C--GC--T-T-C----T-T-G-A-G-G--G---T-GA-----GA-G-A--------G-G-A-A---AG-C------GG--A--ATT----
C-C-A-G-GT--GT-A-G-CG-GT--G---A--A-A---TG-C-GT-AG--AT-A-TC--T-G--G-A-G-G-A-AC-A-CC---AG--C--G--GC-GAA-G--G-C--G-------G--C-T-T-T---CTG--G--CT-C-A--------------------T-T---C-C-T--GA--CG-C-T-G--A-GG--A-G-CG-A--AA-G-C---G-TG--GG-
T--AG-C-G-AA--------CG--GG Bacteria(100);unclassified(100);unclassified(100);unclassified(100);unclassified(100);unclassified(100);unclassified(100);unclassified(100);unclassified(100);

Otu1718 0 0 0 0 0 0 0 0 0 0 0 0 0 0 0 0 0 0 0 0 0 0 0 0 0 0 0 0
0 0 0 0 0 0 0 0 0 3 0 0 0 0 0 0 0 0 0 0 0 0 0 0 0 0 0 0
0 1 0 0 0 0 0 1 0 0 0 0 0 0 0 0 0 0 0 0 0 0 0 0 0 0 0 1
0 0 1 0 0 0 0 0 0 1 0 0 0 0 0 0 0 0 0 0 0 0 0 0 0 0 9

M02255_77_000000000-ADUT3_1_1110_4777_8912 T----AC---GG-AG-GAT--------GCG-A-G------C--G--T---T--AT-C-CGG-AT------TT-A------------------TT------------G-G--G--------G-TA-G-G-C-G------------G--C-CT-G-T-T-----------AA----G-T-
C-A--------G-C-G--G--TG--A-AA-TC--T-A-GG-A-G-----CT-T-AA------C-T-C-C-T-A-A--A-T----T-G--C-C---A--T-------T--GA-T-A---C-------T--G-G--CG--G-G-C----T-T-G-A-G-T--G---T-AG-----AT-G-A--------G-G-T-A---GG-C------GG--A--ATG----C-G-T-G-GT--GT-
A-G-CG-GT--G---G--A-A---TG-C-AT-AG--AT-A-TC--A-C--G-C-A-G-A-AC-T-CC---GA--T--T--GC-GAA-G--G-C--A-------G--C-T-T-A---CTA--A--GG-T-A--------------------C-A---A-C-T--GA--CG-C-T-G--A-AG--C-A-CG-A--AA-G-C---G-TG--GG-T--AT-C-A-AA-----
---CA--GG Bacteria(100);Bacteroidetes(100);Bacteroidia(100);Bacteroidales(100);Porphyromonadaceae(100);Porphyromonas(100);unclassified(100);unclassified(100);unclassified(100);

Otu1719 0 2 0 0 0 0 0 0 0 0 0 0 0 0 0 0 0 0 0 0 0 0 0 0 0 0 0 0
0 0 0 0 0 0 0 0 0 0 0 0 0 0 0 0 0 0 0 0 0 0 0 0 0 0 2 0 0
0 0 0 0 0 0 7 0 0 0 0 2 0 0 0 0 0 0 30 16 6 0 0 0 0 0 0 0
0 0 0 17 0 0 0 0 0 0 0 0 0 0 0 0 0 3 0 1 3 0 0 0 0 0 0

M02255_77_000000000-ADUT3_1_2108_15526_12638 T----AA---GC-AC-CGG--------CTA-A-C------T--A--C---G--TG-C-CAG-CA------GC-C--G-C--GG-TA-------AT--A-----CG-TA-GG-GT-------G-CA-A-G-C-G------------T-TAATC-G-G-A----
-------AT----T-A-C-T--------G-A-T--G--TG--A-AA-TC--C-C-CG-G-G-----CT-T-AA------C-C-T-G-G-G-A--A-C----T-G--C-A---T--T-------T--GT-G-A---C-------T--G-G--CG--G-G-C----T-A-G-A-G-T--A---T-GG-----CA-G-A--------G-G-G-G---GG-T------AG--A--ATT----
C-C-A-C-GT--GT-A-G-CA-GT--G---A--A-A---TG-C-GT-AG--AG-A-TG--T-G--G-A-G-G-A-AT-A-CC---GA--T--G--GC-GAA-G--G-C--A-------G--C-C-C-C---CTG--G--GC-C-A--------------------A-T---A-C-T--GA--CG-C-T-C--A-TG--C-A-CG-A--AA-G-C---G-TG--GG-
G--AG-C-A-AA--------CA--GG Bacteria(100);Proteobacteria(100);Betaproteobacteria(100);unclassified(100);unclassified(100);unclassified(100);unclassified(100);unclassified(100);unclassified(100);

Otu1720 0 0 0 0 0 0 0 0 0 0 0 0 1 0 0 0 0 0 0 0 0 0 0 0 0 0 0 0
0 0 0 0 0 0 0 0 0 0 0 0 0 0 0 0 0 0 0 0 0 0 27 0 0 0 0 0
0 0 0 0 0 0 0 0 0 0 0 0 0 0 0 0 0 0 0 0 0 0 0 0 0 0 0 0
0 0 0 0 0 0 0 0 0 0 0 0 0 0 0 0 0 0 0 0 0 0 0 0 0 0 0
M02255_77_000000000-ADUT3_1_1109_24272_9536 T----AC---GT-AG-GCA--------GCG-A-G------C--G--T---T--GT-T-CGG-AG------TT-A--C-T--GG-GC-------GT--A-----AA-GA-GT-GC-------G-TA-G-G-C-G------------G--T-GT-T-C-C-----------AA----G-T-C-T--------G-T-T--G--TG--A-AA-TC--T-C-CC-G-G-----CT-T-AA------C-T-G-G-G-A-G--G-G----T-G--C-G---G--C-------G--GA-A-A---C-------T--G-G--AA--T-G-C----T-G-G-A-G-T--G---T-GG-----GA-G-A--------G-G-T-A---AG-C------GG--A--ATT----C-C-C-G-GT--GT-A-G-CG-GT--G---A--A-A---TG-C-GT-AG--AT-A-TC--G-G--G-A-G-G-A-AC-A-CC---TG--C--G--GT-GTA-G--A-C--G-------G--C-T-T-A---CTG--G--AC-C-A--------------------T-A---A-C-T--GA--CG-C-T-G--A-GG--C-A-CG-A--AA-G-C---G-TG--GG-T--AG-C-A-AA--------CA--GG Bacteria(100);unclassified(100);unclassified(100);unclassified(100);unclassified(100);unclassified(100);unclassified(100);unclassified(100);unclassified(100);

Otu1721 0 0 0 0 0 0 0 0 0 0 0 0 3 0 0 0 0 0 0 0 0 0 0 0 0 0 0 0
0 0 0 0 0 0 0 0 0 0 0 0 0 0 0 0 0 0 0 0 0 0 0 0 0 0 0 0
0 0 0 0 0 0 0 0 0 0 0 0 0 0 0 0 0 0 0 0 0 0 0 0 0 0 0 0
0 0 0 0 0 0 0 0 0 0 0 0 0 0 0 0 0 0 0 0 0 0 0 0 0 0 0
M02255_76_000000000-ADUNE_1_2115_23858_6147 G----AC---AG-AA-TAA--------GCA-C-C------G--G--C---T--AA---C-T-CT------GT-G--C-C--AG-CA-------GC--C-----GC-G--GT-AA-------T-AC-A-G-A-G------------G--G-TG-C-A-A-----------GC----G-T-T-G--------A-A-T--C--GG--A-AA-TC--C-C-CG-G-G-----CT-C-AA------C-C-T-G-G-G-A--A-C----G-G--C-A---T--C-------C--AA-A-A---C-------T--G-G--CA--A-G-C----T-A-G-A-G-T--A---T-GG-----TA-G-A--------G-G-G-T---AG-T------GG--A--ATT----T-C-C-T-GT--GT-A-G-CG-GT--G---A--A-A---TG-C-GT-AG--AT-A-TA--G-G--A-A-G-G-A-AC-A-CC---AG--T--G--GC-GAA-G--G-C--G-------A--C-T-A-C---CTG--G--AC-T-G--------------------A-T---A-C-T--GA--CA-C-T-G--A-GG--T-G-CG-A--AA-G-C---G-TG--GG-G--AG-C-A-AA--------CA--GG Bacteria(100);Proteobacteria(100);Gammaproteobacteria(100);unclassified(100);unclassified(100);unclassified(100);unclassified(100);unclassified(100);unclassified(100);

Otu1722 0 0 0 0 0 0 0 0 0 0 19 0 0 0 0 0 0 0 0 0 0 0 0 0 0 0 0 0
0 0 0 0 0 0 0 0 0 0 0 0 0 0 0 0 0 0 0 0 0 0 0 0 0 0 0 0
0 0 0 0 0 0 0 0 0 0 0 0 0 0 0 0 0 0 0 0 0 0 0 0 0 0 0 0
0 0 0 0 0 0 0 0 0 0 0 0 0 0 0 0 0 0 0 0 0 0 0 0 0 0 0
M02255_76_000000000-ADUNE_1_1116_26202_13343 .....AC----T-AC-GT---------GCC-A-G------C--A--G---C--CG-C-GGT-AA------TA-C--G-T--AG-GT-------GG--C-----AA-GC-G--TT-------G----T-C-C-G------------G--T-TC-T-T-T-----------AA----G-T-C-T--------G-A-T--G--TG--A-AA-GC--C-C-CC-G-G-----CT-C-AA------C-C-G-G-G-G-A--G------G-GT-C-A---T--T-------G--GA-A-A---C-------T--G-G--AG--A-A-C----T-T-G-A-G-T--G---C-AG-----AA-G-A--------G-G-A-G---AG-T------GG--A--ATT----C-C-A-C-GT--GT-A-G-CG-GT--G---A--A-A---TG-C-GT-AG--AG-A-TC--T-G--G-A-G-G-A-AT-A-CC---GA--T--G--GC-GAA-G--G-C--A-------G--C-C-T-T---CTG--G--CA-T-A--------------------A-T---A-C-T--GA--CA-C-T-G--A-GG--C-T-CG-A--AA-G-C---G-TG--GG-T--AG-C-A-AA--------CA--GG Bacteria(100);Firmicutes(100);Bacilli(100);unclassified(100);unclassified(100);unclassified(100);unclassified(100);unclassified(100);unclassified(100);

Otu1723 0 59 32 0 59 0 0 0 0 0 0 0 0 0 0 0 3 0 0 0 0 0 0 0 0 1 1 1
3 0 0 0 0 7 0 33 0 0 0 0 0 0 0 0 0 0 0 0 0 0 0 22 0 0 0 0 0
0 0 0 0 0 0 0 0 0 0 0 0 0 0 0 0 0 12 0 0 0 0 0 0 0 0 0 0 49
0 0 9 7 50 0 0 0 0 0 0 0 0 34 0 9 0 27 7 0 212 4 0 0 3 107 0
M02255_77_000000000-ADUT3_1_1101_15813_11092 T----AC---GT-AG-GGT--------ACA-A-G------C--G--T---T--GT-C-CGG-AA------TT-A--T-T--GG-GC-------GT--A-----AA-GA-GC-TC-------G-TA-G-G-T-G------------G--T-CT-G-T-C-----------AC----G-T-C-T--------G-C-T--G--TG--G-AA-AC--G-C-AA-G-G-----CT-C-AA------C-C-T-T-G-C-G--C-G----T-G--C-A---G--T-------G--GG-T-A---C-------G--G-G--CG--G-A-C----T-A-G-A-G-T--G---C-AG-----TA-G-G--------G-G-A-G---TC-T------GG--A--ATT----C-C-T-G-GT--GT-A-G-CG-GT--G---A--A-A---TG-C-GC-AG--AT-A-TC--A-G--G-A-G-G-A-AC-A-CC---GG--T--G--GC-GAA-G--G-C--G-------G--G-A-C-T---CTG--G--GC-T-G--------------------T-A---A-C-T--GA--CG-C-T-G--A-GG--A-G-CG-A--AA-G-C---A-TG--GG-G--AG-C-G-AA--------CA--GG Bacteria(100);Actinobacteria(100);Actinobacteria(100);Actinobacteridae(100);Actinomycetales(100);Micrococcineae(100);Brevibacteriaceae(100);Brevibacterium(100);unclassified(100);

Otu1724 0 27 0 0 0 0 0 0 0 0 0 0 0 0 0 0 0 0 0 0 0 0 0 0 0 0 0 0
0 0 0 0 0 0 0 0 0 0 0 0 0 0 0 0 0 0 0 0 0 0 0 0 0 0 0 0
0 0 0 0 0 0 0 0 0 0 0 0 0 0 0 0 0 0 0 0 0 0 0 0 0 0 0 0
0 0 0 0 0 0 0 0 0 0 0 0 0 0 0 0 0 0 0 0 0 0 0 0 0 0 0
M02255_76_000000000-ADUNE_1_1103_23857_13505 .....AC----T-AC-GT---------GCC-A-G------C--A--G---C--CG-C-GGT-AA------TA-C--G-T--AG-GG-------GG--C-----AA----GC-GT---------TG-T-C-C-G------------G--C-AA-T-A-T-----------AA----G-T-C-A--------G-A-T--G--TG--A-AA-GG--T-G-AG-G-G-----CT-C-AA------C-C-C-T-T-A-G--A-C----T-G--C-A---T--C-------T--GA-T-A---C-------T--G-T--AT--A-G-C----T-T-G-A-G-T--G---T-GA-----GA-G-A--------G-G-A-A---AG-C------GG--A--ATT----C-C-T-A-GT--GT-A-G-CG-GT--G---A--A-A---TG-C-GT-AG--AT-A-TT--A-G--G-A-G-G-A-AC-A-CC---AG--T--G--GC-GAA-G--G-C--G-------G--C-T-T-T---CTG--G--CT-C-A--------------------T-A---A-C-T--GA--CG-C-T-G--A-GG--C-G-CG-A--AA-G-C---G-TG--GG-G--AG-C-A-AA--------CA--GG Bacteria(100);Firmicutes(100);Clostridia(100);Clostridiales(100);unclassified(100);unclassified(100);unclassified(100);unclassified(100);unclassified(100);

Otu1725 0 26 0 0 0 0 0 0 8 0 0 0 0 0 0 0 0 0 0 0 0 0 0 0 0 0 0 0
0 0 0 0 0 0 0 0 0 0 0 0 0 0 0 0 0 0 0 0 0 0 0 0 0 0 0 0
0 0 0 0 0 0 0 0 0 0 0 0 0 0 0 0 0 0 0 0 0 0 0 0 0 0 0 0
0 0 0 0 0 0 0 0 0 0 0 0 0 0 0 0 0 0 0 0 0 0 0 1 0 0 0
M02255_76_000000000-ADUNE_1_1103_17569_23723 T----AC---GT-AG-GGG--------GCA-A-G------C--G--T---T--AT-C-CGG-AT------TT-A--C-T--GG-GT-------GT--A-----AA----G--G--------G--A--------------------G-----C-G-G-C----------
AG----G-T-C-A--------G-A-T--G--TG--A-AA-AC--C-C-GG-G-G-----CT-C-AA------C-C-C-C-G-G-G--A-C----T-G--C-A---T--T-------T--GA-A-A---C-------T--G-T--CG--G-A-C----T-A-G-A-G-T--G---C-CG-----GA-G-A--------G-G-T-A---AG-T------GG--A--ATT----C-C-T-
A-GT--GT-A-G-CG-GT--G---A--A-A---TG-C-GT-AG--AT-A-TT--A-G--G-A-G-G-A-AC-A-CC---AG--T--G--GC-GAA-G--G-C--G-------G--C-T-T-A---CTG--G--AC-G-G--------------------T-A---A-C-T--GA--CG-C-T-G--A-GG--C-T-CG-A--AA-G-C---G-TG--GG-G--
AG-C-A-AA--------CA--GG Bacteria(100);Firmicutes(100);Clostridia(100);Clostridiales(100);Lachnospiraceae(100);unclassified(100);unclassified(100);unclassified(100);unclassified(100);

Otu1726 0 0 0 0 0 0 0 0 0 0 14 0 0 0 0 0 0 0 0 0 0 0 0 0 0 0 0 0
0 0 0 0 0 0 0 0 0 0 0 0 0 0 0 0 0 0 0 0 0 0 0 0 0 0 0 0
1 0 0 0 0 0 0 0 0 0 0 0 0 0 0 0 0 0 0 0 0 0 0 0 0 0 0 0
0 0 0 0 0 0 0 0 0 0 0 0 0 0 0 0 0 0 0 0 0 0 0 0 0 0 0
M02255_77_000000000-ADUT3_1_1117_23639_14852 .....AC----T-AC-GT---------GCC-A-G------C---------A--G--C-CGC-GG------TA-A--T-A--CG-TA-------G---G-----TG-GC-AA-GC-------G-TT-G-T-C-C------------G----GA-T-T-T-----------
A----T-T-C-G--------G-A-T--G--TG--A-AA-GC--C-C-CC-G-G-----CT-C-AA------C-C-G-G-G-G-A--G------G-GT-C-A---T--T-------G--GA-A-A---C-------T--G-G--AG--A-A-C----T-T-G-A-G-T--G---C-AG-----AA-G-A--------G-G-A-G---AG-T------GG--A--ATT----C-C-A-
C-GT--GT-A-G-CG-GT--G---A--A-A---TG-C-GT-AG--AT-A-TG--T-G--G-A-G-G-A-AC-A-CC---AG--T--G--GC-GAA-G--G-C--G-------A--C-T-C-T---CTG--G--TC-T-G--------------------T-A---A-C-T--GA--CG-C-T-G--A-GG--C-T-CG-A--AA-G-C---G-TG--GG-G--AG-
C-A-AA--------CA--GG Bacteria(100);Firmicutes(100);Bacilli(100);unclassified(100);unclassified(100);unclassified(100);unclassified(100);unclassified(100);unclassified(100);

Otu1727 0 0 0 0 0 0 0 0 70 0 0 0 0 0 0 0 0 0 0 0 0 0 0 0 0 0 0 0
0 0 0 0 0 0 0 0 0 0 0 0 0 0 0 0 0 0 0 0 0 0 0 0 0 0 0 0
0 0 0 0 0 0 0 0 0 0 0 0 0 0 0 0 0 0 0 0 0 0 0 0 0 0 0 0
0 0 0 0 0 0 0 0 0 0 0 0 0 0 0 0 0 0 0 0 0 0 0 0 0 0 0
M02255_77_000000000-ADUT3_1_1118_24031_15597 T----AC---GT-AG-GTG--------GCA-A-G------C--G--T---T--GT-C-CGG-AT------TT-A--C-T--GG-GT-------GT--A-----AA-GG-GC-GT-------G-CA-G-C-C-G------------G--G-TG-C-G-C-----
------AA----G-T-C-A--------G-A-T--G--TG--A-AA-TC--T-C-AG-G-G-----CT-C-AA------C-C-C-T-G-A-A--A-C----T-G--C-A---T--T-------T--GA-A-A---C-------T--G-T--GC--A-T-C----T-T-G-A-G-T--G---C-CG-----GA-G-A--------G-G-T-A---AT-C------GG--A--ATT----C-
C-T-T-GT--GT-A-G-CG-GT--G---A--A-A---TG-C-GT-AG--AT-A-TA--A-G--G-A-A-G-A-AC-A-CC---AG--T--G--GC-GAA-G--G-C--G-------G--A-T-T-A---CTG--G--AC-G-G--------------------T-A---A-C-T--GA--CG-G-T-G--A-GG--C-G-CG-A--AA-G-C---G-TG--GG-
G--AG-C-A-AA--------CA--GG Bacteria(100);Firmicutes(100);Clostridia(100);Clostridiales(100);Ruminococcaceae(100);unclassified(100);unclassified(100);unclassified(100);unclassified(100);

Otu1728 0 0 0 0 0 0 0 0 44 0 0 0 0 0 0 0 0 0 0 0 0 0 0 0 0 0 0 0
0 0 0 0 0 0 0 0 0 0 0 0 0 0 0 0 0 0 0 0 0 0 0 0 0 0 0 0
0 0 0 0 0 0 0 0 0 0 0 0 0 0 0 0 0 0 0 0 0 0 0 0 0 0 0 0
0 0 0 0 0 0 0 0 0 0 0 0 0 0 0 0 0 0 0 0 0 0 0 0 0 0 0
M02255_77_000000000-ADUT3_1_1119_4405_13948 T----AC---GT-AG-GGA--------GCA-A-G------C--G--T---T--GT-C-CGG-AA------TT-A--C-T--GG-GT-------GT--A-----AA-GG-GT-GC-------G-TA-G-G-C-G------------G--G-TA-T-G-C-----------
AA----G-T-C-A--------T-A-T--G--TG--A-AA-TA--C-C-GG-G-G-----CT-C-AA------C-T-C-C-G-G-G--G-C----T-G--C-A---T--A-------A--GA-A-A---C-------T--G-T--AT--A-T-C----T-T-G-A-G-T--A---C-AG-----GA-G-A--------G-G-T-A---AG-C------GG--A--ATT----C-C-T-
A-GT--GT-A-G-CG-GT--G---A--A-A---TG-C-GT-AG--AT-A-TT--A-G--G-A-G-G-A-AC-A-CC---AG--T--G--GC-GAA-G--G-C--G-------G--C-T-T-A---CTG--G--AC-T-G--------------------A-A---A-C-T--GA--CG-C-T-G--A-GG--C-A-CG-A--AA-G-C---G-TG--GG-G--
AG-C-A-AA--------CA--GG Bacteria(100);Firmicutes(100);Clostridia(100);Clostridiales(100);unclassified(100);unclassified(100);unclassified(100);unclassified(100);unclassified(100);

Otu1729 0 0 0 13 0 0 0 0 0 8 0 0 0 0 0 0 0 0 0 1 0 0 0 0 0 0 0 0
0 0 0 0 0 0 0 0 0 0 0 0 0 0 0 0 0 0 0 0 0 11 0 4 0 0 0 0
0 0 0 0 0 0 0 0 0 0 0 0 0 0 0 0 0 0 0 0 0 0 0 0 0 0 0 0
0 0 0 0 0 0 0 0 0 0 0 0 0 2 0 0 0 0 0 0 0 0 0 0 0 0 0
M02255_76_000000000-ADUNE_1_1115_23507_22399 C----CC---AG-AA-GAA--------GCA-C-C------G--G--C---T--AACT-CTG-TG------CC-A--G-C--AG-CC-------GC--G-----GT-AA-TA-CA-------G-AG-G-G-T-GC-----------A--A-GC-G-T-T---
--------AA----G-T-C-G--------G-A-A--G--TG--A-AA-TC--C-C-AG-G-G-----CT-C-AA------C-C-T-T-G-G-A--A-T----G-G--C-A---C--C-------C--GA-T-A---C-------T--G-G--CA--G-G-C----T-A-G-A-G-T--A---T-GG-----TA-G-A--------G-G-G-G---TG-T------GG--A--ATT----
T-C-C-T-GT--GT-A-G-CG-GT--G---A--A-A---TG-C-GT-AG--AT-A-TA--G-G--A-A-G-G-A-AC-A-TC---AG--T--G--GC-GAA-G--G-C--G-------A--C-A-C-C---CTG--G--AC-T-A--------------------A-T---A-C-T--GA--CA-C-T-G--A-GG--T-G-CG-A--AA-G-C---G-TG--GG-
G--AG-C-A-AA--------CA--GG Bacteria(100);Proteobacteria(100);Gammaproteobacteria(100);Oceanospirillales(100);Oceanospirillaceae(100);Marinomonas(100);unclassified(100);unclassified(100);unclassified(100);

Otu1730 0 0 0 0 0 0 0 0 20 0 0 0 0 0 0 0 0 0 0 0 0 0 0 0 0 0 0 0
0 0 0 0 0 0 0 0 0 0 0 0 0 0 0 0 0 0 0 0 0 0 0 0 0 0 0 0 0
0 0 0 0 0 0 0 0 0 0 0 0 0 0 24 0 0 0 0 0 0 0 0 0 0 0 0 0 0
0 0 0 0 0 0 0 0 0 0 0 0 0 0 0 0 0 0 0 0 0 0 0 0 0 0 0
M02255_77_000000000-ADUT3_1_2113_27176_14055 T----AC---GT-AT-GGA--------GCA-A-G------C--G--T---T--AT-C-CGG-AT------TT-A--C-T--GG-GT-------GT--A-----AA-GG-GA-GT-------G-TA-G-G-C-G------------G--C-AT-G-G-C-----
------AA----G-T-C-T--------G-A-T--G--TG--A-AA-AT--C-C-GG-G-G-----CT-T-AA------C-C-C-C-G-G-A--C-C----T-G--C-A---T--T-------G--GA-A-A---C-------T--G-T--CA--G-G-C----T-G-G-A-G-T--G---T-CG-----GA-G-A--------G-G-T-A---AG-T------GG--A--ATT----C-
C-T-A-GT--GT-A-G-CG-GT--G---A--A-A---TG-C-GT-AG--AT-A-TT--A-G--G-A-G-G-A-AC-A-CC---AG--T--G--GC-GAA-G--G-C--G-------G--C-T-T-A---CTG--G--AC-G-A--------------------C-A---A-C-T--GA--CG-C-T-G--A-GG--C-T-CG-A--AA-G-C---G-TG--GG-
G--AG-C-A-AA--------CA--GG Bacteria(100);Firmicutes(100);Clostridia(100);Clostridiales(100);Lachnospiraceae(100);unclassified(100);unclassified(100);unclassified(100);unclassified(100);

Otu1731 0 175 12 0 0 0 0 0 0 0 0 0 0 0 0 0 0 0 0 0 0 0 0 0 0 0 0 0
0 0 0 0 0 0 0 0 0 0 0 13 0 0 0 0 0 0 0 0 0 0 0 0 0 0 0 0 0
0 0 0 0 0 0 0 0 0 0 0 0 0 0 0 0 0 5 0 0 0 0 0 0 0 0 0 0 0
0 0 0 0 0 0 0 0 0 0 0 0 0 0 0 0 0 0 0 0 0 0 0 0 10 0 0
M02255_76_000000000-ADUNE_1_1106_23211_10947 T----CG---GC-TA-ACT--------CCG-T-GC-----C--A--G---C--AG-C-CGC-GG------TA-A--T-A--CG-GA-------GG--A-----TG-CG-AG-CG-------T-TA-T-C-C-G------------GA-T-TT-A-T-T-----
------AA----G-T-T-A--------G-T-G--G--TT--A-AA-TA--T-T-TG-A-G-----CT-A-AA------C-T-C-A-A-T-T--G-T------G--C-C---A--T-------T--AA-T-A---C-------T--G-G--TA--A-A-C----T-G-G-A-G-T--A---C-AG-----AC-G-A--------G-G-T-A---GG-C------GG--A--ATA----A-
G-T-T-AA--GT-A-G-CG-GT--G---A--A-A---TG-C-AT-AG--AT-A-TA--A-C--T-T-A-G-A-AC-T-CC---GA--T--A--GC-GAA-G--G-C--A-------G--C-T-T-A---CCA--G--AC-T-G--------------------T-A---A-C-T--GA--CG-C-T-G--A-TG--C-A-CG-A--GA-G-C---G-TG--GG-T--
AG-C-G-AA--------CA--GG Bacteria(100);Bacteroidetes(100);Bacteroidia(100);Bacteroidales(100);Porphyromonadaceae(100);Odoribacter(100);unclassified(100);unclassified(100);unclassified(100);

Otu1732 0 0 0 0 0 0 0 0 1 0 0 0 0 0 0 0 0 0 0 0 0 0 0 0 0 0 0 0
0 0 0 0 0 0 0 0 0 0 1 0 0 0 0 0 0 0 0 0 0 0 0 0 0 0 0 0 0
0 0 0 0 0 0 0 0 0 0 0 0 0 0 0 0 0 1 0 2 0 0 0 0 0 0 0 0 0
0 0 0 0 0 0 0 0 0 0 0 0 0 1 0 0 0 0 0 0 0 0 0 0 0 0 12
M02255_77_000000000-ADUT3_1_1113_2783_16749 T----AC---GG-AA-GGT--------CCA-G-G------C--G--T---T--AT-C-CGG-AT------TT-A-------------------TT------------G-G--G--------G-TA-G-G-C-T------------G--G-AG-A-T-T-----------AA----G-T-
G-T--------G-T-T--G--TG--A-AA-TG--T-A-GA-C-G-----CT-C-AA------C-G-T-C-T-G-A--A-T----T-G--C-A---G--C-------G--CA-T-A---C-------T--G-G--TT--T-C-C----T-T-G-A-G-T--A---C-GC-----AC-A-A--------C-G-T-T---GG-C------GG--A--ATT----C-G-T-C-GT--GT-A-
G-CG-GT--G---A--A-A---TG-C-TT-AG--AT-A-TG--A-C--G-A-A-G-A-AC-T-CC---GA--T--T--GC-GAA-G--G-C--A-------G--C-T-G-A---CGG--G--AG-C-G--------------------C-A---A-C-T--GA--CG-C-T-T--A-AG--C-T-CG-A--AG-G-T---G-CG--GG-T--AT-C-A-AA-------
-CA--GG Bacteria(100);Bacteroidetes(100);Bacteroidia(100);Bacteroidales(100);Prevotellaceae(100);Prevotella(100);unclassified(100);unclassified(100);unclassified(100);

Otu1733 0 0 0 0 0 0 0 0 0 0 0 0 0 0 0 0 0 0 0 0 0 0 9 0 17 0 0 0
0 0 0 0 0 0 0 0 0 0 0 0 0 0 0 0 0 0 0 0 0 0 0 0 0 0 0 0 0
0 0 0 0 0 0 0 0 0 0 0 0 0 0 0 0 0 0 0 0 0 0 0 0 0 0 0 0 0
0 0 0 0 0 0 0 0 0 0 0 0 0 0 0 0 0 0 0 1 0 0 0 0 0 0 0
M02255_77_000000000-ADUT3_1_1115_11748_5697 C----TA---AC-TC-CGT--------GCC-A-G------C--A--G---C--CG-C-GGT-AA------TA-C--G-G--AG-GG-------GG--C-----GA-GCGTT-AA-------T-CG-G-A-A-T------------T--A-CT-G-T-T-----------AA-
---G-C-A-A--------G-A-T--G--TG--A-AA-GC--C-C-GG-G-G-----CT-C-AA------C-C-T-C-G-G-A--A-C----A-G--C-A---T--T-------T--TG-A-A---C-------T--G-G--CA--G-A-C----T-A-G-A-G-T--C---T-TG-----TA-G-A--------G-G-G-G---GG-T------AG--A--ATT----T-C-A-G-
GT--GT-A-G-CG-GT--G---A--A-A---TG-C-GT-AG--AG-A-TC--T-G--A-A-G-G-A-AT-A-CC---GG--T--G--GC-GAA-G--G-C--G-------G--C-C-C-C---CTG--G--AC-A-A--------------------A-G---A-C-T--GA--CG-C-T-C--A-GA--T-G-CG-A--AA-G-C---G-TG--GG-G--AG-
C-A-AA--------CA--GG Bacteria(100);Proteobacteria(100);Gammaproteobacteria(100);Vibrionales(100);Vibrionaceae(100);Photobacterium(100);unclassified(100);unclassified(100);unclassified(100);

Otu1734 0 0 0 6 0 0 0 0 0 4 0 0 0 0 0 0 0 0 0 0 0 0 0 0 0 0 0 0
0 0 0 0 0 0 0 0 0 0 0 0 0 0 0 4 0 0 1 0 0 0 0 0 0 0 0 0 0
0 0 0 0 0 0 0 0 1 0 0 0 0 0 0 0 0 0 0 0 0 0 0 0 0 0 0 13 0
10 0 0 0 5 0 0 0 0 0 3 0 0 0 0 0 0 0 0 0 0 0 0 0 0 0 0
M02255_76_000000000-ADUNE_1_1107_27728_11018 T----AC---GG-GG-GGG--------GCA-A-G------T--G--T---T--CT-T-CGG-AA------TG-A--C-T--AG-GC-------GT--A-----AA-GG-GC-AC-------T-GA-A-T-C-G------------G-------G---T--------
----T----G---C------------A-A--G--TT--C-AA-----G-T-CG-C-C-----AA-A-AA------C-C-G-G-C-G-G--A-A----T-G--C-T---C--T-------C--GA-A-A---C-------C--A-A--TT--C-A-C----T-T-G-A-G-T--G---A-GA-----CA-G-A--------G-G-A-G---AG-T------TT--C--ATT----T-C-G-T-
GT--GT-A-G-GG-GT--T---A--A-A---TC-C-GT-AG--AT-C-TA--C-G--A-A-G-G-A-AG-G-CC---AA--A--A--GC-GAA-G--G-C--A-------G--C-T-C-T---TTG--G--GT-C-C--------------------C-T---A-C-T--GA--CG-C-T-G--A-GG--T-G-CG-A--AA-G-C---A-TG--GG-G--AG-C-
A-AA--------CT--GG Bacteria(100);unclassified(100);unclassified(100);unclassified(100);unclassified(100);unclassified(100);unclassified(100);unclassified(100);unclassified(100);

Otu1735 0 0 0 0 0 0 0 0 1 0 0 0 0 0 0 0 0 0 0 0 0 0 0 0 0 0 0 0
0 0 0 0 0 0 0 0 0 0 0 0 0 37 0 0 0 0 0 0 0 0 0 0 0 0 0 0 0
0 0 0 0 0 0 0 0 0 0 0 0 0 0 0 0 0 0 0 0 0 0 0 0 0 0 0 0 0
0 0 0 0 0 0 0 0 0 0 0 0 0 0 0 0 0 0 0 0 0 0 0 0 0 0 0
M02255_77_000000000-ADUT3_1_1110_8140_19981 T----AC---GT-AG-GGC--------GCA-A-G------C--G--T---T--GT-C-CGG-AA------TT-A--T-T--GG-GC-------GT--A-----AA-GA-GC-TC-------G-TA-G-G-C-G------------G--C-CT-G-T-C-----------GC-
---G-T-C-C--------G-C-T--G--TG--A-AA-TC--C-C-GG-G-G-----CT-T-AA------C-C-C-C-G-G-G--C-C----T-G--C-A---G--T-------G--GA-T-A---C-------G--G-G--CA--G-G-C----T-G-G-A-G-G--C---A-GG-----CA-G-G--------G-G-A-G---AA-C------GG--A--ATT----C-C-C-G-
GT--GT-A-G-CG-GT--G---A--A-A---TG-C-GC-AG--AT-A-TC--G-G--G-A-G-G-A-AC-A-CC---GG--T--G--GC-GAA-G--G-C--G-------G--T-T-C-T---CTG--G--GC-C-T--------------------G-T---A-C-T--GA--CG-C-T-G--A-GG--A-G-CG-A--AA-G-C---G-TG--GG-G--AG-
C-A-AA--------CA--GG Bacteria(100);Actinobacteria(100);Actinobacteria(100);Actinobacteridae(100);Actinomycetales(100);Frankineae(100);unclassified(100);unclassified(100);unclassified(100);

Otu1736 0 0 0 0 0 0 0 0 1 1 0 0 0 0 0 0 0 0 1 0 0 0 0 0 0 0 0 0
0 0 0 0 0 0 0 0 0 0 0 0 0 0 0 0 0 0 0 0 0 0 0 0 0 0 0 0 0
0 0 0 0 0 0 0 0 0 0 0 0 0 0 0 0 0 0 0 0 0 0 0 0 0 0 4 0 0
0 0 0 0 0 0 0 0 0 0 0 0 0 0 0 0 0 0 0 1 0 0 0 0 0 1 1
M02255_77_000000000-ADUT3_1_1109_9665_3913 C----TA---AC-TC-CGT--------GCC-A-G------C-----A---G--CC-G-CGG-TA------AT-A--C-G--GA-GG-------GTG-C-----AA-GCGTT-AA-------T-CG-G-A-A-T------------T--A-CT-G-T-C-----------AA-
---G-T-C-G--------G-A-T--G--TG--A-AA-TC--C-C-CG-G-G-----CT-C-AA------C-C-T-G-G-G-A--A-C----T-G--C-A---T--T-------C--GA-A-A---C-------T--G-G--CA--G-G-C----T-A-G-A-G-T--C---T-TG-----TA-G-A--------G-G-G-G---GG-T------AG--A--ATT----C-C-A-G-
GT--GT-A-G-CG-GT--G---A--A-A---TG-C-GT-AG--AG-A-TC--T-G--G-A-G-G-A-AT-A-CC---GG--T--G--GC-GAA-G--G-C--G-------G--C-C-C-C---CTG--G--AC-A-A--------------------A-G---A-C-T--GA--CG-C-T-C--A-GG--T-G-CG-A--AA-G-C---G-TG--GG-G--AG-
C-A-AA--------CA--GG Bacteria(100);Proteobacteria(100);Gammaproteobacteria(100);Enterobacteriales(100);Enterobacteriaceae(100);Enteric_Bacteria_cluster(100);unclassified(100);unclassified(100);unclassified(100);

Otu1737 0 0 1 0 0 0 0 0 0 0 0 0 0 0 0 0 0 0 0 0 0 0 0 0 0 0 0 0
0 0 0 0 0 0 0 0 0 0 0 0 0 0 0 0 0 0 0 0 0 0 0 0 0 0 0 0 0
0 0 0 0 0 0 0 0 0 0 0 0 0 0 0 0 0 0 0 0 0 0 0 0 0 0 0 0 0
0 0 0 0 0 0 0 0 0 0 0 0 0 0 0 0 0 0 0 0 49 0 0 0 0 0 0
M02255_76_000000000-ADUNE_1_1115_9908_20781 GT---TC---GT-GCCAGC--------AGC-C-G------C--G--G---T--AA-G-ACG-AA------CC-G--T-G--CG-AA-------CG--T-----TA-TT-CG-GA-------A-TC-A-C-T-G------------G--G-CG-G-C-C-----------
AC----G-T-C-G--------AGA-G--C--TG--A-AA-TC--C-C-CC-G-G-----CT-C-AA------C-C-G-G-G-G-A--A-G----G-G--G-C---C--T-------C--GA-T-A---C-------G--G-G--TC--G-C-C----T-C-G-A-G-T--G---G-CG-----GA-G-G--------G-G-G-A---GC-C------GG--A--ACT----C-
A-C-G-GT--GG-A-G-CG-GT--G---A--A-A---TG-C-GT-TG--AG-A-TC--G-T--G-A-G-G-A-AC-G-CC---GA--C--G--GC-GAA-A--G-C--A-------G--G-C-T-C---CTG--G--AC-G-C--------------------C-G---A-C-T--GA--CG-T-T-G--A-GG--C-G-CG-A--AA-G-C---C-AG--GG-
G--AG-C-G-AA--------CG--GG Bacteria(100);Planctomycetes(100);Planctomycetacia(100);Planctomycetales(100);Planctomycetaceae(100);Singulisphaera(100);unclassified(100);unclassified(100);unclassified(100);

Otu1738 0 0 0 0 0 0 0 0 0 5 0 0 0 0 0 0 0 0 0 0 0 0 0 0 0 0 0 0
0 0 0 0 0 0 0 0 0 0 0 0 0 0 0 0 0 0 0 0 0 0 0 0 0 0 0 0 0
0 0 0 0 0 0 0 0 0 0 0 0 0 0 0 0 0 0 0 0 0 0 0 0 0 0 0 0 0
0 0 0 0 0 0 0 0 0 0 0 0 0 0 0 0 0 0 0 0 0 0 0 0 0 0 0
M02255_77_000000000-ADUT3_1_2118_11433_15471 G----AC---GG-GG-GGG--------GCA-A-G------T--G--T---T--CT-T-CGG-AA------TG-A--C-T--AG-GC-------GT--A-----AA-GG-GC-AC-------G-GA-G-G-C-G------------G--T-GA-A-T-C---
--------GG----G-T-T-G--------A-A-A--G--GG--A-AA-GT--C-G-CC---A-----AA-A-AC------T-G-G-T-G-G-A--A-T----G-C--T-C---T--C-------G---A-A-A---C-------C--A-A--TT--C-A-C----T-T-G-A-G-T--G---A-GA-----CA-G-A--------G-G-A-G---AG-T------GG--A--ATT----
T-C-G-T-GT--GT-A-G-GG-GT--G---A--A-A---TC-C-GA-AG--AT-C-TA--C-G--A-A-G-G-A-AC-G-CC---AA--A--A--GC-GAA-G--G-C--A-------G--C-T-C-T---CTG--G--GT-C-C--------------------C-T---A-C-C--GA--CG-C-T-G--G-AG--T-G-CG-A--AA-G-C---A-TG--GG-
G--AG-C-G-AA--------CG--GG Bacteria(100);unclassified(100);unclassified(100);unclassified(100);unclassified(100);unclassified(100);unclassified(100);unclassified(100);unclassified(100);

Otu1739 0 0 0 0 0 0 19 0 0 0 0 2 0 0 0 0 0 0 0 0 0 0 0 0 0 0 0 0
0 0 0 0 0 0 0 28 0 0 0 0 0 0 0 0 0 0 0 0 0 0 0 0 0 0 0 0 0
0 0 0 0 0 0 0 0 0 0 0 0 0 0 0 0 0 0 0 0 0 0 0 0 0 0 0 0 0
0 0 0 0 0 0 0 0 0 0 0 0 0 0 0 0 0 0 0 7 0 0 0 0 0 0 0
M02255_77_000000000-ADUT3_1_2117_26030_11995 T----AC---GT-AG-GGT--------GCG-A-G------C--G--T---T--GT-C-CGG-AA------TT-A--C-T--GG-GC-------GT--A-----AA-GA-GC-TC-------G-TA-G-G-C-G------------G--T-TT-G-T-C------
-----GC----G-T-C-G--------T-C-T--G--TG--A-AA-AC--T-C-GG-A-G-----CT-T-AA------C-T-C-C-G-G-G--C-G----T-G--C-A---G--G-------C--GA-T-A---C-------G--G-G--CA--G-A-C----T-T-G-A-G-T--A---C-TA-----CA-G-G--------G-G-A-G---AC-T------GG--A--ATT----C-
C-T-G-GT--GT-A-G-CG-GT--G---A--A-A---TG-C-GC-AG--AT-A-TC--A-G--G-A-G-G-A-AC-A-CC---GG--T--G--GC-GAA-G--G-C--G-------G--G-T-C-T---CTG--G--GT-A-G--------------------T-A---A-C-T--GA--CG-C-T-G--A-GG--A-G-CG-A--AA-G-C---A-TG--GG-
T--AG-C-G-AA--------CA--GG Bacteria(100);Actinobacteria(100);Actinobacteria(100);Actinobacteridae(100);Actinomycetales(100);Corynebacterineae(100);unclassified(100);unclassified(100);unclassified(100);

Otu1740 0 126 58 0 0 0 80 0 66 0 0 0 0 0 0 0 0 0 0 0 0 0 0 9 0 0 0 0
0 357 0 0 0 0 0 0 0 0 0 0 0 0 0 0 0 0 0 0 0 0 0 0 4 0 0 0
0 0 0 0 0 0 0 0 0 0 0 1 0 0 0 0 13 0 0 0 0 0 0 0 0 0 0 0
0 0 0 0 0 18 8 0 0 0 0 90 0 0 0 0 0 0 0 0 0 0 0 0 26 0 0

M02255_77_000000000-ADUT3_1_1101_3174_11382 T----AC---GT-AG-GGG--------GCA-A-G------C--G--T---T--AT-C-CGG-AT------TT-A--C-T--GG-GT-------GT--A-----AA-GG-GA-GC-------G-TA-G-A-C-G------------G--C-TG-T-G-C-----------
AA----G-T-C-T--------G-A-A--G--TG--A-AA-GG--C-A-TG-G-G-----CT-C-AA------C-C-T-G-T-G-G--A-C----T-G--C-T---T--T-------G--GA-A-A---C-------T--G-T--GC--A-G-C----T-A-G-A-G-T--G---T-CG-----GA-G-A--------G-G-T-A---AG-T------GG--A--ATT----C-C-T-
A-GT--GT-A-G-CG-GT--G---A--A-A---TG-C-GT-AG--AT-A-TT--A-G--G-A-G-G-A-AC-A-CC---AG--T--G--GC-GAA-G--G-C--G-------G--C-T-T-A---CTG--G--AC-G-A--------------------T-G---A-C-T--GA--CG-T-T-G--A-GG--C-T-CG-A--AA-G-C---G-TG--GG-G--
AG-C-A-AA--------CA--GG Bacteria(100);Firmicutes(100);Clostridia(100);Clostridiales(100);Lachnospiraceae(100);Dorea(100);unclassified(100);unclassified(100);unclassified(100);

Otu1741 0 0 101 0 0 0 0 0 0 0 0 0 0 0 0 0 0 0 0 0 0 0 0 0 0 0 0 0
0 0 0 0 0 0 0 0 0 0 0 0 0 0 0 0 0 0 0 0 0 0 0 0 0 0 0 0
0 0 0 7 0 0 0 0 0 0 0 0 0 0 0 0 0 0 0 0 0 0 0 0 0 0 0 1
0 0 0 0 0 0 0 0 0 0 0 0 0 0 0 0 0 0 0 0 0 0 0 0 0 0 0

M02255_77_000000000-ADUT3_1_1101_9253_13466 T----AC---GT-AG-GGA--------GCG-A-G------C--G--T---T--AT-C-CGG-AT------TT-A--C-T--GG-GT-------GT--A-----AA-GG-GC-GT-------G-TA-G-G-C-G------------G--G-GA-A-G-C-----------
AA----G-T-C-A--------G-A-T--G--TG--A-AA-AC--C-A-GT-G-G-----CT-C-AA------C-C-A-C-T-G-G--C-C----T-G--C-A---T--T-------T--GA-A-A---C-------T--G-T--TT--T-T-C----T-T-G-A-G-T--G---A-TG-----GA-G-A--------G-G-C-A---GG-C------GG--A--ATT----C-C-G-
T-GT--GT-A-G-CG-GT--G---A--A-A---TG-C-GT-AG--AT-A-TA--C-G--G-A-G-G-A-AC-A-CC---AG--T--G--GC-GAA-G--G-C--G-------G--C-C-T-G---CTG--G--AC-A-T--------------------T-A---A-C-T--GA--CG-C-T-G--A-GG--C-G-CG-A--AA-G-C---G-TG--GG-G--
AG-C-A-AA--------CA--GG Bacteria(100);Firmicutes(100);Clostridia(100);Clostridiales(100);Ruminococcaceae(100);unclassified(100);unclassified(100);unclassified(100);unclassified(100);

Otu1742 0 137 0 0 0 0 0 0 0 0 0 0 0 0 0 0 0 0 0 0 0 0 0 0 0 0 0 0
0 0 0 0 0 0 0 0 0 0 0 0 0 0 0 0 0 0 0 0 0 0 0 0 0 0 0 0
0 0 0 0 0 0 0 0 0 0 0 0 0 0 0 0 0 0 0 0 0 0 0 0 0 0 0 0
0 0 0 0 0 0 0 0 0 0 0 0 0 0 0 0 0 0 0 0 0 0 0 0 0 0 0

M02255_77_000000000-ADUT3_1_1101_6958_15729 T----AC---GT-AG-GTG--------GCA-A-G------C--G--T---T--GT-C-CGG-AT------TT-A--C-T--GG-GT-------GT--A-----AA-GG-GC-GT-------G-TA-G-G-C-G------------G--G-AA-G-A-C-----------
AG----G-T-C-A--------G-A-T--G--TG--A-AA-TG--T-C-GG-G-G-----CT-C-AA------C-C-C-C-G-G-A--A-C----T-G--C-A---T--T-------T--GA-A-A---C-------C--G-T--TT--T-T-C----T-T-G-A-G-T--A---T-CG-----GA-G-A--------G-G-C-A---GG-C------GG--A--ATT----C-C-T-
A-GT--GT-A-G-CG-GT--G---A--A-A---TG-C-GT-AG--AT-A-TT--A-G--G-A-G-G-A-AC-A-CC---AG--T--G--GC-GAA-G--G-C--G-------G--C-C-T-G---CTG--G--AC-G-A--------------------C-A---A-C-T--GA--CG-C-T-G--A-GG--C-G-CG-A--AA-G-C---G-TG--GG-G--
AG-C-A-AA--------CA--GG Bacteria(100);Firmicutes(100);Clostridia(100);Clostridiales(100);unclassified(100);unclassified(100);unclassified(100);unclassified(100);unclassified(100);

Otu1743 0 432 140 0 0 0 404 21 1213 0 0 0 0 0 0 0 0 0 0 0 0 0 0 0 0 0 0 0
0 242 0 24 0 0 0 0 0 0 0 132 0 0 0 0 0 0 0 0 0 0 0 0 0 0 0 0
0 0 0 0 0 0 0 0 0 0 0 0 0 0 0 10 0 0 0 0 0 0 2 0 0 0 0 0
0 0 0 0 0 0 0 0 0 0 0 82 0 0 4 0 0 0 0 0 0 0 26 11 248 0 0

M02255_77_000000000-ADUT3_1_1101_14241_16007 T----AC---GT-AG-GTG--------GCA-A-G------C--G--T---T--AT-C-CGG-AA------TT-A--C-T--GG-GT-------GT--A-----AA-GG-GA-GC-------G-CA-G-G-C-G------------G--G-AT-A-G-C----
-------AA----G-T-C-A--------G-C-T--G--TG--A-AA-AC--T-A-TG-G-G-----CT-C-AA------C-C-C-A-T-A-A--A-C----T-G--C-A---G--T-------T--GA-A-A---C-------T--G-T--TA--T-T-C----T-T-G-A-G-T--G---G-AG-----TA-G-A--------G-G-C-A---AG-C------GG--A--ATT----C-
C-G-A-GT--GT-A-G-CG-GT--G---A--A-A---TG-C-GT-AG--AT-A-TT--C-G--G-A-G-G-A-AC-A-CC---AG--T--G--GC-GAA-G--G-C--G-------G--C-T-T-G---CTG--G--GC-T-C--------------------T-A---A-C-T--GA--CG-C-T-G--A-GG--C-T-CG-A--AA-G-T---G-TG--GG-G-
-AG-C-A-AA--------CA--GG Bacteria(100);Firmicutes(100);Clostridia(100);Clostridiales(100);Ruminococcaceae(100);unclassified(100);unclassified(100);unclassified(100);unclassified(100);

Otu1744 0 0 0 7 0 0 1 0 0 0 0 0 0 0 0 0 0 0 0 0 0 0 0 0 0 0 0 1
0 0 0 0 0 0 0 0 0 0 0 0 0 0 0 0 0 0 0 0 0 0 1 0 0 0 0 0
0 0 0 0 1 0 0 0 0 0 0 0 0 0 0 0 0 0 0 0 0 0 0 0 0 0 0 0
0 0 0 0 1 0 0 0 0 0 0 0 0 0 0 0 0 0 0 0 0 0 0 0 9 0 0

M02255_76_000000000-ADUNE_1_2114_12624_13351 T----GG---GT-TA-ATG--------AAG-C-G------C--C--G---G--CT-A-ACT-AC------GT-G--C-C--AG-CA-------GC--C-----GC-GG-TA-AT-------A-CG-T-A-G-G------------G--C-TG-C-G-A-----
------GC----G-T-C-T--------GTC-C--G--TG--A-AT-TC--C-T-CT-G-G-----CT-C-AG------C-T-G-G-G-G-G--C-G----T-G--C-G---G--T-------G--GG-T-A---C-------G--G-G--CT--G-G-C----T-T-G-A-G-T--G---C-GG-----TA-G-G--------G-G-A-G---AC-T------GG--A--ACT----
C-C-T-G-GT--GT-A-G-CG-GT--G---G--A-A---TG-C-GC-AG--AT-A-TC--A-G--G-A-G-G-A-AC-A-CC---GG--T--G--GC-GAA-G--G-C--G-------G--G-T-C-T---CTG--G--GC-C-G--------------------T-T---A-C-T--GA--CG-C-T-G--A-GG--A-G-CG-A--AA-G-C---G-TG--
GG-G--AG-C-G-AA--------CA--GG Bacteria(100);Actinobacteria(100);Actinobacteria(100);unclassified(100);unclassified(100);unclassified(100);unclassified(100);unclassified(100);unclassified(100);

Otu1745 0 0 0 0 0 0 0 0 0 0 2 0 0 0 0 0 0 0 0 0 0 0 0 0 0 2 5 0
0 0 0 0 0 0 0 0 0 0 0 0 0 0 0 0 0 0 0 0 0 0 0 0 0 0 0 0 0
0 0 0 0 0 0 0 0 0 0 0 0 0 0 0 0 0 0 0 0 0 0 2 0 0 0 0 0 0
4 0 0 0 0 0 0 0 0 0 0 0 0 0 0 0 0 0 0 0 0 0 0 0 0 0 0

M02255_77_000000000-ADUT3_1_2117_20466_11590 T----AC---GT-AG-ATG--------GCA-A-G------C--G--T---T--GT-C-CGG-AT------TT-A--T-T--GG-GC-------GT--A-----AA-GC-GA-GC-------G-CA-G-G-C-G-------------G--T-TC-T-T-T-----------AA----G-T-C-T--------G-A-T--G--TG--A-AA-GA--C-C-CC-A-G-----CT-C-AA------C-T-G-G-G-G-A--A------G-GT-C-A---T--T-------G--GA-A-A---C-------T--G-G--AG--A-A-C----T-T-G-A-G-T--G---C-AG-----AA-G-A--------G-G-A-G---AG-T------GG--A--ATT----C-C-A-T-GT--GT-A-G-CG-GT--G---A--A-A---TG-C-GT-AG--AT-A-TA--T-G--G-A-G-G-A-AC-A-CC---AG--T--G--GC-GAA-G--G-C--G-------A--C-T-C-T---CTG--G--TC-T-G---------------------T-A---A-C-T--GA--CG-C-T-G--A-GG--C-T-CG-A--AA-G-C---G-TG--GG-G--AG-C-A-AA--------CA--GG Bacteria(100);Firmicutes(100);Bacilli(100);Lactobacillales(100);Carnobacteriaceae(100);Carnobacterium(100);unclassified(100);unclassified(100);unclassified(100);

Otu1746 0 0 0 0 0 0 0 0 0 9 0 0 0 0 0 0 0 0 0 0 0 0 0 0 0 0 0 0
0 0 0 0 0 0 0 0 0 0 0 0 0 0 0 0 0 0 0 0 0 0 0 0 0 0 0 0 0
0 0 0 0 0 0 0 0 0 0 0 0 0 0 0 0 0 0 0 0 0 0 0 0 0 0 0 0 0
0 0 0 0 0 0 0 0 0 0 0 0 0 0 0 0 0 0 0 0 0 0 0 0 0 0 0

M02255_76_000000000-ADUNE_1_1106_28608_16556 .....AC----T-AC-GT---------GCC-A-G------C--A--G---C--CG-C-GGT-AA------TA-C--G-T--AG-GT-------GG--C-----AA-GC-G--TT-------G----T-C-C-G-------------G--A-TT-G-A-T-----------AA----G-T-C-T--------G-A-T--G--TG--A-AA-GC--C-T-TC-G-G-----CT-C-AA------C-C-G-A-A-G-A--A-C----T-G--C-A---T--C-------A--GA-A-A---C-------T--G-T--CA--A-T-C----T-T-G-A-G-T--G---C-AG-----AA-G-A--------G-G-A-G---AG-T------GG--A--ACT----C-C-A-T-GT--GT-A-G-CG-GT--G---G--A-A---TG-C-GT-AG--AT-A-TA--T-G--G-A-A-G-A-AC-A-CC---AG--T--G--GC-GAA-G--G-C--G-------G--C-T-C-T---CTG--G--TC-T-G---------------------T-A---A-C-T--GA--CG-C-T-G--A-GG--C-T-CG-A--AA-G-C---A-TG--GG-T--AG-C-G-AA--------CA--GG Bacteria(100);Firmicutes(100);Bacilli(100);Lactobacillales(100);unclassified(100);unclassified(100);unclassified(100);unclassified(100);unclassified(100);

Otu1747 0 0 0 0 0 0 0 0 0 0 1 0 0 0 0 0 0 0 0 0 0 0 0 0 0 0 0 0
2 0 0 63 5 15 0 0 0 0 0 81 0 0 0 11 12 0 0 0 0 0 0 0 0 0 0 0 0
0 19 0 0 0 0 0 1 0 0 0 0 3 0 8 6 19 0 28 23 8 0 0 0 0 50 0 0 3
6 0 0 0 0 21 8 0 0 51 2 268 1 0 5 0 0 0 0 0 0 0 0 0 0 0 0

M02255_77_000000000-ADUT3_1_2104_24045_6228 T----AC---GT-AG-GGG--------GCG-A-G------C--G--T---T--AT-C-CGG-AT------TC-A--T-T--GG-GC-------GT--A-----AA-GC-GC-GC-------G-TA-G-G-C-G-------------G--C-CC-G-G-C-----------AG----G-C-C-G--------G-G-G--G--TC--G-AA-GC--G-G-GG-G-G-----CT-C-AA------C-C-C-C-C-C-G--A------A-G--C-C---C--C-------C--GG-A-A---C-------C--T-C--CGC-G-G-C----T-T-G-G-G-T--C---C-GG-----TA-G-G--------G-G-A-G---GG-T------GG--A--ACA----C-C-C-G-GT--GT-A-G-CG-GT--G---G--A-A---TG-C-GC-AG--AT-A-TC--G-G--G-T-G-G-A-AC-A-CC---GG--T--G--GC-GAA-G--G----C-------GGC--C-C-T---CTG--G--GC-C-G---------------------A-G---A-C-C--GA--CG-C-T-G--A-GG--C-G-CG-A--AA-G-C---T-GG--GG-G--AG-C-G-AA--------CA--GG Bacteria(100);Actinobacteria(100);Actinobacteria(100);Coriobacteridae(100);Coriobacteriales(100);Coriobacterineae(100);Coriobacteriaceae(100);Collinsella(100);unclassified(100);

Otu1748 0 0 0 0 0 0 0 0 0 21 0 0 0 0 0 0 0 0 0 0 0 0 0 0 0 0 0 0
0 0 0 0 0 0 0 0 0 0 0 0 0 0 0 0 0 0 0 0 0 0 0 0 0 0 0 0 0
0 0 0 0 0 0 0 0 0 0 0 0 0 0 0 0 0 0 0 0 0 0 0 0 0 0 0 0 0
0 0 0 0 0 0 0 0 0 0 0 0 0 0 0 0 0 0 0 0 0 0 0 0 0 0 0

M02255_76_000000000-ADUNE_1_1108_27539_19078 T----GG---CT-AA-TCT--------CGT-G-C------C--A--G---C--AG-C-CGC-GG------T--A--A-T--AC-GA-------GG--G-----GT-GC-AA-GC-------GTTA-T-T-C-G-------------G--A-AT-G-C-T-----------AA----G-T-C-A--------A-C-T--G--TT--A-AA-TT--C-C-TC-G-G-----CC-T-AA------C-T-G-A-G-G-A--T-C----A-G--C-G---G--T-------A--GA-A-A---C-------T--G-G--CG--C-G-C----T-T-G-A-G-G--G---T-GA-----GA-G-A--------G-A-G-A---AG-T------GG--A--ATT----C-T-C-G-GA--GT-A-G-CG-GT--A---A--A-A---TG-C-GT-AG--AT-C-TC--G-A--G-A-G-G-A-AC-A-CC---GA--T--G--GC-GAA-G--G-C--A-------G--C-T-T-C---TTG--G--CT-C-A---------------------T-A---C-C-T--GA--CG-T-T-G--A-GG--C-A-CG-A--AA-G-C---G-TG--GG-G--AG-C-A-AA--------CA--GG Bacteria(100);Candidate_division_TM6(100);unclassified(100);unclassified(100);unclassified(100);unclassified(100);unclassified(100);unclassified(100);unclassified(100);

Otu1749 0 333 153 0 0 0 35 63 234 0 0 0 0 0 0 0 0 0 0 0 0 0 0 0 0 0 0 0
0 0 0 0 0 0 0 0 0 0 0 0 0 0 0 0 0 0 0 0 0 0 0 0 0 0 0 0 0
0 0 0 0 0 0 0 0 0 0 0 0 0 0 0 0 0 0 0 0 0 0 0 0 0 0 0 0 0
0 0 0 0 0 0 0 0 0 0 0 0 0 0 0 0 0 0 0 0 0 0 0 0 94 0 0

M02255_77_000000000-ADUT3_1_1101_7862_22325 T----AC---GT-AT-GGA--------GCA-A-G------C--G--T---T--AT-C-CGG-AT------TT-A--C-T--GG-GT-------GT--A-----AA-GG-GA-GT-------G-TA-G-G-T-G-------------G--C-CA-G-G-C-----------AA----G-T-C-A--------G-A-A--G--TG--A-AA-GC--C-C-GG-G-G-----CT-C-AA------C-C-C-C-G-G-G--A-C----T-G--C-T---T--T-------T--GA-A-A---C-------T--G-C--AG--G-G-C----T-A-G-A-G-T--G---C-AG-----GA-G-G--------G-G-C-A---AG-T------GG--A--ATT----C-C-T-A-GT--GT-A-G-CG-GT--G---A--A-A---TG-C-GT-AG--AT-A-TT--A-G--G-A-G-G-A-AC-A-CC---AG--T--G--GC-GAA-G--G-C--G-------G--C-T-T-G---CTG--G--AC-T-G---------------------T-A---A-C-T--GA--CA-C-T-G--A-GG--C-T-CG-A--AA-G-C---G-TG--GG-G--AG-C-A-AA--------CA--GG Bacteria(100);Firmicutes(100);Clostridia(100);Clostridiales(100);Lachnospiraceae(100);Lachnospira(100);unclassified(100);unclassified(100);unclassified(100);

Otu1750 0 55 0 0 0 0 35 0 239 0 0 0 0 0 0 0 0 0 0 0 0 0 0 0 0 0 0 0
0 0 0 0 0 0 0 0 0 0 0 128 0 0 0 0 0 0 0 0 0 0 0 0 0 0 0 0 0
0 0 0 0 0 0 0 0 0 0 0 0 0 0 0 0 0 0 0 0 0 0 0 0 0 0 0 0 0
0 0 0 0 0 0 0 0 0 0 0 0 0 0 0 0 0 0 0 0 0 0 0 0 1 0 0

M02255_77_000000000-ADUT3_1_1102_20459_9615 T----AC---GT-AG-GGG--------GCG-A-G------C--G--T---T--GT-C-CGG-AA------TT-A--C-T--GG-GC-------GT--A-----AA-GG-GT-GC-------G-TA-G-G-C-G------------G--T-TT-G-G-T----------
AA----G-T-T-G--------G-A-T--G--TG--A-AA-TA--C-C-CG-G-G-----CT-T-AA------C-T-T-G-G-G-G--G-C----T-G--C-A---T--C-------C--AA-T-A---C-------T--G-T--CG--G-A-C----T-T-G-A-G-T--G---C-AG-----GA-G-A--------G-G-A-A---AG-C------GG--A--ATT----C-C-T-
A-GT--GT-A-G-CG-GT--G---A--A-A---TG-C-GT-AG--AT-A-TT--A-G--G-A-G-G-A-AC-A-CC---GG--T--G--GC-GAA-G--G-C--G-------G--C-T-T-T---CTG--G--AC-T-G--------------------T-A---A-C-T--GA--CG-C-T-G--A-GG--C-A-CG-A--AA-G-C---G-TG--GG-G--
AG-C-A-AA--------CA--GG Bacteria(100);Firmicutes(100);Clostridia(100);Clostridiales(100);unclassified(100);unclassified(100);unclassified(100);unclassified(100);unclassified(100);

Otu1751 0 395 177 0 0 0 447 95 347 0 0 0 0 0 0 0 0 0 0 0 0 0 0 4 0 0 0 0
0 0 0 0 0 0 0 0 0 0 0 20 9 0 0 49 0 0 0 32 0 0 0 0 0 0 0 0 0
0 0 0 0 0 0 0 0 0 0 0 0 0 0 0 2 0 19 0 0 0 0 0 0 0 0 0 0 0
0 0 0 0 0 0 0 0 0 0 0 0 0 0 0 0 10 0 0 0 0 0 0 16 367 0 0

M02255_77_000000000-ADUT3_1_1102_13770_10071 T----AC---GT-AG-GGG--------GCA-A-G------C--G--T---T--AT-C-CGG-AT------TT-A--C-T--GG-GT-------GT--A-----AA-GG-GA-GC-------G-TA-G-A-C-G------------G--A-CT-G-G-C----
-------AA----G-T-C-T--------G-A-T--G--TG--A-AA-GG--C-G-GG-G-G-----CT-CA-A------C-C-C-C-T-G-G--A-C----T-G--C-A---T--T-------G--GA-A-A---C-------T--G-T--TA--G-T-C----T-T-G-A-G-T--G---C-CG-----GA-G-AG---------G-T-A---AG-C------GG--A--ATT----
C-C-T-A-GT--GT-A-G-CG-GT--G---A--A-A---TG-C-GT-AG--AT-A-TT--A-G--G-A-G-G-A-AC-A-CC---AG--T--G--GC-GAA-G--G-C--G-------G--C-T-T-A---CTG--G--AC-G-G--------------------T-A---A-C-T--GA--CG-T-T-G--A-GG--C-T-CG-A--AA-G-C---G-TG--GG-
G--AG-C-A-AA--------CA--GG Bacteria(100);Firmicutes(100);Clostridia(100);Clostridiales(100);Lachnospiraceae(100);Blautia(100);unclassified(100);unclassified(100);unclassified(100);

Otu1752 0 0 0 0 0 0 0 0 0 0 17 0 0 0 0 0 0 0 0 0 0 0 0 0 0 0 0 0
0 0 0 0 0 0 0 0 0 0 0 0 0 0 0 0 0 0 0 0 0 0 0 0 0 0 0 0 0
0 0 0 0 0 0 0 0 0 0 0 0 0 0 0 0 0 0 0 0 0 0 0 0 0 0 0 0 0
0 0 0 0 0 0 0 0 0 0 0 0 0 0 0 0 0 0 0 0 0 0 0 0 0 0 0

M02255_77_000000000-ADUT3_1_1111_14852_10405 T----AC---GA-AG-GGG--------GCT-A-G------C--G--T---T--GT-T-CGG-AA------TT-A--C-T--GG-GC-------GT--A-----AA-GC-GC-GC-------G-TA-G-G-C-G------------G--G-TA-G-A-T----
-------AA----G-T-T-A--------G-A-G--G--TG--A-AA-TC--C-C-AG-G-G-----CT-C-AA------C-C-C-T-G-G-A--A-C----T-G--C-C---T--T-------T--AA-T-A---C-------T--G-T--CT--A-T-C----T-G-G-A-G-T--A---T-GG-----AA-G-A--------G-G-T-G---AG-T------GG--A--ATT----C-
C-G-A-GT--GT-A-G-AG-GT--G---A--A-A---TT-C-GT-AG--AT-A-TT--C-G--G-A-G-G-A-AC-A-TC---AG--T--G--GC-GAA-G--G-C--G-------G--C-T-C-A---CTG--G--TC-C-A--------------------T-T---A-C-T--GA--CG-C-T-G--A-GG--T-G-CG-A--AA-G-C---G-TG--GG-G-
-AG-C-A-AA--------CA--GG Bacteria(100);Proteobacteria(100);Alphaproteobacteria(100);unclassified(100);unclassified(100);unclassified(100);unclassified(100);unclassified(100);unclassified(100);

Otu1753 0 0 0 0 0 0 0 0 0 0 0 0 3 0 0 0 0 0 0 0 0 0 0 0 0 0 0 0
0 0 0 0 0 0 0 0 0 0 0 0 0 0 0 0 0 0 0 0 0 0 0 0 0 0 0 0 0
0 0 0 0 0 0 0 0 0 0 0 0 0 2 0 0 0 0 0 0 0 0 0 0 0 0 0 0 0
0 0 0 0 0 0 0 0 0 0 0 0 0 0 0 0 0 0 0 0 0 0 0 0 0 0 0

M02255_77_000000000-ADUT3_1_2101_27062_19370 G----AC---AG-AA-TAA--------GCA-C-C------G--G--C---T--AACT-CTG-TG------CC-A--G-C--AG-CC-------GC--G-----GT-AA-TA-CA-------G-AG-G-G-T-G------------C--A-AG-C-G-T----
-------TA----A-T-C-G--------G-A----A--T---T-AC-TC--C-C-CG-G-G-----CT-C-AA------C-C-T-G-G-G-A--A-C----T-G--C-A---T--T-------C--AA-A-A---C-------T--G-A--CT--G-A-C----T-A-G-A-G-T--A---T-GG-----TA-G-A--------G-G-G-T---GG-T------GG--A--ATT----T-
C-C-T-GT--GT-A-G-CG-GT--G---A--A-A---TG-C-GT-AG--AT-A-TA--G-G--A-A-G-G-A-AC-A-CC---AG--T--G--GC-GAA-G--G-C--G-------A--C-C-A-C---CTG--G--AC-T-G--------------------A-T---A-C-T--GA--CA-C-T-G--A-GG--T-G-CG-A--AA-G-C---G-TG--GG-
G--AG-C-A-AA--------CA--GG Bacteria(100);Proteobacteria(100);Gammaproteobacteria(100);unclassified(100);unclassified(100);unclassified(100);unclassified(100);unclassified(100);unclassified(100);

Otu1754 0 0 0 0 0 0 0 0 0 0 0 0 0 5 0 0 0 0 0 0 0 0 0 0 0 0 0 0
0 0 0 0 0 0 0 0 0 0 0 0 0 0 0 0 0 0 0 0 0 0 0 0 0 0 0 0 0
0 0 0 0 0 0 0 0 0 0 0 0 0 0 0 0 0 0 0 0 0 0 0 0 0 0 0 0 0
0 0 0 0 0 0 0 0 0 0 0 0 0 0 0 0 0 0 0 0 0 0 0 0 0 0 0

M02255_76_000000000-ADUNE_1_1116_12548_23972 C----TA---AC-TC-CGT--------GCC-A-G------C-----A---G--CC-G-CGG-TA------AT-A--C-G--GA-GG-------GT--G-----CA--A-GC--G-------T-TA-A-T-C-G------------G--T-AA-G-T-T--------
---AA----G-T-C-A--------G-C-T--G--TG--A-AA-TC--C-C-CG-G-G-----CT-C-AA------C-C-T-G-G-G-A--A-T----T-G--C-A---G--T-------T--GA-A-A---C-------T--G-G--CT--T-G-C----T-A-G-A-G-T--A---T-GG-----TA-G-A--------G-G-G-G---TG-T------GG--A--ATT----T-C-
A-G-GT--GT-A-G-CG-GT--G---A--A-A---TG-C-GT-AG--AT-A-TC--T-G--A-A-G-G-A-AC-A-TC---AG--T--G--GC-GAA-G--G-C--G-------A--C-A-C-C---CTG--G--AC-C-A--------------------A-T---A-C-T--GA--CG-C-T-G--A-GG--T-G-CG-A--AA-G-C---G-TG--GG-G--
AG-C-A-AA--------CA--GG Bacteria(100);Proteobacteria(100);Gammaproteobacteria(100);unclassified(100);unclassified(100);unclassified(100);unclassified(100);unclassified(100);unclassified(100);

Otu1755 65 0 0 0 0 0 0 0 0 0 0 0 0 0 0 0 0 0 0 0 0 0 0 0 0 0 0 0
0 0 0 0 0 0 0 0 0 0 0 0 0 0 0 0 0 0 0 0 0 0 0 0 0 0 0 0
0 0 0 0 0 0 0 0 0 0 0 0 0 0 0 0 0 0 0 0 0 0 0 0 0 0 0 0
0 0 0 0 0 0 0 0 0 0 0 0 0 0 0 0 0 0 0 0 0 0 0 0 0 0 0
M02255_77_000000000-ADUT3_1_1110_10866_17474 T----AC---GA-AG-GGG--------GCT-A-G------C--G--T---T--GC-T-CGG-AA------TT-A--C-T--GG-GC-------GT--A-----AA-GG-GC-GC-------G-TA-G-G-C-G------------G--T-CT-G-C-C----
-------AA----G-T-C-A--------G-G-C--G--TG--A-AA-TT--C-C-TG-G-G-----CT-C-AA------C-C-T-G-G-G-G--A-C----T-G--C-G---C--T-------T--GA-T-A---C-------T--G-G--CG--G-A-C----T-T-G-A-G-G--A---T-GG-----AA-G-A--------G-G-C-T---CG-T------GG--A--ATT----
C-C-C-A-GT--GT-A-G-AG-GT--G---A--A-A---TT-C-GT-AG--AT-A-TT--G-G--G-A-A-G-A-AC-A-CC---GG--T--G--GC-GAA-G--G-C--G-------G--C-G-A-G---CTG--G--TC-C-A--------------------T-T---A-C-T--GA--CG-C-T-G--A-GG--C-G-CG-A--CA-G-C---G-TG--
GG-G--AG-C-A-AA--------CA--GG Bacteria(100);Proteobacteria(100);Alphaproteobacteria(100);Rhodospirillales(100);Acetobacteraceae(100);Roseomonas(100);unclassified(100);unclassified(100);unclassified(100);

Otu1756 0 0 0 1 0 0 0 0 0 0 0 0 0 0 0 0 0 0 0 0 0 0 0 0 0 0 0 0
0 0 0 0 0 0 0 0 0 0 0 0 0 0 0 0 0 0 0 0 0 0 0 0 0 0 0 0
0 0 0 0 0 0 0 0 0 0 0 0 0 0 0 0 0 0 0 0 0 0 0 0 0 0 12 0
0 0 0 0 0 0 0 0 0 0 0 0 0 0 0 0 0 0 0 0 0 0 0 0 0 0 0
M02255_77_000000000-ADUT3_1_2117_16347_12506 T----AC---GT-AG-GGT--------GCG-A-G------C--G--T---T--GT-C-CGG-AA------TT-A--T-T--GG-GC-------GT--A-----AA-GG-GA-TT-------G-TA-G-G-C-G------------G--T-CC-G-T-C-----
------GC----G-T-C-G--------A-A-A--G--TG--A-AA-AC--T-C-AG-G-G-----CT-C-AA------C-C-C-T-G-A-G--C-C----T-G--C-T---T--T-------C--GA-T-A---C-------G--G-G--CG--G-A-C----T-A-G-A-G-G--T---A-TG-----CA-G-G--------G-G-A-G---AA-C------GG--A--ATT----
C-C-T-G-GT--GG-A-G-CG-GT--G---G--A-A---TG-C-GC-AG--AT-A-TC--A-G--G-A-G-G-A-AC-A-CC---GG--T--G--GC-GAA-G--G-C--G-------G--T-T-C-T---CTG--G--GC-A-T--------------------T-A---C-C-T--GA--CG-C-T-G--A-GA--A-T-CG-A--AA-G-C---G-TG--
GG-G--AG-C-A-AA--------CA--GG Bacteria(100);Actinobacteria(100);Actinobacteria(100);Actinobacteridae(100);Actinomycetales(100);Propionibacterineae(100);unclassified(100);unclassified(100);unclassified(100);

Otu1757 0 0 0 0 0 0 1 8 0 0 0 0 0 0 0 0 0 0 0 0 0 0 0 0 0 0 0 0
0 0 0 0 0 0 0 0 0 0 0 0 0 0 0 0 0 0 0 0 0 0 0 0 0 0 0 0
0 0 0 0 0 0 0 0 0 0 0 0 0 0 0 0 0 0 0 0 0 0 0 0 0 0 0 0
0 0 0 0 0 0 0 0 0 0 0 0 0 0 0 0 0 0 0 0 4 0 4 0 0 0 0
M02255_76_000000000-ADUNE_1_2114_26320_16859 T----AC---GTGCC-AGCA-------GCC-G-C------G--G--T---A--AT-A-CGT-AG------GG-T--A-C--TA-GC-------GT--T-----GT-CC-GG-AT-------T-TA---T-T-G------------G--T-TT-G-T-C-------
----GC----G-T-C-T--------G-T-C--G--TG--A-AA-GC--C-T-GT-G-G-----CT-T-AA------C-C-A-T-G-G-G--C-T----T-G--C-G---G--T-------G--GG-T-A---C-------G--G-G--CA--G-G-C----T-T-G-A-G-T--G---C-GG-----TA-G-G--------G-G-A-G---AC-T------GG--A--ATT----C-
C-T-G-GT--GT-A-G-CG-GT--G---G--A-A---TG-C-GC-AG--AT-A-TC--A-G--G-A-G-G-A-AC-A-CC---GG--T--G--GC-GAA-G--G-C--G-------G--G-T-C-T---CTG--G--GC-C-G--------------------T-T---A-C-T--GA--CA-C-T-G--A-GG--A-G-CG-A--AA-G-C---G-TG--GG-
G--AG-C-G-AA--------CA--GG Bacteria(100);Actinobacteria(100);Actinobacteria(100);Actinobacteridae(100);Actinomycetales(100);unclassified(100);unclassified(100);unclassified(100);unclassified(100);

Otu1758 18 0 0 0 0 0 0 0 0 0 0 0 0 0 0 0 0 0 0 0 0 0 0 0 0 0 0 0
0 0 0 0 0 0 0 0 0 0 0 0 0 0 0 0 0 0 0 0 0 0 0 0 0 0 0 0
0 0 0 0 0 0 0 0 0 0 0 0 0 0 0 0 0 0 0 0 0 0 0 0 0 0 0 0
0 0 0 0 0 0 0 0 0 0 0 0 0 0 0 0 0 0 0 0 0 0 0 0 0 0 0
M02255_77_000000000-ADUT3_1_1102_10242_18631 T----AC---GT-AG-GAG--------GCG-A-G------C--G--T---T--GT-C-CGG-AT------TT-A--C-T--GG-GC-------GT--A-----AA-GC-GC-GC-------G-CA-G-G-C-G------------G--C-AT-G-G-C----
-------AA----G-T-A-A--------G-G-T--G--TG--A-AA-TC--T-C-CC-T-G-----CC-T-AA------C-G-G-G-G-A-G--G-G----T-G--C-A---C--T-------T--TA-G-A---C-------T--G-C--CA--A-G-C----T-A-G-A-G-G--A---C-GG-----TA-G-A--------G-G-G-G---GG-T------AG--A--ATT----
G-C-T-G-GT--GT-A-G-CG-GT--G---A--A-A---TG-C-GT-AG--AG-A-TC--A-G--C-A-G-G-A-AT-A-CC---CG--T--G--GC-GAA-G--G-C--G-------G--C-C-C-C---CTG--G--GC-C-G--------------------A-A---C-C-T--GA--CG-C-T-G--A-GG--C-G-CG-A--AG-G-C---T-AG--
GG-G--AG-C-G-AA--------CG--GG Bacteria(100);unclassified(100);unclassified(100);unclassified(100);unclassified(100);unclassified(100);unclassified(100);unclassified(100);unclassified(100);

Otu1759 0 0 0 0 0 0 71 0 53 0 0 0 0 0 0 0 0 0 0 0 0 0 0 0 0 0 0 0
0 0 0 0 0 0 0 0 0 0 0 0 0 0 0 0 0 0 0 0 0 0 0 0 0 0 0 0
0 0 0 0 0 0 0 0 0 0 0 0 0 0 0 0 0 0 0 0 0 0 0 0 0 0 0 0
0 0 0 0 0 9 0 0 0 0 0 0 0 0 0 0 0 0 0 0 0 0 0 0 0 0 0
M02255_77_000000000-ADUT3_1_2103_19432_23475 T----AC---GT-AG-GGG--------GCA-A-G------C--G--T---T--AT-C-CGG-AT------TT-A--C-T--GG-GT-------GT--A-----AA-GG-GA-GC-------G-TA-G-A-C-G------------G--T-AT-G-G-C-----
------AA----G-T-C-T--------G-A-T--G--TG--A-AA-GG--C-C-AG-G-G-----CT-C-AA------C-C-C-T-G-G-G--A-C----T-G--C-A---T--T-------G--GA-A-A---C-------T--G-T--CG--A-A-C----T-A-G-A-G-T--G---T-CG-----GA-G-A--------G-G-C-A---AG-T------GG--A--ATT----
C-C-T-A-GT--GT-A-G-CG-GT--G---A--A-A---TG-C-GT-AG--AT-A-TT--A-G--G-A-G-G-A-AC-A-CC---AG--T--G--GC-GAA-G--G-C--G-------G--C-T-T-G---CTG--G--AC-G-A--------------------T-G---A-C-T--GA--CG-T-T-G--A-GG--C-T-CG-A--AA-G-C---G-TG--GG-
G--AG-C-A-AA--------CA--GG Bacteria(100);Firmicutes(100);Clostridia(100);Clostridiales(100);Lachnospiraceae(100);unclassified(100);unclassified(100);unclassified(100);unclassified(100);

Otu1760 0 0 0 0 0 0 1 0 0 0 0 0 0 0 0 0 0 0 0 0 0 0 0 0 0 0 0 0
0 0 0 0 0 0 0 0 0 0 0 0 0 0 0 0 0 0 0 0 0 0 0 0 0 0 0 0 0
0 0 0 0 0 0 0 0 0 0 0 0 0 0 0 0 0 0 0 0 0 0 0 0 0 0 0 0 0
0 0 0 0 0 0 0 0 0 0 0 0 0 11 0 0 0 0 0 0 0 0 0 0 0 0 0
M02255_76_000000000-ADUNE_1_1101_14849_14271 T----AC---GT-AG-GGG--------GCG-A-G------C--G--T---T--AT-C-CGG-AT------TC-A--T-T--GG-GC-------GT--A-----AA----GC-GC-------------G-C--------------G----CA-C-C------------
AG----G-C-G-G--------G-A-T--C--TC--G-AA-AC--C-G-GG-G-G-----CT-C-AA------C-C-T-C-C-G-G--G------C-G--G-A---T--C-------C--CG-A-A---C-------C--G-G--TG--T-G-C----T-C-G-A-G-T--G---C-GG-----TA-G-A--------G-G-A-A---GT-C------GG--A--ATT----C-C-
C-G-GT--GT-A-G-CG-GT--G---A--A-A---TG-C-GC-AG--AT-A-TC--G-G--G-A-A-G-A-AC-A-CC---GA--T--G--GC-GAA-G--G-C--A-------G--A-C-T-T---CTG--G--GC-C-G--------------------A-C---A-C-T--GA--CG-C-T-C--A-GG--C-G-CG-A--AA-G-C---T-GG--GG-G--
AG-C-G-AA--------CA--GG Bacteria(100);Actinobacteria(100);Actinobacteria(100);Coriobacteridae(100);Coriobacteriales(100);Coriobacterineae(100);Coriobacteriaceae(100);unclassified(100);unclassified(100);

Otu1761 0 0 0 0 0 0 0 0 0 0 0 15 0 0 0 0 0 0 0 0 0 0 0 0 0 0 0 0
0 0 0 0 0 0 0 0 0 0 0 0 0 0 0 0 0 0 0 0 0 0 0 0 0 0 0 0 0
0 0 0 0 0 0 0 0 0 0 0 0 0 0 0 0 0 0 0 0 23 0 0 0 0 0 0 0 0
0 0 0 0 0 0 0 0 0 0 0 0 0 0 0 0 0 0 0 0 0 0 0 0 0 0 0
M02255_77_000000000-ADUT3_1_1116_27458_18070 T----AC---GT-AG-GTG--------GCA-A-G------C--G--T---T--GT-C-CGG-AT------TT-A--C-T--GG-GC-------GT--A-----AA-GG-GA-GC-------G-TA-G-G-C-G------------G--A-TA-C-T-T-----
------AA----G-T-G-G--------G-A-T--G--TG--A-AA-TA--C-C-CG-G-G-----CT-C-AA------C-C-C-G-G-G-T--G-C----T-G--C-A---T--T-------C--CA-A-A---C-------T--G-G--GT--A-T-C----T-A-G-A-G-T--G---T-GG-----GA-G-A--------G-G-A-A---AG-T------GG--A--ATT----
C-C-T-A-GT--GT-A-G-CG-GT--G---A--A-A---TG-C-GT-AG--AT-A-TT--A-G--G-A-A-G-A-AC-A-CC---AG--T--G--GC-GAA-G--G-C--G-------A--C-T-T-T---CTG--G--AC-C-A--------------------T-A---A-C-T--GA--CG-C-T-G--A-GG--C-T-CG-A--AA-G-C---G-TG--GG-
G--AG-C-A-AA--------CA--GG Bacteria(100);Firmicutes(100);Clostridia(100);Clostridiales(100);Clostridiaceae(100);unclassified(100);unclassified(100);unclassified(100);unclassified(100);

Otu1762 0 0 0 0 0 0 0 0 0 0 0 3 0 0 0 0 0 0 0 0 0 0 0 0 0 0 1 0
0 0 0 0 0 0 0 0 0 0 0 0 0 0 0 0 0 0 0 0 0 0 0 0 0 0 0 0 0
0 0 0 0 0 0 0 0 0 0 0 0 0 0 0 0 0 0 0 0 0 0 0 0 0 0 0 0 0
0 0 0 0 0 0 0 0 0 0 0 0 0 0 0 0 0 0 0 0 0 0 0 0 0 0 0
M02255_76_000000000-ADUNE_1_2111_11848_6402 C----GG---CT-AA-CTC--------CGT-G-C------C--A--G---C--AG-C-CGC-GG------TA-A--T-A--CG-GA-------GG--A-----TC-CA-AG-CG-------T-TA-T-C-C-G------------G--A-AT-C-A-T-----------AA--
--G-T-C-A--------G-T-G--G--TG--A-AA-TC--T-C-CC-C-G-----CT-C-AA------C-G-G-G-G-A-A--A-C----G-G--C-C---A--T-------T--GA-T-A---C-------T--G-T--AA--G-G-C----T-T-G-A-A-T--T---A-TT-----AG-G-A--------A-G-T-A---AC-T------AG--A--ATA----T-G-T-A-GT-
-GT-A-G-CG-GT--G---A--A-A---TG-C-TT-AG--AG-A-TT--A-C--A-T-G-G-A-AT-A-CC---AA--T--T--GC-GAA-G--G-C--A-------G--G-T-T-A---CTA--C--TA-A-T--------------------G-G---A-T-T--GA--CG-C-T-G--A-TG--G-A-CG-A--AA-G-C---G-TG--GG-T--AG-C-G-
AA--------CA--GG Bacteria(100);Bacteroidetes(100);Flavobacteria(100);Flavobacteriales(100);Flavobacteriaceae(100);Flavobacterium(100);unclassified(100);unclassified(100);unclassified(100);

Otu1763 0 0 0 0 0 0 21 0 0 0 0 0 0 0 0 0 0 0 0 0 0 0 0 0 0 0 0 0
0 0 0 0 0 0 0 0 0 0 0 0 0 0 0 0 0 0 0 0 0 0 0 0 0 0 0 0 0
0 0 0 0 0 0 0 0 0 0 0 0 0 0 0 0 0 0 0 0 0 0 0 0 0 0 0 0 0
0 0 0 0 0 0 4 0 0 0 0 0 0 0 0 0 0 0 0 0 0 0 0 0 43 0 0
M02255_77_000000000-ADUT3_1_1103_13319_4609 T----AC---GT-AG-GTG--------GCA-A-G------C--G--T---T--GT-C-CGG-AT------TT-A--C-T--GG-GT-------GT--A-----AA-GG-GC-GT-------G-CA-G-C-C-G------------G--G-CC-G-G-C----------
AA----G-T-C-A--------G-A-T--G--TG--A-AA-TC--T-G-GA-G-G-----CT-T-AA------C-C-T-C-C-A-A--A-C----T-G--C-A---T--T-------T--GA-A-A---C-------T--G-T--AG--G-T-C----T-T-G-A-G-T--A---C-CG-----GA-G-A--------G-G-T-T---AT-C------GG--A--ATT----C-C-T-T-
GT--GT-A-G-CG-GT--G---A--A-A---TG-C-GT-AG--AT-A-TA--A-G--G-A-A-G-A-AC-A-CC---AG--T--G--GC-GAA-G--G-C--G-------G--A-T-A-A---CTG--G--AC-G-G--------------------C-A---A-C-T--GA--CG-G-T-G--A-GG--C-G-CG-A--AA-G-C---G-TG--GG-G--AG-
C-A-AA--------CA--GG Bacteria(100);Firmicutes(100);Clostridia(100);Clostridiales(100);Ruminococcaceae(100);Oscillibacter(100);unclassified(100);unclassified(100);unclassified(100);

Otu1764 0 0 0 0 0 0 41 0 0 0 0 0 0 0 0 0 0 0 0 0 0 0 0 0 0 0 0 0
0 0 0 0 0 0 0 0 0 0 0 0 0 0 0 0 0 0 0 0 0 0 0 0 0 0 0 0 0
0 0 0 0 0 0 0 0 0 0 0 0 0 0 0 0 0 0 0 0 0 0 0 0 0 0 0 0 0
0 0 0 0 0 0 0 0 0 0 0 0 0 0 0 0 0 0 0 0 0 0 0 0 0 0 0
M02255_77_000000000-ADUT3_1_1117_17029_15371 T----AC---GT-AG-GTG--------GCA-A-G------C--G--T---T--GT-C-CGG-AT------TT-A--C-T--GG-GT-------GT--A-----AA-GG-GC-GT-------G-CA-G-C-C-G------------G--G-CT-G-G-C-----
------AA----G-T-C-A--------G-G-C--G--TG--A-AA-TC--C-C-AG-G-G-----CT-C-AA------C-C-C-T-G-G-A--A-C----T-G--C-G---T--T-------T--GA-A-A---C-------T--G-C--TG--G-T-C----T-T-G-A-G-T--A---C-CG-----GA-G-A--------G-G-T-C---AT-C------GG--A--ATT----C-
C-T-T-GT--GT-A-G-CG-GT--G---A--A-A---TG-C-GT-AG--AT-A-TA--A-G--G-A-A-G-A-AC-A-CC---AG--T--G--GC-GAA-G--G-C--G-------G--A-T-G-A---CTG--G--AC-G-G--------------------C-A---A-C-T--GA--CG-G-T-G--A-GG--C-G-CG-A--AA-G-C---G-TG--GG-
G--AG-C-A-AA--------CA--GG Bacteria(100);Firmicutes(100);unclassified(100);unclassified(100);unclassified(100);unclassified(100);unclassified(100);unclassified(100);unclassified(100);

Otu1765 0 0 0 0 0 0 1 29 0 0 0 0 0 0 0 0 0 0 0 0 0 0 0 0 0 0 0 0
0 0 0 0 0 0 0 0 0 0 0 0 0 0 0 0 0 0 0 0 0 0 0 0 0 0 0 0 0
0 0 0 0 0 0 0 0 0 0 0 0 0 0 0 0 0 0 0 0 0 0 0 0 0 0 0 0 0
0 0 0 0 0 0 0 0 0 0 0 0 0 0 0 0 0 0 0 0 0 0 0 0 0 0 0
M02255_77_000000000-ADUT3_1_1117_22972_11780 G----AC---AG-AG-GAT--------GCA-A-G------A--G--T---T--AT-C-CGG-AA------TG-A--C-T--GG-GC-------GT--A-----AA-GT-TT-CT-------G-TA-G-G-T-G------------G--C-TT-T-T-C------
-----AA----G-T-C-C--------G-T-C--G--TC--A-AA-TC--C-C-AG-G-G-----CT-C-AA------C-C-C-T-A-G-A--C-A----G-A--C-G---G--T-------G--GA-A-A---C-------T--A-T--CA--A-G-C----T-G-G-A-G-T--A---T-GG-----TA-G-G--------G-G-C-A---GA-G------GG--A--ATT----T-
C-C-G-GT--GG-A-G-CG-AT--G---A--A-A---TG-C-GT-TG--AG-A-TC--G-G--A-A-A-G-A-AC-A-CC---AA--T--G--GC-GAA-A--G-C--T-------C--T-C-T-G---CTG--G--GC-C-G--------------------A-C---A-C-T--GA--CA-C-T-C--A-GA--G-A-TG-A--AA-G-C---T-AG--GG-G-
-AG-A-A-AA--------TG--GG Bacteria(100);unclassified(100);unclassified(100);unclassified(100);unclassified(100);unclassified(100);unclassified(100);unclassified(100);unclassified(100);

Otu1766 130 0 0 0 0 0 0 0 0 0 0 0 0 0 0 0 0 0 0 0 0 0 0 0 0 0 0 0
0 0 0 0 0 0 0 0 0 0 0 0 0 0 0 0 0 0 0 0 0 0 0 0 0 0 0 0 0
0 0 0 0 0 0 0 0 0 0 0 0 0 0 0 0 0 0 0 0 0 0 0 0 0 0 0 0 0
0 0 0 0 0 0 0 0 0 0 0 0 0 0 0 0 0 0 0 0 0 0 0 0 0 0 0
M02255_77_000000000-ADUT3_1_1102_9029_13006 T----AC---GT-AG-GTG--------GCG-A-G------C--G--T---T--GT-C-CGG-AT------TT-A--C-T--GG-GT-------GT--A-----AA-GG-GC-GC-------G-TA-G-G-C-G------------G--G-GA-T-G-C-----------
AA----G-T-C-A--------G-A-T--G--TG--A-AA-TC--T-C-CA-G-G-----CT-C-AA------C-C-T-G-G-A-A--A-C----T-G--C-A---T--T-------T--GA-A-A---C-------T--G-C--AT--T-T-C----T-T-G-A-G-T--A---T-CG-----GA-G-A--------G-G-C-A---GG-C------GG--A--ATT----C-C-G-
T-GT--GT-A-G-CG-GT--G---A--A-A---TG-C-GT-AG--AT-A-TA--C-G--G-A-A-G-A-AC-A-CC---AG--T--G--GC-GAA-G--G-C--G-------G--C-C-T-G---CTG--G--AC-G-A--------------------C-A---A-C-T--GA--CG-C-T-G--A-GG--C-G-CG-A--AA-G-C---G-TG--GG-G--
AG-C-A-AA--------CA--GG Bacteria(100);Firmicutes(100);Clostridia(100);Clostridiales(100);unclassified(100);unclassified(100);unclassified(100);unclassified(100);unclassified(100);

Otu1767 141 0 0 0 0 0 0 0 0 0 0 0 0 0 0 0 0 0 0 0 0 0 0 0 0 0 0 0
0 0 0 0 0 0 0 0 0 0 0 0 0 0 0 0 0 0 0 0 0 0 0 0 0 0 0 0 0
0 0 0 0 0 0 0 0 0 0 0 0 0 0 0 0 0 0 0 0 0 0 0 0 0 0 0 0 0
0 0 0 0 0 0 0 0 0 0 0 0 0 0 0 0 0 0 0 0 0 0 0 0 0 0 0
M02255_77_000000000-ADUT3_1_1107_11795_21241 T----AC---GG-AG-GGG--------GCT-A-G------C--G--T---T--GT-T-CGG-AA------TT-A--C-T--GG-GC-------GT--A-----AA-GC-GC-AC-------G-TA-G-G-C-G------------G--T-TG-C-T-C-----
------AA----G-T-C-A--------G-A-G--G--TG--A-AA-GC--C-T-GG-A-G-----CT-C-AA------C-T-C-C-A-G-A--A-C----T-G--C-C---T--T-------T--GA-A-A---C-------T--A-G--GT--G-A-C----T-T-G-A-A-T--C---C-AG-----GA-G-A--------G-G-T-G---AG-T------GG--A--ATT----C-
C-G-A-GT--GT-A-G-AG-GT--G---A--A-A---TT-C-GT-AG--AT-A-TT--C-G--G-A-A-G-A-AC-A-CC---AG--T--G--GC-GAA-G--G-C--G-------G--C-T-C-A---CTG--G--AC-T-G--------------------G-T---A-T-T--GA--CG-C-T-G--A-GG--T-G-CG-A--AA-G-C---G-TG--GG-
G--AG-C-A-AA--------CA--GG Bacteria(100);Proteobacteria(100);Alphaproteobacteria(100);Sphingomonadales(100);unclassified(100);unclassified(100);unclassified(100);unclassified(100);unclassified(100);

Otu1768 0 0 0 0 0 0 4 92 0 0 0 0 0 0 0 0 0 0 0 0 0 0 0 0 0 0 0 0
0 0 0 0 0 0 0 0 0 0 0 0 0 0 0 0 0 0 0 0 0 0 0 0 0 0 0 0 0
0 0 0 0 0 0 0 0 0 0 0 0 0 0 0 0 0 0 0 0 0 0 0 0 0 0 0 0 0
0 0 0 0 0 0 0 0 0 0 0 0 0 0 0 0 0 0 0 0 0 0 0 0 0 0 0
M02255_77_000000000-ADUT3_1_1111_27853_9522 T----AC---GG-AG-GAT--------CCA-A-G------C--G--T---T--AT-C-CGG-AA------TT-A--C-T--GG-GC-------GT--A-----AA-GA-GTTGC-------G-TA-G-G-C-G------------G--C-AT-T-G-T-----------
AA----G-T-A-G--------G-T-A--G--TG--A-AA-TC--C-T-GG-G-G-----CT-C-AA------C-C-C-T-T-T-A--A-A----C-A--T-T---A--T-------C--TA-A-A---C-------T--G-C--AAA-G---C----T-A-G-A-A-T--A---T-GA-----GA-G-A--------G-G-T-C---GC-T------GG--A--ATT----C-C-T-
A-GT--GT-A-G-GA-GT--G---A--A-A---TC-C-GT-AG--AT-A-TT--A-G--G-A-G-G-A-AC-A-CC---GA--T--G--GC-GTA-G--G-C--A-------G--G-C-G-A---CTG--G--CT-C-A--------------------T-T---A-T-T--GA--CG-C-T-A--A-GG--C-A-CG-A--AA-G-C---G-TG--GG-T--AG-
C-A-AA--------CG--GG Bacteria(100);Candidate_division_TM7(100);unclassified(100);unclassified(100);unclassified(100);unclassified(100);unclassified(100);unclassified(100);unclassified(100);

Otu1769 0 0 0 0 0 0 6 1 92 0 0 0 0 0 0 0 0 0 0 0 0 0 0 0 0 0 0 0
0 0 0 0 0 0 0 0 0 0 0 0 0 0 0 0 0 0 0 0 0 0 0 0 0 0 0 0 0
0 0 0 0 0 0 0 0 0 0 0 0 0 0 0 0 0 0 0 0 0 0 0 0 0 0 0 0 0
0 0 0 0 0 0 0 0 0 0 0 0 0 0 0 0 0 0 0 0 0 0 0 0 0 0 0
M02255_77_000000000-ADUT3_1_1115_17008_22576 T----AC---GT-AG-GGG--------GCA-A-G------C--G--T---T--AT-C-CGG-AT------TT-A--C-T--GG-GT-------GT--A-----AA-GG-GA-GC-------G-TA-G-A-C-G------------G--C-AA-G-G-C----
-------AA----G-T-C-T--------G-A-A--G--TG--A-AA-GC--C-C-GG-T-G-----CT-T-AA------C-G-C-C-G-G-G--A-C----T-G--C-T---T--T-------G--GA-A-A---C-------T--G-T--TT--G-G-C----T-G-G-A-G-T--G---C-CG-----GA-G-A--------G-G-T-A---AG-C------GG--A--ATT----
C-C-T-A-GT--GT-A-G-CG-GT--G---A--A-A---TG-C-GT-AG--AT-A-TT--A-G--G-A-A-G-A-AC-A-CC---AG--T--G--GC-GAA-G--G-C--G-------G--C-T-T-A---CTG--G--AC-G-G--------------------T-A---A-C-T--GA--CG-T-T-G--A-GG--C-T-CG-A--AA-G-C---G-TG--GG-
G--AG-C-A-AA--------CA--GG Bacteria(100);Firmicutes(100);Clostridia(100);Clostridiales(100);Lachnospiraceae(100);unclassified(100);unclassified(100);unclassified(100);unclassified(100);

Otu1770 0 0 0 0 0 0 0 0 0 0 0 24 1 0 0 2 2 12 6 25 0 3 0 2 0 0 1 1
0 0 0 0 0 0 0 0 0 0 0 0 4 0 0 0 0 0 0 0 0 0 0 0 0 0 0 0
0 0 0 0 0 1 7 0 0 2 16 0 0 0 0 0 0 4 0 0 0 0 0 0 0 0 0 0
0 0 0 0 0 6 0 2 2 0 3 0 0 0 2 0 0 0 0 0 0 0 0 0 0 0 0

M02255_77_000000000-ADUT3_1_2103_9878_12175 C----AG---CA-GC-CGC--------GGT-A--------A--T--A---C--AG-A-GGG-TG------CA-A--G-C--GT-TA-------AT--C-----GG-AT-TT-AC-------G-TA-G-G-T-G------------G--C-CA-A-T-T----------AA--
--G-T-C-A--------A-A-T--G--TG--A-AA-TC--C-C-CG-A-G-----CT-T-AA------C-T-T-G-G-G-A--A-T----T-G--C-A---T--T-------C--GA-T-A---C-------T--G-G--TT--G-G-C----T-A-G-A-G-T--A---T-GG-----GA-G-A--------G-G-A-T---GG-T------AG--A--ATT----C-C-A-G-
GT--GT-A-G-CG-GT--G---A--A-A---TG-C-GT-AG--AG-A-TC--T-G--G-A-G-G-A-AT-A-CC---GA--T--G--GC-GAA-G--G-C--A-------G--C-C-A-T---CTG--G--CC-T-A--------------------A-T---A-C-T--GA--CA-C-T-G--A-GG--T-A-CG-A--AA-G-C---A-TG--GG-G--AG-C-
A-AA--------CA--GG Bacteria(100);Proteobacteria(100);Gammaproteobacteria(100);Pseudomonadales(100);Moraxellaceae(100);Acinetobacter(100);unclassified(100);unclassified(100);unclassified(100);

Otu1771 0 0 0 0 0 0 0 0 0 0 0 0 0 3 0 0 0 0 0 0 0 0 0 0 0 0 0 0
0 0 0 0 0 0 0 0 0 0 0 0 0 0 0 0 0 0 0 0 0 0 0 0 0 0 0 0
0 0 0 0 0 0 0 0 0 0 0 0 0 0 0 0 0 0 0 0 0 0 0 0 0 0 0 0
0 0 0 0 0 0 0 0 0 0 0 0 0 0 0 0 0 0 0 0 0 0 0 0 0 0 0

M02255_76_000000000-ADUNE_1_1117_10898_24269 C----AA---CA-AA-AGA--------AGC-A-C------C--G--G---C--TA-A-C-T-CC------GT-G--C-C--AG-CA-------GC--C-----GC-G--GT-AA-------T-AC-G-G-A-G------------G--G-TG-C-A-A------
-----GC----G-T-T-A--------G-A-T--C--GG--A-AA-TC--C-C-CG-G-G-----CT-T-AA------C-C-T-G-G-G-A--A-T----G-G--C-A---T--C-------T--AA-A-A---C-------T--G-G--TC--A-G-C----T-A-G-A-G-T--C---T-TG-----TA-G-A--------G-G-G-G---GG-T------AG--A--ATT----C-
C-A-T-GT--GT-A-G-CG-GT--G---A--A-A---TG-C-GT-AG--AG-A-TG--T-G--G-A-G-G-A-AT-A-CC---GG--T--G--GC-GAA-G--G-C--G-------G--C-C-C-C---CTG--G--AC-A-A--------------------A-G---A-C-T--GA--CG-C-T-C--A-GG--T-G-CG-A--AA-G-C---G-TG--GG-
G--AG-C-A-AA--------CA--GG Bacteria(100);Proteobacteria(100);Gammaproteobacteria(100);Enterobacteriales(100);Enterobacteriaceae(100);unclassified(100);unclassified(100);unclassified(100);unclassified(100);

Otu1772 0 0 0 0 0 0 10 0 0 0 0 0 0 0 0 0 0 0 0 0 0 0 0 0 0 0 0 0
0 0 0 0 0 0 0 0 0 0 0 0 0 0 0 0 0 0 0 0 0 0 0 0 0 0 0 0
0 0 0 0 0 0 0 0 0 0 0 0 0 0 0 0 0 0 0 0 0 0 0 0 0 0 0 0
0 0 0 0 0 0 0 0 0 0 0 0 0 0 0 0 0 0 0 0 0 0 0 0 18 0 0

M02255_77_000000000-ADUT3_1_1116_8002_4121 T----AC---GT-AG-GGG--------GCA-A-G------C--G--T---T--AT-C-CGG-AT------TT-A--C-T--GG-GT-------GT--A-----AA-GG-GA-GC-------G-CA-G-G-T-G------------G--C-AA-G-G-C----------
AA----G-C-C-A--------G-A-A--G--TG--A-AA-AC--C-C-GG-G-G-----CT-T-AA------C-C-C-C-GTG-G--A-T----T-G--C-T---T--T-------T--GG-A-A---C-------T--G-T--CT--G-G-C----T-A-G-A-G-T--G---C-AG-----GA-G-G--------G-G-C-A---GG-C------GG--A--ATT----C-C-
T-A-GT--GT-A-G-CG-GT--G---A--A-A---TG-C-GT-AG--AT-A-TT--A-G--G-A-G-G-A-AC-A-CC---GG--T--G--GC-GAA-G--G-C--G-------G--C-C-T-G---CTG--G--AC-T-G--------------------T-A---A-C-T--GA--CA-C-T-G--A-GG--C-T-CG-A--AA-G-C---G-TG--GG-G--
AG-C-A-AA--------CA--GG Bacteria(100);Firmicutes(100);Clostridia(100);Clostridiales(100);Lachnospiraceae(100);unclassified(100);unclassified(100);unclassified(100);unclassified(100);

Otu1773 0 0 0 0 0 0 1 28 0 0 0 0 0 0 0 0 0 0 0 0 0 0 0 0 0 0 0 0
0 0 0 0 0 0 0 0 0 0 0 0 0 0 0 0 0 0 0 0 0 0 0 0 0 0 0 0
0 0 0 0 0 0 0 0 0 0 0 0 0 0 0 0 0 0 0 0 0 0 0 0 0 0 0 0
0 0 0 0 0 0 0 0 0 0 0 0 0 0 0 0 0 0 0 0 0 0 0 0 0 0 0

M02255_76_000000000-ADUNE_1_1116_16772_6211 .....AC----T-AC-GT---------GCC-A-G------C--A--G---C--CG-C-GGT-AA------TA-C--G-T--AG-GT-------GG--C-----AA-GC-G--TT-------G-TC-C-G---G------------A--T-CT-T-T-T----------AA----G-
T-C-T--------G-A-T--G--TG--A-AA-GC--C-T-TC-G-G-----CT-T-AA------C-C-G-G-A-G-A--A-G----T-G--C-A---T--C-------G--GA-A-A---C-------T--G-G--GA--G-A-C----T-T-G-A-G-T--G---C-AG-----AA-G-A--------G-G-A-C---AG-T------GG--A--ACT----C-C-A-T-GT--
GT-A-G-CG-GT--G---A--A-A---TG-C-GT-AG--AT-A-TA--T-G--G-A-A-G-A-AC-A-CC---AG--T--G--GC-GAA-G--G-C--G-------G--C-T-G-T---CTG--G--TC-T-G--------------------T-A---A-C-T--GA--CG-C-T-G--A-GG--C-T-CG-A--AA-G-T---A-TG--GG-T--AG-C-A-
AA--------CA--GG Bacteria(100);Firmicutes(100);Bacilli(100);Lactobacillales(100);Lactobacillaceae(100);Lactobacillus(100);unclassified(100);unclassified(100);unclassified(100);

Otu1774 8 0 0 0 0 0 0 0 0 0 0 0 0 0 0 0 0 0 0 0 0 0 0 0 0 0 0 3
0 0 0 0 0 0 0 0 0 0 0 0 0 0 0 0 0 0 0 0 0 0 0 0 0 0 0 0
0 0 0 0 0 0 0 0 0 0 0 0 0 0 0 0 0 0 0 0 0 0 0 0 0 0 0 0
0 0 0 0 0 0 0 0 0 0 0 0 0 0 0 0 0 0 0 0 0 0 0 0 0 0 0

M02255_76_000000000-ADUNE_1_2119_12624_16976 .....AC----T-CT-GT---------GCC-A-G------C-----A---G--CC-G-CGG-TA------AT-A--C-A--GA-G--------GT--G-----GC-AA-GC-G--------T-TG-T-T-C-G------------G--A-CT-T-C-T----------
AA----G-T-C-A--------G-A-C--G--TG--A-AA-TC--C-C-CC-G-G-----CT-T-AA------C-C-T-G-G-G-A--A-C----T-G--C-G---T--C-------T--GA-T-A---C-------T--G-G--GA--G-G-C----T-T-G-A-G-T--A---T-GG-----GA-G-A--------G-G-G-A---TG-T------AG--A--ATT----C-C-A-
G-GT--GT-A-G-CG-GT--G---A--A-A---TG-C-GT-AG--AT-A-TC--T-G--G-A-G-G-A-AT-A-CC---GG--T--G--GC-GAA-G--G-C--G-------G--C-A-T-C---CTG--G--AC-C-A--------------------T-A---A-C-T--GA--CG-C-T-G--A-GG--C-G-CG-A--AA-G-C---T-AG--GG-G--
AG-C-A-AA--------CG--GG Bacteria(100);Acidobacteria(100);Acidobacteria(100);Acidobacteriales(100);Acidobacteriaceae(100);uncultured(100);unclassified(100);unclassified(100);unclassified(100);

Otu1775 247 0 0 0 0 0 0 0 0 0 0 0 0 0 0 0 0 0 0 0 0 0 0 0 0 0 0 0
0 0 0 0 0 0 0 0 0 0 0 0 0 0 0 0 0 0 0 0 0 0 0 0 0 0 0 0 0
0 0 0 0 0 0 0 0 0 0 0 0 0 0 0 0 0 0 0 0 0 0 0 0 0 0 0 0 0
0 0 0 0 0 0 0 0 0 0 0 0 0 0 0 0 0 0 0 0 0 0 0 0 0 0 0
M02255_77_000000000-ADUT3_1_1102_11814_8362 T----AC---GG-AG-GGG--------GCT-A-G------C--G--T---T--GT-T-CGG-AA------TT-A--C-T--GG-GC-------GT--A-----AA-GC-GC-AC-------G-TA-G-G-C-G------------G--C-TT-T-G-T-----------AA-
---G-T-T-A--------G-A-G--G--TG--A-AA-GC--C-T-GG-T-G-----CT-C-AA------C-A-T-C-A-G-A--A-C----T-G--C-C---T--T-------T--AA-G-A---C-------T--G-C--AT--C-G-C----T-T-G-A-A-T--C---A-TG-----GA-G-A--------G-G-T-G---GG-T------GG--A--ATT----C-C-G-A-
GT--GT-A-G-AG-GT--G---A--A-A---TT-C-GT-AG--AT-A-TT--C-G--G-A-A-G-A-AC-A-CC---AG--T--G--GC-GAA-G--G-C--G-------G--C-C-C-A---CTG--G--AC-A-T--------------------G-T---A-T-T--GA--CG-C-T-G--A-GG--T-G-CG-A--AA-G-C---G-TG--GG-G--AG-
C-A-AA--------CA--GG Bacteria(100);Proteobacteria(100);Alphaproteobacteria(100);Sphingomonadales(100);Sphingomonadaceae(100);Sphingomonas(100);unclassified(100);unclassified(100);unclassified(100);

Otu1776 0 0 0 0 0 0 4 52 0 0 0 0 0 0 0 0 0 0 0 0 0 0 0 0 0 0 0 0
0 0 0 0 0 0 0 0 0 0 0 0 0 0 0 0 0 0 0 0 0 0 0 0 0 0 0 0 0
0 0 0 0 0 0 0 0 0 0 0 0 0 0 0 0 0 0 0 0 0 0 0 0 0 0 0 0 0
0 0 0 0 0 0 0 0 0 0 0 0 0 0 0 0 0 0 0 0 0 0 0 0 0 0 0
M02255_77_000000000-ADUT3_1_2107_12933_15940 T----AC---GT-AG-GGT--------CCG-A-G------C--G--T---T--GT-C-CGG-AA------TT-A--T-T--GG-GC-------GT--A-----AA-GG-GC-TC-------G-TA-G-G-C-G------------G--T-TT-G-T-C------
-----GC----G-T-C-G--------G-G-A--G--TG--A-AA-AC--C-T-AC-A-G-----CT-T-AA------C-T-G-T-G-G-G--C-T----T-G--C-T---T--T-------C--GA-T-A---C-------G--G-G--CA--G-A-C----T-T-G-A-G-G--C---A-TG-----CA-G-G--------G-G-A-G---AA-C------GG--A--ATT----C-
C-T-G-GT--GT-A-G-CG-GT--G---A--A-A---TG-C-GC-AG--AT-A-TC--A-G--G-A-G-G-A-AC-A-CC---GG--T--G--GC-GAA-G--G-C--G-------G--T-T-C-T---CTG--G--GC-A-T--------------------G-T---T-C-T--GA--CG-C-T-G--A-GG--A-G-CG-A--AA-G-T---G-TG--GG-
G--AG-C-G-AA--------CA--GG Bacteria(100);Actinobacteria(100);Actinobacteria(100);Actinobacteridae(100);Actinomycetales(100);Propionibacterineae(100);Nocardioidaceae(100);Nocardioides(100);unclassified(100);

Otu1777 0 0 0 0 0 0 0 0 0 0 0 0 0 7 0 0 0 0 0 0 0 0 0 0 0 0 0 0
0 0 0 0 0 0 0 0 0 0 0 0 0 0 0 0 0 0 0 0 0 0 0 0 0 0 0 0 0
0 0 0 0 0 0 0 0 0 0 0 0 0 0 0 0 0 0 0 0 0 0 0 0 0 0 0 0 0
0 0 0 0 0 0 0 0 0 0 0 0 0 0 0 0 0 0 0 0 0 0 0 0 0 0 0
M02255_77_000000000-ADUT3_1_1113_11285_15948 T----AC---GT-AG-GTG--------GCG-A-G------C--G--T---T--GT-C-CGG-AA------TT-A--C-T--GG-GT-------GT--A-----AA-GG-GT-GC-------G-TA-G-G-C-G------------G--C-TA-A-T-T-----
------AA----G-T-T-A--------G-T-T--G--TG--A-AA-AC--C-T-TA-G-G-----CT-C-AA------C-T-T-A-A-G-G--A-G----T-G--C-A---A--C-------T--AA-A-A---C-------T--G-G--GT--A-G-C----T-A-G-A-G-T--G---C-AG-----GA-G-A--------G-G-A-T---GA-C------GG--A--ATT----C-
C-C-G-GA--GT-A-G-CG-GT--G---A--A-A---TG-C-GT-AG--AT-A-TC--G-G--G-A-G-G-A-AC-A-CC---AG--T--G--GC-GAA-G--G-C--G-------G--T-C-A-T---CTG--G--AC-T-G--------------------T-A---A-C-T--GA--CG-C-T-G--A-GG--C-A-CG-A--AA-G-C---G-TG--GG-
G--AG-C-A-AA--------CA--GG Bacteria(100);unclassified(100);unclassified(100);unclassified(100);unclassified(100);unclassified(100);unclassified(100);unclassified(100);unclassified(100);

Otu1778 0 0 0 0 0 0 0 1 0 0 0 0 0 0 0 0 0 0 0 0 0 0 0 0 0 0 0 0
0 4 1 0 0 0 0 1 0 0 6 0 0 0 0 1 0 0 0 0 5 0 0 3 0 0 0 0 0
0 0 0 0 0 0 0 0 0 0 0 0 0 0 9 0 0 0 0 0 0 0 0 0 0 8 0 7 0
0 2 0 0 2 0 0 0 0 0 0 0 0 0 0 0 0 0 0 2 0 0 0 0 1 0 0
M02255_76_000000000-ADUNE_1_1105_9936_6260 T----AA---CT-AC-GTG--------CC--A-G------C--A--G---C--CG-C-GGT-AA------TA-C--G-T--AG---------GT--C-----CC-GA-GC-G--------T-TG-T-C-C-G------------G--T-TA-G-A-T-----------AA----
G-T-C-T--------G-A-A--G--TT--A-AA-GG--C-T-GT-G-G-----CT-T-AA------C-C-A-T-A-G-T--A------T-G--C-T---T--T-------G--GA-A-A---C-------T--G-T--TT--A-A-C----T-T-G-A-G-T--G---C-AG-----AA-G-G--------G-G-A-G---AG-T------GG--A--ATT----C-C-A-T-GT--
GT-A-G-CG-GT--G---A--A-A---TG-C-GT-AG--AT-A-TA--T-G--G-A-G-G-A-AC-A-CC---GG--T--G--GC-GAA-A--G-C--G-------G--C-T-C-T---CTG--G--TC-T-G--------------------T-A---A-C-T--GA--CG-C-T-G--A-GG--C-T-CG-A--AA-G-C---G-TG--GG-G--AG-C-A-
AA--------CA--GG Bacteria(100);Firmicutes(100);Bacilli(100);Lactobacillales(100);Streptococcaceae(100);Streptococcus(100);unclassified(100);unclassified(100);unclassified(100);

Otu1779 0 0 0 20 0 0 0 0 0 0 0 0 0 0 0 0 0 0 0 0 0 0 0 0 0 0 0 0
0 0 0 0 0 0 0 0 0 0 0 0 0 0 0 0 0 0 0 0 0 0 0 0 0 0 0 0 0
0 0 0 0 0 0 0 0 0 0 0 0 0 0 0 0 0 0 0 0 0 0 0 0 0 0 0 0 0
0 0 0 0 0 0 0 0 0 0 0 0 0 0 0 0 0 0 0 0 0 0 0 0 0 0 0
M02255_77_000000000-ADUT3_1_2115_22179_14678 T----AC---GT-AG-GGT--------GCA-A-G------C--G--T---T--GT-C-CGG-AA------TT-A--T-T--GG-GC-------GT--A-----AA-GG-GC-TC-------G-TA-G-G-C-G------------G--T-CC-A-T-C------
-----GC----G-T-C-G--------G-G-A--G--TG--A-AA-AG--C-T-GG-G-G-----CT-T-AA------C-C-C-C-A-G-T--C-C----T-G--C-T---T--C-------C--GA-T-A---C-------G--G-G--TG--G-A-C----T-A-G-A-G-G--T---A-GG-----TA-G-G--------G-G-A-G---AT-C------GG--A--ATT----
C-C-T-G-GT--GT-A-G-CG-GT--G---A--A-A---TG-C-GC-AG--AT-A-TC--A-G--G-A-G-G-A-AC-A-CC---GG--T--G--GC-GAA-G--G-C--G-------G--A-T-C-T---CTG--G--GC-C-T--------------------T-A---C-C-T--GA--CG-C-T-G--A-GG--A-G-CG-A--AA-G-C---A-TG--
GG-G--AG-C-A-AA--------CA--GG Bacteria(100);Actinobacteria(100);Actinobacteria(100);Actinobacteridae(100);Actinomycetales(100);unclassified(100);unclassified(100);unclassified(100);unclassified(100);

Otu1780 0 0 0 7 0 0 0 0 0 2 0 0 0 0 0 0 0 0 0 0 0 0 0 0 0 0 0 0
0 0 0 0 0 0 0 0 0 0 0 0 0 0 0 3 0 0 1 0 3 0 0 0 0 0 0 0 0
0 0 0 0 0 0 2 2 0 0 0 0 0 0 0 0 2 0 0 0 0 0 0 0 0 0 0 15 0
10 1 6 0 9 0 0 0 0 0 0 0 0 0 0 0 0 0 0 0 0 0 0 0 0 0 0
M02255_76_000000000-ADUNE_1_2106_24636_9457 T----AC---GG-GG-GGG--------GCA-A-G------T--G--T---T--CT-T-CGG-AA------TG-A--C-T--AG-GC-------GT--A-----AA-GG-GC-A--------G-T--------G------------A--A-TC-G--------------G-----G-T-T-G--------C-A-A--G--TT--C-AA-----G-T-CG-C-C-----AA-A-AA------C-C-G-G-C-G-G--A-A----T-G--C-T---C--T-------C--GA-A-A---C-------C--A-A--TT--C-A-C----T-T-G-A-G-T--G---A-GA-----CA-G-A--------G-G-A-G---AG-T------TT--C--ATT----T-C-G-T-GT--GT-A-G-GG-GT--T---A--A-A---TC-C-GT-AG--AT-C-TA--C-G--A-A-G-G-A-AG-G-CC---AA--A--A--GC-GAA-G--G-C--A-------G--C-T-C-T---TTG--G--GT-C-C--------------------C-T---A-C-T--GA--CG-C-T-G--A-GG--T-G-CG-A--AA-G-C---A-TG--GG-G--AG-C-A-AA--------CT--GG Bacteria(100);unclassified(100);unclassified(100);unclassified(100);unclassified(100);unclassified(100);unclassified(100);unclassified(100);unclassified(100);

Otu1781 179 0 0 0 0 0 0 0 0 0 0 0 0 0 0 0 0 0 0 0 0 0 0 0 0 0 0 0
0 0 0 0 0 0 0 0 0 0 0 0 0 0 0 0 0 0 0 0 0 39 0 0 0 0 0 0 0
0 0 0 0 0 0 0 0 0 0 0 0 0 0 0 0 0 0 0 0 0 0 0 0 0 0 0 0 0
0 0 0 0 0 0 0 0 0 0 0 0 0 0 0 0 0 0 0 0 0 0 0 0 0 0 0
M02255_76_000000000-ADUNE_1_1103_6563_19350 .....AC----T-AC-GT---------GCC-A-G------C--A--G---C--CG-C-GGT-AA------TA-C--G-T--AG-GT-------GG--C-----AA-GC----G--------T-TA-T-C-C-G-------------G--T-TT-T-C-T-----------AA----G-T-C-T--------G-A-T--G--TG--A-AA-GC--C-T-TC-G-G-----CT-T-AA------C-C-G-A-A-G-A--A-G----T-G--C-A---T--C-------G--GA-A-A-A---C-------T--G-G--AT--G-A-C----T-T-G-A-G-T--G---C-AG-----AA-G-A--------G-G-G-C---AG-T------GG--A--ACT----C-C-A-T-GT--GT-A-G-CG-GT--G---G--A-A---TG-C-GT-AG--AT-A-TA--T-G--G-A-A-G-A-AC-A-CC---AG--T--G--GC-GAA-G--G-C--G-------G--C-T-G-C---CTG--G--TC-T-G--------------------T-A---A-C-T--GA--CG-C-T-G--A-GG--C-T-CG-A--AA-G-C---A-TG--GG-T--AG-C-G-AA--------CA--GG Bacteria(100);Firmicutes(100);Bacilli(100);Lactobacillales(100);Lactobacillaceae(100);unclassified(100);unclassified(100);unclassified(100);unclassified(100);

Otu1782 0 0 0 0 0 0 120 8 174 0 0 0 0 0 0 1 0 0 0 0 0 0 0 0 0 0 0 0
0 0 0 0 0 0 0 0 0 0 0 0 0 0 0 0 0 0 0 0 0 0 0 0 0 0 0 0 0
0 0 0 0 0 0 0 0 0 0 0 0 0 0 0 0 0 13 0 0 0 0 0 0 0 0 0 0 0
0 0 0 0 0 0 0 0 0 0 0 0 0 0 0 0 0 0 0 0 0 0 0 0 0 4 0 0
M02255_77_000000000-ADUT3_1_1114_25108_12239 T----AC---GT-AT-GGT--------GCA-A-G------C--G--T---T--AT-C-CGG-AT------TT-A--C-T--GG-GT-------GT--A-----AA-GG-GT-GA-------G-TA-G-G-C-G-------------G--T-CA-T-G-C-----------AA----G-T-C-A--------T-A-T--G--TG--A-AA-TG--T-C-AG-G-G-----CT-T-AA------C-C-T-T-G-G-C--G-C----T-G--C-A---T--A-------A--GA-A-A---C-------T--G-T--AT--G-A-C----T-A-G-A-G-T--G---C-AG-----GA-G-A--------G-G-T-A---AG-C------GG--A--ATT----C-C-T-A-GT--GT-A-G-CG-GT--G---A--A-A---TG-C-GT-AG--AT-A-TT--A-G--G-A-A-G-A-AC-A-CC---GG--T--G--GC-GAA-G--G-C--G-------G--C-T-T-A---CTG--G--AC-T-G--------------------T-T---A-C-T--GA--CG-C-T-G--A-GT--C-A-CG-A--AA-G-C---G-TG--GG-G--AG-C-A-AA--------CA--GG Bacteria(100);Firmicutes(100);Clostridia(100);Clostridiales(100);unclassified(100);unclassified(100);unclassified(100);unclassified(100);unclassified(100);

Otu1783 23 0 0 0 0 0 0 0 0 0 0 0 0 0 0 0 0 0 0 0 0 0 0 0 0 0 0 0
0 0 0 0 0 0 0 0 0 0 0 0 0 0 0 0 0 0 0 0 0 0 0 0 0 0 0 0 0
0 0 0 0 0 0 0 0 0 0 0 0 0 0 0 0 0 0 0 0 0 0 0 0 0 0 0 0 0
0 0 0 0 0 0 0 0 0 0 0 0 0 0 0 0 0 0 0 0 0 0 0 0 0 0 0
M02255_76_000000000-ADUNE_1_1106_17885_9326 T----AA---CT-CT-GTG--------CCA-G-C------A--G--C---C--GC-G-GTA-AT------AC-A--G-A--GG-TC--------C--C-----AA-GC-GT--T-------G-TT-C-G---G-------------A--T--T-G-G-C-----------AA----G-T-T-C--------G-A-T--G--TG--A-AA-GC--C-C-CG-G-G-----CT-T-AA------C-C-C-G-G-G-A--A-T----G-G--C-A---T--T-------G--GA-T-A---C-------T--G-T--TG--A-T-C----T-G-G-A-G-G--G---T-CG-----GA-G-G--------G-G-G-G---AC-T------GG--A--ATT----C-T-T-G-GT--GT-A-G-CA-GT--G---A--A-A---TG-C-GT-AG--AT-A-TC--A-A--G-A-G-G-A-AC-A-CC---AG--T--G--GC-GAA-G--G-C--G-------A--G-T-C-C---CTG--G--AC-G-A--------------------T-A---C-C-T--GA--CA-C-T-G--A-GG--C-A-CG-A--AA-G-C---C-AA--GG-G--AG-C-A-AA--------CA--GG Bacteria(100);unclassified(100);unclassified(100);unclassified(100);unclassified(100);unclassified(100);unclassified(100);unclassified(100);unclassified(100);

Otu1784 0 0 0 25 0 0 0 0 0 0 0 0 0 0 0 0 0 0 0 0 0 0 0 0 0 0 0 0
0 0 0 0 0 0 0 0 0 0 0 0 0 0 0 0 0 0 0 0 0 0 0 0 0 0 0 0 0
0 0 0 0 0 0 0 0 0 0 0 0 0 0 0 0 0 0 0 0 0 0 0 0 0 0 0 0 0
0 0 0 0 0 0 0 0 0 0 0 0 0 0 0 0 0 0 0 0 0 0 0 0 0 0 0
M02255_77_000000000-ADUT3_1_1101_12064_19066 T----AC---GG-AG-GGG--------GCT-A-G------C--G--T---T--GT-C-CGG-AA------TT-A--C-T--GG-GC-------GT--A-----AA-GC-GC-GC-------G-TA-G-G-T-G-------------G--T-CT-G-C-C-----------AA----G-T-C-G--------G-G-G--G--TG--A-AA-GC--C-C-GG-G-G-----CT-C-AA------C-T-C-C-G-G-A--A-T----T-G--C-C---T--C-------C--GA-G-A---C-------T--G-G--CG--G-A-C----T-C-G-A-G-T--T---C-GG-----GA-G-A--------G-G-A-G---AG-C------GG--A--ATT----C-C-C-A-GT--GT-A-G-AG-GT--G---A--A-A---TT-C-GT-AG--AT-A-TT--G-G--G-A-A-G-A-AC-A-CC---GG--T--G--GC-GAA-G--G-C--G-------G--C-T-C-T---CTG--G--CC-C-G--------------------C-A---A-C-T--GA--CG-C-T-G--A-GG--C-G-CG-A--AA-G-C---G-TG--GG-G--AG-C-A-AA--------CA--GG Bacteria(100);Proteobacteria(100);Alphaproteobacteria(100);unclassified(100);unclassified(100);unclassified(100);unclassified(100);unclassified(100);unclassified(100);

Otu1785 0 0 0 0 0 0 0 0 0 0 0 4 0 0 0 0 0 0 0 0 0 0 0 0 0 0 0 42
0 0 0 0 0 0 0 0 0 0 0 0 0 0 0 0 0 0 0 0 0 0 0 0 0 0 0 0 0
0 0 0 0 0 0 0 0 0 0 0 0 0 0 0 0 0 0 0 0 0 0 0 0 0 0 0 0 0
0 0 0 0 0 0 0 0 0 0 0 0 0 0 0 0 0 0 0 0 0 0 0 0 0 0 0 0
M02255_77_000000000-ADUT3_1_1111_26189_19434 T----AC---GG-AG-GGG--------GCT-A-G------C--G--T---T--GT-T-CGG-AA------TT-A--C-T--GG-GC-------GT--A-----AA-GC-GC-GC-------G-TA-G-G-C-G-------------G--C-TT-T-G-T-----
------AA----G-T-C-A--------G-A-G--G--TG--A-AA-GC--C-T-GG-A-G-----CT-C-AA------C-T-C-C-A-G-A--A-C----T-G--C-C---T--T-------T--GA-G-A---C-------T--G-C--AT--C-G-C----T-T-G-A-A-T--C---A-TG-----GA-G-A--------G-G-T-T---AG-T------GG--A--ATT----C-
C-G-A-GT--GT-A-G-AG-GT--G---A--A-A---TT-C-GT-AG--AT-A-TT--C-G--G-A-A-G-A-AC-A-CC---AG--T--G--GC-GAA-G--G-C--G-------A--C-T-A-A---CTG--G--AC-A-T---------------------G-T---A-T-T--GA--CG-C-T-G--A-GG--T-G-CG-A--AA-G-C---G-TG--GG-G-
-AG-C-A-AA--------CA--GG Bacteria(100);Proteobacteria(100);Alphaproteobacteria(100);Sphingomonadales(100);unclassified(100);unclassified(100);unclassified(100);unclassified(100);unclassified(100);

Otu1786 0 0 0 0 0 0 0 0 0 0 0 0 0 4 0 0 0 0 0 0 0 0 0 0 0 0 0 0
0 0 0 0 0 0 0 0 0 0 0 0 0 0 0 0 0 0 0 0 0 0 0 0 0 0 0 0 0
0 0 0 0 0 0 0 0 0 0 0 0 0 0 0 0 0 0 0 0 0 0 0 0 0 0 0 0 0
0 0 0 0 0 0 0 0 0 0 0 0 0 0 0 0 0 0 0 0 0 0 0 0 0 0 0 0
M02255_77_000000000-ADUT3_1_1113_23507_11947 T----AC---GT-AG-GCA--------GCA-A-G------C--G--T---T--GT-T-CGG-AG------TT-A--C-T--GG-GC-------GT--A-----AA-GG-GT-GT-------G-TA-G-G-C-G-------------G--C-CT-T-C-T------
-----AA----G-T-T-T--------G-G-T--G--TG--A-AA-TC--T-C-CC-G-G-----CT-C-AA------C-C-G-G-G-A-G--G-G----T-G--C-G---C--C-------G--AA-G-A---C-------T--G-G--GG--G-G-C----T-A-G-A-G-T--A---T-GG-----GA-T-G--------G-G-A-G---AG-T------AG--A--ATT----
C-C-C-G-GT--GT-A-G-CG-GT--G---A--A-A---TG-C-GT-AG--AT-A-TC--G-G--G-A-G-G-A-AT-A-CC---TG--T--G--GT-GTA-G--A-C--G-------G--C-T-C-T---CTG--G--AC-C-A---------------------T-A---A-C-T--GA--CG-C-T-G--A-GA--C-A-CG-A--AA-G-C---G-TG--GG-
T--AG-C-A-AA--------CA--GG Bacteria(100);Acidobacteria(100);Acidobacteria(100);Acidobacteriales(100);Acidobacteriaceae(100);unclassified(100);unclassified(100);unclassified(100);unclassified(100);

Otu1787 0 0 0 0 0 0 0 109 0 0 0 0 0 0 0 0 0 0 0 0 0 0 0 0 0 0 0 0
0 0 0 0 0 0 0 0 0 0 0 0 0 0 0 0 0 0 0 0 0 0 0 0 0 0 0 0 0
0 0 0 0 0 0 0 0 0 0 0 0 0 0 0 0 0 0 0 0 0 0 3 0 0 0 0 0 0
0 0 0 0 0 0 0 0 0 0 0 0 0 0 0 0 0 0 0 0 0 0 0 0 0 0 0 0
M02255_77_000000000-ADUT3_1_1114_12150_22676 T----AC---GG-AG-GGG--------GCT-A-G------C--G--T---T--AT-T-CGG-AA------TT-A--C-T--GG-GC-------GT--A-----AA-GC-GC-GC-------G-TA-G-G-C-G-------------G--T-TT-T-T-C------
-----AA----G-T-C-A--------G-A-G--G--TG--A-AA-GC--C-C-GG-A-G-----CT-C-AA------C-T-C-C-G-G-A--A-T----T-G--C-C---T--T-------T--GA-A-A---C-------T--G-G--AT--C-A-C----T-A-G-A-A-T--C---A-TG-----GA-G-A--------G-G-T-C---AG-T------GG--A--ATT----C-
C-G-A-GT--GT-A-G-AG-GT--G---A--A-A---TT-C-GT-AG--AT-A-TT--C-G--G-A-A-G-A-AC-A-CC---AG--T--G--GC-GAA-G--G-C--G-------A--C-T-G-A---CTG--G--AC-A-T---------------------G-T---A-T-T--GA--CG-C-T-G--A-GG--T-G-CG-A--AA-G-C---G-TG--GG-
G--AG-C-A-AA--------CA--GG Bacteria(100);Proteobacteria(100);Alphaproteobacteria(100);Sphingomonadales(100);unclassified(100);unclassified(100);unclassified(100);unclassified(100);unclassified(100);

Otu1788 83 0 0 0 0 0 0 0 0 0 0 0 0 0 0 0 0 0 0 0 0 0 0 0 0 0 0 0
0 0 0 0 0 0 0 0 0 0 0 0 0 0 0 0 0 0 0 0 0 0 0 0 0 0 0 0 0
0 0 0 0 0 0 0 0 0 0 0 0 0 0 0 0 0 0 0 0 0 0 0 0 0 0 0 0 0
0 0 0 0 0 0 0 0 0 0 0 0 0 0 0 0 0 0 0 0 0 0 0 0 0 0 0 0
M02255_77_000000000-ADUT3_1_1106_21795_20599 T----AC---GT-AG-GGC--------GCA-A-G------C--G--T---T--GT-C-CGG-AT------TT-A--T-T--GG-GC-------GT--A-----AA-GA-GC-TC-------G-TA-G-G-C-G-------------G--T-TC-G-T-C------
-----GC----G-T-C-T--------G-G-T--G--TG--A-AA-GT--C-C-AT-G-G-----CT-T-AA------C-C-G-T-G-G-A--T-T----G-G--C-G---C--C-------G--GG-T-A---C-------G--G-G--CG--G-G-C----T-G-G-A-G-T--G---C-AG-----TA-G-G--------G-G-A-G---AC-T------GG--A--ATT----
C-T-C-G-GT--GT-A-A-CG-GT--G---G--A-A---TG-T-GT-AG--AT-A-TC--G-G--G-A-A-G-A-AC-A-CC---AA--T--G--GC-GAA-G--G-C--A-------G--G-T-C-T---CTG--G--GC-T-G---------------------T-T---A-C-T--GA--CG-C-T-G--A-GG--A-G-CG-A--AA-G-C---G-TG--GG-
G--AG-C-G-AA--------CA--GG Bacteria(100);Actinobacteria(100);Actinobacteria(100);Actinobacteridae(100);Bifidobacteriales(100);Bifidobacteriaceae(100);unclassified(100);unclassified(100);unclassified(100);

Otu1789 0 0 0 2 0 0 0 1 0 0 0 0 0 0 0 0 0 0 0 0 0 0 0 0 0 0 0 0
0 1 0 0 1 0 0 2 1 0 9 0 0 0 6 9 4 2 0 1 58 0 1 3 1 0 0 0 0
0 0 0 0 0 0 0 0 0 0 0 0 0 1 3 0 1 1 0 0 0 0 0 0 0 0 5 0 0
0 1 1 1 0 0 0 0 0 0 0 3 0 1 0 0 2 0 0 1 0 4 0 0 0 0 0 0
M02255_77_000000000-ADUT3_1_1102_13368_11142 T----AC---GT-AG-GTG--------GCA-A-G------C--G--T---T--AT-C-CGG-AA------TT-A--T-T--GG-GC-------GT--A-----AA-GC-GC-GC-------G-TA-G-G-C-G-------------G--T-TT-T-T-T------
-----AA----G-T-C-T--------G-A-T--G--TG--A-AA-GC--C-C-AC-G-G-----CT-C-AA------C-C-G-T-G-G-A--G------G-GT-C-A---T--T-------G--GA-A-A---C-------T--G-G--AA--A-A-C----T-T-G-A-G-T--G---C-AG-----AA-G-A--------G-G-A-A---AG-T------GG--A--ATT----C-
C-A-T-GT--GT-A-G-CG-GT--G---A--A-A---TG-C-GC-AG--AG-A-TA--T-G--G-A-G-G-A-AC-A-CC---AG--T--G--GC-GAA-G--G-C--G-------A--C-T-T-T---CTG--G--TC-T-G---------------------T-A---A-C-T--GA--CG-C-T-G--A-TG--T-G-CG-A--AA-G-C---G-TG--GG-G-
-AT-C-A-AA--------CA--GG Bacteria(100);Firmicutes(100);Bacilli(100);Bacillales(100);Staphylococcaceae(100);Staphylococcus(100);unclassified(100);unclassified(100);unclassified(100);

Otu1790 0 0 0 0 0 0 0 0 0 0 0 1 0 0 0 3 0 0 0 0 0 0 0 0 0 0 0 0
0 0 0 0 0 0 0 0 0 0 0 0 0 0 0 0 0 0 0 0 0 0 0 0 0 0 0 0
0 0 0 0 0 0 0 0 0 0 0 0 0 0 0 0 0 0 0 0 0 0 0 0 0 0 0 0
0 0 0 0 0 0 0 0 0 0 0 0 0 0 0 0 0 0 0 0 0 0 0 0 0 0 0
M02255_77_000000000-ADUT3_1_1116_12892_16663 T----AA---CC-AG-AAA--------GCC-A-C------G--G--C---T--AA-C-TAC-GT------GC-C--A-G--CA-GC-------CG--C-----GG-TA-AT-AC-------G-TA-G-G-T-G-------------G--C-CA-A-T-T------
-----AA----G-T-C-A--------A-A-T--G--TG--A-AA-TC--C-C-CG-A-G-----CT-T-AA------C-T-T-G-G-G-A--A-T----T-G--C-A---T--T-------C--GA-T-A---C-------T--G-G--TT--G-G-C----T-A-G-A-G-T--A---T-GG-----GA-G-A--------G-G-A-T---GG-T------AG--A--ATT----C-
C-A-G-GT--GT-A-G-CG-GT--G---A--A-A---TG-C-GT-AG--AG-A-TC--T-G--G-A-G-G-A-AT-A-CC---GA--T--G--GC-GAA-G--G-C--A-------G--C-C-A-T---CTG--G--CC-T-A--------------------A-T---A-C-T--GA--CA-C-T-G--A-GG--T-A-CG-A--AA-G-C---A-TG--GG-G-
-AG-C-A-AA--------CA--GG Bacteria(100);Proteobacteria(100);Gammaproteobacteria(100);Pseudomonadales(100);Moraxellaceae(100);Acinetobacter(100);unclassified(100);unclassified(100);unclassified(100);

Otu1791 0 0 0 1 0 0 0 0 0 0 0 0 0 0 0 0 0 0 0 0 0 0 0 0 0 0 0 0
0 0 0 0 0 0 0 0 0 0 0 0 0 0 2 0 0 0 0 0 0 16 0 0 0 0 0 0 0
0 0 0 0 0 0 0 0 0 0 0 0 0 0 0 0 0 0 0 0 0 0 0 0 0 0 0 0
0 0 0 1 0 0 0 0 0 0 0 0 0 0 0 0 0 0 0 0 0 0 0 0 0 0 0
M02255_76_000000000-ADUNE_1_2101_6920_8305 A----AA---GC-CA-CGG--------CTA-A-C------T--A--C---G--TG-C-CAG-CA------GC-C--G-C--GG-TA-------AT--A-----CG-TA-GG-TG-------G-CA-A-G-C-G-------------T--TATC-C-G-G-----------
AA----G-T-C-T--------G-A-T--G--TG--A-AA-GC--C-C-AC-G-G-----CT-C-AA------C-C-G-T-G-G-A--G------G-GT-C-A---T--T-------G--GA-A-A---C-------T--G-G--GA--A-A-C----T-T-G-A-G-T--A---C-AG-----AA-G-A--------G-G-A-A---AG-T------GG--A--ATT----C-C-
A-T-GT--GT-A-G-CG-GT--G---A--A-A---TG-C-GC-AG--AG-A-TA--T-G--G-A-G-G-A-AC-A-CC---AG--T--G--GC-GAA-G--G-C--G-------A--C-T-T-T---CTG--G--TC-T-G--------------------T-A---A-C-T--GA--CG-C-T-G--A-TG--T-G-CG-A--AA-G-C---G-TG--GG-G--
AT-C-A-AA--------CA--GG Bacteria(100);Firmicutes(100);Bacilli(100);Bacillales(100);unclassified(100);unclassified(100);unclassified(100);unclassified(100);unclassified(100);

Otu1792 0 0 0 0 0 0 20 1 0 0 0 0 0 0 0 0 0 0 0 0 0 0 0 0 0 0 0 0
0 0 0 0 0 0 0 0 0 0 0 0 0 0 0 0 0 0 0 0 0 0 0 0 0 0 0 0
0 0 0 0 0 0 0 0 0 0 0 0 0 0 0 0 0 0 0 0 0 0 0 0 0 0 0 0
0 0 0 0 0 0 0 0 0 0 0 0 0 0 0 0 0 0 0 0 0 0 30 0 0 0 0
M02255_77_000000000-ADUT3_1_2101_19775_16466 G----AC---GA-AG-GGG--------GCA-A-G------C--G--T---T--GT-T-CGG-AA------TT-A--C-T--GG-GC-------GT--A-----AA-GG-GC-GC-------G-TA-G-G-C-G-------------G--T-TC-T-T-T----
-------AA----G-T-C-A--------G-G-C--G--TG--A-AA-GC--C-C-TG-G-G-----CT-C-AA------C-C-T-G-G-G-A--G-T----G-G--C-G---C--T-------T--GA-T-A---C-------T--G-G--GG--G-A-C----T-A-G-A-G-A--G---T-GG-----GA-G-A--------G-G-T-T---GG-T------GG--A--ATT---
-C-C-C-A-GT--GT-A-G-AG-GT--G---A--A-A---TT-C-GT-AG--AT-A-TT--G-G--G-A-A-G-A-AC-A-CC---GG--T--G--GC-GAA-G--G-C--G-------G--C-C-A-A---CTG--G--AC-C-A--------------------T-T---T-C-T--GA--CG-C-T-G--A-GG--C-G-CG-A--AA-G-C---G-TG--
GG-G--AG-C-A-AA--------CA--GG Bacteria(100);Proteobacteria(100);Alphaproteobacteria(100);Rhodospirillales(100);unclassified(100);unclassified(100);unclassified(100);unclassified(100);unclassified(100);

Otu1793 0 0 0 2 0 0 0 1 0 17 0 7 0 0 0 0 0 1 0 0 0 1 0 0 0 0 0 0
17 0 0 0 0 0 0 0 0 0 0 0 0 0 0 0 0 0 0 0 0 0 0 0 0 0 0 0
0 0 0 0 0 0 0 0 0 0 0 0 0 0 0 0 0 0 0 2 0 0 0 1 0 0 0 0
0 0 0 0 0 0 0 0 0 0 0 0 0 0 0 0 0 0 0 1 0 11 0 33 0 0 0
M02255_77_000000000-ADUT3_1_1110_20827_5399 C----TA---AC-TC-TGT--------GCC-A-G------C-----A---G--CC-G-CGG-TA------AT-A--C-A--GA-GG-------GTG-C-----AA-GC-GT-TA-------A-TC-G-GAATT-------------A--C-TT-G-A-T-----------AA-
---G-T-C-A--------G-A-T--G--TG--A-AA-TC--C-C-CG-G-G-----CT-T-AA------C-C-T-G-G-G-A--A-C----T-G--C-A---T--C-------T--GA-T-A---C-------T--G-T--TA--G-G-C----T-A-G-A-A-T--A---G-GT-----GA-G-A--------G-G-A-A---GG-T------AG--A--ATT----C-C-A-G-
GT--GT-A-G-CG-GT--G---A--A-A---TG-C-GT-AG--AG-A-TC--T-G--G-A-G-G-A-AT-A-CC---GA--T--G--GC-GAA-G--G-C--A-------G--C-C-T-T---CTG--G--CA-T-C--------------------A-T---A-T-T--GA--CA-C-T-G--A-GG--T-T-CG-A--AA-G-C---G-TG--GG-T--AG-C-
A-AA--------CA--GG Bacteria(100);Proteobacteria(100);Gammaproteobacteria(100);Pseudomonadales(100);Moraxellaceae(100);Psychrobacter(100);unclassified(100);unclassified(100);unclassified(100);

Otu1794 42 0 0 0 0 0 0 0 0 0 0 0 0 0 0 0 0 0 0 0 0 0 0 0 0 0 0 0
0 0 0 0 0 0 0 0 0 0 0 0 0 0 0 0 0 0 0 0 0 0 0 0 0 0 0 0
0 0 0 0 0 0 0 0 0 0 0 0 0 0 0 0 0 0 0 0 0 0 0 0 0 0 0 0
0 0 0 0 0 0 0 0 0 0 0 0 0 0 0 0 0 0 0 0 0 0 0 0 0 0 0
M02255_76_000000000-ADUNE_1_1101_11440_21710 C----GG---CT-AA-CTC--------CGT-G-C------C--A--G---C--AG-C-CGC-GG------TA-A--T-A--CG-GA-------GG--A-----TC-CA-AG-CG-------T-TA-T-C-C-G-------------GA-T-TT-A-T-T------
-----AA----G-T-C-A--------G-A-G--G--TG--A-AA-GG--C-G-GT-A-G-----CT-T-AA------C-T-A-T-C-G-G--A-G----T-G--C-C---T--T-------T--GA-T-A---C-------T--G-A--TG--A-G-C----T-T-G-A-A-T--G---C-AG-----CT-G-A--------G-G-T-A---GG-C------GG--A--ATG----T-
G-A-C-AA--GT-A-G-CG-GT--G---A--A-A---TG-C-AT-AG--AT-A-TG--T-C--A-C-A-G-A-AC-A-CC---GA--T--T--GC-GAA-G--G-C--A-------G--C-T-T-A---CTA--A--AG-T-G--------------------T-G---A-T-T--GA--CG-C-T-G--A-GG--C-A-CG-A--AA-G-C---G-TG--GG-G-
-AT-C-A-AA--------CA--GG Bacteria(100);Bacteroidetes(100);Sphingobacteria(100);Sphingobacteriales(100);Sphingobacteriaceae(100);unclassified(100);unclassified(100);unclassified(100);unclassified(100);

Otu1795 84 0 0 0 0 0 0 0 0 0 0 0 0 0 0 0 0 0 0 0 0 0 0 0 0 0 0 0
0 0 0 0 0 0 0 0 0 0 0 0 0 0 0 0 0 0 0 0 0 0 0 0 0 0 0 0
0 0 0 0 0 0 0 0 0 0 0 0 0 0 0 0 0 0 0 0 0 0 0 0 0 0 0 0
0 0 0 0 0 0 0 0 0 0 0 0 0 0 0 0 0 0 0 0 0 0 0 0 0 0 0
M02255_77_000000000-ADUT3_1_1111_13590_10367 T----AC---AG-AG-GGC--------CCA-A-G------C--G--T---T--AA-T-CGG-AA------TC-A--C-T--GG-GC-------TT--A-----AA-GG-GT-GC-------G-CA-G-G-C-G------------G--G-CC-T-G-T-----
------AA----G-T-G-C--------T-T-T--G--TG--A-AA-TC--C-C-AC-T-G-----CT-C-AA------C-G-G-T-G-G-A--A-T----T-G--C-T---C--G-------G--CA-A-A---C-------T--A-C--AG--G-T-C----T-T-G-A-G-C--C---C-GA-----CA-G-G--------G-G-T-C---AT-C------GG--A--ACG----C-
T-A-G-GT--GG-A-G-CG-GT--G---A--A-A---TG-C-GT-AG--AT-A-TC--T-A--G-C-G-G-A-AC-G-CC---AA--A--G--GT-GAA-G--A-C--A-------G--A-T-G-A---CTG--G--GT-C-G--------------------G-C---G-C-T--GA--CG-C-T-C--A-GG--C-A-CG-A--AA-G-C---G-TG--GG-
G--AG-C-A-AA--------CG--GG Bacteria(100);Planctomycetes(100);Phycisphaerae(100);Phycisphaerales(100);Phycisphaeraceae(100);Phycisphaera(100);unclassified(100);unclassified(100);unclassified(100);

Otu1796 139 0 0 0 0 0 0 0 0 0 0 0 0 0 0 0 0 0 0 0 0 0 0 0 0 0 0 0
1 0 0 0 0 0 0 0 0 0 0 0 0 0 0 0 0 0 0 0 0 0 0 0 0 0 0 0
0 0 0 0 0 0 0 0 0 0 0 0 0 0 0 0 0 0 0 0 0 0 0 0 0 0 0 0
0 0 0 0 0 0 0 0 0 0 0 0 0 0 0 0 0 0 0 0 0 0 0 0 0 0 0
M02255_77_000000000-ADUT3_1_1101_21442_12746 T----AC---GA-AG-GGG--------GCT-A-G------C--G--T---T--GC-T-CGG-AA------TC-A--C-T--GG-GC-------GT--A-----AA-GG-GC-GC-------G-TA-G-G-C-G------------G--A-CT-C-T-T-----
------AA----G-T-C-A--------G-A-G--G--TG--A-AA-GC--C-C-AA-G-G-----CT-C-AA------C-C-T-T-G-G-A--A-T----T-G--C-C---T--T-------T--GA-T-A---C-------T--G-G--GA--G-T-C----T-A-G-A-G-A--C---C-GG-----AA-G-A--------G-G-T-A---AG-T------GG--A--ACT----
G-C-G-A-GT--GT-A-G-AG-GT--G---A--A-A---TT-C-GT-AG--AT-A-TT--C-G--C-A-A-G-A-AC-A-CC---AG--T--G--GC-GAA-G--G-C--G-------G--C-T-T-A---CTG--G--TC-C-G--------------------G-T---T-C-T--GA--CG-C-T-G--A-GG--C-G-CG-A--AA-G-C---G-TG--GG-
G--AG-C-A-AA--------CA--GG Bacteria(100);Proteobacteria(100);Alphaproteobacteria(100);Rhizobiales(100);unclassified(100);unclassified(100);unclassified(100);unclassified(100);unclassified(100);

Otu1797 61 236 281 0 0 13 171 39 457 0 0 0 0 0 0 0 0 1 0 0 0 1 0 0 0 0 0 0
1 0 6 44 0 0 0 0 0 0 0 281 13 0 0 0 0 0 0 0 0 0 0 0 0 0 0 0
0 0 0 0 0 0 0 0 0 0 0 0 1 0 5 3 0 20 0 0 0 0 0 0 0 111 0 0 0
0 30 0 0 0 17 11 5 0 0 0 53 0 0 0 0 23 0 0 0 0 0 26 4 125 0 0
M02255_76_000000000-ADUNE_1_1105_12169_5346 T----CG---GC-TA-ACT--------CCG-T-G------C--C--A---G--CA-G-CCG-CG------GT-A--A-T--AC-GG-------AG--G-----AT-CC-GA-GC-------GTTA-T-C-C-G------------G--A-TG-TAT-T-----------AA-
---G-T-C-A--------G-T-T--G--TG--A-AA-GT--T-T-GC-G-G-----CT-C-AA------C-C-G-T-A-A-A--A-T----T-G--C-A---G--T-------T--GA-T-A---C-------T--G-G--AT--A-T-C----T-T-G-A-G-T--G---C-AG-----TT-G-A--------G-G-C-A---GG-C------GG--A--ATT----C-G-T-G-
GT--GT-A-G-CG-GT--G---A--A-A---TG-C-TT-AG--AT-A-TC--A-C--G-A-A-G-A-AC-T-CC---GA--T--T--GC-GAA-G--G-C--A-------G--C-C-T-G---CTA--A--GC-T-G--------------------C-A---A-C-T--GA--CA-T-T-G--A-GG--C-T-CG-A--AA-G-T---G-TG--GG-T--AT-C-
A-AA--------CA--GG Bacteria(100);Bacteroidetes(100);Bacteroidia(100);Bacteroidales(100);Bacteroidaceae(100);Bacteroides(100);unclassified(100);unclassified(100);unclassified(100);

Otu1798 0 0 0 0 0 0 0 0 0 0 0 0 0 0 0 0 0 0 0 0 0 0 0 0 0 0 0 0
0 0 0 0 0 0 0 0 0 0 0 0 0 0 0 0 0 0 0 0 0 0 0 0 0 0 0 0
0 0 0 0 0 0 0 0 0 0 0 0 0 0 0 0 0 0 0 0 0 3 0 0 0 0 0 0 0
0 0 0 0 0 11 0 0 0 0 0 0 0 0 0 0 0 0 0 0 0 0 0 0 0 0 0
M02255_77_000000000-ADUT3_1_1107_9933_15015 T----AC---GT-AG-GGC--------GCA-A-G------C--G--T---T--GT-C-CGG-AA------TT-A--T-T--GG-GC-------GT--A-----AA-GA-GC-TC-------G-TA-G-G-C-G------------G--T-CT-G-T-C-----------GC-
---G-T-C-T--------G-G-T--G--TG--A-AA-TC--C-C-AT-G-G-----CT-C-AA------C-T-G-T-G-G-G--C-T----T-G--C-A---T--C-------G--GG-T-A---C-------G--G-G--CA--G-A-C----T-A-G-A-G-T--G---C-TG-----TA-G-G--------G-G-A-G---AC-T------GG--A--ATT----C-C-T-G-
GT--GT-A-G-CG-GT--G---G--A-A---TG-C-GC-AG--AT-A-TC--A-G--G-A-G-G-A-AC-A-CC---GA--T--G--GC-GAA-G--G-C--A-------G--G-T-C-T---CTG--G--GC-A-G--------------------T-T---A-C-T--GA--CG-C-T-G--A-GG--A-G-CG-A--AA-G-C---A-TG--GG-G--AG-
C-G-AA--------CA--GG Bacteria(100);Actinobacteria(100);Actinobacteria(100);Actinobacteridae(100);Actinomycetales(100);Micrococcineae(100);unclassified(100);unclassified(100);unclassified(100);

Otu1799 0 0 0 0 0 0 0 26 0 0 0 0 0 0 0 0 0 0 0 0 0 0 0 0 0 0 0 0
0 0 0 0 0 0 0 0 6 0 0 0 0 0 0 0 0 0 0 0 0 0 0 0 0 0 0 0
0 0 0 0 0 0 0 0 0 0 0 0 0 0 0 0 0 0 0 0 0 0 0 0 0 0 0 0
0 0 0 0 0 0 0 0 0 0 0 0 0 0 0 0 0 0 0 0 0 0 0 0 0 0 0
M02255_77_000000000-ADUT3_1_1116_23536_15213 T----AA---GC-AC-CGG--------CTA-A-C------T--A--C---G--TG-C-CAG-CA------GC-C--G-C--GG-TA-------AT--A-----CG-TA-GG-GT-------G-CA-A-G-C-G------------T--TAAT-C-G-G-----
------AA----T-TAC-T--------G-T-T--G--TG--A-AA-TC--C-C-CG-G-G-----CT-T-AA------C-C-T-G-G-G-A--A-T----G-G--C-A---A--T-------G--GA-G-A---C-------T--G-C--AA--G-G-C----T-A-G-A-G-T--T---T-GG-----CA-G-A--------G-G-G-G---GG-T------AG--A--ATT----
C-C-A-C-GT--GT-A-G-CA-GT--G---A--A-A---TG-C-GT-AG--AG-A-TG--T-G--G-A-G-G-A-AC-A-CC---GA--T--G--GC-GAA-G--G-C--A-------G--C-C-C-C---CTG--G--GT-C-A--------------------A-G---A-C-T--GA--CG-C-T-C--A-TG--C-A-CG-A--AA-G-C---G-TG--
GG-G--AG-C-A-AA--------CA--GG Bacteria(100);Proteobacteria(100);Betaproteobacteria(100);Burkholderiales(100);unclassified(100);unclassified(100);unclassified(100);unclassified(100);unclassified(100);

Otu1800 0 0 0 0 0 0 0 0 0 0 0 16 0 0 0 0 0 0 0 0 0 0 0 0 0 0 0 0
0 0 0 0 0 0 0 0 0 0 0 0 0 0 0 0 0 0 0 0 0 0 0 0 0 0 0 0 0
0 0 0 0 0 0 0 0 0 0 0 0 0 0 0 0 0 0 0 0 0 0 0 0 0 0 0 0 0
0 0 0 0 0 0 0 0 0 0 0 0 0 0 0 0 0 0 0 0 0 0 0 0 0 0 0
M02255_77_000000000-ADUT3_1_2109_21268_18833 T----AC---GG-AG-GGG--------GTT-A-G------C--G--T---T--GT-T-CGG-AA------TT-A--C-T--GG-GC-------GT--A-----AA-GC-GC-GC-------G-TA-G-G-C-G------------G--A-TT-A-G-A----
-------AA----G-T-A-T--------G-G-G--G--TG--A-AA-TC--C-C-AG-G-G-----CT-C-AA------C-C-C-T-G-G-A--A-C----T-G--C-C---T--C-------A--TA-A-A---C-------T--A-C--TA--G-T-C----T-A-G-A-G-T--T---C-GA-----GA-G-A--------G-G-T-G---AG-T------GG--A--ATT----
G-T-G-A-GT--GT-A-G-AG-GT--G---A--A-A---TT-C-GT-AG--AT-A-TT--C-A--C-A-G-G-A-AC-A-CC---AG--T--G--GC-GAA-G--G-C--G-------G--C-T-C-A---CTG--G--CT-C-G--------------------A-T---A-C-T--GA--CG-C-T-G--A-GG--T-G-CG-A--AA-G-T---G-TG--GG-
G--AG-C-A-AA--------CA--GG Bacteria(100);Proteobacteria(100);Alphaproteobacteria(100);Rhodobacterales(100);Rhodobacteraceae(100);Roseobacter_clade(100);unclassified(100);unclassified(100);unclassified(100);

Otu1801 0 0 0 0 0 0 14 15 0 18 0 0 0 0 0 0 0 0 0 0 0 0 0 0 0 0 0 0
0 0 0 0 0 0 0 0 0 0 0 0 0 0 0 0 0 0 0 0 0 0 0 0 0 0 0 0 0
0 0 0 0 0 0 0 0 0 0 0 0 0 0 0 0 0 0 0 0 0 0 0 0 0 0 0 0 0
0 0 0 0 0 0 0 0 0 0 0 0 0 0 0 0 0 0 0 0 0 0 6 0 0 0 0
M02255_76_000000000-ADUNE_1_1117_22958_19455 C----TA---AC-TC-CGT--------GCC-A-G------C-----A---G--CC-G-CGG-TA------AT-A--C-G--G--AG-------GG--T-----GC-AA-GC--G-------T-TA-T-T-C-G------------G--C-AA-T-C-T---------
--AA----G-T-C-A--------G-A-T--G--TG--A-AA-GC--C-C-CG-G-G-----CT-T-AA------C-C-T-G-G-G-A--A-C----T-G--C-A---T--T-------T--GA-A-A---C-------T--G-G--GT--G-G-C----T-A-G-A-G-T--A---T-GA-----AA-G-A--------G-G-A-A---AG-C------GG--A--ATT----T-C-
C-A-GT--GT-A-G-CA-GT--G---A--A-A---TG-C-GT-AG--AT-A-TT--G-G--A-A-G-G-A-AC-A-CC---GA--T--G--GC-GAA-G--G-C--A-------G--C-T-T-T---CTG--G--GT-C-G--------------------A-T---A-C-T--GA--CG-C-T-C--A-TG--T-G-CG-A--AA-G-C---G-TG--GG-G--
AG-C-A-AA--------CA--GG Bacteria(100);Proteobacteria(100);Gammaproteobacteria(100);Cardiobacteriales(100);Cardiobacteriaceae(100);Cardiobacterium(100);unclassified(100);unclassified(100);unclassified(100);

Otu1802 0 0 0 0 0 0 41 4 121 0 0 0 0 0 0 0 0 0 0 0 0 0 0 0 0 0 0 0
0 0 0 97 0 0 0 0 0 0 0 0 0 0 0 0 0 0 0 0 0 0 0 0 0 0 0 0 0
0 0 0 0 0 0 0 0 0 0 0 0 0 0 0 0 0 0 0 0 0 0 0 0 0 0 0 0 0
0 0 0 0 0 0 0 0 0 0 0 0 0 0 1 0 0 0 0 0 0 0 0 0 0 0 0
M02255_77_000000000-ADUT3_1_2114_15993_10567 T----AC---GT-AG-GGG--------GCA-A-G------C--G--T---T--GT-C-CGG-AA------TT-A--T-T--GG-GC-------GT--A-----AA-GA-GT-AC-------G-TA-G-G-C-G------------G--T-TT-G-C-T-----
------AA----G-C-G-C--------A-A-G--G--TG--A-AA-GG--C-A-GT-G-G-----CT-T-AA------C-C-A-T-T-G-T--A------A-G--C-C---T--T-------G--CG-A-A---C-------T--G-A--CA--G-A-C----T-T-G-A-G-T--G---C-AG-----GA-G-A--------G-G-A-A---AG-C------GG--A--ATT----
C-C-T-A-GT--GT-A-G-CG-GT--G---A--A-A---TG-C-GT-AG--AT-A-TT--A-G--G-A-G-G-A-AC-A-CC---GG--T--G--GC-GAA-G--G-C--G-------G--C-T-T-T---CTG--G--AC-T-G--------------------T-A---A-C-T--GA--CG-C-T-G--A-GG--T-A-CG-A--AA-G-C---G-TG--GG-
G--AG-C-A-AA--------CA--GG Bacteria(100);Firmicutes(100);Clostridia(100);Clostridiales(100);unclassified(100);unclassified(100);unclassified(100);unclassified(100);unclassified(100);

Otu1803 0 0 0 0 0 0 9 0 0 0 0 0 0 0 0 0 0 0 0 0 0 0 0 0 0 0 0 0
0 0 0 0 0 0 0 0 0 0 0 25 0 0 0 0 0 0 0 0 0 0 0 0 0 0 0 0 0
0 0 0 0 0 0 0 0 0 0 0 0 0 0 0 0 0 0 0 0 0 0 0 0 0 0 0 0 0
0 0 0 0 0 0 0 0 0 0 0 0 0 0 0 0 0 0 0 0 0 0 0 0 0 0 0
M02255_77_000000000-ADUT3_1_1116_20761_16433 T----AC---GT-AG-GGA--------GCA-A-G------C--G--T---T--GT-C-CGG-AT------TT-A--C-T--GG-GT-------GT--A-----AA-GG-GT-GC-------G-TA-G-G-C-G------------G--C-TA-G-G-C----
-------AA----G-T-C-A--------G-A-C--G--TG--A-AA-AC--C-A-TG-G-G-----CT-C-AA------C-C-T-G-T-G-G--A-C----T-G--C-G---T--T-------T--GA-A-A---C-------T--G-T--TT--A-G-C----T-T-G-A-G-T--G---A-AG-----TA-G-A--------G-G-T-A---GG-C------GG--A--ATT----
C-C-C-G-GT--GT-A-G-CG-GT--G---A--A-A---TG-C-GT-AG--AG-A-TC--G-G--G-A-G-G-A-AC-A-CC---AG--T--G--GC-GAA-G--G-C--G-------G--C-C-T-A---CTG--G--GC-T-T--------------------T-A---A-C-T--GA--CG-C-T-G--A-GG--C-A-CG-A--AA-G-C---A-TG--
GG-T--AG-C-A-AA--------CA--GG Bacteria(100);unclassified(100);unclassified(100);unclassified(100);unclassified(100);unclassified(100);unclassified(100);unclassified(100);unclassified(100);
